# Inhibition of the hexamerization of SARS-CoV-2 endoribonuclease and modeling of RNA structures bound to the hexamer

**DOI:** 10.1038/s41598-022-07792-2

**Published:** 2022-03-09

**Authors:** Duy Phuoc Tran, Yuta Taira, Takumi Ogawa, Ryoga Misu, Yoshiki Miyazawa, Akio Kitao

**Affiliations:** School of Life Sciences and Technology, Tokyo Institute of Technology, 2-12-1 Ookayama, Meguro-ku, Tokyo, 152-8550 Japan

**Keywords:** Computational biophysics, Virtual screening

## Abstract

Non-structural protein 15 (Nsp15) of severe acute respiratory syndrome coronavirus 2 (SARS-CoV-2) forms a homo hexamer and functions as an endoribonuclease. Here, we propose that Nsp15 activity may be inhibited by preventing its hexamerization through drug binding. We first explored the stable conformation of the Nsp15 monomer as the global free energy minimum conformation in the free energy landscape using a combination of parallel cascade selection molecular dynamics (PaCS-MD) and the Markov state model (MSM), and found that the Nsp15 monomer forms a more open conformation with larger druggable pockets on the surface. Targeting the pockets with high druggability scores, we conducted ligand docking and identified compounds that tightly bind to the Nsp15 monomer. The top poses with Nsp15 were subjected to binding free energy calculations by dissociation PaCS-MD and MSM (dPaCS-MD/MSM), indicating the stability of the complexes. One of the identified pockets, which is distinctively bound by inosine analogues, may be an alternative binding site to stabilize viral RNA binding and/or an alternative catalytic site. We constructed a stable RNA structure model bound to both UTP and alternative binding sites, providing a reasonable proposed model of the Nsp15/RNA complex.

## Introduction

Coronaviruses possess the largest genome of known RNA viruses^1^ and have attracted significant attention since the severe acute respiratory syndrome coronavirus (SARS-CoV) outbreak in 2002, and the Middle East respiratory syndrome coronavirus (MERS-CoV) affected Arabic countries in 2012^2^. Currently, severe acute respiratory syndrome coronavirus 2 (SARS-CoV-2) is spreading globally and has caused the largest pandemic of the twenty-first century. Understanding the behavior of the SARS-CoV-2 virus at the molecular level would thus help curb the pandemic and prevent coronavirus-related diseases.

The SARS-CoV-2 genome encodes spike, nucleocapsid, membrane, and envelope proteins, as well as 16 non-structural proteins (Nsps)^3^. The functions of the 16 Nsps were poorly understood until recently^4^. The highly conserved nidoviral RNA uridylate-specific endoribonuclease (NendoU) activity of Nsp15 allows evasion of the immune response^5^. By using BLAST with the UniProtKB database^6^, we confirmed that SARS-CoV-2 Nsp15 shares significant amino acid sequence identity (more than 40%) with SARS, MERS, and bat, rat, bovine, shrew, porcine, canine and ferret coronaviruses Nsp15 and several other SARS-CoV-2 proteins can suppress primary interferon production and interferon signaling, thus possibly interfering with the body’s defense against infections^7^. Nsp15 cleaves the polyuridine (polyU) of negative-sense RNAs of beta-CoV mouse hepatitis virus (MHV) strain A59 (MHV-A59) and alpha-CoV porcine epidemic diarrhea virus (PEDV), limits the abundance and length of polyU, and delays the type I interferon response in macrophages^8^. The structure of Nsp15, suggested to be a dimer of homo-trimers, is well-conserved between MERS-CoV, SARS-CoV, MHV, and MERS-CoV^9^. Also, hexamerization was shown to promote nidoviral uridylate-specific endoribonuclease activity^9^. The crystal structures of apo and citrate-bound SARS-CoV-2 Nsp15 confirmed that both forms of the protein are hexamers^10^. Comparison of the apo and UTP-bound states of the Nsp15 hexamer obtained by cryo-EM reconstructions indicates conformational dynamics between these states^11^. These results suggested that SARS-CoV-2 activity can be inhibited by preventing the hexamerization of Nsp15 and intervening NendoU activity through drug binding. Since interface residues in oligomeric proteins tend to be evolutionally conserved^12,13^, targeting pockets around the Nsp15 oligomer interface is a reasonable approach for reducing the possibility of drug resistance by mutation. In addition, a study of 27 SARS-CoV-2 proteins showed that mutation rate ranges are very high for spike, Nsp12 (around 1.0), NS9c, and nucleocapsid (> 0.5), while those for the other proteins are very low, including for Nsp15 (≤ 0.03)^14^. Right after the translation from viral RNA to protein, Nsp15 should exist as a monomer. Then, each monomer should conduct conformational changes to adapt itself to the suitable oligomeric form. Therefore, if ligand binding to Nsp15 monomers occur before oligomerization, Nsp15 activities should be disturbed.

Inheriting the above idea of inhibition, we first explored stable conformations of the SARS-CoV-2 Nsp15 monomer based on the hexameric apo form crystal structure by using an enhanced molecular simulation method, the Parallel Cascade Selection Molecular Dynamics (PaCS-MD) simulation^15^. Analysis of the free energy landscape of the conformational space using the Markov state model (MSM)^16^ indicated that the conformations of the global free energy minimum in the monomeric state significantly differ from that in the hexameric state. We targeted these conformations and identified possible druggable pockets suitable for stabilizing the monomeric conformations through drug binding and inhibiting hexamerization, then virtually screened possible compounds confirmed to stably bind to the proposed pocket by binding free energy calculations. One of the identified pockets was distinctively bound by inosine analogues, suggesting an alternative UTP binding site. By constructing an RNA structure connecting the *prior*-knowledge and nearest alternative UTP binding sites on the Nsp15 hexamer surface, we propose a reasonable structure model of the Nsp15/RNA complex.

## Results and discussion

### Features of the hexameric structure and initial modeling of the monomeric conformation

**Figure 1:**
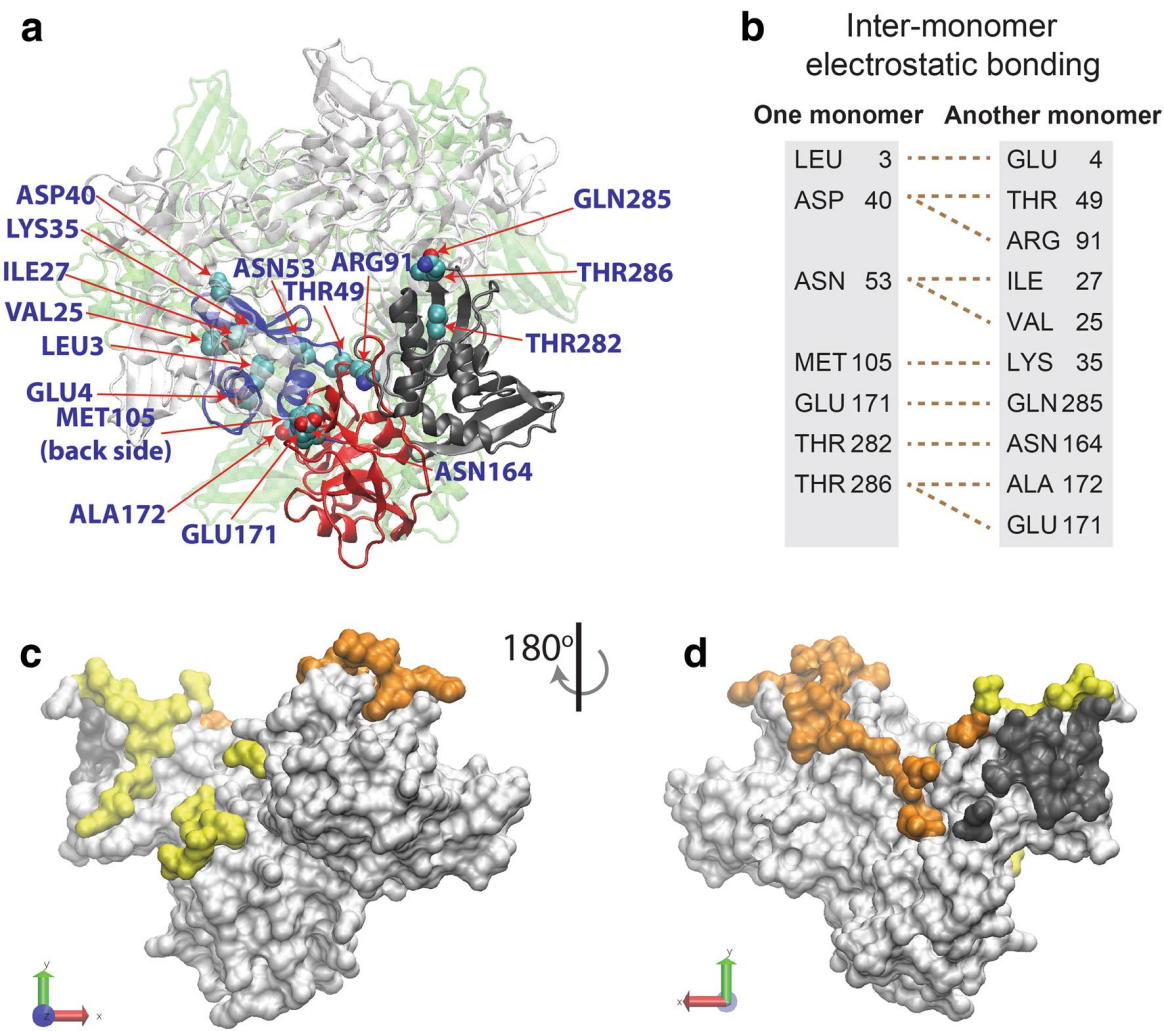
Structural features of the Nsp15 monomer in the apo hexamer. (**a**) A representative monomer structure (N-term domain: blue, Mid domain: red, and C-term domain: grey), the other dimers (transparent white) in the front trimer, and the backside trimers (pale green) are depicted. The residues shown by CPK models are involved in electrostatic bonding (salt bridges and hydrogen bonds) between the pairs of amino acids listed in (**b**). In (**b**), broken lines indicate the pattern of the electrostatic bondings. (**c, d**) The residues that form inter-monomer interfaces. The white area denotes non-interfacial residues, while other colors (yellow, orange, and dark grey) indicate the hexamer interface residues. In (**d**), the protein is rotated 180° around the y axis. The structure images in this paper were created using VMD^60^ unless otherwise specified.

The apo (PDB ID: 6VWW) and citrate-bound (6W01) states of the SARS-CoV-2 Nsp15 hexamer crystal structure form a ring-like complex as a dimer of trimers (Fig. 1a)^10^. The root-mean-square deviation (RMSD) between the apo and citrate-bound monomers is very small (0.026 nm) and thus we focused on the apo hexameric form (“apoH” hereafter). The UTP-bound form determined by cryo-EM (PDB ID: 7K0R^11^) also shows the hexameric form (“UTPH” hereafter). The monomer RMSD value between UTPH and apoH is small (0.037 nm). Each monomer is L-shaped and consists of three domains: the N-terminal domain (residues 1‒68 in blue. “N-term domain” hereafter); the middle domain (residues 69‒202 in red, “Mid domain”); and the C-terminal domain, which contains a NendoU catalytic site (residues 203‒347 in dark grey, “C-term domain”). N-term domain includes a linker to Mid domain in the C-terminal end, and Mid domain contains a linker to C-term domain in the C-terminal end. Nine pairs of residues that form inter-monomer ionic and hydrogen bonds (hereafter “electrostatic bonding”) in the hexamer (Fig. 1b–d) stabilize the hexamer. The monomer uses the large surface area of N-term domain as inter-subunit interfaces that mostly interact with the orange region to make a trimer. The dark grey region mainly interacts with another trimer.

In the initial step, we conducted 5 distinct trials of 1 μs standard molecular dynamics (MD) simulations starting from the monomer structure taken from the apo Nsp15 hexamer crystal structure (PDB ID: 6VWW^10^). The backbone RMSD from the crystal structure plateaus at 0.18 ± 0.06 nm (hereafter, values after ‘±’ indicate standard deviation unless otherwise noted) after 200 ns relaxation (left panel of Fig. S1), showing that the monomer structure changed little within this time scale. However, this does not necessarily mean real convergence over longer time scales. To further examine possible larger conformational changes from the hexameric state, we conducted enhanced conformational sampling in the next step. As shown in the plot of the root-mean-square fluctuation of each residue (RMSF) using the last 500 ns of the 1 μs MD trajectory (right panel of Fig. S1), there were large fluctuations up to 0.3 nm in the end of N-term domain that links to Mid domain.

### Enhanced conformational sampling indicates large domain movement

Proteins often exhibit significant conformational change upon complex formation^17,18^. We employed PaCS-MD to examine conformational differences between the monomeric and hexameric forms of Nsp15. This method is an enhanced conformational sampling simulation that generates conformational transition pathways using cycles of multiple independent MD simulations without applying bias to the system and can be used to observe events whose timescales are longer than that of standard MD^15,19,20^. By integrating the trajectories obtained by PaCS-MD and analyzing them with the MSM^16^, we can obtain various quantities such as the free energy landscape of conformational change^21,22^, binding free energy^23,24^, and association/dissociation rate constants^18,25^. To enhance conformational sampling, PaCS-MD requires a quantity for selecting the initial structures for the next cycle. Here, we employed RMSD_init_, which is defined as the backbone RMSD from the initial structure of each PaCS-MD trial. The use of RMSD_init_ allowed PaCS-MD to significantly enhance conformational changes further from the initial structure, and is here called rmsdPaCS-MD.

**Figure 2:**
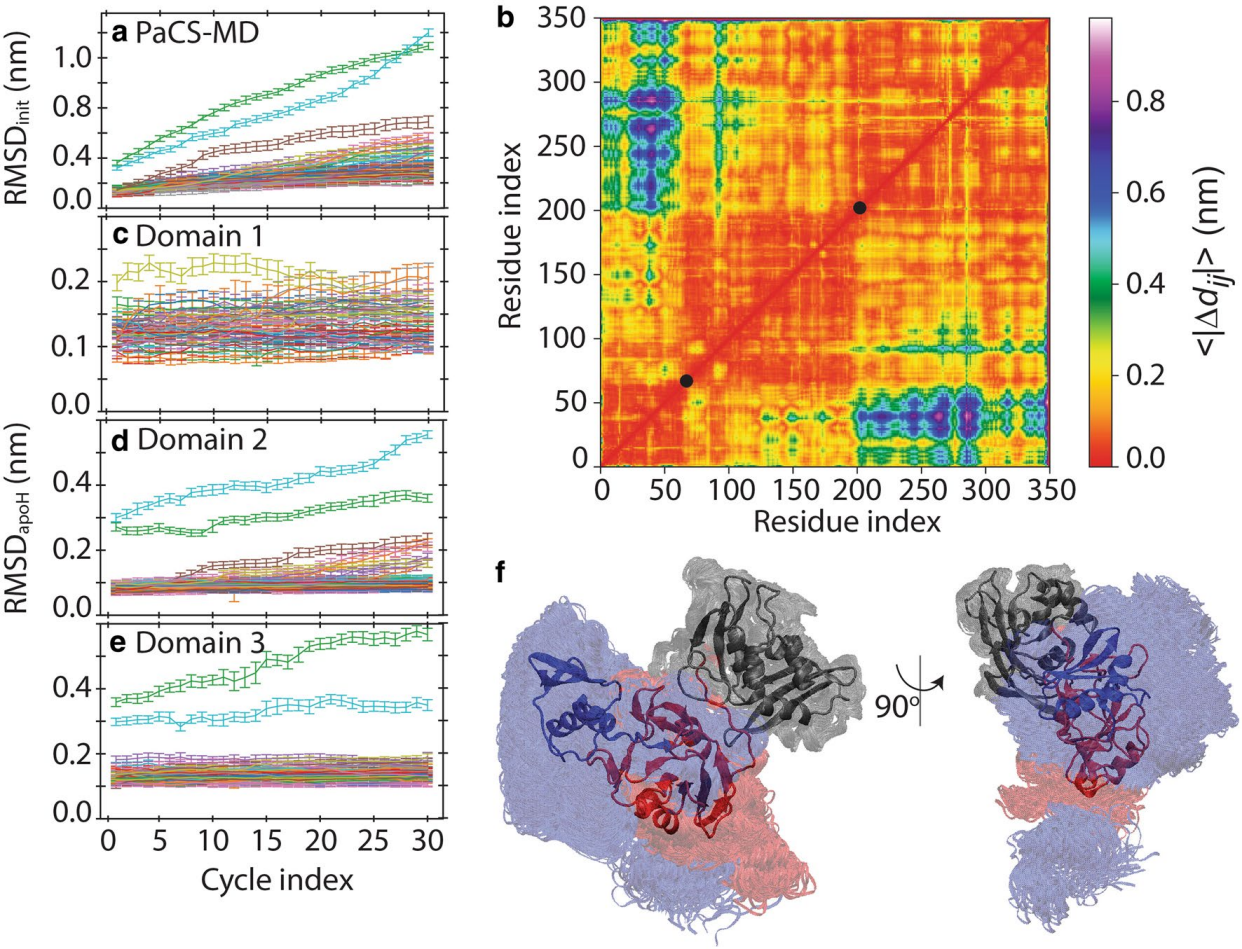
Conformational variation of the Nsp15 monomer. (**a**) Root-mean-square deviation (RMSD_init)_ and its standard deviations (error bars). (**b**) Change in the inter C_α_ distance between the initial and final snapshots of rmsdPaCS-MD averaged over trials, $\langle \left|\Delta {d}_{ij}\right|\rangle$. The color of a point specified by the residue indices *i* and *j* identifies the value of $\langle \left|\Delta {d}_{ij}\right|\rangle ,$ as shown in the color bar on the right. The two filled black circles indicate the borders of the three domains. (**c, d, e**) RMSD of each domain compared to that of the crystal structure (RMSD_apoH_). (**f**) Visualization of the last frames at cycle 30 in all rmsdPaCS-MD trials after performing best-fitting of the backbone atoms of C-term domain. Each domain is specified by a distinct color, as in Fig. 1a.

Thirty independent rmsdPaCS-MD trials were conducted starting from 30 distinct initial structures selected from the aforementioned five 1 μs MDs. For each trial, we employed 30 replicas (the number of MD runs performed in parallel in each cycle), and performed 30 cycles of rmsdPaCS-MD, excluding the initial cycle (cycle 0) that generates the initial conformations of cycle 1. Figure 2a shows RMSD_init_ as a function of the number of cycles. In cycle 1, RMSD_init_ ranged from 0.09 to 0.19 nm except for two cases (green and red) and gradually increased as the cycle evolved, indicating significant enlargement of the sampled conformational space. After 30 cycles, RMSD_init_ reached 0.2‒0.78 nm, except for two cases with RMSD_init_ > 1 nm.

To characterize the conformational space sampled by rmsdPaCS-MD, we first measured the inter-C_α_ distances between residues *i* and *j* of the last snapshot of each of the 30 trials and then calculated the distance changes $\langle \left|\Delta {d}_{ij}\right|\rangle$ compared to the distances in the rmsdPaCS-MD initial conformation (Fig. 2b). Three notable regions with low $\langle \left|\Delta {d}_{ij}\right|\rangle$ values (< 0.1 nm) agreed with the three domains already defined. The RMSD of each domain compared to the crystal structure (RMSD_apoH_) versus the MD cycle index (Fig. 2c‒e) remained around 0.2 nm or lower, except for the aforementioned two cases (cyan and green) in which Mid and C-term domains partially unfolded. Consistent with the small-domain RMSDs shown in Fig. 2c–e, $\langle \left|\Delta {d}_{ij}\right|\rangle$ were mostly less than 0.4 nm in each domain, showing their rigidity. Even in the exceptional cases, N-term domain did not unfold in any of the rmsdPaCS-MD trials, indicating it has the greatest rigidity. Large distance changes between N-term and C-term domains (Fig. 2b) imply large motions between these domains. These motions can also be shown by best-fitting C-term domain and observing the position of N-term domain. These conformations include extremely large movements from the apoH structure (Fig. 2f), many of which may be unfeasible and should be excluded from the following analyses.

### The open form is a stable conformation as a monomer

Although rmsdPaCS-MD implies possible motions of proteins, it does not directly indicate whether the observed motion is plausible. To identify stable conformations of the Nsp15 monomer, we calculated the free energy landscape in conformational space using rmsdPaCS-MD/MSM, in which merged rmsdPaCS-MD trajectories were analyzed by the MSM. rmsdPaCS-MD/MSM can obtain the protein free energy landscape spanned by representative coordinates of protein motion and find the lowest free energy conformation in the landscape^18,22–25^. We calculated the free energy landscape spanned by two collective variables, namely, the two most important time-independent components (TIC 1 and TIC 2) obtained by time-independent component analysis (TICA)^26^. Maximum-likelihood estimation was employed to construct the MSM by using the C_α_ coordinates of all residues projected onto the TICs after performing least-squares fitting of N-term domain. Since this domain exhibited the highest rigidity, this choice mainly focused the analysis on characterizing the possible motions of the other domains. After carefully checking the convergence of k-means clustering and the relation between lag time and the implied timescale via extensive MSM trials, we determined that the best lag time was 50 ps and used this value for further analyses. The evolution of the implied timescale as a function of lag time showed good quality of the MSM (Fig. S2), with 121 highly connected microstates identified. The disconnected states were related to unfolded structures.

**Figure 3:**
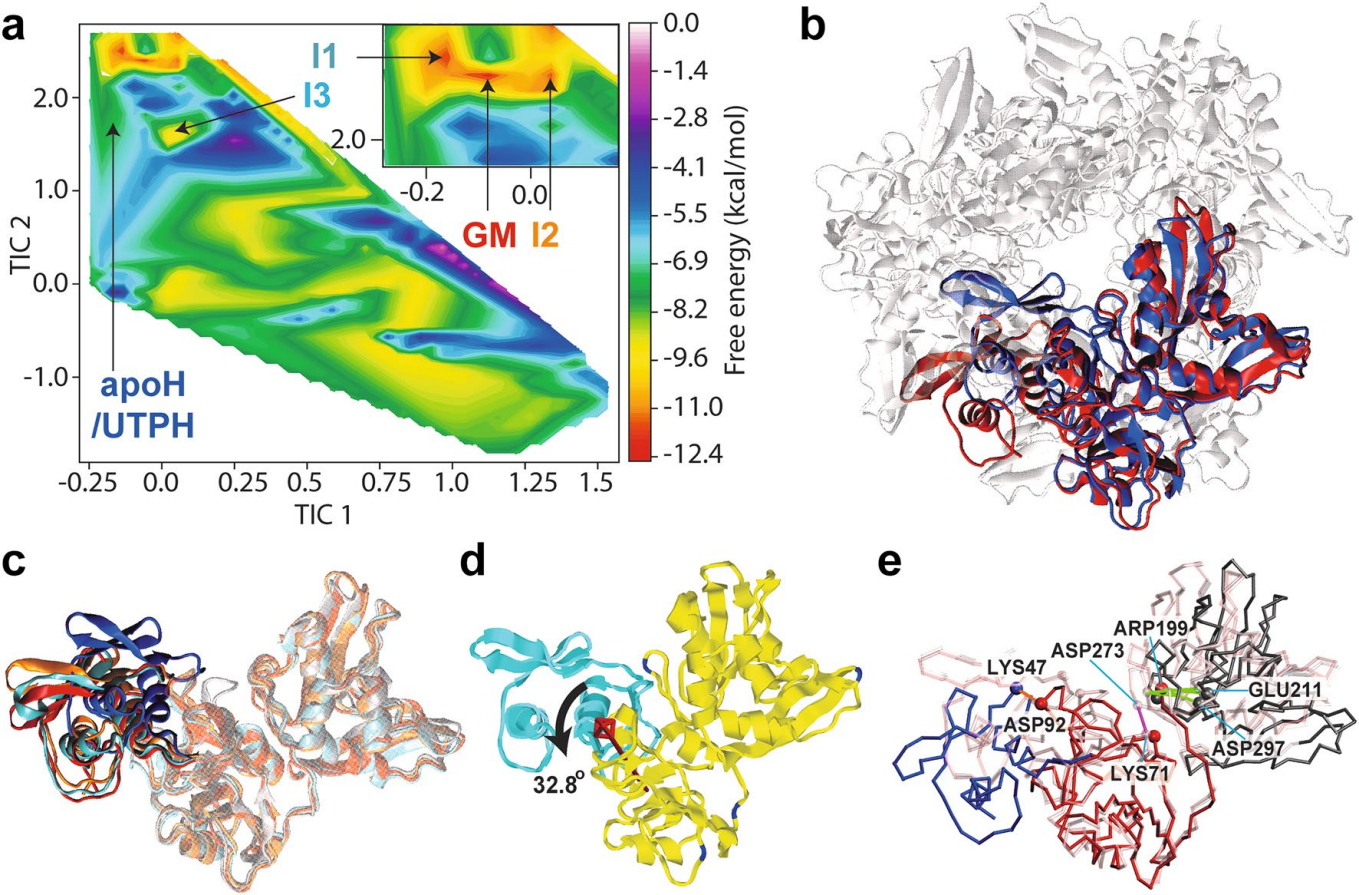
Stable conformation of the Nsp15 monomer. (**a**) Free energy landscape obtained by MSM. The inset shows a close-up view around the global free energy minimum (GM). I1, I2, and I3 represent three intermediates, and apoH indicates the crystal conformation. (**b)** Visualization of GM (red) superimposed onto one monomer of apoH (blue) in the hexameric state by best-fitting Mid and C-term domains. (**c**) Comparison of the GM (red), I1 (cyan), I2 (orange), and I3 (blue) conformations superimposed onto the Mid and C-term domains. (**d**) Result of DynDom3D analysis between GM and apoH, shown by the apoH conformation. The red arrow indicates the rotational axis of the dynamic domain (cyan) rotation, which corresponds to N-term domain. (**e**) Inter-domain salt bridges in the GM, apoH, and UTPH conformations. The apoH conformation is shown in white. N-term (blue), Mid (red), and C-term (dark grey) domains of the GM conformation and UTPH conformation (pink) are superimposed on the apoH conformation (white) by best-fitting Mid domain. The green lines connecting ARG199 and LYS205/ASP297 indicate the salt bridges maintained in all the conformations. The magenta lines between LYS71 and ASP273 represent the salt bridge formed in apoH and UTPH, and the orange line between LYS71 and ASP92 shows the salt bridge established solely in GM. (**d**) was created using Rasmol^61^.

The obtained free energy landscape of the Nsp15 monomer is shown in Fig. 3a. We found multiple free energy minima, global free energy minima (denoted as GM), and three intermediates (I1, I2, and I3), all of which are significantly different from apoH. The C_α_ RMSD between GM and apoH is 0.53 nm if the whole monomer is superimposed. The free energy of the microstate closest to apoH is higher than that of GM by 7.4 kcal/mol, which indicates that the GM structure is significantly more stable than the apoH structure as a monomer. GM, I1 (+ 0.9 kcal/mol compared to GM), and I2 (+ 1.3 kcal/mol) can be considered to belong to the same free energy basin (see the close-up view of the landscape, shown as an inset in Fig. 3a). Of these microstates, the I3 conformation is closest to that of apoH. Figure 3b shows the GM conformation (red) superimposed onto one of the Nsp15 hexamer subunits (blue) by best-fitting Mid and C-term domains and indicates the large relative movement of N-term domain outward of the hexamer ring. Since the GM conformation tends to open the trimer ring, we regard GM as a more “open” conformation compared to apoH. We obtained deeper insights to the structural differences between GM and I1‒I3 by performing least-squares fitting of Mid and C-term domains (Fig. 3c). While N-term domain of GM (red), I1 (green), and M2 (orange) from the same basin highly overlapped, M3 (blue), which is closest to apoH, took a more compact form compared to the other conformations. The GM conformation is more exposed to solvent as a monomer, showing an increase in solvent accessible surface area of 12.0 ± 2.1 nm^2^ compared to apoH.

The conformational difference between the conformations of GM and apoH was further analyzed by the dynamic domain analysis method DynDom3D^27^, which identifies “dynamic domains” from two structures and characterizes the rotation of a dynamic domain relative to others around an axis. First, we applied DynDom3D to the GM and apoH conformations (Fig. 3d). DynDom3D identified two domains: large (yellow) and small (cyan). The large domain consists of the entire C-term domain and most of Mid domain, while the small domain comprises N-term domain (except for the side chain of ASN63 and PRO66‒PRO68) and small fragments of Mid domain (parts of VAL85‒THR99 and GLY101‒THR106). The small domain rotates around the axis shown in Fig. 3d by 32.8° with a negligibly small translation (0.004 nm), yielding a 75.0% closure motion. This result means that conformational change upon hexamer formation can be considered as a rigid-body rotation of one domain relative to the other.

### Electrostatic interactions to stabilize the monomeric conformation

We investigated the conformational difference between the hexameric and monomeric forms by analyzing the change in electrostatic bonding (hydrogen bonds and salt bridges) between apoH and GM within a monomer. The salt bridges are shown in Fig. 3e. Hydrogen bonds formed by ASN63 and PRO66 of N-term domain with their counterparts in Mid domain maintain electrostatic bonding in both apoH and GM. Although ASN63 and PRO66 are situated in N-term domain, they are assigned as part of the large dynamic domain by DynDom3D, indicating that the cyan dynamic domain in Fig. 3d (mostly N-term domain) can rotate while maintaining these interactions. ASN63 exchanged a hydrogen bonding partner with Mid domain from THR84 (apoH) to TYR89 in GM. An additional salt bridge between LYS47 (N-term domain) and ASP92 (Mid domain) is formed in GM, helping stabilize the open N-term domain arrangement relative to Mid domain.

**Figure 4:**
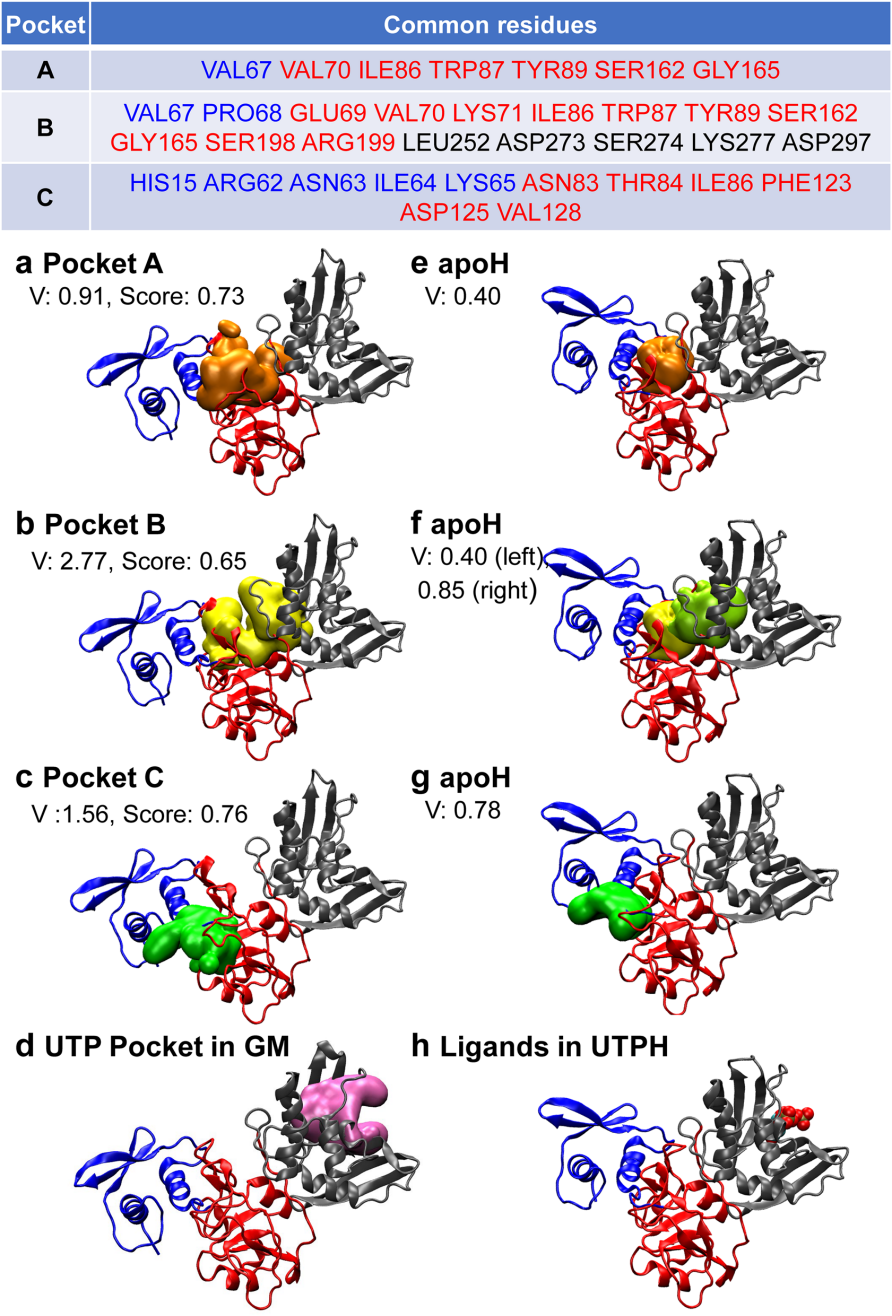
Major druggable pockets around the hexamer interfaces. The table at the top shows the residues common to the pockets and are assigned as Pockets A‒C. The residue names shown in blue, red, and black indicate that they belong to N-term, Mid, or C-term domain, respectively. (**a)** Pockets A (green), (**b**) B (yellow), and (**c**) C (orange) identified for the monomer GM conformation. Druggable score of the pocket (Score) and pocket volume (V. unit: nm^3^) are shown. (**d**) UTP-binding pocket found in GM. (**e**‒**g**) The pockets most overlapped with pockets A‒C in apoH are depicted. (**h**) UTPH structure and 5′-UMP shown as a CPK model. Each domain is specified by a distinct color, as in Fig. 1a.

Between Mid and C-term domains, 12 residue pairs maintain electrostatic bonding, including salt bridges between ARG199 (Mid domain) and GLU211/ASP297 (C-term domain) in both apoH and GM. These many interactions are considered to stabilize the arrangement between Mid and C-term domains, consistent with the result that these two domains are considered as one dynamic domain. However, the contribution of the ARG199‒GLU211/ARG199‒ASP297 interactions may be limited, as ARG199 is situated in the end linker between Mid and C-term domains. Three interactions found in apoH are not formed in GM but two new interactions are created. This result implies that the interactions between Mid and C-term domains are slightly weakened in the monomer. In addition, the salt bridge between LYS71 (Mid domain) and ASP273 (C-term domain) maintained in apoH is lost in GM. This loss contributes to loosening the packing between the two domains around LYS71‒ASP273, consistent with the presence of a larger space in GM, as shown in the next section: Pocket B located between Mid and C-term domains in Fig. 4b in the monomer is significantly larger than the corresponding pocket in apoH in Fig. 4e. As mentioned earlier, the monomer conformation of UTPH is very similar to that of apoH, and the salt bridges in apoH are also maintained in UTPH.

### Druggable pockets around the hexamer interface

Significant conformational difference between GM and apoH/UTPH indicates that large conformational change is essential for oligomerization. If the GM conformation is stabilized by ligand binding, ligand binding should prevent hexamerization. For this purpose, we predicted druggable pockets in the GM conformation using PockDrug. In this method, each pocket is defined by the atoms that form the pocket. We applied this method to 100 structures selected from the GM microstates and identified 150 druggable pockets. The residues that form the pockets are highly overlapped in all 150 cases, and interestingly, TRP89 in Mid domain was without exception part of the pocket. The identified pockets were grouped into 25 clusters using the overlap ratio of the pocket-forming residues. Of the clusters situated around the hexamer interfaces, we selected the three most-populated clusters of pockets (hereafter Pockets A, B, and C), whose populations were 19/150 (12.7%), 16/150 (10.7%), and 13/150 (8.7%), respectively. The table in Fig. 4 shows the residues common in each of Pockets A‒C, and Table S1 provides the PockDrug descriptors. Pocket A (druggable score: 0.73) is mostly formed by Mid domain, while Pocket B (0.65) spreads across all the domains. All the pocket-A-forming residues are included in the pocket-B-forming residues because Pocket B is assigned slightly more open structures. Pocket C (0.76) is mainly formed by the interface between N-term and Mid domains, implying that the binding of a compound to this pocket can prevent conformational change of N-term domain relative to the other domains. Of these pockets, Pocket A is the smallest in volume and Pockets B and C are 3- and 1.5-fold larger compared to Pocket A, respectively (Fig. 4a–c). The ratio of hydrophobic residues is similar between the pockets, but according to “hydrophobic kyte” obtained by PockDrug, Pockets A (− 0.13 ± 0.21) and C (− 0.11 ± 0.32) are less hydrophilic than Pocket B (− 0.43 ± 0.18). Considering the ratio of polar residues, Pocket B showed slightly higher polarity (0.61 ± 0.03) compared to Pockets A and C, whose polarities coincidently have the same value (0.52 ± 0.05). Furthermore, Pocket C is formed by the three positively charged residues HIS15, ARG62, and LYS65. Overall, we judged that Pocket C, whose druggable score is the highest, was a suitable target for subsequent virtual screening and thus we examined it by two screening methods. Furthermore, we conducted virtual screening of Pockets A and B by one screening method. Pockets A, B, and C are distant from the UTP-binding pocket (Fig. 4d). The main mutated residues of Nsp15 are THR34 (residue mutation rate = 0.03), LYS13 (0.01), ARG207 (0.01), and THR115 (0.01)^14^, which are not included in these pockets (Fig. 4).

We also investigated druggable pockets for the monomer conformation of apoH and identified pockets corresponding to Pockets A‒C (Fig. 4e–g). The pockets in apoH were considerably smaller than Pockets A‒C, and Pocket B was divided into two pockets in the crystal form of apoH, indicating that the GM conformation is more suitable as a drug target. PockDrug did not find any pocket around the UTP pocket in the UTPH conformation (Fig. 4h), likely due to the induced fit of the residues surrounding 5′-UMP eliminating any available space.

To understand conservation of the pocket-A- and -C-forming residues across corona viruses, we performed BLAST on Uniprot database, searching for sequences similar to the SARS-CoV-2 NSP15 sequence. Then, the selected sequences whose pairwise sequence identities with the SARS-CoV-2 NSP15 > 40% were subjected to multiple sequence alignment (Fig. S3). We found that TRP110 and TYR112 of pocket A and ASP148 and Gly188 of pocket C are highly conserved across the coronavirus species. Those residues may be considered as key residues for the future drug screening.

### Virtual screening of antiviral compounds to find hexamerization inhibitors

**Figure 5:**
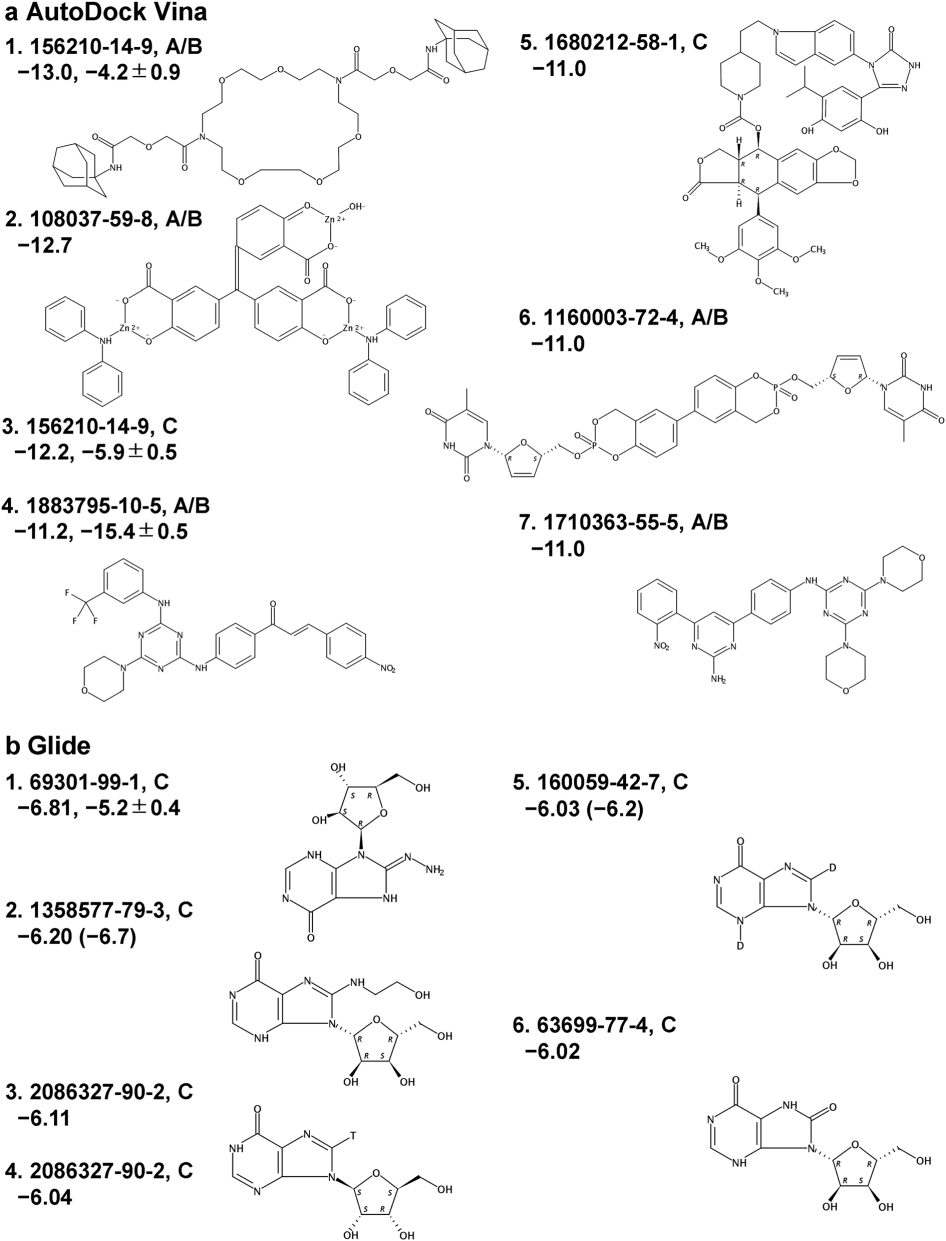
Top compounds identified by virtual screening. These compounds were screened from the antiviral compound library provided by The American Chemical Society (Mar. 2020)^62^ using (**a**) AutoDock Vina^28^ and (**b**) Schrödinger Glide^33^. Their ranking numbers, CAS Registry Number®, pockets (A/B or C), Vina or Glide scores, and standard binding free energies ($\Delta {G}^{o}$) obtained by dPaCS-MD/MDM and 2D structures are shown. In (**b**), Vina scores are also shown in parentheses if AutoDock Vina scores were calculated. The Vina and Glide scores and $\Delta {G}^{o}$ are in kcal/mol. 2D structures were created by SciFinder.

**Figure 6:**
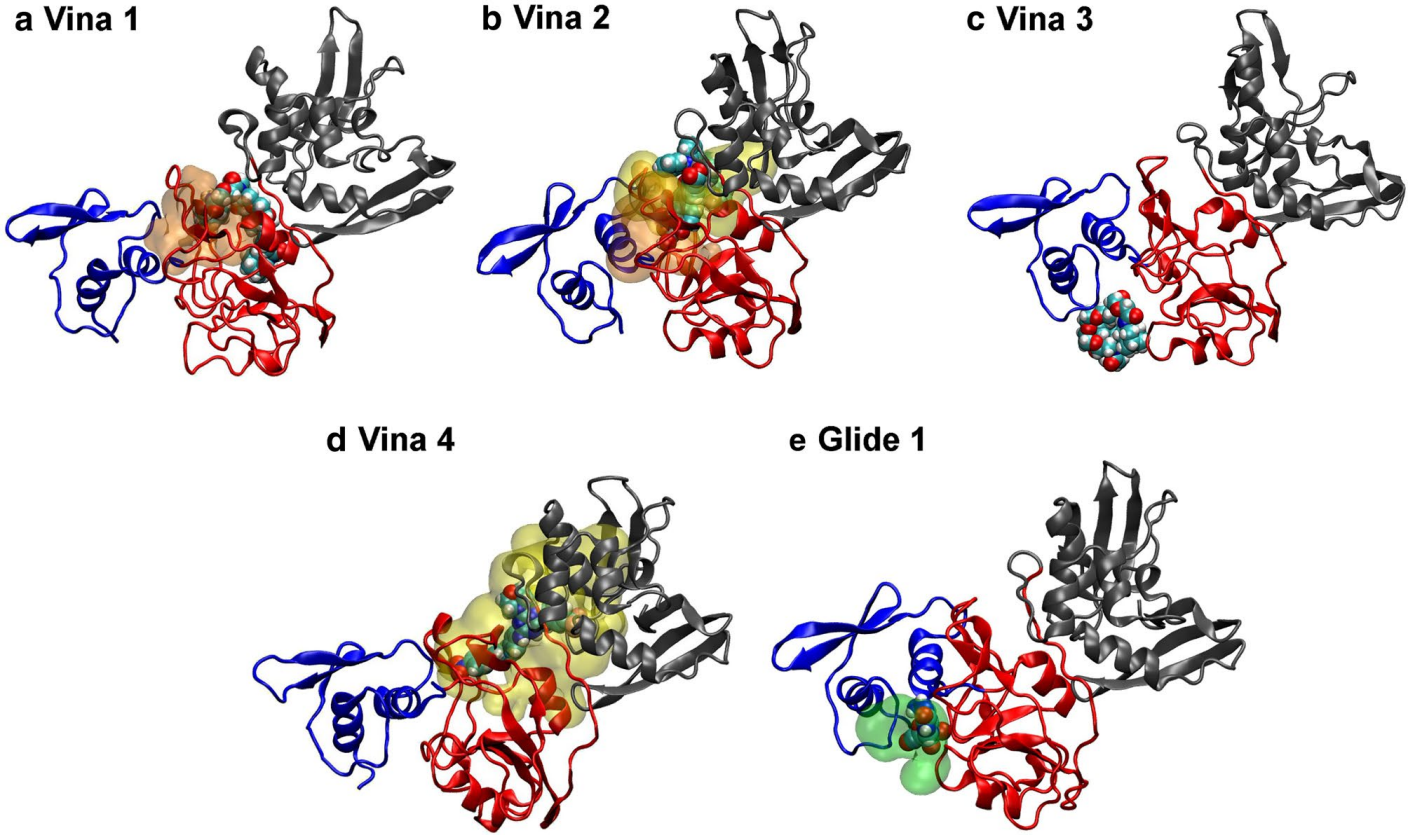
Complex structures of the top poses. (**a–d**) The top 4 poses identified by AutoDock Vina^28^ and (**e**) the top pose identified by Schrödinger Glide^33^. Representative complex structures after free 100 ns MD are shown except for (**b**), in which the output of AutoDock Vina is shown.

We performed ligand docking with 49,430 antiviral compounds provided by The American Chemical Society (CAS COVID-19 Antiviral Candidate Compounds Dataset) by using the docking tool AutoDock Vina^28^, targeting the centers of mass of Pockets A and C as the centers of the docking box. Since the docking box of Pocket A also completely contains Pocket B, docking to Pocket A simultaneously considers docking to Pocket B. Of 49,430 compounds, the screening yielded 22,945 poses per Pocket, except for 26,485 compounds that contain atoms not supported by AutoDock Vina or were not converted into the AutoDock PDBQT format (Table S2). Of these, 7 poses of 6 compounds showed an AutoDock Vina score lower than − 12.0 kcal/mol (Fig. 5a). The compound with the best Vina score (CAS Registry Number®, CAS RN®: 156210-14-9), which was originally designed as an ion-selective electrode^29^, binds to Pockets A/B (pose 1) and C (pose 3). This compound is very flexible and relatively large (805.01 Da) compared to Linpinski’s rule of five (500 Da)^30,31^. The second compound (CAS RN®: 108037-59-8) that tightly binds to Pocket A/B (pose 2) also binds to Pocket C as pose 9 (Table S2). The compound of pose 4 (1883795-10-5), which is much smaller (591.54 Da) compared to the previous two compounds, was reported to have antimicrobial activity^32^. Poses 5–7 have equivalent Vina scores. The compound of pose 7 (1710363-55-5) is the smallest of the compounds shown in Fig. 5a (556.58 Da). Considering Lipinski’s rule, the compounds of poses 4 and 6 are close to 500 Da and the others are much larger. Next, we investigated the stability of poses 1, 3 and 4 with Nsp15 using five 100 ns classical MD simulations (Fig. 6a, c, d). Pose 2 (Fig. 6b) containing three zinc ions was not optimized because classical MD simulation of zinc-containing compounds is not straightforward. Poses 1, 3 and 4 maintained stable binding with Nsp15, and Nsp15 maintained the open conformation that prevents hexamerization. We further examined the standard binding free energy $\Delta G^\circ$ of poses 1, 3, and 4 by dissociation PaCS-MD and MSM (dPaCS-MD/MSM) (Fig. S4). Of these, pose 4 (1883795-10-5) showed the lowest $\Delta G^\circ$ of − 15.4 ± 0.2 kcal/mol ($\Delta W$ = 17.3 ± 0.3 and $\Delta {G}_{V}$ = 1.9 ± 0.2 kcal/mol (the values after ‘±’ show the standard errors), which is equivalent to a dissociation constant *K*_*d*_ of 6.0 pM at 300 K. Although AutoDock Vina scores were very low for poses 1 and 3, the values of $\Delta G^\circ$ were significantly higher (pose 1: $\Delta G^\circ$ =  − 4.2 ± 0.4, $\Delta W$ = 6.0 ± 0.5, and $\Delta {G}_{V}$ = 1.8 ± 0.3 kcal/mol; pose 3: $\Delta G^\circ$ =  − 5.9 ± 0.2, $\Delta W$ = 7.6 ± 0.2, and $\Delta {G}_{V}$ = 1.7 ± 0.2 kcal/mol), suggesting that the binding of this compound (156210-14-9) is not as strong as expected from the AutoDock Vina scores.

We performed ligand docking using the docking tool Glide^33–35^ in the Schrödinger® package, targeting Pocket C that was judged as the best target in the previous section. All the compounds were considered. The screening yielded 225 poses of 24 compounds with Glide scores below − 2.8 kcal/mol (Table S3), which is the default cutoff value of Glide. Of these, 6 poses of 5 compounds showed a Glide score lower than − 6.0 kcal/mol, implying relatively strong binding to Pocket C (Fig. 5b). Interestingly, all 5 compounds are inosine-related compounds and interact with HIS15, ILE64, and LYS65 in N-term domain (Fig. S5). Most of the other compounds are nucleoside analogues (Table S3). The 6 binding poses with Nsp15 maintained stable binding during 100 ns MD simulations (Fig. 6e, and S6), and Nsp15 maintained the open conformation. Vina scores were obtained for the compounds of poses 2 and 5 (values in parentheses in Fig. 5b) which agreed with the corresponding Glide scores, indicating that the Vina and Glide scores are comparable. We further examined the standard binding free energy $\Delta G^\circ$ of the top pose with the compound (69301-99-1) by dPaCS-MD/MSM (Fig. 3d), and obtained a standard binding free energy $\Delta G^\circ$ of − 5.2 ± 0.4 kcal/mol ($\Delta W$ = 6.5 ± 0.3 and $\Delta {G}_{V}$ = 1.3 ± 0.2 kcal/mol) that is similar to the Glide score. These results indicate that the compounds shown in Fig. 5b are significantly weaker binders than the others.

During the revising process of this paper, Schultz et al. validated antiviral activity and selectivity of 122 drugs against SARS-CoV-2 and found that 16 of these are nucleoside analogues, including antivirals remdesivir and molnupiravir approved for COVID-19^36^. As shown above, all five compounds that we found by GLIDE are nucleotide analogues as well. Very recently, Kumar et al. carried out in silico and in vitro screening for active compounds from their in-house libraries targeting to Nsp15 and identified ‘Compound IV’ ((2S,3S)-3-amino-1-(4-(4-(tert-butyl)benzyl)piperazin-1-yl)-4-phenylbutan-2-ol) as the best one in in vitro assays^37^. Of these, functional groups of Compound IV share analogous interactions with Nsp15 in Pocket A/B with those of the compound of pose 4 that shows the lowest $\Delta G^\circ$. Choi et al. used high-throughput assay to screen compounds against Nsp15, and inhibition was confirmed for three hits in vitro and showed that Exebryl-1 (ß-amyloid anti-aggregation molecule for Alzheimer’s therapy) has antiviral activity^38^. They also showed that the most plausible binding residues with Exebryl-1 significantly overlap with those of Pocket B. Interestingly, the compounds that we found by AutoDock Vina shares some similarity in functional groups with Exebryl-1. This suggests the potentials of those predicted compounds as a starting point for further drug developments.

### Possible alternative RNA binding site and RNA structure bound to the Nsp15 hexamer

**Figure 7:**
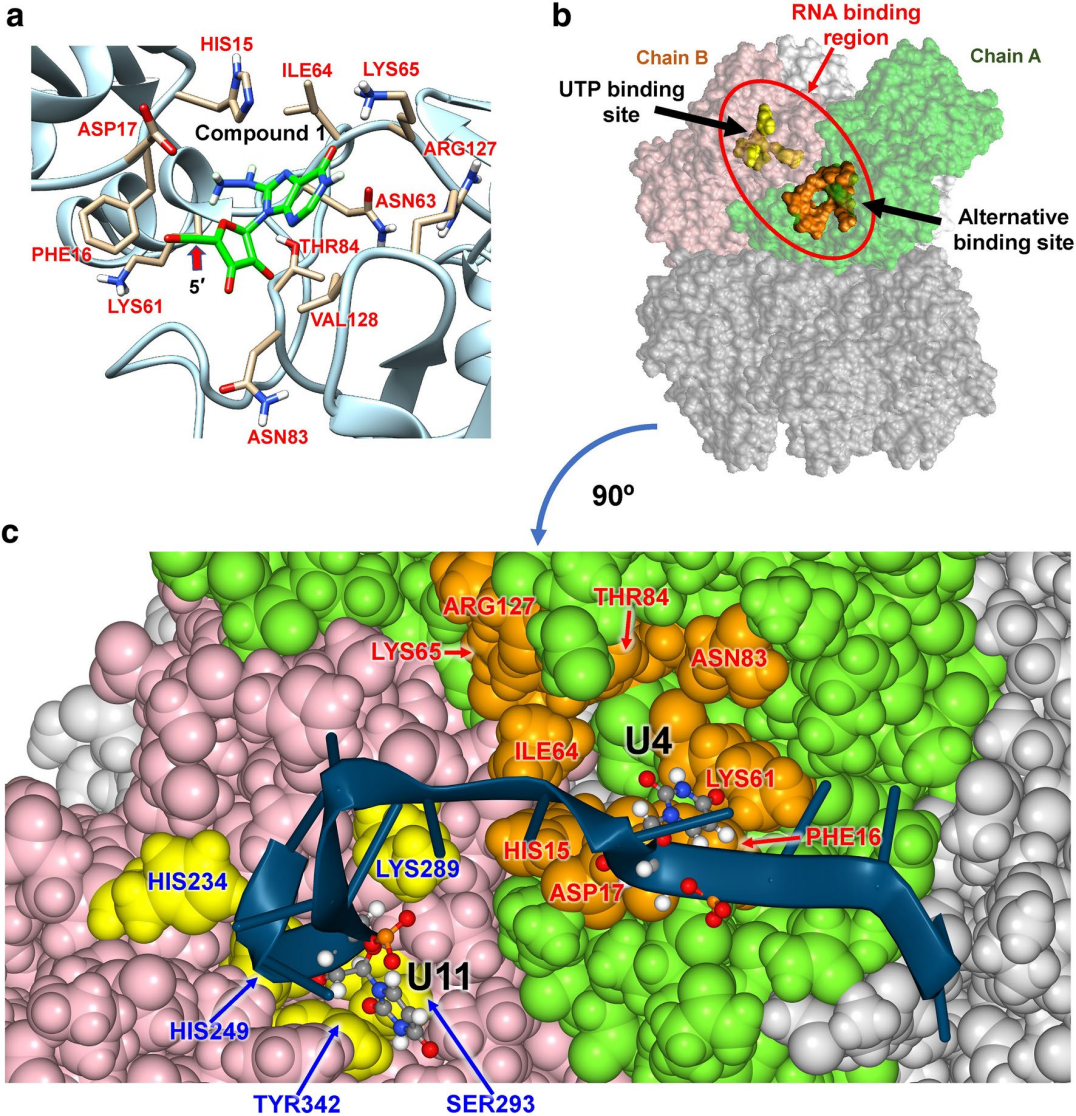
Nucleoside binding in Pocket C and possible RNA structure bound to the Nsp15 hexamer. (**a**) The best compound identified by Glide (CAS RN®: 69301-99-1. 1H-Purine-6,8-dione, 9-β-D-arabinofuranosyl-7,9-dihydro-, 8-hydrazone) and surrounding residues. The orientation is slightly changed from Fig. 6e to better show coordination of the surrounding residues. The 5′-carbon of the ribose ring is indicated by a red arrow. (**b**) Spatial arrangement of the UTP binding site and alternative nucleotide binding site (Pocket C) mapped onto UTPH. The residues shown in yellow are HIS235, HIS250, LYS290, SER294, and TYR343, and those shown in orange are those presented in (**a**). (**c**) A representative tridecaU RNA structure bound to the Nsp15 hexamer after 100 ns equilibration by MD. The coloring is the same as in (**b**). (**a** and **c**) were created using Chimera^63^ and NGLview^64^, respectively.

As described above, the top 6 poses in Pocket C identified by Glide are inosine analogs, which suggests possible binding of other nucleosides and nucleotides. We used PockDrug to examine if space remained around the compounds in the complex structures and found that it did, except for Glide poses 4 and 6 (Fig. 6 and S6), indicating the possibility of improving binding by adding chemical groups to the compounds. Figure 7a shows the coordinated residues around the compound of pose 1. The 5′-end of the ribose ring is exposed to solvent, reserving space for an additional group (e.g., a phosphate group), suggesting that Pocket C may be an alternative nucleotide binding site.

Interestingly, the pocket around this region is partly exposed to solvent even in the hexamer (Fig. 7b), although the pocket has half the volume of the pocket in the monomer (compare Fig. 4c, g). Also, Pocket C of one monomer is situated near the UTP binding site of the nearest monomer in the hexamer (Fig. 7b). If this pocket is an alternative RNA binding site, it can stabilize the viral RNA bound to Nsp15 during NendoU catalysis. Another possibility is that Pocket C has a catalytic site because the residues of Pocket C resemble the catalytic residues of ribonucleases. Histidine, aspartic acid and lysine are typical amino acid residues located around ribonuclease catalytic sites^11^ and HIS15, ASP17, LYS61, and LYS65 in Pocket C are situated near the ribose ring (Fig. 7a).

To further examine this idea, we constructed single-stranded RNA models bound to the Nsp15 hexamer based on UTPH (the UTP-bound hexameric form) and the arrangement of the two binding sites on the Nsp15 hexamer surface (Fig. 7b). First, three RNA chains consisting of tridecaU (5'-(U)_13_–3') were modeled, so that each 11th U (U11) binds to one of the UTP binding sites. We prepared relatively long RNA chains so that one of the RNA residues can fit into the alternative binding site. After simulated annealing, the RNA chain closest to the alternative binding site (the 3^rd^ RNA chain) was selected, and the closest U residue (initially U3, and U4 in the final step) was pulled toward the alternative binding site by Steered MD (SMD). After modeling and equilibration, five independent 100 ns MD simulations of the RNA-bound Nsp15 hexamer were conducted. We confirmed that the RNAs stably bound to Nsp15 during the MDs, and U4 and U11 maintained binding to the alternative and UTP binding sites, respectively (Fig. 7c, and S7). Therefore, single stranded RNA chains consisting of 8 nucleic acids or longer can bind to both pockets. RNA binding is stabilized not only by the interactions of U4 and U11 with the pocket residues but also by the following Nsp15/RNA interactions shown as (Nsp15 residues)/(RNA residue): SER2, ASN5, GLN19/U1; GLU22/U2; GLN245, LYS290/U10; TRP333/U12. These amino acid residues have no overlap with the aforementioned mutated residues, suggesting that the mutations have no direct effect on RNA binding. Interestingly, U4 of the 1^st^ RNA chain, which was not subjected to SMD, also reached near the alternative binding site with a flipped-out uridine group, although the uridine group was not perfectly situated in the pocket. Since RNA binding should be stabilized by binding to the alternative binding site, Nsp15 hexamerization is a key for NendoU catalysis, suggesting that preventing hexamerization may efficiently inhibit the catalytic activation of Nsp15. After initial submission of this paper, Frazier et al. determined the post-cleavage cryo-EM structure of Nsp15, indicating that AUA trinucleotide actually binds to Pocket C. They also showed that mutating HIS15 disrupts RNA cleavage and Nsp15 oligomerizaion^39^. These finding show that our calculation results are consistent with these experiments.

## Conclusion

We proposed that SARS-CoV-2 activity can be prevented by preventing the hexamerization of endoribonuclease Nsp15 with drug binding. We first explored the stable conformation of the Nsp15 monomer as the global free energy minimum conformation by using rmsdPaCS-MD/MSM. Compared to the hexamer form, N-term domain rotates by 32.8°, creating larger druggable pockets on the surface of Nsp15. Targeting the pockets with high druggability scores, we conducted ligand docking and identified compounds that tightly bind to the Nsp15 monomer. Nsp15s complexed with the top compounds were subjected to binding free energy calculations by dPaCS-MD/MSM, indicating the stability of the complexes. The binding of the compounds maintained the open conformation of Nsp15, which can prevent hexamerization. These compounds may provide leads for drug development against COVID-19. Of these, the compound of AutoDock Vina pose 4, whose binding free energy is the lowest and whose molecular weight is relatively low, is the best candidate. Pocket C is suggested to be an alternative binding site to stabilize viral RNA binding and/or an alternative catalytic site. Further, we constructed a structure model of RNA-bound Nsp15 and demonstrated the stability of the complex by MD simulation, thereby proposing a reasonable model of the Nsp15/RNA complex during NendoU activity.

## Methods

### Model preparation, equilibration, and rmsdPaCS-MD

The AMBER ff19SB force field^40^ was used for the protein. Nsp15 monomer was solvated in a 15.9 × 14.7 × 14.7 *nm*^*3*^ box with OPC water molecules^41^. Potassium and chloride ions were added to mimic a 0.15 M ion concentration and charge neutrality. The relaxation simulations were performed using AMBER18^42^ and PaCS-MD simulations were performed using GROMACS 2019.4^43^.

We carried out equilibration as follows. (1) The solvated models were energy-minimized by the steepest descent method followed by the conjugate gradient method with positional restraints applied on the heavy atoms of Nsp15 (force constant: 1000 kJ/mol nm^2^). (2) The system with the same restraints was heated from 0 to 300 K within 1 ns and thermalized at 300 K for another 1 ns using an NVT ensemble simulation. Five different trials were carried out with different randomly-generated initial velocities to provide statistics for the simulations. (3) MD simulation with the NPT ensemble was conducted for the next 100 ps at 300 K with a relaxation time of 0.1 ps for heat bath coupling, and at 1.0 atm with a relaxation time of 2.0 ps for isotropic pressure coupling. (4) The force constant of the positional restraints was reduced by 100 kJ/mol nm^2^ every 100 ps until it vanished (total 0.9 ns). (5) The system was equilibrated for 1 µs using the NPT ensemble. (6) The selected conformations were converted for GROMACS to conduct 30 cycles of PaCS-MD. The equation of motion was integrated using the velocity Verlet method^44^ with bond constraints by the SHAKE method^45^ (steps 2–5) and without bond constraints (PaCS-MD step). The isothermal condition was established by Langevin dynamics^46,47^ in steps 3, 4, and 5, and by the Nosé-Hoover thermostat in the PaCS-MD step. The isobaric condition was achieved using the Berendsen (steps 3, 4)^48^, Monte-Carlo (step 5)^49^, and Parrinello-Rahman barostats (step 6)^50^. The snapshots sampled in the second half (500 ns each) of the 5 runs of step 5 were grouped into 50 clusters. We selected the 30 highest populated structures from step 5 and used them in step 6. Upon conversion from AMBER to GROMACS, we equilibrated the system for 10 ns with 1000 kJ/mol nm^2^ positional restraints of the protein backbone. For each of these structures, 1 ns MD was conducted (hereafter cycle 0), and 30 snapshots of the first rmsdPaCS-MD cycle were selected. We used 30 replicas and 0.1 ns MD simulations for each cycle and recorded the snapshots every 0.5 ps for analysis. All the MD trajectories generated by PaCS-MD, including cycle 0, were merged and used in MSM. The total simulation cost of rmsdPaCS-MD was 2.73 μs MD: [(1 ns MD for cycle 0 + 0.1 ns MD × 30 replicas × 30 cycles) × 30 trials]. Together with the 1.8 μs relaxation MDs, the total computational cost of this step was 4.53 μs. The same timestep, barostat, and thermostat settings were applied to the following MD simulations of the complexes and dPaCS-MD.

### Markov state model

The initial dataset for the MSM was constructed as all C_α_ coordinates of Nsp15 after performing least-squares fitting of N-term domain with the crystal conformation. The dataset was then discretized into 1000 microstates by k-means clustering^51^ with the k-means ++ algorithm^52^. After carefully checking the convergence of the cluster centers over multiple trials with different lag times, the dataset was projected onto the time-lagged independent components space (TIC space)^53^. The original dimension of 1044 was reduced to 562, keeping 95% of the fluctuations in the reduced space. We built the MSM by using maximum-likelihood estimators with a sliding count algorithm to fulfill the detailed balance condition. PyEMMA package^54^ was used to construct the MSM.

### Domain motion analysis

We used DynDom3D for domain motion analysis^27^. Default DynDom3D parameter values were used: grid size 0.4 nm; block factor 2; occupancy 0.6; and minimum domain size 200.

### Prediction of druggable pockets

We used PockDrug-Server^55^ to predict druggable pockets. The pocket estimation method fpocket^56^ was applied to 100 structures randomly selected from the GM microstate with a ligand proximity threshold of 5.5 Å. Pockets with a druggability score greater than 0.5 were regarded as druggable pockets. PockDrug can identify small pockets but we only considered pockets formed by more than 13 residues because smaller pockets tend to be less druggable. Of the identified druggable pockets, those frequently found for different conformers were selected by clustering using pocket similarity measured by the overlap ratio, which was defined as the number of common pocket-forming residues included in both pockets divided by the number of pocket-forming residues included in one pocket or the other. The clustering method UPGMA (unweighted pair group method with arithmetic mean) was employed.

### Binding free energy calculation by dPaCS-MD/MSM

First, we conducted 5 trials of the 100 ns relaxation MD simulations for the aforementioned 4 complexes formed between the selected compounds and Nsp15 protein. Then, we carried out 5 trials of dPaCS-MD for each complex. The initial structure of each trial was taken from the last frame of the 5 MDs. We used 30 replicas in dPaCS-MD, with each replica 100 ps long. We used the AMBER ff19SB force field^40^ for Nsp15 and GAFF2^57^ to determine the ligand force field. The partial charges of the ligand were parameterized using the Gaussian package^58^. The OPC water model was applied in these simulations. The dPaCS-MD trials were carried out until the inter-COM distance was over 7 nm, then we constructed the MSM using the inter-Center of Mass (COM) distances obtained by dPaCS-MD and calculated the volume correction as described in the literature^23,25^ We calculated the standard binding free energy of the best compound as follows:

1 $$\Delta G^\circ =-\Delta W+\Delta {G}_{V}$$

where $\Delta W$ is the free energy difference (potential of mean force: PMF) from the bound state to the unbound state (Fig. S4) and $\Delta {G}_{V}$ is the volume correction of the free energy difference.

### Modeling of RNA structure bound to Nsp15 hexamer

The settings for modeling were the same as those in the aforementioned simulations, unless otherwise specified. For RNA, the DESRES potential^59^ was used. In the following MD simulations, the isothermal condition was established by Langevin dynamics^46,47^ and the force constant for the positional restraints was 100 kcal/molÅ^2^ for steps 2–10, and 13. Modeling was conducted using the following procedure. (1) An RNA model consisting of tridecaU (5'-(U)_13_–3') was constructed by using the 5′-UMP structure of the cryo-EM as U11 and by adding 10 U in the 5′-end and two U in the 3′-end. One of the Nsp15 trimers from the UTP-bound hexamer was employed, and U11 of tridecaU was placed in each UTP-binding pocket. (2) The system was solvated into a 15.8 × 15.2 × 12.9 nm^3^ box and energy minimized. Then, 200 ps MD simulation was conducted using an NVT ensemble at 300 K with positional restraints imposed on the C_α_ atoms of Nsp15 and the heavy atoms of RNA, followed by 10 ns MD using an NPT ensemble with the Berendsen barostat and 40 ns MD with the Monte Carlo barostat. (3) The system was heated to 400 K during 100 ps with positional restraints on the C_α_ atoms of Nsp15 and U11, and (4) 100 ps NVT MD was performed at 400 K. (5) The system was heated to 500 K during 100 ps with the same positional restraints, (6) 100 ps restrained NVT MD was conducted at 500 K, and (7) 2.0 ns MD at 500 K was continued. (8) 100 ps NPT MD at 500 K and 1.0 atm was performed with the Berendsen barostat. (9) 20 ns NPT MD was conducted with the Monte Carlo barostat. (10) Simulated annealing down to 300 K was performed starting from a selected structure whose RNAs were relatively close to the alternative binding site. (11) A structure whose RNA was the closest to the alternative binding site (the 3^rd^ RNA chain) was selected and 100 ns MD was conducted with reduced positional restraints (force constant: 10 kcal/molÅ^2^). (12) Another trimer was added to the system to reconstruct the Nsp15 hexamer with 3 RNA chains, and the system was solvated into a 14.6 × 15.3 × 15.9 nm^3^ box. A relaxation procedure similar to (2) was conducted and then 200 ns NPT MD was performed at 300 K and 1 atm without positional restraints. (13) 10 ns steered MD (SMD) was performed by pulling the O4 atom of U3 (the residue closest to the binding site at this point) toward the NE2 atom of HIS17 of the alternative binding site with positional restraints on Nsp15 and U11. (14). 10 ns MD was conducted by decreasing the force constant by 10 kcal/molÅ^2^ per 1 ns until it vanished. At this stage, positional restraints for the O4 and NE2 were also applied with a force constant of 100 kcal/molÅ^2^. (15) 10 ns MD was performed by reducing the positional restraint for the O4 and NE2, and (16) free MD was conducted for 50 ns. This resulted in U4 of the 3rd RNA chain being the closest to the target pocket. (17). Similar to (13), 10 ns SMD was performed by pulling the O4 of U4 toward the C_α_ atom of LYS67 with the same setting as in (15), so that the RNA tightly bound deeper into the alternative binding site. (18) 10 ns MD was performed by reducing the positional restraints for O4 and NE2, and 19) five independent free MD simulations were conducted for 100 ns.

## Supplementary Information

Supplementary Information 1.

Supplementary Information 2.

Supplementary Information 3.

## Data Availability

The data supporting this study are included in this published article and its Supplementary Information.
