# Supplementary material for: Inhibition of the hexamerization of SARS-CoV-2 endoribonuclease and modeling of RNA structures bound to the hexamer
Source: Sci Rep. 2022 Mar 9;12:3860. doi: 10.1038/s41598-022-07792-2 (PMC8907205; doi:10.1038/s41598-022-07792-2)
Supplement: Supplementary file 1 — Supplementary Information 1. [file 41598_2022_7792_MOESM1_ESM.pdf]

Supplementary information for

Inhibition of the Hexamerization of SARS-CoV-2 Endoribonuclease and Modeling of RNA Structures Bound to the Hexamer

Duy Phuoc Tran*, Yuta Taira+, Takumi Ogawa+, Ryoga Misu, Yoshiki Miyazawa, Akio Kitao*

School of Life Sciences and Technology, Tokyo Institute of Technology

+These authors contributed equally.

*Corresponding authors

School of Life Science and Technology, Tokyo Institute of Technology, 2-12-1, Ookayama, Meguro-ku, Tokyo 152-8550, Japan

Tel: +81-3-5734-3373

Fax: +81-3-5734-3372

E-mail: akitao@bio.titech.ac.jp, tpduy@bio.titech.ac.jp

Supplementary tables ***3***

Table S1. **3**

Table S2. **4**

Table S3. **4**

Supplementary Figures ***5***

Figure S1. **5**

Figure S2. **6**

Figure S3. **7**

Figure S4. **9**

Figure S5. **10**

Figure S6. **11**

Supplementary tables

Table S1.

Features of Pocket A–C characterized by descriptors of PockDrug[1].

Descriptor	Pocket A	Pocket B	Pocket C
Druggability score	0.73±0.09	0.65±0.1	0.76±0.14
Confidence	0.04±0.02	0.07±0.02	0.04±0.02
Surface hull	499.8±84.78	1097.53±135.55	724.22±196.06
Diameter hull	16.95±1.84	28.35±2.57	21.75±3.54
Volume hull	905.13±231.02	2773.7±569.1	1559.13±653.24
Smallest size	10.13±1.12	12.33±1.74	10.89±1.55
Radius cylinder	8.24±0.96	14.01±1.26	10.8±1.78
Nb RES	17.47±1.82	33.06±4.21	23.92±5.28
Hydrophobic residues	0.63±0.03	0.61±0.03	0.69±0.04
Hydrophobic kyte	-0.13±0.21	-0.43±0.18	-0.11±0.32
Polar residues	0.52±0.05	0.61±0.03	0.52±0.05
Aromatic residues	0.12±0.01	0.09±0.01	0.16±0.03
Aliphatic residues	0.3±0.04	0.27±0.03	0.26±0.05
Otyr atom	0.02±0.01	0.02±0.01	0.0±0.0
Ne2 atom	0.0±0.0	0.0±0.0	0.01±0.01
Nlys atom	0.01±0.01	0.03±0.01	0.02±0.01
Ntrp atom	0.0±0.0	0.0±0.0	0.0±0.0
Ooh atom	0.02±0.01	0.03±0.01	0.02±0.01
Nd1 atom	0.0±0.0	0.0±0.0	0.02±0.01

Table S2.

List of 45,890 poses bound to all the identified pockets (22,485 compounds for two pockets) obtained by AutoDock Vina[2] from the antiviral compound library provided by American Chemical Society 2020[3]. See a separate file.

Table S3.

List of 225 poses of 24 compounds bound to Pocket C obtained by Schrödinger Glide package[4] from the antiviral compound library provided by American Chemical Society 2020[3]. See a separate file.

Supplementary Figures

Figure S1.

The root-mean-square deviation (RMSD) of the NSP15 monomer from the crystal structure as the function of MD time (left panel) and the root-mean-square fluctuation (RMSF) of each residue in five distinct 1 μs MD simulations. The calculation is performed by using backbone atoms of NSP15 after performing least square fitting to the initial structure.

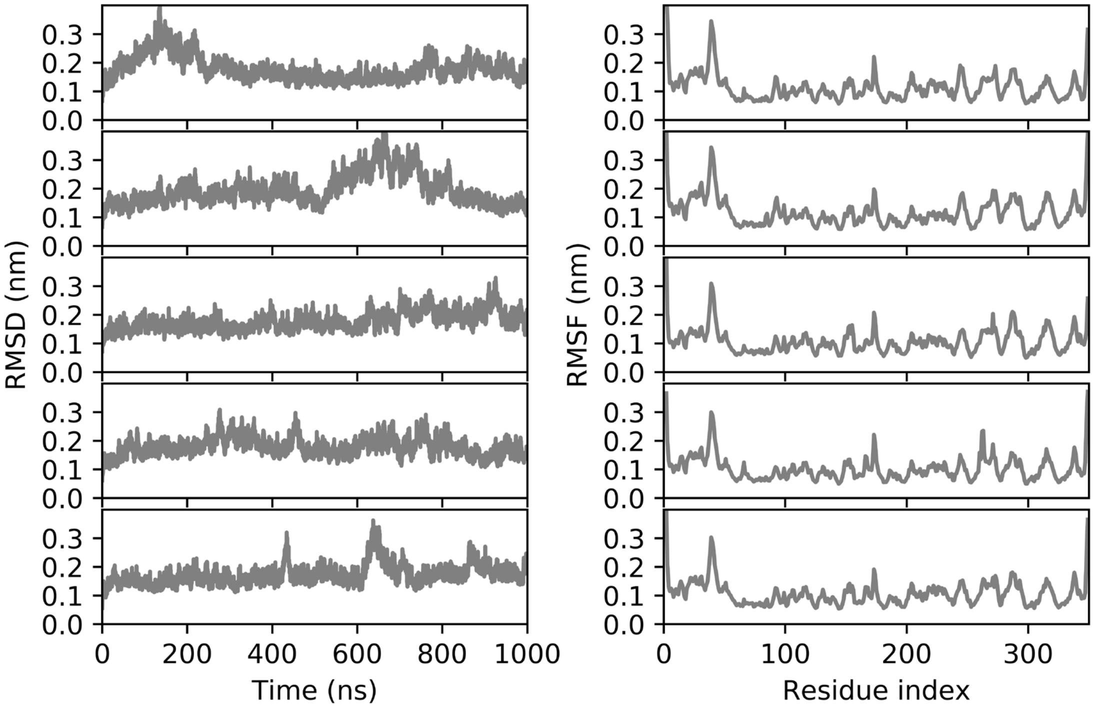

Figure S2.

Implied time scale evolution versus lag time in MSM.

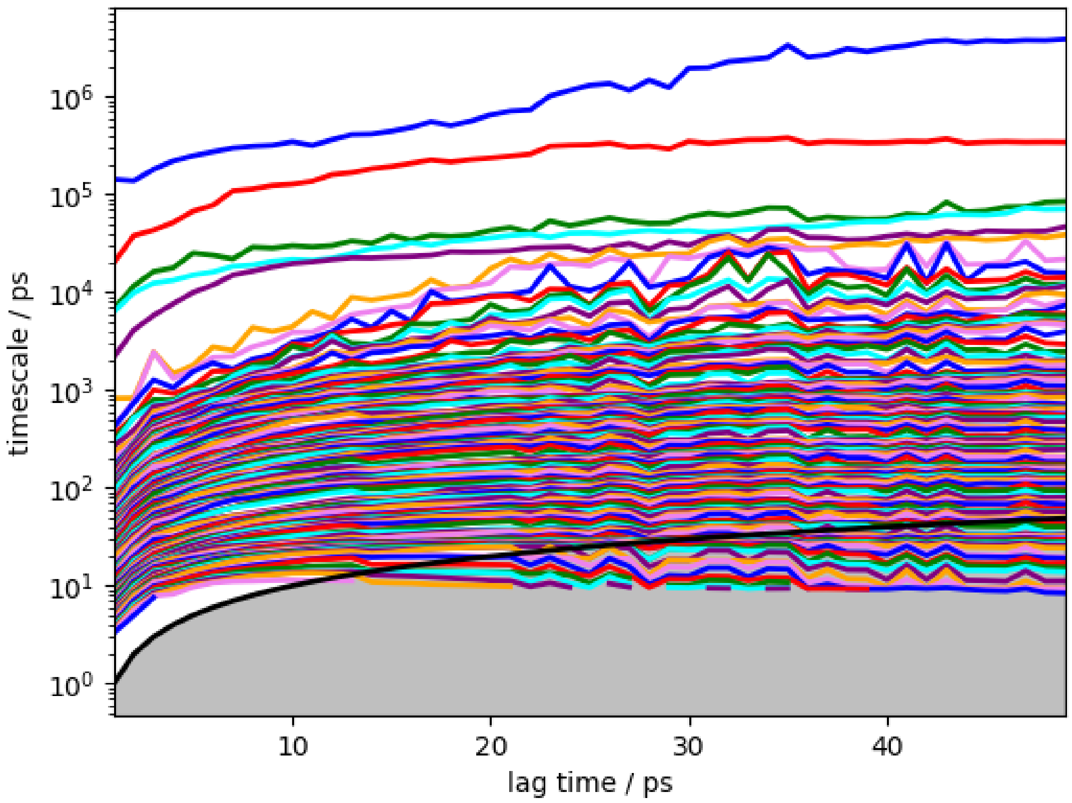

Figure S3.

Multiple sequence alignment between the SARS-CoV2 NSP15 sequence (PDB ID: 6VWW) used in this study and those having similar sequences. Replicase polyprotein 1ab of SARS-CoV (Uniprot ID: P0C6X7), MERS-CoV (Uniprot ID: K9N7C7), murine hepatitis virus (Uniprot ID: P0C6X8), bovine coronavirus (Uniprot ID: P0C6X0), porcine epidemic diarrhea virus (Uniprot ID: P0C6Y4), porcine transmissible gastroenteritis coronavirus (Uniprot ID: P0C6Y5), feline coronavirus (Uniprot ID: Q98VG9), Avian infectious bronchitis virus (Uniprot ID: P0C6Y2), porcine transmissible gastroenteritis coronavirus (Uniprot ID: P18457), breda virus 1 (Uniprot ID: P0C6V8), and berna virus (Uniprot ID: P0C6V7). Light red highlight denotes the Pocket-A-forming residues while light green indicates the Pocket-C-forming residues.

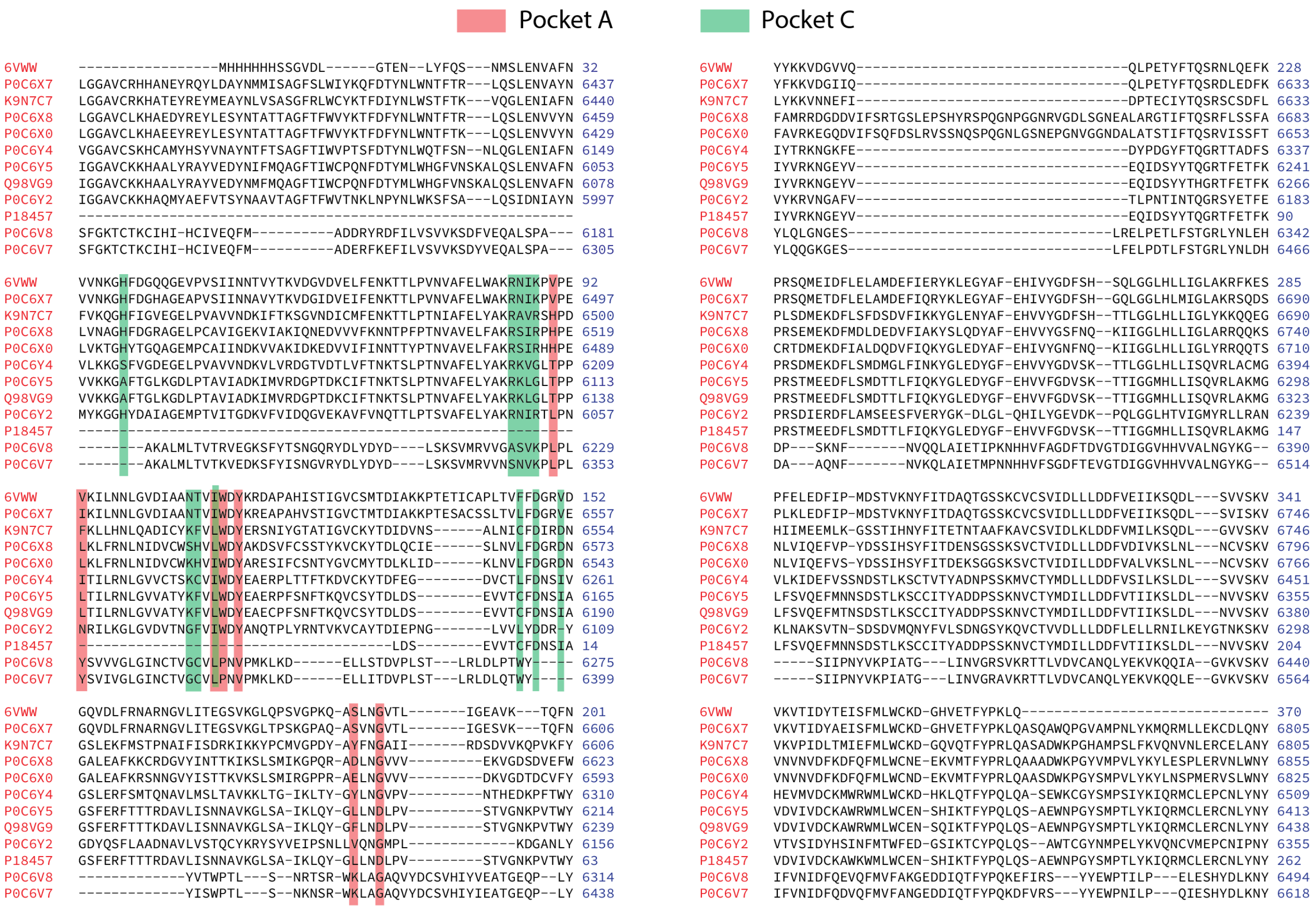

Figure S4.

Potential of mean force (PMF) as a function of the center of distance between Nsp15 and compounds for **a** Glide poses 1, **b** 3, **c** 4, and **d** AutoDock pose 1 obtained by five trials of dPaCS-MD/MSM. In each panel, CAS RN® of the compound and target pocket (A/B or C) are also shown.

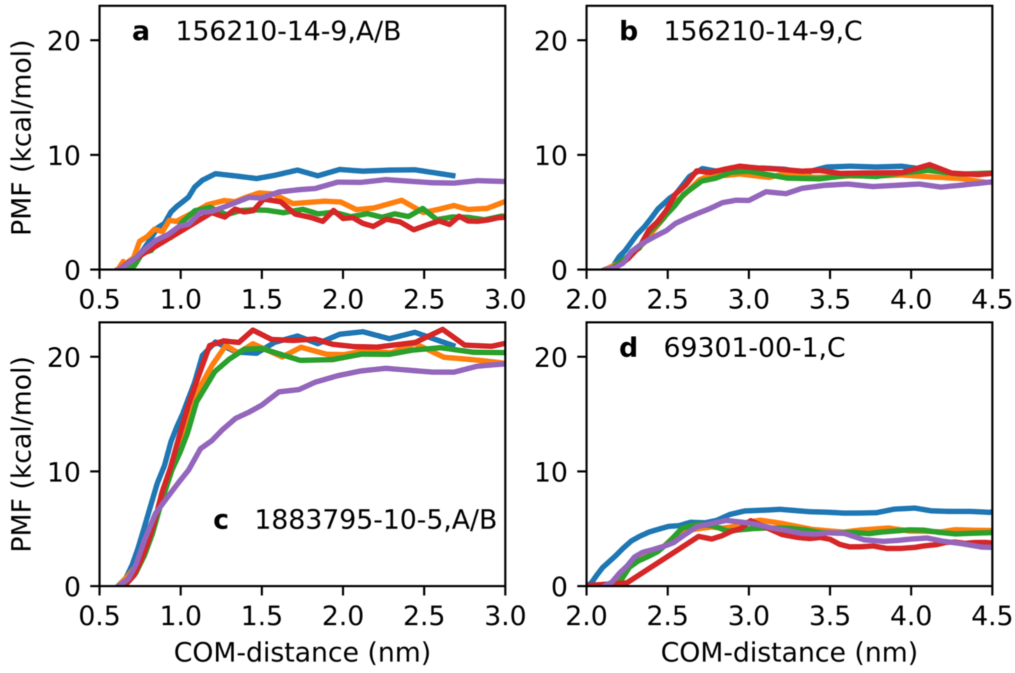

Figure S5.

Interaction map between NSP15 and top 6 compounds bound to Pocket C generated by Schrödinger Glide package[4]. Please note that residue numbers in this figure are shifted by +2.

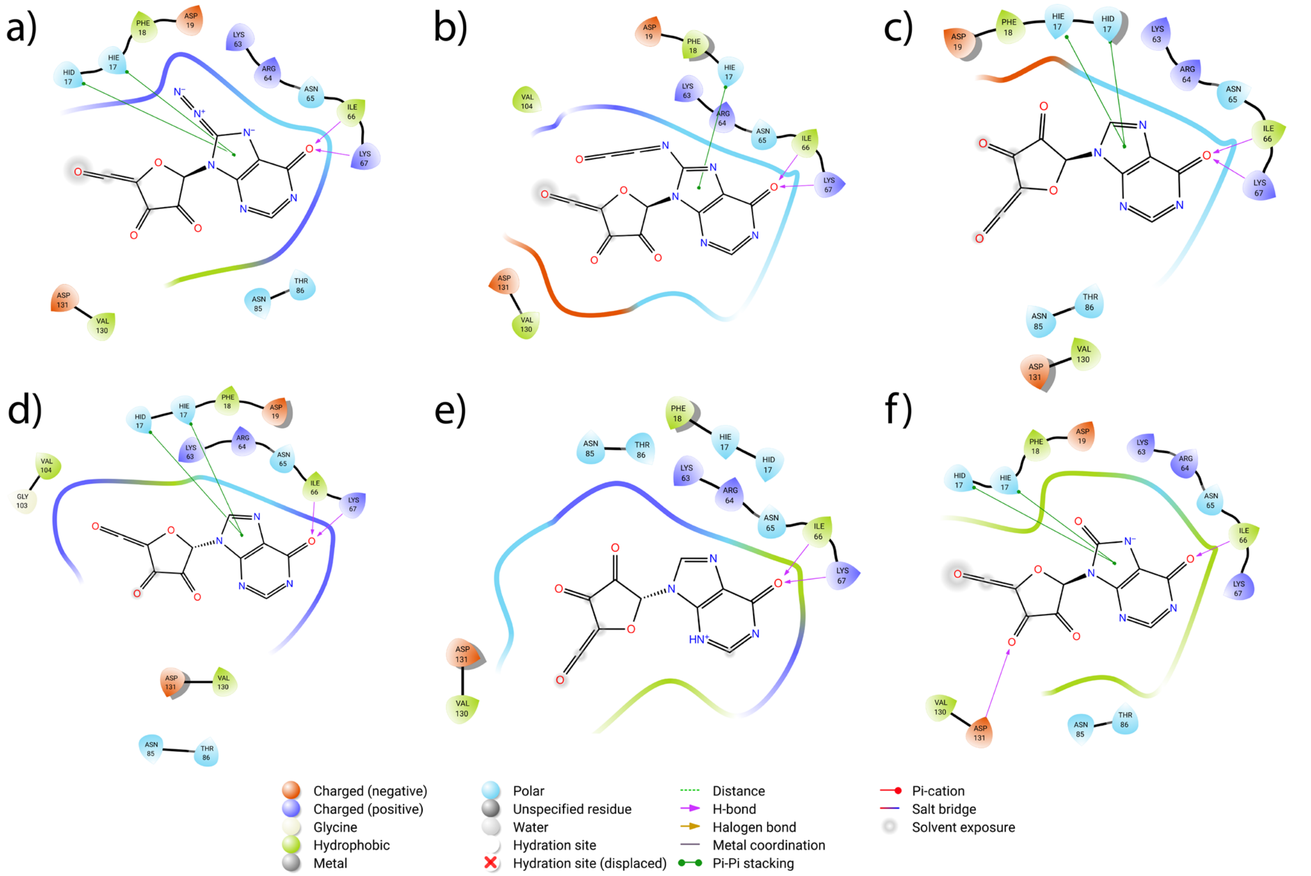

Figure S6.

Compounds of the top 6 poses bound to Pocket C and their complexes with NSP15. These compounds were screened from the antiviral compound library provided by The American Chemical Society (Mar. 2020)[5] using Schrödinger Glide[4]. Their 2D structures, docking scores in kcal/mol, and complex structures after 100 ns MD are shown. **1** Compound 1. 1H-Purine-6,8-dione, 9-β-D-arabinofuranosyl-7,9-dihydro-, 8-hydrazone (CAS RN: 69301-99-1), **2** Compound 2. Inosine, 8-[(2-hydroxyethyl)amino]-(1358577-79-3), **3, 4** Compound 3. 6H-Purin-8-t-6-one, 1,9-dihydro-9-β-L-ribofuranosyl- (2086327-90-2), **5** Compound 4. Inosine-1,8-d2 (160059-42-7), and **6** Compound 5. Inosine, 7,8-dihydro-8-oxo-(63699-77-4). The compound names are provided as the CAS Index Name in the library. 2D conformations of the compounds were obtained using Schrödinger Maestro[4]. The compounds are shown as CPK models. Transparent surfaces represent druggable pockets identified by PockDrug. The structure images were created using VMD[5].

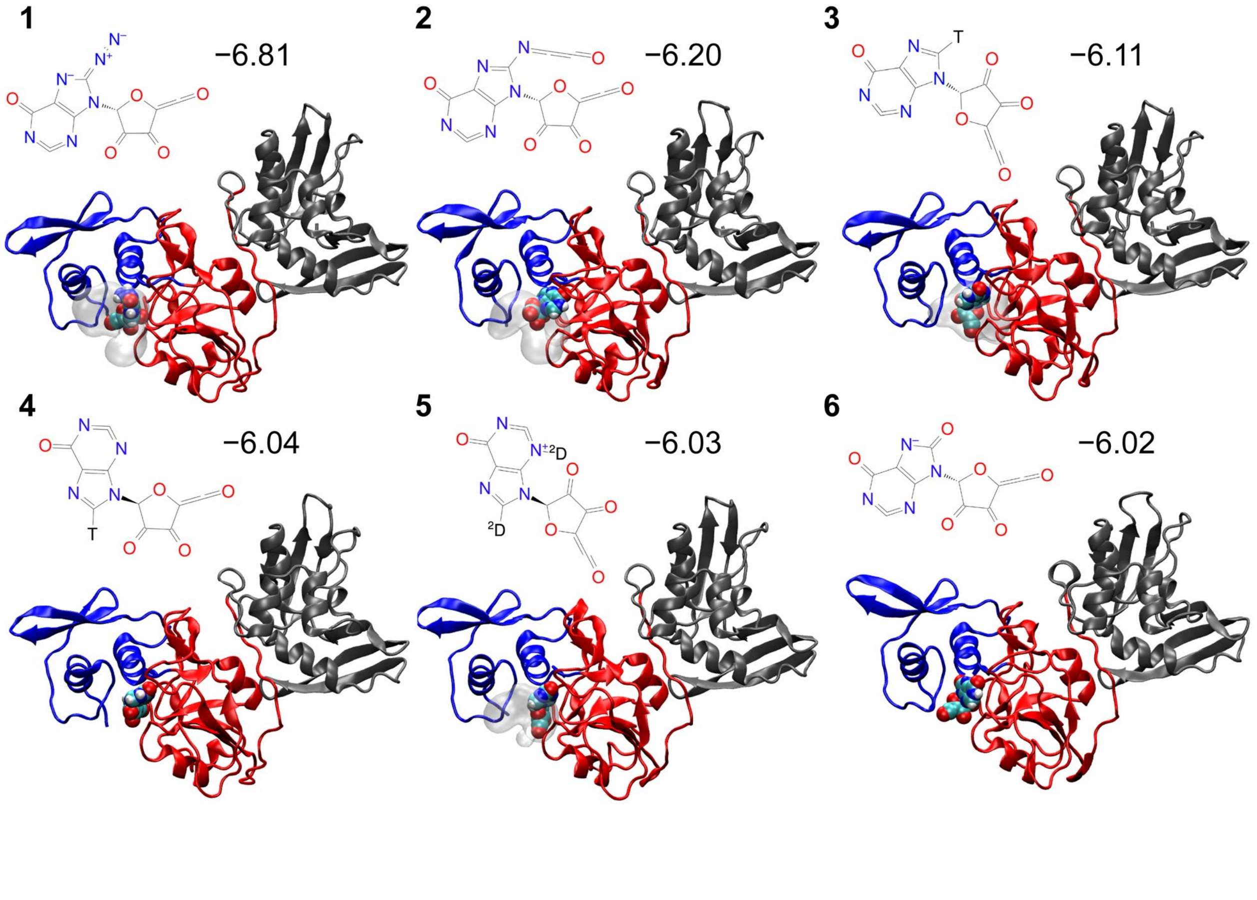

Figure S7.

The root-mean-squared-deviation (RMSD) of the trideca-U RNA structure bound to the Nsp15 hexamer from the initial structure of free MD (Time 0 ns in this plot) calculated for heavy atoms during five independent 100 ns MD simulations. The calculation was done after performing heavy-atom best-fitting of the RNA.

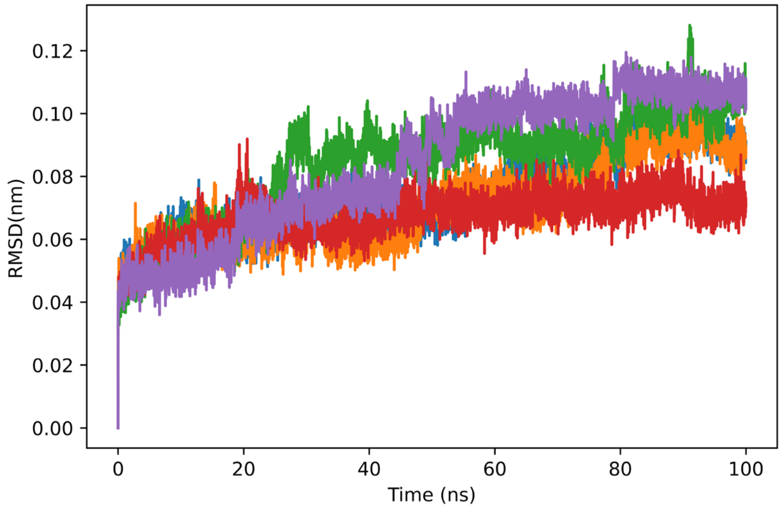

Reference

(1) Borrel, A.; Regad, L.; Xhaard, H.; Petitjean, M.; Camproux, A.-C. PockDrug: A Model for Predicting Pocket Druggability That Overcomes Pocket Estimation Uncertainties. *J. Chem. Inf. Model.* **2015**, *55* (4), 882–895. https://doi.org/10.1021/ci5006004.

(2) Trott, O.; Olson, A. J. AutoDock Vina: Improving the Speed and Accuracy of Docking with a New Scoring Function, Efficient Optimization, and Multithreading. *J. Comput. Chem.* **2009**, *31* (2), NA-NA. https://doi.org/10.1002/jcc.21334.

(3) American Chemical Society. CAS COVID-19 Antiviral Candidate Compounds Dataset. https://www.cas.org/covid-19-sar-dataset.

(4) Richard A. Friesner, *; Robert B. Murphy, †; Matthew P. Repasky, †; Leah L. Frye, ‡; Jeremy R. Greenwood, †; Thomas A. Halgren, †; Paul C. Sanschagrin, † and; Mainz†, D. T. Extra Precision Glide: Docking and Scoring Incorporating a Model of Hydrophobic Enclosure for Protein−Ligand Complexes. **2006**. https://doi.org/10.1021/JM051256O.

(5) Humphrey, W.; Dalke, A.; Schulten, K. VMD: Visual Molecular Dynamics. *J. Mol. Graph.* **1996**, *14* (1), 33–38. https://doi.org/10.1016/0263-7855(96)00018-5.
