# Supplementary material for: Inhibition of the hexamerization of SARS-CoV-2 endoribonuclease and modeling of RNA structures bound to the hexamer
Source: Sci Rep. 2022 Mar 9;12:3860. doi: 10.1038/s41598-022-07792-2 (PMC8907205; doi:10.1038/s41598-022-07792-2)
Supplement: Supplementary file 3 — Supplementary Information 3. [file 41598_2022_7792_MOESM3_ESM.pdf]

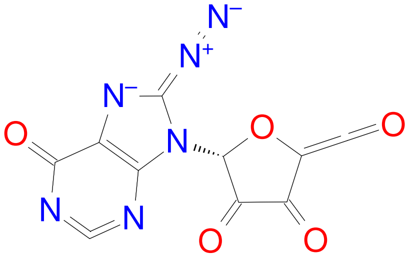

title	1H-Purine-6,8-dione, 9-[
docking score	-6.805
ct format	None
molecular.weight	298.26
cas.index.name	1H-Purine-6,8-dione, 9-[

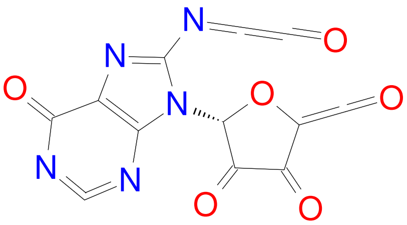

title	Inosine, 8-[(2-hydroxyet
docking score	-6.199
ct format	None
molecular.weight	327.29
cas.index.name	Inosine, 8-[(2-hydroxyet

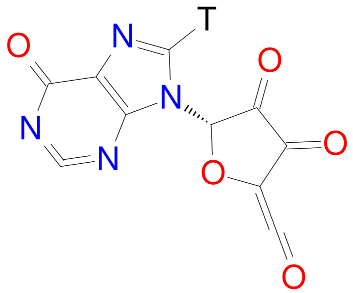

title	6H-Purin-8-t-6-one, 1,9-(
docking score	-6.11
ct format	None
molecular.weight	None
cas.index.name	6H-Purin-8-t-6-one, 1,9-(

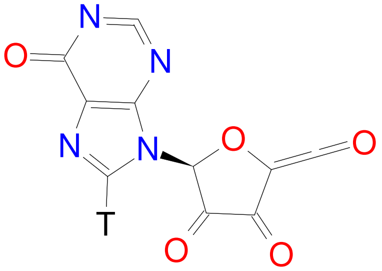

title	6H-Purin-8-t-6-one, 1,9-(
docking score	-6.041
ct format	None
molecular.weight	None
cas.index.name	6H-Purin-8-t-6-one, 1,9-(

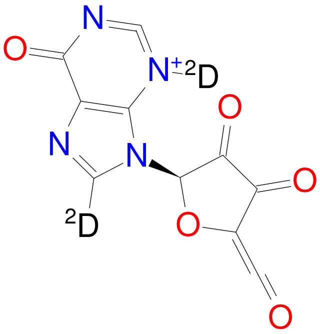

title	Inosine-1,8-d2
docking score	-6.03
ct format	None
molecular.weight	None
cas.index.name	Inosine-1,8-d2

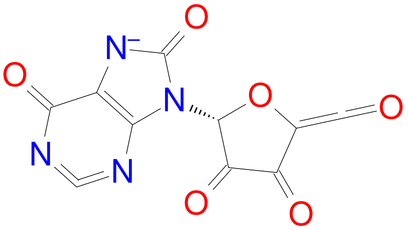

title	7,8-Dihydro-8-oxoinosin
docking score	-6.023
ct format	None
molecular.weight	284.23
cas.index.name	Inosine, 7,8-dihydro-8-o:

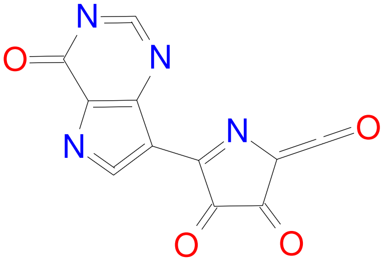

title	4H-Pyrrolo[3,2-d]pyrimi(
docking score	-5.994
ct format	None
molecular.weight	266.25
cas.index.name	4H-Pyrrolo[3,2-d]pyrimi(

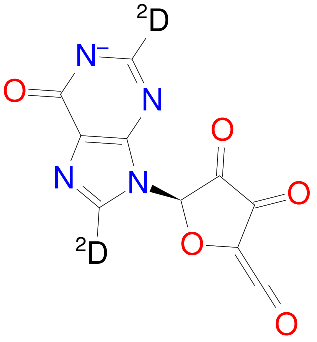

title	Inosine-2,8-d2
docking score	-5.956
ct format	None
molecular.weight	None
cas.index.name	Inosine-2,8-d2

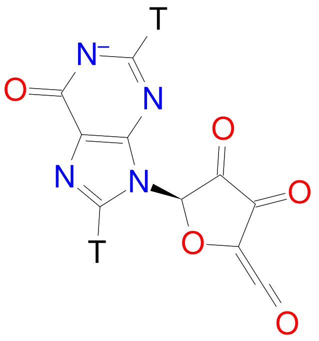

title	Inosine-2,8-t2
docking score	-5.956
ct format	None
molecular.weight	None
cas.index.name	Inosine-2,8-t2

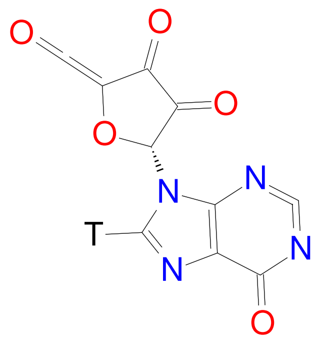

title	6H-Purin-8-t-6-one, 1,9-(
docking score	-5.926
ct format	None
molecular.weight	None
cas.index.name	6H-Purin-8-t-6-one, 1,9-(

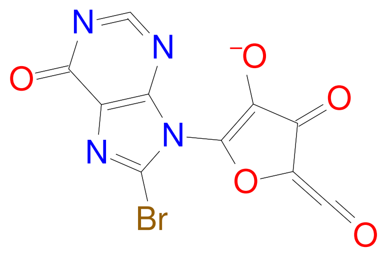

title	
docking score	-5.88
ct format	None
molecular.weight	347.12
cas.index.name	Not Yet Assigned

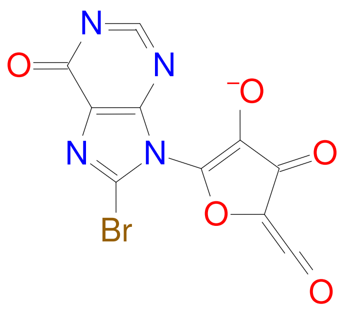

title	
docking score	-5.853
ct format	None
molecular.weight	347.12
cas.index.name	Not Yet Assigned

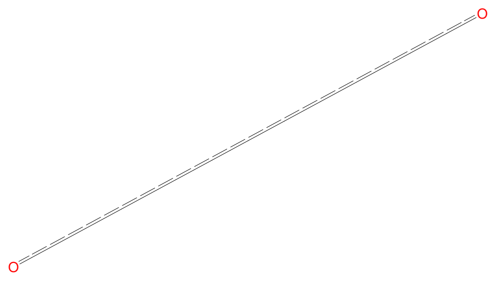

title	1,25-Pentacosanediol
docking score	-5.853
ct format	None
molecular.weight	384.68
cas.index.name	1,25-Pentacosanediol

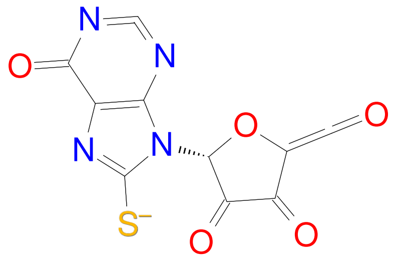

title	Inosine, 7,8-dihydro-8-th
docking score	-5.852
ct format	None
molecular.weight	300.29
cas.index.name	Inosine, 7,8-dihydro-8-th

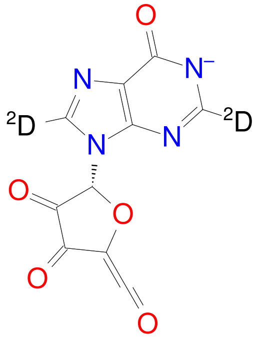

title	Inosine-2,8-d2
docking score	-5.846
ct format	None
molecular.weight	None
cas.index.name	Inosine-2,8-d2

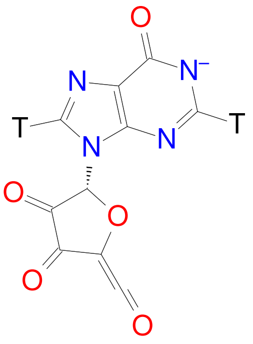

title	Inosine-2,8-t2
docking score	-5.846
ct format	None
molecular.weight	None
cas.index.name	Inosine-2,8-t2

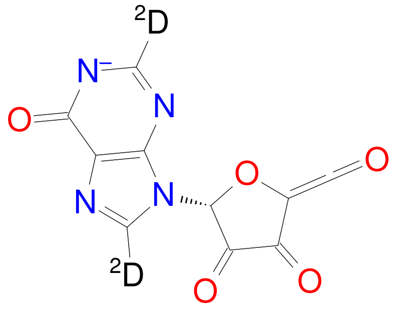

title	Inosine-2,8-d2
docking score	-5.781
ct format	None
molecular.weight	None
cas.index.name	Inosine-2,8-d2

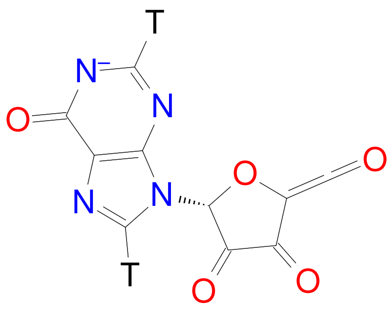

title	Inosine-2,8-t2
docking score	-5.781
ct format	None
molecular.weight	None
cas.index.name	Inosine-2,8-t2

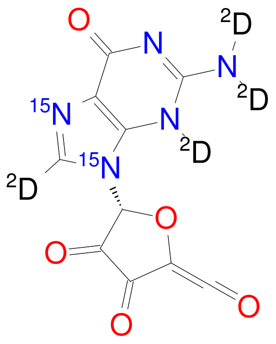

title	Guanosine-N,N,1,8-d4-7
docking score	-5.777
ct format	None
molecular.weight	None
cas.index.name	Guanosine-N,N,1,8-d4-7

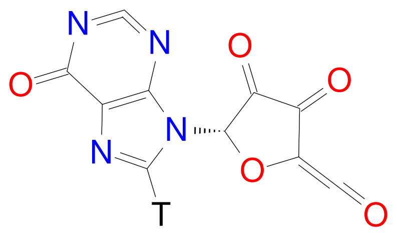

title	6H-Purin-8-t-6-one, 1,9-(
docking score	-5.776
ct format	None
molecular.weight	None
cas.index.name	6H-Purin-8-t-6-one, 1,9-(

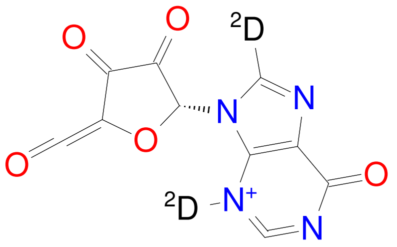

title	Inosine-1,8-d2
docking score	-5.768
ct format	None
molecular.weight	None
cas.index.name	Inosine-1,8-d2

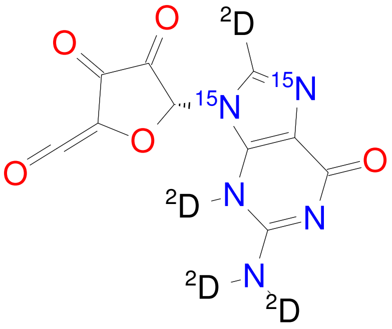

title	Guanosine-N,N,1,8-d4-7
docking score	-5.651
ct format	None
molecular.weight	None
cas.index.name	Guanosine-N,N,1,8-d4-7

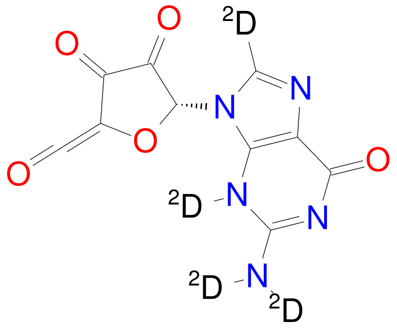

title	Guanosine-N,N,1,8-d4
docking score	-5.649
ct format	None
molecular.weight	None
cas.index.name	Guanosine-N,N,1,8-d4

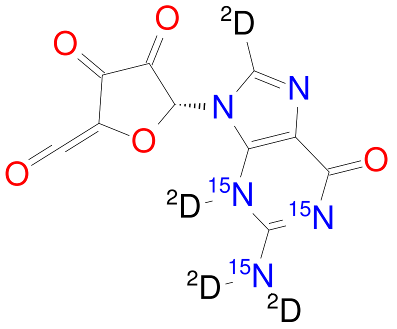

title	Guanosine-N,N,1,8-d4-N
docking score	-5.649
ct format	None
molecular.weight	None
cas.index.name	Guanosine-N,N,1,8-d4-N

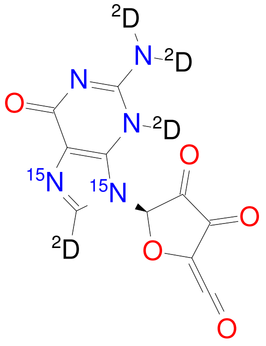

title	Guanosine-N,N,1,8-d4-7
docking score	-5.648
ct format	None
molecular.weight	None
cas.index.name	Guanosine-N,N,1,8-d4-7

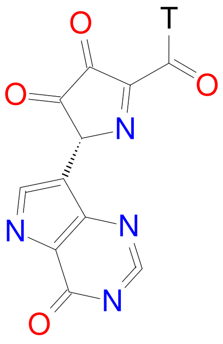

title	4H-Pyrrolo[3,2-d]pyrimic
docking score	-5.628
ct format	None
molecular.weight	None
cas.index.name	4H-Pyrrolo[3,2-d]pyrimic

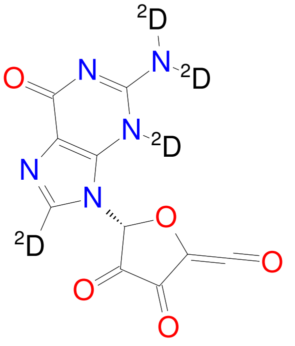

title	Guanosine-N,N,1,8-d4
docking score	-5.598
ct format	None
molecular.weight	None
cas.index.name	Guanosine-N,N,1,8-d4

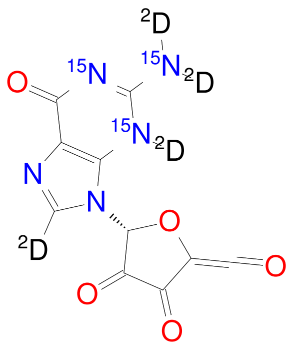

title	Guanosine-N,N,1,8-d4-N
docking score	-5.598
ct format	None
molecular.weight	None
cas.index.name	Guanosine-N,N,1,8-d4-N

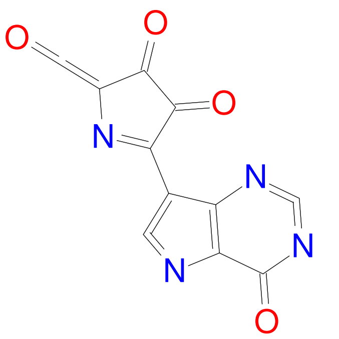

title	4H-Pyrrolo[3,2-d]pyrimic
docking score	-5.595
ct format	None
molecular.weight	266.25
cas.index.name	4H-Pyrrolo[3,2-d]pyrimic

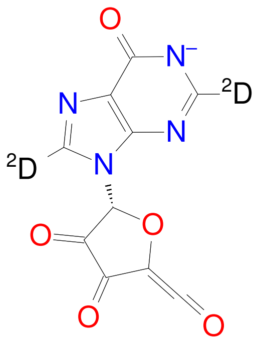

title	Inosine-2,8-d2
docking score	-5.562
ct format	None
molecular.weight	None
cas.index.name	Inosine-2,8-d2

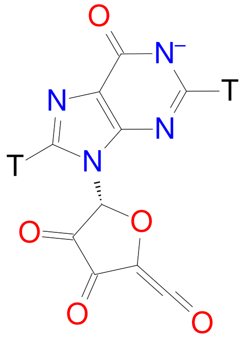

title	Inosine-2,8-t2
docking score	-5.562
ct format	None
molecular.weight	None
cas.index.name	Inosine-2,8-t2

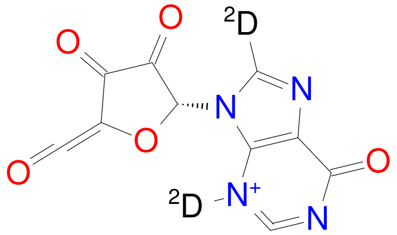

title	Inosine-1,8-d2
docking score	-5.552
ct format	None
molecular.weight	None
cas.index.name	Inosine-1,8-d2

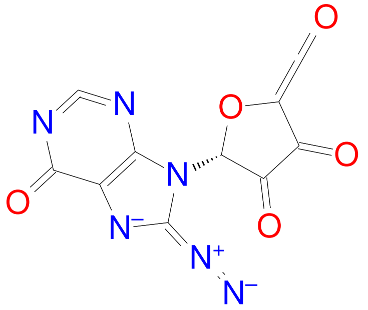

title	1H-Purine-6,8-dione, 9-[
docking score	-5.54
ct format	None
molecular.weight	298.26
cas.index.name	1H-Purine-6,8-dione, 9-[

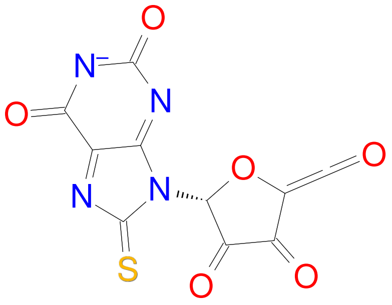

title	Xanthosine, 7,8-dihydro
docking score	-5.472
ct format	None
molecular.weight	316.29
cas.index.name	Xanthosine, 7,8-dihydro

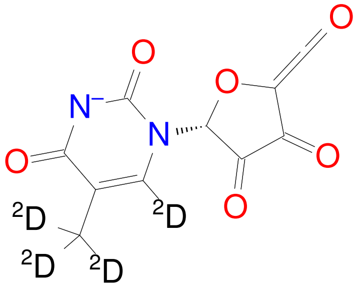

title	Uridine-6-d, 5-(methyl-d:
docking score	-5.434
ct format	None
molecular.weight	None
cas.index.name	Uridine-6-d, 5-(methyl-d:

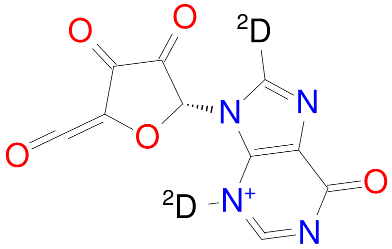

title	Inosine-1,8-d2
docking score	-5.43
ct format	None
molecular.weight	None
cas.index.name	Inosine-1,8-d2

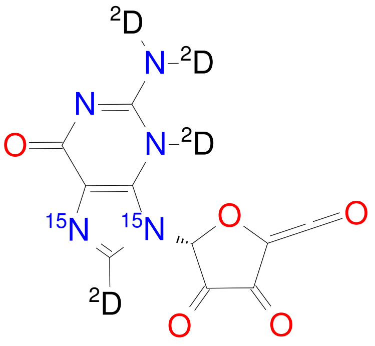

title	Guanosine-N,N,1,8-d4-7
docking score	-5.426
ct format	None
molecular.weight	None
cas.index.name	Guanosine-N,N,1,8-d4-7

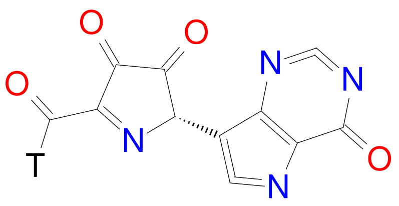

title	4H-Pyrrolo[3,2-d]pyrimic
docking score	-5.423
ct format	None
molecular.weight	None
cas.index.name	4H-Pyrrolo[3,2-d]pyrimic

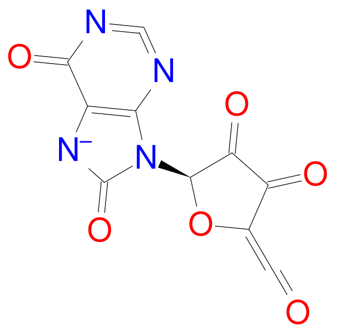

title	7,8-Dihydro-8-oxoinosin
docking score	-5.42
ct format	None
molecular.weight	284.23
cas.index.name	Inosine, 7,8-dihydro-8-o:

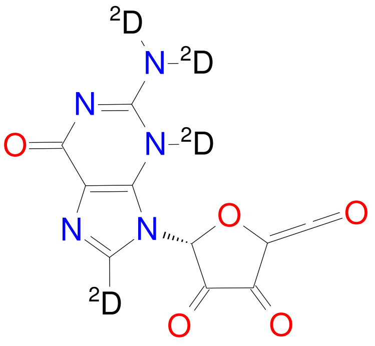

title	Guanosine-N,N,1,8-d4
docking score	-5.418
ct format	None
molecular.weight	None
cas.index.name	Guanosine-N,N,1,8-d4

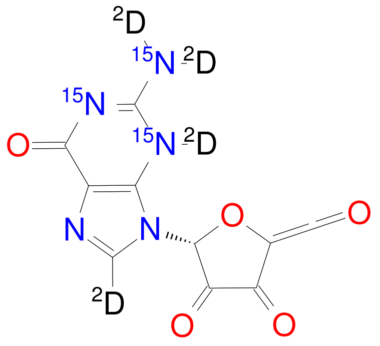

title	Guanosine-N,N,1,8-d4-N
docking score	-5.418
ct format	None
molecular.weight	None
cas.index.name	Guanosine-N,N,1,8-d4-N

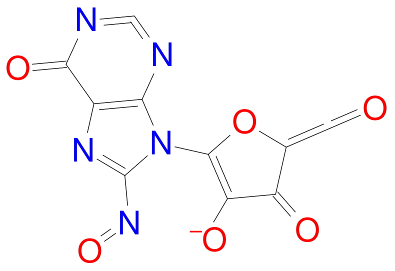

title	1H-Purine-6,8-dione, 7,9
docking score	-5.397
ct format	None
molecular.weight	299.24
cas.index.name	1H-Purine-6,8-dione, 7,9

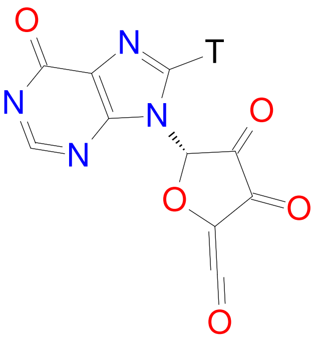

title	6H-Purin-8-t-6-one, 1,9-(
docking score	-5.396
ct format	None
molecular.weight	None
cas.index.name	6H-Purin-8-t-6-one, 1,9-(

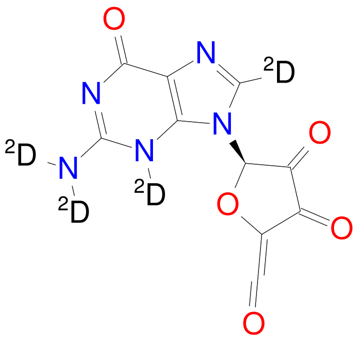

title	Guanosine-N,N,1,8-d4
docking score	-5.388
ct format	None
molecular.weight	None
cas.index.name	Guanosine-N,N,1,8-d4

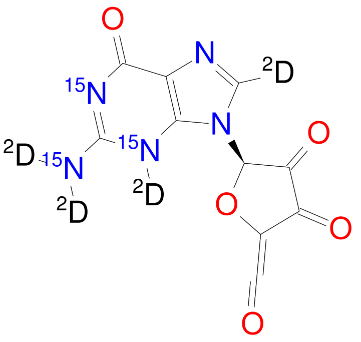

title	Guanosine-N,N,1,8-d4-N
docking score	-5.388
ct format	None
molecular.weight	None
cas.index.name	Guanosine-N,N,1,8-d4-N

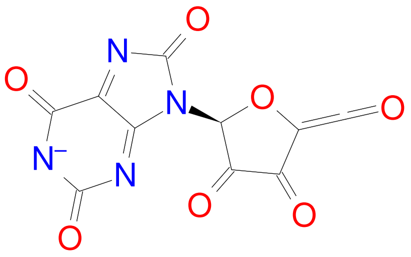

title	7,9-Dihydro-9-β-D-ribofu
docking score	-5.386
ct format	None
molecular.weight	300.22
cas.index.name	1H-Purine-2,6,8(3H)-trio

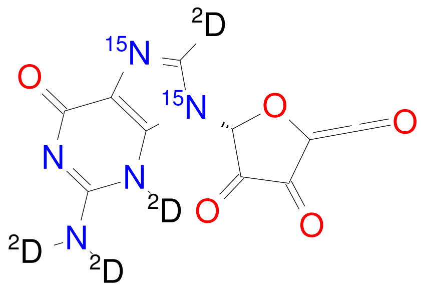

title	Guanosine-N,N,1,8-d4-7
docking score	-5.383
ct format	None
molecular.weight	None
cas.index.name	Guanosine-N,N,1,8-d4-7

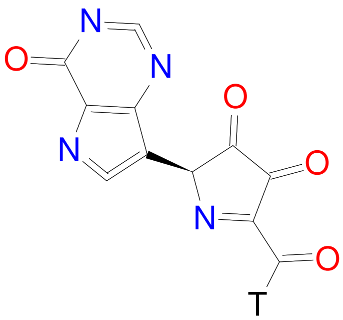

title	4H-Pyrrolo[3,2-d]pyrimi
docking score	-5.342
ct format	None
molecular.weight	None
cas.index.name	4H-Pyrrolo[3,2-d]pyrimi

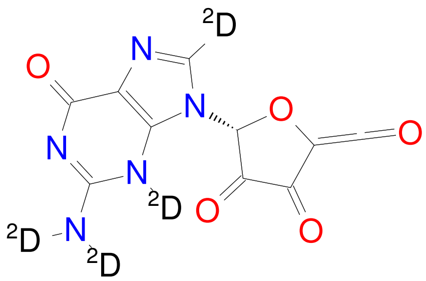

title	Guanosine-N,N,1,8-d4
docking score	-5.333
ct format	None
molecular.weight	None
cas.index.name	Guanosine-N,N,1,8-d4

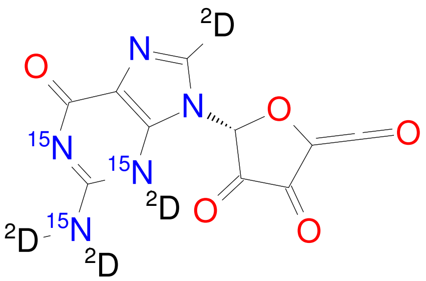

title	Guanosine-N,N,1,8-d4-N
docking score	-5.333
ct format	None
molecular.weight	None
cas.index.name	Guanosine-N,N,1,8-d4-N

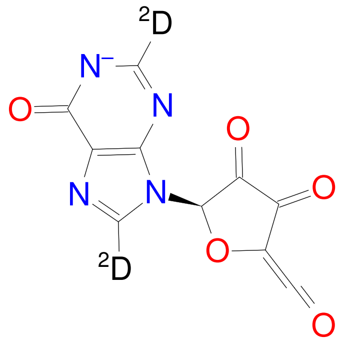

title	Inosine-2,8-d2
docking score	-5.326
ct format	None
molecular.weight	None
cas.index.name	Inosine-2,8-d2

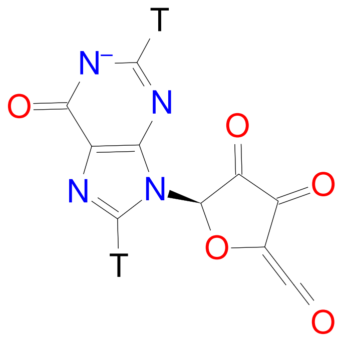

title	Inosine-2,8-t2
docking score	-5.326
ct format	None
molecular.weight	None
cas.index.name	Inosine-2,8-t2

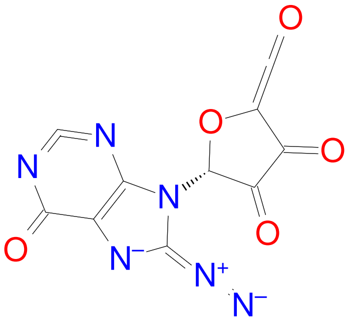

title	1H-Purine-6,8-dione, 9-β
docking score	-5.326
ct format	None
molecular.weight	298.26
cas.index.name	1H-Purine-6,8-dione, 9-β

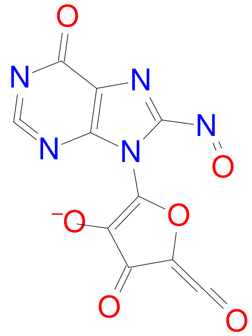

title	1H-Purine-6,8-dione, 7,9
docking score	-5.317
ct format	None
molecular.weight	299.24
cas.index.name	1H-Purine-6,8-dione, 7,9

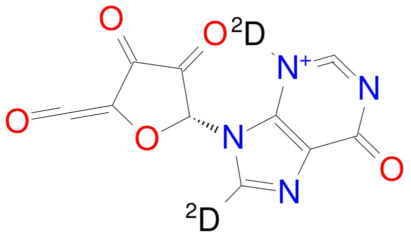

title	Inosine-1,8-d2
docking score	-5.296
ct format	None
molecular.weight	None
cas.index.name	Inosine-1,8-d2

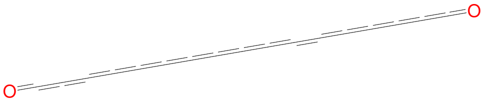

title	1,17-Heptadecanediol
docking score	-5.288
ct format	None
molecular.weight	272.47
cas.index.name	1,17-Heptadecanediol

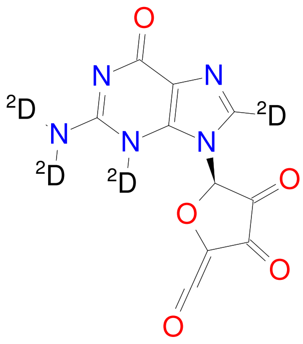

title	Guanosine-N,N,1,8-d4
docking score	-5.288
ct format	None
molecular.weight	None
cas.index.name	Guanosine-N,N,1,8-d4

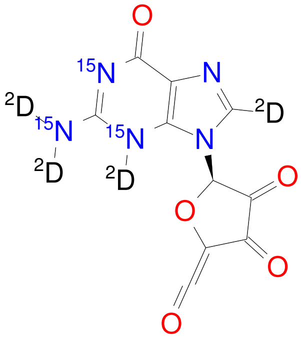

title	Guanosine-N,N,1,8-d4-N
docking score	-5.288
ct format	None
molecular.weight	None
cas.index.name	Guanosine-N,N,1,8-d4-N

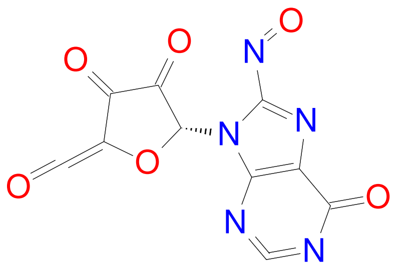

title	Inosine, 8-(hydroxyamir
docking score	-5.27
ct format	None
molecular.weight	299.24
cas.index.name	Inosine, 8-(hydroxyamir

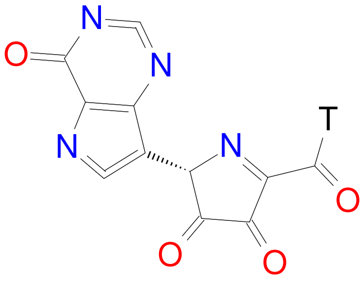

title	4H-Pyrrolo[3,2-d]pyrimic
docking score	-5.269
ct format	None
molecular.weight	None
cas.index.name	4H-Pyrrolo[3,2-d]pyrimic

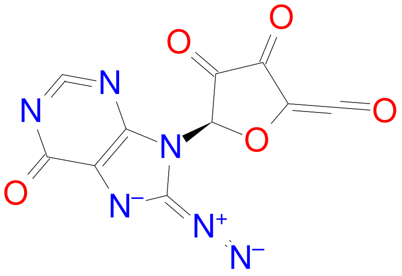

title	1H-Purine-6,8-dione, 9-[
docking score	-5.268
ct format	None
molecular.weight	298.26
cas.index.name	1H-Purine-6,8-dione, 9-[

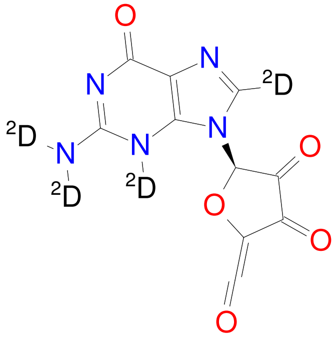

title	Guanosine-N,N,1,8-d4
docking score	-5.239
ct format	None
molecular.weight	None
cas.index.name	Guanosine-N,N,1,8-d4

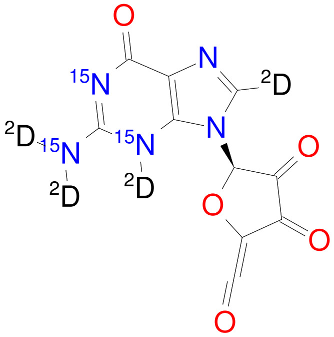

title	Guanosine-N,N,1,8-d4-N
docking score	-5.239
ct format	None
molecular.weight	None
cas.index.name	Guanosine-N,N,1,8-d4-N

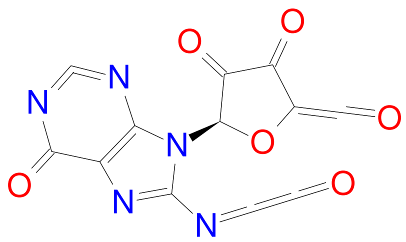

title	Inosine, 8-[(2-hydroxyetl
docking score	-5.226
ct format	None
molecular.weight	327.29
cas.index.name	Inosine, 8-[(2-hydroxyetl

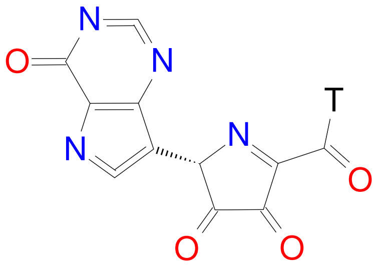

title	4H-Pyrrolo[3,2-d]pyrimic
docking score	-5.212
ct format	None
molecular.weight	None
cas.index.name	4H-Pyrrolo[3,2-d]pyrimic

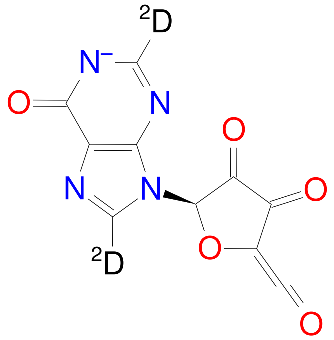

title	Inosine-2,8-d2
docking score	-5.209
ct format	None
molecular.weight	None
cas.index.name	Inosine-2,8-d2

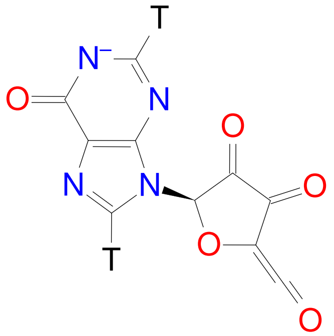

title	Inosine-2,8-t2
docking score	-5.209
ct format	None
molecular.weight	None
cas.index.name	Inosine-2,8-t2

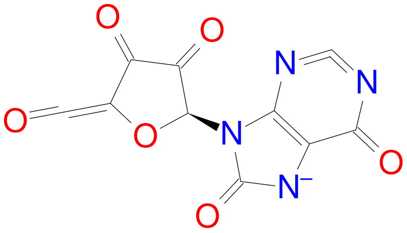

title	7,8-Dihydro-8-oxoinosin
docking score	-5.178
ct format	None
molecular.weight	284.23
cas.index.name	Inosine, 7,8-dihydro-8-o:

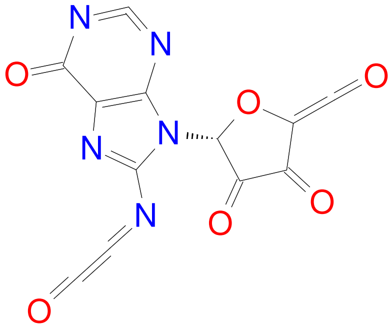

title	Inosine, 8-[(2-hydroxyetl
docking score	-5.176
ct format	None
molecular.weight	327.29
cas.index.name	Inosine, 8-[(2-hydroxyetl

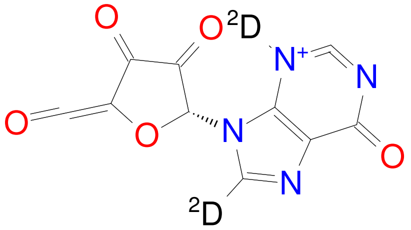

title	Inosine-1,8-d2
docking score	-5.16
ct format	None
molecular.weight	None
cas.index.name	Inosine-1,8-d2

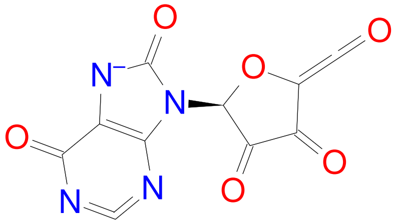

title	7,8-Dihydro-8-oxoinosin
docking score	-5.157
ct format	None
molecular.weight	284.23
cas.index.name	Inosine, 7,8-dihydro-8-o:

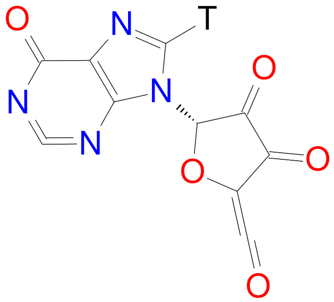

title	6H-Purin-8-t-6-one, 1,9-(
docking score	-5.155
ct format	None
molecular.weight	None
cas.index.name	6H-Purin-8-t-6-one, 1,9-(

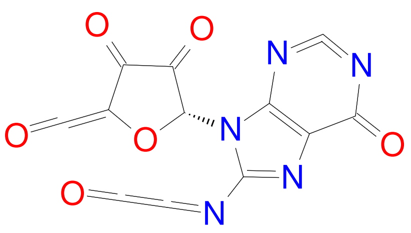

title	Inosine, 8-[(2-hydroxyet
docking score	-5.148
ct format	None
molecular.weight	327.29
cas.index.name	Inosine, 8-[(2-hydroxyet

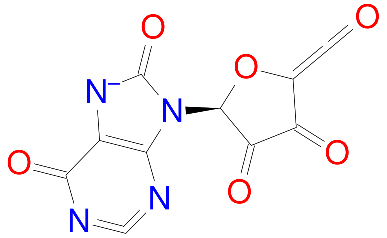

title	7,8-Dihydro-8-oxoinosin
docking score	-5.132
ct format	None
molecular.weight	284.23
cas.index.name	Inosine, 7,8-dihydro-8-o:

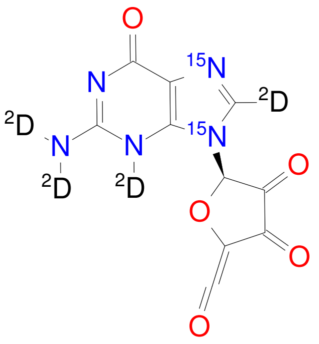

title	Guanosine-N,N,1,8-d4-7
docking score	-5.123
ct format	None
molecular.weight	None
cas.index.name	Guanosine-N,N,1,8-d4-7

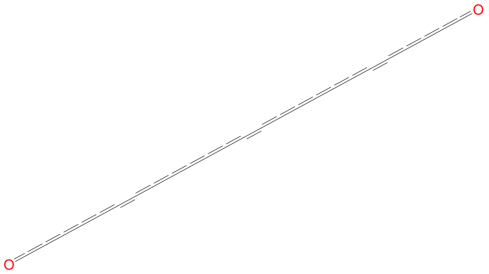

title	1,25-Pentacosanediol
docking score	-5.122
ct format	None
molecular.weight	384.68
cas.index.name	1,25-Pentacosanediol

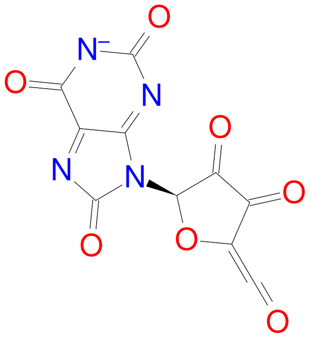

title	7,9-Dihydro-9-β-D-ribofu
docking score	-5.105
ct format	None
molecular.weight	300.22
cas.index.name	1H-Purine-2,6,8(3H)-tric

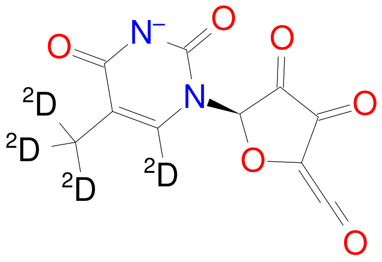

title	Uridine-6-d, 5-(methyl-d:
docking score	-5.096
ct format	None
molecular.weight	None
cas.index.name	Uridine-6-d, 5-(methyl-d:

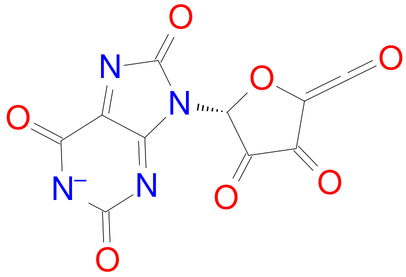

title	7,9-Dihydro-9-β-D-ribofu
docking score	-5.092
ct format	None
molecular.weight	300.22
cas.index.name	1H-Purine-2,6,8(3H)-tric

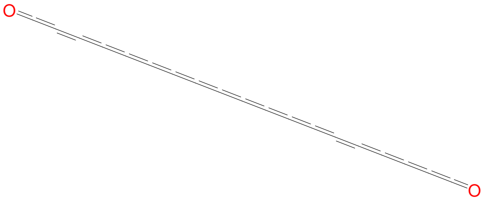

title	1,19-Nonadecanediol
docking score	-5.087
ct format	None
molecular.weight	300.52
cas.index.name	1,19-Nonadecanediol

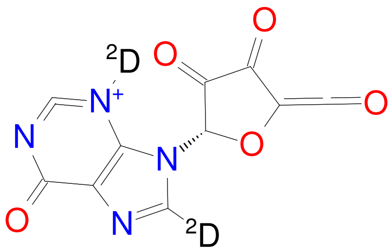

title	Inosine-1,8-d2
docking score	-5.08
ct format	None
molecular.weight	None
cas.index.name	Inosine-1,8-d2

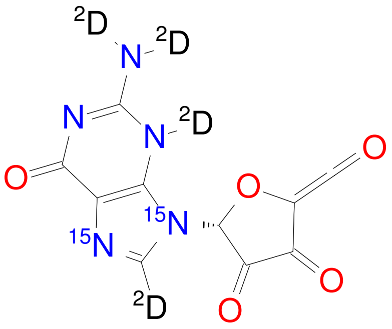

title	Guanosine-N,N,1,8-d4-7
docking score	-5.075
ct format	None
molecular.weight	None
cas.index.name	Guanosine-N,N,1,8-d4-7

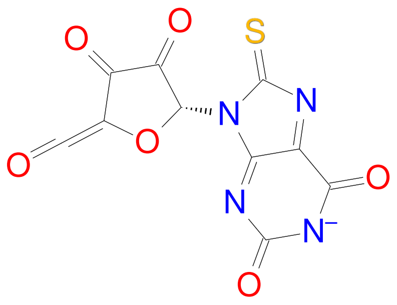

title	Xanthosine, 7,8-dihydro
docking score	-5.054
ct format	None
molecular.weight	316.29
cas.index.name	Xanthosine, 7,8-dihydro

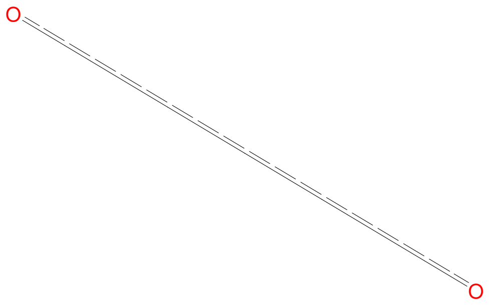

title	1,17-Heptadecanediol
docking score	-5.047
ct format	None
molecular.weight	272.47
cas.index.name	1,17-Heptadecanediol

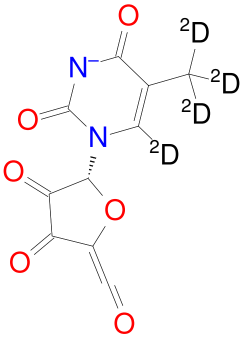

title	Uridine-6-d, 5-(methyl-d:
docking score	-5.035
ct format	None
molecular.weight	None
cas.index.name	Uridine-6-d, 5-(methyl-d:

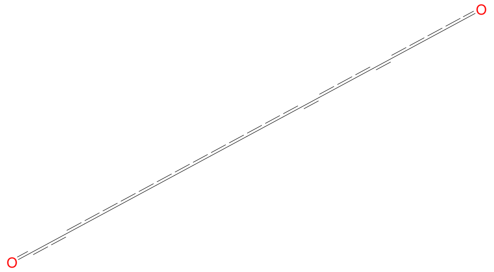

title	1,25-Pentacosanediol
docking score	-5.035
ct format	None
molecular.weight	384.68
cas.index.name	1,25-Pentacosanediol

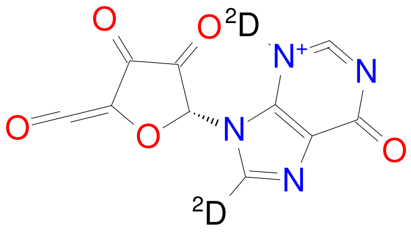

title	Inosine-1,8-d2
docking score	-5.034
ct format	None
molecular.weight	None
cas.index.name	Inosine-1,8-d2

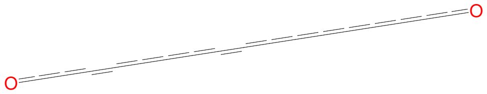

title	1,17-Heptadecanediol
docking score	-5.033
ct format	None
molecular.weight	272.47
cas.index.name	1,17-Heptadecanediol

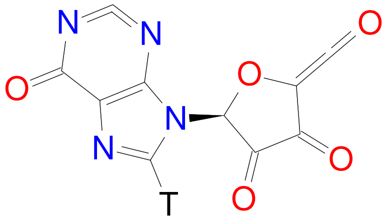

title	6H-Purin-8-t-6-one, 1,9-(
docking score	-5.027
ct format	None
molecular.weight	None
cas.index.name	6H-Purin-8-t-6-one, 1,9-(

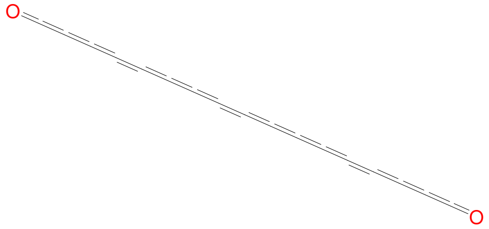

title	1,17-Heptadecanediol
docking score	-5.023
ct format	None
molecular.weight	272.47
cas.index.name	1,17-Heptadecanediol

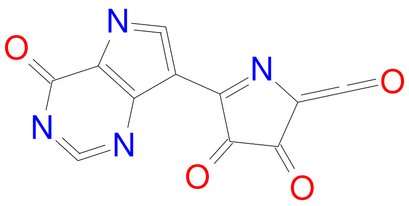

title	4H-Pyrrolo[3,2-d]pyrimi
docking score	-5.018
ct format	None
molecular.weight	266.25
cas.index.name	4H-Pyrrolo[3,2-d]pyrimi

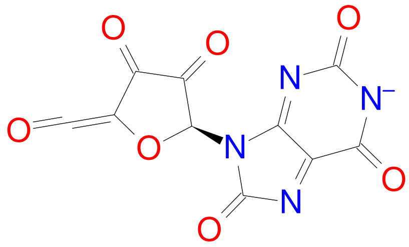

title	7,9-Dihydro-9-β-D-ribofu
docking score	-5.014
ct format	None
molecular.weight	300.22
cas.index.name	1H-Purine-2,6,8(3H)-tric

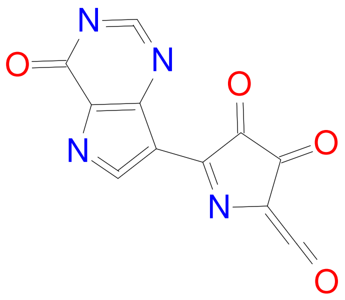

title	4H-Pyrrolo[3,2-d]pyrimi
docking score	-5.013
ct format	None
molecular.weight	266.25
cas.index.name	4H-Pyrrolo[3,2-d]pyrimi

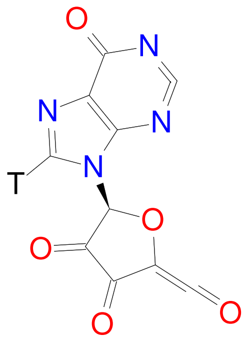

title	6H-Purin-8-t-6-one, 1,9-c
docking score	-5.005
ct format	None
molecular.weight	None
cas.index.name	6H-Purin-8-t-6-one, 1,9-c

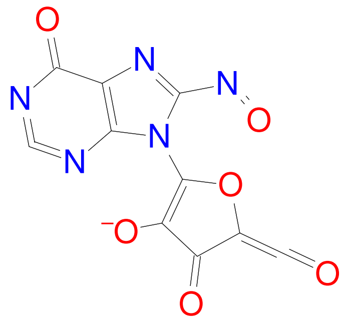

title	1H-Purine-6,8-dione, 7,9
docking score	-4.988
ct format	None
molecular.weight	299.24
cas.index.name	1H-Purine-6,8-dione, 7,9

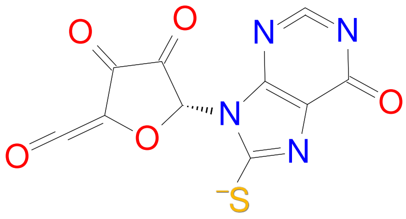

title	Inosine, 7,8-dihydro-8-tł
docking score	-4.986
ct format	None
molecular.weight	300.29
cas.index.name	Inosine, 7,8-dihydro-8-tł

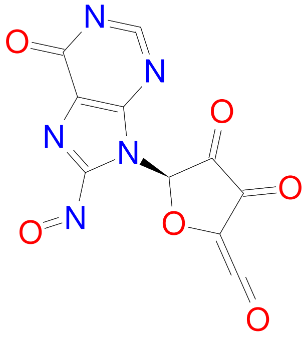

title	Inosine, 8-(hydroxyamir
docking score	-4.979
ct format	None
molecular.weight	299.24
cas.index.name	Inosine, 8-(hydroxyamir

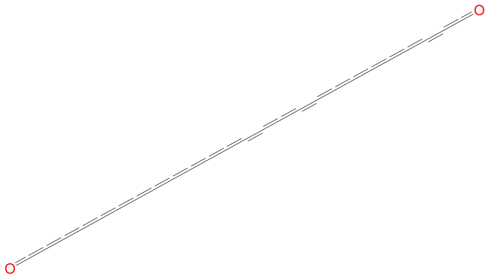

title	1,25-Pentacosanediol
docking score	-4.956
ct format	None
molecular.weight	384.68
cas.index.name	1,25-Pentacosanediol

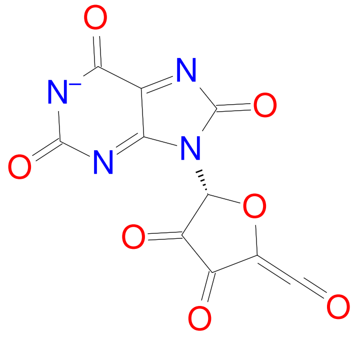

title	7,9-Dihydro-9-β-D-ribofu
docking score	-4.955
ct format	None
molecular.weight	300.22
cas.index.name	1H-Purine-2,6,8(3H)-tric

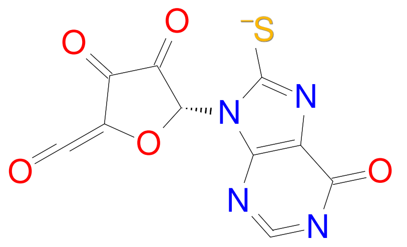

title	Inosine, 7,8-dihydro-8-th
docking score	-4.946
ct format	None
molecular.weight	300.29
cas.index.name	Inosine, 7,8-dihydro-8-th

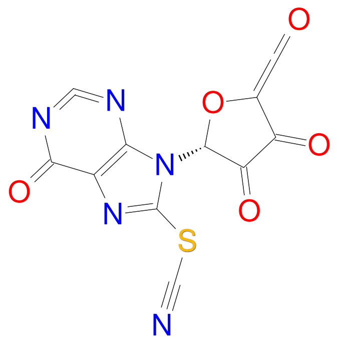

title	Thiocyanic acid, 6-hydro
docking score	-4.938
ct format	None
molecular.weight	325.3
cas.index.name	Thiocyanic acid, 6-hydro

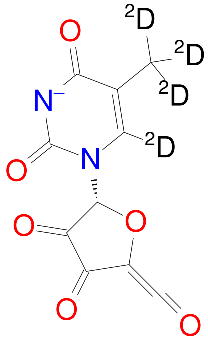

title	Uridine-6-d, 5-(methyl-d:
docking score	-4.923
ct format	None
molecular.weight	None
cas.index.name	Uridine-6-d, 5-(methyl-d:

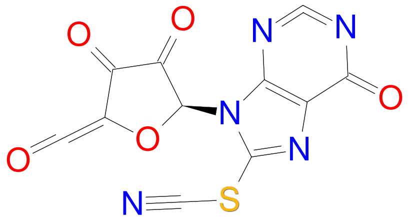

title	Thiocyanic acid, 6-hydro
docking score	-4.921
ct format	None
molecular.weight	325.3
cas.index.name	Thiocyanic acid, 6-hydro

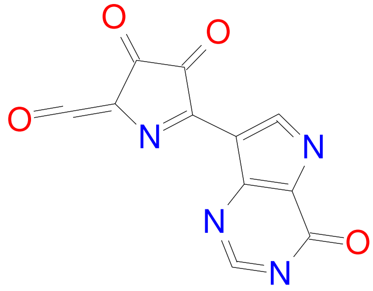

title	4H-Pyrrolo[3,2-d]pyrimic
docking score	-4.909
ct format	None
molecular.weight	266.25
cas.index.name	4H-Pyrrolo[3,2-d]pyrimic

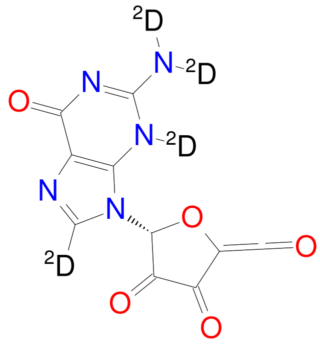

title	Guanosine-N,N,1,8-d4
docking score	-4.891
ct format	None
molecular.weight	None
cas.index.name	Guanosine-N,N,1,8-d4

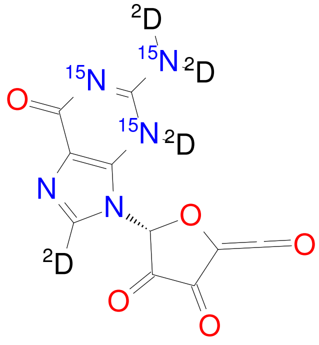

title	Guanosine-N,N,1,8-d4-N
docking score	-4.891
ct format	None
molecular.weight	None
cas.index.name	Guanosine-N,N,1,8-d4-N

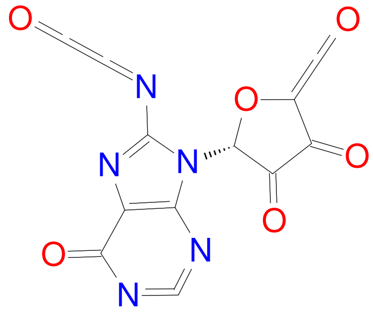

title	Inosine, 8-[(2-hydroxyet
docking score	-4.885
ct format	None
molecular.weight	327.29
cas.index.name	Inosine, 8-[(2-hydroxyet

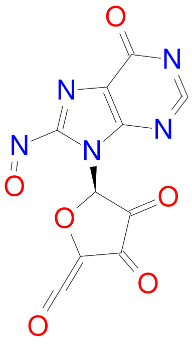

title	Inosine, 8-(hydroxyamir
docking score	-4.874
ct format	None
molecular.weight	299.24
cas.index.name	Inosine, 8-(hydroxyamir

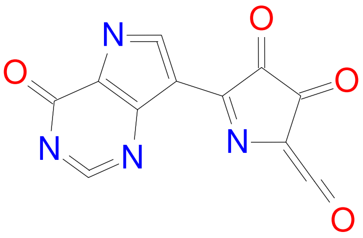

title	4H-Pyrrolo[3,2-d]pyrimic
docking score	-4.87
ct format	None
molecular.weight	266.25
cas.index.name	4H-Pyrrolo[3,2-d]pyrimic

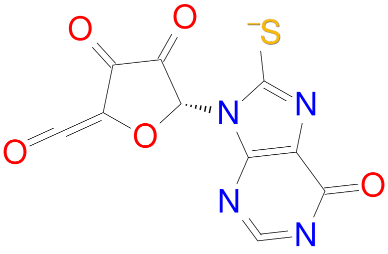

title	Inosine, 7,8-dihydro-8-tł
docking score	-4.869
ct format	None
molecular.weight	300.29
cas.index.name	Inosine, 7,8-dihydro-8-tł

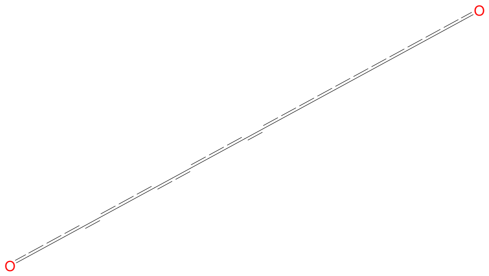

title	1,25-Pentacosanediol
docking score	-4.846
ct format	None
molecular.weight	384.68
cas.index.name	1,25-Pentacosanediol

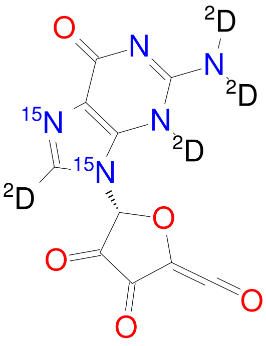

title	Guanosine-N,N,1,8-d4-7
docking score	-4.839
ct format	None
molecular.weight	None
cas.index.name	Guanosine-N,N,1,8-d4-7

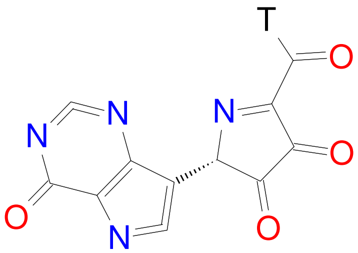

title	4H-Pyrrolo[3,2-d]pyrimic
docking score	-4.832
ct format	None
molecular.weight	None
cas.index.name	4H-Pyrrolo[3,2-d]pyrimic

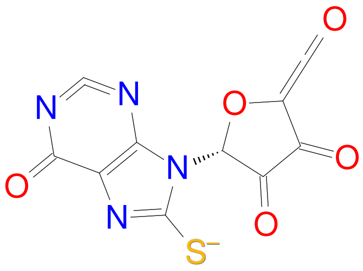

title	Inosine, 7,8-dihydro-8-tł
docking score	-4.83
ct format	None
molecular.weight	300.29
cas.index.name	Inosine, 7,8-dihydro-8-tł

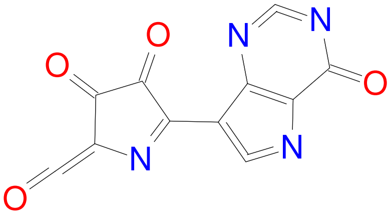

title	4H-Pyrrolo[3,2-d]pyrimic
docking score	-4.805
ct format	None
molecular.weight	266.25
cas.index.name	4H-Pyrrolo[3,2-d]pyrimic

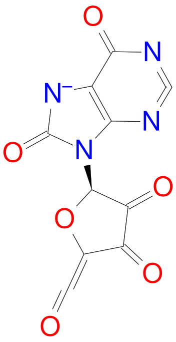

title	7,8-Dihydro-8-oxoinosin
docking score	-4.8
ct format	None
molecular.weight	284.23
cas.index.name	Inosine, 7,8-dihydro-8-o:

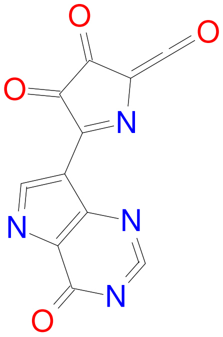

title	4H-Pyrrolo[3,2-d]pyrimic
docking score	-4.781
ct format	None
molecular.weight	266.25
cas.index.name	4H-Pyrrolo[3,2-d]pyrimic

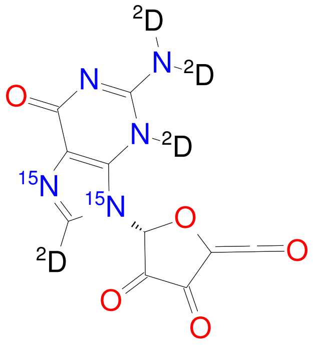

title	Guanosine-N,N,1,8-d4-7
docking score	-4.777
ct format	None
molecular.weight	None
cas.index.name	Guanosine-N,N,1,8-d4-7

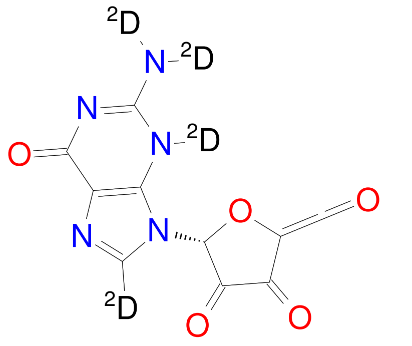

title	Guanosine-N,N,1,8-d4
docking score	-4.747
ct format	None
molecular.weight	None
cas.index.name	Guanosine-N,N,1,8-d4

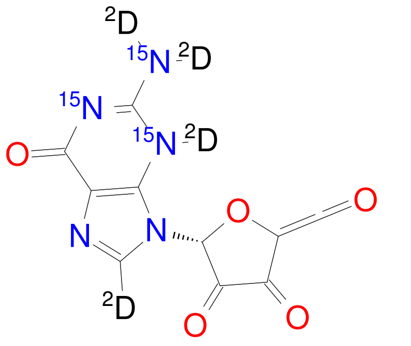

title	Guanosine-N,N,1,8-d4-N
docking score	-4.747
ct format	None
molecular.weight	None
cas.index.name	Guanosine-N,N,1,8-d4-N

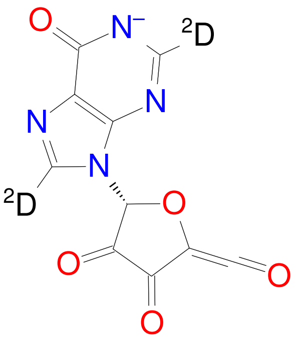

title	Inosine-2,8-d2
docking score	-4.744
ct format	None
molecular.weight	None
cas.index.name	Inosine-2,8-d2

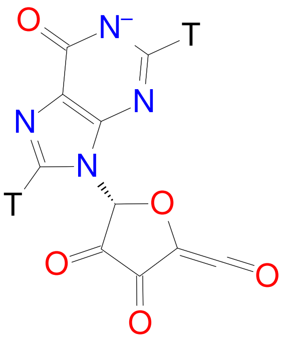

title	Inosine-2,8-t2
docking score	-4.744
ct format	None
molecular.weight	None
cas.index.name	Inosine-2,8-t2

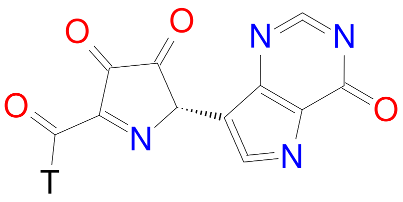

title	4H-Pyrrolo[3,2-d]pyrimi
docking score	-4.742
ct format	None
molecular.weight	None
cas.index.name	4H-Pyrrolo[3,2-d]pyrimi

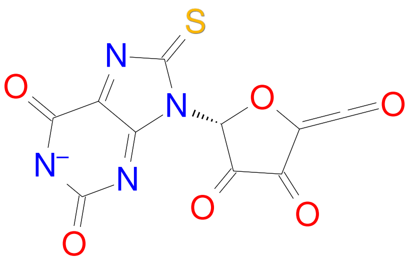

title	Xanthosine, 7,8-dihydro
docking score	-4.741
ct format	None
molecular.weight	316.29
cas.index.name	Xanthosine, 7,8-dihydro

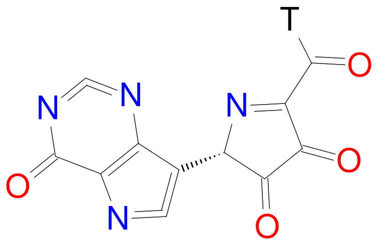

title	4H-Pyrrolo[3,2-d]pyrimi
docking score	-4.736
ct format	None
molecular.weight	None
cas.index.name	4H-Pyrrolo[3,2-d]pyrimi

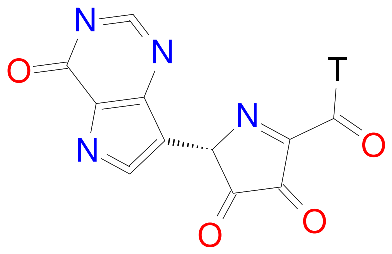

title	4H-Pyrrolo[3,2-d]pyrimi
docking score	-4.726
ct format	None
molecular.weight	None
cas.index.name	4H-Pyrrolo[3,2-d]pyrimi

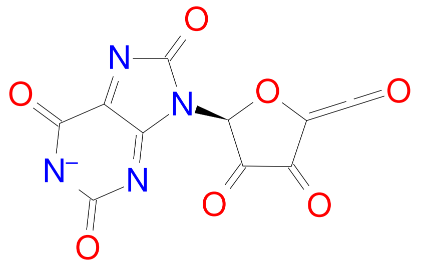

title	7,9-Dihydro-9-β-D-ribofu
docking score	-4.7
ct format	None
molecular.weight	300.22
cas.index.name	1H-Purine-2,6,8(3H)-trio

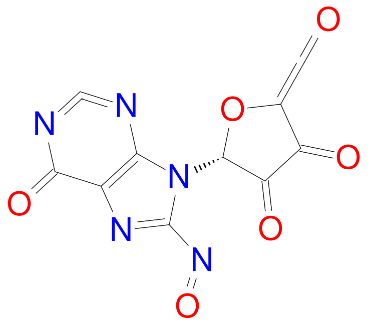

title	Inosine, 8-(hydroxyamir
docking score	-4.69
ct format	None
molecular.weight	299.24
cas.index.name	Inosine, 8-(hydroxyamir

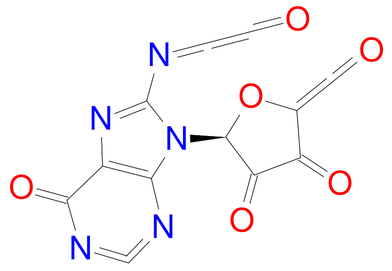

title	Inosine, 8-[(2-hydroxyetl
docking score	-4.686
ct format	None
molecular.weight	327.29
cas.index.name	Inosine, 8-[(2-hydroxyetl

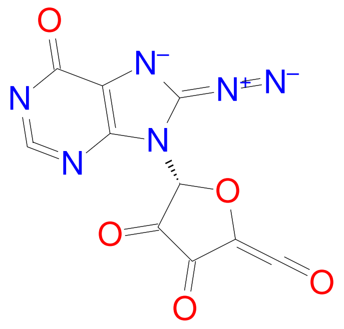

title	1H-Purine-6,8-dione, 9-ß
docking score	-4.686
ct format	None
molecular.weight	298.26
cas.index.name	1H-Purine-6,8-dione, 9-ß

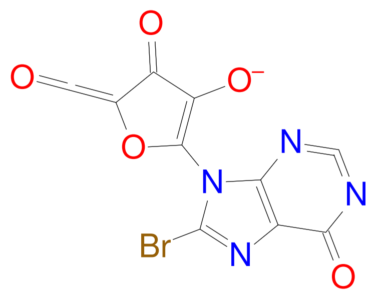

title	
docking score	-4.655
ct format	None
molecular.weight	347.12
cas.index.name	Not Yet Assigned

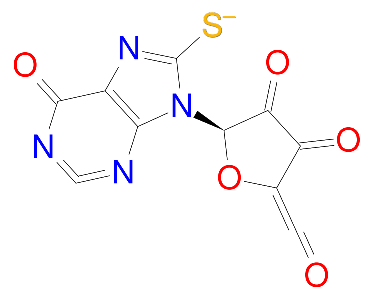

title	Inosine, 7,8-dihydro-8-tl
docking score	-4.629
ct format	None
molecular.weight	300.29
cas.index.name	Inosine, 7,8-dihydro-8-tl

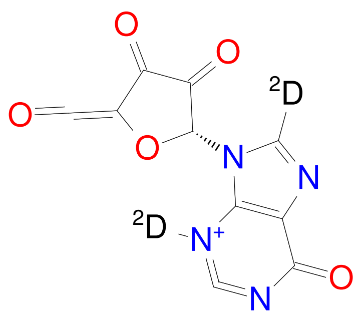

title	Inosine-1,8-d2
docking score	-4.592
ct format	None
molecular.weight	None
cas.index.name	Inosine-1,8-d2

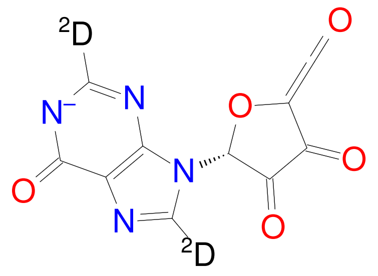

title	Inosine-2,8-d2
docking score	-4.57
ct format	None
molecular.weight	None
cas.index.name	Inosine-2,8-d2

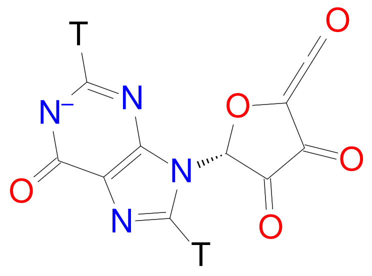

title	Inosine-2,8-t2
docking score	-4.57
ct format	None
molecular.weight	None
cas.index.name	Inosine-2,8-t2

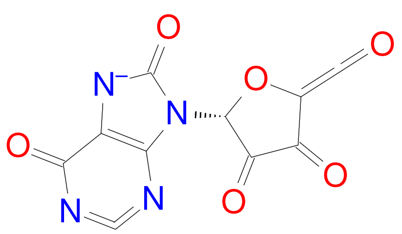

title	7,8-Dihydro-8-oxoinosin
docking score	-4.563
ct format	None
molecular.weight	284.23
cas.index.name	Inosine, 7,8-dihydro-8-o:

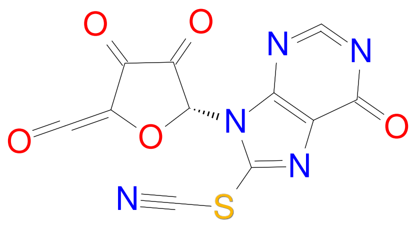

title	Thiocyanic acid, 6-hydro
docking score	-4.552
ct format	None
molecular.weight	325.3
cas.index.name	Thiocyanic acid, 6-hydro

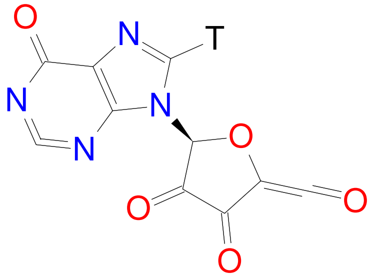

title	6H-Purin-8-t-6-one, 1,9-(
docking score	-4.531
ct format	None
molecular.weight	None
cas.index.name	6H-Purin-8-t-6-one, 1,9-(

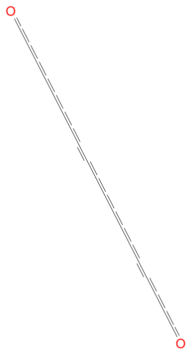

title	1,19-Nonadecanediol
docking score	-4.517
ct format	None
molecular.weight	300.52
cas.index.name	1,19-Nonadecanediol

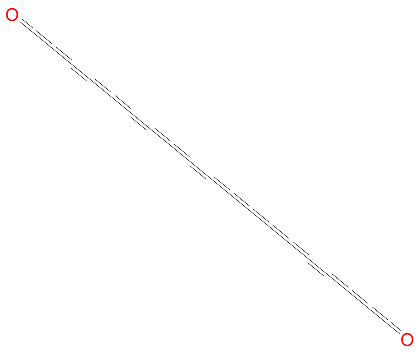

title	1,19-Nonadecanediol
docking score	-4.499
ct format	None
molecular.weight	300.52
cas.index.name	1,19-Nonadecanediol

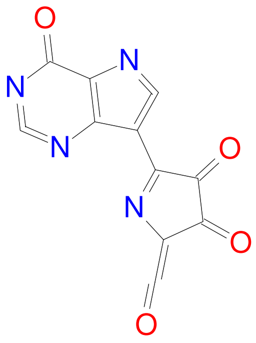

title	4H-Pyrrolo[3,2-d]pyrimic
docking score	-4.499
ct format	None
molecular.weight	266.25
cas.index.name	4H-Pyrrolo[3,2-d]pyrimic

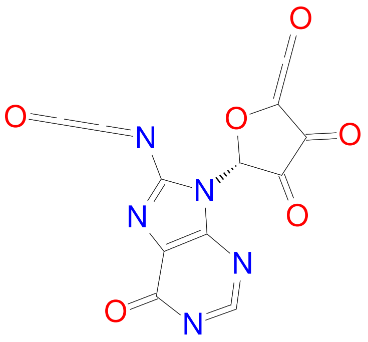

title	Inosine, 8-[(2-hydroxyetl
docking score	-4.49
ct format	None
molecular.weight	327.29
cas.index.name	Inosine, 8-[(2-hydroxyetl

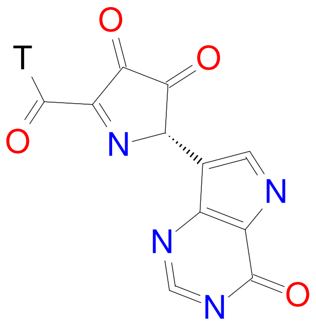

title	4H-Pyrrolo[3,2-d]pyrimic
docking score	-4.475
ct format	None
molecular.weight	None
cas.index.name	4H-Pyrrolo[3,2-d]pyrimic

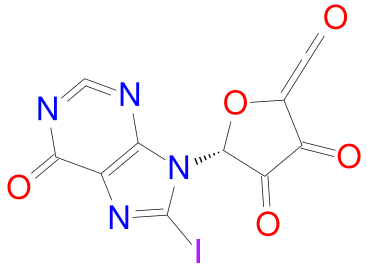

title	Inosine, 8-iodo-
docking score	-4.462
ct format	None
molecular.weight	394.12
cas.index.name	Inosine, 8-iodo-

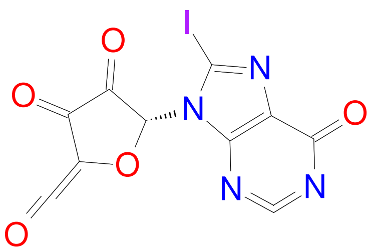

title	Inosine, 8-iodo-
docking score	-4.435
ct format	None
molecular.weight	394.12
cas.index.name	Inosine, 8-iodo-

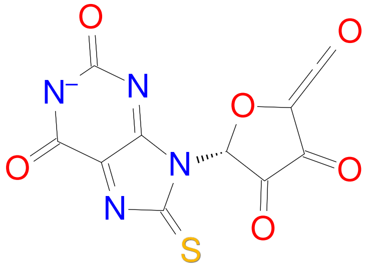

title	Xanthosine, 7,8-dihydro
docking score	-4.427
ct format	None
molecular.weight	316.29
cas.index.name	Xanthosine, 7,8-dihydro

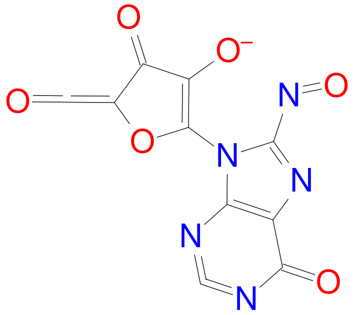

title	1H-Purine-6,8-dione, 7,9
docking score	-4.421
ct format	None
molecular.weight	299.24
cas.index.name	1H-Purine-6,8-dione, 7,9

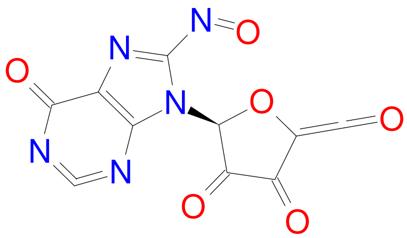

title	Inosine, 8-(hydroxyamir
docking score	-4.408
ct format	None
molecular.weight	299.24
cas.index.name	Inosine, 8-(hydroxyamir

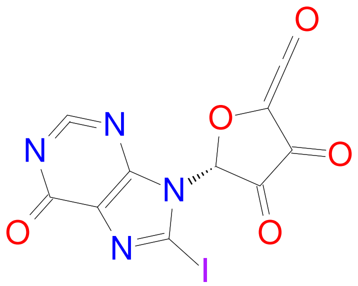

title	Inosine, 8-iodo-
docking score	-4.405
ct format	None
molecular.weight	394.12
cas.index.name	Inosine, 8-iodo-

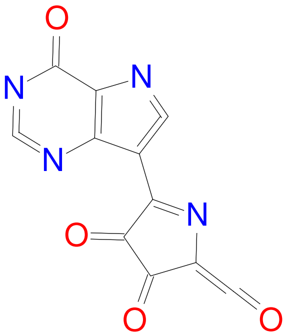

title	4H-Pyrrolo[3,2-d]pyrimi
docking score	-4.403
ct format	None
molecular.weight	266.25
cas.index.name	4H-Pyrrolo[3,2-d]pyrimi

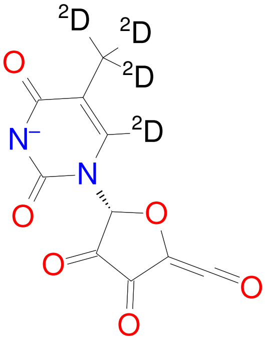

title	Uridine-6-d, 5-(methyl-d
docking score	-4.383
ct format	None
molecular.weight	None
cas.index.name	Uridine-6-d, 5-(methyl-d

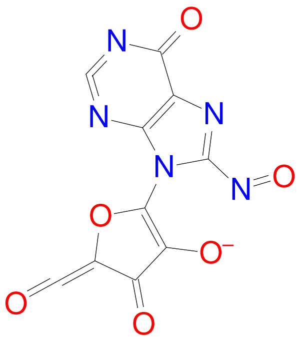

title	1H-Purine-6,8-dione, 7,9
docking score	-4.367
ct format	None
molecular.weight	299.24
cas.index.name	1H-Purine-6,8-dione, 7,9

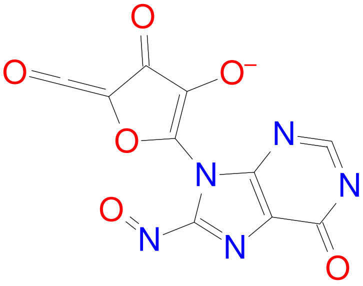

title	1H-Purine-6,8-dione, 7,9
docking score	-4.364
ct format	None
molecular.weight	299.24
cas.index.name	1H-Purine-6,8-dione, 7,9

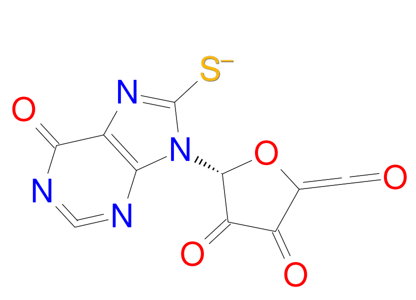

title	Inosine, 7,8-dihydro-8-th
docking score	-4.33
ct format	None
molecular.weight	300.29
cas.index.name	Inosine, 7,8-dihydro-8-th

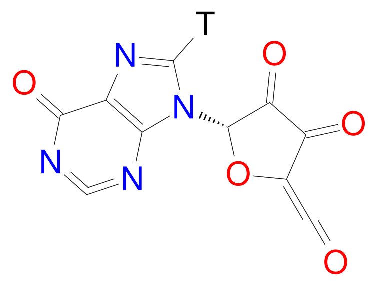

title	6H-Purin-8-t-6-one, 1,9-(
docking score	-4.327
ct format	None
molecular.weight	None
cas.index.name	6H-Purin-8-t-6-one, 1,9-(

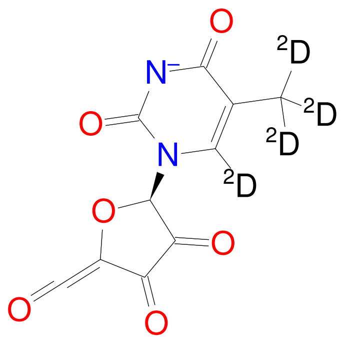

title	Uridine-6-d, 5-(methyl-d:
docking score	-4.322
ct format	None
molecular.weight	None
cas.index.name	Uridine-6-d, 5-(methyl-d:

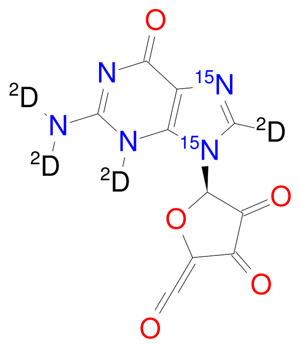

title	Guanosine-N,N,1,8-d4-7
docking score	-4.265
ct format	None
molecular.weight	None
cas.index.name	Guanosine-N,N,1,8-d4-7

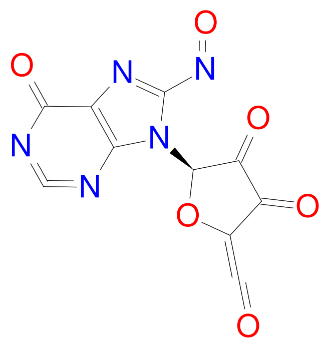

title	Inosine, 8-(hydroxyamir
docking score	-4.252
ct format	None
molecular.weight	299.24
cas.index.name	Inosine, 8-(hydroxyamir

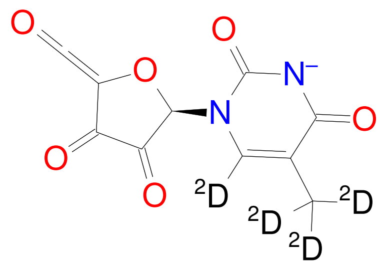

title	Uridine-6-d, 5-(methyl-d:
docking score	-4.248
ct format	None
molecular.weight	None
cas.index.name	Uridine-6-d, 5-(methyl-d:

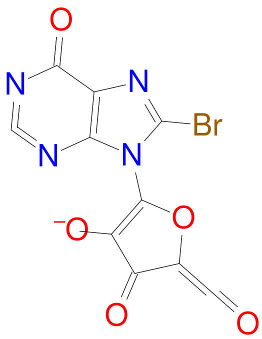

title	
docking score	-4.247
ct format	None
molecular.weight	347.12
cas.index.name	Not Yet Assigned

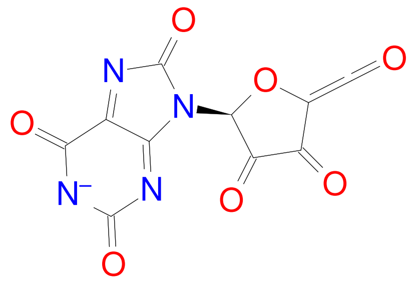

title	7,9-Dihydro-9-β-D-ribofu
docking score	-4.238
ct format	None
molecular.weight	300.22
cas.index.name	1H-Purine-2,6,8(3H)-tric

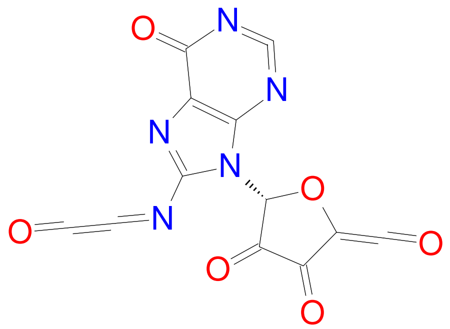

title	Inosine, 8-[(2-hydroxyetl
docking score	-4.212
ct format	None
molecular.weight	327.29
cas.index.name	Inosine, 8-[(2-hydroxyetl

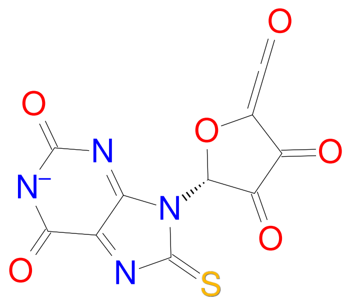

title	Xanthosine, 7,8-dihydro
docking score	-4.204
ct format	None
molecular.weight	316.29
cas.index.name	Xanthosine, 7,8-dihydro

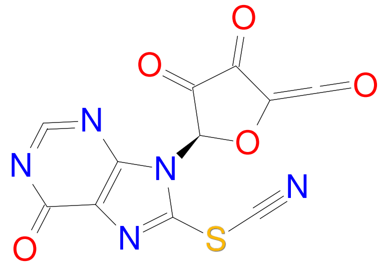

title	Thiocyanic acid, 6-hydr
docking score	-4.193
ct format	None
molecular.weight	325.3
cas.index.name	Thiocyanic acid, 6-hydr

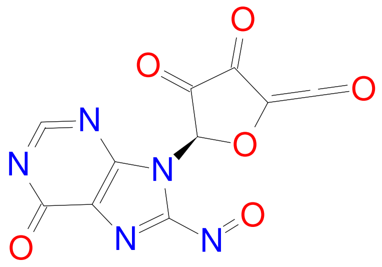

title	Inosine, 8-(hydroxyamir
docking score	-4.141
ct format	None
molecular.weight	299.24
cas.index.name	Inosine, 8-(hydroxyamir

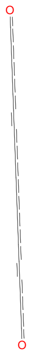

title	1,19-Nonadecanediol
docking score	-4.131
ct format	None
molecular.weight	300.52
cas.index.name	1,19-Nonadecanediol

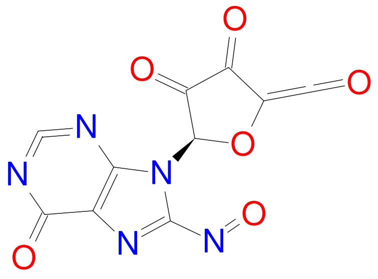

title	Inosine, 8-(hydroxyamir
docking score	-4.092
ct format	None
molecular.weight	299.24
cas.index.name	Inosine, 8-(hydroxyamir

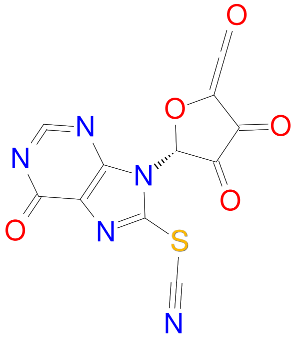

title	Thiocyanic acid, 6-hydr
docking score	-4.089
ct format	None
molecular.weight	325.3
cas.index.name	Thiocyanic acid, 6-hydr

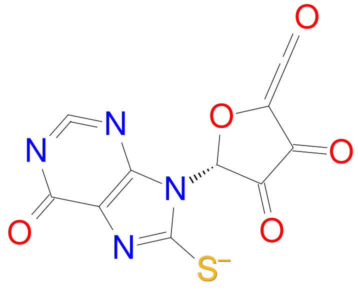

title	Inosine, 7,8-dihydro-8-th
docking score	-4.087
ct format	None
molecular.weight	300.29
cas.index.name	Inosine, 7,8-dihydro-8-th

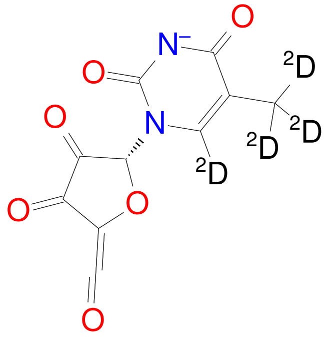

title	Uridine-6-d, 5-(methyl-d
docking score	-4.069
ct format	None
molecular.weight	None
cas.index.name	Uridine-6-d, 5-(methyl-d

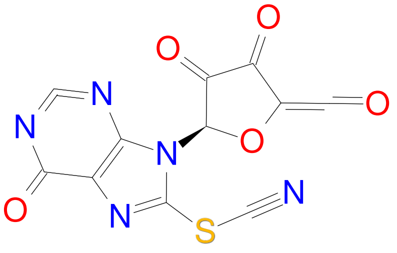

title	Thiocyanic acid, 6-hydr
docking score	-4.067
ct format	None
molecular.weight	325.3
cas.index.name	Thiocyanic acid, 6-hydr

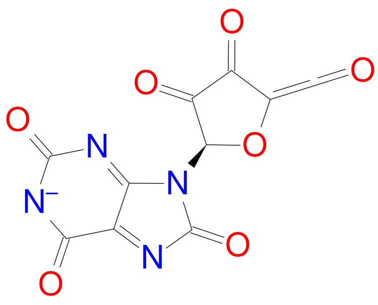

title	7,9-Dihydro-9-β-D-ribofu
docking score	-4.052
ct format	None
molecular.weight	300.22
cas.index.name	1H-Purine-2,6,8(3H)-tric

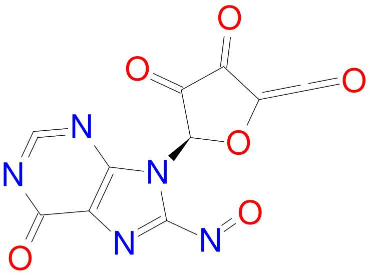

title	Inosine, 8-(hydroxyamir
docking score	-4.046
ct format	None
molecular.weight	299.24
cas.index.name	Inosine, 8-(hydroxyamir

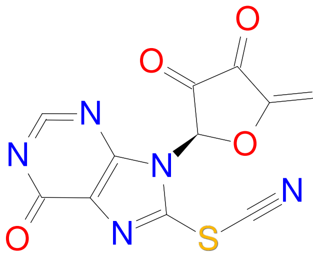

title	Thiocyanic acid, 6-hydrc
docking score	-4.041
ct format	None
molecular.weight	325.3
cas.index.name	Thiocyanic acid, 6-hydrc

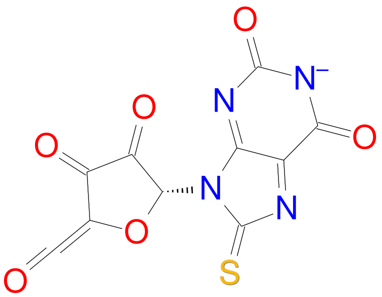

title	Xanthosine, 7,8-dihydro
docking score	-4.037
ct format	None
molecular.weight	316.29
cas.index.name	Xanthosine, 7,8-dihydro

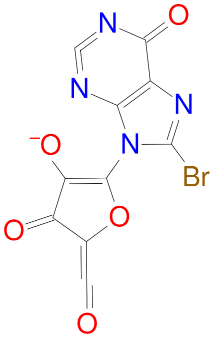

title	
docking score	-4.001
ct format	None
molecular.weight	347.12
cas.index.name	Not Yet Assigned

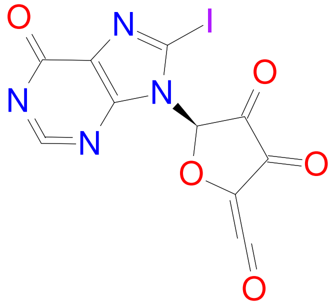

title	Inosine, 8-iodo-
docking score	-3.998
ct format	None
molecular.weight	394.12
cas.index.name	Inosine, 8-iodo-

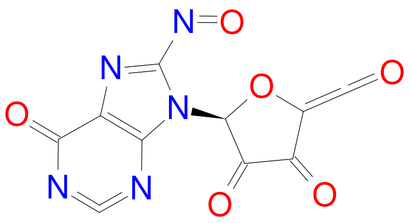

title	Inosine, 8-(hydroxyamir
docking score	-3.986
ct format	None
molecular.weight	299.24
cas.index.name	Inosine, 8-(hydroxyamir

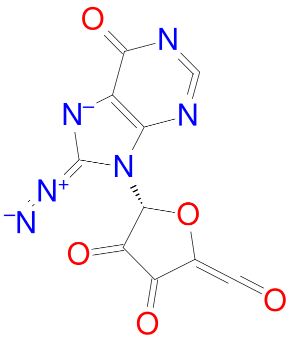

title	1H-Purine-6,8-dione, 9-β
docking score	-3.979
ct format	None
molecular.weight	298.26
cas.index.name	1H-Purine-6,8-dione, 9-β

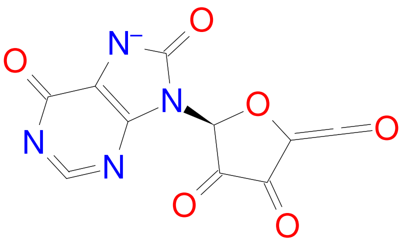

title	7,8-Dihydro-8-oxoinosin
docking score	-3.962
ct format	None
molecular.weight	284.23
cas.index.name	Inosine, 7,8-dihydro-8-o:

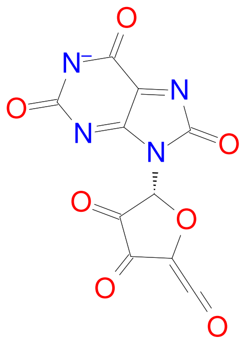

title	7,9-Dihydro-9-β-D-ribofu
docking score	-3.958
ct format	None
molecular.weight	300.22
cas.index.name	1H-Purine-2,6,8(3H)-tric

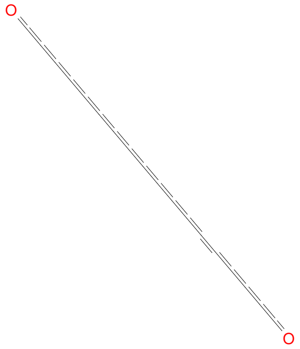

title	1,18-Octadecanediol
docking score	-3.935
ct format	None
molecular.weight	286.49
cas.index.name	1,18-Octadecanediol

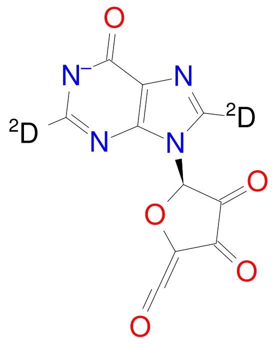

title	Inosine-2,8-d2
docking score	-3.927
ct format	None
molecular.weight	None
cas.index.name	Inosine-2,8-d2

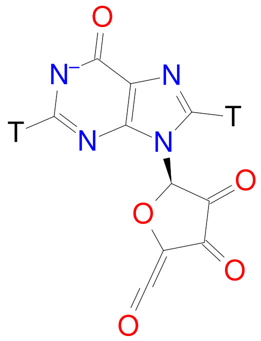

title	Inosine-2,8-t2
docking score	-3.927
ct format	None
molecular.weight	None
cas.index.name	Inosine-2,8-t2

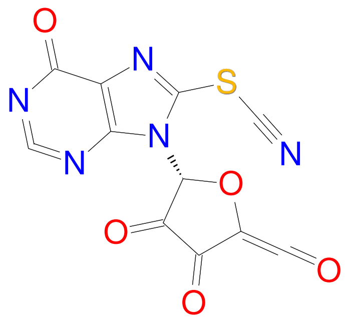

title	Thiocyanic acid, 6-hydrc
docking score	-3.927
ct format	None
molecular.weight	325.3
cas.index.name	Thiocyanic acid, 6-hydrc

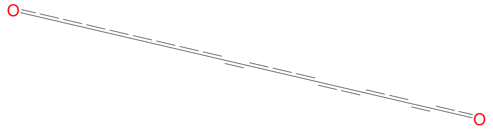

title	1,19-Nonadecanediol
docking score	-3.926
ct format	None
molecular.weight	300.52
cas.index.name	1,19-Nonadecanediol

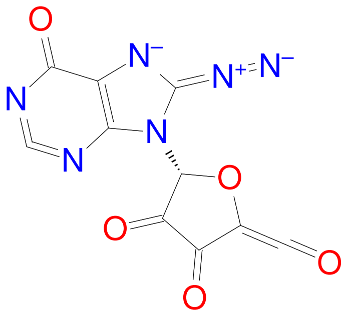

title	1H-Purine-6,8-dione, 9-f
docking score	-3.909
ct format	None
molecular.weight	298.26
cas.index.name	1H-Purine-6,8-dione, 9-f

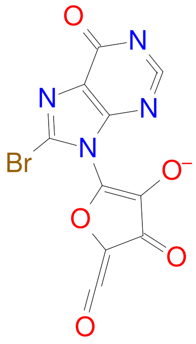

title	
docking score	-3.903
ct format	None
molecular.weight	347.12
cas.index.name	Not Yet Assigned

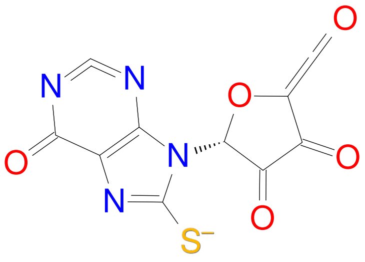

title	Inosine, 7,8-dihydro-8-th
docking score	-3.898
ct format	None
molecular.weight	300.29
cas.index.name	Inosine, 7,8-dihydro-8-th

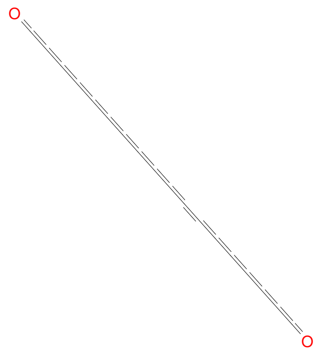

title	1,18-Octadecanediol
docking score	-3.898
ct format	None
molecular.weight	286.49
cas.index.name	1,18-Octadecanediol

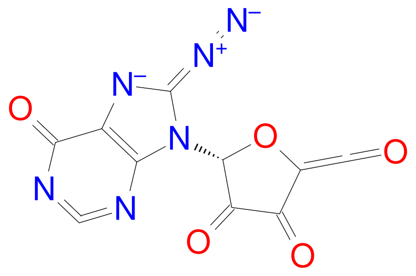

title	1H-Purine-6,8-dione, 9-[
docking score	-3.888
ct format	None
molecular.weight	298.26
cas.index.name	1H-Purine-6,8-dione, 9-[

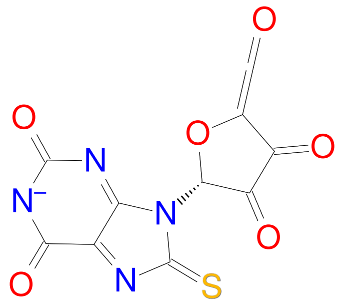

title	Xanthosine, 7,8-dihydro
docking score	-3.88
ct format	None
molecular.weight	316.29
cas.index.name	Xanthosine, 7,8-dihydro

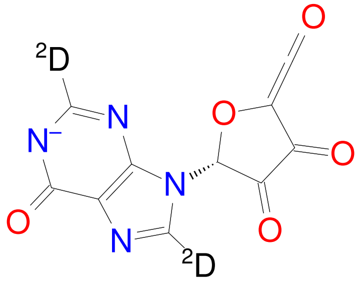

title	Inosine-2,8-d2
docking score	-3.877
ct format	None
molecular.weight	None
cas.index.name	Inosine-2,8-d2

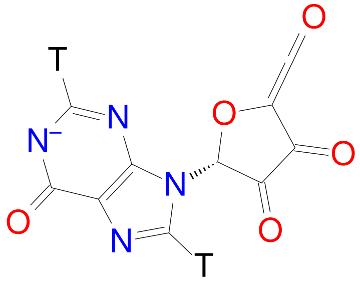

title	Inosine-2,8-t2
docking score	-3.877
ct format	None
molecular.weight	None
cas.index.name	Inosine-2,8-t2

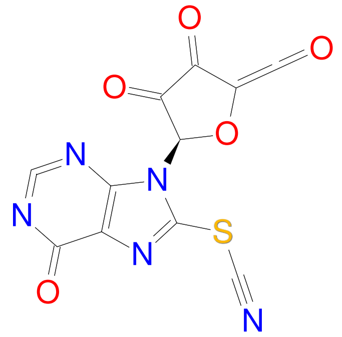

title	Thiocyanic acid, 6-hydr
docking score	-3.864
ct format	None
molecular.weight	325.3
cas.index.name	Thiocyanic acid, 6-hydr

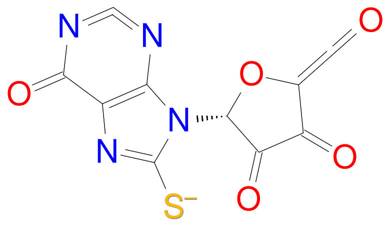

title	Inosine, 7,8-dihydro-8-th
docking score	-3.843
ct format	None
molecular.weight	300.29
cas.index.name	Inosine, 7,8-dihydro-8-th

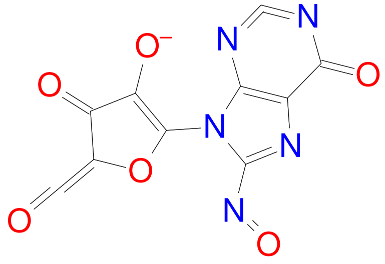

title	1H-Purine-6,8-dione, 7,9
docking score	-3.84
ct format	None
molecular.weight	299.24
cas.index.name	1H-Purine-6,8-dione, 7,9

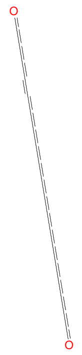

title	1,19-Nonadecanediol
docking score	-3.818
ct format	None
molecular.weight	300.52
cas.index.name	1,19-Nonadecanediol

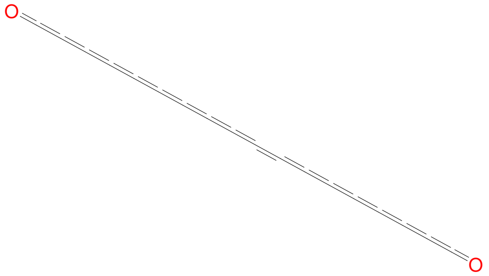

title	1,18-Octadecanediol
docking score	-3.77
ct format	None
molecular.weight	286.49
cas.index.name	1,18-Octadecanediol

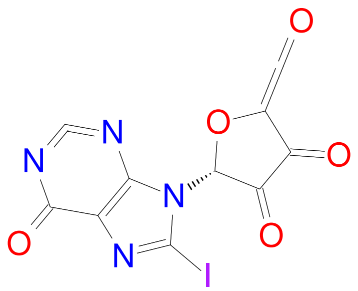

title	Inosine, 8-iodo-
docking score	-3.765
ct format	None
molecular.weight	394.12
cas.index.name	Inosine, 8-iodo-

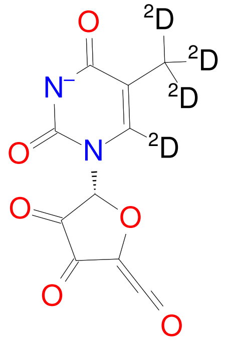

title	Uridine-6-d, 5-(methyl-d[illegible]
docking score	-3.743
ct format	None
molecular.weight	None
cas.index.name	Uridine-6-d, 5-(methyl-d[illegible]

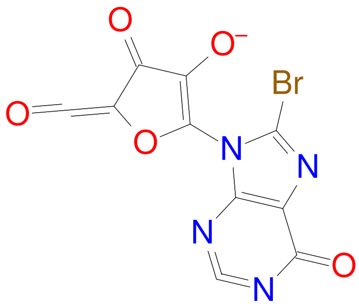

title	
docking score	-3.663
ct format	None
molecular.weight	347.12
cas.index.name	Not Yet Assigned

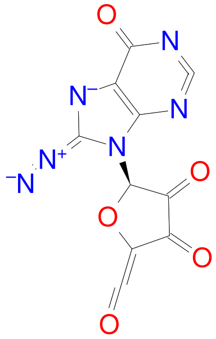

title	1H-Purine-6,8-dione, 9-[illegible]
docking score	-3.658
ct format	None
molecular.weight	298.26
cas.index.name	1H-Purine-6,8-dione, 9-[illegible]

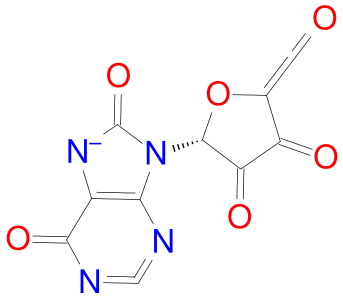

title	7,8-Dihydro-8-oxoinosin
docking score	-3.651
ct format	None
molecular.weight	284.23
cas.index.name	Inosine, 7,8-dihydro-8-o[illegible]

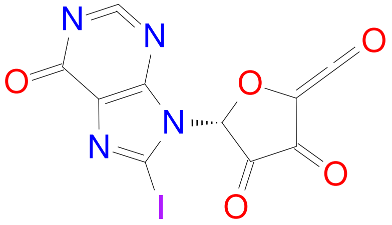

title	Inosine, 8-iodo-
docking score	-3.628
ct format	None
molecular.weight	394.12
cas.index.name	Inosine, 8-iodo-

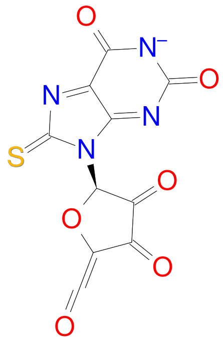

title	Xanthosine, 7,8-dihydro
docking score	-3.624
ct format	None
molecular.weight	316.29
cas.index.name	Xanthosine, 7,8-dihydro

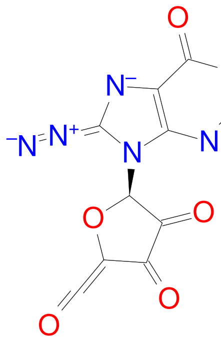

title	1H-Purine-6,8-dione, 9-[illegible]
docking score	-3.621
ct format	None
molecular.weight	298.26
cas.index.name	1H-Purine-6,8-dione, 9-[illegible]

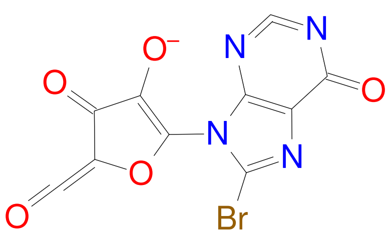

title	
docking score	-3.62
ct format	None
molecular.weight	347.12
cas.index.name	Not Yet Assigned

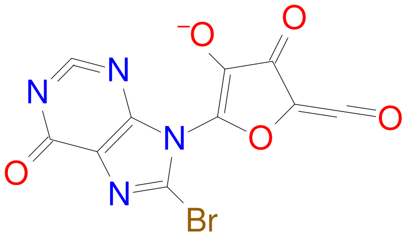

title	
docking score	-3.617
ct format	None
molecular.weight	347.12
cas.index.name	Not Yet Assigned

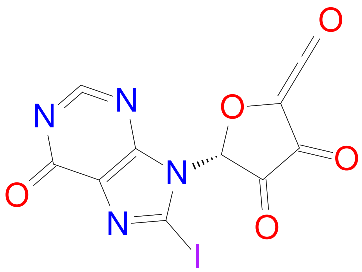

title	Inosine, 8-iodo-
docking score	-3.587
ct format	None
molecular.weight	394.12
cas.index.name	Inosine, 8-iodo-

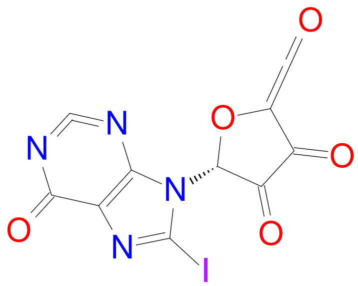

title	Inosine, 8-iodo-
docking score	-3.574
ct format	None
molecular.weight	394.12
cas.index.name	Inosine, 8-iodo-

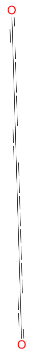

title	1,19-Nonadecanediol
docking score	-3.562
ct format	None
molecular.weight	300.52
cas.index.name	1,19-Nonadecanediol

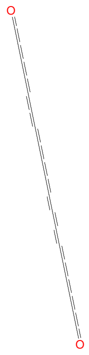

title	1,19-Nonadecanediol
docking score	-3.534
ct format	None
molecular.weight	300.52
cas.index.name	1,19-Nonadecanediol

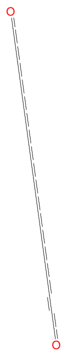

title	1,19-Nonadecanediol
docking score	-3.526
ct format	None
molecular.weight	300.52
cas.index.name	1,19-Nonadecanediol

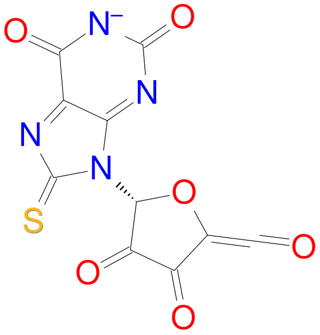

title	Xanthosine, 7,8-dihydro
docking score	-3.478
ct format	None
molecular.weight	316.29
cas.index.name	Xanthosine, 7,8-dihydro

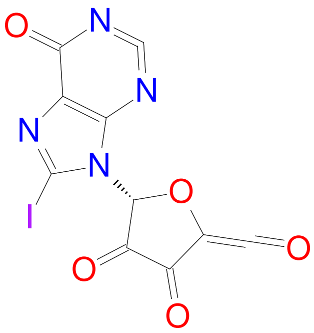

title	Inosine, 8-iodo-
docking score	-3.404
ct format	None
molecular.weight	394.12
cas.index.name	Inosine, 8-iodo-

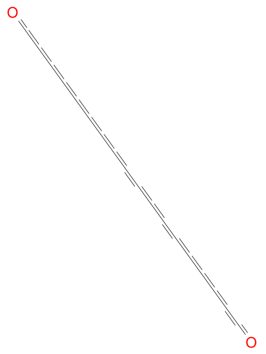

title	1,18-Octadecanediol
docking score	-3.389
ct format	None
molecular.weight	286.49
cas.index.name	1,18-Octadecanediol

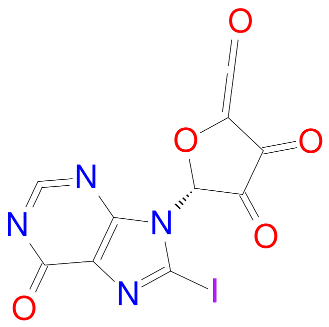

title	Inosine, 8-iodo-
docking score	-3.326
ct format	None
molecular.weight	394.12
cas.index.name	Inosine, 8-iodo-

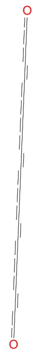

title	1,18-Octadecanediol
docking score	-3.283
ct format	None
molecular.weight	286.49
cas.index.name	1,18-Octadecanediol

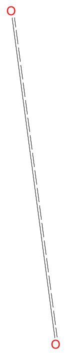

title	1,18-Octadecanediol
docking score	-3.246
ct format	None
molecular.weight	286.49
cas.index.name	1,18-Octadecanediol

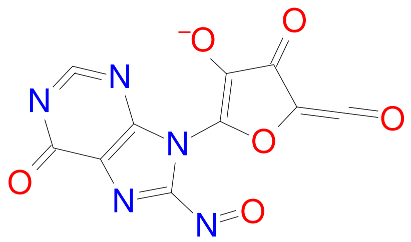

title	1H-Purine-6,8-dione, 7,9
docking score	-3.223
ct format	None
molecular.weight	299.24
cas.index.name	1H-Purine-6,8-dione, 7,9

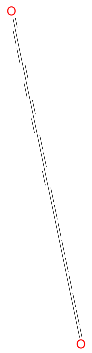

title	1,18-Octadecanediol
docking score	-3.16
ct format	None
molecular.weight	286.49
cas.index.name	1,18-Octadecanediol

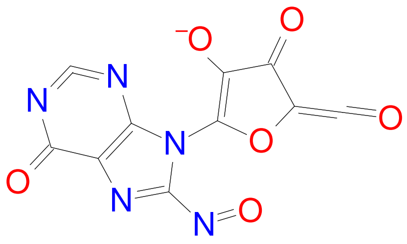

title	1H-Purine-6,8-dione, 7,9
docking score	-3.039
ct format	None
molecular.weight	299.24
cas.index.name	1H-Purine-6,8-dione, 7,9

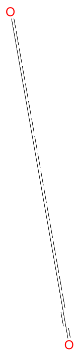

title	1,18-Octadecanediol
docking score	-3.006
ct format	None
molecular.weight	286.49
cas.index.name	1,18-Octadecanediol

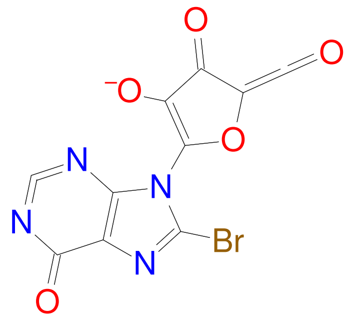

title	
docking score	-2.808
ct format	None
molecular.weight	347.12
cas.index.name	Not Yet Assigned
