# Supplementary material for: Inhibition of the hexamerization of SARS-CoV-2 endoribonuclease and modeling of RNA structures bound to the hexamer
Source: Sci Rep. 2022 Mar 9;12:3860. doi: 10.1038/s41598-022-07792-2 (PMC8907205; doi:10.1038/s41598-022-07792-2)
Supplement: Supplementary file 2 — Supplementary Information 2. [file 41598_2022_7792_MOESM2_ESM.pdf]

Rank	SMILE	CAS number	Molecular name	Formula	MW	Pocket	Affinity
1	O[C@H]1[C@H](c2c3c([nH]c2)c(N)ncn3)N[C@H](CSCCCC)[C@H]1O	156210-14-9	Acetamide, 2,2,Ä≤-[1,4,7,13,16-pentaoxa-10,19-diazacycloheneicosane-10,19-diylb	C42H68N4O11	805.01	A/B	-13
2	C(=O)([C@H](Cc1cnc[nH]1)NC(=O)[C@@H](NC(=O)[C@H](Cc1ccc(O)cc1)NC(=O)[C@@H](NC(=O)OCc1ccccc1)C(C)C)CC	108037-59-8	Zinc, hydroxy[3-[bis(3-carboxy-4-hydroxyphenyl)methylene]-6-oxo-1,4-cyclohexadie	C46H32N2O10Zn3		A/B	-12.7
3	O[C@H]1[C@H](c2c3c([nH]c2)c(N)ncn3)N[C@H](CSCCCC)[C@H]1O	156210-14-9	Acetamide, 2,2,Ä≤-[1,4,7,13,16-pentaoxa-10,19-diazacycloheneicosane-10,19-diylb	C42H68N4O11	805.01	C	-12.2
4	N(c1nc(nc(Nc2ccc(C(=O)C=Cc3cc(N(=O)=O)ccc3)cc2)n1)N1CCOCC1)c1cc(C(F)(F)F)ccc1	1883795-10-5	2-Propen-1-one, 1-[4-[[4-(4-morpholinyl)-6-[[3-(trifluoromethyl)phenyl]amino]-1,	C29H24F3N7O4	591.54	A/B	-11.2
5	O=C1[C@@H]2[C@@H](c3c([C@H](OC(=O)N4CCN(Cc5ccc(cc5)n5c(n[nH]c5=O)c5cc(C(C)C)c(O)cc5O)CC4)[C@H]2CO1)c	1680212-58-1	1-Piperidinecarboxylic acid, 4-[2-[5-[3-[2,4-dihydroxy-5-(1-methylethyl)phenyl]-	C49H51N5O12	901.96	A/B	-11
6	Cc1c2c(cc(c1)c1cc3c(c(C)c1)OP(=O)(OC[C@H]1O[C@H](C=C1)n1c(=O)[nH]c(=O)c(C)c1)OC3)COP(=O)(OC[C@H]1O[C	1160003-72-4	Thymidine, 5,Ä≤,5,Ä≤,Ä≤,Ä≤-O-(2,2,Ä≤-dioxido[6,6,Ä≤-bi-4H-1,3,2-benzodioxaphos	C34H32N4O14P2	782.58	C	-11
7	N(c1nc(nc(n1)N1CCOCC1)N1CCOCC1)c1ccc(cc1)c1cc(nc(N)n1)c1cc(Cl)ccc1	1710363-55-5	1,3,5-Triazin-2-amine, N-[4-[2-amino-6-(2-nitrophenyl)-4-pyrimidinyl]phenyl]-4,6	C27H28N10O4	556.58	C	-11
8	N(c1nc(nc(Nc2ccc(C(=O)C=Cc3cc(Cl)ccc3)cc2)n1)N1CCOCC1)c1cc(C(F)(F)F)ccc1	1883795-11-6	2-Propen-1-one, 1-[4-[[4-(4-morpholinyl)-6-[[3-(trifluoromethyl)phenyl]amino]-1,	C29H24F3N7O4	591.54	A/B	-10.9
9	C(=O)([C@H](Cc1cnc[nH]1)NC(=O)[C@@H](NC(=O)[C@H](Cc1ccc(O)cc1)NC(=O)[C@@H](NC(=O)OCc1ccccc1)C(C)C)CC	108037-59-8	Zinc, hydroxy[3-[bis(3-carboxy-4-hydroxyphenyl)methylene]-6-oxo-1,4-cyclohexadie	C46H32N2O10Zn3		C	-10.9
10	O[C@@H]1[C@H](n2c3c(nc2[3H])c(N)ncn3)O[C@H](COP(=O)(O)O)[C@H]1O	1268815-10-6	Thymidine, 3,Ä≤-azido-3,Ä≤-deoxy-5,Ä≤-O-[7-(3,Ä≤-deoxy-3,Ä≤-fluorothymidin-5,Ä≤	C28H30FN7O14P2		A/B	-10.8
11	S(=O)(=O)(O)c1c2c(c(N)c(N=Nc3ccc(N=Nc4c(N)c5c(c(S(=O)(=O)O)c4)cccc5)cc3)c1)cccc2	17094-98-3	1-Naphthalenesulfonic acid, 3,3,Ä≤-[4,4,Ä≤-biphenylylenebis(sulfonyl-p-phenylen	C44H32N6O10S4	933.02	C	-10.8
12	N(c1nc(nc(n1)N1CCOCC1)N1CCOCC1)c1ccc(cc1)c1cc(nc(N)n1)c1ccc(Br)cc1	1628172-17-7	1,3,5-Triazin-2-amine, N-[4-[2-amino-6-(3-nitrophenyl)-4-pyrimidinyl]phenyl]-4,6	C27H28N10O4	556.58	A/B	-10.7
13	N(c1nc(nc(n1)N1CCOCC1)N1CCOCC1)c1ccc(cc1)c1cc(nc(N)n1)c1ccc(N(CC)CC)cc1	1710363-61-3	1,3,5-Triazin-2-amine, N-[4-[2-amino-6-(3,4-dimethylphenyl)-4-pyrimidinyl]phenyl	C29H33N9O2	539.63	A/B	-10.7
14	C(NC(=O)[C@H](C)N)(C)C12CC3CC(C2)CC(C1)C3	129002-19-3	Aurintricarboxylic acid, 2,6-lutidine uranyl complex	C36H28N2O16U3		C	-10.7
15	C(=O)([C@@H](NC(=O)OC)[C@@H]1C[C@@H]2[C@H](C1)C2)N1[C@H]2[C@H](C2)C[C@H]1c1[nH]c(cn1)c1ccc(cc1)c1ccc	1413124-78-3	Carbamic acid, N,N,Ä≤-[[1,1,Ä≤-biphenyl]-4,4,Ä≤-diylbis[1H-imidazole-5,2-diyl-(	C48H50F4N8O6	910.95	A/B	-10.6
16	C(OC[C@@H]1[C@@H](O[C@H]2[C@H](OC(=O)C)[C@@H](OC(=O)C)[C@H](OC(=O)C)[C@@H](COC(=O)C)O2)C[C@@H](O1)n1	148834-36-0	Thymidine, 3,Ä≤-O-[3-O-benzoyl-2-deoxy-2-(1,3-dihydro-1,3-dioxo-2H-isoindol-2-y	C38H32F3N3O12	779.67	A/B	-10.6
17	O=c1n([C@@H]2O[C@H](CO)[C@@H](O)C2)cc(c(=O)[nH]1)c1ccc(N=Nc2ccc(N(C)C)cc2)cc1	1627963-49-8	3-Pyridinecarbonitrile, 2-amino-4-(3,4-dimethylphenyl)-6-[4-[(4,6-di-4-morpholin	C31H33N9O2	563.65	C	-10.6
18	N(CC(=O)[C@H](Cc1ccccc1)C[C@@H](CN1[C@H](C(=O)NC(C)(C)C)CN(CCCc2cccnc2)CC1)O)[C@H]1c2c(C[C@H]1O)cccc	1237988-76-9	D-erythro-Pentonamide, 2,3,5-trideoxy-N-[(1S,2R)-2,3-dihydro-2-hydroxy-1H-inden-	C36H48N4O5	616.79	A/B	-10.5
19	O=C1N2[C@@H]3[C@H](CO[C@@H]2Cn2c1c(O)c(=O)c(C(=O)NCc1c(F)cc(F)cc1)c2)CCC3	1309560-98-2	2H-Pyrido[1,Ä≤,2,Ä≤:4,5]pyrazino[1,2-a][3,1]benzoxazine-10-carboxamide, N-[(2,4	C29H27F2N3O5	535.54	A/B	-10.5
20	O=C1N2[C@@H]3[C@H](CO[C@@H]2Cn2c1c(O)c(=O)c(C(=O)NCc1c(F)cc(F)cc1)c2)CCC3	1309561-72-5	2H-Pyrido[1,Ä≤,2,Ä≤:4,5]pyrazino[1,2-a][3,1]benzoxazine-10-carboxamide, N-[(2,4	C29H27F2N3O5	535.54	A/B	-10.5
21	O=c1c2c(oc(c1O)c1[nH]ccn1)c(C)cc(OCc1ccc(F)cc1)c2	1312906-78-7	4H-1-Benzopyran-4-one, 6-[(4-fluorophenyl)methoxy]-3-hydroxy-8-methyl-2-(3-pheny	C25H18FN3O4	443.43	A/B	-10.5
22	O=C1C2C(c3c(ccc(c3C2)c2[nH]c(nc2)[C@H]2N(C(=O)[C@@H](NC(=O)OC)C(C)C)CCC2)c2c3c(c(cc2)c2[nH]c(nc2)[C@	1612236-60-8	Carbamic acid, N-[(1S)-1-[[(2S)-2-[5-[3-[1,Ä≤,3,Ä≤-dihydro-7,Ä≤-[2-[(2S)-1-[(2S	C52H66N8O6	899.13	A/B	-10.5
23	[C@@H](Cc1ccccc1)(NC(=O)[C@H](CCN1CCOCC1)NC(=O)N(Cc1nc(C(C)C)sc1)C)CC[C@H](Cc1ccccc1)NC(=O)OCc1cncs1	1865786-70-4	2,5,6,10,13-Pentaazatetradecanedioic acid, 3,12-bis(1,1-dimethylethyl)-8-hydroxy	C40H53N7O5S2.C38H52N6O7		A/B	-10.5
24	O(C(=O)c1ccccc1)[C@H]1[C@@H](O[C@H](COP(=O)(O[C@H]2[C@@H](O[C@H](CO)[C@H]2OC(=O)c2ccccc2)n2nc(C(=O)N	190141-38-9	Adenosine, 1,Ä≤-[3-(aminocarbonyl)-1H-1,2,4-triazol-1-yl]-3,Ä≤-O-benzoyl-P-(2-c	C72H59ClN9O16P	1372.72	A/B	-10.5
25	O[C@@H]1[C@@H](O[C@H](CO)[C@H]1CO)n1c(=O)nc(N)cc1	133701-45-8	Phenanthro[1,10,9,8-opqra]perylene-1,3,4,6,8,13-hexol, 10,11-dimethyl-	C30H18O6	474.46	C	-10.5
26	N(c1nc(nc(n1)N1CCOCC1)N1CCOCC1)c1ccc(cc1)c1cc(nc(N)n1)c1ccc(Br)cc1	1628172-17-7	1,3,5-Triazin-2-amine, N-[4-[2-amino-6-(3-nitrophenyl)-4-pyrimidinyl]phenyl]-4,6	C27H28N10O4	556.58	C	-10.5
27	C(=O)([C@@H](NC(=O)OC)C(C)C)N1[C@@H]([C@@H]2C[C@H]1CC2)c1[nH]c(cn1)c1ccc(cc1)c1ccc(cc1)c1[nH]c(nc1)[	1628172-19-9	1,3,5-Triazin-2-amine, N-[4-[2-amino-6-(4-bromophenyl)-4-pyrimidinyl]phenyl]-4,6	C27H28BrN9O2	590.47	C	-10.5
28	N(c1nc(nc(n1)N1CCOCC1)N1CCOCC1)c1ccc(cc1)c1cc(nc(N)n1)c1ccc(N(CC)CC)cc1	1710363-61-3	1,3,5-Triazin-2-amine, N-[4-[2-amino-6-(3,4-dimethylphenyl)-4-pyrimidinyl]phenyl	C29H33N9O2	539.63	C	-10.5
29	Cc1c2OC3(OCc2c(COC(=O)CCC(=O)OC[C@@H]2[C@@H](N=[N+]=[N-])C[C@@H](O2)n2c(=O)[nH]c(=O)c(C)c2)cn1)CCCC3	1804976-64-4	Not Yet Assigned	C40H40N4O12	768.77	C	-10.5
30	N(=Nc1c2c(c(N=Nc3cc4c(c(S(=O)(=O)O)c3)cccc4S(=O)(=O)O)cc1)cccc2)c1c2c(c(N=Nc3c(C)cc(N)c(C)c3)cc1)ccc	10134-25-5	1,5-Naphthalenedisulfonic acid, 3-[2-[4-[2-[4-[2-(4-amino-2,5-dimethylphenyl)dia	C38H29N7O9S3.3Na		A/B	-10.4
31	N(C(=O)[C@H](Cc1ccccc1)C[C@@H](CN1[C@H](C(=O)NC(C)(C)C)CN(Cc2cc3c(nc2)ocn3)CC1)O)[C@H]1c2c(C[C@H]1O)	1237809-06-1	Not Yet Assigned	C40H49N5O4	663.85	A/B	-10.4
32	O=c1c2c(oc(c1O)c1[nH]ccn1)c(Cl)cc(OCc1ccc(F)cc1)c2	1312907-78-0	4H-1-Benzopyran-4-one, 8-chloro-6-[(4-fluorophenyl)methoxy]-3-hydroxy-2-(3-pheny	C24H15ClFN3O4	463.85	A/B	-10.4
33	C(=O)([C@@H](NC(=O)OC)C(C)C)N1[C@H](c2[nH]c(c3c4c(c(cc3)c3ccc(cc3)c3[nH]c(nc3)[C@H]3N(C(=O)[C@@H](NC	1419385-17-3	Carbamic acid, N-[(1S)-1-cyclohexyl-2-[(2S)-2-[5-[4-[7,Ä≤-[2-[(2S)-1-[(2S)-2-cy	C53H68N8O6	913.16	A/B	-10.4
34	C(OP(=O)(O[C@H]1[C@@H](O[C@H](COP(=O)(O[C@H]2[C@@H](O[C@H](CO)[C@H]2O)n2nc(C(=O)N)nc2)O)[C@H]1O)n1nc	1422459-54-8	Adenosine, adenylyl-(2,Ä≤,Ü5,Ä≤)-1,Ä≤-[3-(aminocarbonyl)-1H-1,2,4-triazol-1-yl	C28H34N14O16P2	884.6	A/B	-10.4
35	CC1(C)COc2c(-c3c(cc(cc3)c3[nH]c(nc3)[C@H]3N(C(=O)[C@@H](NC(=O)OC)C(C)C)CCC3)OC1)ccc(c2)c1[nH]c(nc1)[	1449762-15-5	Carbamic acid, N,N,Ä≤-[(6,7,8,9-tetrahydrodibenzo[b,d][1,6]dioxecin-3,12-diyl)b	C44H56N8O8	824.96	A/B	-10.4
36	C(n1c2c(nc1CC)c(C)cc(C)n2)c1ccc(cc1)c1c(S(=O)(=O)NC(=O)NCCc2ccccc2)ccc(C(CC)(C)C)c1	154553-61-4	[1,1,Ä≤:3,Ä≤,1,Ä≤,Ä≤-Terphenyl]-4,Ä≤-sulfonamide, 4,Ä≤,Ä≤-[(2-ethyl-5,7-dimeth	C38H37N5O3S	643.8	A/B	-10.4
37	N(C(=O)[C@H](Cc1ccccc1)C[C@@H](CN1[C@H](C(=O)NC(C)(C)C)CN(Cc2ccc(Cl)nc2)CC1)O)[C@H]1c2c(C[C@H]1O)ccc	164725-39-7	1-Piperazinepentanamide, 4-[(2-chloro-6-methyl-4-pyridinyl)methyl]-N-(2,3-dihydr	C37H48ClN5O4	662.26	A/B	-10.4
38	N(C(=O)[C@H](Cc1ccccc1)C[C@@H](CN1[C@H](C(=O)NC(C)(C)C)CN(Cc2cccnc2)CC1)O)[C@H]1c2c(C[C@H]1OC(=O)c1c	170227-74-4	Benzoic acid, 4-(4-morpholinylmethyl)-, 1-[[5-[2-[[(1,1-dimethylethyl)amino]carb	C48H60N6O6	817.03	A/B	-10.4
39	O(C(=O)N[C@@H](Cc1ccc(OCc2c(C(=O)O)sc(Oc3ccccc3)n2)cc1)[C@@H](CN(S(=O)(=O)c1cc2c(cc1)ncs2)CC(C)C)O)[	1887206-21-4	2-Pyridinecarboxylic acid, 3-[[4-[(2S,3R)-4-[(6-benzothiazolylsulfonyl)(2-methyl	C41H42F2N4O10S2	852.92	A/B	-10.4
40	O=c1c2c(n(cn2)[C@@H]2O[C@H](COP(=O)(OC[C@@H]3[C@@H](N=[N+]=[N-])C[C@@H](O3)n3c(=O)[nH]c(=O)c(C)c3)O)	197900-97-3	Inosine, 3,Ä≤-azido-3,Ä≤-deoxythymidylyl-(5,Ä≤,Ü5,Ä≤)-2,Ä≤,3,Ä≤-dideoxy-, 5,Ä	C30H34N13O11P		A/B	-10.4
41	O=c1n([C@@H]2O[C@H](COP(=[17O])([18OH])O)[C@@H](O)C2)cc(C)c(=O)[nH]1	10134-26-6	1,5-Naphthalenedisulfonic acid, 3-[2-[4-[2-[4-[2-(4-amino-2,5-dimethylphenyl)dia	C38H29N7O9S3.3Na		C	-10.4
42	C(CCCCCCn1c2-c3c(CN(C(=O)CCNC(=O)CCN4C(=O)CCC4=O)c4c(-c2nn1)cccc4)cccc3)(F)(F)c1c2c(N(c3c(NC2=O)c(C)	1450913-00-4	1-Pyrrolidinepropanamide, N-[3-[1-[7-(11-cyclopropyl-6,11-dihydro-4-methyl-6-oxo	C47H48F2N10O5	870.94	C	-10.4
43	N(c1nc(nc(n1)N1CCOCC1)N1CCOCC1)c1ccc(cc1)c1cc(nc(N)n1)c1cc(C)c(C)cc1	1710363-59-9	1,3,5-Triazin-2-amine, N-[4-[2-amino-6-(3-chlorophenyl)-4-pyrimidinyl]phenyl]-4,	C27H28ClN9O2	546.02	C	-10.4
44	O=C1[C@@]2([C@@H]([C@@H](/C(=C/C(=O)[C@@H]3[C@@]4(N5[C@@H]([C@H]3c3cc(OC)c(O)cc3)CCC5)c3c5c(C4=O)ccc	1807888-53-4	Spiro[acenaphthylene-1(2H),3,Ä≤-[3H]pyrrolizin]-2-one, 2,Ä≤-[(1Z)-3-[(1R,1,Ä≤R,	C53H46N2O8	838.94	C	-10.4
45	O=c1n([C@@H]2O[C@H](COP(=[17O])([18OH])O)[C@@H](O)C2)cc(C)c(=O)[nH]1	10134-26-6	1,5-Naphthalenedisulfonic acid, 3-[2-[4-[2-[4-[2-(4-amino-2,5-dimethylphenyl)dia	C38H29N7O9S3.3Na		A/B	-10.3
46	O(Cc1ccc(F)cc1)c1c2c(oc(c(O)c2=O)c2[nH]ccn2)cc(c1)c1ccccc1	1312903-18-6	4H-1-Benzopyran-4-one, 5-[(4-fluorophenyl)methoxy]-3-hydroxy-7-phenyl-2-(3-pheny	C30H20FN3O4	505.5	A/B	-10.3
47	C(=O)([C@@H](NC(=O)OC)C(C)C)N1[C@H](c2[nH]c(c3c4c(c(cc3)c3c5c(c(cc3)c3[nH]c(nc3)[C@H]3N(C(=O)[C@@H](	1612235-51-4	Carbamic acid, N,N,Ä≤-[(1,Ä≤,1,Ä≤,Ä≤,Ä≤,3,Ä≤,3,Ä≤,Ä≤,Ä≤-tetrahydro[4,Ä≤,4,Ä≤,	C54H70N8O6	927.18	A/B	-10.3
48	N(C(=O)[C@H](Cc1ccccc1)C[C@@H](CN1[C@H](C(=O)NC(C)(C)C)CN(CCCC)CC1)O)[C@H]1c2c(C[C@H]1O)cccc2	164725-21-7	1-Piperazinepentanamide, N-(2,3-dihydro-2-hydroxy-1H-inden-1-yl)-2-[[(1,1-dimeth	C41H50N4O4	662.86	A/B	-10.3
49	C(C(C(NC(=O)C(NC(=O)c1nc2c(cc1)cccc2)CC(=O)N)CSc1ccccc1)O)N1C(C(=O)NC(C)(C)C)CC2C(C1)CCCC2	168394-47-6	Butanediamide, N1-[3-[3-[[(1,1-dimethylethyl)amino]carbonyl]octahydro-2(1H)-isoq	C42H52N6O5S	752.96	A/B	-10.3
50	N(C(=O)C)[C@H]1[C@H](OCCC)C=C(C(=O)OC)C[C@@H]1N=[N+]=[N-]	1872209-11-4	Not Yet Assigned	C35H35N5O8	653.68	A/B	-10.3
51	C=C1[C@H](n2c3c(nc2)c(=O)nc(N)[nH]3)C[C@@H](O)[C@@H]1CO	1883795-13-8	2-Propen-1-one, 1-[4-[[4-(4-morpholinyl)-6-[[3-(trifluoromethyl)phenyl]amino]-1,	C27H23F3N6O2S	552.57	A/B	-10.3
52	N(c1nc(nc(n1)N1CCOCC1)N1CCOCC1)c1ccc(cc1)c1cc(nc(N)n1)c1cc(N(=O)=O)ccc1	1628172-16-6	1,3,5-Triazin-2-amine, N-[4-[2-amino-6-(3-methoxyphenyl)-4-pyrimidinyl]phenyl]-4	C28H31N9O3	541.6	C	-10.3
53	O=c1c2c(n(cc1C(=O)OC[C@@H]1[C@@H](N=[N+]=[N-])C[C@@H](O1)n1c(=O)[nH]c(=O)c(C)c1)C1CC1)cc(c(F)c2)N1CC	1174398-24-3	Thymidine, 3,Ä≤-azido-3,Ä≤-deoxy-, 5,Ä≤-[1-cyclopropyl-7-[3,5-dimethyl-4-[(2-ph	C45H45F2N9O10		A/B	-10.2
54	N(c1nc(nc(Nc2ccc(C(=O)C=Cc3cccs3)cc2)n1)N1CCOCC1)c1cc(C(F)(F)F)ccc1	1883795-12-7	2-Propen-1-one, 3-(3-chlorophenyl)-1-[4-[[4-(4-morpholinyl)-6-[[3-(trifluorometh	C29H24ClF3N6O2	580.99	A/B	-10.2
55	S(=O)(=O)(O)c1c2c(c(N)c(N=Nc3c(C)cccc3)c1)ccc(S(=O)(=O)O)c2.[NaH]	13083-07-3	2,6-Naphthalenedisulfonic acid, 4-[2-[4-[2-(4-amino-1-naphthalenyl)diazenyl]-1-n	C30H21N5O6S2.2Na		C	-10.2
56	C([C@H](OCP(=O)(OCCCO)O)C)n1c2c(nc1)c(N)ncn2	1345852-51-8	2-Naphthalenesulfonic acid, 5-[2-(4-amino-7-sulfo-1-naphthalenyl)diazenyl]-8-[2-	C26H19N5O9S3	641.65	C	-10.2
57	N(C(=O)Nc1ccc(S(=O)(=O)c2ccc(N)cc2)cc1)C12CC3CC(C2)CC(C1)C3	1431218-99-3	2-Naphthalenesulfonic acid, 5-amino-8-[(1E)-2-[2,5-dimethyl-4-[(1E)-2-(3-sulfoph	C24H21N5O6S2	539.58	C	-10.2
58	C([C@H](OP(=O)(O)O)CO)n1c(=O)nc(N)cc1	151408-85-4	1,5-Naphthalenedisulfonic acid, 3-[2-[4-[2-(4-amino-2-methylphenyl)diazenyl]-7-s	C27H21N5O9S3	655.68	C	-10.2
59	C(=O)(C(NC(=O)OC)C(C)C)N1C(c2[nH]c(c3c4c(C5CC4CC5)c(cc3)c3ccc(cc3)c3[nH]c(nc3)C3N(C(=O)C(NC(=O)OC)C(	1560650-21-6	Carbamic acid, N,N,Ä≤-[(1,2,3,4-tetrahydro-1,4-methanonaphthalene-5,8-diyl)bis[	C51H60N8O6	881.07	C	-10.2
60	O(C(=O)OCCCCCC)c1c2n(N([C@H]3N(C2=O)CCOC3)[C@H]2c3c(CSc4c2cccc4)c(F)c(F)cc3)ccc1=O	1985606-92-5	1H-Indole-1-carboxylic acid, 3-[[[(12aR)-12-[(11S)-7,8-difluoro-6,11-dihydrodibe	C35H28F2N4O6S	670.68	C	-10.2
61	O(C[C@H]1O[C@H](CS1)n1c(=O)nc(N)cc1)P1Oc2c(cc(CO)c(O)c2)CO1	1099829-95-4	Not Yet Assigned	C35H36N10O14P2		A/B	-10.1
62	N(C(C)C)(C(C)C)P1Oc2c(cc3c(c2)OP(OC[C@H]2O[C@H](CS2)n2c(=O)nc(N)cc2)OC3)CO1	1099829-99-8	Thymidine, 3,Ä≤-azido-3,Ä≤-deoxy-, 5,Ä≤,5,Ä≤,Ä≤,Ä≤,Ä≤-[1,1,Ä≤,5,5,Ä≤-tetrahydroge	C71H62N16O29S6		A/B	-10.1
63	C(C)(C)(C)c1c2c(cc(c1)c1cc(C(C)(C)C)c3c(c1)COP(=O)(OC[C@H]1O[C@H](C=C1)n1c(=O)[nH]c(=O)c(C)c1)O3)COP	1160003-73-5	Thymidine, 5,Ä≤,5,Ä≤,Ä≤,Ä≤-O-(8,8,Ä≤-dimethyl-2,2,Ä≤-dioxido[6,6,Ä≤-bi-4H-1,3,	C36H36N4O14P2	810.64	A/B	-10.1
64	O=c1n([C@@H]2O[C@H](COC(=O)c3cccnc3)C=C2)cc(C)c(=O)[nH]1	1188424-89-6	7,10-Ethanopyrimido[1,2-a]azepine-2-carboxamide, N-[(4-fluorophenyl)methyl]-4,6,	C23H23FN6O5	482.46	A/B	-10.1
65	[C@H](Cc1ccccc1)(C[C@@H]([C@H](Cc1ccccc1)NC(=O)OCc1ccns1)O)NC(=O)[C@@H](NC(=O)N(Cc1nc(CC)sc1)C)C(C)C	1236407-24-1	2,7,10,12-Tetraazatridecanoic acid, 4-hydroxy-12-methyl-9-(1-methylethyl)-8,11-d	C38H45N7O5S3	776	A/B	-10.1
66	O=C1N2[C@@H]3[C@@H](C[C@H](CC3)c3ccccc3)CO[C@@H]2Cn2c1c(O)c(=O)c(C(=O)NCc1c(F)cc(F)cc1)c2	1309560-96-0	2H-Pyrido[1,Ä≤,2,Ä≤:4,5]pyrazino[1,2-a][3,1]benzoxazine-10-carboxamide, N-[(2,4	C23H23F2N3O5	459.44	A/B	-10.1
67	O=C1N2[C@@H]3[C@@H](C[C@H](CC3)c3ccccc3)CO[C@@H]2Cn2c1c(O)c(=O)c(C(=O)NCc1c(F)cc(F)cc1)c2	1309561-70-3	2H-Pyrido[1,Ä≤,2,Ä≤:4,5]pyrazino[1,2-a][3,1]benzoxazine-10-carboxamide, N-[(2,4	C23H23F2N3O5	459.44	A/B	-10.1
68	O(Cc1ccc(F)cc1)c1c2c(c(Cl)c(c1)c1ccccc1)oc(c(O)c2=O)c1[nH]ccn1	1312905-26-2	4H-1-Benzopyran-4-one, 8-chloro-5-[(4-fluorophenyl)methoxy]-3-hydroxy-7-phenyl-2	C30H19ClFN3O4	539.94	A/B	-10.1
69	N(=C1CCN(c2nc(nc(n2)N2CCOCC2)c2c3c(ccc2)[nH]nc3)CC1)O	1391925-72-6	1H-Indazol-4-amine, N-[4-(1H-indazol-4-yl)-6-(4-morpholinyl)-1,3,5-triazin-2-yl]	C21H19N9O	413.44	A/B	-10.1
70	C(=O)([C@@H](NC(=O)OC)C(C)C)N1[C@H](c2[nH]c(c3c4c(c(cc3)c3c5c(c(cc3)c3[nH]c(nc3)[C@H]3N(C(=O)[C@@H](	1612236-36-8	Carbamic acid, N-[(1S)-1-[[(2S)-2-[5-[8-[1,Ä≤,3,Ä≤-dihydro-7,Ä≤-[2-[(2S)-1-[(2S	C52H66N8O6	899.13	A/B	-10.1
71	O=c1n([C@@H]2O[C@H](CO)[C@@H](O)C2)cc(c(=O)[nH]1)c1ccc(N=Nc2ccc(N(C)C)cc2)cc1	1627963-49-8	3-Pyridinecarbonitrile, 2-amino-4-(3,4-dimethylphenyl)-6-[4-[(4,6-di-4-morpholin	C31H33N9O2	563.65	A/B	-10.1
72	C(CCCCCCCCCCCCCCC/C=C\CCCC)C1=C(O)C(=O)C=C(O)C1=O	1637815-93-0	Adenosine, adenylyl-(2,Ä≤,Ü5,Ä≤)-1,Ä≤-[3-(aminocarbonyl)-1H-1,2,4-triazol-1-yl	C28H36N14O16P2	886.62	A/B	-10.1
73	O=C1N2[C@@H]3[C@@H]4[C@@H](C4)[C@@H](C3)O[C@H]2Cn2c1c(O)c(=O)c(C(=O)N[C@H](C(F)(F)F)c1c(F)cc(F)cc1)c	1644157-73-2	2,12-Methano-1H-cyclopropa[e]pyrido[1,Ä≤,2,Ä≤:4,5]pyrazino[2,1-b][1,3]oxazepine	C23H18F5N3O5	511.4	A/B	-10.1
74	N(C(=O)[C@H](Cc1ccccc1)C[C@@H](CN1[C@H](C(=O)NC(C)(C)C)CN(CCOCc2ccccc2)CC1)O)[C@H]1c2c(C[C@H]1O)cccc	164725-29-5	1-Piperazinepentanamide, N-(2,3-dihydro-2-hydroxy-1H-inden-1-yl)-2-[[(1,1-dimeth	C40H49N5O4	663.85	A/B	-10.1
75	N(c1nc(nc(n1)N1CCOCC1)N1CCOCC1)c1ccc(cc1)c1cc(nc(N)n1)c1cc(C)c(C)cc1	1710363-59-9	1,3,5-Triazin-2-amine, N-[4-[2-amino-6-(3-chlorophenyl)-4-pyrimidinyl]phenyl]-4,	C27H28ClN9O2	546.02	A/B	-10.1
76	S(=O)(=O)(O)c1c2c(c(N)c(N=Nc3c4c(ccc3)cccc4)c1)cccc2	172649-56-8	1-Piperazinepentanamide, 4-[(2-cyclopentyl-4-thiazolyl)methyl]-N-(2,3-dihydro-2-	C39H53N5O4S	687.93	A/B	-10.1
77	C(=O)([C@H](NC(=O)OC)C(C)C)N1[C@H](c2[nH]c(c3c4c(c(cc3)c3ccc(cc3)c3[nH]c(nc3)[C@H]3N(C(=O)[C@H](NC(=	1312608-54-0	Carbamic acid, N-[(1S)-2-methyl-1-[[(2S)-2-[5-[4-[4-[2-[(2S)-1-(2-phenylacetyl)-	C45H47N7O4	749.9	C	-10.1
78	N(C(=O)[C@H](Cc1ccccc1)C[C@@H](CN1[C@H](C(=O)NC(C)(C)C)CN(Cc2ccc(Cl)nc2)CC1)O)[C@H]1c2c(C[C@H]1O)ccc	164725-39-7	1-Piperazinepentanamide, 4-[(2-chloro-6-methyl-4-pyridinyl)methyl]-N-(2,3-dihydr	C37H48ClN5O4	662.26	C	-10.1
79	S(=O)(=O)(O)c1cc2c(cc1N=Nc1c3c(c(N=Nc4cc5c(cc4)cccc5)cc1)cccc3)cc(N)c(S(=O)(=O)O)c2.[LiH]	178609-77-3	2-Naphthalenesulfonic acid, 8-[2-(7-amino-3-sulfo-2-naphthalenyl)diazenyl]-5-[2-	C30H21N5O9S3.3Li		C	-10.1
80	C([C@H](OCP(=O)(O)O)CO)n1c(=O)[nH]c(=[N])cc1	18031-63-5	2-Naphthalenesulfonic acid, 8,8,Ä≤-[p-phenylenebis(azo)]bis[7-hydroxy-	C26H18N4O8S2	578.57	C	-10.1
81	O[C@H]1[C@@H](O[C@H](CO)[C@H]1O)n1nc(C(=O)NS(=O)(=O)c2ccc(C)cc2)nc1	1843232-28-9	Not Yet Assigned	C34H30N8NiO2S2		C	-10.1
82	N(CCCCN(CCCC)CCCC)c1c2c(ncc1C)cc(Cl)cc2	1141510-07-7	D-Glucopyranosiduronic acid, (2R)-3,6-dihydro-6-oxo-2-(2-phenylethyl)-2-propyl-5	C37H41F3N2O11S	778.79	A/B	-10
83	Cc1c2c(cc(c1)c1cc3c(c(C)c1)OP(=O)(OC[C@H]1O[C@H](C=C1)n1c(=O)[nH]c(=O)c(C)c1)OC3)COP(=O)(OC[C@H]1O[C	1160003-72-4	Thymidine, 5,Ä≤,5,Ä≤,Ä≤,Ä≤-O-(2,2,Ä≤-dioxido[6,6,Ä≤-bi-4H-1,3,2-benzodioxaphos	C34H32N4O14P2	782.58	A/B	-10
84	N(=C1c2c(N(CCCC)C1=O)cccc2)NC(=S)[NH2][Cu+]([NH2]C(=S)NN=C1c2c(N(CCCC)C1=O)cccc2)[Cl-]	122359-62-0	2-Isoindolineacetic acid, Œ±-isopropyl-1,3-dioxo-, tetraester with N-(9-Œ≤-D-glu	C76H70N10O21	1459.42	A/B	-10
85	C(NC(=O)[C@H](Cc1ccccc1)N)(C(=O)NCC(=O)O)C12CC3CC(C2)CC(C1)C3	1229574-81-5	L-Phenylalanine, L-tryptophyl-2-tricyclo[3.3.1.13,7]dec-1-ylglycylglycyl-L-pheny	C43H50N6O6	746.89	A/B	-10
86	O=c1c2c(oc(c1O)c1[nH]cnn1)c(C)cc(OCc1ccc(F)cc1)c2	1312906-68-5	4H-1-Benzopyran-4-one, 6-[(4-fluorophenyl)methoxy]-3-hydroxy-8-methyl-2-(5-pheny	C27H19FN2O4	454.45	A/B	-10
87	C(=O)([C@@H](NC(=O)OC)C(C)C)N1[C@@H](C[C@H](C)C1)c1[nH]c(C#Cc2ccc(C#Cc3[nH]c(nc3C)[C@H]3N(C(=O)[C@@H	1313207-56-5	Carbamic acid, N,N,Ä≤-[1,4-phenylenebis[2,1-ethanediyl-1H-imidazole-5,2-diyl[(2S	C40H58N8O6	746.94	A/B	-10
88	C(=O)([C@@H](NC(=O)OC)C(C)C)N1[C@@H](CC2(C1)CC=CC2)c1[nH]c(cn1)c1ccc(cc1)c1ccc(cc1)c1[nH]c(nc1)[C@H]	1351852-94-2	Carbamic acid, N,N,Ä≤-[[1,1,Ä≤-biphenyl]-4,4,Ä≤-diylbis[1H-imidazole-5,2-diyl-1	C46H58N8O8	851	A/B	-10
89	C(=C\C(=O)CCCCC)/c1cc(O)c(O)cc1	1369304-03-9	4-Quinazolinecarboxamide, 2-phenyl-N-(1-tricyclo[3.3.1.13,7]dec-1-ylethyl)-	C27H29N3O	411.54	A/B	-10
90	N(C(=O)[C@H](Cc1ccccc1)C[C@@H](CN1[C@H](C(=O)NC(C)(C)C)CN(Cc2cc3c(cc2)cccc3)CC1)O)[C@H]1c2c(C[C@H]1O	164725-20-6	1-Piperazinepentanamide, 4-([1,1,Ä≤-biphenyl]-4-ylmethyl)-N-(2,3-dihydro-2-hydro	C43H52N4O4	688.9	A/B	-10
91	C(=O)(/C=C(\O)/c1ccccc1)c1c(OC)cc(OC)c(C(=O)C)c1O	170227-77-7	Benzoic acid, 4-(chloromethyl)-, 1-[[5-[2-[[(1,1-dimethylethyl)amino]carbonyl]-4	C44H52ClN5O5	766.37	A/B	-10
92	C(C)(C)(C)c1c2c(cc(c1)c1cc(C(C)(C)C)c3c(c1)COP(=O)(OC[C@H]1O[C@H](C=C1)n1c(=O)[nH]c(=O)c(C)c1)O3)COP	1160003-73-5	Thymidine, 5,Ä≤,5,Ä≤,Ä≤,Ä≤-O-(8,8,Ä≤-dimethyl-2,2,Ä≤-dioxido[6,6,Ä≤-bi-4H-1,3,	C36H36N4O14P2	810.64	C	-10
93	O=c1n(c(cc(=O)[nH]1)[2H])[C@@H]1O[C@H](COP(=O)(O)O)[C@@H](O)C1	116028-33-2	2-Naphthalenesulfonic acid, 7-[2-[4-hydroxy-3-[2-(2-hydroxy-1-naphthalenyl)diaze	C26H18N4O5S	498.51	C	-10
94	C(=O)([C@@H](NC(=O)OC)C(C)C)N1[C@@H](C[C@H](C)C1)c1[nH]c(C#Cc2ccc(C#Cc3[nH]c(nc3C)[C@H]3N(C(=O)[C@@H	1313207-56-5	Carbamic acid, N,N,Ä≤-[1,4-phenylenebis[2,1-ethanediyl-1H-imidazole-5,2-diyl[(2S	C40H58N8O6	746.94	C	-10
95	CC1C2c3c(c(ccc3c3ccc(cc3)c3[nH]c(nc3)[C@H]3N(C(=O)[C@@H](NC(=O)OC)C(C)C)C[C@@H](C)C3)c3[nH]c(nc3)[C@	1560650-26-1	Carbamic acid, N-[2-methyl-1-[[2-[5-[4-[1,2,3,4-tetrahydro-8-[2-[1-[2-[(methoxyc	C45H56N8O6	804.98	C	-10
96	O(Cc1ccncc1)c1c2n(N([C@H]3N(C2=O)CCOC3)[C@H]2c3c(CSc4c2cccc4)c(F)c(F)cc3)ccc1=O	1985606-19-6	1H-[1,4]Oxazino[3,4-c]pyrido[2,1-f][1,2,4]triazine-6,8-dione, 7-[[4-(acetyloxy)p	C33H27F2N3O6S	631.65	C	-10
97	O(c1c2n(N([C@H]3N(C2=O)CCOC3)[C@H]2c3c(CSc4c2cccc4)c(F)c(F)cc3)ccc1=O)C1C=C(C)C(=O)O1	1985606-79-8	1H-[1,4]Oxazino[3,4-c]pyrido[2,1-f][1,2,4]triazine-6,8-dione, 12-[(11S)-7,8-difl	C32H23F2N3O6S	615.6	C	-10
98	C(N1c2c(C(=NNC(=S)Nc3ccccc3)C1=O)cccc2)N1CCN(CC1)c1ccccc1	100847-01-6	Hydrazinecarbothioamide, 2,2,Ä≤-[1,4-piperazinediylbis[methylene(2-oxo-1H-indol-	C36H34N10O2S2	702.85	A/B	-9.9
99	S(=O)(=O)([O-])c1cc2c(c3c1cc(N=N(=O)c1cc4c(cc1)c1c(cc4S(=O)(=O)[O-])[N]4=N[C-]5C(=[O][Cu+2]4[O-]1)N(	1073242-68-8	L-Proline, 1-[3-[2-(2,Ä≤,3,Ä≤-didehydro-3,Ä≤-deoxythymidin-5,Ä≤-O-yl)-2-oxido-4	C32H34N3O10P	651.6	A/B	-9.9
100	O=c1n(c(cc(=O)[nH]1)[2H])[C@@H]1O[C@H](COP(=O)(O)O)[C@@H](O)C1	116028-33-2	2-Naphthalenesulfonic acid, 7-[2-[4-hydroxy-3-[2-(2-hydroxy-1-naphthalenyl)diaze	C26H18N4O5S	498.51	A/B	-9.9
101	CC1(OC(CO)SC1)n1c(=O)nc(N)cc1	1222703-14-1	Not Yet Assigned	C33H46ClN5O4	612.2	A/B	-9.9
102	N(NC(=O)c1ccncc1)c1nc(nc(n1)N1CCOCC1)N1c2c(CCC1)cccc2	1239884-92-4	Isoquinoline, 2,2,Ä≤-[6-(4-morpholinyl)-1,3,5-triazine-2,4-diyl]bis[1,2,3,4-tetr	C25H28N6O	428.53	A/B	-9.9
103	S(=O)(=O)(NCCNCC=Cc1ccc(Br)cc1)c1c2c(ccc1)cncc2.Cl	1309561-76-9	Cyclopenta[d]pyrido[1,Ä≤,2,Ä≤:4,5]pyrazino[2,1-b][1,3]oxazine-9-carboxamide, N-	C22H21F2N3O5	445.42	A/B	-9.9
104	O(Cc1ccc(F)cc1)c1c2c(c(C)c(c1)c1ccccc1)oc(c(O)c2=O)c1[nH]ccn1	1312903-70-0	4H-1-Benzopyran-4-one, 5-[(4-fluorophenyl)methoxy]-3-hydroxy-8-methyl-7-phenyl-2	C31H22FN3O4	519.52	A/B	-9.9
105	O(Cc1ccc(F)cc1)c1c(c(C)c2c(c1)c(=O)c(O)c(o2)c1[nH]ccn1)c1ccccc1	1312916-96-3	4H-1-Benzopyran-4-one, 6-[(4-fluorophenyl)methoxy]-3-hydroxy-8-methyl-7-phenyl-2	C31H22FN3O4	519.52	A/B	-9.9
106	O(C)c1c2c(cc(OCc3ccc(F)cc3)c1c1ccccc1)c(=O)c(O)c(o2)c1ncccn1	1312917-01-3	4H-1-Benzopyran-4-one, 6-[(4-fluorophenyl)methoxy]-3-hydroxy-8-methyl-7-phenyl-2	C32H23FN2O4	518.53	A/B	-9.9
107	O(Cc1ccc(F)cc1)c1c(c(c2c(c1)c(=O)c(O)c(o2)c1ncccn1)c1ccccc1)c1ccccc1	1312918-01-6	4H-1-Benzopyran-4-one, 8-chloro-6-[(4-fluorophenyl)methoxy]-3-hydroxy-7-phenyl-2	C31H20ClFN2O4	538.95	A/B	-9.9
108	O(C(=O)N[C@@H](Cc1ccccc1)[C@@H](CN(S(=O)(=O)c1cc(OCCC=C)ccc1)CCCCC=C)O)[C@@H]1[C@H]2[C@@H](OC1)OCC2	1346988-80-4	2,7,10,12-Tetraazatridecanoic acid, 13-[2-(cyclopentylmethyl)-4-thiazolyl]-4-hyd	C40H52N6O5S2	761.01	A/B	-9.9
109	[C@H](N(C(=O)[C@@H](NC(=O)NCc1nc(C(C)C)sc1)C(C)C)NC(=O)OC(C)(C)C)(Cc1ccccc1)C[C@@H]([C@H](Cc1ccccc1)	1349738-14-2	6H-Dipyrido[3,2-b:2,Ä≤,3,Ä≤-e][1,4]diazepin-6-one, 11-cyclopropyl-8-[2-[[1,3-di	C30H27FN6O5S	602.64	A/B	-9.9
110	O(P(=O)(OC[C@H]1O[C@H]([C@H](O)[C@@H]1O)n1c2c(nc1)c(N)ncn2)O)[C@H]1[C@@H](O[C@H](COP(=O)(O[C@@H]2[C@	1357016-08-0	Adenosine, 5,Ä≤-O-(phosphonomethyl)-Œ≤-D-arabino-adenylyl-(2,Ä≤,Ü5,Ä≤)-adenyly	C41H52N20O25P4	1348.87	A/B	-9.9
111	O=c1c2c(n(cn2)[C@@H]2O[C@H](CO)[C@@H](O)[C@H]2O)[nH]c(n1)n1cc(CCCC)nn1	1384519-82-7	Inosine, 2-[4-[(1,3-dihydro-1,3-dioxo-2H-isoindol-2-yl)methyl]-1H-1,2,3-triazol-	C21H18N8O7	494.42	A/B	-9.9
112	C(=O)([C@@H](NC(=O)OC)C(C)C)N1[C@@H](C[C@@H](C)C1)c1[nH]c(cn1)c1ccc(cc1)c1c2CCc3cc(c(CCc(c1)cc2)cc3)	1415120-18-1	Carbamic acid, N,N,Ä≤-[tricyclo[8.2.2.24,7]hexadeca-4,6,10,12,13,15-hexaene-5,1	C60H68N8O6	997.23	A/B	-9.9
113	Cn1c(nc(NCCNC(=O)/C=C/C(F)(F)F)n1)N1CCOCC1	1463435-52-0	1,3,5-Triazine-2,4-diamine, 6-(4-morpholinyl)-N2-[4-[[4-(4-morpholinyl)-6-(pheny	C30H37N11O3	599.69	A/B	-9.9
114	C(OP(=O)(O[C@H]1[C@@H](O[C@H](CO)[C@H]1OC(=O)c1ccccc1)n1nc(C(=O)N)nc1)OCCc1ccc(N(=O)=O)cc1)[C@@H]1[C	1469875-43-1	Adenosine, 1,Ä≤-[3-(aminocarbonyl)-1H-1,2,4-triazol-1-yl]-3,Ä≤-O-benzoyl-1,Ä≤-d	C40H37N10O14P	912.75	A/B	-9.9
115	C(n1c2c(nc1CC)c(C)cc(C)n2)c1ccc(cc1)c1c(S(=O)(=O)NC(=O)CCc2ccccc2)ccc(OC(C)C)c1	154552-68-8	Benzenepropanamide, N-[[4,Ä≤-[(2-ethyl-5,7-dimethyl-3H-imidazo[4,5-b]pyridin-3-y	C34H33F3N4O4S	650.71	A/B	-9.9
116	C(n1c2c(nc1CC)c(C)cc(C)n2)c1ccc(cc1)c1c(S(=O)(=O)NC(=O)NCCc2ccccc2)ccc(OC(C)C)c1	154553-67-0	[1,1,Ä≤-Biphenyl]-2-sulfonamide, 4,Ä≤-[(2-ethyl-5,7-dimethyl-3H-imidazo[4,5-b]p	C34H34F3N5O4S	665.73	A/B	-9.9

117	C(=O)([C@@H](NC(=O)OC)C(C)C)N1[C@H](c2[nH]c(c3c4c(c(cc3)c3c5c(c(cc3)c3[nH]c(nc3)[C@H]3N(C(=O)[C@@H](	1612236-50-6	Carbamic acid, N-[(1S)-1-[[(2S)-2-[5-[8-[1,Ä≤,3,Ä≤-dihydro-7,Ä≤-[2-[(2S)-1-[(2S	C51H64N8O7	901.1	A/B	-9.9
118	FC1C2c3c(c(ccc3c3c4c(c(cc3)c3[nH]c(nc3)[C@H]3N(C(=O)[C@@H](NC(=O)OC)C(C)C)CCC3)CC3(C4)CCCC3)c3[nH]c(	1612236-67-5	Carbamic acid, N-[(1S)-1-[[(2S)-2-[5-[3-[1,Ä≤,3,Ä≤-dihydro-7,Ä≤-[2-[(2S)-1-[(2S	C52H64N8O7	913.11	A/B	-9.9
119	O[C@H]1[C@@H](O[C@H](COC(=O)CCCCCCC/C=C\CCCCCCCC)[C@H]1O)n1nc(C(=O)N)nc1	162518-60-7	D-erythro-Pentonamide, 2,3,5-trideoxy-N-(2,3-dihydro-2-hydroxy-1H-inden-1-yl)-5-	C42H55N5O11		A/B	-9.9
120	N(c1nc(nc(n1)N1CCOCC1)N1CCOCC1)c1ccc(C(=O)C=Cc2ccco2)cc1	1628172-08-6	2-Propen-1-one, 1-[4-[(4,6-di-4-morpholinyl-1,3,5-triazin-2-yl)amino]phenyl]-3-(	C32H32N6O4	564.63	A/B	-9.9
121	C(=O)([C@@H](NC(=O)OC)C(C)C)N1[C@@H](C[C@@]2(C1)CCOC2)c1[nH]c2c(n1)ccc1c2ccc(c1)c1ccc(cc1)c1[nH]c(nc	1653967-11-3	Carbamic acid, N-[(1S)-1-[[(2S)-2-[2,Ä≤-[(3S,5S)-2-[(2S)-2-[(methoxycarbonyl)ami	C48H56N8O7	857.01	A/B	-9.9
122	O=C1[C@@H]2[C@@H](c3c([C@H](OC(=O)Oc4ccc(N(=O)=O)cc4)[C@H]2CO1)cc1c(c3)OCO1)c1cc(OC)c(OC)c(OC)c1	1680211-74-8	1-Piperidinecarboxylic acid, 4-[2-[3-[2-amino-6-[(ethylamino)carbonyl]thieno[2,3	C45H47N5O11S	865.95	A/B	-9.9
123	[C@H](Cc1ccccc1)(C(=O)N[C@H](/C=C/S(=O)(=O)c1ccccc1)CCCC)NC(=O)N1CCOCC1	170111-48-5	4-Morpholinecarboxamide, N-[1-([1,1,Ä≤-biphenyl]-4-ylmethyl)-2-oxo-2-[[1-(2-phen	C37H39N3O5S	637.79	A/B	-9.9
124	N(C(=O)[C@H](Cc1ccccc1)C[C@@H](CN1[C@H](C(=O)NC(C)(C)C)CN(Cc2cccnc2)CC1)O)[C@H]1c2c(C[C@H]1OC(=O)CCC	170227-69-7	1-Piperazinepentanamide, N-[2-(benzoyloxy)-2,3-dihydro-1H-inden-1-yl]-2-[[(1,1-d	C43H51N5O5	717.9	A/B	-9.9
125	C(=O)(O)c1c2c(sc1NC(=O)c1c(CNc3ccc(C[C@@H]([C@@H](CN(S(=O)(=O)c4cc5c(cc4)ncs5)CC(C)C)O)NC(=O)O[C@@H]	1887206-23-6	5-Thiazolecarboxylic acid, 4-[[4-[(2S,3R)-4-[(6-benzothiazolylsulfonyl)(2-methyl	C39H42N4O11S3	838.97	A/B	-9.9
126	C([C@H](OCP(=O)(OCOC(=O)OC(C)(C)C)OCOC(=O)OC(C)(C)C)C)n1c2c(nc1)c(N)ncn2	201340-99-0	2,4,6,8-Tetraoxa-5-phosphanonanedioic acid, 5-[[(1R)-2-(6-amino-9H-purin-9-yl)-1	C21H34N5O10P	547.5	A/B	-9.9
127	C(N1c2c(C(=NNC(=S)Nc3ccc(C=Cc4ccccc4)cc3)C1=O)cccc2)N1CCCCC1	108075-29-2	1,3,6-Naphthalenetrisulfonic acid, 5-[2-[4-[2-(4-amino-2-methylphenyl)diazenyl]-	C27H21N5O12S4	735.74	C	-9.9
128	C(=O)(OCC)c1c2c(n(C)c1CSc1cc3c(cc1)cccc3)cc(Br)c(O)c2CN(C)C	1131041-15-0	2-Naphthalenesulfonic acid, 5-amino-8-[2-[4-[2-(4-amino-2-sulfophenyl)diazenyl]-	C26H20N6O9S3	656.67	C	-9.9
129	C(OC(COP(=O)(O)O)COP(=O)(O)O)n1c2c(nc1)c(=O)nc(N)[nH]2	1185728-95-3	1,4-Benzenedisulfonic acid, 2-[2-[4-[2-(4-amino-7-sulfo-1-naphthalenyl)diazenyl]	C27H21N5O12S4	735.74	C	-9.9
130	C(#N)c1ccc(c2nc(nc(n2)N2CCOCC2)N2CCOCC2)cc1	1197160-78-3	Urea, N-[4-[[4-(dimethylamino)-1-piperidinyl]carbonyl]phenyl]-N,Ä≤-[4-(4,6-di-4-	C32H41N9O4	615.73	C	-9.9
131	N(=Nc1c2c(c(S(=O)(=O)O)ccc2)c(N)cc1)c1c2c(c(N=Nc3ccccc3)cc1)ccc(S(=O)(=O)O)c2	1345852-33-6	1-Naphthalenesulfonic acid, 5-amino-8-[2-[7-sulfo-4-[2-(3-sulfophenyl)diazenyl]-	C26H19N5O9S3	641.65	C	-9.9
132	C(=O)([C@@H](NC(=O)OC)C(C)C)N1[C@@H]([C@@H]2C[C@H]1CC2)c1[nH]c2c(n1)ccc1c2ccc(c1)c1ccc2c(cc1)c1c(cc2)	1377041-05-8	Carbamic acid, N-[(1S)-2-[(2S)-2-[5-[7-[2-[(2S)-1-[(2S)-2-[(methoxycarbonyl)amir	C48H54N8O7	854.99	C	-9.9
133	C(=O)([C@@H](NC(=O)OC)C(C)C)N1[C@@H]([C@@H]2C[C@H]1CC2)c1[nH]c(cn1)c1c(F)cc(cc1)c1ccc2c(cc1)c1c(cc2)n	1377041-19-4	Carbamic acid, N-[(1S)-2-[(2S)-2-[5-[4-[2-[(2S)-1-[(2S)-2-[(methoxycarbonyl)amir	C48H54N8O7	854.99	C	-9.9
134	O[C@H]1[C@@H](O[C@H](C(=O)N[C@H](Cc2c3c([nH]c2)cccc3)C(=O)O)[C@H]1O)n1nc(C(=O)N)nc1	1414793-73-9	Not Yet Assigned	C29H37N9O7	623.66	C	-9.9
135	C(n1c2c(nc1CC)c(C)cc(C)n2)c1ccc(cc1)c1c(S(=O)(=O)NC(=O)CCc2ccccc2)ccc(C(CC)(C)C)c1	154552-62-2	Benzenepropanamide, N-[[4,Ä≤-[(2-ethyl-5,7-dimethyl-3H-imidazo[4,5-b]pyridin	C38H36N4O3S	628.78	C	-9.9
136	O=C1C2c3c(c(ccc3c3c4c(c(cc3)c3[nH]c(nc3)[C@H]3N(C(=O)[C@@H](NC(=O)OC)C(C)C)CCC3)C3CC4CC3)c3[nH]c(nc3	1627533-41-8	Carbamic acid, N,N,Ä≤-[(1,1,Ä≤,2,2,Ä≤,3,3,Ä≤,4,4,Ä≤-octahydro-9-methyl[5,5,Ä≤-b	C51H64N8O6	885.1	C	-9.9
137	O=C1[C@@H]2[C@@H](c3c([C@H](OC(=O)C(C)(C)C)[C@H]2CO1)cc1c(c3)OCO1)c1cc(OC)c(OC)c(OC)c1	1653967-17-9	Carbamic acid, N-[(1S)-1-[[(2S)-2-[7-[4-[2-[(3S)-2-[(2S)-2-[(methoxycarbonyl)ami	C46H56N8O7	832.99	C	-9.9
138	O=C1c2n(N([C@H]3N1C[C@H](C)OC3)[C@H]1c3c(CSc4c1cccc4)cc(F)cc3)ccc(=O)c2O	1985607-61-1	1H-[1,4]Oxazino[3,4-c]pyrido[2,1-f][1,2,4]triazine-6,8-dione, 12-[(11S)-7,8-difl	C34H25F2N3O7S	657.64	C	-9.9
139	C(=O)([C@@H](NC(=O)OC)C(C)C)N1[C@@H](C[C@H]2[C@@H]1CCC2)c1[nH]c(cn1)c1c2c(c(cc1)c1ccc3c(cc1)c1c(cc3)n	2007901-64-4	Carbamic acid, N-[(1S)-1-[[(2S,4S)-2-[5-[2,3-dihydro-7-[2-[(2S)-1-[(2S)-2-[(meth	C45H53FN8O6	820.95	C	-9.9
140	C(=O)([C@@H](NC(=O)OC)C(C)C)N1[C@@H](CCC1)c1[nH]c(cn1)c1cc(C)c(c(C)c1)c1c(C)cc(cc1C)c1[nH]c(nc1)[C@H	1007882-44-1	Carbamic acid, N-[(1R)-2-oxo-1-phenyl-2-[(2S)-2-[5-[4,Ä≤-[2-[(2S)-1-(2-phenylace	C44H43N7O4	733.86	A/B	-9.8
141	C(=O)([C@H](NC(=O)OC)c1ccccc1)N1[C@@H](CCC1)c1[nH]c(cn1)c1ccc(cc1)c1ccc(cc1)c1[nH]c(nc1)[C@H]1N(C(=O	1009119-85-0	Carbamic acid, N,N,Ä≤-[[1,1,Ä≤-biphenyl]-3,3,Ä≤-diylbis[1H-imidazole-5,2-diyl-(	C46H46N8O6	806.91	A/B	-9.8
142	O=c1n(cc(c(=O)[nH]1)c1ccccc1)[C@@H]1O[C@H](COP(=O)(OP(=O)(O[C@H]2O[C@H](CO)[C@@H](O)[C@H](O)[C@H]2O)	1061717-09-6	1H-Indole-3-acetic acid, 1-(1,3-benzodioxol-5-ylmethyl)-Œ±-oxo-, (5R,5aR,8aR,9R)	C40H33NO12	719.69	A/B	-9.8
143	C(=O)(C(NC(=O)OCC)C)N1[C@@H](CCC1)c1[nH]c(cn1)[C@H]1CC[C@@H](CC1)c1ccc(cc1)c1[nH]c(nc1)[C@H]1N(C(=O)	1240887-36-8	Carbamic acid, N-[(1R)-2-[(2S)-2-[5-[4-[trans-4-[2-[(2S)-1-[(2R)-2-[(methoxycarb	C36H48N8O6	688.82	A/B	-9.8
144	C(=O)([C@@H](NC(=O)OC)C(C)C)N1[C@H](c2[nH]c(c3cc4c(-c5c(cc(cc5)c5[nH]c(nc5)[C@H]5N(C(=O)[C@@H](NC(=O	1246559-80-7	Carbamic acid, N,N,Ä≤-[7,10,13,16,19,22-hexaoxatricyclo[21.3.1.12,6]octacosa-1(2	C50H68N8O12	973.12	A/B	-9.8
145	O(C[C@@H]1[C@@H](N=[N+]=[N-])C[C@@H](O1)n1c(=O)[nH]c(=O)c(C)c1)P1(=O)Oc2c(cc3c(c2)COP(=O)(OC[C@H]2O[	1268815-07-1	Thymidine, 5,Ä≤,5,Ä≤,Ä≤,Ä≤-O-(4,9-dihydro-2,7-dioxidobenzo[1,2-d:4,5-d,Ä≤]bis[1	C28H30N10O14P2		A/B	-9.8
146	C(NC(=O)[C@H](C)N)(C)C12CC3CC(C2)CC(C1)C3	129002-19-3	Aurintricarboxylic acid, 2,6-lutidine uranyl complex	C36H28N2O16U3		A/B	-9.8
147	O=C1N2[C@@H]3[C@H](CO[C@@H]2Cn2c1c(O)c(=O)c(C(=O)NCc1c(F)cc(F)cc1)c2)CCCC3	1309561-14-5	Cyclopenta[d]pyrido[1,Ä≤,2,Ä≤:4,5]pyrazino[2,1-b][1,3]oxazine-9-carboxamide, N-	C22H21F2N3O5	445.42	A/B	-9.8
148	C(=O)([C@H](NC(=O)OC)C(C)C)N1[C@H](c2[nH]c(c3c4c(c(cc3)c3ccc(cc3)c3[nH]c(nc3)[C@H]3N(C(=O)[C@H](NC(=	1312608-54-0	Carbamic acid, N-[(1S)-2-methyl-1-[[(2S)-2-[5-[4-[4-[2-[(2S)-1-(2-phenylacetyl)-	C45H47N7O4	749.9	A/B	-9.8
149	O(Cc1ccc(F)cc1)c1c2c(oc(c(O)c2=O)c2[nH]ccn2)cc(C)c1	1312894-97-5	4H-1-Benzopyran-4-one, 5-[(4-fluorophenyl)methoxy]-3-hydroxy-7-methyl-2-(3-pheny	C25H18FN3O4	443.43	A/B	-9.8
150	O(C)c1c2c(c(OCc3ccc(F)cc3)cc1c1ccccc1)c(=O)c(O)c(o2)c1[nH]ccn1	1312904-47-4	4H-1-Benzopyran-4-one, 5-[(4-fluorophenyl)methoxy]-3-hydroxy-8-methoxy-7-phenyl-	C31H22FN3O5	535.52	A/B	-9.8
151	O(Cc1ccc(F)cc1)c1c2c(c(c(c1)c1ccccc1)c1ccccc1)oc(c(O)c2=O)c1ncccn1	1312905-34-2	4H-1-Benzopyran-4-one, 8-chloro-5-[(4-fluorophenyl)methoxy]-3-hydroxy-7-phenyl-2	C31H20ClFN2O4	538.95	A/B	-9.8
152	O=c1c2c(oc(c1O)c1ncccn1)ccc(OCc1ccc(F)cc1)c2	1312905-85-3	4H-1-Benzopyran-4-one, 5-[(4-fluorophenyl)methoxy]-3-hydroxy-7,8-diphenyl-2-(5-p	C37H25FN2O4	580.6	A/B	-9.8
153	O(Cc1ccc(F)cc1)c1c(c(Cl)c2c(c1)c(=O)c(O)c(o2)c1[nH]ccn1)c1ccccc1	1312917-96-6	4H-1-Benzopyran-4-one, 8-chloro-6-[(4-fluorophenyl)methoxy]-3-hydroxy-7-phenyl-2	C30H19ClFN3O4	539.94	A/B	-9.8
154	O([C@H]1[C@@H](O[C@H](CO)[C@H]1O)n1c2c(nc1)c(=O)nc(N)[nH]2)[C@@H]1O[C@H](COP(=O)(O)O)[C@@H](O)[C@H]1	1312918-51-6	4H-1-Benzopyran-4-one, 6-[(4-fluorophenyl)methoxy]-3-hydroxy-7,8-diphenyl-2-(5-p	C37H25FN2O4	580.6	A/B	-9.8
155	O=C1N2[C@H](c3nc(c([nH]3)CCCCC[C@H](C)[C@@H]1NC(=O)OC)c1ccc(cc1)c1ccc(cc1)c1[nH]c(nc1)[C@H]1N(C(=O)[	1318775-76-6	L-Alaninamide, (2S)-N-benzoyl-2-tricyclo[3.3.1.13,7]dec-1-ylglycyl-N-[(1S)-3-[[	C35H45N5O7S	679.83	A/B	-9.8
156	N(=Nc1c2c(cc(S(=O)(=O)O)cc2)c(N)cc1)c1c2c(c(N=Nc3ccccc3)cc1)ccc(S(=O)(=O)O)c2	1345852-35-8	1-Naphthalenesulfonic acid, 8-amino-5-[2-[4-(2-phenyldiazenyl)-7-sulfo-1-naphtha	C26H19N5O6S2	561.59	A/B	-9.8
157	C(=O)(NC(CC1CC1)C(=O)C(=O)N)[C@@H]1[C@@H]2[C@@H](C2(C)C)CN1C(=O)[C@@H](NC(=O)NC(SCc1ccccc1)(C)C)C(C)	1346776-56-4	Not Yet Assigned	C37H48N4O6S	676.87	A/B	-9.8
158	[C@H](Cc1ccccc1)(C[C@@H]([C@H](Cc1ccccc1)NC(=O)OCc1cncs1)O)NC(=O)[C@H](NC(=O)N(Cc1nc(OC)sc1)C)C(C)C	1346847-45-7	Not Yet Assigned	C39H53N5O4S	687.93	A/B	-9.8
159	C(C)(C)c1n([C@@H]2C[C@@H]3N(C(CC(NC(=O)C4C(CC(F)(F)CC4([2H])[2H])([2H])[2H])c4ccccc4)([2H])[2H])C[C@	1349132-63-3	4-Morpholinecarboxamide, N-[(1R)-2-[[(2E)-3-(1-naphthalenylsulfonyl)-1-(2-phenyl	C35H37N3O5S	611.75	A/B	-9.8
160	O=C1CC2(c3n(c(nn3)c3cc(OC)c(cc3)c3cnc(C)o3)CCC2)Oc2c1ccc(Cl)c2	1388690-68-3	Spiro[2H-1-benzopyran-2,8,Ä≤(5,Ä≤H)-[1,2,4]triazolo[4,3-a]pyridin]-4(3H)-one, 6	C25H22N4O4	442.47	A/B	-9.8
161	N(c1nc(nc(n1)N1CCOCC1)c1c2c([nH]nc2)ccc1)c1ccccc1	1391925-85-1	1-Piperazinecarboxamide, 4-[4-(1H-indazol-4-yl)-6-(4-morpholinyl)-1,3,5-triazin-	C20H25N9O2	423.47	A/B	-9.8
162	C(=O)(O)[C@]12[C@@H]([C@@H]3[C@@](C)(CC1)[C@@]1(C)[C@H](CC3)[C@]3(C)[C@@H](CC1)C(C)(C)[C@@H](OCOC)CC	1399935-96-6	1,4-Benzenediamine, N1-[4-chloro-6-(4-morpholinyl)-1,3,5-triazin-2-yl]-N4-(7-chl	C22H19Cl2N7O	468.34	A/B	-9.8
163	O[C@H]1[C@@H](O[C@H](C(=O)N[C@H](Cc2c3c([nH]c2)cccc3)C(=O)O)[C@H]1O)n1nc(C(=O)N)nc1	1414793-73-9	Not Yet Assigned	C29H37N9O7	623.66	A/B	-9.8
164	O[C@H]1[C@@H](O[C@H](C(=O)N[C@H](Cc2ccccc2)C(=O)N(Cc2[nH]c3c(n2)ccc(C)c3)C)[C@H]1O)n1nc(C(=O)N)nc1	1414801-65-2	Not Yet Assigned	C28H33N9O7	607.62	A/B	-9.8
165	C(CCCCCCn1c2-c3c(CN(C(=O)CCNC(=O)CCN4C(=O)CCC4=O)c4c(-c2nn1)cccc4)cccc3)(F)(F)c1c2c(N(c3c(NC2=O)c(C)	1450913-00-4	1-Pyrrolidinepropanamide, N-[3-[1-[7-(11-cyclopropyl-6,11-dihydro-4-methyl-6-oxo	C47H48F2N10O5	870.94	A/B	-9.8
166	O=c1n([C@@H]2O[C@H](COC(=O)c3ccccc3)SC2)ccc(NC(=O)C)n1	1469975-50-5	Œ≤-D-lyxo-Adenosine, adenylyl-(2,Ä≤,Üí5,Ä≤)-1,Ä≤-[3-(aminocarbonyl)-1H-1,2,4-tr	C28H34N14O16P2	884.6	A/B	-9.8
167	[C@@H](Cc1cc(C)ccc1)(NC(=O)N1CCN(C)CC1)C(=O)N[C@@H](CCc1ccccc1)C=CS(=O)(=O)c1ccccc1	1499175-66-4	1-Piperazinecarboxamide, 4-methyl-N-[(1S)-1-(2-naphthalenylmethyl)-2-[[(1S)-3-(2	C40H42N4O4S	674.85	A/B	-9.8
168	O=C1[C@@H]2[C@@H](c3c([C@H](OCC#C)[C@H]2CO1)cc1c(c3)OCO1)c1cc(OC)c(OC)c(OC)c1	160031-96-9	Pyrido[2,3-d]pyrimidine-2,4(1H,3H)-dione, 3,5,7-triphenyl-1-(2,3,5-tri-O-benzoyl	C51H37N3O9	835.85	A/B	-9.8
169	C(=O)([C@@H](NC(=O)OC)C(C)C)N1[C@H](c2[nH]c(c3c4c(c(cc3)c3c5c(c(cc3)c3[nH]c(nc3)[C@H]3N(C(=O)[C@@H](	1612236-02-8	Carbamic acid, N-[(1S)-1-[[(1S,3S,4R)-3-[5-[7-[1,Ä≤,3,Ä≤-dihydro-7,Ä≤-[2-[(2S)-	C56H72N8O7	969.22	A/B	-9.8
170	C(OCCOC(=O)[C@@H](NC(=S)Nc1ccccc1)C(C)C)n1c2c(nc1)c(=O)nc(N)[nH]2	1612842-73-5	Cyclopenta[c]pyrrole-1-carboxamide, (2R)-2-cyclohexyl-N-(2-pyrazinylcarbonyl)gly	C36H53N7O6	679.85	A/B	-9.8
171	O(C)c1c2n(C[C@@H]3N(C2=O)[C@@H]2C[C@H](O3)CC2)cc(C(=O)NCc2c(F)cc(F)cc2F)c1=O	1616340-67-0	2,5-Methanopyrido[1,Ä≤,2,Ä≤:4,5]pyrazino[2,1-b][1,3]oxazepine-10-carboxamide, N	C22H18F5N3O5	499.39	A/B	-9.8
172	CC1C2c3c(c(ccc3c3c4c(c(cc3)c3[nH]c(nc3)C3N(C(=O)C(NC(=O)OC)C(C)C)CCC3)C3CC4CC3)c3[nH]c(nc3)C3N(C(=O)	1627533-38-3	Carbamic acid, N-[(1S)-2-methyl-1-[[(2S)-2-[5-[1,1,Ä≤,2,2,Ä≤,3,3,Ä≤,4,4,Ä≤-octa	C52H64N8O8	929.11	A/B	-9.8
173	C(=O)([C@@H](NC(=O)OC)C(C)C)N1[C@@H](C[C@H]2[C@@H]1CCCC2)c1[nH]c2-c3c(CCc2n1)cc(cc3)c1ccc(cc1)c1[nH]	1653966-64-3	Carbamic acid, N-[(1S)-1-[[(3S,5S)-3-[7-[4-[2-[(3S,5S)-2-[(2S)-2-[(methoxycarbor	C50H62N8O8	903.08	A/B	-9.8
174	O=C1[C@@H]2[C@@H](c3c([C@H](OC(=O)N4CCC(CCn5c6c(nc5Sc5cc7c(cc5Br)OCO7)c(N)ncn6)CC4)[C@H]2CO1)cc1c(c3	1680212-59-2	1-Piperazinecarboxylic acid, 4-[[4-[3-[2,4-dihydroxy-5-(1-methylethyl)phenyl]-1,	C45H47N5O12	849.88	A/B	-9.8
175	N(c1nc(nc(n1)N1CCOCC1)N1CCOCC1)c1ccc(cc1)c1cc(nc(N)n1)c1cc(Cl)ccc1	1710363-55-5	1,3,5-Triazin-2-amine, N-[4-[2-amino-6-(2-nitrophenyl)-4-pyrimidinyl]phenyl]-4,6	C27H28N10O4	556.58	A/B	-9.8
176	[C@H](C(C)(C)C)(C1=C(O)CC(CCC)(CCC)OC1=O)c1cc(NS(=O)(=O)c2cc(C(F)(F)F)ccn2)ccc1	174484-34-5	2-Pyridinesulfonamide, N-[3-[1-[5,6-dihydro-4-hydroxy-2-oxo-6,6-bis(2-phenylethy	C36H35F3N2O5S	664.73	A/B	-9.8
177	C(Cc1ccccc1)C1(CCc2ccccc2)CC(=C([C@H](C(C)(C)C)c2cc(NS(=O)(=O)c3ccc(N)cn3)ccc2)C(=O)O1)O	174484-61-8	2-Pyridinesulfonamide, 5-cyano-N-[3-[[5,6-dihydro-4-hydroxy-2-oxo-6,6-bis(2-phen	C34H31N3O5S	593.69	A/B	-9.8
178	C(Cc1ccc(F)cc1)[C@]1(CCC)CC(=C([C@@H](C(C)(C)C)c2cc(NS(=O)(=O)c3ccc(C#N)cn3)ccc2)C(=O)O1)O	174590-32-0	2-Pyridinesulfonamide, 5-cyano-N-[3-[1-[6-[2-(4-fluorophenyl)ethyl]-5,6-dihydro-	C33H36FN3O5S	605.72	A/B	-9.8
179	O=c1n([C@@H]2O[C@H](COP(=O)(OP(=O)(OC[C@@H]3[C@@H](N=[N+]=[N-])C[C@@H](O3)n3c(=O)[nH]c(=O)cc3)O)O)[C	175923-27-0	Guanosine 5,Ä≤-(trihydrogen diphosphate), 2,Ä≤,3,Ä≤-dideoxy-, P,Ä≤,Üí5,Ä≤-ester	C20H26N10O11P2	644.43	A/B	-9.8
180	C(=O)([C@@H](NC(=O)OC)C(C)(C)C)N1[C@@H]([C@@H]2C[C@H]1CC2)c1[nH]c(cn1)c1ccc2c(-c3c(C(F)(F)C2(F)F)cc(c	1801161-67-0	Carbamic acid, N,N,Ä≤-[(9,9,10,10-tetrafluoro-9,10-dihydro-2,7-phenanthrenediyl)	C46H52F4N8O6	888.95	A/B	-9.8
181	C(=O)([C@@H](NC(=O)OC)C(C)C)N1[C@H](c2[nH]c(c3c4c(c(cc3)c3ccc(cc3)c3[nH]c(nc3)[C@H]3N(C(=O)[C@@H](NC	1802921-87-4	Carbamic acid, N-[(1S)-2-methyl-1-[[(2S,5S)-2-methyl-5-[5-[4-[1,2,3,4-tetrahydro	C47H60N8O6	833.03	A/B	-9.8
182	O=c1c2c(n(cn2)[C@@H]2C(=C)[C@H](CO)[C@@H](OC(=O)C)C2)[nH]c(N)n1	1860843-01-1	Cyclohexanecarboxylic acid, (1S,2R,4S)-4-[2-[[(1,1-dimethylethoxy)carbonyl]aminc	C43H47N5O6	729.86	A/B	-9.8
183	C(=O)(O)c1c(sc2c1CC(C)(C)CC2)c1c(CNc2ccc(C[C@@H]([C@@H](CN(S(=O)(=O)c3cc4c(cc3)ncs4)CC3CCCC3)O)NC(=O	1887206-53-2	Benzo[b]thiophene-3-carboxylic acid, 2-[2-[[[4-[(2S,3R)-4-[(6-benzothiazolylsulf	C48H53F3N4O9S3	983.15	A/B	-9.8
184	C(=O)([C@H](NC(=O)OC)c1ccccc1)N1[C@@H](CCC1)c1[nH]c(cn1)c1ccc(cc1)c1ccc(cc1)c1[nH]c(nc1)[C@H]1N(C(=O	1009108-67-1	Carbamic acid, N-[(1R)-2-[(2S)-2-[5-[4,Ä≤-[2-[(2S)-1-[2-(4-methylphenyl)acetyl]-	C45H45N7O4	747.88	C	-9.8
185	N(=Nc1c2c(c(N=Nc3c(C)cc(N)cc3)cc1)ccc(S(=O)(=O)O)c2)c1c2c(c(S(=O)(=O)O)cc(S(=O)(=O)O)c2)ccc1S(=O)(=O	108075-27-0	1,3,6-Naphthalenetrisulfonic acid, 5-[2-[4-[2-(4-amino-7-sulfo-1-naphthalenyl)di	C28H23N5O12S4	749.77	C	-9.8
186	O(C[C@H]1O[C@H](CS1)n1c(=O)nc(N)cc1)P1Oc2c(cc(CO)c(O)c2)CO1	1099829-95-4	Not Yet Assigned	C35H36N10O14P2		C	-9.8
187	C(F)(F)c1n(c2c(n1)cccc2)c1nc(nc(N[C@H]2CC[C@H](NC(=O)CO)CC2)n1)N1CCOCC1	1240029-12-2	Propanamide, N-[trans-4-[[4-[2-(difluoromethyl)-1H-benzimidazol-1-yl]-6-(4-morph	C25H32F2N8O2	514.57	C	-9.8
188	C(=O)([C@@H](NC(=O)OC)C(C)C)N1[C@@H]([C@@H]2C[C@H]1CC2)c1[nH]c(cn1)c1ccc2c(-c3c(CC2)cc(cc3)c2[nH]c(nc	1256388-51-8	Carbamic acid, N-[(1S)-1-[[(6S)-6-[5-[9,9-difluoro-7-[2-[(1R,3S,4S)-2-[(2S)-2-[(	C49H54F2N8O6	889	C	-9.8
189	C([C@H](OCP(=O)(OCCCOCCCCCCCCCCCCCCCCCC)O)C)n1c2c(nc1)c(N)ncn2.[KH]	13011-56-8	2,7-Naphthalenedisulfonic acid, 4-[2-[4-[2-(4-amino-1-naphthalenyl)diazenyl]-1-r	C30H21N5O6S2.2Na		C	-9.8
190	Cc1sc2c(ccc(c2n1)c1ccc(cc1)c1[nH]c(nc1)[C@H]1N(C(=O)[C@@H](NC(=O)OC)C(C)C)CCC1)c1[nH]c(nc1)[C@H]1N(C	1312608-56-2	Carbamic acid, N-[2-[(2S)-2-[5-[4-[4-[2-[(2S)-1-[2-[(methoxycarbonyl)amino]acety	C38H40N8O6	704.77	C	-9.8
191	N(=Nc1c2c(c(N=Nc3cc(S(=O)(=O)O)ccc3)cc1)ccc(S(=O)(=O)O)c2)c1c2c(c(N)cc1)cccc2S(=O)(=O)O	1345852-32-5	2-Naphthalenesulfonic acid, 8-[2-(4-amino-6-sulfo-1-naphthalenyl)diazenyl]-5-[2-	C26H19N5O9S3	641.65	C	-9.8
192	N(=Nc1ccc(C=Cc2ccc(N=Nc3c4c(cc(S(=O)(=O)O)c3N)cc(S(=O)(=O)O)cc4)cc2)cc1)c1c2c(cc(S(=O)(=O)O)c1N)cc(S	140942-29-6	2-Naphthalenesulfonic acid, 5,5,Ä≤-[1,2-ethanediylbis[(3-sulfo-4,1-phenylene)azo	C34H28N6O12S4.4Na		C	-9.8
193	CC1(C)COc2c(-c3c(cc(cc3)c3c(CC)nc([nH]3)[C@H]3N(C(=O)[C@@H](NC(=O)OC)C(C)C)CCC3)OC1)ccc(c2)c1c(CC)nc	1449762-21-3	Carbamic acid, N,N,Ä≤-[(7,8-dihydro-7,7-dimethyl-6H-dibenzo[f,h][1,5]dioxonin-3,	C45H58N8O8	838.99	C	-9.8
194	N(c1nc(nc(n1)N1CCOCC1)N1CCOCC1)c1ccc(C(=O)C=Cc2cc(OC)c(OC)c(OC)c2)cc1	1628172-05-3	2-Propen-1-one, 1-[4-[(4,6-di-4-morpholinyl-1,3,5-triazin-2-yl)amino]phenyl]-3-(	C26H27N7O5	517.54	C	-9.8
195	O=C1N([C@H]2O[C@H](CO)[C@@H](O)C2)C[C@H](C)C(=O)N1	1680213-95-9	1-Piperidinecarboxylic acid, 4-[2-[6-amino-8-[(6-bromo-1,3-benzodioxol-5-yl)thic	C42H41BrN6O11S	917.78	C	-9.8
196	C(n1c2c(nc1CC)c(C)cc(C)n2)c1ccc(cc1)c1c(S(=O)(=O)NC(=O)c2c(O)cccc2)cccc1	174844-66-7	Benzamide, N-[[4,Ä≤-[(2-ethyl-5,7-dimethyl-3H-imidazo[4,5-b]pyridin-3-yl)methyl]	C31H30N4O4S		C	-9.8
197	N(c1nc(nc(Nc2ccc(C(=O)C=Cc3cc(Cl)ccc3)cc2)n1)N1CCOCC1)c1cc(C(F)(F)F)ccc1	1883795-11-6	2-Propen-1-one, 1-[4-[[4-(4-morpholinyl)-6-[[3-(trifluoromethyl)phenyl]amino]-1,	C29H24F3N7O4	591.54	C	-9.8
198	N(c1nc(Nc2ccccc2)cc(C)n1)c1c(C)cc(C)cc1C	1985610-22-7	1H-[1,4]Oxazino[3,4-c]pyrido[2,1-f][1,2,4]triazine-6,8-dione, 12-[(11S)-7,8-difl	C25H21F2N3O4S	497.51	C	-9.8
199	C(=O)([C@@H](NC(=O)OC)C(C)C)N1CC2(C[C@H]1c1[nH]c(cn1)c1c3c(c(cc1)c1cc4c(cc1)c1c(cc4)nc([nH]1)[C@H]1N	2007901-77-9	Carbamic acid, N-[(1S)-1-[[(2S)-2-[7-[2,3-dihydro-7-[2-[(8S)-7-[(2S)-2-[(methoxy	C47H56N8O8	861	C	-9.8
200	O(C[C@@H]1[C@@H](N=[N+]=[N-])C[C@@H](O1)n1c(=O)[nH]c(=O)c(C)c1)P1(=O)Oc2c(cc3c(c2)OP(=O)(OC[C@@H]2[C	1099830-36-0	Not Yet Assigned	C28H30FN7O14P2		A/B	-9.7
201	N(=C\1/c2c(N(C)C1=O)ccc(S(=O)(=O)N1CCOCC1)c2)\NC(=S)Nc1c(CC)cccc1	1164492-51-6	Hydrazinecarbothioamide, 2-[1,2-dihydro-1-methyl-5-(4-morpholinylsulfonyl)-2-oxo	C21H23N5O4S2	473.57	A/B	-9.7
202	N(=C\1/c2c(N(Cc3ccccc3)C1=O)ccc(C)c2)\NC(=S)N	1164513-99-8	Hydrazinecarbothioamide, 2-[1,2-dihydro-1-methyl-5-(4-morpholinylsulfonyl)-2-oxo	C22H25N5O4S2	487.6	A/B	-9.7
203	C(=O)(O[C@H](Cc1cc(O)c(O)cc1)C(=O)O)[C@H]1c2c(O[C@@H]1c1cc(O)c(O)cc1)c(O)ccc2/C=C/C(=O)O[C@H](Cc1cc(	1215192-26-9	Benzoic acid, 5-[2-[3-carboxy-4-[[4-(1,1-dimethylethyl)benzoyl]amino]phenyl]ethy	C35H31F3N2O6	632.63	A/B	-9.7
204	C(N=C)(C)C12CC3CC(C2)CC(C1)C3	1222434-12-9	Not Yet Assigned	C33H46FN5O4	595.75	A/B	-9.7
205	N(C(=O)[C@H](Cc1ccccc1)C[C@@H](CN1[C@H](C(=O)NC(C)(C)C)CN(Cc2cn3c(n2)cncc3)CC1)O)[C@H]1c2c(C[C@H]1O)	1237808-95-5	Not Yet Assigned	C37H47N7O4	653.81	A/B	-9.7
206	N(C(=O)[C@H](Cc1ccccc1)C[C@@H](CN1[C@H](C(=O)NC(C)(C)C)CN(Cc2cc3c(nc2)ccs3)CC1)O)[C@H]1c2c(C[C@H]1O)	1237808-96-6	Not Yet Assigned	C37H47N7O4	653.81	A/B	-9.7
207	C(=O)([C@H](NC(=O)OC)c1ccccc1)N1[C@@H](CCC1)c1[nH]c(cn1)C1=CCC(CC1)c1ccc(cc1)c1[nH]c(nc1)[C@H]1N(C(=	1240885-55-5	Carbamic acid, N-[(1R)-2-[(2S)-2-[5-[4-[6-[2-[(2S)-1-[(2R)-2-[(methoxycarbonyl)a	C50H48N8O6	856.97	A/B	-9.7
208	O(Cc1ccc(F)cc1)c1c2c(c(Cl)c(c1)c1ccccc1)oc(c(O)c2=O)c1ncccn1	1312904-56-5	4H-1-Benzopyran-4-one, 5-[(4-fluorophenyl)methoxy]-3-hydroxy-8-methoxy-7-phenyl-	C32H23FN2O5	534.53	A/B	-9.7
209	Cc1c2c(cc(OCc3ccc(F)cc3)c1C)c(=O)c(O)c(o2)c1ncccn1	1312908-83-0	4H-1-Benzopyran-4-one, 6-[(4-fluorophenyl)methoxy]-3-hydroxy-7-methyl-2-(5-pheny	C26H19FN2O4	442.44	A/B	-9.7
210	O=c1c2c(cc(c(OCc3ccc(F)cc3)c2)c2ccccc2)oc(c1O)c1[nH]ccn1	1312916-45-2	4H-1-Benzopyran-4-one, 6-[(4-fluorophenyl)methoxy]-3-hydroxy-7-phenyl-2-(3-pheny	C30H20FN3O4	505.5	A/B	-9.7
211	O([C@@H]1[C@@H](COP2(=O)Oc3c(CO2)cccc3C)O[C@H](C1)n1c(=O)[nH]c(=O)c(I)c1)P1(=O)Oc2c(CO1)cccc2C	1313031-58-1	Uridine, 2,Ä≤-deoxy-5-iodo-3,Ä≤,5,Ä≤-bis-O-(2-oxido-4H-1,3,2-benzodioxaphosphor	C23H21IN2O11P2	690.27	A/B	-9.7
212	[C@@H](NC(=O)c1ccccc1)(C(=O)N[C@H](C(=O)N[C@H](C(=O)C(=O)NCc1ccccc1)C(C)C)C)C12CC3CC(C2)CC(C1)C3	1318773-52-2	Piperazine, 1-[(2S)-N-benzoyl-2-tricyclo[3.3.1.13,7]dec-1-ylglycyl-L-alanyl-(3S)	C35H50N6O6	650.81	A/B	-9.7
213	C(#CC1C(C1)C1)[C@@]1(C(F)(F)F)c2c(NC(=O)O1)ccc(Cl)c2	1322625-54-6	Not Yet Assigned	C37H40D8N4O5		A/B	-9.7
214	S(=O)(=O)(N(C[C@H]([C@H](Cc1ccccc1)NC(=O)O[C@H]1CCOC1)O)CC(C)(C)C)c1ccc(N(=O)=O)cc1	133261-56-0	Guanosine 3,Ä≤-(tetrahydrogen triphosphate), P,Ä≤,Ä≤,Üí5,Ä≤-ester with guanosir	C20H27N10O18P3	788.41	A/B	-9.7
215	C(c1nc(CN(C(=O)N[C@@H](C(=O)N[C@H]([C@H]([C@@H]([C@H](Cc2ccccc2)NC(=O)OCc2cnco2)O)O)Cc2ccccc2)C(C)C)	1350182-18-1	2,7,10,12-Tetraazatridecanoic acid, 13-[2-[(dimethylamino)methyl]-4-thiazolyl]-4	C37H49N7O6S	719.89	A/B	-9.7
216	C(=O)([C@@H](NC(=O)OC)C(C)C)N1C[C@@]2(C[C@H]1c1[nH]c(cn1)c1ccc(cc1)c1ccc(cc1)c1[nH]c(nc1)[C@H]1N(C(=	1351856-00-2	Carbamic acid, N-[(1S)-1-[[(2S,4R)-4-cyclopropyl-2-[5-[4,Ä≤-[2-[(3S,5S)-2-[(2S)-	C47H60N8O7	849.03	A/B	-9.7
217	C(=O)([C@@H](NC(=O)OC)C(C)C)N1C[C@]2(C[C@H]1c1[nH]c(cn1)c1ccc(cc1)c1ccc(cc1)c1[nH]c(nc1)[C@H]1N(C(=O	1351856-02-4	Carbamic acid, N-[(1S)-1-[[(2S,4R)-4-cyclopropyl-2-[5-[4,Ä≤-[2-[(5S,8S)-7-[(2S)-	C46H56N8O8	848.99	A/B	-9.7
218	O=c1n([C@@H]2O[C@H](COP(=O)(NCC(=O)OC)OCC)[C@@H](N=[N+]=[N-])C2)cc(C)c(=O)[nH]1	1361474-13-6	Guanosine, 5,Ä≤-[N-[2-(1H-indol-2-yl)ethyl]carbamate]	C21H23N7O6	469.45	A/B	-9.7
219	N(c1nc(nc(NCCNC(=O)c2ccc(cc2)C2OOC(OO2)c2ccc(C)cc2)n1)N1CCOCC1)c1ccc(C)cc1	1393922-43-4	Benzamide, 4-[6-(4-methylphenyl)-1,2,4,5-tetroxan-3-yl]-N-[2-[[4-(4-morpholinyl)	C35H40N8O6	668.74	A/B	-9.7
220	[C@H](C(=O)N1[C@H]2[C@H](C2)C[C@H]1c1[nH]c(cn1)c1ccc(cc1)c1ccc(cc1)c1[nH]c(nc1)[C@H]1N(C(=O)[C@@H](N	1413115-52-2	Carbamic acid, N,N,Ä≤-[[1,1,Ä≤-biphenyl]-4,4,Ä≤-diylbis[1H-imidazole-5,2-diyl-(	C48H54N8O6	838.99	A/B	-9.7
221	O=c1n(cc(C(CCl)O)c(=O)[nH]1)[C@@H]1O[C@H](CO)[C@@H](O)C1	1421823-01-9	Benzoic acid, 5-[bis(3-carboxy-4-hydroxyphenyl)methyl]-3-[(3-carboxy-4-oxo-2,5-c	C30H20O12	572.47	A/B	-9.7
222	O(C[C@@H]1[C@@H](N=[N+]=[N-])C[C@@H](O1)n1c(=O)[nH]c(=O)c(C)c1)[P@]1(=O)O[C@@H]2[C@H](O[C@H](C2)n2c(	1448853-79-9	Thymidine, [P(R)]-thymidylyl-(3,Ä≤-5,Ä≤,Üí5,Ä≤)-3,Ä≤-azido-3,Ä≤-deoxy-	C20H24N7O10P		A/B	-9.7
223	O[C@H]1[C@H](n2c3c(nc2)c(=O)nc(N)[nH]3)O[C@H](CO)[C@H]1NNC[C@H]1O[C@H](C[C@@H]1O)n1c2c(nc1)c(=O)nc(N	1513874-13-9	Guanosine, 3,Ä≤-O-diphosphinicoguanylyliminomethylene-(3,Ä≤,Üí5,Ä≤)-2,Ä≤,5,Ä≤-d	C21H27N11O8	561.51	A/B	-9.7
224	O=c1c2c(n(cn2)[C@H]2C[C@H](NS(=O)(=O)NC[C@H]3O[C@H](C[C@@H]3O)n3c4c(nc3)c(=O)nc(N)[nH]4)[C@@H](CO)O2	1513874-57-1	Guanosine, 2,Ä≤-deoxy-3,Ä≤-de(phosphinicooxy)guanylyliminosulfonylimino-(3,Ä≤,Ü	C20H26N12O9S	610.56	A/B	-9.7
225	O=c1c2c(n(cn2)[C@@H]2O[C@H](C=O)[C@@H](O)[C@H]2O)[nH]c(N)n1	1513874-60-6	Guanosine, 3,Ä≤-de(phosphinicooxy)guanylyliminosulfonylimino-(3,Ä≤,Üí5,Ä≤)-5,Ä≤	C20H26N12O10S	626.56	A/B	-9.7
226	C(n1c2c(nc1CC)c(C)cc(C)n2)c1ccc(cc1)c1c(S(=O)(=O)NC(=O)CCc2ccccc2)ccc(OCC(F)(F)F)c1	154552-67-7	Benzenepropanamide, N-[[4,Ä≤-[(2-ethyl-5,7-dimethyl-3H-imidazo[4,5-b]pyridin-3-y	C37H41N5O4S	651.82	A/B	-9.7
227	C(=O)(C(NC(=O)OC)C(C)C)N1C(c2[nH]c(c3c4c(C5CC4CC5)c(cc3)c3ccc(cc3)c3[nH]c(nc3)C3N(C(=O)C(NC(=O)OC)C(	1560650-21-6	Carbamic acid, N,N,Ä≤-[(1,2,3,4-tetrahydro-1,4-methanonaphthalene-5,8-diyl)bis[4	C51H60N8O6	881.07	A/B	-9.7
228	C(=O)([C@@H](NC(=O)OC)C(C)C)N1[C@H](c2[nH]c(c3c4c(c(cc3)c3c5c(c(cc3)c3[nH]c(nc3)[C@H]3N(C(=O)[C@@H](	1627533-30-5	Carbamic acid, N,N,Ä≤-[(1,1,Ä≤,2,2,Ä≤,3,3,Ä≤,4,4,Ä≤-octahydro[5,5,Ä≤-bi-1,4-met	C50H60F2N8O6	907.06	A/B	-9.7
229	[C@H]([C@@H](CO)O)(O)[C@H]1[C@H](NC(=O)C(C)C)[C@@H](N)C=C(C(=O)O)O1	162754-90-7	Œ≤-D-arabino-Adenosine, 5,Ä≤-O-phosphonoadenylyl-(2,Ä≤,Üí5,Ä≤)-adenylyl-(2,Ä≤,Ü	C30H38N15O19P3	1005.63	A/B	-9.7
230	N(c1nc(nc(n1)N1CCOCC1)N1CCOCC1)c1ccc(C(=O)C=Cc2cc(OC)c(OC)c(OC)c2)cc1	1628172-05-3	2-Propen-1-one, 1-[4-[(4,6-di-4-morpholinyl-1,3,5-triazin-2-yl)amino]phenyl]-3-(	C26H27N7O5	517.54	A/B	-9.7
231	N(C(=O)[C@H](Cc1ccccc1)C[C@@H](CN1[C@H](C(=O)NC(C)(C)C)CN(Cc2c(C)noc2C)CC1)O)[C@H]1c2c(C[C@H]1O)cccc	164725-31-9	D-erythro-Pentonamide, 5-[(2S)-4-(1H-benzimidazol-2-ylmethyl)-2-[[(1,1-dimethyle	C38H48N6O4	652.83	A/B	-9.7
232	C(C1c2c(CC1O)cccc2)N1CC(C(=O)NC(C)(C)C)N(CC(CC(C(=O)NC2c3c(CC2O)cccc3)Cc2ccccc2)O)CC1	164725-64-8	1-Piperazinepentanamide, N-(2,3-dihydro-2-hydroxy-1H-inden-1-yl)-4-[(2,3-dihydro	C40H52N4O4	652.87	A/B	-9.7
233	C(=O)([C@@H](NC(=O)OC)C(C)C)N1CC2(C[C@H]1c1[nH]c(cn1)c1ccc(cc1)c1cc3c(cc1)c1c(cc3)nc([nH]1)[C@H]1N(C	1653966-60-9	Carbamic acid, N-[(1S)-1-[[(3S,5S)-3-[2,Ä≤-[(6S)-5-[(2S)-2-[(methoxycarbonyl)ami	C50H58N8O7	883.04	A/B	-9.7

234	C(=O)([C@@H](NC(=O)OC)C(C)C)N1C[C@@]2(C[C@H]1c1[nH]c(cn1)c1ccc(cc1)c1ccc(cc1)c1[nH]c(nc1)[C@H]1N(C(=	1653966-95-0	Carbamic acid, N,N,Ä≤-[[7,7,Ä≤-bi-1H-naphth[1,2-d]imidazole]-2,2,Ä≤-diylbis[(3S	C52H62N8O8	927.1	A/B	-9.7
235	N(c1nc(nc(n1)N1CCOCC1)N1CCOCC1)c1ccc(C(=O)/C=C/c2cc(OC)c(OC)cc2)cc1	1710363-29-3	2-Propen-1-one, 1-[4-[(4,6-di-4-morpholinyl-1,3,5-triazin-2-yl)amino]phenyl]-3-(	C26H27N7O5	517.54	A/B	-9.7
236	N(c1nc(nc(n1)N1CCOCC1)N1CCOCC1)c1ccc(C(=O)/C=C/c2ccc(N(CC)CC)cc2)cc1	1710363-35-1	2-Propen-1-one, 3-(3,4-dimethylphenyl)-1-[4-[(4,6-di-4-morpholinyl-1,3,5-triazir	C28H32N6O3	500.59	A/B	-9.7
237	C(Cc1ccccc1)[C@]1(CCC)CC(=C([C@H](CC)c2cc(NS(=O)(=O)c3ccc(C#N)cn3)ccc2)C(=O)O1)O	174484-48-1	3-Pyridinecarboxamide, 6-[[[3-[1-[6-[2-(4-fluorophenyl)ethyl]-5,6-dihydro-4-hydr	C31H34FN3O6S	595.68	A/B	-9.7
238	[C@@H](C(C)(C)C)(C1=C(O)C[C@](CCc2ccccc2)(CCC)OC1=O)c1cc(NS(=O)(=O)c2cc(C(F)(F)F)ccn2)ccc1	174590-24-0	2-Pyridinesulfonamide, N-[3-[1-[5,6-dihydro-4-hydroxy-2-oxo-6-(2-phenylethyl)-6-	C33H37F3N2O5S	630.72	A/B	-9.7
239	[C@@H](COCCCCCCCCCCCCCCCCCC)(COP(=O)(C(=O)O)O)O	179602-70-1	Adenosine 5,Ä≤-(pentahydrogen tetraphosphate), P,Ä≤,Ä≤,Ä≤,Üí5,Ä≤-ester with 9-(	C20H28N10O19P4	836.39	A/B	-9.7
240	FC1(F)c2c(-c3c(cc(cc3)c3[nH]c(nc3)[C@H]3N(C(=O)[C@@H](NC(=O)OC)C(C)C)CCC3)C1(F)F)ccc(c2)c1[nH]c(nc1)	1801161-78-3	Carbamic acid, N-[(1S)-2-methyl-1-[[(2S)-2-[5-[9,9,10,10-tetrafluoro-9,10-dihydr	C44H50F4N8O8	894.91	A/B	-9.7
241	O=C1[C@]2([C@H]([C@H](/C(=C/C(=O)[C@@H]3[C@@]4(N5[C@@H]([C@H]3c3cc(OC)c(O)cc3)CCC5)c3c(NC4=O)ccc(F)c	1807888-30-7	Not Yet Assigned	C45H42F2N4O8	804.83	A/B	-9.7
242	O(P(=O)(OC[C@H]1O[C@H]([C@H](O)[C@@H]1O)n1c2c(nc1)c(N)ncn2)O)[C@H]1[C@@H](O[C@H](COP(=O)(O[C@H]2[C@@	190141-45-8	Adenosine, 1,Ä≤-[3-(aminocarbonyl)-1H-1,2,4-triazol-1-yl]-3,Ä≤-O-benzoyl-1,Ä≤-d	C106H90N16O28P2	2097.89	A/B	-9.7
243	[C@@H](C(=O)N[C@@H](Cc1ccccc1)C[C@@H]([C@H](Cc1ccccc1)NC(=O)COc1c(C)cccc1C)O)(C(C)C)N1C(=O)NNCC1	192725-39-6	1(2H)-Pyrimidineacetamide, N-[(1S,3S,4S)-4-[[2-(2,6-dimethylphenoxy)acetyl]aminc	C37H46N4O6	642.78	A/B	-9.7
244	O(C(=O)OCCCCCC)c1c2n(N([C@H]3N(C2=O)CCOC3)[C@H]2c3c(CSc4c2cccc4)c(F)c(F)cc3)ccc1=O	1985606-92-5	1H-Indole-1-carboxylic acid, 3-[[[(12aR)-12-[(11S)-7,8-difluoro-6,11-dihydrodibe	C35H28F2N4O6S	670.68	A/B	-9.7
245	O=c1n([C@@H]2O[C@H](COP(=O)S)[C@@H](OP(=O)(O)S)C2)cc(C)c(=O)[nH]1	2024571-98-8	Not Yet Assigned	C29H38IN7O18P2	961.5	A/B	-9.7
246	O(C(=O)C)[C@H]1[C@@H](O[C@H](COCC(=O)O)[C@H]1OC(=O)C)n1nc(C(=O)N)nc1	1002336-62-0	1H-1,2,4-Triazole-3-carboxamide, 1-[2,3-O-(methoxymethylene)-5-O-[4-[[[4,6-O-[(S	C31H34N4O13	670.62	C	-9.7
247	C(=O)([C@H](NC(=O)OC)c1ccccc1)N1[C@@H](CCC1)c1[nH]c(cn1)c1ccc(cc1)c1ccc(cc1)c1[nH]c(nc1)[C@H]1N(C(=O	1009108-66-0	Carbamic acid, N-[(1R)-2-[(2S)-2-[5-[4,Ä≤-[2-[(2S)-1-[2-(3-methylphenyl)acetyl]-	C45H45N7O4	747.88	C	-9.7
248	S(=O)(=O)([O-])c1cc2c(c3c1cc(N=N(=O)c1cc4c(cc1)c1c(cc4S(=O)(=O)[O-])[N]4=N[C-]5C(=[O][Cu+2]4[O-]1)N(	1073242-68-8	L-Proline, 1-[3-[2-(2,Ä≤,3,Ä≤-didehydro-3,Ä≤-deoxythymidin-5,Ä≤-O-yl)-2-oxido-4	C32H34N3O10P	651.6	C	-9.7
249	O=c1n([C@@H]2O[C@H](CO)[C@@H](O)C2[2H])cc(C)c(=O)[nH]1	108924-74-9	Naphthionic acid, 3,3,Ä≤-[thiobis[(4-methyl-o-phenylene)azo]]di-, disodium salt	C34H28N6O6S3.2Na		C	-9.7
250	C(=O)([C@H](NC(=O)OC)C(C)C)N1CC2(C[C@H]1c1[nH]c(cn1)c1ccc(cc1)c1ccc(cc1)c1[nH]c(nc1)[C@H]1N(C(=O)[C@	1129634-62-3	Carbamic acid, N,N,Ä≤-[[1,1,Ä≤-biphenyl]-4,4,Ä≤-diylbis[1H-imidazole-5,2-diyl-(	C42H50N8O6	762.9	C	-9.7
251	O=c1n([C@H]2C[C@@H](O[Si](C(C)(C)C)(C)C)[C@@H](C=O)O2)cc(C)c(=O)[nH]1	1182735-51-8	5,Ä≤-Uridylic acid, 2,Ä≤-deoxy-5-iodo-, 3,Ä≤-[4-[(5Œ±,17Œ≤)-3-oxoandrostan-17-y	C32H44IN2O12P	806.58	C	-9.7
252	N(=Nc1ccc(C(=O)N)cc1)c1c2c(cc(S(=O)(=O)O)cc2)c(N)cc1	1238950-64-5	2-Naphthalenesulfonic acid, 8-amino-5-[2-[4-[2-(4-cyanophenyl)diazenyl]-7-sulfo-	C27H18N6O6S2	586.6	C	-9.7
253	C(=O)([C@H](NC(=O)OC)c1ccccc1)N1[C@@H](CCC1)c1[nH]c(cn1)[C@H]1CC[C@@H](CC1)c1ccc(cc1)c1[nH]c([C@@H](	1240890-46-3	Carbamic acid, N-[(1R)-2-[(2S)-2-[5-[trans-4-[4-[2-[(1S)-1-[[(2R)-2-[(methoxycar	C45H52N8O6	800.94	C	-9.7
254	C(=O)([C@H](NC(=O)OC)c1ccccc1)N1[C@@H](CCC1)c1[nH]c(cn1)C1=CC[C@H](CC1)c1ccc(cc1)c1[nH]c(nc1)[C@H]1N	1240891-27-3	Carbamic acid, N-[(1R)-2-[(2S)-2-[5-[3-[trans-4-[2-[(2S)-1-[(2R)-2-[(methoxycarb	C46H52N8O6	812.96	C	-9.7
255	N(C(=O)Nc1ccc(N(C)C)cc1)c1ccc(c2nc(nc(n2)N2CCOCC2)N2C3CCC2COC3)cc1	1254065-96-7	Urea, N-[4-[4-(4-morpholinyl)-6-(3-oxa-8-azabicyclo[3.2.1]oct-8-yl)-1,3,5-triazi	C26H29N7O3	487.55	C	-9.7
256	C(=O)([C@@H](NC(=O)OC)C(C)C)N1CC2(C[C@H]1c1[nH]c(cn1)c1cc3c(cc1)cc(cc3)c1ccc(cc1)c1[nH]c(nc1)[C@H]1N	1256392-10-5	Carbamic acid, N-[(1S)-2-[(2S)-2-[5-[9,9-difluoro-7-[2-[(6S)-5-[(2S)-2-[(methoxy	C45H52F2N8O7	854.94	C	-9.7
257	C(C)n1c2c(c(=O)c(C(=O)O)c1)cc(F)c(c2F)N1CC(C)N(CN2c3c(/C(=N/NC(=S)NO)/C2=O)cc(F)cc3)CC1	1265623-22-0	3-Quinolinecarboxylic acid, 7-[4-[[(3Z)-5-chloro-2,3-dihydro-3-[2-[(methoxyaminc	C28H28ClF2N7O5S	648.08	C	-9.7
258	O(Cc1ccc(F)cc1)c1c2c(c(C)c(c1)c1ccccc1)oc(c(O)c2=O)c1ncccn1	1312903-23-3	4H-1-Benzopyran-4-one, 5-[(4-fluorophenyl)methoxy]-3-hydroxy-7-phenyl-2-(5-pheny	C31H21FN2O4	504.51	C	-9.7
259	C(=O)(OCC)c1c2c(n(C)c1CSc1ccccc1)cc(Br)c(O)c2CN(C)C	1316840-85-3	3-Quinolinecarboxylic acid, 7-[4-[2-[3-[2-(aminothioxomethyl)hydrazinylidene]-5-	C30H31F2N7O5S	639.67	C	-9.7
260	N(=Nc1cc(S(=O)(=O)O)ccc1)c1c2c(c(N=Nc3c4c(cc(S(=O)(=O)O)cc4)c(N)cc3)cc1)ccc(S(=O)(=O)O)c2	1345852-47-2	1-Naphthalenesulfonic acid, 5-amino-8-[2-[4-[2-(3-sulfophenyl)diazenyl]-1-naphth	C26H19N5O6S2	561.59	C	-9.7
261	C(=O)([C@@H](NC(=O)OC)C1CCOCC1)N1[C@H]2[C@H](C2)C[C@H]1c1[nH]c(cn1)c1c2c(c(cc1)c1ccc(cc1)c1[nH]c(nc1	1359868-95-3	Carbamic acid, N-[(1S)-1-[[(1R,3S,5R)-3-[5-[4-[7-[2-[(1R,3S,5R)-2-[(2S)-2-[(meth	C43H50N10O6	802.92	C	-9.7
262	C(=O)([C@@H](NC(=O)OC)C(C)C)N1[C@H]2[C@H](C2)C[C@H]1c1[nH]c2c(n1)ccc1c2ccc(c1)c1cc(F)c(cc1)c1[nH]c(n	1377041-37-6	Carbamic acid, N,N,Ä≤-[2,7-triphenylenediylbis[1H-imidazole-5,2-diyl-(2S)-2,1-py	C50H56N8O8	897.03	C	-9.7
263	C(=O)([C@@H](NC(=O)OC)C(C)C)N1[C@H](c2[nH]c(c3cc4c5c6c(cc(cc6OC4)c4[nH]c(nc4)[C@H]4N(C(=O)[C@@H](NC(	1377043-96-3	Carbamic acid, N-[(1S)-1-[[(1R,3S,5R)-3-[2,Ä≤-[(1R,3S,4S)-2-[(2S)-2-[(methoxycar	C47H52N8O6	824.97	C	-9.7
264	C(CC(=O)CC(CCc1ccc(O)cc1)O)c1cc(OC)c(O)cc1	1388690-73-0	Spiro[2H-1-benzopyran-2,8,Ä≤(5,Ä≤H)-[1,2,4]triazolo[4,3-a]pyridin]-4(3H)-one, 7	C25H21ClN4O4	476.91	C	-9.7
265	O=C1[C@H]2[C@@H](c3c([C@H](OC(=O)OC)[C@H]2CO1)cc1c(c3)OCO1)c1cc(OC)c(OC)c(OC)c1	1453805-45-2	1-Piperazinecarbothioamide, N-(7-chloro-4-quinolinyl)-4-(4,6-di-4-morpholinyl-1,	C25H30ClN9O2S	556.08	C	-9.7
266	C(=O)([C@@H](NC(=O)OC)C(C)C)N1[C@H](c2[nH]c(c3c4c(C5CC4CC5)c(cc3)c3ccc(cc3)c3[nH]c(nc3)[C@H]3N(C(=O)	1560639-05-5	Carbamic acid, N-[(1S)-1-cyclohexyl-2-[(2S)-2-[5-[8-[4-[2-[(2S)-1-[(2S)-2-cycloh	C51H64N8O6	885.1	C	-9.7
267	C(=O)([C@@H](NC(=O)OC)C1CCCCC1)N1[C@H](c2[nH]c(c3c4c(C5(CC4)CCCC5)c(cc3)c3ccc(cc3)c3[nH]c(nc3)[C@H]3	1588403-95-5	Carbamic acid, N-[(1S)-1-[[(2S,4S)-2-[5-[2,Ä≤,3,Ä≤-dihydro-7,Ä≤-[4-[2-[(2S,4S)-	C49H64N8O6	861.08	C	-9.7
268	C(O[C@@H]1CCP(=O)(O)OC1)n1c2c(nc1)c(=O)nc(N)[nH]2	1607795-41-4	Urea, N,N,Ä≤,Ä≤-1,4-phenylenebis[N,Ä≤-(1-tricyclo[3.3.1.13,7]dec-1-ylethyl)-	C32H46N4O2	518.73	C	-9.7
269	O=C1CC2c3c(c(ccc3c3ccc(cc3)c3[nH]c(nc3)[C@H]3N(C(=O)[C@@H](NC(=O)OC)C(C)C)CCC3)c3[nH]c(nc3)[C@H]3N(C	1613055-52-9	Carbamic acid, N-[(1S)-1-[[(2S)-2-[5-[3-[4-[2-[(2S)-1-[(2S)-2-[[(1,1-dimethyleth	C51H68N8O6	889.14	C	-9.7
270	C(#N)c1c(cc(nc1N)c1ccc(Nc2nc(nc(n2)N2CCOCC2)N2CCOCC2)cc1)c1cc(C)c(C)cc1	1627963-44-3	1,3,5-Triazin-2-amine, N-[4-[5-(3,4-dimethylphenyl)-3-isoxazolyl]phenyl]-4,6-di-	C28H31N7O3	513.59	C	-9.7
271	C(=O)([C@@H](NC(=O)OC)C(C)C)N1C[C@]2(C[C@H]1c1[nH]c(cn1)c1ccc(cc1)c1cc3c(cc1)c1c(cc3)nc([nH]1)[C@H]1	1653966-54-1	Carbamic acid, N-[(1S)-1-[[(2S)-2-[7-[4-[2-[(3S,5S)-2-[(2S)-2-[(methoxycarbonyl)	C46H56N8O7	832.99	C	-9.7
272	C(NC(=O)c1ccc(CCOc2cc(OC(F)(F)F)ccc2)cc1)(C)C12CC3CC(C2)CC(C1)C3	166022-38-4	Guanosine, P-thiothymidylyl-(3,Ä≤,Üí5,Ä≤)-2,Ä≤-deoxy-P-thioguanylyl-(3,Ä≤,Üí5,Ä	C30H38N12O15P2S2	932.77	C	-9.7
273	O=c1n(cc(C)c(=O)[nH]1)[C@H]1O[C@H](C(=O)O)[C@@H](O)C1	170790-36-0	Benzenepropanamide, 4-[(2-ethyl-5,7-dimethyl-3H-imidazo[4,5-b]pyridin-3-yl)methy	C32H32N4O3S	552.69	C	-9.7
274	N(c1nc(nc(n1)N1CCOCC1)N1CCOCC1)c1ccc(C(=O)/C=C/c2cc(OC)c(OC)cc2)cc1	1710363-29-3	2-Propen-1-one, 1-[4-[(4,6-di-4-morpholinyl-1,3,5-triazin-2-yl)amino]phenyl]-3-(	C26H27N7O5	517.54	C	-9.7
275	N(c1nc(nc(n1)N1CCOCC1)N1CCOCC1)c1ccc(C(=O)/C=C/c2ccc(N(CC)CC)cc2)cc1	1710363-35-1	2-Propen-1-one, 3-(3,4-dimethylphenyl)-1-[4-[(4,6-di-4-morpholinyl-1,3,5-triazir	C28H32N6O3	500.59	C	-9.7
276	O=c1n([C@@H]2O[C@H](COP(=O)(OCC(COCCCCCCCCCCCCC)OCCCCCCCCCC)O)[C@@H](N=[N+]=[N-])C2)cc(C)c(=O)[nH]1	178152-65-3	D-erythro-Pentonamide, 2,3,5-trideoxy-N-[(1S,2S,3R)-2,3-dihydro-2,3-dihydroxy-1H	C36H47N5O5	629.79	C	-9.7
277	O(Cc1c2c(n(C(=O)OC)c1)cccc2)c1c2n(N([C@H]3N(C2=O)CCOC3)[C@H]2c3c(CSc4c2cccc4)c(F)c(F)cc3)ccc1=O	1985606-91-4	1,2-Pyrrolidinedicarboxylic acid, 1-[[[(12aR)-12-[(11S)-7,8-difluoro-6,11-dihydr	C32H30F2N4O8S	668.66	C	-9.7
278	O(C(=O)O[C@@H]1[C@H]2[C@@H](OC1)OCC2)c1c2n(N([C@H]3N(C2=O)CCOC3)[C@H]2c3c(CSc4c2cccc4)c(F)c(F)cc3)cc	1985607-26-8	Carbonic acid, 1-acetyl-3-azetidinyl (12aR)-12-[(11S)-7,8-difluoro-6,11-dihydroc	C30H26F2N4O7S	624.61	C	-9.7
279	[C@H](CC)(C1=C(O)C[C@@H](OC1=O)c1ccccc1)c1cc(NS(=O)(=O)c2c(C(F)(F)F)cccn2)ccc1	1109235-78-0	2-Pyridinesulfonamide, N-[3-[(1R)-1-[(6S)-5,6-dihydro-4-hydroxy-2-oxo-6-phenyl-2	C26H23F3N2O5S	532.53	A/B	-9.6
280	O=C1C2C(c3c(C(O)C2CO1)cc1c(c3)OCO1)c1cc(OC)c(OC(=O)C)c(OC)c1	114002-77-6	Benzoic acid, 3,3,Ä≤-[(4-oxo-2,5-cyclohexadien-1-ylidene)methylene]bis[6-hydroxy	C21H14O7	378.33	A/B	-9.6
281	C([C@@H](OP(=O)(O)O)CO)n1c2c(nc1)c(N)ncn2	1222444-00-9	Not Yet Assigned	C36H52FN5O4	637.83	A/B	-9.6
282	N(C(=O)[C@H](CC1CCCCC1)C[C@@H](CN1[C@H](C(=O)NC(C)(C)C)CN(Cc2cccnc2)CC1)O)[C@H]1c2c(C[C@H]1O)cccc2	1222674-71-6	Not Yet Assigned	C36H46FN5O4	631.78	A/B	-9.6
283	N(C(=O)[C@H](Cc1ccccc1)C[C@@H](CN1[C@H](C(=O)NC(C)(C)C)CN(Cc2cccnc2)CC1)C)C1c2c(C[C@H]1O)cccc2	1223086-75-6	Not Yet Assigned	C38H47N5O4S	669.88	A/B	-9.6
284	[C@@H](NC(=O)[C@H](Cc1ccccc1)NC(=O)N1CCN(C)CC1)(/C=C/S(=O)(=O)c1ccccc1)c1c(C)cc(C)cc1C	1253912-35-4	1-Piperazinecarboxamide, 4-methyl-N-[(1S)-2-[[(1R,2E)-1-(2-naphthalenyl)-3-(pher	C34H36N4O4S	596.74	A/B	-9.6
285	O(Cc1ccc(F)cc1)c1c2c(c(cc1)c1ccccc1)oc(c(O)c2=O)c1[nH]cnn1	1312894-37-3	4H-1-Benzopyran-4-one, 5-[(4-fluorophenyl)methoxy]-3-hydroxy-8-phenyl-2-(5-pheny	C32H21FN2O4	516.52	A/B	-9.6
286	O(Cc1ccc(F)cc1)c1c2c(c(C)c(c1)c1ccccc1)oc(c(O)c2=O)c1ncccn1	1312903-23-3	4H-1-Benzopyran-4-one, 5-[(4-fluorophenyl)methoxy]-3-hydroxy-7-phenyl-2-(5-pheny	C31H21FN2O4	504.51	A/B	-9.6
287	O=c1c2c(oc(c1O)c1[nH]ccn1)ccc(OCc1ccc(F)cc1)c2	1312906-28-7	4H-1-Benzopyran-4-one, 6-[(4-fluorophenyl)methoxy]-3-hydroxy-2-(3-phenyl-1H-1,2,	C24H16FN3O4	429.4	A/B	-9.6
288	Cc1c2c(cc(OCc3ccc(F)cc3)c1OC)c(=O)c(O)c(o2)c1ncccn1	1312911-92-4	4H-1-Benzopyran-4-one, 7-chloro-6-[(4-fluorophenyl)methoxy]-3-hydroxy-2-(5-pheny	C25H16ClFN2O4	462.86	A/B	-9.6
289	Clc1c2c(cc(OCc3ccc(F)cc3)c1OC)c(=O)c(O)c(o2)c1ncccn1	1312913-93-1	4H-1-Benzopyran-4-one, 7-chloro-6-[(4-fluorophenyl)methoxy]-3-hydroxy-8-methoxy-	C26H18ClFN2O5	492.88	A/B	-9.6
290	S(=O)(=O)(N(C[C@H]([C@H](Cc1ccccc1)NC(=O)O[C@H]1CCOC1)O)CC(C)C)c1ccc(NCOCCOCCOC)cc1	1348885-10-8	Carbamic acid, N-[(1S,2R)-3-[[(4-aminophenyl)sulfonyl](2-methylpropyl)amino]-1-[	C38H46N3O10PS	767.82	A/B	-9.6
291	C(=O)([C@@H](NC(=O)OC)C1CCOCC1)N1[C@@H](CCC1)c1[nH]c(c(Cl)n1)c1ccc(cc1)c1ccc(cc1)c1[nH]c(nc1Cl)[C@H]	1377042-44-8	Carbamic acid, N-[(1S)-2-[(2S)-2-[5-[3-(2-cyclopropylethynyl)-4-[2-[(2S)-1-[(2S)	C49H56N8O7	869.02	A/B	-9.6
292	N(C(C(=O)N1CCN(S(=O)(=O)C)CC1)C)c1nc(nc(n1)N1CCOCC1)c1cc(O)ccc1	1391924-42-7	2(1H)-Pyridinone, 5-[4-[[2-[4-(methylsulfonyl)-1-piperazinyl]-2-oxoethyl]amino]-	C19H26N8O5S	478.53	A/B	-9.6
293	O[C@H]1[C@@H](O[C@H](C(=O)N[C@H](Cc2c3c([nH]c2)cccc3)C(=O)N2CCN(C(=O)N3CCCCC3)CC2)[C@H]1O)n1nc(C(=O)	1414793-67-1	Not Yet Assigned	C28H35N9O8	625.63	A/B	-9.6
294	O[C@H]1[C@@H](O[C@H](C(=O)N[C@@H](C(=O)N2CCN(C(=O)c3c(OC)cccc3)CC2)Cc2ccccc2)[C@H]1O)n1nc(C(=O)N)nc1	1414793-80-8	Not Yet Assigned	C28H29N7O8	591.57	A/B	-9.6
295	O[C@H]1[C@@H](O[C@H](C(=O)N[C@@H](C(=O)N2CCN(CC(=O)Nc3ccccn3)CC2)Cc2ccccc2)[C@H]1O)n1nc(C(=O)N)nc1	1414794-00-5	1H-Indole-3-propanamide, Œ±-[[[(2S,3S,4R,5R)-5-[3-(aminocarbonyl)-1H-1,2,4-triaz	C26H32N8O7	568.58	A/B	-9.6
296	O=C1c2c(N(c3c(N1C)c(C)cc(F)n3)C1CC1)ncc(CCO)c2	1415206-23-3	Carbamic acid, N-[(1S)-2-methyl-1-[[(2S)-octahydro-2-[5-[15-[4-[2-[(2S)-octahydr	C58H72N8O6	977.24	A/B	-9.6
297	O=C1c2c(c(ccc2c2ccc(cc2)c2[nH]c(nc2)[C@H]2N(C(=O)[C@@H](NC(=O)OC)C(C)C)CCC2)c2[nH]c(nc2)[C@H]2N(C(=O	1419385-23-1	Carbamic acid, N-[(1S)-1-[[(2S)-2-[5-[1,Ä≤,3,Ä≤-dihydro-7,Ä≤-[4-[2-[(2S)-1-[(2S	C51H68N8O6	889.14	A/B	-9.6
298	C(OC[C@@H]1[C@@H](OC(=O)c2ccccc2)[C@@H](OP(=O)(OC[C@@H]2[C@@H](OC(=O)c3ccccc3)[C@@H](OP(=O)(OC[C@@H]	1422459-45-7	Adenosine, N-benzoyl-3,Ä≤-O-benzoyl-5,Ä≤-O-[(4-methoxyphenyl)diphenylmethyl]-P-	C92H80N16O25P2	1871.66	A/B	-9.6
299	C([C@H](OCP(=O)(OP(=O)(OCC[N+](C)(C)C)[O-])O)CO)n1c(=O)nc(N)cc1	1422459-57-1	Adenosine, 1,Ä≤-[3-(aminocarbonyl)-1H-1,2,4-triazol-1-yl]-1,Ä≤-de(6-amino-9H-pu	C26H33N13O17P2	861.56	A/B	-9.6
300	N(=Nc1c2c(c(N)cc1)cccc2)c1c2c(c(S(=O)(=O)O)ccc2)c(O)cc1	1432018-34-2	2-Naphthalenesulfonic acid, 5-[2-(1-naphthalenyl)diazenyl]-	C20H14N2O3S	362.4	A/B	-9.6
301	O[C@H]1[C@H](n2c3c(nc2)c(=O)nc(N)[nH]3)O[C@H](CO)[C@H]1ONC[C@H]1O[C@H](C[C@@H]1O)n1c2c(nc1)c(=O)nc(N	1513874-14-0	Guanosine, 3,Ä≤-de(phosphinicooxy)guanylyl-1,2-hydrazinediyl-(3,Ä≤,Üí5,Ä≤)-2,Ä≤	C20H26N12O7	546.5	A/B	-9.6
302	O=c1c2c(n(cn2)[C@H]2C[C@H](NNC[C@H]3O[C@H](C[C@@H]3O)n3c4c(nc3)c(=O)nc(N)[nH]4)[C@@H](CO)O2)[nH]c(N)	1513874-38-8	Guanosine, 2,Ä≤-deoxy-3,Ä≤-O-dephosphinicoguanylyliminomethylene-(3,Ä≤,Üí5,Ä≤)-	C21H27N11O7	545.51	A/B	-9.6
303	C(=O)(NCc1ccc(F)cc1)c1c(OCOC(=O)OC(C)C)c(=O)n(C)c(C(NC(=O)c2oc(C)nn2)(C)C)n1	1538604-08-8	Carbonic acid, cyclohexyl 1-[[4-[[[(4-fluorophenyl)methyl]amino]carbonyl]-1,6-di	C30H37FN6O8	628.65	A/B	-9.6
304	C(n1c2c(nc1CC)c(C)cc(C)n2)c1ccc(cc1)c1c(S(=O)(=O)NC(=O)CCc2ccccc2)ccc(CSC)c1	154552-58-6	Benzenepropanamide, N-[[4,Ä≤-[(2-ethyl-5,7-dimethyl-3H-imidazo[4,5-b]pyridin-3-y	C33H34N4O4S	582.71	A/B	-9.6
305	C(n1c2c(nc1CC)c(C)cc(C)n2)c1ccc(cc1)c1c(S(=O)(=O)NC(=O)c2ccccc2)ccc(CN(CC)CC)c1	154553-24-9	Benzamide, N-[[5-(1,1-dimethylethyl)-4,Ä≤-[(2-ethyl-5,7-dimethyl-3H-imidazo[4,5-	C34H36N4O3S	580.74	A/B	-9.6
306	C(n1c2c(nc1CC)c(C)cc(C)n2)c1ccc(cc1)c1c(S(=O)(=O)NC(C)(C)C)ccc(CCC)c1	154553-68-1	[1,1,Ä≤-Biphenyl]-2-sulfonamide, 4,Ä≤-[(2-ethyl-5,7-dimethyl-3H-imidazo[4,5-b]py	C35H39N5O4S	625.78	A/B	-9.6
307	[C@@H](Cc1ccccc1)(NC(=O)[C@@H](NC(=O)N(Cc1nc(C(C)C)sc1)C)C(C)C)[C@@H]1[C@@H]([C@H](Cc2ccccc2)NC(=O)O	1551453-60-1	2,7,10,12-Tetraazatridec-4-enoic acid, 12-methyl-9-(1-methylethyl)-13-[2-(1-meth	C37H46N6O4S2	702.93	A/B	-9.6
308	CN1C2c3c(C1CC2)c(ccc3c1[nH]c(nc1)[C@H]1N(C(=O)[C@@H](NC(=O)OC)C(C)C)C[C@H](F)C1)c1ccc(cc1)c1[nH]c(nc	1560639-50-0	Carbamic acid, N-[(1S)-1-cyclohexyl-2-[(2S)-2-[5-[8-[4-[2-[(2S)-1-[(2S)-2-cycloh	C51H65N9O6	900.12	A/B	-9.6
309	C(=O)([C@@H](NC(=O)OC)C(C)C)N1[C@H](c2[nH]c(c3c4c(c(cc3)c3ccc(cc3)c3[nH]c(nc3)[C@H]3N(C(=O)[C@@H](NC	1560640-31-4	Carbamic acid, N,N,Ä≤-[(1,2,3,4-tetrahydro-1,4-ethanonaphthalene-5,8-diyl)bis[4,	C54H66N8O6	923.15	A/B	-9.6
310	N(C(CCCN(CC)CC)C)c1c2c(ncc1S(=O)C)cc(Cl)cc2	1611493-60-7	2,5-Methanopyrido[1,Ä≤,2,Ä≤:4,5]pyrazino[2,1-b][1,3]oxazepine-10-carboxamide, 2	C21H18F3N3O5	449.38	A/B	-9.6
311	[C@@H](Cc1ccccc1)(NC(=O)[C@H](CCN1CCOCC1)NC(=O)N(Cc1nc(C(C)C)sc1)C)CC[C@H](Cc1ccccc1)NC(=O)OCc1cncs1	1627204-38-9	Carbamic acid, N-[(1S)-1-[[(2S)-2-[5-[8-[8-[2-[(2S)-4,4-difluoro-1-[(2S)-2-[(met	C51H60F4N8O6	957.07	A/B	-9.6
312	CC12c3c(c(ccc3c3[nH]c(nc3)[C@H]3N(C(=O)[C@@H](NC(=O)OC)C(C)C)CC(C)(C)C3)c3c4c(c(cc3)c3[nH]c(nc3)[C@H	1627533-42-9	Carbamic acid, N,N,Ä≤-[(1,1,Ä≤,2,2,Ä≤,3,3,Ä≤,4,4,Ä≤-octahydro-9-oxo[5,5,Ä≤-bi-	C50H60N8O7	885.06	A/B	-9.6
313	C[C@]12[C@H]([C@H]3[C@@H]([C@]4(C)[C@H](C[C@H]3O)CCCC4)CC2)CC[C@@H]1[C@@H](CCCOS(=O)(=O)O)C	1632197-40-0	1,3,5-Triazine-2,4-diamine, N2-(9-ethyl-9H-carbazol-3-yl)-6-(4-morpholinyl)-N4-p	C27H27N7O	465.55	A/B	-9.6
314	C(OC[C@@H]1[C@@H](OC(=O)c2ccccc2)[C@@H](OP(=O)(OC[C@@H]2[C@@H](OC(=O)c3ccccc3)[C@@H](OP(=O)(OC[C@H]3	1637769-13-1	Adenosine, 1,Ä≤-[3-(aminocarbonyl)-1H-1,2,4-triazol-1-yl]-3,Ä≤-O-benzoyl-1,Ä≤-d	C47H43N10O15P	1018.88	A/B	-9.6
315	O(P(=O)(OC[C@H]1O[C@H]([C@H](OP(=O)(OC[C@H]2O[C@H]([C@H](O)C2)n2c3c(nc2)c(N)ncn3)O)[C@@H]1O)n1nc(C(=	1637815-91-8	Adenosine, 1,Ä≤-[3-(aminocarbonyl)-1H-1,2,4-triazol-1-yl]-1,Ä≤-de(6-amino-9H-pu	C28H36N14O16P2	886.62	A/B	-9.6
316	O=c1n([C@@H]2O[C@H](COP(=O)(C(O)c3ccc(C)cc3)OC[C@H]3O[C@H](C=C3)n3c(=O)[nH]c(=O)c(C)c3)C=C2)cc(C)c(=	164652-01-1	Thymidine, 2,Ä≤,3,Ä≤-didehydro-P,3,Ä≤-dideoxy-P-[hydroxy(4-methoxyphenyl)methyl	C28H31N4O11P	630.54	A/B	-9.6
317	O=c1n([C@@H]2O[C@H](COP(=O)(C(O)c3ccc(C#N)cc3)OC[C@H]3O[C@H](C=C3)n3c(=O)[nH]c(=O)c(C)c3)C=C2)cc(C)c	164652-04-4	Thymidine, P-[(4-chlorophenyl)hydroxymethyl]-2,Ä≤,3,Ä≤-didehydro-P,3,Ä≤-dideoxy	C27H28ClN4O10P	634.96	A/B	-9.6
318	O=c1n([C@@H]2O[C@H](COP(=O)(C(O)c3c(Cl)cccc3Cl)OC[C@H]3O[C@H](C=C3)n3c(=O)[nH]c(=O)c(C)c3)C=C2)cc(C)	164652-09-9	Thymidine, 2,Ä≤,3,Ä≤-didehydro-P,3,Ä≤-dideoxy-P-(hydroxy-4-pyridinylmethyl)thym	C26H28N5O10P	601.5	A/B	-9.6
319	C(C(C)(C)C)(C1=C(O)CC(CCC)(CCC)OC1=O)c1cc(NS(=O)(=O)c2ccc(C#N)cn2)ccc1	174484-09-4	2-Pyridinesulfonamide, N-[3-[1-[6,6-bis[2-(4-fluorophenyl)ethyl]-5,6-dihydro-4-h	C36H33F2N3O5S	657.73	A/B	-9.6
320	N(C(=O)[C@H](Cc1ccccc1)C[C@@H](CN1[C@H](C(=O)NC(C)(C)C)CN(Cc2cc(C(C=NOC)O)c(=O)[nH]c2)CC1)O)[C@H]1c2	179952-08-0	D-erythro-Pentonamide, 2,3,5-trideoxy-5-[(2S)-4-[(2,3-dihydro-2,3-dihydroxyfuro[	C38H49N5O7	687.83	A/B	-9.6
321	C(OCCOC(=O)C)n1c2c(nc1F)c(=O)nc(NC(=O)C)[nH]2	1811520-72-5	Butanedioic acid, 1-[(3R,5aS,6R,8aS,9R,10S,12R,12aR)-decahydro-3,6,9-trimethyl-3	C41H48O15	780.81	A/B	-9.6
322	O=c1n([C@H]2c3c([C@@H](CO)O2)cccc3)cc(C)c(=O)[nH]1	183239-85-2	D-erythro-Pentonamide, 2,3,5-trideoxy-N-[(1S,2R)-2,3-dihydro-2-hydroxy-1H-inden-	C42H55N5O10		A/B	-9.6
323	C(OC[C@@H]1[C@@H](OC(=O)c2ccccc2)C[C@@H](C1=C)n1c2c(nc1)c(=O)nc(NC(=O)OC(C)(C)C)[nH]2)(c1ccccc1)(c1c	1860842-95-0	3-Pyridinecarboxylic acid, (1S,2R,4S)-4-[2-[[(1,1-dimethylethoxy)carbonyl]amino]	C42H40N6O6	724.8	A/B	-9.6
324	O=C1[C@@H]2[C@@H](c3c([C@H](OC(=O)CCCC[C@H]4SC(SCC4)c4c(Cl)cc(Cl)cc4)[C@H]2CO1)cc1c(c3)OCO1)c1cc(OC)	1875123-92-4	1,3-Dithiane-4-pentanoic acid, 2-(2,3-dichlorophenyl)-, (5R,5aR,8aR,9R)-5,5a,6,8	C37H38Cl2O9S2	761.73	A/B	-9.6
325	C(O[C@@H]1[C@@H](COC(c2ccccc2)(c2ccccc2)c2ccccc2)O[C@H]([C@@H]1O)n1nc(C(=O)N)nc1)(c1ccccc1)(c1ccccc1	1899098-55-5	1H-1,2,4-Triazole-3-carboxamide, 1-[2,3,5-tris-O-(triphenylmethyl)-Œ≤-D-ribofura	C65H54N4O5	971.15	A/B	-9.6
326	O(C(=O)OCC1(C(=O)OC)CCCC1)c1c2n(N([C@H]3N(C2=O)CCOC3)[C@H]2c3c(CSc4c2cccc4)c(F)c(F)cc3)ccc1=O	1985607-41-7	Benzoic acid, 2-[[[[(12aR)-12-[(11S)-7,8-difluoro-6,11-dihydrodibenzo[b,e]thiepi	C33H25F2N3O8S	661.63	A/B	-9.6
327	C(=O)(C(=O)Nc1ccc(OC)cc1)c1c(C23CC4CC(C3)CC(C2)C4)[nH]c2c1cccc2	2014403-80-4	1H-Indole-3-acetamide, Œ±-oxo-2-tricyclo[3.3.1.13,7]dec-1-yl-N-[3-(trifluorometh	C27H25F3N2O2	466.49	A/B	-9.6
328	O=c1n([C@@H]2O[C@H](COC(=O)COc3c(OC)ccc(C(=O)c4c(Cl)ccc(Br)c4)c3)C=C2)cc(C)c(=O)[nH]1	2015224-37-8	Acetic acid, 2-[2-methoxy-5-(4-nitrobenzoyl)phenoxy]-, [(2S,5R)-5-(3,4-dihydro-5	C26H23N3O10	537.47	A/B	-9.6
329	C(OC(=O)c1ccc(CO[C@@H]2O[C@H]3[C@@H]([C@H](O)[C@H]2O)O[C@H](OC3)c2ccccc2)cc1)[C@@H]1[C@@H]2[C@H]([C@	1002336-58-4	1H-1,2,4-Triazole-3-carboxamide, 1-[2,3-O-(methoxymethylene)-5-O-[4-[[4,6-O-[(S)	C30H32N4O13	656.59	C	-9.6
330	N(=Nc1c2c(c(N=Nc3cc4c(c(S(=O)(=O)O)c3)cccc4S(=O)(=O)O)cc1)cccc2)c1c2c(c(N=Nc3c(C)cc(N)c(C)c3)cc1)ccc	10134-25-5	1,5-Naphthalenedisulfonic acid, 3-[2-[4-[2-[4-[2-(4-amino-2,5-dimethylphenyl)dia	C38H29N7O9S3.3Na		C	-9.6
331	N(=N\c1cc(S(=O)(=O)O)c(C=Cc2c(S(=O)(=O)O)cc(/N=N/c3c4c(cc(S(=O)(=O)O)cc4)c(N)cc3)cc2)cc1)/c1c2c(cc(S	1052531-25-5	Not Yet Assigned	C34H26N6O18S6.Na		C	-9.6
332	N(=Nc1cc(N=Nc2cc3c(cc2)ccc(S(=O)(=O)O)c3)ccc1O)c1c2c(ccc1O)cccc2	116028-32-1	2-Naphthalenesulfonic acid, 5-[2-[4-hydroxy-3-[2-(2-hydroxy-1-naphthalenyl)diaze	C26H18N4O5S	498.51	C	-9.6
333	O=c1c2c3c(c(c(F)c2)N2CCN(C)CC2)OC[C@H](C)n3cc1C(=O)OC[C@@H]1[C@@H](N=[N+]=[N-])C[C@@H](O1)n1c(=O)[nH	1174398-26-5	Thymidine, 3,Ä≤-azido-3,Ä≤-deoxy-, 5,Ä≤-[9-fluoro-2,3-dihydro-3-methyl-10-(4-me	C28H31FN8O7		C	-9.6
334	N(=Nc1c(S(=O)(=O)O)cc(C)c(S(=O)(=O)O)c1)c1c2c(c(N=Nc3c4c(ccc(S(=O)(=O)O)c4)c(N)cc3)cc1)ccc(S(=O)(=O)	1185727-95-0	1,3,5-Naphthalenetrisulfonic acid, 7-[2-[4-[2-(4-amino-2-methylphenyl)diazenyl]-	C27H21N5O12S4	735.74	C	-9.6
335	C(F)(F)c1n(c2c(n1)cccc2)c1nc(nc(N[C@H]2CC[C@@H](CC2)NC(=O)N(CCN(C)CC1)n1)N1CCOCC1	1240024-62-7	4-Morpholinecarboxamide, N-[trans-4-[[4-[2-(difluoromethyl)-1H-benzimidazol-1-yl	C26H33F2N9O3	557.6	C	-9.6
336	C(=O)([C@@H](NC(=O)OC)C(C)C)N1C[C@H]([C@H]2C[C@@H]1CC2)c1[nH]c(cn1)c1ccc(cc1)c1ccc(cc1)c1[nH]c(nc1)[C	1273591-34-6	Carbamic acid, N-[(1S)-1-[[(2S)-2-[5-[4,Ä≤-[2-[(1S,3S,4R)-2-[(2S)-2-[(methoxycar	C42H50N8O6	762.9	C	-9.6
337	C(=O)([C@@H](NC(=O)OC)C(C)C)N1[C@@H]2[C@H](C2)C[C@H]1c1[nH]c(c(Br)n1)c1ccc(cc1)c1ccc(cc1)c1[nH]c(nc1)	1312336-43-8	Carbamic acid, N-[(1S)-2-[(1R,3S,5R)-3-[7-[4-[5-chloro-2-[(1R,3S,5R)-2-[(2S)-2-[	C48H53ClN8O8	905.44	C	-9.6
338	O(Cc1ccc(F)cc1)c1c2c(cc(O)c2=O)c2[nH]cnn2)c1cccccc1	1312903-08-4	4H-1-Benzopyran-4-one, 5-[(4-fluorophenyl)methoxy]-3-hydroxy-7-phenyl-2-(5-pheny	C32H21FN2O4	516.52	C	-9.6
339	O=C1[C@@H]2[C@@H](c3c([C@H](OC(=O)C)[C@H](CSSC[C@@H](C(=O)NCC(=O)N[C@@H]4c5c(-c6c(CC4)cc(OC)c(OC)c6OC)	1335096-09-7	Inosine, 8-[[4-(2-benzothiazolyl)phenyl]amino]-	C23H20N6O5S	492.51	C	-9.6
340	N(=Nc1cc(S(=O)(=O)O)ccc1)c1c2c(c(N=Nc3c4c(cc(S(=O)(=O)O)cc4)c(N)cc3)cc1)ccc(S(=O)(=O)O)c2	1345852-30-3	2-Naphthalenesulfonic acid, 8-amino-5-[2-[4-[2-(3-sulfophenyl)diazenyl]-1-naphth	C26H19N5O6S2	561.59	C	-9.6
341	N(=Nc1cc2c(S(=O)(=O)O)ccc2c(N)c1)c1c2c(c(N=Nc3c4cc(S(=O)(=O)O)c4)ccc3)cc1)ccc(S(=O)(=O)O)c2	1345852-36-9	2-Naphthalenesulfonic acid, 8-amino-5-[2-[4-(2-phenyldiazenyl)-7-sulfo-1-naphtha	C26H19N5O6S2	561.59	C	-9.6
342	C(C)(C)c1n(c2[C@@H]3N(CC(NC(=O)C4CCC(F)(F)CC4)c4ccccc4)([2H])[2H])C(C)C3)(	1347417-62-9	[1,1,Ä≤-Biphenyl]-2-acetamide, 4,Ä≤-[(2-ethyl-5,7-dimethyl-3H-imidazo[4,5-b]pyr	C31H30N4O3S	538.66	C	-9.6
343	C(=O)([C@@H](NC(=O)OC)C(C)C)N1C[C@H](c2[nH]c(c3c4c(cc3)c3cc5c(cc3)c3[nH]c(nc3)[C@H]3N(C(=O)[C@@H](	1350718-55-6	Carbamic acid, N,N,Ä≤-[[1,1,Ä≤-binaphthalene]-4,4,Ä≤-diylbis[1H-imidazole-5,2-d	C48H54N8O6	838.99	C	-9.6
344	C(=O)([C@@H](NC(=O)OC)C(C)C)N1[C@H]2[C@H](C2)C[C@H]1c1[nH]c(cn1)c1c2c(c(cc1)c1ccc(cc1)c1[nH]c(nc1)[C	1359868-84-0	Carbamic acid, N-[(1S)-2-[(2S)-2-[5-[4-[4-[2-[(2S)-1-[(2S)-2-[(methoxycarbonyl)a	C48H56N8O8	873.01	C	-9.6
345	O(C)c1c2n(cc(C(=O)O)c(=O)c2cc(F)c1N1C[C@H]2[C@@H](C1)CCCN2C2CCNc2C1=O)C1CC1	1372618-24-0	3-Quinolinecarboxylic acid, 7-[(4aS,7aS)-1-[3-[2-(aminothioxomethyl)hydrazinyli	C31H32FN7O5S	633.69	C	-9.6
346	C(=O)([C@@H](NC(=O)OC)C(C)C)N1CCC2(C[C@H]1c1[nH]c3c(n1)cccc3c1ccc(cc1)c1[nH]c(nc1)[C@H]1N(	1377037-04-1	Carbamic acid, N-[(1S)-2-[(1R,3S,4S)-3-[4,5-dihydro-2,Ä≤-[(2S)-1-[(2S)-2-[(metho	C48H56N8O7	857.01	C	-9.6
347	C(=O)([C@@H](NC(=O)OC)C(C)C)N1[C@@H](Cc2cccc2)C[C@H]1c1[nH]c(cn1)c1ccc(cc1)c1ccc(cc1)c1[nH]c(nc1)[C@H]1N(	1377040-17-9	Carbamic acid, N,N,Ä≤-[[9-(1-methylethylidene)-9H-fluorene-2,7-diyl]bis[1H-imida	C44H54N8O6	790.95	C	-9.6
348	C(=O)([C@@H](NC(=O)OC)C(C)C)N1[C@@H]2C[C@H]1CC2]c1[nH]c2-c3c(CCc2n1)cc(cc3)c1cc2c(cc1)c1ccc2	1377040-99-7	Carbamic acid, N-[(1S)-1-[[(2S)-2-[4,Ä≤5,Ä≤-dihydro-2,Ä≤-[(1R,3S,4S)-2-[(2S)-2-	C46H54N8O6	814.97	C	-9.6
349	O=C1[C@H]2[C@@H](c3c([C@@H](O)[C@H]2CO1)cc1c(c3)OCO1)c1cc(OC)c(OC(=O)OC)c(OC)c1	1389335-43-6	Spiro[2H-indene-2,2,Ä≤-pyrrolidine]-1,3-dione, 3,Ä≤-[(1Z)-3-[1,3-dihydro-3,Ä≤-(	C53H42N2O10	866.91	C	-9.6
350	C(C(C)C)n1c2c3c(cc(C(=O)OC)cc3)nc(N)c2nc1	1426342-68-8	Benzoic acid, 2,2,Ä≤-[2,7-naphthalenediylbis(carbonylimino)]bis-	C26H18N2O6	454.43	C	-9.6

351	N(C(CCCN(CC)CC)C)c1c2c(nc(c1)C1CCCCC1)cc(Cl)cc2	153597-34-3	1,3,6-Naphthalenetrisulfonic acid, 7-[2-[4-[2-(4-amino-2-methylphenyl)diazenyl]-	C27H21N5O12S4	735.74	C	-9.6
352	CN1C2c3c(c(ccc3c3[nH]c(nc3)[C@H]3N(C(=O)[C@@H](NC(=O)OC)C(C)C)CCC3)c3ccc(cc3)c3[nH]c(nc3)[C@H]3N(C(=	1560639-67-9	Carbamic acid, N-[(1S)-2-methyl-1-[[(1R,3S,4S)-3-[5-[4-[1,2,3,4-tetrahydro-8-[2-	C49H61N9O6	872.07	C	-9.6
353	C(=O)([C@@H](NC(=O)OC)C(C)C)N1[C@H](c2[nH]c(c3c4c(C5OC4CC5)c(cc3)c3ccc(cc3)c3[nH]c(nc3)[C@H]3N(C(=O)	1560640-94-9	Carbamic acid, N-[(1S)-1-[[(2S,4R)-4-fluoro-2-[5-[8-[4-[2-[(2S,4R)-4-fluoro-1-[(	C44H52F2N8O7	842.93	C	-9.6
354	C(=O)([C@@H](NC(=O)OC)C(C)C)N1[C@H](c2[nH]c(c3c4c(C5(CC4)CCOC5)c(cc3)c3ccc(cc3)c3[nH]c(nc3)[C@H]3N(C	1588404-26-5	Carbamic acid, N-[(1S)-2-methyl-1-[[(2S)-2-[5-[3,Ä≤,4,4,Ä≤,5-tetrahydro-8,Ä≤-[4	C47H60N8O7	849.03	C	-9.6
355	C(=O)([C@@H](NC(=O)OC)C(C)C)N1[C@@H](C[C@@H](OC(=O)N2Cc3c(C2)c(F)ccc3)C1)c1[nH]c(cn1)c1ccc(cc1)c1ccc	163980-26-5	2,7-Naphthalenedisulfonic acid, 4,4,Ä≤-[1,2-ethenediylbis[(3-sulfo-4,1-phenylene	C34H26N6O18S6.6Na		C	-9.6
356	C(=O)([C@@H](NC(=O)OC)C(C)C)N1C[C@]2(C[C@H]1c1[nH]c(cn1)c1ccc(cc1)c1cc3c(cc1)c1c(cc3)nc([nH]1)[C@H]1	1653966-90-5	Carbamic acid, N-[(1S)-1-[[(2S)-2-[7-[4-[2-[(5R,8S)-7-[(2S)-2-[(methoxycarbonyl)	C45H54N8O7	818.96	C	-9.6
357	O=c1n([C@@H]2O[C@H](COP(=O)(O)O)[C@@H](O)C2)cc(Cl)c(N)n1	165967-23-7	Guanosine, 2,Ä≤-deoxy-P-thiocytidylyl-(3,Ä≤,Ü15,Ä≤)-2,Ä≤-deoxy-P-thiocytidylyl-	C28H37N11O14P2S2	877.74	C	-9.6
358	O=C1[C@@H]2[C@@H](c3c([C@H](OC(=O)Oc4ccc(N(=O)=O)cc4)[C@H]2CO1)cc1c(c3)OCO1)c1cc(OC)c(OC)c(OC)c1	1680211-74-8	1-Piperidinecarboxylic acid, 4-[2-[3-[2-amino-6-[(ethylamino)carbonyl]thieno[2,3	C45H47N5O11S	865.95	C	-9.6
359	O=C1[C@]2([C@H]([C@H](/C(=C/C(=O)[C@@H]3[C@@]4(N5[C@@H]([C@H]3c3cc(OC)c(O)cc3)CCC5)c3c(NC4=O)cccc3)/	1807888-22-7	Not Yet Assigned	C45H44N4O8	768.85	C	-9.6
360	N(C(C)C)c1c([nH]c(=O)cc1)N1CCN(C(=O)c2cc3c([nH]2)ccc(NS(=O)(=O)C)c3)CC1	188780-42-9	Methanesulfonamide, N-[2-[[4-[3-amino-6-(sulfooxy)-2-pyridinyl]-1-piperazinyl]ca	C19H22N6O7S2	510.54	C	-9.6
361	N(C(=O)C1CN(C)CCO1)C12CC3(O)CC(C2)CC(C1)C3	195063-36-6	2,7-Naphthalenedisulfonic acid, 4,4,Ä≤-[(3,3,Ä≤-dimethyl[1,1,Ä≤-biphenyl]-4,4,Ä	C34H26N4O14S4.4Na		C	-9.6
362	O(COC(=O)N1[C@H](C(=O)OC)CCC1)c1c2n(N([C@H]3N(C2=O)CCOC3)[C@H]2c3c(CSc4c2cccc4)c(F)c(F)cc3)ccc1=O	1985606-90-3	1,2-Pyrrolidinedicarboxylic acid, 1-[(12aR)-12-[(11S)-7,8-difluoro-6,11-dihydro	C31H28F2N4O7S	638.64	C	-9.6
363	O(C(=O)c1ccc(N(C)C)cc1)c1c2n(N([C@H]3N(C2=O)CCOC3)[C@H]2c3c(CSc4c2cccc4)c(F)c(F)cc3)ccc1=O	1985607-32-6	1H-[1,4]Oxazino[3,4-c]pyrido[2,1-f][1,2,4]triazine-6,8-dione, 12-[(11S)-7,8-difl	C32H27F2N3O5S	603.64	C	-9.6
364	O=C1c2n(N([C@H]3N1C[C@H](C)OC3)[C@H]1c3c(CSc4c1cccc4)c(F)c(F)cc3)ccc(=O)c2O	1985609-82-2	1H-[1,4]Oxazino[3,4-c]pyrido[2,1-f][1,2,4]triazine-6,8-dione, 12-[(11S)-8-fluoro	C25H22FN3O4S	479.52	C	-9.6
365	C(=O)([C@@H](NC(=O)OC)C(C)C)N1[C@H](c2[nH]c3c4c(cc(cc4)c4c5c(cc4)c4[nH]c(nc4)[C@H]4N(C(=O)[C@@H](N	2007900-40-3	Carbamic acid, N-[(1S)-1-[[(2S)-2-[7-[2,3-dihydro-7-[2-[(2S)-1-[(2S)-2-[(methoxy	C45H54N8O6	802.96	C	-9.6
366	S(=O)(=O)(O)c1c2c(c(N)c(N=Nc3ccc(cc3)c3ccc(cc3)c3ccc(N=Nc4c(N)c5c(c(S(=O)(=O)O)c4)cccc5)cc3)c1)cccc2	100043-33-2	1-Naphthalenesulfonic acid, 4-[(4-hydroxy-3,5-dimethylphenyl)azo]-, ion(2-), (E)	C18H14N2O4S		A/B	-9.5
367	C(OC(=O)c1ccc(CO[C@@H]2O[C@H]3[C@@H]([C@H](O)[C@H]2O)O[C@H](OC3)c2ccccc2)cc1)[C@@H]1[C@@H]2[C@H]([C@	1002336-58-4	1H-1,2,4-Triazole-3-carboxamide, 1-[2,3-O-(methoxymethylene)-5-O-[4-[[4,6-O-[(S)	C30H32N4O13	656.59	A/B	-9.5
368	[C@H](Cc1ccccc1)(CC[C@H](Cc1ccccc1)NC(=O)OCc1cncs1)NC(=O)[C@@H](NC(=O)N(Cc1cncs1)C)CCO	1004317-55-8	2,7,10,12-Tetraazatridecanoic acid, 9-(1H-imidazol-5-ylmethyl)-12-methyl-13-[2-(	C38H46N8O4S2	742.95	A/B	-9.5
369	O(C(=O)OCCOCCOC)[C@H]1[C@@H](O[C@H](CO)[C@H]1O)n1nc(C(=O)N)nc1	1053697-88-3	Not Yet Assigned	C34H46F3N5O7	693.75	A/B	-9.5
370	O=C1[C@@H]2[C@@H](c3c([C@H](OC(=O)C(=O)c4c5c(n(Cc6cc7c(cc6)OCO7)c4)cccc5)[C@H]2CO1)cc1c(c3)OCO1)c1cc	1061717-07-4	1H-Indole-3-acetic acid, 1-[(3,4-dichlorophenyl)methyl]-Œ±-oxo-, (5R,5aR,8aR,9R)	C39H31Cl2NO10	744.57	A/B	-9.5
371	C(OCCOC(=O)[C@H](CC(=O)O)N)n1c2c(nc1)c(=O)nc(N)[nH]2	108692-91-7	Furo[3,Ä≤,4,Ä≤:6,7]naphtho[2,3-d]-1,3-dioxole-6,9-dione, 5-[4-(acetyloxy)phenyl	C21H16O7	380.35	A/B	-9.5
372	O([C@H]1[C@@H](O[C@H](CO)[C@H]1O)n1cc(CC)c(=O)[nH]c1=O)[C@H]1[C@@H](O[C@H](CO)[C@H]1O)n1cc(CC)c(=O)[	112714-85-9	[1,1,Ä≤-Biphenyl]-4,4,Ä≤-disulfonic acid, 2,2,Ä≤-bis[2-(2-hydroxy-1-naphthaleny	C32H22N4O8S2.2Na		A/B	-9.5
373	O[C@H]1[C@H](n2c3c(nc2)c(=O)nc(N)[nH]3)O[C@H](COP(=O)(OCCN(C)c2ccc(C=O)cc2)O)[C@H]1O	1132935-63-7	Methanesulfonamide, N-[6-[5-(3,4-dihydro-2,4-dioxo-1(2H)-pyrimidinyl)-3-(1,1-dim	C26H27N3O5S	493.57	A/B	-9.5
374	N(=C\1/c2c(N(C)C1=O)ccc(S(=O)(=O)N1CCOCC1)c2)\NC(=S)Nc1cc(C)cc(C)c1	1164492-63-0	Hydrazinecarbothioamide, 2-[1,2-dihydro-1-methyl-5-(4-morpholinylsulfonyl)-2-oxo	C22H25N5O4S2	487.6	A/B	-9.5
375	O=C1N2c3c(/C/1=N/NC(=S)N)c(C)ccc3C(=CC2(C)C)C	1164493-89-3	Hydrazinecarbothioamide, 2-[1,2-dihydro-1-methyl-5-(4-morpholinylsulfonyl)-2-oxo	C21H23N5O4S2	473.57	A/B	-9.5
376	Nc1ncc(c2nc(n3c4c(cn3)CCCC4)nc(n2)N2CCOCC2)cn1	1174191-79-7	2-Pyridinamine, 5-[4-(4-morpholinyl)-6-(4,5,6,7-tetrahydro-1H-indazol-1-yl)-1,3,	C19H22N8O	378.43	A/B	-9.5
377	O=c1c2c(n(cc1C(=O)OC[C@@H]1[C@@H](N=[N+]=[N-])C[C@@H](O1)n1c(=O)[nH]c(=O)c(C)c1)C1CC1)cc(F)c2)N1CC	1174398-20-9	Thymidine, 3,Ä≤-azido-3,Ä≤-deoxy-, 5,Ä≤-[1-ethyl-6-fluoro-1,4-dihydro-4-oxo-7-[	C34H35FN8O8		A/B	-9.5
378	O(C)c1c2n(cc(C(=O)OC[C@@H]3[C@@H](N=[N+]=[N-])C[C@@H](O3)n3c(=O)[nH]c(=O)c(C)c3)c(=O)c2cc(F)c1N1CC(C	1174398-22-1	Thymidine, 3,Ä≤-azido-3,Ä≤-deoxy-, 5,Ä≤-[1-ethyl-6,8-difluoro-1,4-dihydro-7-[3-	C35H36F2N8O8		A/B	-9.5
379	Cc1n(nc(C(=O)N)n1)[C@@H]1O[C@H](CO)[C@@H](O)C1	1174716-28-9	6H-Purin-6-one, 2-amino-9-[(1S,3R,4S)-3-[[[(4S)-4-(3-chlorophenyl)-2-oxido-1,3,2	C21H23ClN5O6P	507.86	A/B	-9.5
380	C([C@H](OP(=O)(O)O)CO)n1c2c(nc1)c(N)ncn2	1222138-67-1	Not Yet Assigned	C36H45ClFN5O4	666.22	A/B	-9.5
381	C(N(C(=O)N[C@@H](C(=O)N[C@@H](Cc1ccccc1)C[C@@H]([C@H](Cc1ccccc1)NC(=O)OCc1cncs1)O)C(C)C)C)c1nc(sc1)c	1236406-31-7	Not Yet Assigned	C40H45FN6O6S	756.89	A/B	-9.5
382	S(=O)(=O)(N(C[C@H]([C@H](Cc1ccccc1)NC(=O)O[C@H]1CCOC1)O)CC(C)C)c1cc2c(cc1)OCC2	1237809-07-2	Not Yet Assigned	C37H46N6O5	654.8	A/B	-9.5
383	c1(nc(nc(n1)N1CCOCC1)N1Cc2c(CC1)cccc2)N1Cc2c(CC1)cccc2	1239884-86-6	Quinoline, 1,1,Ä≤-[6-(4-morpholinyl)-1,3,5-triazine-2,4-diyl]bis[1,2,3,4-tetrahy	C25H28N6O	428.53	A/B	-9.5
384	C(=O)(Nc1sc(N(=O)=O)cn1)c1c(OC(=O)c2ccccc2)cccc1	1259403-50-3	Adenosine, 5,Ä≤-(2-amino-2-oxoethyl)-5,Ä≤-deoxy-3,Ä≤-O-methyladenylyl-(2,Ä≤,Üí5	C32H43N15O17P2	971.72	A/B	-9.5
385	O(Cc1ccc(F)cc1)c1c2c(c(c(C)c1)c1ccccc1)oc(c(O)c2=O)c1[nH]ccn1	1312896-97-1	4H-1-Benzopyran-4-one, 5-[(4-fluorophenyl)methoxy]-3-hydroxy-7-methyl-8-phenyl-2	C31H22FN3O4	519.52	A/B	-9.5
386	O(Cc1ccc(F)cc1)c1c2c(c(OC)c(Cl)c1)oc(c(O)c2=O)c1ncccn1	1312901-23-7	4H-1-Benzopyran-4-one, 7-chloro-5-[(4-fluorophenyl)methoxy]-3-hydroxy-8-methyl-2	C26H18ClFN2O4	476.88	A/B	-9.5
387	Cc1c2c(cc(OCc3ccc(F)cc3)c1)c(=O)c(O)c(o2)c1ncccn1	1312906-33-4	4H-1-Benzopyran-4-one, 6-[(4-fluorophenyl)methoxy]-3-hydroxy-2-(5-phenyl-1H-imid	C25H17FN2O4	428.41	A/B	-9.5
388	O=c1c2c(c(cc(OCc3ccc(F)cc3)c2)c2ccccc2)oc(c1O)c1[nH]ccn1	1312908-28-3	4H-1-Benzopyran-4-one, 6-[(4-fluorophenyl)methoxy]-3-hydroxy-8-phenyl-2-(3-pheny	C30H20FN3O4	505.5	A/B	-9.5
389	O=c1c2c(oc(c1O)c1[nH]ccn1)cc(C)c(OCc1ccc(F)cc1)c2	1312908-78-3	4H-1-Benzopyran-4-one, 6-[(4-fluorophenyl)methoxy]-3-hydroxy-7-methyl-2-(3-pheny	C25H18FN3O4	443.43	A/B	-9.5
390	Cc1c(c2c(cc1OCc1ccc(F)cc1)c(=O)c(O)c(o2)c1ncccn1)c1ccccc1	1312910-45-4	4H-1-Benzopyran-4-one, 8-chloro-6-[(4-fluorophenyl)methoxy]-3-hydroxy-7-methyl-2	C26H18ClFN2O4	476.88	A/B	-9.5
391	O=c1c2c(cc(OCc3ccc(F)cc3)c2)c2ccccc2)oc(c1O)c1ncccn1	1312915-98-2	4H-1-Benzopyran-4-one, 7-chloro-6-[(4-fluorophenyl)methoxy]-3-hydroxy-8-phenyl-2	C31H20ClFN2O4	538.95	A/B	-9.5
392	C(C)(C)(C)c1c2c(c(F)c(C(C)(C)C)c1)COP(=O)(O[C@@H]1[C@@H](COP3(=O)Oc4c(CO3)c(F)c(C(C)(C)C)cc4C(C)(C)C	1313031-59-2	Uridine, 2,Ä≤-deoxy-5-iodo-3,Ä≤,5,Ä≤-bis-O-(8-methyl-2-oxido-4H-1,3,2-benzodiox	C25H25IN2O11P2	718.32	A/B	-9.5
393	N(=Nc1cc(S(=O)(=O)O)ccc1)c1c2c(c(N=Nc3c4c(ccc(S(=O)(=O)O)c4)c(N)cc3)cc1)ccc(S(=O)(=O)O)c2	1345852-50-7	2-Naphthalenesulfonic acid, 5-[2-(4-amino-6-sulfo-1-naphthalenyl)diazenyl]-8-[2-	C26H19N5O9S3	641.65	A/B	-9.5
394	C(OC(CO)CO)n1c2c(nc1)C(=O)NC(N)N2	1347883-56-0	Carbamic acid, N-[(1S,2R)-2-hydroxy-3-[[[4-[[(4-hydroxyphenyl)nitrophosphinyl]me	C34H41N4O14PS	792.75	A/B	-9.5
395	O=c1n([C@@H]2O[C@H](CO)SC2)cc(C)c(N)n1	137622-85-6	Butanediamide, N1-[(1S,2S)-3-[(3S,4aS,8aS)-3-[[(1,1-dimethylethyl)amino]carbonyl	C38H50N6O5	670.84	A/B	-9.5
396	C(=O)([C@@H](NC(=O)OC)C1CCOCC1)N1[C@@H]([C@@H]2C[C@H]1CC2)c1[nH]c(cn1)c1cc2c(-c3c(CC2)cc(cc3)c2[nH]c	1377036-52-6	Carbamic acid, N-[(1S)-1-[[(2S)-2-[2,Ä≤-[(1R,3S,4S)-2-[(2S)-2-[(methoxycarbonyl)	C46H52N8O6	812.96	A/B	-9.5
397	O(C(=O)NC(C(CN(S(=O)(=O)c1ccc(OC)cc1)CCCP(=O)(O)O)O)Cc1ccccc1)C1C2C(OC1)OCC2	1383434-59-0	Carbamic acid, N-[(1S,2R)-1-[[4-[[2-[bis(phenylmethoxy)phosphinyl]ethyl]amino]ph	C44H56N3O11PS	865.97	A/B	-9.5
398	C(=O)([C@@H](NC(=O)OC)C(C)C)N1[C@H](c2[nH]c(c(n2)c2ccco2)c2ccc(cc2)c2ccc(cc2)c2c(nc([nH]2)[C@H]2N(C(	1388224-09-6	Carbamic acid, N,N,Ä≤-[[1,1,Ä≤-biphenyl]-4,4,Ä≤-diylbis[(4-phenyl-1H-imidazole-	C52H58N8O6	891.07	A/B	-9.5
399	O=C1[C@@H]2[C@@H](c3c([C@@H](O)[C@H]2CO1)cc1c(c3)OCO1)c1cc(OC)c(OC(=O)OC)c(OC)c1	1389335-43-6	Spiro[2H-indene-2,2,Ä≤-pyrrolidine]-1,3-dione, 3,Ä≤-[(1Z)-3-[1,3-dihydro-3,Ä≤-(	C53H42N2O10	866.91	A/B	-9.5
400	Nc1nc(c2c3c([nH]nc3)ccc2)nc(n1)N1CCOCC1	1391925-97-5	2-Piperazinone, 4-[4-(1H-indazol-4-yl)-6-(4-morpholinyl)-1,3,5-triazin-2-yl]-	C18H20N8O2	380.4	A/B	-9.5
401	N(c1nc(nc(NCCNC(=O)c2ccc(cc2)C2OOC(OO2)c2ccc(C)cc2)n1)N1CCOCC1)c1c(C)cccc1	1393922-44-5	Benzamide, N-[2-[[4-[(4-methylphenyl)amino]-6-(4-morpholinyl)-1,3,5-triazin-2-yl	C32H35N7O6	613.66	A/B	-9.5
402	C(#N)[C@@H]1[C@@H](O[C@H](CO)[C@H]1O)n1c(=O)[nH]c(=O)c(C)c1	1408308-05-3	4-Pyrimidinecarboxamide, 1,6-dihydro-5-hydroxy-6-oxo-2-(2-quinolinyl)-N-(1-tricy	C26H28N4O3	444.53	A/B	-9.5
403	C(=O)([C@@H](NC(=O)OC)C(C)C)N1[C@@H](CC2C1CCCC2)c1[nH]c(cn1)c1c2cc(c(c1)CCc1ccc(CC2)cc1)c1ccc(cc1)c1	1415120-49-8	Carbamic acid, N,N,Ä≤-[tricyclo[8.2.2.24,7]hexadeca-4,6,10,12,13,15-hexaene-5,11	C56H64N8O6	945.16	A/B	-9.5
404	C(=O)([C@@H](NC(=O)OC)C(C)C)N1[C@H](c2[nH]c(c3c4c(c(cc3)c3ccc(cc3)c3[nH]c(nc3)[C@H]3N(C(=O)[C@@H](NC	1419384-78-3	Carbamic acid, N-[(1S)-1-[[(2S)-2-[5-[1,Ä≤,1,Ä≤-difluoro-1,Ä≤,3,Ä≤-dihydro-7,Ä≤	C47H58F2N8O6	869.01	A/B	-9.5
405	C(=O)([C@@H](NC(=O)OC)C(C)C)N1[C@H](c2[nH]c(c3c4c(c(cc3)c3ccc(cc3)c3[nH]c(nc3)[C@H]3N(C(=O)[C@@H](NC	1419385-21-9	Carbamic acid, N-[(1S)-1-[[(2S,4R)-4-fluoro-2-[5-[4-[7,Ä≤-[2-[(2S,4R)-4-fluoro-1	C47H58F2N8O6	869.01	A/B	-9.5
406	C(=O)([C@@H](NC(=O)OC)C(C)C)N1[C@H](c2[nH]c(c3ccc(c4c5CC6(Cc5cc4)c4ccc(cc4)c4[nH]c(nc4)[C@H]4N(C(=	1419385-37-7	Carbamic acid, N-[(1S)-1-[[(2S)-2-[5-[4-[1,Ä≤,3,Ä≤-dihydro-7,Ä≤-[2-[(2S)-1-[(2S	C47H58N8O7	847.01	A/B	-9.5
407	C(OC[C@@H]1[C@@H](OC(=O)c2ccccc2)[C@@H](OP(=O)(OC[C@@H]2[C@@H](OC(=O)c3ccccc3)[C@@H](OP(=O)(OC[C@@H]	1422459-38-8	Adenosine, 1,Ä≤-[3-(aminocarbonyl)-1H-1,2,4-triazol-1-yl]-3,Ä≤-O-benzoyl-1,Ä≤-d	C60H53N10O15P	1185.09	A/B	-9.5
408	C(OC[C@@H]1[C@@H](OC(=O)c2ccccc2)[C@@H](OP(=O)(OC[C@@H]2[C@@H](OC(=O)c3ccccc3)[C@@H](OP(=O)(OC[C@@H]	1422459-42-4	Adenosine, 1,Ä≤-[3-(aminocarbonyl)-1H-1,2,4-triazol-1-yl]-3,Ä≤-O-benzoyl-1,Ä≤-d	C92H80N16O25P2	1871.66	A/B	-9.5
409	O(C)c1c2[C@@H]([C@H]3[C@@H](Cc2cc(OC)c1OC)COC3=O)c1cc2c(cc1)OCO2	1422529-90-5	2-Pyridinesulfonamide, 5-cyano-N-[3-[(1R)-1-[(6S)-5,6-dihydro-4-hydroxy-2-oxo-6-	C33H37N3O5S	587.73	A/B	-9.5
410	N(Cc1ccc(C#N)cc1)(c1nc(nc(C#N)n1)N1CCOCC1)C1CCCCC1	1443830-71-4	1,3,5-Triazine-2-carbonitrile, 4-[cyclohexyl[[4-(trifluoromethyl)phenyl]methyl]a	C22H25F3N6O	446.47	A/B	-9.5
411	N(C(C)C)c1c(nccc1)N1CCN(C(=O)c2cc3c([nH]2)ccc(NS(=O)(=O)CCN)c3)CC1	147540-05-4	2H-Isoindole-2-ethanesulfonamide, 1,3-dihydro-N-[2-[[4-[3-[(1-methylethyl)amino]	C31H33N7O5S	615.7	A/B	-9.5
412	C(C#C)(N)C12CC3CC(C2)CC(C1)C3	148313-04-6	Carbamic acid, [9-[2-O-[[2-(4-nitrophenyl)ethoxy]carbonyl]-Œ≤-D-arabinofuranosyl	C28H27N7O12	653.55	A/B	-9.5
413	O[C@H]1[C@H](n2c3c(nc2)c(=O)nc(N)[nH]3)O[C@H](CO)[C@H]1N[Si](NC[C@H]1O[C@H](C[C@@H]1O)n1c2c(nc1)c(=O	1513874-15-1	Guanosine, 3,Ä≤-O-diphosphinicoguanylylimino-(3,Ä≤,Üí5,Ä≤)-2,Ä≤,5,Ä≤-dideoxy-	C20H25N11O8	547.48	A/B	-9.5
414	O[C@H]1[C@H](n2c3c(nc2)c(=O)nc(N)[nH]3)O[C@H](CO)[C@H]1NS(=O)(=O)NC[C@H]1O[C@H]([C@H](O)[C@@H]1O)n1c	1513874-58-2	Guanosine, 2,Ä≤-deoxy-3,Ä≤-de(phosphinicooxy)guanylyliminosulfonylimino-(3,Ä≤,Ü	C20H26N12O8S	594.56	A/B	-9.5
415	C(=O)([C@@H](NC(=O)OC)C(C)C)N1[C@H](c2[nH]c(c3c4c(C5CC4CC5)c(cc3)c3ccc(cc3)c3[nH]c(nc3)[C@H]3N(C(=O)	1560639-05-5	Carbamic acid, N-[(1S)-1-cyclohexyl-2-[(2S)-2-[5-[8-[4-[2-[(2S)-1-[(2S)-2-cycloh	C51H64N8O6	885.1	A/B	-9.5
416	CN1C2c3c(C1CC2)c(ccc3c1[nH]c(nc1)[C@H]1N(C(=O)[C@@H](NC(=O)OC)C(C)C)CC(C)(C)C1)c1ccc(cc1)c1[nH]c(nc1	1560639-55-5	Carbamic acid, N-[(1S)-1-[[(2S,4R)-4-fluoro-2-[5-[8-[4-[2-[(2S,4R)-4-fluoro-1-[(	C45H55F2N9O6	855.97	A/B	-9.5
417	C(=O)([C@@H](NC(=O)OC)C(C)C)N1[C@H](c2[nH]c(c3c4c(C5OC4CC5)c(cc3)c3ccc(cc3)c3[nH]c(nc3)[C@H]3N(C(=O)	1560640-57-4	Carbamic acid, N,N,Ä≤-[(1,4-dihydro-1,4-ethanonaphthalene-5,8-diyl)bis[4,1-pheny	C52H60N8O6	893.08	A/B	-9.5
418	N(c1c2c(c(O)c(CN3CCN(CC)CC3)c1)CCCC2)c1c2c(ncc1)cc(Cl)cc2	156893-82-2	1-Naphthalenol, 4-[(7-chloro-4-quinolinyl)amino]-5,6,7,8-tetrahydro-2-[(4-methyl	C25H29ClN4O	436.98	A/B	-9.5
419	C(/C=C\CCCC(=O)OC(C)C)[C@@H]1[C@@H](/C=C/[C@H](COc2cc(C(F)(F)F)ccc2)O)[C@H](O)C[C@@H]1O	157263-00-8	Benzamide, N-[[4,Ä≤-[(2-ethyl-5,7-dimethyl-3H-imidazo[4,5-b]pyridin-3-yl)methyl]	C30H28N4O3S	524.63	A/B	-9.5
420	C(OCO)n1c2c(nc1)c(=O)nc(N)[nH]2	160729-91-9	D-erythro-Pentonamide, 2,3,5-trideoxy-N-[(1S,2R)-2,3-dihydro-2-hydroxy-1H-inden-	C38H47N5O5	653.81	A/B	-9.5
421	C(O[C@@H]1CCP(=O)(O)OC1)n1c2c(nc1)c(=O)nc(N)[nH]2	1607795-41-4	Urea, N,N,Ä≤,Ä≤-1,4-phenylenebis[N,Ä≤-(1-tricyclo[3.3.1.13,7]dec-1-ylethyl)-	C32H46N4O2	518.73	A/B	-9.5
422	O=C1N2[C@H](Cn3c1c(O)c(=O)c(C(=O)NCc1c(F)cc(F)cc1)c3)O[C@@H]1C[C@H]2C[C@@H]1C	1616339-98-0	2,5-Methanopyrido[1,Ä≤,2,Ä≤:4,5]pyrazino[2,1-b][1,3]oxazepine-10-carboxamide, N	C23H21F2N3O5	457.43	A/B	-9.5
423	O=C1N2[C@@H](Cn3c1c(O)c(=O)c(C(=O)NCc1c(F)cc(F)c(Cl)c1)c3)O[C@@H]1C[C@@H]2CC1	1616340-30-7	2,5-Methanopyrido[1,Ä≤,2,Ä≤:4,5]pyrazino[2,1-b][1,3]oxazepine-10-carboxamide, 2	C21H18F3N3O5	449.38	A/B	-9.5
424	C=C1[C@@H](n2c3c(nc2)c(OC)nc(N)n3)C[C@H](O)[C@H]1CO	1638769-65-9	Phosphoric acid, [(1R,3S,5S)-3-(2-amino-1,6-dihydro-6-oxo-9H-purin-9-yl)-5-hydro	C26H24N5O10P	597.47	A/B	-9.5
425	S(c1nc(nc(Nc2ccc(F)cc2)n1)N1CCOCC1)c1oc(nn1)c1ccc(F)cc1	1642584-18-6	1,3,5-Triazin-2-amine, 4-[[5-(4-fluorophenyl)-1,3,4-oxadiazol-2-yl]thio]-N-(4-me	C22H20FN7O2S	465.5	A/B	-9.5
426	C(=O)([C@@H](NC(=O)OC)C(C)C)N1C[C@]2(C[C@H]1c1[nH]c(cn1)c1ccc(cc1)c1cc3c(cc1)c1c(cc3)nc([nH]1)[C@H]1	1653967-12-4	Carbamic acid, N-[(1S)-1-[[(5S,8S)-8-[7-[4-[2-[(5S,8S)-7-[(2S)-2-[(methoxycarbor	C48H58N8O8	875.02	A/B	-9.5
427	C[C@]12[C@H]([C@H]3[C@@H]([C@]4(C)C(=CC(=O)C=C4)CC3)[C@@H](O)C2)CC(C#N)[C@@H]1C(=O)OC[C@@H]1[C@H](N	168394-48-7	Butanediamide, N1-[3-[3-[[(1,1-dimethylethyl)amino]carbonyl]octahydro-2(1H)-isoq	C38H50N6O5S	702.91	A/B	-9.5
428	S(=O)(=O)(O)c1c2c(c(N)c(N=Nc3ccc(N=Nc4c(N)c5c(c(S(=O)(=O)O)c4)cccc5)cc3)c1)cccc2	17094-98-3	1-Naphthalenesulfonic acid, 3,3,Ä≤-[4,4,Ä≤-biphenylylenebis(sulfonyl-p-phenylen	C44H32N6O10S4	933.02	A/B	-9.5
429	S(=O)(=O)(O)c1c2c(c(N)c(N=Nc3c(C)cc(cc3)c3cc(C)c(N=Nc4c(N)c5c(c(S(=O)(=O)O)c4)cccc5)cc3)c1)cccc2	173291-37-7	Guanosine, 2,Ä≤-deoxyguanylyl-(3,Ä≤,Üí5,Ä≤)-2,Ä≤-O-methyl-	C21H27N10O11P	626.47	A/B	-9.5
430	C(Cc1ccc(F)cc1)C1(CCc2ccc(F)cc2)CC(=C(C(C(C)(C)C)c2cc(NS(=O)(=O)c3ccc(C#N)cn3)ccc2)C(=O)O1)O	174484-05-0	2-Pyridinesulfonamide, 5-cyano-N-[3-[1-[6-[2-(4-fluorophenyl)ethyl]-5,6-dihydro-	C31H32FN3O5S	577.67	A/B	-9.5
431	C(Cc1ccccc1)C1(CCC)CC(=C(C(C(C)(C)C)c2cc(NS(=O)(=O)c3ccc(N)cn3)ccc2)C(=O)O1)O	174484-42-5	2-Pyridinesulfonamide, N-[3-[(1S)-1-[(6S)-5,6-dihydro-4-hydroxy-2-oxo-6-(2-pheny	C31H33F3N2O5S	602.66	A/B	-9.5
432	C(Cc1ccccc1)[C@]1(CCC)CC(=C([C@H](CC)c2cc(NS(=O)(=O)c3ccc(C#N)cn3)ccc2)C(=O)O1)O	174590-27-3	2-Pyridinesulfonamide, N-[3-[(1S)-1-[(6R)-5,6-dihydro-4-hydroxy-2-oxo-6-(2-pheny	C31H33F3N2O5S	602.66	A/B	-9.5
433	O=c1n([C@@H]2O[C@H](COP(=O)(OP(=O)(OP(=O)(OCc3ccc(OC(=O)c4ccc(C)cc4)cc3)OCc3ccc(OC(=O)C)cc3)O)O)C=C2	1883328-99-1	Benzoic acid, 4-(trifluoromethyl)-, 4-[3-[[4-(acetyloxy)phenyl]methoxy]-9-[(2S,5	C34H32F3N2O17P3	890.54	A/B	-9.5
434	C(OC[C@@H]1[C@@H](OC(=O)c2ccccc2)[C@@H](OP(=O)(OC[C@@H]2[C@@H](OC(=O)c3ccccc3)[C@@H](OP(=O)(OC[C@@H]	190141-44-7	Adenosine, adenylyl-(2,Ä≤,Üí5,Ä≤)-1,Ä≤-[3-(aminocarbonyl)-1H-1,2,4-triazol-1-yl	C28H36N14O17P2	902.62	A/B	-9.5
435	O[C@@H]1[C@H](n2c3c(nc2)c(NC(=O)/C=C(/C=C/C=C(/C=C/C2=C(C)CCCC2(C)C)\C)\C)ncn3)O[C@H]2[C@H]1O[Si](C(	1971078-28-0	2-Pyridinamine, 5-[4,6-bis[(2R,6S)-2,6-dimethyl-4-morpholinyl]-1,3,5-triazin-2-y	C21H28F3N7O2	467.49	A/B	-9.5
436	O=c1n([C@@H]2O[C@H](COC(=O)COc3c(OC)ccc(C(=O)c4c(OC)cccc4)c3)C=C2)cc(C)c(=O)[nH]1	2015224-38-9	Acetic acid, 2-[5-(5-bromo-2-chlorobenzoyl)-2-methoxyphenoxy]-, [(2S,5R)-5-(3,4-	C26H22BrClN2O8	605.82	A/B	-9.5
437	C(=O)([C@H](NC(=O)OC)c1ccccc1)N1[C@@H](CCC1)c1[nH]c(cn1)c1cc(C)c(cc1)c1ccc(cc1)c1[nH]c(nc1)[C@H]1N(C	1009113-19-2	Carbamic acid, N,N,Ä≤-[(2,2,Ä≤-dimethyl[1,1,Ä≤-biphenyl]-4,4,Ä≤-diyl)bis[1H-imi	C48H50N8O6	834.96	C	-9.5
438	C(=O)([C@@H](NC(=O)OC)CCN(C)C)N1[C@@H](CCC1)c1[nH]c(cn1)c1ccc(cc1)c1ccc(cc1)c1[nH]c(nc1)[C@H]1N(C(=O	1009115-33-6	Carbamic acid, N,N,Ä≤-[[1,1,Ä≤-biphenyl]-4,4,Ä≤-diylbis[1H-imidazole-5,2-diyl-(	C48H50N8O6	834.96	C	-9.5
439	[C@H](Cc1ccccc1)(C[C@@H]([C@H](Cc1ccccc1)NC(=O)OCc1cncs1)O)NC(=O)[C@@H](NC(=O)N(Cc1nc(C(C)C)sc1)C)C(	10147-22-5	2-Naphthalenesulfonic acid, 7-amino-8-[2-[2-methyl-4-[2-(2-methylphenyl)diazenyl	C24H21N5O3S.Na		C	-9.5
440	N(=N\c1cc(S(=O)(=O)O)c(CCc2c(S(=O)(=O)O)cc(/N=N/c3c4c(cc(S(=O)(=O)O)c3N)cc(S(=O)(=O)O)cc4)cc2)cc1)/c	1053055-72-3	Not Yet Assigned	C34H28N6O12S4	840.88	C	-9.5
441	S(=O)(=O)(O)c1c2c(c(S(=O)(=O)O)cc(/N=N/c3cc(S(=O)(=O)O)c(C=Cc4c(S(=O)(=O)O)cc(/N=N/c5c(N)c6c(c(S(=O)	1053055-78-9	Not Yet Assigned	C34H28N6O18S6	1001.01	C	-9.5
442	O=C1[C@@H]2[C@H](c3c(c(OC)c(OC)c(OC)c3)[C@@H](O)[C@H]2CO1)c1cc2c(cc1)OCO2	1083160-34-2	2,7-Anthracenedisulfonamide, 9,10-dihydro-9,10-bis(hydroxyimino)-N2,N7-bis(1-tri	C38H48N4O6S2	720.94	C	-9.5
443	C(OCCOC(=O)[C@H](CC(=O)O)N)n1c2c(nc1)c(=O)nc(N)[nH]2	108692-91-7	Furo[3,Ä≤,4,Ä≤:6,7]naphtho[2,3-d]-1,3-dioxole-6,9-dione, 5-[4-(acetyloxy)phenyl	C21H16O7	380.35	C	-9.5
444	C(CN1CCCCC1)n1c2c(ccc(S(=O)(=O)N(C[C@H]([C@H](Cc3ccccc3)NC(=O)O[C@@H]3[C@H]4[C@@H](OC3)OCC4)O)CC(C)C	119415-70-2	Naphthionic acid, 3,3,Ä≤-[thiobis(p-phenyleneazo)]di-	C32H24N6O6S3	684.76	C	-9.5
445	O=c1n(c2oc(COP(=O)(O)O)cc2)cc(C)c(=O)[nH]1	1224571-51-0	Guanosine, 8-(1-pyrenyl)-	C26H21N5O5	483.48	C	-9.5
446	O=c1n([C@@H]2O[C@H](COB(O)O)C=C2)cc(C)c(=O)[nH]1	1227637-40-2	2,6-Anthracenedisulfonamide, 9,10-dihydro-9,10-bis(hydroxyimino)-N2,N6-bis(1-tri	C38H48N4O6S2	720.94	C	-9.5
447	C(=O)([C@@H](NC(=O)OC)C(C)C)N1[C@H](c2[nH]c(c3ccc4c(c(ccc4)c4cc(ccc4)c4[nH]c(nc4)[C@H]4N(C(=O)[C@@H](	1228553-46-5	Carbamic acid, N-[(1S)-1-[[(2S)-2-[5-[4-[5-[2-[(2S)-1-[(2S)-2-[(methoxycarbonyl)	C44H52N8O6	788.93	C	-9.5
448	N(C(=O)[C@H](Cc1ccccc1)C[C@@H](CN1[C@H](C(=O)NC(C)(C)C)CN(Cc2cc3c(nc2)ocn3)CC1)O)[C@H]1c2c(C[C@H]1O)	1237809-06-1	Not Yet Assigned	C40H49N5O4	663.85	C	-9.5
449	C(=O)([C@H](NC(=O)OC)C(C)C)N1[C@H](c2[nH]c(c3cc4c(-c5c(C4)cc(cc5)c4[nH]c(nc4)[C@H]4N(C(=O)[C@H](NC(=	1240880-69-6	Carbamic acid, N,N,Ä≤-[9H-fluorene-2,7-diylbis[1H-imidazole-5,2-diyl-(2S)-2,1-py	C41H50N8O8	782.88	C	-9.5
450	C(=O)([C@H](NC(=O)OC)c1ccccc1)N1[C@@H](CCC1)c1[nH]c(cn1)C1=CCC(CC1)c1ccc(cc1)c1[nH]c(nc1)[C@H]1N(C(=	1240885-55-5	Carbamic acid, N-[(1R)-2-[(2S)-2-[5-[4-[6-[2-[(2S)-1-[(2R)-2-[(methoxycarbonyl)a	C50H48N8O6	856.97	C	-9.5
451	O(c1nc(Nc2c(Br)cc(C#N)cc2)nc(N)c1Br)c1c(C)cc(C#N)cc1C	1246661-70-0	Carbamic acid, N,N,Ä≤-[2,6-biphenylenediylbis[1H-imidazole-5,2-diyl-(2S)-2,1-pyr	C40H48N8O6	736.86	C	-9.5
452	C(=O)([C@@H](NC(=O)OC)C(C)C)N1[C@@H](CCC1)c1[nH]c(cn1)c1cc2c(cc1)cc(cc2)c1ccc(cc1)c1[nH]c(nc1)[C@H]1	1256392-91-2	Carbamic acid, N-[(1S)-2-methyl-1-[[(2S)-2-[5-[4,Ä≤-[2-[(6S)-5-[(2S)-3-methyl-1-	C43H54N8O5	762.94	C	-9.5
453	O=c1c2c(n(cc1C(=O)O)C1CC1)cc(F)c2)N1CCN(CN2c3c(/C(=N/NC(=S)NOC)/C2=O)cc(C)cc3)CC1	1265623-19-5	3-Quinolinecarboxylic acid, 1-cyclopropyl-7-[4-[[(3Z)-2,3-dihydro-3-[2-[(hydroxy	C28H28FN7O5S	593.63	C	-9.5
454	C(C)n1c2c(c(=O)c(C(=O)O)c1)cc(F)c(c2F)N1CC(C)N(CN2c3c(/C(=N/NC(=S)NOC)/C2=O)cc(F)cc3)CC1	1265623-23-1	3-Quinolinecarboxylic acid, 1-ethyl-6,8-difluoro-7-[4-[[(3Z)-5-fluoro-2,3-dihydr	C27H26F3N7O5S	617.6	C	-9.5
455	C(C)n1c2c(c(=O)c(C(=O)O)c1)cc(F)c(c2F)N1CC(C)N(CN2c3c(/C(=N/NC(=S)NO)/C2=O)cc(C)cc3)CC1	1265623-24-2	3-Quinolinecarboxylic acid, 1-ethyl-6,8-difluoro-7-[4-[[(3Z)-5-fluoro-2,3-dihydr	C28H28F3N7O5S	631.63	C	-9.5
456	O(C)c1c2n(cc(C(=O)O)c(=O)c2cc(F)c1N1CC(C)N(CN2c3c(/C(=N/NC(=S)NO)/C2=O)cc(Cl)cc3)CC1)C1CC1	1265623-26-4	3-Quinolinecarboxylic acid, 7-[4-[[(3Z)-2,3-dihydro-3-[2-[(methoxyamino)thioxome	C29H31F2N7O5S	627.66	C	-9.5
457	O(Cc1ccc(F)cc1)c1c2c(oc(c(O)c2=O)c2[nH]ccn2)cc(c1)c1ccccc1	1312903-18-6	4H-1-Benzopyran-4-one, 5-[(4-fluorophenyl)methoxy]-3-hydroxy-7-phenyl-2-(3-pheny	C30H20FN3O4	505.5	C	-9.5
458	O=c1c2c(cc(c(OCc3ccc(F)cc3)c2)c2ccccc2)oc(c1O)c1[nH]cnn1	1312916-35-0	4H-1-Benzopyran-4-one, 6-[(4-fluorophenyl)methoxy]-3-hydroxy-7-phenyl-2-(5-pheny	C32H21FN2O4	516.52	C	-9.5
459	O(Cc1ccc(F)cc1)c1cc2c(c(C)c2c(c1)c(=O)c(O)c(o2)c1[nH]cnn1)c1ccccc1	1312916-86-1	4H-1-Benzopyran-4-one, 6-[(4-fluorophenyl)methoxy]-3-hydroxy-8-methyl-7-phenyl-2	C33H23FN2O4	530.55	C	-9.5
460	O=c1n([C@H]2C[C@H](OP3(=O)Oc4c(CO3)cccc4)[C@@H](CO)O2)cc(I)c(=O)[nH]1	1313031-60-5	Uridine, 3,Ä≤,5,Ä≤-bis-O-[6,8-bis(1,1-dimethylethyl)-5-fluoro-2-oxido-4H-1,3,2-]	C39H51F2IN2O11P2	950.68	C	-9.5
461	O(C)c1c2n(cc(C(=O)O)c(=O)c2cc(F)c1N1CC(C)N(CCN2c3c(C(=NNC(=S)N)C2=O)cc(F)cc3)CC1)C1CC1	1316840-83-1	3-Quinolinecarboxylic acid, 7-[4-[2-[3-[2-(aminothioxomethyl)hydrazinylidene]-2,	C30H32FN7O5S	621.68	C	-9.5
462	N(=Nc1cc(S(=O)(=O)O)ccc1)c1c2c(c(N=Nc3c4c(ccc(S(=O)(=O)O)c4)c(N)cc3)cc1)ccc(S(=O)(=O)O)c2	1345852-50-7	2-Naphthalenesulfonic acid, 5-[2-(4-amino-6-sulfo-1-naphthalenyl)diazenyl]-8-[2-	C26H19N5O9S3	641.65	C	-9.5
463	C(n1c2c(nc1CC)c(C)cc(C)n2)c1ccc(cc1)c1c(S(=O)(=O)NC(=O)OCc2ccccc2)ccc(CCCC)c1	1350008-11-5	Benzamide, 2-(2-aminoethyl)-N-[[4,Ä≤-[(2-ethyl-5,7-dimethyl-3H-imidazo[4,5-b]pyr	C32H33N5O3S	567.7	C	-9.5
464	C(=O)([C@@H](NC(=O)OC)C(C)C)N1[C@H](c2[nH]c(c3c4c(c(cc3)c3c5c(c(cc3)c3[nH]c(nc3)[C@H]3N(C(=O)[C@@H](	1350719-08-2	Carbamic acid, N,N,Ä≤-[(2,2,Ä≤,3,Ä≤,3,Ä≤-tetrahydro[4,4,Ä≤-bi-1H-indene]-7,7,Ä≤	C50H62N8O6	871.08	C	-9.5
465	S(=O)(=O)(O)c1c2c(c(N)c(N=Nc3ccc(SC)cc3)c1)cccc2.[NaH]	1357165-21-9	1-Naphthalenesulfonic acid, 4-amino-3-[2-(3,Ä≤-methyl[1,1,Ä≤-biphenyl]-4-yl)dia	C23H19N3O3S.Na		C	-9.5
466	C(C[C@@H](CO)O)n1c2c(nc1)c(=O)nc[nH]2	1372618-37-5	3-Quinolinecarboxylic acid, 7-[(4aS,7aS)-1-[2-[3-[2-(aminothioxomethyl)hydraziny	C32H33F2N7O5S	665.71	C	-9.5
467	O=c1c2c3c4c1cc(cc4ccc3cc(c2)c1[nH]c(nc1)[C@H]1N(C(=O)[C@@H](NC(=O)OC)C(C)C)CCC1)c1[nH]c(nc1)[C@H]1N(	1377035-70-5	Carbamic acid, N-[(1S)-2-methyl-1-[[(2S)-2-[5-[4,5,9,10-tetrahydro-7-[2-[(2S)-1-	C46H56N8O7	832.99	C	-9.5

468	C(=O)([C@@H](NC(=O)OC)C(C)C)N1[C@H](c2[nH]c3c(c4c(c5c3ccc(c5)c3ccc(cc3)c3[nH]c(nc3)[C@H]3N(C(=O)[C@@	1377041-17-2	Carbamic acid, N-[(1S)-1-[[(2S)-2-[9-[4-[2-[(2S)-1-[(2S)-2-[(methoxycarbonyl)ami	C46H52N8O6	812.96	C	-9.5
469	C(=O)([C@@H](NC(=O)OC)C1CCOCC1)N1[C@@H]([C@@H]2C[C@H]1CC2)c1[nH]c(cn1)c1cc(C#CC)c(cc1)c1cc2c(cc1)c1c	1377041-59-2	Carbamic acid, N-[(1S)-2-[(1R,3S,4S)-3-[5-[2-fluoro-4-[2-[(1R,3S,5R)-2-[(2S)-2-[	C47H53FN8O7	860.97	C	-9.5
470	C(=O)([C@@H](NC(=O)OC)C(C)C)N1[C@H]2[C@H](C2)C[C@H]1c1[nH]c(c(Cl)n1)c1ccc(cc1)c1cc2c(cc1)c1c(cc2)nc(	1377043-22-5	Carbamic acid, N-[(1S)-1-[[(1R,3S,5R)-3-[7-[4-[5-chloro-2-[(1R,3S,4S)-2-[(2S)-2-	C45H51ClN8O6	835.39	C	-9.5
471	O=c1n(C2(C[C@H](O)[C@@H](C[3H])O2)[3H])c(c(c(N)n1)[3H])[3H]	1378293-94-7	2-Naphthalenesulfonic acid, 5-amino-8-[2-[3-methyl-4-[2-(4-sulfophenyl)diazenyl]	C23H19N5O6S2	525.56	C	-9.5
472	O=c1c2c(n(cn2)[C@@H]2O[C@H](COP(=O)(Oc3ccccc3)N[C@@H](C(=O)OCC)C)OC2)[nH]c(N)n1	1380386-52-6	Carbamic acid, N,N,Ä≤-[[1,1,Ä≤-binaphthalene]-5,5,Ä≤-diylbis[1H-imidazole-5,2-d	C48H54N8O6	838.99	C	-9.5
473	O=c1c2c(n(cn2)[C@@H]2O[C@H](COP(=O)(OCCCCC#C)O)[C@@H](O)[C@H]2O)[nH]c(N)n1	1407096-78-9	1,4-Benzenedisulfonic acid, 2-[2-[4-[2-(4-amino-5-sulfo-1-naphthalenyl)diazenyl]	C26H19N5O12S4.3Na		C	-9.5
474	C(=O)([C@@H](NC(=O)OC)C(C)C)N1[C@H](c2[nH]c(c3c4c(c(cc3)c3ccc(cc3)c3[nH]c(nc3)[C@H]3N(C(=O)[C@@H](NC	1419385-17-3	Carbamic acid, N-[(1S)-1-cyclohexyl-2-[(2S)-2-[5-[4-[7,Ä≤-[2-[(2S)-1-[(2S)-2-cyc	C53H68N8O6	913.16	C	-9.5
475	C(N1c2c(C(=NNC(=S)N(CC=C)CC=C)C1=O)cc(Cl)cc2)N1CCN(CC1)c1ccc(Cl)cc1	1426583-67-6	Hydrazinecarbothioamide, 2-[1,2-dihydro-5-methyl-2-oxo-1-[(4-phenyl-1-piperaziny	C33H32N6OS	560.71	C	-9.5
476	CC1(C)COc2c(-c3c(cc(cc3)c3[nH]c(nc3)[C@H]3N(C(=O)[C@@H](NC(=O)OC)C(C)C)CCC3)OC1)ccc(c2)c1[nH]c(nc1)[	1449762-15-5	Carbamic acid, N,N,Ä≤-[(6,7,8,9-tetrahydrodibenzo[b,d][1,6]dioxecin-3,12-diyl)b	C44H56N8O8	824.96	C	-9.5
477	C(n1c2c(nc1CC)c(C)cc(C)n2)c1ccc(cc1)c1c(S(=O)(=O)NC(=O)c2ccccc2)ccc(CN(CC)CC)c1	154553-24-9	Benzamide, N-[[5-(1,1-dimethylethyl)-4,Ä≤-[(2-ethyl-5,7-dimethyl-3H-imidazo[4,5-	C34H36N4O3S	580.74	C	-9.5
478	C(n1c2c(nc1CC)c(C)cc(C)n2)c1ccc(cc1)c1c(S(=O)(=O)NC(=O)c2ccccc2)ccc(OC(C)C)c1	154553-27-2	Benzamide, N-[[4,Ä≤-[(2-ethyl-5,7-dimethyl-3H-imidazo[4,5-b]pyridin-3-yl)methyl]	C32H29F3N4O4S	622.66	C	-9.5
479	C(n1c2c(nc1CC)c(C)cc(C)n2)c1ccc(cc1)c1c(S(=O)(=O)NC(=O)NCCc2ccccc2)ccc(CC)c1	154553-50-1	[1,1,Ä≤-Biphenyl]-2-sulfonamide, 4,Ä≤-[(2-ethyl-5,7-dimethyl-3H-imidazo[4,5-b]p	C33H35N5O3S	581.73	C	-9.5
480	C(=O)([C@@H](NC(=O)OC)C(C)C)N1[C@H](c2[nH]c(c3c4c(C5OC4CC5)c(cc3)c3ccc(cc3)c3[nH]c(nc3)[C@H]3N(C(=O)	1560640-57-4	Carbamic acid, N,N,Ä≤-[(1,4-dihydro-1,4-ethanonaphthalene-5,8-diyl)bis[4,1-pheny	C52H60N8O6	893.08	C	-9.5
481	C(=O)(C(NC(=O)OC)C(C)C)N1C(c2[nH]c(c3ccc(c4c5c(c(cc4)c4ccc(cc4)c4[nH]c(nc4)C4N(C(=O)C(NC(=O)OC)C(C)C	1560650-14-7	Carbamic acid, N-[(1S)-2-methyl-1-[[(2S)-2-[5-[4-[1,2,3,4-tetrahydro-8-[2-[(2S)-	C49H58N8O6	855.03	C	-9.5
482	C(=O)([C@@H](NC(=O)OC)C(C)C)N1[C@H](c2[nH]c(c3c4c(C5(CC4)CCCC5)c(cc3)c3ccc(cc3)c3[nH]c(nc3)[C@H]3N(C	1588403-96-6	Carbamic acid, N-[(1S)-1-cyclohexyl-2-[(2S)-2-[5-[4-[4,Ä≤-[2-[(2S)-1-[(2S)-2-cyc	C53H68N8O6	913.16	C	-9.5
483	CC12c3c(c(ccc3c3[nH]c(nc3)[C@H]3N(C(=O)[C@@H](NC(=O)OC)C(C)C)CC(C)(C)C3)c3c4c(c(cc3)c3[nH]c(nc3)[C@H	1627533-42-9	Carbamic acid, N,N,Ä≤-[(1,1,Ä≤,2,2,Ä≤,3,3,Ä≤,4,4,Ä≤-octahydro-9-oxo[5,5,Ä≤-bi-	C50H60N8O7	885.06	C	-9.5
484	N(c1nc(nc(n1)N1CCOCC1)N1CCOCC1)c1ccc(C(=O)C=Cc2ccco2)cc1	1628172-08-6	2-Propen-1-one, 1-[4-[(4,6-di-4-morpholinyl-1,3,5-triazin-2-yl)amino]phenyl]-3-(	C32H32N6O4	564.63	C	-9.5
485	C(=O)([C@@H](NC(=O)OC)C(C)C)N1CC2(C[C@H]1c1[nH]c(cn1)c1ccc(cc1)c1cc3c(cc1)c1c(cc3)nc([nH]1)[C@H]1N(C	1653967-16-8	Carbamic acid, N-[(1S)-1-[[(2S)-2-[7-[4-[2-[(5R,8S)-7-[(2S)-2-[(methoxycarbonyl)	C45H54N8O7	818.96	C	-9.5
486	C(CN1CCCC1)n1c2c(c3c1cccc3)nc1c(c2NC(CCCN(CC)CC)C)ccc(Cl)c1	167105-67-1	Uridine, 2,Ä≤-deoxy-5-(1-pyrenyl)-	C25H20N2O5	428.44	C	-9.5
487	N(C(=O)[C@H](Cc1ccccc1)C[C@@H](CN1[C@H](C(=O)NC(C)(C)C)CN(Cc2cccnc2)CC1)O)[C@H]1c2c(C[C@H]1OC(=O)c1c	170227-74-4	Benzoic acid, 4-(4-morpholinylmethyl)-, 1-[[5-[2-[[(1,1-dimethylethyl)amino]carb	C48H60N6O6	817.03	C	-9.5
488	S(=O)(=O)(O)c1c2c(c(N)c(N=Nc3ccc(S(=O)(=O)c4ccc(cc4)c4ccc(S(=O)(=O)c5ccc(N=Nc6c(N)c7c(c(S(=O)(=O)O)c	17094-96-1	1-Naphthalenesulfonic acid, 3,3,Ä≤-[4,4,Ä≤-biphenylylenebis(thio-p-phenyleneazo	C44H32N6O6S4	869.02	C	-9.5
489	C(C=CC)(N)C12CC3CC(C2)CC(C1)C3	178478-40-5	2H-Naphth[1,8-cd]isothiazole, 2-[[1-[2-(4-methyl-1-piperazinyl)-2-tricyclo[3.3.1	C33H46N4O2S	562.81	C	-9.5
490	C(=O)([C@@H](NC(=O)OC)C(C)(C)C)N1[C@H](c2[nH]c(c3cc4C5(c6c(-c4cc3)ccc(c6)c3[nH]c(nc3)[C@H]3N(C(=O)[C	1800210-26-7	Carbamic acid, N,N,Ä≤-[spiro[9H-fluorene-9,3,Ä≤-oxetane]-2,7-diylbis[1H-imidazo	C49H60N8O7	873.05	C	-9.5
491	O=C1c2n(N([C@H]3N1CCOC3)[C@H]1c3c(CSc4c1cccc4)cccc3)ccc(=O)c2O	1985605-59-1	1H-[1,4]Oxazino[3,4-c]pyrido[2,1-f][1,2,4]triazine-6,8-dione, 12-[(11S)-7,8-difl	C24H19F2N3O4S	483.49	C	-9.5
492	O=C1c2n(N([C@H]3N1CCOC3)[C@H]1c3c(CSc4c1cccc4)c(Cl)ccc3)ccc(=O)c2O	1985605-62-6	1H-[1,4]Oxazino[3,4-c]pyrido[2,1-f][1,2,4]triazine-6,8-dione, 12-[(11S)-8-fluoro	C24H20FN3O4S	465.5	C	-9.5
493	Fc1c2[C@H](N3n4c(C(=O)N5[C@H]3COCC5)c(O)c(=O)cc4)c3c(SCc2ccc1)c(F)ccc3	1985605-70-6	1H-[1,4]Oxazino[3,4-c]pyrido[2,1-f][1,2,4]triazine-6,8-dione, 12-[(11S)-8,9-difl	C24H19F2N3O4S	483.49	C	-9.5
494	Fc1c2[C@H](N3n4c(C(=O)N5[C@H]3COC[C@@H]5C)c(O)c(=O)cc4)c3c(SCc2ccc1)c(F)ccc3	1985605-79-5	1H-[1,4]Oxazino[3,4-c]pyrido[2,1-f][1,2,4]triazine-6,8-dione, 12-[(11S)-7,8-difl	C25H21F2N3O4S	497.51	C	-9.5
495	O(C(=O)C(CC(=O)OC)(C)C)c1c2n(N([C@H]3N(C2=O)CCOC3)[C@H]2c3c(CSc4c2cccc4)c(F)c(F)cc3)ccc1=O	1985606-57-2	1H-[1,4]Oxazino[3,4-c]pyrido[2,1-f][1,2,4]triazine-6,8-dione, 12-[(11S)-7,8-difl	C32H24F2N4O8S	662.62	C	-9.5
496	C(=O)([C@@H](NC(=O)OC)C(C)C)N1[C@@H]([C@H]2[C@H](C2)C1)c1[nH]c(cn1)c1c2c(c(cc1)c1cc3c(cc1)c1c(cc3)nc	2007901-62-2	Carbamic acid, N-[(1S)-1-cyclopropyl-2-[(2S,5S)-2-[5-[2,3-dihydro-7-[2-[(2S,5S)-	C47H56N8O6	829	C	-9.5
497	C(=O)([C@@H](NC(=O)OC)C(C)C)N1[C@H](c2[nH]c(c3cc4c5-c6c(cc(cc6CC4)c4[nH]c(nc4)[C@H]4N(C(=O)[C@@H](NC	2020089-25-0	Carbamic acid, N-[(1S)-1-[[(2S,4R)-4-fluoro-2-[5-[7-[2-[(2S,4R)-4-fluoro-1-[(2S)	C44H52F2N8O6	826.93	C	-9.5
498	C(=O)([C@@H](NC(=O)OC)C(C)C)N1[C@@H](CCC1)c1[nH]c(cn1)c1ccc(cc1)c1ccc(cc1)c1[nH]c(nc1)[C@H]1N(C(=O)C	1009119-89-4	Carbamic acid, N,N,Ä≤-[[1,1,Ä≤-biphenyl]-3,4,Ä≤-diylbis[1H-imidazole-5,2-diyl-(	C46H46N8O6	806.91	A/B	-9.4
499	[C@H](Cc1ccc(C)cc1)(C(=O)N[C@H](/C=C/S(=O)(=O)c1ccccc1)CCc1ccccc1)NC(=O)N1CCN(C)CC1	1011726-28-5	1-Piperazinecarboxamide, 4-methyl-N-[(1S)-2-oxo-2-[[(1S,2E)-1-(2-phenylethyl)-3-	C33H37F3N4O4S	642.73	A/B	-9.4
500	O[C@H]1[C@](n2c3c(n[14cH]2)c(N)ncn3)(O[C@H](CO)[C@H]1O)[2H]	1024149-47-0	Benzoic acid, 3,3,Ä≤-[(3-carboxy-4-oxocyclohexylidene)methylene]bis[6-hydroxy-	C22H18O9	426.37	A/B	-9.4
501	N(C(CCCN(CC)CC)C)c1c2c(ncc1CCC)cc(Cl)cc2	1027058-27-0	Phosphoric acid, 4-[(5R,5aR,9R)-5,5a,6,8,8a,9-hexahydro-9-methoxy-6-oxofuro[3,Ä≤	C36H35O11P	674.63	A/B	-9.4
502	O=c1n([C@H]2C[C@H](OP(=O)(OC[C@H]3O[C@H](C[C@@H]3O)n3c(=O)[nH]c(=O)cc3)O)[C@@H](CO)O2)cc(C)c(=O)[nH]	103167-67-5	2-Naphthalenesulfonic acid, 7-hydroxy-8-[2-[2-hydroxy-5-[2-(1-naphthalenyl)diaze	C26H18N4O5S	498.51	A/B	-9.4
503	O=c1n([C@H]2C[C@H](ON)[C@@H](CO)O2)ccc(=O)[nH]1	103242-83-7	Adenosine, adenylyl-(2,Ä≤,Ü5,Ä≤)-guanylyl-(2,Ä≤,Ü5,Ä≤)-	C30H37N15O17P2	941.65	A/B	-9.4
504	C(OCCOC(=O)[C@H](C(C)C)N)N1C2C(N=C1)C(=O)N=C(N)N2	1041426-20-3	Butanediamide, N1-[3-[3-[[(1,1-dimethylethyl)amino]carbonyl]octahydro-2(1H)-isoq	C39H52N6O5	684.87	A/B	-9.4
505	O=C1[O-][Al+3]2([O]=c3c(C(=O)[O-]2)cc(=C(c2cc(C(=O)[O-])c(O)cc2)c2cc(C(=O)[O-])c(O)cc2)cc3)[O]=c2c1c	1050328-16-9	D-erythro-Pentonamide, 2,3,5-trideoxy-N-[(1S)-2,3-dihydro-2-oxo-1H-inden-1-yl]-5	C36H45N5O4	611.77	A/B	-9.4
506	N(C(=O)[C@H](Cc1ccccc1)C[C@@H](CN1[C@H](C(=O)NC(C)(C)C)CN(Cc2c(F)cccc2F)CC1)O)[C@H]1c2c(C[C@H]1O)ccc	1064475-88-2	Not Yet Assigned	C39H50N4O4	638.84	A/B	-9.4
507	O=c1n([C@@H]2O[C@H](COP3(=O)Oc4c(CO3)cc(Cc3cc5c(cc3)OP(=O)(OC[C@H]3O[C@H](C=C3)n3c(=O)[nH]c(=O)c(C)c	1099829-92-1	Not Yet Assigned	C35H35FN4O14P2	816.62	A/B	-9.4
508	[C@@H](CC)(C1=C(O)C[C@@H](OC1=O)c1ccccc1)c1cc(NS(=O)(=O)c2c(C(F)(F)F)cccn2)ccc1	1109235-81-5	2-Pyridinesulfonamide, N-[3-[(1S)-1-[(6S)-5,6-dihydro-4-hydroxy-2-oxo-6-phenyl-2	C26H23F3N2O5S	532.53	A/B	-9.4
509	C(=O)(C(NC(=O)OC)(C)C)N1[C@@H](CC[C@@H]1C)c1[nH]c(cn1)c1ccc(cc1)c1ccc(cc1)c1[nH]c(nc1)[C@H]1N(C(=O)C	1129634-95-2	Carbamic acid, N,N,Ä≤-[[1,1,Ä≤-biphenyl]-4,4,Ä≤-diylbis[1H-imidazole-5,2-diyl[(	C36H42N8O6	682.77	A/B	-9.4
510	O[C@@H]1[C@H](n2c3c(nc2)c(N)ncn3)O[C@H](COP(=O)(OC)C)[C@H]1O	115561-47-2	Œ≤-D-arabino-Adenosine, P-deoxy-P-methyl-Œ≤-D-arabino-adenylyl-(5,Ä≤,Ü5,Ä≤)-	C21H27N10O9P	594.47	A/B	-9.4
511	C(C)(C)c1n(c2c(n1)cccc2)c1nc(nc(n1)N1CCOCC1)c1cnc(N)nc1	1174191-80-0	2-Pyrimidinamine, 5-[4-(4-morpholinyl)-6-(4,5,6,7-tetrahydro-1H-indazol-1-yl)-1,	C18H21N9O	379.42	A/B	-9.4
512	C(=O)([C@@H](NC(=O)OC)C(C)C)N1[C@H](c2[nH]c3-c4c(cc(cc4)c4cc(F)c(cc4)c4[nH]c(nc4)[C@H]4N(C(=O)[C@@H]	1180670-04-5	Carbamic acid, N,N,Ä≤-[(4,4,Ä≤,5,5,Ä≤-tetrahydro[7,7,Ä≤-bi-1H-naphth[1,2-d]imic	C46H54N8O6	814.97	A/B	-9.4
513	C(=O)([C@@H](NC(=O)OC)C(C)C)N1[C@H]2[C@H](C2)C[C@H]1c1[nH]c2c(n1)ccc1c2ccc(c1)c1cc2c(cc1)c1c(cc2)nc(	1180670-22-7	Carbamic acid, N,N,Ä≤-[(4,5-dihydro[7,7,Ä≤-bi-1H-naphth[1,2-d]imidazole]-2,2,Ä≤	C46H52N8O6	812.96	A/B	-9.4
514	C(OCCOP(=O)(Oc1c2c(ccc1)cccc2)N[C@H](C(=O)OC)C(C)C)n1c2c(nc1)c(=O)nc(N=CN(C)C)[nH]2	1184942-72-0	5,8-Dioxa-3-aza-4-phosphanonanoic acid, 9-[2-[[(dimethylamino)methylene]amino]-1	C28H34N7O7P	611.59	A/B	-9.4
515	[C@](C(=O)N[C@@H](Cc1ccccc1)C[C@@H]([C@H](Cc1ccccc1)NC(=O)C(Oc1c(C([2H])([2H])[2H])c(c(c(c1C([2H])([	1224729-14-9	1(2H)-Pyrimidineacetamide-Œ±-d, N-[(1S,3S,4S)-4-[[2-[2,6-di(methyl-d3)phenoxy-3,	C37H21D27N4O5		A/B	-9.4
516	C(#N)C1=C(N)Oc2c(C1c1cc(F)c(F)cc1)ccn1c2ncc1	1225373-03-4	7H-Imidazo[1,2-a]pyrano[2,3-c]pyridine-8-carbonitrile, 9-amino-7-[4-(trifluorome	C18H11F3N4O	356.3	A/B	-9.4
517	O[C@H]1C(n2c3c(nc2)c(=S)nc[nH]3)O[C@H](CO)[C@H]1O	1225376-22-6	6H-Dipyrido[3,2-b:2,Ä≤,3,Ä≤-e][1,4]diazepin-6-one, 11-cyclopropyl-5,11-dihydro-	C27H26N6O2S	498.6	A/B	-9.4
518	C(=O)([C@@H](NC(=O)OC)C(C)C)N1[C@@H](CCC1)c1[nH]c(cn1)c1ccc(C#Cc2ccc(cc2)c2[nH]c(nc2)[C@H]2N(C(=O)[C	1228965-86-3	Carbamic acid, N-[(1R)-2-oxo-2-[(2S)-2-[5-[4-[2-[4-[2-[(2S)-1-[(2R)-1-oxo-2-pher	C48H47N7O4	785.93	A/B	-9.4
519	C(=O)(OCC)c1c2c(n(C)c1CS(=O)CCCc1ccccc1)cc(Br)c(O)c2CN(C)C	1233233-18-5	Benzoic acid, 3,3,Ä≤-[[3-[(aminooxy)carbonyl]-4-oxo-2,5-cyclohexadien-1-ylidene]	C22H15NO9	437.36	A/B	-9.4
520	N(C(=O)[C@H](Cc1ccccc1)C[C@@H](CN1[C@H](C(=O)NC(C)(C)C)CN(Cc2cc3c(s2)cccc3)CC1)O)[C@H]1c2c(C[C@H]1O)	1237808-91-1	Not Yet Assigned	C39H49N5O5	667.84	A/B	-9.4
521	N(C(=O)[C@H](Cc1ccccc1)C[C@@H](CN1[C@H](C(=O)NC(C)(C)C)CN(Cc2cn3c(cn2)ncc3)CC1)O)[C@H]1c2c(C[C@H]1O)	1237808-93-3	Not Yet Assigned	C39H48N4O4S	668.89	A/B	-9.4
522	N(C(=O)[C@H](Cc1ccccc1)C[C@@H](CN1[C@H](C(=O)NC(C)(C)C)CN(Cc2cc3c(cc2)scc3)CC1)O)[C@H]1c2c(C[C@H]1O)	1237808-99-9	Not Yet Assigned	C40H51N5O4	665.86	A/B	-9.4
523	N(C(=O)[C@H](Cc1ccccc1)C[C@@H](CN1[C@H](C(=O)NC(C)(C)C)CN(Cc2cc3c(s2)cccn3)CC1)O)[C@H]1c2c(C[C@H]1O)	1237809-00-5	Not Yet Assigned	C39H48N4O4S	668.89	A/B	-9.4
524	N(C(=O)[C@H](Cc1ccccc1)C[C@@H](CN1[C@H](C(=O)NC(C)(C)C)CN(Cc2cc3c(nc2)scc3)CC1)O)[C@H]1c2c(C[C@H]1O)	1237809-01-6	Not Yet Assigned	C38H47N5O4S	669.88	A/B	-9.4
525	C(F)(F)c1n(c2c(n1)cccc2)c1nc(nc(N[C@H]2CC[C@@H](CC2)N2CCOCC2)n1)N1CCOCC1	1240023-38-4	Acetamide, 2-bromo-N-[trans-4-[[4-[2-(difluoromethyl)-1H-benzimidazol-1-yl]-6-(4	C23H27BrF2N8O2	565.41	A/B	-9.4
526	[C@@H](NC(=O)[C@@H](NC(=O)N1CCN(C)CC1)CC(C)C)(/C=C/S(=O)(=O)c1ccccc1)c1ccccc1	1253912-39-8	1-Piperazinecarboxamide, N-[(1S)-2-[[(1R,2E)-1-[1,1,Ä≤-biphenyl]-4-yl-3-(phenyls	C36H38N4O4S	622.78	A/B	-9.4
527	[C@@H](NC(=O)[C@@H](NC(=O)N1CCN(C)CC1)CC(C)C)(/C=C/S(=O)(=O)c1ccccc1)c1ccc(cc1)c1ccccc1	1253912-45-6	1-Piperazinecarboxamide, 4-methyl-N-[(1S)-3-methyl-1-[[[(1R,2E)-1-(2-naphthaleny	C31H38N4O4S	562.72	A/B	-9.4
528	C(=O)([C@@H](NC(=O)OC)C(C)C)N1[C@@H](CC2(C1)OCCCO2)c1[nH]c(cn1)c1ccc(cc1)c1ccc(cc1)c1[nH]c(nc1)[C@H]	1272655-03-4	Carbamic acid, N,N,Ä≤-[[1,1,Ä≤-biphenyl]-4,4,Ä≤-diylbis[1H-imidazole-5,2-diyl[(	C50H66N8O10	939.11	A/B	-9.4
529	N(c1nc(nc(N/N=C/c2c(F)cccc2F)n1)N1CCOCC1)c1ccc(F)cc1	1279692-10-2	2-Naphthalenecarboxaldehyde, 2-[4-[(4-fluorophenyl)amino]-6-(4-morpholinyl)-1,3,	C24H22FN7O	443.48	A/B	-9.4
530	O(Cc1ccc(F)cc1)c1c2c(c(cc1)c1ccccc1)oc(c(O)c2=O)c1[nH]ccn1	1312894-47-5	4H-1-Benzopyran-4-one, 5-[(4-fluorophenyl)methoxy]-3-hydroxy-8-phenyl-2-(3-pheny	C30H20FN3O4	505.5	A/B	-9.4
531	O(Cc1ccc(F)cc1)c1c2c(oc(c(O)c2=O)c2ncccn2)c(C)c(C)c1	1312895-02-5	4H-1-Benzopyran-4-one, 5-[(4-fluorophenyl)methoxy]-3-hydroxy-7-methyl-2-(5-pheny	C26H19FN2O4	442.44	A/B	-9.4
532	O(Cc1ccc(F)cc1)c1c2c(c(OC)c(C)c1)oc(c(O)c2=O)c1ncccn1	1312895-52-5	4H-1-Benzopyran-4-one, 5-[(4-fluorophenyl)methoxy]-3-hydroxy-7,8-dimethyl-2-(5-p	C27H21FN2O4	456.46	A/B	-9.4
533	O(Cc1ccc(F)cc1)c1c2c(c(c(C)c1)c1ccccc1)oc(c(O)c2=O)c1ncccn1	1312896-52-8	4H-1-Benzopyran-4-one, 8-chloro-5-[(4-fluorophenyl)methoxy]-3-hydroxy-7-methyl-2	C26H18ClFN2O4	476.88	A/B	-9.4
534	O(Cc1ccc(F)cc1)c1c2c(oc(c(O)c2=O)c2[nH]ccn2)cc(OC)c1	1312897-58-7	4H-1-Benzopyran-4-one, 5-[(4-fluorophenyl)methoxy]-3-hydroxy-7-methoxy-2-(3-pher	C25H18FN3O5	459.43	A/B	-9.4
535	O(Cc1ccc(F)cc1)c1c2c(oc(c(O)c2=O)c2ncccn2)c(C)c(Cl)c1	1312900-73-4	4H-1-Benzopyran-4-one, 7-chloro-5-[(4-fluorophenyl)methoxy]-3-hydroxy-2-(5-pheny	C25H16ClFN2O4	462.86	A/B	-9.4
536	O(Cc1ccc(F)cc1)c1c2c(c(c(Cl)c1)c1ccccc1)oc(c(O)c2=O)c1[nH]ccn1	1312902-68-3	4H-1-Benzopyran-4-one, 7-chloro-5-[(4-fluorophenyl)methoxy]-3-hydroxy-8-phenyl-2	C30H19ClFN3O4	539.94	A/B	-9.4
537	O(Cc1ccc(F)cc1)c1c2c(oc(c(O)c2=O)c2ncccn2)cc(c1)c1ccccc1	1312902-73-0	4H-1-Benzopyran-4-one, 7-chloro-5-[(4-fluorophenyl)methoxy]-3-hydroxy-8-phenyl-2	C31H20ClFN2O4	538.95	A/B	-9.4
538	O=c1c2c(c(cc(OCc3ccc(F)cc3)c2)c2ccccc2)oc(c1O)c1ncccn1	1312907-83-7	4H-1-Benzopyran-4-one, 8-chloro-6-[(4-fluorophenyl)methoxy]-3-hydroxy-2-(5-pheny	C25H16ClFN2O4	462.86	A/B	-9.4
539	O=c1c2c(c(cc(OCc3ccc(F)cc3)c2)c2ccccc2)oc(c1O)c1[nH]cnn1	1312908-18-1	4H-1-Benzopyran-4-one, 6-[(4-fluorophenyl)methoxy]-3-hydroxy-8-phenyl-2-(5-pheny	C32H21FN2O4	516.52	A/B	-9.4
540	O(C)c1c2c(cc(OCc3ccc(F)cc3)c1C)c(=O)c(O)c(o2)c1ncccn1	1312909-45-7	4H-1-Benzopyran-4-one, 6-[(4-fluorophenyl)methoxy]-3-hydroxy-7,8-dimethyl-2-(5-p	C27H21FN2O4	456.46	A/B	-9.4
541	Cc1c(c2c(cc1OCc1ccc(F)cc1)c(=O)c(O)c(o2)c1[nH]c(cn1)c1ccccc1)c1ccccc1	1312910-94-3	4H-1-Benzopyran-4-one, 2-(5-chloro-1H-imidazol-2-yl)-6-[(4-fluorophenyl)methoxy]	C26H18ClFN2O4	476.88	A/B	-9.4
542	O=c1c2c(oc(c1O)c1ncccn1)cc(OC)c(OCc1ccc(F)cc1)c2	1312910-95-4	4H-1-Benzopyran-4-one, 6-[(4-fluorophenyl)methoxy]-3-hydroxy-7-methyl-8-phenyl-2	C32H23FN2O4	518.53	A/B	-9.4
543	O=c1c2c(oc(c1O)c1ncccn1)cc(Cl)c(OCc1ccc(F)cc1)c2	1312911-47-9	4H-1-Benzopyran-4-one, 6-[(4-fluorophenyl)methoxy]-3-hydroxy-7-methoxy-2-(5-pher	C26H19FN2O5	458.44	A/B	-9.4
544	O(C)c1c(c2c(cc1OCc1ccc(F)cc1)c(=O)c(O)c(o2)c1ncccn1)c1ccccc1	1312914-93-4	4H-1-Benzopyran-4-one, 7,8-dichloro-6-[(4-fluorophenyl)methoxy]-3-hydroxy-2-(5-p	C25H15Cl2FN2O4	497.3	A/B	-9.4
545	Clc1c(c2c(cc1OCc1ccc(F)cc1)c(=O)c(O)c(o2)c1[nH]c(OC)nn1)c1ccccc1	1312915-82-4	4H-1-Benzopyran-4-one, 7-chloro-6-[(4-fluorophenyl)methoxy]-3-hydroxy-2-(3-methy	C25H17ClFN3O4	477.87	A/B	-9.4
546	O=C1c2n(C[C@H]3N1[C@H](C)CCO3)cc(C(=O)NCc1c(F)cc(F)cc1)c(=O)c2O.C(#CC1CC1)[C@@]1(C(F)(F)F)c2c(NC(=O)	13195-20-5	1-Naphthalenesulfonic acid, 4-hydroxy-3-[[4,Ä≤-[[4-[(1-hydroxy-4-sulfo-2-naphthy	C44H32N6O8S2.2Na		A/B	-9.4
547	O[C@H]1[C@H](n2c3c(nc2)c(=O)nc[nH]3)O[C@H](COP(=O)(OP(=O)(OP(=O)(O)O)O)O)[C@H]1O	1319715-08-6	2H-Pyrido[1,Ä≤,2,Ä≤:4,5]pyrazino[2,1-b][1,3]oxazine-9-carboxamide, N-[(2,4-difl	C37H48N4O5.C20H19F2N3O5		A/B	-9.4
548	C(OCCOC(=O)c1ccccc1)n1c2c(c(=O)nc(NC(=O)C)[nH]2)nc1	1331749-55-3	2-Naphthalenecarboxaldehyde, 2-[4-[(4-fluorophenyl)amino]-6-(4-morpholinyl)-1,3,	C24H22FN7O	443.48	A/B	-9.4
549	N(=Nc1c2c(c(S(=O)(=O)O)ccc2)c(N)cc1)c1c2c(c(N=Nc3ccccc3)cc1)ccc(S(=O)(=O)O)c2	1345852-33-6	1-Naphthalenesulfonic acid, 5-amino-8-[2-[7-sulfo-4-[2-(3-sulfophenyl)diazenyl]-	C26H19N5O9S3	641.65	A/B	-9.4
550	C(=O)([C@@H](NC(=O)OC)[C@H](CC)C)N1[C@@H](CCC1)c1[nH]c(cn1)c1cc2c(cc1)cc(cc2)c1ccc(cc1)c1[nH]c(nc1)[	1351332-62-1	Carbamic acid, N-[(1R)-2-[(2S)-2-[5-[4-[6-[2-[(2S)-1-[(2S)-2-[(methoxycarbonyl)a	C45H46N8O6	794.9	A/B	-9.4
551	C(F)(F)c1n(c2c(n1)cccc2)c1nc(nc(NC2CCN(C)CC2)n1)N1CCOCC1	1359953-84-6	1H-Benzimidazol-5-amine, N-[4-[2-(difluoromethyl)-1H-benzimidazol-1-yl]-6-(4-mor	C23H21F2N9O	477.47	A/B	-9.4
552	C(=O)([C@@H](NC(=O)OC)C(C)C)N1[C@@H](CCC1)c1[nH]c(cn1)c1ccc(C(=Cc2ccc(cc2)c2[nH]c(nc2)[C@H]2N(C(=O)[	1374421-49-4	Carbamic acid, N,N,Ä≤-[[1-[4-(trifluoromethyl)phenyl]-1,2-ethanediyl]bis[4,1-phe	C49H57F3N8O6	911.02	A/B	-9.4
553	C(=O)([C@@H](NC(=O)OC)C(C)C)N1[C@@H](CCC1)c1[nH]c(cn1)c1ccc(C(Cc2ccc(cc2)c2[nH]c(nc2)[C@H]2N(C(=O)[C	1374421-54-1	Carbamic acid, N,N,Ä≤-[[1-[4-(trifluoromethyl)phenyl]-1,2-ethenediyl]bis[4,1-phe	C49H55F3N8O6	909.01	A/B	-9.4
554	C(=O)([C@@H](NC(=O)OC)C1CCOCC1)N1[C@@H]([C@@H]2C[C@H]1CC2)c1[nH]c(cn1)c1cc2c(-c3c(CC2)cc(cc3)c2[nH]c	1377037-08-5	Carbamic acid, N-[(1S)-2-[(8S)-8-[7-[4-[2-[(2S)-1-[(2S)-2-[(methoxycarbonyl)amir	C46H54N8O9	862.97	A/B	-9.4
555	C(=O)([C@@H](NC(=O)OC)C(C)C)N1[C@H]2[C@H](C2)C[C@H]1c1[nH]c2c(n1)ccc1c2ccc(c1)c1ccc(cc1)c1[nH]c(nc1)	1377037-16-5	Carbamic acid, N-[(1S)-2-[(1R,3S,4S)-3-[4,Ä≤,5,Ä≤-dihydro-2,Ä≤-[(2S)-1-[(2S)-2-	C48H56N8O7	857.01	A/B	-9.4
556	C(=O)([C@@H](NC(=O)OC)C1CCOCC1)N1[C@H](c2[nH]c(c3cc4c(c5c(c6c4cccc6)cc(cc5)c4[nH]c(nc4)[C@H]4N(C(=O)	1377041-29-6	Carbamic acid, N-[(1S)-2-[(1R,3S,4S)-3-[5-[2-fluoro-4-[2-[(2S)-1-[(2S)-2-[(methc	C46H53FN8O7	848.96	A/B	-9.4
557	C(=O)([C@@H](NC(=O)OC)C(C)C)N1[C@H](c2[nH]c(c3cc4c5c6c(cc(cc6OC4)c4[nH]c(nc4)[C@H]4N(C(=O)[C@@H](NC(	1377043-96-3	Carbamic acid, N-[(1S)-1-[[(1R,3S,5R)-3-[2,Ä≤-[(1R,3S,4S)-2-[(2S)-2-[(methoxyca	C47H52N8O6	824.97	A/B	-9.4
558	C(=O)([C@@H](NC(=O)OC)C(C)C)N1[C@@H]([C@@H]2C[C@H]1CC2)c1[nH]c2c(n1)ccc1c2ccc(c1)c1ccc(cc1)c1[nH]c(n	1377604-13-1	Carbamic acid, N-[(1S)-2-[(1R,3S,4S)-3-[7-[4-[2-[(2S)-1-[(2S)-2-[(methoxycarbony	C46H54N8O7	830.97	A/B	-9.4
559	C[C@@]12C(=CC[C@H]3[C@@]1(C)CC[C@@H]1[C@]3(C)CCC(=O)[C@H]1C)[C@H]1[C@@]3(C[C@H]2OC3=O)CCC(C)(C)[C@H]	1377604-20-0	Carbamic acid, N-[(1S)-2-[(1R,3S,4S)-3-[2,Ä≤-[(2S)-1-[(2S)-2-[(methoxycarbonyl)a	C48H54N8O7	854.99	A/B	-9.4
560	OC1CCN(c2nc(nc(n2)N2CCOCC2)c2c3c(ccc2)[nH]nc3)CC1	1391925-86-2	1,3,5-Triazin-2-amine, 4-(1H-indazol-4-yl)-6-(4-morpholinyl)-N-phenyl-	C20H19N7O	373.41	A/B	-9.4
561	N(CCNC(=O)c1ccc(cc1)C1OOC(OO1)c1ccc(C)cc1)c1nc(nc(n1)N1CCOCC1)N1CCN(CC1)c1ccccc1	1393922-42-3	Benzamide, N-[2-[[4-(cyclohexylamino)-6-(4-morpholinyl)-1,3,5-triazin-2-yl]aminc	C31H39N7O6	605.68	A/B	-9.4
562	O[C@H]1[C@@H](O[C@H](C(=O)N[C@@H](C(=O)N2CCN(CC2)c2ccc(Cl)cn2)Cc2ccccc2)[C@H]1O)n1nc(C(=O)N)nc1	1414801-66-3	Not Yet Assigned	C27H30N8O6	562.58	A/B	-9.4
563	C(=C1C=C(Cc2cc(C(=O)O)c(O)cc2)C(=O)C(=C1)C(=O)O)(c1cc(C(=O)O)c(O)cc1)c1cc(C(=O)O)c(O)cc1	141511-26-4	Benzoic acid, 5-[(3-carboxy-4-hydroxyphenyl)(5-carboxy-6-oxo-2,4-cyclohexadien-1	C22H14O9	422.34	A/B	-9.4
564	O=c1n([C@@H]2O[C@H](CO)[C@@H](F)C2)cc(CC)c(N)n1	1416435-27-2	D-erythro-Pentonamide, 2,3,5-trideoxy-N-[(1S,2R)-2,3-dihydro-2-hydroxy-1H-inden-	C36H47N5O6	645.79	A/B	-9.4
565	O=c1n(cc([Se]C(F)(F)F)c(=O)[nH]1)[C@@H]1O[C@H](CO)[C@@H](O)C1	1419385-63-9	Carbamic acid, N-[(1S)-1-[[(2S)-2-[5-[1,Ä≤,3,Ä≤-dihydro-7,Ä≤-[4-[2-[(8S)-7-[(2S	C49H62N8O8	891.07	A/B	-9.4
566	O(C)c1c2n(cc(C(=O)O)c(=O)c2cc(F)c1N1CC(C)N(CN2c3c(C(=NNC(=S)N(CC=C)CC=C)C2=O)cc(F)cc3)CC1)C1CC1	1426583-87-0	3-Quinolinecarboxylic acid, 7-[4-[[5-chloro-3-[2-[(diphenylamino)thioxomethyl]hy	C41H37ClFN7O5S	794.29	A/B	-9.4
567	O=C1[C@@H]2[C@@H](c3c([C@H](OC(=O)c4cc(C)ccc4)[C@H]2CO1)cc1c(c3)OCO1)c1c(Cl)c(OC)c(OC)c(OC)c1	1449601-78-8	1-Naphthaleneacetic acid, (5R,5aR,8aR,9S)-9-(2-chloro-3,4,5-trimethoxyphenyl)-5,	C34H29ClO9	617.04	A/B	-9.4
568	O=c1n([C@@H]2O[C@H](CO)C=C2)cc(I)c(=O)[nH]1	1449762-38-2	Carbamic acid, N,N,Ä≤-[(6,11-dihydrotribenzo[b,d,h][1,6]dioxecin-3,14-diyl)bis[	C48H56N8O8	873.01	A/B	-9.4
569	O=C1[C@H]2[C@@H](c3c([C@H](OC(=O)OC)[C@H]2CO1)cc1c(c3)OCO1)c1cc(OC)c(OC)c(OC)c1	1453805-45-2	1-Piperazinecarbothioamide, N-(7-chloro-4-quinolinyl)-4-(4,6-di-4-morpholinyl-1,	C25H30ClN9O2S	556.08	A/B	-9.4
570	O[C@H]1[C@H](n2c3c(nc2)c(=O)nc(N)[nH]3)O[C@H](CNN[C@@H]2[C@@H](CO)O[C@H](C2)n2c3c(nc2)c(=O)nc(N)[nH]	1513874-26-4	Guanosine, 2,Ä≤-deoxy-3,Ä≤-O-dephosphinicoguanylyliminomethylene-(3,Ä≤,Ü5,Ä≤)-	C21H27N11O8	561.51	A/B	-9.4
571	O[C@H]1[C@H](n2c3c(nc2)c(=O)nc(N)[nH]3)O[C@H](CNO[C@@H]2[C@@H](CO)O[C@H](C2)n2c3c(nc2)c(=O)nc(N)[nH]	1513874-27-5	Guanosine, 2,Ä≤-deoxy-3,Ä≤-de(phosphinicooxy)guanylyl-1,2-hydrazinediyl-(3,Ä≤,Ü	C20H26N12O7	546.5	A/B	-9.4
572	O[C@H]1[C@H](n2c3c(nc2)c(=O)nc(N)[nH]3)O[C@H](CO)[C@H]1N[Si](NC[C@H]1O[C@H]([C@H](O)[C@@H]1O)n1c2c(n	1513874-51-5	Guanosine, 3,Ä≤-de(phosphinicooxy)guanylyl-1,2-hydrazinediyl-(3,Ä≤,Ü5,Ä≤)-5,Ä≤	C20H26N12O8	562.5	A/B	-9.4
573	CN1c2c(N(C1)[C@@H]1O[C@H](CO)[C@@H](O)[C@H]1O)[nH]c(N)nc2=O	153116-69-9	Carbamic acid, [[[[4,Ä≤-[(2-ethyl-5,7-dimethyl-3H-imidazo[4,5-b]pyridin-3-yl)met	C26H27N5O5S	521.59	A/B	-9.4
574	O(C(OC(=O)OCC1CC1)CC)c1c(C(=O)NCc2ccc(F)cc2)nc(C(NC(=O)c2nnc(C)o2)(C)C)n(C)c1=O	1538603-98-3	Carbonic acid, 1-[[4-[[[(4-fluorophenyl)methyl]amino]carbonyl]-1,6-dihydro-1-met	C31H33FN6O8	636.63	A/B	-9.4
575	C(n1c2c(nc1CC)c(C)cc(C)n2)c1ccc(cc1)c1c(S(=O)(=O)NC(=O)CCCCC)ccc(CN(C)C)c1	154551-85-6	Hexanamide, N-[[5-(1,1-dimethylethyl)-4,Ä≤-[(2-ethyl-5,7-dimethyl-3H-imidazo[4,5	C33H42N4O3S	574.78	A/B	-9.4
576	C(n1c2c(nc1CC)c(C)cc(C)n2)c1ccc(cc1)c1c(S(=O)(=O)NC(=O)CCc2ccccc2)ccc(CN(C)C)c1	154552-64-4	Benzenepropanamide, N-[[5-(1,1-dimethylethyl)-4,Ä≤-[(2-ethyl-5,7-dimethyl-3H-imi	C36H40N4O3S	608.79	A/B	-9.4
577	C(n1c2c(nc1CC)c(C)cc(C)n2)c1ccc(cc1)c1c(S(=O)(=O)NC(=O)c2ccccc2)ccc(CCC)c1	154553-12-5	Benzamide, N-[[5-ethyl-4,Ä≤-[(2-ethyl-5,7-dimethyl-3H-imidazo[4,5-b]pyridin-3-y]	C32H32N4O3S	552.69	A/B	-9.4
578	CN1C2c3c(c(ccc3c3[nH]c(nc3)[C@H]3N(C(=O)[C@@H](NC(=O)OC)C(C)C)CCC3)c3ccc(cc3)c3[nH]c(nc3)[C@H]3N(C(=	1560639-67-9	Carbamic acid, N-[(1S)-2-methyl-1-[[(1R,3S,4S)-3-[5-[4-[1,2,3,4-tetrahydro-8-[2-	C49H61N9O6	872.07	A/B	-9.4
579	C(=O)([C@@H](NC(=O)OC)C(C)C)N1[C@H](c2[nH]c(c3c4c(c(cc3)c3c5c(c(cc3)c3[nH]c(nc3)[C@H]3N(C(=O)[C@@H](	1560650-00-1	Carbamic acid, N-[(1S)-2-methyl-1-[[(2S,4R)-4-methyl-2-[5-[4-[1,2,3,4-tetrahydrc	C48H62N8O6	847.06	A/B	-9.4
580	C(=O)([C@@H](NC(=O)OC)C(C)C)N1[C@H](c2[nH]c(c3c4c(c(cc3)c3c5c(c(cc3)c3[nH]c(nc3)[C@H]3N(C(=O)[C@@H](	1560650-13-6	Carbamic acid, N-[(1S)-1-[[(2S)-2-[5-[8-[2,3-dihydro-7-[2-[(2S)-1-[(2S)-2-[(meth	C48H60N8O6	845.04	A/B	-9.4
581	C(=O)([C@@H](NC(=O)OC)C(C)C)N1[C@H](c2[nH]c(c3c4c(C5(CC4)CCOCC5)c(cc3)c3ccc(cc3)c3[nH]c(nc3)[C@H]3N(	1588404-09-4	Carbamic acid, N-[(1S)-1-[[(2S)-2-[5-[2,Ä≤,3,Ä≤-dihydro-7,Ä≤-[4-[2-[(8S)-7-[(2S	C49H62N8O8	891.07	A/B	-9.4
582	C(#C)[C@]1(O)[C@@H](O[C@@](CO)(F)[C@H]1O)n1c2c(nc1)c(=O)nc(N)[nH]2	1613584-82-9	Inosine, 2,Ä≤-deoxyadenylyl-(5,Ä≤,Ü5,Ä≤)-	C20H24N9O10P	581.43	A/B	-9.4
583	O=C1N2[C@H](Cn3c1c(O)c(=O)c(C(=O)NCc1c(F)cc(F)cc1F)c3)O[C@@H]1C[C@H]2CC1	1616340-09-0	2,5-Methanopyrido[1,Ä≤,2,Ä≤:4,5]pyrazino[2,1-b][1,3]oxazepine-10-carboxamide, N	C22H21F2N3O5	445.42	A/B	-9.4
584	O=C1N2[C@@H](Cn3c1c(O)c(=O)c(C(=O)NCc1c(F)cc(F)c(F)c1)c3)O[C@H]1C[C@@H]2CC1	1616340-17-0	2,6-Methanopyrido[1,Ä≤,2,Ä≤:4,5]pyrazino[2,1-b][1,6,3]dioxazocine-11-carboxamide	C21H19F2N3O6	447.39	A/B	-9.4

585	O=C1N2[C@@H](Cn3c1c(O)c(=O)c(C(=O)N[C@H](C)c1c(F)cc(F)cc1F)c3)O[C@H]1C[C@@H]2CC1	1616340-31-8	2,5-Methanopyrido[1,Ä≤,2,Ä≤:4,5]pyrazino[2,1-b][1,3]oxazepine-10-carboxamide, N	C21H18ClF2N3O5	465.83	A/B	-9.4
586	O=C1N2[C@@H](Cn3c1c(O)c(=O)c(C(=O)N[C@H](C(F)(F)F)c1c(F)cc(F)cc1)c3)O[C@H]1C[C@@H]2CC1	1616340-44-3	2,5-Methanopyrido[1,Ä≤,2,Ä≤:4,5]pyrazino[2,1-b][1,3]oxazepine-10-carboxamide, 2	C22H20F3N3O5	463.41	A/B	-9.4
587	O=c1n([C@H]2C[C@H](OP(=O)(S)S)[C@@H](CO)O2)cc(C)c(=O)[nH]1	162008-29-9	Acetamide, 2,2,Ä≤-[1,4,10,13-tetraoxa-7,16-diazacyclooctadecane-7,16-diylbis[(2-	C40H64N4O10	760.96	A/B	-9.4
588	N(c1nc(nc(n1)N1CCOCC1)N1CCOCC1)c1ccc(C(=O)C=Cc2cc(Oc3ccccc3)ccc2)cc1	1628172-07-5	2-Propen-1-one, 3-(4-bromophenyl)-1-[4-[(4,6-di-4-morpholinyl-1,3,5-triazin-2-yl	C26H27BrN6O3	551.43	A/B	-9.4
589	C(=O)([C@@H](NC(=O)OC)C(C)C)N1[C@@H]([C@@H]2C[C@H]1CC2)c1[nH]c(cn1)c1ccc(cc1)c1ccc(cc1)c1[nH]c(nc1)[	1628172-19-9	1,3,5-Triazin-2-amine, N-[4-[2-amino-6-(4-bromophenyl)-4-pyrimidinyl]phenyl]-4,6	C27H28BrN9O2	590.47	A/B	-9.4
590	C(=O)([C@@H](NC(=O)OC)C1CCCCC1)N1[C@@H](CCC1)c1[nH]c(cn1)c1ccc(cc1)c1ccc(cc1)c1[nH]c(nc1)[C@H]1N(C(=	1639871-26-3	Carbamic acid, N,N,Ä≤-[[1,1,Ä≤-biphenyl]-4,4,Ä≤-diylbis[1H-imidazole-5,2-diyl[(	C46H54N8O6	814.97	A/B	-9.4
591	C(P(=O)(OC[C@H]1O[C@H](C=C1)n1c(=O)[nH]c(=O)c(C)c1)OC[C@H]1O[C@H](C=C1)n1c(=O)[nH]c(=O)c(C)c1)(O)c1c	164652-06-6	Thymidine, 2,Ä≤,3,Ä≤-didehydro-P,3,Ä≤-dideoxy-P-[hydroxy(4-nitrophenyl)methyl]t	C27H28N5O12P	645.51	A/B	-9.4
592	O=c1n([C@@H]2O[C@H](COP(=O)(OC[C@H]3O[C@H](C=C3)n3c(=O)[nH]c(=O)c(C)c3)C(CCCCCCC)O)C=C2)cc(C)c(=O)[n	164652-10-2	Thymidine, 2,Ä≤,3,Ä≤-didehydro-P,3,Ä≤-dideoxy-P-[(2,6-dichlorophenyl)hydroxymet	C27H27Cl2N4O10P	669.4	A/B	-9.4
593	N(C(=O)[C@H](Cc1ccccc1)C[C@@H](CN1[C@H](C(=O)NC(C)(C)C)CN(CC2CCCCC2)CC1)O)[C@H]1c2c(C[C@H]1O)cccc2	164725-65-9	1-Piperazinepentanamide, N-(2,3-dihydro-2-hydroxy-1H-inden-1-yl)-4-[(2,3-dihydro	C40H52N4O5	668.86	A/B	-9.4
594	O=c1n([C@@H]2O[C@H](COP(=O)(O)O)[C@@H](O)C2)cc(Cl)c(N)n1	165967-23-7	Guanosine, 2,Ä≤-deoxy-P-thiocytidylyl-(3,Ä≤,Ü5,Ä≤)-2,Ä≤-deoxy-P-thiocytidylyl-	C28H37N11O14P2S2	877.74	A/B	-9.4
595	N(N)C12CC3CC(C2)CC(C1)C3	167773-40-2	Benzamide-carbonyl-11C, N-[[4,Ä≤-[(2-ethyl-5,7-dimethyl-3H-imidazo[4,5-b]pyridir	C30H28N4O3S		A/B	-9.4
596	N(c1nc(nc(n1)N1CCOCC1)N1CCOCC1)c1ccc(C(=O)/C=C/c2c(N(=O)=O)cccc2)cc1	1710363-25-9	2-Propen-1-one, 1-[4-[(4,6-di-4-morpholinyl-1,3,5-triazin-2-yl)amino]phenyl]-3-(	C27H30N6O4	502.56	A/B	-9.4
597	N(c1nc(nc(n1)N1CCOCC1)N1CCOCC1)c1ccc(C(=O)/C=C/c2cc(C)c(C)cc2)cc1	1710363-32-8	2-Propen-1-one, 3-(3-chlorophenyl)-1-[4-[(4,6-di-4-morpholinyl-1,3,5-triazin-2-y	C26H27ClN6O3	506.98	A/B	-9.4
598	C[C@]12[C@H]([C@H]3[C@H](CC2)[C@]2(C)C(CC3)C[C@@H](O)CC2)CC[C@@H]1[C@@](C)(O)[C@@H]1CCCCN1	171781-52-5	Uridine, 2,Ä≤-deoxy-5-[2-(triphenylmethoxy)ethyl]-, 3,Ä≤,5,Ä≤-bis(4-methylbenzo	C46H42N2O8	750.83	A/B	-9.4
599	C(Cc1ccccc1)[C@]1(CCC)CC(=C([C@@H](CC)c2cc(NS(=O)(=O)c3ccc(C(F)(F)F)cn3)ccc2)C(=O)O1)O	174590-26-2	2-Pyridinesulfonamide, N-[3-[(1R)-1-[(6S)-5,6-dihydro-4-hydroxy-2-oxo-6-(2-pheny	C31H33F3N2O5S	602.66	A/B	-9.4
600	C(n1c2c(nc1CC)c(C)cc(C)n2)c1ccc(cc1)c1c(S(=O)(=O)NC(=O)c2cc(O)ccc2)cccc1	174844-69-0	Benzamide, N-[[4,Ä≤-[(2-ethyl-5,7-dimethyl-3H-imidazo[4,5-b]pyridin-3-yl)methyl]	C37H34N4O4S	630.76	A/B	-9.4
601	C(O[C@@H](COC(=O)[C@@H](NC(=O)OCc1ccccc1)C(C)C)COCc1ccccc1)n1c2c(nc1)c(=O)nc(N)[nH]2	175865-56-2	L-Valine, N-[(phenylmethoxy)carbonyl]-, 2-[(2-amino-1,6-dihydro-6-oxo-9H-purin-9	C29H34N6O7	578.62	A/B	-9.4
602	N(C(CCN)(C)C)c1c2c(cc(Cl)cc2)ncc1	177155-68-9	Benzamide, N,N,Ä≤-(methylenedi-4,1-phenylene)bis[2-hydroxy-5-nitro-	C27H20N4O8	528.47	A/B	-9.4
603	C(OP(=S)(Oc1cc2c(cc1)cccc2)N[C@H](C(=O)OC(C)C)C)[C@@]1(C(F)F)O[C@H]([C@@H](O)[C@@H]1O)n1c2c(nc1)c(N)	1803134-23-7	L-Alanine, N-[hydroxy(2-naphthalenyloxy)phosphinyl]-, 1-methylethyl ester, 5,Ä≤-	C27H31F2N6O8P	636.54	A/B	-9.4
604	O=C1[C@]2([C@H]([C@H](/C(=C/C(=O)[C@@H]3[C@@]4(N5[C@@H]([C@H]3c3cc(OC)c(O)cc3)CCC5)c3c(NC4=O)ccc(C)c	1807888-38-5	Not Yet Assigned	C47H48N4O8	796.91	A/B	-9.4
605	O=C1[C@@]2([C@@H]([C@@H](/C(=C/C(=O)[C@@H]3[C@@]4(N5[C@@H]([C@H]3c3cc(OC)c(O)cc3)CCC5)c3c5c(C4=O)ccc	1807888-53-4	Spiro[acenaphthylene-1(2H),3,Ä≤-[3H]pyrrolizin]-2-one, 2,Ä≤-[(1Z)-3-[(1R,1,Ä≤R,	C53H46N2O8	838.94	A/B	-9.4
606	C[C@]1(F)[C@@H](O[C@H](CO[P@](=O)(Oc2ccccc2)N[C@H](C(=O)O)C)[C@H]1O)n1c(=O)[nH]c(=O)cc1	185905-37-7	Cytidine, 2,Ä≤-deoxy-P-methylguanylyl-(3,Ä≤,Ü5,Ä≤)-P-thiothymidylyl-(3,Ä≤,Ü5,	C30H40N10O16P2S	890.71	A/B	-9.4
607	O=c1n([C@@H]2O[C@H](CO)[C@H](S)C2)cc(C)c(=O)[nH]1	186339-93-5	D-erythro-Pentonamide, 2,3,5-trideoxy-N-[(1S,2S,3S)-2,3-dihydro-2,3-dihydroxy-1H	C42H55N5O11		A/B	-9.4
608	N(=Nc1c2c(c(N)cc1)cccc2)c1c2c(cc(S(=O)(=O)O)c1)cc(S(=O)(=O)O)cc2.[NaH]	10114-72-4	2-Naphthalenesulfonic acid, 8-amino-5-[2-[4-[2-[2,4-diamino-5-[2-[3-[2-(2,4-diam	C35H31N13O6S2.2Na		C	-9.4
609	N(=N\c1cc(S(=O)(=O)O)c(CCc2c(S(=O)(=O)O)cc(/N=N/c3c4c(cc(S(=O)(=O)O)c3N)cc(S(=O)(=O)O)cc4)cc2)cc1)/c	1052524-91-0	Not Yet Assigned	C34H28N6O12S4.Na		C	-9.4
610	S(=O)(=O)(O)c1c2c(c(S(=O)(=O)O)cc(/N=N/c3cc(S(=O)(=O)O)c(C=Cc4c(S(=O)(=O)O)cc(/N=N/c5c(N)c6c(c(S(=O)	1052525-21-9	Not Yet Assigned	C34H28N6O18S6.Na		C	-9.4
611	Nc1c2c(cc(S(=O)(=O)O)cc2)ccc1/N=N/c1cc(S(=O)(=O)O)c(/C=C/c2c(S(=O)(=O)O)cc(/N=N/c3c(N)c4c(cc3)cc(S(=	1053056-45-3	Not Yet Assigned	C34H26N6O12S4	838.86	C	-9.4
612	C(=O)([C@H](Cc1cnc[nH]1)NC(=O)[C@@H](NC(=O)[C@H](Cc1ccc(O)cc1)NC(=O)[C@@H](NC(=O)OCc1ccccc1)CC(C)C)[	109014-48-4	2,7-Naphthalenedisulfonic acid, 3,3,Ä≤-[thiobis[(4-methyl-o-phenylene)azo]]bis[4	C34H28N6O12S5.4Na		C	-9.4
613	O=c1n([C@@H]2O[C@H](COP3(=O)Oc4c(CO3)cc(Cc3cc5c(cc3)OP(=O)(OC[C@H]3O[C@H](C=C3)n3c(=O)[nH]c(=O)c(C)c	1099829-92-1	Not Yet Assigned	C35H35FN4O14P2	816.62	C	-9.4
614	O=c1n([C@@H]2O[C@H](COP3(=O)Oc4c(CO3)cc(Cc3cc5c(cc3)OP(=O)(OC[C@@H]3[C@@H](N=[N+]=[N-])C[C@@H](O3)n3	1099829-93-2	Not Yet Assigned	C35H35N7O14P2		C	-9.4
615	N(=Nc1c(CCCC)cccc1)c1c2c(cc(S(=O)(=O)O)cc2)ccc1O	1107652-06-1	3-Pyridinecarboxylic acid, 6,6,Ä≤-dithiobis-, 3,3,Ä≤-bis[(5R,5aR,8aR,9R)-5,5a,6	C56H48N2O18S2	1101.11	C	-9.4
616	C(=O)(OCC)c1c2c(CN(C)C)c(O[C@@H]3O[C@H](C(=O)O)[C@@H](O)[C@H](O)[C@H]3O)c(Br)cc2n(C)c1CSc1ccccc1	1130877-40-5	2-Naphthalenesulfonic acid, 5-amino-8-[2-[4-[2-(4-amino-2-sulfophenyl)diazenyl]-	C26H20N6O9S3.3Na		C	-9.4
617	C(C)n1c2c(c(=O)c(C(=O)O)c1)cc(F)c(c2)N1CCN(Cc2cn(c(=O)[nH]c2=O)[C@H]2C[C@H](N=[N+]=[N-])[C@@H](CO)O2	1174398-27-6	Thymidine, 3,Ä≤-azido-3,Ä≤-deoxy-, 5,Ä≤-[(3S)-9-fluoro-2,3-dihydro-3-methyl-10-	C28H31FN8O7		C	-9.4
618	C(=O)([C@@H](NC(=O)OC)C1CC1)N1[C@H](c2[nH]c(c3cc4c(c5c(o4)cc(cc5)c4[nH]c(nc4)[C@H]4N(C(=O)[C@@H](NC(	1179150-61-8	Carbamic acid, N,N,Ä≤-[3,7-dibenzofurandiylbis[1H-imidazole-5,2-diyl-(2S)-2,1-py	C40H48N8O7	752.86	C	-9.4
619	C(=O)([C@@H](NC(=O)OC)C1CCOCC1)N1[C@H]2[C@H](C2)C[C@H]1c1[nH]c2c(n1)ccc1c2ccc(c1)c1cc(F)c(cc1)c1[nH]	1180670-54-5	Carbamic acid, N-[(1S)-2-[(1R,3S,5R)-3-[2,Ä≤-[(1R,3S,5R)-2-[(2S)-2-[(methoxycarl	C48H52N8O7	852.98	C	-9.4
620	O=c1n([C@@H]2O[C@H](CO[Si](C(C)(C)C)(C)C)[C@@H](N=[N+]=[N-])C2)cc(C)c(=O)[nH]1	1206156-32-2	1-Piperazinecarboxylic acid, 4-methyl-, [[(4R,12aS)-9-[[[(2,4-difluorophenyl)met	C27H31F2N5O7	575.56	C	-9.4
621	C(=O)(N[C@H](C(=O)C(=O)NC1CC1)CCC)[C@H]1N(C(=O)[C@H](NC(=O)[C@H](NC(O)c2cnccn2)C2CCCCC2)C(C)(C)C)CC[	1223091-84-6	Not Yet Assigned	C37H49N5O5	643.82	C	-9.4
622	O(P(=O)(OC[C@H]1O[C@H]([C@H](O)[C@@H]1O)n1nc(C(=O)N)nc1)N[C@H](C(=O)OCc1ccccc1)C)c1c2c(ccc1)cccc2	1228377-63-6	Glycine, N-[hydroxy(1-naphthalenyloxy)phosphinyl]-, phenylmethyl ester, 5,Ä≤-est	C32H34N5O9P	663.61	C	-9.4
623	C(=O)([C@@H](NC(=O)OC)C(C)C)N1[C@@H](CCC1)c1[nH]c(cn1)[C@@H]1CC[C@H](CC1)c1ccc(cc1)c1[nH]c(nc1)[C@H]	1228667-96-6	Carbamic acid, N-[(1R)-2-[(2S)-2-[5-[trans-4-[4-[2-[(2S)-1-[(2R)-2-(dimethylamir	C46H54N8O4	782.97	C	-9.4
624	C(F)(F)c1n(c2c(n1)cccc2)c1nc(nc(N[C@H]2CC[C@H](NC(=O)N3CCOCC3)CC2)n1)N1CCOCC1	1240024-41-2	Urea, N-[trans-4-[[4-[2-(difluoromethyl)-1H-benzimidazol-1-yl]-6-(4-morpholinyl)	C24H31F2N9O2	515.56	C	-9.4
625	C(=O)([C@@H](NC(=O)OC)CC)N1[C@H](c2[nH]c(c3cc4c(-c5c(C4)cc(cc5)c4[nH]c(nc4)[C@H]4N(C(=O)[C@@H](NC(=O	1240880-38-9	Carbamic acid, N,N,Ä≤-[9H-fluorene-2,7-diylbis[1H-imidazole-5,2-diyl-(2S)-2,1-py	C37H42N8O6	694.78	C	-9.4
626	C(=O)([C@H](NC(=O)OC)CC)N1[C@H](c2[nH]c(c3cc4c(-c5c(C4)cc(cc5)c4[nH]c(nc4)[C@H]4N(C(=O)[C@H](NC(=O)O	1240880-47-0	Carbamic acid, N,N,Ä≤-[9H-fluorene-2,7-diylbis[1H-imidazole-5,2-diyl-(2S)-2,1-py	C39H46N8O6	722.83	C	-9.4
627	O=c1n([C@@H]2O[C@H](COP(=O)(Oc3ccccc3)N[C@@H](C(=O)OC)C)[C@@H](N=[N+]=[N-])C2)cc(C)c(=O)[nH]1	1256547-81-5	Carbamic acid, N-[(1S)-2-methyl-1-[[(2S)-2-[5-[6-[4-[2-[(2S,5R)-5-phenyl-2-pyrro	C43H45N7O3	707.86	C	-9.4
628	O=c1c2c(n(cc1C(=O)O)C1CC1)cc(c(F)c2)N1CCN(CN2c3c(/C(=N/NC(=S)NOC)/C2=O)cc(F)cc3)CC1	1265623-17-3	3-Quinolinecarboxylic acid, 1-cyclopropyl-6-fluoro-7-[4-[[(3Z)-5-fluoro-2,3-dihy	C27H25F2N7O5S	597.59	C	-9.4
629	C(=O)([C@@H](NC(=O)OC)C(C)C)N1[C@H]2[C@H](C2)C[C@H]1c1[nH]c(c(C#N)n1)c1ccc(cc1)c1ccc(cc1)c1[nH]c(nc1	1312336-67-6	Carbamic acid, N,N,Ä≤-[[1,1,Ä≤-biphenyl]-4,4,Ä≤-diylbis[(5-fluoro-1H-imidazole-	C46H56F2N8O8	886.98	C	-9.4
630	C(=O)([C@@H](NC(=O)OC)C(C)C)N1[C@H](c2[nH]c(c3c4c(c(cc3)c3ccc(cc3)c3[nH]c(nc3)[C@H]3N(C(=O)Cc5ccccc5	1312608-53-9	Carbamic acid, N-[(1S)-2-methyl-1-[[(2S)-2-[5-[4-[4-[2-[(2S)-1-(2-phenylacetyl)-	C45H47N7O4	749.9	C	-9.4
631	O(Cc1ccc(F)cc1)c1c2c(c(c(c1)c1ccccc1)c1ccccc1)oc(c(O)c2=O)c1nnc(C)[nH]1	1312905-76-2	4H-1-Benzopyran-4-one, 5-[(4-fluorophenyl)methoxy]-3-hydroxy-7,8-diphenyl-2-(1H-	C30H20FN3O4	505.5	C	-9.4
632	O=c1c2c(oc(c1O)c1ncccn1)ccc(OCc1ccc(F)cc1)c2	1312905-85-3	4H-1-Benzopyran-4-one, 5-[(4-fluorophenyl)methoxy]-3-hydroxy-7,8-diphenyl-2-(5-p	C37H25FN2O4	580.6	C	-9.4
633	Cc1c2c(cc(OCc3ccc(F)cc3)c1C)c(=O)c(O)c(o2)c1ncccn1	1312908-83-0	4H-1-Benzopyran-4-one, 6-[(4-fluorophenyl)methoxy]-3-hydroxy-7-methyl-2-(5-pheny	C26H19FN2O4	442.44	C	-9.4
634	O(C)c1c2c(cc(OCc3ccc(F)cc3)c1C)c(=O)c(O)c(o2)c1ncccn1	1312909-45-7	4H-1-Benzopyran-4-one, 6-[(4-fluorophenyl)methoxy]-3-hydroxy-7,8-dimethyl-2-(5-p	C27H21FN2O4	456.46	C	-9.4
635	O(C)c1c2c(cc(OCc3ccc(F)cc3)c1OC)c(=O)c(O)c(o2)c1ncccn1	1312912-93-8	4H-1-Benzopyran-4-one, 7-chloro-6-[(4-fluorophenyl)methoxy]-3-hydroxy-8-methyl-2	C26H18ClFN2O4	476.88	C	-9.4
636	O=c1c2c(cc(c(OCc3ccc(F)cc3)c2)c2ccccc2)oc(c1O)c1[nH]ccn1	1312916-45-2	4H-1-Benzopyran-4-one, 6-[(4-fluorophenyl)methoxy]-3-hydroxy-7-phenyl-2-(3-pheny	C30H20FN3O4	505.5	C	-9.4
637	O(Cc1ccc(F)cc1)c1c(c(Cl)c2c(c1)c(=O)c(O)c(o2)c1[nH]cnn1)c1ccccc1	1312917-86-4	4H-1-Benzopyran-4-one, 8-chloro-6-[(4-fluorophenyl)methoxy]-3-hydroxy-7-phenyl-2	C32H20ClFN2O4	550.96	C	-9.4
638	N(=Nc1cc(C)c(N=Nc2cc(S(=O)(=O)O)ccc2)cc1)c1c2c(c(S(=O)(=O)O)ccc2)c(N)cc1	1345852-38-1	1-Naphthalenesulfonic acid, 8-amino-5-[2-[7-sulfo-4-[2-(7-sulfo-1-naphthalenyl)c	C30H21N5O9S3	691.71	C	-9.4
639	N(=Nc1cc(S(=O)(=O)O)ccc1)c1c2c(c(N=Nc3c4c(c(S(=O)(=O)O)ccc4)c(N)cc3)cc1)ccc(S(=O)(=O)O)c2	1345852-44-9	2-Naphthalenesulfonic acid, 8-amino-5-[2-[4-[2-(4-sulfophenyl)diazenyl]-1-naphth	C26H19N5O6S2	561.59	C	-9.4
640	[C@@H]([C@@H](CN(S(=O)(=O)c1ccc(N)cc1)CC(C)C)OCC[C@@H]1CCOC1)(Cc1ccccc1)NC(=O)O[C@H]1CCOC1	1348000-86-1	6H-Dipyrido[3,2-b:2,Ä≤,3,Ä≤-e][1,4]diazepin-6-one, 2-chloro-11-cyclopropyl-8-[2	C30H27ClN6O5S	619.09	C	-9.4
641	C(n1c2c(nc1CC)c(C)cc(C)n2)c1ccc(cc1)c1c(S(=O)(=O)NC(=O)COCC)ccc(CN2CCCC2)c1	1348325-66-5	Benzoic acid, 4,4,Ä≤-methylenebis[2-[(4-methoxybenzoyl)amino]-	C31H26N2O8	554.55	C	-9.4
642	C(=O)([C@@H](NC(=O)OC)C(C)C)N1[C@@]2(C[C@H](Cl)CC2)c1[nH]c(cn1)c1c2c(c(cc1)c1ccc(cc1)c1[nH]c(nc1)[C@	1350719-00-4	Carbamic acid, N-[(1S)-2-methyl-1-[[(1S,4R)-1-[5-[4-[5,6,7,8-tetrahydro-4-[2-[(1	C48H60N8O6	845.04	C	-9.4
643	C(=O)([C@@H](NC(=O)OC)C(C)C)N1[C@H](c2[nH]c(c3c4c(c(cc3)c3c5c(c(cc3)c3[nH]c(nc3)[C@H]3N(C(=O)[C@@H](	1350719-39-9	Carbamic acid, N,N,Ä≤-[[8,8,Ä≤-biquinoline]-5,5,Ä≤-diylbis[1H-imidazole-5,2-diy	C46H52N10O6	840.97	C	-9.4
644	O[C@H]1[C@@H](O[C@H](COC(=O)CCCCCCC/C=C/CCCCCCCC)[C@H]1O)n1nc(C(=O)N)nc1	1352918-39-8	Uridine 5,Ä≤-(tetrahydrogen triphosphate), 5-[4-(5-amino-2-benzoxazolyl)-2-fluor	C22H22FN4O15P3	694.35	C	-9.4
645	S(=O)(=O)(O)c1c2c(c(N)c(N=Nc3ccc(cc3)c3cc(C)ccc3)c1)cccc2	1357245-71-6	1-Naphthalenesulfonic acid, 4-amino-3-[2-(4,Ä≤-methyl[1,1,Ä≤-biphenyl]-4-yl)dia	C23H19N3O3S	417.48	C	-9.4
646	C(OC(=O)CCC(=O)O[C@H]([C@H](Cc1ccccc1)NC(=O)COc1c(C)cccc1C)C[C@H](Cc1ccccc1)NC(=O)[C@H](C(C)C)N1C(=O	1370468-36-2	Carbamic acid, N,N,Ä≤-[[(6S)-6-phenyl-6H-indolo[1,2-c][1,3]benzoxazine-3,10-diyl	C49H55N9O7	882.02	C	-9.4
647	S(=O)(=O)(O)c1c(ccc(N=Nc2c3c(ccc(S(=O)(=O)O)c3)c(N)cc2)c1)c1c(S(=O)(=O)O)cc(N=Nc2c3c(ccc(S(=O)(=O)O)	1375454-32-2	[1,1,Ä≤-Biphenyl]-2,2,Ä≤-disulfonic acid, 4,4,Ä≤-bis[2-[2,4-diamino-5-sulfo-3-[	C44H34N12O18S6	1211.2	C	-9.4
648	C(=O)([C@@H](NC(=O)OC)C(C)C)N1[C@@H](CCC1)c1[nH]c(cn1)c1cc2c(cc1)cc(cc2)c1cc(ccc1)c1[nH]c(nc1)[C@H]1	1377038-87-3	Carbamic acid, N-[(1R)-2-[(2S)-2-[5-[4-[6-[2-[(2S)-1-[(2S)-2-[(methoxycarbonyl)a	C48H52N8O6	836.98	C	-9.4
649	C(=O)([C@@H](NC(=O)OC)C(C)C)N1[C@H]2[C@H](C2)C[C@H]1c1[nH]c2c(n1)ccc1c2ccc(c1)c1ccc(cc1)c1[nH]c(nc1C	1377043-10-1	Carbamic acid, N-[(1S)-2-[(1R,3S,5R)-3-[7-[4-[2-[(1R,3S,4S)-2-[(2S)-2-[(methoxyc	C47H54N8O7	842.98	C	-9.4
650	C(=O)([C@@H](NC(=O)OC)C(C)C)N1[C@@H]([C@@H]2C[C@H]1CC2)c1[nH]c2c(n1)ccc1c2ccc(c1)c1cc(F)c(cc1)c1[nH]	1377043-55-4	Carbamic acid, N-[(1S)-1-[[(1R,3S,5R)-3-[5-[2-fluoro-4-[2-[(1R,3S,4S)-2-[(2S)-2-	C45H51FN8O6	818.93	C	-9.4
651	C(=O)(O)[C@]12[C@@H]([C@@H]3[C@@](C)(CC1)[C@@]1(C)[C@H](CC3)[C@]3(C)[C@@H](CC1)C(C)(C)[C@@H](OCOC)CC	1399935-96-6	1,4-Benzenediamine, N1-[4-chloro-6-(4-morpholinyl)-1,3,5-triazin-2-yl]-N4-(7-chl	C22H19Cl2N7O	468.34	C	-9.4
652	C(=O)([C@@H](NC(=O)OC)[C@@H]1C[C@@H]2[C@H](C1)C2)N1[C@H]2[C@H](C2)C[C@H]1c1[nH]c(cn1)c1ccc(cc1)c1ccc	1413124-78-3	Carbamic acid, N,N,Ä≤-[[1,1,Ä≤-biphenyl]-4,4,Ä≤-diylbis[1H-imidazole-5,2-diyl-(	C48H50F4N8O6	910.95	C	-9.4
653	O[C@H]1[C@@H](O[C@H](C(=O)N[C@@H](C(=O)N2CCN(CC(=O)Nc3ccccn3)CC2)Cc2ccccc2)[C@H]1O)n1nc(C(=O)N)nc1	1414794-00-5	1H-Indole-3-propanamide, Œ±-[[[(2S,3S,4R,5R)-5-[3-(aminocarbonyl)-1H-1,2,4-triaz	C26H32N8O7	568.58	C	-9.4
654	C(=O)([C@@H](NC(=O)OC)C(C)C)N1[C@H](c2[nH]c(c3c4c(c(cc3)c3ccc(cc3)c3[nH]c(nc3)[C@H]3N(C(=O)[C@@H](NC	1419385-39-9	Carbamic acid, N,N,Ä≤-[(1,Ä≤,3,Ä≤-dihydrospiro[cyclopentane-1,2,Ä≤-[2H]indene]-	C53H64N8O6	909.13	C	-9.4
655	C(OP(=O)(O[C@H]1[C@@H](O[C@H](COP(=O)(O[C@H]2[C@@H](O[C@H](CO)[C@H]2O)n2c3c(nc2)c(N)ncn3)O)[C@H]1O)n	1422459-51-5	Adenosine, 1,Ä≤-[3-(aminocarbonyl)-1H-1,2,4-triazol-1-yl]-1,Ä≤-de(6-amino-9H-pu	C28H34N14O16P2	884.6	C	-9.4
656	O[C@H]1[13C@H]([13c]2c3c([nH]c2)c(=O)nc[nH]3)[15NH][C@H](CO)[C@H]1O	1469875-66-8	Not Yet Assigned	C60H53N10O15P	1185.09	C	-9.4
657	C(n1c2c(nc1CC)c(C)cc(C)n2)c1ccc(cc1)c1c(S(=O)(=O)NC(=O)c2ccccc2)ccc(OCC)c1	154553-20-5	Benzamide, N-[[4,Ä≤-[(2-ethyl-5,7-dimethyl-3H-imidazo[4,5-b]pyridin-3-yl)methyl]	C32H32N4O4S	568.69	C	-9.4
658	C(n1c2c(nc1CC)c(C)cc(C)n2)c1ccc(cc1)c1c(S(=O)(=O)NC(=O)c2ccccc2)ccc(C(C)(C)C)c1	154553-23-8	Benzamide, N-[[5-(1,1-dimethylpropyl)-4,Ä≤-[(2-ethyl-5,7-dimethyl-3H-imidazo[4,5	C35H38N4O3S	594.77	C	-9.4
659	CN1C2c3c(C1CC2)c(ccc3c1[nH]c(nc1)[C@H]1N(C(=O)[C@@H](NC(=O)OC)C(C)C)C[C@H](F)C1)c1ccc(cc1)c1[nH]c(nc	1560639-50-0	Carbamic acid, N-[(1S)-1-cyclohexyl-2-[(2S)-2-[5-[8-[4-[2-[(2S)-1-[(2S)-2-cycloh	C51H65N9O6	900.12	C	-9.4
660	C(=O)([C@@H](NC(=O)OC)C(C)C)N1[C@H](c2[nH]c(c3c4c(c(cc3)c3ccc(cc3)c3[nH]c(nc3)[C@H]3N(C(=O)[C@@H](NC	1560639-90-8	Carbamic acid, N,N,Ä≤-[(1,2,3,4-tetrahydro-1,4-ethanonaphthalene-5,8-diyl)bis[4,	C52H62N8O6	895.1	C	-9.4
661	C(=O)([C@@H](NC(=O)OC)C(C)C)N1[C@H](c2[nH]c(c3c4c(c(cc3)c3ccc(cc3)c3[nH]c(nc3)[C@H]3N(C(=O)[C@@H](NC	1560640-31-4	Carbamic acid, N,N,Ä≤-[(1,2,3,4-tetrahydro-1,4-ethanonaphthalene-5,8-diyl)bis[4,	C54H66N8O6	923.15	C	-9.4
662	CN1C2c3c(C1CC2)c(ccc3c1[nH]c(nc1)[C@H]1N(C(=O)[C@@H](NC(=O)OC)C(C)C)CCC1)c1ccc(cc1)c1[nH]c(nc1)[C@H]	1560649-18-4	Carbamic acid, N-[(1S)-1-[[(2S,4S)-4-fluoro-2-[5-[8-[4-[2-[(2S,4S)-4-fluoro-1-[(	C45H54F2N8O6	840.96	C	-9.4
663	C(=O)([C@@H](NC(=O)OC)C(C)C)N1[C@H](c2[nH]c(c3c4c(c(cc3)c3c5c(c(cc3)c3[nH]c(nc3)[C@H]3N(C(=O)[C@@H](	1627533-30-5	Carbamic acid, N,N,Ä≤-[(1,1,Ä≤,2,2,Ä≤,3,3,Ä≤,4,4,Ä≤-octahydro[5,5,Ä≤-bi-1,4-me	C50H60F2N8O6	907.06	C	-9.4
664	C(=O)([C@@H](NC(=O)OC)C(C)C)N1[C@H](c2[nH]c(c3c4c(c(cc3)c3c5c(c(cc3)c3[nH]c(nc3)[C@H]3N(C(=O)[C@@H](	1627533-95-2	Carbamic acid, N-[(1S)-2-methyl-1-[[(2S)-2-[5-[1,2,3,4-tetrahydro-2-methyl-3-oxo	C50H61N9O7	900.08	C	-9.4
665	O=c1n([C@@H]2O[C@H](COP(=O)(C(O)c3c(N(=O)=O)cc(N(=O)=O)cc3)OC[C@@H]3[C@@H](N=[N+]=[N-])C[C@@H](O3)n3	164652-14-6	Thymidine, 3,Ä≤-azido-P,3,Ä≤-dideoxy-P-[(2,6-dinitrophenyl)hydroxymethyl]thymid	C27H29N12O14P		C	-9.4
666	C(=O)([C@@H](NC(=O)OC)C(C)C)N1C[C@@]2(C[C@H]1c1[nH]c3c(n1)ccc1c3ccc(c1)c1ccc(cc1)c1[nH]c(nc1)[C@H]1N	1653967-03-3	Carbamic acid, N-[(1S)-2-methyl-1-[[(2S,3aS,7aS)-octahydro-2-[1,4,5,6-tetrahydro	C51H66N8O7	903.12	C	-9.4
667	O=C1[C@@H]2[C@@H](c3c([C@H](OC(=O)N4CCN(Cc5ccc(cc5)n5c(n[nH]c5=O)c5cc(C(C)C)c(O)cc5O)CC4)[C@H]2CO1)c	1680212-58-1	1-Piperidinecarboxylic acid, 4-[2-[5-[3-[2,4-dihydroxy-5-(1-methylethyl)phenyl]-	C49H51N5O12	901.96	C	-9.4
668	S(=O)(=O)(O)c1c2c(c(N)c(N=Nc3ccc(cc3)c3ccc(cc3)c3ccc(N=Nc4c(N)c5c(c(S(=O)(=O)O)c4)cccc5)cc3)c1)cccc2	17095-35-1	1-Naphthalenesulfonic acid, 3,3,Ä≤-[1,4-phenylenebis(azo)]bis[4-amino-	C26H20N6O6S2	576.6	C	-9.4
669	N(c1nc(nc(n1)N1CCOCC1)N1CCOCC1)c1ccc(C(=O)/C=C/c2c(N(=O)=O)cccc2)cc1	1710363-25-9	2-Propen-1-one, 1-[4-[(4,6-di-4-morpholinyl-1,3,5-triazin-2-yl)amino]phenyl]-3-(	C27H30N6O4	502.56	C	-9.4
670	O[C@@H]1[C@H](n2c3c(nc2)c(N)nc(F)n3)O[C@H](CO[P@](=O)O[C@@H](CCO2)c2ccncc2)[C@H]1O	1710363-64-6	1,3,5-Triazin-2-amine, N-[4-[2-amino-6-[4-(diethylamino)phenyl]-4-pyrimidinyl]ph	C31H38N10O2	582.7	C	-9.4
671	C(n1c2c(nc1CC)c(C)cc(C)n2)c1ccc(cc1)c1c(S(=O)(=O)NC(=O)c2cc(O)ccc2)cccc1	174844-69-0	Benzamide, N-[[4,Ä≤-[(2-ethyl-5,7-dimethyl-3H-imidazo[4,5-b]pyridin-3-yl)methyl]	C37H34N4O4S	630.76	C	-9.4
672	C(n1c2c(nc1CC)c(C)cc(C)n2)c1ccc(cc1)c1c(S(=O)(=O)NC(=O)c2ccc(OC)cc2)cccc1	174844-72-5	Benzamide, N-[[4,Ä≤-[(2-ethyl-5,7-dimethyl-3H-imidazo[4,5-b]pyridin-3-yl)methyl]	C37H34N4O4S	630.76	C	-9.4
673	C(CN1CCC(CN2c3c4c(S2(=O)=O)cccc4ccc3)CC1)(C12CC3CC(C2)CC(C1)C3)N1CCOCC1	178478-35-8	Ethanone, 2-[4-[(1,1-dioxido-2H-naphth[1,8-cd]isothiazol-2-yl)methyl]-1-piperidi	C28H35N3O3S	493.66	C	-9.4
674	ClC1(Cl)[C@H]2[C@H](O[C@H]1n1c(=O)nc(N)cc1)CO[P@](=O)(OC(C)C)O2	178609-80-8	2,7-Naphthalenedisulfonic acid, 3-amino-6-[2-[4-[2-(2-naphthalenyl)diazenyl]-1-r	C30H21N5O6S2.2Li		C	-9.4
675	O=C1[C@]2([C@H]([C@H](/C(=C/C(=O)[C@@H]3[C@@]4(N5[C@@H]([C@H]3c3cc(OC)c(O)cc3)CCC5)c3c(NC4=O)ccc(I)c	1807888-26-1	Not Yet Assigned	C45H42I2N4O8	1020.65	C	-9.4
676	C(=O)([C@@H](NC(=O)OC)C(C)C)N1[C@@H](C2CC1CC2)c1[nH]c(cn1)c1ccc(cc1)c1ccc(cc1)c1ccc(cc1)c1[nH]c(nc1)	1808296-00-5	Carbamic acid, N-[(1S)-1-[[(2S)-2,5-dihydro-2-[5-[4,Ä≤-[2-[(3S)-2-[(2S)-2-[(metl	C42H50N8O6	762.9	C	-9.4
677	O=C1[C@@H]2[C@@H](c3c([C@H](OC(=O)[C@@H](C)N4C(=O)C5C(C4=O)CCCC5)[C@H]2CO1)cc1c(c3)OCO1)c1cc(OC)c(OC	1809321-45-6	2H-Isoindole-2-acetic acid, 1,3-dihydro-Œ±-methyl-1,3-dioxo-, (5R,5aR,8aR,9R)-5,	C33H29NO11	615.58	C	-9.4
678	O(COC(=O)OC)c1c2n(N([C@H]3N(C2=O)CCOC3)[C@H]2c3c(CSc4c2cccc4)c(F)c(F)cc3)ccc1=O	182959-81-5	1,3,5-Naphthalenetrisulfonic acid, 7-[2-[4-[2-(4-amino-7-sulfo-1-naphthalenyl)di	C30H21N5O15S5	851.84	C	-9.4
679	N(c1nc(nc(Nc2ccc(C(=O)C=Cc3cccs3)cc2)n1)N1CCOCC1)c1cc(C(F)(F)F)ccc1	1883795-12-7	2-Propen-1-one, 3-(3-chlorophenyl)-1-[4-[[4-(4-morpholinyl)-6-[[3-(trifluorometh	C29H24ClF3N6O2	580.99	C	-9.4
680	O(C(=O)c1ccccc1)[C@H]1[C@@H](O[C@H](COP(=O)(O[C@H]2[C@@H](O[C@H](CO)[C@H]2OC(=O)c2ccccc2)n2nc(C(=O)N	190141-38-9	Adenosine, 1,Ä≤-[3-(aminocarbonyl)-1H-1,2,4-triazol-1-yl]-3,Ä≤-O-benzoyl-P-(2-c	C72H59ClN9O16P	1372.72	C	-9.4
681	C(OC[C@@H]1[C@@H](OC(=O)c2ccccc2)[C@@H](OP(=O)(OC[C@@H]2[C@@H](OC(=O)c3ccccc3)[C@@H](OP(=O)(OC[C@@H]	190141-44-7	Adenosine, adenylyl-(2,Ä≤,Ü5,Ä≤)-1,Ä≤-[3-(aminocarbonyl)-1H-1,2,4-triazol-1-yl	C28H36N14O17P2	902.62	C	-9.4
682	Fc1c2[C@H](N3n4c(C(=O)N5[C@H]3COCC5)c(O)c(=O)cc4)c3c(SCc2ccc1)cccc3	1985605-63-7	1H-[1,4]Oxazino[3,4-c]pyrido[2,1-f][1,2,4]triazine-6,8-dione, 12-[(11S)-7-chlorc	C24H20ClN3O4S	481.95	C	-9.4
683	O(P(=O)(N[C@H](C(=O)OCC)C)OC(C)C)c1c2n(N([C@H]3N(C2=O)CCOC3)[C@H]2c3c(CSc4c2cccc4)c(F)c(F)cc3)ccc1=O	1985606-76-5	1H-[1,4]Oxazino[3,4-c]pyrido[2,1-f][1,2,4]triazine-6,8-dione, 12-[(11S)-7,8-difl	C29H23F2N3O7S	595.57	C	-9.4
684	O(COC(=O)O[C@@H](C(=O)OC)C)c1c2n(N([C@H]3N(C2=O)CCOC3)[C@H]2c3c(CSc4c2cccc4)c(F)c(F)cc3)ccc1=O	1985606-96-9	Benzeneacetic acid, Œ±-[[[[[(12aR)-12-[(11S)-7,8-difluoro-6,11-dihydrodibenzo[b,	C35H29F2N3O9S	705.68	C	-9.4
685	O(COC(=O)O[C@@H]1C(C)(C)COC1=O)c1c2n(N([C@H]3N(C2=O)CCOC3)[C@H]2c3c(CSc4c2cccc4)c(F)c(F)cc3)ccc1=O	1985607-01-9	Benzoic acid, 2-[[[[[(12aR)-12-[(11S)-7,8-difluoro-6,11-dihydrodibenzo[b,e]thiep	C34H27F2N3O9S	691.65	C	-9.4
686	O(COC(=O)O[C@H](C(=O)OC)C)c1c2n(N([C@H]3N(C2=O)CCOC3)[C@H]2c3c(CSc4c2cccc4)c(F)c(F)cc3)ccc1=O	1985607-05-3	Benzeneacetic acid, Œ±-[[[[[(12aR)-12-[(11S)-7,8-difluoro-6,11-dihydrodibenzo[b,	C35H29F2N3O9S	705.68	C	-9.4
687	O(C(=O)OCC(=O)OCC)c1c2n(N([C@H]3N(C2=O)CCOC3)[C@H]2c3c(CSc4c2cccc4)c(F)c(F)cc3)ccc1=O	1985607-54-2	Benzeneacetic acid, Œ±-[[[[(12aR)-12-[(11S)-7,8-difluoro-6,11-dihydrodibenzo[b,e	C34H27F2N3O8S	675.66	C	-9.4
688	O(Cc1ccccn1)c1c2n(N([C@H]3N(C2=O)CCOC3)[C@H]2c3c(CSc4c2cccc4)c(F)c(F)cc3)ccc1=O	1985607-59-7	1H-[1,4]Oxazino[3,4-c]pyrido[2,1-f][1,2,4]triazine-6,8-dione, 12-[(11S)-7,8-difl	C30H24F2N4O4S	574.6	C	-9.4
689	C(=O)([C@@H](NC(=O)OC)C(C)C)N1[C@H](c2[nH]c3c4c(cc(cc4)c4c5c(c(cc4)c4[nH]c(nc4Cl)[C@H]4N(C(=O)[C@@H]	2007901-51-9	Carbamic acid, N-[(1S)-2-[(2S,5S)-2-[5-[2,3-dihydro-7-[2-[(2S,5S)-1-[(2S)-2-[(me	C49H60N8O7	873.05	C	-9.4
690	S(Cc1cc2c(oc1=O)cccc2)c1n(c2c(n1)c(=O)nc[nH]2)[C@@H]1O[C@H](CO)[C@@H](O)[C@H]1O	2020089-26-1	Carbamic acid, N-[(1S)-1-[[(2S)-2-[5-[7-[2-[(2S)-4,4-difluoro-1-[(2S)-2-[(methox	C44H50F4N8O6	862.91	C	-9.4
691	O=c1n([C@@H]2O[C@H](COP(=O)(O[C@@H]3[C@@H](CO)O[C@H](C3)n3c(=O)[nH]c(=O)c(C)c3)O)[C@@H](OP(=O)(OC[C@	2024571-97-7	Not Yet Assigned	C29H37IN6O19P2	962.48	C	-9.4
692	[C@@H](Cc1ccccc1)(NC(=O)[C@H](CC(=O)N1CCOCC1)NC(=O)N(Cc1nc(C(C)C)sc1)C)CC[C@H](Cc1ccccc1)NC(=O)OCc1c	1004318-00-6	2,7,10,12-Tetraazatridecanoic acid, 12-methyl-13-[2-(1-methylethyl)-4-thiazolyl]	C41H54N8O6S2	819.05	A/B	-9.3
693	C(=O)([C@H](NC(=O)OC)c1ccccc1)N1[C@@H](CCC1)c1[nH]c(cn1)c1ccc(cc1)c1ccc(cc1)c1[nH]c(nc1)[C@H]1N(C(=O	1009108-67-1	Carbamic acid, N-[(1R)-2-[(2S)-2-[5-[4,Ä≤-[2-[(2S)-1-[2-(4-methylphenyl)acetyl]-	C45H45N7O4	747.88	A/B	-9.3
694	C(=O)([C@H](NC(=O)OC)c1ccccc1)N1[C@@H](CCC1)c1[nH]c(cn1)c1cc(C)c(cc1)c1ccc(cc1)c1[nH]c(nc1)[C@H]1N(C	1009113-19-2	Carbamic acid, N,N,Ä≤-[(2,2,Ä≤-dimethyl[1,1,Ä≤-biphenyl]-4,4,Ä≤-diyl)bis[1H-imi	C48H50N8O6	834.96	A/B	-9.3
695	N(CCCC)[C@H]1[C@H]2[C@H]3[C@@](C)([C@@H]([C@@H](CCCC(C)C)C)CC3)CC[C@@H]2[C@]2(C)[C@@H](Cl)C[C@@H](O)	101609-53-4	Benzenesulfonic acid, 4-[2-(2-hydroxy-4-phenanthrenyl)diazenyl]-	C20H14N2O4S	378.4	A/B	-9.3
696	C(=C\c1ccc(C)cc1)/c1c(Nc2c(C)cc(/C=C/C#N)cc2C)nc(Nc2ccc(C#N)cc2)nc1COC	1037452-83-7	Benzonitrile, 4-[[4-[[4-[(1E)-2-cyanoethenyl]-2,6-dimethylphenyl]amino]-6-(metho	C33H27F3N6O	580.6	A/B	-9.3
697	C(=C\1/C=C(C(=O)O)C(=O)C=C1)(/c1c(O)c(C(=O)O)ccc1)\c1cc(C(=O)O)c(O)cc1	1049974-61-9	Benzoic acid, 5-[(Z)-(3-carboxy-4-hydroxyphenyl)(3-carboxy-4-oxo-2,5-cyclohexadi	C30H20O12	572.47	A/B	-9.3
698	C(=O)(NC(C)(C)C)[C@@H]1CN(C2c3c(CC2)cccc3)CCN1C[C@H](C[C@H](C(=O)N[C@H]1c2c(C[C@H]1O)cccc2)Cc1ccccc1	1064475-92-8	Not Yet Assigned	C44H55N5O5	733.94	A/B	-9.3
699	C(OCCOP(=O)(C)O)n1c2c(nc1)c(=O)nc(N)[nH]2	1085743-78-7	D-glycero-Pentonamide, 2,3,5-trideoxy-N-[(1S,2R)-2,3-dihydro-2-hydroxy-1H-inden-	C36H47N5O4	613.79	A/B	-9.3
700	C(C=C(c1ccccc1)c1ccccc1)N1CCN(C23CC4CC(C3)CC(C2)C4)CC1	108768-11-2	1,2-Ethanediamine, N2-[2-(10,11-dihydro-5H-dibenzo[a,d]cyclohepten-5-ylidene)eth	C35H40N2	488.71	A/B	-9.3
701	O=c1n([C@@H]2O[C@H](COP3(=O)Oc4c(CO3)cc(Cc3cc5c(cc3)OP(=O)(OC[C@H]3O[C@H](C=C3)n3c(=O)[nH]c(=O)c(C)c	1099829-91-0	Not Yet Assigned	C35H36FN7O14P2		A/B	-9.3

702	O=c1c2c(n(cn2)[C@H]2C[C@H](OP(=O)(OCCCOP(=O)(OC[C@H]3O[C@H](C[C@@H]3O)n3c4c(nc3)cnc(N)n4)O)O)[C@@H](	1105693-48-8	Guanosine, 2,Ä≤-deoxyguanylyl-(3,Ä≤,Ü5,Ä≤)-2,Ä≤,6-dideoxy-	C20H25N10O9P	580.45	A/B	-9.3
703	C(#Cc1ccc(F)cc1)c1n(nc(C(=O)N)n1)[C@@H]1O[C@H](CO)[C@@H](O)[C@H]1O	1106736-04-2	1H-1,2,4-Triazole-3-carboxamide, 1-Œ≤-D-ribofuranosyl-5-[2-[3-(trifluoromethyl)p	C17H15F3N4O5	412.32	A/B	-9.3
704	O=c1n([C@@H]2O[C@H](COP(=O)(OP(=O)(O[C@@H]3O[C@@H]([C@@H](C)O)[C@H](O)[C@H]3O)O)O)[C@@H](O)C2)cc(C)c	112475-41-9	Œ≤-D-arabino-3,Ä≤-Adenylic acid, Œ≤-D-arabino-uridylyl-(3,Ä≤,Ü5,Ä≤)-Œ≤-D-arab	C38H48N14O29P4	1288.76	A/B	-9.3
705	C(Cc1ccccc1)[C@]1(CCC)CC(=C([C@H](CC)c2cc(c(NS(=O)(=O)c3ccc(C(F)(F)F)cn3)c(c(c2[2H])[2H])[2H])[2H])C(	1126619-23-5	Not Yet Assigned	C31H27D6F3N2O5S		A/B	-9.3
706	N(=C\1/c2c(N(C)C1=O)ccc(S(=O)(=O)N1CCOCC1)c2)\NC(=S)Nc1c(C)cccc1	1164493-65-5	Hydrazinecarbothioamide, 2-[1,2-dihydro-1-methyl-5-(4-morpholinylsulfonyl)-2-oxo	C22H25N5O4S2	487.6	A/B	-9.3
707	Cc1n(c2nc(nc(n2)N2CCOCC2)c2cc(O)ccc2)ccn1	1174190-66-9	Benzonitrile, 3-[4-(2-methyl-1H-benzimidazol-1-yl)-6-(4-morpholinyl)-1,3,5-triaz	C22H19N7O	397.43	A/B	-9.3
708	C(=O)([C@@H](NC(=O)OC)C1CCOCC1)N1[C@H]2[C@H](C2)C[C@H]1c1[nH]c2c(n1)ccc1c2ccc(c1)c1cc(F)c(cc1)c1[nH]	1180670-54-5	Carbamic acid, N-[(1S)-2-[(1R,3S,5R)-3-[2,Ä≤-[(1R,3S,5R)-2-[(2S)-2-[(methoxycarb	C48H52N8O7	852.98	A/B	-9.3
709	O=c1n([C@H]2O[C@@H](CO)C=C2)ccc(=O)[nH]1	1217860-55-3	Not Yet Assigned	C31H28D5F3N2O6S		A/B	-9.3
710	N(C(=O)[C@H](Cc1ccccc1)C[C@@H](CN1[C@H](C(=O)NC(C)(C)C)CN(Cc2nc3c(s2)cccc3)CC1)O)C1c2c(C[C@H]1O)cccc	1223083-11-1	Not Yet Assigned	C38H47N5O5	653.81	A/B	-9.3
711	C(=O)([C@H](NC(=O)OC)c1ccccc1)N1[C@@H](CCC1)c1[nH]c(cn1)[C@H]1CC[C@@H](CC1)c1ccc(cc1)c1[nH]c(nc1)[C@	1228667-95-5	Carbamic acid, N-[(1R)-2-[(2S)-2-[5-[4-[trans-4-[2-[(2S)-1-[(2R)-2-(dimethylamir	C46H54N8O4	782.97	A/B	-9.3
712	C(NC(=O)[C@H](Cc1ccc(O)cc1)N)(C(=O)NCC(=O)N[C@@H](Cc1ccccc1)C(=O)NCC(=O)O)C12CC3CC(C2)CC(C1)C3	1229574-79-1	L-Methionine, L-tryptophyl-2-tricyclo[3.3.1.13,7]dec-1-ylglycylglycyl-L-phenylal	C39H50N6O6S	730.92	A/B	-9.3
713	[C@H](Cc1ccccc1)(C[C@@H]([C@H](Cc1ccccc1)NC(=O)OCc1cncs1)O)NC(=O)[C@H](NC(=O)N(Cc1nc(CN(C)C)sc1)C)C(	1236407-81-0	2,7,10,12-Tetraazatridecanoic acid, 4-hydroxy-12-methyl-9-(1-methylethyl)-8,11-c	C42H48N6O5S2	781	A/B	-9.3
714	N(C(=O)[C@H](Cc1ccccc1)C[C@@H](CN1[C@H](C(=O)NC(C)(C)C)CN(Cc2ccsc2)CC1)O)[C@H]1c2c(C[C@H]1O)cccc2	1237988-64-5	D-erythro-Pentonamide, 2,3,5-trideoxy-N-[(1S,2R)-2,3-dihydro-2-hydroxy-1H-inden-	C36H48N4O4S	632.86	A/B	-9.3
715	C(F)(F)c1n(c2c(n1)cccc2)c1nc(nc(N[C@H]2CC[C@H](NC(=O)N(CCN(C)C)C)CC2)n1)N1CCOCC1	1240024-62-7	4-Morpholinecarboxamide, N-[trans-4-[[4-[2-(difluoromethyl)-1H-benzimidazol-1-yl	C26H33F2N9O3	557.6	A/B	-9.3
716	C(F)(F)c1n(c2c(n1)cccc2)c1nc(nc(N[C@H]2CC[C@H](N)CC2)n1)N1[C@H](C)COCC1	1240025-19-7	Acetamide, N-[trans-4-[[4-[2-(difluoromethyl)-1H-benzimidazol-1-yl]-6-(4-morphol	C23H28F2N8O2	486.52	A/B	-9.3
717	C(F)(F)c1n(c2c(n1)cccc2)c1nc(nc(N[C@H]2CC[C@H](NC(=O)C(C)C)CC2)n1)N1CCOCC1	1240029-10-0	Propanamide, N-[trans-4-[[4-[2-(difluoromethyl)-1H-benzimidazol-1-yl]-6-(4-morph	C24H30F2N8O2	500.54	A/B	-9.3
718	C(=O)(NC)c1c2c(cc(N(CCOP(=O)(OC[C@H]3O[C@H]([C@H](O)[C@@H]3O)n3nc(C(=O)N)nc3)O)S(=O)(=O)C)c(c2)C2CC2	1246280-90-9	Not Yet Assigned	C33H38FN6O12PS	792.72	A/B	-9.3
719	C(=O)([C@H](NC(=O)OC)c1ccccc1)N1[C@@H](CCC1)c1[nH]c(cn1)c1ccc(cc1)c1ccc(cc1)c1ccc(cc1)c1[nH]c(nc1)[C	1250936-29-8	Carbamic acid, N,N,Ä≤-[1,2-ethanediylbis[4,1-phenylene-1H-imidazole-5,2-diyl-(2S	C48H50N8O6	834.96	A/B	-9.3
720	[C@@H](NC(=O)[C@H](Cc1ccccc1)NC(=O)N1CCN(C)CC1)(/C=C/S(=O)(=O)c1ccccc1)c1ccc2c(cc1)cccc2	1253912-33-2	1-Piperazinecarboxamide, 4-methyl-N-[(1S)-2-[[(1R,2E)-1-(1-naphthalenyl)-3-(pher	C34H36N4O4S	596.74	A/B	-9.3
721	N(C(=O)Nc1ccc(N(C)C)cc1)c1ccc(c2nc(nc(n2)N2CCOCC2)N2C3CCC2COC3)cc1	1254065-96-7	Urea, N-[4-[4-(4-morpholinyl)-6-(3-oxa-8-azabicyclo[3.2.1]oct-8-yl)-1,3,5-triazi	C26H29N7O3	487.55	A/B	-9.3
722	N(C(=O)Nc1ccc(CCN(C)C)cc1)c1ccc(c2nc(N3C4CCC3COC4)nc(n2)N2CCOCC2)cc1	1254065-98-9	Urea, N-[4-(dimethylamino)phenyl]-N,Ä≤-[4-[4-(4-morpholinyl)-6-(3-oxa-8-azabicyc	C28H34N8O3	530.62	A/B	-9.3
723	C(=O)([C@@H](NC(=O)OC)C(S(=O)(=O)C)(C)C)N1[C@@H](CCC1)c1[nH]c(cn1)c1ccc(cc1)c1ccc(cc1)c1[nH]c(nc1)[C	1256386-77-2	Carbamic acid, N-[(1S)-1-[[(2S)-2-[5-[4,Ä≤-[2-[1-[[(2S)-2-[(methoxycarbonyl)amir	C40H50N8O6	738.88	A/B	-9.3
724	O(C)c1c2n(cc(C(=O)O)c(=O)c2cc(F)c1N1CCN(CN2c3c(C(=NNC(=S)N)C2=O)cccc3)CC1)C1CC1	1262891-47-3	2H-Indol-2-one, 3,3,Ä≤-[methylenebis[(1,5-dihydro-5-thioxo-4H-1,2,4-triazole-3,4	C25H16Br2N10O4S2	744.4	A/B	-9.3
725	C(=O)([C@H](Cc1cnc[nH]1)NC(=O)[C@@H](NC(=O)[C@H](Cc1ccc(O)cc1)NC(=O)c1cccnc1)CCCCNC(=O)[C@@H](NC(=O)	127052-34-0	Uridine, 5,Ä≤-O-phosphonoadenylyl-(3,Ä≤,Ü5,Ä≤)-5-methyl-, homopolymer	(C20H27N7O15P2)x		A/B	-9.3
726	N(CCC#N)(CC)c1c2c(ncc1)cc(Cl)cc2	127060-75-7	L-Isoleucine, N-(3-pyridinylcarbonyl)-L-tyrosyl-N6-[N2-[(phenylmethoxy)carbonyl]	C52H69N13O11	1052.18	A/B	-9.3
727	C(=O)([C@@H](NC(=O)OC)C(C)C)N1CC2(C[C@H]1c1[nH]c(cn1)c1ccc(cc1)c1ccc(cc1)c1[nH]c(nc1)[C@H]1N(C(=O)[C	1272655-63-6	Carbamic acid, N-[(1S)-1-[[(2S)-2-[5-[4,Ä≤-[2-[(3S)-2-[(2S)-2-[(methoxycarbonyl)	C44H56N8O7	808.96	A/B	-9.3
728	C([C@H](OCP(=O)(O)O)CO)n1c2c(nc1)c(N)nc(N)n2.[NaH]	1277183-98-8	Furo[3,Ä≤,4,Ä≤:6,7]naphtho[2,3-d]-1,3-dioxol-6(5aH)-one, 5,8,8a,9-tetrahydro-5-	C28H26O7Se	553.46	A/B	-9.3
729	O=C1N2[C@@H]3[C@H](CO[C@@H]2Cn2c1c(O)c(=O)c(C(=O)NCc1c(F)cc(F)cc1)c2)CCCC3	1309560-49-3	2H-Pyrido[1,Ä≤,2,Ä≤:4,5]pyrazino[2,1-b][1,3]oxazine-9-carboxamide, N-[(2,4-difl	C20H19F2N3O5	419.38	A/B	-9.3
730	O(Cc1ccc(F)cc1)c1c2c(c(cc1)c1ccccc1)oc(c(O)c2=O)c1ncc(OC)cn1	1312894-34-0	4H-1-Benzopyran-4-one, 5-[(4-fluorophenyl)methoxy]-3-hydroxy-2-(5-methyl-2-pyrim	C27H19FN2O4	454.45	A/B	-9.3
731	O(Cc1ccc(F)cc1)c1c2c(oc(c(O)c2=O)c2[nH]ccn2)c(C)c(C)c1	1312895-47-8	4H-1-Benzopyran-4-one, 5-[(4-fluorophenyl)methoxy]-3-hydroxy-7,8-dimethyl-2-(3-p	C26H20FN3O4	457.45	A/B	-9.3
732	O(Cc1ccc(F)cc1)c1c2c(oc(c(O)c2=O)c2[nH]ccn2)c(Cl)c(C)c1	1312896-47-1	4H-1-Benzopyran-4-one, 8-chloro-5-[(4-fluorophenyl)methoxy]-3-hydroxy-7-methyl-2	C25H17ClFN3O4	477.87	A/B	-9.3
733	O(Cc1ccc(F)cc1)c1c2c(c(C)c1)c1ccccc1)oc(c(O)c2=O)c1[nH]c(OC)nn1	1312896-94-8	4H-1-Benzopyran-4-one, 5-[(4-fluorophenyl)methoxy]-3-hydroxy-7-methyl-2-(3-methy	C26H20FN3O4	457.45	A/B	-9.3
734	O(Cc1ccc(F)cc1)c1c2c(oc(c(O)c2=O)c2ncccn2)cc(OC)c1	1312897-02-1	4H-1-Benzopyran-4-one, 5-[(4-fluorophenyl)methoxy]-3-hydroxy-7-methyl-8-phenyl-2	C32H23FN2O4	518.53	A/B	-9.3
735	O(Cc1ccc(F)cc1)c1c2c(oc(c(O)c2=O)c2[nH]ccn2)c(C)c(Cl)c1	1312901-18-0	4H-1-Benzopyran-4-one, 7-chloro-5-[(4-fluorophenyl)methoxy]-3-hydroxy-8-methyl-2	C25H17ClFN3O4	477.87	A/B	-9.3
736	O(Cc1ccc(F)cc1)c1c2c(c(c(Cl)c1)c1ccccc1)oc(c(O)c2=O)c1ncccn1	1312902-23-0	4H-1-Benzopyran-4-one, 7,8-dichloro-5-[(4-fluorophenyl)methoxy]-3-hydroxy-2-(5-p	C25H15Cl2FN2O4	497.3	A/B	-9.3
737	O(Cc1ccc(F)cc1)c1c2c(c(c1)c1ccccc1)c1ccccc1)oc(c(O)c2=O)c1[nH]cnn1	1312905-70-6	4H-1-Benzopyran-4-one, 5-[(4-fluorophenyl)methoxy]-3-hydroxy-7,8-diphenyl-2-(5-p	C38H25FN2O4	592.61	A/B	-9.3
738	Cc1c(c2c(cc1OCc1ccc(F)cc1)c(=O)c(O)c(o2)c1ncc(cn1)c1ccccc1)c1ccccc1	1312910-79-4	4H-1-Benzopyran-4-one, 2-(5-chloro-2-pyrimidinyl)-6-[(4-fluorophenyl)methoxy]-3-	C27H18ClFN2O4	488.89	A/B	-9.3
739	Clc1c(c2c(cc1OCc1ccc(F)cc1)c(=O)c(O)c(o2)c1ncc(cn1)c1ccccc1)c1ccccc1	1312915-66-4	4H-1-Benzopyran-4-one, 7-chloro-2-(5-chloro-2-pyrimidinyl)-6-[(4-fluorophenyl)me	C26H15Cl2FN2O4	509.31	A/B	-9.3
740	O=c1n([C@@H]2O[C@H](CO[P@]3(=O)Oc4c(CO3)cccc4C)[C@@H](O)C2)cc(I)c(=O)[nH]1	1313031-55-8	Uridine, 2,Ä≤-deoxy-5-iodo-5,Ä≤-O-[(2R)-2-oxido-4H-1,3,2-benzodioxaphosphorin-2	C16H16IN2O8P	522.19	A/B	-9.3
741	O[C@H]1[C@H](n2c3c(nc2)c(=O)nc[nH]3)O[C@H](COC)[C@H]1O	1348794-70-6	1-Piperazinecarboxamide, 4-methyl-N-[(1S)-2-[[(1S,2E)-3-(2-naphthalenylsulfonyl)	C36H40N4O4S	624.79	A/B	-9.3
742	C(=O)([C@@H](NC(=O)OC)C(C)C)N1[C@@H](C[C@@H](C1)C1CC1)c1[nH]c(cn1)c1ccc(cc1)c1ccc(cc1)c1cc([nH]c1)[C	1351856-06-8	Carbamic acid, N-[(1S)-1-[[(2S,4R)-4-cyclopropyl-2-[5-[4,Ä≤-[2-[(3S)-2-[(2S)-2-	C48H62N8O6	847.06	A/B	-9.3
743	S(=O)(=O)(O)c1c2c(c(N)c(N=Nc3ccc(C#N)cc3)c1)cccc2.[NaH]	1357016-21-7	Œ≤-D-arabino-Adenosine, 5,Ä≤-O-phosphonoadenylyl-(2,Ä≤,Ü5,Ä≤)-adenylyl-(2,Ä≤,Ü	C41H52N20O25P4	1348.87	A/B	-9.3
744	C(F)(F)c1n(c2c(n1)cccc2)c1nc(nc(N[C@H]2CC[C@@](C)(O)CC2)n1)N1CCOCC1	1359952-51-4	Benzamide, N-[4-[2-(difluoromethyl)-1H-benzimidazol-1-yl]-6-(4-morpholinyl)-1,3,	C22H19F2N7O2	451.43	A/B	-9.3
745	C(F)(F)c1n(c2c(n1)cccc2)c1nc(nc(Nc2cc3c(cc2)n(C)cn3)n1)N1CCOCC1	1359953-79-9	1H-Benzimidazol-6-amine, N-[4-[2-(difluoromethyl)-1H-benzimidazol-1-yl]-6-(4-mor	C22H19F2N9O	463.44	A/B	-9.3
746	C(=O)([C@@H](NC(=O)OC)C(C)C)N1[C@@H]([C@@H]2C[C@H]1CC2)c1[nH]c2-c3c(CCc2n1)cc(cc3)c1cc2c(cc1)c1c(cc2	1377040-99-7	Carbamic acid, N-[(1S)-1-[[(2S)-2-[4,Ä≤,5,Ä≤-dihydro-2,Ä≤-[(1R,3S,4S)-2-[(2S)-2	C46H54N8O6	814.97	A/B	-9.3
747	C(=O)([C@@H](NC(=O)OC)C(C)C)N1[C@@H]([C@@H]2C[C@H]1CC2)c1[nH]c2c(n1)ccc1c2ccc(c1)c1ccc(cc1)c1[nH]c(n	1377042-74-4	Carbamic acid, N-[(1S)-1-[[(2S)-2-[2,Ä≤-[(8S)-7-[(2S)-2-[(methoxycarbonyl)amino]	C46H52N8O8	844.95	A/B	-9.3
748	O[C@H]1[C@H](n2c3c(nc2)c(N)ncn3)O[C@H](COP(=O)(OC(=O)N)O)[C@H]1O	1378278-01-3	Guanosine, 2,Ä≤-deoxyguanylyl-(3,Ä≤,Ü5,Ä≤)-	C20H25N10O11P	612.45	A/B	-9.3
749	O=c1n([C@@H]2O[C@H](CO)[C@H]([18F])C2)cc(C)c(=O)[nH]1	1383925-97-0	Guanosine, 2,Ä≤-deoxy-5,Ä≤-O-phosphono-P-thioguanylyl-(3,Ä≤,Ü5,Ä≤)-	C20H26N10O13P2S	708.49	A/B	-9.3
750	C(=O)([C@@H](NC(=O)OC)C(C)C)N1[C@H](c2[nH]c(c(n2)c2cccs2)c2ccc(cc2)c2ccc(cc2)c2c(nc([nH]2)[C@H]2N(C(	1388224-10-9	Carbamic acid, N,N,Ä≤-[[1,1,Ä≤-biphenyl]-4,4,Ä≤-diylbis[[5-(2-furanyl)-1H-imida	C48H54N8O8	870.99	A/B	-9.3
751	OC1c2c(c(ccc2)c2nc(nc(NCC(=O)N(C)C)n2)N2CCOCC2)CC1	1391924-37-0	Urea, N,Ä≤-[1-[4-(6-amino-3-pyridinyl)-6-(4-morpholinyl)-1,3,5-triazin-2-yl]-4-p	C20H29N9O2	427.5	A/B	-9.3
752	N(C(Cc1ccccc1)C(=O)N)c1nc(nc(n1)N1CCOCC1)c1cc(O)ccc1	1391924-56-3	Ethanone, 2-[[4-(3-hydroxyphenyl)-6-(4-morpholinyl)-1,3,5-triazin-2-yl]amino]-1-	C20H27N7O5S	477.54	A/B	-9.3
753	N(CC(=O)O)(C(=O)C)c1nc(nc(n1)N1CCOCC1)c1cc(NC(=O)C)ccc1	1391924-87-0	Ethanone, 2-[[4-(4-aminophenyl)-6-(4-morpholinyl)-1,3,5-triazin-2-yl]amino]-1-[4	C20H28N8O4S	476.55	A/B	-9.3
754	N(CCC(=O)N(C)C)c1nc(nc(n1)N1CCOCC1)c1c2c([nH]nc2)ccc1	1391925-89-5	4-Piperidinamine, 1-[4-(1H-indazol-4-yl)-6-(4-morpholinyl)-1,3,5-triazin-2-yl]-	C19H24N8O	380.45	A/B	-9.3
755	[Ru+3]([NH3])([NH3])([NH3])([NH3])([NH3])[N]1=CN(c2c1c(=O)nc[nH]2)C1OC(CO)C(O)C1	1404118-68-8	Benzamide, N-[5-bromo-6-(4-cyano-2,6-dimethylphenoxy)-2-[(4-cyanophenyl)amino]-4	C27H19BrN6O2	539.38	A/B	-9.3
756	O[C@H]1[C@@H](O[C@H](C(=O)O)[C@H]1O)n1nc(C(=O)N)nc1	1414793-90-0	Not Yet Assigned	C29H33N7O8	607.61	A/B	-9.3
757	C(=C1C(=O)C(=CC=C1)C(=O)O)(c1cc(C(=O)O)c(O)cc1)c1cc(C(=O)O)c(O)cc1	141511-25-3	Benzoic acid, 5-[(3-carboxy-4-hydroxyphenyl)(3-carboxy-4-oxo-2,5-cyclohexadien-1	C30H20O12	572.47	A/B	-9.3
758	C(=O)([C@@H](NC(=O)OC)C(C)C)N1[C@@H](CCC1)c1[nH]c(C#Cc2c3CCc4cc(C#Cc5[nH]c(nc5)[C@H]5N(C(=O)[C@@H](N	1415120-38-5	Carbamic acid, N,N,Ä≤-[tricyclo[8.2.2.24,7]hexadeca-4,6,10,12,13,15-hexaene-5,11	C44H56N8O6	792.97	A/B	-9.3
759	C(=O)([C@@H](NC(=O)OC)C(C)C)N1[C@H](c2[nH]c(c3c4c(c(cc3)c3ccc(cc3)c3[nH]c(nc3)[C@H]3N(C(=O)[C@@H](NC	1419384-99-8	Carbamic acid, N-[(1S)-1-[[(2S,4S)-2-[5-[1,Ä≤,3,Ä≤-dihydro-7,Ä≤-[4-[2-[(2S,4S)-	C50H66N8O6	875.11	A/B	-9.3
760	C(OP(=O)(O[C@H]1[C@@H](O[C@H](COP(=O)(O[C@H]2[C@@H](O[C@H](CO)[C@H]2O)n2c3c(nc2)c(N)ncn3)O)[C@H]1O)n	1422459-51-5	Adenosine, 1,Ä≤-[3-(aminocarbonyl)-1H-1,2,4-triazol-1-yl]-1,Ä≤-de(6-amino-9H-pu	C28H34N14O16P2	884.6	A/B	-9.3
761	C(N1c2c(C(=NNC(=S)N(CC=C)CC=C)C1=O)cc(Cl)cc2)N1CCN(CC1)c1ccc(Cl)cc1	1426583-67-6	Hydrazinecarbothioamide, 2-[1,2-dihydro-5-methyl-2-oxo-1-[(4-phenyl-1-piperaziny	C33H32N6OS	560.71	A/B	-9.3
762	O[C@H]1[C@@H](O[C@H](COP(=O)(OP(=O)(OC[C@H]2O[C@H]([C@H](O)[C@@H]2O)n2nc(C(=O)N)nc2)O)O)[C@H]1O)n1nc	1445905-50-9	Adenosine, 5,Ä≤-O-[hydroxy[[hydroxy(phosphonooxy)phosphinyl]oxy]phosphinyl]guany	C20H28N10O20P4	852.39	A/B	-9.3
763	O=C1[C@H]2[C@@H](c3c([C@H](OC(=O)c4cc(C)ccc4)[C@H]2CO1)cc1c(c3)OCO1)c1c(Br)c(OC)c(OC)c(OC)c1	1449602-01-0	1-Naphthaleneacetic acid, (5R,5aR,8aR,9S)-9-(2-bromo-3,4,5-trimethoxyphenyl)-5,5	C34H29BrO9	661.49	A/B	-9.3
764	O[C@H]1[13C@H]([13c]2c3c([nH]c2)c(=O)nc[nH]3)[15NH][C@H](CO)[C@H]1O	1469875-66-8	Not Yet Assigned	C60H53N10O15P	1185.09	A/B	-9.3
765	C(n1c2c(nc1CC)c(C)cc(C)n2)c1ccc(cc1)c1c(S(=O)(=O)NC(=O)N(CC=C)CC=C)cccc1	148401-53-0	[1,1,Ä≤-Biphenyl]-2-sulfonamide, 4,Ä≤-[(2-ethyl-5,7-dimethyl-3H-imidazo[4,5-b]p	C32H33N5O3S	567.7	A/B	-9.3
766	O=c1n([C@@H]2O[C@H](CO)[C@@H](S)C2)ccc(N)n1	148503-84-8	Inosine 5,Ä≤-(pentahydrogen tetraphosphate), P,Ä≤,Ä≤,Ä≤,Ü5,Ä≤-ester with adenc	C20H27N9O20P4	837.37	A/B	-9.3
767	C(O)[C@@H]1[C@@H](O[C@H](CO)C1)n1c(=O)[nH]c(=O)c(C)c1	15180-30-0	Guanosine, 2,Ä≤-deoxyguanylyl-(3,Ä≤,Ü5,Ä≤)-2,Ä≤-deoxy-	C20H25N10O10P	596.45	A/B	-9.3
768	O=c1n(ccc(N)n1)[C@H]1O[C@@H](COP(=O)(N[C@H](C(=O)OCc2ccccc2)C)N[C@H](C(=O)OCc2ccccc2)C)SC1	1526921-99-2	8-Oxa-3,5-diaza-4-phosphanonanoic acid, 4-[[(2R,5S)-5-[4-(acetylamino)-2-oxo-1(2	C30H36N5O9PS	673.67	A/B	-9.3
769	C(n1c2c(nc1CC)c(C)cc(C)n2)c1ccc(cc1)c1c(S(=O)(=O)NC(=O)NCCCC)ccc(C(CC)(C)C)c1	154552-43-9	[1,1,Ä≤:3,Ä≤,1,Ä≤,Ä≤-Terphenyl]-4,Ä≤-sulfonamide, N-[(butylamino)carbonyl]-4,Ä≤	C34H37N5O3S	595.75	A/B	-9.3
770	C(n1c2c(nc1CC)c(C)cc(C)n2)c1ccc(cc1)c1c(S(=O)(=O)NC(=O)CCc2ccccc2)ccc(OC)c1	154552-57-5	Benzenepropanamide, N-[[4,Ä≤-[(2-ethyl-5,7-dimethyl-3H-imidazo[4,5-b]pyridin-3-y	C36H40N4O3S	608.79	A/B	-9.3
771	C(n1c2c(nc1CC)c(C)cc(C)n2)c1ccc(cc1)c1c(S(=O)(=O)NC(=O)CCc2ccccc2)ccc(SCC)c1	154552-69-9	Benzenepropanamide, N-[[4,Ä≤-[(2-ethyl-5,7-dimethyl-3H-imidazo[4,5-b]pyridin-3-y	C35H38N4O4S	610.77	A/B	-9.3
772	C(n1c2c(nc1CC)c(C)cc(C)n2)c1ccc(cc1)c1c(S(=O)(=O)NC(=O)NCCc2ccccc2)ccc(C(C)C)c1	154553-53-4	[1,1,Ä≤-Biphenyl]-2-sulfonamide, 5-butyl-4,Ä≤-[(2-ethyl-5,7-dimethyl-3H-imidazo	C36H41N5O3S	623.81	A/B	-9.3
773	S(c1nc(nc(Nc2ccc(N(=O)=O)cc2)n1)N1CCOCC1)c1oc(nn1)c1ccc(Cl)cc1	1547295-42-0	1,3,5-Triazin-2-amine, 4-[[5-(4-chlorophenyl)-1,3,4-oxadiazol-2-yl]thio]-N-(4-fl	C21H17ClFN7O2S	485.92	A/B	-9.3
774	C(=O)([C@@H](NC(=O)OC)C(C)C)N1[C@H](c2[nH]c(c3c4c(C5OC4CC5)c(cc3)c3ccc(cc3)c3[nH]c(nc3)[C@H]3N(C(=O)	1560640-89-2	Carbamic acid, N-[(1S)-1-cyclohexyl-2-[(2S)-2-[5-[8-[4-[2-[(2S)-1-[(2S)-2-cycloh	C50H62N8O7	887.08	A/B	-9.3
775	Fc1c(c2c3c(c(cc2)c2[nH]c(nc2)[C@H]2N(C(=O)[C@@H](NC(=O)OC)C(C)C)CCC2)C2CC3CC2)ccc(c1)c1[nH]c(nc1)[C@	1560641-29-3	Carbamic acid, N-[(1S)-1-[[(2S,4S)-2-[5-[4-[1,4-dihydro-8-[2-[(2S,4S)-1-[(2S)-2-	C46H56N8O7	832.99	A/B	-9.3
776	O=C1N2[C@H](Cn3c1c(O)c(=O)c(C(=O)N[C@@H](C)c1c(F)cc(F)cc1)c3)O[C@@H]1C[C@H]2CC1	1616339-79-7	2,5-Methanopyrido[1,Ä≤,2,Ä≤:4,5]pyrazino[2,1-b][1,3]oxazepine-10-carboxamide, N	C22H21F2N3O5	445.42	A/B	-9.3
777	[C@@H](OC(=O)NCCCCCCN=[N+]=[N-])([C@@H](CO)O)[C@H]1[C@H](NC(=O)C)[C@@H](N=C(NC(=O)OC(C)(C)C)NC(=O)OC	1621228-28-1	2-Propen-1-one, 1-[4-[(4,6-di-4-morpholinyl-1,3,5-triazin-2-yl)amino]phenyl]-3-(	C27H30N6O4	502.56	A/B	-9.3
778	N(C(=O)[C@H](Cc1ccccc1)C[C@@H](CN1[C@H](C(=O)NC(C)(C)C)CNCC1)O)[C@H]1c2c([C@H](O)[C@H]1O)cccc2	162518-57-2	D-erythro-Pentonamide, 2,3,5-trideoxy-N-[(1S,2R)-2,3-dihydro-2-hydroxy-1H-inden-	C36H47N5O5	629.79	A/B	-9.3
779	C(=O)([C@@H](NC(=O)OC)C(C)C)N1[C@H](c2[nH]c(c3c4c(c(cc3)c3c5c(c(cc3)c3[nH]c(nc3)[C@H]3N(C(=O)[C@@H](	1627533-21-4	Carbamic acid, N,N,Ä≤-[(1,1,Ä≤,2,2,Ä≤,3,3,Ä≤,4,4,Ä≤-octahydro[5,5,Ä≤-bi-1,4-me	C50H62N8O6	871.08	A/B	-9.3
780	C(=O)([C@@H](NC(=O)OC)C(C)C)N1[C@H](c2[nH]c(c3c4c(c(cc3)c3c5c(c(cc3)c3[nH]c(nc3)[C@H]3N(C(=O)[C@@H](	1627533-95-2	Carbamic acid, N-[(1S)-2-methyl-1-[[(2S)-2-[5-[1,2,3,4-tetrahydro-2-methyl-3-oxc	C50H61N9O7	900.08	A/B	-9.3
781	N(c1nc(nc(n1)N1CCOCC1)N1CCOCC1)c1ccc(cc1)c1cc(nc(N)n1)c1cc(N(=O)=O)ccc1	1628172-16-6	1,3,5-Triazin-2-amine, N-[4-[2-amino-6-(3-methoxyphenyl)-4-pyrimidinyl]phenyl]-4	C28H31N9O3	541.6	A/B	-9.3
782	O(CCCCC[n+]1ccccc1)c1c(OC)cc(CCC(=O)/C=C/CCc2cc(OC)c(OCCCCC[n+]3ccccc3)cc2)cc1	1633020-46-8	Benzeneacetic acid, Œ±-[(3,4-dimethoxyphenyl)methylene]-3,4-dimethoxy-, (5R,5aR,	C41H40O13	740.75	A/B	-9.3
783	C(=O)([C@@H](NC(=O)OC)C(C)C)N1[C@H](c2[nH]c(c3c4c(c(ccc4)c4ccc(cc4)c4[nH]c(nc4)[C@H]4N(C(=O)[C@@H](N	1639870-97-5	Carbamic acid, N,N,Ä≤-[[1,1,Ä≤-biphenyl]-4,4,Ä≤-diylbis[1H-imidazole-5,2-diyl[(	C46H42N8O6	802.88	A/B	-9.3
784	C(=O)([C@@H](NC(=O)OC)C1CCCCC1)N1[C@@H](C=CC1)c1[nH]c(cn1)c1ccc(cc1)c1ccc(cc1)c1[nH]c(nc1)[C@H]1N(C(	1639871-25-2	Carbamic acid, N-[(1S)-1-cyclohexyl-2-[(2S)-2-[5-[4,Ä≤-[2-[(2S)-1-[(2S)-2-cyclol	C46H56N8O6	816.99	A/B	-9.3
785	S(c1nc(nc(Nc2ccc(N(=O)=O)cc2)n1)N1CCOCC1)c1oc(nn1)c1ccc(F)cc1	1642584-19-7	1,3,5-Triazin-2-amine, N-(4-fluorophenyl)-4-[[5-(4-fluorophenyl)-1,3,4-oxadiazol	C21H17F2N7O2S	469.47	A/B	-9.3
786	N(C(=O)[C@H](Cc1ccccc1)C[C@@H](CN1[C@H](C(=O)NC(C)(C)C)CN(CCC2CCN(C(=O)OC(C)(C)C)CC2)CC1)O)[C@H]1c2c	164725-18-2	1-Piperazinepentanamide, N-(2,3-dihydro-2-hydroxy-1H-inden-1-yl)-2-[[(1,1-dimeth	C41H55N5O6	713.91	A/B	-9.3
787	C(C(=O)OC)(N1CC(C(=O)NC(C)(C)C)N(CC(CC(C(=O)NC2c3c(CC2O)cccc3)Cc2ccccc2)O)CC1)c1ccccc1	164725-48-8	1-Piperazinepentanamide, N-(2,3-dihydro-2-hydroxy-1H-inden-1-yl)-2-[[(1,1-dimeth	C40H54N6O5	698.89	A/B	-9.3
788	N(C(=O)C(Cc1ccccc1)CC(CN1C(C(=O)NC(C)(C)C)CN(C(CO)c2ccccc2)CC1)O)C1c2c(CC1O)cccc2	164725-49-9	1-Piperazineacetic acid, 4-[5-[(2,3-dihydro-2-hydroxy-1H-inden-1-yl)amino]-2-hyc	C39H50N4O6	670.84	A/B	-9.3
789	N(C(=O)[C@H](Cc1ccccc1)C[C@@H](CN1[C@H](C(=O)NC(C)(C)C)CN(Cc2cc(Cl)c(Cl)cc2)CC1)O)[C@H]1c2c(C[C@H]1O	164725-57-9	1-Piperazinepentanamide, 4-[[4-(acetylamino)-2-chlorophenyl]methyl]-N-(2,3-dihyc	C39H50ClN5O5	704.3	A/B	-9.3
790	C(=O)([C@@H](NC(=O)OC)[C@@H]1C[C@@H](C)O[C@H](C)C1)N1C[C@@]2(C[C@H]1c1[nH]c(cn1)c1ccc(cc1)c1ccc(cc1)	1653967-07-7	Carbamic acid, N-[(1S)-1-[[(2S)-2-[5-[4-[2-[(3S,5S)-2-[(2S)-2-[(methoxycarbonyl)	C46H56N8O7	832.99	A/B	-9.3
791	C(=O)([C@@H](NC(=O)OC)C(C)C)N1[C@@H](C[C@]2(C1)CCCOC2)c1[nH]c2c(n1)ccc1c2ccc(c1)c1cc2c(cc1)c1c(cc2)n	1653967-10-2	Carbamic acid, N-[(1S)-1-[[(2S)-2-[7-[4-[2-[(3S,5S)-2-[(2S)-2-[(methoxycarbonyl)	C46H56N8O7	832.99	A/B	-9.3
792	O=C1N([C@H]2O[C@H](CO)[C@@H](O)C2)C[C@H](C)C(=O)N1	1680213-95-9	1-Piperidinecarboxylic acid, 4-[2-[6-amino-8-[(6-bromo-1,3-benzodioxol-5-yl)thic	C42H41BrN6O11S	917.78	A/B	-9.3
793	C(=O)([C@H](Cc1cnc[nH]1)NC(=O)[C@@H](NC(=O)[C@H](Cc1ccc(O)cc1)NC(=O)c1cccnc1)CCCCNC(=O)[C@@H](NC(=O)	168565-08-0	L-Proline, 1-[N-[N6-(N2-acetyl-L-arginyl)-N2-[N-(3-pyridinylcarbonyl)-L-tyrosyl]	C40H54N12O9	846.93	A/B	-9.3
794	C(CC(COC(=O)c1cc(Oc2ccccc2)ccc1)(CO)CO)n1c2c(nc1)c(=O)nc(N)[nH]2	172645-17-9	Benzoic acid, 3-phenoxy-, 2-[2-(2-amino-1,6-dihydro-6-oxo-9H-purin-9-yl)ethyl]-2	C37H33N5O8	675.69	A/B	-9.3
795	C(Cc1ccccc1)C1(CCC)CC(=C(C(CC)c2cc(NS(=O)(=O)c3ccc(C(F)(F)F)cn3)ccc2)C(=O)O1)O	174484-76-5	2-Pyridinesulfonamide, 5-amino-N-[3-[1-[6-[2-(4-fluorophenyl)ethyl]-5,6-dihydro-	C30H34FN3O5S	567.67	A/B	-9.3
796	C(OC)[C@@]1(CO)[C@@H](O)C[C@@H](O1)n1c(=O)[nH]c(=O)c(C)c1	179169-45-0	1-Piperazinepentanamide, N-(2,3-dihydro-2,5-dihydroxy-1H-inden-1-yl)-2-[[(1,1-di	C36H47N5O5	629.79	A/B	-9.3
797	FC1(F)c2c(-c3c(cc(cc3)c3[nH]c(nc3)[C@H]3N(C(=O)[C@@H](NC(=O)OC)C(C)(C)C)CCC3)C1(F)F)ccc(c2)c1[nH]c(n	1801161-68-1	Carbamic acid, N,N,Ä≤-[(9,9,10,10-tetrafluoro-9,10-dihydro-2,7-phenanthrenediyl)	C48H56F4N8O6	917	A/B	-9.3
798	O=C1[C@@H]2[C@@H](c3c([C@H](OC(=O)CCCCCCCCCCCCCCCC(=O)O[C@H]4c5c([C@H]([C@@H]6[C@@H]4COC6=O)c4cc(OC	1801429-06-0	2-Naphthalenecarbonitrile, 6-[[2-[(4-cyanophenyl)amino]-4-pyrimidinyl]amino]-5,7	C24H18N6	390.44	A/B	-9.3
799	O=C1[C@]2([C@H]([C@H](/C(=C/C(=O)[C@@H]3[C@@]4(N5[C@@H]([C@H]3c3cc(OC)c(O)cc3)CCC5)c3c(NC4=O)ccc(OC)	1807888-50-1	Not Yet Assigned	C47H48N4O10	828.9	A/B	-9.3
800	O=C1[C@@H]2[C@@H](c3c([C@H](OC(=O)[C@H](Cc4ccccc4)N4C(=O)C5C(C4=O)CCCC5)[C@H]2CO1)cc1c(c3)OCO1)c1cc(	1809321-41-2	2H-Isoindole-2-acetic acid, octahydro-1,3-dioxo-Œ±-(phenylmethyl)-, (5R,5aR,8aR,	C39H39NO11	697.73	A/B	-9.3
801	O=C1[C@@H]2[C@@H](c3c([C@H](OC(=O)C(Cc4ccccc4)N4C(=O)C5C(C4=O)C4OC5CC4)[C@H]2CO1)cc1c(c3)OCO1)c1cc(O	1809325-20-9	2H-Isoindole-2-acetic acid, octahydro-1,3-dioxo-Œ±-(phenylmethyl)-, (5R,5aR,8aR,	C39H39NO11	697.73	A/B	-9.3
802	O=C1[C@@H]2[C@@H](c3c([C@H](OC(=O)C(C)N4C(=O)C5C(C4=O)CCCC5)[C@H]2CO1)cc1c(c3)OCO1)c1cc(OC)c(OC)c(OC	1809325-29-8	4,7-Epoxy-2H-isoindole-2-acetic acid, octahydro-1,3-dioxo-Œ±-(phenylmethyl)-, (5	C39H37NO12	711.71	A/B	-9.3
803	Cl[C@]1(F)[C@@H](O[C@H](CO)[C@H]1O)n1c(=O)[nH]c(=NO)cc1	182069-41-6	Benzenepropanoic acid, Œ≤,Œ≤,2,4-tetramethyl-6-(phenylmethoxy)-, 2-[(2-amino-1,	C29H35N5O6	549.62	A/B	-9.3
804	O=C1[C@@H]2[C@H](c3c(C(=O)[C@H]2CO1)cc1c(c3)OCO1)c1cc2c(cc1)OCO2	1835272-58-6	Carbamic acid, N-[(1S)-1-[[(2S,5S)-2-[5-[1,Ä≤,3,Ä≤-dihydro-7,Ä≤-[4-[2-[(2S,5S)-	C49H64N8O6	861.08	A/B	-9.3
805	O=C1[C@@H]2[C@@H](c3c([C@H](OC(=O)CCCC[C@H]4SC(SCC4)c4ccc(C(F)(F)F)cc4)[C@H]2CO1)cc1c(c3)OCO1)c1cc(O	1875123-85-5	1,3-Dithiane-4-pentanoic acid, 2-[3-(trifluoromethyl)phenyl]-, (5R,5aR,8aR,9R)-5	C38H39F3O9S2	760.84	A/B	-9.3
806	O=c1c2c(n(cn2)[C@@H]2O[C@H](COP(=O)(Nc3c(F)cc(F)cc3)Oc3ccc(Cl)cc3)CC2)[nH]cn1	1922936-30-8	Phosphoramidic acid, N-[[3-(trifluoromethyl)phenyl]methyl]-, 4-chlorophenyl [(2S	C24H22ClF3N5O5P	583.88	A/B	-9.3
807	O(C(=O)OC(CC(=O)OCC)(C)C)c1c2n(N([C@H]3N(C2=O)COC3)[C@H]2c3c(CSc4c2cccc4)c(F)c(F)cc3)ccc1=O	1985607-49-5	Carbamic acid, N,N-dimethyl-, 4-[[[(12aR)-12-[(11S)-7,8-difluoro-6,11-dihydrodik	C34H30F2N4O6S	660.69	A/B	-9.3
808	N(C(=O)Nc1c(OC)cc(Cl)cc1)c1cc2cc(C34CC5CC(C4)CC(C3)C5)[nH]c2cc1	1987854-67-0	Urea, N-(2-methyl-4-nitrophenyl)-N,Ä≤-(2-tricyclo[3.3.1.13,7]dec-1-yl-1H-indol-5	C26H28N4O3	444.53	A/B	-9.3
809	N(C(=O)Nc1cc(OC)cc(OC)c1)c1cc2cc(C34CC5CC(C4)CC(C3)C5)[nH]c2cc1	1987854-70-5	Benzoic acid, 4-methyl-3-[[[(2-tricyclo[3.3.1.13,7]dec-1-yl-1H-indol-5-yl)amino]	C28H31N3O3	457.56	A/B	-9.3
810	N(C(=O)NC1CC1)c1cc2cc(C34CC5CC(C4)CC(C3)C5)[nH]c2cc1	1987854-72-7	Urea, N-(3,4-dimethoxyphenyl)-N,Ä≤-(2-tricyclo[3.3.1.13,7]dec-1-yl-1H-indol-5-y]	C27H31N3O3	445.55	A/B	-9.3
811	C(=O)([C@@H](NC(=O)OC)C(C)C)N1CC2(C[C@H]1c1[nH]c(cn1)c1c3c(c(cc1)c1cc4c(cc1)c1c(cc4)nc([nH]1)[C@H]1N	2007901-27-9	Carbamic acid, N-[(1S)-1-[[(2S)-2-[7-[2,2-difluoro-2,3-dihydro-7-[2-[(2S)-1-[(2S	C45H52F2N8O6	838.94	A/B	-9.3
812	C([C@H](OCP(=O)(OCOC(=O)OCC(C)(C)C)OCOC(=O)OCC(C)(C)C)C)n1c2c(nc1)c(N)ncn2	201341-01-7	2,4,6,8-Tetraoxa-5-phosphanonanedioic acid, 5-[[(1R)-2-(6-amino-9H-purin-9-yl)-1	C21H34N5O10P	547.5	A/B	-9.3
813	C(=O)([C@@H](NC(=O)OC)C(C)C)N1[C@@H](CCC1)c1[nH]c(cn1)c1c(F)cc(cc1)c1ccc(cc1)c1[nH]c(nc1)[C@H]1N(C(=	1009116-11-3	Carbamic acid, N-[(1S)-1-cyclopropyl-2-[(2S)-2-[4-[4,Ä≤-[2-[(2S)-1-[(2S)-2-cyclc	C41H45F3N8O6	802.84	C	-9.3
814	O=c1n([C@H]2C[C@H](OP(=S)(S)S)[C@@H](CO)O2)cc(C)c(=O)[nH]1	10115-26-1	2-Naphthalenesulfonic acid, 8-[2-[4-[2-(2,4-diaminophenyl)diazenyl]-1-naphthaler	C26H20N6O3S.Na		C	-9.3
815	O(c1n/c(=N\c2ccc(C#N)cc2)/n(O)c(N)c1Br)c1c(C)cc(C#N)cc1C	1027961-32-5	1-Naphthalenesulfonic acid, 4-amino-3-[(1E)-2-(4,Ä≤-amino[1,1,Ä≤-biphenyl]-4-yl	C22H18N4O3S	418.47	C	-9.3
816	O=c1n([C@H]2C[C@H](OP(=O)(OC[C@H]3O[C@H](C[C@@H]3O)n3c(=O)[nH]c(=O)cc3)O)[C@@H](CO)O2)cc(C)c(=O)[nH]	103167-67-5	2-Naphthalenesulfonic acid, 7-hydroxy-8-[2-[2-hydroxy-5-[2-(1-naphthalenyl)diaze	C26H18N4O5S	498.51	C	-9.3
817	O(P(=O)(C(=O)O)O)[C@@]1(CC)c2c(c(=O)n3c(-c4c(C3)cc3c(n4)cccc3)c2)COC1=O	1038983-70-8	Phosphinecarboxylic acid, 1-[[(4S)-4-ethyl-3,4,12,14-tetrahydro-4-hydroxy-3,14-c	C21H17N2O9P	472.34	C	-9.3
818	N(=N\c1cc(S(=O)(=O)O)c(C=Cc2c(S(=O)(=O)O)cc(/N=N/c3c4c(cc(S(=O)(=O)O)cc4)c(N)cc3)cc2)cc1)/c1c2c(cc(S	1053056-37-3	Not Yet Assigned	C34H26N6O18S6	998.99	C	-9.3

819	O=c1n(cc(c(=O)[nH]1)c1ccccc1)[C@@H]1O[C@H](COP(=O)(OP(=O)(O[C@H]2O[C@H](CO)[C@@H](O)[C@H](O)[C@H]2O)	1061717-09-6	1H-Indole-3-acetic acid, 1-(1,3-benzodioxol-5-ylmethyl)-Œ±-oxo-, (5R,5aR,8aR,9R)	C40H33NO12	719.69	C	-9.3
820	O=c1n([C@@H]2O[C@H](COP3(=O)Oc4c(CO3)cc(Cc3cc5c(cc3)OP(=O)(OC[C@H]3O[C@H](C=C3)n3c(=O)[nH]c(=O)c(C)c	1099829-91-0	Not Yet Assigned	C35H36FN7O14P2		C	-9.3
821	C(#Cc1cn(C)cn1)c1n(nc(C(=O)N)n1)[C@@H]1O[C@H](CO)[C@@H](O)[C@H]1O	1123187-68-7	1H-1,2,4-Triazole-3-carboxamide, 5-[2-(1-pyrenyl)ethynyl]-1-Œ≤-D-ribofuranosyl-	C26H20N4O5	468.46	C	-9.3
822	C(=O)(N[C@H](C(=O)C(=O)NC1CC1)CCC)[C@@H]1[C@@H]2[C@H](CN1C(=O)[C@@H](NC(=O)[C@@H](NC(=O)c1cnccn1)C1C	112685-86-6	Benzenesulfonic acid, 3-[2-[5-hydroxy-4-[2-(2-hydroxy-1-naphthalenyl)diazenyl]-2	C23H18N4O5S	462.48	C	-9.3
823	C(=O)([C@@H](NC(=O)OC)C)N1CC2(C[C@H]1c1[nH]c(cn1)c1ccc(cc1)c1ccc(cc1)c1[nH]c(nc1)[C@H]1N(C(=O)[C@@H]	1129634-55-4	Carbamic acid, N,N,Ä≤-[[1,1,Ä≤-biphenyl]-4,4,Ä≤-diylbis[1H-imidazole-5,2-diyl-(	C38H42N8O6	706.79	C	-9.3
824	C(=O)([C@@H](NC(=O)OC)CC)N1CC2(C[C@H]1c1[nH]c(cn1)c1ccc(cc1)c1ccc(cc1)c1[nH]c(nc1)[C@H]1N(C(=O)[C@@H	1129634-58-7	Carbamic acid, N,N,Ä≤-[[1,1,Ä≤-biphenyl]-4,4,Ä≤-diylbis[1H-imidazole-5,2-diyl-(	C40H46N8O6	734.84	C	-9.3
825	O[C@H]1[C@H](n2c3c(nc2)c(=O)nc(N)[nH]3)O[C@H](COP(=O)(OCCN(C)c2ccc(C=O)cc2)O)[C@H]1O	1132935-63-7	Methanesulfonamide, N-[6-[5-(3,4-dihydro-2,4-dioxo-1(2H)-pyrimidinyl)-3-(1,1-dim	C26H27N3O5S	493.57	C	-9.3
826	N(CCCCN(CCCC)CCCC)c1c2c(ncc1C)cc(Cl)cc2	1141510-07-7	D-Glucopyranosiduronic acid, (2R)-3,6-dihydro-6-oxo-2-(2-phenylethyl)-2-propyl-5	C37H41F3N2O11S	778.79	C	-9.3
827	C(=O)(N[C@@](C(C1CCC1)([2H])[2H])(C(=O)C(=O)N)[2H])[C@@H]1[C@@H]2[C@@H](C2(C([2H])([2H])[2H])C([2H])	1158082-69-9	3-Azabicyclo[3.1.0]hexane-2-carboxamide, N-[(1S)-3-amino-1-(cyclobutyl-1,2,2,3,3	C27H11D34N5O5		C	-9.3
828	O(C[C@H]1O[C@H](C=C1)n1c(=O)[nH]c(=O)c(C)c1)P1(=O)Oc2c(cc(cc2)c2cc(CO)c(O)cc2)CO1	1160003-74-6	Thymidine, 5,Ä≤,5,Ä≤,Ä≤,Ä≤-O-[8,8,Ä≤-bis(1,1-dimethylethyl)-2,2,Ä≤-dioxido[6,6	C42H48N4O14P2	894.8	C	-9.3
829	CC1(C)c2c(-c3c1cc(cc3)c1[nH]c(nc1)[C@H]1N(C(=O)[C@@H](NC(=O)OC)C(C)C)CCC1)ccc(c2)c1[nH]c(nc1)[C@H]1N	1179150-37-8	Carbamic acid, N,N,Ä≤-[[9-(dimethylamino)-9-methyl-9H-fluorene-2,7-diyl]bis[1H-i	C44H57N9O6	807.98	C	-9.3
830	CC1(C)c2c(-c3c1cc(cc3)c1[nH]c(nc1)[C@H]1N(C(=O)[C@@H](NC(=O)OC)C3CC3)CCC1)ccc(c2)c1[nH]c(nc1)[C@H]1N	1179150-48-1	Carbamic acid, N,N,Ä≤-[(9,9-dimethyl-9H-fluorene-2,7-diyl)bis[1H-imidazole-5,2-d	C43H54N8O6	778.94	C	-9.3
831	S(=O)(=O)(n1c2c(c3c1cc(cc3)c1[nH]c(nc1)[C@H]1N(C(=O)[C@@H](NC(=O)OC)C(C)C)CCC1)ccc(c2)c1[nH]c(nc1)[C	1179150-90-3	Carbamic acid, N,N,Ä≤-[(9-methyl-9H-carbazole-2,7-diyl)bis[1H-imidazole-5,2-diyl	C41H51N9O6	765.9	C	-9.3
832	C(=O)([C@@H](NC(=O)OC)C(C)C)N1[C@H](c2[nH]c(c3cc4c(c5c([nH]4)cc(cc5)c4[nH]c(nc4)[C@H]4N(C(=O)[C@@H](	1179151-07-5	Carbamic acid, N,N,Ä≤-[[9-(phenylsulfonyl)-9H-carbazole-2,7-diyl]bis[1H-imidazol	C46H53N9O8S	892.03	C	-9.3
833	[Si](O[C@@H]1[C@@H](CO[Si](C(C)(C)C)(c2ccccc2)c2ccccc2)O[C@H](C1)n1c(=O)[nH]c(=O)c(C)c1)(C(C)(C)C)(c	1180670-57-8	Carbamic acid, N-[(1S)-2-[(1R,3S,5R)-3-[7-[3-fluoro-4-[2-[(1R,3S,5R)-2-[(2S)-2-[	C46H51FN8O7	846.94	C	-9.3
834	C(NC(=O)c1nnc(C)o1)(C)(C)C1=NC(C(=O)NCc2ccc(F)cc2)C(=O)C(=O)N1C([2H])([2H])[2H]	1190629-38-9	1-Naphthalenesulfonic acid, 4-amino-3-[2-[4-[2-(4-hydroxyphenyl)diazenyl]phenyl]	C22H17N5O4S	447.47	C	-9.3
835	C(#N)C1=C(N)Oc2c(C1c1cc3c(cc1)OCO3)ccn1c2nc(C)c1	1225373-08-9	7H-Imidazo[1,2-a]pyrano[2,3-c]pyridine-8-carbonitrile, 9-amino-7-(1,3-benzodioxo	C18H12N4O3	332.31	C	-9.3
836	O=c1n(cc([C@H](CI)O)c(=O)[nH]1)[C@@H]1O[C@H](CO)[C@@H](O)C1	1238382-72-3	Androsta-5,16-dien-3-ol, 17-(1H-benzimidazol-1-yl)-, 3-(2-phosphonoacetate), (3Œ	C28H35N2O5P	510.56	C	-9.3
837	C(F)(F)c1n(c2c(n1)cccc2)c1nc(nc(NC[C@H]2CC[C@H](N)CC2)n1)N1CCOCC1	1240024-64-9	Urea, N-[trans-4-[[4-[2-(difluoromethyl)-1H-benzimidazol-1-yl]-6-(4-morpholinyl)	C25H33F2N9O3	545.58	C	-9.3
838	[C@H](N(C(=O)[C@@H](NC(=O)OC)C(C)C)C)(C)c1[nH]c(c2cc3c(-c4c(C3)cc(cc4)c3[nH]c(nc3)[C@H]3N(C(=O)[C@@H	1240880-92-5	Carbamic acid, N,N,Ä≤-[9H-fluorene-2,7-diylbis[1H-imidazole-5,2-diyl-(2S)-2,1-py	C45H54N8O8	834.96	C	-9.3
839	C(=O)([C@H](NC(=O)OC)c1ccccc1)N1[C@@H](CCC1)c1[nH]c(cn1)c1cc2c(cc1)cc(cc2)c1ccc(cc1)c1[nH]c(nc1)[C@H	1240885-51-1	Carbamic acid, N-[(1R)-2-[(2S)-2-[5-[4-[7-[2-[(2S)-1-[(2R)-2-[(methoxycarbonyl)a	C50H48N8O6	856.97	C	-9.3
840	C(n1c2c(nc1)c(N)ncn2)[C@H]1COP(=O)(OC[C@@H](C(=O)OC(C)C)N)CO1	1254066-01-7	Urea, N-[4-[4-(4-morpholinyl)-6-(3-oxa-8-azabicyclo[3.2.1]oct-8-yl)-1,3,5-triazi	C24H27N9O3	489.53	C	-9.3
841	C(=O)([C@@H](NC(=O)OC)C(C)C)N1[C@@H](CCC1)c1[nH]c(cn1)c1sc(cc1)c1ccc(cc1)c1[nH]c(nc1)[C@H]1N(C(=O)[C	1256385-46-2	Carbamic acid, N,N,Ä≤-[(5,5-dioxido-3,7-dibenzothiophenediyl)bis[1H-imidazole-5,	C40H48N8O8S	800.92	C	-9.3
842	C(=O)([C@@H](NC(=O)OC)C(C)C)N1[C@H](c2[nH]c(c3ccc(c4c5c(c(ccc5)c5ccc(cc5)c5[nH]c(nc5)[C@H]5N(C(=O)[C	1256387-45-7	Carbamic acid, N,N,Ä≤-[[2,2,Ä≤-binaphthalene]-6,6,Ä≤-diylbis[1H-imidazole-5,2-d	C48H54N8O6	838.99	C	-9.3
843	C(=O)([C@@H](Cc1ccccc1)NC(=O)OC)N1[C@@H](CCC1)c1[nH]c(cn1)c1ccc(cc1)c1cc2c(cc1)cc(cc2)c1[nH]c(nc1)[C	1256389-80-6	Carbamic acid, N-[(1S)-2-[(2S)-2-[5-[4-[6-[2-[(2S)-1-[(2S)-2-[(methoxycarbonyl)a	C48H52N8O6	836.98	C	-9.3
844	N(=C1c2c(N(C(=O)C)C1=O)ccc(Br)c2)n1c(Cc2n(N=C3c4c(N(C(=O)C)C3=O)ccc(Br)c4)c(=S)[nH]n2)n[nH]c1=S	1262891-46-2	2H-Indol-2-one, 3,3,Ä≤-[methylenebis[(1,5-dihydro-5-thioxo-4H-1,2,4-triazole-3,4	C25H18N10O4S2	586.6	C	-9.3
845	C(CCCCC)(P(=O)(O)O)C(=O)O	1263191-47-4	3-Quinolinecarboxylic acid, 7-[4-[2-[3-[2-(aminothioxomethyl)hydrazinylidene]-5-	C29H29F2N7O5S	625.65	C	-9.3
846	O=c1c2c(n(cc1C(=O)O)C1CC1)cc(c(F)c2)N1CCN(CN2c3c(/C(=N/NC(=S)NOC)/C2=O)cc(Cl)cc3)CC1	1265623-15-1	3-Quinolinecarboxylic acid, 7-[4-[[(3Z)-5-chloro-2,3-dihydro-3-[2-[(hydroxyaminc	C27H25ClFN7O5S	614.05	C	-9.3
847	O=c1c2c(n(cc1C(=O)O)C1CC1)cc(c(F)c2)N1CCN(CN2c3c(/C(=N/NC(=S)NO)/C2=O)cc(C)cc3)CC1	1265623-18-4	3-Quinolinecarboxylic acid, 1-cyclopropyl-6-fluoro-7-[4-[[(3Z)-5-fluoro-2,3-dihy	C28H27F2N7O5S	611.62	C	-9.3
848	O=c1c2c(n([14cH]n2)[C@@H]2O[C@H](CO)[C@@H](O)[C@H]2O)[nH]c(N)n1	129318-05-4	1,5-Naphthalenedisulfonic acid, 3-[2-[4-[2-(4-amino-2-methylphenyl)diazenyl]pher	C23H19N5O6S2	525.56	C	-9.3
849	C(=O)([C@@H](NC(=O)OC)C1CCC(F)(F)CC1)N1[C@H]2[C@H](C2)C[C@H]1c1[nH]c(c(Cl)n1)c1ccc(cc1)c1ccc(cc1)c1[	1312336-38-1	Carbamic acid, N-[(1S)-2-[(1R,3S,5R)-3-[5-chloro-4-[4,Ä≤-[5-chloro-2-[(1R,3S,5R)	C47H52Cl2F2N8O7	949.87	C	-9.3
850	O(Cc1ccc(F)cc1)c1c2c(oc(c(O)c2=O)c2[nH]cnn2)c(C)c(C)c1	1312895-37-6	4H-1-Benzopyran-4-one, 5-[(4-fluorophenyl)methoxy]-3-hydroxy-7,8-dimethyl-2-(5-p	C28H21FN2O4	468.48	C	-9.3
851	O(Cc1ccc(F)cc1)c1c2c(c(c(C)c1)c1ccccc1)oc(c(O)c2=O)c1[nH]cnn1	1312896-87-9	4H-1-Benzopyran-4-one, 5-[(4-fluorophenyl)methoxy]-3-hydroxy-7-methyl-8-phenyl-2	C33H23FN2O4	530.55	C	-9.3
852	O(Cc1ccc(F)cc1)c1c2c(c(c(Cl)c1)c1ccccc1)oc(c(O)c2=O)c1[nH]cnn1	1312902-58-1	4H-1-Benzopyran-4-one, 7-chloro-5-[(4-fluorophenyl)methoxy]-3-hydroxy-8-phenyl-2	C32H20ClFN2O4	550.96	C	-9.3
853	O=c1c2c(oc(c1O)c1ncccn1)cc(C)c(OCc1ccc(F)cc1)c2	1312908-33-0	4H-1-Benzopyran-4-one, 6-[(4-fluorophenyl)methoxy]-3-hydroxy-8-phenyl-2-(5-pheny	C31H21FN2O4	504.51	C	-9.3
854	Clc1c2c(cc(OCc3ccc(F)cc3)c1C)c(=O)c(O)c(o2)c1[nH]ccn1	1312910-40-9	4H-1-Benzopyran-4-one, 8-chloro-6-[(4-fluorophenyl)methoxy]-3-hydroxy-7-methyl-2	C25H17ClFN3O4	477.87	C	-9.3
855	Cc1c(c2c(cc1OCc1ccc(F)cc1)c(=O)c(O)c(o2)c1[nH]ccn1)c1ccccc1	1312910-90-9	4H-1-Benzopyran-4-one, 6-[(4-fluorophenyl)methoxy]-3-hydroxy-7-methyl-8-phenyl-2	C31H22FN3O4	519.52	C	-9.3
856	O=c1c2c(oc(c1O)c1ncccn1)cc(OC)c(OCc1ccc(F)cc1)c2	1312910-95-4	4H-1-Benzopyran-4-one, 6-[(4-fluorophenyl)methoxy]-3-hydroxy-7-methyl-8-phenyl-2	C32H23FN2O4	518.53	C	-9.3
857	Clc1c(c2c(cc1OCc1ccc(F)cc1)c(=O)c(O)c(o2)c1[nH]ccn1)c1ccccc1	1312915-88-0	4H-1-Benzopyran-4-one, 7-chloro-6-[(4-fluorophenyl)methoxy]-3-hydroxy-8-phenyl-2	C30H19ClFN3O4	539.94	C	-9.3
858	O=c1c2c(cc(c(OCc3ccc(F)cc3)c2)c2ccccc2)oc(c1O)c1ncccn1	1312915-98-2	4H-1-Benzopyran-4-one, 7-chloro-6-[(4-fluorophenyl)methoxy]-3-hydroxy-8-phenyl-2	C31H20ClFN2O4	538.95	C	-9.3
859	O(C)c1c2c(cc(OCc3ccc(F)cc3)c1c1ccccc1)c(=O)c(O)c(o2)c1[nH]cnn1	1312917-36-4	4H-1-Benzopyran-4-one, 6-[(4-fluorophenyl)methoxy]-3-hydroxy-8-methoxy-7-phenyl-	C33H23FN2O5	546.54	C	-9.3
860	C(#CC1C(Cl)C1)[C@@]1(C(F)(F)F)c2c(NC(=O)O1)ccc(Cl)c2	1322625-54-6	Not Yet Assigned	C37H40D8N4O5		C	-9.3
861	N(=Nc1c2c(c(N=Nc3ccc(S(=O)(=O)O)cc3)cc1)cccc2)c1c2c(cc(S(=O)(=O)O)cc2)c(N)cc1	1345852-43-8	2-Naphthalenesulfonic acid, 8-[2-(4-amino-7-sulfo-1-naphthalenyl)diazenyl]-5-[2-	C26H19N5O9S3	641.65	C	-9.3
862	C(=O)([C@@H](NC(=O)OC)C(C)C)N1[C@@]2(C[C@H](C1)CC2)c1[nH]c(cn1)c1c2c(c(cc1)c1ccc(cc1)c1[nH]c(nc1)[C@	1350718-88-5	Carbamic acid, N,N,Ä≤-[[4,4,Ä≤-bi-1H-indene]-7,7,Ä≤-diylbis[1H-imidazole-5,2-di	C50H58N8O6	867.05	C	-9.3
863	S(=O)(=O)(O)c1c2c(c(N)c(N=Nc3ccc(cc3)c3cc(C)ccc3)c1)cccc2.[NaH]	1357165-20-8	1-Naphthalenesulfonic acid, 4-amino-3-[2-(4,Ä≤-methyl[1,1,Ä≤-biphenyl]-4-yl)dia	C23H19N3O3S.Na		C	-9.3
864	S(=O)(=O)(O)c1c2c(c(N=Nc3ccccc3)ccc2N)ccc1	1357245-72-7	1-Naphthalenesulfonic acid, 4-amino-3-[2-(3,Ä≤-methyl[1,1,Ä≤-biphenyl]-4-yl)dia	C23H19N3O3S	417.48	C	-9.3
865	C(F)(F)c1n(c2c(n1)cccc2)c1nc(nc(Nc2ccc(cc2)n2cccc2)n1)N1CCOCC1	1359956-28-7	1,3,5-Triazin-2-amine, 4-[2-(difluoromethyl)-1H-benzimidazol-1-yl]-6-(4-morpholi	C26H28F2N8O	506.55	C	-9.3
866	C(=O)([C@@H](NC(=O)OC)C(C)C)N1[C@@H](CCC1)c1[nH]c(cn1)c1ccc(cc1)c1ccc(cc1)c1ccc(cc1)c1ccc(cc1)c1[nH]	1375881-14-3	Carbamic acid, N,N,Ä≤-[[1,1,Ä≤:3,Ä≤,1,Ä≤,Ä≤-terphenyl]-4,4,Ä≤,Ä≤-diylbis[1H-im	C46H54N8O6	814.97	C	-9.3
867	C(=O)([C@@H](NC(=O)OC)C(C)C)N1[C@H](c2[nH]c(c3cc4c(c5c(c6c4cccc6)cc(cc5)c4[nH]c(nc4)[C@H]4N(C(=O)[C@	1377035-64-7	Carbamic acid, N,N,Ä≤-[(4,5,9,10-tetrahydro-2,7-pyrenediyl)bis[1H-imidazole-5,2-	C44H54N8O6	790.95	C	-9.3
868	C(=O)([C@@H](NC(=O)OC)C(C)C)N1[C@@H]([C@@H]2C[C@H]1CC2)c1[nH]c(cn1)c1ccc(cc1)c1cc2c(cc1)c1c(cc2)nc([	1377035-72-7	Carbamic acid, N,N,Ä≤-[(4-oxo-4H-cyclopenta[def]phenanthrene-2,6-diyl)bis[1H-im	C43H48N8O7	788.89	C	-9.3
869	C(=O)([C@@H](NC(=O)OC)C1CCOCC1)N1[C@@H]([C@@H]2C[C@H]1CC2)c1[nH]c2c(n1)ccc1c2ccc(c1)c1cc2c(-c3c(CC2)	1377037-10-9	Carbamic acid, N-[(1S)-2-[(1R,3S,4S)-3-[5-[9,10-dihydro-7-[2-[(2S)-1-[(2S)-2-[(m	C46H56N8O7	832.99	C	-9.3
870	C(=O)([C@@H](NC(=O)OC)C(C)C)N1[C@@H]([C@H]2C[C@@H]1CC2)c1[nH]c2c(n1)ccc1c2ccc(c1)c1cc2c(cc1)c1c(cc2)	1377041-96-7	Carbamic acid, N-[(1S)-2-[(1R,3S,4S)-3-[5-[4-[2-[(1R,3S,5R)-2-[(2S)-2-[(methoxyc	C47H54N8O7	842.98	C	-9.3
871	N(=Nc1cc(C)c(N=Nc2ccc(C(=O)O)cc2)cc1)c1c2c(c(N=Nc3ccc(O)cc3)cc1)ccc(S(=O)(=O)O)c2	1378293-75-4	2-Naphthalenesulfonic acid, 5-[2-(4-hydroxyphenyl)diazenyl]-8-[2-[3-methyl-4-[2-	C29H22N6O7S2	630.65	C	-9.3
872	O=C1CC2(c3n(c(nn3)c3cc(OC)c(cc3)c3cnc(C)o3)CCC2)Oc2c1ccc(Cl)c2	1388690-68-3	Spiro[2H-1-benzopyran-2,8,Ä≤(5,Ä≤H)-[1,2,4]triazolo[4,3-a]pyridin]-4(3H)-one, 6	C25H22N4O4	442.47	C	-9.3
873	N(=Nc1cc(S(=O)(=O)O)c(C=Cc2c(S(=O)(=O)O)cc(N=Nc3c4c(cc(S(=O)(=O)O)c3N)cc(S(=O)(=O)O)cc4)cc2)cc1)c1c2	140942-26-3	2-Naphthalenesulfonic acid, 5,5,Ä≤-[1,2-ethenediylbis[(3-sulfo-4,1-phenylene)azo	C34H26N6O12S4.4Na		C	-9.3
874	O[C@H]1[C@@H](O[C@H](C(=O)N[C@H](Cc2c3c([nH]c2)cccc3)C(=O)N2CCN(C(=O)N3CCCCC3)CC2)[C@H]1O)n1nc(C(=O)	1414793-67-1	Not Yet Assigned	C28H35N9O8	625.63	C	-9.3
875	C(=O)([C@@H](NC(=O)OC)C(C)C)N1[C@H](c2[nH]c(c3c4c(c(cc3)c3ccc(cc3)c3[nH]c(nc3)[C@H]3N(C(=O)[C@@H](NC	1419384-99-8	Carbamic acid, N-[(1S)-1-[[(2S,4S)-2-[5-[1,Ä≤,3,Ä≤-dihydro-7,Ä≤-[4-[2-[(2S,4S)-	C50H66N8O6	875.11	C	-9.3
876	C(=O)([C@@H](NC(=O)OC)C1CCCCC1)N1[C@H](c2[nH]c(c3c4c(c(cc3)c3ccc(cc3)c3[nH]c(nc3)[C@H]3N(C(=O)[C@@H]	1419385-03-7	Carbamic acid, N-[(1S)-1-[[(2S,4S)-2-[5-[1,Ä≤,3,Ä≤-dihydro-7,Ä≤-[4-[2-[(2S,4S)-	C49H64N8O6	861.08	C	-9.3
877	N(=C1c2c(N(CN3CCN(CC3)c3cc4c(cc3F)c(=O)c(C(=O)O)cn4CC)C1=O)ccc(C)c2)NC(=S)N(CC=C)CC=C	1426583-83-6	3-Quinolinecarboxylic acid, 7-[4-[[3-[2-[(diphenylamino)thioxomethyl]hydrazinyli	C38H33F2N7O4S	721.77	C	-9.3
878	O(C)c1c2n(cc(C(=O)O)c(=O)c2cc(F)c1N1CC(C)N(CN2c3c(C(=NNC(=S)N(CC=C)CC=C)C2=O)cc(Cl)cc3)CC1)C1CC1	1426583-85-8	3-Quinolinecarboxylic acid, 7-[4-[[3-[2-[(diphenylamino)thioxomethyl]hydrazinyli	C39H36FN7O4S	717.81	C	-9.3
879	O=c1n([C@H]2O[C@@H](CO)SC2)cc(F)c(=O)[nH]1	1437791-52-0	Pyrimidinium, 4-[[4-[(1E)-2-cyanoethenyl]-2,6-dimethylphenyl]amino]-2-[(4-cyanop	C28H26N6O6		C	-9.3
880	N(c1c2c(n(cn2)[C@@H]2C[C@@H](CO)C=C2)nc(N)n1)C1CC1	144334-87-2	Benzoic acid, 3,3,Ä≤-methylenebis[6-[(2-aminobenzoyl)amino]-	C29H24N4O6	524.52	C	-9.3
881	C(=O)(O)c1cc(ccc1NC(=O)c1c(N)cccc1)c1cc(C(=O)O)c(NC(=O)c2c(N)cccc2)cc1	144615-01-0	Benzoic acid, 3,3,Ä≤-methylenebis[6-[(2-aminobenzoyl)amino]-, homopolymer	(C29H24N4O6)x		C	-9.3
882	O[C@H]1[C@H](n2c3c(nc2)c(=O)nc[nH]3)O[C@H](COP(=O)(O)O)[C@H]1O.O[C@H]1[C@@H](O[C@H](COP(=O)(O)O)[C@H	1449239-46-6	1-Naphthalenol, 4-[(7-chloro-4-quinolinyl)amino]-5,6,7,8-tetrahydro-2-[(4-phenyl	C30H31ClN4O	499.05	C	-9.3
883	C(OC[C@@H]1[C@@H](O[C@H]2[C@H](OC(=O)C)[C@@H](OC(=O)C)[C@H](OC(=O)C)[C@@H](COC(=O)C)O2)C[C@@H](O1)n1	148834-36-0	Thymidine, 3,Ä≤-O-[3-O-benzoyl-2-deoxy-2-(1,3-dihydro-1,3-dioxo-2H-isoindol-2-yl	C38H32F3N3O12	779.67	C	-9.3
884	N(=Nc1c(C)cc(N)cc1)c1c2c(c(N=Nc3cc(S(=O)(=O)O)ccc3)cc1)ccc(S(=O)(=O)O)c2	151534-66-6	1,4-Benzenedisulfonic acid, 2-[2-[4-[2-(4-amino-2-methylphenyl)diazenyl]-6-sulfo	C23H19N5O9S3	605.62	C	-9.3
885	C(n1c2c(nc1CC)c(C)cc(C)n2)c1ccc(cc1)c1c(S(=O)(=O)NC(=O)CCc2ccccc2)ccc(OCC(F)(F)F)c1	154552-67-7	Benzenepropanamide, N-[[4,Ä≤-[(2-ethyl-5,7-dimethyl-3H-imidazo[4,5-b]pyridin-3-y	C37H41N5O4S	651.82	C	-9.3
886	C(n1c2c(nc1CC)c(C)cc(C)n2)c1ccc(cc1)c1c(S(=O)(=O)NC(=O)c2c(OCC)cccc2)ccc(C(CC)(C)C)c1	154553-02-3	Benzamide, 2-ethoxy-N-[[4,Ä≤,Ä≤-[(2-ethyl-5,7-dimethyl-3H-imidazo[4,5-b]pyridin	C38H36N4O4S	644.78	C	-9.3
887	C(n1c2c(nc1CC)c(C)cc(C)n2)c1ccc(cc1)c1c(S(=O)(=O)NC(=O)c2ccccc2)ccc(C(CC)(C)C)c1	154553-22-7	Benzamide, N-[[4,Ä≤,Ä≤-[(2-ethyl-5,7-dimethyl-3H-imidazo[4,5-b]pyridin-3-yl)met	C36H32N4O3S	600.73	C	-9.3
888	S(c1nc(nc(Nc2ccc(N(=O)=O)cc2)n1)N1CCOCC1)c1oc(nn1)c1ccc(Cl)cc1	1547295-42-0	1,3,5-Triazin-2-amine, 4-[[5-(4-chlorophenyl)-1,3,4-oxadiazol-2-yl]thio]-N-(4-fl	C21H17ClFN7O2S	485.92	C	-9.3
889	C(=O)([C@@H](NC(=O)OC)C1CCCCC1)N1[C@H](c2[nH]c(c3c4c(C5CC4CC5)c(cc3)c3ccc(cc3)c3[nH]c(nc3)[C@H]3N(C(	1560639-00-0	Carbamic acid, N-[(1S)-2-methyl-1-[[(2S,4S)-4-methyl-2-[5-[4-[1,2,3,4-tetrahydro	C47H60N8O6	833.03	C	-9.3
890	C(=O)([C@@H](NC(=O)OC)C(C)C)N1[C@H](c2[nH]c(c3c4c(C5CC4CC5)c(cc3)c3ccc(cc3)c3[nH]c(nc3)[C@H]3N(C(=O)	1560639-10-2	Carbamic acid, N-[(1S)-1-[[(2S,4R)-4-fluoro-2-[5-[8-[4-[2-[(2S,4R)-4-fluoro-1-[(	C45H54F2N8O6	840.96	C	-9.3
891	C(=O)([C@@H](NC(=O)OC)C(C)C)N1[C@H](c2[nH]c(c3ccc(c4c5c(c(cc4)c4ccc(cc4)c4[nH]c(nc4)[C@H]4N(C(=O)[C@	1560640-25-6	Carbamic acid, N-[(1S)-1-[[(2S)-4,4-dimethyl-2-[5-[4-[1,2,3,4-tetrahydro-8-[2-[(	C50H66N8O6	875.11	C	-9.3
892	C(=O)([C@@H](NC(=O)OC)C(C)C)N1[C@H](c2[nH]c(c3c4c(c(cc3)c3ccc(cc3)c3[nH]c(nc3)[C@H]3N(C(=O)[C@@H](NC	1560640-37-0	Carbamic acid, N-[(1S)-2-methyl-1-[[(1R,3S,4S)-3-[5-[4-[1,2,3,4-tetrahydro-8-[2-	C50H62N8O6	871.08	C	-9.3
893	C(=O)([C@@H](NC(=O)OC)C1CCCCC1)N1[C@H](c2[nH]c(c3c4c(C5OC4CC5)c(cc3)c3ccc(cc3)c3[nH]c(nc3)[C@H]3N(C(	1560640-82-5	Carbamic acid, N-[(1S)-2-methyl-1-[[(2S,4S)-4-methyl-2-[5-[4-[1,2,3,4-tetrahydro	C46H58N8O7	835	C	-9.3
894	CC1c2c(C3C1CCC3)c(ccc2c1[nH]c(nc1)[C@H]1N(C(=O)[C@@H](NC(=O)OC)C(C)C)CCC1)c1ccc(cc1)c1[nH]c(nc1)[C@H	1613056-86-2	Carbamic acid, N-[(1S)-1-[[(2S)-2-[5-[4-[1,2,3,3a,8,8a-hexahydro-7-[2-[(2S)-1-[(	C46H56N8O7	832.99	C	-9.3
895	[C@@H](Cc1ccccc1)(NC(=O)[C@H](CCN1CCOCC1)NC(=O)N(Cc1nc(C(C)C)sc1)C)CC[C@H](Cc1ccccc1)NC(=O)OCc1cncs1	1627204-38-9	Carbamic acid, N-[(1S)-1-[[(2S)-2-[5-[8-[8-[2-[(2S)-4,4-difluoro-1-[(2S)-2-[(met	C51H60F4N8O6	957.07	C	-9.3
896	C[C@]12[C@H]([C@H]3[C@@H]([C@]4(C)[C@H](C[C@H]3O)CCCC4)CC2)CC[C@@H]1[C@@H](CCCOS(=O)(=O)O)C	1632197-40-0	1,3,5-Triazine-2,4-diamine, N2-(9-ethyl-9H-carbazol-3-yl)-6-(4-morpholinyl)-N4-p	C27H27N7O	465.55	C	-9.3
897	S(c1nc(nc(Nc2ccc(N(=O)=O)cc2)n1)N1CCOCC1)c1oc(nn1)c1ccc(F)cc1	1642584-19-7	1,3,5-Triazin-2-amine, N-(4-fluorophenyl)-4-[[5-(4-fluorophenyl)-1,3,4-oxadiazol	C21H17F2N7O2S	469.47	C	-9.3
898	O=C1N2[C@@H]3[C@@H]4[C@@H](C4)[C@@H](C3)O[C@H]2Cn2c1c(O)c(=O)c(C(=O)N[C@H](C(F)(F)F)c1c(F)cc(F)cc1)c	1644157-73-2	2,12-Methano-1H-cyclopropa[e]pyrido[1,Ä≤,2,Ä≤:4,5]pyrazino[2,1-b][1,3]oxazepine	C23H18F5N3O5	511.4	C	-9.3
899	O(C(=O)OCc1ccccc1)[C@H]1[C@@H](O[C@H](COP(=O)(OCc2ccccc2)N2CCCCC2)[C@H]1OC(=O)OCc1ccccc1)n1nc(C(=O)N	1649477-48-4	1-Naphthalenesulfonic acid, 4-amino-3-[2-(4,Ä≤-amino[1,1,Ä≤-biphenyl]-4-yl)diaz	C22H18N4O3S	418.47	C	-9.3
900	C(=O)([C@@H](NC(=O)OC)C(C)C)N1C[C@@]2(C[C@H]1c1[nH]c(cn1)c1ccc(cc1)c1cc3c(cc1)c1c(cc3)nc([nH]1)[C@H]	1653966-79-0	Carbamic acid, N-[(1S)-1-[[(3S,5S)-3-[5-[4-[2-[(1R,3S,5R)-2-[(2S)-2-[(methoxycar	C47H56N8O7	845	C	-9.3
901	C(=O)([C@@H](NC(=O)OC)C(C)C)N1C[C@@]2(C[C@H]1c1[nH]c(cn1)c1ccc(cc1)c1cc3c(cc1)c1c(cc3)nc([nH]1)[C@H]	1653966-87-0	Carbamic acid, N-[(1S)-2-methyl-1-[[(3S,5S)-3-[7-[4-[2-[(2S,3aS,7aS)-octahydro-1	C50H62N8O7	887.08	C	-9.3
902	C(=O)([C@@H](NC(=O)OC)C(C)C)N1C[C@@]2(C[C@H]1c1[nH]c3c(n1)ccc1c3ccc(c1)c1cc3c(cc1)c1c(cc3)nc([nH]1)[	1653966-93-8	Carbamic acid, N-[(1S)-2-methyl-1-[[(3S,5S)-3-[5-[4-[2-[(2S,3aS,7aS)-octahydro-1	C50H62N8O7	887.08	C	-9.3
903	N(c1nc(nc(n1)N1CCOCC1)N1CCOCC1)c1ccc(C(=O)/C=C/c2cc(C)c(C)cc2)cc1	1710363-32-8	2-Propen-1-one, 3-(3-chlorophenyl)-1-[4-[(4,6-di-4-morpholinyl-1,3,5-triazin-2-y	C26H27ClN6O3	506.98	C	-9.3
904	FC1(F)CC2(c3c(-c4c2cc(cc4)c2[nH]c(nc2)[C@H]2N(C(=O)[C@@H](NC(=O)OC)[C@H](CC)C)CCC2)ccc(c3)c2[nH]c(nc	1800210-32-5	Carbamic acid, N,N,Ä≤-[(3,3-difluorospiro[cyclobutane-1,9,Ä≤-[9H]fluorene]-2,Ä≤	C44H52F2N8O6	826.93	C	-9.3
905	C(=O)([C@@H](NC(=O)OC)C(C)C)N1[C@@H]([C@@H]2C[C@H]1CC2)c1[nH]c(cn1)c1cc2c(-c3c(C(F)(F)C2(F)F)cc(cc3)	1801161-65-8	Carbamic acid, N,N,Ä≤-[(9,9,10,10-tetrafluoro-9,10-dihydro-2,7-phenanthrenediyl)	C42H48F4N8O6	836.87	C	-9.3
906	C(OCCOC(=O)C)n1c2c(nc1F)c(=O)nc(NC(=O)C)[nH]2	1811520-72-5	Butanedioic acid, 1-[(3R,5aS,6R,8aS,9R,10S,12R,12aR)-decahydro-3,6,9-trimethyl-3	C41H48O15	780.81	C	-9.3
907	O[C@H]1[C@H](n2c3c(nc2)c(=O)nc(N)[nH]3)O[C@H](COP(=O)(OP(=O)(CP(=O)(OP(=O)(OC[C@H]2O[C@H]([C@H](O)[C	1813562-22-9	Carbonic acid, 2-[[2-[[[[4-[(1E)-2-[4-(dicyanomethylene)-4H-1-benzopyran-2-yl]et	C48H41N3O13S2	931.98	C	-9.3
908	N(C(C)C)c1c(nc(OS(=O)(=O)O)cc1)N1CCN(C(=O)c2[nH]c3c(c2)cc(NS(=O)(=O)C)cc3)CC1	188780-39-4	Œ≤-D-Glucopyranosiduronic acid, 5-[(1-methylethyl)amino]-6-[4-[[5-[(methylsulfon	C28H36N6O10S	648.68	C	-9.3
909	O=C1c2n(N([C@H]3N1[C@H](C)COC3)[C@H]1c3c(CSc4c1cccc4)c(F)c(F)cc3)ccc(=O)c2O	1985605-77-3	1H-[1,4]Oxazino[3,4-c]pyrido[2,1-f][1,2,4]triazine-6,8-dione, 12-[(11S)-7-fluoro	C24H20FN3O4S	465.5	C	-9.3
910	O(C(=O)OCC)c1c2n(N([C@H]3N(C2=O)CCOC3)[C@H]2c3c(CSc4c2cccc4)c(F)c(F)cc3)ccc1=O	1985606-53-8	1H-[1,4]Oxazino[3,4-c]pyrido[2,1-f][1,2,4]triazine-6,8-dione, 12-[(11S)-7,8-difl	C31H25F2N3O4S	573.61	C	-9.3
911	O(COC(=O)OC(C(=O)OC(C)(C)C)(C)C)c1c2n(N([C@H]3N(C2=O)CCOC3)[C@H]2c3c(CSc4c2cccc4)c(F)c(F)cc3)ccc1=O	1985607-02-0	Carbonic acid, [[(12aR)-12-[(11S)-7,8-difluoro-6,11-dihydrodibenzo[b,e]thiepin-1	C32H29F2N3O9S	669.65	C	-9.3
912	O(C(=O)N(C)c1ccccc1)c1c2n(N([C@H]3N(C2=O)CCOC3)[C@H]2c3c(CSc4c2cccc4)c(F)c(F)cc3)ccc1=O	1985607-07-5	Carbonic acid, [[(12aR)-12-[(11S)-7,8-difluoro-6,11-dihydrodibenzo[b,e]thiepin-1	C32H29F2N3O9S	669.65	C	-9.3
913	O(C(=O)OC1CCN(C)CC1)c1c2n(N([C@H]3N(C2=O)CCOC3)[C@H]2c3c(CSc4c2cccc4)c(F)c(F)cc3)ccc1=O	1985607-29-1	Carbonic acid, 4-(acetylamino)phenyl (12aR)-12-[(11S)-7,8-difluoro-6,11-dihydroc	C33H26F2N4O7S	660.64	C	-9.3
914	O([C@@H](C)c1ccccc1)c1c2n(N([C@H]3N(C2=O)CCOC3)[C@H]2c3c(CSc4c2cccc4)c(F)c(F)cc3)ccc1=O	1985607-42-8	Cyclopentanecarboxylic acid, 1-[[[[[(12aR)-12-[(11S)-7,8-difluoro-6,11-dihydrodi	C33H31F2N3O8S	667.68	C	-9.3
915	C(=O)([C@@H](NC(=O)OC)C(C)C)N1[C@H](c2[nH]c3c4c(cc(cc4)c4c5c(c(cc4)c4[nH]c(nc4)[C@H]4N(C(=O)[C@@H](N	2007901-41-7	Carbamic acid, N-[(1S)-1-[[(2S,5S)-2-[7-[4-[2-[(2S)-1-[(2S)-2-cyclohexyl-2-[(met	C50H62N8O6	871.08	C	-9.3
916	C(=O)([C@@H](NC(=O)OC)C(C)C)N1[C@H](c2[nH]c3c4c(cc(cc4)c4c5c(c(cc4)c4[nH]c(nc4)[C@H]4N(C(=O)[C@@H](N	2007901-66-6	Carbamic acid, N-[(1S)-2-methyl-1-[[(2S)-2-[7-[5,6,7,8-tetrahydro-4-[2-[(2S)-1-[	C46H56N8O6	816.99	C	-9.3
917	N(C(=O)COC(=O)Cn1c2c(oc1=O)cccc2)C12CC3CC(C2)CC(C1)C3	1002355-35-2	1H-1,2,4-Triazole-3-carboxamide, 1-[2,3-O-(methoxymethylene)-5-O-[[4-[[4,6-O-[(S	C30H38N4O13	662.64	A/B	-9.2
918	C(=O)([C@H](NC(=O)OC)c1ccccc1)N1[C@@H](CCC1)c1[nH]c(cn1)c1ccc(cc1)c1ccc(cc1)c1[nH]c(nc1)[C@H]1N(C(=O	1009108-66-0	Carbamic acid, N-[(1R)-2-[(2S)-2-[5-[4,Ä≤-[2-[(2S)-1-[2-(3-methylphenyl)acetyl]-	C45H45N7O4	747.88	A/B	-9.2
919	C(=O)([C@H](NC(=O)OC)c1ccccc1)N1[C@@H](CCC1)c1[nH]c(cn1)c1ccc(cc1)c1ccc(cc1)c1[nH]c(nc1)[C@H]1N(C(=O	1009108-72-8	Carbamic acid, N-[(1R)-2-[(2S)-2-[5-[4,Ä≤-[2-[(2S)-1-(2-cyclohexylacetyl)-2-pyrr	C44H49N7O4	739.9	A/B	-9.2
920	C(=O)([C@H](N(C)C)c1ccccc1)N1[C@@H](CCC1)c1[nH]c(cn1)c1ccc(cc1)c1ccc(cc1)c1[nH]c(nc1)[C@H]1N(C(=O)CN	1009108-73-9	Carbamic acid, N-[(1R)-2-oxo-2-[(2S)-2-[5-[4,Ä≤-[2-[(2S)-1-[(2R)-1-oxo-2-phenylp	C45H45N7O4	747.88	A/B	-9.2
921	C(=O)([C@@H](NC(=O)OC)CCN(C)C)N1[C@@H](CCC1)c1[nH]c(cn1)c1ccc(cc1)c1ccc(cc1)c1[nH]c(nc1)[C@H]1N(C(=O	1009115-33-6	Carbamic acid, N,N,Ä≤-[[1,1,Ä≤-biphenyl]-4,4,Ä≤-diylbis[1H-imidazole-5,2-diyl-(	C48H50N8O6	834.96	A/B	-9.2
922	C(CCCc1cc(O)c(O)cc1)c1cc(O)c(O)cc1	10142-99-1	Benzoic acid, 5-[(3-carboxy-4-hydroxyphenyl)(3-carboxy-4-oxo-2,5-cyclohexadien-1	C23H16O9	436.37	A/B	-9.2
923	N(c1nc(Nc2ccc(C#N)cc2)nc(CN2CCN(C)CC2)c1)c1c(C)cc(/C=C/C#N)cc1C	1037444-98-6	Benzonitrile, 4-[[4-[[4-[(1E)-2-cyanoethenyl]-2,6-dimethylphenyl]amino]-6-(4-mor	C27H27N7O	465.55	A/B	-9.2
924	C([C@H](OC[P@@](=O)(N[C@H](C(=O)OC)CC(C)C)Oc1ccccc1)C)n1c2c(nc1)c(N)ncn2	1051375-16-6	2H-Pyrido[1,Ä≤,2,Ä≤:4,5]pyrazino[2,1-b][1,3]oxazine-9-carboxamide, N-[(2,4-difl	C20H19F2N3O5	419.38	A/B	-9.2
925	O=C1[C@@H]2[C@@H](c3c([C@H](O)[C@H]2CO1)cc1c(c3)OCO1)c1cc(OC)c(O)c(O)c1	10566-04-8	Guanosine, guanylyl-(5,Ä≤,Üí5,Ä≤)-	C20H25N10O12P	628.45	A/B	-9.2
926	O=c1n(ccc(N)n1)[C@@H]1O[C@H](COP(=O)(OP(=O)(OP(=O)(O)O)O)O)[C@@H](F)C1	1064476-02-3	Not Yet Assigned	C40H49N5O6	695.85	A/B	-9.2
927	O(C[C@H]1O[C@H](C=C1)n1c(=O)[nH]c(=O)c(C)c1)P1(=O)Oc2c(cc(CCC(=O)N[C@H](C(=O)O)C)cc2)CO1	1073242-65-5	L-Alanine, N-[3-[2-(2,Ä≤,3,Ä≤-didehydro-3,Ä≤-deoxythymidin-5,Ä≤-O-yl)-2-oxido-4	C30H32N3O10P	625.56	A/B	-9.2
928	O=c1n(cc(F)nc1C(=O)N)C1OC(CO)C=C1	1079990-15-0	2-Pyrazinecarboxamide, 4-[5-[[(6-fluoro-3,4-dihydro-2-oxido-2H-pyrazino[2,3-e]-1	C15H11F2N6O7P	456.25	A/B	-9.2
929	Clc1c2c(cc(NC(CCCN(CC)CC)C)cc2)ncc1	108112-22-7	Hydrazinecarbothioamide, 2-[1,2-dihydro-2-oxo-1-(1-piperidinylmethyl)-3H-indol-3	C29H29N5OS	495.64	A/B	-9.2
930	C(=O)(N[C@H](C(=O)C(=O)NC1CC1)CCC)[C@@H]1[C@@H]2[C@H](CN1C(=O)[C@@H](NC(=O)[C@@H](NC(=O)c1c(nc(c(n1)	1134176-05-8	Cyclopenta[c]pyrrole-1-carboxamide-d, (2S)-2-cyclohexyl-N-(2-pyrazinylcarbonyl)g	C36H49D4N7O6		A/B	-9.2
931	C(=O)(N[C@](C(=O)C(=O)NC1CC1)(C(C(C([2H])([2H])[2H])([2H])[2H])([2H])[2H])[2H])[C@@H]1[C@@H]2[C@H](C	1134176-13-8	Cyclopenta[c]pyrrole-1-carboxamide-3,4,5,6-d4, (2S)-2-cyclohexyl-N-(2-pyrazinylc	C36H42D11N7O6		A/B	-9.2
932	N(=Nc1cc(N=Nc2cc3c(cc2)ccc(S(=O)(=O)O)c3)ccc1O)c1c2c(ccc1O)cccc2	116028-32-1	2-Naphthalenesulfonic acid, 5-[2-[4-hydroxy-3-[2-(2-hydroxy-1-naphthalenyl)diaze	C26H18N4O5S	498.51	A/B	-9.2
933	N(=Nc1c(C)cc(cc1)c1cc(C)c(N=Nc2ccc(O)cc2)cc1)c1c2c(cc(S(=O)(=O)O)cc2S(=O)(=O)O)ccc1O	1169930-40-8	D-erythro-Pentonamide, 2,3,5-trideoxy-N-[(1S,2R)-2,3-dihydro-2-hydroxy-1H-inden-	C36H47N5O4	613.79	A/B	-9.2
934	Cc1n(c2nc(nc(n2)N2CCOCC2)c2cc(CO)ccc2)ccn1	1174190-76-1	Benzenemethanol, 3-[4-(2-methyl-1H-benzimidazol-1-yl)-6-(4-morpholinyl)-1,3,5-tr	C22H22N6O2	402.45	A/B	-9.2
935	C(OC(C(O)([2H])[2H])([2H])[2H])n1c2c(nc1)c(=O)nc(N)[nH]2	1185110-50-2	Not Yet Assigned	C22H14O9.Na		A/B	-9.2

936	O=c1n([C@@H]2O[C@H](CO[Si](C(C)(C)C)(C)C)[C@@H](N=[N+]=[N-])C2)cc(C)c(=O)[nH]1	1206156-32-2	1-Piperazinecarboxylic acid, 4-methyl-, [[(4R,12aS)-9-[[[(2,4-difluorophenyl)met	C27H31F2N5O7	575.56	A/B	-9.2
937	C(=O)(N[C@H](C(=O)C(=O)NC1CC1)CCC)[C@H]1N(C(=O)[C@@H](NC(=O)[C@@H](NC(=O)c2cnccn2)C2CCCCC2)CCC(=O)C=	1208119-28-1	Not Yet Assigned	C39H57N7O7	735.91	A/B	-9.2
938	C(N(C(=O)N[C@@H](C(=O)N[C@@H](Cc1ccccc1)C[C@@H]([C@H](Cc1ccccc1)NC(=O)OCc1cncs1)O)C(C)C)C)c1nc(sc1)C	1236407-28-5	2,7,10,12-Tetraazatridecanoic acid, 13-[2-(1-cyclopenten-1-yl)-4-thiazolyl]-4-hy	C39H48N6O5S2	744.97	A/B	-9.2
939	[C@H](Cc1ccccc1)(C[C@@H]([C@H](Cc1ccccc1)NC(=O)OCc1ccns1)O)NC(=O)[C@@H](NC(=O)N(Cc1nc(CCC)sc1)C)C(C)	1236407-63-8	2,7,10,12-Tetraazatridecanoic acid, 4-hydroxy-12-methyl-9-(1-methylethyl)-8,11-c	C42H50N6O6S	766.95	A/B	-9.2
940	N(C(=O)[C@H](Cc1ccccc1)C[C@@H](CN1[C@H](C(=O)NC(C)(C)C)CN(Cc2c3n(ccn3)ccn2)CC1)O)[C@H]1c2c(C[C@H]1O)	1237808-94-4	Not Yet Assigned	C37H47N7O4	653.81	A/B	-9.2
941	N(CC(=O)[C@H](Cc1ccccc1)C[C@@H](CN1[C@H](C(=O)NC(C)(C)C)CN(Cc2c(C)ccnc2C)CC1)O)[C@H]1c2c(C[C@H]1O)cc	1237988-79-2	Not Yet Assigned	C39H53N5O4	655.87	A/B	-9.2
942	[C@@H](C(=O)N[C@@H](Cc1ccccc1)C[C@@H]([C@H](Cc1ccccc1)NC(=O)COc1c(C)cccc1C)O)(C(C)C)N1C(=O)NCCC1.O	1239018-93-9	7,10-Methanopyrimido[1,2-a]azepine-2-carboxamide, N-[(4-fluorophenyl)methyl]-4,6	C24H25FN6O7S	560.55	A/B	-9.2
943	C(F)(F)c1n(c2c(n1)cccc2)c1nc(nc(N[C@H]2CC[C@H](NCC)CC2)n1)N1CCOCC1	1240024-08-1	Methanesulfonamide, N-[trans-4-[[4-[2-(difluoromethyl)-1H-benzimidazol-1-yl]-6-(	C22H28F2N8O3S	522.57	A/B	-9.2
944	C(=O)([C@H](NC(=O)OC)c1ccccc1)N1[C@@H](CCC1)c1[nH]c(cn1)c1ccc(CCCCc2[nH]c(nc2)[C@H]2N(C(=O)[C@H](NC(	1240891-49-9	Carbamic acid, N-[(1R)-2-[(2S)-2-[5-[4-[(1R)-4-[2-[(2S)-1-[(2R)-2-[(methoxycarbo	C46H50N8O6	810.94	A/B	-9.2
945	C(=O)([C@H](NC(=O)OC)c1ccccc1)N1[C@@H](CCC1)c1[nH]c(cn1)C1CCC(=CCCCc2[nH]c(nc2)[C@H]2N(C(=O)[C@H](NC	1240891-98-8	Carbamic acid, N-[(1R)-2-[(2S)-2-[5-[4-[(2E)-4-[2-[(2S)-1-[(2R)-2-[(methoxycarbo	C44H48N8O6	784.9	A/B	-9.2
946	C(=O)([C@@H](NC(=O)OC)C(C)C)N1[C@@H](CCC1)c1[nH]c(C#Cc2ccc(C#Cc3[nH]c(nc3)[C@H]3N(C(=O)[C@@H](NC(=O)	1240893-24-6	Carbamic acid, N-[(1R)-2-[(2S)-2-[5-[4-[2-[2-[(2S)-1-[(2R)-2-[(methoxycarbonyl)a	C42H46N8O6	758.86	A/B	-9.2
947	O[C@H]1[C@H](n2c3c(nc2)c(=O)nc(N)[nH]3)O[C@H](COP(=O)(OP(=O)(O[C@@H]2[C@@H](CO)O[C@H]([C@@H]2O)n2c3c	124705-06-2	Guanosine 5,Ä≤-(trihydrogen diphosphate), P,Ä≤,Üî2,Ä≤-ester with adenosine	C20H26N10O14P2	692.43	A/B	-9.2
948	C(OCCOC(=O)/C=C/c1ccc(O)cc1)n1c2c(c(=O)nc(N)[nH]2)nc1	1253912-50-3	1-Piperazinecarboxamide, N-[(1S)-1-[[[(1R,2E)-1-[1,1,Ä≤-biphenyl]-4-yl-3-(phenyl	C33H40N4O4S	588.76	A/B	-9.2
949	O=c1n([C@@H]2O[C@H](COP(=O)(Oc3ccccc3)N[C@@H](C(=O)OC)C)[C@@H](N=[N+]=[N-])C2)cc(C)c(=O)[nH]1	1256547-81-5	Carbamic acid, N-[(1S)-2-methyl-1-[[(2S)-2-[5-[6-[4-[2-[(2S,5R)-5-phenyl-2-pyrro	C43H45N7O3	707.86	A/B	-9.2
950	C(=O)([C@@H](NC(=O)OC)C(C)C)N1[C@@H]([C@@H]2C[C@H]1CC2)c1[nH]c(cn1)c1ccc(cc1)c1ccc(cc1)c1[nH]c([C@@H	1273591-40-4	Carbamic acid, N-[(1S)-1-[[(1R,3S,4S)-3-[5-[4,Ä≤-[2-[(1S)-1-[[(2S)-2-[(methoxyca	C40H50N8O6	738.88	A/B	-9.2
951	N(Cc1ccc(F)cc1)c1nc(nc(N/N=C/c2c(O)cccc2)n1)N1CCOCC1	1279692-14-6	1H-Indole-2,3-dione, 3-[2-[4-[[(3-chlorophenyl)methyl]amino]-6-(4-morpholinyl)-1	C22H21ClN8O2	464.91	A/B	-9.2
952	C(OC(COC(=O)[C@H](C)N)CO)n1c2c(nc1)c(=O)nc(N)[nH]2	130914-77-1	5,9-Dioxa-2,12-diazatridecanedioic acid, 7-[(2-amino-1,6-dihydro-6-oxo-9H-purin-	C35H43N7O10	721.76	A/B	-9.2
953	C(=O)([C@@H](NC(=O)OC)C1CCOCC1)N1[C@@H](C[C@H](C)C1)c1[nH]c(c(Cl)n1)c1ccc(cc1)c1ccc(cc1)c1[nH]c(nc1C	1312336-26-7	Carbamic acid, N-[(1S)-2-[(2S,4S)-2-[5-[4,Ä≤-[5-chloro-2-[(2S,4S)-1-[(2S)-2-[(me	C46H57ClN8O8	885.45	A/B	-9.2
954	C(OC[C@H](Cn1c(=O)nc(NC(=O)c2ccccc2)cc1)OCP(=O)(OCC)O)(c1ccccc1)(c1ccccc1)c1ccccc1	1312805-81-4	Adenosine, 1,Ä≤-[3-(aminocarbonyl)-1H-1,2,4-triazol-1-yl]-1,Ä≤-de(6-amino-9H-pu	C24H34N12O19P2	856.54	A/B	-9.2
955	O(Cc1ccc(F)cc1)c1c2c(oc(c(O)c2=O)c2ncccn2)c(C)cc1	1312892-20-8	4H-1-Benzopyran-4-one, 5-[(4-fluorophenyl)methoxy]-3-hydroxy-2-(5-phenyl-1H-imid	C25H17FN2O4	428.41	A/B	-9.2
956	O(Cc1ccc(F)cc1)c1c2c(c(cc1)c1ccccc1)oc(c(O)c2=O)c1ncccn1	1312894-02-2	4H-1-Benzopyran-4-one, 8-chloro-5-[(4-fluorophenyl)methoxy]-3-hydroxy-2-(5-pheny	C25H16ClFN2O4	462.86	A/B	-9.2
957	O(Cc1ccc(F)cc1)c1c2c(c(cc1)c1ccccc1)oc(c(O)c2=O)c1ncc(cn1)c1ccccc1	1312894-36-2	4H-1-Benzopyran-4-one, 2-(5-chloro-2-pyrimidinyl)-5-[(4-fluorophenyl)methoxy]-3-	C26H16ClFN2O4	474.87	A/B	-9.2
958	O(Cc1ccc(F)cc1)c1c2c(oc(c(O)c2=O)c2ncccn2)c(Cl)c(C)c1	1312896-02-8	4H-1-Benzopyran-4-one, 5-[(4-fluorophenyl)methoxy]-3-hydroxy-8-methoxy-7-methyl-	C27H21FN2O5	472.46	A/B	-9.2
959	O(Cc1ccc(F)cc1)c1c2c(c(c(Cl)c1)c1ccccc1)oc(c(O)c2=O)c1[nH]c(OC)nn1	1312902-65-0	4H-1-Benzopyran-4-one, 7-chloro-5-[(4-fluorophenyl)methoxy]-3-hydroxy-2-(3-methy	C25H17ClFN3O4	477.87	A/B	-9.2
960	O(Cc1ccc(F)cc1)c1c2c(c(C)c(c1)c1ccccc1)oc(c(O)c2=O)c1[nH]cnn1	1312903-59-5	4H-1-Benzopyran-4-one, 5-[(4-fluorophenyl)methoxy]-3-hydroxy-8-methyl-7-phenyl-2	C33H23FN2O4	530.55	A/B	-9.2
961	Clc1c2c(c(OCc3ccc(F)cc3)cc1c1ccccc1)c(=O)c(O)c(o2)c1ncc(cn1)c1ccccc1	1312905-07-9	4H-1-Benzopyran-4-one, 8-chloro-2-(5-chloro-2-pyrimidinyl)-5-[(4-fluorophenyl)me	C26H15Cl2FN2O4	509.31	A/B	-9.2
962	O=c1c2c(oc(c1O)c1nnc(Cl)[nH]1)c(C)cc(OCc1ccc(F)cc1)c2	1312906-76-5	4H-1-Benzopyran-4-one, 6-[(4-fluorophenyl)methoxy]-3-hydroxy-2-(5-methoxy-1H-1,2	C20H16FN3O5	397.36	A/B	-9.2
963	O=c1c2c(oc(c1O)c1[nH]c(nn1)c1ccccc1)c(C)cc(OCc1ccc(F)cc1)c2	1312906-77-6	4H-1-Benzopyran-4-one, 2-(5-chloro-1H-1,2,4-triazol-3-yl)-6-[(4-fluorophenyl)met	C19H13ClFN3O4	401.78	A/B	-9.2
964	O(C)c1c2c(c(=O)c(O)c(o2)c2ncccn2)cc(OCc2ccc(F)cc2)c1	1312906-83-4	4H-1-Benzopyran-4-one, 6-[(4-fluorophenyl)methoxy]-3-hydroxy-8-methyl-2-(5-pheny	C26H19FN2O4	442.44	A/B	-9.2
965	Clc1c2c(cc(OCc3ccc(F)cc3)c1)c(=O)c(O)c(o2)c1ncccn1	1312907-33-7	4H-1-Benzopyran-4-one, 6-[(4-fluorophenyl)methoxy]-3-hydroxy-8-methoxy-2-(5-pher	C26H19FN2O5	458.44	A/B	-9.2
966	O=c1c2c(c(cc(OCc3ccc(F)cc3)c2)c2ccccc2)oc(c1O)c1[nH]c(OC)nn1	1312908-25-0	4H-1-Benzopyran-4-one, 6-[(4-fluorophenyl)methoxy]-3-hydroxy-2-(3-methyl-1H-1,2,	C25H18FN3O4	443.43	A/B	-9.2
967	O=c1c2c(cc(OCc3ccc(F)cc3)c2)c2ccccc2)oc(c1O)c1[nH]c(Cl)nn1	1312908-26-1	4H-1-Benzopyran-4-one, 6-[(4-fluorophenyl)methoxy]-3-hydroxy-2-(5-methoxy-1H-1,2	C25H18FN3O5	459.43	A/B	-9.2
968	Cc1c(c2c(cc1OCc1ccc(F)cc1)c(=O)c(O)c(o2)c1[nH]c(Cl)cn1)c1ccccc1	1312910-93-2	4H-1-Benzopyran-4-one, 6-[(4-fluorophenyl)methoxy]-3-hydroxy-2-(5-methoxy-1H-imi	C27H21FN2O5	472.46	A/B	-9.2
969	O(C)c1c2c(cc(OCc3ccc(F)cc3)c1OC)c(=O)c(O)c(o2)c1ncccn1	1312912-93-8	4H-1-Benzopyran-4-one, 7-chloro-6-[(4-fluorophenyl)methoxy]-3-hydroxy-8-methyl-2	C26H18ClFN2O4	476.88	A/B	-9.2
970	Clc1c2c(cc(OCc3ccc(F)cc3)c1Cl)c(=O)c(O)c(o2)c1ncccn1	1312914-48-9	4H-1-Benzopyran-4-one, 8-chloro-6-[(4-fluorophenyl)methoxy]-3-hydroxy-7-methoxy-	C26H18ClFN2O5	492.88	A/B	-9.2
971	Clc1c(c2c(cc1OCc1ccc(F)cc1)c(=O)c(O)c(o2)c1[nH]c(C)nn1)c1ccccc1	1312915-80-2	4H-1-Benzopyran-4-one, 7-chloro-6-[(4-fluorophenyl)methoxy]-3-hydroxy-8-phenyl-2	C24H15ClFN3O4	463.85	A/B	-9.2
972	Clc1c(c2c(cc1OCc1ccc(F)cc1)c(=O)c(O)c(o2)c1[nH]c(OC)cn1)c1ccccc1	1312915-92-6	4H-1-Benzopyran-4-one, 7-chloro-6-[(4-fluorophenyl)methoxy]-3-hydroxy-2-(5-methy	C26H18ClFN2O4	476.88	A/B	-9.2
973	[C@@H](NC(=O)c1ccccc1)(C(=O)N[C@H](C(=O)N[C@H](C(=O)C(=O)NCC(=O)N1CCN(C)CC1)C(C)C)C12CC3CC(C2)CC(C	13186-45-3	Benzoic acid, 3,3,Ä≤-[(3-carboxy-4-oxo-2,5-cyclohexadien-1-ylidene)methylene]bis	C22H14O9.3Na		A/B	-9.2
974	O=C1N2[C@H](c3nc(c([nH]3)CCc3nc([C@H](C)[C@@H]1NC(=O)OC)ccc3)c1ccc(cc1)c1ccc(cc1)c1[nH]c(nc1)[C@H]1N	1319189-58-6	Carbamic acid, N-[(12S,13S,18aS)-5,6,13,14,16,17,18,18a-octahydro-3-[4,Ä≤-[2-[(	C47H54N8O6	826.98	A/B	-9.2
975	O[C@H]1[C@H](n2c3c(nc2)c(=NO)nc(Cl)[nH]3)O[C@H](CO)[C@H]1O	133826-32-1	Guanosine 5,Ä≤-(trihydrogen diphosphate), 2,Ä≤-deoxy-, P,Ä≤,Üî5,Ä≤-ester with 2	C20H26N10O13P2	676.43	A/B	-9.2
976	N(=Nc1c2c(c(N=Nc3cc(S(=O)(=O)O)ccc3)cc1)ccc(S(=O)(=O)O)c2)c1c2c(c(N)cc1)cccc2S(=O)(=O)O	1345852-32-5	2-Naphthalenesulfonic acid, 8-[2-(4-amino-6-sulfo-1-naphthalenyl)diazenyl]-5-[2-	C26H19N5O9S3	641.65	A/B	-9.2
977	C(C)(C)c1n([C@@H]2C[C@@H]3N(C(CC(NC(=O)C4CCC(F)(F)CC4)c4ccccc4)([2H])[2H])C[C@@H](C2)CC3)c(C([2H])([	1347417-62-2	[1,1,Ä≤-Biphenyl]-2-acetamide, 4,Ä≤-[(2-ethyl-5,7-dimethyl-3H-imidazo[4,5-b]pyr	C31H30N4O3S	538.66	A/B	-9.2
978	O=C1C2C(N(C=N2)[C@@H]2O[C@H](CO)CC2)NC(=N1)N	1347855-43-9	Benzamide, N-[[4,Ä≤-[(2-ethyl-5,7-dimethyl-3H-imidazo[4,5-b]pyridin-3-yl)methyl]	C30H27N5O5S	569.63	A/B	-9.2
979	C(C(OCP(=O)(C)C)C)n1c2c(nc1)c(N)ncn2	1348646-41-2	Carbamic acid, N-[(1S,2R)-1-[[4-[[bis(4-hydroxyphenyl)phosphinyl]methoxy]phenyl]	C39H47N2O11PS	782.84	A/B	-9.2
980	P(=O)(COc1ccc(S(=O)(=O)N(C[C@H]([C@H](Cc2ccc(N(=O)=O)cc2)NC(=O)O[C@@H]2[C@H]3[C@@H](OC2)OCC3)O)CC(C)	1348806-74-5	Carbamic acid, N-[(1S,2R)-1-[[4-[[bis(4-hydroxyphenoxy)phosphinyl]methoxy]phenyl	C39H47N2O13PS	814.83	A/B	-9.2
981	C(=O)(NC(C)(C)C)[C@H]1N(C[C@H]([C@@H](NC(=O)c2c(C)c(O)ccc2)CSc2ccccc2)O)C[C@]2(C)[C@H](C1)CCCC2	1349030-58-5	Not Yet Assigned	C53H70N12O11	1051.2	A/B	-9.2
982	[C@H](Cc1ccccc1)(C[C@@H]([C@H](Cc1ccccc1)NC(=O)OCc1cccnc1)O)NC(=O)[C@H](N(C(=O)NCc1cscn1)C)C(C)C	1350078-92-0	Not Yet Assigned	C36H53N7O6	679.85	A/B	-9.2
983	C(=O)([C@@H](NC(=O)OC)C(C)C)N1[C@@]2(C[C@H](C1)CC2)c1[nH]c(cn1)c1c2c(c(cc1)c1ccc(cc1)c1[nH]c(nc1)[C@	1350719-00-4	Carbamic acid, N-[(1S)-2-methyl-1-[[(1S,4R)-1-[5-[4-[5,6,7,8-tetrahydro-4-[2-[(1	C48H60N8O6	845.04	A/B	-9.2
984	N(Cc1ccc(cc1)c1ccccn1)(C[C@@H]([C@H](Cc1ccccc1)NC(=O)[C@@H](NC(=O)OC)C(C)(C)C)O)NC(=O)[C@@H](NC(=O)O	1354820-55-5	2,5,6,10,13-Pentaazatetradecanedioic acid, 3-(1,1-dimethylethyl)-8-hydroxy-12-(2	C38H52N6O8	720.85	A/B	-9.2
985	C(F)(F)c1n(c2c(n1)cccc2)c1nc(nc(Nc2c(O)cccc2)n1)N1CCOCC1	1359956-21-0	Phenol, 3-[[4-[2-(difluoromethyl)-1H-benzimidazol-1-yl]-6-(4-morpholinyl)-1,3,5-	C21H19F2N7O2	439.42	A/B	-9.2
986	C(OC(=O)CCC(=O)O[C@H]([C@H](Cc1ccccc1)NC(=O)COc1c(C)cccc1C)C[C@H](Cc1ccccc1)NC(=O)[C@H](C(C)C)N1C(=O	1370468-36-2	Carbamic acid, N,N,Ä≤-[[(6S)-6-phenyl-6H-indolo[1,2-c][1,3]benzoxazine-3,10-diyl	C49H55N9O7	882.02	A/B	-9.2
987	C(=O)([C@@H](NC(=O)OC)C(C)C)N1[C@@H](CCC1)c1[nH]c(cn1)c1ccc(cc1)c1ccc(nc1)c1ccc(cc1)c1[nH]c(nc1)[C@H	1375847-97-4	Benzoic acid, 3,3,Ä≤-[(3-carboxy-4-oxo-2,5-cyclohexadien-1-yl)methylene]bis[6-hy	C22H16O9	424.36	A/B	-9.2
988	C(=O)([C@@H](NC(=O)OC)C1CCOCC1)N1CC2(C[C@H]1c1[nH]c3c(n1)ccc1c3ccc(c1)c1ccc(cc1)c1[nH]c(nc1)[C@H]1N(	1377037-04-1	Carbamic acid, N-[(1S)-2-[(1R,3S,4S)-3-[4,5-dihydro-2,Ä≤-[(2S)-1-[(2S)-2-[(metho	C48H56N8O7	857.01	A/B	-9.2
989	C(=O)([C@@H](NC(=O)OC)C1CCOCC1)N1[C@@H]([C@@H]2C[C@H]1CC2)c1[nH]c2c(n1)ccc1c2ccc(c1)c1ccc2c(-c3c(CC2)	1377037-10-9	Carbamic acid, N-[(1S)-2-[(1R,3S,4S)-3-[5-[9,10-dihydro-7-[2-[(2S)-1-[(2S)-2-[(m	C46H56N8O7	832.99	A/B	-9.2
990	C(=O)([C@@H](NC(=O)OC)C(C)C)N1[C@H](c2[nH]c3c4c(cc(cc4)c4ccc(cc4)c4[nH]c(nc4)[C@H]4N(C(=O)[C@@H](NC(	1377044-95-5	Carbamic acid, N-[(1S)-1-[[(2S)-2-[7-[4-[2-[(2S)-1-[(2S)-2-[(methoxycarbonyl)ami	C50H54N8O6	863.01	A/B	-9.2
991	N(=Nc1cc(C)c(N=Nc2ccc(S(=O)(=O)O)cc2)cc1)c1c2c(c(N=Nc3ccc(O)cc3)cc1)ccc(S(=O)(=O)O)c2	1378293-74-3	2-Naphthalenesulfonic acid, 5-[2-(4-aminophenyl)diazenyl]-8-[2-[3-methyl-4-[2-(4	C29H23N7O6S2	629.67	A/B	-9.2
992	O(C(=O)C)[C@H]1[C@@H](O[C@H](CO)[C@H]1OC(=O)C)n1c2c([nH]c1=O)c(=O)nc(N)[nH]2	1384189-29-0	Phosphoramidic acid, N-(phenylmethyl)-, [(1R,3S,5S)-3-[2-[[(dimethylamino)methyl	C29H34N7O5P	591.6	A/B	-9.2
993	N(CC(=O)N1CCN(S(=O)(=O)C)CC1)c1nc(nc(n1)N1CCOCC1)c1ccc(=O)[nH]c1	1391924-38-1	Acetamide, 2-[[4-(2,3-dihydro-1-hydroxy-1H-inden-4-yl)-6-(4-morpholinyl)-1,3,5-t	C20H26N6O3	398.46	A/B	-9.2
994	N(CC(=O)N1CCN(S(=O)(=O)C)CC1)c1nc(nc(n1)N1CCOCC1)c1cc(O)ccc1	1391924-48-3	Ethanone, 2-[[4-(6-fluoro-3-pyridinyl)-6-(4-morpholinyl)-1,3,5-triazin-2-yl]amin	C19H25FN8O4S	480.52	A/B	-9.2
995	O=C1CCN(c2nc(nc(n2)N2CCOCC2)c2c3c(ccc2)[nH]nc3)CC1	1391925-87-3	4-Piperidinol, 1-[4-(1H-indazol-4-yl)-6-(4-morpholinyl)-1,3,5-triazin-2-yl]-	C19H23N7O2	381.43	A/B	-9.2
996	NC1CCN(c2nc(nc(n2)N2CCOCC2)c2c3c(ccc2)[nH]nc3)CC1	1391925-88-4	4-Piperidinone, 1-[4-(1H-indazol-4-yl)-6-(4-morpholinyl)-1,3,5-triazin-2-yl]-	C19H21N7O2	379.42	A/B	-9.2
997	c1(nc(nc(n1)N1CCOCC1)N1CCNCC1)c1c2c(ccc1)[nH]nc2	1391925-95-3	1H-Indazole, 4-[4-(4-methyl-1-piperazinyl)-6-(4-morpholinyl)-1,3,5-triazin-2-yl]	C19H24N8O	380.45	A/B	-9.2
998	N(c1nc(nc(NCCNC(=O)c2ccc(cc2)C2OOC(OO2)c2ccc(C)cc2)n1)N1CCOCC1)c1ccc(OC)cc1	1393922-46-7	Benzamide, N-[2-[[4-[[4-(1-methylethyl)phenyl]amino]-6-(4-morpholinyl)-1,3,5-tri	C34H39N7O6	641.72	A/B	-9.2
999	C(NC(=O)[C@@H](NC(=O)OC(C)(C)C)CCNC(=O)OC(C)(C)C)(C)C12CC3CC(C2)CC(C1)C3	1398581-53-7	Benzoic acid, 2,2,Ä≤-[carbonylbis(4,1-phenylenecarbonylimino)]bis-	C29H20N2O7	508.48	A/B	-9.2
1000	C(=O)([C@@H](NC(=O)OC)C(C)C)N1[C@H](c2[nH]c(c3c4c(c(cc3)c3ccc(cc3)c3[nH]c(nc3)[C@H]3N(C(=O)[C@@H](NC	1419385-39-9	Carbamic acid, N,N,Ä≤-[(1,Ä≤,3,Ä≤-dihydrospiro[cyclopentane-1,2,Ä≤-[2H]indene]-	C53H64N8O6	909.13	A/B	-9.2
1001	C(N1c2c(C(=NNC(=S)N(CC=C)CC=C)C1=O)cc(C)cc2)N1CCN(CC1)c1ccc(Cl)cc1	1426583-71-2	Hydrazinecarbothioamide, 2-[1-[[4-(4-chlorophenyl)-1-piperazinyl]methyl]-5-fluor	C32H28ClFN6OS	599.12	A/B	-9.2
1002	N(c1c2c(c(O)c(CN3CCC(O)CC3)c1)CCCC2)c1c2c(ncc1)cc(Cl)cc2	1449239-43-3	1-Naphthalenol, 4-[(7-chloro-4-quinolinyl)amino]-2-[(3,5-dimethyl-1-piperidinyl)	C27H32ClN3O	450.02	A/B	-9.2
1003	C(OC[C@@H]1[C@@H](OC(=O)c2ccccc2)[C@@H](OP(=O)(OC[C@@H]2[C@@H](OC(=O)c3ccccc3)[C@@H](OP(=O)(OC[C@@H]	1469875-46-4	Œ≤-D-lyxo-Adenosine, 1,Ä≤-[3-(aminocarbonyl)-1H-1,2,4-triazol-1-yl]-3,Ä≤-O-benz	C40H37N10O14P	912.75	A/B	-9.2
1004	[C@@H](O[C@H](COP(=O)(OP(=O)(CP(=O)(OP(=O)(OC[C@@H](O[C@H](CO)n1c2c(nc1)c(N)ncn2)CO)O)O)O)O)CO)(CO)n	149297-82-5	Inosine 5,Ä≤-(tetrahydrogen triphosphate), P,Ä≤,Ä≤,Üî5,Ä≤-ester with inosine	C20H25N8O18P3	758.38	A/B	-9.2
1005	O=c1c2c(n(cn2)[C@H]2C[C@H](ONCC[C@H]3O[C@H](C[C@@H]3O)n3c4c(nc3)c(=O)nc(N)[nH]4)[C@@H](CO)O2)[nH]c(N	1513874-37-7	Guanosine, 2,Ä≤-deoxy-3,Ä≤-de(phosphinicooxy)guanylyl-1,2-hydrazinediylmethylen	C21H28N12O6	544.52	A/B	-9.2
1006	O=c1c2c(n(cn2)[C@H]2C[C@H](NS(=O)(=O)NC[C@H]3O[C@H]([C@H](O)[C@@H]3O)n3c4c(nc3)c(=O)nc(N)[nH]4)[C@@H	1513874-56-0	Guanosine, 3,Ä≤-de(phosphinicooxy)guanylyliminosulfonylimino-(3,Ä≤,Üî5,Ä≤)-2,Ä≤	C20H26N12O9S	610.56	A/B	-9.2
1007	N(CC(CCCCC)(C)C)c1c2c(cc(Cl)cc2)ncc1	153380-30-4	Butanediamide, N1-[3-[3-[[(1,1-dimethylethyl)amino]carbonyl]octahydro-2(1H)-isoq	C38H48N6O6	684.82	A/B	-9.2
1008	C(=O)(NCc1ccc(F)cc1)c1c(OC(OC(=O)CC(C)C)C(C)C)c(=O)n(C)c(C(NC(=O)c2oc(C)nn2)(C)C)n1	1538604-12-4	4-Pyrimidinecarboxamide, 5-[1-(acetyloxy)-2-methylpropoxy]-N-[(4-fluorophenyl)me	C26H31FN6O7	558.56	A/B	-9.2
1009	C(n1c2c(nc1CC)c(C)cc(C)n2)c1ccc(cc1)c1c(S(=O)(=O)NC(=O)CCc2ccccc2)ccc(C(C)(C)C)c1	154552-63-3	Benzenepropanamide, N-[[5-(1,1-dimethylpropyl)-4,Ä≤-[(2-ethyl-5,7-dimethyl-3H-ir	C37H42N4O3S	622.82	A/B	-9.2
1010	C(n1c2c(nc1CC)c(C)cc(C)n2)c1ccc(cc1)c1c(S(=O)(=O)NC(=O)c2c(Oc3ccccc3)cccc2)ccc(CCC)c1	154552-72-4	Benzamide, N-[[5-ethyl-4,Ä≤-[(2-ethyl-5,7-dimethyl-3H-imidazo[4,5-b]pyridin-3-y]	C38H36N4O4S	644.78	A/B	-9.2
1011	C(n1c2c(nc1CC)c(C)cc(C)n2)c1ccc(cc1)c1c(S(=O)(=O)NC(=O)c2c(Oc3ccccc3)cccc2)ccc(CC(C)C)c1	154552-75-7	Benzamide, N-[[4,Ä≤-[(2-ethyl-5,7-dimethyl-3H-imidazo[4,5-b]pyridin-3-yl)methyl]	C39H38N4O4S	658.81	A/B	-9.2
1012	C(n1c2c(nc1CC)c(C)cc(C)n2)c1ccc(cc1)c1c(S(=O)(=O)NC(=O)c2c(OCC)cccc2)ccc(OCCCC)c1	154552-95-1	Benzamide, 2-ethoxy-N-[[4,Ä≤-[(2-ethyl-5,7-dimethyl-3H-imidazo[4,5-b]pyridin-3-y	C35H38N4O4S	610.77	A/B	-9.2
1013	C(n1c2c(nc1CC)c(C)cc(C)n2)c1ccc(cc1)c1c(S(=O)(=O)NC(=O)c2ccccc2)ccc(OC)c1	154553-17-0	Benzamide, N-[[5-[(dimethylamino)methyl]-4,Ä≤-[(2-ethyl-5,7-dimethyl-3H-imidazo	C33H35N5O3S	581.73	A/B	-9.2
1014	O(C(=O)Nc1ccc(S(=O)(=O)N(C[C@H]([C@H](Cc2ccccc2)N)O)CC(C)C)cc1)[C@H]1[C@@H]2[C@H](OC1)OCC2	1546918-97-1	Carbamic acid, N-[4-[[[(2R,3S)-3-[[[[(3R,3aS,6aR)-hexahydrofuro[2,3-b]furan-3-yl	C34H45N3O11S	703.8	A/B	-9.2
1015	S(c1nc(nc(Nc2ccc(F)cc2)n1)N1CCOCC1)c1oc(nn1)c1ccc(Cl)cc1	1547295-40-8	1,3,5-Triazin-2-amine, 4-[[5-(4-chlorophenyl)-1,3,4-oxadiazol-2-yl]thio]-N-(4-me	C22H20ClN7O2S	481.96	A/B	-9.2
1016	C(=O)([C@@H](NC(=O)OC)C(C)C)N1[C@H](c2[nH]c(c3c4c(C5CC4CC5)c(cc3)c3ccc(cc3)c3[nH]c(nc3)[C@H]3N(C(=O)	1560638-96-1	Carbamic acid, N-[(1S)-1-[[(2S)-2-[5-[8-[4-[2-[(2S)-4,4-difluoro-1-[(2S)-2-[(met	C45H52F4N8O6	876.94	A/B	-9.2
1017	O=C1C2c3c(c(ccc3c3[nH]c(nc3)[C@H]3N(C(=O)[C@@H](NC(=O)OC)C(C)C)CCC3)c3ccc(cc3)c3[nH]c(nc3)[C@H]3N(C(	1560639-40-8	Carbamic acid, N-[(1S)-2-methyl-1-[[(2S)-2-[5-[4-[1,2,3,4-tetrahydro-8-[2-[(2S)-	C46H58N8O6	819	A/B	-9.2
1018	C(=O)([C@@H](NC(=O)OC)C1CCCCC1)N1[C@H](c2[nH]c(c3c4c(C5OC4CC5)c(cc3)c3ccc(cc3)c3[nH]c(nc3)[C@H]3N(C(	1560640-82-5	Carbamic acid, N-[(1S)-2-methyl-1-[[(2S,4S)-4-methyl-2-[5-[4-[1,2,3,4-tetrahydro	C46H58N8O7	835	A/B	-9.2
1019	C(=O)([C@@H](NC(=O)OC)C(C)C)N1[C@H](c2[nH]c(c3c4c(C5OC4CC5)c(cc3)c3ccc(cc3)c3[nH]c(nc3)[C@H]3N(C(=O)	1560640-94-9	Carbamic acid, N-[(1S)-1-[[(2S,4R)-4-fluoro-2-[5-[8-[4-[2-[(2S,4R)-4-fluoro-1-[(	C44H52F2N8O7	842.93	A/B	-9.2
1020	C(=O)(C(NC(=O)OC)C(C)C)N1C(c2[nH]c(c3ccc(c4c5c(c(cc4)c4ccc(cc4)c4[nH]c(nc4)C4N(C(=O)C(NC(=O)OC)C(C)C	1560650-14-7	Carbamic acid, N-[(1S)-2-methyl-1-[[(2S)-2-[5-[4-[1,2,3,4-tetrahydro-8-[2-[(2S)-	C49H58N8O6	855.03	A/B	-9.2
1021	O=c1n([C@@H]2O[C@H](CO)C(C2[3H])[3H])cc(C)c(=O)[nH]1	157695-29-9	Guanosine, 2,Ä≤-deoxy-5,Ä≤-O-phosphonoguanylyl-(3,Ä≤,Üî5,Ä≤)-2,Ä≤,3,Ä≤-dideoxy-	C20H26N10O12P2	660.43	A/B	-9.2
1022	C[C@@]12[C@@H]3[C@H]([C@H]4[C@](C)(CC3)[C@@H]([C@@H](CCCC(C)C)C)CC4)C(=O)C=C1[C@H](O)[C@H](CC2)N1CCC	1609273-15-5	5,Ä≤-Adenylic acid, anhydride with P-(5,Ä≤-guanylylmethyl)phosphonic acid (1:1)	C21H29N10O16P3	770.43	A/B	-9.2
1023	C[C@@]12[C@@H]3[C@H](C[C@H]1[C@H]1[C@H](CC2)[C@]2(C)[C@@H](CC1)CC(NCCN)CC2)O[C@]1([C@H]3C)CC[C@H](C)	1613083-81-0	Carbamic acid, N-[(1S)-1-[[(2S)-2-[5-[4-[1,2,3,3a,8,8a-hexahydro-7-[2-[(2S)-1-[(	C47H60N8O6	833.03	A/B	-9.2
1024	C(OCCOP(=O)(OC[C@@H]1OC(C)(C)OC1)O)n1c2c(nc1)c(=O)nc(N)[nH]2	1616340-94-3	2,5-Methanopyrido[1,Ä≤,2,Ä≤:4,5]pyrazino[2,1-b][1,3]oxazepine-10-carboxamide, 2	C22H20F3N3O5	463.41	A/B	-9.2
1025	N(C(=O)[C@H](Cc1ccc(O)cc1)C[C@@H](CN1[C@H](C(=O)NC(C)(C)C)CN(Cc2c[n+](ccc2)[C@@H]2O[C@H](C(=O)[O-])[	162518-59-4	D-erythro-Pentonamide, 2,3,5-trideoxy-N-[(1S,2R)-2,3-dihydro-2-hydroxy-1H-inden-	C36H47N5O5	629.79	A/B	-9.2
1026	N(C(=O)C)[C@H]1[C@H]([C@@H]([C@@H](CO)O)O)OC(=C[C@@H]1NC(=N)N)C(=O)O.[NaH]	1627533-96-3	Carbamic acid, N,N,Ä≤-[(1,1,Ä≤,2,2,Ä≤,3,3,Ä≤,4,4,Ä≤-octahydro[5,5,Ä≤-bi-1,4-me	C50H58F4N8O6	943.04	A/B	-9.2
1027	C(#N)c1c(cc(nc1N)c1ccc(Nc2nc(nc(n2)N2CCOCC2)N2CCOCC2)cc1)c1cc(C)c(C)cc1	1627963-44-3	1,3,5-Triazin-2-amine, N-[4-[5-(3,4-dimethylphenyl)-3-isoxazolyl]phenyl]-4,6-di-	C28H31N7O3	513.59	A/B	-9.2
1028	C(OC[C@@H]1[C@@H](OC(=O)c2ccccc2)[C@@H](OP(=O)(OC[C@@H]2[C@@H](OC(=O)c3ccccc3)[C@@H](OP(=O)(OC[C@H]3	1637769-15-3	Adenosine, 1,Ä≤-[3-(aminocarbonyl)-1H-1,2,4-triazol-1-yl]-3,Ä≤-O-benzoyl-1,Ä≤-d	C99H86N16O26P2	1977.78	A/B	-9.2
1029	C(=O)([C@@H](NC(=O)OC)C1CCCC1)N1[C@@H](CCC1)c1[nH]c(cn1)c1ccc(cc1)c1ccc(cc1)c1[nH]c(nc1)[C@H]1N(C(=O	1639871-27-4	Carbamic acid, N-[(1S)-1-cyclohexyl-2-[(2S)-2-[5-[4,Ä≤-[2-[(2S)-1-[(2S)-2-cyclop	C45H54N8O6	802.96	A/B	-9.2
1030	O=c1n([C@H]2O[C@@H](COP(=O)(Cl)Cl)SC2)ccc(N)n1	1642584-27-7	1,3,5-Triazin-2-amine, N-(4-chlorophenyl)-4-[[5-(4-fluorophenyl)-1,3,4-oxadiazol	C21H17ClFN7O2S	485.92	A/B	-9.2
1031	O=c1n([C@@H]2O[C@@H](COP(=O)(C(O)c3ccc(Cl)cc3)OC[C@H]3O[C@H](C=C3)n3c(=O)[nH]c(=O)c(C)c3)C=C2)cc(C)c(	164652-03-3	Thymidine, 2,Ä≤,3,Ä≤-didehydro-P,3,Ä≤-dideoxy-P-(hydroxyphenylmethyl)thymidylyl	C27H29N4O10P	600.51	A/B	-9.2
1032	N(C(=O)[C@H](Cc1ccccc1)C[C@@H](CN1[C@H](C(=O)NC(C)(C)C)CN(Cc2c(C)ccnc2C)CC1)O)[C@H]1c2c(C[C@H]1O)ccc	164725-36-4	1-Piperazinepentanamide, N-(2,3-dihydro-2-hydroxy-1H-inden-1-yl)-2-[[(1,1-dimeth	C38H50N4O5	642.83	A/B	-9.2
1033	N(C(=O)[C@H](Cc1ccccc1)C[C@@H](CN1[C@H](C(=O)NC(C)(C)C)CN(Cc2c(Cl)nccc2)CC1)O)[C@H]1c2c(C[C@H]1O)ccc	164725-40-0	D-erythro-Pentonamide, 5-[(2S)-4-[(6-chloro-3-pyridinyl)methyl]-2-[[(1,1-dimethy	C36H46ClN5O4	648.23	A/B	-9.2
1034	N(C(=O)[C@H](Cc1ccccc1)C[C@@H](CN1[C@H](C(=O)NC(C)(C)C)CN(Cc2n(C)ccc2)CC1)O)[C@H]1c2c(C[C@H]1O)cccc2	164725-59-1	3-Pyridinecarboxylic acid, 5-[[4-[5-[(2,3-dihydro-2-hydroxy-1H-inden-1-yl)amino]	C39H51N5O6	685.85	A/B	-9.2
1035	C(=O)([C@@H](NC(=O)OC)C(C)C)N1C[C@@]2(C[C@H]1c1[nH]c3c(n1)ccc1c3ccc(c1)c1ccc(cc1)c1[nH]c(nc1)[C@H]1N	1653967-03-3	Carbamic acid, N-[(1S)-2-methyl-1-[[(2S,3aS,7aS)-octahydro-2-[1,4,5,6-tetrahydro	C51H66N8O7	903.12	A/B	-9.2
1036	C(CC(COC(=O)[C@H](C(C)C)N)COC(=O)[C@H](C(C)C)N)n1c2c(nc1)c(=O)nc(N)[nH]2	169281-94-1	Carbamic acid, [[4,Ä≤-[(2-ethyl-5,7-dimethyl-3H-imidazo[4,5-b]pyridin-3-yl)methy	C35H38N4O4S	610.77	A/B	-9.2
1037	C(CC(COC(=O)[C@@H](NC(=O)OCc1ccccc1)CC(C)C)(CO)CO)n1c2c(nc1)c(=O)nc(N)[nH]2	172645-36-2	L-Alanine, N-[(phenylmethoxy)carbonyl]-, 2-[2-(2-amino-1,6-dihydro-6-oxo-9H-puri	C33H39N7O10	693.7	A/B	-9.2
1038	O[C@H]1[C@H](n2c3c(nc2)c(=O)nc(N)[nH]3)O[C@H](COP(=O)(O[C@@H]2[C@@H](CO)O[C@H](C2)n2c3c(nc2)c(N)ncn3	173291-31-1	Guanosine, 2,Ä≤-deoxy-P-thioguanylyl-(3,Ä≤,Üî5,Ä≤)-	C20H25N10O10PS	628.51	A/B	-9.2
1039	O=c1n([C@@H]2O[C@H](COP(=O)([C@@H](O)c3c(N(=O)=O)cccc3)OC[C@@H]3[C@@H](N=[N+]=[N-])C[C@@H](O3)n3c(=O	173658-24-7	Thymidine, 3,Ä≤-azido-P-[(4-cyanophenyl)hydroxymethyl]-P,3,Ä≤-dideoxythymidylyl	C28H30N11O10P		A/B	-9.2
1040	C(C(C)(C)C)(C1=C(O)CC(CCC2CC2)(CCC2CC2)OC1=O)c1cc(NS(=O)(=O)c2ccc(C#N)cn2)ccc1	174484-13-0	2-Pyridinesulfonamide, 5-cyano-N-[3-[1-[5,6-dihydro-4-hydroxy-2-oxo-6-(2-phenyle	C31H33N3O5S	559.68	A/B	-9.2
1041	C(Cc1ccccc1)C1(CCc2ccccc2)CC(=C([C@@H](C(C)(C)C)c2cc(NS(=O)(=O)c3ccc(N)cn3)ccc2)C(=O)O1)O	174484-63-0	2-Pyridinesulfonamide, 5-amino-N-[3-[1-[5,6-dihydro-4-hydroxy-2-oxo-6,6-bis(2-ph	C37H41N3O5S	639.8	A/B	-9.2
1042	C(Cc1ccc(F)cc1)[C@]1(CCC)CC(=C([C@@H](CC)c2cc(NS(=O)(=O)c3ccc(C#N)cn3)ccc2)C(=O)O1)O	174590-36-4	2-Pyridinesulfonamide, 5-cyano-N-[3-[1-[6-[2-(4-fluorophenyl)ethyl]-5,6-dihydro-	C31H32FN3O5S	577.67	A/B	-9.2
1043	O=c1n([C@@H]2O[C@H](COP(=O)(OC[C@H](OCCCCCCCCCCCC)C)O)[C@@H](N=[N+]=[N-])C2)cc(C)c(=O)[nH]1	178993-30-1	3,Ä≤-Guanylic acid, 2,Ä≤-deoxy-P-thioguanylyl-(3,Ä≤,Üî5,Ä≤)-2,Ä≤-deoxy-	C20H26N10O12P2S	692.49	A/B	-9.2
1044	OC1c2c(OC1SC[C@H](NC(=O)CC[C@@H](C(=O)O)N)C(=O)NCC(=O)O)ncc(CN1C[C@@H](C(=O)NC(C)(C)C)N(C[C@H](C[C@H	179952-06-8	L-Cysteine, N-acetyl-S-[5-[[3-[[(1,1-dimethylethyl)amino]carbonyl]-4-[2,3,5-tric	C43H56N6O9S	833	A/B	-9.2
1045	O=C1[C@]2([C@H]([C@H](/C=C/C(=O)[C@@H]3[C@@]4(N5[C@@H]([C@H]3c3cc(OC)c(O)cc3)CCC5)c3c(NC4=O)c(C)cc(	1807888-42-1	Not Yet Assigned	C49H52N4O8	824.96	A/B	-9.2
1046	O=C1[C@@H]2[C@@H](c3c([C@H](OC(=O)[C@@H](C)N4C(=O)c5c(C4=O)cccc5)[C@H]2CO1)cc1c(c3)OCO1)c1cc(OC)c(OC	1809321-43-4	2H-Isoindole-2-acetic acid, 1,3-dihydro-Œ±-methyl-1,3-dioxo-, (5R,5aR,8aR,9R)-5,	C33H29NO11	615.58	A/B	-9.2
1047	C(C(=O)OC[C@H]1O[C@H](C=C1)n1c(=O)[nH]c(=O)c(C)c1)C1(CC(=O)OCC=C)C(=O)OCO1	1848258-49-0	Carbamic acid, N-[(1S)-1-(1H-indol-3-ylmethyl)-2-oxo-2-[(1-tricyclo[3.3.1.13,7]c	C31H37N3O3	499.64	A/B	-9.2
1048	O=c1c2c(n(cn2)[C@@H]2O[C@H](COP(=O)(Oc3ccc(Cl)cc3)NCc3cc(Cl)ccc3)CC2)[nH]cn1	1922936-28-4	Phosphoramidic acid, N-[(4-nitrophenyl)methyl]-, 4-chlorophenyl [(2S,5R)-5-(1,6-	C23H22ClN6O7P	560.88	A/B	-9.2
1049	N(C(=O)[C@H]1O[C@@H](C(=O)N2CCOCC2)CC1)C12CC3(O)CC(C2)CC(C1)C3	194347-55-2	Œ≤-D-Glucopyranuronic acid, 1-deoxy-1-[2-[[4-[3-[(1-methylethyl)amino]-2-pyridir	C28H36N6O9S	632.69	A/B	-9.2
1050	O=c1n([C@@H]2O[C@H](COP3(=O)Oc4c(CO3)cc(OC)cc4)[C@@H](N=[N+]=[N-])C2)cc(C)c(=O)[nH]1	195885-80-4	Thymidine, 3,Ä≤-azido-3,Ä≤-deoxy-5,Ä≤-O-(2-oxido-4H-1,3,2-benzodioxaphosphorin-	C17H18N5O7P		A/B	-9.2
1051	N(c1nc(Nc2ccc(C#N)cc2)cc(C)n1)c1c(C)cc(C)cc1C	1984820-41-8	Benzonitrile, 2-[[6-methyl-2-[(2,4,6-trimethylphenyl)amino]-4-pyrimidinyl]amino]	C21H21N5	343.42	A/B	-9.2
1052	O(C(=O)C(CC(=O)OC)(C)C)c1c2n(N([C@H]3N(C2=O)CCOC3)[C@H]2c3c(CSc4c2cccc4)c(F)c(F)cc3)ccc1=O	1985606-57-2	1H-[1,4]Oxazino[3,4-c]pyrido[2,1-f][1,2,4]triazine-6,8-dione, 12-[(11S)-7,8-difl	C32H24F2N4O8S	662.62	A/B	-9.2

1053	O(C(=O)OCC(=O)C)c1c2n(N([C@H]3N(C2=O)CCOC3)[C@H]2c3c(CSc4c2cccc4)c(F)c(F)cc3)ccc1=O	1985607-24-6	Carbonic acid, (12aR)-12-[(11S)-7,8-difluoro-6,11-dihydrodibenzo[b,e]thiepin-11-	C30H23F2N3O9S	639.58	A/B	-9.2
1054	N(C(=O)Nc1cc(C(C)(C)C)on1)c1cc2cc(C34CC5CC(C4)CC(C3)C5)[nH]c2cc1	1987854-79-4	Urea, N-2-benzothiazolyl-N,Ä≤-(2-tricyclo[3.3.1.13,7]dec-1-yl-1H-indol-5-yl)-	C26H26N4OS	442.58	A/B	-9.2
1055	C(=O)([C@@H](NC(=O)OC)C(C)C)N1[C@H](c2[nH]c3c4c(cc(cc4)c4c5c(c(cc4)c4[nH]c(nc4)[C@H]4N(C(=O)[C@@H](N	2007901-33-7	Carbamic acid, N-[(1S)-1-[[(2S)-2-[7-[2,3-dihydro-7-[2-[(6S)-5-[(2S)-2-[(methoxy	C47H56N8O6	829	A/B	-9.2
1056	C(=O)([C@@H](NC(=O)OC)C(C)C)N1[C@H](c2[nH]c3c4c(cc(cc4)c4c5c(c(cc4)c4[nH]c(nc4Cl)[C@H]4N(C(=O)[C@@H]	2007901-51-9	Carbamic acid, N-[(1S)-2-[(2S,5S)-2-[5-[2,3-dihydro-7-[2-[(2S,5S)-1-[(2S)-2-[(me	C49H60N8O7	873.05	A/B	-9.2
1057	C(OC(=O)CCNC(=O)OCc1ccccc1)[C@@H]1[C@@H]2[C@H]([C@@H](O1)n1nc(C(=O)N)nc1)OC(OC)O2	1002335-61-6	1H-1,2,4-Triazole-3-carboxamide, 1-[2,3-O-(methoxymethylene)-5-O-[(2S)-1-oxo-2-[	C28H35N5O14	665.6	C	-9.2
1058	C(=O)([C@H](NC(=O)OC)c1ccccc1)N1[C@@H](CCC1)c1[nH]c(cn1)c1ccc(cc1)c1ccc(cc1)c1[nH]c(nc1)[C@H]1N(C(=O	1009107-42-9	Carbamic acid, N-[(1R)-2-[(2S)-2-[5-[4,Ä≤-[2-[(2S)-1-acetyl-2-pyrrolidinyl]-1H-	C38H39N7O4	657.76	C	-9.2
1059	C(=O)([C@H](NC(=O)OC)c1ccccc1)N1[C@@H](CCC1)c1[nH]c(cn1)c1ccc(cc1)c1ccc(cc1)c1[nH]c(nc1)[C@H]1N(C(=O	1009107-47-4	Carbamic acid, N-[(1R)-2-[(2S)-2-[5-[4,Ä≤-[2-[(2S)-1-(2-cyclopropylacetyl)-2-pyr	C41H43N7O4	697.82	C	-9.2
1060	C(=O)([C@H](NC(=O)OC)c1ccccc1)N1[C@@H](CCC1)c1[nH]c(cn1)c1ccc(cc1)c1ccc(cc1)c1[nH]c(nc1)[C@H]1N(C(=O	1009107-61-2	Carbamic acid, N-[(1R)-2-oxo-1-phenyl-2-[(2S)-2-[5-[4,Ä≤-[2-[(2S)-1-[2-(4-pyridi	C43H42N8O4	734.84	C	-9.2
1061	C(=O)([C@@H](NC(=O)OC)C(C)C)N1[C@@H](CCC1)c1[nH]c(cn1)c1ccc(cc1)c1ccc(cc1)c1[nH]c(nc1)[C@H]1N(C(=O)C	1009119-89-4	Carbamic acid, N,N,Ä≤-[[1,1,Ä≤-biphenyl]-3,4,Ä≤-diylbis[1H-imidazole-5,2-diyl-(	C46H46N8O6	806.91	C	-9.2
1062	S(=O)(=O)(O)c1c2c(c(N=Nc3cc(N=Nc4ccc(cc4)c4ccc(N=Nc5c(N)c(S(=O)(=O)O)cc(C)c5N)cc4)c(N)cc3N)cc1)cccc2	101807-81-2	1-Naphthalenesulfonic acid, 4-amino-3-[2-[4-(2-phenylethenyl)phenyl]diazenyl]-	C24H19N3O3S	429.49	C	-9.2
1063	O=c1n([C@@H]2O[C@H](COCP(=O)(OP(=O)(OCCCOCCCCCCCCCCCCCCCCC)O)O)SC2)cc(F)c(N)n1	1026294-95-0	6H-Dipyrido[3,2-b:2,Ä≤,3,Ä≤-e][1,4]diazepin-6-one, 11-cyclopropyl-8-[2-[(1,3-di	C25H24FN5O4S	509.55	C	-9.2
1064	O=c1n([C@H]2C[C@H](ON)[C@@H](CO)O2)ccc(=O)[nH]1	103242-83-7	Adenosine, adenylyl-(2,Ä≤,Ü i5,Ä≤)-guanylyl-(2,Ä≤,Üi5,Ä≤)-	C30H37N15O17P2	941.65	C	-9.2
1065	Nc1c2c(cc(S(=O)(=O)O)cc2)ccc1/N=N/c1cc(S(=O)(=O)O)c(/C=C/c2c(S(=O)(=O)O)cc(/N=N/c3c(N)c4c(cc3)cc(S(=	1052531-67-5	Not Yet Assigned	C34H26N6O12S4.Na		C	-9.2
1066	C(C(OCP(=O)(O)O)C)n1c2c(nc1)c(N)ncn2	107018-93-9	1,5-Naphthalenedisulfonic acid, 3-[2-[4-[2-(4-amino-5-sulfo-1-naphthalenyl)diaze	C30H21N5O12S4	771.77	C	-9.2
1067	C(NS(=O)(=O)c1cc2c(cc1)C(=NO)c1c(C2=NO)cc(S(=O)(=O)NC(C)C23CC4CC(C3)CC(C2)C4)cc1)(C)C12CC3CC(C2)CC(C	1083159-44-7	2,7-Anthracenedisulfonamide, 9,10-dihydro-9,10-dioxo-N2,N7-bis(1-tricyclo[3.3.1.	C38H46N2O6S2	690.91	C	-9.2
1068	O(C[C@@H]1[C@@H](N=[N+]=[N-])C[C@@H](O1)n1c(=O)[nH]c(=O)c(C)c1)P1(=O)Oc2c(cc3c(c2)OP(=O)(OC[C@@H]2[C	1099830-36-0	Not Yet Assigned	C28H30FN7O14P2		C	-9.2
1069	O=c1c2c(n(cn2)[C@@H]2C[C@H](CO)[C@@H](O)C2)[nH]cn1	111586-18-6	1-Naphthalenesulfonic acid, 4-amino-3-[2-(4-sulfo-1-naphthalenyl)diazenyl]-, soc	C20H15N3O6S2.2Na		C	-9.2
1070	C(=O)([C@@H](NC(=O)OC)C(C)C)N1[C@@H](CCC1)c1[nH]c(c(C)n1)c1ccc(cc1)c1ccc(cc1)c1[nH]c(nc1C)[C@H]1N(C(	1129636-27-6	Carbamic acid, N,N,Ä≤-[[1,1,Ä≤-biphenyl]-4,4,Ä≤-diylbis[(4-methyl-1H-imidazole-	C38H46N8O6	710.82	C	-9.2
1071	N(=N\c1ccccc1)/c1c2c(c(/N=N/c3c(C)cc(OCC)cc3)cc1)ccc(S(=O)(=O)O)c2	1135683-38-3	1-Naphthalenesulfonic acid, 4-amino-3-[(1E)-2-[4-[(4-hydroxyphenyl)methyl]phenyl	C23H19N3O4S	433.48	C	-9.2
1072	C(=O)([C@@H](NC(=O)OC)C(C)C)N1[C@H](c2[nH]c(c3cc4C5(c6c(-c4cc3)ccc(c6)c3[nH]c(nc3)[C@H]3N(C(=O)[C@@H	1179150-52-7	Carbamic acid, N,N,Ä≤-[(9,9-dimethyl-9H-fluorene-2,7-diyl)bis[1H-imidazole-5,2-c	C43H50N8O6	774.91	C	-9.2
1073	O=c1n([C@@H]2O[C@H](COP(=O)(OCCCCCCSSCCCCCCO)O)[C@@H](O)C2)cc(C)c(=O)[nH]1	118951-97-6	1-Naphthalenesulfonic acid, 4-amino-3-[2-[3-cyano-4-(2-phenylethenyl)phenyl]diaz	C25H18N4O3S	454.5	C	-9.2
1074	S(=O)(=O)(O)c1c2c(c(N)c(N=Nc3ccc(Sc4ccc(N=Nc5c(N)c6c(c(S(=O)(=O)O)c5)cccc6)cc4)cc3)c1)cccc2	119415-69-9	Naphthionic acid, 3,3,Ä≤-[selenobis(p-phenyleneazo)]di-	C32H24N6O6S2Se	731.66	C	-9.2
1075	N(C(=O)Nc1ccc(C(=O)N2CCC(N(C)C)CC2)cc1)c1ccc(c2nc(nc(n2)N2CCOCC2)N2CCOCC2)cc1	1197160-28-3	Urea, N-[4-[4-(4-morpholinyl)-6-(3-oxa-8-azabicyclo[3.2.1]oct-8-yl)-1,3,5-triazi	C25H28N8O3	488.54	C	-9.2
1076	O(COC(=O)NCCOC)c1c2n(cc(C(=O)NCc3c(F)cc(F)cc3)c1=O)C[C@H]1N(C2=O)[C@H](C)CCO1	1206156-28-6	4-Morpholinecarboxylic acid, [[(4R,12aS)-9-[[[(2,4-difluorophenyl)methyl]amino]c	C26H28F2N4O8	562.52	C	-9.2
1077	C(=O)(O[C@H](Cc1cc(O)c(O)cc1)C(=O)O)[C@H]1c2c(O[C@@H]1c1cc(O)c(O)cc1)c(O)ccc2/C=C/C(=O)O[C@H](Cc1cc(	1215192-26-9	Benzoic acid, 5-[2-[3-carboxy-4-[[4-(1,1-dimethylethyl)benzoyl]amino]phenyl]ethy	C35H31F3N2O6	632.63	C	-9.2
1078	N(C(=O)[C@H](Cc1ccccc1)C[C@@H](CN1[C@H](C(=O)NC(C)(C)C)CN(Cc2nc3c(s2)cccc3)CC1)O)C1c2c(C[C@H]1O)cccc	1223083-11-1	Not Yet Assigned	C38H47N5O5	653.81	C	-9.2
1079	C(=O)([C@@H](NC(=O)OC)C(C)C)N1[C@@H](CCC1)c1[nH]c(cn1)c1ccc(cc1)c1cc2c(cc1)cc(cc2)c1[nH]c(nc1)[C@@H]	1228633-21-3	Carbamic acid, N-[(1S)-2-methyl-1-[[(2S)-2-[5-[6-[4-[2-(2S)-2-pyrrolidinyl-1H-im	C37H41N7O3	631.77	C	-9.2
1080	C(NC(=O)[C@H](Cc1c2c([nH]c1)cccc2)N)(C(=O)NCC(=O)N[C@@H](Cc1ccccc1)C(=O)N[C@@H](Cc1ccccc1)C(=O)O)C12	1229574-80-4	Glycine, L-tyrosyl-2-tricyclo[3.3.1.13,7]dec-1-ylglycylglycyl-L-phenylalanyl-	C34H43N5O7	633.73	C	-9.2
1081	N(=Nc1ccc(C#N)cc1)c1c2c(cc(S(=O)(=O)O)cc2)c(N)cc1	1238840-07-7	Not Yet Assigned	C27H18N6O6S2.2Na		C	-9.2
1082	C(F)(F)c1n(c2c(n1)cccc2)c1nc(nc(N[C@H]2CC[C@H](NC)CC2)n1)N1CCOCC1	1240023-43-1	Acetamide, N-[trans-4-[[4-[2-(difluoromethyl)-1H-benzimidazol-1-yl]-6-(4-morphol	C24H30F2N8O2	500.54	C	-9.2
1083	C(F)(F)c1n(c2c(n1)cccc2)c1nc(nc(N[C@H]2CC[C@H](NC(=O)NCCOC)CC2)n1)N1CCOCC1	1240024-63-8	Urea, N,Ä≤-[trans-4-[[4-[2-(difluoromethyl)-1H-benzimidazol-1-yl]-6-(4-morpholir	C27H38F2N10O2	572.65	C	-9.2
1084	C(F)(F)c1n(c2c(n1)cccc2)c1nc(nc(N[C@H]2CC[C@H](NS(=O)(=O)CC)CC2)n1)N1CCOCC1	1240029-13-3	Acetamide, N-[trans-4-[[4-[2-(difluoromethyl)-1H-benzimidazol-1-yl]-6-(4-morphol	C23H28F2N8O3	502.52	C	-9.2
1085	C(F)(F)c1n(c2c(n1)cccc2)c1nc(nc(N[C@H]2CC[C@H](NC(C(C)O)C)CC2)n1)N1CCOCC1	1240030-52-7	1-Butanol, 3-[[trans-4-[[4-[2-(difluoromethyl)-1H-benzimidazol-1-yl]-6-(4-morphc	C25H34F2N8O2	516.59	C	-9.2
1086	C(F)(F)c1n(c2c(n1)cccc2)c1nc(nc(N[C@H]2CC[C@H](NC(COC)C)CC2)n1)N1CCOCC1	1240030-54-9	2-Butanol, 3-[[trans-4-[[4-[2-(difluoromethyl)-1H-benzimidazol-1-yl]-6-(4-morphc	C25H34F2N8O2	516.59	C	-9.2
1087	C(F)(F)c1n(c2c(n1)cccc2)c1nc(nc(N[C@H]2CC[C@H](NC3CCCCC3)CC2)n1)N1CCOCC1	1240030-55-0	1,4-Cyclohexanediamine, N1-[4-[2-(difluoromethyl)-1H-benzimidazol-1-yl]-6-(4-mor	C25H34F2N8O2	516.59	C	-9.2
1088	C(=O)([C@@H](NC(=O)OC)C(C)C)N1[C@H](c2[nH]c(c3c4cc(-c5cc(c(cc5)c5[nH]c(nc5)[C@H]5N(C(=O)[C@@H](NC(=O	1246559-82-9	Carbamic acid, N,N,Ä≤-[(6,7,9,10,12,13-hexahydrodibenzo[k,m][1,4,7,10]tetraoxacy	C46H60N8O10	885.02	C	-9.2
1089	C(=O)([C@@H](NC(=O)OC)c1ccccc1)N1[C@@H](CCC1)c1[nH]c(cn1)c1ccc(cc1)c1cc2c(cc1)cc(cc2)c1[nH]c(nc1)[C@	1256391-24-8	Carbamic acid, N,N,Ä≤-[(5,10-dihydro[1]benzopyrano[5,4,3-cde][1]benzopyran-2,7-c	C42H50N8O8	794.9	C	-9.2
1090	C(=O)([C@@H](NC(=O)OC)C(C)C)N1CC2(C[C@H]1c1[nH]c(cn1)c1cc3c(-c4c(C3(F)F)cc(cc4)c3[nH]c(nc3)[C@H]3N(C	1256392-07-0	Carbamic acid, N-[(1S)-1-[[(2S)-2-[5-[9,9-difluoro-7-[2-[(6S)-5-[(2S)-2-[(methox	C43H50F2N8O6	812.9	C	-9.2
1091	C(=O)([C@@H](NC(=O)OC)C(C)C)N1[C@@H](CCC1)c1[nH]c(cn1)c1ccc(C#Cc2ccc(cc2)c2[nH]c(nc2)[C@H]2N(C(=O)[C	1256392-21-8	Carbamic acid, N-[(1S)-1-[[(2S)-2-[5-[6-[4-[2-[(2S)-1-[(2S)-2-cyclohexyl-2-[(met	C47H56N8O6	829	C	-9.2
1092	C(OC(=O)c1ccccc1)[C@H]1O[C@H]([C@H]2[C@@H]1OC(C)(C)O2)n1nc(C(=O)N)nc1	1257423-89-4	1H-1,2,4-Triazole-3-carboxamide, 1-[2,3-O-(1-methylethylidene)-5-O-[3,4,5-tris(p	C39H38N4O9	706.74	C	-9.2
1093	O[C@@H]1[C@H](n2c3c(nc2[3H])c(N)ncn3)O[C@H](COP(=O)(O)O)[C@H]1O	1268815-10-6	Thymidine, 3,Ä≤-azido-3,Ä≤-deoxy-5,Ä≤-O-[7-(3,Ä≤-deoxy-3,Ä≤-fluorothymidin-5,Ä≤	C28H30FN7O14P2		C	-9.2
1094	C(=O)([C@@H](NC(=O)O[C@@H]1CCOC1)C(C)C)N1[C@@H]([C@@H]2C[C@H]1CC2)c1[nH]c(cn1)c1ccc(cc1)c1ccc(cc1)c1	1273589-69-7	Carbamic acid, N-[(1S)-2-methyl-1-[[(2S)-2-[5-[4,Ä≤-[2-[(1R,3S,4S)-2-[(2S)-3-met	C50H64N8O8	905.09	C	-9.2
1095	N(=O)(=O)c1c(cccc1)c1nn(c(=S)s1)c1nc(nc(n1)N1CCOCC1)N1CCOCC1	1309437-28-2	1,3,4-Thiadiazole-2(3H)-thione, 3-(4,6-di-4-morpholinyl-1,3,5-triazin-2-yl)-5-(4	C19H20N8O4S2	488.54	C	-9.2
1096	O=c1n([C@@H]2O[C@H](CO[Si](C(C)(C)C)(C)C)[C@@H](N)C2)cc(C)c(=O)[nH]1	1309437-34-0	1,3,4-Thiadiazole-2(3H)-thione, 5-(4-chloro-3-nitrophenyl)-3-(4,6-di-4-morpholir	C19H19ClN8O4S2	522.99	C	-9.2
1097	C(=O)([C@@H](NC(=O)OC)C1CCOCC1)N1[C@H]2[C@H](C2)C[C@H]1c1[nH]c2c(n1)ccc1c2ccc(c1)c1ccc(cc1)c1[nH]c(n	1312336-41-6	Carbamic acid, N-[(1S)-1-[[(1R,3S,5R)-3-[7-[4-[5-chloro-2-[(1R,3S,5R)-2-[(2S)-2-	C44H49ClN8O6	821.36	C	-9.2
1098	C(=O)([C@@H](NC(=O)OC)C1CCOCC1)N1[C@@H](CC[C@@H]1C)c1[nH]c(c(F)n1)c1ccc(cc1)c1ccc(cc1)c1[nH]c(nc1F)[	1312336-66-5	Carbamic acid, N-[(1S)-2-[(2S,5S)-2-[5-[4,Ä≤-[5-fluoro-2-[(2S,5S)-1-[(2S)-2-[(me	C46H57FN8O8	868.99	C	-9.2
1099	O(Cc1ccc(F)cc1)c1c2c(oc(c(O)c2=O)c2[nH]cnn2)c(C)cc1	1312892-56-0	4H-1-Benzopyran-4-one, 5-[(4-fluorophenyl)methoxy]-3-hydroxy-8-methyl-2-(5-pheny	C27H19FN2O4	454.45	C	-9.2
1100	O(Cc1ccc(F)cc1)c1c2c(c(cc1)c1ccccc1)oc(c(O)c2=O)c1[nH]cnn1	1312894-37-3	4H-1-Benzopyran-4-one, 5-[(4-fluorophenyl)methoxy]-3-hydroxy-8-phenyl-2-(5-pheny	C32H21FN2O4	516.52	C	-9.2
1101	O(Cc1ccc(F)cc1)c1c2c(oc(c(O)c2=O)c2[nH]ccn2)c(C)c(C)c1	1312895-47-8	4H-1-Benzopyran-4-one, 5-[(4-fluorophenyl)methoxy]-3-hydroxy-7,8-dimethyl-2-(3-p	C26H20FN3O4	457.45	C	-9.2
1102	O(Cc1ccc(F)cc1)c1c2c(oc(c(O)c2=O)c2[nH]cnn2)c(Cl)c(C)c1	1312896-37-9	4H-1-Benzopyran-4-one, 8-chloro-5-[(4-fluorophenyl)methoxy]-3-hydroxy-7-methyl-2	C27H18ClFN2O4	488.89	C	-9.2
1103	O(Cc1ccc(F)cc1)c1c2c(oc(c(O)c2=O)c2[nH]cnn2)c(C)c(Cl)c1	1312901-08-8	4H-1-Benzopyran-4-one, 7-chloro-5-[(4-fluorophenyl)methoxy]-3-hydroxy-8-methyl-2	C27H18ClFN2O4	488.89	C	-9.2
1104	O(Cc1ccc(F)cc1)c1c2c(oc(c(O)c2=O)c2[nH]cnn2)c(Cl)c(Cl)c1	1312902-08-1	4H-1-Benzopyran-4-one, 7,8-dichloro-5-[(4-fluorophenyl)methoxy]-3-hydroxy-2-(5-p	C26H15Cl2FN2O4	509.31	C	-9.2
1105	O(C)c1c2c(c(OCc3ccc(F)cc3)cc1c1ccccc1)c(=O)c(O)c(o2)c1ncccn1	1312903-78-8	4H-1-Benzopyran-4-one, 5-[(4-fluorophenyl)methoxy]-3-hydroxy-8-methyl-7-phenyl-2	C32H23FN2O4	518.53	C	-9.2
1106	O(Cc1ccc(F)cc1)c1c2c(c(Cl)c(c1)c1ccccc1)oc(c(O)c2=O)c1[nH]ccn1	1312905-26-2	4H-1-Benzopyran-4-one, 8-chloro-5-[(4-fluorophenyl)methoxy]-3-hydroxy-7-phenyl-2	C30H19ClFN3O4	539.94	C	-9.2
1107	O(Cc1ccc(F)cc1)c1c2c(c(c(c1)c1ccccc1)c1ccccc1)oc(c(O)c2=O)c1[nH]cnn1	1312905-70-6	4H-1-Benzopyran-4-one, 5-[(4-fluorophenyl)methoxy]-3-hydroxy-7,8-diphenyl-2-(5-p	C38H25FN2O4	592.61	C	-9.2
1108	O(Cc1ccc(F)cc1)c1c2c(c(c(c1)c1ccccc1)c1ccccc1)oc(c(O)c2=O)c1[nH]c(nn1)c1ccccc1	1312905-79-5	4H-1-Benzopyran-4-one, 2-(5-chloro-1H-1,2,4-triazol-3-yl)-5-[(4-fluorophenyl)met	C30H19ClFN3O4	539.94	C	-9.2
1109	Cc1c2c(cc(OCc3ccc(F)cc3)c1)c(=O)c(O)c(o2)c1ncccn1	1312906-33-4	4H-1-Benzopyran-4-one, 6-[(4-fluorophenyl)methoxy]-3-hydroxy-2-(5-phenyl-1H-imic	C25H17FN2O4	428.41	C	-9.2
1110	O(C)c1c2c(c(=O)c(O)c(o2)c2ncccn2)cc(OCc2ccc(F)cc2)c1	1312906-83-4	4H-1-Benzopyran-4-one, 6-[(4-fluorophenyl)methoxy]-3-hydroxy-8-methyl-2-(5-pheny	C26H19FN2O4	442.44	C	-9.2
1111	O=c1c2c(c(cc(OCc3ccc(F)cc3)c2)c2ccccc2)oc(c1O)c1[nH]ccn1	1312908-28-3	4H-1-Benzopyran-4-one, 6-[(4-fluorophenyl)methoxy]-3-hydroxy-8-phenyl-2-(3-pheny	C30H20FN3O4	505.5	C	-9.2
1112	O=c1c2c(oc(c1O)c1[nH]ccn1)cc(C)c(OCc1ccc(F)cc1)c2	1312908-78-3	4H-1-Benzopyran-4-one, 6-[(4-fluorophenyl)methoxy]-3-hydroxy-7-methyl-2-(3-pheny	C25H18FN3O4	443.43	C	-9.2
1113	Cc1c(c2c(cc1OCc1ccc(F)cc1)c(=O)c(O)c(o2)c1ncccn1)c1ccccc1	1312910-45-4	4H-1-Benzopyran-4-one, 8-chloro-6-[(4-fluorophenyl)methoxy]-3-hydroxy-7-methyl-2	C26H18ClFN2O4	476.88	C	-9.2
1114	Cc1c(c2c(cc1OCc1ccc(F)cc1)c(=O)c(O)c(o2)c1[nH]c(C)nn1)c1ccccc1	1312910-86-3	4H-1-Benzopyran-4-one, 6-[(4-fluorophenyl)methoxy]-3-hydroxy-7-methyl-8-phenyl-2	C25H18FN3O4	443.43	C	-9.2
1115	Cc1c2c(cc(OCc3ccc(F)cc3)c1Cl)c(=O)c(O)c(o2)c1[nH]ccn1	1312912-83-6	4H-1-Benzopyran-4-one, 7-chloro-6-[(4-fluorophenyl)methoxy]-3-hydroxy-8-methyl-2	C25H17ClFN3O4	477.87	C	-9.2
1116	O(C)c1c(c2c(cc1OCc1ccc(F)cc1)c(=O)c(O)c(o2)c1ncccn1)c1ccccc1	1312914-93-4	4H-1-Benzopyran-4-one, 7,8-dichloro-6-[(4-fluorophenyl)methoxy]-3-hydroxy-2-(5-p	C25H15Cl2FN2O4	497.3	C	-9.2
1117	Clc1c(c2c(cc1OCc1ccc(F)cc1)c(=O)c(O)c(o2)c1[nH]c(C)nn1)c1ccccc1	1312915-80-2	4H-1-Benzopyran-4-one, 7-chloro-6-[(4-fluorophenyl)methoxy]-3-hydroxy-8-phenyl-2	C24H15ClFN3O4	463.85	C	-9.2
1118	O(Cc1ccc(F)cc1)c1c(c(C)c2c(c1)c(=O)c(O)c(o2)c1ncccn1)c1ccccc1	1312916-50-9	4H-1-Benzopyran-4-one, 6-[(4-fluorophenyl)methoxy]-3-hydroxy-7-phenyl-2-(5-pheny	C31H21FN2O4	504.51	C	-9.2
1119	O(Cc1ccc(F)cc1)c1c(c(C)c2c(c1)c(=O)c(O)c(o2)c1[nH]ccn1)c1ccccc1	1312916-96-3	4H-1-Benzopyran-4-one, 6-[(4-fluorophenyl)methoxy]-3-hydroxy-8-methyl-7-phenyl-2	C31H22FN3O4	519.52	C	-9.2
1120	O=c1n([C@@H]2O[C@H](COP(=O)(OP(=O)(O)O)OP(=O)(O)O)C=C2)cc(C)c(=O)[nH]1	131466-86-9	1-Naphthalenesulfonic acid, 3-hydroxy-7-[3-[4-(2-phenyldiazenyl)phenyl]-2-triaze	C22H17N5O4S	447.47	C	-9.2
1121	S(=O)(=O)(N(C[C@H]([C@H](Cc1ccccc1)NC(=O)O[C@H]1CCOC1)O)CC(C)(C)C)c1ccc(N(=O)=O)cc1	133261-56-0	Guanosine 3,Ä≤-(tetrahydrogen triphosphate), P,Ä≤,Ä≤,Üi5,Ä≤-ester with guanosir	C20H27N10O18P3	788.41	C	-9.2
1122	C(OC[C@@H]1[C@@H](OP(N(C(C)C)C(C)C)OCCC#N)C[C@@H](O1)n1cc(CCCNC(=O)C(F)(F)F)c(=O)[nH]c1=O)(c1ccc(OC)	1341204-36-1	Carbamic acid, N-[(1S)-2-methyl-1-[[(2S)-2-[5-[4-[(6S,7S,22aS)-1,2,3,5,6,7,8,9,1	C46H60N8O6	821.02	C	-9.2
1123	N(=Nc1c2c(cc(S(=O)(=O)O)cc2)c(N)cc1)c1c2c(c(N=Nc3cc(S(=O)(=O)O)ccc3)cc1)ccc(S(=O)(=O)O)c2	1345852-31-4	1-Naphthalenesulfonic acid, 8-amino-5-[2-[7-sulfo-4-[2-(3-sulfophenyl)diazenyl]-	C26H19N5O9S3	641.65	C	-9.2
1124	N(=Nc1c2c(c(N=Nc3cc(S(=O)(=O)O)ccc3)cc1)cccc2)c1c2c(c(N)cc1)cccc2S(=O)(=O)O	1345852-46-1	1-Naphthalenesulfonic acid, 5-amino-8-[2-[4-(2-phenyldiazenyl)-7-sulfo-1-naphtha	C26H19N5O6S2	561.59	C	-9.2
1125	O=C1C2C(N(C=N2)[C@@H]2O[C@H](CO)CC2)NC(=N1)N	1347855-43-9	Benzamide, N-[[4,Ä≤-[(2-ethyl-5,7-dimethyl-3H-imidazo[4,5-b]pyridin-3-yl)methyl]	C30H27N5O5S	569.63	C	-9.2
1126	C(=O)([C@@H](NC(=O)OC)C(C)C)N1[C@H](c2[nH]c(c3c4c(c(cc3)c3c5c(c(cc3)c3[nH]c(nc3)[C@H]3N(C(=O)[C@@H](	1350719-38-8	Carbamic acid, N,N,Ä≤-[[5,5,Ä≤-biquinoline]-8,8,Ä≤-diylbis[1H-imidazole-5,2-diy	C46H52N10O6	840.97	C	-9.2
1127	C(CCCCCCc1cc(O)c(O)cc1)c1cc(O)c(O)cc1	1351396-15-0	6H-Purin-6-one, 2-amino-9-[(4aR,6S,7aS)-hexahydro-5-methylene-2-phenylcyclopenta	C19H19N5O3	365.39	C	-9.2
1128	C(=O)([C@@H](NC(=O)OC)C(C)C)N1C[C@@]2(C[C@H]1c1[nH]c(cn1)c1ccc(cc1)c1ccc(cc1)c1[nH]c(nc1)[C@H]1N(C(=	1351852-62-4	Carbamic acid, N-[(1S)-1-[[(2S)-4,4-difluoro-2-[5-[4,Ä≤-[2-[(5S,8S)-7-[(2S)-2-[	C43H52F2N8O7	830.92	C	-9.2
1129	C(=O)([C@@H](NC(=O)OC)C(C)C)N1C[C@@]2(C[C@H]1c1[nH]c(cn1)c1c(F)c(F)c(cc1)c1c(F)c(F)c(cc1)c1[nH]c(nc1	1351852-81-7	Carbamic acid, N-[(1S)-1-[[(2S)-2-[5-[3,3,Ä≤-difluoro-4,Ä≤-[2-[(5S,8S)-7-[(2S)-	C43H52F2N8O7	830.92	C	-9.2
1130	C(=O)([C@@H](NC(=O)OC)C(C)C)N1C[C@@]2(C[C@H]1c1[nH]c(cn1)c1c(C)c(C)c(cc1)c1c(C)c(C)c(cc1)c1[nH]c(nc1	1351852-83-9	Carbamic acid, N-[(1S)-1-[[(2S)-2-[5-[4,Ä≤-[2-[(5S,8S)-7-[(2S)-2-[(methoxycarbor	C45H58N8O7	822.99	C	-9.2
1131	Cc1c2c(N(c3c(C(=O)N2)cccn3)C2CC2)nc(Cl)c1	1355573-52-2	5(12H)-Naphthacenone, 1-chloro-7-(2,4-dihydroxy-6-methylphenyl)-2,4,6,9-tetrahyc	C27H21ClO7	492.9	C	-9.2
1132	C(=O)([C@@H](NC(=O)OC)C(C)C)N1[C@H](c2[nH]c(c3c4c(c(cc3)c3ccc(cc3)c3[nH]c(nc3)[C@H]3N(C(=O)[C@@H](NC	1359868-92-0	Carbamic acid, N-[(1S)-2-[(1R,3S,5R)-3-[5-[4-[7-[2-[(1R,3S,5R)-2-[(2S)-2-[(metho	C46H52N10O8S	905.03	C	-9.2
1133	C(=O)([C@@H](NC(=O)OC)C(C)C)N1[C@H](c2[nH]c(c3cc4c5c(c6c(c4cc3)ccc(c6)c3[nH]c(nc3)[C@H]3N(C(=O)[C@@H	1377035-60-3	Carbamic acid, N,N,Ä≤-[dibenzo[f,h]quinoxaline-6,11-diylbis[1H-imidazole-5,2-diy	C44H50N10O6	814.93	C	-9.2
1134	C(=O)([C@@H](NC(=O)OC)C(C)C)N1[C@H](c2[nH]c(c3cc4c5-c6c(cc(cc6CC4)c4[nH]c(nc4)[C@H]4N(C(=O)[C@@H](NC	1377035-62-5	Carbamic acid, N,N,Ä≤-[dibenzo[a,c]phenazine-2,7-diylbis[1H-imidazole-5,2-diyl-	C48H52N10O6	864.99	C	-9.2
1135	C(=O)([C@@H](NC(=O)OC)C(C)C)N1[C@@H]([C@@H]2C[C@H]1CC2)c1[nH]c2c(n1)ccc1c2ccc(c1)c1ccc2c(cc1)c1c(cc2)	1377036-50-4	Carbamic acid, N-[(1S)-2-[(1R,3S,4S)-3-[5-[4-[2-[(2S)-1-[(2S)-2-[(methoxycarbony	C46H54N8O7	830.97	C	-9.2
1136	C(=O)([C@@H](NC(=O)OC)C1CCOCC1)N1[C@@H]([C@@H]2C[C@H]1CC2)c1[nH]c(cn1)c1cc2c(-c3c(CC2)cc(cc3)c2[nH]c	1377036-52-6	Carbamic acid, N-[(1S)-1-[[(2S)-2-[2,Ä≤-[(1R,3S,4S)-2-[(2S)-2-[(methoxycarbonyl)	C46H52N8O6	812.96	C	-9.2
1137	C(=C1c2c(-c3c1cc(cc3)c1[nH]c(nc1)[C@H]1N(C(=O)[C@@H](NC(=O)OC)C(C)C)CCC1)ccc(c2)c1[nH]c(nc1)[C@H]1N(	1377040-05-5	Carbamic acid, N-[(1S)-1-[[(2S)-2-[5-[3-[6-[2-[(2S)-1-[(2S)-2-[(methoxycarbonyl)	C44H52N8O6	788.93	C	-9.2
1138	C(=O)([C@@H](NC(=O)OC)C(C)C)N1[C@H](c2[nH]c(c3cc4c5-c6c(cc(cc6OC4)c4[nH]c(nc4)[C@H]4N(C(=O)[C@@H](NC	1377045-52-7	Carbamic acid, N,N,Ä≤-[(6,6,Ä≤-difluoro[7,7,Ä≤-bi-1H-naphth[1,2-d]imidazole]-2,	C44H48F2N8O6	822.9	C	-9.2
1139	C[C@@]12C(=CC[C@H]3[C@@]1(C)CC[C@@H]1[C@]3(C)CCC(=O)[C@H]1C)[C@H]1[C@@]3(C[C@H]2OC3=O)CCC(C)(C)[C@H]	1377604-20-0	Carbamic acid, N-[(1S)-2-[(1R,3S,4S)-3-[2,Ä≤-[(2S)-1-[(2S)-2-[(methoxycarbonyl)a	C48H54N8O7	854.99	C	-9.2
1140	N(=Nc1cc(C)c(N=Nc2ccc(S(=O)(=O)O)cc2)cc1)c1c2c(ccc(S(=O)(=O)O)c2)c(N)cc1	1378293-93-6	Benzoic acid, 4-[2-[4-[2-(4-amino-7-sulfo-1-naphthalenyl)diazenyl]-2-methylpheny	C24H19N5O5S	489.5	C	-9.2
1141	O=C1C2(C(C(C(=O)/C=C(\O)/C3C4(N5C(C3c3cc(OC)c(O)cc3)CCCC5)c3c(NC4=O)cccc3)C3N2CCCC3)c2cc(OC)c(O)cc2)	1389334-85-3	Spiro[2H-indene-2,3,Ä≤-[3H]pyrrolizine]-1,3-dione, 2,Ä≤-[(1Z)-3-[1,Ä≤,2,Ä≤,5,Ä≤	C47H42N2O10	794.84	C	-9.2
1142	C(OC(=O)c1cccnc1)[C@H]1O[C@H]([C@H]2[C@@H]1OC(C)(C)O2)n1nc(C(=O)N)nc1	1395899-00-9	Dodecanoic acid, 12-amino-, [(2R,5S)-5-[4-[[bis(4-methoxyphenyl)phenylmethyl]ami	C41H51FN4O6S	746.93	C	-9.2
1143	C(NC(=O)[C@@H](NC(=O)OC(C)(C)C)CCNC(=O)OC(C)(C)C)(C)C12CC3CC(C2)CC(C1)C3	1398581-53-7	Benzoic acid, 2,2,Ä≤-[carbonylbis(4,1-phenylenecarbonylimino)]bis-	C29H20N2O7	508.48	C	-9.2
1144	[N]c1n(c2c(n1)c(=O)nc[nH]2)[C@@H]1O[C@H](CO)[C@@H](O)[C@H]1O	1423120-02-8	3-Azabicyclo[3.1.0]hexane-2-carboxamide, N-[(1S)-1-(2-amino-2-oxoacetyl)butyl]-3	C40H68N6O5	713.01	C	-9.2
1145	N(=C1c2c(N(CN3CCN(CC3)c3ccc(OC)cc3)C1=O)ccc(F)c2)NC(=S)N(CC=C)CC=C	1426583-75-6	Hydrazinecarbothioamide, 2-[5-chloro-1,2-dihydro-1-[[4-(4-methoxyphenyl)-1-piper	C33H31ClN6O2S	611.16	C	-9.2
1146	O(C)c1c2n(cc(C(=O)O)c(=O)c2cc(F)c1N1CC(C)N(CN2c3c(C(=NNC(=S)N(CC=C)CC=C)C2=O)cc(F)cc3)CC1)C1CC1	1426583-87-0	3-Quinolinecarboxylic acid, 7-[4-[[5-chloro-3-[2-[(diphenylamino)thioxomethyl]hy	C41H37ClFN7O5S	794.29	C	-9.2
1147	C(=O)([C@@H](NC(=O)OC)C(C)C)N1[C@@H]2c3nc(c([nH]3)COCCCON=C(C)O[C@@H](C2)C1)c1ccc(cc1)c1ccc(cc1)c1[n	1431625-79-4	Carbamic acid, N-[(1S)-1-[[(2S)-2-[5-[4,Ä≤-[(2S,5S)-3-[(2S)-2-[(methoxycarbonyl)	C45H59N9O10S	918.07	C	-9.2
1148	[C@H](OC)([C@H]1[C@H](NC(=O)C)[C@@H](NC(=N)N)C=C(C(=O)O)O1)[C@@H]1OC(CCCCCCC)(OC)OC1	144269-19-2	2-Naphthalenesulfonic acid, 8,8,Ä≤-[[1,1,Ä≤-biphenyl]-4,4,Ä≤-diylbis(azo)]bis[7	C32H24N6O8S2	684.7	C	-9.2
1149	C(=O)(O)c1cc(ccc1NC(=O)c1c(N(=O)=O)cccc1)c1cc(C(=O)O)c(NC(=O)c2c(N(=O)=O)cccc2)cc1	144334-82-7	Benzoic acid, 3,3,Ä≤-methylenebis[6-[(2-nitrobenzoyl)amino]-	C29H20N4O10	584.49	C	-9.2
1150	O(C[C@@H]1[C@@H](N=[N+]=[N-])C[C@@H](O1)n1c(=O)[nH]c(=O)c(C)c1)[P@]1(=O)O[C@@H]2[C@H](O[C@H](C2)n2c(	1448853-79-9	Thymidine, [P(R)]-thymidylyl-(3,Ä≤-5,Ä≤,Üi5,Ä≤)-3,Ä≤-azido-3,Ä≤-deoxy-	C20H24N7O10P		C	-9.2
1151	C(=O)(N[C@H](C(=O)C(=O)NC1CC1)CCC)[C@H]1N(C(=O)[C@@H](NC(=O)[C@@H](NC(=O)c2cn(=O)ccn2)C2CCCCC2)C(C)(	152195-59-0	1,5-Naphthalenedisulfonic acid, 3-[2-[4-[2-[4-[2-(4-amino-5-sulfo-1-naphthalenyl	C40H27N7O15S5.5Na		C	-9.2
1152	C(=O)(NC(C(=O)C(=O)NCC=C)CCCC)[C@@H]1[C@@H]2[C@@H](C2(C)C)CN1C(=O)[C@@H](NC(=O)N[C@@H](CN1C(=O)CCC1=	1528752-67-1	3-Azabicyclo[3.1.0]hexane-2-carboxamide, N-[3-amino-1-(cyclobutylmethyl)-2,3-dic	C36H56N6O7S	716.93	C	-9.2
1153	C(=O)([C@@H](NC(=O)OC)C(C)C)N1[C@H](c2[nH]c(c3c4c(C5CC4CC5)c(cc3)c3ccc(cc3)c3[nH]c(nc3)[C@H]3N(C(=O)	1560639-12-4	Carbamic acid, N-[(1S)-1-[[(2S)-4,4-dimethyl-2-[5-[4-[1,2,3,4-tetrahydro-8-[2-[(	C49H64N8O6	861.08	C	-9.2
1154	CC12c3c(C(C1)CC2)c(ccc3c1[nH]c(nc1)[C@H]1N(C(=O)[C@@H](NC(=O)OC)C(C)C)CCC1)c1ccc(cc1)c1[nH]c(nc1)[C@	1560639-28-2	Carbamic acid, N-[(1S)-2-methyl-1-[[(2S)-2-[5-[1,2,3,4-tetrahydro-8-[4-[2-[(8S)-	C47H58N8O8	863.01	C	-9.2
1155	O=C1C2c3c(C1CC2)c(ccc3c1[nH]c(nc1)[C@H]1N(C(=O)[C@@H](NC(=O)OC)C(C)C)CCC1)c1ccc(cc1)c1[nH]c(nc1)[C@H	1560649-85-5	Carbamic acid, N-[(1S)-1-[[(2S)-2-[5-[4-[1,4-dihydro-8-[2-[(2S)-1-[(2S)-2-[(meth	C45H54N8O6	802.96	C	-9.2
1156	CN1C2c3c(C1C=C2)c(ccc3c1[nH]c(nc1)[C@H]1N(C(=O)[C@@H](NC(=O)OC)C(C)C)CCC1)c1ccc(cc1)c1[nH]c(nc1)[C@H	1560649-87-7	Carbamic acid, N-[(1S)-2-methyl-1-[[(2S)-2-[5-[4-[1,2,3,4-tetrahydro-8-[2-[(2S)-	C45H54N8O7	818.96	C	-9.2
1157	N(c1c2c(c(O)c(CN3CCN(CC)CC3)c1)CCCC2)c1c2c(nccl)cc(Cl)cc2	156893-82-2	1-Naphthalenol, 4-[(7-chloro-4-quinolinyl)amino]-5,6,7,8-tetrahydro-2-[(4-methyl	C25H29ClN4O	436.98	C	-9.2
1158	N(C(CCCN(CC)CC)C)(C(=O)CCCCCN1C(=O)C=CC1=O)c1c2c(nccl)cc(Cl)cc2	1600500-37-5	Benzonitrile, 2-[4-[[[(5R,5aR,8aR,9R)-5,5a,6,8,8a,9-hexahydro-8-oxo-9-(3,4,5-tri	C32H28N4O8	596.59	C	-9.2
1159	C(=O)([C@@H](NC(=O)OC)C(C)C)N1[C@H](c2[nH]c(c3c4c(cc3)c3c5c(c(cc3)c3[nH]c(nc3)[C@H]3N(C(=O)[C@@H](	1612236-50-6	Carbamic acid, N-[(1S)-1-[[(2S)-2-[5-[8-[1,Ä≤,3,Ä≤-dihydro-7,Ä≤-[2-[(2S)-1-[(2S	C51H64N8O7	901.1	C	-9.2
1160	C[C@]1(F)[C@@H](O[C@H](COP(=O)(Oc2ccccc2)N[C@H](C(=O)OC(C)C)C)[C@H]1O)n1c(=O)[nH]c(=O)c(c1[2H])[2H]	162112-43-8	Benzamide, N-[[4,Ä≤-[(2-ethyl-5,7-dimethyl-3H-imidazo[4,5-b]pyridin-3-yl)methyl]	C34H36N4O3S	580.74	C	-9.2
1161	N(C(=O)[C@H](Cc1ccccc1)C[C@@H](CN1[C@H](C(=O)NC(C)(C)C)CNCC1)O)[C@H]1c2c([C@H](O)[C@H]1O)cccc2	162518-57-2	D-erythro-Pentonamide, 2,3,5-trideoxy-N-[(1S,2R)-2,3-dihydro-2-hydroxy-1H-inden-	C36H47N5O5	629.79	C	-9.2
1162	O=c1n([C@H]2C[C@H](OP(=O)(OC[C@H]3O[C@H](C[C@@H]3O)n3c(=O)[nH]c(=O)c(C)c3)F)[C@@H](CO)O2)cc(C)c(=O)[	1648953-56-3	1-Naphthalenesulfonic acid, 4-amino-3-(2-[1,1,Ä≤-biphenyl]-4-yldiazenyl)-	C22H17N3O3S	403.45	C	-9.2
1163	C(=O)([C@@H](NC(=O)OC)C(C)C)N1C[C@@]2(C[C@H]1c1[nH]c(cn1)c1cc3c(cc1)c1c(cc3)cc(cc1)c1[nH]c(nc1)[C@H]	1653966-75-6	Carbamic acid, N-[(1S)-1-[[(2S,3aS,6aS)-hexahydro-2-[7-[4-[2-[(3S,5S)-2-[(2S)-2-	C49H60N8O7	873.05	C	-9.2
1164	C(=O)([C@@H](NC(=O)OC)C(C)C)N1C[C@@]2(C[C@H]1c1[nH]c(cn1)c1ccc(cc1)c1ccc(cc1)c1[nH]c(nc1)[C@H]1N(C(=	1653966-95-0	Carbamic acid, N,N,Ä≤-[[7,7,Ä≤-bi-1H-naphth[1,2-d]imidazole]-2,2,Ä≤-diylbis[(3S	C52H62N8O8	927.1	C	-9.2
1165	C(=O)([C@@H](NC(=O)OC)C(C)C)N1[C@@H](C[C@]2(C1)CCCOC2)c1[nH]c2c(n1)ccc1c2ccc(c1)c1ccc2c(cc1)c1c(cc2)n	1653967-10-2	Carbamic acid, N-[(1S)-1-[[(2S)-2-[7-[4-[2-[(3S,5S)-2-[(2S)-2-[(methoxycarbonyl)	C46H56N8O7	832.99	C	-9.2
1166	C(CN1CCC(CN2c3c4c(S2(=O)=O)cccc4ccc3)CC1)(NC)C12CC3CC(C2)CC(C1)C3	178477-44-6	1-Piperidineethanamine, 4-[(1,1-dioxido-2H-naphth[1,8-cd]isothiazol-2-yl)methyl]	C28H37N3O2S	479.68	C	-9.2
1167	FC1(F)CC2(c3c(-c4c2cc(cc4)c2[nH]c(nc2)[C@H]2N(C(=O)[C@@H](NC(=O)OC)C(C)C)CCC2)ccc(c3)c2[nH]c(nc2)[C@	1800210-29-0	Carbamic acid, N,N,Ä≤-[spiro[9H-fluorene-9,3,Ä≤-oxetane]-2,7-diylbis[1H-imidazo	C43H48N8O7	788.89	C	-9.2
1168	FC1(F)c2c(-c3c(cc(cc3)c3[nH]c(nc3)[C@H]3N(C(=O)[C@@H](NC(=O)OC)C(C)(C)C)CCC3)C1(F)F)ccc(c2)c1[nH]c(n	1801161-68-1	Carbamic acid, N,N,Ä≤-[(9,9,10,10-tetrafluoro-9,10-dihydro-2,7-phenanthrenediyl)	C48H56F4N8O6	917	C	-9.2
1169	C(=O)([C@@H](NC(=O)OC)C(C)C)N1[C@@H](C2CC1CC2)c1[nH]c(cn1)c1ccc(cc1)c1ccc(cc1)c1[nH]c(nc1)[C@H]1N(C(	1808295-77-3	Carbamic acid, N,N,Ä≤-[1,4-phenylenebis[1H-imidazole-5,2-diyl-2,1-pyrrolidinedi	C34H46N8O6	662.78	C	-9.2

1170	C(=O)([C@@H](NC(=O)OC)C(C)C)N1[C@@H](CCC1)c1[nH]c(cn1)c1ccc(cc1)c1ccc(cc1)c1[nH]c(C[C@H]2N(C(=O)[C@@	1808295-82-0	Carbamic acid, N-[(1S)-1-[[(2S)-2-[5-[4,Ä≤-[2-[(3S)-2-[(2S)-2-[(methoxycarbonyl)	C42H52N8O6	764.91	C	-9.2
1171	O=C1[C@@H]2[C@@H](c3c([C@H](OC(=O)[C@@H](Cc4ccccc4)N4C(=O)C5C(C4=O)CCCC5)[C@H]2CO1)cc1c(c3)OCO1)c1cc	1809321-40-1	2H-Isoindole-2-acetic acid, 1,3-dihydro-1,3-dioxo-Œ±-(phenylmethyl)-, (5R,5aR,8a	C39H33NO11	691.68	C	-9.2
1172	O=C1[C@@H]2[C@@H](c3c([C@H](OC(=O)[C@@H](C)N4C(=O)c5c(C4=O)cccc5)[C@H]2CO1)cc1c(c3)OCO1)c1cc(OC)c(OC	1809321-43-4	2H-Isoindole-2-acetic acid, 1,3-dihydro-Œ±-methyl-1,3-dioxo-, (5R,5aR,8aR,9R)-5,	C33H29NO11	615.58	C	-9.2
1173	O=C1[C@@H]2[C@@H](c3c([C@H](OC(=O)[C@H](C)N4C(=O)c5c(C4=O)cccc5)[C@H]2CO1)cc1c(c3)OCO1)c1cc(OC)c(OC)	1809321-44-5	2H-Isoindole-2-acetic acid, 1,3-dihydro-Œ±-methyl-1,3-dioxo-, (5R,5aR,8aR,9R)-5,	C33H29NO11	615.58	C	-9.2
1174	O=C1[C@@H]2[C@@H](c3c([C@H](OC(=O)[C@H](Cc4ccccc4)N4C(=O)C5C(C4=O)C4OC5CC4)[C@H]2CO1)cc1c(c3)OCO1)c1	1809325-26-5	4,7-Epoxy-2H-isoindole-2-acetic acid, octahydro-1,3-dioxo-Œ±-(phenylmethyl)-, (5	C39H37NO12	711.71	C	-9.2
1175	N(c1nc(nc(Nc2ccc(C(=O)C=Cc3ccc(N(=O)=O)cc3)cc2)n1)N1CCOCC1)c1cc(C(F)(F)F)ccc1	1883585-93-0	Urea, N-(2-fluoro-6-methylphenyl)-N,Ä≤-(2-tricyclo[3.3.1.13,7]dec-1-yl-1H-indol-	C26H28FN3O	417.52	C	-9.2
1176	N(C(C)C)c1c(nc(O[C@@H]2O[C@H](C(=O)O)[C@@H](O)[C@H](O)[C@H]2O)cc1)N1CCN(C(=O)c2cc3c([nH]2)ccc(NS(=O)	188780-38-3	Methanesulfonamide, N-[2-[[4-[3-amino-4-(sulfooxy)-2-pyridinyl]-1-piperazinyl]ca	C19H22N6O7S2	510.54	C	-9.2
1177	Nc1n(c2c(n1)c(=O)nc(N)[nH]2)[C@@H]1O[C@H](COP(=O)(OP(=O)(OP(=O)(O)O)O)O)[C@@H](O)C1	188780-44-1	Methanesulfonamide, N-[2-[[4-[1,6-dihydro-3-[(1-methylethyl)amino]-6-oxo-2-pyric	C22H28N6O4S	472.56	C	-9.2
1178	C(=O)(c1n(c2c(c1)cc(NS(=O)(=O)C)cc2)[C@@H]1O[C@H](C(=O)O)[C@@H](O)[C@H](O)[C@H]1O)N1CCN(CC1)c1c(NC(C	194347-54-1	Œ≤-D-Glucopyranosiduronic acid, 2-[[4-[3-[(1-methylethyl)amino]-2-pyridinyl]-1-p	C28H36N6O10S	648.68	C	-9.2
1179	O=C1c2n(N([C@H]3N1CCOC3)[C@H]1c3c(CSc4c1cccc4)cc(F)cc3)ccc(=O)c2O	1985605-60-4	1H-[1,4]Oxazino[3,4-c]pyrido[2,1-f][1,2,4]triazine-6,8-dione, 12-[(11S)-6,11-dih	C24H21N3O4S	447.51	C	-9.2
1180	O(C(=O)N(C)C)c1c2n(N([C@H]3N(C2=O)CCOC3)[C@H]2c3c(CSc4c2cccc4)c(F)c(F)cc3)ccc1=O	1985606-20-9	1H-[1,4]Oxazino[3,4-c]pyrido[2,1-f][1,2,4]triazine-6,8-dione, 12-[(11S)-7,8-difl	C30H24F2N4O4S	574.6	C	-9.2
1181	O(C(=O)OC1CCOCC1)c1c2n(N([C@H]3N(C2=O)CCOC3)[C@H]2c3c(CSc4c2cccc4)c(F)c(F)cc3)ccc1=O	1985607-15-5	Carbonic acid, (12aR)-12-[(11S)-7,8-difluoro-6,11-dihydrodibenzo[b,e]thiepin-11-	C31H29F2N3O7S	625.64	C	-9.2
1182	O(C(=O)OCC(=O)C)c1c2n(N([C@H]3N(C2=O)CCOC3)[C@H]2c3c(CSc4c2cccc4)c(F)c(F)cc3)ccc1=O	1985607-24-6	Carbonic acid, (12aR)-12-[(11S)-7,8-difluoro-6,11-dihydrodibenzo[b,e]thiepin-11-	C30H23F2N3O9S	639.58	C	-9.2
1183	O(P(=O)(OCC)OCC)c1c2n(N([C@H]3N(C2=O)CCOC3)[C@H]2c3c(CSc4c2cccc4)c(F)c(F)cc3)ccc1=O	1985607-33-7	Benzoic acid, 4-(dimethylamino)-, (12aR)-12-[(11S)-7,8-difluoro-6,11-dihydrodibe	C33H28F2N4O5S	630.66	C	-9.2
1184	O(C(=O)OCc1ccc(N=[N+]=[N-])cc1)c1c2n(N([C@H]3N(C2=O)CCOC3)[C@H]2c3c(CSc4c2cccc4)c(F)c(F)cc3)ccc1=O	1985607-47-3	1H-[1,4]Oxazino[3,4-c]pyrido[2,1-f][1,2,4]triazine-6,8-dione, 7-[(4-azidophenyl)	C31H24F2N6O4S		C	-9.2
1185	O(C(=O)O[C@H]1C(C)(C)COC1=O)c1c2n(N([C@H]3N(C2=O)CCOC3)[C@H]2c3c(CSc4c2cccc4)c(F)c(F)cc3)ccc1=O	1985607-51-9	Carbonic acid, (12aR)-12-[(11S)-7,8-difluoro-6,11-dihydrodibenzo[b,e]thiepin-11-	C31H27F2N3O8S	639.62	C	-9.2
1186	N(C(=O)NC12CC3CC(C2)CC(C1)C3)c1cc2cc(C34CC5CC(C4)CC(C3)C5)[nH]c2cc1	1987854-76-1	Urea, N-[(4-fluorophenyl)methyl]-N,Ä≤-(2-tricyclo[3.3.1.13,7]dec-1-yl-1H-indol-5	C26H28FN3O	417.52	C	-9.2
1187	O=c1n([C@@H]2O[C@H](COP(=O)(OP(=O)(OP(=O)(O)O)O)O)[C@@H](OC)C2)cc(C)c(=O)[nH]1	1987854-81-8	Urea, N-2-pyrimidinyl-N,Ä≤-(2-tricyclo[3.3.1.13,7]dec-1-yl-1H-indol-5-yl)-	C23H25N5O	387.48	C	-9.2
1188	C(=O)([C@@H](NC(=O)OC)C(C)C)N1CCC2(C[C@H]1c1[nH]c(cn1)c1c3c(c(cc1)c1cc4c(cc1)c1c(cc4)nc([nH]1)[C@H]1N	2007901-27-9	Carbamic acid, N-[(1S)-1-[[(2S)-2-[7-[2,2-difluoro-2,3-dihydro-7-[2-[(2S)-1-[(2S	C45H52F2N8O6	838.94	C	-9.2
1189	C(=O)([C@@H](NC(=O)OC)C(C)C)N1[C@H](c2[nH]c3c4c(cc(cc4)c4c5c(cc4)c4[nH]c(nc4)[C@H]4N(C(=O)[C@@H](N	2007901-60-0	Carbamic acid, N-[(1S)-1-cyclohexyl-2-[(2S,5S)-2-[5-[2,3-dihydro-7-[2-[(2S,5S)-1	C50H62N8O6	871.08	C	-9.2
1190	O(C)C1c2c(c(ccc2c2[nH]c(nc2)[C@H]2N(C(=O)[C@@H](NC(=O)OC)C(C)C)CCC2)c2cc3c(-c4c(CC3)nc([nH]4)[C@H]3N	2007901-75-7	Carbamic acid, N-[(1S)-1-[[(2S)-2-[7-[4-[2-[(2S)-1-[(2S)-2-cyclohexyl-2-[(methox	C50H62N8O6	871.08	C	-9.2
1191	C(=O)([C@@H](NC(=O)OC)C(C)C)N1CCC2(C[C@H]1c1[nH]c3c(n1)ccc1c3ccc(c1)c1c3c(c(cc1)c1[nH]c(nc1)[C@H]1N(C	2007901-84-8	Carbamic acid, N-[(1S)-1-[[(2S,3S,5S)-5-[5-[2,3-dihydro-7-[2-[(3S)-2-[(2S)-2-[(m	C51H64N8O6	885.1	C	-9.2
1192	[C@H](Cc1ccccc1)(CC[C@H](Cc1ccccc1)NC(=O)OCc1cncs1)NC(=O)[C@@H](NC(=O)N(Cc1nc(C(C)C)sc1)C)CCN(C)C	1004318-01-7	2,7,10,12-Tetraazatridecanoic acid, 12-methyl-13-[2-(1-methylethyl)-4-thiazolyl]	C40H51N7O6S2	790.01	A/B	-9.1
1193	S(=O)(=O)(O)c1c2c(c(N)c(/N=N/c3ccc(cc3)c3ccc(N)cc3)c1)cccc2	1027956-05-3	6H-Purin-6-one, 2-amino-9-[(1S,3R,4S)-4-(benzoyloxy)-3-[(benzoyloxy)methyl]-2-me	C26H23N5O5	485.49	A/B	-9.1
1194	N(c1nc(Nc2ccc(C#N)cc2)nc(CNCCc2ccc(S(=O)(=O)N)cc2)c1)c1c(C)cc(/C=C/C#N)cc1C	1037445-06-9	Benzonitrile, 4-[[4-[[4-[(1E)-2-cyanoethenyl]-2,6-dimethylphenyl]amino]-6-[[[(2,	C29H31N7O2	509.6	A/B	-9.1
1195	N(c1c(C)cc(/C=C/C#N)cc1C)c1nc(Nc2ccc(C#N)cc2)nc(CN(OC)C)c1	1037445-08-1	Benzenesulfonamide, 4-[2-[[[6-[[4-[(1E)-2-cyanoethenyl]-2,6-dimethylphenyl]aminc	C31H30N8O2S	578.69	A/B	-9.1
1196	C(=O)(NCCc1cccs1)c1c(Nc2c(C)cc(/C=C/C#N)cc2C)nc(Nc2ccc(C#N)cc2)nc1COC	1037452-52-0	5-Pyrimidinecarboxamide, N-[2-[4-(aminosulfonyl)phenyl]ethyl]-4-[[4-[(1E)-2-cyar	C33H32N8O4S	636.72	A/B	-9.1
1197	C(C=C)(O)c1c(Nc2c(C)cc(/C=C/C#N)cc2C)nc(Nc2ccc(C#N)cc2)nc1COC	1037452-57-5	5-Pyrimidinecarboxamide, 2-[(4-cyanophenyl)amino]-4-[[4-[(1E)-2-cyano-1-propen-1	C32H30N8O2	558.63	A/B	-9.1
1198	N(Cc1scc(Br)c1)C12CC3CC(C2)CC(C1)C3	103908-26-5	C.I. Mordant Violet 16, trisodium salt	C25H20O9.3Na		A/B	-9.1
1199	N(C(=O)[C@H](Cc1ccccc1)C[C@@H](CN1[C@H](C(=O)NC(C)(C)C)CN(CCN2C(=O)c3c(C2=O)cccc3)CC1)O)[C@H]1c2c(C[	1064476-01-2	Not Yet Assigned	C39H50N4O4	638.84	A/B	-9.1
1200	N(C(=O)[C@H](Cc1ccccc1)CC(CN1[C@H](C(=O)NC(C)(C)C)CN(Cc2cccnc2)CC1)O)[C@H]1c2c(C[C@H]1O)cccc2	1085743-77-6	2-Piperazinecarboxamide, 1-[[[(2S)-3-[[(1S,2R)-2,3-dihydro-2-hydroxy-1H-inden-1-	C35H45N5O4S	631.83	A/B	-9.1
1201	C(NC(=O)c1nnc(C([2H])([2H])[2H])o1)(C)(C)c1nc(C(=O)NCc2ccc(F)cc2)c(O)c(=O)n1C([2H])([2H])[2H]	1100750-85-3	4-Pyrimidinecarboxamide, N-[(4-fluorophenyl)methyl]-1,6-dihydro-5-hydroxy-1-meth	C20H12D9FN6O5		A/B	-9.1
1202	C(NC(=O)c1nnc(C)o1)(C([2H])([2H])[2H])(C([2H])([2H])[2H])c1nc(C(=O)NC(c2c(c(c(F)c(c2[2H])[2H])[2H])[	1100751-02-7	4-Pyrimidinecarboxamide, N-[(4-fluorophenyl-2,3,5,6-d4)methyl]-1,6-dihydro-5-hyc	C20H8D13FN6O5		A/B	-9.1
1203	O=C1[C@@H]2[C@@H](c3c([C@H](OC(=O)CCCSSCCCC(=O)O[C@H]4c5c([C@H]([C@@H]6[C@@H]4COC6=O)c4cc(OC)c(OC)c(	1107175-32-5	2-Naphthalenesulfonic acid, 5-[2-(4-hydroxyphenyl)diazenyl]-6-(2-phenyldiazenyl)	C22H16N4O4S	432.45	A/B	-9.1
1204	[C@@H](CC)(C1=C(O)C[C@H](OC1=O)c1ccccc1)c1cc(NS(=O)(=O)c2c(C(F)(F)F)cccn2)ccc1	1109235-80-4	2-Pyridinesulfonamide, N-[3-[(1R)-1-[(6R)-5,6-dihydro-4-hydroxy-2-oxo-6-phenyl-2	C26H23F3N2O5S	532.53	A/B	-9.1
1205	C(=O)([C@H](NC(=O)OC)c1ccccc1)N1[C@@H](CCC1)c1[nH]c(C(C)n1)c1ccc(cc1)c1ccc(cc1)c1[nH]c(nc1)[C@H]1N(C	1129635-01-3	Carbamic acid, N,N,Ä≤-[[1,1,Ä≤-biphenyl]-4,4,Ä≤-diylbis[1H-imidazole-5,2-diyl[(	C38H46N8O6	710.82	A/B	-9.1
1206	O(P(=O)(OP(=O)(O)O)O)[C@H]1[C@@H](O[C@H](COP(=O)(OP(=O)(O)O)O)[C@H]1O)n1c2c(nc1)c(=O)nc(NC(=O)C(C)C)	1135685-39-0	2-Naphthalenesulfonic acid, 5-[(2E)-2-(2-methyl-4-oxo-2,5-cyclohexadien-1-ylider	C23H18N4O4S	446.48	A/B	-9.1
1207	N(=C\1/c2c(N(C)C1=O)ccc(S(=O)(=O)N1CCOCC1)c2)\NC(=S)Nc1c(C)cccc1C	1164536-63-3	Hydrazinecarbothioamide, 2-[1,2-dihydro-1-methyl-5-(4-morpholinylsulfonyl)-2-oxc	C22H25N5O4S2	487.6	A/B	-9.1
1208	C(N1c2c(/C(=N/NC(=S)Nc3c(C)c(Cl)ccc3)/C1=O)cccc2)N1CCOCC1	1164536-73-5	Hydrazinecarbothioamide, 2-[1,2-dihydro-1-methyl-5-(4-morpholinylsulfonyl)-2-oxc	C22H25N5O4S2	487.6	A/B	-9.1
1209	Cc1n(c2nc(nc(n2)N2CCOCC2)c2cnc(N)nc2)ccn1	1174190-88-5	1H-Indazole, 4-[4-(2-methyl-1H-imidazol-1-yl)-6-(4-morpholinyl)-1,3,5-triazin-2-	C18H18N8O	362.39	A/B	-9.1
1210	c1(c2c(ccc1)[nH]cc2)c1nc(nc(n1)N1CCOCC1)n1ccnc1	1174191-05-9	2-Pyridinamine, 5-[4-(3,5-dimethyl-1H-pyrazol-1-yl)-6-(4-morpholinyl)-1,3,5-tria	C17H20N8O	352.39	A/B	-9.1
1211	C(F)(F)c1n(c2c(n1)cccc2)c1nc(nc(n1)N1CCOCC1)c1cnc(N)nc1	1174191-22-0	2-Pyridinamine, 5-[4-[3-methyl-5-(trifluoromethyl)-1H-pyrazol-1-yl]-6-(4-morphol	C17H17F3N8O	406.37	A/B	-9.1
1212	C(=O)([C@@H](NC(=O)OC)C(C)C)N1[C@H](c2[nH]c3-c4c(cc(cc4)c4ccc(cc4)c4[nH]c(nc4)[C@H]4N(C(=O)[C@@H](NC	1180669-76-4	Carbamic acid, N-[(1S)-1-[[(2S)-2-[8-[3-fluoro-4-[2-[(2S)-1-[(2S)-2-[(methoxycar	C43H53FN8O6	796.93	A/B	-9.1
1213	C(#N)c1ccc(c2nc(nc(n2)N2CCOCC2)N2CCOCC2)cc1	1197160-78-3	Urea, N-[4-[[4-(dimethylamino)-1-piperidinyl]carbonyl]phenyl]-N,Ä≤-[4-(4,6-di-4-	C32H41N9O4	615.73	A/B	-9.1
1214	N(C(=O)[C@H](Cc1ccccc1)C[C@@H](CN1[C@H](C(=O)NC(C)(C)C)CN(Cc2c(S)cccc2)CC1)O)C1c2c(C[C@H]1O)cccc2	1223096-37-4	Not Yet Assigned	C45H63N5O6	770.01	A/B	-9.1
1215	[C@@H](C(=O)N[C@@H](Cc1ccccc1)C[C@@H]([C@](C(c1c(c(c(c(c1[2H])[2H])[2H])[2H])[2H])([2H])[2H])(NC(=O)	1224729-52-5	1(2H)-Pyrimidine-4,5,6-d3-acetamide, N-[(1S,3S,4S)-4-[[2-[2,6-di(methyl-d3)phenc	C37H25D23N4O5		A/B	-9.1
1216	c1(nc(nc(n1)N1CCOCC1)N1CCOCC1)c1cc2c(cc1)[nH]cc2	1225037-85-3	1H-Indazole, 5-(4,6-di-4-morpholinyl-1,3,5-triazin-2-yl)-	C18H21N7O2	367.4	A/B	-9.1
1217	N(Cc1ccccn1)c1nc(nc(n1)N1CCOCC1)c1cc(O)ccc1	1225037-91-1	1H-Indazole, 6-(4,6-di-4-morpholinyl-1,3,5-triazin-2-yl)-	C18H21N7O2	367.4	A/B	-9.1
1218	C(#N)C1=C(N)Oc2c(C1c1c(O)cccc1)ccn1c2ncc1	1225373-09-0	7H-Imidazo[1,2-a]pyrano[2,3-c]pyridine-8-carbonitrile, 9-amino-7-(1,3-benzodioxc	C19H14N4O3	346.34	A/B	-9.1
1219	N(Cc1ccccn1)c1nc(nc(n1)N1CCOCC1)c1cc2c(cc1)nc[nH]2	1225638-99-2	1,3,5-Triazin-2-amine, 4-(1H-indazol-5-yl)-6-(4-morpholinyl)-N-(2-pyridinylmethy	C20H20N8O	388.43	A/B	-9.1
1220	C(=O)([C@H](N(C)C)c1ccccc1)N1[C@@H](CCC1)c1[nH]c(cn1)c1cc2c(cc1)cc(cc2)c1ccc(cc1)c1[nH]c(nc1)[C@H]1N	1228552-24-6	Carbamic acid, N-[(1S)-1-[[(2S)-2-[5-[6-[4-[2-[(2S)-1-[(2R)-2-(dimethylamino)-2-	C47H52N8O4	792.97	A/B	-9.1
1221	C(=O)([C@@H](NC(=O)OC)C(C)C)N1[C@@H](CCC1)c1[nH]c(cn1)[C@@H]1CC[C@H](CC1)c1ccc(cc1)c1[nH]c(nc1)[C@H]	1228667-96-6	Carbamic acid, N-[(1R)-2-[(2S)-2-[5-[trans-4-[4-[2-[(2S)-1-[(2R)-2-(dimethylamir	C46H54N8O4	782.97	A/B	-9.1
1222	C(N(C(=O)N[C@H](C(=O)N[C@@H](Cc1ccccc1)C[C@@H]([C@H](Cc1ccccc1)NC(=O)OCc1cnco1)O)C(C)C)C)c1nc(sc1)c1	1236406-29-3	2,7,10,12-Tetraazatridecanoic acid, 13-[2-(4-fluorophenyl)-4-thiazolyl]-4-hydrox	C40H45FN6O5S2	772.95	A/B	-9.1
1223	C(N(C(=O)N[C@H](C(=O)N[C@@H](Cc1ccccc1)C[C@@H]([C@H](Cc1ccccc1)NC(=O)OCc1ccns1)O)C(C)C)C)c1nc(sc1)c1	1236406-46-4	2,7,10,12-Tetraazatridecanoic acid, 13-(2-ethenyl-4-thiazolyl)-4-hydroxy-12-meth	C36H44N6O6S	688.84	A/B	-9.1
1224	C(N(C(=O)N[C@H](C(=O)N[C@@H](Cc1ccccc1)C[C@@H]([C@H](Cc1ccccc1)NC(=O)OCc1cnco1)O)C(C)C)C)c1nc(sc1)C1	1236406-69-1	Not Yet Assigned	C40H46N6O6S	738.89	A/B	-9.1
1225	N(C(=O)[C@H](Cc1ccccc1)C[C@@H](CN1[C@H](C(=O)NC(C)(C)C)CN(Cc2cn3c(cc2)ncc3)CC1)O)[C@H]1c2c(C[C@H]1O)	1237809-02-7	Not Yet Assigned	C38H47N5O4S	669.88	A/B	-9.1
1226	N(c1nc(nc(NNC(=O)c2ccncc2)n1)N1CCOCC1)c1ccccc1	1239885-09-6	4-Pyridinecarboxylic acid, 2-[4-(3,4-dihydro-1(2H)-quinolinyl)-6-(4-morpholinyl)	C22H24N8O2	432.48	A/B	-9.1
1227	C(=O)([C@H](NC(=O)OC)c1ccccc1)N1[C@@H](CCC1)c1[nH]c(cn1)c1cc2c(cc1)cc(cc2)c1ccc(cc1)c1[nH]c(nc1)[C@H	1240885-51-1	Carbamic acid, N-[(1R)-2-[(2S)-2-[5-[4-[7-[2-[(2S)-1-[(2R)-2-[(methoxycarbonyl)a	C50H48N8O6	856.97	A/B	-9.1
1228	C(=O)([C@@H](NC(=O)OC)CC)N1[C@@H](CCC1)c1[nH]c(cn1)[C@H]1CC[C@@H](CC1)c1ccc(cc1)c1[nH]c(nc1)[C@H]1N(	1240887-40-4	Carbamic acid, N-[2-[(2S)-2-[5-[4-[trans-4-[2-[(2S)-1-[2-[(ethoxycarbonyl)amino]	C38H52N8O6	716.87	A/B	-9.1
1229	C(=O)([C@H](NC(=O)OC)C1CC1)N1[C@@H](CCC1)c1[nH]c(cn1)[C@H]1CC[C@@H](CC1)c1ccc(cc1)c1[nH]c(nc1)[C@H]1	1240887-61-9	Carbamic acid, N-[2-[(2S)-2-[5-[4-[trans-4-[2-[(2S)-1-[2-[(methoxycarbonyl)aminc	C38H52N8O6	716.87	A/B	-9.1
1230	C(=O)N1[C@H]2[C@@H]1[C@@H](CO)O[C@H]2n1c(=O)nc(N)cc1	1241404-87-4	Carbamic acid, N,N,Ä≤-[[1,1,Ä≤-bicyclohexyl]-4,4,Ä≤-diylbis[1H-imidazole-5,2-di	C46H58N8O6	819	A/B	-9.1
1231	O=c1n([C@@H]2O[C@H](CO)[C@@H](O)C2)cc([Te])c(=O)[nH]1	1246559-92-1	Carbamic acid, N,N,Ä≤-[7,10,15,18-tetraoxatricyclo[17.3.1.12,6]tetracosa-1(23),2	C48H64N8O10	913.07	A/B	-9.1
1232	[C@@H](NC(=O)[C@@H](NC(=O)N1CCN(C)CC1)CC(C)C)(/C=C/S(=O)(=O)c1ccccc1)c1cc2c(cc1)cccc2	1253912-43-4	1-Piperazinecarboxamide, 4-methyl-N-[(1S)-3-methyl-1-[[[(1R,2E)-1-(1-naphthaleny	C31H38N4O4S	562.72	A/B	-9.1
1233	C(=O)([C@@H](NC(=O)OC)C(C)C)N1[C@@H](C[C@@H](C1)c1ccccc1)c1[nH]c(cn1)c1ccc(cc1)c1cc2c(cc1)cc(cc2)c1[	1256390-00-7	Carbamic acid, N-[(1S)-1-[[(2S)-2-[5-[6-[4-[2-[(2S,4S)-1-[(2S)-2-[(methoxycarbor	C50H56N8O6	865.03	A/B	-9.1
1234	C(OC(=O)c1ccccc1)[C@H]1O[C@H]([C@H]2[C@@H]1OC(C)(C)O2)n1nc(C(=O)N)nc1	1257423-89-4	1H-1,2,4-Triazole-3-carboxamide, 1-[2,3-O-(1-methylethylidene)-5-O-[3,4,5-tris(p	C39H38N4O9	706.74	A/B	-9.1
1235	C(=O)([C@@H](NC(=O)OC)C(C)C)N1CCC2(C[C@H]1c1[nH]c(cn1)c1ccc(cc1)c1ccc(cc1)c1[nH]c(nc1)[C@H]1N(C(=O)[C	1272655-44-3	Carbamic acid, N-[(1S)-1-[[(2S)-2-[5-[4,Ä≤-[2-[(2R,3R,8S)-7-[(2S)-2-[(methoxycar	C44H56N8O8	824.96	A/B	-9.1
1236	C(=O)([C@@H](NC(=O)OC)CC)N1[C@@H]([C@@H]2C[C@H]1CC2)c1[nH]c(cn1)c1ccc(cc1)c1ccc(cc1)c1[nH]c(nc1)[C@H	1273589-45-9	Carbamic acid, N-[(1S)-2-[(2S)-2-[5-[4,Ä≤-[2-[(1R,3S,4S)-2-[(2S)-2-[(methoxycarb	C38H44N8O6	708.81	A/B	-9.1
1237	O=C1[C@@H]2[C@@H](c3c([C@@H]([Se]c4ccc(C)cc4)[C@H]2CO1)cc1c(c3)OCO1)c1cc(OC)c(O)c(OC)c1	127708-96-7	Furo[3,Ä≤,4,Ä≤:6,7]naphtho[2,3-d]-1,3-dioxol-6(5aH)-one, 5,8,8a,9-tetrahydro-9-	C26H22O6	430.45	A/B	-9.1
1238	C(=O)([C@@H](NC(=O)OC)C(C)C)N1[C@@H](CC(=C)C1)c1[nH]c(cn1)c1cc2c(cc1)cc(cc2)c1ccc(cc1)c1[nH]c(nc1)[C	1306733-97-0	Carbamic acid, N-[(1S)-1-[[(2S,5R)-2-[5-[4-[6-[2-[(2S,5R)-1-[(2S)-2-[(methoxycar	C46H56N8O6	816.99	A/B	-9.1
1239	C(=O)([C@@H](NC(=O)OC)C1CCOCC1)N1[C@H]2[C@H](C2)C[C@H]1c1[nH]c(c(Cl)n1)c1ccc(cc1)c1ccc(cc1)c1[nH]c(n	1312336-39-2	Carbamic acid, N-[(1S)-2-[(1R,3S,5R)-3-[5-[4,Ä≤-[5-chloro-2-[(1R,3S,5R)-2-[(2S)-	C47H53ClF2N8O7	915.42	A/B	-9.1
1240	Cc1sc2c(ccc(c2n1)c1ccc(cc1)c1[nH]c(nc1)[C@H]1N(C(=O)[C@@H](NC(=O)OC)C(C)C)CCC1)c1[nH]c(nc1)[C@H]1N(C	1312608-56-2	Carbamic acid, N-[2-[(2S)-2-[5-[4-[4-[2-[(2S)-1-[2-[(methoxycarbonyl)amino]acety	C38H40N8O6	704.77	A/B	-9.1
1241	O(Cc1ccc(F)cc1)c1c2c(c(cc1)c1ccccc1)oc(c(O)c2=O)c1ncc(C)cn1	1312894-33-9	4H-1-Benzopyran-4-one, 5-[(4-fluorophenyl)methoxy]-3-hydroxy-8-phenyl-2-(2-pyrim	C26H17FN2O4	440.42	A/B	-9.1
1242	O(Cc1ccc(F)cc1)c1c2c(c(cc1)c1ccccc1)oc(c(O)c2=O)c1ncc(Cl)cn1	1312894-35-1	4H-1-Benzopyran-4-one, 5-[(4-fluorophenyl)methoxy]-3-hydroxy-2-(5-methoxy-2-pyri	C27H19FN2O5	470.45	A/B	-9.1
1243	O(Cc1ccc(F)cc1)c1c2c(c(cc1)c1ccccc1)oc(c(O)c2=O)c1[nH]c(cn1)c1ccccc1	1312894-51-1	4H-1-Benzopyran-4-one, 2-(5-chloro-1H-imidazol-2-yl)-5-[(4-fluorophenyl)methoxy]	C25H16ClFN2O4	462.86	A/B	-9.1
1244	O(Cc1ccc(F)cc1)c1c2c(c(c(C)c1)c1ccccc1)oc(c(O)c2=O)c1ncc(OC)cn1	1312896-84-6	4H-1-Benzopyran-4-one, 5-[(4-fluorophenyl)methoxy]-3-hydroxy-7-methyl-2-(5-methy	C28H21FN2O4	468.48	A/B	-9.1
1245	O(Cc1ccc(F)cc1)c1c2c(c(c(C)c1)c1ccccc1)oc(c(O)c2=O)c1ncc(cn1)c1ccccc1	1312896-86-8	4H-1-Benzopyran-4-one, 2-(5-chloro-2-pyrimidinyl)-5-[(4-fluorophenyl)methoxy]-3-	C27H18ClFN2O4	488.89	A/B	-9.1
1246	O(Cc1ccc(F)cc1)c1c2c(c(c(C)c1)c1ccccc1)oc(c(O)c2=O)c1[nH]cnn1	1312896-87-9	4H-1-Benzopyran-4-one, 5-[(4-fluorophenyl)methoxy]-3-hydroxy-7-methyl-8-phenyl-2	C33H23FN2O4	530.55	A/B	-9.1
1247	O(Cc1ccc(F)cc1)c1c2c(c(c(C)c1)c1ccccc1)oc(c(O)c2=O)c1[nH]c(nn1)c1ccccc1	1312896-96-0	4H-1-Benzopyran-4-one, 2-(5-chloro-1H-1,2,4-triazol-3-yl)-5-[(4-fluorophenyl)met	C25H17ClFN3O4	477.87	A/B	-9.1
1248	O(Cc1ccc(F)cc1)c1c2c(oc(c(O)c2=O)c2ncccn2)c(C)c(OC)c1	1312897-63-4	4H-1-Benzopyran-4-one, 5-[(4-fluorophenyl)methoxy]-3-hydroxy-7-methoxy-2-(5-pher	C26H19FN2O5	458.44	A/B	-9.1
1249	O(Cc1ccc(F)cc1)c1c2c(c(OC)c(OC)c1)oc(c(O)c2=O)c1ncccn1	1312898-13-7	4H-1-Benzopyran-4-one, 5-[(4-fluorophenyl)methoxy]-3-hydroxy-7-methoxy-8-methyl-	C27H21FN2O5	472.46	A/B	-9.1
1250	O(C)c1c(c2c(c(OCc3ccc(F)cc3)c1)c(=O)c(O)c(o2)c1ncc(OC)cn1)c1ccccc1	1312899-46-9	4H-1-Benzopyran-4-one, 5-[(4-fluorophenyl)methoxy]-3-hydroxy-7-methoxy-2-(5-meth	C28H21FN2O5	484.48	A/B	-9.1
1251	O(C)c1c(c2c(c(OCc3ccc(F)cc3)c1)c(=O)c(O)c(o2)c1ncc(cn1)c1ccccc1)c1ccccc1	1312899-48-1	4H-1-Benzopyran-4-one, 2-(5-chloro-2-pyrimidinyl)-5-[(4-fluorophenyl)methoxy]-3-	C27H18ClFN2O5	504.89	A/B	-9.1
1252	O(C)c1c(c2c(c(OCc3ccc(F)cc3)c1)c(=O)c(O)c(o2)c1[nH]c(nn1)c1ccccc1)c1ccccc1	1312899-58-3	4H-1-Benzopyran-4-one, 2-(5-chloro-1H-1,2,4-triazol-3-yl)-5-[(4-fluorophenyl)met	C25H17ClFN3O5	493.87	A/B	-9.1
1253	O(Cc1ccc(F)cc1)c1c2c(oc(c(O)c2=O)c2[nH]ccn2)c(Cl)c(Cl)c1	1312902-18-3	4H-1-Benzopyran-4-one, 7,8-dichloro-5-[(4-fluorophenyl)methoxy]-3-hydroxy-2-(3-p	C24H14Cl2FN3O4	498.29	A/B	-9.1
1254	O(Cc1ccc(F)cc1)c1c2c(oc(c(O)c2=O)c2ncc(C)cn2)cc(c1)c1ccccc1	1312903-04-0	4H-1-Benzopyran-4-one, 5-[(4-fluorophenyl)methoxy]-3-hydroxy-7-phenyl-2-(2-pyrim	C26H17FN2O4	440.42	A/B	-9.1
1255	O(Cc1ccc(F)cc1)c1c2c(oc(c(O)c2=O)c2[nH]cnn2)cc(c1)c1ccccc1	1312903-08-4	4H-1-Benzopyran-4-one, 5-[(4-fluorophenyl)methoxy]-3-hydroxy-7-phenyl-2-(5-pheny	C32H21FN2O4	516.52	A/B	-9.1
1256	O(Cc1ccc(F)cc1)c1c2c(oc(c(O)c2=O)c2[nH]c(nn2)c2ccccc2)cc(c1)c1ccccc1	1312903-17-5	4H-1-Benzopyran-4-one, 2-(5-chloro-1H-1,2,4-triazol-3-yl)-5-[(4-fluorophenyl)met	C24H15ClFN3O4	463.85	A/B	-9.1
1257	O(Cc1ccc(F)cc1)c1c2c(oc(c(O)c2=O)c2[nH]c(Cl)cn2)cc(c1)c1ccccc1	1312903-21-1	4H-1-Benzopyran-4-one, 5-[(4-fluorophenyl)methoxy]-3-hydroxy-2-(5-methoxy-1H-imi	C26H19FN2O5	458.44	A/B	-9.1
1258	Cc1c2c(c(OCc3ccc(F)cc3)cc1c1ccccc1)c(=O)c(O)c(o2)c1[nH]c(nn1)c1ccccc1	1312903-69-7	4H-1-Benzopyran-4-one, 2-(5-chloro-1H-1,2,4-triazol-3-yl)-5-[(4-fluorophenyl)met	C25H17ClFN3O4	477.87	A/B	-9.1
1259	O(C)c1c2c(c(OCc3ccc(F)cc3)cc1c1ccccc1)c(=O)c(O)c(o2)c1ncccn1	1312903-78-8	4H-1-Benzopyran-4-one, 5-[(4-fluorophenyl)methoxy]-3-hydroxy-8-methyl-7-phenyl-2	C32H23FN2O4	518.53	A/B	-9.1
1260	O(Cc1ccc(F)cc1)c1c2c(c(Cl)c(c1)c1ccccc1)oc(c(O)c2=O)c1[nH]c(OC)nn1	1312905-20-6	4H-1-Benzopyran-4-one, 8-chloro-5-[(4-fluorophenyl)methoxy]-3-hydroxy-2-(3-methy	C25H17ClFN3O4	477.87	A/B	-9.1
1261	Clc1c2c(c(OCc3ccc(F)cc3)cc1c1ccccc1)c(=O)c(O)c(o2)c1[nH]c(nn1)c1ccccc1	1312905-24-0	4H-1-Benzopyran-4-one, 8-chloro-2-(5-chloro-1H-1,2,4-triazol-3-yl)-5-[(4-fluorop	C24H14Cl2FN3O4	498.29	A/B	-9.1
1262	O(Cc1ccc(F)cc1)c1c2c(c(c(c1)c1ccccc1)c1ccccc1)oc(c(O)c2=O)c1[nH]c(nn1)c1ccccc1	1312905-79-5	4H-1-Benzopyran-4-one, 2-(5-chloro-1H-1,2,4-triazol-3-yl)-5-[(4-fluorophenyl)met	C30H19ClFN3O4	539.94	A/B	-9.1
1263	O=c1c2c(oc(c1O)c1[nH]c(nn1)c1ccccc1)c(Cl)cc(OCc1ccc(F)cc1)c2	1312907-77-9	4H-1-Benzopyran-4-one, 8-chloro-2-(5-chloro-1H-1,2,4-triazol-3-yl)-6-[(4-fluorop	C18H10Cl2FN3O4	422.19	A/B	-9.1
1264	O=c1c2c(c(cc(OCc3ccc(F)cc3)c2)c2ccccc2)oc(c1O)c1[nH]c(C)nn1	1312908-24-9	4H-1-Benzopyran-4-one, 6-[(4-fluorophenyl)methoxy]-3-hydroxy-8-phenyl-2-(1H-1,2,	C24H16FN3O4	429.4	A/B	-9.1
1265	O=c1c2c(c(cc(OCc3ccc(F)cc3)c2)c2ccccc2)oc(c1O)c1[nH]c(cn1)c1ccccc1	1312908-32-9	4H-1-Benzopyran-4-one, 2-(5-chloro-1H-imidazol-2-yl)-6-[(4-fluorophenyl)methoxy]	C25H16ClFN2O4	462.86	A/B	-9.1
1266	O=c1c2c(oc(c1O)c1ncccn1)cc(C)c(OCc1ccc(F)cc1)c2	1312908-33-0	4H-1-Benzopyran-4-one, 6-[(4-fluorophenyl)methoxy]-3-hydroxy-8-phenyl-2-(5-pheny	C31H21FN2O4	504.51	A/B	-9.1
1267	O(C)c1c2c(cc(OCc3ccc(F)cc3)c1C)c(=O)c(O)c(o2)c1[nH]ccn1	1312909-90-2	4H-1-Benzopyran-4-one, 6-[(4-fluorophenyl)methoxy]-3-hydroxy-8-methoxy-7-methyl-	C26H20FN3O5	473.45	A/B	-9.1
1268	O=c1c2c(oc(c1O)c1[nH]ccn1)cc(OC)c(OCc1ccc(F)cc1)c2	1312911-40-2	4H-1-Benzopyran-4-one, 6-[(4-fluorophenyl)methoxy]-3-hydroxy-7-methoxy-2-(3-pher	C25H18FN3O5	459.43	A/B	-9.1
1269	Cc1c2c(cc(OCc3ccc(F)cc3)c1Cl)c(=O)c(O)c(o2)c1ncccn1	1312912-48-3	4H-1-Benzopyran-4-one, 6-[(4-fluorophenyl)methoxy]-3-hydroxy-7-methoxy-8-methyl-	C27H21FN2O5	472.46	A/B	-9.1
1270	O(C)c1c2c(cc(OCc3ccc(F)cc3)c1Cl)c(=O)c(O)c(o2)c1ncccn1	1312913-48-6	4H-1-Benzopyran-4-one, 6-[(4-fluorophenyl)methoxy]-3-hydroxy-7,8-dimethoxy-2-(5-	C27H21FN2O6	488.46	A/B	-9.1
1271	Clc1c(c2c(cc1OCc1ccc(F)cc1)c(=O)c(O)c(o2)c1[nH]c(Cl)cn1)c1ccccc1	1312915-94-8	4H-1-Benzopyran-4-one, 7-chloro-6-[(4-fluorophenyl)methoxy]-3-hydroxy-2-(5-methc	C26H18ClFN2O5	492.88	A/B	-9.1
1272	[C@@H](NC(=O)c1ccccc1)(C(=O)N[C@H](C(=O)N[C@H](C(=O)C(=O)NCc1ccc(S(=O)(=O)N)cc1)C(C)C)C)C12CC3CC(C2)	1318775-18-6	L-Alaninamide, (2S)-N-benzoyl-2-tricyclo[3.3.1.13,7]dec-1-ylglycyl-N-[(1S)-1-(1-	C35H44N4O5	600.75	A/B	-9.1
1273	FC1(F)[C@@H](O[C@H](COP(=O)(Oc2c3c(ccc2)cccc3)N[C@H](C(=O)OC)C)[C@H]1O)n1c(=O)[nH]c(=O)c(I)c1	1321581-01-4	L-Alanine, N-(2,Ä≤-deoxy-2,Ä≤,2,Ä≤-difluoro-5-iodo-P-1-naphthalenyl-5,Ä≤-uridyl	C29H27F2IN3O9P	757.41	A/B	-9.1
1274	N(c1nc(nc(NN=Cc2ccc(NC(=O)C)cc2)n1)N1CCOCC1)c1ccccc1	1331749-46-2	1H-Indole-2,3-dione, 3-[2-[4-[[(3-chlorophenyl)methyl]amino]-6-(4-morpholinyl)-1	C22H21ClN8O2	464.91	A/B	-9.1
1275	N(S(=O)(=O)c1ccc(N)cc1)(C[C@H]([C@H](Cc1ccccc1)NC(=O)O[C@H]1COC1)O)CC(C)(C)C	1332605-79-4	Not Yet Assigned	C35H45N5O4	599.76	A/B	-9.1
1276	C(CC#C)(N)C12CC3CC(C2)CC(C1)C3	133947-44-1	Guanosine, 2,Ä≤-deoxy-N-[9-(2-deoxy-Œ≤-D-erythro-pentofuranosyl)-6,9-dihydro-6-	C20H23N9O8	517.45	A/B	-9.1
1277	[C@H]([C@H](Cc1ccccc1)NC(=O)O[C@H]1CCOC1)(CN(S(=O)(=O)c1ccc(N)cc1)CC(C)C)OC(=O)COC	1346923-13-4	Benzenepropanamide, N-[[4,Ä≤-[(2-ethyl-5,7-dimethyl-3H-imidazo[4,5-b]pyridin-3-y	C37H41N5O3S	635.82	A/B	-9.1
1278	C(P(=O)(OCCCC)OCCCC)(NN(C(=O)c1ccccc1)c1ccc(N(=O)=O)cc1)(C(=O)OCC)C(=O)OCC	1349891-52-6	Not Yet Assigned	C40H47N5O5	677.83	A/B	-9.1
1279	C(=O)([C@@H](NC(=O)OC)C(C)C)N1[C@H](c2[nH]c(c3c4c(c(cc3)c3c5c(c(cc3)c3[nH]c(nc3)[C@H]3N(C(=O)[C@@H](	1350718-51-2	Carbamic acid, N,N,Ä≤-[[4,4,Ä≤-bi-1H-indene]-7,7,Ä≤-diylbis[1H-imidazole-5,2-di	C46H58N8O6	819	A/B	-9.1
1280	C(=O)([C@@H](NC(=O)OC)C(C)C)N1[C@@]2(C[C@H](C1)CC2)c1[nH]c(cn1)c1c2c(c(cc1)c1c3c(c(cc1)c1[nH]c(nc1)[	1350718-87-4	Carbamic acid, N-[(1S)-1-[[(1S,4R)-1-[5-[4-[7-[2-[(1S,4R)-2-[(2S)-2-[(methoxycar	C47H56N8O6	829	A/B	-9.1
1281	C(=O)([C@@H](NC(=O)OC)C(C)C)N1[C@H](c2[nH]c(c3c4c(c(cc3)c3c5c(c(cc3)c3[nH]c(nc3)[C@H]3N(C(=O)[C@@H](	1350719-08-2	Carbamic acid, N,N,Ä≤-[(2,2,Ä≤,3,Ä≤,3,Ä≤-tetrahydro[4,4,Ä≤-bi-1H-indene]-7,7,Ä≤	C50H62N8O6	871.08	A/B	-9.1
1282	C(=O)([C@H](Cc1ccccc1)NC(=O)OC)N1[C@@H](C[C@@H](C1)C1CC1)c1[nH]c(cn1)c1ccc(cc1)c1ccc(cc1)c1[nH]c(nc1	1351854-42-6	Carbamic acid, N-[(1R)-2-[(2S)-2-[5-[4,Ä≤-[2-[(2S,4R)-4-cyclopropyl-1-[(2R)-2-[	C51H54N8O6	875.02	A/B	-9.1
1283	C(=O)([C@@H](NC(=O)OC)C(C)C)N1[C@@H](C[C@]2(C1)CCCOC2)c1[nH]c(cn1)c1ccc(cc1)c1ccc(cc1)c1[nH]c(nc1)[C	1351856-03-5	Carbamic acid, N-[(1S)-1-[[(2S,4R)-4-cyclopropyl-2-[5-[4,Ä≤-[2-[(5R,8S)-7-[(2S)-	C45H55N9O8	849.97	A/B	-9.1
1284	C(=O)([C@@H](NC(=O)OC)C(C)C)N1[C@@H](C=C(C)C1)c1[nH]c(cn1)c1cc2c(cc1)cc(cc2)c1ccc(cc1)c1[nH]c(nc1)[C	1352391-95-7	Carbamic acid, N-[(1S)-1-[[(2S)-2-[5-[4-[6-[2-[(2S)-2,5-dihydro-1-[(2S)-2-[(meth	C45H52N8O6	800.94	A/B	-9.1
1285	C(F)(F)c1n(c2c(n1)cccc2)c1nc(nc(Nc2cc(O)ccc2)n1)N1CCOCC1	1359956-20-9	Phenol, 4-[[4-[2-(difluoromethyl)-1H-benzimidazol-1-yl]-6-(4-morpholinyl)-1,3,5-	C21H19F2N7O2	439.42	A/B	-9.1
1286	C(=O)([C@@H](NC(=O)OC)C(C)C)N1[C@@H]([C@@H]2C[C@H]1CC2)c1[nH]c2c(n1)ccc1c2ccc(c1)c1cc2c(cc1)c1c(cc2)	1377036-50-4	Carbamic acid, N-[(1S)-2-[(1R,3S,4S)-3-[5-[4-[2-[(2S)-1-[(2S)-2-[(methoxycarbony	C46H54N8O7	830.97	A/B	-9.1

1287	C(=O)([C@@H](NC(=O)OC)C(C)C)N1[C@H](c2[nH]c3c(c4c(c5c3ccc(c5)c3ccc(cc3)c3[nH]c(nc3)[C@H]3N(C(=O)[C@@	1377041-15-0	Carbamic acid, N-[(1S)-2-[(2S)-2-[2,Ä≤-[(1R,3S,4S)-2-[(2S)-2-[(methoxycarbonyl)a	C48H54N8O7	854.99	A/B	-9.1
1288	C(=O)([C@@H](NC(=O)OC)C(C)C)N1[C@@H]([C@@H]2C[C@H]1CC2)c1[nH]c(cn1)c1cc2c(cc1)c1c(cc2)cc(cc1)c1[nH]c	1377041-23-0	Carbamic acid, N-[(1S)-2-[(2S)-2-[7-[3-fluoro-4-[2-[(1R,3S,4S)-2-[(2S)-2-[(metho	C46H53FN8O7	848.96	A/B	-9.1
1289	C(=O)([C@@H](NC(=O)OC)C(C)C)N1[C@@H]([C@H]2C[C@@H]1CC2)c1[nH]c2c(n1)ccc1c2ccc(c1)c1cc2c(cc1)c1c(cc2)	1377041-96-7	Carbamic acid, N-[(1S)-2-[(1R,3S,4S)-3-[5-[4-[2-[(1R,3S,5R)-2-[(2S)-2-[(methoxyc	C47H54N8O7	842.98	A/B	-9.1
1290	C(=O)([C@@H](NC(=O)OC)C(C)C)N1[C@H]2[C@H](C2)C[C@H]1c1[nH]c(c(Cl)n1)c1ccc(cc1)c1cc2c(cc1)c1c(cc2)nc(	1377043-22-5	Carbamic acid, N-[(1S)-1-[[(1R,3S,5R)-3-[7-[4-[5-chloro-2-[(1R,3S,4S)-2-[(2S)-2-	C45H51ClN8O6	835.39	A/B	-9.1
1291	C(=O)([C@@H](NC(=O)OC)C(C)C)N1[C@@H](CCC1)c1[nH]c(cn1)c1ccc(C#CC#CC#Cc2[nH]c(nc2)[C@H]2N(C(=O)[C@@H]	1380293-14-0	Carbamic acid, N,N,Ä≤-[(trans,trans)-[1,1,Ä≤-bicyclohexyl]-4,4,Ä≤-diylbis[1H-im	C40H62N8O6	750.97	A/B	-9.1
1292	O[C@H]1[C@H](n2c3c(nc2)c(=O)nc(N)[nH]3)O[C@H](COP(=O)(OP(=O)(CP(=O)(O)O)O)O)[C@H]1O	1391194-42-5	Not Yet Assigned	C20H21FN6O5		A/B	-9.1
1293	C(=O)(NC)N1CCN(c2nc(nc(n2)N2CCOCC2)c2c3c(ccc2)[nH]nc3)CC1	1391925-75-9	4-Piperidinone, 1-[4-(1H-indazol-4-yl)-6-(4-morpholinyl)-1,3,5-triazin-2-yl]-, c	C19H22N8O2	394.43	A/B	-9.1
1294	O=C1CN(c2nc(nc(n2)N2CCOCC2)c2c3c(ccc2)[nH]nc3)CCN1	1391925-96-4	1H-Indazole, 4-[4-(4-morpholinyl)-6-(1-piperazinyl)-1,3,5-triazin-2-yl]-	C18H22N8O	366.42	A/B	-9.1
1295	Nc1ncc(c2nc(nc(n2)N2CCOCC2)N2CCN(C)CC2)cn1	1391926-27-4	2-Pyridinamine, 5-[4-(4-methyl-1-piperazinyl)-6-(4-morpholinyl)-1,3,5-triazin-2-	C18H23F3N8O	424.42	A/B	-9.1
1296	O=C1c2n(C([C@H]3N1[C@H](C)C(C(O3)([2H])[2H])([2H])[2H])([2H])[2H])cc(C(=O)NCc1c(F)cc(F)cc1)c(=O)c2O	1407167-34-3	2H-Pyrido[1,Ä≤,2,Ä≤:4,5]pyrazino[2,1-b][1,3]oxazine-2,3,4,12-d4-9-carboxamide,	C20H12D7F2N3O5		A/B	-9.1
1297	O=C1c2n(C[C@]3(N1[C@H](C([2H])([2H])[2H])C(C(O3)([2H])[2H])([2H])[2H])[2H])cc(C(=O)NCc1c(F)cc(F)cc1)	1407167-43-4	2H-Pyrido[1,Ä≤,2,Ä≤:4,5]pyrazino[2,1-b][1,3]oxazine-2,3,4-d3-9-carboxamide, N-[	C20H10D9F2N3O5		A/B	-9.1
1298	C(=O)([C@@H](NC(=O)OC)C(C)C)N1[C@@H](CCC1)c1[nH]c(cn1)c1ccc(cc1)c1c2CCc3cc(c(CCc(c1)cc2)cc3)c1ccc(cc	1415120-43-2	Carbamic acid, N-[tricyclo[8.2.2.24,7]hexadeca-4,6,10,12,13,15-hexaene-5,11-diyl	C48H56N8O6	841.01	A/B	-9.1
1299	C(=O)([C@H](NC(=O)OC)C(C)C)N1[C@H](CCC1)c1[nH]c(cn1)c1ccc(cc1)c1ccc(cc1)c1[nH]c(nc1)[C@@H]1N(C(=O)[C	1417333-58-4	Carbamic acid, N,N,Ä≤-[[1,1,Ä≤-biphenyl]-4,4,Ä≤-diylbis[1H-imidazole-5,2-diyl-(	C40H50N8O6	738.88	A/B	-9.1
1300	C(OCCn1c2c(nc1)c(N)ncn2)[P@]1(=O)O[C@@H](C[C@H](O1)c1ccccc1)c1cc(Cl)ccc1	1417393-24-8	9H-Purin-6-amine, 9-[2-[[(2R,4R,6R)-4-(3-chlorophenyl)-2-oxido-6-phenyl-1,3,2-di	C23H23ClN5O4P	499.89	A/B	-9.1
1301	C(=O)([C@@H](NC(=O)OC)C(C)C)N1[C@H](c2[nH]c(c3c4c(c(cc3)c3ccc(cc3)c3[nH]c(nc3)[C@H]3N(C(=O)[C@@H](NC	1419384-91-0	Carbamic acid, N-[(1S)-1-[[(2S)-2-[5-[1,Ä≤,3,Ä≤-dihydro-7,Ä≤-[4-[2-[(2S)-1-[(2S	C47H60N8O6	833.03	A/B	-9.1
1302	C(=O)([C@@H](NC(=O)OC)C1CCCCC1)N1[C@H](c2[nH]c(c3c4c(c(cc3)c3ccc(cc3)c3[nH]c(nc3)[C@H]3N(C(=O)[C@@H]	1419385-03-7	Carbamic acid, N-[(1S)-1-[[(2S,4S)-2-[5-[1,Ä≤,3,Ä≤-dihydro-7,Ä≤-[4-[2-[(2S,4S)-	C49H64N8O6	861.08	A/B	-9.1
1303	C(Nc1nc(NCCNC(=O)C=C)nc(NCC#C)n1)(C)C12CC3CC(C2)CC(C1)C3	1422258-95-4	Benzamide, 2-chloro-5-nitro-N-[2-[[4-(2-propyn-1-ylamino)-6-[(1-tricyclo[3.3.1.1	C27H33ClN8O3	553.06	A/B	-9.1
1304	C(C(C)C)n1c2c3c(cc(C(=O)OC)cc3)nc(N)c2nc1	1426342-68-8	Benzoic acid, 2,2,Ä≤-[2,7-naphthalenediylbis(carbonylimino)]bis-	C26H18N2O6	454.43	A/B	-9.1
1305	C(N1c2c(C(=NNC(=S)N(CC=C)CC=C)C1=O)cc(F)cc2)N1CCN(CC1)c1ccccc1	1426583-63-2	Hydrazinecarbothioamide, 2-[5-chloro-1,2-dihydro-2-oxo-1-[(4-phenyl-1-piperaziny	C32H29ClN6OS	581.13	A/B	-9.1
1306	C(N1c2c(C(=NNC(=S)N(CC=C)CC=C)C1=O)cc(C)cc2)N1CCN(CC1)c1ccccc1	1426583-65-4	Hydrazinecarbothioamide, 2-[5-fluoro-1,2-dihydro-2-oxo-1-[(4-phenyl-1-piperaziny	C32H29FN6OS	564.68	A/B	-9.1
1307	N(=C1c2c(N(CN3CCN(CC3)c3ccc(OC)cc3)C1=O)ccc(Cl)c2)NC(=S)N(CC=C)CC=C	1426583-73-4	Hydrazinecarbothioamide, 2-[1-[[4-(4-chlorophenyl)-1-piperazinyl]methyl]-1,2-dih	C33H31ClN6OS	595.16	A/B	-9.1
1308	C(C(=O)NC(=N)N[C@@H]1[C@@H](NC(=O)C)[C@H]([C@@H]([C@@H](CO)O)O)OC(=C1)C(=O)O)C12CC3CC(C2)CC(C1)C3	1442563-11-2	D-glycero-D-galacto-Non-2-enonic acid, 5-(acetylamino)-2,6-anhydro-3,4,5-trideox	C24H28N4O8S	532.57	A/B	-9.1
1309	O=c1n([C@@H]2O[C@H](CO[P@@](=O)(Oc3ccccc3)NCCNC(=O)CC/C=C\C/C=C\C/C=C\C/C=C\C/C=C\C/C=C\CC)[C@@H](N=	1443511-06-5	L-Alanine, N-[(phenylmethoxy)carbonyl]-3-[[(phenylmethoxy)carbonyl]amino]-, (5R,	C41H40N2O13	768.76	A/B	-9.1
1310	N(Cc1ccc(C(F)(F)F)cc1)(c1nc(nc(C#N)n1)N1CCOCC1)C1CCCCC1	1443830-70-3	1,3,5-Triazine-2-carbonitrile, 4-[[(4-chlorophenyl)methyl]cyclohexylamino]-6-(4-	C21H25ClN6O	412.92	A/B	-9.1
1311	Ic1n(c2c(n1)c(=O)nc(N)[nH]2)[C@H]1[C@H](O)[C@H]2[C@H](O1)COP(=O)(O)O2	144510-03-2	Guanosine, 8-[(4-nitrophenyl)thio]-, cyclic 3,Ä≤,5,Ä≤-(hydrogen phosphate)	C16H15N6O9PS	498.36	A/B	-9.1
1312	C(OCCOC(=O)[C@@H](N(C(=O)OCc1ccccc1)C)C(C)C)n1c2c(nc1)c(=O)nc(N)[nH]2	1447584-14-6	L-Valine, 3,Ä≤-ester with 5,Ä≤-O-[(2R,4S)-4-(3-chlorophenyl)-2-oxido-1,3,2-diox	C25H32ClN6O9P	626.98	A/B	-9.1
1313	O=C1[C@@H]2[C@@H](c3c([C@H](OC(=O)c4ccc(N(=O)=O)cc4)[C@H]2CO1)cc1c(c3)OCO1)c1c(Br)c(OC)c(OC)c(OC)c1	1449602-10-1	Furo[3,Ä≤,4,Ä≤:6,7]naphtho[2,3-d]-1,3-dioxol-6(5aH)-one, 5-(2-bromo-3,4,5-trime	C29H24BrNO11	642.41	A/B	-9.1
1314	O(C(=O)OCc1ccccc1)[C@H]1[C@@H](O[C@H](CO[P@@](=O)(OCc2ccccc2)N2CCCCC2)[C@H]1OC(=O)OCc1ccccc1)n1nc(C(	1451063-80-1	1H-1,2,4-Triazole-3-carboxamide, 1-[2,3-bis-O-[(phenylmethoxy)carbonyl]-5-O-[(R)	C36H40N5O11P	749.7	A/B	-9.1
1315	N(c1nc(nc(NCCCNc2c3c(cc(Cl)cc3)ncc2)n1)N1CCOCC1)c1ccccc1	1454694-96-2	1,3,5-Triazine-2,4-diamine, N2-[3-[(7-chloro-4-quinolinyl)amino]propyl]-N4-(4-et	C27H31ClN8O	519.04	A/B	-9.1
1316	N(c1nc(nc(NCCCCOc2nc(nc(Nc3ccccc3)n2)N2CCOCC2)n1)N1CCOCC1)c1ccccc1	1463435-49-5	1,3,5-Triazine-2,4-diamine, 6-(4-morpholinyl)-N2-[5-[[4-(4-morpholinyl)-6-(pheny	C31H39N11O3	613.71	A/B	-9.1
1317	O=c1c2c(n(cn2)[13C@@H]2O[C@H](CO)[C@@H](O)[C@H]2O)[nH]c(N)n1	146849-74-3	Phosphoric acid, 4-[(5R,5aR,8aR,9S)-5,5a,6,8,8a,9-hexahydro-9-hydroxy-6-oxofuro[	C35H33O11P	660.6	A/B	-9.1
1318	[C@H]([C@@H](COC(=O)C)OC(=O)C)(OC(=O)C)[C@H]1[C@H](NC(=O)C)[C@@H](N=C(NC(=O)[C@@H](C)c2ccc(CC(C)C)cc	1469338-36-0	D-glycero-D-galacto-Non-2-enonic acid, 5-(acetylamino)-2,6-anhydro-3,4,5-trideox	C26H32N4O9	544.55	A/B	-9.1
1319	C(OC[C@@H]1[C@@H](OC(=O)c2ccccc2)[C@@H](OP(=O)(OC[C@@H]2[C@H]3[C@H](O3)[C@@H](O2)n2c3c(nc2)c(NC(=O)c	1469875-51-1	Œ≤-D-lyxo-Adenosine, N-benzoyl-3,Ä≤-O-benzoyl-5,Ä≤-O-[(4-methoxyphenyl)diphenyl	C92H80N16O25P2	1871.66	A/B	-9.1
1320	O=C1N(c2c3c(c4c(n2)cccc4)n(CC(C)C)cn3)c2c1cc(Br)cc2	1508303-87-4	7-Azabicyclo[4.2.0]octa-1,3,5-trien-8-one, 7-[1-(2-methylpropyl)-1H-imidazo[4,5-	C21H18N4O	342.39	A/B	-9.1
1321	C(C(C)C)n1c2c(c(Nc3c(C(=O)O)cc(I)cc3)nc3c2cccc3)nc1	1508303-97-6	Benzoic acid, 5-chloro-2-[[1-(2-methylpropyl)-1H-imidazo[4,5-c]quinolin-4-yl]ami	C21H19ClN4O2	394.85	A/B	-9.1
1322	P(=O)(Nc1nc(=O)n(cc1)[C@H]1O[C@@H](CO)SC1)(N[C@H](C(=O)OCc1ccccc1)C)N[C@H](C(=O)OCc1ccccc1)C	1526921-97-0	Not Yet Assigned	C48H57N7O13P2S	1034.02	A/B	-9.1
1323	N(c1c2c(n(cn2)[C@@H]2C[C@H](COP(=O)(N[C@H](C(=O)OCC(C)(C)C)C)N[C@H](C(=O)OCC(C)(C)C)C)C=C2)nc(N)n1)C	1526922-09-7	8-Oxa-3,5-diaza-4-phosphanonanoic acid, 4-[[(1S,4R)-4-[2-amino-6-(cyclopropylami	C34H41N8O6P	688.71	A/B	-9.1
1324	C(=O)(NCc1ccc(F)cc1)c1c(OC(OC(=O)OC2CCCCC2)C)c(=O)n(C)c(C(NC(=O)c2oc(C)nn2)(C)C)n1	1538603-91-6	Carbonic acid, 1-[[4-[[[(4-fluorophenyl)methyl]amino]carbonyl]-1,6-dihydro-1-met	C26H31FN6O8	574.56	A/B	-9.1
1325	C(=O)(NCc1ccc(F)cc1)c1c(OC(OC(=O)c2ccccc2)C)c(=O)n(C)c(C(NC(=O)c2oc(C)nn2)(C)C)n1	1538604-21-5	Cyclopropanecarboxylic acid, 1-[[4-[[[(4-fluorophenyl)methyl]amino]carbonyl]-1,6	C26H29FN6O7	556.54	A/B	-9.1
1326	C(n1c2c(nc1CC)c(C)cc(C)n2)c1ccc(cc1)c1c(S(=O)(=O)NC(=O)CCCCC)ccc(CC)c1	154551-72-1	Hexanamide, N-[[4,Ä≤-[(2-ethyl-5,7-dimethyl-3H-imidazo[4,5-b]pyridin-3-yl)methyl	C30H36N4O3S	532.7	A/B	-9.1
1327	C(n1c2c(nc1CC)c(C)cc(C)n2)c1ccc(cc1)c1c(S(=O)(=O)NC(=O)NCCCC)ccc(CC)c1	154552-32-6	[1,1,Ä≤-Biphenyl]-2-sulfonamide, N-[(butylamino)carbonyl]-4,Ä≤-[(2-ethyl-5,7-di	C29H35N5O3S	533.68	A/B	-9.1
1328	C(n1c2c(nc1CC)c(C)cc(C)n2)c1ccc(cc1)c1c(S(=O)(=O)NC(=O)CCc2ccccc2)ccc(CCC)c1	154552-52-0	Benzenepropanamide, N-[[5-ethyl-4,Ä≤-[(2-ethyl-5,7-dimethyl-3H-imidazo[4,5-b]pyr	C34H36N4O3S	580.74	A/B	-9.1
1329	C(n1c2c(nc1CC)c(C)cc(C)n2)c1ccc(cc1)c1c(S(=O)(=O)NC(=O)CCc2ccccc2)ccc(CCCC)c1	154552-53-1	Benzenepropanamide, N-[[4,Ä≤-[(2-ethyl-5,7-dimethyl-3H-imidazo[4,5-b]pyridin-3-y	C35H38N4O3S	594.77	A/B	-9.1
1330	C(n1c2c(nc1CC)c(C)cc(C)n2)c1ccc(cc1)c1c(S(=O)(=O)NC(=O)CCc2ccccc2)ccc(COC)c1	154552-59-7	Benzenepropanamide, N-[[4,Ä≤-[(2-ethyl-5,7-dimethyl-3H-imidazo[4,5-b]pyridin-3-y	C34H36N4O3S2	612.8	A/B	-9.1
1331	C(n1c2c(nc1CC)c(C)cc(C)n2)c1ccc(cc1)c1c(S(=O)(=O)NC(=O)CCc2ccccc2)ccc(CN2CCOCC2)c1	154552-66-6	Benzenepropanamide, N-[[5-[(diethylamino)methyl]-4,Ä≤-[(2-ethyl-5,7-dimethyl-3H-	C37H43N5O3S	637.83	A/B	-9.1
1332	C(n1c2c(nc1CC)c(C)cc(C)n2)c1ccc(cc1)c1c(S(=O)(=O)NC(=O)c2c(OCC)cccc2)ccc(CC(C)C)c1	154552-96-2	Benzamide, N-[[5-butoxy-4,Ä≤-[(2-ethyl-5,7-dimethyl-3H-imidazo[4,5-b]pyridin-3-y	C36H40N4O5S	640.79	A/B	-9.1
1333	C(n1c2c(nc1CC)c(C)cc(C)n2)c1ccc(cc1)c1c(S(=O)(=O)NC(=O)NCCc2ccccc2)ccc(CC)c1	154553-50-1	[1,1,Ä≤-Biphenyl]-2-sulfonamide, 4,Ä≤-[(2-ethyl-5,7-dimethyl-3H-imidazo[4,5-b]p	C33H35N5O3S	581.73	A/B	-9.1
1334	C(n1c2c(nc1CC)c(C)cc(C)n2)c1ccc(cc1)c1c(S(=O)(=O)NC(=O)NCCc2ccccc2)ccc(OCC(F)(F)F)c1	154553-65-8	[1,1,Ä≤-Biphenyl]-2-sulfonamide, 5-[(diethylamino)methyl]-4,Ä≤-[(2-ethyl-5,7-di	C37H44N6O3S	652.85	A/B	-9.1
1335	S(c1nc(nc(Nc2ccc(N(C)C)cc2)n1)N1CCOCC1)c1oc(nn1)c1ccc(Cl)cc1	1547295-48-6	1,3,5-Triazin-2-amine, 4-[[5-(4-chlorophenyl)-1,3,4-oxadiazol-2-yl]thio]-N-(3-me	C22H20ClN7O2S	481.96	A/B	-9.1
1336	C(=O)([C@@H](NC(=O)OC)C(C)C)N1CC2(C[C@H]1c1[nH]c(cn1)c1ccc(cc1)c1ccc(cc1)c1[nH]c(nc1)[C@H]1N(C(=O)[C	155487-46-0	Guanosine, 2,Ä≤-deoxy-P-thioguanylyl-(3,Ä≤,Üí5,Ä≤)-2,Ä≤-deoxy-	C20H25N10O9PS	612.51	A/B	-9.1
1337	CC12c3c(C(C1)CC2)c(ccc3c1[nH]c(nc1)[C@H]1N(C(=O)[C@@H](NC(=O)OC)C(C)C)CCC1)c1ccc(cc1)c1[nH]c(nc1)[C@	1560639-28-2	Carbamic acid, N-[(1S)-2-methyl-1-[[(2S)-2-[5-[1,2,3,4-tetrahydro-8-[4-[2-[(8S)-	C47H58N8O8	863.01	A/B	-9.1
1338	C(=O)([C@@H](NC(=O)OC)C(C)C)N1[C@H](c2[nH]c(c3c4c(c(cc3)c3ccc(cc3)c3[nH]c(nc3)[C@H]3N(C(=O)[C@@H](NC	1560639-90-8	Carbamic acid, N,N,Ä≤-[(1,2,3,4-tetrahydro-1,4-ethanonaphthalene-5,8-diyl)bis[4,	C52H62N8O6	895.1	A/B	-9.1
1339	C(=O)([C@@H](NC(=O)OC)C(C)C)N1[C@H](c2[nH]c(c3ccc(c4c5c(c(cc4)c4ccc(cc4)c4[nH]c(nc4)[C@H]4N(C(=O)[C@	1560640-47-2	Carbamic acid, N-[(1S)-2-methyl-1-[[(2S)-2-[5-[1,2,3,4-tetrahydro-8-[4-[2-[(8S)-	C48H60N8O8	877.04	A/B	-9.1
1340	CC1C2c3c(C1CC2)c(ccc3c1[nH]c(nc1)[C@H]1N(C(=O)[C@@H](NC(=O)OC)C(C)C)CCC1)c1ccc(cc1)c1[nH]c(nc1)[C@H]	1561048-18-7	Carbamic acid, N-[(1S)-2-methyl-1-[[(2S)-2-[5-[1,2,3,4-tetrahydro-8-[4-[2-[(3S)-	C46H56N8O6	816.99	A/B	-9.1
1341	N(c1c2c(c(O)c(CN3CCN(CCCCC)CC3)c1)CCCC2)c1c2c(ncc1)cc(Cl)cc2	156893-88-8	1-Naphthalenol, 4-[(7-chloro-4-quinolinyl)amino]-5,6,7,8-tetrahydro-2-[[4-(1-met	C28H35ClN4O	479.06	A/B	-9.1
1342	C(=O)([C@@H](NC(=O)OC)C(C)C)N1[C@H](c2[nH]c(c3c4c(C5(CC4)CCCC5)c(cc3)c3ccc(cc3)c3[nH]c(nc3)[C@H]3N(C	1588403-96-6	Carbamic acid, N-[(1S)-1-cyclohexyl-2-[(2S)-2-[5-[4-[4,Ä≤-[2-[(2S)-1-[(2S)-2-cyc	C53H68N8O6	913.16	A/B	-9.1
1343	C(=O)([C@@H](NC(=O)OC)C(C)C)N1[C@H](c2[nH]c(c3c4c(C5(CC4)CCCC5)c(cc3)c3ccc(cc3)c3[nH]c(nc3)[C@H]3N(C	1588403-99-9	Carbamic acid, N-[(1S)-1-[[(2S)-2-[5-[2,Ä≤,3,Ä≤-dihydro-7,Ä≤-[4-[2-[(2S)-1-[(2S	C51H68N8O6	889.14	A/B	-9.1
1344	C(OCCOP(=O)(OCOC(=O)OC(CC)C)OCOC(=O)OC(CC)C)n1c2c(nc1)c(=O)nc(N)[nH]2	1610585-44-8	2,4,6,8-Tetraoxa-5-phosphanonanedioic acid, 5-[2-[(2-amino-1,6-dihydro-6-oxo-9H-	C18H28N5O12P	537.41	A/B	-9.1
1345	O=C1CC2c3c(c(ccc3c3ccc(cc3)c3[nH]c(nc3)[C@H]3N(C(=O)[C@@H](NC(=O)OC)C(C)C)CCC3)c3[nH]c(nc3)[C@H]3N(C	1613055-52-9	Carbamic acid, N-[(1S)-1-[[(2S)-2-[5-[3-[4-[2-[(2S)-1-[(2S)-2-[[(1,1-dimethyleth	C51H68N8O6	889.14	A/B	-9.1
1346	O=C1N2[C@@H](Cn3c1c(O)c(=O)c(C(=O)NCc1c(F)cc(F)cc1)c3)O[C@H]1C[C@@H]2COC1	1616340-16-9	2,6-Methanopyrido[1,Ä≤,2,Ä≤:4,5]pyrazino[2,1-b][1,6,3]dioxazocine-11-carboxamide	C21H19F2N3O6	447.39	A/B	-9.1
1347	O=c1c2c(n(cn2)[C@@H]2O[C@H](COP(=O)(Oc3ccc(Cl)cc3)N3CCN(CC3)c3ccc(Cl)cc3)CC2)[nH]cn1	1618656-26-0	Inosine, 2,Ä≤,3,Ä≤-dideoxy-, 5,Ä≤-[4-chlorophenyl P-[4-(4-nitrophenyl)-1-pipera	C26H27ClN7O7P	615.96	A/B	-9.1
1348	N(C(=O)[C@H](Cc1cccnc1)C[C@@H](CN1[C@H](C(=O)NC(C)(C)C)CN(Cc2c(F)cccc2F)CC1)O)[C@H]1c2c(C[C@H]1O)ccc	162105-24-0	1-Piperazinepentanamide, N-(2,3-dihydro-2-hydroxy-1H-inden-1-yl)-2-[[(1,1-dimeth	C35H46N6O4	614.78	A/B	-9.1
1349	N(C(=O)[C@H](Cc1ccncc1)C[C@@H](CN1[C@H](C(=O)NC(C)(C)C)CN(Cc2cc3c(s2)scc3)CC1)O)[C@H]1c2c(C[C@H]1O)c	162105-32-0	1-Piperazinepentanamide, N-(2,3-dihydro-2-hydroxy-1H-inden-1-yl)-2-[[(1,1-dimeth	C36H45N5O4S2	675.9	A/B	-9.1
1350	O[C@H]1[C@H](O[C@@H](CO)[C@@H]1O)n1c(=O)[nH]c(=O)c(I)c1	1622332-38-0	D-erythro-Pentonamide, 2,3,5-trideoxy-N-[(1S,2R)-2,3-dihydro-2-hydroxy-1H-inden-	C36H47N5O4.xClH		A/B	-9.1
1351	C[C@]1(O)[C@H](n2c3c(nc2)c(=O)nc(N)[nH]3)O[C@H](COP(=O)(N[C@H](C(=O)OCC)C)SCC)[C@H]1O	162679-84-7	Butanediamide, N1-[3-[3-[[(1,1-dimethylethyl)amino]carbonyl]octahydro-2(1H)-isoq	C38H50N6O5	670.84	A/B	-9.1
1352	O=C1C2c3c(c(ccc3c3c4c(c(cc3)c3[nH]c(nc3)[C@H]3N(C(=O)[C@@H](NC(=O)OC)C(C)C)CCC3)C3CC4CC3)c3[nH]c(nc3	1627533-41-8	Carbamic acid, N,N,Ä≤-[(1,1,Ä≤,2,2,Ä≤,3,3,Ä≤,4,4,Ä≤-octahydro-9-methyl[5,5,Ä≤-b	C51H64N8O6	885.1	A/B	-9.1
1353	CN1C2c3c(c(ccc3c3c4c(c(cc3)c3[nH]c(nc3)[C@H]3N(C(=O)[C@@H](NC(=O)OC)C(C)C)CCC3)C3CC4CC3)c3[nH]c(nc3)	1627533-93-0	Carbamic acid, N,N,Ä≤-[(1,1,Ä≤,2,2,Ä≤,3,3,Ä≤,4,4,Ä≤-octahydro-1-methyl[5,5,Ä≤-b	C55H72N8O6	941.21	A/B	-9.1
1354	C(=O)([C@H](NC(=O)OC)c1ccccc1)N1[C@@H](C=CC1)c1[nH]c(cn1)c1ccc(cc1)c1ccc(cc1)c1[nH]c(nc1)[C@H]1N(C(=	1639870-96-4	Carbamic acid, N-[(1R)-2-[(2S)-2,5-dihydro-2-[5-[4,Ä≤-[2-[(2S)-1-[(2R)-2-[(metho	C46H44N8O6	804.89	A/B	-9.1
1355	S(c1nc(nc(Nc2ccc(OC)cc2)n1)N1CCOCC1)c1oc(nn1)c1ccc(F)cc1	1642584-21-1	1,3,5-Triazin-2-amine, 4-[[5-(4-fluorophenyl)-1,3,4-oxadiazol-2-yl]thio]-6-(4-mo	C21H17FN8O4S	496.47	A/B	-9.1
1356	N(c1nc(nc(Sc2oc(nn2)c2ccc(F)cc2)n1)N1CCOCC1)c1ccc(C)ccc1	1642584-22-2	1,3,5-Triazin-2-amine, 4-[[5-(4-fluorophenyl)-1,3,4-oxadiazol-2-yl]thio]-N-(4-me	C22H20FN7O3S	481.5	A/B	-9.1
1357	N(c1nc(nc(Sc2oc(nn2)c2ccc(F)cc2)n1)N1CCOCC1)c1cc(Br)ccc1	1642584-23-3	1,3,5-Triazin-2-amine, 4-[[5-(4-fluorophenyl)-1,3,4-oxadiazol-2-yl]thio]-N-(3-me	C22H20FN7O2S	465.5	A/B	-9.1
1358	N(c1nc(nc(Sc2oc(nn2)c2ccc(F)cc2)n1)N1CCOCC1)c1cc(Cl)ccc1	1642584-25-5	Ethanone, 1-[4-[[4-[[5-(4-fluorophenyl)-1,3,4-oxadiazol-2-yl]thio]-6-(4-morpholi	C23H20FN7O3S	493.51	A/B	-9.1
1359	O=c1n([C@@H]2O[C@H](COP(=O)(C(O)c3ccncc3)OC[C@H]3O[C@H](C=C3)n3c(=O)[nH]c(=O)c(C)c3)C=C2)cc(C)c(=O)[	164652-08-8	Thymidine, 2,Ä≤,3,Ä≤-didehydro-P,3,Ä≤-dideoxy-P-[(2,4-dinitrophenyl)hydroxymeth	C27H27N6O14P	690.51	A/B	-9.1
1360	N(C(=O)[C@H](Cc1ccccc1)C[C@@H](CN1[C@H](C(=O)NC(C)(C)C)CN(CC=Cc2ccccc2)CC1)O)[C@H]1c2c(C[C@H]1O)cccc	164725-33-1	1-Piperazinepentanamide, N-(2,3-dihydro-2-hydroxy-1H-inden-1-yl)-2-[[(1,1-dimeth	C36H49N5O5	631.8	A/B	-9.1
1361	N(C(=O)[C@H](Cc1ccccc1)C[C@@H](CN1[C@H](C(=O)NC(C)(C)C)CN(Cc2c(F)cccc2)CC1)O)[C@H]1c2c(C[C@H]1O)cccc	164725-45-5	1-Piperazinepentanamide, N-(2,3-dihydro-2-hydroxy-1H-inden-1-yl)-2-[[(1,1-dimeth	C37H47FN4O4	630.79	A/B	-9.1
1362	C(=O)([C@@H](NC(=O)OC)C(C)C)N1[C@@H](C[C@H]2[C@@H]1CCCC2)c1[nH]c2c(n1)ccc1c2ccc(c1)c1ccc(cc1)c1[nH]c	1653966-92-7	Carbamic acid, N-[(1S)-1-[[(3S,5S)-3-[5-[4-[2-[(6S)-5-[(2S)-2-[(methoxycarbonyl)	C48H58N8O7	859.02	A/B	-9.1
1363	C(=O)([C@@H](NC(=O)OC)C(C)C)N1C[C@]2(C[C@H]1c1[nH]c3c(n1)ccc1c3ccc(c1)c1cc3c(cc1)c1c(cc3)nc([nH]1)[	1653966-93-8	Carbamic acid, N-[(1S)-2-methyl-1-[[(3S,5S)-3-[5-[4-[2-[(2S,3aS,7aS)-octahydro-1	C50H62N8O7	887.08	A/B	-9.1
1364	N(C(=O)[C@H](Cc1ccccc1)C[C@@H](CN1[C@H](C(=O)NC(C)(C)C)CN(Cc2cccnc2)CC1)O)[C@H]1c2c(C[C@H]1OC(=O)C1c	170227-71-1	1-Piperazinepentanamide, N-[2-(acetyloxy)-2,3-dihydro-1H-inden-1-yl]-2-[[(1,1-di	C38H49N5O5	655.83	A/B	-9.1
1365	O=c1n([C@@H]2O[C@H](COP(=O)([C@@H](O)c3ccc(C#N)cc3)OC[C@@H]3[C@@H](N=[N+]=[N-])C[C@@H](O3)n3c(=O)[nH	173658-23-6	Thymidine, 3,Ä≤-azido-P-[(4-chlorophenyl)hydroxymethyl]-P,3,Ä≤-dideoxythymidyly	C27H30ClN10O10P		A/B	-9.1
1366	C(Cc1ccccc1)C1(CCc2ccccc2)CC(=C([C@H](CC)c2cc(NS(=O)(=O)c3cc(C(F)(F)F)ccn3)ccc2)C(=O)O1)O	174484-32-3	2-Pyridinesulfonamide, N-[3-[1-[5,6-dihydro-4-hydroxy-2-oxo-6,6-bis(2-phenylethy	C36H35F3N2O5S	664.73	A/B	-9.1
1367	O=c1n([C@@H]2O[C@H](COP(=O)(OP(=O)(OCc3ccc(OC(=O)CCCCCCC)cc3)OCc3ccc(OC(=O)CCCC)cc3)O)[C@@H](N=[N+]=	1801838-95-8	Thymidine 5,Ä≤-(trihydrogen diphosphate), 3,Ä≤-azido-3,Ä≤-deoxy-, P,Ä≤-[[4-(ace	C34H32F3N5O14P2		A/B	-9.1
1368	O(c1nc(Nc2ccc(C#N)cc2)nc(N)c1Br)c1c(C)cc(C#N)cc1C	1803132-28-6	2H-Pyrido[1,Ä≤,2,Ä≤:4,5]pyrazino[2,1-b][1,3]oxazine-9-carboxamide, N-[(2,4-difl	C20H19F2N3O5		A/B	-9.1
1369	CC12CC3(CC(C)(C1)CC(C3)C2)N1CSCCSC1	1803330-64-4	Not Yet Assigned	C30H36N14O17P2	926.64	A/B	-9.1
1370	C([C@H](OCCP(=O)(OCC)OCC)CO)n1c2c(nc1)c(N)ncn2	1807796-52-6	L-Alanine, N-[(S)-[[(1R)-2-(6-amino-9H-purin-9-yl)-1-methylethoxy]methyl]phenoxy	C42H42F2N7O7P	825.8	A/B	-9.1
1371	O=C1[C@]2([C@H]([C@H](/C(=C/C(=O)[C@@H]3[C@@]4(N5[C@@H]([C@H]3c3cc(OC)c(O)cc3)CCC5)c3c(NC4=O)ccc(Cl)	1807888-34-1	Not Yet Assigned	C45H42Cl2N4O8	837.74	A/B	-9.1
1372	C(C(C(Cc1cc(O)c(O)cc1)C)C)c1ccc([O])c([O])cc1	1817756-68-5	Benzoic acid, 5-[bis(3-carboxy-4-hydroxyphenyl)methyl]-3-[[3-carboxy-5-[(3-carbo	C45H30O18.C30H20O12.C22H14O9		A/B	-9.1
1373	O=c1n([C@@H]2O[C@H](CO)[C](O)C2)ccc(N)n1	18186-48-6	Guanosine 5,Ä≤-(trihydrogen diphosphate), P,Ä≤-(6-deoxy-Œ±-D-lyxo-hexopyranos-4	C16H23N5O15P2	587.33	A/B	-9.1
1374	Nc1c2c(n(cn2)[C@@H]2O[C@@H](C=O)CC2)ncn1	1831102-39-6	Butanedioic acid, 1-[(3R,5aS,6R,8aS,9R,10R,12R,12aR)-decahydro-3,6,9-trimethyl-3	C41H48O15	780.81	A/B	-9.1
1375	[C@@H](Cc1ccccc1)(NC(=O)N1CCN(CC2CC2)CC1)C(=O)N[C@@H](CCc1ccccc1)/C=C/S(=O)(=O)c1ccccc1	1851276-84-0	1-Piperazinecarboxamide, N-[(1S)-2-oxo-2-[[(1S,2E)-1-(2-phenylethyl)-3-(phenylsu	C37H40N4O4S	636.8	A/B	-9.1
1376	[C@@H](Cc1ccccc1)(NC(=O)N1CCN(C)CC1)C(=O)N[C@H](/C=C/S(=O)(=O)c1ccc(Br)cc1)CCc1ccccc1	1860793-31-2	1-Piperazinecarboxamide, N-[(1S)-2-[[(1S,2E)-3-[(4-chlorophenyl)sulfonyl]-1-(2-p	C32H37ClN4O4S	609.18	A/B	-9.1
1377	C(=O)(CC(=O)c1c(O)ccc(C)c1)c1c(OC)cc(O)cc1	1882055-62-0	6H,7H-[1]Benzopyrano[4,3-b][1]benzopyran-7-one, 10-hydroxy-6-(2H-tetrazol-5-yl)-	C17H10N4O4	334.29	A/B	-9.1
1378	N(c1nc(nc(Nc2ccc(C(=O)C=Cc3ccc(N(=O)=O)cc3)cc2)n1)N1CCOCC1)c1ccc(C(F)(F)F)ccc1	1883585-93-0	Urea, N-(2-fluoro-6-methylphenyl)-N,Ä≤-(2-tricyclo[3.3.1.13,7]dec-1-yl-1H-indol-	C26H28FN3O	417.52	A/B	-9.1
1379	O(C(=O)N[C@@H](Cc1ccc(OCCOc2c(C(=O)O)cc(cc2)c2c(F)cc(F)cc2)cc1)[C@@H](CN(S(=O)(=O)c1ccc2c(cc1)ncs2)CC	1887205-94-8	Carbamic acid, N-[(1S,2R)-3-[(6-benzothiazolylsulfonyl)(2-methylpropyl)amino]-1-	C41H43F2N3O9S2	823.92	A/B	-9.1
1380	O(P(=O)(OC[C@H]1O[C@H]([C@H](O)[C@@H]1O)n1nc(C(=O)N)nc1)O)[C@H]1[C@@H](O[C@H](COP(=O)(O[C@H]2[C@@H](	190141-40-3	Adenosine, N-benzoyl-3,Ä≤-O-benzoyl-5,Ä≤-O-[(4-methoxyphenyl)diphenylmethyl]-P-	C106H90N16O28P2	2097.89	A/B	-9.1
1381	[C@H]([C@@H](COC(=O)C)OC(=O)C)(OC(=O)NCCN=[N+]=[N-])[C@H]1[C@H](NC(=O)C)[C@@H](N=C(NC(=O)OC(C)(C)C)N	1928714-98-0	Benzoic acid, 4-[trans-4-[[(tricyclo[3.3.1.13,7]dec-1-ylamino)carbonyl]amino]cyc	C24H32N2O3	396.52	A/B	-9.1
1382	O[C@@H]1[C@H](n2c3c(nc2)c(NCc2cc(C(F)(F)F)cccc2)ncn3)O[C@H](CO)[C@H]1O	1947401-71-9	9H-Purin-6-amine, 9-Œ≤-D-arabinofuranosyl-N-[[3-(trifluoromethyl)phenyl]methyl]-	C18H18F3N5O4	425.36	A/B	-9.1
1383	F[C@H]1[C@H](n2c3c(nc2)c(=O)nc(N)[nH]3)O[C@H]2[C@H]1OP(=O)(O)OC[C@@H]1[C@@H](OP(=O)(O)OC2)[C@@H](F)[	1951464-81-5	3,Ä≤-Inosinic acid, 2,Ä≤-deoxy-2,Ä≤-fluoroinosinylyl-(3,Ä≤,Üí5,Ä≤)-2,Ä≤-deoxy-	C20H20F2N8O12P2	664.36	A/B	-9.1
1384	O[C@H]1[C@H](n2c3c(nc2)c(=O)nc(NCc2ccc(OCc4ccc(O)cc4)cc2)[nH]3)O[C@H](CO)[C@H]1O	197165-29-0	Retinoic acid, 2,Ä≤-ester with 9-Œ≤-D-arabinofuranosyl-9H-purin-6-amine	C30H39N5O5	549.66	A/B	-9.1
1385	O(COC(=O)O[C@@H](C(=O)OC)C)c1c2n(N([C@H]3N(C2=O)CCOC3)[C@H]2c3c(CSc4c2cccc4)c(F)c(F)cc3)ccc1=O	1985606-96-9	Benzeneacetic acid, Œ±-[[[[(12aR)-12-[(11S)-7,8-difluoro-6,11-dihydrodibenzo[b,	C35H29F2N3O9S	705.68	A/B	-9.1
1386	O(COC(=O)O[C@H](C(=O)OC)C)c1c2n(N([C@H]3N(C2=O)CCOC3)[C@H]2c3c(CSc4c2cccc4)c(F)c(F)cc3)ccc1=O	1985607-05-3	Benzeneacetic acid, Œ±-[[[[(12aR)-12-[(11S)-7,8-difluoro-6,11-dihydrodibenzo[b,	C35H29F2N3O9S	705.68	A/B	-9.1
1387	O=C1[C@@H]2[C@@H](c3c([C@H](OC(=O)c4c5c(ccn4)cccc5)[C@H]2CO1)cc1c(c3)OCO1)c1cc(OC)c(OC)c(OC)c1	1989611-35-9	6-Quinolinecarboxylic acid, 2-methyl-, (5R,5aR,8aR,9R)-5,5a,6,8,8a,9-hexahydro-8	C33H29NO9	583.58	A/B	-9.1
1388	C(=O)([C@@H](NC(=O)OC)[C@H](CC)C)N1[C@H](c2[nH]c3c4c(cc(cc4)c4c5c(c(cc4)c4[nH]c(nc4)[C@H]4N(C(=O)[C@	2007900-74-3	Carbamic acid, N-[(1S)-2-[(2S,5S)-2-[7-[2,3-dihydro-7-[2-[(2S,5S)-1-[(2S)-2-[(me	C51H62N8O8	915.09	A/B	-9.1
1389	C(=O)([C@@H](NC(=O)OC)C(C)C)N1CC2(C[C@H]1c1[nH]c(cn1)c1c3c(c(cc1)c1cc4c(cc1)c1c(cc4)nc([nH]1)[C@H]1N	2007901-77-9	Carbamic acid, N-[(1S)-1-[[(2S)-2-[7-[2,3-dihydro-7-[2-[(8S)-7-[(2S)-2-[(methoxy	C47H56N8O8	861	A/B	-9.1
1390	C(=O)([C@@H](NC(=O)OC)C(C)C)N1C2C(C2)C[C@H]1c1[nH]c2c(n1)ccc1c2ccc(c1)c1c2c(c(cc1)c1[nH]c(nc1)[C@H]1	2007901-81-5	Carbamic acid, N-[(1S)-1-[[(2S,5R)-2-[7-[2,3-dihydro-7-[2-[(2S,4S)-1-[(2S)-2-[(m	C47H52F6N8O6	938.96	A/B	-9.1
1391	C(=O)([C@@H](NC(=O)OC)C(C)C)N1CC2(C[C@H]1c1[nH]c3c(n1)ccc1c3ccc(c1)c1c3c(c(cc1)c1[nH]c(nc1)[C@H]1N(C	2007901-83-7	Carbamic acid, N-[(1S)-1-[[(2S,4S)-2-[5-[2,3-dihydro-7-[2-[(3S)-2-[(2S)-2-[(meth	C47H53F3N8O6	882.97	A/B	-9.1
1392	C(=O)([C@@H](NC(=O)OC)C(C)C)N1[C@H](c2[nH]c3c4c(cc(cc4)c4c5c(c(cc4)c4[nH]c(nc4)[C@H]4N(C(=O)[C@@H](N	2007901-86-0	Carbamic acid, N-[(1S)-1-[[(2S,5S)-2-[5-[2,3-dihydro-7-[2-[(3S)-2-[(2S)-2-[(meth	C51H64N8O6	885.1	A/B	-9.1
1393	C(=O)(C(=O)Nc1c(C)cc(Cl)cc1)c1c([nH]c2c1cccc2)C12CC3CC(C2)CC(C1)C3	2014403-82-6	1H-Indole-3-acetamide, N-(2,4-dimethylphenyl)-Œ±-oxo-2-tricyclo[3.3.1.13,7]dec-1	C28H30N2O2	426.55	A/B	-9.1
1394	C(=O)(C(=O)Nc1c(C)cc(C(=O)OC)cc1)c1c(C23CC4CC(C3)CC(C2)C4)[nH]c2c1cccc2	2014403-84-8	1H-Indole-3-acetamide, N-(2-methyl-4-nitrophenyl)-Œ±-oxo-2-tricyclo[3.3.1.13,7]d	C27H27N3O4	457.52	A/B	-9.1
1395	C(=O)(C(=O)Nc1cc(OC)cc(OC)c1)c1c(C23CC4CC(C3)CC(C2)C4)[nH]c2c1cccc2	2014403-85-9	Benzoic acid, 3-methyl-4-[[2-oxo-2-(2-tricyclo[3.3.1.13,7]dec-1-yl-1H-indol-3-yl	C29H30N2O4	470.56	A/B	-9.1
1396	O=c1n([C@@H]2O[C@H](COC(=O)CCC(=O)Oc3c(OC)ccc(C(=O)c4ccc(N(=O)=O)cc4)c3)C=C2)cc(C)c(=O)[nH]1	2015224-13-0	Butanedioic acid, 1-[[(2S,5R)-5-(3,4-dihydro-5-methyl-2,4-dioxo-1(2H)-pyrimidiny	C28H25FN2O9	552.5	A/B	-9.1
1397	O=C1[C@@H]2[C@@H](c3c([C@@H](SCC)[C@H]2CO1)cc1c(c3)OCO1)c1cc(OC)c(OC)c(OC)c1	2015224-40-3	Acetic acid, 2-[5-(2-chlorobenzoyl)-2-methoxyphenoxy]-, [(2S,5R)-5-(3,4-dihydro-	C26H23ClN2O8	526.92	A/B	-9.1
1398	C(=O)([C@@H](NC(=O)OC)C(C)C)N1[C@H](c2[nH]c(c3cc4c5-c6c(cc(cc6CC4)c4[nH]c(nc4)[C@H]4N(C(=O)[C@@H](NC	2020089-25-0	Carbamic acid, N-[(1S)-1-[[(2S,4R)-4-fluoro-2-[5-[7-[2-[(2S,4R)-4-fluoro-1-[(2S)	C44H52F2N8O6	826.93	A/B	-9.1
1399	C(=O)([C@H](NC(=O)OC)c1ccccc1)N1[C@@H](CCC1)c1[nH]c(cn1)c1ccc(cc1)c1ccc(cc1)c1[nH]c(nc1)[C@H]1N(C(=O	1009107-55-4	Carbamic acid, N-[(1R)-2-[(2S)-2-[5-[4,Ä≤-[2-[(2S)-1-[(2R)-2-(dimethylamino)-2-p	C46H48N8O4	776.92	C	-9.1
1400	C(=O)([C@H](NC(=O)OC)c1ccccc1)N1[C@@H](CCC1)c1[nH]c(cn1)c1ccc(cc1)c1ccc(cc1)c1[nH]c(nc1)[C@H]1N(C(=O	1009108-65-9	Carbamic acid, N-[(1R)-2-[(2S)-2-[5-[4,Ä≤-[2-[(2S)-1-[2-(2-methylphenyl)acetyl]-	C45H45N7O4	747.88	C	-9.1
1401	C(=O)([C@H](NC(=O)OC)c1ccccc1)N1[C@@H](CCC1)c1[nH]c(cn1)c1cc(C)c(cc1)c1c(C)cc(cc1)c1[nH]c(nc1)[C@H]1	1009111-84-5	Carbamic acid, N-[2-oxo-2-[(2S)-2-[5-[4,Ä≤-[2-[(2S)-1-[(2R)-2-phenyl-2-(1-pyrrol	C42H46N8O4	726.87	C	-9.1
1402	O=c1n([C@@H]2O[C@H](CO)[C@@H](O)C2)cc(Br)c(N)n1	1021936-90-2	[1,1,Ä≤-Biphenyl]-2-carboxylic acid, 4,Ä≤-[2-(5-tricyclo[3.3.1.13,7]dec-1-yl-1H	C28H30N2O2	426.55	C	-9.1
1403	N(C(=O)C)c1c2[C@H]([C@@H]3[C@@H]([C@@H](O)c2cc2c1OCO2)COC3=O)c1cc(OC)c(OC)c(OC)c1	1025945-68-9	2-Naphthalenesulfonic acid, 5-amino-8-[(1E)-2-[4-[(1E)-2-[4-[(1E)-2-(4-amino-7-s	C34H26N6O9S3	758.8	C	-9.1

1404	N(=N\c1cc(S(=O)(=O)O)c(C=Cc2c(S(=O)(=O)O)cc(/N=N/c3c4c(cc(S(=O)(=O)O)c3N)cc(S(=O)(=O)O)cc4)cc2)cc1)/	1053056-26-0	Not Yet Assigned	C34H26N6O12S4	838.86	C	-9.1
1405	O=c1n([C@H]2C[C@H](OP(=O)(OP(=O)(OP(=O)(O)O)O)O)[C@@H](CO)O2)cc(C)c(=O)[nH]1	1056950-70-9	Propanoic acid, 2-[[(2,4,5,7-tetranitro-9H-fluoren-9-ylidene)amino]oxy]-, (5R,5a	C38H29N5O18	843.66	C	-9.1
1406	C(=[N+]1c2c(NC(=O)c3c1nccc3)c(C)cc(=O)[nH]2)CC	1061160-26-6	Œ≤-D-Glucopyranuronic acid, 1-(11-cyclopropyl-6,11-dihydro-6-oxo-5H-dipyrido[3,2	C21H20N4O9	472.4	C	-9.1
1407	C[C@]12[C@H]([C@]3(C)[C@@H](CC2)C(C)(C)CCC3)[C@@H](O)CC2=C([C@@H](C[C@@H]([C@H](C(C)(C)O)O)O)C)CC[C@	106812-25-3	1,3,6-Naphthalenetrisulfonic acid, 7-[2-[4-[2-(4-amino-2-methylphenyl)diazenyl]-	C27H21N5O12S4	735.74	C	-9.1
1408	N(CCNCC=C1c2c(CCc3c1cccc3)cccc2)C12CC3CC(C2)CC(C1)C3	108768-09-8	Piperazine, 1-[2-(10,11-dihydro-5H-dibenzo[a,d]cyclohepten-5-ylidene)ethyl]-4-tr	C31H38N2	438.65	C	-9.1
1409	C[C@]12[C@@]3(C=C([C@@H]4[C@@H]1CC[C@@]1(C)[C@H]4CC[C@@H]1[C@@H](CCCC(C)C)C)NC(=S)N3)C[C@@H](OC(=O)C	1092169-36-2	2-Naphthalenesulfonic acid, 6-amino-5-[2-[2,Ä≤,3-dimethyl-4,Ä≤-[2-(6-sulfo-1-na	C34H27N5O6S2.2Na		C	-9.1
1410	[Pt+2]([Cl-])([Cl-])([OH2])[N]1=CN(COCCO)c2c1c(=O)nc(N)[nH]2	1109235-82-6	2-Pyridinesulfonamide, N-[3-[(1S)-1-[(6R)-5,6-dihydro-4-hydroxy-2-oxo-6-phenyl-2	C26H23F3N2O5S	532.53	C	-9.1
1411	Oc1c2[C@H]([C@H]3[C@H]([C@@H](c4c3cc(O)cc4O)c3cc(O)c(O)cc3)c2cc(O)c1)c1cc(O)c(O)cc1	1115371-10-2	Indeno[2,1-a]indene-1,3,6,8-tetrol, 5-(3,4-dihydroxyphenyl)-4b,5,9b,10-tetrahydr	C28H22O7	470.47	C	-9.1
1412	C(N(C(=O)C(=O)c1c2c([nH]c1)cccc2)CC(=O)O)(C(=O)NC1CCCCC1)C12CC3CC(C2)CC(C1)C3	1115371-12-4	Indeno[2,1-a]indene-1,3,6,8-tetrol, 5,10-bis(3,4-dihydroxyphenyl)-4b,5,9b,10-tet	C28H22O8	486.47	C	-9.1
1413	C(=O)([C@@H](NC(=O)OC)C(C)C)N1[C@@H](CCC1)c1[nH]c(c(C)n1)c1ccc(cc1)c1ccc(cc1)c1[nH]c(nc1)[C@H]1N(C(=	1129636-14-1	Carbamic acid, N-[(1S)-2-[(2S)-2-[5-[4,Ä≤-[2-[(2S)-1-[(2S)-2-[(methoxycarbonyl)a	C37H44N8O6	696.8	C	-9.1
1414	O=C1C2C(c3c(C(O)C2CO1)cc1c(c3)OCO1)c1cc(OC)c(OC(=O)C)c(OC)c1	114002-77-6	Benzoic acid, 3,3,Ä≤-[(4-oxo-2,5-cyclohexadien-1-ylidene)methylene]bis[6-hydroxy	C21H14O7	378.33	C	-9.1
1415	C(C)n1c2c(c(=O)c(C(=O)OC[C@@H]3[C@@H](N=[N+]=[N-])C[C@@H](O3)n3c(=O)[nH]c(=O)c(C)c3)c1)cc(F)c(c2F)N1	1174398-21-0	Thymidine, 3,Ä≤-azido-3,Ä≤-deoxy-, 5,Ä≤-[1-cyclopropyl-6-fluoro-1,4-dihydro-4-o	C35H35FN8O8		C	-9.1
1416	O=c1c2c(n(cc1C(=O)O)C1CC1)cc(c(F)c2)N1CCN(Cc2cn(c(=O)[nH]c2=O)[C@H]2C[C@H](N=[N+]=[N-])[C@@H](CO)O2)	1174398-28-7	3-Quinolinecarboxylic acid, 7-[4-[[1-(3-azido-2,3-dideoxy-Œ≤-D-erythro-pentofura	C26H29FN8O7		C	-9.1
1417	C(=O)([C@@H](NC(=O)OC)C(C)(C)C)N1[C@H](c2[nH]c(c3cc4c(c5c(o4)cc(cc5)c4[nH]c(nc4)[C@H]4N(C(=O)[C@@H](	1179150-63-0	Carbamic acid, N,N,Ä≤-[3,7-dibenzofurandiylbis[1H-imidazole-5,2-diyl-(2S)-2,1-py	C40H44N8O7	748.83	C	-9.1
1418	Cn1c2c(c3c1cc(cc3)c1[nH]c(nc1)[C@H]1N(C(=O)[C@@H](NC(=O)OC)C(C)C)CCC1)ccc(c2)c1[nH]c(nc1)[C@H]1N(C(=	1179150-68-5	Carbamic acid, N,N,Ä≤-[3,7-dibenzofurandiylbis[1H-imidazole-5,2-diyl-(2S)-2,1-py	C40H48N8O9	784.86	C	-9.1
1419	C(=O)([C@@H](NC(=O)OC)C(C)C)N1[C@H](c2[nH]c3-c4c(cc(cc4)c4cc(F)c(cc4)c4[nH]c(nc4)[C@H]4N(C(=O)[C@@H]	1180670-04-5	Carbamic acid, N,N,Ä≤-[(4,4,Ä≤,5,5,Ä≤-tetrahydro[7,7,Ä≤-bi-1H-naphth[1,2-d]imic	C46H54N8O6	814.97	C	-9.1
1420	C(=O)([C@@H](NC(=O)OC)C(C)C)N1[C@H]2[C@H](C2)C[C@H]1c1[nH]c2c(n1)ccc1c2ccc(c1)c1ccc2c(cc1)c1c(cc2)nc(	1180670-22-7	Carbamic acid, N,N,Ä≤-[(4,5-dihydro[7,7,Ä≤-bi-1H-naphth[1,2-d]imidazole]-2,2,Ä≤	C46H52N8O6	812.96	C	-9.1
1421	O=c1n([C@@H]2O[C@H](COC(=O)c3cccnc3)C=C2)cc(C)c(=O)[nH]1	1188424-89-6	7,10-Ethanopyrimido[1,2-a]azepine-2-carboxamide, N-[(4-fluorophenyl)methyl]-4,6,	C23H23FN6O5	482.46	C	-9.1
1422	N(=C1c2c(N(CN3CCN(CC3)c3c4c(cc(C(F)(F)F)cc4)ncc3)C1=O)cccc2Br)NC(=S)N	1218813-17-2	Hydrazinecarbothioamide, 2-[4-chloro-1,2-dihydro-2-oxo-1-[[4-[7-(trifluoromethyl	C24H21ClF3N7OS	547.98	C	-9.1
1423	C(N1c2c(C(=NNC(=S)N)C1=O)ccc(Br)c2)N1CCN(CC1)c1c2c(cc(C(F)(F)F)cc2)ncc1	1218813-20-7	Hydrazinecarbothioamide, 2-[6-chloro-1,2-dihydro-2-oxo-1-[[4-[7-(trifluoromethyl	C24H21ClF3N7OS	547.98	C	-9.1
1424	N(C(=O)[C@H](Cc1ccccc1)C[C@@H](CN1[C@H](C(=O)NC(C)(C)C)CN(Cc2cccnc2)CC1)O)[C@H]1c2c(C[C@H]1O)c(F)ccc	1222663-16-2	Not Yet Assigned	C36H46ClN5O4	648.23	C	-9.1
1425	c1(nc(nc(n1)N1CCOCC1)N1CCOCC1)c1cc2c(cc1)[nH]cc2	1225037-85-3	1H-Indazole, 5-(4,6-di-4-morpholinyl-1,3,5-triazin-2-yl)-	C18H21N7O2	367.4	C	-9.1
1426	C(#N)C1=C(N)Oc2c(C1c1ccncc1)ccn1c2ncc1	1225373-21-6	7H-Imidazo[1,2-a]pyrano[2,3-c]pyridine-8-carbonitrile, 9-amino-7-(5-methyl-3-isc	C15H11N5O2	293.28	C	-9.1
1427	C(OP(=O)(Oc1c2c(ccc1)cccc2)N[C@H](C(=O)OCc1ccccc1)C)[C@@H]1[C@@H]2[C@H]([C@@H](O1)n1nc(C(=O)N)nc1)OC	1228343-69-8	3-Azabicyclo[3.1.0]hexane-2-carboxamide, N-[3-amino-1-(cyclopropylmethyl)-2,3-di	C36H53N5O5	635.84	C	-9.1
1428	C(NC(=O)[C@H](Cc1ccccc1)N)(C(=O)NCC(=O)O)C12CC3CC(C2)CC(C1)C3	1229574-81-5	L-Phenylalanine, L-tryptophyl-2-tricyclo[3.3.1.13,7]dec-1-ylglycylglycyl-L-pheny	C43H50N6O6	746.89	C	-9.1
1429	C(F)(F)c1n(c2c(n1)cccc2)c1nc(nc(N[C@H]2CC[C@H](NC(=O)C(C)C)CC2)n1)N1CCOCC1	1240029-10-0	Propanamide, N-[trans-4-[[4-[2-(difluoromethyl)-1H-benzimidazol-1-yl]-6-(4-morph	C24H30F2N8O2	500.54	C	-9.1
1430	C(COCP(=O)([O-])O)n1c2c(nc1)c(N)cnc(N)n2	1240306-16-4	2-Naphthalenesulfonic acid, 5-[(1E)-2-(3-hydroxy-2-naphthalenyl)diazenyl]-	C20H14N2O4S	378.4	C	-9.1
1431	C(=O)([C@@H](NC(=O)OC)C)N1[C@H](c2[nH]c(c3cc4c(-c5c(C4)cc(cc5)c4[nH]c(nc4)[C@H]4N(C(=O)[C@@H](NC(=O)	1240880-36-7	Carbamic acid, N,N,Ä≤-[9H-fluorene-2,7-diylbis[1H-imidazole-5,2-diyl-(2S)-2,1-py	C37H42N8O6	694.78	C	-9.1
1432	C(=O)([C@@H](NC(=O)OC)C1CCOCC1)N1[C@H](c2[nH]c(c3cc4c(-c5c(C4)cc(cc5)c4[nH]c(nc4)[C@H]4N(C(=O)[C@@H]	1240880-80-1	Carbamic acid, N,N,Ä≤-[9H-fluorene-2,7-diylbis[1H-imidazole-5,2-diyl-(2S)-2,1-py	C47H58N8O8	863.01	C	-9.1
1433	C(=O)([C@@H](NC(=O)OC)C(C)C)N1[C@H](c2[nH]c3c(n2)ccc(c3)c2ccc(cc2)c2ccc(cc2)c2[nH]c(nc2)[C@H]2N(C(=O	124118-11-2	2-Naphthalenesulfonic acid, 5,5,Ä≤-(2-nitro-4,4,Ä≤-biphenylylenebisazo)bis[6-am	C32H23N7O8S2	697.7	C	-9.1
1434	[C@@H](NC(=O)[C@@H](NC(=O)N1CCN(C)CC1)CC(C)C)(/C=C/S(=O)(=O)c1ccccc1)c1ccccc1	1253912-39-8	1-Piperazinecarboxamide, N-[(1S)-2-[[(1R,2E)-1-[1,1,Ä≤-biphenyl]-4-yl-3-(phenyls	C36H38N4O4S	622.78	C	-9.1
1435	N(C(=O)Nc1ccc(CCN(C)C)cc1)c1ccc(c2nc(N3C4CCC3COC4)nc(n2)N2CCOCC2)cc1	1254065-98-9	Urea, N-[4-(dimethylamino)phenyl]-N,Ä≤-[4-[4-(4-morpholinyl)-6-(3-oxa-8-azabicyc	C28H34N8O3	530.62	C	-9.1
1436	C(=O)([C@@H](NC(=O)OC)C(C)C)N1[C@@H](CCC1)c1[nH]c(cn1)c1cc2c(cc1)cc(cc2)c1ccc(cc1)c1[nH]c(nc1)[C@H]1	1256384-94-7	Carbamic acid, N,N,Ä≤-[1,4-phenylenebis[1H-imidazole-5,2-diyl-(2S)-2,1-pyrrolid	C34H46N8O6	662.78	C	-9.1
1437	C(=O)([C@@H](NC(=O)OC)C(C)C)N1[C@@H](CCC1)c1[nH]c(cn1)c1ccc(cc1)c1ccc(cc1)c1[nH]c(nc1)[C@H]1N(C(=O)[	1256391-36-2	Carbamic acid, N-[2-[(2S)-2-[5-[4-[6-[2-[(2S)-1-[2-[(methoxycarbonyl)amino]-2-ph	C50H48N8O6	856.97	C	-9.1
1438	C[C@]12[C@H]([C@H]3[C@@H]([C@]4(C)[C@H](CC3)C[C@@H](N)CC4)CC2=O)CC[C@@H]1[C@@H](CCC(=O)NCCN(CC)CC)C	125680-35-5	Cholan-24-amide, 3-(methylamino)-12-oxo-N-(phenylmethyl)-, (3Œ≤,5Œ≤)-	C32H48N2O2	492.74	C	-9.1
1439	O(C)c1c2n(cc(C(=O)O)c(=O)c2cc(F)c1N1CCN(CCN2c3c(C(=NNC(=S)N)C2=O)cc(F)cc3)CC1)C1CC1	1263191-46-3	3-Quinolinecarboxylic acid, 7-[4-[2-[3-[2-(aminothioxomethyl)hydrazinylidene]-2,	C29H30FN7O5S	607.66	C	-9.1
1440	C(C)n1c2c(c(=O)c(C(=O)O)c1)cc(F)c(c2F)N1CC(C)N(CN2c3c(/C(=N/NC(=S)NOC)/C2=O)cc(C)cc3)CC1	1265623-25-3	3-Quinolinecarboxylic acid, 7-[4-[[(3Z)-2,3-dihydro-3-[2-[(hydroxyamino)thioxome	C28H29F2N7O5S	613.64	C	-9.1
1441	S=c1n(nc(s1)c1ccc(Cl)cc1)c1nc(nc(n1)N1CCOCC1)N1CCOCC1	1309437-31-7	1,3,4-Thiadiazole-2(3H)-thione, 3-(4,6-di-4-morpholinyl-1,3,5-triazin-2-yl)-5-(4	C20H22N8O5S2	518.57	C	-9.1
1442	C(=O)([C@@H](NC(=O)OC)C(C)C)N1[C@@H](CC(=C)C1)c1[nH]c(cn1)c1ccc(C=Cc2[nH]c(nc2)[C@H]2N(C(=O)[C@@H](N	1311398-46-5	Carbamic acid, N-[(1S)-1-[[(2S)-2-[5-[6-[2-[(2S)-1-[(2S)-2-[(methoxycarbonyl)ami	C43H50N8O6	774.91	C	-9.1
1443	C(=O)([C@@H](NC(=O)OC)C(C)C)N1[C@@H](C[C@H](C)C1)c1[nH]c(c(Cl)n1)c1ccc(cc1)c1ccc(cc1)c1[nH]c(nc1)[C@	1312336-27-8	Carbamic acid, N-[(1S)-2-[(2S,4S)-2-[5-chloro-4-[4,Ä≤-[5-chloro-2-[(2S,4S)-1-[(	C44H54Cl2N8O7	877.85	C	-9.1
1444	C(=O)([C@@H](NC(=O)OC)C1CCC(F)(F)CC1)N1[C@H]2[C@H](C2)C[C@H]1c1[nH]c(c(Cl)n1)c1ccc(cc1)c1ccc(cc1)c1[	1312336-36-9	Carbamic acid, N-[(1S)-2-[(1R,3S,5R)-3-[5-[4,Ä≤-[5-chloro-2-[(1R,3S,5R)-2-[(2S)-	C48H53ClF4N8O6	949.43	C	-9.1
1445	C(=O)([C@@H](NC(=O)OC)C(C)C)N1[C@H](c2[nH]c(c3c4c(c(cc3)c3ccc(cc3)c3[nH]c(nc3)[C@H]3N(C(=O)[C@@H](NC	1312608-64-2	Carbamic acid, N-[(1S)-1-[[(2S)-2-[5-[4-[7-[2-[(2S)-1-[(2S)-2-[(methoxycarbonyl)	C42H51N9O6	777.91	C	-9.1
1446	O(Cc1ccc(F)cc1)c1c2c(oc(c(O)c2=O)c2[nH]cnn2)c(Cl)cc1	1312893-87-0	4H-1-Benzopyran-4-one, 8-chloro-5-[(4-fluorophenyl)methoxy]-3-hydroxy-2-(5-pheny	C26H16ClFN2O4	474.87	C	-9.1
1447	O(Cc1ccc(F)cc1)c1c2c(c(cc1)c1ccccc1)oc(c(O)c2=O)c1[nH]ccn1	1312894-47-5	4H-1-Benzopyran-4-one, 5-[(4-fluorophenyl)methoxy]-3-hydroxy-8-phenyl-2-(3-pheny	C30H20FN3O4	505.5	C	-9.1
1448	O(Cc1ccc(F)cc1)c1c2c(oc(c(O)c2=O)c2ncccn2)cc(C)c1	1312894-52-2	4H-1-Benzopyran-4-one, 5-[(4-fluorophenyl)methoxy]-3-hydroxy-8-phenyl-2-(5-pheny	C31H21FN2O4	504.51	C	-9.1
1449	O(Cc1ccc(F)cc1)c1c2c(oc(c(O)c2=O)c2[nH]cnn2)cc(C)c1	1312894-87-3	4H-1-Benzopyran-4-one, 5-[(4-fluorophenyl)methoxy]-3-hydroxy-7-methyl-2-(5-pheny	C27H19FN2O4	454.45	C	-9.1
1450	O(Cc1ccc(F)cc1)c1c2c(c(c(C)c1)c1ccccc1)oc(c(O)c2=O)c1nnc(C)[nH]1	1312896-93-7	4H-1-Benzopyran-4-one, 5-[(4-fluorophenyl)methoxy]-3-hydroxy-7-methyl-8-phenyl-2	C25H18FN3O4	443.43	C	-9.1
1451	O(Cc1ccc(F)cc1)c1c2c(oc(c(O)c2=O)c2[nH]cnn2)cc(Cl)c1	1312900-58-5	4H-1-Benzopyran-4-one, 7-chloro-5-[(4-fluorophenyl)methoxy]-3-hydroxy-2-(5-pheny	C26H16ClFN2O4	474.87	C	-9.1
1452	O(Cc1ccc(F)cc1)c1c2c(oc(c(O)c2=O)c2[nH]ccn2)cc(Cl)c1	1312900-68-7	4H-1-Benzopyran-4-one, 7-chloro-5-[(4-fluorophenyl)methoxy]-3-hydroxy-2-(3-pheny	C24H15ClFN3O4	463.85	C	-9.1
1453	O(Cc1ccc(F)cc1)c1c2c(oc(c(O)c2=O)c2[nH]ccn2)c(C)c(Cl)c1	1312901-18-0	4H-1-Benzopyran-4-one, 7-chloro-5-[(4-fluorophenyl)methoxy]-3-hydroxy-8-methyl-2	C25H17ClFN3O4	477.87	C	-9.1
1454	O(Cc1ccc(F)cc1)c1c2c(c(c(Cl)c1)c1ccccc1)oc(c(O)c2=O)c1nnc(C)[nH]1	1312902-64-9	4H-1-Benzopyran-4-one, 7-chloro-5-[(4-fluorophenyl)methoxy]-3-hydroxy-8-phenyl-2	C24H15ClFN3O4	463.85	C	-9.1
1455	O(Cc1ccc(F)cc1)c1c2c(oc(c(O)c2=O)c2ncccn2)cc(c1)c1ccccc1	1312902-73-0	4H-1-Benzopyran-4-one, 7-chloro-5-[(4-fluorophenyl)methoxy]-3-hydroxy-8-phenyl-2	C31H20ClFN2O4	538.95	C	-9.1
1456	O(Cc1ccc(F)cc1)c1c2c(c(Cl)c(c1)c1ccccc1)oc(c(O)c2=O)c1[nH]cnn1	1312905-08-0	4H-1-Benzopyran-4-one, 8-chloro-5-[(4-fluorophenyl)methoxy]-3-hydroxy-7-phenyl-2	C32H20ClFN2O4	550.96	C	-9.1
1457	O(Cc1ccc(F)cc1)c1c2c(c(c(c1)c1ccccc1)c1ccccc1)oc(c(O)c2=O)c1ncccn1	1312905-34-2	4H-1-Benzopyran-4-one, 8-chloro-5-[(4-fluorophenyl)methoxy]-3-hydroxy-7-phenyl-2	C31H20ClFN2O4	538.95	C	-9.1
1458	O=c1c2c(oc(c1O)c1[nH]ccn1)c(C)cc(OCc1ccc(F)cc1)c2	1312906-78-7	4H-1-Benzopyran-4-one, 6-[(4-fluorophenyl)methoxy]-3-hydroxy-8-methyl-2-(3-pheny	C25H18FN3O4	443.43	C	-9.1
1459	O=c1c2c(oc(c1O)c1[nH]ccn1)c(Cl)cc(OCc1ccc(F)cc1)c2	1312907-78-0	4H-1-Benzopyran-4-one, 8-chloro-6-[(4-fluorophenyl)methoxy]-3-hydroxy-2-(3-pheny	C24H15ClFN3O4	463.85	C	-9.1
1460	O=c1c2c(c(cc(OCc3ccc(F)cc3)c2)c2ccccc2)oc(c1O)c1ncccn1	1312907-83-7	4H-1-Benzopyran-4-one, 8-chloro-6-[(4-fluorophenyl)methoxy]-3-hydroxy-2-(5-pheny	C25H16ClFN2O4	462.86	C	-9.1
1461	O=c1c2c(c(cc(OCc3ccc(F)cc3)c2)c2ccccc2)oc(c1O)c1[nH]c(C)nn1	1312908-24-9	4H-1-Benzopyran-4-one, 6-[(4-fluorophenyl)methoxy]-3-hydroxy-8-phenyl-2-(1H-1,2,	C24H16FN3O4	429.4	C	-9.1
1462	Cc1c2c(cc(OCc3ccc(F)cc3)c1C)c(=O)c(O)c(o2)c1[nH]ccn1	1312909-40-2	4H-1-Benzopyran-4-one, 6-[(4-fluorophenyl)methoxy]-3-hydroxy-7,8-dimethyl-2-(3-p	C26H20FN3O4	457.45	C	-9.1
1463	Cc1c2c(cc(OCc3ccc(F)cc3)c1OC)c(=O)c(O)c(o2)c1ncccn1	1312911-92-4	4H-1-Benzopyran-4-one, 7-chloro-6-[(4-fluorophenyl)methoxy]-3-hydroxy-2-(5-pheny	C25H16ClFN2O4	462.86	C	-9.1
1464	O(C)c1c2c(cc(OCc3ccc(F)cc3)c1c1ccccc1)c(=O)c(O)c(o2)c1ncccn1	1312917-01-3	4H-1-Benzopyran-4-one, 6-[(4-fluorophenyl)methoxy]-3-hydroxy-8-methyl-7-phenyl-2	C32H23FN2O4	518.53	C	-9.1
1465	O(Cc1ccc(F)cc1)c1c(c(Cl)c2c(c1)c(=O)c(O)c(o2)c1[nH]ccn1)c1ccccc1	1312917-96-6	4H-1-Benzopyran-4-one, 8-chloro-6-[(4-fluorophenyl)methoxy]-3-hydroxy-7-phenyl-2	C30H19ClFN3O4	539.94	C	-9.1
1466	O(Cc1ccc(F)cc1)c1c(c(c2c(c1)c(=O)c(O)c(o2)c1ncccn1)c1ccccc1)c1ccccc1	1312918-01-6	4H-1-Benzopyran-4-one, 8-chloro-6-[(4-fluorophenyl)methoxy]-3-hydroxy-7-phenyl-2	C31H20ClFN2O4	538.95	C	-9.1
1467	O=c1n([C@@H]2O[C@H](CO[P@@]3(=O)Oc4c(CO3)cccc4)[C@@H](O)C2)cc(I)c(=O)[nH]1	1313031-63-8	Uridine, 3,Ä≤-O-[6,8-bis(1,1-dimethylethyl)-5-fluoro-2-oxido-4H-1,3,2-benzodioxa	C24H31FIN2O8P	652.39	C	-9.1
1468	C(=O)(N)c1nn(cn1)[C@@H]1O[C@H](CO)C=C1	1319189-63-3	Carbamic acid, N-[(12R,13S,18aS)-5,6,13,14,16,17,18,18a-octahydro-3-[4,Ä≤-[2-[(	C46H53N9O6	827.97	C	-9.1
1469	O[C@H]1[C@H](n2c3c(nc2)c(=NO)nc(Cl)[nH]3)O[C@H](CO)[C@H]1O	133826-32-1	Guanosine 5,Ä≤-(trihydrogen diphosphate), 2,Ä≤-deoxy-, P,Ä≤,Ü5,Ä≤-ester with 2	C20H26N10O13P2	676.43	C	-9.1
1470	C(n1c2c(nc1CC)c(C)cc(C)n2)c1ccc(cc1)c1c(C(=O)NS(=O)(=O)c2ccccc2)cccc1	1347431-47-3	Not Yet Assigned	C30H38D5F2N5O		C	-9.1
1471	O(C(=O)N[C@@H](Cc1ccc(OCP(=O)(NC(CC(=O)O)C)O)cc1)[C@@H](CN(S(=O)(=O)c1ccc(OC)cc1)CC(C)C)O)[C@@H]1[C@	1348561-60-3	3-Azabicyclo[3.1.0]hexane-2-carboxamide, N-[3-amino-1-(cyclobutylmethyl)-2,3-dic	C35H54N8O6	682.85	C	-9.1
1472	[C@H](N(C(=O)[C@@H](NC(=O)NCc1nc(C(C)C)sc1)C(C)C)NC(=O)OC(C)(C)C)(Cc1ccccc1)C[C@@H]([C@H](Cc1ccccc1)	1349738-14-2	6H-Dipyrido[3,2-b:2,Ä≤,3,Ä≤-e][1,4]diazepin-6-one, 11-cyclopropyl-8-[2-[[1,3-di	C30H27FN6O5S	602.64	C	-9.1
1473	C(n1c2c(nc1CC)c(C)cc(C)n2)c1ccc(cc1)c1c(S(=O)(=O)NC(=O)NCCOCCO)cccc1	1349746-85-5	Furo[3,Ä≤,4,Ä≤:6,7]naphtho[2,3-d]-1,3-dioxol-6(5aH)-one, 9-(acetyloxy)-5-(5a,6,	C36H42N2O9	646.73	C	-9.1
1474	C(=O)([C@@H](NC(=O)OC)C(C)C)N1[C@H](c2[nH]c(c3c4c(c(cc3)c3c5c(c(cc3)c3[nH]c(nc3)[C@H]3N(C(=O)[C@@H](	1350718-42-1	Carbamic acid, N,N,Ä≤-[(2,2,Ä≤-dimethyl[4,7,Ä≤-bi-1H-indole]-4,Ä≤,7-diyl)bis[1H	C46H56N10O6	845	C	-9.1
1475	C(=O)([C@@H](NC(=O)OC)C(C)C)N1[C@H](c2[nH]c(c3c4c(c(cc3)c3c5c(c(cc3)c3[nH]c(nc3)[C@H]3N(C(=O)[C@@H](	1350718-51-2	Carbamic acid, N,N,Ä≤-[[4,4,Ä≤-bi-1H-indene]-7,7,Ä≤-diylbis[1H-imidazole-5,2-di	C46H58N8O6	819	C	-9.1
1476	C(=O)([C@@H](NC(=O)OC)[C@H](CC)C)N1[C@@H](CCC1)c1[nH]c(cn1)c1cc2c(cc1)cc(cc2)c1ccc(cc1)c1[nH]c(nc1)[	1351332-62-1	Carbamic acid, N-[(1R)-2-[(2S)-2-[5-[4-[6-[2-[(2S)-1-[(2S)-2-[(methoxycarbonyl)a	C45H46N8O6	794.9	C	-9.1
1477	C[C@@]12[C@@H]3[C@H]([C@H]4[C@](C)(C[C@H]3OC(=O)C)[C@@H]([C@@H](CCCC(C)C)C)CC4)C[C@@H](O)[C@@]1(O)C[	1351334-71-8	Carbamic acid, N-[(1S)-1-[[(2S)-2-[[[[6-[4-[2-[(2S)-1-[(2S)-2-[(methoxycarbonyl)	C43H53N7O7	779.92	C	-9.1
1478	C(=O)([C@@H](NC(=O)OC)C(C)C)N1C[C@@]2(C[C@H]1c1[nH]c(cn1)c1ccc(cc1)c1ccc(cc1)c1[nH]c(nc1)[C@H]1N(C(=	1351852-64-6	Carbamic acid, N-[(1S)-1-[[(2S,4R)-4-fluoro-2-[5-[4,Ä≤-[2-[(5S,8S)-7-[(2S)-2-[(n	C43H53FN8O7	812.93	C	-9.1
1479	C(=O)([C@@H](NC(=O)OC)C(C)C)N1C[C@@]2(C[C@H]1c1[nH]c(cn1)c1c(C)cc(cc1)c1cc(C)c(cc1)c1[nH]c(nc1)[C@H]	1351852-82-8	Carbamic acid, N-[(1S)-2-methyl-1-[[(2S)-2-[5-[2,2,Ä≤,3,3,Ä≤-tetrafluoro-4,Ä≤-[	C43H50F4N8O7	866.9	C	-9.1
1480	C(=O)([C@@H](NC(=O)OC)C(C)C)N1C[C@@]2(C[C@H]1c1[nH]c(cn1)c1c(Cl)cc(cc1Cl)c1cc(Cl)c(c(Cl)c1)c1[nH]c(n	1351852-88-4	Carbamic acid, N-[(1S)-1-[[(2S)-2-[5-[3,3,Ä≤-dichloro-4,Ä≤-[2-[(5S,8S)-7-[(2S)-	C43H52Cl2N8O7	863.83	C	-9.1
1481	O=c1n(cc(c(=O)[nH]1)c1c(F)cc(cc1)c1nc2c(o1)cccc2)[C@@H]1O[C@H](COP(=O)(OP(=O)(OP(=O)(O)O)O)O)[C@@H](	1352918-35-4	Uridine 5,Ä≤-(tetrahydrogen triphosphate), 5-[4-(2-benzofuranyl)-2-fluorophenyl]	C23H22FN2O15P3	678.34	C	-9.1
1482	O(P(=O)(COC[C@H]1O[C@H]([C@@H](OP(=O)(OC[C@H]2O[C@H]([C@H](O)[C@@H]2O)n2c3c(nc2)c(N)ncn3)O)[C@@H]1O)	1357016-12-6	Adenosine, 5,Ä≤-O-phosphonoadenylylmethylene-(2,Ä≤,Ü5,Ä≤)-Œ≤-D-arabino-adenyly	C41H52N20O25P4	1348.87	C	-9.1
1483	C(=O)([C@@H](NC(=O)OC)C1CCOCC1)N1[C@H](c2[nH]c(c3c4c(c(cc3)c3ccc(cc3)c3[nH]c(nc3)[C@H]3N(C(=O)[C@@H]	1359868-86-2	Carbamic acid, N-[(1S)-2-[(1R,3S,5R)-3-[5-[4-[2,3-dihydro-7-[2-[(1R,3S,5R)-2-[(2	C49H58N8O8	887.03	C	-9.1
1484	C(=O)([C@@H](NC(=O)OC)C(C)C)N1[C@@H](CCC1)c1[nH]c(cn1)c1ccc(C(Cc2ccc(cc2)c2[nH]c(nc2)[C@H]2N(C(=O)[C	1374421-54-1	Carbamic acid, N,N,Ä≤-[[1-[4-(trifluoromethyl)phenyl]-1,2-ethenediyl]bis[4,1-phe	C49H55F3N8O6	909.01	C	-9.1
1485	C(=O)([C@@H](NC(=O)OC)C1CCOCC1)N1[C@@H]([C@@H]2C[C@H]1CC2)c1[nH]c(cn1)c1c(F)cc(cc1)c1cc2c(cc1)c1c(cc	1377041-25-2	Carbamic acid, N-[(1S)-1-[[(2S)-2-[5-[7-[2-[(1R,3S,4S)-2-[(2S)-2-[(methoxycarbor	C44H52N8O6	788.93	C	-9.1
1486	C(=O)([C@@H](NC(=O)OC)C(C)C)N1[C@@H]([C@@H]2C[C@H]1CC2)c1[nH]c2c(n1)ccc1c2ccc(c1)c1ccc(Cl)c(cc1)c1[nH	1377042-88-0	Carbamic acid, N,N,Ä≤-[(9,10-dihydro-5H-phenanthro[4,5-bcd]pyran-2,7-diyl)bis[1H	C43H52N8O7	792.92	C	-9.1
1487	C(#CC)c1c(c(C#CC)cc(c1)c1[nH]c(nc1)[C@H]1N(C(=O)[C@@H](NC(=O)OC)C(C)C)CCC1)c1cc2c(cc1)c1c(cc2)nc([nH	1377042-92-6	Carbamic acid, N-[(1S)-1-[[(2S)-2-[7-[4-[2-[(2S)-1-[(2S)-2-[(methoxycarbonyl)ami	C45H52N8O6	800.94	C	-9.1
1488	C(=O)([C@@H](NC(=O)OC)C1CCOCC1)N1[C@H](c2[nH]c3c4c(cc(cc4)c4cc5c(cc4)c4c(cc5)nc([nH]4)[C@H]4N(C(=O)[	1377047-55-6	Carbamic acid, N-[(1S)-1-[[(2S)-2-[5-[4-[2-[(2S,4S)-1-[(2S)-2-[(methoxycarbonyl)	C45H56N8O7	820.98	C	-9.1
1489	C(=O)([C@@H](NC(=O)OC)C(C)C)N1[C@H](c2[nH]c3c4c(cc(cc4)c4cc5c(cc4)c4c(cc5)nc([nH]4)[C@H]4N(C(=O)[C@@	1377048-66-2	Carbamic acid, N-[(1S)-2-[(2S,4S)-2-[2,Ä≤-[(2S,4S)-1-[(2S)-2-[(methoxycarbonyl)a	C49H58N8O8	887.03	C	-9.1
1490	N(=Nc1cc(C)c(N=Nc2ccc(S(=O)(=O)O)cc2)cc1)c1c2c(c(N=Nc3ccc(O)cc3)cc1)ccc(S(=O)(=O)O)c2	1378293-74-3	2-Naphthalenesulfonic acid, 5-[2-(4-aminophenyl)diazenyl]-8-[2-[3-methyl-4-[2-(4	C29H23N7O6S2	629.67	C	-9.1
1491	N(C(=O)C)C1C(C(C(CO)O)O)OC(=CC1NC(=N)N)C(=O)O	1381969-10-3	Carbamic acid, N-[(1S)-1-[[(2S)-2-[5-[10-[4-(1,1-dimethylethyl)phenyl]-6-[2-[(2S	C52H62N8O6	895.1	C	-9.1
1492	N(CC(=O)N1CCN(S(=O)(=O)C)CC1)c1nc(nc(n1)N1CCOCC1)c1cc(OC)ccc1	1391924-46-1	Glycine, N-[4-(2,3-dihydro-1-oxo-1H-inden-4-yl)-6-(4-morpholinyl)-1,3,5-triazin-	C18H19N5O4	369.37	C	-9.1
1493	N(CCNC(=O)c1ccc(cc1)C1OOC(OO1)c1ccc(C)cc1)c1nc(nc(n1)N1CCOCC1)N1CCN(CC1)c1ccccc1	1393922-42-3	Benzamide, N-[2-[[4-(cyclohexylamino)-6-(4-morpholinyl)-1,3,5-triazin-2-yl]aminc	C31H39N7O6	605.68	C	-9.1
1494	N(Cc1ccc(Br)cn1)C12CC3CC(C2)CC(C1)C3	140942-35-4	1-Naphthalenesulfonic acid, 7,7,Ä≤-[1,2-ethanediylbis[(3-sulfo-4,1-phenylene)azc	C34H28N6O12S4.4Na		C	-9.1
1495	O[C@H]1[C@@H](O[C@H](C(=O)NCc2cc(on2)c2cc(OC)ccc2)[C@H]1O)n1nc(C(=O)N)nc1	1414801-85-6	Not Yet Assigned	C20H24N6O6	444.44	C	-9.1
1496	C(COCP1(=O)OCC(Oc2ccccc2)CO1)n1c2c(nc1)c(N)ncn2	1417393-27-1	9H-Purin-6-amine, 9-[2-[[(2S,4S,6S)-4-(3-chlorophenyl)-2-oxido-6-phenyl-1,3,2-di	C23H23ClN5O4P	499.89	C	-9.1
1497	C(=O)([C@@H](NC(=O)OC)C(C)C)N1[C@H](c2[nH]c(c3c4c(c(cc3)c3ccc(cc3)c3[nH]c(nc3)[C@H]3N(C(=O)[C@@H](NC	1419384-93-2	Carbamic acid, N-[(1S)-1-[[(2S)-2-[5-[4-[7,Ä≤-[2-[(2S)-4,4-difluoro-1-[(2S)-2-[	C47H56F4N8O6	904.99	C	-9.1
1498	O=C1c2c(c(ccc2c2ccc(cc2)c2[nH]c(nc2)[C@H]2N(C(=O)[C@@H](NC(=O)OC)C(C)C)CCC2)c2[nH]c(nc2)[C@H]2N(C(=O	1419385-23-1	Carbamic acid, N-[(1S)-1-[[(2S)-2-[5-[1,Ä≤,3,Ä≤-dihydro-7,Ä≤-[4-[2-[(2S)-1-[(2S	C51H68N8O6	889.14	C	-9.1
1499	C(OC[C@@H]1[C@@H](OC(=O)c2ccccc2)[C@@H](OP(=O)(OC[C@@H]2[C@@H](OC(=O)c3ccccc3)[C@@H](OP(=O)(OC[C@@H]	1422459-38-8	Adenosine, 1,Ä≤-[3-(aminocarbonyl)-1H-1,2,4-triazol-1-yl]-3,Ä≤-O-benzoyl-1,Ä≤-d	C60H53N10O15P	1185.09	C	-9.1
1500	C(N1c2c(C(=NNC(=S)N(CC=C)CC=C)C1=O)cc(F)cc2)N1CCN(CC1)c1ccccc1	1426583-63-2	Hydrazinecarbothioamide, 2-[5-chloro-1,2-dihydro-2-oxo-1-[(4-phenyl-1-piperaziny	C32H29ClN6OS	581.13	C	-9.1
1501	O=C1N(c2c3c(c4c(n2)cccc4)n(CC(C)C)cn3)c2c1cc(Br)cc2	1508303-87-4	7-Azabicyclo[4.2.0]octa-1,3,5-trien-8-one, 7-[1-(2-methylpropyl)-1H-imidazo[4,5-	C21H18N4O	342.39	C	-9.1
1502	O=c1c2c(n(cn2)[C@@H]2O[C@H](C=O)[C@@H](O)[C@H]2O)[nH]c(N)n1	1513874-60-6	Guanosine, 3,Ä≤-de(phosphinicooxy)guanylyliminosulfonylimino-(3,Ä≤,Ü5,Ä≤)-5,Ä≤	C20H26N12O10S	626.56	C	-9.1
1503	N(C(=O)C)[C@H]1[C@H]([C@@H]([C@@H](CO)O)O)OC(=C[C@@H]1N=[N+]=[N-])C(=O)O	151780-52-8	2-Naphthalenesulfonic acid, 8,8,Ä≤-[1,2-ethenediylbis[(3-sulfo-4,1-phenylene)-2,	C34H26N6O12S4	838.86	C	-9.1
1504	CN1c2c(N(C1)[C@@H]1O[C@H](CO)[C@@H](O)[C@H]1O)[nH]c(N)nc2=O	153116-69-9	Carbamic acid, [[[[4,Ä≤-[(2-ethyl-5,7-dimethyl-3H-imidazo[4,5-b]pyridin-3-yl)met	C26H27N5O5S	521.59	C	-9.1
1505	C(n1c2c(nc1CC)c(C)cc(C)n2)c1ccc(cc1)c1c(S(=O)(=O)NC(=O)NCCCC)ccc(C(CC)(C)C)c1	154552-43-9	[1,1,Ä≤:3,Ä≤,1,Ä≤,Ä≤-Terphenyl]-4,Ä≤-sulfonamide, N-[(butylamino)carbonyl]-4,Ä≤	C34H37N5O3S	595.75	C	-9.1
1506	C(n1c2c(nc1CC)c(C)cc(C)n2)c1ccc(cc1)c1c(S(=O)(=O)NC(=O)CCc2ccccc2)ccc(CN(C)C)c1	154552-64-4	Benzenepropanamide, N-[[5-(1,1-dimethylethyl)-4,Ä≤-[(2-ethyl-5,7-dimethyl-3H-im:	C36H40N4O3S	608.79	C	-9.1
1507	C(n1c2c(nc1CC)c(C)cc(C)n2)c1ccc(cc1)c1c(S(=O)(=O)NC(=O)c2c(Oc3ccccc3)cccc2)ccc(CC)c1	154552-71-3	Benzamide, N-[[4,Ä≤-[(2-ethyl-5,7-dimethyl-3H-imidazo[4,5-b]pyridin-3-yl)methyl]	C37H34N4O4S	630.76	C	-9.1
1508	C(n1c2c(nc1CC)c(C)cc(C)n2)c1ccc(cc1)c1c(S(=O)(=O)NC(=O)c2c(Oc3ccccc3)cccc2)ccc(CC(C)C)c1	154552-75-7	Benzamide, N-[[4,Ä≤-[(2-ethyl-5,7-dimethyl-3H-imidazo[4,5-b]pyridin-3-yl)methyl]	C39H38N4O4S	658.81	C	-9.1
1509	C(n1c2c(nc1CC)c(C)cc(C)n2)c1ccc(cc1)c1c(S(=O)(=O)NC(=O)c2ccccc2)ccc(c1)c1ccccc1	154553-21-6	Benzamide, N-[[5-ethoxy-4,Ä≤-[(2-ethyl-5,7-dimethyl-3H-imidazo[4,5-b]pyridin-3-y	C32H32N4O4S	568.69	C	-9.1
1510	C(n1c2c(nc1CC)c(C)cc(C)n2)c1ccc(cc1)c1c(S(=O)(=O)NC(C)(C)C)ccc(CCC)c1	154553-68-1	[1,1,Ä≤-Biphenyl]-2-sulfonamide, 4,Ä≤-[(2-ethyl-5,7-dimethyl-3H-imidazo[4,5-b]p	C35H39N5O4S	625.78	C	-9.1
1511	C(=O)([C@@H](NC(=O)OC)C(C)C)N1[C@H](c2[nH]c(c3c4c(C5CC4CC5)c(cc3)c3ccc(cc3)c3[nH]c(nc3)[C@H]3N(C(=O)	1560638-77-8	Carbamic acid, N,N,Ä≤-[(1,2,3,4-tetrahydro-1,4-methanonaphthalene-5,8-diyl)bis[4	C51H60N8O6	881.07	C	-9.1
1512	C(=O)([C@@H](NC(=O)OC)C(C)C)N1[C@H](c2[nH]c(c3c4c(C5CC4CC5)c(cc3)c3ccc(cc3)c3[nH]c(nc3)[C@H]3N(C(=O)	1560638-84-7	Carbamic acid, N-[(1S)-2-methyl-1-[[(2S)-2-[5-[4-[1,2,3,4-tetrahydro-8-[2-[(2S)-	C45H56N8O6	804.98	C	-9.1
1513	C(=O)([C@@H](NC(=O)OC)C(C)C)N1[C@H](c2[nH]c(c3c4c(c(cc3)c3ccc(cc3)c3[nH]c(nc3)[C@H]3N(C(=O)[C@@H](NC	1560640-23-4	Carbamic acid, N-[(1S)-1-[[(2S,4R)-4-fluoro-2-[5-[8-[4-[2-[(2S,4R)-4-fluoro-1-[(	C46H56F2N8O6	854.98	C	-9.1
1514	C(=O)([C@@H](NC(=O)OC)C(C)C)N1[C@H](c2[nH]c(c3ccc(c4c5c(c(cc4)c4ccc(cc4)c4[nH]c(nc4)[C@H]4N(C(=O)[C@	1560640-47-2	Carbamic acid, N-[(1S)-2-methyl-1-[[(2S)-2-[5-[1,2,3,4-tetrahydro-8-[4-[2-[(8S)-	C48H60N8O8	877.04	C	-9.1
1515	C(=O)([C@@H](NC(=O)OC)C(C)C)N1[C@H](c2[nH]c(c3c4c(C5CC4C=C5)c(cc3)c3ccc(cc3)c3[nH]c(nc3)[C@H]3N(C(=O	1560649-48-0	Carbamic acid, N-[(1S)-1-[[(2S)-2-[5-[8-[4-[2-[(2S)-4,4-difluoro-1-[(2S)-2-[(met	C45H53F4N9O6	891.95	C	-9.1
1516	C(=O)([C@@H](NC(=O)OC)C(C)C)N1[C@H](c2[nH]c(c3c4c(C5NC4CC5)c(cc3)c3ccc(cc3)c3[nH]c(nc3)[C@H]3N(C(=O)	1560649-93-5	Carbamic acid, N-[(1S)-1-[[(2S)-2-[5-[4-[1,4-dihydro-8-[2-[(2S)-1-[(2S)-2-[(meth	C45H55N9O6	817.97	C	-9.1
1517	N(N=O)c1c2c(c(S(=O)(=O)O)cc1N=Nc1c3c(c(S(=O)(=O)O)cc1)cccc3)cccc2	1598475-05-8	1-Naphthalenesulfonic acid, 4-[2-(2-hydroxy-7-sulfo-1-naphthalenyl)diazenyl]-	C20H14N2O7S2	458.46	C	-9.1
1518	C(=O)([C@@H](NC(=O)OC)C(C)C)N1[C@H](c2[nH]c(c3c4c(c(cc3)c3c5c(c(cc3)c3[nH]c(nc3)[C@H]3N(C(=O)[C@@H](	1612236-36-8	Carbamic acid, N-[(1S)-1-[[(2S)-2-[5-[8-[1,Ä≤,3,Ä≤-dihydro-7,Ä≤-[2-[(2S)-1-[(2S	C52H66N8O6	899.13	C	-9.1
1519	C(=O)([C@@H](NC(=O)OC(C)(C)C)C(C)C)N1[C@H](c2[nH]c(c3c4c(C5C(C4)CC5)c(cc3)c3ccc(cc3)c3[nH]c(nc3)[C@H	1613055-16-5	Carbamic acid, N-[(1S)-2-methyl-1-[[(2S)-2-[5-[4-[2,2a,7,7a-tetrahydro-6-[2-[(2S	C45H56N8O6	804.98	C	-9.1
1520	O(C)c1c2n(C[C@@H]3N(C2=O)[C@@H]2C[C@H](O3)CC2)cc(C(=O)NCc2c(F)cc(F)cc2F)c1=O	1616340-67-0	2,5-Methanopyrido[1,Ä≤,2,Ä≤:4,5]pyrazino[2,1-b][1,3]oxazepine-10-carboxamide, N	C22H18F5N3O5	499.39	C	-9.1

1521	N(c1nc(nc(n1)N1CCOCC1)N1CCOCC1)c1ccc(C(=O)C=Cc2cc(Oc3ccccc3)ccc2)cc1	1628172-07-5	2-Propen-1-one, 3-(4-bromophenyl)-1-[4-[(4,6-di-4-morpholinyl-1,3,5-triazin-2-yl	C26H27BrN6O3	551.43	C	-9.1
1522	S(c1nc(nc(Nc2ccc(F)cc2)n1)N1CCOCC1)c1oc(nn1)c1ccc(F)cc1	1642584-18-6	1,3,5-Triazin-2-amine, 4-[[5-(4-fluorophenyl)-1,3,4-oxadiazol-2-yl]thio]-N-(4-me	C22H20FN7O2S	465.5	C	-9.1
1523	N(c1nc(nc(Oc2ccc(C#N)cc2)n1)N1CCOCC1)c1cc(OC)ccc1	1643332-91-5	Benzonitrile, 4-[[4-[(3-methylphenyl)amino]-6-(4-morpholinyl)-1,3,5-triazin-2-yl	C21H20N6O2	388.42	C	-9.1
1524	N(C(=O)[C@H](Cc1ccccc1)C[C@@H](CN1[C@H](C(=O)NC(C)(C)C)CN(Cc2c(Cl)nccc2)CC1)O)[C@H]1c2c(C[C@H]1O)ccc	164725-40-0	D-erythro-Pentonamide, 5-[(2S)-4-[(6-chloro-3-pyridinyl)methyl]-2-[[(1,1-dimethy	C36H46ClN5O4	648.23	C	-9.1
1525	N(C(=O)[C@H](Cc1ccccc1)C[C@@H](CN1[C@H](C(=O)NC(C)(C)C)CN(Cc2c(F)cc(F)cc2)CC1)O)[C@H]1c2c(C[C@H]1O)c	165946-06-5	1-Piperazinepentanamide, N-(2,3-dihydro-2-hydroxy-1H-inden-1-yl)-2-[[(1,1-dimeth	C35H45N5O6S	663.83	C	-9.1
1526	C(n1c2c(nc1CC)c(C)cc(C)n2)c1ccc(cc1)c1c(S(=O)(=O)NP(=O)(OCC)OCC)cccc1	169281-47-4	Phosphoramidic acid, [[4,Ä≤-[(2-ethyl-5,7-dimethyl-3H-imidazo[4,5-b]pyridin-3-yl	C37H37N4O5PS	680.75	C	-9.1
1527	N(C(=O)[C@H](Cc1ccccc1)C[C@@H](CN1[C@H](C(=O)NC(C)(C)C)CN(Cc2cccnc2)CC1)O)[C@H]1c2c(C[C@H]1OC(=O)CN(	170227-67-5	Butanedioic acid, mono[1-[[5-[2-[[(1,1-dimethylethyl)amino]carbonyl]-4-(3-pyridi	C40H51N5O7	713.86	C	-9.1
1528	C(=O)(/C=C(\O)/c1ccccc1)c1c(OC)cc(OC)c(C(=O)C)c1O	170227-77-7	Benzoic acid, 4-(chloromethyl)-, 1-[[5-[2-[[(1,1-dimethylethyl)amino]carbonyl]-4	C44H52ClN5O5	766.37	C	-9.1
1529	C([C@H](OCP(=O)(OC(C)C)OC(C)C)C)n1c2c(nc1)c(N)nc(N)n2	173253-38-8	Guanosine, 5,Ä≤-[4-(2,5-dihydro-2,5-dioxo-1H-pyrrol-1-yl)benzoate]	C21H18N6O8	482.4	C	-9.1
1530	C(n1c2c(nc1CC)c(C)cc(C)n2)c1ccc(cc1)c1c(S(=O)(=O)NC(=O)c2c(OC)cccc2)cccc1	174844-67-8	Benzamide, N-[[4,Ä≤-[(2-ethyl-5,7-dimethyl-3H-imidazo[4,5-b]pyridin-3-yl)methyl]	C30H28N4O4S	540.63	C	-9.1
1531	C(n1c2c(nc1CC)c(C)cc(C)n2)c1ccc(cc1)c1c(S(=O)(=O)NC(=O)c2cc(OC)ccc2)cccc1	174844-70-3	Benzamide, N-[[4,Ä≤-[(2-ethyl-5,7-dimethyl-3H-imidazo[4,5-b]pyridin-3-yl)methyl]	C30H28N4O4S	540.63	C	-9.1
1532	S(=O)(=O)(O)c1c2c(c(N)c(N=Nc3ccc(N(=Nc4cc(S(=O)(=O)O)c(C=Cc5c(S(=O)(=O)O)cccc5)cc4)=O)cc3)c1)cccc2	180074-89-9	1-Naphthalenesulfonic acid, 4-amino-3-[2-[4-[1-oxido-2-(4-sulfophenyl)diazenyl]p	C22H17N5O7S2	527.53	C	-9.1
1533	O=c1n([C@@H]2O[C@H](COP(=O)(N[C@H](C(=O)O)C)O)C=C2)cc(C)c(=O)[nH]1	180075-13-2	1-Naphthalenesulfonic acid, 4-amino-3-[2-[4-[1-oxido-2-[3-sulfo-4-[2-(2-sulfophe	C30H23N5O10S3	709.73	C	-9.1
1534	C(=O)([C@@H](NC(=O)OC)C(C)(C)C)N1[C@H]([C@@H]2C[C@H]1CC2)c1[nH]c(cn1)c1cc2c(-c3c(C(F)(F)C2(F)F)cc(c	1801161-67-0	Carbamic acid, N,N,Ä≤-[(9,9,10,10-tetrafluoro-9,10-dihydro-2,7-phenanthrenediyl)	C46H52F4N8O6	888.95	C	-9.1
1535	C(=O)([C@@H](NC(=O)OC)C(C)C)N1CC2(C[C@H]1c1[nH]c(cn1)c1cc3c(-c4c(C(F)(F)C3(F)F)cc(cc4)c3[nH]c(nc3)[C	1801161-71-6	Carbamic acid, N,N,Ä≤-[(9,9,10,10-tetrafluoro-9,10-dihydro-2,7-phenanthrenediyl)	C44H52F4N8O6	864.93	C	-9.1
1536	O=C1[C@]2([C@H]([C@H](/C(=C/C(=O)[C@@H]3[C@@]4(N5[C@@H]([C@H]3cc(OC)c(O)cc3)CCC5)c3c(NC4=O)ccc(OC)	1807888-50-1	Not Yet Assigned	C47H48N4O10	828.9	C	-9.1
1537	O=C1[C@@H]2[C@@H](c3c([C@H](OC(=O)C(C)N4C(=O)c5c(C4=O)cccc5)[C@H]2CO1)cc1c(c3)OCO1)c1cc(OC)c(OC)c(OC	1809321-42-3	2H-Isoindole-2-acetic acid, octahydro-1,3-dioxo-Œ±-(phenylmethyl)-, (5R,5aR,8aR,	C39H39NO11	697.73	C	-9.1
1538	O=C1[C@@H]2[C@@H](c3c([C@H](OC(=O)C(C)N4C(=O)C5C(C4=O)CCCC5)[C@H]2CO1)cc1c(c3)OCO1)c1cc(OC)c(OC)c(OC	1809325-29-8	4,7-Epoxy-2H-isoindole-2-acetic acid, octahydro-1,3-dioxo-Œ±-(phenylmethyl)-, (5	C39H37NO12	711.71	C	-9.1
1539	O=C1[C@@H]2[C@@H](c3c([C@H](OC(=O)CCCC[C@H]4SC(SCC4)c4c(OC)cccc4)[C@H]2CO1)cc1c(c3)OCO1)c1cc(OC)c(OC	1875123-86-6	1,3-Dithiane-4-pentanoic acid, 2-[4-(trifluoromethyl)phenyl]-, (5R,5aR,8aR,9R)-5	C38H39F3O9S2	760.84	C	-9.1
1540	C(=O)(CC(=O)c1c(O)ccc(C)c1)c1c(OC)cc(O)cc1	1882055-62-0	6H,7H-[1]Benzopyrano[4,3-b][1]benzopyran-7-one, 10-hydroxy-6-(2H-tetrazol-5-yl)-	C17H10N4O4	334.29	C	-9.1
1541	O(C(=O)OCCS(=O)(=O)C)c1c2n(N([C@H]3N(C2=O)CCOC3)[C@H]2c3c(CSc4c2cccc4)c(F)c(F)cc3)ccc1=O	1985607-27-9	Carbonic acid, (12aR)-12-[(11S)-7,8-difluoro-6,11-dihydrodibenzo[b,e]thiepin-11-	C31H27F2N3O8S	639.62	C	-9.1
1542	O(C(=O)OCCO[Si](C(C)(C)C)(C)C)c1c2n(N([C@H]3N(C2=O)CCOC3)[C@H]2c3c(CSc4c2cccc4)c(F)c(F)cc3)ccc1=O	1985607-44-0	1H-[1,4]Oxazino[3,4-c]pyrido[2,1-f][1,2,4]triazine-6,8-dione, 12-[(11S)-7,8-difl	C32H27F2N3O4S	587.64	C	-9.1
1543	O(C(=O)OC(CC(=O)OCC)(C)C)c1c2n(N([C@H]3N(C2=O)CCOC3)[C@H]2c3c(CSc4c2cccc4)c(F)c(F)cc3)ccc1=O	1985607-49-5	Carbamic acid, N,N-dimethyl-, 4-[[[(12aR)-12-[(11S)-7,8-difluoro-6,11-dihydrodib	C34H30F2N4O6S	660.69	C	-9.1
1544	O(C(=O)O[C@@H](C(=O)OC)c1ccccc1)c1c2n(N([C@H]3N(C2=O)CCOC3)[C@H]2c3c(CSc4c2cccc4)c(F)c(F)cc3)ccc1=O	1985607-52-0	Carbonic acid, (12aR)-12-[(11S)-7,8-difluoro-6,11-dihydrodibenzo[b,e]thiepin-11-	C31H27F2N3O8S	639.62	C	-9.1
1545	O(Cc1c(oc(=O)o1)c1ccccc1)c1c2n(N([C@H]3N(C2=O)CCOC3)[C@H]2c3c(CSc4c2cccc4)c(F)c(F)cc3)ccc1=O	1985607-60-0	1H-[1,4]Oxazino[3,4-c]pyrido[2,1-f][1,2,4]triazine-6,8-dione, 12-[(11S)-7,8-difl	C30H24F2N4O4S	574.6	C	-9.1
1546	N(C(=O)Nc1c(OC)cc(Cl)cc1)c1cc2cc(C34CC5CC(C4)CC(C3)C5)[nH]c2cc1	1987854-67-0	Urea, N-(2-methyl-4-nitrophenyl)-N,Ä≤-(2-tricyclo[3.3.1.13,7]dec-1-yl-1H-indol-5	C26H28N4O3	444.53	C	-9.1
1547	N(C(=O)Nc1cc(C(=O)OC)ccc1C)c1cc2cc(C34CC5CC(C4)CC(C3)C5)[nH]c2cc1	1987854-69-2	Urea, N-(4-chloro-2-methylphenyl)-N,Ä≤-(2-tricyclo[3.3.1.13,7]dec-1-yl-1H-indol-	C26H28ClN3O	433.97	C	-9.1
1548	N(C(=O)Nc1cc(OC)cc(OC)c1)c1cc2cc(C34CC5CC(C4)CC(C3)C5)[nH]c2cc1	1987854-70-5	Benzoic acid, 4-methyl-3-[[[(2-tricyclo[3.3.1.13,7]dec-1-yl-1H-indol-5-yl)amino]	C28H31N3O3	457.56	C	-9.1
1549	N(C(=O)Nc1cc(C(C)(C)C)on1)c1cc2cc(C34CC5CC(C4)CC(C3)C5)[nH]c2cc1	1987854-79-4	Urea, N-2-benzothiazolyl-N,Ä≤-(2-tricyclo[3.3.1.13,7]dec-1-yl-1H-indol-5-yl)-	C26H26N4OS	442.58	C	-9.1
1550	C(=O)([C@@H](NC(=O)OC)[C@H](CC)C)N1[C@H](c2[nH]c3c4c(cc(cc4)c4c5c(c(cc4)c4[nH]c(nc4)[C@H]4N(C(=O)[C@	2007900-74-3	Carbamic acid, N-[(1S)-2-[(2S,5S)-2-[7-[2,3-dihydro-7-[2-[(2S,5S)-1-[(2S)-2-[(me	C51H62N8O8	915.09	C	-9.1
1551	C(=O)([C@@H](NC(=O)OC)C(C)C)N1[C@H](c2[nH]c3c4c(cc(cc4)c4c5c(c(cc4)c4[nH]c(nc4)[C@H]4N(C(=O)[C@@H](N	2007901-33-7	Carbamic acid, N-[(1S)-1-[[(2S)-2-[7-[2,3-dihydro-7-[2-[(6S)-5-[(2S)-2-[(methoxy	C47H56N8O6	829	C	-9.1
1552	O[C@H]1[C@@H](O[C@H](COCC(=O)NCCNC(=O)[C@@H](NC(=O)CO[C@@H]2O[C@H](CO)[C@H](O)[C@H](O)[C@H]2O)CCCCNC	1002334-80-6	1H-1,2,4-Triazole-3-carboxamide, 5,Ä≤,5,Ä≤,Ä≤,Ä≤-O-[[2-[[2-(Œ≤-D-galactopyranos	C31H47N11O19	877.77	A/B	-9
1553	C(C(C(NC(=O)c1c(C)c(O)ccc1)CSc1cc(CC)ccc1O)O)N1C(C(=O)NC(C)(C)C)CC2C(C1)CCCC2	1009057-19-5	3-Isoquinolinecarboxamide, N-(1,1-dimethylethyl)decahydro-2-[2-hydroxy-3-[(3-hyc	C38H49N3O5S	659.88	A/B	-9
1554	C(=O)([C@H](NC(=O)OC(C)(C)C)C(C)C)N1[C@@H](CCC1)c1[nH]c(cn1)c1ccc(cc1)c1ccc(cc1)c1[nH]c(nc1)[C@H]1N(	1009106-27-7	Carbamic acid, N-[(1R)-2-[(2S)-2-[5-[4,Ä≤-[2-[(2S)-1-[(2R)-2-[(methoxycarbonyl)a	C46H46N8O6	806.91	A/B	-9
1555	O([C@H](CC)C=C)[C@H]1[C@H](NC(=O)C)[C@@H](N)CC(=C1)C(=O)O	10211-53-7	2-Naphthalenesulfonic acid, 5-amino-8-[2-[4-[2-[2,4-diamino-5-[2-[3-[2-(2,4-diam	C35H31N13O6S2.2Na		A/B	-9
1556	C(C(C)C)n1c2c(nc1N1CCCC1)c(N)nc1c2cccc1	1026369-91-4	2-Naphthalenesulfonic acid, 8-amino-5-[2-(4,Ä≤-hydroxy[1,1,Ä≤-biphenyl]-4-yl)hy	C22H19N3O4S	421.47	A/B	-9
1557	N(c1nc(Nc2ccc(C#N)cc2)nc(COCC=C)c1)c1c(C)cc(/C=C/C#N)cc1C	1037444-77-1	Benzonitrile, 4-[[4-[[4-[(1E)-2-cyanoethenyl]-2,6-dimethylphenyl]amino]-6-(3-pyr	C27H21N7	443.5	A/B	-9
1558	N(c1c(C)cc(/C=C/C#N)cc1C)c1nc(Nc2ccc(C#N)cc2)nc(CNCC2CC2)c1	1037445-00-3	Benzonitrile, 4-[[4-[[4-[(1E)-2-cyanoethenyl]-2,6-dimethylphenyl]amino]-6-[(4-me	C28H30N8	478.59	A/B	-9
1559	N(c1c(C)cc(/C=C/C#N)cc1C)c1nc(Nc2ccc(C#N)cc2)nc(CNCc2ccco2)c1	1037445-24-1	Benzonitrile, 4-[[4-[[4-[(1E)-2-cyanoethenyl]-2,6-dimethylphenyl]amino]-6-[[(2-p	C29H26N8	486.57	A/B	-9
1560	N(c1c(C)cc(/C=C/C#N)cc1C)c1nc(Nc2ccc(C#N)cc2)nc(CNCC2CCCO2)c1	1037445-28-5	Benzonitrile, 4-[[4-[[4-[(1E)-2-cyanoethenyl]-2,6-dimethylphenyl]amino]-6-[[(3-p	C29H26N8	486.57	A/B	-9
1561	C(=O)(NC1CC1)c1c(Nc2c(C)cc(/C=C/C#N)cc2C)nc(Nc2ccc(C#N)cc2)nc1COC	1037452-37-1	3-Piperidinecarboxamide, 1-[[4-[[4-[(1E)-2-cyanoethenyl]-2,6-dimethylphenyl]amir	C31H32N8O3	564.64	A/B	-9
1562	C(C)N1c2c(NC(=O)c3c1nccc3)c(C)cc(=O)[nH]2	1057218-04-8	Phosphonic acid, P-[[[5-bromo-6-(4-cyano-2,6-dimethylphenoxy)-2-[(4-cyanophenyl)	C35H30BrN6O4P	709.53	A/B	-9
1563	O(C(=O)N[C@@H](Cc1ccc(CNCCP(=O)(Oc2ccccc2)O[C@@H](C(=O)OC)C)cc1)[C@@H](CN(S(=O)(=O)c1ccc(OC)cc1)CC(C	1057218-68-4	Phosphonic acid, P-[[[4-amino-5-bromo-6-(4-cyano-2,6-dimethylphenoxy)-2-pyrimidi	C35H30BrN6O4P	709.53	A/B	-9
1564	O=C1[C@@H]2[C@@H](c3c([C@H](OC(=O)C(=O)c4c5c(n(Cc6cc(Cl)c(Cl)cc6)c4)cccc5)[C@H]2CO1)cc1c(c3)OCO1)c1c	1061717-04-1	1H-Indole-3-acetic acid, 1-[(4-chlorophenyl)methyl]-Œ±-oxo-, (5R,5aR,8aR,9R)-5,5	C39H32ClNO10	710.13	A/B	-9
1565	N(C(=O)[C@H](Cc1ccccc1)C[C@@H](CN1[C@H](C(=O)NC(C)(C)C)CN(C/C=C/c2ccccc2)CC1)O)[C@H]1c2c(C[C@H]1O)cc	1064475-83-7	Not Yet Assigned	C39H50N4O5	654.84	A/B	-9
1566	C(n1c2c(nc1)c(N)ncn2)C1COP(=O)(O)CO1	106931-26-4	4-Quinolinamine, N-[4-[4-[2-amino-5-(4-chlorophenyl)-6-ethyl-4-pyrimidinyl]-1-pi	C30H35Cl2N7	564.55	A/B	-9
1567	O=c1n(C2OC(COP3(=O)Oc4c(C(=O)N3)nc(F)cn4)C=C2)cc(F)nc1C(=O)N	1079990-13-8	Thymidine, 2,Ä≤,3,Ä≤-didehydro-3,Ä≤-deoxy-5,Ä≤-O-(6-fluoro-3,4-dihydro-2-oxido-	C15H13FN5O7P	425.27	A/B	-9
1568	C(NS(=O)(=O)c1cc2c(cc1)C(=NO)c1c(C2=NO)cc(S(=O)(=O)NC(C)C23CC4CC(C3)CC(C2)C4)cc1)(C)C12CC3CC(C2)CC(C	1083159-44-7	2,7-Anthracenedisulfonamide, 9,10-dihydro-9,10-dioxo-N2,N7-bis(1-tricyclo[3.3.1.	C38H46N2O6S2	690.91	A/B	-9
1569	C(NC(=O)c1nnc(C)o1)(C)(C)c1nc(C(=O)NC(c2ccc(F)cc2)([2H])[2H])c(O)c(=O)n1C	1100750-83-1	4-Pyrimidinecarboxamide, N-[(4-fluorophenyl)methyl]-1,6-dihydro-5-hydroxy-1-(met	C20H18D3FN6O5		A/B	-9
1570	C(NC(=O)c1nnc(C([2H])([2H])[2H])o1)(C([2H])([2H])[2H])(C([2H])([2H])[2H])c1nc(C(=O)NCc2ccc(F)cc2)c(O	1100750-84-2	4-Pyrimidinecarboxamide, N-[(4-fluorophenyl)methyl-d2]-1,6-dihydro-5-hydroxy-1-m	C20H19D2FN6O5		A/B	-9
1571	C(NC(=O)c1nnc(C([2H])([2H])[2H])o1)(C)(C)c1nc(C(=O)NC(c2ccc(F)cc2)([2H])[2H])c(O)c(=O)n1C([2H])([2H]	1100750-91-1	4-Pyrimidinecarboxamide, N-[(4-fluorophenyl)methyl]-1,6-dihydro-5-hydroxy-1-(met	C20H9D12FN6O5		A/B	-9
1572	C(NC(=O)c1nnc(C)o1)(C([2H])([2H])[2H])(C([2H])([2H])[2H])c1nc(C(=O)NCc2c(c(c(F)c(c2[2H])[2H])[2H])[2	1100750-95-5	4-Pyrimidinecarboxamide, N-[(4-fluorophenyl-2,3,5,6-d4)methyl]-1,6-dihydro-5-hyc	C20H14D7FN6O5		A/B	-9
1573	C(NC(=O)c1nnc(C([2H])([2H])[2H])o1)(C)(C)c1nc(C(=O)NC(c2c(c(c(F)c(c2[2H])[2H])[2H])[2H])([2H])[2H])c	1100751-00-5	4-Pyrimidinecarboxamide, N-[(4-fluorophenyl-2,3,5,6-d4)methyl]-1,6-dihydro-5-hyc	C20H11D10FN6O5		A/B	-9
1574	C(NC(=O)c1nnc(C)o1)(C([2H])([2H])[2H])(C([2H])([2H])[2H])c1nc(C(=O)NCc2c(c(c(F)c(c2[2H])[2H])[2H])[2	1100751-01-6	4-Pyrimidinecarboxamide, N-[(4-fluorophenyl-2,3,5,6-d4)methyl-d2]-1,6-dihydro-5-	C20H12D9FN6O5		A/B	-9
1575	O[C@@H]1[C@H](n2c3c(nc2)c(N)ncn3)O[C@H](COC(=O)[C@H](C)N2C(=O)c3c(C2=O)cccc3)[C@H]1O	111392-86-0	9H-Purin-6-amine, 9-[5-O-[2-(1,3-dihydro-1,3-dioxo-2H-isoindol-2-yl)-1-oxopropyl	C21H20N6O7	468.42	A/B	-9
1576	C(Cc1ccccc1)[C@]1(CCC)CC(=C([C@H](CC)c2cc(N(S(=O)(=O)c3ccc(C(F)(F)F)cn3)[2H])ccc2)C(=O)O1)O[2H]	1126619-10-0	Not Yet Assigned	C31D33F3N2O5S		A/B	-9
1577	C(C)c1nc(nc(n1)N1CCOCC1)N1CCC(C(=O)NCc2c(C(F)(F)F)cccc2)CC1	1141895-01-3	4-Piperidinecarboxamide, 1-[4-(4-morpholinyl)-1,3,5-triazin-2-yl]-N-[[2-(trifluc	C21H25F3N6O2	450.46	A/B	-9
1578	O=c1n(C2O[C@H](CO)[C@@H](O)C2)ccc(=O)[nH]1	1146971-89-2	Œ≤-D-arabino-Adenosine, 2,Ä≤-O-dephosphinico-5,Ä≤-O-phosphono-Œ≤-D-arabino-ader	C43H56N20O25P4	1376.92	A/B	-9
1579	C(N1c2c(/C(=N/NC(=S)N)/C1=O)cccc2)c1ccccc1	1164453-93-3	Hydrazinecarbothioamide, 2-[1,2-dihydro-5-(4-morpholinylsulfonyl)-2-oxo-1-(2-prc	C23H25N5O4S2	499.61	A/B	-9
1580	C(F)(F)(F)c1n(nc(C)c1)c1nc(nc(n1)N1CCOCC1)c1cnc(N)nc1	1174191-13-9	2-Pyrimidinamine, 5-[4-(4-morpholinyl)-6-[3-(trifluoromethyl)-1H-pyrazol-1-yl]-1	C15H14F3N9O	393.33	A/B	-9
1581	N(=Nc1c2c(cc(S(=O)(=O)O)cc2)ccc1)c1c2c(c(S(=O)(=O)O)ccc2)c(N)cc1	1174191-14-0	2-Pyrimidinamine, 5-[4-[3-methyl-5-(trifluoromethyl)-1H-pyrazol-1-yl]-6-(4-morph	C16H16F3N9O	407.35	A/B	-9
1582	C(F)(F)(F)c1n(nc(C)c1)c1nc(nc(n1)N1CCOCC1)c1ccc(N)nc1	1174191-21-9	2-Pyridinamine, 5-[4-(4-morpholinyl)-6-[3-(trifluoromethyl)-1H-pyrazol-1-yl]-1,3	C16H15F3N8O	392.34	A/B	-9
1583	CN1Cc2c(-c3c(cc(cc3)c3ccc(cc3)c3[nH]c(nc3)[C@H]3N(C(=O)[C@@H](NC(=O)OC)C(C)C)CCC3)Cl)[nH]c(n2)[C@H]1	1180669-80-0	Carbamic acid, N-[(1S)-1-[[(2S)-2-[8-[4-[2-[(2S)-4,4-difluoro-1-[(2S)-2-[(methox	C43H52F2N8O6	814.92	A/B	-9
1584	C(=O)([C@@H](NC(=O)OC)C(C)C)N1[C@H](c2[nH]c3-c4c(cc(cc4)c4ccc(cc4)c4[nH]c(nc4)[C@H]4N(C(=O)[C@@H](NC	1180669-85-5	Carbamic acid, N,N,Ä≤-[(1,1,Ä≤,4,4,Ä≤,5,5,Ä≤,6,6,Ä≤-octahydro[8,8,Ä≤-bibenzo[3	C46H58N8O6	819	A/B	-9
1585	C(=O)([C@@H](NC(=O)OC)C(C)C)N1[C@H](c2[nH]c3-c4c(cc(cc4)c4cc5c(-c6c(CC5)nc([nH]6)[C@H]5N(C(=O)[C@@H]	1180670-02-3	Carbamic acid, N-[(1S)-1-[[(1R,3S,5R)-3-[5-[4-[4,5-dihydro-2-[(1R,3S,5R)-2-[(2S)	C46H53F3N8O6	870.96	A/B	-9
1586	C(=O)([C@@H](NC(=O)OC)C(C)C)N1[C@H](c2[nH]c3-c4c(cc(cc4)c4ccc(cc4)c4[nH]c(nc4)[C@H]4N(C(=O)[C@@H](NC	1180670-09-0	Carbamic acid, N-[(1S)-1-[[(1R,3S,5R)-3-[5-[4-[4,5-dihydro-2-[(1R,3S,5R)-2-[(2S)	C44H51FN8O6	806.92	A/B	-9
1587	N(C(=O)Nc1ccc(C(=O)N2CCC(N(C)C)CC2)cc1)c1ccc(c2nc(nc(n2)N2CCOCC2)N2CCOCC2)cc1	1197160-28-3	Urea, N-[4-[4-(4-morpholinyl)-6-(3-oxa-8-azabicyclo[3.2.1]oct-8-yl)-1,3,5-triazi	C25H28N8O3	488.54	A/B	-9
1588	C(=O)(N[C@H](C(=O)C(=O)NC1CC1)CCC)[C@H]1N(C(=O)[C@@H](NC(=O)[C@@H](NC(=O)c2cnccn2)C2CCCCC2)CNS(=O)(=	1208119-19-0	Cyclopenta[c]pyrrole-1-carboxamide, (2S)-2-cyclohexyl-N-(2-pyrazinylcarbonyl)gly	C36H52N8O7	708.85	A/B	-9
1589	O=c1n([C@@H]2O[C@H](CO)[C@@H](O)C2)ccc([NH-])n1	121135-53-3	Inosine, 3,Ä≤-azido-3,Ä≤-deoxythymidylyl-(5,Ä≤,Ü5,Ä≤)-2,Ä≤,3,Ä≤-dideoxy-	C20H24N9O9P		A/B	-9
1590	C(=O)(NC(C)(C)C)[C@H]1N(C[C@H]([C@H](Cc2ccccc2)NC(=O)[C@@H](NC(=O)c2nc3c(cc2)cccc3)CC(=O)NCCOCCOC)O)	1219093-07-8	Poly(oxy-1,2-ethanediyl), Œ±-[2-[[(3S)-4-[[(1S,2R)-3-[(3S,4aS,8aS)-3-[[(1,1-dime	(C2H4O)nC41H56N6O6		A/B	-9
1591	N(CCCCN(CC)CC)(CCCCN(CC)CC)c1c2c(ncc1)cc(Cl)cc2	1222804-50-3	Hexanamide, N-[[4,Ä≤-[(2-ethyl-5,7-dimethyl-3H-imidazo[4,5-b]pyridin-3-yl)methyl	C34H43N5O3S	601.8	A/B	-9
1592	N(C(=O)[C@H](Cc1ccccc1)C[C@@H](CN1[C@H](C(=O)NC(C)(C)C)CN(Cc2cn(C)c(=O)cc2)CC1)O)C1c2c(C[C@H]1O)cccc	1223089-55-1	1-Piperazinepentanamide, N-[(2R)-2,3-dihydro-2-hydroxy-1H-inden-1-yl]-2-[[(1,1-c	C37H49N5O3	611.82	A/B	-9
1593	C(=O)(N[C@H](C(=O)C(=O)NC1CC1)CCC)[C@H]1N(C(=O)[C@H](NC(=O)[C@H](NC(O)c2cnccn2)C2CCCCC2)C(C)(C)C)CC[	1223091-84-6	Not Yet Assigned	C37H49N5O5	643.82	A/B	-9
1594	[C@@H](C(=O)N[C@@H](Cc1ccccc1)C[C@@H]([C@](C(c1c(c(c(c(c1[2H])[2H])[2H])[2H])[2H])([2H])[2H])(NC(=O)	1224729-45-6	1(2H)-Pyrimidine-4,5,6-d3-acetamide, N-[(1S,3S,4S)-4-[[2-(2,6-dimethylphenoxy)ac	C37H32D16N4O5		A/B	-9
1595	C(F)(F)(F)c1c(cnc(NC(=O)C)c1)c1nc(nc(n1)N1CCOCC1)N1CCOCC1	1225037-82-0	Acetamide, N-[5-(4,6-di-4-morpholinyl-1,3,5-triazin-2-yl)-4-(trifluoromethyl)-2-	C18H21F3N8O3	454.41	A/B	-9
1596	c1(nc(nc(n1)N1CCOCC1)N1CCOCC1)c1cc2c(cc1)nc[nH]2	1225037-86-4	1H-Indole, 5-(4,6-di-4-morpholinyl-1,3,5-triazin-2-yl)-	C19H22N6O2	366.42	A/B	-9
1597	C(#N)C1=C(N)Oc2c(C1c1ccncc1)ccn1c2ncc1	1225373-21-6	7H-Imidazo[1,2-a]pyrano[2,3-c]pyridine-8-carbonitrile, 9-amino-7-(5-methyl-3-isc	C15H11N5O2	293.28	A/B	-9
1598	N(Cc1ccccn1)c1nc(nc(n1)N1CCOCC1)c1c2c(nc[nH]2)ccc1	1225639-00-8	1,3,5-Triazin-2-amine, 4-(1H-benzimidazol-6-yl)-6-(4-morpholinyl)-N-(2-pyridinyl	C20H20N8O	388.43	A/B	-9
1599	O(P(=O)(OC[C@H]1O[C@H]([C@H](O)[C@@H]1O)n1nc(C(=O)N)nc1)N[C@H](C(=O)OCc1ccccc1)C)c1c2c(ccc1)cccc2	1228377-63-6	Glycine, N-[hydroxy(1-naphthalenyloxy)phosphinyl]-, phenylmethyl ester, 5,Ä≤-est	C32H34N5O9P	663.61	A/B	-9
1600	C(NC(=O)[C@H](Cc1c2c([nH]c1)cccc2)N)(C(=O)NCC(=O)N[C@@H](Cc1ccccc1)C(=O)N[C@@H](Cc1ccccc1)C(=O)O)C12	1229574-80-4	Glycine, L-tyrosyl-2-tricyclo[3.3.1.13,7]dec-1-ylglycylglycyl-L-phenylalanyl-	C34H43N5O7	633.73	A/B	-9
1601	C(O[C@@H]([C@H](C)O)CO)n1c2c(nc1)c(=O)nc(N)[nH]2	1235508-38-9	Acetamide, N-[6,9-dihydro-9-[(1S,3R,4S)-2-methylene-4-(phenylmethoxy)-3-[(phenyl	C28H29N5O4	499.56	A/B	-9
1602	[C@H](Cc1ccccc1)([C@@H]([C@@H]([C@H](Cc1ccccc1)NC(=O)OCc1cncs1)O)O)NC(=O)[C@@H](NC(=O)N(Cc1nc(C(C)C)	1236408-47-1	2,7,10,12-Tetraazatridecanoic acid, 4-hydroxy-12-methyl-9-(1-methylethyl)-8,11-c	C41H48N6O5S2	768.99	A/B	-9
1603	[C@H](Cc1ccccc1)(C[C@@H]([C@H](Cc1ccccc1)NC(=O)OCc1cnco1)O)NC(=O)[C@@H](NC(=O)N(Cc1nc(C(C)C)sc1)CC)C	1236409-74-7	2,7,10,12-Tetraazatridecanoic acid, 4-hydroxy-12-methyl-9-(1-methylethyl)-8,11-c	C41H48N6O5S2	768.99	A/B	-9
1604	C(OCCOC(=O)c1ccc(CN2CCOCC2)cc1)n1c2c(nc1)c(=O)nc(N)[nH]2	1237515-04-6	[1,1,Ä≤-Biphenyl]-4-carboxylic acid, 3-[[(4-hydroxy[1,1,Ä≤-biphenyl]-3-yl)carbo:	C26H19NO4	409.43	A/B	-9
1605	N(C(=O)[C@H](Cc1ccccc1)C[C@@H](CN1[C@H](C(=O)NC(C)(C)C)CN(CCc2cc3c([nH]2)cccc3)CC1)O)[C@H]1c2c(C[C@H	1237808-97-7	Not Yet Assigned	C38H47N5O4S	669.88	A/B	-9
1606	N(C(=O)[C@H](Cc1ccccc1)C[C@@H](CN1[C@H](C(=O)NC(C)(C)C)CN(Cc2c3c(ncc2)cccc3)CC1)O)[C@H]1c2c(C[C@H]1O	1237809-04-9	Not Yet Assigned	C38H48N6O4	652.83	A/B	-9
1607	N(C(=O)[C@H](Cc1ccccc1)C[C@@H](CN1[C@H](C(=O)NC(C)(C)C)CN(Cc2cc3c(s2)scc3)CC1)O)[C@H]1c2c(C[C@H]1O)c	1237988-61-2	Not Yet Assigned	C39H53N5O6	687.87	A/B	-9
1608	N(C(=O)[C@H](Cc1ccccc1)C[C@@H](CN1[C@H](C(=O)NC(C)(C)C)CN(Cc2ccc(Br)s2)CC1)O)[C@H]1c2c(C[C@H]1O)cccc	1237989-10-4	D-erythro-Pentonamide, 2,3,5-trideoxy-N-[(1S,2R)-2,3-dihydro-2-hydroxy-1H-inden-	C35H46N4O5	602.76	A/B	-9
1609	O=C1[C@@H]2[C@@H](c3c([C@H](OC(=O)CCCCCCC=CCC=CCC=CCC)[C@H]2CO1)cc1c(c3)OCO1)c1cc(OC)c(OC)c(OC)c1	123824-80-6	1-Piperazinecarboxylic acid, 4-(1,3-benzodioxol-5-ylmethyl)-, 5,5a,6,8,8a,9-hexa	C35H36N2O11	660.67	A/B	-9
1610	C(F)(F)c1n(c2c(n1)cccc2)c1nc(nc(N[C@H]2CC[C@H](NC(=O)CBr)CC2)n1)N1CCOCC1	1240023-37-3	Acetamide, 2-amino-N-[trans-4-[[4-[2-(difluoromethyl)-1H-benzimidazol-1-yl]-6-(4	C23H29F2N9O2	501.53	A/B	-9
1611	C(F)(F)c1n(c2c(n1)cccc2)c1nc(nc(N[C@H]2CC[C@H](NC(=O)N3CCOCC3)CC2)n1)N1CCOCC1	1240024-41-2	Urea, N-[trans-4-[[4-[2-(difluoromethyl)-1H-benzimidazol-1-yl]-6-(4-morpholinyl)	C24H31F2N9O2	515.56	A/B	-9
1612	[C@@H](C(=O)N[C@@H](Cc1ccccc1)C[C@@H]([C@H](Cc1ccccc1)NC(=O)COc1c(C)cccc1C)O)(C(C)C)N1C(=O)NCCC1.O(C	12407-24-8	Cuprate(2-), bis[4-[(4,5-dihydro-3-methyl-5-oxo-1-phenyl-1H-pyrazol-4-yl)azo]-3-	C40H28CuN8O10S2.2Na		A/B	-9
1613	C(=O)([C@@H](NC(=O)OC)C1CCOCC1)N1[C@H](c2[nH]c(c3cc4c(-c5c(C4)cc(cc5)c4[nH]c(nc4)[C@H]4N(C(=O)[C@@H]	1240880-80-1	Carbamic acid, N,N,Ä≤-[9H-fluorene-2,7-diylbis[1H-imidazole-5,2-diyl-(2S)-2,1-py	C47H58N8O8	863.01	A/B	-9
1614	C(=O)([C@@H](NC(=O)OC)[C@H](C)O)N1[C@@H](CCC1)c1[nH]c(cn1)[C@H]1CC[C@@H](CC1)c1ccc(cc1)c1[nH]c(nc1)[	1240887-52-8	Carbamic acid, N-[(1S,2R)-2-hydroxy-1-[[(2S)-2-[5-[4-[trans-4-[2-[(2S)-1-[(2S,3F	C38H52N8O8	748.87	A/B	-9
1615	C(=O)([C@H](NC(=O)OC)c1ccccc1)N1[C@@H](CCC1)c1[nH]c(cn1)[C@H]1CC[C@@H](CC1)c1ccc(cc1)c1[nH]c([C@@H](	1240890-46-3	Carbamic acid, N-[(1R)-2-[(2S)-2-[5-[trans-4-[4-[2-[(1S)-1-[[(2R)-2-[(methoxycar	C45H52N8O6	800.94	A/B	-9
1616	C(=O)([C@H](NC(=O)OC)c1ccccc1)N1[C@@H](CCC1)c1[nH]c(cn1)C1=CC[C@@H](CC1)c1ccc(cc1)c1[nH]c(nc1)[C@H]1	1240891-47-7	Carbamic acid, N-[(1R)-2-[(2S)-2-[5-[4-[(1S)-4-[2-[(2S)-1-[(2R)-2-[(methoxycarbc	C46H50N8O6	810.94	A/B	-9
1617	[C@@H](NC(=O)[C@H](Cc1ccccc1)NC(=O)N1CCN(C)CC1)(/C=C/S(=O)(=O)c1ccccc1)c1ccc(cc1)c1ccccc1	1253912-37-6	1-Piperazinecarboxamide, 4-methyl-N-[(1S)-2-oxo-1-(phenylmethyl)-2-[[(1R,2E)-3-(	C33H40N4O4S	588.76	A/B	-9
1618	C(=O)([C@@H](NC(=O)OC)C(C)C)N1CC2(C[C@H]1c1[nH]c(cn1)c1cc3c(cc1)cc(cc3)c1ccc(cc1)c1[nH]c(nc1)[C@H]1N	1256392-10-5	Carbamic acid, N-[(1S)-2-[(2S)-2-[5-[9,9-difluoro-7-[2-[(6S)-5-[(2S)-2-[(methoxy	C45H52F2N8O7	854.94	A/B	-9
1619	O=c1c2c(n(cn2)[C@@H]2O[C@H](COP(=O)(OP(=O)(OP(=O)(O)O)O)O)[C@@H](O)C2)[nH]c(NCCCCCC)n1	127860-13-3	Guanosine, 5,Ä≤-[3-(fluorosulfonyl)benzoate]	C17H16FN5O8S	469.4	A/B	-9
1620	Cn1c(c2c([nH]c(n2)[C@H]2N(C(=O)[C@@H](NC(=O)OC)C3CCOCC3)CCC2)c2ccc(cc2)c2ccc(cc2)c2[nH]c(nc2)[C@H]2N	1312337-05-5	Carbamic acid, N-[(1S)-1-[[(2S)-2-[5-[4,Ä≤-[2-[(2S)-1-[(2S)-2-[(methoxycarbonyl)	C44H54N10O6	818.96	A/B	-9
1621	C(=O)([C@@H](NC(=O)OC)[C@@H]1CCCOC1)N1[C@H]2[C@H](C2)C[C@H]1c1[nH]c(cn1)c1ccc(cc1)c1ccc(cc1)c1[nH]c(	1312338-49-0	Carbamic acid, N-[(1S)-2-[(1R,3S,5R)-3-[5-[4,Ä≤-[2-[(1R,3S,5R)-2-[(2S)-2-(4,4-di	C47H54F2N8O7	880.98	A/B	-9
1622	Cc1oc2c(ccc(c2c1)c1ccc(cc1)c1[nH]c(nc1)[C@@]1(C)N(C(=O)[C@@H](NC(=O)OC)C(C)C)CCC1)c1[nH]c(nc1)[C@@]1	1312608-84-6	Carbamic acid, N,N,Ä≤-[3,8-phenanthridinediylbis[1H-imidazole-5,2-diyl-(2S)-2,1-	C41H49N9O6	763.88	A/B	-9
1623	O(Cc1ccc(F)cc1)c1c2c(oc(c(O)c2=O)c2[nH]c(OC)nn2)ccc1	1312892-07-1	4H-1-Benzopyran-4-one, 5-[(4-fluorophenyl)methoxy]-3-hydroxy-2-(5-phenyl-2-pyrim	C26H17FN2O4	440.42	A/B	-9
1624	O(Cc1ccc(F)cc1)c1c2c(oc(c(O)c2=O)c2[nH]ccn2)ccc1	1312892-15-1	4H-1-Benzopyran-4-one, 5-[(4-fluorophenyl)methoxy]-3-hydroxy-2-(3-phenyl-1H-1,2,	C24H16FN3O4	429.4	A/B	-9
1625	O(Cc1ccc(F)cc1)c1c2c(oc(c(O)c2=O)c2[nH]cnn2)c(C)cc1	1312892-56-0	4H-1-Benzopyran-4-one, 5-[(4-fluorophenyl)methoxy]-3-hydroxy-8-methyl-2-(5-pheny	C27H19FN2O4	454.45	A/B	-9
1626	O(Cc1ccc(F)cc1)c1c2c(oc(c(O)c2=O)c2ncccn2)c(OC)cc1	1312892-84-4	4H-1-Benzopyran-4-one, 5-[(4-fluorophenyl)methoxy]-3-hydroxy-8-methyl-2-(5-pheny	C26H19FN2O4	442.44	A/B	-9
1627	O(Cc1ccc(F)cc1)c1c2c(oc(c(O)c2=O)c2[nH]cnn2)c(OC)cc1	1312893-37-0	4H-1-Benzopyran-4-one, 5-[(4-fluorophenyl)methoxy]-3-hydroxy-8-methoxy-2-(5-pher	C27H19FN2O5	470.45	A/B	-9
1628	O(Cc1ccc(F)cc1)c1c2c(oc(c(O)c2=O)c2[nH]cnn2)c(Cl)cc1	1312893-87-0	4H-1-Benzopyran-4-one, 8-chloro-5-[(4-fluorophenyl)methoxy]-3-hydroxy-2-(5-pheny	C26H16ClFN2O4	474.87	A/B	-9
1629	O(Cc1ccc(F)cc1)c1c2c(c(cc1)c1ccccc1)oc(c(O)c2=O)c1[nH]c(C)nn1	1312894-43-1	4H-1-Benzopyran-4-one, 5-[(4-fluorophenyl)methoxy]-3-hydroxy-8-phenyl-2-(1H-1,2,	C24H16FN3O4	429.4	A/B	-9
1630	O(Cc1ccc(F)cc1)c1c2c(c(cc1)c1ccccc1)oc(c(O)c2=O)c1[nH]c(OC)nn1	1312894-44-2	4H-1-Benzopyran-4-one, 5-[(4-fluorophenyl)methoxy]-3-hydroxy-2-(3-methyl-1H-1,2,	C25H18FN3O4	443.43	A/B	-9
1631	O(Cc1ccc(F)cc1)c1c2c(c(cc1)c1ccccc1)oc(c(O)c2=O)c1[nH]c(nn1)c1ccccc1	1312894-46-4	4H-1-Benzopyran-4-one, 2-(5-chloro-1H-1,2,4-triazol-3-yl)-5-[(4-fluorophenyl)met	C24H15ClFN3O4	463.85	A/B	-9
1632	O(Cc1ccc(F)cc1)c1c2c(c(cc1)c1ccccc1)oc(c(O)c2=O)c1[nH]c(OC)cn1	1312894-49-7	4H-1-Benzopyran-4-one, 5-[(4-fluorophenyl)methoxy]-3-hydroxy-2-(5-methyl-1H-imic	C26H19FN2O4	442.44	A/B	-9
1633	O(Cc1ccc(F)cc1)c1c2c(c(cc1)c1ccccc1)oc(c(O)c2=O)c1[nH]c(Cl)cn1	1312894-50-0	4H-1-Benzopyran-4-one, 5-[(4-fluorophenyl)methoxy]-3-hydroxy-2-(5-methoxy-1H-imi	C26H19FN2O5	458.44	A/B	-9
1634	O(Cc1ccc(F)cc1)c1c2c(oc(c(O)c2=O)c2[nH]c(OC)nn2)c(C)c(C)c1	1312895-44-5	4H-1-Benzopyran-4-one, 5-[(4-fluorophenyl)methoxy]-3-hydroxy-7,8-dimethyl-2-(3-m	C21H18FN3O4	395.38	A/B	-9
1635	O(Cc1ccc(F)cc1)c1c2c(oc(c(O)c2=O)c2[nH]c(OC)cn2)c(C)c(C)c1	1312895-49-0	4H-1-Benzopyran-4-one, 5-[(4-fluorophenyl)methoxy]-3-hydroxy-7,8-dimethyl-2-(5-m	C22H19FN2O4	394.4	A/B	-9
1636	O(Cc1ccc(F)cc1)c1c2c(oc(c(O)c2=O)c2[nH]c(OC)cn2)c(Cl)c(C)c1	1312896-49-3	4H-1-Benzopyran-4-one, 8-chloro-5-[(4-fluorophenyl)methoxy]-3-hydroxy-7-methyl-2	C21H16ClFN2O4	414.81	A/B	-9
1637	O(Cc1ccc(F)cc1)c1c2c(c(c(C)c1)c1ccccc1)oc(c(O)c2=O)c1ncc(C)cn1	1312896-83-5	4H-1-Benzopyran-4-one, 5-[(4-fluorophenyl)methoxy]-3-hydroxy-7-methyl-8-phenyl-2	C27H19FN2O4	454.45	A/B	-9

1638	O(Cc1ccc(F)cc1)c1c2c(c(c(C)c1)c1ccccc1)oc(c(O)c2=O)c1nnc(C)[nH]1	1312896-93-7	4H-1-Benzopyran-4-one, 5-[(4-fluorophenyl)methoxy]-3-hydroxy-7-methyl-8-phenyl-2	C25H18FN3O4	443.43	A/B	-9
1639	O(Cc1ccc(F)cc1)c1c2c(c(c(C)c1)c1ccccc1)oc(c(O)c2=O)c1nnc(Cl)[nH]1	1312896-95-9	4H-1-Benzopyran-4-one, 5-[(4-fluorophenyl)methoxy]-3-hydroxy-2-(5-methoxy-1H-1,2	C26H20FN3O5	473.45	A/B	-9
1640	O(Cc1ccc(F)cc1)c1c2c(c(OC)c(OC)c1)oc(c(O)c2=O)c1[nH]ccn1	1312898-59-1	4H-1-Benzopyran-4-one, 5-[(4-fluorophenyl)methoxy]-3-hydroxy-7,8-dimethoxy-2-(3-	C26H20FN3O6	489.45	A/B	-9
1641	O(C)c1c(c2c(c(OCc3ccc(F)cc3)c1)c(=O)c(O)c(o2)c1ncccn1)c1ccccc1	1312899-14-1	4H-1-Benzopyran-4-one, 8-chloro-5-[(4-fluorophenyl)methoxy]-3-hydroxy-7-methoxy-	C26H18ClFN2O5	492.88	A/B	-9
1642	O(Cc1ccc(F)cc1)c1c2c(oc(c(O)c2=O)c2[nH]ccn2)cc(Cl)c1	1312900-68-7	4H-1-Benzopyran-4-one, 7-chloro-5-[(4-fluorophenyl)methoxy]-3-hydroxy-2-(3-pheny	C24H15ClFN3O4	463.85	A/B	-9
1643	O(Cc1ccc(F)cc1)c1c2c(c(c(Cl)c1)c1ccccc1)oc(c(O)c2=O)c1ncc(OC)cn1	1312902-55-8	4H-1-Benzopyran-4-one, 7-chloro-5-[(4-fluorophenyl)methoxy]-3-hydroxy-2-(5-methy	C27H18ClFN2O4	488.89	A/B	-9
1644	O(Cc1ccc(F)cc1)c1c2c(c(c(Cl)c1)c1ccccc1)oc(c(O)c2=O)c1[nH]cnn1	1312902-58-1	4H-1-Benzopyran-4-one, 7-chloro-5-[(4-fluorophenyl)methoxy]-3-hydroxy-8-phenyl-2	C32H20ClFN2O4	550.96	A/B	-9
1645	O(Cc1ccc(F)cc1)c1c2c(c(c(Cl)c1)c1ccccc1)oc(c(O)c2=O)c1nnc(Cl)[nH]1	1312902-66-1	4H-1-Benzopyran-4-one, 7-chloro-5-[(4-fluorophenyl)methoxy]-3-hydroxy-2-(5-metho	C25H17ClFN3O5	493.87	A/B	-9
1646	O(Cc1ccc(F)cc1)c1c2c(c(c(Cl)c1)c1ccccc1)oc(c(O)c2=O)c1[nH]c(nn1)c1ccccc1	1312902-67-2	4H-1-Benzopyran-4-one, 7-chloro-2-(5-chloro-1H-1,2,4-triazol-3-yl)-5-[(4-fluorop	C24H14Cl2FN3O4	498.29	A/B	-9
1647	O(Cc1ccc(F)cc1)c1c2c(oc(c(O)c2=O)c2[nH]c(C)nn2)cc(c1)c1ccccc1	1312903-14-2	4H-1-Benzopyran-4-one, 5-[(4-fluorophenyl)methoxy]-3-hydroxy-7-phenyl-2-(1H-1,2,	C24H16FN3O4	429.4	A/B	-9
1648	O(Cc1ccc(F)cc1)c1c2c(oc(c(O)c2=O)c2[nH]c(cn2)c2ccccc2)cc(c1)c1ccccc1	1312903-22-2	4H-1-Benzopyran-4-one, 2-(5-chloro-1H-imidazol-2-yl)-5-[(4-fluorophenyl)methoxy]	C25H16ClFN2O4	462.86	A/B	-9
1649	Cc1c2c(c(OCc3ccc(F)cc3)cc1c1ccccc1)c(=O)c(O)c(o2)c1ncc(C)cn1	1312903-54-0	4H-1-Benzopyran-4-one, 5-[(4-fluorophenyl)methoxy]-3-hydroxy-8-methyl-7-phenyl-2	C27H19FN2O4	454.45	A/B	-9
1650	O(Cc1ccc(F)cc1)c1c2c(c(C)c(c1)c1ccccc1)oc(c(O)c2=O)c1[nH]c(OC)nn1	1312903-67-5	4H-1-Benzopyran-4-one, 5-[(4-fluorophenyl)methoxy]-3-hydroxy-8-methyl-2-(3-methy	C26H20FN3O4	457.45	A/B	-9
1651	O(Cc1ccc(F)cc1)c1c2c(c(c(c1)c1ccccc1)c1ccccc1)oc(c(O)c2=O)c1nnc(C)[nH]1	1312905-76-2	4H-1-Benzopyran-4-one, 5-[(4-fluorophenyl)methoxy]-3-hydroxy-7,8-diphenyl-2-(1H-	C30H20FN3O4	505.5	A/B	-9
1652	O(C)c1c2c(cc(OCc3ccc(F)cc3)c1)c(=O)c(O)c(o2)c1[nH]cnn1	1312907-18-8	4H-1-Benzopyran-4-one, 6-[(4-fluorophenyl)methoxy]-3-hydroxy-8-methoxy-2-(5-pher	C27H19FN2O5	470.45	A/B	-9
1653	Clc1c2c(cc(OCc3ccc(F)cc3)c1)c(=O)c(O)c(o2)c1[nH]c(OC)nn1	1312907-75-7	4H-1-Benzopyran-4-one, 8-chloro-6-[(4-fluorophenyl)methoxy]-3-hydroxy-2-(3-methy	C19H13ClFN3O4	401.78	A/B	-9
1654	O=c1c2c(c(cc(OCc3ccc(F)cc3)c2)c2ccccc2)oc(c1O)c1ncc(C)cn1	1312908-14-7	4H-1-Benzopyran-4-one, 6-[(4-fluorophenyl)methoxy]-3-hydroxy-8-phenyl-2-(2-pyrim	C26H17FN2O4	440.42	A/B	-9
1655	O=c1c2c(c(cc(OCc3ccc(F)cc3)c2)c2ccccc2)oc(c1O)c1ncc(OC)cn1	1312908-15-8	4H-1-Benzopyran-4-one, 6-[(4-fluorophenyl)methoxy]-3-hydroxy-2-(5-methyl-2-pyrim	C27H19FN2O4	454.45	A/B	-9
1656	O=c1c2c(c(cc(OCc3ccc(F)cc3)c2)c2ccccc2)oc(c1O)c1[nH]c(nn1)c1ccccc1	1312908-27-2	4H-1-Benzopyran-4-one, 2-(5-chloro-1H-1,2,4-triazol-3-yl)-6-[(4-fluorophenyl)met	C24H15ClFN3O4	463.85	A/B	-9
1657	O=c1c2c(c(cc(OCc3ccc(F)cc3)c2)c2ccccc2)oc(c1O)c1[nH]c(Cl)cn1	1312908-31-8	4H-1-Benzopyran-4-one, 6-[(4-fluorophenyl)methoxy]-3-hydroxy-2-(5-methoxy-1H-imi	C26H19FN2O5	458.44	A/B	-9
1658	Cc1c2c(cc(OCc3ccc(F)cc3)c1C)c(=O)c(O)c(o2)c1[nH]ccn1	1312909-40-2	4H-1-Benzopyran-4-one, 6-[(4-fluorophenyl)methoxy]-3-hydroxy-7,8-dimethyl-2-(3-p	C26H20FN3O4	457.45	A/B	-9
1659	Clc1c2c(cc(OCc3ccc(F)cc3)c1C)c(=O)c(O)c(o2)c1[nH]ccn1	1312910-40-9	4H-1-Benzopyran-4-one, 8-chloro-6-[(4-fluorophenyl)methoxy]-3-hydroxy-7-methyl-2	C25H17ClFN3O4	477.87	A/B	-9
1660	Clc1c2c(cc(OCc3ccc(F)cc3)c1Cl)c(=O)c(O)c(o2)c1[nH]ccn1	1312914-83-2	4H-1-Benzopyran-4-one, 7,8-dichloro-6-[(4-fluorophenyl)methoxy]-3-hydroxy-2-(3-p	C24H14Cl2FN3O4	498.29	A/B	-9
1661	Clc1c(c2c(cc1OCc1ccc(F)cc1)c(=O)c(O)c(o2)c1[nH]c(nn1)c1ccccc1)c1ccccc1	1312915-86-8	4H-1-Benzopyran-4-one, 7-chloro-2-(5-chloro-1H-1,2,4-triazol-3-yl)-6-[(4-fluorop	C24H14Cl2FN3O4	498.29	A/B	-9
1662	Clc1c(c2c(cc1OCc1ccc(F)cc1)c(=O)c(O)c(o2)c1[nH]ccn1)c1ccccc1	1312915-88-0	4H-1-Benzopyran-4-one, 7-chloro-6-[(4-fluorophenyl)methoxy]-3-hydroxy-8-phenyl-2	C30H19ClFN3O4	539.94	A/B	-9
1663	O(Cc1ccc(F)cc1)c1c(c(C)c2c(c1)c(=O)c(O)c(o2)c1[nH]c(OC)nn1)c1ccccc1	1312916-93-0	4H-1-Benzopyran-4-one, 6-[(4-fluorophenyl)methoxy]-3-hydroxy-8-methyl-2-(3-methy	C26H20FN3O4	457.45	A/B	-9
1664	O(Cc1ccc(F)cc1)c1c(c(C)c2c(c1)c(=O)c(O)c(o2)c1[nH]c(OC)cn1)c1ccccc1	1312916-98-5	4H-1-Benzopyran-4-one, 6-[(4-fluorophenyl)methoxy]-3-hydroxy-8-methyl-2-(5-methy	C27H21FN2O4	456.46	A/B	-9
1665	O(Cc1ccc(F)cc1)c1c(c(Cl)c2c(c1)c(=O)c(O)c(o2)c1[nH]c(OC)nn1)c1ccccc1	1312917-93-3	4H-1-Benzopyran-4-one, 8-chloro-6-[(4-fluorophenyl)methoxy]-3-hydroxy-2-(3-methy	C25H17ClFN3O4	477.87	A/B	-9
1666	O(Cc1ccc(F)cc1)c1c(c(Cl)c2c(c1)c(=O)c(O)c(o2)c1[nH]c(OC)cn1)c1ccccc1	1312917-98-8	4H-1-Benzopyran-4-one, 8-chloro-6-[(4-fluorophenyl)methoxy]-3-hydroxy-2-(5-methy	C26H18ClFN2O4	476.88	A/B	-9
1667	C(OC(=O)CCC(=O)OC(C)c1c(non1=O)c1ccccc1)[C@@H]1[C@@H]2[C@H]([C@@H](O1)n1c(=O)[nH]c(=O)c(I)c1)OC(C)(C	1314187-72-8	Uridine, 5-iodo-2,Ä≤,3,Ä≤-O-(1-methylethylidene)-, 5,Ä≤-[4-[(2-oxido-4-phenyl-1	C25H25IN4O11	684.39	A/B	-9
1668	N(=Nc1c2c(c(S(=O)(=O)O)ccc2)c(N)cc1)c1c2c(c(N=Nc3c4c(ccc(S(=O)(=O)O)c4)ccc3)cc1)ccc(S(=O)(=O)O)c2	1345852-36-9	2-Naphthalenesulfonic acid, 8-amino-5-[2-[4-(2-phenyldiazenyl)-7-sulfo-1-naphtha	C26H19N5O6S2	561.59	A/B	-9
1669	N(=Nc1cc(S(=O)(=O)O)ccc1)c1c2c(c(N=Nc3c4c(cc(S(=O)(=O)O)cc4)c(N)cc3)cc1)ccc(S(=O)(=O)O)c2	1345852-47-2	1-Naphthalenesulfonic acid, 5-amino-8-[2-[4-[2-(3-sulfophenyl)diazenyl]-1-naphth	C26H19N5O6S2	561.59	A/B	-9
1670	C([C@H](OCP(=O)(OCCCO)O)C)n1c2c(nc1)c(N)ncn2	1345852-51-8	2-Naphthalenesulfonic acid, 5-[2-(4-amino-7-sulfo-1-naphthalenyl)diazenyl]-8-[2-	C26H19N5O9S3	641.65	A/B	-9
1671	O(C(=O)NC(C(CN(S(=O)(=O)c1ccc(OC)cc1)CC(C)C)O)Cc1ccccc1)[C@@H]1C2C(OC1)OCC2O	1346867-70-6	Not Yet Assigned	C40H57N7O6S	763.99	A/B	-9
1672	N(Cc1ccc(cc1)c1ccccn1)(NC(=O)[C@@H](NC(=O)O)C(C)(C)C)C[C@@H]([C@H](Cc1ccccc1)NC(=O)[C@@H](NC(=O)OC)C	1346943-89-2	Not Yet Assigned	C33H47N7O6	637.77	A/B	-9
1673	C(N(C(=O)N[C@@H](C(=O)N[C@@H](Cc1ccccc1)C[C@@H]([C@H](Cc1ccccc1)NC(=O)OCc1cncs1)O)C(C)C)C)c1nc(sc1)C	1346997-82-7	2,7,10,12-Tetraazatridecanoic acid, 13-[2-(cyclopentylmethyl)-4-thiazolyl]-4-hyc	C40H52N6O5S2	761.01	A/B	-9
1674	N(Cc1c(C(C)C)scn1)(C(=O)N[C@H](C(=O)N[C@@H](Cc1ccccc1)C[C@@H]([C@H](Cc1ccccc1)NC(=O)OCc1cncs1)O)C)C1	1347004-03-8	2,7,10,12-Tetraazatridecanoic acid, 13-[2-(1-cyclopenten-1-yl)-4-thiazolyl]-4-hy	C39H48N6O5S2	744.97	A/B	-9
1675	C(n1c2c(nc1CC)c(C)cc(C)n2)c1ccc(cc1)c1c(S(=O)(=O)NS(=O)(=O)C)ccc(CCCC)c1	1347438-63-4	[1,1,Ä≤-Biphenyl]-2-carboxamide, 4,Ä≤-[(2-ethyl-5,7-dimethyl-3H-imidazo[4,5-b]p	C30H28N4O3S	524.63	A/B	-9
1676	O=c1n([C@@H]2O[C@H](COC(=O)CCCCCCC)C=C2)cc(C)c(=O)[nH]1	1347650-44-5	3H-Imidazo[4,5-b]pyridine-5-carboxylic acid, 3-[[2,Ä≤-[[(butoxycarbonyl)amino]su	C28H30N4O6S	550.63	A/B	-9
1677	C(n1c2c(nc1CC)c(C)cc(C)n2)c1ccc(cc1)c1c(S(=O)(=O)NC(=O)COCCC)ccc(CN2CCCC2)c1	1348325-66-5	Benzoic acid, 4,4,Ä≤-methylenebis[2-[(4-methoxybenzoyl)amino]-	C31H26N2O8	554.55	A/B	-9
1678	C(=O)(N[C@@H](CC1CCC1)C(=O)C(=O)NCc1sc(c(C(=O)N(C)C)n1)c1ccccc1)[C@@H]1[C@@H]2[C@@H](C2(C)C)CN1C(=O)	1348364-52-2	Butanediamide, N1-[(1R,2S)-3-[(3S,4aS,8aS)-3-[[(1,1-dimethylethyl)amino]carbonyl	C38H52N6O4	656.86	A/B	-9
1679	N(C(C)C)c1c(nccc1)N1CCN(C(O)c2cc3c([nH]2)ccc(NS(=O)(=O)C)c3)CC1	1348419-20-4	1,4-Cyclohexanediamine, N4-(7-chloro-4-quinolinyl)-N1,N1-bis[4-(4-fluorophenyl)b	C35H40ClF2N3	576.16	A/B	-9
1680	C([C@H]([C@H](Cc1ccccc1)NC(=O)[C@@H](NC(=O)c1nc2c(cc1)cccc2)CC(=O)N)O)N1[C@H]([C@H](NC(C)(C)C)O)C[C@	1349059-15-9	Not Yet Assigned	C36H48N6O4	628.8	A/B	-9
1681	O=c1n([C@H]2C[C@H](C#C)[C@@H](CO)O2)cc(C)c(=O)[nH]1	1349525-48-9	[1,1,Ä≤-Biphenyl]-2-sulfonamide, N-[(butylamino)carbonyl]-4,Ä≤-[(2-ethyl-5,7-di	C33H42N6O3S	602.79	A/B	-9
1682	C(=O)(NC(C(=O)C(=O)N)CCCC)[C@@H]1[C@@H]2[C@@H](C2(C)C)CN1C(=O)[C@@H](NC(=O)N[C@H](CN1C(=O)CCC1)C(C)(	1349786-53-3	2,7,10,12-Tetraazatridecanoic acid, 13-[2-(4-chlorophenyl)-4-thiazolyl]-4-hydrox	C40H45ClN6O5S2	789.41	A/B	-9
1683	C(=O)([C@@H](NC(=O)OC)C(C)C)N1[C@@]2(C[C@H](Cl)CC2)c1[nH]c(cn1)c1c2c(c(cc1)c1ccc(cc1)c1[nH]c(nc1)[C@	1350718-94-3	Carbamic acid, N-[(1S)-1-[[(1S,4R)-1-[5-[4-[7-[2-[(1S,4R)-2-[(2S)-2-[(methoxycar	C47H56N8O6S	861.06	A/B	-9
1684	C(=O)([C@@H](NC(=O)OC)C(C)C)N1[C@@]2(C[C@H](Cl)CC2)c1[nH]c(cn1)c1c2c(c(cc1)c1ccc(cc1)c1[nH]c(nc1)[C@	1350718-98-7	Carbamic acid, N-[(1S)-1-[[(1S,4R)-1-[5-[4-[7-[2-[(1S,4R)-2-[(2S)-2-[(methoxycar	C47H56N8O6S	861.06	A/B	-9
1685	C(=O)([C@@H](NC(=O)OC)C(C)C)N1[C@@]2(C[C@H](Cl)CC2)c1[nH]c(cn1)c1c2c(c(cc1)c1ccc(cc1)c1[nH]c(nc1)[C@	1350719-06-0	Carbamic acid, N-[(1S)-1-[[(1S,4R)-1-[5-[4-[7-[2-[(1S,4R)-2-[(2S)-2-[(methoxycar	C46H56N10O6	845	A/B	-9
1686	C(=O)([C@H](NC(=O)OC)c1ccccc1)N1[C@@H](CCC1)c1[nH]c(cn1)c1cc(C)c(cc1)c1cc2c(cc1)cc(cc2)c1[nH]c(nc1)[	1351329-94-6	Carbamic acid, N-[(1S,2R)-2-hydroxy-1-[[(2S)-2-[5-[4-[6-[2-[(2S)-1-[(2S,3R)-3-hy	C42H48N8O8	792.88	A/B	-9
1687	C(=O)([C@@H](NC(=O)OC)C(C)C)N1[C@@H](CCC1)c1[nH]c(cn1)c1cc2c(cc1)cc(cc2)c1c(C)cc(cc1)c1[nH]c(nc1)[C@	1351330-45-4	Carbamic acid, N-[(1R)-2-[(2S)-2-[5-[4-[6-[2-[(2S)-1-[(2S)-2-[(methoxycarbonyl)a	C48H52N8O6	836.98	A/B	-9
1688	C(CCCCCCc1cc(O)c(O)cc1)c1cc(O)c(O)cc1	1351396-15-0	6H-Purin-6-one, 2-amino-9-[(4aR,6S,7aS)-hexahydro-5-methylene-2-phenylcyclopenta	C19H19N5O3	365.39	A/B	-9
1689	C(=O)([C@@H](NC(=O)OC)C(C)C)N1C[C@@]2(C[C@H]1c1[nH]c(cn1)c1ccc(cc1)c1ccc(cc1)c1[nH]c(nc1)[C@@]1(C)N(	1351851-22-3	Carbamic acid, N-[(1S)-2-[(5S,8S)-8-[5-[4,Ä≤-[2-[(2S)-1-[(2S)-2-[(methoxycarbony	C45H56N8O8	836.97	A/B	-9
1690	C(=O)([C@@H](NC(=O)OC)C(C)C)N1[C@@H](C[C@@H](Cl)C1CC1)c1[nH]c(cn1)c1ccc(cc1)c1ccc(cc1)c1[nH]c(nc1)[C	1351855-45-2	Carbamic acid, N-[(1S)-1-[[(2S,4R)-4-cyclopropyl-2-[5-[4,Ä≤-[2-[(2S)-4,4-difluo	C43H52F2N8O6	814.92	A/B	-9
1691	C(=O)([C@@H](NC(=O)OC)C(C)C)N1[C@@H](CC2(Cl)CCOCC2)c1[nH]c(cn1)c1ccc(cc1)c1ccc(cc1)c1[nH]c(nc1)[C@H]	1351856-04-6	Carbamic acid, N-[(1S)-1-[[(2S,4R)-4-cyclopropyl-2-[5-[4,Ä≤-[2-[(3S,5S)-2-[(2S)-	C47H60N8O7	849.03	A/B	-9
1692	O=c1n(cc(c(=O)[nH]1)c1c(F)cc(cc1)c1nc2c(o1)cccc2)[C@@H]1O[C@H](COP(=O)(OP(=O)(OP(=O)(O)O)O)O)[C@@H](	1352918-35-4	Uridine 5,Ä≤-(tetrahydrogen triphosphate), 5-[4-(2-benzofuranyl)-2-fluorophenyl]	C23H22FN2O15P3	678.34	A/B	-9
1693	Oc1c2c(cc(O)cc2cc2c1C(=O)c1c(C2(C)C)c(Cl)c(O)cc1O)c1c(C)cc(O)cc1O	1355359-12-4	Thymidine, 3,Ä≤-azido-3,Ä≤-deoxy-3-[(1E,3Z)-5-[1-[(4-fluorophenyl)methyl]-1H-1,	C24H23FN8O6		A/B	-9
1694	C(=O)([C@@H](NC(=O)OC)C(C)C)N1[C@H]2[C@H](C2)C[C@H]1c1[nH]c(cn1)c1c2c(c(cc1)c1ccc(cc1)c1[nH]c(nc1)[C	1359868-94-2	Carbamic acid, N-[(1S)-2-[(2S,5S)-2-[5-[4-[7-[2-[(2S,5S)-1-[(2S)-2-[(methoxycarb	C46H56N10O8S	909.06	A/B	-9
1695	C(F)(F)c1n(c2c(n1)cccc2)c1nc(nc(NCCS(=O)(=O)N2CCOCC2)n1)N1CCOCC1	1359953-58-4	Cyclohexanol, 4-[[4-[2-(difluoromethyl)-1H-benzimidazol-1-yl]-6-(4-morpholinyl)-	C21H25F2N7O2	445.47	A/B	-9
1696	C(F)(F)c1n(c2c(n1)cccc2)c1nc(nc(Nc2ccc(OC)cc2)n1)N1CCOCC1	1359956-22-1	Phenol, 2-[[4-[2-(difluoromethyl)-1H-benzimidazol-1-yl]-6-(4-morpholinyl)-1,3,5-	C21H19F2N7O2	439.42	A/B	-9
1697	C(F)(F)c1n(c2c(n1)cccc2)c1nc(nc(Nc2ccc(cc2)n2cccc2)n1)N1CCOCC1	1359956-28-7	1,3,5-Triazin-2-amine, 4-[2-(difluoromethyl)-1H-benzimidazol-1-yl]-6-(4-morpholi	C26H28F2N8O	506.55	A/B	-9
1698	O(S(=O)(=O)c1c(C)cc(C)cc1C)c1c(C(=O)NCc2ccc(F)cc2)nc(C(NC(=O)c2nnc(C)o2)(C)C)n(C)c1=O	1370588-52-5	4-Pyrimidinecarboxamide, N-[(4-fluorophenyl)methyl]-1,6-dihydro-1-methyl-2-[1-me	C21H23FN6O7S	522.51	A/B	-9
1699	O=C1OC2(c3c1ccc(cc3)N1C(=O)C(SCCN[C@@H]3[C@@H](NC(=O)C)[C@H](OC(CC)CC)C=C(C(=O)OCC)C3)CC1=O)c1c(Oc3c2	1373244-20-2	1-Cyclohexene-1-carboxylic acid, 4-(acetylamino)-5-[[[(3,Ä≤,6,Ä≤-dihydroxy-3-ox	C37H39N3O10	685.72	A/B	-9
1700	C(=O)([C@@H](NC(=O)OC)C(C)C)N1[C@@H](CCC1)c1[nH]c(cn1)c1ccc(C(=Cc2ccc(cc2)c2[nH]c(nc2)[C@H]2N(C(=O)[	1374421-55-2	Carbamic acid, N,N,Ä≤-[[1-[4-(1,1-dimethylethyl)phenyl]-1,2-ethanediyl]bis[4,1-p	C52H66N8O6	899.13	A/B	-9
1701	C[C@@]12[C@@H]([C@]3(C)[C@@H](CC2)C(C)(C)[C@@H](O)CC3)CC[C@H]2[C@@]1(C)CC[C@]1(C)C2=C[C@@](CO)(C)CC1	1374421-59-6	Carbamic acid, N,N,Ä≤-[[1-[4-(1,1-dimethylethyl)phenyl]-1,2-ethenediyl]bis[4,1-p	C52H64N8O6	897.11	A/B	-9
1702	C(=O)([C@@H](NC(=O)OC)C(C)C)N1[C@@H]([C@@H]2C[C@H]1CC2)c1[nH]c2c(n1)ccc1c2ccc(c1)c1cc(F)c(cc1)c1[nH]	1377043-55-4	Carbamic acid, N-[(1S)-1-[[(1R,3S,5R)-3-[5-[2-fluoro-4-[2-[(1R,3S,4S)-2-[(2S)-2-	C45H51FN8O6	818.93	A/B	-9
1703	O[C@]1([C@H](n2c3c(nc2)c(=O)nc[nH]3)O[C@H](CO)[C@H]1O)[2H]	1377051-25-6	Carbamic acid, N,N,Ä≤-[(6-fluoro[7,7,Ä≤-bi-1H-naphth[1,2-d]imidazole]-2,2,Ä≤-di	C44H49FN8O6	804.91	A/B	-9
1704	O=c1c2c(n(cn2)[C@@H]2O[C@H](CO)[C@@H](O)C2)[nH]c(n1)n1cc(nn1)c1ccccc1	1384519-84-9	Inosine, 2-[4-(4-fluorophenyl)-1H-1,2,3-triazol-1-yl]-	C18H16FN7O5	429.36	A/B	-9
1705	C(=O)([C@@H](NC(=O)OC)C(C)C)N1[C@@H](C[C@H](N=[N+]=[N-])C1)c1[nH]c(cn1)c1ccc(cc1)c1ccc(cc1)c1[nH]c(n	1388224-11-0	Carbamic acid, N,N,Ä≤-[[1,1,Ä≤-biphenyl]-4,4,Ä≤-diylbis[[5-(2-thienyl)-1H-imida	C48H54N8O6S2	903.12	A/B	-9
1706	O=C1C2(C(C(C(=O)/C=C(\O)/C3C4(N5C(C3c3cc(OC)c(O)cc3)CCCC5)c3c(NC4=O)cccc3)C3N2CCCC3)c2cc(OC)c(O)cc2)	1389334-85-3	Spiro[2H-indene-2,3,Ä≤-[3H]pyrrolizine]-1,3-dione, 2,Ä≤-[(1Z)-3-[1,Ä≤,2,Ä≤,5,Ä≤	C47H42N2O10	794.84	A/B	-9
1707	N(c1nc(nc(n1)N1CCOCC1)c1sc(N)nc1)c1nccs1	1391918-17-4	Acetic acid, 2-[[1-[4-[[[(4-fluorophenyl)methyl]amino]carbonyl]-1,6-dihydro-5-hy	C20H23FN6O6	462.43	A/B	-9
1708	Nc1ncc(c2nc(nc(n2)N2CCOCC2)N2CCNC(=O)CC2)cn1	1391924-14-3	Benzamide, 3-[[4-(2-amino-5-pyrimidinyl)-6-(4-morpholinyl)-1,3,5-triazin-2-yl]am	C18H19N9O2	393.4	A/B	-9
1709	Nc1ncc(c2nc(nc(n2)N2CCOCC2)N2CCNCC2)cn1	1391924-44-9	1-Propanone, 2-[[4-(3-hydroxyphenyl)-6-(4-morpholinyl)-1,3,5-triazin-2-yl]amino]	C21H29N7O5S	491.56	A/B	-9
1710	N(CC(=O)N1CCN(S(=O)(=O)C)CC1)c1nc(nc(n1)N1CCOCC1)c1cc(OC)ccc1	1391924-46-1	Glycine, N-[4-(2,3-dihydro-1-oxo-1H-inden-4-yl)-6-(4-morpholinyl)-1,3,5-triazin-	C18H19N5O4	369.37	A/B	-9
1711	N(CCC(=O)N(C)C)c1nc(nc(n1)N1CCOCC1)c1ccc(N)nc1	1391924-57-4	Benzenepropanamide, Œ±-[[4-(3-hydroxyphenyl)-6-(4-morpholinyl)-1,3,5-triazin-2-y	C22H24N6O3	420.46	A/B	-9
1712	N(CCC(=O)O)c1nc(nc(n1)N1CCOCC1)c1c2c(ccc1)[nH]nc2	1391925-92-0	1,3,5-Triazin-2-amine, 4-(1H-indazol-4-yl)-6-(4-morpholinyl)-N-(tetrahydro-2H-py	C19H23N7O2	381.43	A/B	-9
1713	C(Nc1c(F)cn(c(=O)n1)[C@H]1O[C@@H](COC(=O)CCCCCCCCCCCNC(=O)OCC2c3c(-c4c2cccc4)cccc3)SC1)(c1ccc(OC)cc1	1395898-97-1	Œ≤-Alanine, N-[(9H-fluoren-9-ylmethoxy)carbonyl]-, [(2R,5S)-5-[4-[[bis(4-methoxy	C47H43FN4O8S	842.93	A/B	-9
1714	O=C1c2n(C([C@H]3N1[C@H](C)CCO3)([2H])[2H])cc(C(=O)NC(c1c(F)cc(F)cc1)([2H])[2H])c(=O)c2O	1407166-89-5	2H-Pyrido[1,Ä≤,2,Ä≤:4,5]pyrazino[2,1-b][1,3]oxazine-2,3,4,12-d4-9-carboxamide,	C20H7D12F2N3O5		A/B	-9
1715	O=C1c2n(C([C@H]3N1[C@](C([2H])([2H])[2H])(C(C(O3)([2H])[2H])([2H])[2H])[2H])([2H])[2H])cc(C(=O)NCc1c	1407167-20-7	2H-Pyrido[1,Ä≤,2,Ä≤:4,5]pyrazino[2,1-b][1,3]oxazine-2,3,4,12-d4-9-carboxamide,	C20H8D11F2N3O5		A/B	-9
1716	O=C1c2n(C[C@H]3N1[C@](C)(C(C(O3)([2H])[2H])([2H])[2H])[2H])cc(C(=O)NCc1c(F)cc(F)cc1)c(=O)c2O	1407167-24-1	2H-Pyrido[1,Ä≤,2,Ä≤:4,5]pyrazino[2,1-b][1,3]oxazine-2,3-d2-9-carboxamide, N-[(2	C20H12D7F2N3O5		A/B	-9
1717	O=C1c2n(C[C@H]3N1[C@](C([2H])([2H])[2H])(CCO3)[2H])cc(C(=O)NCc1c(F)cc(F)cc1)c(=O)c2O	1407167-26-3	2H-Pyrido[1,Ä≤,2,Ä≤:4,5]pyrazino[2,1-b][1,3]oxazine-2,3-d2-9-carboxamide, N-[(2	C20H15D4F2N3O5		A/B	-9
1718	O=C1c2n(C([C@H]3N1[C@H](C([2H])([2H])[2H])C(C(O3)([2H])[2H])([2H])[2H])([2H])[2H])cc(C(=O)NCc1c(F)cc	1407167-30-9	2H-Pyrido[1,Ä≤,2,Ä≤:4,5]pyrazino[2,1-b][1,3]oxazine-4-d-9-carboxamide, N-[(2,4-	C20H18DF2N3O5		A/B	-9
1719	O=C1c2n(C([C@H]3N1[C@](C)(CCO3)[2H])([2H])[2H])cc(C(=O)NCc1c(F)cc(F)cc1)c(=O)c2O	1407167-40-1	2H-Pyrido[1,Ä≤,2,Ä≤:4,5]pyrazino[2,1-b][1,3]oxazine-12-d-9-carboxamide, N-[(2,4	C20H14D5F2N3O5		A/B	-9
1720	O=C1c2n(C([C@]3(N1[C@H](C)CCO3)[2H])([2H])[2H])cc(C(=O)NCc1c(F)cc(F)cc1)c(=O)c2O	1407167-41-2	2H-Pyrido[1,Ä≤,2,Ä≤:4,5]pyrazino[2,1-b][1,3]oxazine-4,12-d2-9-carboxamide, N-[(	C20H16D3F2N3O5		A/B	-9
1721	O=C1c2n(C[C@]3(N1[C@](C([2H])([2H])[2H])(C(C(O3)([2H])[2H])([2H])[2H])[2H])[2H])cc(C(=O)NCc1c(F)cc(F	1407167-42-3	2H-Pyrido[1,Ä≤,2,Ä≤:4,5]pyrazino[2,1-b][1,3]oxazine-12-d-9-carboxamide, N-[(2,4	C20H16D3F2N3O5		A/B	-9
1722	O=C1c2n(C[C@]3(N1[C@H](C)C(C(O3)([2H])[2H])([2H])[2H])[2H])cc(C(=O)NCc1c(F)cc(F)cc1)c(=O)c2O	1407167-45-6	2H-Pyrido[1,Ä≤,2,Ä≤:4,5]pyrazino[2,1-b][1,3]oxazine-2,3,4-d3-9-carboxamide, N-[	C20H13D6F2N3O5		A/B	-9
1723	O=C1c2n(C[C@]3(N1[C@](C([2H])([2H])[2H])(CCO3)[2H])[2H])cc(C(=O)NCc1c(F)cc(F)cc1)c(=O)c2O	1407167-46-7	2H-Pyrido[1,Ä≤,2,Ä≤:4,5]pyrazino[2,1-b][1,3]oxazine-2,3-d2-9-carboxamide, N-[(2	C20H14D5F2N3O5		A/B	-9
1724	O=C1c2n(C[C@]3(N1[C@H](C([2H])([2H])[2H])CCO3)[2H])cc(C(=O)NCc1c(F)cc(F)cc1)c(=O)c2O	1407167-47-8	2H-Pyrido[1,Ä≤,2,Ä≤:4,5]pyrazino[2,1-b][1,3]oxazine-4-d-9-carboxamide, N-[(2,4-	C20H14D5F2N3O5		A/B	-9
1725	O=C1c2n(C[C@]3(N1[C@](C)(CCO3)[2H])[2H])cc(C(=O)NCc1c(F)cc(F)cc1)c(=O)c2O	1407167-48-9	2H-Pyrido[1,Ä≤,2,Ä≤:4,5]pyrazino[2,1-b][1,3]oxazine-9-carboxamide, N-[(2,4-difl	C20H15D4F2N3O5		A/B	-9
1726	O=C1c2n(C([C@]3(N1[C@](C)(C(C(O3)([2H])[2H])([2H])[2H])[2H])[2H])([2H])[2H])cc(C(=O)NCc1c(F)cc(F)cc1	1407167-51-4	2H-Pyrido[1,Ä≤,2,Ä≤:4,5]pyrazino[2,1-b][1,3]oxazine-2,3,12-d3-9-carboxamide, N-	C20H9D10F2N3O5		A/B	-9
1727	O=C1c2n(C([C@]3(N1[C@H](C)C(C(O3)([2H])[2H])([2H])[2H])[2H])([2H])[2H])cc(C(=O)NCc1c(F)cc(F)cc1)c(=O	1407167-52-5	2H-Pyrido[1,Ä≤,2,Ä≤:4,5]pyrazino[2,1-b][1,3]oxazine-2,3,4,12-d4-9-carboxamide,	C20H11D8F2N3O5		A/B	-9
1728	O=C1c2n(C([C@]3(N1[C@](C([2H])([2H])[2H])(CCO3)[2H])[2H])([2H])[2H])cc(C(=O)NCc1c(F)cc(F)cc1)c(=O)c2	1407167-53-6	2H-Pyrido[1,Ä≤,2,Ä≤:4,5]pyrazino[2,1-b][1,3]oxazine-2,3,12-d3-9-carboxamide, N-	C20H12D7F2N3O5		A/B	-9
1729	O=C1c2n(C([C@]3(N1[C@H](C([2H])([2H])[2H])CCO3)[2H])([2H])[2H])cc(C(=O)NCc1c(F)cc(F)cc1)c(=O)c2O	1407167-54-7	2H-Pyrido[1,Ä≤,2,Ä≤:4,5]pyrazino[2,1-b][1,3]oxazine-4,12-d2-9-carboxamide, N-[(	C20H12D7F2N3O5		A/B	-9
1730	O=C1c2n(C([C@]3(N1[C@](C)(CCO3)[2H])[2H])([2H])[2H])cc(C(=O)NCc1c(F)cc(F)cc1)c(=O)c2O	1407167-55-8	2H-Pyrido[1,Ä≤,2,Ä≤:4,5]pyrazino[2,1-b][1,3]oxazine-12-d-9-carboxamide, N-[(2,4	C20H13D6F2N3O5		A/B	-9
1731	[C@@H](Cc1ccccc1)(NC(=O)[C@H](CCN1CCOCC1)NC(=O)N(Cc1nc(C(C)C)sc1)C)CC[C@H](Cc1ccccc1)NC(=O)OCc1cncs1	1407167-56-9	2H-Pyrido[1,Ä≤,2,Ä≤:4,5]pyrazino[2,1-b][1,3]oxazine-4,12-d2-9-carboxamide, N-[(	C20H15D4F2N3O5		A/B	-9
1732	C(NC(=O)c1[nH]c(nc(=O)c1O)c1nc2c(cc1)cccc2)(C)C12CC3CC(C2)CC(C1)C3	1408305-01-0	4-Pyrimidinecarboxamide, 1,6-dihydro-5-hydroxy-6-oxo-2-(2-pyridinyl)-N-(1-tricyc	C22H26N4O3	394.47	A/B	-9
1733	[C@H](C(=O)N1[C@@H](CC[C@@H]1C)c1[nH]c(cn1)c1ccc(cc1)c1ccc(cc1)c1[nH]c(nc1)[C@H]1N(C(=O)[C@@H](NC(=O	1412816-57-9	Carbamic acid, N,N,Ä≤-[[1,1,Ä≤-biphenyl]-4,4,Ä≤-diylbis[1H-imidazole-5,2-diyl[(	C46H54N8O6	814.97	A/B	-9
1734	C(=C1C=C(C(=O)O)C(=O)C=C1)(c1cc(Cc2cc(C(=O)O)c(O)cc2)c(O)c(C(=O)O)c1)c1cc(C(=O)O)c(O)cc1	141511-21-9	Benzoic acid, 3-[(3-carboxy-4-hydroxyphenyl)(3-carboxy-4-oxo-2,5-cyclohexadien-1	C22H14O9	422.34	A/B	-9
1735	C(=O)([C@@H](NC(=O)OC)C(C)C)N1CC2(C[C@H]1c1[nH]c(cn1)c1ccc(cc1)c1c3CCc4cc(c(CCc(c1)c3)cc4)c1ccc(cc1	1415119-56-0	Carbamic acid, N-[(1S)-1-[[(2S)-2-[5-[11-[4-[2-[(2S)-1-[(2S)-2-[(methoxycarbonyl	C50H60N8O6	869.06	A/B	-9
1736	C(=O)([C@@H](NC(=O)OC)C(C)C)N1[C@H](CCC1)c1[nH]c(cn1)c1ccc(cc1)c1ccc(cc1)c1[nH]c(nc1)[C@H]1N(C(=O)[C	1417333-60-8	Carbamic acid, N,N,Ä≤-[[1,1,Ä≤-biphenyl]-4,4,Ä≤-diylbis[1H-imidazole-5,2-diyl-(	C40H50N8O6	738.88	A/B	-9
1737	C(=O)([C@@H](NC(=O)OC)[C@H](CC)C)N1[C@H](c2[nH]c(c3c4c(cc3)c3ccc(cc3)c3[nH]c(nc3)[C@H]3N(C(=O)[C@@	1419519-53-1	Carbamic acid, N-[(1S)-1-[[(3S)-3-[5-[1,Ä≤,3,Ä≤-dihydro-7,Ä≤-[4-[2-[(3S)-2-[(2S	C49H60N8O6	857.05	A/B	-9
1738	N(=C1c2c(N(CN3CCN(CC3)c3ccc(OC)cc3)C1=O)ccc(C)c2)NC(=S)N(CC=C)CC=C	1426583-77-8	Hydrazinecarbothioamide, 2-[5-fluoro-1,2-dihydro-1-[[4-(4-methoxyphenyl)-1-piper	C33H31FN6O2S	594.7	A/B	-9
1739	N(=C1c2c(N(CN3CCN(CC3)c3cc4c(cc3F)c(=O)c(C(=O)O)cn4CC)C1=O)ccc(Cl)c2)NC(=S)N(CC=C)CC=C	1426583-79-0	Hydrazinecarbothioamide, 2-[1,2-dihydro-1-[[4-(4-methoxyphenyl)-1-piperazinyl]me	C34H34N6O2S	590.74	A/B	-9
1740	N(C(=O)Nc1ccc(S(=O)(=O)c2ccc(N)cc2)cc1)C12CC3CC(C2)CC(C1)C3	1431218-99-3	2-Naphthalenesulfonic acid, 5-amino-8-[(1E)-2-[2,5-dimethyl-4-[(1E)-2-(3-sulfoph	C24H21N5O6S2	539.58	A/B	-9
1741	C(=O)([C@@H](NC(=O)OC)C(C)C)N1[C@H]2c3nc(c([nH]3)COCCCCO[C@H](C2)C1)c1ccc(cc1)c1ccc(cc1)c1[nH]c(nc1)	1431625-83-0	Carbamic acid, N-[(1S)-1-[[(2S)-2-[5-[4,Ä≤-[(2S,5S)-3-[(2S)-2-[(methoxycarbonyl)	C46H59N9O9	882.02	A/B	-9
1742	S(=O)(=O)(O)c1c2c(c(S(=O)(=O)O)ccc2)cc(N=Nc2c(N)c(C)c(N)cc2)c1	1431626-03-7	Carbamic acid, N-[(1S)-1-[[(2S)-2-[5-[4,Ä≤-[(2S,5S)-3-[(2S)-2-[(methoxycarbonyl)	C45H58N8O8	838.99	A/B	-9
1743	C[C@]1(F)[C@@H](O[C@@](CO[P@](=O)(Oc2cc3c(cc2)cccc3)N[C@H](C(=O)OC(C)C)C)(F)[C@H]1O)n1c(=O)[nH]c(=O)	1443648-70-1	L-Alanine, N-[[P(S),2,Ä≤R]-5-bromo-2,Ä≤-deoxy-2,Ä≤-fluoro-4,Ä≤-C-fluoro-2,Ä≤-me	C26H29BrF2N3O9P	676.4	A/B	-9
1744	N(Cc1ccc(Cl)cc1)(c1nc(nc(C#N)n1)N1CCOCC1)C1CCCCC1	1443830-69-0	1,3,5-Triazine-2-carbonitrile, 4-[cyclohexyl[(4-fluorophenyl)methyl]amino]-6-(4-	C21H25FN6O	396.46	A/B	-9
1745	O(c1nc(Nc2ccc(C#N)cc2)nc(N)c1Br)c1c(C)cc(C#N)cc1C.C(=O)(C)O	1445668-98-3	Formic acid, compd. with 4-[[6-amino-5-bromo-2-[(4-cyanophenyl)amino]-4-pyrimidi	C20H15BrN6O.CH2O2		A/B	-9
1746	O=c1n(cc(F)c(n1)n1cncn1)[C@H]1O[C@@H](CO)SC1	1446690-23-8	Benzamide, 4-[[4-[[4-[(1E)-3-amino-3-oxo-1-propen-1-yl]-2,6-dimethylphenyl]aminc	C22H22N6O2	402.45	A/B	-9
1747	O(c1nc(Nc2ccc(C#N)cc2)nc(Cl)c1Br)c1c(C)cc(C)cc1C	1448853-80-2	Thymidine, [P(S)]-thymidylyl-(3,Ä≤-5,Ä≤,Ü5,Ä≤)-3,Ä≤-azido-3,Ä≤-deoxy-	C20H24N7O10P		A/B	-9
1748	O=C1[C@@H]2[C@@H](c3c([C@H](OC(=O)c4cc(N(=O)=O)c(Cl)cc4)[C@H]2CO1)cc1c(c3)OCO1)c1c(Cl)c(OC)c(OC)c(OC	1449601-81-3	Furo[3,Ä≤,4,Ä≤:6,7]naphtho[2,3-d]-1,3-dioxol-6(5aH)-one, 5-(2-chloro-3,4,5-trim	C29H24ClNO11	597.95	A/B	-9
1749	N(c1c(C)cc(/C=C/C#N)cc1C)c1nc(Nc2ccc(C#N)cc2)ncc1.C(C)(C)O.Cl	1469778-99-1	Benzonitrile, 4-[[4-[[4-[(1E)-2-cyanoethenyl]-2,6-dimethylphenyl]amino]-2-pyrimi	C22H18N6.C3H8O.ClH		A/B	-9
1750	C(OC[C@@H]1[C@@H](OC(=O)c2ccccc2)[C@@H](OP(=O)(OC[C@@H]2[C@@H](OC(=O)c3ccccc3)[C@@H](OP(=O)(OC[C@@H]	1469875-50-0	Œ≤-D-lyxo-Adenosine, 1,Ä≤-[3-(aminocarbonyl)-1H-1,2,4-triazol-1-yl]-3,Ä≤-O-benz	C92H80N16O25P2	1871.66	A/B	-9
1751	C(n1c2c(nc1CC)c(C)cc(C)n2)c1ccc(cc1)c1c(S(=O)(=O)NC(=O)OCCC)cccc1	148401-87-0	Carbamic acid, [[4,Ä≤-[(2-ethyl-5,7-dimethyl-3H-imidazo[4,5-b]pyridin-3-yl)methy	C28H30N4O4S	518.63	A/B	-9
1752	C(n1c2c(nc1CC)c(C)cc(C)n2)c1ccc(cc1)c1c(S(=O)(=O)NC(=O)NCC2CC2)cccc1	148401-91-6	[1,1,Ä≤-Biphenyl]-2-sulfonamide, 4,Ä≤-[(2-ethyl-5,7-dimethyl-3H-imidazo[4,5-b]p	C27H31N5O3S	505.63	A/B	-9
1753	O=c1n([C@@H]2O[C@H](CO)[C@@H](O)C2)cc(CNOC)c(=O)[nH]1	1485692-24-7	2H-Pyrido[1,Ä≤,2,Ä≤:4,5]pyrazino[2,1-b][1,3]oxazine-9-carboxamide, N-[(2,4-difl	C20H19F2N3O6	435.38	A/B	-9
1754	[N+](#N)c1c2c(c(N=Nc3ccc(S(=O)(=O)O)cc3)cc1)ccc(S(=O)(=O)O)c2	1504558-52-4	Benzonitrile, 4-[[6-[[4-[(1E)-2-cyanoethenyl]-2,6-dimethylphenyl]amino]-9-[(4-me	C31H26N8O	526.59	A/B	-9

1755	C(NCC(COc1c(C(=O)Nc2ccc(C)cc2)cccc1)O)(C)C12CC3CC(C2)CC(C1)C3	1510804-54-2	Benzamide, 5-chloro-2-[2-hydroxy-3-[(1-tricyclo[3.3.1.13,7]dec-1-ylethyl)amino]p	C33H39ClN2O3	547.13	A/B	-9
1756	O=c1c2c(n(cn2)[C@H]2C[C@H](N[Si](NC[C@H]3O[C@H]([C@H](O)[C@@H]3O)n3c4c(nc3)c(=O)nc(N)[nH]4)(C)C)[C@@	1513874-28-6	Guanosine, 2,Ä≤-deoxy-3,Ä≤-O-dephosphinicoguanylylimino-(3,Ä≤,Ü5,Ä≤)-5,Ä≤-deox	C20H25N11O8	547.48	A/B	-9
1757	O[C@H]1[C@H](n2c3c(nc2)c(=O)nc(N)[nH]3)O[C@H](CO)[C@H]1NNC[C@H]1O[C@H]([C@H](O)[C@@H]1O)n1c2c(nc1)c(	1513874-50-4	Guanosine, 3,Ä≤-O-dephosphinicoguanylyliminomethylene-(3,Ä≤,Ü5,Ä≤)-5,Ä≤-deoxy-	C21H27N11O9	577.51	A/B	-9
1758	[C@H](O)([C@H]1[C@H](NC(=O)C)[C@@H](NC(=N)N)C=C(C(=O)OC)O1)[C@H]1COC(=O)O1	151559-13-6	2-Naphthalenesulfonic acid, 8-[2-(4-amino-2-methylphenyl)diazenyl]-5-[2-(3-sulfo	C23H19N5O6S2	525.56	A/B	-9
1759	C(=O)(NC(C(=O)C(=O)NCC=C)CCCC)[C@@H]1[C@@H]2[C@@H](C2(C)C)CN1C(=O)[C@@H](NC(=O)N[C@@H](CN1C(=O)CCC1=	1528752-67-1	3-Azabicyclo[3.1.0]hexane-2-carboxamide, N-[3-amino-1-(cyclobutylmethyl)-2,3-dio	C36H56N6O7S	716.93	A/B	-9
1760	O(C(=O)CCC(=O)C)[C@@H]1[C@H](n2c3c(nc2)c(NC(=O)c2ccccc2)ncn3)O[C@H](CO)[C@H]1O	1541908-17-1	Adenosine, uridylyl-(3,Ä≤,Ü5,Ä≤)-adenylyl-(3,Ä≤,Ü5,Ä≤)-[uridylyl-(5,Ä≤,Ü3,Ä≤	C67H83N27O46P6	2188.37	A/B	-9
1761	C(n1c2c(nc1CC)c(C)cc(C)n2)c1ccc(cc1)c1c(S(=O)(=O)NC(=O)NCCCC)ccc(CCC)c1	154552-33-7	[1,1,Ä≤-Biphenyl]-2-sulfonamide, N-[(butylamino)carbonyl]-5-ethyl-4,Ä≤-[(2-ethy	C30H37N5O3S	547.71	A/B	-9
1762	C(n1c2c(nc1CC)c(C)cc(C)n2)c1ccc(cc1)c1c(S(=O)(=O)NC(=O)NCCCC)ccc(OCCCC)c1	154552-36-0	[1,1,Ä≤-Biphenyl]-2-sulfonamide, N-[(butylamino)carbonyl]-4,Ä≤-[(2-ethyl-5,7-di	C31H39N5O3S	561.74	A/B	-9
1763	C(n1c2c(nc1CC)c(C)cc(C)n2)c1ccc(cc1)c1c(S(=O)(=O)NC(=O)NCCCC)ccc(CSC)c1	154552-39-3	[1,1,Ä≤-Biphenyl]-2-sulfonamide, N-[(butylamino)carbonyl]-4,Ä≤-[(2-ethyl-5,7-di	C29H35N5O4S	549.68	A/B	-9
1764	C(n1c2c(nc1CC)c(C)cc(C)n2)c1ccc(cc1)c1c(S(=O)(=O)NC(=O)CCc2ccccc2)ccc(OCC)c1	154552-60-0	Benzenepropanamide, N-[[4,Ä≤-[(2-ethyl-5,7-dimethyl-3H-imidazo[4,5-b]pyridin-3-y	C34H36N4O4S	596.74	A/B	-9
1765	C(n1c2c(nc1CC)c(C)cc(C)n2)c1ccc(cc1)c1c(S(=O)(=O)NC(=O)c2c(Oc3ccccc3)cccc2)ccc(CCCC)c1	154552-73-5	Benzamide, N-[[4,Ä≤-[(2-ethyl-5,7-dimethyl-3H-imidazo[4,5-b]pyridin-3-yl)methyl]	C39H38N4O4S	658.81	A/B	-9
1766	C(n1c2c(nc1CC)c(C)cc(C)n2)c1ccc(cc1)c1c(S(=O)(=O)NC(=O)c2c(OCC)cccc2)ccc(C)c1	154552-80-4	Benzamide, N-[[4,Ä≤-[(2-ethyl-5,7-dimethyl-3H-imidazo[4,5-b]pyridin-3-yl)methyl]	C38H36N4O5S	660.78	A/B	-9
1767	C(n1c2c(nc1CC)c(C)cc(C)n2)c1ccc(cc1)c1c(S(=O)(=O)NC(=O)c2c(OCC)cccc2)ccc(OC)c1	154552-97-3	Benzamide, 2-ethoxy-N-[[4,Ä≤-[(2-ethyl-5,7-dimethyl-3H-imidazo[4,5-b]pyridin-3-y	C36H40N4O4S	624.79	A/B	-9
1768	C(n1c2c(nc1CC)c(C)cc(C)n2)c1ccc(cc1)c1c(S(=O)(=O)NC(=O)c2ccccc2)ccc(COC)c1	154553-19-2	Benzamide, N-[[4,Ä≤-[(2-ethyl-5,7-dimethyl-3H-imidazo[4,5-b]pyridin-3-yl)methyl]	C32H32N4O3S2	584.75	A/B	-9
1769	C(n1c2c(nc1CC)c(C)cc(C)n2)c1ccc(cc1)c1c(S(=O)(=O)NC(=O)c2ccccc2)ccc(C(CC)(C)C)c1	154553-22-7	Benzamide, N-[[4,Ä≤,Ä≤-[(2-ethyl-5,7-dimethyl-3H-imidazo[4,5-b]pyridin-3-yl)met	C36H32N4O3S	600.73	A/B	-9
1770	C(n1c2c(nc1CC)c(C)cc(C)n2)c1ccc(cc1)c1c(S(=O)(=O)NC(=O)c2ccccc2)ccc(SCC)c1	154553-28-3	Benzamide, N-[[4,Ä≤-[(2-ethyl-5,7-dimethyl-3H-imidazo[4,5-b]pyridin-3-yl)methyl]	C33H34N4O4S	582.71	A/B	-9
1771	C(n1c2c(nc1CC)c(C)cc(C)n2)c1ccc(cc1)c1c(S(=O)(=O)NC(=O)COCCC)ccc(OCCCC)c1	154553-34-1	Acetamide, N-[[4,Ä≤-[(2-ethyl-5,7-dimethyl-3H-imidazo[4,5-b]pyridin-3-yl)methyl]	C31H38N4O4S	562.72	A/B	-9
1772	C(n1c2c(nc1CC)c(C)cc(C)n2)c1ccc(cc1)c1c(S(=O)(=O)NC(=O)NCCc2ccccc2)ccc(CC(C)C)c1	154553-55-6	[1,1,Ä≤-Biphenyl]-2-sulfonamide, 5-butoxy-4,Ä≤-[(2-ethyl-5,7-dimethyl-3H-imidaz	C36H41N5O4S	639.81	A/B	-9
1773	C(n1c2c(nc1CC)c(C)cc(C)n2)c1ccc(cc1)c1c(S(=O)(=O)NC(=O)NCCc2ccccc2)ccc(CSC)c1	154553-57-8	[1,1,Ä≤-Biphenyl]-2-sulfonamide, 4,Ä≤-[(2-ethyl-5,7-dimethyl-3H-imidazo[4,5-b]p	C33H35N5O4S	597.73	A/B	-9
1774	C(n1c2c(nc1CC)c(C)cc(C)n2)c1ccc(cc1)c1c(S(=O)(=O)NC(=O)NCCc2ccccc2)ccc(COC)c1	154553-58-9	[1,1,Ä≤-Biphenyl]-2-sulfonamide, 4,Ä≤-[(2-ethyl-5,7-dimethyl-3H-imidazo[4,5-b]p	C34H37N5O3S2	627.82	A/B	-9
1775	C(n1c2c(nc1CC)c(C)cc(C)n2)c1ccc(cc1)c1c(S(=O)(=O)NC(=O)NCCc2ccccc2)ccc(c1)c1ccccc1	154553-60-3	[1,1,Ä≤-Biphenyl]-2-sulfonamide, 5-ethoxy-4,Ä≤-[(2-ethyl-5,7-dimethyl-3H-imidaz	C34H37N5O4S	611.75	A/B	-9
1776	S(c1nc(nc(Nc2ccc(OC)cc2)n1)N1CCOCC1)c1oc(nn1)c1ccc(Cl)cc1	1547295-44-2	1,3,5-Triazin-2-amine, 4-[[5-(4-chlorophenyl)-1,3,4-oxadiazol-2-yl]thio]-6-(4-mo	C21H17ClN8O4S	512.93	A/B	-9
1777	N(c1nc(nc(Sc2oc(nn2)c2ccc(Cl)cc2)n1)N1CCOCC1)c1cc(C)ccc1	1547295-46-4	1,3,5-Triazin-2-amine, N-(4-chlorophenyl)-4-[[5-(4-chlorophenyl)-1,3,4-oxadiazol	C21H17Cl2N7O2S	502.38	A/B	-9
1778	N(c1nc(nc(Sc2oc(nn2)c2ccc(Cl)cc2)n1)N1CCOCC1)c1cc(Br)ccc1	1547295-50-0	1,3,5-Triazin-2-amine, N-(3-chlorophenyl)-4-[[5-(4-chlorophenyl)-1,3,4-oxadiazol	C21H17Cl2N7O2S	502.38	A/B	-9
1779	C(=O)([C@@H](NC(=O)OC)C(C)C)N1[C@H](c2[nH]c(c3c4c(C5CC4CC5)c(cc3)c3ccc(cc3)c3[nH]c(nc3)[C@H]3N(C(=O)	1560639-10-2	Carbamic acid, N-[(1S)-1-[[(2S,4R)-4-fluoro-2-[5-[8-[4-[2-[(2S,4R)-4-fluoro-1-[(	C45H54F2N8O6	840.96	A/B	-9
1780	N(c1c2c(c(O)c(CN3CCN(CCC)CC3)c1)CCCC2)c1c2c(ncc1)cc(Cl)cc2	156893-83-3	1-Naphthalenol, 4-[(7-chloro-4-quinolinyl)amino]-2-[(4-ethyl-1-piperazinyl)methy	C26H31ClN4O	451	A/B	-9
1781	N(c1c2c(c(O)c(CN3CCN(C(C)C)CC3)c1)CCCC2)c1c2c(ncc1)cc(Cl)cc2	156893-84-4	1-Naphthalenol, 4-[(7-chloro-4-quinolinyl)amino]-5,6,7,8-tetrahydro-2-[(4-propyl	C27H33ClN4O	465.03	A/B	-9
1782	Cc1n(c(=O)[nH]c(=O)c1C)[C@H]1O[C@H](CO)[C@@H](O)C1	156893-90-2	1-Naphthalenol, 4-[(7-chloro-4-quinolinyl)amino]-5,6,7,8-tetrahydro-2-[[4-(3-met	C29H37ClN4O	493.08	A/B	-9
1783	Oc1n(C2OC(CO)C(O)C2)cc(C)c(=O)n1	157038-13-6	Carbamic acid, [[2-[4-[(2-ethyl-5,7-dimethyl-3H-imidazo[4,5-b]pyridin-3-yl)methy	C34H40N4O4S	600.77	A/B	-9
1784	C(O[P@]1(=O)O[C@@H](CCO1)c1cc(Cl)ccc1)[C@@H]1[C@@H]2[C@H]([C@@H](O1)n1nc(C(=O)N)nc1)OC(=O)O2	1578257-10-9	1H-1,2,4-Triazole-3-carboxamide, 1-[5-O-[(2R,4S)-4-(3-chlorophenyl)-2-oxido-1,3,	C17H20ClN4O8P	474.79	A/B	-9
1785	CN1CC2(c3c(CC2)c(ccc3c2ccc(cc2)c2[nH]c(nc2)[C@H]2N(C(=O)[C@@H](NC(=O)OC)C(C)C)CCC2)c2[nH]c(nc2)[C@H]	1588404-18-5	Carbamic acid, N-[(1S)-1-[[(2S)-2-[5-[4-[4-[2-[(2S)-4,4-difluoro-1-[(2S)-2-[(met	C47H56F4N8O7	920.99	A/B	-9
1786	C(OC(=O)[C@H]1N(C(=O)OC(C)(C)C)CCC1)[C@H]1O[C@H]([C@H]2[C@@H]1OC(C)(C)O2)n1nc(C(=O)N)nc1	160522-65-6	L-Tryptophan, N-[(1,1-dimethylethoxy)carbonyl]-, ester with 1-[2,3-O-(1-methylet	C27H34N6O8	570.59	A/B	-9
1787	C(=O)([C@@H](NC(=O)OC)[C@H]1C[C@H](O[C@H](C)C1)C1CC1)N1[C@@H](CC[C@@H]1C)c1[nH]c(cn1)c1ccc(cc1)c1ccc	1608494-79-6	Carbamic acid, N,N,Ä≤-[[1,1,Ä≤-biphenyl]-4,4,Ä≤-diylbis[1H-imidazole-5,2-diyl[(	C54H70N8O8	959.18	A/B	-9
1788	O=C1N2[C@H](Cn3c1c(O)c(=O)c(C(=O)NCc1c(F)cc(F)cc1)c3)O[C@@H]1C[C@H]2CC1	1611493-56-1	2,5-Methanopyrido[1,Ä≤,2,Ä≤:4,5]pyrazino[2,1-b][1,3]oxazepine-10-carboxamide, N	C21H19F2N3O5	431.39	A/B	-9
1789	O=c1n([C@H]2C[C@@H](ON)[C@@H](CO)O2)cc(C)c(=O)[nH]1	1613740-97-8	2H-Isoindole-2-acetic acid, 1,3-dihydro-Œ±-(1-methylethyl)-1,3-dioxo-, 2-[(2-ami	C21H22N6O6	454.44	A/B	-9
1790	O=c1c2c(n(cn2)[C@@H]2O[C@H](COP(=O)(Oc3ccc(Cl)cc3)NCCn3ccnc3)CC2)[nH]cn1	1618656-18-0	Inosine, 2,Ä≤,3,Ä≤-dideoxy-, 5,Ä≤-[4-chlorophenyl N-[2-(1H-indol-3-yl)ethyl]pho	C26H26ClN6O5P	568.95	A/B	-9
1791	O=c1c2c(n(cn2)[C@@H]2O[C@H](COP(=O)(Oc3ccc(Cl)cc3)NCc3cccnc3)CC2)[nH]cn1	1618656-20-4	Inosine, 2,Ä≤,3,Ä≤-dideoxy-, 5,Ä≤-[4-chlorophenyl N-[2-(3,4-dihydroxyphenyl)eth	C24H25ClN5O7P	561.91	A/B	-9
1792	N(C(=O)[C@H](Cc1ccncc1)C[C@@H](CN1[C@H](C(=O)NC(C)(C)C)CN(Cc2cccnc2)CC1)O)[C@H]1c2c(C[C@H]1O)cccc2	162105-23-9	1-Piperazinepentanamide, N-(2,3-dihydro-2-hydroxy-1H-inden-1-yl)-2-[[(1,1-dimeth	C35H46N6O4	614.78	A/B	-9
1793	[C@@H](C(CC)CC)(NC(=O)C)[C@H]1[C@H](NC(=N)N)C[C@H](C(=O)OCCC(=O)OC[C@H]2N[C@H]([C@H](O)[C@@H]2O)c2c3	1638370-19-0	Cyclopentanecarboxylic acid, 3-[(1S)-1-(acetylamino)-2-ethylbutyl]-4-[(aminoimir	C28H43N9O8	633.7	A/B	-9
1794	N(C(=O)C)[C@H]1[C@H]([C@@H]([C@@H](CO)O)O)OC(=C[C@@H]1NC(=N)N)S(=O)(=O)O	164124-56-5	Cytidine, 2,Ä≤-deoxyguanylyl-(3,Ä≤,Ü5,Ä≤)-2,Ä≤,3,Ä≤-didehydro-2,Ä≤,3,Ä≤-dideo	C19H23N8O9P	538.41	A/B	-9
1795	O(c1nc(nc(Nc2ccccc2)n1)N1CCOCC1)c1ccc(C#N)cc1	1643332-87-9	Benzonitrile, 4-[[4-[(3-chloro-4-fluorophenyl)amino]-6-(4-morpholinyl)-1,3,5-tri	C20H16ClFN6O2	426.83	A/B	-9
1796	N(c1nc(nc(Oc2ccc(C#N)cc2)n1)N1CCOCC1)c1cc(OC)ccc1	1643332-91-5	Benzonitrile, 4-[[4-[(3-methylphenyl)amino]-6-(4-morpholinyl)-1,3,5-triazin-2-yl	C21H20N6O2	388.42	A/B	-9
1797	C(P(=O)(OC[C@H]1O[C@H](C=C1)n1c(=O)[nH]c(=O)c(C)c1)OC[C@H]1O[C@H](C=C1)n1c(=O)[nH]c(=O)c(C)c1)(O)c1c	164652-07-7	Thymidine, 2,Ä≤,3,Ä≤-didehydro-P,3,Ä≤-dideoxy-P-[hydroxy(2-nitrophenyl)methyl]t	C27H28N5O12P	645.51	A/B	-9
1798	O=c1n([C@@H]2O[C@H](COP(=O)(C(O)c3c(N(=O)=O)cc(N(=O)=O)cc3)OC[C@@H]3[C@@H](N=[N+]=[N-])C[C@@H](O3)n3	164652-14-6	Thymidine, 3,Ä≤-azido-P,3,Ä≤-dideoxy-P-[(2,6-dinitrophenyl)hydroxymethyl]thymid	C27H29N12O14P		A/B	-9
1799	N(C(=O)[C@H](Cc1ccccc1)C[C@@H](CN1[C@H](C(=O)NC(C)(C)C)CN(CCCc2cccnc2)CC1)O)[C@H]1c2c(C[C@H]1O)cccc2	164725-12-6	1-Piperazinepentanamide, N-(2,3-dihydro-2-hydroxy-1H-inden-1-yl)-2-[[(1,1-dimeth	C36H47N5O4	613.79	A/B	-9
1800	N(C(=O)[C@H](Cc1ccccc1)C[C@@H](CN1[C@H](C(=O)NC(C)(C)C)CN(CCN2CCOCC2)CC1)O)[C@H]1c2c(C[C@H]1O)cccc2	164725-23-9	1-Piperazinepentanamide, N-(2,3-dihydro-2-hydroxy-1H-inden-1-yl)-2-[[(1,1-dimeth	C37H55N5O4	633.86	A/B	-9
1801	N(C(=O)[C@H](Cc1ccccc1)C[C@@H](CN1[C@H](C(=O)NC(C)(C)C)CN(Cc2cc(C)nc(Cl)c2)CC1)O)[C@H]1c2c(C[C@H]1O)	164725-38-6	1-Piperazinepentanamide, N-(2,3-dihydro-2-hydroxy-1H-inden-1-yl)-2-[[(1,1-dimeth	C38H51N5O4	641.84	A/B	-9
1802	N(C(=O)[C@H](Cc1ccccc1)C[C@@H](CN1[C@H](C(=O)NC(C)(C)C)CN(Cc2cnc(C)cn2)CC1)O)[C@H]1c2c(C[C@H]1O)cccc	164725-42-2	1-Piperazinepentanamide, N-(2,3-dihydro-2-hydroxy-1H-inden-1-yl)-2-[[(1,1-dimeth	C37H49N5O4	627.82	A/B	-9
1803	N(C(=O)[C@H](Cc1ccccc1)C[C@@H](CN1[C@H](C(=O)NC(C)(C)C)CN(Cc2ccc(nc2)N2CCOCC2)CC1)O)[C@H]1c2c(C[C@H]	164725-47-7	1-Piperazinepentanamide, 4-[(2-bromophenyl)methyl]-N-(2,3-dihydro-2-hydroxy-1H-i	C37H47BrN4O4	691.7	A/B	-9
1804	C(=O)([C@@H](NC(=O)OC)C(C)C)N1C[C@@]2(C[C@H]1c1[nH]c(cn1)c1ccc(cc1)c1cc3c(cc1)c1c(cc3)nc([nH]1)[C@H]	1653966-87-0	Carbamic acid, N-[(1S)-2-methyl-1-[[(3S,5S)-3-[7-[4-[2-[(2S,3aS,7aS)-octahydro-1	C50H62N8O7	887.08	A/B	-9
1805	C(=O)([C@@H](NC(=O)OC)C(C)C)N1C[C@]2(C[C@H]1c1[nH]c(cn1)c1ccc(cc1)c1cc3c(cc1)c1c(cc3)nc([nH]1)[C@H]1	1653966-90-5	Carbamic acid, N-[(1S)-1-[[(2S)-2-[7-[4-[2-[(5R,8S)-7-[(2S)-2-[(methoxycarbonyl)	C45H54N8O7	818.96	A/B	-9
1806	C(CN1CCCC1)n1c2c(c3c1cccc3)nc1c(c2NC(CCCN(CC)CC)C)ccc(Cl)c1	167105-67-1	Uridine, 2,Ä≤-deoxy-5-(1-pyrenyl)-	C25H20N2O5	428.44	A/B	-9
1807	C(n1c2c(nc1CC)c(C)cc(C)n2)c1ccc(cc1)c1c(S(=O)(=O)NC(=O)OCCCC)ccc(C(C)C)c1	169281-57-6	Carbamic acid, [[4,Ä≤-[(2-ethyl-5,7-dimethyl-3H-imidazo[4,5-b]pyridin-3-yl)methy	C33H41N5O4S	603.77	A/B	-9
1808	[C@H](Cc1ccccc1)(C(=O)N[C@H](/C=C/S(=O)(=O)c1ccccc1)CCCCN)NC(=O)N1CCOCC1	170111-94-1	4-Morpholinecarboxamide, N-[2-oxo-2-[[1-(2-phenylethyl)-5-(phenylsulfonyl)-2,4-p	C33H37N3O5S	587.73	A/B	-9
1809	O=c1n(cc(C)c(=O)[nH]1)[C@H]1O[C@H](C(=O)O)[C@@H](O)C1	170790-36-0	Benzenepropanamide, 4-[(2-ethyl-5,7-dimethyl-3H-imidazo[4,5-b]pyridin-3-yl)methy	C32H32N4O3S	552.69	A/B	-9
1810	O=C1[C@@H]2[C@@H](c3c([C@H](OC(=O)Cc4c5c([nH]c4C)cccc5)[C@H]2CO1)cc1c(c3)OCO1)c1cc(OC)c(OC)c(OC)c1	1715004-91-3	1H-Indole-3-carboxylic acid, (5R,5aR,8aR,9R)-5,5a,6,8,8a,9-hexahydro-8-oxo-9-(3,	C31H27NO9	557.55	A/B	-9
1811	C(Oc1cc(C)ccc1)c1c2c(N(c3c(C(=O)N2)cccn3)C2CC2)ncc1	172326-06-6	6H-Dipyrido[3,2-b:2,Ä≤,3,Ä≤-e][1,4]diazepin-6-one, 11-cyclopropyl-5,11-dihydro-	C22H20N4O2	372.42	A/B	-9
1812	C(CCn1c2c(nc1)c(=O)nc(N)[nH]2)(COC(=O)c1cc(Oc2ccccc2)ccc1)(COC(=O)c1cc(Oc2ccccc2)ccc1)CO	172645-16-8	Benzoic acid, 3-phenoxy-, 2-[2-(2-amino-1,6-dihydro-6-oxo-9H-purin-9-yl)ethyl]-2	C50H41N5O10	871.89	A/B	-9
1813	C(Cc1ccccc1)[C@]1(CCC(F)(F)F)CC(=C([C@@H](C(C)(C)C)c2cc(NS(=O)(=O)c3ccc(N)cn3)ccc2)C(=O)O1)O	174484-20-9	2-Pyridinesulfonamide, 5-amino-N-[3-[1-[5,6-dihydro-4-hydroxy-2-oxo-6-(2-phenyle	C32H36F3N3O5S	631.71	A/B	-9
1814	C(Cc1ccccc1)C1(CCc2ccccc2)CC(=C([C@@H](CC)c2cc(NS(=O)(=O)c3ccc(C(F)(F)F)cn3)ccc2)C(=O)O1)O	174484-31-2	2-Pyridinesulfonamide, N-[3-[1-[5,6-dihydro-4-hydroxy-2-oxo-6,6-bis(2-phenylethy	C36H35F3N2O5S	664.73	A/B	-9
1815	C(F)(F)(F)c1nc(no1)c1cc(C)c(OCCCc2ccncc2)c(C)c1	174484-81-2	2-Pyridinesulfonamide, N-[3-[1-[5,6-dihydro-4-hydroxy-2-oxo-6-(2-phenylethyl)-6-	C31H33F3N2O5S	602.66	A/B	-9
1816	C(n1c2c(nc1CC)c(C)cc(C)n2)c1ccc(cc1)c1c(S(=O)(=O)NC(=O)c2cc(OC)ccc2)cccc1	174844-70-3	Benzamide, N-[[4,Ä≤-[(2-ethyl-5,7-dimethyl-3H-imidazo[4,5-b]pyridin-3-yl)methyl]	C30H28N4O4S	540.63	A/B	-9
1817	O=c1n([C@@H]2O[C@H](CO[P@@]3(=O)Oc4c(CO3)cccc4)C=C2)cc(C)c(=O)[nH]1	174848-83-0	Thymidine, 2,Ä≤,3,Ä≤-didehydro-3,Ä≤-deoxy-5,Ä≤-O-[(2S)-6-methyl-2-oxido-4H-1,3,	C18H19N2O7P	406.33	A/B	-9
1818	O=c1n([C@@H]2O[C@H](COP(=O)(OP(=O)(OC[C@H]3O[C@H](CC3)n3c(=O)nc(N)cc3)O)O)[C@@H](N=[N+]=[N-])C2)cc(C	175923-17-8	Inosine 5,Ä≤-(trihydrogen diphosphate), 2,Ä≤,3,Ä≤-dideoxy-, P,Ä≤,Ü5,Ä≤-ester w	C20H25N9O12P2		A/B	-9
1819	C(=O)([C@@H](NC(=O)OC)C(C)C)N1[C@@H]([C@@H]2C[C@H]1CC2)c1[nH]c(cn1)c1ccc2c(-c3c(C(F)(F)C2(F)F)cc(cc3)	1801161-65-8	Carbamic acid, N,N,Ä≤-[(9,9,10,10-tetrafluoro-9,10-dihydro-2,7-phenanthrenediyl)	C42H48F4N8O6	836.87	A/B	-9
1820	O=c1n([C@@H]2O[C@H](COC(=O)CCC(=O)OCc3c4c(c(C)nc3)OC(C)OC4)[C@@H](N=[N+]=[N-])C2)cc(C)c(=O)[nH]1	1805775-76-1	Thymidine, 3,Ä≤-azido-3,Ä≤-deoxy-, 5,Ä≤-[4-[(8,Ä≤-methylspiro[cyclohexane-1,2,Ä	C28H34N6O9		A/B	-9
1821	O[C@H]1[C@H](n2c3c(nc2)c(N)ncn3)O[C@H](COS(=O)(=O)C)[C@H]1O	1808012-11-4	Phosphonic acid, P-[[(1S)-1-[4-[[(4-methoxyphenyl)diphenylmethyl]amino]-2-oxo-1	C54H50N3O7P	883.96	A/B	-9
1822	O=C1[C@@H]2[C@@H](c3c([C@H](OC(=O)[C@@H](Cc4ccccc4)N4C(=O)C5C(C4=O)CCCC5)[C@H]2CO1)cc1c(c3)OCO1)c1cc	1809321-40-1	2H-Isoindole-2-acetic acid, 1,3-dihydro-1,3-dioxo-Œ±-(phenylmethyl)-, (5R,5aR,8a	C39H33NO11	691.68	A/B	-9
1823	O=c1n([C@@H]2O[C@H](CO[P@@](=O)(Oc3c(Br)cc(Br)cc3)N[C@H](C(=O)OC)C)C=C2)cc(C)c(=O)[nH]1	184031-31-0	L-Alanine, N-[(S)-2,Ä≤,3,Ä≤-didehydro-3,Ä≤-deoxy-P-phenyl-5,Ä≤-thymidylyl]-, ph	C26H28N3O8P	541.49	A/B	-9
1824	N(=Nc1c(C)cc(S(=O)(=O)O)cc1)c1c2c(ccc1O)cccc2	185213-44-9	2H-Indeno[1,2-d]oxazole, 3,3a,8,8a-tetrahydro-2,2-dimethyl-3-[2,3,5-trideoxy-5-[	C39H51N5O4	653.85	A/B	-9
1825	O=C1[C@@H]2[C@@H](c3c([C@H](OC(=O)CCCC[C@H]4SC(SCC4)c4c(OC)cccc4)[C@H]2CO1)cc1c(c3)OCO1)c1cc(OC)c(OC	1875123-86-6	1,3-Dithiane-4-pentanoic acid, 2-[4-(trifluoromethyl)phenyl]-, (5R,5aR,8aR,9R)-5	C38H39F3O9S2	760.84	A/B	-9
1826	O=C1[C@@H]2[C@@H](c3c([C@H](O[C@H]4[C@H](OC(=O)c5ccccc5)[C@@H](OC(=O)c5ccccc5)[C@@H](OC(=O)c5ccccc5)	1883269-84-8	Furo[3,Ä≤,4,Ä≤:6,7]naphtho[2,3-d]-1,3-dioxol-6(5aH)-one, 5,8,8a,9-tetrahydro-9-	C48H42O15	858.84	A/B	-9
1827	O=c1n([C@@H]2O[C@H](COP3(=O)Oc4c(CO3)cc(Cl)cc4)C=C2)cc(C)c(=O)[nH]1	188548-77-8	Thymidine, 2,Ä≤,3,Ä≤-didehydro-3,Ä≤-deoxy-5,Ä≤-O-(6-nitro-2-oxido-4H-1,3,2-benz	C17H16N3O9P	437.3	A/B	-9
1828	O[C@H]1[C@H](n2c3c(nc2)c(N)ncn3)O[C@H](COP(=O)(O)O)[C@H]1O	1887206-54-3	Benzo[b]thiophene-3-carboxylic acid, 2-[2-[[[4-[(2S,3R)-4-[(6-benzothiazolylsulf	C49H55F3N4O9S3	997.17	A/B	-9
1829	O=[14c]1n(C2OC(CO)C=C2)cc(F)c(=O)[nH]1	189075-02-3	Uridine, 2,Ä≤-deoxy-Œ≤-L-guanylyl-(3,Ä≤,Ü5,Ä≤)-2,Ä≤-deoxy-5-fluoro-	C19H23FN7O11P	575.4	A/B	-9
1830	C(OC[C@@H]1[C@@H](OC(=O)c2ccccc2)[C@@H](OP(=O)(OC[C@@H]2[C@@H](OC(=O)c3ccccc3)[C@@H](OP(=O)(OC[C@@H]	190141-42-5	Adenosine, adenylyl-(2,Ä≤,Ü5,Ä≤)-adenylyl-(2,Ä≤,Ü5,Ä≤)-1,Ä≤-[3-(aminocarbonyl	C28H36N14O17P2	902.62	A/B	-9
1831	C(F)(F)c1c(cnc(N)n1)c1nc(nc(n1)N1CCOCC1)N1[C@@H](C)COCC1	1927839-32-4	1H-1,2,4-Triazole-3-carboxamide, 1-Œ≤-D-ribofuranosyl-5-[(1E)-2-[4-(trifluoromet	C17H17F3N4O5	414.34	A/B	-9
1832	Clc1c2c(c3c(nc([nH]3)[C@H]3N(C(=O)[C@@H](NC(=O)OC)C(C)C)CCC3)c1)ccc(c2)c1ccc(cc1)c1[nH]c(nc1)[C@H]1N	1949770-71-1	Carbamic acid, N-[(1S)-1-[[(2S)-2-[7-[4-[2-[(2S)-1-[(2S)-2-[(methoxycarbonyl)ami	C48H61N9O8	892.05	A/B	-9
1833	O=c1c2c(n(cn2)[C@@H]2O[C@H](CO)[C@@H](O)[C@H]2O)[nH]c([15NH2])[15n]1	1971915-59-9	Guanosine, N-[[4-[(4-hydroxyphenyl)methoxy]phenyl]methyl]-	C24H25N5O7	495.48	A/B	-9
1834	N(=[N+]=[N-])[C@]1(CO)O[C@H]([C@@H](O)[C@@H]1O)n1c2c(nc1)c(N)ncn2	1972596-90-9	L-Alanine, N-[[P(S),2,Ä≤R]-3,Ä≤-O-[(2R)-2-(acetylamino)-3-[(triphenylmethyl)thi	C46H50FN4O11PS	916.95	A/B	-9
1835	O[C@H]1[C@H](n2c3c(nc2)c(=O)nc(N)[nH]3)O[C@H](COc2c(F)c(F)c(N(=O)=O)c(F)c2F)[C@H]1O	197900-98-4	Inosine, 3,Ä≤-azido-3,Ä≤-deoxythymidylyl-(5,Ä≤,Ü5,Ä≤)-1-(2-cyanoethyl)-2,Ä≤,3,	C23H27N10O9P		A/B	-9
1836	N(c1c(C#N)cccc1)c1nc(Nc2cc(C)cc(C)c2)ncc1	1984373-26-3	Benzonitrile, 2-[[2-[(2,4-dimethylphenyl)amino]-6-methyl-4-pyrimidinyl]amino]-	C20H19N5	329.4	A/B	-9
1837	O(Cc1c2c(n(C(=O)OC)c1)cccc2)c1c2n(N([C@H]3N(C2=O)CCOC3)[C@H]2c3c(CSc4c2cccc4)c(F)c(F)cc3)ccc1=O	1985606-91-4	1,2-Pyrrolidinedicarboxylic acid, 1-[[[(12aR)-12-[(11S)-7,8-difluoro-6,11-dihydr	C32H30F2N4O8S	668.66	A/B	-9
1838	O(Cc1ccc(OC(=O)N(C)C)cc1)c1c2n(N([C@H]3N(C2=O)CCOC3)[C@H]2c3c(CSc4c2cccc4)c(F)c(F)cc3)ccc1=O	1985607-48-4	Carbonic acid, (4-azidophenyl)methyl ((12aR)-12-[(11S)-7,8-difluoro-6,11-dihydro	C32H24F2N6O6S		A/B	-9
1839	O(C(=O)O[C@H](C(=O)OC)c1ccccc1)c1c2n(N([C@H]3N(C2=O)CCOC3)[C@H]2c3c(CSc4c2cccc4)c(F)c(F)cc3)ccc1=O	1985607-53-1	Benzeneacetic acid, Œ±-[[[[(12aR)-12-[(11S)-7,8-difluoro-6,11-dihydrodibenzo[b,e	C34H27F2N3O8S	675.66	A/B	-9
1840	N(C(=O)NCCCCC)c1cc2cc(C34CC5CC(C4)CC(C3)C5)[nH]c2cc1	1987854-74-9	Urea, N-(phenylmethyl)-N,Ä≤-(2-tricyclo[3.3.1.13,7]dec-1-yl-1H-indol-5-yl)-	C26H29N3O	399.53	A/B	-9
1841	N(C(=O)Nc1ncccn1)c1cc2cc(C34CC5CC(C4)CC(C3)C5)[nH]c2cc1	1987854-80-7	Urea, N-[5-(1,1-dimethylethyl)-3-isoxazolyl]-N,Ä≤-(2-tricyclo[3.3.1.13,7]dec-1-y	C26H32N4O2	432.56	A/B	-9
1842	O=C1[C@@H]2[C@@H](c3c([C@H](OC(=O)c4nc5c(nc4)cccc5)[C@H]2CO1)cc1c(c3)OCO1)c1cc(OC)c(OC)c(OC)c1	1989611-36-0	1-Isoquinolinecarboxylic acid, (5R,5aR,8aR,9R)-5,5a,6,8,8a,9-hexahydro-8-oxo-9-(	C32H27NO9	569.56	A/B	-9
1843	O=C1[C@@H]2[C@@H](c3c([C@H](OC(=O)c4cn(c5c(c4=O)cc(F)c(Cl)c5)C4CC4)[C@H]2CO1)cc1c(c3)OCO1)c1cc(OC)c(	1989611-37-1	2-Quinoxalinecarboxylic acid, (5R,5aR,8aR,9R)-5,5a,6,8,8a,9-hexahydro-8-oxo-9-(3	C31H26N2O9	570.55	A/B	-9
1844	C(F)(F)c1n(c2c(n1)cccc2)c1nc(nc(n1)N1CCOCC1)N1C[C@H](C(=O)O)CCC1	2001083-69-6	3-Piperidinecarboxylic acid, 1-[4-[2-(difluoromethyl)-1H-benzimidazol-1-yl]-6-(4	C21H23F2N7O3	459.45	A/B	-9
1845	C(=O)([C@@H](NC(=O)OC)C(C)C)N1[C@H](c2[nH]c3c4c(cc(cc4)c4c5c(c(cc4)c4[nH]c(nc4)[C@H]4N(C(=O)[C@@H](N	2007900-40-3	Carbamic acid, N-[(1S)-1-[[(2S)-2-[7-[2,3-dihydro-7-[2-[(2S)-1-[(2S)-2-[(methoxy	C45H54N8O6	802.96	A/B	-9
1846	C(=O)([C@@H](NC(=O)OC)C(C)C)N1[C@H](c2[nH]c(c3c4c(c(cc3)c3cc5c(cc3)c3c(cc5)nc([nH]3)[C@H]3N(C(=O)[C@	2007901-08-6	Carbamic acid, N-[(1S)-1-[[(2S)-2-[7-[2,3-dihydro-7-[2-[(3S)-2-[(2S)-2-[(methoxy	C47H56N8O6	829	A/B	-9
1847	C(=O)([C@@H](NC(=O)OC)C(C)C)N1[C@H](c2[nH]c3c4c(cc(cc4)c4c5c(c(cc4)c4[nH]c(nc4)[C@H]4N(C(=O)[C@@H](N	2007901-50-8	Carbamic acid, N-[(1S)-1-[[(2S)-2-[4-cyclopropyl-5-[2,3-dihydro-7-[2-[(2S)-1-[(2	C48H58N8O6	843.02	A/B	-9
1848	C(=O)([C@@H](NC(=O)OC)C(C)C)N1CC2(C[C@H]1c1[nH]c3c(n1)ccc1c3ccc(c1)c1c3c(cc1)c1[nH]c(nc1)[C@H]1N(C	2007901-84-8	Carbamic acid, N-[(1S)-1-[[(2S,3S,5S)-5-[5-[2,3-dihydro-7-[2-[(3S)-2-[(2S)-2-[(m	C51H64N8O6	885.1	A/B	-9
1849	O[C@H]1[C@H](n2c3c(nc2)c(=O)nc(N)[nH]3)O[C@H](COP(=O)(O)O)[C@H]1OP(=O)(OC[C@H]1O[C@H]([C@H](O)[C@@H]	2013599-59-0	2-Pyridinesulfonamide, 5-(difluorofluoro-18F-methyl)-N-[3-[(1R)-1-[(6R)-5,6-dihy	C31H33F3N2O5S		A/B	-9
1850	C(COCP(=O)(O)O)n1c2c(nc1)c(=S)nc(N)[nH]2	20137-01-3	Adenosine, 5,Ä≤-O-phosphonoguanylyl-(3,Ä≤,Ü5,Ä≤)-	C20H26N10O14P2	692.43	A/B	-9
1851	O=c1n([C@@H]2O[C@H](COP(=O)(O[C@@H]3[C@@H](CO)O[C@H](C3)n3c(=O)[nH]c(=O)c(C)c3)O)[C@@H](OP(=O)(OC[C@	2024571-97-7	Not Yet Assigned	C29H37IN6O19P2	962.48	A/B	-9
1852	[Si](OC[C@H]1O[C@H]([C@H](O)[C@@H]1O)n1nc(C(=O)N)nc1)(C(C)C)(C(C)C)C(C)C	1002335-65-0	1H-1,2,4-Triazole-3-carboxamide, 1-[2,3-O-(methoxymethylene)-5-O-[1-oxo-3-[[2-[[	C28H35N5O14	665.6	C	-9
1853	C(=O)([C@@H](NC(=O)OC)C(C)C)N1[C@@H](CCC1)c1[nH]c(cn1)c1cc(C)c(c(C)c1)c1c(C)cc(cc1C)c1[nH]c(nc1)[C@H	1007882-44-1	Carbamic acid, N-[(1R)-2-oxo-1-phenyl-2-[(2S)-2-[5-[4,Ä≤-[2-[(2S)-1-(2-phenylace	C44H43N7O4	733.86	C	-9
1854	C(=O)([C@H](N(CC)CC)c1ccccc1)N1[C@@H](CCC1)c1[nH]c(cn1)c1ccc(cc1)c1ccc(cc1)c1[nH]c(nc1)[C@H]1N(C(=O)	1009107-40-7	Carbamic acid, N-[(1R)-2-[(2S)-2-[5-[4,Ä≤-[2-[(2S)-1-[2-[[(1,1-dimethylethoxy)ca	C43H48N8O6	772.89	C	-9
1855	C(=O)([C@H](NC(=O)OC)c1ccccc1)N1[C@@H](CCC1)c1[nH]c(cn1)c1ccc(cc1)c1ccc(cc1)c1[nH]c(nc1)[C@H]1N(C(=O	1009108-72-8	Carbamic acid, N-[(1R)-2-[(2S)-2-[5-[4,Ä≤-[2-[(2S)-1-(2-cyclohexylacetyl)-2-pyrr	C44H49N7O4	739.9	C	-9
1856	C(=O)([C@H](NC(=O)OC)C(C)C)N1[C@@H]2[C@@H](C2)C[C@H]1c1[nH]c(cn1)c1ccc(cc1)c1ccc(cc1)c1[nH]c(nc1)[C@	1009118-20-0	[1,1,Ä≤-Biphenyl]-2-carboxylic acid, 4,4,Ä≤-bis[2-[(2S)-1-[(2S)-2-cyclopropyl-2-	C41H46N8O8	778.85	C	-9
1857	C(=O)(C(NC(=O)OC)C(C)C)N1[C@H](CCC1)c1[nH]c(cn1)c1ccc(cc1)c1ccc(cc1)c1[nH]c(nc1)[C@@H]1N(C(=O)C(NC(=	1009119-30-5	Carbamic acid, N-[(1S)-1-methyl-2-oxo-2-[(2S)-2-[5-[4,Ä≤-[2-(2S)-2-pyrrolidinyl-	C31H35N7O3	553.65	C	-9
1858	C(=C\1/C=C(C(=O)O)C(=O)C=C1)(/c1c(O)c(C(=O)O)ccc1)\c1cc(C(=O)O)c(O)cc1	1049974-61-9	Benzoic acid, 5-[(Z)-(3-carboxy-4-hydroxyphenyl)(3-carboxy-4-oxo-2,5-cyclohexadi	C30H20O12	572.47	C	-9
1859	S(=O)(=O)(O)c1c2c(c(N)c(/N=N/c3cc(S(=O)(=O)O)c(C=Cc4c(S(=O)(=O)O)cc(/N=N/c5c(N)c6c(c(S(=O)(=O)O)c5)c	1052528-88-7	Not Yet Assigned	C34H26N6O18S6.Na		C	-9
1860	C(=O)(NC(C)(C)C)[C@@H]1CN(C2c3c(CC2)cccc3)CCN1C[C@H](C[C@H](C(=O)N[C@H]1c2c(C[C@H]1O)cccc2)Cc1ccccc1	1064475-92-8	Not Yet Assigned	C44H55N5O5	733.94	C	-9
1861	N(C(=O)[C@H](Cc1ccccc1)C[C@@H](CN1[C@H](C(=O)NC(C)(C)C)CN(CCN2C(=O)c3c(C2=O)cccc3)CC1)O)[C@H]1c2c(C[	1064476-01-2	Not Yet Assigned	C39H50N4O4	638.84	C	-9
1862	N(C(CCCN(CC)CC)C)c1c2c(nc3c1cccc3)cc(Cl)c(N(=O)=O)c2	1071752-50-5	1-Naphthalenesulfonic acid, 4-[(1E)-2-(2-hydroxy-1-naphthalenyl)diazenyl]-	C20H14N2O4S	378.4	C	-9
1863	O=C1[C@@H]2[C@@H](c3c([C@H](OC(=O)c4ccc(SSc5ccc(C(=O)O[C@H]6c7c([C@H]([C@@H]8[C@@H]6COC8=O)c6cc(OC)c	1107652-05-0	Benzoic acid, 4,4,Ä≤-dithiobis-, 1,1,Ä≤-bis[(5R,5aR,8aR,9R)-5,5a,6,8,8a,9-hexah	C58H50O18S2	1099.14	C	-9
1864	[C@H](CC)(C1=C(O)C[C@H](OC1=O)c1ccccc1)c1cc(NS(=O)(=O)c2c(C(F)(F)F)cccn2)ccc1	1109235-69-9	2-Pyridinesulfonamide, N-[3-[1-(5,6-dihydro-4-hydroxy-2-oxo-6-phenyl-2H-pyran-3-	C28H27F3N2O5S	560.58	C	-9
1865	O=c1n([C@@H]2O[C@H](COP(=O)(OP(=O)(O[C@@H]3O[C@@H]([C@@H](C)O)[C@H](O)[C@H]3O)O)O)[C@@H](O)C2)cc(C)c	112475-41-9	Œ≤-D-arabino-3,Ä≤-Adenylic acid, Œ≤-D-arabino-uridylyl-(3,Ä≤,Ü5,Ä≤)-Œ≤-D-arab	C38H48N14O29P4	1288.76	C	-9
1866	C(=O)(CNC(=O)OC)N1CC2(C[C@H]1c1[nH]c(cn1)c1ccc(cc1)c1ccc(cc1)c1[nH]c(nc1)[C@H]1N(C(=O)CNC(=O)OC)CC3(	1129634-51-0	Carbamic acid, N,N,Ä≤-[[1,1,Ä≤-biphenyl]-4,4,Ä≤-diylbis[1H-imidazole-5,2-diyl-(	C44H54N8O6	790.95	C	-9
1867	O=c1c2c3c(c(F)c2)N2CCN(C)CC2)OCC(C)n3cc1C(=O)OC[C@@H]1[C@@H](N=[N+]=[N-])C[C@@H](O1)n1c(=O)[nH]c(=	1174398-25-4	Thymidine, 3,Ä≤-azido-3,Ä≤-deoxy-, 5,Ä≤-[1-cyclopropyl-7-(4-ethyl-1-piperazinyl	C29H33FN8O6		C	-9
1868	C(C)n1c2c(c(=O)c(C(=O)O)c1)cc(F)c(c2F)N1CC(C)N(Cc2cn(c(=O)[nH]c2=O)[C@H]2C[C@H](N=[N+]=[N-])[C@@H](C	1174398-29-8	3-Quinolinecarboxylic acid, 7-[4-[[1-(3-azido-2,3-dideoxy-Œ≤-D-erythro-pentofura	C27H29FN8O7		C	-9
1869	OC1c2c(-c3c1cc(cc3)c1[nH]c(nc1)[C@H]1N(C(=O)[C@@H](NC(=O)OC)C(C)C)CCC1)ccc(c2)c1[nH]c(nc1)[C@H]1N(C(	1179149-84-8	Carbamic acid, N,N,Ä≤-[(9-oxo-9H-fluorene-2,7-diyl)bis[1H-imidazole-5,2-diyl-(2S	C41H48N8O7	764.87	C	-9
1870	C(=O)([C@@H](NC(=O)OC)C(C)C)N1[C@@H](c2[nH]c(c3cc4c(cc5c(o4)cc(cc5)c4[nH]c(nc4)[C@@H]4N(C(=O)[C@@H](NC(	1179150-57-2	Carbamic acid, N,N,Ä≤-[spiro[cyclobutane-1,9,Ä≤-[9H]fluorene]-2,Ä≤,7-diylbis	C44H54N8O6	790.95	C	-9
1871	C(COC)C1c2c(-c3c1cc(cc3)c1[nH]c(nc1)[C@H]1N(C(=O)[C@@H](NC(=O)OC)C(C)C)CCC1)ccc(c2)c1[nH]c(nc1)[C@H]	1179151-18-8	Carbamic acid, N,N,Ä≤-[9H-carbazole-2,7-diylbis[1H-imidazole-5,2-diyl-(2S)-2,1-p	C40H49N9O6	751.87	C	-9

1872	N(CCCN)(CCC)c1c2c(cc(Cl)cc2)ncc1	1179151-35-9	Carbamic acid, N,N,Ä≤-[[9-(2-methoxyethyl)-9H-fluorene-2,7-diyl]bis[1H-imidazole	C44H56N8O7	808.96	C	-9
1873	C(=O)([C@@H](NC(=O)OC)C(C)C)N1[C@H](c2[nH]c3-c4c(cc(cc4)c4cc(F)c(cc4)c4[nH]c(nc4)[C@H]4N(C(=O)[C@@H]	1180669-72-0	Carbamic acid, N-[(1S)-2-methyl-1-[[(1R,3S,5R)-3-[5-[4-[1,4,5,6-tetrahydro-2-[(1	C45H54N8O6	802.96	C	-9
1874	S(=O)(=O)(/C=C/C[C@@H](NC(=O)[C@@H](NC(=O)N1CCNCC1)CC(C)C)CCc1ccccc1)c1ccccc1	1222468-14-5	Not Yet Assigned	C35H45ClN6O4	649.22	C	-9
1875	N(C(=O)[C@H](CC1CCCCC1)C[C@@H](CN1[C@H](C(=O)NC(C)(C)C)CN(Cc2cccnc2)CC1)O)[C@H]1c2c(C[C@H]1O)cccc2	1222674-71-6	Not Yet Assigned	C36H46FN5O4	631.78	C	-9
1876	C(F)(F)(F)c1c(c2nc(nc(n2)N2CCOCC2)N2CCOCC2)ccc(N)n1	1225037-83-1	Acetamide, N-[5-(4,6-di-4-morpholinyl-1,3,5-triazin-2-yl)-4-(trifluoromethyl)-2-	C19H22F3N7O3	453.42	C	-9
1877	O=c1n([C@H]2C[C@H](OP(=O)(OCCCCCCCCCCCCCCCC)OCCC#N)[C@@H](CO)O2)cc(C)c(=O)[nH]1	1228633-22-4	Carbamic acid, N-[(1S)-2-methyl-1-[[(2S)-2-[5-[4-[6-[2-(2S)-2-pyrrolidinyl-1H-im	C37H41N7O3	631.77	C	-9
1878	C(=O)([C@H](NC(=O)OC)c1ccccc1)N1[C@@H](CCC1)c1[nH]c(cn1)c1ccc(cc1)[C@H]1CC[C@@H](CC1)c1[nH]c(nc1)[C@	1228667-93-3	Carbamic acid, N-[(1S)-2-[(2S)-2-[5-[4-[trans-4-[2-[(2S)-1-[(2S)-2-[(methoxycarb	C46H52N8O6	812.96	C	-9
1879	C(=O)([C@H](NC(=O)OC)c1ccccc1)N1[C@@H](CCC1)c1[nH]c(cn1)c1ccc(C#Cc2ccc(cc2)c2[nH]c(nc2)[C@H]2N(C(=O)	1228965-84-1	Carbamic acid, N-[(1R)-2-oxo-2-[(2S)-2-[5-[4-[2-[4-[2-[(2S)-1-[(2R)-1-oxo-2-phen	C47H45N7O4	771.9	C	-9
1880	N(C(=O)[C@H](Cc1ccccc1)C[C@@H](CN1[C@H](C(=O)NC(C)(C)C)CN(Cc2c3c(ncc2)cccc3)CC1)O)[C@H]1c2c(C[C@H]1O	1237809-04-9	Not Yet Assigned	C38H48N6O4	652.83	C	-9
1881	N(CC(=O)[C@H](Cc1ccccc1)C[C@@H](CN1[C@H](C(=O)NC(C)(C)C)CN(CCCc2cccnc2)CC1)O)[C@H]1c2c(C[C@H]1O)cccc	1237988-76-9	D-erythro-Pentonamide, 2,3,5-trideoxy-N-[(1S,2R)-2,3-dihydro-2-hydroxy-1H-inden-	C36H48N4O5	616.79	C	-9
1882	N(NC(=O)c1ccncc1)c1nc(nc(n1)N1CCOCC1)N1c2c(CCC1)cccc2	1239884-92-4	Isoquinoline, 2,2,Ä≤-[6-(4-morpholinyl)-1,3,5-triazine-2,4-diyl]bis[1,2,3,4-tet	C25H28N6O	428.53	C	-9
1883	C(=O)([C@@H](NC(=O)OC)C(C)C)N1[C@@H](CCC1)c1[nH]c(cn1)c1ccc(cc1)c1ccc([C@@H](NC(=O)[C@H]2N(C(=O)[C@@	1240427-38-6	L-Prolinamide, N-(methoxycarbonyl)-L-alanyl-N-[(1S)-1-[4,Ä≤-[2-[(2S)-1-[(2S)-2-	C36H45N7O7	687.79	C	-9
1884	C(=O)([C@@H](NC(=O)OC)C(C)C)N1[C@@H](CCC1)c1[nH]c(cn1)c1ccc(cc1)c1ccc(C(=O)NC[C@H]2N(C(=O)[C@@H](NC(	1240427-43-3	Carbamic acid, N-[(1S)-2-[(2S)-2-[[[4,Ä≤-[2-[(2S)-1-[(2S)-2-[(methoxycarbonyl)a	C35H43N7O7	673.76	C	-9
1885	C(=O)([C@@H](NC(=O)O[C@@H]1CCOC1)C(C)C)N1[C@H](c2[nH]c(c3cc4c(-c5c(C4)cc(cc5)c4[nH]c(nc4)[C@H]4N(C(=	1240880-78-7	Carbamic acid, N,N,Ä≤-[9H-fluorene-2,7-diylbis[1H-imidazole-5,2-diyl-(2S)-2,1-py	C49H62N8O8	891.07	C	-9
1886	C(=O)([C@H](NC(=O)OC)C)N1[C@@H](CCC1)c1[nH]c(cn1)[C@H]1CC[C@@H](CC1)c1ccc(cc1)c1[nH]c(nc1)[C@H]1N(C(	1240886-31-0	Carbamic acid, N-[(1R)-2-[(2S)-2-[5-[4-[4-[2-[(2S)-1-[(2R)-2-[(methoxycarbonyl)a	C46H50N8O6	810.94	C	-9
1887	C(=O)([C@@H](NC(=O)OC)[C@@H](C)O)N1[C@@H](CCC1)c1[nH]c(cn1)[C@H]1CC[C@@H](CC1)c1ccc(cc1)c1[nH]c(nc1)	1240887-46-0	Carbamic acid, N-[(1R)-1-[[(2S)-2-[5-[4-[trans-4-[2-[(2S)-1-[(2R)-2-[(methoxycar	C38H52N8O6	716.87	C	-9
1888	C(=O)([C@H](NC(=O)OC)c1ccccc1)N1[C@@H](CCC1)c1[nH]c(cn1)C1=CC[C@@H](CC1)c1ccc(cc1)c1[nH]c(nc1)[C@H]1	1240891-47-7	Carbamic acid, N-[(1R)-2-[(2S)-2-[5-[4-[(1S)-4-[2-[(2S)-1-[(2R)-2-[(methoxycarbo	C46H50N8O6	810.94	C	-9
1889	C(=O)([C@H](NC(=O)OC)c1ccccc1)N1[C@@H](CCC1)c1[nH]c(cn1)c1ccc(C[C@H]2[C@@H](Cc3[nH]c(nc3)[C@H]3N(C(=	1240891-78-4	Carbamic acid, N-[(1R)-2-[(2S)-2-[5-[4-[(2Z)-4-[2-[(2S)-1-[(2R)-2-[(methoxycarbo	C44H48N8O6	784.9	C	-9
1890	C(=O)([C@@H](NC(=O)OC)C(C)C)N1[C@@H](CCC1)c1[nH]c(cn1)C1CCC(CC1)c1ccc(cc1)c1[nH]c(nc1)[C@H]1N(C(=O)[	1246956-03-5	Carbamic acid, N,N,Ä≤-[[1,1,Ä≤-bicyclohexyl]-4,4,Ä≤-diylbis[1H-imidazole-5,2-di	C36H54N8O6	694.86	C	-9
1891	C(=O)([C@@H](NC(=O)OC)C(C)C)N1[C@H]2[C@H](C2)C[C@H]1c1[nH]c(cn1)c1ccc(C#Cc2ccc(cc2)c2[nH]c(nc2)[C@H]	1250937-05-3	Carbamic acid, N,N,Ä≤-[[1,1,Ä≤:4,Ä≤,1,Ä≤,Ä≤-terphenyl]-4,4,Ä≤,Ä≤-diylbis[1H-im	C52H50N8O6	883	C	-9
1892	O=s1c2c(c3c1cc(cc3)c1[nH]c(nc1)[C@H]1N(C(=O)[C@@H](NC(=O)OC)C(C)C)CCC1)ccc(c2)c1[nH]c(nc1)[C@H]1N(C(	1256387-82-2	Carbamic acid, N,N,Ä≤-[3,7-dibenzothiophenediylbis[1H-imidazole-5,2-diyl-(2S)-2,	C40H48N8O6S	768.92	C	-9
1893	C(=O)([C@@H](NC(=O)OC)C(C)C)N1CC2(C[C@H]1c1[nH]c(cn1)c1ccc(cc1)c1ccc(cc1)c1[nH]c(nc1)[C@H]1N(C(=O)[C	1256387-83-3	Carbamic acid, N,N,Ä≤-[(5-oxido-3,7-dibenzothiophenediyl)bis[1H-imidazole-5,2-di	C40H48N8O7S	784.92	C	-9
1894	C(=O)([C@@H](NC(=O)OC)C(C)C)N1[C@H](c2[nH]c(c3cc4c(-c5c(cc(cc5)c5[nH]c(nc5)[C@H]5N(C(=O)[C@@H](NC(=O	1256388-66-5	Carbamic acid, N-[(1S)-1-[[(2S)-2-[5-[9,10-dihydro-7-[2-[(1R,3S,4S)-2-[(2S)-2-[(	C44H54N8O6	790.95	C	-9
1895	C(=O)([C@@H](NC(=O)OC)C(C)C)N1[C@@H]([C@@H]2C[C@H]1CC2)c1[nH]c(cn1)c1ccc(C#Cc2ccc(cc2)c2[nH]c(nc2)[C	1256388-88-1	Carbamic acid, N-[(1S)-1-[[(2S)-2-[5-[7-[2-[(1R,3S,4S)-2-[(2S)-2-[(methoxycarbon	C42H50N8O7	778.9	C	-9
1896	C(=O)([C@@H](NC(=O)OC)C(C)C)N1[C@@H](C[C@@H](Cc2ccccc2)C1)c1[nH]c(cn1)c1ccc(cc1)c1cc2c(cc1)cc(cc2)c1	1256390-02-9	Carbamic acid, N-[(1S)-1-[[(2S)-2-[5-[6-[4-[2-[(2S,4R)-1-[(2S)-2-[(methoxycarbon	C50H56N8O6	865.03	C	-9
1897	O=c1c2c(n(cc1C(=O)O)C1CC1)cc(c(F)c2)N1CCN(CN2c3c(/C(=N/NC(=S)NO)/C2=O)cc(Cl)cc3)CC1	1265623-14-0	3-Quinolinecarboxylic acid, 7-[4-[[(3Z)-2,3-dihydro-3-[2-[(hydroxyamino)thioxome	C27H28FN7O5S	581.62	C	-9
1898	C(C)n1c2c(c(=O)c(C(=O)O)c1)cc(F)c(c2F)N1CC(C)N(CN2c3c(/C(=N/NC(=S)NOC)/C2=O)cc(Cl)cc3)CC1	1265623-21-9	3-Quinolinecarboxylic acid, 7-[4-[[(3Z)-5-chloro-2,3-dihydro-3-[2-[(hydroxyamino	C27H26ClF2N7O5S	634.05	C	-9
1899	O(C)c1c2n(cc(C(=O)O)c(=O)c2cc(F)c1N1CC(C)N(CN2c3c(/C(=N/NC(=S)NO)/C2=O)cc(C)cc3)CC1)C1CC1	1265623-30-0	3-Quinolinecarboxylic acid, 1-cyclopropyl-6-fluoro-7-[4-[[(3Z)-5-fluoro-2,3-dihy	C30H31F2N7O6S	655.67	C	-9
1900	C([C@H](OCP(=O)(OCCOCCCCCCCCCCCCCCCCC)O)CO)n1c(=O)nc(N)cc1	1265623-33-3	3-Quinolinecarboxylic acid, 7-[4-[[(3Z)-2,3-dihydro-3-[2-[(methoxyamino)thioxome	C28H30FN7O5S	595.65	C	-9
1901	O(C[C@@H]1[C@@H](N=[N+]=[N-])C[C@@H](O1)n1c(=O)[nH]c(=O)c(C)c1)P1(=O)Oc2c(cc3c(c2)COP(=O)(OC[C@H]2O[	1268815-07-1	Thymidine, 5,Ä≤,5,Ä≤,Ä≤,Ä≤-O-(4,9-dihydro-2,7-dioxidobenzo[1,2-d:4,5-d,Ä≤]bis[1	C28H30N10O14P2		C	-9
1902	C(=O)([C@@H](NC(=O)OC)CC)N1[C@@H]([C@@H]2C[C@H]1CC2)c1[nH]c(cn1)c1ccc(cc1)c1ccc(cc1)c1[nH]c(nc1)[C@H	1273589-45-9	Carbamic acid, N-[(1S)-2-[(2S)-2-[5-[4,Ä≤-[2-[(1R,3S,4S)-2-[(2S)-2-[(methoxycarb	C38H44N8O6	708.81	C	-9
1903	C(=O)([C@@H](NC(=O)OC)CCC)N1[C@@H]([C@@H]2C[C@H]1CC2)c1[nH]c(cn1)c1ccc(cc1)c1ccc(cc1)c1[nH]c(nc1)[C@	1273589-56-2	Carbamic acid, N-[(1S)-1-[[(2S)-2-[5-[4,Ä≤-[2-[(1R,3S,4S)-2-[(2S)-2-[(methoxycar	C42H48N8O6	760.88	C	-9
1904	C[C@@]1(N(C(=O)[C@@H](NC(=O)OC)C(C)C)[C@H]2C[C@@H]1CC2)c1[nH]c(cn1)c1ccc(cc1)c1ccc(cc1)c1[nH]c(nc1)[	1273591-31-3	Carbamic acid, N-[(1S)-1-[[(2S)-2-[5-[4,Ä≤-[2-[(1S,3S,4R)-2-[(2S)-2-[(methoxycar	C42H50N8O7	778.9	C	-9
1905	C(=C\C(=O)CC(=O)CCc1ccccc1)/c1cc(OC)c(O)cc1	128589-94-6	1-Naphthalenesulfonic acid, 4-[2-(2-hydroxy-1-naphthalenyl)diazenyl]-, ion(1-)	C20H13N2O4S		C	-9
1906	S=c1n(nc(s1)c1cc(N(=O)=O)c(OC)cc1)c1nc(nc(n1)N1CCOCC1)N1CCOCC1	1309437-30-6	1,3,4-Thiadiazole-2(3H)-thione, 3-(4,6-di-4-morpholinyl-1,3,5-triazin-2-yl)-5-(3	C19H20N8O4S2	488.54	C	-9
1907	S=c1n(c2nc(nc(n2)N2CCOCC2)N2CCOCC2)nc(s1)c1cc(N(=O)=O)ccc1Cl	1309437-32-8	1,3,4-Thiadiazole-2(3H)-thione, 5-(4-chlorophenyl)-3-(4,6-di-4-morpholinyl-1,3,5	C19H20ClN7O2S2	477.99	C	-9
1908	O=C1N2[C@@H]3[C@H](CO[C@@H]2Cn2c1c(O)c(=O)c(C(=O)NCc1c(F)cc(F)cc1)c2)CCC3	1309561-72-5	2H-Pyrido[1,Ä≤,2,Ä≤:4,5]pyrazino[1,2-a][3,1]benzoxazine-10-carboxamide, N-[(2,4	C29H27F2N3O5	535.54	C	-9
1909	C(=O)([C@@H](NC(=O)OC)C(C)C)N1[C@@H](CC(=C)C1)c1[nH]c(cn1)c1cc2c(cc1)cc(C=Cc1[nH]c(nc1)[C@H]1N(C(=O)	1311398-58-9	Carbamic acid, N-[(1S)-1-[[(2S)-2-[5-[2-[6-[2-[(2S)-1-[(2S)-2-[(methoxycarbonyl)	C41H52N8O6	752.9	C	-9
1910	C(=O)([C@@H](NC(=O)OC)C(C)C)N1[C@@H](CCC1)c1[nH]c(c(Br)n1)c1ccc(cc1)c1ccc(cc1)c1[nH]c(nc1Br)[C@H]1N(	1312335-95-7	Carbamic acid, N,N,Ä≤-[[1,1,Ä≤-biphenyl]-4,4,Ä≤-diylbis[(5-chloro-1H-imidazole-	C42H48Cl2N8O6	831.79	C	-9
1911	C(=O)([C@@H](NC(=O)OC)C1CCOCC1)N1[C@H]2[C@H](C2)C[C@H]1c1[nH]c(c(Cl)n1)c1ccc(cc1)c1ccc(cc1)c1[nH]c(n	1312336-08-5	Carbamic acid, N-[(1S)-2-[(1R,3S,5R)-3-[5-[4,Ä≤-[5-chloro-2-[(1R,3S,5R)-2-[(2S)-	C44H51ClN8O7	839.38	C	-9
1912	C(=O)([C@@H](NC(=O)OC)C(C)C)N1[C@H](c2[nH]c(c(n2)c2ccc(cc2)c2ccc(cc2)c2c(nc([nH]2)[C@H]2N(C(=O)[C@@H	1312336-97-2	Carbamic acid, N-[(1S)-2-[(2S)-2-[4-cyclopropyl-5-[4,Ä≤-[2-[(2S)-1-[(2S)-2-[(met	C47H58N8O8	863.01	C	-9
1913	C(=O)([C@@H](NC(=O)OC)C1CCOCC1)N1[C@@H](C[C@H](C)C1)c1[nH]c(cn1)c1ccc(cc1)c1ccc(cc1)c1[nH]c(nc1)[C@H	1312338-32-1	Carbamic acid, N,N,Ä≤-[[1,1,Ä≤-biphenyl]-4,4,Ä≤-diylbis[1H-imidazole-5,2-diyl[(	C46H58N8O8	851	C	-9
1914	C(=O)([C@@H](NC(=O)OC)C(C)C)N1[C@H](c2[nH]c(c3c4c(c(cc3)c3ccc(cc3)c3[nH]c(nc3)[C@H]3N(C(=O)[C@@H](NC	1312608-45-9	Carbamic acid, N-[(1S)-1-[[(2S)-2-[5-[4-[2,3-dihydro-7-[2-[(2S)-1-[(2S)-2-[(meth	C43H54N8O6	778.94	C	-9
1915	O[C@H]1[C@H](n2c3c(nc2)c(N)ncn3)O[C@H](COP(=O)(CCP(=O)(O)O)O)[C@H]1O	1312609-79-2	Carbamic acid, N-[(1S)-2-methyl-1-[[(2S)-2-[5-[4-[4-[2-(2S)-2-pyrrolidinyl-1H-im	C37H41N7O3	631.77	C	-9
1916	O(Cc1ccc(F)cc1)c1c2c(oc(c(O)c2=O)c2[nH]c(OC)nn2)ccc1	1312892-07-1	4H-1-Benzopyran-4-one, 5-[(4-fluorophenyl)methoxy]-3-hydroxy-2-(5-phenyl-2-pyrim	C26H17FN2O4	440.42	C	-9
1917	O(Cc1ccc(F)cc1)c1c2c(oc(c(O)c2=O)c2[nH]ccn2)c(C)cc1	1312892-69-5	4H-1-Benzopyran-4-one, 5-[(4-fluorophenyl)methoxy]-3-hydroxy-8-methyl-2-(3-pheny	C25H18FN3O4	443.43	C	-9
1918	O(Cc1ccc(F)cc1)c1c2c(c(cc1)c1ccccc1)oc(c(O)c2=O)c1[nH]c(C)nn1	1312894-43-1	4H-1-Benzopyran-4-one, 5-[(4-fluorophenyl)methoxy]-3-hydroxy-8-phenyl-2-(1H-1,2,	C24H16FN3O4	429.4	C	-9
1919	O(Cc1ccc(F)cc1)c1c2c(oc(c(O)c2=O)c2ncccn2)c(C)c(C)c1	1312895-02-5	4H-1-Benzopyran-4-one, 5-[(4-fluorophenyl)methoxy]-3-hydroxy-7-methyl-2-(5-pheny	C26H19FN2O4	442.44	C	-9
1920	O(Cc1ccc(F)cc1)c1c2c(oc(c(O)c2=O)c2[nH]ccn2)c(Cl)c(C)c1	1312896-47-1	4H-1-Benzopyran-4-one, 8-chloro-5-[(4-fluorophenyl)methoxy]-3-hydroxy-7-methyl-2	C25H17ClFN3O4	477.87	C	-9
1921	O(Cc1ccc(F)cc1)c1c2c(c(c(C)c1)c1ccccc1)oc(c(O)c2=O)c1[nH]cc(C)n1	1312896-98-2	4H-1-Benzopyran-4-one, 5-[(4-fluorophenyl)methoxy]-3-hydroxy-2-(1H-imidazol-2-yl	C26H19FN2O4	442.44	C	-9
1922	O(C)c1c(c2c(c(OCc3ccc(F)cc3)c1)c(=O)c(O)c(o2)c1[nH]cnn1)c1ccccc1	1312899-49-2	4H-1-Benzopyran-4-one, 5-[(4-fluorophenyl)methoxy]-3-hydroxy-7-methoxy-8-phenyl-	C33H23FN2O5	546.54	C	-9
1923	O(C)c1c(c2c(c(OCc3ccc(F)cc3)c1)c(=O)c(O)c(o2)c1[nH]ccn1)c1ccccc1	1312899-59-4	4H-1-Benzopyran-4-one, 5-[(4-fluorophenyl)methoxy]-3-hydroxy-7-methoxy-8-phenyl-	C31H22FN3O5	535.52	C	-9
1924	O(Cc1ccc(F)cc1)c1c2c(oc(c(O)c2=O)c2[nH]ccn2)c(Cl)c(Cl)c1	1312902-18-3	4H-1-Benzopyran-4-one, 7,8-dichloro-5-[(4-fluorophenyl)methoxy]-3-hydroxy-2-(3-p	C24H14Cl2FN3O4	498.29	C	-9
1925	O(Cc1ccc(F)cc1)c1c2c(c(c(Cl)c1)c1ccccc1)oc(c(O)c2=O)c1[nH]cc(C)n1	1312902-69-4	4H-1-Benzopyran-4-one, 7-chloro-5-[(4-fluorophenyl)methoxy]-3-hydroxy-2-(1H-imic	C25H16ClFN2O4	462.86	C	-9
1926	O(Cc1ccc(F)cc1)c1c2c(c(C)c(c1)c1ccccc1)oc(c(O)c2=O)c1[nH]cnn1	1312903-59-5	4H-1-Benzopyran-4-one, 5-[(4-fluorophenyl)methoxy]-3-hydroxy-8-methyl-7-phenyl-2	C33H23FN2O4	530.55	C	-9
1927	O(Cc1ccc(F)cc1)c1c2c(c(C)c(c1)c1ccccc1)oc(c(O)c2=O)c1[nH]c(C)nn1	1312903-66-4	4H-1-Benzopyran-4-one, 5-[(4-fluorophenyl)methoxy]-3-hydroxy-8-methyl-7-phenyl-2	C25H18FN3O4	443.43	C	-9
1928	O(Cc1ccc(F)cc1)c1c2c(c(C)c(c1)c1ccccc1)oc(c(O)c2=O)c1[nH]ccn1	1312903-70-0	4H-1-Benzopyran-4-one, 5-[(4-fluorophenyl)methoxy]-3-hydroxy-8-methyl-7-phenyl-2	C31H22FN3O4	519.52	C	-9
1929	O(Cc1ccc(F)cc1)c1c2c(c(c(c1)c1ccccc1)c1ccccc1)oc(c(O)c2=O)c1[nH]ccn1	1312905-80-8	4H-1-Benzopyran-4-one, 5-[(4-fluorophenyl)methoxy]-3-hydroxy-7,8-diphenyl-2-(3-p	C36H24FN3O4	581.59	C	-9
1930	O=c1c2c(c(cc(OCc3ccc(F)cc3)c2)c2ccccc2)oc(c1O)c1[nH]cnn1	1312908-18-1	4H-1-Benzopyran-4-one, 6-[(4-fluorophenyl)methoxy]-3-hydroxy-8-phenyl-2-(5-pheny	C32H21FN2O4	516.52	C	-9
1931	Cc1c(c2c(cc1OCc1ccc(F)cc1)c(=O)c(O)c(o2)c1[nH]cnn1)c1ccccc1	1312910-80-7	4H-1-Benzopyran-4-one, 6-[(4-fluorophenyl)methoxy]-3-hydroxy-7-methyl-8-phenyl-2	C33H23FN2O4	530.55	C	-9
1932	O=c1c2c(oc(c1O)c1ncccn1)cc(Cl)c(OCc1ccc(F)cc1)c2	1312911-47-9	4H-1-Benzopyran-4-one, 6-[(4-fluorophenyl)methoxy]-3-hydroxy-7-methoxy-2-(5-pher	C26H19FN2O5	458.44	C	-9
1933	O=c1c2c(oc(c1O)c1[nH]ccn1)cc(Cl)c(OCc1ccc(F)cc1)c2	1312911-82-2	4H-1-Benzopyran-4-one, 7-chloro-6-[(4-fluorophenyl)methoxy]-3-hydroxy-2-(3-pheny	C24H15ClFN3O4	463.85	C	-9
1934	Cc1c2c(cc(OCc3ccc(F)cc3)c1OC)c(=O)c(O)c(o2)c1[nH]ccn1	1312912-38-1	4H-1-Benzopyran-4-one, 6-[(4-fluorophenyl)methoxy]-3-hydroxy-7-methoxy-8-methyl-	C26H20FN3O5	473.45	C	-9
1935	Cc1c2c(cc(OCc3ccc(F)cc3)c1Cl)c(=O)c(O)c(o2)c1ncccn1	1312912-48-3	4H-1-Benzopyran-4-one, 6-[(4-fluorophenyl)methoxy]-3-hydroxy-7-methoxy-8-methyl-	C27H21FN2O5	472.46	C	-9
1936	Cc1c2c(cc(OCc3ccc(F)cc3)c1Cl)c(=O)c(O)c(o2)c1[nH]cnn1	1312912-63-2	4H-1-Benzopyran-4-one, 7-chloro-6-[(4-fluorophenyl)methoxy]-3-hydroxy-8-methyl-2	C27H18ClFN2O4	488.89	C	-9
1937	Clc1c2c(cc(OCc3ccc(F)cc3)c1Cl)c(=O)c(O)c(o2)c1[nH]cnn1	1312914-63-8	4H-1-Benzopyran-4-one, 7,8-dichloro-6-[(4-fluorophenyl)methoxy]-3-hydroxy-2-(5-p	C26H15Cl2FN2O4	509.31	C	-9
1938	Clc1c2c(cc(OCc3ccc(F)cc3)c1Cl)c(=O)c(O)c(o2)c1[nH]ccn1	1312914-83-2	4H-1-Benzopyran-4-one, 7,8-dichloro-6-[(4-fluorophenyl)methoxy]-3-hydroxy-2-(3-p	C24H14Cl2FN3O4	498.29	C	-9
1939	Clc1c(c2c(cc1OCc1ccc(F)cc1)c(=O)c(O)c(o2)c1ncccn1)c1ccccc1	1312915-53-9	4H-1-Benzopyran-4-one, 6-[(4-fluorophenyl)methoxy]-3-hydroxy-7-methoxy-8-phenyl-	C32H23FN2O5	534.53	C	-9
1940	O(Cc1ccc(F)cc1)c1c(cc2c(c1)c(=O)c(O)c(o2)c1[nH]c(C)nn1)c1ccccc1	1312916-41-8	4H-1-Benzopyran-4-one, 6-[(4-fluorophenyl)methoxy]-3-hydroxy-7-phenyl-2-(1H-1,2,	C24H16FN3O4	429.4	C	-9
1941	O(Cc1ccc(F)cc1)c1c(c(c2c(c1)c(=O)c(O)c(o2)c1[nH]c(nn1)c1ccccc1)c1ccccc1)c1ccccc1	1312918-45-8	4H-1-Benzopyran-4-one, 2-(5-chloro-1H-1,2,4-triazol-3-yl)-6-[(4-fluorophenyl)met	C30H19ClFN3O4	539.94	C	-9
1942	N(=Nc1c2c(c(S(=O)(=O)O)cc1)c(N)ccc2)c1c2c(c(N=Nc3ccc(S(=O)(=O)O)cc3)cc1)ccc(S(=O)(=O)O)c2	1345852-39-2	1-Naphthalenesulfonic acid, 8-amino-5-[2-[3-methyl-4-[2-(3-sulfophenyl)diazenyl]	C23H19N5O6S2	525.56	C	-9
1943	N(=Nc1c2c(c(N=Nc3ccccc3)cc1)ccc(S(=O)(=O)O)c2)c1c2c(c(N)cc1)cccc2S(=O)(=O)O	1345852-45-0	1-Naphthalenesulfonic acid, 8-amino-5-[2-[6-sulfo-4-[2-(3-sulfophenyl)diazenyl]-	C26H19N5O9S3	641.65	C	-9
1944	C(N(C(=O)N[C@H](C(=O)N[C@@H](Cc1ccccc1)C[C@@H]([C@H](Cc1ccccc1)NC(=O)OCc1cncs1)O)C(C)C)C)c1nc(sc1)C1	1348088-23-2	Benzamide, 4-chloro-N-[[4,Ä≤-[(2-ethyl-5,7-dimethyl-3H-imidazo[4,5-b]pyridin-3-y	C30H27ClN4O3S	559.08	C	-9
1945	C(=O)([C@@H](NC(=O)OC)C(C)C)N1[C@H](c2[nH]c(c3c4c(c(cc3)c3c5c(c(cc3)c3[nH]c(nc3)[C@H]3N(C(=O)[C@@H](	1350718-53-4	Carbamic acid, N,N,Ä≤-[(5,5,Ä≤,6,6,Ä≤,7,7,Ä≤,8,8,Ä≤-octahydro[1,1,Ä≤-binaphtha	C48H62N8O6	847.06	C	-9
1946	C(=O)([C@@H](NC(=O)OC)C(C)C)N1[C@@]2(C[C@H](C1)CC2)c1[nH]c(cn1)c1c2c(c(cc1)c1ccc(cc1)c1[nH]c(nc1)[C@	1350718-92-1	Carbamic acid, N-[(1S)-1-[[(1S,4R)-1-[5-[4-[7-[2-[(1S,4R)-2-[(2S)-2-[(methoxycar	C47H57N9O6	844.01	C	-9
1947	C(=O)([C@@H](NC(=O)OC)C(C)C)N1[C@@]2(C[C@H](C1)CC2)c1[nH]c(cn1)c1c2c(c(cc1)c1ccc(cc1)c1[nH]c(nc1)[C@	1350718-95-4	Carbamic acid, N-[(1S)-1-[[(1S,4R)-1-[5-[4-[4-[2-[(1S,4R)-2-[(2S)-2-[(methoxycar	C48H56N8O6	841.01	C	-9
1948	C(=O)([C@@H](NC(=O)OC)C(C)C)N1[C@H](c2[nH]c(c3c4c(c(cc3)c3c5c(c(cc3)c3[nH]c(nc3)[C@H]3N(C(=O)[C@@H](	1350719-40-2	Carbamic acid, N,N,Ä≤-[[7,7,Ä≤-bi-1H-indole]-4,4,Ä≤-diylbis[1H-imidazole-5,2-di	C44H52N10O6	816.95	C	-9
1949	C(=O)([C@@H](NC(=O)OC)C(C)C)N1[C@@H](CCC1)c1[nH]c(cn1)c1ccc2c(cc1)cc(cc2)c1c(C)cc(cc1)c1[nH]c(nc1)[C@	1351330-45-4	Carbamic acid, N-[(1R)-2-[(2S)-2-[5-[4-[6-[2-[(2S)-1-[(2S)-2-[(methoxycarbonyl)a	C48H52N8O6	836.98	C	-9
1950	C(=O)([C@@H](NC(=O)OC)[C@@H](CC)C)N1[C@@H](CCC1)c1[nH]c(cn1)c1cc2c(cc1)cc(cc2)c1ccc(cc1)c1[nH]c(nc1)	1351330-62-5	Carbamic acid, N-[(1R)-2-[(2S)-2-[5-[6-[4-[2-[(2S)-1-[(2S)-2-[(methoxycarbonyl)a	C48H52N8O6	836.98	C	-9
1951	C(=O)([C@@H](NC(=O)OC)C(C)C)N1[C@@H](CCC1)c1[nH]c(cn1)c1ccc(cc1)c1cc2c(cc1)cc(cc2)c1[nH]c([C@@H](NC(	1351331-61-7	Carbamic acid, N-[(1S)-1-cyclopentyl-2-[(2S)-2-[5-[4-[6-[2-[(2S)-1-[(2S)-2-cyclo	C48H56N8O6	841.01	C	-9
1952	C(=O)([C@@H](NC(=O)OC)C(C)C)N1C[C@@]2(C[C@H]1c1[nH]c(cn1)c1ccc(cc1)c1ccc(cc1)c1[nH]c(nc1)[C@H]1N(C(=	1351852-66-8	Carbamic acid, N-[(1S)-1-[[(2S,4S)-4-fluoro-2-[5-[4,Ä≤-[2-[(5S,8S)-7-[(2S)-2-[(r	C43H53FN8O7	812.93	C	-9
1953	C(=O)([C@@H](NC(=O)OC)C(C)C)N1[C@@H](C[C@]2(C1)CCCO2)c1[nH]c(c(CO)n1)c1ccc(cc1)c1ccc(cc1)c1[nH]c(nc1	1351852-77-1	Carbamic acid, N-[(1S)-1-[[(2S)-2-[5-chloro-4-[4,Ä≤-[2-[(5S,8S)-7-[(2S)-2-[(meth	C43H53ClN8O7	829.38	C	-9
1954	C(=O)([C@@H](NC(=O)OC)C(C)C)N1[C@@H]([C@@H]2[C@@H](C2)C1)c1[nH]c(cn1)c1ccc(cc1)c1ccc(cc1)c1[nH]c(nc1	1351852-98-6	Carbamic acid, N-[(1S)-1-[[(5S,8S)-8-[5-[4,Ä≤-[2-[(2S,4S)-1-[(2S)-2-[(methoxycar	C44H56N8O7	808.96	C	-9
1955	C(=O)([C@@H](NC(=O)OC)C(C)C)N1CC2(C[C@H]1c1[nH]c(cn1)c1ccc(cc1)c1ccc(cc1)c1[nH]c(nc1)[C@H]1N(C(=O)[C	1351853-23-0	Carbamic acid, N-[(1S)-1-[[(2S)-2-[5-[4,Ä≤-[2-[(5S,8S)-7-[(2S)-2-[(methoxycarbon	C42H51N9O8	809.91	C	-9
1956	C(F)(F)c1n(c2c(n1)cccc2)c1nc(nc(Nc2ccc(N(C)C)cc2)n1)N1CCOCC1	1359956-24-3	1,3,5-Triazin-2-amine, 4-[2-(difluoromethyl)-1H-benzimidazol-1-yl]-N-(3-methoxyp	C22H21F2N7O2	453.44	C	-9
1957	C(=O)([C@@H](NC(=O)OC)C1CCOCC1)N1[C@@H]([C@@H]2C[C@H]1CC2)c1[nH]c(cn1)c1cc2c(-c3c(CC2)cc(cc3)c2[nH]c	1377037-08-5	Carbamic acid, N-[(1S)-2-[(8S)-8-[7-[4-[2-[(2S)-1-[(2S)-2-[(methoxycarbonyl)amir	C46H54N8O9	862.97	C	-9
1958	C(=O)([C@@H](NC(=O)OC)C(C)C)N1[C@@H]([C@H]2C[C@@H]1CC2)c1[nH]c2c(n1)ccc1c2ccc(c1)c1cc2c(cc1)c1c(cc2)	1377042-05-1	Carbamic acid, N-[(1S)-2-[(2S)-2-[2,Ä≤-[(1S,3S,4R)-2-[(2S)-2-[(methoxycarbonyl)a	C48H54N8O7	854.99	C	-9
1959	C(#CC)c1c(ccc(c1)c1[nH]c(nc1)[C@H]1N(C(=O)[C@@H](NC(=O)OC)C(C)C)CCC1)c1c(C#CC)cc(cc1)c1[nH]c(nc1)[C@	1377042-68-6	Carbamic acid, N-[(1S)-2-[(2S)-2-[5-chloro-4-[4,Ä≤-[5-chloro-2-[(2S)-1-[(2S)-2-	C42H50Cl2N8O7	849.8	C	-9
1960	C(=O)([C@@H](NC(=O)OC)C(C)C)N1[C@H](c2[nH]c3c4c(cc(cc4)c4c(C#CC)cc(cc4)c4[nH]c(nc4)[C@H]4N(C(=O)[C@@	1377042-90-4	Carbamic acid, N-[(1S)-1-[[(2S)-2-[5-[2-chloro-4-[2-[(1R,3S,4S)-2-[(2S)-2-[(meth	C44H51ClN8O6	823.38	C	-9
1961	C(=O)([C@@H](NC(=O)OC)C(C)C)N1CC2(C[C@H]1c1[nH]c3c(n1)ccc1c3ccc(c1)c1ccc(cc1)c1[nH]c(nc1)[C@H]1N(C(=	1377043-41-8	Carbamic acid, N-[(1S)-1-[[(2S)-2-[5-chloro-4-[4-[2-[(1R,3S,4S)-2-[(2S)-2-[(meth	C44H51ClN8O6	823.38	C	-9
1962	C(=O)([C@@H](NC(=O)OC)C(C)C)N1[C@H](c2[nH]c(c3cc4c5-c6c(cc(cc6OC4)c4[nH]c(nc4)[C@H]4N(C(=O)[C@@H](NC	1377045-94-7	Carbamic acid, N-[(1S)-1-[[(2S,4S)-2-[2,Ä≤-[(1R,3S,5R)-2-[(2S)-2-[(methoxycarbon	C47H54N8O7	842.98	C	-9
1963	O[C@]1([C@H](n2c3c(nc2)c(=O)nc[nH]3)O[C@H](CO)[C@H]1O)[2H]	1377051-25-6	Carbamic acid, N,N,Ä≤-[(6-fluoro[7,7,Ä≤-bi-1H-naphth[1,2-d]imidazole]-2,2,Ä≤-di	C44H49FN8O6	804.91	C	-9
1964	C(=O)([C@@H](NC(=O)OC)C1CCOCC1)N1[C@@H]([C@@H]2C[C@H]1CC2)c1[nH]c2c(n1)ccc1c2ccc(c1)c1ccc(cc1)c1[nH]	1377604-11-9	Carbamic acid, N-[(1S)-1-[[(2S)-2-[5-[4-[2-[(1R,3S,4S)-2-[(2S)-2-[(methoxycarbon	C44H52N8O6	788.93	C	-9
1965	N(=Nc1cc(C)c(N=Nc2ccc(C(=O)O)cc2)cc1)c1c2c(ccc(S(=O)(=O)O)c2)c(N)cc1	1378293-82-3	Benzoic acid, 4-[2-[4-[2-[4-[2-(4-hydroxyphenyl)diazenyl]-7-sulfo-1-naphthalenyl	C30H22N6O6S	594.6	C	-9
1966	O[C@H]1[C@@H](O[C@@H](CO)[C@H]1O)n1c(=O)nc(N)c(F)c1	1382649-70-8	Carbamic acid, N,N,Ä≤-[[3-(4-methoxyphenyl)-1,2-cyclopropanediyl]bis[4,1-phenyle	C50H60N8O7	885.06	C	-9
1967	O[C@H]1[C@H](n2c3c(nc2)c(=O)nc(N)[nH]3)O[C@H](COP(=O)(O)O)[C@H]1OP(=O)(OC[C@H]1O[C@H]([C@H](O)[C@@H]	1383925-90-3	Guanosine, 2,Ä≤-deoxy-5,Ä≤-O-phosphonoguanylyl-(3,Ä≤,Üí5,Ä≤)-	C20H26N10O14P2	692.43	C	-9
1968	C(=O)([C@@H](NC(=O)OC)C(C)C)N1[C@H](c2[nH]c(c(n2)c2ccco2)c2ccc(cc2)c2ccc(cc2)c2c(nc([nH]2)[C@H]2N(C(	1388224-09-6	Carbamic acid, N,N,Ä≤-[[1,1,Ä≤-biphenyl]-4,4,Ä≤-diylbis[(4-phenyl-1H-imidazole-	C52H58N8O6	891.07	C	-9
1969	CN1C2(C(C(C(=O)/C=C(\O)/C3C4(c5c(NC4=O)cccc5)N(C)CC3c3cc(O)c(OC)cc3)C1)c1cc(OC)c(O)cc1)c1c(NC2=O)ccc	1389335-22-1	Spiro[2H-indene-2,3,Ä≤(2,Ä≤H)-indolizine]-1,3-dione, 2,Ä≤-[(1Z)-3-[1,Ä≤,5,Ä≤,6,	C49H46N2O10	822.9	C	-9
1970	N(C(=O)C)[C@H]1[C@H]([C@@H]([C@@H](CO)O)O)OC(=C[C@@H]1NC(=N)N)C(=O)O.N	1391054-30-0	[9,9,Ä≤-Bi-6H-dipyrido[3,2-b:2,Ä≤,3,Ä≤-e][1,4]diazepine]-6,6,Ä≤-dione, 11,11,Ä≤	C30H26N8O2	530.58	C	-9
1971	C(=O)(NC)N1CCN(c2nc(nc(n2)N2CCOCC2)c2c3c(ccc2)[nH]nc3)CC1	1391925-75-9	4-Piperidinone, 1-[4-(1H-indazol-4-yl)-6-(4-morpholinyl)-1,3,5-triazin-2-yl]-, c	C19H22N8O2	394.43	C	-9
1972	NC1CCN(c2nc(nc(n2)N2CCOCC2)c2c3c(ccc2)[nH]nc3)CC1	1391925-88-4	4-Piperidinone, 1-[4-(1H-indazol-4-yl)-6-(4-morpholinyl)-1,3,5-triazin-2-yl]-	C19H21N7O2	379.42	C	-9
1973	O=C1CN(c2nc(nc(n2)N2CCOCC2)c2c3c(ccc2)[nH]nc3)CCN1	1391925-96-4	1H-Indazole, 4-[4-(4-morpholinyl)-6-(1-piperazinyl)-1,3,5-triazin-2-yl]-	C18H22N8O	366.42	C	-9
1974	[C@H](C(=O)N1[C@H]2[C@H](C2)C[C@H]1c1[nH]c(cn1)c1ccc(cc1)c1ccc(cc1)c1[nH]c(nc1)[C@H]1N(C(=O)[C@@H](N	1412813-73-0	Carbamic acid, N,N,Ä≤-[[1,1,Ä≤-biphenyl]-4,4,Ä≤-diylbis[1H-imidazole-5,2-diyl-(	C48H54N8O8	870.99	C	-9
1975	C(=O)([C@@H](NC(=O)OC)[C@@H]1CC2(OCC1)CCCC2)N1[C@@H](CC[C@@H]1C)c1[nH]c(cn1)c1ccc(cc1)c1ccc(cc1)c1[n	1412816-31-9	Carbamic acid, N,N,Ä≤-[[1,1,Ä≤-biphenyl]-4,4,Ä≤-diylbis[1H-imidazole-5,2-diyl[(	C54H70N8O8	959.18	C	-9
1976	C(=O)([C@H](NC(=O)OC)C1CC(=C)C1)N1[C@@H](CC[C@@H]1C)c1[nH]c(cn1)c1ccc(cc1)c1ccc(cc1)c1[nH]c(nc1)[C@H	1412816-49-9	Carbamic acid, N,N,Ä≤-[[1,1,Ä≤-biphenyl]-4,4,Ä≤-diylbis[1H-imidazole-5,2-diyl[(	C46H54N8O6	814.97	C	-9
1977	O[C@H]1[C@@H](O[C@H](C(=O)N[C@H](Cc2ccccc2)C(=O)N(Cc2[nH]c3c(n2)ccc(C)c3)C)[C@H]1O)n1nc(C(=O)N)nc1	1414801-65-2	Not Yet Assigned	C28H33N9O7	607.62	C	-9
1978	C(=O)(Nc1c(C(=O)O)cccc1)c1cc2c(ccc(C(=O)Nc3c(C(=O)O)cccc3)c2)cc1	1426342-67-7	Benzoic acid, 2,2,Ä≤-[[1,1,Ä≤-biphenyl]-4,4,Ä≤-diylbis(carbonylimino)]bis-	C28H20N2O6	480.47	C	-9
1979	C(N1c2c(C(=NNC(=S)N(CC=C)CC=C)C1=O)cc(C)cc2)N1CCN(CC1)c1ccc(Cl)cc1	1426583-71-2	Hydrazinecarbothioamide, 2-[1-[[4-(4-chlorophenyl)-1-piperazinyl]methyl]-5-fluor	C32H28ClFN6OS	599.12	C	-9
1980	N(=C1c2c(N(CN3CCN(CC3)c3ccc(OC)cc3)C1=O)ccc(C)c2)NC(=S)N(CC=C)CC=C	1426583-77-8	Hydrazinecarbothioamide, 2-[5-fluoro-1,2-dihydro-1-[[4-(4-methoxyphenyl)-1-piper	C33H31FN6O2S	594.7	C	-9
1981	N(=C1c2c(N(CN3CCN(CC3)c3cc4c(cc3F)c(=O)c(C(=O)O)cn4CC)C1=O)ccc(Cl)c2)NC(=S)N(CC=C)CC=C	1426583-79-0	Hydrazinecarbothioamide, 2-[1,2-dihydro-1-[[4-(4-methoxyphenyl)-1-piperazinyl]me	C34H34N6O2S	590.74	C	-9
1982	C(c1c2c(N(c3c(C(=O)N2)cccn3)C2CC2)ncc1)n1cc(C[C@@H](C(=O)N[C@H](C(=O)N[C@H](C(=O)N[C@H](C(=O)N[C@H](	1426583-91-6	3-Quinolinecarboxylic acid, 1-cyclopropyl-7-[4-[[3-[2-[(diphenylamino)thioxometh	C42H40FN7O5S	773.87	C	-9
1983	O(P(=O)(OC[C@H]1O[C@H]([C@H](O)[C@@H]1O)n1c2c(nc1)c(N)ncn2)O)[C@H]1[C@@H](O[C@H](COP(=O)(OP(=O)(OP(=	1445874-20-3	Guanosine, 5,Ä≤-O-(1,3,5,5-tetrahydroxy-1,3,5-trioxido-2-oxa-1,3,5-triphosphaper	C21H30N10O20P4	866.41	C	-9
1984	O=c1n([C@H]2O[C@H](CO)C=C2)cc(I)c(=O)[nH]1	1449762-38-2	Carbamic acid, N,N,Ä≤-[(6,11-dihydrotribenzo[b,d,h][1,6]dioxecin-3,14-diyl)bis[	C48H56N8O8	873.01	C	-9
1985	O=c1nc(n(cn2)[C@H]2C[C@H](N[Si](NC[C@H]3O[C@@H]([C@H]3O)n3c4c(nc3)c(=O)nc(N)[nH]4)(C)C)[C@	1513874-28-6	Guanosine, 2,Ä≤-deoxy-3,Ä≤-O-phosphinicoguanylylimino-(3,Ä≤,Üí5,Ä≤)-5,Ä≤-deox	C20H25N11O8	547.48	C	-9
1986	C(O)[C@@H]1[C@@H](O[C@@H](C1)n1c(=O)[nH]c(=O)c(C)c1	15180-30-0	Guanosine, 2,Ä≤-deoxyguanylyl-(3,Ä≤,Üí5,Ä≤)-2,Ä≤-deoxy-	C20H25N10O10P	596.45	C	-9
1987	C(n1c2c(nc1CC)c(C)cc(C)n2)c1ccc(cc1)c1c(S(=O)(=O)NC(=O)CCc2ccccc2)ccc(CCC)c1	154552-52-0	Benzenepropanamide, N-[[5-ethyl-4,Ä≤-[(2-ethyl-5,7-dimethyl-3H-imidazo[4,5-b]py	C34H36N4O3S	580.74	C	-9
1988	C(n1c2c(nc1CC)c(C)cc(C)n2)c1ccc(cc1)c1c(S(=O)(=O)NC(=O)CCc2ccccc2)ccc(CCCC)c1	154552-53-1	Benzenepropanamide, N-[[4,Ä≤-[(2-ethyl-5,7-dimethyl-3H-imidazo[4,5-b]pyridin-3-y	C35H38N4O3S	594.77	C	-9

1989	C(n1c2c(nc1CC)c(C)cc(C)n2)c1ccc(cc1)c1c(S(=O)(=O)NC(=O)c2c(Oc3ccccc3)cccc2)ccc(COC)c1	154552-77-9	Benzamide, N-[[4,Ä≤-[(2-ethyl-5,7-dimethyl-3H-imidazo[4,5-b]pyridin-3-yl)methyl]	C40H40N4O4S	672.84	C	-9
1990	C(n1c2c(nc1CC)c(C)cc(C)n2)c1ccc(cc1)c1c(S(=O)(=O)NC(=O)COCCC)ccc(C)c1	154553-29-4	Benzamide, N-[[4,Ä≤-[(2-ethyl-5,7-dimethyl-3H-imidazo[4,5-b]pyridin-3-yl)methyl]	C32H32N4O3S2	584.75	C	-9
1991	C(n1c2c(nc1CC)c(C)cc(C)n2)c1ccc(cc1)c1c(S(=O)(=O)NC(=O)NCCc2ccccc2)ccc(COC)c1	154553-58-9	[1,1,Ä≤-Biphenyl]-2-sulfonamide, 4,Ä≤-[(2-ethyl-5,7-dimethyl-3H-imidazo[4,5-b]p	C34H37N5O3S2	627.82	C	-9
1992	C(n1c2c(nc1CC)c(C)cc(C)n2)c1ccc(cc1)c1c(S(=O)(=O)NC(=O)NCCc2ccccc2)ccc(C(CC)(C)C)c1	154553-61-4	[1,1,Ä≤:3,Ä≤,1,Ä≤,Ä≤-Terphenyl]-4,Ä≤-sulfonamide, 4,Ä≤,Ä≤-[(2-ethyl-5,7-dimeth	C38H37N5O3S	643.8	C	-9
1993	O(C(=O)N[C@@H](Cc1ccccc1)[C@@H](CN(S(=O)(=O)c1ccc(NC(=O)C)cc1)CC(C)C)O)[C@@H]1[C@H]2[C@@H](OC1)OCC2	1546919-06-5	Carbamic acid, N-[(1S,2R)-3-[[[4-[[[[(3S,3aR,6aS)-hexahydrofuro[2,3-b]furan-3-yl	C41H53N3O15S	859.94	C	-9
1994	C(=O)([C@@H](NC(=O)OC)C(C)C)N1[C@H](c2[nH]c(c3c4c(C5CC4CC5)c(cc3)c3ccc(cc3)c3[nH]c(nc3)[C@H]3N(C(=O)	1560649-05-9	Carbamic acid, N-[(1S)-1-[[(2S)-2-[5-[8-[2-fluoro-4-[2-[(2S)-1-[(2S)-2-[(methoxy	C45H55FN8O6	822.97	C	-9
1995	CN1C2(c3c(CC2)c(ccc3c2ccc(cc2)c2[nH]c(nc2)[C@H]2N(C(=O)[C@@H](NC(=O)OC)C(C)C)CCC2)c2[nH]c(nc2)[C@H]2	1588404-23-2	Carbamic acid, N-[(1S)-1-[[(2S)-2-[5-[4-[2,3-dihydro-4-[2-[(2S)-1-[(2S)-2-[(meth	C47H61N9O6	848.04	C	-9
1996	FC1C2c3c(c(ccc3c3c4c(c(cc3)c3[nH]c(nc3)[C@H]3N(C(=O)[C@@H](NC(=O)OC)C(C)C)CCC3)CC3(C4)CCCC3)c3[nH]c(	1612236-67-5	Carbamic acid, N-[(1S)-1-[[(2S)-2-[5-[3-[1,Ä≤,3,Ä≤-dihydro-7,Ä≤-[2-[(2S)-1-[(2S	C52H64N8O7	913.11	C	-9
1997	O=C1N2[C@@H](Cn3c1c(O)c(=O)c(C(=O)NCc1c(F)cc(F)c(Cl)c1)c3)O[C@H]1C[C@@H]2CC1	1616340-30-7	2,5-Methanopyrido[1,Ä≤,2,Ä≤:4,5]pyrazino[2,1-b][1,3]oxazepine-10-carboxamide, 2	C21H18F3N3O5	449.38	C	-9
1998	O=C1N2[C@@H](Cn3c1c(O)c(=O)c(C(=O)N[C@H](C)c1c(F)cc(F)cc1F)c3)O[C@H]1C[C@@H]2CC1	1616340-31-8	2,5-Methanopyrido[1,Ä≤,2,Ä≤:4,5]pyrazino[2,1-b][1,3]oxazepine-10-carboxamide, N	C21H18ClF2N3O5	465.83	C	-9
1999	N(C(=O)[C@H](Cc1ccncc1)C[C@@H](CN1[C@H](C(=O)NC(C)(C)C)CN(Cc2cc3c(s2)scc3)CC1)O)[C@H]1c2c(C[C@H]1O)c	162105-32-0	1-Piperazinepentanamide, N-(2,3-dihydro-2-hydroxy-1H-inden-1-yl)-2-[[(1,1-dimeth	C36H45N5O4S2	675.9	C	-9
2000	[C@@H](OC(=O)NCCCCCCN=[N+]=[N-])([C@@H](CO)O)[C@H]1[C@H](NC(=O)C)[C@@H](N=C(NC(=O)OC(C)(C)C)NC(=O)OC	1621228-28-1	2-Propen-1-one, 1-[4-[(4,6-di-4-morpholinyl-1,3,5-triazin-2-yl)amino]phenyl]-3-(	C27H30N6O4	502.56	C	-9
2001	[C@H]([C@@H](CO)O)(O)[C@H]1[C@H](NC(=O)C(C)C)[C@@H](N)C=C(C(=O)O)O1	162754-90-7	Œ≤-D-arabino-Adenosine, 5,Ä≤-O-phosphonoadenylyl-(2,Ä≤,Üí5,Ä≤)-adenylyl-(2,Ä≤,Ü	C30H38N15O19P3	1005.63	C	-9
2002	C(=O)([C@@H](NC(=O)OC)C(C)C)N1[C@H](c2[nH]c(c3c4c(c(ccc4)c4ccc(cc4)c4[nH]c(nc4)[C@H]4N(C(=O)[C@@H](N	1639870-98-6	Carbamic acid, N-[(1S)-1-[[(2S)-2,5-dihydro-2-[5-[4-[5-[2-[(2S)-1-[(2S)-2-[(meth	C44H50N8O6	786.92	C	-9
2003	N(c1nc(nc(Oc2ccc(C#N)cc2)n1)N1CCOCC1)c1cc(Br)ccc1	1643332-92-6	Benzonitrile, 4-[[4-[(3-methoxyphenyl)amino]-6-(4-morpholinyl)-1,3,5-triazin-2-y	C21H20N6O3	404.42	C	-9
2004	C(P(=O)(OC[C@H]1O[C@H](C=C1)n1c(=O)[nH]c(=O)c(C)c1)OC[C@H]1O[C@H](C=C1)n1c(=O)[nH]c(=O)c(C)c1)(O)c1c	164652-06-6	Thymidine, 2,Ä≤,3,Ä≤-didehydro-P,3,Ä≤-dideoxy-P-[hydroxy(4-nitrophenyl)methyl]t	C27H28N5O12P	645.51	C	-9
2005	O=c1n([C@@H]2O[C@H](COP(=O)(OC[C@H]3O[C@H](C=C3)n3c(=O)[nH]c(=O)c(C)c3)C(CCCCCCC)O)C=C2)cc(C)c(=O)[n	164652-10-2	Thymidine, 2,Ä≤,3,Ä≤-didehydro-P,3,Ä≤-dideoxy-P-[(2,6-dichlorophenyl)hydroxymet	C27H27Cl2N4O10P	669.4	C	-9
2006	N(C(=O)[C@H](Cc1ccccc1)C[C@@H](CN1[C@H](C(=O)NC(C)(C)C)CN(Cc2c(Br)cccc2)CC1)O)[C@H]1c2c(C[C@H]1O)ccc	164725-46-6	1-Piperazinepentanamide, N-(2,3-dihydro-2-hydroxy-1H-inden-1-yl)-2-[[(1,1-dimeth	C37H47FN4O4	630.79	C	-9
2007	C(#CC1CC1)[C@@]1(C(F)(F)F)c2c(N(CCCN3C(=O)c4c(C3=O)cccc4)C(=O)O1)ccc(Cl)c2	1648921-57-6	1-Naphthalenesulfonic acid, 4-amino-3-(2-[1,1,Ä≤-biphenyl]-4-yldiazenyl)-, sodiu	C22H17N3O3S.Na		C	-9
2008	C(=O)([C@@H](NC(=O)OC)C(C)C)N1[C@@H](C[C@@]2(C1)CCOC2)c1[nH]c2c(n1)ccc1c2ccc(c1)c1ccc(cc1)c1[nH]c(nc	1653967-11-3	Carbamic acid, N-[(1S)-1-[[(2S)-2-[2,Ä≤-[(3S,5S)-2-[(2S)-2-[(methoxycarbonyl)ami	C48H56N8O7	857.01	C	-9
2009	C(=O)([C@@H](NC(=O)OC)C(C)C)N1C[C@]2(C[C@H]1c1[nH]c3c(n1)ccc1c3ccc(c1)c1ccc(cc1)c1[nH]c(nc1)[C@H]1N(	1653967-13-5	Carbamic acid, N-[(1S)-1-[[(2S)-2-[7-[4-[2-[(5S,8S)-7-[(2S)-2-[(methoxycarbonyl)	C45H54N8O7	818.96	C	-9
2010	[C@H](Cc1ccccc1)(C(=O)N[C@H](/C=C/S(=O)(=O)c1ccccc1)CCCC)NC(=O)N1CCOCC1	170111-48-5	4-Morpholinecarboxamide, N-[1-([1,1,Ä≤-biphenyl]-4-ylmethyl)-2-oxo-2-[[1-(2-pher	C37H39N3O5S	637.79	C	-9
2011	O=c1n([C@@H]2O[C@H](CO[P@@]3(=O)Oc4c(CO3)cc(C)cc4)C=C2)cc(C)c(=O)[nH]1	174844-73-6	Benzamide, N-[[4,Ä≤-[(2-ethyl-5,7-dimethyl-3H-imidazo[4,5-b]pyridin-3-yl)methyl]	C31H30N4O4S	554.66	C	-9
2012	N(C(=O)[C@H](Cc1ccccc1)C[C@@H](CN1[C@H](C(=O)NC(C)(C)C)CN(Cc2cc(C(C=NOC)O)c(=O)[nH]c2)CC1)O)[C@H]1c2	179952-08-0	D-erythro-Pentonamide, 2,3,5-trideoxy-5-[(2S)-4-[(2,3-dihydro-2,3-dihydroxyfuro[	C38H49N5O7	687.83	C	-9
2013	C(=O)([C@@H](NC(=O)OC)[C@H](CC)C)N1[C@H](c2[nH]c(c3cc4C5(c6c(-c4cc3)ccc(c6)c3[nH]c(nc3)[C@H]3N(C(=O)	1800210-24-5	Carbamic acid, N,N,Ä≤-[spiro[9H-fluorene-9,3,Ä≤-oxetane]-2,7-diylbis[1H-imidazo	C43H52N8O7	792.92	C	-9
2014	O=C1[C@@H]2[C@@H](c3c([C@H](OC(=O)CCCCCCCCCCCCCCCC(=O)O[C@H]4c5c([C@H]([C@@H]6[C@@H]4COC6=O)c4cc(OC	1801429-06-0	2-Naphthalenecarbonitrile, 6-[[2-[(4-cyanophenyl)amino]-4-pyrimidinyl]amino]-5,7	C24H18N6	390.44	C	-9
2015	C(=O)([C@@H](NC(=O)OC)C1CCCCC1)N1[C@@H](CCC1)c1[nH]c(cn1)c1ccc(cc1)c1ccc(cc1)c1[nH]c(cc1)[C@H]1N(C(=	1807681-03-3	Carbamic acid, N-[(1S)-2-methyl-1-[[(2S)-2-[5-[4,Ä≤-[2,4,5,6-tetrahydro-3-[(2S)-	C44H55N7O6	777.95	C	-9
2016	O=C1[C@]2([C@H]([C@H](/C(=C/C(=O)[C@@H]3[C@@]4(N5[C@@H]([C@H]3c3cc(OC)c(O)cc3)CCC5)c3c(NC4=O)c(C)cc(	1807888-42-1	Not Yet Assigned	C49H52N4O8	824.96	C	-9
2017	C(=O)([C@@H](NC(=O)OC)C(C)C)N1[C@H](CCC1)c1[nH]c(cn1)c1ccc(cc1)c1[nH]c(nc1)[C@H]1N(C(=O)[C@@H](NC(=O	1808295-76-2	Acetamide, N,N,Ä≤-[[1,1,Ä≤-biphenyl]-4,4,Ä≤-diylbis[1H-imidazole-5,2-diyl[(2S)-	C40H46N8O4	702.84	C	-9
2018	F[C@H]1[C@@H](O[C@H](CO)[C@H]1O)n1c(=O)nc(N)c(C)c1	1824690-74-5	1-Piperazinecarbothioamide, N-[4-amino-6-(4-morpholinyl)-1,3,5-triazin-2-yl]-4-(	C21H24ClN9OS	485.99	C	-9
2019	[Si](OC[C@@H]1O[C@@H](CS1)n1c(=O)nc(NC(=O)c2cccnc2)cc1)(C(C)(C)C)(c1ccccc1)c1ccccc1	18268-54-7	1-Naphthalenesulfonic acid, 4-[2-(2-hydroxy-1-naphthalenyl)diazenyl]-	C20H14N2O4S	378.4	C	-9
2020	O=c1n([C@@H]2O[C@H](CO)[C@H](S)C2)cc(C)c(=O)[nH]1	186339-93-5	D-erythro-Pentonamide, 2,3,5-trideoxy-N-[(1S,2S,3S)-2,3-dihydro-2,3-dihydroxy-1H	C42H55N5O11		C	-9
2021	O=C1[C@@H]2[C@@H](c3c([C@H](OC(=O)CN4c5c(C(=O)C4=O)cc(C)cc5)[C@H]2CO1)cc1c(c3)OCO1)c1cc(OC)c(OC)c(OC	1864833-30-6	1H-Indole-1-acetic acid, 4-bromo-2,3-dihydro-2,3-dioxo-, (5R,5aR,8aR,9R)-5,5a,6,	C32H26BrNO11	680.45	C	-9
2022	O=C1[C@@H]2[C@@H](c3c([C@H](OC(=O)CN4c5c(C(=O)C4=O)cccc5F)[C@H]2CO1)cc1c(c3)OCO1)c1cc(OC)c(OC)c(OC)c	1864833-36-2	1H-Indole-1-acetic acid, 6-chloro-2,3-dihydro-2,3-dioxo-, (5R,5aR,8aR,9R)-5,5a,6	C32H26ClNO11	636	C	-9
2023	O=c1c2c(-c3c(OC2c2[nH]nnn2)cccc3)oc2c1ccc(O)c2	188066-01-5	1-Naphthalenesulfonic acid, 4-amino-3-[2-(4-sulfophenyl)diazenyl]-	C16H13N3O6S2	407.42	C	-9
2024	N(C(=O)Nc1c(C)cccc1F)c1cc2cc(C34CC5CC(C4)CC(C3)C5)[nH]c2cc1	1883585-92-9	Urea, N-(2-methylphenyl)-N,Ä≤-(2-tricyclo[3.3.1.13,7]dec-1-yl-1H-indol-5-yl)-	C26H29N3O	399.53	C	-9
2025	N(c1nc(nc(Nc2ccc(C(=O)C=Cc3cc(N(=O)=O)ccc3)cc2)n1)N1CCOCC1)c1cc(C(F)(F)F)ccc1	1883795-10-5	2-Propen-1-one, 1-[4-[[4-(4-morpholinyl)-6-[[3-(trifluoromethyl)phenyl]amino]-1,	C29H24F3N7O4	591.54	C	-9
2026	C(=O)(c1cc2c([nH]1)ccc(NS(=O)(=O)C)c2)N1CCN(CC1)c1c(N)ccc(=O)[nH]1	188780-40-7	Methanesulfonamide, N-[2-[[4-[3-[(1-methylethyl)amino]-6-(sulfooxy)-2-pyridinyl]	C22H28N6O7S2	552.62	C	-9
2027	O=c1n([C@@H]2O[C@H](COC(=O)C(=C)C)[C@@H](N=[N+]=[N-])C2)cc(C)c(=O)[nH]1	1934287-13-4	Carbamic acid, N-[(1S)-1-[[(2S)-2-[5-[6-[2-[(2S)-1-[(2S)-2-[(methoxycarbonyl)ami	C43H52N8O6	776.92	C	-9
2028	O=C1[C@@H]2[C@@H](c3c([C@H](OC(=O)Cl)[C@H]2CO1)cc1c(c3)OCO1)c1cc(OC)c(O)c(OC)c1	19591-05-0	1-Naphthalenesulfonic acid, 3,3,Ä≤-[(2,2,2-trichloroethylidene)bis(p-phenyleneaz	C34H25Cl3N6O6S2	784.09	C	-9
2029	O=c1c2c(n(cn2)[C@@H]2O[C@H](CO)[C@@H](O)[C@H]2O)[nH]c([15NH2])[15n]1	1971915-59-9	Guanosine, N-[[4-[(4-hydroxyphenyl)methoxy]phenyl]methyl]-	C24H25N5O7	495.48	C	-9
2030	N(C(=O)C)[C@@H]1[C@@H](NC(=N)NC(=O)Cn2cc(nn2)c2cc(C(=O)OCC)ccc2)C=C(C(=O)O)O[C@H]1[C@@H]([C@@H](CO)O	1982351-44-9	D-glycero-D-galacto-Non-2-enonic acid, 5-(acetylamino)-4-[[[[2-[4-(3-aminophenyl	C22H28N8O8	532.51	C	-9
2031	O(CC(=O)OC(C)(C)C)c1c2n(N([C@H]3N(C2=O)CCOC3)[C@H]2c3c(CSc4c2cccc4)c(F)c(F)cc3)ccc1=O	1985606-84-5	1H-[1,4]Oxazino[3,4-c]pyrido[2,1-f][1,2,4]triazine-6,8-dione, 12-[(11S)-7,8-difl	C27H21F2N3O7S	569.53	C	-9
2032	O(P(=O)(N[C@H](C(=O)OC)CC(C)C)OCC)c1c2n(N([C@H]3N(C2=O)CCOC3)[C@H]2c3c(CSc4c2cccc4)c(F)c(F)cc3)ccc1=	1985606-88-9	Butanedioic acid, 2,2-dimethyl-, 1-[[[(12aR)-12-[(11S)-7,8-difluoro-6,11-dihydro	C32H31F2N3O8S	655.67	C	-9
2033	O(P(=O)(N[C@H](C(=O)OC)C(C)(C)C)OCC)c1c2n(N([C@H]3N(C2=O)CCOC3)[C@H]2c3c(CSc4c2cccc4)c(F)c(F)cc3)ccc	1985607-18-8	Cyclopentaneacetic acid, 1-[[[[(12aR)-12-[(11S)-7,8-difluoro-6,11-dihydrodibenzo	C34H33F2N3O8S	681.7	C	-9
2034	O(COC(=O)OCC(C(=O)OCC)(C)C)c1c2n(N([C@H]3N(C2=O)CCOC3)[C@H]2c3c(CSc4c2cccc4)c(F)c(F)cc3)ccc1=O	1985607-37-1	Carbonic acid, [[(12aR)-12-[(11S)-7,8-difluoro-6,11-dihydrodibenzo[b,e]thiepin-1	C30H29F2N3O7S	613.63	C	-9
2035	O(Cc1ccc(N=[N+]=[N-])cc1)c1c2n(N([C@H]3N(C2=O)CCOC3)[C@H]2c3c(CSc4c2cccc4)c(F)c(F)cc3)ccc1=O	1985607-46-2	Benzoic acid, 2-(acetyloxy)-, (12aR)-12-[(11S)-7,8-difluoro-6,11-dihydrodibenzo[	C33H25F2N3O7S	645.63	C	-9
2036	N(C(=O)c1c(Cl)cccc1)c1cc2cc(C34CC5CC(C4)CC(C3)C5)[nH]c2cc1	1987854-63-6	Benzamide, 4-chloro-N-(2-tricyclo[3.3.1.13,7]dec-1-yl-1H-indol-5-yl)-	C25H25ClN2O	404.93	C	-9
2037	N(C(=O)Nc1ncccn1)c1cc2cc(C34CC5CC(C4)CC(C3)C5)[nH]c2cc1	1987854-80-7	Urea, N-[5-(1,1-dimethylethyl)-3-isoxazolyl]-N,Ä≤-(2-tricyclo[3.3.1.13,7]dec-1-y	C26H32N4O2	432.56	C	-9
2038	C(=O)([C@@H](NC(=O)OC)C(C)C)N1[C@H](c2[nH]c(c3c4c(c(cc3)c3cc5c(cc3)c3c(cc5)nc([nH]3)[C@H]3N(C(=O)[C@	2007901-08-6	Carbamic acid, N-[(1S)-1-[[(2S)-2-[7-[2,3-dihydro-7-[2-[(3S)-2-[(2S)-2-[(methoxy	C47H56N8O6	829	C	-9
2039	C(=O)([C@@H](NC(=O)OC)C(C)C)N1[C@H](c2[nH]c3c4c(cc(cc4)c4c5c(c(cc4)c4[nH]c(nc4)[C@H]4N(C(=O)[C@@H](N	2007901-63-3	Carbamic acid, N-[(1S)-1-[[(2S)-2-[7-[2,3-dihydro-7-[2-[(1R,2S,5S)-3-[(2S)-2-[(m	C46H54N8O6	814.97	C	-9
2040	O(Cc1ccccc1)[C@@H]1[C@@H](NC(=O)CCC(=O)OC[C@H]2O[C@H]([C@H](O)[C@@H]2O)n2nc(C(=O)N)nc2)C(OCc2ccccc2)	1002336-72-2	D-Galactopyranoside, phenylmethyl 2-[(3-carboxy-1-oxopropyl)amino]-2-deoxy-3,4,6	C48H53N5O13	907.96	A/B	-8.9
2041	C(=O)([C@H](NC(=O)OC)c1ccccc1)N1[C@@H](CCC1)c1[nH]c(cn1)c1ccc(cc1)c1ccc(cc1)c1[nH]c(nc1)[C@H]1N(C(=O	1007882-36-1	Carbamic acid, N-[(1R)-2-[(2S)-2-[5-[4,Ä≤-[2-[(1S)-1-[[(2R)-2-[(methoxycarbonyl)	C45H46N8O6	794.9	A/B	-8.9
2042	C(=O)([C@H](NC(=O)OC)c1ccccc1)N1[C@@H](CCC1)c1[nH]c(cn1)c1ccc(cc1)c1ccc(cc1)c1[nH]c(nc1)[C@H]1N(C(=O	1009107-55-4	Carbamic acid, N-[(1R)-2-[(2S)-2-[5-[4,Ä≤-[2-[(2S)-1-[(2R)-2-(dimethylamino)-2-p	C46H48N8O4	776.92	A/B	-8.9
2043	C(=O)([C@H](NC(=O)OC)c1ccccc1)N1[C@@H](CCC1)c1[nH]c(cn1)c1ccc(cc1)c1ccc(cc1)c1[nH]c(nc1)[C@H]1N(C(=O	1009107-61-2	Carbamic acid, N-[(1R)-2-oxo-1-phenyl-2-[(2S)-2-[5-[4,Ä≤-[2-[(2S)-1-[2-(4-pyridi	C43H42N8O4	734.84	A/B	-8.9
2044	C(=O)([C@H](NC(=O)OC)c1ccccc1)N1[C@@H](CCC1)c1[nH]c(cn1)c1ccc(cc1)c1ccc(cc1)c1[nH]c(nc1)[C@H]1N(C(=O	1009107-67-8	Carbamic acid, N-[(1R)-2-[(2S)-2-[5-[4,Ä≤-[2-[(2S)-1-[(2S)-2-(acetylamino)-1-oxo	C41H44N8O5	728.84	A/B	-8.9
2045	C(=O)([C@H](NC(=O)OC)c1ccccc1)N1[C@@H](CCC1)c1[nH]c(cn1)c1ccc(cc1)c1ccc(cc1)c1[nH]c(nc1)[C@H]1N(C(=O	1009107-71-4	Carbamic acid, N-[(1R)-2-oxo-1-phenyl-2-[(2S)-2-[5-[4,Ä≤-[2-[(2S)-1-[2-(1-pyrrol	C42H46N8O4	726.87	A/B	-8.9
2046	C(=O)([C@H](NC(=O)OC)c1ccccc1)N1[C@@H](CCC1)c1[nH]c(cn1)c1ccc(cc1)c1ccc(cc1)c1[nH]c(nc1)[C@H]1N(C(=O	1009108-65-9	Carbamic acid, N-[(1R)-2-[(2S)-2-[5-[4,Ä≤-[2-[(2S)-1-[2-(2-methylphenyl)acetyl]-	C45H45N7O4	747.88	A/B	-8.9
2047	O=c1n([C@H]2C[C@H](OP(=S)(S)S)[C@@H](CO)O2)cc(C)c(=O)[nH]1	10115-26-1	2-Naphthalenesulfonic acid, 8-[2-[4-[2-(2,4-diaminophenyl)diazenyl]-1-naphthaler	C26H20N6O3S.Na		A/B	-8.9
2048	O=c1n([C@@H]2O[C@H](CO)[C@@H](O)C2)cc(Br)c(N)n1	1021936-90-2	[1,1,Ä≤-Biphenyl]-2-carboxylic acid, 4,Ä≤-[2-(5-tricyclo[3.3.1.13,7]dec-1-yl-1H	C28H30N2O2	426.55	A/B	-8.9
2049	O=C1[C@@H]2[C@@H](c3c([C@@H](OCCSC)C2CO1)cc1c(c3)OCO1)c1cc(OC)c(O)c(OC)c1	1027506-86-0	Benzonitrile, 4-[[2-[(4-cyanophenyl)amino]-4-pyrimidinyl]amino]-2,6-dimethyl-	C20H16N6	340.38	A/B	-8.9
2050	N(c1cc(C(O)c2nccs2)nc(Nc2ccc(C#N)cc2)n1)c1c(C)cc(/C=C/C#N)cc1C	1037444-93-1	Benzonitrile, 4-[[4-[[4-[(1E)-2-cyanoethenyl]-2,6-dimethylphenyl]amino]-6-(2-thi	C26H19N7OS	477.54	A/B	-8.9
2051	O[C@@H]1[C@H](n2c3c(nc2)c(N)ncn3)O[C@H](COC2CCCCO2)[C@H]1O	1040031-42-2	9H-Purin-6-amine, 9-[5-O-(triphenylmethyl)-Œ≤-D-arabinofuranosyl]-	C29H27N5O4	509.56	A/B	-8.9
2052	O=C1[C@@H]2[C@@H](c3c(C[C@@H]2C(=O)O1)cc1c(c3)OCO1)c1cc(OC)c(OC)c(OC)c1	1049986-97-1	Benzoic acid, 3,3,Ä≤-[[5-(dihydroxymethylene)-4,6-dioxo-2-cyclohexen-1-ylidene]r	C24H18O12	498.39	A/B	-8.9
2053	N(=N\c1cc(S(=O)(=O)O)c(CCc2c(S(=O)(=O)O)cc(/N=N/c3c4c(cc(S(=O)(=O)O)cc4)c(N)cc3)cc2)cc1)/c1c2c(cc(S(	1052524-73-8	Not Yet Assigned	C34H28N6O18S6.Na		A/B	-8.9
2054	N(=N\c1cc(S(=O)(=O)O)c(CCc2c(S(=O)(=O)O)cc(/N=N/c3c4c(cc(S(=O)(=O)O)c3N)cc(S(=O)(=O)O)cc4)cc2)cc1)/c	1052524-91-0	Not Yet Assigned	C34H28N6O12S4.Na		A/B	-8.9
2055	S(=O)(=O)(O)c1c2c(c(N)c(/N=N/c3cc(S(=O)(=O)O)c(C=Cc4c(S(=O)(=O)O)cc(/N=N/c5c(N)c6c(c(S(=O)(=O)O)c5)c	1052528-88-7	Not Yet Assigned	C34H26N6O18S6.Na		A/B	-8.9
2056	O[C@H]1[C@H](n2c3c(nc2)c(=O)nc(N)[nH]3)O[C@H](COP(=O)(OP(=O)(OP(=O)(OC[C@H]2O[C@H]([C@H](O)[C@@H]2O)	10527-46-5	Guanosine 5,Ä≤-(pentahydrogen tetraphosphate), P,Ä≤,Ä≤,Ä≤,Üí5,Ä≤-ester with ade	C20H28N10O20P4	852.39	A/B	-8.9
2057	O=c1n([C@H]2C[C@H](N=[N+]=[N-])[C@@H](CS)O2)cc(C)c(=O)[nH]1	1054661-48-1	Not Yet Assigned	C22H27N9O11	593.5	A/B	-8.9
2058	C(=O)(NC(CC1(O)CCC1)C(=O)C(=O)N)C1[C@@H]2[C@@H](C2(C)C)CN1C(=O)[C@@H](NC(=O)NC(C)(C)C)C(C)(C)C	1056452-34-6	Benzoic acid, 3,3,Ä≤-methylenebis[6-[[(4-methylphenyl)methyl]amino]-	C31H30N2O4	494.58	A/B	-8.9
2059	N(C(=O)[C@H](Cc1ccccc1)C[C@@H](CN1[C@H](C(=O)NC(C)(C)C)CN(C(CO)c2ccccc2)CC1)O)[C@H]1c2c(C[C@H]1O)ccc	1064475-89-3	Not Yet Assigned	C37H46F2N4O4	648.78	A/B	-8.9
2060	C(OCCOC(=O)[C@H](NC(=O)[C@@H](C(C)C)N)C(C)C)n1c2c(nc1)c(=O)nc(N)[nH]2	1064707-07-8	4-Pyrimidinecarboxamide, N-[(4-fluorophenyl)methyl]-1,6-dihydro-5-hydroxy-1-meth	C20H22FN7O4	443.43	A/B	-8.9
2061	C(OCCO)n1c2c(c(=O)nc(NC(=O)C(CCC)CCC)[nH]2)nc1	1067461-91-9	2-Propenoic acid, 3-phenyl-, 2-[[1,6-dihydro-6-oxo-2-[[(2E)-1-oxo-3-phenyl-2-pro	C26H23N5O5	485.49	A/B	-8.9
2062	O=c1n([C@@H]2O[C@H](CO[Si](C(C)(C)C)(C)C)SC2)ccc(NC(=O)CCCCCCCCCCCCC)n1	1099829-81-8	Thymidine, 2,Ä≤,3,Ä≤-didehydro-3,Ä≤-deoxy-5,Ä≤-O-[6-[[4-hydroxy-3-(hydroxymethy	C25H25N2O8P	512.45	A/B	-8.9
2063	C(NC(=O)c1nnc(C([2H])([2H])[2H])o1)(C)(C)c1nc(C(=O)NCc2c(c(c(F)c(c2[2H])[2H])[2H])[2H])c(O)c(=O)n1C	1100750-94-4	4-Pyrimidinecarboxamide, N-[(4-fluorophenyl)methyl-d2]-1,6-dihydro-5-hydroxy-1-(	C20H7D14FN6O5		A/B	-8.9
2064	C(NC(=O)c1nnc(C)o1)(C)(C)c1nc(C(=O)NC(c2c(c(c(F)c(c2[2H])[2H])[2H])[2H])([2H])[2H])c(O)c(=O)n1C([2H]	1100751-03-8	4-Pyrimidinecarboxamide, N-[(4-fluorophenyl-2,3,5,6-d4)methyl-d2]-1,6-dihydro-5-	C20H9D12FN6O5		A/B	-8.9
2065	C(NC(=O)c1nnc(C)o1)(C([2H])([2H])[2H])(C([2H])([2H])[2H])c1nc(C(=O)NC(c2c(c(c(F)c(c2[2H])[2H])[2H])[	1100751-06-1	4-Pyrimidinecarboxamide, N-[(4-fluorophenyl-2,3,5,6-d4)methyl-d2]-1,6-dihydro-5-	C20H9D12FN6O5		A/B	-8.9
2066	[Pt+2]([Cl-])([Cl-])([OH2])[N]1=CN(COCCO)c2c1c(=O)nc(N)[nH]2	1109235-82-6	2-Pyridinesulfonamide, N-[3-[(1S)-1-[(6R)-5,6-dihydro-4-hydroxy-2-oxo-6-phenyl-2	C26H23F3N2O5S	532.53	A/B	-8.9
2067	C(Cc1ccccc1)[C@]1(CCC)C(C(=C(=C([C@H](CC)c2cc(NS(=O)(=O)c3ccc(C(F)(F)F)cn3)ccc2)C(=O)O1)O)([2H])[2H]	1126619-13-3	Not Yet Assigned	C31H31D2F3N2O5S		A/B	-8.9
2068	N(=Nc1c2c(ccc(S(=O)(=O)O)c2)c(N)cc1)c1c2c(c(N=Nc3c(S(=O)(=O)O)cc(N)cc3)cc1)ccc(S(=O)(=O)O)c2	1131041-13-8	2-Naphthalenesulfonic acid, 8-[2-(4-amino-2-methylphenyl)diazenyl]-5-[2-(4-aminc	C23H20N6O6S2	540.57	A/B	-8.9
2069	O=c1c2c(n(cn2)[C@@H]2O[C@H](CO)[C@@H](O)[C@H]2O)[nH]c(NCc2c3c(cc4c2cccc4)cccc3)n1	1134793-87-5	Guanosine, N-(1-naphthalenylmethyl)-	C21H21N5O5	423.42	A/B	-8.9
2070	O(c1nc(Nc2cc(cc(C#N)cc2[2H])[2H])nc(N)c1Br)c1c(C([2H])([2H])[2H])c(c(C#N)c(c1C([2H])([2H])[2H])[2H])[	1142095-62-2	Benzonitrile-2,3,5,6-d4, 4-[[4-amino-5-bromo-6-[4-cyano-2,6-di(methyl-d3)phenoxy	C20H3BrD12N6O		A/B	-8.9
2071	O(c1nc(Nc2cc(C#N)c(c2)[2H])[2H])nc(N)c1Br)c1c(C)c(c(C#N)c(c1C)[2H])[2H]	1142095-87-1	Benzonitrile-2,6-d2, 4-[[6-amino-5-bromo-2-[(4-cyanophenyl-2,6-d2)amino]-4-pyrim	C20H11BrD4N6O		A/B	-8.9
2072	O(c1nc(Nc2ccc(C#N)cc2)nc(N)c1Br)c1c(C([2H])([2H])[2H])cc(C#N)cc1C([2H])([2H])[2H]	1142096-03-4	Benzonitrile-2,6-d2, 4-[[4-amino-5-bromo-6-(4-cyano-2,6-dimethylphenoxy)-2-pyrim	C20H13BrD2N6O		A/B	-8.9
2073	O[C@H]1[C@H](n2c3c(nc2)c(=O)nc(N)[nH]3)O[C@H](CO)[C@@]1(C)O	115298-25-4	2,7-Naphthalenedisulfonic acid, 4,4,Ä≤-[9,10-anthracenediylbis(2,1-ethenediyl-4,	C50H36N6O12S4.4Na		A/B	-8.9
2074	O=c1n(C2OC(COP(=O)(Cl)Cl)C=C2)cc(C)c(=O)[nH]1	1172604-20-4	D-erythro-Pentonamide, 2,3,5-trideoxy-N-[(1S)-2,3-dihydro-2-oxo-1H-inden-1-yl]-5	C30H40N4O4	520.66	A/B	-8.9
2075	Cc1n(c2c(n1)cccc2)c1nc(nc(n1)N1CCOCC1)c1cc(C#N)ccc1	1174190-65-8	Phenol, 3-[4-(2-methyl-1H-benzimidazol-1-yl)-6-(4-morpholinyl)-1,3,5-triazin-2-y	C21H20N6O2	388.42	A/B	-8.9
2076	C(=O)([C@@H](NC(=O)OC)C(C)(C)C)N1[C@H](c2[nH]c(c3ccc4c(c5c(o4)cc(cc5)c4[nH]c(nc4)[C@H]4N(C(=O)[C@@H](	1179150-63-0	Carbamic acid, N,N,Ä≤-[3,7-dibenzofurandiylbis[1H-imidazole-5,2-diyl-(2S)-2,1-py	C40H44N8O7	748.83	A/B	-8.9
2077	C(COC)C1c2c(-c3c1cc(cc3)c1[nH]c(nc1)[C@H]1N(C(=O)[C@@H](NC(=O)OC)C(C)C)CCC1)ccc(c2)c1[nH]c(nc1)[C@H]	1179151-18-8	Carbamic acid, N,N,Ä≤-[9H-carbazole-2,7-diylbis[1H-imidazole-5,2-diyl-(2S)-2,1-p	C40H49N9O6	751.87	A/B	-8.9
2078	C(=O)([C@@H](NC(=O)OC)C(C)C)N1[C@H](c2[nH]c3-c4c(cc(cc4)c4ccc(cc4)c4[nH]c(nc4)[C@H]4N(C(=O)[C@@H](NC	1180668-68-1	Carbamic acid, N-[(1S)-2-methyl-1-[[(2S)-2-[5-[4-[1,4,5,6-tetrahydro-2-[(2S)-1-[	C43H54N8O6	778.94	A/B	-8.9
2079	C(=O)([C@@H](NC(=O)OC)C(C)C)N1[C@H](c2[nH]c3-c4c(cc(cc4)c4ccc(cc4)c4c(CCC(F)(F)F)nc([nH]4)[C@H]4N(C(	1180669-64-0	Carbamic acid, N-[(1S)-2-methyl-1-[[(2S)-2-[5-[4-[1,4,5,6-tetrahydro-2-[(2S)-1-[	C45H55F3N8O6	860.96	A/B	-8.9
2080	C(=O)([C@@H](NC(=O)OC)C(C)C)N1[C@H]2[C@H](C2)C[C@H]1c1[nH]c(c(CC(F)(F)F)n1)c1ccc(cc1)c1cc2c(-c3c(CC2	1180669-99-1	Carbamic acid, N-[(1S)-1-[[(1R,3S,5R)-3-[4,5-dihydro-7-[4-[2-[(1R,3S,5R)-2-[(2S)	C44H52N8O6	788.93	A/B	-8.9
2081	C(=O)([C@@H](NC(=O)OC)C(C)C)N1[C@H](c2[nH]c3c4c(cc(cc4)c4cc(F)c(cc4)c4[nH]c(nc4)[C@H]4N(C(=O)[C@@H](	1180670-23-8	Carbamic acid, N,N,Ä≤-[[7,7,Ä≤-bi-1H-naphth[1,2-d]imidazole]-2,2,Ä≤-diylbis[(1R	C46H50N8O6	810.94	A/B	-8.9
2082	C(=O)([C@@H](NC(=O)OC)C(C)C)N1[C@H](c2[nH]c3c4c(cc(cc4)c4cccc(cc4)c4[nH]c(nc4)[C@H]4N(C(=O)[C@@H](NC(	1180670-28-3	Carbamic acid, N-[(1S)-1-[[(1R,3S,5R)-3-[7-[3-fluoro-4-[2-[(1R,3S,5R)-2-[(2S)-2-	C44H49FN8O6	804.91	A/B	-8.9
2083	C(=O)([C@@H](NC(=O)OC)C(C)C)N1[C@H](c2[nH]c3c4c(cc(cc4)c4cccc(cc4)c4[nH]c(nc4)[C@H]4N(C(=O)[C@@H](NC(	1180670-34-1	Carbamic acid, N-[(1S)-1-[[(2S)-4,4-difluoro-2-[7-[4-[2-[(2S)-1-[(2S)-2-[(methox	C42H48F2N8O6	798.88	A/B	-8.9
2084	N(=Nc1c(S(=O)(=O)O)cc(C)c(S(=O)(=O)O)c1)c1c2c(c(N=Nc3c4c(ccc(S(=O)(=O)O)c4)c(N)cc3)cc1)ccc(S(=O)(=O)	1185727-95-0	1,3,5-Naphthalenetrisulfonic acid, 7-[2-[4-[2-(4-amino-2-methylphenyl)diazenyl]-	C27H21N5O12S4	735.74	A/B	-8.9
2085	O=c1c2c(n(cn2)[C@@H]2O[C@H](COP(=O)=O)[C@@H](O)C2)[nH]c(N)n1	118662-86-5	Uridine, cytidylyl-(3,Ä≤,Üí5,Ä≤)-guanylyl-(3,Ä≤,Üí5,Ä≤)-cytidylyl-(3,Ä≤,Üí5,Ä≤	C37H48N13O27P3	1199.77	A/B	-8.9
2086	N(=C1c2c(N(CN3CCOCC3)C1=O)ccc(Cl)c2)NC(=S)NC1CCCCC1	118989-43-8	Hydrazinecarbothioamide, 2-[1-[[4-(4-chlorophenyl)-1-piperazinyl]methyl]-1,2-dih	C27H33ClN6OS	525.11	A/B	-8.9
2087	C(NC(=O)c1nnc(C)o1)(C)(C)C1=NC(C(=O)NCc2ccc(F)cc2)C(=O)C(=O)N1C([2H])([2H])[2H]	1190629-38-9	1-Naphthalenesulfonic acid, 4-amino-3-[2-[4-[2-(4-hydroxyphenyl)diazenyl]phenyl]	C22H17N5O4S	447.47	A/B	-8.9
2088	O(COC(=O)N1CCOCC1)c1c2n(C[C@H]3N(C2=O)[C@H](C)CCO3)cc(C(=O)NCc2c(F)cc(F)cc2)c1=O	1206156-27-5	1-Pyrrolidinecarboxylic acid, [[(4R,12aS)-9-[[[(2,4-difluorophenyl)methyl]amino]	C26H28F2N4O7	546.52	A/B	-8.9
2089	[C@H](C(=O)N[C@@H](Cc1ccccc1)C[C@H]([C@H](Cc1ccccc1)NC(=O)COc1c(C)cccc1C)O)(C(C)C)N1C(=O)NCCC1	1217819-15-2	Not Yet Assigned	C31H29D4F3N2O5S		A/B	-8.9
2090	O=C1[C@@H]2[C@@H](c3c([C@H](OC(=O)CCC(=O)NCCCCNC(=O)[C@@H](C)c4cc(C(=O)c5ccccc5)ccc4)[C@H]2CO1)cc1c(	1222083-91-1	Butanoic acid, 4-[[4-[[(2S)-2-(6-methoxy-2-naphthalenyl)-1-oxopropyl]amino]butyl	C44H48N2O12	796.86	A/B	-8.9
2091	C(OCCO)N1c2c(c(=O)nc(NC)[nH]2)NC1	1222524-60-8	Not Yet Assigned	C36H46FN5O4	631.78	A/B	-8.9
2092	N(C(=O)[C@H](Cc1ccccc1)C[C@@H](CN1[C@H](C(=O)NC(C)(C)C)CN(Cc2c(S)cc(Br)cc2)CC1)O)C1c2c(C[C@H]1O)cccc	1223078-67-8	Not Yet Assigned	C40H51N5O4	665.86	A/B	-8.9
2093	C(F)(F)(F)c1c(c2nc(nc(n2)N2CCOCC2)N2CCOCC2)ccc(N)n1	1225037-83-1	Acetamide, N-[5-(4,6-di-4-morpholinyl-1,3,5-triazin-2-yl)-4-(trifluoromethyl)-2-	C19H22F3N7O3	453.42	A/B	-8.9
2094	c1(nc(nc(n1)N1CCOCC1)N1CCOCC1)c1c2c(ccc1)nc[nH]2	1225037-87-5	1H-Benzimidazole, 6-(4,6-di-4-morpholinyl-1,3,5-triazin-2-yl)-	C18H21N7O2	367.4	A/B	-8.9
2095	C(#N)C1=C(N)Oc2c(C1c1cc3c(cc1)OCO3)ccn1c2nc(C)c1	1225373-08-9	7H-Imidazo[1,2-a]pyrano[2,3-c]pyridine-8-carbonitrile, 9-amino-7-(1,3-benzodioxc	C18H12N4O3	332.31	A/B	-8.9
2096	O=c1n([C@@H]2O[C@H](CO)CC2)ccc(=S)[nH]1	1225639-01-9	1,3,5-Triazin-2-amine, 4-(1H-benzimidazol-7-yl)-6-(4-morpholinyl)-N-(2-pyridinyl	C20H20N8O	388.43	A/B	-8.9
2097	C(CC(=O)CC(CCCCC)O)c1cc(O)c(O)cc1	1228554-23-1	Carbamic acid, N-[(1S)-1-[[(2S)-2-[5-[3-[6-[2-[(2S)-1-[(2S)-2-[(methoxycarbonyl)	C44H52N8O6	788.93	A/B	-8.9
2098	C(=O)([C@@H](NC(=O)OC)C(C)C)N1[C@@H](CCC1)c1[nH]c(cn1)c1ccc(cc1)c1cc2c(cc1)cc(cc2)c1[nH]c(nc1)[C@@H]	1228633-21-3	Carbamic acid, N-[(1S)-2-methyl-1-[[(2S)-2-[5-[6-[4-[2-(2S)-2-pyrrolidinyl-1H-im	C37H41N7O3	631.77	A/B	-8.9
2099	C(=O)([C@@H](NC(=O)OC)c1ccccc1)N1[C@@H](CCC1)c1[nH]c(cn1)[C@H]1CC[C@@H](CC1)c1ccc(cc1)c1[nH]c(nc1)[C	1228667-92-2	Carbamic acid, N-[(1R)-2-[(2S)-2-[5-[4-[trans-4-[2-[(2S)-1-[(2R)-2-[(methoxycarb	C46H52N8O6	812.96	A/B	-8.9
2100	C(=O)([C@@H](NC(=O)OC(C)(C)C)C(C)C)N1[C@@H](CCC1)c1[nH]c(cn1)c1ccc(C#Cc2ccc(cc2)c2[nH]c(nc2)[C@H]2N(	1228964-85-9	Carbamic acid, N,N,Ä≤-[1,2-ethynediylbis[4,1-phenylene-1H-imidazole-5,2-diyl-(2S	C48H46N8O6	830.93	A/B	-8.9
2101	C(=O)([C@@H](NC(=O)OC)c1ccccc1)N1[C@@H](CCC1)c1[nH]c(cn1)c1ccc2c(cc1)cc(C#Cc1[nH]c(nc1)[C@H]1N(C(=O)[	1228967-99-4	Carbamic acid, N-[(1R)-2-[(2S)-2-[5-[2-[6-[2-[(2S)-1-[(2S)-2-[(methoxycarbonyl)a	C43H46N8O6	770.88	A/B	-8.9
2102	C(NC(=O)[C@H](Cc1ccccc1)N)C(=O)NCC(=O)N[C@@H](Cc1ccccc1)C(=O)N[C@@H](Cc1ccccc1)C(=O)O)C12CC3CC(C2)C	1229574-76-8	L-Phenylalanine, L-tyrosyl-2-tricyclo[3.3.1.13,7]dec-1-ylglycylglycyl-L-phenylal	C41H49N5O7	723.86	A/B	-8.9
2103	[C@H](Cc1ccccc1)(C[C@@H]([C@H](Cc1ccccc1)NC(=O)OCc1ccno1)O)NC(=O)[C@@H](NC(=O)N(C)Cc1nc(Cc2nccs2)sc1)C	1236406-76-0	Not Yet Assigned	C40H45ClN6O6S	773.34	A/B	-8.9
2104	[C@H](Cc1ccccc1)(C[C@@H]([C@H](Cc1ccccc1)NC(=O)OCc1ccno1)O)NC(=O)[C@@H](NC(=O)N(C)Cc1nc(C(C)C)sc1)CC(	1236406-95-3	2,7,10,12-Tetraazatridecanoic acid, 4-hydroxy-12-methyl-9-(1-methylethyl)-8,11-d	C42H50N6O5S2	783.01	A/B	-8.9
2105	C(N(C(=O)N[C@H](C(=O)N[C@@H](Cc1ccccc1)C[C@@H]([C@H](Cc1ccccc1)NC(=O)OCc1cncs1)O)C(C)C)C)c1nc(sc1)C1	1236408-07-3	2,7,10,12-Tetraazatridecanoic acid, 4-hydroxy-12-methyl-9-(1-methylethyl)-8,11-d	C41H48N6O6S	752.92	A/B	-8.9

2106	C(N(C(=O)N[C@H](C(=O)N[C@@H](Cc1ccccc1)C[C@@H]([C@H](Cc1ccccc1)NC(=O)OCc1cnco1)O)C(C)C)C)c1nc(sc1)C1	1236408-73-3	2,7,10,12-Tetraazatridecanoic acid, 4-hydroxy-12-methyl-9-(1-methylethyl)-8,11-c	C39H51N7O5S2	762	A/B	-8.9
2107	N(C(=O)c1cc(ccc1O)c1ccccc1)c1c(C(=O)O)ccc(c1)c1ccccc1	1237482-27-7	2,7,10,12-Tetraazatridecanoic acid, 13-[2-(2-cyclopenten-1-yl)-4-thiazolyl]-4-hy	C39H48N6O5S2	744.97	A/B	-8.9
2108	N(C(=O)[C@H](Cc1ccccc1)C[C@@H](CN1[C@H](C(=O)NC(C)(C)C)CN(Cc2c(C)ccs2)CC1)O)[C@H]1c2c(C[C@H]1O)cccc2	1237988-63-4	D-erythro-Pentonamide, 2,3,5-trideoxy-N-[(1S,2R)-2,3-dihydro-2-hydroxy-1H-inden-	C37H46N4O4S2	674.92	A/B	-8.9
2109	N(CC(=O)[C@H](Cc1ccccc1)C[C@@H](CN1[C@H](C(=O)NC(C)(C)C)CN(CCCN2CCCCC2)CC1)O)[C@H]1c2c(C[C@H]1O)cccc	1237988-69-0	D-erythro-Pentonamide, 2,3,5-trideoxy-N-[(1S,2R)-2,3-dihydro-2-hydroxy-1H-inden-	C35H46N4O4S	618.83	A/B	-8.9
2110	[C@H](Cc1ccccc1)(C[C@@H]([C@H](Cc1ccccc1)NC(=O)OC(C)c1cncs1)O)NC(=O)[C@@H](NC(=O)N(Cc1nc(C(C)C)sc1)C	1237989-15-9	Not Yet Assigned	C37H48ClN5O4	662.26	A/B	-8.9
2111	C(F)(F)c1n(c2c(n1)cccc2)c1nc(nc(N[C@H]2CC[C@H](N)CC2)n1)N1CCOCC1	1240023-44-2	1,4-Cyclohexanediamine, N1-[4-[2-(difluoromethyl)-1H-benzimidazol-1-yl]-6-(4-mor	C22H28F2N8O	458.51	A/B	-8.9
2112	C(F)(F)c1n(c2c(n1)cccc2)c1nc(nc(N[C@H]2CC[C@H](N(CC)CC)CC2)n1)N1CCOCC1	1240024-26-3	1,4-Cyclohexanediamine, N1-[4-[2-(difluoromethyl)-1H-benzimidazol-1-yl]-6-(4-mor	C23H30F2N8O	472.53	A/B	-8.9
2113	C(F)(F)c1n(c2c(n1)cccc2)c1nc(nc(N[C@H]2CC[C@H](NC(=O)NCCOC)CC2)n1)N1CCOCC1	1240024-63-8	Urea, N,Ä≤-[trans-4-[[4-[2-(difluoromethyl)-1H-benzimidazol-1-yl]-6-(4-morpholi	C27H38F2N10O2	572.65	A/B	-8.9
2114	C(=O)([C@@H](NC(=O)OC)C)N1[C@@H](CCC1)c1[nH]c(cn1)c1ccc(cc1)c1ccc([C@@H](NC(=O)[C@H]2N(C(=O)[C@@H](N	1240427-24-0	Carbamic acid, N-[(1S)-1-[[(2S)-2-[[[[4,Ä≤-[2-[(1S)-1-[[(2S)-2-[(methoxycarbony]	C37H49N7O7	703.83	A/B	-8.9
2115	N(C(=O)c1ccc(/C=C/c2ccc(C(=O)Nc3c(C(=O)O)cc(C)cc3)cc2)cc1)c1c(C(=O)O)cc(C)cc1	1240502-75-3	Benzoic acid, 2,2,Ä≤-[(1E)-1,2-ethenediylbis([1,1,Ä≤-biphenyl]-4,Ä≤,4-diylcarbo	C42H30N2O6	658.7	A/B	-8.9
2116	C(=O)([C@H](NC(=O)OC)CC)N1[C@@H](CCC1)c1[nH]c(cn1)[C@H]1CC[C@@H](CC1)c1ccc(cc1)c1[nH]c(nc1)[C@H]1N(C	1240887-45-9	Carbamic acid, N-[(1S)-1-[[(2S)-2-[5-[4-[trans-4-[2-[(2S)-1-[(2S)-2-[(methoxycar	C38H52N8O6	716.87	A/B	-8.9
2117	C(=O)([C@@H](N(C(=O)OC)C)C(C)C)N1[C@@H](CCC1)c1[nH]c(cn1)[C@H]1CC[C@@H](CC1)c1ccc(cc1)c1[nH]c(nc1)[C	1240887-70-0	Carbamic acid, N-[(1R)-1-[[(2S)-2-[5-[4-[trans-4-[2-[(2S)-1-[(2R)-2-[(methoxycar	C40H56N8O6	744.92	A/B	-8.9
2118	Nc1c2c(n(cn2)[C@H]2[C@@H](O)[C@H](O)[C@@H](CO)O2)nc(Cc2ccccc2)n1	125339-07-3	9H-Purin-6-amine, 9-Œ≤-D-arabinofuranosyl-2-phenyl-	C16H17N5O4	343.34	A/B	-8.9
2119	[C@@H](NC(=O)[C@H](Cc1ccccc1)NC(=O)N1CCN(C)CC1)(/C=C/S(=O)(=O)c1ccccc1)c1ccccc1	1253912-09-2	4-Morpholinecarboxamide, N-[(1S)-2-oxo-1-(phenylmethyl)-2-[[(1R,2E)-1-phenyl-3-(	C29H31N3O5S	533.64	A/B	-8.9
2120	[C@@H](NC(=O)[C@H](Cc1ccccc1)NC(=O)N1CCN(C)CC1)(/C=C/S(=O)(=O)c1ccccc1)c1c2c(ccc1)cccc2	1253912-31-0	1-Piperazinecarboxamide, 4-methyl-N-[(1S)-2-oxo-1-(phenylmethyl)-2-[[(1R,2E)-1-p	C30H34N4O4S	546.68	A/B	-8.9
2121	C(n1c2c(nc1)c(N)ncn2)[C@H]1COP(=O)(OC[C@@H](C(=O)OC(C)C)N)CO1	1254066-01-7	Urea, N-[4-[4-(4-morpholinyl)-6-(3-oxa-8-azabicyclo[3.2.1]oct-8-yl)-1,3,5-triazi	C24H27N9O3	489.53	A/B	-8.9
2122	O(c1nc(Nc2ccc(C#N)cc2)nc(N)c1Br)c1c(C)cc(C#N)cc1C.S(=O)(=O)(O)c1ccc(C)cc1	1255672-00-4	Benzonitrile, 4-[[6-amino-5-bromo-2-[(4-cyanophenyl)amino]-4-pyrimidinyl]oxy]-3,	C20H15BrN6O.xBrH		A/B	-8.9
2123	C(=O)([C@@H](NC(=O)OC)C(C)C)N1[C@@H]([C@@H]2C[C@H]1CC2)c1[nH]c(cn1)c1cc2c(-c3c(CC2)cc(cc3)c2[nH]c(nc	1256388-51-8	Carbamic acid, N-[(1S)-1-[[(6S)-6-[5-[9,9-difluoro-7-[2-[(1R,3S,4S)-2-[(2S)-2-[(	C49H54F2N8O6	889	A/B	-8.9
2124	C(=O)([C@@H](NC(=O)OC)C(C)C)N1[C@@H](C[C@H](C1)c1ccccc1)c1[nH]c(cn1)c1ccc(cc1)c1cc2c(cc1)cc(cc2)c1[n	1256389-81-7	Carbamic acid, N-[(1R)-2-[(2S)-2-[5-[4-[6-[2-[(2S)-1-[(2S)-2-[(methoxycarbonyl)a	C48H52N8O6	836.98	A/B	-8.9
2125	C(=O)([C@@H](NC(=O)OC)c1ccccc1)N1[C@@H](CCC1)c1[nH]c(cn1)c1cc2c(cc1)cc(cc2)c1ccc(cc1)c1[nH]c(nc1)[C@	1256391-32-8	Carbamic acid, N-[(1R)-2-[(2S)-2-[5-[4-[6-[2-[(2S)-1-[(2S)-2-[(methoxycarbonyl)a	C47H50N8O6	822.95	A/B	-8.9
2126	C(=O)([C@@H](NC(=O)OC)C(C)C)N1[C@@H](CCC1)c1[nH]c(cn1)c1ccc(cc1)c1ccc(cc1)c1[nH]c(nc1)[C@H]1N(C(=O)[	1256391-36-2	Carbamic acid, N-[2-[(2S)-2-[5-[4-[6-[2-[(2S)-1-[2-[(methoxycarbonyl)amino]-2-ph	C50H48N8O6	856.97	A/B	-8.9
2127	C(=O)([C@@H](NC(=O)OC)C(C)C)N1CC2(C[C@H]1c1[nH]c(cn1)c1cc3c(-c4c(C3(F)F)cc(cc4)c3[nH]c(nc3)[C@H]3N(C	1256392-07-0	Carbamic acid, N-[(1S)-1-[[(2S)-2-[5-[9,9-difluoro-7-[2-[(6S)-5-[(2S)-2-[(methox	C43H50F2N8O6	812.9	A/B	-8.9
2128	C(=O)([C@@H](NC(=O)OC)C(C)C)N1[C@@H](CCC1)c1[nH]c(cn1)c1ccc(cc1)c1cc2c(cc1)cc(cc2)c1[nH]c(nc1)[C@H]1	1261070-49-8	Carbamic acid, N-[(1S)-2-[(2S)-2-[5-[6-[4-[2-[(2S)-1-[(2S)-2-[(methoxycarbonyl)a	C42H48N8O6	760.88	A/B	-8.9
2129	C(F)(F)c1n(c2c(n1)cccc2)c1nc(nc(N(C)c2ccccc2)n1)N1CCOCC1	1261281-10-0	1,3,5-Triazin-2-amine, 4-[2-(difluoromethyl)-1H-benzimidazol-1-yl]-6-(4-morpholi	C21H19F2N7O	423.42	A/B	-8.9
2130	C(F)(F)c1n(c2c(n1)cccc2)c1nc(nc(N(Cc2ccccc2)C)n1)N1CCOCC1	1261281-12-2	1,3,5-Triazin-2-amine, 4-[2-(difluoromethyl)-1H-benzimidazol-1-yl]-6-(4-morpholi	C22H21F2N7O	437.45	A/B	-8.9
2131	N(=C1c2c(N(C(=O)C)C1=O)ccc(Br)c2)n1c(Cc2n(N=C3c4c(N(C(=O)C)C3=O)ccc(Br)c4)c(=S)[nH]n2)n[nH]c1=S	1262891-46-2	2H-Indol-2-one, 3,3,Ä≤-[methylenebis[(1,5-dihydro-5-thioxo-4H-1,2,4-triazole-3,4	C25H18N10O4S2	586.6	A/B	-8.9
2132	N(=C\1/c2c(N(CN3CCN(CC3)c3cc4c(cc3F)c(=O)c(C(=O)O)cn4CC)C1=O)ccc(C)c2)\NC(=S)NOC	1265623-32-2	3-Quinolinecarboxylic acid, 1-cyclopropyl-7-[4-[[(3Z)-2,3-dihydro-3-[2-(methoxy	C31H34FN7O6S	651.71	A/B	-8.9
2133	O=c1n([C@@H]2O[C@H](COP3(=O)Oc4c(CO3)cc3c(c4)COP(=O)(OC[C@@H]4[C@@H](N=[N+]=[N-])C[C@@H](O4)n4c(=O)[	1268669-59-5	Guanosine 5,Ä≤-(tetrahydrogen triphosphate), N-methyl-, P,Ä≤,Ä≤,Ü15,Ä≤-ester wi	C21H29N10O18P3	802.43	A/B	-8.9
2134	C(=O)([C@@H](NC(=O)OC)C(C)C)N1C[C@@]2(C[C@H]1c1[nH]c(cn1)c1ccc(cc1)c1ccc(cc1)c1[nH]c(nc1)[C@H]1N(C(=	1272654-98-4	Carbamic acid, N-[(1S)-1-[[(2S)-2-[5-[4,Ä≤-[2-[(3S,7S,9S)-2-[(2S)-2-[(methoxycar	C45H58N8O8	838.99	A/B	-8.9
2135	N(c1nc(nc(C#N)n1)N1CCOCC1)(C1CCCCC1)c1ccccc1	1275581-81-1	1,3,5-Triazine-2-carbonitrile, 4-(4-morpholinyl)-6-[(4-propylcyclohexyl)[[3-(tri	C25H31F3N6O	488.55	A/B	-8.9
2136	N(c1nc(nc(N/N=C/c2c(O)cccc2)n1)N1CCOCC1)c1ccc(F)cc1	1279692-11-3	Benzaldehyde, 2,6-difluoro-, 2-[4-[(4-fluorophenyl)amino]-6-(4-morpholinyl)-1,3,	C20H18F3N7O	429.4	A/B	-8.9
2137	N(=C\1/c2c(NC1=O)cccc2)/Nc1nc(nc(NCc2ccccc2)n1)N1CCOCC1	1279692-12-4	Benzaldehyde, 2-hydroxy-, 2-[4-[(4-fluorophenyl)amino]-6-(4-morpholinyl)-1,3,5-t	C20H20FN7O2	409.42	A/B	-8.9
2138	N(=C\1/c2c(NC1=O)cccc2)\Nc1nc(nc(NCc2cc(Cl)ccc2)n1)N1CCOCC1	1279692-13-5	1H-Indole-2,3-dione, 3-[2-[4-(4-morpholinyl)-6-[(phenylmethyl)amino]-1,3,5-triaz	C22H22N8O2	430.46	A/B	-8.9
2139	N(S(=O)(=O)c1ccc(C)cc1)c1c2c(n(C[C@H](OCP(=O)(O)O)C)cn2)ncn1	1306734-25-7	Carbamic acid, N-[(1S)-1-[[(2S,4R)-2-[5-[4-[6-[2-[(2S,4R)-1-[(2S)-2-[(methoxycar	C46H56N8O6	816.99	A/B	-8.9
2140	S(=O)(=O)(O)c1c2c(c(N)c(N=Nc3c(C)cccc3)c1)ccc(S(=O)(=O)O)c2.[NaH]	13083-07-3	2,6-Naphthalenedisulfonic acid, 4-[2-[4-[2-(4-amino-1-naphthalenyl)diazenyl]-1-r	C30H21N5O6S2.2Na		A/B	-8.9
2141	C(=O)([C@@H](NC(=O)OC)C1CCOCC1)N1[C@@H](CC[C@@H]1C)c1[nH]c(c(Cl)n1)c1ccc(cc1)c1ccc(cc1)c1[nH]c(nc1Cl	1312336-18-7	Carbamic acid, N,N,Ä≤-[[1,1,Ä≤-biphenyl]-4,4,Ä≤-diylbis[(5-chloro-1H-imidazole-	C46H56Cl2N8O8	919.89	A/B	-8.9
2142	C(=O)([C@@H](NC(=O)OC)C1CCC(F)(F)CC1)N1[C@H]2[C@H](C2)C[C@H]1c1[nH]c(c(Cl)n1)c1ccc(cc1)c1ccc(cc1)c1[	1312336-36-9	Carbamic acid, N-[(1S)-2-[(1R,3S,5R)-3-[5-[4,Ä≤-[5-chloro-2-[(1R,3S,5R)-2-[(2S)-	C48H53ClF4N8O6	949.43	A/B	-8.9
2143	C(=O)([C@@H](NC(=O)OC)C(C)C)N1[C@H]2[C@H](C2)C[C@H]1c1[nH]c(c(C#N)n1)c1ccc(cc1)c1ccc(cc1)c1[nH]c(nc1	1312336-67-6	Carbamic acid, N,N,Ä≤-[[1,1,Ä≤-biphenyl]-4,4,Ä≤-diylbis[(5-fluoro-1H-imidazole-	C46H56F2N8O8	886.98	A/B	-8.9
2144	C(=O)([C@@H](NC(=O)OC)C1CCOCC1)N1[C@H](c2[nH]c(c(n2)c2ccc(cc2)c2ccc(cc2)c2[nH]c(nc2)[C@H]2N(C(=O)[C@	1312336-96-1	Carbamic acid, N-[(1S)-2-[(2S,5S)-2-[7-[4-[5-chloro-2-[(2S,5S)-1-[(2S)-2-[(metho	C48H57ClN8O8	909.47	A/B	-8.9
2145	C(=O)([C@@H](NC(=O)OC)C1CCC(F)(F)CC1)N1[C@H]2[C@H](C2)C[C@H]1c1[nH]c(cn1)c1ccc(cc1)c1ccc(cc1)c1[nH]c	1312338-46-7	Carbamic acid, N,N,Ä≤-[[1,1,Ä≤-biphenyl]-4,4,Ä≤-diylbis[1H-imidazole-5,2-diyl-(	C48H54F4N8O6	914.99	A/B	-8.9
2146	O(Cc1ccc(F)cc1)c1c2c(oc(c(O)c2=O)c2[nH]ccn2)c(C)cc1	1312892-69-5	4H-1-Benzopyran-4-one, 5-[(4-fluorophenyl)methoxy]-3-hydroxy-8-methyl-2-(3-pheny	C25H18FN3O4	443.43	A/B	-8.9
2147	O(Cc1ccc(F)cc1)c1c2c(oc(c(O)c2=O)c2[nH]ccn2)c(Cl)cc1	1312893-97-2	4H-1-Benzopyran-4-one, 8-chloro-5-[(4-fluorophenyl)methoxy]-3-hydroxy-2-(3-pheny	C24H15ClFN3O4	463.85	A/B	-8.9
2148	O(Cc1ccc(F)cc1)c1c2c(c(cc1)c1ccccc1)oc(c(O)c2=O)c1[nH]c(Cl)nn1	1312894-45-3	4H-1-Benzopyran-4-one, 5-[(4-fluorophenyl)methoxy]-3-hydroxy-2-(5-methoxy-1H-1,2	C25H18FN3O5	459.43	A/B	-8.9
2149	O(Cc1ccc(F)cc1)c1c2c(oc(c(O)c2=O)c2ncccn2)cc(C)c1	1312894-52-2	4H-1-Benzopyran-4-one, 5-[(4-fluorophenyl)methoxy]-3-hydroxy-8-phenyl-2-(5-pheny	C31H21FN2O4	504.51	A/B	-8.9
2150	O(Cc1ccc(F)cc1)c1c2c(oc(c(O)c2=O)c2[nH]c(nn2)c2ccccc2)c(C)c(C)c1	1312895-46-7	4H-1-Benzopyran-4-one, 2-(5-chloro-1H-1,2,4-triazol-3-yl)-5-[(4-fluorophenyl)met	C20H15ClFN3O4	415.8	A/B	-8.9
2151	O(C)c1c2c(c(OCc3ccc(F)cc3)cc1C)c(=O)c(O)c(o2)c1[nH]cnn1	1312895-87-6	4H-1-Benzopyran-4-one, 5-[(4-fluorophenyl)methoxy]-3-hydroxy-8-methoxy-7-methyl-	C28H21FN2O5	484.48	A/B	-8.9
2152	O(C)c1c2c(c(OCc3ccc(F)cc3)cc1C)c(=O)c(O)c(o2)c1[nH]ccn1	1312895-97-8	4H-1-Benzopyran-4-one, 5-[(4-fluorophenyl)methoxy]-3-hydroxy-8-methoxy-7-methyl-	C26H20FN3O5	473.45	A/B	-8.9
2153	O(Cc1ccc(F)cc1)c1c2c(oc(c(O)c2=O)c2[nH]c(nn2)c2ccccc2)c(Cl)c(C)c1	1312896-46-0	4H-1-Benzopyran-4-one, 8-chloro-2-(5-chloro-1H-1,2,4-triazol-3-yl)-5-[(4-fluorop	C19H12Cl2FN3O4	436.22	A/B	-8.9
2154	O(Cc1ccc(F)cc1)c1c2c(c(c(C)c1)c1ccccc1)oc(c(O)c2=O)c1[nH]c(OC)cn1	1312896-99-3	4H-1-Benzopyran-4-one, 5-[(4-fluorophenyl)methoxy]-3-hydroxy-7-methyl-2-(5-methy	C27H21FN2O4	456.46	A/B	-8.9
2155	O(Cc1ccc(F)cc1)c1c2c(oc(c(O)c2=O)c2[nH]ccn2)c(C)c(OC)c1	1312898-08-0	4H-1-Benzopyran-4-one, 5-[(4-fluorophenyl)methoxy]-3-hydroxy-7-methoxy-8-methyl-	C26H20FN3O5	473.45	A/B	-8.9
2156	O(Cc1ccc(F)cc1)c1c2c(oc(c(O)c2=O)c2[nH]ccn2)c(Cl)c(OC)c1	1312899-09-4	4H-1-Benzopyran-4-one, 8-chloro-5-[(4-fluorophenyl)methoxy]-3-hydroxy-7-methoxy-	C25H17ClFN3O5	493.87	A/B	-8.9
2157	O(Cc1ccc(F)cc1)c1c2c(c(c(Cl)c1)c1ccccc1)oc(c(O)c2=O)c1ncc(C)cn1	1312902-54-7	4H-1-Benzopyran-4-one, 7-chloro-5-[(4-fluorophenyl)methoxy]-3-hydroxy-8-phenyl-2	C26H16ClFN2O4	474.87	A/B	-8.9
2158	O(Cc1ccc(F)cc1)c1c2c(c(c(Cl)c1)c1ccccc1)oc(c(O)c2=O)c1nnc(C)[nH]1	1312902-64-9	4H-1-Benzopyran-4-one, 7-chloro-5-[(4-fluorophenyl)methoxy]-3-hydroxy-8-phenyl-2	C24H15ClFN3O4	463.85	A/B	-8.9
2159	O(Cc1ccc(F)cc1)c1c2c(oc(c(O)c2=O)c2[nH]c(OC)nn2)cc(c1)c1ccccc1	1312903-15-3	4H-1-Benzopyran-4-one, 5-[(4-fluorophenyl)methoxy]-3-hydroxy-2-(3-methyl-1H-1,2,	C25H18FN3O4	443.43	A/B	-8.9
2160	O(Cc1ccc(F)cc1)c1c2c(oc(c(O)c2=O)c2[nH]c(C)cn2)cc(c1)c1ccccc1	1312903-19-7	4H-1-Benzopyran-4-one, 5-[(4-fluorophenyl)methoxy]-3-hydroxy-2-(1H-imidazol-2-yl	C25H17FN2O4	428.41	A/B	-8.9
2161	O(Cc1ccc(F)cc1)c1c2c(oc(c(O)c2=O)c2[nH]c(OC)cn2)cc(c1)c1ccccc1	1312903-20-0	4H-1-Benzopyran-4-one, 5-[(4-fluorophenyl)methoxy]-3-hydroxy-2-(5-methyl-1H-imic	C26H19FN2O4	442.44	A/B	-8.9
2162	O(Cc1ccc(F)cc1)c1c2c(c(C)c(c1)c1ccccc1)oc(c(O)c2=O)c1ncc(OC)cn1	1312903-55-1	4H-1-Benzopyran-4-one, 5-[(4-fluorophenyl)methoxy]-3-hydroxy-8-methyl-2-(5-methy	C28H21FN2O4	468.48	A/B	-8.9
2163	O(Cc1ccc(F)cc1)c1c2c(c(C)c(c1)c1ccccc1)oc(c(O)c2=O)c1[nH]c(C)nn1	1312903-66-4	4H-1-Benzopyran-4-one, 5-[(4-fluorophenyl)methoxy]-3-hydroxy-8-methyl-7-phenyl-2	C25H18FN3O4	443.43	A/B	-8.9
2164	O(Cc1ccc(F)cc1)c1c2c(c(C)c(c1)c1ccccc1)oc(c(O)c2=O)c1[nH]c(Cl)nn1	1312903-68-6	4H-1-Benzopyran-4-one, 5-[(4-fluorophenyl)methoxy]-3-hydroxy-2-(5-methoxy-1H-1,2	C26H20FN3O5	473.45	A/B	-8.9
2165	Cc1c2c(c(OCc3ccc(F)cc3)cc1c1ccccc1)c(=O)c(O)c(o2)c1[nH]c(cn1)c1ccccc1	1312903-76-6	4H-1-Benzopyran-4-one, 2-(5-chloro-1H-imidazol-2-yl)-5-[(4-fluorophenyl)methoxy]	C26H18ClFN2O4	476.88	A/B	-8.9
2166	O(C)c1c2c(c(OCc3ccc(F)cc3)cc1c1ccccc1)c(=O)c(O)c(o2)c1[nH]c(OC)nn1	1312904-42-9	4H-1-Benzopyran-4-one, 5-[(4-fluorophenyl)methoxy]-3-hydroxy-8-methoxy-2-(3-meth	C26H20FN3O5	473.45	A/B	-8.9
2167	Clc1c2c(c(OCc3ccc(F)cc3)cc1c1ccccc1)c(=O)c(O)c(o2)c1ncc(C)cn1	1312905-04-6	4H-1-Benzopyran-4-one, 8-chloro-5-[(4-fluorophenyl)methoxy]-3-hydroxy-7-phenyl-2	C26H16ClFN2O4	474.87	A/B	-8.9
2168	O(Cc1ccc(F)cc1)c1c2c(c(Cl)c(c1)c1ccccc1)oc(c(O)c2=O)c1[nH]c(C)nn1	1312905-19-3	4H-1-Benzopyran-4-one, 8-chloro-5-[(4-fluorophenyl)methoxy]-3-hydroxy-7-phenyl-2	C24H15ClFN3O4	463.85	A/B	-8.9
2169	Clc1c2c(c(OCc3ccc(F)cc3)cc1c1ccccc1)c(=O)c(O)c(o2)c1[nH]c(cn1)c1ccccc1	1312905-32-0	4H-1-Benzopyran-4-one, 8-chloro-2-(5-chloro-1H-imidazol-2-yl)-5-[(4-fluorophenyl	C25H15Cl2FN2O4	497.3	A/B	-8.9
2170	O(Cc1ccc(F)cc1)c1c2c(c(c(c1)c1ccccc1)c1ccccc1)oc(c(O)c2=O)c1[nH]ccn1	1312905-80-8	4H-1-Benzopyran-4-one, 5-[(4-fluorophenyl)methoxy]-3-hydroxy-7,8-diphenyl-2-(3-p	C36H24FN3O4	581.59	A/B	-8.9
2171	O(C)c1c2c(c(=O)c(O)c(o2)c2nnc(Cl)[nH]2)cc(OCc2ccc(F)cc2)c1	1312907-26-8	4H-1-Benzopyran-4-one, 6-[(4-fluorophenyl)methoxy]-3-hydroxy-8-methoxy-2-(5-meth	C20H16FN3O6	413.36	A/B	-8.9
2172	O(C)c1c2c(cc(OCc3ccc(F)cc3)c1)c(=O)c(O)c(o2)c1[nH]c(nn1)c1ccccc1	1312907-27-9	4H-1-Benzopyran-4-one, 2-(5-chloro-1H-1,2,4-triazol-3-yl)-6-[(4-fluorophenyl)met	C19H13ClFN3O5	417.77	A/B	-8.9
2173	O=c1c2c(oc(c1O)c1nnc(Cl)[nH]1)c(Cl)cc(OCc1ccc(F)cc1)c2	1312907-76-8	4H-1-Benzopyran-4-one, 8-chloro-6-[(4-fluorophenyl)methoxy]-3-hydroxy-2-(5-metho	C19H13ClFN3O5	417.77	A/B	-8.9
2174	O=c1c2c(c(cc(OCc3ccc(F)cc3)c2)c2ccccc2)oc(c1O)c1ncc(cn1)c1ccccc1	1312908-17-0	4H-1-Benzopyran-4-one, 2-(5-chloro-2-pyrimidinyl)-6-[(4-fluorophenyl)methoxy]-3-	C26H16ClFN2O4	474.87	A/B	-8.9
2175	Cc1c2c(cc(OCc3ccc(F)cc3)c1C)c(=O)c(O)c(o2)c1[nH]c(OC)cn1	1312909-42-4	4H-1-Benzopyran-4-one, 6-[(4-fluorophenyl)methoxy]-3-hydroxy-7,8-dimethyl-2-(5-m	C22H19FN2O4	394.4	A/B	-8.9
2176	Clc1c2c(cc(OCc3ccc(F)cc3)c1C)c(=O)c(O)c(o2)c1[nH]c(OC)cn1	1312910-42-1	4H-1-Benzopyran-4-one, 8-chloro-6-[(4-fluorophenyl)methoxy]-3-hydroxy-7-methyl-2	C21H16ClFN2O4	414.81	A/B	-8.9
2177	Cc1c(c2c(cc1OCc1ccc(F)cc1)c(=O)c(O)c(o2)c1ncc(C)cn1)c1ccccc1	1312910-76-1	4H-1-Benzopyran-4-one, 6-[(4-fluorophenyl)methoxy]-3-hydroxy-7-methyl-8-phenyl-2	C27H19FN2O4	454.45	A/B	-8.9
2178	Cc1c(c2c(cc1OCc1ccc(F)cc1)c(=O)c(O)c(o2)c1[nH]cnn1)c1ccccc1	1312910-80-7	4H-1-Benzopyran-4-one, 6-[(4-fluorophenyl)methoxy]-3-hydroxy-7-methyl-8-phenyl-2	C33H23FN2O4	530.55	A/B	-8.9
2179	Cc1c(c2c(cc1OCc1ccc(F)cc1)c(=O)c(O)c(o2)c1[nH]ccn1)c1ccccc1	1312910-90-9	4H-1-Benzopyran-4-one, 6-[(4-fluorophenyl)methoxy]-3-hydroxy-7-methyl-8-phenyl-2	C31H22FN3O4	519.52	A/B	-8.9
2180	Cc1c(c2c(cc1OCc1ccc(F)cc1)c(=O)c(O)c(o2)c1[nH]c(C)cn1)c1ccccc1	1312910-91-0	4H-1-Benzopyran-4-one, 6-[(4-fluorophenyl)methoxy]-3-hydroxy-2-(1H-imidazol-2-yl	C26H19FN2O4	442.44	A/B	-8.9
2181	Cc1c2c(cc(OCc3ccc(F)cc3)c1Cl)c(=O)c(O)c(o2)c1[nH]ccn1	1312912-83-6	4H-1-Benzopyran-4-one, 7-chloro-6-[(4-fluorophenyl)methoxy]-3-hydroxy-8-methyl-2	C25H17ClFN3O4	477.87	A/B	-8.9
2182	Clc1c(c2c(cc1OCc1ccc(F)cc1)c(=O)c(O)c(o2)c1ncc(OC)cn1)c1ccccc1	1312915-62-0	4H-1-Benzopyran-4-one, 7-chloro-6-[(4-fluorophenyl)methoxy]-3-hydroxy-2-(5-methy	C27H18ClFN2O4	488.89	A/B	-8.9
2183	Clc1c(c2c(cc1OCc1ccc(F)cc1)c(=O)c(O)c(o2)c1[nH]c(C)cn1)c1ccccc1	1312915-90-4	4H-1-Benzopyran-4-one, 7-chloro-6-[(4-fluorophenyl)methoxy]-3-hydroxy-2-(1H-imic	C25H16ClFN2O4	462.86	A/B	-8.9
2184	O(Cc1ccc(F)cc1)c1c(c(C)c2c(c1)c(=O)c(O)c(o2)c1ncccn1)c1ccccc1	1312916-50-9	4H-1-Benzopyran-4-one, 6-[(4-fluorophenyl)methoxy]-3-hydroxy-7-phenyl-2-(5-pheny	C31H21FN2O4	504.51	A/B	-8.9
2185	Cc1c2c(cc(OCc3ccc(F)cc3)c1c1ccccc1)c(=O)c(O)c(o2)c1[nH]c(nn1)c1ccccc1	1312916-95-2	4H-1-Benzopyran-4-one, 2-(5-chloro-1H-1,2,4-triazol-3-yl)-6-[(4-fluorophenyl)met	C25H17ClFN3O4	477.87	A/B	-8.9
2186	O(Cc1ccc(F)cc1)c1c(c(C)c2c(c1)c(=O)c(O)c(o2)c1[nH]c(C)cn1)c1ccccc1	1312916-97-4	4H-1-Benzopyran-4-one, 6-[(4-fluorophenyl)methoxy]-3-hydroxy-2-(1H-imidazol-2-yl	C26H19FN2O4	442.44	A/B	-8.9
2187	O(Cc1ccc(F)cc1)c1c(c(C)c2c(c1)c(=O)c(O)c(o2)c1[nH]c(Cl)cn1)c1ccccc1	1312916-99-6	4H-1-Benzopyran-4-one, 6-[(4-fluorophenyl)methoxy]-3-hydroxy-2-(5-methoxy-1H-imi	C27H21FN2O5	472.46	A/B	-8.9
2188	O(Cc1ccc(F)cc1)c1c(c(Cl)c2c(c1)c(=O)c(O)c(o2)c1[nH]c(C)cn1)c1ccccc1	1312917-97-7	4H-1-Benzopyran-4-one, 8-chloro-6-[(4-fluorophenyl)methoxy]-3-hydroxy-2-(1H-imic	C25H16ClFN2O4	462.86	A/B	-8.9
2189	O(Cc1ccc(F)cc1)c1c(c(Cl)c2c(c1)c(=O)c(O)c(o2)c1[nH]c(Cl)cn1)c1ccccc1	1312917-99-9	4H-1-Benzopyran-4-one, 8-chloro-6-[(4-fluorophenyl)methoxy]-3-hydroxy-2-(5-metho	C26H18ClFN2O5	492.88	A/B	-8.9
2190	S(Cc1cc2c(oc1=O)ccc(F)c2)c1n(c2c(n1)c(=O)nc(N)[nH]2)[C@@H]1O[C@H](CO)[C@@H](O)[C@H]1O	1328880-82-5	Guanosine, 8-[[(2-oxo-2H-1-benzopyran-3-yl)methyl]thio]-	C20H19N5O7S	473.46	A/B	-8.9
2191	N(=C1c2c(NC1=O)cccc2)Nc1nc(nc(NCc2cc(Cl)ccc2)n1)N1CCOCC1	1331749-45-1	1H-Indole-2,3-dione, 3-[2-[4-(4-morpholinyl)-6-[(phenylmethyl)amino]-1,3,5-triaz	C22H22N8O2	430.46	A/B	-8.9
2192	N(Cc1ccc(F)cc1)c1nc(nc(NN=Cc2c(O)cccc2)n1)N1CCOCC1	1331749-49-5	Acetamide, N-[4-[[2-[4-(4-morpholinyl)-6-(phenylamino)-1,3,5-triazin-2-yl]hydraz	C22H24N8O2	432.48	A/B	-8.9
2193	[C@H]([C@H](Cc1ccccc1)NC(=O)O[C@H]1COCC1)(CN(S(=O)(=O)c1ccc(N(=O)=O)cc1)CCC)OP(=O)(O)O	1332579-80-2	Phosphoric acid, 2,6-diethoxy-4-[(5R,5aR,8aR,9S)-5,5a,6,8,8a,9-hexahydro-9-hydrc	C37H37O11P	688.66	A/B	-8.9
2194	O=c1n([C@H]2O[C@H](COP(=O)(O)O)[C@@H](OP(=O)(O)S)C2)cc(C)c(=O)[nH]1	134567-07-0	Thymidine, thymidylyl-(3,Ä≤,Ü15,Ä≤)-4-thio-	C20H27N4O11PS	562.49	A/B	-8.9
2195	N(=Nc1c2c(c(S(=O)(=O)O)ccc2)c(N)cc1)c1c2c(c(N=Nc3cc(S(=O)(=O)O)ccc3)cc1)ccc(S(=O)(=O)O)c2	1345852-30-3	2-Naphthalenesulfonic acid, 8-amino-5-[2-[4-[2-(3-sulfophenyl)diazenyl]-1-naphth	C26H19N5O6S2	561.59	A/B	-8.9
2196	N(=Nc1c2c(cc(S(=O)(=O)O)cc2)c(N)cc1)c1c2c(c(N=Nc3cc(S(=O)(=O)O)ccc3)cc1)ccc(S(=O)(=O)O)c2	1345852-31-4	1-Naphthalenesulfonic acid, 8-amino-5-[2-[7-sulfo-4-[2-(3-sulfophenyl)diazenyl]-	C26H19N5O9S3	641.65	A/B	-8.9
2197	N(=Nc1c2c(c(N=Nc3ccccc3)cc1)ccc(S(=O)(=O)O)c2)c1c2c(c(N)cc1)cccc2S(=O)(=O)O	1345852-45-0	1-Naphthalenesulfonic acid, 8-amino-5-[2-[6-sulfo-4-[2-(3-sulfophenyl)diazenyl]-	C26H19N5O9S3	641.65	A/B	-8.9
2198	[C@H](Cc1ccccc1)(C[C@@H]([C@H](Cc1ccccc1)NC(=O)OCc1cncs1)O)NC(=O)[C@H](N(C(=O)NCc1nc(C(C)C)sc1)C)C	1346960-17-5	Not Yet Assigned	C33H47N5O5	593.76	A/B	-8.9
2199	[C@@H]([C@@H](CN(S(=O)(=O)c1ccc(N)cc1)CC(C)C)OCC[C@@H]1CCOC1)(Cc1ccccc1)NC(=O)O[C@H]1CCOC1	1348000-86-1	6H-Dipyrido[3,2-b:2,Ä≤,3,Ä≤-e][1,4]diazepin-6-one, 2-chloro-11-cyclopropyl-8-[2	C30H27ClN6O5S	619.09	A/B	-8.9
2200	N(c1c2c(ncc1)cc(Cl)cc2)[C@H]1CC[C@@H](N(CCCCc2ccc(F)cc2)CCCCc2ccc(F)cc2)CC1	1348404-69-2	Pentanamide, N-[[4,Ä≤-[(2-ethyl-5,7-dimethyl-3H-imidazo[4,5-b]pyridin-3-yl)methy	C32H40N4O3S	560.75	A/B	-8.9
2201	C([C@H](OCP(=O)(OC(=O)COC(C)C)OC(=O)COC(C)C)C)n1c2c(nc1)c(N)ncn2	1348622-87-6	Not Yet Assigned	C37H51N5O4	629.83	A/B	-8.9
2202	S(=O)(=O)(/C=C/C(NC(=O)[C@@H](Cc1ccccc1)NC(=O)N1CCOCC1)CCc1ccccc1)c1c2c(ccc1)cccc2	1349132-58-6	3H-Imidazo[4,5-b]pyridine-5-carboxylic acid, 3-[[2,Ä≤-[[(butoxycarbonyl)amino]su	C31H36N4O6S	592.71	A/B	-8.9
2203	C[C@@]12[C@]3([C@]4(C3)[C@@H](CC2)C(C)(C)[C@@H](O)CC4)CC=C2[C@@]1(C)C[C@@H](O)[C@]1(C(=O)O)[C@H]2CC(	1349694-41-2	Not Yet Assigned	C41H57N7O6	743.93	A/B	-8.9
2204	C([C@H]([C@H](Cc1ccccc1)NC(=O)[C@@H](NC(=O)C1=NC2C(C=C1)C=CC=C2)CC(=O)N)O)N1[C@H](C(=O)NC(C)(C)C)CC2	1350270-71-1	2,7,10,12-Tetraazatridecanoic acid, 13-[2-(2-cyclopenten-1-yl)-4-thiazolyl]-4-hy	C39H48N6O5S2	744.97	A/B	-8.9
2205	C(=O)([C@@H](NC(=O)OC)C(C)C)N1[C@@]2(C[C@H](C1)CC2)c1[nH]c(cn1)c1c2c(c(cc1)c1c3c(c(cc1)c1[nH]c(nc1)[	1350718-90-9	Carbamic acid, N-[(1S)-1-[[(1S,4R)-1-[5-[4-[7-[2-[(1S,4R)-2-[(2S)-2-[(methoxycar	C46H56N10O6	845	A/B	-8.9
2206	C(=O)([C@@H](NC(=O)OC)C(C)C)N1[C@@]2(C[C@H](C1)CC2)c1[nH]c(cn1)c1c2c(c(cc1)c1ccc(cc1)c1[nH]c(nc1)[C@	1350719-05-9	Carbamic acid, N-[(1S)-1-[[(1S,4R)-1-[5-[4-[7-[2-[(1S,4R)-2-[(2S)-2-[(methoxycar	C46H55N9O6S	862.05	A/B	-8.9
2207	C(=O)([C@@H](NC(=O)OC)C(C)C)N1[C@@]2(C[C@H](C1)CC2)c1[nH]c(cn1)c1c2c(c(cc1)c1c3c(c(cc1)c1[nH]c(nc1)[	1350719-07-1	Carbamic acid, N-[(1S)-1-[[(1S,4R)-1-[5-[4-[7-[2-[(1S,4R)-2-[(2S)-2-[(methoxycar	C47H57N9O6	844.01	A/B	-8.9
2208	C(=O)([C@@H](NC(=O)O[C@@H]1CCOC1)C(C)C)N1C[C@@]2(C[C@H]1c1[nH]c(cn1)c1ccc(cc1)c1ccc(cc1)c1[nH]c(nc1)	1351851-14-3	Carbamic acid, N-[(1S)-1-[[(5S,8S)-8-[5-[4,Ä≤-[2-[(2S)-1-[(2S)-2-[(methoxycarbon	C47H60N8O8	865.03	A/B	-8.9
2209	C(=O)([C@@H](NC(=O)OC)C(C)C)N1C[C@@]2(C[C@H]1c1[nH]c(cn1)c1ccc(cc1)c1ccc(cc1)c1[nH]c(nc1)[C@H]1N(C(=	1351852-69-1	Carbamic acid, N-[(1S)-1-[[(2S,5S)-2-[5-[4,Ä≤-[2-[(5S,8S)-7-[(2S)-2-[(methoxycar	C49H58N8O7	871.03	A/B	-8.9
2210	C(=O)([C@@H](Cc1ccccc1)NC(=O)OC)N1[C@@H](C[C@@H](C1)C1CC1)c1[nH]c(cn1)c1ccc(cc1)c1ccc(cc1)c1[nH]c(nc	1351854-21-1	Carbamic acid, N-[(1S)-2-[(2S)-2-[5-[4,Ä≤-[2-[(2S,4R)-4-cyclopropyl-1-[(2S)-2-[	C49H50N8O6	846.97	A/B	-8.9
2211	C(=O)([C@@H](NC(=O)OC)C(C)C)N1[C@@H](C[C@@H](C1)C1CC1)c1[nH]c(cn1)c1ccc(cc1)c1ccc(cc1)c1[nH]c([C@@H]	1351855-91-8	Carbamic acid, N-[(1S)-1-[[(2S,4R)-4-cyclopropyl-2-[5-[4,Ä≤-[2-[(3S,6S)-1,1-dif]	C45H54F2N8O6	840.96	A/B	-8.9
2212	C(F)(F)c1n(c2c(n1)cccc2)c1nc(nc(NCCS(=O)C2CCCC2)n1)N1CCOCC1	1359951-56-6	1,3,5-Triazin-2-amine, 4-[2-(difluoromethyl)-1H-benzimidazol-1-yl]-6-(4-morpholi	C21H26F2N8O	444.48	A/B	-8.9
2213	C(F)(F)c1n(c2c(n1)cccc2)c1nc(nc(NCCSC2CCCC2)n1)N1CCOCC1	1359952-73-0	1,3,5-Triazin-2-amine, N-cyclohexyl-4-[2-(difluoromethyl)-1H-benzimidazol-1-yl]-	C21H25F2N7O	429.47	A/B	-8.9
2214	N(C12CC3CC(C2)CC(C1)C3)c1nc(nc(n1)N1CCOCC1)n1c2c(nc1C(F)F)cccc2	1359953-54-0	1,3,5-Triazin-2-amine, 4-[2-(difluoromethyl)-1H-benzimidazol-1-yl]-N-(2-methoxyp	C22H21F2N7O2	453.44	A/B	-8.9
2215	O=c1n([C@H]2O[C@@H](CO[Si](C(C)(C)C)(C)C)SC2)cc(F)c(N)n1	1365246-83-8	Not Yet Assigned	C37H44FN11O15S		A/B	-8.9
2216	C(=O)([C@@H](NC(=O)OC)C(C)C)N1[C@H](c2[nH]c(c3cc4c5-c6c(cc(cc6CC4)c4[nH]c(nc4)[C@H]4N(C(=O)[C@@H](NC	1377035-62-5	Carbamic acid, N,N,Ä≤-[dibenzo[a,c]phenazine-2,7-diylbis[1H-imidazole-5,2-diyl-	C48H52N10O6	864.99	A/B	-8.9
2217	C(=O)([C@@H](NC(=O)OC)C(C)C)N1[C@@H]([C@@H]2C[C@H]1CC2)c1[nH]c(cn1)c1cc2c(-c3c(CC2)cc(cc3)c2[nH]c(nc	1377036-90-2	Carbamic acid, N-[(1S)-2-[(2S)-2-[7-[4-[2-[(1R,3S,4S)-2-[(2S)-2-[(methoxycarbony	C46H54N8O7	830.97	A/B	-8.9
2218	C(=O)([C@H](NCC)c1ccccc1)N1[C@@H](CCC1)c1[nH]c(cn1)c1ccc(cc1)c1cc2c(cc1)cc(cc2)c1[nH]c(nc1)[C@H]1N(C	1377038-38-4	Carbamic acid, N-[(1R)-2-[(2S)-2-[5-[4-[6-[2-[(2S)-1-[(2S)-2-[(methoxycarbonyl)a	C48H50N8O6	834.96	A/B	-8.9
2219	C(=O)([C@@H](NC(=O)OC)C1CCOCC1)N1[C@@H]([C@@H]2C[C@H]1CC2)c1[nH]c(cn1)c1c(F)cc(cc1)c1cc2c(cc1)c1c(cc	1377041-25-2	Carbamic acid, N-[(1S)-1-[[(2S)-2-[5-[7-[2-[(1R,3S,4S)-2-[(2S)-2-[(methoxycarbor	C44H52N8O6	788.93	A/B	-8.9
2220	C(=O)([C@@H](NC(=O)OC)C(C)C)N1[C@@H]([C@H]2C[C@@H]1CC2)c1[nH]c2c(n1)ccc1c2ccc(c1)c1cc2c(cc1)c1c(cc2)	1377042-05-1	Carbamic acid, N-[(1S)-2-[(2S)-2-[2,Ä≤-[(1S,3S,4R)-2-[(2S)-2-[(methoxycarbonyl)a	C48H54N8O7	854.99	A/B	-8.9
2221	C(=O)([C@@H](NC(=O)OC)C1CCOCC1)N1[C@H]2[C@H](C2)C[C@H]1c1[nH]c2c(n1)ccc1c2ccc(c1)c1ccc(cc1)c1[nH]c(n	1377042-94-8	Carbamic acid, N-[(1S)-1-[[(2S)-2-[7-[4-[2-[(2S)-1-[(2S)-2-[(methoxycarbonyl)ami	C48H54N8O6	838.99	A/B	-8.9
2222	C(=O)([C@@H](NC(=O)OC)C(C)C)N1[C@H](c2[nH]c3c4c(cc(cc4)c4cc5c(cc4)c4c(cc5)nc([nH]4)[C@H]4N(C(=O)[C@@	1377044-64-8	Carbamic acid, N-[(1S)-1-[[(2S,3aS,6aS)-hexahydro-2-[2,Ä≤-[(2S)-1-[(2S)-2-[(meth	C47H54N8O6	826.98	A/B	-8.9

2223	C(=O)([C@@H](NC(=O)OC)C(C)C)N1[C@H](c2[nH]c(c3cc4c5-c6c(cc(cc6OC4)c4[nH]c(nc4)[C@H]4N(C(=O)[C@@H](NC	1377045-52-7	Carbamic acid, N,N,Ä≤-[(6,6,Ä≤-difluoro[7,7,Ä≤-bi-1H-naphth[1,2-d]imidazole]-2,	C44H48F2N8O6	822.9	A/B	-8.9
2224	N(C(=O)C)[C@H]1[C@H]([C@@H]([C@@H](CO)O)O)OC(=C[C@H]1NC(=O)C)C(=O)O	1380086-96-3	Furo[3,Ä≤,4,Ä≤:6,7]naphtho[2,3-d]-1,3-dioxol-6(5aH)-one, 5,8,8a,9-tetrahydro-9-	C22H22O5	366.41	A/B	-8.9
2225	C([C@H](OCP(=O)(O)O)C)n1c2c(nc1)c(=O)nc(N)[nH]2	1382469-36-4	2-Pyridinesulfonamide, N-[3-[(1R)-1-[(2R)-3,4-dihydro-6-hydroxy-4-oxo-2-(2-pheny	C31H33F3N2O5S	602.66	A/B	-8.9
2226	O(C(=O)N[C@@H](Cc1ccc(NCCP(=O)(OCc2ccccc2)OCc2ccccc2)cc1)[C@@H](CN(S(=O)(=O)c1ccc(OC)cc1)CC(C)C)O)[C	1383434-05-6	Propanoic acid, 2-[[[2-[[4-[(2S,3R)-2-[[[[(3R,3aS,6aR)-hexahydrofuro[2,3-b]furar	C41H56N3O13PS	861.93	A/B	-8.9
2227	O[C@H]1[C@H](n2c3c(nc2)c(=O)nc(N)[nH]3)O[C@H](COP(=O)(O)O)[C@H]1OP(=O)(OC[C@H]1O[C@H]([C@H](O)[C@@H]	1383925-90-3	Guanosine, 2,Ä≤-deoxy-5,Ä≤-O-phosphonoguanylyl-(3,Ä≤,Üí5,Ä≤)-	C20H26N10O14P2	692.43	A/B	-8.9
2228	O[C@H]1[C@H](n2c3c(nc2)c(=O)nc(N)[nH]3)O[C@H](COP(=O)(O[C@@H]2[C@@H](COP(=O)(O)O)O[C@H](C2)n2c3c(nc2	1383925-94-7	Guanosine, 5,Ä≤-O-phosphono-P-thioguanylyl-(3,Ä≤,Üí5,Ä≤)-	C20H26N10O14P2S	724.49	A/B	-8.9
2229	O(C(=O)N[C@@H](Cc1ccc(C(=O)OC)cc1)[C@@H](CN(S(=O)(=O)c1ccc(OC)cc1)CC(C)C)O)[C@@H]1[C@H]2[C@@H](OC1)O	1384843-99-5	9H-Purin-6-amine, 2-fluoro-9-[5-O-[[(2-methylphenyl)methoxy][(phenylmethyl)aminc	C25H28FN6O6P	558.5	A/B	-8.9
2230	CN1C2(C(C(C(=O)/C=C(\O)/C3C4(c5c(NC4=O)cccc5)N(C)CC3c3cc(O)c(OC)cc3)C1)c1cc(OC)c(O)cc1)c1c(NC2=O)ccc	1389335-22-1	Spiro[2H-indene-2,3,Ä≤(2,Ä≤H)-indolizine]-1,3-dione, 2,Ä≤-[(1Z)-3-[1,Ä≤,5,Ä≤,6,	C49H46N2O10	822.9	A/B	-8.9
2231	N(CC(=O)N1CCN(S(=O)(=O)C)CC1)c1nc(nc(n1)N1CCOCC1)c1ccc(F)nc1	1391924-47-2	Ethanone, 2-[[4-(3-methoxyphenyl)-6-(4-morpholinyl)-1,3,5-triazin-2-yl]amino]-1-	C21H29N7O5S	491.56	A/B	-8.9
2232	N([C@@H](CCSC)C(=O)O)c1nc(nc(n1)N1CCOCC1)c1cc(O)ccc1	1391925-02-2	Benzamide, 3-[4-[(2-amino-2-oxoethyl)amino]-6-(4-morpholinyl)-1,3,5-triazin-2-yl	C16H19N7O3	357.37	A/B	-8.9
2233	C(F)(F)(F)c1c(c2nc(nc(n2)N2CCOCC2)N2CCN(S(=O)(=O)N)CC2)cnc(N)c1	1391926-24-1	2-Pyridinamine, 5-[4-(4-morpholinyl)-6-(1-piperazinyl)-1,3,5-triazin-2-yl]-4-(tr	C17H21F3N8O	410.4	A/B	-8.9
2234	C(F)(F)(F)c1c(c2nc(nc(n2)N2CCOCC2)N2CCN(C)CC2)cnc(N)c1	1391926-26-3	2-Pyridinamine, 5-[4-[4-(methylsulfonyl)-1-piperazinyl]-6-(4-morpholinyl)-1,3,5-	C18H23F3N8O3S	488.49	A/B	-8.9
2235	N(c1c(C)cc(/C=C/C#N)cc1C)c1nc(Nc2ccc(C#N)cc2)ncc1.S(=O)(=O)(O)c1ccccc1	1399384-94-1	Butanedioic acid, compd. with 4-[[4-[[4-[(1E)-2-cyanoethenyl]-2,6-dimethylphenyl	C22H18N6.C4H6O4		A/B	-8.9
2236	O=C1c2n(C[C@H]3N1[C@H](C([2H])([2H])[2H])C(C(O3)([2H])[2H])([2H])[2H])cc(C(=O)NCc1c(F)cc(F)cc1)c(=O)	1407167-23-0	2H-Pyrido[1,Ä≤,2,Ä≤:4,5]pyrazino[2,1-b][1,3]oxazine-2,3,4-d3-9-carboxamide, N-[	C20H11D8F2N3O5		A/B	-8.9
2237	O=C1c2n(C[C@H]3N1[C@H](C([2H])([2H])[2H])CCO3)cc(C(=O)NCc1c(F)cc(F)cc1)c(=O)c2O	1407167-27-4	2H-Pyrido[1,Ä≤,2,Ä≤:4,5]pyrazino[2,1-b][1,3]oxazine-4-d-9-carboxamide, N-[(2,4-	C20H15D4F2N3O5		A/B	-8.9
2238	O=C1c2n(C([C@H]3N1[C@](C)(C(C(O3)([2H])[2H])([2H])[2H])[2H])([2H])[2H])cc(C(=O)NCc1c(F)cc(F)cc1)c(=O	1407167-32-1	2H-Pyrido[1,Ä≤,2,Ä≤:4,5]pyrazino[2,1-b][1,3]oxazine-2,3,12-d3-9-carboxamide, N-	C20H10D9F2N3O5		A/B	-8.9
2239	O=C1c2n(C[C@]3(N1[C@H](C)CCO3)[2H])cc(C(=O)NCc1c(F)cc(F)cc1)c(=O)c2O	1407167-49-0	2H-Pyrido[1,Ä≤,2,Ä≤:4,5]pyrazino[2,1-b][1,3]oxazine-4-d-9-carboxamide, N-[(2,4-	C20H17D2F2N3O5		A/B	-8.9
2240	C(OC[C@@H]1[C@@H](OC(=O)c2ccccc2)[C@@H](O)[C@@H](O1)n1nc(C(=O)N)nc1)(c1ccc(OC)cc1)(c1ccc(OC)cc1)c1cc	140891-07-2	1H-1,2,4-Triazole-3-carboxamide, 1-[2-O-benzoyl-5-O-[bis(4-methoxyphenyl)phenylm	C36H34N4O8	650.68	A/B	-8.9
2241	N(=Nc1ccc(C=Cc2ccc(N=Nc3c4c(cc(S(=O)(=O)O)c3N)cc(S(=O)(=O)O)cc4)cc2)cc1)c1c2c(cc(S(=O)(=O)O)c1N)cc(S	140942-29-6	2-Naphthalenesulfonic acid, 5,5,Ä≤-[1,2-ethanediylbis[(3-sulfo-4,1-phenylene)azc	C34H28N6O12S4.4Na		A/B	-8.9
2242	C(=O)([C@@H](NC(=O)OC)C1CCC(=O)CC1)N1[C@H]2[C@H](C2)C[C@H]1c1[nH]c(cn1)c1ccc(cc1)c1ccc(cc1)c1[nH]c(n	1412812-59-9	Carbamic acid, N,N,Ä≤-[[1,1,Ä≤-biphenyl]-4,4,Ä≤-diylbis[1H-imidazole-5,2-diyl[(	C50H62N8O8	903.08	A/B	-8.9
2243	O[C@H]1[C@@H](O[C@H](C(=O)N[C@H](Cc2c3c([nH]c2)cccc3)C(=O)N2CCN(C(=O)N3CCOCC3)CC2)[C@H]1O)n1nc(C(=O)	1414793-63-7	Not Yet Assigned	C27H32N8O7	580.59	A/B	-8.9
2244	N(=C1c2c(N(CN3CCN(CC3)c3cc4c(cc3F)c(=O)c(C(=O)O)cn4CC)C1=O)ccc(F)c2)NC(=S)N(CC=C)CC=C	1426583-81-4	3-Quinolinecarboxylic acid, 7-[4-[[5-chloro-3-[2-[(diphenylamino)thioxomethyl]hy	C38H33ClFN7O4S	738.23	A/B	-8.9
2245	O(C)c1c2n(cc(C(=O)O)c(=O)c2cc(F)c1N1CC(C)N(CN2c3c(C(=NNC(=S)N(CC=C)CC=C)C2=O)cc(C)cc3)CC1)C1CC1	1426583-89-2	3-Quinolinecarboxylic acid, 1-cyclopropyl-7-[4-[[3-[2-[(diphenylamino)thioxometh	C41H37F2N7O5S	777.84	A/B	-8.9
2246	O[C@H]1[C@@H](O[C@H](COP(=O)(Oc2ccccc2)N[C@H](C(=O)OCc2ccccc2)C)[C@H]1O)n1c(=O)[nH]c(=O)c(I)c1	1427057-11-1	L-Alanine, N-(5-iodo-P-1-naphthalenyl-5,Ä≤-uridylyl)-, phenylmethyl ester	C29H29IN3O10P	737.43	A/B	-8.9
2247	[C@H](Cc1ccccc1)(C(=O)N/C(=C/CS(=O)(=O)c1ccccc1)/CCc1ccccc1)NC(=O)N1CCN(C)CC1	1428441-83-1	4-Morpholinecarboxamide, N-[(1S)-2-oxo-2-[[(1Z)-1-(2-phenylethyl)-3-(phenylsulfc	C31H35N3O5S	561.69	A/B	-8.9
2248	O=c1n([C@@H]2C[C@@H](CO)[C@H](CO)O2)ccc(N)n1	14350-38-0	Alanine, N-[1-[N-[N-[N-[N-[N2-carboxy-N5-(nitroamidino)-L-ornithyl]-L-valyl]-L-t	C54H71N13O13	1110.22	A/B	-8.9
2249	O=c1n([C@H]2O[C@@H](CO)SC2)cc(F)c(=O)[nH]1	1437791-52-0	Pyrimidinium, 4-[[4-[(1E)-2-cyanoethenyl]-2,6-dimethylphenyl]amino]-2-[(4-cyanop	C28H26N6O6		A/B	-8.9
2250	N(Cc1ccc(cc1)c1ccccn1)(C[C@@H]([C@H](Cc1ccccc1)NC(=O)[C@@H](NC(=O)OCO)C(C)(C)C)O)NC(=O)[C@@H](NC(=O)	1443765-35-2	2,5,6,10,13-Pentaazatetradecanedioic acid, 3,12-bis(1,1-dimethylethyl)-8-hydroxy	C38H52N6O8	720.85	A/B	-8.9
2251	C(CCCCC(=O)NCCCOc1c(C)cn(c(=O)n1)[C@@H]1O[C@H](CO)C=C1)N1c2c(N(c3c(C1=O)cccn3)C1CC1)nccc2C	1445477-57-5	6H-Dipyrido[3,2-b:2,Ä≤,3,Ä≤-e][1,4]diazepin-6-one, 11-[2-[[4-[2-[[1,2-dihydro-1	C32H32N6O5S2	644.76	A/B	-8.9
2252	O(c1nc(Nc2ccc(C#N)cc2)nc(N)c1Br)c1c(C)cc(C#N)cc1C.S(=O)(=O)(O)O.CO	1445668-94-9	Benzonitrile, 4-[[6-amino-5-bromo-2-[(4-cyanophenyl)amino]-4-pyrimidinyl]oxy]-3,	C20H15BrN6O.CH4O3S		A/B	-8.9
2253	N(c1c2c(ncc1)cc(Cl)cc2)c1c(O)c(CN2CCOCC2)cc(CN2CCOCC2)c1	145431-61-4	Phenol, 2-[(7-chloro-4-quinolinyl)amino]-4,6-bis[(3,5-dimethyl-1-piperidinyl)met	C31H41ClN4O	521.14	A/B	-8.9
2254	C([C@@H](OCP(=O)(OP(=O)(O)O)O)CO)n1c2c(nc1)c(N)ncn2	1458666-15-3	Benzonitrile, 4-[[4-[[4-[(1Z)-2-cyanoethenyl]-2,6-dimethylphenyl]amino]-6-(4-mor	C25H24N8O	452.51	A/B	-8.9
2255	[14C@H]([14CH2][14c]1[14cH][14cH][14cH][14cH][14cH]1)([14C](=O)N[C@@H](CCc1ccccc1)/C=C/S(=O)(=O)c1cc	1465783-86-1	1-Piperazinecarboxamide, 4-methyl-N-[(1S)-2-oxo-2-[[(1S,2E)-1-(2-phenylethyl)-3-	C32H38N4O4S		A/B	-8.9
2256	C(OP(=O)(O[C@H]1[C@@H](O[C@H](COP(=O)(O[C@H]2[C@@H](O[C@H](CO)[C@H]2O)n2c3c(nc2)c(N)ncn3)O)[C@H]1O)n	1469975-49-2	Œ≤-D-lyxo-Adenosine, 1,Ä≤-[3-(aminocarbonyl)-1H-1,2,4-triazol-1-yl]-1,Ä≤-de(6-a	C28H34N14O16P2	884.6	A/B	-8.9
2257	O=c1n(ccc(N)n1)[C@H]1O[C@@H](C(=O)O[C@@H]2[C@@H](C(C)C)CC[C@H](C)C2)SC1	147104-00-5	Thymidine, 2,Ä≤,3,Ä≤-didehydro-3,Ä≤-deoxythymidylyl-(5,Ä≤,Üí5,Ä≤)-2,Ä≤,3,Ä≤-di	C20H23N4O10P	510.39	A/B	-8.9
2258	N(C(C)C)c1c(nccc1)N1CCN(C(=O)c2cc3c([nH]2)ccc(NS(=O)(=O)CCN2CCOCC2)c3)CC1	147539-93-3	1-Piperidineethanesulfonamide, N-[2-[[4-[3-[(1-methylethyl)amino]-2-pyridinyl]-1	C28H39N7O3S	553.72	A/B	-8.9
2259	O(C(=O)c1ccccc1)[C@@H]1[C@H]([C@H](O[C@@H]2[C@@H](CO)O[C@H](C2)n2cc(C(F)(F)F)c(=O)[nH]c2=O)O[C@H]2[C	148834-35-9	Thymidine, 3,Ä≤-O-[2,3-di-O-benzoyl-4,6-O-(phenylmethylene)-Œ≤-D-glucopyranosyl	C37H33F3N2O12	754.66	A/B	-8.9
2260	N(C(=O)[C@H](Cc1ccccc1)C[C@@H](CN1[C@H](C(=O)NC(C)(C)C)CNCC1)O)[C@H]1c2c(C[C@H]1O)cccc2	150283-74-2	L-Phenylalanine, N-[1-(carboxycarbonyl)-L-prolyl]-, Œ±-(phenylmethyl) ester, 5,Ä	C33H35N7O9		A/B	-8.9
2261	N(=C1c2c(N(C)C1=O)ccc(Cl)c2Cl)NC(=S)Nc1ccc(C)cc1	1507366-78-0	Benzonitrile, 4-[[5-amino-2-[[4-[(1E)-2-cyanoethenyl]-2,6-dimethylphenyl]amino]-	C22H19N7	381.43	A/B	-8.9
2262	O[C@H]1[C@H](n2c3c(nc2)c(=O)nc(N)[nH]3)O[C@H](CCNO[C@@H]2[C@@H](CO)O[C@H](C2)n2c3c(nc2)c(=O)nc(N)[nH	1513874-25-3	Guanosine, 2,Ä≤-deoxy-3,Ä≤-de(phosphinicooxy)guanylyl-1,2-hydrazinediylmethylen	C21H28N12O7	560.52	A/B	-8.9
2263	O(C(OC(=O)CC)C(C)C)c1c(C(=O)NCc2ccc(F)cc2)nc(C(NC(=O)c2nnc(C)o2)(C)C)n(C)c1=O	1538604-26-0	4-Pyrimidinecarboxamide, 5-[1-(benzoyloxy)-2-methylpropoxy]-N-[(4-fluorophenyl)m	C31H33FN6O7	620.63	A/B	-8.9
2264	C(n1c2c(nc1CC)c(C)cc(C)n2)c1ccc(cc1)c1c(S(=O)(=O)NC(=O)CCc2ccccc2)ccc(C(C)C)c1	154552-54-2	Benzenepropanamide, N-[[5-butyl-4,Ä≤-[(2-ethyl-5,7-dimethyl-3H-imidazo[4,5-b]pyr	C36H40N4O3S	608.79	A/B	-8.9
2265	C(n1c2c(nc1CC)c(C)cc(C)n2)c1ccc(cc1)c1c(S(=O)(=O)NC(=O)CCc2ccccc2)ccc(CC(C)C)c1	154552-56-4	Benzenepropanamide, N-[[5-butoxy-4,Ä≤-[(2-ethyl-5,7-dimethyl-3H-imidazo[4,5-b]py	C36H40N4O4S	624.79	A/B	-8.9
2266	C(n1c2c(nc1CC)c(C)cc(C)n2)c1ccc(cc1)c1c(S(=O)(=O)NC(=O)CCc2ccccc2)ccc(c1)c1ccccc1	154552-61-1	Benzenepropanamide, N-[[5-ethoxy-4,Ä≤-[(2-ethyl-5,7-dimethyl-3H-imidazo[4,5-b]py	C34H36N4O4S	596.74	A/B	-8.9
2267	C(n1c2c(nc1CC)c(C)cc(C)n2)c1ccc(cc1)c1c(S(=O)(=O)NC(=O)c2c(OCC)cccc2)ccc(CCC)c1	154552-92-8	Benzamide, 2-ethoxy-N-[[5-ethyl-4,Ä≤-[(2-ethyl-5,7-dimethyl-3H-imidazo[4,5-b]pyr	C34H36N4O4S	596.74	A/B	-8.9
2268	C(n1c2c(nc1CC)c(C)cc(C)n2)c1ccc(cc1)c1c(S(=O)(=O)NC(=O)c2ccccc2)ccc(OCCC)c1	154553-15-8	Benzamide, N-[[4,Ä≤-[(2-ethyl-5,7-dimethyl-3H-imidazo[4,5-b]pyridin-3-yl)methyl]	C33H34N4O3S	566.71	A/B	-8.9
2269	C(n1c2c(nc1CC)c(C)cc(C)n2)c1ccc(cc1)c1c(S(=O)(=O)NC(=O)c2ccccc2)ccc(OCC(F)(F)F)c1	154553-25-0	Benzamide, N-[[5-[(diethylamino)methyl]-4,Ä≤-[(2-ethyl-5,7-dimethyl-3H-imidazo[4	C35H39N5O3S	609.78	A/B	-8.9
2270	C(=O)([C@@H](NC(=O)OC)C(C)C)N1[C@H](c2[nH]c(c3c4c(C5CC4CC5)c(cc3)c3ccc(cc3)c3[nH]c(nc3)[C@H]3N(C(=O)	1560638-77-8	Carbamic acid, N,N,Ä≤-[(1,2,3,4-tetrahydro-1,4-methanonaphthalene-5,8-diyl)bis[4	C51H60N8O6	881.07	A/B	-8.9
2271	C(=O)([C@@H](NC(=O)OC)C(C)C)N1[C@H](c2[nH]c(c3ccc(c4c5c(c(cc4)c4ccc(cc4)c4[nH]c(nc4)[C@H]4N(C(=O)[C@	1560639-80-6	Carbamic acid, N-[(1S)-2-methyl-1-[[(2S)-2-[5-[1,2,3,4-tetrahydro-8-[4-[2-[(8S)-	C47H59N9O8	878.03	A/B	-8.9
2272	C(=O)([C@@H](NC(=O)OC)C(C)C)N1[C@H](c2[nH]c(c3c4c(c(cc3)c3ccc(cc3)c3[nH]c(nc3)[C@H]3N(C(=O)[C@@H](NC	1560640-23-4	Carbamic acid, N-[(1S)-1-[[(2S,4R)-4-fluoro-2-[5-[8-[4-[2-[(2S,4R)-4-fluoro-1-[(	C46H56F2N8O6	854.98	A/B	-8.9
2273	CN1C2c3c(C1CC2)c(ccc3c1[nH]c(nc1)[C@H]1N(C(=O)[C@@H](NC(=O)OC)C(C)C)CC(F)(F)C1)c1ccc(cc1)c1[nH]c(nc1	1560649-39-9	Carbamic acid, N-[(1S)-2-methyl-1-[[(2S)-2-[5-[4-[1,2,3,4-tetrahydro-8-[2-[(2S)-	C45H57N9O6	819.99	A/B	-8.9
2274	CC1C2c3c(c(ccc3c3ccc(cc3)c3[nH]c(nc3)[C@H]3N(C(=O)[C@@H](NC(=O)OC)C(C)C)C[C@@H](C)C3)c3[nH]c(nc3)[C@	1560650-26-1	Carbamic acid, N-[2-methyl-1-[[2-[5-[4-[1,2,3,4-tetrahydro-8-[2-[1-[2-[(methoxyc	C45H56N8O6	804.98	A/B	-8.9
2275	O[C@H]1[C@H](n2c3c(nc2)c(=O)nc(N)[nH]3)O[C@H](COP(=O)(O[C@H]2[C@@H](O)[C@@H](O[C@@H]2CO)n2c3c(nc2)c(	158614-79-0	Guanosine, 5,Ä≤-O-phosphonouridylyl-(3,Ä≤,Üí5,Ä≤)-adenylyl-(3,Ä≤,Üí5,Ä≤)-, homc	(C29H37N12O22P3)x		A/B	-8.9
2276	C(=O)([C@@H](NC(=O)OC)C(C)C)N1[C@H](c2[nH]c(c3c4C5(CCc4c(cc3)c3ccc(cc3)c3[nH]c(nc3)[C@H]3N(C(=O)[C@@	1588403-84-2	Carbamic acid, N-[(1S)-1-[[(2S)-2-[5-[2,Ä≤,3,Ä≤-dihydro-7,Ä≤-[4-[2-[(2S)-1-[(2S	C47H60N8O6	833.03	A/B	-8.9
2277	CN1C2(c3c(CC2)c(ccc3c2ccc(cc2)c2[nH]c(nc2)[C@H]2N(C(=O)[C@@H](NC(=O)OC)C(C)C)CCC2)c2[nH]c(nc2)[C@H]2	1588404-23-2	Carbamic acid, N-[(1S)-1-[[(2S)-2-[5-[4-[2,3-dihydro-4-[2-[(2S)-1-[(2S)-2-[(meth	C47H61N9O6	848.04	A/B	-8.9
2278	O=C1[C@@H]2[C@@H](c3c([C@H](OCc4cn(nn4)c4ccc(Cl)cc4)[C@H]2CO1)cc1c(c3)OCO1)c1cc(OC)c(OC)c(OC)c1	1600500-25-1	Furo[3,Ä≤,4,Ä≤:6,7]naphtho[2,3-d]-1,3-dioxol-6(5aH)-one, 5,8,8a,9-tetrahydro-9-	C32H31N3O9	601.6	A/B	-8.9
2279	C(=O)([C@@H](NC(=O)OC(C)(C)C)C(C)C)N1[C@H](c2[nH]c(c3c4c(C5C(C4)CC5)c(cc3)c3ccc(cc3)c3[nH]c(nc3)[C@H	1613055-16-5	Carbamic acid, N-[(1S)-2-methyl-1-[[(2S)-2-[5-[4-[2,2a,7,7a-tetrahydro-6-[2-[(2S	C45H56N8O6	804.98	A/B	-8.9
2280	O[C@H]1[C@@H](O[C@@H](CO)[C@@H]1O)n1c(=O)nc(N)c(F)c1	1623001-35-3	L-Valine, N,N-bis[(phenylmethoxy)carbonyl]-, 2-[(2-amino-1,6-dihydro-6-oxo-9H-pu	C30H34N6O9	622.63	A/B	-8.9
2281	O=c1n([C@@H]2O[C@H](COP(=O)([O-])[O-])C=C2)cc(C)c(=O)[nH]1	1624279-16-8	Pentanamide, N-[[5-ethyl-4,Ä≤-[(2-ethyl-5,7-dimethyl-3H-imidazo[4,5-b]pyridin-3-	C30H36N4O3S	532.7	A/B	-8.9
2282	N(=N\c1cc(S(=O)(=O)O)c(/C=C/c2c(S(=O)(=O)O)cc(/N=N/c3c4c(cc(S(=O)(=O)O)c3N)cc(S(=O)(=O)O)cc4)cc2)cc1	1639790-17-2	Not Yet Assigned	C31H53N5O5	575.78	A/B	-8.9
2283	C(=O)([C@H](NC(=O)OC)C1CCCCC1)N1[C@@H](C=CC1)c1[nH]c(cn1)c1ccc(cc1)c1ccc(cc1)c1[nH]c(nc1)[C@H]1N(C(=	1639871-23-0	Carbamic acid, N-[(1S)-1-cyclohexyl-2-[(2S)-2,5-dihydro-2-[5-[4,Ä≤-[2-[(2S)-1-[	C43H52N8O6	776.92	A/B	-8.9
2284	N(c1nc(nc(Oc2ccc(C#N)cc2)n1)N1CCOCC1)c1cc(Cl)c(F)cc1	1643332-86-8	Benzonitrile, 4-[[4-[(4-fluorophenyl)amino]-6-(4-morpholinyl)-1,3,5-triazin-2-yl	C20H17FN6O2	392.39	A/B	-8.9
2285	O=c1n([C@@H]2O[C@H](COP(=O)(C(O)c3ccc(N(=O)=O)cc3)OC[C@H]3O[C@H](C=C3)n3c(=O)[nH]c(=O)c(C)c3)C=C2)cc	164652-05-5	Thymidine, P-[(4-cyanophenyl)hydroxymethyl]-2,Ä≤,3,Ä≤-didehydro-P,3,Ä≤-dideoxyt	C28H28N5O10P	625.52	A/B	-8.9
2286	N(C(=O)[C@H](Cc1ccccc1)C[C@@H](CN1[C@H](C(=O)NC(C)(C)C)CN(Cc2c(O)c(C(C)(C)C)cc(C(C)(C)C)c2)CC1)O)[C@	164725-10-4	1-Piperazinepentanamide, N-(2,3-dihydro-2-hydroxy-1H-inden-1-yl)-2-[[(1,1-dimeth	C38H57N5O4	647.89	A/B	-8.9
2287	N(C(=O)[C@H](Cc1ccccc1)C[C@@H](CN1[C@H](C(=O)NC(C)(C)C)CN(CCOCCOCCOC)CC1)O)[C@H]1c2c(C[C@H]1O)cccc2	164725-25-1	1-Piperazinepentanamide, N-(2,3-dihydro-2-hydroxy-1H-inden-1-yl)-2-[[(1,1-dimeth	C36H47N5O4	613.79	A/B	-8.9
2288	N(C(=O)[C@H](Cc1ccccc1)C[C@@H](CN1[C@H](C(=O)NC(C)(C)C)CN(Cc2[nH]c3c(n2)cccc3)CC1)O)[C@H]1c2c(C[C@H]	164725-30-8	1-Piperazinepentanamide, N-(2,3-dihydro-2-hydroxy-1H-inden-1-yl)-2-[[(1,1-dimeth	C39H52N4O5	656.85	A/B	-8.9
2289	N(C(=O)[C@H](Cc1ccccc1)C[C@@H](CN1[C@H](C(=O)NC(C)(C)C)CN(Cc2cc(F)ccc2)CC1)O)[C@H]1c2c(C[C@H]1O)cccc	164725-43-3	1-Piperazinepentanamide, N-(2,3-dihydro-2-hydroxy-1H-inden-1-yl)-2-[[(1,1-dimeth	C36H48N6O4	628.8	A/B	-8.9
2290	N(C(=O)[C@H](Cc1ccccc1)C[C@@H](CN1[C@H](C(=O)NC(C)(C)C)CN(Cc2cc(C(=O)OC)cnc2C)CC1)O)[C@H]1c2c(C[C@H]	164725-58-0	D-erythro-Pentonamide, 2,3,5-trideoxy-5-[(2S)-4-[(3,4-dichlorophenyl)methyl]-2-[	C37H46Cl2N4O4	681.69	A/B	-8.9
2291	N(C(=O)C(Cc1ccccc1)CC(CN1C(C(=O)NC(C)(C)C)CN(CC2c3c(CC2)cccc3)CC1)O)C1c2c(CC1O)cccc2	164725-63-7	1-Piperazinepentanamide, N-(2,3-dihydro-2-hydroxy-1H-inden-1-yl)-2-[[(1,1-dimeth	C37H48N4O4	612.8	A/B	-8.9
2292	C(COCP(=O)(OCCC)O)n1c2c(nc1)c(N)ncn2	164725-67-1	1-Piperazinepentanamide, 4-(cyclohexylmethyl)-N-(2,3-dihydro-2-hydroxy-1H-inden-	C37H54N4O4	618.85	A/B	-8.9
2293	C(=O)([C@@H](NC(=O)OC)C(C)C)N1[C@@H](C[C@H]2[C@@H]1CCC2)c1[nH]c2c(n1)ccc1c2ccc(c1)c1ccc(cc1)c1[nH]c(	1653966-67-6	Carbamic acid, N-[(1S)-1-[[(2S,3aS,7aS)-2-[4,5-dihydro-7-[4-[2-[(3S,5S)-2-[(2S)-	C50H64N8O7	889.09	A/B	-8.9
2294	C(=O)([C@@H](NC(=O)OC)C(C)C)N1C[C@@]2(C[C@H]1c1[nH]c(cn1)c1cc3c(cc1)c1c(cc3)cc(cc1)c1[nH]c(nc1)[C@H]	1653966-75-6	Carbamic acid, N-[(1S)-1-[[(2S,3aS,6aS)-hexahydro-2-[7-[4-[2-[(3S,5S)-2-[(2S)-2-	C49H60N8O7	873.05	A/B	-8.9
2295	C(=O)([C@@H](NC(=O)OC)C(C)C)N1C[C@@]2(C[C@H]1c1[nH]c(cn1)c1ccc(cc1)c1cc3c(cc1)c1c(cc3)nc([nH]1)[C@H]	1653966-79-0	Carbamic acid, N-[(1S)-1-[[(3S,5S)-3-[5-[4-[2-[(1R,3S,5R)-2-[(2S)-2-[(methoxycar	C47H56N8O7	845	A/B	-8.9
2296	N(C(=O)[C@H](Cc1ccccc1)C[C@@H](CN1[C@H](C(=O)NC(C)(C)C)CN(Cc2scc(Br)c2)CC1)O)[C@H]1c2c(C[C@H]1O)cccc	165946-08-7	1-Piperazinepentanamide, 4-[(2,4-difluorophenyl)methyl]-N-(2,3-dihydro-2-hydroxy	C37H46F2N4O4	648.78	A/B	-8.9
2297	N(C(=O)[C@H](Cc1ccccc1)C[C@@H](CN1[C@H](C(=O)NC(C)(C)C)CN(Cc2cccs2)CC1)O)[C@H]1c2c(C[C@H]1O)cccc2	165946-09-8	D-erythro-Pentonamide, 5-[(2S)-4-[(4-bromo-2-thienyl)methyl]-2-[[(1,1-dimethylet	C35H45BrN4O4S	697.73	A/B	-8.9
2298	O(C(=O)N[C@@H](Cc1ccc(OC)cc1)[C@@H](CN(S(=O)(=O)c1cc2c(cc1)nc(NC(C)C)s2)CC(C)C)O)[C@@H]1[C@H]2[C@@H]	1672661-56-1	Carbamic acid, N-[(1S,2R)-2-hydroxy-3-[(2-methylpropyl)[[2-(4-piperidinylamino)-	C33H43F2N5O7S2	723.85	A/B	-8.9
2299	C(n1c2c(nc1CC)c(C)cc(C)n2)c1ccc(cc1)c1c(S(=O)(=O)NP(=O)(OCc2ccccc2)OCc2ccccc2)cccc1	169176-86-7	1-Piperazinepentanamide, N-(2,3-dihydro-2-hydroxy-1H-inden-1-yl)-2-[[(1,1-dimeth	C37H49N5O4	627.82	A/B	-8.9
2300	C(n1c2c(nc1CC)c(C)cc(C)n2)c1ccc(cc1)c1c(S(=O)(=O)NC(=O)OCCCC)ccc(CC(C)C)c1	169281-91-8	Carbamic acid, [[4,Ä≤-[(2-ethyl-5,7-dimethyl-3H-imidazo[4,5-b]pyridin-3-yl)methy	C29H34N4O4S	534.67	A/B	-8.9
2301	O(c1c(Br)c(Oc2c(C)cc(C#N)cc2C)nc(Nc2ccc(C#N)cc2)n1)c1c(C)cc(C#N)cc1C	1704729-90-7	Benzamide, 4-[[6-amino-2-[[4-(aminocarbonyl)phenyl]amino]-5-bromo-4-pyrimidinyl]	C20H19BrN6O3	471.31	A/B	-8.9
2302	C(CC(COC(=O)[C@H]1N(C(=O)OCc2ccccc2)CCC1)(CO)CO)n1c2c(nc1)c(=O)nc(N)[nH]2	172645-41-9	1,2-Pyrrolidinedicarboxylic acid, 2,2,Ä≤-[2-[2-(2-amino-1,6-dihydro-6-oxo-9H-pu	C37H43N7O10	745.78	A/B	-8.9
2303	N(C(=O)[C@H](Cc1ccccc1)C[C@@H](CN1[C@H](C(=O)NC(C)(C)C)CN(Cc2nc(sc2)C2CCCC2)CC1)O)[C@H]1c2c(C[C@H]1O	172649-55-7	1-Piperazinepentanamide, N-(2,3-dihydro-2-hydroxy-1H-inden-1-yl)-2-[[(1,1-dimeth	C36H49N5O5S	663.87	A/B	-8.9
2304	[Sn](CCCC)(CCCC)(CCCC)c1cn(c(=O)[nH]c1=O)[C@@H]1O[C@H](CO)[C@@H](O)C1	172669-12-4	1-Naphthalenesulfonic acid, 8-amino-5-[2-(1-naphthalenyl)diazenyl]-	C20H15N3O3S	377.42	A/B	-8.9
2305	C(Cc1ccc(F)cc1)[C@@]1(CCC)CC(=C([C@@H](CC)c2cc(NS(=O)(=O)c3ccc(C#N)cn3)ccc2)C(=O)O1)O	174484-73-2	2-Pyridinesulfonamide, 5-cyano-N-[3-[1-[6-[2-(4-fluorophenyl)ethyl]-5,6-dihydro-	C31H32FN3O5S	577.67	A/B	-8.9
2306	C(n1c2c(nc1CC)c(C)cc(C)n2)c1ccc(cc1)c1c(S(=O)(=O)NC(=O)c2cc(O[11CH3])ccc2)cccc1	174844-65-6	Benzamide, N-[[4,Ä≤-[(2-ethyl-5,7-dimethyl-3H-imidazo[4,5-b]pyridin-3-yl)methyl]	C31H30N4O4S		A/B	-8.9
2307	C(n1c2c(nc1CC)c(C)cc(C)n2)c1ccc(cc1)c1c(S(=O)(=O)NC(=O)c2c(O)cccc2)cccc1	174844-66-7	Benzamide, N-[[4,Ä≤-[(2-ethyl-5,7-dimethyl-3H-imidazo[4,5-b]pyridin-3-yl)methyl]	C31H30N4O4S		A/B	-8.9
2308	C(O[C@H](COC(=O)[C@@H](NC(=O)OCc1ccccc1)C(C)C)COCc1ccccc1)n1c2c(nc1)c(=O)nc(N)[nH]2	175865-55-1	L-Valine, N-[(phenylmethoxy)carbonyl]-, 2-[(2-amino-1,6-dihydro-6-oxo-9H-purin-9	C29H34N6O7	578.62	A/B	-8.9
2309	O=c1c2c(n(cn2)[C@@H]2O[C@H](COP(=O)(OP(=O)(OC[C@H]3O[C@H](CC3)n3c(=O)nc(N)cc3)O)O)CC2)[nH]cn1	175923-22-5	Inosine 5,Ä≤-(trihydrogen diphosphate), 2,Ä≤,3,Ä≤-dideoxy-, P,Ä≤,Üí5,Ä≤-ester w	C20H24N8O11P2	614.4	A/B	-8.9
2310	[C@H](Cc1ccccc1)(C[C@@H]([C@H](Cc1ccccc1)NC(=O)OCc1cncs1)O)NC(=O)[C@@H](NC(=O)N(Cc1nc(C(C)(C)O)sc1)C	176590-65-1	Furo[3,Ä≤,4,Ä≤:6,7]naphtho[2,3-d]-1,3-dioxole-5,8-dione, 9-[4-(acetyloxy)phenyl	C21H16O7	380.35	A/B	-8.9
2311	Oc1c2c(c(c3c1C(=O)OC3)c1cc3c(cc1)OCO3)cc(OC)c(OC)c2	177994-55-7	1-Piperazinepentanamide, N-(2,3-dihydro-2-hydroxy-3-oxo-1H-inden-1-yl)-2-[[(1,1-	C36H45N5O5	627.77	A/B	-8.9
2312	O(c1nc(nc(Nc2ccc(C#N)cc2)n1)N1CCOCC1)c1c(C)cc(C#N)cc1C	1799294-47-5	Benzonitrile, 4-[[4-(4-morpholinyl)-6-(2,4,6-trimethylphenoxy)-1,3,5-triazin-2-y	C23H24N6O2	416.48	A/B	-8.9
2313	C(C)(C)(C)c1ccc(N2[C@@H](CC[C@H]2c2ccc(NC(=O)[C@H]3N(C(=O)[C@@H](NC(=O)OC)C(C)C)CCC3)cc2)c2ccc(NC(=O	1799294-65-7	Benzonitrile, 4-[[4-[(4-cyanophenyl)amino]-6-(4-morpholinyl)-1,3,5-triazin-2-yl]	C23H21N7O2	427.46	A/B	-8.9
2314	C(C[C@H](C[C@H](CCc1cc(O)c(O)cc1)O)O)c1cc(O)c(O)cc1	1801161-79-4	Carbamic acid, N-[(1S)-2-methyl-1-[[(2S)-2-[5-[9,9,10,10-tetrafluoro-9,10-dihydr	C44H52F4N8O7	880.93	A/B	-8.9
2315	O(C(C)(C)C)c1c(C(=O)NCc2ccc(F)cc2)nc(C(NC(=O)c2nnc(C)o2)(C)C)n(C)c1=O	1801912-40-2	Carbonic acid, 4-[[[(4-fluorophenyl)methyl]amino]carbonyl]-1,6-dihydro-1-methyl-	C28H27FN6O7	578.55	A/B	-8.9
2316	O=C1[C@]2([C@H]([C@H](/C(=C/C(=O)[C@@H]3[C@@]4(N5[C@@H]([C@H]3c3cc(OC)c(O)cc3)CCC5)c3c(NC4=O)ccc(I)c	1807888-26-1	Not Yet Assigned	C45H42I2N4O8	1020.65	A/B	-8.9
2317	C(=O)([C@@H](NC(=O)OC)C(C)C)N1C(C2CC1CC2)c1[nH]c(cn1)c1ccc(cc1)c1ccc(cc1)c1[nH]c(nc1)[C@H]1N(C(=O)[C	1808935-13-8	Carbamic acid, N,N,Ä≤-[[1,1,Ä≤-biphenyl]-4,4,Ä≤-diylbis[1H-imidazole-5,2-diyl-2	C44H54N8O6	790.95	A/B	-8.9
2318	O=C1[C@@H]2[C@@H](c3c([C@H](OC(=O)C(C)N4C(=O)c5c(C4=O)cccc5)[C@H]2CO1)cc1c(c3)OCO1)c1cc(OC)c(OC)c(OC	1809321-42-3	2H-Isoindole-2-acetic acid, octahydro-1,3-dioxo-Œ±-(phenylmethyl)-, (5R,5aR,8aR,	C39H39NO11	697.73	A/B	-8.9
2319	O=C1[C@@H]2[C@@H](c3c([C@H](OC(=O)[C@H](Cc4ccccc4)N4C(=O)C5C(C4=O)C4OC5CC4)[C@H]2CO1)cc1c(c3)OCO1)c1	1809325-26-5	4,7-Epoxy-2H-isoindole-2-acetic acid, octahydro-1,3-dioxo-Œ±-(phenylmethyl)-, (5	C39H37NO12	711.71	A/B	-8.9
2320	C[C@@]12[C@@H]3[C@]4([C@H]5[C@@H]([C@@H](C3)C(=C4)C(C)C)C(=O)CC/C/5=N\O)CC[C@H]1[C@@](C(=O)OC)(C)CCC	1810049-73-0	Furo[3,Ä≤,4,Ä≤:6,7]naphtho[2,3-d]-1,3-dioxol-6(5aH)-one, 5-(1,3-benzodioxol-5-y	C20H16O7	368.34	A/B	-8.9
2321	O=C1[C@@H]2[C@H](c3c([C@H](O)[C@H]2CO1)cc1c(c3)OCO1)c1ccc2c(cc1)OCO2	1835316-38-5	Furo[3,Ä≤,4,Ä≤:6,7]naphtho[2,3-d]-1,3-dioxole-5,8-dione, 9-(1,3-benzodioxol-5-y	C20H14O7	366.32	A/B	-8.9
2322	C(C(C(=C)C)O)c1c(OC)c(C(=O)/C=C/c2c(OC)cc(OC)cc2)ccc1OC	183541-58-4	Guanosine, 2,Ä≤-deoxyguanylyl-(5,Ä≤,Üí5,Ä≤)-2,Ä≤-deoxy-	C20H25N10O10P	596.45	A/B	-8.9
2323	O=c1n([C@@H]2O[C@H](COC(=O)CC(CC(=O)O)(C(=O)O)O)C=C2)cc(C)c(=O)[nH]1	184834-50-2	Thymidine, 2,Ä≤,3,Ä≤-didehydro-3,Ä≤-deoxy-, 5,Ä≤-[2,3-bis(phenylmethyl) 2-hydrc	C30H30N2O10	578.57	A/B	-8.9
2324	C(OC[C@@H]1[C@@H](OC(=O)c2cccnc2)C[C@@H](C1=C)n1c2c(nc1)c(=O)nc(NC(=O)OC(C)(C)C)[nH]2)(c1ccccc1)(c1c	1860842-94-9	Acetic acid, 2-ethoxy-, (1S,2R,4S)-4-[2-[[(1,1-dimethylethoxy)carbonyl]amino]-1,	C40H43N5O7	705.8	A/B	-8.9
2325	O=C1[C@@H]2[C@@H](c3c([C@H](O[C@H]4[C@H](OC(=O)c5ccccc5)[C@@H](OC(=O)c5ccccc5)[C@H](COC(=O)c5ccccc5)	1883269-83-7	Furo[3,Ä≤,4,Ä≤:6,7]naphtho[2,3-d]-1,3-dioxol-6(5aH)-one, 5,8,8a,9-tetrahydro-9-	C48H42O15	858.84	A/B	-8.9
2326	O=c1n([C@@H]2O[C@H](COP3(=O)Oc4c(CO3)cccc4)C=C2)cc(C)c(=O)[nH]1	188548-78-9	Thymidine, 5,Ä≤-O-(6-chloro-2-oxido-4H-1,3,2-benzodioxaphosphorin-2-yl)-2,Ä≤,3,	C17H16ClN2O7P	426.74	A/B	-8.9
2327	O=c1n(c2c(c(cc(n2)c2ccc(Cl)cc2)c2ccc(F)cc2)c(=O)n1c1ccccc1)[C@H]1[C@H](OC(=O)c2ccccc2)[C@H](OC(=O)c2	190319-84-7	Pyrido[2,3-d]pyrimidine-2,4(1H,3H)-dione, 3,7-bis(4-chlorophenyl)-5-phenyl-1-(2,	C51H35Cl2N3O9	904.74	A/B	-8.9
2328	O=c1c2c(n(cn2)[C@@H]2O[C@H](COP(=O)(Oc3ccc(Cl)cc3)N3CCN(CC3)c3ccc(F)cc3)CC2)[nH]cn1	1922936-31-9	Phosphoramidic acid, N-(2,4-difluorophenyl)-, 4-chlorophenyl [(2S,5R)-5-(1,6-dih	C22H19ClF2N5O5P	537.84	A/B	-8.9
2329	[C@@H](C(=O)N[C@@H](Cc1ccccc1)C[C@@H]([C@H](Cc1ccccc1)NC(=O)COc1c(C)cccc1C)O)(C(C)C)N1C(=S)NCC1	192725-23-8	1-Imidazolidineacetamide, Œ±-(1,1-dimethylethyl)-N-[(1S,3S,4S)-4-[[2-(2,6-dimeth	C37H48N4O5	628.8	A/B	-8.9
2330	C(=C1C=C(C(=O)[O-][Zr+2](=O)([OH-])([OH2])([OH2])[OH2])C(=O)C=C1)(c1cc2c(cc1)[O-][Zr+2](=O)([NH2]c1c	1969-54-6	Thymidine, thymidylyl-(3,Ä≤,Üí5,Ä≤)-	C20H27N4O12P	546.42	A/B	-8.9
2331	N(c1c(C#N)cccc1)c1nc(Nc2c(C)cc(C)cc2)nc(C)c1	1984373-08-1	Benzonitrile, 2-[[4-[(2,4-dimethylphenyl)amino]-6-methyl-2-pyrimidinyl]amino]-	C20H19N5	329.4	A/B	-8.9
2332	O([C@H](C)c1ccccc1)c1c2n(N([C@H]3N(C2=O)CCOC3)[C@H]2c3c(CSc4c2cccc4)c(F)c(F)cc3)ccc1=O	1985607-43-9	1H-[1,4]Oxazino[3,4-c]pyrido[2,1-f][1,2,4]triazine-6,8-dione, 12-[(11S)-7,8-difl	C32H27F2N3O4S	587.64	A/B	-8.9
2333	N(C(=O)Nc1cc(C(=O)OC)ccc1C)c1cc2cc(C34CC5CC(C4)CC(C3)C5)[nH]c2cc1	1987854-69-2	Urea, N-(4-chloro-2-methylphenyl)-N,Ä≤-(2-tricyclo[3.3.1.13,7]dec-1-yl-1H-indol-	C26H28ClN3O	433.97	A/B	-8.9
2334	N(C(=O)NC12CC3CC(C2)CC(C1)C3)c1cc2cc(C34CC5CC(C4)CC(C3)C5)[nH]c2cc1	1987854-76-1	Urea, N-[(4-fluorophenyl)methyl]-N,Ä≤-(2-tricyclo[3.3.1.13,7]dec-1-yl-1H-indol-5	C26H28FN3O	417.52	A/B	-8.9
2335	O=C1[C@@H]2[C@@H](c3c([C@H](OC(=O)c4c5c(n(C)n4)cccc5)[C@H]2CO1)cc1c(c3)OCO1)c1cc(OC)c(OC)c(OC)c1	1989611-39-3	Benzo[b]thiophene-2-carboxylic acid, (5R,5aR,8aR,9R)-5,5a,6,8,8a,9-hexahydro-8-c	C31H26O9S	574.6	A/B	-8.9
2336	C(=O)([C@@H](NC(=O)OC)C(C)C)N1[C@H](c2[nH]c3-c4c(cc(cc4)c4c5c(c(cc4)c4[nH]c(nc4)[C@H]4N(C(=O)[C@@H](	2007900-97-0	Carbamic acid, N-[(1S)-1-[[(2S,4R)-2-[5-[2,3-dihydro-7-[2-[(2S)-1-[(2S)-2-[(meth	C45H53FN8O6	820.95	A/B	-8.9
2337	C(=O)([C@@H](NC(=O)OC)C(C)C)N1[C@H](c2[nH]c3c4c(cc(cc4)c4c5c(c(cc4)c4[nH]c(nc4)[C@H]4N(C(=O)[C@@H](N	2007901-63-3	Carbamic acid, N-[(1S)-1-[[(2S)-2-[7-[2,3-dihydro-7-[2-[(1R,2S,5S)-3-[(2S)-2-[(m	C46H54N8O6	814.97	A/B	-8.9
2338	O(C)C1c2c(c(ccc2c2[nH]c(nc2)[C@H]2N(C(=O)[C@@H](NC(=O)OC)C(C)C)CCC2)c2cc3c(-c4c(CC3)nc([nH]4)[C@H]3N	2007901-75-7	Carbamic acid, N-[(1S)-1-[[(2S)-2-[7-[4-[2-[(2S)-1-[(2S)-2-cyclohexyl-2-[(methox	C50H62N8O6	871.08	A/B	-8.9
2339	Cc1c2c(COP(=O)(OC[C@H]3O[C@H](C=C3)n3c(=O)[nH]c(=O)c(C)c3)O2)cc(C)c1	201166-00-9	Thymidine, 2,Ä≤,3,Ä≤-didehydro-3,Ä≤-deoxy-5,Ä≤-O-(8-methyl-2-oxido-4H-1,3,2-ber	C18H19N2O7P	406.33	A/B	-8.9

2340	C(=O)(C(=O)Nc1ccc(C)cc1)c1c(C23CC4CC(C3)CC(C2)C4)[nH]c2c1cccc2	2014403-77-9	1H-Indole-3-acetamide, Œ±-oxo-N-phenyl-2-tricyclo[3.3.1.13,7]dec-1-yl-	C26H26N2O2	398.5	A/B	-8.9
2341	C(=O)(C(=O)Nc1cc(C(F)(F)F)ccc1)c1c(C23CC4CC(C3)CC(C2)C4)[nH]c2c1cccc2	2014403-79-1	1H-Indole-3-acetamide, N-(2-methoxyphenyl)-Œ±-oxo-2-tricyclo[3.3.1.13,7]dec-1-yl	C27H28N2O3	428.52	A/B	-8.9
2342	C(=O)(C(=O)Nc1c(F)cc(Br)cc1)c1c([nH]c2c1cccc2)C12CC3CC(C2)CC(C1)C3	2014403-89-3	1H-Indole-3-acetamide, N-(3-chloro-4-fluorophenyl)-Œ±-oxo-2-tricyclo[3.3.1.13,7]	C26H24ClFN2O2	450.93	A/B	-8.9
2343	O=c1n([C@@H]2O[C@H](COC(=O)CCC(=O)Oc3c(OC)ccc(C(=O)c4c(OC)cccc4)c3)C=C2)cc(C)c(=O)[nH]1	2015224-14-1	Butanedioic acid, 1-[[(2S,5R)-5-(3,4-dihydro-5-methyl-2,4-dioxo-1(2H)-pyrimidiny	C28H25N3O11	579.51	A/B	-8.9
2344	[C@H](C(C)C)(CO)n1c2c(cc(Cc3cc(cc(Cl)c3F)N3CC[C@@H](O)C3)c(OC)c2)c(=O)c(C(=O)O)c1	1000161-59-0	3-Quinolinecarboxylic acid, 6-[[3-chloro-2-fluoro-5-(2-oxo-1-piperidinyl)phenyl]	C28H30ClFN2O6	545	C	-8.9
2345	[C@H](C(C)C)(CO)n1c2c(cc(Cc3cc(cc(Cl)c3F)c3nccs3)c(OC)c2)c(=O)c(C(=O)O)c1	1000161-85-2	3-Quinolinecarboxylic acid, 6-[[3-chloro-2-fluoro-5-(2-pyridinyl)phenyl]methyl]-	C28H26ClFN2O5	524.97	C	-8.9
2346	O[C@H]1[C@@H](O[C@H](COC(=O)CCCCO[C@@H]2O[C@H](CO)[C@H](O)[C@H](O)[C@H]2O)[C@H]1O)n1nc(C(=O)N)nc1	1002334-91-9	1H-1,2,4-Triazole-3-carboxamide, 1-[3-O-[[4-[[2-(Œ≤-D-galactopyranosyloxy)acetyl	C23H31N5O12	569.52	C	-8.9
2347	C(OC(=O)C(CO[C@H]1[C@H](OCc2ccccc2)[C@@H](OCc2ccccc2)[C@@H](OCc2ccccc2)[C@@H](COCc2ccccc2)O1)(C)C)[C	1002336-41-5	1H-1,2,4-Triazole-3-carboxamide, 1-[2,3-O-(methoxymethylene)-5-O-[(2S)-2-methyl-	C27H34N4O13	622.58	C	-8.9
2348	N(C(=O)COC(=O)Cn1c2c(oc1=O)cccc2)C12CC3CC(C2)CC(C1)C3	1002355-35-2	1H-1,2,4-Triazole-3-carboxamide, 1-[2,3-O-(methoxymethylene)-5-O-[[4-[[4,6-O-[(S	C30H38N4O13	662.64	C	-8.9
2349	C(=O)([C@H](N1CCCC1)c1ccccc1)N1[C@@H](CCC1)c1[nH]c(cn1)c1ccc(cc1)c1ccc(cc1)c1[nH]c(nc1)[C@H]1N(C(=O)	1009111-82-3	Carbamic acid, N-[(1S)-1-methyl-2-oxo-2-[(2S)-2-[5-[4,Ä≤-[2-[(2S)-1-[(2R)-2-pher	C43H48N8O4	740.89	C	-8.9
2350	C(=O)([C@@H](NC(=O)OCCC=C)C(C)C)N1[C@@H](CCC1)c1[nH]c(cn1)c1ccc(cc1)c1ccc(cc1)c1[nH]c(nc1)[C@H]1N(C(	1009113-22-7	Carbamic acid, N,N,Ä≤-[(2-methyl[1,1,Ä≤-biphenyl]-4,4,Ä≤-diyl)bis[1H-imidazole-	C47H48N8O6	820.93	C	-8.9
2351	C(=O)([C@@H](Nc1cncnc1)C(C)C)N1[C@@H](CCC1)c1[nH]c(cn1)c1ccc(cc1)c1ccc(cc1)c1[nH]c(nc1)[C@H]1N(C(=O)	1009114-17-3	Carbamic acid, N-[(1S)-1-methyl-2-[(2S)-2-[5-[4,Ä≤-[2-[(2S)-1-[(2S)-3-methyl-1-c	C41H47N9O4	729.87	C	-8.9
2352	C(=O)([C@H](Cc1cccnc1)NC(=O)OC)N1[C@@H](CCC1)c1[nH]c(cn1)c1ccc(cc1)c1ccc(cc1)c1[nH]c(nc1)[C@H]1N(C(=	1009114-63-9	Carbamic acid, N-[(1S)-2-[(2S)-2-[5-[4,Ä≤-[2-[(2S)-1-[[cis-4-(diethylamino)cyclc	C42H54N8O4	734.93	C	-8.9
2353	C(=O)([C@H](NC(=O)OC)c1ccccc1)N1[C@@H](CCC1)c1[nH]c(cn1)c1cc(ccc1)c1ccc(cc1)c1[nH]c(nc1)[C@H]1N(C(=O	1009119-85-0	Carbamic acid, N,N,Ä≤-[[1,1,Ä≤-biphenyl]-3,3,Ä≤-diylbis[1H-imidazole-5,2-diyl-(	C46H46N8O6	806.91	C	-8.9
2354	Nc1c2c(c(S(=O)(=O)O)cc1N=Nc1ccc(N(=O)=O)cc1)ccc(S(=O)(=O)O)c2.[NaH]	10114-95-1	2,7-Naphthalenedisulfonic acid, 4-[2-(4-amino-1-naphthalenyl)diazenyl]-, sodium	C20H15N3O6S2.2Na		C	-8.9
2355	C(=O)(N[C@H](C(=O)C(=O)NC1CC1)CCC)[C@@H]1[C@@H]2[C@H](CN1C(=O)[C@@H](NC(=O)[C@@H](NC(=O)c1cnccn1)C1C	1015080-35-9	Butanediamide, N1-[(1S,2R)-3-[(3S,4aS,8aS)-3-[[(1,1-dimethylethyl)amino]carbonyl	C38H50N6O5.C27H45N5O5		C	-8.9
2356	C(=O)([C@@H](NC(=O)OC(C)(C)C)C(C)(C)O)N1[C@@H](CCC1)c1[nH]c(cn1)c1ccc(cc1)c1ccc(cc1)c1[nH]c(nc1)[C@H	1017607-51-0	1-Pyrrolidinecarboxylic acid, 2-[5-[4,Ä≤-[2-[(2S)-1-[(2S)-2-[(methoxycarbonyl)ar	C36H43N7O5	653.77	C	-8.9
2357	O([C@H](CC)C=C)[C@H]1[C@H](NC(=O)C)[C@@H](N)CC(=C1)C(=O)O	10211-53-7	2-Naphthalenesulfonic acid, 5-amino-8-[2-[4-[2-[2,4-diamino-5-[2-[3-[2-(2,4-diam	C35H31N13O6S2.2Na		C	-8.9
2358	C(=C1CC(C(=O)O)C(=O)CC1)(c1cc(C(=O)O)c(O)cc1)c1cc(C(=O)O)c(O)cc1	10241-23-3	2,6-Naphthalenedisulfonic acid, 4-[2-(4-amino-1-naphthalenyl)diazenyl]-, sodium	C20H15N3O6S2.2Na		C	-8.9
2359	C(C(C)C)n1c2c(nc1N1CCCC1)c(N)nc1c2cccc1	1026369-91-4	2-Naphthalenesulfonic acid, 8-amino-5-[2-(4,Ä≤-hydroxy[1,1,Ä≤-biphenyl]-4-yl)hy	C22H19N3O4S	421.47	C	-8.9
2360	C(NC(=O)Oc1cc([C@@H](N(C)C)C)ccc1)(C)C12CC3CC(C2)CC(C1)C3	1046462-11-6	Guanosine, 1-[(11-cyclopropyl-6,11-dihydro-6-oxo-5H-dipyrido[3,2-b:2,Ä≤,3,Ä≤-e]	C25H25N9O5	531.52	C	-8.9
2361	S(=O)(=O)(O)c1c2c(c(S(=O)(=O)O)cc(/N=N/c3cc(S(=O)(=O)O)c(CCc4c(S(=O)(=O)O)cc(/N=N/c5c(N)c6c(c(S(=O)(	1052521-54-6	Not Yet Assigned	C32H24N6O14S5.Na		C	-8.9
2362	O=c1n([C@@H]2O[C@H](CO)[C@@H](O)C2)cc(CCCF)c(=O)[nH]1	1058093-85-8	Not Yet Assigned	C37H58N6O6	682.89	C	-8.9
2363	O=C1[C@@H]2[C@@H](c3c([C@H](OC(=O)C(=O)c4c5c(n(Cc6cc7c(cc6)OCO7)c4)cccc5)[C@H]2CO1)cc1c(c3)OCO1)c1cc	1061717-07-4	1H-Indole-3-acetic acid, 1-[(3,4-dichlorophenyl)methyl]-Œ±-oxo-, (5R,5aR,8aR,9R)	C39H31Cl2NO10	744.57	C	-8.9
2364	C(C(C=CC(=O)CCc1ccccc1)O)c1cc(OC)c(O)cc1	1092440-51-1	2-Naphthalenesulfonic acid, 6-amino-5-[2-[2,Ä≤,3-dimethyl-4,Ä≤-[2-(6-sulfo-1-na	C34H27N5O6S2	665.74	C	-8.9
2365	C(C)(C)(C)c1c2c(cc(C(OC(=O)C(C)C)OC(=O)C(C)C)c1)COP(=O)(OC[C@H]1O[C@H](C=C1)n1c(=O)[nH]c(=O)c(C)c1)O	1095170-77-6	Thymidine, 5,Ä≤-O-[6-acetyl-8-(1,1-dimethylethyl)-2-oxido-4H-1,3,2-benzodioxaph	C23H27N2O8P	490.44	C	-8.9
2366	O=C1[C@@H]2[C@@H](c3c([C@H](OC(=O)CCCSSCCCC(=O)O[C@H]4c5c([C@H]([C@@H]6[C@@H]4COC6=O)c4cc(OC)c(OC)c(	1107175-32-5	2-Naphthalenesulfonic acid, 5-[2-(4-hydroxyphenyl)diazenyl]-6-(2-phenyldiazenyl)	C22H16N4O4S	432.45	C	-8.9
2367	[C@@H](CC)(C1=C(O)C[C@H](OC1=O)c1ccccc1)c1cc(NS(=O)(=O)c2c(C(F)(F)F)cccn2)ccc1	1109235-80-4	2-Pyridinesulfonamide, N-[3-[(1R)-1-[(6R)-5,6-dihydro-4-hydroxy-2-oxo-6-phenyl-2	C26H23F3N2O5S	532.53	C	-8.9
2368	O[C@@H]1[C@H](n2c3c(nc2)c(N)ncn3)O[C@H](CO)[C@H]1OP(=O)(OCCOC(CO)n1c2c(nc1)c(N)ncn2)O	111392-87-1	9H-Purin-6-amine, 9-[5-O-[2-(1,3-dihydro-1,3-dioxo-2H-isoindol-2-yl)-1-oxopropyl	C21H20N6O7	468.42	C	-8.9
2369	O([C@H]1[C@@H](O[C@H](CO)[C@H]1O)n1cc(CC)c(=O)[nH]c1=O)[C@H]1[C@@H](O[C@H](CO)[C@H]1O)n1cc(CC)c(=O)[	112714-85-9	[1,1,Ä≤-Biphenyl]-4,4,Ä≤-disulfonic acid, 2,2,Ä≤-bis[2-(2-hydroxy-1-naphthaleny	C32H22N4O8S2.2Na		C	-8.9
2370	C(=O)([C@@H](NC(=O)OC)CC)N1[C@H]2[C@H](C2)C[C@H]1c1[nH]c(cn1)c1ccc(cc1)c1ccc(cc1)c1[nH]c(nc1)[C@H]1N	1129633-33-5	Carbamic acid, N,N,Ä≤-[[1,1,Ä≤-biphenyl]-4,4,Ä≤-diylbis[1H-imidazole-5,2-diyl-(	C38H42N8O6	706.79	C	-8.9
2371	O(P(=O)(OP(=O)(O)O)O)[C@H]1[C@@H](O[C@H](COP(=O)(OP(=O)(O)O)O)[C@H]1O)n1c2c(nc1)c(=O)nc(NC(=O)C(C)C)	1135685-39-0	2-Naphthalenesulfonic acid, 5-[(2E)-2-(2-methyl-4-oxo-2,5-cyclohexadien-1-ylider	C23H18N4O4S	446.48	C	-8.9
2372	C(C)c1nc(nc(n1)N1CCOCC1)N1CCC(C(=O)NCc2c(C(F)(F)F)cccc2)CC1	1141895-01-3	4-Piperidinecarboxamide, 1-[4-(4-morpholinyl)-1,3,5-triazin-2-yl]-N-[[2-(trifluc	C21H25F3N6O2	450.46	C	-8.9
2373	S(=O)(=O)(O)c1c(cc(C)c(N=Nc2c(N)c3c(c(S(=O)(=O)O)c2)cccc3)c1)c1c(S(=O)(=O)O)cc(N=Nc2c(N)c3c(c(S(=O)(	114813-89-7	1-Naphthalenesulfonic acid, 4-amino-3-[2-(4-sulfophenyl)diazenyl]-, sodium salt	C16H13N3O6S2.2Na		C	-8.9
2374	O(C)c1c2n(cc(C(=O)OC[C@@H]3[C@@H](N=[N+]=[N-])C[C@@H](O3)n3c(=O)[nH]c(=O)c(C)c3)c(=O)c2cc(F)c1N1CC(C	1174398-22-1	Thymidine, 3,Ä≤-azido-3,Ä≤-deoxy-, 5,Ä≤-[1-ethyl-6,8-difluoro-1,4-dihydro-7-[3-	C35H36F2N8O8		C	-8.9
2375	O=c1c2c(n(cc1C(=O)OC[C@@H]1[C@@H](N=[N+]=[N-])C[C@@H](O1)n1c(=O)[nH]c(=O)c(C)c1)C1CC1)cc(c(F)c2)N1CC	1174398-24-3	Thymidine, 3,Ä≤-azido-3,Ä≤-deoxy-, 5,Ä≤-[1-cyclopropyl-7-[3,5-dimethyl-4-[(2-ph	C45H45F2N9O10		C	-8.9
2376	CN1Cc2c(-c3c(cc(cc3)c3[nH]c(nc3)[C@H]3N(C(=O)[C@@H](NC(=O)OC)C(C)C)CCC3)C1)ccc(c2)c1[nH]c(nc1)[C@H]1	1179150-04-9	Carbamic acid, N,N,Ä≤-[9H-fluorene-2,7-diylbis[1H-imidazole-5,2-diyl-(2S)-2,1-py	C41H50N8O6	750.89	C	-8.9
2377	CN1Cc2c(-c3c(cc(cc3)c3ccc(cc3)c3[nH]c(nc3)[C@H]3N(C(=O)[C@@H](NC(=O)OC)C(C)C)CCC3)C1)[nH]c(n2)[C@H]1	1180669-80-0	Carbamic acid, N-[(1S)-1-[[(2S)-2-[8-[4-[2-[(2S)-4,4-difluoro-1-[(2S)-2-[(methox	C43H52F2N8O6	814.92	C	-8.9
2378	C(=O)([C@@H](NC(=O)OC)C(C)C)N1[C@H](c2[nH]c3-c4c(cc(cc4)c4cc5c(-c6c(CC5)nc([nH]6)[C@H]5N(C(=O)[C@@H]	1180670-02-3	Carbamic acid, N-[(1S)-1-[[(1R,3S,5R)-3-[5-[4-[4,5-dihydro-2-[(1R,3S,5R)-2-[(2S)	C46H53F3N8O6	870.96	C	-8.9
2379	C(=O)([C@@H](NC(=O)OC)C(C)C)N1[C@H](c2[nH]c3c4c(cc(cc4)c4cc(F)c(cc4)c4[nH]c(nc4)[C@H]4N(C(=O)[C@@H](	1180670-23-8	Carbamic acid, N,N,Ä≤-[[7,7,Ä≤-bi-1H-naphth[1,2-d]imidazole]-2,2,Ä≤-diylbis[(1R	C46H50N8O6	810.94	C	-8.9
2380	O=c1c2c(n(cn2)[C@@H]2O[C@H](COP(=O)=O)[C@@H](O)C2)[nH]c(N)n1	118662-86-5	Uridine, cytidylyl-(3,Ä≤,Üí5,Ä≤)-guanylyl-(3,Ä≤,Üí5,Ä≤)-cytidylyl-(3,Ä≤,Üí5,Ä≤	C37H48N13O27P3	1199.77	C	-8.9
2381	O(COC(=O)N1CCOCC1)c1c2n(C[C@H]3N(C2=O)[C@H](C)CCO3)cc(C(=O)NCc2c(F)cc(F)cc2)c1=O	1206156-27-5	1-Pyrrolidinecarboxylic acid, [[(4R,12aS)-9-[[[(2,4-difluorophenyl)methyl]amino]	C26H28F2N4O7	546.52	C	-8.9
2382	C(N1c2c(C(=NNC(=S)N)C1=O)cccc2)N1CCN(CC1)c1c2c(cc(C(F)(F)F)cc2)ncc1	1218813-21-8	Hydrazinecarbothioamide, 2-[6-bromo-1,2-dihydro-2-oxo-1-[[4-[7-(trifluoromethyl)	C24H21BrF3N7OS	592.43	C	-8.9
2383	N(C(=O)[C@H](Cc1ccccc1)C[C@@H](CN1[C@H](C(=O)NC(C)(C)C)CN(Cc2cccnc2)CC1)C)C1c2c(C[C@H]1O)cccc2	1223086-75-6	Not Yet Assigned	C38H47N5O4S	669.88	C	-8.9
2384	C(CC(=O)CC(CCCCC)O)c1cc(O)c(O)cc1	1228554-23-1	Carbamic acid, N-[(1S)-1-[[(2S)-2-[5-[3-[6-[2-[(2S)-1-[(2S)-2-[(methoxycarbonyl)	C44H52N8O6	788.93	C	-8.9
2385	C(=O)([C@H](NC(=O)OC)c1ccccc1)N1[C@@H](CCC1)c1[nH]c(cn1)[C@@H]1CC[C@H](CC1)c1ccc(cc1)c1[nH]c(nc1)[C@	1228670-77-6	Carbamic acid, N-[(1S)-2-methyl-1-[[(2S)-2-[5-[4-[trans-4-[2-[(2S)-1-[(2R)-2-phe	C46H60N8O4	789.02	C	-8.9
2386	C(F)(F)c1n(c2c(n1)cccc2)c1nc(nc(N[C@H]2CC[C@@H](CC2)N2CCOCC2)n1)N1CCOCC1	1240023-38-4	Acetamide, 2-bromo-N-[trans-4-[[4-[2-(difluoromethyl)-1H-benzimidazol-1-yl]-6-(4	C23H27BrF2N8O2	565.41	C	-8.9
2387	C(F)(F)c1n(c2c(n1)cccc2)c1nc(nc(N[C@H]2CC[C@H](NCC)CC2)n1)N1CCOCC1	1240024-08-1	Methanesulfonamide, N-[trans-4-[[4-[2-(difluoromethyl)-1H-benzimidazol-1-yl]-6-(	C22H28F2N8O3S	522.57	C	-8.9
2388	C(F)(F)c1n(c2c(n1)cccc2)c1nc(nc(N[C@H]2CC[C@H](NC(CCO)C)CC2)n1)N1CCOCC1	1240030-51-6	1-Propanol, 2-[[trans-4-[[4-[2-(difluoromethyl)-1H-benzimidazol-1-yl]-6-(4-morph	C24H32F2N8O2	502.56	C	-8.9
2389	C(=O)([C@@H](NC(=O)OC)C(C)C)N1[C@@H](CCC1)c1[nH]c(cn1)c1ccc(cc1)c1ccc([C@H](NC(=O)[C@H]2N(C(=O)[C@@H	1240427-41-1	L-Prolinamide, N-(methoxycarbonyl)-L-alanyl-N-[(1R)-1-[4,Ä≤-[2-[(2S)-1-[(2S)-2-	C36H45N7O7	687.79	C	-8.9
2390	C(=O)([C@@H](NC(=O)OC)C(C)C)N1[C@@H](CCC1)c1[nH]c(C#Cc2ccc(C#Cc3[nH]c(nc3)[C@H]3N(C(=O)[C@@H](NC(=O)	1240893-24-6	Carbamic acid, N-[(1R)-2-[(2S)-2-[5-[4-[2-[2-[(2S)-1-[(2R)-2-[(methoxycarbonyl)a	C42H46N8O6	758.86	C	-8.9
2391	[C@H](Nc1nc(C(F)(F)F)c(C(=O)N2CCS(=O)(=O)CC2)cn1)(C)C12CC3CC(C2)CC(C1)C3	1247824-40-3	Benzoic acid, 2-[(4-cyclohexylbenzoyl)amino]-5-(2-cyclohexylethyl)-	C28H35NO3	433.58	C	-8.9
2392	O([C@@H]1[C@H](n2c3c(nc2)c(N)nc(F)n3)O[C@H](CO)[C@H]1O)c1nc2n(cnc2c(N)n1)[C@@H]1O[C@H](CO)[C@@H](O)[	1256093-56-7	9H-Purin-6-amine, 9-[3-O-(6-amino-9-Œ≤-D-arabinofuranosyl-9H-purin-2-yl)-Œ≤-D-a	C20H23FN10O8	550.46	C	-8.9
2393	C(=O)([C@H](NC(=O)OC)C(C)C)N1[C@H](CCC1)c1[nH]c(C#Cc2ccc(C#Cc3[nH]c(nc3)[C@H]3N(C(=O)[C@@H](NC(=O)OC	1256387-55-9	Carbamic acid, N,N,Ä≤-[1,5-naphthalenediylbis[4,1-phenylene-1H-imidazole-5,2-diy	C50H56N8O6	865.03	C	-8.9
2394	S(=O)(=O)(O)c1c2c(c(N)c(/N=N/c3cc(ccc3C)c3cc(C)c(/N=N/c4c(N)c5c(c(S(=O)(=O)O)c4)cccc5)cc3)c1)cccc2	1261070-51-2	Carbamic acid, N-[(1S)-1-[[(2S)-2-[5-[4-[6-[2-[(2S)-1-[2-[(methoxycarbonyl)aminc	C41H46N8O6	746.85	C	-8.9
2395	O=c1n([C@H]2C[C@H](O[P@@](=O)(OC[C@H]3O[C@H](C[C@@H]3O)n3c(=O)[nH]c(=O)c(C)c3)OCOC(=O)C)[C@@H](CO)O2	1261161-48-1	1-Naphthalenesulfonic acid, 3,3,Ä≤-[(3,Ä≤,4-dimethyl[1,1,Ä≤-biphenyl]-3,4,Ä≤-di	C34H28N6O6S2	680.75	C	-8.9
2396	O[C@H]1[C@@H]2[C@@H]([C@]3(C)[C@H](C1)C[C@H](O)[C@H](O)C3)C[C@H](O)[C@@]1(C)[C@H]2CC[C@@H]1[C@@H](CC	126934-30-3	Guanosine 5,Ä≤-(tetrahydrogen triphosphate), P,Ä≤,Ä≤,Üí3,Ä≤-ester with adenosir	C20H27N10O17P3	772.41	C	-8.9
2397	C(=O)([C@H](Cc1cnc[nH]1)NC(=O)[C@@H](NC(=O)[C@H](Cc1ccc(O)cc1)NC(=O)c1cccnc1)CCCCNC(=O)[C@@H](NC(=O)	127052-34-0	Uridine, 5,Ä≤-O-phosphonoadenylyl-(3,Ä≤,Üí5,Ä≤)-5-methyl-, homopolymer	(C20H27N7O15P2)x		C	-8.9
2398	N(c1nc(nc(N/N=C/c2c(F)cccc2F)n1)N1CCOCC1)c1ccc(F)cc1	1279692-10-2	2-Naphthalenecarboxaldehyde, 2-[4-[(4-fluorophenyl)amino]-6-(4-morpholinyl)-1,3,	C24H22FN7O	443.48	C	-8.9
2399	N(S(=O)(=O)c1ccc(C)cc1)c1c2c(n(C[C@H](OCP(=O)(O)O)C)cn2)ncn1	1306734-25-7	Carbamic acid, N-[(1S)-1-[[(2S,4R)-2-[5-[4-[6-[2-[(2S,4R)-1-[(2S)-2-[(methoxycar	C46H56N8O6	816.99	C	-8.9
2400	O=C1N2[C@@H]3[C@H](CO[C@@H]2Cn2c1c(O)c(=O)c(C(=O)NCc1c(F)cc(F)cc1)c2)CCC3	1309560-98-2	2H-Pyrido[1,Ä≤,2,Ä≤:4,5]pyrazino[1,2-a][3,1]benzoxazine-10-carboxamide, N-[(2,4	C29H27F2N3O5	535.54	C	-8.9
2401	C(=O)([C@@H](NC(=O)OC)C(C)C)N1[C@@H](C[C@H](C)C1)c1[nH]c(c(Cl)n1)c1ccc(cc1)c1ccc(cc1)c1[nH]c(nc1)[C@	1312336-24-5	Carbamic acid, N-[(1S)-2-[(2S,5S)-2-[5-[4,Ä≤-[5-chloro-2-[(2S,5S)-1-[(2S)-2-[(me	C46H57ClN8O8	885.45	C	-8.9
2402	C(=O)(Cc1ccccc1)N1[C@H](c2[nH]c(c3c4c(c(cc3)c3ccc(cc3)c3[nH]c(nc3)[C@H]3N(C(=O)[C@@H](NC(=O)OC)C(C)C	1312608-49-3	Carbamic acid, N-[(1S)-1-[[(2S)-2-[5-[4-[7-[2-[(2S)-1-[(2S)-2-[(methoxycarbonyl)	C43H53N9O6	791.94	C	-8.9
2403	C[C@]1(c2[nH]c(c3c4c(c(cc3)c3ccc(cc3)c3[nH]c(nc3)[C@H]3N(C(=O)[C@@H](NC(=O)OC)C(C)C)CCC3)cccc4)cn2)N	1312608-78-8	Carbamic acid, N-[(1S)-1-[[(2S)-2-[5-[2,3-dihydro-7-[4-[2-[(2S)-1-[(2S)-2-[(meth	C44H56N8O6	792.97	C	-8.9
2404	C[C@]1(c2[nH]c(c3c4c(c(cc3)c3ccc(cc3)c3[nH]c(nc3)[C@@]3(C)N(C(=O)[C@@H](NC(=O)OC)C(C)C)CCC3)CCCC4)cn	1312608-79-9	Carbamic acid, N-[(1S)-1-[[(2S)-2-[5-[4-[4-[2-[(2S)-1-[(2S)-2-[(methoxycarbonyl)	C45H54N8O6	802.96	C	-8.9
2405	O(Cc1ccc(F)cc1)c1c2c(oc(c(O)c2=O)c2[nH]cnn2)c(OC)cc1	1312893-37-0	4H-1-Benzopyran-4-one, 5-[(4-fluorophenyl)methoxy]-3-hydroxy-8-methoxy-2-(5-pher	C27H19FN2O5	470.45	C	-8.9
2406	O(Cc1ccc(F)cc1)c1c2c(oc(c(O)c2=O)c2[nH]ccn2)c(Cl)cc1	1312893-97-2	4H-1-Benzopyran-4-one, 8-chloro-5-[(4-fluorophenyl)methoxy]-3-hydroxy-2-(3-pheny	C24H15ClFN3O4	463.85	C	-8.9
2407	O(Cc1ccc(F)cc1)c1c2c(c(c(C)c1)c1ccccc1)oc(c(O)c2=O)c1[nH]ccn1	1312896-97-1	4H-1-Benzopyran-4-one, 5-[(4-fluorophenyl)methoxy]-3-hydroxy-7-methyl-8-phenyl-2	C31H22FN3O4	519.52	C	-8.9
2408	O(Cc1ccc(F)cc1)c1c2c(oc(c(O)c2=O)c2ncccn2)cc(OC)c1	1312897-02-1	4H-1-Benzopyran-4-one, 5-[(4-fluorophenyl)methoxy]-3-hydroxy-7-methyl-8-phenyl-2	C32H23FN2O4	518.53	C	-8.9
2409	O(Cc1ccc(F)cc1)c1c2c(oc(c(O)c2=O)c2[nH]cnn2)c(C)c(OC)c1	1312897-98-5	4H-1-Benzopyran-4-one, 5-[(4-fluorophenyl)methoxy]-3-hydroxy-7-methoxy-8-methyl-	C28H21FN2O5	484.48	C	-8.9
2410	O(Cc1ccc(F)cc1)c1c2c(oc(c(O)c2=O)c2[nH]ccn2)c(C)c(OC)c1	1312898-08-0	4H-1-Benzopyran-4-one, 5-[(4-fluorophenyl)methoxy]-3-hydroxy-7-methoxy-8-methyl-	C26H20FN3O5	473.45	C	-8.9
2411	O(Cc1ccc(F)cc1)c1c2c(oc(c(O)c2=O)c2[nH]cnn2)c(Cl)c(OC)c1	1312898-99-9	4H-1-Benzopyran-4-one, 8-chloro-5-[(4-fluorophenyl)methoxy]-3-hydroxy-7-methoxy-	C27H18ClFN2O5	504.89	C	-8.9
2412	O(C)c1c(c2c(c(OCc3ccc(F)cc3)c1)c(=O)c(O)c(o2)c1nnc(C)[nH]1)c1ccccc1	1312899-55-0	4H-1-Benzopyran-4-one, 5-[(4-fluorophenyl)methoxy]-3-hydroxy-7-methoxy-8-phenyl-	C25H18FN3O5	459.43	C	-8.9
2413	O(Cc1ccc(F)cc1)c1c2c(oc(c(O)c2=O)c2ncccn2)cc(Cl)c1	1312899-64-1	4H-1-Benzopyran-4-one, 5-[(4-fluorophenyl)methoxy]-3-hydroxy-7-methoxy-8-phenyl-	C32H23FN2O5	534.53	C	-8.9
2414	O(Cc1ccc(F)cc1)c1c2c(oc(c(O)c2=O)c2ncccn2)c(C)c(Cl)c1	1312900-73-4	4H-1-Benzopyran-4-one, 7-chloro-5-[(4-fluorophenyl)methoxy]-3-hydroxy-2-(5-pheny	C25H16ClFN2O4	462.86	C	-8.9
2415	O(Cc1ccc(F)cc1)c1c2c(c(c(Cl)c1)c1ccccc1)oc(c(O)c2=O)c1ncc(C)cn1	1312902-54-7	4H-1-Benzopyran-4-one, 7-chloro-5-[(4-fluorophenyl)methoxy]-3-hydroxy-8-phenyl-2	C26H16ClFN2O4	474.87	C	-8.9
2416	O(Cc1ccc(F)cc1)c1c2c(c(c(Cl)c1)c1ccccc1)oc(c(O)c2=O)c1[nH]ccn1	1312902-68-3	4H-1-Benzopyran-4-one, 7-chloro-5-[(4-fluorophenyl)methoxy]-3-hydroxy-8-phenyl-2	C30H19ClFN3O4	539.94	C	-8.9
2417	O(C)c1c2c(c(OCc3ccc(F)cc3)cc1c1ccccc1)c(=O)c(O)c(o2)c1[nH]cnn1	1312904-30-5	4H-1-Benzopyran-4-one, 5-[(4-fluorophenyl)methoxy]-3-hydroxy-8-methoxy-7-phenyl-	C33H23FN2O5	546.54	C	-8.9
2418	Clc1c2c(c(OCc3ccc(F)cc3)cc1c1ccccc1)c(=O)c(O)c(o2)c1ncc(C)cn1	1312905-04-6	4H-1-Benzopyran-4-one, 8-chloro-5-[(4-fluorophenyl)methoxy]-3-hydroxy-7-phenyl-2	C26H16ClFN2O4	474.87	C	-8.9
2419	O(Cc1ccc(F)cc1)c1c2c(c(Cl)c(c1)c1ccccc1)oc(c(O)c2=O)c1[nH]c(C)nn1	1312905-19-3	4H-1-Benzopyran-4-one, 8-chloro-5-[(4-fluorophenyl)methoxy]-3-hydroxy-7-phenyl-2	C24H15ClFN3O4	463.85	C	-8.9
2420	O(Cc1ccc(F)cc1)c1c2c(c(c(c1)c1ccccc1)c1ccccc1)oc(c(O)c2=O)c1nnc(Cl)[nH]1	1312905-78-4	4H-1-Benzopyran-4-one, 5-[(4-fluorophenyl)methoxy]-3-hydroxy-2-(5-methoxy-1H-1,2	C31H22FN3O5	535.52	C	-8.9
2421	O(Cc1ccc(F)cc1)c1c2c(c(c(c1)c1ccccc1)c1ccccc1)oc(c(O)c2=O)c1[nH]cc(C)n1	1312905-81-9	4H-1-Benzopyran-4-one, 5-[(4-fluorophenyl)methoxy]-3-hydroxy-2-(1H-imidazol-2-yl	C31H21FN2O4	504.51	C	-8.9
2422	O(Cc1ccc(F)cc1)c1c2c(c(c(c1)c1ccccc1)c1ccccc1)oc(c(O)c2=O)c1[nH]c(OC)cn1	1312905-82-0	4H-1-Benzopyran-4-one, 5-[(4-fluorophenyl)methoxy]-3-hydroxy-2-(5-methyl-1H-imic	C32H23FN2O4	518.53	C	-8.9
2423	O(Cc1ccc(F)cc1)c1c2c(c(c(c1)c1ccccc1)c1ccccc1)oc(c(O)c2=O)c1[nH]c(cn1)c1ccccc1	1312905-84-2	4H-1-Benzopyran-4-one, 2-(5-chloro-1H-imidazol-2-yl)-5-[(4-fluorophenyl)methoxy]	C31H20ClFN2O4	538.95	C	-8.9
2424	O=c1c2c(oc(c1O)c1[nH]ccn1)ccc(OCc1ccc(F)cc1)c2	1312906-28-7	4H-1-Benzopyran-4-one, 6-[(4-fluorophenyl)methoxy]-3-hydroxy-2-(3-phenyl-1H-1,2,	C24H16FN3O4	429.4	C	-8.9
2425	O=c1c2c(oc(c1O)c1[nH]cnn1)c(C)cc(OCc1ccc(F)cc1)c2	1312906-68-5	4H-1-Benzopyran-4-one, 6-[(4-fluorophenyl)methoxy]-3-hydroxy-8-methyl-2-(5-pheny	C27H19FN2O4	454.45	C	-8.9
2426	O(C)c1c2c(cc(OCc3ccc(F)cc3)c1C)c(=O)c(O)c(o2)c1[nH]ccn1	1312909-90-2	4H-1-Benzopyran-4-one, 6-[(4-fluorophenyl)methoxy]-3-hydroxy-8-methoxy-7-methyl-	C26H20FN3O5	473.45	C	-8.9
2427	Cc1c(c2c(cc1OCc1ccc(F)cc1)c(=O)c(O)c(o2)c1[nH]c(nn1)c1ccccc1)c1ccccc1	1312910-89-6	4H-1-Benzopyran-4-one, 2-(5-chloro-1H-1,2,4-triazol-3-yl)-6-[(4-fluorophenyl)met	C25H17ClFN3O4	477.87	C	-8.9
2428	Clc1c2c(cc(OCc3ccc(F)cc3)c1OC)c(=O)c(O)c(o2)c1ncccn1	1312913-93-1	4H-1-Benzopyran-4-one, 7-chloro-6-[(4-fluorophenyl)methoxy]-3-hydroxy-8-methoxy-	C26H18ClFN2O5	492.88	C	-8.9
2429	Clc1c2c(cc(OCc3ccc(F)cc3)c1OC)c(=O)c(O)c(o2)c1[nH]ccn1	1312914-38-7	4H-1-Benzopyran-4-one, 8-chloro-6-[(4-fluorophenyl)methoxy]-3-hydroxy-7-methoxy-	C25H17ClFN3O5	493.87	C	-8.9
2430	O(C)c1c(c2c(cc1OCc1ccc(F)cc1)c(=O)c(O)c(o2)c1[nH]c(C)nn1)c1ccccc1	1312915-35-7	4H-1-Benzopyran-4-one, 6-[(4-fluorophenyl)methoxy]-3-hydroxy-7-methoxy-8-phenyl-	C25H18FN3O5	459.43	C	-8.9
2431	O(C)c1c(c2c(cc1OCc1ccc(F)cc1)c(=O)c(O)c(o2)c1[nH]ccn1)c1ccccc1	1312915-43-7	4H-1-Benzopyran-4-one, 6-[(4-fluorophenyl)methoxy]-3-hydroxy-7-methoxy-8-phenyl-	C31H22FN3O5	535.52	C	-8.9
2432	Clc1c(c2c(cc1OCc1ccc(F)cc1)c(=O)c(O)c(o2)c1[nH]cnn1)c1ccccc1	1312915-68-6	4H-1-Benzopyran-4-one, 7-chloro-6-[(4-fluorophenyl)methoxy]-3-hydroxy-8-phenyl-2	C32H20ClFN2O4	550.96	C	-8.9
2433	Clc1c(c2c(cc1OCc1ccc(F)cc1)c(=O)c(O)c(o2)c1[nH]c(nn1)c1ccccc1)c1ccccc1	1312915-86-8	4H-1-Benzopyran-4-one, 7-chloro-2-(5-chloro-1H-1,2,4-triazol-3-yl)-6-[(4-fluorop	C24H14Cl2FN3O4	498.29	C	-8.9
2434	O=c1c2c(cc(c(OCc3ccc(F)cc3)c2)c2ccccc2)oc(c1O)c1ncc(C)cn1	1312916-31-6	4H-1-Benzopyran-4-one, 6-[(4-fluorophenyl)methoxy]-3-hydroxy-7-phenyl-2-(2-pyrim	C26H17FN2O4	440.42	C	-8.9
2435	O(Cc1ccc(F)cc1)c1c(c(Cl)c2c(c1)c(=O)c(O)c(o2)c1ncccn1)c1ccccc1	1312917-51-3	4H-1-Benzopyran-4-one, 6-[(4-fluorophenyl)methoxy]-3-hydroxy-8-methoxy-7-phenyl-	C32H23FN2O5	534.53	C	-8.9
2436	O(Cc1ccc(F)cc1)c1c(c(c2c(c1)c(=O)c(O)c(o2)c1[nH]c(OC)nn1)c1ccccc1)c1ccccc1	1312918-43-6	4H-1-Benzopyran-4-one, 6-[(4-fluorophenyl)methoxy]-3-hydroxy-2-(3-methyl-1H-1,2,	C31H22FN3O4	519.52	C	-8.9
2437	O(Cc1ccc(F)cc1)c1c(c(c2c(c1)c(=O)c(O)c(o2)c1[nH]c(OC)cn1)c1ccccc1)c1ccccc1	1312918-48-1	4H-1-Benzopyran-4-one, 6-[(4-fluorophenyl)methoxy]-3-hydroxy-2-(5-methyl-1H-imic	C32H23FN2O4	518.53	C	-8.9
2438	N(=Nc1c2cc(S(=O)(=O)O)cc2c(N)cc1)c1c2c(c(N=Nc3ccccc3)c(S(=O)(=O)O)c2	1345852-35-8	1-Naphthalenesulfonic acid, 8-amino-5-[2-[4-(2-phenyldiazenyl)-7-sulfo-1-naphtha	C26H19N5O6S2	561.59	C	-8.9
2439	[C@H](Cc1ccccc1)[C@@H]([C@H](Cc1ccccc1)NC(=O)OCc1cncs1)NC(=O)[C@H](N(C(=O)NCc1nc(C(C)C)sc1)C)C	1346960-17-5	Not Yet Assigned	C33H47N5O5	593.76	C	-8.9
2440	O(C(=O)N[C@@H](Cc1cccnc1)CN(S(=O)(=O)OCP(=O)(N)O)CC(C)C)O)[C@@H]1[C@H]2[C@@H](OC	1347876-93-0	Furo[3,Ä≤,4,Ä≤:6,7]naphtho[2,3-d]-1,3-dioxol-6(5aH)-one, 9-(acetyloxy)-5-(5a,6,	C36H42N2O11	678.73	C	-8.9
2441	OC1c2c(ccc(S(=O)(=N)N(CC(C(Cc3ccccc3)NC(=O)OC3COC4OCCC34)O)CC(C)C)c2)OC1O	1348244-45-0	Not Yet Assigned	C35H38N4O5S	626.77	C	-8.9
2442	[C@H](Cc1ccccc1)[C@@H]([C@H](Cc1cncs1)NC(=O)OCc1cncs1)NC(=O)[C@@H](N(Cc1nc(C(C)C)sc1)C(C	1348967-32-7	Not Yet Assigned	C38H48N4O5	640.81	C	-8.9
2443	C(=O)([C@@H](NC(=O)OC)C(C)C)N1[C@@H](c2[nH]c3c4c(c(cc3)c3c5c(cc3)[nH]c(nc3)[C@H]3N(C(=O)[C@@H](NC(=O)	1350719-37-7	Carbamic acid, N,N,Ä≤-[4,4,Ä≤-bibenzo[b]thiophene]-7,7,Ä≤-diylbis[1H-imidazole	C44H50N8O6S2	851.05	C	-8.9
2444	C(=O)([C@@H](NC(=O)OC)[C@@H](C)O)N1[C@@H](CCC1)c1[nH]c(cn1)c1cc2c(cc1)cc(cc2)c1ccc(cc1)c1[nH]c(nc1)[	1351329-92-4	Carbamic acid, N-[(1R)-1-[[(2S)-2-[5-[4-[6-[2-[(2S)-1-[(2R)-2-[(methoxycarbonyl)	C44H52N8O6	788.93	C	-8.9
2445	C(=O)([C@H](NC(=O)OC)c1ccccc1)N1[C@@H](CCC1)c1[nH]c(cn1)c1ccc(cc1)c1cc2c(cc(cc2)c1[nH]c(nc1)[C@H	1351332-55-2	Carbamic acid, N-[(1S)-1-[[(2S)-2-[5-[3-cyano-4-[6-[2-[(2S)-1-[(2S)-2-[(methoxyc	C45H51N9O6	813.94	C	-8.9
2446	C(=O)([C@@H](NC(=O)OC)C(C)C)N1C[C@@]2(C[C@H]1c1[nH]c(cn1)c1ccc(F)c(c1)c1ccc(F)c(cc1)c1[nH]c(nc1)[C@H]	1351852-79-3	Carbamic acid, N-[(1S)-1-[[(2S)-2-[5-[4,Ä≤-[5-chloro-2-[(5S,8S)-7-[(2S)-2-[(meth	C45H53ClN8O7	829.38	C	-8.9
2447	C(=O)([C@@H](NC(=O)OC)C(C)C)N1[C@H]([C@@H](C)C1CC1)c1[nH]c(cn1)c1ccc(cc1)c1ccc(cc1)c1[nH]c(nc1)[C	1351854-43-7	Carbamic acid, N-[(1S)-2-[(2S)-2-[5-[4,Ä≤-[2-[(2S,4R)-4-cyclopropyl-1-[(2S)-2-[	C51H54N8O6	875.02	C	-8.9
2448	C(F)(F)c1n(c2c(n1)cccc2)c1nc(nc(NC2CCN(CC2)n1)N1CCOCC1	1359953-84-6	1H-Benzimidazol-5-amine, N-[4-[2-(difluoromethyl)-1H-benzimidazol-1-yl]-6-(4-mor	C23H21F2N9O	477.47	C	-8.9
2449	C(=O)([C@@H](NC(=O)OC)C(C)C)N1[C@@H]([C@H](C(F)(F)F)C1)c1[nH]c(cn1)c1ccc(cc1)c1ccc(cc1)c1[nH]c(n	1361249-31-1	3-Pyrrolidinecarboxylic acid, 5,5,Ä≤-([1,1,Ä≤-biphenyl]-4,4,Ä≤-diyldi-1H-imidazol	C40H50N8O12S2	899	C	-8.9
2450	C(=O)([C@@H](NC(=O)OC)C1CCOCC1)N1[C@H](c2[nH]c(c3cc4c5-c6c(cc(cc6CC4)c4[nH]c(nc4)[C@H]4N(C(=O)[C@@H]	1377035-68-1	Carbamic acid, N,N,Ä≤-[2,7-triphenylenediylbis[1H-imidazole-5,2-diyl-(2S)-2,1-py	C46H52N8O6	812.96	C	-8.9
2451	C(=O)([C@@H](NC(=O)OC)C1CCOCC1)N1[C@H](C[C@@H]([C@@H]2C[C@H]2C)C1)c1[nH]c(cn1)c1ccc(cc1)c1ccc(cc1)[C@H	1377036-32-2	Carbamic acid, N-[(1S)-2-[7-[4-[2-[(1R,3S,4S)-2-[(2S)-2-[(methoxycarbo	C44H52N8O6	788.93	C	-8.9
2452	C(=O)([C@@H](NC(=O)OC)C(C)C)N1[C@H]2[C@H](C2)C[C@H]1c1[nH]c2c(n1)ccc1c2ccc(cc1)c1ccc(cc1)c1[nH]c(nc1)	1377037-16-5	Carbamic acid, N-[(1S)-2-[(1R,3S,4S)-3-[4,Ä≤,5,Ä≤-dihydro-2,Ä≤-[(2S)-1-[(2S)-2-	C48H56N8O7	857.01	C	-8.9
2453	C(=O)([C@H](NC(=O)OC)c1ccccc1)N1[C@@H](C[C@H](C)C1)c1[nH]c(cn1)c1ccc(cc1)c1ccc2c(cc(cc2)c1[nH]c(n	1377037-48-3	Carbamic acid, N-[(1S)-1-[[(2S)-2-[5-[6-[4-[2-[2,3-dihydro-2-[(2S)-2-[(methoxyca	C48H52N8O6	836.98	C	-8.9
2454	C(=O)([C@@H](NC(=O)OC)C1CCOCC1)N1[C@H](c2[nH]c(c3cc4c(c5c(c6c4cccc6)cc5)c4[nH]c(nc4)[C@H]4N(C(=O	1377041-01-4	Carbamic acid, N-[(1S)-2-[(2S)-2-[4,Ä≤,5,Ä≤-dihydro-2,Ä≤-[(1R,3S,4S)-2-[(2S)-2-	C48H56N8O7	857.01	C	-8.9
2455	C(=O)([C@@H](NC(=O)OC)C(C)C)N1[C@H]2[C@H](C2)C[C@H]1c1[nH]c2c(n1)ccc1c2ccc2ccc(cc1)c1ccc(cc1)c1[nH]c(nc1)	1377041-78-5	Carbamic acid, N-[(1S)-1-[[(2S)-2-[5-[4-[2-[(1R,3S,4S)-2-[(2S)-2-[(methoxycar	C45H54N8O6	802.96	C	-8.9
2456	C(=O)([C@@H](NC(=O)OC)C(C)C)N1[C@H](c2[nH]c3c4c(cc(cc4)c4cc5c(cc4)c4c(cc5)nc([nH]4)[C@H]4N(C(=O)[C@@	1377044-04-6	Carbamic acid, N,N,Ä≤-[5H-phenanthro[4,5-bcd]pyran-2,7-diylbis[1H-imidazole-5,2-	C43H50N8O7	790.91	C	-8.9

2457	C(=O)([C@@H](NC(=O)OC)C(C)C)N1[C@H]2[C@H](C2)C[C@H]1c1[nH]c2c(n1)ccc1c2ccc(c1)c1cc2c(cc1)c1c(cc2)nc(	1377045-61-8	Carbamic acid, N,N,Ä≤-[(5,10-dihydro[1]benzopyrano[5,4,3-cde][1]benzopyran-2,7-c	C44H54N8O8	822.95	C	-8.9
2458	O[C@H]1[C@H](n2c3c(nc2)c(N)ncn3)O[C@H](COP(=O)(OC(=O)N)O)[C@H]1O	1378278-01-3	Guanosine, 2,Ä≤-deoxyguanylyl-(3,Ä≤,Ü15,Ä≤)-	C20H25N10O11P	612.45	C	-8.9
2459	N(C(=O)C)[C@H]1[C@H]([C@@H]([C@@H](CO)O)O)OC(=C[C@@H]1NC(=N)N)C(=O)OC	1378393-44-2	Carbamic acid, N-[(1S)-1-[[(2S)-2-[5-[6-[4-[2-[(2S)-1-[(2R)-2-amino-2-phenylacet	C45H48N8O4	764.91	C	-8.9
2460	C(=O)([C@@H](NC(=O)OC)C(C)C)N1[C@@H](CCC1)c1[nH]c(cn1)c1ccc(C#CC#Cc2[nH]c(nc2)[C@H]2N(C(=O)[C@@H](NC	1380292-75-0	Carbamic acid, N-[(1S)-1-[[(2R)-2-[5-(4,Ä≤-cyclohexyl[1,1,Ä≤-biphenyl]-4-yl)-1H	C32H40N4O3	528.68	C	-8.9
2461	N(CCC(=O)N(C)C)c1nc(nc(n1)N1CCOCC1)c1c2c([nH]nc2)ccc1	1391925-89-5	4-Piperidinamine, 1-[4-(1H-indazol-4-yl)-6-(4-morpholinyl)-1,3,5-triazin-2-yl]-	C19H24N8O	380.45	C	-8.9
2462	N(c1nc(nc(n1)N1CCOCC1)c1cnc(N)s1)c1cc(C(=O)N(C)C)ccc1	1391926-09-2	Benzamide, 4-[[4-(2-amino-5-thiazolyl)-6-(4-morpholinyl)-1,3,5-triazin-2-yl]amir	C19H22N8O2S	426.5	C	-8.9
2463	Nc1ncc(c2nc(nc(n2)N2CCOCC2)N2CCN(C)CC2)cn1	1391926-27-4	2-Pyridinamine, 5-[4-(4-methyl-1-piperazinyl)-6-(4-morpholinyl)-1,3,5-triazin-2-	C18H23F3N8O	424.42	C	-8.9
2464	N(c1nc(nc(NCCNC(=O)c2ccc(cc2)C2OOC(OO2)c2ccc(C)cc2)n1)N1CCOCC1)c1ccc(C(C)C)cc1	1393922-45-6	Benzamide, N-[2-[[4-[(2-methylphenyl)amino]-6-(4-morpholinyl)-1,3,5-triazin-2-yl	C32H35N7O6	613.66	C	-8.9
2465	C(#N)[C@@H]1[C@@H](O[C@H](CO)[C@H]1O)n1c(=O)[nH]c(=O)c(C)c1	1408308-05-3	4-Pyrimidinecarboxamide, 1,6-dihydro-5-hydroxy-6-oxo-2-(2-quinolinyl)-N-(1-tricy	C26H28N4O3	444.53	C	-8.9
2466	N(=Nc1cc(S(=O)(=O)O)c(CCc2c(S(=O)(=O)O)cc(N=Nc3c4c(cc(S(=O)(=O)O)cc4)c(N)cc3)cc2)cc1)c1c2c(cc(S(=O)(	140942-27-4	2,7-Naphthalenedisulfonic acid, 4,4,Ä≤-[1,2-ethenediylbis[(3-sulfo-4,1-phenylene	C34H26N6O18S6.6Na		C	-8.9
2467	[C@H](C(=O)N1[C@H]2[C@H](C2)C[C@H]1c1[nH]c(cn1)c1ccc(cc1)c1ccc(cc1)c1[nH]c(nc1)[C@H]1N(C(=O)[C@@H](N	1413115-52-2	Carbamic acid, N,N,Ä≤-[[1,1,Ä≤-biphenyl]-4,4,Ä≤-diylbis[1H-imidazole-5,2-diyl-(	C48H54N8O6	838.99	C	-8.9
2468	O[C@H]1[C@@H](O[C@H](C(=O)N[C@@H](C(=O)N2CCN(C(=O)c3c(OC)cccc3)CC2)Cc2ccccc2)[C@H]1O)n1nc(C(=O)N)nc1	1414793-80-8	Not Yet Assigned	C28H29N7O8	591.57	C	-8.9
2469	C(=O)([C@@H](NC(=O)OC)C(C)C)N1[C@@H](CC2C1CCCC2)c1[nH]c(cn1)c1c2cc(c(c1)CCc1ccc(CC2)cc1)c1ccc(cc1)c1	1415120-49-8	Carbamic acid, N,N,Ä≤-[tricyclo[8.2.2.24,7]hexadeca-4,6,10,12,13,15-hexaene-5,1]	C56H64N8O6	945.16	C	-8.9
2470	C(N1c2c(C(=NNC(=S)N(CC=C)CC=C)C1=O)cc(F)cc2)N1CCN(CC1)c1ccc(Cl)cc1	1426583-69-8	Hydrazinecarbothioamide, 2-[5-chloro-1-[[4-(4-chlorophenyl)-1-piperazinyl]methyl	C32H28Cl2N6OS	615.58	C	-8.9
2471	N(=C1c2c(N(CN3CCN(CC3)c3cc4c(cc3F)c(=O)c(C(=O)O)cn4CC)C1=O)ccc(F)c2)NC(=S)N(CC=C)CC=C	1426583-81-4	3-Quinolinecarboxylic acid, 7-[4-[[5-chloro-3-[2-[(diphenylamino)thioxomethyl]hy	C38H33ClFN7O4S	738.23	C	-8.9
2472	C[C@]1(F)[C@@H](O[C@@](CO[P@](=O)(Oc2cc3c(cc2)cccc3)N[C@H](C(=O)OC(C)C)C)(F)[C@H]1O)n1c(=O)[nH]c(=O)	144356-85-4	[1,1,Ä≤-Biphenyl]-3,3,Ä≤-dicarboxylic acid, 4,4,Ä≤-bis[(2-aminobenzoyl)amino]-	C28H22N4O6	510.5	C	-8.9
2473	C(=O)(OCC)c1c2c(n(C)c1CSc1ccccc1)cc(Br)c(O)c2[13CH2][15N](C)C	144615-02-1	[1,1,Ä≤-Biphenyl]-3,3,Ä≤-dicarboxylic acid, 4,4,Ä≤-bis[(2-aminobenzoyl)amino]-,	(C28H22N4O6)x		C	-8.9
2474	C(=O)([C@@H](NC(=O)OC)C(C)C)N1[C@H](c2[nH]c(c3cc4OCC5(CC5)COc5c(-c4cc3)ccc(c5)c3[nH]c(nc3)[C@H]3N(C(	1449762-28-0	Carbamic acid, N,N,Ä≤-[(7,8-dihydro-7,7-dimethyl-6H-dibenzo[f,h][1,5]dioxonin-3,	C51H66N8O8	919.12	C	-8.9
2475	N(c1nc(nc(NCCCCOc2nc(nc(Nc3ccccc3)n2)N2CCOCC2)n1)N1CCOCC1)c1ccccc1	1463435-49-5	1,3,5-Triazine-2,4-diamine, 6-(4-morpholinyl)-N2-[5-[[4-(4-morpholinyl)-6-(pheny	C31H39N11O3	613.71	C	-8.9
2476	O=c1c2c(n(cn2)[13C@@H]2O[C@H](CO)[C@@H](O)[C@H]2O)[nH]c(N)n1	146849-74-3	Phosphoric acid, 4-[(5R,5aR,8aR,9S)-5,5a,6,8,8a,9-hexahydro-9-hydroxy-6-oxofuro[	C35H33O11P	660.6	C	-8.9
2477	N(C(=O)C)[C@@H]1[C@@H](NC(=N)NC(=O)[C@@H](C)c2cc3c(cc2)cc(OC)cc3)C=C(C(=O)O)O[C@H]1[C@@H]([C@@H](CO)	1469338-33-7	Not Yet Assigned	C26H32N4O9	544.55	C	-8.9
2478	C(OC[C@@H]1[C@@H](OC(=O)c2ccccc2)[C@@H](OP(=O)(OC[C@@H]2[C@@H](OC(=O)c3ccccc3)[C@@H](OP(=O)(OC[C@@H]	1469875-46-4	Œ≤-D-lyxo-Adenosine, 1,Ä≤-[3-(aminocarbonyl)-1H-1,2,4-triazol-1-yl]-3,Ä≤-O-benz	C40H37N10O14P	912.75	C	-8.9
2479	O=c1c2c(n(cn2)[C@H]2C[C@H](O[Si](C(C)C)(C(C)C)C(C)C)[C@@H](CO[Si](C(C)C)(C(C)C)C(C)C)O2)[nH]c(NC(=O)	14721-48-3	2-Naphthalenesulfonic acid, 8,8,Ä≤-[(3,3,Ä≤-dimethyl[1,1,Ä≤-biphenyl]-4,4,Ä≤-di	C34H28N6O6S2	680.75	C	-8.9
2480	C(=O)(c1[nH]c2c(c1)cc(NS(=O)(=O)N1CCNCC1)cc2)N1CCN(CC1)c1c(NC(C)C)cccn1	147540-10-1	1-Piperazinesulfonamide, N-[2-[[4-[3-[(1-methylethyl)amino]-2-pyridinyl]-1-piper	C32H40N8O3S	616.78	C	-8.9
2481	N(C(C)C)c1c(nccc1)N1CCN(C(=O)c2[nH]c3c(c2)ccc(NS(=O)(=O)C)c3)CC1	147920-16-9	Methanesulfonamide, 1,1,1-trifluoro-N-[2-[[4-[3-[(1-methylethyl)amino]-2-pyridir	C22H25F3N6O3S	510.53	C	-8.9
2482	O(C(=O)c1ccccc1)[C@@H]1[C@H]([C@H](O[C@@H]2[C@@H](CO)O[C@H](C2)n2cc(C(F)(F)F)c(=O)[nH]c2=O)O[C@H]2[C	148834-35-9	Thymidine, 3,Ä≤-O-[2,3-di-O-benzoyl-4,6-O-(phenylmethylene)-Œ≤-D-glucopyranosyl	C37H33F3N2O12	754.66	C	-8.9
2483	[C@H](O)([C@H]1[C@H](NC(=O)C)[C@@H](NC(=N)N)C=C(C(=O)OC)O1)[C@H]1COC(=O)O1	151559-13-6	2-Naphthalenesulfonic acid, 8-[2-(4-amino-2-methylphenyl)diazenyl]-5-[2-(3-sulfo	C23H19N5O6S2	525.56	C	-8.9
2484	C(n1c2c(nc1CC)c(C)cc(C)n2)c1ccc(cc1)c1c(S(=O)(=O)NC(=O)CCc2ccccc2)ccc(C(C)C)c1	154552-54-2	Benzenepropanamide, N-[[5-butyl-4,Ä≤-[(2-ethyl-5,7-dimethyl-3H-imidazo[4,5-b]py	C36H40N4O3S	608.79	C	-8.9
2485	C(n1c2c(nc1CC)c(C)cc(C)n2)c1ccc(cc1)c1c(S(=O)(=O)NC(=O)CCc2ccccc2)ccc(C(C)(C)C)c1	154552-63-3	Benzenepropanamide, N-[[5-(1,1-dimethylpropyl)-4,Ä≤-[(2-ethyl-5,7-dimethyl-3H-ir	C37H42N4O3S	622.82	C	-8.9
2486	C(n1c2c(nc1CC)c(C)cc(C)n2)c1ccc(cc1)c1c(S(=O)(=O)NC(=O)CCc2ccccc2)ccc(CN(CC)CC)c1	154552-65-5	Benzenepropanamide, N-[[5-[(dimethylamino)methyl]-4,Ä≤-[(2-ethyl-5,7-dimethyl-3H	C35H39N5O3S	609.78	C	-8.9
2487	C(n1c2c(nc1CC)c(C)cc(C)n2)c1ccc(cc1)c1c(S(=O)(=O)NC(=O)CCc2ccccc2)ccc(SCC)c1	154552-69-9	Benzenepropanamide, N-[[4,Ä≤-[(2-ethyl-5,7-dimethyl-3H-imidazo[4,5-b]pyridin-3-y	C35H38N4O4S	610.77	C	-8.9
2488	C(n1c2c(nc1CC)c(C)cc(C)n2)c1ccc(cc1)c1c(S(=O)(=O)NC(=O)c2c(Oc3ccccc3)cccc2)ccc(CCC)c1	154552-72-4	Benzamide, N-[[5-ethyl-4,Ä≤-[(2-ethyl-5,7-dimethyl-3H-imidazo[4,5-b]pyridin-3-y]	C38H36N4O4S	644.78	C	-8.9
2489	C(n1c2c(nc1CC)c(C)cc(C)n2)c1ccc(cc1)c1c(S(=O)(=O)NC(=O)c2c(Oc3ccccc3)cccc2)ccc(C(C)C)c1	154552-74-6	Benzamide, N-[[5-butyl-4,Ä≤-[(2-ethyl-5,7-dimethyl-3H-imidazo[4,5-b]pyridin-3-y]	C40H40N4O4S	672.84	C	-8.9
2490	C(n1c2c(nc1CC)c(C)cc(C)n2)c1ccc(cc1)c1c(S(=O)(=O)NC(=O)c2ccccc2)ccc(SCC)c1	154553-28-3	Benzamide, N-[[4,Ä≤-[(2-ethyl-5,7-dimethyl-3H-imidazo[4,5-b]pyridin-3-yl)methyl]	C33H34N4O4S	582.71	C	-8.9
2491	C(n1c2c(nc1CC)c(C)cc(C)n2)c1ccc(cc1)c1c(S(=O)(=O)NC(=O)NCCc2ccccc2)ccc(OC(C)C)c1	154553-67-0	[1,1,Ä≤-Biphenyl]-2-sulfonamide, 4,Ä≤-[(2-ethyl-5,7-dimethyl-3H-imidazo[4,5-b]p	C34H34F3N5O4S	665.73	C	-8.9
2492	O(C)[C@H]1[C@@H](O[C@H](COP(=O)(O)O)[C@H]1O)n1c2c(nc1)c(=O)nc[nH]2	1547295-52-2	1,3,5-Triazin-2-amine, N-(3-bromophenyl)-4-[5-(4-chlorophenyl)-1,3,4-oxadiazol-	C21H17BrClN7O2S	546.83	C	-8.9
2493	[C@@H](Cc1ccccc1)(NC(=O)[C@@H](NC(=O)N(Cc1nc(C(C)C)sc1)C)C(C)C)[C@@H]1[C@@H]([C@H](Cc2ccccc2)NC(=O)O	1551453-60-1	2,7,10,12-Tetraazatridec-4-enoic acid, 12-methyl-9-(1-methylethyl)-13-[2-(1-meth	C37H46N6O4S2	702.93	C	-8.9
2494	C(=O)([C@@H](NC(=O)OC)C(C)C)N1[C@H](c2[nH]c(c3c4c(C5OC4CC5)c(cc3)c3ccc(cc3)c3[nH]c(nc3)[C@H]3N(C(=O)	1560640-64-3	Carbamic acid, N-[(1S)-2-methyl-1-[[(2S)-2-[5-[4-[1,2,3,4-tetrahydro-8-[2-[(2S)-	C44H54N8O7	806.95	C	-8.9
2495	C(=O)([C@@H](NC(=O)OC)C(C)C)N1[C@H](c2[nH]c(c3c4c(cc3)c3ccc(cc3)c3[nH]c(nc3)[C@H]3N(C(=O)[C@@H](NC	1560649-96-8	Carbamic acid, N-[(1S)-2-methyl-1-[[(2S)-2-[5-[4-[1,2,3,4-tetrahydro-8-[2-[(2S)-	C44H55N9O6	805.96	C	-8.9
2496	C(=O)([C@@H](NC(=O)OC)C(C)C)N1[C@H](c2[nH]c(c3c4c(c(cc3)c3c5c(c(cc3)c3[nH]c(nc3)[C@H]3N(C(=O)[C@@H](	1560650-13-6	Carbamic acid, N-[(1S)-1-[[(2S)-2-[5-[8-[2,3-dihydro-7-[2-[(2S)-1-[(2S)-2-[(meth	C48H60N8O6	845.04	C	-8.9
2497	C(=O)([C@@H](NC(=O)OC)C(C)C)N1CC2(C[C@H]1c1[nH]c(cn1)c1ccc(cc1)c1ccc(cc1)c1[nH]c(nc1)[C@H]1N(C(=O)[C	1571138-18-5	Carbamic acid, N-[(1S)-1-[[(2S)-2-[5-[4,Ä≤-[2-[(3R)-2-[(2S)-2-[(methoxycarbonyl)	C44H57N9O6	807.98	C	-8.9
2498	CN1CC2(c3c(CC2)c(ccc3c2ccc(cc2)c2[nH]c(nc2)[C@H]2N(C(=O)[C@@H](NC(=O)OC)C(C)C)CCC2)c2[nH]c(nc2)[C@H]	1588404-18-5	Carbamic acid, N-[(1S)-1-[[(2S)-2-[5-[4-[4-[2-[(2S)-4,4-difluoro-1-[(2S)-2-[(met	C47H56F4N8O7	920.99	C	-8.9
2499	O=C1[C@@H]2[C@@H](c3c([C@H](OCc4cn(nn4)c4ccc(OC)cc4)[C@H]2CO1)cc1c(c3)OCO1)c1cc(OC)c(OC)c(OC)c1	1600500-23-9	Furo[3,Ä≤,4,Ä≤:6,7]naphtho[2,3-d]-1,3-dioxol-6(5aH)-one, 9-[[1-(2-fluorophenyl)	C31H28FN3O8	589.57	C	-8.9
2500	C(OC(=O)[C@H]1N(C(=O)OC(C)(C)C)CCC1)[C@H]1O[C@H]([C@H]2[C@@H]1OC(C)(C)O2)n1nc(C(=O)N)nc1	160522-65-6	L-Tryptophan, N-[(1,1-dimethylethoxy)carbonyl]-, ester with 1-[2,3-O-(1-methylet	C27H34N6O8	570.59	C	-8.9
2501	O=C1N2[C@H](Cn3c1c(O)c(=O)c(C(=O)NCc1c(F)cc(F)cc1)c3)O[C@@H]1C[C@H]2CC1	1611493-56-1	2,5-Methanopyrido[1,Ä≤,2,Ä≤:4,5]pyrazino[2,1-b][1,3]oxazepine-10-carboxamide, N	C21H19F2N3O5	431.39	C	-8.9
2502	N(C(CCCN(CC)CC)C)c1c2c(ncc1S(=O)C)cc(Cl)cc2	1611493-60-7	2,5-Methanopyrido[1,Ä≤,2,Ä≤:4,5]pyrazino[2,1-b][1,3]oxazepine-10-carboxamide, 2	C21H18F3N3O5	449.38	C	-8.9
2503	N(C(=O)c1cn2c(c(O)c1=O)C(=O)N1[C@H](C2)O[C@@H]2C[C@H]1CC2)C1(CC1)c1c(F)cc(F)cc1	1616339-81-1	2,5-Methanopyrido[1,Ä≤,2,Ä≤:4,5]pyrazino[2,1-b][1,3]oxazepine-10-carboxamide, N	C22H21F2N3O5	445.42	C	-8.9
2504	[C@H](C(=O)N1[C@@H]2[C@@H](C2)C[C@H]1C#N)(N)C12CC3(OC(=O)[C@@H](Cc4ccccc4)N)CC(C2)CC(C1)C3	1616609-30-3	L-Phenylalanine, 3-[(1S)-1-amino-2-[(1S,3S,5S)-3-cyano-2-azabicyclo[3.1.0]hex-2-	C27H34N4O3	462.58	C	-8.9
2505	O(CCCCCC(=O)NO)c1ccc(c2nc(nc(n2)N2CCOCC2)N2CCOCC2)cc1	1619257-66-7	Pentanamide, 5-[4-(4,6-di-4-morpholinyl-1,3,5-triazin-2-yl)phenoxy]-N-hydroxy-	C22H30N6O5	458.51	C	-8.9
2506	C([C@H](OCP(=O)(Cc1ccccc1)O)C)n1c2c(nc1)c(N)ncn2	1619257-73-6	Octanediamide, N1-[4-(4,6-di-4-morpholinyl-1,3,5-triazin-2-yl)phenyl]-N8-hydroxy	C25H35N7O5	513.59	C	-8.9
2507	N(C(=O)[C@H](Cc1ccccc1)C[C@@H](CN1[C@H](C(=O)NC(C)(C)C)CN(Cc2cccnc2)CC1)O)[C@H]1c2c([C@H](O)[C@H]1O)	162518-55-0	D-erythro-Pentonamide, 2,3,5-trideoxy-N-[(1S,2S,3S)-2,3-dihydro-2,3-dihydroxy-1H	C36H47N5O6	645.79	C	-8.9
2508	CN1C2c3c(c(ccc3c3c4c(c(cc3)c3[nH]c(nc3)[C@H]3N(C(=O)[C@@H](NC(=O)OC)C(C)C)CCC3)C3CC4CC3)c3[nH]c(nc3)	1627533-93-0	Carbamic acid, N,N,Ä≤-[(1,1,Ä≤,2,2,Ä≤,3,3,Ä≤,4,4,Ä≤-octahydro-1-methyl[5,5,Ä≤-]	C55H72N8O6	941.21	C	-8.9
2509	N(C(=O)C)[C@H]1[C@H]([C@@H]([C@@H](CO)O)O)OC(=C[C@@H]1NC(=N)N)C(=O)O.[NaH]	1627533-96-3	Carbamic acid, N,N,Ä≤-[(1,1,Ä≤,2,2,Ä≤,3,3,Ä≤,4,4,Ä≤-octahydro[5,5,Ä≤-bi-1,4-me	C50H58F4N8O6	943.04	C	-8.9
2510	C(P(=O)(OCC)OCC)(C/C=C(/CC/C=C(/CC/C=C(/CCC=C(C)C)\C)\C)\C)(C/C=C(/CC/C=C(/CCC=C(C)C)\C)\C)C(=O)OCC	1627841-80-8	1-Naphthalenesulfonic acid, 8-amino-5-[2-[2,5-dimethyl-4-[2-(3-sulfophenyl)diaze	C24H21N5O6S2	539.58	C	-8.9
2511	Nc1c2c(n(cn2)[C@@H]2O[C@H](CO)[C@H](O)[C@H]2O)nc(Cl)n1	1628452-99-2	1-Naphthalenediazonium, 4-[2-(8-hydroxy-6-sulfo-2-naphthalenyl)diazenyl]-7-sulfo	C20H13N4O7S2		C	-8.9
2512	N(C(=O)[C@H](Cc1ccccc1)C[C@@H](CN1[C@H](C(=O)NC(C)(C)C)CN(CCOCc2ccccc2)CC1)O)[C@H]1c2c(C[C@H]1O)cccc	164725-29-5	1-Piperazinepentanamide, N-(2,3-dihydro-2-hydroxy-1H-inden-1-yl)-2-[[(1,1-dimeth	C40H49N5O4	663.85	C	-8.9
2513	C(=O)([C@@H](NC(=O)OC)C(C)C)N1CC2(C[C@H]1c1[nH]c(cn1)c1ccc(cc1)c1cc3c(cc1)c1c(cc3)nc([nH]1)[C@H]1N(C	1653966-60-9	Carbamic acid, N-[(1S)-1-[[(3S,5S)-3-[2,Ä≤-[(6S)-5-[(2S)-2-[(methoxycarbonyl)am	C50H58N8O7	883.04	C	-8.9
2514	C(=O)([C@@H](NC(=O)OC)C(C)C)N1[C@@H](C[C@H]2[C@@H]1CCCC2)c1[nH]c2-c3c(CCc2n1)cc(cc3)c1ccc(cc1)c1[nH]	1653966-64-3	Carbamic acid, N-[(1S)-1-[[(3S,5S)-3-[7-[4-[2-[(3S,5S)-2-[(2S)-2-[(methoxycarbor	C50H62N8O8	903.08	C	-8.9
2515	C(=O)([C@@H](NC(=O)OC)C(C)C)N1[C@H]2[C@H](C2)C[C@H]1c1[nH]c2c(n1)ccc1c2ccc(c1)c1ccc(cc1)c1[nH]c(nc1)	1653966-77-8	Carbamic acid, N-[(1S)-2-methyl-1-[[(3S,5S)-3-[5-[7-[2-[(2S,3aS,7aS)-octahydro-1	C50H62N8O7	887.08	C	-8.9
2516	C(=O)([C@@H](NC(=O)OC)C(C)C)N1CC2(C[C@H]1c1[nH]c3c(n1)ccc1c3ccc(c1)c1ccc(cc1)c1[nH]c(nc1)[C@H]1N(C(=	1653966-91-6	Carbamic acid, N-[(1S)-1-[[(2S)-2-[7-[4-[2-[(3S,5R)-2-[(2S)-2-[(methoxycarbonyl)	C46H56N8O7	832.99	C	-8.9
2517	O(C(OC(=O)OCCOC)C)c1c(C(=O)NCc2ccc(F)cc2)nc(C(NC(=O)c2nnc(C)o2)(C)C)n(C)c1=O	16699-28-8	Guanosine, 8-(9H-fluoren-2-ylamino)-	C23H22N6O5	462.46	C	-8.9
2518	C(C)(C)c1n([C@H]2C[C@H]3N(CC[C@H](NC(=O)C4CC(F)(F)CNC4)c4ccccc4)[C@@H](C2)CC3)c(C)nn1	1673569-89-5	2,8-Diazaspiro[4.5]decane-3-carboxylic acid, 8-[2-amino-6-[(1-tricyclo[3.3.1.13,	C25H38N6O2	454.61	C	-8.9
2519	O=C1[C@@H]2[C@@H](c3c([C@H](OC(=O)N4CCC(CCn5c6c(nc5Sc5cc7c(cc5Br)OCO7)c(N)ncn6)CC4)[C@H]2CO1)cc1c(c3	1680212-59-2	1-Piperazinecarboxylic acid, 4-[[4-[3-[2,4-dihydroxy-5-(1-methylethyl)phenyl]-1,	C45H47N5O12	849.88	C	-8.9
2520	N(c1nc(nc(n1)N1CCOCC1)N1CCOCC1)c1ccc(C(=O)/C=C/c2cc(Cl)ccc2)cc1	1710363-31-7	2-Propen-1-one, 3-(3,4-dimethoxyphenyl)-1-[4-[(4,6-di-4-morpholinyl-1,3,5-triazi	C28H32N6O5	532.59	C	-8.9
2521	O=c1n(c2c([nH]1)c(=O)nc(N)[nH]2)[C@H]1[C@H](O)[C@H]2[C@H](O1)COP(=O)(C)O2	173366-68-2	Guanosine, cyclic 3,Ä≤,5,Ä≤-(P-phenylphosphonate)	C16H16N5O6P	405.3	C	-8.9
2522	C(Cc1ccccc1)[C@]1(CCC(F)(F)F)CC(=C([C@@H](C(C)(C)C)c2cc(NS(=O)(=O)c3ccc(C#N)cn3)ccc2)C(=O)O1)O	174484-16-3	2-Pyridinesulfonamide, 5-cyano-N-[3-[1-[5,6-dihydro-4-hydroxy-2-oxo-6-(2-phenyle	C33H34F3N3O5S	641.7	C	-8.9
2523	C(n1c2c(nc1CC)c(C)cc(C)n2)c1ccc(cc1)c1c(S(=O)(=O)NC(=O)c2cc(O[11CH3])ccc2)cccc1	174844-65-6	Benzamide, N-[[4,Ä≤-[(2-ethyl-5,7-dimethyl-3H-imidazo[4,5-b]pyridin-3-yl)methyl]	C31H30N4O4S		C	-8.9
2524	N(c1c(CO)cc(C=CC#N)cc1C)c1nc(Nc2ccc(C#N)cc2)ncc1	179817-97-1	2-Naphthalenesulfonic acid, 5,5,Ä≤-[(3,3,Ä≤-dimethyl[1,1,Ä≤-biphenyl]-4,4,Ä≤-di	C34H26N4O8S2	682.72	C	-8.9
2525	FC1(F)c2c(-c3c(cc(cc3)c3[nH]c(nc3)[C@H]3N(C(=O)[C@@H](NC(=O)OC)C(C)C)CCC3)C1(F)F)ccc(c2)c1[nH]c(nc1)	1801161-78-3	Carbamic acid, N-[(1S)-2-methyl-1-[[(2S)-2-[5-[9,9,10,10-tetrafluoro-9,10-dihydr	C44H50F4N8O8	894.91	C	-8.9
2526	Cc1c2c(cc(C)c1Nc1nc(Nc3ccc(C#N)cc3)ncc1)cc(C#N)cc2	1801429-05-9	2-Naphthalenecarbonitrile, 6-[[2-[(4-cyanophenyl)amino]-4-pyrimidinyl]amino]-5-m	C23H16N6	376.41	C	-8.9
2527	C(=O)([C@@H](NC(=O)OC)C(C)C)N1[C@H](c2[nH]c(c3c4c(c(cc3)c3ccc(cc3)c3[nH]c(nc3)[C@H]3N(C(=O)[C@@H](NC	1802921-87-4	Carbamic acid, N-[(1S)-2-methyl-1-[[(2S,5S)-2-methyl-5-[5-[4-[1,2,3,4-tetrahydro	C47H60N8O6	833.03	C	-8.9
2528	O=C1[C@@H]2[C@@H](c3c([C@H](OC(=O)[C@@H](Cc4ccccc4)N4C(=O)c5c(C4=O)cccc5)[C@H]2CO1)cc1c(c3)OCO1)c1cc	1809321-37-6	2H-Isoindole-2-acetic acid, 1,3-dihydro-1,3-dioxo-Œ±-(phenylmethyl)-, (5R,5aR,8a	C39H33NO11	691.68	C	-8.9
2529	C(OC[C@@H]1[C@@H](OC(=O)C=Cc2ccccc2)C[C@@H](C1=C)n1c2c(nc1)c(=O)nc(NC(=O)OC(C)(C)C)[nH]2)(c1ccccc1)(	1860842-96-1	Carbamic acid, N-[9-[(1S,3R,4S)-4-(benzoyloxy)-2-methylene-3-[(triphenylmethoxy)	C43H41N5O6	723.82	C	-8.9
2530	O=c1c2c(n(cn2)[C@@H]2C(=C)[C@H](CO)[C@@H](OC(=O)C)C2)[nH]c(N)n1	1860843-01-1	Cyclohexanecarboxylic acid, (1S,2R,4S)-4-[2-[[(1,1-dimethylethoxy)carbonyl]aminc	C43H47N5O6	729.86	C	-8.9
2531	[C@@H](Cc1ccccc1)(NC(=O)[C@H](CCN1CCOCC1)NC(=O)N(Cc1nc(C(C)C)sc1)C)CC[C@H](Cc1ccccc1)NC(=O)OCc1cncs1	1865786-70-4	2,5,6,10,13-Pentaazatetradecanedioic acid, 3,12-bis(1,1-dimethylethyl)-8-hydroxy	C40H53N7O5S2.C38H52N6O7		C	-8.9
2532	O=C1[C@@H]2[C@@H](c3c([C@H](OC(=O)CCCC[C@H]4SC(SCC4)c4ccc(C(F)(F)F)cc4)[C@H]2CO1)cc1c(c3)OCO1)c1cc(O	1875123-85-5	1,3-Dithiane-4-pentanoic acid, 2-[3-(trifluoromethyl)phenyl]-, (5R,5aR,8aR,9R)-5	C38H39F3O9S2	760.84	C	-8.9
2533	C=C1[C@H](n2c3c(nc2)c(=O)nc(N)[nH]3)C[C@@H](O)[C@@H]1CO	1883795-13-8	2-Propen-1-one, 1-[4-[[4-(4-morpholinyl)-6-[[3-(trifluoromethyl)phenyl]amino]-1,	C27H23F3N6O2S	552.57	C	-8.9
2534	C(=O)(O)c1c(sc2c1CC(C)(C)CC2)c1c(CNc2ccc(C[C@@H]([C@@H](CN(S(=O)(=O)c3cc4c(cc3)ncs4)CC3CCCC3)O)NC(=O	1887206-53-2	Benzo[b]thiophene-3-carboxylic acid, 2-[2-[[[4-[(2S,3R)-4-[(6-benzothiazolylsulf	C48H53F3N4O9S3	983.15	C	-8.9
2535	O[C@H]1[C@H](n2c3c(nc2)c(N)ncn3)O[C@H](COP(=O)(O)O)[C@H]1O	1887206-54-3	Benzo[b]thiophene-3-carboxylic acid, 2-[2-[[[4-[(2S,3R)-4-[(6-benzothiazolylsulf	C49H55F3N4O9S3	997.17	C	-8.9
2536	N(=Nc1ccc(cc1)c1ccc(N=Nc2c(N)c3c(c(S(=O)(=O)O)c2)cccc3)cc1)c1c2c(cc(S(=O)(=O)O)cc2O)ccc1N.[NaH]	193612-20-3	2,7-Naphthalenedisulfonic acid, 4,4,Ä≤-[(3,3,Ä≤-dimethyl[1,1,Ä≤-biphenyl]-4,4,Ä	C34H26N4O14S4	842.85	C	-8.9
2537	O=c1n([C@@H]2O[C@H](CO)[C@@H](O)C2)cc(/C=C/CN)c(=O)[nH]1	195826-13-2	Guanosine, 8-[(2-amino-6,7-dihydro-6-oxo-1H-purin-8-yl)oxy]-	C15H16N10O7	448.35	C	-8.9
2538	O[C@H]1[C@H](n2c3c(nc2)c(=O)nc(NCc2ccc(OCc4ccc(O)cc4)cc2)[nH]3)O[C@H](CO)[C@H]1O	197165-29-0	Retinoic acid, 2,Ä≤-ester with 9-Œ≤-D-arabinofuranosyl-9H-purin-6-amine	C30H39N5O5	549.66	C	-8.9
2539	O[C@H]1[C@H](n2c3c(nc2)c(=O)nc(N)[nH]3)O[C@H](COc2c(F)c(=O)[nH]c(O)c2F)[C@H]1O	197903-82-5	Guanosine, 5,Ä≤-O-(2,3,5,6-tetrafluoro-4-nitrophenyl)-	C16H12F4N6O7	476.3	C	-8.9
2540	O(C(=O)C)c1c2n(N([C@H]3N(C2=O)CCOC3)[C@H]2c3c(CSc4c2cccc4)c(F)c(F)cc3)ccc1=O	1985606-15-2	Propanoic acid, 2-[[[[[(12aR)-12-[(11S)-7,8-difluoro-6,11-dihydrodibenzo[b,e]thi	C31H29F2N3O9S	657.64	C	-8.9
2541	O(COC(=O)O[C@H](C(=O)OC)c1ccccc1)c1c2n(N([C@H]3N(C2=O)CCOC3)[C@H]2c3c(CSc4c2cccc4)c(F)c(F)cc3)ccc1=O	1985607-04-2	Propanoic acid, 2-[[[[[(12aR)-12-[(11S)-7,8-difluoro-6,11-dihydrodibenzo[b,e]thi	C32H31F2N3O9S	671.66	C	-8.9
2542	O(C(=O)OCCOC)c1c2n(N([C@H]3N(C2=O)CCOC3)[C@H]2c3c(CSc4c2cccc4)c(F)c(F)cc3)ccc1=O	1985607-08-6	Carbamic acid, N-methyl-N-phenyl-, (12aR)-12-[(11S)-7,8-difluoro-6,11-dihydrodib	C32H26F2N4O5S	616.63	C	-8.9
2543	O(C(=O)O[C@@H]1CCOC1)c1c2n(N([C@H]3N(C2=O)CCOC3)[C@H]2c3c(CSc4c2cccc4)c(F)c(F)cc3)ccc1=O	1985607-12-2	Carbonic acid, (12aR)-12-[(11S)-7,8-difluoro-6,11-dihydrodibenzo[b,e]thiepin-11-	C29H25F2N3O7S	597.59	C	-8.9
2544	O(Cc1cccnc1)c1c2n(N([C@H]3N(C2=O)CCOC3)[C@H]2c3c(CSc4c2cccc4)c(F)c(F)cc3)ccc1=O	1985607-58-6	Propanoic acid, 2-[[[[(12aR)-12-[(11S)-7,8-difluoro-6,11-dihydrodibenzo[b,e]thie	C33H33F2N3O8S	669.69	C	-8.9
2545	C(=O)([C@@H](NC(=O)OC)C(C)C)N1[C@H](c2[nH]c3c4c(cc(cc4)c4c5c(c(cc4)c4[nH]c(nc4)[C@H]4N(C(=O)[C@@H](N	2007900-85-6	Carbamic acid, N-[(1S)-1-[[(2S,5S)-2-[5-[2,3-dihydro-7-[2-[(2S,5S)-1-[(2S)-2-[(m	C48H60N8O6	845.04	C	-8.9
2546	C(=O)([C@@H](NC(=O)OC)C(C)C)N1[C@@H](C2CC1CC2)c1[nH]c(cn1)c1c2c(c(cc1)c1cc3c(cc1)c1c(cc3)nc([nH]1)[C	2007901-01-9	Carbamic acid, N-[(1S)-1-[[(2S)-2-[7-[2,3-dihydro-7-[2-[(2S)-1-[(2S)-2-[(methoxy	C45H56N8O6	804.98	C	-8.9
2547	C(=O)([C@@H](NC(=O)OC)C(C)C)N1[C@H](c2[nH]c3c4c(cc(cc4)c4c5c(c(cc4)c4[nH]c(nc4)[C@H]4N(C(=O)[C@@H](N	2007901-52-0	Carbamic acid, N-[(1S)-1-[[(2S,5S)-2-[5-chloro-4-[2,3-dihydro-7-[2-[(2S,5S)-1-[(	C47H57ClN8O6	865.46	C	-8.9
2548	C(=O)([C@@H](NC(=O)OC)C(C)C)N1[C@H](c2[nH]c3c4c(cc(cc4)c4c5c(c(cc4)c4[nH]c(nc4)[C@H]4N(C(=O)[C@@H](N	2007901-74-6	Carbamic acid, N-[(1S)-2-methyl-1-[[(5S)-2-methyl-5-[7-[5,6,7,8-tetrahydro-4-[2-	C47H58N8O6	831.01	C	-8.9
2549	C(=O)([C@@H](NC(=O)OC)C(C)C)N1[C@H](c2[nH]c3c4c(cc(cc4)c4c5c(c(cc4)c4[nH]c(nc4)[C@H]4N(C(=O)[C@@H](N	2007901-88-2	Carbamic acid, N-[(1S)-1-[[(2S,4S)-2-[7-[2,3-dihydro-7-[2-[(2S,4S)-1-[(2S)-2-[(m	C49H62N8O8	891.07	C	-8.9
2550	O=c1c2c(n(cn2)[C@H]2O[C@@](CO)(C)OC2)[nH]c(N)n1	2007901-91-7	Carbamic acid, N-[(1S)-1-[[(2S,4S)-4-[(difluoromethoxy)methyl]-2-[5-[2,3-dihydro	C49H60F2N8O8	927.05	C	-8.9
2551	O(C(=O)C)[C@H]1[C@@H](O[C@H](COCC(=O)O)[C@H]1OC(=O)C)n1nc(C(=O)N)nc1	1002336-62-0	1H-1,2,4-Triazole-3-carboxamide, 1-[2,3-O-(methoxymethylene)-5-O-[4-[[[4,6-O-[(S	C31H34N4O13	670.62	A/B	-8.8
2552	C=C1[C@@H](n2c3c(nc2)c(=O)nc(N)[nH]3)C[C@H](OC(=O)[C@@H](NC(=O)OCc2ccccc2)C(C)C)[C@H]1CO	1003853-24-4	L-Valine, N-[(phenylmethoxy)carbonyl]-, [(1R,3S,5S)-3-(2-amino-1,6-dihydro-6-oxo	C25H30N6O6	510.54	A/B	-8.8
2553	C=C1[C@@H](n2c3c(nc2)c(=O)nc(N)[nH]3)C[C@H](OC(=O)[C@@H](NC(=O)OCc2ccccc2)C(C)C)[C@H]1COC(=O)[C@@H](	1003853-25-5	L-Valine, N-[(phenylmethoxy)carbonyl]-, (1S,2R,4S)-4-(2-amino-1,6-dihydro-6-oxo-	C25H30N6O6	510.54	A/B	-8.8
2554	N(=C1c2c(N(CN(CC)CC)C1=O)cccc2)NC(=S)Nc1ccccc1	100847-02-7	Hydrazinecarbothioamide, 2-[1,2-dihydro-2-oxo-1-[(4-phenyl-1-piperazinyl)methyl]	C26H26N6OS	470.59	A/B	-8.8
2555	C(=O)([C@H](NC(=O)OC)c1ccccc1)N1[C@@H](CCC1)c1[nH]c(cn1)c1ccc(cc1)c1ccc(cc1)c1[nH]c(nc1)[C@H]1N(C(=O	1009107-42-9	Carbamic acid, N-[(1R)-2-[(2S)-2-[5-[4,Ä≤-[2-[(2S)-1-acetyl-2-pyrrolidinyl]-1H-	C38H39N7O4	657.76	A/B	-8.8
2556	C(=O)([C@H](NC(=O)OC)c1ccccc1)N1[C@@H](CCC1)c1[nH]c(cn1)c1ccc(cc1)c1ccc(cc1)c1[nH]c(nc1)[C@H]1N(C(=O	1009107-47-4	Carbamic acid, N-[(1R)-2-[(2S)-2-[5-[4,Ä≤-[2-[(2S)-1-(2-cyclopropylacetyl)-2-pyr	C41H43N7O4	697.82	A/B	-8.8
2557	C(=O)([C@H](NC(=O)OC)c1ccccc1)N1[C@@H](CCC1)c1[nH]c(cn1)c1ccc(cc1)c1ccc(cc1)c1[nH]c(nc1)[C@H]1N(C(=O	1009107-54-3	Carbamic acid, N-[(1R)-2-[(2S)-2-[5-[4,Ä≤-[2-[(2S)-1-[2-(acetylamino)acetyl]-2-p	C40H42N8O5	714.81	A/B	-8.8
2558	C(=O)([C@H](N1CCCC1)c1ccccc1)N1[C@@H](CCC1)c1[nH]c(cn1)c1ccc(cc1)c1ccc(cc1)c1[nH]c(nc1)[C@H]1N(C(=O)	1009111-82-3	Carbamic acid, N-[(1S)-1-methyl-2-oxo-2-[(2S)-2-[5-[4,Ä≤-[2-[(2S)-1-[(2R)-2-pher	C43H48N8O4	740.89	A/B	-8.8
2559	C(=O)([C@@H](NC(=O)OC)C(C)C)N1[C@@H](CCC1)c1[nH]c(cn1)c1ccc(cc1)c1ccc(cc1)c1[nH]c(nc1)[C@H]1N(C(=O)[	1009114-58-2	Carbamic acid, N-[(1S)-1-[[(2S)-2-[5-[4,Ä≤-[2-[(2S)-1-[[cis-4-[(methoxycarbonyl)	C42H52N8O6	764.91	A/B	-8.8
2560	C(C)(C)(F)[C@@H]1n2c3c4c(nc(N)c3nc2C(CCC)OC1)cccc4	1010105-81-3	8H-[1,4]Oxazino[4,Ä≤,3,Ä≤:1,2]imidazo[4,5-c]quinolin-6-amine, 8-ethyl-11-(1-flu	C18H21FN4O	328.38	A/B	-8.8
2561	[C@@H](Cc1cc(C(F)(F)F)ccc1)(NC(=O)N1CCN(C)CC1)C(=O)N[C@@H](CCc1ccccc1)/C=C/S(=O)(=O)c1ccccc1	1011726-27-4	1-Piperazinecarboxamide, 4-methyl-N-[(1S)-1-[(3-methylphenyl)methyl]-2-oxo-2-[[(	C33H40N4O4S	588.76	A/B	-8.8
2562	S(=O)(=O)(c1cc2c(cc1)NC(=O)C2)N1CCC(CN)CC1	10181-47-2	1-Naphthalenesulfonic acid, 4-[2-[2,4-diamino-5-[2-[4,Ä≤-[2-(2,6-diamino-3-methy	C35H30N10O6S2.2Na		A/B	-8.8
2563	N(C(=O)C)c1c2[C@H]([C@@H]3[C@@H]([C@@H](O)c2cc2c1OCO2)COC3=O)c1cc(OC)c(OC)c(OC)c1	1025945-68-9	2-Naphthalenesulfonic acid, 5-amino-8-[(1E)-2-[4-[(1E)-2-[4-[(1E)-2-(4-amino-7-s	C34H26N6O9S3	758.8	A/B	-8.8
2564	[C@@H](COC)(N1[C@@H](C)CN(CC1)C1(C)CCN(C(=O)c2c(C)ncnc2)CC1)c1ccc(C(F)(F)F)cc1	1026042-89-6	Benzonitrile, 4-[[2-[(2-cyanophenyl)amino]-4-pyrimidinyl]amino]-3,5-dimethyl-	C20H16N6	340.38	A/B	-8.8
2565	C(COP(=O)(COCCCO)O)n1c2c(nc1)c(N)ncn2	1027922-68-4	Not Yet Assigned	C20H21FN5O9PS	557.45	A/B	-8.8
2566	N(c1nc(Nc2ccc(C#N)cc2)nc(CNCc2cccnc2)c1)c1c(C)cc(/C=C/C#N)cc1C	1037445-26-3	Benzonitrile, 4-[[4-[[4-[(1E)-2-cyanoethenyl]-2,6-dimethylphenyl]amino]-6-[[(2-f	C28H25N7O	475.54	A/B	-8.8
2567	N(c1c(C)cc(/C=C/C#N)cc1C)c1nc(Nc2ccc(C#N)cc2)nc(CNCc2cccs2)c1	1037445-34-3	Benzonitrile, 4-[[4-[[4-[(1E)-2-cyanoethenyl]-2,6-dimethylphenyl]amino]-6-[[(4-p	C29H26N8	486.57	A/B	-8.8
2568	N(c1c(C)cc(/C=C/C#N)cc1C)c1nc(Nc2ccc(C#N)cc2)nc(CCl)c1	1037445-59-2	4-Pyrimidinecarboxamide, 6-[[4-[(1E)-2-cyanoethenyl]-2,6-dimethylphenyl]amino]-2	C25H23N7O2	453.5	A/B	-8.8
2569	C(=O)(NCC=C)c1c(Nc2c(C)cc(/C=C/C#N)cc2C)nc(Nc2ccc(C#N)cc2)nc1COC	1037452-39-3	5-Pyrimidinecarboxamide, 4-[[4-[(1E)-2-cyanoethenyl]-2,6-dimethylphenyl]amino]-2	C28H27N7O2	493.56	A/B	-8.8
2570	N(c1c(C(=O)N2CCC(O)CC2)c(COC)nc(Nc2ccc(C#N)cc2)n1)c1c(C)cc(/C=C/C#N)cc1C	1037452-45-1	Benzonitrile, 4-[[4-[[4-[(1E)-2-cyanoethenyl]-2,6-dimethylphenyl]amino]-6-(metho	C30H32N8O2	536.63	A/B	-8.8
2571	N(c1c(C(=O)N2CCOCC2)c(COC)nc(Nc2ccc(C#N)cc2)n1)c1c(C)cc(/C=C/C#N)cc1C	1037452-47-3	Benzonitrile, 4-[[4-[[4-[(1E)-2-cyanoethenyl]-2,6-dimethylphenyl]amino]-5-[(4-hy	C30H31N7O3	537.61	A/B	-8.8
2572	C(=C1C(=O)C(=C(O)O)C(=O)C=C1)(c1c(O)c(C(=O)O)c(O)c(C)c1)c1c(O)c(C(=O)O)c(O)c(C)c1	1049976-37-5	Benzoic acid, 3,3,Ä≤-[(3-carboxy-4-hydroxy-6-oxo-2,4-cyclohexadien-1-ylidene)met	C24H18O12	498.39	A/B	-8.8
2573	S(=O)(=O)(O)c1c2c(c(S(=O)(=O)O)cc(/N=N/c3cc(S(=O)(=O)O)c(CCc4c(S(=O)(=O)O)cc(/N=N/c5c(N)c6c(c(S(=O)(	1052521-54-6	Not Yet Assigned	C32H24N6O14S5.Na		A/B	-8.8

2574	C(=O)(Nc1c(ccnc1)N1C[C@@H](N)CCC1)c1nc(C23CC4CC(C3)CC(C2)C4)ccc1N	10527-47-6	Guanosine 5,Ä≤-(tetrahydrogen triphosphate), P,Ä≤,Ä≤,Ü15,Ä≤-ester with adenosir	C20H27N10O17P3	772.41	A/B	-8.8
2575	N(=N\c1cc(S(=O)(=O)O)c(CCc2c(S(=O)(=O)O)cc(/N=N/c3c4c(cc(S(=O)(=O)O)cc4)c(N)cc3)cc2)cc1)/c1c2c(cc(S(	1053055-44-9	Not Yet Assigned	C32H24N6O14S5	876.89	A/B	-8.8
2576	O=c1n(ccc(N)n1)C1CS(=O)CO1	1055970-12-1	4-Pyrimidinecarbonitrile, 6-[[(1E)-1-(cyanomethylene)-2,3-dihydro-4,6-dimethyl-1	C25H19N7	417.47	A/B	-8.8
2577	O=c1n([C@H]2C[C@H](OP(=O)(OP(=O)(OP(=O)(O)O)O)O)[C@@H](CO)O2)cc(C)c(=O)[nH]1	1056950-70-9	Propanoic acid, 2-[[(2,4,5,7-tetranitro-9H-fluoren-9-ylidene)amino]oxy]-, (5R,5a	C38H29N5O18	843.66	A/B	-8.8
2578	N(C(CCCN(CCOP(=O)(N)O)CC)C)c1c2c(ncc1)cc(Cl)cc2	1057218-76-4	Propanoic acid, 2-[[[2-[[[4-[(2S,3R)-2-[[[[(3R,3aS,6aR)-hexahydrofuro[2,3-b]fura	C41H56N3O13PS	861.93	A/B	-8.8
2579	O[C@H]1[C@H](n2c3c(nc2)c(=O)nc(C#C)[nH]3)O[C@H](COP(=O)(O)O)[C@H]1O	1072329-82-8	Thymidine, 2,Ä≤,3,Ä≤-didehydro-3,Ä≤-deoxy-5,Ä≤-O-[2-oxido-6-[5-oxo-5-(phenylmet	C29H31N2O9P	582.54	A/B	-8.8
2580	O(C[C@H]1O[C@H](C=C1)n1c(=O)[nH]c(=O)c(C)c1)P1(=O)Oc2c(cc(CCC(=O)N3[C@H](C(=O)OC)CCC3)cc2)CO1	1073242-66-6	L-Alanine, N-[3-[2-(2,Ä≤,3,Ä≤-didehydro-3,Ä≤-deoxythymidin-5,Ä≤-O-yl)-2-oxido-4	C23H26N3O10P	535.44	A/B	-8.8
2581	N(CCNCC=C1c2c(CCc3c1cccc3)cccc2)C12CC3CC(C2)CC(C1)C3	108768-09-8	Piperazine, 1-[2-(10,11-dihydro-5H-dibenzo[a,d]cyclohepten-5-ylidene)ethyl]-4-tr	C31H38N2	438.65	A/B	-8.8
2582	N(C12CC3CC(C2)CC(C1)C3)c1nc(NC2CCCCCC2)nc(NCC(=O)O)n1	1091628-79-3	1,2-Propanediol, 3-[[4-(cycloheptylamino)-6-(tricyclo[3.3.1.13,7]dec-1-ylamino)-	C23H38N6O2	430.59	A/B	-8.8
2583	C(NC(=O)c1nnc(C)o1)(C([2H])([2H])[2H])(C([2H])([2H])[2H])c1nc(C(=O)NCc2ccc(F)cc2)c(O)c(=O)n1C([2H])(	1100750-87-5	4-Pyrimidinecarboxamide, N-[(4-fluorophenyl)methyl-d2]-1,6-dihydro-5-hydroxy-1-m	C20H16D5FN6O5		A/B	-8.8
2584	C(NC(=O)c1nnc(C([2H])([2H])[2H])o1)(C([2H])([2H])[2H])(C([2H])([2H])[2H])c1nc(C(=O)NCc2ccc(F)cc2)c(O	1100750-90-0	4-Pyrimidinecarboxamide, N-[(4-fluorophenyl)methyl-d2]-1,6-dihydro-5-hydroxy-1-(	C20H16D5FN6O5		A/B	-8.8
2585	C(NC(=O)c1nnc(C)o1)(C)(C)c1nc(C(=O)NC(c2c(c(c(F)c(c2[2H])[2H])[2H])[2H])([2H])[2H])c(O)c(=O)n1C	1100750-97-7	4-Pyrimidinecarboxamide, N-[(4-fluorophenyl-2,3,5,6-d4)methyl]-1,6-dihydro-5-hyc	C20H14D7FN6O5		A/B	-8.8
2586	C(#Cc1cc(C(F)(F)F)ccc1)c1n(nc(C(=O)N)n1)[C@@H]1O[C@H](CO)[C@@H](O)[C@H]1O	1106736-03-1	1H-1,2,4-Triazole-3-carboxamide, 1-Œ≤-D-ribofuranosyl-5-[2-[4-(trifluoromethyl)p	C17H15F3N4O5	412.32	A/B	-8.8
2587	O=C1[C@@H]2[C@@H](c3c([C@H](OC(=O)c4ccc(SSc5ccc(C(=O)O[C@H]6c7c([C@H]([C@@H]8[C@@H]6COC8=O)c6cc(OC)c	1107652-05-0	Benzoic acid, 4,4,Ä≤-dithiobis-, 1,1,Ä≤-bis[(5R,5aR,8aR,9R)-5,5a,6,8,8a,9-hexah	C58H50O18S2	1099.14	A/B	-8.8
2588	N(=Nc1c(CCCC)cccc1)c1c2c(cc(S(=O)(=O)O)cc2)ccc1O	1107652-06-1	3-Pyridinecarboxylic acid, 6,6,Ä≤-dithiobis-, 3,3,Ä≤-bis[(5R,5aR,8aR,9R)-5,5a,6	C56H48N2O18S2	1101.11	A/B	-8.8
2589	O[C@@H]1[C@H](n2c3c(nc2)c(N)ncn3)O[C@H](CO)[C@H]1OP(=O)(OCCOC(CO)n1c2c(nc1)c(N)ncn2)O	111392-87-1	9H-Purin-6-amine, 9-[5-O-[2-(1,3-dihydro-1,3-dioxo-2H-isoindol-2-yl)-1-oxopropyl	C21H20N6O7	468.42	A/B	-8.8
2590	C(Cc1ccccc1)[C@]1(C(C(C([2H])([2H])[2H])([2H])[2H])([2H])[2H])CC(=C([C@H](CC)c2cc(NS(=O)(=O)c3ccc(C(	1126619-19-9	Not Yet Assigned	C31H29D4F3N2O5S		A/B	-8.8
2591	C(=O)([C@@H](NC(=O)OC)C)N1CC2(C[C@H]1c1[nH]c(cn1)c1ccc(cc1)c1ccc(cc1)c1[nH]c(nc1)[C@H]1N(C(=O)[C@@H]	1129634-55-4	Carbamic acid, N,N,Ä≤-[[1,1,Ä≤-biphenyl]-4,4,Ä≤-diylbis[1H-imidazole-5,2-diyl-(	C38H42N8O6	706.79	A/B	-8.8
2592	O=c1n2[C@@H]3C[C@H]([C@@H](CO)O3)OP(=O)(O)OC[C@H]3O[C@H](n4cc(CCc(c2)c(=O)[nH]1)c(=O)[nH]c4=O)C[C@@H	1134943-90-0	3-Quinolinecarboxylic acid, 7-[4-[[(4S)-6-chloro-4-(2-cyclopropylethynyl)-2-oxo-	C33H29ClF4N4O6	689.05	A/B	-8.8
2593	C(Cc1ccccc1)[C@]1(CCC)CC(=C([C@H](CC)c2cc(N(S(=O)(=O)c3ccc(C(F)(F)F)cn3)O)ccc2)C(=O)O1)O	1141510-04-4	2-Pyridinesulfonamide, N-[3-[(1R)-1-[(6R)-5,6-dihydro-4-hydroxy-2-oxo-6-(2-pheny	C31H33F3N2O5S		A/B	-8.8
2594	O(c1nc(Nc2c(c(c(C#N)c(c2[2H])[2H])[2H])[2H])nc(N)c1Br)c1c(C([2H])([2H])[2H])c(c(C#N)c(c1C([2H])([2H]	1142065-19-7	Methanimidamide, N,Ä≤-[1,2-dihydro-1-[(2S,5R)-2-(hydroxymethyl)-1,3-oxathiolan-5	C23H22F2N4O3S	472.51	A/B	-8.8
2595	O(c1nc(Nc2cc(c(C#N)c(c2)[2H])[2H])nc(N)c1Br)c1c(C([2H])([2H])[2H])c(c(C#N)c(c1C([2H])([2H])[2H])[2H]	1142095-64-4	Benzonitrile-2,6-d2, 4-[[6-amino-5-bromo-2-[(4-cyanophenyl-2,6-d2)amino]-4-pyrim	C20H5BrD10N6O		A/B	-8.8
2596	O(c1nc(Nc2ccc(C#N)cc2)nc(N)c1Br)c1c(C([2H])([2H])[2H])c(c(C#N)c(c1C([2H])([2H])[2H])[2H])[2H]	1142095-71-3	Benzonitrile-2,3,5,6-d4, 4-[[4-amino-5-bromo-6-[4-cyano-2,6-di(methyl-d3)phenoxy	C20H5BrD10N6O		A/B	-8.8
2597	O(c1nc(Nc2c(cc(C#N)cc2[2H])[2H])nc(N)c1Br)c1c(C([2H])([2H])[2H])cc(C#N)cc1C([2H])([2H])[2H]	1142095-74-6	Benzonitrile-2,6-d2, 4-[[6-amino-5-bromo-2-[(4-cyanophenyl)amino]-4-pyrimidinyl]	C20H7BrD8N6O		A/B	-8.8
2598	O(c1nc(Nc2cc(c(C#N)c(c2)[2H])[2H])nc(N)c1Br)c1c(C([2H])([2H])[2H])cc(C#N)cc1C([2H])([2H])[2H]	1142095-77-9	Benzonitrile-3,5-d2, 4-[[4-amino-5-bromo-6-[4-cyano-2,6-di(methyl-d3)phenoxy]-2-	C20H7BrD8N6O		A/B	-8.8
2599	O(c1nc(Nc2c(c(c(C#N)c(c2[2H])[2H])[2H])[2H])nc(N)c1Br)c1c(C)cc(C#N)cc1C	1142095-90-6	Benzonitrile-2,6-d2, 4-[[6-amino-5-bromo-2-[(4-cyanophenyl-3,5-d2)amino]-4-pyrim	C20H11BrD4N6O		A/B	-8.8
2600	O(c1nc(Nc2c(cc(C#N)cc2[2H])[2H])nc(N)c1Br)c1c(C)cc(C#N)cc1C	1142095-96-2	Benzonitrile-2,6-d2, 4-[[6-amino-5-bromo-2-[(4-cyanophenyl)amino]-4-pyrimidinyl]	C20H13BrD2N6O		A/B	-8.8
2601	O(c1nc(Nc2cc(c(C#N)c(c2)[2H])[2H])nc(N)c1Br)c1c(C)cc(C#N)cc1C	1142096-00-1	Benzonitrile-3,5-d2, 4-[[4-amino-5-bromo-6-(4-cyano-2,6-dimethylphenoxy)-2-pyrim	C20H13BrD2N6O		A/B	-8.8
2602	C(=O)(N[C@@H](C(C1CCC1)([2H])[2H])C(=O)C(=O)N)[C@@H]1[C@@H]2[C@@H](C2(C)C)CN1C(=O)[C@@H](NC(=O)NC(C)	1158083-20-5	3-Azabicyclo[3.1.0]hexane-2-carboxamide, N-[(1S)-3-amino-1-(cyclobutylmethyl-d2)	C27H33D12N5O5		A/B	-8.8
2603	N(=C1c2c(N(CN3CCCC3)C1=O)ccc(OC)c2)NC(=S)Nc1c(C)cccn1	1159013-67-8	Hydrazinecarbothioamide, 2-[1-[(diphenylamino)methyl]-1,2-dihydro-5-methoxy-2-ox	C29H26N6O2S	522.62	A/B	-8.8
2604	C(OCCOC(=O)[C@@H](NC(=O)CNC(=O)OC(C)(C)C)C(C)C)n1c2c(nc1)c(=O)nc(N)[nH]2	1160857-03-3	L-Valine, N-[(1,1-dimethylethoxy)carbonyl]-L-phenylalanyl-, 2-[(2-amino-1,6-dihy	C27H37N7O7	571.63	A/B	-8.8
2605	N(=C\1/c2c(N(C)C1=O)ccc(S(=O)(=O)N1CCOCC1)c2)\NC(=S)Nc1cc(C)c(C)cc1	1164513-40-9	Hydrazinecarbothioamide, 2-[1,2-dihydro-1-methyl-5-(4-morpholinylsulfonyl)-2-oxo	C21H23N5O4S2	473.57	A/B	-8.8
2606	Cc1n(c2c(n1)cccc2)c1nc(nc(n1)N1CCOCC1)c1cc(CO)ccc1	1174190-72-7	Phenol, 4-[4-(2-methyl-1H-benzimidazol-1-yl)-6-(4-morpholinyl)-1,3,5-triazin-2-y	C21H20N6O2	388.42	A/B	-8.8
2607	C(C)(C)c1n(c2nc(nc(n2)N2CCOCC2)c2cc(O)ccc2)ccn1	1174190-92-1	2-Pyridinamine, 5-[4-(2-methyl-1H-benzimidazol-1-yl)-6-(4-morpholinyl)-1,3,5-tri	C20H20N8O	388.43	A/B	-8.8
2608	C(CCC)c1n(c2nc(nc(n2)N2CCOCC2)c2cnc(N)nc2)ccn1	1174191-63-9	1H-Pyrrolo[2,3-b]pyridine, 4-[4-(2-ethyl-1H-imidazol-1-yl)-6-(4-morpholinyl)-1,3	C19H20N8O	376.42	A/B	-8.8
2609	Nc1ccc(c2nc(n3c4c(cn3)CCCC4)nc(n2)N2CCOCC2)cn1	1174191-65-1	1H-Pyrrolo[2,3-b]pyridine, 4-[4-(4-morpholinyl)-6-(2-propyl-1H-imidazol-1-yl)-1,	C20H22N8O	390.44	A/B	-8.8
2610	C([C@H](OCP(=O)(OCCCOCCCCCCCCCCCCCCCCCC)O)C)n1c2c(nc1)c(N)ncn2.Cc1c2c(N(c3c(C(=O)N2)cccn3)C2CC2)ncc1	1174386-11-8	Phosphonic acid, P-[[(1R)-2-(6-amino-9H-purin-9-yl)-1-methylethoxy]methyl]-, mor	C28H52N5O5P.C20H15BrN6O		A/B	-8.8
2611	C(=O)([C@@H](NC(=O)OC)C(C)C)N1[C@H](c2[nH]c3-c4c(cc(cc4)c4ccc(cc4)c4[nH]c(nc4)[C@H]4N(C(=O)[C@@H](NC	1180668-73-8	Carbamic acid, N-[(1S)-1-[[(2S)-2-[7-[4-[2-[(2S)-4,4-difluoro-1-[(2S)-2-[(methox	C42H50F2N8O6	800.89	A/B	-8.8
2612	C(=O)([C@@H](NC(=O)OC)C(C)C)N1[C@H]2[C@@H](C2)C[C@H]1c1[nH]c2-c3c(cc(cc3)c3ccc(cc3)c3[nH]c(nc3)[C@H]3	1180669-66-2	Carbamic acid, N-[(1S)-2-methyl-1-[[(2S)-2-[5-[4-[1,4,5,6-tetrahydro-2-[(2S)-1-[	C46H57F3N8O6	874.99	A/B	-8.8
2613	C(=O)([C@@H](NC(=O)OC)C(C)C)N1[C@H]2[C@@H](C2)C[C@H]1c1[nH]c2c(n1)ccc1c2ccc(c1)c1ccc(F)c(cc1)c1[nH]c(n	1180670-26-1	Carbamic acid, N-[(1S)-1-[[(2S)-2-[7-[3-fluoro-4-[2-[(2S)-1-[(2S)-2-[(methoxycar	C42H49FN8O6	780.89	A/B	-8.8
2614	C(OCCOP(=O)(Oc1ccccc1)N[C@H](C(=O)OC(C)C)C)n1c2c(nc1)c(=O)nc(N)[nH]2	1184942-47-9	5,8-Dioxa-3-aza-4-phosphanonanoic acid, 9-(2-amino-1,6-dihydro-6-oxo-9H-purin-9-	C24H29N6O7P	544.5	A/B	-8.8
2615	N(C(=O)[C@@H](C[C@@H](CN1[C@H](C(=O)NC(C)(C)C)CN(Cc2cccnc2)CC1)O[Si](C(C)(C)C)(C)C)Cc1ccccc1)[C@@H]1	1185758-21-7	D-erythro-Pentonamide, 2,3,5-trideoxy-N-[(8S,8aS)-3a,8a-dihydro-2,2-dimethyl-8H-	C39H51N5O5	669.85	A/B	-8.8
2616	O(Cc1ccccc1)[C@@H]1[C@H](OCc2ccccc2)[C@@H](OCc2ccccc2)[C@H](OCCCCCCC(=O)OC[C@H]2O[C@H]([C@H](O)[C@@H	1186192-17-5	1H-1,2,4-Triazole-3-carboxamide, 1-[5-O-[3-[[2-(acetylamino)-2-deoxy-3,4,6-tris-	C40H47N5O12	789.83	A/B	-8.8
2617	N(=C(NC(=O)OC(C)(C)C)NC(=O)OC(C)(C)C)[C@@H]1[C@@H](NC(=O)C)[C@H]([C@@H]([C@@H](CO)O)O)OC(=C1)C(=O)OC	1192409-11-2	Not Yet Assigned	C38H57N5O15	823.88	A/B	-8.8
2618	O(COC(=O)NCCOC)c1c2n(cc(C(=O)NCc3c(F)cc(F)cc3)c1=O)C[C@H]1N(C2=O)[C@H](C)CCO1	1206156-28-6	4-Morpholinecarboxylic acid, [[(4R,12aS)-9-[[[(2,4-difluorophenyl)methyl]amino]c	C26H28F2N4O8	562.52	A/B	-8.8
2619	N(C(=O)CCCC(=O)OCC)C12CC3CC(C2)CC(C1)C3	121373-33-9	Furo[3,Ä≤,4,Ä≤:6,7]naphtho[2,3-d]-1,3-dioxol-6(5aH)-one, 5,8,8a,9-tetrahydro-9-	C26H22O6	430.45	A/B	-8.8
2620	S(=O)(=O)(/C=C/C[C@@H](NC(=O)[C@@H](NC(=O)N1CCNCC1)CC(C)C)CCc1ccccc1)c1ccccc1	1222468-14-5	Not Yet Assigned	C35H45ClN6O4	649.22	A/B	-8.8
2621	N(C(=O)[C@H](Cc1ccccc1)C[C@@H](CN1[C@H](C(=O)NC(C)(C)C)CN(Cc2c3c(on2)cccc3)CC1)O)C1c2c(C[C@H]1O)cccc	1223082-59-4	Not Yet Assigned	C37H47BrN4O4S	723.76	A/B	-8.8
2622	C(=O)(N[C@H](C(=O)C(=O)NC1CC1)CCC)[C@@H]1[C@H](CCN1C(=O)[C@H](NC(=O)[C@H](NC(O)c1cnccn1)C1CCCCC1)C(C	1223101-23-2	Not Yet Assigned	C40H52N4O4	652.87	A/B	-8.8
2623	[C@@H](C(=O)N[C@@H](Cc1ccccc1)C[C@@H]([C@H](Cc1ccccc1)NC(=O)COc1c(C([2H])([2H])[2H])c(c(c1C([2H])(	1224729-54-7	1(2H)-Pyrimidineacetamide, N-[(1S,3S,4S)-4-[[2-[2,6-di(methyl-d3)phenoxy-3,4,5-c	C37H31D17N4O5		A/B	-8.8
2624	c1(nc(nc(n1)N1CCOCC1)N1CCOCC1)c1c2c(ccc1)[nH]nc2	1225037-89-7	1H-Indole, 4-(4,6-di-4-morpholinyl-1,3,5-triazin-2-yl)-	C19H22N6O2	366.42	A/B	-8.8
2625	c1(nc(nc(n1)N1CCOCC1)N1CCOCC1)c1cc2c(cc1)cn[nH]2	1225037-90-0	1H-Indazole, 4-(4,6-di-4-morpholinyl-1,3,5-triazin-2-yl)-	C18H21N7O2	367.4	A/B	-8.8
2626	C(#N)C1=C(N)Oc2c(C1c1cccs1)ccn1c2ncc1	1225373-04-5	7H-Imidazo[1,2-a]pyrano[2,3-c]pyridine-8-carbonitrile, 9-amino-7-(3,4-difluoroph	C17H10F2N4O	324.28	A/B	-8.8
2627	C(#N)C1=C(N)Oc2c(C1c1ccc(cc1)N1CCN(C)CC1)ccn1c2ncc1	1225373-25-0	7H-Imidazo[1,2-a]pyrano[2,3-c]pyridine-8-carbonitrile, 9-amino-7-[4-(methylsulfo	C18H14N4O3S	366.39	A/B	-8.8
2628	C(OP(=O)(Oc1c2c(ccc1)cccc2)N[C@@H](Cc1ccccc1)C(=O)OCc1ccccc1)[C@@H]1[C@@H]2[C@H]([C@@H](O1)n1nc(C(=O	1228377-61-4	L-Alanine, N-[hydroxy(1-naphthalenyloxy)phosphinyl]-, phenylmethyl ester, 5,Ä≤-e	C33H36N5O9P	677.64	A/B	-8.8
2629	C(=O)([C@H](N(C)C)c1ccccc1)N1[C@@H](CCC1)c1[nH]c(cn1)c1ccc(cc1)c1cc2c(cc1)cc(cc2)c1[nH]c(nc1)[C@H]1N	1228505-86-9	2H-1,2,3-Triazole-2-carboxamide, 4,5-diphenyl-N-tricyclo[3.3.1.13,7]dec-1-yl-	C25H26N4O	398.5	A/B	-8.8
2630	C(=O)([C@@H](NC(=O)OC)C)N1[C@H](c2[nH]c(C34CCC(CC3)(CC4)c3ccc(cc3)c3[nH]c(nc3)[C@H]3N(C(=O)[C@@H](NC	1228670-94-7	Not Yet Assigned	C40H40D16N8O6		A/B	-8.8
2631	C(=O)([C@H](NC(=O)OC)c1ccccc1)N1[C@@H](CCC1)c1[nH]c(cn1)c1ccc(C#Cc2ccc(cc2)c2[nH]c(nc2)[C@H]2N(C(=O)	1228965-84-1	Carbamic acid, N-[(1R)-2-oxo-2-[(2S)-2-[5-[4-[2-[4-[2-[(2S)-1-[(2R)-1-oxo-2-pher	C47H45N7O4	771.9	A/B	-8.8
2632	[C@H](Cc1ccccc1)(C[C@@H]([C@H](Cc1ccccc1)NC(=O)OCc1ccns1)O)NC(=O)[C@@H](NC(=O)N(Cc1nc(CN2CCCC2)sc1)C	1236406-89-5	2,7,10,12-Tetraazatridecanoic acid, 4-hydroxy-12-methyl-9-(1-methylethyl)-13-[2-	C38H48N6O5S2	732.95	A/B	-8.8
2633	[C@H](Cc1ccccc1)(C[C@@H]([C@H](Cc1ccccc1)NC(=O)OCc1ccno1)O)NC(=O)[C@@H](NC(=O)N(Cc1nc(CCC)sc1)C(C)	1236408-83-5	Not Yet Assigned	C35H44N6O7S	692.82	A/B	-8.8
2634	[C@H](Cc1ccccc1)([C@@H]([C@@H]([C@H](Cc1ccccc1)NC(=O)OCc1ccns1)O)O)NC(=O)[C@@H](NC(=O)N(Cc1nc(C(C)C)	1236408-93-7	2,7,10,12-Tetraazatridecanoic acid, 13-[2-(4-fluorophenyl)-4-thiazolyl]-4-hydrox	C40H45FN6O5S2	772.95	A/B	-8.8
2635	N(CC(=O)[C@H](Cc1ccccc1)C[C@@H](CN1[C@H](C(=O)NC(C)(C)C)CN(Cc2c(CO)c(O)c(C)nc2)CC1)O)[C@H]1c2c(C[C@H	1237988-60-1	Not Yet Assigned	C37H48ClN5O4	662.26	A/B	-8.8
2636	N(C(=O)[C@H](Cc1ccccc1)C[C@@H](CN1[C@H](C(=O)NC(C)(C)C)CN(Cc2ccc(C)o2)CC1)O)[C@H]1c2c(C[C@H]1O)cccc2	1237988-75-8	D-erythro-Pentonamide, 2,3,5-trideoxy-N-[(1S,2R)-2,3-dihydro-2-hydroxy-1H-inden-	C37H46N4O4S2	674.92	A/B	-8.8
2637	N(CC(=O)[C@H](Cc1ccccc1)C[C@@H](CN1[C@H](C(=O)NC(C)(C)C)CN(Cc2ccc(C)nc2)CC1)O)[C@H]1c2c(C[C@H]1O)ccc	1237989-12-6	Not Yet Assigned	C38H50N4O4	626.83	A/B	-8.8
2638	O=c1n([C@H]2C[C@H](O[P@@](=O)(OC[C@H]3O[C@H](C[C@@H]3O)n3c(=O)nc(N)cc3)OC)[C@@H](CO)O2)cc(C)c(=O)[nH	1238049-01-8	2,7,10,12-Tetraazatridecanoic acid, 4-hydroxy-13-[2-(3-methoxyphenyl)-4-thiazoly	C41H48N6O6S2	784.99	A/B	-8.8
2639	C(F)(F)c1n(c2c(n1)cccc2)c1nc(nc(N[C@H]2CC[C@H](NC(=O)CC)CC2)n1)N1CCOCC1	1240027-38-6	1,4-Cyclohexanediamine, N1-[4-[2-(difluoromethyl)-1H-benzimidazol-1-yl]-6-[(3R)-	C22H28F2N8O	458.51	A/B	-8.8
2640	O(C(CC)CC)[C@@H]1[C@H](NC(=O)C)[C@H](N)CC(=C1)C(=O)OCC	1240030-58-3	1,4-Cyclohexanediamine, N1-cyclohexyl-N4-[4-[2-(difluoromethyl)-1H-benzimidazol-	C27H36F2N8O	526.62	A/B	-8.8
2641	C(=O)([C@@H](NC(=O)OC)C)N1[C@@H](CCC1)c1[nH]c(cn1)c1ccc(cc1)c1ccc(C(=O)NC[C@H]2N(C(=O)[C@@H](NC(=O)O	1240427-42-2	L-Prolinamide, N-(methoxycarbonyl)-L-valyl-N-[(1R)-1-[4,Ä≤-[2-[(2S)-1-[(2S)-2-[	C40H53N7O7	743.89	A/B	-8.8
2642	C(=O)([C@@H](NC(=O)OC)CC)N1[C@H](c2[nH]c(c3cc4c(-c5c(C4)cc(cc5)c4[nH]c(nc4)[C@H]4N(C(=O)[C@@H](NC(=O	1240880-38-9	Carbamic acid, N,N,Ä≤-[9H-fluorene-2,7-diylbis[1H-imidazole-5,2-diyl-(2S)-2,1-py	C37H42N8O6	694.78	A/B	-8.8
2643	C(=O)([C@H](NC(=O)OC)C)N1[C@@H](CCC1)c1[nH]c(cn1)[C@H]1CC[C@@H](CC1)c1ccc(cc1)c1[nH]c(nc1)[C@H]1N(C(	1240886-31-0	Carbamic acid, N-[(1R)-2-[(2S)-2-[5-[4-[4-[2-[(2S)-1-[(2R)-2-[(methoxycarbonyl)a	C46H50N8O6	810.94	A/B	-8.8
2644	C(=O)([C@H](Cc1ccccc1)NC(=O)OC)N1[C@@H](CCC1)c1[nH]c(cn1)[C@H]1CC[C@@H](CC1)c1ccc(cc1)c1[nH]c(nc1)[C	1240888-30-5	Carbamic acid, N-[(1R)-2-[(2S)-2-[5-[4-[trans-4-[2-[(2S)-1-[(2R)-2-[(methoxycarb	C48H56N8O6	841.01	A/B	-8.8
2645	C(=O)([C@H](NC(=O)OC)c1ccccc1)N1[C@@H](CCC1)c1[nH]c(cn1)C1=CC[C@H](CC1)c1ccc(cc1)c1[nH]c(nc1)[C@H]1N	1240891-27-3	Carbamic acid, N-[(1R)-2-[(2S)-2-[5-[3-[trans-4-[2-[(2S)-1-[(2R)-2-[(methoxycarb	C46H52N8O6	812.96	A/B	-8.8
2646	O=c1n([C@H]2O[C@@H](C(=O)OC[C@@H]3COC(C)(C)O3)SC2)ccc(N)n1	1241964-77-1	Thymidine 5,Ä≤-(trihydrogen diphosphate), 3,Ä≤-deoxy-3,Ä≤-fluoro-2-thio-, P,Ä≤,	C20H26FN7O12P2S		A/B	-8.8
2647	C(=O)(NC)c1c2c(cc(N(CCOC(=O)CCC(=O)OC[C@H]3O[C@H]([C@H](O)[C@@H]3O)n3nc(C(=O)N)nc3)S(=O)(=O)C)c(c2)C	1246280-91-0	Not Yet Assigned	C30H34FN6O12PS	752.66	A/B	-8.8
2648	O(c1c(CO)cc(C#N)cc1CO)c1nc(Nc2ccc(C#N)cc2)nc(N)c1Br	1246818-40-5	Not Yet Assigned	C20H13BrClN5O		A/B	-8.8
2649	C(=O)([C@@H](NC(=O)OC)C(C)C)N1[C@@H](CCC1)c1[nH]c(cn1)c1ccc(cc1)c1ccc(C#Cc2ccc(cc2)c2[nH]c(nc2)[C@H]	1250941-31-1	Carbamic acid, N-[(1S)-2-methyl-1-[[(2S)-2-[5-[3-[2-[3-[2-(2S)-2-pyrrolidinyl-1H	C35H39N7O3	605.73	A/B	-8.8
2650	C(=O)([C@@H](NC(=O)OC)C(C)C)N1[C@@H](CCC1)c1[nH]c(cn1)c1ccc(cc1)c1ccc(cc1)c1[nH]c(nc1)[C@H]1N(C(=O)[	1256388-28-9	Carbamic acid, N-[(1S)-1-[[(2S)-2-[5-[4,Ä≤-[2-[(6S)-5-[(2S)-2-[(methoxycarbonyl)	C42H52N8O6	764.91	A/B	-8.8
2651	C(=O)([C@H](NC(=O)OC)c1ccccc1)N1[C@@H](CCC1)c1[nH]c(cn1)c1cc2c(cc1)cc(cc2)c1ccc(cc1)c1[nH]c(nc1)[C@H	1256391-33-9	Carbamic acid, N-[(1S)-2-[(2S)-2-[5-[6-[4-[2-[(2S)-1-[(2S)-2-[(methoxycarbonyl)a	C47H50N8O6	822.95	A/B	-8.8
2652	O[C@H]1[C@@H](O[C@H](COC(=O)c2ccc(N)cc2)[C@H]1O)n1nc(C(=O)N)nc1	1257423-92-9	1H-1,2,4-Triazole-3-carboxamide, 1-[5-O-[3,4,5-tris(phenylmethoxy)benzoyl]-Œ≤-D-	C36H34N4O9	666.68	A/B	-8.8
2653	C(=O)([C@@H](NC(=O)OC)C(C)C)N1[C@H](c2[nH]c(c3ccc([C@H]4N([C@H](CC4)c4ccc(cc4)c4[nH]c(nc4)[C@H]4N(C(	1258226-87-7	L-Prolinamide, 2,2,Ä≤-[[(2S,5S)-1-[4-(1,1-dimethylethyl)phenyl]-2,5-pyrrolidinec	C50H67N7O8	894.11	A/B	-8.8
2654	C(=O)([C@@H](NC(=O)OC)CC)N1[C@@H](CCC1)c1[nH]c(cn1)c1ccc(C#Cc2[nH]c(nc2)[C@H]2N(C(=O)[C@@H](NC(=O)OC	1265531-60-9	Carbamic acid, N-[(1S)-2-[(2S)-2-[5-[4-[2-[2-[(2S)-1-[(2S)-2-[(methoxycarbonyl)a	C32H38N8O6	630.69	A/B	-8.8
2655	O(C)c1c2n(cc(C(=O)O)c(=O)c2cc(F)c1N1CC(C)N(CN2c3c(/C(=N/NC(=S)NO)/C2=O)cc(C)cc3)CC1)C1CC1	1265623-30-0	3-Quinolinecarboxylic acid, 1-cyclopropyl-6-fluoro-7-[4-[[(3Z)-5-fluoro-2,3-dihy	C30H31F2N7O6S	655.67	A/B	-8.8
2656	C(=O)([C@@H](NC(=O)OC)C(C)C)N1[C@@H]([C@@H]2C[C@H]1CC2)c1[nH]c(cn1)c1c(CC)c(CC)c(cc1)c1c(CC)c(CC)c(c	1273591-48-2	Carbamic acid, N-[(1S)-1-[[(2S)-2-[5-[4,Ä≤-[2-[(1R,3S,4S)-2-[(2S)-2-[(methoxycar	C46H60N8O6	821.02	A/B	-8.8
2657	N(c1nc(nc(N/N=C/c2cc3c(cc2)cccc3)n1)N1CCOCC1)c1ccc(F)cc1	1279692-09-9	1-Naphthalenecarboxaldehyde, 2-[4-[(4-fluorophenyl)amino]-6-(4-morpholinyl)-1,3,	C24H22FN7O	443.48	A/B	-8.8
2658	O(C(=O)N[C@H]([C@@H](CN(S(=O)(=O)c1ccc(N)cc1)CC(C)C)O)Cc1ccccc1)[C@@H]1[C@H]2[C@@H](OC1)OC[C@@H]2OCC	1292810-61-7	Carbamic acid, N-[(1S,2R)-3-[[(4-aminophenyl)sulfonyl](2-methylpropyl)amino]-2-h	C33H41N3O8S	639.76	A/B	-8.8
2659	C(=O)([C@@H](NC(=O)OC)C1CCOCC1)N1[C@H]2[C@H](C2)C[C@H]1c1[nH]c2c(n1)ccc1c2ccc(c1)c1ccc(cc1)c1[nH]c(n	1312336-41-6	Carbamic acid, N-[(1S)-1-[[(1R,3S,5R)-3-[7-[4-[5-chloro-2-[(1R,3S,5R)-2-[(2S)-2-	C44H49ClN8O6	821.36	A/B	-8.8
2660	C(=O)([C@@H](NC(=O)OC)C(C)C)N1[C@H]2[C@H](C2)C[C@H]1c1[nH]c(n1)c1cnoc1)c1ccc(cc1)c1ccc(cc1)c1[nH]c	1312337-06-6	Carbamic acid, N-[(1S)-2-[(2S)-2-[5-[4,Ä≤-[2-[(2S)-1-[(2S)-2-[(methoxycarbonyl)a	C48H58N10O8	903.04	A/B	-8.8
2661	O[C@H]1[C@H](n2c3c(nc2)c(N)ncn3)O[C@H](COP(=O)(CCP(=O)(O)O)O)[C@H]1O	1312609-79-2	Carbamic acid, N-[(1S)-2-methyl-1-[[(2S)-2-[5-[4-[4-[2-(2S)-2-pyrrolidinyl-1H-im	C37H41N7O3	631.77	A/B	-8.8
2662	O(Cc1ccc(F)cc1)c1c2c(oc(c(O)c2=O)c2[nH]c(nn2)c2ccccc2)c(C)cc1	1312892-66-2	4H-1-Benzopyran-4-one, 2-(5-chloro-1H-1,2,4-triazol-3-yl)-5-[(4-fluorophenyl)met	C19H13ClFN3O4	401.78	A/B	-8.8
2663	O(Cc1ccc(F)cc1)c1c2c(oc(c(O)c2=O)c2ncccn2)c(Cl)cc1	1312893-52-9	4H-1-Benzopyran-4-one, 5-[(4-fluorophenyl)methoxy]-3-hydroxy-8-methoxy-2-(5-pher	C26H19FN2O5	458.44	A/B	-8.8
2664	O(Cc1ccc(F)cc1)c1c2c(oc(c(O)c2=O)c2[nH]c(nn2)c2ccccc2)c(Cl)cc1	1312893-96-1	4H-1-Benzopyran-4-one, 8-chloro-2-(5-chloro-1H-1,2,4-triazol-3-yl)-5-[(4-fluorop	C18H10Cl2FN3O4	422.19	A/B	-8.8
2665	O(Cc1ccc(F)cc1)c1c2c(c(cc1)c1ccccc1)oc(c(O)c2=O)c1[nH]c(C)cn1	1312894-48-6	4H-1-Benzopyran-4-one, 5-[(4-fluorophenyl)methoxy]-3-hydroxy-2-(1H-imidazol-2-yl	C25H17FN2O4	428.41	A/B	-8.8
2666	O(Cc1ccc(F)cc1)c1c2c(oc(c(O)c2=O)c2ncc(cn2)c2ccccc2)c(C)c(C)c1	1312895-36-5	4H-1-Benzopyran-4-one, 2-(5-chloro-2-pyrimidinyl)-5-[(4-fluorophenyl)methoxy]-3-	C22H16ClFN2O4	426.82	A/B	-8.8
2667	O(Cc1ccc(F)cc1)c1c2c(oc(c(O)c2=O)c2[nH]cnn2)c(C)c(C)c1	1312895-37-6	4H-1-Benzopyran-4-one, 5-[(4-fluorophenyl)methoxy]-3-hydroxy-7,8-dimethyl-2-(5-p	C28H21FN2O4	468.48	A/B	-8.8
2668	O(Cc1ccc(F)cc1)c1c2c(oc(c(O)c2=O)c2[nH]c(Cl)nn2)c(C)c(C)c1	1312895-45-6	4H-1-Benzopyran-4-one, 5-[(4-fluorophenyl)methoxy]-3-hydroxy-2-(5-methoxy-1H-1,2	C21H18FN3O5	411.38	A/B	-8.8
2669	O(Cc1ccc(F)cc1)c1c2c(oc(c(O)c2=O)c2[nH]c(C)cn2)c(C)c(C)c1	1312895-48-9	4H-1-Benzopyran-4-one, 5-[(4-fluorophenyl)methoxy]-3-hydroxy-2-(1H-imidazol-2-yl	C21H17FN2O4	380.37	A/B	-8.8
2670	O(Cc1ccc(F)cc1)c1c2c(oc(c(O)c2=O)c2[nH]c(Cl)cn2)c(C)c(C)c1	1312895-50-3	4H-1-Benzopyran-4-one, 5-[(4-fluorophenyl)methoxy]-3-hydroxy-2-(5-methoxy-1H-imi	C22H19FN2O5	410.4	A/B	-8.8
2671	O(Cc1ccc(F)cc1)c1c2c(oc(c(O)c2=O)c2[nH]c(OC)nn2)c(Cl)c(C)c1	1312896-44-8	4H-1-Benzopyran-4-one, 8-chloro-5-[(4-fluorophenyl)methoxy]-3-hydroxy-7-methyl-2	C20H15ClFN3O4	415.8	A/B	-8.8
2672	O(Cc1ccc(F)cc1)c1c2c(c(c(C)c1)c1ccccc1)oc(c(O)c2=O)c1ncc(Cl)cn1	1312896-85-7	4H-1-Benzopyran-4-one, 5-[(4-fluorophenyl)methoxy]-3-hydroxy-2-(5-methoxy-2-pyri	C28H21FN2O5	484.48	A/B	-8.8
2673	O(Cc1ccc(F)cc1)c1c2c(c(c(C)c1)c1ccccc1)oc(c(O)c2=O)c1[nH]cc(C)n1	1312896-98-2	4H-1-Benzopyran-4-one, 5-[(4-fluorophenyl)methoxy]-3-hydroxy-2-(1H-imidazol-2-yl	C26H19FN2O4	442.44	A/B	-8.8
2674	O(Cc1ccc(F)cc1)c1c2c(c(c(C)c1)c1ccccc1)oc(c(O)c2=O)c1[nH]c(cn1)c1ccccc1	1312897-01-0	4H-1-Benzopyran-4-one, 2-(5-chloro-1H-imidazol-2-yl)-5-[(4-fluorophenyl)methoxy]	C26H18ClFN2O4	476.88	A/B	-8.8
2675	O(C)c1c(c2c(c(OCc3ccc(F)cc3)c1)c(=O)c(O)c(o2)c1[nH]cnn1)c1ccccc1	1312899-49-2	4H-1-Benzopyran-4-one, 5-[(4-fluorophenyl)methoxy]-3-hydroxy-7-methoxy-8-phenyl-	C33H23FN2O5	546.54	A/B	-8.8
2676	O(C)c1c(c2c(c(OCc3ccc(F)cc3)c1)c(=O)c(O)c(o2)c1nnc(Cl)[nH]1)c1ccccc1	1312899-57-2	4H-1-Benzopyran-4-one, 5-[(4-fluorophenyl)methoxy]-3-hydroxy-7-methoxy-2-(5-meth	C26H20FN3O6	489.45	A/B	-8.8
2677	O(C)c1c(c2c(c(OCc3ccc(F)cc3)c1)c(=O)c(O)c(o2)c1[nH]ccn1)c1ccccc1	1312899-59-4	4H-1-Benzopyran-4-one, 5-[(4-fluorophenyl)methoxy]-3-hydroxy-7-methoxy-8-phenyl-	C31H22FN3O5	535.52	A/B	-8.8
2678	O(Cc1ccc(F)cc1)c1c2c(oc(c(O)c2=O)c2ncc(cn2)c2ccccc2)c(C)c(Cl)c1	1312901-07-7	4H-1-Benzopyran-4-one, 7-chloro-2-(5-chloro-2-pyrimidinyl)-5-[(4-fluorophenyl)me	C21H13Cl2FN2O4	447.24	A/B	-8.8
2679	O(Cc1ccc(F)cc1)c1c2c(oc(c(O)c2=O)c2[nH]c(C)nn2)c(C)c(Cl)c1	1312901-14-6	4H-1-Benzopyran-4-one, 7-chloro-5-[(4-fluorophenyl)methoxy]-3-hydroxy-8-methyl-2	C19H13ClFN3O4	401.78	A/B	-8.8
2680	O(Cc1ccc(F)cc1)c1c2c(oc(c(O)c2=O)c2[nH]c(OC)cn2)c(C)c(Cl)c1	1312901-20-4	4H-1-Benzopyran-4-one, 7-chloro-5-[(4-fluorophenyl)methoxy]-3-hydroxy-8-methyl-2	C21H16ClFN2O4	414.81	A/B	-8.8
2681	O(C)c1c2c(c(OCc3ccc(F)cc3)cc1Cl)c(=O)c(O)c(o2)c1[nH]ccn1	1312901-68-0	4H-1-Benzopyran-4-one, 7-chloro-5-[(4-fluorophenyl)methoxy]-3-hydroxy-8-methoxy-	C25H17ClFN3O5	493.87	A/B	-8.8
2682	O(Cc1ccc(F)cc1)c1c2c(oc(c(O)c2=O)c2[nH]c(C)nn2)c(Cl)c(Cl)c1	1312902-14-9	4H-1-Benzopyran-4-one, 7,8-dichloro-5-[(4-fluorophenyl)methoxy]-3-hydroxy-2-(1H-	C18H10Cl2FN3O4	422.19	A/B	-8.8
2683	O(Cc1ccc(F)cc1)c1c2c(oc(c(O)c2=O)c2[nH]c(nn2)c2ccccc2)c(Cl)c(Cl)c1	1312902-17-2	4H-1-Benzopyran-4-one, 7,8-dichloro-2-(5-chloro-1H-1,2,4-triazol-3-yl)-5-[(4-flu	C18H9Cl3FN3O4	456.64	A/B	-8.8
2684	O(Cc1ccc(F)cc1)c1c2c(oc(c(O)c2=O)c2[nH]c(OC)cn2)c(Cl)c(Cl)c1	1312902-20-7	4H-1-Benzopyran-4-one, 7,8-dichloro-5-[(4-fluorophenyl)methoxy]-3-hydroxy-2-(5-m	C20H13Cl2FN2O4	435.23	A/B	-8.8
2685	O(Cc1ccc(F)cc1)c1c2c(oc(c(O)c2=O)c2ncc(OC)cn2)cc(c1)c1ccccc1	1312903-05-1	4H-1-Benzopyran-4-one, 5-[(4-fluorophenyl)methoxy]-3-hydroxy-2-(5-methyl-2-pyrim	C27H19FN2O4	454.45	A/B	-8.8
2686	O(Cc1ccc(F)cc1)c1c2c(oc(c(O)c2=O)c2ncc(cn2)c2ccccc2)cc(c1)c1ccccc1	1312903-07-3	4H-1-Benzopyran-4-one, 2-(5-chloro-2-pyrimidinyl)-5-[(4-fluorophenyl)methoxy]-3-	C26H16ClFN2O4	474.87	A/B	-8.8
2687	O(Cc1ccc(F)cc1)c1c2c(oc(c(O)c2=O)c2[nH]c(Cl)nn2)cc(c1)c1ccccc1	1312903-16-4	4H-1-Benzopyran-4-one, 5-[(4-fluorophenyl)methoxy]-3-hydroxy-2-(5-methoxy-1H-1,2	C25H18FN3O5	459.43	A/B	-8.8
2688	Cc1c2c(c(OCc3ccc(F)cc3)cc1c1ccccc1)c(=O)c(O)c(o2)c1ncc(Cl)cn1	1312903-56-2	4H-1-Benzopyran-4-one, 5-[(4-fluorophenyl)methoxy]-3-hydroxy-2-(5-methoxy-2-pyri	C28H21FN2O5	484.48	A/B	-8.8
2689	O(C)c1c2c(c(OCc3ccc(F)cc3)cc1c1ccccc1)c(=O)c(O)c(o2)c1ncc(C)cn1	1312904-24-7	4H-1-Benzopyran-4-one, 5-[(4-fluorophenyl)methoxy]-3-hydroxy-8-methoxy-7-phenyl-	C27H19FN2O5	470.45	A/B	-8.8
2690	O(C)c1c2c(c(OCc3ccc(F)cc3)cc1c1ccccc1)c(=O)c(O)c(o2)c1[nH]c(nn1)c1ccccc1	1312904-46-3	4H-1-Benzopyran-4-one, 2-(5-chloro-1H-1,2,4-triazol-3-yl)-5-[(4-fluorophenyl)met	C25H17ClFN3O5	493.87	A/B	-8.8

2691	O(Cc1ccc(F)cc1)c1c2c(c(Cl)c(c1)c1ccccc1)oc(c(O)c2=O)c1ncc(OC)cn1	1312905-05-7	4H-1-Benzopyran-4-one, 8-chloro-5-[(4-fluorophenyl)methoxy]-3-hydroxy-2-(5-methy	C27H18ClFN2O4	488.89	A/B	-8.8
2692	Clc1c2c(c(OCc3ccc(F)cc3)cc1c1ccccc1)c(=O)c(O)c(o2)c1ncc(Cl)cn1	1312905-06-8	4H-1-Benzopyran-4-one, 8-chloro-5-[(4-fluorophenyl)methoxy]-3-hydroxy-2-(5-metho	C27H18ClFN2O5	504.89	A/B	-8.8
2693	O(Cc1ccc(F)cc1)c1c2c(c(Cl)c(c1)c1ccccc1)oc(c(O)c2=O)c1[nH]c(Cl)nn1	1312905-22-8	4H-1-Benzopyran-4-one, 8-chloro-5-[(4-fluorophenyl)methoxy]-3-hydroxy-2-(5-metho	C25H17ClFN3O5	493.87	A/B	-8.8
2694	O(Cc1ccc(F)cc1)c1c2c(c(Cl)c(c1)c1ccccc1)oc(c(O)c2=O)c1[nH]c(OC)cn1	1312905-28-4	4H-1-Benzopyran-4-one, 8-chloro-5-[(4-fluorophenyl)methoxy]-3-hydroxy-2-(5-methy	C26H18ClFN2O4	476.88	A/B	-8.8
2695	O(Cc1ccc(F)cc1)c1c2c(c(c(c1)c1ccccc1)c1ccccc1)oc(c(O)c2=O)c1[nH]cc(C)n1	1312905-81-9	4H-1-Benzopyran-4-one, 5-[(4-fluorophenyl)methoxy]-3-hydroxy-2-(1H-imidazol-2-yl	C31H21FN2O4	504.51	A/B	-8.8
2696	Oc1c(oc2c(c1=O)cc(OCc1ccc(F)cc1)cc2)c1[nH]c(nn1)c1ccccc1	1312906-27-6	4H-1-Benzopyran-4-one, 2-(5-chloro-1H-1,2,4-triazol-3-yl)-6-[(4-fluorophenyl)met	C18H11ClFN3O4	387.75	A/B	-8.8
2697	O=c1c2c(oc(c1O)c1nnc(C)[nH]1)c(C)cc(OCc1ccc(F)cc1)c2	1312906-74-3	4H-1-Benzopyran-4-one, 6-[(4-fluorophenyl)methoxy]-3-hydroxy-8-methyl-2-(1H-1,2,	C19H14FN3O4	367.33	A/B	-8.8
2698	O=c1c2c(c(cc(OCc3ccc(F)cc3)c2)c2ccccc2)oc(c1O)c1[nH]c(OC)cn1	1312908-30-7	4H-1-Benzopyran-4-one, 6-[(4-fluorophenyl)methoxy]-3-hydroxy-2-(5-methyl-1H-imic	C26H19FN2O4	442.44	A/B	-8.8
2699	Cc1c2c(cc(OCc3ccc(F)cc3)c1C)c(=O)c(O)c(o2)c1[nH]c(OC)nn1	1312909-37-7	4H-1-Benzopyran-4-one, 6-[(4-fluorophenyl)methoxy]-3-hydroxy-7,8-dimethyl-2-(3-m	C21H18FN3O4	395.38	A/B	-8.8
2700	O=c1c2c(oc(c1O)c1[nH]ccn1)cc(Cl)c(OCc1ccc(F)cc1)c2	1312911-82-2	4H-1-Benzopyran-4-one, 7-chloro-6-[(4-fluorophenyl)methoxy]-3-hydroxy-2-(3-pheny	C24H15ClFN3O4	463.85	A/B	-8.8
2701	Clc1c2c(cc(OCc3ccc(F)cc3)c1OC)c(=O)c(O)c(o2)c1[nH]c(OC)cn1	1312914-42-3	4H-1-Benzopyran-4-one, 8-chloro-6-[(4-fluorophenyl)methoxy]-3-hydroxy-7-methoxy-	C21H16ClFN2O5	430.81	A/B	-8.8
2702	Clc1c2c(cc(OCc3ccc(F)cc3)c1Cl)c(=O)c(O)c(o2)c1[nH]c(cn1)c1ccccc1	1312914-91-2	4H-1-Benzopyran-4-one, 7,8-dichloro-2-(5-chloro-1H-imidazol-2-yl)-6-[(4-fluoroph	C19H10Cl3FN2O4	455.65	A/B	-8.8
2703	O(C)c1c(c2c(cc1OCc1ccc(F)cc1)c(=O)c(O)c(o2)c1ncc(OC)cn1)c1ccccc1	1312915-12-0	4H-1-Benzopyran-4-one, 6-[(4-fluorophenyl)methoxy]-3-hydroxy-7-methoxy-2-(5-meth	C28H21FN2O5	484.48	A/B	-8.8
2704	O(C)c1c(c2c(cc1OCc1ccc(F)cc1)c(=O)c(O)c(o2)c1ncc(cn1)c1ccccc1)c1ccccc1	1312915-17-5	4H-1-Benzopyran-4-one, 2-(5-chloro-2-pyrimidinyl)-6-[(4-fluorophenyl)methoxy]-3-	C27H18ClFN2O5	504.89	A/B	-8.8
2705	O(C)c1c(c2c(cc1OCc1ccc(F)cc1)c(=O)c(O)c(o2)c1[nH]c(OC)nn1)c1ccccc1	1312915-37-9	4H-1-Benzopyran-4-one, 6-[(4-fluorophenyl)methoxy]-3-hydroxy-7-methoxy-2-(3-meth	C26H20FN3O5	473.45	A/B	-8.8
2706	O(C)c1c(c2c(cc1OCc1ccc(F)cc1)c(=O)c(O)c(o2)c1[nH]ccn1)c1ccccc1	1312915-43-7	4H-1-Benzopyran-4-one, 6-[(4-fluorophenyl)methoxy]-3-hydroxy-7-methoxy-8-phenyl-	C31H22FN3O5	535.52	A/B	-8.8
2707	O(C)c1c(c2c(cc1OCc1ccc(F)cc1)c(=O)c(O)c(o2)c1[nH]c(Cl)cn1)c1ccccc1	1312915-49-3	4H-1-Benzopyran-4-one, 6-[(4-fluorophenyl)methoxy]-3-hydroxy-7-methoxy-2-(5-meth	C27H21FN2O6	488.46	A/B	-8.8
2708	Clc1c(c2c(cc1OCc1ccc(F)cc1)c(=O)c(O)c(o2)c1ncccn1)c1ccccc1	1312915-53-9	4H-1-Benzopyran-4-one, 6-[(4-fluorophenyl)methoxy]-3-hydroxy-7-methoxy-8-phenyl-	C32H23FN2O5	534.53	A/B	-8.8
2709	Clc1c(c2c(cc1OCc1ccc(F)cc1)c(=O)c(O)c(o2)c1ncc(C)cn1)c1ccccc1	1312915-60-8	4H-1-Benzopyran-4-one, 7-chloro-6-[(4-fluorophenyl)methoxy]-3-hydroxy-8-phenyl-2	C26H16ClFN2O4	474.87	A/B	-8.8
2710	Clc1c(c2c(cc1OCc1ccc(F)cc1)c(=O)c(O)c(o2)c1[nH]cnn1)c1ccccc1	1312915-68-6	4H-1-Benzopyran-4-one, 7-chloro-6-[(4-fluorophenyl)methoxy]-3-hydroxy-8-phenyl-2	C32H20ClFN2O4	550.96	A/B	-8.8
2711	O=c1c2c(cc(c(OCc3ccc(F)cc3)c2)c2ccccc2)oc(c1O)c1ncc(C)cn1	1312916-31-6	4H-1-Benzopyran-4-one, 6-[(4-fluorophenyl)methoxy]-3-hydroxy-7-phenyl-2-(2-pyrim	C26H17FN2O4	440.42	A/B	-8.8
2712	O(Cc1ccc(F)cc1)c1c(c(C)c2c(c1)c(=O)c(O)c(o2)c1[nH]c(Cl)nn1)c1ccccc1	1312916-94-1	4H-1-Benzopyran-4-one, 6-[(4-fluorophenyl)methoxy]-3-hydroxy-2-(5-methoxy-1H-1,2	C26H20FN3O5	473.45	A/B	-8.8
2713	Cc1c2c(cc(OCc3ccc(F)cc3)c1c1ccccc1)c(=O)c(O)c(o2)c1[nH]c(cn1)c1ccccc1	1312917-00-2	4H-1-Benzopyran-4-one, 2-(5-chloro-1H-imidazol-2-yl)-6-[(4-fluorophenyl)methoxy]	C26H18ClFN2O4	476.88	A/B	-8.8
2714	O(Cc1ccc(F)cc1)c1c(c(Cl)c2c(c1)c(=O)c(O)c(o2)c1[nH]c(C)nn1)c1ccccc1	1312917-92-2	4H-1-Benzopyran-4-one, 8-chloro-6-[(4-fluorophenyl)methoxy]-3-hydroxy-7-phenyl-2	C24H15ClFN3O4	463.85	A/B	-8.8
2715	O(Cc1ccc(F)cc1)c1c(c(Cl)c2c(c1)c(=O)c(O)c(o2)c1[nH]c(Cl)nn1)c1ccccc1	1312917-94-4	4H-1-Benzopyran-4-one, 8-chloro-6-[(4-fluorophenyl)methoxy]-3-hydroxy-2-(5-metho	C25H17ClFN3O5	493.87	A/B	-8.8
2716	Clc1c2c(cc(OCc3ccc(F)cc3)c1c1ccccc1)c(=O)c(O)c(o2)c1[nH]c(nn1)c1ccccc1	1312917-95-5	4H-1-Benzopyran-4-one, 8-chloro-2-(5-chloro-1H-1,2,4-triazol-3-yl)-6-[(4-fluorop	C24H14Cl2FN3O4	498.29	A/B	-8.8
2717	O=c1n([C@@H]2O[C@@H](CO)[C@H](O)C2)cc(C)c(=O)[nH]1	1316315-43-1	Benzonitrile, 4-[[5-bromo-6-chloro-2-[(4-cyanophenyl)amino]-4-pyrimidinyl]oxy]-3	C20H7BrClD6N5O		A/B	-8.8
2718	O=C1N2[C@H](c3nc(c([nH]3)CCc3cc([C@@H](C)[C@@H]1NC(=O)OC)ccc3)c1ccc(cc1)c1ccc(cc1)c1[nH]c(nc1)[C@H]1N	1319189-34-8	Carbamic acid, N-[(10S,11S,16aS)-5,6,7,8,9,10,11,12,14,15,16,16a-dodecahydro-3-[	C44H56N8O6	792.97	A/B	-8.8
2719	C(C(=O)N[C@@H](Cc1ccccc1)C[C@@H]([C@H](Cc1ccccc1)NC(=O)COc1c(C)cccc1C)O)(C(C([2H])([2H])[2H])(C([2H]	132246-55-0	3,Ä≤-Thymidylic acid, diphenyl ester, 5,Ä≤-(diphenyl phosphate)	C34H32N2O11P2	706.57	A/B	-8.8
2720	N(c1nc(nc(NN=Cc2cc3c(cc2)cccc3)n1)N1CCOCC1)c1ccc(F)cc1	1331749-54-2	1-Naphthalenecarboxaldehyde, 2-[4-[(4-fluorophenyl)amino]-6-(4-morpholinyl)-1,3,	C24H22FN7O	443.48	A/B	-8.8
2721	[C@H](Cc1ccccc1)([C@@H]([C@@H]([C@H](Cc1cncs1)NC(=O)OCc1cncs1)O)O)NC(=O)[C@@H](NC(=O)N(Cc1nc(C(C)C)o	1346884-94-3	Carbamic acid, N-[[4,Ä≤-[(2-ethyl-5,7-dimethyl-3H-imidazo[4,5-b]pyridin-3-yl)met	C31H36N4O4S	560.71	A/B	-8.8
2722	[C@@H]([C@@H]([C@@H]([C@H](Cc1ccccc1)NC(=O)OC(C)(C)C)O)O)(Cc1ccccc1)NC(=O)[C@@H](NC(=O)N(Cc1csc(C)n1	1347013-87-9	4-Morpholinecarboxamide, N-[(1S)-1-[(4-fluorophenyl)methyl]-2-oxo-2-[[(1S,2E)-1-	C31H34FN3O5S	579.68	A/B	-8.8
2723	[C@@H](Cc1ccccc1)(C[C@@H]([C@H](Cc1ccccc1)NC(=O)OCc1cncs1)O)NC(=O)[C@H](NC(=O)N(Cc1cc(C(C)C)sc1)C)C	1348325-80-3	Acetamide, N-[[4,Ä≤-[(2-ethyl-5,7-dimethyl-3H-imidazo[4,5-b]pyridin-3-yl)methyl]	C33H41N5O4S	603.77	A/B	-8.8
2724	N(C(CCN(CCO)CC)C)c1c2c(ncc1)cc(Cl)cc2	1348817-22-0	Butanoic acid, 4-[[[[4-[(2S,3R)-2-[[[(3R,3aS,6aR)-hexahydrofuro[2,3-b]furan-3-y	C41H56N3O13PS	861.93	A/B	-8.8
2725	N(Cc1c(C(C)C)scn1)(C(=O)N[C@H](C(=O)N[C@@H](Cc1ccccc1)C[C@@H]([C@H](Cc1ccccc1)NC(=O)OCc1cncs1)O)C(C)	1348981-13-4	Benzamide, 2-ethoxy-N-[[4,Ä≤-[(2-ethyl-5,7-dimethyl-3H-imidazo[4,5-b]pyridin-3-y	C37H41N5O4S	651.82	A/B	-8.8
2726	C(CC)c1c(OC)ccc(C(=O)/C=C/c2cc(O)c(O)cc2)c1O	1349347-15-4	2,7,10,12-Tetraazatridecanoic acid, 13-[2-(3-cyclohexen-1-yl)-4-thiazolyl]-4-hyc	C40H50N6O5S2	758.99	A/B	-8.8
2727	N(C(=O)[C@H](Cc1ccccc1)C[C@@H](CN1[C@H](C(=O)NC(C)(C)C)CN(Cc2cccnc2)CC1)O)C1c2c(C[C@H]1OCCCCCC/C=C\C	1349476-74-9	Pentanamide, N-[[4,Ä≤-[(2-ethyl-5,7-dimethyl-3H-imidazo[4,5-b]pyridin-3-yl)methy	C31H38N4O3S	546.72	A/B	-8.8
2728	[C@@H](OCCNC(=O)CC[C@H](NC(=O)[C@@H](NC(=O)[C@@H](NC(=O)[C@@H](NC(=O)[C@H](CCC(=O)NCCCCC)N)CCC(=O)NC	1349504-35-3	1-Piperazinecarboxamide, N-[(1R)-1-[[[(1S,2E)-3-[[(1R)-1,4-dihydro-1-naphthaleny	C33H44N4O4S	592.79	A/B	-8.8
2729	C(COCP(=O)(O)O)n1c2c(nc1N)c(N)nc(N)n2	1349516-03-5	Butanoic acid, 4-[[[4,Ä≤-[(2-ethyl-5,7-dimethyl-3H-imidazo[4,5-b]pyridin-3-yl)me	C27H28N4O5S	520.6	A/B	-8.8
2730	C(=O)([C@@H](NC(=O)OC)C(C)C)N1[C@H](c2[nH]c(c3c4c(c(cc3)c3c5c(c(cc3)c3[nH]c(nc3)[C@H]3N(C(=O)[C@@H](	1350718-53-4	Carbamic acid, N,N,Ä≤-[(5,5,Ä≤,6,6,Ä≤,7,7,Ä≤,8,8,Ä≤-octahydro[1,1,Ä≤-binaphtha	C48H62N8O6	847.06	A/B	-8.8
2731	C(=O)([C@@H](NC(=O)OC)C(C)C)N1[C@@]2(C[C@H](C1)CC2)c1[nH]c(cn1)c1c2c(c(cc1)c1ccc(cc1)c1[nH]c(nc1)[C@	1350718-88-5	Carbamic acid, N,N,Ä≤-[[4,4,Ä≤-bi-1H-indene]-7,7,Ä≤-diylbis[1H-imidazole-5,2-di	C50H58N8O6	867.05	A/B	-8.8
2732	C(=O)([C@@H](NC(=O)OC)C(C)C)N1[C@@]2(C[C@H](C1)CC2)c1[nH]c(cn1)c1c2c(c(cc1)c1ccc(cc1)c1[nH]c(nc1)[C@	1350718-96-5	Carbamic acid, N-[(1S)-1-[[(1S,4R)-1-[5-[8-[4-[2-[(1S,4R)-2-[(2S)-2-[(methoxycar	C47H55N9O6	842	A/B	-8.8
2733	[C@@H](C(=O)N1[C@@H](CCC1)c1[nH]c(cn1)c1ccc(cc1)c1ccc(cc1)c1[nH]c(nc1)[C@H]1N(C(=O)[C@H](C(C(F)(F)F)	1350719-42-4	Carbamic acid, N,N,Ä≤-[(2,2,Ä≤,3,3,Ä≤-tetrahydro[4,4,Ä≤-bi-1H-indene]-7,7,Ä≤-di	C47H60N8O6	833.03	A/B	-8.8
2734	C(#N)[C@]1(O[C@H](COP(=O)(Oc2ccccc2)N[C@H](C(=O)OC(C)C)C)[C@@H](O)[C@@]1(C)O)c1n2c(cc1)c(N)ncn2	1350719-49-1	Carbamic acid, N,N,Ä≤-[(2,2,Ä≤,3,3,Ä≤-tetrahydro[4,4,Ä≤-bi-1H-indene]-7,7,Ä≤-di	C48H62N8O6	847.06	A/B	-8.8
2735	C(=O)([C@@H](NC(=O)OC)C1CCOCC1)N1C[C@@]2(C[C@H]1c1[nH]c(cn1)c1ccc(cc1)c1ccc(cc1)c1[nH]c(nc1)[C@H]1N(	1351851-16-5	Carbamic acid, N-[(1S)-1-[[(5S,8S)-8-[5-[4,Ä≤-[2-[(2S)-1-[(2S)-2-[(methoxycarbor	C46H58N8O8	851	A/B	-8.8
2736	C(=O)([C@@H](NC(=O)OC)C(C)C)N1C[C@@]2(C[C@H]1c1[nH]c(cn1)c1ccc(cc1)c1ccc(cc1)c1[nH]c(nc1)[C@H]1N(C(=	1351852-64-6	Carbamic acid, N-[(1S)-1-[[(2S,4R)-4-fluoro-2-[5-[4,Ä≤-[2-[(5S,8S)-7-[(2S)-2-[(	C43H53FN8O7	812.93	A/B	-8.8
2737	C(=O)([C@@H](NC(=O)OC)C(C)C)N1C[C@@]2(C[C@H]1c1[nH]c(cn1)c1c(CC)c(CC)c(cc1)c1c(CC)c(CC)c(cc1)c1[nH]c	1351852-84-0	Carbamic acid, N-[(1S)-1-[[(2S)-2-[5-[4,Ä≤-[2-[(5S,8S)-7-[(2S)-2-[(methoxycarbor	C47H62N8O7	851.04	A/B	-8.8
2738	C(=O)([C@@H](NC(=O)OC)C(C)C)N1C[C@@]2(C[C@H]1c1[nH]c(cn1)c1ccc(cc1)c1ccc(cc1)c1[nH]c(nc1)[C@H]1N(C(=	1351852-90-8	Carbamic acid, N-[(1S)-1-[[(5S,8S)-8-[5-[4,Ä≤-[2-[(1R,3S,4S)-2-[(2S)-2-[(methox	C45H56N8O7	820.98	A/B	-8.8
2739	C(=O)([C@@H](NC(=O)OC)C(C)C)N1CC2(C[C@H]1c1[nH]c(cn1)c1ccc(cc1)c1ccc(cc1)c1[nH]c(nc1)[C@H]1N(C(=O)[C	1351853-23-0	Carbamic acid, N-[(1S)-1-[[(2S)-2-[5-[4,Ä≤-[2-[(5S,8S)-7-[(2S)-2-[(methoxycarbor	C42H51N9O8	809.91	A/B	-8.8
2740	C(=O)([C@H](NC(=O)OC)c1ccccc1)N1[C@@H](C[C@@H](C1)C1CC1)c1[nH]c(cn1)c1ccc(cc1)c1ccc(cc1)c1[nH]c(nc1)	1351853-26-3	Carbamic acid, N-[(1S)-1-[[(2S)-2-[5-[4,Ä≤-[2-[(2R,5S,8S)-7-[(2S)-2-[(methoxyca	C44H56N8O7	808.96	A/B	-8.8
2741	C(=O)([C@H](NC(=O)OC)C)N1[C@@H](C[C@@H](C1)C1CC1)c1[nH]c(cn1)c1ccc(cc1)c1ccc(cc1)c1[nH]c(nc1)[C@H]1N	1351853-28-5	Carbamic acid, N-[(1R)-2-[(2S)-2-[5-[4,Ä≤-[2-[(2S,4R)-4-cyclopropyl-1-[(2R)-2-[	C49H50N8O6	846.97	A/B	-8.8
2742	C(#N)[C@]1(O[C@H](CO[P@](=O)(Oc2ccccc2)N[C@H](C(=O)OCC)C)[C@@H](O)[C@@]1(C)O)c1n2c(cc1)c(N)nc(F)n2	1354823-51-0	L-Alanine, N-[(S)-hydroxy(1-naphthalenyloxy)phosphinyl]-, ethyl ester, 6-ester w	C28H31N6O8P	610.55	A/B	-8.8
2743	O=c1n(cc(C)c(=O)n1/C=C/C(=C/C(=O)c1cn(Cc2ccc(F)cc2)nn1)/O)[C@H]1C[C@H](N=[N+]=[N-])[C@@H](CO)O1	1355359-11-3	Thymidine, 3,Ä≤-azido-3,Ä≤-deoxy-3-[(1E,3Z)-5-[1-[(4-fluorophenyl)methyl]-1H-te	C23H22FN9O6		A/B	-8.8
2744	O(P(=O)(COC[C@H]1O[C@H]([C@@H](OP(=O)(OC[C@H]2O[C@H]([C@H](O)[C@@H]2O)n2c3c(nc2)c(N)ncn3)O)[C@@H]1O)	1357016-12-6	Adenosine, 5,Ä≤-O-phosphonoadenylylmethylene-(2,Ä≤,Ü5,Ä≤)-Œ≤-D-arabino-adenyly	C41H52N20O25P4	1348.87	A/B	-8.8
2745	C(=O)([C@@H](NC(=O)OC)C1CCOCC1)N1[C@H]2[C@H](C2)C[C@H]1c1[nH]c(cn1)c1c2c(c(cc1)c1ccc(cc1)c1[nH]c(nc1	1359868-95-3	Carbamic acid, N-[(1S)-1-[[(1R,3S,5R)-3-[5-[4-[7-[2-[(1R,3S,5R)-2-[(2S)-2-[(meth	C43H50N10O6	802.92	A/B	-8.8
2746	C(F)(F)c1n(c2c(n1)cccc2)c1nc(nc(N(C)c2ccncc2)n1)N1CCOCC1	1359956-29-8	1,3,5-Triazin-2-amine, 4-[2-(difluoromethyl)-1H-benzimidazol-1-yl]-6-(4-morpholi	C25H22F2N8O	488.49	A/B	-8.8
2747	C(=O)([C@@H](NC(=O)OC)C(C)C)N1[C@@H](C[C@H](SC)C1)c1[nH]c(cn1)c1ccc(cc1)c1ccc(cc1)c1[nH]c(nc1)[C@H]1	1361249-17-3	1H-1,2,3-Triazole-4-carboxylic acid, 1,1,Ä≤-[[1,1,Ä≤-biphenyl]-4,4,Ä≤-diylbis[1	C48H56N14O10	989.05	A/B	-8.8
2748	O=c1c2c(n(cn2)[C@H]2C[C@H](O[As](=O)(OC[C@H]3O[C@H](C[C@@H]3O)n3c4c(nc3)c(=O)nc(N)[nH]4)O)[C@@H](CO)	1370588-53-6	Benzenesulfonic acid, 2,4,6-trimethyl-, 4-[[[(4-fluorophenyl)methyl]amino]carbor	C29H31FN6O7S	626.66	A/B	-8.8
2749	C(=O)([C@@H](NC(=O)OC)C(C)C)N1[C@@H]([C@@H]2C[C@H]1CC2)c1[nH]c(cn1)c1ccc(cc1)c1cc2c(cc1)c1c(cc2)nc([	1377036-84-4	Carbamic acid, N-[(1S)-2-[(2S)-2-[5-[9,10-dihydro-7-[2-[(1R,3S,4S)-2-[(2S)-2-[(m	C48H58N8O8	875.02	A/B	-8.8
2750	C(=O)([C@H](NC(=O)OC)c1ccccc1)N1[C@@H](C[C@H](C)C1)c1[nH]c(cn1)c1ccc(cc1)c1cc2c(cc1)cc(cc2)c1[nH]c(n	1377037-48-3	Carbamic acid, N-[(1S)-1-[[(2S)-2-[5-[6-[4-[2-[2,3-dihydro-2-[(2S)-2-[(methoxyca	C48H52N8O6	836.98	A/B	-8.8
2751	C(=O)([C@@H](NC(=O)OC)C(C)C)N1[C@@H](CC(=C)C1)c1[nH]c(cn1)c1cc2c(cc1)cc(cc2)c1ccc(cc1)c1[nH]c(nc1)[C	1377037-83-6	Carbamic acid, N-[(1R)-2-[(2S,4S)-2-[5-[4-[6-[2-[(2S)-1-[(2S)-2-[(methoxycarbony	C48H52N8O6	836.98	A/B	-8.8
2752	C(=O)(C(N(C)C)c1ccccc1)N1[C@@H](CCC1)c1[nH]c(cn1)c1ccc(cc1)c1cc2c(cc1)cc(cc2)c1[nH]c(nc1)[C@H]1N(C(=	1377037-87-0	Carbamic acid, N-[(1R)-2-[(2S)-2-[5-[4-[6-[2-[(2S)-1-[(2S)-2-[(methoxycarbonyl)a	C48H50N8O6	834.96	A/B	-8.8
2753	C(=O)([C@@H](NC(=O)OC)C(C)C)N1[C@H](c2[nH]c3c4c(cc(cc4)c4c(C#CC5CC5)cc(cc4)c4[nH]c(nc4)[C@H]4N(C(=O)	1377042-41-5	Carbamic acid, N-[(1S)-1-[[(2S)-2-[7-[2-(2-cyclopropylethynyl)-4-[2-[(2S)-1-[(2S	C47H54N8O6	826.98	A/B	-8.8
2754	C(=O)([C@@H](NC(=O)OC)C(C)C)N1[C@@H]([C@@H]2C[C@H]1CC2)c1[nH]c2c(n1)ccc1c2ccc(c1)c1cc(Cl)c(cc1)c1[nH	1377042-88-0	Carbamic acid, N,N,Ä≤-[(9,10-dihydro-5H-phenanthro[4,5-bcd]pyran-2,7-diyl)bis[1H	C43H52N8O7	792.92	A/B	-8.8
2755	C(#CC)c1c(c(C#CC)cc(c1)c1[nH]c(nc1)[C@H]1N(C(=O)[C@@H](NC(=O)OC)C(C)C)CCC1)c1cc2c(cc1)c1c(cc2)nc([nH	1377042-92-6	Carbamic acid, N-[(1S)-1-[[(2S)-2-[7-[4-[2-[(2S)-1-[(2S)-2-[(methoxycarbonyl)ami	C45H52N8O6	800.94	A/B	-8.8
2756	C(=O)([C@@H](NC(=O)OC)C(C)C)N1[C@H](c2[nH]c3c4c(cc(cc4)c4cc5c(cc4)c4c(cc5)nc([nH]4)[C@H]4N(C(=O)[C@@	1377043-66-7	Carbamic acid, N-[(1S)-1-[[(1R,3S,5R)-3-[5-chloro-4-[2-fluoro-4-[2-[(1R,3S,5R)-2	C44H48ClFN8O6	839.35	A/B	-8.8
2757	C(=O)([C@@H](NC(=O)OC)C(C)C)N1[C@H](c2[nH]c3c4c(cc(cc4)c4ccc(cc4)c4[nH]c(nc4)[C@H]4N(C(=O)[C@@H](NC(	1377044-28-4	Carbamic acid, N-[(1S)-1-[[(2S)-2-[2,Ä≤-[(2S,4S)-4-cyano-1-[(2S)-2-[(methoxycarb	C45H49N9O6	811.93	A/B	-8.8
2758	O[C@H]1[C@@H](O[C@@H](CO)[C@H]1O)n1c(=O)nc(N)c(F)c1	1382649-70-8	Carbamic acid, N,N,Ä≤-[[3-(4-methoxyphenyl)-1,2-cyclopropanediyl]bis[4,1-phenyle	C50H60N8O7	885.06	A/B	-8.8
2759	C(=O)([C@@H](NC(=O)OC)C(C)C)N1[C@@H](CCC1)c1[nH]c(cn1)c1ccc(cc1)c1ccc(cc1)c1[nH]c(nc1)[C@H]1N(C[C@@H	1383566-38-8	Carbamic acid, N,N,Ä≤-[[4,4,Ä≤-bibenzothiazole]-7,7,Ä≤-diylbis[1H-imidazole-5,2	C44H52N10O6S2	881.08	A/B	-8.8
2760	[C@H](Cc1ccccc1)(CC[C@H](Cc1ccccc1)NC(=O)OCc1cnc[nH]1)NC(=O)[C@@H](NC(=O)N(Cc1nc(C(C)C)sc1)C)C(C)C	1383570-20-4	2,7,10,12-Tetraazatridecanoic acid, 12-methyl-9-(1-methylethyl)-13-[2-(1-methyle	C38H51N7O4S	701.92	A/B	-8.8
2761	C(CC(=O)CC(CCc1ccc(O)cc1)O)c1ccc(OC)c(O)cc1	1388690-73-0	Spiro[2H-1-benzopyran-2,8,Ä≤(5,Ä≤H)-[1,2,4]triazolo[4,3-a]pyridin]-4(3H)-one, 7	C25H21ClN4O4	476.91	A/B	-8.8
2762	O=C1C2(C(C(C(=O)/C=C(\O)/C3C4(N5C(C3c3cc(OC)c(O)cc3)CSC5)c3c(NC4=O)ccc(N(=O)=O)c3)C3N2CSC3)c2cc(OC)c	1389320-78-8	Spiro[2H-indene-2,5,Ä≤-[3H,5H]pyrrolo[1,2-c]thiazole]-1,3-dione, 6,Ä≤-[(1Z)-3-[	C45H38N2O10S2	830.92	A/B	-8.8
2763	O=c1n([C@H]2O[C@@H](CO)SC2)ccc(NC(=O)c2cnccn2)n1	1391853-62-5	Uridine, cytidylyl-(3,Ä≤,Ü5,Ä≤)-cytidylyl-(3,Ä≤,Ü5,Ä≤)-guanylyl-(3,Ä≤,Ü5,Ä≤	C37H48N13O27P3	1199.77	A/B	-8.8
2764	S(=O)(=O)(C)N1CCN(c2nc(nc(n2)N2CCOCC2)c2cnc(N)nc2)CC1	1391924-28-9	Benzamide, 3-[[4-(6-amino-3-pyridinyl)-6-(4-morpholinyl)-1,3,5-triazin-2-yl]amir	C21H24N8O2	420.47	A/B	-8.8
2765	N(C(C(=O)O)(C)C)c1nc(nc(n1)N1CCOCC1)c1c2c([nH]nc2)ccc1	1391926-00-3	Propanamide, 2-[[4-(1H-indazol-4-yl)-6-(4-morpholinyl)-1,3,5-triazin-2-yl]amino]	C17H20N8O2	368.39	A/B	-8.8
2766	C(F)(F)(F)c1c(c2nc(nc(n2)N2CCOCC2)N2CCN(S(=O)(=O)C)CC2)cnc(N)c1	1391926-25-2	1-Piperazinesulfonamide, 4-[4-[6-amino-4-(trifluoromethyl)-3-pyridinyl]-6-(4-mor	C17H22F3N9O3S	489.48	A/B	-8.8
2767	C(OCCCCN)n1c2c(c(=O)nc(N)[nH]2)nc1	1391926-65-0	Benzamide, 4-[[4-(6-amino-3-pyridinyl)-6-(4-morpholinyl)-1,3,5-triazin-2-yl]amir	C21H24N8O2	420.47	A/B	-8.8
2768	N(CCNC(=O)c1ccc(cc1)C1OOC(OO1)c1ccc(C)cc1)c1nc(nc(n1)N1CCOCC1)N1CCCCC1	1393922-40-1	Benzamide, N-[2-[(4,6-di-4-morpholinyl-1,3,5-triazin-2-yl)amino]ethyl]-4-[6-(4-m	C29H35N7O7	593.63	A/B	-8.8
2769	C(=O)(O)c1c(NC(=O)c2ccc(C(=O)c3ccc(C(=O)Nc4c(C(=O)O)cccc4)cc3)cc2)cccc1	1398310-29-6	Guanosine, 3,Ä≤-O-dephosphinicothymidylyl-(Œ±,Ü8)-2,Ä≤-deoxy-	C20H25N7O9	507.45	A/B	-8.8
2770	N(c1c(C)cc(/C=C/C#N)cc1C)c1nc(Nc2ccc(C#N)cc2)ncc1.C(=C\C(=O)O)\C(=O)O	1399384-92-9	Benzonitrile, 4-[[4-[[4-[(1E)-2-cyanoethenyl]-2,6-dimethylphenyl]amino]-2-pyrimi	C22H18N6.CH4O3S		A/B	-8.8
2771	O=C1c2n(C[C@H]3N1[C@](C)(C(C(O3)([2H])[2H])([2H])[2H])[2H])cc(C(=O)NC(c1c(F)cc(F)cc1)([2H])[2H])c(=O	1407166-92-0	2H-Pyrido[1,Ä≤,2,Ä≤:4,5]pyrazino[2,1-b][1,3]oxazine-2,3-d2-9-carboxamide, N-[(2	C20H10D9F2N3O5		A/B	-8.8
2772	O=C1c2n(C[C@]3(N1[C@H](C([2H])([2H])[2H])C(C(O3)([2H])[2H])([2H])[2H])[2H])cc(C(=O)NC(c1c(F)cc(F)cc1	1407167-06-9	2H-Pyrido[1,Ä≤,2,Ä≤:4,5]pyrazino[2,1-b][1,3]oxazine-2,3,4-d3-9-carboxamide, N-[	C20H8D11F2N3O5		A/B	-8.8
2773	O=C1c2n(C[C@]3(N1[C@](C([2H])([2H])[2H])(CCO3)[2H])[2H])cc(C(=O)NC(c1c(F)cc(F)cc1)([2H])[2H])c(=O)c2	1407167-09-2	2H-Pyrido[1,Ä≤,2,Ä≤:4,5]pyrazino[2,1-b][1,3]oxazine-2,3-d2-9-carboxamide, N-[(2	C20H12D7F2N3O5		A/B	-8.8
2774	O=C1c2n(C([C@H]3N1[C@H](C)CCO3)([2H])[2H])cc(C(=O)NCc1c(F)cc(F)cc1)c(=O)c2O	1407167-21-8	2H-Pyrido[1,Ä≤,2,Ä≤:4,5]pyrazino[2,1-b][1,3]oxazine-2,3,4,12-d4-9-carboxamide,	C20H9D10F2N3O5		A/B	-8.8
2775	O=C1c2n(C[C@H]3N1[C@](C)(CCO3)[2H])cc(C(=O)NCc1c(F)cc(F)cc1)c(=O)c2O	1407167-28-5	2H-Pyrido[1,Ä≤,2,Ä≤:4,5]pyrazino[2,1-b][1,3]oxazine-9-carboxamide, N-[(2,4-difl	C20H16D3F2N3O5		A/B	-8.8
2776	O=C1c2n(C([C@H]3N1[C@](C([2H])([2H])[2H])(CCO3)[2H])([2H])[2H])cc(C(=O)NCc1c(F)cc(F)cc1)c(=O)c2O	1407167-36-5	2H-Pyrido[1,Ä≤,2,Ä≤:4,5]pyrazino[2,1-b][1,3]oxazine-2,3,12-d3-9-carboxamide, N-	C20H13D6F2N3O5		A/B	-8.8
2777	O=C1c2n(C[C@]3(N1[C@](C)(C(C(O3)([2H])[2H])([2H])[2H])[2H])[2H])cc(C(=O)NCc1c(F)cc(F)cc1)c(=O)c2O	1407167-44-5	2H-Pyrido[1,Ä≤,2,Ä≤:4,5]pyrazino[2,1-b][1,3]oxazine-2,3-d2-9-carboxamide, N-[(2	C20H11D8F2N3O5		A/B	-8.8
2778	O=C1c2n(C([C@]3(N1[C@H](C([2H])([2H])[2H])C(C(O3)([2H])[2H])([2H])[2H])[2H])([2H])[2H])cc(C(=O)NCc1c	1407167-50-3	2H-Pyrido[1,Ä≤,2,Ä≤:4,5]pyrazino[2,1-b][1,3]oxazine-9-carboxamide, N-[(2,4-difl	C20H18DF2N3O5		A/B	-8.8
2779	O(C(=O)C1=CN(C)C=CC1)[C@H]1[C@@H](O[C@H](COC(=O)c2ccccc2)[C@H]1OC(=O)c1ccccc1)n1nc(C(=O)N)nc1	140891-47-0	1H-1,2,4-Triazole-3-carboxamide, 1-[2,5-di-O-benzoyl-3-O-[(1,4-dihydro-1-methyl-	C29H27N5O8	573.55	A/B	-8.8
2780	C(Nc1c(C(=O)N)cccc1)(C(=O)N)c1c(Cl)cccc1Cl	141027-80-7	1,5-Naphthalenedisulfonic acid, 3,3,Ä≤-[1,2-ethenediylbis[(3-sulfo-4,1-phenylene	C34H26N6O18S6.6Na		A/B	-8.8
2781	[C@H](C(=O)N1[C@@H](CC[C@@H]1C)c1[nH]c(cn1)c1ccc(cc1)c1ccc(cc1)c1[nH]c(nc1)[C@H]1N(C(=O)[C@@H](NC(=O	1412813-79-6	Carbamic acid, N,N,Ä≤-[[1,1,Ä≤-biphenyl]-4,4,Ä≤-diylbis[1H-imidazole-5,2-diyl-(	C48H48F6N8O6	946.94	A/B	-8.8
2782	C(=O)([C@@H](NC(=O)OC)[C@@H]1CC2(OCC1)CCCC2)N1[C@@H](CC[C@@H]1C)c1[nH]c(cn1)c1ccc(cc1)c1ccc(cc1)c1[n	1412816-31-9	Carbamic acid, N,N,Ä≤-[[1,1,Ä≤-biphenyl]-4,4,Ä≤-diylbis[1H-imidazole-5,2-diyl[(	C54H70N8O8	959.18	A/B	-8.8
2783	C(OC(CO)CO)n1c2c(c(=O)nc(NCN(CCO)CCO)[nH]2)nc1	1415032-08-4	Benzenesulfonamide, 4-[[[[6,9-dihydro-9-[[2-hydroxy-1-(hydroxymethyl)ethoxy]meth	C20H24N8O7S	520.52	A/B	-8.8
2784	C(=O)([C@@H](NC(=O)OC)C(C)C)N1[C@H](c2[nH]c(c3c4CCc5cc(c(CCc(c3)cc4)cc5)c3[nH]c(nc3)[C@H]3N(C(=O)[C@	1415120-35-2	Carbamic acid, N,N,Ä≤-[tricyclo[8.2.2.24,7]hexadeca-4,6,10,12,13,15-hexaene-5,1!	C56H64N8O6	945.16	A/B	-8.8
2785	C(OCCn1c2c(nc1)c(N)ncn2)[P@@]1(=O)O[C@@H](C[C@H](O1)c1ccccc1)c1cc(Cl)ccc1	1417393-26-0	9H-Purin-6-amine, 9-[2-[[(2S,4R,6R)-4-(3-chlorophenyl)-2-oxido-6-phenyl-1,3,2-di	C23H23ClN5O4P	499.89	A/B	-8.8
2786	C(COCP1(=O)OCC(Oc2ccccc2)CO1)n1c2c(nc1)c(N)ncn2	1417393-27-1	9H-Purin-6-amine, 9-[2-[[(2S,4S,6S)-4-(3-chlorophenyl)-2-oxido-6-phenyl-1,3,2-di	C23H23ClN5O4P	499.89	A/B	-8.8
2787	C(=O)([C@@H](NC(=O)OC)C(C)C)N1[C@H](c2[nH]c(c3c4c(c(cc3)c3ccc(cc3)c3[nH]c(nc3)[C@H]3N(C(=O)[C@@H](NC	1419385-61-7	Carbamic acid, N-[(1S)-1-[[(2S)-2-[5-[1,Ä≤,3,Ä≤-dihydro-7,Ä≤-[4-[2-[(2S)-1-[(2S	C48H62N8O6	847.06	A/B	-8.8
2788	C(N1c2c(C(=NNC(=S)N(CC=C)CC=C)C1=O)cc(F)cc2)N1CCN(CC1)c1ccc(Cl)cc1	1426583-69-8	Hydrazinecarbothioamide, 2-[5-chloro-1-[[4-(4-chlorophenyl)-1-piperazinyl]methyl	C32H28Cl2N6OS	615.58	A/B	-8.8
2789	N(=C1c2c(N(CN3CCN(CC3)c3ccc4c(cc3F)c(=O)c(C(=O)O)cn4CC)C1=O)ccc(C)c2)NC(=S)N(CC=C)CC=C	1426583-83-6	3-Quinolinecarboxylic acid, 7-[4-[[3-[2-[(diphenylamino)thioxomethyl]hydrazinyli	C38H33F2N7O4S	721.77	A/B	-8.8
2790	N(C(=O)C)[C@H]1[C@H]([C@@H]([C@@H](CO)O)O)OC(=C[C@@H]1NC(=N)NC(=O)CSc1cc2c(cc1)cccc2)C(=O)O	1442563-10-1	D-glycero-D-galacto-Non-2-enonic acid, 5-(acetylamino)-2,6-anhydro-3,4,5-trideox	C25H30N4O8	514.53	A/B	-8.8
2791	N(c1c2c(c(O)c(CN3C(C)CCCC3)c1)CCCC2)c1c2c(ncc1)cc(Cl)cc2	1449239-41-1	1-Naphthalenol, 4-[(7-chloro-4-quinolinyl)amino]-2-[(3,5-dimethyl-1-piperazinyl)	C26H31ClN4O	451	A/B	-8.8
2792	O[C@H]1[C@H](n2c3c(nc2)c(=O)nc[nH]3)O[C@H](COP(=O)(O)O)[C@H]1O.O[C@H]1[C@@H](O[C@H](COP(=O)(O)O)[C@H	1449239-46-6	1-Naphthalenol, 4-[(7-chloro-4-quinolinyl)amino]-5,6,7,8-tetrahydro-2-[(4-phenyl	C30H31ClN4O	499.05	A/B	-8.8
2793	N(CCCNc1nc(nc(Nc2ccc(CC)cc2)n1)N1CCOCC1)c1c2c(ncc1)cc(Cl)cc2	1454694-92-8	1,3,5-Triazine-2,4-diamine, N2-[2-[(7-chloro-4-quinolinyl)amino]ethyl]-6-(4-morp	C24H25ClN8O	476.96	A/B	-8.8
2794	N(c1nc(nc(Nc2ccc(C#N)cc2)n1)N1CCOCC1)c1c(C)cc(/C=C\C#N)cc1C	1458666-14-2	Benzonitrile, 4-[[4-[[4-[(1E)-2-cyanoethenyl]-2,6-dimethylphenyl]amino]-6-(4-mor	C25H24N8O	452.51	A/B	-8.8
2795	N(c1c2c(ncc1)cc(Cl)cc2)c1cc(cc(CN2CC(C)CCC2)c1O)c1ccc(Cl)cc1	146298-43-3	[1,1,Ä≤-Biphenyl]-4-ol, 4,Ä≤-chloro-3-[(7-chloro-4-quinolinyl)amino]-5-(1-piper	C27H25Cl2N3O	478.41	A/B	-8.8
2796	N(c1c2c(ncc1)cc(Cl)cc2)c1cc(cc(CN2CCC(C)CC2)c1O)c1ccc(Cl)cc1	146298-44-4	[1,1,Ä≤-Biphenyl]-4-ol, 4,Ä≤-chloro-3-[(7-chloro-4-quinolinyl)amino]-5-[(3-meth	C28H27Cl2N3O	492.44	A/B	-8.8
2797	O[C@H]1[C@@H](O[C@H](CO)[C@H]1O)n1c(=O)[nH]c(=O)c(C)c1	146298-45-5	[1,1,Ä≤-Biphenyl]-4-ol, 4,Ä≤-chloro-3-[(7-chloro-4-quinolinyl)amino]-5-[(4-meth	C28H27Cl2N3O	492.44	A/B	-8.8
2798	O[C@H]1[C@H](n2c3c(nc2)c(=O)nc(N)[nH]3)O[C@H](COP(=O)(CP(=O)(OP(=O)(O)O)O)O)[C@H]1O	1499175-77-7	4-Morpholinecarboxamide, N-[(1S)-2-[[(1S)-1-[(4-hydroxyphenyl)methyl]-3-(phenyls	C30H33N3O6S	563.66	A/B	-8.8
2799	O=c1n([C@@H]2O[C@H](COC(=O)C(=O)N3[C@H](C(=O)N[C@@H](Cc4ccccc4)C(=O)OCc4ccccc4)CCC3)[C@@H](N=[N+]=[N	1502822-78-7	Propanoic acid, 2-hydroxy-, compd. with 4-[[4-[[4-[(1E)-2-cyanoethenyl]-2,6-dime	C22H18N6.C3H6O3		A/B	-8.8
2800	[Si](OC[C@H]1O[C@H](CS1)n1c(=O)nc(N)c(F)c1)(C(C)(C)C)(c1ccccc1)c1ccccc1	151720-22-8	Adenosine, P-(carboxymethyl)-P,2,Ä≤-dideoxyadenylyl-(3,Ä≤,Ü5,Ä≤)-2,Ä≤-deoxy-	C22H27N10O9P	606.49	A/B	-8.8
2801	C(=O)(N[C@H](C(=O)C(=O)NC1CC1)CCC)[C@H]1N(C(=O)[C@@H](NC(=O)[C@@H](NC(=O)c2cn(=O)ccn2)C2CCCCC2)C(C)(	152195-59-0	1,5-Naphthalenedisulfonic acid, 3-[2-[4-[2-[4-[2-(4-amino-5-sulfo-1-naphthalenyl	C40H27N7O15S5.5Na		A/B	-8.8
2802	O(C(OC(=O)C)C)c1c(C(=O)NCc2ccc(F)cc2)nc(C(NC(=O)c2nnc(C)o2)(C)C)n(C)c1=O	1538604-15-7	Cyclopentanecarboxylic acid, 1-[[4-[[[(4-fluorophenyl)methyl]amino]carbonyl]-1,6	C30H37FN6O7	612.65	A/B	-8.8
2803	C(n1c2c(nc1CC)c(C)cc(C)n2)c1ccc(cc1)c1c(S(=O)(=O)NC(=O)CCCCC)ccc(COC)c1	154551-80-1	Hexanamide, N-[[4,Ä≤-[(2-ethyl-5,7-dimethyl-3H-imidazo[4,5-b]pyridin-3-yl)methy]	C31H38N4O3S2	578.79	A/B	-8.8
2804	C(n1c2c(nc1CC)c(C)cc(C)n2)c1ccc(cc1)c1c(S(=O)(=O)NC(=O)NCCCC)ccc(COC)c1	154552-40-6	[1,1,Ä≤-Biphenyl]-2-sulfonamide, N-[(butylamino)carbonyl]-4,Ä≤-[(2-ethyl-5,7-di	C30H37N5O3S2	579.78	A/B	-8.8
2805	C(n1c2c(nc1CC)c(C)cc(C)n2)c1ccc(cc1)c1c(S(=O)(=O)NC(=O)NCCCC)ccc(OCC)c1	154552-41-7	[1,1,Ä≤-Biphenyl]-2-sulfonamide, N-[(butylamino)carbonyl]-4,Ä≤-[(2-ethyl-5,7-di	C30H37N5O4S	563.71	A/B	-8.8
2806	C(n1c2c(nc1CC)c(C)cc(C)n2)c1ccc(cc1)c1c(S(=O)(=O)NC(=O)NCCCC)ccc(C(C)(C)C)c1	154552-44-0	[1,1,Ä≤-Biphenyl]-2-sulfonamide, N-[(butylamino)carbonyl]-5-(1,1-dimethylpropyl)	C33H43N5O3S	589.79	A/B	-8.8
2807	C(n1c2c(nc1CC)c(C)cc(C)n2)c1ccc(cc1)c1c(S(=O)(=O)NC(=O)CCc2ccccc2)ccc(OCCCC)c1	154552-55-3	Benzenepropanamide, N-[[4,Ä≤-[(2-ethyl-5,7-dimethyl-3H-imidazo[4,5-b]pyridin-3-y	C35H38N4O3S	594.77	A/B	-8.8

2808	C(n1c2c(nc1CC)c(C)cc(C)n2)c1ccc(cc1)c1c(S(=O)(=O)NC(=O)c2c(OCC)cccc2)ccc(C(C)C)c1	154552-94-0	Benzamide, N-[[5-butyl-4,Ä≤-[(2-ethyl-5,7-dimethyl-3H-imidazo[4,5-b]pyridin-3-yl	C36H40N4O4S	624.79	A/B	-8.8
2809	C(n1c2c(nc1CC)c(C)cc(C)n2)c1ccc(cc1)c1c(S(=O)(=O)NC(=O)c2c(OCC)cccc2)ccc(COC)c1	154552-99-5	Benzamide, 2-ethoxy-N-[[4,Ä≤-[(2-ethyl-5,7-dimethyl-3H-imidazo[4,5-b]pyridin-3-y	C34H36N4O4S2	628.8	A/B	-8.8
2810	C(n1c2c(nc1CC)c(C)cc(C)n2)c1ccc(cc1)c1c(S(=O)(=O)NC(=O)c2c(OCC)cccc2)ccc(OCC)c1	154553-00-1	Benzamide, 2-ethoxy-N-[[4,Ä≤-[(2-ethyl-5,7-dimethyl-3H-imidazo[4,5-b]pyridin-3-y	C34H36N4O5S	612.74	A/B	-8.8
2811	C(n1c2c(nc1CC)c(C)cc(C)n2)c1ccc(cc1)c1c(S(=O)(=O)NC(=O)c2ccccc2)ccc(CC)c1	154553-11-4	Benzamide, N-[[4,Ä≤-[(2-ethyl-5,7-dimethyl-3H-imidazo[4,5-b]pyridin-3-yl)methyl]	C31H30N4O3S	538.66	A/B	-8.8
2812	C(n1c2c(nc1CC)c(C)cc(C)n2)c1ccc(cc1)c1c(S(=O)(=O)NC(=O)c2ccccc2)ccc(C(C)(C)C)c1	154553-23-8	Benzamide, N-[[5-(1,1-dimethylpropyl)-4,Ä≤-[(2-ethyl-5,7-dimethyl-3H-imidazo[4,5	C35H38N4O3S	594.77	A/B	-8.8
2813	S(c1nc(nc(Nc2ccc(Cl)cc2)n1)N1CCOCC1)c1oc(nn1)c1ccc(Cl)cc1	1547295-45-3	1,3,5-Triazin-2-amine, 4-[[5-(4-chlorophenyl)-1,3,4-oxadiazol-2-yl]thio]-N-(4-me	C22H20ClN7O3S	497.96	A/B	-8.8
2814	O(C)[C@H]1[C@@H](O[C@H](COP(=O)(O)O)[C@H]1O)n1c2c(nc1)c(=O)nc[nH]2	1547295-52-2	1,3,5-Triazin-2-amine, N-(3-bromophenyl)-4-[[5-(4-chlorophenyl)-1,3,4-oxadiazol-	C21H17BrClN7O2S	546.83	A/B	-8.8
2815	C(=O)([C@@H](NC(=O)OC)C1CCCCC1)N1[C@H](c2[nH]c(c3c4c(C5CC4CC5)c(cc3)c3ccc(cc3)c3[nH]c(nc3)[C@H]3N(C(	1560639-00-0	Carbamic acid, N-[(1S)-2-methyl-1-[[(2S,4S)-4-methyl-2-[5-[4-[1,2,3,4-tetrahydro	C47H60N8O6	833.03	A/B	-8.8
2816	C(=O)([C@@H](NC(=O)OC)C(C)C)N1[C@H](c2[nH]c(c3c4c(c(cc3)c3ccc(cc3)c3[nH]c(nc3)[C@H]3N(C(=O)[C@@H](NC	1560640-05-2	Carbamic acid, N-[(1S)-1-[[(2S)-2-[5-[8-[4-[2-[(2S)-4,4-difluoro-1-[(2S)-2-[(met	C46H54F4N8O6	890.96	A/B	-8.8
2817	C(=O)([C@@H](NC(=O)OC)C(C)C)N1[C@H](c2[nH]c(c3c4c(C5OC4CC5)c(cc3)c3ccc(cc3)c3[nH]c(nc3)[C@H]3N(C(=O)	1560640-96-1	Carbamic acid, N-[(1S)-1-[[(2S)-4,4-dimethyl-2-[5-[4-[1,2,3,4-tetrahydro-8-[2-[(	C48H62N8O7	863.06	A/B	-8.8
2818	C(=O)([C@@H](NC(=O)OC)C(C)C)N1[C@H](c2[nH]c(c3c4c(C5CC4CC5)c(cc3)c3ccc(cc3)c3[nH]c(nc3)[C@H]3N(C(=O)	1560649-05-9	Carbamic acid, N-[(1S)-1-[[(2S)-2-[5-[8-[2-fluoro-4-[2-[(2S)-1-[(2S)-2-[(methoxy	C45H55FN8O6	822.97	A/B	-8.8
2819	C(=O)([C@@H](NC(=O)OC)C1CCCCC1)N1[C@H](c2[nH]c(c3c4c(C5(CC4)CCCC5)c(cc3)c3ccc(cc3)c3[nH]c(nc3)[C@H]3	1588403-95-5	Carbamic acid, N-[(1S)-1-[[(2S,4S)-2-[5-[2,Ä≤,3,Ä≤-dihydro-7,Ä≤-[4-[2-[(2S,4S)-	C49H64N8O6	861.08	A/B	-8.8
2820	C(=O)([C@@H](NC(=O)OC)C(C)C)N1[C@H](c2[nH]c(c3c4c(C5(CC4)CCOC5)c(cc3)c3ccc(cc3)c3[nH]c(nc3)[C@H]3N(C	1588404-26-5	Carbamic acid, N-[(1S)-2-methyl-1-[[(2S)-2-[5-[3,Ä≤,4,4,Ä≤,5-tetrahydro-8,Ä≤-[4	C47H60N8O7	849.03	A/B	-8.8
2821	N(C(CCCN(CC)CC)C)(C(=O)CCCCCN1C(=O)C=CC1=O)c1c2c(ncc1)cc(Cl)cc2	1600500-37-5	Benzonitrile, 2-[4-[[[(5R,5aR,8aR,9R)-5,5a,6,8,8a,9-hexahydro-8-oxo-9-(3,4,5-tri	C32H28N4O8	596.59	A/B	-8.8
2822	C(=O)([C@@H](NC(=O)OC)C(C)C)N1C(CN(C)CC1)c1[nH]c(cn1)c1ccc(cc1)c1ccc(cc1)c1[nH]c(nc1)[C@H]1N(C(=O)[C	1616889-50-9	Carbamic acid, N-[(1S)-2-methyl-1-[[(2S)-2-[5-[4,Ä≤-[2-[(2S)-octahydro-1-[(2S)-2	C44H57N9O6	807.98	A/B	-8.8
2823	O=c1c2c(n(cn2)[C@@H]2O[C@H](COP(=O)(Oc3ccc(Cl)cc3)NCc3ccc(F)cc3)CC2)[nH]cn1	1618656-22-6	Inosine, 2,Ä≤,3,Ä≤-dideoxy-, 5,Ä≤-[4-chlorophenyl N-[(2-chlorophenyl)methyl]pho	C23H22Cl2N5O5P	550.33	A/B	-8.8
2824	O=c1c2c(n(cn2)[C@@H]2O[C@H](COP(=O)(Oc3ccc(Cl)cc3)N3CCN(CC3)c3ncccn3)CC2)[nH]cn1	1618656-27-1	Inosine, 2,Ä≤,3,Ä≤-dideoxy-, 5,Ä≤-[4-chlorophenyl P-[4-(2-chlorophenyl)-1-piper	C26H27Cl2N6O5P	605.41	A/B	-8.8
2825	O[C@H]1[C@H]2[C@H]3[C@@](C)([C@@H]([C@@H](CCC(=O)OC)C)CC3)[C@@H](O)C[C@@H]2[C@]2(C)[C@H](Cl)C[C@H](O	1618656-29-3	Inosine, 2,Ä≤,3,Ä≤-dideoxy-, 5,Ä≤-[4-chlorophenyl P-[4-(2-pyridinyl)-1-piperazi	C25H27ClN7O5P	571.95	A/B	-8.8
2826	N(C(=O)[C@H](Cc1ccncc1)C[C@@H](CN1[C@H](C(=O)NC(C)(C)C)CN(Cc2c(F)cccc2F)CC1)O)[C@H]1c2c(C[C@H]1O)ccc	162105-27-3	1-Piperazinepentanamide, 4-[(2,6-difluorophenyl)methyl]-N-(2,3-dihydro-2-hydroxy	C36H45F2N5O4	649.77	A/B	-8.8
2827	S=c1n([C@H]2O[C@@H](CO)CC2)ccc(N)n1	162105-33-1	1-Piperazinepentanamide, N-(2,3-dihydro-2-hydroxy-1H-inden-1-yl)-2-[[(1,1-dimeth	C36H45N5O4S2	675.9	A/B	-8.8
2828	C(n1c2c(nc1CC)c(C)cc(C)n2)c1ccc(cc1)c1c(S(=O)(=O)NC(=O)CCCC)ccc(CC)c1	1624279-15-7	Pentanamide, N-[[4,Ä≤-[(2-ethyl-5,7-dimethyl-3H-imidazo[4,5-b]pyridin-3-yl)methy	C28H32N4O3S	504.64	A/B	-8.8
2829	N(c1nc(nc(n1)N1CCOCC1)c1ccc(OC)cc1)c1c(Cl)cccc1	1627734-67-1	1,3,5-Triazin-2-amine, 4-(4-methoxyphenyl)-6-(4-morpholinyl)-N-(4-nitrophenyl)-	C20H20N6O4	408.41	A/B	-8.8
2830	O(C)c1ccc(c2nc(nc(n2)N2CCOCC2)N2CCN(CC2)c2ccc(OC)cc2)cc1	1627734-80-8	1,3,5-Triazine, 2-[4-(4-fluorophenyl)-1-piperazinyl]-4-(4-methoxyphenyl)-6-(4-mo	C24H27FN6O2	450.51	A/B	-8.8
2831	C(=O)(OC(C)(C)C)N1[C@H]([C@H]2[C@@H]([C@H]1COC(=O)[C@@H]1[C@@H](O)[C@@H]([C@H](C(CC)CC)NC(=O)C)[C@H]	1638370-12-3	Cyclopentanecarboxylic acid, 3-[(1S)-1-(acetylamino)-2-ethylbutyl]-4-[(aminoimir	C26H41N9O6	575.66	A/B	-8.8
2832	C(OCCOP(=O)(Oc1cc2c(cc1)OCO2)Oc1cc2c(cc1)OCO2)n1c2c(nc1)c(=O)nc(N)[nH]2	1638769-61-5	Phosphoric acid, 2-[(2-amino-1,6-dihydro-6-oxo-9H-purin-9-yl)methoxy]ethyl bis(1	C22H20N5O10P	545.4	A/B	-8.8
2833	O([P@@](=O)(OC[C@H]1O[C@H]([C@](C)(F)[C@@H]1O)n1c(=O)[nH]c(=O)cc1)N[C@H](C(=O)OC(C)C)C)c1c2c3c(n(C)c	1639286-23-9	L-Alanine, N-[[P(S),2,Ä≤R]-P-9H-carbazol-2-yl-2,Ä≤-deoxy-2,Ä≤-fluoro-2,Ä≤-methy	C28H32FN4O9P	618.55	A/B	-8.8
2834	O=c1n([C@@H]2O[C@H](CO)[C@@H](O)C2)cc(COC(C(C)C)C(C)C)c(=O)[nH]1	1639286-29-5	L-Alanine, N-[[P(S),2,Ä≤R]-2,Ä≤-deoxy-2,Ä≤-fluoro-P-(1-fluoro-9-methyl-9H-carba	C29H33F2N4O9P	650.56	A/B	-8.8
2835	C(=C\C(=O)CC(=O)/C=C/c1cc(O)ccc1)/c1cc(O)c(O)cc1	1639871-35-4	Carbamic acid, N-[(1R)-2-[(2S)-2,5-dihydro-2-[5-[4,Ä≤-[2-[(2S)-1-[(2S)-2-[(metho	C43H46N8O6	770.88	A/B	-8.8
2836	C[C@]12[C@@H]([C@@H]3[C@](C)([C@@]4(C)[C@H](CC3)[C@]3(C)[C@@H](CC4)C(C)(C)[C@@H](O)CC3)CC1)[C@@H](CC	1642320-07-7	9H-Purin-6-amine, 2-fluoro-9-[5-O-[(1-naphthalenyloxy)[[2-[[(5Z,8Z,11Z,14Z,17Z)-	C42H53FN7O7P	817.89	A/B	-8.8
2837	S(c1nc(nc(Nc2ccc(C(=O)C)cc2)n1)N1CCOCC1)c1oc(nn1)c1ccc(F)cc1	1642584-24-4	1,3,5-Triazin-2-amine, N-(3-bromophenyl)-4-[[5-(4-fluorophenyl)-1,3,4-oxadiazol-	C21H17BrFN7O2S	530.37	A/B	-8.8
2838	S(c1nc(nc(Nc2ccc(Cl)cc2)n1)N1CCOCC1)c1oc(nn1)c1ccc(F)cc1	1642584-26-6	1,3,5-Triazin-2-amine, N-(3-chlorophenyl)-4-[[5-(4-fluorophenyl)-1,3,4-oxadiazol	C21H17ClFN7O2S	485.92	A/B	-8.8
2839	O=c1n([C@@H]2O[C@H](COP(=O)(C(O)c3ccccc3)OC[C@H]3O[C@H](C=C3)n3c(=O)[nH]c(=O)c(C)c3)C=C2)cc(C)c(=O)[	164652-02-2	Thymidine, 2,Ä≤,3,Ä≤-didehydro-P,3,Ä≤-dideoxy-P-[hydroxy(4-methylphenyl)methyl]	C28H31N4O10P	614.54	A/B	-8.8
2840	O=c1n([C@@H]2O[C@H](COP(=O)([O-])[O-])C=C2)ccc(=O)[nH]1	164652-22-6	Thymidine, P-deoxy-P-[hydroxy(2-nitrophenyl)methyl]thymidylyl-(5,Ä≤,Ü5,Ä≤)-2,Ä	C27H30N5O13P	663.53	A/B	-8.8
2841	N(C(=O)[C@H](Cc1ccccc1)C[C@@H](CN1[C@H](C(=O)NC(C)(C)C)CN(Cc2ccccn2)CC1)O)[C@H]1c2c(C[C@H]1O)cccc2	164725-11-5	1-Piperazinepentanamide, 4-[[3,5-bis(1,1-dimethylethyl)-2-hydroxyphenyl]methyl]-	C45H64N4O5	741.01	A/B	-8.8
2842	C(c1c2c(c(C)nc1)OC(C)(C)OC2)N1C[C@@H](C(=O)NC(C)(C)C)N(C[C@H](C[C@H](C(=O)N[C@@H]2c3c(C[C@H]2O)cccc3)	164725-17-1	1-Piperazinepentanamide, N-(2,3-dihydro-2-hydroxy-1H-inden-1-yl)-2-[[(1,1-dimeth	C40H54N6O5	698.89	A/B	-8.8
2843	N(C(=O)[C@H](Cc1ccccc1)C[C@@H](CN1[C@H](C(=O)NC(C)(C)C)CN(Cc2ccc(cc2)c2ccccc2)CC1)O)[C@H]1c2c(C[C@H]	164725-19-3	1-Piperidinecarboxylic acid, 4-[2-[4-[5-[(2,3-dihydro-2-hydroxy-1H-inden-1-yl)am	C42H63N5O6	733.98	A/B	-8.8
2844	N(C(=O)[C@H](Cc1ccccc1)C[C@@H](CN1[C@H](C(=O)NC(C)(C)C)CN(Cc2nc3c(cc2)cccc3)CC1)O)[C@H]1c2c(C[C@H]1O	164725-28-4	D-erythro-Pentonamide, 2,3,5-trideoxy-N-[(1S,2R)-2,3-dihydro-2-hydroxy-1H-inden-	C39H52N4O4	640.85	A/B	-8.8
2845	N(C(=O)[C@H](Cc1ccccc1)C[C@@H](CN1[C@H](C(=O)NC(C)(C)C)CN(Cc2c(C)nc(C)cc2)CC1)O)[C@H]1c2c(C[C@H]1O)c	164725-37-5	1-Piperazinepentanamide, N-(2,3-dihydro-2-hydroxy-1H-inden-1-yl)-2-[[(1,1-dimeth	C38H51N5O4	641.84	A/B	-8.8
2846	N(C(=O)[C@H](Cc1ccccc1)C[C@@H](CN1[C@H](C(=O)NC(C)(C)C)CN(Cc2nc(C)ccc2)CC1)O)[C@H]1c2c(C[C@H]1O)cccc	164725-41-1	1-Piperazinepentanamide, 4-[(2-chloro-3-pyridinyl)methyl]-N-(2,3-dihydro-2-hydro	C36H46ClN5O4	648.23	A/B	-8.8
2847	N(C(=O)[C@H](Cc1ccccc1)C[C@@H](CN1[C@H](C(=O)NC(C)(C)C)CN(Cc2cc(C)ccc2)CC1)O)[C@H]1c2c(C[C@H]1O)cccc	164725-55-7	1-Piperazinepentanamide, N-(2,3-dihydro-2-hydroxy-1H-inden-1-yl)-2-[[(1,1-dimeth	C38H50N4O4	626.83	A/B	-8.8
2848	[C@H](Cc1ccccc1)(C[C@@H]([C@H](Cc1ccccc1)NC(=O)OCc1cncs1)O)NC(=O)[C@@H](NC(=O)N(Cc1coc(C)n1)C)C(C)C	165315-15-1	2,4,7,12-Tetraazatridecan-13-oic acid, 1-(2-ethyl-4-oxazolyl)-10-hydroxy-2-methy	C36H46N6O6S	690.85	A/B	-8.8
2849	C(=O)([C@@H](NC(=O)OC)C(C)C)N1C[C@]2(C[C@H]1c1[nH]c(cn1)c1ccc(cc1)c1cc3c(cc1)c1c(cc3)nc([nH]1)[C@H]1	1653966-54-1	Carbamic acid, N-[(1S)-1-[[(2S)-2-[7-[4-[2-[(3S,5S)-2-[(2S)-2-[(methoxycarbonyl)	C46H56N8O7	832.99	A/B	-8.8
2850	C(=O)([C@@H](NC(=O)OC)C(C)C)N1[C@H]2[C@H](C2)C[C@H]1c1[nH]c2c(n1)ccc1c2ccc(c1)c1ccc(cc1)c1[nH]c(nc1)	1653966-77-8	Carbamic acid, N-[(1S)-2-methyl-1-[[(3S,5S)-3-[5-[7-[2-[(2S,3aS,7aS)-octahydro-1	C50H62N8O7	887.08	A/B	-8.8
2851	C(=O)([C@@H](NC(=O)OC)C(C)C)N1[C@@H]2[C@@H](C2)C[C@H]1c1[nH]c(cn1)c1ccc(cc1)c1ccc2c(cc1)c1c(cc2)nc([n	1653966-83-6	Carbamic acid, N-[(1S)-1-[[(3S,5S)-3-[5-[4-[2-[(1S,3S,5S)-2-[(2S)-2-[(methoxycar	C47H56N8O7	845	A/B	-8.8
2852	C(Nc1c2c(n(=O)c(C(C)(C)C)n1)CCCC2)(C12CC3CC(C2)CC(C1)C3)c1ccccc1	1658454-72-8	4-Quinazolinamine, 5,6,7,8-tetrahydro-2-methyl-N-(phenyltricyclo[3.3.1.13,7]dec-	C26H33N3O	403.56	A/B	-8.8
2853	C([C@H]([C@H](NC(=O)c1ccc2c(cc1)cccc2)CSc1ccccc1)O)N1[C@H](C(=O)NC(C)(C)C)C[C@H]2[C@@H](C1)CCCC2	1665278-93-2	3-Isoquinolinecarboxamide, N-(1,1-dimethylethyl)decahydro-2-[(2S,3R)-2-hydroxy-3	C35H45N3O3S	587.82	A/B	-8.8
2854	O(C(=O)N[C@@H](Cc1ccccc1)[C@@H](CN(S(=O)(=O)c1ccc2c(cc1)nc(S(=O)(=O)C)s2)CC(C)C)O)[C@@H]1[C@H]2[C@@H]	1672661-50-5	Carbamic acid, N-[(1S,2R)-3-[[[2-[(1-cyclopentyl-4-piperidinyl)amino]-6-benzothi	C38H51F2N5O7S2	791.97	A/B	-8.8
2855	O=c1n([C@@H]2O[C@H](COC(=O)C(=O)N[C@H](C(=O)N[C@@H](Cc3ccccc3)C(=O)N[C@H](C(=O)N[C@@H]([C@@H](C)O)C(	1676209-42-9	Cyclopenta[c]pyrrole-1-carboxamide, (2S)-2-cyclohexyl-N-(2-pyrazinylcarbonyl)gly	C36H55N7O7	697.86	A/B	-8.8
2856	[17O]=c1c2c(n(cn2)[C@@H]2O[C@H](CO)[C@@H](O)[C@H]2O)[nH]c(N)n1	168565-10-4	L-Proline, 1-[N-[N6-[N2-[(phenylmethoxy)carbonyl]-L-arginyl]-N2-[N-(3-pyridinylc	C46H58N12O10	939.03	A/B	-8.8
2857	C(n1c2c(nc1CC)c(C)cc(C)n2)c1ccc(cc1)c1c(S(=O)(=O)NC(=O)OCCCC)ccc(CN(C)C)c1	169281-58-7	Carbamic acid, [[4,Ä≤-[(2-ethyl-5,7-dimethyl-3H-imidazo[4,5-b]pyridin-3-yl)methy	C31H38N4O4S	562.72	A/B	-8.8
2858	O[C@@H]1[C@H](n2c3c(nc2)c(N)nc(F)n3)O[C@H](CO[P@]2(=O)O[C@@H](CCO2)c2cccnc2)[C@H]1O	1710363-64-6	1,3,5-Triazin-2-amine, N-[4-[2-amino-6-[4-(diethylamino)phenyl]-4-pyrimidinyl]ph	C31H38N10O2	582.7	A/B	-8.8
2859	S(=O)(=O)(O)c1c2c(c(S(=O)(=O)O)cc(N=Nc3c(C)cc([N+]#N)c(C)c3)c2)cc(N=Nc2c(C)cc([N+]#N)c(C)c2)c1	1715922-33-0	Benzenediazonium, 4,4,Ä≤-[(4,8-disulfo-2,6-naphthalenediyl)bis(2,1-diazenediyl)	C26H22N8O6S2.2Cl		A/B	-8.8
2860	C(c1c2c(N(c3c(C(=O)N2)cccn3)C2CC2)ncc1)c1cc(OC)ccc1O	172326-17-9	6H-Dipyrido[3,2-b:2,Ä≤,3,Ä≤-e][1,4]diazepin-6-one, 11-cyclopropyl-5,11-dihydro-	C21H18N4O2	358.39	A/B	-8.8
2861	N(C(=O)[C@H](Cc1ccccc1)C[C@@H](CN1[C@H](C(=O)NC(C)(C)C)CN(Cc2nc(C(C)C)cs2)CC1)O)[C@H]1c2c(C[C@H]1O)c	172649-46-6	1-Piperazinepentanamide, N-(2,3-dihydro-2-hydroxy-1H-inden-1-yl)-2-[[(1,1-dimeth	C37H51N5O4S	661.9	A/B	-8.8
2862	O=c1c2c(n(cn2)[C@H]2C[C@H](OP(=O)(OC[C@H]3O[C@H]([C@H](O)[C@@H]3O)n3c4c(nc3)c(=O)nc(N)[nH]4)S)[C@@H]	173291-30-0	Guanosine, P-thioguanylyl-(3,Ä≤,Ü5,Ä≤)-	C20H25N10O11PS	644.51	A/B	-8.8
2863	O=c1n([C@@H]2O[C@H](COP(=O)(OC[C@@H]3[C@@H](N=[N+]=[N-])C[C@@H](O3)n3c(=O)[nH]c(=O)c(C)c3)OCc3cc(N(=	173606-89-8	Thymidine, 3,Ä≤-azido-P-[(4-cyanophenyl)methyl]-3,Ä≤-deoxythymidylyl-(5,Ä≤,Ü5,	C28H30N11O10P		A/B	-8.8
2864	O=c1n([C@@H]2O[C@H](COP(=O)([C@@H](O)c3ccc(C)cc3)OC[C@@H]3[C@@H](N=[N+]=[N-])C[C@@H](O3)n3c(=O)[nH]c	173658-20-3	Thymidine, 3,Ä≤-azido-P,3,Ä≤-dideoxy-P-[hydroxy(4-methoxyphenyl)methyl]thymidyl	C28H33N10O11P		A/B	-8.8
2865	[C@H](P(=O)(OC[C@@H]1[C@@H](N=[N+]=[N-])C[C@@H](O1)n1c(=O)[nH]c(=O)c(C)c1)OC[C@@H]1[C@@H](N=[N+]=[N-	173658-28-1	Thymidine, 3,Ä≤-azido-P,3,Ä≤-dideoxy-P-[(2,4-dinitrophenyl)hydroxymethyl]thymid	C27H29N12O14P		A/B	-8.8
2866	C(F)(F)(F)c1nc(no1)c1cc(C)c(OCCCc2cc(C(=O)O)no2)c(C)c1	173899-99-5	3-Isoxazolecarboxylic acid, 5-[3-[2,6-dimethyl-4-[5-(trifluoromethyl)-1,2,4-oxac	C24H20F3N3O5	487.43	A/B	-8.8
2867	[C@H](C(C)(C)C)(C1=C(O)C[C@](CCc2ccccc2)(CCC)OC1=O)c1cc(NS(=O)(=O)c2ccccn2)ccc1	174484-21-0	2-Pyridinesulfonamide, 5-amino-N-[3-[1-[5,6-dihydro-4-hydroxy-2-oxo-6-(2-phenyle	C32H36F3N3O5S	631.71	A/B	-8.8
2868	[C@H](C(C)(C)C)(C1=C(O)C[C@](CCc2ccccc2)(CCC)OC1=O)c1cc(NS(=O)(=O)c2cc(C(F)(F)F)ccn2)ccc1	174484-38-9	2-Pyridinesulfonamide, N-[3-[1-[5,6-dihydro-4-hydroxy-2-oxo-6-(2-phenylethyl)-6-	C33H37F3N2O5S	630.72	A/B	-8.8
2869	C(Cc1ccccc1)[C@]1(CCC)CC(=C([C@H](CC)c2cc(NS(=O)(=O)c3ccc(C(F)(F)F)cn3)ccc2)C(=O)O1)O	174484-40-3	2-Pyridinesulfonamide, N-[3-[1-[5,6-dihydro-4-hydroxy-2-oxo-6-(2-phenylethyl)-6-	C33H37F3N2O5S	630.72	A/B	-8.8
2870	C(Cc1ccccc1)C1(CCc2ccccc2)CC(=C([C@@H](C(C)(C)C)c2cc(NS(=O)(=O)c3ccc(C#N)cn3)ccc2)C(=O)O1)O	174484-67-4	2-Pyridinesulfonamide, 5-cyano-N-[3-[1-[5,6-dihydro-4-hydroxy-2-oxo-6,6-bis(2-ph	C38H39N3O5S	649.8	A/B	-8.8
2871	[C@@H](C(C)(C)C)(C1=C(O)C[C@](CCc2ccccc2)(CCC)OC1=O)c1cc(NS(=O)(=O)c2ccc(C(F)(F)F)cn2)ccc1	174590-22-8	2-Pyridinesulfonamide, N-[3-[1-[5,6-dihydro-4-hydroxy-2-oxo-6-(2-phenylethyl)-6-	C33H37F3N2O5S	630.72	A/B	-8.8
2872	F[C@H]1[C@H](O[C@@H](COP(=O)(O)O)[C@@H]1O)n1c(=O)[nH]c(=O)c(C)c1	174590-37-5	2-Pyridinesulfonamide, 5-cyano-N-[3-[1-[6-[2-(4-fluorophenyl)ethyl]-5,6-dihydro-	C31H32FN3O5S	577.67	A/B	-8.8
2873	C(n1c2c(nc1CC)c(C)cc(C)n2)c1ccc(cc1)c1c(S(=O)(=O)NC(=O)c2ccc(OC)cc2)cccc1	174844-72-5	Benzamide, N-[[4,Ä≤-[(2-ethyl-5,7-dimethyl-3H-imidazo[4,5-b]pyridin-3-yl)methyl]	C37H34N4O4S	630.76	A/B	-8.8
2874	O=c1c2c(n(cn2)[C@@H]2O[C@H](COP(=O)(OP(=O)(OC[C@H]3O[C@H](CC3)n3c4c(nc3)c(=O)nc[nH]4)O)O)CC2)[nH]cn1	175923-21-4	Inosine 5,Ä≤-(trihydrogen diphosphate), 2,Ä≤,3,Ä≤-dideoxy-, P,Ä≤,Ü5,Ä≤-ester w	C20H25N9O10P2	613.41	A/B	-8.8
2875	O=c1n(ccc(N)n1)[C@@H]1O[C@H](COP(=O)(OP(=O)(OC[C@H]2O[C@H](CC2)n2c(=O)nc(N)cc2)O)O)CC1	175923-23-6	Inosine 5,Ä≤-(trihydrogen diphosphate), 2,Ä≤,3,Ä≤-dideoxy-, P,Ä≤,Ü5,Ä≤-ester w	C19H25N7O11P2	589.39	A/B	-8.8
2876	O=c1c2c(n(cn2)[C@@H]2O[C@H](COP(=O)(OP(=O)(OC[C@H]3O[C@H](CC3)n3c4c(nc3)c(=O)nc(N)[nH]4)O)O)CC2)[nH]	175923-25-8	Adenosine 5,Ä≤-(trihydrogen diphosphate), 2,Ä≤,3,Ä≤-dideoxy-, P,Ä≤,Ü5,Ä≤-ester	C19H26N8O10P2	588.4	A/B	-8.8
2877	O=c1n([C@@H]2O[C@H](COP(=O)(OCC(COCCCCCCCCCCCCCC)OCCCCCCCCCCC)O)[C@@H](N=[N+]=[N-])C2)cc(C)c(=O)[nH]1	178152-65-3	D-erythro-Pentonamide, 2,3,5-trideoxy-N-[(1S,2S,3R)-2,3-dihydro-2,3-dihydroxy-1H	C36H47N5O5	629.79	A/B	-8.8
2878	O=c1n([C@@H]2O[C@H](COP(=O)(N[C@H](C(=O)O)C)O)C=C2)cc(C)c(=O)[nH]1	180075-13-2	1-Naphthalenesulfonic acid, 4-amino-3-[2-[4-[1-oxido-2-[3-sulfo-4-[2-(2-sulfophe	C30H23N5O10S3	709.73	A/B	-8.8
2879	C(C#N)C1(CN(S(=O)(=O)CC)C1)n1cc(cn1)c1c2c(ncn1)[nH]cc2	1803132-01-5	2,5,6,10,13-Pentaazatetradecanedioic acid, 3,12-bis(1,1-dimethylethyl)-8-hydroxy	C38H52N6O7		A/B	-8.8
2880	C(OP(=S)(Oc1c2c(ccc1)cccc2)N[C@H](C(=O)OC(C)C)C)[C@@]1(C(F)F)O[C@H]([C@@H](O)[C@@H]1O)n1c2c(nc1)c(N)	1803134-21-5	L-Alanine, N-[hydroxy(1-naphthalenyloxy)phosphinyl]-, 1-methylethyl ester, 5,Ä≤-	C27H31F2N6O8P	636.54	A/B	-8.8
2881	O=C1[C@]2([C@H]([C@H](/C(=C/C(=O)[C@@H]3[C@@]4(N5[C@@H]([C@H]3c3cc(OC)c(O)cc3)CCC5)c3c(NC4=O)cccc3)/	1807888-22-7	Not Yet Assigned	C45H44N4O8	768.85	A/B	-8.8
2882	O=C1[C@@H]2[C@@H](c3c([C@H](OC(=O)[C@@H](Cc4ccccc4)N4C(=O)C5C(C4=O)C4OC5CC4)[C@H]2CO1)cc1c(c3)OCO1)c	1809325-23-2	4,7-Epoxy-2H-isoindole-2-acetic acid, octahydro-1,3-dioxo-Œ±-(phenylmethyl)-, (5	C39H37NO12	711.71	A/B	-8.8
2883	N(c1c2c(n(cn2)[C@@H]2C[C@H](COC(=O)CCC(=O)O)C=C2)nc(N)n1)C1CC1	1821201-88-0	Acetamide, 2-[[4-(1H-indazol-4-yl)-6-(4-morpholinyl)-1,3,5-triazin-2-yl]amino]-N	C18H22N8O2	382.42	A/B	-8.8
2884	C[C@@]12[C@@H]3[C@H]([C@H]4[C@](C)(CC3)[C@@H]([C@@H](C[C@H]([C@H](C(C)C)O)O)C)CC4)C[C@@H](O)[C@H]1C[	1826091-91-1	Carbamic acid, N-[(1S,2R)-3-[[[2-(cyclopropylamino)-6-benzothiazolyl]sulfonyl][(	C33H39FN4O8S2	702.81	A/B	-8.8
2885	C(COCP(=O)(O)O)n1c2c(c(Nc3c4c(ccc3)cccc4)nc(N)n2)nc1	184350-28-5	Phosphonic acid, [[2-[2-amino-6-[(diphenylmethyl)amino]-9H-purin-9-yl]ethoxy]met	C21H23N6O4P	454.42	A/B	-8.8
2886	O=c1n([C@@H]2O[C@H](COP(=O)(OP(=O)(OP(=O)(OCc3ccc(OC(=O)CCCCCCC)cc3)OCc3ccc(OC(=O)C)cc3)O)O)[C@@H](N	1883329-09-6	Not Yet Assigned	C34H33F3N5O17P3		A/B	-8.8
2887	N(C(=O)Nc1c(C)cccc1F)c1cc2cc(C34CC5CC(C4)CC(C3)C5)[nH]c2cc1	1883585-92-9	Urea, N-(2-methylphenyl)-N,Ä≤-(2-tricyclo[3.3.1.13,7]dec-1-yl-1H-indol-5-yl)-	C26H29N3O	399.53	A/B	-8.8
2888	O=c1n([C@@H]2O[C@H](CO)[C@H](O)C2)cc(Cl)c(=O)[nH]1	188548-80-3	Thymidine, 2,Ä≤,3,Ä≤-didehydro-3,Ä≤-deoxy-5,Ä≤-O-(6-methyl-2-oxido-4H-1,3,2-ber	C18H19N2O7P	406.33	A/B	-8.8
2889	C(=O)(O)c1c2c(sc1NC(=O)c1c(CNc3ccc(C[C@@H]([C@@H](CN(S(=O)(=O)c4cc5c(cc4)ncs5)CC4CCC4)O)NC(=O)O[C@@H	1887206-40-7	Benzo[b]thiophene-3-carboxylic acid, 2-[[2-[[[4-[(2S,3R)-4-[(6-benzothiazolylsul	C49H57N5O10S3	972.2	A/B	-8.8
2890	C(=O)(O)c1c(sc2c1CC(C)(C)CC2)c1c(CNc2ccc(C[C@@H]([C@@H](CN(S(=O)(=O)c3cc4c(cc3)ncs4)CC(C)C)O)NC(=O)O	1887206-43-0	Benzo[b]thiophene-3-carboxylic acid, 2-[[2-[[[4-[(2S,3R)-4-[(6-benzothiazolylsul	C48H55N5O10S3	958.17	A/B	-8.8
2891	C(=O)(O)c1c(sc2c1CC(C)(C)CC2)c1c(CNc2ccc(C[C@@H]([C@@H](CN(S(=O)(=O)c3cc4c(cc3)ncs4)CC3CCC3)O)NC(=O)	1887206-52-1	Benzo[b]thiophene-3-carboxylic acid, 2-[2-[[[4-[(2S,3R)-4-[(6-benzothiazolylsulf	C47H53F3N4O9S3	971.14	A/B	-8.8
2892	C(OC[C@@H]1[C@@H](OC(=O)c2ccccc2)[C@@H](OP(=O)(OC[C@@H]2[C@@H](OC(=O)c3ccccc3)[C@@H](OP(=O)(OC[C@@H]	190141-39-0	Adenosine, 1,Ä≤-[3-(aminocarbonyl)-1H-1,2,4-triazol-1-yl]-3,Ä≤-O-benzoyl-P-(2-c	C52H43ClN9O15P	1100.38	A/B	-8.8
2893	O(P(=O)(OC[C@H]1O[C@H]([C@H](OP(=O)(OC[C@H]2O[C@H]([C@H](O)[C@@H]2O)n2c3c(nc2)c(N)ncn3)O)[C@@H]1O)n1	190141-43-6	Adenosine, N-benzoyl-3,Ä≤-O-benzoyl-P-(2-chlorophenyl)-5,Ä≤-O-[(4-methoxyphenyl	C102H82Cl2N14O24P2	2020.68	A/B	-8.8
2894	O=c1n(c2c(c(cc(n2)c2ccc(Cl)cc2)c2ccccc2)c(=O)n1c1ccc(Cl)cc1)[C@H]1[C@H](OC(=O)c2ccccc2)[C@H](OC(=O)c	190319-83-6	Pyrido[2,3-d]pyrimidine-2,4(1H,3H)-dione, 7-(4-chlorophenyl)-3,5-diphenyl-1-(2,3	C51H36ClN3O9	870.3	A/B	-8.8
2895	O=c1c2c(n(cn2)[C@@H]2O[C@H](COP(=O)(Oc3ccc(Cl)cc3)NCc3cc(C(F)(F)F)ccc3)CC2)[nH]cn1	1922936-29-5	Phosphoramidic acid, N-[(3-chlorophenyl)methyl]-, 4-chlorophenyl [(2S,5R)-5-(1,6	C23H22Cl2N5O5P	550.33	A/B	-8.8
2896	O=c1n(ccc(=O)[nH]1)[C@@H]1O[C@H](COS(=O)(=O)O)[C@@H](O)C1	1934276-49-9	2-Pentenoic acid, 4-[[(2R,5S)-2-[(4-fluorophenyl)methyl]-6-methyl-5-[[(5-methyl-	C33H41FN4O7	624.7	A/B	-8.8
2897	O=c1n([C@@H]2O[C@H](COC(=O)C(=C)C)[C@@H](N=[N+]=[N-])C2)cc(C)c(=O)[nH]1	1934287-13-4	Carbamic acid, N-[(1S)-1-[[(2S)-2-[5-[6-[2-[(2S)-1-[(2S)-2-[(methoxycarbonyl)ami	C43H52N8O6	776.92	A/B	-8.8
2898	O=c1n([C@H]2O[C@@H](CO)SC2)cc(F)c(NCNc2c(F)cn(c(=O)n2)[C@H]2O[C@@H](CO)SC2)n1	1962114-96-0	Not Yet Assigned	C17H20F2N6O6S2	506.5	A/B	-8.8
2899	C=C1[C@@H](n2c3c(nc2)C(O)N=C(N)N3)C[C@H](O)[C@H]1CO	1973418-89-1	Acetamide, N-[9-[(1S,3R,4S)-3-[(acetyloxy)methyl]-2-methylene-4-(phenylmethoxy)c	C23H25N5O5	451.48	A/B	-8.8
2900	N(C(=O)C)[C@H]1[C@H]([C@@H]([C@@H](CO)O)O)OC(=C[C@@H]1NC(=N)NC(=O)Cn1cc(nn1)c1cc(N)ccc1)C(=O)O	1982351-43-8	D-glycero-D-galacto-Non-2-enonic acid, 5-(acetylamino)-2,6-anhydro-3,4,5-trideox	C22H26FN7O8	535.48	A/B	-8.8
2901	N(c1nc(Nc2ccc(C#N)cc2)nc(C)c1)c1c(C)cc(C)cc1C	1984393-31-8	Benzonitrile, 2-[[2-[(3,5-dimethylphenyl)amino]-4-pyrimidinyl]amino]-	C19H17N5	315.37	A/B	-8.8
2902	N(c1c(CC)cccc1CC)c1nc(Nc2c(C#N)cccc2)ncc1	1984577-95-8	Benzonitrile, 2-[[2-[(2,4,6-trimethylphenyl)amino]-4-pyrimidinyl]amino]-	C20H19N5	329.4	A/B	-8.8
2903	O(COC(=O)O[C@@H]1C(C)(C)COC1=O)c1c2n(N([C@H]3N(C2=O)CCOC3)[C@H]2c3c(CSc4c2cccc4)c(F)c(F)cc3)ccc1=O	1985607-01-9	Benzoic acid, 2-[[[[[(12aR)-12-[(11S)-7,8-difluoro-6,11-dihydrodibenzo[b,e]thiep	C34H27F2N3O9S	691.65	A/B	-8.8
2904	O([C@@H]1C(C)cccc1)c1c2n(N([C@H]3N(C2=O)CCOC3)[C@H]2c3c(CSc4c2cccc4)c(F)c(F)cc3)ccc1=O	1985607-42-8	Cyclopentanecarboxylic acid, 1-[[[[(12aR)-12-[(11S)-7,8-difluoro-6,11-dihydrodi	C33H31F2N3O8S	667.68	A/B	-8.8
2905	O(C(=O)OCc1ccc(N=[N+]=[N-])cc1)c1c2n(N([C@H]3N(C2=O)CCOC3)[C@H]2c3c(CSc4c2cccc4)c(F)c(F)cc3)ccc1=O	1985607-47-3	1H-[1,4]Oxazino[3,4-c]pyrido[2,1-f][1,2,4]triazine-6,8-dione, 7-[(4-azidophenyl)	C31H24F2N6O4S		A/B	-8.8
2906	N(C(=O)Nc1ccc(C#N)cc1)c1cc2cc(C34CC5CC(C4)CC(C3)C5)[nH]c2cc1	1987854-66-9	Urea, N-(4-cyanophenyl)-N,Ä≤-(2-tricyclo[3.3.1.13,7]dec-1-yl-1H-indol-5-yl)-	C26H26N4O	410.51	A/B	-8.8
2907	N(C(=O)NCCc1cccs1)c1ccc2cc(C34CC5CC(C4)CC(C3)C5)[nH]c2cc1	1987854-77-2	Urea, N-tricyclo[3.3.1.13,7]dec-1-yl-N,Ä≤-(2-tricyclo[3.3.1.13,7]dec-1-yl-1H-ind	C29H37N3O	443.62	A/B	-8.8
2908	N(c1nc(NCCCc2ccccc2)ncc1)c1cc(C)cc(C)c1	1989060-14-1	Benzonitrile, 2-[[2-[(2,5-dimethylphenyl)amino]-4-pyrimidinyl]amino]-	C19H17N5	315.37	A/B	-8.8
2909	O=C1[C@@H]2[C@@H](c3c([C@H](OC(=O)c4cc5c(s4)cccc5)[C@H]2CO1)cc1c(c3)OCO1)c1cc(OC)c(OC)c(OC)c1	1989611-38-2	3-Quinolinecarboxylic acid, 7-chloro-1-cyclopropyl-6-fluoro-1,4-dihydro-4-oxo-,	C35H29ClFNO10	678.06	A/B	-8.8
2910	C(=O)([C@@H](NC(=O)OC)C(C)C)N1[C@H](c2[nH]c3c4cc(cc4)c4c5c(cc4)c4[nH]c(nc4)[C@H]4N(C(=O)[C@@H](N	2007901-52-0	Carbamic acid, N-[(1S)-1-[[(2S,5S)-2-[5-chloro-4-[2,3-dihydro-7-[2-[(2S,5S)-1-[	C47H57ClN8O6	865.46	A/B	-8.8
2911	C(=O)([C@@H](NC(=O)OC)C(C)C)N1[C@H](c2[nH]c3c4cc(cc4)c4c5c(cc4)c4[nH]c(nc4)[C@H]4N(C(=O)[C@@H](N	2007901-60-0	Carbamic acid, N-[(1S)-1-cyclohexyl-2-[(2S,5S)-2-[5-[2,3-dihydro-7-[2-[(2S,5S)-1	C50H62N8O6	871.08	A/B	-8.8
2912	C(=O)([C@@H](NC(=O)OC)C(C)C)N1[C@H](c2[nH]c3c4cc(cc4)c4c5c(cc4)c4[nH]c(nc4)[C@H]4N(C(=O)[C@@H](N	2007901-74-6	Carbamic acid, N-[(1S)-2-methyl-1-[[(5S)-2-methyl-5-[7-[5,6,7,8-tetrahydro-4-[2-	C47H58N8O6	831.01	A/B	-8.8
2913	C(=O)(C(=O)Nc1cc(OC)cccc1)c1c(C23CC4CC(C3)CC(C2)C4)[nH]c2c1cccc2	2014403-78-0	1H-Indole-3-acetamide, N-(4-methylphenyl)-Œ±-oxo-2-tricyclo[3.3.1.13,7]dec-1-yl-	C27H28N2O2	412.52	A/B	-8.8
2914	C(=O)(C(=O)Nc1ccc(Br)cc1F)c1c([nH]c2c1cccc2)C12CC3CC(C2)CC(C1)C3	2014403-90-6	1H-Indole-3-acetamide, N-(4-bromo-2-fluorophenyl)-Œ±-oxo-2-tricyclo[3.3.1.13,7]d	C26H24BrFN2O2	495.38	A/B	-8.8
2915	C(=O)(C(=O)Nc1ncccc1)c1c(C23CC4CC(C3)CC(C2)C4)[nH]c2c1cccc2	2014403-94-0	1H-Indole-3-acetamide, Œ±-oxo-N-2-pyridinyl-2-tricyclo[3.3.1.13,7]dec-1-yl-	C25H25N3O2	399.48	A/B	-8.8
2916	O=c1n([C@H]2O[C@@H](COP(=O)(O[C@@H]3O[C@H](CS3)n3c(=O)nc(N)cc3)O)SC2)cc(N)n1	2020072-75-5	Not Yet Assigned	C22H33N8O8PS2	632.65	A/B	-8.8
2917	S(Cc1cc2c(oc1=O)ccc2)c1n(c2nc1c(=O)nc[nH]2)[C@@H]1O[C@H](CO)[C@@H](O)[C@H]1O	2021230-53-3	Not Yet Assigned	C21H20N4O8S	488.47	A/B	-8.8
2918	[C@H](C(C)C)(CO)n1c2c(cc(Cc3cccc(Cl)c3F)N3CCSCC3)c(OC)c2)c(=O)c(C(=O)O)c1	1000161-77-2	3-Quinolinecarboxylic acid, 6-[(3-chloro-2,4-difluoro-5-(2-oxo-1-pyrrolidinyl)ph	C27H27ClF2N2O6	548.96	C	-8.8
2919	S(=O)(=O)(O)c1c2c(c(N=Nc3ccc(c3)ccc(c3)c3)O)ccc2)cc[illegible]	100043-33-2	1-Naphthalenesulfonic acid, 4-[(4-hydroxy-3,5-dimethylphenyl)azo]-, ion(2-), (E)	C18H14N2O4S		C	-8.8
2920	[C@H](Cc1ccccc1)(CC[C@H](Cc1ccccc1)NC(=O)OCc1cncs1)NC(=O)[C@@H](NC(=O)N(Cc1nc(C(C)C)sc1)C)CCN(C)C	1004318-01-7	2,7,10,12-Tetraazatridecanoic acid, 12-methyl-13-[2-(1-methylethyl)-4-thiazolyl]	C40H51N7O6S2	790.01	C	-8.8
2921	C(=O)([C@H](NC(=O)OC)c1ccccc1)N1[C@@H](CCC1)c1[nH]c(cn1)c1ccc(cc1)c1ccc(cc1)c1[nH]c(nc1)[C@H]1N(C(=O	1009107-54-3	Carbamic acid, N-[(1R)-2-[(2S)-2-[5-[4,Ä≤-[2-[(2S)-1-[2-(acetylamino)acetyl]-2-p	C40H42N8O5	714.81	C	-8.8
2922	C(=O)([C@H](NC(=O)OC)c1ccccc1)N1[C@@H](CCC1)c1[nH]c(cn1)c1ccc(cc1)c1ccc(cc1)c1[nH]c(nc1)[C@H]1N(C(=O	1009107-66-7	Carbamic acid, N-[(1R)-2-[(2S)-2-[5-[4,Ä≤-[2-[(2S)-1-[(2R)-2-(acetylamino)-1-oxo	C41H44N8O5	728.84	C	-8.8
2923	C(=O)([C@H](NC(=O)OC)c1ccccc1)N1[C@@H](CCC1)c1[nH]c(cn1)c1ccc(cc1)c1ccc(cc1)c1[nH]c(nc1)[C@H]1N(C(=O	1009107-71-4	Carbamic acid, N-[(1R)-2-oxo-1-phenyl-2-[(2S)-2-[5-[4,Ä≤-[2-[(2S)-1-[2-(1-pyrro	C42H46N8O4	726.87	C	-8.8
2924	C(=O)([C@@H](NC(=O)OC)COC)N1[C@@H](CCC1)c1[nH]c(cn1)c1ccc(cc1)c1ccc(cc1)c1[nH]c(nc1)[C@H]1N(C(=O)[C	1009115-39-2	Carbamic acid, N,N,Ä≤-[[1,1,Ä≤-biphenyl]-4,4,Ä≤-diylbis[1H-imidazole-5,2-diyl-(	C48H50N8O6	834.96	C	-8.8

2925	C(=O)([C@@H](NC(=O)OC)C1CC1)N1[C@@H]2[C@@H](C2)C[C@H]1c1[nH]c(cn1)c1ccc(cc1)c1ccc(cc1)c1[nH]c(nc1)[C	1009118-25-5	Carbamic acid, N,N,Ä≤-[[1,1,Ä≤-biphenyl]-4,4,Ä≤-diylbis[1H-imidazole-5,2-diyl-(	C40H46N8O6	734.84	C	-8.8
2926	[C@@H]([C@H](CN(Cc1ccc(cc1)c1ccccn1)NC(=O)[C@@H](NC(=O)OC)C(C)(C)C)OC(=O)N)(Cc1ccccc1)NC(=O)[C@@H](N	1023756-62-8	1(2H,6H)-Pyrimidineacetamide, 3-(aminocarbonyl)-N-[(1S,3S,4S)-4-[[2-(2,6-dimethy	C38H49N5O6	671.83	C	-8.8
2927	O[C@H]1[C@@H](O[C@H](COC(=O)CCCCC(=O)OC=C)[C@H]1O)n1nc(C(=O)N)nc1	102905-04-4	Guanosine, 2,Ä≤-deoxyadenylyl-(3,Ä≤,Üí5,Ä≤)-	C20H25N10O10P	596.45	C	-8.8
2928	C([C@@H]([C@H](Cc1cc(O)c(O)cc1)C)C)c1cc(O)c(O)cc1	10318-59-9	Uridine, thymidylyl-(3,Ä≤,Üí5,Ä≤)-2,Ä≤-deoxy-	C19H25N4O12P	532.4	C	-8.8
2929	C(=O)(Nc1c(ccnc1)N1C[C@@H](N)CCC1)c1nc(C23CC4CC(C3)CC(C2)C4)ccc1N	10527-47-6	Guanosine 5,Ä≤-(tetrahydrogen triphosphate), P,Ä≤,Ä≤,Üí5,Ä≤-ester with adenosir	C20H27N10O17P3	772.41	C	-8.8
2930	N(=N\c1cc(S(=O)(=O)O)c(CCc2c(S(=O)(=O)O)cc(/N=N/c3c4c(cc(S(=O)(=O)O)cc4)c(N)cc3)cc2)cc1)/c1c2c(cc(S(	1053055-44-9	Not Yet Assigned	C32H24N6O14S5	876.89	C	-8.8
2931	S(=O)(=O)(O)c1c2c(c(N)c(/N=N/c3cc(S(=O)(=O)O)c(C=Cc4c(S(=O)(=O)O)cc(/N=N/c5c(N)c6c(c(S(=O)(=O)O)c5)c	1053056-10-2	Not Yet Assigned	C34H26N6O18S6	998.99	C	-8.8
2932	O=c1n(ccc(N)n1)C1CS(=O)CO1	1055970-12-1	4-Pyrimidinecarbonitrile, 6-[[(1E)-1-(cyanomethylene)-2,3-dihydro-4,6-dimethyl-1	C25H19N7	417.47	C	-8.8
2933	O=c1n(ccc(N)n1)[C@@H]1O[C@H](COP(=O)(OP(=O)(OP(=O)(O)O)O)O)[C@@H](F)C1	1064476-02-3	Not Yet Assigned	C40H49N5O6	695.85	C	-8.8
2934	C(=O)([C@@H](NC(=O)OC)C(C)C)N1[C@@H](CCC1)c1[nH]c(cn1)c1ccc(cc1)c1ccc(cc1)c1[nH]c([C@H](NC(=O)[C@@H]	1087346-58-4	Carbamic acid, N-[(1S)-2-[(2S)-2-[5-[4,Ä≤-[2-[(1S)-1-[[(2S)-2-[(methoxycarbonyl)	C36H44N8O6	684.78	C	-8.8
2935	O=C1c2c(c3c(c(OC)c2CO1)cc1c(c3)OCO1)c1cc(OC)c(OC)c(OC)c1	1126642-23-6	5(12H)-Naphthacenone, 7-(2,4-dihydroxy-6-methylphenyl)-2,4,6,9-tetrahydroxy-12,1	C27H22O7	458.46	C	-8.8
2936	N(=Nc1c(O)cc(N=Nc2cc(S(=O)(=O)O)ccc2)c(C)c1)c1c2c(ccc1O)cccc2	112685-63-9	Benzenesulfonic acid, 4-[2-[5-hydroxy-4-[2-(2-hydroxy-1-naphthalenyl)diazenyl]-2	C23H18N4O5S	462.48	C	-8.8
2937	C(=O)([C@@H](NC(=O)OC)C)N1[C@@H](CC[C@@H]1C)c1[nH]c(cn1)c1ccc(cc1)c1ccc(cc1)c1[nH]c(nc1)[C@H]1N(C(=O	1129634-98-5	Carbamic acid, N,N,Ä≤-[[1,1,Ä≤-biphenyl]-4,4,Ä≤-diylbis[1H-imidazole-5,2-diyl[(	C40H50N8O6	738.88	C	-8.8
2938	C(=O)([C@@H](NC(=O)OC)C)N1[C@H](c2[nH]c(c(C)n2)c2ccc(cc2)c2ccc(cc2)c2[nH]c(nc2C)[C@H]2N(C(=O)[C@@H](	1129636-25-4	Carbamic acid, N,N,Ä≤-[[1,1,Ä≤-biphenyl]-4,4,Ä≤-diylbis[(4-methyl-1H-imidazole-	C48H50N8O6	834.96	C	-8.8
2939	C(CC)c1c([nH]c(n1)[C@H]1N(C(=O)[C@@H](NC(=O)OC)C(C)C)CCC1)c1ccc(cc1)c1ccc(cc1)c1[nH]c(nc1)[C@H]1N(C(	1129636-44-7	Carbamic acid, N-[(1S)-2-[(2S)-2-[5-[4,Ä≤-[2-[(2S)-1-[(2S)-2-[(methoxycarbonyl)a	C39H48N8O6	724.85	C	-8.8
2940	N(=Nc1c2c(ccc(S(=O)(=O)O)c2)c(N)cc1)c1c2c(c(N=Nc3c(S(=O)(=O)O)cc(N)cc3)cc1)ccc(S(=O)(=O)O)c2.[NaH]	1130877-32-5	2-Naphthalenesulfonic acid, 8-[2-(4-amino-2-methylphenyl)diazenyl]-5-[2-(4-aminc	C23H20N6O6S2.2Na		C	-8.8
2941	C(=O)(N([C@H](C(=O)C(=O)N(C1CC1)[2H])CCC)[2H])[C@@H]1[C@@H]2[C@H](CN1C(=O)[C@@H](N(C(=O)[C@@H](N(C(=	1134176-03-6	Cyclopenta[c]pyrrole-1-carboxamide-N,3,4,5,6-d5, (2S)-2-(cyclohexyl-1,2,2,3,3,4,	C36D53N7O6		C	-8.8
2942	N(=C1c2c(N(CN3CCCC3)C1=O)ccc(Cl)c2)NC(=S)Nc1ccccn1	1148024-22-9	Hydrazinecarbothioamide, 2-[5-chloro-1-[(diphenylamino)methyl]-1,2-dihydro-2-oxc	C27H21ClN6OS	513.01	C	-8.8
2943	C(=CCCO)(c1cc(O)c(O)cc1)c1cc(O)c(O)cc1	114813-90-0	[1,1,Ä≤-Biphenyl]-2,2,Ä≤-disulfonic acid, 4,4,Ä≤-bis[2-(1-amino-4-sulfo-2-napht	C34H28N6O12S4.4Na		C	-8.8
2944	O=c1c2c(n(cn2)[C@@H]2O[C@H](CO)[C@H](O)C2)[nH]c(N)n1	1160003-80-4	Thymidine, 2,Ä≤,3,Ä≤-didehydro-3,Ä≤-deoxy-5,Ä≤-O-[6-[4-hydroxy-3-(hydroxymethyl	C24H23N2O9P	514.42	C	-8.8
2945	N(=Nc1c(C)cc(cc1)c1cc(C)c(N=Nc2ccc(O)cc2)cc1)c1c2c(cc(S(=O)(=O)O)cc2S(=O)(=O)O)ccc1O	1169930-40-8	D-erythro-Pentonamide, 2,3,5-trideoxy-N-[(1S,2R)-2,3-dihydro-2-hydroxy-1H-inden-	C36H47N5O4	613.79	C	-8.8
2946	Cc1n(c2nc(nc(n2)N2CCOCC2)c2cnc(N)nc2)ccn1	1174190-88-5	1H-Indazole, 4-[4-(2-methyl-1H-imidazol-1-yl)-6-(4-morpholinyl)-1,3,5-triazin-2-	C18H18N8O	362.39	C	-8.8
2947	N(=Nc1c2c(cc(S(=O)(=O)O)cc2)ccc1)c1c2c(c(S(=O)(=O)O)ccc2)c(N)cc1	1174191-14-0	2-Pyrimidinamine, 5-[4-[3-methyl-5-(trifluoromethyl)-1H-pyrazol-1-yl]-6-(4-morph	C16H16F3N9O	407.35	C	-8.8
2948	C(C)(C)c1n(c2c(n1)cccc2)c1nc(nc(n1)N1CCOCC1)c1cnc(N)nc1	1174191-80-0	2-Pyrimidinamine, 5-[4-(4-morpholinyl)-6-(4,5,6,7-tetrahydro-1H-indazol-1-yl)-1,	C18H21N9O	379.42	C	-8.8
2949	N(C)(C)C1c2c(-c3c1cc(cc3)c1[nH]c(nc1)[C@H]1N(C(=O)[C@@H](NC(=O)OC)C(C)C)CCC1)ccc(c2)c1[nH]c(nc1)[C@H	1179149-86-0	Carbamic acid, N,N,Ä≤-[(9-hydroxy-9H-fluorene-2,7-diyl)bis[1H-imidazole-5,2-diy]	C41H50N8O7	766.89	C	-8.8
2950	C(=O)([C@@H](NC(=O)OC)C(C)(C)O)N1[C@H](c2[nH]c(c3cc4c(c5c(o4)cc(cc5)c4[nH]c(nc4)[C@H]4N(C(=O)[C@@H](	1179150-66-3	Carbamic acid, N,N,Ä≤-[3,7-dibenzofurandiylbis[1H-imidazole-5,2-diyl-(2S)-2,1-py	C42H52N8O7	780.91	C	-8.8
2951	C(=O)([C@@H](NC(=O)OC)C(C)C)N1[C@H](c2[nH]c3-c4c(cc(cc4)c4ccc(cc4)c4c(CC(F)(F)F)nc([nH]4)[C@H]4N(C(=	1180669-62-8	Carbamic acid, N-[(1S)-1-[[(2S)-2-[5-[4-[4,5-dihydro-2-[(2S)-1-[(2S)-2-[(methoxy	C42H52N8O7	780.91	C	-8.8
2952	C(=O)([C@@H](NC(=O)OC)C(C)C)N1[C@H]2[C@H](C2)C[C@H]1c1[nH]c2-c3c(CCc2n1)cc(cc3)c1cc(F)c(cc1)c1[nH]c(	1180670-05-6	Carbamic acid, N-[(1S)-1-[[(2S)-2-[5-[4-[4,5-dihydro-2-[(2S)-1-[(2S)-2-[(methoxy	C42H51FN8O6	782.9	C	-8.8
2953	C(=O)([C@@H](NC(=O)OC)C(C)C)N1[C@H](c2[nH]c3-c4c(cc(cc4)c4ccc(cc4)c4[nH]c(nc4)[C@H]4N(C(=O)[C@@H](NC	1180670-09-0	Carbamic acid, N-[(1S)-1-[[(1R,3S,5R)-3-[5-[4-[4,5-dihydro-2-[(1R,3S,5R)-2-[(2S)	C44H51FN8O6	806.92	C	-8.8
2954	C(=O)([C@@H](NC(=O)OC)C(C)C)N1[C@H](c2[nH]c3-c4c(cc(cc4)c4ccc(cc4)c4[nH]c(nc4)[C@H]4N(C(=O)[C@@H](NC	1180670-36-3	Carbamic acid, N-[(1S)-1-[[(2S)-2-[7-[4-[2-[(2S)-4,4-difluoro-1-[(2S)-2-[(methox	C42H46F4N8O6	834.86	C	-8.8
2955	O=C1[C@@H]2[C@@H](c3c([C@@H](C(=O)C)[C@H]2CO1)cc1c(c3)OCO1)c1cc(OC)c(OC)c(OC)c1	118584-07-9	2-Naphthalenesulfonic acid, 5-(2-[1,1,Ä≤-biphenyl]-4-yldiazenyl)-6-hydroxy-	C22H16N2O4S	404.44	C	-8.8
2956	S(=O)(=O)(O)c1c2c(c(N)c(N=Nc3cc(C)c(cc3)c3c(C)cc(N=Nc4c(N)c5c(c(S(=O)(=O)O)c4)cccc5)cc3)c1)cccc2	1207449-63-5	1,2-Propanediamine, N2-(7-chloro-4-quinolinyl)-N1-(4,6-di-4-morpholinyl-1,3,5-tr	C23H29ClN8O2	484.98	C	-8.8
2957	O(C(CC)CC)[C@H]1[C@@H](NC(=O)C)[C@@H](N)CC(=C1)C(=O)O	121521-90-2	3-Benzofurancarboxylic acid, 4-[(1E)-3-[(1R)-1-carboxy-2-(3,4-dihydroxyphenyl)et	C36H30O16	718.61	C	-8.8
2958	N(C(CC(C(N(C(=O)C)CC)([2H])[2H])([2H])[2H])C)c1c2c(cc(Cl)cc2)ncc1	1216544-93-2	Methanesulfonamide, N-[2-[[4-[6-fluoro-3-[(1-methylethyl)amino]-2-pyridinyl]-1-p	C22H27FN6O3S	474.55	C	-8.8
2959	N(Cc1ccc(/C=C/C(=O)NO)cc1)C12CC3(C1)CC(C2)CC(C1)C3	1221411-83-1	2-Propenamide, 3-[4-[[(3,5-dimethyltricyclo[3.3.1.13,7]dec-1-yl)amino]methyl]phe	C22H30N2O2	354.49	C	-8.8
2960	O(C)c1c(OC)cc(CC/C=C/C(=O)CCc2cc(OC)c(O)cc2)cc1	1221411-93-3	Benzamide, N-(2-aminophenyl)-4-[(tricyclo[3.3.1.13,7]dec-1-ylamino)methyl]-	C24H29N3O	375.51	C	-8.8
2961	C([C@H]([C@H](Cc1ccccc1)NC(=O)[C@@H](NC(=O)c1cc2c(nc1)cccc2)CC(=O)N)O)N1[C@H](C(=O)NC(C)(C)C)C[C@H]2	1222083-97-7	1-Piperazinebutanoic acid, 4-[2-[1-(4-chlorobenzoyl)-5-methoxy-2-methyl-1H-indol	C49H48ClN3O13	922.37	C	-8.8
2962	C([C@H](OP(=O)(O)O)CO)n1c2c(nc1)c(N)ncn2	1222138-67-1	Not Yet Assigned	C36H45ClFN5O4	666.22	C	-8.8
2963	N(C(=O)[C@H](Cc1ccccc1)C[C@@H](CN1[C@H](C(=O)NC(C)(C)C)CN(Cc2cccnc2)CC1)O)(N)C1c2c(C[C@H]1O)cccc2	1223069-42-8	Not Yet Assigned	C36H45F2N5O4	649.77	C	-8.8
2964	N(C(=O)[C@H](Cc1ccccc1)C[C@@H](CN1[C@H](C(=O)NC(C)(C)C)CN(Cc2c(S)cc(Br)cc2)CC1)O)C1c2c(C[C@H]1O)cccc	1223078-67-8	Not Yet Assigned	C40H51N5O4	665.86	C	-8.8
2965	N(Cc1ccccn1)c1nc(nc(n1)N1CCOCC1)c1c2c(nc[nH]2)ccc1	1225639-00-8	1,3,5-Triazin-2-amine, 4-(1H-benzimidazol-6-yl)-6-(4-morpholinyl)-N-(2-pyridinyl	C20H20N8O	388.43	C	-8.8
2966	C(=O)([C@@H](NC(=O)OC)C(C)C)N1[C@H](c2[nH]c(c3c4c(c(ccc4)c4ccc(cc4)c4[nH]c(nc4)[C@H]4N(C(=O)[C@@H](N	1228552-32-6	Carbamic acid, N-[(1S)-1-[[(2S)-2-[5-[4-[6-[2-[(2S)-1-[(2R)-2-(dimethylamino)-2-	C47H52N8O4	792.97	C	-8.8
2967	C(=O)([C@@H](NC(=O)OC)C(C)C)N1[C@H](c2[nH]c(C34CCC(CC3)(CC4)c3ccc(cc3)c3[nH]c(nc3)[C@H]3N(C(=O)[C@@H	1228668-06-1	Carbamic acid, N-[(1S)-2-[(2S)-2-[5-[4-[trans-4-[2-[(2S)-1-[(2S)-2-[(methoxycarb	C36H48N8O6	688.82	C	-8.8
2968	C(=O)([C@H](NC(=O)OC)c1ccccc1)N1[C@@H](CCC1)c1[nH]c(cn1)c1ccc(C#Cc2ccc(cc2)c2[nH]c(nc2)[C@H]2N(C(=O)	1228965-10-3	Carbamic acid, N-[(1R)-2-[(2S)-2-[5-[4-[2-[4-[2-[(2S)-1-[(2R)-2-[(methoxycarbony	C49H48N8O6	844.96	C	-8.8
2969	C(NC(=O)[C@H](Cc1ccccc1)N)(C(=O)NCC(=O)N[C@@H](Cc1ccccc1)C(=O)N[C@@H](CCSC)C(=O)O)C12CC3CC(C2)CC(C1)	1229574-77-9	L-Phenylalanine, L-phenylalanyl-2-tricyclo[3.3.1.13,7]dec-1-ylglycylglycyl-L-phe	C41H49N5O6	707.86	C	-8.8
2970	C(N(C(=O)N[C@H](C(=O)N[C@@H](Cc1ccccc1)C[C@@H]([C@H](Cc1ccccc1)NC(=O)OCc1ccns1)O)C(C)C)C)c1nc(sc1)c1	1236406-46-4	2,7,10,12-Tetraazatridecanoic acid, 13-(2-ethenyl-4-thiazolyl)-4-hydroxy-12-meth	C36H44N6O6S	688.84	C	-8.8
2971	[C@@H](C(=O)N[C@@H](Cc1ccccc1)C[C@@H]([C@H](Cc1ccccc1)NC(=O)COc1c(C)cccc1C)O)(C(C)C)N1C(=O)NCCC1.O	1239018-93-9	7,10-Methanopyrimido[1,2-a]azepine-2-carboxamide, N-[(4-fluorophenyl)methyl]-4,6	C24H25FN6O7S	560.55	C	-8.8
2972	C(F)(F)c1n(c2c(n1)cccc2)c1nc(nc(N[C@H]2CC[C@H](NC(CO)C)CC2)n1)N1CCOCC1	1240030-39-0	1,4-Cyclohexanediamine, N1-[4-[2-(difluoromethyl)-1H-benzimidazol-1-yl]-6-(4-mor	C24H32F2N8O2	502.56	C	-8.8
2973	O(C(CC)CC)[C@@H]1[C@H](NC(=O)C)[C@H](N)CC(=C1)C(=O)OCC	1240030-58-3	1,4-Cyclohexanediamine, N1-cyclohexyl-N4-[4-[2-(difluoromethyl)-1H-benzimidazol-	C27H36F2N8O	526.62	C	-8.8
2974	C(=O)([C@@H](NC(=O)OC)C(C)C)N1[C@@H](CCC1)c1[nH]c(cn1)c1ccc(cc1)c1ccc(CNC(=O)[C@H]2N(C(=O)[C@@H](NC(	1240427-49-9	L-Prolinamide, N-(methoxycarbonyl)-L-alanyl-N-[[4,Ä≤-[2-[(2S)-1-[(2S)-2-[(methox	C35H43N7O7	673.76	C	-8.8
2975	CC1=NN(C2=[O][Cu+2]345([N](=NC12)c1c([O-]4)cc(S(=O)(=O)[O-])c2c1cccc2)[N](=NC1C(=[O]5)N(N=C1C)c1cccc	12407-23-7	Cobaltate(2-), bis[4-[(4,5-dihydro-3-methyl-5-oxo-1-phenyl-1H-pyrazol-4-yl)azo]-	C40H28CoN8O10S2.2Na		C	-8.8
2976	C(=O)([C@@H](NC(=O)OC)C(C)(C)O)N1[C@H](c2[nH]c(c3cc4c(-c5c(C4)cc(cc5)c4[nH]c(nc4)[C@H]4N(C(=O)[C@@H]	1240880-66-3	Carbamic acid, N,N,Ä≤-[9H-fluorene-2,7-diylbis[1H-imidazole-5,2-diyl-(2S)-2,1-py	C41H46N8O6	746.85	C	-8.8
2977	C(=O)(C(NC(=O)OCC)C)N1[C@@H](CCC1)c1[nH]c(cn1)[C@H]1CC[C@@H](CC1)c1ccc(cc1)c1[nH]c(nc1)[C@H]1N(C(=O)	1240887-36-8	Carbamic acid, N-[(1R)-2-[(2S)-2-[5-[4-[trans-4-[2-[(2S)-1-[(2R)-2-[(methoxycarb	C36H48N8O6	688.82	C	-8.8
2978	C(c1c2c(N(c3c(C(=O)N2)cccn3)C2CC2)ncc1)n1cc(C[C@@H](C(=O)N2[C@H](C(=O)N[C@H](C(=O)N[C@@H](Cc3ccc(O)c	1246392-09-5	L-Arginine, L-alanyl-2-[(11-cyclopropyl-6,11-dihydro-6-oxo-5H-dipyrido[3,2-b:2,Ä	C46H60N14O8	937.06	C	-8.8
2979	O=c1n([C@@H]2C=C(CO)[C@@H](CO)O2)cc(C)c(=O)[nH]1	1255935-58-0	Carbamic acid, N,N,Ä≤-[(1,2,3,5,6,7-hexahydro-s-indacene-2,6-diyl)bis[1H-imidaz	C40H54N8O6	742.91	C	-8.8
2980	Nc1c2c(n(cn2)[C@@H]2O[C@H](CO)[C@@H](O)[C@@H]2O)nc(O[C@@H]2[C@@H](CO)O[C@H]([C@H]2O)n2c3c(nc2)c(N)nc	1256093-55-6	9H-Purin-6-amine, 9-[5-O-(6-amino-9-Œ≤-D-arabinofuranosyl-9H-purin-2-yl)-Œ≤-D-a	C20H23FN10O8	550.46	C	-8.8
2981	C(=O)([C@@H](NC(=O)OC)C(C)C)N1[C@@H](CCC1)c1[nH]c(cn1)c1cc2c(cc1)cc(cc2)c1[nH]c(nc1)[C@H]1N(C(=O)[C@	1256387-39-9	Carbamic acid, N-[(1S)-1-[[(2S)-2-[5-[4-[4-[2-[(2S)-1-[(2S)-2-[(methoxycarbonyl)	C44H52N8O6	788.93	C	-8.8
2982	C(=O)([C@@H](NC(=O)OC)C(C)C)N1[C@@H](C[C@@H](C1)c1ccccc1)c1[nH]c(cn1)c1ccc(cc1)c1cc2c(cc1)cc(cc2)c1[	1256390-00-7	Carbamic acid, N-[(1S)-1-[[(2S)-2-[5-[6-[4-[2-[(2S,4S)-1-[(2S)-2-[(methoxycarbor	C50H56N8O6	865.03	C	-8.8
2983	C(=O)([C@@H](NC(=O)OC)C(C)C)N1C(c2c(Cl)cccc2)c1[nH]c(cn1)c1ccc(cc1)c1ccc(cc1)c1[nH]c(nc1)[C@H]1N(C(=	1256393-09-5	Carbamic acid, N-[2-[(2S)-2-[5-[6-[4-[2-[(2S)-1-[(2S)-2-[(methoxycarbonyl)amino]	C47H50N8O6	822.95	C	-8.8
2984	C[C@]12[C@H]3[C@]4([C@]5(C4)[C@@H](CC3)[C@H](C)C(=O)C=C5)CC[C@]1(C)[C@@H]([C@@H]([C@H](C(=O)C(=C)[C@	1256393-24-4	Carbamic acid, N-[(1S)-1-[[(2S)-2-[5-[6-[4-[2-[(2S)-1-[(2R)-2-cyclohexyl-2-[(met	C47H56N8O6	829	C	-8.8
2985	C(=O)(NC(C(C1(C(C(C1([2H])[2H])([2H])[2H])([2H])[2H])[2H])([2H])[2H])C(=O)C(=O)N)[C@@H]1[C@@H]2[C@@H	1256750-80-7	3-Azabicyclo[3.1.0]hexane-2-carboxamide, N-[3-amino-1-(cyclobutyl-1,2,2,3,3,4,4-	C27H20D25N5O5		C	-8.8
2986	C[C@]12[C@H]([C@H]3[C@@H]([C@]4(C)[C@H](CC3)C[C@@H](NC)CC4)CC2=O)CC[C@@H]1[C@@H](CCC(=O)NCc1ccccc1)C	125680-34-4	Cholan-24-amide, 3-amino-12-oxo-N-(phenylmethyl)-, (3Œ≤,5Œ≤)-	C31H46N2O2	478.71	C	-8.8
2987	O[C@H]1[C@H](n2c3c(nc2)c(=O)nc(N)[nH]3)O[C@H](COP(=O)(OCC)OCC)[C@H]1O	1258228-38-4	Carbamic acid, N,N,Ä≤-[(1-phenyl-2,5-pyrrolidinediyl)bis[4,1-phenylene-1H-imida:	C50H61N9O6	884.08	C	-8.8
2988	C[C@]12[C@H]([C@H]3[C@@H]([C@]4(C)C(CC3)C[C@H](O)CC4)C[C@@H]2O)CC[C@@H]1[C@@H](CCC(=O)NCC(=O)O)C	1266398-02-0	[1,1,Ä≤-Biphenyl]-2,2,Ä≤-disulfonic acid, 4,4,Ä≤-bis[(1E)-2-(2-hydroxy-1-naphth	C34H26N4O8S2.2Na		C	-8.8
2989	O=c1n([C@@H]2O[C@H](COP(=O)(O)S)CC2)ccc(=O)[nH]1	1266476-63-4	[1,1,Ä≤-Biphenyl]-2,2,Ä≤-disulfonic acid, 4,4,Ä≤-bis[(1E)-2-(2-hydroxy-1-naphth	C34H26N4O8S2	682.72	C	-8.8
2990	C(=O)([C@@H](NC(=O)OC)C(C)C)N1CC2(C[C@H]1c1[nH]c(cn1)c1ccc(cc1)c1ccc(cc1)c1[nH]c(nc1)[C@H]1N(C(=O)[C	1272655-63-6	Carbamic acid, N-[(1S)-1-[[(2S)-2-[5-[4,Ä≤-[2-[(3S)-2-[(2S)-2-[(methoxycarbonyl)	C44H56N8O7	808.96	C	-8.8
2991	C(=O)([C@@H](NC(=O)OC)C(C)C)N1CC2(C[C@H]1c1[nH]c(cn1)c1ccc(cc1)c1ccc(cc1)c1[nH]c(nc1)[C@H]1N(C(=O)[C	1272656-46-8	Carbamic acid, N-[(1S)-1-[[(2S)-2-[5-[4,Ä≤-[2-[6-[(2S)-2-[(methoxycarbonyl)aminc	C42H53N9O6	779.93	C	-8.8
2992	C(=O)([C@@H](NC(=O)OC)C(C)C)N1[C@@H]([C@@H]2C[C@H]1CC2)c1[nH]c(cn1)c1ccc(cc1)c1ccc(cc1)c1[nH]c(nc1)[	1273591-10-8	Carbamic acid, N-[(1S)-1-[[(2S,4S)-4-fluoro-2-[5-[4,Ä≤-[2-[(1R,3S,4S)-2-[(2S)-2-	C42H51FN8O6	782.9	C	-8.8
2993	N(Cc1ccc(F)cc1)c1nc(nc(N/N=C/c2c(O)cccc2)n1)N1CCOCC1	1279692-14-6	1H-Indole-2,3-dione, 3-[2-[4-[[(3-chlorophenyl)methyl]amino]-6-(4-morpholinyl)-1	C22H21ClN8O2	464.91	C	-8.8
2994	C(=O)([C@@H](NC(=O)OC)C(C)C)N1[C@@H](CC(=C)C1)c1[nH]c(cn1)c1c(C)c(C)c(cc1)c1c(C)c(C)c(cc1)c1[nH]c(nc	1311398-23-8	Carbamic acid, N-[(1S)-1-[[(2S)-2-[5-[4,Ä≤-[2-[(2S)-1-[(2S)-2-[(methoxycarbonyl)	C43H54N8O6	778.94	C	-8.8
2995	C(=O)([C@@H](NC(=O)OC)C(C)C)N1[C@@H](CC(=C)C1)c1[nH]c(cn1)c1c(Cl)cc(cc1)c1cc(Cl)c(cc1)c1[nH]c(nc1)[C	1311398-25-0	Carbamic acid, N-[(1S)-1-[[(2S)-2-[5-[4,Ä≤-[2-[(2S)-1-[(2S)-2-[(methoxycarbonyl)	C43H48F6N8O6	886.88	C	-8.8
2996	C(=O)([C@@H](NC(=O)OC)C1CCOCC1)N1[C@@H](CC[C@@H]1C)c1[nH]c(c(Cl)n1)c1ccc(cc1)c1ccc(cc1)c1[nH]c(nc1Cl	1312336-18-7	Carbamic acid, N,N,Ä≤-[[1,1,Ä≤-biphenyl]-4,4,Ä≤-diylbis[(5-chloro-1H-imidazole-	C46H56Cl2N8O8	919.89	C	-8.8
2997	C(=O)([C@@H](NC(=O)OC)C1CCOCC1)N1[C@@H](C[C@H](C)C1)c1[nH]c(c(Cl)n1)c1ccc(cc1)c1ccc(cc1)c1[nH]c(nc1C	1312336-26-7	Carbamic acid, N-[(1S)-2-[(2S,4S)-2-[5-[4,Ä≤-[5-chloro-2-[(2S,4S)-1-[(2S)-2-[(me	C46H57ClN8O8	885.45	C	-8.8
2998	C(=O)([C@@H](NC(=O)OC)C(C)C)N1[C@H](c2[nH]c3c4c(cc(cc4)c4ccc(cc4)c4[nH]c(nc4Cl)[C@H]4N(C(=O)[C@@H](N	1312336-52-9	Carbamic acid, N-[(1S)-1-[[(1R,3S,5R)-3-[5-bromo-4-[4-[5-chloro-2-[(1R,3S,5R)-2-	C44H48BrClN8O6	900.26	C	-8.8
2999	C(=O)([C@@H](NC(=O)OC)C1CCOCC1)N1[C@H]2[C@H](C2)C[C@H]1c1[nH]c(c(C#N)n1)c1ccc(cc1)c1ccc(cc1)c1[nH]c(	1312336-68-7	Carbamic acid, N-[(1S)-1-[[(1R,3S,5R)-3-[5-[4,Ä≤-[5-cyano-2-[(1R,3S,5R)-2-[(2S)-	C43H49N9O6	787.91	C	-8.8
3000	Cn1c(c2c([nH]c(n2)[C@H]2N(C(=O)[C@@H](NC(=O)OC)C3CCOCC3)CCC2)c2ccc(cc2)c2ccc(cc2)c2[nH]c(nc2)[C@H]2N	1312337-03-3	Carbamic acid, N,N,Ä≤-[[1,1,Ä≤-biphenyl]-4,4,Ä≤-diylbis[(4-cyclopropyl-1H-imida	C52H62N8O8	927.1	C	-8.8
3001	C(=O)([C@@H](NC(=O)OC)C1CCOCC1)N1[C@H]2[C@H](C2)C[C@H]1c1[nH]c(c(n1)c1cnoc1)c1ccc(cc1)c1ccc(cc1)c1[n	1312337-07-7	Carbamic acid, N-[(1S)-1-[[(1R,3S,5R)-3-[5-(4-isoxazolyl)-4-[4,Ä≤-[2-[(1R,3S,5R)	C45H51N9O7	829.94	C	-8.8
3002	C(=O)([C@@H](NC(=O)OC)[C@@H]1CCCOC1)N1[C@H]2[C@H](C2)C[C@H]1c1[nH]c(cn1)c1ccc(cc1)c1ccc(cc1)c1[nH]c(	1312338-49-0	Carbamic acid, N-[(1S)-2-[(1R,3S,5R)-3-[5-[4,Ä≤-[2-[(1R,3S,5R)-2-[(2S)-2-(4,4-di	C47H54F2N8O7	880.98	C	-8.8
3003	Cc1sc2c(ccc(c2c1)c1ccc(cc1)c1[nH]c(nc1)[C@H]1N(C(=O)[C@@H](NC(=O)OC)C(C)C)CCC1)c1[nH]c(nc1)[C@H]1N(C	1312608-58-4	Carbamic acid, N-[(1S)-1-[[(2S)-2-[5-[4-[7-[2-[(2S)-1-[(2S)-2-[(methoxycarbonyl)	C42H50N8O6S	794.96	C	-8.8
3004	C(OC[C@H](Cn1c(=O)nc(NC(=O)c2ccccc2)cc1)OCP(=O)(OCC)O)(c1ccccc1)(c1ccccc1)c1ccccc1	1312805-81-4	Adenosine, 1,Ä≤-[3-(aminocarbonyl)-1H-1,2,4-triazol-1-yl]-1,Ä≤-de(6-amino-9H-pu	C24H34N12O19P2	856.54	C	-8.8
3005	O(Cc1ccc(F)cc1)c1c2c(oc(c(O)c2=O)c2ncccn2)c(C)cc1	1312892-20-8	4H-1-Benzopyran-4-one, 5-[(4-fluorophenyl)methoxy]-3-hydroxy-2-(5-phenyl-1H-imic	C25H17FN2O4	428.41	C	-8.8
3006	O(Cc1ccc(F)cc1)c1c2c(oc(c(O)c2=O)c2ncccn2)c(Cl)c(C)c1	1312896-02-8	4H-1-Benzopyran-4-one, 5-[(4-fluorophenyl)methoxy]-3-hydroxy-8-methoxy-7-methyl-	C27H21FN2O5	472.46	C	-8.8
3007	O(Cc1ccc(F)cc1)c1c2c(c(C)c1)c1ccccc1)oc(c(O)c2=O)c1ncc(C)cn1	1312896-83-5	4H-1-Benzopyran-4-one, 5-[(4-fluorophenyl)methoxy]-3-hydroxy-7-methyl-8-phenyl-2	C27H19FN2O4	454.45	C	-8.8
3008	O(Cc1ccc(F)cc1)c1c2c(oc(c(O)c2=O)c2[nH]cnn2)cc(OC)c1	1312897-48-5	4H-1-Benzopyran-4-one, 5-[(4-fluorophenyl)methoxy]-3-hydroxy-7-methoxy-2-(5-pher	C27H19FN2O5	470.45	C	-8.8
3009	O(C)c1c2c(c(OCc3ccc(F)cc3)cc1Cl)c(=O)c(O)c(o2)c1[nH]cnn1	1312901-58-8	4H-1-Benzopyran-4-one, 7-chloro-5-[(4-fluorophenyl)methoxy]-3-hydroxy-8-methoxy-	C27H18ClFN2O5	504.89	C	-8.8
3010	O(Cc1ccc(F)cc1)c1c2c(oc(c(O)c2=O)c2ncc(cn2)c2ccccc2)cc(c1)c1ccccc1	1312903-07-3	4H-1-Benzopyran-4-one, 2-(5-chloro-2-pyrimidinyl)-5-[(4-fluorophenyl)methoxy]-3-	C26H16ClFN2O4	474.87	C	-8.8
3011	O(Cc1ccc(F)cc1)c1c2c(c(Cl)c(c1)c1ccccc1)oc(c(O)c2=O)c1ncccn1	1312904-56-5	4H-1-Benzopyran-4-one, 5-[(4-fluorophenyl)methoxy]-3-hydroxy-8-methoxy-7-phenyl-	C32H23FN2O5	534.53	C	-8.8
3012	O(Cc1ccc(F)cc1)c1c2c(c(c(c1)c1ccccc1)c1ccccc1)oc(c(O)c2=O)c1ncc(C)cn1	1312905-66-0	4H-1-Benzopyran-4-one, 5-[(4-fluorophenyl)methoxy]-3-hydroxy-7,8-diphenyl-2-(2-p	C32H21FN2O4	516.52	C	-8.8
3013	O(Cc1ccc(F)cc1)c1c2c(c(c(c1)c1ccccc1)c1ccccc1)oc(c(O)c2=O)c1ncc(OC)cn1	1312905-67-1	4H-1-Benzopyran-4-one, 5-[(4-fluorophenyl)methoxy]-3-hydroxy-2-(5-methyl-2-pyrim	C33H23FN2O4	530.55	C	-8.8
3014	O(Cc1ccc(F)cc1)c1c2c(c(c(c1)c1ccccc1)c1ccccc1)oc(c(O)c2=O)c1[nH]c(OC)nn1	1312905-77-3	4H-1-Benzopyran-4-one, 5-[(4-fluorophenyl)methoxy]-3-hydroxy-2-(3-methyl-1H-1,2,	C31H22FN3O4	519.52	C	-8.8
3015	O(C)c1c2c(cc(OCc3ccc(F)cc3)c1)c(=O)c(O)c(o2)c1[nH]ccn1	1312907-28-0	4H-1-Benzopyran-4-one, 6-[(4-fluorophenyl)methoxy]-3-hydroxy-8-methoxy-2-(3-pher	C25H18FN3O5	459.43	C	-8.8
3016	Clc1c2c(cc(OCc3ccc(F)cc3)c1)c(=O)c(O)c(o2)c1ncccn1	1312907-33-7	4H-1-Benzopyran-4-one, 6-[(4-fluorophenyl)methoxy]-3-hydroxy-8-methoxy-2-(5-pher	C26H19FN2O5	458.44	C	-8.8
3017	O=c1c2c(oc(c1O)c1[nH]cnn1)c(Cl)cc(OCc1ccc(F)cc1)c2	1312907-68-8	4H-1-Benzopyran-4-one, 8-chloro-6-[(4-fluorophenyl)methoxy]-3-hydroxy-2-(5-pheny	C26H16ClFN2O4	474.87	C	-8.8
3018	Cc1c2c(cc(OCc3ccc(F)cc3)c1C)c(=O)c(O)c(o2)c1[nH]cnn1	1312909-30-0	4H-1-Benzopyran-4-one, 6-[(4-fluorophenyl)methoxy]-3-hydroxy-7,8-dimethyl-2-(5-p	C28H21FN2O4	468.48	C	-8.8
3019	Clc1c2c(cc(OCc3ccc(F)cc3)c1C)c(=O)c(O)c(o2)c1ncccn1	1312909-95-7	4H-1-Benzopyran-4-one, 6-[(4-fluorophenyl)methoxy]-3-hydroxy-8-methoxy-7-methyl-	C27H21FN2O5	472.46	C	-8.8
3020	Clc1c2c(cc(OCc3ccc(F)cc3)c1C)c(=O)c(O)c(o2)c1[nH]cnn1	1312910-30-7	4H-1-Benzopyran-4-one, 8-chloro-6-[(4-fluorophenyl)methoxy]-3-hydroxy-7-methyl-2	C27H18ClFN2O4	488.89	C	-8.8
3021	Cc1c(c2c(cc1OCc1ccc(F)cc1)c(=O)c(O)c(o2)c1[nH]c(OC)nn1)c1ccccc1	1312910-87-4	4H-1-Benzopyran-4-one, 6-[(4-fluorophenyl)methoxy]-3-hydroxy-7-methyl-2-(3-methy	C26H20FN3O4	457.45	C	-8.8
3022	O=c1c2c(oc(c1O)c1[nH]cnn1)cc(OC)c(OCc1ccc(F)cc1)c2	1312911-30-0	4H-1-Benzopyran-4-one, 6-[(4-fluorophenyl)methoxy]-3-hydroxy-7-methoxy-2-(5-pher	C27H19FN2O5	470.45	C	-8.8
3023	O=c1c2c(oc(c1O)c1[nH]cnn1)cc(Cl)c(OCc1ccc(F)cc1)c2	1312911-62-8	4H-1-Benzopyran-4-one, 7-chloro-6-[(4-fluorophenyl)methoxy]-3-hydroxy-2-(5-pheny	C26H16ClFN2O4	474.87	C	-8.8
3024	O(C)c1c2c(cc(OCc3ccc(F)cc3)c1Cl)c(=O)c(O)c(o2)c1[nH]ccn1	1312913-83-9	4H-1-Benzopyran-4-one, 7-chloro-6-[(4-fluorophenyl)methoxy]-3-hydroxy-8-methoxy-	C25H17ClFN3O5	493.87	C	-8.8
3025	Clc1c2c(cc(OCc3ccc(F)cc3)c1Cl)c(=O)c(O)c(o2)c1ncccn1	1312914-48-9	4H-1-Benzopyran-4-one, 8-chloro-6-[(4-fluorophenyl)methoxy]-3-hydroxy-7-methoxy-	C26H18ClFN2O5	492.88	C	-8.8
3026	Clc1c(c2c(cc1OCc1ccc(F)cc1)c(=O)c(O)c(o2)c1[nH]c(OC)nn1)c1ccccc1	1312915-82-4	4H-1-Benzopyran-4-one, 7-chloro-6-[(4-fluorophenyl)methoxy]-3-hydroxy-2-(3-methy	C25H17ClFN3O4	477.87	C	-8.8
3027	O(Cc1ccc(F)cc1)c1c(c(c2c(c1)c(=O)c(O)c(o2)c1[nH]c(cn1)c1ccccc1)c1ccccc1)c1ccccc1	1312918-50-5	4H-1-Benzopyran-4-one, 2-(5-chloro-1H-imidazol-2-yl)-6-[(4-fluorophenyl)methoxy]	C31H20ClFN2O4	538.95	C	-8.8
3028	C(CC(=O)C[C@H](CCc1ccc(O)cc1)O)c1cc(O)c(O)cc1	132-34-3	2,7-Naphthalenedisulfonic acid, 3-amino-4-[2-[4,Ä≤-[2-(2-amino-6-sulfo-1-naphtha	C34H28N6O9S3	760.82	C	-8.8
3029	N(c1nc(nc(NN=Cc2ccc(NC(=O)C)cc2)n1)N1CCOCC1)c1ccccc1	1331749-46-2	1H-Indole-2,3-dione, 3-[2-[4-[[(3-chlorophenyl)methyl]amino]-6-(4-morpholinyl)-1	C22H21ClN8O2	464.91	C	-8.8
3030	O=c1n([C@@H]2O[C@H](COP(=O)(N[C@@H](Cc3cnc[nH]3)C(=O)OC)Oc3ccc(N(=O)=O)cc3)C=C2)cc(C)c(=O)[nH]1	1333491-94-3	L-Proline, 1-[2,Ä≤,3,Ä≤-didehydro-3,Ä≤-deoxy-P-(4-nitrophenyl)-5,Ä≤-thymidylyl]	C28H29N4O10P	612.52	C	-8.8
3031	N(=Nc1c2c(ccc(S(=O)(=O)O)c2)c(N)cc1)c1c2c(c(N=Nc3cc(S(=O)(=O)O)ccc3)cc1)ccc(S(=O)(=O)O)c2	1345852-42-7	1-Naphthalenesulfonic acid, 8-amino-4-[2-[7-sulfo-4-[2-(4-sulfophenyl)diazenyl]-	C26H19N5O9S3	641.65	C	-8.8
3032	O(C(=O)NC(C(CN(S(=O)(=O)c1ccc(OC)cc1)CC(C)C)O)Cc1ccccc1)[C@@H]1C2C(OC1)OCC2O	1346867-70-6	Not Yet Assigned	C40H57N7O6S	763.99	C	-8.8
3033	O(C(=O)N[C@@H](Cc1ccc(N(C)C)cc1)[C@@H](CN(S(=O)(=O)c1ccc(OCP(=O)(OCC)N(C)C)cc1)CC(C)C)O)[C@@H]1[C@H]	1347069-23-1	Spiro[3-azabicyclo[3.1.0]hexane-6,1,Ä≤-cyclopropane]-2-carboxamide, N-[3-amino-1	C37H53N5O6	663.85	C	-8.8
3034	C(=O)([C@@H](NC(=O)OC)C(C)C)N1[C@@]2(C[C@H](C1)CC2)c1[nH]c(cn1)c1c2c(c(cc1)c1ccc(cc1)c1[nH]c(nc1)[C@	1350719-05-9	Carbamic acid, N-[(1S)-1-[[(1S,4R)-1-[5-[4-[7-[2-[(1S,4R)-2-[(2S)-2-[(methoxycar	C46H55N9O6S	862.05	C	-8.8
3035	C[C@]1(c2[nH]c(c3c4c(c(cc3)c3c5c(c(cc3)c3[nH]c(nc3)[C@H]3N(C(=O)[C@@H](NC(=O)OC)C(C)C)CCC3)CCC5)CCC4	1350719-41-3	Carbamic acid, N,N,Ä≤-[[4,4,Ä≤-bi-1H-indole]-7,7,Ä≤-diylbis[1H-imidazole-5,2-di	C44H52N10O6	816.95	C	-8.8
3036	C(#N)[C@]1(O[C@H](COP(=O)(Oc2ccccc2)N[C@H](C(=O)OC(C)C)C)[C@@H](O)[C@@]1(C)O)c1n2c(cc1)c(N)ncn2	1350719-49-1	Carbamic acid, N,N,Ä≤-[(2,2,Ä≤,3,3,Ä≤-tetrahydro[4,4,Ä≤-bi-1H-indene]-7,7,Ä≤-di	C48H62N8O6	847.06	C	-8.8
3037	C(=O)([C@@H](NC(=O)OC)C(C)C)N1C[C@@]2(C[C@H]1c1[nH]c(cn1)c1ccc(cc1)c1ccc(cc1)c1[nH]c(nc1)[C@H]1N(C(=	1351852-61-3	Carbamic acid, N-[(1S)-1-[[(5S,8S)-8-[5-[4,Ä≤-[2-[(2S)-1-[(2S)-2-[(methoxycarbor	C44H56N8O7	808.96	C	-8.8
3038	C(=O)([C@@H](NC(=O)OC)C(C)C)N1C[C@@]2(C[C@H]1c1[nH]c(cn1)c1ccc(cc1)c1ccc(cc1)c1[nH]c(nc1)[C@H]1N(C(=	1351852-68-0	Carbamic acid, N-[(1S)-1-[[(2S,4S)-4-cyclopropyl-2-[5-[4,Ä≤-[2-[(5S,8S)-7-[(2S)-	C46H58N8O7	835	C	-8.8
3039	C(=O)([C@@H](NC(=O)OC)C(C)C)N1C[C@@]2(C[C@H]1c1[nH]c(cn1)c1ccc(cc1)c1ccc(cc1)c1[nH]c(nc1)[C@H]1N(C(=	1351852-97-5	Carbamic acid, N-[(1S)-1-[[(5S,8S)-8-[5-[4,Ä≤-[2-[(6S)-5-[(2S)-2-[(methoxycarbor	C45H56N8O7	820.98	C	-8.8
3040	C(=O)([C@@H](NC(=O)OC)C(C)C)N1[C@@H](C[C@]2(C1)CCCCO2)c1[nH]c(cn1)c1ccc(cc1)c1ccc(cc1)c1[nH]c(nc1)[C	1351853-16-1	Carbamic acid, N-[(1S)-1-[[(2S)-2-[5-[4,Ä≤-[2-[(5S,8S)-7-[(2S)-2-[(methoxycarbor	C43H54N8O7	794.94	C	-8.8
3041	C(=O)([C@@H](NC(=O)O[C@@H]1CCOC1)C(C)C)N1[C@@H](C[C@@H](C1)C1CC1)c1[nH]c(cn1)c1ccc(cc1)c1ccc(cc1)c1[	1351854-09-5	Carbamic acid, N-[(1S)-1-[[(2S)-2-[5-[4,Ä≤-[2-[(2S,4R)-4-cyclopropyl-1-[(2S)-3-r	C51H66N8O8	919.12	C	-8.8

3042	Fc1cc(c2nc3c(o2)ccc(N)c3)ccc1c1cn(c(=O)[nH]c1=O)[C@@H]1O[C@H](COP(=O)(OP(=O)(OP(=O)(O)O)O)O)[C@@H](O	1352918-37-6	Uridine 5,Ä≤-(tetrahydrogen triphosphate), 5-[4-(2-benzoxazolyl)-2-fluorophenyl]	C22H21FN3O15P3	679.33	C	-8.8
3043	C(F)(F)c1n(c2c(n1)cccc2)c1nc(nc(Nc2cc3c(cc2)n(C)cn3)n1)N1CCOCC1	1359953-79-9	1H-Benzimidazol-6-amine, N-[4-[2-(difluoromethyl)-1H-benzimidazol-1-yl]-6-(4-mor	C22H19F2N9O	463.44	C	-8.8
3044	C(F)(F)c1n(c2c(n1)cccc2)c1nc(nc(NCC2CCN(C)CC2)n1)N1CCOCC1	1359955-63-7	Ethanone, 1-[4-[[4-[2-(difluoromethyl)-1H-benzimidazol-1-yl]-6-(4-morpholinyl)-1	C22H26F2N8O2	472.49	C	-8.8
3045	O(CCOC)c1c(c(OCCOC)cc(c1)c1[nH]c(nc1)[C@H]1N(C(=O)[C@@H](NC(=O)OC)C(C)C)CCC1)c1ccc(cc1)c1[nH]c(nc1)[	1367573-33-8	Carbamic acid, N-[(1S)-1-[[(2S)-2-[5-[4-[2,3,5,6,8,9-hexahydro-14-[2-[(2S)-1-[(2	C46H60N8O10	885.02	C	-8.8
3046	O(C)c1c2n(cc(C(=O)O)c(=O)c2cc(F)c1N1C[C@@H]2[C@H](C1)CCCN2CCN1c2c(C(=NNC(=S)N)C1=O)cc(F)cc2)C1CC1	1372618-36-4	3-Quinolinecarboxylic acid, 7-[(4aS,7aS)-1-[2-[3-[2-(aminothioxomethyl)hydraziny	C32H34FN7O5S	647.72	C	-8.8
3047	C(=O)([C@@H](NC(=O)OC)C(C)C)N1[C@@H]([C@@H]2C[C@H]1CC2)c1[nH]c(cn1)c1cc2c(-c3c(CC2)cc(cc3)c2[nH]c(nc	1377036-90-2	Carbamic acid, N-[(1S)-2-[(2S)-2-[7-[4-[2-[(1R,3S,4S)-2-[(2S)-2-[(methoxycarbony	C46H54N8O7	830.97	C	-8.8
3048	C(=O)([C@@H](NC(=O)OC)C(C)C)N1[C@@H](CC(=C)C1)c1[nH]c(cn1)c1cc2c(cc1)cc(cc2)c1ccc(cc1)c1[nH]c(nc1)[C	1377037-83-6	Carbamic acid, N-[(1R)-2-[(2S,4S)-2-[5-[4-[6-[2-[(2S)-1-[(2S)-2-[(methoxycarbony	C48H52N8O6	836.98	C	-8.8
3049	C(=O)([C@H](NC(=O)OC)c1ccccc1)N1[C@@H](CCC1)c1[nH]c(cn1)c1ccc(cc1)c1cc2c(cc1)cc(cc2)c1[nH]c(nc1)[C@H]	1377040-83-9	Carbamic acid, N,N,Ä≤-[2,7-phenanthrenediylbis[1H-imidazole-5,2-diyl-(2S)-2,1-py	C42H50N8O6	762.9	C	-8.8
3050	C(=O)([C@@H](NC(=O)OC)C(C)C)N1[C@H](c2[nH]c3c(c4c(c5c3ccc(c5)c3ccc(cc3)c3[nH]c(nc3)[C@H]3N(C(=O)[C@@	1377041-15-0	Carbamic acid, N-[(1S)-2-[(2S)-2-[2,Ä≤-[(1R,3S,4S)-2-[(2S)-2-[(methoxycarbonyl)a	C48H54N8O7	854.99	C	-8.8
3051	C(=O)([C@@H](NC(=O)OC)C(C)C)N1[C@@H](CCC1)c1[nH]c(cn1)c1cc(C#CC)c(cc1)c1ccc(cc1)c1[nH]c(nc1)[C@H]1N(	1377042-11-9	Carbamic acid, N-[(1S)-1-[[(1R,3S,5R)-3-[5-[4-[2-[(1R,3S,4S)-2-[(2S)-2-[(methoxy	C45H52N8O6	800.94	C	-8.8
3052	C(=O)([C@@H](NC(=O)OC)C(C)C)N1[C@H]2[C@H](C2)C[C@H]1c1[nH]c2c(n1)ccc1c2ccc(c1)c1cc(F)c(cc1)c1[nH]c(n	1377043-58-7	Carbamic acid, N-[(1S)-2-[(1R,3S,5R)-3-[5-[2-fluoro-4-[2-[(1R,3S,4S)-2-[(2S)-2-[	C47H53FN8O7	860.97	C	-8.8
3053	C(=O)([C@@H](NC(=O)OC)C(C)C)N1[C@H](c2[nH]c3c4c(cc(cc4)c4ccc5c(cc4)c4c(cc5)nc([nH]4)[C@H]4N(C(=O)[C@@	1377043-66-7	Carbamic acid, N-[(1S)-1-[[(1R,3S,5R)-3-[5-chloro-4-[2-fluoro-4-[2-[(1R,3S,5R)-2	C44H48ClFN8O6	839.35	C	-8.8
3054	C(=O)([C@@H](NC(=O)OC)C(C)C)N1[C@H](c2[nH]c3c4c(cc(cc4)c4ccc5c(cc4)c4c(cc5)nc([nH]4)[C@H]4N(C(=O)[C@@	1377047-25-0	Carbamic acid, N-[(1S)-1-[[(2S)-2-[2,Ä≤-[(2S,4S)-4-[(difluoromethoxy)methyl]-1-	C46H52F2N8O7	866.95	C	-8.8
3055	C(=O)([C@@H](NC(=O)OC)C(C)C)N1[C@H](c2[nH]c(c3c4c(c(ccc4)c4c5c(c(ccc5)c5[nH]c(nc5)[C@H]5N(C(=O)[C@@H]	1380343-66-7	Carbamic acid, N-[(1R)-1-[[(2S)-2-[5-(4-[1,1,Ä≤-biphenyl]-4-ylcyclohexyl)-1H-im	C32H40N4O3	528.68	C	-8.8
3056	O=c1n([C@@H]2O[C@H](CO)[C@H]([18F])C2)cc(C)c(=O)[nH]1	1383925-97-0	Guanosine, 2,Ä≤-deoxy-5,Ä≤-O-phosphono-P-thioguanylyl-(3,Ä≤,Üí5,Ä≤)-	C20H26N10O13P2S	708.49	C	-8.8
3057	O(C)c1c2c([C@H]([C@@H]3[C@@H]([C@H]2O)COC3=O)c2cc(OC)c(OC)cc2)cc2c1OCO2	138709-78-1	2-Naphthalenesulfonic acid, 5-[2-(4-amino-2-methylphenyl)diazenyl]-8-[2-(4-sulfo	C23H19N5O6S2	525.56	C	-8.8
3058	CN1C2(C(C(C(=O)/C=C(\O)/C3C4(c5c(NC4=O)ccc(N(=O)=O)c5)N(C)CC3c3cc(OC)c(O)cc3)C1)c1cc(OC)c(O)cc1)c1c(	1389335-39-0	Spiro[3H-indole-3,2,Ä≤-pyrrolidin]-2(1H)-one, 5-chloro-3,Ä≤-[(1Z)-3-[5-chloro-1	C51H42Cl2N4O8	909.81	C	-8.8
3059	O=C1C2(C(C(C(=O)/C=C(\O)/C3C4(NC(C3c3cc(OC)c(O)cc3)c3ccccc3)C(=O)c3c(C4=O)cccc3)C(N2)c2ccccc2)c2cc(O	1389335-42-5	Spiro[2H-indene-2,2,Ä≤-pyrrolidine]-1,3-dione, 4,Ä≤-[(2Z)-3-hydroxy-3-[4,Ä≤-(4-	C43H38N2O10	742.77	C	-8.8
3060	O=C1CCN(c2nc(nc(n2)N2CCOCC2)c2c3c(ccc2)[nH]nc3)CC1	1391925-87-3	4-Piperidinol, 1-[4-(1H-indazol-4-yl)-6-(4-morpholinyl)-1,3,5-triazin-2-yl]-	C19H23N7O2	381.43	C	-8.8
3061	C(#N)[C@@H]1[C@@H](O[C@H](CO)C1)n1c(=O)nc(N)cc1	1412009-05-2	2-Naphthalenesulfonic acid, 6-hydroxy-5-[2-[2-methyl-4-[2-(4-sulfophenyl)diazeny	C23H18N4O7S2	526.54	C	-8.8
3062	[C@H](C(=O)N1[C@@H](CC[C@@H]1C)c1[nH]c(cn1)c1ccc(cc1)c1ccc(cc1)c1[nH]c(nc1)[C@H]1N(C(=O)[C@@H](NC(=O	1412819-62-5	Carbamic acid, N,N,Ä≤-[[1,1,Ä≤-biphenyl]-4,4,Ä≤-diylbis[1H-imidazole-5,2-diyl[(	C46H54N8O6	814.97	C	-8.8
3063	C(=O)([C@@H](NC(=O)OC(C)(C)C)[C@@H]1C[C@@H]2[C@H](C1)C2)N1[C@H]2[C@H](C2)C[C@H]1c1[nH]c(cn1)c1ccc(cc	1413115-58-8	Carbamic acid, N,N,Ä≤-[[1,1,Ä≤-biphenyl]-4,4,Ä≤-diylbis[1H-imidazole-5,2-diyl-(	C46H50N8O8	842.94	C	-8.8
3064	C(=O)([C@@H](NC(=O)OC)C(C)C)N1[C@H](c2[nH]c(c3c4cc(c(c3)CCc3ccc(CC4)cc3)c3ccc(cc3)c3[nH]c(nc3)[C@H]3	1415119-53-7	Carbamic acid, N,N,Ä≤-[bicyclo[1.1.1]pentane-1,3-diylbis[4,1-phenylene-1H-imidaz	C45H56N8O6	804.98	C	-8.8
3065	O=c1n(cc(C(CCl)O)c(=O)[nH]1)[C@@H]1O[C@H](CO)[C@@H](O)C1	1421823-01-9	Benzoic acid, 5-[bis(3-carboxy-4-hydroxyphenyl)methyl]-3-[(3-carboxy-4-oxo-2,5-c	C30H20O12	572.47	C	-8.8
3066	C(OP(=O)(O[C@H]1[C@@H](O[C@H](COP(=O)(O[C@H]2[C@@H](O[C@H](CO)[C@H]2O)n2nc(C(=O)N)nc2)O)[C@H]1O)n1nc	1422459-54-8	Adenosine, adenylyl-(2,Ä≤,Üí5,Ä≤)-1,Ä≤-[3-(aminocarbonyl)-1H-1,2,4-triazol-1-yl	C28H34N14O16P2	884.6	C	-8.8
3067	C(N1c2c(C(=NNC(=S)N(CC=C)CC=C)C1=O)cc(C)cc2)N1CCN(CC1)c1ccccc1	1426583-65-4	Hydrazinecarbothioamide, 2-[5-fluoro-1,2-dihydro-2-oxo-1-[(4-phenyl-1-piperaziny	C32H29FN6OS	564.68	C	-8.8
3068	N(=C1c2c(N(CN3CCN(CC3)c3ccc(OC)cc3)C1=O)ccc(Cl)c2)NC(=S)N(CC=C)CC=C	1426583-73-4	Hydrazinecarbothioamide, 2-[1-[[4-(4-chlorophenyl)-1-piperazinyl]methyl]-1,2-dih	C33H31ClN6OS	595.16	C	-8.8
3069	O(C)c1c2n(cc(C(=O)O)c(=O)c2cc(F)c1N1CC(C)N(CN2c3c(C(=NNC(=S)N(CC=C)CC=C)C2=O)cc(C)cc3)CC1)C1CC1	1426583-89-2	3-Quinolinecarboxylic acid, 1-cyclopropyl-7-[4-[[3-[2-[(diphenylamino)thioxometh	C41H37F2N7O5S	777.84	C	-8.8
3070	C(CCCCCCCCC#C)CCCCCCCCCO	1439932-29-2	2-Naphthalenesulfonic acid, 6-hydroxy-5-[2-[4-(2-phenyldiazenyl)phenyl]diazenyl]	C22H16N4O4S	432.45	C	-8.8
3071	C(CCCCC(=O)NCCCOc1c(C)cn(c(=O)n1)[C@@H]1O[C@H](CO)C=C1)N1c2c(N(c3c(C1=O)cccn3)C1CC1)nccc2C	1445477-57-5	6H-Dipyrido[3,2-b:2,Ä≤,3,Ä≤-e][1,4]diazepin-6-one, 11-[2-[[4-[2-[[1,2-dihydro-1	C32H32N6O5S2	644.76	C	-8.8
3072	N(c1c2c(c(O)c(CN3CCN(CC3)c3ccccc3)c1)CCCC2)c1c2c(ncc1)cc(Cl)cc2	1449239-44-4	4-Piperidinol, 1-[[4-[(7-chloro-4-quinolinyl)amino]-5,6,7,8-tetrahydro-1-hydroxy	C25H28ClN3O2	437.96	C	-8.8
3073	N(c1c(C)cc(/C=C/C#N)cc1C)c1nc(Nc2ccc(C#N)cc2)ncc1.C(C)(C)O.Cl	1469778-99-1	Benzonitrile, 4-[[4-[[4-[(1E)-2-cyanoethenyl]-2,6-dimethylphenyl]amino]-2-pyrimi	C22H18N6.C3H8O.ClH		C	-8.8
3074	C(OC[C@@H]1[C@@H](OC(=O)c2ccccc2)[C@@H](OP(=O)(OC[C@@H]2[C@H]3[C@H](O3)[C@@H](O2)n2c3c(nc2)c(NC(=O)c	1469875-51-1	Œ≤-D-lyxo-Adenosine, N-benzoyl-3,Ä≤-O-benzoyl-5,Ä≤-O-[(4-methoxyphenyl)diphenyl	C92H80N16O25P2	1871.66	C	-8.8
3075	C(=C\C(=O)c1c(OC)cccc1O)/c1c(OC)ccc(CCCCCCCCC)c1	149731-74-8	Uridine, thymidylyl-(3,Ä≤,Üí5,Ä≤)-2,Ä≤-deoxy-5,6-dihydro-6-hydroxy-, (6S)-	C19H27N4O13P	550.41	C	-8.8
3076	O=C1C2C(c3c(C([Se]C(C(=O)O)C)C2CO1)cc1c(c3)OCO1)c1cc(OC)c(OC)c(OC)c1	150891-68-2	Acetic acid, 2-[[5,5a,6,8,8a,9-hexahydro-8-oxo-9-(3,4,5-trimethoxyphenyl)furo[3,	C24H24O9Se	535.4	C	-8.8
3077	O=C1C2C(c3c(C([Se]C(C(=O)O)CC)C2CO1)cc1c(c3)OCO1)c1cc(OC)c(OC)c(OC)c1	150891-69-3	Propanoic acid, 2-[[5,5a,6,8,8a,9-hexahydro-8-oxo-9-(3,4,5-trimethoxyphenyl)furo	C25H26O9Se	549.43	C	-8.8
3078	O[C@H]1[C@H](n2c3c(nc2)c(=O)nc(N)[nH]3)O[C@H](CO)[C@H]1NNC[C@H]1O[C@H](C[C@@H]1O)n1c2c(nc1)c(=O)nc(N	1513874-13-9	Guanosine, 3,Ä≤-O-diphosphinicoguanylyliminomethylene-(3,Ä≤,Üí5,Ä≤)-2,Ä≤,5,Ä≤-d	C21H27N11O8	561.51	C	-8.8
3079	O[C@H]1[C@H](n2c3c(nc2)c(=O)nc(N)[nH]3)O[C@H](CNO[C@@H]2[C@@H](CO)O[C@H](C2)n2c3c(nc2)c(=O)nc(N)[nH]	1513874-27-5	Guanosine, 2,Ä≤-deoxy-3,Ä≤-de(phosphinicooxy)guanylyl-1,2-hydrazinediyl-(3,Ä≤,Ü	C20H26N12O7	546.5	C	-8.8
3080	O=c1c2c(n(cn2)[C@H]2C[C@H](NS(=O)(=O)NC[C@H]3O[C@H]([C@H](O)[C@@H]3O)n3c4c(nc3)c(=O)nc(N)[nH]4)[C@@H	1513874-56-0	Guanosine, 3,Ä≤-de(phosphinicooxy)guanylyliminosulfonylimino-(3,Ä≤,Üí5,Ä≤)-2,Ä≤	C20H26N12O9S	610.56	C	-8.8
3081	O=c1c2c(n(cn2)[C@H]2C[C@H](NS(=O)(=O)NC[C@H]3O[C@H](C[C@@H]3O)n3c4c(nc3)c(=O)nc(N)[nH]4)[C@@H](CO)O2	1513874-57-1	Guanosine, 2,Ä≤-deoxy-3,Ä≤-de(phosphinicooxy)guanylyliminosulfonylimino-(3,Ä≤,Ü	C20H26N12O9S	610.56	C	-8.8
3082	C(n1c2c(nc1CC)c(C)cc(C)n2)c1ccc(cc1)c1c(S(=O)(=O)NC(=O)CCc2ccccc2)ccc(OC)c1	154552-57-5	Benzenepropanamide, N-[[4,Ä≤-[(2-ethyl-5,7-dimethyl-3H-imidazo[4,5-b]pyridin-3-y	C36H40N4O3S	608.79	C	-8.8
3083	C(n1c2c(nc1CC)c(C)cc(C)n2)c1ccc(cc1)c1c(S(=O)(=O)NC(=O)CCc2ccccc2)ccc(c1)c1cccccc1	154552-61-1	Benzenepropanamide, N-[[5-ethoxy-4,Ä≤-[(2-ethyl-5,7-dimethyl-3H-imidazo[4,5-b]py	C34H36N4O4S	596.74	C	-8.8
3084	C(n1c2c(nc1CC)c(C)cc(C)n2)c1ccc(cc1)c1c(S(=O)(=O)NC(=O)c2c(OCC)cccc2)ccc(CC)c1	154552-91-7	Benzamide, 2-ethoxy-N-[[4,Ä≤-[(2-ethyl-5,7-dimethyl-3H-imidazo[4,5-b]pyridin-3-y	C33H34N4O4S	582.71	C	-8.8
3085	O=C1C2c3c(c(ccc3c3[nH]c(nc3)[C@H]3N(C(=O)[C@@H](NC(=O)OC)C(C)C)CCC3)c3ccc(cc3)c3[nH]c(nc3)[C@H]3N(C(	1560639-42-0	Carbamic acid, N-[(1S)-2-methyl-1-[[(2S)-2-[5-[4-[1,2,3,4-tetrahydro-5-[2-[(2S)-	C46H57N9O7	848	C	-8.8
3086	CN1C2c3c(C1CC2)c(ccc3c1[nH]c(nc1)[C@H]1N(C(=O)[C@@H](NC(=O)OC)C(C)C)CC(C)(C)C1)c1ccc(cc1)c1[nH]c(nc1	1560639-55-5	Carbamic acid, N-[(1S)-1-[[(2S,4R)-4-fluoro-2-[5-[8-[4-[2-[(2S,4R)-4-fluoro-1-[(	C45H55F2N9O6	855.97	C	-8.8
3087	C(=O)([C@@H](NC(=O)OC)C(C)C)N1[C@H](c2[nH]c(c3c4c(c(cc3)c3ccc(cc3)c3[nH]c(nc3)[C@H]3N(C(=O)[C@@H](NC	1560639-95-3	Carbamic acid, N-[(1S)-2-methyl-1-[[(2S)-2-[5-[4-[1,2,3,4-tetrahydro-8-[2-[(2S)-	C46H58N8O6	819	C	-8.8
3088	C(=O)([C@@H](NC(=O)OC)C(C)C)N1[C@H](c2[nH]c(c3c4c(c(cc3)c3c5c(c(cc3)c3[nH]c(nc3)[C@H]3N(C(=O)[C@@H](	1560650-00-1	Carbamic acid, N-[(1S)-2-methyl-1-[[(2S,4R)-4-methyl-2-[5-[4-[1,2,3,4-tetrahydro	C48H62N8O6	847.06	C	-8.8
3089	O[C@H]1[C@H](n2c3c(nc2)c(=O)nc(N)[nH]3)O[C@H](COP(=O)(O[C@H]2[C@@H](O)[C@@H](O[C@@H]2CO)n2c3c(nc2)c(	158614-79-0	Guanosine, 5,Ä≤-O-phosphonouridylyl-(3,Ä≤,Üí5,Ä≤)-adenylyl-(3,Ä≤,Üí5,Ä≤)-, homo	(C29H37N12O22P3)x		C	-8.8
3090	N(C(=O)C)[C@H]1[C@H](OC(CC)CC)C=C(C(=O)OCC)C[C@@H]1NC(=S)NC(=O)OCc1ccccc1	1588414-25-8	Carbamic acid, N-[(1S)-1-[[(3S)-3-[5-[2,Ä≤,3,Ä≤-dihydro-7,Ä≤-[4-[2-[(3S)-2-[(2S	C51H64N8O6	885.1	C	-8.8
3091	O=C1[C@@H]2[C@@H](c3c([C@H](OCc4cn(nn4)c4c(I)cccc4)[C@H]2CO1)cc1c(c3)OCO1)c1cc(OC)c(OC)c(OC)c1	1600500-31-9	Furo[3,Ä≤,4,Ä≤:6,7]naphtho[2,3-d]-1,3-dioxol-6(5aH)-one, 5,8,8a,9-tetrahydro-9-	C31H29N3O9	587.58	C	-8.8
3092	O=C1[C@@H]2[C@@H](c3c([C@H](OCc4cn(nn4)c4cc(Br)ccc4)[C@H]2CO1)cc1c(c3)OCO1)c1cc(OC)c(OC)c(OC)c1	1600500-33-1	Furo[3,Ä≤,4,Ä≤:6,7]naphtho[2,3-d]-1,3-dioxol-6(5aH)-one, 5,8,8a,9-tetrahydro-9-	C31H28IN3O8	697.47	C	-8.8
3093	C(OCO)n1c2c(nc1)c(=O)nc(N)[nH]2	160729-91-9	D-erythro-Pentonamide, 2,3,5-trideoxy-N-[(1S,2R)-2,3-dihydro-2-hydroxy-1H-inden-	C38H47N5O5	653.81	C	-8.8
3094	O=C1N2[C@H](Cn3c1c(O)c(=O)c(C(=O)N[C@@H](C)c1c(F)cc(F)cc1)c3)O[C@@H]1C[C@H]2CC1	1616339-79-7	2,5-Methanopyrido[1,Ä≤,2,Ä≤:4,5]pyrazino[2,1-b][1,3]oxazepine-10-carboxamide, N	C22H21F2N3O5	445.42	C	-8.8
3095	O=C1N2[C@H](Cn3c1c(O)c(=O)c(C(=O)NCc1c(F)cc(F)cc1F)c3)O[C@@H]1C[C@H]2CC1	1616340-09-0	2,5-Methanopyrido[1,Ä≤,2,Ä≤:4,5]pyrazino[2,1-b][1,3]oxazepine-10-carboxamide, N	C22H21F2N3O5	445.42	C	-8.8
3096	C(=O)([C@@H](NC(=O)OC)C(C)C)N1[C@@H](CCC1)c1[nH]c(cn1)c1ccc(cc1)c1ccc(cc1)c1[nH]c(CN2C(=O)[C@@H](C(C	1616889-51-0	Carbamic acid, N-[(1S)-1-[[(2S)-2-[5-[4,Ä≤-[2-[1-[(2S)-2-[(methoxycarbonyl)amino	C41H53N9O6	767.92	C	-8.8
3097	N(C(=O)[C@H](Cc1ccncc1)C[C@@H](CN1[C@H](C(=O)NC(C)(C)C)CN(Cc2cccnc2)CC1)O)[C@H]1c2c(C[C@H]1O)cccc2	162105-23-9	1-Piperazinepentanamide, N-(2,3-dihydro-2-hydroxy-1H-inden-1-yl)-2-[[(1,1-dimeth	C35H46N6O4	614.78	C	-8.8
3098	O[C@H]1[C@@H](O[C@H](COC(=O)CCCCCCC/C=C\CCCCCCCC)[C@H]1O)n1nc(C(=O)N)nc1	162518-60-7	D-erythro-Pentonamide, 2,3,5-trideoxy-N-(2,3-dihydro-2-hydroxy-1H-inden-1-yl)-5-	C42H55N5O11		C	-8.8
3099	C(C)n1c2c(c3c1cccc3)cc(Nc1nc(nc(Nc3ccccc3)n1)N1CCOCC1)cc2	1632197-39-7	9H-Carbazol-3-amine, N-[4-chloro-6-(4-morpholinyl)-1,3,5-triazin-2-yl]-9-ethyl-	C21H21ClN6O	408.88	C	-8.8
3100	O=C1[C@@H]2[C@@H](c3c([C@H](OC(=O)/C(=C/c4cc(OC)c(OC)c(OC)c4)/c4cc(OC)ccc4)[C@H]2CO1)cc1c(c3)OCO1)c1	1633020-41-3	Benzeneacetic acid, 3,4-dimethoxy-Œ±-[[4-methoxy-3-(phenylmethoxy)phenyl]methyle	C47H44O13	816.84	C	-8.8
3101	C(=O)(C1(NC(=O)OC)CC1)N1[C@@H](CCC1)c1[nH]c(cn1)c1ccc(cc1)c1ccc(cc1)c1[nH]c(nc1)[C@H]1N(C(=O)[C@@H](	1639870-46-4	2H-Isoindole-2-carboxylic acid, 4-fluoro-1,3-dihydro-, (3R,5S)-1-[(2S)-2-[[(1,1-	C52H62FN9O8	960.1	C	-8.8
3102	C(=O)([C@@H](NC(=O)OC)C(C)C)N1[C@H](c2[nH]c(c3c4c(c(ccc4)c4ccc(cc4)c4[nH]c(nc4)[C@H]4N(C(=O)[C@@H](N	1639870-97-5	Carbamic acid, N,N,Ä≤-[[1,1,Ä≤-biphenyl]-4,4,Ä≤-diylbis[1H-imidazole-5,2-diyl[(	C46H42N8O6	802.88	C	-8.8
3103	C(=C\C(=O)CC(=O)/C=C/c1cc(O)ccc1)/c1cc(O)c(O)cc1	1639871-35-4	Carbamic acid, N-[(1R)-2-[(2S)-2,5-dihydro-2-[5-[4,Ä≤-[2-[(2S)-1-[(2S)-2-[(metho	C43H46N8O6	770.88	C	-8.8
3104	O=c1n([C@@H]2O[C@H](COP(=O)(C(O)c3ccncc3)OC[C@@H]3[C@@H](N=[N+]=[N-])C[C@@H](O3)n3c(=O)[nH]c(=O)c(C)	164652-15-7	Thymidine, 3,Ä≤-azido-P,3,Ä≤-dideoxy-P-[(2,4-dinitrophenyl)hydroxymethyl]thymid	C27H29N12O14P		C	-8.8
3105	N(C(=O)[C@H](Cc1ccccc1)C[C@@H](CN1[C@H](C(=O)NC(C)(C)C)CN(CCC2CCN(C(=O)OC(C)(C)C)CC2)CC1)O)[C@H]1c2c	164725-18-2	1-Piperazinepentanamide, N-(2,3-dihydro-2-hydroxy-1H-inden-1-yl)-2-[[(1,1-dimeth	C41H55N5O6	713.91	C	-8.8
3106	N(C(=O)[C@H](Cc1ccccc1)C[C@@H](CN1[C@H](C(=O)NC(C)(C)C)CN(Cc2cc3c(cc2)cccc3)CC1)O)[C@H]1c2c(C[C@H]1O	164725-20-6	1-Piperazinepentanamide, 4-([1,1,Ä≤-biphenyl]-4-ylmethyl)-N-(2,3-dihydro-2-hydro	C43H52N4O4	688.9	C	-8.8
3107	N(C(=O)[C@H](Cc1ccccc1)C[C@@H](CN1[C@H](C(=O)NC(C)(C)C)CN(CCN2CCOCC2)CC1)O)[C@H]1c2c(C[C@H]1O)cccc2	164725-23-9	1-Piperazinepentanamide, N-(2,3-dihydro-2-hydroxy-1H-inden-1-yl)-2-[[(1,1-dimeth	C37H55N5O4	633.86	C	-8.8
3108	N(C(=O)[C@H](Cc1ccccc1)C[C@@H](CN1[C@H](C(=O)NC(C)(C)C)CN(Cc2cnc(C)cn2)CC1)O)[C@H]1c2c(C[C@H]1O)cccc	164725-42-2	1-Piperazinepentanamide, N-(2,3-dihydro-2-hydroxy-1H-inden-1-yl)-2-[[(1,1-dimeth	C37H49N5O4	627.82	C	-8.8
3109	C(=O)([C@@H](NC(=O)OC)C(C)C)N1CC2(C[C@H]1c1[nH]c3c(n1)ccc1c3ccc(c1)c1cc3c(cc1)c1c(cc3)nc([nH]1)[C@H]	1653966-58-5	Carbamic acid, N-[(1S)-1-[[(3S,5S)-3-[7-[4-[2-[(1R,3S,5R)-2-[(2S)-2-[(methoxycar	C47H56N8O7	845	C	-8.8
3110	C(=O)([C@@H](NC(=O)OC)C(C)C)N1C[C@@]2(C[C@H]1c1[nH]c3c(n1)ccc1c3ccc(c1)c1ccc(cc1)c1[nH]c(nc1)[C@H]1N	1653966-61-0	Carbamic acid, N-[(1S)-1-[[(3S,5S)-3-[7-[4-[2-[(6S)-5-[(2S)-2-[(methoxycarbonyl)	C48H58N8O7	859.02	C	-8.8
3111	C(=O)([C@@H](NC(=O)OC)C(C)C)N1[C@@H](C[C@H]2[C@@H]1CCC2)c1[nH]c2c(n1)ccc1c2ccc(c1)c1ccc(cc1)c1[nH]c(	1653966-67-6	Carbamic acid, N-[(1S)-1-[[(2S,3aS,7aS)-2-[4,5-dihydro-7-[4-[2-[(3S,5S)-2-[(2S)-	C50H64N8O7	889.09	C	-8.8
3112	C(=O)(NC(C)(C)C)[C@H]1N(C[C@H]([C@@H](NC(=O)c2c(C)c(OC(=O)C)ccc2)CSc2ccccc2)OC(=O)C)C[C@@H]2[C@H](C1	1665278-95-4	3-Isoquinolinecarboxamide, N-(1,1-dimethylethyl)decahydro-2-[(2S,3S)-2-hydroxy-3	C35H45N3O3S	587.82	C	-8.8
3113	O=c1n([C@H]2O[C@@H](CO)[C@H](O)C2)cc(/C=C/Br)c(=O)[nH]1	16672-98-3	1-Naphthalenesulfonic acid, 7-amino-3-hydroxy-4-[2-(2-hydroxy-1-naphthalenyl)dia	C20H15N3O5S	409.42	C	-8.8
3114	C(n1c2c(nc1CC)c(C)cc(C)n2)c1c(F)cc(cc1)c1c(S(=O)(=O)NC(=O)OCCCC)ccc(CCC)c1	169281-61-2	Carbamic acid, [[4,Ä≤,Ä≤-[(2-ethyl-5,7-dimethyl-3H-imidazo[4,5-b]pyridin-3-yl)m	C34H36N4O4S	596.74	C	-8.8
3115	S(=O)(=O)(O)c1c2c(c(N)c(N=Nc3c(C)cc(cc3)c3cc(C)c(N=Nc4c(N)c5c(c(S(=O)(=O)O)c4)cccc5)cc3)c1)cccc2	173291-37-7	Guanosine, 2,Ä≤-deoxyguanylyl-(3,Ä≤,Üí5,Ä≤)-2,Ä≤-O-methyl-	C21H27N10O11P	626.47	C	-8.8
3116	C(Cc1ccc(F)cc1)C1(CCc2ccc(F)cc2)CC(=C(C(CC)c2cc(NS(=O)(=O)c3ccc(C#N)cn3)ccc2)C(=O)O1)O	174484-07-2	2-Pyridinesulfonamide, N-[3-[1-[6,6-bis[2-(4-fluorophenyl)ethyl]-5,6-dihydro-4-h	C38H37F2N3O5S	685.78	C	-8.8
3117	C(Cc1ccccc1)[C@]1(CCC(F)(F)F)CC(=C([C@@H](C(C)(C)C)c2cc(NS(=O)(=O)c3ccc(N)cn3)ccc2)C(=O)O1)O	174484-20-9	2-Pyridinesulfonamide, 5-amino-N-[3-[1-[5,6-dihydro-4-hydroxy-2-oxo-6-(2-phenyle	C32H36F3N3O5S	631.71	C	-8.8
3118	C(Cc1ccccc1)C1(CCc2ccccc2)CC(=C([C@H](C(C)(C)C)c2cc(NS(=O)(=O)c3ccc(N)cn3)ccc2)C(=O)O1)O	174484-61-8	2-Pyridinesulfonamide, 5-cyano-N-[3-[[5,6-dihydro-4-hydroxy-2-oxo-6,6-bis(2-pher	C34H31N3O5S	593.69	C	-8.8
3119	C(Cc1ccccc1)[C@@]1(CCC)CC(=C([C@H](CC)c2cc(NS(=O)(=O)c3ccc(C(F)(F)F)cn3)ccc2)C(=O)O1)O	174590-25-1	2-Pyridinesulfonamide, N-[3-[1-[5,6-dihydro-4-hydroxy-2-oxo-6-(2-phenylethyl)-6-	C33H37F3N2O5S	630.72	C	-8.8
3120	N(C(CCN)(C)C)c1c2c(cc(Cl)cc2)ncc1	177155-68-9	Benzamide, N,N,Ä≤-(methylenedi-4,1-phenylene)bis[2-hydroxy-5-nitro-	C27H20N4O8	528.47	C	-8.8
3121	C[C@]12[C@H]([C@H]3[C@@H]([C@]4(C)[C@H](C[C@H]3O)C[C@H](O)CC4)CC2)CC[C@@H]1[C@@H](CCC(=O)N[C@@H](C(=	177780-28-8	5,Ä≤-Thymidylic acid, 3,Ä≤-azido-3,Ä≤-deoxy-, mono[2-(1,4,7,10,13-pentaoxacyclo	C27H38N5O12P		C	-8.8
3122	O[C@H]1[C@@H]2[C@@H]([C@]3(C)[C@H](C1)C[C@H](OC(=O)N[C@H](C(=O)OC[C@H]1O[C@H]([C@H](O)[C@@H]1O)n1nc(	1777812-93-7	L-Glutamic acid, N-[(3Œ±,5Œ≤,7Œ±)-3,7-dihydroxy-24-oxocholan-24-yl]-, 1,Üí5,Ä≤	C37H57N5O11	747.88	C	-8.8
3123	C(OC(C(O)([2H])[2H])([2H])[2H])n1c2c([nH]c(N)nc2=O)nc1	179472-47-0	1-Naphthalenesulfonic acid, 3,3,Ä≤-[1,4-phenylenebis(azo)]bis[4-amino-, disodiur	C26H20N6O6S2.2Na		C	-8.8
3124	CC12CC3(CC(C)(C1)CC(C3)C2)N1CSCCSC1	1803330-64-4	Not Yet Assigned	C30H36N14O17P2	926.64	C	-8.8
3125	O=C1[C@]2([C@H]([C@H](/C(=C/C(=O)[C@@H]3[C@@]4(N5[C@@H]([C@H]3c3cc(OC)c(O)cc3)CCC5)c3c(NC4=O)ccc(F)c	1807888-30-7	Not Yet Assigned	C45H42F2N4O8	804.83	C	-8.8
3126	O=C1[C@@H]2[C@@H](c3c([C@H](OC(=O)[C@@H](C)N4C(=O)C5C(C4=O)C4OC5CC4)[C@H]2CO1)cc1c(c3)OCO1)c1cc(OC)c	1809325-33-4	4,7-Epoxy-2H-isoindole-2-acetic acid, octahydro-Œ±-methyl-1,3-dioxo-, (5R,5aR,8a	C33H33NO12	635.61	C	-8.8
3127	O=C1[C@@H]2[C@@H](c3c([C@H](OC(=O)[C@H](C)N4C(=O)C5C(C4=O)C4OC5CC4)[C@H]2CO1)cc1c(c3)OCO1)c1cc(OC)c(	1809325-34-5	4,7-Epoxy-2H-isoindole-2-acetic acid, octahydro-Œ±-methyl-1,3-dioxo-, (5R,5aR,8a	C33H33NO12	635.61	C	-8.8
3128	C(C[C@@H](C/C=C/Cc1ccc(O)cc1)O)c1cc(OC)c(O)cc1	1822365-49-0	2H-Pyrido[1,Ä≤,2,Ä≤:4,5]pyrazino[2,1-b][1,3]oxazine-9-carboxamide, N-[(2,4-difl	C26H29F2N3O10	581.52	C	-8.8
3129	Nc1c2c(n(cn2)[C@@H]2O[C@@H](C=O)CC2)ncn1	1831102-39-6	Butanedioic acid, 1-[(3R,5aS,6R,8aS,9R,10R,12R,12aR)-decahydro-3,6,9-trimethyl-3	C41H48O15	780.81	C	-8.8
3130	C(OC[C@@H]1[C@@H](OC(=O)CC)C[C@@H](C1=C)n1c2c(nc1)c(=O)nc(NC(=O)OC(C)(C)C)[nH]2)(c1ccccc1)(c1ccccc1)	1860842-89-2	Carbamic acid, N-[9-[(1S,3R,4S)-4-(acetyloxy)-2-methylene-3-[(triphenylmethoxy)m	C38H39N5O6	661.75	C	-8.8
3131	O(C(=O)N[C@@H](Cc1ccc(OCc2c(C(=O)O)sc(Oc3ccccc3)n2)cc1)[C@@H](CN(S(=O)(=O)c1ccc2c(cc1)ncs2)CC(C)C)O)[	1887206-21-4	2-Pyridinecarboxylic acid, 3-[[4-[(2S,3R)-4-[(6-benzothiazolylsulfonyl)(2-methyl	C41H42F2N4O10S2	852.92	C	-8.8
3132	O=c1n(c2c(c(cc(n2)c2ccc(Cl)cc2)c2ccc(F)cc2)c(=O)n1c1ccccc1)[C@H]1[C@H](OC(=O)c2ccccc2)[C@H](OC(=O)c2	190319-84-7	Pyrido[2,3-d]pyrimidine-2,4(1H,3H)-dione, 3,7-bis(4-chlorophenyl)-5-phenyl-1-(2,	C51H35Cl2N3O9	904.74	C	-8.8
3133	N([Si](C)(C)Cl)C12CC3CC(C2)CC(C1)C3	190319-86-9	Pyrido[2,3-d]pyrimidine-2,4(1H,3H)-dione, 3,7-bis(4-chlorophenyl)-5-(4-fluorophe	C51H34Cl2FN3O9	922.73	C	-8.8
3134	[C@@H](C(=O)N[C@@H](Cc1ccccc1)C[C@@H]([C@H](Cc1ccccc1)NC(=O)COc1c(C)cccc1C)O)(C(C)C)N1C(=O)NNCC1	192725-39-6	1(2H)-Pyrimidineacetamide, N-[(1S,3S,4S)-4-[[2-(2,6-dimethylphenoxy)acetyl]amino	C37H46N4O6	642.78	C	-8.8
3135	C(=O)([C@@H](NC(=O)OC)C(C)C)N1[C@H](c2[nH]c3c4c(cc(cc4)c4cccc(cc4)c4[nH]c(nc4)[C@H]4N(C(=O)[C@@H](NC(	1949770-75-5	Carbamic acid, N-[(1S)-1-[[(2S)-2-[5-chloro-7-[4-[2-[(2S)-1-[(2S)-3-methyl-2-[[[	C48H57ClN8O6	877.47	C	-8.8
3136	C(=O)([C@@H](NC(=O)OC)C(C)C)N1[C@H](c2[nH]c3c4c(cc(cc4)c4cccc(cc4)c4[nH]c(nc4)[C@H]4N(C(=O)[C@@H](NC(	1949770-76-6	Carbamic acid, N-[(1S)-1-[[(2S)-2-[7-[4-[2-[(2S,4R)-4-amino-1-[(2S)-2-[(methoxyc	C42H51N9O6	777.91	C	-8.8
3137	C(=C1C=C(C(=O)[O-][Zr+2](=O)([OH-])([OH2])([OH2])[OH2])C(=O)C=C1)(c1cc2c(cc1)[O-][Zr+2](=O)([NH2]c1c	1969-54-6	Thymidine, thymidylyl-(3,Ä≤,Üí5,Ä≤)-	C20H27N4O12P	546.42	C	-8.8
3138	C(F)(F)(F)c1c(c2nc(nc(n2)N2C[C@H](C)O[C@H](C)C2)N2C[C@H](C)O[C@H](C)C2)cnc(N)c1	1971057-59-6	Carbamic acid, N-[4-[[[(2R,3S)-3-[[[[(3R,3aS,6aR)-hexahydrofuro[2,3-b]furan-3-yl	C34H45N3O11S	703.8	C	-8.8
3139	S(=O)(=O)(c1cc2c(cc1)NC(=O)C2)N1CC(O)C(O)C1	197903-84-7	Guanosine, 5,Ä≤-O-(4-cyano-2,3,6-trifluoro-5-hydroxyphenyl)-	C17H13F3N6O6	454.32	C	-8.8
3140	N(C(=O)C)[C@H]1[C@H]([C@@H]([C@@H](CO)O)O)OC(=C[C@@H]1NC(=N)NC(=O)Cn1cc(nn1)c1cc(Cl)ccc1)C(=O)O	1982351-41-6	D-glycero-D-galacto-Non-2-enonic acid, 5-(acetylamino)-4-[[[[2-[4-(2-aminophenyl	C22H28N8O8	532.51	C	-8.8
3141	Fc1c2[C@H](N3n4c(C(=O)N5[C@H]3COCC5)c(O)c(=O)cc4)c3c(SCc2ccc1)c(Cl)ccc3	1985605-73-9	1H-[1,4]Oxazino[3,4-c]pyrido[2,1-f][1,2,4]triazine-6,8-dione, 12-[(11S)-9-chloro	C24H20ClN3O4S	481.95	C	-8.8
3142	O(COC(=O)O[C@@H](C(=O)OC)c1ccccc1)c1c2n(N([C@H]3N(C2=O)CCOC3)[C@H]2c3c(CSc4c2cccc4)c(F)c(F)cc3)ccc1=	1985606-95-8	Propanoic acid, 3-[[[[[(12aR)-12-[(11S)-7,8-difluoro-6,11-dihydrodibenzo[b,e]thi	C32H31F2N3O9S	671.66	C	-8.8
3143	O(COC(=O)OCC(=O)OCC)c1c2n(N([C@H]3N(C2=O)CCOC3)[C@H]2c3c(CSc4c2cccc4)c(F)c(F)cc3)ccc1=O	1985606-99-2	Propanoic acid, 3-[[[[[(12aR)-12-[(11S)-7,8-difluoro-6,11-dihydrodibenzo[b,e]thi	C30H27F2N3O9S	643.61	C	-8.8
3144	O(C(=O)O[C@@H](COC)C)c1c2n(N([C@H]3N(C2=O)CCOC3)[C@H]2c3c(CSc4c2cccc4)c(F)c(F)cc3)ccc1=O	1985607-13-3	Carbonic acid, (12aR)-12-[(11S)-7,8-difluoro-6,11-dihydrodibenzo[b,e]thiepin-11-	C29H25F2N3O7S	597.59	C	-8.8
3145	O(C(=O)OCC1(C(=O)OC)CCCC1)c1c2n(N([C@H]3N(C2=O)CCOC3)[C@H]2c3c(CSc4c2cccc4)c(F)c(F)cc3)ccc1=O	1985607-41-7	Benzoic acid, 2-[[[[(12aR)-12-[(11S)-7,8-difluoro-6,11-dihydrodibenzo[b,e]thiepi	C33H25F2N3O8S	661.63	C	-8.8
3146	O([C@H](C)c1ccccc1)c1c2n(N([C@H]3N(C2=O)CCOC3)[C@H]2c3c(CSc4c2cccc4)c(F)c(F)cc3)ccc1=O	1985607-43-9	1H-[1,4]Oxazino[3,4-c]pyrido[2,1-f][1,2,4]triazine-6,8-dione, 12-[(11S)-7,8-difl	C32H27F2N3O4S	587.64	C	-8.8
3147	O(Cc1ccc(OC(=O)N(C)C)cc1)c1c2n(N([C@H]3N(C2=O)CCOC3)[C@H]2c3c(CSc4c2cccc4)c(F)c(F)cc3)ccc1=O	1985607-48-4	Carbonic acid, (4-azidophenyl)methyl ((12aR)-12-[(11S)-7,8-difluoro-6,11-dihydro	C32H24F2N6O6S		C	-8.8
3148	O(C(=O)O[C@H](C(=O)OC)c1ccccc1)c1c2n(N([C@H]3N(C2=O)CCOC3)[C@H]2c3c(CSc4c2cccc4)c(F)c(F)cc3)ccc1=O	1985607-53-1	Benzeneacetic acid, Œ±-[[[[(12aR)-12-[(11S)-7,8-difluoro-6,11-dihydrodibenzo[b,e	C34H27F2N3O8S	675.66	C	-8.8
3149	N(C(=O)c1c(Cl)nccc1)c1cc2cc(C34CC5CC(C4)CC(C3)C5)[nH]c2cc1	1987854-64-7	Benzamide, 2-chloro-N-(2-tricyclo[3.3.1.13,7]dec-1-yl-1H-indol-5-yl)-	C25H25ClN2O	404.93	C	-8.8
3150	O=C1[C@@H]2[C@@H](c3c([C@H](OC(=O)c4cn(c5c(c4=O)cc(F)c(Cl)c5)C4CC4)[C@H]2CO1)cc1c(c3)OCO1)c1cc(OC)c(	1989611-37-1	2-Quinoxalinecarboxylic acid, (5R,5aR,8aR,9R)-5,5a,6,8,8a,9-hexahydro-8-oxo-9-(3	C31H26N2O9	570.55	C	-8.8
3151	[C@@H]([C@H](Cc1ccccc1)NC(=O)COc1c(C)cccc1C)(C[C@H](Cc1ccccc1)NC(=O)[C@H](C(C)C)N1C(=O)NCCC1)OC(=O)[	2001083-78-7	3-Piperidinecarboxylic acid, 1-[4-[2-(difluoromethyl)-1H-benzimidazol-1-yl]-6-(4	C21H23F2N7O3	459.45	C	-8.8
3152	FC1(F)Cc2c(ccc2c2cc3c(cc2)c2cc(cc3)nc([nH]2)[C@H]2N(C(=O)[C@@H](NC(=O)OC)C(C)C)CCC2)c2[nH]c(nc2)[C@	2007901-15-5	Carbamic acid, N-[(1S)-1-[[(2S)-2-[7-[5-[2-[(2S)-1-[(2S)-2-[(methoxycarbonyl)ami	C44H52N8O6	788.93	C	-8.8
3153	C(=O)([C@@H](NC(=O)OC)C(C)C)N1[C@H](c2[nH]c3c4cc(cc(cc4)c4[nH]c(nc4)[C@H]4N(C(=O)[C@@H](N	2007901-40-6	Carbamic acid, N-[(1S)-2-methyl-1-[[(2S,5S)-2-methyl-5-[7-[5,6,7,8-tetrahydro-4-	C47H58N8O6	831.01	C	-8.8
3154	C(=O)(C(=O)Nc1ccccc(c1)cc1c(C23CC4CC(C3)CC(C2)C4)[nH]c2c1cccc2	2014403-76-8	1H-Indole-3-acetamide, N-[2-(1H-indol-2-yl)ethyl]-Œ±-oxo-2-tricyclo[3.3.1.13,7]	C30H31N3O2	465.59	C	-8.8
3155	C(=O)(C(=O)Nc1ccccc1)c1c(C23CC4CC(C3)CC(C2)C4)[nH]c2c1cccc2	2014403-77-9	1H-Indole-3-acetamide, Œ±-oxo-N-phenyl-2-tricyclo[3.3.1.13,7]dec-1-yl-	C26H26N2O2	398.5	C	-8.8
3156	C(=O)(C(=O)Nc1c(C)cc(c(=O)OC)cc1)c1c(C23CC4CC(C3)CC(C2)C4)[nH]c2c1cccc2	2014403-84-8	1H-Indole-3-acetamide, N-(2-methyl-4-nitrophenyl)-Œ±-oxo-2-tricyclo[3.3.1.13,7]	C27H27N3O4	457.52	C	-8.8
3157	[C@H](C(=O)N1c2c(CC1)cccc2)(N)C12CC3(O)CC(C2)CC(C1)C3	1000689-64-4	Ethanone, 2-amino-2-(3-hydroxytricyclo[3.3.1.13,7]dec-1-yl)-1-(2H-isoindol-2-yl)	C20H24N2O2	324.42	A/B	-8.7
3158	O[C@H]1[C@@H](O[C@H](COCC(=O)NCCCC(=O)N[C@H]([C@H]([C@H]([C@@H](CO)O)O)O)C=O)[C@H]1O)n1nc(C(=O)N)nc1	1002334-98-6	1H-1,2,4-Triazole-3-carboxamide, 1-[5-O-[4-[(Œ≤-D-galactopyranosyloxy)methyl]ber	C22H28N4O12	540.48	A/B	-8.7

3159	C(OC(=O)[C@@H](NC(=O)OCc1ccccc1)C)[C@@H]1[C@@H]2[C@H]([C@@H](O1)n1nc(C(=O)N)nc1)OC(OC)O2	1002335-53-6	1H-1,2,4-Triazole-3-carboxamide, 1-[2,3-O-(methoxymethylene)-5-O-[[[2-[[2-[[4,6-	C28H36N6O14	680.62	A/B	-8.7
3160	C(=O)([C@@H](N=C(NC#N)N(C)C)C(C)C)N1[C@@H](CCC1)c1[nH]c(cn1)c1ccc(cc1)c1ccc(cc1)c1[nH]c(nc1)[C@H]1N(	1007884-55-0	Carbamic acid, N-[(1S)-1-[[(2S)-2-[5-[4,Ä≤-[2-[(2S)-1-[(2S)-2-[(3-amino-1H-1,2,4	C40H50N12O4	762.9	A/B	-8.7
3161	C(N1c2c(C(=NNC(=S)Nc3ccccc3)C1=O)cccc2)N1CCN(CN2c3c(C(=NNC(=S)Nc4ccccc4)C2=O)cccc3)CC1	1008402-87-6	2H-Isoindole-2-acetic acid, octahydro-Œ±-methyl-1,3-dioxo-, 2-oxo-2-(tricyclo[3.	C23H32N2O5	416.51	A/B	-8.7
3162	C(=O)([C@H](NC(=O)OC)c1ccccc1)N1[C@@H](CCC1)c1[nH]c(cn1)c1ccc(cc1)c1ccc(cc1)c1[nH]c(nc1)[C@H]1N(C(=O	1009057-21-9	3-Isoquinolinecarboxamide, 2-[3-[(3,4-dihydroxy-2-methylbenzoyl)amino]-2-hydroxy	C33H47N3O5S	597.81	A/B	-8.7
3163	C(=O)([C@H](NC(=O)OC)c1ccccc1)N1[C@@H](CCC1)c1[nH]c(cn1)c1ccc(cc1)c1ccc(cc1)c1[nH]c(nc1)[C@H]1N(C(=O	1009107-66-7	Carbamic acid, N-[(1R)-2-[(2S)-2-[5-[4,Ä≤-[2-[(2S)-1-[(2R)-2-(acetylamino)-1-oxo	C41H44N8O5	728.84	A/B	-8.7
3164	C(=O)([C@H](NC(=O)OC)c1ccccc1)N1[C@@H](CCC1)c1[nH]c(cn1)c1ccc(cc1)c1ccc(cc1)c1[nH]c(nc1)[C@H]1N(C(=O	1009107-88-3	Carbamic acid, N-[(1R)-2-[(2S)-2-[5-[4,Ä≤-[2-[(2S)-1-[(2S)-2-(dimethylamino)-1-c	C41H46N8O4	714.86	A/B	-8.7
3165	C(=O)([C@H](NC(=O)OC)c1ccccc1)N1[C@@H](CCC1)c1[nH]c(cn1)c1ccc(cc1)c1ccc(cc1)c1[nH]c(nc1)[C@H]1N(C(=O	1009107-96-3	Carbamic acid, N-[(1R)-2-[(2S)-2-[5-[4,Ä≤-[2-[(2S)-1-[(2S)-2-amino-1-oxopropyl]-	C39H42N8O4	686.8	A/B	-8.7
3166	C(=O)([C@H](N(C)C)c1ccccc1)N1[C@@H](CCC1)c1[nH]c(cn1)c1ccc(cc1)c1ccc(cc1)c1[nH]c(nc1)[C@H]1N(C(=O)[C	1009110-76-2	Carbamic acid, N-[2-[(2S)-2-[5-[4,Ä≤-[2-[(2S)-1-[(2R)-2-(dimethylamino)-2-phenyl	C40H44N8O4	700.83	A/B	-8.7
3167	C(=O)([C@@H](NC(=O)OC)C(C)C)N1[C@@H](CCC1)c1[nH]c(cn1)c1ccc(cc1)c1ccc(cc1)c1[nH]c(nc1)[C@H]1N(C(=O)C	1009113-42-1	Carbamic acid, N-[(1S)-2-methyl-1-[[(2S)-2-[5-[4,Ä≤-[2-[(2S)-1-[2-[(phenylmethyl	C42H48N8O4	728.88	A/B	-8.7
3168	C(=O)([C@@H](NC(=O)OC)C1CC1)N1[C@@H](C[C@@H](O)C1)c1[nH]c(cn1)c1ccc(cc1)c1ccc(cc1)c1[nH]c(nc1)[C@H]1	1009115-58-5	Carbamic acid, N,N,Ä≤-[[1,1,Ä≤-biphenyl]-4,4,Ä≤-diylbis[1H-imidazole-5,2-diyl[(	C40H50N8O8	770.87	A/B	-8.7
3169	C(=O)([C@@H](NC(=O)OCCF)C(C)C)N1[C@@H](CCC1)c1[nH]c(cn1)c1ccc(cc1)c1ccc(cc1)c1[nH]c(nc1)[C@H]1N(C(=O	1009115-68-7	Carbamic acid, N,N,Ä≤-[[1,1,Ä≤-biphenyl]-4,4,Ä≤-diylbis[1H-imidazole-5,2-diyl[(	C40H48F2N8O6	774.86	A/B	-8.7
3170	O=c1n(ccc(N)n1)[C@H]1O[C@@H](COC(=O)O[C@H]2[C@H](C(C)C)CC[C@@H](C)C2)SC1	1011726-31-0	1-Piperazinecarboxamide, 4-methyl-N-[(1S)-2-oxo-2-[[(1S,2E)-1-(2-phenylethyl)-3-	C31H37N5O4S	575.72	A/B	-8.7
3171	O=c1c2c(n(c(n2)[2H])[C@@H]2O[C@H](CO)[C@@H](O)C2)[nH]c(N)n1	1013423-59-0	2,7,10,12-Tetraazatridecanoic acid, 4-hydroxy-12-methyl-9-(1-methylethyl)-13-[2-	C37H48N6O5S2.C31H33F3N2O5S		A/B	-8.7
3172	C(=O)(N[C@H](C(=O)C(=O)NC1CC1)CCC)[C@@H]1[C@@H]2[C@H](CN1C(=O)[C@@H](NC(=O)[C@@H](NC(=O)c1cnccn1)C1C	1015080-09-7	Carbamic acid, N-[[3-[[[[3-methoxy-4-(5-oxazolyl)phenyl]amino]carbonyl]amino]phe	C36H53N7O6.C23H24N4O6		A/B	-8.7
3173	O(c1c(CCC)cc(C#N)cc1C)c1nc(Nc2ccc(C#N)cc2)ncc1Br	1022904-33-1	Benzonitrile, 4-[[2-[(4-cyanophenyl)amino]-5-ethenyl-4-pyrimidinyl]amino]-3,5-di	C22H18N6	366.42	A/B	-8.7
3174	C(OCCO)n1c2c(nc1)cncn2	1025875-79-9	Benzonitrile, 4-[[6-bromo-2-[(4-cyanophenyl)amino]-4-pyrimidinyl]amino]-3,5-dime	C20H15BrN6	419.28	A/B	-8.7
3175	Cc1c2c(n(cn2)c2c(C)cc(/C=C/C#N)cc2C)nc(Nc2ccc(C#N)cc2)n1	1025963-35-2	1,3,4-Oxadiazole-2-carboxamide, N-[1-[(4Z)-4-[[[(4-fluorophenyl)methyl]amino]hyc	C20H21FN6O5	444.42	A/B	-8.7
3176	O=C1c2c(N(c3c(N1C)c(C)cc(F)n3)C1CC1)ncc(CCOc1c3c(NS(=O)(=O)C3)ccc1)c2	1026294-86-9	Benzonitrile, 4-[[4-[[4-[(1E)-2-cyanoethenyl]-2,6-dimethylphenyl]amino]-5-[(3-th	C27H23N7S	477.58	A/B	-8.7
3177	[C@@H](Cc1ccccc1)(NC(=O)N1CCN(C)CC1)C(=O)N[C@@H](CCc1ccccc1)CC	1027582-36-0	2-Naphthalenesulfonic acid, 3,8-diamino-5-[(1E)-2-[4,Ä≤-[(1E)-2-(4-amino-6-sulfc	C32H25N7O6S2	667.71	A/B	-8.7
3178	O(c1nc(Nc2ccc(C#N)cc2)nc(N)c1Br)c1c(C)cc(C#N)cc1C.Cl	1030633-38-5	Benzonitrile, 4-[[6-amino-5-bromo-2-[(4-cyanophenyl)amino]-4-pyrimidinyl]oxy]-3,	C20H15BrN6O.BrH		A/B	-8.7
3179	O(c1nc(Nc2ccc(C#N)cc2)nc(N)c1Br)c1c(C)cc(C#N)cc1C.C(=C\C(=O)O)\C(=O)O	1030633-39-6	Benzonitrile, 4-[[6-amino-5-bromo-2-[(4-cyanophenyl)amino]-4-pyrimidinyl]oxy]-3,	C20H15BrN6O.ClH		A/B	-8.7
3180	C(C[C@H](NC(=O)c1ccccc1)c1ccccc1)(N1[C@@H]2C[C@@H](C[C@H]1CC2)n1c(C(C)C)nnc1C([2H])([2H])[2H])([2H])	1033698-65-5	Benzamide, N-[(1S)-3-[(3-exo)-3-[3-(methyl-d3)-5-(1-methylethyl)-4H-1,2,4-triazc	C29H34D3N5O		A/B	-8.7
3181	N(c1c(C)cc(/C=C/C#N)cc1C)c1nc(Nc2ccc(C#N)cc2)nc(C(=O)NCc2ccncc2)c1	1037444-87-3	4-Pyrimidinecarboxamide, 6-[[4-[(1E)-2-cyanoethenyl]-2,6-dimethylphenyl]amino]-N	C26H22N8O	462.51	A/B	-8.7
3182	N(c1c(C)cc(/C=C/C#N)cc1C)c1nc(Nc2ccc(C#N)cc2)nc(C(=O)NCc2ccco2)c1	1037444-89-5	4-Pyrimidinecarboxamide, 6-[[4-[(1E)-2-cyanoethenyl]-2,6-dimethylphenyl]amino]-2	C29H24N8O	500.55	A/B	-8.7
3183	N(c1c(C(=O)NCC2CCCO2)c(COC)nc(Nc2ccc(C#N)cc2)n1)c1c(C)cc(/C=C/C#N)cc1C	1037452-05-3	5-Pyrimidinecarboxamide, 4-[[4-[(1E)-2-cyanoethenyl]-2,6-dimethylphenyl]amino]-2	C31H28N8O2	544.61	A/B	-8.7
3184	C(OC[C@H](Cn1cc(c(NC(=O)c2ccccc2)nc1=O)c1ccc(Cl)cc1)OCP(=O)(OC(C)C)OC(C)C)(c1ccccc1)(c1ccccc1)c1cccc	1041364-75-3	Phosphonic acid, P-[[(1S)-1-[[4-(benzoylamino)-5-(4-nitrophenyl)-2-oxo-1(2H)-pyr	C46H47N4O9P	830.86	A/B	-8.7
3185	N(Cc1csc(C)n1)C12CC3CC(C2)CC(C1)C3	104281-93-8	Furo[3,Ä≤,4,Ä≤:6,7]naphtho[2,3-d]-1,3-dioxole-6,8-dione, 5-(3,4-dimethoxyphenyl	C21H18O7	382.36	A/B	-8.7
3186	N(C(=O)[C@@H](C[C@@H](CN1[C@H](C(=O)NC(C)(C)C)CN(Cc2cccnc2)CC1)OC(=O)CCCCCNC(=O)[C@@H](NC(=O)OC(C)(C	1050631-45-2	D-erythro-Pentonamide, 2,3,5-trideoxy-N-[(1S,2R)-2-[[6-[[(2S)-2-[[(1,1-dimethyle	C52H75N7O8	926.19	A/B	-8.7
3187	N(=N\c1cc(S(=O)(=O)O)c(C=Cc2c(S(=O)(=O)O)cc(/N=N/c3c4c(cc(S(=O)(=O)O)c3N)cc(S(=O)(=O)O)cc4)cc2)cc1)/	1053056-26-0	Not Yet Assigned	C34H26N6O12S4	838.86	A/B	-8.7
3188	Nc1c2c(cc(S(=O)(=O)O)cc2)ccc1/N=N/c1cc(S(=O)(=O)O)c(/C=C/c2c(S(=O)(=O)O)cc(/N=N/c3c(N)c4c(cc3)cc(S(=	1053056-45-3	Not Yet Assigned	C34H26N6O12S4	838.86	A/B	-8.7
3189	[C@@H]([C@@H](CN(S(=O)(=O)c1ccc(N)cc1)CC(C)C)OP(=O)(O)O)(Cc1ccccc1)NC(=O)OC1COCOC1	1054636-47-3	Not Yet Assigned	C52H69N13O11	1052.18	A/B	-8.7
3190	C(OCCOP(=O)(N[C@@H](Cc1ccccc1)C(=O)OCc1ccccc1)Oc1ccccc1)n1c2c(nc1)c(=O)nc(N)[nH]2	1059581-02-0	5,8-Dioxa-3-aza-4-phosphanonanoic acid, 9-(2-amino-1,6-dihydro-6-oxo-9H-purin-9-	C22H25N6O7P	516.44	A/B	-8.7
3191	C=c1c2=NC(=O)c3c(N(c2ncc1)C1CC1)nccc3	106104-89-6	2-Naphthalenesulfonic acid, 8-[2-(4-amino-2-methylphenyl)diazenyl]-	C17H15N3O3S	341.38	A/B	-8.7
3192	N(C(=O)CCCl)c1c2c(c(S(=O)(=O)O)cc1N=Nc1c(C)cc(cc1)c1cc(C)c(N=Nc3c(NC(=O)CCCl)c4c(c(S(=O)(=O)O)c3)ccc	1061719-17-2	Uridine 5,Ä≤-(trihydrogen diphosphate), 5-(4-methoxyphenyl)-, P,Ä≤-Œ±-D-glucopy	C22H30N2O18P2	672.42	A/B	-8.7
3193	N(c1c(C)cc(C(CC)O)cc1C)c1nc(Nc2ccc(C#N)cc2)ncc1	1064399-73-0	Benzonitrile, 4-[[4-[[4-[(1Z)-2-cyano-1-methyl-1-propen-1-yl]-2,6-dimethylphenyl	C24H22N6	394.47	A/B	-8.7
3194	OC1C(c2c(Cl)cccc2)N1C[C@@H](C(=O)NC(C)(C)C)N(C[C@H](C[C@H](C(=O)N[C@H]2c3c(C[C@H]2O)cccc3)Cc2ccccc2)	1064475-81-5	Not Yet Assigned	C41H56N6O5	712.92	A/B	-8.7
3195	C(NC(=O)c1cnco1)(C)(C)c1nc(C(=O)NCc2ccc(F)cc2)c(O)c(=O)n1C	1064707-04-5	2-Pyrimidinecarboxamide, N-[1-[4-[[[(4-fluorophenyl)methyl]amino]carbonyl]-1,6-c	C21H21FN6O4	440.43	A/B	-8.7
3196	O(C[C@H]1O[C@H](C=C1)n1c(=O)[nH]c(=O)c(C)c1)P1(=O)Oc2c(cc(CCC(=O)N[C@@H](C(=O)OC)C)cc2)CO1	1073242-61-1	Glycine, N-[3-[2-(2,Ä≤,3,Ä≤-didehydro-3,Ä≤-deoxythymidin-5,Ä≤-O-yl)-2-oxido-4H-	C29H30N3O10P	611.54	A/B	-8.7
3197	O(C[C@H]1O[C@H](C=C1)n1c(=O)[nH]c(=O)c(C)c1)P1(=O)Oc2c(cc(CCC(=O)N3[C@H](C(=O)OCc4ccccc4)CCC3)cc2)CO	1073242-67-7	L-Proline, 1-[3-[2-(2,Ä≤,3,Ä≤-didehydro-3,Ä≤-deoxythymidin-5,Ä≤-O-yl)-2-oxido-4	C26H30N3O10P	575.5	A/B	-8.7
3198	[C@@H](NC(=O)N1CCN(C)CC1)(C(=O)N[C@H](C=CS(=O)(=O)c1ccccc1)CCc1ccccc1)CCONC(=N)N	1076088-93-1	1-Piperazinecarboxamide, N-[(1S)-1-[[[(aminoiminomethyl)amino]oxy]methyl]-2-oxo-	C27H37N7O5S	571.69	A/B	-8.7
3199	C(=O)([C@@H](NC(=O)OC)C(C)C)N1[C@@H](CCC1)c1[nH]c(cn1)c1ccc(cc1)c1ccc(cc1)c1[nH]c([C@@H](NC(=O)[C@@H	1087346-73-3	Carbamic acid, N-[(1S)-2-[(2S)-2-[5-[4,Ä≤-[2-[(1R)-1-[[(2S)-2-[(methoxycarbonyl)	C34H40N8O6	656.73	A/B	-8.7
3200	C(C(C=CC(=O)CCc1ccccc1)O)c1cc(OC)c(O)cc1	1092440-51-1	2-Naphthalenesulfonic acid, 6-amino-5-[2-[2,Ä≤,3-dimethyl-4,Ä≤-[2-(6-sulfo-1-nap	C34H27N5O6S2	665.74	A/B	-8.7
3201	C(NC(=O)c1nnc(C)o1)(C)(C)c1nc(C(=O)NCc2ccc(F)cc2)c(O)c(=O)n1C([2H])([2H])[2H]	1100750-82-0	4-Pyrimidinecarboxamide, N-[(4-fluorophenyl)methyl]-1,6-dihydro-5-hydroxy-1-meth	C20H15D6FN6O5		A/B	-8.7
3202	C(NC(=O)c1nnc(C)o1)(C)(C)c1nc(C(=O)NCc2c(c(F)c(c2[2H])[2H])[2H])[2H])c(O)c(=O)n1C([2H])([2H])[2H]	1100750-96-6	4-Pyrimidinecarboxamide, N-[(4-fluorophenyl-2,3,5,6-d4)methyl]-1,6-dihydro-5-hyc	C20H11D10FN6O5		A/B	-8.7
3203	C(NC(=O)c1nnc(C([2H])([2H])[2H])o1)(C([2H])([2H])[2H])(C([2H])([2H])[2H])c1nc(C(=O)NCc2c(c(F)c(c2[	1100750-98-8	4-Pyrimidinecarboxamide, N-[(4-fluorophenyl-2,3,5,6-d4)methyl-d2]-1,6-dihydro-5-	C20H15D6FN6O5		A/B	-8.7
3204	C(NC(=O)c1nnc(C([2H])([2H])[2H])o1)(C([2H])([2H])[2H])(C([2H])([2H])[2H])c1nc(C(=O)NCc2c(c(F)c(c2[	1100751-04-9	4-Pyrimidinecarboxamide, N-[(4-fluorophenyl-2,3,5,6-d4)methyl-d2]-1,6-dihydro-5-	C20H12D9FN6O5		A/B	-8.7
3205	C(COCO)N1c2c(C(=NNC(=S)N)C1=O)cccc2	112362-61-5	Thymidine, Œ±,Œ±,Œ±-trifluoro-5,Ä≤-O-(triphenylmethyl)-, 3,Ä≤-benzenecarbothioa	C36H29F3N2O5S	658.69	A/B	-8.7
3206	C(=O)([C@@H](NC(=O)OC)C)N1[C@@H](CCC1)c1[nH]c(c(C)n1)c1ccc(cc1)c1ccc(cc1)c1[nH]c(nc1)[C@H]1N(C(=O)[C	1129636-11-8	Carbamic acid, N-[(1R)-2-[(2S)-2-[5-[4,Ä≤-[2-[(2S)-1-[(2R)-2-[(methoxycarbonyl)a	C47H48N8O6	820.93	A/B	-8.7
3207	C(=O)(N[C@H](C(=O)C(=O)NC1CC1)CCC)[C@@H]1[C@@H]2[C@H](CN1C(=O)[C@@H](NC(=O)[C@@H](NC(=O)c1cnccn1)C1(	1134176-07-0	Cyclopenta[c]pyrrole-1-carboxamide, (2S)-2-cyclohexyl-N-(2-pyrazinyl-3,5,6-d3-ca	C36H50D3N7O6		A/B	-8.7
3208	C(=O)(N[C@H](C(=O)C(=O)NC1(C(C1([2H])[2H])([2H])[2H])[2H])CCC)[C@@H]1[C@@H]2[C@H](CN1C(=O)[C@@H](NC(	1134176-15-0	Cyclopenta[c]pyrrole-1-carboxamide, (2S)-2-cyclohexyl-N-(2-pyrazinylcarbonyl)gly	C36H45D8N7O6		A/B	-8.7
3209	C(Cc1ccccc1)[C@]1(CCC)CC(=C([C@H](CC)[13c]2[13cH][13c](NS(=O)(=O)c3ccc(C(F)(F)F)cn3)[13cH][13cH][13c	1141509-98-9	2-Pyridinesulfonamide, N-[3-[(1R)-1-[(6R)-5,6-dihydro-4-hydroxy-2-oxo-6-(2-pheny	C31H33F3N2O5S		A/B	-8.7
3210	O(c1nc(Nc2c(c(c(C#N)c(c2[2H])[2H])[2H])[2H])nc(N)c1Br)c1c(C([2H])([2H])[2H])cc(C#N)cc1C([2H])([2H])[	1142095-68-8	Benzonitrile-2,6-d2, 4-[[6-amino-5-bromo-2-[(4-cyanophenyl-3,5-d2)amino]-4-pyrim	C20H5BrD10N6O		A/B	-8.7
3211	O(c1nc(Nc2c(c(c(C#N)c(c2[2H])[2H])[2H])[2H])nc(N)c1Br)c1c(C)c(c(C#N)c(c1C)[2H])[2H]	1142095-80-4	Benzonitrile-2,6-d2, 4-[[4-amino-5-bromo-6-[4-cyano-2,6-di(methyl-d3)phenoxy]-2-	C20H7BrD8N6O		A/B	-8.7
3212	O(c1nc(Nc2cc(cc(C#N)cc2[2H])[2H])nc(N)c1Br)c1c(C)c(c(C#N)c(c1C)[2H])[2H]	1142095-83-7	Benzonitrile-2,3,5,6-d4, 4-[[4-amino-5-bromo-6-(4-cyano-2,6-dimethylphenoxy-3,5-	C20H9BrD6N6O		A/B	-8.7
3213	O(c1nc(Nc2ccc(C#N)cc2)nc(Cl)c1Br)c1c(C([2H])([2H])[2H])c(c(C#N)c(c1C([2H])([2H])[2H])[2H])[2H]	1142096-06-7	Benzonitrile, 4-[[6-amino-5-bromo-2-[(4-cyanophenyl)amino]-4-pyrimidinyl]oxy]-3,	C20H9BrD6N6O		A/B	-8.7
3214	C(C)(C)[Si]1(C(C)C)O[Si](C(C)C)(C(C)C)OC[C@@H]2[C@@H](O1)[C@](O)([C@@H](O2)n1c2c(nc1)c(N)ncn2)[2H]	1142096-20-5	Benzonitrile-2,6-d2, 4-[[5-bromo-6-chloro-2-[(4-cyanophenyl)amino]-4-pyrimidinyl	C20H5BrClD8N5O		A/B	-8.7
3215	[C@H](Cc1ccccc1)(C[C@@H]([C@H](Cc1ccccc1)NC(=O)OCc1cncs1)O)NC(=O)[C@@H](NC(=O)N(Cc1nc(C(=C)C)sc1)C)C	1156504-12-9	2,7,10,12-Tetraazatridecanoic acid, 4-hydroxy-9-(1-methylethyl)-13-[2-(1-methyle	C36H46N6O5S2	706.92	A/B	-8.7
3216	Cc1n(c2nc(nc(n2)N2CCOCC2)c2cc(Cl)ccc2)ccn1	1174190-85-2	2-Pyrimidinamine, 5-[4-(2-methyl-1H-benzimidazol-1-yl)-6-(4-morpholinyl)-1,3,5-t	C19H19N9O	389.41	A/B	-8.7
3217	c1(nc(nc(n1)n1ccnc1)N1CCOCC1)c1ccc2c(cc1)[nH]cc2	1174190-99-8	1H-Indole, 6-[4-(1H-imidazol-1-yl)-6-(4-morpholinyl)-1,3,5-triazin-2-yl]-	C18H17N7O	347.37	A/B	-8.7
3218	Cc1n(c2nc(nc(n2)N2CCOCC2)c2cnc(NC)nc2)nc(C)c1	1174191-01-5	1H-Indole, 5-[4-(1H-imidazol-1-yl)-6-(4-morpholinyl)-1,3,5-triazin-2-yl]-	C18H17N7O	347.37	A/B	-8.7
3219	C(F)(F)(F)c1nn(c2nc(nc(n2)N2CCOCC2)c2ccc(N)nc2)cc1	1174191-20-8	2-Pyridinamine, 5-[4-(3-methyl-1H-pyrazol-1-yl)-6-(4-morpholinyl)-1,3,5-triazin-	C16H18N8O	338.37	A/B	-8.7
3220	Fc1c2c(c(NC(=O)OCc3ccccc3)c(F)c1N1CC(C)N(OC(=O)Cc3ccccc3)C(C)C1)c(=O)c(C(=O)OC[C@@H]1[C@@H](N=[N+]=[	1174398-23-2	Thymidine, 3,Ä≤-azido-3,Ä≤-deoxy-, 5,Ä≤-[1-cyclopropyl-6-fluoro-1,4-dihydro-8-m	C37H39FN8O9		A/B	-8.7
3221	N(C)(C)C1(C)c2c(-c3c1cc(cc3)c1[nH]c(nc1)[C@H]1N(C(=O)[C@@H](NC(=O)OC)C(C)C)CCC1)ccc(c2)c1[nH]c(nc1)[	1179150-25-4	Carbamic acid, N,N,Ä≤-[(5,7-dihydrodibenz[c,e]oxepin-3,9-diyl)bis[1H-imidazole-5	C46H56N8O9	864.98	A/B	-8.7
3222	C(=O)([C@@H](NC(=O)OC)C1CC1)N1[C@H](c2[nH]c(c3cc4c(c5c(o4)cc(cc5)c4[nH]c(nc4)[C@H]4N(C(=O)[C@@H](NC(	1179150-61-8	Carbamic acid, N,N,Ä≤-[3,7-dibenzofurandiylbis[1H-imidazole-5,2-diyl-(2S)-2,1-py	C40H48N8O7	752.86	A/B	-8.7
3223	C(=O)([C@@H](NC(=O)OC)C(C)C)N1[C@H]2[C@H](C2)C[C@H]1c1[nH]c2-c3c(CCc2n1)cc(cc3)c1cc(F)c(cc1)c1[nH]c(	1180670-05-6	Carbamic acid, N-[(1S)-1-[[(2S)-2-[5-[4-[4,5-dihydro-2-[(2S)-1-[(2S)-2-[(methoxy	C42H51FN8O6	782.9	A/B	-8.7
3224	C(OCCOP(=O)(Oc1ccccc1)N[C@H](C(=O)OCc1ccccc1)C(C)C)n1c2c(nc1)c(=O)nc(N)[nH]2	1184942-52-6	5,8-Dioxa-3-aza-4-phosphanonanoic acid, 9-(2-amino-1,6-dihydro-6-oxo-9H-purin-9-	C25H29N6O7P	556.51	A/B	-8.7
3225	N(C(=O)c1nnc(C)o1)C12c3n(CC(CC1)CC2)c(=O)c(O)c(C(=O)NCc1ccc(F)cc1)n3	1188424-53-4	6,9-Ethano-4H-pyrido[1,2-a]pyrimidine-2-carboxamide, N-[(4-fluorophenyl)methyl]-	C22H21FN6O5	468.44	A/B	-8.7
3226	C(C(OCP(=O)(OCC)OCC)([2H])[2H])(n1c2c(nc1)c(N)ncn2)([2H])[2H]	118989-49-4	Hydrazinecarbothioamide, 2-[5-chloro-1-[[4-(4-chlorophenyl)-1-piperazinyl]methyl	C26H30Cl2N6OS	545.53	A/B	-8.7
3227	O(C(=O)N[C@@H](Cc1ccccc1)[C@@H](CN(S(=O)(=O)c1c(OCCCCC=C)cc(OC)cc1)CC=C)O)[C@@H]1[C@H]2[C@@H](OC1)OC	1190374-97-0	Uridine, 2,Ä≤-deoxy-5-(3,7-dimethyl-1,9-dioxo-1H,9H,10H-dipyrano[4,3-b:3,Ä≤,4,Ä	C26H24N2O12	556.47	A/B	-8.7
3228	N(=C(NC(=O)OC(C)(C)C)NC(=O)OC(C)(C)C)[C@@H]1[C@@H](NC(=O)C)[C@H]([C@@H]([C@@H](COC(=O)CC[C@@H](Cc2cc	1192538-89-8	Not Yet Assigned	C38H57N5O14	807.88	A/B	-8.7
3229	O(COC(=O)OCCN1CCOCC1)c1c2n(C[C@H]3N(C2=O)[C@H](C)CCO3)cc(C(=O)NCc2c(F)cc(F)cc2)c1=O	1206102-01-3	Carbonic acid, [[(4R,12aS)-9-[[[(2,4-difluorophenyl)methyl]amino]carbonyl]-3,4,6	C24H26F2N3O12P	617.45	A/B	-8.7
3230	O[C@H]1[C@@H](O[C@H](COP(=O)(OP(=O)(OP(=O)(O)O)O)O)[C@H]1O)n1cc(/C=C/CNC(=O)CNC(=O)[C@@H](NC(=O)OCc2	1206813-87-7	Nitrous acid, (2S)-2-[[(2S)-4-amino-1,4-dioxo-2-[(2-quinolinylcarbonyl)amino]but	C38H49N7O6	699.84	A/B	-8.7
3231	C(C[C@H](NC(=O)C1CCC(F)(F)CC1)c1ccc(O)cc1)N1C2CC(CC1CC2)n1c(C(C([2H])([2H])[2H])C([2H])([2H])[2H])nn	1217503-37-1	L-Alanine, N-[9-[2-deoxy-5-O-[hydroxy[[hydroxy(phosphonooxy)phosphinyl]oxy]phosp	C13H20N5O15P3	579.24	A/B	-8.7
3232	N(C(=O)[C@H](Cc1ccccc1)C[C@@H](CN1[C@H](C(=O)NC(C)(C)C)CN(Cc2cccnc2)CC1)O)[C@H]1c2c(C[C@H]1O)cccc2.S	1217525-37-5	Not Yet Assigned	C36H47N5O4	613.79	A/B	-8.7
3233	C([C@H]([C@H](Cc1ccccc1)NC(=O)[C@@H](NC(=O)c1cc2c(nc1)cccc2)CC(=O)N)O)N1[C@H](C(=O)NC(C)(C)C)C[C@H]2	1222083-97-7	1-Piperazinebutanoic acid, 4-[2-[1-(4-chlorobenzoyl)-5-methoxy-2-methyl-1H-indol	C49H48ClN3O13	922.37	A/B	-8.7
3234	N(C(=O)[C@H](Cc1ccccc1)C[C@@H](CN1[C@H](C(=O)NC(C)(C)C)CN(Cc2cccnc2)CC1)O)(N)C1c2c(C[C@H]1O)cccc2	1223069-42-8	Not Yet Assigned	C36H45F2N5O4	649.77	A/B	-8.7
3235	C(=O)(N[C@H](C(=O)C(=O)NC1CC1)CCC)[C@H]1N(C(=O)[C@H](NC(=O)[C@H](NC(O)c2cnccn2)C2CCCCC2)C(C)(C)C)CC[	1223096-77-2	Not Yet Assigned	C37H48N4O4S	644.87	A/B	-8.7
3236	[C@](C(=O)N[C@@H](C(c1c(c(c(c(c1[2H])[2H])[2H])[2H])[2H])([2H])[2H])C[C@@H]([C@H](Cc1ccccc1)NC(=O)CO	1224729-06-9	1(2H)-Pyrimidineacetamide-Œ±-d, N-[(1S,3S,4S)-4-[[2-[2,6-di(methyl-d3)phenoxy-3,	C37H16D32N4O5		A/B	-8.7
3237	[C@@H](C(=O)N[C@@H](C(c1c(c(c(c(c1[2H])[2H])[2H])[2H])[2H])([2H])[2H])C[C@@H]([C@H](Cc1ccccc1)NC(=O)	1224729-22-9	1(2H)-Pyrimidineacetamide, N-[(1S,3S,4S)-4-[[2-[2,6-di(methyl-d3)phenoxy-3,4,5-c	C37H22D26N4O5		A/B	-8.7
3238	[C@](C(=O)N[C@@H](Cc1ccccc1)C[C@@H]([C@H](Cc1ccccc1)NC(=O)COc1c(C([2H])([2H])[2H])c(c(c(c1C([2H])([2	1224729-30-9	1(2H)-Pyrimidineacetamide-Œ±-d, N-[(1S,3S,4S)-4-[[2-[2,6-di(methyl-d3)phenoxy-3,	C37H23D25N4O5		A/B	-8.7
3239	c1(nc(nc(n1)N1CCOCC1)N1CCOCC1)c1cccnc1	1225037-33-1	Benzonitrile, 3-(4,6-di-4-morpholinyl-1,3,5-triazin-2-yl)-	C18H20N6O2	352.39	A/B	-8.7
3240	c1(nc(nc(n1)N1CCOCC1)N1CCOCC1)c1c2c(ccc1)[nH]cc2	1225037-88-6	1H-Benzimidazole, 7-(4,6-di-4-morpholinyl-1,3,5-triazin-2-yl)-	C18H21N7O2	367.4	A/B	-8.7
3241	O[C@H]1[C@@H](O[C@H](COP(=O)(N[C@H](C(=O)O)C)O)[C@H]1O)n1nc(C(=O)N)nc1	1228377-66-9	Glycine, N-[hydroxy(1-naphthalenyloxy)phosphinyl]-, phenylmethyl ester, 5,Ä≤-est	C27H28N5O9P	597.51	A/B	-8.7
3242	C(=O)([C@@](NC(=O)OC)(C(C([2H])([2H])[2H])(C([2H])([2H])[2H])[2H])[2H])N1[C@H](c2[nH]c(C34CCC(CC3)(C	1228671-78-0	Carbamic acid, N-[(1R)-2-[(2S)-2-[5-[4-[4-[2-[(2S)-1-[(2S)-2-[(methoxycarbonyl)a	C40H54N8O6	742.91	A/B	-8.7
3243	C(=O)([C@@H](NC(=O)OC)C(C)C)N1[C@@H](CCC1)c1[nH]c(cn1)c1ccc(C#Cc2[nH]c(nc2)[C@H]2N(C(=O)[C@@H](NC(=O	1228964-54-2	Carbamic acid, N-[(1R)-2-[(2S)-2-[5-[4-[2-[2-[(2S)-1-[(2R)-2-[(methoxycarbonyl)a	C42H42N8O6	754.83	A/B	-8.7
3244	C(=O)([C@@H](NC(=O)OC)C(C)C)N1[C@@H](CCC1)c1[nH]c(cn1)c1ccc(cc1)c1ccc(C#Cc2[nH]c(nc2)[C@H]2N(C(=O)[C	1228966-44-6	Carbamic acid, N-[(1R)-2-[(2S)-2-[5-[2-[4,Ä≤-[2-[(2S)-1-[(2R)-2-(dimethylamino)-	C48H48N8O4	800.95	A/B	-8.7
3245	C(NC(=O)[C@H](Cc1ccccc1)N)(C(=O)NCC(=O)N[C@@H](Cc1ccccc1)C(=O)N[C@@H](CCSC)C(=O)O)C12CC3CC(C2)CC(C1)	1229574-77-9	L-Phenylalanine, L-phenylalanyl-2-tricyclo[3.3.1.13,7]dec-1-ylglycylglycyl-L-phe	C41H49N5O6	707.86	A/B	-8.7
3246	C(CC)(CC)c1nc(CN(C(=O)N[C@H](C(=O)N[C@@H](Cc2ccccc2)C[C@@H]([C@H](Cc2ccccc2)NC(=O)OCc2cnco2)O)C(C)C)	1236406-32-8	2,7,10,12-Tetraazatridecanoic acid, 4-hydroxy-12-methyl-9-(1-methylethyl)-8,11-c	C40H46N6O5S2	754.96	A/B	-8.7
3247	[C@H](Cc1ccccc1)(C[C@@H]([C@H](Cc1ccccc1)NC(=O)OCc1ccns1)O)NC(=O)[C@@H](NC(=O)N(Cc1nc(OC(C)C)sc1)C)C	1236406-35-1	Not Yet Assigned	C41H48N6O7S	768.92	A/B	-8.7
3248	[C@H](Cc1ccccc1)(C[C@@H]([C@H](Cc1ccccc1)NC(=O)OCc1ccns1)O)NC(=O)[C@@H](NC(=O)N(Cc1nc(OC)sc1)C)C(C)C	1236406-66-8	2,7,10,12-Tetraazatridecanoic acid, 13-[2-(1,2-dimethyl-1-propen-1-yl)-4-thiazol	C39H50N6O5S2	746.98	A/B	-8.7
3249	C(CC)(CC)c1nc(CN(C(=O)N[C@H](C(=O)N[C@@H](Cc2ccccc2)C[C@@H]([C@H](Cc2ccccc2)NC(=O)OCc2ccns2)O)C(C)C)	1236407-19-4	Not Yet Assigned	C36H46N6O6S	690.85	A/B	-8.7
3250	[C@H](Cc1ccccc1)(C[C@@H]([C@H](Cc1ccccc1)NC(=O)OCc1cnco1)O)NC(=O)[C@@H](NC(=O)N(Cc1nc(CC=C)sc1)C)C(C	1236407-29-6	2,7,10,12-Tetraazatridecanoic acid, 13-(2-cyclohexyl-4-thiazolyl)-4-hydroxy-12-m	C40H52N6O5S2	761.01	A/B	-8.7
3251	[C@H](Cc1ccccc1)([C@H]([C@@H]([C@H](Cc1ccccc1)NC(=O)OCc1ccno1)O)O)NC(=O)[C@@H](NC(=O)N(Cc1nc(C(C)C)s	1236407-97-8	2,7,10,12-Tetraazatridecanoic acid, 13-[2-(1-cyclohexen-1-yl)-4-thiazolyl]-4-hyc	C40H50N6O5S2	758.99	A/B	-8.7
3252	[C@H](Cc1ccccc1)(C[C@@H]([C@H](Cc1ccccc1)NC(=O)OCc1cnco1)O)NC(=O)[C@@H](NC(=O)N(Cc1nc(CC(C)C)sc1)C)C	1236409-01-0	2,7,10,12-Tetraazatridecanoic acid, 13-(2-cyclopentyl-4-thiazolyl)-4-hydroxy-12-	C39H50N6O6S	730.92	A/B	-8.7
3253	N(CC(=O)[C@H](Cc1ccccc1)C[C@@H](CN1[C@H](C(=O)NC(C)(C)C)CN(Cc2ccccn2)CC1)O)[C@H]1c2c(C[C@H]1O)cccc2	1237988-91-8	Not Yet Assigned	C38H51N5O4	641.84	A/B	-8.7
3254	O=c1n(cc([C@H](CI)O)c(=O)[nH]1)[C@@H]1O[C@H](CO)[C@@H](O)C1	1238382-72-3	Androsta-5,16-dien-3-ol, 17-(1H-benzimidazol-1-yl)-, 3-(2-phosphonoacetate), (3Œ	C28H35N2O5P	510.56	A/B	-8.7
3255	C(O)[C@]12[C@@H]([C@@H]3[C@](C)([C@@]4(C)[C@H](CC3)[C@]3(C)[C@@H](CC4)C(C)(C)[C@@H](O)CC3)CC1)[C@H](	1239885-21-2	4-Pyridinecarboxylic acid, 2-[4-(4-morpholinyl)-6-(phenylamino)-1,3,5-triazin-2-	C19H20N8O2	392.41	A/B	-8.7
3256	C(F)(F)c1n(c2c(n1)cccc2)c1nc(nc(NC2Cc3c(CC2)nc[nH]3)n1)N1CCOCC1	1240023-56-6	1,4-Cyclohexanediamine, N1-[4-[2-(difluoromethyl)-1H-benzimidazol-1-yl]-6-(4-mor	C21H26F2N8O	444.48	A/B	-8.7
3257	C(F)(F)c1n(c2c(n1)cccc2)c1nc(nc(N[C@H]2CC[C@H](NS(=O)(=O)C)CC2)n1)N1CCOCC1	1240023-59-9	1H-Benzimidazol-6-amine, N-[4-[2-(difluoromethyl)-1H-benzimidazol-1-yl]-6-(4-mor	C22H23F2N9O	467.47	A/B	-8.7
3258	C(F)(F)c1n(c2c(n1)cccc2)c1nc(nc(N[C@H]2CC[C@H](NC(=O)CO)CC2)n1)N1CCOCC1	1240029-12-2	Propanamide, N-[trans-4-[[4-[2-(difluoromethyl)-1H-benzimidazol-1-yl]-6-(4-morph	C25H32F2N8O2	514.57	A/B	-8.7
3259	C(F)(F)c1n(c2c(n1)cccc2)c1nc(nc(N[C@H]2CC[C@H](NCCOC)CC2)n1)N1CCOCC1	1240030-37-8	1,4-Cyclohexanediamine, N1-[4-[2-(difluoromethyl)-1H-benzimidazol-1-yl]-6-(4-mor	C25H34F2N8O	500.59	A/B	-8.7
3260	C(F)(F)c1n(c2c(n1)cccc2)c1nc(nc(N[C@H]2CC[C@H](NC(CCO)C)CC2)n1)N1CCOCC1	1240030-51-6	1-Propanol, 2-[[trans-4-[[4-[2-(difluoromethyl)-1H-benzimidazol-1-yl]-6-(4-morph	C24H32F2N8O2	502.56	A/B	-8.7
3261	C(COCP(=O)([O-])O)n1c2c(nc1)cnc(N)n2	1240306-16-4	2-Naphthalenesulfonic acid, 5-[(1E)-2-(3-hydroxy-2-naphthalenyl)diazenyl]-	C20H14N2O4S	378.4	A/B	-8.7
3262	C(=O)([C@@H](NC(=O)OC)C(C)(C)O)N1[C@H](c2[nH]c(c3cc4c(-c5c(C4)cc(cc5)c4[nH]c(nc4)[C@H]4N(C(=O)[C@@H]	1240880-66-3	Carbamic acid, N,N,Ä≤-[9H-fluorene-2,7-diylbis[1H-imidazole-5,2-diyl-(2S)-2,1-py	C41H46N8O6	746.85	A/B	-8.7
3263	C(=O)([C@@H](Cc1ccccc1)NC(=O)OC)N1[C@@H](CCC1)c1[nH]c(cn1)[C@H]1CC[C@@H](CC1)c1ccc(cc1)c1[nH]c(nc1)[	1240888-13-4	Carbamic acid, N-[(1S)-2-[(2S)-2-[5-[4-[trans-4-[2-[(2S)-1-[(2S)-2-[(methoxycarb	C46H54N10O6	842.98	A/B	-8.7
3264	C(=O)([C@H](NC(=O)OC)c1ccccc1)N1[C@@H](CCC1)c1[nH]c(cn1)c1ccc(ccc1)[C@@H]1CC[C@H](CC1)c1[nH]c(nc1)[C@	1240891-19-3	Carbamic acid, N-[(1R)-2-[(2S)-2-[5-[4-[cis-4-[2-[(2S)-1-[(2R)-2-[(methoxycarbor	C46H52N8O6	812.96	A/B	-8.7
3265	C(=O)([C@H](NC(=O)OC)c1ccccc1)N1[C@@H](CCC1)c1[nH]c(cn1)c1cccc(C[C@H]2[C@H](Cc3[nH]c(nc3)[C@H]3N(C(=O	1240891-89-7	Carbamic acid, N-[(1R)-2-[(2S)-2-[5-[4-[[(1S,2R)-2-[[2-[(2S)-1-[(2R)-2-[(methoxy	C48H56N8O6	841.01	A/B	-8.7
3266	C(c1c2c(N(c3c(C(=O)N2)cccn3)C2CC2)ncc1)n1cc(C[C@@H](C(=O)N2[C@H](C(=O)N[C@H](C(=O)N[C@@H](Cc3ccc(O)c	1246392-09-5	L-Arginine, L-alanyl-2-[(11-cyclopropyl-6,11-dihydro-6-oxo-5H-dipyrido[3,2-b:2,Ä	C46H60N14O8	937.06	A/B	-8.7
3267	C(NCCCC[C@@H](C(=O)N[C@H](C(=O)N[C@H](C(=O)N[C@H](C(=O)N[C@H](C(=O)N[C@@H](CCCCN)C(=O)O)C)CO)CO)C)NC	1246392-10-8	L-Arginine, L-arginyl-1-[(11-cyclopropyl-6,11-dihydro-6-oxo-5H-dipyrido[3,2-b:2,	C82H122N24O18	1732	A/B	-8.7
3268	C(c1c2c(N(c3c(C(=O)N2)cccn3)C2CC2)ncc1)n1cc(C[C@@H](C(=O)N[C@H](C(=O)NCC(=O)N[C@H](C(=O)N[C@@H](Cc2c	1246392-12-0	L-Arginine, L-leucyl-L-leucyl-L-valyl-L-valyl-L-tyrosyl-L-prolyl-2-[(11-cyclopro	C77H107N19O15	1538.79	A/B	-8.7
3269	C(=O)([C@@H](NC(=O)OC)C(C)C)N1[C@H](c2[nH]c(c3c4cc(-c5cc(c(cc5)c5[nH]c(nc5)[C@H]5N(C(=O)[C@@H](NC(=O	1246559-82-9	Carbamic acid, N,N,Ä≤-[(6,7,9,10,12,13-hexahydrodibenzo[k,m][1,4,7,10]tetraoxacy	C46H60N8O10	885.02	A/B	-8.7
3270	O=[13c]1[15n](C2OC(CO)C=C2)cc(F)c(N)[15n]1	1246815-68-8	Benzonitrile, 4-[[6-amino-5-bromo-2-[(4-cyanophenyl)amino]-4-pyrimidinyl]oxy]-3-	C20H15BrN6O2	451.28	A/B	-8.7
3271	C(=O)([C@@H](NC(=O)OC)C(C)C)N1[C@H]2[C@H](C2)C[C@H]1c1[nH]c(cn1)c1ccc(C#Cc2ccc(cc2)c2[nH]c(nc2)[C@H]	1250937-05-3	Carbamic acid, N,N,Ä≤-[[1,1,Ä≤:4,Ä≤,1,Ä≤,Ä≤-terphenyl]-4,4,Ä≤,Ä≤-diylbis[1H-im	C52H50N8O6	883	A/B	-8.7
3272	N(C(=O)Nc1cncnc1)c1ccc(c2nc(N3C4CCC3COC4)nc(n2)N2CCOCC2)cc1	1254065-99-0	Urea, N-[4-[2-(dimethylamino)ethyl]phenyl]-N,Ä≤-[4-[4-(4-morpholinyl)-6-(3-oxa-8	C30H38N8O3	558.67	A/B	-8.7
3273	C(N(C[C@@H]([C@H](Cc1ccccc1)NC(=O)[C@@H](NC(=O)OC(C)(C)C)C(C)(C)C)O)NC(=O)[C@H](C(C)(C)C)N)c1ccc(cc1	1255196-49-6	2,5,6,10,13-Pentaazatetradecanedioic acid, 3,12-bis(1,1-dimethylethyl)-8-hydroxy	C47H62N6O7	823.03	A/B	-8.7
3274	C(=O)(C(NC(=O)OC)C(C)C)N1C(CCC1)c1[nH]c(cn1)C1Cc2c(C1)cc1c(c2)CC(C1)c1[nH]c(nc1)C1N(C(=O)C(NC(=O)OC)	1255935-55-7	Carbamic acid, N,N,Ä≤-[2,6-anthracenediylbis[1H-imidazole-5,2-diyl-(2S)-2,1-pyr	C42H50N8O6	762.9	A/B	-8.7
3275	Nc1c2c(n(cn2)[C@@H]2O[C@H](CO)[C@@H](O)[C@@H]2O)nc(O[C@@H]2[C@@H](CO)O[C@H]([C@H]2O)n2c3c(nc2)c(N)nc	1256093-55-6	9H-Purin-6-amine, 9-[5-O-(6-amino-9-Œ≤-D-arabinofuranosyl-9H-purin-2-yl)-Œ≤-D-a	C20H23FN10O8	550.46	A/B	-8.7

3276	O([C@@H]1[C@H](n2c3c(nc2)c(N)nc(F)n3)O[C@H](CO)[C@H]1O)c1nc2n(cnc2c(N)n1)[C@@H]1O[C@H](CO)[C@@H](O)[	1256093-56-7	9H-Purin-6-amine, 9-[3-O-(6-amino-9-Œ≤-D-arabinofuranosyl-9H-purin-2-yl)-Œ≤-D-a	C20H23FN10O8	550.46	A/B	-8.7
3277	C(=O)([C@@H](NC(=O)OC)C(C)C)N1[C@@H](c2c(Cl)cccc2)c1[nH]c(cn1)c1ccc(cc1)c1ccc(cc1)c1[nH]c(nc1)[C@H]1	1256390-05-2	Carbamic acid, N-[(1S)-1-[[(2S)-2-[5-[6-[4-[2-[(2S,4R)-1-[(2S)-2-[(methoxycarbor	C51H58N8O6	879.06	A/B	-8.7
3278	C(=O)([C@H](NC(=O)OC)C1CCCCC1)N1[C@@H](CCC1)c1[nH]c(cn1)c1ccc(cc1)c1cc2c(cc1)cc(cc2)c1[nH]c(nc1)[C@H	1256393-17-5	Carbamic acid, N-[(1S)-1-[[(2S)-2-[5-[4,Ä≤-[2-[2,3-dihydro-2-[(2S)-2-[(methoxyca	C44H50N8O6	786.92	A/B	-8.7
3279	C(=O)([C@@H](NC(=O)OC)C(C)C)N1[C@@H](CCC1)c1[nH]c(cn1)c1ccc(cc1)c1cc2c(cc1)cc(cc2)c1[nH]c(nc1)[C@H]1	1261070-47-6	Carbamic acid, N-[(1S)-1-[[(2S)-2-[5-[4-[6-[2-[(2S)-1-[(2S)-2-[(methoxycarbonyl)	C42H48N8O6	760.88	A/B	-8.7
3280	C(F)(F)c1n(c2c(n1)cccc2)c1nc(nc(NCc2cccnc2)n1)N1CCOCC1	1261281-17-7	1,3,5-Triazin-2-amine, 4-[2-(difluoromethyl)-1H-benzimidazol-1-yl]-6-(4-morpholi	C21H20F2N8O	438.43	A/B	-8.7
3281	C(C(=O)N[C@@H](Cc1ccccc1)C[C@@H]([C@H](Cc1ccccc1)NC(=O)COc1c(C)cccc1C)O)(C(C([2H])([2H])[2H])C([2H])	1261281-21-3	1,3,5-Triazin-2-amine, 4-[2-(difluoromethyl)-1H-benzimidazol-1-yl]-6-(4-morpholi	C20H18F2N8O	424.41	A/B	-8.7
3282	N(=C1c2c(N(C(=O)C)C1=O)cccc2)n1c(Cc2n(N=C3c4c(N(C(=O)C)C3=O)cccc4)c(=S)[nH]n2)n[nH]c1=S	1262891-45-1	2H-Indol-2-one, 3,3,Ä≤-[methylenebis[(1,5-dihydro-5-thioxo-4H-1,2,4-triazole-3,4	C23H18N10O2S2	530.58	A/B	-8.7
3283	O=c1n([C@@H]2O[C@H](COP(=O)(O)S)CC2)ccc(=O)[nH]1	1266476-63-4	[1,1,Ä≤-Biphenyl]-2,2,Ä≤-disulfonic acid, 4,4,Ä≤-bis[(1E)-2-(2-hydroxy-1-naphth	C34H26N4O8S2	682.72	A/B	-8.7
3284	S(=O)(=O)(O)c1c2c(cc(N)c1N=Nc1ccc(cc1)c1ccc(N=Nc3c(S(=O)(=O)O)c4c(cc3N)cccc4)cc1)cccc2	1266777-28-9	2,7-Naphthalenedisulfonic acid, 3-amino-4-[(1E)-2-[4,Ä≤-[(1E)-2-[4-[[(4-methylph	C35H27N5O9S3.2Na		A/B	-8.7
3285	O=c1n([C@@H]2O[C@H](CO[P@]3(=O)Oc4c(CO3)cccc4C)[C@@H](N=[N+]=[N-])C2)cc(C)c(=O)[nH]1	1270965-56-4	Thymidine, 2,Ä≤,3,Ä≤-didehydro-3,Ä≤-deoxy-5,Ä≤-O-[(2R)-8-methyl-2-oxido-4H-1,3,	C18H19N2O7P	406.33	A/B	-8.7
3286	C(=O)([C@@H](NC(=O)OC)C(C)C)N1[C@@H]([C@H]2C3(C[C@@H]1C2)CCCC3)c1[nH]c(cn1)c1ccc(cc1)c1ccc(cc1)c1[nH	1273591-32-4	Carbamic acid, N-[(1S)-1-[[(2S)-2-[5-[4,Ä≤-[2-[(1R,3S,4S)-2-[(2S)-2-[(methoxycar	C43H54N8O6	778.94	A/B	-8.7
3287	C(=O)([C@@H](NC(=O)OC)C(C)C)N1[C@@H]([C@@H]2C[C@H]1CC2)c1[nH]c(cn1)c1c(F)c(F)c(cc1)c1c(F)c(F)c(cc1)c	1273591-45-9	Carbamic acid, N-[(1S)-1-[[(2S)-2-[5-[3,3,Ä≤-difluoro-4,Ä≤-[2-[(1R,3S,4S)-2-[(2	C42H50F2N8O6	800.89	A/B	-8.7
3288	C(=O)([C@@H](NC(=O)OC)C(C)C)N1[C@@H]([C@@H]2C[C@H]1CC2)c1[nH]c(cn1)c1c(C)c(C)c(cc1)c1c(C)c(C)c(cc1)c	1273591-47-1	Carbamic acid, N-[(1S)-1-[[(2S)-2-[5-[4,Ä≤-[2-[(1R,3S,4S)-2-[(2S)-2-[(methoxycar	C44H56N8O6	792.97	A/B	-8.7
3289	O=c1c2c(c(ncc2)n2c(=O)cccc2)oc(c1c1ccc(cc1)C1(N)CCC1)c1ccccc1	1279087-27-2	4H-Pyrano[2,3-c]pyridin-4-one, 3-[4-(1-aminocyclobutyl)phenyl]-2-phenyl-8-(4H-1,	C26H21N5O2	435.48	A/B	-8.7
3290	O=c1c(c(oc2c1ccnc2n1ccnc1)c1ccccc1)c1ccc(cc1)C1(N)CCC1	1279087-29-4	4H-Pyrano[2,3-c]pyridin-4-one, 3-[4-(1-aminocyclobutyl)phenyl]-8-(2-oxo-1(2H)-py	C29H23N3O3	461.51	A/B	-8.7
3291	O=c1n(cc([C@H](C(=O)O)O)c(=O)[nH]1)[C@@H]1O[C@H](CO)[C@@H](O)[C@H]1O	1279087-34-1	4H-Pyrano[2,3-c]pyridin-4-one, 3-[4-(1-aminocyclobutyl)phenyl]-8-(1H-imidazol-1-	C27H22N4O2	434.49	A/B	-8.7
3292	C(=O)([C@@H](NC(=O)OC)C(C)C)N1[C@@H](CC(=C)C1)c1[nH]c(cn1)c1ccc(cc1)c1ccc(cc1)c1[nH]c([C@@H](N(C(=O)	1311398-40-9	Carbamic acid, N-[(1S)-1-[[(2S)-4-(difluoromethylene)-2-[5-[4,Ä≤-[2-[(2S)-1-[(2S	C42H48F2N8O6	798.88	A/B	-8.7
3293	C(=O)([C@@H](NC(=O)OC)C1CCOCC1)N1[C@H]2[C@H](C2)C[C@H]1c1[nH]c(c(Cl)n1)c1ccc(cc1)c1ccc(cc1)c1[nH]c(n	1312336-02-9	Carbamic acid, N-[(1S)-2-[(1R,3S,5R)-3-[5-[4,Ä≤-[5-chloro-2-[(1R,3S,5R)-2-[(2S)-	C46H53ClN8O8	881.41	A/B	-8.7
3294	C(=O)([C@@H](NC(=O)OC)C1CCOCC1)N1[C@H]2[C@H](C2)C[C@H]1c1[nH]c(c(Cl)n1)c1ccc(cc1)c1ccc(cc1)c1[nH]c(n	1312336-15-4	Carbamic acid, N-[(1S)-2-[(1R,3S,5R)-3-[5-chloro-4-[4,Ä≤-[5-chloro-2-[(1R,3S,5R)	C45H50Cl2N8O7	885.83	A/B	-8.7
3295	C(=O)([C@@H](NC(=O)OC)C1CCC(F)(F)CC1)N1[C@H]2[C@H](C2)C[C@H]1c1[nH]c(c(Cl)n1)c1ccc(cc1)c1ccc(cc1)c1[	1312336-38-1	Carbamic acid, N-[(1S)-2-[(1R,3S,5R)-3-[5-chloro-4-[4,Ä≤-[5-chloro-2-[(1R,3S,5R)	C47H52Cl2F2N8O7	949.87	A/B	-8.7
3296	C(=O)([C@@H](NC(=O)OC)C1CCOCC1)N1[C@H]2[C@H](C2)C[C@H]1c1[nH]c(c(n1)c1cnoc1)c1ccc(cc1)c1ccc(cc1)c1[n	1312337-07-7	Carbamic acid, N-[(1S)-1-[[(1R,3S,5R)-3-[5-(4-isoxazolyl)-4-[4,Ä≤-[2-[(1R,3S,5R)	C45H51N9O7	829.94	A/B	-8.7
3297	C[C@]1(c2[nH]c(c3c4c(c(cc3)c3ccc(cc3)c3[nH]c(nc3)[C@@]3(C)N(C(=O)[C@@H](NC(=O)OC)C(C)C)CCC3)CCCC4)cn	1312608-79-9	Carbamic acid, N-[(1S)-1-[[(2S)-2-[5-[4-[4-[2-[(2S)-1-[(2S)-2-[(methoxycarbonyl)	C45H54N8O6	802.96	A/B	-8.7
3298	C(=O)([C@@H](NC(=O)OC)C(C)C)N1[C@H](c2[nH]c(c3cc4c(c5c(cc(cc5)c5[nH]c(nc5)[C@H]5N(C(=O)[C@@H](NC(=O)	1312608-80-2	Carbamic acid, N-[(1S)-2-methyl-1-[[(2S)-2-methyl-2-[5-[4-[5,6,7,8-tetrahydro-4-	C46H60N8O6	821.02	A/B	-8.7
3299	O(P(=O)(OC[C@H]1O[C@H]([C@H](O)[C@@H]1O)n1nc(C(=O)N)nc1)O)[C@H]1[C@@H](O[C@H](COP(=O)(O[C@H]2[C@@H](	1312805-80-3	Adenosine, 1,Ä≤-[3-(aminocarbonyl)-1H-1,2,4-triazol-1-yl]-1,Ä≤-de(6-amino-9H-pu	C16H23N8O12P	550.37	A/B	-8.7
3300	O(Cc1ccc(F)cc1)c1c2c(oc(c(O)c2=O)c2ncc(OC)cn2)ccc1	1312892-04-8	4H-1-Benzopyran-4-one, 5-[(4-fluorophenyl)methoxy]-3-hydroxy-2-(5-methyl-2-pyrim	C21H15FN2O4	378.35	A/B	-8.7
3301	O(Cc1ccc(F)cc1)c1c2c(oc(c(O)c2=O)c2ncc(cn2)c2ccccc2)ccc1	1312892-06-0	4H-1-Benzopyran-4-one, 2-(5-chloro-2-pyrimidinyl)-5-[(4-fluorophenyl)methoxy]-3-	C20H12ClFN2O4	398.77	A/B	-8.7
3302	O(Cc1ccc(F)cc1)c1c2c(oc(c(O)c2=O)c2ncc(cn2)c2ccccc2)c(C)cc1	1312892-55-9	4H-1-Benzopyran-4-one, 2-(5-chloro-2-pyrimidinyl)-5-[(4-fluorophenyl)methoxy]-3-	C21H14ClFN2O4	412.8	A/B	-8.7
3303	O(Cc1ccc(F)cc1)c1c2c(oc(c(O)c2=O)c2[nH]c(OC)nn2)c(C)cc1	1312892-63-9	4H-1-Benzopyran-4-one, 5-[(4-fluorophenyl)methoxy]-3-hydroxy-8-methyl-2-(3-methy	C20H16FN3O4	381.36	A/B	-8.7
3304	O(Cc1ccc(F)cc1)c1c2c(oc(c(O)c2=O)c2[nH]ccn2)c(OC)cc1	1312893-47-2	4H-1-Benzopyran-4-one, 5-[(4-fluorophenyl)methoxy]-3-hydroxy-8-methoxy-2-(3-pher	C25H18FN3O5	459.43	A/B	-8.7
3305	O(Cc1ccc(F)cc1)c1c2c(oc(c(O)c2=O)c2[nH]c(OC)nn2)c(Cl)cc1	1312893-94-9	4H-1-Benzopyran-4-one, 8-chloro-5-[(4-fluorophenyl)methoxy]-3-hydroxy-2-(3-methy	C19H13ClFN3O4	401.78	A/B	-8.7
3306	O(Cc1ccc(F)cc1)c1c2c(oc(c(O)c2=O)c2[nH]c(OC)nn2)cc(C)c1	1312894-94-2	4H-1-Benzopyran-4-one, 5-[(4-fluorophenyl)methoxy]-3-hydroxy-7-methyl-2-(3-methy	C20H16FN3O4	381.36	A/B	-8.7
3307	O(Cc1ccc(F)cc1)c1c2c(oc(c(O)c2=O)c2ncc(OC)cn2)c(C)c(C)c1	1312895-34-3	4H-1-Benzopyran-4-one, 5-[(4-fluorophenyl)methoxy]-3-hydroxy-7,8-dimethyl-2-(5-m	C23H19FN2O4	406.41	A/B	-8.7
3308	O(Cc1ccc(F)cc1)c1c2c(oc(c(O)c2=O)c2[nH]c(C)nn2)c(C)c(C)c1	1312895-43-4	4H-1-Benzopyran-4-one, 5-[(4-fluorophenyl)methoxy]-3-hydroxy-7,8-dimethyl-2-(1H-	C20H16FN3O4	381.36	A/B	-8.7
3309	O(Cc1ccc(F)cc1)c1c2c(oc(c(O)c2=O)c2[nH]c(cn2)c2ccccc2)c(C)c(C)c1	1312895-51-4	4H-1-Benzopyran-4-one, 2-(5-chloro-1H-imidazol-2-yl)-5-[(4-fluorophenyl)methoxy]	C21H16ClFN2O4	414.81	A/B	-8.7
3310	O(Cc1ccc(F)cc1)c1c2c(oc(c(O)c2=O)c2ncc(OC)cn2)c(Cl)c(C)c1	1312896-34-6	4H-1-Benzopyran-4-one, 8-chloro-5-[(4-fluorophenyl)methoxy]-3-hydroxy-7-methyl-2	C22H16ClFN2O4	426.82	A/B	-8.7
3311	O(Cc1ccc(F)cc1)c1c2c(oc(c(O)c2=O)c2ncc(cn2)c2ccccc2)c(Cl)c(C)c1	1312896-36-8	4H-1-Benzopyran-4-one, 8-chloro-2-(5-chloro-2-pyrimidinyl)-5-[(4-fluorophenyl)me	C21H13Cl2FN2O4	447.24	A/B	-8.7
3312	O(Cc1ccc(F)cc1)c1c2c(oc(c(O)c2=O)c2[nH]c(C)nn2)c(Cl)c(C)c1	1312896-43-7	4H-1-Benzopyran-4-one, 8-chloro-5-[(4-fluorophenyl)methoxy]-3-hydroxy-7-methyl-2	C19H13ClFN3O4	401.78	A/B	-8.7
3313	O(Cc1ccc(F)cc1)c1c2c(oc(c(O)c2=O)c2[nH]c(Cl)nn2)c(Cl)c(C)c1	1312896-45-9	4H-1-Benzopyran-4-one, 8-chloro-5-[(4-fluorophenyl)methoxy]-3-hydroxy-2-(5-metho	C20H15ClFN3O5	431.8	A/B	-8.7
3314	O(Cc1ccc(F)cc1)c1c2c(oc(c(O)c2=O)c2[nH]c(C)cn2)c(Cl)c(C)c1	1312896-48-2	4H-1-Benzopyran-4-one, 8-chloro-5-[(4-fluorophenyl)methoxy]-3-hydroxy-2-(1H-imic	C20H14ClFN2O4	400.79	A/B	-8.7
3315	O(Cc1ccc(F)cc1)c1c2c(oc(c(O)c2=O)c2[nH]c(Cl)cn2)c(Cl)c(C)c1	1312896-50-6	4H-1-Benzopyran-4-one, 8-chloro-5-[(4-fluorophenyl)methoxy]-3-hydroxy-2-(5-metho	C21H16ClFN2O5	430.81	A/B	-8.7
3316	O(Cc1ccc(F)cc1)c1c2c(oc(c(O)c2=O)c2[nH]c(cn2)c2ccccc2)c(Cl)c(C)c1	1312896-51-7	4H-1-Benzopyran-4-one, 8-chloro-2-(5-chloro-1H-imidazol-2-yl)-5-[(4-fluorophenyl	C20H13Cl2FN2O4	435.23	A/B	-8.7
3317	O(C)c1c(c2c(c(OCc3ccc(F)cc3)c1)c(=O)c(O)c(o2)c1ncc(C)cn1)c1ccccc1	1312899-45-8	4H-1-Benzopyran-4-one, 5-[(4-fluorophenyl)methoxy]-3-hydroxy-7-methoxy-8-phenyl-	C27H19FN2O5	470.45	A/B	-8.7
3318	O(C)c1c(c2c(c(OCc3ccc(F)cc3)c1)c(=O)c(O)c(o2)c1[nH]c(OC)nn1)c1ccccc1	1312899-56-1	4H-1-Benzopyran-4-one, 5-[(4-fluorophenyl)methoxy]-3-hydroxy-7-methoxy-2-(3-meth	C26H20FN3O5	473.45	A/B	-8.7
3319	O(Cc1ccc(F)cc1)c1c2c(oc(c(O)c2=O)c2[nH]c(OC)nn2)c(C)c(Cl)c1	1312901-15-7	4H-1-Benzopyran-4-one, 7-chloro-5-[(4-fluorophenyl)methoxy]-3-hydroxy-8-methyl-2	C20H15ClFN3O4	415.8	A/B	-8.7
3320	O(Cc1ccc(F)cc1)c1c2c(oc(c(O)c2=O)c2[nH]c(Cl)nn2)c(C)c(Cl)c1	1312901-16-8	4H-1-Benzopyran-4-one, 7-chloro-5-[(4-fluorophenyl)methoxy]-3-hydroxy-2-(5-metho	C20H15ClFN3O5	431.8	A/B	-8.7
3321	O(Cc1ccc(F)cc1)c1c2c(oc(c(O)c2=O)c2[nH]c(nn2)c2ccccc2)c(C)c(Cl)c1	1312901-17-9	4H-1-Benzopyran-4-one, 7-chloro-2-(5-chloro-1H-1,2,4-triazol-3-yl)-5-[(4-fluorop	C19H12Cl2FN3O4	436.22	A/B	-8.7
3322	O(Cc1ccc(F)cc1)c1c2c(c(c(Cl)c1)c1ccccc1)oc(c(O)c2=O)c1[nH]cc(C)n1	1312902-69-4	4H-1-Benzopyran-4-one, 7-chloro-5-[(4-fluorophenyl)methoxy]-3-hydroxy-2-(1H-imic	C25H16ClFN2O4	462.86	A/B	-8.7
3323	O(Cc1ccc(F)cc1)c1c2c(c(c(Cl)c1)c1ccccc1)oc(c(O)c2=O)c1[nH]c(OC)cn1	1312902-70-7	4H-1-Benzopyran-4-one, 7-chloro-5-[(4-fluorophenyl)methoxy]-3-hydroxy-2-(5-methy	C26H18ClFN2O4	476.88	A/B	-8.7
3324	O(Cc1ccc(F)cc1)c1c2c(c(c(Cl)c1)c1ccccc1)oc(c(O)c2=O)c1[nH]c(cn1)c1ccccc1	1312902-72-9	4H-1-Benzopyran-4-one, 7-chloro-2-(5-chloro-1H-imidazol-2-yl)-5-[(4-fluorophenyl	C25H15Cl2FN2O4	497.3	A/B	-8.7
3325	Cc1c2c(c(OCc3ccc(F)cc3)cc1c1ccccc1)c(=O)c(O)c(o2)c1ncc(cn1)c1ccccc1	1312903-58-4	4H-1-Benzopyran-4-one, 2-(5-chloro-2-pyrimidinyl)-5-[(4-fluorophenyl)methoxy]-3-	C27H18ClFN2O4	488.89	A/B	-8.7
3326	O(C)c1c2c(c(OCc3ccc(F)cc3)cc1c1ccccc1)c(=O)c(O)c(o2)c1[nH]cnn1	1312904-30-5	4H-1-Benzopyran-4-one, 5-[(4-fluorophenyl)methoxy]-3-hydroxy-8-methoxy-7-phenyl-	C33H23FN2O5	546.54	A/B	-8.7
3327	O(Cc1ccc(F)cc1)c1c2c(c(Cl)c(c1)c1ccccc1)oc(c(O)c2=O)c1[nH]cnn1	1312905-08-0	4H-1-Benzopyran-4-one, 8-chloro-5-[(4-fluorophenyl)methoxy]-3-hydroxy-7-phenyl-2	C32H20ClFN2O4	550.96	A/B	-8.7
3328	O=c1c2c(oc(c1O)c1nnc(Cl)[nH]1)ccc(OCc1ccc(F)cc1)c2	1312906-26-5	4H-1-Benzopyran-4-one, 6-[(4-fluorophenyl)methoxy]-3-hydroxy-2-(5-methoxy-1H-1,2	C19H14FN3O5	383.33	A/B	-8.7
3329	O=c1c2c(oc(c1O)c1ncc(Cl)cn1)c(C)cc(OCc1ccc(F)cc1)c2	1312906-66-3	4H-1-Benzopyran-4-one, 6-[(4-fluorophenyl)methoxy]-3-hydroxy-2-(5-methoxy-2-pyri	C22H17FN2O5	408.38	A/B	-8.7
3330	O(C)c1c2c(cc(OCc3ccc(F)cc3)c1)c(=O)c(O)c(o2)c1[nH]c(cn1)c1ccccc1	1312907-32-6	4H-1-Benzopyran-4-one, 2-(5-chloro-1H-imidazol-2-yl)-6-[(4-fluorophenyl)methoxy]	C20H14ClFN2O5	416.79	A/B	-8.7
3331	O=c1c2c(oc(c1O)c1nnc(C)[nH]1)c(Cl)cc(OCc1ccc(F)cc1)c2	1312907-74-6	4H-1-Benzopyran-4-one, 8-chloro-6-[(4-fluorophenyl)methoxy]-3-hydroxy-2-(1H-1,2,	C18H11ClFN3O4	387.75	A/B	-8.7
3332	Cc1c2c(cc(OCc3ccc(F)cc3)c1C)c(=O)c(O)c(o2)c1[nH]cnn1	1312909-30-0	4H-1-Benzopyran-4-one, 6-[(4-fluorophenyl)methoxy]-3-hydroxy-7,8-dimethyl-2-(5-p	C28H21FN2O4	468.48	A/B	-8.7
3333	Cc1c2c(cc(OCc3ccc(F)cc3)c1C)c(=O)c(O)c(o2)c1[nH]c(nn1)c1ccccc1	1312909-39-9	4H-1-Benzopyran-4-one, 2-(5-chloro-1H-1,2,4-triazol-3-yl)-6-[(4-fluorophenyl)met	C20H15ClFN3O4	415.8	A/B	-8.7
3334	Cc1c2c(cc(OCc3ccc(F)cc3)c1C)c(=O)c(O)c(o2)c1[nH]c(Cl)cn1	1312909-43-5	4H-1-Benzopyran-4-one, 6-[(4-fluorophenyl)methoxy]-3-hydroxy-2-(5-methoxy-1H-imi	C22H19FN2O5	410.4	A/B	-8.7
3335	Cc1c2c(cc(OCc3ccc(F)cc3)c1C)c(=O)c(O)c(o2)c1[nH]c(cn1)c1ccccc1	1312909-44-6	4H-1-Benzopyran-4-one, 2-(5-chloro-1H-imidazol-2-yl)-6-[(4-fluorophenyl)methoxy]	C21H16ClFN2O4	414.81	A/B	-8.7
3336	Clc1c2c(cc(OCc3ccc(F)cc3)c1C)c(=O)c(O)c(o2)c1[nH]c(OC)nn1	1312910-37-4	4H-1-Benzopyran-4-one, 8-chloro-6-[(4-fluorophenyl)methoxy]-3-hydroxy-7-methyl-2	C20H15ClFN3O4	415.8	A/B	-8.7
3337	Clc1c2c(cc(OCc3ccc(F)cc3)c1C)c(=O)c(O)c(o2)c1[nH]c(Cl)cn1	1312910-43-2	4H-1-Benzopyran-4-one, 8-chloro-6-[(4-fluorophenyl)methoxy]-3-hydroxy-2-(5-metho	C21H16ClFN2O5	430.81	A/B	-8.7
3338	Clc1c2c(cc(OCc3ccc(F)cc3)c1C)c(=O)c(O)c(o2)c1[nH]c(cn1)c1ccccc1	1312910-44-3	4H-1-Benzopyran-4-one, 8-chloro-2-(5-chloro-1H-imidazol-2-yl)-6-[(4-fluorophenyl	C20H13Cl2FN2O4	435.23	A/B	-8.7
3339	Cc1c(c2c(cc1OCc1ccc(F)cc1)c(=O)c(O)c(o2)c1ncc(Cl)cn1)c1ccccc1	1312910-78-3	4H-1-Benzopyran-4-one, 6-[(4-fluorophenyl)methoxy]-3-hydroxy-2-(5-methoxy-2-pyri	C28H21FN2O5	484.48	A/B	-8.7
3340	O=c1c2c(oc(c1O)c1[nH]c(nn1)c1ccccc1)cc(Cl)c(OCc1ccc(F)cc1)c2	1312911-80-0	4H-1-Benzopyran-4-one, 7-chloro-2-(5-chloro-1H-1,2,4-triazol-3-yl)-6-[(4-fluorop	C18H10Cl2FN3O4	422.19	A/B	-8.7
3341	Cc1c2c(cc(OCc3ccc(F)cc3)c1Cl)c(=O)c(O)c(o2)c1[nH]cnn1	1312912-63-2	4H-1-Benzopyran-4-one, 7-chloro-6-[(4-fluorophenyl)methoxy]-3-hydroxy-8-methyl-2	C27H18ClFN2O4	488.89	A/B	-8.7
3342	Cc1c2c(cc(OCc3ccc(F)cc3)c1Cl)c(=O)c(O)c(o2)c1[nH]c(OC)nn1	1312912-77-8	4H-1-Benzopyran-4-one, 7-chloro-6-[(4-fluorophenyl)methoxy]-3-hydroxy-8-methyl-2	C20H15ClFN3O4	415.8	A/B	-8.7
3343	Cc1c2c(cc(OCc3ccc(F)cc3)c1Cl)c(=O)c(O)c(o2)c1[nH]c(Cl)cn1	1312912-89-2	4H-1-Benzopyran-4-one, 7-chloro-6-[(4-fluorophenyl)methoxy]-3-hydroxy-2-(5-metho	C21H16ClFN2O5	430.81	A/B	-8.7
3344	Clc1c2c(cc(OCc3ccc(F)cc3)c1Cl)c(=O)c(O)c(o2)c1ncc(cn1)c1ccccc1	1312914-61-6	4H-1-Benzopyran-4-one, 7,8-dichloro-2-(5-chloro-2-pyrimidinyl)-6-[(4-fluoropheny	C20H10Cl3FN2O4	467.66	A/B	-8.7
3345	Clc1c2c(cc(OCc3ccc(F)cc3)c1Cl)c(=O)c(O)c(o2)c1[nH]c(C)cn1	1312914-85-4	4H-1-Benzopyran-4-one, 7,8-dichloro-6-[(4-fluorophenyl)methoxy]-3-hydroxy-2-(1H-	C19H11Cl2FN2O4	421.21	A/B	-8.7
3346	O(C)c1c(c2c(cc1OCc1ccc(F)cc1)c(=O)c(O)c(o2)c1[nH]c(cn1)c1ccccc1)c1ccccc1	1312915-51-7	4H-1-Benzopyran-4-one, 2-(5-chloro-1H-imidazol-2-yl)-6-[(4-fluorophenyl)methoxy]	C26H18ClFN2O5	492.88	A/B	-8.7
3347	Clc1c(c2c(cc1OCc1ccc(F)cc1)c(=O)c(O)c(o2)c1ncc(Cl)cn1)c1ccccc1	1312915-64-2	4H-1-Benzopyran-4-one, 7-chloro-6-[(4-fluorophenyl)methoxy]-3-hydroxy-2-(5-metho	C27H18ClFN2O5	504.89	A/B	-8.7
3348	O(Cc1ccc(F)cc1)c1c(c(C)c2c(c1)c(=O)c(O)c(o2)c1[nH]c(C)nn1)c1ccccc1	1312916-92-9	4H-1-Benzopyran-4-one, 6-[(4-fluorophenyl)methoxy]-3-hydroxy-8-methyl-7-phenyl-2	C25H18FN3O4	443.43	A/B	-8.7
3349	Clc1c2c(cc(OCc3ccc(F)cc3)c1c1ccccc1)c(=O)c(O)c(o2)c1[nH]c(cn1)c1ccccc1	1312918-00-5	4H-1-Benzopyran-4-one, 8-chloro-2-(5-chloro-1H-imidazol-2-yl)-6-[(4-fluorophenyl	C25H15Cl2FN2O4	497.3	A/B	-8.7
3350	C(=O)(N)c1nn(cn1)[C@@H]1O[C@H](CO)C=C1	1319189-63-3	Carbamic acid, N-[(12R,13S,18aS)-5,6,13,14,16,17,18,18a-octahydro-3-[4,Ä≤-[2-[(	C46H53N9O6	827.97	A/B	-8.7
3351	N(c1nc(nc(NN=Cc2c3c(ccc2)cccc3)n1)N1CCOCC1)c1ccc(F)cc1	1331749-53-1	1H-Pyrrole-3-carboxaldehyde, 2-methyl-, 2-[4-[(4-fluorophenyl)amino]-6-(4-morphc	C19H21FN8O	396.42	A/B	-8.7
3352	N(c1c(C([2H])([2H])[2H])c(c(/C=C/C#N)c(c1C([2H])([2H])[2H])[2H])[2H])c1nc(Nc2c(cc(C#N)cc2[2H])[2H])n	1331866-44-4	Benzonitrile-3,5-d2, 4-[[4-[[4-[(1E)-2-cyanoethenyl]-2-methyl-6-(methyl-d3)pheny	C22H11D7N6		A/B	-8.7
3353	N(c1c(C)c(c(/C=C/C#N)c(c1C)[2H])[2H])c1nc(Nc2c(c(C#N)c(c2[2H])[2H])[2H])[2H])ncc1	1331866-54-6	Benzonitrile-2,6-d2, 4-[[6-[[4-[(1E)-2-cyanoethenyl]-2,6-di(methyl-d3)phenyl-3,5	C22H6D12N6		A/B	-8.7
3354	N(c1c(C([2H])([2H])[2H])cc(/C=C/C#N)cc1C)c1nc(Nc2ccc(C#N)cc2)nc(c1[2H])[2H]	1331866-88-6	Benzonitrile, 4-[[6-[[4-[(1E)-2-cyanoethenyl]-2,6-dimethylphenyl]amino]-2-pyrimi	C22H16D2N6		A/B	-8.7
3355	O=c1n([C@@H]2O[C@H](COP(=O)(Nc3ccccc3)O)[C@@H](N=[N+]=[N-])C2)cc(C)c(=O)[nH]1	1332981-16-4	2,5,6,10,13-Pentaazatetradecanedioic acid, 3,12-bis(1,1-dimethylethyl)-8-hydroxy	C38H52N6O7	704.86	A/B	-8.7
3356	O=c1c2c(n(cn2)[C@H]2[C@H](O)[C@H](COCc3ccccc3)[C@@H](COCc3ccccc3)O2)[nH]c(N)n1	133713-79-8	Guanosine, N-acetyl-3,Ä≤-deoxy-3,Ä≤-[(phenylmethoxy)methyl]-	C20H23N5O6	429.43	A/B	-8.7
3357	O([P@](=O)(Oc1ccccc1)N[C@H](C(=O)OC(C)C)C)[C@@H]1[C@@H](CO[P@](=O)(Oc2ccccc2)N[C@H](C(=O)OC(C)C)C)O[	133734-21-1	Guanosine, N-acetyl-3,Ä≤-deoxy-3,Ä≤-[(phenylmethoxy)methyl]-, 2,Ä≤-acetate	C22H25N5O7	471.46	A/B	-8.7
3358	C(OC[C@@H]1[C@@H](OP(N(C(C)C)C(C)C)OCCC#N)C[C@@H](O1)n1cc(CCCNC(=O)C(F)(F)F)c(=O)[nH]c1=O)(c1ccc(OC)	1341204-36-1	Carbamic acid, N-[(1S)-2-methyl-1-[[(2S)-2-[5-[4-[(6S,7S,22aS)-1,2,3,5,6,7,8,9,1	C46H60N8O6	821.02	A/B	-8.7
3359	[C@H](Cc1ccccc1)(C[C@@H]([C@H](Cc1ccccc1)NC(=O)OCc1cnco1)O)NC(=O)[C@@H](NC(=O)N(Cc1nc(CN(C)C)sc1)C)C	1346833-82-6	2,7,10,12-Tetraazatridecanoic acid, 13-[2-(1-cyclopenten-1-yl)-4-thiazolyl]-4-hy	C39H48N6O5S2	744.97	A/B	-8.7
3360	C(=C\1/c2c(NC1=O)ccc(S(=O)(=O)N(C[C@H]([C@H](Cc1ccccc1)NC(=O)O[C@@H]1[C@H]3[C@@H](OC1)OCC3)O)CC(C)C)	1346975-24-3	2,7,10,12-Tetraazatridecanoic acid, 4-hydroxy-9,10-dimethyl-13-[2-(1-methylethyl	C35H44N6O5S2	692.89	A/B	-8.7
3361	C(n1c2c(nc1CC)c(C)cc(C)n2)c1ccc(cc1)c1c(S(=O)(=O)NC(=O)COCCCC)ccc(CN2CCCC2)c1	1347556-32-4	Carbamic acid, N-[[4,Ä≤-[(2-ethyl-5,7-dimethyl-3H-imidazo[4,5-b]pyridin-3-yl)met	C30H33F3N4O5S	618.67	A/B	-8.7
3362	C[C@@]12[C@@H]([C@@H]3[C@@](C)([C@@H](O)C2)CC[C@H]3C(=C)C)CC[C@H]2[C@@]1(C)CC(=O)[C@@H]1[C@]2(C)CC[C	1347680-78-7	Acetamide, 2-ethoxy-N-[[4,Ä≤-[(2-ethyl-5,7-dimethyl-3H-imidazo[4,5-b]pyridin-3-y	C32H39N5O4S	589.75	A/B	-8.7
3363	C(n1c2c(nc1CC)c(C)cc(C)n2)c1ccc(cc1)c1c(S(=O)(=O)NC(=O)OC(C)(C)C)cccc1	1348027-53-1	Not Yet Assigned	C40H55N7O6	729.91	A/B	-8.7
3364	OC1c2c(ccc(S(=O)(=O)N(CC(C(Cc3ccccc3)NC(=O)OC3C4C(OC3)OCC4)O)CC(C)C)c2)OC1O	1348244-45-0	Not Yet Assigned	C35H38N4O5S	626.77	A/B	-8.7
3365	OC1=C(O)C(CO)OC1n1c(=O)[nH]c(=O)c(C)c1	1348376-43-1	Not Yet Assigned	C40H57N7O6S	763.99	A/B	-8.7
3366	C=C1[C@@H](n2c3c(nc2)c(=O)nc(N)[nH]3)C[C@H](O)C1COP(=O)(OP(=O)(O)O)O	1348546-68-8	Not Yet Assigned	C37H51N7O6	689.84	A/B	-8.7
3367	C(n1c2c(nc1CC)c(C)cc(C)n2)c1ccc(cc1)c1c(S(=O)(=O)NC(=O)CCOCC)cccc1	1348779-04-3	4-Morpholinecarboxamide, N-[(1R)-1-[(4-fluorophenyl)methyl]-2-oxo-2-[[(2E)-1-(2-	C31H34FN3O5S	579.68	A/B	-8.7
3368	[C@H](Cc1ccccc1)(C[C@@H]([C@H](Cc1cncs1)NC(=O)OCc1cncs1)O)NC(=O)[C@@H](NC(=O)N(Cc1nc(C(C)C)sc1)C)C(C	1348967-32-7	Not Yet Assigned	C38H48N4O5	640.81	A/B	-8.7
3369	N(C(=O)[C@H](Cc1ccccc1)C[C@@H](CN1[C@H](C(=O)NC(C)(C)C)CN(C(=O)c2nc3c(cc2)cccc3)CC1)O)C1c2c(C[C@H]1O	1349876-44-3	Not Yet Assigned	C36H47N5O4	613.79	A/B	-8.7
3370	O=c1n([C@@H]2O[C@H](CO)[C@@H](O)C2)cc(CO)c(=O)[nH]1	1350123-87-3	Carbamic acid, N-[(1S,2R)-3-[[(4-aminophenyl)sulfonyl](2-methylpropyl)amino]-1-[	C38H46N3O12PS	799.82	A/B	-8.7
3371	C(=O)([C@@H](NC(=O)OC)C(C)C)N1[C@H](c2[nH]c(c3c4c(c(cc3)c3c5c(c(cc3)c3[nH]c(nc3)[C@H]3N(C(=O)[C@@H](	1350718-42-1	Carbamic acid, N,N,Ä≤-[(2,2,Ä≤-dimethyl[4,7,Ä≤-bi-1H-indole]-4,Ä≤,7-diyl)bis[1H	C46H56N10O6	845	A/B	-8.7
3372	C(=O)([C@@H](NC(=O)OC)C(C)C)N1[C@@]2(C[C@H](C1)CC2)c1[nH]c(cn1)c1c2c(c(cc1)c1ccc(cc1)c1[nH]c(nc1)[C@	1350718-91-0	Carbamic acid, N,N,Ä≤-[[4,4,Ä≤-bibenzothiazole]-7,7,Ä≤-diylbis[1H-imidazole-5,2	C46H52N10O6S2	905.1	A/B	-8.7
3373	C(=O)([C@@H](NC(=O)OC)C(C)C)N1[C@@]2(C[C@H](C1)CC2)c1[nH]c(cn1)c1c2c(c(cc1)c1ccc(cc1)c1[nH]c(nc1)[C@	1350718-92-1	Carbamic acid, N-[(1S)-1-[[(1S,4R)-1-[5-[4-[7-[2-[(1S,4R)-2-[(2S)-2-[(methoxycar	C47H57N9O6	844.01	A/B	-8.7
3374	C(=O)([C@@H](NC(=O)OC)C(C)C)N1[C@@]2(C[C@H](C1)CC2)c1[nH]c(cn1)c1c2c(c(cc1)c1ccc(cc1)c1[nH]c(nc1)[C@	1350718-95-4	Carbamic acid, N-[(1S)-1-[[(1S,4R)-1-[5-[4-[4-[2-[(1S,4R)-2-[(2S)-2-[(methoxycar	C48H56N8O6	841.01	A/B	-8.7
3375	C(=O)([C@@H](NC(=O)OC)C(C)C)N1[C@@]2(C[C@H](C1)CC2)c1[nH]c(cn1)c1c2c(c(cc1)c1ccc(cc1)c1[nH]c(nc1)[C@	1350718-99-8	Carbamic acid, N-[(1S)-1-[[(1S,4R)-1-[5-[5-[4-[2-[(1S,4R)-2-[(2S)-2-[(methoxycar	C47H55N9O6	842	A/B	-8.7
3376	C(#N)[C@]1(O[C@H](COP(=O)(Oc2ccc(Cl)cc2)N[C@H](C(=O)OCCCCCC)C)[C@@H](O)[C@@]1(C)O)c1n2c(cc1)c(N)ncn	1350735-62-4	L-Alanine, N-(hydroxyphenoxyphosphinyl)-, cyclopentyl ester, 6-ester with 2-C-(4	C27H33N6O8P	600.56	A/B	-8.7
3377	C(=O)([C@H](NC(=O)OC)c1ccccc1)N1[C@@H](CCC1)c1[nH]c(cn1)c1cc2c(cc1)cc(cc2)c1c(C)cc(cc1)c1[nH]c(nc1)[	1351330-60-3	Carbamic acid, N-[(1S)-1-[[(2S)-2-[5-[6-[4-[2-[(2S)-1-[(2S)-2-[(methoxycarbonyl)	C45H54N8O6	802.96	A/B	-8.7
3378	C(=O)([C@H](C(CC)CC)NC(=O)OC)N1[C@@H](CCC1)c1[nH]c(cn1)c1cc2c(cc1)cc(cc2)c1ccc(cc1)c1[nH]c(nc1)[C@H]	1351331-15-1	Carbamic acid, N-[(1S)-1-cyclopropyl-2-[(2S)-2-[5-[4-[6-[2-[(2S)-1-[(2S)-2-cyclc	C44H48N8O6	784.9	A/B	-8.7
3379	C(=O)([C@@H](NC(=O)OC)C(C)C)N1C[C@@]2(C[C@H]1c1[nH]c(cn1)c1ccc(cc1)c1ccc(cc1)c1[nH]c(nc1)[C@H]1N(C(=	1351852-67-9	Carbamic acid, N-[(1S)-1-[[(2S,5R)-2-[5-[4,Ä≤-[2-[(5S,8S)-7-[(2S)-2-[(methoxycar	C49H58N8O7	871.03	A/B	-8.7
3380	C(=O)([C@@H](NC(=O)OC)C(C)C)N1[C@H]2[C@H](C2)C[C@H]1c1[nH]c(cn1)c1ccc(cc1)c1ccc(cc1)c1[nH]c(nc1)[C@H	1351852-95-3	Carbamic acid, N-[(1S)-1-[[(5S,8S)-8-[5-[4,Ä≤-[2-[(3S)-2-[(2S)-2-[(methoxycarbor	C47H58N8O7	847.01	A/B	-8.7
3381	C(=O)([C@@H](NC(=O)OC)C(C)C)N1CC2(C[C@H]1c1[nH]c(cn1)c1ccc(cc1)c1ccc(cc1)c1[nH]c(nc1)[C@H]1N(C(=O)[C	1351852-96-4	Carbamic acid, N-[(1S)-1-[[(5S,8S)-8-[5-[4,Ä≤-[2-[(1R,3S,5R)-2-[(2S)-2-[(methoxy	C44H54N8O7	806.95	A/B	-8.7
3382	C(=O)([C@@H](NC(=O)OC)C(C)C)N1C[C@]2(C[C@H]1c1[nH]c(cn1)c1ccc(cc1)c1ccc(cc1)c1[nH]c(nc1)[C@H]1N(C(=O	1351853-25-2	Carbamic acid, N-[(1S)-1-[[(2S)-2-[5-[4,Ä≤-[2-[(2R,4R,7S)-6-[(2S)-2-[(methoxycar	C43H54N8O7	794.94	A/B	-8.7
3383	C(=O)([C@@H](NC(=O)OC)C(C)C)N1[C@@H](C=C(C)C1)c1[nH]c(cn1)c1ccc(C#Cc2ccc(cc2)c2[nH]c(nc2)[C@H]2N(C(=	1352391-62-8	Carbamic acid, N-[(1S)-1-[[(2S)-2-[5-[6-[4-[2-[(2S)-2,5-dihydro-1-[(2S)-2-[(meth	C45H52N8O6	800.94	A/B	-8.7
3384	C(=O)([C@@H](NC(=O)OC)[C@H]1C[C@@H](C)O[C@@H](C)C1)N1[C@H](c2[nH]c(c3c4c(c(cc3)c3ccc(cc3)c3[nH]c(nc3	1359868-97-5	Carbamic acid, N-[(1S)-2-[(2S,5S)-2-[5-[4-[7-[2-[(2S,5S)-1-[(2S)-2-[(methoxycarb	C47H58N10O8	891.03	A/B	-8.7
3385	C(F)(F)c1n(c2c(n1)cccc2)c1nc(nc(NCC2CCN(C)CC2)n1)N1CCOCC1	1359955-63-7	Ethanone, 1-[4-[[4-[2-(difluoromethyl)-1H-benzimidazol-1-yl]-6-(4-morpholinyl)-1	C22H26F2N8O2	472.49	A/B	-8.7
3386	C(F)(F)c1n(c2c(n1)cccc2)c1nc(nc(Nc2cc(OC)ccc2)n1)N1CCOCC1	1359956-23-2	1,3,5-Triazin-2-amine, 4-[2-(difluoromethyl)-1H-benzimidazol-1-yl]-N-(4-methoxyp	C22H21F2N7O2	453.44	A/B	-8.7
3387	C(F)(F)c1n(c2c(n1)cccc2)c1nc(nc(Nc2ccc(N(C)C)cc2)n1)N1CCOCC1	1359956-24-3	1,3,5-Triazin-2-amine, 4-[2-(difluoromethyl)-1H-benzimidazol-1-yl]-N-(3-methoxyp	C22H21F2N7O2	453.44	A/B	-8.7
3388	C(OC(C=C)CO)n1c2c(nc1)c(=O)nc(N)[nH]2	1360627-56-0	1,3,5-Triazine-2,4-diamine, N2-(4-chlorophenyl)-6-(4-morpholinyl)-N4-(4-nitrophe	C19H18ClN7O3	427.84	A/B	-8.7
3389	C(C[C@@H](CO)O)n1c2c(nc1)c(=O)nc[nH]2	1372618-37-5	3-Quinolinecarboxylic acid, 7-[(4aS,7aS)-1-[2-[3-[2-(aminothioxomethyl)hydraziny	C32H33F2N7O5S	665.71	A/B	-8.7
3390	C(=O)([C@@H](NC(=O)OC)C(C)C)N1[C@@H](CCC1)c1[nH]c(cn1)c1ccc(cc1)c1ccc(cc1)c1ccc(cc1)c1ccc(cc1)c1[nH]	1375881-14-3	Carbamic acid, N,N,Ä≤-[[1,1,Ä≤:3,Ä≤,1,Ä≤,Ä≤-terphenyl]-4,4,Ä≤,Ä≤-diylbis[1H-im	C46H54N8O6	814.97	A/B	-8.7
3391	O=c1c2c3c4c1cc(cc4ccc3cc(c2)c1[nH]c(nc1)[C@H]1N(C(=O)[C@@H](NC(=O)OC)C(C)C)CCC1)c1[nH]c(nc1)[C@H]1N(	1377035-70-5	Carbamic acid, N-[(1S)-2-methyl-1-[[(2S)-2-[5-[4,5,9,10-tetrahydro-7-[2-[(2S)-1-	C46H56N8O7	832.99	A/B	-8.7
3392	C(=O)([C@@H](NC(=O)OC)C1CCOCC1)N1[C@@H]([C@@H]2C[C@H]1CC2)c1[nH]c2-c3c(CCc2n1)cc(cc3)c1cc2c(cc1)c1c(	1377036-98-0	Carbamic acid, N-[(1S)-2-[(2S)-2-[5-[9,10-dihydro-7-[2-[(1R,3S,4S)-2-[(2S)-2-[(m	C46H56N8O7	832.99	A/B	-8.7

3393	C(=O)([C@@H](NC(=O)OC)C(C)C)N1[C@@H](CCC1)c1[nH]c(cn1)c1cc2c(cc1)cc(cc2)c1cc(ccc1)c1[nH]c(nc1)[C@H]1	1377038-87-3	Carbamic acid, N-[(1R)-2-[(2S)-2-[5-[4-[6-[2-[(2S)-1-[(2S)-2-[(methoxycarbonyl)a	C48H52N8O6	836.98	A/B	-8.7
3394	C(=O)([C@@H](NC(=O)OC)C1CCOCC1)N1[C@H](c2[nH]c(c3cc4c(c5c(c6c4cccc6)cc(cc5)c4[nH]c(nc4)[C@H]4N(C(=O)	1377041-01-4	Carbamic acid, N-[(1S)-2-[(2S)-2-[4,Ä≤,5,Ä≤-dihydro-2,Ä≤-[(1R,3S,4S)-2-[(2S)-2-	C48H56N8O7	857.01	A/B	-8.7
3395	C(=O)([C@@H](NC(=O)OC)C(C)C)N1[C@@H]([C@@H]2C[C@H]1CC2)c1[nH]c2c(n1)ccc1c2ccc(c1)c1cc2c(cc1)c1c(cc2)	1377041-05-8	Carbamic acid, N-[(1S)-2-[(2S)-2-[5-[7-[2-[(2S)-1-[(2S)-2-[(methoxycarbonyl)amir	C48H54N8O7	854.99	A/B	-8.7
3396	C(=O)([C@@H](NC(=O)OC)C(C)C)N1[C@H]2[C@H](C2)C[C@H]1c1[nH]c2c(n1)ccc1c2ccc(c1)c1cc(F)c(cc1)c1[nH]c(n	1377041-37-6	Carbamic acid, N,N,Ä≤-[2,7-triphenylenediylbis[1H-imidazole-5,2-diyl-(2S)-2,1-py	C50H56N8O8	897.03	A/B	-8.7
3397	C(=O)([C@@H](NC(=O)OC)C(C)C)N1[C@@H](CCC1)c1[nH]c(cn1)c1cc(C#CC)c(cc1)c1ccc(cc1)c1[nH]c(nc1)[C@H]1N(	1377042-11-9	Carbamic acid, N-[(1S)-1-[[(1R,3S,5R)-3-[5-[4-[2-[(1R,3S,4S)-2-[(2S)-2-[(methoxy	C45H52N8O6	800.94	A/B	-8.7
3398	C(=O)([C@@H](NC(=O)OC)C(C)C)N1[C@H](c2[nH]c3c4c(cc(cc4)c4cc5c(cc4)c4c(cc5)nc([nH]4)[C@H]4N(C(=O)[C@@	1377044-04-6	Carbamic acid, N,N,Ä≤-[5H-phenanthro[4,5-bcd]pyran-2,7-diylbis[1H-imidazole-5,2-	C43H50N8O7	790.91	A/B	-8.7
3399	C(=O)([C@@H](NC(=O)OC)C1CCOCC1)N1[C@@H]([C@@H]2C[C@H]1CC2)c1[nH]c2c(n1)ccc1c2ccc(c1)c1cc2c(cc1)c1c(c	1377604-14-2	Carbamic acid, N-[(1S)-2-[(2S)-2-[5-[4-[2-[(1R,3S,4S)-2-[(2S)-2-[(methoxycarbony	C46H54N8O7	830.97	A/B	-8.7
3400	C(=O)([C@@H](NC(=O)OC)C(C)C)N1[C@@H](CCC1)c1[nH]c(cn1)c1ccc(cc1)c1ccc(cc1)C1CCCCC1	1380290-12-9	Carbamic acid, N-[(1S)-2-methyl-1-[[(2S)-2-(5-[1,1,Ä≤:4,Ä≤,1,Ä≤,Ä≤-terphenyl]-4	C32H34N4O3	522.64	A/B	-8.7
3401	N(CC(=O)N1CCN(S(=O)(=O)C)CC1)c1nc(nc(n1)N1CCOCC1)c1ccc(N)cc1	1391924-85-8	L-Phenylalanine, N-[4-(3-hydroxyphenyl)-6-(4-morpholinyl)-1,3,5-triazin-2-yl]-	C22H23N5O4	421.45	A/B	-8.7
3402	N(c1nc(nc(n1)N1CCOCC1)c1c2c([nH]nc2)ccc1)C1CCOCC1	1391925-90-8	Propanamide, 3-[[4-(1H-indazol-4-yl)-6-(4-morpholinyl)-1,3,5-triazin-2-yl]amino]	C19H24N8O2	396.45	A/B	-8.7
3403	N(CC(=O)O)c1nc(nc(n1)N1CCOCC1)c1c2c([nH]nc2)ccc1	1391926-01-4	Alanine, N-[4-(1H-indazol-4-yl)-6-(4-morpholinyl)-1,3,5-triazin-2-yl]-2-methyl-	C18H21N7O3	383.4	A/B	-8.7
3404	C(=O)([C@@H](NC(=O)OC)[C@H]1C[C@@H](C)O[C@H](C)C1)N1[C@@H](CC[C@@H]1C)c1[nH]c(cn1)c1ccc(cc1)c1ccc(cc	1393644-45-5	4-Pyrimidinecarboxamide, N-[(4-fluorophenyl)methyl]-1,6-dihydro-5-hydroxy-1-meth	C20H20FN6O5		A/B	-8.7
3405	O=C1c2n(C([C@H]3N1[C@](C([2H])([2H])[2H])(C(C(O3)([2H])[2H])([2H])[2H])[2H])([2H])[2H])cc(C(=O)NC(c1	1407166-88-4	2H-Pyrido[1,Ä≤,2,Ä≤:4,5]pyrazino[2,1-b][1,3]oxazine-2,3,4,12-d4-9-carboxamide,	C20H6D13F2N3O5		A/B	-8.7
3406	O=C1c2n(C([C@H]3N1[C@](C)(C(C(O3)([2H])[2H])([2H])[2H])[2H])([2H])[2H])cc(C(=O)NC(c1c(F)cc(F)cc1)([2	1407166-99-7	2H-Pyrido[1,Ä≤,2,Ä≤:4,5]pyrazino[2,1-b][1,3]oxazine-2,3,12-d3-9-carboxamide, N-	C20H8D11F2N3O5		A/B	-8.7
3407	O=C1c2n(C[C@]3(N1[C@](C)(C(C(O3)([2H])[2H])([2H])[2H])[2H])[2H])cc(C(=O)NC(c1c(F)cc(F)cc1)([2H])[2H]	1407167-07-0	2H-Pyrido[1,Ä≤,2,Ä≤:4,5]pyrazino[2,1-b][1,3]oxazine-2,3-d2-9-carboxamide, N-[(2	C20H9D10F2N3O5		A/B	-8.7
3408	O=C1c2n(C[C@]3(N1[C@](C)(CCO3)[2H])[2H])cc(C(=O)NC(c1c(F)cc(F)cc1)([2H])[2H])c(=O)c2O	1407167-11-6	2H-Pyrido[1,Ä≤,2,Ä≤:4,5]pyrazino[2,1-b][1,3]oxazine-9-carboxamide, N-[(2,4-difl	C20H13D6F2N3O5		A/B	-8.7
3409	O=C1c2n(C[C@H]3N1[C@](C([2H])([2H])[2H])(C(C(O3)([2H])[2H])([2H])[2H])[2H])cc(C(=O)NCc1c(F)cc(F)cc1)	1407167-22-9	2H-Pyrido[1,Ä≤,2,Ä≤:4,5]pyrazino[2,1-b][1,3]oxazine-12-d-9-carboxamide, N-[(2,4	C20H17D2F2N3O5		A/B	-8.7
3410	O=C1c2n(C[C@H]3N1[C@H](C)C(C(O3)([2H])[2H])([2H])[2H])cc(C(=O)NCc1c(F)cc(F)cc1)c(=O)c2O	1407167-25-2	2H-Pyrido[1,Ä≤,2,Ä≤:4,5]pyrazino[2,1-b][1,3]oxazine-2,3,4-d3-9-carboxamide, N-[	C20H14D5F2N3O5		A/B	-8.7
3411	C(NC(=O)c1[nH]c(nc(=O)c1O)c1ccccn1)(C)C12CC3CC(C2)CC(C1)C3	1408304-98-2	4-Pyrimidinecarboxamide, 1,6-dihydro-5-hydroxy-6-oxo-2-(2-pyrazinyl)-N-(1-tricyc	C21H25N5O3	395.45	A/B	-8.7
3412	O(C(=O)c1ccccc1)[C@H]1[C@@H](O[C@H](COC(=O)c2ccccc2)[C@H]1OC(=O)C1=CN(C)C=CC1)n1nc(C(=O)N)nc1	140891-44-7	1H-1,2,4-Triazole-3-carboxamide, 1-[3,5-di-O-benzoyl-2-O-(3-pyridinylcarbonyl)-Œ	C28H23N5O8	557.51	A/B	-8.7
3413	C(OC[C@H]1O[C@H]([C@H](OC(=O)CCCCCCCCCC)[C@@H]1O)n1nc(C(=O)N)nc1)(c1ccc(OC)cc1)(c1ccc(OC)cc1)c1cccc	140891-48-1	1H-1,2,4-Triazole-3-carboxamide, 1-[3,5-di-O-benzoyl-2-O-[(1,4-dihydro-1-methyl-	C29H27N5O8	573.55	A/B	-8.7
3414	O[C@H]1[C@@H](O[C@H](C(=O)NCc2cc(on2)c2cc(OC)ccc2)[C@H]1O)n1nc(C(=O)N)nc1	1414801-85-6	Not Yet Assigned	C20H24N6O6	444.44	A/B	-8.7
3415	O[C@H]1[C@@H](O[C@H](C(=O)Nc2ccc(Oc3ccccc3)nc2)[C@H]1O)n1nc(C(=O)N)nc1	1414801-96-9	Not Yet Assigned	C19H20N6O7	444.4	A/B	-8.7
3416	C(OC(CO)CO)n1c2c(nc1)c(=O)nc(NCNc1ccc(S(=O)(=O)NC3C(OC)C(OC)CN=N3)cc1)[nH]2	1415032-05-1	Benzenesulfonamide, 4-[[[[6,9-dihydro-9-[[2-hydroxy-1-(hydroxymethyl)ethoxy]meth	C20H23N9O6S	517.52	A/B	-8.7
3417	C=C1[C@@H](n2c3c(nc2)c(=O)nc(N[C@H](C(=O)O)C)[nH]3)C[C@H](O)[C@H]1CO	141511-27-5	Benzoic acid, 5-[[3-[bis(3-carboxy-4-hydroxyphenyl)methylene]-5-carboxy-6-oxo-1,	C30H20O12	572.47	A/B	-8.7
3418	C(=O)(NC)c1c(=O)[nH]cc(F)n1	1418741-56-6	Thiourea, N-[4-[[6-(1,1-dimethylethyl)-3-(methylthio)-5-oxo-1,2,4-triazin-4(5H)-	C26H30N10O2S2	578.71	A/B	-8.7
3419	O=c1c2c(n(cn2)[C@@H]2O[C@H](COP(=O)(O)O)[C@@](O)([C@H]2O)[2H])[nH]c(N)n1	142217-81-0	6H-Purin-6-one, 2-amino-1,9-dihydro-9-[(1S,3R,4S)-2-methylene-4-(phenylmethoxy)-	C26H27N5O3	457.52	A/B	-8.7
3420	[N]c1n(c2c(n1)c(=O)nc[nH]2)[C@@H]1O[C@H](CO)[C@@H](O)[C@H]1O	1423120-02-8	3-Azabicyclo[3.1.0]hexane-2-carboxamide, N-[(1S)-1-(2-amino-2-oxoacetyl)butyl]-3	C40H68N6O5	713.01	A/B	-8.7
3421	C(=O)(Nc1c(C(=O)O)cccc1)c1cc2c(ccc(C(=O)Nc3c(C(=O)O)cccc3)c2)cc1	1426342-67-7	Benzoic acid, 2,2,Ä≤-[[1,1,Ä≤-biphenyl]-4,4,Ä≤-diylbis(carbonylimino)]bis-	C28H20N2O6	480.47	A/B	-8.7
3422	[CH2]c1c2c(N(c3c(C(=O)N2)cccn3)C2CC2)ncc1	1426656-16-7	Not Yet Assigned	C63H90N16O15S	1343.55	A/B	-8.7
3423	C(=O)(OCC)C1=C(N)OC(=CC1c1ccccc1)c1[nH]c2c(n1)cccc2	1427057-13-3	L-Alanine, N-(5-iodo-P-1-naphthalenyl-5,Ä≤-uridylyl)-, methyl ester	C23H25IN3O10P	661.34	A/B	-8.7
3424	O=c1n(cc(C(F)(F)F)c(=O)n1CC(F)(F)c1ccccc1)[C@@H]1O[C@H](COP(=O)(OP(=O)(O)O)O)[C@@H](O)[C@H]1O	1427676-62-7	Uridine 5,Ä≤-(trihydrogen diphosphate), 3-[2-(2,4-difluorophenyl)ethyl]-5-(trif	C18H19F5N2O12P2	612.29	A/B	-8.7
3425	C([C@H](OCP(=O)(OP(=O)(Oc1ccccc1)CO[C@@H](Cn1c2c(nc1)c(N)ncn2)C)Oc1ccccc1)C)n1c2c(nc1)c(N)ncn2	1428441-88-6	1-Piperazinecarboxamide, 4-methyl-N-[(1S)-2-oxo-1-(phenylmethyl)-2-[[(1E)-1-(phe	C31H36N4O4S	560.71	A/B	-8.7
3426	N(=Nc1ccc(N=Nc2ccccc2)cc1)c1c2c(cc(S(=O)(=O)O)cc2)ccc1O	1439923-16-6	4-Piperidinecarboxamide, 1-[4-methyl-6-(4-morpholinyl)-1,3,5-triazin-2-yl]-N-[[2	C22H27F3N6O2	464.48	A/B	-8.7
3427	[C@H](Cc1ccccc1)(C[C@@H]([C@H](Cc1ccccc1)NC(=O)OC(C)(C)C)O)NC(=O)[C@@H](NC(=O)N(Cc1csc(N)n1)C)C(C)C	144142-20-1	2,4,7,12-Tetraazatridecan-13-oic acid, 10-hydroxy-2-methyl-5-(1-methylethyl)-1-(	C37H46N6O5S	686.86	A/B	-8.7
3428	C[C@]1(F)[C@@H](O[C@@](CO[P@](=O)(Oc2ccccc2)N[C@H](C(=O)OC(C)C)C)(F)[C@H]1O)n1c(=O)[nH]c(=O)cc1	1443648-67-6	L-Alanine, N-[[P(S),2,Ä≤R]-2,Ä≤-deoxy-2,Ä≤-fluoro-4,Ä≤-C-fluoro-2,Ä≤-methyl-P-1	C26H30F2N3O9P	597.5	A/B	-8.7
3429	O(c1nc(Nc2ccc(C#N)cc2)nc(N)c1Br)c1c(C)cc(C#N)cc1C.C(CCC(=O)O)CC(=O)O	1445668-95-0	Benzonitrile, 4-[[6-amino-5-bromo-2-[(4-cyanophenyl)amino]-4-pyrimidinyl]oxy]-3,	C20H15BrN6O.CH4O.H2O4S		A/B	-8.7
3430	O(c1nc(Nc2ccc(C#N)cc2)nc(N)c1Br)c1c(C)cc(C#N)cc1C.CO	1445669-00-0	Benzonitrile, 4-[[6-amino-5-bromo-2-[(4-cyanophenyl)amino]-4-pyrimidinyl]oxy]-3,	C20H15BrN6O.1/2CH4O		A/B	-8.7
3431	O(c1nc(Nc2ccc(C#N)cc2)nc(N)c1Br)c1c(C)cc(C#N)cc1C.C(#N)C	1445669-01-1	Benzonitrile, 4-[[6-amino-5-bromo-2-[(4-cyanophenyl)amino]-4-pyrimidinyl]oxy]-3,	C20H15BrN6O.CH4O		A/B	-8.7
3432	O(P(=O)(OC[C@H]1O[C@H]([C@H](O)[C@@H]1O)n1c2c(nc1)c(N)ncn2)O)[C@H]1[C@@H](O[C@H](COP(=O)(OP(=O)(OP(=	1445874-20-3	Guanosine, 5,Ä≤-O-(1,3,5,5-tetrahydroxy-1,3,5-trioxido-2-oxa-1,3,5-triphosphaper	C21H30N10O20P4	866.41	A/B	-8.7
3433	O[C@H]1[C@H](n2c3c(nc2)c(N)ncn3)O[C@H](COP(=O)(O[As](=O)(O[As](=O)(O)O)O)O)[C@H]1O	144604-17-1	Furo[3,Ä≤,4,Ä≤:6,7]naphtho[2,3-d]-1,3-dioxole-6,9-dione, 5,5a,8,8a-tetrahydro-5	C20H14O8	382.32	A/B	-8.7
3434	O(C(=O)N[C@@H](Cc1ccccc1)[C@@H](CN(S(=O)(=O)c1ccc(NC(=O)NC)cc1)CC(C)C)O)[C@@H]1[C@H]2[C@@H](OC1)OCC2	1450913-01-5	1-Pyrrolidinepropanamide, N-[3-[1-[7-(11-cyclopropyl-6,11-dihydro-4-methyl-6-oxc	C47H48F2N10O5	870.94	A/B	-8.7
3435	N(c1c2c(ncc1)cc(Cl)cc2)c1cc(cc(CN2CCCCC2)c1O)c1ccc(Cl)cc1	146298-42-2	[1,1,Ä≤-Biphenyl]-4-ol, 4,Ä≤-chloro-3-[(7-chloro-4-quinolinyl)amino]-5-(1-pyrro	C26H23Cl2N3O	464.39	A/B	-8.7
3436	O[C@]12[C@](C)([C@@]3(C)[C@H](CC2)[C@]2(C)[C@@H](CC3)C(C)(C)C(=O)CC2)CC[C@]2(C(=O)O)[C@H]1CC(C)(C)CC	1467662-11-8	Uridine, 2,Ä≤-deoxy-5-iodo-3,Ä≤-O-(2,3,5-tri-O-benzoyl-Œ≤-D-ribofuranosyl)-	C35H31IN2O12	798.53	A/B	-8.7
3437	C(=O)(c1[nH]c2c(c1)cc(NS(=O)(=O)CCN(C)C)cc2)N1CCN(CC1)c1c(NC(C)C)cccn1	147540-00-9	4-Pyridineethanesulfonamide, N-[2-[[4-[3-[(1-methylethyl)amino]-2-pyridinyl]-1-p	C28H33N7O3S	547.67	A/B	-8.7
3438	C(=O)(c1[nH]c2c(c1)cc(NS(=O)(=O)N1CCNCC1)cc2)N1CCN(CC1)c1c(NC(C)C)cccn1	147540-10-1	1-Piperazinesulfonamide, N-[2-[[4-[3-[(1-methylethyl)amino]-2-pyridinyl]-1-piper	C32H40N8O3S	616.78	A/B	-8.7
3439	C(=O)(c1cc2c([nH]1)ccc(N)c2)N1CCN(CC1)c1c(NC(CC)(C)C)cccn1	147540-12-3	4-Morpholinesulfonamide, N-[2-[[4-[3-[(1-methylethyl)amino]-2-pyridinyl]-1-piper	C25H33N7O4S	527.64	A/B	-8.7
3440	C(=O)(c1cc2c([nH]1)ccc(NS(=O)(=O)CCCN1CCOCC1)c2)N1CCN(CC1)c1c(NC(C)(C)C)cccn1	147540-35-0	1-Piperidinepropanesulfonamide, N-[2-[[4-[3-[(1,1-dimethylethyl)amino]-2-pyridir	C30H43N7O3S	581.77	A/B	-8.7
3441	C(n1c2c(nc1CC)c(C)cc(C)n2)c1ccc(cc1)c1c(S(=O)(=O)NC(=O)OC)cccc1	148401-77-8	Carbamic acid, [[4,Ä≤-[(2-ethyl-7-methyl-3H-imidazo[4,5-b]pyridin-3-yl)methyl][1	C30H28N4O4S	540.63	A/B	-8.7
3442	C(n1c2c(nc1CC)c(C)cc(C)n2)c1ccc(cc1)c1c(S(=O)(=O)NC(=O)NCCC)cccc1	148401-90-5	[1,1,Ä≤-Biphenyl]-2-sulfonamide, N-[(ethylamino)carbonyl]-4,Ä≤-[(2-ethyl-5,7-di	C26H29N5O3S	491.61	A/B	-8.7
3443	[C@@H](O[C@H](COP(=O)(OP(=O)(NP(=O)(OP(=O)(OC[C@@H](O[C@H](CO)n1c2c(nc1)c(N)ncn2)CO)O)O)O)O)CO)(CO)n	1493431-16-5	Isohypophosphoric acid, P,Ä≤-[(7S,9R)-9-(2-amino-1,6-dihydro-6-oxo-9H-purin-9-yl	C21H34N10O19P4	854.45	A/B	-8.7
3444	N(c1c(N(=O)=O)cnc(Nc2c(C)cc(/C=C/C#N)cc2C)n1)c1ccc(C#N)cc1	1504553-90-5	Benzonitrile, 4-[[6-[[4-[(1E)-2-cyanoethenyl]-2,6-dimethylphenyl]amino]-9H-purir	C23H18N8	406.44	A/B	-8.7
3445	N(c1c2c(n(Cc3ccc(OC)cc3)cn2)nc(Nc2ccc(C#N)cc2)n1)c1c(C)cc(/C=C/C#N)cc1C	1504556-70-0	Benzonitrile, 4-[[2-[[4-[(1E)-2-cyanoethenyl]-2,6-dimethylphenyl]amino]-5-nitro-	C22H17N7O2	411.42	A/B	-8.7
3446	O=c1c2c(n(cn2)[C@H]2C[C@H](N[Si](NC[C@H]3O[C@H](C[C@@H]3O)n3c4c(nc3)c(=O)nc(N)[nH]4)(C)C)[C@@H](CO)O	1513874-39-9	Guanosine, 2,Ä≤-deoxy-3,Ä≤-de(phosphinicooxy)guanylyl-1,2-hydrazinediyl-(3,Ä≤,Ü	C20H26N12O6	530.5	A/B	-8.7
3447	C([C@H](OCP(=O)(OCc1c(C)cccc1)O)C)n1c2c(nc1)c(N)ncn2	151488-21-0	Carbamic acid, [[3-[4-[(2-ethyl-5,7-dimethyl-3H-imidazo[4,5-b]pyridin-3-yl)methy	C31H40N4O4S2	596.8	A/B	-8.7
3448	C(OCCOC(=O)CCC(=O)N)n1c2c(nc1)c(=O)nc(N)[nH]2	1520867-34-8	L-Valine, N-[(phenylmethoxy)carbonyl]-L-valyl-L-prolyl-, 2-[(2-amino-1,6-dihydrc	C31H42N8O8	654.71	A/B	-8.7
3449	O[C@H]1[C@@H](O[C@H](COP(=O)(N[C@@H](C(=O)OCC(C)(C)C)C)N[C@H](C(=O)OCC(C)(C)C)C)[C@H]1O)n1nc(C(=O)N)	1526922-05-3	L-Alanine, N,N,Ä≤-phosphinicobis-, 1,1,Ä≤-bis(phenylmethyl) ester, 5,Ä≤-ester w	C28H35N6O10P	646.59	A/B	-8.7
3450	O=C1c2c(N(C)C1=O)ccc(S(=O)(=O)N1CCC(C)CC1)c2	1529815-12-0	1H-Indole-2,3-dione, 5-[(4-methyl-1-piperidinyl)sulfonyl]-1-(2-naphthalenylmethy	C25H24N2O4S	448.53	A/B	-8.7
3451	C(=O)(NCc1ccc(F)cc1)c1c(OC(OC(=O)OC)CC)c(=O)n(C)c(C(NC(=O)c2oc(C)nn2)(C)C)n1	1538603-99-4	Carbonic acid, cyclopropylmethyl 1-[[4-[[[(4-fluorophenyl)methyl]amino]carbonyl]	C28H33FN6O8	600.6	A/B	-8.7
3452	O(C(OC(=O)OCC1CC1)C(C)C)c1c(C(=O)NCc2ccc(F)cc2)nc(C(NC(=O)c2nnc(C)o2)(C)C)n(C)c1=O	1538604-02-2	Carbonic acid, cyclohexyl 1-[[4-[[[(4-fluorophenyl)methyl]amino]carbonyl]-1,6-di	C31H39FN6O8	642.68	A/B	-8.7
3453	O(C(OC(=O)CC)C)c1c(C(=O)NCc2ccc(F)cc2)nc(C(NC(=O)c2nnc(C)o2)(C)C)n(C)c1=O	1538604-16-8	4-Pyrimidinecarboxamide, 5-[1-(acetyloxy)ethoxy]-N-[(4-fluorophenyl)methyl]-1,6-	C24H27FN6O7	530.51	A/B	-8.7
3454	C(=O)(NCc1ccc(F)cc1)c1c(OC(OC(=O)C)CC)c(=O)n(C)c(C(NC(=O)c2oc(C)nn2)(C)C)n1	1538604-22-6	4-Pyrimidinecarboxamide, 5-[1-(benzoyloxy)ethoxy]-N-[(4-fluorophenyl)methyl]-1,6	C29H29FN6O7	592.58	A/B	-8.7
3455	N(C(=O)CCN)C12CC3(C)CC(C)(C1)CC(C2)C3	154237-39-5	Cyclopentanepropanamide, N-[[4,Ä≤-[(2-ethyl-5,7-dimethyl-3H-imidazo[4,5-b]pyridi	C31H36N4O3S	544.71	A/B	-8.7
3456	C(n1c2c(nc1CC)c(C)cc(C)n2)c1ccc(cc1)c1c(S(=O)(=O)NC(=O)CCCCC)ccc(OCC)c1	154551-81-2	Hexanamide, N-[[4,Ä≤-[(2-ethyl-5,7-dimethyl-3H-imidazo[4,5-b]pyridin-3-yl)methyl	C31H38N4O4S	562.72	A/B	-8.7
3457	C(n1c2c(nc1CC)c(C)cc(C)n2)c1ccc(cc1)c1c(S(=O)(=O)NC(=O)CCCCC)ccc(c1)c1ccccc1	154551-82-3	Hexanamide, N-[[5-ethoxy-4,Ä≤-[(2-ethyl-5,7-dimethyl-3H-imidazo[4,5-b]pyridin-3-	C31H38N4O4S	562.72	A/B	-8.7
3458	C(n1c2c(nc1CC)c(C)cc(C)n2)c1ccc(cc1)c1c(S(=O)(=O)NC(=O)CCCCC)ccc(C(C)(C)C)c1	154551-84-5	Hexanamide, N-[[5-(1,1-dimethylpropyl)-4,Ä≤-[(2-ethyl-5,7-dimethyl-3H-imidazo[4,	C34H44N4O3S	588.8	A/B	-8.7
3459	C(n1c2c(nc1CC)c(C)cc(C)n2)c1ccc(cc1)c1c(S(=O)(=O)NC(=O)CCCCC)ccc(OCC(F)(F)F)c1	154551-88-9	Hexanamide, N-[[4,Ä≤-[(2-ethyl-5,7-dimethyl-3H-imidazo[4,5-b]pyridin-3-yl)methyl	C34H43N5O4S	617.8	A/B	-8.7
3460	C(n1c2c(nc1CC)c(C)cc(C)n2)c1ccc(cc1)c1c(S(=O)(=O)NC(=O)COCC)ccc(CN(C)C)c1	154552-25-7	Acetamide, N-[[5-(1,1-dimethylethyl)-4,Ä≤-[(2-ethyl-5,7-dimethyl-3H-imidazo[4,5-	C31H38N4O4S	562.72	A/B	-8.7
3461	C(n1c2c(nc1CC)c(C)cc(C)n2)c1ccc(cc1)c1c(S(=O)(=O)NC(=O)NCCCC)ccc(OC)c1	154552-38-2	[1,1,Ä≤-Biphenyl]-2-sulfonamide, N-[(butylamino)carbonyl]-4,Ä≤-[(2-ethyl-5,7-di	C32H41N5O3S	575.76	A/B	-8.7
3462	C(n1c2c(nc1CC)c(C)cc(C)n2)c1ccc(cc1)c1c(S(=O)(=O)NC(=O)NCCCC)ccc(CN(CC)CC)c1	154552-46-2	[1,1,Ä≤-Biphenyl]-2-sulfonamide, N-[(butylamino)carbonyl]-5-[(dimethylamino)meth	C31H40N6O3S	576.75	A/B	-8.7
3463	C(n1c2c(nc1CC)c(C)cc(C)n2)c1ccc(cc1)c1c(S(=O)(=O)NC(=O)CCc2ccccc2)ccc(C)c1	154552-50-8	[1,1,Ä≤-Biphenyl]-2-sulfonamide, N-[(butylamino)carbonyl]-4,Ä≤-[(2-ethyl-5,7-di	C31H39N5O4S	577.74	A/B	-8.7
3464	C(n1c2c(nc1CC)c(C)cc(C)n2)c1ccc(cc1)c1c(S(=O)(=O)NC(=O)c2c(Oc3ccccc3)cccc2)ccc(C)c1	154552-70-2	Benzenepropanamide, N-[[4,Ä≤-[(2-ethyl-5,7-dimethyl-3H-imidazo[4,5-b]pyridin-3-y	C34H36N4O3S2	612.8	A/B	-8.7
3465	C(n1c2c(nc1CC)c(C)cc(C)n2)c1ccc(cc1)c1c(S(=O)(=O)NC(=O)c2c(Oc3ccccc3)cccc2)ccc(COC)c1	154552-77-9	Benzamide, N-[[4,Ä≤-[(2-ethyl-5,7-dimethyl-3H-imidazo[4,5-b]pyridin-3-yl)methyl]	C40H40N4O4S	672.84	A/B	-8.7
3466	C(n1c2c(nc1CC)c(C)cc(C)n2)c1ccc(cc1)c1c(S(=O)(=O)NC(=O)c2c(OCC)cccc2)ccc(SCC)c1	154553-09-0	Benzamide, 2-ethoxy-N-[[4,Ä≤-[(2-ethyl-5,7-dimethyl-3H-imidazo[4,5-b]pyridin-3-y	C35H38N4O5S	626.77	A/B	-8.7
3467	C(n1c2c(nc1CC)c(C)cc(C)n2)c1ccc(cc1)c1c(S(=O)(=O)NC(=O)c2ccccc2)ccc(C)c1	154553-10-3	Benzamide, 2-ethoxy-N-[[4,Ä≤-[(2-ethyl-5,7-dimethyl-3H-imidazo[4,5-b]pyridin-3-y	C34H36N4O4S2	628.8	A/B	-8.7
3468	C(n1c2c(nc1CC)c(C)cc(C)n2)c1ccc(cc1)c1c(S(=O)(=O)NC(=O)c2ccccc2)ccc(CCCC)c1	154553-13-6	Benzamide, N-[[4,Ä≤-[(2-ethyl-5,7-dimethyl-3H-imidazo[4,5-b]pyridin-3-yl)methyl]	C33H34N4O3S	566.71	A/B	-8.7
3469	C(n1c2c(nc1CC)c(C)cc(C)n2)c1ccc(cc1)c1c(S(=O)(=O)NC(=O)c2ccccc2)ccc(C(C)C)c1	154553-14-7	Benzamide, N-[[5-butyl-4,Ä≤-[(2-ethyl-5,7-dimethyl-3H-imidazo[4,5-b]pyridin-3-yl	C34H36N4O3S	580.74	A/B	-8.7
3470	C(n1c2c(nc1CC)c(C)cc(C)n2)c1ccc(cc1)c1c(S(=O)(=O)NC(=O)COCCC)ccc(C)c1	154553-29-4	Benzamide, N-[[4,Ä≤-[(2-ethyl-5,7-dimethyl-3H-imidazo[4,5-b]pyridin-3-yl)methyl]	C32H32N4O3S2	584.75	A/B	-8.7
3471	C(n1c2c(nc1CC)c(C)cc(C)n2)c1ccc(cc1)c1c(S(=O)(=O)NC(=O)NCCc2ccccc2)ccc(OCC)c1	154553-59-0	[1,1,Ä≤-Biphenyl]-2-sulfonamide, 4,Ä≤-[(2-ethyl-5,7-dimethyl-3H-imidazo[4,5-b]py	C34H37N5O4S	611.75	A/B	-8.7
3472	N(=C1c2c(N(C)C1=O)cccc2)NC(=S)N(CC=C)CC=C	1551454-39-7	2,5,8,10-Tetraazaundecanoic acid, 10-methyl-11-[2-(1-methylethyl)-4-thiazolyl]-7	C38H49N7O5S2	747.97	A/B	-8.7
3473	CN1C2c3c(C1CC2)c(ccc3c1[nH]c(nc1)[C@H]1N(C(=O)[C@@H](NC(=O)OC)C2CCCCC2)CCC1)c1ccc(cc1)c1[nH]c(nc1)[C	1560639-47-5	Carbamic acid, N-[(1S)-2-methyl-1-[[(2S,4S)-4-methyl-2-[5-[4-[1,2,3,4-tetrahydrc	C47H61N9O6	848.04	A/B	-8.7
3474	C(=O)([C@@H](NC(=O)OC)C(C)C)N1[C@H](c2[nH]c(c3c4c(c(cc3)c3ccc(cc3)c3[nH]c(nc3)[C@H]3N(C(=O)[C@@H](NC	1560639-95-3	Carbamic acid, N-[(1S)-2-methyl-1-[[(2S)-2-[5-[4-[1,2,3,4-tetrahydro-8-[2-[(2S)-	C46H58N8O6	819	A/B	-8.7
3475	C(=O)([C@@H](NC(=O)OC)C(C)C)N1[C@H](c2[nH]c(c3c4c(c(cc3)c3ccc(cc3)c3[nH]c(nc3)[C@H]3N(C(=O)[C@@H](NC	1560640-37-0	Carbamic acid, N-[(1S)-2-methyl-1-[[(1R,3S,4S)-3-[5-[4-[1,2,3,4-tetrahydro-8-[2-	C50H62N8O6	871.08	A/B	-8.7
3476	C(=O)([C@@H](NC(=O)OC)C(C)C)N1[C@H](c2[nH]c(c3c4c(C5CC4C=C5)c(cc3)c3ccc(cc3)c3[nH]c(nc3)[C@H]3N(C(=O	1560649-48-0	Carbamic acid, N-[(1S)-1-[[(2S)-2-[5-[8-[4-[2-[(2S)-4,4-difluoro-1-[(2S)-2-[(met	C45H53F4N9O6	891.95	A/B	-8.7
3477	CN1C2c3c(C1C=C2)c(ccc3c1[nH]c(nc1)[C@H]1N(C(=O)[C@@H](NC(=O)OC)C(C)C)CCC1)c1ccc(cc1)c1[nH]c(nc1)[C@H	1560649-87-7	Carbamic acid, N-[(1S)-2-methyl-1-[[(2S)-2-[5-[4-[1,2,3,4-tetrahydro-8-[2-[(2S)-	C45H54N8O7	818.96	A/B	-8.7
3478	C(=O)([C@@H](NC(=O)OC)C(C)C)N1[C@H](c2[nH]c(c3c4c(c(cc3)c3ccc(cc3)c3[nH]c(nc3)[C@H]3N(C(=O)[C@@H](NC	1561047-25-3	Carbamic acid, N-[(1S)-2-methyl-1-[[(2S,4S)-4-methyl-2-[5-[4-[1,2,3,4-tetrahydrc	C48H62N8O6	847.06	A/B	-8.7
3479	O=c1n([C@H]2O[C@@H](COC(=O)C)SC2)ccc(NC(=O)C)n1	158614-82-5	3,Ä≤-Guanylic acid, P-thioguanylyl-(3,Ä≤,Üí5,Ä≤)-	C20H26N10O14P2S	724.49	A/B	-8.7
3480	O=c1n([C@H]2O[C@@H](COC(=O)C)SC2)ccc(N)n1	158703-48-1	Guanosine, 2,Ä≤-deoxy-P-thioguanylyl-(3,Ä≤,Üí5,Ä≤)-2,Ä≤-deoxy-P-thioguanylyl-(3	C30H37N15O14P2S2	957.78	A/B	-8.7
3481	C(=O)([C@@H](NC(=O)OC)C(C)C)N1[C@H](c2[nH]c(c3c4c(C5(CCC4)CCCO5)c(cc3)c3ccc(cc3)c3[nH]c(nc3)[C@H]3N(	1588404-25-4	Carbamic acid, N-[(1S)-1-[[(2S)-2-[5-[4-[3,4-dihydro-5-[2-[(2S)-1-[(2S)-2-[(meth	C48H63N9O6	862.07	A/B	-8.7
3482	C(=O)([C@@H](NC(=O)OC)C(C)C)N1[C@H](c2[nH]c(c3c4c(C5(CC4)CCCC5)c(cc3)c3ccc(cc3)c3[nH]c(nc3)[C@H]3N(C	1588404-27-6	Carbamic acid, N-[(1S)-2-methyl-1-[[(2S)-2-[5-[2,Ä≤,3,Ä≤,4,5-tetrahydro-7,Ä≤-[4	C46H58N8O7	835	A/B	-8.7
3483	O=C1[C@@H]2[C@@H](c3c([C@H](OCc4cn(nn4)c4ccc(Br)cc4)[C@H]2CO1)cc1c(c3)OCO1)c1cc(OC)c(OC)c(OC)c1	1600500-19-3	Furo[3,Ä≤,4,Ä≤:6,7]naphtho[2,3-d]-1,3-dioxol-6(5aH)-one, 5,8,8a,9-tetrahydro-9-	C32H28F3N3O8	639.58	A/B	-8.7
3484	O[C@@H]1[C@H](n2c3c(nc2)c(N)ncn3)O[C@H](COP(=O)(O[C@@H]2[C@@H](COP(=O)(O[C@H]3[C@H](O)[C@@H](O[C@@H]	160186-54-9	Œ≤-D-arabino-Adenosine, Œ≤-D-arabino-adenylyl-(3,Ä≤,Üí5,Ä≤)-Œ≤-D-arabino-adenyl	C80H97N40O46P7	2571.68	A/B	-8.7
3485	O=C1[C@@H]2[C@@H](c3c([C@@H](O)[C@H]2CO1)cc1c(c3)OCO1)c1cc2c(cc1)OCO2	1610886-41-3	Furo[3,Ä≤,4,Ä≤:6,7]naphtho[2,3-d]-1,3-dioxol-6(5aH)-one, 5-(1,3-benzodioxol-5-y	C20H16O7	368.34	A/B	-8.7
3486	O=C1[C@@H]2[C@@H](c3c([C@H](O)[C@H]2CO1)cc1c(c3)OCO1)c1ccc(Br)cc1	1610886-60-6	Furo[3,Ä≤,4,Ä≤:6,7]naphtho[2,3-d]-1,3-dioxol-6(5aH)-one, 5,8,8a,9-tetrahydro-9-	C23H18O5	374.39	A/B	-8.7
3487	O[C@H]1[C@@H](O[C@H](COP(=O)(OC(OC(=O)c2ccccc2)C)OC(OC(=O)c2ccccc2)C)[C@H]1O)n1c(=O)c(C(=O)N)nc(F)c1	1613031-62-1	2-Pyrazinecarboxamide, 4-[5-O-[bis[1-(2,2-dimethyl-1-oxopropoxy)ethoxy]phosphiny	C24H37FN3O13P	625.53	A/B	-8.7
3488	N(C(=O)c1cn2c(c(O)c1=O)C(=O)N1[C@H](C2)O[C@@H]2C[C@H]1CC2)C1(CC1)c1c(F)cc(F)cc1	1616339-81-1	2,5-Methanopyrido[1,Ä≤,2,Ä≤:4,5]pyrazino[2,1-b][1,3]oxazepine-10-carboxamide, N	C22H21F2N3O5	445.42	A/B	-8.7
3489	C(F)[C@]1(F)[C@@H](O[C@H](COP(=O)(Oc2ccccc2)N[C@H](C(=O)OC(C)C)C)[C@H]1O)n1c(=O)[nH]c(=O)cc1	162189-89-1	Thymidine, P-(2-aminoethyl)-P-deoxythymidylyl-(3,Ä≤,Üí5,Ä≤)-, (S)-	C22H32N5O11P	573.49	A/B	-8.7
3490	N(c1nc(nc(n1)N1CCOCC1)N1CCOCC1)c1ccc(cc1)c1cc(nc(N)n1)c1cc(OC)ccc1	1628172-09-7	2-Propen-1-one, 1-[4-[(4,6-di-4-morpholinyl-1,3,5-triazin-2-yl)amino]phenyl]-3-(	C24H26N6O4	462.5	A/B	-8.7
3491	C[C@]1(F)[C@@H](O[C@H](COP(=O)(Oc2c(cccc2)c2ccccc2)N[C@H](C(=O)OC(C)C)C)[C@H]1O)n1c(=O)[nH]c(=O)cc1	1628724-49-1	L-Alanine, N-[(2,Ä≤R)-P-[1,1,Ä≤-biphenyl]-3-yl-2,Ä≤-deoxy-2,Ä≤-fluoro-2,Ä≤-meth	C28H33FN3O9P	605.55	A/B	-8.7
3492	N(c1nc(nc(Nc2c(O)cccc2)n1)N1CCOCC1)c1nc(OC)cc(OC)n1	1631751-08-0	Phenol, 2-[[4-[(4,6-dimethoxy-2-pyrimidinyl)amino]-6-(4-morpholinyl)-1,3,5-triaz	C19H21N9O6	471.43	A/B	-8.7
3493	C(=O)([C@@H](NC(=O)OC(C)(C)C)C(C)C)N1[C@@H](C[C@@H](OC(=O)N2Cc3c(C2)c(F)ccc3)C1)c1[nH]c(cn1)c1ccc(cc	1639870-44-2	2H-Isoindole-2-carboxylic acid, 4-fluoro-1,3-dihydro-, (3R,5S)-1-[(2S)-2-[(methc	C49H56FN9O8	918.02	A/B	-8.7
3494	N(c1c(C)cc(/C=C/C#N)cc1C)c1nc(Nc2ccc(C#N)cc2)nc(OCCCN2CCOCC2)c1	1642572-60-8	Carbamic acid, N-[(1S,2R)-2-hydroxy-3-[(2-methylpropyl)[(2-methyl-6-quinolinyl)s	C31H39N3O7S	597.72	A/B	-8.7
3495	N(c1nc(nc(Oc2ccc(C#N)cc2)n1)N1CCOCC1)c1ccc(OC)cc1	1643332-88-0	Benzonitrile, 4-[[4-(4-morpholinyl)-6-(phenylamino)-1,3,5-triazin-2-yl]oxy]-	C20H18N6O2	374.4	A/B	-8.7
3496	O=c1n([C@@H]2O[C@H](COP(=O)(C(O)c3c(N(=O)=O)cccc3)OC[C@@H]3[C@@H](N=[N+]=[N-])C[C@@H](O3)n3c(=O)[nH]	164652-12-4	Thymidine, 3,Ä≤-azido-P,3,Ä≤-dideoxy-P-[hydroxy(4-nitrophenyl)methyl]thymidylyl-	C27H30N11O12P		A/B	-8.7
3497	N(C(=O)[C@H](Cc1ccccc1)C[C@@H](CN1[C@H](C(=O)NC(C)(C)C)CN(CC)CC1)O)[C@@H]1c2c(C[C@@H]1O)cccc2	164725-13-7	1-Piperazinepentanamide, N-(2,3-dihydro-2-hydroxy-1H-inden-1-yl)-2-[[(1,1-dimeth	C38H51N5O4	641.84	A/B	-8.7
3498	N(C(=O)[C@H](Cc1ccccc1)C[C@@H](CN1[C@H](C(=O)NC(C)(C)C)CN(CCCc2ccccc2)CC1)O)[C@@H]1c2c(C[C@@H]1O)cccc2	164725-27-3	1-Piperazinepentanamide, N-(2,3-dihydro-2-hydroxy-1H-inden-1-yl)-2-[[(1,1-dimeth	C37H49N5O4	627.82	A/B	-8.7
3499	N(C(=O)[C@H](Cc1ccccc1)C[C@@H](CN1[C@H](C(=O)NC(C)(C)C)CN(CC2CCCCO2)CC1)O)[C@@H]1c2c(C[C@@H]1O)cccc2	164725-50-2	1-Piperazinepentanamide, N-(2,3-dihydro-2-hydroxy-1H-inden-1-yl)-2-[[(1,1-dimeth	C38H50N4O5	642.83	A/B	-8.7
3500	N(C(=O)[C@H](Cc1ccccc1)C[C@@H](CN1[C@H](C(=O)NC(C)(C)C)CN(Cc2cc(C)c(NC(=O)NC)cc2)CC1)O)[C@@H]1c2c(C[C	164725-56-8	1-Piperazinepentanamide, N-(2,3-dihydro-2-hydroxy-1H-inden-1-yl)-2-[[(1,1-dimeth	C38H50N4O4	626.83	A/B	-8.7
3501	C(=O)([C@@H](NC(=O)OC)C(C)C)N1CC2(CC2)C[C@H]1c1[nH]c(cn1)c1ccc2cc(ccc2c1)c1ccc(cc1)c1[nH]c(nc1)[C@H]1N(C(=	1653966-91-6	Carbamic acid, N-[(1S)-1-[[(2S)-2-[7-[4-[2-[(3S,5R)-2-[(2S)-2-[(methoxycarbonyl)	C46H56N8O7	832.99	A/B	-8.7
3502	O=C1[C@@H]2[C@@H](c3c([C@@H](OC(=O)C(C)(C)C)[C@H]2CO1)cc1c(c3)OCO1)c1cc(OC)c(OC)c(OC)c1	1653967-17-9	Carbamic acid, N-[(1S)-1-[[(2S)-2-[7-[4-[2-[(3S)-2-[(2S)-2-[(methoxycarbonyl)ami	C46H56N8O7	832.99	A/B	-8.7
3503	N(C(=O)[C@H](Cc1ccccc1)C[C@@H](CN1[C@H](C(=O)NC(C)(C)C)CN(Cc2cc(F)cc(F)c2)CC1)O)[C@@H]1c2c(C[C@@H]1O)c	165946-06-5	1-Piperazinepentanamide, N-(2,3-dihydro-2-hydroxy-1H-inden-1-yl)-2-[[(1,1-dimeth	C35H45N5O6S	663.83	A/B	-8.7
3504	C(NC(=O)c1ccc(CCOc2cc(F)(F)F)ccc2)cc1)C12CC3CC(C2)CC(C1)C3	166022-38-4	Guanosine, P-thiothymidylyl-(3,Ä≤,Üí5,Ä≤)-2,Ä≤-deoxy-P-thioguanylyl-(3,Ä≤,Üí5,Ä≤	C30H38N12O15P2S2	932.77	A/B	-8.7
3505	O=c1n([C@@H]2O[C@H](COP(=O)(C(O)c3ccc(Cl)cc3)OC[C@@H]3[C@@H](N=[N+]=[N-])C[C@@H](O3)n3c(=O)[nH]c(=O)	166318-76-9	Thymidine, 3,Ä≤-azido-P,3,Ä≤-dideoxy-P-(hydroxyphenylmethyl)thymidylyl-(5,Ä≤,Üí	C27H31N10O10P		A/B	-8.7
3506	C(=O)(NC(C)(C)C)[C@H]1N(C[C@@H]([C@@H](NC(=O)c2c(C)c(OC(=O)C)ccc2)CSc2ccccc2)OC(=O)C)C[C@@H]2[C@@H]1	1665278-95-4	3-Isoquinolinecarboxamide, N-(1,1-dimethylethyl)decahydro-2-[(2S,3S)-2-hydroxy-3	C35H45N3O5S	587.82	A/B	-8.7
3507	C(n1c2c(nc1CC)c(C)cc(C)n2)c1ccc(cc1)c1c(S(=O)(=O)NC(=O)OCCCC)ccc(CSC)c1	169281-59-8	Carbamic acid, [[5-[(dimethylamino)methyl]-4,Ä≤-[(2-ethyl-5,7-dimethyl-3H-imidaz	C31H39N5O4S	577.74	A/B	-8.7
3508	C(n1c2c(nc1CC)c(C)cc(C)n2)c1ccc(cc1)c1c(S(=O)(=O)NC(=O)OCCCC)ccc(CCCC)c1	169281-92-9	Carbamic acid, N-[[4,Ä≤-[(2-ethyl-5,7-dimethyl-3H-imidazo[4,5-b]pyridin-3-yl)met	C32H40N4O4S	576.75	A/B	-8.7
3509	C(n1c2c(nc1CC)c(C)cc(C)n2)c1ccc(cc1)c1c(S(=O)(=O)NC(=O)OCCCC)ccc(Cc2ccccc2)c1	169281-93-0	Carbamic acid, [[5-butyl-4,Ä≤-[(2-ethyl-5,7-dimethyl-3H-imidazo[4,5-b]pyridin-3-	C32H40N4O4S	576.75	A/B	-8.7

3510	S(=O)(=O)(/C=C/[C@@H](NC(=O)[C@H](Cc1ccccc1)NC(=O)N1CCOCC1)CCc1ccccc1)c1ccc2c(cc1)cccc2	170111-56-5	4-Morpholinecarboxamide, N-[2-[[3-[(4-bromophenyl)sulfonyl]-1-(2-phenylethyl)-2-	C31H34BrN3O5S	640.59	A/B	-8.7
3511	P(=O)(C(C(=O)OC)(CO)O)(OC(C)C)OC(C)C	170207-61-1	Pentonamide, 2,3,5-trideoxy-N-(2,3-dihydro-2-hydroxy-1H-inden-1-yl)-5-[2-[[(1,1-	C36H47N5O4	613.79	A/B	-8.7
3512	O=C1N2[C@H]3C[C@@H]([C@@H](CO)O3)OP(=O)(O)OC[C@H]3O[C@@H](n4cc(C[C@](C)(C2)C(=O)N1)c(=O)[nH]c4=O)C[C	1715998-47-2	Benzenediazonium, 4,4,Ä≤-[(4,8-disulfo-2,6-naphthalenediyl)bis(2,1-diazenediyl)]	C26H22N8O6S2		A/B	-8.7
3513	C(Oc1ccc(N)cc1)c1c2c(N(c3c(C(=O)N2)cccn3)C2CC2)ncc1	172326-10-2	6H-Dipyrido[3,2-b:2,Ä≤,3,Ä≤-e][1,4]diazepin-6-one, 4-[(2-chlorophenoxy)methyl]-	C21H17ClN4O2	392.84	A/B	-8.7
3514	C(CC(COC(=O)[C@H]1N(C(=O)OCc2ccccc2)CCC1)(COC(=O)[C@H]1N(C(=O)OCc2ccccc2)CCC1)CO)n1c2c(nc1)c(=O)nc(N	172645-40-8	L-Leucine, N-[(phenylmethoxy)carbonyl]-, 2-[2-(2-amino-1,6-dihydro-6-oxo-9H-puri	C39H51N7O10	777.86	A/B	-8.7
3515	N(C(=O)[C@H](Cc1ccccc1)C[C@@H](CN1[C@H](C(=O)NC(C)(C)C)CN(Cc2cnco2)CC1)O)[C@H]1c2c(C[C@H]1O)cccc2	172649-47-7	1-Piperazinepentanamide, N-(2,3-dihydro-2-hydroxy-1H-inden-1-yl)-2-[[(1,1-dimeth	C37H51N5O4S	661.9	A/B	-8.7
3516	N(C(=O)[C@H](Cc1ccccc1)C[C@@H](CN1[C@H](C(=O)NC(C)(C)C)CN(Cc2cnc(C)s2)CC1)O)[C@H]1c2c(C[C@H]1O)cccc2	172649-50-2	1-Piperazinepentanamide, N-(2,3-dihydro-2-hydroxy-1H-inden-1-yl)-2-[[(1,1-dimeth	C34H45N5O4S	619.82	A/B	-8.7
3517	O[C@H]1[C@@H](O[C@H](COP(=O)(OP(=O)(O[C@@H]2[C@H](NC(=O)C)[C@@H](O)[C@H](O)[C@@H](CO)O2)O)O)[C@H]1O)	173167-80-1	Uridine 5,Ä≤-(trihydrogen diphosphate), 5-[3-[(4-benzoylbenzoyl)amino]propyl]-	C26H29N3O14P2	669.47	A/B	-8.7
3518	C([C@H](OCP(=O)(OC(C)C)OC(C)C)C)n1c2c(nc1)c(N)nc(N)n2	173253-38-8	Guanosine, 5,Ä≤-[4-(2,5-dihydro-2,5-dioxo-1H-pyrrol-1-yl)benzoate]	C21H18N6O8	482.4	A/B	-8.7
3519	O=c1n([C@@H]2O[C@H](COP(=O)([C@H](O)c3ccccc3)OC[C@@H]3[C@@H](N=[N+]=[N-])C[C@@H](O3)n3c(=O)[nH]c(=O)	173658-31-6	Thymidine, 3,Ä≤-azido-P,3,Ä≤-dideoxy-P-[hydroxy(4-methylphenyl)methyl]thymidyly	C28H33N10O10P		A/B	-8.7
3520	[C@@H](C(C)(C)C)(C1=C(O)C[C@@](CCc2ccccc2)(CCC)OC1=O)c1cc(NS(=O)(=O)c2ccc(C(F)(F)F)cn2)ccc1	174484-37-8	2-Pyridinesulfonamide, N-[3-[1-[5,6-dihydro-4-hydroxy-2-oxo-6-(2-phenylethyl)-6-	C33H37F3N2O5S	630.72	A/B	-8.7
3521	C(Cc1ccccc1)[C@]1(CCC)CC(=C([C@@H](CC)c2cc(NS(=O)(=O)c3ccc(C#N)cn3)ccc2)C(=O)O1)O	174590-28-4	2-Pyridinesulfonamide, 5-cyano-N-[3-[(1R)-1-[(6S)-5,6-dihydro-4-hydroxy-2-oxo-6-	C31H33N3O5S	559.68	A/B	-8.7
3522	C(OC(COC(=O)[C@H](C(C)C)N)CO)n1c2c(nc1)c(=O)nc(N)[nH]2.Cl	175865-58-4	L-Valine, N-[(phenylmethoxy)carbonyl]-, 2-[(2-amino-1,6-dihydro-6-oxo-9H-purin-9	C29H34N6O7	578.62	A/B	-8.7
3523	C(=N)(NC(=O)N)N1CCOCC1	175923-30-5	Guanosine 5,Ä≤-(trihydrogen diphosphate), 2,Ä≤,3,Ä≤-dideoxy-, P,Ä≤,Üi5,Ä≤-ester	C20H26N10O12P2		A/B	-8.7
3524	C([C@@H]([C@H]([C@H](Cc1ccccc1)NC(=O)[C@@H](NC(=O)c1nc2c(cc1)cccc2)CC(=O)N)O)O)N1[C@H](C(=O)NC(C)(C)	176586-81-5	L-Arabinitol, 2-[[4-amino-1,4-dioxo-2-[(2-quinolinylcarbonyl)amino]butyl]amino]-	C39H52N6O6	700.87	A/B	-8.7
3525	P(=O)(O)(O)[C@@]1(C(=O)O)O[C@@H]([C@@H]([C@@H](CO)O)O)[C@H](NC(=O)C)[C@@H](O)C1	177738-32-8	Thymidine, P-thiothymidylyl-(3,Ä≤,Üi5,Ä≤)-5,Ä≤-thio-	C20H27N4O10PS2	578.55	A/B	-8.7
3526	C(O[P@@](=O)(Oc1ccccc1)N[C@@H](C(=O)OC(C)C)C)[C@H]1C2([C@H]([C@](C)(O)[C@@H]1O)n1c3c(nc1)c(=O)nc(N)[	1781231-59-1	D-Alanine, N-[(R)-[[(4R,5R,6S,7R)-7-(2-amino-1,6-dihydro-6-oxo-9H-purin-9-yl)-5,	C26H35N6O8P	590.57	A/B	-8.7
3527	C(=O)([C@@H](NC(=O)OC)C(C)(C)C)N1[C@H](c2[nH]c(c3cc4C5(c6c(-c4cc3)ccc(c6)c3[nH]c(nc3)[C@H]3N(C(=O)[C	1800210-26-7	Carbamic acid, N,N,Ä≤-[spiro[9H-fluorene-9,3,Ä≤-oxetane]-2,7-diylbis[1H-imidazo	C49H60N8O7	873.05	A/B	-8.7
3528	FC1(F)CC2(c3c(-c4c2cc(cc4)c2[nH]c(nc2)[C@H]2N(C(=O)[C@@H](NC(=O)OC)C(C)C)CCC2)ccc(c3)c2[nH]c(nc2)[C@	1800210-29-0	Carbamic acid, N,N,Ä≤-[spiro[9H-fluorene-9,3,Ä≤-oxetane]-2,7-diylbis[1H-imidazo	C43H48N8O7	788.89	A/B	-8.7
3529	Cc1c2c(cc(C)c1Nc1nc(Nc3ccc(C#N)cc3)ncc1)cc(C#N)cc2	1801429-05-9	2-Naphthalenecarbonitrile, 6-[[2-[(4-cyanophenyl)amino]-4-pyrimidinyl]amino]-5-m	C23H16N6	376.41	A/B	-8.7
3530	C(OP(=O)(Oc1cc2c(cc1)cccc2)N[C@H](C(=O)OC(C)C)C)[C@@]1(C(F)F)O[C@H]([C@@H](O)[C@@H]1O)n1c2c(nc1)c(N)	1803134-22-6	L-Alanine, N-[hydroxy(1-naphthalenyloxy)phosphinothioyl]-, 1-methylethyl ester,	C27H31F2N6O7PS	652.61	A/B	-8.7
3531	C(OP(=S)(Oc1ccc(OC)cc1)Oc1ccc(OC)cc1)[C@@]1(C(F)F)O[C@H]([C@@H](O)[C@@H]1O)n1c2c(nc1)c(N)ncn2	1803134-29-3	9H-Purin-6-amine, 9-[5-O-[bis(4-methoxyphenoxy)phosphinyl]-4-C-(difluoromethyl)-	C25H26F2N5O9P	609.47	A/B	-8.7
3532	C(=O)([C@@H](NC(=O)OC)C(C)C)N1[C@@H](CCC1)c1[nH]c(cn1)c1ccc(cc1)c1ccc(cc1)c1[nH]c(C[C@H]2N(C(=O)[C@@	1808295-82-0	Carbamic acid, N-[(1S)-1-[[(2S)-2-[5-[4,Ä≤-[2-[(3S)-2-[(2S)-2-[(methoxycarbonyl)	C42H52N8O6	764.91	A/B	-8.7
3533	C(=O)([C@@H](NC(=O)OC)C(C)C)N1[C@@H](C2CC1CC2)c1[nH]c(cn1)c1ccc(cc1)c1ccc(cc1)c1[nH]c(nc1)C1N(C(=O)[	1808935-14-9	Carbamic acid, N-[(1S)-1-[[(2S)-2-[5-[4,Ä≤-[2-[2-[(2S)-2-[(methoxycarbonyl)amino	C42H52N8O6	764.91	A/B	-8.7
3534	O=C1[C@@H]2[C@@H](c3c([C@H](OC(=O)[C@@H](Cc4ccccc4)N4C(=O)c5c(C4=O)cccc5)[C@H]2CO1)cc1c(c3)OCO1)c1cc	1809321-37-6	2H-Isoindole-2-acetic acid, 1,3-dihydro-1,3-dioxo-Œ±-(phenylmethyl)-, (5R,5aR,8a	C39H33NO11	691.68	A/B	-8.7
3535	O=C1[C@@H]2[C@@H](c3c([C@H](OC(=O)[C@H](Cc4ccccc4)N4C(=O)c5c(C4=O)cccc5)[C@H]2CO1)cc1c(c3)OCO1)c1cc(	1809321-39-8	2H-Isoindole-2-acetic acid, 1,3-dihydro-1,3-dioxo-Œ±-(phenylmethyl)-, (5R,5aR,8a	C39H33NO11	691.68	A/B	-8.7
3536	O=C1[C@@H]2[C@@H](c3c([C@H](OC(=O)[C@@H](C)N4C(=O)C5C(C4=O)CCCC5)[C@H]2CO1)cc1c(c3)OCO1)c1cc(OC)c(OC	1809321-45-6	2H-Isoindole-2-acetic acid, 1,3-dihydro-Œ±-methyl-1,3-dioxo-, (5R,5aR,8aR,9R)-5,	C33H29NO11	615.58	A/B	-8.7
3537	O=C1[C@@H]2[C@@H](c3c([C@H](OC(=O)CCCCCC(=O)OC(COP(=O)(OCC(CO)O)O)COC(=O)CCCCCC(=O)O[C@H]4c5c([C@H]	1825370-94-2	Butanedioic acid, 1,1,Ä≤-[1-[[[(2-aminoethoxy)hydroxyphosphinyl]oxy]methyl]-1,2-	C57H62NO26P	1208.07	A/B	-8.7
3538	S(=O)(=O)(N(C[C@@H]([C@H](Cc1ccccc1)NC(=O)O[C@H]1CCOC1)O)CC(C)C)c1ccc(OC(F)(F)F)cc1	184490-24-2	Carbamic acid, [3-[(cyclopentylmethyl)[[4-(trifluoromethoxy)phenyl]sulfonyl]amir	C28H35F3N2O7S	600.65	A/B	-8.7
3539	[C@@H](Cc1ccccc1)(NC(=O)N1CCN(C)CC1)C(=O)N[C@H](/C=C/S(=O)(=O)c1ccc(Cl)cc1)CCc1ccccc1	1860793-30-1	1-Piperazinecarboxamide, 4-methyl-N-[(1S)-2-[[(1S,2E)-3-[(4-methylphenyl)sulfony	C33H40N4O4S	588.76	A/B	-8.7
3540	C(OP(=O)(Oc1ccc(OC)cc1)Oc1ccc(OC)cc1)[C@@]1(C=C)O[C@H]([C@H](O)[C@@H]1O)n1c2c(nc1)c(=O)nc(N)[nH]2	1892589-92-2	Guanosine, 4,Ä≤-C-ethenyl-5,Ä≤-O-(2-oxido-4H-1,3,2-benzodioxaphosphorin-2-yl)-	C19H20N5O8P	477.36	A/B	-8.7
3541	[C@H](Cc1ccccc1)(C[C@@H]([C@H](Cc1ccccc1)NC(=O)OCc1cncs1)O)NC(=O)[C@@H](NC(=O)N(Cc1nc(C(C)C)sc1)C)C	190649-37-7	2,7,10,12-Tetraazatridecanoic acid, 4,12-dihydroxy-9-(1-methylethyl)-13-[2-(1-me	C36H46N6O6S2	722.92	A/B	-8.7
3542	N(c1c(C)cc(C)cc1C)c1nc(NC(C)c2ccccc2)ccn1	1907643-95-1	2-Pyridinecarbonitrile, 5-[[4-(4-morpholinyl)-6-[(2,4,6-trimethylphenyl)amino]-1	C22H24N8O	416.48	A/B	-8.7
3543	[C@@H](C(=O)N[C@H](C[C@@H]([C@H](Cc1ccccc1)NC(=O)COc1c(C)cccc1C)O)C(C)C)(C(C)C)N1C(=O)NC(=O)CC1	192725-41-0	1(2H)-Pyrimidineacetamide, N-[(1R,3S,4S)-4-[[2-(2,6-dimethylphenoxy)acetyl]aminc	C33H48N4O5	580.76	A/B	-8.7
3544	C(=C(\CCc1cc(OC)c(O)cc1)/O)(/C(=O)CCc1cc(OC)c(O)cc1)\N=Nc1ccc(C(=O)NCC(=O)O)cc1	1927874-94-9	Benzoic acid, 4-[2-[(1E)-2-hydroxy-4-(4-hydroxy-3-methoxyphenyl)-1-[3-(4-hydroxy	C28H28N2O8	520.53	A/B	-8.7
3545	[C@H](Cc1ccc(F)cc1)(C(=O)N[C@@H](C[C@H]1C(=O)NCC1)/C=C/C(=O)OC1CCC1)CC(=O)[C@@H](NC(=O)c1noc(C)c1)C(	1934276-48-8	2-Pentenoic acid, 4-[[(2R,5S)-2-[(4-fluorophenyl)methyl]-6-methyl-5-[[(5-methyl-	C36H41FN4O7	660.73	A/B	-8.7
3546	O(C(=O)c1ccccc1)[C@H]1[C@@H](O[C@H](COP(=O)(O[C@H]2[C@@H](O[C@H](CO)[C@H]2OC(=O)c2ccccc2)n2nc(C(=O)N	193898-65-6	1H-1,2,4-Triazole-3-carboxamide, 1-[3-O-benzoyl-5-O-[bis(4-methoxyphenyl)phenylm	C44H42N5O13P	879.8	A/B	-8.7
3547	[C@](N)(c1cc2c(n3c(nc2c2cc(Cl)ccc2)nnn3)cc1)(c1n(C)cnc1)c1ccc(Cl)cc1.[C@H](Cc1ccccc1)(C[C@@H]([C@H](	1942697-59-7	2-Naphthalenesulfonic acid, 5-[(1Z)-2-(3,4-dicyanophenyl)diazenyl]-6-hydroxy-	C18H10N4O4S	378.36	A/B	-8.7
3548	C(=O)([C@@H](NC(=O)OC)C(C)C)N1[C@H](c2[nH]c3c4c(cc(cc4)c4cc(c(cc4)c4[nH]c(nc4)[C@H]4N(C(=O)[C@@H](NC	1949770-69-7	Carbamic acid, N-[(1S)-1-[[(2S)-2-[7-[4-[2-[(2S)-1-[(2S)-2-[(methoxycarbonyl)ami	C43H52N8O6	776.92	A/B	-8.7
3549	O=c1n([C@@H]2O[C@H](CO)[C@@H](O)C2)cc(/C=C/CN)c(=O)[nH]1	195826-13-2	Guanosine, 8-[(2-amino-6,7-dihydro-6-oxo-1H-purin-8-yl)oxy]-	C15H16N10O7	448.35	A/B	-8.7
3550	C[C@]1(F)[C@@H](O[C@H](CO[P@](=O)(Oc2ccccc2)N[C@H](C(=O)OC(C)C)C)[C@H]1OC(=O)CNC(=O)C(C)S)n1c(=O)[nH	1972595-62-2	L-Alanine, N-[[P(S),2,Ä≤R]-2,Ä≤-deoxy-3,Ä≤-O-[[[7,7,Ä≤-dimethoxy-4,Ä≤-(methoxy	C42H45FN3O19P	945.79	A/B	-8.7
3551	O[C@H]1[C@H](n2c3c(nc2)c(=O)nc(N)[nH]3)O[C@H](COc2c(F)c(F)c(C#N)c(O)c2F)[C@H]1O	197903-83-6	Guanosine, 5,Ä≤-O-(3,5-difluoro-1,2-dihydro-6-hydroxy-2-oxo-4-pyridinyl)-	C15H14F2N6O7	428.3	A/B	-8.7
3552	N(C(=O)C)[C@H]1[C@H]([C@@H]([C@@H](CO)O)O)OC(=C[C@@H]1NC(=N)NC(=O)Cn1cc(nn1)c1c(N)cccc1)C(=O)O	1982351-40-5	D-glycero-D-galacto-Non-2-enonic acid, 5-(acetylamino)-2,6-anhydro-3,4,5-trideox	C23H29N7O9	547.52	A/B	-8.7
3553	N(C(=O)C)[C@H]1[C@H]([C@@H]([C@@H](CO)O)O)OC(=C[C@@H]1NC(=N)NC(=O)Cn1cc(nn1)c1ccc(C)cc1)C(=O)O	1982351-48-3	D-glycero-D-galacto-Non-2-enonic acid, 5-(acetylamino)-4-[[[[2-[4-(4-aminophenyl	C22H28N8O8	532.51	A/B	-8.7
3554	C[C@]1(F)[C@@H](O[C@H](CO[P@](=O)(Oc2ccccc2)N[C@H](C(=O)OC(C)C)C)[C@H]1OC(=O)NCCCN1CCOCC1)n1c(=O)[nH	1982359-83-0	L-Alanine, N-[[P(S),2,Ä≤R]-2,Ä≤-deoxy-2,Ä≤-fluoro-2,Ä≤-methyl-P-phenyl-3,Ä≤-O-[	C31H35FN5O10P	687.61	A/B	-8.7
3555	O=C1c2c(c3c(c(O)c2C(=O)O1)cc(OC)c(OC)c3)c1ccc2c(cc1)OCO2	1982359-88-5	L-Alanine, N-[[P(S),2,Ä≤R]-2,Ä≤-deoxy-2,Ä≤-fluoro-2,Ä≤-methyl-P-phenyl-3,Ä≤-O-[	C32H38FN6O10P	716.65	A/B	-8.7
3556	O(C(=O)c1ccc(N(C)C)cc1)c1c2n(N([C@H]3N(C2=O)CCOC3)[C@H]2c3c(CSc4c2cccc4)c(F)c(F)cc3)ccc1=O	1985607-32-6	1H-[1,4]Oxazino[3,4-c]pyrido[2,1-f][1,2,4]triazine-6,8-dione, 12-[(11S)-7,8-difl	C32H27F2N3O5S	603.64	A/B	-8.7
3557	N(C(=O)c1ccc(OC)cc1)c1ccc2cc(C34CC5CC(C4)CC(C3)C5)[nH]c2cc1	1987854-61-4	Benzamide, N-(2-tricyclo[3.3.1.13,7]dec-1-yl-1H-indol-5-yl)-	C25H26N2O	370.49	A/B	-8.7
3558	N(C(=O)Nc1cc(OC)c(OC)cc1)c1ccc2cc(C34CC5CC(C4)CC(C3)C5)[nH]c2cc1	1987854-71-6	Urea, N-(3,5-dimethoxyphenyl)-N,Ä≤-(2-tricyclo[3.3.1.13,7]dec-1-yl-1H-indol-5-yl	C27H31N3O3	445.55	A/B	-8.7
3559	N(CCCN(CCCCN(CCCN)CCCN)CCCN)c1c2c(ncc1)cc(Cl)cc2	1989611-40-6	1H-Indazole-3-carboxylic acid, 1-methyl-, (5R,5aR,8aR,9R)-5,5a,6,8,8a,9-hexahydr	C31H28N2O9	572.56	A/B	-8.7
3560	O=c1c2c(n(cn2)[C@@H]2O[C@H](CO)[C@@H](O)C2)[nH]c(NC)n1	1991063-63-8	Benzonitrile, 2-[[2-[(2,4-dimethylphenyl)amino]-4-pyrimidinyl]amino]-	C19H17N5	315.37	A/B	-8.7
3561	C(=O)([C@@H](NC(=O)OC)C(C)C)N1[C@H](c2[nH]c3c4c(cc(cc4)c4c5c(c(cc4)c4[nH]c(nc4)[C@H]4N(C(=O)[C@@H](N	2007901-65-5	Carbamic acid, N-[(1S)-1-[[(2S,3aS,6aS)-2-[5-[2,3-dihydro-7-[2-[(2S)-1-[(2S)-2-[	C48H58N8O6	843.02	A/B	-8.7
3562	C(=O)(C(=O)Nc1c(C)cc(N(=O)=O)cc1)c1c(C23CC4CC(C3)CC(C2)C4)[nH]c2c1cccc2	2014403-83-7	1H-Indole-3-acetamide, N-(4-chloro-2-methylphenyl)-Œ±-oxo-2-tricyclo[3.3.1.13,7]	C27H27ClN2O2	446.97	A/B	-8.7
3563	[C@H](C(C)C)(CO)n1c2c(cc(Cc3cc(cc(Cl)c3F)N3C(=O)CCC3)c(OC)c2)c(=O)c(C(=O)O)c1	1000161-54-5	3-Quinolinecarboxylic acid, 6-[[3-chloro-2-fluoro-5-(2-oxo-1-pyrrolidinyl)phenyl	C27H28ClFN2O6	530.97	C	-8.7
3564	[C@H](C(C)C)(CO)n1c2c(cc(Cc3cc(cc(Cl)c3F)N3C(=O)CCCC3)c(OC)c2)c(=O)c(C(=O)O)c1	1000161-58-9	3-Quinolinecarboxylic acid, 6-[[3-chloro-2-fluoro-5-(1-piperidinyl)phenyl]methyl	C28H32ClFN2O5	531.02	C	-8.7
3565	[C@H](C(C)C)(CO)n1c2c(cc(Cc3cc(cc(Cl)c3F)N3CC[C@@H](F)C3)c(OC)c2)c(=O)c(C(=O)O)c1	1000161-64-7	3-Quinolinecarboxylic acid, 6-[[3-chloro-5-(3,3-difluoro-1-pyrrolidinyl)-2-fluor	C27H28ClF3N2O5	552.97	C	-8.7
3566	[C@H](C(C)C)(CO)n1c2c(cc(Cc3cc(c(F)c(Cl)c3F)N3CCOCC3)c(OC)c2)c(=O)c(C(=O)O)c1	1000161-66-9	3-Quinolinecarboxylic acid, 6-[[3-chloro-2-fluoro-5-[(3S)-3-fluoro-1-pyrrolidiny	C27H29ClF2N2O5	534.98	C	-8.7
3567	[C@H](C(C)C)(CO)n1c2c(cc(Cc3cc(cc(Cl)c3F)N3CCC(O)CC3)c(OC)c2)c(=O)c(C(=O)O)c1	1000161-79-4	3-Quinolinecarboxylic acid, 6-[[3-chloro-2-fluoro-5-(4-thiomorpholinyl)phenyl]me	C27H30ClFN2O5S	549.05	C	-8.7
3568	[C@H](C(C)C)(CO)n1c2c(cc(Cc3cc(cc(Cl)c3F)c3ccccn3)c(OC)c2)c(=O)c(C(=O)O)c1	1000161-82-9	3-Quinolinecarboxylic acid, 6-[[3-chloro-2-fluoro-5-(4-hydroxy-1-piperidinyl)phe	C28H32ClFN2O6	547.01	C	-8.7
3569	C(COC[P@](=O)(Oc1ccccc1)O[C@H](C(=O)OCC)C)n1c2c(nc1)c(N)ncn2	1000689-65-5	Ethanone, 2-amino-1-(2,3-dihydro-1H-indol-1-yl)-2-(3-hydroxytricyclo[3.3.1.13,7]	C20H26N2O2	326.43	C	-8.7
3570	O[C@H]1[C@@H](O[C@H](CO)[C@H]1OCc1ccc(NC(=O)CO[C@@H]2O[C@H](CO)[C@H](O)[C@H](O)[C@H]2O)cc1)n1nc(C(=O	1002334-90-8	1H-1,2,4-Triazole-3-carboxamide, 1-[2-O-[[4-[[2-(Œ≤-D-galactopyranosyloxy)acety]	C23H31N5O12	569.52	C	-8.7
3571	C(=O)([C@@H](N=C(NC#N)N(C)C)C(C)C)N1[C@@H](CCC1)c1[nH]c(cn1)c1ccc(cc1)c1ccc(cc1)c1[nH]c(nc1)[C@H]1N(	1007884-55-0	Carbamic acid, N-[(1S)-1-[[(2S)-2-[5-[4,Ä≤-[2-[(2S)-1-[(2S)-2-[(3-amino-1H-1,2,4	C40H50N12O4	762.9	C	-8.7
3572	C(=O)([C@@H](NC(=O)OC)C(C)C)N1[C@@H](CCC1)c1[nH]c(cn1)c1ccc(cc1)c1ccc(cc1)c1[nH]c(nc1)[C@@H]1CCCN1	1007884-59-4	1-Pyrrolidinecarboxylic acid, 2-[5-[4,Ä≤-[2-[(2S)-1-[(2S)-2-[(methoxycarbonyl)ar	C38H47N7O5	681.82	C	-8.7
3573	C(=O)([C@H](NC(=O)OC)c1ccccc1)N1[C@@H](CCC1)c1[nH]c(cn1)c1ccc(cc1)c1ccc(cc1)c1[nH]c(nc1)[C@H]1N(C(=O	1009107-44-1	Carbamic acid, N-[(1R)-2-oxo-2-[(2S)-2-[5-[4,Ä≤-[2-[(2S)-1-(1-oxopropyl)-2-pyrro	C39H41N7O4	671.79	C	-8.7
3574	C(=O)([C@H](NC(=O)OC)c1ccccc1)N1[C@@H](CCC1)c1[nH]c(cn1)c1ccc(cc1)c1ccc(cc1)c1[nH]c(nc1)[C@H]1N(C(=O	1009107-67-8	Carbamic acid, N-[(1R)-2-[(2S)-2-[5-[4,Ä≤-[2-[(2S)-1-[(2S)-2-(acetylamino)-1-oxc	C41H44N8O5	728.84	C	-8.7
3575	C(=O)([C@H](NC(=O)OC)c1ccccc1)N1[C@@H](CCC1)c1[nH]c(cn1)c1ccc(cc1)c1ccc(cc1)c1[nH]c(nc1)[C@H]1N(C(=O	1009107-89-4	1-Pyrrolidinecarboxylic acid, 2-[5-[4,Ä≤-[2-[(2S)-1-[(2R)-2-[(methoxycarbonyl)ar	C38H39N7O5	673.76	C	-8.7
3576	C(=O)([C@H](N(C)C)c1ccccc1)N1[C@@H](CCC1)c1[nH]c(cn1)c1ccc(cc1)c1ccc(cc1)c1[nH]c(nc1)[C@H]1N(C(=O)CN	1009108-73-9	Carbamic acid, N-[(1R)-2-oxo-2-[(2S)-2-[5-[4,Ä≤-[2-[(2S)-1-[(2R)-1-oxo-2-phenylp	C45H45N7O4	747.88	C	-8.7
3577	C(=O)([C@@H](NC(=O)OC)C(C)C)N1[C@@H](CCC1)c1[nH]c(cn1)c1ccc(cc1)c1ccc(cc1)c1[nH]c(nc1)[C@H]1N(C(=O)C	1009113-42-1	Carbamic acid, N-[(1S)-2-methyl-1-[[(2S)-2-[5-[4,Ä≤-[2-[(2S)-1-[2-[(phenylmethyl	C42H48N8O4	728.88	C	-8.7
3578	C(=O)([C@H](NCC)c1ccccc1)N1[C@@H](CCC1)c1[nH]c(cn1)c1ccc(cc1)c1ccc(cc1)c1[nH]c(nc1)[C@H]1N(C(=O)[C@@	1009113-73-8	Carbamic acid, N,N,Ä≤-[[1,1,Ä≤-biphenyl]-4,4,Ä≤-diylbis[1H-imidazole-5,2-diyl-(	C44H54N8O8	822.95	C	-8.7
3579	C(=O)([C@H](Cc1cnn[nH]1)NC(=O)OC)N1[C@@H](CCC1)c1[nH]c(cn1)c1ccc(cc1)c1ccc(cc1)c1[nH]c(nc1)[C@H]1N(C	1009114-20-8	Carbamic acid, N-[(1S)-2-methyl-1-[[(2S)-2-[5-[4,Ä≤-[2-[(2S)-1-[(2S)-3-methyl-1-	C42H50N10O4	758.91	C	-8.7
3580	C(=O)(N1[C@@H](CCC1)c1[nH]c(cn1)c1ccc(cc1)c1ccc(cc1)c1[nH]c(nc1)[C@H]1N(C(=O)[C@@H](NC(=O)OC)C)CCC1)	1009114-60-6	Carbamic acid, N-[(1S)-1-[[(2S)-2-[5-[4,Ä≤-[2-[(2S)-1-[[cis-4-(diethylamino)cycl	C44H58N8O4	762.98	C	-8.7
3581	C(=O)([C@H](Cc1ccc(CNC(=O)C)cc1)NC(=O)OC)N1[C@@H](CCC1)c1[nH]c(cn1)c1ccc(cc1)c1ccc(cc1)c1[nH]c(nc1)[	1009115-02-9	Carbamic acid, N-[(1S)-2-[(2S)-2-[5-[4,Ä≤-[2-[(2S)-1-[(2S)-3-(4-hydroxyphenyl)-2	C42H46N8O7	774.86	C	-8.7
3582	C(=O)([C@@H](NC(=O)OC)C(C)C)N1[C@@H]([C@@H](O)CC1)c1[nH]c(cn1)c1ccc(cc1)c1ccc(cc1)c1[nH]c(nc1)[C@H]1	1009115-81-4	Carbamic acid, N-[(1S)-1-[[(2S)-2-[5-[4,Ä≤-[2-[(2S)-4,4-difluoro-1-[(2S)-2-[(met	C40H48F2N8O6	774.86	C	-8.7
3583	C(=O)([C@@H](NC(=O)OC)C(C)C)N1[C@H](c2[nH]c(c(C(F)(F)F)n2)c2ccc(cc2)c2ccc(cc2)c2[nH]c(nc2)[C@H]2N(C(	1009115-84-7	Carbamic acid, N-[(1S)-2-[(2S)-2-[5-[4,Ä≤-[2-[(2R,3S)-3-hydroxy-1-[(2S)-2-[(meth	C36H42N8O7	698.77	C	-8.7
3584	C(=O)([C@H](NC(=O)OC)C)N1[C@@H](CCC1)c1[nH]c(cn1)c1ccc(cc1)c1ccc(cc1)c1[nH]c(nc1)[C@H]1N(C(=O)[C@H](	1009117-01-4	Carbamic acid, N,N,Ä≤-[[1,1,Ä≤-biphenyl]-4,4,Ä≤-diylbis[1H-imidazole-5,2-diyl-(	C36H42N8O6	682.77	C	-8.7
3585	P(=O)(OCC)(OCC)c1nc2c(oc1=O)[nH]c(=O)[nH]c2=O	1029630-44-1	Not Yet Assigned	C34H24N6O6S2.2Na		C	-8.7
3586	O[C@H]1[C@H](n2c3c(n[13cH]2)c(N)n[13cH]n3)O[C@H](COP(=O)(OP(=O)(OP(=O)(O)O)O)O)[C@H]1O	1053056-91-9	Not Yet Assigned	C34H26N6O12S4	838.86	C	-8.7
3587	O(C(=O)OCCOCCOC)[C@H]1[C@@H](O[C@H](CO)[C@H]1O)n1nc(C(=O)N)nc1	1053697-88-3	Not Yet Assigned	C34H46F3N5O7	693.75	C	-8.7
3588	C(=O)(NC(CC1(O)CCC1)C(=O)C(=O)N)C1[C@@H]2[C@@H](C2(C)C)CN1C(=O)[C@@H](NC(=O)NC(C)(C)C)C(C)(C)C	1056452-34-6	Benzoic acid, 3,3,Ä≤-methylenebis[6-[[(4-methylphenyl)methyl]amino]-	C31H30N2O4	494.58	C	-8.7
3589	O(C(=O)NC(Cc1ccccc1)C(CN(S(=O)(=O)c1ccc(OC)cc1)CCCP(=O)(OCc1ccccc1)COCc1ccccc1)O)C1C2C(OC1)OCC2	105785-96-4	5,Ä≤-Uridylic acid, 2,Ä≤-deoxy-5-(1,4-dihydro-3-methyl-1,4-dioxo-2-naphthalenyl	C20H19N2O10P	478.35	C	-8.7
3590	N(c1c(C)cc(C(CC)O)cc1C)c1nc(Nc2ccc(C#N)cc2)ncc1	1064399-73-0	Benzonitrile, 4-[[4-[[4-[(1Z)-2-cyano-1-methyl-1-propen-1-yl]-2,6-dimethylphenyl	C24H22N6	394.47	C	-8.7
3591	O[C@H]1[C@H](n2c3c(nc2)c(=O)nc(C#C)[nH]3)O[C@H](COP(=O)(O)O)[C@H]1O	1072329-82-8	Thymidine, 2,Ä≤,3,Ä≤-didehydro-3,Ä≤-deoxy-5,Ä≤-O-[2-oxido-6-[5-oxo-5-(phenylmet	C29H31N2O9P	582.54	C	-8.7
3592	O(C[C@H]1O[C@H](C=C1)n1c(=O)[nH]c(=O)c(C)c1)P1(=O)Oc2c(cc(CCC(=O)N[C@@H](C(=O)OC)C)cc2)CO1	1073242-61-1	Glycine, N-[3-[2-(2,Ä≤,3,Ä≤-didehydro-3,Ä≤-deoxythymidin-5,Ä≤-O-yl)-2-oxido-4H-	C29H30N3O10P	611.54	C	-8.7
3593	O=c1n(C2OC(COP3(=O)Oc4c(C(=O)N3)nc(F)cn4)C=C2)cc(F)nc1C(=O)N	1079990-13-8	Thymidine, 2,Ä≤,3,Ä≤-didehydro-3,Ä≤-deoxy-5,Ä≤-O-(6-fluoro-3,4-dihydro-2-oxido-	C15H13FN5O7P	425.27	C	-8.7
3594	O=c1n(cc(F)nc1C(=O)N)C1OC(CO)C=C1	1079990-15-0	2-Pyrazinecarboxamide, 4-[5-[[(6-fluoro-3,4-dihydro-2-oxido-2H-pyrazino[2,3-e]-1	C15H11F2N6O7P	456.25	C	-8.7
3595	C(=O)([C@@H](NC(=O)OC)C(C)C)N1[C@@H](CCC1)c1[nH]c(cn1)c1ccc(cc1)c1ccc(cc1)c1[nH]c([C@H](NC(=O)[C@@H]	1087346-62-0	Carbamic acid, N-[(1S)-2-[(2S)-2-[5-[4,Ä≤-[2-[(1R)-1-[[(2S)-2-[(methoxycarbonyl)	C36H44N8O6	684.78	C	-8.7
3596	C(=O)([C@@H](NC(=O)OC)C(C)C)N1[C@@H](CCC1)c1[nH]c(cn1)c1ccc(cc1)c1ccc(cc1)c1[nH]c([C@@H](NC(=O)[C@@H	1087346-73-3	Carbamic acid, N-[(1S)-2-[(2S)-2-[5-[4,Ä≤-[2-[(1R)-1-[[(2S)-2-[(methoxycarbonyl)	C34H40N8O6	656.73	C	-8.7
3597	C(C=C(c1ccccc1)c1ccccc1)N1CCN(C23CC4CC(C3)CC(C2)C4)CC1	108768-11-2	1,2-Ethanediamine, N2-[2-(10,11-dihydro-5H-dibenzo[a,d]cyclohepten-5-ylidene)eth	C35H40N2	488.71	C	-8.7
3598	Clc1n(c2c(n1)c(N[C@@H]1CCOC1)ncn2)[C@H]1[C@@H](OC(=O)C)[C@H](OC(=O)C)[C@@H](COC(=O)C)O1	109840-28-0	1-Naphthalenesulfonic acid, 8-[2-[2-methyl-4-[2-(2-methyl-4-sulfophenyl)diazenyl	C24H20N4O6S2.2Na		C	-8.7
3599	O=c1n([C@@H]2O[C@H](CO[Si](C(C)(C)C)(C)C)SC2)ccc(NC(=O)CCCCCCCCCCCCC)n1	1099829-81-8	Thymidine, 2,Ä≤,3,Ä≤-didehydro-3,Ä≤-deoxy-5,Ä≤-O-[6-[[4-hydroxy-3-(hydroxymethy	C25H25N2O8P	512.45	C	-8.7
3600	O=c1c2c(n(cn2)[C@H]2C[C@H](OP(=O)(OCCCOP(=O)(OC[C@H]3O[C@H](C[C@@H]3O)n3c4c(nc3)cnc(N)n4)O)O)[C@@H](	1105693-48-8	Guanosine, 2,Ä≤-deoxyguanylyl-(3,Ä≤,Üi5,Ä≤)-2,Ä≤,6-dideoxy-	C20H25N10O9P	580.45	C	-8.7
3601	C(#Cc1cccs1)c1n(nc(C(=O)N)n1)[C@@H]1O[C@H](CO)[C@@H](O)[C@H]1O	1106736-09-7	1H-1,2,4-Triazole-3-carboxamide, 5-[2-(2-fluorophenyl)ethynyl]-1-Œ≤-D-ribofuranc	C16H15FN4O5	362.31	C	-8.7
3602	[C@H](CC)(C1=C(O)C[C@@H](OC1=O)c1ccccc1)c1cc(NS(=O)(=O)c2c(C(F)(F)F)cccn2)ccc1	1109235-78-0	2-Pyridinesulfonamide, N-[3-[(1R)-1-[(6S)-5,6-dihydro-4-hydroxy-2-oxo-6-phenyl-2	C26H23F3N2O5S	532.53	C	-8.7
3603	[C@@H](CC)(C1=C(O)C[C@@H](OC1=O)c1ccccc1)c1cc(NS(=O)(=O)c2c(C(F)(F)F)cccn2)ccc1	1109235-81-5	2-Pyridinesulfonamide, N-[3-[(1S)-1-[(6S)-5,6-dihydro-4-hydroxy-2-oxo-6-phenyl-2	C26H23F3N2O5S	532.53	C	-8.7
3604	C(Cc1ccccc1)[C@]1(CCC)C(C(=C([C@H](CC)c2cc(N(S(=O)(=O)c3ccc(C(F)(F)F)cn3)[2H])ccc2)C(=O)O1)O[2H])([2	1126619-14-4	Not Yet Assigned	C31H31D2F3N2O5S		C	-8.7
3605	N(=Nc1c2c(ccc(S(=O)(=O)O)c2)c(N)cc1)c1c2c(c(N=Nc3c(S(=O)(=O)O)cc(N)cc3)cc1)ccc(S(=O)(=O)O)c2	1131041-13-8	2-Naphthalenesulfonic acid, 8-[2-(4-amino-2-methylphenyl)diazenyl]-5-[2-(4-aminc	C23H20N6O6S2	540.57	C	-8.7
3606	N(CC(C)C)(C1CCC2(CC1)OCC(C)(C)OO2)C1CCC(Nc2c3c(ncc2)cc(Cl)cc3)CC1	1159893-66-9	1,4-Cyclohexanediamine, N4-(7-chloro-4-quinolinyl)-N1-(cyclopropylmethyl)-N1-(3,	C29H40ClN3O3	514.1	C	-8.7
3607	N(CCCCC)(C1CCC2(CC1)OCC(C)(C)OO2)C1CCC(Nc2c3c(ncc2)cc(Cl)cc3)CC1	1159893-67-0	1,4-Cyclohexanediamine, N4-(7-chloro-4-quinolinyl)-N1-(3,3-dimethyl-1,2,5-trioxa	C29H42ClN3O3	516.12	C	-8.7
3608	N(=C\1/c2c(N(Cc3ccccc3)C1=O)ccc(C)c2)\NC(=S)N	1164513-99-8	Hydrazinecarbothioamide, 2-[1,2-dihydro-1-methyl-5-(4-morpholinylsulfonyl)-2-oxc	C22H25N5O4S2	487.6	C	-8.7
3609	Cc1n(c2nc(nc(n2)N2CCOCC2)c2cnc(NC)nc2)nc(C)c1	1174191-01-5	1H-Indole, 5-[4-(1H-imidazol-1-yl)-6-(4-morpholinyl)-1,3,5-triazin-2-yl]-	C18H17N7O	347.37	C	-8.7
3610	c1(c2c(ccc1)[nH]cc2)c1nc(nc(n1)N1CCOCC1)n1ccnc1	1174191-05-9	2-Pyridinamine, 5-[4-(3,5-dimethyl-1H-pyrazol-1-yl)-6-(4-morpholinyl)-1,3,5-tria	C17H20N8O	352.39	C	-8.7
3611	C(CCC)c1n(c2nc(nc(n2)N2CCOCC2)c2cnc(N)nc2)ccn1	1174191-63-9	1H-Pyrrolo[2,3-b]pyridine, 4-[4-(2-ethyl-1H-imidazol-1-yl)-6-(4-morpholinyl)-1,3	C19H20N8O	376.42	C	-8.7
3612	O=c1c2c(n(cc1C(=O)OC[C@@H]1[C@@H](N=[N+]=[N-])C[C@@H](O1)n1c(=O)[nH]c(=O)c(C)c1)C1CC1)cc(c(F)c2)N1CC	1174398-20-9	Thymidine, 3,Ä≤-azido-3,Ä≤-deoxy-, 5,Ä≤-[1-ethyl-6-fluoro-1,4-dihydro-4-oxo-7-[	C34H35FN8O8		C	-8.7
3613	C(=O)([C@@H](NC(=O)OC)C(C)C)N1[C@H](c2[nH]c(c3cc4c(-c5c(C4)cc(cc5)c4[nH]c(nc4)[C@H]4N(C(=O)[C@@H](NC	1179149-92-8	Carbamic acid, N,N,Ä≤-[[9-(dimethylamino)-9H-fluorene-2,7-diyl]bis[1H-imidazole-	C43H55N9O6	793.95	C	-8.7
3614	C(=O)([C@@H](NC(=O)OC)C(C)C)N1[C@H](c2[nH]c(c3cc4c(-c5c(cc(cc5)c5[nH]c(nc5)[C@H]5N(C(=O)[C@@H](NC(=O	1179150-14-1	Carbamic acid, N,N,Ä≤-[(6,7-dihydro-6-methyl-5H-dibenz[c,e]azepine-3,9-diyl)bis	C43H55N9O6	793.95	C	-8.7
3615	C(=O)([C@@H](NC(=O)OC)C(C)C)N1[C@H](c2[nH]c3-c4c(cc(cc4)c4ccc(cc4)c4[nH]c(nc4)[C@H]4N(C(=O)[C@@H](NC	1180669-76-4	Carbamic acid, N-[(1S)-1-[[(2S)-2-[8-[3-fluoro-4-[2-[(2S)-1-[(2S)-2-[(methoxycar	C43H53FN8O6	796.93	C	-8.7
3616	O=c1n([C@@H]2O[C@H](COP(=O)(Nc3cc(C(=O)Nc4cc(C(=O)Nc5cc(C(=O)NCCC#N)n(C)c5)n(C)c4)n(C)c3)OC(C)C)C=C2	1190064-46-0	Thymidine, 2,Ä≤,3,Ä≤-didehydro-3,Ä≤-deoxy-, 5,Ä≤-[1-methylethyl N-[5-[[[5-[[[5-	C40H50N13O10P	903.88	C	-8.7
3617	N[C@H]1[C@H](n2c3c(nc2)c(=O)nc(N)[nH]3)O[C@H](COP(=O)(O)n2ccnc2)C1	1190980-51-8	D-erythro-Pentonamide, 2,3,5-trideoxy-N-[(1R,2R,3S)-2,3-dihydro-2,3-dihydroxy-1H	C36H47N5O5	629.79	C	-8.7
3618	O=C1[C@@H]2[C@@H](c3c([C@@H](OC(=O)[C@@H](NC(=O)OC[C@H]4O[C@H](C=C4)n4c(=O)[nH]c(=O)c(C)c4)CC(C)C)[C	1191242-94-0	L-Valine, N-carboxy-, 1-[(3aR,4S,9R,9aR)-1,3,3a,4,9,9a-hexahydro-6,7-dimethoxy-1	C39H45N3O14	779.79	C	-8.7
3619	S(=O)(=O)(O)c1c2c(c(N)c(N=Nc3cc(Br)c(cc3)c3c(Br)cc(N=Nc4c(N)c5c(c(S(=O)(=O)O)c4)cccc5)cc3)c1)cccc2	119697-61-9	Naphthionic acid, 3,3,Ä≤-(2,2,Ä≤-dichloro-4,4,Ä≤-biphenylylenebisazo)di-	C32H22Cl2N6O6S2	721.59	C	-8.7
3620	C(=O)(NC(CC1CC1)C(=O)C(=O)NCC=C)[C@@H]1[C@@H]2[C@@H](C2(C)C)CN1C(=O)[C@@H](NC(=O)N[C@@H](CN1C(=O)CC(	1208257-86-6	3-Azabicyclo[3.1.0]hexane-2-carboxamide, 3-[(2S)-2-[[[[(1R)-1-[(4,4-dimethyl-2-c	C38H60N6O6	696.92	C	-8.7
3621	N(C(=O)CCCC(=O)OCC)C12CC3CC(C2)CC(C1)C3	121373-33-9	Furo[3,Ä≤,4,Ä≤:6,7]naphtho[2,3-d]-1,3-dioxol-6(5aH)-one, 5,8,8a,9-tetrahydro-9-	C26H22O6	430.45	C	-8.7
3622	C(OC[C@@H]1[C@@H](OP(N(C(C)C)C(C)C)OCCC#N)C[C@@H](O1)n1cc(/C=C/c2cc3c(-c4c(C3)cccc4)cc2)c(=O)[nH]c1=	121499-18-1	1-Naphthalenesulfonamide, 4-amino-3-[2-[4-[[4-[2-[1-amino-4-(aminosulfonyl)-2-na	C32H26N8O6S3	714.79	C	-8.7
3623	C(#CC1CC1(C1([2H])[2H])([2H])[2H])[C@@]1(C(F)(F)F)c2c(NC(=O)O1)c(O)cc(Cl)c2	1217755-54-8	Not Yet Assigned	C36H47N5O4	613.79	C	-8.7
3624	[C@H](Cc1ccccc1)([C@@H](CN(S(=O)(=O)c1ccc(N)cc1)C(C)(C)C)O)NC(=O)O[C@H]1CC(OC1([2H])[2H])([2H])[2H]	1217778-78-3	2-Pyridinesulfonamide, N-[3-[(1R)-1-[(2R)-3,4-dihydro-6-hydroxy-4-oxo-2-(2-pheny	C31H33F3N2O5S	602.66	C	-8.7
3625	C(N1c2c(C(=NNC(=S)N)C1=O)ccc(Cl)c2)N1CCN(CC1)c1c2c(cc(C(F)(F)F)cc2)ncc1	1218813-19-4	Hydrazinecarbothioamide, 2-[4-bromo-1,2-dihydro-2-oxo-1-[[4-[7-(trifluoromethyl)	C24H21BrF3N7OS	592.43	C	-8.7
3626	C(N=C)C12CC3CC(C2)CC(C1)C3	1222434-12-9	Not Yet Assigned	C33H46FN5O4	595.75	C	-8.7

3627	C([C@@H](OP(=O)(O)O)CO)n1c2c(nc1)c(N)ncn2	1222444-00-9	Not Yet Assigned	C36H52FN5O4	637.83	C	-8.7
3628	[C@H]([C@@H](CO)O)(O)[C@H]1[C@H](NC=O)[C@@H](NC(=N)N)C=C(C(=O)O)O1	1222518-93-5	Not Yet Assigned	C35H45FN6O4	632.77	C	-8.7
3629	N(Cc1ccccn1)c1nc(nc(n1)N1CCOCC1)c1cc2c(cc1)nc[nH]2	1225638-99-2	1,3,5-Triazin-2-amine, 4-(1H-indazol-5-yl)-6-(4-morpholinyl)-N-(2-pyridinylmethy	C20H20N8O	388.43	C	-8.7
3630	C(=O)([C@@H](NC(=O)OC)C(C)C)N1[C@@H](CCC1)c1[nH]c(cn1)[C@@]1(C)CC[C@@H](CC1)c1ccc(cc1)c1[nH]c(nc1)[C	1228670-79-8	Carbamic acid, N-[(1R)-2-[(2S)-2-[5-[4-[trans-4-[2-[(2S)-1-[(2S)-2-[(methoxycark	C43H54N8O6	778.94	C	-8.7
3631	C(=O)([C@@H](NC(=O)OC)C(C)C)N1[C@H](c2[nH]c(C34CCC(CC3)(CC4)c3ccc(cc3)c3[nH]c(nc3)[C@H]3N(C(=O)[C@H]	1228671-77-9	Carbamic acid, N-[(1S)-2-[(2S)-2-[5-[4-[4-[2-[(2S)-1-[(2S)-2-[(methoxycarbonyl)a	C40H54N8O6	742.91	C	-8.7
3632	C(=O)([C@@](NC(=O)OC)(C(C([2H])([2H])[2H])(C([2H])([2H])[2H])[2H])[2H])N1[C@H](c2[nH]c(C34CCC(CC3)(C	1228671-78-0	Carbamic acid, N-[(1R)-2-[(2S)-2-[5-[4-[4-[2-[(2S)-1-[(2S)-2-[(methoxycarbonyl)a	C40H54N8O6	742.91	C	-8.7
3633	C(=O)([C@H](N(C)C)c1ccccc1)N1[C@@H](CCC1)c1[nH]c(cn1)c1ccc(cc1)c1ccc(C#Cc2[nH]c(nc2)[C@H]2N(C(=O)[C@	1228966-39-9	Carbamic acid, N-[(1R)-2-[(2S)-2-[5-[2-[4,Ä≤-[2-[(2S)-1-[(2R)-2-[(methoxycarbony	C48H46N8O6	830.93	C	-8.7
3634	N(C(=O)[C@H](Cc1ccccc1)C[C@@H](CN1[C@H](C(=O)NC(C)(C)C)CN(Cc2cn3c(n2)cncc3)CC1)O)[C@H]1c2c(C[C@H]1O)	1237808-95-5	Not Yet Assigned	C37H47N7O4	653.81	C	-8.7
3635	N(C(=O)[C@H](Cc1ccccc1)C[C@@H](CN1[C@H](C(=O)NC(C)(C)C)CN(Cc2cc3c(cc2)scc3)CC1)O)[C@H]1c2c(C[C@H]1O)	1237808-99-9	Not Yet Assigned	C40H51N5O4	665.86	C	-8.7
3636	N(C(=O)[C@H](Cc1ccccc1)C[C@@H](CN1[C@H](C(=O)NC(C)(C)C)CN(Cc2cc3c(nc2)scc3)CC1)O)[C@H]1c2c(C[C@H]1O)	1237809-01-6	Not Yet Assigned	C38H47N5O4S	669.88	C	-8.7
3637	C[C@]12[C@H]([C@H]3[C@H](CC2)[C@]2(C)C(=CC3)C[C@@H](OC(=O)CP(=O)(O)O)CC2)CC=C1n1c2c(nc1)cccc2	1238382-70-1	Androsta-5,16-dien-3-ol, 17-(3-pyridinyl)-, 3-(2-phosphonoacetate), (3Œ≤)-	C26H34NO5P	471.53	C	-8.7
3638	c1(nc(nc(n1)N1CCOCC1)N1Cc2c(CC1)cccc2)N1Cc2c(CC1)cccc2	1239884-86-6	Quinoline, 1,1,Ä≤-[6-(4-morpholinyl)-1,3,5-triazine-2,4-diyl]bis[1,2,3,4-tetrahy	C25H28N6O	428.53	C	-8.7
3639	C(O)[C@]12[C@@H]([C@@H]3[C@](C)([C@@]4(C)[C@H](CC3)[C@]3(C)[C@@H](CC4)C(C)(C)[C@@H](O)CC3)CC1)[C@H](	1239885-21-2	4-Pyridinecarboxylic acid, 2-[4-(4-morpholinyl)-6-(phenylamino)-1,3,5-triazin-2-	C19H20N8O2	392.41	C	-8.7
3640	C(=O)([C@@H](NC(=O)OC)CCC)N1[C@H](c2[nH]c(c3cc4c(-c5c(C4)cc(cc5)c4[nH]c(nc4)[C@H]4N(C(=O)[C@@H](NC(=	1240880-57-2	Carbamic acid, N,N,Ä≤-[9H-fluorene-2,7-diylbis[1H-imidazole-5,2-diyl-(2S)-2,1-py	C41H46N8O6	746.85	C	-8.7
3641	C(=O)([C@H](NC(=O)OC)c1ccccc1)N1[C@@H](CCC1)c1[nH]c(cn1)c1cc2c(cc1)ccc(c2)c1ccc(cc1)c1[nH]c(nc1)[C@H	1240883-73-1	Carbamic acid, N-[(1S)-1-[[(2S)-2-[5-[7-[2-[(1S)-1-[[(2S)-2-[(methoxycarbonyl)am	C40H50N8O6	738.88	C	-8.7
3642	C(=O)([C@H](NC(=O)OC)c1ccccc1)N1[C@@H](CCC1)c1[nH]c(cn1)C1CCC(CC1)c1ccc(cc1)c1[nH]c(nc1)[C@H]1N(C(=O	1241379-00-9	Carbamic acid, N-[(1S)-1-[[(2S)-2-[5-[4,Ä≤-[2-[(2S)-1-[(2S)-2-[(methoxycarbonyl)	C44H52N8O6	788.93	C	-8.7
3643	C(=O)([C@H](NC(=O)OC)c1ccccc1)N1[C@@H](CCC1)c1[nH]c(cn1)C1CCC(CC1)C1CCC(CC1)c1[nH]c(nc1)[C@H]1N(C(=O	1241404-86-3	Carbamic acid, N-[(1R)-2-[(2S)-2-[5-[4-[4-[2-[(2S)-1-[(2R)-2-[(methoxycarbonyl)a	C46H52N8O6	812.96	C	-8.7
3644	O[C@H]1[C@@H](O[C@H](COP(=O)(OP(=O)(O[C@H]2O[C@H](CO)[C@H](O)[C@H](O)[C@H]2O)O)O)[C@H]1O)n1cc(c(=O)[	1242712-28-2	Uridine 5,Ä≤-(trihydrogen diphosphate), 5-phenyl-, P,Ä≤-Œ±-D-galactopyranosyl e	C21H28N2O17P2	642.4	C	-8.7
3645	N(C(=O)c1ccc(cc1)C1CCCCC1)c1c(C(=O)O)cc(CCC2CCCCC2)cc1	1247823-91-1	Benzoic acid, 5-cyclohexyl-2-[(4-cyclohexylbenzoyl)amino]-	C26H31NO3	405.53	C	-8.7
3646	[C@@H](NC(=O)[C@H](Cc1ccccc1)NC(=O)N1CCN(C)CC1)(/C=C/S(=O)(=O)c1ccccc1)c1c(C)cc(C)cc1C	1253912-35-4	1-Piperazinecarboxamide, 4-methyl-N-[(1S)-2-[[(1R,2E)-1-(2-naphthalenyl)-3-(pher	C34H36N4O4S	596.74	C	-8.7
3647	C(OCCOC(=O)/C=C/c1ccc(O)cc1)n1c2c(c(=O)nc(N)[nH]2)nc1	1253912-50-3	1-Piperazinecarboxamide, N-[(1S)-1-[[[(1R,2E)-1-[1,1,Ä≤-biphenyl]-4-yl-3-(phenyl	C33H40N4O4S	588.76	C	-8.7
3648	N(C(=O)Nc1cncnc1)c1ccc(c2nc(N3C4CCC3COC4)nc(n2)N2CCOCC2)cc1	1254065-99-0	Urea, N-[4-[2-(dimethylamino)ethyl]phenyl]-N,Ä≤-[4-[4-(4-morpholinyl)-6-(3-oxa-8	C30H38N8O3	558.67	C	-8.7
3649	C(=O)([C@@H](NC(=O)OC)C(C)C)N1[C@@H]([C@@H]2C[C@H]1CC2)c1[nH]c(cn1)c1ccc(cc1)c1ccc(cc1)c1[nH]c(nc1)[	1256383-58-0	Carbamic acid, N-[(1S)-1-[[(2S)-2-[5-[4,Ä≤-[2-[(1R,3S,4S)-2-[(2S)-2-[(methoxycar	C42H52N8O6	764.91	C	-8.7
3650	C(=O)([C@@H](NC(=O)OC)C(C)C)N1[C@@H](CCC1)c1[nH]c(cn1)c1ccc(cc1)c1ccc(cc1)c1[nH]c(CN2C(=O)[C@H](N)CC	1256385-55-3	Carbamic acid, N-[(1S)-1-[[(2S)-2-[5-[4,Ä≤-(1H-imidazol-5-yl)[1,1,Ä≤-biphenyl]-	C29H32N6O3	512.6	C	-8.7
3651	C(=O)([C@@H](NC(=O)OC)C(C)C)N1[C@@H](CCC1)c1[nH]c(cn1)c1ccc(cc1)c1ccc(cc1)c1[nH]c(nc1)[C@H]1N(C(=O)[	1256388-28-9	Carbamic acid, N-[(1S)-1-[[(2S)-2-[5-[4,Ä≤-[2-[(6S)-5-[(2S)-2-[(methoxycarbonyl)	C42H52N8O6	764.91	C	-8.7
3652	C(=O)([C@@H](NC(=O)OC)C(C)C)N1[C@@H](c2c(Cl)cccc2)c1[nH]c(cn1)c1ccc(cc1)c1ccc(cc1)c1[nH]c(nc1)[C@H]1	1256390-05-2	Carbamic acid, N-[(1S)-1-[[(2S)-2-[5-[6-[4-[2-[(2S,4R)-1-[(2S)-2-[(methoxycarbor	C51H58N8O6	879.06	C	-8.7
3653	C(=O)([C@@H](NC(=O)OC)C)N1[C@@H](CC[C@@H]1c1ccccc1)c1[nH]c(cn1)c1ccc(cc1)c1cc2c(cc1)cc(cc2)c1[nH]c(n	1256390-56-3	Carbamic acid, N,N,Ä≤-[[1,1,Ä≤-biphenyl]-4,4,Ä≤-diylbis[1H-imidazole-5,2-diyl-(	C44H54N8O10	854.95	C	-8.7
3654	C(=O)([C@H](NC(=O)OC)c1ccccc1)N1[C@@H](CCC1)c1[nH]c(cn1)c1ccc(cc1)c1cc2c(cc1)cc(cc2)c1[nH]c(nc1)[C@H	1256391-31-7	Carbamic acid, N-[(1S)-2-[(2S)-2-[5-[4-[6-[2-[(2S)-1-[(2S)-2-[(methoxycarbonyl)a	C47H50N8O6	822.95	C	-8.7
3655	C(=O)([C@@H](NC(=O)OC)c1ccccc1)N1[C@@H](CCC1)c1[nH]c(cn1)c1cc2c(cc1)cc(cc2)c1ccc(cc1)c1[nH]c(nc1)[C@	1256391-32-8	Carbamic acid, N-[(1R)-2-[(2S)-2-[5-[4-[6-[2-[(2S)-1-[(2S)-2-[(methoxycarbonyl)a	C47H50N8O6	822.95	C	-8.7
3656	C(=O)(C(NC(=O)OC)c1ccccc1)N1[C@@H](CCC1)c1[nH]c(cn1)c1cc2c(cc1)cc(cc2)c1ccc(cc1)c1[nH]c(nc1)[C@H]1N(	1256391-34-0	Carbamic acid, N-[(1R)-2-[(2S)-2-[5-[6-[4-[2-[(2S)-1-[(2S)-2-[(methoxycarbonyl)a	C47H50N8O6	822.95	C	-8.7
3657	C(=O)([C@@H](NC(=O)OC)C1CCCCC1)N1[C@@H](CCC1)c1[nH]c(cn1)c1ccc(cc1)c1cc2c(cc1)cc(cc2)c1[nH]c(nc1)[C@	1256392-16-1	Carbamic acid, N-[(1S)-1-[[(2S)-2-[5-[6-[4-[2-[(2S)-1-[(2S)-2-[(methoxycarbonyl)	C45H52N8O6	800.94	C	-8.7
3658	C(=O)([C@@H](NC(=O)OC)C(C)C)N1[C@@H](C[C@@H](COC(F)F)C1)c1[nH]c(cn1)c1ccc(cc1)c1ccc(cc1)c1[nH]c(nc1)	1256393-01-7	Carbamic acid, N-[2-[(2S)-2-[5-[4-[6-[2-[(2S)-1-[(2S)-2-[(methoxycarbonyl)amino]	C47H50N8O6	822.95	C	-8.7
3659	C(=O)([C@H](NC(=O)OC)C1CCCCC1)N1[C@@H](CCC1)c1[nH]c(cn1)c1ccc(cc1)c1cc2c(cc1)cc(cc2)c1[nH]c(nc1)[C@H	1256393-17-5	Carbamic acid, N-[(1S)-1-[[(2S)-2-[5-[4,Ä≤-[2-[2,3-dihydro-2-[(2S)-2-[(methoxyca	C44H50N8O6	786.92	C	-8.7
3660	C(=O)(NC(CC1CCC1)(C(=O)C(=O)N)[2H])[C@@H]1[C@@H]2[C@@H](C2(C([2H])([2H])[2H])C([2H])([2H])[2H])CN1C(	1256750-70-5	3-Azabicyclo[3.1.0]hexane-2-carboxamide, N-[3-amino-1-(cyclobutylmethyl-d2)-2,3-	C27H18D27N5O5		C	-8.7
3661	C(=O)([C@@H](NC(=O)OC)C(C)C)N1[C@@H](CCC1)c1[nH]c(cn1)c1ccc(cc1)c1cc2c(cc1)cc(cc2)c1[nH]c(nc1)[C@H]1	1261070-49-8	Carbamic acid, N-[(1S)-2-[(2S)-2-[5-[6-[4-[2-[(2S)-1-[(2S)-2-[(methoxycarbonyl)a	C42H48N8O6	760.88	C	-8.7
3662	C(F)(F)c1n(c2c(n1)cccc2)c1nc(nc(NCc2ccccn2)n1)N1CCOCC1	1261281-13-3	1,3,5-Triazin-2-amine, 4-[2-(difluoromethyl)-1H-benzimidazol-1-yl]-N-methyl-6-(4	C23H23F2N7O	451.47	C	-8.7
3663	O(C)c1c2n(cc(C(=O)O)c(=O)c2cc(F)c1N1CCN(CN2c3c(C(=NNC(=S)N)C2=O)cccc3)CC1)C1CC1	1262891-47-3	2H-Indol-2-one, 3,3,Ä≤-[methylenebis[(1,5-dihydro-5-thioxo-4H-1,2,4-triazole-3,4	C25H16Br2N10O4S2	744.4	C	-8.7
3664	O=C1[C@@H]2[C@@H](c3c([C@H](O[C@@H]4O[C@H]5[C@@H]([C@H](O)[C@H]4O)O[C@H](C)OC5)[C@H]2CO1)cc1c(c3)OCO	126453-82-5	Furo[3,Ä≤,4,Ä≤:6,7]naphtho[2,3-d]-1,3-dioxol-6(5aH)-one, 9-[(4,6-O-ethylidene-Œ	C30H34O13	602.58	C	-8.7
3665	N(=C\1/c2c(N(CN3CCN(CC3)c3cc4c(cc3F)c(=O)c(C(=O)O)cn4CC)C1=O)ccc(Cl)c2)\NC(=S)NOC	1265623-10-6	3-Quinolinecarboxylic acid, 7-[4-[[(3Z)-5-chloro-2,3-dihydro-3-[2-[(hydroxyamino	C26H25ClFN7O5S	602.04	C	-8.7
3666	N(=C\1/c2c(N(CN3CCN(CC3)c3cc4c(cc3F)c(=O)c(C(=O)O)cn4CC)C1=O)ccc(F)c2)\NC(=S)NOC	1265623-12-8	3-Quinolinecarboxylic acid, 1-ethyl-6-fluoro-7-[4-[[(3Z)-5-fluoro-2,3-dihydro-3-	C26H25F2N7O5S	585.58	C	-8.7
3667	N(=C\1/c2c(N(CN3CCN(CC3)c3cc4c(cc3F)c(=O)c(C(=O)O)cn4CC)C1=O)ccc(C)c2)\NC(=S)NO	1265623-13-9	3-Quinolinecarboxylic acid, 1-ethyl-6-fluoro-7-[4-[[(3Z)-5-fluoro-2,3-dihydro-3-	C27H27F2N7O5S	599.61	C	-8.7
3668	C(C)n1c2c(c(=O)c(C(=O)O)c1)cc(F)c(c2F)N1CC(C)N(CN2c3c(/C(=N/NC(=S)NO)/C2=O)cc(Cl)cc3)CC1	1265623-20-8	3-Quinolinecarboxylic acid, 1-cyclopropyl-7-[4-[[(3Z)-2,3-dihydro-3-[2-[(methoxy	C29H30FN7O5S	607.66	C	-8.7
3669	O(C)c1c2n(cc(C(=O)O)c(=O)c2cc(F)c1N1CC(C)N(CN2c3c(/C(=N/NC(=S)NOC)/C2=O)cc(C)cc3)CC1)C1CC1	1265623-31-1	3-Quinolinecarboxylic acid, 1-cyclopropyl-7-[4-[[(3Z)-2,3-dihydro-3-[2-[(hydroxy	C30H32FN7O6S	637.68	C	-8.7
3670	S(=O)(=O)(O)c1c2c(cc(N)c1N=Nc1ccc(cc1)c1ccc(N=Nc3c(S(=O)(=O)O)c4c(cc3N)cccc4)cc1)cccc2	1266777-28-9	2,7-Naphthalenedisulfonic acid, 3-amino-4-[(1E)-2-[4,Ä≤-[(1E)-2-[4-[[(4-methylph	C35H27N5O9S3.2Na		C	-8.7
3671	O=C1[C@@H]2[C@@H](c3c([C@H](O[C@H]4O[C@H](CO)[C@H](O)[C@H](O)[C@H]4O)[C@H]2CO1)cc1c(c3)OCO1)c1ccc(OC)	1266976-34-4	2,7-Naphthalenedisulfonic acid, 3-amino-4-[(1E)-2-[4,Ä≤-[(1E)-2-[4-[[(4-methylph	C35H27N5O9S3	757.81	C	-8.7
3672	C(=O)([C@@H](NC(=O)OC)C(C)C)N1CCC2(C[C@H]1c1[nH]c(cn1)c1ccc(cc1)c1ccc(cc1)c1[nH]c(nc1)[C@H]1N(C(=O)[C	1272655-35-2	Carbamic acid, N-[(1S)-1-[[(2S)-2-[5-[4,Ä≤-[2-[(3S)-2-[(2S)-2-[(methoxycarbonyl)	C43H54N8O8	810.94	C	-8.7
3673	C(=O)([C@@H](NC(=O)OC)C(C)C)N1C(CC2(C1)CCNCC2)c1[nH]c(cn1)c1ccc(cc1)c1ccc(cc1)c1[nH]c(nc1)[C@H]1N(C(	1272656-09-3	Carbamic acid, N,N,Ä≤-[[1,1,Ä≤-biphenyl]-4,4,Ä≤-diylbis[1H-imidazole-5,2-diyl-(	C48H62N8O8	879.05	C	-8.7
3674	C(F)(F)C1C2CC3(N)CC1CC(C2)C3	1272656-56-0	Carbamic acid, N-[(1S)-1-[[(2S)-2-[5-[4,Ä≤-[2-[(7S)-2,2-difluoro-6-[(2S)-2-[(met	C43H52F2N8O6	814.92	C	-8.7
3675	C(=O)([C@@H](NC(=O)OC)C(C)C)N1[C@@H]([C@@H]2C[C@H]1CC2)c1[nH]c(cn1)c1ccc(cc1)c1ccc(cc1)c1[nH]c(nc1)[	1273591-08-4	Carbamic acid, N-[(1S)-1-[[(2S,4R)-4-fluoro-2-[5-[4,Ä≤-[2-[(1R,3S,4S)-2-[(2S)-2-	C42H51FN8O6	782.9	C	-8.7
3676	C(=O)([C@@H](NC(=O)OC)C(C)C)N1[C@@H]([C@@H]2C[C@H]1CC2)c1[nH]c(cn1)c1c(C)c(C)c(cc1)c1c(C)c(C)c(cc1)c	1273591-47-1	Carbamic acid, N-[(1S)-1-[[(2S)-2-[5-[4,Ä≤-[2-[(1R,3S,4S)-2-[(2S)-2-[(methoxycar	C44H56N8O6	792.97	C	-8.7
3677	S(=O)(=O)(O)c1c2c(c(S(=O)(=O)O)ccc2)cc(N=Nc2ccc(N=Nc3c(C)cc(N)cc3)cc2)c1	129318-04-3	1,3,5-Naphthalenetrisulfonic acid, 7-[2-[4-[2-(4-amino-2-methylphenyl)diazenyl]p	C23H19N5O9S3	605.62	C	-8.7
3678	C(=O)([C@@H](NC(=O)OC)C)N1[C@@H](CCC1)c1[nH]c(CCc2ccc(CCc3[nH]c(nc3)[C@H]3N(C(=O)[C@@H](NC(=O)OC)C)C	1307303-77-0	Carbamic acid, N,N,Ä≤-[1,4-phenylenebis[2,1-ethanediyl-1H-imidazole-5,2-diyl-(2S	C44H50N8O6	786.92	C	-8.7
3679	S=c1n(nc(CCl)s1)c1nc(nc(n1)N1CCOCC1)N1CCOCC1	1309437-25-9	1,3,4-Thiadiazole-2(3H)-thione, 3-(4,6-di-4-morpholinyl-1,3,5-triazin-2-yl)-5-ph	C19H21N7O2S2	443.55	C	-8.7
3680	S=c1n(c2nc(nc(n2)N2CCOCC2)N2CCOCC2)nc(s1)c1cc(N(=O)=O)c(Cl)cc1	1309437-33-9	1,3,4-Thiadiazole-2(3H)-thione, 5-(2-chloro-5-nitrophenyl)-3-(4,6-di-4-morpholir	C19H19ClN8O4S2	522.99	C	-8.7
3681	O=C1N2[C@@H]3[C@@H](C[C@H](CC3)c3ccccc3)CO[C@@H]2Cn2c1c(O)c(=O)c(C(=O)NCc1c(F)cc(F)cc1)c2	1309560-96-0	2H-Pyrido[1,Ä≤,2,Ä≤:4,5]pyrazino[1,2-a][3,1]benzoxazine-10-carboxamide, N-[(2,4	C23H23F2N3O5	459.44	C	-8.7
3682	O=C1N2[C@@H]3[C@H](CO[C@@H]2Cn2c1c(O)c(=O)c(C(=O)NCc1c(F)cc(F)cc1)c2)CCCC3	1309561-14-5	Cyclopenta[d]pyrido[1,Ä≤,2,Ä≤:4,5]pyrazino[2,1-b][1,3]oxazine-9-carboxamide, N-	C22H21F2N3O5	445.42	C	-8.7
3683	O=C1N2[C@@H]3[C@@H](C[C@H](CC3)c3ccccc3)CO[C@@H]2Cn2c1c(O)c(=O)c(C(=O)NCc1c(F)cc(F)cc1)c2	1309561-70-3	2H-Pyrido[1,Ä≤,2,Ä≤:4,5]pyrazino[1,2-a][3,1]benzoxazine-10-carboxamide, N-[(2,4	C23H23F2N3O5	459.44	C	-8.7
3684	C(=O)([C@@H](NC(=O)OC)C(C)C)N1[C@@H](CC(=C)C1)c1[nH]c(cn1)c1c(C)cc(cc1)c1cc(C)c(cc1)c1[nH]c(nc1)[C@H	1311398-22-7	Carbamic acid, N-[(1S)-2-methyl-1-[[(2S)-4-methylene-2-[5-[2,2,Ä≤,3,3,Ä≤-tetraf	C41H46F4N8O6	822.85	C	-8.7
3685	C(=O)([C@@H](NC(=O)OC)C(C)C)N1[C@@H](CC(=C)C1)c1[nH]c(cn1)c1c(Cl)cc(cc1Cl)c1cc(Cl)c(c(Cl)c1)c1[nH]c(	1311398-27-2	Carbamic acid, N-[(1S)-1-[[(2S)-2-[5-[3,3,Ä≤-dichloro-4,Ä≤-[2-[(2S)-1-[(2S)-2-[	C41H48Cl2N8O6	819.78	C	-8.7
3686	C(=O)([C@@H](NC(=O)OC)C(C)C)N1[C@@H]([C@@H]2C[C@H]1CC2)c1[nH]c(cn1)c1ccc(cc1)c1ccc(cc1)c1[nH]c(nc1)[	1311398-28-3	Carbamic acid, N-[(1S)-2-methyl-1-[[(2S)-4-methylene-2-[5-[3,3,Ä≤,5,5,Ä≤-tetrac	C41H46Cl4N8O6	888.67	C	-8.7
3687	C(=O)([C@@H](NC(=O)OC)C(C)C)N1[C@@H](CC(=C)C1)c1[nH]c(cn1)c1ccc(C=Cc2ccc(cc2)c2[nH]c(nc2)[C@H]2N(C(=	1311398-49-8	Carbamic acid, N-[(1S)-1-[[(2S)-2-[5-[4-[trans-4-[2-[(2S)-1-[(2S)-2-[(methoxycar	C41H56N8O6	756.93	C	-8.7
3688	C(=O)([C@@H](NC(=O)OC)C(C)C)N1[C@H]2[C@H](C2)C[C@H]1c1[nH]c(c(Cl)n1)c1ccc(cc1)c1ccc(cc1)c1[nH]c(nc1C	1312335-94-6	Carbamic acid, N-[(1S)-1-[[(1R,3S,5R)-3-[5-[4,Ä≤-[5-chloro-2-[(1R,3S,5R)-2-[(2S)	C42H49ClN8O6	797.34	C	-8.7
3689	C(=O)([C@@H](NC(=O)OC)C1CC1)N1[C@H]2[C@H](C2)C[C@H]1c1[nH]c(c(Cl)n1)c1ccc(cc1)c1ccc(cc1)c1[nH]c(nc1C	1312336-13-2	Carbamic acid, N-[(1S)-2-[(1R,3S,5R)-3-[5-chloro-4-[4,Ä≤-[5-chloro-2-[(1R,3S,5R)	C44H50Cl2N8O7	873.82	C	-8.7
3690	C(=O)([C@@H](NC(=O)OC)C1CCC(F)(F)CC1)N1[C@H]2[C@H](C2)C[C@H]1c1[nH]c(c(Cl)n1)c1ccc(cc1)c1ccc(cc1)c1[	1312336-37-0	Carbamic acid, N,N,Ä≤-[[1,1,Ä≤-biphenyl]-4,4,Ä≤-diylbis[(5-chloro-1H-imidazole-	C48H52Cl2F4N8O6	983.88	C	-8.7
3691	C(=O)([C@@H](NC(=O)OC)C(C)C)N1[C@H]2[C@H](C2)C[C@H]1c1[nH]c2c(n1)ccc1c2ccc(c1)c1ccc(cc1)c1[nH]c(nc1C	1312336-40-5	Carbamic acid, N-[(1S)-2-[(1R,3S,5R)-3-[5-chloro-4-[4,Ä≤-[2-[(1R,3S,5R)-2-[(2S)-	C47H53ClF2N8O7	915.42	C	-8.7
3692	Cn1c(c2c([nH]c(n2)[C@H]2N(C(=O)[C@@H](NC(=O)OC)C(C)C)CCC2)c2ccc(cc2)c2ccc(cc2)c2[nH]c(nc2)[C@H]2N(C(	1312336-61-0	Carbamic acid, N-[(1S)-1-[[(2S)-2-[5-[4,Ä≤-[2-[(2S)-1-[(2S)-2-[(methoxycarbonyl)	C44H56N8O6	792.97	C	-8.7
3693	C(=O)([C@@H](NC(=O)OC)C(C)C)N1[C@@H](CC[C@@H]1C)c1[nH]c(c(F)n1)c1ccc(cc1)c1ccc(cc1)c1[nH]c(nc1)[C@H]	1312336-64-3	Carbamic acid, N-[(1S)-2-[(1R,3S,5R)-3-[5-[4,Ä≤-[5-fluoro-2-[(1R,3S,5R)-2-[(2S)-	C46H53FN8O8	864.96	C	-8.7
3694	C(=O)([C@@H](NC(=O)OC)[C@@H]1CCCOC1)N1[C@H]2[C@H](C2)C[C@H]1c1[nH]c(cn1)c1ccc(cc1)c1ccc(cc1)c1[nH]c(n	1312338-72-9	Carbamic acid, N,N,Ä≤-[[1,1,Ä≤-biphenyl]-4,4,Ä≤-diylbis[1H-imidazole-5,2-diyl-(	C46H54N8O8	846.97	C	-8.7
3695	Cc1[nH]c2c(ccc(c2c1)c1ccc(cc1)c1[nH]c(nc1)[C@H]1N(C(=O)[C@@H](NC(=O)OC)C(C)C)CCC1)c1[nH]c(nc1)[C@H]1	1312609-57-6	Carbamic acid, N-[(1S)-1-[[(2S)-2-[5-[4-[7-[2-[(2S)-1-[(2S)-2-[(methoxycarbonyl)	C45H56N8O7	820.98	C	-8.7
3696	O(Cc1ccc(F)cc1)c1c2c(oc(c(O)c2=O)c2[nH]ccn2)c(OC)cc1	1312893-47-2	4H-1-Benzopyran-4-one, 5-[(4-fluorophenyl)methoxy]-3-hydroxy-8-methoxy-2-(3-pher	C25H18FN3O5	459.43	C	-8.7
3697	O(Cc1ccc(F)cc1)c1c2c(oc(c(O)c2=O)c2ncccn2)c(Cl)cc1	1312893-52-9	4H-1-Benzopyran-4-one, 5-[(4-fluorophenyl)methoxy]-3-hydroxy-8-methoxy-2-(5-pher	C26H19FN2O5	458.44	C	-8.7
3698	O(Cc1ccc(F)cc1)c1c2c(oc(c(O)c2=O)c2[nH]ccn2)cc(C)c1	1312894-97-5	4H-1-Benzopyran-4-one, 5-[(4-fluorophenyl)methoxy]-3-hydroxy-7-methyl-2-(3-pheny	C25H18FN3O4	443.43	C	-8.7
3699	O(Cc1ccc(F)cc1)c1c2c(c(OC)c(C)c1)oc(c(O)c2=O)c1ncccn1	1312895-52-5	4H-1-Benzopyran-4-one, 5-[(4-fluorophenyl)methoxy]-3-hydroxy-7,8-dimethyl-2-(5-p	C27H21FN2O4	456.46	C	-8.7
3700	O(C)c1c2c(c(OCc3ccc(F)cc3)cc1C)c(=O)c(O)c(o2)c1[nH]cnn1	1312895-87-6	4H-1-Benzopyran-4-one, 5-[(4-fluorophenyl)methoxy]-3-hydroxy-8-methoxy-7-methyl-	C28H21FN2O5	484.48	C	-8.7
3701	O(Cc1ccc(F)cc1)c1c2c(c(c(C)c1)c1ccccc1)oc(c(O)c2=O)c1ncccn1	1312896-52-8	4H-1-Benzopyran-4-one, 8-chloro-5-[(4-fluorophenyl)methoxy]-3-hydroxy-7-methyl-2	C26H18ClFN2O4	476.88	C	-8.7
3702	O(Cc1ccc(F)cc1)c1c2c(c(c(C)c1)c1ccccc1)oc(c(O)c2=O)c1[nH]c(OC)nn1	1312896-94-8	4H-1-Benzopyran-4-one, 5-[(4-fluorophenyl)methoxy]-3-hydroxy-7-methyl-2-(3-methy	C26H20FN3O4	457.45	C	-8.7
3703	O(Cc1ccc(F)cc1)c1c2c(c(c(C)c1)c1ccccc1)oc(c(O)c2=O)c1nnc(Cl)[nH]1	1312896-95-9	4H-1-Benzopyran-4-one, 5-[(4-fluorophenyl)methoxy]-3-hydroxy-2-(5-methoxy-1H-1,2	C26H20FN3O5	473.45	C	-8.7
3704	O(Cc1ccc(F)cc1)c1c2c(c(c(C)c1)c1ccccc1)oc(c(O)c2=O)c1[nH]cc(Cl)n1	1312897-00-9	4H-1-Benzopyran-4-one, 5-[(4-fluorophenyl)methoxy]-3-hydroxy-2-(5-methoxy-1H-imi	C27H21FN2O5	472.46	C	-8.7
3705	O(Cc1ccc(F)cc1)c1c2c(oc(c(O)c2=O)c2ncccn2)c(C)c(OC)c1	1312897-63-4	4H-1-Benzopyran-4-one, 5-[(4-fluorophenyl)methoxy]-3-hydroxy-7-methoxy-2-(5-pher	C26H19FN2O5	458.44	C	-8.7
3706	O(Cc1ccc(F)cc1)c1c2c(oc(c(O)c2=O)c2[nH]ccn2)c(Cl)c(OC)c1	1312899-09-4	4H-1-Benzopyran-4-one, 8-chloro-5-[(4-fluorophenyl)methoxy]-3-hydroxy-7-methoxy-	C25H17ClFN3O5	493.87	C	-8.7
3707	O(Cc1ccc(F)cc1)c1c2c(c(c(Cl)c1)c1ccccc1)oc(c(O)c2=O)c1[nH]c(OC)nn1	1312902-65-0	4H-1-Benzopyran-4-one, 7-chloro-5-[(4-fluorophenyl)methoxy]-3-hydroxy-2-(3-methy	C25H17ClFN3O4	477.87	C	-8.7
3708	O(Cc1ccc(F)cc1)c1c2c(oc(c(O)c2=O)c2ncc(OC)cn2)cc(c1)c1ccccc1	1312903-05-1	4H-1-Benzopyran-4-one, 5-[(4-fluorophenyl)methoxy]-3-hydroxy-2-(5-methyl-2-pyrim	C27H19FN2O4	454.45	C	-8.7
3709	O(Cc1ccc(F)cc1)c1c2c(oc(c(O)c2=O)c2[nH]c(C)nn2)cc(c1)c1ccccc1	1312903-14-2	4H-1-Benzopyran-4-one, 5-[(4-fluorophenyl)methoxy]-3-hydroxy-7-phenyl-2-(1H-1,2,	C24H16FN3O4	429.4	C	-8.7
3710	O(Cc1ccc(F)cc1)c1c2c(c(C)c(c1)c1ccccc1)oc(c(O)c2=O)c1[nH]c(C)cn1	1312903-71-1	4H-1-Benzopyran-4-one, 5-[(4-fluorophenyl)methoxy]-3-hydroxy-2-(1H-imidazol-2-yl	C26H19FN2O4	442.44	C	-8.7
3711	O(C)c1c2c(c(OCc3ccc(F)cc3)cc1c1ccccc1)c(=O)c(O)c(o2)c1[nH]ccn1	1312904-47-4	4H-1-Benzopyran-4-one, 5-[(4-fluorophenyl)methoxy]-3-hydroxy-8-methoxy-7-phenyl-	C31H22FN3O5	535.52	C	-8.7
3712	O=c1c2c(oc(c1O)c1nnc(C)[nH]1)ccc(OCc1ccc(F)cc1)c2	1312906-19-6	4H-1-Benzopyran-4-one, 6-[(4-fluorophenyl)methoxy]-3-hydroxy-2-(5-phenyl-2-pyrim	C26H17FN2O4	440.42	C	-8.7
3713	O=c1c2c(c(cc(OCc3ccc(F)cc3)c2)c2ccccc2)oc(c1O)c1[nH]c(OC)nn1	1312908-25-0	4H-1-Benzopyran-4-one, 6-[(4-fluorophenyl)methoxy]-3-hydroxy-2-(3-methyl-1H-1,2,	C25H18FN3O4	443.43	C	-8.7
3714	O=c1c2c(oc(c1O)c1[nH]cnn1)cc(C)c(OCc1ccc(F)cc1)c2	1312908-68-1	4H-1-Benzopyran-4-one, 6-[(4-fluorophenyl)methoxy]-3-hydroxy-7-methyl-2-(5-pheny	C27H19FN2O4	454.45	C	-8.7
3715	O=c1c2c(oc(c1O)c1[nH]ccn1)cc(OC)c(OCc1ccc(F)cc1)c2	1312911-40-2	4H-1-Benzopyran-4-one, 6-[(4-fluorophenyl)methoxy]-3-hydroxy-7-methoxy-2-(3-pher	C25H18FN3O5	459.43	C	-8.7
3716	O(C)c1c(c2c(cc1OCc1ccc(F)cc1)c(=O)c(O)c(o2)c1[nH]cnn1)c1ccccc1	1312915-20-0	4H-1-Benzopyran-4-one, 6-[(4-fluorophenyl)methoxy]-3-hydroxy-7-methoxy-8-phenyl-	C33H23FN2O5	546.54	C	-8.7
3717	Clc1c(c2c(cc1OCc1ccc(F)cc1)c(=O)c(O)c(o2)c1[nH]c(Cl)nn1)c1ccccc1	1312915-84-6	4H-1-Benzopyran-4-one, 7-chloro-6-[(4-fluorophenyl)methoxy]-3-hydroxy-2-(5-metho	C25H17ClFN3O5	493.87	C	-8.7
3718	O(Cc1ccc(F)cc1)c1c(cc2c(c1)c(=O)c(O)c(o2)c1[nH]c(C)cn1)c1ccccc1	1312916-46-3	4H-1-Benzopyran-4-one, 6-[(4-fluorophenyl)methoxy]-3-hydroxy-2-(1H-imidazol-2-yl	C25H17FN2O4	428.41	C	-8.7
3719	O(C)c1c2c(cc(OCc3ccc(F)cc3)c1c1ccccc1)c(=O)c(O)c(o2)c1[nH]ccn1	1312917-46-6	4H-1-Benzopyran-4-one, 6-[(4-fluorophenyl)methoxy]-3-hydroxy-8-methoxy-7-phenyl-	C31H22FN3O5	535.52	C	-8.7
3720	O([C@@H]1[C@@H](COP2(=O)Oc3c(CO2)cccc3)O[C@H](Cl)n1c(=O)[nH]c(=O)c(I)c1)P1(=O)Oc2c(CO1)cccc2	1313031-57-0	Uridine, 5,Ä≤-O-[(2R)-6,8-bis(1,1-dimethylethyl)-5-fluoro-2-oxido-4H-1,3,2-benzo	C24H31FIN2O8P	652.39	C	-8.7
3721	O=C1c2n(C[C@H]3N1[C@H](C)CCO3)cc(C(=O)NCc1c(F)cc(F)cc1)c(=O)c2O.C(#CC1CC1)[C@@]1(C(F)(F)F)c2c(NC(=O)	13195-20-5	1-Naphthalenesulfonic acid, 4-hydroxy-3-[[4,Ä≤-[[4-[(1-hydroxy-4-sulfo-2-naphthy	C44H32N6O8S2.2Na		C	-8.7
3722	N(=C1c2c(NC1=O)cccc2)Nc1nc(nc(NCc2cc(Cl)ccc2)n1)N1CCOCC1	1331749-45-1	1H-Indole-2,3-dione, 3-[2-[4-(4-morpholinyl)-6-[(phenylmethyl)amino]-1,3,5-triaz	C22H22N8O2	430.46	C	-8.7
3723	C(C)(C)c1n([C@@H]2C[C@@H]3N(C(CC(NC(=O)C4C(CC(F)(F)CC4([2H])[2H])([2H])[2H])c4ccccc4)([2H])[2H])C[C@	1349132-63-3	4-Morpholinecarboxamide, N-[(1R)-2-[[(2E)-3-(1-naphthalenylsulfonyl)-1-(2-phenyl	C35H37N3O5S	611.75	C	-8.7
3724	C(#CCC)C1(C(F)(F)F)c2c(NC(=O)O1)cc(F)c(Cl)c2	1350203-30-3	2,7,10,12-Tetraazatridecanoic acid, 13-[2-(cyclohexylmethyl)-4-thiazolyl]-4-hydr	C41H54N6O6S	758.97	C	-8.7
3725	Cc1[nH]c2c(ccc(c2n1)c1[nH]c(nc1)[C@H]1N(C(=O)[C@@H](NC(=O)OC)C(C)C)CCC1)c1c2c(c(cc1)c1[nH]c(nc1)[C@H	1350718-39-6	Carbamic acid, N,N,Ä≤-[[4,4,Ä≤-bi-1H-benzimidazole]-7,7,Ä≤-diylbis[1H-imidazole	C42H50N12O6	818.92	C	-8.7
3726	Cc1cc2c(ccc(c2[nH]1)c1[nH]c(nc1)[C@H]1N(C(=O)[C@@H](NC(=O)OC)C(C)C)CCC1)c1c2c(c(cc1)c1[nH]c(nc1)[C@H	1350718-41-0	Carbamic acid, N,N,Ä≤-[[4,7,Ä≤-bi-1H-indole]-4,Ä≤,7-diylbis[1H-imidazole-5,2-di	C44H52N10O6	816.95	C	-8.7
3727	C(=O)([C@@H](NC(=O)OC)C(C)C)N1[C@@]2(C[C@H](C1)CC2)c1[nH]c(cn1)c1c2c(c(cc1)c1ccc(cc1)c1[nH]c(nc1)[C@	1350718-96-5	Carbamic acid, N-[(1S)-1-[[(1S,4R)-1-[5-[8-[4-[2-[(1S,4R)-2-[(2S)-2-[(methoxycar	C47H55N9O6	842	C	-8.7
3728	C(=O)([C@@H](NC(=O)OC)C(C)C)N1[C@@]2(C[C@H](C1)CC2)c1[nH]c(cn1)c1c2c(c(cc1)c1ccc(cc1)c1[nH]c(nc1)[C@	1350718-98-7	Carbamic acid, N-[(1S)-1-[[(1S,4R)-1-[5-[4-[7-[2-[(1S,4R)-2-[(2S)-2-[(methoxycar	C47H56N8O6S	861.06	C	-8.7
3729	C(=O)([C@@H](NC(=O)OC)C(C)C)N1[C@H](c2[nH]c(c3c4c(c(cc3)c3c5c(c(cc3)c3[nH]c(nc3)[C@H]3N(C(=O)[C@@H](	1350719-34-4	Carbamic acid, N,N,Ä≤-[[7,7,Ä≤-bibenzofuran]-4,4,Ä≤-diylbis[1H-imidazole-5,2-di	C44H50N8O8	818.92	C	-8.7
3730	C(=O)([C@H](NC(=O)OC)c1ccccc1)N1[C@@H](CCC1)c1[nH]c(cn1)c1cc2c(cc1)cc(cc2)c1c(C)cc(cc1)c1[nH]c(nc1)[	1351330-60-3	Carbamic acid, N-[(1S)-1-[[(2S)-2-[5-[6-[4-[2-[(2S)-1-[(2S)-2-[(methoxycarbonyl)	C45H54N8O6	802.96	C	-8.7
3731	C(=O)([C@@H](NC(=O)OC)C(C)C)N1[C@@H](C[C@]2(C1)CC=CCO2)c1[nH]c(cn1)c1ccc(cc1)c1ccc(cc1)c1[nH]c(nc1)[	1351853-17-2	Carbamic acid, N-[(1S)-1-[[(2S)-2-[5-[4,Ä≤-[2-[(3S,5S)-2-[(2S)-2-[(methoxycarbor	C44H56N8O7	808.96	C	-8.7
3732	C(=O)([C@@H](NC(=O)OC)C(C)C)N1C[C@@]2(C[C@H]1c1[nH]c(cn1)c1ccc(cc1)c1ccc(cc1)c1[nH]c(nc1)[C@H]1N(C(=	1351853-22-9	Carbamic acid, N-[(1S)-1-[[(2S)-2-[5-[4,Ä≤-[2-[(5S,8S)-7-[(2S)-2-[(methoxycarbor	C43H52N8O8	808.92	C	-8.7
3733	C(=O)([C@H](Cc1ccccc1)NC(=O)OC)N1[C@@H](C[C@@H](C1)C1CC1)c1[nH]c(cn1)c1ccc(cc1)c1ccc(cc1)c1[nH]c(nc1	1351854-42-6	Carbamic acid, N-[(1R)-2-[(2S)-2-[5-[4,Ä≤-[2-[(2S,4R)-4-cyclopropyl-1-[(2R)-2-[	C51H54N8O6	875.02	C	-8.7
3734	C(=O)([C@@H](NC(=O)OC)C(C)C)N1[C@@H](CCC1)c1[nH]c(cn1)c1ccc(C#Cc2ccc(cc2)c2[nH]c(nc2)[C@H]2N(C(=O)[C	1352392-95-0	Carbamic acid, N-[(1S)-1-[[(2S)-2,5-dihydro-4-methyl-2-[5-[4-[2-[4-[2-(2S)-2-pyr	C36H39N7O3	617.74	C	-8.7
3735	O=c1c2c(n(cn2)[C@H]2[C@H](O)[C@H](O)[C@@H](CO)O2)[nH]c(NC2CCCC2)n1	1353757-89-7	Guanosine, N-[(1E)-2-(3-ethynylbenzoyl)-3-oxo-1-buten-1-yl]-	C23H21N5O7	479.44	C	-8.7
3736	O=C1N(C(SC1)c1ccc(Cl)cc1)c1[nH]c(nn1)N1CCOCC1	1354021-14-9	4-Thiazolidinone, 5-methyl-2-(4-methylphenyl)-3-[3-(4-morpholinyl)-1H-1,2,4-tria	C17H21N5O2S	359.45	C	-8.7
3737	O(P(=O)(OC[C@H]1O[C@H]([C@H](O)[C@@H]1O)n1c2c(nc1)c(N)ncn2)O)[C@H]1[C@@H](O[C@H](COP(=O)(O[C@@H]2[C@	1357016-08-0	Adenosine, 5,Ä≤-O-(phosphonomethyl)-Œ≤-D-arabino-adenylyl-(2,Ä≤,Ü15,Ä≤)-adenyly	C41H52N20O25P4	1348.87	C	-8.7
3738	S(=O)(=O)(O)c1c2c(c(N)c(N=Nc3ccc(cc3)c3ccc(C)cc3)c1)cccc2	1357193-89-5	1-Naphthalenesulfonic acid, 5-[2-(4-amino-6-sulfo-1-naphthalenyl)diazenyl]-	C20H15N3O6S2	457.48	C	-8.7
3739	C(=O)([C@@H](NC(=O)OC)C1CCOCC1)N1[C@H]2[C@H](C2)C[C@H]1c1[nH]c(cn1)c1c2c(c(cc1)c1ccc(cc1)c1[nH]c(nc1	1359868-85-1	Carbamic acid, N-[(1S)-1-[[(1R,3S,5R)-3-[5-[4-[2,3-dihydro-7-[2-[(1R,3S,5R)-2-[(	C45H54N8O6	802.96	C	-8.7
3740	C(F)(F)c1n(c2c(n1)cccc2)c1nc(nc(Nc2c(O)cccc2)n1)N1CCOCC1	1359956-21-0	Phenol, 3-[[4-[2-(difluoromethyl)-1H-benzimidazol-1-yl]-6-(4-morpholinyl)-1,3,5-	C21H19F2N7O2	439.42	C	-8.7
3741	C[C@@]12[C@@H]([C@]3(C)[C@@H](CC2)C(C)(C)[C@@H](O)CC3)CC[C@H]2[C@@]1(C)CC[C@]1(C)C2=C[C@@](CO)(C)CC1	1374421-59-6	Carbamic acid, N,N,Ä≤-[[1-[4-(1,1-dimethylethyl)phenyl]-1,2-ethenediyl]bis[4,1-p	C52H64N8O6	897.11	C	-8.7
3742	C(=O)([C@H](NCC)c1ccccc1)N1[C@@H](CCC1)c1[nH]c(cn1)c1ccc(cc1)c1cc2c(cc1)cc(cc2)c1[nH]c(nc1)[C@H]1N(C	1377038-38-4	Carbamic acid, N-[(1R)-2-[(2S)-2-[5-[4-[6-[2-[(2S)-1-[(2S)-2-[(methoxycarbonyl)a	C48H50N8O6	834.96	C	-8.7
3743	C(=O)([C@@H](NC(=O)OC)C(C)C)N1[C@H]2[C@H](C2)C[C@H]1c1[nH]c2c(n1)ccc1c2ccc(c1)c1ccc2c(cc1)c1c(cc2)nc(	1377043-75-8	Carbamic acid, N-[(1S)-1-[[(2S)-2-[2,Ä≤-[(2S,4S)-1-[(2S)-2-[(methoxycarbonyl)ami	C46H54N8O7	830.97	C	-8.7

3744	C(=O)([C@@H](NC(=O)OC)C(C)C)N1[C@H](c2[nH]c3c4c(cc(cc4)c4cc5c(cc4)c4c(cc5)nc([nH]4)[C@H]4N(C(=O)[C@@	1377044-64-8	Carbamic acid, N-[(1S)-1-[[(2S,3aS,6aS)-hexahydro-2-[2,Ä≤-[(2S)-1-[(2S)-2-[(meth	C47H54N8O6	826.98	C	-8.7
3745	N(C(=O)C)[C@H]1[C@H]([C@@H]([C@@H](CO)O)O)OC(=C[C@H]1NC(=O)C)C(=O)O	1380086-96-3	Furo[3,Ä≤,4,Ä≤:6,7]naphtho[2,3-d]-1,3-dioxol-6(5aH)-one, 5,8,8a,9-tetrahydro-9-	C22H22O5	366.41	C	-8.7
3746	O[C@H]1[C@H](n2c3c(nc2)c(=O)nc(N)[nH]3)O[C@H](COP(=O)(O[C@@H]2[C@@H](COP(=O)(O)O)O[C@H](C2)n2c3c(nc2	1383925-94-7	Guanosine, 5,Ä≤-O-phosphono-P-thioguanylyl-(3,Ä≤,Ü í5,Ä≤)-	C20H26N10O14P2S	724.49	C	-8.7
3747	O=c1c2c(n(cn2)[C@@H]2O[C@H](CO)[C@@H](O)[C@H]2O)[nH]c(n1)n1cc(CCCC)nn1	1384519-82-7	Inosine, 2-[4-[(1,3-dihydro-1,3-dioxo-2H-isoindol-2-yl)methyl]-1H-1,2,3-triazol-	C21H18N8O7	494.42	C	-8.7
3748	O=c1c2c(n(cn2)[C@@H]2O[C@H](CO)[C@@H](O)C2)[nH]c(n1)n1cc(nn1)c1ccc(OC)cc1	1384519-85-0	Inosine, 2,Ä≤-deoxy-2-(4-phenyl-1H-1,2,3-triazol-1-yl)-	C18H17N7O4	395.37	C	-8.7
3749	O=c1c2c(n(cn2)[C@@H]2O[C@H](CO)[C@@H](O)C2)[nH]c(n1)n1cc(CO)nn1	1384519-86-1	Inosine, 2,Ä≤-deoxy-2-[4-(4-methoxyphenyl)-1H-1,2,3-triazol-1-yl]-	C19H19N7O5	425.4	C	-8.7
3750	C(=O)([C@@H](NC(=O)OC)C(C)C)N1[C@H](c2[nH]c(c(n2)c2cccs2)c2ccc(cc2)c2ccc(cc2)c2c(nc([nH]2)[C@H]2N(C(	1388224-10-9	Carbamic acid, N,N,Ä≤-[[1,1,Ä≤-biphenyl]-4,4,Ä≤-diylbis[[5-(2-furanyl)-1H-imida	C48H54N8O8	870.99	C	-8.7
3751	C(=O)([C@@H](NC(=O)OC)C(C)C)N1[C@@H](CCC1)c1[nH]c(c(I)n1)c1ccc(cc1)c1ccc(cc1)c1[nH]c(nc1I)[C@H]1N(C(	1388224-13-2	Carbamic acid, N,N,Ä≤-[[1,1,Ä≤-biphenyl]-4,4,Ä≤-diylbis[1H-imidazole-5,2-diyl[(	C40H48N14O6		C	-8.7
3752	N(CC(=O)O)(C(=O)C)c1nc(nc(n1)N1CCOCC1)c1cc(NC(=O)C)ccc1	1391924-87-0	Ethanone, 2-[[4-(4-aminophenyl)-6-(4-morpholinyl)-1,3,5-triazin-2-yl]amino]-1-[4	C20H28N8O4S	476.55	C	-8.7
3753	N(=C1CCN(c2nc(nc(n2)N2CCOCC2)c2c3c(ccc2)[nH]nc3)CC1)O	1391925-72-6	1H-Indazol-4-amine, N-[4-(1H-indazol-4-yl)-6-(4-morpholinyl)-1,3,5-triazin-2-yl]	C21H19N9O	413.44	C	-8.7
3754	N(c1nc(nc(NCCNC(=O)c2ccc(cc2)C2OOC(OO2)c2ccc(C)cc2)n1)N1CCOCC1)C1CCCCC1	1393922-41-2	Benzamide, 4-[6-(4-methylphenyl)-1,2,4,5-tetroxan-3-yl]-N-[2-[[4-(4-morpholinyl)	C30H37N7O6	591.66	C	-8.7
3755	[C@@H](COCP(=O)(OCOC(=O)OC(C)C)OCOC(=O)OC(C)C)(C)n1c2c(nc1)c(N)ncn2	1397776-19-0	Thymidine, thymidylyl-(3,Ä≤,Ü í5,Ä≤)-Œ±-hydroxy-	C20H27N4O13P	562.42	C	-8.7
3756	N(=Nc1c2c(c(S(=O)(=O)O)ccc2)c(N)cc1)c1c2c(c(N=Nc3c(S(=O)(=O)O)ccc(S(=O)(=O)O)c3)cc1)ccc(S(=O)(=O)O)c	1407096-76-7	1,4-Benzenedisulfonic acid, 2-[2-[4-[2-(4-amino-5-sulfo-1-naphthalenyl)diazenyl]	C26H19N5O12S4.3Na		C	-8.7
3757	C(NC(=O)c1[nH]c(nc(=O)c1O)c1nc2c(cc1)cccc2)(C)C12CC3CC(C2)CC(C1)C3	1408305-01-0	4-Pyrimidinecarboxamide, 1,6-dihydro-5-hydroxy-6-oxo-2-(2-pyridinyl)-N-(1-tricyc	C22H26N4O3	394.47	C	-8.7
3758	S(=O)(=O)(O)c1c2c(ccc(N=Nc3cc(S(=O)(=O)O)c(CCc4c(S(=O)(=O)O)cc(N=Nc5c(N)c6c(cc5)cccc6S(=O)(=O)O)cc4)	140942-33-2	2-Naphthalenesulfonic acid, 6,6,Ä≤-[1,2-ethenediylbis[(3-sulfo-4,1-phenylene)az	C34H26N6O12S4.4Na		C	-8.7
3759	C(=C1C(=O)C(=CC=C1)C(=O)O)(c1cc(C(=O)O)c(O)cc1)c1cc(C(=O)O)c(O)cc1	141511-25-3	Benzoic acid, 5-[(3-carboxy-4-hydroxyphenyl)(3-carboxy-4-oxo-2,5-cyclohexadien-1	C30H20O12	572.47	C	-8.7
3760	C(=O)([C@@H](NC(=O)OC)C(C)C)N1[C@H](CCC1)c1[nH]c(cn1)c1ccc(cc1)c1ccc(cc1)c1[nH]c(nc1)[C@H]1N(C(=O)[C	1416037-82-5	Carbamic acid, N-[(1S)-1-[[(2S)-2-[5-[4,Ä≤-[2-[(2R,3R)-3-hydroxy-1-[(2S)-2-[(met	C40H50N8O7	754.87	C	-8.7
3761	C(=O)([C@H](NC(=O)OC)C(C)C)N1[C@@H](CCC1)c1[nH]c(cn1)c1ccc(cc1)c1ccc(cc1)c1[nH]c(nc1)[C@H]1N(C(=O)[C	1417333-67-5	Carbamic acid, N,N,Ä≤-[[1,1,Ä≤-biphenyl]-4,4,Ä≤-diylbis[1H-imidazole-5,2-diyl-2	C40H50N8O6	738.88	C	-8.7
3762	C(=O)([C@@H](NC(=O)OC)[C@H](CC)C)N1[C@H](c2[nH]c(c3c4c(c(cc3)c3ccc(cc3)c3[nH]c(nc3)[C@H]3N(C(=O)[C@@	1419519-53-1	Carbamic acid, N-[(1S)-1-[[(3S)-3-[5-[1,Ä≤,3,Ä≤-dihydro-7,Ä≤-[4-[2-[(3S)-2-[(2S	C49H60N8O6	857.05	C	-8.7
3763	N(=Nc1c2c(c(N)cc1)cccc2)c1c2c(c(S(=O)(=O)O)ccc2)c(O)cc1	1432018-34-2	2-Naphthalenesulfonic acid, 5-[2-(1-naphthalenyl)diazenyl]-	C20H14N2O3S	362.4	C	-8.7
3764	N(Cc1sc(cc1)c1ccccc1)C12CC3(O)CC(C2)CC(C1)C3	1443123-24-7	[1,1,Ä≤-Biphenyl]-4-carboxylic acid, 4,Ä≤-[[(3-hydroxytricyclo[3.3.1.13,7]dec-1	C25H29NO3	391.5	C	-8.7
3765	S(c1n(c2c(n1)c(=O)nc(N)[nH]2)[C@@H]1O[C@H]2[C@H]([C@H]1O)OP(=O)(O)OC2)c1c(OC)cccc1	144510-00-9	Guanosine, 8-(phenylthio)-, cyclic 3,Ä≤,5,Ä≤-(hydrogen phosphate)	C16H16N5O7PS	453.37	C	-8.7
3766	C(=O)([C@@H](NC(=O)OC)C(C)C)N1[C@H](c2[nH]c(c3cc4c(-c5c(cc(cc5)c5[nH]c(nc5)[C@H]5N(C(=O)[C@@H](NC(=O	1449762-33-7	Carbamic acid, N,N,Ä≤-[spiro[cyclopropane-1,7,Ä≤(8,Ä≤H)-[6H]dibenzo[f,h][1,5]di	C45H56N8O8	836.97	C	-8.7
3767	O(C(=O)N[C@@H](Cc1ccccc1)[C@@H](CN(S(=O)(=O)c1ccc(NC(=O)NC)cc1)CC(C)C)O)[C@@H]1[C@H]2[C@@H](OC1)OCC2	1450913-01-5	1-Pyrrolidinepropanamide, N-[3-[1-[7-(11-cyclopropyl-6,11-dihydro-4-methyl-6-oxo	C47H48F2N10O5	870.94	C	-8.7
3768	O=C1[C@@H]2[C@@H](c3c([C@@H](O)[C@H]2CO1)cc1c(c3)OCO1)c1cc(OC)c(OP(=O)(OCc2ccccc2)OCc2ccccc2)c(OC)c1	146849-73-2	Phosphoric acid, 4-(5,5a,6,8,8a,9-hexahydro-9-hydroxy-6-oxofuro[3,Ä≤,4,Ä≤:6,7]n	C33H29O11P	632.55	C	-8.7
3769	N(C(CC)(C)C)c1c(nccc1)N1CCN(C(=O)c2cc3c([nH]2)ccc(NS(=O)(=O)C)c3)CC1	147540-14-5	1-Piperazinesulfonamide, N-[2-[[4-[3-[(1,1-dimethylpropyl)amino]-2-pyridinyl]-1-	C28H40N8O3S	568.73	C	-8.7
3770	N(Cc1ccc(C#N)cc1)(C[C@@H]([C@H](Cc1ccccc1)NC(=O)[C@@H](NC(=O)OC)C(C)C)O)NC(=O)[C@@H](NC(=O)OC)C(C)C	14926-00-2	[1,1,Ä≤-Biphenyl]-3,3,Ä≤-dicarboxylic acid, 4,4,Ä≤-bis(benzoylamino)-	C28H20N2O6	480.47	C	-8.7
3771	C(C#C)n1c2c3c(nc(N)c2nc1)cccc3	149821-01-2	Uridine, thymidylyl-(3,Ä≤,Ü í5,Ä≤)-2,Ä≤-deoxy-5,6-dihydro-6-hydroxy-, (6R)-	C19H27N4O13P	550.41	C	-8.7
3772	O[C@H]1[C@H](n2c3c(nc2)c(=O)nc(N)[nH]3)O[C@H](CCNO[C@@H]2[C@@H](CO)O[C@H](C2)n2c3c(nc2)c(=O)nc(N)[nH	1513874-25-3	Guanosine, 2,Ä≤-deoxy-3,Ä≤-de(phosphinicooxy)guanylyl-1,2-hydrazinediylmethylen	C21H28N12O7	560.52	C	-8.7
3773	O[C@H]1[C@H](n2c3c(nc2)c(=O)nc(N)[nH]3)O[C@H](CNN[C@@H]2[C@@H](CO)O[C@H](C2)n2c3c(nc2)c(=O)nc(N)[nH]	1513874-26-4	Guanosine, 2,Ä≤-deoxy-3,Ä≤-O-dephosphinicoguanylyliminomethylene-(3,Ä≤,Ü í5,Ä≤)-	C21H27N11O8	561.51	C	-8.7
3774	O=c1c2c(n(cn2)[C@H]2C[C@H](NNC[C@H]3O[C@H](C[C@@H]3O)n3c4c(nc3)c(=O)nc(N)[nH]4)[C@@H](CO)O2)[nH]c(N)	1513874-38-8	Guanosine, 2,Ä≤-deoxy-3,Ä≤-O-dephosphinicoguanylyliminomethylene-(3,Ä≤,Ü í5,Ä≤)-	C21H27N11O7	545.51	C	-8.7
3775	C(n1c2c(nc1CC)c(C)cc(C)n2)c1ccc(cc1)c1c(S(=O)(=O)NC(=O)CCc2ccccc2)ccc(COC)c1	154552-59-7	Benzenepropanamide, N-[[4,Ä≤-[(2-ethyl-5,7-dimethyl-3H-imidazo[4,5-b]pyridin-3-y	C34H36N4O3S2	612.8	C	-8.7
3776	C(n1c2c(nc1CC)c(C)cc(C)n2)c1ccc(cc1)c1c(S(=O)(=O)NC(=O)c2c(Oc3ccccc3)cccc2)ccc(CCCC)c1	154552-73-5	Benzamide, N-[[4,Ä≤-[(2-ethyl-5,7-dimethyl-3H-imidazo[4,5-b]pyridin-3-yl)methyl]	C39H38N4O4S	658.81	C	-8.7
3777	O=C1C2c3c(c(ccc3c3[nH]c(nc3)[C@H]3N(C(=O)[C@@H](NC(=O)OC)C(C)C)CCC3)c3ccc(cc3)c3[nH]c(nc3)[C@H]3N(C(	1560639-40-8	Carbamic acid, N-[(1S)-2-methyl-1-[[(2S)-2-[5-[4-[1,2,3,4-tetrahydro-8-[2-[(2S)-	C46H58N8O6	819	C	-8.7
3778	CN1C2c3c(C1CC2)c(ccc3c1[nH]c(nc1)[C@H]1N(C(=O)[C@@H](NC(=O)OC)C(C)C)C[C@@H](C)C1)c1ccc(cc1)c1[nH]c(n	1560639-43-1	Carbamic acid, N-[(1S)-1-[[(2S)-2-[5-[4-[3,4-dihydro-5-[2-[(2S)-1-[(2S)-2-[(meth	C45H54N8O8	834.96	C	-8.7
3779	C(=O)([C@@H](NC(=O)OC)C(C)C)N1[C@H](c2[nH]c(c3c4c(C5OC4CC5)c(cc3)c3ccc(cc3)c3[nH]c(nc3)[C@H]3N(C(=O)	1560640-89-2	Carbamic acid, N-[(1S)-1-cyclohexyl-2-[(2S)-2-[5-[8-[4-[2-[(2S)-1-[(2S)-2-cycloh	C50H62N8O7	887.08	C	-8.7
3780	CN1C2c3c(C1CC2)c(ccc3c1[nH]c(nc1)[C@H]1N(C(=O)[C@@H](NC(=O)OC)C(C)C)CC(F)(F)C1)c1ccc(cc1)c1[nH]c(nc1	1560649-39-9	Carbamic acid, N-[(1S)-2-methyl-1-[[(2S)-2-[5-[4-[1,2,3,4-tetrahydro-8-[2-[(2S)-	C45H57N9O6	819.99	C	-8.7
3781	C(=O)([C@@H](NC(=O)OC)C(C)C)N1CC2(C[C@@H]1c1[nH]c(cn1)c1ccc(cc1)c1ccc(cc1)c1[nH]c(nc1)[C@H]1N(C(=O)[	1571138-38-9	Carbamic acid, N-[(1S)-1-[[(2S)-2-[5-[4,Ä≤-[2-[(7S)-6-[(2S)-2-[(methoxycarbonyl)	C42H53N9O6	779.93	C	-8.7
3782	O=c1n([C@H]2O[C@@H](COC(=O)C)SC2)ccc(NC(=O)C)n1	158614-82-5	3,Ä≤-Guanylic acid, P-thioguanylyl-(3,Ä≤,Ü í5,Ä≤)-	C20H26N10O14P2S	724.49	C	-8.7
3783	O=c1n([C@H]2O[C@@H](COC(=O)C)SC2)ccc(N)n1	158703-48-1	Guanosine, 2,Ä≤-deoxy-P-thioguanylyl-(3,Ä≤,Ü í5,Ä≤)-2,Ä≤-deoxy-P-thioguanylyl-(3	C30H37N15O14P2S2	957.78	C	-8.7
3784	C(=O)([C@@H](NC(=O)OC)C(C)C)N1[C@H](c2[nH]c(c3c4C5(CCc4c(cc3)c3ccc(cc3)c3[nH]c(nc3)[C@H]3N(C(=O)[C@@	1588403-84-2	Carbamic acid, N-[(1S)-1-[[(2S)-2-[5-[2,Ä≤,3,Ä≤-dihydro-7,Ä≤-[4-[2-[(2S)-1-[(2S	C47H60N8O6	833.03	C	-8.7
3785	C(=O)([C@@H](NC(=O)OC)C(C)C)N1[C@H](c2[nH]c(c3c4c(C5(CC4)CCCC5)c(cc3)c3ccc(cc3)c3[nH]c(nc3)[C@H]3N(C	1588403-99-9	Carbamic acid, N-[(1S)-1-[[(2S)-2-[5-[2,Ä≤,3,Ä≤-dihydro-7,Ä≤-[4-[2-[(2S)-1-[(2S	C51H68N8O6	889.14	C	-8.7
3786	C(=O)([C@@H](NC(=O)OC)C(C)C)N1[C@H](c2[nH]c(c3c4c(C5(CC4)CCCC5)c(cc3)c3ccc(cc3)c3[nH]c(nc3)[C@H]3N(C	1588404-27-6	Carbamic acid, N-[(1S)-2-methyl-1-[[(2S)-2-[5-[2,Ä≤,3,Ä≤,4,5-tetrahydro-7,Ä≤-[4	C46H58N8O7	835	C	-8.7
3787	C(n1c2c(nc1CC)c(C)cc(C)n2)c1ccc(cc1)c1c(S(=O)(=O)NC(=O)CCC)cccc1	158963-52-1	Acetamide, N-[[4,Ä≤-[(2-ethyl-5,7-dimethyl-3H-imidazo[4,5-b]pyridin-3-yl)methyl]	C25H26N4O3S	462.56	C	-8.7
3788	N(C(CCCN(CC)CC)C)c1c2c(nc3c1cc(Cl)cc3)cc(Cl)cc2	1598475-07-0	1-Naphthalenesulfonic acid, 4-(nitrosoamino)-3-[2-(4-sulfo-1-naphthalenyl)diazer	C20H14N4O7S2	486.48	C	-8.7
3789	O(CCCCC(=O)NO)c1ccc(c2nc(nc(n2)N2CCOCC2)N2CCOCC2)cc1	1619257-65-6	Butanamide, 4-[4-(4,6-di-4-morpholinyl-1,3,5-triazin-2-yl)phenoxy]-N-hydroxy-	C21H28N6O5	444.48	C	-8.7
3790	N(C(=O)[C@H](Cc1ccccc1)C[C@@H](CN1[C@H](C(=O)NC(C)(C)C)CN(Cc2cn(=O)ccc2)CC1)O)[C@@H]1c2c(C[C@H]1O)ccc	162518-56-1	D-erythro-Pentonamide, 2,3,5-trideoxy-N-[(1S,2S,3S)-2,3-dihydro-2,3-dihydroxy-1H	C36H47N5O5	629.79	C	-8.7
3791	N(c1nc(nc(n1)N1CCOCC1)N1CCOCC1)c1ccc(cc1)c1cc(nc(N)n1)c1cc(OC)ccc1	1628172-09-7	2-Propen-1-one, 1-[4-[(4,6-di-4-morpholinyl-1,3,5-triazin-2-yl)amino]phenyl]-3-(	C24H26N6O4	462.5	C	-8.7
3792	C=C1[C@@H](n2c3c(nc2)c(OC)nc(N)n3)C[C@H](O)[C@H]1CO	1638769-65-9	Phosphoric acid, [(1R,3S,5S)-3-(2-amino-1,6-dihydro-6-oxo-9H-purin-9-yl)-5-hydro	C26H24N5O10P	597.47	C	-8.7
3793	C(=O)([C@H](NC(=O)OC)c1ccccc1)N1[C@@H](CCC1)c1[nH]c(cn1)c1ccc(cc1)c1ccc(cc1)c1[nH]c(nc1)[C@H]1N(C(=O	1639870-84-0	Carbamic acid, N-[(1S)-1-[[(2S)-2,5-dihydro-2-[5-[4,Ä≤-[2-[(2S)-1-[(2S)-2-[(meth	C41H50N8O6	750.89	C	-8.7
3794	C(=O)([C@H](NC(=O)OC)c1ccccc1)N1[C@@H](CCC1)c1[nH]c(cn1)c1ccc(cc1)c1ccc(cc1)c1[nH]c(nc1)[C@H]1N(C(=O	1639870-95-3	Carbamic acid, N-[(1R)-2-[(2S)-2-[5-[4,Ä≤-[2-[(2S)-2,5-dihydro-1-[(2S)-2-[(metho	C43H46N8O6	770.88	C	-8.7
3795	N(c1nc(nc(Oc2ccc(C#N)cc2)n1)N1CCOCC1)c1cc(C)ccc1	1643332-90-4	Benzonitrile, 4-[[4-[(4-chlorophenyl)amino]-6-(4-morpholinyl)-1,3,5-triazin-2-yl	C20H17ClN6O2	408.84	C	-8.7
3796	C(=O)([C@@H](NC(=O)OC)C(C)C)N1[C@@H](CCC1)c1[nH]c(cn1)c1ccc(cc1)c1ccc(cc1)c1[nH]c([C@@H](NC(=O)OC(C)	1645563-56-9	Carbamic acid, N,N,Ä≤-[9H-fluorene-2,7-diylbis[1H-imidazole-5,2-diyl[(2S,5S)-5-m	C43H54N8O6	778.94	C	-8.7
3797	O=c1n([C@@H]2O[C@H](COP(=O)(C(O)c3ccc(Cl)cc3)OC[C@H]3O[C@H](C=C3)n3c(=O)[nH]c(=O)c(C)c3)C=C2)cc(C)c(	164652-03-3	Thymidine, 2,Ä≤,3,Ä≤-didehydro-P,3,Ä≤-dideoxy-P-(hydroxyphenylmethyl)thymidylyl	C27H29N4O10P	600.51	C	-8.7
3798	O=c1n([C@@H]2O[C@H](COP(=O)(C(O)c3ccc(N(=O)=O)cc3)OC[C@H]3O[C@H](C=C3)n3c(=O)[nH]c(=O)c(C)c3)C=C2)cc	164652-05-5	Thymidine, P-[(4-cyanophenyl)hydroxymethyl]-2,Ä≤,3,Ä≤-didehydro-P,3,Ä≤-dideoxyt	C28H28N5O10P	625.52	C	-8.7
3799	N(C(=O)[C@H](Cc1ccccc1)C[C@@H](CN1[C@H](C(=O)NC(C)(C)C)CN(Cc2c(nccc2)N2CCOCC2)CC1)O)[C@@H]1c2c(C[C@H]	164725-16-0	1-Piperazinepentanamide, N-(2,3-dihydro-2-hydroxy-1H-inden-1-yl)-2-[[(1,1-dimeth	C37H49N5O4	627.82	C	-8.7
3800	N(C(=O)[C@H](Cc1ccccc1)C[C@@H](CN1[C@H](C(=O)NC(C)(C)C)CN(CCOCOCCOC)CC1)O)[C@@H]1c2c(C[C@H]1O)cccc2	164725-25-1	1-Piperazinepentanamide, N-(2,3-dihydro-2-hydroxy-1H-inden-1-yl)-2-[[(1,1-dimeth	C36H47N5O4	613.79	C	-8.7
3801	N(C(=O)[C@H](Cc1ccccc1)C[C@@H](CN1[C@H](C(=O)NC(C)(C)C)CN(Cc2cc(Cl)c(Cl)cc2)CC1)O)[C@@H]1c2c(C[C@H]1O	164725-57-9	1-Piperazinepentanamide, 4-[[4-(acetylamino)-2-chlorophenyl]methyl]-N-(2,3-dihyd	C39H50ClN5O5	704.3	C	-8.7
3802	C(COCP(=O)(OCCC)O)n1c2c(nc1)c(N)ncn2	164725-67-1	1-Piperazinepentanamide, 4-(cyclohexylmethyl)-N-(2,3-dihydro-2-hydroxy-1H-inden-	C37H54N4O4	618.85	C	-8.7
3803	C(=O)([C@@H](NC(=O)OC)C(C)C)N1[C@@H]2[C@@H](C2)C[C@H]1c1[nH]c(cn1)c1ccc(cc1)c1ccc2c(cc1)c1c(cc2)nc([n	1653966-83-6	Carbamic acid, N-[(1S)-1-[[(3S,5S)-3-[5-[4-[2-[(1S,3S,5S)-2-[(2S)-2-[(methoxycar	C47H56N8O7	845	C	-8.7
3804	C(=O)([C@@H](NC(=O)OC)C(C)C)N1[C@@H](C[C@H]2[C@@H]1CCCC2)c1[nH]c2c(n1)ccc1c2ccc(c1)c1ccc(cc1)c1[nH]c	1653966-92-7	Carbamic acid, N-[(1S)-1-[[(3S,5S)-3-[5-[4-[2-[(6S)-5-[(2S)-2-[(methoxycarbonyl)	C48H58N8O7	859.02	C	-8.7
3805	C(=O)([C@@H](NC(=O)OC)C(C)C)N1C[C@]2(C[C@H]1c1[nH]c(cn1)c1ccc(cc1)c1cc3c(cc1)c1c(cc3)nc([nH]1)[C@H]1	1653967-12-4	Carbamic acid, N-[(1S)-1-[[(5S,8S)-8-[7-[4-[2-[(5S,8S)-7-[(2S)-2-[(methoxycarbon	C48H58N8O8	875.02	C	-8.7
3806	O(C(=O)N[C@@H](Cc1ccccc1)[C@@H](CN(S(=O)(=O)c1cc2c(cc1)nc(NC1CCNCC1)s2)CC(C)C)O)[C@@H]1[C@H]2[C@@H](	1672661-55-0	Carbamic acid, N-[(1S,2R)-3-[[[2-(cyclohexylamino)-6-benzothiazolyl]sulfonyl](2-	C34H44F2N4O7S2	722.86	C	-8.7
3807	C[C@]12[C@H]([C@H]3[C@@H]([C@]4(C)C(=CC(=O)C=C4)CC3)[C@@H](O)C2)CC(C#N)[C@@H]1C(=O)OC[C@@H]1[C@@H](N	168394-48-7	Butanediamide, N1-[3-[3-[[(1,1-dimethylethyl)amino]carbonyl]octahydro-2(1H)-isoq	C38H50N6O5S	702.91	C	-8.7
3808	[C@H](Cc1ccc(O)cc1)(C(=O)N[C@H](/C=C/S(=O)(=O)c1ccccc1)CCc1ccccc1)NC(=O)N1CCOCC1	170111-44-1	4-Morpholinecarboxamide, N-[1-methyl-2-oxo-2-[[1-(2-phenylethyl)-3-(phenylsulfor	C32H37N3O5S	575.72	C	-8.7
3809	S(=O)(=O)(O)c1c2c(c(S(=O)(=O)O)cc(N=Nc3c(C)cc([N+]#N)c(C)c3)c2)cc(N=Nc2c(C)cc([N+]#N)c(C)c2)c1	1715922-33-0	Benzenediazonium, 4,4,Ä≤-[(4,8-disulfo-2,6-naphthalenediyl)bis(2,1-diazenediyl)]	C26H22N8O6S2.2Cl		C	-8.7
3810	N(=Nc1c2c(ccc1)cccc2)c1c2c(c(S(=O)(=O)O)ccc2)c(N)cc1	172669-10-2	1-Naphthalenesulfonic acid, 4-amino-3-[2-(1-naphthalenyl)diazenyl]-	C20H15N3O3S	377.42	C	-8.7
3811	[Sn](CCCC)(CCCC)(CCCC)c1cn(c(=O)[nH]c1=O)[C@@H]1O[C@H](CO)[C@@H](O)C1	172669-12-4	1-Naphthalenesulfonic acid, 8-amino-5-[2-(1-naphthalenyl)diazenyl]-	C20H15N3O3S	377.42	C	-8.7
3812	O[C@H]1[C@H](n2c3c(nc2)c(=O)nc(N)[nH]3)O[C@H](COP(=O)(O[C@@H]2[C@@H](CO)O[C@H](C2)n2c3c(nc2)c(N)ncn3	173291-31-1	Guanosine, 2,Ä≤-deoxy-P-thioguanylyl-(3,Ä≤,Ü í5,Ä≤)-	C20H25N10O10PS	628.51	C	-8.7
3813	O(C)[C@H]1[C@H](n2c3c(nc2)c(=O)nc(N)[nH]3)O[C@H](COP(=O)(O[C@@H]2[C@@H](CO)O[C@H](C2)n2c3c(nc2)c(=O)	173291-32-2	Guanosine, 2,Ä≤-deoxy-P-thioadenylyl-(3,Ä≤,Ü í5,Ä≤)-	C20H25N10O9PS	612.51	C	-8.7
3814	C(Cc1ccc(F)cc1)C1(CCC)CC(=C(C(CC)c2cc(NS(=O)(=O)c3ccc(C#N)cn3)ccc2)C(=O)O1)O	174484-03-8	2-Pyridinesulfonamide, 5-cyano-N-[3-[1-[6-[2-(4-fluorophenyl)ethyl]-5,6-dihydro-	C33H36FN3O5S	605.72	C	-8.7
3815	C(Cc1ccccc1)[C@@]1(CCC(F)(F)F)CC(=C([C@H](C(C)(C)C)c2cc(NS(=O)(=O)c3ccc(N)cn3)ccc2)C(=O)O1)O	174484-17-4	2-Pyridinesulfonamide, 5-cyano-N-[3-[1-[5,6-dihydro-4-hydroxy-2-oxo-6-(2-phenyle	C33H34F3N3O5S	641.7	C	-8.7
3816	C(Cc1ccccc1)C1(CCC)CC(=C(C(CC)c2cc(NS(=O)(=O)c3ccc(C(F)(F)F)cn3)ccc2)C(=O)O1)O	174484-76-5	2-Pyridinesulfonamide, 5-amino-N-[3-[1-[6-[2-(4-fluorophenyl)ethyl]-5,6-dihydro-	C30H34FN3O5S	567.67	C	-8.7
3817	C(n1c2c(nc1CC)c(C)cc(C)n2)c1ccc(cc1)c1c(S(=O)(=O)NC(=O)c2cc(OCc3ccccc3)ccc2)cccc1	174844-68-9	Benzamide, N-[[4,Ä≤-[(2-ethyl-5,7-dimethyl-3H-imidazo[4,5-b]pyridin-3-yl)methyl]	C31H30N4O4S	554.66	C	-8.7
3818	C(CN1CCC(CN2c3c4c(S2(=O)=O)cccc4ccc3)CC1)(C12CC3CC(C2)CC(C1)C3)N1CCN(C)CC1	178478-39-2	2H-Naphth[1,8-cd]isothiazole, 2-[[1-[2-(4-morpholinyl)-2-tricyclo[3.3.1.13,7]dec	C32H43N3O3S	549.77	C	-8.7
3819	C(=O)([C@@H](NC(=O)OC)C1CC1)N1[C@H](c2[nH]c(c3cc4C5(c6c(-c4cc3)ccc(c6)c3[nH]c(nc3)[C@H]3N(C(=O)[C@@H	1800210-28-9	Carbamic acid, N,N,Ä≤-[spiro[9H-fluorene-9,3,Ä≤-oxetane]-2,7-diylbis[1H-imidazo	C45H56N8O7	820.98	C	-8.7
3820	C(C[C@H](C[C@H](CCc1cc(O)c(O)cc1)O)O)c1cc(O)c(O)cc1	1801161-79-4	Carbamic acid, N-[(1S)-2-methyl-1-[[(2S)-2-[5-[9,9,10,10-tetrafluoro-9,10-dihydr	C44H52F4N8O7	880.93	C	-8.7
3821	C(=O)([C@@H](NC(=O)OC)C(C)C)N1C(C2CC1CC2)c1[nH]c(cn1)c1ccc(cc1)c1ccc(cc1)c1[nH]c(nc1)C1N(C(=O)[C@@H]	1808935-10-5	Carbamic acid, N-[(1S)-2-[(3S)-3-[5-[4,Ä≤-[2-[(3S)-2-[(2S)-2-[(methoxycarbonyl)a	C42H50N8O6	762.9	C	-8.7
3822	N(C=C1C=CC(=N1)C)C12CC3CC(C2)CC(C1)C3	1808935-22-9	Carbamic acid, N,N,Ä≤-[9H-fluorene-2,7-diylbis[1H-imidazole-5,2-diyl-(3S)-2-azab	C45H54N8O6	802.96	C	-8.7
3823	O=C1[C@@H]2[C@@H](c3c([C@H](OC(=O)[C@H](Cc4ccccc4)N4C(=O)c5c(C4=O)cccc5)[C@H]2CO1)cc1c(c3)OCO1)c1cc(	1809321-39-8	2H-Isoindole-2-acetic acid, 1,3-dihydro-1,3-dioxo-Œ±-(phenylmethyl)-, (5R,5aR,8a	C39H33NO11	691.68	C	-8.7
3824	C(=O)([C@]12C(C)(C)[C@](C)(C(=O)O1)CC2)N1c2c([C@@](C#CC3CC3)(C(F)(F)F)OC1=O)cc(Cl)cc2	181362-85-6	Guanosine, adenylyloxyphosphinicomethylenephosphinicooxyphosphinico-(5,Ä≤,Ü í5,Ä	C21H30N10O19P4	850.41	C	-8.7
3825	C[C@@]12[C@@H]3[C@H]([C@H]4[C@](C)(CC3)[C@@H]([C@@H](C[C@H]([C@H](C(C)C)O)O)C)CC4)C[C@@H](O)[C@H]1C[	1826091-91-1	Carbamic acid, N-[(1S,2R)-3-[[[2-(cyclopropylamino)-6-benzothiazolyl]sulfonyl][(	C33H39FN4O8S2	702.81	C	-8.7
3826	O=c1n([C@H]2c3c([C@@H](CO)O2)cccc3)cc(C)c(=O)[nH]1	183239-85-2	D-erythro-Pentonamide, 2,3,5-trideoxy-N-[(1S,2R)-2,3-dihydro-2-hydroxy-1H-inden-	C42H55N5O10		C	-8.7
3827	O(N(=O)=O)C12CC3(C)CC(N)(C1)CC(C2)C3	183506-06-1	Guanosine, 2,Ä≤-deoxy-P-thioadenylyl-(3,Ä≤,Ü í5,Ä≤)-2,Ä≤-deoxy-	C20H25N10O8PS	596.51	C	-8.7
3828	C(OC[C@@H]1[C@@H](OC(=O)C)C[C@@H](C1=C)n1c2c(nc1)c(=O)nc(NC(=O)OC(C)(C)C)[nH]2)(c1ccccc1)(c1ccccc1)c	1860842-88-1	Carbamic acid, N-[6,9-dihydro-9-[(1S,3R,4S)-4-hydroxy-2-methylene-3-[(triphenylm	C36H37N5O5	619.71	C	-8.7
3829	C(OC[C@@H]1[C@@H](OC(=O)c2cccnc2)C[C@@H](C1=C)n1c2c(nc1)c(=O)nc(NC(=O)OC(C)(C)C)[nH]2)(c1ccccc1)(c1c	1860842-94-9	Acetic acid, 2-ethoxy-, (1S,2R,4S)-4-[2-[[(1,1-dimethylethoxy)carbonyl]amino]-1,	C40H43N5O7	705.8	C	-8.7
3830	O=C1[C@@H]2[C@@H](c3c([C@H](OC(=O)CN4c5c(C(=O)C4=O)cc(Br)cc5)[C@H]2CO1)cc1c(c3)OCO1)c1cc(OC)c(OC)c(O	1864833-32-8	1H-Indole-1-acetic acid, 5-chloro-2,3-dihydro-2,3-dioxo-, (5R,5aR,8aR,9R)-5,5a,6	C32H26ClNO11	636	C	-8.7
3831	[C@@H](C(CC)CC)(NC(=O)C)[C@@H]1[C@H](OC(=O)CCCCCCCCCCCCCCCCCN2CCCN(Cc3ccc(CN4CCCNCCNCCCNCC4)cc3)CCNC	1876408-76-2	1,4,8,11-Tetraazacyclotetradecane-1-pentanoic acid, 11-[[4-(1,4,8,11-tetraazacyc	C48H88N12O5	913.29	C	-8.7
3832	C(CC1CC1)[C@@]1(C(F)(F)F)c2c(NC(=O)O1)ccc(Cl)c2	188560-02-3	Inosine 5,Ä≤-(pentahydrogen tetraphosphate), P,Ä≤,Ä≤,Ä≤,Ü í5,Ä≤-ester with inosi	C20H26N8O21P4	838.36	C	-8.7
3833	O(C(=O)N[C@@H](Cc1ccc(OCc2c(C(=O)O)nc(cc2)c2cc(F)cc(F)cc2)cc1)[C@@H](CN(S(=O)(=O)c1cc2c(cc1)ncs2)CC(C	1887206-12-3	Benzo[b]thiophene-3-carboxylic acid, 2-[2-[4-[(2S,3R)-4-[(6-benzothiazolylsulfor	C41H51N3O9S3	826.05	C	-8.7
3834	C(=O)(c1[nH]c2c(c1)cc(NS(=O)(=O)C)cc2)N1CCN(CC1)c1nc(OS(=O)(=O)O)ccc1N	188780-41-8	Methanesulfonamide, N-[2-[[4-(3-amino-1,6-dihydro-6-oxo-2-pyridinyl)-1-piperazir	C19H22N6O4S	430.48	C	-8.7
3835	C[C@@]12[C@@H]3[C@H]([C@H]4[C@](C)(CC3)[C@@H]([C@@H](CCC(=O)O)C)CC4)C(=O)C(CC)[C@@H]1C[C@H](O)CC2	1892540-21-4	Ethanesulfonic acid, 2-[[(3Œ±,5Œ≤,7Œ≤)-7-amino-3-hydroxy-24-oxocholan-24-yl]ami	C26H46N2O5S	498.72	C	-8.7
3836	C(OC[C@@H]1[C@@H](OC(=O)c2ccccc2)[C@@H](OP(=O)(OC[C@@H]2[C@@H](OC(=O)c3ccccc3)[C@@H](OP(=O)(OC[C@@H]	190141-42-5	Adenosine, adenylyl-(2,Ä≤,Ü í5,Ä≤)-adenylyl-(2,Ä≤,Ü í5,Ä≤)-1,Ä≤-[3-(aminocarbonyl	C28H36N14O17P2	902.62	C	-8.7
3837	O=c1c2c(n(cn2)[C@@H]2O[C@H](COP(=O)(F)[O-])[C@@H](O)[C@H]2O)[nH]c(N)n1	190141-46-9	Adenosine, 1,Ä≤-[3-(aminocarbonyl)-1H-1,2,4-triazol-1-yl]-1,Ä≤-de(6-amino-9H-pu	C28H36N14O17P2	902.62	C	-8.7
3838	O=c1n(c2c(c(cc(n2)c2ccc(Cl)cc2)c2ccccc2)c(=O)n1c1ccc(Cl)cc1)[C@H]1[C@H](OC(=O)c2ccccc2)[C@H](OC(=O)c	190319-83-6	Pyrido[2,3-d]pyrimidine-2,4(1H,3H)-dione, 7-(4-chlorophenyl)-3,5-diphenyl-1-(2,3	C51H36ClN3O9	870.3	C	-8.7
3839	C(=O)([C@@H](NC(=O)OC)C(C)C)N1[C@@H]([C@@H]2C[C@H]1CC2)c1[nH]c2c(n1)ccc1c2ccc(c1)c1ccc(cc1)c1[nH]c(n	1949770-80-2	Carbamic acid, N-[(1S)-1-[[(2S,4R)-4-methoxy-2-[5-[4-[2-[(1R,3S,4S)-2-[(2S)-2-[(	C45H54N8O7	818.96	C	-8.7
3840	N(C(=O)C)[C@H]1[C@H]([C@@H]([C@@H](CO)O)O)OC(=C[C@@H]1NC(=N)NC(=O)Cn1cc(nn1)c1cc(F)ccc1)C(=O)O	1982351-42-7	D-glycero-D-galacto-Non-2-enonic acid, 5-(acetylamino)-2,6-anhydro-4-[[[2-[4-(3	C22H26ClN7O8	551.94	C	-8.7
3841	C[C@]1(F)[C@@H](O[C@H](CO[P@](=O)(Oc2ccccc2)N[C@H](C(=O)OC(C)C)C)[C@H]1OC(=O)CN1CCOCC1)n1c(=O)[nH]c(	1982351-52-9	D-glycero-D-galacto-Non-2-enonic acid, 5-(acetylamino)-2,6-anhydro-3,4,5-trideox	C27H31N7O9	597.58	C	-8.7
3842	O=C1c2n(N([C@H]3N1CCOC3)[C@H]1c3c(CSc4c1cccc4)ccc(Cl)c3)ccc(=O)c2O	1985605-72-8	1H-[1,4]Oxazino[3,4-c]pyrido[2,1-f][1,2,4]triazine-6,8-dione, 12-[(11S)-9-fluoro	C24H20FN3O4S	465.5	C	-8.7
3843	Fc1c2[C@H](N3n4c(C(=O)N5[C@H]3COC[C@@H]5C)c(O)c(=O)cc4)c3c(SCc2ccc1)cccc3	1985605-86-4	1H-[1,4]Oxazino[3,4-c]pyrido[2,1-f][1,2,4]triazine-6,8-dione, 12-[(11S)-8-fluoro	C25H22FN3O4S	479.52	C	-8.7
3844	O(C(OC(=O)OC1CCCCC1)C)c1c2n(N([C@H]3N(C2=O)CCOC3)[C@H]2c3c(CSc4c2cccc4)c(F)c(F)cc3)ccc1=O	1985606-81-2	Butanedioic acid, 2-methyl-, 1-[[[(12aR)-12-[(11S)-7,8-difluoro-6,11-dihydrodibe	C31H29F2N3O8S	641.64	C	-8.7
3845	O(COC(=O)Oc1c(C(=O)OC)cccc1)c1c2n(N([C@H]3N(C2=O)CCOC3)[C@H]2c3c(CSc4c2cccc4)c(F)c(F)cc3)ccc1=O	1985607-00-8	Acetic acid, 2-[[[[[(12aR)-12-[(11S)-7,8-difluoro-6,11-dihydrodibenzo[b,e]thiepi	C30H27F2N3O9S	643.61	C	-8.7
3846	O(COC(=O)OC(C(=O)OCC)(C)C)c1c2n(N([C@H]3N(C2=O)CCOC3)[C@H]2c3c(CSc4c2cccc4)c(F)c(F)cc3)ccc1=O	1985607-03-1	Propanoic acid, 2-[[[[[(12aR)-12-[(11S)-7,8-difluoro-6,11-dihydrodibenzo[b,e]thi	C34H35F2N3O9S	699.72	C	-8.7
3847	O(P(=O)(NC(C(=O)OC)(C)C)OCC)c1c2n(N([C@H]3N(C2=O)CCOC3)[C@H]2c3c(CSc4c2cccc4)c(F)c(F)cc3)ccc1=O	1985607-20-2	Cyclohexanecarboxylic acid, 1-[[[[(12aR)-12-[(11S)-7,8-difluoro-6,11-dihydrodibe	C34H37F2N4O8PS	730.72	C	-8.7
3848	O(C(=O)OCc1c(C)oc(=O)o1)c1c2n(N([C@H]3N(C2=O)CCOC3)[C@H]2c3c(CSc4c2cccc4)c(F)c(F)cc3)ccc1=O	1985607-23-5	Cyclohexanepropanoic acid, Œ±-[[[[(12aR)-12-[(11S)-7,8-difluoro-6,11-dihydrodibe	C36H41F2N4O8PS	758.77	C	-8.7
3849	O(C(=O)Oc1ccc(NC(=O)C)cc1)c1c2n(N([C@H]3N(C2=O)CCOC3)[C@H]2c3c(CSc4c2cccc4)c(F)c(F)cc3)ccc1=O	1985607-28-0	Carbonic acid, (12aR)-12-[(11S)-7,8-difluoro-6,11-dihydrodibenzo[b,e]thiepin-11-	C28H25F2N3O8S2	633.64	C	-8.7
3850	O(COC(=O)OC1(CC(=O)OC)CCCC1)c1c2n(N([C@H]3N(C2=O)CCOC3)[C@H]2c3c(CSc4c2cccc4)c(F)c(F)cc3)ccc1=O	1985607-35-9	Phosphoric acid, (12aR)-12-[(11S)-7,8-difluoro-6,11-dihydrodibenzo[b,e]thiepin-1	C32H32F2N3O9PS	703.65	C	-8.7
3851	O(C(=O)OC(C(=O)OCC)(C)C)c1c2n(N([C@H]3N(C2=O)CCOC3)[C@H]2c3c(CSc4c2cccc4)c(F)c(F)cc3)ccc1=O	1985607-38-2	Propanoic acid, 3-[[[[[(12aR)-12-[(11S)-7,8-difluoro-6,11-dihydrodibenzo[b,e]thi	C33H33F2N3O9S	685.69	C	-8.7
3852	N(C(=O)c1ccc(Cl)cc1)c1cc2cc(C34CC5CC(C4)CC(C3)C5)[nH]c2cc1	1987854-62-5	Benzamide, 4-methoxy-N-(2-tricyclo[3.3.1.13,7]dec-1-yl-1H-indol-5-yl)-	C26H28N2O2	400.51	C	-8.7
3853	N(C(=O)Nc1c(C)cc(N(=O)=O)cc1)c1cc2cc(C34CC5CC(C4)CC(C3)C5)[nH]c2cc1	1987854-66-9	Urea, N-(4-cyanophenyl)-N,Ä≤-(2-tricyclo[3.3.1.13,7]dec-1-yl-1H-indol-5-yl)-	C26H26N4O	410.51	C	-8.7
3854	N(C(=O)NC1CC1)c1cc2cc(C34CC5CC(C4)CC(C3)C5)[nH]c2cc1	1987854-72-7	Urea, N-(3,4-dimethoxyphenyl)-N,Ä≤-(2-tricyclo[3.3.1.13,7]dec-1-yl-1H-indol-5-y	C27H31N3O3	445.55	C	-8.7
3855	N(C(=O)NCCc1cccs1)c1cc2cc(C34CC5CC(C4)CC(C3)C5)[nH]c2cc1	1987854-77-2	Urea, N-tricyclo[3.3.1.13,7]dec-1-yl-N,Ä≤-(2-tricyclo[3.3.1.13,7]dec-1-yl-1H-ind	C29H37N3O	443.62	C	-8.7
3856	O=C1[C@@H]2[C@@H](c3c([C@H](OC(=O)c4c5c(ccn4)cccc5)[C@H]2CO1)cc1c(c3)OCO1)c1cc(OC)c(OC)c(OC)c1	1989611-35-9	6-Quinolinecarboxylic acid, 2-methyl-, (5R,5aR,8aR,9R)-5,5a,6,8,8a,9-hexahydro-8	C33H29NO9	583.58	C	-8.7
3857	O=C1[C@@H]2[C@@H](c3c([C@H](OC(=O)c4nc5c(nc4)cccc5)[C@H]2CO1)cc1c(c3)OCO1)c1cc(OC)c(OC)c(OC)c1	1989611-36-0	1-Isoquinolinecarboxylic acid, (5R,5aR,8aR,9R)-5,5a,6,8,8a,9-hexahydro-8-oxo-9-(	C32H27NO9	569.56	C	-8.7
3858	O=C1[C@@H]2[C@@H](c3c([C@H](OC(=O)c4cc5c(s4)cccc5)[C@H]2CO1)cc1c(c3)OCO1)c1cc(OC)c(OC)c(OC)c1	1989611-38-2	3-Quinolinecarboxylic acid, 7-chloro-1-cyclopropyl-6-fluoro-1,4-dihydro-4-oxo-,	C35H29ClFNO10	678.06	C	-8.7
3859	N(c1c(CC)cccc1C)c1nc(Nc2ccc(C#N)cc2)nc(C)c1	1992226-48-8	Benzonitrile, 2-[[2-[(2,6-dimethylphenyl)amino]-6-methyl-4-pyrimidinyl]amino]-	C20H19N5	329.4	C	-8.7
3860	C(=O)([C@@H](NC(=O)OC)C1CCOCC1)N1[C@H](c2[nH]c3c4c(cc(cc4)c4c5c(c(cc4)c4[nH]c(nc4)[C@H]4N(C(=O)[C@@H	2007900-49-2	Carbamic acid, N-[(1S)-1-[[(2S,5S)-2-[7-[2,3-dihydro-7-[2-[(2S,5S)-1-[(2S)-2-[(m	C47H58N8O6	831.01	C	-8.7

3861	C(=O)([C@@H](NC(=O)OC)C(C)C)N1[C@H](c2[nH]c3c4c(cc(cc4)c4c5c(c(cc4)c4[nH]c(nc4)[C@H]4N(C(=O)[C@@H](N	2007900-91-4	Carbamic acid, N-[(1S)-1-[[(2S,4S)-4-[(difluoromethoxy)methyl]-2-[5-[2,3-dihydro	C48H58F2N8O7	897.02	C	-8.7
3862	C(=O)([C@@H](NC(=O)OC)C(C)C)N1[C@H](c2[nH]c3c4c(cc(cc4)c4c5c(c(cc4)c4[nH]c(nc4)[C@H]4N(C(=O)[C@@H](N	2007901-50-8	Carbamic acid, N-[(1S)-1-[[(2S)-2-[4-cyclopropyl-5-[2,3-dihydro-7-[2-[(2S)-1-[(2	C48H58N8O6	843.02	C	-8.7
3863	C([C@H](OCP(=O)(OCOC(=O)OC(C)(C)C)OCOC(=O)OC(C)(C)C)C)n1c2c(nc1)c(N)ncn2	201340-99-0	2,4,6,8-Tetraoxa-5-phosphanonanedioic acid, 5-[[(1R)-2-(6-amino-9H-purin-9-yl)-1	C21H34N5O10P	547.5	C	-8.7
3864	O[C@H]1[C@H](n2c3c(nc2)c(=O)nc(N)[nH]3)O[C@H](COP(=O)(O)O)[C@H]1OP(=O)(OC[C@H]1O[C@H]([C@H](O)[C@@H]	2013599-59-0	2-Pyridinesulfonamide, 5-(difluorofluoro-18F-methyl)-N-[3-[(1R)-1-[(6R)-5,6-dihy	C31H33F3N2O5S		C	-8.7
3865	C(=O)(C(=O)Nc1cc(OC)cc(OC)c1)c1c(C23CC4CC(C3)CC(C2)C4)[nH]c2c1cccc2	2014403-85-9	Benzoic acid, 3-methyl-4-[[2-oxo-2-(2-tricyclo[3.3.1.13,7]dec-1-yl-1H-indol-3-yl	C29H30N2O4	470.56	C	-8.7
3866	C(=O)(C(=O)Nc1cncnc1)c1c(C23CC4CC(C3)CC(C2)C4)[nH]c2c1cccc2	2014403-94-0	1H-Indole-3-acetamide, Œ±-oxo-N-2-pyridinyl-2-tricyclo[3.3.1.13,7]dec-1-yl-	C25H25N3O2	399.48	C	-8.7
3867	C(COC[P@](=O)(Oc1ccccc1)O[C@H](C(=O)OCC)C)n1c2c(nc1)c(N)ncn2	1000689-65-5	Ethanone, 2-amino-1-(2,3-dihydro-1H-indol-1-yl)-2-(3-hydroxytricyclo[3.3.1.13,7]	C20H26N2O2	326.43	A/B	-8.6
3868	N(c1nc(nc(n1)N1CCOCC1)N1CCOCC1)c1ccc(C(=O)OC)cc1	10021-57-5	Uridine, inosinylyl-(3,Ä≤,Üí5,Ä≤)-	C19H23N6O13P	574.39	A/B	-8.6
3869	C(C(C(NC(=O)c1c(C)c(O)c(O)cc1)CSc1cc(C)ccc1)O)N1C(C(=O)NC(C)(C)C)CC2C(C1)CCCC2	1009057-20-8	3-Isoquinolinecarboxamide, N-(1,1-dimethylethyl)-2-[4-[(5-ethyl-2-hydroxyphenyl)	C34H49N3O5S	611.84	A/B	-8.6
3870	C(=O)([C@H](NC(=O)OC)c1ccccc1)N1[C@@H](CCC1)c1[nH]c(cn1)c1ccc(cc1)c1ccc(cc1)c1[nH]c(nc1)[C@H]1N(C(=O	1009107-39-4	Carbamic acid, N-[(1R)-2-[(2S)-2-[5-[4,Ä≤-[2-[(2S)-1-(2-aminoacetyl)-2-pyrrolid	C38H40N8O4	672.78	A/B	-8.6
3871	C(=O)([C@H](NC(=O)OC)c1ccccc1)N1[C@@H](CCC1)c1[nH]c(cn1)c1ccc(cc1)c1ccc(cc1)c1[nH]c(nc1)[C@H]1N(C(=O	1009107-44-1	Carbamic acid, N-[(1R)-2-oxo-2-[(2S)-2-[5-[4,Ä≤-[2-[(2S)-1-(1-oxopropyl)-2-pyrr	C39H41N7O4	671.79	A/B	-8.6
3872	C(=O)([C@H](NC(=O)OC)c1ccccc1)N1[C@@H](CCC1)c1[nH]c(cn1)c1ccc(cc1)c1ccc(cc1)c1[nH]c(nc1)[C@H]1N(C(=O	1009107-50-9	Carbamic acid, N-[(1R)-2-[(2S)-2-[5-[4,Ä≤-[2-[(2S)-1-[2-(dimethylamino)acetyl]-2	C40H44N8O4	700.83	A/B	-8.6
3873	C(=O)([C@H](NC(=O)OC)c1ccccc1)N1[C@@H](CCC1)c1[nH]c(cn1)c1ccc(cc1)c1ccc(cc1)c1[nH]c(nc1)[C@H]1N(C(=O	1009107-51-0	Carbamic acid, N-[(1R)-2-[(2S)-2-[5-[4,Ä≤-[2-[(2S)-1-[(2S)-2-(dimethylamino)-2-p	C46H48N8O4	776.92	A/B	-8.6
3874	C(=O)([C@H](NC(=O)OC)c1ccccc1)N1[C@@H](CCC1)c1[nH]c(cn1)c1ccc(cc1)c1ccc(cc1)c1[nH]c(nc1)[C@H]1N(C(=O	1009107-53-2	Carbamic acid, N-[(1R)-2-[(2S)-2-[5-[4,Ä≤-[2-[(2S)-1-[2-[(methoxycarbonyl)amino]	C40H42N8O6	730.81	A/B	-8.6
3875	C(=O)([C@H](NC(=O)OC)c1ccccc1)N1[C@@H](CCC1)c1[nH]c(cn1)c1ccc(cc1)c1ccc(cc1)c1[nH]c(nc1)[C@H]1N(C(=O	1009107-89-4	1-Pyrrolidinecarboxylic acid, 2-[5-[4,Ä≤-[2-[(2S)-1-[(2R)-2-[(methoxycarbonyl)am	C38H39N7O5	673.76	A/B	-8.6
3876	C(=O)([C@@H](NC(=O)OC)C(C)C)N1[C@@H](CCC1)c1[nH]c(cn1)c1c(F)cc(cc1)c1cc(F)c(cc1)c1[nH]c(nc1)[C@H]1N(	1009116-58-8	Carbamic acid, N-[(1S)-1-cyclopropyl-2-[(2S)-2-[5-[3,3,Ä≤-difluoro-4,Ä≤-[2-[(2S	C40H46F2N8O6	772.84	A/B	-8.6
3877	N(=Nc1c2c(c(N)cc1)cccc2)c1c2c(cc(S(=O)(=O)O)c1)cc(S(=O)(=O)O)cc2.[NaH]	10114-72-4	2-Naphthalenesulfonic acid, 8-amino-5-[2-[4-[2-[2,4-diamino-5-[2-[3-[2-(2,4-diam	C35H31N13O6S2.2Na		A/B	-8.6
3878	O=c1c2c(n(cn2)[C@@H]2O[C@H](COP(=O)(OP(=O)(O[C@@H]3[C@@H](O)C[C@H](N)[C@@H](C)O3)O)O)[C@@H](O)[C@H]2	1012868-99-3	Thymidine, 3-[6-[(3-cyano-4-fluorobenzoyl)amino]hexyl]-Œ±,Œ±,Œ±-trifluoro-	C24H26F4N4O6	542.48	A/B	-8.6
3879	C(=O)(N[C@H](C(=O)C(=O)NC1CC1)CCC)[C@@H]1[C@@H]2[C@H](CN1C(=O)[C@@H](NC(=O)[C@@H](NC(=O)c1cnccn1)C1C	1015080-35-9	Butanediamide, N1-[(1S,2R)-3-[(3S,4aS,8aS)-3-[[(1,1-dimethylethyl)amino]carbonyl	C38H50N6O5.C27H45N5O5		A/B	-8.6
3880	N(C(=O)Nc1cc(S(=O)(=O)NC)ccc1)C12CC3CC(C2)CC(C1)C3	1015252-36-4	Benzenesulfonamide, 3-[[(tricyclo[3.3.1.13,7]dec-1-ylamino)carbonyl]amino]-	C17H23N3O3S	349.45	A/B	-8.6
3881	C(#CC1CC1)[C@@]1(C(F)(F)F)c2c(N(CCCO[Si](C(C)(C)C)(C)C)C(=O)O1)ccc(Cl)c2	1023756-31-1	1(2H,6H)-Pyrimidineacetamide, N-[(1S,3S,4S)-3-(acetyloxy)-4-[[2-(2,6-dimethylphe	C42H50Cl3N5O8	859.23	A/B	-8.6
3882	[C@@H]([C@H](C[C@H](Cc1ccccc1)NC(=O)[C@H](C(C)C)N1C(=O)NCCC1)OC(=O)N)(Cc1ccccc1)NC(=O)COc1c(C)cccc1C	1023756-59-3	1(2H)-Pyrimidineacetamide, N-[(1S,3S,4S)-3-[2-[(1,1-dimethylethyl)amino]-2-oxoet	C43H59N5O6	741.96	A/B	-8.6
3883	N(C(=O)Nc1ccc(Cl)cc1)c1nc(nc(NC(=S)NCCc2cccs2)n1)N1CCOCC1	1025365-10-9	Urea, N-(3-chlorophenyl)-N,Ä≤-[4-(4-morpholinyl)-6-[[[[2-(2-thienyl)ethyl]amino]	C21H23ClN8O2S2	519.04	A/B	-8.6
3884	O=c1n([C@@H]2O[C@H](COCP(=O)(OP(=O)(OCCCOCCCCCCCCCCCCCCCCC)O)O)SC2)cc(F)c(N)n1	1026294-95-0	6H-Dipyrido[3,2-b:2,Ä≤,3,Ä≤-e][1,4]diazepin-6-one, 11-cyclopropyl-8-[2-[(1,3-di	C25H24FN5O4S	509.55	A/B	-8.6
3885	O=c1c2c(n(cn2)[C@H]2C[C@H](C(=C)C)[C@@H](CO)O2)[nH]cn1	1027072-86-1	Methanone, (4,6-dimethyl-1-oxido-5-pyrimidinyl)[(3S)-4-[4-[(1R)-2-methoxy-1-[4-(	C28H38F3N5O3	549.63	A/B	-8.6
3886	Cc1c2c(N(c3c(C(=O)N2)cccn3)C2CC2)ncc1C(C#N)N1CCCC1	1027379-02-7	Not Yet Assigned	C40H56N8O6	744.92	A/B	-8.6
3887	O(c1nc(Nc2ccc(C#N)cc2)nc(N)c1Br)c1c(C)cc(C#N)cc1C.S(=O)(=O)(O)c1ccc(C)cc1	1030633-40-9	Benzonitrile, 4-[[6-amino-5-bromo-2-[(4-cyanophenyl)amino]-4-pyrimidinyl]oxy]-3,	C20H15BrN6O.xC4H4O4		A/B	-8.6
3888	S(=O)(=O)(O)c1c2c(c(N)c(N=Nc3ccc(C=Cc4ccc(N=Nc5c(N)c6c(c(S(=O)(=O)O)c5)cccc6)cc4)cc3)c1)cccc2.[NaH]	1030633-43-2	Benzonitrile, 4-[[6-amino-5-bromo-2-[(4-cyanophenyl)amino]-4-pyrimidinyl]oxy]-3,	C20H15BrN6O.xH2O4S		A/B	-8.6
3889	C([C@@H]([C@H](Cc1cc(O)c(O)cc1)C)C)c1cc(O)c(O)cc1	10318-59-9	Uridine, thymidylyl-(3,Ä≤,Üí5,Ä≤)-2,Ä≤-deoxy-	C19H25N4O12P	532.4	A/B	-8.6
3890	N(c1c(C)cc(C=CC#N)cc1C)c1nc(NC2CCNCC2)ncc1	1033955-12-2	Benzonitrile, 4-[[6-amino-5-bromo-2-(4-piperidinylamino)-4-pyrimidinyl]oxy]-3,5-	C18H21BrN6O	417.3	A/B	-8.6
3891	N(c1nc(Nc2ccc(C#N)cc2)nc(C(=O)NCCOC)c1)c1c(C)cc(/C=C/C#N)cc1C	1037444-81-7	Benzonitrile, 4-[[4-[[4-[(1E)-2-cyanoethenyl]-2,6-dimethylphenyl]amino]-6-[hydro	C30H26N6O2	502.57	A/B	-8.6
3892	N(c1cc(C(=O)c2nccs2)nc(Nc2ccc(C#N)cc2)n1)c1c(C)cc(/C=C/C#N)cc1C	1037444-91-9	4-Pyrimidinecarboxamide, 6-[[4-[(1E)-2-cyanoethenyl]-2,6-dimethylphenyl]amino]-2	C28H23N7O2	489.53	A/B	-8.6
3893	N(c1c(C)cc(/C=C/C#N)cc1C)c1nc(Nc2ccc(C#N)cc2)nc(CNCCc2ccccn2)c1	1037445-12-7	Benzonitrile, 4-[[4-[[4-[(1E)-2-cyanoethenyl]-2,6-dimethylphenyl]amino]-6-(1H-im	C26H22N8	446.51	A/B	-8.6
3894	N(c1nc(Nc2ccc(C#N)cc2)nc(CNCc2ccncc2)c1)c1c(C)cc(/C=C/C#N)cc1C	1037445-32-1	Benzonitrile, 4-[[4-[[4-[(1E)-2-cyanoethenyl]-2,6-dimethylphenyl]amino]-6-[[[3-(	C30H34N8O	522.64	A/B	-8.6
3895	N(c1nc(Nc2ccc(C#N)cc2)nc(Cl)c1)c1c(C)cc(/C=C/C#N)cc1C	1037445-42-3	Benzonitrile, 4-[[4-[[4-[(1E)-2-cyanoethenyl]-2,6-dimethylphenyl]amino]-6-[(hydr	C23H19N7O	409.44	A/B	-8.6
3896	C(=O)(NCCNC(=O)C)c1c(Nc2c(C)cc(/C=C/C#N)cc2C)nc(Nc2ccc(C#N)cc2)nc1COC	1037452-12-2	5-Pyrimidinecarboxamide, 4-[[4-[(1E)-2-cyanoethenyl]-2,6-dimethylphenyl]amino]-2	C31H28N8O2	544.61	A/B	-8.6
3897	N(c1c(C(=O)N(CCN(C)C)C)c(COC)nc(Nc2ccc(C#N)cc2)n1)c1c(C)cc(/C=C/C#N)cc1C	1037452-33-7	5-Pyrimidinecarboxamide, 4-[[4-[(1E)-2-cyanoethenyl]-2,6-dimethylphenyl]amino]-2	C31H28N8O2	544.61	A/B	-8.6
3898	C(=O)(NCCc1ccc(S(=O)(=O)N)cc1)c1c(Nc2c(C)cc(/C=C/C#N)cc2C)nc(Nc2ccc(C#N)cc2)nc1COC	1037452-49-5	Benzonitrile, 4-[[4-[[4-[(1E)-2-cyanoethenyl]-2,6-dimethylphenyl]amino]-6-(metho	C29H29N7O3	523.59	A/B	-8.6
3899	C(=O)(NCc1ccncc1)c1c(Nc2c(C)cc(/C=C(/C#N)\C)cc2C)nc(Nc2ccc(C#N)cc2)nc1COC	1037452-54-2	5-Pyrimidinecarboxamide, 4-[[4-[(1E)-2-cyanoethenyl]-2,6-dimethylphenyl]amino]-2	C31H29N7O2S	563.67	A/B	-8.6
3900	C(OC[C@H](Cn1cc(c(N)nc1=O)c1ccccc1)OCP(=O)(OC(C)C)OC(C)C)(c1ccccc1)(c1ccccc1)c1ccccc1	1041364-78-6	Phosphonic acid, P-[[(1S)-1-[[4-(benzoylamino)-5-(4-chlorophenyl)-2-oxo-1(2H)-py	C46H47ClN3O7P	820.31	A/B	-8.6
3901	C(OC(CC)CO)n1c2c(nc1)c(=O)nc(N)[nH]2	1041873-77-1	1(2H)-Pyrimidineacetamide, N-[1-(2,4-cyclohexadien-1-ylidenemethyl)-4-[[2-(2,6-c	C37H48N4O5	628.8	A/B	-8.6
3902	C(c1c2c(N(c3c(C(=O)N2)cccn3)C2CC2)ncc1)n1c2c(nc1)c(=O)nc(N)[nH]2	1046462-04-7	6H-Dipyrido[3,2-b:2,Ä≤,3,Ä≤-e][1,4]diazepin-6-one, 4-[(2-amino-1,6-dihydro-6-ox	C20H17N9O2	415.41	A/B	-8.6
3903	C(=C1C=C(C(=O)O)C(=CC1=O)O)(c1cc(C(=O)O)c(O)c(C)c1O)c1cc(C(=O)O)c(O)c(C)c1O	1049975-68-9	Benzoic acid, 3-[(Z)-(3-carboxy-4-hydroxyphenyl)(3-carboxy-4-oxo-2,5-cyclohexadi	C22H14O9	422.34	A/B	-8.6
3904	[C@@H](Cc1ccccc1)(NC(=O)[C@H](Cc1cncnc1)NC(=O)N(Cc1nc(C(C)C)sc1)C)CC[C@H](Cc1ccccc1)NC(=O)OCc1cncs1	1051461-45-0	2,7,10,12-Tetraazatridecanoic acid, 12-methyl-13-[2-(1-methylethyl)-4-thiazolyl]	C40H47N7O4S2	753.98	A/B	-8.6
3905	C(Cc1ccccc1)(NC(=O)C(NC(=O)N(Cc1nc(C(C)C)sc1)C)CCNCC(F)(F)CCC(Cc1ccccc1)NC(=O)OCc1cncs1	1051464-20-0	2,7,10,12-Tetraazatridecanoic acid, 9-[2-[(aminocarbonyl)amino]ethyl]-12-methyl-	C37H48N8O5S2	748.96	A/B	-8.6
3906	N(=N\c1cc(S(=O)(=O)O)c(CCc2c(S(=O)(=O)O)cc(/N=N/c3c4c(cc(S(=O)(=O)O)c3N)cc(S(=O)(=O)O)cc4)cc2)cc1)/c	1053055-72-3	Not Yet Assigned	C34H28N6O12S4	840.88	A/B	-8.6
3907	O=c1n(ccc(N)n1)[C@@H]1O[C@H](COP(=O)(OC(=O)O)O)CC1	1053612-93-3	Not Yet Assigned	C15H17N10O7P	480.33	A/B	-8.6
3908	O=c1c(cn(c(=O)[nH]1)[C@@H]1O[C@H](CO)[C@@H](O)C1)C1=C(C)C(=O)C(=C(C)C1=O)C	105785-85-1	Uridine, 2,Ä≤-deoxy-5-(4,5-dimethyl-3,6-dioxo-1,4-cyclohexadien-1-yl)-	C17H18N2O7	362.33	A/B	-8.6
3909	N(C(=O)[C@H](Cc1ccccc1)C[C@@H](CN1[C@H](C(=O)NC(C)(C)C)CN(CCc2ccccn2)CC1)O)[C@H]1c2c(C[C@H]1O)cccc2	1064475-90-6	Not Yet Assigned	C38H50N4O5	642.83	A/B	-8.6
3910	N(C(=O)[C@H](Cc1ccccc1)C[C@@H](CN1[C@H](C(=O)NC(C)(C)C)CN(Cc2c(OCc3ccccc3)nc(C)cc2)CC1)O)[C@H]1c2c(C	1064475-91-7	Not Yet Assigned	C37H49N5O4	627.82	A/B	-8.6
3911	C[C@]1(F)[C@@H](O[C@H](COP(=O)(Oc2ccccc2)N[C@@H](C(=O)OCCCC)C)[C@H]1O)n1c(=O)[nH]c(=O)cc1	1064684-69-0	L-Alanine, N-[[P(R),2,Ä≤R]-2,Ä≤-deoxy-2,Ä≤-fluoro-2,Ä≤-methyl-P-phenyl-5,Ä≤-uri	C25H33FN3O9P	569.52	A/B	-8.6
3912	N(c1c2c(n(cn2)[C@@H]2C[C@H](CO[Si](C(C)C)(C(C)C)C(C)C)C=C2)nc(N)n1)C1CC1	1067882-07-8	Not Yet Assigned	C39H50N4O6	670.84	A/B	-8.6
3913	Cc1c2c(cc(CCC(=O)OCOC(=O)C(C)(C)C)c1)COP(=O)(OC[C@H]1O[C@H](C=C1)n1c(=O)[nH]c(=O)c(C)c1)O2	1070377-34-2	Benzonitrile, 4-[[6-chloro-2-[(4-cyanophenyl)amino]-4-pyrimidinyl]oxy]-3,5-dimet	C20H14ClN5O	375.81	A/B	-8.6
3914	C(C)(C)(C)c1c2c(cc(C(OC(=O)C(C)C)OC(=O)C(C)C)c1)CO[P@@](=O)(OC[C@H]1O[C@H](C=C1)n1c(=O)[nH]c(=O)c(C)	1072917-36-2	Thymidine, 5,Ä≤-O-[(2R)-6-[bis(2-methyl-1-oxopropoxy)methyl]-8-(1,1-dimethylethy	C30H39N2O11P	634.61	A/B	-8.6
3915	[C@@H](NC(=O)N1CCN(C)CC1)(C(=O)N[C@H](C=CS(=O)(=O)c1ccccc1)CCc1ccccc1)CCCNC(=N)NO	1076088-57-7	1-Piperazinecarboxamide, N-[(1S)-3-[[(hydroxyamino)iminomethyl]amino]-1-[[[(1S)-	C28H39N7O5S	585.72	A/B	-8.6
3916	O=C1[C@@H]2[C@H](c3c(c(OC)c(OC)c(OC)c3)[C@@H](O)[C@H]2CO1)c1cc2c(cc1)OCO2	1083160-34-2	2,7-Anthracenedisulfonamide, 9,10-dihydro-9,10-bis(hydroxyimino)-N2,N7-bis(1-tri	C38H48N4O6S2	720.94	A/B	-8.6
3917	N(C(=O)OC(C(=O)OCC=C)SC(=S)N(C)C)(C(CCCN(CC)CC)C)c1c2c(ncc1)cc(Cl)cc2	108440-56-8	Furo[3,Ä≤,4,Ä≤:6,7]naphtho[2,3-d]-1,3-dioxole-6,9-dione, 5-[4-(acetyloxy)phenyl	C21H16O7	380.35	A/B	-8.6
3918	C[C@]12[C@@]3(C=C([C@@H]4[C@@H]1CC[C@@]1(C)[C@H]4CC[C@@H]1[C@@H](CCCC(C)C)C)NC(=S)N3)C[C@@H](OC(=O)C	1092169-36-2	2-Naphthalenesulfonic acid, 6-amino-5-[2-[2,Ä≤,3-dimethyl-4,Ä≤-[2-(6-sulfo-1-na	C34H27N5O6S2.2Na		A/B	-8.6
3919	N(Cc1ccc(cc1)c1ccccn1)(C[C@@H]([C@H](Cc1ccccc1)NC(=O)[C@@H](NC(=O)OC)C(C([2H])([2H])[2H])(C([2H])([2	1092540-50-5	2,5,6,10,13-Pentaazatetradecanedioic acid, 3,12-bis(1,1-dimethylethyl)-8-hydroxy	C38H46D6N6O7		A/B	-8.6
3920	C(NC(=O)c1nnc(C)o1)(C([2H])([2H])[2H])(C([2H])([2H])[2H])c1nc(C(=O)NC(c2ccc(F)cc2)([2H])[2H])c(O)c(=	1100750-88-6	4-Pyrimidinecarboxamide, N-[(4-fluorophenyl)methyl]-1,6-dihydro-5-hydroxy-1-(met	C20H12D9FN6O5		A/B	-8.6
3921	C(NC(=O)c1nnc(C([2H])([2H])[2H])o1)(C([2H])([2H])[2H])(C([2H])([2H])[2H])c1nc(C(=O)NC(c2ccc(F)cc2)([	1100750-93-3	4-Pyrimidinecarboxamide, N-[(4-fluorophenyl)methyl-d2]-1,6-dihydro-5-hydroxy-1-(	C20H10D11FN6O5		A/B	-8.6
3922	C(NC(=O)c1nnc(C([2H])([2H])[2H])o1)(C)(C)c1nc(C(=O)NCc2c(c(c(F)c(c2[2H])[2H])[2H])[2H])c(O)c(=O)n1C(	1100750-99-9	4-Pyrimidinecarboxamide, N-[(4-fluorophenyl-2,3,5,6-d4)methyl]-1,6-dihydro-5-hyd	C20H8D13FN6O5		A/B	-8.6
3923	C(NC(=O)c1nnc(C([2H])([2H])[2H])o1)(C)(C)c1nc(C(=O)NC(c2c(c(c(F)c(c2[2H])[2H])[2H])[2H])([2H])[2H])c	1100751-05-0	4-Pyrimidinecarboxamide, N-[(4-fluorophenyl-2,3,5,6-d4)methyl]-1,6-dihydro-5-hyd	C20H5D16FN6O5		A/B	-8.6
3924	C(NC(=O)c1nnc(C([2H])([2H])[2H])o1)(C([2H])([2H])[2H])(C([2H])([2H])[2H])c1nc(C(=O)NC(c2c(c(c(F)c(c2	1100751-07-2	4-Pyrimidinecarboxamide, N-[(4-fluorophenyl-2,3,5,6-d4)methyl-d2]-1,6-dihydro-5-	C20H6D15FN6O5		A/B	-8.6
3925	O[C@@H]1[C@H](n2c3c(nc2)c(N)ncn3)O[C@](C#C)(CO)[C@H]1O	1100751-08-3	4-Pyrimidinecarboxamide, N-[(4-fluorophenyl-2,3,5,6-d4)methyl-d2]-1,6-dihydro-5-	C20H3D18FN6O5		A/B	-8.6
3926	N(=C1c2c(N(CCCCCC)C1=O)cccc2)NC(=S)Nc1ccccc1	1104302-17-1	Hydrazinecarbothioamide, 2-[1-(2-cyclohexylethyl)-1,2-dihydro-2-oxo-3H-indol-3-y	C23H26N4OS	406.54	A/B	-8.6
3927	O=c1n([C@H]2C[C@H](OC(OCc3ccccc3)C)[C@@H](CO)O2)cc(C(F)(F)F)c(=O)[nH]1	110604-75-6	Thymidine, Œ±,Œ±,Œ±-trifluoro-3,Ä≤,5,Ä≤-bis-O-[1-(phenylmethoxy)ethyl]-	C28H31F3N2O7	564.55	A/B	-8.6
3928	[C@H](CC)(C1=C(O)C[C@H](OC1=O)c1ccccc1)c1cc(NS(=O)(=O)c2c(C(F)(F)F)cccn2)ccc1	1109235-69-9	2-Pyridinesulfonamide, N-[3-[1-(5,6-dihydro-4-hydroxy-2-oxo-6-phenyl-2H-pyran-3-	C28H27F3N2O5S	560.58	A/B	-8.6
3929	O[C@H]1[C@@H](O[C@H](CO)[C@H]1OC(=O)CCCCCCCCCCC)n1c(=O)[nH]c(=O)c(I)c1	111072-10-7	9H-Purin-6-amine, 9-[5-O-[4-(fluorosulfonyl)benzoyl]-Œ≤-D-arabinofuranosyl]-	C17H16FN5O7S	453.4	A/B	-8.6
3930	C(#Cc1n(nc(C(=O)N)n1)[C@@H]1O[C@H](CO)[C@@H](O)[C@H]1O)c1c2c3c4c(cc2)cccc4ccc3cc1	1123187-67-6	1H-1,2,4-Triazole-3-carboxamide, 1-Œ≤-D-ribofuranosyl-5-[2-[2-(trifluoromethyl)p	C17H15F3N4O5	412.32	A/B	-8.6
3931	C(Cc1ccccc1)[C@]1(CCC)C(C(=C([C@H](CC)c2cc(N(S(=O)(=O)c3ccc(C(F)(F)F)cn3)[2H])ccc2)C(=O)O1)O[2H])([2	1126619-14-4	Not Yet Assigned	C31H31D2F3N2O5S		A/B	-8.6
3932	C(=O)([C@@H](NC(=O)OC)CC)N1[C@@H](CC[C@@H]1C)c1[nH]c(cn1)c1ccc(cc1)c1ccc(cc1)c1[nH]c(nc1)[C@H]1N(C(=	1129634-89-4	Carbamic acid, N,N,Ä≤-[[1,1,Ä≤-biphenyl]-4,4,Ä≤-diylbis[1H-imidazole-5,2-diyl[(	C48H50N8O6	834.96	A/B	-8.6
3933	C(C(OC)OC)c1c([nH]c(n1)[C@H]1N(C(=O)[C@@H](NC(=O)OC)C(C)C)CCC1)c1ccc(cc1)c1ccc(cc1)c1[nH]c(nc1)[C@H]	1129636-33-4	Carbamic acid, N-[(1S)-1-[[(2S)-2-[4-(1,3-dioxan-2-ylmethyl)-5-[4,Ä≤-[2-[(2S)-1-	C45H58N8O8	838.99	A/B	-8.6
3934	C(CCCS)(C)N1c2c(NC(=O)c3c1nccc3)c(C)ccn2	1139875-29-8	Not Yet Assigned	C30H35N7O4S	589.71	A/B	-8.6
3935	[C@H]([C@H](Cc1ccccc1)NC(=O)O[C@H]1CCOC1)(CN(S(=O)(=O)c1ccc(N)cc1)CC(C)C)OP(=O)(O)O.[C@H](Cc1ccccc1)	1141895-79-5	4-Piperidinecarboxamide, 1-[4-ethyl-6-(4-morpholinyl)-1,3,5-triazin-2-yl]-N-[[2-	C23H29F3N6O2	478.51	A/B	-8.6
3936	O[C@@H]1[C@H](n2c3c(nc2)c(N)ncn3)O[C@H](COP(=O)O)[C@H]1O	1155353-27-7	Guanosine 5,Ä≤-(trihydrogen diphosphate), P,Ä≤-(4,6-dideoxy-Œ±-D-xylo-hexopyran	C16H25N5O14P2	573.34	A/B	-8.6
3937	O(C[C@H]1O[C@H](C=C1)n1c(=O)[nH]c(=O)c(C)c1)P1(=O)Oc2c(cc(cc2)c2cc(CO)c(O)cc2)CO1	1160003-74-6	Thymidine, 5,Ä≤,5,Ä≤,Ä≤,Ä≤-O-[8,8,Ä≤-bis(1,1-dimethylethyl)-2,2,Ä≤-dioxido[6,6	C42H48N4O14P2	894.8	A/B	-8.6
3938	OC1C(n2c3c(nc2)c(NCC=C)ncn3)OC(CO)C1O	1164118-04-0	Thymidine, [P(S)]-P-thiothymidylyl-(3,Ä≤,Üí5,Ä≤)-5,Ä≤-thio-	C20H27N4O10PS2	578.55	A/B	-8.6
3939	C(=O)(OCC)c1c2c(n(CC)c1CSc1ccccc1)cc(Br)c(O)c2CN(C)C	116688-15-4	2,7-Naphthalenedisulfonic acid, 4-[2-(2,4-dimethylphenyl)diazenyl]-, sodium salt	C18H16N2O6S2.2Na		A/B	-8.6
3940	O=C1[C@@H]2[C@@H](c3c([C@@H](O)[C@H]2CO1)c(O)c1c(c3)OCO1)c1cc(OC)c(O)c(OC)c1	117124-49-9	Carbonic acid, 4-(5,5a,6,8,8a,9-hexahydro-9,10-dihydroxy-6-oxofuro[3,Ä≤,4,Ä≤:6,	C29H26O11	550.51	A/B	-8.6
3941	C(C)n1c2c(c(=O)c(C(=O)OC[C@@H]3[C@@H](N=[N+]=[N-])C[C@@H](O3)n3c(=O)[nH]c(=O)c(C)c3)c1)cc(F)c(c2F)N1	1174398-21-0	Thymidine, 3,Ä≤-azido-3,Ä≤-deoxy-, 5,Ä≤-[1-cyclopropyl-6-fluoro-1,4-dihydro-4-o	C35H35FN8O8		A/B	-8.6
3942	O=c1c2c3c(c(F)c2)N2CCN(C)CC2)OC[C@H](C)n3cc1C(=O)OC[C@@H]1[C@@H](N=[N+]=[N-])C[C@@H](O1)n1c(=O)[nH	1174398-26-5	Thymidine, 3,Ä≤-azido-3,Ä≤-deoxy-, 5,Ä≤-[9-fluoro-2,3-dihydro-3-methyl-10-(4-me	C28H31FN8O7		A/B	-8.6
3943	CN1Cc2c(-c3c(cc(cc3)c3[nH]c(nc3)[C@H]3N(C(=O)[C@@H](NC(=O)OC)C(C)C)CCC3)C1)ccc(c2)c1[nH]c(nc1)[C@H]1	1179150-04-9	Carbamic acid, N,N,Ä≤-[9H-fluorene-2,7-diylbis[1H-imidazole-5,2-diyl-(2S)-2,1-py	C41H50N8O6	750.89	A/B	-8.6
3944	C(=O)([C@@H](NC(=O)OC)C(C)C)N1[C@H](c2[nH]c3-c4c(cc(cc4)c4ccc(cc4)c4c(CC(F)(F)F)nc([nH]4)[C@H]4N(C(=	1180669-62-8	Carbamic acid, N-[(1S)-1-[[(2S)-2-[5-[4-[4,5-dihydro-2-[(2S)-1-[(2S)-2-[(methoxy	C42H52N8O7	780.91	A/B	-8.6
3945	C(=O)([C@@H](NC(=O)OC)C(C)C)N1[C@H](c2[nH]c3-c4c(cc(cc4)c4ccc(F)c(cc4)c4[nH]c(nc4)[C@H]4N(C(=O)[C@@H]	1180669-72-0	Carbamic acid, N-[(1S)-2-methyl-1-[[(1R,3S,5R)-3-[5-[4-[1,4,5,6-tetrahydro-2-[(1	C45H54N8O6	802.96	A/B	-8.6
3946	C(=O)([C@@H](NC(=O)OC)C(C)C)N1[C@H]2[C@H](C2)C[C@H]1c1[nH]c2c(n1)ccc1c2ccc(c1)c1ccc(cc1)c1[nH]c(nc1)	1180670-15-8	Carbamic acid, N-[(1S)-1-[[(2S)-2-[7-[4-[2-[(2S)-1-[(2S)-2-[(methoxycarbonyl)ami	C42H50N8O6	762.9	A/B	-8.6
3947	C(=O)([C@@H](NC(=O)OC)C(C)C)N1[C@H](c2[nH]c3c4c(cc(cc4)c4cc5c(cc4)c4c(cc5)nc([nH]4)[C@H]4N(C(=O)[C@@	1180670-18-1	Carbamic acid, N-[(1S)-1-[[(1R,3S,5R)-3-[7-[4-[2-[(1R,3S,5R)-2-[(2S)-2-[(methoxy	C44H50N8O6	786.92	A/B	-8.6
3948	O(P(=O)(N[C@H](C(=O)OC(C)C)C)OCCOCn1c2c(nc1)c(=O)nc(N)[nH]2)c1c2c(ccc1)cccc2	1184942-46-8	5,8-Dioxa-3-aza-4-phosphanonanoic acid, 9-(2-amino-1,6-dihydro-6-oxo-9H-purin-9-	C25H31N6O7P	558.52	A/B	-8.6
3949	O(P(=O)(N[C@H](C(=O)OCC)C(C)C)OCCOCn1c2c(nc1)c(=O)nc(N)[nH]2)c1c2c(ccc1)cccc2	1184942-54-8	5,8-Dioxa-3-aza-4-phosphanonanoic acid, 9-(2-amino-1,6-dihydro-6-oxo-9H-purin-9-	C24H29N6O7P	544.5	A/B	-8.6
3950	Cc1c2c(N(c3c(C(=O)N2)cccn3)C2(C(C2([2H])[2H])([2H])[2H])[2H])ncc1	1189671-48-4	Not Yet Assigned	C20H15BrN6O		A/B	-8.6
3951	C(N1c2c(C(=NNC(=S)NC3CCCCC3)C1=O)cc(C)cc2)N1CCN(CC1)c1ccc(Cl)cc1	118989-42-7	Hydrazinecarbothioamide, N-cyclohexyl-2-[1,2-dihydro-5-methyl-2-oxo-1-[(4-phenyl	C27H34N6OS	490.66	A/B	-8.6
3952	N[C@H]1[C@H](n2c3c(nc2)c(=O)nc(N)[nH]3)O[C@H](COP(=O)(O)n2ccnc2)C1	1190980-51-8	D-erythro-Pentonamide, 2,3,5-trideoxy-N-[(1R,2R,3S)-2,3-dihydro-2,3-dihydroxy-1H	C36H47N5O5	629.79	A/B	-8.6
3953	N(C(=O)C)[C@H]1[C@H](OC(CC)CC)C=C(C(=O)OC(OC(=O)[C@H](C(C)C)N)C)C[C@@H]1NC(=N)N	1192537-63-5	Not Yet Assigned	C41H63N5O12	817.97	A/B	-8.6
3954	O=C1c2c(NC1=O)ccc(S(=O)(=O)Nc1ccccc1C)c2	1199574-54-3	1H-Indole-5-sulfonamide, 2,3-dihydro-N-(2-methylphenyl)-2,3-dioxo-	C15H12N2O4S	316.33	A/B	-8.6
3955	O[C@H]1[C@@H](O[C@H](COP(=O)(C(P(=O)(O)O)(F)F)O)[C@H]1O)n1c(=O)[nH]c(=O)c(I)c1	1199574-56-5	1H-Indole-5-sulfonamide, 2,3-dihydro-N-(4-methylphenyl)-2,3-dioxo-	C15H12N2O4S	316.33	A/B	-8.6
3956	S(=O)(=O)(O)c1c2c(c(N)c(N=Nc3cc(C)c(cc3)c3c(C)cc(N=Nc4c(N)c5c(c(S(=O)(=O)O)c4)cccc5)cc3)c1)cccc2	1207449-63-5	1,2-Propanediamine, N2-(7-chloro-4-quinolinyl)-N1-(4,6-di-4-morpholinyl-1,3,5-tr	C23H29ClN8O2	484.98	A/B	-8.6
3957	C(=O)(N[C@H](C(C(=O)NC1CC1)O)CCC)[C@H]1N(C(=O)[C@@H](NC(=O)[C@@H](NC(=O)c2cnccn2)C2CCCCC2)CCC(=O)C=C	1208119-21-4	Cyclopenta[c]pyrrole-1-carboxamide, 2-[(2S)-2-[[(2S)-2-cyclohexyl-2-[(2-pyraziny	C37H52N8O7	720.86	A/B	-8.6
3958	N(C(=O)[C@H](C(C)C)N)C12CC3CC(C2)CC(C1)C3	1210068-16-8	Glycine, L-Œ≥-glutamyl-S-(11-cyclopropyl-6,11-dihydro-4-methyl-6-oxo-5H-dipyrid	C25H29N7O7S	571.61	A/B	-8.6
3959	O=C1[C@@H]2[C@@H](c3c([C@H](OC(=O)CCC(=O)NCCCCCNC(=O)Cc4c5c(n(C(=O)c6ccc(Cl)cc6)c4C)ccc(OC)c5)[C@H]2	1222083-95-5	Butanoic acid, 4-[[[4-[[[5-(dimethylamino)-1-naphthalenyl]sulfonyl]amino]phenyl]	C45H45N3O12S	851.92	A/B	-8.6
3960	N(C(=O)[C@H](Cc1ccccc1)C[C@@H](CN1[C@H](C(=O)NC(C)(C)C)CN(Cc2cccnc2)CC1)O)[C@H]1c2c(C[C@H]1O)c(F)ccc	1222663-16-2	Not Yet Assigned	C36H46ClN5O4	648.23	A/B	-8.6
3961	N(C(=O)C)[C@H]1[C@H]([C@@H]([C@@H](CO)O)O)OC(=C[C@@H]1NC(N)N)C(=O)O	1222681-91-5	Not Yet Assigned	C36H53N5O4	619.84	A/B	-8.6
3962	N(C(=O)[C@H](Cc1ccccc1)C[C@@H](CN1[C@H](C(=O)NC(C)(C)C)CN(Cc2cc(C(NC(=O)OC(C)(C)C)(C)C)ccc2)CC1)O)C1	1223096-15-8	Not Yet Assigned	C34H47F3N4O4	632.76	A/B	-8.6
3963	[C@@H](C(=O)N[C@@H](C(c1ccccc1)([2H])[2H])C[C@@H]([C@H](Cc1ccccc1)NC(=O)COc1c(C([2H])([2H])[2H])cccc	1224729-66-1	1(2H)-Pyrimidineacetamide, N-[(1S,3S,4S)-4-[[2-(2,6-dimethylphenoxy)acetyl]aminc	C37H46D2N4O5		A/B	-8.6
3964	Fc1cc(c2nc(nc(n2)N2CCOCC2)N2CCOCC2)cnc1	1225037-39-7	2-Pyridinamine, 5-(4,6-di-4-morpholinyl-1,3,5-triazin-2-yl)-4-(trifluoromethyl)-	C17H20F3N7O2	411.38	A/B	-8.6
3965	C(#N)C1=C(N)Oc2c(C1c1ccc(C(F)(F)F)cc1)ccn1c2ncc1	1225373-02-3	7H-Imidazo[1,2-a]pyrano[2,3-c]pyridine-8-carbonitrile, 9-amino-7-[3-(trifluorome	C18H11F3N4O	356.3	A/B	-8.6
3966	C(=O)([C@@H](NC(=O)OC)C(C)C)N1[C@H](c2[nH]c(c3c4c(c(ccc4)c4ccc(cc4)c4[nH]c(nc4)[C@H]4N(C(=O)[C@@H](N	1228552-32-6	Carbamic acid, N-[(1S)-1-[[(2S)-2-[5-[4-[6-[2-[(2S)-1-[(2R)-2-(dimethylamino)-2-	C47H52N8O4	792.97	A/B	-8.6
3967	C(=O)([C@H](NC(=O)OC)c1ccccc1)N1[C@@H](CCC1)c1[nH]c(cn1)c1ccc(cc1)c1ccc(C#Cc2[nH]c(nc2)[C@H]2N(C(=O)	1228966-26-4	Carbamic acid, N,N,Ä≤-[1,4-phenylenebis[2,1-ethynediyl-1H-imidazole-5,2-diyl-(2S	C44H42N8O6	778.85	A/B	-8.6
3968	O=C1c2c(NC1=O)ccc(S(=O)(=O)N1CC(F)(F)C1)c2	1234339-96-8	1H-Indole-2,3-dione, 5-[(3,3-difluoro-1-pyrrolidinyl)sulfonyl]-	C12H10F2N2O4S	316.28	A/B	-8.6
3969	O=C1N(c2c(C1=NNC(=S)NCC)cccc2)[C@H]1[C@H](OC(=O)C)[C@H](OC(=O)C)[C@@H](COC(=O)C)O1	1234340-16-9	1H-Indole-2,3-dione, 5-[(3,3-difluoro-1-azetidinyl)sulfonyl]-	C11H8F2N2O4S	302.25	A/B	-8.6
3970	O[C@H]1[C@H](n2c3c(nc2)c(=O)nc(N)[nH]3)O[C@H](COP(=O)(OP(=O)(OC[C@H]([C@H]([C@H](C=O)O)O)O)O)O)[C@H]	123466-60-4	Guanosine 5,Ä≤-(trihydrogen diphosphate), P,Ä≤-Œ±-D-xylopyranosyl ester	C15H23N5O15P2	575.32	A/B	-8.6
3971	S(=O)(=O)(NC(=O)OCCCC)c1c(cc(CC(C)C)s1)c1ccc(C(=O)n2cc(C(F)(F)F)nc2)cc1	1236352-56-9	4-Pyrimidinecarboxamide, N-[[4-(fluoro-18F)phenyl]methyl]-1,6-dihydro-5-hydroxy-	C20H21FN6O5		A/B	-8.6
3972	C(N(C(=O)N[C@H](C(=O)N[C@@H](Cc1ccccc1)C[C@@H]([C@H](Cc1ccccc1)NC(=O)OCc1ccns1)O)C(C)C)C)c1nc(sc1)c1	1236406-28-2	2,7,10,12-Tetraazatridecanoic acid, 13-[2,2,Ä≤-bithiazol]-4-yl-4-hydroxy-12-meth	C37H43N7O5S3	761.98	A/B	-8.6
3973	[C@H](Cc1ccccc1)(C[C@@H]([C@H](Cc1ccccc1)NC(=O)OCc1cncs1)O)NC(=O)[C@H](NC(=O)N(Cc1nc(C(=C(C)C)C)sc1)	1236406-60-2	2,7,10,12-Tetraazatridecanoic acid, 13-[2-(cyclohexylmethyl)-4-thiazolyl]-4-hydr	C41H54N6O5S2	775.03	A/B	-8.6
3974	Clc1c(c2nc(CN(C(=O)N[C@@H](C(=O)N[C@@H](Cc3ccccc3)C[C@@H]([C@H](Cc3ccccc3)NC(=O)OCc3cncs3)O)C(C)C)C)	1236406-79-3	2,7,10,12-Tetraazatridecanoic acid, 4-hydroxy-12-methyl-9-(1-methylethyl)-8,11-c	C38H45N7O6S2	759.94	A/B	-8.6
3975	[C@H](Cc1ccccc1)(C[C@@H]([C@H](Cc1ccccc1)NC(=O)OCc1ccno1)O)NC(=O)[C@@H](NC(=O)N(Cc1nc(CC=C)sc1)C)C(C	1236406-82-8	Not Yet Assigned	C37H43N7O6S2	745.91	A/B	-8.6
3976	[C@H](Cc1ccccc1)(C[C@@H]([C@H](Cc1ccccc1)NC(=O)OCc1ccns1)O)NC(=O)[C@@H](NC(=O)N(Cc1nc(CC2CCCCC2)sc1)	1236407-16-1	2,7,10,12-Tetraazatridecanoic acid, 13-[2-(cyclopentylmethyl)-4-thiazolyl]-4-hyd	C40H52N6O6S	744.94	A/B	-8.6
3977	C(CC)(CC)c1nc(CN(C(=O)N[C@H](C(=O)N[C@@H](Cc2ccccc2)C[C@@H]([C@H](Cc2ccccc2)NC(=O)OCc2ccno2)O)C(C)C)	1236408-18-6	2,7,10,12-Tetraazatridecanoic acid, 4-hydroxy-12-methyl-9-(1-methylethyl)-13-[2-	C38H48N6O5S2	732.95	A/B	-8.6

3978	[C@H](Cc1ccccc1)(C[C@@H]([C@H](Cc1ccccc1)NC(=O)OCc1cncs1)O)NC(=O)[C@H](NC(=O)N(Cc1nc(C=C)sc1)C)C(C)C	1236409-50-9	2,7,10,12-Tetraazatridecanoic acid, 13-(2-cyclopropyl-4-thiazolyl)-4-hydroxy-12-	C37H46N6O5S2	718.93	A/B	-8.6
3979	C(N(C(=O)N[C@@H](C(=O)N[C@@H](Cc1ccccc1)C[C@@H]([C@H](Cc1ccccc1)NC(=O)OCc1cncs1)O)C(C)C)C)c1nc(sc1)c	1238048-99-1	2,7,10,12-Tetraazatridecanoic acid, 13-[2,2,Ä≤-bithiazol]-4-yl-4-hydroxy-12-meth	C37H43N7O5S3	761.98	A/B	-8.6
3980	C=C1[C@@H](n2c3c(nc2)c(=O)nc(N)[nH]3)C[C@H]2[C@H]1CO[Si](C(C)(C)C)(C(C)(C)C)O2	123808-83-3	Cytidine, [P(S)]-P-methylthymidylyl-(3,Ä≤,Ü5,Ä≤)-2,Ä≤-deoxy-	C20H28N5O11P	545.44	A/B	-8.6
3981	O=C1[C@@H]2[C@@H](c3c([C@H](OC(=O)N4CCN(Cc5cc6c(cc5)OCO6)CC4)[C@H]2CO1)cc1c(c3)OCO1)c1cc(OC)c(OC)c(O	123824-79-3	[1,4,Ä≤-Bipiperidine]-1,Ä≤-carboxylic acid, 5,5a,6,8,8a,9-hexahydro-8-oxo-9-(3,	C33H40N2O9	608.68	A/B	-8.6
3982	C(F)(F)c1n(c2c(n1)cccc2)c1nc(nc(N[C@H]2CC[C@H](NC(=O)NC)CC2)n1)N1CCOCC1	1240023-33-9	1,4-Cyclohexanediamine, N4-[4-[2-(difluoromethyl)-1H-benzimidazol-1-yl]-6-(4-mor	C23H30F2N8O	472.53	A/B	-8.6
3983	C(F)(F)c1n(c2c(n1)cccc2)c1nc(nc(N[C@H]2CC[C@H](NC(=O)CN)CC2)n1)N1CCOCC1	1240023-34-0	Urea, N-[trans-4-[[4-[2-(difluoromethyl)-1H-benzimidazol-1-yl]-6-(4-morpholinyl)	C23H29F2N9O2	501.53	A/B	-8.6
3984	C(F)(F)c1n(c2c(n1)cccc2)c1nc(nc(N[C@H]2CC[C@H](NC)CC2)n1)N1CCOCC1	1240023-43-1	Acetamide, N-[trans-4-[[4-[2-(difluoromethyl)-1H-benzimidazol-1-yl]-6-(4-morphol	C24H30F2N8O2	500.54	A/B	-8.6
3985	C(=O)([C@@H](NC(=O)OC)C)N1[C@H](c2[nH]c(c3cc4c(-c5c(C4)cc(cc5)c4[nH]c(nc4)[C@H]4N(C(=O)[C@@H](NC(=O)	1240880-36-7	Carbamic acid, N,N,Ä≤-[9H-fluorene-2,7-diylbis[1H-imidazole-5,2-diyl-(2S)-2,1-py	C37H42N8O6	694.78	A/B	-8.6
3986	C(=O)([C@H](NC(=O)OC)c1ccccc1)N1[C@@H](CCC1)c1[nH]c(cn1)c1ccc(C/C=C/Cc2[nH]c(nc2)[C@H]2N(C(=O)[C@H](	1240891-96-6	Carbamic acid, N-[(1R)-2-[(2S)-2-[5-[4-[(1E)-4-[2-[(2S)-1-[(2R)-2-[(methoxycarbo	C44H48N8O6	784.9	A/B	-8.6
3987	O=c1n(cc([C@H](OCCCCCC)c2ccc(N(=O)=O)cc2)c(=O)[nH]1)[C@@H]1O[C@H](CO)[C@@H](O)[C@H]1O	1246746-61-1	Benzonitrile, 4-[[6-amino-5-bromo-2-[(2-bromo-4-cyanophenyl)amino]-4-pyrimidinyl	C20H14Br2N6O	514.17	A/B	-8.6
3988	C(=O)([C@@H](NC(=O)OC)C(C)C)N1[C@@H](CCC1)c1[nH]c(cn1)C1=CCC(CC1)c1ccc(cc1)c1[nH]c(nc1)[C@H]1N(C(=O)	1246852-25-4	Carbamic acid, N,N,Ä≤-[[1,1,Ä≤-bi-3-cyclohexen]-4,4,Ä≤-diylbis[1H-imidazole-5,2	C36H50N8O6	690.83	A/B	-8.6
3989	C(=O)([C@@H](NC(=O)OC)C(C)C)N1[C@@H](CC[C@@H]1C)c1[nH]c(cn1)c1ccc(C#Cc2ccc(cc2)c2[nH]c(nc2)[C@H]2N(C	1250938-96-5	Carbamic acid, N,N,Ä≤-[(1S,2S)-1,2-cyclopropanediylbis[4,1-phenylene-1H-imidazol	C45H58N8O6	806.99	A/B	-8.6
3990	O(c1nc(Nc2ccc(C#N)cc2)nc(N)c1Br)c1c(C)cc(C#N)cc1C.S(=O)(=O)(O)c1ccccc1	1253183-22-0	Benzonitrile, 4-[[6-amino-5-bromo-2-[(4-cyanophenyl)amino]-4-pyrimidinyl]oxy]-3,	C20H15BrN6O.H3O4P		A/B	-8.6
3991	O(c1nc(Nc2ccc(C#N)cc2)nc(N)c1Br)c1c(C)cc(C#N)cc1C.S(=O)(=O)(CC)O	1253183-23-1	Benzonitrile, 4-[[6-amino-5-bromo-2-[(4-cyanophenyl)amino]-4-pyrimidinyl]oxy]-3,	C20H15BrN6O.C6H6O3S		A/B	-8.6
3992	O=c1n([C@@H]2C=C(CO)[C@@H](CO)O2)cc(C)c(=O)[nH]1	1255935-58-0	Carbamic acid, N,N,Ä≤-[(1,2,3,5,6,7-hexahydro-s-indacene-2,6-diyl)bis[1H-imidaz	C40H54N8O6	742.91	A/B	-8.6
3993	C(=O)([C@@H](NC(=O)OC)C(C)C)N1[C@@H](CCC1)c1[nH]c(cn1)c1ccc(cc1)c1ccc(cc1)c1ccc(cc1)c1[nH]c(nc1)[C@H	1256384-64-1	Carbamic acid, N-[(1S)-1-[[(2S)-2-[5-[5,7-dihydro-9-[2-[(1R,3S,4S)-2-[(2S)-2-[(m	C44H54N8O7	806.95	A/B	-8.6
3994	C(=O)([C@@H](NC(=O)OC)c1ccccc1)N1[C@@H](CCC1)c1[nH]c(cn1)c1ccc(cc1)c1cc2c(cc1)cc(cc2)c1[nH]c(nc1)[C@	1256391-24-8	Carbamic acid, N,N,Ä≤-[(5,10-dihydro[1]benzopyrano[5,4,3-cde][1]benzopyran-2,7-	C42H50N8O8	794.9	A/B	-8.6
3995	C(=O)(C(NC(=O)OC)c1ccccc1)N1[C@@H](CCC1)c1[nH]c(cn1)c1cc2c(cc1)cc(cc2)c1ccc(cc1)c1[nH]c(nc1)[C@H]1N(	1256391-34-0	Carbamic acid, N-[(1R)-2-[(2S)-2-[5-[6-[4-[2-[(2S)-1-[(2S)-2-[(methoxycarbonyl)a	C47H50N8O6	822.95	A/B	-8.6
3996	C(=O)([C@@H](NC(=O)OC)C(C)C)N1[C@@H](CCC1)c1[nH]c(cn1)c1ccc(C#Cc2ccc(cc2)c2[nH]c(nc2)[C@H]2N(C(=O)[C	1256392-21-8	Carbamic acid, N-[(1S)-1-[[(2S)-2-[5-[6-[4-[2-[(2S)-1-[(2S)-2-cyclohexyl-2-[(met	C47H56N8O6	829	A/B	-8.6
3997	C(=O)([C@@H](NC(=O)OC)C(C)C)N1[C@@H](C[C@@H](COC(F)F)C1)c1[nH]c(cn1)c1ccc(cc1)c1ccc(cc1)c1[nH]c(nc1)	1256393-01-7	Carbamic acid, N-[2-[(2S)-2-[5-[4-[6-[2-[(2S)-1-[(2S)-2-[(methoxycarbonyl)amino]	C47H50N8O6	822.95	A/B	-8.6
3998	O=c1n([C@H]2C[C@H](O[P@@](=O)(OC[C@H]3O[C@H](C[C@@H]3O)n3c(=O)[nH]c(=O)c(C)c3)OCOC(=O)C)[C@@H](CO)O2	1261161-48-1	1-Naphthalenesulfonic acid, 3,3,Ä≤-[(3,Ä≤,4-dimethyl[1,1,Ä≤-biphenyl]-3,4,Ä≤-di	C34H28N6O6S2	680.75	A/B	-8.6
3999	C(F)(F)c1n(c2c(n1)cccc2)c1nc(nc(NCc2ccccn2)n1)N1CCOCC1	1261281-13-3	1,3,5-Triazin-2-amine, 4-[2-(difluoromethyl)-1H-benzimidazol-1-yl]-N-methyl-6-(	C23H23F2N7O	451.47	A/B	-8.6
4000	C(F)(F)c1n(c2c(n1)cccc2)c1nc(nc(Nc2ccccn2)n1)N1CCOCC1	1261281-19-9	1,3,5-Triazin-2-amine, 4-[2-(difluoromethyl)-1H-benzimidazol-1-yl]-6-(4-morpholi	C21H20F2N8O	438.43	A/B	-8.6
4001	C(F)(F)c1n(c2c(n1)cccc2)c1nc(nc(Nc2ccncc2)n1)N1CCOCC1	1261281-20-2	1,3,5-Triazin-2-amine, 4-[2-(difluoromethyl)-1H-benzimidazol-1-yl]-6-(4-morpholi	C20H18F2N8O	424.41	A/B	-8.6
4002	N(C(CCCNC(C([2H])([2H])[2H])([2H])[2H])C)c1c2c(ncc1)cc(Cl)cc2	1261392-42-0	Not Yet Assigned	C20H15BrN6O		A/B	-8.6
4003	O=c1n([C@H]2O[C@H](COP(=O)(O)O)[C@@H](O)C2)cc(C)c(=O)[nH]1	1261395-89-4	Not Yet Assigned	C20H19D2FN6O5		A/B	-8.6
4004	C(Nc1nc(=O)n(cc1)[C@H]1O[C@@H](CO)SC1)(c1ccccc1)(c1ccccc1)c1ccccc1	126168-31-8	9H-Purin-6-amine, 9-[5-O-(diphenoxyphosphinyl)-Œ≤-D-arabinofuranosyl]-	C22H22N5O7P	499.41	A/B	-8.6
4005	O(C)c1c2n(cc(C(=O)O)c(=O)c2cc(F)c1N1CCN(CCN2c3c(C(=NNC(=S)N)C2=O)cccc3)CC1)C1CC1	1263191-35-0	3-Quinolinecarboxylic acid, 7-[4-[[3-[2-(aminothioxomethyl)hydrazinylidene]-2,3-	C28H28FN7O5S	593.63	A/B	-8.6
4006	O(C(=O)N[C@@H](Cc1ccccc1)[C@@H](CN(S(=O)(=O)c1ccc(OC)cc1)CC(C)C)O)[C@@H]1[C@H]2[C@@H](OC1)OC[C@H]2OC	1265531-61-0	Carbamic acid, N-[(1S)-1-[[(2S)-2-[5-[4-[2-[2-[(2S)-1-[(2S)-2-[(methoxycarbonyl)	C34H42N8O6	658.75	A/B	-8.6
4007	O=c1c2c(n(cc1C(=O)O)C1CC1)cc(c(F)c2)N1CCN(CN2c3c(/C(=N/NC(=S)NOC)/C2=O)cc(Cl)cc3)CC1	1265623-15-1	3-Quinolinecarboxylic acid, 7-[4-[[(3Z)-5-chloro-2,3-dihydro-3-[2-[(hydroxyamino	C27H25ClFN7O5S	614.05	A/B	-8.6
4008	O=c1c2c(n(cc1C(=O)O)C1CC1)cc(c(F)c2)N1CCN(CN2c3c(/C(=N/NC(=S)NOC)/C2=O)cc(C)cc3)CC1	1265623-19-5	3-Quinolinecarboxylic acid, 1-cyclopropyl-7-[4-[[(3Z)-2,3-dihydro-3-[2-[(hydroxy	C28H28FN7O5S	593.63	A/B	-8.6
4009	C(C)n1c2c(c(=O)c(C(=O)O)c1)cc(F)c(c2F)N1CC(C)N(CN2c3c(/C(=N/NC(=S)NOC)/C2=O)cc(Cl)cc3)CC1	1265623-21-9	3-Quinolinecarboxylic acid, 7-[4-[[(3Z)-5-chloro-2,3-dihydro-3-[2-[(hydroxyamino	C27H26ClF2N7O5S	634.05	A/B	-8.6
4010	O(C)c1c2n(cc(C(=O)O)c(=O)c2cc(F)c1N1CC(C)N(CN2c3c(/C(=N/NC(=S)NO)/C2=O)cc(Cl)cc3)CC1)C1CC1	1265623-26-4	3-Quinolinecarboxylic acid, 7-[4-[[(3Z)-2,3-dihydro-3-[2-[(methoxyamino)thioxome	C29H31F2N7O5S	627.66	A/B	-8.6
4011	O(C)c1c2n(cc(C(=O)O)c(=O)c2cc(F)c1N1CC(C)N(CN2c3c(/C(=N/NC(=S)NO)/C2=O)cc(F)cc3)CC1)C1CC1	1265623-28-6	3-Quinolinecarboxylic acid, 7-[4-[[(3Z)-5-chloro-2,3-dihydro-3-[2-[(methoxyamino	C30H31ClFN7O6S	672.13	A/B	-8.6
4012	C(=O)([C@@H](NC(=O)OC)C(C)C)N1CC2(C[C@H]1c1[nH]c(cn1)c1ccc(cc1)c1ccc(cc1)c1[nH]c(nc1)[C@H]1N(C(=O)[C	1272656-46-8	Carbamic acid, N-[(1S)-1-[[(2S)-2-[5-[4,Ä≤-[2-[6-[(2S)-2-[(methoxycarbonyl)amino	C42H53N9O6	779.93	A/B	-8.6
4013	C(=O)([C@H](NC(=O)OC)C)N1[C@@H]([C@@H]2C[C@H]1CC2)c1[nH]c(cn1)c1ccc(cc1)c1ccc(cc1)c1[nH]c(nc1)[C@H]1	127337-48-8	Benzoic acid, 4-[2-(4-amino-6-sulfo-1-naphthalenyl)diazenyl]-	C17H13N3O5S	371.37	A/B	-8.6
4014	C(=O)([C@@H](NC(=O)O[C@@H]1CCOC1)C(C)C)N1[C@@H]([C@@H]2C[C@H]1CC2)c1[nH]c(cn1)c1ccc(cc1)c1ccc(cc1)c1	1273589-69-7	Carbamic acid, N-[(1S)-2-methyl-1-[[(2S)-2-[5-[4,Ä≤-[2-[(1R,3S,4S)-2-[(2S)-3-met	C50H64N8O8	905.09	A/B	-8.6
4015	C(=O)([C@@H](NC(=O)OC)C(C)C)N1[C@@H]([C@@H]2C[C@H]1CC2)c1[nH]c(cn1)c1ccc(cc1)c1ccc(cc1)c1[nH]c(nc1)[	1273589-77-7	Carbamic acid, N-[(1S)-2-[(2S)-2-[5-[4,Ä≤-[2-[(1R,3S,4S)-2-[(2S)-2-[(methoxycar	C46H56N8O8	848.99	A/B	-8.6
4016	C(=O)([C@@H](NC(=O)OC)C(C)C)N1[C@@H]([C@@H]2C[C@H]1CC2)c1[nH]c(cn1)c1ccc(cc1)c1ccc(cc1)c1[nH]c(nc1)[	1273591-10-8	Carbamic acid, N-[(1S)-1-[[(2S,4S)-4-fluoro-2-[5-[4,Ä≤-[2-[(1R,3S,4S)-2-[(2S)-2-	C42H51FN8O6	782.9	A/B	-8.6
4017	C(=O)([C@@H](NC(=O)OC)C(C)C)N1[C@@H]([C@@H]2C[C@H]1CC2)c1[nH]c(cn1)c1ccc(cc1)c1ccc(cc1)c1[nH]c(nc1)[	1273591-13-1	Carbamic acid, N-[(1S)-1-[[(2S,5S)-2-[5-[4,Ä≤-[2-[(1R,3S,4S)-2-[(2S)-2-[(methoxy	C48H56N8O6	841.01	A/B	-8.6
4018	C(=O)([C@@H](NC(=O)OC)C(C)C)N1[C@@H]([C@@H]2C[C@H]1[C@@H](OC)C2)c1[nH]c(cn1)c1ccc(cc1)c1ccc(cc1)c1[n	1273591-22-2	Carbamic acid, N-[(1S)-1-[[(2S)-2-[5-[4,Ä≤-[2-[(1S,3S,4R,6S)-6-hydroxy-2-[(2S)-2	C42H52N8O7	780.91	A/B	-8.6
4019	C(=O)([C@@H](NC(=O)OC)C(C)C)N1[C@@H]([C@@H]2C[C@H]1CC2)c1[nH]c(cn1)c1c(Cl)cc(cc1Cl)c1cc(Cl)c(c(Cl)c1	1273591-52-8	Carbamic acid, N-[(1S)-1-[[(2S)-2-[5-[3,3,Ä≤-dichloro-4,Ä≤-[2-[(1R,3S,4S)-2-[(2	C42H50Cl2N8O6	833.8	A/B	-8.6
4020	C(N(NC(=O)[C@@H](NC(=O)OCC)C(C)(C)C)C[C@@H]([C@H](Cc1ccccc1)NC(=O)[C@@H](NC(=O)OCC)C(C)(C)C)O)c1ccc(	1292296-11-7	2,5,6,10,13-Pentaazatetradecanedioic acid, 3,12-bis(1,1-dimethylethyl)-8-hydroxy	C38H52N6O7	704.86	A/B	-8.6
4021	C(=O)([C@@H](NC(=O)OC)CC)N1[C@@H](C[C@H](C)C1)c1[nH]c(cn1)c1cc2c(cc1)cc(cc2)c1ccc(cc1)c1[nH]c(nc1)[C	1306734-13-3	Carbamic acid, N-[(1S)-1-[[(2S,4S)-2-[5-[4-[6-[2-[(2S,4S)-1-[(2S)-2-[(methoxycar	C46H56N8O6	816.99	A/B	-8.6
4022	N(=C=S)C12CC3(O)CC(C2)CC(C1)C3	130831-29-7	1,7-Naphthalenedisulfonic acid, 4-amino-3-[2-(2-methylphenyl)diazenyl]-, sodium	C17H15N3O6S2.2Na		A/B	-8.6
4023	C(=O)([C@@H](NC(=O)OC)C1CCOCC1)N1[C@@H](C[C@H](C)C1)c1[nH]c(c(Cl)n1)c1ccc(cc1)c1ccc(cc1)c1[nH]c(nc1)	1312336-28-9	Carbamic acid, N-[(1S)-2-[(2S,4S)-2-[5-[4,Ä≤-[5-chloro-2-[(2S,4S)-1-[(2S)-2-[(me	C44H55ClN8O7	843.41	A/B	-8.6
4024	C(=O)([C@@H](NC(=O)OC)C(C)C)N1[C@H]2[C@H](C2)C[C@H]1c1[nH]c(c(F)n1)c1ccc(cc1)c1ccc(cc1)c1[nH]c(nc1)[	1312336-62-1	Carbamic acid, N-[(1S)-1-[[(2S)-2-[4-[4,Ä≤-[2-[(2S)-1-[(2S)-2-[(methoxycarbonyl)	C44H54N10O6	818.96	A/B	-8.6
4025	C(=O)([C@@H](NC(=O)OC)[C@@H]1CCCOC1)N1[C@H]2[C@H](C2)C[C@H]1c1[nH]c(c(Cl)n1)c1ccc(cc1)c1ccc(cc1)c1[n	1312336-70-1	Carbamic acid, N,N,Ä≤-[[1,1,Ä≤-biphenyl]-4,4,Ä≤-diylbis[(5-fluoro-1H-imidazole-	C46H52F2N8O8	882.95	A/B	-8.6
4026	C(=O)([C@@H](NC(=O)OC)C(C)C)N1[C@@H](C[C@@H]2[C@H]1C2)c1[nH]c(n1)C1CC1)c1ccc(cc1)c1ccc(cc1)c1c(nc(	1312337-01-1	Carbamic acid, N-[(1S)-2-[(1R,3S,5R)-3-[4-cyclopropyl-5-[4,Ä≤-[2-[(1R,3S,5R)-2-	C49H58N8O8	887.03	A/B	-8.6
4027	Cn1c(c2c([nH]c(n2)[C@H]2N(C(=O)[C@@H](NC(=O)OC)C(C)C)CCC2)c2ccc(cc2)c2ccc(cc2)c2[nH]c(nc2)[C@H]2N(C(	1312337-04-4	Carbamic acid, N-[(1S)-2-[(2S)-2-[4-[4,Ä≤-[2-[(2S)-1-[(2S)-2-[(methoxycarbonyl)a	C48H58N10O8	903.04	A/B	-8.6
4028	N(c1c(C([2H])([2H])[2H])cc(/C=C/C#N)cc1C([2H])([2H])[2H])c1nc(Nc2ccc(C#N)cc2)ncc1	1312424-25-1	Benzonitrile, 4-[[4-[[4-[(1E)-2-cyanoethenyl]-2-methyl-6-(methyl-d3)phenyl]amino	C22H15D3N6		A/B	-8.6
4029	Cc1[nH]c2c(ccc(c2n1)c1ccc(cc1)c1[nH]c(nc1)[C@H]1N(C(=O)[C@@H](NC(=O)OC)C(C)C)CCC1)c1[nH]c(nc1)[C@H]1	1312608-33-5	Carbamic acid, N-[(1S)-1-[[(2S)-2-[5-[4-[7-[2-[(2S)-1-[(2S)-2-[(methoxycarbonyl)	C41H50N10O6	778.9	A/B	-8.6
4030	C(=O)(Cc1ccccc1)N1[C@H](c2[nH]c(c3c4c(c(cc3)c3ccc(cc3)c3[nH]c(nc3)[C@H]3N(C(=O)[C@@H](NC(=O)OC)C(C)C	1312608-49-3	Carbamic acid, N-[(1S)-1-[[(2S)-2-[5-[4-[7-[2-[(2S)-1-[(2S)-2-[(methoxycarbonyl)	C43H53N9O6	791.94	A/B	-8.6
4031	C(=O)(CNC(=O)OC)N1[C@H](c2[nH]c(c3c4c(c(cc3)c3ccc(cc3)c3[nH]c(nc3)[C@H]3N(C(=O)CNC(=O)OC)CCC3)cccc4)	1312608-55-1	Carbamic acid, N-[(1R)-1-[[(2S)-2-[5-[4-[4-[2-[(2S)-1-[(2R)-2-[(methoxycarbonyl)	C44H52N8O6	788.93	A/B	-8.6
4032	C(=O)([C@@H](NC(=O)OC)C(C)C)N1[C@H](c2[nH]c(c3c4c(c(cc3)c3ccc(cc3)c3[nH]c(nc3)[C@H]3N(C(=O)[C@@H](NC	1312608-61-9	Carbamic acid, N-[(1S)-1-[[(2S)-2-[5-[4-[7-[2-[(2S)-1-[(2S)-2-[(methoxycarbonyl)	C42H50N8O7	778.9	A/B	-8.6
4033	O(Cc1ccc(F)cc1)c1c2c(oc(c(O)c2=O)c2ncc(OC)cn2)c(C)cc1	1312892-53-7	4H-1-Benzopyran-4-one, 5-[(4-fluorophenyl)methoxy]-3-hydroxy-8-methyl-2-(5-methy	C22H17FN2O4	392.38	A/B	-8.6
4034	O(Cc1ccc(F)cc1)c1c2c(oc(c(O)c2=O)c2nnc(C)[nH]2)c(C)cc1	1312892-62-8	4H-1-Benzopyran-4-one, 5-[(4-fluorophenyl)methoxy]-3-hydroxy-8-methyl-2-(1H-1,2,	C19H14FN3O4	367.33	A/B	-8.6
4035	O(Cc1ccc(F)cc1)c1c2c(oc(c(O)c2=O)c2ncc(cn2)c2ccccc2)c(OC)cc1	1312893-36-9	4H-1-Benzopyran-4-one, 2-(5-chloro-2-pyrimidinyl)-5-[(4-fluorophenyl)methoxy]-3-	C21H14ClFN2O5	428.8	A/B	-8.6
4036	O(Cc1ccc(F)cc1)c1c2c(oc(c(O)c2=O)c2[nH]c(nn2)c2ccccc2)c(OC)cc1	1312893-46-1	4H-1-Benzopyran-4-one, 2-(5-chloro-1H-1,2,4-triazol-3-yl)-5-[(4-fluorophenyl)met	C19H13ClFN3O5	417.77	A/B	-8.6
4037	O(Cc1ccc(F)cc1)c1c2c(oc(c(O)c2=O)c2ncc(OC)cn2)c(Cl)cc1	1312893-84-7	4H-1-Benzopyran-4-one, 8-chloro-5-[(4-fluorophenyl)methoxy]-3-hydroxy-2-(5-methy	C21H14ClFN2O4	412.8	A/B	-8.6
4038	O(Cc1ccc(F)cc1)c1c2c(oc(c(O)c2=O)c2nnc(C)[nH]2)c(Cl)cc1	1312893-93-8	4H-1-Benzopyran-4-one, 8-chloro-5-[(4-fluorophenyl)methoxy]-3-hydroxy-2-(1H-1,2,	C18H11ClFN3O4	387.75	A/B	-8.6
4039	O(Cc1ccc(F)cc1)c1c2c(oc(c(O)c2=O)c2ncc(C)cn2)cc(C)c1	1312894-83-9	4H-1-Benzopyran-4-one, 5-[(4-fluorophenyl)methoxy]-3-hydroxy-7-methyl-2-(2-pyrim	C21H15FN2O4	378.35	A/B	-8.6
4040	O(Cc1ccc(F)cc1)c1c2c(oc(c(O)c2=O)c2ncc(C)cn2)c(C)c(C)c1	1312895-33-2	4H-1-Benzopyran-4-one, 5-[(4-fluorophenyl)methoxy]-3-hydroxy-7,8-dimethyl-2-(2-p	C22H17FN2O4	392.38	A/B	-8.6
4041	O(Cc1ccc(F)cc1)c1c2c(oc(c(O)c2=O)c2ncc(C)cn2)c(Cl)c(C)c1	1312896-33-5	4H-1-Benzopyran-4-one, 8-chloro-5-[(4-fluorophenyl)methoxy]-3-hydroxy-7-methyl-2	C21H14ClFN2O4	412.8	A/B	-8.6
4042	O(Cc1ccc(F)cc1)c1c2c(c(c(C)c1)c1ccccc1)oc(c(O)c2=O)c1[nH]cc(Cl)n1	1312897-00-9	4H-1-Benzopyran-4-one, 5-[(4-fluorophenyl)methoxy]-3-hydroxy-2-(5-methoxy-1H-imi	C27H21FN2O5	472.46	A/B	-8.6
4043	O(Cc1ccc(F)cc1)c1c2c(oc(c(O)c2=O)c2ncc(C)cn2)cc(OC)c1	1312897-44-1	4H-1-Benzopyran-4-one, 5-[(4-fluorophenyl)methoxy]-3-hydroxy-7-methoxy-2-(2-pyri	C21H15FN2O5	394.35	A/B	-8.6
4044	O(Cc1ccc(F)cc1)c1c2c(oc(c(O)c2=O)c2[nH]cnn2)cc(OC)c1	1312897-48-5	4H-1-Benzopyran-4-one, 5-[(4-fluorophenyl)methoxy]-3-hydroxy-7-methoxy-2-(5-pher	C27H19FN2O5	470.45	A/B	-8.6
4045	O(Cc1ccc(F)cc1)c1c2c(oc(c(O)c2=O)c2[nH]c(OC)nn2)cc(OC)c1	1312897-55-4	4H-1-Benzopyran-4-one, 5-[(4-fluorophenyl)methoxy]-3-hydroxy-7-methoxy-2-(3-meth	C20H16FN3O5	397.36	A/B	-8.6
4046	O(Cc1ccc(F)cc1)c1c2c(oc(c(O)c2=O)c2[nH]c(C)nn2)c(C)c(OC)c1	1312898-04-6	4H-1-Benzopyran-4-one, 5-[(4-fluorophenyl)methoxy]-3-hydroxy-7-methoxy-8-methyl-	C20H16FN3O5	397.36	A/B	-8.6
4047	O(Cc1ccc(F)cc1)c1c2c(oc(c(O)c2=O)c2[nH]c(C)nn2)c(Cl)c(OC)c1	1312899-05-0	4H-1-Benzopyran-4-one, 8-chloro-5-[(4-fluorophenyl)methoxy]-3-hydroxy-7-methoxy-	C19H13ClFN3O5	417.77	A/B	-8.6
4048	O(C)c1c(c2c(c(OCc3ccc(F)cc3)c1)c(=O)c(O)c(o2)c1[nH]cc(Cl)n1)c1ccccc1	1312899-62-9	4H-1-Benzopyran-4-one, 5-[(4-fluorophenyl)methoxy]-3-hydroxy-7-methoxy-2-(5-meth	C27H21FN2O6	488.46	A/B	-8.6
4049	O(Cc1ccc(F)cc1)c1c2c(oc(c(O)c2=O)c2[nH]c(OC)nn2)cc(Cl)c1	1312900-65-4	4H-1-Benzopyran-4-one, 7-chloro-5-[(4-fluorophenyl)methoxy]-3-hydroxy-2-(3-methy	C19H13ClFN3O4	401.78	A/B	-8.6
4050	O(Cc1ccc(F)cc1)c1c2c(oc(c(O)c2=O)c2[nH]c(nn2)c2ccccc2)cc(Cl)c1	1312900-67-6	4H-1-Benzopyran-4-one, 7-chloro-2-(5-chloro-1H-1,2,4-triazol-3-yl)-5-[(4-fluorop	C18H10Cl2FN3O4	422.19	A/B	-8.6
4051	O(Cc1ccc(F)cc1)c1c2c(oc(c(O)c2=O)c2ncc(OC)cn2)c(C)c(Cl)c1	1312901-05-5	4H-1-Benzopyran-4-one, 7-chloro-5-[(4-fluorophenyl)methoxy]-3-hydroxy-8-methyl-2	C22H16ClFN2O4	426.82	A/B	-8.6
4052	O(Cc1ccc(F)cc1)c1c2c(oc(c(O)c2=O)c2[nH]c(C)cn2)c(C)c(Cl)c1	1312901-19-1	4H-1-Benzopyran-4-one, 7-chloro-5-[(4-fluorophenyl)methoxy]-3-hydroxy-2-(1H-imic	C20H14ClFN2O4	400.79	A/B	-8.6
4053	O(Cc1ccc(F)cc1)c1c2c(oc(c(O)c2=O)c2[nH]c(Cl)cn2)c(C)c(Cl)c1	1312901-21-5	4H-1-Benzopyran-4-one, 7-chloro-5-[(4-fluorophenyl)methoxy]-3-hydroxy-2-(5-metho	C21H16ClFN2O5	430.81	A/B	-8.6
4054	O(Cc1ccc(F)cc1)c1c2c(oc(c(O)c2=O)c2[nH]c(cn2)c2ccccc2)c(C)c(Cl)c1	1312901-22-6	4H-1-Benzopyran-4-one, 7-chloro-2-(5-chloro-1H-imidazol-2-yl)-5-[(4-fluorophenyl	C20H13Cl2FN2O4	435.23	A/B	-8.6
4055	O(Cc1ccc(F)cc1)c1c2c(oc(c(O)c2=O)c2ncc(OC)cn2)c(Cl)c(Cl)c1	1312902-05-8	4H-1-Benzopyran-4-one, 7,8-dichloro-5-[(4-fluorophenyl)methoxy]-3-hydroxy-2-(5-m	C21H13Cl2FN2O4	447.24	A/B	-8.6
4056	O(Cc1ccc(F)cc1)c1c2c(oc(c(O)c2=O)c2[nH]c(OC)nn2)c(Cl)c(Cl)c1	1312902-15-0	4H-1-Benzopyran-4-one, 7,8-dichloro-5-[(4-fluorophenyl)methoxy]-3-hydroxy-2-(3-m	C19H12Cl2FN3O4	436.22	A/B	-8.6
4057	O(Cc1ccc(F)cc1)c1c2c(oc(c(O)c2=O)c2[nH]c(Cl)nn2)c(Cl)c(Cl)c1	1312902-16-1	4H-1-Benzopyran-4-one, 7,8-dichloro-5-[(4-fluorophenyl)methoxy]-3-hydroxy-2-(5-m	C19H12Cl2FN3O5	452.22	A/B	-8.6
4058	O(Cc1ccc(F)cc1)c1c2c(oc(c(O)c2=O)c2[nH]c(C)cn2)c(Cl)c(Cl)c1	1312902-19-4	4H-1-Benzopyran-4-one, 7,8-dichloro-5-[(4-fluorophenyl)methoxy]-3-hydroxy-2-(1H-	C19H11Cl2FN2O4	421.21	A/B	-8.6
4059	O(Cc1ccc(F)cc1)c1c2c(oc(c(O)c2=O)c2[nH]c(Cl)cn2)c(Cl)c(Cl)c1	1312902-21-8	4H-1-Benzopyran-4-one, 7,8-dichloro-5-[(4-fluorophenyl)methoxy]-3-hydroxy-2-(5-m	C20H13Cl2FN2O5	451.23	A/B	-8.6
4060	O(Cc1ccc(F)cc1)c1c2c(oc(c(O)c2=O)c2[nH]c(cn2)c2ccccc2)c(Cl)c(Cl)c1	1312902-22-9	4H-1-Benzopyran-4-one, 7,8-dichloro-2-(5-chloro-1H-imidazol-2-yl)-5-[(4-fluoroph	C19H10Cl3FN2O4	455.65	A/B	-8.6
4061	O(Cc1ccc(F)cc1)c1c2c(c(c(Cl)c1)c1ccccc1)oc(c(O)c2=O)c1[nH]cc(Cl)n1	1312902-71-8	4H-1-Benzopyran-4-one, 7-chloro-5-[(4-fluorophenyl)methoxy]-3-hydroxy-2-(5-metho	C26H18ClFN2O5	492.88	A/B	-8.6
4062	O(Cc1ccc(F)cc1)c1c2c(oc(c(O)c2=O)c2ncc(Cl)cn2)cc(c1)c1ccccc1	1312903-06-2	4H-1-Benzopyran-4-one, 5-[(4-fluorophenyl)methoxy]-3-hydroxy-2-(5-methoxy-2-pyri	C27H19FN2O5	470.45	A/B	-8.6
4063	O(Cc1ccc(F)cc1)c1c2c(c(C)c(c1)c1ccccc1)oc(c(O)c2=O)c1[nH]c(OC)cn1	1312903-72-2	4H-1-Benzopyran-4-one, 5-[(4-fluorophenyl)methoxy]-3-hydroxy-8-methyl-2-(5-methy	C27H21FN2O4	456.46	A/B	-8.6
4064	O(C)c1c2c(c(OCc3ccc(F)cc3)cc1c1ccccc1)c(=O)c(O)c(o2)c1ncc(OC)cn1	1312904-26-9	4H-1-Benzopyran-4-one, 5-[(4-fluorophenyl)methoxy]-3-hydroxy-8-methoxy-2-(5-meth	C28H21FN2O5	484.48	A/B	-8.6
4065	O(Cc1ccc(F)cc1)c1c2c(c(Cl)c(c1)c1ccccc1)oc(c(O)c2=O)c1[nH]c(C)cn1	1312905-27-3	4H-1-Benzopyran-4-one, 8-chloro-5-[(4-fluorophenyl)methoxy]-3-hydroxy-2-(1H-imic	C25H16ClFN2O4	462.86	A/B	-8.6
4066	Oc1c(oc2c(c1=O)cc(OCc1ccc(F)cc1)cc2)c1[nH]c(OC)nn1	1312906-25-4	4H-1-Benzopyran-4-one, 6-[(4-fluorophenyl)methoxy]-3-hydroxy-2-(3-methyl-1H-1,2,	C19H14FN3O4	367.33	A/B	-8.6
4067	Cc1c2c(cc(OCc3ccc(F)cc3)c1)c(=O)c(O)c(o2)c1[nH]c(OC)cn1	1312906-80-1	4H-1-Benzopyran-4-one, 6-[(4-fluorophenyl)methoxy]-3-hydroxy-8-methyl-2-(5-methy	C21H17FN2O4	380.37	A/B	-8.6
4068	O=c1c2c(oc(c1O)c1[nH]c(cn1)c1ccccc1)c(C)cc(OCc1ccc(F)cc1)c2	1312906-82-3	4H-1-Benzopyran-4-one, 2-(5-chloro-1H-imidazol-2-yl)-6-[(4-fluorophenyl)methoxy]	C20H14ClFN2O4	400.79	A/B	-8.6
4069	O(C)c1c2c(cc(OCc3ccc(F)cc3)c1)c(=O)c(O)c(o2)c1[nH]ccn1	1312907-28-0	4H-1-Benzopyran-4-one, 6-[(4-fluorophenyl)methoxy]-3-hydroxy-8-methoxy-2-(3-pher	C25H18FN3O5	459.43	A/B	-8.6
4070	O=c1c2c(oc(c1O)c1[nH]cnn1)c(Cl)cc(OCc1ccc(F)cc1)c2	1312907-68-8	4H-1-Benzopyran-4-one, 8-chloro-6-[(4-fluorophenyl)methoxy]-3-hydroxy-2-(5-pheny	C26H16ClFN2O4	474.87	A/B	-8.6
4071	O=c1c2c(c(cc(OCc3ccc(F)cc3)c2)c2ccccc2)oc(c1O)c1ncc(Cl)cn1	1312908-16-9	4H-1-Benzopyran-4-one, 6-[(4-fluorophenyl)methoxy]-3-hydroxy-2-(5-methoxy-2-pyri	C27H19FN2O5	470.45	A/B	-8.6
4072	O=c1c2c(c(cc(OCc3ccc(F)cc3)c2)c2ccccc2)oc(c1O)c1[nH]c(C)cn1	1312908-29-4	4H-1-Benzopyran-4-one, 6-[(4-fluorophenyl)methoxy]-3-hydroxy-2-(1H-imidazol-2-yl	C25H17FN2O4	428.41	A/B	-8.6
4073	O=c1c2c(oc(c1O)c1[nH]c(nn1)c1ccccc1)cc(C)c(OCc1ccc(F)cc1)c2	1312908-77-2	4H-1-Benzopyran-4-one, 2-(5-chloro-1H-1,2,4-triazol-3-yl)-6-[(4-fluorophenyl)met	C19H13ClFN3O4	401.78	A/B	-8.6
4074	Cc1c2c(cc(OCc3ccc(F)cc3)c1C)c(=O)c(O)c(o2)c1ncc(OC)cn1	1312909-27-5	4H-1-Benzopyran-4-one, 6-[(4-fluorophenyl)methoxy]-3-hydroxy-7,8-dimethyl-2-(5-m	C23H19FN2O4	406.41	A/B	-8.6
4075	Cc1c2c(cc(OCc3ccc(F)cc3)c1C)c(=O)c(O)c(o2)c1ncc(cn1)c1ccccc1	1312909-29-7	4H-1-Benzopyran-4-one, 2-(5-chloro-2-pyrimidinyl)-6-[(4-fluorophenyl)methoxy]-3-	C22H16ClFN2O4	426.82	A/B	-8.6
4076	Cc1c2c(cc(OCc3ccc(F)cc3)c1C)c(=O)c(O)c(o2)c1[nH]c(C)cn1	1312909-41-3	4H-1-Benzopyran-4-one, 6-[(4-fluorophenyl)methoxy]-3-hydroxy-2-(1H-imidazol-2-yl	C21H17FN2O4	380.37	A/B	-8.6
4077	O(C)c1c2c(cc(OCc3ccc(F)cc3)c1C)c(=O)c(O)c(o2)c1ncc(OC)cn1	1312909-77-5	4H-1-Benzopyran-4-one, 6-[(4-fluorophenyl)methoxy]-3-hydroxy-8-methoxy-7-methyl-	C23H19FN2O5	422.41	A/B	-8.6
4078	Clc1c2c(cc(OCc3ccc(F)cc3)c1C)c(=O)c(O)c(o2)c1ncccn1	1312909-95-7	4H-1-Benzopyran-4-one, 6-[(4-fluorophenyl)methoxy]-3-hydroxy-8-methoxy-7-methyl-	C27H21FN2O5	472.46	A/B	-8.6
4079	Clc1c2c(cc(OCc3ccc(F)cc3)c1C)c(=O)c(O)c(o2)c1ncc(OC)cn1	1312910-27-2	4H-1-Benzopyran-4-one, 8-chloro-6-[(4-fluorophenyl)methoxy]-3-hydroxy-7-methyl-2	C22H16ClFN2O4	426.82	A/B	-8.6
4080	Clc1c2c(cc(OCc3ccc(F)cc3)c1C)c(=O)c(O)c(o2)c1[nH]c(nn1)c1ccccc1	1312910-39-6	4H-1-Benzopyran-4-one, 8-chloro-2-(5-chloro-1H-1,2,4-triazol-3-yl)-6-[(4-fluorop	C19H12Cl2FN3O4	436.22	A/B	-8.6
4081	Cc1c(c2c(cc1OCc1ccc(F)cc1)c(=O)c(O)c(o2)c1[nH]c(nn1)c1ccccc1)c1ccccc1	1312910-89-6	4H-1-Benzopyran-4-one, 2-(5-chloro-1H-1,2,4-triazol-3-yl)-6-[(4-fluorophenyl)met	C25H17ClFN3O4	477.87	A/B	-8.6
4082	O=c1c2c(oc(c1O)c1ncc(OC)cn1)cc(OC)c(OCc1ccc(F)cc1)c2	1312911-27-5	4H-1-Benzopyran-4-one, 6-[(4-fluorophenyl)methoxy]-3-hydroxy-7-methoxy-2-(5-meth	C22H17FN2O5	408.38	A/B	-8.6
4083	O=c1c2c(oc(c1O)c1[nH]c(nn1)c1ccccc1)cc(OC)c(OCc1ccc(F)cc1)c2	1312911-39-9	4H-1-Benzopyran-4-one, 2-(5-chloro-1H-1,2,4-triazol-3-yl)-6-[(4-fluorophenyl)met	C19H13ClFN3O5	417.77	A/B	-8.6
4084	O=c1c2c(oc(c1O)c1nnc(C)[nH]1)cc(Cl)c(OCc1ccc(F)cc1)c2	1312911-74-2	4H-1-Benzopyran-4-one, 7-chloro-6-[(4-fluorophenyl)methoxy]-3-hydroxy-2-(1H-1,2,	C18H11ClFN3O4	387.75	A/B	-8.6
4085	O=c1c2c(oc(c1O)c1[nH]c(OC)nn1)cc(Cl)c(OCc1ccc(F)cc1)c2	1312911-76-4	4H-1-Benzopyran-4-one, 7-chloro-6-[(4-fluorophenyl)methoxy]-3-hydroxy-2-(3-methy	C19H13ClFN3O4	401.78	A/B	-8.6
4086	Cc1c2c(cc(OCc3ccc(F)cc3)c1OC)c(=O)c(O)c(o2)c1[nH]ccn1	1312912-38-1	4H-1-Benzopyran-4-one, 6-[(4-fluorophenyl)methoxy]-3-hydroxy-7-methoxy-8-methyl-	C26H20FN3O5	473.45	A/B	-8.6
4087	Cc1c2c(cc(OCc3ccc(F)cc3)c1Cl)c(=O)c(O)c(o2)c1ncc(C)cn1	1312912-55-2	4H-1-Benzopyran-4-one, 7-chloro-6-[(4-fluorophenyl)methoxy]-3-hydroxy-8-methyl-2	C21H14ClFN2O4	412.8	A/B	-8.6
4088	Cc1c2c(cc(OCc3ccc(F)cc3)c1Cl)c(=O)c(O)c(o2)c1ncc(OC)cn1	1312912-57-4	4H-1-Benzopyran-4-one, 7-chloro-6-[(4-fluorophenyl)methoxy]-3-hydroxy-8-methyl-2	C22H16ClFN2O4	426.82	A/B	-8.6
4089	Cc1c2c(cc(OCc3ccc(F)cc3)c1Cl)c(=O)c(O)c(o2)c1[nH]c(Cl)nn1	1312912-79-0	4H-1-Benzopyran-4-one, 7-chloro-6-[(4-fluorophenyl)methoxy]-3-hydroxy-2-(5-metho	C20H15ClFN3O5	431.8	A/B	-8.6
4090	Cc1c2c(cc(OCc3ccc(F)cc3)c1Cl)c(=O)c(O)c(o2)c1[nH]c(nn1)c1ccccc1	1312912-81-4	4H-1-Benzopyran-4-one, 7-chloro-2-(5-chloro-1H-1,2,4-triazol-3-yl)-6-[(4-fluorop	C19H12Cl2FN3O4	436.22	A/B	-8.6
4091	Cc1c2c(cc(OCc3ccc(F)cc3)c1Cl)c(=O)c(O)c(o2)c1[nH]c(OC)cn1	1312912-87-0	4H-1-Benzopyran-4-one, 7-chloro-6-[(4-fluorophenyl)methoxy]-3-hydroxy-8-methyl-2	C21H16ClFN2O4	414.81	A/B	-8.6
4092	O(C)c1c2c(cc(OCc3ccc(F)cc3)c1OC)c(=O)c(O)c(o2)c1[nH]ccn1	1312913-38-4	4H-1-Benzopyran-4-one, 6-[(4-fluorophenyl)methoxy]-3-hydroxy-7,8-dimethoxy-2-(3-	C26H20FN3O6	489.45	A/B	-8.6
4093	O(C)c1c2c(cc(OCc3ccc(F)cc3)c1Cl)c(=O)c(O)c(o2)c1[nH]ccn1	1312913-83-9	4H-1-Benzopyran-4-one, 7-chloro-6-[(4-fluorophenyl)methoxy]-3-hydroxy-8-methoxy-	C25H17ClFN3O5	493.87	A/B	-8.6
4094	Clc1c2c(cc(OCc3ccc(F)cc3)c1OC)c(=O)c(O)c(o2)c1[nH]c(cn1)c1ccccc1	1312914-46-7	4H-1-Benzopyran-4-one, 8-chloro-2-(5-chloro-1H-imidazol-2-yl)-6-[(4-fluorophenyl	C20H13Cl2FN2O5	451.23	A/B	-8.6

4095	Clc1c2c(cc(OCc3ccc(F)cc3)c1Cl)c(=O)c(O)c(o2)c1[nH]cnn1	1312914-63-8	4H-1-Benzopyran-4-one, 7,8-dichloro-6-[(4-fluorophenyl)methoxy]-3-hydroxy-2-(5-	C26H15Cl2FN2O4	509.31	A/B	-8.6
4096	Clc1c2c(cc(OCc3ccc(F)cc3)c1Cl)c(=O)c(O)c(o2)c1[nH]c(nn1)c1ccccc1	1312914-81-0	4H-1-Benzopyran-4-one, 7,8-dichloro-2-(5-chloro-1H-1,2,4-triazol-3-yl)-6-[(4-flu	C18H9Cl3FN3O4	456.64	A/B	-8.6
4097	O(C)c1c(c2c(cc1OCc1ccc(F)cc1)c(=O)c(O)c(o2)c1ncc(C)cn1)c1ccccc1	1312915-10-8	4H-1-Benzopyran-4-one, 6-[(4-fluorophenyl)methoxy]-3-hydroxy-7-methoxy-8-phenyl-	C27H19FN2O5	470.45	A/B	-8.6
4098	O=c1c2c(cc(c(OCc3ccc(F)cc3)c2)c2ccccc2)oc(c1O)c1[nH]c(OC)nn1	1312916-42-9	4H-1-Benzopyran-4-one, 6-[(4-fluorophenyl)methoxy]-3-hydroxy-2-(3-methyl-1H-1,2,	C25H18FN3O4	443.43	A/B	-8.6
4099	O=c1c2c(cc(c(OCc3ccc(F)cc3)c2)c2ccccc2)oc(c1O)c1[nH]c(OC)cn1	1312916-47-4	4H-1-Benzopyran-4-one, 6-[(4-fluorophenyl)methoxy]-3-hydroxy-2-(5-methyl-1H-imic	C26H19FN2O4	442.44	A/B	-8.6
4100	O(Cc1ccc(F)cc1)c1c(c(c2c(c1)c(=O)c(O)c(o2)c1ncc(cn1)c1ccccc1)c1ccccc1)c1ccccc1	1312918-35-6	4H-1-Benzopyran-4-one, 2-(5-chloro-2-pyrimidinyl)-6-[(4-fluorophenyl)methoxy]-3-	C32H20ClFN2O4	550.96	A/B	-8.6
4101	O(Cc1ccc(F)cc1)c1c(c(c2c(c1)c(=O)c(O)c(o2)c1[nH]c(OC)nn1)c1ccccc1)c1ccccc1	1312918-43-6	4H-1-Benzopyran-4-one, 6-[(4-fluorophenyl)methoxy]-3-hydroxy-2-(3-methyl-1H-1,2,	C31H22FN3O4	519.52	A/B	-8.6
4102	O(Cc1ccc(F)cc1)c1c(c(c2c(c1)c(=O)c(O)c(o2)c1[nH]c(nn1)c1ccccc1)c1ccccc1)c1ccccc1	1312918-45-8	4H-1-Benzopyran-4-one, 2-(5-chloro-1H-1,2,4-triazol-3-yl)-6-[(4-fluorophenyl)met	C30H19ClFN3O4	539.94	A/B	-8.6
4103	O(Cc1ccc(F)cc1)c1c(c(c2c(c1)c(=O)c(O)c(o2)c1[nH]ccn1)c1ccccc1)c1ccccc1	1312918-46-9	4H-1-Benzopyran-4-one, 6-[(4-fluorophenyl)methoxy]-3-hydroxy-7,8-diphenyl-2-(3-	C36H24FN3O4	581.59	A/B	-8.6
4104	O(Cc1ccc(F)cc1)c1c(c(c2c(c1)c(=O)c(O)c(o2)c1[nH]c(OC)cn1)c1ccccc1)c1ccccc1	1312918-48-1	4H-1-Benzopyran-4-one, 6-[(4-fluorophenyl)methoxy]-3-hydroxy-2-(5-methyl-1H-imic	C32H23FN2O4	518.53	A/B	-8.6
4105	O(Cc1ccc(F)cc1)c1c(c(c2c(c1)c(=O)c(O)c(o2)c1[nH]c(cn1)c1ccccc1)c1ccccc1)c1ccccc1	1312918-50-5	4H-1-Benzopyran-4-one, 2-(5-chloro-1H-imidazol-2-yl)-6-[(4-fluorophenyl)methoxy]	C31H20ClFN2O4	538.95	A/B	-8.6
4106	[14C](=O)([C@@H](NC(=O)OC)C(C)C)N1[C@@H](C[C@H](C)C1)c1[nH]c(C#Cc2ccc(C#Cc3[nH]c(nc3)[C@H]3N([14C](=	1313207-89-4	Carbamic acid, N,N,Ä≤-[2,6-naphthalenediylbis[1H-imidazole-5,2-diyl[(2S,4S)-4-me	C40H52N8O6	740.89	A/B	-8.6
4107	O[C@H]1[C@@H](O[C@H](COC(=O)CCC(=O)OCc2c(non2=O)c2ccccc2)[C@H]1O)n1c(=O)[nH]c(=O)c(I)c1	1314187-74-0	Uridine, 5-iodo-2,Ä≤,3,Ä≤-O-(1-methylethylidene)-, 5,Ä≤-[4-[1-(2-oxido-4-phenyl	C26H27IN4O11	698.42	A/B	-8.6
4108	O=c1n([C@@H]2O[C@H](COP(=O)(OP(=O)(O)O)OP(=O)(O)O)C=C2)cc(C)c(=O)[nH]1	131466-86-9	1-Naphthalenesulfonic acid, 3-hydroxy-7-[3-[4-(2-phenyldiazenyl)phenyl]-2-triaze	C22H17N5O4S	447.47	A/B	-8.6
4109	C(Cn1c2c(nc1)c(=O)nc(N)[nH]2)C1(OC)COP(=O)(O)OC1	1321581-02-5	L-Alanine, N-(2,Ä≤-deoxy-2,Ä≤,2,Ä≤-difluoro-5-iodo-P-1-naphthalenyl-5,Ä≤-uridyl	C23H23F2IN3O9P	681.32	A/B	-8.6
4110	C(CCCc1cc(O)c(O)cc1)(O)c1cc(O)c(O)cc1	1330828-95-9	Benzoic acid, 4-[[4-(4-morpholinyl)-6-(phenylamino)-1,3,5-triazin-2-yl]amino]-,	C21H22N6O3	406.44	A/B	-8.6
4111	N(c1c(C([2H])([2H])[2H])c(c(/C=C/C#N)c(c1C([2H])([2H])[2H])[2H])[2H])c1nc(Nc2c(c(c(C#N)c(c2[2H])[2H]	1331866-56-8	Benzonitrile-2,3,5,6-d4, 4-[[4-[[4-[(1E)-2-cyanoethenyl]-2-methyl-6-(methyl-d3)	C22H9D9N6		A/B	-8.6
4112	N(c1c(C([2H])([2H])[2H])c(c(/C=C/C#N)c(c1C([2H])([2H])[2H])[2H])[2H])c1nc(Nc2c(c(c(C#N)c(c2[2H])[2H]	1331866-59-1	Benzonitrile-2,3,5,6-d4, 4-[[6-[[4-[(1E)-2-cyanoethenyl]-2-methyl-6-(methyl-d3)	C22H7D11N6		A/B	-8.6
4113	N(c1c(C([2H])([2H])[2H])c(c(/C(=C(/C#N)\[2H])/[2H])c(c1C([2H])([2H])[2H])[2H])c1nc(Nc2c(cc(C#N)	1331866-71-7	Benzonitrile-3,5-d2, 4-[[4-[[4-[(1E)-2-cyanoethenyl-1,2-d2]-2-methyl-6-(methyl-	C22H9D9N6		A/B	-8.6
4114	N(c1c(C)cc(/C=C/C#N)cc1C)c1nc(Nc2cc(c(C#N)c(c2)[2H])[2H])ncc1	1331866-96-6	Benzonitrile-3,5-d2, 4-[[6-[[4-[(1E)-2-cyanoethenyl]-2,6-di(methyl-d3)phenyl]ami	C22H8D10N6		A/B	-8.6
4115	O=c1n([C@@H]2O[C@H](CO[P@@](=O)(Oc3c(C)cccc3)N[C@H](C(=O)OCc3ccccc3)C)C=C2)cc(C)c(=O)[nH]1	1334335-87-3	L-Alanine, N-[[P(S)]-2,Ä≤,3,Ä≤-didehydro-3,Ä≤-deoxy-P-(2-methylphenyl)-5,Ä≤-thy	C27H30N3O8P	555.52	A/B	-8.6
4116	O=c1c2c(n(cn2)[C@H]2C[C@@H](CO)CO2)[nH]c(N)n1	133713-80-1	Guanosine, 3,Ä≤-deoxy-3,Ä≤-[(phenylmethoxy)methyl]-5,Ä≤-O-(phenylmethyl)-	C25H27N5O5	477.51	A/B	-8.6
4117	N(C(=O)C)[C@H]1[C@H](OC(CC)CC)C=C(C(=O)OC(OC(=O)[C@@H](C(C)C)N)C)C[C@@H]1NC(=N)N	1338231-75-6	2H-Isoindole-2-acetic acid, 1,3-dihydro-Œ±-(1-methylethyl)-1,3-dioxo-, 1-[[[(3R,	C40H57N5O12	799.91	A/B	-8.6
4118	N(S(=O)(=O)c1ccc(NC(=O)COCCN(CCOC)C)cc1)(C[C@H]([C@H](Cc1ccccc1)NC(=O)O[C@H]1CCOC1)O)CC(C)C	1347009-19-1	Spiro[3-azabicyclo[3.1.0]hexane-6,1,Ä≤-cyclopentane]-2-carboxamide, N-[3-amino-	C37H61N5O7S	719.97	A/B	-8.6
4119	C(N(C(=O)N[C@H](C(=O)N[C@@H](Cc1ccccc1)C[C@@H]([C@H](Cc1ccccc1)NC(=O)OCc1cncs1)O)C(C)C)C)c1nc(sc1)C1	1347061-31-7	Carbamic acid, N-[(1S,2R)-1-[[4-[[bis[[(1S)-2-oxo-1-(phenylmethyl)butyl]amino]ph	C51H67N4O12PS	991.14	A/B	-8.6
4120	[C@H](Cc1ccccc1)(C[C@@H]([C@H](Cc1ccccc1)NC(=O)OCc1cncs1)O)NC(=O)[C@@H](NC(=O)N(Cc1nc(C/C(=C/C)/C)sc	1347078-41-4	2,7,10,12-Tetraazatridecanoic acid, 4-hydroxy-12-methyl-9-(1-methylethyl)-8,11-	C38H49N7O5S2	747.97	A/B	-8.6
4121	C(n1c2c(nc1CC)c(C)cc(C)n2)c1ccc(cc1)c1c(S(=O)(=O)NC(=O)OCc2ccccc2)ccc(OCC)c1	1347083-02-6	2,7,10,12-Tetraazatridecanoic acid, 4-hydroxy-12-methyl-13-[2-[(2E)-2-methyl-2-	C39H50N6O5S2	746.98	A/B	-8.6
4122	C(CC)c1c(OC)ccc(C(=O)/C=C/c2ccc(O)cc2)c1O	1348029-87-7	Carbamic acid, N-[[4,Ä≤-[(2-ethyl-5,7-dimethyl-3H-imidazo[4,5-b]pyridin-3-yl)me	C28H32N4O4S	520.64	A/B	-8.6
4123	C(=O)(N[C@H](C(=O)C(=O)NC(CC)CC)CCC)[C@@H]1[C@@H]2[C@H](CN1C(=O)[C@@H](NC(=O)[C@@H](NC(=O)c1cnccn1)C	1348057-62-4	Carbamic acid, N-[[4,Ä≤-[(2-ethyl-5,7-dimethyl-3H-imidazo[4,5-b]pyridin-3-yl)me	C30H34N4O4S2	578.75	A/B	-8.6
4124	C([C@H]([C@H](Cc1ccccc1)NC(=O)[C@@H](NC(=O)c1nc2c(cc1)cccc2)CC(=O)NC(=O)[C@@H](NC(=O)CN)C(C)C)O)N1[C	1348437-32-0	Benzonitrile, 4-[[6-amino-5-bromo-2-[(4-ethynylphenyl)amino]-4-pyrimidinyl]oxy]-	C21H16BrN5O	434.29	A/B	-8.6
4125	N(C(=O)[C@H](Cc1ccccc1)C[C@H](CN1[C@@H](C(=O)NC(C)(C)C)CN(C(=O)Cc2ccccc2)CC1)O)[C@H]1c2c(C[C@H]1O)cc	1348955-75-8	Not Yet Assigned	C30H34D9F2N5O		A/B	-8.6
4126	[C@H](Cc1cncs1)(C[C@@H]([C@H](Cc1ccccc1)NC(=O)OCc1cncs1)O)NC(=O)[C@H](N(C(=O)NCc1nc(C(C)C)sc1)C)C(C)	1349128-70-6	Carbamic acid, N-[[4,Ä≤-[[5-(dimethylamino)-2-ethyl-7-methyl-3H-imidazo[4,5-b]p	C27H31N5O4S	521.63	A/B	-8.6
4127	S(=O)(=O)(/C=C/[C@@H](NC(=O)[C@H](NC(=O)N1CCN(C)CC1)CC(C)C)CCc1ccccc1)[C@H]1c2c(CC=C1)cccc2	1349498-10-7	2,7,10,12-Tetraazatridecanoic acid, 13-[2-(cyclohexylmethyl)-4-thiazolyl]-4-hydr	C41H54N6O6S	758.97	A/B	-8.6
4128	O(C)c1c2OC3(C(Oc2cc(c1)[C@@H]1c2c([C@H](OC(=O)C)[C@@H]4[C@@H]1C(=O)OC4)cc1c(c2)OCO1)(CCCC3)N1CCCC1)N	1349746-51-5	Carbamic acid, N-[[5-ethoxy-4,Ä≤-[(2-ethyl-5,7-dimethyl-3H-imidazo[4,5-b]pyridi	C30H36N4O5S	564.7	A/B	-8.6
4129	[C@H]([C@H](Cc1ccccc1)NC(=O)O[C@H]1CCOC1)(CN(S(=O)(=O)C1CC=C(N)C=C1)CC(C)C)OP(=O)(O)O	1349982-75-7	2,7,10,12-Tetraazatridecanoic acid, 13-(2-cyclohexyl-4-thiazolyl)-4-hydroxy-9-(	C39H50N6O5S2	746.98	A/B	-8.6
4130	C(=O)(N[C@H](C(=O)C(=O)NC1CC1)CCC)[C@@H]1[C@@H]2[C@H](CN1C(=O)[C@@H](NC(=O)[C@@H](NC(=O)c1ncccn1)C1C	1350075-96-5	Carbamic acid, N-[[4,Ä≤-[(2-ethyl-5,7-dimethyl-3H-imidazo[4,5-b]pyridin-3-yl)me	C32H40N4O4S	576.75	A/B	-8.6
4131	O=c1n(ccc(N)n1)[C@H]1O[C@@H](COC(=O)CCCCOC(=O)[C@H]([C@H](CC)C)N)SC1	1350080-03-3	2,7,10,12-Tetraazatridecanoic acid, 4-hydroxy-10-methyl-9-(1-methylethyl)-8,11-	C36H44N6O5S	672.84	A/B	-8.6
4132	C(CC(COC(=O)C)COC(=O)C)N1c2c(NC1)cnc(N)n2	1350231-70-7	Not Yet Assigned	C36H49N7O6	675.82	A/B	-8.6
4133	C(=O)([C@@H](NC(=O)OC)C(C)C)N1[C@@]2(C[C@H](C1)CC2)c1[nH]c(cn1)c1ccc(cc1)c1ccc(cc1)c1[nH]c(nc1)[C@H]	1350718-83-0	Carbamic acid, N,N,Ä≤-[[1,1,Ä≤-biphenyl]-4,4,Ä≤-diylbis[1H-imidazole-5,2-diyl-(	C44H54N8O6	790.95	A/B	-8.6
4134	C(=O)([C@@H](NC(=O)OC)C(C)C)N1[C@@]2(C[C@H](C1)CC2)c1[nH]c(cn1)c1c2c(c(cc1)c1ccc(cc1)c1[nH]c(nc1)[C@	1350719-01-5	Carbamic acid, N-[(1S)-1-[[(1S,4R)-1-[5-[4-[2,3-dihydro-7-[2-[(1S,4R)-2-[(2S)-2-	C47H58N8O6	831.01	A/B	-8.6
4135	C(=O)([C@@H](NC(=O)OC)C(C)C)N1C[C@@]2(C[C@H]1c1[nH]c(cn1)c1c(F)c(F)c(cc1)c1c(F)c(F)c(cc1)c1[nH]c(nc1	1351852-81-7	Carbamic acid, N-[(1S)-1-[[(2S)-2-[5-[3,3,Ä≤-difluoro-4,Ä≤-[2-[(5S,8S)-7-[(2S)-	C43H52F2N8O7	830.92	A/B	-8.6
4136	C(=O)([C@@H](NC(=O)OC)[C@@H](C)O)N1[C@@H](C[C@@H](C1)C1CC1)c1[nH]c(cn1)c1ccc(cc1)c1ccc(cc1)c1[nH]c(n	1351853-92-3	Carbamic acid, N-[(1R)-1-[[(2S)-2-[5-[4,Ä≤-[2-[(2S,4R)-4-cyclopropyl-1-[(2R)-2-	C41H50N8O6	750.89	A/B	-8.6
4137	C(=O)([C@@H](NC(=O)OC)C(C)C)N1[C@@H](C[C@@H](C1)C1CC1)c1[nH]c(cn1)c1ccc(cc1)c1ccc(cc1)c1[nH]c(nc1)[C	1351854-43-7	Carbamic acid, N-[(1S)-2-[(2S)-2-[5-[4,Ä≤-[2-[(2S,4R)-4-cyclopropyl-1-[(2S)-2-[	C51H54N8O6	875.02	A/B	-8.6
4138	C(=O)([C@@H](NC(=O)OC)C(C)C)N1[C@@H](C[C@@H](C1)C1CC1)c1[nH]c(cn1)c1c(CC)c(CC)c(cc1)c1c(CC)c(CC)c(cc	1351855-67-8	Carbamic acid, N-[(1S)-1-[[(2S)-2-[5-[4,Ä≤-[2-[(2S,4R)-4-cyclopropyl-1-[(2S)-2-	C47H62N8O6	835.05	A/B	-8.6
4139	C(=O)(N[C@H](CC1CC(O)C1)C(=O)C(=O)N)[C@@H]1[C@@H]2[C@@H](C2(C)C)CN1C(=O)[C@@H](NC(=O)NC(C)(C)C)C(C)(	1351992-77-2	Cyclopenta[c]pyrrole-1-carboxamide, (2S)-2-cyclohexyl-N-(2-pyrazinylcarbonyl)gly	C35H53N7O6	667.84	A/B	-8.6
4140	C(=O)([C@@H](NC(=O)OC)C(C)C)N1[C@@H](C=C(C1)C1CC1)c1[nH]c(cn1)c1ccc(C#Cc2ccc(cc2)c2[nH]c(nc2)[C@H]2N	1352391-70-8	Carbamic acid, N-[(1S)-1-[[(2S)-4-cyclopropyl-2,5-dihydro-2-[5-[4-[6-[2-[(2S)-1-	C47H54N8O6	826.98	A/B	-8.6
4141	N(c1c2c(n(cn2)[C@@H]2C[C@H](COC(=O)CNC(=O)CNC(=O)OCc3ccccc3)C=C2)nc(N)n1)C1CC1	1353547-59-7	Glycine, N-[(phenylmethoxy)carbonyl]-, [(1S,4R)-4-[2-amino-6-(cyclopropylamino)-	C24H27N7O4	477.52	A/B	-8.6
4142	C(CCNC(=O)C(=C)C)(P(=O)(O)O)C(=O)O	1354695-82-1	6H-Purin-6-one, 2-amino-1,9-dihydro-9-[(1R,3S,4R)-2-methylene-4-(phenylmethoxy)-	C26H27N5O3	457.52	A/B	-8.6
4143	C(C1C(=O)N(N=C1C)c1ccccc1)(c1c(O)n(nc1C)c1ccccc1)c1cn(c(=O)[nH]c1=O)[C@H]1O[C@@H](CO)[C@H](O)C1	1356470-37-5	2,4(1H,3H)-Pyrimidinedione, 1-(3,5-di-O-acetyl-2-deoxy-Œ≤-L-erythro-pentofuranos	C34H34N6O9	670.67	A/B	-8.6
4144	C(=O)(N[C@H](C(=O)C(=O)NC1CC1)CCC)[C@@H]1[C@@H]2[C@H](CN1C(=O)[C@H](NC(=O)[C@H](NC(=O)c1cnccn1)C1CCC	1358069-57-4	Carbamic acid, N,N,Ä≤-[2,6-biphenylenediylbis[1H-imidazole-5,2-diyl[(2S)-4,4-dir	C44H56N8O6	792.97	A/B	-8.6
4145	C(=O)([C@@H](NC(=O)OC)C1CCOCC1)N1[C@H]2[C@H](C2)C[C@H]1c1[nH]c(cn1)c1c2c(c(cc1)c1ccc(cc1)c1[nH]c(nc1	1359868-91-9	Carbamic acid, N-[(1S)-1-[[(1R,3S,5R)-3-[5-[4-[7-[2-[(1R,3S,5R)-2-[(2S)-2-[(meth	C42H48N10O6S	820.96	A/B	-8.6
4146	C(F)(F)c1n(c2c(n1)cccc2)c1nc(nc(NCCS(=O)(=O)C)n1)N1CCOCC1	1359953-72-2	Ethanesulfonamide, 2-[[4-[2-(difluoromethyl)-1H-benzimidazol-1-yl]-6-(4-morpholi	C23H24F2N8O3S	530.55	A/B	-8.6
4147	C(F)(F)c1n(c2c(n1)cccc2)c1nc(nc(NCc2ccc(N(C)C)cc2)n1)N1CCOCC1	1359955-99-9	1,3,5-Triazin-2-amine, 4-[2-(difluoromethyl)-1H-benzimidazol-1-yl]-N-[(1-methyl-	C20H21F2N9O	441.44	A/B	-8.6
4148	N(C12CC3CC(C2)CC(C1)C3)c1ncc(C(=O)NO)cn1	1375454-41-3	[1,1,Ä≤-Biphenyl]-2,2,Ä≤-disulfonic acid, 4,4,Ä≤-bis[2-(4-amino-7-sulfo-1-napht	C32H24N6O12S4	812.83	A/B	-8.6
4149	C(=O)([C@@H](NC(=O)OC)C1CCOCC1)N1[C@@H]([C@@H]2C[C@H]1CC2)c1[nH]c(cn1)c1ccc(cc1)c1ccc(cc1)c1[nH]c(nc	1377036-32-2	Carbamic acid, N-[(1S)-1-[[(2S)-2-[7-[4-[2-[(1R,3S,4S)-2-[(2S)-2-[(methoxycarbor	C44H52N8O6	788.93	A/B	-8.6
4150	C(=O)([C@H](NC(=O)OC)c1cc(C)ccc1)N1[C@@H](CCC1)c1[nH]c(cn1)c1ccc(cc1)c1cc2c(cc1)cc(cc2)c1[nH]c(nc1)[	1377038-49-7	Carbamic acid, N-[(1S)-1-[[(2S)-2-[5-[6-[4-[2-[(2S)-1-[(2R)-2-(ethylamino)-2-phe	C47H52N8O4	792.97	A/B	-8.6
4151	C(=O)([C@H](NC(=O)OC)c1ccccc1)N1[C@@H](CCC1)c1[nH]c(cn1)c1ccc(cc1)c1cc2c(cc1)cc(cc2)c1[nH]c(nc1)[C@H	1377040-83-9	Carbamic acid, N,N,Ä≤-[2,7-phenanthrenediylbis[1H-imidazole-5,2-diyl-(2S)-2,1-py	C42H50N8O6	762.9	A/B	-8.6
4152	C(=O)([C@@H](NC(=O)OC)C1CCOCC1)N1[C@@H]([C@@H]2C[C@H]1CC2)c1[nH]c(cn1)c1cc(C#CC)c(cc1)c1cc2c(cc1)c1c	1377041-59-2	Carbamic acid, N-[(1S)-2-[(1R,3S,4S)-3-[5-[2-fluoro-4-[2-[(1R,3S,5R)-2-[(2S)-2-[	C47H53FN8O7	860.97	A/B	-8.6
4153	C(=O)([C@@H](NC(=O)OC)C(C)C)N1[C@@H]([C@@H]2C[C@H]1CC2)c1[nH]c(cn1)c1cc(C#CC)c(cc1)c1ccc(cc1)c1[nH]c	1377041-62-7	Carbamic acid, N-[(1S)-2-[(1R,3S,4S)-3-[5-[4-[2-[(2S)-1-[(2S)-2-[(methoxycarbony	C49H56N8O7	869.02	A/B	-8.6
4154	C(=O)([C@@H](NC(=O)OC)C(C)C)N1CC2(C[C@H]1c1[nH]c3c(n1)ccc1c3ccc(c1)c1ccc(cc1)c1[nH]c(nc1)[C@H]1N(C(=	1377043-41-8	Carbamic acid, N-[(1S)-1-[[(2S)-2-[5-chloro-4-[4-[2-[(1R,3S,4S)-2-[(2S)-2-[(meth	C44H51ClN8O6	823.38	A/B	-8.6
4155	Fc1c2c(c3c(nc([nH]3)[C@H]3N(C(=O)[C@@H](NC(=O)OC)C(C)C)CCC3)cc2)ccc1c1cc2c(cc1)c1c(cc2)nc([nH]1)[C@H	1377049-84-7	Carbamic acid, N-[(1R)-2-[(2S,4S)-2-[5-[1,11-dihydro-2-[(2S,5S)-1-[(2S)-2-[(meth	C49H54N8O8	883	A/B	-8.6
4156	O=c1c2c(n(cn2)[C@@H]2O[C@H](COP(=O)(Oc3ccccc3)N[C@@H](C(=O)OCC)C)OC2)[nH]c(N)n1	1380386-52-6	Carbamic acid, N,N,Ä≤-[[1,1,Ä≤-binaphthalene]-5,5,Ä≤-diylbis[1H-imidazole-5,2-d	C48H54N8O6	838.99	A/B	-8.6
4157	O=c1c2c(n(cn2)[C@@H]2O[C@H](CO)[C@@H](O)[C@H]2O)[nH]c(n1)n1cc(nn1)c1ccc(C)cc1	1384519-78-1	Inosine, 2-(4-phenyl-1H-1,2,3-triazol-1-yl)-	C18H17N7O5	411.37	A/B	-8.6
4158	O=c1c2c(n(cn2)[C@@H]2O[C@H](CO)[C@@H](O)[C@H]2O)[nH]c(n1)n1cc(nn1)c1ccc(OC)cc1	1384519-79-2	Inosine, 2-[4-(4-methylphenyl)-1H-1,2,3-triazol-1-yl]-	C19H19N7O5	425.4	A/B	-8.6
4159	O(C)c1c2c([C@H]([C@@H]3[C@@H]([C@H]2O)COC3=O)c2cc(OC)c(OC)c(OC)cc2)cc2c1OCO2	138709-78-1	2-Naphthalenesulfonic acid, 5-[2-(4-amino-2-methylphenyl)diazenyl]-8-[2-(4-sulfc	C23H19N5O6S2	525.56	A/B	-8.6
4160	O=C1C2(C(C(C(=O)/C=C(\O)/C3C4(c5c(NC4=O)cccc5)NC(C3c3cc(OC)c(O)cc3)c3ccccc3)C(N2)c2ccccc2)c2cc(OC)c(	1389335-36-7	Spiro[3H-indole-3,2,Ä≤-pyrrolidin]-2(1H)-one, 4,Ä≤-[(2Z)-3-[1,2-dihydro-4,Ä≤-(3	C41H40N4O8	716.78	A/B	-8.6
4161	Nc1sc(c2nc(nc(n2)N2CCOCC2)N2CCOCC2)cn1	1391924-08-5	Benzamide, 3-[[4-(2-amino-5-pyrimidinyl)-6-(4-morpholinyl)-1,3,5-triazin-2-yl]am	C20H23N9O2	421.46	A/B	-8.6
4162	Fc1ccc(c2nc(nc(n2)N2CCOCC2)N2CCNCC2)cn1	1391924-89-2	Benzamide, 3-[4-[[2-(dimethylamino)-2-oxoethyl]amino]-6-(4-morpholinyl)-1,3,5-tr	C18H23N7O3	385.42	A/B	-8.6
4163	N([C@H](C(=O)O)C)c1nc(nc(n1)N1CCOCC1)c1cc(NS(=O)(=O)C)ccc1	1391925-04-4	Acetamide, 2-[[4-[3-(difluoromethyl)phenyl]-6-(4-morpholinyl)-1,3,5-triazin-2-yl	C18H22F2N6O2	392.4	A/B	-8.6
4164	N(CC(=O)N(C)C)c1nc(nc(n1)N1CCOCC1)c1ccc(N)nc1	1391925-34-0	Glycine, N-[4-(4-morpholinyl)-6-(3-nitrophenyl)-1,3,5-triazin-2-yl]-	C15H16N6O5	360.32	A/B	-8.6
4165	O(C(=O)CCCC)[C@H]1[C@@H](O[C@H](COC(=O)C2=CN(C)C=CC2)[C@H]1OC(=O)CCCC)n1nc(C(=O)N)nc1	139621-19-5	1H-1,2,4-Triazole-3-carboxamide, 1-[2,3-di-O-benzoyl-5-O-[(1,4-dihydro-1-methyl-	C29H27N5O8	573.55	A/B	-8.6
4166	O([Si](C(C)(C)C)(C)C)[C@H]1[C@@H](O[C@H](CO)[C@H]1O[Si](C(C)(C)C)(C)C)n1c(=O)[nH]c(=O)c(C)c1	139621-32-2	3-Pyridinecarboxamide, N-[[1-[2,3-di-O-acetyl-5-O-(3-pyridinylcarbonyl)-Œ≤-D-rit	C24H22N6O9	538.47	A/B	-8.6
4167	S(=O)(=O)(O)c1c2c(c(N=Nc3ccc(O)cc3)cc1)cccc2	139630-70-9	1H-1,2,4-Triazole-3-carboxamide, 1-[2,3-O-cyclopentylidene-5-O-(3-pyridinylcarbc	C19H21N5O6	415.4	A/B	-8.6
4168	O[C@H]1[C@H](n2c3c(nc2)c(N)ncn3)O[C@H](C[Se]CC#C)[C@H]1O	1397199-07-3	Furo[3,Ä≤,4,Ä≤:6,7]naphtho[2,3-d]-1,3-dioxol-6(5aH)-one, 5-(1,3-benzodioxol-5-y	C21H18O8	398.36	A/B	-8.6
4169	N(c1c(C)cc(/C=C/C#N)cc1C)c1nc(Nc2ccc(C#N)cc2)ncc1.C(C(=O)O)C(=O)O	1399384-96-3	Ethanesulfonic acid, compd. with 4-[[4-[[4-[(1E)-2-cyanoethenyl]-2,6-dimethylphe	C22H18N6.C2H6O3S		A/B	-8.6
4170	O=C1c2n(C[C@H]3N1[C@](C([2H])([2H])[2H])(C(C(O3)([2H])[2H])([2H])[2H])[2H])cc(C(=O)NC(c1c(F)cc(F)cc1	1407166-90-8	2H-Pyrido[1,Ä≤,2,Ä≤:4,5]pyrazino[2,1-b][1,3]oxazine-12-d-9-carboxamide, N-[(2,4	C20H15D4F2N3O5		A/B	-8.6
4171	O=C1c2n(C[C@H]3N1[C@H](C)C(C(O3)([2H])[2H])([2H])[2H])cc(C(=O)NC(c1c(F)cc(F)cc1)([2H])[2H])c(=O)c2O	1407166-93-1	2H-Pyrido[1,Ä≤,2,Ä≤:4,5]pyrazino[2,1-b][1,3]oxazine-2,3,4-d3-9-carboxamide, N-[	C20H12D7F2N3O5		A/B	-8.6
4172	O=C1c2n(C[C@H]3N1[C@](C([2H])([2H])[2H])(CCO3)[2H])cc(C(=O)NC(c1c(F)cc(F)cc1)([2H])[2H])c(=O)c2O	1407166-94-2	2H-Pyrido[1,Ä≤,2,Ä≤:4,5]pyrazino[2,1-b][1,3]oxazine-2,3-d2-9-carboxamide, N-[(2	C20H13D6F2N3O5		A/B	-8.6
4173	O=C1c2n(C[C@H]3N1[C@H](C([2H])([2H])[2H])CCO3)cc(C(=O)NC(c1c(F)cc(F)cc1)([2H])[2H])c(=O)c2O	1407166-95-3	2H-Pyrido[1,Ä≤,2,Ä≤:4,5]pyrazino[2,1-b][1,3]oxazine-4-d-9-carboxamide, N-[(2,4-	C20H13D6F2N3O5		A/B	-8.6
4174	O=C1c2n(C[C@H]3N1[C@](C)(CCO3)[2H])cc(C(=O)NC(c1c(F)cc(F)cc1)([2H])[2H])c(=O)c2O	1407166-96-4	2H-Pyrido[1,Ä≤,2,Ä≤:4,5]pyrazino[2,1-b][1,3]oxazine-9-carboxamide, N-[(2,4-difl	C20H14D5F2N3O5		A/B	-8.6
4175	O=C1c2n(C([C@H]3N1[C@H](C)C(C(O3)([2H])[2H])([2H])[2H])([2H])[2H])cc(C(=O)NC(c1c(F)cc(F)cc1)([2H])[2	1407167-00-3	2H-Pyrido[1,Ä≤,2,Ä≤:4,5]pyrazino[2,1-b][1,3]oxazine-2,3,4,12-d4-9-carboxamide,	C20H10D9F2N3O5		A/B	-8.6
4176	O=C1c2n(C([C@H]3N1[C@](C([2H])([2H])[2H])(CCO3)[2H])([2H])[2H])cc(C(=O)NC(c1c(F)cc(F)cc1)([2H])[2H])	1407167-01-4	2H-Pyrido[1,Ä≤,2,Ä≤:4,5]pyrazino[2,1-b][1,3]oxazine-2,3,12-d3-9-carboxamide, N-	C20H11D8F2N3O5		A/B	-8.6
4177	O=C1c2n(C([C@H]3N1[C@H](C([2H])([2H])[2H])CCO3)([2H])[2H])cc(C(=O)NC(c1c(F)cc(F)cc1)([2H])[2H])c(=O)	1407167-02-5	2H-Pyrido[1,Ä≤,2,Ä≤:4,5]pyrazino[2,1-b][1,3]oxazine-4,12-d2-9-carboxamide, N-[(	C20H11D8F2N3O5		A/B	-8.6
4178	O=C1c2n(C([C@]3(N1[C@H](C)CCO3)[2H])([2H])[2H])cc(C(=O)NC(c1c(F)cc(F)cc1)([2H])[2H])c(=O)c2O	1407167-04-7	2H-Pyrido[1,Ä≤,2,Ä≤:4,5]pyrazino[2,1-b][1,3]oxazine-4,12-d2-9-carboxamide, N-[(	C20H14D5F2N3O5		A/B	-8.6
4179	O=C1c2n(C[C@]3(N1[C@](C([2H])([2H])[2H])(C(C(O3)([2H])[2H])([2H])[2H])[2H])[2H])cc(C(=O)NC(c1c(F)cc(	1407167-05-8	2H-Pyrido[1,Ä≤,2,Ä≤:4,5]pyrazino[2,1-b][1,3]oxazine-12-d-9-carboxamide, N-[(2,4	C20H14D5F2N3O5		A/B	-8.6
4180	O=C1c2n(C[C@]3(N1[C@H](C)CCO3)[2H])cc(C(=O)NC(c1c(F)cc(F)cc1)([2H])[2H])c(=O)c2O	1407167-12-7	2H-Pyrido[1,Ä≤,2,Ä≤:4,5]pyrazino[2,1-b][1,3]oxazine-4-d-9-carboxamide, N-[(2,4-	C20H15D4F2N3O5		A/B	-8.6
4181	O=C1c2n(C([C@]3(N1[C@H](C([2H])([2H])[2H])C(C(O3)([2H])[2H])([2H])[2H])[2H])([2H])[2H])cc(C(=O)NC(c1	1407167-13-8	2H-Pyrido[1,Ä≤,2,Ä≤:4,5]pyrazino[2,1-b][1,3]oxazine-9-carboxamide, N-[(2,4-difl	C20H16D3F2N3O5		A/B	-8.6
4182	O=C1c2n(C([C@]3(N1[C@](C([2H])([2H])[2H])(CCO3)[2H])[2H])([2H])[2H])cc(C(=O)NC(c1c(F)cc(F)cc1)([2H])	1407167-16-1	2H-Pyrido[1,Ä≤,2,Ä≤:4,5]pyrazino[2,1-b][1,3]oxazine-2,3,12-d3-9-carboxamide, N-	C20H10D9F2N3O5		A/B	-8.6
4183	O=C1c2n(C([C@]3(N1[C@H](C([2H])([2H])[2H])CCO3)[2H])([2H])[2H])cc(C(=O)NC(c1c(F)cc(F)cc1)([2H])[2H])	1407167-17-2	2H-Pyrido[1,Ä≤,2,Ä≤:4,5]pyrazino[2,1-b][1,3]oxazine-4,12-d2-9-carboxamide, N-[(	C20H10D9F2N3O5		A/B	-8.6
4184	O=C1c2n(C([C@]3(N1[C@](C)(CCO3)[2H])[2H])([2H])[2H])cc(C(=O)NC(c1c(F)cc(F)cc1)([2H])[2H])c(=O)c2O	1407167-18-3	2H-Pyrido[1,Ä≤,2,Ä≤:4,5]pyrazino[2,1-b][1,3]oxazine-12-d-9-carboxamide, N-[(2,4	C20H11D8F2N3O5		A/B	-8.6
4185	O=C1c2n(C([C@]3(N1[C@](C([2H])([2H])[2H])(C(C(O3)([2H])[2H])([2H])[2H])[2H])[2H])([2H])[2H])cc(C(=O)	1407167-19-4	2H-Pyrido[1,Ä≤,2,Ä≤:4,5]pyrazino[2,1-b][1,3]oxazine-4,12-d29-carboxamide, N-[(2	C20H13D6F2N3O5		A/B	-8.6
4186	O=C1c2n(C([C@H]3N1[C@H](C([2H])([2H])[2H])CCO3)([2H])[2H])cc(C(=O)NCc1c(F)cc(F)cc1)c(=O)c2O	1407167-38-7	2H-Pyrido[1,Ä≤,2,Ä≤:4,5]pyrazino[2,1-b][1,3]oxazine-4,12-d2-9-carboxamide, N-[(	C20H13D6F2N3O5		A/B	-8.6
4187	C(OC[C@@H]1[C@@H](OC(=O)C(C)(C)C)[C@@H](O)[C@@H](O1)n1nc(C(=O)N)nc1)(c1ccc(OC)cc1)(c1ccc(OC)cc1)c1cc	140891-23-2	1H-1,2,4-Triazole-3-carboxamide, 1-[5-O-[bis(4-methoxyphenyl)phenylmethyl]-2-O-	C34H38N4O8	630.69	A/B	-8.6
4188	N(Cc1ccc(Br)cn1)C12CC3CC(C2)CC(C1)C3	140942-35-4	1-Naphthalenesulfonic acid, 7,7,Ä≤-[1,2-ethanediylbis[(3-sulfo-4,1-phenylene)az	C34H28N6O12S4.4Na		A/B	-8.6
4189	[C@H](C(=O)N1[C@@H](CC[C@@H]1C)c1[nH]c(cn1)c1c(F)cc(cc1)c1ccc(cc1)c1[nH]c(nc1)[C@H]1N(C(=O)[C@@H](NC	1412814-65-3	Carbamic acid, N,N,Ä≤-[(3-methyl[1,1,Ä≤-biphenyl]-4,4,Ä≤-diyl)bis[1H-imidazole-	C51H64N8O8	917.1	A/B	-8.6
4190	O[C@H]1[C@@H](O[C@H](C(=O)N[C@H](Cc2ccccc2)C(=O)O)[C@H]1O)n1nc(C(=O)N)nc1	1414802-06-4	Not Yet Assigned	C19H18N6O6	426.38	A/B	-8.6
4191	C(=O)([C@@H](NC(=O)OC)C(C)C)N1[C@H](c2[nH]c(c3c4ccc(c3)CCc3cc(cc(CC4)cc3)c3ccc(cc3)c3[nH]c(nc3)[C@H]3	1415119-55-9	Carbamic acid, N,N,Ä≤-[tricyclo[8.2.2.24,7]hexadeca-4,6,10,12,13,15-hexaene-5,1	C50H60N8O6	869.06	A/B	-8.6
4192	C(N(NC(=O)[C@@H](NC(=O)OC([2H])([2H])[2H])C(C([2H])([2H])[2H])(C([2H])([2H])[2H])C([2H])([2H])[2H])C	1415634-42-2	2,5,6,10,13-Pentaazatetradecanedioic acid, 3-[1,1-di(methyl-d3)ethyl-2,2,2-d3]-	C38H40D12N6O8		A/B	-8.6
4193	C(F)(F)c1n(c2c(n1)cccc2)c1nc(nc(n1)N1CCOCC1)N1CCN(C(=O)[C@H](C)N)CC1	1416956-30-3	Acetamide, N-[2-[4-[4-[2-(difluoromethyl)-1H-benzimidazol-1-yl]-6-(4-morpholinyl	C23H27F2N9O3	515.52	A/B	-8.6
4194	C(F)(F)c1n(c2c(n1)cccc2)c1nc(nc(n1)N1CCOCC1)N1CCN(C(=O)CCN)CC1	1416956-32-5	1-Propanone, 2-amino-1-[4-[4-[2-(difluoromethyl)-1H-benzimidazol-1-yl]-6-(4-morp	C22H27F2N9O2	487.51	A/B	-8.6
4195	C(=O)([C@H](NC(=O)OC)C(C)C)N1[C@H](CCC1)c1[nH]c(cn1)c1ccc(cc1)c1ccc(cc1)c1[nH]c(nc1)[C@H]1N(C(=O)[C@	1417333-65-3	Carbamic acid, N,N,Ä≤-[[1,1,Ä≤-biphenyl]-4,4,Ä≤-diylbis[1H-imidazole-5,2-diyl-2	C40H50N8O6	738.88	A/B	-8.6
4196	C(COCP1(=O)OCC(OC(=O)C)CO1)n1c2c(nc1)c(N)ncn2	1417393-20-4	9H-Purin-6-amine, 9-[2-[[5-(4-nitrophenyl)-2-oxido-1,3,2-dioxaphosphorinan-2-yl]	C17H19N6O6P	434.34	A/B	-8.6
4197	[C@H](Cc1ccccc1)(C(=O)N[C@H](CCc1ccccc1)C=CS(=O)(=O)c1ccccc1)NC(=O)N1CCN(C)CC1	1428441-71-7	4-Morpholinecarboxamide, N-[(1S)-2-oxo-2-[[(1R)-1-(2-phenylethyl)-3-(phenylsulfc	C31H35N3O5S	561.69	A/B	-8.6
4198	[C@H](Cc1ccccc1)(C(=O)N/C(=C/CS(=O)(=O)c1ccccc1)/CCc1ccccc1)NC(=O)N1CCOCC1	1428441-81-9	1-Piperazinecarboxamide, 4-methyl-N-[(1S)-2-oxo-1-(phenylmethyl)-2-[[(1S)-1-(phe	C31H36N4O4S	560.71	A/B	-8.6
4199	C(=O)([C@@H](NC(=O)OC)C(C)C)N1[C@@H]2c3nc(c([nH]3)COCCCON=C(C)O[C@@H](C2)C1)c1ccc(cc1)c1ccc(cc1)c1[n	1431625-79-4	Carbamic acid, N-[(1S)-1-[[(2S)-2-[5-[4,Ä≤-[(2S,5S)-3-[(2S)-2-[(methoxycarbonyl)	C45H59N9O10S	918.07	A/B	-8.6
4200	C(OCCOC(=O)[C@@](C(C([2H])([2H])[2H])(C([2H])([2H])[2H])[2H])(N)[2H])n1c2c(nc1)c(=O)nc(N)[nH]2.Cl	1432060-78-0	Not Yet Assigned	C37H41D7N4O5		A/B	-8.6
4201	N(C(=O)C)[C@H]1[C@H]([C@@H]([C@@H](CO)O)O)OC(=C[C@@H]1NC(=N)NC(=O)Cc1c(OC)cccc1)C(=O)O	1442563-08-7	D-glycero-D-galacto-Non-2-enonic acid, 5-(acetylamino)-2,6-anhydro-3,4,5-trideox	C24H28N4O8	500.5	A/B	-8.6
4202	N(c1c2c(n(cn2)[C@@H]2C[C@@H](CO)C=C2)nc(N)n1)C1CC1	144334-87-2	Benzoic acid, 3,3,Ä≤-methylenebis[6-[(2-aminobenzoyl)amino]-	C29H24N4O6	524.52	A/B	-8.6
4203	C([C@H](OCP(=O)(O)O)C)n1c2c(nc1)c(Cl)nc(N)n2	1443648-71-2	L-Alanine, N-[[P(S),2,Ä≤R]-5-bromo-2,Ä≤-deoxy-2,Ä≤-fluoro-4,Ä≤-C-fluoro-2,Ä≤-me	C26H29BrF2N3O9P	676.4	A/B	-8.6
4204	S(c1n(c2c(n1)c(=O)nc(N)[nH]2)[C@H]1[C@H](O)[C@H]2[C@H](O1)COP(=O)(O)O2)c1ccc(OC)cc1	144509-93-3	Guanosine, 8-[(2-hydroxyphenyl)thio]-, cyclic 3,Ä≤,5,Ä≤-(hydrogen phosphate)	C16H16N5O8PS	469.37	A/B	-8.6
4205	S(c1n(c2c(n1)c(=O)nc(N)[nH]2)[C@H]1[C@H](O)[C@H]2[C@H](O1)COP(=O)(O)O2)c1ccc(F)cc1	144509-95-5	Guanosine, 8-[(4-methoxyphenyl)thio]-, cyclic 3,Ä≤,5,Ä≤-(hydrogen phosphate)	C17H18N5O8PS	483.39	A/B	-8.6
4206	O(c1nc(Nc2ccc(C#N)cc2)nc(N)c1Br)c1c(C)cc(C#N)cc1C.CO	1445668-99-4	Benzonitrile, 4-[[6-amino-5-bromo-2-[(4-cyanophenyl)amino]-4-pyrimidinyl]oxy]-3,	C20H15BrN6O.C2H4O2		A/B	-8.6
4207	C(NC(=O)N1CCN(CC1)c1c(N(=O)=O)ccc(F)c1F)(C)C12CC3CC(C2)CC(C1)C3	1446259-44-4	1-Piperazinecarboxamide, N-(1-tricyclo[3.3.1.13,7]dec-1-ylethyl)-4-(2,4,5-triflu	C23H30F3N3O	421.5	A/B	-8.6
4208	O=C1[C@@H]2[C@@H](c3c([C@H](OC(=O)c4ccc(Cl)cc4)[C@H]2CO1)cc1c(c3)OCO1)c1c(Br)c(OC)c(OC)c(OC)c1	1449602-06-5	Benzoic acid, 3-chloro-, (5R,5aR,8aR,9S)-9-(2-bromo-3,4,5-trimethoxyphenyl)-5,5a	C29H24BrClO9	631.85	A/B	-8.6
4209	CC1(C)COc2c(-c3c(cc(cc3)c3c(CC)nc([nH]3)[C@H]3N(C(=O)[C@@H](NC(=O)OC)C(C)CCC3)OC1)ccc(c2)c1c(CC)nc	1449762-21-3	Carbamic acid, N,N,Ä≤-[(7,8-dihydro-7,7-dimethyl-6H-dibenzo[f,h][1,5]dioxonin-3,	C45H58N8O8	838.99	A/B	-8.6
4210	N(CCCCNc1nc(nc(NCCO)n1)N1CCOCC1)c1c2c(ncc1)cc(Cl)cc2	1454694-97-3	1,3,5-Triazine-2,4-diamine, N2-[3-[(7-chloro-4-quinolinyl)amino]propyl]-6-(4-mor	C25H27ClN8O	490.99	A/B	-8.6
4211	O=c1n(cc(CC#C)c(=O)[nH]1)[C@@H]1O[C@H](CO)[C@@H](O)[C@H]1O	146356-53-8	Furo[3,Ä≤,4,Ä≤:6,7]naphtho[2,3-d]-1,3-dioxol-6(5aH)-one, 5-[3,5-dimethoxy-4-(ph	C28H26O7	474.5	A/B	-8.6

4212	O=c1c2c(n(cn2)[C@H]2C[C@H](O[Si](C(C)C)(C(C)C)C(C)C)[C@@H](CO[Si](C(C)C)(C(C)C)C(C)C)O2)[nH]c(NC(=O)	14721-48-3	2-Naphthalenesulfonic acid, 8,8,Ä≤-[(3,3,Ä≤-dimethyl[1,1,Ä≤-biphenyl]-4,4,Ä≤-di	C34H28N6O6S2	680.75	A/B	-8.6
4213	P(=O)(CC(=O)OCCC)(OC(C)C)OC(C)C	1473359-00-0	Benzeneacetic acid, Œ±-[[[5-[(2-amino-1,6-dihydro-6-oxo-9H-purin-9-yl)methoxy]-2	C20H25N6O8P	508.42	A/B	-8.6
4214	C(OC[C@H]1N(C(=O)OC(C)(C)C)[C@H]([C@H](O)[C@@H]1OC(=O)[C@@H](NC(=O)OC(C)(C)C)C(C)C)c1c2c([nH]c1)c(N)	1474023-35-2	L-Valine, N-[(1,1-dimethylethoxy)carbonyl]-, (2S,3S,4R,5R)-2-(4-amino-5H-pyrrolo	C45H54N6O8	806.95	A/B	-8.6
4215	C(n1c2c(nc1CC)c(C)cc(C)n2)c1ccc(cc1)c1c(S(=O)(=O)NC(=O)OCC2CC2)cccc1	148401-86-9	Carbamic acid, [[4,Ä≤-[(2-ethyl-5,7-dimethyl-3H-imidazo[4,5-b]pyridin-3-yl)methy	C31H30N4O4S	554.66	A/B	-8.6
4216	C(n1c2c(nc1CC)c(C)cc(C)n2)c1ccc(cc1)c1c(S(=O)(=O)NC(=O)NCC)cccc1	148401-89-2	[1,1,Ä≤-Biphenyl]-2-sulfonamide, 4,Ä≤-[(2-ethyl-5,7-dimethyl-3H-imidazo[4,5-b]p	C25H27N5O3S	477.58	A/B	-8.6
4217	O[C@H]1[C@H](n2c3c(nc2)c(N)ncn3)O[C@H](COP(=O)(OP(=O)(OP(=O)(OP(=O)(OC[C@H]2O[C@H]([C@H](O)[C@@H]2O)	148402-13-5	[1,1,Ä≤-Biphenyl]-2-sulfonamide, N-[(dimethylamino)carbonyl]-4,Ä≤-[(2-ethyl-5,7	C26H29N5O3S	491.61	A/B	-8.6
4218	C(#N)[C@]1(C)[C@@H](O[C@H](CO)[C@H]1O)n1c(=O)nc(NC(=O)C)cc1	149099-88-7	L-Isoleucine, 3-(iodo-125I)-N-(3-pyridinylcarbonyl)-L-tyrosyl-N6-[N2-[(phenylmet	C52H68IN13O11		A/B	-8.6
4219	C(=C\C(=O)c1c(OC)cccc1O)/c1c(OC)ccc(CCCCCCCCC)c1	149731-74-8	Uridine, thymidylyl-(3,Ä≤,Üí5,Ä≤)-2,Ä≤-deoxy-5,6-dihydro-6-hydroxy-, (6S)-	C19H27N4O13P	550.41	A/B	-8.6
4220	C([C@H]([C@@H](NC(=O)[C@@H](NC(=O)c1nc2c(cc1)cccc2)[C@@H]1CCSC1)Cc1ccccc1)O)N1[C@H](C(=O)NC(C)(C)C	150378-17-9	D-erythro-Pentonamide, 2,3,5-trideoxy-N-[(1S,2R)-2,3-dihydro-2-hydroxy-1H-inden-	C36H47N5O4	613.79	A/B	-8.6
4221	O=c1n([C@@H]2O[C@H](CO)[C@@H](O)C2)cc(C#C[Si](C)(C)C)c(=O)[nH]1	151345-35-6	Uridine 5,Ä≤-(tetrahydrogen triphosphate), 5-[(1E)-3-[[[3,Ä≤,6,Ä≤-bis(dimethyla	C37H40N5O18P3	935.66	A/B	-8.6
4222	C(n1c2c(nc1CC)c(C)cc(C)n2)c1ccc(cc1)c1c(S(=O)(=O)NC(=O)OCCCCC)sc(CC(C)C)c1	151488-11-8	Carbamic acid, N-[[3-[4-[(2-ethyl-5,7-dimethyl-3H-imidazo[4,5-b]pyridin-3-yl)met	C30H38N4O4S2	582.78	A/B	-8.6
4223	N(C(=O)C)[C@H]1[C@H]([C@@H]([C@@H](CO)O)O)OC(=C[C@@H]1N=[N+]=[N-])C(=O)O	151780-52-8	2-Naphthalenesulfonic acid, 8,8,Ä≤-[1,2-ethenediylbis[(3-sulfo-4,1-phenylene)-2,	C34H26N6O12S4	838.86	A/B	-8.6
4224	C(=O)(NC(C(=O)C(=O)NCC=C)CCCC)[C@@H]1[C@@H]2[C@@H](C2(C)C)CN1C(=O)[C@@H](NC(=O)N[C@@H](CN1C(=O)CC(C)	1528752-81-9	3-Azabicyclo[3.1.0]hexane-2-carboxamide, 3-[(2S)-2-[[[[(1R)-1-[(2,6-dioxo-1-pipe	C36H58N6O7	686.88	A/B	-8.6
4225	C(=O)(NCc1ccc(F)cc1)c1c(OC(OC(=O)C)C(C)C)c(=O)n(C)c(C(NC(=O)c2oc(C)nn2)(C)C)n1	1538604-11-3	Propanoic acid, 2,2-dimethyl-, [[4-[[[(4-fluorophenyl)methyl]amino]carbonyl]-1,6	C26H31FN6O7	558.56	A/B	-8.6
4226	O(C(OC(=O)C(C)C)C)c1c(C(=O)NCc2ccc(F)cc2)nc(C(NC(=O)c2nnc(C)o2)(C)C)n(C)c1=O	1538604-19-1	Cyclopropanecarboxylic acid, 1-[[4-[[[(4-fluorophenyl)methyl]amino]carbonyl]-1,6	C27H31FN6O7	570.57	A/B	-8.6
4227	O(C(OC(=O)c1ccccc1)C(C)C)c1c(C(=O)NCc2ccc(F)cc2)nc(C(NC(=O)c2nnc(C)o2)(C)C)n(C)c1=O	1538604-25-9	4-Pyrimidinecarboxamide, 5-[1-(benzoyloxy)propoxy]-N-[(4-fluorophenyl)methyl]-1,	C30H31FN6O7	606.6	A/B	-8.6
4228	C(n1c2c(nc1CC)c(C)cc(C)n2)c1ccc(cc1)c1c(S(=O)(=O)NC(=O)CCCCC)ccc(CCC)c1	154551-73-2	Hexanamide, N-[[5-ethyl-4,Ä≤-[(2-ethyl-5,7-dimethyl-3H-imidazo[4,5-b]pyridin-3-y	C31H38N4O3S	546.72	A/B	-8.6
4229	C(n1c2c(nc1CC)c(C)cc(C)n2)c1ccc(cc1)c1c(S(=O)(=O)NC(=O)CCCCC)ccc(OC)c1	154551-78-7	Hexanamide, N-[[4,Ä≤-[(2-ethyl-5,7-dimethyl-3H-imidazo[4,5-b]pyridin-3-yl)methyl	C33H42N4O3S	574.78	A/B	-8.6
4230	C(n1c2c(nc1CC)c(C)cc(C)n2)c1ccc(cc1)c1c(S(=O)(=O)NC(=O)CCCCC)ccc(C(CC)(C)C)c1	154551-83-4	Hexanamide, N-[[4,Ä≤,Ä≤-[(2-ethyl-5,7-dimethyl-3H-imidazo[4,5-b]pyridin-3-yl)me	C35H38N4O3S	594.77	A/B	-8.6
4231	C(n1c2c(nc1CC)c(C)cc(C)n2)c1ccc(cc1)c1c(S(=O)(=O)NC(=O)CCCCC)ccc(CN2CCOCC2)c1	154551-87-8	Hexanamide, N-[[5-[(diethylamino)methyl]-4,Ä≤-[(2-ethyl-5,7-dimethyl-3H-imidazo	C34H45N5O3S	603.82	A/B	-8.6
4232	C(n1c2c(nc1CC)c(C)cc(C)n2)c1ccc(cc1)c1c(S(=O)(=O)NC(=O)CCCCC)ccc(OC(C)C)c1	154551-89-0	Hexanamide, N-[[4,Ä≤-[(2-ethyl-5,7-dimethyl-3H-imidazo[4,5-b]pyridin-3-yl)methyl	C31H35F3N4O4S	616.69	A/B	-8.6
4233	C(n1c2c(nc1CC)c(C)cc(C)n2)c1ccc(cc1)c1c(S(=O)(=O)NC(=O)COCCC)ccc(C(C)(C)C)c1	154552-04-2	Acetamide, 2-butoxy-N-[[5-(1,1-dimethylpropyl)-4,Ä≤-[(2-ethyl-5,7-dimethyl-3H-in	C34H44N4O4S	604.8	A/B	-8.6
4234	C(n1c2c(nc1CC)c(C)cc(C)n2)c1ccc(cc1)c1c(S(=O)(=O)NC(=O)COCC)ccc(C(C)(C)C)c1	154552-24-6	Acetamide, N-[[5-(1,1-dimethylpropyl)-4,Ä≤-[(2-ethyl-5,7-dimethyl-3H-imidazo[4,5	C32H40N4O4S	576.75	A/B	-8.6
4235	C(n1c2c(nc1CC)c(C)cc(C)n2)c1ccc(cc1)c1c(S(=O)(=O)NC(=O)NCCCC)ccc(CCCC)c1	154552-34-8	[1,1,Ä≤-Biphenyl]-2-sulfonamide, N-[(butylamino)carbonyl]-4,Ä≤-[(2-ethyl-5,7-di	C31H39N5O3S	561.74	A/B	-8.6
4236	C(n1c2c(nc1CC)c(C)cc(C)n2)c1ccc(cc1)c1c(S(=O)(=O)NC(=O)NCCCC)ccc(c1)c1ccccc1	154552-42-8	[1,1,Ä≤-Biphenyl]-2-sulfonamide, N-[(butylamino)carbonyl]-5-ethoxy-4,Ä≤-[(2-eth	C30H37N5O4S	563.71	A/B	-8.6
4237	C(n1c2c(nc1CC)c(C)cc(C)n2)c1ccc(cc1)c1c(S(=O)(=O)NC(=O)CCc2ccccc2)ccc(C(CC)(C)C)c1	154552-62-2	Benzenepropanamide, N-[[4,Ä≤,Ä≤-[(2-ethyl-5,7-dimethyl-3H-imidazo[4,5-b]pyridin	C38H36N4O3S	628.78	A/B	-8.6
4238	C(n1c2c(nc1CC)c(C)cc(C)n2)c1ccc(cc1)c1c(S(=O)(=O)NC(=O)c2c(Oc3ccccc3)cccc2)ccc(C(C)C)c1	154552-74-6	Benzamide, N-[[5-butyl-4,Ä≤-[(2-ethyl-5,7-dimethyl-3H-imidazo[4,5-b]pyridin-3-yl	C40H40N4O4S	672.84	A/B	-8.6
4239	C(n1c2c(nc1CC)c(C)cc(C)n2)c1ccc(cc1)c1c(S(=O)(=O)NC(=O)c2c(OCC)cccc2)ccc(c1)c1ccccc1	154553-01-2	Benzamide, 2-ethoxy-N-[[5-ethoxy-4,Ä≤-[(2-ethyl-5,7-dimethyl-3H-imidazo[4,5-b]py	C34H36N4O5S	612.74	A/B	-8.6
4240	C(n1c2c(nc1CC)c(C)cc(C)n2)c1ccc(cc1)c1c(S(=O)(=O)NC(=O)c2c(OCC)cccc2)ccc(OC(C)C)c1	154553-06-7	Benzamide, N-[[5-[(diethylamino)methyl]-4,Ä≤-[(2-ethyl-5,7-dimethyl-3H-imidazo[4	C37H43N5O4S	653.83	A/B	-8.6
4241	C(n1c2c(nc1CC)c(C)cc(C)n2)c1ccc(cc1)c1c(S(=O)(=O)NC(=O)c2ccccc2)ccc(CN(C)C)c1	154553-16-9	Benzamide, N-[[5-butoxy-4,Ä≤-[(2-ethyl-5,7-dimethyl-3H-imidazo[4,5-b]pyridin-3-y	C34H36N4O4S	596.74	A/B	-8.6
4242	C(n1c2c(nc1CC)c(C)cc(C)n2)c1ccc(cc1)c1c(S(=O)(=O)NC(=O)COCCC)ccc(C(C)(C)C)c1	154553-42-1	Acetamide, N-[[5-(1,1-dimethylpropyl)-4,Ä≤-[(2-ethyl-5,7-dimethyl-3H-imidazo[4,5	C33H42N4O4S	590.78	A/B	-8.6
4243	C(n1c2c(nc1CC)c(C)cc(C)n2)c1ccc(cc1)c1c(S(=O)(=O)NC(=O)COCCC)ccc(OC(C)C)c1	154553-46-5	Acetamide, N-[[4,Ä≤-[(2-ethyl-5,7-dimethyl-3H-imidazo[4,5-b]pyridin-3-yl)methyl]	C30H33F3N4O5S	618.67	A/B	-8.6
4244	C(=O)([C@@H](NC(=O)OC)C(C)C)N1[C@@H](C[C@H](O)C1)c1[nH]c(cn1)c1ccc(cc1)c1ccc(cc1)c1[nH]c(nc1)[C@H]1N	1555813-40-5	Carbamic acid, N-[(1S)-1-[[(5S)-2-hydroxy-5-[5-[4,Ä≤-[2-[(8S)-7-[(2S)-2-[(methox	C42H52N8O9	812.91	A/B	-8.6
4245	C(=O)([C@@H](NC(=O)OC)C(C)C)N1[C@H](c2[nH]c(c3c4c(C5CC4CC5)c(cc3)c3ccc(cc3)c3[nH]c(nc3)[C@H]3N(C(=O)	1560639-12-4	Carbamic acid, N-[(1S)-1-[[(2S)-4,4-dimethyl-2-[5-[4-[1,2,3,4-tetrahydro-8-[2-[(	C49H64N8O6	861.08	A/B	-8.6
4246	C(=O)([C@@H](NC(=O)OC)C(C)C)N1[C@H](c2[nH]c(c3ccc(c4c5c(c(cc4)c4ccc(cc4)c4[nH]c(nc4)[C@H]4N(C(=O)[C@	1560640-25-6	Carbamic acid, N-[(1S)-1-[[(2S)-4,4-dimethyl-2-[5-[4-[1,2,3,4-tetrahydro-8-[2-[(	C50H66N8O6	875.11	A/B	-8.6
4247	C(=O)([C@@H](NC(=O)OC)C(C)C)N1[C@H](c2[nH]c(c3c4c(c(cc3)c3ccc(cc3)c3[nH]c(nc3)[C@H]3N(C(=O)[C@@H](NC	1560649-96-8	Carbamic acid, N-[(1S)-2-methyl-1-[[(2S)-2-[5-[4-[1,2,3,4-tetrahydro-8-[2-[(2S)-	C44H55N9O6	805.96	A/B	-8.6
4248	O=C1C2C(c3c(C(SCC)C2CO1)cc1c(c3)OCO1)c1cc(OC)c(OC)c(OC)c1	156350-44-6	Carbonic acid, 4-[(5R,5aR,8aR)-5,5a,6,8,8a,9-hexahydro-6,9-dioxofuro[3,Ä≤,4,Ä≤:	C29H24O10	532.49	A/B	-8.6
4249	C([C@H](OC[P@](=O)(Oc1ccc(C(=O)OCC)cc1)NC(C(=O)OC(C)C)(C)C)C)n1c2c(nc1)c(N)ncn2	1571076-35-1	Alanine, N-[(S)-[[(1R)-2-(6-amino-9H-purin-9-yl)-1-methylethoxy]methyl][4-(trifl	C23H30F3N6O5P	558.49	A/B	-8.6
4250	C(=O)([C@@H](NC(=O)OC)C(C)C)N1CC2(C[C@H]1c1[nH]c(cn1)c1ccc(cc1)c1ccc(cc1)c1[nH]c(nc1)[C@H]1N(C(=O)[C	1571138-18-5	Carbamic acid, N-[(1S)-1-[[(2S)-2-[5-[4,Ä≤-[2-[(3R)-2-[(2S)-2-[(methoxycarbonyl)	C44H57N9O6	807.98	A/B	-8.6
4251	C(=O)([C@@H](NC(=O)OC)C(C)C)N1CC2(C[C@H]1c1[nH]c(cn1)c1ccc(cc1)c1ccc(cc1)c1[nH]c(nc1)[C@H]1N(C(=O)[C	1571138-33-4	2,6-Diazaspiro[3.4]octane-2-carboxylic acid, 6-[(2S)-2-[(methoxycarbonyl)amino]-	C47H61N9O8	880.04	A/B	-8.6
4252	C(=O)([C@@H](NC(=O)OC)C(C)C)N1CC2(C[C@@H]1c1[nH]c(cn1)c1ccc(cc1)c1ccc(cc1)c1[nH]c(nc1)[C@H]1N(C(=O)[	1571138-38-9	Carbamic acid, N-[(1S)-1-[[(2S)-2-[5-[4,Ä≤-[2-[(7S)-6-[(2S)-2-[(methoxycarbonyl)	C42H53N9O6	779.93	A/B	-8.6
4253	C(=O)([C@@H](NC(=O)OC)C(C)C)N1CC2(C[C@@H]1c1[nH]c(cn1)c1ccc(cc1)c1ccc(cc1)c1[nH]c(nc1)[C@H]1N(C(=O)[C	1571138-42-5	Carbamic acid, N-[(1S)-1-[[(2S)-2-[5-[4,Ä≤-[2-[(7S)-2-acetyl-6-[(2S)-2-[(methoxy	C44H55N9O7	821.96	A/B	-8.6
4254	O=c1n([C@@H]2O[C@H](COP(=O)(Oc3ccc(SC)cc3)Oc3ccc(SC)cc3)[C@@H](N=[N+]=[N-])C2)cc(C)c(=O)[nH]1	158171-92-7	5,Ä≤-Thymidylic acid, 3,Ä≤-azido-3,Ä≤-deoxy-, bis(4-cyanophenyl) ester	C24H20N7O7P		A/B	-8.6
4255	O=c1n([C@@H]2O[C@H](COP(=O)(Oc3ccc(I)cc3)Oc3ccc(I)cc3)[C@@H](N=[N+]=[N-])C2)cc(C)c(=O)[nH]1	158171-94-9	5,Ä≤-Thymidylic acid, 3,Ä≤-azido-3,Ä≤-deoxy-, bis[4-(trifluoromethyl)phenyl] es	C24H20F6N5O7P		A/B	-8.6
4256	O[C@H]1[C@H](n2c3c(nc2)c(N)ncn3)O[C@H](COP(=O)(O[C@@H]2[C@@H](COP(=O)(O)O)O[C@H]([C@@H]2O)n2c(=O)[nH	158614-78-9	Guanosine, 5,Ä≤-O-phosphonoadenylyl-(3,Ä≤,Üí5,Ä≤)-cytidylyl-(3,Ä≤,Üí5,Ä≤)-, hom	(C29H38N13O21P3)x		A/B	-8.6
4257	O=C1[C@@H]2[C@@H](c3c([C@H](OCc4cn(nn4)c4c(O)cccc4)[C@H]2CO1)cc1c(c3)OCO1)c1cc(OC)c(OC)c(OC)c1	1600500-29-5	Furo[3,Ä≤,4,Ä≤:6,7]naphtho[2,3-d]-1,3-dioxol-6(5aH)-one, 9-[[1-(3-chloro-4-fluo	C31H27ClFN3O8	624.01	A/B	-8.6
4258	O=C1[C@@H]2[C@@H](c3c([C@H](OCc4cn(nn4)c4cc(Br)ccc4)[C@H]2CO1)cc1c(c3)OCO1)c1cc(OC)c(OC)c(OC)c1	1600500-33-1	Furo[3,Ä≤,4,Ä≤:6,7]naphtho[2,3-d]-1,3-dioxol-6(5aH)-one, 5,8,8a,9-tetrahydro-9-	C31H28IN3O8	697.47	A/B	-8.6
4259	O=C1[C@@H]2[C@@H](c3c([C@H](O)[C@H]2CO1)cc1c(c3)OCO1)c1cc2c(cc1)cccc2	1610886-44-6	Furo[3,Ä≤,4,Ä≤:6,7]naphtho[2,3-d]-1,3-dioxol-6(5aH)-one, 5-(1,3-benzodioxol-5-y	C20H16O7	368.34	A/B	-8.6
4260	O=C1[C@@H]2[C@@H](c3c([C@@H](O)[C@H]2CO1)cc1c(c3)OCO1)c1cc2c(cc1)cccc2	1610886-58-2	Furo[3,Ä≤,4,Ä≤:6,7]naphtho[2,3-d]-1,3-dioxol-6(5aH)-one, 5,8,8a,9-tetrahydro-9-	C23H18O5	374.39	A/B	-8.6
4261	[C@H](C(=O)N1[C@@H]2[C@@H](C2)C[C@H]1C#N)(N)C12CC3(OC(=O)[C@@H](CO)NC)CC(C2)CC(C1)C3	1616608-73-1	L-Serine, N-methyl-, 3-[(1S)-1-amino-2-[(1S,3S,5S)-3-cyano-2-azabicyclo[3.1.0]he	C22H32N4O4	416.51	A/B	-8.6
4262	[C@H](C(=O)N1[C@@H]2[C@@H](C2)C[C@H]1C#N)(N)C12CC3(OC(=O)[C@@H](CC(=O)O)N)CC(C2)CC(C1)C3	1616609-11-0	L-Aspartic acid, 1-[3-[(1S)-1-amino-2-[(1S,3S,5S)-3-cyano-2-azabicyclo[3.1.0]hex	C22H30N4O5	430.5	A/B	-8.6
4263	C(=O)(N[C@@H](CC1CCC1)C(=O)C(=O)N)[C@@H]1[C@@H]2[C@@H](C2(C)C)CN1C(=O)[C@@H](NC(=O)NC1(Cc2ccccc2)CCC	1616696-00-4	3-Azabicyclo[3.1.0]hexane-2-carboxamide, N-[(1S)-3-amino-1-(cyclobutylmethyl)-2,	C35H51N5O5	621.81	A/B	-8.6
4264	C([C@H](OC[P@](=O)(OP(=O)(CO[C@@H](Cn1c2c(nc1)c(N)ncn2)C)O)NCCCC)C)n1c2c(nc1)c(N)ncn2	1619914-57-6	Amidodiphosphonic acid, P,P,Ä≤-bis[[(1R)-2-(6-amino-9H-purin-9-yl)-1-methyletho	C25H33N11O6P2	645.55	A/B	-8.6
4265	N(C(=O)[C@H](Cc1cccnc1)C[C@@H](CN1[C@H](C(=O)NC(C)(C)C)CN(Cc2cccnc2)CC1)O)[C@H]1c2c(C[C@H]1O)cccc2	162105-22-8	D-erythro-Pentonamide, 2,3,5-trideoxy-N-[(1S,2R)-2,3-dihydro-2-hydroxy-1H-inden-	C36H45N5O4S2	675.9	A/B	-8.6
4266	N(C(=O)[C@H](Cc1ccncc1)C[C@@H](CN1[C@H](C(=O)NC(C)(C)C)CN(Cc2cc3c(s2)ccs3)CC1)O)[C@H]1c2c(C[C@H]1O)c	162105-31-9	1-Piperazinepentanamide, N-(2,3-dihydro-2-hydroxy-1H-inden-1-yl)-2-[[(1,1-dimeth	C36H45N5O4S2	675.9	A/B	-8.6
4267	C[C@]1(F)[C@@H](O[C@H](COP(=O)(Oc2ccccc2)N[C@H](C(=O)OCC(C)C)C)[C@H]1O)n1c(=O)[nH]c(=O)c(c1[2H])[2H]	1621154-32-2	L-Alanine, N-[(2,Ä≤R)-2,Ä≤-deoxy-2,Ä≤-fluoro-2,Ä≤-methyl-P-phenyl-5,Ä≤-uridylyl	C26H27D2FN3O9P		A/B	-8.6
4268	F[C@H]1[C@@H](O[C@H](CO)[C@H]1O)n1c(=O)c(C(=O)N)nc(F)c1	1621883-56-4	2-Pyrazinecarboxamide, 4-[2-deoxy-2-fluoro-3,5-bis-O-(phenylmethyl)-Œ≤-D-ribofur	C24H23F2N3O5	471.45	A/B	-8.6
4269	N(C(=O)[C@H](Cc1ccccc1)C[C@@H](CN1[C@H](C(=O)NC(C)(C)C)CN(Cc2cn(=O)ccc2)CC1)O)[C@H]1c2c(C[C@H]1O)ccc	162518-56-1	D-erythro-Pentonamide, 2,3,5-trideoxy-N-[(1S,2S,3S)-2,3-dihydro-2,3-dihydroxy-1H	C36H47N5O5	629.79	A/B	-8.6
4270	N(c1nc(nc(n1)N1CCOCC1)c1ccc(OC)cc1)c1c(C)cccc1	1627734-69-3	1,3,5-Triazin-2-amine, N-(3-chlorophenyl)-4-(4-methoxyphenyl)-6-(4-morpholinyl)-	C20H20ClN5O2	397.86	A/B	-8.6
4271	N(c1nc(nc(n1)N1CCOCC1)c1ccc(OC)cc1)c1cc(C)ccc1	1627734-70-6	1,3,5-Triazin-2-amine, 4-(4-methoxyphenyl)-N-(2-methylphenyl)-6-(4-morpholinyl)-	C21H23N5O2	377.44	A/B	-8.6
4272	C(=O)([C@@H](NC(=O)OC)C(C)C)N1[C@@H](CCC1)c1[nH]c(cn1)c1ccc(C#CC#Cc2[nH]c(nc2)[C@H]2N(C(=O)[C@H](NC(	1627734-81-9	1,3,5-Triazine, 2-(4-methoxyphenyl)-4-[4-(4-methoxyphenyl)-1-piperazinyl]-6-(4-m	C25H30N6O3	462.54	A/B	-8.6
4273	O=C1[C@@H]2[C@@H](c3c([C@H](OC(=O)c4c(N(=O)=O)cc(OC)cc4)[C@H]2CO1)cc1c(c3)OCO1)c1cc(OC)c(OC)c(OC)c1	1633020-32-2	Furo[3,Ä≤,4,Ä≤:6,7]naphtho[2,3-d]-1,3-dioxol-6(5aH)-one, 5,8,8a,9-tetrahydro-9-	C29H25NO11	563.51	A/B	-8.6
4274	O=C1[C@@H]2[C@@H](c3c([C@H](OC(=O)c4c(OC)c(OC)ccc4)[C@H]2CO1)cc1c(c3)OCO1)c1cc(OC)c(OC)c(OC)c1	1633020-35-5	Benzeneacetic acid, Œ±-[(3,4-dimethoxyphenyl)methylene]-4-methoxy-, (5R,5aR,8aR,	C40H38O12	710.72	A/B	-8.6
4275	O=C1[C@@H]2[C@@H](c3c([C@H](OC(=O)/C(=C/c4cc(OCc5ccccc5)c(OC)cc4)/c4cc(OC)c(OC)cc4)[C@H]2CO1)cc1c(c3	1633020-40-2	Benzeneacetic acid, 3,4-dimethoxy-Œ±-[[3-methoxy-4-(phenylmethoxy)phenyl]methyle	C47H44O13	816.84	A/B	-8.6
4276	O=C1[C@@H]2[C@@H](c3c([C@H](OC(=O)/C(=C/c4cc(OC)c(OC)c(OC)c4)/c4cc(OC)ccc4)[C@H]2CO1)cc1c(c3)OCO1)c1	1633020-41-3	Benzeneacetic acid, 3,4-dimethoxy-Œ±-[[4-methoxy-3-(phenylmethoxy)phenyl]methyle	C47H44O13	816.84	A/B	-8.6
4277	[C@@H](C(CC)CC)(NC(=O)C)[C@H]1[C@H](NC(=N)N)C[C@H](C(=O)OCCCC(=O)OC[C@H]2N[C@H]([C@H](O)[C@@H]2O)c2c	1638370-20-3	Cyclopentanecarboxylic acid, 3-[(1S)-1-(acetylamino)-2-ethylbutyl]-4-[(aminoimir	C29H45N9O8	647.72	A/B	-8.6
4278	O=c1c2c(n(cn2)[C@@H]2C(=C)[C@H](COP(=O)(Oc3c4c(OCO4)ccc3)N[C@H](C(=O)OC(C)C)C)[C@@H](O)C2)[nH]c(N)n1	1638770-00-9	L-Alanine, N-[(S)-[[(1R,3S,5S)-3-(2-amino-1,6-dihydro-6-oxo-9H-purin-9-yl)-5-hyd	C25H31N6O9P	590.52	A/B	-8.6
4279	O=c1c2c(n(cn2)[C@@H]2C(=C)[C@H](CO[P@@](=O)(Oc3c4c(OCO4)ccc3)N[C@H](C(=O)OC(C)C)C)[C@@H](O)C2)[nH]c(	1638770-02-1	L-Alanine, N-[[[(1R,3S,5S)-3-(2-amino-1,6-dihydro-6-oxo-9H-purin-9-yl)-5-hydroxy	C25H31N6O9P	590.52	A/B	-8.6
4280	C(=O)([C@@H](NC(=O)NC)C(C)C)N1[C@@H](C=CC1)c1[nH]c(cn1)c1ccc(cc1)c1ccc(cc1)c1[nH]c(nc1)[C@H]1N(C(=O)	1639871-05-8	Carbamic acid, N-[(1S)-1-[[(2S)-2-[5-[4,Ä≤-[2-[(2S)-1-[(2S)-2-[[(dimethylamino)c	C41H51N9O5	749.9	A/B	-8.6
4281	N(c1nc(nc(Oc2ccc(C#N)cc2)n1)N1CCOCC1)c1cc(C)ccc1	1643332-90-4	Benzonitrile, 4-[[4-[(4-chlorophenyl)amino]-6-(4-morpholinyl)-1,3,5-triazin-2-yl	C20H17ClN6O2	408.84	A/B	-8.6
4282	C(=O)([C@@H](NC(=O)OC)C(C)C)N1[C@@H](CC[C@H]1C)c1[nH]c(cn1)c1ccc(cc1)c1ccc(cc1)c1[nH]c(nc1)[C@H]1N(C	1645563-32-1	Carbamic acid, N,N,Ä≤-[[1,1,Ä≤-biphenyl]-4,4,Ä≤-diylbis[1H-imidazole-5,2-diyl-(	C44H54N8O6	790.95	A/B	-8.6
4283	C(=O)([C@@H](NC(=O)OC)C(C)C)N1[C@@H](C[C@H](C)[C@@H]1C)c1[nH]c(cn1)c1ccc(cc1)c1ccc(cc1)c1[nH]c(nc1)[	1645563-39-8	Carbamic acid, N,N,Ä≤-[[1,1,Ä≤-biphenyl]-4,4,Ä≤-diylbis[1H-imidazole-5,2-diyl[(	C42H54N8O6	766.93	A/B	-8.6
4284	O=c1n([C@@H]2O[C@H](COP(=O)(C(O)c3ccc(N(=O)=O)cc3)OC[C@@H]3[C@@H](N=[N+]=[N-])C[C@@H](O3)n3c(=O)[nH]	164652-11-3	Thymidine, 2,Ä≤,3,Ä≤-didehydro-P,3,Ä≤-dideoxy-P-(1-hydroxyoctyl)thymidylyl-(5,Ä	C28H39N4O10P	622.6	A/B	-8.6
4285	N(C(=O)[C@H](Cc1ccccc1)C[C@@H](CN1[C@H](C(=O)NC(C)(C)C)CN(Cc2c(Br)cccc2)CC1)O)[C@H]1c2c(C[C@H]1O)ccc	164725-46-6	1-Piperazinepentanamide, N-(2,3-dihydro-2-hydroxy-1H-inden-1-yl)-2-[[(1,1-dimeth	C37H47FN4O4	630.79	A/B	-8.6
4286	C[C@@]12[C@@H]3[C@H]([C@H]4[C@](C)(CC3)[C@@H]([C@H](CCC(=O)O)C)CC4)[C@@H](O)[C@@H](O)[C@@H]1C[C@H](O	1649938-34-0	1H-1,2,4-Triazole-3-carboxamide, 1-[2,3-bis-O-[(phenylmethoxy)carbonyl]-5-O-[(ph	C36H40N5O11P	749.7	A/B	-8.6
4287	C(=O)([C@@H](NC(=O)OC)C(C)C)N1[C@@H](C[C@]2(C1)CCCOC2)c1[nH]c2c(n1)ccc1c2ccc(c1)c1ccc(cc1)c1[nH]c(nc	1653966-63-2	Carbamic acid, N-[(1S)-1-[[(2S,3aS,6aS)-hexahydro-2-[5-[4-[2-[(3S,5S)-2-[(2S)-2-	C49H60N8O7	873.05	A/B	-8.6
4288	C(=O)([C@@H](NC(=O)OC)C(C)C)N1C[C@]2(C[C@H]1c1[nH]c(cn1)c1ccc(cc1)c1cc3c(-c4c(CCC3)nc([nH]4)[C@H]3N	1653967-01-1	Carbamic acid, N-[(1S)-1-[[(2S,3aS,6aS)-hexahydro-2-[5-[4,Ä≤-[2-[(3S,5S)-2-[(2S)	C47H60N8O7	849.03	A/B	-8.6
4289	C(=O)([C@@H](NC(=O)OC)C(C)C)N1CC2(C[C@H]1c1[nH]c(cn1)c1ccc(cc1)c1cc3c(cc1)c1c(cc3)nc([nH]1)[C@H]1N(C	1653967-16-8	Carbamic acid, N-[(1S)-1-[[(2S)-2-[7-[4-[2-[(5R,8S)-7-[(2S)-2-[(methoxycarbonyl)	C45H54N8O7	818.96	A/B	-8.6
4290	O=c1n([C@@H]2O[C@H](COP(=O)(C(O)c3ccccc3)OC[C@@H]3[C@@H](N=[N+]=[N-])C[C@@H](O3)n3c(=O)[nH]c(=O)c(C)	166318-75-8	Thymidine, 3,Ä≤-azido-P,3,Ä≤-dideoxy-P-[hydroxy(4-methylphenyl)methyl]thymidyly	C28H33N10O10P		A/B	-8.6
4291	O=C1[C@@H]2[C@@H](c3c([C@@H](O)[C@H]2CO1)cc1c(c3)OCO1)c1cc(OC)c(OC(=O)COCC)c(OC)c1	166586-21-6	Acetic acid, phenoxy-, 4-[(5R,5aR,8aR,9S)-5,5a,6,8,8a,9-hexahydro-9-hydroxy-6-ox	C29H26O10	534.51	A/B	-8.6
4292	O=C1[C@@H]2[C@@H](c3c([C@@H](O)[C@H]2CO1)cc1c(c3)OCO1)c1cc(OC)c(OC(=O)CSc2ccccc2)c(OC)c1	166586-32-9	Acetic acid, [(phenylmethyl)thio]-, 4-(5,5a,6,8,8a,9-hexahydro-9-hydroxy-6-oxofu	C30H28O9S	564.6	A/B	-8.6
4293	O(C)c1c2C(C3C(C(O)c2ccc1OC)COC3=O)c1ccc2c(cc1)OCO2	1668551-15-2	Furo[3,Ä≤,4,Ä≤:6,7]naphtho[2,3-d]-1,3-dioxol-6(5aH)-one, 5-(1,3-benzodioxol-5-y	C20H16O7	368.34	A/B	-8.6
4294	O=C1[C@@H]2[C@@H](c3c([C@H](OC(=O)N(CCN(C(=O)OCCC#Cc4c5c(n(Cc6c(C)c(OC)c(C)cn6)c4)nc(N)nc5Cl)C)C)[C@	1680212-41-2	Carbonic acid, (5R,5aR,8aR,9R)-5,5a,6,8,8a,9-hexahydro-8-oxo-9-(3,4,5-trimethoxy	C29H25NO12	579.51	A/B	-8.6
4295	C(n1c2c(nc1CC)c(C)cc(C)n2)c1ccc(cc1)c1c(S(=O)(=O)NC(=O)OCc2ccccc2)ccc(CCC)c1	169281-54-3	Carbamic acid, [[5-ethyl-4,Ä≤-[(2-ethyl-5,7-dimethyl-3H-imidazo[4,5-b]pyridin-3-	C33H34N4O4S	582.71	A/B	-8.6
4296	C(n1c2c(nc1CC)c(C)cc(C)n2)c1ccc(cc1)c1c(S(=O)(=O)NC(=O)OCC)ccc(CCC)c1	169281-55-4	Carbamic acid, [[4,Ä≤-[(2-ethyl-5,7-dimethyl-3H-imidazo[4,5-b]pyridin-3-yl)methy	C34H36N4O4S	596.74	A/B	-8.6
4297	C(n1c2c(nc1CC)c(C)cc(C)n2)c1ccc(cc1)c1c(S(=O)(=O)NC(=O)OCCCC)ccc(c1)c1ccccc1	169281-60-1	Carbamic acid, [[4,Ä≤-[(2-ethyl-5,7-dimethyl-3H-imidazo[4,5-b]pyridin-3-yl)methy	C30H36N4O4S2	580.76	A/B	-8.6
4298	N(C(=O)[C@H](Cc1ccccc1)C[C@@H](CN1[C@H](C(=O)NC(C)(C)C)CN(Cc2cccnc2)CC1)O)[C@H]1c2c(C[C@H]1OC(=O)CN(	170227-67-5	Butanedioic acid, mono[1-[[5-[2-[[(1,1-dimethylethyl)amino]carbonyl]-4-(3-pyridi	C40H51N5O7	713.86	A/B	-8.6
4299	C(OP(=O)(OP(=O)(OP(=O)(O)O)O)S)[C@@]12O[C@H]([C@@H]([C@@H]1O)CC2)n1c2c(nc1)c(=O)nc(N)[nH]2	1706943-59-0	Benzamide, 4-[[6-amino-5-bromo-2-[(4-cyanophenyl)amino]-4-pyrimidinyl]oxy]-3,5-d	C20H17BrN6O2	453.29	A/B	-8.6
4300	O[C@@H]1[C@H](n2c3c(nc2)c(N)nc(F)n3)O[C@H](CO[P@@]2(=O)O[C@@H](CCO2)c2ccncc2)[C@H]1O	1710392-33-8	9H-Purin-6-amine, 2-fluoro-9-[5-O-[(2R,4S)-2-oxido-4-(4-pyridinyl)-1,3,2-dioxaph	C18H20FN6O7P	482.36	A/B	-8.6
4301	N(C(CC#C)CC)c1c2c(ncc1)cc(Cl)cc2	1710392-36-1	9H-Purin-6-amine, 2-fluoro-9-[5-O-[(2S,4S)-2-oxido-4-(4-pyridinyl)-1,3,2-dioxaph	C18H20FN6O7P	482.36	A/B	-8.6
4302	O=c1c2c(n(cn2)[C@@H]2O[C@H](COP(=O)(OC[C@@H]3[C@@H](N=[N+]=[N-])C[C@@H](O3)n3c(=O)[nH]c(=O)c(C)c3)OC	171284-27-8	Inosine, P-[[4-(acetyloxy)phenyl]ethyl]-3,Ä≤-azido-3,Ä≤-deoxythymidylyl-(5,Ä≤,Ü	C29H32N9O11P		A/B	-8.6
4303	O=C1[C@@H]2[C@@H](c3c([C@H](OC(=O)c4c5c([nH]c4)cccc5)[C@H]2CO1)cc1c(c3)OCO1)c1cc(OC)c(OC)c(OC)c1	1715004-90-2	1H-Indene-2-propanoic acid, (5R,5aR,8aR,9R)-5,5a,6,8,8a,9-hexahydro-8-oxo-9-(3,4	C34H32O9	584.61	A/B	-8.6
4304	O=c1n([C@@H]2O[C@H](CO)[C@@H](O)C2)cc/c(=N\O)/[nH]1	171868-08-9	4-Morpholinecarboxamide, N-[2-oxo-2-[[1-(2-phenylethyl)-3-(phenylsulfonyl)-2-pro	C31H35N3O5S	561.69	A/B	-8.6
4305	O([Si](C(C)(C)C)(C)C)[C@@H]1[C@@H](O[C@H](CO[Si](C(C)(C)C)(C)C)[C@H]1O[P@](=O)(CCOCn1c2c(nc1)c(N)ncn	172293-60-6	9H-Purin-6-amine, 9-[3-O-[[2-[(6-amino-9H-purin-9-yl)methoxy]ethyl]hydroxyphosph	C18H23N10O7P	522.41	A/B	-8.6
4306	C(Oc1c(Cl)cccc1)c1c2c(N(c3c(C(=O)N2)cccn3)C2CC2)ncc1	172326-09-9	6H-Dipyrido[3,2-b:2,Ä≤,3,Ä≤-e][1,4]diazepin-6-one, 11-cyclopropyl-5,11-dihydro-	C21H18N4O3	374.39	A/B	-8.6
4307	C(=C)c1c2c(N(c3c(C(=O)N2)cccn3)C2CC2)ncc1	172326-19-1	6H-Dipyrido[3,2-b:2,Ä≤,3,Ä≤-e][1,4]diazepin-6-one, 11-cyclopropyl-5,11-dihydro-	C22H20N4O2	372.42	A/B	-8.6
4308	C(CC)(C1=C(O)CC(CCC)(CCC)OC1=O)c1cc(NS(=O)(=O)c2ccc(C(F)(F)F)cn2)ccc1	174484-23-2	2-Pyridinesulfonamide, N-[3-[1-[5,6-dihydro-4-hydroxy-2-oxo-6-(2-phenylethyl)-6-	C32H38N2O5S	562.72	A/B	-8.6
4309	C(Cc1ccccc1)C1(CCc2ccccc2)CC(=C(C(CC)c2cc(NS(=O)(=O)c3ccc(C#N)cn3)ccc2)C(=O)O1)O	174484-43-6	2-Pyridinesulfonamide, 5-amino-N-[3-[1-[5,6-dihydro-4-hydroxy-2-oxo-6-(2-phenyle	C32H39N3O5S	577.73	A/B	-8.6
4310	[C@H](CC)(C1=C(O)CC(CCC)(CCC)OC1=O)c1cc(NS(=O)(=O)c2ccc(C#N)cn2)ccc1	174484-68-5	2-Pyridinesulfonamide, 5-cyano-N-[3-[1-[5,6-dihydro-4-hydroxy-2-oxo-6,6-bis(2-ph	C38H39N3O5S	649.8	A/B	-8.6
4311	[C@H](C(C)(C)C)(C1=C(O)C[C@@](CCc2ccccc2)(CCC)OC1=O)c1cc(NS(=O)(=O)c2cc(C(F)(F)F)ccn2)ccc1	174590-23-9	2-Pyridinesulfonamide, N-[3-[1-[5,6-dihydro-4-hydroxy-2-oxo-6-(2-phenylethyl)-6-	C33H37F3N2O5S	630.72	A/B	-8.6
4312	C(n1c2c(nc1CC)c(C)cc(C)n2)c1ccc(cc1)c1c(S(=O)(=O)NC(=O)c2c(OC)cccc2)cccc1	174844-67-8	Benzamide, N-[[4,Ä≤-[(2-ethyl-5,7-dimethyl-3H-imidazo[4,5-b]pyridin-3-yl)methyl]	C30H28N4O4S	540.63	A/B	-8.6
4313	C(n1c2c(nc1CC)c(C)cc(C)n2)c1ccc(cc1)c1c(S(=O)(=O)NC(=O)c2ccc(OCc3ccccc3)cc2)cccc1	174844-71-4	Benzamide, N-[[4,Ä≤-[(2-ethyl-5,7-dimethyl-3H-imidazo[4,5-b]pyridin-3-yl)methyl]	C31H30N4O4S	554.66	A/B	-8.6
4314	O=c1n([C@@H]2O[C@H](CO[P@@]3(=O)Oc4c(CO3)cc(C)cc4)C=C2)cc(C)c(=O)[nH]1	174844-73-6	Benzamide, N-[[4,Ä≤-[(2-ethyl-5,7-dimethyl-3H-imidazo[4,5-b]pyridin-3-yl)methyl]	C31H30N4O4S	554.66	A/B	-8.6
4315	O=c1n([C@@H]2O[C@H](CO)[C@@H](O)C2)c[13cH][13c](=O)[nH]1	174848-84-1	Thymidine, 2,Ä≤,3,Ä≤-didehydro-3,Ä≤-deoxy-5,Ä≤-O-[(2S)-2-oxido-4H-1,3,2-benzodi	C17H17N2O7P	392.3	A/B	-8.6
4316	C[C@@H]1[C@@H](O[C@H](CO)[C@H]1O)n1cc(C#C)c(=O)[nH]c1=O	175776-04-2	3-Isoquinolinecarboxamide, N-(1,1-dimethylethyl)decahydro-2-[2-hydroxy-3-[(3-hyd	C32H45N3O4S	567.78	A/B	-8.6
4317	O=c1c2c(n(cn2)[C@@H]2O[C@H](COP(=O)(OP(=O)(OC[C@@H]3[C@@H](N=[N+]=[N-])C[C@@H](O3)n3c(=O)[nH]c(=O)c(	175923-16-7	Inosine 5,Ä≤-(trihydrogen diphosphate), 2,Ä≤,3,Ä≤-dideoxy-, P,Ä≤,Üí5,Ä≤-ester w	C19H23FN6O13P2	624.36	A/B	-8.6
4318	O=c1n(cc(C(F)(F)F)c(=O)[nH]1)[C@H]1C[C@H](OC(=S)c2ccc(C)cc2)[C@@H](COC(=S)c2ccc(C)cc2)O1	177165-79-6	L-Aspartic acid, 4,Üí3,Ä≤-ester with Œ±,Œ±,Œ±-trifluorothymidine 5,Ä≤-(4-chloro	C21H19ClF3N3O8S	565.9	A/B	-8.6
4319	O=c1n([C@H]2O[C@@H](CO)[C@H](F)C2)cc(C)c(N)n1	1773498-62-6	1,3-Oxathiolane-2-carboxylic acid, 5-[4-(benzoylamino)-5-fluoro-2-oxo-1(2H)-pyri	C25H30FN3O5S	503.59	A/B	-8.6
4320	O[C@H]1[C@@H]2[C@@H]([C@]3(C)[C@H](Cl)C[C@H](OC(=O)N[C@H](C(=O)OC[C@H]1O[C@H]([C@H](O)[C@@H]1O)n1nc(	1777812-93-7	L-Glutamic acid, N-[(3Œ±,5Œ≤,7Œ±)-3,7-dihydroxy-24-oxocholan-24-yl]-, 1,Üí5,Ä≤	C37H57N5O11	747.88	A/B	-8.6
4321	C(O[P@@](=O)(Oc1ccccc1)N[C@@H](C(=O)OC(C)C)C)[C@H]1C2([C@H]([C@@H](F)[C@@H]1O)n1c3c(nc1)c(=O)nc(N)[n	1781231-55-7	D-Alanine, N-[(R)-[[(4R,5R,6S,7R)-7-(2-amino-1,6-dihydro-6-oxo-9H-purin-9-yl)-5,	C25H33N6O8P	576.54	A/B	-8.6
4322	C=C1[C@@H](n2c3c(nc2)c(=O)nc(N)[nH]3)[C@](C)(O)[C@H](O)[C@H]1CO[P@](=O)(Oc1ccccc1)N[C@@H](C(=O)OC(C)	1782088-36-1	D-Alanine, N-[(S)-[[(1R,3R,4S,5R)-3-(2-amino-1,6-dihydro-6-oxo-9H-purin-9-yl)-4,	C25H33N6O8P	576.54	A/B	-8.6
4323	C(O[P@](=S)(Oc1ccccc1)N[C@@H](C(=O)OC(C)C)C)[C@H]1C2([C@H]([C@](C)(F)[C@@H]1O)n1c3c(nc1)c(=O)nc(N)[n	1782090-67-8	D-Alanine, N-[(S)-[[(4R,5R,6S,7R)-7-(2-amino-1,6-dihydro-6-oxo-9H-purin-9-yl)-5,	C26H35N6O7PS	606.63	A/B	-8.6
4324	S(=O)(=O)(O)c1cc2c(cc1N=Nc1c3c(c(N=Nc4cc5c(cc4)cccc5)cc1)cccc3)cc(N)c(S(=O)(=O)O)c2.[LiH]	178609-77-3	2-Naphthalenesulfonic acid, 8-[2-(7-amino-3-sulfo-2-naphthalenyl)diazenyl]-5-[2-	C30H21N5O9S3.3Li		A/B	-8.6
4325	C(=O)(NCCN)[C@]12[C@@H]([C@@H]3[C@@](C)(CC1)[C@@]1(C)[C@H](CC3)[C@]3(C)[C@@H](CC1)C(C)(C)[C@@H](O)CC	178693-60-2	Benzoic acid, 3-[2-(4-amino-6-sulfo-1-naphthalenyl)diazenyl]-	C17H13N3O5S	371.37	A/B	-8.6
4326	N(C(=O)[C@H](Cc1ccccc1)C[C@@H](CN1[C@H](C(=O)NC(C)(C)C)CN(Cc2cccnc2)CC1)O)[C@H]1c2c(C[C@H]1O)cc(O)cc	179169-44-9	1-Piperazinepentanamide, N-(2,3-dihydro-2,6-dihydroxy-1H-inden-1-yl)-2-[[(1,1-di	C36H47N5O5	629.79	A/B	-8.6
4327	N(=Nc1c(C)cc(cc1)c1cc(C)c(N=Nc2c3c(cc(S(=O)(=O)O)cc3)ccc2O)cc1)c1c2c(cc(S(=O)(=O)O)cc2)ccc1O	1798014-18-2	1(2H)-Pyrimidineacetamide, N-[(1R,3S,4R)-4-[[2-(2,6-dimethylphenoxy)acetyl]aminc	C37H48N4O5	628.8	A/B	-8.6
4328	C([C@H](OCP(=O)(Oc1cc2c(cc1)CCCC2)N[C@H](C(=O)OC(C)C)C)n1c2c(nc1)c(N)ncn2	1802665-26-4	L-Alanine, N-[[[(1R)-2-(6-amino-9H-purin-9-yl)-1-methylethoxy]methyl][(5,6,7,8-t	C25H35N6O5P	530.56	A/B	-8.6

4329	C(F)(F)[C@]1(CO)O[C@H](C(F)(F)[C@@H]1O)n1c2c(nc1)c(=O)nc(N)[nH]2	1803132-34-4	Benzonitrile, 4-[[6-amino-5-bromo-2-[(4-cyanophenyl)amino]-4-pyrimidinyl]oxy]-3,	C20H15BrN6O		A/B	-8.6
4330	[C@@H](COC)(N1[C@@H](C)CN(CC1)C1(C)CCN(C(=O)c2c(C)ncnc2C)CC1)c1ccc(C(F)(F)F)cc1	1803132-99-1	4-Pyrimidinecarboxamide, N-[(4-fluorophenyl)methyl]-1,6-dihydro-5-hydroxy-1-meth	C20H21FN6O5		A/B	-8.6
4331	C(OP(=O)(N[C@H](C(=O)OC(C)C)C)N[C@H](C(=O)OC(C)C)C)[C@@]1(C(F)F)O[C@H]([C@@H](O)[C@@H]1O)n1c2c(nc1)c	1803134-24-8	L-Alanine, N-[hydroxy(2-naphthalenyloxy)phosphinothioyl]-, 1-methylethyl ester,	C27H31F2N6O7PS	652.61	A/B	-8.6
4332	Cc1c2OC3(OCc2c(COC(=O)CCC(=O)OC[C@@H]2[C@@H](N=[N+]=[N-])C[C@@H](O2)n2c(=O)[nH]c(=O)c(C)c2)cn1)CCCCC	1805775-75-0	Thymidine, 3,Ä≤-azido-3,Ä≤-deoxy-, 5,Ä≤-[4-[(8,Ä≤-methylspiro[cyclopentane-1,2,	C27H32N6O9		A/B	-8.6
4333	O[C@H]1[C@H](n2c3c(nc2)c(=O)nc(N)[nH]3)O[C@H](COP(=O)(OP(=O)(OP(=O)(O[C@@H]2[C@@H](O)[C@H](O)[C@H](O	181427-83-8	Inosine 5,Ä≤-(trihydrogen diphosphate), P,Ä≤-(6-deoxy-Œ≤-L-galactopyranosyl) es	C16H24N4O15P2	574.33	A/B	-8.6
4334	O=c1c2c(n(cn2)[C@@H]2O[C@H](COP(=O)(OC[C@H]3O[C@H](C[C@@H]3O)n3c4c(nc3)c(=O)nc(N)[nH]4)O)[C@@H](O)C2	1835316-42-1	Furo[3,Ä≤,4,Ä≤:6,7]naphtho[2,3-d]-1,3-dioxol-6(5aH)-one, 5-(1,3-benzodioxol-5-y	C20H16O7	368.34	A/B	-8.6
4335	C(=O)(N[C@@H](Cc1ccccc1)C(=O)N[C@H](/C=C/S(=O)(=O)c1ccccc1)CCc1ccccc1)N1CCN(CC1)C1CCCC1	1851279-69-0	1-Piperazinecarboxamide, 4-(1-methylethyl)-N-[(1S)-2-oxo-2-[[(1S,2E)-1-(2-phenyl	C34H42N4O4S	602.79	A/B	-8.6
4336	O[C@H]1[C@@H](O[C@H](COC(=O)[C@H](NC(=O)OCc2ccccc2)C(C)C)[C@H]1O)n1nc(C(=O)N)nc1	1857374-70-9	Not Yet Assigned	C24H31N5O8	517.53	A/B	-8.6
4337	O[C@H]1[C@@H]2[C@@H]([C@]3(C)[C@H](C1)C[C@H](OC(=O)N[C@H](C(=O)OC[C@@H]1[C@@H]4[C@H]([C@@H](O1)n1nc(	1857375-16-6	1H-1,2,4-Triazole-3-carboxamide, 1-[5-O-[(2S)-2-amino-3-methyl-1-oxobutyl]-2,3-C	C20H25N5O6	431.44	A/B	-8.6
4338	O=c1c2c(n(cn2)[C@H]2C[C@H](OP(=O)(OC[C@@H]3[C@@H](OP(=O)(OC[C@H]4O[C@H](C[C@@H]4O)n4c(=O)nc(N)cc4)S)	185897-02-3	D-erythro-Pentonamide, 2,3,5-trideoxy-N-[(1S,2R)-2,3-dihydro-2-hydroxy-1H-inden-	C36H41D6N5O4		A/B	-8.6
4339	S(=O)(=O)(/C=C/[C@@H](NC(=O)[C@H](Cc1ccccc1)NC(=O)N1CCN(C)CC1)CCc1ccccc1)c1ccc(C(F)(F)F)cc1	1860793-32-3	1-Piperazinecarboxamide, N-[(1S)-2-[[(1S,2E)-3-[(4-bromophenyl)sulfonyl]-1-(2-ph	C32H37BrN4O4S	653.63	A/B	-8.6
4340	C(OC[C@@H]1[C@@H](OC(=O)CC)C[C@@H](C1=C)n1c2c(nc1)c(=O)nc(NC(=O)OC(C)(C)C)[nH]2)(c1ccccc1)(c1ccccc1)	1860842-89-2	Carbamic acid, N-[9-[(1S,3R,4S)-4-(acetyloxy)-2-methylene-3-[(triphenylmethoxy)m	C38H39N5O6	661.75	A/B	-8.6
4341	O=C1[C@@H]2[C@@H](c3c([C@H](OC(=O)CN4c5c(C(=O)C4=O)cc(Cl)cc5)[C@H]2CO1)cc1c(c3)OCO1)c1cc(OC)c(OC)c(O	1864833-31-7	1H-Indole-1-acetic acid, 2,3-dihydro-5-methyl-2,3-dioxo-, (5R,5aR,8aR,9R)-5,5a,6	C33H29NO11	615.58	A/B	-8.6
4342	O=c1n(cc(C(=O)OCCC)c(=O)[nH]1)[C@@H]1O[C@H](CO)[C@@H](O)C1	1872202-55-5	1-Piperazinecarbothioamide, N-[4-amino-6-(4-morpholinyl)-1,3,5-triazin-2-yl]-4-[	C23H29ClN10OS	529.06	A/B	-8.6
4343	O=C1[C@@H]2[C@@H](c3c([C@H](OC(=O)CCCC[C@H]4SC(SCC4)c4c(Cl)cccc4)[C@H]2CO1)cc1c(c3)OCO1)c1cc(OC)c(OC	1875123-89-9	1,3-Dithiane-4-pentanoic acid, 2-[4-(1-methylethyl)phenyl]-, (5R,5aR,8aR,9R)-5,5	C40H46O9S2	734.92	A/B	-8.6
4344	O=c1n([C@@H]2O[C@H](COP(=O)(OP(=O)(OP(=O)(OCc3ccc(OC(=O)c4ccc(C)cc4)cc3)OCc3ccc(OC(=O)CCCC)cc3)O)O)C	1883329-04-1	Benzoic acid, 4-(trifluoromethyl)-, 4-[9-[(2S,5R)-5-(3,4-dihydro-5-methyl-2,4-di	C37H38F3N2O17P3	932.62	A/B	-8.6
4345	O=c1c(c2c3c(cnc2)cccc3)cn(c(=O)[nH]1)[C@@H]1O[C@H](CO)[C@@H](O)[C@H]1O	1883514-21-3	Not Yet Assigned	C23H23N3O9	485.44	A/B	-8.6
4346	N(C(=O)Nc1c(C)cccc1)c1cc2cc(C34CC5CC(C4)CC(C3)C5)[nH]c2cc1	1883585-91-8	Urea, N-phenyl-N,Ä≤-(2-tricyclo[3.3.1.13,7]dec-1-yl-1H-indol-5-yl)-	C25H27N3O	385.5	A/B	-8.6
4347	N(C(C)C)c1c(nc(OS(=O)(=O)O)cc1)N1CCN(C(=O)c2[nH]c3c(c2)cc(NS(=O)(=O)C)cc3)CC1	188780-39-4	Œ≤-D-Glucopyranosiduronic acid, 5-[(1-methylethyl)amino]-6-[4-[[5-[(methylsulfor	C28H36N6O10S	648.68	A/B	-8.6
4348	O=c1c2c(n(cn2)[C@@H]2C[C@@H](OP(=O)(OC[C@H]3O[C@H](C[C@@H]3O)n3c(=O)[nH]c(=O)c(F)c3)O)[C@H](CO)O2)[n	189074-92-8	Œ≤-L-Cytidine, 2,Ä≤-deoxy-5-fluorouridylyl-(3,Ä≤,Üí5,Ä≤)-2,Ä≤-deoxy-	C18H23FN5O11P	535.37	A/B	-8.6
4349	N(c1c(C)cc(C)cc1C)c1nc(NCc2cc(C)ccc2)ccn1	1899098-83-9	9H-Purin-6-amine, 9-[5-O-[bis(4-methoxyphenyl)phenylmethyl]-Œ≤-D-arabinofuranosy	C31H30FN5O6	587.6	A/B	-8.6
4350	C(OC[C@@H]1[C@@H](OC(=O)c2ccccc2)[C@@H](OP(=O)(OCCc2ccc(N(=O)=O)cc2)O)[C@@H](O1)n1nc(C(=O)N)nc1)(c1c	190141-32-3	1H-1,2,4-Triazole-3-carboxamide, 1-[3-O-benzoyl-2-O-[(2-chlorophenoxy)(2-cyanoet	C44H39ClN5O10P	864.23	A/B	-8.6
4351	O=c1n(c2c(c(cc(n2)c2ccc(Cl)cc2)c2ccco2)c(=O)n1c1ccccc1)[C@H]1[C@H](OC(=O)c2ccccc2)[C@H](OC(=O)c2cccc	190316-59-7	Guanidine, N-[4-(11-cyclopropyl-6,11-dihydro-6-oxo-5H-dipyrido[3,2-b:2,Ä≤,3,Ä≤-(	C19H23N7O	365.43	A/B	-8.6
4352	N([Si](C)(C)Cl)C12CC3CC(C2)CC(C1)C3	190319-86-9	Pyrido[2,3-d]pyrimidine-2,4(1H,3H)-dione, 3,7-bis(4-chlorophenyl)-5-(4-fluorophe	C51H34Cl2FN3O9	922.73	A/B	-8.6
4353	N(c1nc(nc(Nc2ccc(F)cc2)n1)N1CCOCC1)c1ccc(C#N)nc1	1907643-77-9	2-Pyridinecarbonitrile, 5-[[4-[(4-methylphenyl)amino]-6-(4-morpholinyl)-1,3,5-tr	C20H20N8O	388.43	A/B	-8.6
4354	C(#N)[C@]1(O[C@H](COP(=O)(N[C@@H](Cc2ccccc2)C(=O)OC(C)C)Oc2ccccc2)[C@@H](O)[C@H]1O)c1n2c(cc1)c(N)ncn	1911578-84-1	Not Yet Assigned	C25H29N6O8P	572.51	A/B	-8.6
4355	C(#N)[C@]1(O[C@H](CO[P@](=O)(Oc2ccccc2)N[C@H](C(=O)OCC(C)(C)C)C)[C@@H](O)[C@H]1O)c1n2c(cc1)c(N)ncn2	1911578-87-4	Not Yet Assigned	C22H25N6O8P	532.44	A/B	-8.6
4356	C(#N)[C@@]1([C@H](O[Si](C(C)(C)C)(C)C)[C@H](O[Si](C(C)(C)C)(C)C)[C@@H](CO[P@](=O)(Oc2ccccc2)N[C@H](C	1911578-95-4	Not Yet Assigned	C25H31N6O8P	574.52	A/B	-8.6
4357	O=c1c2c(n(cn2)[C@@H]2O[C@H](COP(=O)(Oc3ccc(Cl)cc3)NCCO)CC2)[nH]cn1	1922936-32-0	Phosphonic acid, P-[4-(4-fluorophenyl)-1-piperazinyl]-, 4-chlorophenyl [(2S,5R)-	C26H27ClFN6O5P	588.95	A/B	-8.6
4358	O(CCOCc1ccccc1)[C@H]1[C@H](n2c3c(nc2)c(=O)nc(N)[nH]3)O[C@H](CO)[C@H]1O	1922936-34-2	Phosphoramidic acid, N-(4-fluoro-3-nitrophenyl)-, 4-chlorophenyl [(2S,5R)-5-(1,6	C22H19ClFN6O7P	564.85	A/B	-8.6
4359	N(c1c(CC)cccc1CC)c1nc(Nc2ccc(C#N)cc2)cc(C)n1	1923559-48-1	Benzonitrile, 2-[[2-[(4-cyanophenyl)amino]-6-methyl-4-pyrimidinyl]amino]-	C19H14N6	326.35	A/B	-8.6
4360	C(=C(\CCc1cc(OC)c(O)cc1)/O)(/C(=O)CCc1cc(OC)c(O)cc1)\N=Nc1ccc(C(=O)O)cc1	1927857-62-2	2-Pyrimidinamine, 4-(difluoromethyl)-5-[4-[(3S)-3-methyl-4-morpholinyl]-6-(4-mor	C17H22F2N8O2	408.41	A/B	-8.6
4361	O[C@H]1[C@@H](O[C@H](COC(=O)CCCCCO[C@H]2[C@H](O)[C@@H](CO)O[C@H](O)[C@@H]2O)[C@H]1O)n1nc(C(=O)N)nc1	1927879-77-3	Glycine, N-[4-[2-[(1E)-2-hydroxy-4-(4-hydroxy-3-methoxyphenyl)-1-[3-(4-hydroxy-3	C30H31N3O9	577.58	A/B	-8.6
4362	N(c1nc(nc(n1)c1cccs1)N1CCOCC1)c1ccc(OC)cc1	1929549-99-4	1,3,5-Triazin-2-amine, 4-(4-morpholinyl)-N-(4-nitrophenyl)-6-phenyl-	C19H18N6O3	378.38	A/B	-8.6
4363	C(=O)([C@@H](NC(=O)OC([2H])([2H])[2H])C(C)C)N1[C@@H](CCC1)c1[nH]c(c(n1)[2H])c1cc(c(c(c1[2H])[2H])c1	1939212-07-3	Carbamic acid, N-[(1S)-1-[[(2S)-2-[5-[4,Ä≤-[2-[(2S)-1-[(2S)-2-[(methoxycarbonyl)	C40H41D9N8O6		A/B	-8.6
4364	N(C(=O)[C@H]1[C@H](N(C)C(=O)CO1)c1cnn(C)c1)C12CC3CC(C2)CC(C1)C3	194159-24-5	L-Valine, N-[(phenylmethoxy)carbonyl]-, 2-[(2-amino-1,6-dihydro-6-oxo-9H-purin-9	C25H32N6O8	544.56	A/B	-8.6
4365	C(=O)(NC(CC1CCC1)C(=O)C(=O)N)[C@@H]1[C@@H]2[C@@H](C2(C)C)CN1C(=O)[C@@H](NC(=O)NC(C)(C)C)C(C)(C)C.O	1942918-72-0	2,7,10,12-Tetraazatridecanoic acid, 4-hydroxy-12-methyl-9-(1-methylethyl)-13-[2-	C37H48N6O5S2.C25H18Cl2N8		A/B	-8.6
4366	C(=O)(c1n(c2c(c1)cc(NS(=O)(=O)C)cc2)[C@@H]1O[C@H](C(=O)O)[C@@H](O)[C@H](O)[C@H]1O)N1CCN(CC1)c1c(NC(C	194347-54-1	Œ≤-D-Glucopyranosiduronic acid, 2-[[4-[3-[(1-methylethyl)amino]-2-pyridinyl]-1-p	C28H36N6O10S	648.68	A/B	-8.6
4367	O[C@@H]1[C@H](n2c3c(nc2)c(NCc2cc(C(F)(F)F)c(F)cc2)ncn3)O[C@H](CO)[C@H]1O	1947401-74-2	9H-Purin-6-amine, 9-Œ≤-D-arabinofuranosyl-N-[[4-chloro-3-(trifluoromethyl)phenyl	C18H17ClF3N5O4	459.81	A/B	-8.6
4368	O[C@@H]1[C@H](n2c3c(nc2)c(NCCCCO)ncn3)O[C@H](C(=O)NCC)[C@H]1O	1949770-81-3	Carbamic acid, N-[(1S)-1-[[(2S,4R)-2-[5-[4-[2-[(1R,3S,4S)-2-[(2S)-2-[(methoxycar	C47H58N8O8	863.01	A/B	-8.6
4369	C(SC(C(=O)NCC(=O)OCOC(=O)O[C@@H]1[C@@H](CO[P@](=O)(Oc2ccccc2)N[C@H](C(=O)OC(C)C)C)O[C@H]([C@]1(C)F)n	1972595-95-1	L-Alanine, N-[[P(S),2,Ä≤R]-3,Ä≤-O-[[(2R)-2-(acetylamino)-3-[(triphenylmethyl)th	C47H52FN4O12PS	946.97	A/B	-8.6
4370	S(=O)(=O)(c1cc2c(cc1)NC(=O)C2)N1CC(O)C(O)C1	197903-84-7	Guanosine, 5,Ä≤-O-(4-cyano-2,3,6-trifluoro-5-hydroxyphenyl)-	C17H13F3N6O6	454.32	A/B	-8.6
4371	N(C(=O)C)[C@H]1[C@H]([C@@H]([C@@H](CO)O)O)OC(=C[C@@H]1NC(=N)NC(=O)CCn1cc(nn1)c1ccccc1)C(=O)O	1982351-34-7	D-glycero-D-galacto-Non-2-enonic acid, 5-(acetylamino)-2,6-anhydro-3,4,5-trideox	C22H27N7O8	517.49	A/B	-8.6
4372	N(C(=O)C)[C@@H]1[C@@H](NC(=N)NC(=O)Cn2cc(nn2)c2cc3c(cc2)cc(OC)cc3)C=C(C(=O)O)O[C@H]1[C@@H]([C@@H](CO	1982351-51-8	D-glycero-D-galacto-Non-2-enonic acid, 5-(acetylamino)-2,6-anhydro-3,4,5-trideox	C22H26FN7O8	535.48	A/B	-8.6
4373	O=C1c2n(N([C@H]3N1CCOC3)[C@H]1c3c(CSc4c1cccc4)c(F)cc(F)c3)ccc(=O)c2O	1985605-68-2	1H-[1,4]Oxazino[3,4-c]pyrido[2,1-f][1,2,4]triazine-6,8-dione, 12-[(11S)-10-fluor	C24H20FN3O4S	465.5	A/B	-8.6
4374	Fc1c2[C@H](N3n4c(c(OCOC(=O)OC)c(=O)cc4)C(=O)N4[C@H]3COCC4)c3c(SCc2ccc1)cccc3	1985606-31-2	1H-[1,4]Oxazino[3,4-c]pyrido[2,1-f][1,2,4]triazine-6,8-dione, 7-[(acetyloxy)meth	C27H24FN3O6S	537.56	A/B	-8.6
4375	O(P(=O)(N[C@H](C(=O)OCC)C)OC(C)C)c1c2n(N([C@H]3N(C2=O)CCOC3)[C@H]2c3c(CSc4c2cccc4)c(F)c(F)cc3)ccc1=O	1985606-76-5	1H-[1,4]Oxazino[3,4-c]pyrido[2,1-f][1,2,4]triazine-6,8-dione, 12-[(11S)-7,8-difl	C29H23F2N3O7S	595.57	A/B	-8.6
4376	O(c1c2n(N([C@H]3N(C2=O)CCOC3)[C@H]2c3c(CSc4c2cccc4)c(F)c(F)cc3)ccc1=O)C1C=C(C)C(=O)O1	1985606-79-8	1H-[1,4]Oxazino[3,4-c]pyrido[2,1-f][1,2,4]triazine-6,8-dione, 12-[(11S)-7,8-difl	C32H23F2N3O6S	615.6	A/B	-8.6
4377	O(C(OC(=O)OCC)C)c1c2n(N([C@H]3N(C2=O)CCOC3)[C@H]2c3c(CSc4c2cccc4)c(F)c(F)cc3)ccc1=O	1985606-82-3	Carbonic acid, cyclohexyl 1-[[(12aR)-12-[(11S)-7,8-difluoro-6,11-dihydrodibenzo[	C33H33F2N3O7S	653.69	A/B	-8.6
4378	O(P(=O)(OC(C(=O)OC)(C)C)OCC)c1c2n(N([C@H]3N(C2=O)CCOC3)[C@H]2c3c(CSc4c2cccc4)c(F)c(F)cc3)ccc1=O	1985607-30-4	Carbonic acid, (12aR)-12-[(11S)-7,8-difluoro-6,11-dihydrodibenzo[b,e]thiepin-11-	C31H30F2N4O6S	624.65	A/B	-8.6
4379	O=c1c2c(n(cn2)[C@H]2O[C@@H](COP(=O)(OP(=O)(OP(=O)(O)O)O)O)[C@H](O)C2)[nH]c(N)n1	198630-55-6	L-erythro-Pentonamide, 2,3,5-trideoxy-N-[(1S,2R)-2,3-dihydro-2-hydroxy-1H-inden-	C30H42N4O4	522.68	A/B	-8.6
4380	N(C(=O)c1c(Cl)ncccl)c1cc2cc(C34CC5CC(C4)CC(C3)C5)[nH]c2cc1	1987854-64-7	Benzamide, 2-chloro-N-(2-tricyclo[3.3.1.13,7]dec-1-yl-1H-indol-5-yl)-	C25H25ClN2O	404.93	A/B	-8.6
4381	FC1(F)Cc2c(c(ccc2c2cc3c(cc2)c2c(cc3)nc([nH]2)[C@H]2N(C(=O)[C@@H](NC(=O)OC)C(C)C)CCC2)c2[nH]c(nc2)[C@	2007901-15-5	Carbamic acid, N-[(1S)-1-[[(2S)-2-[7-[5-[2-[(2S)-1-[(2S)-2-[(methoxycarbonyl)ami	C44H52N8O6	788.93	A/B	-8.6
4382	C(=O)([C@@H](NC(=O)OC)C(C)C)N1[C@@H](C[C@H]2[C@@H]1CCC2)c1[nH]c(cn1)c1c2c(c(cc1)c1cc3c(cc1)c1c(cc3)n	2007901-64-4	Carbamic acid, N-[(1S)-1-[[(2S,4S)-2-[5-[2,3-dihydro-7-[2-[(2S)-1-[(2S)-2-[(meth	C45H53FN8O6	820.95	A/B	-8.6
4383	C(=O)([C@@H](NC(=O)OC)C(C)C)N1[C@@H](CC[C@@H]1C(F)(F)F)c1[nH]c2c(n1)ccc1c2ccc(c1)c1c2c(cc(c1)c1[nH]c	2007901-79-1	Carbamic acid, N-[(1S)-1-[[(2S,5R)-2-[7-[2,3-dihydro-7-[2-[(7S)-6-[(2S)-2-[(meth	C49H57F3N8O6	911.02	A/B	-8.6
4384	O=c1n(C2OC(CO)C(O)C2)ccc(N)n1	2014403-96-2	1H-Indole-3-acetamide, N-[5-(1,1-dimethylethyl)-3-isoxazolyl]-Œ±-oxo-2-tricyclo[	C27H31N3O3	445.55	A/B	-8.6
4385	[C@H](C(C)C)(CO)n1c2c(cc(Cc3cc(cc(Cl)c3F)N3CCC(F)(F)C3)c(OC)c2)c(=O)c(C(=O)O)c1	1000161-63-6	3-Quinolinecarboxylic acid, 6-[[5-(4-acetyl-1-piperazinyl)-3-chloro-2-fluoropher	C29H33ClFN3O6	574.04	C	-8.6
4386	[C@H](C(C)C)(CO)n1c2c(cc(Cc3cc(cc(Cl)c3F)N3CC[C@H](F)C3)c(OC)c2)c(=O)c(C(=O)O)c1	1000161-65-8	3-Quinolinecarboxylic acid, 6-[[3-chloro-2-fluoro-5-[(3R)-3-fluoro-1-pyrrolidiny	C27H29ClF2N2O5	534.98	C	-8.6
4387	C(N(C[C@@H]([C@H](Cc1ccc(OCP(=O)(OCC)OCC)cc1)NC(=O)[C@@H](NC(=O)OC)C(C)(C)C)O)NC(=O)[C@@H](NC(=O)OC)	1003853-26-6	L-Valine, N-[(phenylmethoxy)carbonyl]-, [(1R,3S,5S)-3-(2-amino-1,6-dihydro-6-oxo	C38H45N7O9	743.81	C	-8.6
4388	S(=O)(=O)([O-])c1c2c3c([N]4=N[C-]5C(=[O][Cu+2]4[O-]3)N(N=C5C)c3ccc(S(=O)(=O)[O-])cc3)cc(S(=O)(=O)[O-	1007166-40-6	Benzenesulfonic acid, 4-[2-[4-[2-[2-(2-hydroxyethyl)-5-methylphenyl]diazenyl]-1-	C25H22N4O4S	474.53	C	-8.6
4389	C(=O)([C@@H](NC(=O)OC)C(C)C)N1[C@@H](CCC1)c1[nH]c(cn1)c1ccc(C(=O)O)c(cc1)c1ccc(cc1)c1[nH]c(nc1)[C@H]1	1007884-60-7	Carbamic acid, N-[(1S)-2-methyl-1-[[(2S)-2-[5-[4,Ä≤-[2-[(2S)-2-pyrrolidinyl]-1H-	C33H39N7O3	581.71	C	-8.6
4390	C(C(C(NC(=O)c1c(C)c(O)ccc1)CSc1cc(CC)ccc1O)O)N1C(C(=O)NC(C)(C)C)CC2C(C1)CCCC2	1009057-19-5	3-Isoquinolinecarboxamide, N-(1,1-dimethylethyl)decahydro-2-[2-hydroxy-3-[(3-hyc	C38H49N3O5S	659.88	C	-8.6
4391	C(=O)([C@H](NC(=O)OC(C)(C)C)C(C)C)N1[C@@H](CCC1)c1[nH]c(cn1)c1ccc(cc1)c1ccc(cc1)c1[nH]c(nc1)[C@H]1N(	1009106-27-7	Carbamic acid, N-[(1R)-2-[(2S)-2-[5-[4,Ä≤-[2-[(2S)-1-[(2R)-2-[(methoxycarbonyl)a	C46H46N8O6	806.91	C	-8.6
4392	C(=O)([C@H](NC(=O)OC)c1ccccc1)N1[C@@H](CCC1)c1[nH]c(cn1)c1ccc(cc1)c1ccc(cc1)c1[nH]c(nc1)[C@H]1N(C(=O	1009107-31-6	Acetamide, N,N,Ä≤-[[1,1,Ä≤-biphenyl]-4,4,Ä≤-diylbis[1H-imidazole-5,2-diyl-(2S)-	C40H50N8O4	706.88	C	-8.6
4393	C(=O)([C@H](NC(=O)OC)c1ccccc1)N1[C@@H](CCC1)c1[nH]c(cn1)c1ccc(cc1)c1ccc(cc1)c1[nH]c(nc1)[C@H]1N(C(=O	1009107-50-9	Carbamic acid, N-[(1R)-2-[(2S)-2-[5-[4,Ä≤-[2-[(2S)-1-[2-(dimethylamino)acetyl]-2	C40H44N8O4	700.83	C	-8.6
4394	C(=O)([C@H](NC(=O)OC)c1ccccc1)N1[C@@H](CCC1)c1[nH]c(cn1)c1ccc(cc1)c1ccc(cc1)c1[nH]c(nc1)[C@H]1N(C(=O	1009107-53-2	Carbamic acid, N-[(1R)-2-[(2S)-2-[5-[4,Ä≤-[2-[(2S)-1-[2-[(methoxycarbonyl)amino]	C40H42N8O6	730.81	C	-8.6
4395	C(=O)([C@H](NC(=O)OC)c1ccccc1)N1[C@@H](CCC1)c1[nH]c(cn1)c1ccc(cc1)c1ccc(cc1)c1[nH]c(nc1)[C@H]1N(C(=O	1009107-96-3	Carbamic acid, N-[(1R)-2-[(2S)-2-[5-[4,Ä≤-[2-[(2S)-1-[(2S)-2-amino-1-oxopropyl]-	C39H42N8O4	686.8	C	-8.6
4396	C(=O)([C@H](NC1=NCCN1)C(C)C)N1[C@@H](CCC1)c1[nH]c(cn1)c1ccc(cc1)c1ccc(cc1)c1[nH]c(nc1)[C@H]1N(C(=O)[	1009114-04-8	Carbamic acid, N-[(1S)-2-[(2S)-2-[5-[4,Ä≤-[2-[(2S)-1-[(2S)-2-[(4,5-dihydro-1H-ir	C39H48N10O4	720.86	C	-8.6
4397	C(=O)(N1[C@@H](CCC1)c1[nH]c(cn1)c1ccc(cc1)c1ccc(cc1)c1[nH]c(nc1)[C@H]1N(C(=O)[C@@H](NC(=O)OC)C(C)C	1009114-59-3	Carbamic acid, N-[(1S)-1-[[(2S)-2-[5-[4,Ä≤-[2-[(2S)-1-[[trans-4-[(methoxycarbony	C42H52N8O6	764.91	C	-8.6
4398	C(=O)([C@@H](Cc1ccccc1)NC(=O)OC)N1[C@@H](CCC1)c1[nH]c(cn1)c1ccc(cc1)c1ccc(cc1)c1[nH]c(nc1)[C@H]1N(C(	1009115-12-1	Carbamic acid, N,N,Ä≤-[[1,1,Ä≤-biphenyl]-4,4,Ä≤-diylbis[1H-imidazole-5,2-diyl-(	C40H44N14O6	816.87	C	-8.6
4399	C(=O)([C@@H](NC(=O)OC)C(C)C)N1[C@@H](C[C@@H](O)C1)c1[nH]c(cn1)c1ccc(cc1)c1ccc(cc1)c1[nH]c(nc1)[C@H]1	1009115-57-4	Carbamic acid, N,N,Ä≤-[[1,1,Ä≤-biphenyl]-4,4,Ä≤-diylbis[1H-imidazole-5,2-diyl[(	C36H42N8O8	714.77	C	-8.6
4400	C(=O)([C@@H](NC(=O)OC)C1CC1)N1[C@H](c2[nH]c(c(C(F)(F)F)n2)c2ccc(cc2)c2ccc(cc2)c2[nH]c(nc2)[C@H]2N(C(	1009116-09-9	Carbamic acid, N-[(1S)-1-[[(2S)-2-[4-[4,Ä≤-[2-[(2S)-1-[(2S)-2-[(methoxycarbonyl)	C41H49F3N8O6	806.87	C	-8.6
4401	C(=O)([C@@H](NC(=O)OC(C)(C)C)N1[C@@H](CCC1)c1[nH]c(cn1)c1ccc(cc1)c1ccc(cc1)c1[nH]c(nc1)[C@H]1N(C(=	1009119-10-1	Carbamic acid, N-[(1S)-1-[[(2S)-2-[5-[4,Ä≤-[2-[(2S)-1-[2-[[(1,1-dimethylethoxy)c	C41H52N8O6	752.9	C	-8.6
4402	C(=O)(C(NC(=O)OC)C(C)C)N1[C@H](CCC1)c1[nH]c(cn1)c1ccc(cc1)c1ccc(cc1)c1[nH]c(nc1)[C@H]1N(C(=O)C(NC(=O	1009119-60-1	Carbamic acid, N,N,Ä≤-[[1,1,Ä≤-biphenyl]-4,4,Ä≤-diylbis[1H-imidazole-5,2-diyl-(	C40H50N8O6	738.88	C	-8.6
4403	C(C)(C)(O)[C@@H]1n2c3c4c(nc(N)c3nc2C(CCC)OC1)cccc4	1010105-85-7	8H-[1,4]Oxazino[4,Ä≤,3,Ä≤:1,2]imidazo[4,5-c]quinoline-11-methanol, 6-amino-8-et	C18H22N4O2	326.39	C	-8.6
4404	S(=O)(=O)(c1cc2c(cc1)NC(=O)C2)N1CCC(CN)CC1	10181-47-2	1-Naphthalenesulfonic acid, 4-[2-[2,4-diamino-5-[2-[4,Ä≤-[2-(2,6-diamino-3-methy	C35H30N10O6S2.2Na		C	-8.6
4405	N(Cc1cc(F)ccc1)C12CC3CC(C2)CC(C1)C3	101947-58-4	Uridine, 2,Ä≤-deoxy-5-ethyl-, 3,Ä≤-tricyclo[3.3.1.13,7]decane-1-carboxylate	C22H30N2O6	418.48	C	-8.6
4406	N(C(=O)Nc1ccccc1)c1nc(nc(NC(=S)NCCc2cccs2)n1)N1CCOCC1	1025166-72-6	Benzenesulfonic acid, 2,2,Ä≤-(1,2-ethynediyl)bis[5-[2-(4-amino-1-naphthalenyl)di	C34H24N6O6S2	676.72	C	-8.6
4407	C(OCCOC(=O)C)n1c2c(nc1)c(=O)nc(N)[nH]2	1027276-25-0	1-Naphthalenesulfonic acid, 4-amino-3-[(1E)-2-[4,Ä≤-[(1E)-2-(2-amino-6-sulfo-1-r	C32H24N6O6S2	652.7	C	-8.6
4408	[C@@H](Cc1ccccc1)(NC(=O)N1CCN(C)CC1)C(=O)N[C@@H](CCc1ccccc1)CC	1027582-36-0	2-Naphthalenesulfonic acid, 3,8-diamino-5-[(1E)-2-[4,Ä≤-[(1E)-2-(4-amino-6-sulfo	C32H25N7O6S2	667.71	C	-8.6
4409	C(c1c2c(N(c3c(C(=O)N2)cccn3)C2CC2)ncc1)n1c2-c(c(N)nc1)ncn2	1046462-06-9	Adenosine, 1-[(11-cyclopropyl-6,11-dihydro-6-oxo-5H-dipyrido[3,2-b:2,Ä≤,3,Ä≤-e]	C25H25N9O4	515.52	C	-8.6
4410	N(=N\c1cc(S(=O)(=O)O)c(CCc2c(S(=O)(=O)O)cc(/N=N/c3c4c(cc(S(=O)(=O)O)cc4)c(N)cc3)cc2)cc1)/c1c2c(cc(S(	1052524-73-8	Not Yet Assigned	C34H28N6O18S6.Na		C	-8.6
4411	O=c1n([C@@H]2O[C@H](COP(=O)OCCCCCC)C=C2)cc(C)c(=O)[nH]1	1052538-42-7	Not Yet Assigned	C32H24N6O14S5.Na		C	-8.6
4412	O=c1n([C@H]2C[C@H](N=[N+]=[N-])[C@@H](CS)O2)cc(C)c(=O)[nH]1	1054661-48-1	Not Yet Assigned	C22H27N9O11	593.5	C	-8.6
4413	O=C1[C@@H]2[C@@H](c3c([C@H](OC(=O)C(=O)c4c5c(n(Cc6cc(Cl)c(Cl)cc6)c4)cccc5)[C@H]2CO1)cc1c(c3)OCO1)c1c	1061717-04-1	1H-Indole-3-acetic acid, 1-[(4-chlorophenyl)methyl]-Œ±-oxo-, (5R,5aR,8aR,9R)-5,5	C39H32ClNO10	710.13	C	-8.6
4414	N(c1c(C(C)C)cc(C=CC#N)cc1)c1nc(Nc2ccc(C#N)cc2)ncc1	1066549-60-7	Benzonitrile, 4-[[4-[[4-[(1E)-2-cyano-1-methyl-1-propen-1-yl]-2,3,6-trimethylphe	C25H24N6	408.5	C	-8.6
4415	N(c1c2c(n(cn2)[C@@H]2C[C@H](CO[Si](C(C)C)(C(C)C)C(C)C)C=C2)nc(N)n1)C1CC1	1067882-07-8	Not Yet Assigned	C39H50N4O6	670.84	C	-8.6
4416	O(C[C@H]1O[C@H](C=C1)n1c(=O)[nH]c(=O)c(C)c1)P1(=O)Oc2c(cc(CCC(=O)N[C@H](C(=O)OC)C)cc2)CO1	1073242-62-2	D-Alanine, N-[3-[2-(2,Ä≤,3,Ä≤-didehydro-3,Ä≤-deoxythymidin-5,Ä≤-O-yl)-2-oxido-4	C24H28N3O10P	549.47	C	-8.6
4417	O(C[C@H]1O[C@H](C=C1)n1c(=O)[nH]c(=O)c(C)c1)P1(=O)Oc2c(cc(CCC(=O)N3[C@H](C(=O)OCc4ccccc4)CCC3)cc2)CO	1073242-67-7	L-Proline, 1-[3-[2-(2,Ä≤,3,Ä≤-didehydro-3,Ä≤-deoxythymidin-5,Ä≤-O-yl)-2-oxido-4	C26H30N3O10P	575.5	C	-8.6
4418	[C@H](Cc1cc(C(=N)N)ccc1)(C(=O)N[C@H](C=CS(=O)(=O)c1ccccc1)CCc1ccccc1)NC(=O)N1CCN(C)CC1	1076088-66-8	1-Piperazinecarboxamide, N-[(1S)-1-[[3-(aminomethyl)phenyl]methyl]-2-oxo-2-[[(1S	C33H41N5O4S	603.77	C	-8.6
4419	C(C)(C)(C)c1c2c(cc(C(=C)OC(=O)C)c1)COP(=O)(OC[C@H]1O[C@H](C=C1)n1c(=O)[nH]c(=O)c(C)c1)O2	1095170-80-1	Thymidine, 5,Ä≤-O-[6-[1-(acetyloxy)ethenyl]-8-methyl-2-oxido-4H-1,3,2-benzodioxa	C22H23N2O9P	490.4	C	-8.6
4420	[C@@H](C(CC)CC)(NC(=O)C)[C@H]1[C@H](NC(=N)N)C[C@H](C(=O)O)[C@H]1O.O	111590-59-1	6H-Purin-6-one, 2-amino-1,9-dihydro-9-[3-hydroxy-4-[[(4-methoxyphenyl)diphenylme	C31H31N5O4	537.61	C	-8.6
4421	C(=O)([C@@H](NC(=O)OC)C1CC1)N1[C@H]2[C@H](C2)C[C@H]1c1[nH]c(cn1)c1ccc(cc1)c1ccc(cc1)c1[nH]c(nc1)[C@H	1129633-62-0	Carbamic acid, N,N,Ä≤-[[1,1,Ä≤-biphenyl]-4,4,Ä≤-diylbis[1H-imidazole-5,2-diyl-(	C40H46N8O6	734.84	C	-8.6
4422	C(=O)([C@@H](NC(=O)OC)CC)N1[C@@H](CC[C@@H]1C)c1[nH]c(cn1)c1ccc(cc1)c1ccc(cc1)c1[nH]c(nc1)[C@H]1N(C(=	1129634-89-4	Carbamic acid, N,N,Ä≤-[[1,1,Ä≤-biphenyl]-4,4,Ä≤-diylbis[1H-imidazole-5,2-diyl[(	C48H50N8O6	834.96	C	-8.6
4423	C(=O)([C@H](NC(=O)OC)c1ccccc1)N1[C@@H](CCC1)c1[nH]c(c(C)n1)c1ccc(cc1)c1ccc(cc1)c1[nH]c(nc1)[C@H]1N(C	1129635-01-3	Carbamic acid, N,N,Ä≤-[[1,1,Ä≤-biphenyl]-4,4,Ä≤-diylbis[1H-imidazole-5,2-diyl[(	C38H46N8O6	710.82	C	-8.6
4424	C(=O)([C@@H](NC(=O)OC)C)N1[C@@H](CCC1)c1[nH]c(c(C)n1)c1ccc(cc1)c1ccc(cc1)c1[nH]c(nc1)[C@H]1N(C(=O)[C	1129636-11-8	Carbamic acid, N-[(1R)-2-[(2S)-2-[5-[4,Ä≤-[2-[(2S)-1-[(2R)-2-[(methoxycarbonyl)a	C47H48N8O6	820.93	C	-8.6
4425	C(Cc1ccccc1)[C@]1(CCC)CC(=C([C@H](CC)[13c]2[13cH][13c](NS(=O)(=O)c3ccc(C(F)(F)F)cn3)[13cH][13cH][13c	1141509-98-9	2-Pyridinesulfonamide, N-[3-[(1R)-1-[(6R)-5,6-dihydro-4-hydroxy-2-oxo-6-(2-pheny	C31H33F3N2O5S		C	-8.6
4426	C(=O)(N)c1nn(cn1)[C@@H]1O[C@H](CO)CC1	114371-63-0	Hydrazinecarbothioamide, 2-[1-(3,4-dihydro-4-oxo-3-phenyl-1-phthalazinyl)-1,2-di	C23H16N6O2S	440.48	C	-8.6
4427	O=c1n([C@H]2C[C@H](O[P@](=O)(SC[C@H]3O[C@H](C[C@@H]3O)n3c(=O)[nH]c(=O)c(C)c3)S)[C@@H](CO)O2)cc(C)c(=	1164118-00-6	Thymidine, [P(R)]-P-thiothymidylyl-(3,Ä≤,Üí5,Ä≤)-5,Ä≤-thio-	C20H27N4O10PS2	578.55	C	-8.6
4428	C(O)C1(CO)C(Cn2c3c(nc2)c(=O)nc(N)[nH]3)C1	1166382-14-4	Uridine 5,Ä≤-(tetrahydrogen triphosphate), 2,Ä≤-deoxy-5-(1,1,2,2,3,3,3-heptaflu	C12H14F7N2O14P3	636.15	C	-8.6
4429	Cc1n(c2c(n1)cccc2)c1nc(nc(n1)N1CCOCC1)c1cc(C#N)ccc1	1174190-65-8	Phenol, 3-[4-(2-methyl-1H-benzimidazol-1-yl)-6-(4-morpholinyl)-1,3,5-triazin-2-y	C21H20N6O2	388.42	C	-8.6
4430	Cc1n(c2c(n1)cccc2)c1nc(nc(n1)N1CCOCC1)c1cc(CO)ccc1	1174190-72-7	Phenol, 4-[4-(2-methyl-1H-benzimidazol-1-yl)-6-(4-morpholinyl)-1,3,5-triazin-2-y	C21H20N6O2	388.42	C	-8.6
4431	Cc1nn(c2nc(nc(n2)N2CCOCC2)c2ccc(N)nc2)cc1	1174191-16-2	1-Naphthalenesulfonic acid, 8-amino-5-[2-(6-sulfo-1-naphthalenyl)diazenyl]-	C20H15N3O6S2	457.48	C	-8.6
4432	C(F)(F)(F)c1nn(c2nc(nc(n2)N2CCOCC2)c2ccc(N)nc2)cc1	1174191-20-8	2-Pyridinamine, 5-[4-(3-methyl-1H-pyrazol-1-yl)-6-(4-morpholinyl)-1,3,5-triazin-	C16H18N8O	338.37	C	-8.6
4433	Cc1n(nc(C(=O)N)n1)[C@@H]1O[C@H](CO)[C@@H](O)C1	1174716-28-9	6H-Purin-6-one, 2-amino-9-[(1S,3R,4S)-3-[[[(4S)-4-(3-chlorophenyl)-2-oxido-1,3,2	C21H23ClN5O6P	507.86	C	-8.6
4434	O(C(N[C@H]([C@@H](CN(S(=O)(=O)c1ccc(N)cc1)CC(C)C)O)Cc1ccccc1)O)[C@@H]1[C@H]2[C@@H](OC1)OCC2	1177956-82-9	Not Yet Assigned	C36H45N5O4	611.77	C	-8.6
4435	N(C)(C)C1(C)c2c(-c3c1cc(cc3)c1[nH]c(nc1)[C@H]1N(C(=O)[C@@H](NC(=O)OC)C(C)C)CCC1)ccc(c2)c1[nH]c(nc1)[	1179150-25-4	Carbamic acid, N,N,Ä≤-[(5,7-dihydrodibenz[c,e]oxepin-3,9-diyl)bis[1H-imidazole-5	C46H56N8O9	864.98	C	-8.6
4436	C(=O)([C@@H](NC(=O)OC)C(C)C)N1[C@H]2[C@H](C2)C[C@H]1c1[nH]c2-c3c(CCc2n1)cc(cc3)c1cc2c(-c3c(CC2)nc([n	1180670-03-4	Carbamic acid, N,N,Ä≤-[(4,4,Ä≤,5,5,Ä≤-tetrahydro[7,7,Ä≤-bi-1H-naphth[1,2-d]imic	C44H54N8O6	790.95	C	-8.6
4437	C(=O)([C@@H](NC(=O)OC)C(C)C)N1[C@H](c2[nH]c3c4c(cc(cc4)c4cc5c(cc4)c4c(cc5)nc([nH]4)[C@H]4N(C(=O)[C@@	1180670-18-1	Carbamic acid, N-[(1S)-1-[[(1R,3S,5R)-3-[7-[4-[2-[(1R,3S,5R)-2-[(2S)-2-[(methoxy	C44H50N8O6	786.92	C	-8.6
4438	C(=O)([C@@H](NC(=O)OC)C(C)C)N1[C@H](c2[nH]c3c4c(cc(cc4)c4cccc(cc4)c4[nH]c(nc4)[C@H]4N(C(=O)[C@@H](NC(	1180670-28-3	Carbamic acid, N-[(1S)-1-[[(1R,3S,5R)-3-[7-[3-fluoro-4-[2-[(1R,3S,5R)-2-[(2S)-2-	C44H49FN8O6	804.91	C	-8.6
4439	C(=O)([C@@H](NC(=O)OC)C1CCOCC1)N1[C@H]2[C@H](C2)C[C@H]1c1[nH]c2c(n1)ccc1c2ccc(c1)c1ccc2c(cc1)c1c(cc2)	1180670-49-8	Carbamic acid, N-[(1S)-2-[(2S)-2-[2,Ä≤-[(2S)-1-[(2S)-2-[(methoxycarbonyl)amino]-	C46H52N8O7	828.95	C	-8.6
4440	C(=C1C=C(C(=O)O)C(=O)C=C1)(c1cc(C(=O)O)c(O)cc1)c1ccc(C(=O)O)c(O)cc1.[NaH]	1184957-98-9	2-Naphthalenesulfonic acid, 5-amino-8-[2-(4-amino-2,5-dimethylphenyl)diazenyl]-	C18H18N4O3S	370.43	C	-8.6
4441	N(CCN1CCOCC1)c1nc(nc(NCCCNc2c3c(ncc2)cc(Cl)cc3)n1)N1CCOCC1	1190366-32-5	1,3-Propanediamine, N1-(7-chloro-4-quinolinyl)-N3-[4-(4-methyl-1-piperazinyl)-6-	C24H32ClN9O	498.02	C	-8.6
4442	N(=C(NC(=O)OC(OC(=O)[C@H](C(C)C)N)C)NC(=O)OC(OC(=O)[C@H](C(C)C)N)C)[C@@H]1[C@@H](NC(=O)C)[C@H](OC(CC	1192537-89-5	Not Yet Assigned	C36H46N6O15	802.78	C	-8.6
4443	O[C@H]1[C@@H](O[C@H](COP(=O)(C(P(=O)(O)O)(F)F)O)[C@H]1O)n1c(=O)[nH]c(=O)c(I)c1	1199574-56-5	1H-Indole-5-sulfonamide, 2,3-dihydro-N-(4-methylphenyl)-2,3-dioxo-	C15H12N2O4S	316.33	C	-8.6
4444	C(#N)C1=C(N)Oc2c(C1c1cn(c(=O)[nH]c1=O)[C@H]1C[C@H](OC(=O)C)[C@@H](COC(=O)C)O1)c(=O)n(C)c(C)c2	1217551-22-8	L-Phenylalanine, N-acetyl-L-phenylalanyl-N-(1-tricyclo[3.3.1.13,7]dec-1-ylethyl)	C36H46N4O6	630.77	C	-8.6
4445	N(Cc1ccc(/C=C/C(=O)NO)cc1)C12CC3(CO)CC(C2)CC(C1)C3	1221411-85-3	2-Propenamide, 3-[4-[[(3-chlorotricyclo[3.3.1.13,7]dec-1-yl)amino]methyl]phenyl]	C20H25ClN2O2	360.88	C	-8.6

4446	N(Cc1ccc(C(=O)Nc2c(N)cccc2)cc1)C12CC3CC(C2)CC(C1)C3	1221411-87-5	2-Propenamide, N-hydroxy-3-[4-[[[3-(hydroxymethyl)tricyclo[3.3.1.13,7]dec-1-yl]a	C21H28N2O3	356.46	C	-8.6
4447	c1(nc(nc(n1)N1CCOCC1)N1CCOCC1)c1c2c(ccc1)[nH]nc2	1225037-89-7	1H-Indole, 4-(4,6-di-4-morpholinyl-1,3,5-triazin-2-yl)-	C19H22N6O2	366.42	C	-8.6
4448	O=c1n([C@@H]2O[C@H](CO)CC2)ccc(=S)[nH]1	1225639-01-9	1,3,5-Triazin-2-amine, 4-(1H-benzimidazol-7-yl)-6-(4-morpholinyl)-N-(2-pyridinyl	C20H20N8O	388.43	C	-8.6
4449	C(=O)([C@H](N(C)C)c1ccccc1)N1[C@@H](CCC1)c1[nH]c(cn1)[C@@H]1CC[C@H](CC1)c1ccc(cc1)c1[nH]c(nc1)[C@H]1	1228669-17-7	Carbamic acid, N-[(1S)-1-[[(2S)-2-[5-[4-[4-[2-[(2S)-1-[(2S)-2-[(methoxycarbonyl)	C42H58N8O6	770.96	C	-8.6
4450	C(=O)([C@H](NC(=O)OC)c1ccccc1)N1[C@@H](CCC1)c1[nH]c(cn1)c1ccc(cc1)[C@@H]1CC[C@H](CC1)c1[nH]c(nc1)[C@	1228670-78-7	Carbamic acid, N-[(1R)-2-[(2S)-2-[5-[trans-4-[4-[2-[(2S)-1-[(2S)-2-[(methoxycarb	C43H54N8O6	778.94	C	-8.6
4451	C(=O)([C@H](NC(=O)OC)C)N1[C@H](c2[nH]c(C34CCC(CC3)(CC4)c3ccc(cc3)c3[nH]c(nc3)[C@H]3N(C(=O)[C@@H](NC(	1228671-52-0	Carbamic acid, N-[(1S)-2-[(2S)-2-[5-[4-[4-[2-[(2S)-1-[(2S)-2-[(methoxycarbonyl)a	C40H54N8O6	742.91	C	-8.6
4452	C(=O)([C@H](N(CC)CC)c1ccccc1)N1[C@@H](CCC1)c1[nH]c(cn1)c1ccc(C#Cc2ccc(cc2)c2[nH]c(nc2)[C@H]2N(C(=O)[	1228967-09-6	Carbamic acid, N-[(1S)-2-methyl-1-[[(2S)-2-[5-[4-[2-[4-[2-[(2S)-1-[(2R)-2-phenyl	C47H52N8O4	792.97	C	-8.6
4453	O=C1N(c2c(C1=NNC(=S)NCC)cccc2)[C@H]1[C@H](OC(=O)C)[C@H](OC(=O)C)[C@@H](COC(=O)C)O1	1234340-16-9	1H-Indole-2,3-dione, 5-[(3,3-difluoro-1-azetidinyl)sulfonyl]-	C11H8F2N2O4S	302.25	C	-8.6
4454	N(C(=O)[C@H](Cc1ccccc1)C[C@@H](CN1[C@H](C(=O)NC(C)(C)C)CN(Cc2cn3c(cn2)ncc3)CC1)O)[C@H]1c2c(C[C@H]1O)	1237808-93-3	Not Yet Assigned	C39H48N4O4S	668.89	C	-8.6
4455	N(C(=O)[C@H](Cc1ccccc1)C[C@@H](CN1[C@H](C(=O)NC(C)(C)C)CN(Cc2cc3c(nc2)ccs3)CC1)O)[C@H]1c2c(C[C@H]1O)	1237808-96-6	Not Yet Assigned	C37H47N7O4	653.81	C	-8.6
4456	N(CC(=O)[C@H](Cc1ccccc1)C[C@@H](CN1[C@H](C(=O)NC(C)(C)C)CN(Cc2c(C)ccnc2C)CC1)O)[C@H]1c2c(C[C@H]1O)cc	1237988-79-2	Not Yet Assigned	C39H53N5O4	655.87	C	-8.6
4457	O=C1[C@@H]2[C@@H](c3c([C@H](OC(=O)N4CCN(Cc5cc6c(cc5)OCO6)CC4)[C@H]2CO1)cc1c(c3)OCO1)c1cc(OC)c(OC)c(O	123824-79-3	[1,4,Ä≤-Bipiperidine]-1,Ä≤-carboxylic acid, 5,5a,6,8,8a,9-hexahydro-8-oxo-9-(3,	C33H40N2O9	608.68	C	-8.6
4458	N(c1nc(nc(NNC(=O)c2ccncc2)n1)N1CCOCC1)c1ccccc1	1239885-09-6	4-Pyridinecarboxylic acid, 2-[4-(3,4-dihydro-1(2H)-quinolinyl)-6-(4-morpholinyl)	C22H24N8O2	432.48	C	-8.6
4459	C(F)(F)c1n(c2c(n1)cccc2)c1nc(nc(N[C@H]2CC[C@H](NS(=O)(=O)C)CC2)n1)N1CCOCC1	1240023-59-9	1H-Benzimidazol-6-amine, N-[4-[2-(difluoromethyl)-1H-benzimidazol-1-yl]-6-(4-mor	C22H23F2N9O	467.47	C	-8.6
4460	C(=O)([C@@H](N(C(=O)OC)C)C(C)C)N1[C@H](c2[nH]c(c3cc4c(-c5c(C4)cc(cc5)c4[nH]c(nc4)[C@H]4N(C(=O)[C@@H]	1240880-73-2	Carbamic acid, N,N,Ä≤-[9H-fluorene-2,7-diylbis[1H-imidazole-5,2-diyl-(2S)-2,1-py	C41H50N8O6	750.89	C	-8.6
4461	C(=O)([C@@H](NC(=O)OC1CCOCC1)C(C)C)N1[C@H](c2[nH]c(c3cc4c(-c5c(C4)cc(cc5)c4[nH]c(nc4)[C@H]4N(C(=O)[C	1240880-74-3	Carbamic acid, N,N,Ä≤-[9H-fluorene-2,7-diylbis[1H-imidazole-5,2-diyl-(2S)-2,1-py	C43H54N8O6	778.94	C	-8.6
4462	C(=O)([C@@H](NC(=O)OC)C(C)C)N1[C@@H](CCC1)c1[nH]c(cn1)[C@H]1CC[C@@H](CC1)c1ccc(cc1)c1[nH]c([C@@H](N(	1240890-58-7	Carbamic acid, N-[(1R)-2-[[(1S)-1-[5-[4-[trans-4-[2-[(2S)-1-[2-[(methoxycarbonyl	C39H48N8O6	724.85	C	-8.6
4463	C(=O)([C@H](NC(=O)OC)c1ccccc1)N1[C@@H](CCC1)c1[nH]c(cn1)c1ccc(ccc1)[C@@H]1CC[C@H](CC1)c1[nH]c(nc1)[C@	1240891-19-3	Carbamic acid, N-[(1R)-2-[(2S)-2-[5-[4-[cis-4-[2-[(2S)-1-[(2R)-2-[(methoxycarbor	C46H52N8O6	812.96	C	-8.6
4464	C(=O)([C@H](NC(=O)OC)c1ccccc1)N1[C@@H](CCC1)c1[nH]c(cn1)C1CCC(=CCCCc2[nH]c(nc2)[C@H]2N(C(=O)[C@H](NC	1240891-98-8	Carbamic acid, N-[(1R)-2-[(2S)-2-[5-[4-[(2E)-4-[2-[(2S)-1-[(2R)-2-[(methoxycarbo	C44H48N8O6	784.9	C	-8.6
4465	C(=O)([C@@H](NC(=O)OC)C(C)C)N1[C@@H](CCC1)c1[nH]c(CCc2cc3c(cc2)cc(CCc2[nH]c(nc2)[C@H]2N(C(=O)[C@@H](	1240893-43-9	Carbamic acid, N,N,Ä≤-[1,4-phenylenebis[2,1-ethanediyl-1H-imidazole-5,2-diyl-(2S	C38H54N8O6	718.89	C	-8.6
4466	O=C1[C@@H]2[C@@H](c3c([C@H](OC(=O)CCCCCCCC=CCC=CCCCCC)[C@H]2CO1)cc1c(c3)OCO1)c1cc(OC)c(OC)c(OC)c1	1240893-60-0	Carbamic acid, N,N,Ä≤-[2,6-naphthalenediylbis[2,1-ethanediyl-1H-imidazole-5,2-di	C42H56N8O6	768.94	C	-8.6
4467	C(=O)N1[C@H]2[C@@H]1[C@@H](CO)O[C@H]2n1c(=O)nc(N)cc1	1241404-87-4	Carbamic acid, N,N,Ä≤-[[1,1,Ä≤-bicyclohexyl]-4,4,Ä≤-diylbis[1H-imidazole-5,2-di	C46H58N8O6	819	C	-8.6
4468	C(C(C(Cc1cc(O)c(O)cc1)C)C)c1cc(O)c([O])cc1	1242712-29-3	Uridine 5,Ä≤-(trihydrogen diphosphate), 5-(4-methoxyphenyl)-, P,Ä≤-Œ±-D-galacto	C22H30N2O18P2	672.42	C	-8.6
4469	O=c1n([C@H]2C[C@H](OCC)[C@@H](CO)O2)cc(C)c(N)n1	1246852-30-1	Carbamic acid, N-[(1S)-2-[(2S)-2-[5-[4-[4-[2-[(2S)-1-[(2S)-2-[(methoxycarbonyl)a	C36H46N8O6	686.8	C	-8.6
4470	C(=O)([C@@H](NC(=O)OC)C(C)C)N1[C@H](c2[nH]c(c3ccc([C@@H]4[C@H](C4)c4ccc(cc4)c4[nH]c(nc4)[C@H]4N(C(=O	1250938-88-5	Carbamic acid, N,N,Ä≤-[(1R,2R)-1,2-cyclopropanediylbis[4,1-phenylene-1H-imidazol	C45H54N8O6	802.96	C	-8.6
4471	C(=O)(C(NC(=O)OC)C(C)C)N1C(CCC1)c1[nH]c(cn1)C1Cc2c(C1)cc1c(c2)CC(C1)c1[nH]c(nc1)C1N(C(=O)C(NC(=O)OC)	1255935-55-7	Carbamic acid, N,N,Ä≤-[2,6-anthracenediylbis[1H-imidazole-5,2-diyl-(2S)-2,1-pyr	C42H50N8O6	762.9	C	-8.6
4472	C(=O)([C@@H](NC(=O)OC)C(C)C)N1[C@@H]([C@@H]2C[C@H]1CC2)c1[nH]c(cn1)c1cc2c(-c3c(COC2)cc(cc3)c2[nH]c(n	1256384-41-4	Carbamic acid, N,N,Ä≤-[(6,7-dihydro-6-methyl-5-oxo-5H-dibenz[c,e]azepine-3,9-diy	C43H53N9O7	807.94	C	-8.6
4473	C(=O)([C@@H](NC(=O)OC)C(C)C)N1[C@@H](CCC1)c1[nH]c(cn1)c1ccc(cc1)c1ccc(cc1)c1ccc(cc1)c1[nH]c(nc1)[C@H	1256384-64-1	Carbamic acid, N-[(1S)-1-[[(2S)-2-[5-[5,7-dihydro-9-[2-[(1R,3S,4S)-2-[(2S)-2-[(m	C44H54N8O7	806.95	C	-8.6
4474	C(=O)([C@@H](NC(=O)OC)C(C)C)N1[C@@H](C[C@H](Cl)c1ccccc1)c1[nH]c(cn1)c1ccc(cc1)c1cc2c(cc1)cc(cc2)c1[n	1256389-81-7	Carbamic acid, N-[(1R)-2-[(2S)-2-[5-[4-[6-[2-[(2S)-1-[(2S)-2-[(methoxycarbonyl)a	C48H52N8O6	836.98	C	-8.6
4475	C(=O)([C@@H](NC(=O)OC)C(C)C)N1CC2(C[C@H]1c1[nH]c(cn1)c1ccc(cc1)c1ccc(cc1)c1[nH]c(nc1)[C@H]1N(C(=O)[C	1256390-33-6	Carbamic acid, N-[(1S)-1-[[(2S)-2-[5-[4,Ä≤-[2-[(1R)-2,3-dihydro-2-[(2S)-2-[(meth	C44H50N8O6	786.92	C	-8.6
4476	C(=O)([C@H](NC(=O)OC)c1ccccc1)N1[C@@H](CCC1)c1[nH]c(cn1)c1cc2c(cc1)cc(cc2)c1ccc(cc1)c1[nH]c(nc1)[C@H	1256391-33-9	Carbamic acid, N-[(1S)-2-[(2S)-2-[5-[6-[4-[2-[(2S)-1-[(2S)-2-[(methoxycarbonyl)a	C47H50N8O6	822.95	C	-8.6
4477	C(=O)(Nc1sc(N(=O)=O)cn1)c1cc(OC(=O)c2ccccc2)cccc1	1259403-50-3	Adenosine, 5,Ä≤-(2-amino-2-oxoethyl)-5,Ä≤-deoxy-3,Ä≤-O-methyladenylyl-(2,Ä≤,Üí5	C32H43N15O17P2	971.72	C	-8.6
4478	C(=O)([C@@H](NC(=O)OC)C(C)C)N1[C@@H](CCC1)c1[nH]c(cn1)c1ccc(cc1)c1cc2c(cc1)cc(cc2)c1[nH]c(nc1)[C@H]1	1261070-50-1	Carbamic acid, N-[2-[(2S)-2-[5-[6-[4-[2-[(2S)-1-[(2S)-2-[(methoxycarbonyl)amino]	C43H50N8O6	774.91	C	-8.6
4479	N(=C\1/c2c(N(CN3CCN(CC3)c3cc4c(cc3F)c(=O)c(C(=O)O)cn4CC)C1=O)ccc(F)c2)\NC(=S)NO	1265623-11-7	3-Quinolinecarboxylic acid, 7-[4-[[(3Z)-5-chloro-2,3-dihydro-3-[2-[(methoxyaminc	C27H27ClFN7O5S	616.06	C	-8.6
4480	O=c1c2c(n(cc1C(=O)O)C1CC1)cc(c(F)c2)N1CCN(CN2c3c(/C(=N/NC(=S)NO)/C2=O)cc(F)cc3)CC1	1265623-16-2	3-Quinolinecarboxylic acid, 7-[4-[[(3Z)-5-chloro-2,3-dihydro-3-[2-[(methoxyaminc	C28H27ClFN7O5S	628.07	C	-8.6
4481	O(C)c1c2n(cc(C(=O)O)c(=O)c2cc(F)c1N1CC(C)N(CN2c3c(/C(=N/NC(=S)NOC)/C2=O)cc(F)cc3)CC1)C1CC1	1265623-29-7	3-Quinolinecarboxylic acid, 1-cyclopropyl-6-fluoro-7-[4-[[(3Z)-5-fluoro-2,3-dihy	C29H29F2N7O6S	641.65	C	-8.6
4482	C(=O)([C@@H](NC(=O)OC)C(C)C)N1[C@@H](CC2(C1)OCCCO2)c1[nH]c(cn1)c1ccc(cc1)c1ccc(cc1)c1[nH]c(nc1)[C@H]	1272655-03-4	Carbamic acid, N,N,Ä≤-[[1,1,Ä≤-biphenyl]-4,4,Ä≤-diylbis[1H-imidazole-5,2-diyl[(	C50H66N8O10	939.11	C	-8.6
4483	C(=O)([C@@H](NC(=O)OC)C)N1[C@@H]([C@@H]2C[C@H]1CC2)c1[nH]c(cn1)c1ccc(cc1)c1ccc(cc1)c1[nH]c(nc1)[C@H]	1273589-44-8	Carbamic acid, N-[(1R)-2-[(2S)-2-[5-[4,Ä≤-[2-[(1R,3S,4S)-2-[(2R)-2-[(methoxycarb	C38H44N8O6	708.81	C	-8.6
4484	C(=O)([C@H](NC(=O)OC)CC)N1[C@@H]([C@@H]2C[C@H]1CC2)c1[nH]c(cn1)c1ccc(cc1)c1ccc(cc1)c1[nH]c(nc1)[C@H]	1273589-50-6	Carbamic acid, N-[(1S)-1-[[(2S)-2-[5-[4,Ä≤-[2-[(1R,3S,4S)-2-[(2S)-2-[(methoxyca	C40H48N8O6	736.86	C	-8.6
4485	C(=O)([C@@H](NC(=O)OC)C(C)C)N1[C@@H]([C@@H]2C[C@H]1CC2)c1[nH]c(cn1)c1ccc(cc1)c1ccc(cc1)c1[nH]c(nc1)[	1273591-06-2	Carbamic acid, N-[(1S)-1-[[(2S)-4,4-difluoro-2-[5-[4,Ä≤-[2-[(1R,3S,4S)-2-[(2S)-2	C42H50F2N8O6	800.89	C	-8.6
4486	C(=O)([C@@H](NC(=O)OC)C(C)C)N1[C@@H]([C@@H]2C[C@H]1[C@@H](OC)C2)c1[nH]c(cn1)c1ccc(cc1)c1ccc(cc1)c1[n	1273591-22-2	Carbamic acid, N-[(1S)-1-[[(2S)-2-[5-[4,Ä≤-[2-[(1S,3S,4R,6S)-6-hydroxy-2-[(2S)-2	C42H52N8O7	780.91	C	-8.6
4487	C(=O)([C@@H](NC(=O)OC)C(C)C)N1[C@@H]([C@@H]2C[C@H]1CC2)c1[nH]c(cn1)c1c(F)cc(cc1)c1cc(F)c(cc1)c1[nH]c	1273591-42-6	Carbamic acid, N-[(1S)-1-[[[(1S)-1-[5-[4,Ä≤-[2-[(1R,3S,4S)-2-[(2S)-2-[(methoxyca	C43H56N8O6	780.95	C	-8.6
4488	N(c1nc(nc(N/N=C/c2cc3c(cc2)cccc3)n1)N1CCOCC1)c1ccc(F)cc1	1279692-09-9	1-Naphthalenecarboxaldehyde, 2-[4-[(4-fluorophenyl)amino]-6-(4-morpholinyl)-1,3,	C24H22FN7O	443.48	C	-8.6
4489	N(=C\1/c2c(NC1=O)cccc2)/Nc1nc(nc(NCc2ccccc2)n1)N1CCOCC1	1279692-12-4	Benzaldehyde, 2-hydroxy-, 2-[4-[(4-fluorophenyl)amino]-6-(4-morpholinyl)-1,3,5-t	C20H20FN7O2	409.42	C	-8.6
4490	C(=O)([C@@H](NC(=O)OC)C(C)C)N1[C@@H](C[C@@H](C)C1)c1[nH]c(cn1)c1cc2c(cc1)cc(cc2)c1ccc(cc1)c1[nH]c(nc	1306734-19-9	Carbamic acid, N-[(1S)-1-[[(2S,4S)-2-[5-[4-[6-[2-[(2S,4S)-1-[(2S)-2-[(methoxycar	C44H52N8O6	788.93	C	-8.6
4491	C(=O)([C@@H](NC(=O)OC)C(C)C)N1[C@@H](CCC1)c1[nH]c(/C=C/c2ccc(/C=C/c3[nH]c(nc3)[C@H]3N(C(=O)[C@@H](NC	1307303-72-5	Carbamic acid, N,N,Ä≤-[1,4-phenylenebis[(1E)-2,1-ethenediyl-1H-imidazole-5,2-diy	C34H42N8O6	658.75	C	-8.6
4492	S=c1n(nc(s1)c1cc(N(=O)=O)ccc1)c1nc(nc(n1)N1CCOCC1)N1CCOCC1	1309437-29-3	1,3,4-Thiadiazole-2(3H)-thione, 3-(4,6-di-4-morpholinyl-1,3,5-triazin-2-yl)-5-(2	C19H20N8O4S2	488.54	C	-8.6
4493	S(=O)(=O)(NCCNCC=Cc1ccc(Br)cc1)c1c2c(ccc1)cncc2.Cl	1309561-76-9	Cyclopenta[d]pyrido[1,Ä≤,2,Ä≤:4,5]pyrazino[2,1-b][1,3]oxazine-9-carboxamide, N-	C22H21F2N3O5	445.42	C	-8.6
4494	C(=O)([C@@H](NC(=O)OC)C1CCOCC1)N1[C@H]2[C@H](C2)C[C@H]1c1[nH]c(c(Cl)n1)c1ccc(cc1)c1ccc(cc1)c1[nH]c(n	1312336-02-9	Carbamic acid, N-[(1S)-2-[(1R,3S,5R)-3-[5-[4,Ä≤-[5-chloro-2-[(1R,3S,5R)-2-[(2S)-	C46H53ClN8O8	881.41	C	-8.6
4495	C(=O)([C@@H](NC(=O)OC)C1CCCC1)N1[C@H]2[C@H](C2)C[C@H]1c1[nH]c(c(Cl)n1)c1ccc(cc1)c1ccc(cc1)c1[nH]c(nc1	1312336-14-3	Carbamic acid, N-[(1S)-2-[(1R,3S,5R)-3-[5-chloro-4-[4,Ä≤-[5-chloro-2-[(1R,3S,5R)	C44H48Cl2N8O7	871.81	C	-8.6
4496	C(=O)([C@@H](NC(=O)OC)C1CCOCC1)N1[C@H]2[C@H](C2)C[C@H]1c1[nH]c(c(Cl)n1)c1ccc(cc1)c1ccc(cc1)c1[nH]c(n	1312336-15-4	Carbamic acid, N-[(1S)-2-[(1R,3S,5R)-3-[5-chloro-4-[4,Ä≤-[5-chloro-2-[(1R,3S,5R)	C45H50Cl2N8O7	885.83	C	-8.6
4497	C(=O)([C@@H](NC(=O)OC)[C@@H]1CCCOC1)N1[C@H]2[C@H](C2)C[C@H]1c1[nH]c(c(Cl)n1)c1ccc(cc1)c1ccc(cc1)c1[n	1312336-70-1	Carbamic acid, N,N,Ä≤-[[1,1,Ä≤-biphenyl]-4,4,Ä≤-diylbis[(5-fluoro-1H-imidazole-	C46H52F2N8O8	882.95	C	-8.6
4498	C(=O)([C@@H](NC(=O)OC)[C@H]1CCCOC1)N1[C@H]2[C@H](C2)C[C@H]1c1[nH]c(c(Cl)n1)c1ccc(cc1)c1ccc(cc1)c1[nH	1312336-75-6	Carbamic acid, N-[(1S)-2-[(1R,3S,5R)-3-[5-[4,Ä≤-[5-chloro-2-[(1R,3S,5R)-2-[(2S)-	C46H53ClN8O8	881.41	C	-8.6
4499	Cn1c(c2c([nH]c(n2)[C@H]2N(C(=O)[C@@H](NC(=O)OC)C(C)C)CCC2)c2ccc(cc2)c2ccc(cc2)c2[nH]c(nc2)[C@H]2N(C(	1312337-04-4	Carbamic acid, N-[(1S)-2-[(2S)-2-[4-[4,Ä≤-[2-[(2S)-1-[(2S)-2-[(methoxycarbonyl)a	C48H58N10O8	903.04	C	-8.6
4500	Cn1c(c2c([nH]c(n2)[C@H]2N(C(=O)[C@@H](NC(=O)OC)C3CCOCC3)CCC2)c2ccc(cc2)c2ccc(cc2)c2[nH]c(nc2)[C@H]2N	1312337-05-5	Carbamic acid, N-[(1S)-1-[[(2S)-2-[5-[4,Ä≤-[2-[(2S)-1-[(2S)-2-[(methoxycarbonyl)	C44H54N10O6	818.96	C	-8.6
4501	C(=O)([C@@H](NC(=O)OC)C1CCOCC1)N1[C@@H](C[C@H](C)C1)c1[nH]c(cn1)c1ccc(cc1)c1ccc(cc1)c1[nH]c(nc1)[C@H	1312338-23-0	Carbamic acid, N-[(1S)-2-[(1R,3S,5R)-3-[5-[4,Ä≤-[2-[(1R,3S,5R)-2-[(2S)-2-[(metho	C44H52N8O7	804.93	C	-8.6
4502	C(=O)([C@@H](NC(=O)OC)C(C)C)N1[C@H](c2[nH]c(c3c4c(c(cc3)c3ccc(cc3)c3[nH]c(nc3)[C@H]3N(C(=O)[C@@H](NC	1312608-46-0	Carbamic acid, N-[(1S)-1-[[(2S)-2-[5-[4-[7-[2-[(2S)-1-[(2S)-2-[(methoxycarbonyl)	C41H50N8O8	782.88	C	-8.6
4503	C(=O)([C@@H](NC(=O)OC)C(C)C)N1[C@H](c2[nH]c(c3c4c(c(cc3)c3ccc(cc3)c3[nH]c(nc3)[C@H]3N(C(=O)[C@@H](NC	1312608-47-1	Carbamic acid, N-[(1S)-2-methyl-1-[[(2S)-2-[5-[4-[5,6,7,8-tetrahydro-4-[2-[(2S)-	C44H56N8O6	792.97	C	-8.6
4504	C(=O)([C@@H](NC(=O)OC)C(C)C)N1[C@H](c2[nH]c(c3c4c(c(cc3)c3ccc(cc3)c3[nH]c(nc3)[C@H]3N(C(=O)[C@@H](NC	1312608-69-7	Carbamic acid, N-[(1S)-1-[[(2S)-2-[5-[5-[4-[2-[(2S)-1-[(2S)-2-[(methoxycarbonyl)	C43H51N9O6	789.92	C	-8.6
4505	C(=O)([C@@H](NC(=O)OC)C(C)C)N1[C@H](c2[nH]c(c3cc4c(c5c(cc(cc5)c5[nH]c(nc5)[C@H]5N(C(=O)[C@@H](NC(=O)	1312608-80-2	Carbamic acid, N-[(1S)-2-methyl-1-[[(2S)-2-methyl-2-[5-[4-[5,6,7,8-tetrahydro-4-	C46H60N8O6	821.02	C	-8.6
4506	O(Cc1ccc(F)cc1)c1c2c(c(cc1)c1ccccc1)oc(c(O)c2=O)c1[nH]c(OC)nn1	1312894-44-2	4H-1-Benzopyran-4-one, 5-[(4-fluorophenyl)methoxy]-3-hydroxy-2-(3-methyl-1H-1,2,	C25H18FN3O4	443.43	C	-8.6
4507	O(Cc1ccc(F)cc1)c1c2c(c(cc1)c1ccccc1)oc(c(O)c2=O)c1[nH]c(nn1)c1ccccc1	1312894-46-4	4H-1-Benzopyran-4-one, 2-(5-chloro-1H-1,2,4-triazol-3-yl)-5-[(4-fluorophenyl)met	C24H15ClFN3O4	463.85	C	-8.6
4508	O(Cc1ccc(F)cc1)c1c2c(oc(c(O)c2=O)c2[nH]c(C)nn2)c(C)c(C)c1	1312895-43-4	4H-1-Benzopyran-4-one, 5-[(4-fluorophenyl)methoxy]-3-hydroxy-7,8-dimethyl-2-(1H-	C20H16FN3O4	381.36	C	-8.6
4509	O(Cc1ccc(F)cc1)c1c2c(oc(c(O)c2=O)c2[nH]c(C)cn2)c(C)c(C)c1	1312895-48-9	4H-1-Benzopyran-4-one, 5-[(4-fluorophenyl)methoxy]-3-hydroxy-2-(1H-imidazol-2-yl	C21H17FN2O4	380.37	C	-8.6
4510	O(C)c1c2c(c(OCc3ccc(F)cc3)cc1C)c(=O)c(O)c(o2)c1[nH]ccn1	1312895-97-8	4H-1-Benzopyran-4-one, 5-[(4-fluorophenyl)methoxy]-3-hydroxy-8-methoxy-7-methyl-	C26H20FN3O5	473.45	C	-8.6
4511	O(Cc1ccc(F)cc1)c1c2c(oc(c(O)c2=O)c2[nH]c(C)nn2)c(Cl)c(C)c1	1312896-43-7	4H-1-Benzopyran-4-one, 8-chloro-5-[(4-fluorophenyl)methoxy]-3-hydroxy-7-methyl-2	C19H13ClFN3O4	401.78	C	-8.6
4512	O(Cc1ccc(F)cc1)c1c2c(c(c(C)c1)c1ccccc1)oc(c(O)c2=O)c1[nH]c(nn1)c1ccccc1	1312896-96-0	4H-1-Benzopyran-4-one, 2-(5-chloro-1H-1,2,4-triazol-3-yl)-5-[(4-fluorophenyl)met	C25H17ClFN3O4	477.87	C	-8.6
4513	O(Cc1ccc(F)cc1)c1c2c(c(OC)c(OC)c1)oc(c(O)c2=O)c1[nH]cnn1	1312898-49-9	4H-1-Benzopyran-4-one, 5-[(4-fluorophenyl)methoxy]-3-hydroxy-7,8-dimethoxy-2-(5-	C28H21FN2O6	500.47	C	-8.6
4514	O(Cc1ccc(F)cc1)c1c2c(c(OC)c(OC)c1)oc(c(O)c2=O)c1[nH]ccn1	1312898-59-1	4H-1-Benzopyran-4-one, 5-[(4-fluorophenyl)methoxy]-3-hydroxy-7,8-dimethoxy-2-(3-	C26H20FN3O6	489.45	C	-8.6
4515	O(C)c1c(c2c(c(OCc3ccc(F)cc3)c1)c(=O)c(O)c(o2)c1[nH]c(OC)nn1)c1ccccc1	1312899-56-1	4H-1-Benzopyran-4-one, 5-[(4-fluorophenyl)methoxy]-3-hydroxy-7-methoxy-2-(3-meth	C26H20FN3O5	473.45	C	-8.6
4516	O(C)c1c(c2c(c(OCc3ccc(F)cc3)c1)c(=O)c(O)c(o2)c1[nH]c(nn1)c1ccccc1)c1ccccc1	1312899-58-3	4H-1-Benzopyran-4-one, 2-(5-chloro-1H-1,2,4-triazol-3-yl)-5-[(4-fluorophenyl)met	C25H17ClFN3O5	493.87	C	-8.6
4517	O(Cc1ccc(F)cc1)c1c2c(oc(c(O)c2=O)c2[nH]c(C)nn2)c(C)c(Cl)c1	1312901-14-6	4H-1-Benzopyran-4-one, 7-chloro-5-[(4-fluorophenyl)methoxy]-3-hydroxy-8-methyl-2	C19H13ClFN3O4	401.78	C	-8.6
4518	O(Cc1ccc(F)cc1)c1c2c(c(OC)c(Cl)c1)oc(c(O)c2=O)c1ncccn1	1312901-23-7	4H-1-Benzopyran-4-one, 7-chloro-5-[(4-fluorophenyl)methoxy]-3-hydroxy-8-methyl-2	C26H18ClFN2O4	476.88	C	-8.6
4519	O(Cc1ccc(F)cc1)c1c2c(oc(c(O)c2=O)c2[nH]c(C)nn2)c(Cl)c(Cl)c1	1312902-14-9	4H-1-Benzopyran-4-one, 7,8-dichloro-5-[(4-fluorophenyl)methoxy]-3-hydroxy-2-(1H-	C18H10Cl2FN3O4	422.19	C	-8.6
4520	O(Cc1ccc(F)cc1)c1c2c(c(c(Cl)c1)c1ccccc1)oc(c(O)c2=O)c1nnc(Cl)[nH]1	1312902-66-1	4H-1-Benzopyran-4-one, 7-chloro-5-[(4-fluorophenyl)methoxy]-3-hydroxy-2-(5-metho	C25H17ClFN3O5	493.87	C	-8.6
4521	O(Cc1ccc(F)cc1)c1c2c(c(c(Cl)c1)c1ccccc1)oc(c(O)c2=O)c1[nH]cc(Cl)n1	1312902-71-8	4H-1-Benzopyran-4-one, 7-chloro-5-[(4-fluorophenyl)methoxy]-3-hydroxy-2-(5-metho	C26H18ClFN2O5	492.88	C	-8.6
4522	O(Cc1ccc(F)cc1)c1c2c(oc(c(O)c2=O)c2ncc(Cl)cn2)cc(c1)c1ccccc1	1312903-06-2	4H-1-Benzopyran-4-one, 5-[(4-fluorophenyl)methoxy]-3-hydroxy-2-(5-methoxy-2-pyri	C27H19FN2O5	470.45	C	-8.6
4523	O(Cc1ccc(F)cc1)c1c2c(oc(c(O)c2=O)c2[nH]c(OC)nn2)cc(c1)c1ccccc1	1312903-15-3	4H-1-Benzopyran-4-one, 5-[(4-fluorophenyl)methoxy]-3-hydroxy-2-(3-methyl-1H-1,2,	C25H18FN3O4	443.43	C	-8.6
4524	O(Cc1ccc(F)cc1)c1c2c(oc(c(O)c2=O)c2[nH]c(OC)cn2)cc(c1)c1ccccc1	1312903-20-0	4H-1-Benzopyran-4-one, 5-[(4-fluorophenyl)methoxy]-3-hydroxy-2-(5-methyl-1H-imic	C26H19FN2O4	442.44	C	-8.6
4525	O(Cc1ccc(F)cc1)c1c2c(c(C)c(c1)c1ccccc1)oc(c(O)c2=O)c1ncc(OC)cn1	1312903-55-1	4H-1-Benzopyran-4-one, 5-[(4-fluorophenyl)methoxy]-3-hydroxy-8-methyl-2-(5-methy	C28H21FN2O4	468.48	C	-8.6
4526	O(Cc1ccc(F)cc1)c1c2c(c(Cl)c(c1)c1ccccc1)oc(c(O)c2=O)c1[nH]c(C)cn1	1312905-27-3	4H-1-Benzopyran-4-one, 8-chloro-5-[(4-fluorophenyl)methoxy]-3-hydroxy-2-(1H-imic	C25H16ClFN2O4	462.86	C	-8.6
4527	O(Cc1ccc(F)cc1)c1c2c(c(c(c1)c1ccccc1)c1ccccc1)oc(c(O)c2=O)c1ncc(Cl)cn1	1312905-68-2	4H-1-Benzopyran-4-one, 5-[(4-fluorophenyl)methoxy]-3-hydroxy-2-(5-methoxy-2-pyri	C33H23FN2O5	546.54	C	-8.6
4528	O(C)c1c2c(cc(OCc3ccc(F)cc3)c1)c(=O)c(O)c(o2)c1[nH]cnn1	1312907-18-8	4H-1-Benzopyran-4-one, 6-[(4-fluorophenyl)methoxy]-3-hydroxy-8-methoxy-2-(5-pher	C27H19FN2O5	470.45	C	-8.6
4529	O=c1c2c(c(cc(OCc3ccc(F)cc3)c2)c2ccccc2)oc(c1O)c1[nH]c(nn1)c1ccccc1	1312908-27-2	4H-1-Benzopyran-4-one, 2-(5-chloro-1H-1,2,4-triazol-3-yl)-6-[(4-fluorophenyl)met	C24H15ClFN3O4	463.85	C	-8.6
4530	O=c1c2c(c(cc(OCc3ccc(F)cc3)c2)c2ccccc2)oc(c1O)c1[nH]c(OC)cn1	1312908-30-7	4H-1-Benzopyran-4-one, 6-[(4-fluorophenyl)methoxy]-3-hydroxy-2-(5-methyl-1H-imic	C26H19FN2O4	442.44	C	-8.6
4531	O=c1c2c(c(cc(OCc3ccc(F)cc3)c2)c2ccccc2)oc(c1O)c1[nH]c(cn1)c1ccccc1	1312908-32-9	4H-1-Benzopyran-4-one, 2-(5-chloro-1H-imidazol-2-yl)-6-[(4-fluorophenyl)methoxy]	C25H16ClFN2O4	462.86	C	-8.6
4532	Clc1c2c(cc(OCc3ccc(F)cc3)c1C)c(=O)c(O)c(o2)c1[nH]c(C)nn1	1312910-36-3	4H-1-Benzopyran-4-one, 8-chloro-6-[(4-fluorophenyl)methoxy]-3-hydroxy-7-methyl-2	C19H13ClFN3O4	401.78	C	-8.6
4533	Cc1c(c2c(cc1OCc1ccc(F)cc1)c(=O)c(O)c(o2)c1[nH]c(Cl)nn1)c1ccccc1	1312910-88-5	4H-1-Benzopyran-4-one, 6-[(4-fluorophenyl)methoxy]-3-hydroxy-2-(5-methoxy-1H-1,2	C26H20FN3O5	473.45	C	-8.6
4534	Cc1c(c2c(cc1OCc1ccc(F)cc1)c(=O)c(O)c(o2)c1[nH]c(OC)cn1)c1ccccc1	1312910-92-1	4H-1-Benzopyran-4-one, 6-[(4-fluorophenyl)methoxy]-3-hydroxy-7-methyl-2-(5-methy	C27H21FN2O4	456.46	C	-8.6
4535	Cc1c(c2c(cc1OCc1ccc(F)cc1)c(=O)c(O)c(o2)c1[nH]c(cn1)c1ccccc1)c1ccccc1	1312910-94-3	4H-1-Benzopyran-4-one, 2-(5-chloro-1H-imidazol-2-yl)-6-[(4-fluorophenyl)methoxy]	C26H18ClFN2O4	476.88	C	-8.6
4536	Cc1c2c(cc(OCc3ccc(F)cc3)c1OC)c(=O)c(O)c(o2)c1[nH]cnn1	1312912-17-6	4H-1-Benzopyran-4-one, 6-[(4-fluorophenyl)methoxy]-3-hydroxy-7-methoxy-8-methyl-	C28H21FN2O5	484.48	C	-8.6
4537	Cc1c2c(cc(OCc3ccc(F)cc3)c1Cl)c(=O)c(O)c(o2)c1[nH]c(C)nn1	1312912-75-6	4H-1-Benzopyran-4-one, 7-chloro-6-[(4-fluorophenyl)methoxy]-3-hydroxy-8-methyl-2	C19H13ClFN3O4	401.78	C	-8.6
4538	O(C)c1c2c(cc(OCc3ccc(F)cc3)c1Cl)c(=O)c(O)c(o2)c1[nH]cnn1	1312913-63-5	4H-1-Benzopyran-4-one, 7-chloro-6-[(4-fluorophenyl)methoxy]-3-hydroxy-8-methoxy-	C27H18ClFN2O5	504.89	C	-8.6
4539	Clc1c2c(cc(OCc3ccc(F)cc3)c1OC)c(=O)c(O)c(o2)c1[nH]cnn1	1312914-18-3	4H-1-Benzopyran-4-one, 8-chloro-6-[(4-fluorophenyl)methoxy]-3-hydroxy-7-methoxy-	C27H18ClFN2O5	504.89	C	-8.6
4540	Clc1c2c(cc(OCc3ccc(F)cc3)c1Cl)c(=O)c(O)c(o2)c1[nH]c(C)nn1	1312914-75-2	4H-1-Benzopyran-4-one, 7,8-dichloro-6-[(4-fluorophenyl)methoxy]-3-hydroxy-2-(1H-	C18H10Cl2FN3O4	422.19	C	-8.6
4541	Clc1c(c2c(cc1OCc1ccc(F)cc1)c(=O)c(O)c(o2)c1ncc(C)cn1)c1ccccc1	1312915-60-8	4H-1-Benzopyran-4-one, 7-chloro-6-[(4-fluorophenyl)methoxy]-3-hydroxy-8-phenyl-2	C26H16ClFN2O4	474.87	C	-8.6
4542	Clc1c(c2c(cc1OCc1ccc(F)cc1)c(=O)c(O)c(o2)c1[nH]c(OC)cn1)c1ccccc1	1312915-92-6	4H-1-Benzopyran-4-one, 7-chloro-6-[(4-fluorophenyl)methoxy]-3-hydroxy-2-(5-methy	C26H18ClFN2O4	476.88	C	-8.6
4543	Clc1c(c2c(cc1OCc1ccc(F)cc1)c(=O)c(O)c(o2)c1[nH]c(cn1)c1ccccc1)c1ccccc1	1312915-96-0	4H-1-Benzopyran-4-one, 7-chloro-2-(5-chloro-1H-imidazol-2-yl)-6-[(4-fluorophenyl	C25H15Cl2FN2O4	497.3	C	-8.6
4544	O=c1c2c(cc(OCc3ccc(F)cc3)c2)oc(c1O)c1ncc(OC)cn1	1312916-32-7	4H-1-Benzopyran-4-one, 6-[(4-fluorophenyl)methoxy]-3-hydroxy-2-(5-methyl-2-pyrim	C27H19FN2O4	454.45	C	-8.6
4545	O([C@H]1[C@@H](O[C@H](CO)[C@H]1O)n1c2c(nc1)c(=O)nc(N)[nH]2)[C@H]1O[C@H](COP(=O)(O)O)[C@@H](O)[C@H]1[	1312918-51-6	4H-1-Benzopyran-4-one, 6-[(4-fluorophenyl)methoxy]-3-hydroxy-7,8-diphenyl-2-(5-p	C37H25FN2O4	580.6	C	-8.6
4546	O([C@@H]1[C@@H](COP2(=O)Oc3c(CO2)cccc3C)O[C@@H](Cl)n1c(=O)[nH]c(=O)c(I)c1)P1(=O)Oc2c(CO1)cccc2C	1313031-58-1	Uridine, 2,Ä≤-deoxy-5-iodo-3,Ä≤,5,Ä≤-bis-O-(2-oxido-4H-1,3,2-benzodioxaphosphor	C23H21IN2O11P2	690.27	C	-8.6
4547	O=C1N2[C@H](c3nc(c[nH]3)CCc3cc([C@H](C)[C@@H]1NC(=O)OC)ccc3)c1ccc(cc1)c1ccc(cc1)c1[nH]c(nc1)[C@H]1N	1319189-34-8	Carbamic acid, N-[(10S,11S,16aS)-5,6,7,8,9,10,11,12,14,15,16,16a-dodecahydro-3-[	C44H56N8O6	792.97	C	-8.6
4548	C(OCCOC(=O)c1ccccc1)n1c2c(nc(NC(=O)C)[nH]2)nc1	1331749-55-3	2-Naphthalenecarboxaldehyde, 2-[4-[(4-fluorophenyl)amino]-6-(4-morpholinyl)-1,3,	C24H22FN7O	443.48	C	-8.6
4549	C=C1[C@@H](n2c3c(nc2)c(=O)nc(N)[nH]3)C[C@@H](O)[C@@H]1CO	1333141-31-3	3-Quinolinecarboxylic acid, 6-[(3-chloro-2-fluorophenyl)methyl]-1,4-dihydro-5-hy	C22H21ClFNO5	433.86	C	-8.6
4550	O=c1n([C@@H]2O[C@H](CO[P@@](=O)(Oc3ccc(C)cc3)N[C@H](C(=O)OCc3ccccc3)C)C=C2)cc(C)c(=O)[nH]1	1334335-89-5	L-Alanine, N-[[P(S)]-2,Ä≤,3,Ä≤-didehydro-3,Ä≤-deoxy-P-(4-methylphenyl)-5,Ä≤-thy	C27H30N3O8P	555.52	C	-8.6
4551	C(=O)(NC(C(=O)C(=O)NCCC)CCC)[C@@H]1[C@@H]2[C@H](CN1C(=O)[C@@H](NC(=O)[C@H](NC(=O)c1cnccn1)C(C)C)C(	1346829-38-6	1(2H)-Pyrimidineacetamide, N-[(1R,3S,4S)-4-[[2-(2,6-dimethylphenoxy)acetyl]amino	C38H52N4O3	612.84	C	-8.6
4552	[C@H](Cc1ccccc1)([C@@H]([C@@H](Cc1ccccc1)NC(=O)COc1c(C)cccc1C)O)NC(=O)[C@@H](NC(=O)N(C)Cc1nc(C)C(C)o	1346884-94-3	Carbamic acid, N-[[4,Ä≤-(2-ethyl-5,7-dimethyl-3H-imidazo[4,5-b]pyridin-3-yl)met	C31H36N4O4S	560.71	C	-8.6
4553	O=C1N([C@H]2O[C@@H](CO)SC2)C=CC(N)N1	1347245-30-0	Methanesulfonamide, N-[2-[4-[4-[(1-methylethyl)amino]-2-pyridinyl]-1-piperazinyl	C22H28N6O3S	456.56	C	-8.6
4554	C(C)(C)c1n([C@@H]2C[C@@H]3N(C(CC(NC(=O)C4CC(C(F)(F)C(C4)([2H])[2H])([2H])[2H])[2H])[2H]c4ccccc4)([2H])[2H])[2H])C[	1348320-90-0	2-Piperazinecarboxamide, 1-[(2S,4R)-6-[(1S,2R)-2,3-dihydro-2-hydroxy-1H-inden-1-	C37H48N4O4	612.8	C	-8.6
4555	OC1=C(O)C(CO)OC1n1c(=O)[nH]c(=O)c(C)c1	1348376-43-1	Not Yet Assigned	C40H57N7O6S	763.99	C	-8.6
4556	C(=O)([C@@H](NC(=O)OC)C(C)C)N1[C@H](c2[nH]c(c3c4c(cc(c3)c3c5c(cc(cc3)c3[nH]c(nc3)[C@H]3N(C(=O)[C@@H](	1350718-40-9	Carbamic acid, N,N,Ä≤-[(2,2,Ä≤-dimethyl[4,4,Ä≤-bi-1H-benzimidazole]-7,7,Ä≤-diyl	C44H54N12O6	846.98	C	-8.6
4557	Cc1nc2c(ccc(c2s1)c1[nH]c(nc1)[C@H]1N(C(=O)[C@@H](NC(=O)OC)C(C)C)CCC1)c1c2c(c(cc1)c1[nH]c(nc1)[C@H]1N	1350718-43-2	Carbamic acid, N,N,Ä≤-[[4,7-bibenzothiazole]-4,Ä≤,7-diylbis[1H-imidazole-5,2	C42H48N10O6S2	853.02	C	-8.6
4558	C(=O)([C@@H](NC(=O)OC)C(C)C)N1[C@@]2(C[C@H](Cl)CC2)C1c1[nH]c(cn1)c1ccc(cc1)c1ccc(cc1)c1[nH]c(nc1)[C@@]	1350718-61-4	Carbamic acid, N,N,Ä≤-[[4,4,Ä≤-bibenzothiazole]-7,7,Ä≤-diylbis[1H-imidazole-5,2	C42H48N10O6S2	853.02	C	-8.6
4559	C(=O)([C@@H](NC(=O)OC)C(C)C)N1[C@@]2(C[C@H](Cl)CC2)C1c1[nH]c(cn1)c1ccc2c(cc1)c1ccc(cc1)c1[nH]c(nc1)[	1350718-87-4	Carbamic acid, N-[(1S)-1-[[(1S,4R)-1-[5-[4-[7-[2-[(1S,4R)-2-[(2S)-2-[(methoxycar	C47H56N8O6	829	C	-8.6
4560	C(=O)([C@@H](NC(=O)OC)C(C)C)N1[C@@]2(C[C@H](Cl)CC2)C1c1[nH]c(cn1)c1cc2c(cc1)c1ccc(cc1)c1[nH]c(nc1)[	1350718-90-9	Carbamic acid, N-[(1S)-1-[[(1S,4R)-1-[5-[4-[7-[2-[(1S,4R)-2-[(2S)-2-[(methoxycar	C46H56N10O6	845	C	-8.6
4561	C(=O)([C@@H](NC(=O)OC)C(C)C)N1[C@@]2(C[C@H](Cl)CC2)C1c1[nH]c(cn1)c1cc2c(cc1)c1cccc(cc1)c1[nH]c(nc1)[C@	1350718-99-8	Carbamic acid, N-[(1S)-1-[[(1S,4R)-1-[5-[5-[4-[2-[(1S,4R)-2-[(2S)-2-[(methoxycar	C47H55N9O6	842	C	-8.6
4562	C(=O)([C@@H](NC(=O)OC)C(C)C)N1[C@@]2(C[C@H](Cl)CC2)C1c1[nH]c(cn1)c1cc2c(cc1)c1cccc(cc1)c1[nH]c(nc1)[C@	1350719-01-5	Carbamic acid, N-[(1S)-1-[[(1S,4R)-1-[5-[4-[2,3-dihydro-7-[2-[(1S,4R)-2-[(2S)-2-	C47H58N8O6	831.01	C	-8.6

4563	C(=O)([C@@H](NC(=O)OC)C(C)C)N1[C@@]2(C[C@H](Cl)CC2)c1[nH]c(cn1)c1c2c(c(cc1)c1c3c(c(cc1)c1[nH]c(nc1)[	1350719-07-1	Carbamic acid, N-[(1S)-1-[[(1S,4R)-1-[5-[4-[7-[2-[(1S,4R)-2-[(2S)-2-[(methoxycar	C47H57N9O6	844.01	C	-8.6
4564	C(=O)([C@@H](NC(=O)OC)C(C)C)N1[C@H](c2[nH]c(c3c4c(cc3)c3c5c(cc3)c3[nH]c(nc3)[C@H]3N(C(=O)[C@@H](	1350719-36-6	Carbamic acid, N,N,Ä≤-[[4,4,Ä≤-bibenzofuran]-7,7,Ä≤-diylbis[1H-imidazole-5,2-di	C44H50N8O8	818.92	C	-8.6
4565	C(=O)([C@@H](NC(=O)OC)CC#CC)N1[C@@H](CCC1)c1[nH]c(cn1)c1ccc(cc1)c1cc2c(cc1)cc(cc2)c1[nH]c(nc1)[C@H]1	1351332-82-5	Carbamic acid, N-[(1S)-1-[[(2S)-2-[5-[6-[4-[2-[(2S)-1-[(2S)-2-[(methoxycarbonyl)	C44H50N8O6	786.92	C	-8.6
4566	C(=O)([C@@H](N(C(=O)OC)C)C(C)C)N1C[C@@]2(C[C@H]1c1[nH]c(cn1)c1ccc(cc1)c1ccc(cc1)c1[nH]c(nc1)[C@H]1N(	1351851-12-1	Carbamic acid, N-[(1S)-1-[[(2S)-2-[5-[4,Ä≤-[2-[(5S,8S)-7-[(2S)-2-[(methoxycarbor	C43H54N8O7	794.94	C	-8.6
4567	C(=O)([C@@H](NC(=O)OC)C(C)C)N1C[C@@]2(C[C@H]1c1[nH]c(cn1)c1ccc(cc1)c1ccc(cc1)c1[nH]c(nc1)[C@H]1N(C(=	1351852-65-7	Carbamic acid, N-[(1S)-1-[[(5S,8S)-8-[5-[4,Ä≤-[2-[(2S,5S)-1-[(2S)-2-[(methoxycar	C44H56N8O7	808.96	C	-8.6
4568	C(=O)([C@@H](NC(=O)OC)C(C)C)N1C[C@@]2(C[C@H]1c1[nH]c(cn1)c1ccc(cc1)c1ccc(cc1)c1[nH]c(nc1Cl)[C@H]1N(C	1351852-76-0	Carbamic acid, N-[(1S)-1-[[(2S)-2-[5-(hydroxymethyl)-4-[4,Ä≤-[2-[(5S,8S)-7-[(2S)	C44H56N8O8	824.96	C	-8.6
4569	C(=O)([C@@H](NC(=O)OC)C(C)C)N1C[C@@]2(C[C@H]1c1[nH]c(c(Cl)n1)c1ccc(cc1)c1ccc(cc1)c1[nH]c(nc1)[C@H]1N	1351852-78-2	Carbamic acid, N-[(1S)-1-[[(2S)-2-[5-[4,Ä≤-[5-(hydroxymethyl)-2-[(5S,8S)-7-[(2S)	C44H56N8O8	824.96	C	-8.6
4570	C(=O)([C@@H](NC(=O)OC)C(C)C)N1C[C@]2(C[C@H]1c1[nH]c(cn1)c1ccc(cc1)c1ccc(cc1)c1[nH]c(nc1)[C@H]1N(C(=O	1351853-00-3	Carbamic acid, N-[(1S)-1-[[(5S,8S)-8-[5-[4,Ä≤-[2-[(3S,6S)-1,1-difluoro-5-[(2S)-2	C45H54F2N8O7	856.96	C	-8.6
4571	C(=O)([C@@H](NC(=O)OC)C(C)C)N1[C@@H]([C@@H]2C[C@H]1CC2)c1[nH]c(cn1)c1ccc(cc1)c1ccc(cc1)c1[nH]c(nc1)[	1351855-76-9	Carbamic acid, N-[(1S)-1-[[(2S,4R)-4-cyclopropyl-2-[5-[3,3,Ä≤,5,5,Ä≤-tetrachlor	C43H50Cl4N8O6	916.72	C	-8.6
4572	C(=O)([C@@H](NC(=O)OC)C(C)C)N1[C@@H](C=C(C)C1)c1[nH]c(cn1)c1ccc(C#Cc2ccc(cc2)c2[nH]c(nc2)[C@H]2N(C(=	1352391-62-8	Carbamic acid, N-[(1S)-1-[[(2S)-2-[5-[6-[4-[2-[(2S)-2,5-dihydro-1-[(2S)-2-[(meth	C45H52N8O6	800.94	C	-8.6
4573	OC1[C@@H](O[C@H](CO)[C@@H]1O)n1nc(C(=O)N)nc1	1355346-33-6	5,Ä≤-Inosinic acid, 2,Ä≤,3,Ä≤-didehydro-2,Ä≤,3,Ä≤-dideoxy-, 5,Ä≤-ester with 1-(	C18H21N8O10P	540.38	C	-8.6
4574	S(=O)(=O)(O)c1c2c(c(N)c(N=Nc3ccc(cc3)c3ccc(C)cc3)c1)cccc2.[NaH]	1357165-04-8	1-Naphthalenesulfonic acid, 4-amino-3-[2-(4-cyanophenyl)diazenyl]-, sodium salt	C17H12N4O3S.Na		C	-8.6
4575	C(=O)([C@@H](NC(=O)OC)C1CCOCC1)N1[C@H](c2[nH]c(c3c4c(c(cc3)c3ccc(cc3)c3[nH]c(nc3)[C@H]3N(C(=O)[C@@H]	1359868-96-4	Carbamic acid, N-[(1S)-2-[(1R,3S,5R)-3-[5-[4-[7-[2-[(1R,3S,5R)-2-[(2S)-2-[(metho	C47H54N10O8	886.99	C	-8.6
4576	C(F)(F)c1n(c2c(n1)cccc2)c1nc(nc(Nc2ccc(OC)cc2)n1)N1CCOCC1	1359956-22-1	Phenol, 2-[[4-[2-(difluoromethyl)-1H-benzimidazol-1-yl]-6-(4-morpholinyl)-1,3,5-	C21H19F2N7O2	439.42	C	-8.6
4577	C(F)(F)c1n(c2c(n1)cccc2)c1nc(nc(N(C)c2ccncc2)n1)N1CCOCC1	1359956-29-8	1,3,5-Triazin-2-amine, 4-[2-(difluoromethyl)-1H-benzimidazol-1-yl]-6-(4-morpholi	C25H22F2N8O	488.49	C	-8.6
4578	C(=C\C(=O)CCCCC)/c1cc(O)c(O)cc1	1369304-03-9	4-Quinazolinecarboxamide, 2-phenyl-N-(1-tricyclo[3.3.1.13,7]dec-1-ylethyl)-	C27H29N3O	411.54	C	-8.6
4579	C(=O)([C@@H](NC(=O)OC)C(C)C)N1[C@H]2[C@H](C2)C[C@H]1c1[nH]c(c(Cl)n1)c1ccc(cc1)c1ccc(cc1)c1[nH]c(nc1)	1373165-76-4	Carbamic acid, N-[(1S)-1-[[(2S,4S)-2-[5-chloro-4-[4,Ä≤-[2-[(1R,3S,5R)-2-[(2S)-2-	C42H51ClN8O7	815.36	C	-8.6
4580	N(C12CC3CC(C2)CC(C1)C3)c1ncc(C(=O)NO)cn1	1375454-41-3	[1,1,Ä≤-Biphenyl]-2,2,Ä≤-disulfonic acid, 4,4,Ä≤-bis[2-(4-amino-7-sulfo-1-napht	C32H24N6O12S4	812.83	C	-8.6
4581	C(=O)([C@@H](NC(=O)OC)C1CCOCC1)N1[C@@H]([C@@H]2C[C@H]1CC2)c1[nH]c2-c3c(CCc2n1)cc(cc3)c1ccc2c(cc1)c1c(	1377036-98-0	Carbamic acid, N-[(1S)-2-[(2S)-2-[5-[9,10-dihydro-7-[2-[(1R,3S,4S)-2-[(2S)-2-[(m	C46H56N8O7	832.99	C	-8.6
4582	C(=O)([C@H](NC(=O)OC)c1ccccc1)N1[C@@H](CC(=C)C1)c1[nH]c(cn1)c1ccc(cc1)c1cc2c(cc1)cc(cc2)c1[nH]c(nc1)	1377037-91-6	Carbamic acid, N-[(1S)-1-[[(2S)-2-[5-[6-[4-[2-[(2S)-1-[2-(dimethylamino)-2-pheny	C47H52N8O4	792.97	C	-8.6
4583	C(=O)([C@@H](NC(=O)OC)C(C)C)N1[C@H](c2[nH]c(c3cc4c(c5c(cc(cc5)c5[nH]c(nc5)[C@H]5N(C(=O)[C@@H](NC(=O)	1377040-67-9	Carbamic acid, N,N,Ä≤-[(9-cyano-2,7-phenanthrenediyl)bis[1H-imidazole-5,2-diyl-	C43H49N9O6	787.91	C	-8.6
4584	C(=O)([C@@H](NC(=O)OC)C1CCOCC1)N1[C@H](c2[nH]c(c3cc4c(c5c(c6c4cccc6)cc(cc5)c4[nH]c(nc4)[C@H]4N(C(=O)	1377041-29-6	Carbamic acid, N-[(1S)-2-[(1R,3S,4S)-3-[5-[2-fluoro-4-[2-[(2S)-1-[(2S)-2-[(metho	C46H53FN8O7	848.96	C	-8.6
4585	C(=O)([C@@H](NC(=O)OC)C1CCOCC1)N1[C@@H](CCC1)c1[nH]c(c(Cl)n1)c1ccc(cc1)c1ccc(cc1)c1[nH]c(nc1Cl)[C@H]	1377042-52-8	Carbamic acid, N,N,Ä≤-[[1,1,Ä≤-biphenyl]-4,4,Ä≤-diylbis[(5-chloro-1H-imidazole-	C44H52Cl2N8O8	891.84	C	-8.6
4586	C(=O)([C@@H](NC(=O)OC)C(C)C)N1[C@@H]([C@@H]2C[C@H]1CC2)c1[nH]c2c(n1)ccc1c2ccc(c1)c1ccc(cc1)c1[nH]c(n	1377042-74-4	Carbamic acid, N-[(1S)-1-[[(2S)-2-[2,Ä≤-[(8S)-7-[(2S)-2-[(methoxycarbonyl)amino]	C46H52N8O8	844.95	C	-8.6
4587	C(=O)([C@@H](NC(=O)OC)C(C)C)N1[C@@H]([C@@H]2C[C@H]1CC2)c1[nH]c2c(n1)ccc1c2ccc(c1)c1ccc(cc1)c1[nH]c(n	1377043-30-5	Carbamic acid, N-[(1S)-1-[[(2S)-2-[7-[4-[5-chloro-2-[(1R,3S,5R)-2-[(2S)-2-[(meth	C43H49ClN8O6	809.35	C	-8.6
4588	C(=O)([C@@H](NC(=O)OC)C(C)C)N1[C@H]2[C@H](C2)C[C@H]1c1[nH]c(cn1)c1c(F)cc(cc1)c1cc2c(cc1)c1c(cc2)nc([	1377043-47-4	Carbamic acid, N-[(1S)-1-[[(2S)-2-[7-[4-[5-chloro-2-[(1R,3S,4S)-2-[(2S)-2-[(meth	C44H51ClN8O6	823.38	C	-8.6
4589	C(=O)([C@@H](NC(=O)OC)C(C)C)N1[C@@H](CCC1)c1[nH]c(cn1)c1ccc(cc1)c1ccc(cc1)C1CCCCC1	1380290-12-9	Carbamic acid, N-[(1S)-2-methyl-1-[[(2S)-2-(5-[1,1,Ä≤:4,Ä≤,1,Ä≤,Ä≤-terphenyl]-4	C32H34N4O3	522.64	C	-8.6
4590	C(=O)([C@@H](NC(=O)OC)C(C)C)N1[C@@H](CCC1)c1[nH]c(cn1)c1ccc(cc1)c1ccc(cc1)c1[nH]c(NC(=O)OC)nc1	1383565-91-0	Carbamic acid, N-[(1S)-1-[[(2S)-2-[5-[4,Ä≤-[2-(acetylamino)-1H-imidazol-5-yl][1,	C31H35N7O4	569.65	C	-8.6
4591	O=C1C2(C(C(C(=O)/C=C(\O)/C3C4(N5C(C3c3cc(OC)c(O)cc3)CSC5)c3c(NC4=O)ccc(N(=O)=O)c3)C3N2CSC3)c2cc(OC)c	1389320-78-8	Spiro[2H-indene-2,5,Ä≤-[3H,5H]pyrrolo[1,2-c]thiazole]-1,3-dione, 6,Ä≤-[(1Z)-3-[	C45H38N2O10S2	830.92	C	-8.6
4592	S(=O)(=O)(C)N1CCN(c2nc(nc(n2)N2CCOCC2)c2cnc(N)nc2)CC1	1391924-28-9	Benzamide, 3-[[4-(6-amino-3-pyridinyl)-6-(4-morpholinyl)-1,3,5-triazin-2-yl]amir	C21H24N8O2	420.47	C	-8.6
4593	C[C@@H]1[C@H]2[C@@]34[C@H](O[C@@H]1OC[C@@H]1[C@@H](N=[N+]=[N-])C[C@@H](O1)n1c(=O)[nH]c(=O)c(C)c1)O[C	1392513-41-5	Thymidine, 3,Ä≤-azido-3-[[3-[(7-chloro-4-quinolinyl)amino]phenyl]methyl]-3,Ä≤-de	C26H24ClN7O4		C	-8.6
4594	O=c1n(ccc(N)n1)[C@@H]1O[C@H](CO[Si](C)(C)C)[C@@H](S)C1	1402045-22-0	Guanosine 5,Ä≤-(trihydrogen diphosphate), P,Ä≤-D-arabinopyranosyl ester	C15H23N5O15P2	575.32	C	-8.6
4595	C(NC(=O)c1[nH]c(nc(=O)c1O)c1ccccn1)(C)C12CC3CC(C2)CC(C1)C3	1408304-98-2	4-Pyrimidinecarboxamide, 1,6-dihydro-5-hydroxy-6-oxo-2-(2-pyrazinyl)-N-(1-tricyc	C21H25N5O3	395.45	C	-8.6
4596	Nc1c2c(cc(S(=O)(=O)O)cc2)ccc1N=Nc1cc(S(=O)(=O)O)c(C=Cc2c(S(=O)(=O)O)cc(N=Nc3c(N)c4c(cc3)cc(S(=O)(=O)	140942-32-1	2,7-Naphthalenedisulfonic acid, 4,4,Ä≤-[1,2-ethenediylbis(4,1-phenyleneazo)]bis	C34H26N6O12S4.4Na		C	-8.6
4597	S(=O)(=O)(O)c1c2c(c(S(=O)(=O)O)cc(N=Nc3cc(S(=O)(=O)O)c(C=Cc4c(S(=O)(=O)O)cc(N=Nc5c(N)c6c(c(S(=O)(=O)	14102-77-3	Isopodophyllotoxin, 3,Ä≤,4,Ä≤,5,Ä≤-tridemethoxy-, (¬±)-	C19H16O5	324.33	C	-8.6
4598	C(Nc1c(C(=O)N)cccc1)(C(=O)N)c1c(Cl)cccc1Cl	141027-80-7	1,5-Naphthalenedisulfonic acid, 3,3,Ä≤-[1,2-ethenediylbis[(3-sulfo-4,1-phenylene	C34H26N6O18S6.6Na		C	-8.6
4599	O[C@H]1[C@@H](O[C@H](C(=O)N[C@H](Cc2c3c([nH]c2)cccc3)C(=O)N2CCN(C(=O)N3CCOCC3)CC2)[C@H]1O)n1nc(C(=O)	1414793-63-7	Not Yet Assigned	C27H32N8O7	580.59	C	-8.6
4600	O[C@H]1[C@@H](O[C@H](C(=O)N[C@H](Cc2ccccc2)C(=O)O)[C@H]1O)n1nc(C(=O)N)nc1	1414802-06-4	Not Yet Assigned	C19H18N6O6	426.38	C	-8.6
4601	[C@@H](Cc1ccccc1)(C[C@@H]([C@H](Cc1ccccc1)NC(=O)OCc1cncs1)O)NC(=O)[C@@H](NC(=O)N(Cc1nc(C(C)C)sc1)C)C	1414808-96-0	Not Yet Assigned	C21H23N7O6	469.45	C	-8.6
4602	C(=O)([C@@H](NC(=O)OC)C(C)C)N1[C@@H](CCC1)c1[nH]c(C#Cc2c3CCc4cc(C#Cc5[nH]c(nc5)[C@H]5N(C(=O)[C@@H](N	1415120-38-5	Carbamic acid, N,N,Ä≤-[tricyclo[8.2.2.24,7]hexadeca-4,6,10,12,13,15-hexaene-5,11	C44H56N8O6	792.97	C	-8.6
4603	C(F)(F)c1n(c2c(n1)cccc2)c1nc(nc(n1)N1CCOCC1)N1CCN(C(=O)[C@@H](NC)C)CC1	1416956-39-2	1-Propanone, 3-amino-1-[4-[4-[2-(difluoromethyl)-1H-benzimidazol-1-yl]-6-(4-morp	C22H27F2N9O2	487.51	C	-8.6
4604	C(OCCn1c2c(nc1)c(N)ncn2)[P@]1(=O)O[C@@H](C[C@H](O1)c1ccccc1)c1cc(Cl)ccc1	1417393-24-8	9H-Purin-6-amine, 9-[2-[[(2R,4R,6R)-4-(3-chlorophenyl)-2-oxido-6-phenyl-1,3,2-di	C23H23ClN5O4P	499.89	C	-8.6
4605	N(c1nc(nc(NC(=O)Nc2ccccc2)n1)N1CCOCC1)n1c(SC)nnc(C(C)(C)C)c1=O	1418540-48-3	Urea, N-(4-chlorophenyl)-N,Ä≤-[4-[[6-(1,1-dimethylethyl)-3-(methylthio)-5-oxo-1,	C22H27ClN10O3S	547.03	C	-8.6
4606	C(=O)([C@@H](NC(=O)OC)C(C)C)N1[C@H](c2[nH]c(c3c4c(c(cc3)c3ccc(cc3)c3[nH]c(nc3)[C@H]3N(C(=O)[C@@H](NC	1419384-91-0	Carbamic acid, N-[(1S)-1-[[(2S)-2-[5-[1,Ä≤,3,Ä≤-dihydro-7,Ä≤-[4-[2-[(2S)-1-[(2S	C47H60N8O6	833.03	C	-8.6
4607	C(=O)([C@@H](NC(=O)OC)C(C)C)N1[C@H](c2[nH]c(c3c4c(c(cc3)c3ccc(cc3)c3[nH]c(nc3)[C@H]3N(C(=O)[C@@H](NC	1419385-21-9	Carbamic acid, N-[(1S)-1-[[(2S,4R)-4-fluoro-2-[5-[4-[7,Ä≤-[2-[(2S,4R)-4-fluoro-1	C47H58F2N8O6	869.01	C	-8.6
4608	C(=O)([C@@H](NC(=O)OC)C(C)C)N1[C@H](c2[nH]c(c3c4c(c(cc3)c3ccc(cc3)c3[nH]c(nc3)[C@H]3N(C(=O)[C@@H](NC	1419385-61-7	Carbamic acid, N-[(1S)-1-[[(2S)-2-[5-[1,Ä≤,3,Ä≤-dihydro-7,Ä≤-[4-[2-[(2S)-1-[(2S	C48H62N8O6	847.06	C	-8.6
4609	O(C)c1c2n(cc(C(=O)O)c(=O)c2cc(F)c1N1CC(C)N(CN2c3c(C(=NNC(=S)N(c4ccccc4)c4ccccc4)C2=O)cc(Cl)cc3)CC1)C	1426583-86-9	3-Quinolinecarboxylic acid, 7-[4-[[5-chloro-3-[2-[(di-2-propen-1-ylamino)thioxom	C35H37ClFN7O5S	722.23	C	-8.6
4610	O(C)c1c2n(cc(C(=O)O)c(=O)c2cc(F)c1N1CC(C)N(CN2c3c(C(=NNC(=S)N(c4ccccc4)c4ccccc4)C2=O)cc(C)cc3)CC1)C1	1426583-90-5	3-Quinolinecarboxylic acid, 1-cyclopropyl-7-[4-[[3-[2-[(di-2-propen-1-ylamino)th	C36H40FN7O5S	701.81	C	-8.6
4611	N(C(=O)Nc1ncccn1)C12CC3CC(C2)CC(C1)C3	1431287-78-3	Urea, N-[4-[(4-aminophenyl)sulfonyl]phenyl]-N,Ä≤-tricyclo[3.3.1.13,7]dec-1-yl-	C23H27N3O3S	425.54	C	-8.6
4612	S(=O)(=O)(O)c1c2c(c(S(=O)(=O)O)ccc2)cc(N=Nc2c(N)c(C)c(N)cc2)c1	1431626-03-7	Carbamic acid, N-[(1S)-1-[[(2S)-2-[5-[4,Ä≤-[(2S,5S)-3-[(2S)-2-[(methoxycarbonyl)	C45H58N8O8	838.99	C	-8.6
4613	C[C@]12[C@@]3(C)[C@@H]([C@H](CC3)[C@]3(C)CCC(=O)O3)[C@H](O)C[C@@H]1[C@]1(C)[C@@H](CC2)C(C)(C)[C@@H](	1431852-69-5	Dammar-25-ene-3,12-diol, 20,24-epoxy-, 3-acetate, (3Œ≤,12Œ≤,24S)-	C32H52O4	500.75	C	-8.6
4614	[C@@H](C(=O)N[C@@H](Cc1ccccc1)C[C@@H]([C@H](Cc1ccccc1)NC(=O)COc1c(C)cccc1C)O)(C(C([2H])([2H])[2H])(C	1432018-35-3	1-Naphthalenesulfonic acid, 5-[2-(4-amino-1-naphthalenyl)diazenyl]-8-hydroxy-	C20H15N3O4S	393.42	C	-8.6
4615	N(=Nc1ccc(N=Nc2ccccc2)cc1)c1c2c(cc(S(=O)(=O)O)cc2)ccc1O	1439923-16-6	4-Piperidinecarboxamide, 1-[4-methyl-6-(4-morpholinyl)-1,3,5-triazin-2-yl]-N-[[2	C22H27F3N6O2	464.48	C	-8.6
4616	N(C(=O)C)[C@H]1[C@H]([C@@H]([C@@H](CO)O)O)OC(=C[C@@H]1NC(=N)NC(=O)Cc1c2c(ccc1)cccc2)C(=O)O	1442563-07-6	D-glycero-D-galacto-Non-2-enonic acid, 5-(acetylamino)-2,6-anhydro-3,4,5-trideox	C24H28N4O8	500.5	C	-8.6
4617	N(Cc1cc(ccc1)c1ccco1)C12CC3CC(C2)CC(C1)C3	1443121-99-0	[1,1,Ä≤-Biphenyl]-3-carbonitrile, 4-fluoro-4,Ä≤-[(tricyclo[3.3.1.13,7]dec-1-ylar	C24H25FN2	360.47	C	-8.6
4618	C(Oc1ccc(NCCCC#Cc2nc(=O)n(cc2)[C@@H]2O[C@H](CO)SC2)cc1)c1c2c(N(c3c(C(=O)N2)cccn3)C2CC2)ncc1	1445477-86-0	Thymidine, 4-S-[2-[[(11-cyclopropyl-6,11-dihydro-6-oxo-5H-dipyrido[3,2-b:2,Ä≤,3,	C27H29N7O4S	547.63	C	-8.6
4619	C(Oc1c(C)cn(c(=O)n1)[C@@H]1O[C@H](CO)[C@@H](O)C1)c1c2c(N(c3c(C(=O)N2)cccn3)C2CC2)ncc1	1449678-53-8	Thymidine, 3-[(11-cyclopropyl-6,11-dihydro-6-oxo-5H-dipyrido[3,2-b:2,Ä≤,3,Ä≤-e]	C25H26N6O6	506.51	C	-8.6
4620	O=C1[C@@H]2[C@H](c3c(C(=O)[C@@H]2CO1)cc(OC)c(OC)c3)c1cc(OC)c(OC)c(OC)c1	146237-09-4	Furo[3,Ä≤,4,Ä≤:6,7]naphtho[2,3-d]-1,3-dioxole-5,8-dione, 9-(3,4-dimethoxyphenyl	C21H18O7	382.36	C	-8.6
4621	S(=O)(=O)(O)c1c2c(c(N=Nc3c(C)cc(cc3)c3cc(C)c(N=Nc4c5c(c(S(=O)(=O)O)ccc5)c(N)cc4)cc3)ccc2N)ccc1	14678-28-5	2-Naphthalenesulfonic acid, 5,5,Ä≤-[(3,3,Ä≤-dimethyl[1,1,Ä≤-biphenyl]-4,4,Ä≤-di	C34H28N6O6S2	680.75	C	-8.6
4622	C(OP(=O)(O[C@H]1[C@@H](O[C@H](COP(=O)(O[C@H]2[C@@H](O[C@H](CO)[C@H]2O)n2c3c(nc2)c(N)ncn3)O)[C@H]1O)n	1469975-49-2	Œ≤-D-lyxo-Adenosine, 1,Ä≤-[3-(aminocarbonyl)-1H-1,2,4-triazol-1-yl]-1,Ä≤-de(6-a	C28H34N14O16P2	884.6	C	-8.6
4623	N(C(C)C)c1c(ncccc1)N1CCN(C(=O)c2cc3c([nH]2)ccc(NS(=O)(=O)CCN)c3)CC1	147540-05-4	2H-Isoindole-2-ethanesulfonamide, 1,3-dihydro-N-[2-[[4-[3-[(1-methylethyl)amino]	C31H33N7O5S	615.7	C	-8.6
4624	C([C@H]([C@H](Cc1ccccc1)NC(=O)[C@@H](NC(=O)c1nc2c(cc1)cccc2)CC(=O)N)O)N1[C@H](C(=O)NC(C)(C)C)C[C@@H]	147920-19-2	Methanesulfonamide, N-methyl-N-[2-[[4-[3-[(1-methylethyl)amino]-2-pyridinyl]-1-p	C23H30N6O3S	470.59	C	-8.6
4625	C(n1c2c(nc1CC)c(C)cc(C)n2)c1ccc(cc1)c1c(S(=O)(=O)NC(=O)OCC2CC2)cccc1	148401-86-9	Carbamic acid, [[4,Ä≤-[(2-ethyl-5,7-dimethyl-3H-imidazo[4,5-b]pyridin-3-yl)methy	C31H30N4O4S	554.66	C	-8.6
4626	O=c1n([C@@H]2O[C@H](CO)[C@@H](S)C2)ccc(N)n1	148503-84-8	Inosine 5,Ä≤-(pentahydrogen tetraphosphate), P,Ä≤,Ä≤,Ä≤,Ü5,Ä≤-ester with adenc	C20H27N9O20P4	837.37	C	-8.6
4627	O=c1n([C@@H]2O[C](CO)[C@@H](O)C2)cc(C)c(=O)[nH]1	1506558-84-4	Carbamic acid, N,N,Ä≤-[(1Z)-1,2-ethenediylbis[3,1-phenylene-1H-imidazole-5,2-diy	C42H52N8O6	764.91	C	-8.6
4628	O=C1N(c2c3c(c4c(n2)cccc4)n(CC(C)C)cn3)c2c1cc(Cl)cc2	1508303-90-9	7-Azabicyclo[4.2.0]octa-1,3,5-trien-8-one, 4-chloro-7-[1-(2-methylpropyl)-1H-imi	C21H17ClN4O	376.84	C	-8.6
4629	O=c1c2c(n(cn2)[C@H]2C[C@H](ONCC[C@H]3O[C@H](C[C@@H]3O)n3c4c(nc3)c(=O)nc(N)[nH]4)[C@@H](CO)O2)[nH]c(N	1513874-37-7	Guanosine, 2,Ä≤-deoxy-3,Ä≤-de(phosphinicooxy)guanylyl-1,2-hydrazinediylmethylen	C21H28N12O6	544.52	C	-8.6
4630	O=c1c2c(n(cn2)[C@H]2C[C@H](N[Si](NC[C@H]3O[C@H](C[C@@H]3O)n3c4c(nc3)c(=O)nc(N)[nH]4)(C)C)[C@@H](CO)O	1513874-39-9	Guanosine, 2,Ä≤-deoxy-3,Ä≤-de(phosphinicooxy)guanylyl-1,2-hydrazinediyl-(3,Ä≤,Ü	C20H26N12O6	530.5	C	-8.6
4631	O=c1n([C@@H]2O[C@H](COP(=O)(N[C@H](C(=O)OCc3ccccc3)C)N[C@H](C(=O)OCc3ccccc3)C)C=C2)cc(C)c(=O)[nH]1	1526902-53-3	9H-Purin-6-amine, 9-Œ≤-D-arabinofuranosyl-8-cyclohexyl-2-fluoro-	C16H22FN5O4	367.38	C	-8.6
4632	O=C1c2c(N(C)C1=O)ccc(S(=O)(=O)N1CCC(C)CC1)c2	1529815-12-0	1H-Indole-2,3-dione, 5-[(4-methyl-1-piperidinyl)sulfonyl]-1-(2-naphthalenylmethy	C25H24N2O4S	448.53	C	-8.6
4633	C(n1c2c(nc1CC)c(C)cc(C)n2)c1ccc(cc1)c1c(S(=O)(=O)NC(=O)NCCCC)ccc(OCCCC)c1	154552-36-0	[1,1,Ä≤-Biphenyl]-2-sulfonamide, N-[(butylamino)carbonyl]-4,Ä≤-[(2-ethyl-5,7-dir	C31H39N5O3S	561.74	C	-8.6
4634	C(n1c2c(nc1CC)c(C)cc(C)n2)c1ccc(cc1)c1c(S(=O)(=O)NC(=O)CCc2ccccc2)ccc(OCC)c1	154552-60-0	Benzenepropanamide, N-[[4,Ä≤-[(2-ethyl-5,7-dimethyl-3H-imidazo[4,5-b]pyridin-3-y	C34H36N4O4S	596.74	C	-8.6
4635	C(n1c2c(nc1CC)c(C)cc(C)n2)c1ccc(cc1)c1c(S(=O)(=O)NC(=O)c2c(OCC)cccc2)ccc(C)c1	154552-80-4	Benzamide, N-[[4,Ä≤-[(2-ethyl-5,7-dimethyl-3H-imidazo[4,5-b]pyridin-3-yl)methyl]	C38H36N4O5S	660.78	C	-8.6
4636	C(n1c2c(nc1CC)c(C)cc(C)n2)c1ccc(cc1)c1c(S(=O)(=O)NC(=O)c2c(OCC)cccc2)ccc(C(C)(C)C)c1	154553-03-4	Benzamide, N-[[5-(1,1-dimethylpropyl)-4,Ä≤-[(2-ethyl-5,7-dimethyl-3H-imidazo[4,5	C37H42N4O4S	638.82	C	-8.6
4637	C(=O)([C@@H](NC(=O)OC)C(C)C)N1CC2(C[C@H]1c1[nH]c(cn1)c1ccc(cc1)c1ccc(cc1)c1[nH]c(nc1)[C@H]1N(C(=O)[C	155487-46-0	Guanosine, 2,Ä≤-deoxy-P-thioguanylyl-(3,Ä≤,Ü5,Ä≤)-2,Ä≤-deoxy-	C20H25N10O9PS	612.51	C	-8.6
4638	C(=O)([C@@H](NC(=O)OC)C(C)C)N1[C@@H](C[C@@H](O)C1)c1[nH]c(cn1)c1ccc(cc1)c1ccc(cc1)c1[nH]c(nc1)[C@H]1N	1555813-40-5	Carbamic acid, N-[(1S)-1-[[(5S)-2-hydroxy-5-[5-[4,Ä≤-[2-[(8S)-7-[(2S)-2-[(methox	C42H52N8O9	812.91	C	-8.6
4639	C(=O)([C@@H](NC(=O)OC)C(C)C)N1[C@H](c2[nH]c(c3c4c(c(cc3)c3ccc(cc3)c3[nH]c(nc3)[C@H]3N(C(=O)[C@@H](NC	1560640-20-1	Carbamic acid, N-[(1S)-1-cyclohexyl-2-[(2S)-2-[5-[8-[4-[2-[(2S)-1-[(2S)-2-cycloh	C52H66N8O6	899.13	C	-8.6
4640	O[C@H]1[C@H]2[C@H]3[C@@](C)([C@@H]([C@@H](CCC(=O)NCCS(=O)O)C)CC3)[C@@H](O)C[C@@H]2[C@]2(C)[C@H](C1)C	1562395-48-5	1-Pyrrolidinecarboxylic acid, 2-[5-[4,Ä≤-[2-[(2S)-1-[(2R)-2-[(methoxycarbonyl)ar	C38H47N7O5	681.82	C	-8.6
4641	C(=O)([C@@H](NC(=O)OC)C(C)C)N1CC2(C[C@H]1c1[nH]c(cn1)c1ccc(cc1)c1ccc(cc1)c1[nH]c(nc1)[C@H]1N(C(=O)[C	1571138-06-1	Carbamic acid, N-[(1S)-1-[[(2S)-2-[5-[4,Ä≤-[2-[(3S)-2-[(2S)-2-[(methoxycarbonyl)	C44H56N8O8S	857.03	C	-8.6
4642	S(=O)(=O)(O)c1c2c(cc(S(=O)(=O)O)c1)ccc(N=Nc1c(cc(N)cc1)c1ccccc1)c2	157437-00-8	1,3-Naphthalenedisulfonic acid, 7-[2-(6-amino[1,1,Ä≤-biphenyl]-3-yl)diazenyl]-	C22H17N3O6S2	483.52	C	-8.6
4643	O(C(=O)C)[C@H]1[C@@H](O[C@H](CO[P@]2(=O)O[C@@H](CCO2)c2cc(Cl)ccc2)[C@H]1OC(=O)C)n1nc(C(=O)N)nc1	1578257-11-0	Not Yet Assigned	C18H18ClN4O9P	500.78	C	-8.6
4644	O=c1n([C@@H]2O[C@H](COP(=O)(Oc3ccc(I)cc3)Oc3ccc(I)cc3)[C@@H](N=[N+]=[N-])C2)cc(C)c(=O)[nH]1	158171-94-9	5,Ä≤-Thymidylic acid, 3,Ä≤-azido-3,Ä≤-deoxy-, bis[4-(trifluoromethyl)phenyl] es	C24H20F6N5O7P		C	-8.6
4645	C(=C\C=C\c1cc(O)c(O)cc1)/c1cc(O)c(O)cc1	158629-14-2	Retinoic acid, [5-(4-amino-2-oxo-1(2H)-pyrimidinyl)-1,3-oxathiolan-2-yl]methyl e	C28H37N3O4S	511.68	C	-8.6
4646	C(=O)([C@@H](NC(=O)OC)C(C)C)N1[C@@H](c2[nH]c(c3c4c(C5(CC4)CCCC5)c(cc3)c3ccc(cc3)c3[nH]c(nc3)[C@H]3N(C	1588403-87-5	Carbamic acid, N-[(1S)-1-[[(2S)-2-[5-[2,Ä≤,3,Ä≤-dihydro-4,Ä≤-[4-[2-[(2S)-1-[(2S	C47H60N8O6	833.03	C	-8.6
4647	C(=O)([C@@H](NC(=O)OC)C(C)C)N1[C@@H](c2[nH]c(c3c4c(C5(CC4)CCCC5)c(cc3)c3ccc(cc3)c3[nH]c(nc3)[C@H]3N(C	1588403-98-8	Carbamic acid, N-[(1S)-1-[[(2S,4S)-4-fluoro-2-[5-[4-[4-[2-[(2S,4S)-4-fluoro-	C47H58F2N8O6	869.01	C	-8.6
4648	O=C1[C@@H]2[C@H](c3c(C(=O)[C@@H]2CO1)cc(OC)c(OC)c3)OC(=O)c1cc(OC)c(OC)c(OC)c1	1600500-12-6	Furo[3,Ä≤,4,Ä≤:6,7]naphtho[2,3-d]-1,3-dioxol-6(5aH)-one, 5,8,8a,9-tetrahydro-9-	C31H29N3O8	571.58	C	-8.6
4649	C(=O)([C@@H](NC(=O)OC)C(C)C)N1[C@@H](c2[nH]c(c3c4c(cc3)c3cc5c(cc3)c3[nH]c(nc3)[C@H]3N(C(=O)[C@@H](	1612236-02-8	Carbamic acid, N-[(1S)-1-[[(1S,3S,4R)-3-[5-[7-[1,Ä≤,3,Ä≤-dihydro-7,Ä≤-[2-[(2S)-	C56H72N8O7	969.22	C	-8.6
4650	O=C1N2[C@@H](Cn3c1c(O)c(=O)c(C(=O)N[C@H](C(F)(F)F)c1c(F)cc(F)cc1)c3)O[C@@H]1C[C@@H]2CC1	1616340-44-3	2,5-Methanopyrido[1,Ä≤,2,Ä≤:4,5]pyrazino[2,1-b][1,3]oxazepine-10-carboxamide, 2	C22H20F3N3O5	463.41	C	-8.6
4651	O=c1nc2c(n(cn2)[C@@H]2O[C@H](COP(=O)(Oc3ccc(Cl)cc3)NCCc3c[nH]c4c3cccc4)CC2)[nH]cn1	1618656-18-0	Inosine, 2,Ä≤,3,Ä≤-dideoxy-, 5,Ä≤-[N-[2-(1H-indol-3-yl)ethyl]pho	C26H26ClN6O5P	568.95	C	-8.6
4652	O=c1n([C@@H]2C[C@H](OP(=O)(S)S)[C@@H](CO)O2)cc(C)c(=O)[nH]1	162008-29-9	Acetamide, 2,2,Ä≤-[1,4,10,13-tetraoxa-7,16-diazacyclooctadecane-7,16-diylbis(2-	C40H64N4O10	760.96	C	-8.6
4653	C(F)[C@]1(F)[C@@H](O[C@H](COP(=O)(Oc2ccccc2)N[C@H](C(=O)OC(C)C)C)[C@H]1O)n1cc(C)c(=O)[nH]c1=O	162189-89-1	Thymidine, P-(2-aminoethyl)-P-deoxythymidylyl-(3,Ä≤,Ü5,Ä≤)-, (S)-	C22H32N5O11P	573.49	C	-8.6
4654	C[C@]1(O)[C@@H](n2c3nc(=O)nc(N)[nH]3)O[C@H](COP(=O)(N[C@H](C(=O)OCC)C)SCC)[C@H]1O	162679-84-7	Butanediamide, N1-[3-[3-[[(1,1-dimethylethyl)amino]carbonyl]octahydro-2(1H)-isoq	C38H50N6O5	670.84	C	-8.6
4655	O=C1N2[C@H](C(=O)N[C@H](NCCN)[C@H](O)C[C@H](NC(=O)CCCCCCCC[C@H](C[C@H](CC)C)C)C(=O)N[C@@H]([C@H](	1628026-65-2	Uridine, 2,Ä≤-deoxy-5-[4-[2-[4-(dimethylamino)phenyl]diazenyl]phenyl]-	C23H25N5O5	451.48	C	-8.6
4656	N(c1nc(nc(n1)N1CCOCC1)N1CCOCC1)c1ccc(C(=O)C=Cc2ccc(Br)cc2)cc1	1628172-06-4	2-Propen-1-one, 1-[4-[(4,6-di-4-morpholinyl-1,3,5-triazin-2-yl)amino]phenyl]-3-(	C29H34N6O6	562.62	C	-8.6
4657	[N+](#N)c1c2c(N=Nc3cc4ccc(S(=O)(=O)O)cc4O)ccc(S(=O)(=O)O)c2	1628452-95-8	2-Naphthalenesulfonic acid, 6-[2-(4-amino-6-sulfo-1-naphthalenyl)diazenyl]-4-hy	C20H15N3O7S2	473.48	C	-8.6
4658	C(CCCCCCCCCCCCCCC/C=C\CCCCCCC)C1=C(O)C(=O)C=C(O)C1=O	1637815-93-0	Adenosine, adenylyl-(2,Ä≤,Ü5,Ä≤)-1,Ä≤-N-[3-(aminocarbonyl)-1H-1,2,4-triazol-1	C28H36N14O16P2	886.62	C	-8.6
4659	[C@@H](C(CC)CC)(NC(=O)C)[C@H]1[C@H](NC(=N)N)C[C@H](C(=O)OCCCC(=O)OC[C@H]2N[C@H]([C@H](O)[C@@H]2O)c2	1638370-20-3	Cyclopentanecarboxylic acid, 3-[(1S)-1-(acetylamino)-2-ethylbutyl]-4-[(aminoimir	C29H45N9O8	647.72	C	-8.6
4660	C[C@]1(F)[C@@H](O[C@H](CO[P@](=O)(Oc2ccc3c(cc2)OCO3)N[C@H](C(=O)OC(C)C)C)[C@H]1O)n1c(=O)[nH]c(=O)cc1	1638769-83-1	5,8-Dioxa-3-aza-4-phosphanonanoic acid, 9-(2-amino-1,6-dihydro-6-oxo-9H-purin-9-	C24H31N6O9P	578.51	C	-8.6
4661	O=c1n([C@@H]2O[C@H](CO)[C@@H](O)C2)cc(COC(C(C)C)C(C)(C)C)c(=O)[nH]1	1639286-29-5	L-Alanine, N-[[P(S),2,Ä≤R]-2,Ä≤-deoxy-2,Ä≤-fluoro-P-(1-fluoro-9-methyl-9H-carba	C29H33F2N4O9P	650.56	C	-8.6
4662	C(=O)([C@@H](NC(=O)OC(C)(C)C)C(C)C)N1[C@@H](C[C@@H](OC(=O)N2Cc3c(F)ccc3C2)C1)c1[nH]c(cn1)c1ccc(cc	1639870-44-2	2H-Isoindole-2-carboxylic acid, 4-fluoro-1,3-dihydro-, (3R,5S)-1-[(2S)-2-[(metho	C49H56FN9O8	918.02	C	-8.6
4663	C(=O)([C@H](NC(=O)OC)C1CCCCC1)N1[C@@H](C=CC1)c1[nH]c(cn1)c1ccc(cc1)c1ccc(cc1)c1[nH]c(nc1)[C@H]1N(C(	1639871-23-0	Carbamic acid, N-[(1S)-1-cyclohexyl-2-[(2S)-2,5-dihydro-2-[5-[4,Ä≤-[2-[(2S)-1-[	C43H52N8O6	776.92	C	-8.6
4664	S(c1nc(nc(Nc2ccc(C(=O)C)cc2)n1)N1CCOCC1)c1oc(nn1)c1ccc(F)cc1	1642584-24-4	1,3,5-Triazin-2-amine, N-(3-bromophenyl)-4-[[5-(4-fluorophenyl)-1,3,4-oxadiazol-	C21H17BrFN7O2S	530.37	C	-8.6
4665	O=c1n([C@@H]2O[C@H](COP(=O)(C(O)c3ccc(C)cc3)OC[C@@H]3O[C@H](C=C3)n3c(=O)[nH]c(=O)c(C)c3)C=C2)cc(C)c(=	164652-01-1	Thymidine, 2,Ä≤,3,Ä≤-didehydro-P,3,Ä≤-dideoxy-P-[hydroxy(4-methoxyphenyl)methyl	C28H31N4O11P	630.54	C	-8.6
4666	O=c1n([C@@H]2O[C@H](COP(=O)(C(O)c3ccccc3)OC[C@@H]3O[C@H](C=C3)n3c(=O)[nH]c(=O)c(C)c3)C=C2)cc(C)c(=O)[	164652-02-2	Thymidine, 2,Ä≤,3,Ä≤-didehydro-P,3,Ä≤-dideoxy-P-[hydroxy(4-methylphenyl)methyl]	C28H31N4O10P	614.54	C	-8.6
4667	O=c1n([C@@H]2O[C@H](COP(=O)(C(O)c3ccc(C#N)cc3)OC[C@@H]3O[C@H](C=C3)n3c(=O)[nH]c(=O)c(C)c3)C=C2)cc(C)c(	164652-04-4	Thymidine, P-[(4-chlorophenyl)hydroxymethyl]-2,Ä≤,3,Ä≤-didehydro-P,3,Ä≤-dideoxy	C27H28ClN4O10P	634.96	C	-8.6
4668	O=c1n([C@@H]2O[C@H](COP(=O)(C(O)c3ccnc3)OC[C@@H]3O[C@H](C=C3)n3c(=O)[nH]c(=O)c(C)c3)C=C2)cc(C)c(=O)[	164652-08-8	Thymidine, 2,Ä≤,3,Ä≤-didehydro-P,3,Ä≤-dideoxy-P-[(2,4-dinitrophenyl)hydroxymeth	C27H27N6O14P	690.51	C	-8.6
4669	N(C(=O)[C@H](Cc1ccccc1)C[C@@H](CN1[C@H](C(=O)NC(C)(C)C)CN(CC(C)C)CC1)O)[C@H]1c2c(C[C@H]1O)cccc2	164725-13-7	1-Piperazinepentanamide, N-(2,3-dihydro-2-hydroxy-1H-inden-1-yl)-2-[[(1,1-dimeth	C38H51N5O4	641.84	C	-8.6
4670	N(C(=O)[C@H](Cc1ccccc1)C[C@@H](CN1[C@H](C(=O)NC(C)(C)C)CN(Cc2cc(F)ccc2)CC1)O)[C@H]1c2c(C[C@H]1O)cccc	164725-43-3	1-Piperazinepentanamide, N-(2,3-dihydro-2-hydroxy-1H-inden-1-yl)-2-[[(1,1-dimeth	C36H48N6O4	628.8	C	-8.6
4671	N(C(=O)[C@H](Cc1ccccc1)C[C@@H](CN1[C@H](C(=O)NC(C)(C)C)CN(Cc2cccs2)CC1)O)[C@H]1c2c(C[C@H]1O)cccc2	164725-54-6	1-Piperazinepentanamide, N-(2,3-dihydro-2-hydroxy-1H-inden-1-yl)-2-[[(1,1-dimeth	C36H52N4O5	620.82	C	-8.6
4672	N(C(=O)C(Cc1ccccc1)CC(CN1C(C(=O)NC(C)(C)C)CN(CC2c3cc(OC)ccc3)CC1)O)C1c2c(CC1O)cccc2	164725-63-7	1-Piperazinepentanamide, N-(2,3-dihydro-2-hydroxy-1H-inden-1-yl)-2-[[(1,1-dimeth	C37H48N4O4	612.8	C	-8.6
4673	C(P(=O)([O-])[O-])C(=O)[O-]	164927-34-8	Thymidine, P-deoxy-P-fluorothymidylyl-(3,Ä≤,Ü5,Ä≤)-	C20H26FN4O11P	548.41	C	-8.6
4674	C(COCP(=O)(N[C@@H](Cc1ccccc1)C(=O)OC(C)C)Oc1ccccc1)n1cnc2c(nc1)c(N)ncn2	165133-86-8	Methanesulfonamide, N-[2-[[4-(3-amino-2-pyridinyl)-1-piperazinyl]carbonyl]-1H-ir	C19H22N6O3S	414.48	C	-8.6
4675	C(=O)([C@@H](NC(=O)OC)C(C)C)N1[C@@H]([C@@]2(C1)CCCC2)c1[nH]c2c(n1)ccc1c2ccc(cc1)c1ccc(cc1)c1[nH]c(nc	1653966-63-2	Carbamic acid, N-[(1S)-1-[[(2S,3aS,6aS)-hexahydro-2-[5-[4-[2-[(3S,5S)-2-[(2S)-2-	C49H60N8O7	873.05	C	-8.6
4676	N(C(=O)[C@H](Cc1ccccc1)C[C@@H](CN1[C@H](C(=O)NC(C)(C)C)CN(Cc2ccsc2)CC1)O)[C@H]1c2c(C[C@H]1O)cccc2	165946-09-8	D-erythro-Pentonamide, 5-[(2S)-4-[(4-bromo-2-thienyl)methyl]-2-[[(1,1-dimethylet	C35H45BrN4O4S	697.73	C	-8.6
4677	O=c1n([C@@H]2O[C@H](COP(=O)(C(O)c3cc(N(=O)=O)ccc3)OC[C@@H]3[C@@H](N=[N+]=[N-])C[C@@H](O3)n3c(=O)[nH]	166318-78-1	Thymidine, 3,Ä≤-azido-P-[(4-cyanophenyl)hydroxymethyl]-P,3,Ä≤-dideoxythymidylyl	C28H30N11O10P		C	-8.6
4678	C(C(C(NC(=O)C(NC(=O)c1nc2c(cc1)cccc2)CC(=O)N)CSc1ccccc1)O)N1C(C(=O)NC(C)(C)C)CC2C(C1)CCCC2	168394-47-6	Butanediamide, N1-[3-[3-[[(1,1-dimethylethyl)amino]carbonyl]octahydro-2(1H)-isoc	C42H52N6O5S	752.96	C	-8.6
4679	[C@H]([C@H](CO)O)(O)[C@H]1[C@H](NC(=O)C)[C@@H](N)C[C@H](C(=O)O)O1	169046-93-9	[1,1,Ä≤-Biphenyl]-4-acetic acid, 2-fluoro-Œ±-methyl-, 4-(2-amino-1,6-dihydro-6-	C25H26FN5O4	479.5	C	-8.6

4680	N(C(=O)[C@H](Cc1ccccc1)C[C@@H](CN1[C@H](C(=O)NC(C)(C)C)CN(Cc2cccnc2)CC1)O)[C@H]1c2c(C[C@H]1OC(=O)c1c	170227-71-1	1-Piperazinepentanamide, N-[2-(acetyloxy)-2,3-dihydro-1H-inden-1-yl]-2-[[(1,1-di	C38H49N5O5	655.83	C	-8.6
4681	O=c1n(cc(C)c(=O)[nH]1)[C@@H]1[C@@H](O)C[C@H](CO)O1	17039-11-1	2,7-Naphthalenedisulfonic acid, 4,4,Ä≤-[(3-sulfo[1,1,Ä≤-biphenyl]-4,4,Ä≤-diyl)b	C32H24N6O15S5	892.89	C	-8.6
4682	O=c1n([C@@H]2OC(=CC2)CO[Si](C(C)(C)C)(C)C)cc(C)c(=O)[nH]1	1704730-70-0	Benzonitrile, 4,4,Ä≤-[[5-bromo-2-[(4-cyanophenyl)amino]-4,6-pyrimidinediyl]bis(	C29H21BrN6O2	565.42	C	-8.6
4683	N(=O)(=O)c1c(c2cc(nc(N)n2)c2ccc(Nc3nc(nc(n3)N3CCOCC3)N3CCOCC3)cc2)cccc1	1710363-37-3	2-Propen-1-one, 3-[4-(diethylamino)phenyl]-1-[4-[(4,6-di-4-morpholinyl-1,3,5-tri	C30H37N7O3	543.66	C	-8.6
4684	O=c1n([C@@H]2O[C@H](COP(=O)([C@@H](O)c3c(N(=O)=O)cccc3)OC[C@@H]3[C@@H](N=[N+]=[N-])C[C@@H](O3)n3c(=O	173658-24-7	Thymidine, 3,Ä≤-azido-P-[(4-cyanophenyl)hydroxymethyl]-P,3,Ä≤-dideoxythymidylyl	C28H30N11O10P		C	-8.6
4685	O=c1n([C@@H]2O[C@H](COP(=O)([C@@H](O)c3c(N(=O)=O)cc(N(=O)=O)cc3)OC[C@@H]3[C@@H](N=[N+]=[N-])C[C@@H](	173658-27-0	Thymidine, 3,Ä≤-azido-P,3,Ä≤-dideoxy-P-[hydroxy(4-nitrophenyl)methyl]thymidylyl	C27H30N11O12P		C	-8.6
4686	[C@H](P(=O)(OC[C@@H]1[C@@H](N=[N+]=[N-])C[C@@H](O1)n1c(=O)[nH]c(=O)c(C)c1)OC[C@@H]1[C@@H](N=[N+]=[N-	173658-28-1	Thymidine, 3,Ä≤-azido-P,3,Ä≤-dideoxy-P-[(2,4-dinitrophenyl)hydroxymethyl]thymid	C27H29N12O14P		C	-8.6
4687	N(=Nc1ccc(S(=O)(=O)O)cc1)c1c2c(cc(C(C)(C)C)cc2)ccc1O	173658-39-4	Thymidine, 3,Ä≤-azido-P,3,Ä≤-dideoxy-P-[(2,6-dinitrophenyl)hydroxymethyl]thymid	C27H29N12O14P		C	-8.6
4688	C(Cc1ccc(F)cc1)C1(CCC)CC(=C(C(C(C)(C)C)c2cc(NS(=O)(=O)c3ccc(C#N)cn3)ccc2)C(=O)O1)O	174484-01-6	2-Pyridinesulfonamide, 5-cyano-N-[3-[1-[5,6-dihydro-4-hydroxy-2-oxo-6-(2-phenyle	C33H37N3O5S	587.73	C	-8.6
4689	C(Cc1ccccc1)C1(CCc2ccccc2)CC(=C([C@@H](CC)c2cc(NS(=O)(=O)c3cc(C(F)(F)F)ccn3)ccc2)C(=O)O1)O	174484-33-4	2-Pyridinesulfonamide, N-[3-[1-[5,6-dihydro-4-hydroxy-2-oxo-6,6-bis(2-phenylethy	C36H35F3N2O5S	664.73	C	-8.6
4690	C(Cc1ccccc1)[C@]1(CCC)CC(=C([C@H](CC)c2cc(NS(=O)(=O)c3ccc(C(F)(F)F)cn3)ccc2)C(=O)O1)O	174484-40-3	2-Pyridinesulfonamide, N-[3-[1-[5,6-dihydro-4-hydroxy-2-oxo-6-(2-phenylethyl)-6-	C33H37F3N2O5S	630.72	C	-8.6
4691	C(Cc1ccccc1)[C@]1(CCC)CC(=C([C@H](CC)c2cc(NS(=O)(=O)c3ccc(C#N)cn3)ccc2)C(=O)O1)O	174484-48-1	3-Pyridinecarboxamide, 6-[[[3-[1-[6-[2-(4-fluorophenyl)ethyl]-5,6-dihydro-4-hydr	C31H34FN3O6S	595.68	C	-8.6
4692	C(Cc1ccccc1)C1(CCc2ccccc2)CC(=C(Cc2cc(NS(=O)(=O)c3ccc(C#N)cn3)ccc2)C(=O)O1)O	174484-58-3	2-Pyridinesulfonamide, 5-cyano-N-[3-[1-[6-[2-(4-fluorophenyl)ethyl]-5,6-dihydro-	C33H36FN3O5S	605.72	C	-8.6
4693	C(Cc1ccccc1)C1(CCc2ccccc2)CC(=C([C@@H](C(C)(C)C)c2cc(NS(=O)(=O)c3ccc(N)cn3)ccc2)C(=O)O1)O	174484-63-0	2-Pyridinesulfonamide, 5-amino-N-[3-[1-[5,6-dihydro-4-hydroxy-2-oxo-6,6-bis(2-ph	C37H41N3O5S	639.8	C	-8.6
4694	C(Cc1ccccc1)C1(CCc2ccccc2)CC(=C([C@H](C(C)(C)C)c2cc(NS(=O)(=O)c3ccc(C#N)cn3)ccc2)C(=O)O1)O	174484-64-1	2-Pyridinesulfonamide, 5-amino-N-[3-[1-[5,6-dihydro-4-hydroxy-2-oxo-6,6-bis(2-ph	C37H41N3O5S	639.8	C	-8.6
4695	[C@H](C(C)(C)C)(C1=C(O)C[C@@](CCc2ccccc2)(CCC)OC1=O)c1cc(NS(=O)(=O)c2cc(C(F)(F)F)ccn2)ccc1	174590-23-9	2-Pyridinesulfonamide, N-[3-[1-[5,6-dihydro-4-hydroxy-2-oxo-6-(2-phenylethyl)-6-	C33H37F3N2O5S	630.72	C	-8.6
4696	[C@@H](C(C)(C)C)(C1=C(O)C[C@](CCc2ccccc2)(CCC)OC1=O)c1cc(NS(=O)(=O)c2cc(C(F)(F)F)ccn2)ccc1	174590-24-0	2-Pyridinesulfonamide, N-[3-[1-[5,6-dihydro-4-hydroxy-2-oxo-6-(2-phenylethyl)-6-	C33H37F3N2O5S	630.72	C	-8.6
4697	Nc1c2c(n(cn2)[C@@H]2O[C@H](COP(=O)(OP(=O)(OC[C@H]3O[C@H](CC3)n3c4c(nc3)c(=O)nc[nH]4)O)O)CC2)ncn1	175923-19-0	Adenosine 5,Ä≤-(trihydrogen diphosphate), 2,Ä≤,3,Ä≤-dideoxy-, P,Ä≤,Ü5,Ä≤-ester	C20H26N10O11P2		C	-8.6
4698	P(=O)(O)(O)[C@@]1(C(=O)O)O[C@@H]([C@@H]([C@@H](CO)O)O)[C@H](NC(=O)C)[C@@H](O)C1	177738-32-8	Thymidine, P-thiothymidylyl-(3,Ä≤,Ü5,Ä≤)-5,Ä≤-thio-	C20H27N4O10PS2	578.55	C	-8.6
4699	N(C(=O)c1ccc(F)cc1)c1n2c(nccc2nn1)N1CCOCC1	1778635-63-4	Benzamide, N-[5-(4-morpholinyl)-1,2,4-triazolo[4,3-c]pyrimidin-3-yl]-4-(trifluor	C17H15F3N6O2	392.34	C	-8.6
4700	Oc1c2c(c(c3c1C(=O)OC3)c1cc3c(cc1)OCO3)cc(OC)c(OC)c2	177994-55-7	1-Piperazinepentanamide, N-(2,3-dihydro-2-hydroxy-3-oxo-1H-inden-1-yl)-2-[[(1,1-	C36H45N5O5	627.77	C	-8.6
4701	C(CN1CCC(CN2c3c4c(S2(=O)=O)cccc4ccc3)CC1)(N)C12CC3CC(C2)CC(C1)C3	178477-38-8	2H-Naphth[1,8-cd]isothiazole-2-butanamine, N-methyl-N-[2-(1-pyrrolidinyl)-2-tric	C31H43N3O2S	521.76	C	-8.6
4702	C(CN1CCC(CN2c3c4c(S2(=O)=O)cccc4ccc3)CC1)(N(CC)CC)C12CC3CC(C2)CC(C1)C3	178477-48-0	2H-Naphth[1,8-cd]isothiazole, 2-[[1-[2-(1-pyrrolidinyl)-2-tricyclo[3.3.1.13,7]de	C32H43N3O2S	533.77	C	-8.6
4703	S(=O)(=O)(O)c1c2c(c(N)c(N=Nc3ccc(N=Nc4c(N)c5c(c(S(=O)(=O)O)c4)cccc5)cc3)c1)cccc2.[NaH]	179472-46-9	1-Naphthalenesulfonic acid, 3,3,Ä≤-[9H-fluorene-2,7-diylbis(azo)]bis[4-amino-, (	C33H24N6O6S2.2Na		C	-8.6
4704	O(C(=O)N[C@@H](Cc1cc(ccc1)c1cc(OC)ccc1)[C@@H](CN(S(=O)(=O)c1ccc(OC)cc1)CC(C)C)O)[C@@H]1[C@H]2[C@@H](	1800006-53-4	Carbamic acid, N-[(1S,2R)-1-[(3,Ä≤,5,Ä≤-dimethyl[1,1,Ä≤-biphenyl]-3-yl)methyl]-	C36H46N2O8S	666.82	C	-8.6
4705	C(CC(NC(=O)C1CCC(F)(F)CC1)c1cccs1)N1[C@H]2C[C@H](C[C@@H]1CC2)n1c(C(C)C)nnc1C	1800532-32-4	1,3-Naphthalenedisulfonic acid, 7-amino-8-[2-(4-sulfo-1-naphthalenyl)diazenyl]-,	C20H15N3O9S3.3Na		C	-8.6
4706	O([Si](C(C)(C)C)(C)C)c1c(C(=O)NCc2ccc(F)cc2)nc(C(NC(=O)c2nnc(C)o2)(C)C)n(C)c1=O	1801912-44-6	4-Pyrimidinecarboxamide, N-[(4-fluorophenyl)methyl]-1,6-dihydro-1-methyl-2-[1-me	C25H29FN6O6	528.53	C	-8.6
4707	O=C1[C@]2([C@H]([C@H](/C(=C/C(=O)[C@@H]3[C@@]4(N5[C@@H]([C@H]3c3cc(OC)c(O)cc3)CCC5)c3c(NC4=O)ccc(C)c	1807888-38-5	Not Yet Assigned	C47H48N4O8	796.91	C	-8.6
4708	C(=O)([C@@H](NC(=O)OC)C(C)C)N1[C@@H](C2CC1CC2)c1[nH]c(cn1)c1ccc(cc1)c1ccc(cc1)c1[nH]c(nc1)[C@H]1N(C(	1808295-91-1	Carbamic acid, N-[(1S)-1-[[(2S)-2-[5-[4,Ä≤-[2-[(3S,5S,6R)-5,6-dihydroxy-2-[(2S)-	C42H52N8O8	796.91	C	-8.6
4709	C(=O)([C@@H](NC(=O)OC)C(C)C)N1[C@@H](C2CC1CC2)c1[nH]c(cn1)c1ccc(/C=C/c2ccc(cc2)c2[nH]c(nc2)[C@H]2N(C	1808296-04-9	Carbamic acid, N-[(1S)-1-[[(2S)-2-[5-[4,Ä≤,Ä≤-[2-[(3S)-2-[(2S)-2-[(methoxycarbo	C48H56N8O6	841.01	C	-8.6
4710	N(C(=O)C)[C@H]1[C@H]([C@@H]([C@@H](CO)O)O)OC(=C[C@@H]1N[11C]#N)C(=O)O	1808296-08-3	Carbamic acid, N-[(1S)-1-[[(2S)-2-[5-[4,Ä≤-[2-[(2S,6S)-1-[(2S)-2-[(methoxycarbon	C42H54N8O6	766.93	C	-8.6
4711	O=C1[C@@H]2[C@@H](c3c([C@H](OC(=O)C(C)N4C(=O)C5C(C4=O)C4OC5CC4)[C@H]2CO1)cc1c(c3)OCO1)c1cc(OC)c(OC)c	1809325-31-2	2H-Isoindole-2-acetic acid, octahydro-Œ±-methyl-1,3-dioxo-, (5R,5aR,8aR,9R)-5,5a	C33H35NO11	621.63	C	-8.6
4712	O=C1[C@@H]2[C@H](c3c(C(=O)[C@H]2CO1)cc1c(c3)OCO1)c1cc2c(cc1)OCO2	1835272-58-6	Carbamic acid, N-[(1S)-1-[[(2S,5S)-2-[5-[1,Ä≤,3,Ä≤-dihydro-7,Ä≤-[4-[2-[(2S,5S)-	C49H64N8O6	861.08	C	-8.6
4713	O=c1c2c(n(cn2)[C@@H]2O[C@H](COP(=O)(OC[C@H]3O[C@H](C[C@@H]3O)n3c4c(nc3)c(=O)nc(N)[nH]4)O)[C@@H](O)C2	1835316-42-1	Furo[3,Ä≤,4,Ä≤:6,7]naphtho[2,3-d]-1,3-dioxol-6(5aH)-one, 5-(1,3-benzodioxol-5-y	C20H16O7	368.34	C	-8.6
4714	O[C@@H]1[C@@H]2[C@@H]([C@]3(C)[C@H](C1)C[C@H](O)CC3)CC[C@@]1(C)[C@H]2CC[C@@H]1[C@@H](CCC(=O)N[C@@H](	1854104-55-4	L-Cysteine, N-[(3Œ±,5Œ≤,7Œ±)-3,7-dihydroxy-24-oxocholan-24-yl]-	C27H45NO5S	495.71	C	-8.6
4715	C(OC[C@@H]1[C@@H](OC(=O)COCC)C[C@@H](C1=C)n1c2c(nc1)c(=O)nc(NC(=O)OC(C)(C)C)[nH]2)(c1ccccc1)(c1ccccc	1860842-93-8	3-Butenoic acid, (1S,2R,4S)-4-[2-[[(1,1-dimethylethoxy)carbonyl]amino]-1,6-dihyd	C40H41N5O6	687.78	C	-8.6
4716	C(OC[C@@H]1[C@@H](OC(=O)c2ccccc2)C[C@@H](C1=C)n1c2c(nc1)c(=O)nc(NC(=O)OC(C)(C)C)[nH]2)(c1ccccc1)(c1c	1860842-95-0	3-Pyridinecarboxylic acid, (1S,2R,4S)-4-[2-[[(1,1-dimethylethoxy)carbonyl]amino]	C42H40N6O6	724.8	C	-8.6
4717	O([Si](CNCCN)(C)C)[Si](CNCCN)(C)C.C(=C1C=C(C(=O)O)C(=O)C=C1)(c1cc(C(=O)O)c(O)cc1)c1cc(C(=O)O)c(O)cc1	1864833-37-3	1H-Indole-1-acetic acid, 7-fluoro-2,3-dihydro-2,3-dioxo-, (5R,5aR,8aR,9R)-5,5a,6	C32H26FNO11	619.55	C	-8.6
4718	O=c1n([C@@H]2O[C@H](COP(=O)(OP(=O)(OP(=O)(OCc3ccc(OC(=O)CCCCCCC)cc3)OCc3ccc(OC(=O)C)cc3)O)O)C=C2)cc(	1883329-00-7	Benzoic acid, 4-methyl-, 4-[3-[[4-(acetyloxy)phenyl]methoxy]-9-[(2S,5R)-5-(3,4-d	C34H35N2O17P3	836.57	C	-8.6
4719	N(C(=O)Nc1c(C)cccc1)c1cc2cc(C34CC5CC(C4)CC(C3)C5)[nH]c2cc1	1883585-91-8	Urea, N-phenyl-N,Ä≤-(2-tricyclo[3.3.1.13,7]dec-1-yl-1H-indol-5-yl)-	C25H27N3O	385.5	C	-8.6
4720	O(C)c1c2[C@@H]([C@@H]3[C@@H](Cc2cc2c1OCO2)COC3=O)c1cc(OC)c(OC)c(C)c1	1913279-97-6	Furo[3,Ä≤,4,Ä≤:6,7]naphtho[2,3-d]-1,3-dioxol-6(5aH)-one, 5-(2,6-dimethoxyphenyl	C21H20O6	368.38	C	-8.6
4721	C(=O)([C@@H](NC(=O)OC)C(C)C)N1[C@@H](CCC1)c1[nH]c(cn1)c1c(c(c(c(c1[2H])[2H])c1c(c(c(c(c1[2H])[2H])c1	1939212-01-7	Carbamic acid, N,N,Ä≤-[[1,1,Ä≤-biphenyl]-4,4,Ä≤-diyl-2,3,5,6-d4-bis[1H-imidazol	C40H40D10N8O6		C	-8.6
4722	Clc1c2c(c3c(nc([nH]3)[C@H]3N(C(=O)[C@@H](NC(=O)OCCOC)C(C)C)CCC3)c1)ccc(c2)c1ccc(cc1)c1[nH]c(nc1)[C@H	1949770-73-3	Carbamic acid, N-[(1S)-1-[[(2S)-2-[5-chloro-7-[4-[2-[(2S)-1-[(2S)-3-methyl-2-[[(	C46H57ClN8O6	853.45	C	-8.6
4723	C(=O)([C@@H](NC(=O)OC)C(C)C)N1[C@@H]([C@@H]2C[C@H]1CC2)c1[nH]c2c(n1)ccc1c2ccc(c1)c1ccc(cc1)c1[nH]c(n	1949770-78-8	Carbamic acid, N-[(1S)-1-[[(2S)-2-[7-[4-[2-[(2S,4R)-4-[(methoxycarbonyl)amino]-1	C44H53N9O8	835.95	C	-8.6
4724	[C@@H](Cc1ccccc1)(NC(=O)[C@H](CCN1CCOCC1)NC(=O)N(Cc1nc(C(C)C)sc1)C)CC[C@H](Cc1ccccc1)NC(=O)OCc1cncs1	1964472-16-9	2,7,10,12-Tetraazatridecanoic acid, 12-methyl-13-[2-(1-methylethyl)-4-thiazolyl]	C40H53N7O5S2.C23H16O6		C	-8.6
4725	O[C@H]1[C@H](n2c3c(nc2)c(=O)nc(N)[nH]3)O[C@H](COc2c(F)c(F)c(C#N)c(O)c2F)[C@H]1O	197903-83-6	Guanosine, 5,Ä≤-O-(3,5-difluoro-1,2-dihydro-6-hydroxy-2-oxo-4-pyridinyl)-	C15H14F2N6O7	428.3	C	-8.6
4726	N(C(=O)C)[C@H]1[C@H]([C@@H]([C@@H](CO)O)O)OC(=C[C@@H]1NC(=N)NC(=O)Cn1cc(nn1)c1c(OC)cccc1)C(=O)O	1982351-39-2	D-glycero-D-galacto-Non-2-enonic acid, 5-(acetylamino)-2,6-anhydro-4-[[[[2-[4-(1	C22H31N7O8	521.52	C	-8.6
4727	N(C(=O)C)[C@H]1[C@H]([C@@H]([C@@H](CO)O)O)OC(=C[C@@H]1NC(=N)NC(=O)Cn1cc(nn1)c1ccc(C)cc1)C(=O)O	1982351-48-3	D-glycero-D-galacto-Non-2-enonic acid, 5-(acetylamino)-4-[[[[2-[4-(4-aminophenyl	C22H28N8O8	532.51	C	-8.6
4728	O(COC(=O)OCC(C(=O)OC)(C)C)c1c2n(N([C@H]3N(C2=O)CCOC3)[C@H]2c3c(CSc4c2cccc4)c(F)c(F)cc3)ccc1=O	1985606-94-7	Carbonic acid, (12aR)-12-[(11S)-7,8-difluoro-6,11-dihydrodibenzo[b,e]thiepin-11-	C30H29F2N3O6S	597.63	C	-8.6
4729	O(COC(=O)OCC(=O)OC)c1c2n(N([C@H]3N(C2=O)CCOC3)[C@H]2c3c(CSc4c2cccc4)c(F)c(F)cc3)ccc1=O	1985606-97-0	Propanoic acid, 2-[[[[[(12aR)-12-[(11S)-7,8-difluoro-6,11-dihydrodibenzo[b,e]thi	C30H27F2N3O9S	643.61	C	-8.6
4730	O(COC(=O)O[C@H]1C(C)(C)COC1=O)c1c2n(N([C@H]3N(C2=O)CCOC3)[C@H]2c3c(CSc4c2cccc4)c(F)c(F)cc3)ccc1=O	1985607-06-4	Propanoic acid, 2-[[[[[(12aR)-12-[(11S)-7,8-difluoro-6,11-dihydrodibenzo[b,e]thi	C30H27F2N3O9S	643.61	C	-8.6
4731	O(COC(=O)OC(C)(C)C)c1c2n(N([C@H]3N(C2=O)CCOC3)[C@H]2c3c(CSc4c2cccc4)c(F)c(F)cc3)ccc1=O	1985607-36-0	Cyclopentaneacetic acid, 1-[[[[[(12aR)-12-[(11S)-7,8-difluoro-6,11-dihydrodibenz	C34H33F2N3O9S	697.7	C	-8.6
4732	O(C(=O)OCC(C(=O)OC)(C)C)c1c2n(N([C@H]3N(C2=O)CCOC3)[C@H]2c3c(CSc4c2cccc4)c(F)c(F)cc3)ccc1=O	1985607-39-3	Propanoic acid, 2-[[[[(12aR)-12-[(11S)-7,8-difluoro-6,11-dihydrodibenzo[b,e]thie	C31H29F2N3O8S	641.64	C	-8.6
4733	O(C(=O)O[C@@H](C(=O)OC)C)c1c2n(N([C@H]3N(C2=O)CCOC3)[C@H]2c3c(CSc4c2cccc4)c(F)c(F)cc3)ccc1=O	1985607-56-4	Propanoic acid, 2-[[[[(12aR)-12-[(11S)-7,8-difluoro-6,11-dihydrodibenzo[b,e]thie	C29H25F2N3O8S	613.59	C	-8.6
4734	N(C(=O)Nc1cc(OC)c(OC)cc1)c1cc2cc(C34CC5CC(C4)CC(C3)C5)[nH]c2cc1	1987854-71-6	Urea, N-(3,5-dimethoxyphenyl)-N,Ä≤-(2-tricyclo[3.3.1.13,7]dec-1-yl-1H-indol-5-y	C27H31N3O3	445.55	C	-8.6
4735	N(C(=O)NCCCCC)c1cc2cc(C34CC5CC(C4)CC(C3)C5)[nH]c2cc1	1987854-74-9	Urea, N-(phenylmethyl)-N,Ä≤-(2-tricyclo[3.3.1.13,7]dec-1-yl-1H-indol-5-yl)-	C26H29N3O	399.53	C	-8.6
4736	N(CCCN(CCCCN(CCCN)CCCN)CCCN)c1c2c(ncc1)cc(Cl)cc2	1989611-40-6	1H-Indazole-3-carboxylic acid, 1-methyl-, (5R,5aR,8aR,9R)-5,5a,6,8,8a,9-hexahydr	C31H28N2O9	572.56	C	-8.6
4737	C(=O)([C@@H](NC(=O)OC)C(C)C)N1[C@H](c2[nH]c3-c4c(cc(cc4)c4c5c(c(cc4)c4[nH]c(nc4)[C@H]4N(C(=O)[C@@H](	2007900-97-0	Carbamic acid, N-[(1S)-1-[[(2S,4R)-2-[5-[2,3-dihydro-7-[2-[(2S)-1-[(2S)-2-[(meth	C45H53FN8O6	820.95	C	-8.6
4738	C(=O)([C@@H](NC(=O)OC)C(C)C)N1[C@@H](CC[C@@H]1C(F)(F)F)c1[nH]c2c(n1)ccc1c2ccc(c1)c1c2c(c(cc1)c1[nH]c	2007901-79-1	Carbamic acid, N-[(1S)-1-[[(2S,5R)-2-[7-[2,3-dihydro-7-[2-[(7S)-6-[(2S)-2-[(meth	C49H57F3N8O6	911.02	C	-8.6
4739	C(=O)([C@@H](NC(=O)OC)C(C)C)N1CC2(C[C@H]1c1[nH]c3c(n1)ccc1c3ccc(c1)c1c3c(c(cc1)c1[nH]c(nc1)[C@H]1N(C	2007901-83-7	Carbamic acid, N-[(1S)-1-[[(2S,4S)-2-[5-[2,3-dihydro-7-[2-[(3S)-2-[(2S)-2-[(meth	C47H53F3N8O6	882.97	C	-8.6
4740	C(C(C)C)n1c2c(c(=NN)[nH]c3c2cccc3)nc1	2010108-58-2	Not Yet Assigned	C44H46Cl2Cu2N8O2S2		C	-8.6
4741	C([C@H](OCP(=O)(OCOC(=O)OCC(C)(C)C)OCOC(=O)OCC(C)(C)C)C)n1c2c(nc1)c(N)ncn2	201341-01-7	2,4,6,8-Tetraoxa-5-phosphanonanedioic acid, 5-[[(1R)-2-(6-amino-9H-purin-9-yl)-1	C21H34N5O10P	547.5	C	-8.6
4742	C(=O)(C(=O)Nc1c(OC)cccc1)c1c(C23CC4CC(C3)CC(C2)C4)[nH]c2c1cccc2	2014403-78-0	1H-Indole-3-acetamide, N-(4-methylphenyl)-Œ±-oxo-2-tricyclo[3.3.1.13,7]dec-1-yl-	C27H28N2O2	412.52	C	-8.6
4743	C(=O)(C(=O)Nc1ccc(OC)cc1)c1c(C23CC4CC(C3)CC(C2)C4)[nH]c2c1cccc2	2014403-80-4	1H-Indole-3-acetamide, Œ±-oxo-2-tricyclo[3.3.1.13,7]dec-1-yl-N-[3-(trifluorometh	C27H25F3N2O2	466.49	C	-8.6
4744	C(=O)(C(=O)Nc1c(C)cc(Cl)cc1)c1c([nH]c2c1cccc2)C12CC3CC(C2)CC(C1)C3	2014403-82-6	1H-Indole-3-acetamide, N-(2,4-dimethylphenyl)-Œ±-oxo-2-tricyclo[3.3.1.13,7]dec-1	C28H30N2O2	426.55	C	-8.6
4745	O=c1n([C@@H]2O[C@H](COP(=O)S)[C@@H](OP(=O)(O)S)C2)cc(C)c(=O)[nH]1	2024571-98-8	Not Yet Assigned	C29H38IN7O18P2	961.5	C	-8.6
4746	S(=O)(=O)(O)c1c2c(c(N)c(N=Nc3ccc(cc3)c3ccc(cc3)c3ccc(cc3)c3ccc(N=Nc4c(N)c5c(c(S(=O)(=O)O)c4)cccc5)cc	1000606-67-6	1-Naphthalenesulfonic acid, 3,3,Ä≤-[[1,1,Ä≤:4,Ä≤,1,Ä≤,Ä≤-terphenyl]-4,4,Ä≤,Ä≤-	C38H28N6O6S2.2Na		A/B	-8.5
4747	O=[14c]1n(cc(c(=O)[nH]1)[2H])[C@@H]1O[C@H](COP(=O)(O)O)[C@@H](O)C1	1000606-70-1	1-Naphthalenesulfonic acid, 3,3,Ä≤-[[1,1,Ä≤:4,Ä≤,1,Ä≤:4,Ä≤,Ä≤,1,Ä≤,Ä≤,Ä≤-q	C44H32N6O6S2.2Na		A/B	-8.5
4748	O[C@H]1[C@@H](O[C@H](CO)[C@H]1OCc1ccc(NC(=O)CO[C@@H]2O[C@H](CO)[C@H](O)[C@H](O)[C@H]2O)cc1)n1ncC(=O	1002334-90-8	1H-1,2,4-Triazole-3-carboxamide, 1-[2-O-[[4-[[2-(Œ≤-D-galactopyranosyloxy)acety	C23H31N5O12	569.52	A/B	-8.5
4749	O[C@H]1[C@@H](O[C@H](COC(=O)CCCCO[C@@H]2O[C@H](CO)[C@H](O)[C@H](O)[C@H]2O)[C@H]1O)n1nc(C(=O)N)nc1	1002334-91-9	1H-1,2,4-Triazole-3-carboxamide, 1-[3-O-[[4-[[2-(Œ≤-D-galactopyranosyloxy)acety	C23H31N5O12	569.52	A/B	-8.5
4750	O(Cc1ccccc1)[C@H]1[C@@H](OCc2ccccc2)[C@@H](COCc2ccccc2)O[C@@H](OCCCOC(=O)OC[C@H]2O[C@H]([C@H](O)[C@@	1002335-15-0	1H-1,2,4-Triazole-3-carboxamide, 1-[2,3-O-(methoxymethylene)-5-O-[[3-[[2,3,4,6-t	C48H54N4O14	910.96	A/B	-8.5
4751	C(OC(=O)C(CO[C@H]1[C@H](OCc2ccccc2)[C@@H](OCc2ccccc2)[C@@H](OCc2ccccc2)[C@@H](COCc2ccccc2)O1)(C)C)[C	1002336-41-5	1H-1,2,4-Triazole-3-carboxamide, 1-[2,3-O-(methoxymethylene)-5-O-[(2S)-2-methyl-	C27H34N4O13	622.58	A/B	-8.5
4752	C(OC(=O)C1CCC(O[C@@H]2O[C@H]3[C@@H]([C@H](O)[C@H]2O)O[C@H](OC3)c2ccccc2)CC1)[C@@H]1[C@@H]2[C@H]([C@@	1002355-31-8	1H-1,2,4-Triazole-3-carboxamide, 1-[5-O-[[4-(Œ≤-D-galactopyranosyloxy)cyclohexy	C21H32N4O12	532.5	A/B	-8.5
4753	[C@H](Cc1ccccc1)(CC[C@H](Cc1ccccc1)NC(=O)OCc1cncs1)NC(=O)[C@@H](NC(=O)N(Cc1nc(C(C)C)sc1)C)CC	1004316-42-0	2,7,10,12-Tetraazatridecanoic acid, 9,12-dimethyl-13-[2-(1-methylethyl)-4-thiazo	C35H44N6O4S2	676.89	A/B	-8.5
4754	[C@H](Cc1ccccc1)(NC(=O)[C@@H](NC(=O)N(Cc1nc(C(C)C)sc1)C)CCNS(=O)(=O)C)CC[C@@H](Cc1ccccc1)NC(=O)OCc1c	1004317-01-4	2,7,10,12-Tetraazatridecanoic acid, 9-[2-(acetylamino)ethyl]-12-methyl-13-[2-(1-	C38H49N7O5S2	747.97	A/B	-8.5
4755	N(Cc1nc(C(C)C)sc1)(C(=O)NC(C(=O)NC(Cc1ccccc1)CCC(Cc1ccccc1)NC(=O)OCc1cncs1)C(C)C)C1CC1	1004318-42-6	2,7,10,12-Tetraazatridecanoic acid, 9-ethyl-12-methyl-13-[2-(1-methylethyl)-4-th	C36H46N6O4S2	690.92	A/B	-8.5
4756	C(=O)([C@H](N(CC)CC)c1ccccc1)N1[C@@H](CCC1)c1[nH]c(cn1)c1ccc(cc1)c1ccc(cc1)c1[nH]c(nc1)[C@H]1N(C(=O)	1009107-40-7	Carbamic acid, N-[(1R)-2-[(2S)-2-[5-[4,Ä≤-[2-[(2S)-1-[2-[[(1,1-dimethylethoxy)ca	C43H48N8O6	772.89	A/B	-8.5
4757	C(=O)([C@H](NC(=O)OC)c1ccccc1)N1[C@@H](CCC1)c1[nH]c(cn1)c1cc(C)c(cc1)c1c(C)cc(cc1)c1[nH]c(nc1)[C@H]1	1009111-84-5	Carbamic acid, N-[2-oxo-2-[(2S)-2-[5-[4,Ä≤-[2-[(2S)-1-[(2R)-2-phenyl-2-(1-pyrrol	C42H46N8O4	726.87	A/B	-8.5
4758	C(=O)(N1[C@@H](CCC1)c1[nH]c(cn1)c1ccc(cc1)c1ccc(cc1)c1[nH]c(nc1)[C@H]1N(C(=O)[C@@H](NC(=O)OC)C(C)C)C	1009114-59-3	Carbamic acid, N-[(1S)-1-[[(2S)-2-[5-[4,Ä≤-[2-[(2S)-1-[[trans-4-[(methoxycarbony	C42H52N8O6	764.91	A/B	-8.5
4759	C(=O)([C@H](Cc1ccc(CNC(=O)C)cc1)NC(=O)OC)N1[C@@H](CCC1)c1[nH]c(cn1)c1ccc(cc1)c1ccc(cc1)c1[nH]c(nc1)[	1009115-02-9	Carbamic acid, N-[(1S)-2-[(2S)-2-[5-[4,Ä≤-[2-[(2S)-1-[(2S)-3-(4-hydroxyphenyl)-2	C42H46N8O7	774.86	A/B	-8.5
4760	C(=O)([C@@H](Cc1ccccc1)NC(=O)OC)N1[C@@H](CCC1)c1[nH]c(cn1)c1ccc(cc1)c1ccc(cc1)c1[nH]c(nc1)[C@H]1N(C(	1009115-12-1	Carbamic acid, N,N,Ä≤-[[1,1,Ä≤-biphenyl]-4,4,Ä≤-diylbis[1H-imidazole-5,2-diyl-(	C40H44N14O6	816.87	A/B	-8.5
4761	C(=O)([C@@H](NC(=O)OC)COC)N1[C@@H](CCC1)c1[nH]c(cn1)c1ccc(cc1)c1ccc(cc1)c1[nH]c(nc1)[C@H]1N(C(=O)[C	1009115-39-2	Carbamic acid, N,N,Ä≤-[[1,1,Ä≤-biphenyl]-4,4,Ä≤-diylbis[1H-imidazole-5,2-diyl-(	C48H50N8O6	834.96	A/B	-8.5
4762	C(=O)([C@@H](NC(=O)OC)C(C)C)N1[C@@H](C[C@@H](O)C1)c1[nH]c(cn1)c1ccc(cc1)c1ccc(cc1)c1[nH]c(nc1)[C@H]1	1009115-57-4	Carbamic acid, N,N,Ä≤-[[1,1,Ä≤-biphenyl]-4,4,Ä≤-diylbis[1H-imidazole-5,2-diyl[(	C36H42N8O8	714.77	A/B	-8.5
4763	C(=O)([C@@H](NC(=O)OC)C(C)C)N1[C@@H](CCC1)c1[nH]c(cn1)c1ccc(cc1)c1ccc(cc1)c1[nH]c(nc1)[C@H]1N(C(=O)C	1009117-84-3	Carbamic acid, N-[(1R)-2-[(2S)-2-[5-[4,Ä≤-[2-[(2S)-1-[(2S)-2-[(methoxycarbonyl)a	C43H48N8O6	772.89	A/B	-8.5
4764	C(=O)([C@@H](NC(=O)OC(C)(C)C)C(C)C)N1[C@@H](CCC1)c1[nH]c(cn1)c1ccc(cc1)c1ccc(cc1)c1[nH]c(nc1)[C@H]1N	1009119-15-6	Carbamic acid, N,N,Ä≤-[[1,1,Ä≤-biphenyl]-4,4,Ä≤-diylbis[1H-imidazole-5,2-diyl-(	C42H54N8O6	766.93	A/B	-8.5
4765	C(C)(C)[C@@H]1n2c3c(nc2C(CCC)OC1)c(N)nc1c3cccc1	1010105-77-7	8H-[1,4]Oxazino[4,Ä≤,3,Ä≤:1,2]imidazo[4,5-c]quinolin-6-amine, 8-ethyl-10,11-dih	C18H22N4O	310.39	A/B	-8.5
4766	[C@H](Cc1cccnc1)(C(=O)N[C@@H](CCc1ccccc1)/C=C/S(=O)(=O)c1ccccc1)NC(=O)N1CCN(C)CC1	1011726-29-6	1-Piperazinecarboxamide, 4-methyl-N-[(1S)-1-[(4-methylphenyl)methyl]-2-oxo-2-[[(	C33H40N4O4S	588.76	A/B	-8.5
4767	C(=O)(NC(CC1CCC1)C(=O)C(=O)N)[C@@H]1[C@@H]2[C@@H](C2(C)C)CN1C(=O)[C@@H](NC(=O)N[C@H](COC(=O)NC)C(C)C	1025806-17-0	Not Yet Assigned	C21H20FN5O8S	521.48	A/B	-8.5
4768	O=c1n(cc(C)c(=O)[nH]1)[C@H]1C(=C[C@@H](CO)O1)O	1025971-16-7	Benzonitrile, 4-[[9-[4-[(1E)-2-cyanoethenyl]-2,6-dimethylphenyl]-6-methyl-9H-pur	C24H19N7	405.45	A/B	-8.5
4769	O[C@H]1[C@@H](O[C@H](COC(=O)CCCCC(=O)OC=C)[C@H]1O)n1nc(C(=O)N)nc1	102905-04-4	Guanosine, 2,Ä≤-deoxyadenylyl-(3,Ä≤,Ü5,Ä≤)-	C20H25N10O10P	596.45	A/B	-8.5
4770	O[C@H]1[C@H](n2c3c(nc2)c(N)ncn3)O[C@H](CO)[C@H]1OC	1029874-46-1	Not Yet Assigned	C34H24N6O6S2.2Na		A/B	-8.5
4771	O=c1n(cc(C#C)c(=O)[nH]1)[C@H]1C[C@H](N=[N+]=[N-])[C@@H](CO)O1	10300-41-1	Thymidine, 2,Ä≤-deoxyuridylyl-(3,Ä≤,Ü5,Ä≤)-	C19H25N4O12P	532.4	A/B	-8.5
4772	O(c1nc(Nc2ccc(C#N)cc2)nc(N)c1Br)c1c(C)cc(C#N)cc1C.C(=C\C(=O)O)/C(=O)O	1030633-41-0	Benzonitrile, 4-[[6-amino-5-bromo-2-[(4-cyanophenyl)amino]-4-pyrimidinyl]oxy]-3,	C20H15BrN6O.C7H8O3S		A/B	-8.5
4773	O(c1nc(Nc2ccc(C#N)cc2)nc(N)c1Br)c1c(C)cc(C#N)cc1C.S(=O)(=O)(O)O	1030633-42-1	Benzonitrile, 4-[[6-amino-5-bromo-2-[(4-cyanophenyl)amino]-4-pyrimidinyl]oxy]-3,	C20H15BrN6O.xC4H4O4		A/B	-8.5
4774	C(CC(=O)CC(=O)CCc1cc(O)ccc1)c1ccc(O)cc1	1030827-56-5	Not Yet Assigned	C34H26N6O6S2.2Na		A/B	-8.5
4775	N(c1nc(Nc2ccc(C#N)cc2)nc(C(=O)NCCC#N)c1)c1c(C)cc(/C=C/C#N)cc1C	1037444-85-1	4-Pyrimidinecarboxamide, 6-[[4-[(1E)-2-cyanoethenyl]-2,6-dimethylphenyl]amino]-2	C25H22FN7O	455.49	A/B	-8.5
4776	N(c1c(C)cc(/C=C/C#N)cc1C)c1nc(Nc2ccc(C#N)cc2)nc(CN2CCOCC2)c1	1037444-95-3	Benzonitrile, 4-[[4-[[4-[(1E)-2-cyanoethenyl]-2,6-dimethylphenyl]amino]-6-(hydro	C26H21N7OS	479.56	A/B	-8.5
4777	N(c1nc(Nc2ccc(C#N)cc2)nc(C(=O)N(OC)C)c1)c1c(C)cc(/C=C/C#N)cc1C	1037445-57-0	4-Pyrimidinecarboxylic acid, 6-[[4-[(1E)-2-cyanoethenyl]-2,6-dimethylphenyl]amir	C23H18N6O2	410.43	A/B	-8.5
4778	N(c1c(c(COC)nc(Nc2ccc(C#N)cc2)n1)c1ccncc1)c1c(C)cc(/C=C/C#N)cc1C	1037451-93-6	Benzonitrile, 4-[[4-[[4-[(1E)-2-cyanoethenyl]-2,6-dimethylphenyl]amino]-6-(metho	C29H25N7O	487.56	A/B	-8.5
4779	C(=O)(NCCOC)c1c(Nc2c(C)cc(/C=C/C#N)cc2C)nc(Nc2ccc(C#N)cc2)nc1COC	1037452-07-5	5-Pyrimidinecarboxamide, 4-[[4-[(1E)-2-cyanoethenyl]-2,6-dimethylphenyl]amino]-2	C30H31N7O3	537.61	A/B	-8.5
4780	O[C@H]1[C@@H](O[C@H](COC(=O)CCCCCCCCC(=O)OC(C)n2c(=O)[nH]c(=O)cc2)[C@H]1O)n1nc(C(=O)N)nc1	1038531-29-1	1H-1,2,4-Triazole-3-carboxamide, 1-[5-O-[6-[1-(3,4-dihydro-2,4-dioxo-1(2H)-pyrim	C20H26N6O10	510.45	A/B	-8.5
4781	C(c1c2c(N(c3c(C(=O)N2)cccn3)C2CC2)ncc1)n1c(=O)c2c(nc1N)n(cn2)[C@@H]1O[C@H](CO)[C@@H](O)C1	1046462-10-5	Guanosine, 6-O-[(11-cyclopropyl-6,11-dihydro-6-oxo-5H-dipyrido[3,2-b:2,Ä≤,3,Ä≤-(	C25H25N9O5	531.52	A/B	-8.5
4782	C(CC(=O)CC(=O)C)c1cc(O)c(O)cc1	105037-92-1	Naphtho[2,3-c]furan-1(3H)-one, 9-(1,3-benzodioxol-5-yl)-4,7-dihydroxy-6-methoxy-	C20H14O7	366.32	A/B	-8.5
4783	C(Cc1ccccc1)(CCC(Cc1ccccc1)NC(=O)OCc1cncs1)NC(=O)C(CCN1C(=O)OCC1)NC(=O)N(Cc1nc(C(C)C)sc1)C	1051464-63-1	2,7,10,12-Tetraazatridecanoic acid, 9-[2-(2,5-dioxo-1-pyrrolidinyl)ethyl]-12-met	C40H49N7O6S2	787.99	A/B	-8.5
4784	[C@@H](Cc1ccccc1)(NC(=O)[C@H](CCN1CCOCC1)NC(=O)N(Cc1sc(C(C)C)nc1)C)CC[C@H](Cc1ccccc1)NC(=O)OCc1cncs1	1051465-36-1	14-Thia-2,7,13-triazapentadecanoic acid, 12-imino-9-[[methyl[[2-(1-methylethyl)	C38H50N8O6S3	811.05	A/B	-8.5
4785	S(=O)(=O)(O)c1c2c(c(S(=O)(=O)O)cc(/N=N/c3cc(S(=O)(=O)O)c(C=Cc4c(S(=O)(=O)O)cc(/N=N/c5c(N)c6c(c(S(=O)	1052525-21-9	Not Yet Assigned	C34H28N6O18S6.Na		A/B	-8.5
4786	N(=N\c1cc(S(=O)(=O)O)c(C=Cc2c(S(=O)(=O)O)cc(/N=N/c3c4c(cc(S(=O)(=O)O)c3N)cc(S(=O)(=O)O)cc4)cc2)cc1)/	1052530-88-7	Not Yet Assigned	C34H26N6O12S4.Na		A/B	-8.5
4787	N(=N\c1cc(S(=O)(=O)O)c(C=Cc2c(S(=O)(=O)O)cc(/N=N/c3c4c(cc(S(=O)(=O)O)cc4)c(N)cc3)cc2)cc1)/c1c2c(cc(S	1052531-25-5	Not Yet Assigned	C34H26N6O18S6.Na		A/B	-8.5
4788	Nc1c2c(cc(S(=O)(=O)O)cc2)ccc1/N=N/c1cc(S(=O)(=O)O)c(/C=C/c2c(S(=O)(=O)O)cc(/N=N/c3c(N)c4c(cc3)cc(S(=	1052531-67-5	Not Yet Assigned	C34H26N6O12S4.Na		A/B	-8.5
4789	N(=N\c1cc(S(=O)(=O)O)c(C=Cc2c(S(=O)(=O)O)cc(/N=N/c3c4c(cc(S(=O)(=O)O)cc4)c(N)cc3)cc2)cc1)/c1c2c(cc(S	1053056-37-3	Not Yet Assigned	C34H26N6O18S6	998.99	A/B	-8.5
4790	N(c1c(C)cc(/C(=C(\C#N)/C)/C)cc1C)c1nc(Nc2ccc(C#N)cc2)ncc1	1064399-72-9	Benzonitrile, 4-[[4-[[4-(1-hydroxyethyl)-2,6-dimethylphenyl]amino]-2-pyrimidinyl	C21H21N5O	359.42	A/B	-8.5
4791	[C@H](C(=O)N[C@@H](CCc1ccccc1)C=CS(=O)(=O)c1ccccc1)(NC(=O)N1CCN(C)CC1)CCNC(=N)NO	1076088-56-6	1-Piperazinecarboxamide, N-[(1S)-3-[(aminoiminomethyl)amino]-1-[[[(1S)-1-(2-pher	C28H39N7O4S	569.72	A/B	-8.5
4792	[C@@H](NC(=O)N1CCN(C)CC1)(C(=O)N[C@H](C=CS(=O)(=O)c1ccccc1)CCc1ccccc1)CCCNC(=N)NN(=O)=O	1076088-61-3	1-Piperazinecarboxamide, N-[(1S)-3-[[imino(nitroamino)methyl]amino]-1-[[[(1S)-1-	C28H38N8O6S	614.72	A/B	-8.5
4793	[C@H](Cc1cc(C(=N)N)ccc1)(C(=O)N[C@H](C=CS(=O)(=O)c1ccccc1)CCc1ccccc1)NC(=O)N1CCN(C)CC1	1076088-66-8	1-Piperazinecarboxamide, N-[(1S)-1-[[3-(aminomethyl)phenyl]methyl]-2-oxo-2-[[(1S	C33H41N5O4S	603.77	A/B	-8.5
4794	[C@@H](NC(=O)N1CCN(C)CC1)(C(=O)N[C@H](C=CS(=O)(=O)c1ccccc1)CCc1ccccc1)CCNNC(=N)N	1076088-88-4	1-Piperazinecarboxamide, N-[(1S)-1-[[2-(aminoiminomethyl)hydrazinyl]methyl]-2-ox	C27H38N8O4S	570.71	A/B	-8.5
4795	N(C(=O)[C@H](Cc1ccccc1)CS(=O)CN1[C@H](C(=O)NC(C)(C)C)CN(Cc2cccnc2)CC1)[C@H]1c2c(C[C@H]1O)cccc2	1085743-76-5	2-Piperazinecarboxamide, 1-[[[(2S)-3-[[(1S,2R)-2,3-dihydro-2-hydroxy-1H-inden-1-	C35H45N5O3S	615.83	A/B	-8.5
4796	C(=O)([C@@H](NC(=O)OC)C(C)C)N1[C@@H](CCC1)c1[nH]c(cn1)c1ccc(cc1)c1ccc(cc1)c1[nH]c([C@H](NC(=O)[C@@H]	1087346-58-4	Carbamic acid, N-[(1S)-2-[(2S)-2-[5-[4,Ä≤-[2-[(1S)-1-[[(2S)-2-[(methoxycarbonyl)	C36H44N8O6	684.78	A/B	-8.5

4797	N(C12CC3CC(C2)CC(C1)C3)c1nc(NCCCCCCO)nc(NCCCO)n1	1091628-81-7	Glycine, N-[4-(cycloheptylamino)-6-(tricyclo[3.3.1.13,7]dec-1-ylamino)-1,3,5-tri	C22H34N6O2	414.54	A/B	-8.5
4798	N(=O)(=O)c1n(c2c(n1)c(=O)nc(N)[nH]2)[C@H]1[C@H](OC(=O)C)[C@H](OC(=O)C)[C@@H](COC(=O)C)O1	1095412-77-3	2H-Isoindole-2-acetic acid, 5,6-dichloro-1,3-dihydro-1,3-dioxo-, 2-oxo-2-(tricyc	C22H22Cl2N2O5	465.33	A/B	-8.5
4799	O=c1c2c(n(cn2)[C@@H]2O[C@H](COP(=O)(OP(=O)(Oc3c(C=O)cccc3)O)O)[C@@H](O)[C@H]2O)[nH]c(N)n1	109606-33-9	5,Ä≤-Guanylic acid, mono(2-formylphenyl) ester	C17H18N5O9P	467.33	A/B	-8.5
4800	O=c1c2c(n(cn2)[C@@H]2O[C@H](COP(=O)(OP(=O)(OP(=O)(Oc3c(C=O)cccc3)O)O)O)[C@@H](O)[C@H]2O)[nH]c(N)n1	109606-34-0	Guanosine 5,Ä≤-(trihydrogen diphosphate), P,Ä≤-(2-formylphenyl) ester	C17H19N5O12P2	547.31	A/B	-8.5
4801	C(NC(=O)c1nnc(C([2H])([2H])[2H])o1)(C)(C)c1nc(C(=O)NC(c2ccc(F)cc2)([2H])[2H])c(O)c(=O)n1C	1100750-86-4	4-Pyrimidinecarboxamide, N-[(4-fluorophenyl)methyl]-1,6-dihydro-5-hydroxy-1-(met	C20H15D6FN6O5		A/B	-8.5
4802	[C@H](O[C@H](COP(=O)(OP(=O)(O)O)O)CO)(CO)n1c2c(nc1)c(=O)nc(N)[nH]2	1101448-32-1	Thymidine 5,Ä≤-(trihydrogen diphosphate), 3,Ä≤-azido-3,Ä≤-deoxy-, P,Ä≤,P,Ä≤-bis	C28H31N5O14P2		A/B	-8.5
4803	O=c1n(cc(C(F)(F)F)c(=O)[nH]1)[C@@H]1O[C@H](COCOC(C)c2ccccc2)[C@@H](O)C1	110604-85-8	Thymidine, 3,Ä≤-O-[2,2-dimethyl-1-(phenylmethoxy)propyl]-Œ±,Œ±,Œ±-trifluoro-	C22H27F3N2O6	472.45	A/B	-8.5
4804	C(#Cc1cccs1)c1n(nc(C(=O)N)n1)[C@@H]1O[C@H](CO)[C@@H](O)[C@H]1O	1106736-09-7	1H-1,2,4-Triazole-3-carboxamide, 5-[2-(2-fluorophenyl)ethynyl]-1-Œ≤-D-ribofuranc	C16H15FN4O5	362.31	A/B	-8.5
4805	N(=Nc1ccc(CCCCCC)cc1)c1c2c(cc(S(=O)(=O)O)cc2)ccc1O	1107694-03-0	2-Naphthalenesulfonic acid, 5-[2-(3-ethylphenyl)diazenyl]-6-hydroxy-	C18H16N2O4S	356.4	A/B	-8.5
4806	O[C@@H]1[C@@H](O[C@H](COP(=O)(OP(=O)(OP(=O)(O)O)O)O)[C@H]1O)n1cc(CCc2ccccc2)c(=O)[nH]c1=O	112066-16-7	Furo[3,Ä≤,4,Ä≤:6,7]naphtho[2,3-d]-1,3-dioxol-6(5aH)-one, 5,8,8a,9-tetrahydro-9-	C21H20O8	400.38	A/B	-8.5
4807	C(#Cc1ccccc1)c1n(nc(C(=O)N)n1)[C@@H]1O[C@H](CO)[C@@H](O)[C@H]1O	1123187-47-2	1H-1,2,4-Triazole-3-carboxamide, 1-Œ≤-D-ribofuranosyl-5-[2-[4-(trifluoromethyl)p	C17H19F3N4O5	416.35	A/B	-8.5
4808	C(#Cc1ccc(C)cc1)c1n(nc(C(=O)N)n1)[C@@H]1O[C@H](CO)[C@@H](O)[C@H]1O	1123187-63-2	1H-1,2,4-Triazole-3-carboxamide, 5-(2-phenylethynyl)-1-Œ≤-D-ribofuranosyl-	C16H16N4O5	344.32	A/B	-8.5
4809	C(#Cc1ccc(Cl)cc1)c1n(nc(C(=O)N)n1)[C@@H]1O[C@H](CO)[C@@H](O)[C@H]1O	1123187-65-4	1H-1,2,4-Triazole-3-carboxamide, 5-[2-(4-methylphenyl)ethynyl]-1-Œ≤-D-ribofuranc	C17H18N4O5	358.35	A/B	-8.5
4810	C(Cc1c(c(c(c(c1[2H])[2H])[2H])[2H])[2H])[C@]1(CCC)CC(=C([C@H](CC)c2cc(NS(=O)(=O)c3ccc(C(F)(F)F)cn3)c	1126619-16-6	Not Yet Assigned	C31H29D4F3N2O5S		A/B	-8.5
4811	C(=O)([C@@H](NC(=O)OC(C)(C)C)C(C)C)N1[C@H]2[C@H](C2)C[C@H]1c1[nH]c(cn1)c1ccc(cc1)c1ccc(cc1)c1[nH]c(n	1129634-03-2	Carbamic acid, N-[(1S)-1-[[(1R,3S,5R)-3-[5-[4,Ä≤-[2-[(1R,3S,5R)-2-acetyl-2-azabi	C37H41N7O4	647.77	A/B	-8.5
4812	C(=O)([C@@H](NC(=O)OC)C(C)C)N1[C@@H](CC[C@@H]1C)c1[nH]c(cn1)c1ccc(cc1)c1ccc(cc1)c1[nH]c(nc1)[C@H]1N(	1129634-66-7	Carbamic acid, N,N,Ä≤-[[1,1,Ä≤-biphenyl]-4,4,Ä≤-diylbis[1H-imidazole-5,2-diyl-(	C44H54N8O6	790.95	A/B	-8.5
4813	C(=O)([C@@H](NC(=O)OC)C(C)C)N1[C@@H](CCC1)c1[nH]c(c(C)n1)c1ccc(cc1)c1ccc(cc1)c1[nH]c(nc1C)[C@H]1N(C(	1129636-27-6	Carbamic acid, N,N,Ä≤-[[1,1,Ä≤-biphenyl]-4,4,Ä≤-diylbis[(4-methyl-1H-imidazole-	C38H46N8O6	710.82	A/B	-8.5
4814	C(=O)(N[C@H](C(=O)C(=O)NC1CC1)CCC)[C@@H]1[C@@H]2[C@H](CN1C(=O)[C@@](NC(=O)[C@@H](NC(=O)c1cnccn1)C1CC	1134176-09-2	Cyclopenta[c]pyrrole-1-carboxamide, (2S)-2-(cyclohexyl-1,2,2,3,3,4,4,5,5,6,6-d11	C36H42D11N7O6		A/B	-8.5
4815	O([C@]1([C@@](O[C@](C(O[2H])([2H])[2H])([C@]1(O[2H])[2H])[2H])(n1nc(C(=N[2H])N([2H])[2H])nc1[2H])[2H	1134793-88-6	Guanosine, N-(9-anthracenylmethyl)-	C25H23N5O5	473.48	A/B	-8.5
4816	O(c1nc(Nc2ccc(C#N)cc2)nc(N)c1Br)c1c(C)c(c(C#N)c(c1C)[2H])[2H]	1142095-93-9	Benzonitrile-2,3,5,6-d4, 4-[[4-amino-5-bromo-6-(4-cyano-2,6-dimethylphenoxy)-2-p	C20H11BrD4N6O		A/B	-8.5
4817	C(=O)(N)c1nn(cn1)[C@@H]1O[C@H](CO)CC1	114371-63-0	Hydrazinecarbothioamide, 2-[1-(3,4-dihydro-4-oxo-3-phenyl-1-phthalazinyl)-1,2-di	C23H16N6O2S	440.48	A/B	-8.5
4818	O=c1n([C@@H]2O[C@H](CO)[C@@H](O)C2)cc(C([2H])([2H])[2H])c(N)n1	1160591-56-9	1,3,5-Triazin-2-amine, N-[4-(3,4-dimethoxyphenyl)-6-(methylthio)-2-pyrimidinyl]-	C24H30N8O4S	526.61	A/B	-8.5
4819	N(=Nc1ccc(cc1)c1ccc(cc1)c1ccc(N=Nc2c3c(cc(S(=O)(=O)O)cc3S(=O)(=O)O)ccc2N)cc1)c1c2c(cc(S(=O)(=O)O)cc2	116200-19-2	1-Naphthalenesulfonic acid, 3,3,Ä≤-[[1,1,Ä≤:4,Ä≤,1,Ä≤,Ä≤-terphenyl]-4,4,Ä≤,Ä≤-	C38H28N6O8S2.2Li		A/B	-8.5
4820	Cc1n(c2nc(nc(n2)N2CCOCC2)c2cnc(N)nc2)c2c(n1)cccc2	1174190-83-0	Benzenemethanol, 3-[4-(2-methyl-1H-imidazol-1-yl)-6-(4-morpholinyl)-1,3,5-triazi	C18H20N6O2	352.39	A/B	-8.5
4821	Cc1n(c2nc(nc(n2)N2CCOCC2)c2ccc(N)nc2)nc(C)c1	1174191-04-8	2-Pyrimidinamine, 5-[4-(3,5-dimethyl-1H-pyrazol-1-yl)-6-(4-morpholinyl)-1,3,5-tr	C17H21N9O	367.41	A/B	-8.5
4822	C(F)(F)(F)c1nn(c2nc(nc(n2)N2CCOCC2)c2cnc(N)nc2)cc1	1174191-12-8	2-Pyrimidinamine, 5-[4-(3-methyl-1H-pyrazol-1-yl)-6-(4-morpholinyl)-1,3,5-triazi	C15H17N9O	339.36	A/B	-8.5
4823	C(C)c1n(c2c(n1)cccc2)c1nc(nc(n1)N1CCOCC1)c1cnc(N)nc1	1174191-29-7	2-Pyrimidinamine, 5-[4-[2-(difluoromethyl)-1H-benzimidazol-1-yl]-6-(4-morpholiny	C19H17F2N9O	425.39	A/B	-8.5
4824	C(CC#N)c1n(c2nc(nc(n2)N2CCOCC2)c2cnc(N)nc2)ccn1	1174191-46-8	2-Pyrimidinamine, 5-[4-(2-ethyl-1H-benzimidazol-1-yl)-6-(4-morpholinyl)-1,3,5-tr	C20H21N9O	403.44	A/B	-8.5
4825	C(=O)([C@@H](NC(=O)OC)C(C)C)N1[C@H](c2[nH]c(c3cc4c(c5c([nH]4)cc(cc5)c4[nH]c(nc4)[C@H]4N(C(=O)[C@@H](	1179151-07-5	Carbamic acid, N,N,Ä≤-[[9-(phenylsulfonyl)-9H-carbazole-2,7-diyl]bis[1H-imidazo]	C46H53N9O8S	892.03	A/B	-8.5
4826	C(=O)([C@@H](NC(=O)OC)C(C)C)N1[C@H](c2[nH]c3c4c(cc(cc4)c4ccc(cc4)c4[nH]c(nc4)[C@H]4N(C(=O)[C@@H](NC(	1180670-32-9	Carbamic acid, N-[(1S)-1-[[(2S)-2-[7-[4-[2-[(2S)-4,4-difluoro-1-[(2S)-2-[(methox	C42H48F2N8O6	798.88	A/B	-8.5
4827	C(=O)([C@@H](NC(=O)OC)C1CCOCC1)N1[C@H]2[C@H](C2)C[C@H]1c1[nH]c2c(n1)ccc1c2ccc(c1)c1cc2c(cc1)c1c(cc2)	1180670-49-8	Carbamic acid, N-[(1S)-2-[(2S)-2-[2,Ä≤-[(2S)-1-[(2S)-2-[(methoxycarbonyl)amino]-	C46H52N8O7	828.95	A/B	-8.5
4828	C(C#N)C1(CN(S(=O)(=O)c2cc(F)ccc2)C1)n1cc(cn1)c1c2c(ncn1)[nH]cc2	1187594-47-3	3-Azetidineacetonitrile, 1-[(4-fluorophenyl)sulfonyl]-3-[4-(7H-pyrrolo[2,3-d]pyr	C20H16FN7O2S	437.45	A/B	-8.5
4829	O=c1n([C@@H]2O[C@H](COP(=O)(OCCCCCCSSCCCCCCO)O)[C@@H](O)C2)cc(C)c(=O)[nH]1	118951-97-6	1-Naphthalenesulfonic acid, 4-amino-3-[2-[3-cyano-4-(2-phenylethenyl)phenyl]diaz	C25H18N4O3S	454.5	A/B	-8.5
4830	C(N1c2c(C(=NNC(=S)NC3CCCCC3)C1=O)cc(Cl)cc2)N1CCN(CC1)c1ccc(Cl)cc1	118989-48-3	Hydrazinecarbothioamide, 2-[5-chloro-1,2-dihydro-2-oxo-1-[(4-phenyl-1-piperaziny	C26H31ClN6OS	511.08	A/B	-8.5
4831	O=c1n([C@@H]2O[C@H](COP(=O)(Nc3cc(C(=O)Nc4cc(C(=O)Nc5cc(C(=O)NCCC#N)n(C)c5)n(C)c4)n(C)c3)OC(C)C)C=C2	1190064-46-0	Thymidine, 2,Ä≤,3,Ä≤-didehydro-3,Ä≤-deoxy-, 5,Ä≤-[1-methylethyl N-[5-[[[5-[[[5-	C40H50N13O10P	903.88	A/B	-8.5
4832	[C@@H](C(OC([2H])([2H])[2H])([2H])[2H])(N1[C@@H](C)CN(CC1)C1(C)CCN(C(=O)c2c(C([2H])([2H])[2H])ncnc2C	1191293-06-7	Methanone, [4,6-di(methyl-d3)-5-pyrimidinyl][4-[(3S)-4-[(1R)-2-(methoxy-d3)-1-[4	C28H29D9F3N5O2		A/B	-8.5
4833	[C@](C(OC([2H])([2H])[2H])([2H])[2H])(N1[C@@H](C)CN(CC1)C1(C)CCN(C(=O)c2c(C([2H])([2H])[2H])ncnc2C([	1191293-15-8	Methanone, (4,6-dimethyl-5-pyrimidinyl)[4-[(3S)-4-[(1R)-2-(methoxy-d3)-1-[4-(tri	C28H35D3F3N5O2		A/B	-8.5
4834	C[C@]12[C@H]([C@H]3[C@H](C[C@@H]2O)[C@]2(C)[C@H](CC3)C[C@@](CN)(O)CC2)CC[C@@H]1[C@@H](CCC(=O)OC)C	1191293-21-6	Methanone, (4,6-dimethyl-5-pyrimidinyl)[4-[(3S)-4-[(1R)-2-methoxy-1-[4-(trifluor	C28H36D2F3N5O2		A/B	-8.5
4835	C([C@H](OCP(=O)(OC[C@H](NC(=O)[C@@H](C(C)C)N)C(=O)OC(C)C)O)CO)n1c(=O)nc(N)cc1	1192315-56-2	Benzoic acid, 3,3,Ä≤-thiobis[6-[(4-butylbenzoyl)amino]-	C36H36N2O6S	624.75	A/B	-8.5
4836	C(CN1CCCCC1)n1c2c(ccc(S(=O)(=O)N(C[C@H]([C@H](Cc3ccccc3)NC(=O)O[C@@H]3[C@H]4[C@@H](OC3)OCC4)O)CC(C)C	119415-70-2	Naphthionic acid, 3,3,Ä≤-[thiobis(p-phenyleneazo)]di-	C32H24N6O6S3	684.76	A/B	-8.5
4837	O(COC(=O)N1CCN(C)CC1)c1c2n(C[C@H]3N(C2=O)[C@H](C)CCO3)cc(C(=O)NCc2c(F)cc(F)cc2)c1=O	1206156-30-0	Carbamic acid, N-[2-(4-morpholinyl)ethyl]-, [[(4R,12aS)-9-[[[(2,4-difluorophenyl	C28H33F2N5O8	605.59	A/B	-8.5
4838	N(Cc1c(CN(Cc2ccccc2)C)cccc1)c1c2c(ncc1)cc(Cl)cc2	120746-51-2	1-Naphthalenesulfonic acid, 3,3,Ä≤-[(2,2,Ä≤-dimethyl[1,1,Ä≤-biphenyl]-4,4,Ä≤-di	C34H28N6O6S2	680.75	A/B	-8.5
4839	O=c1n([C@@H]2O[C@H](CO)C=C2)cc(Cl)c(=O)[nH]1	1208119-31-6	Cyclopenta[c]pyrrole-1-carboxamide, 2-[(2S)-2-[[(2S)-2-cyclohexyl-2-[(2-pyraziny	C38H53N7O7	719.87	A/B	-8.5
4840	O=c1n([C@@H]2O[C@H](COP(=O)(O)O)[C@@H](O)C2)cc(C)c(=O)[nH]1	1212463-68-7	Furo[3,Ä≤,4,Ä≤:6,7]naphtho[2,3-d]-1,3-dioxol-6(5aH)-one, 5,8,8a,9-tetrahydro-5-	C21H20O7	384.38	A/B	-8.5
4841	O=C1[C@@H]2[C@@H](c3c([C@H](OC(=O)[C@@H](NC(=O)OC(C)(C)C)CSSC[C@@H](C(=O)O[C@H]4c5c([C@H]([C@@H]6[C@	1213264-39-1	11-Oxa-5,6-dithia-2,9-diazatridecanoic acid, 3-[[[(5R,5aR,8aR,9R)-5,5a,6,8,8a,9-	C58H69N3O18S3	1192.37	A/B	-8.5
4842	O=c1c2c(n(cn2)[C@@H]2O[C@H](CO)[C@@H](F)C2)[nH]cn1	1213264-42-6	L-Cystine, N,N,Ä≤-bis[(1,1-dimethylethoxy)carbonyl]-, 1,1,Ä≤-bis[(5R,5aR,8aR,9R	C60H68N2O22S2	1233.31	A/B	-8.5
4843	C(OC[C@@H]1[C@@H](OP(N(C(C)C)C(C)C)OCCC#N)C[C@@H](O1)n1cc(/C=C/c2cc3c(-c4c(C3)cccc4)cc2)c(=O)[nH]c1=	121499-18-1	1-Naphthalenesulfonamide, 4-amino-3-[2-[4-[[4-[2-[1-amino-4-(aminosulfonyl)-2-na	C32H26N8O6S3	714.79	A/B	-8.5
4844	O=c1c2c(n(cn2)[C@@H]2O[C@H](COP(=O)(OP(=O)(OP(=O)(O)O)O)O)[C@@H](O)C2)[nH]c(N[C@H](C(=O)O)C)n1	1217503-36-0	D-Alanine, N-[9-[2-deoxy-5-O-[hydroxy[[hydroxy(phosphonooxy)phosphinyl]oxy]phosp	C13H20N5O15P3	579.24	A/B	-8.5
4845	C(#N)C1=C(N)Oc2c(C1c1cn(c(=O)[nH]c1=O)[C@H]1C[C@H](OC(=O)C)[C@@H](COC(=O)C)O1)c(=O)n(C)c(C)c2	1217551-22-8	L-Phenylalanine, N-acetyl-L-phenylalanyl-N-(1-tricyclo[3.3.1.13,7]dec-1-ylethyl)	C36H46N4O6	630.77	A/B	-8.5
4846	C(=O)(NC(C)(C)C)[C@H]1N(C[C@H]([C@@H](NC(=O)c2c(C([2H])([2H])[2H])c(O)ccc2)CSc2ccccc2)O)C[C@@H]2[C@H	1217628-64-2	1(2H)-Pyrimidineacetamide, N-[(1R,3S,4S)-4-[[2-(2,6-dimethylphenoxy)acetyl]aminc	C37H48N4O5	628.8	A/B	-8.5
4847	C(#CC1C(C1([2H])[2H])([2H])[2H])[C@@]1(C(F)(F)F)c2cc(NC(=O)O1)c(O)cc(Cl)c2	1217755-54-8	Not Yet Assigned	C36H47N5O4	613.79	A/B	-8.5
4848	Cc1n([C@H]2C[C@H]3N(CC[C@H](NC(=O)C4CCC(F)(F)CC4)c4cc(F)ccc4)[C@@H](C2)CC3)c(C)nn1	1221188-59-5	Cyclobutanecarboxamide, N-[(1S)-3-[(3-exo)-3-(3,5-dimethyl-4H-1,2,4-triazol-4-yl	C25H32F3N5O	475.55	A/B	-8.5
4849	C(C1(CC1)c1ccccc1)N1C[C@@H](C(=O)NC(C)(C)C)N(C[C@H](C[C@H](C(=O)NC2c3c(C[C@H]2O)cccc3)Cc2ccccc2)O)CC	1223101-05-0	Not Yet Assigned	C33H48FN5O4	597.76	A/B	-8.5
4850	[C@@H](C(=O)N[C@@H](Cc1ccccc1)C[C@@H]([C@](C(c1c(c(c(c(c1[2H])[2H])[2H])[2H])[2H])([2H])[2H])(NC(=O)	1224729-47-8	1(2H)-Pyrimidine-4,5,6-d3-acetamide, N-[(1S,3S,4S)-4-[[2-[2,6-di(methyl-d3)phenc	C37H31D17N4O5		A/B	-8.5
4851	c1(nc(nc(n1)N1CCOCC1)N1CCOCC1)c1cc2c(cc1)[nH]nc2	1225037-84-2	2-Pyridinamine, 5-(4,6-di-4-morpholinyl-1,3,5-triazin-2-yl)-6-(trifluoromethyl)-	C17H20F3N7O2	411.38	A/B	-8.5
4852	C(#N)C1=C(N)Oc2c(C1c1ccccc1)ccn1c2ncc1	1225373-22-7	7H-Imidazo[1,2-a]pyrano[2,3-c]pyridine-8-carbonitrile, 9-amino-7-(4-pyridinyl)-	C16H11N5O	289.29	A/B	-8.5
4853	C(#N)C1=C(N)Oc2c(C1c1cc(CC(=C)C)c(C)c(OC)c1)ccn1c2nc(C)c1	1225373-26-1	7H-Imidazo[1,2-a]pyrano[2,3-c]pyridine-8-carbonitrile, 9-amino-7-[4-(4-methyl-1-	C22H22N6O	386.45	A/B	-8.5
4854	C(=O)(NC(CC1CC1)C(=O)C(=O)N)[C@@H]1[C@@H]2[C@@H](C2(C)C)CN1C(=O)[C@@H](NC(=O)NC1(CN)CCCC1)C(C)(C)C	1228343-60-9	3-Azabicyclo[3.1.0]hexane-2-carboxamide, N-[3-amino-1-(cyclopropylmethyl)-2,3-di	C30H49N5O5	559.74	A/B	-8.5
4855	C(=O)([C@H](NC(=O)OC)C)N1[C@H](c2[nH]c(C34CCC(CC3)(CC4)c3ccc(cc3)c3[nH]c(nc3)[C@H]3N(C(=O)[C@@H](NC(	1228671-52-0	Carbamic acid, N-[(1S)-2-[(2S)-2-[5-[4-[4-[2-[(2S)-1-[(2S)-2-[(methoxycarbonyl)a	C40H54N8O6	742.91	A/B	-8.5
4856	C(=O)([C@H](NC(=O)OC)c1ccccc1)N1[C@@H](CCC1)c1[nH]c(cn1)c1ccc(C#Cc2ccc(cc2)c2[nH]c(nc2)[C@H]2N(C(=O)	1228965-10-3	Carbamic acid, N-[(1R)-2-[(2S)-2-[5-[4-[2-[4-[2-[(2S)-1-[(2R)-2-[(methoxycarbony	C49H48N8O6	844.96	A/B	-8.5
4857	C(=C1C=C(C(=O)O)NC(=O)C=C1)(c1cc(C(=O)O)c(O)cc1)c1cc(C(=O)O)c(O)cc1	1233224-95-7	2-Pyridinesulfonamide, N-[3-[(1R)-1-[(6R)-5,6-dihydro-4-hydroxy-2-oxo-6-(2-pheny	C28H27F3N2O5S	560.58	A/B	-8.5
4858	N(S(=O)(=O)c1ccc(N(=O)=O)cc1)(C[C@H]([C@H](Cc1ccccc1)NC(=O)O[C@@H]1[C@@H](COCC(=O)OC(C)(C)C)OCC1)O)C	1233853-05-8	1H-Benzotriazole-1-carboxamide, N-tricyclo[3.3.1.13,7]dec-1-yl-	C17H20N4O	296.37	A/B	-8.5
4859	[C@@H](OC(=O)[C@H]1N(C(=O)C(=O)C(CC)(C)C)CCCC1)(CCc1cc(OC)c(OC)cc1)c1ccc(OCCNC(=O)COC[C@@H]2[C@@H](OC	1233885-48-7	Not Yet Assigned	C60H79N5O17S	1174.36	A/B	-8.5
4860	[C@H](Cc1ccccc1)(C[C@@H]([C@H](Cc1ccccc1)NC(=O)OCc1cnco1)O)NC(=O)[C@@H](NC(=O)N(Cc1nc(CCC)sc1)C)C(C)	1236406-23-7	2,7,10,12-Tetraazatridecanoic acid, 13-[2-(1-cyclohexen-1-yl)-4-thiazolyl]-4-hyc	C40H50N6O5S2	758.99	A/B	-8.5
4861	[C@H](Cc1ccccc1)(C[C@@H]([C@H](Cc1ccccc1)NC(=O)OCc1cncs1)O)NC(=O)[C@H](NC(=O)N(Cc1nc(CC2CCCCC2)sc1)C	1236406-59-9	2,7,10,12-Tetraazatridecanoic acid, 13-[2-(1,1-dimethylethyl)-4-thiazolyl]-4-hyc	C38H50N6O5S2	734.97	A/B	-8.5
4862	[C@H](Cc1ccccc1)(C[C@@H]([C@H](Cc1ccccc1)NC(=O)OCc1cnco1)O)NC(=O)[C@@H](NC(=O)N(Cc1nc(OC(C)C)sc1)C)C	1236407-12-7	Not Yet Assigned	C37H48N6O7S	720.88	A/B	-8.5
4863	[C@H](Cc1ccccc1)(C[C@@H]([C@H](Cc1ccccc1)NC(=O)OCc1ccno1)O)NC(=O)[C@@H](NC(=O)N(Cc1nc(CN(C)C)sc1)C)C	1236408-25-5	2,7,10,12-Tetraazatridecanoic acid, 13-[2-(1-ethylpropyl)-4-thiazolyl]-4-hydroxy	C39H52N6O5S2	749	A/B	-8.5
4864	C(N(C(=O)N[C@@H](C(=O)N[C@@H](Cc1ccccc1)C[C@@H]([C@H](Cc1ccccc1)NC(=O)OCc1cncs1)O)C(C)C)C)c1nc(sc1)c	1236408-92-6	2,7,10,12-Tetraazatridecanoic acid, 4-hydroxy-12-methyl-9-(1-methylethyl)-8,11-c	C42H50N6O5S2	783.01	A/B	-8.5
4865	[C@H](Cc1ccccc1)(C[C@@H]([C@H](Cc1ccccc1)NC(=O)OCc1ccns1)O)NC(=O)[C@@H](NC(=O)N(Cc1nc(C(C)C)sc1)C)C	1236409-33-8	2,7,10,12-Tetraazatridecanoic acid, 4-hydroxy-12-methyl-9-(1-methylethyl)-8,11-c	C42H48N6O5S2	781	A/B	-8.5
4866	[C@H](Cc1ccccc1)(C[C@@H]([C@H](Cc1ccccc1)NC(=O)OCc1ccns1)O)NC(=O)[C@@H](NC(=O)N(Cc1nc(/C=C/C)sc1)C)C	1236409-76-9	2,7,10,12-Tetraazatetradecanoic acid, 4-hydroxy-9-(1-methylethyl)-12-[[2-(1-meth	C38H50N6O6S	718.91	A/B	-8.5
4867	N(C(=O)[C@H](Cc1ccccc1)C[C@@H](CN1[C@H](C(=O)NC(C)(C)C)CN(Cc2cc3c(s2)ccs3)CC1)O)[C@H]1c2c(C[C@H]1O)c	1237988-73-6	Not Yet Assigned	C39H59N5O4	661.92	A/B	-8.5
4868	N(CC(=O)[C@H](Cc1ccccc1)C[C@@H](CN1[C@H](C(=O)NC(C)(C)C)CN(Cc2ccccc2)CC1)O)[C@H]1c2c(C[C@H]1O)cccc2	1237989-11-5	Not Yet Assigned	C35H45BrN4O4S	697.73	A/B	-8.5
4869	N(CC(=O)[C@H](Cc1ccccc1)C[C@@H](CN1[C@H](C(=O)NC(C)(C)C)CN(Cc2c(Cl)nccc2)CC1)O)[C@H]1c2c(C[C@H]1O)cc	1237989-13-7	Not Yet Assigned	C38H51N5O4	641.84	A/B	-8.5
4870	C[C@]12[C@H]([C@H]3[C@@H]([C@]4(C)C(=CC3)C[C@@H](OC(=O)CP(=O)(O)O)CC4)CC2)CC=C1c1cccnc1	123824-93-1	Carbonic acid, 5,5a,6,8,8a,9-hexahydro-8-oxo-9-(3,4,5-trimethoxyphenyl)furo[3,Ä≤	C29H26O10	534.51	A/B	-8.5
4871	C(F)(F)c1n(c2c(n1)cccc2)c1nc(nc(N[C@H]2CC[C@H](N(C(=O)C)C)CC2)n1)N1CCOCC1	1240023-39-5	1,3,5-Triazin-2-amine, 4-[2-(difluoromethyl)-1H-benzimidazol-1-yl]-6-(4-morpholi	C25H32F2N8O2	514.57	A/B	-8.5
4872	C(F)(F)c1n(c2c(n1)cccc2)c1nc(nc(NC[C@H]2CC[C@H](N)CC2)n1)N1CCOCC1	1240024-64-9	Urea, N-[trans-4-[[4-[2-(difluoromethyl)-1H-benzimidazol-1-yl]-6-(4-morpholinyl)	C25H33F2N9O3	545.58	A/B	-8.5
4873	C(F)(F)c1n(c2c(n1)cccc2)c1nc(nc(N[C@H]2CC[C@H](NS(=O)(=O)CC)CC2)n1)N1CCOCC1	1240029-13-3	Acetamide, N-[trans-4-[[4-[2-(difluoromethyl)-1H-benzimidazol-1-yl]-6-(4-morphol	C23H28F2N8O3	502.52	A/B	-8.5
4874	C(F)(F)c1n(c2c(n1)cccc2)c1nc(nc(N[C@H]2CC[C@H](NCC(C)C)CC2)n1)N1CCOCC1	1240029-86-0	Ethanesulfonamide, N-[trans-4-[[4-[2-(difluoromethyl)-1H-benzimidazol-1-yl]-6-(4	C23H30F2N8O3S	536.6	A/B	-8.5
4875	C(F)(F)c1n(c2c(n1)cccc2)c1nc(nc(N[C@H]2CC[C@H](NC(COC)C)CC2)n1)N1CCOCC1	1240030-54-9	2-Butanol, 3-[[trans-4-[[4-[2-(difluoromethyl)-1H-benzimidazol-1-yl]-6-(4-morphc	C25H34F2N8O2	516.59	A/B	-8.5
4876	C(=O)([C@@H](NC(=O)OC)C(C)C)N1[C@@H](CCC1)c1[nH]c(cn1)c1ccc(cc1)c1ccc([C@@H](NC(=O)[C@H]2N(C(=O)[C@@	1240427-38-6	L-Prolinamide, N-(methoxycarbonyl)-L-alanyl-N-[(1S)-1-[4,Ä≤-[2-[(2S)-1-[(2S)-2-	C36H45N7O7	687.79	A/B	-8.5
4877	C(=O)([C@@H](NC(=O)O[C@@H]1CCOC1)C(C)C)N1[C@H](c2[nH]c(c3cc4c(-c5c(C4)cc(cc5)c4[nH]c(nc4)[C@H]4N(C(=	1240880-78-7	Carbamic acid, N,N,Ä≤-[9H-fluorene-2,7-diylbis[1H-imidazole-5,2-diyl-(2S)-2,1-py	C49H62N8O8	891.07	A/B	-8.5
4878	[C@H](N(C(=O)[C@@H](NC(=O)OC)C(C)C)C)(C)c1[nH]c(c2cc3c(-c4c(C3)cc(cc4)c3[nH]c(nc3)[C@H]3N(C(=O)[C@@H	1240880-92-5	Carbamic acid, N,N,Ä≤-[9H-fluorene-2,7-diylbis[1H-imidazole-5,2-diyl-(2S)-2,1-py	C45H54N8O8	834.96	A/B	-8.5
4879	C(=O)([C@H](NC(=O)OC)c1ccccc1)N1[C@@H](CCC1)c1[nH]c(cn1)c1cc2c(cc1)ccc(c2)c1ccc(cc1)c1[nH]c(nc1)[C@H	1240883-73-1	Carbamic acid, N-[(1S)-1-[[(2S)-2-[5-[7-[2-[(1S)-1-[[(2S)-2-[(methoxycarbonyl)am	C40H50N8O6	738.88	A/B	-8.5
4880	C(=O)([C@@H](NC(=O)OC)[C@@H](C)O)N1[C@@H](CCC1)c1[nH]c(cn1)[C@H]1CC[C@@H](CC1)c1ccc(cc1)c1[nH]c(nc1)	1240887-46-0	Carbamic acid, N-[(1R)-1-[[(2S)-2-[5-[4-[trans-4-[2-[(2S)-1-[(2R)-2-[(methoxycar	C38H52N8O6	716.87	A/B	-8.5
4881	C(=O)([C@@H](NC(=O)OC)C(C)C)N1[C@@H](CCC1)c1[nH]c(cn1)[C@H]1CC[C@@H](CC1)c1ccc(cc1)c1[nH]c([C@@H](N(	1240890-53-2	Carbamic acid, N-[(1R)-2-[(2S)-2-[5-[trans-4-[4-[2-[(1S)-1-[[(2S)-2-[(methoxycar	C42H54N8O6	766.93	A/B	-8.5
4882	C(=O)([C@H](NC(=O)OC)c1ccccc1)N1[C@@H](CCC1)c1[nH]c(cn1)C1CCC(CC1)c1ccc(cc1)c1[nH]c(nc1)[C@H]1N(C(=O	1241379-00-9	Carbamic acid, N-[(1S)-1-[[(2S)-2-[5-[4,Ä≤-[2-[(2S)-1-[(2S)-2-[(methoxycarbonyl)	C44H52N8O6	788.93	A/B	-8.5
4883	O[C@H]1[C@@H](O[C@H](COP(=O)(OP(=O)(O[C@H]2O[C@H](CO)[C@H](O)[C@H](O)[C@H]2O)O)O)[C@H]1O)n1cc(c(=O)[	1242712-28-2	Uridine 5,Ä≤-(trihydrogen diphosphate), 5-phenyl-, P,Ä≤-Œ±-D-galactopyranosyl e	C21H28N2O17P2	642.4	A/B	-8.5
4884	C(C(C(Cc1cc(O)c(O)cc1)C)C)c1cc(O)c([O])cc1	1242712-29-3	Uridine 5,Ä≤-(trihydrogen diphosphate), 5-(4-methoxyphenyl)-, P,Ä≤-Œ±-D-galacto	C22H30N2O18P2	672.42	A/B	-8.5
4885	O(C)[C@H]1[C@@H](c2c(Cl)cc(C(F)(F)F)cc2)N1[C@@H](C)CN(CC1)C1(C)CCN(C(=O)c2c(C)ncnc2C)CC1	1246280-93-2	Not Yet Assigned	C37H41FN6O12S	812.82	A/B	-8.5
4886	O(c1nc(Nc2c(Br)cc(C#N)cc2)nc(N)c1Br)c1c(C)cc(C#N)cc1C	1246661-70-0	Carbamic acid, N,N,Ä≤-[2,6-biphenylenediylbis[1H-imidazole-5,2-diyl-(2S)-2,1-pyr	C40H48N8O6	736.86	A/B	-8.5
4887	C(OC(C(OC(=O)C)([2H])[2H])(C(O)([2H])[2H])[2H])n1c2c(nc1)c(=O)nc(N)[nH]2	1246818-67-6	Benzonitrile, 4-[[6-amino-5-bromo-2-[(4-cyanophenyl)amino]-4-pyrimidinyl]oxy]-3,	C20H15BrN6O3	467.28	A/B	-8.5
4888	O[C@H]1[C@H](n2c3c(nc2)c(=O)nc(N)[nH]3)O[C@H](CO)[C@@H]1OP(=O)(O)O	1246995-20-9	3-Pyridinecarboxamide, compd. with 4-[[6-amino-5-bromo-2-[(4-cyanophenyl)amino]-	C20H15BrN6O.C6H6N2O		A/B	-8.5
4889	O=c1n([C@@H]2O[C@H](CO)C=C2)cc(Cl)c(N)n1	124705-10-8	Guanosine 5,Ä≤-(trihydrogen diphosphate), P,Ä≤,Üì3,Ä≤-ester with adenosine	C20H26N10O14P2	692.43	A/B	-8.5
4890	[C@H](Nc1nc(C(F)(F)F)c(C(=O)N2CCS(=O)(=O)CC2)cn1)(C)C12CC3CC(C2)CC(C1)C3	1247824-40-3	Benzoic acid, 2-[(4-cyclohexylbenzoyl)amino]-5-(2-cyclohexylethyl)-	C28H35NO3	433.58	A/B	-8.5
4891	C[C@]1(F)[C@@H](O[C@H](COP(=O)(Oc2ccccc2)N[C@H](C(=O)Oc2ccccc2)C)[C@H]1O)n1c(=O)[nH]c(=O)cc1	1250829-92-5	L-Alanine, N-[(2,Ä≤R)-2,Ä≤-deoxy-2,Ä≤-fluoro-2,Ä≤-methyl-P-phenyl-5,Ä≤-uridylyl]	C25H33FN3O9P	569.52	A/B	-8.5
4892	C(=O)([C@@H](NC(=O)OC)C(C)C)N1[C@H]2[C@H](C2)C[C@H]1c1[nH]c(cn1)c1ccc(cc1)[C@H]1[C@@H](C1)c1ccc(cc1)	1250938-81-8	Carbamic acid, N,N,Ä≤-[(1S,2S)-1,2-cyclopropanediylbis[4,1-phenylene-1H-imidazol	C45H54N8O6	802.96	A/B	-8.5
4893	C(#CC1CC1)[C@@]1(C(F)(F)F)c2cc(NC(=O)O1)ccc(Cl)c2.O	1253183-24-2	Ethanesulfonic acid, compd. with 4-[[6-amino-5-bromo-2-[(4-cyanophenyl)amino]-4-	C20H15BrN6O.C2H6O3S		A/B	-8.5
4894	C(N(NC(=O)[C@@H](NC(=O)OCc1ccccc1)C(C)(C)C)C[C@@H]([C@H](Cc1ccccc1)NC(=O)[C@@H](NC(=O)OC(C)(C)C)C(C)	1255196-42-9	Not Yet Assigned	C73H96N6O13	1265.58	A/B	-8.5
4895	O=c1n([C@@H]2O[C@H](COP(=O)(C)Cl)[C@@H](O)C2)cc(C)c(=O)[nH]1	1255672-02-6	Benzonitrile, 4-[[6-amino-5-bromo-2-[(4-cyanophenyl)amino]-4-pyrimidinyl]oxy]-3,	C20H15BrN6O.xC7H8O3S		A/B	-8.5
4896	C(=O)([C@@H](NC(=O)OC)C(C)C)N1[C@@H](CCC1)c1[nH]c(cn1)c1cc2c(cc1)cc(cc2)c1[nH]c(nc1)[C@H]1N(C(=O)[C@	1256387-39-9	Carbamic acid, N-[(1S)-1-[[(2S)-2-[5-[4-[4-[2-[(2S)-1-[(2S)-2-[(methoxycarbonyl)	C44H52N8O6	788.93	A/B	-8.5
4897	O=s1c2c(c3c1cc(cc3)c1[nH]c(nc1)[C@H]1N(C(=O)[C@@H](NC(=O)OC)C(C)C)CCC1)ccc(c2)c1[nH]c(nc1)[C@H]1N(C(	1256387-82-2	Carbamic acid, N,N,Ä≤-[3,7-dibenzothiophenediylbis[1H-imidazole-5,2-diyl-(2S)-2,	C40H48N8O6S	768.92	A/B	-8.5
4898	C(=O)([C@H](Cc1ccccc1)NC(=O)OC)N1[C@@H](CCC1)c1[nH]c(cn1)c1ccc(cc1)c1cc2c(cc1)cc(cc2)c1[nH]c(nc1)[C@	1256389-48-6	Carbamic acid, N-[(1S)-1-[[(2S,4S)-4-cyano-2-[5-[4-[6-[2-[(2S)-1-[(2S)-2-[(methc	C45H51N9O6	813.94	A/B	-8.5
4899	C(=O)([C@@H](Cc1ccccc1)NC(=O)OC)N1[C@@H](CCC1)c1[nH]c(cn1)c1ccc(cc1)c1cc2c(cc1)cc(cc2)c1[nH]c(nc1)[C	1256389-80-6	Carbamic acid, N-[(1S)-2-[(2S)-2-[5-[4-[6-[2-[(2S)-1-[(2S)-2-[(methoxycarbonyl)a	C48H52N8O6	836.98	A/B	-8.5
4900	C(=O)([C@@H](NC(=O)OC)C(C)C)N1[C@@H](C[C@@H](Cc2ccccc2)C1)c1[nH]c(cn1)c1ccc(cc1)c1cc2c(cc1)cc(cc2)c1	1256390-02-9	Carbamic acid, N-[(1S)-1-[[(2S)-2-[5-[6-[4-[2-[(2S,4R)-1-[(2S)-2-[(methoxycarbor	C50H56N8O6	865.03	A/B	-8.5
4901	C(=O)([C@@H](NC(=O)OC)C(C)C)N1CC2(C[C@H]1c1[nH]c(cn1)c1ccc(cc1)c1ccc(cc1)c1[nH]c(nc1)[C@H]1N(C(=O)[C	1256390-33-6	Carbamic acid, N-[(1S)-1-[[(2S)-2-[5-[4,Ä≤-[2-[(1R)-2,3-dihydro-2-[(2S)-2-[(meth	C44H50N8O6	786.92	A/B	-8.5
4902	C(=O)([C@@H](NC(=O)OC)C)N1[C@@H](CC[C@@H]1c1ccccc1)c1[nH]c(cn1)c1ccc(cc1)c1ccc2c(cc1)cc(cc2)c1[nH]c(n	1256390-56-3	Carbamic acid, N,N,Ä≤-[[1,1,Ä≤-biphenyl]-4,4,Ä≤-diylbis[1H-imidazole-5,2-diyl-(	C44H54N8O10	854.95	A/B	-8.5
4903	C(=O)([C@@H](NC(=O)OC)[C@H](CC)C)N1[C@@H](CCC1)c1[nH]c(cn1)c1cc2c(cc1)cc(C#Cc1[nH]c(nc1)[C@H]1N(C(=O	1256390-70-1	Carbamic acid, N-[(1S)-2-[(2S,5R)-2-[5-[4-[6-[2-[(2S)-1-[(2S)-2-[(methoxycarbony	C48H52N8O6	836.98	A/B	-8.5
4904	C(=O)([C@H](NC(=O)OC)c1ccccc1)N1[C@@H](CCC1)c1[nH]c(cn1)c1ccc(cc1)c1cc2c(cc1)cc(cc2)c1[nH]c(nc1)[C@H	1256391-31-7	Carbamic acid, N-[(1S)-2-[(2S)-2-[5-[4-[6-[2-[(2S)-1-[(2S)-2-[(methoxycarbonyl)a	C47H50N8O6	822.95	A/B	-8.5
4905	C[C@]12[C@H]3[C@]4([C@]5(C4)[C@@H](CC3)[C@H](C)C(=O)C=C5)CC[C@]1(C)[C@@H]([C@@H]([C@H](C(=O)C(=C)[C@	1256393-24-4	Carbamic acid, N-[(1S)-1-[[(2S)-2-[5-[6-[4-[2-[(2S)-1-[(2R)-2-cyclohexyl-2-[(met	C47H56N8O6	829	A/B	-8.5
4906	C(=O)([C@@H](NC(=O)OC)C(C)C)N1[C@@H](CCC1)c1[nH]c(cn1)c1ccc(cc1)c1ccc2c(cc1)cc(cc2)c1[nH]c(nc1)[C@H]1	1261070-48-7	Carbamic acid, N-[(1S)-1-[[(2S)-2-[5-[6-[4-[2-[(2S)-1-[(2S)-2-[(methoxycarbonyl)	C43H50N8O6	774.91	A/B	-8.5
4907	S(=O)(=O)(O)c1c2c(c(N)c(/N=N/c3cc(ccc3C)c3cc(C)c(/N=N/c4c(N)c5c(c(S(=O)(=O)O)c4)cccc5)cc3)c1)cccc2	1261070-51-2	Carbamic acid, N-[(1S)-1-[[(2S)-2-[5-[4-[6-[2-[(2S)-1-[2-[(methoxycarbonyl)aminc	C41H46N8O6	746.85	A/B	-8.5
4908	O=c1n([C@H]2C[C@H](O[P@@](=O)(OC[C@H]3O[C@H](C[C@@H]3O)n3c(=O)[nH]c(=O)c(C)c3)SSCC)[C@@H](CO)O2)cc(C	1261162-91-7	Not Yet Assigned	C23H31N4O14P	618.48	A/B	-8.5
4909	O=c1n([C@H]2C[C@H](O[P@](=O)(OC[C@H]3O[C@H](C[C@@H]3O)n3c(=O)[nH]c(=O)c(C)c3)SSCC)[C@@H](CO)O2)cc(C)	1261163-57-8	Thymidine, [P(R)]-P-deoxy-P-(ethyldithio)thymidylyl-(3,Ä≤,Üì5,Ä≤)-	C22H31N4O11PS2	622.61	A/B	-8.5
4910	C(F)(F)c1n(c2c(n1)cccc2)c1nc(nc(NCc2ccccc2)n1)N1CCOCC1	1261281-11-1	1,3,5-Triazin-2-amine, 4-[2-(difluoromethyl)-1H-benzimidazol-1-yl]-N-methyl-6-(4	C22H21F2N7O	437.45	A/B	-8.5
4911	C(F)(F)c1n(c2c(n1)cccc2)c1nc(nc(NCc2ccncc2)n1)N1CCOCC1	1261281-18-8	1,3,5-Triazin-2-amine, 4-[2-(difluoromethyl)-1H-benzimidazol-1-yl]-6-(4-morpholi	C21H20F2N8O	438.43	A/B	-8.5
4912	O=c1c2c(n(cc1C(=O)O)C1CC1)cc(c(F)c2)N1CCN(CN2c3c(/C(=N/NC(=S)NO)/C2=O)cc(F)cc3)CC1	1265623-16-2	3-Quinolinecarboxylic acid, 7-[4-[[(3Z)-5-chloro-2,3-dihydro-3-[2-[(methoxyaminc	C28H27ClFN7O5S	628.07	A/B	-8.5
4913	C(C)n1c2c(c(=O)c(C(=O)O)c1)cc(F)c(c2F)N1CC(C)N(CN2c3c(/C(=N/NC(=S)NO)/C2=O)cc(Cl)cc3)CC1	1265623-20-8	3-Quinolinecarboxylic acid, 1-cyclopropyl-7-[4-[[(3Z)-2,3-dihydro-3-[2-[(methoxy	C29H30FN7O5S	607.66	A/B	-8.5

4914	C(C)n1c2c(c(=O)c(C(=O)O)c1)cc(F)c(c2F)N1CC(C)N(CN2c3c(/C(=N/NC(=S)NOC)/C2=O)cc(F)cc3)CC1	1265623-23-1	3-Quinolinecarboxylic acid, 1-ethyl-6,8-difluoro-7-[4-[[(3Z)-5-fluoro-2,3-dihydr	C27H26F3N7O5S	617.6	A/B	-8.5
4915	O(C)c1c2n(cc(C(=O)O)c(=O)c2cc(F)c1N1CC(C)N(CN2c3c(/C(=N/NC(=S)NOC)/C2=O)cc(F)cc3)CC1)C1CC1	1265623-29-7	3-Quinolinecarboxylic acid, 1-cyclopropyl-6-fluoro-7-[4-[[(3Z)-5-fluoro-2,3-dihy	C29H29F2N7O6S	641.65	A/B	-8.5
4916	O[C@H]1[C@@H]2[C@@H]([C@]3(C)[C@H](C1)C[C@H](O)[C@H](O)C3)C[C@H](O)[C@@]1(C)[C@H]2CC[C@@H]1[C@@H](CC	126934-30-3	Guanosine 5,Ä≤-(tetrahydrogen triphosphate), P,Ä≤,Ä≤,Üi3,Ä≤-ester with adenosir	C20H27N10O17P3	772.41	A/B	-8.5
4917	Cc1c2c(CO[P@](=O)(OC[C@H]3O[C@H](C=C3)n3c(=O)[nH]c(=O)c(C)c3)O2)cc(C)c1	1270965-83-7	Thymidine, 2,Ä≤,3,Ä≤-didehydro-3,Ä≤-deoxy-5,Ä≤-O-[(2S)-6,8-dimethyl-2-oxido-4H-	C19H21N2O7P	420.35	A/B	-8.5
4918	C(=O)([C@@H](NC(=O)OC)C(C)C)N1C[C@@]2(C[C@H]1c1[nH]c(cn1)c1ccc(cc1)c1ccc(cc1)c1[nH]c(nc1)[C@H]1N(C(=	127246-67-7	9H-Purin-6-amine, 9-[2,3-bis-O-[(4-methylphenyl)sulfonyl]-Œ≤-D-arabinofuranosyl]	C24H25N5O8S2	575.61	A/B	-8.5
4919	C(=O)([C@@H](NC(=O)OC)C(C)C)N1C(CC2(C1)CCNCC2)c1[nH]c(cn1)c1ccc(cc1)c1ccc(cc1)c1[nH]c(nc1)[C@H]1N(C(	1272656-09-3	Carbamic acid, N,N,Ä≤-[[1,1,Ä≤-biphenyl]-4,4,Ä≤-diylbis[1H-imidazole-5,2-diyl-(	C48H62N8O8	879.05	A/B	-8.5
4920	C(=O)([C@@H](NC(=O)OC)C)N1[C@@H]([C@@H]2C[C@H]1CC2)c1[nH]c(cn1)c1ccc(cc1)c1ccc(cc1)c1[nH]c(nc1)[C@H]	1273589-44-8	Carbamic acid, N-[(1R)-2-[(2S)-2-[5-[4,Ä≤-[2-[(1R,3S,4S)-2-[(2R)-2-[(methoxycarb	C38H44N8O6	708.81	A/B	-8.5
4921	C(=O)([C@@H](NC(=O)OC)C1CCOCC1)N1[C@@H]([C@@H]2C[C@H]1CC2)c1[nH]c(cn1)c1ccc(cc1)c1ccc(cc1)c1[nH]c(nc	1273589-71-1	Carbamic acid, N-[(1S)-2-methyl-1-[[(2S)-2-[5-[4,Ä≤-[2-[(1R,3S,4S)-2-[(2S)-3-met	C48H60N8O8	877.04	A/B	-8.5
4922	C[C@@]1(N(C(=O)[C@@H](NC(=O)OC)C(C)C)[C@H]2C[C@@H]1CC2)c1[nH]c(cn1)c1ccc(cc1)c1ccc(cc1)c1[nH]c(nc1)[	1273591-31-3	Carbamic acid, N-[(1S)-1-[[(2S)-2-[5-[4,Ä≤-[2-[(1S,3S,4R)-2-[(2S)-2-[(methoxycar	C42H50N8O7	778.9	A/B	-8.5
4923	C(=O)([C@@H](NC(=O)OC)C(C)C)N1[C@@H]([C@@H]2C[C@H]1C=C2)c1[nH]c(cn1)c1ccc(cc1)c1ccc(cc1)c1[nH]c(nc1)	1273591-33-5	Carbamic acid, N-[(1S)-1-[[(1S,3S,4R)-3-[5-[4,Ä≤-[2-[(2S)-1-[(2S)-2-[(methoxycar	C46H58N8O6	819	A/B	-8.5
4924	C(=O)([C@@H](NC(=O)OC)C(C)C)N1[C@@H]([C@@H]2C[C@H]1CC2)c1[nH]c(cn1)c1c(C)cc(cc1)c1cc(C)c(cc1)c1[nH]c	1273591-46-0	Carbamic acid, N-[(1S)-2-methyl-1-[[(2S)-2-[5-[2,2,Ä≤,3,3,Ä≤-tetrafluoro-4,Ä≤-[	C42H48F4N8O6	836.87	A/B	-8.5
4925	N(Cc1ccccc1)c1nc(nc(N/N=C/c2c(O)cccc2)n1)N1CCOCC1	1279692-16-8	Benzaldehyde, 2-hydroxy-, 2-[4-[[(4-fluorophenyl)methyl]amino]-6-(4-morpholinyl)	C21H22FN7O2	423.44	A/B	-8.5
4926	O=c1n([C@H]2O[C@H](CO)[C@H](O)C2)cc(C)c(=O)[nH]1	128098-56-6	1-Naphthalenesulfonic acid, 6-hydroxy-5-[2-(4-sulfophenyl)diazenyl]-	C16H12N2O7S2	408.41	A/B	-8.5
4927	N(=C1c2c(N(CCNc3c4c(ncc3)cc(Cl)cc4)C1=O)c(C)cc(Cl)c2)NC(=S)N	1283084-56-9	Hydrazinecarbothioamide, 2-[5-bromo-1-[2-[(7-chloro-4-quinolinyl)amino]ethyl]-1,	C21H18BrClN6OS	517.83	A/B	-8.5
4928	N(=C1c2c(N(CCNc3c4c(cc(Cl)cc4)ncc3)C1=O)ccc(F)c2)NC(=S)N	1283084-57-0	Hydrazinecarbothioamide, 2-[5-chloro-1-[2-[(7-chloro-4-quinolinyl)amino]ethyl]-1	C21H18Cl2N6OS	473.38	A/B	-8.5
4929	N(CCCN)(CC(C)C)c1c2c(ncc1)cc(Cl)cc2	129232-80-0	Guanosine, 5,Ä≤-[4-(bromomethyl)benzoate]	C18H18BrN5O6	480.27	A/B	-8.5
4930	C(=C\C(=C)C)/c1c(OC)c(C(=O)/C=C/c2ccc(OC)cc2)ccc1OC	129266-61-1	2-Naphthalenesulfonic acid, 5-[2-(4-aminophenyl)diazenyl]-8-[2-(4-sulfophenyl)di	C22H17N5O6S2	511.53	A/B	-8.5
4931	O=c1c2c(n([14cH]n2)[C@@H]2O[C@H](CO)[C@@H](O)[C@H]2O)[nH]c(N)n1	129318-05-4	1,5-Naphthalenedisulfonic acid, 3-[2-[4-[2-(4-amino-2-methylphenyl)diazenyl]pher	C23H19N5O6S2	525.56	A/B	-8.5
4932	O=c1n([C@H]2C[C@H](OP(=O)(OCCC(C)O)O)[C@@H](CO)O2)cc(C)c(=O)[nH]1	1300033-19-5	4H-1-Benzopyran-4-one, 2-(2-quinazolinyl)-, oxime	C17H11N3O2	289.29	A/B	-8.5
4933	C(=C\C(=O)c1ccccc1)/c1c(OC)c(C=O)c(O)c(C)c1O	130272-39-8	Guanosine 5,Ä≤-(trihydrogen diphosphate), P,Ä≤-Œ±-D-arabinopyranosyl ester	C15H23N5O15P2	575.32	A/B	-8.5
4934	C([C@@H](OCP(=O)(O)O)CF)n1c2c(c(NC)ncn2)nc1	1305288-48-5	Acetamide, N-methyl-2-[[4-(4-morpholinyl)-6-(phenylamino)-1,3,5-triazin-2-yl]thi	C26H34N6O2S	494.65	A/B	-8.5
4935	C(=O)([C@@H](NC(=O)OC)C(C)C)N1[C@@H](CC[C@H]1C)c1[nH]c(cn1)c1cc2c(cc1)cc(cc2)c1ccc(cc1)c1[nH]c(nc1)[	1306733-02-7	Carbamic acid, N-[(1S)-1-[[(2S,5S)-2-[5-[4-[6-[2-[(2S,5S)-1-[(2S)-2-[(methoxycar	C46H56N8O6	816.99	A/B	-8.5
4936	N(C(=O)NCCC(O)c1ccncc1)C12CC3CC(C2)CC(C1)C3	131107-83-0	1-Naphthalenesulfonic acid, 4-amino-3-[2-(2-methylphenyl)diazenyl]-	C17H15N3O3S	341.38	A/B	-8.5
4937	C(=O)([C@@H](NC(=O)OC)C(C)C)N1[C@@H](CC(=C)C1)c1[nH]c(cn1)c1ccc(C=Cc2[nH]c(nc2)[C@H]2N(C(=O)[C@@H](N	1311398-46-5	Carbamic acid, N-[(1S)-1-[[(2S)-2-[5-[6-[2-[(2S)-1-[(2S)-2-[(methoxycarbonyl)ami	C43H50N8O6	774.91	A/B	-8.5
4938	C(=O)([C@@H](NC(=O)OC)C1CC1)N1[C@H]2[C@H](C2)C[C@H]1c1[nH]c(c(Cl)n1)c1ccc(cc1)c1ccc(cc1)c1[nH]c(nc1C	1312336-04-1	Carbamic acid, N,N,Ä≤-[[1,1,Ä≤-biphenyl]-4,4,Ä≤-diylbis[(5-chloro-1H-imidazole-	C46H52Cl2N8O8	915.86	A/B	-8.5
4939	C(=O)([C@@H](NC(=O)OC)C(C)C)N1[C@H]2[C@H](C2)C[C@H]1c1[nH]c2c(n1)ccc1c2ccc(c1)c1ccc(cc1)c1[nH]c(nc1C	1312336-40-5	Carbamic acid, N-[(1S)-2-[(1R,3S,5R)-3-[5-chloro-4-[4,Ä≤-[2-[(1R,3S,5R)-2-[(2S)-	C47H53ClF2N8O7	915.42	A/B	-8.5
4940	C(=O)([C@@H](NC(=O)OC)C(C)C)N1[C@H](c2[nH]c3c4c(cc(cc4)c4ccc(cc4)c4[nH]c(nc4Cl)[C@H]4N(C(=O)[C@@H](N	1312336-52-9	Carbamic acid, N-[(1S)-1-[[(1R,3S,5R)-3-[5-bromo-4-[4-[5-chloro-2-[(1R,3S,5R)-2-	C44H48BrClN8O6	900.26	A/B	-8.5
4941	C(=O)([C@@H](NC(=O)OC)C1CCOCC1)N1[C@@H](CC[C@@H]1C)c1[nH]c(c(F)n1)c1ccc(cc1)c1ccc(cc1)c1[nH]c(nc1F)[	1312336-66-5	Carbamic acid, N-[(1S)-2-[(2S,5S)-2-[5-[4,Ä≤-[5-fluoro-2-[(2S,5S)-1-[(2S)-2-[(me	C46H57FN8O8	868.99	A/B	-8.5
4942	C(=O)([C@@H](NC(=O)OC)C1CCOCC1)N1[C@H]2[C@H](C2)C[C@H]1c1[nH]c(c(C#N)n1)c1ccc(cc1)c1ccc(cc1)c1[nH]c(	1312336-68-7	Carbamic acid, N-[(1S)-1-[[(1R,3S,5R)-3-[5-[4,Ä≤-[5-cyano-2-[(1R,3S,5R)-2-[(2S)-	C43H49N9O6	787.91	A/B	-8.5
4943	C(=O)([C@H](C(COC)COC)NC(=O)OC)N1[C@H]2[C@H](C2)C[C@H]1c1[nH]c(c(Cl)n1)c1ccc(cc1)c1ccc(cc1)c1[nH]c(n	1312337-08-8	Carbamic acid, N-[(1S)-2-[(1R,3S,5R)-3-[5-(4-isoxazolyl)-4-[4,Ä≤-[2-[(1R,3S,5R)-	C49H55N9O9	914.02	A/B	-8.5
4944	N(c1c(C[2H])cc(/C=C/C#N)cc1C([2H])[2H])c1nc(Nc2ccc(C#N)cc2)ncc1	1312424-29-5	Benzonitrile, 4-[[4-[[4-[(1E)-2-cyanoethenyl]-2-(methyl-d2)-6-(methyl-d3)phenyl]	C22H13D5N6		A/B	-8.5
4945	C(=O)([C@@H](NC(=O)OC)C(C)C)N1[C@H](c2[nH]c(c3c4c(c(cc3)c3ccc(cc3)c3[nH]c(nc3)[C@H]3N(C(=O)[C@@H](NC	1312608-38-0	Carbamic acid, N-[(1S)-1-[[(2S)-2-[5-[4-[4-[2-[(2S)-1-[(2S)-2-[(methoxycarbonyl)	C42H51N9O6S	809.98	A/B	-8.5
4946	C(=O)([C@@H](NC(=O)OC)C(C)C)N1[C@H](c2[nH]c(c3c4c(c(cc3)c3ccc(cc3)c3[nH]c(nc3)[C@@]3(C)N(C(=O)[C@@H]	1312608-77-7	Carbamic acid, N-[(1S)-1-[[(2S)-2-[5-[4-[7-[2-[(2S)-1-[(2S)-2-[(methoxycarbonyl)	C44H55N9O6	805.96	A/B	-8.5
4947	Nc1n(c2c(n1)c(=O)nc[nH]2)C1OC(CO)CC1	1312612-07-9	Carbamic acid, N-[(1S)-1-[[(2S)-2-[5-[4-[4-[2-[(2S)-1-[(2S)-2-[(methoxycarbonyl)	C46H56N8O6	816.99	A/B	-8.5
4948	P(=O)(OC[C@H](NC(=O)[C@H](C)N)C(=O)OC)(CO[C@@H](Cn1c(=O)nc(N)cc1)CO)OCC	1312772-88-5	Tricyclo[3.3.1.13,7]decan-1-amine, N-[[4-[6-(4-methylphenyl)-1,2,4,5-tetroxan-3-	C26H31NO4	421.53	A/B	-8.5
4949	O(Cc1ccc(F)cc1)c1c2c(oc(c(O)c2=O)c2[nH]c(nn2)c2ccccc2)ccc1	1312892-14-0	4H-1-Benzopyran-4-one, 2-(5-chloro-1H-1,2,4-triazol-3-yl)-5-[(4-fluorophenyl)met	C18H11ClFN3O4	387.75	A/B	-8.5
4950	O(Cc1ccc(F)cc1)c1c2c(oc(c(O)c2=O)c2ncc(C)cn2)c(C)cc1	1312892-52-6	4H-1-Benzopyran-4-one, 5-[(4-fluorophenyl)methoxy]-3-hydroxy-8-methyl-2-(2-pyrim	C21H15FN2O4	378.35	A/B	-8.5
4951	O(Cc1ccc(F)cc1)c1c2c(oc(c(O)c2=O)c2nnc(Cl)[nH]2)c(C)cc1	1312892-64-0	4H-1-Benzopyran-4-one, 5-[(4-fluorophenyl)methoxy]-3-hydroxy-2-(5-methoxy-1H-1,2	C20H16FN3O5	397.36	A/B	-8.5
4952	O(Cc1ccc(F)cc1)c1c2c(oc(c(O)c2=O)c2[nH]c(OC)cn2)c(C)cc1	1312892-75-3	4H-1-Benzopyran-4-one, 5-[(4-fluorophenyl)methoxy]-3-hydroxy-8-methyl-2-(5-methy	C21H17FN2O4	380.37	A/B	-8.5
4953	O(Cc1ccc(F)cc1)c1c2c(oc(c(O)c2=O)c2[nH]c(cn2)c2ccccc2)c(C)cc1	1312892-81-1	4H-1-Benzopyran-4-one, 2-(5-chloro-1H-imidazol-2-yl)-5-[(4-fluorophenyl)methoxy]	C20H14ClFN2O4	400.79	A/B	-8.5
4954	O(Cc1ccc(F)cc1)c1c2c(oc(c(O)c2=O)c2ncc(OC)cn2)c(OC)cc1	1312893-34-7	4H-1-Benzopyran-4-one, 5-[(4-fluorophenyl)methoxy]-3-hydroxy-8-methoxy-2-(5-meth	C22H17FN2O5	408.38	A/B	-8.5
4955	O(C)c1c2c(c(OCc3ccc(F)cc3)cc1)c(=O)c(O)c(o2)c1[nH]c(OC)nn1	1312893-44-9	4H-1-Benzopyran-4-one, 5-[(4-fluorophenyl)methoxy]-3-hydroxy-8-methoxy-2-(3-meth	C20H16FN3O5	397.36	A/B	-8.5
4956	O(C)c1c2c(c(OCc3ccc(F)cc3)cc1)c(=O)c(O)c(o2)c1[nH]c(OC)cn1	1312893-49-4	4H-1-Benzopyran-4-one, 5-[(4-fluorophenyl)methoxy]-3-hydroxy-8-methoxy-2-(5-meth	C21H17FN2O5	396.37	A/B	-8.5
4957	O(Cc1ccc(F)cc1)c1c2c(oc(c(O)c2=O)c2[nH]c(Cl)cn2)c(OC)cc1	1312893-50-7	4H-1-Benzopyran-4-one, 5-[(4-fluorophenyl)methoxy]-3-hydroxy-8-methoxy-2-(5-meth	C21H17FN2O6	412.37	A/B	-8.5
4958	O(Cc1ccc(F)cc1)c1c2c(oc(c(O)c2=O)c2ncc(C)cn2)c(Cl)cc1	1312893-83-6	4H-1-Benzopyran-4-one, 8-chloro-5-[(4-fluorophenyl)methoxy]-3-hydroxy-2-(2-pyrim	C20H12ClFN2O4	398.77	A/B	-8.5
4959	O(Cc1ccc(F)cc1)c1c2c(oc(c(O)c2=O)c2ncc(cn2)c2ccccc2)c(Cl)cc1	1312893-86-9	4H-1-Benzopyran-4-one, 8-chloro-2-(5-chloro-2-pyrimidinyl)-5-[(4-fluorophenyl)me	C20H11Cl2FN2O4	433.22	A/B	-8.5
4960	O(Cc1ccc(F)cc1)c1c2c(oc(c(O)c2=O)c2nnc(Cl)[nH]2)c(Cl)cc1	1312893-95-0	4H-1-Benzopyran-4-one, 8-chloro-5-[(4-fluorophenyl)methoxy]-3-hydroxy-2-(5-metho	C19H13ClFN3O5	417.77	A/B	-8.5
4961	O(Cc1ccc(F)cc1)c1c2c(oc(c(O)c2=O)c2[nH]c(OC)cn2)c(Cl)cc1	1312893-99-4	4H-1-Benzopyran-4-one, 8-chloro-5-[(4-fluorophenyl)methoxy]-3-hydroxy-2-(5-methy	C20H14ClFN2O4	400.79	A/B	-8.5
4962	O(Cc1ccc(F)cc1)c1c2c(oc(c(O)c2=O)c2[nH]c(cn2)c2ccccc2)c(Cl)cc1	1312894-01-1	4H-1-Benzopyran-4-one, 8-chloro-2-(5-chloro-1H-imidazol-2-yl)-5-[(4-fluorophenyl	C19H11Cl2FN2O4	421.21	A/B	-8.5
4963	O(Cc1ccc(F)cc1)c1c2c(oc(c(O)c2=O)c2nnc(Cl)[nH]2)cc(C)c1	1312894-95-3	4H-1-Benzopyran-4-one, 5-[(4-fluorophenyl)methoxy]-3-hydroxy-2-(5-methoxy-1H-1,2	C20H16FN3O5	397.36	A/B	-8.5
4964	O(Cc1ccc(F)cc1)c1c2c(oc(c(O)c2=O)c2[nH]c(nn2)c2ccccc2)cc(C)c1	1312894-96-4	4H-1-Benzopyran-4-one, 2-(5-chloro-1H-1,2,4-triazol-3-yl)-5-[(4-fluorophenyl)met	C19H13ClFN3O4	401.78	A/B	-8.5
4965	O(Cc1ccc(F)cc1)c1c2c(oc(c(O)c2=O)c2[nH]cc(C)n2)cc(C)c1	1312894-98-6	4H-1-Benzopyran-4-one, 5-[(4-fluorophenyl)methoxy]-3-hydroxy-2-(1H-imidazol-2-yl	C20H15FN2O4	366.34	A/B	-8.5
4966	O(Cc1ccc(F)cc1)c1c2c(c(OC)c(C)c1)oc(c(O)c2=O)c1[nH]c(C)nn1	1312895-93-4	4H-1-Benzopyran-4-one, 5-[(4-fluorophenyl)methoxy]-3-hydroxy-8-methoxy-7-methyl-	C20H16FN3O5	397.36	A/B	-8.5
4967	O(Cc1ccc(F)cc1)c1c2c(c(OC)c(C)c1)oc(c(O)c2=O)c1[nH]c(OC)nn1	1312895-94-5	4H-1-Benzopyran-4-one, 5-[(4-fluorophenyl)methoxy]-3-hydroxy-8-methoxy-7-methyl-	C21H18FN3O5	411.38	A/B	-8.5
4968	O(Cc1ccc(F)cc1)c1c2c(c(OC)c(C)c1)oc(c(O)c2=O)c1[nH]c(nn1)c1ccccc1	1312895-96-7	4H-1-Benzopyran-4-one, 2-(5-chloro-1H-1,2,4-triazol-3-yl)-5-[(4-fluorophenyl)met	C20H15ClFN3O5	431.8	A/B	-8.5
4969	O(Cc1ccc(F)cc1)c1c2c(oc(c(O)c2=O)c2[nH]c(nn2)c2ccccc2)c(C)c(OC)c1	1312898-07-9	4H-1-Benzopyran-4-one, 2-(5-chloro-1H-1,2,4-triazol-3-yl)-5-[(4-fluorophenyl)met	C20H15ClFN3O5	431.8	A/B	-8.5
4970	O(Cc1ccc(F)cc1)c1c2c(oc(c(O)c2=O)c2[nH]c(OC)cn2)c(C)c(OC)c1	1312898-10-4	4H-1-Benzopyran-4-one, 5-[(4-fluorophenyl)methoxy]-3-hydroxy-7-methoxy-8-methyl-	C22H19FN2O5	410.4	A/B	-8.5
4971	O(Cc1ccc(F)cc1)c1c2c(oc(c(O)c2=O)c2[nH]c(nn2)c2ccccc2)c(Cl)c(OC)c1	1312899-08-3	4H-1-Benzopyran-4-one, 8-chloro-2-(5-chloro-1H-1,2,4-triazol-3-yl)-5-[(4-fluorop	C19H12Cl2FN3O5	452.22	A/B	-8.5
4972	O(Cc1ccc(F)cc1)c1c2c(oc(c(O)c2=O)c2[nH]c(OC)cn2)c(Cl)c(OC)c1	1312899-11-8	4H-1-Benzopyran-4-one, 8-chloro-5-[(4-fluorophenyl)methoxy]-3-hydroxy-7-methoxy-	C21H16ClFN2O5	430.81	A/B	-8.5
4973	O(C)c1c(c2c(c(OCc3ccc(F)cc3)c1)c(=O)c(O)c(o2)c1nnc(C)[nH]1)c1ccccc1	1312899-55-0	4H-1-Benzopyran-4-one, 5-[(4-fluorophenyl)methoxy]-3-hydroxy-7-methoxy-8-phenyl-	C25H18FN3O5	459.43	A/B	-8.5
4974	O(C)c1c(c2c(c(OCc3ccc(F)cc3)c1)c(=O)c(O)c(o2)c1[nH]c(OC)cn1)c1ccccc1	1312899-61-8	4H-1-Benzopyran-4-one, 5-[(4-fluorophenyl)methoxy]-3-hydroxy-7-methoxy-2-(5-meth	C27H21FN2O5	472.46	A/B	-8.5
4975	O(C)c1c(c2c(c(OCc3ccc(F)cc3)c1)c(=O)c(O)c(o2)c1[nH]c(cn1)c1ccccc1)c1ccccc1	1312899-63-0	4H-1-Benzopyran-4-one, 2-(5-chloro-1H-imidazol-2-yl)-5-[(4-fluorophenyl)methoxy]	C26H18ClFN2O5	492.88	A/B	-8.5
4976	O(Cc1ccc(F)cc1)c1c2c(oc(c(O)c2=O)c2ncccn2)cc(Cl)c1	1312899-64-1	4H-1-Benzopyran-4-one, 5-[(4-fluorophenyl)methoxy]-3-hydroxy-7-methoxy-8-phenyl-	C32H23FN2O5	534.53	A/B	-8.5
4977	O(Cc1ccc(F)cc1)c1c2c(oc(c(O)c2=O)c2ncc(C)cn2)cc(Cl)c1	1312900-54-1	4H-1-Benzopyran-4-one, 7-chloro-5-[(4-fluorophenyl)methoxy]-3-hydroxy-2-(2-pyrim	C20H12ClFN2O4	398.77	A/B	-8.5
4978	O(Cc1ccc(F)cc1)c1c2c(oc(c(O)c2=O)c2ncc(OC)cn2)cc(Cl)c1	1312900-55-2	4H-1-Benzopyran-4-one, 7-chloro-5-[(4-fluorophenyl)methoxy]-3-hydroxy-2-(5-methy	C21H14ClFN2O4	412.8	A/B	-8.5
4979	O(C)c1c2c(c(OCc3ccc(F)cc3)cc1Cl)c(=O)c(O)c(o2)c1[nH]cnn1	1312901-58-8	4H-1-Benzopyran-4-one, 7-chloro-5-[(4-fluorophenyl)methoxy]-3-hydroxy-8-methoxy-	C27H18ClFN2O5	504.89	A/B	-8.5
4980	O(Cc1ccc(F)cc1)c1c2c(oc(c(O)c2=O)c2ncccn2)c(Cl)c(Cl)c1	1312901-73-7	4H-1-Benzopyran-4-one, 7-chloro-5-[(4-fluorophenyl)methoxy]-3-hydroxy-8-methoxy-	C26H18ClFN2O5	492.88	A/B	-8.5
4981	O(Cc1ccc(F)cc1)c1c2c(oc(c(O)c2=O)c2ncc(cn2)c2ccccc2)c(Cl)c(Cl)c1	1312902-07-0	4H-1-Benzopyran-4-one, 7,8-dichloro-2-(5-chloro-2-pyrimidinyl)-5-[(4-fluorophen	C20H10Cl3FN2O4	467.66	A/B	-8.5
4982	O(Cc1ccc(F)cc1)c1c2c(oc(c(O)c2=O)c2[nH]cnn2)c(Cl)c(Cl)c1	1312902-08-1	4H-1-Benzopyran-4-one, 7,8-dichloro-5-[(4-fluorophenyl)methoxy]-3-hydroxy-2-(5-p	C26H15Cl2FN2O4	509.31	A/B	-8.5
4983	O(Cc1ccc(F)cc1)c1c2c(c(C)c(c1)c1ccccc1)oc(c(O)c2=O)c1[nH]c(Cl)cn1	1312903-74-4	4H-1-Benzopyran-4-one, 5-[(4-fluorophenyl)methoxy]-3-hydroxy-2-(5-methoxy-1H-imi	C27H21FN2O5	472.46	A/B	-8.5
4984	O(C)c1c2c(c(OCc3ccc(F)cc3)cc1c1ccccc1)c(=O)c(O)c(o2)c1ncc(Cl)cn1	1312904-28-1	4H-1-Benzopyran-4-one, 5-[(4-fluorophenyl)methoxy]-3-hydroxy-8-methoxy-2-(5-meth	C28H21FN2O6	500.47	A/B	-8.5
4985	O(C)c1c2c(c(OCc3ccc(F)cc3)cc1c1ccccc1)c(=O)c(O)c(o2)c1nnc(C)[nH]1	1312904-40-7	4H-1-Benzopyran-4-one, 5-[(4-fluorophenyl)methoxy]-3-hydroxy-8-methoxy-7-phenyl-	C25H18FN3O5	459.43	A/B	-8.5
4986	O(C)c1c2c(c(OCc3ccc(F)cc3)cc1c1ccccc1)c(=O)c(O)c(o2)c1[nH]cc(C)n1	1312904-49-6	4H-1-Benzopyran-4-one, 5-[(4-fluorophenyl)methoxy]-3-hydroxy-2-(1H-imidazol-2-yl	C26H19FN2O5	458.44	A/B	-8.5
4987	O(C)c1c2c(c(OCc3ccc(F)cc3)cc1c1ccccc1)c(=O)c(O)c(o2)c1[nH]c(OC)cn1	1312904-51-0	4H-1-Benzopyran-4-one, 5-[(4-fluorophenyl)methoxy]-3-hydroxy-8-methoxy-2-(5-meth	C27H21FN2O5	472.46	A/B	-8.5
4988	O(Cc1ccc(F)cc1)c1c2c(c(Cl)c(c1)c1ccccc1)oc(c(O)c2=O)c1[nH]c(Cl)cn1	1312905-30-8	4H-1-Benzopyran-4-one, 8-chloro-5-[(4-fluorophenyl)methoxy]-3-hydroxy-2-(5-metho	C26H18ClFN2O5	492.88	A/B	-8.5
4989	O=c1c2c(oc(c1O)c1nnc(C)[nH]1)ccc(OCc1ccc(F)cc1)c2	1312906-19-6	4H-1-Benzopyran-4-one, 6-[(4-fluorophenyl)methoxy]-3-hydroxy-2-(5-phenyl-2-pyrim	C26H17FN2O4	440.42	A/B	-8.5
4990	O=c1c2c(oc(c1O)c1[nH]cc(Cl)n1)ccc(OCc1ccc(F)cc1)c2	1312906-31-2	4H-1-Benzopyran-4-one, 6-[(4-fluorophenyl)methoxy]-3-hydroxy-2-(5-methoxy-1H-imi	C20H15FN2O5	382.34	A/B	-8.5
4991	Cc1c2c(cc(OCc3ccc(F)cc3)c1)c(=O)c(O)c(o2)c1ncc(OC)cn1	1312906-65-2	4H-1-Benzopyran-4-one, 6-[(4-fluorophenyl)methoxy]-3-hydroxy-8-methyl-2-(5-methy	C22H17FN2O4	392.38	A/B	-8.5
4992	O(C)c1c2c(cc(OCc3ccc(F)cc3)c1)c(=O)c(O)c(o2)c1ncc(Cl)cn1	1312907-16-6	4H-1-Benzopyran-4-one, 6-[(4-fluorophenyl)methoxy]-3-hydroxy-8-methoxy-2-(5-meth	C22H17FN2O6	424.38	A/B	-8.5
4993	Clc1c2c(cc(OCc3ccc(F)cc3)c1)c(=O)c(O)c(o2)c1[nH]c(OC)cn1	1312907-80-4	4H-1-Benzopyran-4-one, 8-chloro-6-[(4-fluorophenyl)methoxy]-3-hydroxy-2-(5-methy	C20H14ClFN2O4	400.79	A/B	-8.5
4994	O=c1c2c(oc(c1O)c1[nH]cc(Cl)n1)c(Cl)cc(OCc1ccc(F)cc1)c2	1312907-81-5	4H-1-Benzopyran-4-one, 8-chloro-6-[(4-fluorophenyl)methoxy]-3-hydroxy-2-(5-metho	C20H14ClFN2O5	416.79	A/B	-8.5
4995	O=c1c2c(oc(c1O)c1[nH]c(cn1)c1ccccc1)c(Cl)cc(OCc1ccc(F)cc1)c2	1312907-82-6	4H-1-Benzopyran-4-one, 8-chloro-2-(5-chloro-1H-imidazol-2-yl)-6-[(4-fluorophenyl	C19H11Cl2FN2O4	421.21	A/B	-8.5
4996	O=c1c2c(oc(c1O)c1[nH]c(OC)nn1)cc(C)c(OCc1ccc(F)cc1)c2	1312908-75-0	4H-1-Benzopyran-4-one, 6-[(4-fluorophenyl)methoxy]-3-hydroxy-7-methyl-2-(3-methy	C20H16FN3O4	381.36	A/B	-8.5
4997	O=c1c2c(oc(c1O)c1[nH]cc(Cl)n1)cc(C)c(OCc1ccc(F)cc1)c2	1312908-81-8	4H-1-Benzopyran-4-one, 6-[(4-fluorophenyl)methoxy]-3-hydroxy-2-(5-methoxy-1H-imi	C21H17FN2O5	396.37	A/B	-8.5
4998	Cc1c2c(cc(OCc3ccc(F)cc3)c1C)c(=O)c(O)c(o2)c1ncc(C)cn1	1312909-26-4	4H-1-Benzopyran-4-one, 6-[(4-fluorophenyl)methoxy]-3-hydroxy-7,8-dimethyl-2-(2-p	C22H17FN2O4	392.38	A/B	-8.5
4999	Cc1c2c(cc(OCc3ccc(F)cc3)c1C)c(=O)c(O)c(o2)c1[nH]c(C)nn1	1312909-36-6	4H-1-Benzopyran-4-one, 6-[(4-fluorophenyl)methoxy]-3-hydroxy-7,8-dimethyl-2-(1H-	C20H16FN3O4	381.36	A/B	-8.5
5000	Cc1c2c(cc(OCc3ccc(F)cc3)c1C)c(=O)c(O)c(o2)c1[nH]c(Cl)nn1	1312909-38-8	4H-1-Benzopyran-4-one, 6-[(4-fluorophenyl)methoxy]-3-hydroxy-2-(5-methoxy-1H-1,2	C21H18FN3O5	411.38	A/B	-8.5
5001	O(C)c1c2c(cc(OCc3ccc(F)cc3)c1C)c(=O)c(O)c(o2)c1[nH]c(nn1)c1ccccc1	1312909-89-9	4H-1-Benzopyran-4-one, 2-(5-chloro-1H-1,2,4-triazol-3-yl)-6-[(4-fluorophenyl)met	C20H15ClFN3O5	431.8	A/B	-8.5
5002	Clc1c2c(cc(OCc3ccc(F)cc3)c1C)c(=O)c(O)c(o2)c1ncc(cn1)c1ccccc1	1312910-29-4	4H-1-Benzopyran-4-one, 8-chloro-2-(5-chloro-2-pyrimidinyl)-6-[(4-fluorophenyl)me	C21H13Cl2FN2O4	447.24	A/B	-8.5
5003	Clc1c2c(cc(OCc3ccc(F)cc3)c1C)c(=O)c(O)c(o2)c1[nH]c(C)nn1	1312910-36-3	4H-1-Benzopyran-4-one, 8-chloro-6-[(4-fluorophenyl)methoxy]-3-hydroxy-7-methyl-2	C19H13ClFN3O4	401.78	A/B	-8.5
5004	Clc1c2c(cc(OCc3ccc(F)cc3)c1C)c(=O)c(O)c(o2)c1[nH]c(C)cn1	1312910-41-0	4H-1-Benzopyran-4-one, 8-chloro-6-[(4-fluorophenyl)methoxy]-3-hydroxy-2-(1H-imic	C20H14ClFN2O4	400.79	A/B	-8.5
5005	Cc1c(c2c(cc1OCc1ccc(F)cc1)c(=O)c(O)c(o2)c1[nH]c(C)nn1)c1ccccc1	1312910-86-3	4H-1-Benzopyran-4-one, 6-[(4-fluorophenyl)methoxy]-3-hydroxy-7-methyl-8-phenyl-2	C25H18FN3O4	443.43	A/B	-8.5
5006	Cc1c(c2c(cc1OCc1ccc(F)cc1)c(=O)c(O)c(o2)c1[nH]c(OC)cn1)c1ccccc1	1312910-92-1	4H-1-Benzopyran-4-one, 6-[(4-fluorophenyl)methoxy]-3-hydroxy-7-methyl-2-(5-methy	C27H21FN2O4	456.46	A/B	-8.5
5007	O=c1c2c(oc(c1O)c1nnc(C)[nH]1)cc(OC)c(OCc1ccc(F)cc1)c2	1312911-36-6	4H-1-Benzopyran-4-one, 6-[(4-fluorophenyl)methoxy]-3-hydroxy-7-methoxy-2-(1H-1,2	C19H14FN3O5	383.33	A/B	-8.5
5008	O=c1c2c(oc(c1O)c1[nH]c(OC)nn1)cc(OC)c(OCc1ccc(F)cc1)c2	1312911-37-7	4H-1-Benzopyran-4-one, 6-[(4-fluorophenyl)methoxy]-3-hydroxy-7-methoxy-2-(3-meth	C20H16FN3O5	397.36	A/B	-8.5
5009	O=c1c2c(oc(c1O)c1ncc(OC)cn1)cc(Cl)c(OCc1ccc(F)cc1)c2	1312911-56-0	4H-1-Benzopyran-4-one, 7-chloro-6-[(4-fluorophenyl)methoxy]-3-hydroxy-2-(5-methy	C21H14ClFN2O4	412.8	A/B	-8.5
5010	O=c1c2c(oc(c1O)c1[nH]c(OC)cn1)cc(Cl)c(OCc1ccc(F)cc1)c2	1312911-86-6	4H-1-Benzopyran-4-one, 7-chloro-6-[(4-fluorophenyl)methoxy]-3-hydroxy-2-(5-methy	C20H14ClFN2O4	400.79	A/B	-8.5
5011	O=c1c2c(oc(c1O)c1[nH]c(cn1)c1ccccc1)cc(Cl)c(OCc1ccc(F)cc1)c2	1312911-90-2	4H-1-Benzopyran-4-one, 7-chloro-2-(5-chloro-1H-imidazol-2-yl)-6-[(4-fluorophenyl	C19H11Cl2FN2O4	421.21	A/B	-8.5
5012	Cc1c2c(cc(OCc3ccc(F)cc3)c1OC)c(=O)c(O)c(o2)c1ncc(cn1)c1ccccc1	1312912-15-4	4H-1-Benzopyran-4-one, 2-(5-chloro-2-pyrimidinyl)-6-[(4-fluorophenyl)methoxy]-3-	C22H16ClFN2O5	442.82	A/B	-8.5
5013	Cc1c2c(cc(OCc3ccc(F)cc3)c1OC)c(=O)c(O)c(o2)c1[nH]c(OC)nn1	1312912-32-5	4H-1-Benzopyran-4-one, 6-[(4-fluorophenyl)methoxy]-3-hydroxy-7-methoxy-8-methyl-	C21H18FN3O5	411.38	A/B	-8.5
5014	Cc1c2c(cc(OCc3ccc(F)cc3)c1OC)c(=O)c(O)c(o2)c1[nH]c(nn1)c1ccccc1	1312912-36-9	4H-1-Benzopyran-4-one, 2-(5-chloro-1H-1,2,4-triazol-3-yl)-6-[(4-fluorophenyl)met	C20H15ClFN3O5	431.8	A/B	-8.5
5015	Cc1c2c(cc(OCc3ccc(F)cc3)c1Cl)c(=O)c(O)c(o2)c1ncc(cn1)c1ccccc1	1312912-61-0	4H-1-Benzopyran-4-one, 7-chloro-2-(5-chloro-2-pyrimidinyl)-6-[(4-fluorophenyl)me	C21H13Cl2FN2O4	447.24	A/B	-8.5
5016	Cc1c2c(cc(OCc3ccc(F)cc3)c1Cl)c(=O)c(O)c(o2)c1[nH]c(C)cn1	1312912-85-8	4H-1-Benzopyran-4-one, 7-chloro-6-[(4-fluorophenyl)methoxy]-3-hydroxy-2-(1H-imic	C20H14ClFN2O4	400.79	A/B	-8.5
5017	Cc1c2c(cc(OCc3ccc(F)cc3)c1Cl)c(=O)c(O)c(o2)c1[nH]c(cn1)c1ccccc1	1312912-91-6	4H-1-Benzopyran-4-one, 7-chloro-2-(5-chloro-1H-imidazol-2-yl)-6-[(4-fluorophenyl	C20H13Cl2FN2O4	435.23	A/B	-8.5
5018	O(C)c1c2c(cc(OCc3ccc(F)cc3)c1Cl)c(=O)c(O)c(o2)c1ncc(OC)cn1	1312913-57-7	4H-1-Benzopyran-4-one, 7-chloro-6-[(4-fluorophenyl)methoxy]-3-hydroxy-8-methoxy-	C22H16ClFN2O5	442.82	A/B	-8.5
5019	Clc1c2c(cc(OCc3ccc(F)cc3)c1OC)c(=O)c(O)c(o2)c1[nH]c(C)nn1	1312914-30-9	4H-1-Benzopyran-4-one, 8-chloro-6-[(4-fluorophenyl)methoxy]-3-hydroxy-7-methoxy-	C19H13ClFN3O5	417.77	A/B	-8.5
5020	Clc1c2c(cc(OCc3ccc(F)cc3)c1OC)c(=O)c(O)c(o2)c1[nH]ccn1	1312914-38-7	4H-1-Benzopyran-4-one, 8-chloro-6-[(4-fluorophenyl)methoxy]-3-hydroxy-7-methoxy-	C25H17ClFN3O5	493.87	A/B	-8.5
5021	Clc1c2c(cc(OCc3ccc(F)cc3)c1OC)c(=O)c(O)c(o2)c1[nH]c(Cl)cn1	1312914-44-5	4H-1-Benzopyran-4-one, 8-chloro-6-[(4-fluorophenyl)methoxy]-3-hydroxy-7-methoxy-	C21H16ClFN2O6	446.81	A/B	-8.5
5022	Clc1c2c(cc(OCc3ccc(F)cc3)c1Cl)c(=O)c(O)c(o2)c1ncc(OC)cn1	1312914-57-0	4H-1-Benzopyran-4-one, 7,8-dichloro-6-[(4-fluorophenyl)methoxy]-3-hydroxy-2-(5-m	C21H13Cl2FN2O4	447.24	A/B	-8.5
5023	Clc1c2c(cc(OCc3ccc(F)cc3)c1Cl)c(=O)c(O)c(o2)c1[nH]c(C)nn1	1312914-75-2	4H-1-Benzopyran-4-one, 7,8-dichloro-6-[(4-fluorophenyl)methoxy]-3-hydroxy-2-(1H-	C18H10Cl2FN3O4	422.19	A/B	-8.5
5024	Clc1c2c(cc(OCc3ccc(F)cc3)c1Cl)c(=O)c(O)c(o2)c1[nH]c(OC)nn1	1312914-77-4	4H-1-Benzopyran-4-one, 7,8-dichloro-6-[(4-fluorophenyl)methoxy]-3-hydroxy-2-(3-m	C19H12Cl2FN3O4	436.22	A/B	-8.5
5025	Clc1c2c(cc(OCc3ccc(F)cc3)c1Cl)c(=O)c(O)c(o2)c1[nH]c(Cl)nn1	1312914-79-6	4H-1-Benzopyran-4-one, 7,8-dichloro-6-[(4-fluorophenyl)methoxy]-3-hydroxy-2-(5-m	C19H12Cl2FN3O5	452.22	A/B	-8.5
5026	Clc1c2c(cc(OCc3ccc(F)cc3)c1Cl)c(=O)c(O)c(o2)c1[nH]c(OC)cn1	1312914-87-6	4H-1-Benzopyran-4-one, 7,8-dichloro-6-[(4-fluorophenyl)methoxy]-3-hydroxy-2-(5-m	C20H13Cl2FN2O4	435.23	A/B	-8.5
5027	Clc1c2c(cc(OCc3ccc(F)cc3)c1Cl)c(=O)c(O)c(o2)c1[nH]c(Cl)cn1	1312914-89-8	4H-1-Benzopyran-4-one, 7,8-dichloro-6-[(4-fluorophenyl)methoxy]-3-hydroxy-2-(5-m	C20H13Cl2FN2O5	451.23	A/B	-8.5
5028	O(C)c1c(c2c(cc1OCc1ccc(F)cc1)c(=O)c(O)c(o2)c1ncc(Cl)cn1)c1ccccc1	1312915-14-2	4H-1-Benzopyran-4-one, 6-[(4-fluorophenyl)methoxy]-3-hydroxy-7-methoxy-2-(5-meth	C28H21FN2O6	500.47	A/B	-8.5
5029	O(C)c1c(c2c(cc1OCc1ccc(F)cc1)c(=O)c(O)c(o2)c1[nH]c(Cl)nn1)c1ccccc1	1312915-39-1	4H-1-Benzopyran-4-one, 6-[(4-fluorophenyl)methoxy]-3-hydroxy-7-methoxy-2-(5-meth	C26H20FN3O6	489.45	A/B	-8.5
5030	O(C)c1c(c2c(cc1OCc1ccc(F)cc1)c(=O)c(O)c(o2)c1[nH]c(nn1)c1ccccc1)c1ccccc1	1312915-41-5	4H-1-Benzopyran-4-one, 2-(5-chloro-1H-1,2,4-triazol-3-yl)-6-[(4-fluorophenyl)met	C25H17ClFN3O5	493.87	A/B	-8.5

5031	O(C)c1c(c2c(cc1OCc1ccc(F)cc1)c(=O)c(O)c(o2)c1[nH]c(C)cn1)c1ccccc1	1312915-45-9	4H-1-Benzopyran-4-one, 6-[(4-fluorophenyl)methoxy]-3-hydroxy-2-(1H-imidazol-2-yl	C26H19FN2O5	458.44	A/B	-8.5
5032	O=c1c2c(cc(c(OCc3ccc(F)cc3)c2)c2ccccc2)oc(c1O)c1[nH]cnn1	1312916-35-0	4H-1-Benzopyran-4-one, 6-[(4-fluorophenyl)methoxy]-3-hydroxy-7-phenyl-2-(5-pheny	C32H21FN2O4	516.52	A/B	-8.5
5033	O(Cc1ccc(F)cc1)c1c(cc2c(c1)c(=O)c(O)c(o2)c1[nH]c(C)nn1)c1ccccc1	1312916-41-8	4H-1-Benzopyran-4-one, 6-[(4-fluorophenyl)methoxy]-3-hydroxy-7-phenyl-2-(1H-1,2,	C24H16FN3O4	429.4	A/B	-8.5
5034	O(Cc1ccc(F)cc1)c1c(cc2c(c1)c(=O)c(O)c(o2)c1[nH]c(C)cn1)c1ccccc1	1312916-46-3	4H-1-Benzopyran-4-one, 6-[(4-fluorophenyl)methoxy]-3-hydroxy-2-(1H-imidazol-2-yl	C25H17FN2O4	428.41	A/B	-8.5
5035	Cc1c2c(cc(OCc3ccc(F)cc3)c1c1ccccc1)c(=O)c(O)c(o2)c1ncc(C)cn1	1312916-82-7	4H-1-Benzopyran-4-one, 6-[(4-fluorophenyl)methoxy]-3-hydroxy-8-methyl-7-phenyl-2	C27H19FN2O4	454.45	A/B	-8.5
5036	O(C)c1c(c(OCc2ccc(F)cc2)cc2c1oc(c(O)c2=O)c1ncc(C)cn1)c1ccccc1	1312917-32-0	4H-1-Benzopyran-4-one, 6-[(4-fluorophenyl)methoxy]-3-hydroxy-8-methoxy-7-phenyl-	C27H19FN2O5	470.45	A/B	-8.5
5037	O(C)c1c(c(OCc2ccc(F)cc2)cc2c1oc(c(O)c2=O)c1[nH]c(C)nn1)c1ccccc1	1312917-42-2	4H-1-Benzopyran-4-one, 6-[(4-fluorophenyl)methoxy]-3-hydroxy-8-methoxy-7-phenyl-	C25H18FN3O5	459.43	A/B	-8.5
5038	O(C)c1c2c(cc(OCc3ccc(F)cc3)c1c1ccccc1)c(=O)c(O)c(o2)c1[nH]c(OC)nn1	1312917-43-3	4H-1-Benzopyran-4-one, 6-[(4-fluorophenyl)methoxy]-3-hydroxy-8-methoxy-2-(3-meth	C26H20FN3O5	473.45	A/B	-8.5
5039	O(C)c1c(c(OCc2ccc(F)cc2)cc2c1oc(c(O)c2=O)c1[nH]c(nn1)c1ccccc1)c1ccccc1	1312917-45-5	4H-1-Benzopyran-4-one, 2-(5-chloro-1H-1,2,4-triazol-3-yl)-6-[(4-fluorophenyl)met	C25H17ClFN3O5	493.87	A/B	-8.5
5040	O(C)c1c2c(cc(OCc3ccc(F)cc3)c1c1ccccc1)c(=O)c(O)c(o2)c1[nH]c(OC)cn1	1312917-48-8	4H-1-Benzopyran-4-one, 6-[(4-fluorophenyl)methoxy]-3-hydroxy-8-methoxy-2-(5-meth	C27H21FN2O5	472.46	A/B	-8.5
5041	O(Cc1ccc(F)cc1)c1c(c(c2c(c1)c(=O)c(O)c(o2)c1[nH]c(Cl)cn1)c1ccccc1)c1ccccc1	1312918-49-2	4H-1-Benzopyran-4-one, 6-[(4-fluorophenyl)methoxy]-3-hydroxy-2-(5-methoxy-1H-imi	C32H23FN2O5	534.53	A/B	-8.5
5042	C(=O)([C@@H](NC(=O)OC)C(C)C)N1[C@@H]([C@@H]2C[C@H]1CC2)c1[nH]c(cn1)c1ccc(cc1)c1ccc(cc1)c1[nH]c(nc1)[	1321849-44-8	Carbamic acid, N,N,Ä≤-[[1,1,Ä≤-biphenyl]-4,4,Ä≤-diylbis[1H-imidazole-5,2-diyl-(	C46H58N8O6	819	A/B	-8.5
5043	O=c1n([C@H]2O[C@@H](C(=O)O[C@H]3[C@H](C(C)C)CC[C@@H](C)C3)SC2)cc(F)c(NC(=O)C)n1	1321849-66-4	Carbamic acid, N-[(1S)-2-[(1R,3S,4S)-3-[5-[4,Ä≤-[2-[(1R,3S,4S)-2-[(2S)-2-[(meth	C43H52N8O6	776.92	A/B	-8.5
5044	O=c1c2c(n(cn2)[C@@H]2C(=C)[C@H](COCOC)[C@@H](OCOC)C2)[nH]c(N)n1	1328880-85-8	Guanosine, 8-[[(6-methyl-2-oxo-2H-1-benzopyran-3-yl)methyl]thio]-	C21H21N5O7S	487.49	A/B	-8.5
5045	N(c1c(C([2H])([2H])[2H])cc(/C=C/C#N)cc1C([2H])([2H])[2H])c1nc(Nc2cc(c(C#N)c(c2)[2H])[2H])ncc1	1331866-98-8	Benzonitrile-2,6-d2, 4-[[4-[[4-[(1E)-2-cyanoethenyl]-2-methyl-6-(methyl-d3)pheny	C22H13D5N6		A/B	-8.5
5046	O=c1n([C@@H]2O[C@H](COP(=O)(N[C@@H](Cc3ccc(O)cc3)C(=O)OC)Oc3ccc(N(=O)=O)cc3)C=C2)cc(C)c(=O)[nH]1	1333491-88-5	Benzeneacetic acid, Œ±-[[[[(2S,5R)-5-(3,4-dihydro-5-methyl-2,4-dioxo-1(2H)-pyrim	C26H27N4O10P	586.49	A/B	-8.5
5047	O=c1n([C@@H]2O[C@H](CO[P@@](=O)(Oc3ccc(C)cc3)N[C@H](C(=O)OCc3ccccc3)C)C=C2)cc(C)c(=O)[nH]1	1334335-89-5	L-Alanine, N-[[P(S)]-2,Ä≤,3,Ä≤-didehydro-3,Ä≤-deoxy-P-(4-methylphenyl)-5,Ä≤-thy	C27H30N3O8P	555.52	A/B	-8.5
5048	N(c1c2c(n(cn2)[C@@H]2C[C@H](COC(=O)C(CCSSCCC(C(=O)OC[C@H]3C[C@H](C=C3)n3c4c(nc3)c(NC3CC3)nc(N)n4)C)C	1334335-90-8	L-Alanine, N-[[P(R)]-2,Ä≤,3,Ä≤-didehydro-3,Ä≤-deoxy-P-(4-methylphenyl)-5,Ä≤-thy	C27H30N3O8P	555.52	A/B	-8.5
5049	O=C1[C@@H]2[C@@H](c3c([C@H](OC(=O)[C@H](CSSC[C@@H](C(=O)NCC(=O)N[C@@H]4c5c(-c6c(CC4)cc(OC)c(OC)c6OC)	1335096-09-7	Inosine, 8-[[4-(2-benzothiazolyl)phenyl]amino]-	C23H20N6O5S	492.51	A/B	-8.5
5050	C(F)(F)(F)c1n(c2c(n1)cccc2)c1nc(nc(n1)N1CCOCC1)N1CCOCC1	133656-54-9	Thymidine, 3,Ä≤-azido-3,Ä≤-deoxy-P-[4-(methylsulfonyl)phenyl]thymidylyl-(5,Ä≤,Ü	C27H31N10O12PS		A/B	-8.5
5051	N(C(=O)C)[C@H]1[C@H](OC(CC)CC)C=C(C(=O)OC(OC(=O)[C@@H](C(C)C)N)c2ccccc2)C[C@@H]1NC(=N)N	1338231-81-4	2H-Isoindole-2-acetic acid, 1,3-dihydro-Œ±-(1-methylethyl)-1,3-dioxo-, [[[(3R,4R	C45H59N5O12	861.98	A/B	-8.5
5052	N(C(=O)C)[C@@H]1[C@@H](N=C(NC(=O)OC(C)(C)C)NC(=O)OC(C)(C)C)CC(=C[C@H]1OC(CC)CC)C(=O)OC(C(C)C)Br	1338231-94-9	2H-Isoindole-2-acetic acid, 1,3-dihydro-Œ±-(1-methylethyl)-5-nitro-1,3-dioxo-, 1	C42H60N6O14	872.96	A/B	-8.5
5053	C(=O)(N[C@H](C(=O)C(=O)NC1CC1)CCC)[C@@H]1[C@@H]2[C@H](CN1C(=O)[C@@H](NC(=O)[C@@H](NC(=O)c1cnccn1)C1C	1346928-08-2	Not Yet Assigned	C40H55N4O12PS	846.92	A/B	-8.5
5054	[C@@H]([C@H](Cc1ccccc1)NC(=O)COc1c(C)cccc1C)(C[C@@H](NC(=O)[C@H](C(C)(C)C)N1C(=O)NCCC1)Cc1ccccc1)OCC	1347056-95-4	2,7,10,12-Tetraazatridecanoic acid, 13-[2-(2E)-2-buten-1-yl-4-thiazolyl]-4-hydro	C38H48N6O5S2	732.95	A/B	-8.5
5055	C(n1c2c(nc1CC)c(C)cc(N(C)C)n2)c1ccc(cc1)c1c(S(=O)(=O)NC(=O)OCC)ccc(CC)c1	1347110-16-0	Carbamic acid, N-[[5-ethoxy-4,Ä≤-[(2-ethyl-5,7-dimethyl-3H-imidazo[4,5-b]pyridin	C33H34N4O5S	598.71	A/B	-8.5
5056	[C@H](Cc1ccccc1)(C[C@@H]([C@H](Cc1ccccc1)NC(=O)OCc1cncs1)O)N(C(=O)[C@@H](NC(=O)NCc1nc(C(C)C)sc1)C(C)	1347117-89-8	Carbamic acid, N-[[4,Ä≤-[[5-(dimethylamino)-2-ethyl-7-methyl-3H-imidazo[4,5-b]py	C29H35N5O4S	549.68	A/B	-8.5
5057	C(N(C(=O)N[C@H](C(=O)N[C@@H](Cc1ccccc1)C[C@@H]([C@H](Cc1ccccc1)NC(=O)OCc1cncs1)O)C(C)C)C)c1nc(sc1)C1	1348088-23-2	Benzamide, 4-chloro-N-[[4,Ä≤-[(2-ethyl-5,7-dimethyl-3H-imidazo[4,5-b]pyridin-3-y	C30H27ClN4O3S	559.08	A/B	-8.5
5058	C(OCCO)N1c2c(N(C)C1)c(=O)nc(N)[nH]2	1348102-87-3	2,7,10,12-Tetraazatridecanoic acid, 13-[2-(3-cyclohexen-1-yl)-4-thiazolyl]-4-hyd	C40H50N6O5S2	758.99	A/B	-8.5
5059	C(C(OCP(=O)(O)O)C)n1c2c(nc1N)c(N)nc(N)n2	1348833-85-1	Carbamic acid, N-[[4,Ä≤-[(2-ethyl-5,7-dimethyl-3H-imidazo[4,5-b]pyridin-3-yl)met	C31H37FN4O4S	580.71	A/B	-8.5
5060	C(OCCOC(=O)CCCOC(=O)[C@H](C(C)C)N)n1c2c(nc1)c(=O)nc(N)[nH]2	1349133-63-6	Not Yet Assigned	C30H37D6F2N5O		A/B	-8.5
5061	C(=O)(N[C@@H](C(=O)C(=O)NCC1CCCCC1)CCC)[C@@H]1[C@@H]2[C@@H](CN1C(=O)[C@@H](NC(=O)[C@@H](NC(=O)c1cncc	1349390-22-2	2,7,10,12-Tetraazatridecanoic acid, 4-hydroxy-12-methyl-9-(1-methylethyl)-8,11-d	C39H51N7O6S	745.93	A/B	-8.5
5062	C(n1c2c(nc1CC)c(C)cc(C)n2)c1ccc(cc1)c1c(S(=O)(=O)NC(=O)OCc2ccccc2)ccc(CCCC)c1	1350008-11-5	Benzamide, 2-(2-aminoethyl)-N-[[4,Ä≤-[(2-ethyl-5,7-dimethyl-3H-imidazo[4,5-b]py	C32H33N5O3S	567.7	A/B	-8.5
5063	Cc1cc2c(ccc(c2[nH]1)c1[nH]c(nc1)[C@H]1N(C(=O)[C@@H](NC(=O)OC)C(C)C)CCC1)c1c2c(c(cc1)c1[nH]c(nc1)[C@H	1350718-41-0	Carbamic acid, N,N,Ä≤-[[4,7,Ä≤-bi-1H-indole]-4,Ä≤,7-diylbis[1H-imidazole-5,2-di	C44H52N10O6	816.95	A/B	-8.5
5064	C(=O)([C@@H](NC(=O)OC)C(C)C)N1[C@H](c2[nH]c(c3c4c(c(cc3)c3c5c(c(cc3)c3[nH]c(nc3)[C@H]3N(C(=O)[C@@H](	1350718-46-5	Carbamic acid, N,N,Ä≤-[(2,2,Ä≤-dimethyl[4,7,Ä≤-bibenzo[b]thiophene]-4,Ä≤,7-diyl	C46H54N8O6S2	879.1	A/B	-8.5
5065	C(=O)([C@@H](NC(=O)OC)C(C)C)N1[C@H](c2[nH]c(c3c4c(c(cc3)c3c5c(c(cc3)c3[nH]c(nc3)[C@H]3N(C(=O)[C@@H](	1350719-38-8	Carbamic acid, N,N,Ä≤-[[5,5,Ä≤-biquinoline]-8,8,Ä≤-diylbis[1H-imidazole-5,2-diy	C46H52N10O6	840.97	A/B	-8.5
5066	C(=O)([C@@H](NC(=O)OC)[C@@H](CC)C)N1[C@@H](CCC1)c1[nH]c(cn1)c1cc2c(cc1)cc(cc2)c1ccc(cc1)c1[nH]c(nc1)	1351330-62-5	Carbamic acid, N-[(1R)-2-[(2S)-2-[5-[6-[4-[2-[(2S)-1-[(2S)-2-[(methoxycarbonyl)a	C48H52N8O6	836.98	A/B	-8.5
5067	C(=O)([C@@H](NC(=O)OC)C(C)C)N1[C@@H](CCC1)c1[nH]c(cn1)c1ccc(cc1)c1cc2c(cc1)cc(cc2)c1[nH]c([C@@H](NC(	1351331-61-7	Carbamic acid, N-[(1S)-1-cyclopentyl-2-[(2S)-2-[5-[4-[6-[2-[(2S)-1-[(2S)-2-cyclo	C48H56N8O6	841.01	A/B	-8.5
5068	C(=O)([C@@H](NC(=O)OC)C(C)C)N1C[C@@]2(C[C@H]1c1[nH]c(cn1)c1ccc(cc1)c1ccc(cc1)c1[nH]c(nc1)[C@H]1N(C(=	1351852-65-7	Carbamic acid, N-[(1S)-1-[[(5S,8S)-8-[5-[4,Ä≤-[2-[(2S,5S)-1-[(2S)-2-[(methoxyca	C44H56N8O7	808.96	A/B	-8.5
5069	C(=O)([C@@H](NC(=O)OC)C(C)C)N1C[C@@]2(C[C@H]1c1[nH]c(cn1)c1c(C)c(C)c(cc1)c1c(C)c(C)c(cc1)c1[nH]c(nc1	1351852-83-9	Carbamic acid, N-[(1S)-1-[[(2S)-2-[5-[4,Ä≤-[2-[(5S,8S)-7-[(2S)-2-[(methoxycarbo	C45H58N8O7	822.99	A/B	-8.5
5070	C(=O)([C@@H](NC(=O)OC)C(C)C)N1C[C@@]2(C[C@H]1c1[nH]c(cn1)c1c(Cl)cc(cc1)c1cc(Cl)c(cc1)c1[nH]c(nc1)[C@	1351852-86-2	Carbamic acid, N-[(1S)-1-[[(2S)-2-[5-[4,Ä≤-[2-[(5S,8S)-7-[(2S)-2-[(methoxycarbo	C45H52F6N8O7	930.93	A/B	-8.5
5071	C(=O)([C@@H](NC(=O)OC)C(C)C)N1[C@@H](C[C@@]2(C1)CCCO2)c1[nH]c(cn1)c1ccc(cc1)c1ccc(cc1)c1[nH]c(nc1)[C	1351852-93-1	Carbamic acid, N,N,Ä≤-[[1,1,Ä≤-biphenyl]-4,4,Ä≤-diylbis[1H-imidazole-5,2-diyl-(	C46H58N8O8	851	A/B	-8.5
5072	FC1(F)[C@@]2(C[C@H](N(C(=O)[C@@H](NC(=O)OC)C(C)C)C2)c2[nH]c(cn2)c2ccc(cc2)c2ccc(cc2)c2[nH]c(nc2)[C@H	1351852-99-7	Carbamic acid, N-[(1S)-1-[[(5S,8S)-8-[5-[4,Ä≤-[2-[(1S,2S,5R)-3-[(2S)-2-[(methoxy	C44H54N8O7	806.95	A/B	-8.5
5073	FC1(F)[C@@]2(C[C@H](N(C(=O)[C@@H](NC(=O)OC)C(C)C)C2)c2[nH]c(cn2)c2ccc(cc2)c2ccc(cc2)c2[nH]c(nc2)[C@H	1351853-18-3	Carbamic acid, N-[(1S)-1-[[(2S)-2-[5-[4,Ä≤-[2-[(3S,5S)-2-[(2S)-2-[(methoxycarbo	C44H54N8O7	806.95	A/B	-8.5
5074	C(=O)([C@@H](NC(=O)OC)C(C)C)N1[C@@H](C=C(C)C1)c1[nH]c(cn1)c1cc2c(cc1)cc(C#Cc1[nH]c(nc1)[C@H]1N(C(=O)	1352391-96-8	Carbamic acid, N-[(1S)-1-[[(2S)-2-[5-[4-[6-[2-[(2S)-2,5-dihydro-1-[(2S)-2-[(meth	C46H52N8O6	812.96	A/B	-8.5
5075	C(=O)([C@@H](NC(=O)OC)C(C)C)N1[C@@H](CCC1)c1[nH]c(cn1)c1ccc(C#Cc2ccc(cc2)c2[nH]c(nc2)[C@@H]2C=C(C)CN	1352392-65-4	Carbamic acid, N-[(1S)-1-[[(2S)-4-cyclopropyl-2-[5-[4-[6-[2-[(2S)-2,5-dihydro-1-	C48H54N8O6	838.99	A/B	-8.5
5076	O=c1n(cc(C)c(=O)n1C(=O)OCc1ccccc1)[C@@H]1O[C@H](CO)C=C1	1353369-73-9	1(2H)-Pyrimidinecarboxylic acid, 3-(2-deoxy-Œ≤-L-erythro-pentofuranosyl)-3,6-dih	C19H19F3N2O7	444.36	A/B	-8.5
5077	O=c1n(cc(I)c(=O)n1C(=O)OC1CCCC1)[C@@H]1O[C@H](CO)[C@@H](O)C1	1353386-83-0	Thymidine, 3-[(cyclopentyloxy)carbonyl]-Œ±,Œ±,Œ±-trifluoro-	C16H19F3N2O7	408.33	A/B	-8.5
5078	O[C@H]1[C@@H](O[C@H](COP(=O)(N[C@@H](Cc2ccc(O)cc2)C(=O)OC)Oc2ccc(N(=O)=O)cc2)[C@H]1O)n1nc(C(=O)N)nc1	1353639-69-6	Not Yet Assigned	C24H27N6O12P	622.48	A/B	-8.5
5079	O[C@H]1[C@@H](O[C@H](COP(=O)(N[C@@H](Cc2cnc[nH]2)C(=O)OC)Oc2ccc(N(=O)=O)cc2)[C@H]1O)n1nc(C(=O)N)nc1	1353639-72-1	L-Phenylalanine, N-[hydroxy(4-nitrophenoxy)phosphinyl]-, methyl ester, 5,Ä≤-este	C24H27N6O11P	606.48	A/B	-8.5
5080	OC1[C@@H](O[C@H](CO)[C@@H]1O)n1nc(C(=O)N)nc1	1355346-33-6	5,Ä≤-Inosinic acid, 2,Ä≤,3,Ä≤-didehydro-2,Ä≤,3,Ä≤-dideoxy-, 5,Ä≤-ester with 1-(	C18H21N8O10P	540.38	A/B	-8.5
5081	S(=O)(=O)(O)c1c2c(c(N)c(N=Nc3ccc(cc3)c3ccc(C)cc3)c1)cccc2	1357193-89-5	1-Naphthalenesulfonic acid, 5-[2-(4-amino-6-sulfo-1-naphthalenyl)diazenyl]-	C20H15N3O6S2	457.48	A/B	-8.5
5082	C(=O)([C@@H](NC(=O)OC)C(C)C)N1[C@H]2[C@H](C2)C[C@H]1c1[nH]c(cn1)c1c2c(c(cc1)c1ccc(cc1)c1[nH]c(nc1)[C	1359868-84-0	Carbamic acid, N-[(1S)-2-[(2S)-2-[5-[4-[4-[2-[(2S)-1-[(2S)-2-[(methoxycarbonyl)a	C48H56N8O8	873.01	A/B	-8.5
5083	C(=O)([C@@H](NC(=O)OC)C1CCOCC1)N1[C@H](c2[nH]c(c3c4c(c(cc3)c3ccc(cc3)c3[nH]c(nc3)[C@H]3N(C(=O)[C@@H]	1359868-86-2	Carbamic acid, N-[(1S)-2-[(1R,3S,5R)-3-[5-[4-[2,3-dihydro-7-[2-[(1R,3S,5R)-2-[(2	C49H58N8O8	887.03	A/B	-8.5
5084	C(F)(F)c1n(c2c(n1)cccc2)c1nc(nc(N[C@@H]2CC[C@@H](O)CC2)n1)N1CCOCC1	1359953-57-3	1,3,5-Triazin-2-amine, 4-[2-(difluoromethyl)-1H-benzimidazol-1-yl]-6-(4-morpholi	C25H29F2N7O	481.54	A/B	-8.5
5085	C(F)(F)c1n(c2c(n1)cccc2)c1nc(nc(NCC2(O)CCN(C)CC2)n1)N1CCOCC1	1359955-65-9	1,3,5-Triazin-2-amine, 4-[2-(difluoromethyl)-1H-benzimidazol-1-yl]-N-[(1-methyl-	C22H28F2N8O	458.51	A/B	-8.5
5086	C(F)(F)c1n(c2c(n1)cccc2)c1nc(nc(NCCN2CCCCC2)n1)N1CCOCC1	1359955-85-3	1,3,5-Triazin-2-amine, 4-[2-(difluoromethyl)-1H-benzimidazol-1-yl]-6-(4-morpholi	C21H25F2N7O2	445.47	A/B	-8.5
5087	C(F)(F)c1n(c2c(n1)cccc2)c1nc(nc(NCCN2CCC(O)CC2)n1)N1CCOCC1	1359955-91-1	Ethanone, 2-[[4-[2-(difluoromethyl)-1H-benzimidazol-1-yl]-6-(4-morpholinyl)-1,3,	C22H26F2N8O2	472.49	A/B	-8.5
5088	C(F)(F)c1n(c2c(n1)cccc2)c1nc(nc(N(CCCO)C)n1)N1CCOCC1	1359956-03-8	1,3,5-Triazin-2-amine, 4-[2-(difluoromethyl)-1H-benzimidazol-1-yl]-N-[[4-(dimeth	C24H26F2N8O	480.51	A/B	-8.5
5089	C(F)(F)c1n(c2c(n1)cccc2)c1nc(nc(Nc2ccc(cc2)N2CCCCC2)n1)N1CCOCC1	1359956-25-4	1,4-Benzenediamine, N4-[4-[2-(difluoromethyl)-1H-benzimidazol-1-yl]-6-(4-morphol	C23H24F2N8O	466.49	A/B	-8.5
5090	N(c1nc(nc(Nc2ccc(Cl)cc2)n1)N1CCOCC1)c1ccc(N(=O)=O)cc1	1360627-48-0	1,3,5-Triazine-2,4-diamine, 6-(4-morpholinyl)-N2-(4-nitrophenyl)-N4-phenyl-	C19H19N7O3	393.4	A/B	-8.5
5091	C(=O)([C@@H](NC(=O)OC)C(C)C)N1[C@@H](C[C@H](C(F)(F)F)C1)c1[nH]c(cn1)c1ccc(cc1)c1ccc(cc1)c1[nH]c(nc1C	1361249-41-3	Carbamic acid, N,N,Ä≤-[[1,1,Ä≤-biphenyl]-4,4,Ä≤-diylbis[(5-chloro-1H-imidazole-	C42H46Cl2F6N8O6	943.76	A/B	-8.5
5092	C(=O)([C@@H](NC(=O)OC)C(C)C)N1[C@@H](C[C@H](SC)C1)c1[nH]c(cn1)c1ccc(cc1)c1ccc(cc1)c1[nH]c(nc1Br)[C@H	1373165-57-1	Carbamic acid, N-[(1S)-1-[[(2S)-2-[5-[4,Ä≤-[5-fluoro-2-[(2S)-1-[(2S)-2-[(methoxy	C40H49FN8O6	756.87	A/B	-8.5
5093	C(=O)([C@@H](NC(=O)OC)C(C)C)N1[C@H](c2[nH]c(c3cc4c(c5c(c6c4cccc6)cc(cc5)c4[nH]c(nc4)[C@H]4N(C(=O)[C@	1377035-64-7	Carbamic acid, N,N,Ä≤-[(4,5,9,10-tetrahydro-2,7-pyrenediyl)bis[1H-imidazole-5,2-	C44H54N8O6	790.95	A/B	-8.5
5094	C(=O)([C@@H](NC(=O)OC)C(C)C)N1[C@@H]([C@@H]2C[C@H]1CC2)c1[nH]c(cn1)c1ccc(cc1)c1cc2c(cc1)c1c(cc2)nc([	1377035-72-7	Carbamic acid, N,N,Ä≤-[(4-oxo-4H-cyclopenta[def]phenanthrene-2,6-diyl)bis[1H-imi	C43H48N8O7	788.89	A/B	-8.5
5095	C(=O)([C@@H](NC(=O)OC)C1CCOCC1)N1[C@@H]([C@@H]2C[C@H]1CC2)c1[nH]c(cn1)c1ccc(cc1)c1cc2c(cc1)c1c(cc2)n	1377036-48-0	Carbamic acid, N-[(1S)-2-[(1R,3S,4S)-3-[5-[4,Ä≤-[2-[(2S)-1-[(2S)-2-[(methoxycarb	C44H54N8O7	806.95	A/B	-8.5
5096	C(=O)([C@@H](NC(=O)OC)C(C)C)N1[C@@H](Cc2c1cccc2)c1[nH]c(cn1)c1ccc(cc1)c1ccc(cc1)c1[nH]c(nc1)[C@H]1N(	1377040-17-9	Carbamic acid, N,N,Ä≤-[[9-(1-methylethylidene)-9H-fluorene-2,7-diyl]bis[1H-imida	C44H54N8O6	790.95	A/B	-8.5
5097	C(=O)([C@@H](NC(=O)OC)C(C)C)N1[C@H](c2[nH]c(c3cc4c(c5c(cc(cc5)c5[nH]c(nc5)[C@H]5N(C(=O)[C@@H](NC(=O)	1377040-67-9	Carbamic acid, N,N,Ä≤-[(9-cyano-2,7-phenanthrenediyl)bis[1H-imidazole-5,2-diyl-	C43H49N9O6	787.91	A/B	-8.5
5098	C(=O)([C@@H](NC(=O)OC)C(C)C)N1[C@H]2[C@H](C2)C[C@H]1c1[nH]c2c(n1)ccc1c2ccc(c1)c1ccc(cc1)c1[nH]c(nc1)	1377041-78-5	Carbamic acid, N-[(1S)-1-[[(2S)-2-[5-[4,Ä≤-[2-[(1R,3S,4S)-2-[(2S)-2-[(methoxyca	C45H54N8O6	802.96	A/B	-8.5
5099	C(=O)([C@@H](NC(=O)OC)C(C)C)N1[C@H](c2[nH]c3c4c(cc(cc4)c4c(C#CC5CC5)cc(cc4)c4[nH]c(nc4)[C@H]4N(C(=O)	1377042-38-0	Carbamic acid, N-[(1R)-2-[(2S)-2-[5-[4,Ä≤-[2-[(2S)-1-[(2S)-2-[(methoxycarbonyl)a	C46H50N8O6	810.94	A/B	-8.5
5100	C(=O)([C@@H](NC(=O)OC)C(C)C)N1[C@@H]([C@@H]2C[C@H]1CC2)c1[nH]c2c(n1)ccc1c2ccc(c1)c1ccc(cc1)c1[nH]c(n	1377043-30-5	Carbamic acid, N-[(1S)-1-[[(2S)-2-[7-[4-[5-chloro-2-[(1R,3S,5R)-2-[(2S)-2-[(meth	C43H49ClN8O6	809.35	A/B	-8.5
5101	N(C(=O)N[C@H]1CC[C@@H](OCC=C)CC1)C12CC3CC(C2)CC(C1)C3	1378287-04-7	Urea, N-[4-(2-oxo-2-phenylethyl)phenyl]-N,Ä≤-tricyclo[3.3.1.13,7]dec-1-yl-	C25H28N2O2	388.5	A/B	-8.5
5102	C(OCC[Si](C)(C)C)n1c2c(nc1)c(=O)nc(Cl)[nH]2	138152-16-6	Benzenesulfonic acid, 4-[2-(4-hydroxy-1-naphthalenyl)diazenyl]-, ion(2-)	C16H10N2O4S		A/B	-8.5
5103	[C@@H](Cc1ccccc1)(NC(=O)[C@H](CCN1CCOCC1)NC(=O)N(C)CC[C@H](Cc1ccccc1)NC(=O)OCc1cncs1	1383569-80-9	Carbamic acid, N-[(1R,4R)-4-[[(2S)-2-[[(4-methyl-1-piperazinyl)carbonyl]amino]-4	C37H51N7O5S	705.91	A/B	-8.5
5104	[C@H](Cc1ccccc1)(CC[C@H](Cc1ccccc1)NC(=O)OCc1cnco1)NC(=O)[C@@H](NC(=O)N(Cc1nc(C(C)C)sc1)C)C(C)C	1383570-17-9	2,7,10,12-Tetraazatridecanoic acid, 12-methyl-9-(1-methylethyl)-13-[2-(1-methyle	C39H50N6O4S	698.92	A/B	-8.5
5105	C(=O)(N(Cc1nc(C(C)C)sc1)C)N1[C@H](C(=O)N[C@@H](Cc2ccccc2)CC[C@H](Cc2ccccc2)NC(=O)OCc2cncs2)C[C@@H](C	1383620-51-6	Carbamic acid, N-[(1R,4R)-4-[[[(4R)-3-[[methyl[[2-(1-methylethyl)-4-thiazolyl]me	C36H44N6O4S3	720.97	A/B	-8.5
5106	O[C@H]1[C@H](n2c3c(nc2)c(=O)nc(N)[nH]3)O[C@H](COP(=O)(O[C@@H]2[C@@H](COP(=O)(O)O)O[C@H](C2)n2c3c(nc2	1383782-59-9	2-Propen-1-one, 1,1,Ä≤-(4,6-dimethoxy-1,3-phenylene)bis[3-(3-hydroxyphenyl)-	C26H22O6	430.45	A/B	-8.5
5107	C(O)[C@H]1[C@H](n2c3c(nc2[3H])c(=O)nc(N)[nH]3)C[C@@H]1CO	1388224-22-3	Carbamic acid, N,N,Ä≤-[[1,1,Ä≤-biphenyl]-4,4,Ä≤-diylbis[(5-fluoro-1H-imidazole-	C40H48F2N8O6	774.86	A/B	-8.5
5108	N([C@@H](Cc1ccccc1)C(=O)O)c1nc(nc(n1)N1CCOCC1)c1cc(O)ccc1	1391924-72-3	2(1H)-Pyridinone, 5-[4-(4-morpholinyl)-6-(1-piperazinyl)-1,3,5-triazin-2-yl]-	C16H21N7O2	343.38	A/B	-8.5
5109	N(CC(=O)O)c1nc(nc(n1)N1CCOCC1)c1ccc(=O)[nH]c1	1391925-40-8	L-Valine, N-[4-(3-hydroxyphenyl)-6-(4-morpholinyl)-1,3,5-triazin-2-yl]-	C18H23N5O4	373.41	A/B	-8.5
5110	N(C(C(=O)O)(C)C)c1nc(nc(n1)N1CCOCC1)c1cc(O)ccc1	1391925-52-2	L-Alanine, N-[4-(3-hydroxyphenyl)-6-(4-morpholinyl)-1,3,5-triazin-2-yl]-	C16H19N5O4	345.35	A/B	-8.5
5111	N(c1nc(nc(n1)N1CCOCC1)c1sc(N)nc1)c1ccc(C(=O)N(C)C)cc1	1391926-02-5	Glycine, N-[4-(1H-indazol-4-yl)-6-(4-morpholinyl)-1,3,5-triazin-2-yl]-	C16H17N7O3	355.35	A/B	-8.5
5112	O=C1[C@@H]2[C@H](c3c([C@@H](OCc4ccccc4)[C@H]2CO1)cc1c(c3)OCO1)c1cc(OC)c(OC)c(OC)c1	1392513-42-6	Thymidine, 3,Ä≤-azido-5,Ä≤-O-[(3R,5aS,6R,8aS,9R,10S,12R,12aR)-decahydro-3,6,9-t	C25H35N5O8		A/B	-8.5
5113	N(C(=N)Nc1c(F)cc(F)cc1)C12CC3CC(C2)CC(C1)C3	139291-67-1	Guanidine, N-tricyclo[3.3.1.13,7]dec-1-yl-N,Ä≤-[2-(trifluoromethyl)phenyl]-	C18H22F3N3	337.38	A/B	-8.5
5114	C(=C\1/c2c(NC1=O)ccc(S(=O)(=O)N(C[C@H]([C@H](Cc1ccccc1)NC(=O)O[C@@H]1[C@H]3[C@@H](OC1)OCC3)O)CC(C)C)	1393922-47-8	Benzamide, N-[2-[[4-[(4-methoxyphenyl)amino]-6-(4-morpholinyl)-1,3,5-triazin-2-y	C32H35N7O7	629.66	A/B	-8.5
5115	C(OC[C@@H]1[C@@H](OC(=O)C(C)C)[C@@H](OC(=O)C(C)C)[C@@H](O1)n1nc(C(=O)NC(=O)C(C)C)nc1)(c1ccc(OC)cc1)(	139621-26-4	1H-1,2,4-Triazole-3-carboxamide, 1-(2,3,5-tri-O-benzoyl-Œ≤-D-ribofuranosyl)-	C29H24N4O8	556.52	A/B	-8.5
5116	[C@@H](COCP(=O)(OCOC(=O)OC(C)C)OCOC(=O)OC(C)C)(C)n1c2c(nc1)c(N)ncn2	1397776-19-0	Thymidine, thymidylyl-(3,Ä≤,Ü5,Ä≤)-Œ±-hydroxy-	C20H27N4O13P	562.42	A/B	-8.5
5117	N(c1c(C)cc(/C=C/C#N)cc1C)c1nc(Nc2ccc(C#N)cc2)ncc1.Br	1399384-89-4	Benzonitrile, 4-[[4-[[4-[(1E)-2-cyanoethenyl]-2,6-dimethylphenyl]amino]-2-pyrimi	C22H18N6.H3O4P		A/B	-8.5
5118	N(c1c(C)cc(/C=C/C#N)cc1C)c1nc(Nc2ccc(C#N)cc2)ncc1.S(=O)(=O)(O)c1ccc(C)cc1	1399384-90-7	Benzonitrile, 4-[[4-[[4-[(1E)-2-cyanoethenyl]-2,6-dimethylphenyl]amino]-2-pyrimi	C22H18N6.BrH		A/B	-8.5
5119	O=C1c2n(C([C@H]3N1[C@](C)(CCO3)[2H])([2H])[2H])cc(C(=O)NC(c1c(F)cc(F)cc1)([2H])[2H])c(=O)c2O	1407167-03-6	2H-Pyrido[1,Ä≤,2,Ä≤:4,5]pyrazino[2,1-b][1,3]oxazine-12-d-9-carboxamide, N-[(2,4	C20H12D7F2N3O5		A/B	-8.5
5120	O(C(=O)C(C)(C)C)[C@H]1[C@@H](O[C@H](COC(=O)c2ccccc2)[C@H]1OC(=O)c1cccnc1)n1nc(C(=O)N)nc1	140891-33-4	1H-1,2,4-Triazole-3-carboxamide, 1-[5-O-benzoyl-3-O-(2,2-dimethyl-1-oxopropyl)-2	C26H27N5O8	537.52	A/B	-8.5
5121	O(C(=O)c1cccnc1)[C@H]1[C@@H](O[C@H](COC(=O)c2ccccc2)[C@H]1OC(=O)c1ccccc1)n1nc(C(=O)N)nc1	140891-43-6	1H-1,2,4-Triazole-3-carboxamide, 1-[2,5-di-O-benzoyl-3-O-(3-pyridinylcarbonyl)-Œ	C28H23N5O8	557.51	A/B	-8.5
5122	N(=Nc1cc(S(=O)(=O)O)c(C=Cc2c(S(=O)(=O)O)cc(N=Nc3c4c(cc(S(=O)(=O)O)c3N)cc(S(=O)(=O)O)cc4)cc2)cc1)c1c2	140942-26-3	2-Naphthalenesulfonic acid, 5,5,Ä≤-[1,2-ethenediylbis[(3-sulfo-4,1-phenylene)azo	C34H26N6O12S4.4Na		A/B	-8.5
5123	[C@H](C(=O)N1[C@@H](CC[C@@H]1C)c1[nH]c(cn1)c1c(C)cc(cc1)c1ccc(cc1)c1[nH]c(nc1)[C@H]1N(C(=O)[C@@H](NC	1412813-87-6	Carbamic acid, N,N,Ä≤-[[1,1,Ä≤-biphenyl]-4,4,Ä≤-diylbis[1H-imidazole-5,2-diyl[(	C48H52F6N8O6	950.97	A/B	-8.5
5124	O[C@H]1[C@@H](O[C@H](C(=O)Nc2ccc(C(=O)N3CCCCC3)cc2)[C@H]1O)n1nc(C(=O)N)nc1	1414801-76-5	Not Yet Assigned	C26H29ClN8O6	585.01	A/B	-8.5
5125	N(C(=O)C(F)(F)F)[C@H]1[C@H](OC(CC)CC)C=C(C(=O)OCCC2CCCCC2)C[C@@H]1N	1415566-94-7	Benzamide, 2-(benzoyloxy)-3,5-bis(1-methylethyl)-N-(5-nitro-2-thiazolyl)-	C23H23N3O5S	453.51	A/B	-8.5
5126	C(N(NC(=O)[C@@H](NC(=O)OC)C(C([2H])([2H])[2H])(C([2H])([2H])[2H])C([2H])([2H])[2H])C[C@@H]([C@H](Cc1	1415634-45-5	2,5,6,10,13-Pentaazatetradecanedioic acid, 3-[1,1-di(methyl-d3)ethyl-2,2,2-d3]-8	C38H43D9N6O8		A/B	-8.5
5127	C[C@]12[C@H]3[C@]4([C@]5(C4)[C@@H](CC3)[C@H](C)C(=O)CC5)CC[C@]1(C)[C@@H]([C@@H](CC[C@@H](C(=O)C)C)C)	1415665-49-4	Thymidine 5,Ä≤-(tetrahydrogen triphosphate), 2,Ä≤,3,Ä≤-didehydro-3,Ä≤-deoxy-, P	C20H25N4O16P3	670.35	A/B	-8.5
5128	N(c1nc(nc(NC(=O)Nc2ccc(F)cc2)n1)N1CCOCC1)n1c(SC)nnc(C(C)(C)C)c1=O	1418554-15-0	Urea, N-[4-[[6-(1,1-dimethylethyl)-3-(methylthio)-5-oxo-1,2,4-triazin-4(5H)-yl]a	C23H30N10O3S	526.61	A/B	-8.5
5129	N(C(=S)Nc1nc(nc(Nn2c(SC)nnc(C(C)(C)C)c2=O)n1)N1CCOCC1)c1c2c(ccc1)cccc2	1418741-55-5	Thiourea, N-[4-[[6-(1,1-dimethylethyl)-3-(methylthio)-5-oxo-1,2,4-triazin-4(5H)-	C22H27N11O4S2	573.65	A/B	-8.5
5130	O=c1n([C@H]2C[C@H](N=[N+]=[N-])[C@@H](C#C)O2)cc(C)c(=O)[nH]1	1421058-64-1	Tricyclo[3.3.1.13,7]decane-1-carboxylic acid, (5R,5aR,8aR,9R)-5,5a,6,8,8a,9-hexa	C33H36O9	576.63	A/B	-8.5
5131	N(C(=O)N1Cc2c(Cl)cccc2)C12CC3CC(C2)CC(C1)C3	1422361-26-9	1,3-Oxathiolane-2-carboxylic acid, 5-[5-fluoro-2-oxo-4-(1H-1,2,4-triazol-1-yl)-1	C20H26FN5O4S	451.51	A/B	-8.5
5132	C(N1c2c(C(=NNC(=S)N(CC=C)CC=C)C1=O)cc(Cl)cc2)N1CCN(CC1)c1ccccc1	1426583-61-0	Hydrazinecarbothioamide, 2-[1,2-dihydro-5-methyl-1-(4-morpholinylmethyl)-2-oxo-3	C27H27N5O2S	485.6	A/B	-8.5
5133	C(c1c2c(N(c3c(C(=O)N2)cccn3)C2CC2)ncc1)n1cc(C[C@@H](C(=O)N[C@H](C(=O)N[C@H](C(=O)N[C@H](C(=O)N[C@H](	1426583-91-6	3-Quinolinecarboxylic acid, 1-cyclopropyl-7-[4-[[3-[2-[(diphenylamino)thioxometh	C42H40FN7O5S	773.87	A/B	-8.5
5134	C(=O)(OCC)C1=C(N)OC(=CC1c1cc(OCC)ccc1)c1[nH]c2c(n1)cccc2	1426813-54-8	4H-Pyran-3-carboxylic acid, 2-amino-6-(1H-benzimidazol-2-yl)-4-(3-nitrophenyl)-,	C21H18N4O5	406.39	A/B	-8.5
5135	C(=O)(OCC)C1=C(N)OC(=CC1c1c(Br)cccc1)c1[nH]c2c(n1)cccc2	1426814-82-5	4H-Pyran-3-carboxylic acid, 2-amino-6-(1H-benzimidazol-2-yl)-4-(3-methylphenyl)-	C22H21N3O3	375.42	A/B	-8.5
5136	[C@@H](C(=O)N[C@@H](Cc1ccccc1)C[C@@H]([C@H](Cc1ccccc1)NC(=O)COc1c(C)cccc1C)O)(C(C)C)N1C(=O)NCCC1.C(O	1427788-93-9	Œ≤-Cyclodextrin, compd. with (Œ±S)-N-[(1S,3S,4S)-4-[[2-(2,6-dimethylphenoxy)acet	C42H70O35.C37H48N4O5		A/B	-8.5
5137	OC1C(n2c3c(nc2)c(N)ncn3)OC(CO)C1O	1427788-94-0	Œ≥-Cyclodextrin, compd. with (Œ±S)-N-[(1S,3S,4S)-4-[[2-(2,6-dimethylphenoxy)acet	C48H80O40.C37H48N4O5		A/B	-8.5
5138	[C@H](Cc1ccccc1)(C(=O)N/C(=C\CS(=O)(=O)c1ccccc1)/CCc1ccccc1)NC(=O)N1CCN(C)CC1	1428441-86-4	1-Piperazinecarboxamide, 4-methyl-N-[(1S)-2-oxo-2-[[(1E)-1-(2-phenylethyl)-3-(ph	C32H38N4O4S	574.73	A/B	-8.5
5139	O(C(=O)CCCCCCCCCCCCCCC)[C@H]1[C@@H](O[C@H](COC(=O)CCCCCCCCCCCCCCC)[C@H]1OC(=O)CCCCCCCCCCCCCCC)n1nc(C	143395-85-1	L-Phenylalaninamide, 1-[3-[[4-amino-1,4-dioxo-2-[(2-quinolinylcarbonyl)amino]but	C45H56N8O7	820.98	A/B	-8.5
5140	O[C@H]1[C@H](n2c3c(nc2)c(=O)nc(N)[nH]3)O[C@H](COP(=O)(OP(=O)(O[C@H]2O[C@H](CO)[C@@H](O)[C@@H](O)[C@H	1438405-17-4	Guanosine 5,Ä≤-(trihydrogen diphosphate), P,Ä≤-(4-deoxy-Œ±-D-xylo-hexopyranosyl	C16H25N5O15P2	589.34	A/B	-8.5
5141	S(c1n(c2c(n1)c(=O)nc(N)[nH]2)[C@H]1[C@H](O)[C@H]2[C@H](O1)COP(=O)(O)O2)c1ccc(N(=O)=O)cc1	144510-01-0	Guanosine, 8-[(2-methoxyphenyl)thio]-, cyclic 3,Ä≤,5,Ä≤-(hydrogen phosphate)	C17H18N5O8PS	483.39	A/B	-8.5
5142	C(=O)(O)c1cc(ccc1NC(=O)c1c(N)cccc1)c1cc(C(=O)O)c(NC(=O)c2c(N)cccc2)cc1	144615-01-0	Benzoic acid, 3,3,Ä≤-methylenebis[6-[(2-aminobenzoyl)amino]-, homopolymer	(C29H24N4O6)x		A/B	-8.5
5143	O=C1[C@@H]2[C@@H](c3c([C@H](OC(=O)c4ccc(Br)cc4)[C@H]2CO1)cc1c(c3)OCO1)c1c(Br)c(OC)c(OC)c(OC)c1	1449602-08-7	Benzoic acid, 3-bromo-, (5R,5aR,8aR,9S)-9-(2-bromo-3,4,5-trimethoxyphenyl)-5,5a,	C29H24Br2O9	676.3	A/B	-8.5
5144	O=C1[C@@H]2[C@@H](c3c([C@H](OC(=O)c4cc(N(=O)=O)ccc4)[C@H]2CO1)cc1c(c3)OCO1)c1c(Br)c(OC)c(OC)c(OC)c1	1449602-09-8	Benzoic acid, 4-bromo-, (5R,5aR,8aR,9S)-9-(2-bromo-3,4,5-trimethoxyphenyl)-5,5a,	C29H24Br2O9	676.3	A/B	-8.5
5145	P(=O)(OCC)(OCC)c1c(c2c(oc1=O)cccc2)c1ccccc1	1452007-28-1	Benzonitrile, 4-[[4-chloro-6-[(2,4,6-trimethylphenyl)amino]-2-pyrimidinyl]amino]	C20H18ClN5	363.84	A/B	-8.5
5146	N(C(=O)N)C12CC3(CC(C2)CC(C3)C1)c1ccc(C)cc1	1459693-44-7	Glycine, N-[[[3-(4-methylphenyl)tricyclo[3.3.1.13,7]dec-1-yl]amino]carbonyl]-	C20H26N2O3	342.43	A/B	-8.5
5147	N(C(=O)OCC)C12CC3(CC)CC(C2)CC(C1)C3	1460306-55-1	Benzonitrile, 3-hydroxy-4-[[4-[(2,4,6-trimethylphenyl)amino]-2-pyrimidinyl]aminc	C20H19N5O	345.4	A/B	-8.5

5148	S(=O)(=O)(O)c1c2c(c(N=Nc3c(C)cc(cc3)c3cc(C)c(N=Nc4c5c(c(S(=O)(=O)O)ccc5)c(N)cc4)cc3)ccc2N)ccc1	14678-28-5	2-Naphthalenesulfonic acid, 5,5,Ä≤-[(3,3,Ä≤-dimethyl[1,1,Ä≤-biphenyl]-4,4,Ä≤-di	C34H28N6O6S2	680.75	A/B	-8.5
5149	O=C1[C@@H]2[C@@H](c3c([C@@H](O)[C@H]2CO1)cc1c(c3)OCO1)c1cc(OC)c(OP(=O)(OCc2ccccc2)OCc2ccccc2)c(OC)c1	146849-73-2	Phosphoric acid, 4-(5,5a,6,8,8a,9-hexahydro-9-hydroxy-6-oxofuro[3,Ä≤,4,Ä≤:6,7]na	C33H29O11P	632.55	A/B	-8.5
5150	N(C(=O)C)[C@H]1[C@H]([C@@H]([C@@H](COC(=O)[C@@H](C)c2cc3c(cc2)cc(OC)cc3)O)O)OC(=C[C@@H]1NC(=N)N)C(=O	1469338-32-6	D-glycero-D-galacto-Non-2-enonic acid, 5-(acetylamino)-2,6-anhydro-4-[[bis[[(1,1	C38H52N4O13	772.84	A/B	-8.5
5151	C(=O)(c1cc2c([nH]1)ccc(NS(=O)(=O)C(F)(F)F)c2)N1CCN(CC1)c1c(NC(C)C)cccn1	147920-12-5	1H-Indole-6-carbonitrile, 2-[[4-[3-[(1-methylethyl)amino]-2-pyridinyl]-1-piperaz	C22H24N6O	388.47	A/B	-8.5
5152	C(n1c2c(nc1CC)c(C)ccn2)c1ccc(cc1)c1c(S(=O)(=O)NC(=O)OC=C)cccc1	148401-75-6	Carbamic acid, [[4,Ä≤-[(2-ethyl-7-methyl-3H-imidazo[4,5-b]pyridin-3-yl)methyl][	C26H28N4O4S	492.59	A/B	-8.5
5153	C(n1c2c(nc1CC)c(C)cc(C)n2)c1ccc(cc1)c1c(S(=O)(=O)NC(=O)NCC=C)cccc1	148401-92-7	[1,1,Ä≤-Biphenyl]-2-sulfonamide, N-[[(cyclopropylmethyl)amino]carbonyl]-4,Ä≤-[(	C28H31N5O3S	517.64	A/B	-8.5
5154	C(n1c2c(nc1CC)c(C)cc(C)n2)c1ccc(cc1)c1c(S(=O)(=O)NC(=O)N(C)C)cccc1	148401-93-8	[1,1,Ä≤-Biphenyl]-2-sulfonamide, 4,Ä≤-[(2-ethyl-5,7-dimethyl-3H-imidazo[4,5-b]p	C27H29N5O3S	503.62	A/B	-8.5
5155	C(#N)[C@]1(C)[C@H]2[C@H](O[C@H]1n1c(=O)nc(N)cc1)COP(=O)(OC1CCC1)O2	1491023-21-2	Cytidine, 2,Ä≤-cyano-2,Ä≤-deoxy-2,Ä≤-methyl-, cyclic 3,Ä≤,5,Ä≤-(phenyl phosphat	C17H17N4O6P	404.31	A/B	-8.5
5156	N(C12CC3CC(C2)CC(C1)C3)c1scc(C)n1	1497240-17-1	2,7,10,12-Tetraazatridecanoic acid, 4-hydroxy-12-methyl-9-(1-methylethyl)-13-[2-	C37H48N6O5S2.2ClH		A/B	-8.5
5157	C(C#C)n1c2c3c(nc(N)c2nc1)cccc3	149821-01-2	Uridine, thymidylyl-(3,Ä≤,Ü5,Ä≤)-2,Ä≤-deoxy-5,6-dihydro-6-hydroxy-, (6R)-	C19H27N4O13P	550.41	A/B	-8.5
5158	C([C@H]([C@@H](NC(=O)[C@@H](NC(=O)c1nc2c(cc1)cccc2)C1CCCC1)Cc1ccccc1)O)N1[C@H](C(=O)NC(C)(C)C)C[C@H]	150323-38-9	D-erythro-Pentonamide, 2,3,5-trideoxy-N-[(1S,2R)-2,3-dihydro-2-hydroxy-1H-inden-	C30H42N4O4	522.68	A/B	-8.5
5159	O=C1C2C(c3c(C([Se]CC(=O)O)C2CO1)cc1c(c3)OCO1)c1cc(OC)c(OC)c(OC)c1	150891-67-1	Acetic acid, 2-[[5,5a,6,8,8a,9-hexahydro-8-oxo-9-(3,4,5-trimethoxyphenyl)furo[3,	C26H28O9Se	563.46	A/B	-8.5
5160	O=C1[C@@H]2[C@@H](c3c([C@@H]([Se]CC(=O)O)[C@H]2CO1)cc1c(c3)OCO1)c1cc(OC)c(O)c(OC)c1	152999-29-6	Acetic acid, [[5,5a,6,8,8a,9-hexahydro-9-(4-hydroxy-3,5-dimethoxyphenyl)-8-oxofu	C25H26O9Se	549.43	A/B	-8.5
5161	O=C1[C@@H]2[C@@H](c3c([C@H](O[C@H]4O[C@H](CO)[C@@H](O)[C@H](O)[C@@H]4O)[C@H]2CO1)cc1c(c3)OCO1)c1cc(O	153459-54-2	L-Alanine, N-[[9,10-dihydro-12-[[(1-tricyclo[3.3.1.13,7]dec-1-ylethyl)amino]carb	C34H40N2O4	540.69	A/B	-8.5
5162	C(=O)(NCc1ccc(F)cc1)c1c(OC(OC(=O)OCc2ccccc2)C)c(=O)n(C)c(C(NC(=O)c2oc(C)nn2)(C)C)n1	1538603-92-7	Carbonic acid, cyclohexyl 1-[[4-[[[(4-fluorophenyl)methyl]amino]carbonyl]-1,6-di	C29H35FN6O8	614.62	A/B	-8.5
5163	O(C(OC(=O)OC(C)CC)c1c(C(=O)NCc2ccc(F)cc2)nc(C(NC(=O)c2nnc(C)o2)(C)C)n(C)c1=O	1538603-96-1	Carbonic acid, butyl 1-[[4-[[[(4-fluorophenyl)methyl]amino]carbonyl]-1,6-dihydro	C27H33FN6O8	588.58	A/B	-8.5
5164	C(n1c2c(nc1CC)c(C)cc(C)n2)c1ccc(cc1)c1c(S(=O)(=O)NC(=O)CCCCC)ccc(CCCC)c1	154551-74-3	Hexanamide, N-[[4,Ä≤-[(2-ethyl-5,7-dimethyl-3H-imidazo[4,5-b]pyridin-3-yl)methyl	C32H40N4O3S	560.75	A/B	-8.5
5165	C(n1c2c(nc1CC)c(C)cc(C)n2)c1ccc(cc1)c1c(S(=O)(=O)NC(=O)CCCCC)ccc(SCC)c1	154551-90-3	Hexanamide, N-[[4,Ä≤-[(2-ethyl-5,7-dimethyl-3H-imidazo[4,5-b]pyridin-3-yl)methyl	C32H40N4O4S	576.75	A/B	-8.5
5166	C(n1c2c(nc1CC)c(C)cc(C)n2)c1ccc(cc1)c1c(S(=O)(=O)NC(=O)COCCCC)ccc(CCC)c1	154551-93-6	Acetamide, 2-butoxy-N-[[5-ethyl-4,Ä≤-[(2-ethyl-5,7-dimethyl-3H-imidazo[4,5-b]pyr	C31H38N4O4S	562.72	A/B	-8.5
5167	C(n1c2c(nc1CC)c(C)cc(C)n2)c1ccc(cc1)c1c(S(=O)(=O)NC(=O)COCCCC)ccc(OC(C)C)c1	154552-09-7	Acetamide, 2-butoxy-N-[[4,Ä≤-[(2-ethyl-5,7-dimethyl-3H-imidazo[4,5-b]pyridin-3-y	C31H35F3N4O5S	632.69	A/B	-8.5
5168	C(n1c2c(nc1CC)c(C)cc(C)n2)c1ccc(cc1)c1c(S(=O)(=O)NC(=O)COCC)ccc(CC)c1	154552-12-2	Acetamide, 2-ethoxy-N-[[4,Ä≤-[(2-ethyl-5,7-dimethyl-3H-imidazo[4,5-b]pyridin-3-y	C28H32N4O4S	520.64	A/B	-8.5
5169	C(n1c2c(nc1CC)c(C)cc(C)n2)c1ccc(cc1)c1c(S(=O)(=O)NC(=O)COCC)ccc(OCCCC)c1	154552-16-6	Acetamide, 2-ethoxy-N-[[4,Ä≤-[(2-ethyl-5,7-dimethyl-3H-imidazo[4,5-b]pyridin-3-y	C30H36N4O4S	548.7	A/B	-8.5
5170	C(n1c2c(nc1CC)c(C)cc(C)n2)c1ccc(cc1)c1c(S(=O)(=O)NC(=O)COCC)ccc(OC)c1	154552-18-8	Acetamide, 2-ethoxy-N-[[4,Ä≤-[(2-ethyl-5,7-dimethyl-3H-imidazo[4,5-b]pyridin-3-y	C31H38N4O4S	562.72	A/B	-8.5
5171	C(n1c2c(nc1CC)c(C)cc(C)n2)c1ccc(cc1)c1c(S(=O)(=O)NC(=O)COCC)ccc(CSC)c1	154552-19-9	Acetamide, 2-ethoxy-N-[[4,Ä≤-[(2-ethyl-5,7-dimethyl-3H-imidazo[4,5-b]pyridin-3-y	C28H32N4O5S	536.64	A/B	-8.5
5172	C(n1c2c(nc1CC)c(C)cc(C)n2)c1ccc(cc1)c1c(S(=O)(=O)NC(=O)COCC)ccc(OC(C)C)c1	154552-29-1	Acetamide, 2-ethoxy-N-[[4,Ä≤-[(2-ethyl-5,7-dimethyl-3H-imidazo[4,5-b]pyridin-3-y	C29H31F3N4O5S	604.64	A/B	-8.5
5173	C(n1c2c(nc1CC)c(C)cc(C)n2)c1ccc(cc1)c1c(S(=O)(=O)NC(=O)NCCCC)ccc(CN(C)C)c1	154552-45-1	[1,1,Ä≤-Biphenyl]-2-sulfonamide, N-[(butylamino)carbonyl]-5-(1,1-dimethylethyl)-	C32H41N5O3S	575.76	A/B	-8.5
5174	C(n1c2c(nc1CC)c(C)cc(C)n2)c1ccc(cc1)c1c(S(=O)(=O)NC(=O)NCCCC)ccc(OCC(F)(F)F)c1	154552-47-3	[1,1,Ä≤-Biphenyl]-2-sulfonamide, N-[(butylamino)carbonyl]-5-[(diethylamino)methy	C33H44N6O3S	604.81	A/B	-8.5
5175	C(n1c2c(nc1CC)c(C)cc(C)n2)c1ccc(cc1)c1c(S(=O)(=O)NC(=O)c2c(Oc3ccccc3)cccc2)ccc(CC)c1	154552-71-3	Benzamide, N-[[4,Ä≤-[(2-ethyl-5,7-dimethyl-3H-imidazo[4,5-b]pyridin-3-yl)methyl]	C37H34N4O4S	630.76	A/B	-8.5
5176	C(n1c2c(nc1CC)c(C)cc(C)n2)c1ccc(cc1)c1c(S(=O)(=O)NC(=O)c2c(OCC)cccc2)ccc(CC)c1	154552-91-7	Benzamide, 2-ethoxy-N-[[4,Ä≤-[(2-ethyl-5,7-dimethyl-3H-imidazo[4,5-b]pyridin-3-y	C33H34N4O4S	582.71	A/B	-8.5
5177	C(n1c2c(nc1CC)c(C)cc(C)n2)c1ccc(cc1)c1c(S(=O)(=O)NC(=O)c2c(OCC)cccc2)ccc(CSC)c1	154552-98-4	Benzamide, 2-ethoxy-N-[[4,Ä≤-[(2-ethyl-5,7-dimethyl-3H-imidazo[4,5-b]pyridin-3-y	C33H34N4O5S	598.71	A/B	-8.5
5178	C(n1c2c(nc1CC)c(C)cc(C)n2)c1ccc(cc1)c1c(S(=O)(=O)NC(=O)c2ccccc2)ccc(OCC)c1	154553-20-5	Benzamide, N-[[4,Ä≤-[(2-ethyl-5,7-dimethyl-3H-imidazo[4,5-b]pyridin-3-yl)methyl]	C32H32N4O4S	568.69	A/B	-8.5
5179	C(n1c2c(nc1CC)c(C)cc(C)n2)c1ccc(cc1)c1c(S(=O)(=O)NC(=O)COCCC)ccc(OC)c1	154553-36-3	Acetamide, N-[[5-[(dimethylamino)methyl]-4,Ä≤-[(2-ethyl-5,7-dimethyl-3H-imidazo	C31H39N5O4S	577.74	A/B	-8.5
5180	C(n1c2c(nc1CC)c(C)cc(C)n2)c1ccc(cc1)c1c(S(=O)(=O)NC(=O)COCCC)ccc(COC)c1	154553-38-5	Acetamide, N-[[4,Ä≤-[(2-ethyl-5,7-dimethyl-3H-imidazo[4,5-b]pyridin-3-yl)methyl]	C30H36N4O4S2	580.76	A/B	-8.5
5181	C(n1c2c(nc1CC)c(C)cc(C)n2)c1ccc(cc1)c1c(S(=O)(=O)NC(=O)COCCC)ccc(C(CC)(C)C)c1	154553-41-0	Acetamide, N-[[4,Ä≤,Ä≤-[(2-ethyl-5,7-dimethyl-3H-imidazo[4,5-b]pyridin-3-yl)met	C34H36N4O4S	596.74	A/B	-8.5
5182	C(n1c2c(nc1CC)c(C)cc(C)n2)c1ccc(cc1)c1c(S(=O)(=O)NC(=O)COCCC)ccc(OCC(F)(F)F)c1	154553-45-4	Acetamide, N-[[4,Ä≤-[(2-ethyl-5,7-dimethyl-3H-imidazo[4,5-b]pyridin-3-yl)methyl]	C33H41N5O5S	619.77	A/B	-8.5
5183	C(n1c2c(nc1CC)c(C)cc(C)n2)c1ccc(cc1)c1c(S(=O)(=O)NC(=O)COCCC)ccc(SCC)c1	154553-47-6	Acetamide, N-[[4,Ä≤-[(2-ethyl-5,7-dimethyl-3H-imidazo[4,5-b]pyridin-3-yl)methyl]	C31H38N4O5S	578.72	A/B	-8.5
5184	C(n1c2c(nc1CC)c(C)cc(C)n2)c1ccc(cc1)c1c(S(=O)(=O)NC(=O)COCC)ccc(SCC)c1	154553-48-7	Acetamide, N-[[4,Ä≤-[(2-ethyl-5,7-dimethyl-3H-imidazo[4,5-b]pyridin-3-yl)methyl]	C30H36N4O4S2	580.76	A/B	-8.5
5185	O=c1n([C@@H]2O[C@H](COC(=O)CCC(=O)N3[C@H](C(=O)N[C@H](Cc4ccccc4)C(=O)OCc4ccccc4)CCC3)[C@@H](N=[N+]=[	154566-67-3	D-Phenylalanine, N-(1-carboxy-L-prolyl)-, 1-(phenylmethyl) ester, ester with 3,Ä	C32H35N7O8		A/B	-8.5
5186	N(CC(CNC)C)c1c2c(ncc1)cc(Cl)cc2	154814-39-8	2,7-Naphthalenedisulfonic acid, 4-amino-3-[2-[4,Ä≤-[2-(2,4-diaminophenyl)diazeny	C37H33N9O9S3.3Na		A/B	-8.5
5187	CN1C2c3c(C1CC2)c(ccc3c1[nH]c(nc1)[C@H]1N(C(=O)[C@@H](NC(=O)OC)C(C)C)C[C@@H](C)C1)c1ccc(cc1)c1[nH]c(n	1560639-43-1	Carbamic acid, N-[(1S)-1-[[(2S)-2-[5-[4-[3,4-dihydro-5-[2-[(2S)-1-[(2S)-2-[(meth	C45H54N8O8	834.96	A/B	-8.5
5188	CN1C2c3c(C1CC2)c(ccc3c1[nH]c(nc1)[C@H]1N(C(=O)[C@@H](NC(=O)OC)C(C)C)[C@H]2C[C@@H]1CC2)c1ccc(cc1)c1[n	1560639-57-7	Carbamic acid, N-[(1S)-1-[[(2S)-4,4-dimethyl-2-[5-[4-[1,2,3,4-tetrahydro-8-[2-[(	C49H65N9O6	876.1	A/B	-8.5
5189	C(=O)([C@@H](NC(=O)OC)C1CCCCC1)N1[C@H](c2[nH]c(c3c4c(c(cc3)c3ccc(cc3)c3[nH]c(nc3)[C@H]3N(C(=O)[C@@H]	1560640-11-0	Carbamic acid, N-[(1S)-2-methyl-1-[[(2S,4S)-4-methyl-2-[5-[4-[1,2,3,4-tetrahydro	C48H62N8O6	847.06	A/B	-8.5
5190	O[C@H]1[C@H](n2c3c(nc2)c(N)nc(SCC#C)n3)O[C@H](CO)[C@H]1O	1561048-25-6	Carbamic acid, N-[(1S)-2-methyl-1-[[(2S)-2-[5-[4-[1,2,3,4-tetrahydro-8-[2-[(2S)-	C46H58N8O6	819	A/B	-8.5
5191	N(c1c2c(c(O)c(CN3CCN(CCC(C)C)CC3)c1)CCCC2)c1c2c(ncc1)cc(Cl)cc2	156893-89-9	1-Naphthalenol, 4-[(7-chloro-4-quinolinyl)amino]-5,6,7,8-tetrahydro-2-[(4-pentyl	C29H37ClN4O	493.08	A/B	-8.5
5192	S(=O)(=O)(O)c1c2c(cc(S(=O)(=O)O)c1)ccc(N=Nc1cc(c(N)cc1)c1ccccc1)c2	157392-84-2	Thymidine 5,Ä≤-(trihydrogen diphosphate), P-methyl P,Ä≤,P,Ä≤-diphenyl ester	C23H26N2O11P2	568.41	A/B	-8.5
5193	S(=O)(=O)(O)c1c2c(cc(S(=O)(=O)O)c1)ccc(N=Nc1c(cc(N)cc1)c1ccccc1)c2	157437-00-8	1,3-Naphthalenedisulfonic acid, 7-[2-(6-amino[1,1,Ä≤-biphenyl]-3-yl)diazenyl]-	C22H17N3O6S2	483.52	A/B	-8.5
5194	O=c1n([C@@H]2O[C@H](COP(=O)(OC[C@@H]3O[C@@H](CS3)n3c(=O)nc(N)cc3)COc3ccccc3)[C@@H](N=[N+]=[N-])C2)cc	1579273-91-8	Thymidine, 3,Ä≤-azido-3,Ä≤-deoxy-, 5,Ä≤,5,Ä≤,Ä≤,Ä≤-[P-(azidomethyl)phosphonate	C21H26N13O9P		A/B	-8.5
5195	C(=C\C=C\c1cc(O)c(O)cc1)/c1cc(O)c(O)cc1	158629-14-2	Retinoic acid, [5-(4-amino-2-oxo-1(2H)-pyrimidinyl)-1,3-oxathiolan-2-yl]methyl e	C28H37N3O4S	511.68	A/B	-8.5
5196	C(=O)([C@@H](NC(=O)OC)C(C)C)N1[C@H](c2[nH]c(c3c4c(C5(CC4)CCCC5)c(cc3)c3ccc(cc3)c3[nH]c(nc3)[C@H]3N(C	1588403-87-5	Carbamic acid, N-[(1S)-1-[[(2S)-2-[5-[2,Ä≤,3,Ä≤-dihydro-4,Ä≤-[4-[2-[(2S)-1-[(2S	C47H60N8O6	833.03	A/B	-8.5
5197	N(C(=O)C)[C@H]1[C@H](OC(CC)CC)C=C(C(=O)OCC)C[C@@H]1NC(=S)NC(=O)OCc1ccccc1	1588414-25-8	Carbamic acid, N-[(1S)-1-[[(3S)-3-[5-[2,Ä≤,3,Ä≤-dihydro-7,Ä≤-[4-[2-[(3S)-2-[(2S	C51H64N8O6	885.1	A/B	-8.5
5198	C(n1c2c(nc1CC)c(C)cc(C)n2)c1ccc(cc1)c1c(S(=O)(=O)NC(=O)CCC)cccc1	158963-52-1	Acetamide, N-[[4,Ä≤-[(2-ethyl-5,7-dimethyl-3H-imidazo[4,5-b]pyridin-3-yl)methyl]	C25H26N4O3S	462.56	A/B	-8.5
5199	O[C@@H]1[C@H](n2c3c(nc2)c(N)nc(OCC)n3)O[C@H](COP(=O)(O)O)[C@H]1O	158963-55-4	Cyclopropanecarboxamide, N-[[4,Ä≤-[(2-ethyl-5,7-dimethyl-3H-imidazo[4,5-b]pyridi	C27H28N4O3S	488.6	A/B	-8.5
5200	N(=Cc1cc(C(F)(F)F)ccc1)C12CC3CC(C2)CC(C1)C3	160013-75-2	Tricyclo[3.3.1.13,7]decan-1-amine, N-[[2-(trifluoromethyl)phenyl]methylene]-	C18H20F3N	307.35	A/B	-8.5
5201	C(OCCOP(=O)(OCOC(=O)CC)OCOC(=O)CC)n1c2c(nc1)c(=O)nc(N)[nH]2	161056-99-1	Uridine 5,Ä≤-(trihydrogen diphosphate), 5-iodo-, P,Ä≤-anhydride with [(2,5,6-tr	C19H25IN5O16P3	799.25	A/B	-8.5
5202	O[C@H]1[C@H](n2c3c(nc2)c(=O)nc(N)[nH]3)O[C@H](COP(=O)(OP(=O)(OP(=O)(O)O)O)OC)[C@H]1O	1610778-50-1	Guanosine, 5,Ä≤-(hydrogen P-ethylphosphonate), anhydride with diphosphoric acid	C12H20N5O13P3	535.23	A/B	-8.5
5203	O[C@H]1[C@@H](O[C@H](COP(=O)(OC(OC(=O)CC)C(C)C)OC(OC(=O)CC)C(C)C)[C@H]1O)n1c(=O)c(C(=O)N)nc(F)c1	1613031-63-2	2-Pyrazinecarboxamide, 4-[5-O-[bis[1-(benzoyloxy)ethoxy]phosphinyl]-Œ≤-D-ribofur	C28H29FN3O13P	665.51	A/B	-8.5
5204	O[C@H]1[C@@H](O[C@H](COP(=O)(Oc2c(OCC)cccc2)Oc2c(OCC)cccc2)[C@H]1O)n1c(=O)c(C(=O)N)nc(F)c1	1613031-68-7	2-Pyrazinecarboxamide, 4-[5-O-(diphenoxyphosphinyl)-Œ≤-D-ribofuranosyl]-6-fluor	C22H21FN3O9P	521.39	A/B	-8.5
5205	O[C@H]1[C@@H](O[C@H](COP(=O)(Oc2ccccc2)N[C@@H](C(=O)OC(C)C)C)[C@H]1O)n1c(=O)c(C(=O)N)nc(F)c1	1613031-79-0	L-Alanine, N-(hydroxyphenoxyphosphinyl)-, cyclohexyl ester, 5,Ä≤-ester with 6-fl	C25H32FN4O10P	598.51	A/B	-8.5
5206	O=c1n([C@@H]2O[C@H](CO)C(=O)C2)ccc(N)n1	161363-12-8	Guanosine, 2,Ä≤-deoxy-N-[3-(trifluoromethyl)phenyl]-	C17H16F3N5O4	411.34	A/B	-8.5
5207	O[C@H]1[C@H](n2c3c(nc2)c(=O)nc[nH]3)O[C@H](COC(=O)[C@H](CO)N)[C@H]1O	1616409-51-8	L-Phenylalanine, 1,1,Ä≤-[2-[(2-amino-1,6-dihydro-6-oxo-9H-purin-9-yl)methoxy]-1,	C27H31N7O6	549.58	A/B	-8.5
5208	[C@H](C(=O)N1[C@@H]2[C@@H](C2)C[C@H]1C#N)(N)C12CC3(OC(=O)C(C)N4CCCC4)CC(C2)CC(C1)C3	1616608-20-8	Cyclopentanecarboxylic acid, 1-amino-, 3-[(1S)-1-amino-2-[(1S,3S,5S)-3-cyano-2-a	C24H34N4O3	426.55	A/B	-8.5
5209	C([C@H](OCP(=O)(Cc1ccccc1)O)C)n1c2c(nc1)c(N)ncn2	1619257-73-6	Octanediamide, N1-[4-(4,6-di-4-morpholinyl-1,3,5-triazin-2-yl)phenyl]-N8-hydroxy	C25H35N7O5	513.59	A/B	-8.5
5210	N(C(=O)[C@H](Cc1cccnc1)C[C@@H](CN1[C@H](C(=O)NC(C)(C)C)CN(Cc2cc3c(s2)scc3)CC1)O)[C@H]1c2c(C[C@H]1O)c	1620896-08-3	Not Yet Assigned	C31H34N4O10S	654.69	A/B	-8.5
5211	C[C@]1(F)[C@@H](O[C@H](C(OP(=O)(N[C@H](C(=O)OC(C)C)C)OC(C)C)[C@H]1O)n1c(=O)[nH]c(=O)c(c1[2H])[2H]	1621166-94-6	L-Alanine, N-[(4-fluorophenoxy)hydroxyphosphinyl]-, ethyl ester, 5,Ä≤-ester wit	C22H26D2F2N3O9P		A/B	-8.5
5212	N(C(=O)[C@H](Cc1ccccc1)C[C@@H](CN1[C@H](C(=O)NC(C)(C)C)CN(Cc2cccnc2)CC1)O)[C@H]1c2c([C@H](O)[C@H]1O)	162518-55-0	D-erythro-Pentonamide, 2,3,5-trideoxy-N-[(1S,2S,3S)-2,3-dihydro-2,3-dihydroxy-1H	C36H47N5O6	645.79	A/B	-8.5
5213	N(c1nc(nc(n1)N1CCOCC1)c1ccc(OC)cc1)c1cc(Cl)ccc1	1627734-68-2	1,3,5-Triazin-2-amine, N-(2-chlorophenyl)-4-(4-methoxyphenyl)-6-(4-morpholinyl)-	C20H20ClN5O2	397.86	A/B	-8.5
5214	C(C)(C)N1CCN(c2nc(nc(n2)N2CCOCC2)c2ccc(OC)cc2)CC1	1627734-76-2	1,3,5-Triazine, 2-[4-(3-chlorophenyl)-1-piperazinyl]-4-(4-methoxyphenyl)-6-(4-mo	C24H27ClN6O2	466.96	A/B	-8.5
5215	C[C@]1(F)[C@@H](O[C@H](COP(=O)(Oc2cc(ccc2)c2ccccc2)N[C@H](C(=O)OC(C)C)C)[C@H]1O)n1c(=O)[nH]c(=O)cc1	1628724-48-0	L-Alanine, N-[(2,Ä≤R)-P-[1,1,Ä≤-biphenyl]-4-yl-2,Ä≤-deoxy-2,Ä≤-fluoro-2,Ä≤-meth	C28H33FN3O9P	605.55	A/B	-8.5
5216	N(c1nc(nc(Nc2cc(Cl)c(Cl)cc2)n1)N1CCOCC1)c1nc(OC)cc(OC)n1	1631751-05-7	1,3,5-Triazine-2,4-diamine, N2-(4,6-dimethoxy-2-pyrimidinyl)-6-(4-morpholinyl)-N	C19H21N9O5	455.43	A/B	-8.5
5217	N(c1nc(nc(Nc2c(O)cc(N(=O)=O)cc2)n1)N1CCOCC1)c1nc(OC)cc(OC)n1	1631751-07-9	1,3,5-Triazine-2,4-diamine, N2-(4,6-dimethoxy-2-pyrimidinyl)-6-(4-morpholinyl)-N	C19H21N9O5	455.43	A/B	-8.5
5218	N(c1nc(nc(Nc2nc(OC)cc(OC)n2)n1)N1CCOCC1)c1c(O)ccc(Cl)c1	1631751-14-8	1,3,5-Triazine-2,4-diamine, N2-(4,6-dimethoxy-2-pyrimidinyl)-N4-(2,4-dinitropher	C19H20N10O7	500.42	A/B	-8.5
5219	N(c1nc(nc(Nc2cc(Cl)c(F)cc2)n1)N1CCOCC1)c1nc(OC)cc(OC)n1	1631751-15-9	Phenol, 4-chloro-2-[[4-[(4,6-dimethoxy-2-pyrimidinyl)amino]-6-(4-morpholinyl)-1,	C19H21ClN8O4	460.87	A/B	-8.5
5220	O=c1n(ccc(N)n1)[C@H]1O[C@@H](COP(=O)(Oc2cc3c(cc2)OCO3)Oc2cc3c(cc2)OCO3)SC1	1638769-62-6	Phosphoric acid, 2-[(2-amino-1,6-dihydro-6-oxo-9H-purin-9-yl)methoxy]ethyl bis(1	C22H20N5O10P	545.4	A/B	-8.5
5221	C[C@]1(F)[C@@H](O[C@H](CO[P@](=O)(Oc2cc3c(cc2)OCO3)N[C@H](C(=O)OC(C)C)C)[C@H]1O)n1c(=O)[nH]c(=O)cc1	1638769-83-1	5,8-Dioxa-3-aza-4-phosphanonanoic acid, 9-(2-amino-1,6-dihydro-6-oxo-9H-purin-9-	C24H31N6O9P	578.51	A/B	-8.5
5222	O=c1n([C@@H]2O[C@H](CO[P@](=O)(Oc3cc4c(cc3)OCO4)N[C@H](C(=O)OC(C)C)C)SC2)ccc(N)n1	1638769-89-7	L-Alanine, N-[[P(S),2,Ä≤R]-P-1,3-benzodioxol-5-yl-2,Ä≤-deoxy-2,Ä≤-fluoro-2,Ä≤-m	C26H33FN3O11P	613.53	A/B	-8.5
5223	O=c1c2c(n(cn2)[C@@H]2C(=C)[C@H](CO[P@](=O)(Oc3c4c(OCO4)ccc3)N[C@H](C(=O)OC(C)C)C)[C@@H](O)C2)[nH]c(N	1638770-04-3	L-Alanine, N-[(R)-[[(1R,3S,5S)-3-(2-amino-1,6-dihydro-6-oxo-9H-purin-9-yl)-5-hyd	C25H31N6O9P	590.52	A/B	-8.5
5224	C(OCCOP(=O)(Oc1cc2c(cc1)OCO2)N[C@H](C(=O)OC)C)n1c2c(nc1)c(=O)nc(N)[nH]2	1638770-06-5	L-Alanine, N-[(S)-[[(1R,3S,5S)-3-(2-amino-1,6-dihydro-6-oxo-9H-purin-9-yl)-5-hyd	C25H31N6O9P	590.52	A/B	-8.5
5225	C(=O)(C1=C(C(P(=O)(OC)OC)C(=O)OC)C(=O)N(C1c1ccccc1)c1ccccc1)c1ccoc1	1638949-52-6	5,Ä≤-Guanylic acid, N-[[(4-hydroxyphenyl)thio]methyl]-	C17H20N5O9PS	501.41	A/B	-8.5
5226	C(=O)([C@@H](NC(=O)OC)C(C)C)N1[C@@H](C[C@@H](OC(=O)N2Cc3c(C2)c(F)ccc3)C1)c1[nH]c(cn1)c1ccc(cc1)c1ccc	163980-26-5	2,7-Naphthalenedisulfonic acid, 4,4,Ä≤-[1,2-ethenediylbis[(3-sulfo-4,1-phenylene	C34H26N6O18S6.6Na		A/B	-8.5
5227	C(=O)([C@H](NC(=O)OC)c1ccccc1)N1[C@@H](CCC1)c1[nH]c(cn1)c1ccc(cc1)c1ccc(cc1)c1[nH]c(nc1)[C@H]1N(C(=O	1639870-95-3	Carbamic acid, N-[(1R)-2-[(2S)-2-[5-[4,Ä≤-[2-[(2S)-2,5-dihydro-1-[(2S)-2-[(metho	C43H46N8O6	770.88	A/B	-8.5
5228	C(=O)([C@@H](NC(=O)OC)C(C)C)N1[C@H](c2[nH]c(c3c4c(c(ccc4)c4ccc(cc4)c4[nH]c(nc4)[C@H]4N(C(=O)[C@@H](N	1639870-98-6	Carbamic acid, N-[(1S)-1-[[(2S)-2,5-dihydro-2-[5-[4-[5-[2-[(2S)-1-[(2S)-2-[(meth	C44H50N8O6	786.92	A/B	-8.5
5229	O(c1nc(nc(Nc2ccc(F)cc2)n1)N1CCOCC1)c1ccc(C#N)cc1	1643332-84-6	Benzonitrile, 4-[[4-[(4-methylphenyl)amino]-6-(4-morpholinyl)-1,3,5-triazin-2-yl	C21H20N6O2	388.42	A/B	-8.5
5230	O=c1n([C@@H]2O[C@H](COP(=O)(C(O)c3ccncc3)OC[C@@H]3[C@@H](N=[N+]=[N-])C[C@@H](O3)n3c(=O)[nH]c(=O)c(C)	164652-15-7	Thymidine, 3,Ä≤-azido-P,3,Ä≤-dideoxy-P-[(2,4-dinitrophenyl)hydroxymethyl]thymid	C27H29N12O14P		A/B	-8.5
5231	C(P(=O)(OC[C@@H]1[C@@H](N=[N+]=[N-])C[C@@H](O1)n1c(=O)[nH]c(=O)c(C)c1)OC[C@H]1O[C@H](C=C1)n1c(=O)[nH	164652-19-1	Thymidine, 3,Ä≤-azido-P,3,Ä≤-dideoxy-P-[hydroxy(4-nitrophenyl)methyl]thymidylyl	C27H29N8O12P		A/B	-8.5
5232	C(P(=O)(OC[C@H]1O[C@H](CC1)n1c(=O)[nH]c(=O)c(C)c1)OC[C@H]1O[C@H](C=C1)n1c(=O)[nH]c(=O)c(C)c1)(O)c1c(	164652-20-4	Thymidine, 3,Ä≤-azido-P,3,Ä≤-dideoxy-P-[(2,6-dinitrophenyl)hydroxymethyl]thymid	C27H28N9O14P		A/B	-8.5
5233	N(C(=O)[C@H](Cc1ccccc1)C[C@@H](CN1[C@H](C(=O)NC(C)(C)C)CN(Cc2c(nccc2)N2CCOCC2)CC1)O)[C@H]1c2c(C[C@H]	164725-16-0	1-Piperazinepentanamide, N-(2,3-dihydro-2-hydroxy-1H-inden-1-yl)-2-[[(1,1-dimeth	C37H49N5O4	627.82	A/B	-8.5
5234	N(C(=O)[C@H](Cc1ccccc1)C[C@@H](CN1[C@H](C(=O)NC(C)(C)C)CN(Cc2c(C)cccc2)CC1)O)[C@H]1c2c(C[C@H]1O)cccc	164725-54-6	1-Piperazinepentanamide, N-(2,3-dihydro-2-hydroxy-1H-inden-1-yl)-2-[[(1,1-dimeth	C36H52N4O5	620.82	A/B	-8.5
5235	N(C(=O)[C@H](Cc1ccccc1)C[C@@H](CN1[C@H](C(=O)NC(C)(C)C)CN(Cc2ccccc2)CC1)O)[C@H]1c2c(C[C@H]1O)cccc2	164725-61-5	1-Piperazinepentanamide, N-(2,3-dihydro-2-hydroxy-1H-inden-1-yl)-2-[[(1,1-dimeth	C35H47N5O4S	633.84	A/B	-8.5
5236	C(=O)([C@@H](NC(=O)OC)C(C)C)N1[C@H]2[C@H](C2)C[C@H]1c1[nH]c(cn1)c1ccc(cc1)c1ccc2c(cc1)c1c(cc2)nc([nH]	1653966-56-3	Carbamic acid, N-[(1S)-1-[[(2S)-2-[7-[4-[2-[(3S,5R)-2-[(2S)-2-[(methoxycarbonyl)	C46H56N8O7	832.99	A/B	-8.5
5237	C(=O)([C@@H](NC(=O)OC)C(C)C)N1C[C@]2(C[C@H]1c1[nH]c3c(n1)ccc1c3ccc(c1)c1ccc(cc1)c1[nH]c(nc1)[C@H]1N(	1653967-13-5	Carbamic acid, N-[(1S)-1-[[(2S)-2-[7-[4-[2-[(5S,8S)-7-[(2S)-2-[(methoxycarbonyl)	C45H54N8O7	818.96	A/B	-8.5
5238	C([C@@H](CCO)CO)n1c2c(nc1)c(=O)[nH]c(=O)[nH]2	1658454-73-9	4-Quinazolinamine, 2-(1,1-dimethylethyl)-5,6,7,8-tetrahydro-N-(phenyltricyclo[3.	C29H39N3O	445.64	A/B	-8.5
5239	C(COCP(=O)(OCC(Cl)(Cl)Cl)OC)n1c2c(nc1)c(N)ncn2	165946-10-1	1-Piperazinepentanamide, N-(2,3-dihydro-2-hydroxy-1H-inden-1-yl)-2-[[(1,1-dimeth	C35H46N4O4S	618.83	A/B	-8.5
5240	O=c1n(ccc(N)n1)[C@@H]1O[C@H](COCC)[C@@H](O)C1	1661023-45-5	Benzamide, N-(1-tricyclo[3.3.1.13,7]dec-1-ylethyl)-4-[2-[3-(trifluoromethoxy)phe	C28H32F3NO3	487.55	A/B	-8.5
5241	O=c1n([C@@H]2O[C@H](COP(=O)(C(O)c3ccc(C#N)cc3)OC[C@@H]3[C@@H](N=[N+]=[N-])C[C@@H](O3)n3c(=O)[nH]c(=O	166318-77-0	Thymidine, 3,Ä≤-azido-P-[(4-chlorophenyl)hydroxymethyl]-P,3,Ä≤-dideoxythymidyly	C27H30ClN10O10P		A/B	-8.5
5242	O=c1n([C@@H]2O[C@H](COP(=O)(OC[C@@H]3[C@@H](N=[N+]=[N-])C[C@@H](O3)n3c(=O)[nH]c(=O)c(C)c3)OCc3ccccc3	166318-79-2	Thymidine, 3,Ä≤-azido-P,3,Ä≤-dideoxy-P-[hydroxy(3-nitrophenyl)methyl]thymidylyl	C27H30N11O12P		A/B	-8.5
5243	C(=O)(NC(C)(C)C)[C@H]1N(C[C@H]([C@@H](Cc2ccccc2)NC(=O)c2c(C)c(O)ccc2)O)C[C@@H]2[C@H](C1)CCCC2	1665279-32-2	3-Isoquinolinecarboxamide, N-(1,1-dimethylethyl)decahydro-2-[(2S,3S)-2-hydroxy-3	C32H45N3O4	535.72	A/B	-8.5
5244	C([C@@H](CNC(=O)c1c(C)c(O)ccc1)O)N1[C@H](C(=O)NC(C)(C)C)C[C@H]2[C@@H](C1)CCCC2	1665279-38-8	3-Isoquinolinecarboxamide, N-(1,1-dimethylethyl)decahydro-2-[(2S,3R)-2-hydroxy-3	C32H45N3O4	535.72	A/B	-8.5
5245	C(OCCOCn1c2c(nc1)c(=O)nc(N)[nH]2)n1c2c(c(=O)nc(N)[nH]2)nc1	166762-89-6	Benzamide, N-[9-[[2-(benzoyloxy)ethoxy]methyl]-6,9-dihydro-6-oxo-1H-purin-2-yl]-	C22H19N5O5	433.42	A/B	-8.5
5246	O(C(=O)N[C@@H](Cc1ccc(OC)cc1)[C@@H](CN(S(=O)(=O)c1cc2c(cc1)nc(NC(C)C)o2)CC(C)C)O)[C@@H]1[C@H]2[C@@H]	1672661-45-8	Carbamic acid, N-[(1S,2R)-2-hydroxy-3-[[[2-[(1-methylethyl)amino]-6-benzoxazolyl	C31H40F2N4O8S	666.73	A/B	-8.5
5247	O(C(=O)N[C@@H](Cc1ccccc1)[C@@H](CN(S(=O)(=O)c1cc2c(cc1)nc(NC1CCNCC1)s2)CC(C)C)O)[C@@H]1[C@H]2[C@@H](	1672661-55-0	Carbamic acid, N-[(1S,2R)-3-[[[2-(cyclohexylamino)-6-benzothiazolyl]sulfonyl](2-	C34H44F2N4O7S2	722.86	A/B	-8.5
5248	O(C(=O)N[C@@H](Cc1ccccc1)[C@@H](CN(S(=O)(=O)c1ccc(N)cc1)CC(C)(C)F)O)[C@@H]1[C@H]2[C@@H](OC1)OCC2(F)F	1672661-57-2	Carbamic acid, N-[(1S,2R)-2-hydroxy-1-[(4-methoxyphenyl)methyl]-3-[[[2-[(1-methy	C32H42F2N4O8S2	712.82	A/B	-8.5
5249	O(C)c1c2C(C3C(Cc2cc2c1OCO2)COC3=O)c1cc(OC)c(O)cc1	167759-55-9	Furo[3,Ä≤,4,Ä≤:6,7]naphtho[2,3-d]-1,3-dioxol-6(5aH)-one, 5,8,8a,9-tetrahydro-5-	C22H22O8	414.41	A/B	-8.5
5250	C(CC(COC(=O)C(C)c1ccc(F)c(cc1)c1ccccc1)CO)n1c2c(nc1)c(=O)nc(N)[nH]2	169046-87-1	[1,1,Ä≤-Biphenyl]-4-acetic acid, 2-fluoro-Œ±-methyl-, 2-[(2-amino-1,6-dihydro-6-	C23H22FN5O4	451.45	A/B	-8.5
5251	[C@H]([C@@H](CO)O)(O)[C@H]1[C@H](NC(=O)C)[C@@H](N)C[C@@H](C(=O)O)O1	169046-93-9	[1,1,Ä≤-Biphenyl]-4-acetic acid, 2-fluoro-Œ±-methyl-, 4-(2-amino-1,6-dihydro-6-o	C25H26FN5O4	479.5	A/B	-8.5
5252	C(n1c2c(nc1CC)c(C)cc(C)n2)c1ccc(cc1)c1c(S(=O)(=O)NC(=O)OCCCC)ccc(CCC)c1	169281-52-1	Carbamic acid, [[5-ethyl-4,Ä≤-[(2-ethyl-5,7-dimethyl-3H-imidazo[4,5-b]pyridin-3-	C30H36N4O4S	548.7	A/B	-8.5
5253	O=c1n([C@H]2O[C@@H](COP(N(C(C)C)C(C)C)OP(OP(OC[C@@H]3O[C@@H](CS3)n3c(=O)nc(N)cc3)N(C(C)C)C(C)C)Cl)SC	1701446-54-9	Not Yet Assigned	C30H49ClF2N7O9P3S	850.19	A/B	-8.5
5254	O=c1n([C@@H]2OC(=CC2)CO[Si](C(C)(C)C)(C)C)cc(C)c(=O)[nH]1	1704730-70-0	Benzonitrile, 4,4,Ä≤-[[5-bromo-2-[(4-cyanophenyl)amino]-4,6-pyrimidinediyl]bis(	C29H21BrN6O2	565.42	A/B	-8.5
5255	S(=O)(=O)(O)c1c2c(c(N)c(N=Nc3ccc(S(=O)(=O)c4ccc(cc4)c4ccc(S(=O)(=O)c5ccc(N=Nc6c(N)c7c(c(S(=O)(=O)O)c	17094-96-1	1-Naphthalenesulfonic acid, 3,3,Ä≤-[4,4,Ä≤-biphenylylenebis(thio-p-phenyleneazo	C44H32N6O6S4	869.02	A/B	-8.5
5256	C(Oc1ccc(C)cc1)c1c2c(N(c3c(C(=O)N2)cccn3)C2CC2)ncc1	172326-07-7	6H-Dipyrido[3,2-b:2,Ä≤,3,Ä≤-e][1,4]diazepin-6-one, 11-cyclopropyl-5,11-dihydro-	C22H20N4O2	372.42	A/B	-8.5
5257	C(CCn1c2c(nc1)c(=O)nc(N)[nH]2)(COC(=O)[C@H](Cc1ccccc1)NC(=O)OCc1ccccc1)(COC(=O)C)COC(=O)C	172645-42-0	1,2-Pyrrolidinedicarboxylic acid, 2-[4-(2-amino-1,6-dihydro-6-oxo-9H-purin-9-yl)	C24H30N6O7	514.53	A/B	-8.5
5258	[15NH2]c1c2c(n(c[15n]2)[C@@H]2O[C@H](CO)[C@@H](O)C2)ncn1	173167-81-2	Uridine 5,Ä≤-(trihydrogen diphosphate), 5-[3-[(4-benzoylbenzoyl)amino]propyl]-,	C34H42N4O19P2	872.66	A/B	-8.5
5259	O=c1n([C@@H]2O[C@H](COP(=O)([C@H](O)c3c(N(=O)=O)cccc3)OC[C@@H]3[C@@H](N=[N+]=[N-])C[C@@H](O3)n3c(=O)	173658-34-9	Thymidine, 3,Ä≤-azido-P-[(4-cyanophenyl)hydroxymethyl]-P,3,Ä≤-dideoxythymidylyl	C28H30N11O10P		A/B	-8.5
5260	O=c1n([C@@H]2O[C@H](COP(=O)([C@H](O)c3c(N(=O)=O)cc(N(=O)=O)cc3)OC[C@@H]3[C@@H](N=[N+]=[N-])C[C@@H](O	173658-37-2	Thymidine, 3,Ä≤-azido-P,3,Ä≤-dideoxy-P-[hydroxy(4-nitrophenyl)methyl]thymidylyl	C27H30N11O12P		A/B	-8.5
5261	N(=Nc1ccc(S(=O)(=O)O)cc1)c1c2c(cc(C(C)(C)C)cc2)ccc1O	173658-39-4	Thymidine, 3,Ä≤-azido-P,3,Ä≤-dideoxy-P-[(2,6-dinitrophenyl)hydroxymethyl]thymid	C27H29N12O14P		A/B	-8.5
5262	C(Cc1ccccc1)[C@@]1(CCC(F)(F)F)CC(=C([C@H](C(C)(C)C)c2cc(NS(=O)(=O)c3ccc(N)cn3)ccc2)C(=O)O1)O	174484-17-4	2-Pyridinesulfonamide, 5-cyano-N-[3-[1-[5,6-dihydro-4-hydroxy-2-oxo-6-(2-phenyle	C33H34F3N3O5S	641.7	A/B	-8.5
5263	C(Cc1ccccc1)[C@@]1(CCC)CC(=C([C@@H](CC)c2cc(NS(=O)(=O)c3ccc(C(F)(F)F)cn3)ccc2)C(=O)O1)O	174484-41-4	2-Pyridinesulfonamide, N-[3-[(1R)-1-[(6R)-5,6-dihydro-4-hydroxy-2-oxo-6-(2-pheny	C31H33F3N2O5S	602.66	A/B	-8.5
5264	C(Cc1ccccc1)[C@@]1(CCC)CC(=C([C@@H](CC)c2cc(NS(=O)(=O)c3ccc(C#N)cn3)ccc2)C(=O)O1)O	174484-51-6	2-Pyridinesulfonamide, 5-cyano-N-[3-[(1R)-1-[(6R)-5,6-dihydro-4-hydroxy-2-oxo-6-	C31H33N3O5S	559.68	A/B	-8.5

5265	C(Cc1ccccc1)C1(CCc2ccccc2)CC(=C(C(C(C)(C)C)c2cc(NS(=O)(=O)c3ccc(C#N)cn3)ccc2)C(=O)O1)O	174484-52-7	2-Pyridinesulfonamide, 5-cyano-N-[3-[(1S)-1-[(6S)-5,6-dihydro-4-hydroxy-2-oxo-6-	C31H33N3O5S	559.68	A/B	-8.5
5266	C(Cc1ccc(F)cc1)[C@@]1(CCC)CC(=C([C@@H](C(C)(C)C)c2cc(NS(=O)(=O)c3ccc(C#N)cn3)ccc2)C(=O)O1)O	174484-57-2	2-Pyridinesulfonamide, 5-cyano-N-[3-[1-[6-[2-(4-fluorophenyl)ethyl]-5,6-dihydro-	C33H36FN3O5S	605.72	A/B	-8.5
5267	C(Cc1ccccc1)[C@@]1(CCC(F)(F)F)CC(=C([C@@H](C(C)(C)C)c2cc(NS(=O)(=O)c3ccc(N)cn3)ccc2)C(=O)O1)O	174590-18-2	2-Pyridinesulfonamide, 5-amino-N-[3-[1-[5,6-dihydro-4-hydroxy-2-oxo-6-(2-phenyle	C32H36F3N3O5S	631.71	A/B	-8.5
5268	[C@@H](C(C)(C)C)(C1=C(O)C[C@](CCc2ccccc2)(CCC)OC1=O)c1cc(NS(=O)(=O)c2ccccn2)ccc1	174590-20-6	2-Pyridinesulfonamide, N-[3-[1-[5,6-dihydro-4-hydroxy-2-oxo-6-(2-phenylethyl)-6-	C32H38N2O5S	562.72	A/B	-8.5
5269	C(Cc1ccccc1)[C@@]1(CCC)CC(=C([C@H](CC)c2cc(NS(=O)(=O)c3ccc(C(F)(F)F)cn3)ccc2)C(=O)O1)O	174590-25-1	2-Pyridinesulfonamide, N-[3-[1-[5,6-dihydro-4-hydroxy-2-oxo-6-(2-phenylethyl)-6-	C33H37F3N2O5S	630.72	A/B	-8.5
5270	O=c1n([C@@H]2O[C@H](CO[P@]3(=O)Oc4c(CO3)cc(C)cc4)C=C2)cc(C)c(=O)[nH]1	174717-70-5	Carbamic acid, N-[[4,Ä≤-[(2-ethyl-5,7-dimethyl-3H-imidazo[4,5-b]pyridin-3-yl)met	C28H32N4O4S	520.64	A/B	-8.5
5271	O=c1n([C@@H]2O[C@H](CO[P@]3(=O)Oc4c(CO3)cccc4)C=C2)cc(C)c(=O)[nH]1	174746-71-5	Thymidine, 2,Ä≤,3,Ä≤-didehydro-3,Ä≤-deoxy-5,Ä≤-O-[(2R)-6-methyl-2-oxido-4H-1,3,	C18H19N2O7P	406.33	A/B	-8.5
5272	O=c1n(cc(C(F)(F)F)c(=O)[nH]1)[C@H]1C[C@H](OS(=O)(=O)C)[C@@H](COC(=S)c2ccc(Cl)cc2)O1	177165-73-0	Thymidine, Œ±,Œ±,Œ±-trifluoro-, 5,Ä≤-(4-chlorobenzenecarbothioate) 3,Ä≤-(2-thio	C22H16ClF3N2O6S2	560.95	A/B	-8.5
5273	O=c1n([C@H]2C[C@H](OC(=S)c3ccccc3)[C@@H](COC(=S)c3ccccc3)O2)cc(C(F)(F)F)c(=O)[nH]1	177165-80-9	Thymidine, Œ±,Œ±,Œ±-trifluoro-, 3,Ä≤,5,Ä≤-bis(4-methylbenzenecarbothioate)	C26H23F3N2O5S2	564.6	A/B	-8.5
5274	C(CN1CCC(CN2c3c4c(S2(=O)=O)cccc4ccc3)CC1)(NC)C12CC3CC(C2)CC(C1)C3	178477-44-6	1-Piperidineethanamine, 4-[(1,1-dioxido-2H-naphth[1,8-cd]isothiazol-2-yl)methyl]	C28H37N3O2S	479.68	A/B	-8.5
5275	C(CN1CCC(CN2c3c4c(S2(=O)=O)cccc4ccc3)CC1)(C12CC3CC(C2)CC(C1)C3)N1CCOCC1	178478-35-8	Ethanone, 2-[4-[(1,1-dioxido-2H-naphth[1,8-cd]isothiazol-2-yl)methyl]-1-piperidi	C28H35N3O3S	493.66	A/B	-8.5
5276	N(c1c(CO)cc(C=CC#N)cc1C)c1nc(Nc2ccc(C#N)cc2)ncc1	179817-97-1	2-Naphthalenesulfonic acid, 5,5,Ä≤-[(3,3,Ä≤-dimethyl[1,1,Ä≤-biphenyl]-4,4,Ä≤-di	C34H26N4O8S2	682.72	A/B	-8.5
5277	O([C@H](OC(=O)OCC)C)c1c(C(=O)NCc2ccc(F)cc2)nc(C(NC(=O)c2nnc(C)o2)(C)C)n(C)c1=O	1798830-21-3	Carbonic acid, ethyl (1S)-1-[[4-[[[(4-fluorophenyl)methyl]amino]carbonyl]-1,6-di	C25H29FN6O8	560.53	A/B	-8.5
5278	N(C(=O)[C@H](Cc1ccccc1)C[C@@H](CN1[C@H](C(=O)NC(C)(C)C)CN(Cc2cc3c(nc2)OC(O)C3O)CC1)O)[C@H]1c2c(C[C@H	179952-07-9	Glycine, N-[S-[5-[[3-[[(1,1-dimethylethyl)amino]carbonyl]-4-[2,3,5-trideoxy-N-(2	C48H64N8O12S	977.13	A/B	-8.5
5279	C[C@]1(F)[C@@H](O[C@H](CO[P@](=O)(Oc2cc3c(cc2)OCCO3)N[C@H](C(=O)OC(C)C)C)[C@H]1O)n1c(=O)[nH]c(=O)cc1	1800478-35-6	L-Alanine, N-[[P(S),2,Ä≤R]-2,Ä≤-deoxy-2,Ä≤-fluoro-2,Ä≤-methyl-P-(5,6,7,8-tetrah	C26H35FN3O9P	583.54	A/B	-8.5
5280	O([Si](C(C)(C)C)(C)C)c1c(C(=O)NCc2ccc(F)cc2)nc(C(NC(=O)c2nnc(C)o2)(C)C)n(C)c1=O	1801912-44-6	4-Pyrimidinecarboxamide, N-[(4-fluorophenyl)methyl]-1,6-dihydro-1-methyl-2-[1-me	C25H29FN6O6	528.53	A/B	-8.5
5281	C[C@]1(Cl)[C@@H](O[C@H](COP(=O)(Oc2ccccc2)N[C@@H](C(=O)OC(C)C)C)[C@H]1O)n1c(=O)[nH]c(=O)cc1	1802921-99-8	Carbamic acid, N-[(1S)-1-[[(2S,4S)-4-(methoxymethyl)-2-[5-[4-[1,2,3,4-tetrahydro	C48H62N8O7	863.06	A/B	-8.5
5282	C(OP1(=S)Oc2c(CO1)cccc2)[C@@]1(C(F)F)O[C@H]([C@@H](O)[C@@H]1O)n1c2c(nc1)c(N)ncn2	1803134-27-1	9H-Purin-6-amine, 9-[4-C-(difluoromethyl)-5-O-(2-oxido-4H-1,3,2-benzodioxaphosph	C18H18F2N5O7P	485.34	A/B	-8.5
5283	C(F)(F)[C@@]12[C@H]([C@H](O)[C@@H](O1)n1c3c(nc1)c(N)ncn3)OP(=O)(OC(C)C)OC2	1803134-30-6	9H-Purin-6-amine, 9-[5-O-[bis(4-methoxyphenoxy)phosphinothioyl]-4-C-(difluoromet	C25H26F2N5O8PS	625.54	A/B	-8.5
5284	O=C1[C@]2([C@H]([C@H](/C(=C/C(=O)[C@@H]3[C@@]4(N5[C@@H]([C@H]3c3cc(OC)c(O)cc3)CCC5)c3c(N(C)C4=O)cccc	1807888-46-5	Not Yet Assigned	C47H48N4O8	796.91	A/B	-8.5
5285	C(=O)([C@@H](NC(=O)OC)C(C)C)N1[C@@H](CCC1)c1[nH]c(cn1)c1ccc(cc1)c1ccc(cc1)c1[nH]c([C@@H](NC(=O)[C@@H	1808295-95-5	Carbamic acid, N-[(1S)-1-[[(2S)-2-[5-[4,Ä≤-[2-[(7S)-6-[(2S)-2-[(methoxycarbonyl)	C43H55N9O6	793.95	A/B	-8.5
5286	O=C1[C@@H]2[C@@H](c3c([C@H](OC(=O)[C@H](C)N4C(=O)c5c(C4=O)cccc5)[C@H]2CO1)cc1c(c3)OCO1)c1cc(OC)c(OC)	1809321-44-5	2H-Isoindole-2-acetic acid, 1,3-dihydro-Œ±-methyl-1,3-dioxo-, (5R,5aR,8aR,9R)-5,	C33H29NO11	615.58	A/B	-8.5
5287	C(C[C@@H](C/C=C/Cc1ccc(O)cc1)O)c1cc(OC)c(O)cc1	1822365-49-0	2H-Pyrido[1,Ä≤,2,Ä≤:4,5]pyrazino[2,1-b][1,3]oxazine-9-carboxamide, N-[(2,4-difl	C26H29F2N3O10	581.52	A/B	-8.5
5288	C(COCCl)n1c2c(c(N)ncn2)nc1	1823109-75-6	D-glycero-D-galacto-Non-2-enonic acid, 5-(acetylamino)-4-[(aminoiminomethyl)amir	C19H21ClFN3O4S.C12H20N4O7		A/B	-8.5
5289	C(OC(C[18F])CO)n1c2c(nc1)c(=O)nc(N)[nH]2	1838684-56-2	1-Piperazinecarboxamide, N-[(1S)-4-[[imino(methoxyamino)methyl]amino]-1-[[[(1S,2	C30H43N7O5S	613.77	A/B	-8.5
5290	C(OC[C@@H]1[C@@H](OC(=O)COCC)C[C@@H](C1=C)n1c2c(nc1)c(=O)nc(NC(=O)OC(C)(C)C)[nH]2)(c1ccccc1)(c1ccccc	1860842-93-8	3-Butenoic acid, (1S,2R,4S)-4-[2-[[(1,1-dimethylethoxy)carbonyl]amino]-1,6-dihyd	C40H41N5O6	687.78	A/B	-8.5
5291	C(OC[C@@H]1[C@@H](OC(=O)C=Cc2ccccc2)C[C@@H](C1=C)n1c2c(nc1)c(=O)nc(NC(=O)OC(C)(C)C)[nH]2)(c1ccccc1)(	1860842-96-1	Carbamic acid, N-[9-[(1S,3R,4S)-4-(benzoyloxy)-2-methylene-3-[(triphenylmethoxy)	C43H41N5O6	723.82	A/B	-8.5
5292	O=C1[C@@H]2[C@@H](c3c([C@H](OC(=O)CN4c5c(C(=O)C4=O)c(Br)ccc5)[C@H]2CO1)cc1c(c3)OCO1)c1cc(OC)c(OC)c(O	1864833-29-3	1H-Indole-1-acetic acid, 4-chloro-2,3-dihydro-2,3-dioxo-, (5R,5aR,8aR,9R)-5,5a,6	C32H26ClNO11	636	A/B	-8.5
5293	O=C1[C@@H]2[C@@H](c3c([C@H](OC(=O)CCCC[C@H]4SC(SCC4)c4cc(OC)c(OC)cc4)[C@H]2CO1)cc1c(c3)OCO1)c1cc(OC)	1875123-87-7	1,3-Dithiane-4-pentanoic acid, 2-(2-methoxyphenyl)-, (5R,5aR,8aR,9R)-5,5a,6,8,8a	C38H42O10S2	722.86	A/B	-8.5
5294	O=C1[C@@H]2[C@@H](c3c([C@H](OC(=O)CCCC[C@H]4SC(SCC4)c4cc(Br)ccc4)[C@H]2CO1)cc1c(c3)OCO1)c1cc(OC)c(OC	1875123-93-5	1,3-Dithiane-4-pentanoic acid, 2-(2,4-dichlorophenyl)-, (5R,5aR,8aR,9R)-5,5a,6,8	C37H38Cl2O9S2	761.73	A/B	-8.5
5295	O=c1n([C@@H]2O[C@H](COP(=O)(OP(=O)(OP(=O)(OCc3ccc(OC(=O)CCCCCCC)cc3)OCc3ccc(OC(=O)CCCC)cc3)O)O)[C@@H	1883329-13-2	Not Yet Assigned	C37H39F3N5O17P3		A/B	-8.5
5296	O[C@H]1[C@@H](O[C@H](CO)[C@H]1O)n1cc(c(=O)[nH]c1=O)c1cc(ncc1)N1CCN(C)CC1	1883514-10-0	Not Yet Assigned	C17H17N3O6	359.33	A/B	-8.5
5297	O(C(=O)N[C@@H](Cc1ccc(OCc2c(C(=O)O)nc(cc2)c2c(F)cc(F)cc2)cc1)[C@@H](CN(S(=O)(=O)c1cc2c(cc1)ncs2)CC(C	1887206-12-3	Benzo[b]thiophene-3-carboxylic acid, 2-[2-[4-[(2S,3R)-4-[(6-benzothiazolylsulfor	C41H51N3O9S3	826.05	A/B	-8.5
5298	C(=O)(O)c1c2c(sc1NC(=O)c1c(CNc3ccc(C[C@@H]([C@@H](CN(S(=O)(=O)c4cc5c(cc4)ncs5)CC4CCCC4)O)NC(=O)O[C@@	1887206-36-1	Benzo[b]thiophene-3-carboxylic acid, 2-[[2-[[[4-[(2S,3R)-4-[(6-benzothiazolylsul	C47H55N5O10S3	946.16	A/B	-8.5
5299	O(P(=O)(OC[C@@H]1[C@@H](OC(=O)c2ccccc2)[C@@H](OC(=O)c2ccccc2)[C@@H](O1)n1nc(C(=O)N)nc1)OCCc1ccc(N(=O	190141-35-6	Adenosine, N-benzoyl-3,Ä≤-O-benzoyl-5,Ä≤-O-[(4-methoxyphenyl)diphenylmethyl]-P-	C74H63N10O18P	1411.32	A/B	-8.5
5300	O=c1c2c(n(cn2)[C@@H]2O[C@H](COP(=O)(F)[O-])[C@@H](O)[C@H]2O)[nH]c(N)n1	190141-46-9	Adenosine, 1,Ä≤-[3-(aminocarbonyl)-1H-1,2,4-triazol-1-yl]-1,Ä≤-de(6-amino-9H-pu	C28H36N14O17P2	902.62	A/B	-8.5
5301	O=c1n(c2c(c(cc(n2)c2ccccc2)c2ccco2)c(=O)n1c1ccccc1)[C@H]1[C@H](OC(=O)c2ccccc2)[C@H](OC(=O)c2ccccc2)[	190319-80-3	Pyrido[2,3-d]pyrimidine-2,4(1H,3H)-dione, 3,7-bis(4-chlorophenyl)-5-(2-furanyl)-	C49H33Cl2N3O10	894.71	A/B	-8.5
5302	O=c1n(c2c(c(cc(n2)c2ccc(Cl)cc2)c2ccccc2)c(=O)n1c1ccccc1)[C@H]1[C@H](OC(=O)c2ccccc2)[C@H](OC(=O)c2ccc	190319-82-5	Pyrido[2,3-d]pyrimidine-2,4(1H,3H)-dione, 3-(4-chlorophenyl)-5-(2-furanyl)-7-phe	C49H34ClN3O10	860.26	A/B	-8.5
5303	N(c1nc(nc(Nc2ccccc2)n1)N1CCOCC1)c1ccc(C#N)nc1	1907643-78-0	2-Pyridinecarbonitrile, 5-[[4-[(4-fluorophenyl)amino]-6-(4-morpholinyl)-1,3,5-tr	C19H17FN8O	392.39	A/B	-8.5
5304	C(=O)(N[C@@H](CC1CCC1)C(=O)C(=O)N)[C@H]1[C@H]2[C@H](C2(C)C)CN1C(=O)[C@@H](NC(=O)NCS(=O)(=O)N(C(CF)(C	1919044-30-6	2-Pyrimidinamine, 5-(4,6-di-4-morpholinyl-1,3,5-triazin-2-yl)-4-(trifluoromethyl	C16H19F3N8O2	412.37	A/B	-8.5
5305	C[C@]12[C@H]([C@H]3[C@@H]([C@]4(C)[C@H](CC3)CCCC4)CC2)CC[C@@H]1[C@@H](CCC(=O)NCCNCCNCCN)C	193898-72-5	Adenosine, 1,Ä≤-[3-(aminocarbonyl)-1H-1,2,4-triazol-1-yl]-3,Ä≤-O-benzoyl-1,Ä≤-d	C54H47N10O17P	1138.98	A/B	-8.5
5306	N(Cc1c(Cl)cc(F)cc1)c1c2c(n(cn2)[C@@H]2O[C@H](CO)[C@@H](O)[C@@H]2O)ncn1	1947401-68-4	9H-Purin-6-amine, 9-Œ≤-D-arabinofuranosyl-N-[[3-fluoro-5-(trifluoromethyl)pheny]	C18H17F4N5O4	443.35	A/B	-8.5
5307	O[C@@H]1[C@H](n2c3c(nc2)c(NCc2cc(C(F)(F)F)c(Cl)cc2)ncn3)O[C@H](CO)[C@H]1O	1947401-73-1	9H-Purin-6-amine, 9-Œ≤-D-arabinofuranosyl-N-[[4-(trifluoromethyl)phenyl]methyl]-	C18H18F3N5O4	425.36	A/B	-8.5
5308	O[C@@H]1[C@H](n2c3c(nc2)c(NCc2cc(C(F)(F)F)cc(C(F)(F)F)c2)ncn3)O[C@H](CO)[C@H]1O	1947401-75-3	9H-Purin-6-amine, 9-Œ≤-D-arabinofuranosyl-N-[[4-fluoro-3-(trifluoromethyl)pheny]	C18H17F4N5O4	443.35	A/B	-8.5
5309	Clc1c2c(c3c(nc([nH]3)[C@H]3N(C(=O)[C@@H](NC(=O)OC(C)C)C(C)C)CCC3)c1)ccc(c2)c1ccc(cc1)c1[nH]c(nc1)[C@	1949770-72-2	Carbamic acid, N-[(1S)-1-[[(2S)-2-[5-chloro-7-[4-[2-[(2S)-1-[(2S)-2-[(methoxycar	C42H49ClN8O6	797.34	A/B	-8.5
5310	C(=O)([C@@H](NC(=O)OC)C(C)C)N1[C@H](c2[nH]c3c4c(cc(cc4)c4ccc(cc4)c4[nH]c(nc4)[C@H]4N(C(=O)[C@@H](NC(	1949770-77-7	Carbamic acid, N-[(1S)-1-[[(2S)-2-[7-[4-[2-[(2S,4R)-4-(acetylamino)-1-[(2S)-2-[	C44H53N9O7	819.95	A/B	-8.5
5311	N(C(=O)C1CN(C)CCO1)C12CC3(O)CC(C2)CC(C1)C3	195063-36-6	2,7-Naphthalenedisulfonic acid, 4,4,Ä≤-[(3,3,Ä≤-dimethyl[1,1,Ä≤-biphenyl]-4,4,Ä	C34H26N4O14S4.4Na		A/B	-8.5
5312	O[C@H]1[C@@H](O[C@H](CO[P@@]2(=O)Oc3c(CO2)cccc3)[C@H]1O)n1c(=O)[nH]c(=NO)cc1	1955522-21-0	Uridine, 5,Ä≤-O-[(2R)-2-oxido-4H-1,3,2-benzodioxaphosphorin-2-yl]-, 4-oxime	C16H18N3O9P	427.3	A/B	-8.5
5313	C[C@]1(F)[C@@H](O[C@H](CO[P@](=O)(Oc2ccccc2)N[C@H](C(=O)OC(C)C)C)[C@H]1OCSC)n1c(=O)[nH]c(=O)cc1	1972595-88-2	L-Alanine, N-[[P(S),2,Ä≤R]-2,Ä≤-deoxy-2,Ä≤-fluoro-2,Ä≤-methyl-3,Ä≤-O-[2-[[1-oxo	C46H50FN4O11PS	916.95	A/B	-8.5
5314	O[C@H]1[C@H](n2c3c(nc2)c(=O)nc(N)[nH]3)O[C@H](COc2c(F)c(=O)[nH]c(O)c2F)[C@H]1O	197903-82-5	Guanosine, 5,Ä≤-O-(2,3,5,6-tetrafluoro-4-nitrophenyl)-	C16H12F4N6O7	476.3	A/B	-8.5
5315	N(c1c(C#N)cccc1)c1nc(Nc2c(C)cc(C)cc2)cc(C)n1	1984303-61-8	Benzonitrile, 2-[[2-[[2-methyl-6-(1-methylethyl)phenyl]amino]-4-pyrimidinyl]amir	C21H21N5	343.42	A/B	-8.5
5316	N(c1c(C#N)cccc1)c1nc(Nc2ccc(C(C)C)cc2)ncc1	1984395-04-1	Benzonitrile, 4-[[4-methyl-6-[(2,4,6-trimethylphenyl)amino]-2-pyrimidinyl]amino]	C21H21N5	343.42	A/B	-8.5
5317	O(C(=O)CCC)c1c2n(N([C@H]3N(C2=O)CCOC3)[C@H]2c3c(CSc4c2cccc4)c(F)c(F)cc3)ccc1=O	1985606-49-2	1-Piperazinecarboxylic acid, 4-methyl-, (12aR)-12-[(11S)-7,8-difluoro-6,11-dihyc	C30H29F2N5O5S	609.64	A/B	-8.5
5318	O(C(=O)OCC)c1c2n(N([C@H]3N(C2=O)CCOC3)[C@H]2c3c(CSc4c2cccc4)c(F)c(F)cc3)ccc1=O	1985606-53-8	1H-[1,4]Oxazino[3,4-c]pyrido[2,1-f][1,2,4]triazine-6,8-dione, 12-[(11S)-7,8-difl	C31H25F2N3O4S	573.61	A/B	-8.5
5319	O(C(=O)N(C)c1ccccc1)c1c2n(N([C@H]3N(C2=O)CCOC3)[C@H]2c3c(CSc4c2cccc4)c(F)c(F)cc3)ccc1=O	1985607-07-5	Carbonic acid, [[(12aR)-12-[(11S)-7,8-difluoro-6,11-dihydrodibenzo[b,e]thiepin-1	C32H29F2N3O9S	669.65	A/B	-8.5
5320	O(P(=O)(NC1(C(=O)OC)CC1)OCC)c1c2n(N([C@H]3N(C2=O)CCOC3)[C@H]2c3c(CSc4c2cccc4)c(F)c(F)cc3)ccc1=O	1985607-21-3	Not Yet Assigned	C31H33F2N4O8PS	690.65	A/B	-8.5
5321	O(C(=O)OC1CCN(C)CC1)c1c2n(N([C@H]3N(C2=O)CCOC3)[C@H]2c3c(CSc4c2cccc4)c(F)c(F)cc3)ccc1=O	1985607-29-1	Carbonic acid, 4-(acetylamino)phenyl (12aR)-12-[(11S)-7,8-difluoro-6,11-dihydroc	C33H26F2N4O7S	660.64	A/B	-8.5
5322	O(C(=O)O[C@H]1C(C)(C)COC1=O)c1c2n(N([C@H]3N(C2=O)CCOC3)[C@H]2c3c(CSc4c2cccc4)c(F)c(F)cc3)ccc1=O	1985607-51-9	Carbonic acid, (12aR)-12-[(11S)-7,8-difluoro-6,11-dihydrodibenzo[b,e]thiepin-11-	C31H27F2N3O8S	639.62	A/B	-8.5
5323	O(C(=O)O[C@@H](C(=O)OC)c1ccccc1)c1c2n(N([C@H]3N(C2=O)CCOC3)[C@H]2c3c(CSc4c2cccc4)c(F)c(F)cc3)ccc1=O	1985607-52-0	Carbonic acid, (12aR)-12-[(11S)-7,8-difluoro-6,11-dihydrodibenzo[b,e]thiepin-11-	C31H27F2N3O8S	639.62	A/B	-8.5
5324	O(Cc1cccnc1)c1c2n(N([C@H]3N(C2=O)CCOC3)[C@H]2c3c(CSc4c2cccc4)c(F)c(F)cc3)ccc1=O	1985607-58-6	Propanoic acid, 2-[[[[(12aR)-12-[(11S)-7,8-difluoro-6,11-dihydrodibenzo[b,e]thie	C33H33F2N3O8S	669.69	A/B	-8.5
5325	O(Cc1c(oc(=O)o1)c1ccccc1)c1c2n(N([C@H]3N(C2=O)CCOC3)[C@H]2c3c(CSc4c2cccc4)c(F)c(F)cc3)ccc1=O	1985607-60-0	1H-[1,4]Oxazino[3,4-c]pyrido[2,1-f][1,2,4]triazine-6,8-dione, 12-[(11S)-7,8-difl	C30H24F2N4O4S	574.6	A/B	-8.5
5326	C(F)(F)c1n(c2c(n1)cccc2)c1nc(nc(n1)N1CCOCC1)N1CCN(CC1)N1[C@@H](C(=O)O)CCC1	2001083-61-8	Propanoic acid, 2-[[4-[4-[2-(difluoromethyl)-1H-benzimidazol-1-yl]-6-(4-morpholi	C22H27F2N9O3	503.51	A/B	-8.5
5327	C(=O)([C@@H](NC(=O)OC)C1CCOCC1)N1[C@H](c2[nH]c3c4c(cc(cc4)c4c5c(c(cc4)c4[nH]c(nc4)[C@H]4N(C(=O)[C@@H	2007900-49-2	Carbamic acid, N-[(1S)-1-[[(2S,5S)-2-[7-[2,3-dihydro-7-[2-[(2S,5S)-1-[(2S)-2-[(m	C47H58N8O6	831.01	A/B	-8.5
5328	C(=O)([C@@H](NC(=O)OC)C(C)C)N1[C@H](c2[nH]c3c4c(cc(cc4)c4c5c(c(cc4)c4[nH]c(nc4)[C@H]4N(C(=O)[C@@H](N	2007901-41-7	Carbamic acid, N-[(1S)-1-[[(2S,5S)-2-[7-[4-[2-[(2S)-1-[(2S)-2-cyclohexyl-2-[(met	C50H62N8O6	871.08	A/B	-8.5
5329	C(=O)([C@@H](NC(=O)OC)C(C)C)N1[C@@H]([C@H]2[C@H](C2)C1)c1[nH]c(cn1)c1c2c(c(cc1)c1cc3c(cc1)c1c(cc3)nc	2007901-62-2	Carbamic acid, N-[(1S)-1-cyclopropyl-2-[(2S,5S)-2-[5-[2,3-dihydro-7-[2-[(2S,5S)-	C47H56N8O6	829	A/B	-8.5
5330	C(=O)([C@@H](NC(=O)OC)C(C)C)N1[C@H](c2[nH]c3c4c(cc(cc4)c4c5c(c(cc4)c4[nH]c(nc4)[C@H]4N(C(=O)[C@@H](N	2007901-66-6	Carbamic acid, N-[(1S)-2-methyl-1-[[(2S)-2-[7-[5,6,7,8-tetrahydro-4-[2-[(2S)-1-[	C46H56N8O6	816.99	A/B	-8.5
5331	C(=O)(C(=O)Nc1cccnc1)c1c(C23CC4CC(C3)CC(C2)C4)[nH]c2c1cccc2	2014403-93-9	1H-Indole-3-acetamide, Œ±-oxo-N-3-pyridinyl-2-tricyclo[3.3.1.13,7]dec-1-yl-	C25H25N3O2	399.48	A/B	-8.5
5332	N(=Nc1ccc(S(=O)(=O)[O-])cc1)c1c2cc(cc(S(=O)(=O)[O-])cc2)ccc1O	201594-20-9	Furo[3,Ä≤,4,Ä≤:6,7]naphtho[2,3-d]-1,3-dioxole-5,8-dione, 9-(3,4-dihydroxy-5-met	C20H16O8	384.34	A/B	-8.5
5333	O=c1n([C@H]2C/C(=N/OC(=O)c3ccccc3)/[C@@H](CO)O2)cc(C)c(=O)[nH]1	201600-68-2	2,4(1H,3H)-Pyrimidinedione, 1-[(2R,4E,5S)-4-[(benzoyloxy)imino]tetrahydro-5-(hyc	C17H17N3O6	359.33	A/B	-8.5
5334	[C@H](C(C)C)(CO)n1c2c(cc(Cc3cc(cc(Cl)c3F)N3C(=O)OCC3)c(OC)c2)c(=O)c(C(=O)O)c1	1000161-55-6	3-Quinolinecarboxylic acid, 6-[[3-chloro-2-fluoro-5-(1-pyrrolidinyl)phenyl]methy	C27H30ClFN2O5	516.99	C	-8.5
5335	[C@H](C(C)C)(CO)n1c2c(cc(Cc3cc(cc(Cl)c3F)N3CCOCC3)c(OC)c2)c(=O)c(C(=O)O)c1	1000161-56-7	3-Quinolinecarboxylic acid, 6-[[3-chloro-2-fluoro-5-(2-oxo-3-oxazolidinyl)phenyl]	C26H26ClFN2O7	532.95	C	-8.5
5336	[C@H](C(C)C)(CO)n1c2c(cc(Cc3cc(cc(Cl)c3F)N3CCCCC3)c(OC)c2)c(=O)c(C(=O)O)c1	1000161-57-8	3-Quinolinecarboxylic acid, 6-[[3-chloro-2-fluoro-5-(4-morpholinyl)phenyl]methyl	C27H30ClFN2O6	532.99	C	-8.5
5337	[C@H](C(C)C)(CO)n1c2c(cc(Cc3cc(cc(Cl)c3F)N3CC[C@H](O)C3)c(OC)c2)c(=O)c(C(=O)O)c1	1000161-60-3	3-Quinolinecarboxylic acid, 6-[[3-chloro-2-fluoro-5-[(3R)-3-hydroxy-1-pyrrolidir	C27H30ClFN2O6	532.99	C	-8.5
5338	[C@H](C(C)C)(CO)n1c2c(cc(Cc3cc(cc(Cl)c3F)N3N(C)C(=O)CC3)c(OC)c2)c(=O)c(C(=O)O)c1	1000161-61-4	3-Quinolinecarboxylic acid, 6-[[3-chloro-2-fluoro-5-[(3S)-3-hydroxy-1-pyrrolidir	C27H30ClFN2O6	532.99	C	-8.5
5339	[C@H](C(C)C)(CO)n1c2c(cc(Cc3cc(cc(Cl)c3F)N3CCN(C(=O)C)CC3)c(OC)c2)c(=O)c(C(=O)O)c1	1000161-62-5	3-Quinolinecarboxylic acid, 6-[[3-chloro-2-fluoro-5-(2-methyl-3-oxo-1-pyrazolidi	C27H29ClFN3O6	545.99	C	-8.5
5340	[C@H](C(OC)(C)C)(CO)n1c2c(cc(Cc3cc(cc(Cl)c3F)N3CCOCC3)c(OC)c2)c(=O)c(C(=O)O)c1	1000161-86-3	3-Quinolinecarboxylic acid, 6-[[3-chloro-2-fluoro-5-(2-thiazolyl)phenyl]methyl]-	C26H24ClFN2O5S	531	C	-8.5
5341	O=c1c2c(n(cn2)[C@@H]2C[C@H](CO)[C@@H](O)C2)[nH]c(N)n1	1000162-06-0	3-Quinolinecarboxylic acid, 6-[[3-chloro-2-fluoro-5-(5-methyl-1,2,4-oxadiazol-3-	C26H25ClFN3O6	529.94	C	-8.5
5342	[C@H](C(=O)N1c2c(CC1)cccc2)(N)C12CC3(O)CC(C2)CC(C1)C3	1000689-64-4	Ethanone, 2-amino-2-(3-hydroxytricyclo[3.3.1.13,7]dec-1-yl)-1-(2H-isoindol-2-yl)	C20H24N2O2	324.42	C	-8.5
5343	C(OC(=O)C1CCC(O[C@@H]2O[C@H]3[C@@H]([C@H](O)[C@H]2O)O[C@H](OC3)c2ccccc2)CC1)[C@@H]1[C@@H]2[C@H]([C@@	1002355-31-8	1H-1,2,4-Triazole-3-carboxamide, 1-[5-O-[[4-(Œ≤-D-galactopyranosyloxy)cyclohexy]	C21H32N4O12	532.5	C	-8.5
5344	C(=O)([C@@H](NC(=O)OC)C(C)C)N1[C@@H](CCC1)c1[nH]c(cn1)c1ccc(cc1)c1ccc(cc1)c1[nH]c(nc1)[C@H]1N(C(=O)O	1007884-58-3	Carbamic acid, N-[(1S)-1-[[(2S)-2-[5-[4,Ä≤-[2-[(2S)-1-[(2S)-2-[[(cyanoamino)(dir	C42H53N11O4	775.94	C	-8.5
5345	C(=O)([C@@H](NC(=O)OC)C(C)C)N1[C@@H](CCC1)c1[nH]c(cn1)c1cc(CO)c(cc1)c1ccc(cc1)c1[nH]c(nc1)[C@H]1N(C(	1007885-23-5	Carbamic acid, N-[(1S)-1-[[(2S)-2-[5-[2,Ä≤-(aminocarbonyl)-4,Ä≤-[2-[(2S)-1-[(2S	C41H51N9O7	781.9	C	-8.5
5346	C(=O)([C@H](NC(=O)OC)c1ccccc1)N1[C@@H](CCC1)c1[nH]c(cn1)c1ccc(cc1)c1ccc(cc1)c1[nH]c(nc1)[C@H]1N(C(=O	1009107-39-4	Carbamic acid, N-[(1R)-2-[(2S)-2-[5-[4,Ä≤-[2-[(2S)-1-(2-aminoacetyl)-2-pyrrolidi	C38H40N8O4	672.78	C	-8.5
5347	C(=O)([C@H](NC(=O)OC)c1ccccc1)N1[C@@H](CCC1)c1[nH]c(cn1)c1ccc(cc1)c1ccc(cc1)c1[nH]c(nc1)[C@H]1N(C(=O	1009107-88-3	Carbamic acid, N-[(1R)-2-[(2S)-2-[5-[4,Ä≤-[2-[(2S)-1-[(2S)-2-(dimethylamino)-1-c	C41H46N8O4	714.86	C	-8.5
5348	C(=O)([C@H](N(C)C)c1ccccc1)N1[C@@H](CCC1)c1[nH]c(cn1)c1ccc(cc1)c1ccc(cc1)c1[nH]c(nc1)[C@H]1N(C(=O)[C	1009110-76-2	Carbamic acid, N-[2-[(2S)-2-[5-[4,Ä≤-[2-[(2S)-1-[(2R)-2-(dimethylamino)-2-pheny]	C40H44N8O4	700.83	C	-8.5
5349	C(=O)([C@@H](NC(=O)OC1CCOCC1)C(C)C)N1[C@@H](CCC1)c1[nH]c(cn1)c1ccc(cc1)c1ccc(cc1)c1[nH]c(nc1)[C@H]1N	1009113-82-9	Carbamic acid, N-[(1S)-1-[[(2S)-2-[5-[4,Ä≤-[2-[(2S)-1-[(2S)-2-[(methoxycarbonyl)	C43H54N8O7	794.94	C	-8.5
5350	C(=O)([C@@H](NC(=O)CC)C(C)C)N1[C@@H](CCC1)c1[nH]c(cn1)c1ccc(cc1)c1ccc(cc1)c1[nH]c(nc1)[C@H]1N(C(=O)[	1009113-87-4	Acetamide, N,N,Ä≤-[[1,1,Ä≤-biphenyl]-4,4,Ä≤-diylbis[1H-imidazole-5,2-diyl-(2S)-	C40H50N8O4	706.88	C	-8.5
5351	C(=O)([C@@H](Nc1cccnc1)C(C)C)N1[C@@H](CCC1)c1[nH]c(cn1)c1ccc(cc1)c1ccc(cc1)c1[nH]c(nc1)[C@H]1N(C(=O)	1009114-16-2	Carbamic acid, N-[(1S)-2-methyl-1-[[(2S)-2-[5-[4,Ä≤-[2-[(2S)-1-[(2S)-3-methyl-1-	C43H51N9O4	757.92	C	-8.5
5352	C(=O)([C@H](Cc1cnn[nH]1)NC(=O)OC)N1[C@@H](CCC1)c1[nH]c(cn1)c1ccc(cc1)c1ccc(cc1)c1[nH]c(nc1)[C@H]1N(C	1009114-22-0	Carbamic acid, N-[(1S)-2-[(2S)-2-[5-[4,Ä≤-[2-[(2S)-1-[(2S)-2-[(methoxycarbonyl)a	C38H43N11O6	749.82	C	-8.5
5353	C(=O)([C@@H](NC(=O)OC)C(C)C)N1[C@@H](CCC1)c1[nH]c(cn1)c1ccc(cc1)c1ccc(cc1)c1[nH]c(nc1)[C@H]1N(C(=O)[	1009114-58-2	Carbamic acid, N-[(1S)-1-[[(2S)-2-[5-[4,Ä≤-[2-[(2S)-1-[[cis-4-[(methoxycarbonyl)	C42H52N8O6	764.91	C	-8.5
5354	C(=O)([C@@H](NC(=O)OC)C(C)C)N1[C@@H](CCC1)c1[nH]c(cn1)c1ccc(cc1)c1ccc(cc1)c1[nH]c(nc1)[C@H]1N(C(=O)[	1009115-03-0	Carbamic acid, N-[(1S)-1-[[(2S)-2-[5-[4,Ä≤-[2-[(2S)-1-[(2S)-3-[4-[(acetylamino)r	C47H55N9O7	858	C	-8.5
5355	C(=O)([C@@H](NC(=O)OC)C(C)C)N1[C@@H](C[C@H](O)C1)c1[nH]c(cn1)c1ccc(cc1)c1ccc(cc1)c1[nH]c(nc1)[C@H]1N	1009115-61-0	Carbamic acid, N,N,Ä≤-[[1,1,Ä≤-biphenyl]-4,4,Ä≤-diylbis[1H-imidazole-5,2-diyl[(	C36H42N8O8	714.77	C	-8.5
5356	C(=O)([C@H](N(CC)CC)c1ccccc1)N1[C@@H](CCC1)c1[nH]c(cn1)c1ccc(cc1)c1ccc(cc1)c1[nH]c(nc1)[C@H]1N(C(=O)	1009117-53-6	Carbamic acid, N-[2-[(2S)-2-[5-[4,Ä≤-[2-[(2S)-1-[(2R)-2-(diethylamino)-2-phenyla	C42H48N8O4	728.88	C	-8.5
5357	C(=O)([C@@H](NC(=O)OC)C(C)C)N1[C@@H](CCC1)c1[nH]c(cn1)c1ccc(cc1)c1ccc(cc1)c1[nH]c(nc1)[C@H]1N(C(=O)[	1009117-77-4	Carbamic acid, N-[(1S)-1-[[(2S)-2-[5-[4,Ä≤-[2-[(2S)-1-[2-[(methoxycarbonyl)aminc	C37H44N8O6	696.8	C	-8.5
5358	C(=O)([C@@H](NC(=O)OC)C(C)(C)O)N1[C@@H](CCC1)c1[nH]c(cn1)c1ccc(cc1)c1ccc(cc1)c1[nH]c(nc1)[C@H]1N(C(=	1009118-10-8	Carbamic acid, N-[(1S)-1-[[(2S)-2-[5-[4,Ä≤-[2-[(2S)-1-[(2S)-2-amino-3-hydroxy-3-	C38H48N8O5	696.84	C	-8.5
5359	C(=O)([C@@H](NC(=O)OC(C)(C)C)C(C)C)N1[C@@H](CCC1)c1[nH]c(cn1)c1ccc(cc1)c1ccc(cc1)c1[nH]c(nc1)[C@H]1N	1009119-15-6	Carbamic acid, N,N,Ä≤-[[1,1,Ä≤-biphenyl]-4,4,Ä≤-diylbis[1H-imidazole-5,2-diyl-(	C42H54N8O6	766.93	C	-8.5
5360	O=C1[C@@H]2[C@@H](c3c([C@@H](O)[C@H]2CO1)cc1c(c3)OCO1)c1cc(OC)c(OC(=O)C(Cl)Cl)c(OC)c1	1016316-67-8	2-Naphthalenesulfonic acid, 5-[2-(2,3-dimethylphenyl)diazenyl]-6-hydroxy-	C18H16N2O4S	356.4	C	-8.5
5361	O[C@H]1[C@@H](O[C@H](CO)[C@H]1O)n1c(=O)[nH]c(=O)c(I)c1	102394-26-3	1-Naphthalenesulfonic acid, 7-hydroxy-8-[2-[4-(2-phenyldiazenyl)phenyl]diazenyl]	C22H16N4O4S	432.45	C	-8.5
5362	O=C1[C@@H]2[C@@H](c3c([C@](C(O)C4C5CCCC4CCC5)(O)[C@H]2CO1)cc1c(c3)OCO1)c1cc(OC)c(OC)c(OC)c1	1026020-95-0	Uridine 5,Ä≤-(tetrahydrogen triphosphate), 2,Ä≤-deoxy-5-(3-nitrophenyl)-	C15H18N3O16P3	589.24	C	-8.5
5363	O=c1n(cc(C#C)c(=O)[nH]1)[C@H]1C[C@H](N=[N+]=[N-])[C@@H](CO)O1	10300-41-1	Thymidine, 2,Ä≤-deoxyuridylyl-(3,Ä≤,Ü5,Ä≤)-	C19H25N4O12P	532.4	C	-8.5
5364	C(C[C@H](NC(=O)c1ccccc1)c1ccccc1)N1[C@@H]2C[C@@H](C[C@H]1CC2)n1c(C(C)C)nnc1C([2H])([2H])[2H]	1033698-64-4	Benzamide, N-[(1S)-3-[(3-exo)-3-[3-methyl-5-(1-methylethyl)-4H-1,2,4-triazol-4-y	C29H35D2N5O		C	-8.5
5365	N(c1cc(C(O)c2nccs2)nc(Nc2ccc(C#N)cc2)n1)c1c(C)cc(/C=C/C#N)cc1C	1037444-93-1	Benzonitrile, 4-[[4-[[4-[(1E)-2-cyanoethenyl]-2,6-dimethylphenyl]amino]-6-(2-thi	C26H19N7OS	477.54	C	-8.5
5366	N(c1c(C)cc(/C=C/C#N)cc1C)c1nc(Nc2ccc(C#N)cc2)nc(CN2CCOCC2)c1	1037444-95-3	Benzonitrile, 4-[[4-[[4-[(1E)-2-cyanoethenyl]-2,6-dimethylphenyl]amino]-6-(hydro	C26H21N7OS	479.56	C	-8.5
5367	C(c1c2c(N(c3c(C(=O)N2)cccn3)C2CC2)ncc1)n1c2c(nc1)c(=O)nc(N)[nH]2	1046462-04-7	6H-Dipyrido[3,2-b:2,Ä≤,3,Ä≤-e][1,4]diazepin-6-one, 4-[(2-amino-1,6-dihydro-6-ox	C20H17N9O2	415.41	C	-8.5
5368	N(C(=O)[C@H](Cc1ccccc1)C[C@@H](CN1[C@H](C(=O)NC(C)(C)C)CN(Cc2cccnc2)CC1)O)[C@H]1c2c(CC1=O)cccc2	1050328-14-7	D-erythro-Pentonamide, 2,3,5-trideoxy-N-[(1R)-2,3-dihydro-2-oxo-1H-inden-1-yl]-5	C36H45N5O4	611.77	C	-8.5
5369	O=C1[O-][Al+3]2([O]=c3c(C(=O)[O-]2)cc(=C(c2cc(C(=O)[O-])c(O)cc2)c2cc(C(=O)[O-])c(O)cc2)cc3)[O]=c2c1c	1050328-16-9	D-erythro-Pentonamide, 2,3,5-trideoxy-N-[(1S)-2,3-dihydro-2-oxo-1H-inden-1-yl]-5	C36H45N5O4	611.77	C	-8.5
5370	N(=N\c1cc(S(=O)(=O)O)c(C=Cc2c(S(=O)(=O)O)cc(/N=N/c3c4c(cc(S(=O)(=O)O)c3N)cc(S(=O)(=O)O)cc4)cc2)cc1)/	1052530-88-7	Not Yet Assigned	C34H26N6O12S4.Na		C	-8.5
5371	C(=O)(NC(C(=O)C(=O)N)CC(F)(F)F)C1[C@@H]2[C@@H](C2(C)C)CN1C(=O)[C@@H](NC(=O)NC(C)(C)C)C(C)(C)C	1056467-70-9	3-Azabicyclo[3.1.0]hexane-2-carboxamide, N-[3-amino-1-[(3,3-difluorocyclobutyl)m	C27H43F2N5O5	555.66	C	-8.5
5372	N(C(=O)C1Oc2c(N(C(=O)C)C1)cccc2)C12CC3CC(C2)CC(C1)C3	1062227-15-9	Benzeneacetic acid, 3,4-dimethoxy-, (5R,5aR,8aR,9R)-5,5a,6,8,8a,9-hexahydro-8-ox	C32H32O11	592.59	C	-8.5
5373	C(C)(C)(C)c1c2c(cc(C(OC(=O)C)OC(=O)C)c1)CO[P@@](=O)(OC[C@H]1O[C@H](C=C1)n1c(=O)[nH]c(=O)c(C)c1)O2	1072917-33-9	Thymidine, 5,Ä≤-O-[(2R)-6-[bis(acetyloxy)methyl]-8-(1,1-dimethylethyl)-2-oxido-4	C26H31N2O11P	578.5	C	-8.5
5374	C(C)(C)(C)c1c2c(cc(C(OC(=O)C(C)C)OC(=O)C(C)C)c1)CO[P@](=O)(OC[C@H]1O[C@H](C=C1)n1c(=O)[nH]c(=O)c(C)c	1072917-35-1	Thymidine, 5,Ä≤-O-[6-[bis(2-methyl-1-oxopropoxy)methyl]-8-methyl-2-oxido-4H-1,3,	C27H33N2O11P	592.53	C	-8.5
5375	N(C(=O)[C@H](Cc1ccccc1)CC(CN1[C@H](C(=O)NC(C)(C)C)CN(Cc2cccnc2)CC1)O)[C@H]1c2c(C[C@H]1O)cccc2	1085743-77-6	2-Piperazinecarboxamide, 1-[[[(2S)-3-[[(1S,2R)-2,3-dihydro-2-hydroxy-1H-inden-1-	C35H45N5O4S	631.83	C	-8.5
5376	O=c1n(cc(c(=O)[nH]1)c1ccc(Br)cc1)[C@@H]1O[C@H](CO)[C@@H](O)C1	108664-89-7	Uridine, 2,Ä≤-deoxy-5-[4-(phenylmethoxy)phenyl]-	C22H22N2O6	410.42	C	-8.5
5377	Cc1c2c(cc(C(=O)C)c1)COP(=O)(OC[C@H]1O[C@H](C=C1)n1c(=O)[nH]c(=O)c(C)c1)O2	1095170-75-4	Thymidine, 5,Ä≤-O-(6-acetyl-2-oxido-4H-1,3,2-benzodioxaphosphorin-2-yl)-2,Ä≤,3,.	C19H19N2O8P	434.34	C	-8.5
5378	C(c1cc2c(OP(OC[C@H]3O[C@H](C=C3)n3c(=O)[nH]c(=O)c(C)c3)OC2)cc1)c1cc(CO)c(O)cc1	1099829-79-4	Not Yet Assigned	C23H24N3O7PS	517.49	C	-8.5
5379	C(NC(=O)c1nnc(C([2H])([2H])[2H])o1)(C)(C)c1nc(C(=O)NC(c2c(c(c(F)c(c2[2H])[2H])[2H])[2H])([2H])[2H])c	1100751-00-5	4-Pyrimidinecarboxamide, N-[(4-fluorophenyl-2,3,5,6-d4)methyl]-1,6-dihydro-5-hyc	C20H11D10FN6O5		C	-8.5
5380	C(n1c2c(c(N)nc(N)n2)nc1)[C@H]1COP(=O)(O)CO1	1105693-49-9	3,Ä≤-Guanylic acid, 2,Ä≤-deoxy-, mono[3-(phosphonooxy)propyl] ester, P,Ä≤,Ü5,Ä	C23H32N10O13P2	718.51	C	-8.5
5381	C(Cc1ccccc1)[C@]1(CCC)C(C(=C([C@H](CC)c2cc(NS(=O)(=O)c3ccc(C(F)(F)F)cn3)ccc2)C(=O)O1)O)([2H])[2H]	1126619-13-3	Not Yet Assigned	C31H31D2F3N2O5S		C	-8.5

5382	C(Cc1ccccc1)[C@]1(C(C(C([2H])([2H])[2H])([2H])[2H])([2H])[2H])CC(=C([C@H](CC)c2cc(NS(=O)(=O)c3ccc(C(	1126619-19-9	Not Yet Assigned	C31H29D4F3N2O5S		C	-8.5
5383	Oc1c2c(cc(O)cc2cc2c1C(=O)c1c(C2(C)C)cc(O)cc1O)c1c(C)cc(O)cc1O	1126619-27-9	Not Yet Assigned	C31H30D3F3N2O5S		C	-8.5
5384	C(=O)([C@@H](NC(=O)OC)C(C)C)N1[C@@H](CC[C@@H]1C)c1[nH]c(cn1)c1ccc(cc1)c1ccc(cc1)c1[nH]c(nc1)[C@H]1N(	1129634-66-7	Carbamic acid, N,N,Ä≤-[[1,1,Ä≤-biphenyl]-4,4,Ä≤-diylbis[1H-imidazole-5,2-diyl-(	C44H54N8O6	790.95	C	-8.5
5385	C(=O)(C(NC(=O)OC)(C)C)N1[C@@H](CC[C@@H]1C)c1[nH]c(cn1)c1ccc(cc1)c1ccc(cc1)c1[nH]c(nc1)[C@H]1N(C(=O)C	1129634-95-2	Carbamic acid, N,N,Ä≤-[[1,1,Ä≤-biphenyl]-4,4,Ä≤-diylbis[1H-imidazole-5,2-diyl[(	C36H42N8O6	682.77	C	-8.5
5386	C(#CC1(C(C1([2H])[2H])([2H])[2H])[2H])[C@@]1(C(F)(F)F)c2c(N(C(=O)O1)[2H])c(c(Cl)c2[2H])[2H])[2H]	1132640-84-6	L-Valine, N-[(9H-fluoren-9-ylmethoxy)carbonyl]-, 2-[(2-amino-1,6-dihydro-6-oxo-9	C28H30N6O6	546.57	C	-8.5
5387	O=c1c2c(n(cn2)[C@@H]2O[C@H](CO)[C@@H](O)[C@H]2O)[nH]c(NCc2c3c(cc4c2cccc4)cccc3)n1	1134793-87-5	Guanosine, N-(1-naphthalenylmethyl)-	C21H21N5O5	423.42	C	-8.5
5388	O=c1n2[C@@H]3C[C@H]([C@@H](CO)O3)OP(=O)(O)OC[C@H]3O[C@H](n4cc(CCc(c2)c(=O)[nH]1)c(=O)[nH]c4=O)C[C@@H	1134943-90-0	3-Quinolinecarboxylic acid, 7-[4-[[(4S)-6-chloro-4-(2-cyclopropylethynyl)-2-oxo-	C33H29ClF4N4O6	689.05	C	-8.5
5389	O=c1n([C@@H]2O[C@H](COP(=O)(NC(=O)[C@@H](NC(=O)C)C)O)C=C2)cc(C)c(=O)[nH]1	1147100-84-2	Thymidine, 2,Ä≤,3,Ä≤-didehydro-3,Ä≤-deoxy-, 5,Ä≤-[hydrogen N-[[(2S)-1-acetyl-2-	C17H23N4O8P	442.36	C	-8.5
5390	O=c1c2c(N(C[N]2)[C@@H]2O[C@H](CO)[C@@H](O)C2)[nH]c(N)n1	115561-54-1	Œ≤-D-arabino-Adenosine, P-deoxy-P-methyl-Œ≤-D-arabino-adenylyl-(3,Ä≤,Ü'3,Ä≤)-	C21H27N10O9P	594.47	C	-8.5
5391	O=c1n([C@@H]2O[C@H](CO)[C@@H](O)C2)cc(C([2H])([2H])[2H])c(N)n1	1160591-56-9	1,3,5-Triazin-2-amine, N-[4-(3,4-dimethoxyphenyl)-6-(methylthio)-2-pyrimidinyl]-	C24H30N8O4S	526.61	C	-8.5
5392	O=C1[C@@H]2[C@@H](c3c([C@@H](OC)[C@H]2CO1)c(O)c1c(c3)OCO1)c1cc(OC)c(O)c(OC)c1	117124-08-0	1-Naphthalenesulfonic acid, 8-amino-7-[2-[4,Ä≤-[2-[5-(1,1-dimethylethyl)-2-hydro	C34H33N5O4S.Na		C	-8.5
5393	N(CCCCNC(=O)C(C)C)c1c2c(ncc1)cc(Cl)cc2	1172131-68-8	4-Pyrimidinecarboxamide, 5-(benzoyloxy)-N-[(4-fluorophenyl)methyl]-1,6-dihydro-1	C27H25FN6O6	548.52	C	-8.5
5394	O(C)c1c2n(cc(C(=O)O)c(=O)c2cc(F)c1N1CC(C)N(Cc2cn(c(=O)[nH]c2=O)[C@H]2C[C@H](N=[N+]=[N-])[C@@H](CO)O2	1174398-30-1	3-Quinolinecarboxylic acid, 7-[4-[[1-(3-azido-2,3-dideoxy-Œ≤-D-erythro-pentofura	C27H30F2N8O7		C	-8.5
5395	C(=O)(NC(C)(C)C)[C@H]1N(C[C@H]([C@@H](NC(=O)c2c(C)c(OC(=O)c3ccncc3)ccc2)CSc2ccccc2)OC(=O)c2ccncc2)C[	1174398-31-2	3-Quinolinecarboxylic acid, 7-[4-[[1-(3-azido-2,3-dideoxy-Œ≤-D-erythro-pentofura	C29H33FN8O8		C	-8.5
5396	C(=O)([C@@H](NC(=O)OC)C(C)C)N1[C@H]2[C@H](C2)C[C@H]1c1[nH]c(c(CC(F)(F)F)n1)c1ccc(cc1)c1cc2c(-c3c(CC2	1180669-99-1	Carbamic acid, N-[(1S)-1-[[(1R,3S,5R)-3-[4,5-dihydro-7-[4-[2-[(1R,3S,5R)-2-[(2S)	C44H52N8O6	788.93	C	-8.5
5397	C(=O)([C@@H](NC(=O)OC)C(C)C)N1[C@H]2[C@H](C2)C[C@H]1c1[nH]c2-c3c(CCc2n1)cc(cc3)c1cc2c(cc1)c1c(cc2)nc	1180670-19-2	Carbamic acid, N,N,Ä≤-[[7,7,Ä≤-bi-1H-naphth[1,2-d]imidazole]-2,2,Ä≤-diylbis[(2S	C44H50N8O6	786.92	C	-8.5
5398	C(=O)([C@@H](NC(=O)OC)C1CCOCC1)N1[C@H](c2[nH]c3c4c(cc(cc4)c4cc5c(cc4)c4c(cc5)nc([nH]4)[C@H]4N(C(=O)[	1180670-38-5	Carbamic acid, N-[(1S)-2-methyl-1-[[(2S)-2-[5-[4-[4,5,6,7-tetrahydro-2-[(2S)-1-[	C44H56N8O6	792.97	C	-8.5
5399	C(N1c2c(C(=NNC(=S)NC3CCCCC3)C1=O)cc(C)cc2)N1CCN(CC1)c1ccc(Cl)cc1	118989-42-7	Hydrazinecarbothioamide, N-cyclohexyl-2-[1,2-dihydro-5-methyl-2-oxo-1-[(4-phenyl	C27H34N6OS	490.66	C	-8.5
5400	C(OCCOC(C)C)n1c2c(nc1)c(=O)nc(N)[nH]2	1190884-56-0	4-Pyrimidinecarboxamide, N-[(4-fluorophenyl)methyl]-1,4,5,6-tetrahydro-1-(methyl	C20H18D3FN6O5		C	-8.5
5401	C[C@]1(F)[C@H](n2c3c(nc2)c(=O)nc(N)[nH]3)O[C@H]2[C@H]1O[P@@](=O)(OC1CCCCC1)OC2	1199809-52-3	Guanosine, 2,Ä≤-deoxy-2,Ä≤-fluoro-2,Ä≤-methyl-, cyclic 3,Ä≤,5,Ä≤-[cyclopentyl	C16H21FN5O6P	429.34	C	-8.5
5402	O=c1n([C@H]2C[C@H](N=[N+]=[N-])[C@@H](CO)O2)cc(C)c(=O)n1CCCCn1c(=O)n(cc(C)c1=O)[C@@H]1O[C@H](CO)C=C1	1215171-12-2	Thymidine, 3,3,Ä≤,Ä≤-[1,4-phenylenebis(methylene)]bis[3,Ä≤-azido-3,Ä≤-deoxy-	C28H32N10O8		C	-8.5
5403	N(C(=O)[C@H](Cc1ccccc1)C[C@@H](CN1[C@H](C(=O)NC(C)(C)C)CN(Cc2cccnc2)CC1)O)[C@H]1c2c(C[C@H]1O)cccc2.S	1217525-37-5	Not Yet Assigned	C36H47N5O4	613.79	C	-8.5
5404	C(C([2H])([2H])[2H])(C([2H])([2H])[2H])c1n(C2CC3N(CC[C@H](NC(=O)C4CCC(F)(F)CC4)c4ccccc4)C(C2)CC3)c(C	1217535-55-1	Cyclohexanecarboxamide, 4,4-difluoro-N-[(1S)-3-[3-[3-(hydroxymethyl)-5-(1-methyl	C29H41F2N5O2	529.66	C	-8.5
5405	N(=C1c2c(N(CN3CCN(CC3)c3c4c(ncc3)cc(Cl)cc4)C1=O)cccc2Br)NC(=S)N	1218813-09-2	Hydrazinecarbothioamide, 2-[4-chloro-1-[[4-(7-chloro-4-quinolinyl)-1-piperazinyl	C23H21Cl2N7OS	514.43	C	-8.5
5406	O=c1n([C@@H]2O[C@H](CO)CC2)ccc(N=CN(C)C)n1	1218813-22-9	Hydrazinecarbothioamide, 2-[1,2-dihydro-2-oxo-1-[[4-[7-(trifluoromethyl)-4-quinc	C24H22F3N7OS	513.54	C	-8.5
5407	N(Cc1ccc(/C=C/C(=O)NO)cc1)C12CC3(C)CC(C)(C1)CC(C2)C3	1221411-80-8	2-Propenamide, N-hydroxy-3-[4-[[(3-hydroxytricyclo[3.3.1.13,7]dec-1-yl)amino]met	C20H26N2O3	342.43	C	-8.5
5408	O=C1[C@@H]2[C@@H](c3c([C@H](OC(=O)CCC(=O)NCCCCNC(=O)[C@@H](C)c4cc(C(=O)c5ccccc5)ccc4)[C@H]2CO1)cc1c(	1222083-91-1	Butanoic acid, 4-[[4-[[(2S)-2-(6-methoxy-2-naphthalenyl)-1-oxopropyl]amino]butyl	C44H48N2O12	796.86	C	-8.5
5409	N(C(=O)[C@H](Cc1ccccc1)C[C@@H](CN1[C@H](C(=O)NC(C)(C)C)CN(Cc2cn(C)c(=O)cc2)CC1)O)C1c2c(C[C@H]1O)cccc	1223089-55-1	1-Piperazinepentanamide, N-[(2R)-2,3-dihydro-2-hydroxy-1H-inden-1-yl]-2-[[(1,1-c	C37H49N5O3	611.82	C	-8.5
5410	c1(nc(nc(n1)N1CCOCC1)N1CCOCC1)c1cc2c(cc1)[nH]nc2	1225037-84-2	2-Pyridinamine, 5-(4,6-di-4-morpholinyl-1,3,5-triazin-2-yl)-6-(trifluoromethyl)-	C17H20F3N7O2	411.38	C	-8.5
5411	c1(nc(nc(n1)N1CCOCC1)N1CCOCC1)c1c2c(ccc1)[nH]cc2	1225037-88-6	1H-Benzimidazole, 7-(4,6-di-4-morpholinyl-1,3,5-triazin-2-yl)-	C18H21N7O2	367.4	C	-8.5
5412	N(C(=O)C)[C@H]1[C@H]([C@@H]([C@@H](CO)O)O)OC(=C[C@@H]1NC#N)C(=O)OC	1228215-36-8	Carbamic acid, N-[(1S)-1-[[(2S)-2-[5-[4,Ä≤-[2-[(2S)-4-ethylidene-1-[(2S)-2-[(met	C42H52N8O6	764.91	C	-8.5
5413	C(OP(=O)(Oc1c2c(ccc1)cccc2)N[C@@H](Cc1ccccc1)C(=O)OCc1ccccc1)[C@@H]1[C@@H]2[C@H]([C@@H](O1)n1nc(C(=O	1228377-61-4	L-Alanine, N-[hydroxy(1-naphthalenyloxy)phosphinyl]-, phenylmethyl ester, 5,Ä≤-e	C33H36N5O9P	677.64	C	-8.5
5414	C(=O)([C@H](N(C)C)c1ccccc1)N1[C@@H](CCC1)c1[nH]c(cn1)c1cc2c(cc1)cc(cc2)c1ccc(cc1)c1[nH]c(nc1)[C@H]1N	1228552-24-6	Carbamic acid, N-[(1S)-1-[[(2S)-2-[5-[6-[4-[2-[(2S)-1-[(2R)-2-(dimethylamino)-2-	C47H52N8O4	792.97	C	-8.5
5415	C(=O)([C@H](NC(=O)OC(C)(C)C)C(C)C)N1[C@@H](CCC1)c1[nH]c(cn1)[C@H]1CC[C@@H](CC1)c1ccc(cc1)c1[nH]c(nc1	1228662-82-5	Cholan-24-oic acid, 3-[(3,Ä≤-azido-3,Ä≤-deoxy-5,Ä≤-thymidylyl)oxy]-12-hydroxy-,	C44H62N9O12P		C	-8.5
5416	C(=O)([C@H](NC(=O)OC)c1ccccc1)N1[C@@H](CCC1)c1[nH]c(cn1)[C@H]1CC[C@@H](CC1)c1ccc(cc1)c1[nH]c(nc1)[C@	1228667-95-5	Carbamic acid, N-[(1R)-2-[(2S)-2-[5-[4-[trans-4-[2-[(2S)-1-[(2R)-2-(dimethylamir	C46H54N8O4	782.97	C	-8.5
5417	C(=O)(/C=C/c1ccc(O)cc1)c1c(OC)cc(O)c(C(=O)/C=C/c2ccc(O)cc2)c1O	1228672-14-7	Carbamic acid, N,N,Ä≤-[(1E)-1,2-ethenediylbis[1H-imidazole-5,2-diyl-(2S)-2,1-py	C48H48N8O6	832.94	C	-8.5
5418	Cn1nc(nn1)c1ccc(CN(C[C@@H]([C@H](Cc2ccccc2)NC(=O)[C@@H](NC(=O)OC)C(C)C)O)NC(=O)[C@@H](NC(=O)OC)C(C)(	122931-60-6	2,7-Naphthalenedisulfonic acid, 4,4,Ä≤-[(3,3,Ä≤-disulfo[1,1,Ä≤-biphenyl]-4,4,Ä≤	C32H24N6O18S6	972.95	C	-8.5
5419	C(OCCOC(=O)c1ccc(CN2CCOCC2)cc1)n1c2c(nc1)c(=O)nc(N)[nH]2	1237515-04-6	[1,1,Ä≤-Biphenyl]-4-carboxylic acid, 3-[[(4-hydroxy[1,1,Ä≤-biphenyl]-3-yl)carbo	C26H19NO4	409.43	C	-8.5
5420	N(C(=O)[C@H](Cc1ccccc1)C[C@@H](CN1[C@H](C(=O)NC(C)(C)C)CN(Cc2cc3c(s2)cccc3)CC1)O)[C@H]1c2c(C[C@H]1O)	1237808-91-1	Not Yet Assigned	C39H49N5O5	667.84	C	-8.5
5421	N(C(=O)[C@H](Cc1ccccc1)C[C@@H](CN1[C@H](C(=O)NC(C)(C)C)CN(CCc2cc3c([nH]2)cccc3)CC1)O)[C@H]1c2c(C[C@H	1237808-97-7	Not Yet Assigned	C38H47N5O4S	669.88	C	-8.5
5422	N(C(=O)[C@H](Cc1ccccc1)C[C@@H](CN1[C@H](C(=O)NC(C)(C)C)CN(Cc2cn3c(cc2)ncc3)CC1)O)[C@H]1c2c(C[C@H]1O)	1237809-02-7	Not Yet Assigned	C38H47N5O4S	669.88	C	-8.5
5423	C(F)(F)c1n(c2c(n1)cccc2)c1nc(nc(N[C@H]2CC[C@H](NC(=O)CN)CC2)n1)N1CCOCC1	1240023-34-0	Urea, N-[trans-4-[[4-[2-(difluoromethyl)-1H-benzimidazol-1-yl]-6-(4-morpholinyl)	C23H29F2N9O2	501.53	C	-8.5
5424	C(F)(F)c1n(c2c(n1)cccc2)c1nc(nc(N[C@H]2CC[C@H](N(C(=O)C)C)CC2)n1)N1CCOCC1	1240023-39-5	1,3,5-Triazin-2-amine, 4-[2-(difluoromethyl)-1H-benzimidazol-1-yl]-6-(4-morpholi	C25H32F2N8O2	514.57	C	-8.5
5425	C(F)(F)c1n(c2c(n1)cccc2)c1nc(nc(N[C@H]2CC[C@H](NC(=O)CC)CC2)n1)N1CCOCC1	1240027-38-6	1,4-Cyclohexanediamine, N1-[4-[2-(difluoromethyl)-1H-benzimidazol-1-yl]-6-[(3R)-	C22H28F2N8O	458.51	C	-8.5
5426	C(=O)([C@@](NC(=O)OC)(C(C([2H])([2H])[2H])(C([2H])([2H])[2H])[2H])[2H])N1[C@@H](CCC1)c1[nH]c(cn1)c1c	1240427-02-4	Carbamic acid, N-[(1S)-1-[[(2S)-2-[5-[3-[[4-[2-[(2S)-1-[(2S)-2-[(methoxycarbonyl	C40H50N8O6S	770.94	C	-8.5
5427	C(=O)([C@H](NC(=O)OC)c1ccccc1)N1[C@@H](CCC1)c1[nH]c(cn1)c1ccc(CCCCc2[nH]c(nc2)[C@H]2N(C(=O)[C@H](NC(	1240891-49-9	Carbamic acid, N-[(1R)-2-[(2S)-2-[5-[4-[(1R)-4-[2-[(2S)-1-[(2R)-2-[(methoxycarbo	C46H50N8O6	810.94	C	-8.5
5428	C(=O)([C@H](NC(=O)OC)c1ccccc1)N1[C@@H](CCC1)c1[nH]c(cn1)c1ccc(C[C@H]2[C@H](Cc3[nH]c(nc3)[C@H]3N(C(=O	1240891-89-7	Carbamic acid, N-[(1R)-2-[(2S)-2-[5-[4-[[(1S,2R)-2-[[2-[(2S)-1-[(2R)-2-[(methoxy	C48H56N8O6	841.01	C	-8.5
5429	C(=O)(NC)c1c2cc(cc(N(CCOC(=O)CCC(=O)OC[C@H]3O[C@H]([C@H](O)[C@@H]3O)n3nc(C(=O)N)nc3)S(=O)(=O)C)c(c2)C	1246280-91-0	Not Yet Assigned	C30H34FN6O12PS	752.66	C	-8.5
5430	O[C@H]1[C@H](n2c3c(nc2)c(=O)nc(N)[nH]3)O[C@H](COP(=O)(OP(=O)(O[C@@H]2[C@@H](CO)O[C@H]([C@@H]2O)n2c3c	124705-06-2	Guanosine 5,Ä≤-(trihydrogen diphosphate), P,Ä≤,Üí2,Ä≤-ester with adenosine	C20H26N10O14P2	692.43	C	-8.5
5431	C(=O)([C@@H](NC(=O)OC)C(C)C)N1[C@@H](CCC1)c1[nH]c(cn1)c1ccc(cc1)c1ccc(C#Cc2ccc(cc2)c2[nH]c(nc2)[C@H]	1250941-31-1	Carbamic acid, N-[(1S)-2-methyl-1-[[(2S)-2-[5-[3-[2-[3-[2-(2S)-2-pyrrolidinyl-1H	C35H39N7O3	605.73	C	-8.5
5432	[C@@H](NC(=O)[C@H](Cc1ccccc1)NC(=O)N1CCN(C)CC1)(/C=C/S(=O)(=O)c1ccccc1)c1ccc2c(cc1)cccc2	1253912-33-2	1-Piperazinecarboxamide, 4-methyl-N-[(1S)-2-[[(1R,2E)-1-(1-naphthalenyl)-3-(pher	C34H36N4O4S	596.74	C	-8.5
5433	[C@@H](NC(=O)[C@@H](NC(=O)N1CCN(C)CC1)CC(C)C)(/C=C/S(=O)(=O)c1ccccc1)c1ccc2c(cc1)cccc2	1253912-43-4	1-Piperazinecarboxamide, 4-methyl-N-[(1S)-3-methyl-1-[[[(1R,2E)-1-(1-naphthaleny	C31H38N4O4S	562.72	C	-8.5
5434	C(=O)([C@@H](NC(=O)OC)C(C)C)N1[C@@H](CCC1)c1[nH]c(cn1)c1ccc(cc1)c1[nH]c(nc1)[C@H]1N(C(=O)[C@@H](NC(=	1256384-87-8	Carbamic acid, N,N,Ä≤-[2,5-thiophenediylbis[4,1-phenylene-1H-imidazole-5,2-diyl-	C44H52N8O6S	821	C	-8.5
5435	O=S1(=O)c2c(-c3c1cc(cc3)c1[nH]c(nc1)[C@H]1N(C(=O)[C@@H](NC(=O)OC)C(C)C)CCC1)ccc(c2)c1[nH]c(nc1)[C@H]	1256385-04-2	Carbamic acid, N-[(1S)-1-[[(2S)-2-[5-[4-[6-[2-[(2S)-1-[(2S)-2-[(methoxycarbonyl)	C44H52N8O6	788.93	C	-8.5
5436	C(=O)([C@@H](NC(=O)OC)C(C)C)N1[C@@H](CCC1)c1[nH]c(cn1)c1ccc(cc1)c1ccc(cc1)c1[nH]c(CN2C(=O)[C@@H](N)C	1256385-98-4	Carbamic acid, N-[(1S)-1-[[(2S)-2-[5-[4,Ä≤-[2-[[(3R)-3-amino-2-oxo-1-piperidiny]	C35H42N8O4	638.76	C	-8.5
5437	N(C(=O)[C@@H](NC(=O)OC)C(C)C)C1(CC1)c1[nH]c(cn1)c1ccc(cc1)c1ccc(cc1)c1[nH]c(nc1)[C@H]1N(C(=O)[C@@H](	1256386-32-9	Carbamic acid, N-[(1S)-1-[[(2S)-2-[5-[4-[2-[4-[2-[(2S)-1-[(2S)-2-[(methoxycarbor	C42H51N9O6	777.91	C	-8.5
5438	C(=O)([C@@H](NC(=O)OC)C(C)C)N1[C@@H](CCC1)c1[nH]c(cn1)c1ccc(/C=C/c2ccc(cc2)c2[nH]c(nc2)[C@H]2N(C(=O)	1256387-42-4	Carbamic acid, N,N,Ä≤-[2,6-naphthalenediylbis[1H-imidazole-5,2-diyl-(2S)-2,1-py	C38H48N8O6	712.84	C	-8.5
5439	C(=O)([C@@H](NC(=O)OC)C(C)C)N1[C@@H](CC(=C)C1)c1[nH]c(cn1)c1ccc(cc1)c1cc2c(cc1)cc(cc2)c1[nH]c(nc1)[C	1256392-14-9	Carbamic acid, N-[(1S)-1-[[(6S)-6-[5-[4-[6-[2-[(6S)-5-[(2S)-2-[(methoxycarbonyl)	C48H56N8O6	841.01	C	-8.5
5440	C(=O)([C@@H](NC(=O)OC)C(C)C)N1[C@@H](CCC1)c1[nH]c(cn1)c1ccc(cc1)c1cc2c(cc1)cc(cc2)c1[nH]c(nc1)[C@H]1	1261070-48-7	Carbamic acid, N-[(1S)-1-[[(2S)-2-[5-[6-[4-[2-[(2S)-1-[(2S)-2-[(methoxycarbonyl)	C43H50N8O6	774.91	C	-8.5
5441	O(C)c1c2n(cc(C(=O)O)c(=O)c2cc(F)c1N1CCN(CCN2c3c(C(=NNC(=S)N)C2=O)cccc3)CC1)C1CC1	1263191-35-0	3-Quinolinecarboxylic acid, 7-[4-[[3-[2-(aminothioxomethyl)hydrazinylidene]-2,3-	C28H28FN7O5S	593.63	C	-8.5
5442	O(C)c1c2n(cc(C(=O)O)c(=O)c2cc(F)c1N1CC(C)N(CN2c3c(/C(=N/NC(=S)NO)/C2=O)cc(F)cc3)CC1)C1CC1	1265623-28-6	3-Quinolinecarboxylic acid, 7-[4-[[(3Z)-5-chloro-2,3-dihydro-3-[2-[(methoxyaminc	C30H31ClFN7O6S	672.13	C	-8.5
5443	S(=O)(=O)(O)c1c(cc(C)c(/N=N/c2c3c(ccc2O)cccc3)c1)c1c(S(=O)(=O)O)cc(/N=N/c2c3c(ccc2O)cccc3)c(C)c1	1266475-22-2	Glycine, N-[(3Œ±,12Œ±)-3,12-dihydroxy-24-oxocholan-24-yl]-	C26H43NO5	449.62	C	-8.5
5444	O=c1n([C@@H]2O[C@H](COP3(=O)Oc4c(CO3)cc3c(c4)COP(=O)(OC[C@@H]4[C@@H](N=[N+]=[N-])C[C@@H](O4)n4c(=O)[	1268669-59-5	Guanosine 5,Ä≤-(tetrahydrogen triphosphate), N-methyl-, P,Ä≤,Ä≤,Üí5,Ä≤-ester wi	C21H29N10O18P3	802.43	C	-8.5
5445	Cc1c2c(CO[P@@](=O)(OC[C@H]3O[C@H](C=C3)n3c(=O)[nH]c(=O)c(C)c3)O2)cc(C)c1	1270965-60-0	Thymidine, 3,Ä≤-azido-3,Ä≤-deoxy-5,Ä≤-O-[(2R)-8-methyl-2-oxido-4H-1,3,2-benzodi	C18H20N5O7P		C	-8.5
5446	C(=O)([C@@H](NC(=O)OC)C(C)C)N1CC2(CC1c1[nH]c(cn1)c1ccc(cc1)c1ccc(cc1)c1[nH]c(nc1)[C@H]1N(C(=O)[C@@H]	1272656-28-6	Carbamic acid, N-[(1S)-1-[[(2S)-2-[5-[4,Ä≤-[2-[2-[(2S)-2-[(methoxycarbonyl)aminc	C44H57N9O6	807.98	C	-8.5
5447	C(=O)([C@@H](NC(=O)OC)C1CCOCC1)N1[C@@H]([C@@H]2C[C@H]1CC2)c1[nH]c(cn1)c1ccc(cc1)c1ccc(cc1)c1[nH]c(nc	1273589-71-1	Carbamic acid, N-[(1S)-2-methyl-1-[[(2S)-2-[5-[4,Ä≤-[2-[(1R,3S,4S)-2-[(2S)-3-met	C48H60N8O8	877.04	C	-8.5
5448	C(=O)([C@@H](NC(=O)OC)C(C)C)N1[C@@H]([C@H]2[C@H](OC)C[C@@H]1C2)c1[nH]c(cn1)c1ccc(cc1)c1ccc(cc1)c1[nH	1273591-17-5	Carbamic acid, N-[(1S)-1-[[(2S)-2-[5-[4,Ä≤-[2-[(1S,3S,4S,5R)-5-hydroxy-2-[(2S)-2	C42H52N8O7	780.91	C	-8.5
5449	C(=O)([C@@H](NC(=O)OC)C(C)C)N1[C@H]2[C@H](C2)C[C@H]1c1[nH]c(cn1)c1ccc(cc1)c1ccc(cc1)c1[nH]c(nc1)[C@H	1273591-35-7	Carbamic acid, N-[(1S)-1-[[(2S)-2-[5-[4,Ä≤-[2-[(1S,3R,4R)-2-[(2S)-2-[(methoxycar	C42H52N8O6	764.91	C	-8.5
5450	C(=O)([C@@H](NC(=O)OC)C(C)C)N1[C@@H]([C@@H]2C[C@H]1CC2)c1[nH]c(cn1)c1c(CC)c(CC)c(cc1)c1c(CC)c(CC)c(c	1273591-48-2	Carbamic acid, N-[(1S)-1-[[(2S)-2-[5-[4,Ä≤-[2-[(1R,3S,4S)-2-[(2S)-2-[(methoxycar	C46H60N8O6	821.02	C	-8.5
5451	C(=O)([C@@H](NC(=O)OC)C(C)C)N1[C@@H]([C@@H]2C[C@H]1CC2)c1[nH]c(cn1)c1c(Cl)cc(cc1Cl)c1cc(Cl)c(c(Cl)c1	1273591-52-8	Carbamic acid, N-[(1S)-1-[[(2S)-2-[5-[3,3,Ä≤-dichloro-4,Ä≤-[2-[(1R,3S,4S)-2-[(2	C42H50Cl2N8O6	833.8	C	-8.5
5452	C(=O)([C@@H](NC(=O)OC)C(C)C)N1[C@@H]([C@@H]2C[C@H]1CC2)c1[nH]c(cn1)[C@@H]1CC[C@H](CC1)c1ccc(cc1)c1[n	1273591-53-9	Carbamic acid, N-[(1S)-2-methyl-1-[[(2S)-2-[5-[3,3,Ä≤,5,5,Ä≤-tetrachloro-4,Ä≤-[	C42H48Cl4N8O6	902.69	C	-8.5
5453	C(=O)([C@@H](NC(=O)OC)C(C)C)N1[C@@H]([C@@H]2C[C@H]1CC2)c1[nH]c(cn1)c1ccc(C=Cc2ccc(cc2)c2[nH]c(nc2)[C	1273591-77-7	Carbamic acid, N-[(1S)-1-[[(2S)-2-[5-[4-[trans-4-[2-[(1R,3S,4S)-2-[(2S)-2-[(meth	C42H58N8O6	770.96	C	-8.5
5454	C(=O)([C@@H](NC(=O)OC)C(C)C)N1[C@@H](CCC1)c1[nH]c(cn1)[C@@H]1CC[C@H](CC1)c1ccc(NC[C@H]2N(C(=O)[C@@H]	1275019-75-4	Carbamic acid, N-[(1S)-1-[[(2S)-2-[5-[4,Ä≤-[[(2R)-2-[[[(2S)-2-[(methoxycarbonyl)	C39H51N7O7	729.86	C	-8.5
5455	O=C1[C@@H]2[C@@H](c3c([C@@H]([Se]c4ccc(C)cc4)[C@H]2CO1)cc1c(c3)OCO1)c1cc(OC)c(O)c(OC)c1	127708-96-7	Furo[3,Ä≤,4,Ä≤:6,7]naphtho[2,3-d]-1,3-dioxol-6(5aH)-one, 5,8,8a,9-tetrahydro-9-	C26H22O6	430.45	C	-8.5
5456	C([C@H](OCP(=O)(O)O)CO)n1c2c(nc1)c(N)nc(N)n2.[NaH]	1277183-98-8	Furo[3,Ä≤,4,Ä≤:6,7]naphtho[2,3-d]-1,3-dioxol-6(5aH)-one, 5,8,8a,9-tetrahydro-5-	C28H26O7Se	553.46	C	-8.5
5457	O=c1c(c(oc2c1ccnc2n1ccnc1)c1ccccc1)c1ccc(cc1)C1(N)CCC1	1279087-29-4	4H-Pyrano[2,3-c]pyridin-4-one, 3-[4-(1-aminocyclobutyl)phenyl]-8-(2-oxo-1(2H)-py	C29H23N3O3	461.51	C	-8.5
5458	O[C@H]1[C@H](n2c3c(nc2)c(N)ncn3)O[C@H](COP(=O)(OCC(=O)O)O)[C@H]1O	1285535-41-2	1,3,5-Triazin-2-amine, 4,6-di-4-morpholinyl-N-[4-(4-nitrophenyl)-2-thiazolyl]-	C20H22N8O4S	470.5	C	-8.5
5459	C(=C\C(=O)c1ccccc1)/c1c(OC)c(C=O)c(O)c(C)c1O	130272-39-8	Guanosine 5,Ä≤-(trihydrogen diphosphate), P,Ä≤-Œ±-D-arabinopyranosyl ester	C15H23N5O15P2	575.32	C	-8.5
5460	C(=O)([C@@H](NC(=O)OC)C)N1[C@@H](CCC1)c1[nH]c(C#Cc2ccc(C#Cc3[nH]c(nc3)[C@H]3N(C(=O)[C@@H](NC(=O)OC)C	1307303-78-1	Carbamic acid, N,N,Ä≤-[1,4-phenylenebis[2,1-ethanediyl-1H-imidazole-5,2-diyl-(2S	C34H46N8O6	662.78	C	-8.5
5461	C(=O)([C@@H](NC(=O)OC)C(C)C)N1[C@@H](CC(=C)C1)c1[nH]c(cn1)c1c(C(F)(F)F)cc(cc1)c1cc(C(F)(F)F)c(cc1)c1	1311398-24-9	Carbamic acid, N-[(1S)-1-[[(2S)-2-[5-[4,Ä≤-[2-[(2S)-1-[(2S)-2-[(methoxycarbonyl)	C45H58N8O6	806.99	C	-8.5
5462	C(=O)([C@@H](NC(=O)OC)C(C)C)N1[C@@H](CC(=C)C1)c1[nH]c(cn1)c1ccc(cc1)c1ccc(cc1)c1[nH]c(nc1)[C@H]1N(C(	1311398-31-8	Carbamic acid, N-[(1S)-1-[[(2S)-2-[5-[4,Ä≤-[2-[(2S)-1-[(2S)-2-[(methoxycarbonyl)	C41H50N8O6	750.89	C	-8.5
5463	C(=O)([C@@H](NC(=O)OC)C(C)C)N1[C@@H](CC[C@@H]1C)c1[nH]c(cn1)c1ccc(cc1)c1ccc(cc1)c1[nH]c(nc1)[C@H]1N(	1311398-35-2	Carbamic acid, N-[(1S)-1-[[(2S)-2-[5-[4,Ä≤-[2-[(1R,3S,5R)-2-[(2S)-2-[(methoxycar	C42H50N8O6	762.9	C	-8.5
5464	C(=O)([C@@H](NC(=O)OC)C1CCOCC1)N1[C@@H](CC[C@@H]1C)c1[nH]c(c(Cl)n1)c1ccc(cc1)c1ccc(cc1)c1[nH]c(nc1Cl	1312336-16-5	Carbamic acid, N-[(1S)-2-[(1R,3S,5R)-3-[5-chloro-4-[4,Ä≤-[5-chloro-2-[(1R,3S,5R)	C44H50Cl2N8O8	889.82	C	-8.5
5465	C(=O)([C@@H](NC(=O)OC)C1CCOCC1)N1[C@@H](C[C@H](C)C1)c1[nH]c(c(Cl)n1)c1ccc(cc1)c1ccc(cc1)c1[nH]c(nc1)	1312336-28-9	Carbamic acid, N-[(1S)-2-[(2S,4S)-2-[5-[4,Ä≤-[5-chloro-2-[(2S,4S)-1-[(2S)-2-[(me	C44H55ClN8O7	843.41	C	-8.5
5466	C(=O)([C@@H](NC(=O)OC)C(C)C)N1[C@@H](C[C@H](C)C1)c1[nH]c(c(Cl)n1)c1ccc(cc1)c1ccc(cc1)c1[nH]c(nc1Cl)[	1312336-29-0	Carbamic acid, N-[(1S)-2-[(2S,4S)-2-[5-chloro-4-[4,Ä≤-[2-[(2S,4S)-1-[(2S)-2-[(me	C44H55ClN8O7	843.41	C	-8.5
5467	C(=O)([C@@H](NC(=O)OC)C(C)C)N1[C@@H](CCC1)c1[nH]c(c(I)n1)c1ccc(cc1)c1ccc(cc1)c1[nH]c(nc1)[C@H]1N(C(=	1312336-48-3	Carbamic acid, N-[(1S)-1-[[(2S)-2-[5-[4,Ä≤-[5-bromo-2-[(2S)-1-[(2S)-2-[(methoxy	C40H49BrN8O6	817.77	C	-8.5
5468	C(=O)([C@@H](NC(=O)OC)C(C)C)N1[C@H]2[C@H](C2)C[C@H]1c1[nH]c2c(n1)cc(Cl)c1c2ccc(c1)c1ccc(cc1)c1[nH]c(	1312336-50-7	Carbamic acid, N-[(1S)-1-[[(2S)-2-[5-[4,Ä≤-[5-iodo-2-[(2S)-1-[(2S)-2-[(methoxyca	C40H49IN8O6	864.77	C	-8.5
5469	C(c1c([nH]c(n1)[C@H]1N(C(=O)[C@@H](NC(=O)OC)C(C)C)CCC1)c1ccc(cc1)c1ccc(cc1)c1[nH]c(nc1)[C@H]1N(C(=O)	1312336-59-6	Carbamic acid, N-[(1S)-2-[(2S)-2-[5-[4,Ä≤-[2-[(2S)-1-[(2S)-2-[(methoxycarbonyl)a	C47H58N8O8	863.01	C	-8.5
5470	C(=O)([C@@H](NC(=O)OC)C1CCOCC1)N1[C@H]2[C@H](C2)C[C@H]1c1[nH]c(c(F)n1)c1ccc(cc1)c1ccc(cc1)c1[nH]c(nc	1312336-63-2	Carbamic acid, N-[(1S)-1-[[(1R,3S,5R)-3-[5-[4,Ä≤-[5-fluoro-2-[(1R,3S,5R)-2-[(2S)	C42H49FN8O6	780.89	C	-8.5
5471	C(=O)([C@@H](NC(=O)OC)C1CCOCC1)N1[C@H](c2[nH]c(c(n2)c2ccc(cc2)c2ccc(cc2)c2[nH]c(nc2)[C@H]2N(C(=O)[C@	1312336-96-1	Carbamic acid, N-[(1S)-2-[(2S,5S)-2-[7-[4-[5-chloro-2-[(2S,5S)-1-[(2S)-2-[(metho	C48H57ClN8O8	909.47	C	-8.5
5472	C(=O)([C@H](C(COC)COC)NC(=O)OC)N1[C@H]2[C@H](C2)C[C@H]1c1[nH]c(c(Cl)n1)c1ccc(cc1)c1ccc(cc1)c1[nH]c(n	1312337-08-8	Carbamic acid, N-[(1S)-2-[(1R,3S,5R)-3-[5-(4-isoxazolyl)-4-[4,Ä≤-[2-[(1R,3S,5R)-	C49H55N9O9	914.02	C	-8.5
5473	C(=O)([C@@H](NC(=O)OC)C1CCC(F)(F)CC1)N1[C@H]2[C@H](C2)C[C@H]1c1[nH]c(cn1)c1ccc(cc1)c1ccc(cc1)c1[nH]c	1312338-46-7	Carbamic acid, N,N,Ä≤-[[1,1,Ä≤-biphenyl]-4,4,Ä≤-diylbis[1H-imidazole-5,2-diyl-(	C48H54F4N8O6	914.99	C	-8.5
5474	Cc1[nH]c2c(ccc(c2n1)c1ccc(cc1)c1[nH]c(nc1)[C@H]1N(C(=O)[C@@H](NC(=O)OC)C(C)C)CCC1)c1[nH]c(nc1)[C@H]1	1312608-33-5	Carbamic acid, N-[(1S)-1-[[(2S)-2-[5-[4-[7-[2-[(2S)-1-[(2S)-2-[(methoxycarbonyl)	C41H50N10O6	778.9	C	-8.5
5475	Cc1cc2c(ccc(c2[nH]1)c1ccc(cc1)c1[nH]c(nc1)[C@H]1N(C(=O)[C@@H](NC(=O)OC)C(C)C)CCC1)c1[nH]c(nc1)[C@H]1	1312608-35-7	Carbamic acid, N-[(1S)-1-[[(2S)-2-[5-[4-[4-[2-[(2S)-1-[(2S)-2-[(methoxycarbonyl)	C42H51N9O6	777.91	C	-8.5
5476	C(=O)([C@@H](NC(=O)OC)C(C)C)N1[C@H](c2[nH]c(c3c4c(c(cc3)c3ccc(cc3)c3[nH]c(nc3)[C@H]3N(C(=O)[C@@H](NC	1312608-38-0	Carbamic acid, N-[(1S)-1-[[(2S)-2-[5-[4-[4-[2-[(2S)-1-[(2S)-2-[(methoxycarbonyl)	C42H51N9O6S	809.98	C	-8.5
5477	Cc1cc2c(ccc(c2o1)c1ccc(cc1)c1[nH]c(nc1)[C@H]1N(C(=O)[C@@H](NC(=O)OC)C(C)C)CCC1)c1[nH]c(nc1)[C@H]1N(C	1312608-43-7	Carbamic acid, N-[(1S)-1-[[(2S)-2-[5-[4-[4-[2-[(2S)-1-[(2S)-2-[(methoxycarbonyl)	C42H50N8O7	778.9	C	-8.5
5478	Cc1c2c(c(ccc2c2ccc(cc2)c2[nH]c(nc2)[C@H]2N(C(=O)[C@@H](NC(=O)OC)C(C)C)CCC2)c2[nH]c(nc2)[C@H]2N(C(=O)	1312608-48-2	Carbamic acid, N-[(1S)-1-[[(2S)-2-[5-[4-[2,3-dihydro-8-[2-[(2S)-1-[(2S)-2-[(meth	C42H52N8O8	796.91	C	-8.5
5479	Cn1c2c(ccc(c2cc1)c1ccc(cc1)c1[nH]c(nc1)[C@H]1N(C(=O)[C@@H](NC(=O)OC)C(C)C)CCC1)c1[nH]c(nc1)[C@H]1N(C	1312608-70-0	Carbamic acid, N-[(1S)-1-[[(2S)-2-[5-[8-[4-[2-[(2S)-1-[(2S)-2-[(methoxycarbonyl)	C43H51N9O6	789.92	C	-8.5
5480	C(=O)([C@@H](NC(=O)OC)C(C)C)N1[C@H](c2[nH]c(c3c4c(c(cc3)c3ccc(cc3)c3[nH]c(nc3)[C@@]3(C)N(C(=O)[C@@H]	1312608-77-7	Carbamic acid, N-[(1S)-1-[[(2S)-2-[5-[4-[7-[2-[(2S)-1-[(2S)-2-[(methoxycarbonyl)	C44H55N9O6	805.96	C	-8.5
5481	C(=O)([C@@H](NC(=O)OC)C(C)C)N1[C@H](c2[nH]c(c3ccc(c4c5c(c(cc4)c4[nH]c(nc4)[C@@H]4CCCN4)cccc5)cc3)cn2	1312609-62-3	Carbamic acid, N-[(1S)-1-[[(2S)-2-[5-[4-[7-[2-[(2S)-1-[(2S)-2-[(methoxycarbonyl)	C43H53N9O6	791.94	C	-8.5
5482	O(Cc1ccc(F)cc1)c1c2c(oc(c(O)c2=O)c2[nH]ccn2)ccc1	1312892-15-1	4H-1-Benzopyran-4-one, 5-[(4-fluorophenyl)methoxy]-3-hydroxy-2-(3-phenyl-1H-1,2,	C24H16FN3O4	429.4	C	-8.5
5483	O(Cc1ccc(F)cc1)c1c2c(oc(c(O)c2=O)c2nnc(C)[nH]2)c(C)cc1	1312892-62-8	4H-1-Benzopyran-4-one, 5-[(4-fluorophenyl)methoxy]-3-hydroxy-8-methyl-2-(1H-1,2,	C19H14FN3O4	367.33	C	-8.5
5484	O(Cc1ccc(F)cc1)c1c2c(oc(c(O)c2=O)c2ncccn2)c(OC)cc1	1312892-84-4	4H-1-Benzopyran-4-one, 5-[(4-fluorophenyl)methoxy]-3-hydroxy-8-methyl-2-(5-pheny	C26H19FN2O4	442.44	C	-8.5
5485	O(Cc1ccc(F)cc1)c1c2c(c(cc1)c1ccccc1)oc(c(O)c2=O)c1ncc(Cl)cn1	1312894-35-1	4H-1-Benzopyran-4-one, 5-[(4-fluorophenyl)methoxy]-3-hydroxy-2-(5-methoxy-2-pyri	C27H19FN2O5	470.45	C	-8.5
5486	O(Cc1ccc(F)cc1)c1c2c(c(cc1)c1ccccc1)oc(c(O)c2=O)c1[nH]c(OC)cn1	1312894-49-7	4H-1-Benzopyran-4-one, 5-[(4-fluorophenyl)methoxy]-3-hydroxy-2-(5-methyl-1H-imic	C26H19FN2O4	442.44	C	-8.5
5487	O(Cc1ccc(F)cc1)c1c2c(c(cc1)c1ccccc1)oc(c(O)c2=O)c1[nH]c(cn1)c1ccccc1	1312894-51-1	4H-1-Benzopyran-4-one, 2-(5-chloro-1H-imidazol-2-yl)-5-[(4-fluorophenyl)methoxy]	C25H16ClFN2O4	462.86	C	-8.5
5488	O(Cc1ccc(F)cc1)c1c2c(oc(c(O)c2=O)c2nnc(C)[nH]2)cc(C)c1	1312894-93-1	4H-1-Benzopyran-4-one, 5-[(4-fluorophenyl)methoxy]-3-hydroxy-7-methyl-2-(1H-1,2,	C19H14FN3O4	367.33	C	-8.5
5489	O(Cc1ccc(F)cc1)c1c2c(c(OC)c(C)c1)oc(c(O)c2=O)c1[nH]c(C)nn1	1312895-93-4	4H-1-Benzopyran-4-one, 5-[(4-fluorophenyl)methoxy]-3-hydroxy-8-methoxy-7-methyl-	C20H16FN3O5	397.36	C	-8.5
5490	O(Cc1ccc(F)cc1)c1c2c(oc(c(O)c2=O)c2[nH]c(nn2)c2ccccc2)c(Cl)c(C)c1	1312896-46-0	4H-1-Benzopyran-4-one, 8-chloro-2-(5-chloro-1H-1,2,4-triazol-3-yl)-5-[(4-fluorop	C19H12Cl2FN3O4	436.22	C	-8.5
5491	O(Cc1ccc(F)cc1)c1c2c(oc(c(O)c2=O)c2ncccn2)c(Cl)c(OC)c1	1312898-64-8	4H-1-Benzopyran-4-one, 5-[(4-fluorophenyl)methoxy]-3-hydroxy-7,8-dimethoxy-2-(5-	C27H21FN2O6	488.46	C	-8.5
5492	O(C)c1c(c2c(c(OCc3ccc(F)cc3)c1)c(=O)c(O)c(o2)c1ncc(OC)cn1)c1ccccc1	1312899-46-9	4H-1-Benzopyran-4-one, 5-[(4-fluorophenyl)methoxy]-3-hydroxy-7-methoxy-2-(5-meth	C28H21FN2O5	484.48	C	-8.5
5493	O(Cc1ccc(F)cc1)c1c2c(c(OC)c(Cl)c1)oc(c(O)c2=O)c1[nH]c(C)nn1	1312901-64-6	4H-1-Benzopyran-4-one, 7-chloro-5-[(4-fluorophenyl)methoxy]-3-hydroxy-8-methoxy-	C19H13ClFN3O5	417.77	C	-8.5
5494	O(C)c1c2c(c(OCc3ccc(F)cc3)cc1Cl)c(=O)c(O)c(o2)c1[nH]ccn1	1312901-68-0	4H-1-Benzopyran-4-one, 7-chloro-5-[(4-fluorophenyl)methoxy]-3-hydroxy-8-methoxy-	C25H17ClFN3O5	493.87	C	-8.5
5495	O(Cc1ccc(F)cc1)c1c2c(c(c(Cl)c1)c1ccccc1)oc(c(O)c2=O)c1ncccn1	1312902-23-0	4H-1-Benzopyran-4-one, 7,8-dichloro-5-[(4-fluorophenyl)methoxy]-3-hydroxy-2-(5-p	C25H15Cl2FN2O4	497.3	C	-8.5
5496	O(Cc1ccc(F)cc1)c1c2c(c(c(Cl)c1)c1ccccc1)oc(c(O)c2=O)c1ncc(cn1)c1ccccc1	1312902-57-0	4H-1-Benzopyran-4-one, 7-chloro-2-(5-chloro-2-pyrimidinyl)-5-[(4-fluorophenyl)me	C26H15Cl2FN2O4	509.31	C	-8.5
5497	O(Cc1ccc(F)cc1)c1c2c(c(c(Cl)c1)c1ccccc1)oc(c(O)c2=O)c1[nH]c(nn1)c1ccccc1	1312902-67-2	4H-1-Benzopyran-4-one, 7-chloro-2-(5-chloro-1H-1,2,4-triazol-3-yl)-5-[(4-fluorop	C24H14Cl2FN3O4	498.29	C	-8.5
5498	O(Cc1ccc(F)cc1)c1c2c(oc(c(O)c2=O)c2ncc(C)cn2)cc(c1)c1ccccc1	1312903-04-0	4H-1-Benzopyran-4-one, 5-[(4-fluorophenyl)methoxy]-3-hydroxy-7-phenyl-2-(2-pyrim	C26H17FN2O4	440.42	C	-8.5

5499	O(Cc1ccc(F)cc1)c1c2c(oc(c(O)c2=O)c2[nH]c(nn2)c2ccccc2)cc(c1)c1ccccc1	1312903-17-5	4H-1-Benzopyran-4-one, 2-(5-chloro-1H-1,2,4-triazol-3-yl)-5-[(4-fluorophenyl)met	C24H15ClFN3O4	463.85	C	-8.5
5500	Cc1c2c(c(OCc3ccc(F)cc3)cc1c1ccccc1)c(=O)c(O)c(o2)c1ncc(cn1)c1ccccc1	1312903-58-4	4H-1-Benzopyran-4-one, 2-(5-chloro-2-pyrimidinyl)-5-[(4-fluorophenyl)methoxy]-3-	C27H18ClFN2O4	488.89	C	-8.5
5501	O(Cc1ccc(F)cc1)c1c2c(c(Cl)c(c1)c1ccccc1)oc(c(O)c2=O)c1ncc(OC)cn1	1312905-05-7	4H-1-Benzopyran-4-one, 8-chloro-5-[(4-fluorophenyl)methoxy]-3-hydroxy-2-(5-methy	C27H18ClFN2O4	488.89	C	-8.5
5502	Clc1c2c(c(OCc3ccc(F)cc3)cc1c1ccccc1)c(=O)c(O)c(o2)c1ncc(cn1)c1ccccc1	1312905-07-9	4H-1-Benzopyran-4-one, 8-chloro-2-(5-chloro-2-pyrimidinyl)-5-[(4-fluorophenyl)me	C26H15Cl2FN2O4	509.31	C	-8.5
5503	O(Cc1ccc(F)cc1)c1c2c(c(c(c1)c1ccccc1)c1ccccc1)oc(c(O)c2=O)c1[nH]cc(Cl)n1	1312905-83-1	4H-1-Benzopyran-4-one, 5-[(4-fluorophenyl)methoxy]-3-hydroxy-2-(5-methoxy-1H-imi	C32H23FN2O5	534.53	C	-8.5
5504	O=c1c2c(oc(c1O)c1nnc(C)[nH]1)c(C)cc(OCc1ccc(F)cc1)c2	1312906-74-3	4H-1-Benzopyran-4-one, 6-[(4-fluorophenyl)methoxy]-3-hydroxy-8-methyl-2-(1H-1,2,	C19H14FN3O4	367.33	C	-8.5
5505	O=c1c2c(oc(c1O)c1nnc(C)[nH]1)c(Cl)cc(OCc1ccc(F)cc1)c2	1312907-74-6	4H-1-Benzopyran-4-one, 8-chloro-6-[(4-fluorophenyl)methoxy]-3-hydroxy-2-(1H-1,2,	C18H11ClFN3O4	387.75	C	-8.5
5506	O=c1c2c(c(cc(OCc3ccc(F)cc3)c2)c2ccccc2)oc(c1O)c1ncc(C)cn1	1312908-14-7	4H-1-Benzopyran-4-one, 6-[(4-fluorophenyl)methoxy]-3-hydroxy-8-phenyl-2-(2-pyrim	C26H17FN2O4	440.42	C	-8.5
5507	O=c1c2c(oc(c1O)c1nnc(C)[nH]1)cc(C)c(OCc1ccc(F)cc1)c2	1312908-74-9	4H-1-Benzopyran-4-one, 6-[(4-fluorophenyl)methoxy]-3-hydroxy-7-methyl-2-(1H-1,2,	C19H14FN3O4	367.33	C	-8.5
5508	O=c1c2c(oc(c1O)c1[nH]cc(Cl)n1)cc(C)c(OCc1ccc(F)cc1)c2	1312908-81-8	4H-1-Benzopyran-4-one, 6-[(4-fluorophenyl)methoxy]-3-hydroxy-2-(5-methoxy-1H-imi	C21H17FN2O5	396.37	C	-8.5
5509	Cc1c2c(cc(OCc3ccc(F)cc3)c1C)c(=O)c(O)c(o2)c1[nH]c(C)nn1	1312909-36-6	4H-1-Benzopyran-4-one, 6-[(4-fluorophenyl)methoxy]-3-hydroxy-7,8-dimethyl-2-(1H-	C20H16FN3O4	381.36	C	-8.5
5510	Cc1c2c(cc(OCc3ccc(F)cc3)c1C)c(=O)c(O)c(o2)c1[nH]c(OC)cn1	1312909-42-4	4H-1-Benzopyran-4-one, 6-[(4-fluorophenyl)methoxy]-3-hydroxy-7,8-dimethyl-2-(5-m	C22H19FN2O4	394.4	C	-8.5
5511	O(C)c1c2c(cc(OCc3ccc(F)cc3)c1C)c(=O)c(O)c(o2)c1[nH]cnn1	1312909-80-0	4H-1-Benzopyran-4-one, 6-[(4-fluorophenyl)methoxy]-3-hydroxy-8-methoxy-7-methyl-	C28H21FN2O5	484.48	C	-8.5
5512	Cc1c(c2c(cc1OCc1ccc(F)cc1)c(=O)c(O)c(o2)c1[nH]c(C)cn1)c1ccccc1	1312910-91-0	4H-1-Benzopyran-4-one, 6-[(4-fluorophenyl)methoxy]-3-hydroxy-2-(1H-imidazol-2-yl	C26H19FN2O4	442.44	C	-8.5
5513	Cc1c(c2c(cc1OCc1ccc(F)cc1)c(=O)c(O)c(o2)c1[nH]c(Cl)cn1)c1ccccc1	1312910-93-2	4H-1-Benzopyran-4-one, 6-[(4-fluorophenyl)methoxy]-3-hydroxy-2-(5-methoxy-1H-imi	C27H21FN2O5	472.46	C	-8.5
5514	O=c1c2c(oc(c1O)c1nnc(C)[nH]1)cc(Cl)c(OCc1ccc(F)cc1)c2	1312911-74-2	4H-1-Benzopyran-4-one, 7-chloro-6-[(4-fluorophenyl)methoxy]-3-hydroxy-2-(1H-1,2,	C18H11ClFN3O4	387.75	C	-8.5
5515	Cc1c2c(cc(OCc3ccc(F)cc3)c1Cl)c(=O)c(O)c(o2)c1[nH]c(OC)cn1	1312912-87-0	4H-1-Benzopyran-4-one, 7-chloro-6-[(4-fluorophenyl)methoxy]-3-hydroxy-8-methyl-2	C21H16ClFN2O4	414.81	C	-8.5
5516	O(C)c1c2c(cc(OCc3ccc(F)cc3)c1OC)c(=O)c(O)c(o2)c1[nH]ccn1	1312913-38-4	4H-1-Benzopyran-4-one, 6-[(4-fluorophenyl)methoxy]-3-hydroxy-7,8-dimethoxy-2-(3-	C26H20FN3O6	489.45	C	-8.5
5517	O(C)c1c2c(cc(OCc3ccc(F)cc3)c1Cl)c(=O)c(O)c(o2)c1ncccn1	1312913-48-6	4H-1-Benzopyran-4-one, 6-[(4-fluorophenyl)methoxy]-3-hydroxy-7,8-dimethoxy-2-(5-	C27H21FN2O6	488.46	C	-8.5
5518	O(C)c1c(c2c(cc1OCc1ccc(F)cc1)c(=O)c(O)c(o2)c1[nH]c(OC)nn1)c1ccccc1	1312915-37-9	4H-1-Benzopyran-4-one, 6-[(4-fluorophenyl)methoxy]-3-hydroxy-7-methoxy-2-(3-meth	C26H20FN3O5	473.45	C	-8.5
5519	O(C)c1c(c2c(cc1OCc1ccc(F)cc1)c(=O)c(O)c(o2)c1[nH]c(nn1)c1ccccc1)c1ccccc1	1312915-41-5	4H-1-Benzopyran-4-one, 2-(5-chloro-1H-1,2,4-triazol-3-yl)-6-[(4-fluorophenyl)met	C25H17ClFN3O5	493.87	C	-8.5
5520	Clc1c(c2c(cc1OCc1ccc(F)cc1)c(=O)c(O)c(o2)c1[nH]c(Cl)cn1)c1ccccc1	1312915-94-8	4H-1-Benzopyran-4-one, 7-chloro-6-[(4-fluorophenyl)methoxy]-3-hydroxy-2-(5-metho	C26H18ClFN2O5	492.88	C	-8.5
5521	O(Cc1ccc(F)cc1)c1c(c(c2c(c1)c(=O)c(O)c(o2)c1[nH]c(C)nn1)c1ccccc1)c1ccccc1	1312918-42-5	4H-1-Benzopyran-4-one, 6-[(4-fluorophenyl)methoxy]-3-hydroxy-7,8-diphenyl-2-(1H-	C30H20FN3O4	505.5	C	-8.5
5522	O(Cc1ccc(F)cc1)c1c(c(c2c(c1)c(=O)c(O)c(o2)c1[nH]ccn1)c1ccccc1)c1ccccc1	1312918-46-9	4H-1-Benzopyran-4-one, 6-[(4-fluorophenyl)methoxy]-3-hydroxy-7,8-diphenyl-2-(3-p	C36H24FN3O4	581.59	C	-8.5
5523	O=C1N2[C@H](c3nc(c([nH]3)CCc3nc([C@H](C)[C@@H]1NC(=O)OC)ccc3)c1ccc(cc1)c1ccc(cc1)c1[nH]c(nc1)[C@H]1N	1319189-58-6	Carbamic acid, N-[(12S,13S,18aS)-5,6,13,14,16,17,18,18a-octahydro-3-[4,Ä≤-[2-[(	C47H54N8O6	826.98	C	-8.5
5524	N(c1nc(nc(NN=Cc2cc3c(cc2)cccc3)n1)N1CCOCC1)c1ccc(F)cc1	1331749-54-2	1-Naphthalenecarboxaldehyde, 2-[4-[(4-fluorophenyl)amino]-6-(4-morpholinyl)-1,3,	C24H22FN7O	443.48	C	-8.5
5525	N(CCC#C)c1c2c(cc(Cl)cc2)ncc1	134071-76-4	1-Naphthalenesulfonic acid, 3,3,Ä≤-[(3,3,Ä≤-dibutyl[1,1,Ä≤-biphenyl]-4,4,Ä≤-diy	C40H40N6O6S2.2Na		C	-8.5
5526	O=c1n([C@@H]2O[C@H](COC(=O)OC/C=C\COC(=O)OC(ON(=O)=O)C)[C@@H](N=[N+]=[N-])C2)cc(C)c(=O)[nH]1	1344730-84-2	Thymidine, 2,Ä≤,3,Ä≤-didehydro-3,Ä≤-deoxy-, 5,Ä≤-[hydrogen N-[[(2S)-1-(tripheny	C34H35N4O7P	642.64	C	-8.5
5527	N(S(=O)(=O)c1ccc(NC(=O)CCCC)cc1)(C[C@H]([C@@H](Cc1ccccc1)NC(=O)O[C@H]1CCOC1)O)CC(C)C	1346795-73-0	Not Yet Assigned	C36H49N5O4	615.81	C	-8.5
5528	C(n1c2c(nc1CC)c(C)cc(C)n2)c1ccc(cc1)c1c(CC(=O)NS(=O)(=O)c2ccccc2)cccc1	1347365-79-0	Not Yet Assigned	C30H34D9F2N5O		C	-8.5
5529	C(n1c2c(nc1CC)c(C)cc(C)n2)c1ccc(cc1)c1c(S(=O)(=O)NS(=O)(=O)C)ccc(CCCC)c1	1347438-63-4	[1,1,Ä≤-Biphenyl]-2-carboxamide, 4,Ä≤-[(2-ethyl-5,7-dimethyl-3H-imidazo[4,5-b]p	C30H28N4O3S	524.63	C	-8.5
5530	N(C(=O)[C@H](Cc1ccccc1)C[C@H](CN1[C@@H](C(=O)NC(C)(C)C)CN(C(=O)Cc2ccccc2)CC1)O)[C@H]1c2c(C[C@H]1O)cc	1348955-75-8	Not Yet Assigned	C30H34D9F2N5O		C	-8.5
5531	C(n1c2c(nc1CC)c(C)cc(N(C)C)n2)c1ccc(cc1)c1c(S(=O)(=O)NC(=O)OCC)cccc1	1349123-79-0	3-Azabicyclo[3.1.0]hexane-2-carboxamide, N-[3-amino-1-(cyclobutylmethyl)-2,3-dic	C38H60N6O7	712.92	C	-8.5
5532	N(CC(=O)[C@H](Cc1ccccc1)C[C@@H](CN1[C@H](C(=O)NC(C)(C)C)CN(Cc2cccnc2)CC1)O)[C@H]1c2c(C[C@H]1O)cccc2	1349205-89-5	Not Yet Assigned	C41H57N5O5	699.92	C	-8.5
5533	O=c1n([C@@H]2O[C@H](CO)[C@@H](O)C2)cc(CO)c(=O)[nH]1	1350123-87-3	Carbamic acid, N-[(1S,2R)-3-[[(4-aminophenyl)sulfonyl](2-methylpropyl)amino]-1-[	C38H46N3O12PS	799.82	C	-8.5
5534	C(c1nc(CN(C(=O)N[C@@H](C(=O)N[C@H]([C@H]([C@@H]([C@H](Cc2ccccc2)NC(=O)OCc2cnco2)O)O)Cc2ccccc2)C(C)C)	1350182-18-1	2,7,10,12-Tetraazatridecanoic acid, 13-[2-[(dimethylamino)methyl]-4-thiazolyl]-4	C37H49N7O6S	719.89	C	-8.5
5535	C(=O)([C@@H](NC(=O)OC)C(C)C)N1[C@@]2(C[C@H](C1)CC2)c1[nH]c(cn1)c1c2c(c(cc1)c1ccc(cc1)c1[nH]c(nc1)[C@	1350718-94-3	Carbamic acid, N-[(1S)-1-[[(1S,4R)-1-[5-[4-[7-[2-[(1S,4R)-2-[(2S)-2-[(methoxycar	C47H56N8O6S	861.06	C	-8.5
5536	C(=O)([C@@H](NC(=O)OC)C(C)C)N1[C@@H](CCC1)c1[nH]c(cn1)c1ccc(cc1)c1cc2c(cc1)cc(cc2)c1[nH]c(nc1)[C@H]1	1351332-50-7	Carbamic acid, N-[(1S)-1-[[(2S)-2-[5-[4-[6-[2-[(1S)-1-[[(2S)-2-[(methoxycarbonyl	C42H50N8O6	762.9	C	-8.5
5537	C(=O)([C@@H](NC(=O)OC)C(C)C)N1[C@@H](CCC1)c1[nH]c(cn1)c1ccc(cc1)c1cc2c(cc1)cc(cc2)c1[nH]c(nc1)[C@H]1	1351332-53-0	Carbamic acid, N-[(1S)-1-[[(2S)-2-[5-[4-[6-[2-[(2S)-1-[(2S)-2-[(methoxycarbonyl)	C45H54N8O6	802.96	C	-8.5
5538	C(=O)([C@@H](NC(=O)OC)C(C)C)N1[C@@H](CCC1)c1[nH]c(cn1)c1ccc(cc1)c1cc2c(cc1)cc(C(=O)NC[C@H]1N(C(=O)[C	1351334-63-8	Carbamic acid, N-[(1S)-1-[[(2S)-2-[[[4-[6-[2-[(2S)-1-[(2S)-2-[(methoxycarbonyl)a	C43H53N7O7	779.92	C	-8.5
5539	C(=O)([C@@H](NC(=O)OC)C(C)C)N1C[C@@]2(C[C@H]1c1[nH]c(cn1)c1ccc(cc1)c1ccc(cc1)c1c(CO)nc([nH]1)[C@H]1N	1351852-75-9	Carbamic acid, N-[(1S)-1-[[(2S)-2-[5-[4,Ä≤-[2-[(5S,8S)-7-[(2S)-2-[(methoxycarbor	C45H58N8O8	838.99	C	-8.5
5540	C(=O)([C@@H](NC(=O)OC)C(C)C)N1[C@@H](CC2(C1)CC=CC2)c1[nH]c(cn1)c1ccc(cc1)c1ccc(cc1)c1[nH]c(nc1)[C@H]	1351852-94-2	Carbamic acid, N,N,Ä≤-[[1,1,Ä≤-biphenyl]-4,4,Ä≤-diylbis[1H-imidazole-5,2-diyl-1	C46H58N8O8	851	C	-8.5
5541	FC1(F)[C@@]2(C[C@H](N(C(=O)[C@@H](NC(=O)OC)C(C)C)C2)c2[nH]c(cn2)c2ccc(cc2)c2ccc(cc2)c2[nH]c(nc2)[C@H	1351853-18-3	Carbamic acid, N-[(1S)-1-[[(2S)-2-[5-[4,Ä≤-[2-[(3S,5S)-2-[(2S)-2-[(methoxycarbor	C44H54N8O7	806.95	C	-8.5
5542	C(=O)([C@H](NC(=O)OC)C)N1[C@@H](C[C@@H](C1)C1CC1)c1[nH]c(cn1)c1ccc(cc1)c1ccc(cc1)c1[nH]c(nc1)[C@H]1N	1351853-28-5	Carbamic acid, N-[(1R)-2-[(2S)-2-[5-[4,Ä≤-[2-[(2S,4R)-4-cyclopropyl-1-[(2R)-2-[	C49H50N8O6	846.97	C	-8.5
5543	C(=O)(C(NC(=O)OCC)C)N1[C@@H](C[C@@H](C1)C1CC1)c1[nH]c(cn1)c1ccc(cc1)c1ccc(cc1)c1[nH]c(nc1)[C@H]1N(C(	1351853-86-5	Carbamic acid, N-[(1S)-2-[(2S)-2-[5-[4,Ä≤-[2-[(2S,4R)-4-cyclopropyl-1-[(2S)-2-[	C39H46N8O6	722.83	C	-8.5
5544	FC1(F)[C@@]2(C[C@H](N(C(=O)[C@@H](NC(=O)OC)C(C)C)C2)c2[nH]c(cn2)c2ccc(cc2)c2ccc(cc2)c2[nH]c(nc2)[C@H	1351855-90-7	Carbamic acid, N-[(1S)-1-[[(2S,4R)-4-cyclopropyl-2-[5-[4,Ä≤-[2-[(1S,2S,5R)-3-[(	C44H54N8O6	790.95	C	-8.5
5545	C(=O)([C@@H](NC(=O)OC)C(C)C)N1[C@@H](C=C(C1)C1CC1)c1[nH]c(cn1)c1ccc(cc1)c1ccc2c(cc1)cc(cc2)c1[nH]c(nc	1352392-64-3	Carbamic acid, N-[(1S)-1-[[(2S)-2-[5-[4-[2-[6-[2-[(2S)-2,5-dihydro-1-[(2S)-2-[(m	C47H52N8O6	824.97	C	-8.5
5546	C(C(OCP(=O)(O)O)C#C)n1c2c(nc1)c(N)ncn2	1352476-95-9	4-Pyrimidinecarboxamide, N-[(4-fluorophenyl)methyl]-1,4,5,6-tetrahydro-1-methyl-	C20H21FN6O5	444.42	C	-8.5
5547	C(=O)([C@@H](NC(=O)OC)[C@H]1C[C@@H](C)O[C@H](C)C1)N1[C@H](c2[nH]c(c3c4c(c(cc3)c3ccc(cc3)c3[nH]c(nc3)	1359868-88-4	Carbamic acid, N-[(1S)-2-[(2S,5S)-2-[5-[4-[2,3-dihydro-7-[2-[(2S,5S)-1-[(2S)-2-[	C49H62N8O8	891.07	C	-8.5
5548	C(=O)([C@@H](NC(=O)OC)C1CCOCC1)N1[C@H]2[C@H](C2)C[C@H]1c1[nH]c(cn1)c1c2c(c(cc1)c1ccc(cc1)c1[nH]c(nc1	1359868-91-9	Carbamic acid, N-[(1S)-1-[[(1R,3S,5R)-3-[5-[4-[7-[2-[(1R,3S,5R)-2-[(2S)-2-[(meth	C42H48N10O6S	820.96	C	-8.5
5549	C(F)(F)c1n(c2c(n1)cccc2)c1nc(nc(N(CCCO)C)n1)N1CCOCC1	1359956-03-8	1,3,5-Triazin-2-amine, 4-[2-(difluoromethyl)-1H-benzimidazol-1-yl]-N-[[4-(dimeth	C24H26F2N8O	480.51	C	-8.5
5550	C(F)(F)c1n(c2c(n1)cccc2)c1nc(nc(N(C)c2ccc(OC)cc2)n1)N1CCOCC1	1359956-34-5	1,3,5-Triazin-2-amine, 4-[2-(difluoromethyl)-1H-benzimidazol-1-yl]-N-methyl-6-(4	C21H20F2N8O	438.43	C	-8.5
5551	C(=O)([C@@H](NC(=O)OC)[C@H]1C[C@@H](C)O[C@H](C)C1)N1[C@H](c2[nH]c(c3c4c(c(cc3)c3ccc(cc3)c3[nH]c(nc3)	1359956-35-6	1,3,5-Triazin-2-amine, 4-[2-(difluoromethyl)-1H-benzimidazol-1-yl]-N-(4-methoxyp	C23H23F2N7O2	467.47	C	-8.5
5552	[C@@H]([C@H](Cc1ccccc1)NC(=O)COc1c(C)cccc1C)(C[C@H](Cc1ccccc1)NC(=O)[C@H](C(C)C)N1C(=O)NCCC1)OC(=O)C	1370516-54-3	Acetic acid, 2-(carboxymethoxy)-, 1-[(2R)-3,6-dihydro-6-oxo-2-(2-phenylethyl)-2-	C35H37F3N2O9S	718.74	C	-8.5
5553	C(=O)([C@@H](NC(=O)OC)C(C)(C)C)N1[C@@H](C[C@H](SC)C1)c1[nH]c(cn1)c1ccc(cc1)c1c(F)cc(cc1)c1[nH]c(nc1C	1373165-86-6	Carbamic acid, N-[(1S)-1-[[(2S)-2-[5-chloro-4-[4,Ä≤-[2-[(1R,3S,5R)-2-[(2S)-2-[(r	C41H49ClN8O6	785.33	C	-8.5
5554	C(=O)([C@@H](NC(=O)OC)C(C)C)N1[C@@H](CCC1)c1[nH]c(cn1)c1ccc(C(=Cc2ccc(cc2)c2[nH]c(nc2)[C@H]2N(C(=O)[	1374421-49-4	Carbamic acid, N,N,Ä≤-[[1-[4-(trifluoromethyl)phenyl]-1,2-ethanediyl]bis[4,1-phe	C49H57F3N8O6	911.02	C	-8.5
5555	C(=O)([C@@H](NC(=O)OC)C1CCOCC1)N1[C@@H]([C@@H]2C[C@H]1CC2)c1[nH]c(cn1)c1ccc(cc1)c1ccc2c(cc1)c1c(cc2)n	1377036-48-0	Carbamic acid, N-[(1S)-2-[(1R,3S,4S)-3-[5-[4,Ä≤-[2-[(2S)-1-[(2S)-2-[(methoxycarl	C44H54N8O7	806.95	C	-8.5
5556	C(=O)([C@@H](NC(=O)OC)C(C)C)N1C(c2c(C1)cccc2)c1[nH]c(cn1)c1ccc(cc1)c1cc2c(cc1)cc(cc2)c1[nH]c(nc1)[C@	1377037-22-3	Carbamic acid, N-[(1S)-1-[[(1R,3S,5R)-3-[7-[4-[2-[(1R,3S,4S)-2-[(2S)-2-[(methoxy	C45H52N8O6	800.94	C	-8.5
5557	C(=O)(C(N(C)C)c1ccccc1)N1[C@@H](CCC1)c1[nH]c(cn1)c1ccc(cc1)c1cc2c(cc1)cc(cc2)c1[nH]c(nc1)[C@H]1N(C(=	1377037-87-0	Carbamic acid, N-[(1R)-2-[(2S)-2-[5-[4-[6-[2-[(2S)-1-[(2S)-2-[(methoxycarbonyl)a	C48H50N8O6	834.96	C	-8.5
5558	C(=O)([C@@H](NC(=O)OC)C(C)C)N1[C@@H]([C@@H]2C[C@H]1CC2)c1[nH]c2c(n1)ccc1c2ccc(c1)c1ccc(cc1)c1[nH]c(n	1377042-08-4	Carbamic acid, N-[(1S)-1-[[(2S)-2-[2,Ä≤-[(1S,3S,4R)-2-[(2S)-2-[(methoxycarbonyl)	C46H52N8O6	812.96	C	-8.5
5559	C(=O)([C@@H](NC(=O)OC)C1CCOCC1)N1[C@@H](CCC1)c1[nH]c(c(Cl)n1)c1ccc(cc1)c1ccc(cc1)c1[nH]c(nc1Cl)[C@H]	1377042-44-8	Carbamic acid, N-[(1S)-2-[(2S)-2-[5-[3-(2-cyclopropylethynyl)-4-[2-[(2S)-1-[(2S)	C49H56N8O7	869.02	C	-8.5
5560	C(=O)([C@@H](NC(=O)OC)C1CCOCC1)N1[C@H]2[C@H](C2)C[C@H]1c1[nH]c2c(n1)ccc1c2ccc(c1)c1ccc(cc1)c1[nH]c(n	1377042-94-8	Carbamic acid, N-[(1S)-1-[[(2S)-2-[7-[4-[2-[(2S)-1-[(2S)-2-[(methoxycarbonyl)ami	C48H54N8O6	838.99	C	-8.5
5561	C(=O)([C@@H](NC(=O)OC)C(C)C)N1[C@@H]([C@@H]2C[C@H]1CC2)c1[nH]c(c(Cl)n1)c1ccc(cc1)c1ccc2c(cc1)c1c(cc2)	1377043-44-1	Carbamic acid, N-[(1S)-1-[[(6S)-6-[7-[4-[2-[(1R,3S,4S)-2-[(2S)-2-[(methoxycarbor	C46H54N8O6	814.97	C	-8.5
5562	C(=O)([C@@H](NC(=O)OC)C(C)C)N1[C@H](c2[nH]c3c4c(cc(cc4)c4cc5c(cc4)c4c(cc5)nc([nH]4)[C@H]4N(C(=O)[C@@	1377043-72-5	Carbamic acid, N-[(1S)-1-[[(2S)-2-[2,Ä≤-[(2S,4R)-1-[(2S)-2-[(methoxycarbonyl)am:	C46H54N8O7	830.97	C	-8.5
5563	C(=O)([C@@H](NC(=O)OC)C(C)C)N1[C@@H](C[C@H]2[C@@H]1CCC2)c1[nH]c2c(n1)ccc1c2ccc(c1)c1ccc2c(cc1)c1c(cc2	1377044-31-9	Carbamic acid, N-[(1S)-1-[[(2S)-2-[5-[4-[2-[(2S,4S)-1-[(2S)-2-[(methoxycarbonyl)	C44H54N8O7	806.95	C	-8.5
5564	C(=O)([C@@H](NC(=O)OC)C(C)C)N1[C@@H]([C@@H]2C[C@H]1CC2)c1[nH]c2c(n1)ccc1c2ccc(c1)c1ccc(cc1)c1[nH]c(n	1377604-13-1	Carbamic acid, N-[(1S)-2-[(1R,3S,4S)-3-[7-[4-[2-[(2S)-1-[(2S)-2-[(methoxycarbony	C46H54N8O7	830.97	C	-8.5
5565	C(=O)([C@@H](NC(=O)OC)C(C)C)N1[C@@H](CCC1)c1[nH]c(cn1)c1ccc(cc1)c1ccc(cc1)c1ccccc1	1380289-77-9	Carbamic acid, N,N,Ä≤-[(trans,trans)-[1,1,Ä≤-bicyclohexyl]-4,4,Ä≤-diylbis[1H-im	C40H62N8O6	750.97	C	-8.5
5566	C(=O)([C@@H](NC(=O)OC)C(C)C)N1[C@@H](CCC1)c1[nH]c(cn1)c1ccc(cc1)c1sc(C#Cc2[nH]c(nc2)[C@H]2N(C(=O)[C@	1380290-14-1	Carbamic acid, N-[(1S)-1-[[(2S)-2-[5-(4,Ä≤-cyclohexyl[1,1,Ä≤-biphenyl]-4-yl)-1H	C32H40N4O3	528.68	C	-8.5
5567	O[C@H]1[C@H](n2c3c(nc2)c(=O)nc(N)[nH]3)O[C@H](COP(=O)(O[C@@H]2[C@@H](COP(=O)(O)O)O[C@H](C2)n2c3c(nc2	1383782-59-9	2-Propen-1-one, 1,1,Ä≤-(4,6-dimethoxy-1,3-phenylene)bis[3-(3-hydroxyphenyl)-	C26H22O6	430.45	C	-8.5
5568	O=c1c2c(n(cn2)[C@@H]2O[C@H](CO)[C@@H](O)[C@H]2O)[nH]c(n1)n1cc(nn1)c1ccc(OC)cc1	1384519-79-2	Inosine, 2-[4-(4-methylphenyl)-1H-1,2,3-triazol-1-yl]-	C19H19N7O5	425.4	C	-8.5
5569	N(C(C(=O)N)C)c1nc(nc(n1)N1CCOCC1)c1c2c([nH]nc2)ccc1	1391925-99-7	1-Propanol, 2-[[4-(1H-indazol-4-yl)-6-(4-morpholinyl)-1,3,5-triazin-2-yl]amino]-	C18H23N7O2	369.42	C	-8.5
5570	O=C1[C@@H]2[C@H](c3c([C@@H](OCc4ccccc4)[C@H]2CO1)cc1c(c3)OCO1)c1cc(OC)c(OC)c(OC)c1	1392513-42-6	Thymidine, 3,Ä≤-azido-5,Ä≤-O-[(3R,5aS,6R,8aS,9R,10S,12R,12aR)-decahydro-3,6,9-t	C25H35N5O8		C	-8.5
5571	N(C(=N)Nc1c(F)cc(F)cc1)C12CC3CC(C2)CC(C1)C3	139291-67-1	Guanidine, N-tricyclo[3.3.1.13,7]dec-1-yl-N,Ä≤-[2-(trifluoromethyl)phenyl]-	C18H22F3N3	337.38	C	-8.5
5572	N(CCNC(=O)c1ccc(cc1)C1OOC(OO1)c1ccc(C)cc1)c1nc(nc(n1)N1CCOCC1)N1CCCCC1	1393922-40-1	Benzamide, N-[2-[(4,6-di-4-morpholinyl-1,3,5-triazin-2-yl)amino]ethyl]-4-[6-(4-m	C29H35N7O7	593.63	C	-8.5
5573	N(c1nc(nc(NCCNC(=O)c2ccc(cc2)C2OOC(OO2)c2ccc(C)cc2)n1)N1CCOCC1)c1ccc(C)cc1	1393922-43-4	Benzamide, 4-[6-(4-methylphenyl)-1,2,4,5-tetroxan-3-yl]-N-[2-[[4-(4-morpholinyl)	C35H40N8O6	668.74	C	-8.5
5574	O=c1n([C@H]2O[C@@H](COC(=O)CCCCCCCCCCCC)SC2)cc(F)c(N)n1	1395898-93-7	Dodecanoic acid, 12-(ethylthio)-, [(2R,5S)-5-[4-[[bis(4-methoxyphenyl)phenylmeth	C43H54FN3O6S2	792.03	C	-8.5
5575	N(c1c(C)cc(/C=C/C#N)cc1C)c1nc(Nc2ccc(C#N)cc2)ncc1.S(=O)(=O)(O)c1ccccc1	1399384-94-1	Butanedioic acid, compd. with 4-[[4-[[4-[(1E)-2-cyanoethenyl]-2,6-dimethylphenyl	C22H18N6.C4H6O4		C	-8.5
5576	[Ru+3]([NH3])([NH3])([NH3])([NH3])([NH3])[N]1=CN(c2c1c(=O)nc[nH]2)C1OC(CO)C(O)C1	1404118-68-8	Benzamide, N-[5-bromo-6-(4-cyano-2,6-dimethylphenoxy)-2-[(4-cyanophenyl)amino]-4	C27H19BrN6O2	539.38	C	-8.5
5577	O=C1c2n(C([C@H]3N1[C@H](C)CCO3)([2H])[2H])cc(C(=O)NC(c1c(F)cc(F)cc1)([2H])[2H])c(=O)c2O	1407166-89-5	2H-Pyrido[1,Ä≤,2,Ä≤:4,5]pyrazino[2,1-b][1,3]oxazine-2,3,4,12-d4-9-carboxamide,	C20H7D12F2N3O5		C	-8.5
5578	C(=O)([C@@H](NC(=O)OC)[C@H]1CC2(OCC1)CCCC2)N1[C@@H](CC[C@@H]1C)c1[nH]c(cn1)c1ccc(cc1)c1ccc(cc1)c1[nH	1412816-01-3	Carbamic acid, N,N,Ä≤-[[1,1,Ä≤-biphenyl]-4,4,Ä≤-diylbis[1H-imidazole-5,2-diyl[(	C52H66N8O8	931.13	C	-8.5
5579	O[C@H]1[C@@H](O[C@H](C(=O)O)[C@H]1O)n1nc(C(=O)N)nc1	1414793-90-0	Not Yet Assigned	C29H33N7O8	607.61	C	-8.5
5580	O[C@H]1[C@@H](O[C@H](C(=O)Nc2ccc(C(=O)N3CCCCC3)cc2)[C@H]1O)n1nc(C(=O)N)nc1	1414801-76-5	Not Yet Assigned	C26H29ClN8O6	585.01	C	-8.5
5581	O[C@H]1[C@@H](O[C@H](C(=O)Nc2ccc(Oc3ccccc3)nc2)[C@H]1O)n1nc(C(=O)N)nc1	1414801-96-9	Not Yet Assigned	C19H20N6O7	444.4	C	-8.5
5582	C(=O)([C@@H](NC(=O)OC)C(C)C)N1[C@H](c2[nH]c(c3c4ccc(c3)CCc3c(cc(CC4)cc3)c3ccc(cc3)c3[nH]c(nc3)[C@H]3	1415119-55-9	Carbamic acid, N,N,Ä≤-[tricyclo[8.2.2.24,7]hexadeca-4,6,10,12,13,15-hexaene-5,1!	C50H60N8O6	869.06	C	-8.5
5583	C(=O)([C@@H](NC(=O)OC)C(C)C)N1CC2(C[C@H]1c1[nH]c(cn1)c1ccc(cc1)c1c3CCc4cc(c(CCc(c1)cc3)cc4)c1ccc(cc1	1415119-56-0	Carbamic acid, N-[(1S)-1-[[(2S)-2-[5-[11-[4-[2-[(2S)-1-[(2S)-2-[(methoxycarbonyl	C50H60N8O6	869.06	C	-8.5
5584	C(=O)([C@@H](NC(=O)OC)C(C)C)N1[C@@H](C[C@@H](C)C1)c1[nH]c(cn1)c1ccc(cc1)c1c2CCc3cc(cc(CCc(c1)cc2)cc3)	1415120-18-1	Carbamic acid, N,N,Ä≤-[tricyclo[8.2.2.24,7]hexadeca-4,6,10,12,13,15-hexaene-5,1:	C60H68N8O6	997.23	C	-8.5
5585	C(=O)([C@@H](NC(=O)OC)C(C)C)N1[C@H](c2[nH]c(c3ccc(c4c5cc(c(c4)CCc4ccc(CC5)cc4)c4ccc(cc4)c4[nH]c(nc4)	1415120-20-5	Carbamic acid, N,N,Ä≤-[tricyclo[8.2.2.24,7]hexadeca-4,6,10,12,13,15-hexaene-5,1:	C58H68N8O6	973.21	C	-8.5
5586	O=C1c2c(N(c3c(N1C)c(C)cc(F)n3)C1CC1)ncc(CCO)c2	1415206-23-3	Carbamic acid, N-[(1S)-2-methyl-1-[[(2S)-octahydro-2-[5-[15-[4-[2-[(2S)-octahydr	C58H72N8O6	977.24	C	-8.5
5587	C(=O)([C@H](NC(=O)OC)C(C)C)N1[C@H](CCC1)c1[nH]c(cn1)c1ccc(cc1)c1ccc(cc1)c1[nH]c(nc1)[C@H]1N(C(=O)[C@	1417333-65-3	Carbamic acid, N,N,Ä≤-[[1,1,Ä≤-biphenyl]-4,4,Ä≤-diylbis[1H-imidazole-5,2-diyl-2	C40H50N8O6	738.88	C	-8.5
5588	C(=O)([C@@H](NC(=O)OC)C(C)C)N1[C@H](c2[nH]c(c3c4c(c(cc3)c3ccc(cc3)c3[nH]c(nc3)[C@H]3N(C(=O)[C@@H](NC	1419384-78-3	Carbamic acid, N-[(1S)-1-[[(2S)-2-[5-[1,Ä≤,1,Ä≤-difluoro-1,Ä≤,3,Ä≤-dihydro-7,Ä≤	C47H58F2N8O6	869.01	C	-8.5
5589	N(=C1c2c(N(CN3CCCC3)C1=O)ccc(Cl)c2)NC(=S)Nc1ccc(Cl)cc1	1421478-71-8	3-Quinolinecarboxylic acid, 6-[(3-chloro-2-fluorophenyl)methyl]-1-(1-cyclohexyl-	C26H27ClFNO5	487.95	C	-8.5
5590	O=C1C2C(c3c(C(O)C2CO1)cc1c(c3)OCO1)c1ccccc1	14254-91-2	Picropodophyllin, 3,Ä≤,4,Ä≤,5,Ä≤-tridemethoxy-, DL-	C19H16O5	324.33	C	-8.5
5591	C[C@]12[C@@]3(C)[C@@H]([C@H](CC3)[C@@]3(C)O[C@H](C(=C)C)CC3)[C@H](O)C[C@@H]1[C@]1(C)[C@@H](CC2)C(C)(	1431852-65-1	Dammar-25-ene-3,12-diol, 20,24-epoxy-, (3Œ≤,12Œ≤,24R)-	C30H50O3	458.72	C	-8.5
5592	C[C@@]12[C@@H]([C@]3(C)[C@@H](CC2)C(C)(C)[C@@H](O)CC3)C[C@@H](O)[C@H]2[C@@]1(C)CC[C@@H]2[C@@]1(C)OC(	1431852-66-2	Dammar-25-ene-3,12-diol, 20,24-epoxy-, (3Œ≤,12Œ≤,24S)-	C30H50O3	458.72	C	-8.5
5593	C([C@H](OCP(=O)(OCCCO)O)CO)n1c(=O)nc(N)cc1	1431852-70-8	18-Norcholan-24-oic acid, 3-(acetyloxy)-12,20-dihydroxy-4,4,8,14-tetramethyl-, (	C29H46O5	474.67	C	-8.5
5594	C(=O)(O)c1c(NC(=O)c2c(N)cccc2)ccc(Cc2cc(C(=O)O)c(NC(=O)c3c(N)cccc3)cc2)c1	144334-83-8	[1,1,Ä≤-Biphenyl]-3,3,Ä≤-dicarboxylic acid, 4,4,Ä≤-bis[(2-nitrobenzoyl)amino]-	C28H18N4O10	570.46	C	-8.5
5595	O=c1n(cc(F)c(n1)n1ncnc1)[C@H]1O[C@@H](CO)SC1	1446690-23-8	Benzamide, 4-[[4-[[4-[(1E)-3-amino-3-oxo-1-propen-1-yl]-2,6-dimethylphenyl]aminc	C22H22N6O2	402.45	C	-8.5
5596	N(c1c2c(ncc1)cc(Cl)cc2)c1c(O)c(CN2CC(C)CC(C)C2)cc(CN2CC(C)CC(C)C2)c1	145431-60-3	Phenol, 2-[(7-chloro-4-quinolinyl)amino]-4,6-bis[(4-methyl-1-piperidinyl)methyl]	C29H37ClN4O	493.08	C	-8.5
5597	[C@H]([C@@H](COC(=O)C)OC(=O)C)(OC(=O)C)[C@H]1[C@H](NC(=O)C)[C@@H](N=C(NC(=O)[C@@H](C)c2ccc(CC(C)C)cc	1469338-36-0	D-glycero-D-galacto-Non-2-enonic acid, 5-(acetylamino)-2,6-anhydro-3,4,5-trideo>	C26H32N4O9	544.55	C	-8.5
5598	C(OP(=O)(O[C@H]1[C@@H](O[C@H](CO)[C@H]1OC(=O)c1ccccc1)n1nc(C(=O)N)nc1)OCCc1ccc(N(=O)=O)cc1)[C@@H]1[C	1469875-43-1	Adenosine, 1,Ä≤-[3-(aminocarbonyl)-1H-1,2,4-triazol-1-yl]-3,Ä≤-O-benzoyl-1,Ä≤-d	C40H37N10O14P	912.75	C	-8.5
5599	C(=O)(c1[nH]c2c(c1)cc(NS(=O)(=O)CCN(C)C)cc2)N1CCN(CC1)c1c(NC(C)C)cccn1	147540-00-9	4-Pyridineethanesulfonamide, N-[2-[[4-[3-[(1-methylethyl)amino]-2-pyridinyl]-1-p	C28H33N7O3S	547.67	C	-8.5
5600	C(=O)(c1[nH]c2c(c1)cc(NS(=O)(=O)N1CCN(Cc3ccccc3)CC1)cc2)N1CCN(CC1)c1c(NC(C)C)cccn1	147540-09-8	1-Piperazinesulfonamide, 4-methyl-N-[2-[[4-[3-[(1-methylethyl)amino]-2-pyridinyl	C26H36N8O3S	540.68	C	-8.5
5601	C(=O)(c1cc2c([nH]1)ccc(N)c2)N1CCN(CC1)c1c(NC(CC)(C)C)cccn1	147540-12-3	4-Morpholinesulfonamide, N-[2-[[4-[3-[(1-methylethyl)amino]-2-pyridinyl]-1-piper	C25H33N7O4S	527.64	C	-8.5
5602	C(=O)(c1cc2c([nH]1)ccc(NS(=O)(=O)CCCN1CCCCC1)c2)N1CCN(CC1)c1c(NC(C)(C)C)cccn1	147540-34-9	1-Piperidinepropanesulfonamide, N-[2-[[4-[3-[(1-methylethyl)amino]-2-pyridinyl]-	C29H41N7O3S	567.75	C	-8.5
5603	C(=O)(c1cc2c([nH]1)ccc(N(S(=O)(=O)C)C)c2)N1CCN(CC1)c1c(NC(C)C)cccn1	147920-17-0	Methanesulfonamide, N-[2-[[4-[3-[(1-methylethyl)amino]-2-pyridinyl]-1-piperaziny	C22H28N6O3S	456.56	C	-8.5
5604	C(n1c2c(nc1CC)c(C)cc(C)n2)c1ccc(cc1)c1c(S(=O)(=O)NC(=O)NC)cccc1	148401-88-1	Carbamic acid, [[4,Ä≤-[(2-ethyl-5,7-dimethyl-3H-imidazo[4,5-b]pyridin-3-yl)methy	C27H30N4O4S	506.62	C	-8.5
5605	C(n1c2c(nc1CC)c(C)cc(C)n2)c1ccc(cc1)c1c(S(=O)(=O)NC(=O)NCC2CC2)cccc1	148401-91-6	[1,1,Ä≤-Biphenyl]-2-sulfonamide, 4,Ä≤-[(2-ethyl-5,7-dimethyl-3H-imidazo[4,5-b]p	C27H31N5O3S	505.63	C	-8.5
5606	C(=O)([C@@H](NC(=O)OC)C(C)C)N1[C@@H](C[C@H](COC)C1)c1[nH]c(cn1)c1ccc(cc1)c1ccc(cc1)c1[nH]c(nc1)[C@H]	1491957-08-4	Carbamic acid, N-[(1S)-2-[(2S,5S)-2-[5-[4,Ä≤-[2-[(2S,4S)-1-[(2S)-2-[(methoxycarl	C47H60N8O9	881.03	C	-8.5
5607	[C@@H](Cc1cc(C)ccc1)(NC(=O)N1CCN(C)CC1)C(=O)N[C@@H](CCc1ccccc1)C=CS(=O)(=O)c1ccccc1	1499175-66-4	1-Piperazinecarboxamide, 4-methyl-N-[(1S)-1-(2-naphthalenylmethyl)-2-[[(1S)-3-(2	C40H42N4O4S	674.85	C	-8.5
5608	C([C@H]([C@@H](NC(=O)[C@@H](NC(=O)c1nc2c(cc1)cccc2)[C@@H]1CCSC1)Cc1ccccc1)O)N1[C@H](C(=O)NC(C)(C)C)C	150378-17-9	D-erythro-Pentonamide, 2,3,5-trideoxy-N-[(1S,2R)-2,3-dihydro-2-hydroxy-1H-inden-	C36H47N5O4	613.79	C	-8.5
5609	[N+](#N)c1c2c(c(N=Nc3ccc(S(=O)(=O)O)cc3)cc1)ccc(S(=O)(=O)O)c2	1504558-52-4	Benzonitrile, 4-[[6-[[4-[(1E)-2-cyanoethenyl]-2,6-dimethylphenyl]amino]-9-[(4-me	C31H26N8O	526.59	C	-8.5
5610	C(OCCOC(=O)C)n1c2c(nc1)c(=O)nc[nH]2	150557-91-8	Inosine, 2,Ä≤-deoxy-2-[4-[2-(trifluoromethyl)phenyl]-1-piperazinyl]-	C21H23F3N6O4	480.44	C	-8.5
5611	O[C@H]1[C@H](n2c3c(nc2)c(=O)nc(N)[nH]3)O[C@H](CO)[C@H]1ONC[C@H]1O[C@H](C[C@@H]1O)n1c2c(nc1)c(=O)nc(N	1513874-14-0	Guanosine, 3,Ä≤-de(phosphinicooxy)guanylyl-1,2-hydrazinediyl-(3,Ä≤,Ü5,Ä≤)-2,Ä≤	C20H26N12O7	546.5	C	-8.5
5612	C(=O)(NCc1ccc(F)cc1)c1c(OC(OC(=O)C)CC)c(=O)n(C)c(C(NC(=O)c2oc(C)nn2)(C)C)n1	1538604-22-6	4-Pyrimidinecarboxamide, 5-[1-(benzoyloxy)ethoxy]-N-[(4-fluorophenyl)methyl]-1,6	C29H29FN6O7	592.58	C	-8.5
5613	C(n1c2c(nc1CC)c(C)cc(C)n2)c1ccc(cc1)c1c(S(=O)(=O)NC(=O)COCCCC)ccc(C(CC)(C)C)c1	154552-03-1	Acetamide, 2-butoxy-N-[[4,Ä≤,Ä≤-[(2-ethyl-5,7-dimethyl-3H-imidazo[4,5-b]pyridin	C35H38N4O4S	610.77	C	-8.5
5614	C(n1c2c(nc1CC)c(C)cc(C)n2)c1ccc(cc1)c1c(S(=O)(=O)NC(=O)COCC)ccc(C(CC)(C)C)c1	154552-23-5	Acetamide, 2-ethoxy-N-[[4,Ä≤,Ä≤-[(2-ethyl-5,7-dimethyl-3H-imidazo[4,5-b]pyridin	C33H34N4O4S	582.71	C	-8.5
5615	C(n1c2c(nc1CC)c(C)cc(C)n2)c1ccc(cc1)c1c(S(=O)(=O)NC(=O)NCCCC)ccc(CN(C)C)c1	154552-45-1	[1,1,Ä≤-Biphenyl]-2-sulfonamide, N-[(butylamino)carbonyl]-5-(1,1-dimethylethyl)-	C32H41N5O3S	575.76	C	-8.5

5616	C(n1c2c(nc1CC)c(C)cc(C)n2)c1ccc(cc1)c1c(S(=O)(=O)NC(=O)c2c(OCC)cccc2)ccc(CCC)c1	154552-92-8	Benzamide, 2-ethoxy-N-[[5-ethyl-4,Ä≤-[(2-ethyl-5,7-dimethyl-3H-imidazo[4,5-b]py	C34H36N4O4S	596.74	C	-8.5
5617	C(n1c2c(nc1CC)c(C)cc(C)n2)c1ccc(cc1)c1c(S(=O)(=O)NC(=O)c2c(OCC)cccc2)ccc(CCCC)c1	154552-93-9	Benzamide, 2-ethoxy-N-[[4,Ä≤-[(2-ethyl-5,7-dimethyl-3H-imidazo[4,5-b]pyridin-3-y	C35H38N4O4S	610.77	C	-8.5
5618	C(n1c2c(nc1CC)c(C)cc(C)n2)c1ccc(cc1)c1c(S(=O)(=O)NC(=O)c2c(OCC)cccc2)ccc(OCCCC)c1	154552-95-1	Benzamide, 2-ethoxy-N-[[4,Ä≤-[(2-ethyl-5,7-dimethyl-3H-imidazo[4,5-b]pyridin-3-y	C35H38N4O4S	610.77	C	-8.5
5619	C(n1c2c(nc1CC)c(C)cc(C)n2)c1ccc(cc1)c1c(S(=O)(=O)NC(=O)c2ccccc2)ccc(CC)c1	154553-11-4	Benzamide, N-[[4,Ä≤-[(2-ethyl-5,7-dimethyl-3H-imidazo[4,5-b]pyridin-3-yl)methyl]	C31H30N4O3S	538.66	C	-8.5
5620	C(n1c2c(nc1CC)c(C)cc(C)n2)c1ccc(cc1)c1c(S(=O)(=O)NC(=O)c2ccccc2)ccc(OCCCC)c1	154553-15-8	Benzamide, N-[[4,Ä≤-[(2-ethyl-5,7-dimethyl-3H-imidazo[4,5-b]pyridin-3-yl)methyl]	C33H34N4O3S	566.71	C	-8.5
5621	C(n1c2c(nc1CC)c(C)cc(C)n2)c1ccc(cc1)c1c(S(=O)(=O)NC(=O)NCCc2ccccc2)ccc(OC)c1	154553-56-7	[1,1,Ä≤-Biphenyl]-2-sulfonamide, 4,Ä≤-[(2-ethyl-5,7-dimethyl-3H-imidazo[4,5-b]p	C36H41N5O3S	623.81	C	-8.5
5622	C(n1c2c(nc1CC)c(C)cc(C)n2)c1ccc(cc1)c1c(S(=O)(=O)NC(=O)NCCc2ccccc2)ccc(OCC)c1	154553-59-0	[1,1,Ä≤-Biphenyl]-2-sulfonamide, 4,Ä≤-[(2-ethyl-5,7-dimethyl-3H-imidazo[4,5-b]p	C34H37N5O4S	611.75	C	-8.5
5623	CC1C2c3c(C1CC2)c(ccc3c1[nH]c(nc1)[C@H]1N(C(=O)[C@@H](NC(=O)OC)C(C)C)CCC1)c1ccc(cc1)c1[nH]c(nc1)[C@H]	1561048-18-7	Carbamic acid, N-[(1S)-2-methyl-1-[[(2S)-2-[5-[1,2,3,4-tetrahydro-8-[4-[2-[(3S)-	C46H56N8O6	816.99	C	-8.5
5624	O[C@H]1[C@H](n2c3c(nc2)c(N)nc(SCC#C)n3)O[C@H](CO)[C@H]1O	1561048-25-6	Carbamic acid, N-[(1S)-2-methyl-1-[[(2S)-2-[5-[4-[1,2,3,4-tetrahydro-8-[2-[(2S)-	C46H58N8O6	819	C	-8.5
5625	C(=O)([C@@H](NC(=O)OC)C(C)C)N1[C@H](CC2(C1)CCNCC2)c1[nH]c(cn1)c1ccc(cc1)c1ccc(cc1)c1[nH]c(nc1)[C@H]1	1571138-15-2	Carbamic acid, N-[(1S)-1-[[(2S)-2-[5-[4,Ä≤-[2-[(3S)-2-[(2S)-2-[(methoxycarbonyl)	C44H57N9O6	807.98	C	-8.5
5626	O=c1n([C@@H]2O[C@H](COP(=O)(Oc3ccc(SC)cc3)Oc3ccc(SC)cc3)[C@@H](N=[N+]=[N-])C2)cc(C)c(=O)[nH]1	158171-92-7	5,Ä≤-Thymidylic acid, 3,Ä≤-azido-3,Ä≤-deoxy-, bis(4-cyanophenyl) ester	C24H20N7O7P		C	-8.5
5627	C(=O)(OC[n+]1cc(C(=O)N)ccc1)N1c2c(N(c3c(C1=O)cccn3)C1CC1)ncc c2C	1598376-36-3	1-Naphthalenesulfonic acid, 4-(nitrosoamino)-3-[2-(4-sulfo-1-naphthalenyl)diazer	C20H14N4O7S2.2Na		C	-8.5
5628	C(=O)([C@@H](NC(=O)OC)[C@@H]1C[C@@H](O[C@@H](C)C1)C1CC1)N1[C@@H](CC[C@@H]1C)c1[nH]c(cn1)c1ccc(cc1)c1	1608494-81-0	Carbamic acid, N,N,Ä≤-[[1,1,Ä≤-biphenyl]-4,4,Ä≤-diylbis[1H-imidazole-5,2-diyl[(	C54H70N8O8	959.18	C	-8.5
5629	C(OCCOP(=O)(OCOC(=O)OC1CCCCC1)OCOC(=O)OC1CCCCC1)n1c2c(nc1)c(=O)nc(N)[nH]2	1610585-47-1	2,4,6,8-Tetraoxa-5-phosphanonanedioic acid, 5-[2-[(2-amino-1,6-dihydro-6-oxo-9H-	C22H36N5O12P	593.52	C	-8.5
5630	C(=O)([C@@H](NC(=O)OC)C(C)C)N1[C@H](c2[nH]c(c3c4c(c(cc3)c3c5c(c(cc3)c3[nH]c(nc3)[C@H]3N(C(=O)[C@@H](	1612235-51-4	Carbamic acid, N,N,Ä≤-[(1,Ä≤,1,Ä≤,Ä≤,Ä≤,3,Ä≤,3,Ä≤,Ä≤,Ä≤-tetrahydro[4,Ä≤,4,Ä≤,	C54H70N8O6	927.18	C	-8.5
5631	O[C@H]1[C@@H](O[C@H](COP(=O)(OC(OC(=O)c2ccccc2)C)OC(OC(=O)c2ccccc2)C)[C@H]1O)n1c(=O)c(C(=O)N)nc(F)c1	1613031-62-1	2-Pyrazinecarboxamide, 4-[5-O-[bis[1-(2,2-dimethyl-1-oxopropoxy)ethoxy]phosphiny	C24H37FN3O13P	625.53	C	-8.5
5632	O=C1N2[C@@H](Cn3c1c(O)c(=O)c(C(=O)NCc1c(F)cc(F)cc1)c3)O[C@H]1C[C@@H]2COC1	1616340-16-9	2,6-Methanopyrido[1,Ä≤,2,Ä≤:4,5]pyrazino[2,1-b][1,6,3]dioxazocine-11-carboxamid	C21H19F2N3O6	447.39	C	-8.5
5633	O=C1N2[C@@H](Cn3c1c(O)c(=O)c(C(=O)NCc1c(F)cc(F)c(F)c1)c3)O[C@H]1C[C@@H]2CC1	1616340-17-0	2,6-Methanopyrido[1,Ä≤,2,Ä≤:4,5]pyrazino[2,1-b][1,6,3]dioxazocine-11-carboxamid	C21H19F2N3O6	447.39	C	-8.5
5634	C(OCCOP(=O)(OC[C@@H]1OC(C)(C)OC1)O)n1c2c(nc1)c(=O)nc(N)[nH]2	1616340-94-3	2,5-Methanopyrido[1,Ä≤,2,Ä≤:4,5]pyrazino[2,1-b][1,3]oxazepine-10-carboxamide, 2	C22H20F3N3O5	463.41	C	-8.5
5635	[C@H](C(=O)N1[C@@H]2[C@@H](C2)C[C@H]1C#N)(N)C12CC3(OC(=O)CN(C)C)CC(C2)CC(C1)C3	1616608-23-1	1-Pyrrolidineacetic acid, Œ±-methyl-, 3-[(1S)-1-amino-2-[(1S,3S,5S)-3-cyano-2-az	C25H36N4O3	440.58	C	-8.5
5636	C(=O)(N[C@H](C(=O)C(=O)N)CCC)[C@H]1N(C(=O)[C@@H](NC(=O)[C@@H](NC(=O)c2cnccn2)C2CCCCC2)C(C)(C)C[C@H	1616696-01-5	3-Azabicyclo[3.1.0]hexane-2-carboxamide, N-[(1S)-3-amino-1-(cyclobutylmethyl)-2,	C36H53N5O5	635.84	C	-8.5
5637	[Sn](CCCC)(CCCC)(CCCC)c1cn(c(=O)[nH]c1=O)[C@H]1O[C@@H](CO)[C@H](O)C1	1616949-74-6	1(2H)-Pyrimidineacetamide, N-[(1S,3S,4S)-4-[[2-(2,6-dimethylphenoxy)acetyl]aminc	C37H46F2N4O5	664.78	C	-8.5
5638	O(CCCC(=O)NO)c1cc(c2nc(nc(n2)N2CCOCC2)N2CCOCC2)ccc1	1619257-68-9	Heptanamide, 7-[4-(4,6-di-4-morpholinyl-1,3,5-triazin-2-yl)phenoxy]-N-hydroxy-	C24H34N6O5	486.56	C	-8.5
5639	O(CCCCCC(=O)NO)c1cc(c2nc(nc(n2)N2CCOCC2)N2CCOCC2)ccc1	1619257-70-3	Pentanamide, 5-[3-(4,6-di-4-morpholinyl-1,3,5-triazin-2-yl)phenoxy]-N-hydroxy-	C22H30N6O5	458.51	C	-8.5
5640	O=c1n([C@@H]2O[C@H](COP(=O)(Nc3cc(C(=O)Nc4cc(C(=O)Nc5cc(C(=O)NCCC(=N)N)n(C)c5)n(C)c4)n(C)c3)OC3CCCCC	1620662-77-2	5,Ä≤-Thymidylic acid, 3,Ä≤-azido-3,Ä≤-deoxy-, 5,Ä≤-[cyclohexyl N-[5-[[[5-[[[5-[	C37H46N13O9P		C	-8.5
5641	N(C(=O)[C@H](Cc1cccnc1)C[C@@H](CN1[C@H](C(=O)NC(C)(C)C)CN(Cc2c(F)cccc2F)CC1)O)[C@H]1c2c(C[C@H]1O)ccc	162105-24-0	1-Piperazinepentanamide, N-(2,3-dihydro-2-hydroxy-1H-inden-1-yl)-2-[[(1,1-dimeth	C35H46N6O4	614.78	C	-8.5
5642	S=c1n([C@H]2O[C@@H](CO)CC2)ccc(N)n1	162105-33-1	1-Piperazinepentanamide, N-(2,3-dihydro-2-hydroxy-1H-inden-1-yl)-2-[[(1,1-dimeth	C36H45N5O4S2	675.9	C	-8.5
5643	N(C(=O)[C@H](Cc1ccc(O)cc1)C[C@@H](CN1[C@H](C(=O)NC(C)(C)C)CN(Cc2cccnc2)CC1)O)[C@H]1c2c(C[C@H]1O)cccc	162518-58-3	D-erythro-Pentonamide, 2,3,5-trideoxy-N-[(1S,2S,3S)-2,3-dihydro-2,3-dihydroxy-1H	C30H42N4O5	538.68	C	-8.5
5644	C(=O)([C@@H](NC(=O)OC)C(C)C)N1[C@H](c2[nH]c(c3c4c(c(cc3)c3c5c(c(cc3)c3[nH]c(nc3)[C@H]3N(C(=O)[C@@H](	1627533-21-4	Carbamic acid, N,N,Ä≤-[(1,1,Ä≤,2,2,Ä≤,3,3,Ä≤,4,4,Ä≤-octahydro[5,5,Ä≤-bi-1,4-me	C50H62N8O6	871.08	C	-8.5
5645	N(c1nc(nc(n1)N1CCOCC1)c1ccc(OC)cc1)c1c(Cl)cccc1	1627734-67-1	1,3,5-Triazin-2-amine, 4-(4-methoxyphenyl)-6-(4-morpholinyl)-N-(4-nitrophenyl)-	C20H20N6O4	408.41	C	-8.5
5646	N(c1nc(nc(n1)N1CCOCC1)c1ccc(OC)cc1)c1cc(C)ccc1	1627734-70-6	1,3,5-Triazin-2-amine, 4-(4-methoxyphenyl)-N-(2-methylphenyl)-6-(4-morpholinyl)-	C21H23N5O2	377.44	C	-8.5
5647	O(P(=O)(OC[C@H]1O[C@H]([C@H](OP(=O)(OC[C@H]2O[C@H]([C@H](O)C2)n2c3c(nc2)c(N)ncn3)O)[C@@H]1O)n1nc(C(=	1637815-91-8	Adenosine, 1,Ä≤-[3-(aminocarbonyl)-1H-1,2,4-triazol-1-yl]-1,Ä≤-de(6-amino-9H-pu	C28H36N14O16P2	886.62	C	-8.5
5648	C(=O)([C@@H](NC(=O)OC)C(C)C)N1[C@@H](C=CC1)c1[nH]c(cn1)c1ccc(cc1)c1ccc(cc1)c1[nH]c(nc1)[C@H]1N(C(=O)	1639870-81-7	Carbamic acid, N-[(1S)-1-[[(2S)-2,5-dihydro-2-[5-[4,Ä≤-[2-[(2S)-1-[[1-[(methoxy	C39H44N8O6	720.82	C	-8.5
5649	C(=O)([C@@H](NC(=O)N(C)C)C(C)C)N1[C@@H](C=CC1)c1[nH]c(cn1)c1ccc(cc1)c1ccc(cc1)c1[nH]c(nc1)[C@H]1N(C(	1639870-99-7	Carbamic acid, N-[(1S)-1-[[(2S)-2,5-dihydro-2-[5-[5-[4-[2-[(2S)-1-[(2S)-2-[(meth	C44H50N8O6	786.92	C	-8.5
5650	C(=O)([C@@H](NC(=O)OC)C1CCCCC1)N1[C@@H](C=CC1)c1[nH]c(cn1)c1ccc(cc1)c1ccc(cc1)c1[nH]c(nc1)[C@H]1N(C(	1639871-25-2	Carbamic acid, N-[(1S)-1-cyclohexyl-2-[(2S)-2-[5-[4,Ä≤-[2-[(2S)-1-[(2S)-2-cyclo	C46H56N8O6	816.99	C	-8.5
5651	C(=O)([C@@H](NC(=O)OC)C(C)C)N1[C@@H](CC(O)C1)c1[nH]c(cn1)c1ccc(cc1)c1ccc(cc1)c1[nH]c(nc1)[C@H]1N(C(=	1639871-28-5	Carbamic acid, N-[(1S)-1-cyclopentyl-2-[(2S)-2-[5-[4,Ä≤-[2-[(2S)-1-[(2S)-2-cycl	C44H52N8O6	788.93	C	-8.5
5652	N(c1nc(nc(Sc2oc(nn2)c2ccc(F)cc2)n1)N1CCOCC1)c1cc(Br)ccc1	1642584-23-3	1,3,5-Triazin-2-amine, 4-[[5-(4-fluorophenyl)-1,3,4-oxadiazol-2-yl]thio]-N-(3-me	C22H20FN7O2S	465.5	C	-8.5
5653	S(c1nc(nc(Nc2ccc(Cl)cc2)n1)N1CCOCC1)c1oc(nn1)c1ccc(F)cc1	1642584-26-6	1,3,5-Triazin-2-amine, N-(3-chlorophenyl)-4-[[5-(4-fluorophenyl)-1,3,4-oxadiazol	C21H17ClFN7O2S	485.92	C	-8.5
5654	O=c1n([C@@H]2O[C@H](COP(=O)(C(O)c3c(N(=O)=O)cccc3)OC[C@H]3O[C@H](C=C3)n3c(=O)[nH]c(=O)c(C)c3)[C@@H](	164652-21-5	Thymidine, P,3,Ä≤-dideoxy-P-[hydroxy(2-nitrophenyl)methyl]thymidylyl-(5,Ä≤,Ü5,.	C27H30N5O12P	647.53	C	-8.5
5655	N(C(=O)[C@H](Cc1ccccc1)C[C@@H](CN1[C@H](C(=O)NC(C)(C)C)CN(CCCc2cccnc2)CC1)O)[C@H]1c2c(C[C@H]1O)cccc2	164725-12-6	1-Piperazinepentanamide, N-(2,3-dihydro-2-hydroxy-1H-inden-1-yl)-2-[[(1,1-dimeth	C36H47N5O4	613.79	C	-8.5
5656	N(C(=O)[C@H](Cc1ccccc1)C[C@@H](CN1[C@H](C(=O)NC(C)(C)C)CN(Cc2nc3c(cc2)cccc3)CC1)O)[C@H]1c2c(C[C@H]1O	164725-28-4	D-erythro-Pentonamide, 2,3,5-trideoxy-N-[(1S,2R)-2,3-dihydro-2-hydroxy-1H-inden-	C39H52N4O4	640.85	C	-8.5
5657	N(C(=O)[C@H](Cc1ccccc1)C[C@@H](CN1[C@H](C(=O)NC(C)(C)C)CN(Cc2c(C)ccnc2C)CC1)O)[C@H]1c2c(C[C@H]1O)ccc	164725-36-4	1-Piperazinepentanamide, N-(2,3-dihydro-2-hydroxy-1H-inden-1-yl)-2-[[(1,1-dimeth	C38H50N4O5	642.83	C	-8.5
5658	N(C(=O)[C@H](Cc1ccccc1)C[C@@H](CN1[C@H](C(=O)NC(C)(C)C)CN(Cc2csc(C)n2)CC1)O)[C@H]1c2c(C[C@H]1O)cccc2	164725-60-4	1-Piperazinepentanamide, N-(2,3-dihydro-2-hydroxy-1H-inden-1-yl)-2-[[(1,1-dimeth	C36H49N5O4	615.81	C	-8.5
5659	C(=O)([C@@H](NC(=O)OC)C(C)C)N1C[C@@]2(C[C@H]1c1[nH]c(cn1)c1ccc(cc1)c1ccc(cc1)c1[nH]c(nc1)[C@H]1N(C(=	1653966-96-1	Carbamic acid, N-[(1S)-2-methyl-1-[[(3S,5S)-3-[5-[4,Ä≤-[2-[(2S,3aS,7aS)-octahyd	C48H62N8O7	863.06	C	-8.5
5660	C(=O)([C@@H](NC(=O)OC)[C@@H]1C[C@@H](C)O[C@H](C)C1)N1C[C@@]2(C[C@H]1c1[nH]c(cn1)c1ccc(cc1)c1ccc(cc1)	1653967-07-7	Carbamic acid, N-[(1S)-1-[[(2S)-2-[5-[4-[2-[(3S,5S)-2-[(2S)-2-[(methoxycarbonyl)	C46H56N8O7	832.99	C	-8.5
5661	C(=O)([C@@H](NC(=O)OC)C(C)C)N1C[C@@]2(C[C@H]1c1[nH]c(cn1)c1ccc(cc1)c1cc3c(cc1)c1c(cc3)nc([nH]1)[C@H]	1653967-14-6	Carbamic acid, N-[(1S)-1-[[(2S)-2-[5-[4-[2-[(5S,8S)-7-[(2S)-2-[(methoxycarbonyl)	C45H54N8O7	818.96	C	-8.5
5662	C(=O)([C@@H](NC(=O)OC)C(C)C)N1C[C@]2(C[C@H]1c1[nH]c(cn1)c1ccc(cc1)c1cc3c(cc1)c1c(cc3)nc([nH]1)[C@H]1	1653967-15-7	Carbamic acid, N-[(1S)-1-[[(2S)-2-[7-[4-[2-[(5S,8S)-7-[(2S)-2-[(methoxycarbonyl)	C45H54N8O7	818.96	C	-8.5
5663	C(Nc1c2c(n(=O)c(C)n1)CCCC2)(C12CC3CC(C2)CC(C1)C3)c1ccccc1	1658454-65-9	4-Quinazolinamine, 5,6,7,8-tetrahydro-2-methyl-N-(1-tricyclo[3.3.1.13,7]dec-1-yl	C21H31N3O	341.49	C	-8.5
5664	O(C(=O)N[C@H]([C@@H](CN(S(=O)(=O)c1cc2c(cc1)nc(N(C)C)s2)CC(C)C)O)Cc1ccccc1)[C@@H]1[C@H]2[C@@H](OC1)O	1672661-51-6	Carbamic acid, N-[(1S,2R)-2-hydroxy-3-[(2-methylpropyl)[[2-(methylsulfonyl)-6-be	C29H35F2N3O9S3	703.79	C	-8.5
5665	C([C@H](NC(=O)N1CCOCC1)C(=O)N[C@H](/C=C/S(=O)(=O)c1ccccc1)CCc1ccccc1)c1ccc(cc1)c1ccccc1	170111-45-2	4-Morpholinecarboxamide, N-[(1S)-1-[(4-hydroxyphenyl)methyl]-2-oxo-2-[[(1S,2E)-1	C31H35N3O6S	577.69	C	-8.5
5666	N(C(=O)[C@H](Cc1ccccc1)C[C@@H](CN1[C@H](C(=O)NC(C)(C)C)CN(Cc2cccnc2)CC1)O)[C@H]1c2c(C[C@H]1OC(=O)c1c	170227-75-5	Benzoic acid, 4-[(4-methyl-1-piperazinyl)methyl]-, 1-[[5-[2-[[(1,1-dimethylethyl	C49H63N7O5	830.07	C	-8.5
5667	O=c1c2c(n(cn2)[C@@H]2O[C@H](COP(=O)(OC[C@@H]3[C@@H](N=[N+]=[N-])C[C@@H](O3)n3c(=O)[nH]c(=O)c(C)c3)OC	171284-27-8	Inosine, P-[[4-(acetyloxy)phenyl]ethyl]-3,Ä≤-azido-3,Ä≤-deoxythymidylyl-(5,Ä≤,Ü	C29H32N9O11P		C	-8.5
5668	S(=O)(=O)(O)c1c2c(c(N)c(N=Nc3c4c(ccc3)cccc4)c1)cccc2	172649-56-8	1-Piperazinepentanamide, 4-[(2-cyclopentyl-4-thiazolyl)methyl]-N-(2,3-dihydro-2-	C39H53N5O4S	687.93	C	-8.5
5669	[15NH2]c1c2c(n(c[15n]2)[C@@H]2O[C@H](CO)[C@@H](O)C2)ncn1	173167-81-2	Uridine 5,Ä≤-(trihydrogen diphosphate), 5-[3-[(4-benzoylbenzoyl)amino]propyl]-,	C34H42N4O19P2	872.66	C	-8.5
5670	O[C@H]1[C@H](n2c3c(nc2)c(=O)nc(N)[nH]3)O[C@H]2[C@H]1OP(=O)(OC2)c1ccccc1	173324-63-5	1,3-Naphthalenedisulfonic acid, 6-hydroxy-5-[2-[4-(2-phenyldiazenyl)phenyl]diaze	C22H16N4O7S2	512.52	C	-8.5
5671	[C@H](C(C)(C)C)(C1=C(O)C[C@](CCc2ccccc2)(CCC)OC1=O)c1cc(NS(=O)(=O)c2ccccn2)ccc1	174484-21-0	2-Pyridinesulfonamide, 5-amino-N-[3-[1-[5,6-dihydro-4-hydroxy-2-oxo-6-(2-phenyle	C32H36F3N3O5S	631.71	C	-8.5
5672	C(Cc1ccccc1)[C@@]1(CCC)CC(=C([C@@H](CC)c2cc(NS(=O)(=O)c3ccc(C(F)(F)F)cn3)ccc2)C(=O)O1)O	174484-41-4	2-Pyridinesulfonamide, N-[3-[(1R)-1-[(6R)-5,6-dihydro-4-hydroxy-2-oxo-6-(2-pheny	C31H33F3N2O5S	602.66	C	-8.5
5673	C(Cc1ccc(F)cc1)[C@@]1(CCC)CC(=C([C@@H](C(C)(C)C)c2cc(NS(=O)(=O)c3ccc(C#N)cn3)ccc2)C(=O)O1)O	174484-57-2	2-Pyridinesulfonamide, 5-cyano-N-[3-[1-[6-[2-(4-fluorophenyl)ethyl]-5,6-dihydro-	C33H36FN3O5S	605.72	C	-8.5
5674	C(n1c2c(nc1CC)c(C)cc(C)n2)c1ccc(cc1)c1c(S(=O)(=O)NC(=O)c2ccc(OCc3ccccc3)cc2)cccc1	174844-71-4	Benzamide, N-[[4,Ä≤-[(2-ethyl-5,7-dimethyl-3H-imidazo[4,5-b]pyridin-3-yl)methyl]	C31H30N4O4S	554.66	C	-8.5
5675	C(=NO)(CN1CCC(CN2c3c4c(S2(=O)=O)cccc4ccc3)CC1)C12CC3CC(C2)CC(C1)C3	178477-54-8	1-Piperidineethanamine, 4-[(1,1-dioxido-2H-naphth[1,8-cd]isothiazol-2-yl)methyl]	C32H43N3O2S	533.77	C	-8.5
5676	C(OC)[C@@]1(CO)[C@@H](O)C[C@@H](O1)n1c(=O)[nH]c(=O)c(C)c1	179169-45-0	1-Piperazinepentanamide, N-(2,3-dihydro-2,5-dihydroxy-1H-inden-1-yl)-2-[[(1,1-di	C36H47N5O5	629.79	C	-8.5
5677	[C@H](C(=O)N[C@H](Cc1ccccc1)C[C@@H]([C@@H](Cc1ccccc1)NC(=O)COc1c(C)cccc1C)O)(C(C)C)N1C(=O)NCCC1	1797984-95-2	1(2H)-Pyrimidineacetamide, N-[(1R,3S,4R)-4-[[2-(2,6-dimethylphenoxy)acetyl]aminc	C37H46N4O6	642.78	C	-8.5
5678	C(=O)(OC)n1c(ncc1c1ccc(cc1)c1ccc(cc1)c1n(C(=O)OC)c(nc1)[C@H]1N(C(=O)[C@@H](NC(=O)OC)C(C)C)CCC1)[C@H]	1800502-87-7	Carbamic acid, N-[(1S)-1-[[(2S)-2-[1-(3-cyclopentyl-1-oxopropyl)-5-[4,Ä≤-[1-(3-	C56H74N8O8	987.24	C	-8.5
5679	O=C1[C@@H]2C(c3c(C(=O)[C@H]2CO1)cc1c(c3)OCO1)c1ccc2c(cc1)OCO2	1807621-83-5	Furo[3,Ä≤,4,Ä≤:6,7]naphtho[2,3-d]-1,3-dioxol-6(5aH)-one, 5-(1,3-benzodioxol-5-y	C20H16O7	368.34	C	-8.5
5680	C(=O)([C@@H](NC(=O)OC)C(C)C)N1[C@H](c2c3c(c([nH]2)c2ccc(cc2)c2ccc(cc2)c2[nH]c(nc2)[C@H]2N(C(=O)[C@@H	1807622-11-2	Furo[3,Ä≤,4,Ä≤:6,7]naphtho[2,3-d]-1,3-dioxole-5,8-dione, 9-(1,3-benzodioxol-5-y	C20H14O7	366.32	C	-8.5
5681	O=C1[C@]2([C@H]([C@H](/C(=C/C(=O)[C@@H]3[C@@]4(N5[C@@H]([C@H]3c3cc(OC)c(O)cc3)CCC5)c3c(NC4=O)ccc(Cl)	1807888-34-1	Not Yet Assigned	C45H42Cl2N4O8	837.74	C	-8.5
5682	O=C1[C@]2([C@H]([C@H](/C(=C/C(=O)[C@@H]3[C@@]4(N5[C@@H]([C@H]3c3cc(OC)c(O)cc3)CCC5)c3c(N(C)C4=O)cccc	1807888-46-5	Not Yet Assigned	C47H48N4O8	796.91	C	-8.5
5683	O=C1[C@@H]2[C@@H](c3c([C@H](OC(=O)[C@H](C)N4C(=O)C5C(C4=O)CCCC5)[C@H]2CO1)cc1c(c3)OCO1)c1cc(OC)c(OC)	1809321-46-7	2H-Isoindole-2-acetic acid, octahydro-Œ±-methyl-1,3-dioxo-, (5R,5aR,8aR,9R)-5,5a	C33H35NO11	621.63	C	-8.5
5684	O[C@H]1[C@@H](O[C@H](COC(=O)[C@H](Cc2ccccc2)NC(=O)OC(C)(C)C)[C@H]1O)n1nc(C(=O)N)nc1	1809518-39-5	Not Yet Assigned	C20H27N10O15P3	740.41	C	-8.5
5685	O[C@H]1[C@H](n2c3c(nc2)c(=O)nc[nH]3)O[C@H](COP(=O)(OP(=O)(O[C@@H]2[C@@H](O)[C@H](O)[C@H](O)[C@H](C)O	181427-77-0	Xanthosine 5,Ä≤-(trihydrogen diphosphate), P,Ä≤-(6-deoxy-Œ≤-L-galactopyranosyl)	C16H24N4O16P2	590.33	C	-8.5
5686	O=C1[C@@H]2[C@@H](c3c([C@H](OC(=O)CCC(=O)OC(COP(=O)(OCC[N+](C)(C)C)[O-])COC(=O)CCC(=O)O[C@H]4c5c([C@	1825370-91-9	Hexanedioic acid, 1-[(5R,5aR,8aR,9R)-5,5a,6,8,8a,9-hexahydro-8-oxo-9-(3,4,5-trim	C59H64O23	1141.13	C	-8.5
5687	O=C1[C@@H]2[C@@H](c3c([C@H](OC(=O)CCCCCC(=O)OC(COP(=O)(OCC(CO)O)O)COC(=O)CCCCCC(=O)O[C@H]4c5c([C@H]	1825370-94-2	Butanedioic acid, 1,1,Ä≤-[1-[[[(2-aminoethoxy)hydroxyphosphinyl]oxy]methyl]-1,2-	C57H62NO26P	1208.07	C	-8.5
5688	O=C1[C@@H]2[C@@H](c3c([C@H](OC(=O)CCCCC(=O)OC(COP(=O)(OCC(C(=O)OCc4ccccc4)NC(=O)OCc4ccccc4)OCc4ccccc	1825370-97-5	4,6,10-Trioxa-5-phosphahexadecanedioic acid, 2-amino-8-[[6-[[(5R,5aR,8aR,9R)-5,5	C62H70NO28P	1308.18	C	-8.5
5689	O=C1[C@@H]2[C@H](c3c([C@H](O)[C@H]2CO1)cc1c(c3)OCO1)c1ccc2c(cc1)OCO2	1835316-38-5	Furo[3,Ä≤,4,Ä≤:6,7]naphtho[2,3-d]-1,3-dioxole-5,8-dione, 9-(1,3-benzodioxol-5-y	C20H14O7	366.32	C	-8.5
5690	N(C(C)(C)C)c1c(nccc1)N1CCN(C(=O)c2cc3c([nH]2)ccc(NS(=O)(=O)C)c3)CC1	184574-94-5	Methanesulfonamide, N-[2-[[4-[3-(ethylamino)-2-pyridinyl]-1-piperazinyl]carbonyl	C21H26N6O3S	442.53	C	-8.5
5691	C([C@H](OCP(=O)(OCOC(=O)OC(C)C)OCOC(=O)OC(C)C)C)n1c2c(nc1)c(N)ncn2.P(=O)(O)(O)O	184574-95-6	Methanesulfonamide, N-[2-[[4-[3-[(1,1-dimethylethyl)amino]-2-pyridinyl]-1-pipera	C23H30N6O3S	470.59	C	-8.5
5692	C(OC[C@H]1C(=C)[C@H](C[C@@H]1O)n1c2c(nc1)c(=O)nc(NC(=O)OC(C)(C)C)[nH]2)(c1ccccc1)(c1ccccc1)c1ccccc1	1860842-87-0	6H-Purin-6-one, 2-amino-1,9-dihydro-9-[(1S,3R,4S)-4-hydroxy-2-methylene-3-[(trip	C31H29N5O3	519.59	C	-8.5
5693	C(OC[C@@H]1[C@@H](OC(=O)CCC)C[C@@H](C1=C)n1c2c(nc1)c(=O)nc(NC(=O)OC(C)(C)C)[nH]2)(c1ccccc1)(c1ccccc1	1860842-90-5	Carbamic acid, N-[6,9-dihydro-9-[(1S,3R,4S)-2-methylene-4-(1-oxopropoxy)-3-[(tri	C39H41N5O6	675.77	C	-8.5
5694	O=C1[C@@H]2[C@@H](c3c([C@H](OC(=O)CN4c5c(C(=O)C4=O)c(Cl)ccc5)[C@H]2CO1)cc1c(c3)OCO1)c1cc(OC)c(OC)c(O	1864833-28-2	1H-Indole-1-acetic acid, 2,3-dihydro-2,3-dioxo-, (5R,5aR,8aR,9R)-5,5a,6,8,8a,9-h	C32H27NO11	601.56	C	-8.5
5695	O=C1[C@@H]2[C@@H](c3c([C@H](OC(=O)CN4c5c(C(=O)C4=O)c(Br)ccc5)[C@H]2CO1)cc1c(c3)OCO1)c1cc(OC)c(OC)c(O	1864833-29-3	1H-Indole-1-acetic acid, 4-chloro-2,3-dihydro-2,3-dioxo-, (5R,5aR,8aR,9R)-5,5a,6	C32H26ClNO11	636	C	-8.5
5696	O=C1[C@@H]2[C@@H](c3c([C@H](OC(=O)CN4c5c(C(=O)C4=O)cc(F)cc5)[C@H]2CO1)cc1c(c3)OCO1)c1cc(OC)c(OC)c(OC	1864833-34-0	1H-Indole-1-acetic acid, 6-bromo-2,3-dihydro-2,3-dioxo-, (5R,5aR,8aR,9R)-5,5a,6,	C32H26BrNO11	680.45	C	-8.5
5697	O=C1[C@@H]2[C@@H](c3c([C@H](OC(=O)CN4c5c(C(=O)C4=O)ccc(Cl)c5)[C@H]2CO1)cc1c(c3)OCO1)c1cc(OC)c(OC)c(O	1864833-35-1	1H-Indole-1-acetic acid, 5-fluoro-2,3-dihydro-2,3-dioxo-, (5R,5aR,8aR,9R)-5,5a,6	C32H26FNO11	619.55	C	-8.5
5698	O=c1n(cc(C(=O)OCCC)c(=O)[nH]1)[C@@H]1O[C@H](CO)[C@@H](O)C1	1872202-55-5	1-Piperazinecarbothioamide, N-[4-amino-6-(4-morpholinyl)-1,3,5-triazin-2-yl]-4-[	C23H29ClN10OS	529.06	C	-8.5
5699	O=C1[C@@H]2[C@@H](c3c([C@H](OC(=O)CCCC[C@H]4SC(SCC4)c4ccc(C)cc4)[C@H]2CO1)cc1c(c3)OCO1)c1cc(OC)c(OC)	1875123-83-3	1,3-Dithiane-4-pentanoic acid, 2-(2-methylphenyl)-, (5R,5aR,8aR,9R)-5,5a,6,8,8a,	C38H42O9S2	706.86	C	-8.5
5700	O=C1[C@@H]2[C@@H](c3c([C@H](OC(=O)CCCC[C@H]4SC(SCC4)c4cc(C(F)(F)F)ccc4)[C@H]2CO1)cc1c(c3)OCO1)c1cc(O	1875123-84-4	1,3-Dithiane-4-pentanoic acid, 2-(4-methylphenyl)-, (5R,5aR,8aR,9R)-5,5a,6,8,8a,	C38H42O9S2	706.86	C	-8.5
5701	O=C1[C@@H]2[C@@H](c3c([C@H](OC(=O)CCCC[C@H]4SC(SCC4)c4c(Cl)cc(Cl)cc4)[C@H]2CO1)cc1c(c3)OCO1)c1cc(OC)	1875123-92-4	1,3-Dithiane-4-pentanoic acid, 2-(2,3-dichlorophenyl)-, (5R,5aR,8aR,9R)-5,5a,6,8	C37H38Cl2O9S2	761.73	C	-8.5
5702	[C@@H](C(CC)CC)(NC(=O)C)[C@@H]1[C@H](OC(=O)CCCCCN2CCCN(Cc3ccc(CN4CCCNCCNCCCNCC4)cc3)CCNCCCNCC2)[C@@H	1876408-81-9	1,4,8,11-Tetraazacyclotetradecane-1-butanoic acid, 11-[[4-(1,4,8,11-tetraazacycl	C47H86N12O5	899.26	C	-8.5
5703	C(=O)(O)c1c2c(sc1NC(=O)c1c(CNc3ccc(C[C@@H]([C@@H](CN(S(=O)(=O)c4cc5c(cc4)ncs5)CC(C)C)O)NC(=O)O[C@@H]	1887206-23-6	5-Thiazolecarboxylic acid, 4-[[4-[(2S,3R)-4-[(6-benzothiazolylsulfonyl)(2-methyl	C39H42N4O11S3	838.97	C	-8.5
5704	C(OP(=O)(Oc1ccc(OC)cc1)Oc1ccc(OC)cc1)[C@@]1(C=C)O[C@H]([C@H](O)[C@@H]1O)n1c2c(nc1)c(=O)nc(N)[nH]2	1892589-92-2	Guanosine, 4,Ä≤-C-ethenyl-5,Ä≤-O-(2-oxido-4H-1,3,2-benzodioxaphosphorin-2-yl)-	C19H20N5O8P	477.36	C	-8.5
5705	C(OC[C@@H]1[C@@H](OC(=O)c2ccccc2)[C@@H](OP(=O)(OC[C@@H]2[C@@H](OC(=O)c3ccccc3)[C@@H](OP(=O)(OC[C@@H]	190141-39-0	Adenosine, 1,Ä≤-[3-(aminocarbonyl)-1H-1,2,4-triazol-1-yl]-3,Ä≤-O-benzoyl-P-(2-c	C52H43ClN9O15P	1100.38	C	-8.5
5706	N(c1nc(nc(Nc2ccc(C#N)nc2)n1)N1CCOCC1)c1c(C)cc(C)cc1C	1907643-94-0	2-Pyridinecarbonitrile, 5-[[4-[(3-methoxyphenyl)amino]-6-(4-morpholinyl)-1,3,5-t	C20H20N8O2	404.43	C	-8.5
5707	O=c1c2c(n(cn2)[C@@H]2O[C@H](COP(=O)(Oc3ccc(Cl)cc3)NCc3cc(Cl)ccc3)CC2)[nH]cn1	1922936-28-4	Phosphoramidic acid, N-[(4-nitrophenyl)methyl]-, 4-chlorophenyl [(2S,5R)-5-(1,6-	C23H22ClN6O7P	560.88	C	-8.5
5708	N(CCCCCCl)c1c2c(cc(Cl)cc2)ncc1	19289-79-3	1-Naphthalenesulfonic acid, 4-[2-(1-naphthalenyl)diazenyl]-	C20H14N2O3S	362.4	C	-8.5
5709	C(=O)([C@@H](NC(=O)OC)C(C)C)N1[C@H](c2[nH]c3c4c(cc(cc4)c4cc(c(cc4)c4[nH]c(nc4)[C@H]4N(C(=O)[C@@H](NC	1949770-69-7	Carbamic acid, N-[(1S)-1-[[(2S)-2-[7-[4-[2-[(2S)-1-[(2S)-2-[(methoxycarbonyl)ami	C43H52N8O6	776.92	C	-8.5
5710	Clc1c2c(c3c(nc([nH]3)[C@H]3N(C(=O)[C@@H](NC(=O)OC(C)C)C(C)C)CCC3)c1)ccc(c2)c1ccc(cc1)c1[nH]c(nc1)[C@	1949770-72-2	Carbamic acid, N-[(1S)-1-[[(2S)-2-[5-chloro-7-[4-[2-[(2S)-1-[(2S)-2-[(methoxycar	C42H49ClN8O6	797.34	C	-8.5
5711	C(=O)([C@@H](NC(=O)OC)C(C)C)N1[C@@H]([C@@H]2C[C@H]1CC2)c1[nH]c2c(n1)ccc1c2ccc(c1)c1ccc(cc1)c1[nH]c(n	1949770-79-9	Carbamic acid, N-[(1S)-1-[[(2S,4R)-4-hydroxy-2-[5-[4-[2-[(1R,3S,4S)-2-[(2S)-2-[(	C44H52N8O7	804.93	C	-8.5
5712	C=C1[C@@H](n2c3c(nc2)C(O)N=C(N)N3)C[C@H](O)[C@H]1CO	1973418-89-1	Acetamide, N-[9-[(1S,3R,4S)-3-[(acetyloxy)methyl]-2-methylene-4-(phenylmethoxy)c	C23H25N5O5	451.48	C	-8.5
5713	N(C(=O)C)[C@H]1[C@H]([C@@H]([C@@H](CO)O)O)OC(=C[C@@H]1NC(=N)NC(=O)CCn1cc(nn1)c1ccccc1)C(=O)O	1982351-34-7	D-glycero-D-galacto-Non-2-enonic acid, 5-(acetylamino)-2,6-anhydro-3,4,5-trideox	C22H27N7O8	517.49	C	-8.5
5714	N(C(=O)C)[C@H]1[C@H]([C@@H]([C@@H](CO)O)O)OC(=C[C@@H]1NC(=N)NC(=O)Cn1cc(nn1)c1cc(N)ccc1)C(=O)O	1982351-43-8	D-glycero-D-galacto-Non-2-enonic acid, 5-(acetylamino)-2,6-anhydro-3,4,5-trideox	C22H26FN7O8	535.48	C	-8.5
5715	N(C(=O)C)[C@@H]1[C@@H](NC(=N)NC(=O)Cn2cc(nn2)c2cc3c(cc2)cc(OC)cc3)C=C(C(=O)O)O[C@H]1[C@@H]([C@@H](CO	1982351-51-8	D-glycero-D-galacto-Non-2-enonic acid, 5-(acetylamino)-2,6-anhydro-3,4,5-trideox	C22H26FN7O8	535.48	C	-8.5
5716	O(C(=O)OCCO)c1c2n(N([C@H]3N(C2=O)CCOC3)[C@H]2c3c(CSc4c2cccc4)c(F)c(F)cc3)ccc1=O	1985606-17-4	Propanoic acid, 2-[[[[(12aR)-12-[(11S)-7,8-difluoro-6,11-dihydrodibenzo[b,e]thie	C30H27F2N3O8S	627.61	C	-8.5
5717	O(P(=O)(N[C@H](C(=O)OC)C(C)C)OCC)c1c2n(N([C@H]3N(C2=O)CCOC3)[C@H]2c3c(CSc4c2cccc4)c(F)c(F)cc3)ccc1=O	1985606-21-0	Carbamic acid, N,N-dimethyl-, (12aR)-12-[(11S)-7,8-difluoro-6,11-dihydrodibenzo[	C27H24F2N4O5S	554.57	C	-8.5
5718	O(COC(=O)C)c1c2n(N([C@H]3N(C2=O)CCOC3)[C@H]2c3c(CSc4c2cccc4)c(F)c(F)cc3)ccc1=O	1985606-24-3	3,5,8-Trioxa-4-phosphanonanoic acid, 4-[[(12aR)-12-[(11S)-7,8-difluoro-6,11-dihy	C30H28F2N3O11PS	707.59	C	-8.5
5719	O(COC(=O)OC)c1c2n(N([C@H]3N(C2=O)CCOC3)[C@H]2c3c(CSc4c2cccc4)cc(F)cc3)ccc1=O	1985606-39-0	1H-[1,4]Oxazino[3,4-c]pyrido[2,1-f][1,2,4]triazine-6,8-dione, 7-[(acetyloxy)meth	C27H24FN3O6S	537.56	C	-8.5
5720	O(COC(=O)C(CC(=O)OC)(C)C)c1c2n(N([C@H]3N(C2=O)CCOC3)[C@H]2c3c(CSc4c2cccc4)c(F)c(F)cc3)ccc1=O	1985606-87-8	Carbamic acid, N-[[[(12aR)-12-[(11S)-7,8-difluoro-6,11-dihydrodibenzo[b,e]thiepi	C29H28F2N4O6S	598.62	C	-8.5
5721	O(P(=O)(N[C@@H](CC1CCCCC1)C(=O)OC)OCC)c1c2n(N([C@H]3N(C2=O)CCOC3)[C@H]2c3c(CSc4c2cccc4)c(F)c(F)cc3)c	1985607-22-4	Cyclopropanecarboxylic acid, 1-[[[[(12aR)-12-[(11S)-7,8-difluoro-6,11-dihydrodib	C31H31F2N4O8PS	688.64	C	-8.5
5722	N(C(=O)[C@@H](Cc1ccccc1)C[C@H](CN1[C@H](C(=O)NC(C)(C)C)CNCC1)O)[C@H]1c2c(C[C@H]1O)cccc2	1985868-86-7	Benzonitrile, 4-[[4-[(2,5-dimethylphenyl)amino]-6-methyl-2-pyrimidinyl]amino]-	C20H19N5	329.4	C	-8.5
5723	N(C(=O)Nc1ccc(C#N)cc1)c1ccc2cc(C34CC5CC(C4)CC(C3)C5)[nH]c2cc1	1987854-65-8	3-Pyridinecarboxamide, 2-chloro-N-(2-tricyclo[3.3.1.13,7]dec-1-yl-1H-indol-5-yl)	C24H24ClN3O	405.92	C	-8.5
5724	N(C(=O)Nc1nc2c(s1)cccc2)c1cc2cc(C34CC5CC(C4)CC(C3)C5)[nH]c2cc1	1987854-78-3	Urea, N-[2-(2-thienyl)ethyl]-N,Ä≤-(2-tricyclo[3.3.1.13,7]dec-1-yl-1H-indol-5-yl)	C25H29N3OS	419.58	C	-8.5
5725	N(c1nc(NCCCc2ccccc2)ncc1)c1c(C)cc(C)cc1	1989060-14-1	Benzonitrile, 2-[[2-[(2,5-dimethylphenyl)amino]-4-pyrimidinyl]amino]-	C19H17N5	315.37	C	-8.5
5726	C(F)(F)c1n(c2c(n1)cccc2)c1nc(nc(n1)N1CCOCC1)N1CCN(CC1)N1[C@@H](C(=O)O)CCC1	2001083-61-8	Propanoic acid, 2-[[4-[4-[2-(difluoromethyl)-1H-benzimidazol-1-yl]-6-(4-morpholi	C22H27F2N9O3	503.51	C	-8.5
5727	C(F)(F)c1n(c2c(n1)cccc2)c1nc(nc(n1)N1CCOCC1)N1CCN(CC1)[C@@H]1CC[C@H](N)CC1	2001083-62-9	2-Pyrrolidinecarboxylic acid, 1-[4-[4-[2-(difluoromethyl)-1H-benzimidazol-1-yl]-	C24H29F2N9O3	529.54	C	-8.5
5728	C(=O)(C(=O)Nc1c(C)cc(C)cc1)c1c([nH]c2c1cccc2)C12CC3CC(C2)CC(C1)C3	2014403-81-5	1H-Indole-3-acetamide, N-(4-methoxyphenyl)-Œ±-oxo-2-tricyclo[3.3.1.13,7]dec-1-yl	C27H28N2O3	428.52	C	-8.5
5729	C(=O)(C(=O)Nc1ccccn1)c1c(C23CC4CC(C3)CC(C2)C4)[nH]c2c1cccc2	2014403-93-9	1H-Indole-3-acetamide, Œ±-oxo-N-3-pyridinyl-2-tricyclo[3.3.1.13,7]dec-1-yl-	C25H25N3O2	399.48	C	-8.5
5730	O=c1n([C@@H]2O[C@H](COC(=O)COc3c(OC)ccc(C(=O)c4ccc(Br)cc4)c3)C=C2)cc(C)c(=O)[nH]1	2015224-35-6	Acetic acid, 2-[5-(4-fluorobenzoyl)-2-methoxyphenoxy]-, [(2S,5R)-5-(3,4-dihydro-	C26H23FN2O8	510.47	C	-8.5
5731	S(Cc1cc2c(oc1=O)ccc(C)c2)c1n(c2c(n1)c(=O)nc[nH]2)[C@@H]1O[C@H](CO)[C@@H](O)[C@H]1O	2021230-53-3	Not Yet Assigned	C21H20N4O8S	488.47	C	-8.5
5732	N(c1c2c(cc(Cl)cc2)ncc1)c1ccc(CN(CC)CC)c(C)cc1	100100-62-7	Uridine, 2,Ä≤-deoxy-5-iodo-, cyclic 3,Ä≤,5,Ä≤-(phenylmethyl phosphate)	C16H16IN2O7P	506.19	A/B	-8.4

5733	C(OC(=O)CCNC(=O)OCc1ccccc1)[C@@H]1[C@@H]2[C@H]([C@@H](O1)n1nc(C(=O)N)nc1)OC(OC)O2	1002335-61-6	1H-1,2,4-Triazole-3-carboxamide, 1-[2,3-O-(methoxymethylene)-5-O-[(2S)-1-oxo-2-[	C28H35N5O14	665.6	A/B	-8.4
5734	[Si](OC[C@H]1O[C@H]([C@H](O)[C@@H]1O)n1nc(C(=O)N)nc1)(C(C)C)(C(C)C)C(C)C	1002335-65-0	1H-1,2,4-Triazole-3-carboxamide, 1-[2,3-O-(methoxymethylene)-5-O-[1-oxo-3-[[2-[	C28H35N5O14	665.6	A/B	-8.4
5735	C(Cc1ccccc1)(CCC(Cc1ccccc1)NC(=O)OCc1cncs1)NC(=O)C(NC(=O)N(Cc1cncs1)C)CO	1004318-68-6	2,7,10,12-Tetraazatridecanoic acid, 9-(1-hydroxyethyl)-12-methyl-8,11-dioxo-3,6-	C33H40N6O5S2	664.84	A/B	-8.4
5736	O(C(=O)N[C@@H](Cc1ccccc1)[C@@H](CN(S(=O)(=O)c1ccc(OCc2ccccc2)cc1)CC(C)C)N)[C@@H]1[C@H]2[C@@H](OC1)OC	1005324-65-1	Carbamic acid, N-[(1S,2R)-3-[[[4-(acetylamino)phenyl]sulfonyl](2-methylpropyl)am	C29H40N4O7S	588.72	A/B	-8.4
5737	S(=O)(=O)([O-])c1c2c3c([N]4=N[C-]5C(=[O][Cu+2]4[O-]3)N(N=C5C)c3ccc(S(=O)(=O)[O-])cc3)cc(S(=O)(=O)[O-	1007166-40-6	Benzenesulfonic acid, 4-[2-[4-[2-[2-(2-hydroxyethyl)-5-methylphenyl]diazenyl]-1-	C25H22N4O4S	474.53	A/B	-8.4
5738	C(=O)([C@H](NC(=O)OC)c1ccccc1)N1[C@@H](CCC1)c1[nH]c(cn1)c1ccc(cc1)c1ccc(cc1)c1[nH]c(nc1)[C@H]1N(C(=O	1009107-87-2	Carbamic acid, N-[(1R)-2-[(2S)-2-[5-[4,Ä≤-[2-[(2S)-1-[(2S)-2-[(methoxycarbonyl)a	C41H44N8O6	744.84	A/B	-8.4
5739	C(=O)([C@@H](NC(=O)OCC=C)C(C)C)N1[C@@H](CCC1)c1[nH]c(cn1)c1ccc(cc1)c1ccc(cc1)c1[nH]c(nc1)[C@H]1N(C(	1009113-22-7	Carbamic acid, N,N,Ä≤-[(2-methyl[1,1,Ä≤-biphenyl]-4,4,Ä≤-diyl)bis[1H-imidazole-	C47H48N8O6	820.93	A/B	-8.4
5740	C(=O)([C@@H](NC(=O)OC)C1CCOCC1)N1[C@@H](CCC1)c1[nH]c(cn1)c1ccc(cc1)c1ccc(cc1)c1[nH]c(nc1)[C@H]1N(C(=	1009113-72-7	Carbamic acid, N,N,Ä≤-[[1,1,Ä≤-biphenyl]-4,4,Ä≤-diylbis[1H-imidazole-5,2-diyl-(	C42H54N8O6	766.93	A/B	-8.4
5741	C(=O)([C@@H](NC(=O)O[C@H]1CCOC1)C(C)C)N1[C@@H](CCC1)c1[nH]c(cn1)c1ccc(cc1)c1ccc(cc1)c1[nH]c(nc1)[C@H	1009113-75-0	Carbamic acid, N-[(1S)-2-[(2S)-2-[5-[4,Ä≤-[2-[(2S)-1-[(2R)-2-(ethylamino)-2-pher	C41H46N8O4	714.86	A/B	-8.4
5742	C(=O)([C@H](NC1=NCCN1)C(C)C)N1[C@@H](CCC1)c1[nH]c(cn1)c1ccc(cc1)c1ccc(cc1)c1[nH]c(nc1)[C@H]1N(C(=O)[	1009114-04-8	Carbamic acid, N-[(1S)-2-[(2S)-2-[5-[4,Ä≤-[2-[(2S)-1-[(2S)-2-[(4,5-dihydro-1H-ir	C39H48N10O4	720.86	A/B	-8.4
5743	C(=O)([C@@H](Nc1cccnc1)C(C)C)N1[C@@H](CCC1)c1[nH]c(cn1)c1ccc(cc1)c1ccc(cc1)c1[nH]c(nc1)[C@H]1N(C(=O)	1009114-16-2	Carbamic acid, N-[(1S)-2-methyl-1-[[(2S)-2-[5-[4,Ä≤-[2-[(2S)-1-[(2S)-3-methyl-1-	C43H51N9O4	757.92	A/B	-8.4
5744	C(=O)([C@H](Cc1cccnc1)NC(=O)OC)N1[C@@H](CCC1)c1[nH]c(cn1)c1ccc(cc1)c1ccc(cc1)c1[nH]c(nc1)[C@H]1N(C(=	1009114-63-9	Carbamic acid, N-[(1S)-2-[(2S)-2-[5-[4,Ä≤-[2-[(2S)-1-[[cis-4-(diethylamino)cycl(	C42H54N8O4	734.93	A/B	-8.4
5745	C(=O)([C@H](Cc1ccc(O)cc1)NC(=O)OC)N1[C@@H](CCC1)c1[nH]c(cn1)c1ccc(cc1)c1ccc(cc1)c1[nH]c(nc1)[C@H]1N(	1009115-00-7	Carbamic acid, N-[(1S)-1-[[(2S)-2-[5-[4,Ä≤-[2-[(2S)-1-[(2S)-3-(4-hydroxyphenyl)-	C44H50N8O7	802.92	A/B	-8.4
5746	C(=O)([C@H](NC(=O)OC)CC(C)C)N1[C@@H](C[C@@H](F)C1)c1[nH]c(cn1)c1ccc(cc1)c1ccc(cc1)c1[nH]c(nc1)[C@H]1	1009115-63-2	Carbamic acid, N,N,Ä≤-[[1,1,Ä≤-biphenyl]-4,4,Ä≤-diylbis[1H-imidazole-5,2-diyl[(	C40H48F2N8O6	774.86	A/B	-8.4
5747	C(=O)([C@@H](NC(=O)OC)C(C)C)N1[C@@H](CC(F)(F)C1)c1[nH]c(cn1)c1ccc(cc1)c1ccc(cc1)c1[nH]c(nc1)[C@H]1N(	1009115-76-7	Carbamic acid, N,N,Ä≤-[[1,1,Ä≤-biphenyl]-4,4,Ä≤-diylbis[1H-imidazole-5,2-diyl[(	C40H46F4N8O6	810.84	A/B	-8.4
5748	C(=O)([C@@H](NC(=O)OC)C(C)C)N1[C@@H](CCC1)c1[nH]c(cn1)c1c(F)cc(cc1)c1ccc(cc1)c1[nH]c(nc1)[C@H]1N(C(=	1009116-11-3	Carbamic acid, N-[(1S)-1-cyclopropyl-2-[(2S)-2-[4-[4,Ä≤-[2-[(2S)-1-[(2S)-2-cycl(	C41H45F3N8O6	802.84	A/B	-8.4
5749	C(=O)([C@@H](NC(=O)OC)C(C)(C)O)N1[C@@H](CCC1)c1[nH]c(cn1)c1ccc(cc1)c1ccc(cc1)c1[nH]c(nc1)[C@H]1N(C(=	1009116-88-4	Carbamic acid, N,N,Ä≤-[[1,1,Ä≤-biphenyl]-4,4,Ä≤-diylbis[1H-imidazole-5,2-diyl-(	C46H46N8O6	806.91	A/B	-8.4
5750	C(=O)([C@H](N(CC)CC)c1ccccc1)N1[C@@H](CCC1)c1[nH]c(cn1)c1ccc(cc1)c1ccc(cc1)c1[nH]c(nc1)[C@H]1N(C(=O)	1009117-51-4	Carbamic acid, N-[(1R)-2-[(2S)-2-[5-[4,Ä≤-[2-[(2S)-1-[(2R)-2-(diethylamino)-2-pl	C48H52N8O4	804.98	A/B	-8.4
5751	C(=O)([C@@H](NC(=O)OC)CCC)N1[C@@H](CCC1)c1[nH]c(cn1)c1ccc(cc1)c1ccc(cc1)c1[nH]c(nc1)[C@H]1N(C(=O)[C@	1009117-99-0	Carbamic acid, N-[(1S)-2-[(2S)-2-[5-[4,Ä≤-[2-[(2S)-1-[(2S)-2-cyclopropyl-2-[(met	C38H44N8O6	708.81	A/B	-8.4
5752	C(=O)([C@H](NC(=O)OC)C(C)C)N1[C@@H]2[C@@H](C2)C[C@H]1c1[nH]c(cn1)c1ccc(cc1)c1ccc(cc1)c1[nH]c(nc1)[C@	1009118-20-0	[1,1,Ä≤-Biphenyl]-2-carboxylic acid, 4,4,Ä≤-bis[2-[(2S)-1-[(2S)-2-cyclopropyl-2	C41H46N8O8	778.85	A/B	-8.4
5753	[C@H](Cc1ccccc1)(C[C@@H]([C@H](Cc1ccccc1)NC(=O)OCc1cncs1)O)NC(=O)C(NC(=O)NC)C(C)C	10108-97-1	Guanosine 5,Ä≤-(trihydrogen diphosphate), P,Ä≤-Œ≤-D-glucopyranosyl ester	C16H25N5O16P2	605.34	A/B	-8.4
5754	N(C(=O)Nc1ccc(S(=O)(=O)NC(=O)C)cc1)C12CC3CC(C2)CC(C1)C3	1015252-57-9	Benzenesulfonamide, N-methyl-3-[[(tricyclo[3.3.1.13,7]dec-1-ylamino)carbonyl]ami	C18H25N3O3S	363.47	A/B	-8.4
5755	O=C1[C@@H]2[C@@H](c3c([C@@H](O)[C@H]2CO1)cc1c(c3)OCO1)c1cc(OC)c(OC(=O)C(Br)Br)c(OC)c1	101648-62-8	Acetic acid, 2,2-dichloro-, 4-[(5R,5aR,8aR,9S)-5,5a,6,8,8a,9-hexahydro-9-hydroxy	C23H20Cl2O9	511.31	A/B	-8.4
5756	O=C1[C@@H]2[C@@H](c3c([C@@H](O)[C@H]2CO1)cc1c(c3)OCO1)c1cc(OC)c(O)c(O)c1	102306-96-7	Furo[3,Ä≤,4,Ä≤:6,7]naphtho[2,3-d]-1,3-dioxol-6(8H)-one, 5,9-dihydro-5-(4-hydrox	C21H18O7	382.36	A/B	-8.4
5757	C(#CC1CC1)[C@@]1(C(F)(F)F)c2c(N(CCO[Si](C(C)(C)C)(C)C)C(=O)O1)ccc(Cl)c2	1023756-55-9	Benzoic acid, 4-[[[4-[(4S)-6-chloro-4-(2-cyclopropylethynyl)-2-oxo-4-(trifluorom	C30H25ClF3N3O7	631.98	A/B	-8.4
5758	C(=C1CC(C(=O)O)C(=O)CC1)(c1cc(C(=O)O)c(O)cc1)c1cc(C(=O)O)c(O)cc1	10241-23-3	2,6-Naphthalenedisulfonic acid, 4-[2-(4-amino-1-naphthalenyl)diazenyl]-, sodium	C20H15N3O6S2.2Na		A/B	-8.4
5759	N(C(CCCN(CC)CC)C)c1c2c(ncc1C)cc(Cl)c(C)c2	1024656-58-3	2H-1,2,3-Triazole, 4-(4-chlorophenyl)-5-phenyl-2-tricyclo[3.3.1.13,7]dec-1-yl-	C24H24ClN3	389.92	A/B	-8.4
5760	N(C(=O)Nc1c(Cl)cccc1)c1nc(nc(NC(=S)NCCc2cccs2)n1)N1CCOCC1	1025365-03-0	Urea, N-[4-(4-morpholinyl)-6-[[[[2-(2-thienyl)ethyl]amino]thioxomethyl]amino]-1,	C21H23N9O4S2	529.6	A/B	-8.4
5761	O=C1[C@@H]2[C@@H](c3c([C@H](O)[C@H]2OC1)cc1c(c3)OCO1)c1cc(OC)c(OC)c(OC)c1	1027583-83-0	1-Piperazinecarboxamide, N-[(1S)-2-[[(1R)-1-ethyl-3-phenylpropyl]amino]-2-oxo-1-	C26H36N4O2	436.59	A/B	-8.4
5762	S(=O)(=O)(O)c1c2c(c(N=Nc3cc(C)ccc3)cc1)cccc2	102768-80-9	1-Naphthalenesulfonic acid, 4-[2-(2-methylphenyl)diazenyl]-	C17H14N2O3S	326.37	A/B	-8.4
5763	O=c1n(C2OC(CO)SC2)ccc(NCN(C)C)n1	1027931-59-4	Cyclopenta[c]pyrrole-1-carboxamide, 2-[(2S)-2-[[(2S)-2-cyclohexyl-2-[(2-pyraziny	C36H52N6O6	664.83	A/B	-8.4
5764	C(OCCOC(COCc1ccccc1)COCc1ccccc1)n1c2c(nc1)c(=O)nc(NC(=O)C)[nH]2	103025-12-3	Acetamide, N-[6,9-dihydro-6-oxo-9-[[3-(phenylmethoxy)-1-[(phenylmethoxy)methyl]p	C26H29N5O5	491.54	A/B	-8.4
5765	O[C@H]1[C@H](n2c3c(nc2)c(=O)nc(NC(=O)C=Cc2ccccc2)[nH]3)O[C@H](COP(=O)(O)O)[C@H]1O	1032827-65-8	5,Ä≤-Guanylic acid, N-(2-aminobenzoyl)-	C17H19N6O9P	482.34	A/B	-8.4
5766	N(CCCN(CCC1)CCC1)c1c2c(cc(Cl)cc2)ncc1	1033909-19-1	Uridine 5,Ä≤-(tetrahydrogen triphosphate), 5-(1H-indol-5-ylmethyl)-2,Ä≤-O-methy	C19H24N3O15P3	627.33	A/B	-8.4
5767	C(OC1CO[32P](=O)(O)OC1)n1c2c(n[14cH]1)c(=O)nc(N)[nH]2	1035343-48-6	5-Thiazolecarboxamide, N-(2-chloro-6-methylphenyl)-2-[[4-methyl-6-(4-morpholinyl	C19H20ClN7O2S	445.93	A/B	-8.4
5768	N(c1nc(Nc2ccc(C#N)cc2)nc(C(=O)NCCF)c1)c1c(C)cc(/C=C/C#N)cc1C	1037444-83-9	4-Pyrimidinecarboxamide, 6-[[4-[(1E)-2-cyanoethenyl]-2,6-dimethylphenyl]amino]-2	C26H25N7O2	467.52	A/B	-8.4
5769	N(c1c(C)cc(/C=C/C#N)cc1C)c1nc(Nc2ccc(C#N)cc2)nc(CN(CC#N)C)c1	1037445-14-9	Benzonitrile, 4-[[4-[[4-[(1E)-2-cyanoethenyl]-2,6-dimethylphenyl]amino]-6-[[[2-(	C30H28N8	500.6	A/B	-8.4
5770	N(c1c(C)cc(/C=C/C#N)cc1C)c1nc(Nc2ccc(C#N)cc2)nc(CNCCNC(=O)C)c1	1037445-16-1	Benzonitrile, 4-[[4-[[4-[(1E)-2-cyanoethenyl]-2,6-dimethylphenyl]amino]-6-[[(cya	C26H24N8	448.52	A/B	-8.4
5771	N(c1c(C)cc(/C=C/C#N)cc1C)c1nc(Nc2ccc(C#N)cc2)nc(C=O)c1	1037445-53-6	Benzonitrile, 4-[[4-[[4-[(1E)-2-cyanoethenyl]-2,6-dimethylphenyl]amino]-6-(hydro	C23H20N6O	396.44	A/B	-8.4
5772	N(c1c(C(=O)NCCc2ccccn2)c(COC)nc(Nc2ccc(C#N)cc2)n1)c1c(C)cc(/C=C/C#N)cc1C	1037452-21-3	5-Pyrimidinecarboxamide, 4-[[4-[(1E)-2-cyanoethenyl]-2,6-dimethylphenyl]amino]-2	C31H34N8O3	566.65	A/B	-8.4
5773	C(=O)(c1c(Nc2c(C)cc(/C=C/C#N)cc2C)nc(Nc2ccc(C#N)cc2)nc1COC)N1CCN(CCO)CC1	1037452-25-7	5-Pyrimidinecarboxamide, 4-[[4-[(1E)-2-cyanoethenyl]-2,6-dimethylphenyl]amino]-N	C28H26N8O2	506.56	A/B	-8.4
5774	N(c1c(C(C)O)c(COC)nc(Nc2ccc(C#N)cc2)n1)c1c(C)cc(/C=C/C#N)cc1C	1037452-67-7	Benzonitrile, 4-[[4-[[4-[(1E)-2-cyanoethenyl]-2,6-dimethylphenyl]amino]-5-(1-hyc	C27H26N6O2	466.53	A/B	-8.4
5775	C(=CC(=O)N)c1c(Nc2c(C)cc(/C=C/C#N)cc2C)nc(Nc2ccc(C#N)cc2)nc1COC	1037452-77-9	2-Propenamide, 3-[4-[[4-[(1E)-2-cyanoethenyl]-2,6-dimethylphenyl]amino]-2-[(4-cy	C29H29N7O2	507.59	A/B	-8.4
5776	N(c1c(/C=C/c2ccc(OC)cc2)c(COC)nc(Nc2ccc(C#N)cc2)n1)c1c(C)cc(/C=C/C#N)cc1C	1037452-85-9	Benzonitrile, 4-[[4-[[4-[(1E)-2-cyanoethenyl]-2,6-dimethylphenyl]amino]-6-(metho	C33H30N6O	526.63	A/B	-8.4
5777	C(=C)(OCC)c1c(Nc2c(C)cc(/C=C/C#N)cc2C)nc(Nc2ccc(C#N)cc2)nc1COC	1037452-87-1	Benzonitrile, 4-[[4-[[4-[(1E)-2-cyanoethenyl]-2,6-dimethylphenyl]amino]-6-(metho	C33H30N6O2	542.63	A/B	-8.4
5778	O=C1[C@@H]2[C@@H](c3c([C@H](OC(=O)CCCCCNC(=O)CCCC[C@H]4[C@@H]5[C@H](CS4)NC(=O)N5)[C@H]2CO1)cc1c(c3)O	1041171-06-5	Thiazolium, 5-[6-hydroxy-14-methyl-11-(1-methylethyl)-15-[2-(1-methylethyl)-4-th	C38H51N6O5S2		A/B	-8.4
5779	[C@@H]([C@H](Cc1ccccc1)NC(=O)COc1c(C)cccc1C)(C[C@H](Cc1ccccc1)NC(=O)[C@H](C(C)C)N1C(=O)NCCC1)OC(=O)[	104264-48-4	Furo[3,Ä≤,4,Ä≤:6,7]naphtho[2,3-d]-1,3-dioxol-6(5aH)-one, 5,8,8a,9-tetrahydro-9-	C21H20O8	400.38	A/B	-8.4
5780	C(Oc1c2c(n(cn2)[C@@H]2O[C@H](CO)[C@@H](O)C2)nc(N)n1)c1c2c(N(c3c(C(=O)N2)cccn3)C2CC2)ncc1	1046462-09-2	Cytidine, 3-[(11-cyclopropyl-6,11-dihydro-6-oxo-5H-dipyrido[3,2-b:2,Ä≤,3,Ä≤-e][	C24H25N7O5	491.5	A/B	-8.4
5781	O=C1c2c(c3c(c(O)c2CO1)cc1c(c3)OCO1)c1cc(OC(C)C)c(OC(C)C)cc1	104757-06-4	Furo[3,Ä≤,4,Ä≤:6,7]naphtho[2,3-d]-1,3-dioxol-6(8H)-one, 9-hydroxy-5-(3-methoxyp	C20H14O6	350.32	A/B	-8.4
5782	S(=O)(=O)(O)c1c2c(c(N)c(/N=N/c3cc(S(=O)(=O)O)c(C=Cc4c(S(=O)(=O)O)cc(/N=N/c5c(N)c6c(c(S(=O)(=O)O)c5)c	1053056-10-2	Not Yet Assigned	C34H26N6O18S6	998.99	A/B	-8.4
5783	O[C@H]1[C@H](n2c3c(n[13cH]2)c(N)n[13cH]n3)O[C@H](COP(=O)(OP(=O)(OP(=O)(O)O)O)O)[C@H]1O	1053056-91-9	Not Yet Assigned	C34H26N6O12S4	838.86	A/B	-8.4
5784	O(C(=O)NC(Cc1ccccc1)C(CN(S(=O)(=O)c1ccc(OC)cc1)CC(CCCCP(=O)(O)O)(C)C)O)C1C2C(OC1)OCC2	1057218-36-6	14-Oxa-2,6-diaza-13-phosphapentadecanoic acid, 4-hydroxy-6-[(4-methoxyphenyl)sul	C46H59N2O11PS	879.01	A/B	-8.4
5785	O(C(=O)N[C@@H](Cc1ccc(OCP(=O)(OC(C)C)O)cc1)[C@@H](CN(S(=O)(=O)c1ccc(OC)cc1)CC(C)C)O)[C@@H]1[C@H]2[C@	1057961-13-3	Carbamic acid, N-[(1S,2R)-2-hydroxy-3-[[(4-methoxyphenyl)sulfonyl](2-methylpropy	C39H53N2O12PS	804.88	A/B	-8.4
5786	N(=Nc1c(C)cc(N)cc1)c1c2c(ccc(S(=O)(=O)O)c2)ccc1	106104-88-5	1-Naphthalenesulfonic acid, 5-[2-(4-amino-2-methylphenyl)diazenyl]-	C17H15N3O3S	341.38	A/B	-8.4
5787	S(=O)(=O)([O-])c1c2c(c([N])c(N=Nc3ccc(cc3)c3ccc(N=Nc4c(N)c5c(c(S(=O)(=O)[O-])c4)cccc5)cc3)c1)cccc2	1061609-16-2	1-Naphthalenesulfonic acid, 4-amino-3-[2-[4,Ä≤-[2-(1-amino-2-naphthalenyl)diazer	C32H23N6O3S		A/B	-8.4
5788	C(=C\C(=O)CC(=O)/C=C/c1ccccc1)/c1cc(O)c(O)cc1	1061609-19-5	Imidogen, [2-[2-[4,Ä≤-[2-(1-amino-4-sulfo-2-naphthalenyl)diazenyl][1,1,Ä≤-biphe	C32H20N6O6S2		A/B	-8.4
5789	O=c1n([C@@H]2O[C@@](CO)(C)C=C2)ccc(=O)[nH]1	106423-44-3	1-Naphthalenesulfonic acid, 3-[2-[4-[2-(4-amino-6-sulfo-1-naphthalenyl)diazenyl]	C32H25N9O6S2.2Na		A/B	-8.4
5790	O[C@H]1[C@@H](O[C@H](COC(=O)CCCCC(=O)OC(C)n2cc(N(=O)=O)nc2)[C@H]1O)n1nc(C(=O)N)nc1	1070681-23-0	4H-1,3,2-Benzodioxaphosphorin-6-propanoic acid, 2-[[(2S,5R)-5-(3,4-dihydro-5-met	C27H33N2O11P	592.53	A/B	-8.4
5791	O=c1c2c(n(cn2)[C@@H]2O[C@H](COCP(=O)(OCC)OCC)CC2)[nH]cn1	1071050-15-1	Not Yet Assigned	C38H36N3O7P	677.68	A/B	-8.4
5792	O(C[C@H]1O[C@H](C=C1)n1c(=O)[nH]c(=O)c(C)c1)P1(=O)Oc2c(cc(CCC(=O)N[C@H](C(=O)OC)C)cc2)CO1	1073242-62-2	D-Alanine, N-[3-[2-(2,Ä≤,3,Ä≤-didehydro-3,Ä≤-deoxythymidin-5,Ä≤-O-yl)-2-oxido-4	C24H28N3O10P	549.47	A/B	-8.4
5793	[15NH2]C12CC3CC(C2)CC(C1)C3	107458-94-6	Alanine, N-[1-[N-[N-[N-[N-(N2-carboxy-L-arginyl)-L-valyl]-L-tyrosyl]-L-valyl]-L-	C54H72N12O11	1065.22	A/B	-8.4
5794	O[C@@H]1[C@H](n2c3c(nc2)c(N)ncn3)O[C@H](COP(=O)(OCC(OC(=O)CCCCCCCCCCCCCCC)COC(=O)CCCCCCCCCCCCCCC)O)[	107713-17-7	Alaninamide, carboxynitro-L-arginyl-L-valyl-L-tyrosyl-L-valyl-L-histidyl-L-proly	C53H70N14O12	1095.21	A/B	-8.4
5795	C(N1c2c(C(=NNC(=S)Nc3ccc(C=Cc4ccccc4)cc3)C1=O)cccc2)N1CCCCC1	108075-29-2	1,3,6-Naphthalenetrisulfonic acid, 5-[2-[4-[2-(4-amino-2-methylphenyl)diazenyl]-	C27H21N5O12S4	735.74	A/B	-8.4
5796	O=C1[C@H]2[C@H](c3c(C(=O)[C@@H]2CO1)cc1c(c3)OCO1)c1ccc(OC(=O)C)cc1	108440-52-4	Furo[3,Ä≤,4,Ä≤:6,7]naphtho[2,3-d]-1,3-dioxole-5,8-dione, 5a,6,8a,9-tetrahydro-9	C19H14O6	338.31	A/B	-8.4
5797	O=c1n(cc(c(=O)[nH]1)c1ccc(Br)cc1)[C@@H]1O[C@H](CO)[C@@H](O)C1	108664-89-7	Uridine, 2,Ä≤-deoxy-5-[4-(phenylmethoxy)phenyl]-	C22H22N2O6	410.42	A/B	-8.4
5798	C(=O)([C@@H](NC(=O)OC)C)N1[C@H](c2[nH]c(cn2)c2ccc(cc2)c2ccc(cc2)c2[nH]c([C@H](NC(=O)[C@@H](NC(=O)OC)	1087346-70-0	Carbamic acid, N-[(1S)-1-[[(2S)-2-[5-[4,Ä≤-[2-[(1R)-1-[[(2S)-2-[(methoxycarbony]	C38H48N8O6	712.84	A/B	-8.4
5799	Nc1c2c(cc(S(=O)(=O)O)c1N=Nc1c(Sc3c(N=Nc4c(S(=O)(=O)O)cc5c(c4N)ccc(S(=O)(=O)O)c5)ccc(C)c3)cc(C)cc1)cc	109010-93-7	3,5-Cyclohexadiene-1,2-dione, 5-[(5R,5aR,8aR,9S)-5,5a,6,8,8a,9-hexahydro-9-hydro	C20H16O8	384.34	A/B	-8.4
5800	Cc1c2c(cc(C(=O)C)c1)COP(=O)(OC[C@H]1O[C@H](C=C1)n1c(=O)[nH]c(=O)c(C)c1)O2	1095170-75-4	Thymidine, 5,Ä≤-O-(6-acetyl-2-oxido-4H-1,3,2-benzodioxaphosphorin-2-yl)-2,Ä≤,3,.	C19H19N2O8P	434.34	A/B	-8.4
5801	O=c1n([C@@H]2O[C@H](COP3(=O)Oc4c(CO3)cc(Cc3cc5c(cc3)OP(=O)(OC[C@@H]3[C@@H](N=[N+]=[N-])C[C@@H](O3)n3	1099829-93-2	Not Yet Assigned	C35H35N7O14P2		A/B	-8.4
5802	O=c1n([C@@H]2O[C@H](COC(=O)CCC(=O)OC[C@H]3O[C@H](C[C@@H]3F)n3c(=O)[nH]c(=O)c(C)c3)[C@@H](N=[N+]=[N-]	1099830-13-3	Not Yet Assigned	C22H30N4O7P2S	556.51	A/B	-8.4
5803	O=c1n([C@@H]2O[C@H](COC(=O)CCC(=O)OC[C@H]3O[C@H](CS3)n3c(=O)nc(N)cc3)[C@@H](N=[N+]=[N-])C2)cc(C)c(=O	1099830-21-3	Thymidine, 3,Ä≤-azido-3,Ä≤-deoxy-, 5,Ä≤-(hydrogen butanedioate), 5,Ä≤-ester wit	C24H28FN7O10		A/B	-8.4
5804	O=c1n([C@H]2C[C@H](OC(OCc3ccccc3)C(C)(C)C)[C@@H](CO)O2)cc(C(F)(F)F)c(=O)[nH]1	110604-84-7	Thymidine, 5,Ä≤-O-[2,2-dimethyl-1-(phenylmethoxy)propyl]-Œ±,Œ±,Œ±-trifluoro-	C22H27F3N2O6	472.45	A/B	-8.4
5805	C(#Cc1ccc(OC)cc1)c1n(nc(C(=O)N)n1)[C@@H]1O[C@H](CO)[C@@H](O)[C@H]1O	110671-98-2	1-Naphthalenesulfonic acid, 3,3,Ä≤-[methylenebis(4,1-phenyleneazo)]bis[4-amino-,	C33H26N6O6S2.2Na		A/B	-8.4
5806	C(#Cc1c(F)cccc1)c1n(nc(C(=O)N)n1)[C@@H]1O[C@H](CO)[C@@H](O)[C@H]1O	1106736-06-4	1H-1,2,4-Triazole-3-carboxamide, 5-[2-(4-fluorophenyl)ethynyl]-1-Œ≤-D-ribofuran(	C16H15FN4O5	362.31	A/B	-8.4
5807	N(=Nc1c(CCCCCCC)cccc1)c1c2c(cc(S(=O)(=O)O)cc2)ccc1O	1107693-99-1	2-Naphthalenesulfonic acid, 5-[2-(2-butylphenyl)diazenyl]-6-hydroxy-	C20H20N2O4S	384.45	A/B	-8.4
5808	Oc1c2[C@H]([C@H]3[C@H]([C@@H](c4c3cc(O)cc4O)c3cc(O)c(O)cc3)c2cc(O)c1)c1cc(O)c(O)cc1	1115371-10-2	Indeno[2,1-a]indene-1,3,6,8-tetrol, 5-(3,4-dihydroxyphenyl)-4b,5,9b,10-tetrahydr	C28H22O7	470.47	A/B	-8.4
5809	O=c1c2c(N(C[N]2)[C@@H]2O[C@H](CO)[C@@H](O)[C@H]2O)[nH]c(N)n1	1120346-89-5	5,Ä≤-Cytidylic acid, 5,Ä≤-ester with 4-amino-1-Œ≤-D-ribofuranosyl-2(1H)-pyrimid	C19H26N5O12P	547.41	A/B	-8.4
5810	O=C1c2c(c3c(c(OC)c2CO1)cc1c(c3)OCO1)c1cc(OC)c(OC)c(OC)c1	1126642-23-6	5(12H)-Naphthacenone, 7-(2,4-dihydroxy-6-methylphenyl)-2,4,6,9-tetrahydroxy-12,1	C27H22O7	458.46	A/B	-8.4
5811	N[C@H]1C[C@@H]2C[C@H](C1)CCC2	112727-02-3	Uridine, 2,Ä≤,2,Ä≤,Ä≤,Ä≤-oxybis[2,Ä≤-deoxy-5-ethyl-	C22H30N4O11	526.49	A/B	-8.4
5812	C(=O)([C@@H](NC(=O)OC)C)N1[C@@H](CC[C@@H]1C)c1[nH]c(cn1)c1ccc(cc1)c1ccc(cc1)c1[nH]c(nc1)[C@H]1N(C(=O	1129634-98-5	Carbamic acid, N,N,Ä≤-[[1,1,Ä≤-biphenyl]-4,4,Ä≤-diylbis[1H-imidazole-5,2-diyl[(	C40H50N8O6	738.88	A/B	-8.4
5813	C(CC)c1c([nH]c(n1)[C@H]1N(C(=O)[C@@H](NC(=O)OC)C(C)C)CCC1)c1ccc(cc1)c1ccc(cc1)c1[nH]c(nc1)[C@H]1N(C(	1129636-44-7	Carbamic acid, N-[(1S)-2-[(2S)-2-[5-[4,Ä≤-[2-[(2S)-1-[(2S)-2-[(methoxycarbonyl)a	C39H48N8O6	724.85	A/B	-8.4
5814	N(=Nc1c2c(ccc(S(=O)(=O)O)c2)c(N)cc1)c1c2c(c(N=Nc3c(S(=O)(=O)O)cc(N)cc3)cc1)ccc(S(=O)(=O)O)c2.[NaH]	1130877-32-5	2-Naphthalenesulfonic acid, 8-[2-(4-amino-2-methylphenyl)diazenyl]-5-[2-(4-aminc	C23H20N6O6S2.2Na		A/B	-8.4
5815	N(C(=O)C([2H])([2H])[2H])([C@H]1[C@]([C@@]([C@@](C(O[2H])([2H])[2H])(O[2H])[2H])(O[2H])[2H])(OC(=C[C	113296-27-8	5,Ä≤-Guanylic acid, mono[2-[(4-formylphenyl)methylamino]ethyl] ester	C20H25N6O9P	524.42	A/B	-8.4
5816	N(=N\c1ccccc1)/c1c2c(c(/N=N/c3c(C)cc(OCC)cc3)cc1)ccc(S(=O)(=O)O)c2	1135683-38-3	1-Naphthalenesulfonic acid, 4-amino-3-[(1E)-2-[4-[(4-hydroxyphenyl)methyl]phenyl	C23H19N3O4S	433.48	A/B	-8.4
5817	N(=N\c1ccccc1)/c1c2c(c(N/N=C\3/C(=CC(=O)C=C3)C)cc1)ccc(S(=O)(=O)O)c2	1135683-63-4	2-Naphthalenesulfonic acid, 5-[(1E)-2-(4-ethoxy-2-methylphenyl)diazenyl]-8-[(1E)	C25H22N4O4S	474.53	A/B	-8.4
5818	C(C)(C)c1n([C@@H]2C[C@@H]3N(CC[C@H](NC(=O)C4CCC(F)(F)CC4)c4ccccc4)[C@H](C2)CC3)nc(C)n1	1147100-86-4	Thymidine, 2,Ä≤,3,Ä≤-didehydro-3,Ä≤-deoxy-, 5,Ä≤-[hydrogen N-[(2S)-2-(acetylami	C21H25N4O8P	492.42	A/B	-8.4
5819	C(=CCCO)(c1cc(O)c(O)cc1)c1cc(O)c(O)cc1	114813-90-0	[1,1,Ä≤-Biphenyl]-2,2,Ä≤-disulfonic acid, 4,4,Ä≤-bis[2-(1-amino-4-sulfo-2-napht	C34H28N6O12S4.4Na		A/B	-8.4
5820	O=c1c2c(n(cn2)[C@@H]2O[C@H](CO)[C@H](O)C2)[nH]c(N)n1	1160003-80-4	Thymidine, 2,Ä≤,3,Ä≤-didehydro-3,Ä≤-deoxy-5,Ä≤-O-[6-[4-hydroxy-3-(hydroxymethyl	C24H23N2O9P	514.42	A/B	-8.4
5821	N(=C1c2c(N(Cc3ccccc3)C1=O)ccc(C)c2)NC(=S)Nc1ccc(C)cc1	1161792-75-1	Hydrazinecarbothioamide, 2-[5-fluoro-1,2-dihydro-2-oxo-1-(phenylmethyl)-3H-indol	C23H19FN4OS	418.49	A/B	-8.4
5822	O=c1n([C@H]2C[C@H](O[P@](=O)(SC[C@H]3O[C@H](C[C@@H]3O)n3c(=O)[nH]c(=O)c(C)c3)S)[C@@H](CO)O2)cc(C)c(=	1164118-00-6	Thymidine, [P(R)]-P-thiothymidylyl-(3,Ä≤,Üí5,Ä≤)-5,Ä≤-thio-	C20H27N4O10PS2	578.55	A/B	-8.4
5823	Cc1n(c2nc(nc(n2)N2CCOCC2)c2cc(O)ccc2)c2c(n1)cccc2	1174126-22-7	1,3,5-Triazin-2-amine, 4-ethoxy-6-(4-morpholinyl)-N-[3-(trifluoromethyl)phenyl]-	C16H18F3N5O2	369.34	A/B	-8.4
5824	Cc1n(c2nc(nc(n2)N2CCOCC2)c2c3c([nH]nc3)ccc2)ccn1	1174190-87-4	1,3,5-Triazine, 2-(3-chlorophenyl)-4-(2-methyl-1H-imidazol-1-yl)-6-(4-morpholiny	C17H17ClN6O	356.81	A/B	-8.4
5825	C(C)(C)c1n(ccn1)c1nc(nc(n1)N1CCOCC1)c1cnc(N)nc1	1174190-93-2	Phenol, 3-[4-[2-(1-methylethyl)-1H-imidazol-1-yl]-6-(4-morpholinyl)-1,3,5-triazi	C19H22N6O2	366.42	A/B	-8.4
5826	c1(nc(nc(n1)n1ccnc1)N1CCOCC1)c1cc2c(cc1)cc[nH]2	1174190-96-5	2-Pyrimidinamine, 4-methyl-5-[4-[2-(1-methylethyl)-1H-imidazol-1-yl]-6-(4-morphc	C18H23N9O	381.43	A/B	-8.4
5827	Cc1nn(c2nc(nc(n2)N2CCOCC2)c2cnc(N)nc2)cc1	1174191-06-0	1H-Indole, 4-[4-(1H-imidazol-1-yl)-6-(4-morpholinyl)-1,3,5-triazin-2-yl]-	C18H17N7O	347.37	A/B	-8.4
5828	C(C)n1c2c(c(=O)c(C(=O)O)c1)cc(F)c(c2)N1CCN(Cc2cn(c(=O)[nH]c2=O)[C@H]2C[C@H](N=[N+]=[N-])[C@@H](CO)O2	1174398-27-6	Thymidine, 3,Ä≤-azido-3,Ä≤-deoxy-, 5,Ä≤-[(3S)-9-fluoro-2,3-dihydro-3-methyl-10-	C28H31FN8O7		A/B	-8.4
5829	C([C@H](OCP1(=O)OC(CCO1)c1cc(Cl)ccc1)C)n1c2c(nc1)c(N)ncn2	1174716-09-6	9H-Purin-6-amine, 9-[(2R)-2-[[(2S,4S)-4-(3-chlorophenyl)-2-oxido-1,3,2-dioxaphos	C18H21ClN5O4P	437.82	A/B	-8.4
5830	O(C(N[C@H]([C@@H](CN(S(=O)(=O)c1ccc(N)cc1)CC(C)C)O)Cc1ccccc1)O)[C@@H]1[C@H]2[C@@H](OC1)OCC2	1177956-82-9	Not Yet Assigned	C36H45N5O4	611.77	A/B	-8.4
5831	CC1(C)c2c(-c3c1cc(cc3)c1[nH]c(nc1)[C@H]1N(C(=O)[C@@H](NC(=O)OC)C3CC3)CCC1)ccc(c2)c1[nH]c(nc1)[C@H]1N	1179150-48-1	Carbamic acid, N,N,Ä≤-[(9,9-dimethyl-9H-fluorene-2,7-diyl)bis[1H-imidazole-5,2-c	C43H54N8O6	778.94	A/B	-8.4
5832	C(=O)([C@@H](NC(=O)OC)C(C)C)N1[C@H](c2[nH]c(c3cc4C5(c6c(-c4cc3)ccc(c6)c3[nH]c(nc3)[C@H]3N(C(=O)[C@@H	1179150-52-7	Carbamic acid, N,N,Ä≤-[(9,9-dimethyl-9H-fluorene-2,7-diyl)bis[1H-imidazole-5,2-c	C43H50N8O6	774.91	A/B	-8.4
5833	O=c1c2c(n(cn2)[C@@H]2OC(=CO)[C@@H](O)C2)[nH]cn1	1179324-28-7	Furo[3,Ä≤,4,Ä≤:6,7]naphtho[2,3-d]-1,3-dioxol-6(5aH)-one, 5,8,8a,9-tetrahydro-9-	C20H18O5	338.35	A/B	-8.4
5834	C(=O)([C@@H](NC(=O)OC)C(C)C)N1[C@H](c2[nH]c3-c4c(cc(cc4)c4ccc(cc4)c4c(CCC(F)(F)F)nc([nH]4)[C@@H]4N(C(	1180669-94-6	Carbamic acid, N-[(1S)-1-[[(2S)-2-[4,5-dihydro-7-[4-[2-[(2S)-1-[(2S)-2-[(methoxy	C42H52N8O6	764.91	A/B	-8.4
5835	C(=O)([C@@H](NC(=O)OC)C(C)C)N1[C@H](c2[nH]c3c4c(cc(cc4)c4ccc(cc4)c4[nH]c(nc4)[C@H]4N(C(=O)[C@@H](NC(	1180670-13-6	Carbamic acid, N-[(1S)-1-[[(2S)-2-[5-[4-[2-[(2S)-4,4-difluoro-1-[(2S)-2-[(methox	C42H50F2N8O6	800.89	A/B	-8.4
5836	O(P(=O)(N[C@H](C(=O)OCC)C)OCCOCn1c2c(nc1)c(=O)nc(N)[nH]2)c1c2c(ccc1)cccc2	1184942-44-6	5,8-Dioxa-3-aza-4-phosphanonanoic acid, 9-(2-amino-1,6-dihydro-6-oxo-9H-purin-9-	C24H29N6O7P	544.5	A/B	-8.4
5837	C(OCCOP(=O)(Oc1ccccc1)NC(C(=O)OCc1ccccc1)(C)C)n1c2c(nc1)c(=O)nc(N=CN(C)C)[nH]2	1184942-71-9	5,8-Dioxa-3-aza-4-phosphanonanoic acid, 9-[2-[[(dimethylamino)methylene]amino]-1	C32H36N7O7P	661.64	A/B	-8.4
5838	C(C#N)C1(CN(S(=O)(=O)c2c(F)cccc2)C1)n1cc(cn1)c1c2c(ncn1)[nH]cc2	1187594-49-5	3-Azetidineacetonitrile, 1-[(3-fluorophenyl)sulfonyl]-3-[4-(7H-pyrrolo[2,3-d]pyr	C20H16FN7O2S	437.45	A/B	-8.4
5839	O=c1n([C@@H]2O[C@H](COP(=O)(Nc3cc(C(=O)Nc4cc(C(=O)NCCC(=N)N)n(C)c4)n(C)c3)OC(C)C)C=C2)cc(C)c(=O)[nH]	1190064-43-7	Thymidine, 2,Ä≤,3,Ä≤-didehydro-3,Ä≤-deoxy-, 5,Ä≤-[1-methylethyl N-[5-[[(3-aminc	C22H32N7O7P	537.51	A/B	-8.4
5840	C[C@@]12[C@@H]3[C@H]([C@H]4[C@](C)(CC3)[C@@H]([C@@H](CCCC(C)C)C)CC4)CCC1=CC1=C(C2)C(=O)CC(C)(C)O1	1191242-97-3	L-Phenylalanine, N-carboxy-, 1-[(3aR,4S,9R,9aR)-1,3,3a,4,9,9a-hexahydro-6,7-dime	C43H45N3O14	827.83	A/B	-8.4
5841	[C@](C(OC([2H])([2H])[2H])([2H])[2H])(N1[C@@H](C)CN(CC1)C1(C)CCN(C(=O)c2c(C)ncnc2C)CC1)(c1ccc(C(F)(F	1191293-08-9	Methanone, [4,6-di(methyl-d3)-5-pyrimidinyl][4-[(3S)-4-[(1R)-2-(methoxy-d3)-1-[4	C28H27D11F3N5O2		A/B	-8.4
5842	[C@](COC([2H])([2H])[2H])(N1[C@@H](C)CN(CC1)C1(C)CCN(C(=O)c2c(C)ncnc2C)CC1)(c1ccc(C(F)(F)F)cc1)[2H]	1191293-11-4	Methanone, (4,6-dimethyl-5-pyrimidinyl)[4-[(3S)-4-[(1R)-2-(methoxy-d3)-1-[4-(tri	C28H33D5F3N5O2		A/B	-8.4
5843	N(=C(NC(=O)OC(C)(C)C)NC(=O)OC(C)(C)C)[C@@H]1[C@@H](NC(=O)C)[C@H]([C@@H]([C@@H](COC(=O)CC[C@@H](Cc2cc	1192538-87-6	Not Yet Assigned	C38H57N5O14	807.88	A/B	-8.4
5844	N(=C(NC(=O)OC(C)(C)C)NC(=O)OC(C)(C)C)[C@@H]1[C@@H](NC(=O)C)[C@H]([C@@H]([C@@H](COC(=O)[C@@H](N)c2ccc	1192539-03-9	Not Yet Assigned	C35H51N5O14	765.8	A/B	-8.4
5845	O=C1c2c(ccc(S(=O)(=O)Nc3ccc(C)cc3)c2)NC1=O	1199574-55-4	1H-Indole-5-sulfonamide, 2,3-dihydro-2,3-dioxo-N-phenyl-	C14H10N2O4S	302.31	A/B	-8.4
5846	C[C@]1(O)[C@H](n2c3c(nc2)c(=O)nc(N)[nH]3)O[C@H](COP(=O)(OCCSC(=O)C(CO)(C)C)NC(C)C)[C@H]1O	1202498-41-6	Guanosine, 2,Ä≤-C-methyl-, 5,Ä≤-(hydrogen phosphoramidate)	C11H17N6O7P	376.26	A/B	-8.4
5847	C(=O)(NC(CC1CCC1)C(=O)C(=O)NCC=C)[C@@H]1[C@@H]2[C@@H](C2(C)C)CN1C(=O)[C@@H](NC(=O)N[C@@H](CN1C(=O)CC	1208257-84-4	3-Azabicyclo[3.1.0]hexane-2-carboxamide, 3-[(2S)-2-[[[[(1R)-1-[(4,4-dimethyl-2-c	C38H64N6O6	700.95	A/B	-8.4
5848	N(C(=O)COC(=O)CN1C(=O)CCCCC1)C12CC3CC(C2)CC(C1)C3	1209652-72-1	1-Imidazolidineacetic acid, 4,4-dimethyl-2,5-dioxo-, 2-[[(4-fluorophenyl)methyl]	C26H32FN3O5	485.55	A/B	-8.4
5849	C(OCCOP(=O)(OCCOCn1c2c(nc1)c(=O)nc(N)[nH]2)O)n1c2c(nc1)c(=O)nc(N)[nH]2	121117-73-5	Phosphoric acid, mono[2-[(2-amino-1,6-dihydro-6-oxo-9H-purin-9-yl)methoxy]ethyl]	C18H23N10O7P	522.41	A/B	-8.4

5850	O=c1c2C(C(=C(N)Oc2cc(C)n1CC)C#N)c1cn(c(=O)[nH]c1=O)[C@H]1C[C@H](OC(=O)C)[C@@H](COC(=O)C)O1	1217597-32-4	Uridine, 5-(2-amino-3-cyano-5,6-dihydro-7-methyl-5-oxo-4H-pyrano[3,2-c]pyridin-4	C23H23N5O9	513.46	A/B	-8.4
5851	C([C@](OCP(=O)(O)O)(C([2H])([2H])[2H])[2H])(n1c2c(nc1)c(N)ncn2)([2H])[2H]	1217703-38-2	6H-Furo[3,Ä≤,4,Ä≤:6,7]naphtho[2,3-c]-1,2-dioxol-6-one, 3,5,5a,8,8a,9-hexahydro-	C22H22O8	414.41	A/B	-8.4
5852	[C@H](Cc1ccccc1)([C@@H](CN(S(=O)(=O)c1ccc(N)cc1)C(C)(C)C)O)NC(=O)O[C@H]1CC(OC1([2H])[2H])([2H])[2H]	1217778-78-3	2-Pyridinesulfonamide, N-[3-[(1R)-1-[(2R)-3,4-dihydro-6-hydroxy-4-oxo-2-(2-pheny	C31H33F3N2O5S	602.66	A/B	-8.4
5853	O=c1n([C@@H]2O[C@H](CO)CC2)ccc(N=CN(C)C)n1	1218813-22-9	Hydrazinecarbothioamide, 2-[1,2-dihydro-2-oxo-1-[[4-[7-(trifluoromethyl)-4-quino	C24H22F3N7OS	513.54	A/B	-8.4
5854	[C@](C(=O)N[C@@H](C(c1c(c(c(c(c1[2H])[2H])[2H])[2H])[2H])([2H])[2H])C[C@@H]([C@H](Cc1ccccc1)NC(=O)CO	1224729-10-5	1(2H)-Pyrimidineacetamide-Œ±-d, N-[(1S,3S,4S)-4-[[2-[2,6-di(methyl-d3)phenoxy-3,	C37H24D24N4O5		A/B	-8.4
5855	[C@@H](C(=O)N[C@@H](C(c1c(c(c(c(c1[2H])[2H])[2H])[2H])[2H])([2H])[2H])C[C@@H]([C@H](Cc1ccccc1)NC(=O)	1224729-25-2	1(2H)-Pyrimidine-4,5,6-d3-acetamide, N-[(1S,3S,4S)-4-[[2-(2,6-dimethylphenoxy)ac	C37H33D15N4O5		A/B	-8.4
5856	[C@](C(=O)N[C@@H](Cc1ccccc1)C[C@@H]([C@](C(c1c(c(c(c(c1[2H])[2H])[2H])[2H])[2H])([2H])[2H])(NC(=O)CO	1224729-29-6	1(2H)-Pyrimidine-4,5,6-d3-acetamide-Œ±-d, N-[(1S,3S,4S)-4-[[2-(2,6-dimethylpheno	C37H26D22N4O5		A/B	-8.4
5857	[C@](C(=O)N[C@@H](Cc1ccccc1)C[C@@H]([C@H](Cc1ccccc1)NC(=O)COc1c(C)cccc1C)O)(C(C([2H])([2H])[2H])(C([	1224729-34-3	1(2H)-Pyrimidineacetamide-Œ±-d, N-[(1S,3S,4S)-4-[[2-[2,6-di(methyl-d3)phenoxy-3,	C37H31D17N4O5		A/B	-8.4
5858	[C@@H](C(=O)N[C@@H](Cc1ccccc1)C[C@@H]([C@H](Cc1ccccc1)NC(=O)C(Oc1c(C)cccc1C)([2H])[2H])O)(C(C)C)N1C(	1224729-50-3	1(2H)-Pyrimidineacetamide, N-[(1S,3S,4S)-4-[[2-[2,6-di(methyl-d3)phenoxy-3,4,5-d	C37H37D11N4O5		A/B	-8.4
5859	C(#N)C1=C(N)Oc2c(C1c1cc(OC)c(OC)c(Br)c1)ccn1c2ncc1	1225373-10-3	7H-Imidazo[1,2-a]pyrano[2,3-c]pyridine-8-carbonitrile, 9-amino-7-(2-hydroxypheny	C17H12N4O2	304.3	A/B	-8.4
5860	C(#N)C1=C(N)Oc2c(C1c1cc(C(C)(C)C)c(O)c(C(C)(C)C)c1)ccn1c2ncc1	1225373-27-2	7H-Imidazo[1,2-a]pyrano[2,3-c]pyridine-8-carbonitrile, 9-amino-7-[3-methoxy-4-me	C24H24N4O2	400.47	A/B	-8.4
5861	O(P(=O)(O)O)[C@H]1[C@@H](O[C@H](CO)[C@H]1O)n1c2c(nc1)c(=O)nc(NC)[nH]2	122591-44-0	Œ≤-D-arabino-Adenosine, Œ≤-D-arabino-uridylyl-(3,Ä≤,Ü í5,Ä≤)-Œ≤-D-arabino-adenyl	C57H70N21O40P5	1844.15	A/B	-8.4
5862	N(C(=O)C)[C@H]1[C@H]([C@@H]([C@@H](CO)O)O)OC(=C[C@@H]1NC#N)C(=O)OC	1228215-36-8	Carbamic acid, N-[(1S)-1-[[(2S)-2-[5-[4,Ä≤-[2-[(2S)-4-ethylidene-1-[(2S)-2-[(met	C42H52N8O6	764.91	A/B	-8.4
5863	C(=O)([C@H](NC(=O)OC)c1ccccc1)N1[C@@H](CCC1)c1[nH]c(cn1)c1ccc(cc1)[C@H]1CC[C@@H](CC1)c1[nH]c(nc1)[C@	1228667-93-3	Carbamic acid, N-[(1S)-2-[(2S)-2-[5-[4-[trans-4-[2-[(2S)-1-[(2S)-2-[(methoxycarb	C46H52N8O6	812.96	A/B	-8.4
5864	C(=O)([C@@H](NC(=O)OC)[C@H](CC)C)N1[C@@H](CCC1)c1[nH]c(cn1)[C@@H]1CC[C@H](CC1)c1ccc(cc1)c1[nH]c(nc1)	1228670-69-6	Carbamic acid, N-[(1S)-1-cyclopropyl-2-[(2S)-2-[5-[4-[trans-4-[2-[(2S)-1-[(2S)-2	C40H52N8O6	740.89	A/B	-8.4
5865	C(=O)([C@H](NC(=O)OC)c1ccccc1)N1[C@@H](CCC1)c1[nH]c(cn1)c1ccc(cc1)[C@@H]1CC[C@H](CC1)c1[nH]c(nc1)[C@	1228670-78-7	Carbamic acid, N-[(1R)-2-[(2S)-2-[5-[trans-4-[4-[2-[(2S)-1-[(2S)-2-[(methoxycarb	C43H54N8O6	778.94	A/B	-8.4
5866	C(=O)([C@@H](NC(=O)OC)C(C)C)N1[C@@H](CCC1)c1[nH]c(cn1)[C@@]1(C)CC[C@@H](CC1)c1ccc(cc1)c1[nH]c(nc1)[C	1228670-79-8	Carbamic acid, N-[(1R)-2-[(2S)-2-[5-[4-[trans-4-[2-[(2S)-1-[(2S)-2-[(methoxycarb	C43H54N8O6	778.94	A/B	-8.4
5867	C(=O)([C@H](N(C)C)c1ccccc1)N1[C@@H](CCC1)c1[nH]c(cn1)c1ccc(cc1)c1ccc(C#Cc2[nH]c(nc2)[C@H]2N(C(=O)[C@	1228966-39-9	Carbamic acid, N-[(1R)-2-[(2S)-2-[5-[2-[4,Ä≤-[2-[(2S)-1-[(2R)-2-[(methoxycarbony	C48H46N8O6	830.93	A/B	-8.4
5868	N(Cc1ccc(cc1)c1ccccn1)(C[C@@H]([C@H](Cc1ccccc1)NC(=O)[C@@H](NC(=O)OC(C)(C)C)C(C)(C)C)O)NC(=O)[C@@H](	1233885-50-1	D-erythro-Pentitol, 1,4-anhydro-2-deoxy-5-O-[2-[[2-[3-[(1R)-3-(3,4-dimethoxypher	C60H81N5O15S	1144.37	A/B	-8.4
5869	C(N(C(=O)N[C@H](C(=O)N[C@@H](Cc1ccccc1)C[C@@H]([C@H](Cc1ccccc1)NC(=O)OCc1ccns1)O)C(C)C)C)c1nc(sc1)C1	1236406-41-9	Not Yet Assigned	C40H52N6O6S	744.94	A/B	-8.4
5870	C(N(C(=O)N[C@H](C(=O)N[C@@H](Cc1ccccc1)C[C@@H]([C@H](Cc1ccccc1)NC(=O)OCc1ccns1)O)C(C)C)C)c1nc(sc1)C1	1236406-84-0	2,7,10,12-Tetraazatridecanoic acid, 4-hydroxy-12-methyl-9-(1-methylethyl)-8,11-c	C37H46N6O6S	702.86	A/B	-8.4
5871	[C@H](Cc1ccccc1)(C[C@@H]([C@H](Cc1ccccc1)NC(=O)OCc1cnco1)O)NC(=O)[C@@H](NC(=O)N(Cc1nc(C=C(C)C)sc1)C)	1236407-39-8	Not Yet Assigned	C38H45N7O6S2	759.94	A/B	-8.4
5872	C(CC)(CC)c1nc(CN(C(=O)N[C@@H](C(=O)N[C@@H](Cc2ccccc2)C[C@@H]([C@H](Cc2ccccc2)NC(=O)OCc2cncs2)O)C(C)C	1236408-24-4	2,7,10,12-Tetraazatridecanoic acid, 4-hydroxy-12-methyl-9-(1-methylethyl)-8,11-c	C40H46N6O6S	738.89	A/B	-8.4
5873	[C@H](Cc1ccccc1)(C[C@@H]([C@H](Cc1ccccc1)NC(=O)OCc1cncs1)O)NC(=O)[C@H](NC(=O)N(Cc1nc(CC2=CCCC2)sc1)C	1236408-27-7	2,7,10,12-Tetraazatridecanoic acid, 13-[2-[(dimethylamino)methyl]-4-thiazolyl]-4	C37H49N7O6S	719.89	A/B	-8.4
5874	[C@H](Cc1ccccc1)([C@@H]([C@@H]([C@H](Cc1ccccc1)NC(=O)OCc1cncs1)O)O)NC(=O)[C@@H](NC(=O)N(Cc1nc(C(C)C)	1236409-09-8	2,7,10,12-Tetraazatridecanoic acid, 4-hydroxy-13-[2-[(2-methoxy-4-thiazolyl)meth	C39H47N7O6S3	806.03	A/B	-8.4
5875	N(CC(=O)[C@H](Cc1ccccc1)C[C@@H](CN1[C@H](C(=O)NC(C)(C)C)CN(CCc2ccccn2)CC1)O)[C@H]1c2c(C[C@H]1O)cccc2	1237988-97-4	Not Yet Assigned	C37H49N5O4	627.82	A/B	-8.4
5876	C(F)(F)c1n(c2c(n1)cccc2)c1nc(nc(N[C@H]2CC[C@H](NC(=O)C)CC2)n1)N1CCOCC1	1240024-75-2	1,3,5-Triazin-2-amine, N-[(trans-4-aminocyclohexyl)methyl]-4-[2-(difluoromethyl)	C22H28F2N8O	458.51	A/B	-8.4
5877	C(=O)([C@H](NC(=O)OC)c1ccccc1)N1[C@@H](CCC1)c1[nH]c(cn1)[C@H]1CC[C@@H](CC1)c1ccc(cc1)c1[nH]c([C@@H](	1240888-31-6	Carbamic acid, N-[(1S)-2-[(2S)-2-[5-[4-[trans-4-[2-[(2S)-1-[(2S)-2-[(methoxycarb	C48H56N8O6	841.01	A/B	-8.4
5878	O=c1c2c(n(cn2)[C@H]2[C@H](O)[C@@H](CO)C2)[nH]c(N)n1	1244855-96-6	Furo[3,Ä≤,4,Ä≤:6,7]naphtho[2,3-d]-1,3-dioxol-6(5aH)-one, 5,8,8a,9-tetrahydro-5-	C21H20O7	384.38	A/B	-8.4
5879	C(Nc1c2c(n3c(n1)c(S(=O)(=O)c1ccc(C)cc1)nn3)ccs2)(C)C12CC3CC(C2)CC(C1)C3	1246178-71-1	Thieno[2,3-e][1,2,3]triazolo[1,5-a]pyrimidin-5-amine, 3-(phenylsulfonyl)-N-(1-tr	C25H27N5O2S2	493.64	A/B	-8.4
5880	C(=O)([C@@H](NC(=O)OC)C(C)C)N1[C@@H]([C@@H]2C[C@H]1CC2)c1[nH]c(cn1)c1ccc(cc1)c1ccc(cc1)c1[nH]c(nc1)[	1256382-61-2	3-Pyridinecarboxamide, 1,4-dihydro-4-oxo-1,6-diphenyl-N-(1-tricyclo[3.3.1.13,7]d	C30H32N2O2	452.59	A/B	-8.4
5881	C(=O)([C@@H](NC(=O)OC)C(C)C)N1[C@H](c2[nH]c(c3ccc(c4c5c(c(ccc5)c5ccc(cc5)c5[nH]c(nc5)[C@H]5N(C(=O)[C	1256387-45-7	Carbamic acid, N,N,Ä≤-[[2,2,Ä≤-binaphthalene]-6,6,Ä≤-diylbis[1H-imidazole-5,2-d	C48H54N8O6	838.99	A/B	-8.4
5882	C(=O)([C@H](NC(=O)OC)C(C)C)N1[C@H](CCC1)c1[nH]c(C#Cc2ccc(C#Cc3[nH]c(nc3)[C@H]3N(C(=O)[C@@H](NC(=O)OC	1256387-55-9	Carbamic acid, N,N,Ä≤-[1,5-naphthalenediylbis[4,1-phenylene-1H-imidazole-5,2-diy	C50H56N8O6	865.03	A/B	-8.4
5883	C(=O)([C@@H](NC(=O)OC)C(C)C)N1[C@@H]([C@@H]2C[C@H]1CC2)c1[nH]c(cn1)c1ccc(C#Cc2ccc(cc2)c2[nH]c(nc2)[C	1256388-88-1	Carbamic acid, N-[(1S)-1-[[(2S)-2-[5-[7-[2-[(1R,3S,4S)-2-[(2S)-2-[(methoxycarbor	C42H50N8O7	778.9	A/B	-8.4
5884	C(=O)([C@@H](NC(=O)OC)C(C)C)N1[C@@H](C[C@H](C#N)C1)c1[nH]c(cn1)c1ccc(cc1)c1cc2c(cc1)cc(cc2)c1[nH]c(n	1256389-41-9	Carbamic acid, N-[(1S)-1-[[(2S,4R)-4-cyano-2-[5-[4-[6-[2-[(2S)-1-[(2S)-2-[(metho	C45H51N9O6	813.94	A/B	-8.4
5885	C[C@]12[C@H]([C@H]3[C@@H]([C@]4(C)[C@H](CC3)C[C@@H](NC)CC4)CC2=O)CC[C@@H]1[C@@H](CCC(=O)NCc1ccccc1)C	125680-34-4	Cholan-24-amide, 3-amino-12-oxo-N-(phenylmethyl)-, (3Œ≤,5Œ≤)-	C31H46N2O2	478.71	A/B	-8.4
5886	O=c1n([C@H]2C[C@H](O[P@](=O)(OC[C@H]3O[C@H](C[C@@H]3O)n3c(=O)[nH]c(=O)c(C)c3)OCOC(=O)C)[C@@H](CO)O2)	1261162-87-1	Thymidine, [P(R)]-P-[(acetyloxy)methyl]thymidylyl-(3,Ä≤,Ü í5,Ä≤)-	C23H31N4O14P	618.48	A/B	-8.4
5887	O=c1c2c(n(cn2)[C@@H]2O[C@H](COP(=O)(O)O)[C@@H](O)[C@H]2O)[nH]c(N[C@@H]2C(=O)OCC2)n1	1261453-87-5	5,Ä≤-Guanylic acid, N-[(3R)-tetrahydro-5-hydroxy-2-oxo-2H-pyran-3-yl]-	C15H20N5O11P	477.32	A/B	-8.4
5888	O=c1n([C@@H]2O[C@H](COC(=O)CCC)C=C2)cc(C)c(=O)[nH]1	1262278-49-8	Diphosphoric acid, P,P,Ä≤-bis[[(2R,5S)-5-(4-amino-2-oxo-1(2H)-pyrimidinyl)-1,3-c	C16H22N6O11P2S2	600.46	A/B	-8.4
5889	O=c1c2c(n(cc1C(=O)O)C1CC1)cc(c(F)c2)N1CCN(CN2c3c(/C(=N/NC(=S)NO)/C2=O)cc(Cl)cc3)CC1	1265623-14-0	3-Quinolinecarboxylic acid, 7-[4-[[(3Z)-2,3-dihydro-3-[2-[(hydroxyamino)thioxome	C27H28FN7O5S	581.62	A/B	-8.4
5890	O=c1c2c(n(cc1C(=O)O)C1CC1)cc(c(F)c2)N1CCN(CN2c3c(/C(=N/NC(=S)NO)/C2=O)cc(C)cc3)CC1	1265623-18-4	3-Quinolinecarboxylic acid, 1-cyclopropyl-6-fluoro-7-[4-[[(3Z)-5-fluoro-2,3-dihy	C28H27F2N7O5S	611.62	A/B	-8.4
5891	C(C)n1c2c(c(=O)c(C(=O)O)c1)cc(F)c(c2F)N1CC(C)N(CN2c3c(/C(=N/NC(=S)NO)/C2=O)cc(C)cc3)CC1	1265623-24-2	3-Quinolinecarboxylic acid, 1-ethyl-6,8-difluoro-7-[4-[[(3Z)-5-fluoro-2,3-dihydr	C28H28F3N7O5S	631.63	A/B	-8.4
5892	C[C@]12[C@H]([C@H]3[C@@H]([C@]4(C)C(CC3)C[C@H](O)CC4)C[C@@H]2O)CC[C@@H]1[C@@H](CCC(=O)NCC(=O)O)C	1266398-02-0	[1,1,Ä≤-Biphenyl]-2,2,Ä≤-disulfonic acid, 4,4,Ä≤-bis[(1E)-2-(2-hydroxy-1-naphth	C34H26N4O8S2.2Na		A/B	-8.4
5893	N(=N\c1ccc(cc1)c1ccc(/N=N/c2ccc(OS(=O)(=O)c3ccc(C)cc3)cc2)cc1)/c1c2c(cc(S(=O)(=O)O)c1N)cc(S(=O)(=O)O	1266974-36-0	1-Naphthalenesulfonic acid, 2,2,Ä≤-[[1,1,Ä≤-biphenyl]-4,4,Ä≤-diylbis(2,1-diazen	C32H24N6O6S2	652.7	A/B	-8.4
5894	O=C1[C@@H]2[C@@H](c3c([C@H](O[C@H]4O[C@H](CO)[C@H](O)[C@H](O)[C@H]4O)[C@H]2CO1)cc1c(c3)OCO1)c1cc(OC)	1266976-34-4	2,7-Naphthalenedisulfonic acid, 3-amino-4-[(1E)-2-[4,Ä≤-[(1E)-2-[4-[[(4-methylpl	C35H27N5O9S3	757.81	A/B	-8.4
5895	C(=O)([C@@H](NC(=O)OC)C(C)C)N1[C@@H]([C@@H]2C[C@H]1CC2)c1[nH]c(cn1)c1ccc(cc1)c1ccc(cc1)c1[nH]c([C@@H	1273591-38-0	Carbamic acid, N,N,Ä≤-[[1,1,Ä≤-biphenyl]-4,4,Ä≤-diylbis[1H-imidazole-5,2-diyl-(	C44H54N8O6	790.95	A/B	-8.4
5896	C(=O)([C@@H](NC(=O)OC)C(C)C)N1[C@@H]([C@@H]2C[C@H]1CC2)c1[nH]c(cn1)[C@@H]1CC[C@H](CC1)c1ccc(cc1)c1[n	1273591-53-9	Carbamic acid, N-[(1S)-2-methyl-1-[[(2S)-2-[5-[3,3,Ä≤,5,5,Ä≤-tetrachloro-4,Ä≤-[	C42H48Cl4N8O6	902.69	A/B	-8.4
5897	N(Cc1cc(Cl)ccc1)c1nc(nc(N/N=C/c2c(O)cccc2)n1)N1CCOCC1	1279692-17-9	Benzaldehyde, 2-hydroxy-, 2-[4-(4-morpholinyl)-6-[(phenylmethyl)amino]-1,3,5-tri	C21H23N7O2	405.45	A/B	-8.4
5898	O=c1n(cc(C[Se]C(C)C)c(=O)[nH]1)[C@@H]1O[C@H](CO)[C@@H](O)C1	1279692-18-0	Benzaldehyde, 2-hydroxy-, 2-[4-[[(3-chlorophenyl)methyl]amino]-6-(4-morpholinyl)	C21H22ClN7O2	439.9	A/B	-8.4
5899	O[C@H]1[C@H](n2c3c(nc2)c(N)ncn3)O[C@H](COP(=O)(OCCC(=O)O)O)[C@H]1O	1285535-41-2	1,3,5-Triazin-2-amine, 4,6-di-4-morpholinyl-N-[4-(4-nitrophenyl)-2-thiazolyl]-	C20H22N8O4S	470.5	A/B	-8.4
5900	C(=O)([C@@H](NC(=O)OC)C(C)C)N1[C@@H](CC(=NOC)C1)c1[nH]c(cn1)c1ccc(cc1)c1ccc(cc1)c1[nH]c(nc1)[C@H]1N(	1311398-32-9	Carbamic acid, N-[(1S)-1-[[(2S)-2-[5-[4,Ä≤-[2-[(2S)-1-[(2S)-2-[(methoxycarbonyl)	C42H50N8O6	762.9	A/B	-8.4
5901	C(=O)([C@@H](NC(=O)OC)C(C)C)N1[C@@H](C[C@H](C)C1)c1[nH]c(cn1)c1ccc(cc1)c1ccc(cc1)c1[nH]c(nc1)[C@H]1N	1311398-37-4	Carbamic acid, N-[(1S)-1-[[(2S)-2-[5-[4,Ä≤-[2-[(6S)-5-[(2S)-2-[(methoxycarbonyl)	C43H52N8O6	776.92	A/B	-8.4
5902	C(=O)([C@@H](NC(=O)OC)C(C)C)N1[C@@H](CC(=C)C1)c1[nH]c(cn1)c1cc2c(cc1)cc1c(c2)ccc(c1)c1[nH]c(nc1)[C@H	1311398-44-3	Carbamic acid, N-[(1S)-1-[[(2S)-2-[5-[6-[2-[(2S)-1-[(2S)-2-[(methoxycarbonyl)ami	C39H48N8O6	724.85	A/B	-8.4
5903	O=c1n([C@@H]2O[C@H](CO)[C@@H](Cl)C2)cc(C)c(N)n1	1311398-60-3	Carbamic acid, N-[(1S)-1-[[(2S)-2-[5-[2-[6-[2-[(2S)-1-[(2S)-2-[(methoxycarbonyl)	C41H48N8O6	748.87	A/B	-8.4
5904	C(=O)([C@@H](NC(=O)OC)C1CCC1)N1[C@H]2[C@H](C2)C[C@H]1c1[nH]c(c(Cl)n1)c1ccc(cc1)c1ccc(cc1)c1[nH]c(nc1	1312336-14-3	Carbamic acid, N-[(1S)-2-[(1R,3S,5R)-3-[5-chloro-4-[4,Ä≤-[5-chloro-2-[(1R,3S,5R)	C44H48Cl2N8O7	871.81	A/B	-8.4
5905	C(=O)([C@@H](NC(=O)OC)C1CCC(F)(F)CC1)N1[C@H]2[C@H](C2)C[C@H]1c1[nH]c(c(Cl)n1)c1ccc(cc1)c1ccc(cc1)c1[	1312336-33-6	Carbamic acid, N,N,Ä≤-[[1,1,Ä≤-biphenyl]-4,4,Ä≤-diylbis[(5-chloro-1H-imidazole-	C44H56Cl2N8O6	863.87	A/B	-8.4
5906	C(=O)([C@@H](NC(=O)OC)C1CCC(F)(F)CC1)N1[C@H]2[C@H](C2)C[C@H]1c1[nH]c(c(Cl)n1)c1ccc(cc1)c1ccc(cc1)c1[	1312336-37-0	Carbamic acid, N,N,Ä≤-[[1,1,Ä≤-biphenyl]-4,4,Ä≤-diylbis[(5-chloro-1H-imidazole-	C48H52Cl2F4N8O6	983.88	A/B	-8.4
5907	C(=O)([C@@H](NC(=O)OC)C(C)C)N1[C@@H](CCC1)c1[nH]c(c(I)n1)c1ccc(cc1)c1ccc(cc1)c1[nH]c(nc1)[C@H]1N(C(=	1312336-48-3	Carbamic acid, N-[(1S)-1-[[(2S)-2-[5-[4,Ä≤-[5-bromo-2-[(2S)-1-[(2S)-2-[(methoxy	C40H49BrN8O6	817.77	A/B	-8.4
5908	Cn1c(c2c([nH]c(n2)[C@H]2N(C(=O)[C@@H](NC(=O)OC)C(C)C)CCC2)c2ccc(cc2)c2ccc(cc2)c2[nH]c(nc2)[C@H]2N(C(	1312336-61-0	Carbamic acid, N-[(1S)-1-[[(2S)-2-[5-[4,Ä≤-[2-[(2S)-1-[(2S)-2-[(methoxycarbonyl)	C44H56N8O6	792.97	A/B	-8.4
5909	C(=O)([C@@H](NC(=O)OC)C(C)C)N1[C@@H](CC[C@@H]1C)c1[nH]c(c(F)n1)c1ccc(cc1)c1ccc(cc1)c1[nH]c(nc1)[C@H]	1312336-64-3	Carbamic acid, N-[(1S)-2-[(1R,3S,5R)-3-[5-[4,Ä≤-[5-fluoro-2-[(1R,3S,5R)-2-[(2S)-	C46H53FN8O8	864.96	A/B	-8.4
5910	C(=O)([C@@H](NC(=O)OC)C1CCOCC1)N1[C@@H](C[C@H](C)C1)c1[nH]c(cn1)c1ccc(cc1)c1ccc(cc1)c1[nH]c(nc1)[C@H	1312338-32-1	Carbamic acid, N,N,Ä≤-[[1,1,Ä≤-biphenyl]-4,4,Ä≤-diylbis[1H-imidazole-5,2-diyl[(	C46H58N8O8	851	A/B	-8.4
5911	Cc1c2c(c(ccc2c2ccc(cc2)c2[nH]c(nc2)[C@H]2N(C(=O)[C@@H](NC(=O)OC)C(C)C)CCC2)c2[nH]c(nc2)[C@@]2(C)N(C(	1312608-76-6	Carbamic acid, N-[(1S)-1-[[(2S)-2-[5-[4-[7-[2-[(2S)-1-[(2S)-2-[(methoxycarbonyl)	C44H55N9O6	805.96	A/B	-8.4
5912	C[C@]1(c2[nH]c(c3c4c(c(cc3)c3ccc(cc3)c3[nH]c(nc3)[C@H]3N(C(=O)[C@@H](NC(=O)OC)C(C)C)CCC3)cccc4)cn2)N	1312608-78-8	Carbamic acid, N-[(1S)-1-[[(2S)-2-[5-[2,3-dihydro-7-[4-[2-[(2S)-1-[(2S)-2-[(meth	C44H56N8O6	792.97	A/B	-8.4
5913	C(=O)([C@@H](NC(=O)OC)C(C)C)N1[C@@H](CCC1)c1[nH]c(cn1)c1c(N)c(N)c(cc1)c1ccc(cc1)c1[nH]c(nc1)[C@H]1N(	1312610-46-0	Carbamic acid, N-[(1S)-1-[[(2S)-2-[5-[4-[7-[2-[(2S)-1-[(2S)-2-[(methoxycarbonyl)	C40H48N10O6S	796.94	A/B	-8.4
5914	O(Cc1ccc(F)cc1)c1c2c(oc(c(O)c2=O)c2[nH]cc(Cl)n2)ccc1	1312892-18-4	4H-1-Benzopyran-4-one, 5-[(4-fluorophenyl)methoxy]-3-hydroxy-2-(5-methoxy-1H-imi	C20H15FN2O5	382.34	A/B	-8.4
5915	O(Cc1ccc(F)cc1)c1c2c(oc(c(O)c2=O)c2[nH]cc(C)n2)c(C)cc1	1312892-72-0	4H-1-Benzopyran-4-one, 5-[(4-fluorophenyl)methoxy]-3-hydroxy-2-(1H-imidazol-2-yl	C20H15FN2O4	366.34	A/B	-8.4
5916	O(Cc1ccc(F)cc1)c1c2c(oc(c(O)c2=O)c2[nH]cc(Cl)n2)c(C)cc1	1312892-78-6	4H-1-Benzopyran-4-one, 5-[(4-fluorophenyl)methoxy]-3-hydroxy-2-(5-methoxy-1H-imi	C21H17FN2O5	396.37	A/B	-8.4
5917	O(Cc1ccc(F)cc1)c1c2c(oc(c(O)c2=O)c2[nH]c(C)nn2)c(OC)cc1	1312893-43-8	4H-1-Benzopyran-4-one, 5-[(4-fluorophenyl)methoxy]-3-hydroxy-8-methoxy-2-(1H-1,2	C19H14FN3O5	383.33	A/B	-8.4
5918	O(Cc1ccc(F)cc1)c1c2c(oc(c(O)c2=O)c2[nH]c(cn2)c2ccccc2)c(OC)cc1	1312893-51-8	4H-1-Benzopyran-4-one, 2-(5-chloro-1H-imidazol-2-yl)-5-[(4-fluorophenyl)methoxy]	C20H14ClFN2O5	416.79	A/B	-8.4
5919	O(Cc1ccc(F)cc1)c1c2c(oc(c(O)c2=O)c2ncc(Cl)cn2)c(Cl)cc1	1312893-85-8	4H-1-Benzopyran-4-one, 8-chloro-5-[(4-fluorophenyl)methoxy]-3-hydroxy-2-(5-metho	C21H14ClFN2O5	428.8	A/B	-8.4
5920	O(Cc1ccc(F)cc1)c1c2c(oc(c(O)c2=O)c2ncc(cn2)c2ccccc2)cc(C)c1	1312894-86-2	4H-1-Benzopyran-4-one, 2-(5-chloro-2-pyrimidinyl)-5-[(4-fluorophenyl)methoxy]-3-	C21H14ClFN2O4	412.8	A/B	-8.4
5921	O(Cc1ccc(F)cc1)c1c2c(oc(c(O)c2=O)c2[nH]cnn2)cc(C)c1	1312894-87-3	4H-1-Benzopyran-4-one, 5-[(4-fluorophenyl)methoxy]-3-hydroxy-7-methyl-2-(5-pheny	C27H19FN2O4	454.45	A/B	-8.4
5922	O(Cc1ccc(F)cc1)c1c2c(oc(c(O)c2=O)c2[nH]c(OC)cn2)cc(C)c1	1312894-99-7	4H-1-Benzopyran-4-one, 5-[(4-fluorophenyl)methoxy]-3-hydroxy-7-methyl-2-(5-methy	C21H17FN2O4	380.37	A/B	-8.4
5923	O(Cc1ccc(F)cc1)c1c2c(c(OC)c(C)c1)oc(c(O)c2=O)c1[nH]c(Cl)nn1	1312895-95-6	4H-1-Benzopyran-4-one, 5-[(4-fluorophenyl)methoxy]-3-hydroxy-8-methoxy-2-(5-meth	C21H18FN3O6	427.38	A/B	-8.4
5924	O(Cc1ccc(F)cc1)c1c2c(oc(c(O)c2=O)c2ncc(Cl)cn2)c(Cl)c(C)c1	1312896-35-7	4H-1-Benzopyran-4-one, 8-chloro-5-[(4-fluorophenyl)methoxy]-3-hydroxy-2-(5-metho	C22H16ClFN2O5	442.82	A/B	-8.4
5925	O(Cc1ccc(F)cc1)c1c2c(oc(c(O)c2=O)c2[nH]cnn2)c(Cl)c(C)c1	1312896-37-9	4H-1-Benzopyran-4-one, 8-chloro-5-[(4-fluorophenyl)methoxy]-3-hydroxy-7-methyl-2	C27H18ClFN2O4	488.89	A/B	-8.4
5926	O(Cc1ccc(F)cc1)c1c2c(oc(c(O)c2=O)c2ncc(OC)cn2)cc(OC)c1	1312897-45-2	4H-1-Benzopyran-4-one, 5-[(4-fluorophenyl)methoxy]-3-hydroxy-7-methoxy-2-(5-meth	C22H17FN2O5	408.38	A/B	-8.4
5927	O(Cc1ccc(F)cc1)c1c2c(oc(c(O)c2=O)c2[nH]c(nn2)c2ccccc2)cc(OC)c1	1312897-57-6	4H-1-Benzopyran-4-one, 2-(5-chloro-1H-1,2,4-triazol-3-yl)-5-[(4-fluorophenyl)met	C19H13ClFN3O5	417.77	A/B	-8.4
5928	O(Cc1ccc(F)cc1)c1c2c(oc(c(O)c2=O)c2ncc(OC)cn2)c(C)c(OC)c1	1312897-95-2	4H-1-Benzopyran-4-one, 5-[(4-fluorophenyl)methoxy]-3-hydroxy-7-methoxy-8-methyl-	C23H19FN2O5	422.41	A/B	-8.4
5929	O(Cc1ccc(F)cc1)c1c2c(oc(c(O)c2=O)c2[nH]cnn2)c(C)c(OC)c1	1312897-98-5	4H-1-Benzopyran-4-one, 5-[(4-fluorophenyl)methoxy]-3-hydroxy-7-methoxy-8-methyl-	C28H21FN2O5	484.48	A/B	-8.4
5930	O(Cc1ccc(F)cc1)c1c2c(oc(c(O)c2=O)c2[nH]c(OC)nn2)c(C)c(OC)c1	1312898-05-7	4H-1-Benzopyran-4-one, 5-[(4-fluorophenyl)methoxy]-3-hydroxy-7-methoxy-8-methyl-	C21H18FN3O5	411.38	A/B	-8.4
5931	O(Cc1ccc(F)cc1)c1c2c(oc(c(O)c2=O)c2[nH]c(Cl)nn2)c(C)c(OC)c1	1312898-06-8	4H-1-Benzopyran-4-one, 5-[(4-fluorophenyl)methoxy]-3-hydroxy-7-methoxy-2-(5-meth	C21H18FN3O6	427.38	A/B	-8.4
5932	O(Cc1ccc(F)cc1)c1c2c(oc(c(O)c2=O)c2[nH]c(C)cn2)c(C)c(OC)c1	1312898-09-1	4H-1-Benzopyran-4-one, 5-[(4-fluorophenyl)methoxy]-3-hydroxy-2-(1H-imidazol-2-yl	C21H17FN2O5	396.37	A/B	-8.4
5933	O(Cc1ccc(F)cc1)c1c2c(oc(c(O)c2=O)c2[nH]c(Cl)cn2)c(C)c(OC)c1	1312898-11-5	4H-1-Benzopyran-4-one, 5-[(4-fluorophenyl)methoxy]-3-hydroxy-7-methoxy-2-(5-meth	C22H19FN2O6	426.39	A/B	-8.4
5934	O(Cc1ccc(F)cc1)c1c2c(oc(c(O)c2=O)c2[nH]c(cn2)c2ccccc2)c(C)c(OC)c1	1312898-12-6	4H-1-Benzopyran-4-one, 2-(5-chloro-1H-imidazol-2-yl)-5-[(4-fluorophenyl)methoxy]	C21H16ClFN2O5	430.81	A/B	-8.4
5935	O(Cc1ccc(F)cc1)c1c2c(c(OC)c(OC)c1)oc(c(O)c2=O)c1[nH]cnn1	1312898-49-9	4H-1-Benzopyran-4-one, 5-[(4-fluorophenyl)methoxy]-3-hydroxy-7,8-dimethoxy-2-(5-	C28H21FN2O6	500.47	A/B	-8.4
5936	O(Cc1ccc(F)cc1)c1c2c(oc(c(O)c2=O)c2ncccn2)c(Cl)c(OC)c1	1312898-64-8	4H-1-Benzopyran-4-one, 5-[(4-fluorophenyl)methoxy]-3-hydroxy-7,8-dimethoxy-2-(5-	C27H21FN2O6	488.46	A/B	-8.4
5937	O(Cc1ccc(F)cc1)c1c2c(oc(c(O)c2=O)c2ncc(OC)cn2)c(Cl)c(OC)c1	1312898-96-6	4H-1-Benzopyran-4-one, 8-chloro-5-[(4-fluorophenyl)methoxy]-3-hydroxy-7-methoxy-	C22H16ClFN2O5	442.82	A/B	-8.4
5938	O(Cc1ccc(F)cc1)c1c2c(oc(c(O)c2=O)c2ncc(cn2)c2ccccc2)c(Cl)c(OC)c1	1312898-98-8	4H-1-Benzopyran-4-one, 8-chloro-2-(5-chloro-2-pyrimidinyl)-5-[(4-fluorophenyl)me	C21H13Cl2FN2O5	463.24	A/B	-8.4
5939	O(Cc1ccc(F)cc1)c1c2c(oc(c(O)c2=O)c2[nH]cnn2)c(Cl)c(OC)c1	1312898-99-9	4H-1-Benzopyran-4-one, 8-chloro-5-[(4-fluorophenyl)methoxy]-3-hydroxy-7-methoxy-	C27H18ClFN2O5	504.89	A/B	-8.4
5940	O(Cc1ccc(F)cc1)c1c2c(oc(c(O)c2=O)c2[nH]c(OC)nn2)c(Cl)c(OC)c1	1312899-06-1	4H-1-Benzopyran-4-one, 8-chloro-5-[(4-fluorophenyl)methoxy]-3-hydroxy-7-methoxy-	C20H15ClFN3O5	431.8	A/B	-8.4
5941	O(Cc1ccc(F)cc1)c1c2c(oc(c(O)c2=O)c2[nH]c(Cl)nn2)c(Cl)c(OC)c1	1312899-07-2	4H-1-Benzopyran-4-one, 8-chloro-5-[(4-fluorophenyl)methoxy]-3-hydroxy-7-methoxy-	C20H15ClFN3O6	447.8	A/B	-8.4
5942	O(Cc1ccc(F)cc1)c1c2c(oc(c(O)c2=O)c2[nH]c(C)cn2)c(Cl)c(OC)c1	1312899-10-7	4H-1-Benzopyran-4-one, 8-chloro-5-[(4-fluorophenyl)methoxy]-3-hydroxy-2-(1H-imic	C20H14ClFN2O5	416.79	A/B	-8.4
5943	O(Cc1ccc(F)cc1)c1c2c(oc(c(O)c2=O)c2[nH]c(Cl)cn2)c(Cl)c(OC)c1	1312899-12-9	4H-1-Benzopyran-4-one, 8-chloro-5-[(4-fluorophenyl)methoxy]-3-hydroxy-7-methoxy-	C21H16ClFN2O6	446.81	A/B	-8.4
5944	O(C)c1c(c2c(c(OCc3ccc(F)cc3)c1)c(=O)c(O)c(o2)c1ncc(Cl)cn1)c1ccccc1	1312899-47-0	4H-1-Benzopyran-4-one, 5-[(4-fluorophenyl)methoxy]-3-hydroxy-7-methoxy-2-(5-meth	C28H21FN2O6	500.47	A/B	-8.4
5945	O(C)c1c(c2c(c(OCc3ccc(F)cc3)c1)c(=O)c(O)c(o2)c1[nH]cc(C)n1)c1ccccc1	1312899-60-7	4H-1-Benzopyran-4-one, 5-[(4-fluorophenyl)methoxy]-3-hydroxy-2-(1H-imidazol-2-yl	C26H19FN2O5	458.44	A/B	-8.4
5946	O(Cc1ccc(F)cc1)c1c2c(oc(c(O)c2=O)c2ncc(cn2)c2ccccc2)cc(Cl)c1	1312900-57-4	4H-1-Benzopyran-4-one, 7-chloro-2-(5-chloro-2-pyrimidinyl)-5-[(4-fluorophenyl)me	C20H11Cl2FN2O4	433.22	A/B	-8.4
5947	O(Cc1ccc(F)cc1)c1c2c(oc(c(O)c2=O)c2[nH]cnn2)cc(Cl)c1	1312900-58-5	4H-1-Benzopyran-4-one, 7-chloro-5-[(4-fluorophenyl)methoxy]-3-hydroxy-2-(5-pheny	C26H16ClFN2O4	474.87	A/B	-8.4
5948	O(Cc1ccc(F)cc1)c1c2c(oc(c(O)c2=O)c2nnc(C)[nH]2)cc(Cl)c1	1312900-64-3	4H-1-Benzopyran-4-one, 7-chloro-5-[(4-fluorophenyl)methoxy]-3-hydroxy-2-(1H-1,2,	C18H11ClFN3O4	387.75	A/B	-8.4
5949	O(Cc1ccc(F)cc1)c1c2c(oc(c(O)c2=O)c2[nH]cc(C)n2)cc(Cl)c1	1312900-69-8	4H-1-Benzopyran-4-one, 7-chloro-5-[(4-fluorophenyl)methoxy]-3-hydroxy-2-(1H-imic	C19H12ClFN2O4	386.76	A/B	-8.4
5950	O(Cc1ccc(F)cc1)c1c2c(oc(c(O)c2=O)c2ncc(C)cn2)c(C)c(Cl)c1	1312901-04-4	4H-1-Benzopyran-4-one, 7-chloro-5-[(4-fluorophenyl)methoxy]-3-hydroxy-8-methyl-2	C21H14ClFN2O4	412.8	A/B	-8.4
5951	O(Cc1ccc(F)cc1)c1c2c(oc(c(O)c2=O)c2[nH]cnn2)c(C)c(Cl)c1	1312901-08-8	4H-1-Benzopyran-4-one, 7-chloro-5-[(4-fluorophenyl)methoxy]-3-hydroxy-8-methyl-2	C27H18ClFN2O4	488.89	A/B	-8.4
5952	O(Cc1ccc(F)cc1)c1c2c(oc(c(O)c2=O)c2ncc(C)cn2)c(Cl)c(Cl)c1	1312902-04-7	4H-1-Benzopyran-4-one, 7,8-dichloro-5-[(4-fluorophenyl)methoxy]-3-hydroxy-2-(2-p	C20H11Cl2FN2O4	433.22	A/B	-8.4
5953	O(Cc1ccc(F)cc1)c1c2c(c(c(Cl)c1)c1ccccc1)oc(c(O)c2=O)c1ncc(cn1)c1ccccc1	1312902-57-0	4H-1-Benzopyran-4-one, 7-chloro-2-(5-chloro-2-pyrimidinyl)-5-[(4-fluorophenyl)me	C26H15Cl2FN2O4	509.31	A/B	-8.4
5954	O(C)c1c2c(c(OCc3ccc(F)cc3)cc1c1ccccc1)c(=O)c(O)c(o2)c1ncc(cn1)c1ccccc1	1312904-29-2	4H-1-Benzopyran-4-one, 2-(5-chloro-2-pyrimidinyl)-5-[(4-fluorophenyl)methoxy]-3-	C27H18ClFN2O5	504.89	A/B	-8.4
5955	O(C)c1c2c(c(OCc3ccc(F)cc3)cc1c1ccccc1)c(=O)c(O)c(o2)c1nnc(Cl)[nH]1	1312904-45-2	4H-1-Benzopyran-4-one, 5-[(4-fluorophenyl)methoxy]-3-hydroxy-8-methoxy-2-(5-meth	C26H20FN3O6	489.45	A/B	-8.4
5956	O(C)c1c2c(c(OCc3ccc(F)cc3)cc1c1ccccc1)c(=O)c(O)c(o2)c1[nH]cc(Cl)n1	1312904-52-1	4H-1-Benzopyran-4-one, 5-[(4-fluorophenyl)methoxy]-3-hydroxy-8-methoxy-2-(5-meth	C27H21FN2O6	488.46	A/B	-8.4
5957	O(C)c1c2c(c(OCc3ccc(F)cc3)cc1c1ccccc1)c(=O)c(O)c(o2)c1[nH]c(cn1)c1ccccc1	1312904-53-2	4H-1-Benzopyran-4-one, 2-(5-chloro-1H-imidazol-2-yl)-5-[(4-fluorophenyl)methoxy]	C26H18ClFN2O5	492.88	A/B	-8.4
5958	O(Cc1ccc(F)cc1)c1c2c(c(c(c1)c1ccccc1)c1ccccc1)oc(c(O)c2=O)c1ncc(OC)cn1	1312905-67-1	4H-1-Benzopyran-4-one, 5-[(4-fluorophenyl)methoxy]-3-hydroxy-2-(5-methyl-2-pyrim	C33H23FN2O4	530.55	A/B	-8.4
5959	O(Cc1ccc(F)cc1)c1c2c(c(c(c1)c1ccccc1)c1ccccc1)oc(c(O)c2=O)c1[nH]c(OC)cn1	1312905-82-0	4H-1-Benzopyran-4-one, 5-[(4-fluorophenyl)methoxy]-3-hydroxy-2-(5-methyl-1H-imic	C32H23FN2O4	518.53	A/B	-8.4
5960	O=c1c2c(oc(c1O)c1ncc(OC)cn1)ccc(OCc1ccc(F)cc1)c2	1312906-16-3	4H-1-Benzopyran-4-one, 6-[(4-fluorophenyl)methoxy]-3-hydroxy-2-(5-methyl-2-pyrim	C21H15FN2O4	378.35	A/B	-8.4
5961	Oc1c(oc2c(c1=O)cc(OCc1ccc(F)cc1)cc2)c1[nH]c(cn1)c1ccccc1	1312906-32-3	4H-1-Benzopyran-4-one, 2-(5-chloro-1H-imidazol-2-yl)-6-[(4-fluorophenyl)methoxy]	C19H12ClFN2O4	386.76	A/B	-8.4
5962	Cc1c2c(cc(OCc3ccc(F)cc3)c1)c(=O)c(O)c(o2)c1[nH]c(OC)nn1	1312906-75-4	4H-1-Benzopyran-4-one, 6-[(4-fluorophenyl)methoxy]-3-hydroxy-8-methyl-2-(3-methy	C20H16FN3O4	381.36	A/B	-8.4
5963	O(C)c1c2c(c(=O)c(O)c(o2)c2[nH]c(OC)cn2)cc(OCc2ccc(F)cc2)c1	1312907-30-4	4H-1-Benzopyran-4-one, 6-[(4-fluorophenyl)methoxy]-3-hydroxy-8-methoxy-2-(5-meth	C21H17FN2O5	396.37	A/B	-8.4
5964	O=c1c2c(oc(c1O)c1ncc(Cl)cn1)c(Cl)cc(OCc1ccc(F)cc1)c2	1312907-66-6	4H-1-Benzopyran-4-one, 8-chloro-6-[(4-fluorophenyl)methoxy]-3-hydroxy-2-(5-metho	C21H14ClFN2O5	428.8	A/B	-8.4
5965	O=c1c2c(oc(c1O)c1ncc(cn1)c1ccccc1)c(Cl)cc(OCc1ccc(F)cc1)c2	1312907-67-7	4H-1-Benzopyran-4-one, 8-chloro-2-(5-chloro-2-pyrimidinyl)-6-[(4-fluorophenyl)me	C20H11Cl2FN2O4	433.22	A/B	-8.4
5966	O=c1c2c(oc(c1O)c1ncc(C)cn1)cc(C)c(OCc1ccc(F)cc1)c2	1312908-64-7	4H-1-Benzopyran-4-one, 6-[(4-fluorophenyl)methoxy]-3-hydroxy-7-methyl-2-(2-pyrim	C21H15FN2O4	378.35	A/B	-8.4

5967	O=c1c2c(oc(c1O)c1ncc(OC)cn1)cc(C)c(OCc1ccc(F)cc1)c2	1312908-65-8	4H-1-Benzopyran-4-one, 6-[(4-fluorophenyl)methoxy]-3-hydroxy-7-methyl-2-(5-methy	C22H17FN2O4	392.38	A/B	-8.4
5968	O=c1c2c(oc(c1O)c1[nH]cnn1)cc(C)c(OCc1ccc(F)cc1)c2	1312908-68-1	4H-1-Benzopyran-4-one, 6-[(4-fluorophenyl)methoxy]-3-hydroxy-7-methyl-2-(5-pheny	C27H19FN2O4	454.45	A/B	-8.4
5969	O=c1c2c(oc(c1O)c1[nH]c(OC)cn1)cc(C)c(OCc1ccc(F)cc1)c2	1312908-80-7	4H-1-Benzopyran-4-one, 6-[(4-fluorophenyl)methoxy]-3-hydroxy-7-methyl-2-(5-methy	C21H17FN2O4	380.37	A/B	-8.4
5970	O=c1c2c(oc(c1O)c1[nH]c(cn1)c1ccccc1)cc(C)c(OCc1ccc(F)cc1)c2	1312908-82-9	4H-1-Benzopyran-4-one, 2-(5-chloro-1H-imidazol-2-yl)-6-[(4-fluorophenyl)methoxy]	C20H14ClFN2O4	400.79	A/B	-8.4
5971	O(C)c1c2c(cc(OCc3ccc(F)cc3)c1C)c(=O)c(O)c(o2)c1[nH]c(C)nn1	1312909-86-6	4H-1-Benzopyran-4-one, 6-[(4-fluorophenyl)methoxy]-3-hydroxy-8-methoxy-7-methyl-	C20H16FN3O5	397.36	A/B	-8.4
5972	O(C)c1c2c(cc(OCc3ccc(F)cc3)c1C)c(=O)c(O)c(o2)c1[nH]c(OC)nn1	1312909-87-7	4H-1-Benzopyran-4-one, 6-[(4-fluorophenyl)methoxy]-3-hydroxy-8-methoxy-7-methyl-	C21H18FN3O5	411.38	A/B	-8.4
5973	O(C)c1c2c(cc(OCc3ccc(F)cc3)c1C)c(=O)c(O)c(o2)c1[nH]c(OC)cn1	1312909-92-4	4H-1-Benzopyran-4-one, 6-[(4-fluorophenyl)methoxy]-3-hydroxy-8-methoxy-7-methyl-	C22H19FN2O5	410.4	A/B	-8.4
5974	Clc1c2c(cc(OCc3ccc(F)cc3)c1C)c(=O)c(O)c(o2)c1ncc(C)cn1	1312910-26-1	4H-1-Benzopyran-4-one, 8-chloro-6-[(4-fluorophenyl)methoxy]-3-hydroxy-7-methyl-2	C21H14ClFN2O4	412.8	A/B	-8.4
5975	Clc1c2c(cc(OCc3ccc(F)cc3)c1C)c(=O)c(O)c(o2)c1[nH]cnn1	1312910-30-7	4H-1-Benzopyran-4-one, 8-chloro-6-[(4-fluorophenyl)methoxy]-3-hydroxy-7-methyl-2	C27H18ClFN2O4	488.89	A/B	-8.4
5976	Cc1c(c2c(cc1OCc1ccc(F)cc1)c(=O)c(O)c(o2)c1ncc(OC)cn1)c1ccccc1	1312910-77-2	4H-1-Benzopyran-4-one, 6-[(4-fluorophenyl)methoxy]-3-hydroxy-7-methyl-2-(5-methy	C28H21FN2O4	468.48	A/B	-8.4
5977	Cc1c(c2c(cc1OCc1ccc(F)cc1)c(=O)c(O)c(o2)c1[nH]c(Cl)nn1)c1ccccc1	1312910-88-5	4H-1-Benzopyran-4-one, 6-[(4-fluorophenyl)methoxy]-3-hydroxy-2-(5-methoxy-1H-1,2	C26H20FN3O5	473.45	A/B	-8.4
5978	O=c1c2c(oc(c1O)c1ncc(C)cn1)cc(OC)c(OCc1ccc(F)cc1)c2	1312911-26-4	4H-1-Benzopyran-4-one, 6-[(4-fluorophenyl)methoxy]-3-hydroxy-7-methoxy-2-(2-pyri	C21H15FN2O5	394.35	A/B	-8.4
5979	O=c1c2c(oc(c1O)c1[nH]cnn1)cc(OC)c(OCc1ccc(F)cc1)c2	1312911-30-0	4H-1-Benzopyran-4-one, 6-[(4-fluorophenyl)methoxy]-3-hydroxy-7-methoxy-2-(5-pher	C27H19FN2O5	470.45	A/B	-8.4
5980	O=c1c2c(oc(c1O)c1[nH]c(cn1)c1ccccc1)cc(OC)c(OCc1ccc(F)cc1)c2	1312911-45-7	4H-1-Benzopyran-4-one, 2-(5-chloro-1H-imidazol-2-yl)-6-[(4-fluorophenyl)methoxy]	C20H14ClFN2O5	416.79	A/B	-8.4
5981	O=c1c2c(oc(c1O)c1ncc(C)cn1)cc(Cl)c(OCc1ccc(F)cc1)c2	1312911-54-8	4H-1-Benzopyran-4-one, 7-chloro-6-[(4-fluorophenyl)methoxy]-3-hydroxy-2-(2-pyrim	C20H12ClFN2O4	398.77	A/B	-8.4
5982	O=c1c2c(oc(c1O)c1ncc(cn1)c1ccccc1)cc(Cl)c(OCc1ccc(F)cc1)c2	1312911-60-6	4H-1-Benzopyran-4-one, 7-chloro-2-(5-chloro-2-pyrimidinyl)-6-[(4-fluorophenyl)me	C20H11Cl2FN2O4	433.22	A/B	-8.4
5983	O=c1c2c(oc(c1O)c1[nH]cc(C)n1)cc(Cl)c(OCc1ccc(F)cc1)c2	1312911-84-4	4H-1-Benzopyran-4-one, 7-chloro-6-[(4-fluorophenyl)methoxy]-3-hydroxy-2-(1H-imic	C19H12ClFN2O4	386.76	A/B	-8.4
5984	O=c1c2c(oc(c1O)c1[nH]cc(Cl)n1)cc(Cl)c(OCc1ccc(F)cc1)c2	1312911-88-8	4H-1-Benzopyran-4-one, 7-chloro-6-[(4-fluorophenyl)methoxy]-3-hydroxy-2-(5-metho	C20H14ClFN2O5	416.79	A/B	-8.4
5985	Cc1c2c(cc(OCc3ccc(F)cc3)c1OC)c(=O)c(O)c(o2)c1ncc(C)cn1	1312912-09-6	4H-1-Benzopyran-4-one, 6-[(4-fluorophenyl)methoxy]-3-hydroxy-7-methoxy-8-methyl-	C22H17FN2O5	408.38	A/B	-8.4
5986	Cc1c2c(cc(OCc3ccc(F)cc3)c1OC)c(=O)c(O)c(o2)c1[nH]cnn1	1312912-17-6	4H-1-Benzopyran-4-one, 6-[(4-fluorophenyl)methoxy]-3-hydroxy-7-methoxy-8-methyl-	C28H21FN2O5	484.48	A/B	-8.4
5987	Cc1c2c(cc(OCc3ccc(F)cc3)c1OC)c(=O)c(O)c(o2)c1[nH]c(C)nn1	1312912-30-3	4H-1-Benzopyran-4-one, 6-[(4-fluorophenyl)methoxy]-3-hydroxy-7-methoxy-8-methyl-	C20H16FN3O5	397.36	A/B	-8.4
5988	Cc1c2c(cc(OCc3ccc(F)cc3)c1OC)c(=O)c(O)c(o2)c1[nH]c(OC)cn1	1312912-42-7	4H-1-Benzopyran-4-one, 6-[(4-fluorophenyl)methoxy]-3-hydroxy-7-methoxy-8-methyl-	C22H19FN2O5	410.4	A/B	-8.4
5989	Cc1c2c(cc(OCc3ccc(F)cc3)c1OC)c(=O)c(O)c(o2)c1[nH]c(cn1)c1ccccc1	1312912-46-1	4H-1-Benzopyran-4-one, 2-(5-chloro-1H-imidazol-2-yl)-6-[(4-fluorophenyl)methoxy]	C21H16ClFN2O5	430.81	A/B	-8.4
5990	Cc1c2c(cc(OCc3ccc(F)cc3)c1Cl)c(=O)c(O)c(o2)c1[nH]c(C)nn1	1312912-75-6	4H-1-Benzopyran-4-one, 7-chloro-6-[(4-fluorophenyl)methoxy]-3-hydroxy-8-methyl-2	C19H13ClFN3O4	401.78	A/B	-8.4
5991	O(C)c1c2c(cc(OCc3ccc(F)cc3)c1Cl)c(=O)c(O)c(o2)c1[nH]c(C)nn1	1312913-75-9	4H-1-Benzopyran-4-one, 7-chloro-6-[(4-fluorophenyl)methoxy]-3-hydroxy-8-methoxy-	C19H13ClFN3O5	417.77	A/B	-8.4
5992	O(C)c1c2c(cc(OCc3ccc(F)cc3)c1Cl)c(=O)c(O)c(o2)c1[nH]c(OC)nn1	1312913-77-1	4H-1-Benzopyran-4-one, 7-chloro-6-[(4-fluorophenyl)methoxy]-3-hydroxy-8-methoxy-	C20H15ClFN3O5	431.8	A/B	-8.4
5993	Clc1c2c(cc(OCc3ccc(F)cc3)c1OC)c(=O)c(O)c(o2)c1ncc(C)cn1	1312914-10-5	4H-1-Benzopyran-4-one, 8-chloro-6-[(4-fluorophenyl)methoxy]-3-hydroxy-7-methoxy-	C21H14ClFN2O5	428.8	A/B	-8.4
5994	Clc1c2c(cc(OCc3ccc(F)cc3)c1OC)c(=O)c(O)c(o2)c1[nH]c(nn1)c1ccccc1	1312914-36-5	4H-1-Benzopyran-4-one, 8-chloro-2-(5-chloro-1H-1,2,4-triazol-3-yl)-6-[(4-fluorop	C19H12Cl2FN3O5	452.22	A/B	-8.4
5995	Clc1c2c(cc(OCc3ccc(F)cc3)c1Cl)c(=O)c(O)c(o2)c1ncc(Cl)cn1	1312914-59-2	4H-1-Benzopyran-4-one, 7,8-dichloro-6-[(4-fluorophenyl)methoxy]-3-hydroxy-2-(5-m	C21H13Cl2FN2O5	463.24	A/B	-8.4
5996	O(C)c1c(c2c(cc1OCc1ccc(F)cc1)c(=O)c(O)c(o2)c1[nH]cnn1)c1ccccc1	1312915-20-0	4H-1-Benzopyran-4-one, 6-[(4-fluorophenyl)methoxy]-3-hydroxy-7-methoxy-8-phenyl-	C33H23FN2O5	546.54	A/B	-8.4
5997	O(C)c1c(c2c(cc1OCc1ccc(F)cc1)c(=O)c(O)c(o2)c1[nH]c(OC)cn1)c1ccccc1	1312915-47-1	4H-1-Benzopyran-4-one, 6-[(4-fluorophenyl)methoxy]-3-hydroxy-7-methoxy-2-(5-meth	C27H21FN2O5	472.46	A/B	-8.4
5998	Clc1c(c2c(cc1OCc1ccc(F)cc1)c(=O)c(O)c(o2)c1[nH]c(Cl)nn1)c1ccccc1	1312915-84-6	4H-1-Benzopyran-4-one, 7-chloro-6-[(4-fluorophenyl)methoxy]-3-hydroxy-2-(5-metho	C25H17ClFN3O5	493.87	A/B	-8.4
5999	Clc1c(c2c(cc1OCc1ccc(F)cc1)c(=O)c(O)c(o2)c1[nH]c(cn1)c1ccccc1)c1ccccc1	1312915-96-0	4H-1-Benzopyran-4-one, 7-chloro-2-(5-chloro-1H-imidazol-2-yl)-6-[(4-fluorophenyl	C25H15Cl2FN2O4	497.3	A/B	-8.4
6000	O(Cc1ccc(F)cc1)c1c(cc2c(c1)c(=O)c(O)c(o2)c1[nH]c(Cl)nn1)c1ccccc1	1312916-43-0	4H-1-Benzopyran-4-one, 6-[(4-fluorophenyl)methoxy]-3-hydroxy-2-(5-methoxy-1H-1,2	C25H18FN3O5	459.43	A/B	-8.4
6001	O=c1c2c(cc(c(OCc3ccc(F)cc3)c2)c2ccccc2)oc(c1O)c1[nH]c(nn1)c1ccccc1	1312916-44-1	4H-1-Benzopyran-4-one, 2-(5-chloro-1H-1,2,4-triazol-3-yl)-6-[(4-fluorophenyl)met	C24H15ClFN3O4	463.85	A/B	-8.4
6002	O(Cc1ccc(F)cc1)c1c(cc2c(c1)c(=O)c(O)c(o2)c1[nH]c(Cl)cn1)c1ccccc1	1312916-48-5	4H-1-Benzopyran-4-one, 6-[(4-fluorophenyl)methoxy]-3-hydroxy-2-(5-methoxy-1H-imi	C26H19FN2O5	458.44	A/B	-8.4
6003	O(Cc1ccc(F)cc1)c1c(c(C)c2c(c1)c(=O)c(O)c(o2)c1ncc(OC)cn1)c1ccccc1	1312916-83-8	4H-1-Benzopyran-4-one, 6-[(4-fluorophenyl)methoxy]-3-hydroxy-8-methyl-2-(5-methy	C28H21FN2O4	468.48	A/B	-8.4
6004	O(C)c1c2c(cc(OCc3ccc(F)cc3)c1c1ccccc1)c(=O)c(O)c(o2)c1[nH]ccn1	1312917-46-6	4H-1-Benzopyran-4-one, 6-[(4-fluorophenyl)methoxy]-3-hydroxy-8-methoxy-7-phenyl-	C31H22FN3O5	535.52	A/B	-8.4
6005	O(C)c1c(c(OCc2ccc(F)cc2)cc2c1oc(c(O)c2=O)c1[nH]c(Cl)cn1)c1ccccc1	1312917-49-9	4H-1-Benzopyran-4-one, 6-[(4-fluorophenyl)methoxy]-3-hydroxy-8-methoxy-2-(5-meth	C27H21FN2O6	488.46	A/B	-8.4
6006	O(Cc1ccc(F)cc1)c1c(c(Cl)c2c(c1)c(=O)c(O)c(o2)c1ncccn1)c1ccccc1	1312917-51-3	4H-1-Benzopyran-4-one, 6-[(4-fluorophenyl)methoxy]-3-hydroxy-8-methoxy-7-phenyl-	C32H23FN2O5	534.53	A/B	-8.4
6007	O(Cc1ccc(F)cc1)c1c(c(Cl)c2c(c1)c(=O)c(O)c(o2)c1ncc(OC)cn1)c1ccccc1	1312917-83-1	4H-1-Benzopyran-4-one, 8-chloro-6-[(4-fluorophenyl)methoxy]-3-hydroxy-2-(5-methy	C27H18ClFN2O4	488.89	A/B	-8.4
6008	O(Cc1ccc(F)cc1)c1c(c(Cl)c2c(c1)c(=O)c(O)c(o2)c1[nH]cnn1)c1ccccc1	1312917-86-4	4H-1-Benzopyran-4-one, 8-chloro-6-[(4-fluorophenyl)methoxy]-3-hydroxy-7-phenyl-2	C32H20ClFN2O4	550.96	A/B	-8.4
6009	O(Cc1ccc(F)cc1)c1c(c(c2c(c1)c(=O)c(O)c(o2)c1ncc(C)cn1)c1ccccc1)c1ccccc1	1312918-32-3	4H-1-Benzopyran-4-one, 6-[(4-fluorophenyl)methoxy]-3-hydroxy-7,8-diphenyl-2-(2-p	C32H21FN2O4	516.52	A/B	-8.4
6010	O(Cc1ccc(F)cc1)c1c(c(c2c(c1)c(=O)c(O)c(o2)c1[nH]c(Cl)nn1)c1ccccc1)c1ccccc1	1312918-44-7	4H-1-Benzopyran-4-one, 6-[(4-fluorophenyl)methoxy]-3-hydroxy-2-(5-methoxy-1H-1,2	C31H22FN3O5	535.52	A/B	-8.4
6011	O(Cc1ccc(F)cc1)c1c(c(c2c(c1)c(=O)c(O)c(o2)c1[nH]c(C)cn1)c1ccccc1)c1ccccc1	1312918-47-0	4H-1-Benzopyran-4-one, 6-[(4-fluorophenyl)methoxy]-3-hydroxy-2-(1H-imidazol-2-yl	C31H21FN2O4	504.51	A/B	-8.4
6012	C(=O)([C@@H](NC(=O)OC)C(C)C)N1[C@@H](C[C@H](C)C1)c1[nH]c(C#Cc2ccc(C#Cc3[nH]c(nc3)[C@H]3N(C(=O)[C@H](	1313207-70-3	Carbamic acid, N,N,Ä≤-[1,4-phenylenebis[2,1-ethynediyl-1H-imidazole-5,2-diyl[(2S	C40H50N8O6	738.88	A/B	-8.4
6013	N(=C1c2c(N(Cc3ccc(F)cc3)C1=O)ccc(F)c2)NC(=S)Nc1ccc(Cl)cc1	1323295-74-4	Hydrazinecarbothioamide, 2-[5-fluoro-1-[(4-fluorophenyl)methyl]-1,2-dihydro-2-ox	C23H18F2N4OS	436.48	A/B	-8.4
6014	S(Cc1cc2c(oc1=O)cccc2)c1n(c2c(n1)c(=O)nc(N)[nH]2)[C@@H]1O[C@H](CO)[C@@H](O)[C@H]1O	1328839-28-6	1,3,4-Oxadiazole-2-carboxylic acid, 5-methyl-, 4-[[[(4-fluorophenyl)methyl]aminc	C24H23FN8O7	554.49	A/B	-8.4
6015	N(Cc1cc(Cl)ccc1)c1nc(nc(NN=Cc2c(O)cccc2)n1)N1CCOCC1	1331749-50-8	Benzaldehyde, 2-hydroxy-, 2-[4-[[(4-fluorophenyl)methyl]amino]-6-(4-morpholinyl)	C21H22FN7O2	423.44	A/B	-8.4
6016	N(c1nc(nc(NN=Cc2c(C)[nH]cc2)n1)N1CCOCC1)c1ccc(F)cc1	1331749-52-0	Benzaldehyde, 2-hydroxy-, 2-[4-[[(3-chlorophenyl)methyl]amino]-6-(4-morpholinyl)	C21H22ClN7O2	439.9	A/B	-8.4
6017	N(c1c(C)c(c(/C=C/C#N)c(c1C)[2H])[2H])c1nc(Nc2ccc(C#N)cc2)nc(c1[2H])[2H]	1331866-39-7	Benzonitrile, 4-[[4-[[4-[(1E)-2-cyanoethenyl]-2,6-di(methyl-d3)phenyl-3,5-d2]ami	C22H10D8N6		A/B	-8.4
6018	N(c1c(C)c(c(/C=C/C#N)c(c1C)[2H])[2H])c1nc(Nc2c(cc(C#N)cc2[2H])[2H])nc(c1[2H])[2H]	1331866-45-5	Benzonitrile-3,5-d2, 4-[[4-[[4-[(1E)-2-cyanoethenyl]-2,6-di(methyl-d3)phenyl-3,5	C22H8D10N6		A/B	-8.4
6019	N(c1c(C([2H])([2H])[2H])c(c(/C(=C(/C#N)\[2H])/[2H])c(c1C([2H])([2H])[2H])[2H])[2H])c1nc(Nc2c(c(c(C#N	1331866-86-4	Benzonitrile-2,3,5,6-d4, 4-[[6-[[4-[(1E)-2-cyanoethenyl-1,2-d2]-2-methyl-6-(meth	C22H5D13N6		A/B	-8.4
6020	N(c1c(C([2H])([2H])[2H])cc(/C(=C(/C#N)\[2H])/[2H])cc1C([2H])([2H])[2H])c1nc(Nc2c(cc(C#N)cc2[2H])[2H]	1331867-18-5	Benzonitrile-3,5-d2, 4-[[6-[[4-[(1E)-2-cyanoethenyl-1,2-d2]-2-methyl-6-(methyl-c	C22H9D9N6		A/B	-8.4
6021	O[C@H]1[C@H](n2c3c(nc2)c(=O)nc(N)[nH]3)O[C@H](CO)[C@H]1OP(=O)(OP(=O)(OP(=O)(OC[C@H]1O[C@H]([C@H](O)[	133261-55-9	Guanosine 3,Ä≤-(hexahydrogen pentaphosphate), P,Ä≤,Ä≤,Ä≤,Ä≤,Ü5,Ä≤-ester with	C20H29N10O24P5	948.37	A/B	-8.4
6022	[C@H](C(C)C)(CO)n1c2c(c(O)c(Cc3c(F)c(Cl)ccc3)cc2)c(=O)c(C(=O)O)c1	1333117-18-2	Benzonitrile, 4-[[4-[(4-azido-2,6-dimethylphenyl)amino]-2-pyrimidinyl]amino]-	C19H16N8		A/B	-8.4
6023	O=c1n([C@@H]2O[C@H](COP(=O)(N[C@@H](Cc3cnc[nH]3)C(=O)OC)Oc3ccc(N(=O)=O)cc3)C=C2)cc(C)c(=O)[nH]1	1333491-94-3	L-Proline, 1-[2,Ä≤,3,Ä≤-didehydro-3,Ä≤-deoxy-P-(4-nitrophenyl)-5,Ä≤-thymidylyl]	C28H29N4O10P	612.52	A/B	-8.4
6024	C([C@H](OCP(=O)(O)O)C)n1c2c(nc1)c(N)nc(F)n2	1333491-95-4	L-Histidine, N-[2,Ä≤,3,Ä≤-didehydro-3,Ä≤-deoxy-P-(4-nitrophenyl)-5,Ä≤-thymidyly	C23H25N6O10P	576.45	A/B	-8.4
6025	C(OCCO)n1c2c(nc1)c(=O)nc([NH-])[nH]2	133416-30-5	1,3-Propanedione, 2-[(2,4-dimethoxyphenyl)methylene]-1-(2-hydroxy-5-methylphenyl	C25H22O5	402.44	A/B	-8.4
6026	Fc1c(c2ccc(C[C@@H]([C@@H](CN(S(=O)(=O)c3ccc(OC)cc3)CC(C)C)O)NC(=O)O[C@H]3CCOC3)cc2)cccc1	1335386-68-9	Carbamic acid, N-[(1S,2R)-1-[(3,Ä≤-fluoro[1,1,Ä≤-biphenyl]-4-yl)methyl]-2-hydro	C32H39FN2O7S	614.72	A/B	-8.4
6027	C([C@@H]([C@@H](CN(S(=O)(=O)c1ccc(OC)cc1)CC(C)C)O)NC(=O)O[C@H]1CCOC1)c1ccc(cc1)c1ccc(C(=O)C)cc1	1335386-74-7	[1,1,Ä≤-Biphenyl]-3-carboxylic acid, 4,Ä≤-[(2S,3R)-3-hydroxy-4-[[(4-methoxyphen	C34H42N2O9S	654.77	A/B	-8.4
6028	O=c1n(cc(CO)c(=O)[nH]1)[C@H]1O[C@H](CO)CC1	1336889-75-8	1H-Benzimidazole, 1-(4,6-di-4-morpholinyl-1,3,5-triazin-2-yl)-2-(trifluoromethyl	C19H20F3N7O2	435.4	A/B	-8.4
6029	C(OCCO)n1c2c(c(=O)nc(N)n2C)nc1	1338468-06-6	2-Propen-1-one, 1-[2-hydroxy-4-methoxy-3-[(2E,6E)-3,7,11-trimethyl-2,6,10-dodeca	C31H38O4	474.63	A/B	-8.4
6030	C(N)(C12CC3CC(C2)CC(C1)C3)C1CC1	1342898-19-4	Benzenepropanamide, Œ±-amino-N-[(1S)-2-[(1S,3S,5S)-3-cyano-2-azabicyclo[3.1.0]he	C27H34N4O3	462.58	A/B	-8.4
6031	C(n1c2c(nc1)c(N)ncn2)[C@H]1COP(=O)(Oc2ccccc2)CO1	1345345-61-0	1-Naphthalenol, 4-[(7-chloro-4-quinolinyl)amino]-5,6,7,8-tetrahydro-2-[(4-methyl	C26H30ClN3O	435.99	A/B	-8.4
6032	N(=Nc1c2c(c(S(=O)(=O)O)cc1)c(N)ccc2)c1c2c(c(N=Nc3ccc(S(=O)(=O)O)cc3)cc1)ccc(S(=O)(=O)O)c2	1345852-39-2	1-Naphthalenesulfonic acid, 8-amino-5-[2-[3-methyl-4-[2-(3-sulfophenyl)diazenyl]	C23H19N5O6S2	525.56	A/B	-8.4
6033	N(=Nc1c2c(c(N=Nc3cc(S(=O)(=O)O)ccc3)cc1)cccc2)c1c2c(c(N)cc1)cccc2S(=O)(=O)O	1345852-46-1	1-Naphthalenesulfonic acid, 5-amino-8-[2-[4-(2-phenyldiazenyl)-7-sulfo-1-naphtha	C26H19N5O6S2	561.59	A/B	-8.4
6034	[C@H]([C@H](Cc1ccccc1)NC(=O)O[C@H]1CCOC1)(CN(S(=O)(=O)c1ccc(NC(=O)CCCC)cc1)CC(C)C)OC(=O)CCCC	1346769-35-4	Benzamide, N-[[4,Ä≤-[(2-ethyl-5,7-dimethyl-3H-imidazo[4,5-b]pyridin-3-yl)methyl]	C35H37N5O3S	607.77	A/B	-8.4
6035	[C@H](Cc1ccccc1)(C[C@@H]([C@H](Cc1ccccc1)NC(=O)OCc1cncs1)O)NC(=O)[C@@H](NC(=O)N(Cc1nc(C/C=C/C)sc1)C)	1347053-78-4	2,7,10,12-Tetraazatridecanoic acid, 13-(2-amino-4-thiazolyl)-5-hydroxy-12-methyl	C34H43N7O5S2	693.88	A/B	-8.4
6036	C(N(C(=O)N[C@H](C(=O)N[C@@H](Cc1ccccc1)C[C@@H]([C@H](Cc1ccccc1)NC(=O)OCc1cncs1)O)C(C)C)C)c1nc(sc1)C1	1347062-99-0	2,7,10,12-Tetraazatridecanoic acid, 13-[2-(3-cyclopenten-1-yl)-4-thiazolyl]-4-hy	C39H48N6O5S2	744.97	A/B	-8.4
6037	C(n1c2c(nc1CC)c(C)cc(C)n2)c1ccc(cc1)c1c(C(=O)NS(=O)(=O)c2ccccc2)cccc1	1347431-47-3	Not Yet Assigned	C30H38D5F2N5O		A/B	-8.4
6038	C(C)(C)c1n([C@@H]2C[C@@H]3N(CCC(NC(=O)C4CCC(F)(F)CC4)c4ccccc4)C[C@@H](C2)CC3)c(C([2H])([2H])[2H])nn1	1347562-45-1	Acetamide, 2-butoxy-N-[[4,Ä≤-[(2-ethyl-5,7-dimethyl-3H-imidazo[4,5-b]pyridin-3-y	C34H43N5O4S	617.8	A/B	-8.4
6039	C(n1c2c(nc1CC)c(C)cc(C(=O)O)n2)c1ccc(cc1)c1c(S(=O)(=O)NC(=O)OCCCC)cccc1	1347625-83-5	Not Yet Assigned	C30H33D10F2N5O		A/B	-8.4
6040	O[C@@H]1[C@H](n2c3c(nc2)c(N)nc(F)n3)O[C@H](COP(=O)(OC(COCCCCCCCCCCCCCCCCC)OCC(F)(F)F)O)[C@H]1O	1348218-13-2	2,7,10,12-Tetraazatridecanoic acid, 4-hydroxy-12-methyl-9-(1-methylethyl)-8,11-c	C39H52N6O5S2	749	A/B	-8.4
6041	O(C(=O)N[C@@H](Cc1ccc(OCP(=O)(NC(CC(=O)O)C)O)cc1)[C@@H](CN(S(=O)(=O)c1ccc(OC)cc1)CC(C)C)O)[C@@H]1[C@	1348561-60-3	3-Azabicyclo[3.1.0]hexane-2-carboxamide, N-[3-amino-1-(cyclobutylmethyl)-2,3-dic	C35H54N8O6	682.85	A/B	-8.4
6042	O=c1n([C@H]2O[C@@H](COC(=O)CCC(=O)OC(COC(=O)[C@H](C(C)C)N)COC(=O)[C@H](C(C)C)N)SC2)ccc(N)n1	1348563-29-0	Butanoic acid, 3-[[[[4-[(2S,3R)-2-[[[[(3R,3aS,6aR)-hexahydrofuro[2,3-b]furan-3-y	C33H48N3O13PS	757.79	A/B	-8.4
6043	C(C)(C)c1n([C@@H]2C[C@@H]3N(CCC(NC(=O)C4C(C(C(F)(F)C(C4([2H])[2H])([2H])[2H])([2H])[2H])([2H])[2H])c	1348608-01-4	Not Yet Assigned	C36H49N5O4	615.81	A/B	-8.4
6044	C(C[C@H](NC(=O)CC(F)(F)F)c1ccccc1)N1C2CC(CC1CC2)n1c(C(C)C)nnc1C	1348923-92-1	2,7,10,12-Tetraazatridecanoic acid, 4-hydroxy-10-methyl-9-(1-methylethyl)-12-[2-	C37H48N6O5S2	720.94	A/B	-8.4
6045	O(C(=O)N[C@@H](Cc1ccc(OCP(=O)(OCCCC)OCC)cc1)[C@@H](CN(S(=O)(=O)c1ccc(OC)cc1)CC(C)C)O)[C@@H]1[C@H]2[C	1349212-13-0	Not Yet Assigned	C37H49N5O4	627.82	A/B	-8.4
6046	C(=O)(NC(CC1CC1)C(=O)C(=O)N)[C@@H]1[C@@H]2[C@@H](C2(C)C)CN1C(=O)[C@@H](NC(=O)N[C@H](CNS(=O)(=O)c1ccc	1349400-99-2	Not Yet Assigned	C40H61N7O6	735.96	A/B	-8.4
6047	C([C@H]([C@H](Cc1ccccc1)NC(=O)[C@@H](NC(=O)c1nc2c(cc1)cccc2)CC(=O)NC(=O)[C@@H](NC(=O)[C@H](C(C)C)N)C	1349618-97-8	3H-Imidazo[4,5-b]pyridine-5-carboxylic acid, 3-[[2,Ä≤-[[[(1,1-dimethylethoxy)ca	C28H30N4O6S	550.63	A/B	-8.4
6048	[C@H](Cc1ccccc1)(C[C@@H]([C@H](Cc1ccccc1)NC(=O)OCc1cncs1)O)NC(=O)[C@H](NC(=O)N(Cc1nc(N(C)C)sc1)C)C(C	1349680-80-3	Carbamic acid, N-[[5-[(diethylamino)methyl]-4,Ä≤-[(2-ethyl-5,7-dimethyl-3H-imida	C33H43N5O4S	605.79	A/B	-8.4
6049	C(=O)(NC(C(=O)C(=O)NC1CC1)CCC)[C@@H]1[C@@H]2[C@H](CN1C(=O)[C@@H](NC(=O)[C@@H](NC(=O)c1cnccn1)C1CCCCC	1350050-13-3	2,7,10,12-Tetraazatridecanoic acid, 4-hydroxy-12-methyl-9-(1-methylethyl)-13-[2-	C38H50N6O5S2	734.97	A/B	-8.4
6050	O(C(=O)N[C@@H](Cc1ccc(OCP(=O)(NC(C(=O)OCC)C)OCCCC)cc1)[C@@H](CN(S(=O)(=O)c1ccc(OC)cc1)CC(C)C)O)[C@@H	1350099-52-3	Not Yet Assigned	C36H47N5O4	613.79	A/B	-8.4
6051	C(#CCC)C1(C(F)(F)F)c2cc(NC(=O)O1)cc(F)c(Cl)c2	1350203-30-3	2,7,10,12-Tetraazatridecanoic acid, 13-[2-(cyclohexylmethyl)-4-thiazolyl]-4-hydr	C41H54N6O6S	758.97	A/B	-8.4
6052	C(=O)([C@@H](NC(=O)OC)C(C)C)N1[C@H](c2[nH]c(c3c4c(c(cc3)c3c5c(c(cc3)c3[nH]c(nc3)[C@H]3N(C(=O)[C@@H](	1350718-44-3	Carbamic acid, N,N,Ä≤-[(2,2,Ä≤-dimethyl[4,7,Ä≤-bibenzothiazole]-4,Ä≤,7-diyl)bis	C44H52N10O6S2	881.08	A/B	-8.4
6053	C(=O)([C@@H](NC(=O)OC)C(C)C)N1[C@H](c2[nH]c(c3c4c(c(cc3)c3c5c(c(cc3)c3[nH]c(nc3)[C@H]3N(C(=O)[C@@H](	1350719-39-9	Carbamic acid, N,N,Ä≤-[[8,8,Ä≤-biquinoline]-5,5,Ä≤-diylbis[1H-imidazole-5,2-diy	C46H52N10O6	840.97	A/B	-8.4
6054	C[C@]1(c2[nH]c(c3c4c(c(cc3)c3c5c(c(cc3)c3[nH]c(nc3)[C@H]3N(C(=O)[C@@H](NC(=O)OC)C(C)C)CCC3)CCC5)CCC4	1350719-41-3	Carbamic acid, N,N,Ä≤-[[4,4,Ä≤-bi-1H-indole]-7,7,Ä≤-diylbis[1H-imidazole-5,2-di	C44H52N10O6	816.95	A/B	-8.4
6055	C(N1c2c(C(=Nn3c(Cc4ccccc4)n[nH]c3=S)C1=O)cc(Br)cc2)N1CCCCC1	1350822-78-4	2H-Indol-2-one, 5-chloro-3-[[1,5-dihydro-3-(phenylmethyl)-5-thioxo-4H-1,2,4-tria	C23H23ClN6OS	466.99	A/B	-8.4
6056	C(=O)([C@@H](NC(=O)OC)C(C)C)N1[C@@H](CCC1)c1[nH]c(cn1)c1ccc(C#N)c(cc1)c1cc2c(cc1)cc(cc2)c1[nH]c(nc1)[	1351332-54-1	Carbamic acid, N-[(1S)-1-[[(3R)-3-[5-[6-[4-[2-[(2S)-1-[(2S)-2-[(methoxycarbonyl)	C44H52N8O6S	821	A/B	-8.4
6057	C(=O)([C@@H](NC(=O)OC)C(C)C)N1[C@@H](CCC1)c1[nH]c(cn1)c1cc2c(cc1)cc(cc2)c1ccc(C(=O)NC[C@H]2N(C(=O)[C	1351332-85-8	Carbamic acid, N-[(1S)-1-[[(2S)-2-[5-[4-[6-[2-[(2S)-1-[(2S)-2-[(methoxycarbonyl)	C45H50N8O6	798.93	A/B	-8.4
6058	C(=O)([C@@H](NC(=O)OC)C(C)(C)O)N1C[C@@]2(C[C@H]1c1[nH]c(cn1)c1ccc(cc1)c1ccc(cc1)c1[nH]c(nc1)[C@H]1N(	1351851-07-4	Carbamic acid, N-[(1S)-1-[[(2S)-2-[5-[4,Ä≤-[2-[(5S,8S)-7-[(2S)-2-cyclopropyl-2-	C43H52N8O7	792.92	A/B	-8.4
6059	C(=O)([C@@H](NC(=O)OC)C(C)C)N1C[C@@]2(C[C@H]1c1[nH]c(cn1)c1ccc(cc1)c1ccc(cc1)c1c(COC)nc([nH]1)[C@H]1	1351852-72-6	1H-Imidazole-5-carboxylic acid, 4-[4,Ä≤-[2-[(5S,8S)-7-[(2S)-2-[(methoxycarbonyl)	C45H56N8O9	852.97	A/B	-8.4
6060	C(=O)([C@@H](NC(=O)OC)C(C)C)N1C[C@@]2(C[C@H]1c1[nH]c(cn1)c1c(C)cc(cc1)c1cc(C)c(cc1)c1[nH]c(nc1)[C@H]	1351852-82-8	Carbamic acid, N-[(1S)-2-methyl-1-[[(2S)-2-[5-[2,2,Ä≤,3,3,Ä≤-tetrafluoro-4,Ä≤-[	C43H50F4N8O7	866.9	A/B	-8.4
6061	C(=O)([C@@H](NC(=O)OC)C(C)C)N1C[C@@]2(C[C@H]1c1[nH]c(cn1)c1c(Cl)cc(cc1Cl)c1cc(Cl)c(c(Cl)c1)c1[nH]c(n	1351852-88-4	Carbamic acid, N-[(1S)-1-[[(2S)-2-[5-[3,3,Ä≤-dichloro-4,Ä≤-[2-[(5S,8S)-7-[(2S)-	C43H52Cl2N8O7	863.83	A/B	-8.4
6062	C(=O)([C@@H](NC(=O)OC)C(C)C)N1[C@@H](C[C@]2(C1)CC=CCO2)c1[nH]c(cn1)c1ccc(cc1)c1ccc(cc1)c1[nH]c(nc1)[	1351853-17-2	Carbamic acid, N-[(1S)-1-[[(2S)-2-[5-[4,Ä≤-[2-[(3S,5S)-2-[(2S)-2-[(methoxycarbor	C44H56N8O7	808.96	A/B	-8.4
6063	C(=O)([C@@H](NC(=O)OC)C(C)C)N1C[C@]2(C[C@H]1c1[nH]c(cn1)c1ccc(cc1)c1ccc(cc1)c1[nH]c(nc1)[C@H]1N(C(=O	1351853-20-7	Carbamic acid, N-[(1S)-1-[[(2S)-2-[5-[4,Ä≤-[2-[(3S)-2-[(2S)-2-[(methoxycarbonyl)	C44H54N8O6	790.95	A/B	-8.4
6064	C(=O)([C@H](NC(=O)OC)C1CC1)N1[C@@H](C[C@@H](C1)C1CC1)c1[nH]c(cn1)c1ccc(cc1)c1ccc(cc1)c1[nH]c(nc1)[C@	1351854-00-6	Carbamic acid, N-[2-[(2S)-2-[5-[4,Ä≤-[2-[(2S,4R)-4-cyclopropyl-1-[2-[(methoxyca	C41H50N8O6	750.89	A/B	-8.4
6065	C(=O)([C@H](NC(=O)OC)C(C)C)N1[C@@H](C[C@@H](C1)C1CC1)c1[nH]c(cn1)c1ccc(cc1)c1ccc(cc1)c1[nH]c(nc1)[C@	1351854-03-9	Carbamic acid, N-[(1S)-1-[[(2S)-2-[5-[4,Ä≤-[2-[(2S,4R)-4-cyclopropyl-1-[(2S)-3-h	C43H54N8O8	810.94	A/B	-8.4
6066	C(=O)([C@@H](NC(=O)OC)c1ccccc1)N1[C@@H](C[C@@H](C1)C1CC1)c1[nH]c(cn1)c1ccc(cc1)c1ccc(cc1)c1[nH]c(nc1	1351854-17-5	Carbamic acid, N-[(1S)-2-[(2S)-2-[5-[4,Ä≤-[2-[(2S,4R)-4-cyclopropyl-1-[(2S)-2-[	C47H58N8O8	863.01	A/B	-8.4
6067	C(=O)([C@@H](NC(=O)OC)C(C)C)N1[C@@H](C[C@@H](C1)C1CC1)c1[nH]c(cn1)c1c(Cl)cc(cc1)c1cc(Cl)c(cc1)c1[nH]	1351855-70-3	Carbamic acid, N-[(1S)-1-[[(2S)-2-[5-[4,Ä≤-[2-[(2S,4R)-4-cyclopropyl-1-[(2S)-2-	C45H52F6N8O6	914.93	A/B	-8.4
6068	C(=O)([C@@H](NC(=O)OC)C(C)C)N1[C@@H](C[C@@H](C1)C1CC1)c1[nH]c(cn1)c1ccc(cc1)c1ccc(cc1)c1[nH]c(nc1)[C	1351855-95-2	Carbamic acid, N-[(1S)-1-[[(2S,4R)-4-cyclobutyl-2-[5-[4,Ä≤-[2-[(2S,4R)-4-cyclopr	C47H60N8O6	833.03	A/B	-8.4
6069	C(=O)(N[C@H](C(C(=O)NC1CC1)O)CCC)[C@@H]1[C@@H]2[C@H](CN1C(=O)[C@@H](NC(=O)[C@@H](NC(=O)c1cnccn1)C1CC	1351992-75-0	Cyclopenta[c]pyrrole-1-carboxamide, (2S)-2-cyclohexyl-N-(2-pyrazinylcarbonyl)gly	C35H51N7O6	665.82	A/B	-8.4
6070	C(=O)([C@@H](NC(=O)OC)C(C)C)N1[C@@H](C=C(C)C1)c1[nH]c(cn1)c1cc2c(cc1)cc(C#Cc1ccc(cc1)c1[nH]c(nc1)[C@	1352392-61-0	Carbamic acid, N-[(1S)-1-[[(2S)-4-ethyl-2,5-dihydro-2-[5-[6-[2-[2-[(2S)-1-[(2S)-	C42H50N8O6	762.9	A/B	-8.4
6071	O[C@H]1[C@@H](O[C@H](COC(=O)CCCCCCC/C=C/CCCCCCCC)[C@H]1O)n1nc(C(=O)N)nc1	1352918-39-8	Uridine 5,Ä≤-(tetrahydrogen triphosphate), 5-[4-(5-amino-2-benzoxazolyl)-2-fluo	C22H22FN4O15P3	694.35	A/B	-8.4
6072	O=C1N(C(SC1)c1cc(OC)c(OC)cc1)c1[nH]c(nn1)N1CCOCC1	1354021-16-1	4-Thiazolidinone, 2-(4-chlorophenyl)-5-methyl-3-[3-(4-morpholinyl)-1H-1,2,4-tria	C16H18ClN5O2S	379.86	A/B	-8.4
6073	O=C1c2c(c3c(c(O)c2CO1)cc1c(c3)OCO1)c1c(OC)ccc(OC)c1	1354632-21-5	Furo[3,Ä≤,4,Ä≤:6,7]naphtho[2,3-d]-1,3-dioxol-6(8H)-one, 5-(3,5-dimethoxyphenyl)	C21H16O7	380.35	A/B	-8.4
6074	C(#N)[C@]1(O[C@H](CO[P@](=O)(Oc2ccc(Cl)cc2)N[C@H](C(=O)OCCCCCCC)C)[C@@H](O)[C@@]1(C)O)c1n2c(cc1)c(N)	1354823-49-6	L-Alanine, N-[(S)-hydroxyphenoxyphosphinyl]-, cyclopentyl ester, 6-ester with 2-	C27H33N6O8P	600.56	A/B	-8.4
6075	C(#N)[C@]1(O[C@H](COP(=O)(Oc2ccccc2)N[C@H](C(=O)OCC)C)[C@@H](O)[C@H]1O)c1n2c(cc1)c(N)ncn2	1355050-11-1	L-Alanine, N-(hydroxyphenoxyphosphinyl)-, 2-ethylbutyl ester, 6-ester with 2-C-(	C27H35N6O8P	602.58	A/B	-8.4
6076	C(=O)([C@@H](NC(=O)OC)C(C)C)N1[C@H](c2[nH]c(c3c4c(c(cc3)c3ccc(cc3)c3[nH]c(nc3)[C@H]3N(C(=O)[C@@H](NC	1359868-92-0	Carbamic acid, N-[(1S)-2-[(1R,3S,5R)-3-[5-[4-[7-[2-[(1R,3S,5R)-2-[(2S)-2-[(metho	C46H52N10O8S	905.03	A/B	-8.4
6077	C(=O)([C@@H](NC(=O)OC)C1CCOCC1)N1[C@H](c2[nH]c(c3c4c(c(cc3)c3ccc(cc3)c3[nH]c(nc3)[C@H]3N(C(=O)[C@@H]	1359868-96-4	Carbamic acid, N-[(1S)-2-[(1R,3S,5R)-3-[5-[4-[7-[2-[(1R,3S,5R)-2-[(2S)-2-[(metho	C47H54N10O8	886.99	A/B	-8.4
6078	C(F)(F)c1n(c2c(n1)cccc2)c1nc(nc(N[C@H]2CC[C@](C)(O)CC2)n1)N1CCOCC1	1359952-69-4	Cyclohexanol, 4-[[4-[2-(difluoromethyl)-1H-benzimidazol-1-yl]-6-(4-morpholinyl)-	C22H27F2N7O2	459.49	A/B	-8.4
6079	C(F)(F)c1n(c2c(n1)cccc2)c1nc(nc(NCC2CCCO2)n1)N1CCOCC1	1359952-70-7	Cyclohexanol, 4-[[4-[2-(difluoromethyl)-1H-benzimidazol-1-yl]-6-(4-morpholinyl)-	C22H27F2N7O2	459.49	A/B	-8.4
6080	C(F)(F)c1n(c2c(n1)cccc2)c1nc(nc(Nc2cc3c(cc2)nc[nH]3)n1)N1CCOCC1	1359953-76-6	1H-Benzimidazole, 2-(difluoromethyl)-1-[4-(4-morpholinyl)-6-(4-pyridinylthio)-1,	C20H17F2N7OS	441.46	A/B	-8.4
6081	C(=O)([C@@H](NC(=O)OC)[C@H]1C[C@@H](C)O[C@H](C)C1)N1[C@H](c2[nH]c(c3c4c(c(cc3)c3ccc(cc3)c3[nH]c(nc3)	1359956-35-6	1,3,5-Triazin-2-amine, 4-[2-(difluoromethyl)-1H-benzimidazol-1-yl]-N-(4-methoxyp	C23H23F2N7O2	467.47	A/B	-8.4
6082	O=c1n([C@@H]2O[C@H](COC(=O)CC[C@@H](C(=O)OC[C@@H]3O[C@@H](CS3)n3c(=O)nc(N)cc3)NC(=O)CCC(=O)OC[C@H]3O	1365246-82-7	L-Glutamic acid, N-acetyl-, 1-[[(2R,5S)-5-(4-amino-2-oxo-1(2H)-pyrimidinyl)-1,3-	C25H31N9O10S		A/B	-8.4
6083	N(=[N+]=[N-])[C@]1(CO)O[C@H]([C@H](O)[C@@H]1O)n1nc(C(=O)N)nc1	1365257-27-7	1H-1,2,4-Triazole-3-carboxamide, 1-(4-C-azido-3,5-di-O-benzoyl-Œ≤-D-ribofuranosy	C22H19N7O7		A/B	-8.4

6084	[C@@H]([C@H](Cc1ccccc1)NC(=O)COc1c(C)cccc1C)(C[C@H](Cc1ccccc1)NC(=O)[C@H](C(C)C)N1C(=O)NCCC1)OC(=O)C	1370516-54-3	Acetic acid, 2-(carboxymethoxy)-, 1-[(2R)-3,6-dihydro-6-oxo-2-(2-phenylethyl)-2-	C35H37F3N2O9S	718.74	A/B	-8.4
6085	S(=O)(=O)(O)c1c(ccc(N=Nc2c3c(ccc(S(=O)(=O)O)c3)c(N)cc2)c1)c1c(S(=O)(=O)O)cc(N=Nc2c3c(ccc(S(=O)(=O)O)	1375454-32-2	[1,1,Ä≤-Biphenyl]-2,2,Ä≤-disulfonic acid, 4,4,Ä≤-bis[2-[2,4-diamino-5-sulfo-3-[	C44H34N12O18S6	1211.2	A/B	-8.4
6086	C(#N)c1c2c(c3c(cc(cc3)c3[nH]c(nc3)[C@H]3N(C(=O)[C@@H](NC(=O)OC)C(C)C)CCC3)c1)ccc(c2)c1[nH]c(nc1)[C@H	1377040-53-3	Carbamic acid, N,N,Ä≤-[[1,1,Ä≤-biphenyl]-4,4,Ä≤-diylbis[1H-imidazole-5,2-diyl[(	C48H50N8O6	834.96	A/B	-8.4
6087	C(=O)([C@@H](NC(=O)OC)C(C)C)N1[C@H]2[C@H](C2)C[C@H]1c1[nH]c2c(n1)ccc1c2ccc(c1)c1cc(F)c(cc1)c1[nH]c(n	1377041-55-8	Carbamic acid, N-[(1S)-1-[[(1R,3S,5R)-3-[7-[3-fluoro-4-[2-[(1R,3S,4S)-2-[(2S)-2-	C45H51FN8O6	818.93	A/B	-8.4
6088	C(=O)([C@@H](NC(=O)OC)[C@H](CC)C)N1[C@H](c2[nH]c(c3cc4c5-c6c(cc(cc6OC4)c4[nH]c(nc4)[C@H]4N(C(=O)[C@@	1377042-18-6	Carbamic acid, N,N,Ä≤-[[2-(1-propyn-1-yl)[1,1,Ä≤-biphenyl]-4,4,Ä≤-diyl]bis[1H-i	C43H52N8O6	776.92	A/B	-8.4
6089	C(=O)([C@@H](NC(=O)OC)C(C)C)N1[C@H](c2[nH]c(c3cc4c5-c6c(cc(cc6OC4)c4[nH]c(nc4)[C@H]4N(C(=O)[C@@H](NC	1377042-82-4	Carbamic acid, N-[(1S)-2-[(1R,3S,5R)-3-[5-[4-[2-[(1R,3S,4S)-2-[(2S)-2-[(methoxyc	C47H54N8O7	842.98	A/B	-8.4
6090	C(=O)([C@@H](NC(=O)OC)C(C)C)N1[C@H](c2[nH]c3c4c(cc(cc4)c4c(C#CC)cc(cc4)c4[nH]c(nc4)[C@H]4N(C(=O)[C@@	1377042-90-4	Carbamic acid, N-[(1S)-1-[[(2S)-2-[5-[2-chloro-4-[2-[(1R,3S,4S)-2-[(2S)-2-[(meth	C44H51ClN8O6	823.38	A/B	-8.4
6091	C(=O)([C@@H](NC(=O)OC)C(C)C)N1[C@H](c2[nH]c3c4c(cc(cc4)c4cc5c(cc4)c4c(cc5)nc([nH]4)[C@H]4N(C(=O)[C@@	1377046-95-1	Carbamic acid, N,N,Ä≤-[(5,10-dihydro[1]benzopyrano[5,4,3-cde][1]benzopyran-2,7-d	C44H54N8O8	822.95	A/B	-8.4
6092	N(C(=O)Nc1ccc(SC)cc1)C12CC3CC(C2)CC(C1)C3	1378286-89-5	Urea, N-[3-(difluoromethoxy)phenyl]-N,Ä≤-tricyclo[3.3.1.13,7]dec-1-yl-	C18H22F2N2O2	336.38	A/B	-8.4
6093	N(C(=O)C)[C@H]1[C@H]([C@@H]([C@@H](CO)O)O)OC(=C[C@@H]1NC(=N)N)C(=O)OC	1378393-44-2	Carbamic acid, N-[(1S)-1-[[(2S)-2-[5-[6-[4-[2-[(2S)-1-[(2R)-2-amino-2-phenylacet	C45H48N8O4	764.91	A/B	-8.4
6094	C=C1[C@@H](n2c3c(nc2)c(=O)nc(N)[nH]3)C[C@H](O)[C@H]1COP(=O)(NCc1ccccc1)OCc1ccccc1	1384189-22-3	Phosphoramidic acid, N-(phenylmethyl)-, [(1S,4R)-4-[2-amino-6-(cyclopropylamino)	C28H32N7O3P	545.57	A/B	-8.4
6095	C=C1[C@@H](n2c3c(nc2)c(=O)nc(N=CN(C)C)[nH]3)C[C@H](O)[C@H]1CO	1384189-23-4	Phosphoramidic acid, N-(phenylmethyl)-, [(1R,3S,5S)-3-(2-amino-1,6-dihydro-6-oxo	C26H29N6O5P	536.52	A/B	-8.4
6096	[C@H]([C@@H](COC(=O)C)OC(=O)C)(OC(=O)C)[C@H]1[C@H](NC(=O)C)[C@@H](N=C(NC(=O)OC(C)(C)C)NC(=O)OC(C)(C)	138474-05-2	Guanosine, 2,Ä≤-deoxy-5-iodocytidylyl-(3,Ä≤,Üí5,Ä≤)-2,Ä≤-deoxy-	C19H24IN8O10P	682.32	A/B	-8.4
6097	N(c1c(C)cc(/C=C/C#N)cc1C)c1nc(Nc2ccc(C#N)cc2)ncc1.C(CC(=O)O)C(=O)O	1388223-94-6	Propanedioic acid, compd. with 4-[[4-[[4-[(1E)-2-cyanoethenyl]-2,6-dimethylpheny	C22H18N6.xC3H4O4		A/B	-8.4
6098	O=C1C2(C(C(C(=O)/C=C(\O)/C3C4(N5C(C3c3cc(OC)c(O)cc3)CCC5)c3c(NC4=O)ccc(N(=O)=O)c3)C3N2CCC3)c2cc(OC)c	1389334-81-9	Spiro[3H-indole-3,3,Ä≤-[3H]pyrrolizin]-2(1H)-one, 5-chloro-2,Ä≤-[(1Z)-3-[5-chlo	C45H42Cl2N4O8	837.74	A/B	-8.4
6099	O=C1C2(C(C(C(=O)/C=C(\O)/C3C4(NC(C3c3cc(OC)c(O)cc3)c3ccccc3)C(=O)c3c(C4=O)cccc3)C(N2)c2ccccc2)c2cc(O	1389335-42-5	Spiro[2H-indene-2,2,Ä≤-pyrrolidine]-1,3-dione, 4,Ä≤-[(2Z)-3-hydroxy-3-[4,Ä≤-(4-	C43H38N2O10	742.77	A/B	-8.4
6100	N([C@H](C(=O)N(C)C)C)c1nc(nc(n1)N1CCOCC1)c1ccc(N)nc1	1391925-27-1	Benzoic acid, 3-[4-[(carboxymethyl)amino]-6-(4-morpholinyl)-1,3,5-triazin-2-yl]-	C16H17N5O5	359.34	A/B	-8.4
6101	CN1CCN(c2nc(nc(n2)N2CCOCC2)c2c3c(ccc2)[nH]nc3)CC1	1391925-94-2	Œ≤-Alanine, N-[4-(1H-indazol-4-yl)-6-(4-morpholinyl)-1,3,5-triazin-2-yl]-	C17H19N7O3	369.38	A/B	-8.4
6102	N(c1nc(nc(NCCNC(=O)c2ccc(cc2)C2OOC(OO2)c2ccc(C)cc2)n1)N1CCOCC1)C1CCCCC1	1393922-41-2	Benzamide, 4-[6-(4-methylphenyl)-1,2,4,5-tetroxan-3-yl]-N-[2-[[4-(4-morpholinyl)	C30H37N7O6	591.66	A/B	-8.4
6103	C(OC[C@@H]1[C@@H](OC(=O)CCCC)[C@@H](OC(=O)CCCC)[C@@H](O1)n1nc(C(=O)N)nc1)(c1ccc(OC)cc1)(c1ccc(OC)cc1	139620-97-6	1H-1,2,4-Triazole-3-carboxamide, 1-[2,3-di-O-benzoyl-5-O-[bis(4-methoxyphenyl)ph	C43H38N4O9	754.78	A/B	-8.4
6104	O(C(=O)CCCC)[C@H]1[C@@H](O[C@H](CO)[C@H]1OC(=O)CCCC)n1nc(C(=O)N)nc1	139621-03-7	1H-1,2,4-Triazole-3-carboxamide, 1-(2,3-di-O-benzoyl-Œ≤-D-ribofuranosyl)-	C22H20N4O7	452.42	A/B	-8.4
6105	O(C(=O)CCCC)[C@H]1[C@@H](O[C@H](COC(=O)c2cccnc2)[C@H]1OC(=O)CCCC)n1nc(C(=O)N)nc1	139621-07-1	1H-1,2,4-Triazole-3-carboxamide, 1-[2,3-di-O-benzoyl-5-O-(3-pyridinylcarbonyl)-Œ	C28H23N5O8	557.51	A/B	-8.4
6106	O=C1c2n(C([C@]3(N1[C@](C([2H])([2H])[2H])(C(C(O3)([2H])[2H])([2H])[2H])[2H])[2H])([2H])[2H])cc(C(=O)	1407166-87-3	2H-Pyrido[1,Ä≤,2,Ä≤:4,5]pyrazino[2,1-b][1,3]oxazine-2,3,4,12-d4-9-carboxamide,	C20H3D16F2N3O5		A/B	-8.4
6107	O=C1c2n(C[C@H]3N1[C@H](C)CCO3)cc(C(=O)NC(c1c(F)cc(F)cc1)([2H])[2H])c(=O)c2O	1407166-97-5	2H-Pyrido[1,Ä≤,2,Ä≤:4,5]pyrazino[2,1-b][1,3]oxazine-4-d-9-carboxamide, N-[(2,4-	C20H16D3F2N3O5		A/B	-8.4
6108	C(=O)(NC(CC1CCC1)C(=O)C(=O)N)[C@@H]1[C@@H]2[C@@H](C2(C)C)CN1C(=O)C(NC(=O)NC(C)(C)C)C(C)(C)C	1407494-85-2	Carbamic acid, N,N,Ä≤-[[1,1,Ä≤-biphenyl]-4,4,Ä≤-diylbis[1H-imidazole-5,2-diyl-2	C40H50N8O6		A/B	-8.4
6109	O(C(=O)C(C)(C)C)[C@H]1[C@@H](O[C@H](CO)[C@H]1OC(=O)c1cccnc1)n1nc(C(=O)N)nc1	140891-26-5	1H-1,2,4-Triazole-3-carboxamide, 1-[5-O-[bis(4-methoxyphenyl)phenylmethyl]-3-O-(	C40H41N5O9	735.78	A/B	-8.4
6110	O(C(=O)c1ccccc1)[C@H]1[C@@H](O[C@H](CO)[C@H]1OC(=O)C1=CN(C)C=CC1)n1nc(C(=O)N)nc1	140891-40-3	1H-1,2,4-Triazole-3-carboxamide, 1-[2-O-benzoyl-3-O-(3-pyridinylcarbonyl)-Œ≤-D-r	C21H19N5O7	453.4	A/B	-8.4
6111	C(=O)([C@@H](NC(=O)OC(C)(C)C)[C@@H]1C[C@@H]2[C@H](C1)C2)N1[C@H]2[C@H](C2)C[C@H]1c1[nH]c(cn1)c1ccc(cc	1413115-58-8	Carbamic acid, N,N,Ä≤-[[1,1,Ä≤-biphenyl]-4,4,Ä≤-diylbis[1H-imidazole-5,2-diyl-(	C46H50N8O8	842.94	A/B	-8.4
6112	[C@@H](Cc1ccccc1)(C[C@@H]([C@H](Cc1ccccc1)NC(=O)OCc1cncs1)O)NC(=O)[C@@H](NC(=O)N(Cc1nc(C(C)C)sc1)C)C	1414808-96-0	Not Yet Assigned	C21H23N7O6	469.45	A/B	-8.4
6113	[C@H](Cc1ccccc1)(C[C@H]([C@H](Cc1ccccc1)NC(=O)OCc1cncs1)O)NC(=O)[C@@H](NC(=O)N(Cc1nc(C(C)C)sc1)C)C(C	1414933-80-4	2,7,10,12-Tetraazatridecanoic acid, 4-hydroxy-12-methyl-9-(1-methylethyl)-13-[2-	C37H48N6O5S2	720.94	A/B	-8.4
6114	C(N(NC(=O)[C@@H](NC(=O)OC)C(C([2H])([2H])[2H])(C([2H])([2H])[2H])C([2H])([2H])[2H])C([C@@H]([C@H](Cc	1415634-51-3	2,5,6,10,13-Pentaazatetradecanedioic-7,7-d2 acid, 3-(1,1-dimethylethyl)-8-hydrox	C38H48D4N6O8		A/B	-8.4
6115	O=c1n([C@@H]2O[C@H](COP(=O)(OP(=O)(OP(=O)(OC[C@H]3O[C@H](C=C3)n3c(=O)[nH]c(=O)c(C)c3)O)O)O)C=C2)cc(C	1415665-48-3	Thymidine 5,Ä≤-(trihydrogen diphosphate), 2,Ä≤,3,Ä≤-didehydro-3,Ä≤-deoxy-, P,Ä≤	C20H24N4O13P2	590.37	A/B	-8.4
6116	C(F)(F)c1n(c2c(n1)cccc2)c1nc(nc(n1)N1CCOCC1)N1CCN(C(=O)CNC(=O)C)CC1	1416956-29-0	Ethanone, 2-amino-1-[4-[4-[2-(difluoromethyl)-1H-benzimidazol-1-yl]-6-(4-morphol	C21H25F2N9O2	473.48	A/B	-8.4
6117	C(OCCn1c2c(nc1)c(N)ncn2)[P@]1(=O)O[C@H](C[C@@H](O1)c1ccccc1)c1cc(Cl)ccc1	1417393-23-7	9H-Purin-6-amine, 9-[2-[[4-(3-chlorophenyl)-2-oxido-6-phenyl-1,3,2-dioxaphosphor	C23H23ClN5O4P	499.89	A/B	-8.4
6118	C(OCCn1c2c(nc1)c(N)ncn2)[P@@]1(=O)O[C@H](C[C@@H](O1)c1ccccc1)c1cc(Cl)ccc1	1417393-25-9	9H-Purin-6-amine, 9-[2-[[(2R,4S,6S)-4-(3-chlorophenyl)-2-oxido-6-phenyl-1,3,2-di	C23H23ClN5O4P	499.89	A/B	-8.4
6119	N(c1nc(nc(NC(=O)Nc2ccccc2)n1)N1CCOCC1)n1c(SC)nnc(C(C)(C)C)c1=O	1418540-48-3	Urea, N-(4-chlorophenyl)-N,Ä≤-[4-[[6-(1,1-dimethylethyl)-3-(methylthio)-5-oxo-1,	C22H27ClN10O3S	547.03	A/B	-8.4
6120	C(=O)([C@@H](NC(=O)OC)C(C)C)N1[C@H](c2[nH]c(c3c4c(c(cc3)c3ccc(cc3)c3[nH]c(nc3)[C@H]3N(C(=O)[C@@H](NC	1419384-93-2	Carbamic acid, N-[(1S)-1-[[(2S)-2-[5-[4-[7,Ä≤-[2-[(2S)-4,4-difluoro-1-[(2S)-2-[	C47H56F4N8O6	904.99	A/B	-8.4
6121	C([C@H](OCP(=O)(OCOC(=O)C(C)(C)C)OCOC(=O)C(C)(C)C)C)n1c2c(c(N=C)ncn2)nc1	14254-92-3	Epipicropodophyllin, 3,Ä≤,4,Ä≤,5,Ä≤-tridemethoxy-, DL-	C19H16O5	324.33	A/B	-8.4
6122	C(N1c2c(C(=NNC(=S)N(CC=C)CC=C)C1=O)cc(F)cc2)N1CCOCC1	1426583-57-4	Hydrazinecarbothioamide, 2-[5-chloro-1,2-dihydro-1-(4-morpholinylmethyl)-2-oxo-3	C26H24ClN5O2S	506.02	A/B	-8.4
6123	O(C)c1c2n(cc(C(=O)O)c(=O)c2cc(F)c1N1CC(C)N(CN2c3c(C(=NNC(=S)N(CC=C)CC=C)C2=O)cc(Cl)cc3)CC1)C1CC1	1426583-85-8	3-Quinolinecarboxylic acid, 7-[4-[[3-[2-[(diphenylamino)thioxomethyl]hydrazinyli	C39H36FN7O4S	717.81	A/B	-8.4
6124	C(=O)([C@@H](NC(=O)OC)C(C)C)N1[C@@H](CCC1)c1[nH]c(cn1)c1ccc(cc1)c1ccc(CO)cc1	1426938-72-8	Carbamic acid, N-[(1S)-1-[[(2S)-2-[5-[4,Ä≤-(aminocarbonyl)[1,1,Ä≤-biphenyl]-4-y	C27H31N5O4	489.57	A/B	-8.4
6125	C(=O)(OCC)C1=C(N)OC(=CC1c1ccc(C)cc1)c1[nH]c2c(n1)cccc2	1427084-38-5	4H-Pyran-3-carboxylic acid, 2-amino-6-(1H-benzimidazol-2-yl)-4-(3-bromophenyl)-,	C21H18BrN3O3	440.29	A/B	-8.4
6126	O[C@H]1[C@H](n2c3c(nc2)c(=O)nc(N)[nH]3)O[C@H](COP(=O)(O[Sn](C)(C)C)O[Sn](C)(C)C)[C@H]1O	1427676-63-8	Uridine 5,Ä≤-(trihydrogen diphosphate), 3-(2,2-difluoro-2-phenylethyl)-5-(triflu	C18H19F5N2O12P2	612.29	A/B	-8.4
6127	C[C@]12[C@@]3(C)[C@@H]([C@H](CC3)[C@@]3(C)O[C@@H](C(=C)C)CC3)[C@H](O)C[C@@H]1[C@]1(C)[C@@H](CC2)C(C)	1431635-54-9	1,5-Naphthalenedisulfonic acid, 3-[2-(2,4-diamino-5-methylphenyl)diazenyl]-	C17H16N4O6S2	436.46	A/B	-8.4
6128	C([C@H]([C@H](Cc1ccccc1)NC(=O)[C@@H](NC(=O)c1nc2c(cc1)cccc2)CC(=O)N)OC(=O)CCCCO[C@@H]1O[C@H](CO)[C@@	1438393-27-1	Guanosine 5,Ä≤-(trihydrogen diphosphate), P,Ä≤-(2,6-dideoxy-Œ±-D-arabino-hexopy	C16H25N5O14P2	573.34	A/B	-8.4
6129	O[C@H]1[C@H](n2c3c(nc2)c(=O)nc(N)[nH]3)O[C@H](COP(=O)(OP(=O)(O[C@H]2O[C@H](CO)C[C@H](O)[C@H]2O)O)O)[	1438395-53-9	D-erythro-Pentonamide, 2,3,5-trideoxy-N-[(1S,2R)-2,3-dihydro-2-hydroxy-1H-inden-	C37H49N5O4S	659.88	A/B	-8.4
6130	[C@H](Cc1ccccc1)(C[C@@H]([C@H](Cc1ccccc1)NC(=O)OCc1cccnc1)O)NC(=O)[C@@H](NC(=O)N(Cc1csc(C)n1)C)C(C)C	144142-14-3	2,4,7,12-Tetraazatridecan-13-oic acid, 1-(2-amino-4-thiazolyl)-10-hydroxy-2-meth	C36H45N7O5S	687.85	A/B	-8.4
6131	N(Cc1cc(ccc1)c1ccco1)C12CC3CC(C2)CC(C1)C3	1443121-99-0	[1,1,Ä≤-Biphenyl]-3-carbonitrile, 4-fluoro-4,Ä≤-[(tricyclo[3.3.1.13,7]dec-1-ylam	C24H25FN2	360.47	A/B	-8.4
6132	C(=O)(O)c1cc(ccc1NC(=O)c1c(N(=O)=O)cccc1)c1cc(C(=O)O)c(NC(=O)c2c(N(=O)=O)cccc2)cc1	144334-82-7	Benzoic acid, 3,3,Ä≤-methylenebis[6-[(2-nitrobenzoyl)amino]-	C29H20N4O10	584.49	A/B	-8.4
6133	S(c1n(c2c(n1)c(=O)nc(N)[nH]2)[C@@H]1O[C@H]2[C@H]([C@H]1O)OP(=O)(O)OC2)c1c(N)cccc1	144509-86-4	Guanosine, 8-[(2,4-dihydroxyphenyl)thio]-, cyclic 3,Ä≤,5,Ä≤-(hydrogen phosphate	C16H16N5O9PS	485.37	A/B	-8.4
6134	S(c1n(c2c(n1)c(=O)nc(N)[nH]2)[C@H]1[C@H](O)[C@H]2[C@H](O1)COP(=O)(O)O2)c1ccc(N)cc1	144509-88-6	Guanosine, 8-[(4-hydroxyphenyl)thio]-, cyclic 3,Ä≤,5,Ä≤-(hydrogen phosphate)	C16H16N5O8PS	469.37	A/B	-8.4
6135	S(c1n(c2c(n1)c(=O)nc(N)[nH]2)[C@H]1[C@H](O)[C@H]2[C@H](O1)COP(=O)(O)O2)c1cc(Cl)ccc1	144509-89-7	Guanosine, 8-[(4-aminophenyl)thio]-, cyclic 3,Ä≤,5,Ä≤-(hydrogen phosphate)	C16H17N6O7PS	468.38	A/B	-8.4
6136	O(c1nc(Nc2ccc(C#N)cc2)nc(N)c1Br)c1c(C)cc(C#N)cc1C.C(=O)O	1445668-97-2	1,3,5-Benzenetricarboxylic acid, compd. with 4-[[6-amino-5-bromo-2-[(4-cyanopher	C20H15BrN6O.C9H6O6.H2O		A/B	-8.4
6137	C(=O)(OCC)c1c2c(n(C)c1CSc1ccccc1)cc(Br)c(O)c2[13CH2][15N](C)C	144615-02-1	[1,1,Ä≤-Biphenyl]-3,3,Ä≤-dicarboxylic acid, 4,4,Ä≤-bis[(2-aminobenzoyl)amino]-,	(C28H22N4O6)x		A/B	-8.4
6138	O=c1c2c(n(cn2)[C@H]2C[C@H](O[Si](C(C)(C)C)(C)C)[C@@H](C=O)O2)[nH]c(N)n1	1446515-17-8	1H-Indole-2,3-dione, 5-[(4-methyl-1-piperazinyl)sulfonyl]-	C13H15N3O4S	309.34	A/B	-8.4
6139	O=C1[C@@H]2[C@@H](c3c([C@H](OC(=O)c4cc(N(=O)=O)ccc4)[C@H]2CO1)cc1c(c3)OCO1)c1c(Cl)c(OC)c(OC)c(OC)c1	1449601-80-2	Benzoic acid, 3-chloro-, (5R,5aR,8aR,9S)-9-(2-chloro-3,4,5-trimethoxyphenyl)-5,5	C29H24Cl2O9	587.4	A/B	-8.4
6140	O=C1[C@@H]2[C@@H](c3c([C@H](OC(=O)c4cc(F)ccc4)[C@H]2CO1)cc1c(c3)OCO1)c1c(Br)c(OC)c(OC)c(OC)c1	1449602-03-2	Benzoic acid, 4-methyl-, (5R,5aR,8aR,9S)-9-(2-bromo-3,4,5-trimethoxyphenyl)-5,5a	C30H27BrO9	611.43	A/B	-8.4
6141	O=C1[C@@H]2[C@@H](c3c([C@H](OC(=O)c4ccc(F)cc4)[C@H]2CO1)cc1c(c3)OCO1)c1c(Br)c(OC)c(OC)c(OC)c1	1449602-04-3	Benzoic acid, 3-fluoro-, (5R,5aR,8aR,9S)-9-(2-bromo-3,4,5-trimethoxyphenyl)-5,5a	C29H24BrFO9	615.4	A/B	-8.4
6142	O=C1[C@@H]2[C@@H](c3c([C@H](OC(=O)c4cc(Br)ccc4)[C@H]2CO1)cc1c(c3)OCO1)c1c(Br)c(OC)c(OC)c(OC)c1	1449602-07-6	Benzoic acid, 4-chloro-, (5R,5aR,8aR,9S)-9-(2-bromo-3,4,5-trimethoxyphenyl)-5,5a	C29H24BrClO9	631.85	A/B	-8.4
6143	O=C1[C@@H]2[C@@H](c3c([C@H](OC(=O)c4ccc(C#N)cc4)[C@H]2CO1)cc1c(c3)OCO1)c1c(Br)c(OC)c(OC)c(OC)c1	1449602-11-2	Furo[3,Ä≤,4,Ä≤:6,7]naphtho[2,3-d]-1,3-dioxol-6(5aH)-one, 5-(2-bromo-3,4,5-trime	C29H24BrNO11	642.41	A/B	-8.4
6144	C(Oc1c(C)cn(c(=O)n1)[C@@H]1O[C@H](CO)[C@@H](O)C1)c1c2c(N(c3c(C(=O)N2)cccn3)C2CC2)ncc1	1449678-53-8	Thymidine, 3-[(11-cyclopropyl-6,11-dihydro-6-oxo-5H-dipyrido[3,2-b:2,Ä≤,3,Ä≤-e]	C25H26N6O6	506.51	A/B	-8.4
6145	Cc1c(C23CC4CC(C3)CC(C2)C4)[nH]cn1	145004-87-1	Carbamic acid, [[4,Ä≤-[(2-ethyl-5,7-dimethyl-3H-imidazo[4,5-b]pyridin-3-yl)methy	C26H28N4O4S	492.59	A/B	-8.4
6146	Cc1c(C23CC4CC(C3)CC(C2)C4)[nH]c(n1)c1ccco1	145031-90-9	1H-Imidazole, 4-methyl-2-phenyl-5-tricyclo[3.3.1.13,7]dec-1-yl-	C20H24N2	292.42	A/B	-8.4
6147	Cc1c2c(N(c3c(C(=O)N2)cccn3)C2CC2)nc(C(CCCCCn2c3-c4c(CN(C(=O)CCNC(=O)CCN5C(=O)CCC5=O)c5c(-c3nn2)cccc	1450912-99-8	1-Pyrrolidinepropanamide, N-[3-[1-[7-(11-cyclopropyl-6,11-dihydro-4-methyl-6-oxo	C47H48F2N10O5	870.94	A/B	-8.4
6148	N(c1c2c(ncc1)cc(Cl)cc2)c1c(O)c(CN2CCC(C)CC2)cc(CN2CCC(C)CC2)c1	145431-59-0	Phenol, 2-[(7-chloro-4-quinolinyl)amino]-4,6-bis[(3-methyl-1-piperidinyl)methyl]	C29H37ClN4O	493.08	A/B	-8.4
6149	N(CCNc1c2c(ncc1)cc(Cl)cc2)c1nc(nc(NCCCO)n1)N1CCOCC1	1454694-99-5	1-Propanol, 3-[[4-[[4-[(7-chloro-4-quinolinyl)amino]butyl]amino]-6-(4-morpholiny	C23H31ClN8O2	487	A/B	-8.4
6150	N(C(C)C)c1c(nccc1)N1CCN(C(=O)c2cc3c([nH]2)ccc(NS(=O)(=O)CCN2CCCCC2)c3)CC1	147539-88-6	1-Pyrrolidineethanesulfonamide, N-[2-[[4-[3-[(1-methylethyl)amino]-2-pyridinyl]-	C27H37N7O3S	539.69	A/B	-8.4
6151	N(C(C)C)c1c(nccc1)N1CCN(C(=O)c2[nH]c3c(c2)ccc(NS(=O)(=O)C)c3)CC1	147920-16-9	Methanesulfonamide, 1,1,1-trifluoro-N-[2-[[4-[3-[(1-methylethyl)amino]-2-pyridir	C22H25F3N6O3S	510.53	A/B	-8.4
6152	C(n1c2c(nc1CC)c(C)ccn2)c1ccc(cc1)c1c(S(=O)(=O)NC(=O)OC)cccc1	148401-69-8	Benzoic acid, anhydride with [[4,Ä≤-[(2-butyl-3H-imidazo[4,5-b]pyridin-3-yl)meth	C31H28N4O5S	568.64	A/B	-8.4
6153	C(n1c2c(nc1CC)c(C)cc(C)n2)c1ccc(cc1)c1c(S(=O)(=O)NC(=O)NC)cccc1	148401-88-1	Carbamic acid, [[4,Ä≤-[(2-ethyl-5,7-dimethyl-3H-imidazo[4,5-b]pyridin-3-yl)methy	C27H30N4O4S	506.62	A/B	-8.4
6154	O(C(=O)c1ccccc1)[C@H]1[C@H]2[C@H](O[C@@H](O[C@@H]3[C@@H](CO)O[C@H](C3)n3cc(C(F)(F)F)c(=O)[nH]c3=O)[C	148834-34-8	Thymidine, Œ±,Œ±,Œ±-trifluoro-3,Ä≤-O-[2,3,4,6-tetrakis-O-(phenylmethyl)-Œ≤-D-gl	C44H45F3N2O10	818.83	A/B	-8.4
6155	C(C(C(Cc1ccccc1)NC(=O)C(NC(=O)c1nc2c(cc1)cccc2)CC(=O)N)O)N1C(C(=O)N[C@H](C(=O)N[C@H](C(=O)N[C@@H](CC	1504664-35-0	1-Naphthalenediazonium, 7-sulfo-4-[2-(4-sulfophenyl)diazenyl]-	C16H11N4O6S2		A/B	-8.4
6156	C(C(C)C)n1c2c(c(Nc3c(C(=O)O)cccc3Br)nc3c2cccc3)nc1	1508303-93-2	Benzoic acid, 2-[[1-(2-methylpropyl)-1H-imidazo[4,5-c]quinolin-4-yl]amino]-	C21H20N4O2	360.41	A/B	-8.4
6157	C([C@H](OP(=O)(O)O)CO)n1c(=O)nc(N)cc1	151408-85-4	1,5-Naphthalenedisulfonic acid, 3-[2-[4-[2-(4-amino-2-methylphenyl)diazenyl]-7-s	C27H21N5O9S3	655.68	A/B	-8.4
6158	N(=Nc1c(C)cc(N)cc1)c1c2c(c(N=Nc3cc(S(=O)(=O)O)ccc3)cc1)ccc(S(=O)(=O)O)c2	151534-66-6	1,4-Benzenedisulfonic acid, 2-[2-[4-[2-(4-amino-2-methylphenyl)diazenyl]-6-sulfo	C23H19N5O9S3	605.62	A/B	-8.4
6159	C(=O)(NC(C(=O)C(=O)NCCC)CCCC)[C@@H]1[C@@H]2[C@@H](C2(C)C)CN1C(=O)[C@@H](NC(=O)N[C@@H](CN1C(=O)CC(C)(	1528752-98-8	3-Azabicyclo[3.1.0]hexane-2-carboxamide, 3-[(2S)-2-[[[(1R)-1-[(4,4-dimethyl-2,6	C37H62N6O7	702.92	A/B	-8.4
6160	O=c1n(ccc(NC(=O)CCCCCCCCCCCCCCC)n1)[C@@H]1O[C@H](COP(=O)(O)O)CC1	152999-31-0	Propanoic acid, 2-[[5,5a,6,8,8a,9-hexahydro-9-(4-hydroxy-3,5-dimethoxyphenyl)-8-	C24H24O9Se	535.4	A/B	-8.4
6161	O[C@@H]1c2c([C@H]([C@@H]3[C@@H]1COC3)c1cc(OC)c(O)c(OC)c1)cc1c(c2)OCO1	153230-73-0	Carbonic acid, 4-[5,5a,6,8,8a,9-hexahydro-9-hydroxy-6-methoxyfuro[3,Ä≤,4,Ä≤:6,7	C30H30O10	550.55	A/B	-8.4
6162	C(=O)(NCc1ccc(F)cc1)c1c(OC(OC(=O)OCc2ccccc2)CC)c(=O)n(C)c(C(NC(=O)c2oc(C)nn2)(C)C)n1	1538603-97-2	Carbonic acid, 1-[[4-[[[(4-fluorophenyl)methyl]amino]carbonyl]-1,6-dihydro-1-met	C27H33FN6O8	588.58	A/B	-8.4
6163	C(=O)(NCc1ccc(F)cc1)c1c(OC(OC(=O)OCc2ccccc2)C(C)C)c(=O)n(C)c(C(NC(=O)c2oc(C)nn2)(C)C)n1	1538604-04-4	Carbonic acid, ethyl 1-[[4-[[[(4-fluorophenyl)methyl]amino]carbonyl]-1,6-dihydro	C26H31FN6O8	574.56	A/B	-8.4
6164	O(C(OC(=O)OCC)C(C)C)c1c(C(=O)NCc2ccc(F)cc2)nc(C(NC(=O)c2nnc(C)o2)(C)C)n(C)c1=O	1538604-06-6	Carbonic acid, 1-[[4-[[[(4-fluorophenyl)methyl]amino]carbonyl]-1,6-dihydro-1-met	C26H31FN6O8	574.56	A/B	-8.4
6165	O(C(OC(=O)C1CCCC1)C(C)C)c1c(C(=O)NCc2ccc(F)cc2)nc(C(NC(=O)c2nnc(C)o2)(C)C)n(C)c1=O	1538604-14-6	Cyclopropanecarboxylic acid, 1-[[4-[[[(4-fluorophenyl)methyl]amino]carbonyl]-1,6	C28H33FN6O7	584.6	A/B	-8.4
6166	C(=O)(NCc1ccc(F)cc1)c1c(OC(OC(=O)C(C)C)CC)c(=O)n(C)c(C(NC(=O)c2oc(C)nn2)(C)C)n1	1538604-17-9	4-Pyrimidinecarboxamide, N-[(4-fluorophenyl)methyl]-1,6-dihydro-1-methyl-2-[1-me	C25H29FN6O7	544.53	A/B	-8.4
6167	C(n1c2c(nc1CC)c(C)cc(C)n2)c1ccc(cc1)c1c(S(=O)(=O)NC(=O)CCCCC)ccc1	154551-77-6	Hexanamide, N-[[5-butoxy-4,Ä≤-[(2-ethyl-5,7-dimethyl-3H-imidazo[4,5-b]pyridin-3-	C33H42N4O4S	590.78	A/B	-8.4
6168	C(n1c2c(nc1CC)c(C)cc(C)n2)c1ccc(cc1)c1c(S(=O)(=O)NC(=O)COCCCC)ccc(OC)c1	154551-98-1	Acetamide, 2-butoxy-N-[[4,Ä≤-[(2-ethyl-5,7-dimethyl-3H-imidazo[4,5-b]pyridin-3-y	C33H42N4O4S	590.78	A/B	-8.4
6169	C(n1c2c(nc1CC)c(C)cc(C)n2)c1ccc(cc1)c1c(S(=O)(=O)NC(=O)COCCCC)ccc(OCC(F)(F)F)c1	154552-08-6	Acetamide, 2-butoxy-N-[[4,Ä≤-[(2-ethyl-5,7-dimethyl-3H-imidazo[4,5-b]pyridin-3-y	C34H43N5O5S	633.8	A/B	-8.4
6170	C(n1c2c(nc1CC)c(C)cc(C)n2)c1ccc(cc1)c1c(S(=O)(=O)NC(=O)COCCCC)ccc(CN(CC)CC)c1	154552-26-8	Acetamide, N-[[5-[(dimethylamino)methyl]-4,Ä≤-[(2-ethyl-5,7-dimethyl-3H-imidazo	C30H37N5O4S	563.71	A/B	-8.4
6171	C(n1c2c(nc1CC)c(C)cc(C)n2)c1ccc(cc1)c1c(S(=O)(=O)NC(=O)NCCCC)ccc(OC(C)C)c1	154552-49-5	[1,1,Ä≤-Biphenyl]-2-sulfonamide, N-[(butylamino)carbonyl]-4,Ä≤-[(2-ethyl-5,7-di	C30H34F3N5O4S	617.68	A/B	-8.4
6172	C(n1c2c(nc1CC)c(C)cc(C)n2)c1ccc(cc1)c1c(S(=O)(=O)NC(=O)c2ccccc2)ccc(CN(CC)CC)c1	154552-65-5	Benzenepropanamide, N-[[5-[(dimethylamino)methyl]-4,Ä≤-[(2-ethyl-5,7-dimethyl-3H	C35H39N5O3S	609.78	A/B	-8.4
6173	C(n1c2c(nc1CC)c(C)cc(C)n2)c1ccc(cc1)c1c(S(=O)(=O)NC(=O)c2ccccc2)ccc(CSC)c1	154553-18-1	Benzamide, N-[[4,Ä≤-[(2-ethyl-5,7-dimethyl-3H-imidazo[4,5-b]pyridin-3-yl)methyl]	C31H30N4O4S	554.66	A/B	-8.4
6174	C(n1c2c(nc1CC)c(C)cc(C)n2)c1ccc(cc1)c1c(S(=O)(=O)NC(=O)c2ccccc2)ccc(OCC)c1	154553-21-6	Benzamide, N-[[5-ethoxy-4,Ä≤-[(2-ethyl-5,7-dimethyl-3H-imidazo[4,5-b]pyridin-3-y	C32H32N4O4S	568.69	A/B	-8.4
6175	C(n1c2c(nc1CC)c(C)cc(C)n2)c1ccc(cc1)c1c(S(=O)(=O)NC(=O)COCC)ccc(CC)c1	154553-30-7	Acetamide, N-[[4,Ä≤-[(2-ethyl-5,7-dimethyl-3H-imidazo[4,5-b]pyridin-3-yl)methyl]	C29H34N4O4S	534.67	A/B	-8.4
6176	C(n1c2c(nc1CC)c(C)cc(C)n2)c1ccc(cc1)c1c(S(=O)(=O)NC(=O)COCC)ccc(C(C)C)c1	154553-33-0	Acetamide, N-[[5-butyl-4,Ä≤-[(2-ethyl-5,7-dimethyl-3H-imidazo[4,5-b]pyridin-3-y	C32H40N4O4S	576.75	A/B	-8.4
6177	C(n1c2c(nc1CC)c(C)cc(C)n2)c1ccc(cc1)c1c(S(=O)(=O)NC(=O)COCC)ccc(OCC)c1	154553-40-9	Acetamide, N-[[5-ethoxy-4,Ä≤-[(2-ethyl-5,7-dimethyl-3H-imidazo[4,5-b]pyridin-3-y	C30H36N4O5S	564.7	A/B	-8.4
6178	C(n1c2c(nc1CC)c(C)cc(C)n2)c1ccc(cc1)c1c(S(=O)(=O)NC(=O)COCC)ccc(CN(CC)CC)c1	154553-43-2	Acetamide, N-[5-(1,1-dimethylethyl)-4,Ä≤-[(2-ethyl-5,7-dimethyl-3H-imidazo[4,5-	C32H40N4O4S	576.75	A/B	-8.4
6179	O=C1C2c3c(cccc3c3[nH]c(nc3)[C@H]3N(C(=O)[C@@H](NC(=O)OC)C(C)C)CCC3)c3ccc(cc3)c3[nH]c(nc3)[C@H]3N(C(	1560639-42-0	Carbamic acid, N-[(1S)-2-methyl-1-[[(2S)-2-[5-[4-[1,2,3,4-tetrahydro-5-[2-[(2S)-	C46H57N9O7	848	A/B	-8.4
6180	C(=O)([C@@H](NC(=O)OC)C(C)C)N1[C@H](c2[nH]c(c3cc4c(C5OC4CC5)c(cc3)c3ccc(cc3)c3[nH]c(nc3)[C@H]3N(C(=O)[C	1560640-64-3	Carbamic acid, N-[(1S)-2-methyl-1-[[(2S)-2-[5-[4-[1,2,3,4-tetrahydro-8-[2-[(2S)-	C44H54N8O7	806.95	A/B	-8.4
6181	C(=O)([C@@H](NC(=O)OC)C(C)C)N1[C@H](c2[nH]c(c3c4c(C5NC4CC5)ccc3)c3ccc(cc3)c3[nH]c(nc3)[C@H]3N(C(=O)[C	1560649-93-5	Carbamic acid, N-[(1S)-1-[[(2S)-2-[5-[4-[1,4-dihydro-8-[2-[(2S)-1-[(2S)-2-[(meth	C45H55N9O6	817.97	A/B	-8.4
6182	[illegible]	156893-85-5	1-Naphthalenol, 4-[(7-chloro-4-quinolinyl)amino]-5,6,7,8-tetrahydro-2-[[4-(1-met	C27H33ClN4O	465.03	A/B	-8.4
6183	C([C@H](OC[P@](=O)(Oc1cccnc1)NC(C(=O)OC(C)C)(C)C)C)n1c2c(nc1)c(N)ncn2	1571076-30-6	Alanine, N-[(S)-[[(1R)-2-(6-amino-9H-purin-9-yl)-1-methylethoxy]methyl](2-naphth	C26H33N6O5P	540.55	A/B	-8.4
6184	O[C@H]1[C@@H](O[C@H](COP2(=O)O[C@H](CCO2)c2ccc(Cl)cc2)[C@H]1O)n1nc(C(=O)N)nc1	1578257-08-5	1H-1,2,4-Triazole-3-carboxamide, 1-[5-O-[(4S)-4-(3-chlorophenyl)-2-oxido-1,3,2-d	C17H20ClN4O8P	474.79	A/B	-8.4
6185	O(C(=O)CC)[C@H]1[C@@H](O[C@H](CO[P@2](=O)O[C@@H](CCO2)c2cc(Cl)ccc2)[C@H]1OC(=O)CC)n1nc(C(=O)N)nc1	1578257-13-2	Not Yet Assigned	C23H28ClN4O10P	586.92	A/B	-8.4
6186	O[C@H]1[C@H](n2c3c(nc2)c(=O)nc(N)[nH]3)O[C@H](COP(=O)(OP(=O)(O[C@@H]2[C@H](O)[C@@H](O)[C@H](O)[C@@H	15839-70-0	Guanosine 5,Ä≤-(trihydrogen diphosphate), P,Ä≤-(6-deoxy-Œ≤-L-galactopyranosyl)	C16H25N5O15P2	589.34	A/B	-8.4
6187	C(n1c2c(nc1CC)c(C)cc(C)n2)c1ccc(cc1)c1c(S(=O)(=O)NC(=O)CC)cccc1	158963-54-3	Propanamide, N-[[4,Ä≤-[(2-ethyl-5,7-dimethyl-3H-imidazo[4,5-b]pyridin-3-yl)methy	C27H30N4O3S	490.62	A/B	-8.4
6188	N(N=c1c2c(c(S(=O)(=O)O)cc1N=Nc1c3c(c(S(=O)(=O)O)ccc3)cccc2	1598475-05-8	1-Naphthalenesulfonic acid, 4-[2-(2-hydroxy-7-sulfo-1-naphthalenyl)diazenyl]-	C20H14N2O7S2	458.46	A/B	-8.4
6189	O=C1[C@@H]2[C@@H](c3c([C@H](OCc4cn(nn4)c4c(F)cccc4)[C@H]2CO1)cc1c(c3)OCO1)c1cc(OC)c(OC)c(OC)c1	1600500-21-7	Furo[3,Ä≤,4,Ä≤:6,7]naphtho[2,3-d]-1,3-dioxol-6(5aH)-one, 9-[[1-(4-bromophenyl)-	C31H28BrN3O8	650.47	A/B	-8.4
6190	O=C1[C@@H]2[C@@H](c3c([C@H](OCc4cn(nn4)c4ccc(OC)cc4)[C@H]2CO1)cc1c(c3)OCO1)c1cc(OC)c(OC)c(OC)c1	1600500-23-9	Furo[3,Ä≤,4,Ä≤:6,7]naphtho[2,3-d]-1,3-dioxol-6(5aH)-one, 9-[[1-(2-fluorophenyl)	C31H28FN3O8	589.57	A/B	-8.4
6191	O=C1[C@@H]2[C@@H](c3c([C@H](OCc4cn(nn4)c4cc(Cl)c(F)cc4)[C@H]2CO1)cc1c(c3)OCO1)c1cc(OC)c(OC)c(OC)c1	1600500-27-3	Furo[3,Ä≤,4,Ä≤:6,7]naphtho[2,3-d]-1,3-dioxol-6(5aH)-one, 9-[[1-(4-chlorophenyl)-	C31H28ClN3O8	606.02	A/B	-8.4
6192	O=C1[C@@H]2[C@@H](c3c([C@H](OCc4cn(nn4)c4c(I)cccc4)[C@H]2CO1)cc1c(c3)OCO1)c1cc(OC)c(OC)c(OC)c1	1600500-31-9	Furo[3,Ä≤,4,Ä≤:6,7]naphtho[2,3-d]-1,3-dioxol-6(5aH)-one, 5,8,8a,9-tetrahydro-9-	C31H29N3O9	587.58	A/B	-8.4
6193	O=C1[C@@H]2[C@@H](c3c([C@H](OCc4cn(nn4)c4c(C#N)cccc4)[C@H]2CO1)cc1c(c3)OCO1)c1cc(OC)c(OC)c(OC)c1	1600500-35-3	Furo[3,Ä≤,4,Ä≤:6,7]naphtho[2,3-d]-1,3-dioxol-6(5aH)-one, 9-[[1-(3-bromophenyl)-	C31H28BrN3O8	650.47	A/B	-8.4
6194	C(COC[P@@](=O)(Oc1ccccc1)N[C@H](C(=O)OCC(C)C)C)n1c2c(nc1)c(N)ncn2	1607007-18-0	Diphosphonic acid, P,P,Ä≤-bis[[(1R)-2-(6-amino-9H-purin-9-yl)-1-methylethoxy]met	C18H26N10O7P2	556.41	A/B	-8.4
6195	O[C@H]1[C@H](n2c3c(nc2)c(=O)nc(N)[nH]3)O[C@H](COP(=O)(OP(=O)(O[C@@H]2[C@H](O)[C@H](O)[C@@H](O)[C@H]	1608090-20-5	7-Deazaguanosine, 2,Ä≤-deoxy-5-iodocytidylyl-(3,Ä≤,Üí5,Ä≤)-2,Ä≤-deoxy-7-iodo-	C20H24I2N7O10P	807.23	A/B	-8.4
6196	O=c1n([C@@H]2O[C@H](CO[P@@](=O)(N[C@@H](Cc3ccccc3)C(=O)OCCC#N)[C@@H](N=[N+]=[N-])C2)cc(C)c(=O)[nH]	160999-64-4	9H-Purin-6-amine, 9-Œ≤-D-arabinofuranosyl-N-[(4-nitrophenyl)methyl]-	C17H18N6O6	402.36	A/B	-8.4
6197	C(OCCOC(=O)[C@@H](NC(=O)Nc1cc(C(F)(F)F)c(Cl)cc1)C(C)C)n1c2c(nc1)c(=O)nc([nH]2)N	1612850-51-7	L-Valine, N-[[(3-chloro-4-fluorophenyl)amino]carbonyl]-, 2-[(2-amino-1,6-dihydro	C20H23ClFN7O5	495.89	A/B	-8.4
6198	O[C@H]1[C@@H](O[C@H](COP(=O)(OCOC(=O)OCC)OCOC(=O)OCC)[C@H]1O)n1cc(nc(C(=O)N)c1=O)F	1613031-57-4	2-Pyrazinecarboxamide, 4-[5-O-[bis[[(cyclohexylcarbonyl)oxy]methoxy]phosphinyl]-	C26H37FN3O13P	649.56	A/B	-8.4
6199	CC1c2c(C3C1CCC3)c(ccc2c1[nH]c(nc1)[C@H]1N(C(=O)[C@@H](NC(=O)OC)C(C)C)CCC1)c1ccc(cc1)c1[nH]c(nc1)[C@H	1613056-86-2	Carbamic acid, N-[(1S)-1-[[(2S)-2-[5-[4-[1,2,3a,8,8a-hexahydro-7-[2-[(2S)-1-[(	C46H56N8O7	832.99	A/B	-8.4
6200	[C@H](C(=O)N1[C@@H]2[C@H](C2)C[C@H]1C#N)(N)C12CC3(OC(=O)[C@@H](CCC(=O)N)N)CC(C2)CC(C1)C3	1616609-18-7	L-Glutamine, 3-[(S)-1-amino-2-[(1R,3S,5S)-3-cyano-2-azabicyclo[3.1.0]hex-2-yl]-	C23H33N5O4	443.54	A/B	-8.4

6201	[C@H](C(=O)N1[C@@H]2[C@@H](C2)C[C@H]1C#N)(N)C12CC3(OC(=O)[C@H]([C@H](C)O)N)CC(C2)CC(C1)C3	1616609-31-4	D-Phenylalanine, 3-[(1S)-1-amino-2-[(1S,3S,5S)-3-cyano-2-azabicyclo[3.1.0]hex-2-	C27H34N4O3	462.58	A/B	-8.4
6202	[C@H](C(=O)N1[C@@H]2[C@@H](C2)C[C@H]1C#N)(N)C12CC3(OC(=O)[C@@H](NC)C)CC(C2)CC(C1)C3	1616609-39-2	Cyclobutanecarboxylic acid, 1-amino-, 3-[(1S)-1-amino-2-[(1S,3S,5S)-3-cyano-2-az	C23H32N4O3	412.53	A/B	-8.4
6203	[C@H](C(=O)N1[C@@H]2[C@@H](C2)C[C@H]1C#N)(N)C12CC3(OC(=O)[C@H](C(C)C)N)CC(C2)CC(C1)C3	1616609-43-8	D-Alanine, N,N-dimethyl-, 3-[(1S)-1-amino-2-[(1S,3S,5S)-3-cyano-2-azabicyclo[3.1	C23H34N4O3	414.54	A/B	-8.4
6204	[C@H](C(=O)N1[C@@H]2[C@@H](C2)C[C@H]1C#N)(N)C12CC3(OC(=O)[C@@H](CC(C)C)N)CC(C2)CC(C1)C3	1616609-46-1	L-Leucine, 3-[(1S)-1-amino-2-[(1S,3S,5S)-3-cyano-2-azabicyclo[3.1.0]hex-2-yl]-2-	C24H36N4O3	428.57	A/B	-8.4
6205	C(=O)(N[C@H](C(=O)C(=O)N)CCC)[C@H]1N(C(=O)[C@@H](NC(=O)[C@@H](NC(=O)c2cnccn2)C2CCCCC2)C(C)(C)C)C[C@H	1616696-01-5	3-Azabicyclo[3.1.0]hexane-2-carboxamide, N-[(1S)-3-amino-1-(cyclobutylmethyl)-2,	C36H53N5O5	635.84	A/B	-8.4
6206	O(C(=O)N[C@H]([C@@H](CN(S(=O)(=O)c1ccc(OC)cc1)CC(C)C)O)Cc1ccccc1)[C@@H]1[C@H]2[C@@H](OC1)OCC2(F)F	1616728-87-0	Cyclopenta[c]pyrrole-1-carboxamide, (2S)-2-cyclohexyl-N-(2-pyrazinylcarbonyl)gly	C36H55N7O6	681.87	A/B	-8.4
6207	O=c1c2c(n(cn2)[C@@H]2O[C@H](COP(=O)(N(CCCl)CCCl)Oc3ccc(Cl)cc3)CC2)[nH]cn1	1618656-23-7	Inosine, 2,Ä≤,3,Ä≤-dideoxy-, 5,Ä≤-[4-chlorophenyl N-[(4-fluorophenyl)methyl]pho	C23H22ClFN5O5P	533.88	A/B	-8.4
6208	N(C(=O)[C@H](Cc1cccnc1)C[C@@H](CN1[C@H](C(=O)NC(C)(C)C)CN(Cc2scc(Br)c2)CC1)O)[C@H]1c2c(C[C@H]1O)cccc	162105-28-4	1-Piperazinepentanamide, 4-[(2,6-difluorophenyl)methyl]-N-(2,3-dihydro-2-hydroxy	C36H45F2N5O4	649.77	A/B	-8.4
6209	C[C@]1(F)[C@@H](O[C@H](C(OP(=O)(Oc2ccccc2)N[C@H](C(=O)OC(C)C(C)C)C)[C@H]1O)n1c(=O)[nH]c(=O)c(c1[2H	1621166-15-1	L-Alanine, N-(hydroxyphenoxyphosphinyl)-, 2-methylpropyl ester, 5,Ä≤-ester with	C24H31D2FN3O9P		A/B	-8.4
6210	N(CCCNc1nc(ccn1)N1CCN(C)CC1)c1nc(nc(n1)N1CCOCC1)N1CCOCC1	1623752-47-5	1,2-Ethanediamine, N1-(4,6-di-4-morpholinyl-1,3,5-triazin-2-yl)-N2-[4-(4-methyl-	C22H35N11O2	485.59	A/B	-8.4
6211	N(CCCCNc1nc(ccn1)N1CCN(C)CC1)c1nc(nc(n1)N1CCOCC1)N1CCOCC1	1623752-48-6	1,3-Propanediamine, N1-(4,6-di-4-morpholinyl-1,3,5-triazin-2-yl)-N3-[4-(4-methyl	C23H37N11O2	499.61	A/B	-8.4
6212	N(c1nc(nc(n1)N1CCOCC1)c1ccc(OC)cc1)c1ccc(N(=O)=O)cc1	1627734-66-0	1,3,5-Triazin-2-amine, 4-(4-methoxyphenyl)-6-(4-morpholinyl)-N-(3-nitrophenyl)-	C20H20N6O4	408.41	A/B	-8.4
6213	C(P(=O)(OCC)OCC)(C/C=C(/CC/C=C(/CCC=C(C)C)\C)\C)(C/C=C(/CC/C=C(/CCC=C(C)C)\C)\C)C(=O)OCC	1627841-80-8	1-Naphthalenesulfonic acid, 8-amino-5-[2-[2,5-dimethyl-4-[2-(3-sulfophenyl)diaze	C24H21N5O6S2	539.58	A/B	-8.4
6214	C[C@]1(F)[C@@H](O[C@H](COP(=O)(Oc2ccc(C#Cc3ccccc3)cc2)N[C@H](C(=O)OC(C)C)C)[C@H]1O)n1c(=O)[nH]c(=O)c	1628724-51-5	L-Alanine, N-[(2,Ä≤R)-2,Ä≤-deoxy-2,Ä≤-fluoro-2,Ä≤-methyl-P-[2-(phenylmethyl)phe	C29H35FN3O9P	619.58	A/B	-8.4
6215	O=c1n([C@@H]2O[C@H](COP(=O)(Oc3ccccc3)NCCNC(=O)CC/C=C\C/C=C\C/C=C\C/C=C\C/C=C\C/C=C\CC)[C@@H](N=[N+]	1628724-89-9	L-Alanine, N-[(2,Ä≤R)-2,Ä≤-deoxy-2,Ä≤-fluoro-2,Ä≤-methyl-P-(4,Ä≤-methyl[1,1,Ä≤	C29H35FN3O9P	619.58	A/B	-8.4
6216	N(c1nc(nc(Nc2ccc(N(=O)=O)cc2)n1)N1CCOCC1)c1nc(OC)cc(OC)n1	1631751-04-6	1,3,5-Triazine-2,4-diamine, N2-(4,6-dimethoxy-2-pyrimidinyl)-N4-(4-methylphenyl)	C20H24N8O3	424.46	A/B	-8.4
6217	N(c1nc(nc(Nc2cc(N(=O)=O)ccc2)n1)N1CCOCC1)c1nc(OC)cc(OC)n1	1631751-06-8	1,3,5-Triazine-2,4-diamine, N2-(3,4-dichlorophenyl)-N4-(4,6-dimethoxy-2-pyrimidi	C19H20Cl2N8O3	479.32	A/B	-8.4
6218	N(c1nc(nc(Nc2c(Cl)cccc2)n1)N1CCOCC1)c1nc(OC)cc(OC)n1	1631751-09-1	Phenol, 2-[[4-[(4,6-dimethoxy-2-pyrimidinyl)amino]-6-(4-morpholinyl)-1,3,5-triaz	C19H22N8O4	426.43	A/B	-8.4
6219	N(c1nc(nc(Nc2cc(Cl)ccc2)n1)N1CCOCC1)c1nc(OC)cc(OC)n1	1631751-10-4	1,3,5-Triazine-2,4-diamine, N2-(2-chlorophenyl)-N4-(4,6-dimethoxy-2-pyrimidinyl)	C19H21ClN8O3	444.87	A/B	-8.4
6220	O=C1[C@@H]2[C@@H](c3c([C@H](OC(=O)/C(=C/c4cc(OC)c(OCc5ccccc5)cc4)/c4cc(OC)c(OC)cc4)[C@H]2CO1)cc1c(c3	1633020-39-9	2-Propynoic acid, 3-phenyl-, (5R,5aR,8aR,9R)-5,5a,6,8,8a,9-hexahydro-8-oxo-9-(3,	C31H26O9	542.53	A/B	-8.4
6221	O=C1[C@@H]2[C@@H](c3c([C@H](OC(=O)/C(=C/c4cc(OC)c(OC)c(OC)c4)/c4ccc(OC)cc4)[C@H]2CO1)cc1c(c3)OCO1)c1	1633020-44-6	Benzeneacetic acid, Œ±-[[4-(acetyloxy)-3-methoxyphenyl]methylene]-3,4-dimethoxy-	C42H40O14	768.76	A/B	-8.4
6222	[C@@H](C(CC)CC)(NC(=O)C)[C@H]1[C@H](NC(=N)N)C[C@H](C(=O)OCC(=O)OC[C@H]2N[C@H]([C@H](O)[C@@H]2O)c2c3c	1638370-18-9	1-Cyclohexene-1-carboxylic acid, 4-(acetylamino)-5-amino-3-(1-ethylpropoxy)-, [(	C25H37N7O6	531.6	A/B	-8.4
6223	C[C@]1(F)[C@@H](O[C@H](CO[P@](=O)(Oc2cc3c(cc2)OCO3)N[C@H](C(=O)OC2CCCCC2)C)[C@H]1O)n1c(=O)[nH]c(=O)c	1638769-88-6	L-Alanine, N-[[P(S),2,Ä≤R]-P-1,3-benzodioxol-5-yl-2,Ä≤-deoxy-2,Ä≤-fluoro-2,Ä≤-m	C23H29FN3O11P	573.46	A/B	-8.4
6224	C(=O)([C@@H](NC(=O)OC)C(C)C)N1[C@@H](C=CC1)c1[nH]c(cn1)c1ccc(cc1)c1ccc(cc1)c1[nH]c(nc1)[C@H]1N(C(=O)	1639870-81-7	Carbamic acid, N-[(1S)-1-[[(2S)-2,5-dihydro-2-[5-[4,Ä≤-[2-[(2S)-1-[[1-[(methoxyc	C39H44N8O6	720.82	A/B	-8.4
6225	C(#N)[C@]1(O[C@H](COP(=O)(N[C@H](C(=O)O)C)O)[C@@H](O)[C@@]1(C)O)c1n2c(cc1)c(N)ncn2	1643332-93-7	Benzonitrile, 4-[[4-[(3-bromophenyl)amino]-6-(4-morpholinyl)-1,3,5-triazin-2-yl]	C20H17BrN6O2	453.29	A/B	-8.4
6226	C(=O)([C@@H](NC(=O)OC)C(C)C)N1[C@@H](CCC1)c1[nH]c(cn1)c1ccc(cc1)c1ccc(cc1)c1[nH]c([C@@H](NC(=O)OC(C)	1645563-56-9	Carbamic acid, N,N,Ä≤-[9H-fluorene-2,7-diylbis[1H-imidazole-5,2-diyl[(2S,5S)-5-m	C43H54N8O6	778.94	A/B	-8.4
6227	N(C(=O)[C@H](Cc1ccccc1)C[C@@H](CN1[C@H](C(=O)NC(C)(C)C)CN(Cc2ccncc2)CC1)O)[C@H]1c2c(C[C@H]1O)cccc2	164725-24-0	1-Piperazinepentanamide, N-(2,3-dihydro-2-hydroxy-1H-inden-1-yl)-2-[[(1,1-dimeth	C36H53N5O5	635.84	A/B	-8.4
6228	N(C(=O)[C@H](Cc1ccccc1)C[C@@H](CN1[C@H](C(=O)NC(C)(C)C)CN(Cc2csc(C)n2)CC1)O)[C@H]1c2c(C[C@H]1O)cccc2	164725-60-4	1-Piperazinepentanamide, N-(2,3-dihydro-2-hydroxy-1H-inden-1-yl)-2-[[(1,1-dimeth	C36H49N5O4	615.81	A/B	-8.4
6229	C(C(C)C)n1c2c(nc1C(OC(=O)C)c1ccccc1)c(N)nc1c2cccc1	165070-57-5	Benzenesulfonic acid, 4-[2-(2-hydroxy-1-naphthalenyl)diazenyl]-3,5-dimethyl-	C18H16N2O4S	356.4	A/B	-8.4
6230	C(N(C(=O)N[C@H](C(=O)N[C@@H](Cc1ccccc1)C[C@@H]([C@H](Cc1ccccc1)NC(=O)OCc1cncs1)O)C(C)C)C)c1nc(sc1)C1	165315-19-5	2,4,7,12-Tetraazatridecan-13-oic acid, 10-hydroxy-2-methyl-5-(1-methylethyl)-3,6	C38H49N7O5S2	747.97	A/B	-8.4
6231	[C@H](Cc1ccccc1)(C[C@@H]([C@H](Cc1ccccc1)NC(=O)OCc1cncs1)O)NC(=O)[C@@H](NC(=O)N(Cc1nc(CC(C)C)sc1)C)C	165315-31-1	2,4,7,12-Tetraazatridecan-13-oic acid, 1-(2-cyclopentyl-4-thiazolyl)-10-hydroxy-	C39H50N6O5S2	746.98	A/B	-8.4
6232	C(=O)([C@@H](NC(=O)OC)C(C)C)N1C[C@@]2(C[C@H]1c1[nH]c3c(n1)ccc1c3ccc(c1)c1ccc(cc1)c1[nH]c(nc1)[C@H]1N	1653966-61-0	Carbamic acid, N-[(1S)-1-[[(3S,5S)-3-[7-[4-[2-[(6S)-5-[(2S)-2-[(methoxycarbonyl)	C48H58N8O7	859.02	A/B	-8.4
6233	C(=O)([C@@H](NC(=O)OC)C(C)C)N1C[C@@]2(C[C@H]1c1[nH]c(cn1)c1ccc(cc1)c1ccc(cc1)c1[nH]c(nc1)[C@H]1N(C(=	1653966-96-1	Carbamic acid, N-[(1S)-2-methyl-1-[[(3S,5S)-3-[5-[4,Ä≤-[2-[(2S,3aS,7aS)-octahydr	C48H62N8O7	863.06	A/B	-8.4
6234	N(C(=O)[C@H](Cc1ccccc1)C[C@@H](CN1[C@H](C(=O)NC(C)(C)C)CN(S(=O)(=O)c2cccnc2)CC1)O)[C@H]1c2c(C[C@H]1O	1659319-41-1	Thymidine, 3,Ä≤-azido-3,Ä≤-deoxy-, 5,Ä≤-[[(2R,5S)-5-(4-amino-2-oxo-1(2H)-pyrimi	C19H24N9O9PS		A/B	-8.4
6235	O=c1n([C@@H]2O[C@H](COP(=O)(C(O)c3cc(N(=O)=O)ccc3)OC[C@@H]3[C@@H](N=[N+]=[N-])C[C@@H](O3)n3c(=O)[nH]	166318-78-1	Thymidine, 3,Ä≤-azido-P-[(4-cyanophenyl)hydroxymethyl]-P,3,Ä≤-dideoxythymidylyl	C28H30N11O10P		A/B	-8.4
6236	O[C@H]1[C@H](n2c3c(nc2)c(=O)nc(N)[nH]3)O[C@H](COP(=O)(OP(=O)(O[C@H]2O[C@H](CO)[C@@H](O)[C@H](O)C2)O)	1668551-18-5	Furo[3,Ä≤,4,Ä≤:6,7]naphtho[2,3-d]-1,3-dioxol-6(5aH)-one, 5-(3,4-dimethoxyphenyl	C21H20O7	384.38	A/B	-8.4
6237	O=C1[C@@H]2[C@@H](c3c([C@H](OC(=O)CCC(=O)OC(COC(=O)CCC(=O)O[C@H]4c5c([C@H]([C@@H]6[C@@H]4COC6=O)c4cc	1669412-77-4	Butanedioic acid, 1,1,Ä≤-[1-(dimethylamino)-1,2-ethanediyl] 4,4,Ä≤-bis[(5R,5aR,	C57H61NO22	1112.09	A/B	-8.4
6238	O(C(=O)N[C@@H](Cc1ccccc1)[C@@H](CN(S(=O)(=O)c1ccc(OC)cc1)CC(C)C)O)[C@@H]1[C@@H]2[C@H](OC1)OC(F)(F)C2	1672661-69-6	Carbamic acid, N-[(1S,2R)-2-hydroxy-3-[[(4-methoxyphenyl)sulfonyl](2-methylpropy	C28H36F2N2O8S	598.66	A/B	-8.4
6239	C([C@H](NC(=O)N1CCOCC1)C(=O)N[C@H](/C=C/S(=O)(=O)c1ccccc1)CCc1ccccc1)c1ccc(cc1)c1ccccc1	170111-45-2	4-Morpholinecarboxamide, N-[(1S)-1-[(4-hydroxyphenyl)methyl]-2-oxo-2-[[(1S,2E)-1	C31H35N3O6S	577.69	A/B	-8.4
6240	N(c1nc(nc(n1)N1CCOCC1)N1CCOCC1)c1ccc(C(=O)/C=C/c2cc(Cl)ccc2)cc1	1710363-31-7	2-Propen-1-one, 3-(3,4-dimethoxyphenyl)-1-[4-[(4,6-di-4-morpholinyl-1,3,5-triazi	C28H32N6O5	532.59	A/B	-8.4
6241	O=c1c2c(n(cn2)[C@@H]2O[C@H](COP(=O)(OC[C@@H]3[C@@H](N=[N+]=[N-])C[C@@H](O3)n3c(=O)[nH]c(=O)c(C)c3)OC	171284-25-6	Thymidine, 3,Ä≤-azido-3,Ä≤-deoxy-P-[[4-(2,2-dimethyl-1-oxopropoxy)phenyl]methyl	C32H39N10O12P		A/B	-8.4
6242	O=C1[C@@H]2[C@@H](c3c([C@H](OC(=O)c4cccs4)[C@H]2CO1)cc1c(c3)OCO1)c1cc(OC)c(OC)c(OC)c1	1715004-92-4	1H-Indole-3-acetic acid, 2-methyl-, (5R,5aR,8aR,9R)-5,5a,6,8,8a,9-hexahydro-8-ox	C33H31NO9	585.6	A/B	-8.4
6243	C(Oc1c(O)cccc1)c1c2c(N(c3c(C(=O)N2)cccn3)C2CC2)ncc1	172326-08-8	6H-Dipyrido[3,2-b:2,Ä≤,3,Ä≤-e][1,4]diazepin-6-one, 11-cyclopropyl-5,11-dihydro-	C22H20N4O2	372.42	A/B	-8.4
6244	C(Oc1ccc(NCC)cc1)c1c2c(N(c3c(C(=O)N2)cccn3)C2CC2)ncc1	172326-11-3	6H-Dipyrido[3,2-b:2,Ä≤,3,Ä≤-e][1,4]diazepin-6-one, 4-[(4-aminophenoxy)methyl]-1	C21H19N5O2	373.41	A/B	-8.4
6245	C(c1c2c(N(c3c(C(=O)N2)cccn3)C2CC2)ncc1)c1ccccc1	172326-15-7	6H-Dipyrido[3,2-b:2,Ä≤,3,Ä≤-e][1,4]diazepin-6-one, 11-cyclopropyl-5,11-dihydro-	C21H17N5O4	403.39	A/B	-8.4
6246	N(C(=O)[C@H](Cc1ccccc1)C[C@@H](CN1[C@H](C(=O)NC(C)(C)C)CN(Cc2nc(COC)sc2)CC1)O)[C@H]1c2c(C[C@H]1O)ccc	172649-52-4	1-Piperazinepentanamide, N-(2,3-dihydro-2-hydroxy-1H-inden-1-yl)-4-[[2-(dimethyl	C36H50N6O4S	662.89	A/B	-8.4
6247	O[C@H]1[C@H](n2c3c(nc2)c(=O)nc(N)[nH]3)O[C@H](COP(=O)(O[C@H]2[C@@H](O)[C@@H](O[C@@H]2CO)n2c3c(nc2)c(	17332-08-0	3,Ä≤-Adenylic acid, guanylyl-(3,Ä≤,Üí5,Ä≤)-	C20H26N10O14P2	692.43	A/B	-8.4
6248	O=c1n([C@@H]2O[C@H](COP(=O)(OC[C@@H]3[C@@H](N=[N+]=[N-])C[C@@H](O3)n3c(=O)[nH]c(=O)c(C)c3)OCc3ccc(Cl	173606-87-6	Thymidine, 3,Ä≤-azido-3,Ä≤-deoxy-P-[(4-methylphenyl)methyl]thymidylyl-(5,Ä≤,Üí5	C28H33N10O10P		A/B	-8.4
6249	O=c1n([C@@H]2O[C@H](COP(=O)([C@@H](O)c3ccc(OC)cc3)OC[C@@H]3[C@@H](N=[N+]=[N-])C[C@@H](O3)n3c(=O)[nH]	173606-90-1	Thymidine, 3,Ä≤-azido-3,Ä≤-deoxy-P-[(3-nitrophenyl)methyl]thymidylyl-(5,Ä≤,Üí5,	C27H30N11O12P		A/B	-8.4
6250	O=c1n([C@@H]2O[C@H](COP(=O)([C@@H](O)c3c(N(=O)=O)cc(N(=O)=O)cc3)OC[C@@H]3[C@@H](N=[N+]=[N-])C[C@@H](	173658-27-0	Thymidine, 3,Ä≤-azido-P,3,Ä≤-dideoxy-P-[hydroxy(4-nitrophenyl)methyl]thymidylyl	C27H30N11O12P		A/B	-8.4
6251	O=c1n([C@@H]2O[C@H](COP(=O)([C@H](O)c3ccc(C)cc3)OC[C@@H]3[C@@H](N=[N+]=[N-])C[C@@H](O3)n3c(=O)[nH]c(	173658-30-5	Thymidine, 3,Ä≤-azido-P,3,Ä≤-dideoxy-P-[hydroxy(4-methoxyphenyl)methyl]thymidyl	C28H33N10O11P		A/B	-8.4
6252	C(Cc1ccc(F)cc1)C1(CCC)CC(=C(C(CC)c2cc(NS(=O)(=O)c3ccc(C#N)cn3)ccc2)C(=O)O1)O	174484-03-8	2-Pyridinesulfonamide, 5-cyano-N-[3-[1-[6-[2-(4-fluorophenyl)ethyl]-5,6-dihydro-	C33H36FN3O5S	605.72	A/B	-8.4
6253	C(Cc1ccccc1)C1(CCc2ccccc2)CC(=C([C@H](CC)c2cc(NS(=O)(=O)c3ccc(C(F)(F)F)cn3)ccc2)C(=O)O1)O	174484-30-1	2-Pyridinesulfonamide, N-[3-[1-(5,6-dihydro-4-hydroxy-2-oxo-6,6-dipropyl-2H-pyra	C28H35F3N2O5S	568.65	A/B	-8.4
6254	C(Cc1ccc(F)cc1)C1(CCC)CC(=C(C(CC)c2cc(NS(=O)(=O)c3ccc(C(=O)N)cn3)ccc2)C(=O)O1)O	174484-47-0	2-Pyridinesulfonamide, 5-cyano-N-[3-[1-[5,6-dihydro-4-hydroxy-2-oxo-6,6-bis(2-ph	C36H35N3O5S	621.75	A/B	-8.4
6255	[C@H](C(C)(C)C)(C1=C(O)C[C@@](CCc2ccccc2)(CCC)OC1=O)c1cc(NS(=O)(=O)c2ccccn2)ccc1	174590-19-3	2-Pyridinesulfonamide, 5-amino-N-[3-[1-[5,6-dihydro-4-hydroxy-2-oxo-6-(2-phenyle	C32H36F3N3O5S	631.71	A/B	-8.4
6256	O=c1c2c(n(cn2)[C@@H]2O[C@H](COP(=O)(OP(=O)(OC[C@H]3O[C@H](C[C@@H]3O)n3c(=O)[nH]c(=O)c(F)c3)O)O)CC2)[	175923-15-6	Thymidine 5,Ä≤-(trihydrogen diphosphate), 3,Ä≤-azido-3,Ä≤-deoxy-, P,Ä≤,Üí5,Ä≤-e	C19H24FN7O14P2		A/B	-8.4
6257	[C@H](Cc1ccccc1)(C[C@@H]([C@H](Cc1ccccc1)NC(=O)OCc1cncs1)O)NC(=O)[C@@H](NC(=O)NC)C(C)C	176655-56-4	2,7,10,12-Tetraazatridecanoic acid, 4-hydroxy-13-[2-(1-hydroxy-1-methylethyl)-4-	C37H48N6O6S2	736.94	A/B	-8.4
6258	O=c1n(cc(C(F)(F)F)c(=O)[nH]1)[C@@H]1O[C@H](COC(=S)c2ccc(Cl)cc2)[C@@H](O)C1	177165-83-2	Thymidine, Œ±,Œ±,Œ±-trifluoro-, 3,Ä≤,5,Ä≤-bis(4-chlorobenzenecarbothioate)	C24H17Cl2F3N2O5S2	605.43	A/B	-8.4
6259	C(O[P@@](=S)(Oc1ccccc1)N[C@@H](C(=O)OC(C)C)C)[C@H]1C2([C@H]([C@@H](F)[C@@H]1O)n1c3c(nc1)c(=O)nc(N)[n	1781232-55-0	D-Alanine, N-[(R)-[[(4R,5R,6S,7R)-7-(2-amino-1,6-dihydro-6-oxo-9H-purin-9-yl)-5,	C25H33N6O7PS	592.6	A/B	-8.4
6260	C(=O)(NC(C)(C)C)[C@@H]1N(C[C@H]([C@@H](NC(=O)c2c(C)c(O)ccc2)CSc2ccccc2)O)C[C@@H]2[C@H](C1)CCCC2	1796934-12-7	2,7,10,12-Tetraazatridecanoic acid, 4-hydroxy-13-[2-(1-hydroxy-1-methylethyl)-4-	C37H48N6O6S2	736.94	A/B	-8.4
6261	C([C@H](OCP(=O)(OCOC(=O)OC(C)C)OCOC(=O)OC(C)C)C)n1c2c(nc1)c(N)ncn2.C(=O)(O)c1ccc(=O)oc1	1799419-19-4	L-Prolinamide, 2,2,Ä≤-[[(2S,5S)-1-[4-(1,1-dimethylethyl)phenyl]-2,5-pyrrolidine	C50H67N7O8.C40H43N7O7S.C37H48N6O5S2		A/B	-8.4
6262	O(C(=O)N[C@@H](Cc1cc(ccc1)c1c(OC)cccc1)[C@@H](CN(S(=O)(=O)c1ccc(OC)cc1)CC(C)C)O)[C@@H]1[C@H]2[C@@H](	1800006-54-5	Carbamic acid, N-[(1S,2R)-2-hydroxy-1-[(3,Ä≤-methoxy[1,1,Ä≤-biphenyl]-3-yl)meth	C35H44N2O9S	668.8	A/B	-8.4
6263	FC1(F)CC2(c3c(-c4c2cc(cc4)c2[nH]c(nc2)[C@H]2N(C(=O)[C@@H](NC(=O)OC)[C@H](CC)C)CCC2)ccc(c3)c2[nH]c(nc	1800210-32-5	Carbamic acid, N,N,Ä≤-[(3,3-difluorospiro[cyclobutane-1,9,Ä≤-[9H]fluorene]-2,Ä≤	C44H52F2N8O6	826.93	A/B	-8.4
6264	C[C@]1(F)[C@@H](O[C@H](CO[P@](=O)(Oc2ccc(cc2)C2CC2)N[C@H](C(=O)OC(C)C)C)[C@H]1O)n1c(=O)[nH]c(=O)cc1	1800478-04-9	L-Alanine, N-[[P(S),2,Ä≤R]-P-(3-cyclopropylphenyl)-2,Ä≤-deoxy-2,Ä≤-fluoro-2,Ä≤-	C25H33FN3O9P	569.52	A/B	-8.4
6265	C[C@]1(F)[C@@H](O[C@H](CO[P@@](=O)(Oc2ccc(cc2)C2CC2)N[C@H](C(=O)OC(C)C)C)[C@H]1O)n1c(=O)[nH]c(=O)cc1	1800478-06-1	L-Alanine, N-[[P(S),2,Ä≤R]-P-(4-cyclopropylphenyl)-2,Ä≤-deoxy-2,Ä≤-fluoro-2,Ä≤-	C25H33FN3O9P	569.52	A/B	-8.4
6266	C[C@]1(F)[C@@H](O[C@H](CO[P@@](=O)(Oc2ccc(C#Cc3ccccc3)cc2)N[C@H](C(=O)OC(C)C)C)[C@H]1O)n1c(=O)[nH]c(	1800478-16-3	L-Alanine, N-[[P(S),2,Ä≤R]-2,Ä≤-deoxy-2,Ä≤-fluoro-2,Ä≤-methyl-P-[4-(2-phenyleth	C30H33FN3O9P	629.57	A/B	-8.4
6267	C[C@]1(F)[C@@H](O[C@H](CO[P@](=O)(Oc2cc3c(cc2)CCC3)N[C@H](C(=O)OC(C)C)C)[C@H]1O)n1c(=O)[nH]c(=O)cc1	1800478-34-5	L-Alanine, N-[[P(S),2,Ä≤R]-2,Ä≤-deoxy-P-(2,3-dihydro-1H-inden-5-yl)-2,Ä≤-fluoro	C25H33FN3O9P	569.52	A/B	-8.4
6268	C[C@]1(F)[C@@H](O[C@H](CO[P@](=O)(Oc2cc3c(cc2)CCC3)N[C@H](C(=O)OC(C)C)C)[C@H]1OC(=O)C)n1c(=O)[nH]c(	1800478-61-8	L-Alanine, N-[[P(S),2,Ä≤R]-3,Ä≤-O-acetyl-2,Ä≤-deoxy-P-(2,3-dihydro-1H-inden-5-y	C27H35FN3O10P	611.55	A/B	-8.4
6269	C[C@]1(F)[C@@H](O[C@H](CO[P@](=O)(Oc2ccc(cc2)C2(F)COC2)N[C@H](C(=O)OC(C)C)C)[C@H]1O)n1c(=O)[nH]c(=O)	1800478-79-8	L-Alanine, N-[[P(S),2,Ä≤R]-2,Ä≤-deoxy-2,Ä≤-fluoro-P-[4-(3-hydroxy-3-oxetanyl)ph	C25H33FN3O11P	601.52	A/B	-8.4
6270	C(=O)(OC)n1c(ncc1c1ccc(cc1)c1ccc(cc1)c1n(C(=O)OC)c(nc1)[C@H]1N(C(=O)[C@@H](NC(=O)OC)C(C)C)CCC1)[C@H]	1800502-87-7	Carbamic acid, N-[(1S)-1-[[(2S)-2-[1-(3-cyclopentyl-1-oxopropyl)-5-[4,Ä≤-[1-(3-c	C56H74N8O8	987.24	A/B	-8.4
6271	C(=O)(n1c(ncc1c1ccc(cc1)c1ccc(cc1)c1n(C(=O)c2ccccc2)c(nc1)[C@H]1N(C(=O)[C@@H](NC(=O)OC)C(C)C)CCC1)[C	1800502-94-6	Carbamic acid, N-[(1S)-1-[[(2S)-2-[1-[2-(acetyloxy)acetyl]-5-[4,Ä≤-[1-[2-(acetyl	C48H58N8O12	939.02	A/B	-8.4
6272	C(NC(=O)c1oc(C)nn1)(C)(C)c1nc(C(=O)NCc2ccc(F)cc2)c(OCC=C)c(=O)n1C	1801912-41-3	4-Pyrimidinecarboxamide, 5-(1,1-dimethylethoxy)-N-[(4-fluorophenyl)methyl]-1,6-d	C24H29FN6O5	500.52	A/B	-8.4
6273	C(CCCCCCCCCCCCCCCC)CCCC(CCC)O	1802141-49-6	2H-Pyrido[1,Ä≤,2,Ä≤:4,5]pyrazino[2,1-b][1,3]oxazine-9-carboxamide, N-[(2,4-difl	C22H23F2N3O5	447.43	A/B	-8.4
6274	O=C1[C@@H]2C(c3c(C(=O)[C@H]2CO1)cc1c(c3)OCO1)c1cc2c(cc1)OCO2	1807621-83-5	Furo[3,Ä≤,4,Ä≤:6,7]naphtho[2,3-d]-1,3-dioxol-6(5aH)-one, 5-(1,3-benzodioxol-5-y	C20H16O7	368.34	A/B	-8.4
6275	C(=O)([C@@H](NC(=O)OC)C(C)C)N1[C@H](c2c3c(c([nH]2)c2ccc(cc2)c2ccc(cc2)c2[nH]c(nc2)[C@H]2N(C(=O)[C@@H	1807622-11-2	Furo[3,Ä≤,4,Ä≤:6,7]naphtho[2,3-d]-1,3-dioxole-5,8-dione, 9-(1,3-benzodioxol-5-y	C20H14O7	366.32	A/B	-8.4
6276	C(=O)([C@@H](NC(=O)OC)C(C)C)N1[C@@H](C2CC1[C@@H](O)[C@H]2O)c1[nH]c(cn1)c1ccc(cc1)c1ccc(cc1)c1[nH]c(n	1808295-90-0	Carbamic acid, N-[(1S)-1-[[(2S)-2-[5-[4,Ä≤-[2-[(3S,6R)-6-hydroxy-2-[(2S)-2-[(met	C42H52N8O7	780.91	A/B	-8.4
6277	C(=O)([C@@H](NC(=O)OC)C(C)C)N1[C@@H](C2CC1CC2)c1[nH]c(cn1)c1ccc(cc1)c1ccc(cc1)c1[nH]c(nc1)[C@H]1N(C(	1808295-91-1	Carbamic acid, N-[(1S)-1-[[(2S)-2-[5-[4,Ä≤-[2-[(3S,5S,6R)-5,6-dihydroxy-2-[(2S)-	C42H52N8O8	796.91	A/B	-8.4
6278	C(=O)([C@@H](NC(=O)OC)C(C)C)N1[C@@H](C2CC1CC2)c1[nH]c(cn1)c1ccc(/C=C/c2ccc(cc2)c2[nH]c(nc2)[C@H]2N(C	1808296-04-9	Carbamic acid, N-[(1S)-1-[[(2S)-2-[5-[4,Ä≤,Ä≤-[2-[(3S)-2-[(2S)-2-[(methoxycarbo	C48H56N8O6	841.01	A/B	-8.4
6279	C(=O)([C@@H](NC(=O)OC)C(C)C)N1C(C2CC1CC2)c1[nH]c(cn1)c1cc(ccc1)c1ccc(cc1)c1[nH]c(nc1)[C@H]1N(C(=O)[C	1808935-16-1	Carbamic acid, N-[(1S)-1-[[hexahydro-2-[5-[4,Ä≤-[2-[(3S)-2-[(2S)-2-[(methoxycarb	C44H56N8O6	792.97	A/B	-8.4
6280	O=C1[C@@H]2[C@@H](c3c([C@H](OC(=O)[C@@H](C)N4C(=O)C5C(C4=O)C4OC5CC4)[C@H]2CO1)cc1c(c3)OCO1)c1cc(OC)c	1809325-33-4	4,7-Epoxy-2H-isoindole-2-acetic acid, octahydro-Œ±-methyl-1,3-dioxo-, (5R,5aR,8a	C33H33NO12	635.61	A/B	-8.4
6281	C(=O)([C@]12C(C)(C)[C@](C)(C(=O)O1)CC2)N1c2c([C@@](C#CC3CC3)(C(F)(F)F)OC1=O)cc(Cl)cc2	181362-85-6	Guanosine, adenylyloxyphosphinicomethylenephosphinicooxyphosphinico-(5,Ä≤,Üí5,Ä≤	C21H30N10O19P4	850.41	A/B	-8.4
6282	O(N(=O)=O)C12CC3(C)CC(N)(C1)CC(C2)C3	183506-06-1	Guanosine, 2,Ä≤-deoxy-P-thioadenylyl-(3,Ä≤,Üí5,Ä≤)-2,Ä≤-deoxy-	C20H25N10O8PS	596.51	A/B	-8.4
6283	O=C1[C@@H]2[C@@H](c3c([C@](CCCC)(O)[C@H]2CO1)cc1c(c3)OCO1)c1cc(OC)c(OC)c(OC)c1	183986-37-0	Carbonic acid, 4-[5,5a,6,8,8a,9-hexahydro-9-hydroxy-6-oxo-9-(phenylethynyl)furo[	C37H30O10	634.63	A/B	-8.4
6284	[P@](=O)(N([C@@H]([C@H](CC)C)C(=O)OC)C)(Oc1ccccc1)OC[C@H]1O[C@H](C=C1)n1c(=O)[nH]c(=O)c(C)c1	184031-35-4	L-Alanine, N-[2,Ä≤,3,Ä≤-didehydro-3,Ä≤-deoxy-P-(2,4-dibromophenyl)-5,Ä≤-thymidy	C26H26Br2N3O8P	699.28	A/B	-8.4
6285	[C@@H](Cc1ccccc1)(NC(=O)N1CCN(C)CC1)C(=O)N[C@H](/C=C/S(=O)(=O)c1ccc(C)cc1)CCc1ccccc1	1860793-29-8	1-Piperazinecarboxamide, N-[(1S)-2-[[(1S,2E)-3-[(4-methoxyphenyl)sulfonyl]-1-(2-	C33H40N4O5S	604.76	A/B	-8.4
6286	C[C@]1(F)[C@@H](O[C@H](CO[P@](=O)(Oc2ccccc2)N[C@H](C(=O)OC(C)C)C)[C@H]1OC(=O)[C@H](Cc1ccc(O)cc1)N)n1	1863084-42-7	L-Tryptophan, 3,Ä≤-ester with N-[[P(S),2,Ä≤R]-2,Ä≤-deoxy-2,Ä≤-fluoro-2,Ä≤-methy	C33H39FN5O10P	715.66	A/B	-8.4
6287	O=C1[C@@H]2[C@@H](c3c([C@H](OC(=O)CN4c5c(C(=O)C4=O)cccc5F)[C@H]2CO1)cc1c(c3)OCO1)c1cc(OC)c(OC)c(OC)c	1864833-36-2	1H-Indole-1-acetic acid, 6-chloro-2,3-dihydro-2,3-dioxo-, (5R,5aR,8aR,9R)-5,5a,6	C32H26ClNO11	636	A/B	-8.4
6288	[C@@H](Cc1ccccc1)(NC(=O)[C@H](CCN1CCOCC1)NC(=O)N(Cc1nc(C(C)C)sc1)C)CC[C@H](Cc1ccccc1)NC(=O)OCc1cncs1	18657-20-0	Guanosine 5,Ä≤-(trihydrogen diphosphate), P,Ä≤-(6-deoxy-Œ±-D-talopyranosyl) est	C16H25N5O15P2	589.34	A/B	-8.4
6289	O=C1[C@@H]2[C@@H](c3c([C@H](OC(=O)CCCC[C@H]4SC(SCC4)C4CCCCC4)[C@H]2CO1)cc1c(c3)OCO1)c1cc(OC)c(OC)c(O	1875123-95-7	1,3-Dithiane-4-pentanoic acid, 2-(3-hydroxy-4-methoxyphenyl)-, (5R,5aR,8aR,9R)-5	C38H42O11S2	738.86	A/B	-8.4
6290	C[C@]1(F)[C@@H](O[C@H](CO[P@](=O)(Oc2ccccc2)N[C@H](C(=O)OC(C)C)C)[C@H]1O)n1c(=O)n(CCOC(=O)CNC(=O)C(C	1882854-54-7	L-Alanine, N-[[P(S),2,Ä≤R]-2,Ä≤-deoxy-2,Ä≤-fluoro-5-[1-[[2-[(2-mercapto-1-oxopr	C29H40FN4O12PS	718.68	A/B	-8.4
6291	O=C1[C@@H]2[C@@H](c3c([C@H](O[C@H]4[C@H](OC(=O)c5ccccc5)[C@@H](OC(=O)c5ccccc5)[C@H](OC(=O)c5ccccc5)[	1882902-03-5	1,3,5-Triazine-2,4-diamine, N2,N4-bis(3,4-dichlorophenyl)-6-(4-morpholinyl)-	C19H16Cl4N6O	486.18	A/B	-8.4
6292	O=c1n(cc(c(=O)[nH]1)c1cc(Cl)ccc1)[C@@H]1O[C@H](CO)[C@@H](O)C1	1884694-82-9	Not Yet Assigned	C15H20N7O7P	441.34	A/B	-8.4
6293	O=c1n([C@@H]2O[C@H](COP3(=O)Oc4c(CO3)cc(C)cc4)C=C2)cc(C)c(=O)[nH]1	188548-79-0	Thymidine, 2,Ä≤,3,Ä≤-didehydro-3,Ä≤-deoxy-5,Ä≤-O-(2-oxido-4H-1,3,2-benzodioxaph	C17H17N2O7P	392.3	A/B	-8.4
6294	C(CC)n1c2c3c(ncc2nc1N)cccc3	191594-64-6	2,5,6,10,13-Pentaazatetradecanedioic acid, 6-([1,1,Ä≤-biphenyl]-4-ylmethyl)-3,12	C39H53N5O7	703.87	A/B	-8.4
6295	O[C@@H]1[C@H](n2c3c(nc2)c(N)ncn3)O[C@H](COP(=O)(OP(=O)(NCCN)O)O)[C@H]1O	192625-72-2	9H-Purin-6-amine, 9-[5-O-[[[2-[(4-aminophenyl)amino]ethyl]amino]hydroxyphosphiny	C18H25N8O6P	480.41	A/B	-8.4
6296	[C@@H](C(=O)N[C@@H](Cc1ccccc1)C[C@@H]([C@H](Cc1ccccc1)NC(=O)COc1c(C)cccc1C)O)(C(C)C)N1C(=O)NCCC1	192725-16-9	1-Imidazolidineacetamide, N-[(1S,3S,4S)-4-[[2-(2,6-dimethylphenoxy)acetyl]amino]	C36H46N4O5	614.77	A/B	-8.4
6297	C(=C\c1c(F)cccc1)/c1n(nc(C(=O)N)n1)[C@@H]1O[C@H](CO)[C@@H](O)[C@H]1O	1927839-26-6	1H-1,2,4-Triazole-3-carboxamide, 5-[(1Z)-2-(2-fluorophenyl)ethenyl]-1-Œ≤-D-ribo	C16H17FN4O5	364.33	A/B	-8.4
6298	C(=C\c1ccc(C(F)(F)F)cc1)/c1n(nc(C(=O)N)n1)[C@@H]1O[C@H](CO)[C@@H](O)[C@H]1O	1927839-30-2	1H-1,2,4-Triazole-3-carboxamide, 1-Œ≤-D-ribofuranosyl-5-[(1Z)-2-[4-(trifluoromet	C17H17F3N4O5	414.34	A/B	-8.4
6299	C(OCCCCCCO[C@H]1[C@H](O[C@H]2[C@@H]1OC(C)(C)O2)[C@H]1COC(C)(C)O1)[C@@H]1[C@@H]2[C@H]([C@@H](O1)n1nc(	192865-93-3	1H-1,2,4-Triazole-3-carboxamide, 1-[2,3-O-(1-methylethylidene)-Œ≤-D-ribofuranos	C29H44N4O12	640.68	A/B	-8.4
6300	O[C@H]1[C@@H](O[C@H](COCCCCCCO[C@H]2[C@H](O)[C@@H](CO)O[C@H](O)[C@@H]2O)[C@H]1O)n1nc(C(=O)N)nc1	192865-94-4	1H-1,2,4-Triazole-3-carboxamide, 1-[5-O-[6-[1,2:5,6-bis-O-(1-methylethylidene)-Œ	C29H46N4O11	626.7	A/B	-8.4
6301	N(CCCCCCl)c1c2c(cc(Cl)cc2)ncc1	19289-79-3	1-Naphthalenesulfonic acid, 4-[2-(1-naphthalenyl)diazenyl]-	C20H14N2O3S	362.4	A/B	-8.4
6302	O[C@@H]1[C@H](n2c3c(nc2)c(NCc2c(O)c(OC)ccc2)ncn3)O[C@H](CO)[C@H]1O	1947400-91-0	1,3-Benzenediol, 5-[[(9-Œ≤-D-arabinofuranosyl-9H-purin-6-yl)amino]methyl]-	C17H19N5O6	389.36	A/B	-8.4
6303	N(Cc1cc(C(F)(F)F)cc(F)c1)c1c2c(n(cn2)[C@@H]2O[C@H](CO)[C@@H](O)[C@@H]2O)ncn1	1947401-67-3	9H-Purin-6-amine, 9-Œ≤-D-arabinofuranosyl-N-[[4-[(trifluoromethyl)thio]phenyl]me	C18H18F3N5O4S	457.43	A/B	-8.4
6304	N(Cc1c(O)cc(Cl)cc1)c1c2c(n(cn2)[C@@H]2O[C@H](CO)[C@@H](O)[C@@H]2O)ncn1	1947402-06-3	Phenol, 2-[[(9-Œ≤-D-arabinofuranosyl-9H-purin-6-yl)amino]methyl]-6-chloro-	C17H18ClN5O5	407.81	A/B	-8.4
6305	C(=O)([C@@H](NC(=O)OC)C(C)C)N1[C@H](c2[nH]c3c4c(cc(cc4)c4cc(C)c(cc4)c4[nH]c(nc4)[C@H]4N(C(=O)[C@@H](	1949770-68-6	Carbamic acid, N-[(1S)-1-[[(2S)-2-[7-[3-hydroxy-4-[2-[(2S)-1-[(2S)-2-[(methoxyca	C42H50N8O7	778.9	A/B	-8.4
6306	C(=O)([C@@H](NC(=O)OC)C(C)C)N1[C@H](c2[nH]c3c4c(cc(cc4)c4cc(OCCN5CCOCC5)c(cc4)c4[nH]c(nc4)[C@H]4N(C(	1949770-70-0	Carbamic acid, N-[(1S)-1-[[(2S)-2-[7-[3-cyclopropyl-4-[2-[(2S)-1-[(2S)-2-[(metho	C45H54N8O6	802.96	A/B	-8.4
6307	O(C(=O)C)[C@H]1[C@H](OC(=O)C)[C@@H](COC(=O)C)O[C@@H](SCCOP(=O)(OC[C@@H]2[C@@H](N=[N+]=[N-])C[C@@H](O	195514-63-7	2-Piperidinecarboxylic acid, 1-[(2S)-1-oxo-2-(3,4,5-trimethoxyphenyl)butyl]-, 2,	C78H98N4O20	1411.63	A/B	-8.4
6308	O=c1n([C@@H]2O[C@H](COP3(=O)Oc4c(CO3)cc(Cl)cc4)[C@@H](N=[N+]=[N-])C2)cc(C)c(=O)[nH]1	195885-78-0	Thymidine, 3,Ä≤-azido-3,Ä≤-deoxy-5,Ä≤-O-(6-nitro-2-oxido-4H-1,3,2-benzodioxapho	C17H17N6O9P		A/B	-8.4
6309	O=c1n([C@H]2O[C@@H](COCNc3c(F)cn(c(=O)n3)[C@H]3O[C@@H](CO)SC3)SC2)cc(F)c(N)n1	1962114-95-9	Not Yet Assigned	C17H22FN8O6PS	516.44	A/B	-8.4
6310	C[C@]1(F)[C@@H](O[C@H](CO[P@](=O)(Oc2ccccc2)N[C@H](C(=O)OC(C)C)C)[C@H]1OC(=O)[C@@H](NC(=O)C(C)S)C)n1	1972595-80-4	L-Alanine, N-[[P(S),2,Ä≤R]-2,Ä≤-deoxy-3,Ä≤-O-[[[4,Ä≤-(ethoxycarbonyl)-7,7,Ä≤-di	C43H47FN3O19P	959.81	A/B	-8.4
6311	N(C(=O)C)[C@H]1[C@H]([C@@H]([C@@H](CO)O)O)OC(=C[C@@H]1NC(=N)NC(=O)Cn1cc(nn1)c1ccc(Cl)cc1)C(=O)O	1982351-49-4	D-glycero-D-galacto-Non-2-enonic acid, 5-(acetylamino)-2,6-anhydro-3,4,5-trideox	C23H29N7O8	531.52	A/B	-8.4
6312	C[C@]1(F)[C@@H](O[C@H](CO[P@](=O)(Oc2ccccc2)N[C@H](C(=O)OC(C)C)C)[C@H]1OC(=O)CCCn1c2c(cc1)cccn2)n1c(	1982359-81-8	L-Alanine, N-[[P(S),2,Ä≤R]-2,Ä≤-deoxy-2,Ä≤-fluoro-2,Ä≤-methyl-P-phenyl-3,Ä≤-O-[	C31H35FN5O10P	687.61	A/B	-8.4
6313	N(c1nc(Nc2c(C)cc(C)cc2C)ncc1)c1c(C#N)cccc1	1984414-47-2	Benzonitrile, 2-[[4-[[2-methyl-6-(1-methylethyl)phenyl]amino]-2-pyrimidinyl]amir	C21H21N5	343.42	A/B	-8.4
6314	O(Cc1ccncc1)c1c2n(N([C@H]3N(C2=O)CCOC3)[C@H]2c3c(CSc4c2cccc4)c(F)c(F)cc3)ccc1=O	1985606-19-6	1H-[1,4]Oxazino[3,4-c]pyrido[2,1-f][1,2,4]triazine-6,8-dione, 7-[[4-(acetyloxy)p	C33H27F2N3O6S	631.65	A/B	-8.4
6315	O(C(=O)N(C)C)c1c2n(N([C@H]3N(C2=O)CCOC3)[C@H]2c3c(CSc4c2cccc4)c(F)c(F)cc3)ccc1=O	1985606-20-9	1H-[1,4]Oxazino[3,4-c]pyrido[2,1-f][1,2,4]triazine-6,8-dione, 12-[(11S)-7,8-difl	C30H24F2N4O4S	574.6	A/B	-8.4
6316	O(C(=O)C(C)C)c1c2n(N([C@H]3N(C2=O)CCOC3)[C@H]2c3c(CSc4c2cccc4)c(F)c(F)cc3)ccc1=O	1985606-46-9	1H-[1,4]Oxazino[3,4-c]pyrido[2,1-f][1,2,4]triazine-6,8-dione, 12-[(11S)-7,8-difl	C27H23F2N3O5S	539.55	A/B	-8.4
6317	O(CC(=O)OC(C)(C)C)c1c2n(N([C@H]3N(C2=O)CCOC3)[C@H]2c3c(CSc4c2cccc4)c(F)c(F)cc3)ccc1=O	1985606-84-5	1H-[1,4]Oxazino[3,4-c]pyrido[2,1-f][1,2,4]triazine-6,8-dione, 12-[(11S)-7,8-difl	C27H21F2N3O7S	569.53	A/B	-8.4

6318	O(C(=O)OCCOC)c1c2n(N([C@H]3N(C2=O)CCOC3)[C@H]2c3c(CSc4c2cccc4)c(F)c(F)cc3)ccc1=O	1985607-08-6	Carbamic acid, N-methyl-N-phenyl-, (12aR)-12-[(11S)-7,8-difluoro-6,11-dihydrodib	C32H26F2N4O5S	616.63	A/B	-8.4
6319	O(C(=O)OCCO[Si](C(C)(C)C)(C)C)c1c2n(N([C@H]3N(C2=O)CCOC3)[C@H]2c3c(CSc4c2cccc4)c(F)c(F)cc3)ccc1=O	1985607-44-0	1H-[1,4]Oxazino[3,4-c]pyrido[2,1-f][1,2,4]triazine-6,8-dione, 12-[(11S)-7,8-difl	C32H27F2N3O4S	587.64	A/B	-8.4
6320	O(Cc1ccccn1)c1c2n(N([C@H]3N(C2=O)CCOC3)[C@H]2c3c(CSc4c2cccc4)c(F)c(F)cc3)ccc1=O	1985607-59-7	1H-[1,4]Oxazino[3,4-c]pyrido[2,1-f][1,2,4]triazine-6,8-dione, 12-[(11S)-7,8-difl	C30H24F2N4O4S	574.6	A/B	-8.4
6321	N(c1nc(Nc2ccc(C#N)cc2)nc(C)c1)c1c(C)ccc(C)c1	1985844-18-5	2,4-Pyrimidinediamine, 6-methyl-N4-phenyl-N2-(2,4,6-trimethylphenyl)-	C20H22N4	318.42	A/B	-8.4
6322	O=[13c]1[15n]([13C@H]2O[13C@H]([13CH2]O)[13C@@H](O)[13CH2]2)[13cH][13c]([13CH3])c(=O)[15nH]1	1987876-49-2	2,5,6,10,13-Pentaazatetradecanedioic acid, 3,12-bis(1,1-dimethylethyl)-8-hydroxy	C36H48N6O7	676.8	A/B	-8.4
6323	C(#C)[C@@]1(F)[C@@H](O[C@H](COP(=O)(Oc2ccccc2)N[C@@H](C(=O)OC(C)C)[C@H]1O)n1c(=O)[nH]c(=O)cc1	1988382-09-7	Benzonitrile, 2-[[2-[(4-methylphenyl)amino]-4-pyrimidinyl]amino]-	C18H15N5	301.35	A/B	-8.4
6324	N(C(CC(C)Cl)C)c1c2c(ncc1)cc(Cl)cc2	1992274-15-3	Benzonitrile, 2-[[2-[(2-ethyl-6-methylphenyl)amino]-6-methyl-4-pyrimidinyl]aminc	C21H21N5	343.42	A/B	-8.4
6325	O[C@H]1[C@@H](O[C@H](CO[P@](=O)(OP(=O)(O[P@@](=O)(OC[C@H]2O[C@H]([C@H](O)[C@@H]2O)n2nc(C(=O)N)nc2)S)	2001001-61-0	Not Yet Assigned	C16H25N8O16P3S2	742.47	A/B	-8.4
6326	C(F)(F)c1n(c2c(n1)cccc2)c1nc(nc(n1)N1CCOCC1)N1CCN(CC1)[C@@H]1CC[C@H](N)CC1	2001083-62-9	2-Pyrrolidinecarboxylic acid, 1-[4-[4-[2-(difluoromethyl)-1H-benzimidazol-1-yl]-	C24H29F2N9O3	529.54	A/B	-8.4
6327	C(=O)([C@@H](NC(=O)OC)C(C)C)N1[C@@H](C2CC1CC2)c1[nH]c(cn1)c1c2c(c(cc1)c1cc3c(cc1)c1c(cc3)nc([nH]1)[C	2007901-01-9	Carbamic acid, N-[(1S)-1-[[(2S)-2-[7-[2,3-dihydro-7-[2-[(2S)-1-[(2S)-2-[(methoxy	C45H56N8O6	804.98	A/B	-8.4
6328	C(=O)([C@@H](NC(=O)OC)C(C)C)N1[C@H](c2[nH]c3c4c(cc(cc4)c4c5c(cc4)c4[nH]c(nc4)[C@H]4N(C(=O)[C@@H](N	2007901-40-6	Carbamic acid, N-[(1S)-2-methyl-1-[[(2S,5S)-2-methyl-5-[7-[5,6,7,8-tetrahydro-4-	C47H58N8O6	831.01	A/B	-8.4
6329	C(=O)([C@@H](NC(=O)OC)C(C)C)N1[C@@H](CC2(C1)OCCO2)c1[nH]c(cn1)c1c2c(c(cc1)c1cc3c(cc1)c1c(cc3)nc([nH]	2007901-76-8	Carbamic acid, N-[(1S)-1-[[(2S)-2-[5-[7-[4,5-dihydro-2-[(2S)-1-[(2S)-2-[(methoxy	C46H58N8O7	835	A/B	-8.4
6330	C[C@]1(F)[C@@H](O[C@H](CO[P@](=O)(Oc2ccccc2)N[C@H](C(=O)OC(C)C)C)[C@H]1OCC=C)n1c(=O)[nH]c(=O)cc1	2011008-65-2	L-Alanine, N-[[P(S),2,Ä≤R]-2,Ä≤-deoxy-2,Ä≤-fluoro-2,Ä≤-methyl-P-phenyl-3,Ä≤-O-	C29H35FN3O9P	619.58	A/B	-8.4
6331	C([C@H](OCP(=O)(OCOC(=O)OC(C)C)OCOC(=O)OC(C)C)C)n1c2c(nc1)c(N)ncn2.C(=C\C(=O)O)/C(=O)O	2021230-56-6	Not Yet Assigned	C21H21N5O7S	487.49	A/B	-8.4
6332	[C@H](Cc1ccccc1)(C[C@@H]([C@H](Cc1ccccc1)NC(=O)OCc1cncs1)O)NC(=O)CCNC(=O)N(Cc1nc(C(C)C)sc1)C	202816-84-0	2,7,11,13-Tetraazatetradecanoic acid, 5-hydroxy-13-methyl-14-[2-(1-methylethyl)-	C35H44N6O5S2	692.89	A/B	-8.4
6333	[C@H](Cc1ccccc1)(C[C@@H]([C@H](Cc1ccccc1)NC(=O)[C@@H](NC(=O)N(CCc1nc(C(C)C)sc1)CC)C)O)NC(=O)OCc1cncs	202816-98-6	2,7,10,12-Tetraazatetradecanoic acid, 5-hydroxy-9,12-dimethyl-14-[2-(1-methyleth	C36H46N6O5S2	706.92	A/B	-8.4
6334	O[C@H]1[C@@H](O[C@H](COC(=O)c2ccc(CO[C@@H]3O[C@H](CO)[C@H](O)[C@H](O)[C@H]3O)cc2)[C@H]1O)n1nc(C(=O)N	1002334-97-5	1H-1,2,4-Triazole-3-carboxamide, 1-[5-O-[4-(Œ≤-D-galactopyranosyloxy)benzoyl]-Œ	C21H26N4O12	526.45	C	-8.4
6335	C(OC(=O)OCCCO[C@H]1[C@H](OCc2ccccc2)[C@@H](OCc2ccccc2)[C@@H](OCc2ccccc2)[C@@H](COCc2ccccc2)O1)[C@@H]	1002335-14-9	1H-1,2,4-Triazole-3-carboxamide, 1-[5-O-[1-oxo-3-[[2,3,4,6-tetrakis-O-(phenylmet	C45H50N4O12	838.9	C	-8.4
6336	C(=O)([C@@H](Nc1[nH]c(N)nn1)C(C)C)N1[C@@H](CCC1)c1[nH]c(cn1)c1ccc(cc1)c1ccc(cc1)c1[nH]c(nc1)[C@H]1N(	1007884-53-8	Carbamic acid, N-[(1S)-1-[[(2S)-2-[5-[4,Ä≤-[2-[(2S)-1-[(2S)-2-amino-3-methyl-1-c	C38H48N8O4	680.84	C	-8.4
6337	C(=O)([C@@H](NC(=O)OC)C(C)C)N1[C@@H](CCC1)c1[nH]c(cn1)c1cc(CN(C)C)c(cc1)c1ccc(cc1)c1[nH]c(nc1)[C@H]1	1007885-30-4	Carbamic acid, N-[(1S)-1-[[(2S)-2-[5-[2,Ä≤-(hydroxymethyl)-4,Ä≤-[2-[(2S)-1-[(2S	C41H52N8O7	768.9	C	-8.4
6338	C(C(C(NC(=O)c1c(C)c(O)c(O)cc1)CSc1cc(C)ccc1)O)N1C(C(=O)NC(C)(C)C)CC2C(C1)CCCC2	1009057-20-8	3-Isoquinolinecarboxamide, N-(1,1-dimethylethyl)-2-[4-[(5-ethyl-2-hydroxyphenyl)	C34H49N3O5S	611.84	C	-8.4
6339	C(=O)([C@H](NC(=O)OC)c1ccccc1)N1[C@@H](CCC1)c1[nH]c(cn1)c1ccc(cc1)c1ccc(cc1)c1[nH]c(nc1)[C@H]1N(C(=O	1009107-41-8	Carbamic acid, N-[(1S)-2-[(2S)-2-[5-[4,Ä≤-[2-[(2S)-1-[(2R)-2-(diethylamino)-2-ph	C43H50N8O4	742.91	C	-8.4
6340	C(=O)([C@H](NC(=O)OC)c1ccccc1)N1[C@@H](CCC1)c1[nH]c(cn1)c1ccc(cc1)c1ccc(cc1)c1[nH]c(nc1)[C@H]1N(C(=O	1009107-51-0	Carbamic acid, N-[(1R)-2-[(2S)-2-[5-[4,Ä≤-[2-[(2S)-1-[(2S)-2-(dimethylamino)-2-p	C46H48N8O4	776.92	C	-8.4
6341	C(=O)([C@H](NC(=O)OC)c1ccccc1)N1[C@@H](CCC1)c1[nH]c(cn1)c1ccc(cc1)c1ccc(cc1)c1[nH]c(nc1)[C@H]1N(C(=O	1009107-87-2	Carbamic acid, N-[(1R)-2-[(2S)-2-[5-[4,Ä≤-[2-[(2S)-1-[(2S)-2-[(methoxycarbonyl)a	C41H44N8O6	744.84	C	-8.4
6342	C(=O)([C@H](N1CCCC1)c1ccccc1)N1[C@@H](CCC1)c1[nH]c(cn1)c1ccc(cc1)c1ccc(cc1)c1[nH]c(nc1)[C@H]1N(C(=O)	1009110-77-3	Carbamic acid, N-[(1S)-2-[(2S)-2-[5-[4,Ä≤-[2-[(2S)-1-[(2R)-2-(dimethylamino)-2-p	C41H46N8O4	714.86	C	-8.4
6343	C[C@@]1(N(C(=O)[C@@H](NC(=O)OC)C(C)C)CCC1)c1[nH]c(cn1)c1ccc(cc1)c1ccc(cc1)c1[nH]c(nc1)[C@H]1N(C(=O)[	1009113-48-7	Carbamic acid, N-[(1S)-2-[(2S)-2-[5-[4,Ä≤-[2-[(2S)-1-[(2S)-2-[(methoxycarbonyl)a	C40H48N8O7	752.86	C	-8.4
6344	C(=O)([C@@H](NC(=O)O[C@H]1CCOC1)C(C)C)N1[C@@H](CCC1)c1[nH]c(cn1)c1ccc(cc1)c1ccc(cc1)c1[nH]c(nc1)[C@H	1009113-75-0	Carbamic acid, N-[(1S)-2-[(2S)-2-[5-[4,Ä≤-[2-[(2S)-1-[(2R)-2-(ethylamino)-2-pher	C41H46N8O4	714.86	C	-8.4
6345	C(=O)([C@@H](NC(=O)C)C(C)C)N1[C@@H](CCC1)c1[nH]c(cn1)c1ccc(cc1)c1ccc(cc1)c1[nH]c(nc1)[C@H]1N(C(=O)[C	1009113-86-3	Carbamic acid, N-[(1S)-2-[(2S)-2-[5-[4,Ä≤-[2-[(2S)-1-[(2S)-2-[(methoxycarbonyl)a	C42H52N8O7	780.91	C	-8.4
6346	C(=O)([C@@H](NC(=O)OC)C(C)C)N1[C@@H](CCC1)c1[nH]c(cn1)c1ccc(cc1)c1ccc(cc1)c1[nH]c(nc1)[C@H]1N(C(=O)[	1009114-23-1	Carbamic acid, N-[(1S)-2-[(2S)-2-[5-[4,Ä≤-[2-[(2S)-1-[(2S)-2-[(methoxycarbonyl)a	C40H47N11O6	777.87	C	-8.4
6347	C(=O)([C@H](Cc1ccncc1)NC(=O)OC)N1[C@@H](CCC1)c1[nH]c(cn1)c1ccc(cc1)c1ccc(cc1)c1[nH]c(nc1)[C@H]1N(C(=	1009114-74-2	Carbamic acid, N-[(1S)-2-[(2S)-2-[5-[4,Ä≤-[2-[(2S)-1-[(2S)-2-[(methoxycarbonyl)a	C41H45N9O6	759.85	C	-8.4
6348	C(=O)([C@@H](NC(=O)OC)[C@@H](C)O)N1[C@@H](CCC1)c1[nH]c(cn1)c1ccc(cc1)c1ccc(cc1)c1[nH]c(nc1)[C@H]1N(C	1009116-92-0	Carbamic acid, N,N,Ä≤-[[1,1,Ä≤-biphenyl]-4,4,Ä≤-diylbis[1H-imidazole-5,2-diyl-(	C40H50N8O8	770.87	C	-8.4
6349	C(=O)([C@@H](NC(=O)OC)C(C)C)N1[C@H](CCC1)c1[nH]c(cn1)c1ccc(cc1)c1ccc(cc1)c1[nH]c(nc1)[C@H]1N(C(=O)[C	1009117-26-3	Carbamic acid, N,N,Ä≤-[[1,1,Ä≤-biphenyl]-4,4,Ä≤-diylbis[1H-imidazole-5,2-diyl-(	C40H50N8O6	738.88	C	-8.4
6350	C(=O)([C@H](N(CC)CC)c1ccccc1)N1[C@@H](CCC1)c1[nH]c(cn1)c1ccc(cc1)c1ccc(cc1)c1[nH]c(nc1)[C@H]1N(C(=O)	1009117-51-4	Carbamic acid, N-[(1R)-2-[(2S)-2-[5-[4,Ä≤-[2-[(2S)-1-[(2R)-2-(diethylamino)-2-ph	C48H52N8O4	804.98	C	-8.4
6351	C(=O)([C@H](N(CC)CC)c1ccccc1)N1[C@@H](CCC1)c1[nH]c(cn1)c1ccc(cc1)c1ccc(cc1)c1[nH]c(nc1)[C@H]1N(C(=O)	1009117-56-9	Carbamic acid, N-[(1R)-2-[(2S)-2-[5-[4,Ä≤-[2-[(2S)-1-[(2R)-2-(diethylamino)-2-ph	C43H50N8O4	742.91	C	-8.4
6352	C(=O)([C@@H](NC(=O)OC)C(C)C)N1[C@@H](CCC1)c1[nH]c(cn1)c1ccc(cc1)c1ccc(cc1)c1[nH]c(nc1)[C@H]1N(C(=O)[	1009117-85-4	Carbamic acid, N-[(1S)-2-methyl-1-[[(2S)-2-[5-[4,Ä≤-[2-[(2S)-1-(3-methyl-1-oxobu	C38H47N7O4	665.82	C	-8.4
6353	C(=O)([C@@H](NC(=O)OC)COC)N1[C@@H](CCC1)c1[nH]c(cn1)c1ccc(cc1)c1ccc(cc1)c1[nH]c(nc1)[C@H]1N(C(=O)[C@	1009118-05-1	Carbamic acid, N-[(1S)-2-[(2S)-2-[5-[4,Ä≤-[2-[(2S)-1-[(2R)-2-(ethylmethylamino)-	C42H48N8O4	728.88	C	-8.4
6354	C(=O)([C@@H](NC(=O)OC)C(C)C)N1[C@H]2[C@H](C2)C[C@H]1c1[nH]c(cn1)c1ccc(cc1)c1ccc(cc1)c1[nH]c(nc1)[C@H	1009118-29-9	Carbamic acid, N,N,Ä≤-[[1,1,Ä≤-biphenyl]-4,4,Ä≤-diylbis[1H-imidazole-5,2-diyl-(	C38H42N8O6	706.79	C	-8.4
6355	N(C(CCN(C)C)C)c1c2c(ncc1)cc(Cl)cc2	102345-56-2	4-Quinolinamine, 7-chloro-N-[4-[4-(7-chloro-4-quinolinyl)hexahydro-1H-1,4-diazep	C28H31Cl2N5	508.49	C	-8.4
6356	[C@@H](COC)(N1[C@@H](C)CN(CC1)C1(C)CCN(C(=O)c2c(C)ncnc2)CC1)c1ccc(C(F)(F)F)cc1	1026042-89-6	Benzonitrile, 4-[[2-[(2-cyanophenyl)amino]-4-pyrimidinyl]amino]-3,5-dimethyl-	C20H16N6	340.38	C	-8.4
6357	S(=O)(CC[Si](C)(C)C)C1=C[C@@H](CO)O[C@H]1n1c(=O)[nH]c(=O)c(C)c1	1026776-36-2	Adenosine, guanylyl-(3,Ä≤,Üí5,Ä≤)-2,Ä≤-deoxy-	C20H25N10O10P	596.45	C	-8.4
6358	O=c1c2c(n(cn2)[C@H]2C[C@H](C(=C)C)[C@@H](CO)O2)[nH]cn1	1027072-86-1	Methanone, (4,6-dimethyl-1-oxido-5-pyrimidinyl)[(3S)-4-[4-[(1R)-2-methoxy-1-[4-(	C28H38F3N5O3	549.63	C	-8.4
6359	N(c1nc(Nc2ccc(C#N)cc2)nc(COCC=C)c1)c1c(C)cc(/C=C/C#N)cc1C	1037444-77-1	Benzonitrile, 4-[[4-[[4-[(1E)-2-cyanoethenyl]-2,6-dimethylphenyl]amino]-6-(3-pyr	C27H21N7	443.5	C	-8.4
6360	N(c1c(C)cc(/C=C/C#N)cc1C)c1nc(Nc2ccc(C#N)cc2)nc(CN(OC)C)c1	1037445-08-1	Benzenesulfonamide, 4-[2-[[[6-[[4-[(1E)-2-cyanoethenyl]-2,6-dimethylphenyl]aminc	C31H30N8O2S	578.69	C	-8.4
6361	C(C=C)(O)c1c(Nc2c(C)cc(/C=C/C#N)cc2C)nc(Nc2ccc(C#N)cc2)nc1COC	1037452-57-5	5-Pyrimidinecarboxamide, 2-[(4-cyanophenyl)amino]-4-[[4-[(1E)-2-cyano-1-propen-1	C32H30N8O2	558.63	C	-8.4
6362	O=c1n2c(-c3c(C2)cc2c(n3)ccc(OP(=O)(C(=O)O)O)c2)cc2c1COC(=O)[C@@]2(CC)O	1038983-64-0	Phosphinecarboxylic acid, 1-[2-[(2S,4S)-4-[(3-amino-2,3,6-trideoxy-Œ±-L-lyxo-hex	C28H30NO15P	651.51	C	-8.4
6363	C(c1c2c(N(c3c(C(=O)N2)cccn3)C2CC2)ncc1)n1c(=O)n(ccc1=N)[C@@H]1O[C@H](CO)[C@@H](O)C1	1046462-08-1	6H-Dipyrido[3,2-b:2,Ä≤,3,Ä≤-e][1,4]diazepin-6-one, 4-[(6-amino-9H-purin-9-yl)me	C20H17N9O	399.41	C	-8.4
6364	C(c1c2c(N(c3c(C(=O)N2)cccn3)C2CC2)ncc1)n1c(=O)c2c(nc1N)n(cn2)[C@@H]1O[C@H](CO)[C@@H](O)C1	1046462-10-5	Guanosine, 6-O-[(11-cyclopropyl-6,11-dihydro-6-oxo-5H-dipyrido[3,2-b:2,Ä≤,3,Ä≤-(	C25H25N9O5	531.52	C	-8.4
6365	C([C@H](OC[P@@](=O)(N[C@H](C(=O)OC)CC(C)C)Oc1ccccc1)C)n1c2c(nc1)c(N)ncn2	1051375-16-6	2H-Pyrido[1,Ä≤,2,Ä≤:4,5]pyrazino[2,1-b][1,3]oxazine-9-carboxamide, N-[(2,4-difl	C20H19F2N3O5	419.38	C	-8.4
6366	S(=O)(=O)(O)c1c2c(c(S(=O)(=O)O)cc(/N=N/c3ccc(S(=O)(=O)c4ccc(/N=N/c5c(N)c6c(c(S(=O)(=O)O)c5)cccc6S(=O	1052534-64-1	Not Yet Assigned	C34H26N6O12S4.Na		C	-8.4
6367	N(=Nc1c(C)cc(N)cc1)c1c2c(c(S(=O)(=O)O)ccc2)ccc1	106104-87-4	1-Naphthalenesulfonic acid, 7-[2-(4-amino-2-methylphenyl)diazenyl]-	C17H15N3O3S	341.38	C	-8.4
6368	OC1C(c2c(Cl)cccc2)N1C[C@@H](C(=O)NC(C)(C)C)N(C[C@H](C[C@H](C(=O)N[C@H]2c3c(C[C@H]2O)cccc3)Cc2ccccc2)	1064475-81-5	Not Yet Assigned	C41H56N6O5	712.92	C	-8.4
6369	N(C(=O)[C@H](Cc1ccccc1)C[C@@H](CN1[C@H](C(=O)NC(C)(C)C)CN(C/C=C/c2ccccc2)CC1)O)[C@H]1c2c(C[C@H]1O)cc	1064475-83-7	Not Yet Assigned	C39H50N4O5	654.84	C	-8.4
6370	N(C(=O)[C@H](Cc1ccccc1)C[C@@H](CN1[C@H](C(=O)NC(C)(C)C)CN(Cc2c(OCc3ccccc3)nc(C)cc2)CC1)O)[C@H]1c2c(C	1064475-91-7	Not Yet Assigned	C37H49N5O4	627.82	C	-8.4
6371	C(n1c2c(nc1)c(N)ncn2)C1COP(=O)(O)CO1	106931-26-4	4-Quinolinamine, N-[4-[4-[2-amino-5-(4-chlorophenyl)-6-ethyl-4-pyrimidinyl]-1-pi	C30H35Cl2N7	564.55	C	-8.4
6372	O(C[C@H]1O[C@H](C=C1)n1c(=O)[nH]c(=O)c(C)c1)P1(=O)Oc2c(cc(CCCCC(=O)OCc3ccccc3)cc2)CO1	1072329-81-7	Thymidine, 5,Ä≤-O-[6-(4-carboxybutyl)-2-oxido-4H-1,3,2-benzodioxaphosphorin-2-yl	C22H25N2O9P	492.42	C	-8.4
6373	O(C[C@H]1O[C@H](C=C1)n1c(=O)[nH]c(=O)c(C)c1)P1(=O)Oc2c(cc(CCC(=O)NCC(=O)OCc3ccccc3)cc2)CO1	1073242-60-0	Glycine, N-[3-[2-(2,Ä≤,3,Ä≤-didehydro-3,Ä≤-deoxythymidin-5,Ä≤-O-yl)-2-oxido-4H-	C23H26N3O10P	535.44	C	-8.4
6374	[C@@H](NC(=O)N1CCN(C)CC1)(C(=O)N[C@H](C=CS(=O)(=O)c1ccccc1)CCc1ccccc1)CCCNC(=N)NO	1076088-57-7	1-Piperazinecarboxamide, N-[(1S)-3-[[(hydroxyamino)iminomethyl]amino]-1-[[[(1S)-	C28H39N7O5S	585.72	C	-8.4
6375	C(OCCOP(=O)(C)O)n1c2c(nc1)c(=O)nc(N)[nH]2	1085743-78-7	D-glycero-Pentonamide, 2,3,5-trideoxy-N-[(1S,2R)-2,3-dihydro-2-hydroxy-1H-inden-	C36H47N5O4	613.79	C	-8.4
6376	O=c1c2c(n(cn2)[C@H]2C[C@@H](CO)[C@@H](O)[C@@H]2O)[nH]c(N)n1	1087346-93-7	Carbamic acid, N-[(1S)-2-[(2S)-2-[5-[4,Ä≤-[2-[(1R)-1-[[(2S)-2-[(methoxycarbonyl)	C41H46N8O6	746.85	C	-8.4
6377	O=c1n([C@@H]2O[C@H](COP(=O)(OP(=O)(OP(=O)(O)O)O)O)[C@H](N=[N+]=[N-])C2)cc(C)c(=O)[nH]1	108988-58-5	1,3-Naphthalenedisulfonic acid, 8,8,Ä≤-[thiobis[(4-methyl-o-phenylene)azo]]bis[7	C34H28N6O12S5.4Na		C	-8.4
6378	N(C(=O)COC(=O)CN1C(=O)c2c(C1=O)cc(Cl)c(Cl)c2)C12CC3CC(C2)CC(C1)C3	1095170-81-2	Thymidine, 5,Ä≤-O-[6-[1-(acetyloxy)ethenyl]-8-(1,1-dimethylethyl)-2-oxido-4H-1,3	C25H29N2O9P	532.48	C	-8.4
6379	C(NC(=O)c1nnc(C)o1)(C)(C)c1nc(C(=O)NC(c2ccc(F)cc2)([2H])[2H])c(O)c(=O)n1C	1100750-83-1	4-Pyrimidinecarboxamide, N-[(4-fluorophenyl)methyl]-1,6-dihydro-5-hydroxy-1-(met	C20H18D3FN6O5		C	-8.4
6380	C(NC(=O)c1nnc(C([2H])([2H])[2H])o1)(C)(C)c1nc(C(=O)NC(c2ccc(F)cc2)([2H])[2H])c(O)c(=O)n1C	1100750-86-4	4-Pyrimidinecarboxamide, N-[(4-fluorophenyl)methyl]-1,6-dihydro-5-hydroxy-1-(met	C20H15D6FN6O5		C	-8.4
6381	[C@H](O[C@H](COP(=O)(OP(=O)(O)O)O)CO)(CO)n1c2c(nc1)c(=O)nc(N)[nH]2	1101448-32-1	Thymidine 5,Ä≤-(trihydrogen diphosphate), 3,Ä≤-azido-3,Ä≤-deoxy-, P,Ä≤,P,Ä≤-bis	C28H31N5O14P2		C	-8.4
6382	C(#Cc1ccc(OC)cc1)c1n(nc(C(=O)N)n1)[C@@H]1O[C@H](CO)[C@@H](O)[C@H]1O	110671-98-2	1-Naphthalenesulfonic acid, 3,3,Ä≤-[methylenebis(4,1-phenyleneazo)]bis[4-amino-,	C33H26N6O6S2.2Na		C	-8.4
6383	C(=O)(C=Cc1cc(OC)c(OC)cc1)c1c(OC)c(C)c(OC)cc1	111797-50-3	1-Naphthalenesulfonic acid, 7-[2-(2-hydroxy-1-naphthalenyl)diazenyl]-	C20H14N2O4S	378.4	C	-8.4
6384	C(Cc1c(c(c(c(c1[2H])[2H])[2H])[2H])[2H])[C@]1(CCC)CC(=C([C@H](CC)c2cc(NS(=O)(=O)c3ccc(C(F)(F)F)cn3)c	1126619-16-6	Not Yet Assigned	C31H29D4F3N2O5S		C	-8.4
6385	C(=O)([C@@H](NC(=O)OC(C)(C)C)C(C)C)N1[C@H]2[C@H](C2)C[C@H]1c1[nH]c(cn1)c1ccc(cc1)c1ccc(cc1)c1[nH]c(n	1129634-03-2	Carbamic acid, N-[(1S)-1-[[(1R,3S,5R)-3-[5-[4,Ä≤-[2-[(1R,3S,5R)-2-acetyl-2-azabi	C37H41N7O4	647.77	C	-8.4
6386	C(=O)([C@H](NC(=O)OC)C(C)C)N1[C@@H](CC[C@@H]1C)c1[nH]c(cn1)c1ccc(cc1)c1ccc(cc1)c1[nH]c(nc1)[C@H]1N(C	1129634-85-0	Carbamic acid, N,N,Ä≤-[[1,1,Ä≤-biphenyl]-4,4,Ä≤-diylbis[1H-imidazole-5,2-diyl[(	C42H54N8O6	766.93	C	-8.4
6387	C(C(OC)OC)c1c([nH]c(n1)[C@H]1N(C(=O)[C@@H](NC(=O)OC)C(C)C)CCC1)c1ccc(cc1)c1ccc(cc1)c1[nH]c(nc1)[C@H]	1129636-33-4	Carbamic acid, N-[(1S)-1-[[(2S)-2-[4-(1,3-dioxan-2-ylmethyl)-5-[4,Ä≤-[2-[(2S)-1-	C45H58N8O8	838.99	C	-8.4
6388	O([C@]1([C@@](O[C@](C(O[2H])([2H])[2H])([C@]1(O[2H])[2H])[2H])(n1nc(C(=N[2H])N([2H])[2H])nc1[2H])[2H	1134793-88-6	Guanosine, N-(9-anthracenylmethyl)-	C25H23N5O5	473.48	C	-8.4
6389	O(C)c1c2n(cc(C(=O)O)c(=O)c2cc(F)c1N1CCN(CN2c3c([C@@](C#CC4CC4)(C(F)(F)F)OC2=O)cc(Cl)cc3)CC1)C1CC1	1134943-89-7	3-Quinolinecarboxylic acid, 7-[4-[[(4S)-6-chloro-4-(2-cyclopropylethynyl)-2-oxo-	C32H27ClF4N4O5	659.03	C	-8.4
6390	N(=N\c1ccccc1)/c1c2c(c(N/N=C\3/C(=CC(=O)C=C3)C)cc1)ccc(S(=O)(=O)O)c2	1135683-63-4	2-Naphthalenesulfonic acid, 5-[(1E)-2-(4-ethoxy-2-methylphenyl)diazenyl]-8-[(1E)	C25H22N4O4S	474.53	C	-8.4
6391	[C@H]([C@H](Cc1ccccc1)NC(=O)O[C@H]1CCOC1)(CN(S(=O)(=O)c1ccc(N)cc1)CC(C)C)OP(=O)(O)O.[C@H](Cc1ccccc1)	1141895-79-5	4-Piperidinecarboxamide, 1-[4-ethyl-6-(4-morpholinyl)-1,3,5-triazin-2-yl]-N-[[2-	C23H29F3N6O2	478.51	C	-8.4
6392	C([C@H](OCP(=O)(O)O)CO)n1c2c(nc1)c(N)ncn2.[NaH]	1152269-31-2	3-Azabicyclo[3.1.0]hexane-2-carboxamide, N-[3-amino-1-(cyclobutylmethyl)-2,3-dic	C33H55N5O6	617.82	C	-8.4
6393	C(=O)(N[C@@](CC1(C(C(C1([2H])[2H])([2H])[2H])([2H])[2H])[2H])(C(=O)C(=O)N)[2H])[C@@H]1[C@@H]2[C@@H](	1158083-26-1	3-Azabicyclo[3.1.0]hexane-2-carboxamide, N-[(1S)-3-amino-1-(cyclobutyl-1,2,2,3,3	C27H35D10N5O5		C	-8.4
6394	N(=C\1/c2c(N(C)C1=O)ccc(S(=O)(=O)N1CCOCC1)c2)\NC(=S)Nc1c(C)cccc1	1164493-65-5	Hydrazinecarbothioamide, 2-[1,2-dihydro-1-methyl-5-(4-morpholinylsulfonyl)-2-oxc	C22H25N5O4S2	487.6	C	-8.4
6395	Cc1n(c2nc(nc(n2)N2CCOCC2)c2c3c([nH]nc3)ccc2)ccn1	1174190-87-4	1,3,5-Triazine, 2-(3-chlorophenyl)-4-(2-methyl-1H-imidazol-1-yl)-6-(4-morpholiny	C17H17ClN6O	356.81	C	-8.4
6396	C(C)(C)c1n(c2nc(nc(n2)N2CCOCC2)c2cc(O)ccc2)ccn1	1174190-92-1	2-Pyridinamine, 5-[4-(2-methyl-1H-benzimidazol-1-yl)-6-(4-morpholinyl)-1,3,5-tri	C20H20N8O	388.43	C	-8.4
6397	Cc1nn(c2nc(nc(n2)N2CCOCC2)c2cnc(N)nc2)cc1	1174191-06-0	1H-Indole, 4-[4-(1H-imidazol-1-yl)-6-(4-morpholinyl)-1,3,5-triazin-2-yl]-	C18H17N7O	347.37	C	-8.4
6398	Nc1ncc(c2nc(n3c4c(cn3)CCCC4)nc(n2)N2CCOCC2)cn1	1174191-79-7	2-Pyridinamine, 5-[4-(4-morpholinyl)-6-(4,5,6,7-tetrahydro-1H-indazol-1-yl)-1,3,	C19H22N8O	378.43	C	-8.4
6399	C(=O)([C@@H](NC(=O)OC)C(C)C)N1[C@H](c2[nH]c3-c4c(cc(cc4)c4ccc(cc4)c4[nH]c(nc4)[C@H]4N(C(=O)[C@@H](NC	1180668-73-8	Carbamic acid, N-[(1S)-1-[[(2S)-2-[7-[4-[2-[(2S)-4,4-difluoro-1-[(2S)-2-[(methox	C42H50F2N8O6	800.89	C	-8.4
6400	C(=O)([C@@H](NC(=O)OC)C(C)C)N1[C@H]2[C@H](C2)C[C@H]1c1[nH]c2-c3c(cc(cc3)c3ccc(cc3)c3[nH]c(nc3)[C@H]3	1180669-66-2	Carbamic acid, N-[(1S)-2-methyl-1-[[(2S)-2-[5-[4-[1,4,5,6-tetrahydro-2-[(2S)-1-[	C46H57F3N8O6	874.99	C	-8.4
6401	C(=O)([C@@H](NC(=O)OC)C(C)C)N1[C@H](c2[nH]c3-c4c(cc(cc4)c4ccc(cc4)c4[nH]c(nc4)[C@H]4N(C(=O)[C@@H](NC	1180669-85-5	Carbamic acid, N,N,Ä≤-[(1,1,Ä≤,4,4,Ä≤,5,5,Ä≤,6,6,Ä≤-octahydro[8,8,Ä≤-bibenzo[3	C46H58N8O6	819	C	-8.4
6402	C(=O)([C@@H](NC(=O)OC)C(C)C)N1[C@H]2[C@H](C2)C[C@H]1c1[nH]c2c(n1)ccc1c2ccc(c1)c1cc(F)c(cc1)c1[nH]c(n	1180670-26-1	Carbamic acid, N-[(1S)-1-[[(2S)-2-[7-[3-fluoro-4-[2-[(2S)-1-[(2S)-2-[(methoxycar	C42H49FN8O6	780.89	C	-8.4
6403	C(=O)([C@@H](NC(=O)OC)C(C)C)N1[C@H](c2[nH]c3c4c(cc(cc4)c4ccc(cc4)c4[nH]c(nc4)[C@H]4N(C(=O)[C@@H](NC(	1180670-32-9	Carbamic acid, N-[(1S)-1-[[(2S)-2-[7-[4-[2-[(2S)-4,4-difluoro-1-[(2S)-2-[(methox	C42H48F2N8O6	798.88	C	-8.4
6404	C(=O)([C@@H](NC(=O)OC)C(C)C)N1[C@H](c2[nH]c3c4c(cc(cc4)c4ccc(cc4)c4[nH]c(nc4)[C@H]4N(C(=O)[C@@H](NC(	1180670-34-1	Carbamic acid, N-[(1S)-1-[[(2S)-4,4-difluoro-2-[7-[4-[2-[(2S)-1-[(2S)-2-[(methox	C42H48F2N8O6	798.88	C	-8.4
6405	C(OC(C(O)([2H])[2H])([2H])[2H])n1c2c(nc1)c(=O)nc(N)[nH]2	1185110-50-2	Not Yet Assigned	C22H14O9.Na		C	-8.4
6406	C(COCP(=O)(O)O)n1c2c(nc1)c(C)ncn2	1185514-90-2	2-Naphthalenesulfonic acid, 8-[(1E)-2-phenyldiazenyl]-6-[(1E)-2-[4-[(1E)-2-(4-su	C28H20N6O6S2	600.62	C	-8.4
6407	O(Cc1ccccc1)[C@@H]1[C@H](OCc2ccccc2)[C@@H](OCc2ccccc2)[C@H](OCCCCCCC(=O)OC[C@H]2O[C@H]([C@H](O)[C@@H	1186192-17-5	1H-1,2,4-Triazole-3-carboxamide, 1-[5-O-[3-[[2-(acetylamino)-2-deoxy-3,4,6-tris-	C40H47N5O12	789.83	C	-8.4
6408	O=c1n([C@@H]2O[C@H](COC(=O)OCCOCCOCCOC)[C@@H](N=[N+]=[N-])C2)cc(C)c(=O)[nH]1	1186380-69-7	Methanone, (4,6-dimethyl-5-pyrimidinyl)[4-[(3S)-4-[(1R)-2-ethoxy-2,3-dihydro-5-(	C30H40F3N5O2	559.67	C	-8.4
6409	N(C(=O)c1nnc(C)o1)C12c3n(CC(CC1)CC2)c(=O)c(O)c(C(=O)NCc1ccc(F)cc1)n3	1188424-53-4	6,9-Ethano-4H-pyrido[1,2-a]pyrimidine-2-carboxamide, N-[(4-fluorophenyl)methyl]-	C22H21FN6O5	468.44	C	-8.4
6410	N(=C1c2c(N(CN3CCOCC3)C1=O)ccc(Cl)c2)NC(=S)NC1CCCCC1	118989-43-8	Hydrazinecarbothioamide, 2-[1-[[4-(4-chlorophenyl)-1-piperazinyl]methyl]-1,2-dih	C27H33ClN6OS	525.11	C	-8.4
6411	N(CCN1CCOCC1)c1nc(nc(NCCNc2c3c(ncc2)cc(Cl)cc3)n1)N1CCOCC1	1190366-22-3	1,2-Ethanediamine, N1-(7-chloro-4-quinolinyl)-N2-[4-(4-methyl-1-piperazinyl)-6-(	C23H30ClN9O	484	C	-8.4
6412	O=C1[C@@H]2[C@@H](c3c([C@@H](OC(=O)[C@@H](NC(=O)OC[C@H]4O[C@H](C=C4)n4c(=O)[nH]c(=O)c(C)c4)C(C)C)[C@	1191242-93-9	L-Alanine, N-carboxy-, 1-[(3aR,4S,9R,9aR)-1,3,3a,4,9,9a-hexahydro-6,7-dimethoxy-	C37H41N3O14	751.73	C	-8.4
6413	O=c1c2c(n(cn2)[C@@]2(O[C@H](CO)[C@@H](O)C2[3H])[3H])[nH]c(N)n1	119505-86-1	1-Naphthalenesulfonic acid, 4-amino-3-[2-[4,Ä≤-[2-[5-(1,1-dimethylethyl)-2-hydrc	C32H29N5O4S.Na		C	-8.4
6414	O(COC(=O)N1CCN(C)CC1)c1c2n(C[C@H]3N(C2=O)[C@H](C)CCO3)cc(C(=O)NCc2c(F)cc(F)cc2)c1=O	1206156-30-0	Carbamic acid, N-[2-(4-morpholinyl)ethyl]-, [(4R,12aS)-9-[[[(2,4-difluorophenyl]	C28H33F2N5O8	605.59	C	-8.4
6415	N(C(CNc1nc(nc(n1)N1CCOCC1)N1CCOCC1)C)c1c2c(ncc1)cc(Cl)cc2	1207449-62-4	1,2-Ethanediamine, N1-(7-chloro-4-quinolinyl)-N2-(4,6-di-4-morpholinyl-1,3,5-tri	C22H27ClN8O2	470.96	C	-8.4
6416	N(Cc1c(CN(Cc2ccccc2)C)cccc1)c1c2c(ncc1)cc(Cl)cc2	120746-51-2	1-Naphthalenesulfonic acid, 3,3,Ä≤-[(2,2,Ä≤-dimethyl[1,1,Ä≤-biphenyl]-4,4,Ä≤-di	C34H28N6O6S2	680.75	C	-8.4
6417	C([C@](OCP(=O)(O)O)(C([2H])([2H])[2H])[2H])(n1c2c(nc1)c(N)ncn2)([2H])[2H]	1217703-38-2	6H-Furo[3,Ä≤,4,Ä≤:6,7]naphtho[2,3-c]-1,2-dioxol-6-one, 3,5,5a,8,8a,9-hexahydro-	C22H22O8	414.41	C	-8.4
6418	C(N1c2c(C(=NNC(=S)N)C1=O)ccc(Br)c2)N1CCN(CC1)c1c2c(ncc1)cc(Cl)cc2	1218813-12-7	Hydrazinecarbothioamide, 2-[6-chloro-1-[[4-(7-chloro-4-quinolinyl)-1-piperazinyl	C23H21Cl2N7OS	514.43	C	-8.4
6419	N(=C1c2c(N(CN3CCN(CC3)c3c4c(cc(C(F)(F)F)cc4)ncc3)C1=O)cccc2Cl)NC(=S)N	1218813-14-9	Hydrazinecarbothioamide, 2-[6-bromo-1-[[4-(7-chloro-4-quinolinyl)-1-piperazinyl]	C23H21BrClN7OS	558.88	C	-8.4
6420	N(C(=O)C)[C@H]1[C@H]([C@@H]([C@@H](CO)O)O)OC(=C[C@@H]1NC(N)N)C(=O)O	1222681-91-5	Not Yet Assigned	C36H53N5O4	619.84	C	-8.4
6421	[C@](C(=O)N[C@@H](C(c1c(c(c(c(c1[2H])[2H])[2H])[2H])[2H])([2H])[2H])C[C@@H]([C@](C(c1c(c(c(c(c1[2H])	1224728-97-5	1(2H)-Pyrimidine-4,5,6-d3-acetamide-Œ±-d, N-[(1S,3S,4S)-4-[[2-(2,6-dimethylphenc	C37H17D31N4O5		C	-8.4
6422	[C@](C(=O)N[C@@H](C(c1c(c(c(c(c1[2H])[2H])[2H])[2H])[2H])([2H])[2H])C[C@@H]([C@H](Cc1ccccc1)NC(=O)C(	1224729-01-4	1(2H)-Pyrimidine-4,5,6-d3-acetamide-Œ±-d, N-[(1S,3S,4S)-4-[[2-(2,6-dimethylphenc	C37H25D23N4O5		C	-8.4
6423	[C@@H](C(=O)N[C@@H](Cc1ccccc1)C[C@@H]([C@](C(c1c(c(c(c(c1[2H])[2H])[2H])[2H])[2H])([2H])[2H])(NC(=O)	1224729-45-6	1(2H)-Pyrimidine-4,5,6-d3-acetamide, N-[(1S,3S,4S)-4-[[2-(2,6-dimethylphenoxy)ac	C37H32D16N4O5		C	-8.4
6424	[C@@H](C(=O)N[C@@H](Cc1ccccc1)C[C@@H]([C@](C(c1c(c(c(c(c1[2H])[2H])[2H])[2H])[2H])([2H])[2H])(NC(=O)	1224729-52-5	1(2H)-Pyrimidine-4,5,6-d3-acetamide, N-[(1S,3S,4S)-4-[[2-[2,6-di(methyl-d3)phenc	C37H25D23N4O5		C	-8.4
6425	[C@@H](C(=O)N[C@@H](C(c1ccccc1)([2H])[2H])C[C@@H]([C@H](C(c1ccccc1)([2H])[2H])NC(=O)COc1c(C)cccc1C)O	1224729-62-7	1(2H)-Pyrimidine-4,5,6-d3-acetamide, N-[(1S,3S,4S)-4-[[2-[2,6-di(methyl-d3)phenc	C37H34D14N4O5		C	-8.4
6426	C(=O)([C@@H](NC(=O)OC)c1ccccc1)N1[C@@H](CCC1)c1[nH]c(cn1)[C@H]1CC[C@@H](CC1)c1ccc(cc1)c1[nH]c(nc1)[C	1228667-92-2	Carbamic acid, N-[(1R)-2-[(2S)-2-[5-[4-[trans-4-[2-[(2S)-1-[(2R)-2-[(methoxycarb	C46H52N8O6	812.96	C	-8.4
6427	C(=O)([C@@H](NC(=O)OC)C(C)C)N1[C@H](c2[nH]c(C34CCC(CC3)(CC4)c3ccc(cc3)c3[nH]c(nc3)[C@H]3N(C(=O)[C@@H	1228671-53-1	Carbamic acid, N-[(1R)-2-[(2S)-2-[5-[4-[4-[2-[(2S)-1-[(2S)-2-[(methoxycarbonyl)a	C40H54N8O6	742.91	C	-8.4
6428	C(=O)([C@@H](NC(=O)OC(C)(C)C)C(C)C)N1[C@@H](CCC1)c1[nH]c(cn1)c1ccc(C#Cc2ccc(cc2)c2[nH]c(nc2)[C@H]2N(	1228964-85-9	Carbamic acid, N,N,Ä≤-[1,2-ethynediylbis[4,1-phenylene-1H-imidazole-5,2-diyl-(2S	C48H46N8O6	830.93	C	-8.4
6429	C(=O)([C@@H](NC(=O)OC)[C@@H](C)O)N1[C@@H](CCC1)c1[nH]c(cn1)c1ccc(C#Cc2ccc(cc2)c2[nH]c(nc2)[C@H]2N(C(	1228966-10-6	Carbamic acid, N-[(1R)-2-[(2S)-2-[5-[4-[2-[4-[2-[(1S)-1-[[(2R)-2-[(methoxycarbor	C48H48N8O6	832.94	C	-8.4
6430	C(=O)([C@@H](NC(=O)OC)c1ccccc1)N1[C@@H](CCC1)c1[nH]c(cn1)c1ccc2c(cc1)cc(C#Cc1[nH]c(nc1)[C@H]1N(C(=O)[	1228967-99-4	Carbamic acid, N-[(1R)-2-[(2S)-2-[5-[2-[6-[2-[(2S)-1-[(2S)-2-[(methoxycarbonyl)a	C43H46N8O6	770.88	C	-8.4
6431	C(CC)(CC)c1nc(CN(C(=O)N[C@@H](C(=O)N[C@@H](Cc2ccccc2)C[C@@H]([C@H](Cc2ccccc2)NC(=O)OCc2cncs2)O)C(C)C	1236408-24-4	2,7,10,12-Tetraazatridecanoic acid, 4-hydroxy-12-methyl-9-(1-methylethyl)-8,11-c	C40H46N6O6S	738.89	C	-8.4
6432	N(C(=O)[C@H](Cc1ccccc1)C[C@@H](CN1[C@H](C(=O)NC(C)(C)C)CN(Cc2c3n(ccn3)ccn2)CC1)O)[C@H]1c2c(C[C@H]1O)	1237808-94-4	Not Yet Assigned	C37H47N7O4	653.81	C	-8.4
6433	N(C(=O)[C@H](Cc1ccccc1)C[C@@H](CN1[C@H](C(=O)NC(C)(C)C)CN(Cc2ccc3c(s2)cccn3)CC1)O)[C@H]1c2c(C[C@H]1O)	1237809-00-5	Not Yet Assigned	C39H48N4O4S	668.89	C	-8.4
6434	N(C(=O)[C@H](Cc1ccccc1)C[C@@H](CN1[C@H](C(=O)NC(C)(C)C)CN(Cc2ccsc2)CC1)O)[C@H]1c2c(C[C@H]1O)cccc2	1237988-64-5	D-erythro-Pentonamide, 2,3,5-trideoxy-N-[(1S,2R)-2,3-dihydro-2-hydroxy-1H-inden-	C36H48N4O4S	632.86	C	-8.4

6435	C=C1[C@@H](n2c3c(nc2)c(=O)nc(N)[nH]3)C[C@H]2[C@H]1CO[Si](C(C)(C)C)(C(C)(C)C)O2	123808-83-3	Cytidine, [P(S)]-P-methylthymidylyl-(3,Ä≤,Üí5,Ä≤)-2,Ä≤-deoxy-	C20H28N5O11P	545.44	C	-8.4
6436	C(=O)([C@@H](NC(=O)OC)CC=C)N1[C@H](c2[nH]c(c3cc4c(-c5c(C4)cc(cc5)c4[nH]c(nc4)[C@H]4N(C(=O)[C@@H](NC(	1240880-49-2	Carbamic acid, N,N,Ä≤-[9H-fluorene-2,7-diylbis[1H-imidazole-5,2-diyl-(2S)-2,1-py	C39H46N8O6	722.83	C	-8.4
6437	C(=O)([C@H](Cc1ccccc1)NC(=O)OC)N1[C@@H](CCC1)c1[nH]c(cn1)[C@H]1CC[C@@H](CC1)c1ccc(cc1)c1[nH]c(nc1)[C	1240888-30-5	Carbamic acid, N-[(1R)-2-[(2S)-2-[5-[4-[trans-4-[2-[(2S)-1-[(2R)-2-[(methoxycarb	C48H56N8O6	841.01	C	-8.4
6438	C(=O)([C@H](NC(=O)OC)c1ccccc1)N1[C@@H](CCC1)c1[nH]c(cn1)[C@H]1CC[C@@H](CC1)c1ccc(cc1)c1[nH]c([C@@H](	1240888-31-6	Carbamic acid, N-[(1S)-2-[(2S)-2-[5-[4-[trans-4-[2-[(2S)-1-[(2S)-2-[(methoxycarb	C48H56N8O6	841.01	C	-8.4
6439	C(=O)(CNC(=O)OC)N1[C@@H](CCC1)c1[nH]c(cn1)[C@H]1CC[C@@H](CC1)c1ccc(cc1)c1[nH]c([C@@H](N(C(=O)[C@H](N	1240890-54-3	Carbamic acid, N-[(1R)-2-[[(1S)-1-[5-[4-[trans-4-[2-[(2S)-1-[(2S)-2-[(methoxycar	C42H54N8O6	766.93	C	-8.4
6440	C(=O)([C@H](NC(=O)OC)c1ccccc1)N1[C@@H](CCC1)c1[nH]c(cn1)c1ccc(C/C=C\Cc2[nH]c(nc2)[C@H]2N(C(=O)[C@H](	1240891-75-1	Carbamic acid, N-[(1R)-2-[(2S)-2-[5-[4-[4-[2-[(2S)-1-[(2R)-2-[(methoxycarbonyl)a	C44H50N8O6	786.92	C	-8.4
6441	C(=O)([C@H](NC(=O)OC)c1ccccc1)N1[C@@H](CCC1)c1[nH]c(cn1)c1ccc(C/C=C/Cc2[nH]c(nc2)[C@H]2N(C(=O)[C@H](	1240891-96-6	Carbamic acid, N-[(1R)-2-[(2S)-2-[5-[4-[(1E)-4-[2-[(2S)-1-[(2R)-2-[(methoxycarbo	C44H48N8O6	784.9	C	-8.4
6442	C(Nc1c2c(n3c(n1)c(S(=O)(=O)c1ccc(C)cc1)nn3)ccs2)(C)C12CC3CC(C2)CC(C1)C3	1246178-71-1	Thieno[2,3-e][1,2,3]triazolo[1,5-a]pyrimidin-5-amine, 3-(phenylsulfonyl)-N-(1-tr	C25H27N5O2S2	493.64	C	-8.4
6443	C(=O)(NC)c1c2c(cc(N(CCOP(=O)(OC[C@H]3O[C@H]([C@H](O)[C@@H]3O)n3nc(C(=O)N)nc3)O)S(=O)(=O)C)c(c2)C2CC2	1246280-90-9	Not Yet Assigned	C33H38FN6O12PS	792.72	C	-8.4
6444	C(=O)([C@@H](NC(=O)OC)C)N1[C@@H](CCC1)c1[nH]c(cn1)C1=CCC(CC1)C1CCC(=CC1)c1[nH]c(nc1)[C@H]1N(C(=O)[C@	1246852-24-3	Carbamic acid, N,N,Ä≤-[[1,1,Ä≤-bi-3-cyclohexen]-4,4,Ä≤-diylbis[1H-imidazole-5,2	C40H58N8O6	746.94	C	-8.4
6445	C(=O)([C@@H](NC(=O)OC)C(C)C)N1[C@@H](CCC1)c1[nH]c(cn1)C1=CCC(CC1)c1ccc(cc1)c1[nH]c(nc1)[C@H]1N(C(=O)	1246852-25-4	Carbamic acid, N,N,Ä≤-[[1,1,Ä≤-bi-3-cyclohexen]-4,4,Ä≤-diylbis[1H-imidazole-5,2	C36H50N8O6	690.83	C	-8.4
6446	O=c1n([C@@H]2O[C@H](CO)C=C2)cc(Cl)c(N)n1	124705-10-8	Guanosine 5,Ä≤-(trihydrogen diphosphate), P,Ä≤,Üí3,Ä≤-ester with adenosine	C20H26N10O14P2	692.43	C	-8.4
6447	C(=O)([C@@H](NC(=O)OC)C(C)C)N1[C@H]2[C@H](C2)C[C@H]1c1[nH]c(cn1)c1ccc(cc1)[C@H]1[C@@H](C1)c1ccc(cc1)	1250938-81-8	Carbamic acid, N,N,Ä≤-[(1S,2S)-1,2-cyclopropanediylbis[4,1-phenylene-1H-imidazol	C45H54N8O6	802.96	C	-8.4
6448	Nc1c2c(n(cn2)[C@H]2[C@@H](O)[C@H](O)[C@@H](CO)O2)nc(Cc2ccccc2)n1	125339-07-3	9H-Purin-6-amine, 9-Œ≤-D-arabinofuranosyl-2-phenyl-	C16H17N5O4	343.34	C	-8.4
6449	[C@@H](NC(=O)[C@H](Cc1ccccc1)NC(=O)N1CCN(C)CC1)(/C=C/S(=O)(=O)c1ccccc1)c1ccc(cc1)c1ccccc1	1253912-37-6	1-Piperazinecarboxamide, 4-methyl-N-[(1S)-2-oxo-1-(phenylmethyl)-2-[[(1R,2E)-3-(	C33H40N4O4S	588.76	C	-8.4
6450	[C@@H](NC(=O)[C@@H](NC(=O)N1CCN(C)CC1)CC(C)C)(/C=C/S(=O)(=O)c1ccccc1)c1ccc(cc1)c1ccccc1	1253912-45-6	1-Piperazinecarboxamide, 4-methyl-N-[(1S)-3-methyl-1-[[[(1R,2E)-1-(2-naphthaleny	C31H38N4O4S	562.72	C	-8.4
6451	C(=O)([C@@H](NC(=O)OC)C(C)C)N1[C@@H](CCC1)c1[nH]c(cn1)c1cc(C#N)c(cc1)c1ccc(cc1)c1[nH]c(nc1)[C@H]1N(C	1256387-69-5	L-Prolinamide, N-(methoxycarbonyl)-L-valyl-N-[4,Ä≤,Ä≤-[2-[(2S)-1-[(2S)-2-[(meth	C44H53N7O7	791.93	C	-8.4
6452	C(=O)([C@@H](NC(=O)OC)C(C)C)N1CC2(C[C@H]1c1[nH]c(cn1)c1cc3c(-c4c(C3(F)F)cc(cc4)c3cc4c(cc3)nc([nH]4)[	1256388-44-9	Carbamic acid, N-[(1S)-1-[[(2S)-2-[5-[4,Ä≤-[2-[(1R)-2-hydroxy-1-[[(2S)-2-[(metho	C38H48N8O7	728.84	C	-8.4
6453	C(=O)([C@@H](NC(=O)OC)C(C)C)N1[C@@H]([C@@H]2C[C@H]1CC2)c1[nH]c(cn1)c1cc2c(cc1)c1c(o2)cc(cc1)c1[nH]c(	1256388-72-3	Carbamic acid, N,N,Ä≤-[6H-dibenzo[b,d]pyran-3,8-diylbis[1H-imidazole-5,2-diyl-(2	C41H50N8O7	766.89	C	-8.4
6454	C(=O)([C@@H](NC(=O)OC)C(C)C)N1[C@@H](C[C@@H](C#N)C1)c1[nH]c(cn1)c1ccc(cc1)c1cc2c(cc1)cc(cc2)c1[nH]c(	1256389-37-3	Carbamic acid, N,N,Ä≤-[(3-cyano[1,1,Ä≤-biphenyl]-4,4,Ä≤-diyl)bis[1H-imidazole-5	C41H49N9O6	763.88	C	-8.4
6455	C(=O)([C@@H](NC(=O)OC)C(C)C)N1[C@@H](C[C@H](C#N)C1)c1[nH]c(cn1)c1ccc(cc1)c1cc2c(cc1)cc(cc2)c1[nH]c(n	1256389-41-9	Carbamic acid, N-[(1S)-1-[[(2S,4R)-4-cyano-2-[5-[4-[6-[2-[(2S)-1-[(2S)-2-[(metho	C45H51N9O6	813.94	C	-8.4
6456	C(=O)([C@@H](NC(=O)OC)[C@H](CC)C)N1[C@@H](CCC1)c1[nH]c(cn1)c1cc2c(cc1)cc(C#Cc1[nH]c(nc1)[C@H]1N(C(=O	1256390-70-1	Carbamic acid, N-[(1S)-2-[(2S,5R)-2-[5-[4-[6-[2-[(2S)-1-[(2S)-2-[(methoxycarbony	C48H52N8O6	836.98	C	-8.4
6457	C(=O)(C(NC(=O)OC)c1ccccc1)N1[C@@H](CCC1)c1[nH]c(cn1)c1ccc(cc1)c1cc2c(cc1)cc(cc2)c1[nH]c(nc1)[C@H]1N(	1256392-99-0	Carbamic acid, N-[(1S)-1-[[(2S)-2-[5-[6-[4-[2-[(2S)-1-[(2S)-2-(methoxyamino)-3-m	C43H52N8O5	760.92	C	-8.4
6458	C(=O)(NC(C(C1CCC1)([2H])[2H])C(=O)C(=O)N)[C@@H]1[C@@H]2[C@@H](C2(C([2H])([2H])[2H])C([2H])([2H])[2H]	1256750-98-7	3-Azabicyclo[3.1.0]hexane-2-carboxamide, N-[3-amino-1-(cyclobutylmethyl-d2)-2,3-	C27H36D9N5O5		C	-8.4
6459	C(F)(F)c1n(c2c(n1)cccc2)c1nc(nc(NCc2ccccc2)n1)N1CCOCC1	1261281-11-1	1,3,5-Triazin-2-amine, 4-[2-(difluoromethyl)-1H-benzimidazol-1-yl]-N-methyl-6-(4	C22H21F2N7O	437.45	C	-8.4
6460	O(C)c1c2n(cc(C(=O)O)c(=O)c2cc(F)c1N1CC(C)N(CN2c3c(/C(=N/NC(=S)NOC)/C2=O)cc(Cl)cc3)CC1)C1CC1	1265623-27-5	3-Quinolinecarboxylic acid, 7-[4-[[(3Z)-5-chloro-2,3-dihydro-3-[2-[(hydroxyamino	C29H29ClFN7O6S	658.1	C	-8.4
6461	N(=C\1/c2c(N(CN3CCN(CC3)c3cc4c(cc3F)c(=O)c(C(=O)O)cn4CC)C1=O)ccc(C)c2)\NC(=S)NOC	1265623-32-2	3-Quinolinecarboxylic acid, 1-cyclopropyl-7-[4-[[(3Z)-2,3-dihydro-3-[2-[(methoxy	C31H34FN7O6S	651.71	C	-8.4
6462	O[C@@H]1c2n(c3c(n2)c(=O)nc(N)[nH]3)[C@@H]2O[C@H]1[C@@H](O)[C@H]2O	1266324-50-8	1-Naphthalenesulfonic acid, 4-amino-3-[(1E)-2-[4,Ä≤-[(1E)-2-(2-amino-8-hydroxy-6	C32H24N6O7S2	668.7	C	-8.4
6463	C(=O)([C@@H](NC(=O)OC)C1CC1)N1[C@@H]([C@@H]2C[C@H]1CC2)c1[nH]c(cn1)c1ccc(cc1)c1ccc(cc1)c1[nH]c(nc1)[	1273589-59-5	Carbamic acid, N-[(1S)-1-[[(2S)-2-[5-[4,Ä≤-[2-[(1R,3S,4S)-2-[(2S)-2-[(methoxycar	C42H52N8O6	764.91	C	-8.4
6464	C(=O)([C@@H](NC(=O)OC)C(C)C)N1[C@@H]([C@@H]2C[C@H]1C[C@H]2O)c1[nH]c(cn1)c1ccc(cc1)c1ccc(cc1)c1[nH]c(	1273591-14-2	Carbamic acid, N-[(1S)-1-[[(2S,4S)-4-hydroxy-2-[5-[4,Ä≤-[2-[(1R,3S,4S)-2-[(2S)-2	C42H52N8O7	780.91	C	-8.4
6465	C(=O)([C@@H](NC(=O)OC)C(C)C)N1[C@@H](C2CCC1CC2)c1[nH]c(cn1)c1ccc(cc1)c1ccc(cc1)c1[nH]c(nc1)[C@H]1N(C	1273591-18-6	Carbamic acid, N-[(1S)-1-[[(2S)-2-[5-[4,Ä≤-[2-[(1S,3S,4S,5R)-5-methoxy-2-[(2S)-2	C43H54N8O7	794.94	C	-8.4
6466	C(=O)([C@@H](NC(=O)OC)C(C)C)N1[C@@H]([C@@H]2C[C@H]1CC2)c1[nH]c(cn1)c1c(C)cc(cc1)c1cc(C)c(cc1)c1[nH]c	1273591-46-0	Carbamic acid, N-[(1S)-2-methyl-1-[[(2S)-2-[5-[2,2,Ä≤,3,3,Ä≤-tetrafluoro-4,Ä≤-[	C42H48F4N8O6	836.87	C	-8.4
6467	C(=O)([C@@H](NC(=O)OC)C(C)C)N1[C@@H]([C@@H]2C[C@H]1CC2)c1[nH]c(cn1)c1c(Cl)cc(cc1)c1cc(Cl)c(cc1)c1[nH	1273591-50-6	Carbamic acid, N-[(1S)-1-[[(2S)-2-[5-[4,Ä≤-[2-[(1R,3S,4S)-2-[(2S)-2-[(methoxycar	C44H50F6N8O6	900.91	C	-8.4
6468	O=c1n([C@H]2C[C@H](OP(=O)(OCCC(C)O)O)[C@@H](CO)O2)cc(C)c(=O)[nH]1	1300033-19-5	4H-1-Benzopyran-4-one, 2-(2-quinazolinyl)-, oxime	C17H11N3O2	289.29	C	-8.4
6469	O=C1N2[C@@H]3[C@H](CO[C@@H]2Cn2c1c(O)c(=O)c(C(=O)NCc1c(F)cc(F)cc1)c2)CCCC3	1309560-49-3	2H-Pyrido[1,Ä≤,2,Ä≤:4,5]pyrazino[2,1-b][1,3]oxazine-9-carboxamide, N-[(2,4-difl	C20H19F2N3O5	419.38	C	-8.4
6470	C(=O)([C@@H](NC(=O)OC)C(C)C)N1[C@@H](CC(=C)C1)c1[nH]c(cn1)c1c(F)c(F)c(cc1)c1c(F)c(F)c(cc1)c1[nH]c(nc	1311398-21-6	Carbamic acid, N-[(1S)-1-[[(2S)-2-[5-[3,3,Ä≤-difluoro-4,Ä≤-[2-[(2S)-1-[(2S)-2-[	C41H48F2N8O6	786.87	C	-8.4
6471	C(=O)([C@@H](NC(=O)OC)C(C)C)N1[C@@H](CCC1)c1[nH]c(c(Cl)n1)c1ccc(cc1)c1ccc(cc1)c1[nH]c(nc1)[C@H]1N(C(	1312335-96-8	Carbamic acid, N,N,Ä≤-[[1,1,Ä≤-biphenyl]-4,4,Ä≤-diylbis[(5-bromo-1H-imidazole-2	C40H48Br2N8O6	896.67	C	-8.4
6472	C(=O)([C@@H](NC(=O)OC)C(C)C)N1[C@@H](CCC1)c1[nH]c(c(Br)n1)c1ccc(cc1)c1ccc(cc1)c1[nH]c(nc1)[C@H]1N(C(	1312336-45-0	Carbamic acid, N,N,Ä≤-[[1,1,Ä≤-biphenyl]-4,4,Ä≤-diylbis[(5-bromo-1H-imidazole-4	C42H48Br2N8O6	920.69	C	-8.4
6473	C(=O)([C@@H](NC(=O)OC)C(C)C)N1[C@H]2[C@H](C2)C[C@H]1c1[nH]c(c(F)n1)c1ccc(cc1)c1ccc(cc1)c1[nH]c(nc1)[	1312336-62-1	Carbamic acid, N-[(1S)-1-[[(2S)-2-[4-[4,Ä≤-[2-[(2S)-1-[(2S)-2-[(methoxycarbonyl)	C44H54N10O6	818.96	C	-8.4
6474	C(=O)([C@@H](NC(=O)OC)C1CCOCC1)N1[C@H]2[C@H](C2)C[C@H]1c1[nH]c(c(n1)c1ccc(cc1)c1ccc(cc1)c1c(nc([nH]1	1312337-02-2	Carbamic acid, N,N,Ä≤-[[1,1,Ä≤-biphenyl]-4,4,Ä≤-diylbis[(5-cyclopropyl-1H-imida	C48H58N8O6	843.02	C	-8.4
6475	C(=O)([C@@H](NC(=O)OC)C(C)C)N1[C@@H](C[C@H](C)C1)c1[nH]c(cn1)c1ccc(cc1)c1ccc(cc1)c1[nH]c(nc1)[C@H]1N	1312338-34-3	Carbamic acid, N-[(1S)-2-[(2S,4S)-2-[5-[4,Ä≤-[2-[(2S,4S)-1-[(2S)-2-[(methoxycarb	C44H56N8O7	808.96	C	-8.4
6476	C(=O)([C@@H](NC(=O)OC)C(C)C)N1[C@H](c2[nH]c(c3c4c(c(cc3)c3ccc(cc3)c3[nH]c(nc3)[C@H]3N(C(=O)[C@@H](NC	1312608-44-8	Carbamic acid, N-[(1S)-1-[[(2S)-2-[5-[4-[4-[2-[(2S)-1-[(2S)-2-[(methoxycarbonyl)	C43H52N8O7	792.92	C	-8.4
6477	C(=O)([C@@H](NC(=O)OC)C(C)C)N1[C@H](c2[nH]c(c3c4c(c(cc3)c3ccc(cc3)c3[nH]c(nc3)[C@H]3N(C(=O)[C@@H](NC	1312608-61-9	Carbamic acid, N-[(1S)-1-[[(2S)-2-[5-[4-[7-[2-[(2S)-1-[(2S)-2-[(methoxycarbonyl)	C42H50N8O7	778.9	C	-8.4
6478	C[C@]1(c2[nH]c(c3c4c(c(cc3)c3ccc(cc3)c3[nH]c(nc3)[C@@]3(C)N(C(=O)[C@@H](NC(=O)OC)C(C)C)CCC3)ncs4)cn2	1312608-72-2	Carbamic acid, N-[(1S)-1-[[(2S)-2-[5-[4-[7-[2-[(2S)-1-[(2S)-2-[(methoxycarbonyl)	C43H53N9O6	791.94	C	-8.4
6479	Cc1oc2c(ccc(c2c1)c1ccc(cc1)c1[nH]c(nc1)[C@@]1(C)N(C(=O)[C@@H](NC(=O)OC)C(C)C)CCC1)c1[nH]c(nc1)[C@@]1	1312608-84-6	Carbamic acid, N,N,Ä≤-[3,8-phenanthridinediylbis[1H-imidazole-5,2-diyl-(2S)-2,1-	C41H49N9O6	763.88	C	-8.4
6480	Nc1n(c2c(n1)c(=O)nc[nH]2)C1OC(CO)CC1	1312612-07-9	Carbamic acid, N-[(1S)-1-[[(2S)-2-[5-[4-[4-[2-[(2S)-1-[(2S)-2-[(methoxycarbonyl)	C46H56N8O6	816.99	C	-8.4
6481	O(Cc1ccc(F)cc1)c1c2c(oc(c(O)c2=O)c2nnc(C)[nH]2)c(Cl)cc1	1312893-93-8	4H-1-Benzopyran-4-one, 8-chloro-5-[(4-fluorophenyl)methoxy]-3-hydroxy-2-(1H-1,2,	C18H11ClFN3O4	387.75	C	-8.4
6482	O(Cc1ccc(F)cc1)c1c2c(c(cc1)c1ccccc1)oc(c(O)c2=O)c1ncccn1	1312894-02-2	4H-1-Benzopyran-4-one, 8-chloro-5-[(4-fluorophenyl)methoxy]-3-hydroxy-2-(5-pheny	C25H16ClFN2O4	462.86	C	-8.4
6483	O(Cc1ccc(F)cc1)c1c2c(c(cc1)c1ccccc1)oc(c(O)c2=O)c1[nH]c(Cl)nn1	1312894-45-3	4H-1-Benzopyran-4-one, 5-[(4-fluorophenyl)methoxy]-3-hydroxy-2-(5-methoxy-1H-1,2	C25H18FN3O5	459.43	C	-8.4
6484	O(Cc1ccc(F)cc1)c1c2c(c(cc1)c1ccccc1)oc(c(O)c2=O)c1[nH]c(Cl)cn1	1312894-50-0	4H-1-Benzopyran-4-one, 5-[(4-fluorophenyl)methoxy]-3-hydroxy-2-(5-methoxy-1H-imi	C26H19FN2O5	458.44	C	-8.4
6485	O(Cc1ccc(F)cc1)c1c2c(oc(c(O)c2=O)c2ncc(C)cn2)cc(C)c1	1312894-83-9	4H-1-Benzopyran-4-one, 5-[(4-fluorophenyl)methoxy]-3-hydroxy-7-methyl-2-(2-pyrim	C21H15FN2O4	378.35	C	-8.4
6486	O(Cc1ccc(F)cc1)c1c2c(oc(c(O)c2=O)c2ncc(OC)cn2)cc(C)c1	1312894-84-0	4H-1-Benzopyran-4-one, 5-[(4-fluorophenyl)methoxy]-3-hydroxy-7-methyl-2-(5-methy	C22H17FN2O4	392.38	C	-8.4
6487	O(Cc1ccc(F)cc1)c1c2c(oc(c(O)c2=O)c2[nH]c(Cl)cn2)c(C)c(C)c1	1312895-50-3	4H-1-Benzopyran-4-one, 5-[(4-fluorophenyl)methoxy]-3-hydroxy-2-(5-methoxy-1H-imi	C22H19FN2O5	410.4	C	-8.4
6488	O(Cc1ccc(F)cc1)c1c2c(c(c(C)c1)c1ccccc1)oc(c(O)c2=O)c1[nH]c(OC)cn1	1312896-99-3	4H-1-Benzopyran-4-one, 5-[(4-fluorophenyl)methoxy]-3-hydroxy-7-methyl-2-(5-methy	C27H21FN2O4	456.46	C	-8.4
6489	O(Cc1ccc(F)cc1)c1c2c(oc(c(O)c2=O)c2[nH]ccn2)cc(OC)c1	1312897-58-7	4H-1-Benzopyran-4-one, 5-[(4-fluorophenyl)methoxy]-3-hydroxy-7-methoxy-2-(3-pher	C25H18FN3O5	459.43	C	-8.4
6490	O(Cc1ccc(F)cc1)c1c2c(oc(c(O)c2=O)c2[nH]c(nn2)c2ccccc2)c(C)c(OC)c1	1312898-07-9	4H-1-Benzopyran-4-one, 2-(5-chloro-1H-1,2,4-triazol-3-yl)-5-[(4-fluorophenyl)met	C20H15ClFN3O5	431.8	C	-8.4
6491	O(Cc1ccc(F)cc1)c1c2c(oc(c(O)c2=O)c2[nH]c(C)nn2)c(Cl)c(OC)c1	1312899-05-0	4H-1-Benzopyran-4-one, 8-chloro-5-[(4-fluorophenyl)methoxy]-3-hydroxy-7-methoxy-	C19H13ClFN3O5	417.77	C	-8.4
6492	O(C)c1c(c2c(c(OCc3ccc(F)cc3)c1)c(=O)c(O)c(o2)c1ncc(cn1)c1ccccc1)c1ccccc1	1312899-48-1	4H-1-Benzopyran-4-one, 2-(5-chloro-2-pyrimidinyl)-5-[(4-fluorophenyl)methoxy]-3-	C27H18ClFN2O5	504.89	C	-8.4
6493	O(C)c1c(c2c(c(OCc3ccc(F)cc3)c1)c(=O)c(O)c(o2)c1[nH]cc(C)n1)c1ccccc1	1312899-60-7	4H-1-Benzopyran-4-one, 5-[(4-fluorophenyl)methoxy]-3-hydroxy-2-(1H-imidazol-2-yl	C26H19FN2O5	458.44	C	-8.4
6494	O(C)c1c(c2c(c(OCc3ccc(F)cc3)c1)c(=O)c(O)c(o2)c1[nH]c(OC)cn1)c1ccccc1	1312899-61-8	4H-1-Benzopyran-4-one, 5-[(4-fluorophenyl)methoxy]-3-hydroxy-7-methoxy-2-(5-meth	C27H21FN2O5	472.46	C	-8.4
6495	O(C)c1c(c2c(c(OCc3ccc(F)cc3)c1)c(=O)c(O)c(o2)c1[nH]cc(Cl)n1)c1ccccc1	1312899-62-9	4H-1-Benzopyran-4-one, 5-[(4-fluorophenyl)methoxy]-3-hydroxy-7-methoxy-2-(5-meth	C27H21FN2O6	488.46	C	-8.4
6496	O(Cc1ccc(F)cc1)c1c2c(oc(c(O)c2=O)c2nnc(C)[nH]2)cc(Cl)c1	1312900-64-3	4H-1-Benzopyran-4-one, 7-chloro-5-[(4-fluorophenyl)methoxy]-3-hydroxy-2-(1H-1,2,	C18H11ClFN3O4	387.75	C	-8.4
6497	O(Cc1ccc(F)cc1)c1c2c(oc(c(O)c2=O)c2[nH]cc(C)n2)cc(Cl)c1	1312900-69-8	4H-1-Benzopyran-4-one, 7-chloro-5-[(4-fluorophenyl)methoxy]-3-hydroxy-2-(1H-imid	C19H12ClFN2O4	386.76	C	-8.4
6498	O(Cc1ccc(F)cc1)c1c2c(oc(c(O)c2=O)c2ncccn2)c(Cl)c(Cl)c1	1312901-73-7	4H-1-Benzopyran-4-one, 7-chloro-5-[(4-fluorophenyl)methoxy]-3-hydroxy-8-methoxy-	C26H18ClFN2O5	492.88	C	-8.4
6499	O(Cc1ccc(F)cc1)c1c2c(oc(c(O)c2=O)c2[nH]c(Cl)nn2)cc(c1)c1ccccc1	1312903-16-4	4H-1-Benzopyran-4-one, 5-[(4-fluorophenyl)methoxy]-3-hydroxy-2-(5-methoxy-1H-1,2	C25H18FN3O5	459.43	C	-8.4
6500	O(Cc1ccc(F)cc1)c1c2c(oc(c(O)c2=O)c2[nH]c(cn2)c2ccccc2)cc(c1)c1ccccc1	1312903-22-2	4H-1-Benzopyran-4-one, 2-(5-chloro-1H-imidazol-2-yl)-5-[(4-fluorophenyl)methoxy]	C25H16ClFN2O4	462.86	C	-8.4
6501	O(Cc1ccc(F)cc1)c1c2c(c(C)c(c1)c1ccccc1)oc(c(O)c2=O)c1[nH]c(OC)nn1	1312903-67-5	4H-1-Benzopyran-4-one, 5-[(4-fluorophenyl)methoxy]-3-hydroxy-8-methyl-2-(3-methy	C26H20FN3O4	457.45	C	-8.4
6502	O(Cc1ccc(F)cc1)c1c2c(c(C)c(c1)c1ccccc1)oc(c(O)c2=O)c1[nH]c(Cl)nn1	1312903-68-6	4H-1-Benzopyran-4-one, 5-[(4-fluorophenyl)methoxy]-3-hydroxy-2-(5-methoxy-1H-1,2	C26H20FN3O5	473.45	C	-8.4
6503	Cc1c2c(c(OCc3ccc(F)cc3)cc1c1ccccc1)c(=O)c(O)c(o2)c1[nH]c(nn1)c1ccccc1	1312903-69-7	4H-1-Benzopyran-4-one, 2-(5-chloro-1H-1,2,4-triazol-3-yl)-5-[(4-fluorophenyl)met	C25H17ClFN3O4	477.87	C	-8.4
6504	O(C)c1c2c(c(OCc3ccc(F)cc3)cc1c1ccccc1)c(=O)c(O)c(o2)c1nnc(C)[nH]1	1312904-40-7	4H-1-Benzopyran-4-one, 5-[(4-fluorophenyl)methoxy]-3-hydroxy-8-methoxy-7-phenyl-	C25H18FN3O5	459.43	C	-8.4
6505	O=c1c2c(c(cc(OCc3ccc(F)cc3)c2)c2ccccc2)oc(c1O)c1[nH]c(Cl)nn1	1312908-26-1	4H-1-Benzopyran-4-one, 6-[(4-fluorophenyl)methoxy]-3-hydroxy-2-(5-methoxy-1H-1,2	C25H18FN3O5	459.43	C	-8.4
6506	O=c1c2c(c(cc(OCc3ccc(F)cc3)c2)c2ccccc2)oc(c1O)c1[nH]c(C)cn1	1312908-29-4	4H-1-Benzopyran-4-one, 6-[(4-fluorophenyl)methoxy]-3-hydroxy-2-(1H-imidazol-2-yl	C25H17FN2O4	428.41	C	-8.4
6507	O=c1c2c(c(cc(OCc3ccc(F)cc3)c2)c2ccccc2)oc(c1O)c1[nH]c(Cl)cn1	1312908-31-8	4H-1-Benzopyran-4-one, 6-[(4-fluorophenyl)methoxy]-3-hydroxy-2-(5-methoxy-1H-imi	C26H19FN2O5	458.44	C	-8.4
6508	O=c1c2c(oc(c1O)c1ncc(OC)cn1)cc(C)c(OCc1ccc(F)cc1)c2	1312908-65-8	4H-1-Benzopyran-4-one, 6-[(4-fluorophenyl)methoxy]-3-hydroxy-7-methyl-2-(5-methy	C22H17FN2O4	392.38	C	-8.4
6509	O=c1c2c(oc(c1O)c1[nH]c(OC)nn1)cc(C)c(OCc1ccc(F)cc1)c2	1312908-75-0	4H-1-Benzopyran-4-one, 6-[(4-fluorophenyl)methoxy]-3-hydroxy-7-methyl-2-(3-methy	C20H16FN3O4	381.36	C	-8.4
6510	O=c1c2c(oc(c1O)c1nnc(Cl)[nH]1)cc(C)c(OCc1ccc(F)cc1)c2	1312908-76-1	4H-1-Benzopyran-4-one, 6-[(4-fluorophenyl)methoxy]-3-hydroxy-2-(5-methoxy-1H-1,2	C20H16FN3O5	397.36	C	-8.4
6511	O=c1c2c(oc(c1O)c1[nH]c(cn1)c1ccccc1)cc(C)c(OCc1ccc(F)cc1)c2	1312908-82-9	4H-1-Benzopyran-4-one, 2-(5-chloro-1H-imidazol-2-yl)-6-[(4-fluorophenyl)methoxy]	C20H14ClFN2O4	400.79	C	-8.4
6512	Cc1c2c(cc(OCc3ccc(F)cc3)c1C)c(=O)c(O)c(o2)c1ncc(C)cn1	1312909-26-4	4H-1-Benzopyran-4-one, 6-[(4-fluorophenyl)methoxy]-3-hydroxy-7,8-dimethyl-2-(2-p	C22H17FN2O4	392.38	C	-8.4
6513	Cc1c2c(cc(OCc3ccc(F)cc3)c1C)c(=O)c(O)c(o2)c1[nH]c(OC)nn1	1312909-37-7	4H-1-Benzopyran-4-one, 6-[(4-fluorophenyl)methoxy]-3-hydroxy-7,8-dimethyl-2-(3-m	C21H18FN3O4	395.38	C	-8.4
6514	Cc1c2c(cc(OCc3ccc(F)cc3)c1C)c(=O)c(O)c(o2)c1[nH]c(C)cn1	1312909-41-3	4H-1-Benzopyran-4-one, 6-[(4-fluorophenyl)methoxy]-3-hydroxy-2-(1H-imidazol-2-yl	C21H17FN2O4	380.37	C	-8.4
6515	O(C)c1c2c(cc(OCc3ccc(F)cc3)c1C)c(=O)c(O)c(o2)c1[nH]c(C)nn1	1312909-86-6	4H-1-Benzopyran-4-one, 6-[(4-fluorophenyl)methoxy]-3-hydroxy-8-methoxy-7-methyl-	C20H16FN3O5	397.36	C	-8.4
6516	Clc1c2c(cc(OCc3ccc(F)cc3)c1C)c(=O)c(O)c(o2)c1ncc(C)cn1	1312910-26-1	4H-1-Benzopyran-4-one, 8-chloro-6-[(4-fluorophenyl)methoxy]-3-hydroxy-7-methyl-2	C21H14ClFN2O4	412.8	C	-8.4
6517	Cc1c(c2c(cc1OCc1ccc(F)cc1)c(=O)c(O)c(o2)c1ncc(C)cn1)c1ccccc1	1312910-76-1	4H-1-Benzopyran-4-one, 6-[(4-fluorophenyl)methoxy]-3-hydroxy-7-methyl-8-phenyl-2	C27H19FN2O4	454.45	C	-8.4
6518	Cc1c(c2c(cc1OCc1ccc(F)cc1)c(=O)c(O)c(o2)c1ncc(OC)cn1)c1ccccc1	1312910-77-2	4H-1-Benzopyran-4-one, 6-[(4-fluorophenyl)methoxy]-3-hydroxy-7-methyl-2-(5-methy	C28H21FN2O4	468.48	C	-8.4
6519	Cc1c(c2c(cc1OCc1ccc(F)cc1)c(=O)c(O)c(o2)c1ncc(cn1)c1ccccc1)c1ccccc1	1312910-79-4	4H-1-Benzopyran-4-one, 2-(5-chloro-2-pyrimidinyl)-6-[(4-fluorophenyl)methoxy]-3-	C27H18ClFN2O4	488.89	C	-8.4
6520	O=c1c2c(oc(c1O)c1[nH]c(OC)nn1)cc(Cl)c(OCc1ccc(F)cc1)c2	1312911-76-4	4H-1-Benzopyran-4-one, 7-chloro-6-[(4-fluorophenyl)methoxy]-3-hydroxy-2-(3-methy	C19H13ClFN3O4	401.78	C	-8.4
6521	Cc1c2c(cc(OCc3ccc(F)cc3)c1Cl)c(=O)c(O)c(o2)c1[nH]c(C)cn1	1312912-85-8	4H-1-Benzopyran-4-one, 7-chloro-6-[(4-fluorophenyl)methoxy]-3-hydroxy-2-(1H-imic	C20H14ClFN2O4	400.79	C	-8.4
6522	Cc1c2c(cc(OCc3ccc(F)cc3)c1Cl)c(=O)c(O)c(o2)c1[nH]c(cn1)c1ccccc1	1312912-91-6	4H-1-Benzopyran-4-one, 7-chloro-2-(5-chloro-1H-imidazol-2-yl)-6-[(4-fluorophenyl	C20H13Cl2FN2O4	435.23	C	-8.4
6523	O(C)c1c2c(cc(OCc3ccc(F)cc3)c1Cl)c(=O)c(O)c(o2)c1[nH]c(C)nn1	1312913-75-9	4H-1-Benzopyran-4-one, 7-chloro-6-[(4-fluorophenyl)methoxy]-3-hydroxy-8-methoxy-	C19H13ClFN3O5	417.77	C	-8.4
6524	O(C)c1c(c2c(cc1OCc1ccc(F)cc1)c(=O)c(O)c(o2)c1ncc(C)cn1)c1ccccc1	1312915-10-8	4H-1-Benzopyran-4-one, 6-[(4-fluorophenyl)methoxy]-3-hydroxy-7-methoxy-8-phenyl-	C27H19FN2O5	470.45	C	-8.4
6525	O(C)c1c(c2c(cc1OCc1ccc(F)cc1)c(=O)c(O)c(o2)c1[nH]c(C)cn1)c1ccccc1	1312915-45-9	4H-1-Benzopyran-4-one, 6-[(4-fluorophenyl)methoxy]-3-hydroxy-2-(1H-imidazol-2-yl	C26H19FN2O5	458.44	C	-8.4
6526	O(C)c1c(c2c(cc1OCc1ccc(F)cc1)c(=O)c(O)c(o2)c1[nH]c(cn1)c1ccccc1)c1ccccc1	1312915-51-7	4H-1-Benzopyran-4-one, 2-(5-chloro-1H-imidazol-2-yl)-6-[(4-fluorophenyl)methoxy]	C26H18ClFN2O5	492.88	C	-8.4
6527	Clc1c(c2c(cc1OCc1ccc(F)cc1)c(=O)c(O)c(o2)c1ncc(OC)cn1)c1ccccc1	1312915-62-0	4H-1-Benzopyran-4-one, 7-chloro-6-[(4-fluorophenyl)methoxy]-3-hydroxy-2-(5-methy	C27H18ClFN2O4	488.89	C	-8.4
6528	Clc1c(c2c(cc1OCc1ccc(F)cc1)c(=O)c(O)c(o2)c1[nH]c(C)cn1)c1ccccc1	1312915-90-4	4H-1-Benzopyran-4-one, 7-chloro-6-[(4-fluorophenyl)methoxy]-3-hydroxy-2-(1H-imic	C25H16ClFN2O4	462.86	C	-8.4
6529	O=c1c2c(cc(c(OCc3ccc(F)cc3)c2)c2ccccc2)oc(c1O)c1ncc(cn1)c1ccccc1	1312916-34-9	4H-1-Benzopyran-4-one, 2-(5-chloro-2-pyrimidinyl)-6-[(4-fluorophenyl)methoxy]-3-	C26H16ClFN2O4	474.87	C	-8.4
6530	O(Cc1ccc(F)cc1)c1c(c(C)c2c(c1)c(=O)c(O)c(o2)c1ncc(OC)cn1)c1ccccc1	1312916-83-8	4H-1-Benzopyran-4-one, 6-[(4-fluorophenyl)methoxy]-3-hydroxy-8-methyl-2-(5-methy	C28H21FN2O4	468.48	C	-8.4
6531	Cc1c2c(cc(OCc3ccc(F)cc3)c1c1ccccc1)c(=O)c(O)c(o2)c1ncc(Cl)cn1	1312916-84-9	4H-1-Benzopyran-4-one, 6-[(4-fluorophenyl)methoxy]-3-hydroxy-2-(5-methoxy-2-pyri	C28H21FN2O5	484.48	C	-8.4
6532	O=c1n([C@@H]2O[C@H](CO[P@]3(=O)Oc4c(CO3)cccc4C)[C@@H](O)C2)cc(I)c(=O)[nH]1	1313031-55-8	Uridine, 2,Ä≤-deoxy-5-iodo-5,Ä≤-O-[(2R)-2-oxido-4H-1,3,2-benzodioxaphosphorin-2-	C16H16IN2O8P	522.19	C	-8.4
6533	[14C](=O)([C@@H](NC(=O)OC)C(C)C)N1[C@@H](C[C@H](C)C1)c1[nH]c(C#Cc2ccc(C#Cc3[nH]c(nc3)[C@H]3N([14C](=	1313207-89-4	Carbamic acid, N,N,Ä≤-[2,6-naphthalenediylbis[1H-imidazole-5,2-diyl[(2S,4S)-4-me	C40H52N8O6	740.89	C	-8.4
6534	C(Cn1c2c(nc1)c(=O)nc(N)[nH]2)C1(OC)COP(=O)(O)OC1	1321581-02-5	L-Alanine, N-(2,Ä≤-deoxy-2,Ä≤,2,Ä≤-difluoro-5-iodo-P-1-naphthalenyl-5,Ä≤-uridyl	C23H23F2IN3O9P	681.32	C	-8.4
6535	S(Cc1cc2c(oc1=O)ccc(F)c2)c1n(c2c(n1)c(=O)nc(N)[nH]2)[C@@H]1O[C@H](CO)[C@@H](O)[C@H]1O	1328880-82-5	Guanosine, 8-[[(2-oxo-2H-1-benzopyran-3-yl)methyl]thio]-	C20H19N5O7S	473.46	C	-8.4
6536	S(Cc1cc2c(oc1=O)ccc(C)c2)c1n(c2c(n1)c(=O)nc(N)[nH]2)[C@@H]1O[C@H](CO)[C@@H](O)[C@H]1O	1328880-83-6	Guanosine, 8-[[(6-fluoro-2-oxo-2H-1-benzopyran-3-yl)methyl]thio]-	C20H18FN5O7S	491.45	C	-8.4
6537	O=c1c2c(n(cn2)[C@@H]2C(=C)[C@H](COCOC)[C@@H](OCOC)C2)[nH]c(N)n1	1328880-85-8	Guanosine, 8-[[(6-methyl-2-oxo-2H-1-benzopyran-3-yl)methyl]thio]-	C21H21N5O7S	487.49	C	-8.4
6538	N(Cc1ccc(F)cc1)c1nc(nc(NN=Cc2c(O)cccc2)n1)N1CCOCC1	1331749-49-5	Acetamide, N-[4-[[2-[4-(4-morpholinyl)-6-(phenylamino)-1,3,5-triazin-2-yl]hydraz	C22H24N8O2	432.48	C	-8.4
6539	Fc1cc(ccc1)c1ccc(C[C@@H]([C@@H](CN(S(=O)(=O)c2ccc(OC)cc2)CC(C)C)O)NC(=O)O[C@H]2CCOC2)cc1	1335386-65-6	Carbamic acid, N-[(1S,2R)-1-[(4,Ä≤-fluoro[1,1,Ä≤-biphenyl]-4-yl)methyl]-2-hydro:	C32H39FN2O7S	614.72	C	-8.4
6540	[C@H](C(=O)N1[C@@H]2[C@@H](C2)C[C@H]1C#N)(NC(=O)[C@@H]1CCCN1)C12CC3(O)CC(C2)CC(C1)C3	1339955-30-4	Not Yet Assigned	C26H37N5O4	483.6	C	-8.4
6541	S(=O)(=O)(O)c1c2c(c(N)c(N=Nc3c(CCCC)cc(cc3)c3cc(CCCC)c(N=Nc4c(N)c5c(c(S(=O)(=O)O)c4)cccc5)cc3)c1)ccc	134071-75-3	1-Naphthalenesulfonic acid, 3,3,Ä≤-[(3,3,Ä≤-dipropyl[1,1,Ä≤-biphenyl]-4,4,Ä≤-di	C38H36N6O6S2.2Na		C	-8.4
6542	C(=O)(NC(CC1CC1)C(=O)C(=O)N)[C@@H]1[C@@H]2[C@@H](C2(C)C)CN1C(=O)[C@@H](NC(=O)NC(SCc1ccccc1)(C)C)C(C)	1346776-56-4	Not Yet Assigned	C37H48N4O6S	676.87	C	-8.4
6543	N(S(=O)(=O)c1ccc(NC(=O)COCCN(CCOC)C)cc1)(C[C@H]([C@H](Cc1ccccc1)NC(=O)O[C@H]1CCOC1)O)CC(C)C	1347009-19-1	Spiro[3-azabicyclo[3.1.0]hexane-6,1,Ä≤-cyclopentane]-2-carboxamide, N-[3-amino-1	C37H61N5O7S	719.97	C	-8.4
6544	[C@@H]([C@@H]([C@@H]([C@H](Cc1ccccc1)NC(=O)OC(C)(C)C)O)O)(Cc1ccccc1)NC(=O)[C@@H](NC(=O)N(Cc1csc(C)n1	1347013-87-9	4-Morpholinecarboxamide, N-[(1S)-1-[(4-fluorophenyl)methyl]-2-oxo-2-[[(1S,2E)-1-	C31H34FN3O5S	579.68	C	-8.4
6545	N(C(=O)[C@@H](C[C@@H](CN1[C@H](C(=O)NC(C)(C)C)CN(Cc2cccnc2)CC1)OC(=O)CCCCOc1ccc(C[C@@H](C(=O)O)N)cc1	1348247-15-3	Carbamic acid, N-[3-[[(2,3-dihydro-2,3-dihydroxy-5-benzofuranyl)sulfonyl](2-meth	C29H38N2O10S	606.68	C	-8.4
6546	C(=O)(O)c1c(NC(=O)c2ccc(OC)cc2)cc(Cc2cc(NC(=O)c3ccc(OC)cc3)c(C(=O)O)cc2)cc1	1348323-89-6	Not Yet Assigned	C30H37D6F2N5O		C	-8.4
6547	C([C@H]([C@H](Cc1ccccc1)NC(=O)[C@@H](NC(=O)c1nc2c(cc1)cccc2)CC(=O)NC(=O)[C@@H](NC(=O)CN)C(C)C)O)N1[C	1348524-51-5	Benzamide, 2-(2-aminoethyl)-N-[[4,Ä≤-[(2-ethyl-5,7-dimethyl-3H-imidazo[4,5-b]pyr	C32H33N5O3S	567.7	C	-8.4
6548	C(n1c2c(nc1CC)c(C)cc(C)n2)c1ccc(cc1)c1c(S(=O)(=O)NC(=O)CCOCC)cccc1	1348779-04-3	4-Morpholinecarboxamide, N-[(1R)-1-[(4-fluorophenyl)methyl]-2-oxo-2-[[(2E)-1-(2-	C31H34FN3O5S	579.68	C	-8.4
6549	C(=O)(NC(CC1CC1)C(=O)C(=O)N)[C@@H]1[C@@H]2[C@@H](C2(C)C)CN1C(=O)[C@@H](NC(=O)N[C@H](CNS(=O)(=O)c1ccc	1349400-99-2	Not Yet Assigned	C40H61N7O6	735.96	C	-8.4
6550	C(=O)(NC(C(=O)C(=O)NC1CC1)CCC)[C@@H]1[C@@H]2[C@H](CN1C(=O)[C@@H](NC(=O)[C@@H](NC(=O)c1cnccn1)C(C)C)C	1349603-61-7	Not Yet Assigned	C27H34O11	534.55	C	-8.4
6551	Cc1cc2c(ccc(c2s1)c1[nH]c(nc1)[C@H]1N(C(=O)[C@@H](NC(=O)OC)C(C)C)CCC1)c1c2c(c(cc1)c1[nH]c(nc1)[C@H]1N	1350718-45-4	Carbamic acid, N,N,Ä≤-[[4,7,Ä≤-bibenzothiophene]-4,Ä≤,7-diylbis[1H-imidazole-5,	C44H50N8O6S2	851.05	C	-8.4

6552	C(=O)([C@@H](NC(=O)OC)C(C)C)N1[C@@]2(C[C@H](C1)CC2)c1[nH]c(cn1)c1c2c(c(cc1)c1ccc(cc1)c1[nH]c(nc1)[C@	1350719-06-0	Carbamic acid, N-[(1S)-1-[[(1S,4R)-1-[5-[4-[7-[2-[(1S,4R)-2-[(2S)-2-[(methoxycar	C46H56N10O6	845	C	-8.4
6553	C(=O)([C@@H](NC(=O)OC)C(C)C)N1[C@H](c2[nH]c(c3c4c(c(cc3)c3c5c(c(cc3)c3[nH]c(nc3)[C@H]3N(C(=O)[C@@H](	1350719-33-3	Carbamic acid, N,N,Ä≤-[[7,7,Ä≤-bibenzothiazole]-4,4,Ä≤-diylbis[1H-imidazole-5,2	C42H48N10O6S2	853.02	C	-8.4
6554	C(=O)([C@@H](NC(=O)OC)C(C)C)N1[C@H](c2[nH]c(c3c4c(c(cc3)c3c5c(c(cc3)c3[nH]c(nc3)[C@H]3N(C(=O)[C@@H](	1350719-35-5	Carbamic acid, N,N,Ä≤-[[7,7,Ä≤-bibenzo[b]thiophene]-4,4,Ä≤-diylbis[1H-imidazole	C44H50N8O6S2	851.05	C	-8.4
6555	C(=O)([C@H](NC(=O)OC)c1ccccc1)N1[C@@H](CCC1)c1[nH]c(cn1)c1cc(C)c(cc1)c1cc2c(cc1)cc(cc2)c1[nH]c(nc1)[	1351329-94-6	Carbamic acid, N-[(1S,2R)-2-hydroxy-1-[[(2S)-2-[5-[4-[6-[2-[(2S)-1-[(2S,3R)-3-hy	C42H48N8O8	792.88	C	-8.4
6556	C(=O)([C@@H](NC(=O)OC)C(C)C)N1C[C@@]2(C[C@H]1c1[nH]c(cn1)c1ccc(cc1)c1ccc(cc1)c1[nH]c(nc1)[C@H]1N(C(=	1351851-08-5	Carbamic acid, N-[(1S)-1-[[(2S)-2-[5-[4,Ä≤-[2-[(5S,8S)-7-[(2S)-3-hydroxy-2-[(met	C43H54N8O8	810.94	C	-8.4
6557	C(=O)([C@@H](NC(=O)OC)C(C)C)N1C[C@@]2(C[C@H]1c1[nH]c(cn1)c1ccc(cc1)c1ccc(cc1)c1c(COC)nc([nH]1)[C@H]1	1351852-72-6	1H-Imidazole-5-carboxylic acid, 4-[4,Ä≤-[2-[(5S,8S)-7-[(2S)-2-[(methoxycarbonyl)	C45H56N8O9	852.97	C	-8.4
6558	C(=O)([C@@H](NC(=O)OC)C(C)C)N1C[C@@]2(C[C@H]1c1[nH]c(c(C(=O)OC)n1)c1ccc(cc1)c1ccc(cc1)c1[nH]c(nc1)[C	1351852-73-7	Carbamic acid, N-[(1S)-1-[[(2S)-2-[4-[4,Ä≤-[2-[(5S,8S)-7-[(2S)-2-[(methoxycarbor	C45H58N8O8	838.99	C	-8.4
6559	C(=O)([C@@H](NC(=O)OC)C(C)C)N1C[C@@]2(C[C@H]1c1[nH]c(cn1)c1c(Cl)cc(cc1)c1cc(Cl)c(cc1)c1[nH]c(nc1)[C@	1351852-86-2	Carbamic acid, N-[(1S)-1-[[(2S)-2-[5-[4,Ä≤-[2-[(5S,8S)-7-[(2S)-2-[(methoxycarbor	C45H52F6N8O7	930.93	C	-8.4
6560	FC1(F)[C@@]2(C[C@H](N(C(=O)[C@@H](NC(=O)OC)C(C)C)C2)c2[nH]c(cn2)c2ccc(cc2)c2ccc(cc2)c2[nH]c(nc2)[C@H	1351852-99-7	Carbamic acid, N-[(1S)-1-[[(5S,8S)-8-[5-[4,Ä≤-[2-[(1S,2S,5R)-3-[(2S)-2-[(methoxy	C44H54N8O7	806.95	C	-8.4
6561	C(=O)([C@@H](NC(=O)OC)C)N1[C@@H](C[C@@H](C1)C1CC1)c1[nH]c(cn1)c1ccc(cc1)c1ccc(cc1)c1[nH]c(nc1)[C@H]1	1351853-85-4	Carbamic acid, N-[(1R)-2-[(2S)-2-[5-[4,Ä≤-[2-[(2S,4R)-4-cyclopropyl-1-[(2R)-2-[	C39H46N8O6	722.83	C	-8.4
6562	C(=O)([C@H](NC(=O)OC)C1CC1)N1[C@@H](C[C@@H](C1)C1CC1)c1[nH]c(cn1)c1ccc(cc1)c1ccc(cc1)c1[nH]c(nc1)[C@	1351854-00-6	Carbamic acid, N-[2-[(2S)-2-[5-[4,Ä≤-[2-[(2S,4R)-4-cyclopropyl-1-[2-[(methoxycar	C41H50N8O6	750.89	C	-8.4
6563	C(=O)([C@@H](NC(=O)OC)C(C)C)N1[C@@H](C[C@@H](C1)C1CC1)c1[nH]c(cn1)c1ccc(cc1)c1ccc(cc1)c1[nH]c(nc1)[C	1351855-88-3	Carbamic acid, N-[(1S)-1-[[(2S,4R)-4-cyclopropyl-2-[5-[4,Ä≤-[2-[(6S)-5-[(2S)-2-	C45H56N8O6	804.98	C	-8.4
6564	C(=O)([C@@H](NC(=O)OC)C(C)C)N1[C@@H](C[C@@H](C1)C1CC1)c1[nH]c(cn1)c1ccc(cc1)c1ccc(cc1)c1cc([nH]c1)[C	1351856-06-8	Carbamic acid, N-[(1S)-1-[[(2S,4R)-4-cyclopropyl-2-[5-[4,Ä≤-[2-[(3S)-2-[(2S)-2-	C48H62N8O6	847.06	C	-8.4
6565	C(=O)(N[C@H](C(C(=O)NC1CC1)O)CCC)[C@@H]1[C@@H]2[C@H](CN1C(=O)[C@@H](NC(=O)[C@@H](NC(=O)c1cnccn1)C1CC	1351992-75-0	Cyclopenta[c]pyrrole-1-carboxamide, (2S)-2-cyclohexyl-N-(2-pyrazinylcarbonyl)gly	C35H51N7O6	665.82	C	-8.4
6566	O=c1n(cc(C(F)(F)F)c(=O)n1C(=O)CCCC=CCC=CCC=CCC=CCC=CCCC)[C@@H]1O[C@H](CO)[C@@H](O)C1	1353249-45-2	Thymidine, 2,Ä≤,3,Ä≤-didehydro-3,Ä≤-deoxy-3-(1-oxo-5,8,11,14,17-heneicosapentae	C31H42N2O5	522.68	C	-8.4
6567	C(OCCP(=O)(OC(C)C)OC(C)C)n1c2c(nc1)c(=O)nc(N)[nH]2	1356470-38-6	2,4(1H,3H)-Pyrimidinedione, 1-(2-deoxy-Œ≤-L-erythro-pentofuranosyl)-5-[(4,5-dihy	C30H30N6O7	586.6	C	-8.4
6568	C(=O)([C@@H](NC(=O)OC)C1CCOCC1)N1[C@H](c2[nH]c(c3c4c(c(cc3)c3ccc(cc3)c3[nH]c(nc3)[C@H]3N(C(=O)[C@@H]	1359868-93-1	Carbamic acid, N-[(1S)-1-[[(2S,5S)-2-[5-[4-[7-[2-[(2S,5S)-1-[(2S)-2-[(methoxycar	C42H52N10O6S	824.99	C	-8.4
6569	C(F)(F)c1n(c2c(n1)cccc2)c1nc(nc(N[C@@H]2CC[C@@H](O)CC2)n1)N1CCOCC1	1359953-57-3	1,3,5-Triazin-2-amine, 4-[2-(difluoromethyl)-1H-benzimidazol-1-yl]-6-(4-morpholi	C25H29F2N7O	481.54	C	-8.4
6570	C(=O)([C@@H](NC(=O)OC)C(C)C)N1[C@@H](C[C@H](S(=O)(=O)O)C1)c1[nH]c(cn1)c1ccc(cc1)c1ccc(cc1)c1[nH]c(nc	1361249-30-0	3-Pyrrolidinesulfonic acid, 1-[(2S)-2-[(methoxycarbonyl)amino]-3-methyl-1-oxobut	C41H52N8O9S2	865.03	C	-8.4
6571	C(C)(C)(C)c1c2c(c(F)c(C(C)(C)C)c1)COP(=O)(OC[C@H]1O[C@H](C[C@@H]1O)n1c(=O)[nH]c(=O)c(I)c1)O2	1364916-50-6	1H-1,2,4-Triazole-3-carboxamide, 1-[5-O-[[5-(dimethylamino)-1-naphthalenyl]sulfo	C20H23N5O7S	477.49	C	-8.4
6572	N(C(=O)C)[C@H]1[C@H](OC(CC)CC)C=C(C(=O)OCC)C[C@@H]1NCCS	1373244-21-3	1-Cyclohexene-1-carboxylic acid, 4-(acetylamino)-5-[[2-[[1-(3,Ä≤,6,Ä≤-dihydroxy	C42H45N3O11S	799.89	C	-8.4
6573	C(=O)([C@@H](NC(=O)OC)C(C)C)N1[C@@H](CCC1)c1[nH]c(cn1)c1ccc(C(=Cc2ccc(cc2)c2[nH]c(nc2)[C@H]2N(C(=O)[	1374421-55-2	Carbamic acid, N,N,Ä≤-[[1-[4-(1,1-dimethylethyl)phenyl]-1,2-ethanediyl]bis[4,1-p	C52H66N8O6	899.13	C	-8.4
6574	C(=O)([C@@H](NC(=O)OC)C(C)C)N1[C@@H](CCC1)c1[nH]c(cn1)c1ccc(cc1)c1ccc(nc1)c1ccc(cc1)c1[nH]c(nc1)[C@H	1375847-97-4	Benzoic acid, 3,3,Ä≤-[(3-carboxy-4-oxo-2,5-cyclohexadien-1-yl)methylene]bis[6-hy	C22H16O9	424.36	C	-8.4
6575	C(=O)([C@H](NC(=O)OC)c1cc(C)ccc1)N1[C@@H](CCC1)c1[nH]c(cn1)c1ccc(cc1)c1cc2c(cc1)cc(cc2)c1[nH]c(nc1)[	1377038-49-7	Carbamic acid, N-[(1S)-1-[[(2S)-2-[5-[6-[4-[2-[(2S)-1-[(2R)-2-(ethylamino)-2-phe	C47H52N8O4	792.97	C	-8.4
6576	C(#N)c1c2c(c3c(cc(cc3)c3[nH]c(nc3)[C@H]3N(C(=O)[C@@H](NC(=O)OC)C(C)C)CCC3)c1)ccc(c2)c1[nH]c(nc1)[C@H	1377040-53-3	Carbamic acid, N,N,Ä≤-[[1,1,Ä≤-biphenyl]-4,4,Ä≤-diylbis[1H-imidazole-5,2-diyl[(	C48H50N8O6	834.96	C	-8.4
6577	C(=O)([C@@H](NC(=O)OC)C(C)C)N1[C@@H]([C@@H]2C[C@H]1CC2)c1[nH]c(cn1)c1cc2c(cc1)c1c(cc2)cc(cc1)c1[nH]c	1377041-23-0	Carbamic acid, N-[(1S)-2-[(2S)-2-[7-[3-fluoro-4-[2-[(1R,3S,4S)-2-[(2S)-2-[(metho	C46H53FN8O7	848.96	C	-8.4
6578	C(=O)([C@@H](NC(=O)OC)C(C)C)N1[C@H](c2[nH]c3c4c(cc(cc4)c4c(C#CC5CC5)cc(cc4)c4[nH]c(nc4)[C@H]4N(C(=O)	1377042-38-0	Carbamic acid, N-[(1R)-2-[(2S)-2-[5-[4,Ä≤-[2-[(2S)-1-[(2S)-2-[(methoxycarbonyl)a	C46H50N8O6	810.94	C	-8.4
6579	C(=O)([C@@H](NC(=O)OC)C(C)C)N1[C@H](c2[nH]c3c4c(cc(cc4)c4c(C#CC5CC5)cc(cc4)c4[nH]c(nc4)[C@H]4N(C(=O)	1377042-41-5	Carbamic acid, N-[(1S)-1-[[(2S)-2-[7-[2-(2-cyclopropylethynyl)-4-[2-[(2S)-1-[(2S	C47H54N8O6	826.98	C	-8.4
6580	C(=O)([C@@H](NC(=O)OC)C(C)C)N1[C@H](c2[nH]c3c4c(cc(cc4)c4ccc(cc4)c4[nH]c(nc4)[C@H]4N(C(=O)[C@@H](NC(	1377044-95-5	Carbamic acid, N-[(1S)-1-[[(2S)-2-[7-[4-[2-[(2S)-1-[(2S)-2-[(methoxycarbonyl)ami	C50H54N8O6	863.01	C	-8.4
6581	N(C(=O)N[C@H]1CC[C@@H](OCC=C)CC1)C12CC3CC(C2)CC(C1)C3	1378287-04-7	Urea, N-[4-(2-oxo-2-phenylethyl)phenyl]-N,Ä≤-tricyclo[3.3.1.13,7]dec-1-yl-	C25H28N2O2	388.5	C	-8.4
6582	C(=O)([C@@H](NC(=O)OC)C(C)C)N1[C@H](CCC1)c1[nH]c(cn1)c1ccc(cc1)c1ccc(cc1)C1CCCCC1	1380292-73-8	Carbamic acid, N-[(1S)-1-[[(2S)-2-[5-[4-(2-[1,1,Ä≤-biphenyl]-4-ylethynyl)phenyl]	C34H34N4O3	546.66	C	-8.4
6583	C[C@@]1(N(C(=O)[C@@H](NC(=O)OC)C(C)C)CCC1)c1[nH]c(cn1)c1ccc(cc1)c1ccc(cc1)c1[nH]c(nc1)[C@@]1(C)N(C(=	1383566-02-6	Carbamic acid, N-[(1S)-1-[[(2S)-2-[5-[4,Ä≤-[2-[(methoxycarbonyl)amino]-1H-imidaz	C31H35N7O5	585.65	C	-8.4
6584	F[C@@H]1[C@@H](O[C@H](COC(=O)C)[C@H]1OC(=O)C)n1cc(CC)c(=O)[nH]c1=O	1383566-40-2	Carbamic acid, N-[(1S)-1-[[(2S)-2-[5-[4,Ä≤-(2-amino-1H-imidazol-5-yl)[1,1,Ä≤-bi	C29H33N7O3	527.62	C	-8.4
6585	[C@@H]([C@@H](CO)O)(OC)[C@H]1[C@H](NC(=O)C)[C@@H](N[13C](=[15NH])[15NH2])C=C(C(=O)O)O1	1391048-05-7	Cyclohexanecarboxamide, 4-chloro-4-fluoro-N-[(1S)-3-[3-[3-methyl-5-(1-methylethy	C29H41ClFN5O	530.12	C	-8.4
6586	Nc1sc(c2nc(nc(n2)N2CCOCC2)N2CCOCC2)cn1	1391924-08-5	Benzamide, 3-[[4-(2-amino-5-pyrimidinyl)-6-(4-morpholinyl)-1,3,5-triazin-2-yl]am	C20H23N9O2	421.46	C	-8.4
6587	N(CC(=O)N(C)C)c1nc(nc(n1)N1CCOCC1)c1cc(C(=O)N)ccc1	1391924-88-1	Glycine, N-acetyl-N-[4-[3-(acetylamino)phenyl]-6-(4-morpholinyl)-1,3,5-triazin-2	C19H22N6O5	414.42	C	-8.4
6588	N(c1nc(nc(n1)N1CCOCC1)c1c2c([nH]nc2)ccc1)c1ccccc1	1391925-85-1	1-Piperazinecarboxamide, 4-[4-(1H-indazol-4-yl)-6-(4-morpholinyl)-1,3,5-triazin-	C20H25N9O2	423.47	C	-8.4
6589	C[C@]12[C@H]([C@H]3[C@H](CC2)[C@]2(C)[C@@H](CC3)C[C@@H](O)CC2)C[C@H](OC)[C@@H]1C(C)C1CCCCN1	1393727-92-8	Not Yet Assigned	C26H45NO	387.64	C	-8.4
6590	C(=C\1/c2c(NC1=O)ccc(S(=O)(=O)N(C[C@H]([C@H](Cc1ccccc1)NC(=O)O[C@@H]1[C@H]3[C@@H](OC1)OCC3)O)CC(C)C)	1393922-47-8	Benzamide, N-[2-[[4-[(4-methoxyphenyl)amino]-6-(4-morpholinyl)-1,3,5-triazin-2-y	C32H35N7O7	629.66	C	-8.4
6591	O[C@H]1[C@H](n2c3c(nc2)c(N)ncn3)O[C@H](C[Se]CC#C)[C@H]1O	1397199-07-3	Furo[3,Ä≤,4,Ä≤:6,7]naphtho[2,3-d]-1,3-dioxol-6(5aH)-one, 5-(1,3-benzodioxol-5-y	C21H18O8	398.36	C	-8.4
6592	N(c1c(C)cc(/C=C/C#N)cc1C)c1nc(Nc2ccc(C#N)cc2)ncc1.S(=O)(=O)(C)O	1399384-91-8	Benzonitrile, 4-[[4-[[4-[(1E)-2-cyanoethenyl]-2,6-dimethylphenyl]amino]-2-pyrimi	C22H18N6.C7H8O3S		C	-8.4
6593	N(c1c(C)cc(/C=C/C#N)cc1C)c1nc(Nc2ccc(C#N)cc2)ncc1.C(CC(=O)O)C(=O)O	1399384-93-0	Benzonitrile, 4-[[4-[[4-[(1E)-2-cyanoethenyl]-2,6-dimethylphenyl]amino]-2-pyrimi	C22H18N6.C4H4O4		C	-8.4
6594	C(OCCOP(=O)(NCCCCCC)NCCCCCC)n1c2c(nc1)c(=O)nc(N)[nH]2	1400636-38-5	4-Piperidinecarboxylic acid, 1-[(2,3-dihydro-2,3-dioxo-1H-indol-5-yl)sulfonyl]-	C14H14N2O6S	338.34	C	-8.4
6595	O=C1c2n(C[C@H]3N1[C@](C)(CCO3)[2H])cc(C(=O)NC(c1c(F)cc(F)cc1)([2H])[2H])c(=O)c2O	1407166-96-4	2H-Pyrido[1,Ä≤,2,Ä≤:4,5]pyrazino[2,1-b][1,3]oxazine-9-carboxamide, N-[(2,4-difl	C20H14D5F2N3O5		C	-8.4
6596	O=C1c2n(C([C@H]3N1[C@H](C)CCO3)([2H])[2H])cc(C(=O)NCc1c(F)cc(F)cc1)c(=O)c2O	1407167-21-8	2H-Pyrido[1,Ä≤,2,Ä≤:4,5]pyrazino[2,1-b][1,3]oxazine-2,3,4,12-d4-9-carboxamide,	C20H9D10F2N3O5		C	-8.4
6597	O=C1c2n(C[C@H]3N1[C@](C)(CCO3)[2H])cc(C(=O)NCc1c(F)cc(F)cc1)c(=O)c2O	1407167-28-5	2H-Pyrido[1,Ä≤,2,Ä≤:4,5]pyrazino[2,1-b][1,3]oxazine-9-carboxamide, N-[(2,4-difl	C20H16D3F2N3O5		C	-8.4
6598	C(=O)(NC(CC1CCC1)C(=O)C(=O)N)[C@@H]1[C@@H]2[C@@H](C2(C)C)CN1C(=O)C(NC(=O)NC(C)(C)C)C(C)(C)C	1407494-85-2	Carbamic acid, N,N,Ä≤-[[1,1,Ä≤-biphenyl]-4,4,Ä≤-diylbis[1H-imidazole-5,2-diyl-2	C40H50N8O6		C	-8.4
6599	[C@H](C(=O)N1[C@@H](CC[C@@H]1C)c1[nH]c(cn1)c1ccc(cc1)c1ccc(cc1)c1[nH]c(nc1)[C@H]1N(C(=O)[C@@H](NC(=O	1412813-79-6	Carbamic acid, N,N,Ä≤-[[1,1,Ä≤-biphenyl]-4,4,Ä≤-diylbis[1H-imidazole-5,2-diyl-(	C48H48F6N8O6	946.94	C	-8.4
6600	O[C@H]1[C@@H](O[C@H](C(=O)N[C@@H](C(=O)N2CCN(CC2)c2ccc(Cl)cn2)Cc2ccccc2)[C@H]1O)n1nc(C(=O)N)nc1	1414801-66-3	Not Yet Assigned	C27H30N8O6	562.58	C	-8.4
6601	C=C1[C@@H](n2c3c(nc2)c(=O)nc(N[C@H](C(=O)O)C)[nH]3)C[C@H](O)[C@H]1CO	141511-27-5	Benzoic acid, 5-[[3-[bis(3-carboxy-4-hydroxyphenyl)methylene]-5-carboxy-6-oxo-1,	C30H20O12	572.47	C	-8.4
6602	C(OCCn1c2c(nc1)c(N)ncn2)[P@@]1(=O)O[C@H](C[C@@H](O1)c1ccccc1)c1cc(Cl)ccc1	1417393-25-9	9H-Purin-6-amine, 9-[2-[[(2R,4S,6S)-4-(3-chlorophenyl)-2-oxido-6-phenyl-1,3,2-di	C23H23ClN5O4P	499.89	C	-8.4
6603	N(c1nc(nc(NC(=O)Nc2ccc(F)cc2)n1)N1CCOCC1)n1c(SC)nnc(C(C)(C)C)c1=O	1418554-15-0	Urea, N-[4-[[6-(1,1-dimethylethyl)-3-(methylthio)-5-oxo-1,2,4-triazin-4(5H)-yl]a	C23H30N10O3S	526.61	C	-8.4
6604	N(C(=S)Nc1nc(nc(Nn2c(SC)nnc(C(C)(C)C)c2=O)n1)N1CCOCC1)c1c2c(ccc1)cccc2	1418741-55-5	Thiourea, N-[4-[[6-(1,1-dimethylethyl)-3-(methylthio)-5-oxo-1,2,4-triazin-4(5H)-	C22H27N11O4S2	573.65	C	-8.4
6605	C(c1cc(C=C2C=C(C(=O)O)C(=O)C=C2)c(O)c(C(=O)O)c1)(c1cc(C(=O)O)c(O)cc1)c1cc(C(=O)O)c(O)cc1	1421610-13-0	1,3-Propanediamine, N1-(7-chloro-4-quinolinyl)-N3-(4,6-di-4-morpholinyl-1,3,5-tr	C23H29ClN8O2	484.98	C	-8.4
6606	C(O)[C@@]12[C@H](O)[C@@H]([C@H](O1)n1nc(C(=O)N)nc1)OC2	1422500-60-4	4-Quinazolinamine, N-[(3-amino-3-oxetanyl)methyl]-2-(2,3-dihydro-1,1-dioxido-1,4	C22H25N5O3S	439.53	C	-8.4
6607	C(=O)(OCC)C1=C(N)OC(=CC1c1cc(OCC)ccc1)c1[nH]c2c(n1)cccc2	1426813-54-8	4H-Pyran-3-carboxylic acid, 2-amino-6-(1H-benzimidazol-2-yl)-4-(3-nitrophenyl)-,	C21H18N4O5	406.39	C	-8.4
6608	C(=O)([C@@H](NC(=O)OC)C(C)C)N1[C@@H](CCC1)c1[nH]c(cn1)c1ccc(cc1)c1ccc(CO)cc1	1426938-72-8	Carbamic acid, N-[(1S)-1-[[(2S)-2-[5-[4,Ä≤-(aminocarbonyl)[1,1,Ä≤-biphenyl]-4-y	C27H31N5O4	489.57	C	-8.4
6609	C[C@]12[C@H]([C@H]3[C@H](CC2)[C@@H]2[C@@H](CC3)C[C@@](C(F)(F)F)(O)CC2)CC[C@@H]1[C@@H](CCC(C)(C)O)C	1427208-74-9	19,26,27-Trinorergostane-3,24-diol, 3-(trifluoromethyl)-, (3Œ±,5Œ±)-	C26H43F3O2	444.61	C	-8.4
6610	[C@H](Cc1ccccc1)(C(=O)N/C(=C/CS(=O)(=O)c1ccccc1)/CCc1ccccc1)NC(=O)N1CCN(C)CC1	1428441-83-1	4-Morpholinecarboxamide, N-[(1S)-2-oxo-2-[[(1Z)-1-(2-phenylethyl)-3-(phenylsulfo	C31H35N3O5S	561.69	C	-8.4
6611	N(C(=O)C)[C@H]1[C@H]([C@@H]([C@@H](CO)O)O)OC(=C[C@@H]1NC(=N)NC(=O)Cc1c(OC)cccc1)C(=O)O	1442563-08-7	D-glycero-D-galacto-Non-2-enonic acid, 5-(acetylamino)-2,6-anhydro-3,4,5-trideox	C24H28N4O8	500.5	C	-8.4
6612	N(C(=O)C)[C@H]1[C@H]([C@@H]([C@@H](CO)O)O)OC(=C[C@@H]1NC(=N)NCc1ccccc1)C(=O)O	1442563-32-7	D-glycero-D-galacto-Non-2-enonic acid, 5-(acetylamino)-2,6-anhydro-3,4,5-trideox	C24H30N4O7	486.52	C	-8.4
6613	S(c1n(c2c(n1)c(=O)nc(N)[nH]2)[C@@H]1O[C@H]2[C@H]([C@H]1O)OP(=O)(O)OC2)c1ccccc1	144509-99-9	Guanosine, 8-[(3-methoxyphenyl)thio]-, cyclic 3,Ä≤,5,Ä≤-(hydrogen phosphate)	C17H18N5O8PS	483.39	C	-8.4
6614	O=C1[C@@H]2[C@@H](c3c([C@H](OC(=O)c4cc(C)ccc4)[C@H]2CO1)cc1c(c3)OCO1)c1c(Cl)c(OC)c(OC)c(OC)c1	1449601-78-8	1-Naphthaleneacetic acid, (5R,5aR,8aR,9S)-9-(2-chloro-3,4,5-trimethoxyphenyl)-5,	C34H29ClO9	617.04	C	-8.4
6615	C(=O)([C@@H](NC(=O)OC)C(C)C)N1[C@H](c2[nH]c(c(n2)C2CC2)c2cc3c(-c4c(cc(cc4)c4c(nc([nH]4)[C@H]4N(C(=O)	1449762-26-8	Carbamic acid, N,N,Ä≤-[(7,8-dihydro-7,7-dimethyl-6H-dibenzo[f,h][1,5]dioxonin-3,	C49H66N8O8	895.1	C	-8.4
6616	O=c1n([C@@H]2O[C@H](CO)SC2)cc(F)c(=O)[nH]1	1452579-97-3	Carbonothioic dihydrazide, 2-[1,2-dihydro-2-oxo-1-(phenylmethyl)-3H-indol-3-ylic	C23H19N5OS	413.49	C	-8.4
6617	N(c1c2c(ncc1)cc(Cl)cc2)c1cc(O)c(CN2C(C)CCCC2)cc(CN2C(C)CCCC2)c1	145431-57-8	Phenol, 2-[(7-chloro-4-quinolinyl)amino]-4,6-bis(1-piperidinylmethyl)-	C27H33ClN4O	465.03	C	-8.4
6618	O=c1[15n]([C@@H]2O[C@H](CO)[C@@H](O)C2)cc(C=O)c(=O)[15nH]1	1456866-47-9	1-Pyrrolidinecarboxylic acid, 2-[5-[4,Ä≤-[2-[(2S)-1-[(2S)-2-[(methoxycarbonyl)ar	C39H49N7O5	695.85	C	-8.4
6619	O=c1n(ccc(N)n1)[C@H]1O[C@@H](C(=O)O[C@@H]2[C@@H](C(C)C)CC[C@H](C)C2)SC1	147104-00-5	Thymidine, 2,Ä≤,3,Ä≤-didehydro-3,Ä≤-deoxythymidylyl-(5,Ä≤,Üí5,Ä≤)-2,Ä≤,3,Ä≤-di	C20H23N4O10P	510.39	C	-8.4
6620	N(C(C)C)c1c(nccc1)N1CCN(C(=O)c2cc3c([nH]2)ccc(NS(=O)(=O)CCN2CCCC2)c3)CC1	147539-87-5	Ethanesulfonamide, N-[2-[[4-[3-[(1-methylethyl)amino]-2-pyridinyl]-1-piperazinyl	C23H30N6O3S	470.59	C	-8.4
6621	N(C(C)C)c1c(nccc1)N1CCN(C(=O)c2cc3c([nH]2)ccc(NS(=O)(=O)CCN2CCOCC2)c3)CC1	147539-93-3	1-Piperidineethanesulfonamide, N-[2-[[4-[3-[(1-methylethyl)amino]-2-pyridinyl]-1	C28H39N7O3S	553.72	C	-8.4
6622	C(=O)(c1[nH]c2c(c1)cc(NS(=O)(=O)N1CCN(C)CC1)cc2)N1CCN(CC1)c1c(NC(C)C)cccn1	147540-06-5	Ethanesulfonamide, 2-amino-N-[2-[[4-[3-[(1-methylethyl)amino]-2-pyridinyl]-1-pip	C23H31N7O3S	485.6	C	-8.4
6623	C(=O)(c1[nH]c2c(c1)cc(NS(=O)(=O)N1CCOCC1)cc2)N1CCN(CC1)c1c(NC(C)C)cccn1	147540-11-2	1-Piperazinesulfonamide, N-[2-[[4-[3-[(1-methylethyl)amino]-2-pyridinyl]-1-piper	C25H34N8O3S	526.65	C	-8.4
6624	C(=O)(c1cc2c([nH]1)ccc(NS(=O)(=O)CCCN1CCOCC1)c2)N1CCN(CC1)c1c(NC(C)(C)C)cccn1	147540-35-0	1-Piperidinepropanesulfonamide, N-[2-[[4-[3-[(1,1-dimethylethyl)amino]-2-pyridir	C30H43N7O3S	581.77	C	-8.4
6625	C(n1c2c(nc1CC)c(C)cc(C)n2)c1ccc(cc1)c1c(S(=O)(=O)NC(=O)N(CC=C)CC=C)cccc1	148401-53-0	[1,1,Ä≤-Biphenyl]-2-sulfonamide, 4,Ä≤-[(2-ethyl-5,7-dimethyl-3H-imidazo[4,5-b]p	C32H33N5O3S	567.7	C	-8.4
6626	C(n1c2c(nc1CC)c(C)ccn2)c1ccc(cc1)c1c(S(=O)(=O)NC(=O)OC)cccc1	148401-69-8	Benzoic acid, anhydride with [[4,Ä≤-[(2-butyl-3H-imidazo[4,5-b]pyridin-3-yl)metl	C31H28N4O5S	568.64	C	-8.4
6627	C(n1c2c(nc1CC)c(C)cc(C)n2)c1ccc(cc1)c1c(S(=O)(=O)NC(=O)NCC)cccc1	148401-89-2	[1,1,Ä≤-Biphenyl]-2-sulfonamide, 4,Ä≤-[(2-ethyl-5,7-dimethyl-3H-imidazo[4,5-b]p	C25H27N5O3S	477.58	C	-8.4
6628	C([C@H]([C@@H](NC(=O)[C@@H](NC(=O)c1nc2c(cc1)cccc2)C1CCCC1)Cc1ccccc1)O)N1[C@H](C(=O)NC(C)(C)C)C[C@H]	150323-38-9	D-erythro-Pentonamide, 2,3,5-trideoxy-N-[(1S,2R)-2,3-dihydro-2-hydroxy-1H-inden-	C30H42N4O4	522.68	C	-8.4
6629	C(C(C(Cc1ccccc1)NC(=O)C(NC(=O)c1nc2c(cc1)cccc2)CC(=O)N)O)N1C(C(=O)N[C@H](C(=O)N[C@H](C(=O)N[C@@H](CC	1504664-35-0	1-Naphthalenediazonium, 7-sulfo-4-[2-(4-sulfophenyl)diazenyl]-	C16H11N4O6S2		C	-8.4
6630	[Si](OC[C@H]1O[C@H](CS1)n1c(=O)nc(N)c(F)c1)(C(C)(C)C)(c1ccccc1)c1ccccc1	151720-22-8	Adenosine, P-(carboxymethyl)-P,2,Ä≤-dideoxyadenylyl-(3,Ä≤,Üí5,Ä≤)-2,Ä≤-deoxy-	C22H27N10O9P	606.49	C	-8.4
6631	O[C@H]1[C@H](n2c3c(nc2)c(=O)nc(N)[nH]3)O[C@H](COP(=O)(OP(=O)(OC2C(O)C(O)C(O)C(C)O2)O)O)[C@H]1O	1523571-59-6	Guanosine 5,Ä≤-(trihydrogen diphosphate), P,Ä≤-hexopyranosyl ester	C16H25N5O16P2	605.34	C	-8.4
6632	C[C@@]12[C@@H]3[C@H]([C@H]4[C@](C)(CC3)[C@@H]([C@@H](CCCC(C)C)C)CC4)CC[C@H]1[C@H](CC=C)[C@H](O)CC2	1527517-44-7	5,Ä≤-Guanylic acid, mono[1,2-dihydro-3,6-dimethyl-2-oxo-4-pyridinyl] ester	C17H21N6O9P	484.36	C	-8.4
6633	C(=O)(NC(C(=O)C(=O)NCCC)CCCC)[C@@H]1[C@@H]2[C@@H](C2(C)C)CN1C(=O)[C@@H](NC(=O)N[C@@H](CN1C(=O)CC(C)(	1528752-98-8	3-Azabicyclo[3.1.0]hexane-2-carboxamide, 3-[(2S)-2-[[[[(1R)-1-[(4,4-dimethyl-2,6	C37H62N6O7	702.92	C	-8.4
6634	O=C1[C@@H]2[C@@H](c3c([C@H](O[C@H]4O[C@H](CO)[C@@H](O)[C@H](O)[C@@H]4O)[C@H]2CO1)cc1c(c3)OCO1)c1cc(O	153459-54-2	L-Alanine, N-[[9,10-dihydro-12-[[(1-tricyclo[3.3.1.13,7]dec-1-ylethyl)amino]carl	C34H40N2O4	540.69	C	-8.4
6635	N(C(=O)[C@H]1N2C[C@@H]([C@H]1CC)Oc1c(nc3c(n1)cc(OC)cc3)C(F)(F)CCCC[C@H]1[C@H](OC(=O)N[C@@H](C(C)(C)C	1535208-26-4	Furo[3,Ä≤,4,Ä≤:6,7]naphtho[2,3-d]-1,3-dioxol-6(5aH)-one, 5,8,8a,9-tetrahydro-9-	C28H32O13	576.55	C	-8.4
6636	C(=O)(NCc1ccc(F)cc1)c1c(OC(OC(=O)OCc2ccccc2)C)c(=O)n(C)c(C(NC(=O)c2oc(C)nn2)(C)C)n1	1538603-92-7	Carbonic acid, cyclohexyl 1-[[4-[[[(4-fluorophenyl)methyl]amino]carbonyl]-1,6-di	C29H35FN6O8	614.62	C	-8.4
6637	N(C(=O)CCN)C12CC3(C)CC(C)(C1)CC(C2)C3	154237-39-5	Cyclopentanepropanamide, N-[[4,Ä≤-[(2-ethyl-5,7-dimethyl-3H-imidazo[4,5-b]pyrid:	C31H36N4O3S	544.71	C	-8.4
6638	C(n1c2c(nc1CC)c(C)cc(C)n2)c1ccc(cc1)c1c(S(=O)(=O)NC(=O)c2c(OCC)cccc2)ccc(CSC)c1	154552-98-4	Benzamide, 2-ethoxy-N-[[4,Ä≤-[(2-ethyl-5,7-dimethyl-3H-imidazo[4,5-b]pyridin-3-y	C33H34N4O5S	598.71	C	-8.4
6639	C(n1c2c(nc1CC)c(C)cc(C)n2)c1ccc(cc1)c1c(S(=O)(=O)NC(=O)c2c(OCC)cccc2)ccc(COC)c1	154552-99-5	Benzamide, 2-ethoxy-N-[[4,Ä≤-[(2-ethyl-5,7-dimethyl-3H-imidazo[4,5-b]pyridin-3-y	C34H36N4O4S2	628.8	C	-8.4
6640	C(n1c2c(nc1CC)c(C)cc(C)n2)c1ccc(cc1)c1c(S(=O)(=O)NC(=O)c2ccccc2)ccc(CCC)c1	154553-12-5	Benzamide, N-[[5-ethyl-4,Ä≤-[(2-ethyl-5,7-dimethyl-3H-imidazo[4,5-b]pyridin-3-y]	C32H32N4O3S	552.69	C	-8.4
6641	C(n1c2c(nc1CC)c(C)cc(C)n2)c1ccc(cc1)c1c(S(=O)(=O)NC(=O)c2ccccc2)ccc(CCCC)c1	154553-13-6	Benzamide, N-[[4,Ä≤-[(2-ethyl-5,7-dimethyl-3H-imidazo[4,5-b]pyridin-3-yl)methyl]	C33H34N4O3S	566.71	C	-8.4
6642	C(n1c2c(nc1CC)c(C)cc(C)n2)c1ccc(cc1)c1c(S(=O)(=O)NC(=O)c2ccccc2)ccc(C(C)C)c1	154553-14-7	Benzamide, N-[[5-butyl-4,Ä≤-[(2-ethyl-5,7-dimethyl-3H-imidazo[4,5-b]pyridin-3-y]	C34H36N4O3S	580.74	C	-8.4
6643	C(n1c2c(nc1CC)c(C)cc(C)n2)c1ccc(cc1)c1c(S(=O)(=O)NC(=O)NCCc2ccccc2)ccc(C(C)C)c1	154553-53-4	[1,1,Ä≤-Biphenyl]-2-sulfonamide, 5-butyl-4,Ä≤-[(2-ethyl-5,7-dimethyl-3H-imidazo	C36H41N5O3S	623.81	C	-8.4
6644	C(n1c2c(nc1CC)c(C)cc(C)n2)c1ccc(cc1)c1c(S(=O)(=O)NC(=O)NCCc2ccccc2)ccc(OCCCC)c1	154553-54-5	[1,1,Ä≤-Biphenyl]-2-sulfonamide, 4,Ä≤-[(2-ethyl-5,7-dimethyl-3H-imidazo[4,5-b]p	C35H39N5O3S	609.78	C	-8.4
6645	S(c1nc(nc(Nc2ccc(OC)cc2)n1)N1CCOCC1)c1oc(nn1)c1ccc(Cl)cc1	1547295-44-2	1,3,5-Triazin-2-amine, 4-[[5-(4-chlorophenyl)-1,3,4-oxadiazol-2-yl]thio]-6-(4-mo	C21H17ClN8O4S	512.93	C	-8.4
6646	S(c1nc(nc(Nc2ccc(N(C)C)cc2)n1)N1CCOCC1)c1oc(nn1)c1ccc(Cl)cc1	1547295-48-6	1,3,5-Triazin-2-amine, 4-[[5-(4-chlorophenyl)-1,3,4-oxadiazol-2-yl]thio]-N-(3-me	C22H20ClN7O2S	481.96	C	-8.4
6647	N(c1nc(nc(Sc2oc(nn2)c2ccc(Cl)cc2)n1)N1CCOCC1)c1cc(Br)ccc1	1547295-50-0	1,3,5-Triazin-2-amine, N-(3-chlorophenyl)-4-[[5-(4-chlorophenyl)-1,3,4-oxadiazol	C21H17Cl2N7O2S	502.38	C	-8.4
6648	O=C1C2=C(Cc3c([C@H]2c2cc(OC)c(O)c(OC)c2)cc2c(c3)OCO2)CO1	1552276-36-4	Benzo[h]quinoline-2-propanoic acid, 4-chloro-Œ±-phosphono-	C16H13ClNO5P	365.7	C	-8.4
6649	C(=O)([C@@H](NC(=O)OC)C(C)C)N1[C@H](c2[nH]c(c3c4c(C5CC4CC5)c(cc3)c3ccc(cc3)c3[nH]c(nc3)[C@H]3N(C(=O)	1560638-96-1	Carbamic acid, N-[(1S)-1-[[(2S)-2-[5-[8-[4-[2-[(2S)-4,4-difluoro-1-[(2S)-2-[(met	C45H52F4N8O6	876.94	C	-8.4
6650	C(=O)([C@@H](NC(=O)OC)C(C)C)N1[C@H](c2[nH]c(c3c4c(c(cc3)c3ccc(cc3)c3[nH]c(nc3)[C@H]3N(C(=O)[C@@H](NC	1560640-05-2	Carbamic acid, N-[(1S)-1-[[(2S)-2-[5-[8-[4-[2-[(2S)-4,4-difluoro-1-[(2S)-2-[(met	C46H54F4N8O6	890.96	C	-8.4
6651	C(=O)([C@@H](NC(=O)OC)C1CCCCC1)N1[C@H](c2[nH]c(c3c4c(c(cc3)c3ccc(cc3)c3[nH]c(nc3)[C@H]3N(C(=O)[C@@H]	1560640-11-0	Carbamic acid, N-[(1S)-2-methyl-1-[[(2S,4S)-4-methyl-2-[5-[4-[1,2,3,4-tetrahydrc	C48H62N8O6	847.06	C	-8.4
6652	C(=O)([C@@H](NC(=O)OC)C(C)C)N1[C@H](c2[nH]c(c3c4c(C5OC4CC5)c(cc3)c3ccc(cc3)c3[nH]c(nc3)[C@H]3N(C(=O)	1560640-96-1	Carbamic acid, N-[(1S)-1-[[(2S)-4,4-dimethyl-2-[5-[4-[1,2,3,4-tetrahydro-8-[2-[(	C48H62N8O7	863.06	C	-8.4
6653	Fc1c(c2c3c(c(cc2)c2[nH]c(nc2)[C@H]2N(C(=O)[C@@H](NC(=O)OC)C(C)C)CCC2)C2CC3CC2)ccc(c1)c1[nH]c(nc1)[C@	1560641-29-3	Carbamic acid, N-[(1S)-1-[[(2S,4S)-2-[5-[4-[1,4-dihydro-8-[2-[(2S,4S)-1-[(2S)-2-	C46H56N8O7	832.99	C	-8.4
6654	Cc1n(c(=O)[nH]c(=O)c1C)[C@H]1O[C@H](CO)[C@@H](O)C1	156893-90-2	1-Naphthalenol, 4-[(7-chloro-4-quinolinyl)amino]-5,6,7,8-tetrahydro-2-[[4-(3-met	C29H37ClN4O	493.08	C	-8.4
6655	C(/C=C\CCCC(=O)OC(C)C)[C@@H]1[C@@H](/C=C/[C@H](COc2cc(C(F)(F)F)ccc2)O)[C@H](O)C[C@@H]1O	157263-00-8	Benzamide, N-[[4,Ä≤-[(2-ethyl-5,7-dimethyl-3H-imidazo[4,5-b]pyridin-3-yl)methyl]	C30H28N4O3S	524.63	C	-8.4
6656	O=c1n([C@@H]2O[C@H](CO)C(C2[3H])[3H])cc(C)c(=O)[nH]1	157695-29-9	Guanosine, 2,Ä≤-deoxy-5,Ä≤-O-phosphonoguanylyl-(3,Ä≤,Üí5,Ä≤)-2,Ä≤,3,Ä≤-dideoxy-	C20H26N10O12P2	660.43	C	-8.4
6657	C(=O)([C@@H](NC(=O)OC)C(C)C)N1[C@H](c2[nH]c(c3c4c(C5(CC4)CCOCC5)c(cc3)c3ccc(cc3)c3[nH]c(nc3)[C@H]3N(	1588404-09-4	Carbamic acid, N-[(1S)-1-[[(2S)-2-[5-[2,Ä≤,3,Ä≤-dihydro-7,Ä≤-[4-[2-[(8S)-7-[(2S	C49H62N8O8	891.07	C	-8.4
6658	O=C1[C@@H]2[C@@H](c3c([C@H](OCc4cn(nn4)c4c(O)cccc4)[C@H]2CO1)cc1c(c3)OCO1)c1cc(OC)c(OC)c(OC)c1	1600500-29-5	Furo[3,Ä≤,4,Ä≤:6,7]naphtho[2,3-d]-1,3-dioxol-6(5aH)-one, 9-[[1-(3-chloro-4-fluo	C31H27ClFN3O8	624.01	C	-8.4
6659	O[C@H]1[C@H](n2c3c(nc2)c(=O)nc(N)[nH]3)O[C@H](COP(=O)(OP(=O)(O[C@@H]2[C@@H](O)[C@H](O)[C@@H](O)[C@H]	1608090-20-5	7-Deazaguanosine, 2,Ä≤-deoxy-5-iodocytidylyl-(3,Ä≤,Üí5,Ä≤)-2,Ä≤-deoxy-7-iodo-	C20H24I2N7O10P	807.23	C	-8.4
6660	C(=O)([C@@H](NC(=O)OC)[C@H]1C[C@H](O[C@H](C)C1)C1CC1)N1[C@@H](CC[C@@H]1C)c1[nH]c(cn1)c1ccc(cc1)c1ccc	1608494-79-6	Carbamic acid, N,N,Ä≤-[[1,1,Ä≤-biphenyl]-4,4,Ä≤-diylbis[1H-imidazole-5,2-diyl[(	C54H70N8O8	959.18	C	-8.4
6661	O=C1[C@@H]2[C@@H](c3c([C@@H](O)[C@H]2CO1)cc1c(c3)OCO1)c1cc2c(cc1)cccc2	1610886-58-2	Furo[3,Ä≤,4,Ä≤:6,7]naphtho[2,3-d]-1,3-dioxol-6(5aH)-one, 5,8,8a,9-tetrahydro-9-]	C23H18O5	374.39	C	-8.4
6662	C(#C)[C@]1(O)[C@@H](O[C@@](CO)(F)[C@H]1O)n1c2c(nc1)c(=O)nc(N)[nH]2	1613584-82-9	Inosine, 2,Ä≤-deoxyadenylyl-(5,Ä≤,Üí5,Ä≤)-	C20H24N9O10P	581.43	C	-8.4
6663	O=C1N2[C@H](Cn3c1c(O)c(=O)c(C(=O)NCc1c(F)cc(F)cc1)c3)O[C@@H]1C[C@H]2C[C@@H]1C	1616339-98-0	2,5-Methanopyrido[1,Ä≤,2,Ä≤:4,5]pyrazino[2,1-b][1,3]oxazepine-10-carboxamide, N	C23H21F2N3O5	457.43	C	-8.4
6664	C[C@@]12[C@@H]3[C@](C)([C@H](CC=O)[C@]([C@H](C(=O)O)C)(C)CC3)CC[C@@]1(C)[C@H]1[C@@](C)(CC2)CCC(C)(C)	1616968-79-6	Carbamic acid, N,N,Ä≤-[1,3-cyclobutanediylbis[4,1-phenylene-1H-imidazol-5,2-diy]	C44H56N8O6	792.97	C	-8.4
6665	O=c1c2c(n(cn2)[C@@H]2O[C@H](COP(=O)(Oc3ccc(Cl)cc3)N3CCN(CC3)c3ccc(Cl)cc3)CC2)[nH]cn1	1618656-26-0	Inosine, 2,Ä≤,3,Ä≤-dideoxy-, 5,Ä≤-[4-chlorophenyl P-[4-(4-nitrophenyl)-1-pipera	C26H27ClN7O7P	615.96	C	-8.4
6666	O[C@H]1[C@H]2[C@H]3[C@@](C)([C@@H]([C@@H](CCC(=O)OC)C)CC3)[C@@H](O)C[C@@H]2[C@]2(C)[C@H](Cl)C[C@H](O	1618656-29-3	Inosine, 2,Ä≤,3,Ä≤-dideoxy-, 5,Ä≤-[4-chlorophenyl P-[4-(2-pyridinyl)-1-piperazi	C25H27ClN7O5P	571.95	C	-8.4
6667	O(CCCCCCC(=O)NO)c1ccc(c2nc(nc(n2)N2CCOCC2)N2CCOCC2)cc1	1619257-67-8	Hexanamide, 6-[4-(4,6-di-4-morpholinyl-1,3,5-triazin-2-yl)phenoxy]-N-hydroxy-	C23H32N6O5	472.54	C	-8.4
6668	O(CCCCC(=O)NO)c1cc(c2nc(nc(n2)N2CCOCC2)N2CCOCC2)ccc1	1619257-69-0	Butanamide, 4-[3-(4,6-di-4-morpholinyl-1,3,5-triazin-2-yl)phenoxy]-N-hydroxy-	C21H28N6O5	444.48	C	-8.4

6669	O(CCCCCCC(=O)NO)c1cc(c2nc(nc(n2)N2CCOCC2)N2CCOCC2)ccc1	1619257-71-4	Hexanamide, 6-[3-(4,6-di-4-morpholinyl-1,3,5-triazin-2-yl)phenoxy]-N-hydroxy-	C23H32N6O5	472.54	C	-8.4
6670	C[C@]12[C@@H]3[C@](C)([C@@]4(C)C(=CC3=O)[C@H]3[C@@](C)(CC4)CC[C@@](C(=O)O)(C)C3)CC[C@H]2C(C)(C)C(=O)	1620662-80-7	5,Ä≤-Thymidylic acid, 2,Ä≤,3,Ä≤-didehydro-3,Ä≤-deoxy-, 5,Ä≤-[cyclohexyl N-[5-[	C37H48N11O9P	821.82	C	-8.4
6671	N(C(=O)[C@H](Cc1ccncc1)C[C@@H](CN1[C@H](C(=O)NC(C)(C)C)CN(Cc2cc3c(s2)ccs3)CC1)O)[C@H]1c2c(C[C@H]1O)c	162105-31-9	1-Piperazinepentanamide, N-(2,3-dihydro-2-hydroxy-1H-inden-1-yl)-2-[[(1,1-dimeth	C36H45N5O4S2	675.9	C	-8.4
6672	NC1(C23CC4CC(C3)CC(C2)C4)CCCCC1	1621336-59-1	3-Azabicyclo[3.1.0]hexane-2-carboxamide, N-[3-amino-1-(cyclobutylmethyl)-2,3-dic	C35H56N6O6	656.86	C	-8.4
6673	O[C@H]1[C@H](O[C@@H](CO)[C@@H]1O)n1c(=O)[nH]c(=O)c(I)c1	1622332-38-0	D-erythro-Pentonamide, 2,3,5-trideoxy-N-[(1S,2R)-2,3-dihydro-2-hydroxy-1H-inden-	C36H47N5O4.xClH		C	-8.4
6674	N(C(=O)[C@H](Cc1ccc(O)cc1)C[C@@H](CN1[C@H](C(=O)NC(C)(C)C)CN(Cc2c[n+](ccc2)[C@@H]2O[C@H](C(=O)[O-])[	162518-59-4	D-erythro-Pentonamide, 2,3,5-trideoxy-N-[(1S,2R)-2,3-dihydro-2-hydroxy-1H-inden-	C36H47N5O5	629.79	C	-8.4
6675	CC1C2c3c(c(ccc3c3c4c(c(cc3)c3[nH]c(nc3)C3N(C(=O)C(NC(=O)OC)C(C)C)CCC3)C3CC4CC3)c3[nH]c(nc3)C3N(C(=O)	1627533-38-3	Carbamic acid, N-[(1S)-2-methyl-1-[[(2S)-2-[5-[1,1,Ä≤,2,2,Ä≤,3,3,Ä≤,4,4,Ä≤-octa	C52H64N8O8	929.11	C	-8.4
6676	C([C@H](OCP(=O)(Cl)Cl)C)n1c2c(nc1)c(N)ncn2	1630726-94-1	1-Naphthalenesulfonic acid, 3,3,Ä≤-[(2,2,Ä≤-dimethyl[1,1,Ä≤-biphenyl]-4,4,Ä≤-di	C34H28N6O6S2.Na		C	-8.4
6677	C[C@@]12[C@@H]([C@]3(C)[C@@H](CC2)C(C)(C)C(=O)CC3)CC[C@H]2[C@@]1(C)CC[C@@H]2[C@](CCC(C(C)(C)O)O)(C)O	1632374-49-2	1H-Thieno[3,4-d]imidazole-4-pentanoic acid, hexahydro-2-oxo-, (5R,5aR,8aR,9R)-5,	C32H36N2O10S	640.7	C	-8.4
6678	O=C1[C@@H]2[C@@H](c3c([C@H](OC(=O)c4c(N(=O)=O)cc(OC)cc4)[C@H]2CO1)cc1c(c3)OCO1)c1cc(OC)c(OC)c(OC)c1	1633020-32-2	Furo[3,Ä≤,4,Ä≤:6,7]naphtho[2,3-d]-1,3-dioxol-6(5aH)-one, 5,8,8a,9-tetrahydro-9-	C29H25NO11	563.51	C	-8.4
6679	O=C1[C@@H]2[C@@H](c3c([C@H](OC(=O)C#CC)[C@H]2CO1)cc1c(c3)OCO1)c1cc(OC)c(OC)c(OC)c1	1633020-37-7	Benzoic acid, 2-amino-5-nitro-, (5R,5aR,8aR,9R)-5,5a,6,8,8a,9-hexahydro-8-oxo-9-	C29H26N2O11	578.52	C	-8.4
6680	[C@@H](C(CC)CC)(NC(=O)C)[C@H]1[C@H](NC(=N)N)C[C@H](C(=O)OCCCCC(=O)OC[C@H]2N[C@H]([C@H](O)[C@@H]2O)c2	1638370-21-4	Cyclopentanecarboxylic acid, 3-[(1S)-1-(acetylamino)-2-ethylbutyl]-4-[(aminoimir	C30H47N9O8	661.75	C	-8.4
6681	C(OCCOP(=O)(Oc1cc2c(cc1)OCO2)Oc1cc2c(cc1)OCO2)n1c2c(nc1)c(=O)nc(N)[nH]2	1638769-61-5	Phosphoric acid, 2-[(2-amino-1,6-dihydro-6-oxo-9H-purin-9-yl)methoxy]ethyl bis(1	C22H20N5O10P	545.4	C	-8.4
6682	C(=O)([C@@H](NC(=O)NC)C(C)C)N1[C@@H](C=CC1)c1[nH]c(cn1)c1ccc(cc1)c1ccc(cc1)c1[nH]c(nc1)[C@H]1N(C(=O)	1639871-05-8	Carbamic acid, N-[(1S)-1-[[(2S)-2-[5-[4,Ä≤-[2-[(2S)-1-[(2S)-2-[[(dimethylamino)c	C41H51N9O5	749.9	C	-8.4
6683	C(=O)([C@@H](NC(=O)OC)C1CCCC1)N1[C@@H](C=CC1)c1[nH]c(cn1)c1ccc(cc1)c1ccc(cc1)c1[nH]c(nc1)[C@H]1N(C(=	1639871-20-7	Carbamic acid, N-[(1S)-1-[[(2S)-2-[5-[4,Ä≤-[2-[(2S)-1-[(2S)-2-[[(dimethylamino)c	C41H51N9O5	749.9	C	-8.4
6684	C(=O)([C@@H](NC(=O)OC)C1CCCCC1)N1[C@@H](CCC1)c1[nH]c(cn1)c1ccc(cc1)c1ccc(cc1)c1[nH]c(nc1)[C@H]1N(C(=	1639871-26-3	Carbamic acid, N,N,Ä≤-[[1,1,Ä≤-biphenyl]-4,4,Ä≤-diylbis[1H-imidazole-5,2-diyl[(	C46H54N8O6	814.97	C	-8.4
6685	C(=O)([C@@H](NC(=O)OC)C(C)C)N1[C@H](c2[nH]c(c3cc4c(-c5c(cc(cc5)c5[nH]c(nc5)[C@H]5N(C(=O)[C@@H](NC(=O	1645563-52-5	Carbamic acid, N,N,Ä≤-[[1,1,Ä≤-biphenyl]-4,4,Ä≤-diylbis[1H-imidazole-5,2-diyl[(	C46H58N8O6	819	C	-8.4
6686	O=c1n([C@@H]2O[C@H](COP(=O)(C(O)c3c(Cl)cccc3Cl)OC[C@H]3O[C@H](C=C3)n3c(=O)[nH]c(=O)c(C)c3)C=C2)cc(C)	164652-09-9	Thymidine, 2,Ä≤,3,Ä≤-didehydro-P,3,Ä≤-dideoxy-P-(hydroxy-4-pyridinylmethyl)thym	C26H28N5O10P	601.5	C	-8.4
6687	N(C(=O)[C@H](Cc1ccccc1)C[C@@H](CN1[C@H](C(=O)NC(C)(C)C)CN(CCCC)CC1)O)[C@H]1c2c(C[C@H]1O)cccc2	164725-21-7	1-Piperazinepentanamide, N-(2,3-dihydro-2-hydroxy-1H-inden-1-yl)-2-[[(1,1-dimeth	C41H50N4O4	662.86	C	-8.4
6688	N(C(=O)[C@H](Cc1ccccc1)C[C@@H](CN1[C@H](C(=O)NC(C)(C)C)CN(Cc2[nH]c3c(n2)cccc3)CC1)O)[C@H]1c2c(C[C@H]	164725-30-8	1-Piperazinepentanamide, N-(2,3-dihydro-2-hydroxy-1H-inden-1-yl)-2-[[(1,1-dimeth	C39H52N4O5	656.85	C	-8.4
6689	N(C(=O)[C@H](Cc1ccccc1)C[C@@H](CN1[C@H](C(=O)NC(C)(C)C)CN(Cc2c(C)noc2C)CC1)O)[C@H]1c2c(C[C@H]1O)cccc	164725-31-9	D-erythro-Pentonamide, 5-[(2S)-4-(1H-benzimidazol-2-ylmethyl)-2-[[(1,1-dimethyle	C38H48N6O4	652.83	C	-8.4
6690	N(C(=O)[C@H](Cc1ccccc1)C[C@@H](CN1[C@H](C(=O)NC(C)(C)C)CN(Cc2cc(C)nc(Cl)c2)CC1)O)[C@H]1c2c(C[C@H]1O)	164725-38-6	1-Piperazinepentanamide, N-(2,3-dihydro-2-hydroxy-1H-inden-1-yl)-2-[[(1,1-dimeth	C38H51N5O4	641.84	C	-8.4
6691	C(C(=O)OC)(N1CC(C(=O)NC(C)(C)C)N(CC(CC(C(=O)NC2c3c(CC2O)cccc3)Cc2ccccc2)O)CC1)c1ccccc1	164725-48-8	1-Piperazinepentanamide, N-(2,3-dihydro-2-hydroxy-1H-inden-1-yl)-2-[[(1,1-dimeth	C40H54N6O5	698.89	C	-8.4
6692	N(C(=O)[C@H](Cc1ccccc1)C[C@@H](CN1[C@H](C(=O)NC(C)(C)C)CN(Cc2c(Cl)cc(NC(=O)C)cc2)CC1)O)[C@H]1c2c(C[C	164725-56-8	1-Piperazinepentanamide, N-(2,3-dihydro-2-hydroxy-1H-inden-1-yl)-2-[[(1,1-dimeth	C38H50N4O4	626.83	C	-8.4
6693	C(C1c2c(CC1O)cccc2)N1CC(C(=O)NC(C)(C)C)N(CC(CC(C(=O)NC2c3c(CC2O)cccc3)Cc2ccccc2)O)CC1	164725-64-8	1-Piperazinepentanamide, N-(2,3-dihydro-2-hydroxy-1H-inden-1-yl)-4-[(2,3-dihydro	C40H52N4O4	652.87	C	-8.4
6694	O=c1n([C@@H]2O[C@H](COP(=O)(C(O)c3ccc(Cl)cc3)OC[C@@H]3[C@@H](N=[N+]=[N-])C[C@@H](O3)n3c(=O)[nH]c(=O)	166318-76-9	Thymidine, 3,Ä≤-azido-P,3,Ä≤-dideoxy-P-(hydroxyphenylmethyl)thymidylyl-(5,Ä≤,Üi	C27H31N10O10P		C	-8.4
6695	C([C@H]([C@H](NC(=O)c1cc2c(cc1)cccc2)CSc1ccccc1)O)N1[C@H](C(=O)NC(C)(C)C)C[C@H]2[C@@H](C1)CCCC2	1665278-93-2	3-Isoquinolinecarboxamide, N-(1,1-dimethylethyl)decahydro-2-[(2S,3R)-2-hydroxy-3	C35H45N3O3S	587.82	C	-8.4
6696	O(C(=O)N[C@@H](Cc1ccccc1)[C@@H](CN(S(=O)(=O)c1cc2c(cc1)nc(NC1CCN(CC1)C1CCCC1)s2)CC(C)C)O)[C@@H]1[C@H	1672661-49-2	Carbamic acid, N-[(1S,2R)-3-[[[2-(cyclopropylamino)-6-benzothiazolyl]sulfonyl](2	C31H38F2N4O7S2	680.78	C	-8.4
6697	C(=O)([C@H](Cc1cnc[nH]1)NC(=O)[C@@H](NC(=O)[C@H](Cc1ccc(O)cc1)NC(=O)c1cccnc1)CCCCNC(=O)[C@@H](NC(=O)	168565-08-0	L-Proline, 1-[N-[N6-(N2-acetyl-L-arginyl)-N2-[N-(3-pyridinylcarbonyl)-L-tyrosyl]	C40H54N12O9	846.93	C	-8.4
6698	N(=Nc1ccc(CO)cc1)c1n(c2c(n1)c(=O)nc(N)[nH]2)[C@@H]1O[C@H](CO)[C@@H](O)[C@H]1O	168701-66-4	Benzoic acid, 4-[(2-amino-6,9-dihydro-6-oxo-9-Œ≤-D-ribofuranosyl-1H-purin-8-yl)a	C17H17N7O7	431.36	C	-8.4
6699	S(=O)(=O)(O)c1c2c(c(S(=O)(=O)O)cc(N=Nc3c(C)cc([N+]#N)c(C)c3)c2)cc(N=Nc2c(C)cc([N+]#N)c(C)c2)c1.[Cl-]	1715922-32-9	1,5-Naphthalenedisulfonic acid, 3,7-bis[2-(4-amino-2,5-dimethylphenyl)diazenyl]-	C26H26N6O6S2	582.65	C	-8.4
6700	C(CC(COC(=O)c1cc(Oc2ccccc2)ccc1)(CO)CO)n1c2c(nc1)c(=O)nc(N)[nH]2	172645-17-9	Benzoic acid, 3-phenoxy-, 2-[2-(2-amino-1,6-dihydro-6-oxo-9H-purin-9-yl)ethyl]-2	C37H33N5O8	675.69	C	-8.4
6701	O=c1c2c(n(cn2)[C@@H]2O[C@H](CO)[C@@H](O)C2)[nH]c(N=CN(C)C)n1	1733-14-8	1-Naphthalenesulfonic acid, 3,3,Ä≤-[(3-methylene[1,1,Ä≤-biphenyl]-4,4,Ä≤-diyl)b	C34H28N6O6S2	680.75	C	-8.4
6702	N(=Nc1ccc(N=Nc2ccccc2)cc1)c1c2c(c(S(=O)(=O)O)cc(S(=O)(=O)O)c2)ccc1O	17332-09-1	3,Ä≤-Guanylic acid, guanylyl-(3,Ä≤,Üi5,Ä≤)-	C20H26N10O15P2	708.43	C	-8.4
6703	O=c1n([C@@H]2O[C@H](COP(=O)(OC[C@@H]3[C@@H](N=[N+]=[N-])C[C@@H](O3)n3c(=O)[nH]c(=O)c(C)c3)OCc3cc(N(=	173606-89-8	Thymidine, 3,Ä≤-azido-P-[(4-cyanophenyl)methyl]-3,Ä≤-deoxythymidylyl-(5,Ä≤,Üi5,	C28H30N11O10P		C	-8.4
6704	O=c1n([C@@H]2O[C@H](COP(=O)([C@H](O)c3ccc(OC)cc3)OC[C@@H]3[C@@H](N=[N+]=[N-])C[C@@H](O3)n3c(=O)[nH]c	173658-29-2	Thymidine, 3,Ä≤-azido-P,3,Ä≤-dideoxy-P-[(2,6-dinitrophenyl)hydroxymethyl]thymid	C27H29N12O14P		C	-8.4
6705	O=c1n([C@@H]2O[C@H](COP(=O)([C@H](O)c3ccc(C)cc3)OC[C@@H]3[C@@H](N=[N+]=[N-])C[C@@H](O3)n3c(=O)[nH]c(	173658-30-5	Thymidine, 3,Ä≤-azido-P,3,Ä≤-dideoxy-P-[hydroxy(4-methoxyphenyl)methyl]thymidyl	C28H33N10O11P		C	-8.4
6706	O=c1n([C@@H]2O[C@H](COP(=O)([C@H](O)c3ccccc3)OC[C@@H]3[C@@H](N=[N+]=[N-])C[C@@H](O3)n3c(=O)[nH]c(=O)	173658-31-6	Thymidine, 3,Ä≤-azido-P,3,Ä≤-dideoxy-P-[hydroxy(4-methylphenyl)methyl]thymidyly	C28H33N10O10P		C	-8.4
6707	C(F)(F)(F)c1nc(no1)c1cc(C)c(OCCCc2cc(C(=O)O)no2)c(C)c1	173899-99-5	3-Isoxazolecarboxylic acid, 5-[3-[2,6-dimethyl-4-[5-(trifluoromethyl)-1,2,4-oxad	C24H20F3N3O5	487.43	C	-8.4
6708	C(CC)(C1=C(O)CC(CCC)(CCC)OC1=O)c1cc(NS(=O)(=O)c2ccc(C(F)(F)F)cn2)ccc1	174484-23-2	2-Pyridinesulfonamide, N-[3-[1-[5,6-dihydro-4-hydroxy-2-oxo-6-(2-phenylethyl)-6-	C32H38N2O5S	562.72	C	-8.4
6709	C(Cc1ccccc1)[C@@]1(CCC)CC(=C([C@@H](CC)c2cc(NS(=O)(=O)c3ccc(C#N)cn3)ccc2)C(=O)O1)O	174484-51-6	2-Pyridinesulfonamide, 5-cyano-N-[3-[(1R)-1-[(6R)-5,6-dihydro-4-hydroxy-2-oxo-6-	C31H33N3O5S	559.68	C	-8.4
6710	C(OC[C@@H]1[C@@H](C=C[C@H](P(=O)(OC(C)C)OC(C)C)O1)n1c(=O)nc(NC(=O)c2ccccc2)cc1)(c1ccccc1)(c1ccccc1)c	174564-65-9	3-Quinolinecarboxamide, N-[1,2-dihydro-1-[2-(hydroxymethyl)-1,3-oxathiolan-5-yl]	C18H16N4O4S	384.41	C	-8.4
6711	C(Cc1ccccc1)[C@]1(CCC(F)(F)F)CC(=C([C@H](C(C)C)C)c2cc(NS(=O)(=O)c3ccc(N)cn3)ccc2)C(=O)O1)O	174590-15-9	2-Pyridinesulfonamide, 5-cyano-N-[3-[1-[5,6-dihydro-4-hydroxy-2-oxo-6-(2-phenyle	C33H34F3N3O5S	641.7	C	-8.4
6712	C(Cc1ccc(F)cc1)[C@@]1(CCC)CC(=C([C@H](CC)c2cc(NS(=O)(=O)c3ccc(C#N)cn3)ccc2)C(=O)O1)O	174590-33-1	2-Pyridinesulfonamide, 5-cyano-N-[3-[1-[6-[2-(4-fluorophenyl)ethyl]-5,6-dihydro-	C33H36FN3O5S	605.72	C	-8.4
6713	C[C@@H]1[C@@H](O[C@H](CO)[C@H]1O)n1cc(C#C)c(=O)[nH]c1=O	175776-04-2	3-Isoquinolinecarboxamide, N-(1,1-dimethylethyl)decahydro-2-[2-hydroxy-3-[(3-hyc	C32H45N3O4S	567.78	C	-8.4
6714	O=c1n([C@H]2O[C@@H](C(=O)O[C@H]3[C@H](C(C)C)CC[C@@H](C)C3)SC2)cc(F)c(NC(=O)c2ccccc2)n1	1773498-59-1	1,3-Oxathiolane-2-carboxylic acid, 5-[2-oxo-4-[(phenylmethyl)amino]-1(2H)-pyrimi	C25H33N3O4S	471.61	C	-8.4
6715	O(c1nc(Nc2ccc(C#N)cc2)nc(N)c1Br)c1c(C)cc(C#N)cc1C	1803132-28-6	2H-Pyrido[1,Ä≤,2,Ä≤:4,5]pyrazino[2,1-b][1,3]oxazine-9-carboxamide, N-[(2,4-difl	C20H19F2N3O5		C	-8.4
6716	C(OP1(=S)Oc2c(CO1)cccc2)[C@@]1(C(F)F)O[C@H]([C@@H](O)[C@@H]1O)n1c2c(nc1)c(N)ncn2	1803134-27-1	9H-Purin-6-amine, 9-[4-C-(difluoromethyl)-5-O-(2-oxido-4H-1,3,2-benzodioxaphosph	C18H18F2N5O7P	485.34	C	-8.4
6717	C([C@H](OCP(=O)(OCC)OCC)C)n1c2c(nc1)c(N)ncn2	1805821-44-6	Cyclopenta[c]pyrrole-1-carboxamide, (2S)-2-cyclohexyl-N-(2-pyrazinylcarbonyl)gly	C36H55N7O6	681.87	C	-8.4
6718	C(=O)(C(NC(=O)OC)C(C)C)N1C(c2[nH]c(c3cc4c(-c5c(cc(cc5)c5[nH]c(nc5)C5N(C(=O)C(NC(=O)OC)C(C)C)CCC5)CC4	1807613-51-9	Carbamic acid, N,N,Ä≤-[[(4aR)-9,10-dihydro-2,7-phenanthrenediyl]bis[1H-imidazole	C42H52N8O6	764.91	C	-8.4
6719	O=C1[C@@H]2C(c3c([C@H](O)[C@H]2CO1)cc1c(c3)OCO1)c1cc2c(cc1)OCO2	1807613-52-0	Carbamic acid, N,N,Ä≤-[[(4aS)-9,10-dihydro-2,7-phenanthrenediyl]bis[1H-imidazole	C42H52N8O6	764.91	C	-8.4
6720	C(=O)([C@@H](NC(=O)OC)C(C)C)N1[C@@H](C2CC1CC2)c1[nH]c(cn1)c1ccc(cc1)c1ccc(cc1)c1[nH]c(nc1)[C@H]1N(C(	1808295-99-9	Carbamic acid, N,N,Ä≤-[[1,1,Ä≤-biphenyl]-4,4,Ä≤-diylbis[1H-imidazole-5,2-diyl(h	C44H58N8O6	794.98	C	-8.4
6721	C(=O)([C@@H](NC(=O)OC)C(C)C)N1[C@@H](CCC1)c1[nH]c(cn1)c1ccc(cc1)c1ccc(cc1)c1[nH]c(nc1)[C@H]1N(C(=O)[	1808296-07-2	Carbamic acid, N-[(1S)-1-[[(2S)-2-[5-[4-[(1E)-2-[4-[2-[(3S)-2-[(2S)-2-[(methoxyc	C44H54N8O6	790.95	C	-8.4
6722	O=c1c2c(n(cn2)[C@@H]2O[C@H](COP(=O)(O[C@@H]3[C@@H](COP(=O)(O)O)O[C@H](C3)n3c4c(nc3)c(N)ncn4)O)[C@@H]	1809325-35-6	4,7-Epoxy-2H-isoindole-2-acetic acid, octahydro-Œ±-methyl-1,3-dioxo-, (5R,5aR,8a	C33H33NO12	635.61	C	-8.4
6723	C(=O)([C@@H](NC(=O)OC)C(C)C)N1[C@@H](C[C@H](SC)C1)c1[nH]c(c(Cl)n1)c1ccc(cc1)c1ccc(cc1)c1[nH]c(nc1Cl)	1819985-23-3	Carbamic acid, N-[(1S)-1-[[(2S)-2-[5-[4,Ä≤-[5-chloro-2-[(2S,4S)-1-[(2S)-2-[(meth	C41H51ClN8O7S	835.41	C	-8.4
6724	Nc1c2c(n(cn2)[C@@H]2O[C@H](CO[C@@H](P(=O)(O)O)C(=O)O)[C@@H](O)[C@H]2O)nc(I)n1	1821044-04-5	Adenosine, N-benzoyl-5,Ä≤-O-(carboxyphosphonomethyl)-2-iodo-	C19H19IN5O10P	635.26	C	-8.4
6725	N(c1c2c(n(cn2)[C@@H]2C[C@H](COC(=O)CCC(=O)O)C=C2)nc(N)n1)C1CC1	1821201-88-0	Acetamide, 2-[[4-(1H-indazol-4-yl)-6-(4-morpholinyl)-1,3,5-triazin-2-yl]amino]-N	C18H22N8O2	382.42	C	-8.4
6726	C(COCP(=O)(OCCN=[N+]=[N-])O)n1c2c(nc1)c(N)ncn2	1831055-61-8	L-Alanine, N-(S)-phosphono-, 1-ethyl ester, cyclic (P,Üi3,Ä≤,5,Ä≤)-ester with 2	C23H26FN6O9P	580.46	C	-8.4
6727	[C@@H](NC(=O)N1CCN(C)CC1)(C(=O)N[C@H](/C=C/S(=O)(=O)c1ccccn1)CCc1ccccc1)CCCNC(=N)N	1838684-41-5	1-Piperazinecarboxamide, N-[(1S)-4-[[(aminoiminomethyl)amino]oxy]-1-[[[(1S,2E)-1	C29H41N7O5S	599.74	C	-8.4
6728	N(C)C1=[S][Ni+2]234([N](=c5c(=[O]3)n(Cc3ccccc3)c3c5cccc3)[N-]C(=[S]4)NC)[N](=c3c(=[O]2)n(Cc2ccccc2)c	1843232-27-8	Not Yet Assigned	C22H22N8NiO2S2		C	-8.4
6729	C(C(=O)OC[C@H]1O[C@H](C=C1)n1c(=O)[nH]c(=O)c(C)c1)C1(CC(=O)OCC=C)C(=O)OCO1	1848258-49-0	Carbamic acid, N-[(1S)-1-(1H-indol-3-ylmethyl)-2-oxo-2-[(1-tricyclo[3.3.1.13,7]c	C31H37N3O3	499.64	C	-8.4
6730	N(=Nc1c(c(c(S(=O)(=O)O)c(c1[2H])[2H])[2H])[2H])c1c2c(ccc1O)cccc2	1860793-33-4	1-Piperazinecarboxamide, 4-methyl-N-[(1S)-2-oxo-2-[[(1S,2E)-1-(2-phenylethyl)-3-	C33H37F3N4O4S	642.73	C	-8.4
6731	O=C1[C@@H]2[C@@H](c3c([C@H](OC(=O)CN4c5c(C(=O)C4=O)cc(Cl)cc5)[C@H]2CO1)cc1c(c3)OCO1)c1cc(OC)c(OC)c(O	1864833-31-7	1H-Indole-1-acetic acid, 2,3-dihydro-5-methyl-2,3-dioxo-, (5R,5aR,8aR,9R)-5,5a,6	C33H29NO11	615.58	C	-8.4
6732	O=C1[C@@H]2[C@@H](c3c([C@H](OC(=O)CCCC[C@H]4SC(SCC4)c4cc(OC)c(OC)cc4)[C@H]2CO1)cc1c(c3)OCO1)c1cc(OC)	1875123-87-7	1,3-Dithiane-4-pentanoic acid, 2-(2-methoxyphenyl)-, (5R,5aR,8aR,9R)-5,5a,6,8,8a	C38H42O10S2	722.86	C	-8.4
6733	O=C1[C@@H]2[C@@H](c3c([C@H](OC(=O)CCCC[C@H]4SC(SCC4)c4c(Cl)c(Cl)ccc4)[C@H]2CO1)cc1c(c3)OCO1)c1cc(OC)	1875123-91-3	1,3-Dithiane-4-pentanoic acid, 2-(4-chlorophenyl)-, (5R,5aR,8aR,9R)-5,5a,6,8,8a,	C37H39ClO9S2	727.28	C	-8.4
6734	O=c1n([C@@H]2O[C@H](CO)[C@H](O)C2)cc(Cl)c(=O)[nH]1	188548-80-3	Thymidine, 2,Ä≤,3,Ä≤-didehydro-3,Ä≤-deoxy-5,Ä≤-O-(6-methyl-2-oxido-4H-1,3,2-ber	C18H19N2O7P	406.33	C	-8.4
6735	C(=O)(O)c1c(sc2c1CC(C)(C)CC2)c1c(CNc2ccc(C[C@@H]([C@@H](CN(S(=O)(=O)c3cc4c(cc3)ncs4)CC3CCC3)O)NC(=O)	1887206-52-1	Benzo[b]thiophene-3-carboxylic acid, 2-[2-[[[4-[(2S,3R)-4-[(6-benzothiazolylsulf	C47H53F3N4O9S3	971.14	C	-8.4
6736	O=[14c]1n(C2OC(CO)C=C2)cc(F)c(=O)[nH]1	189075-02-3	Uridine, 2,Ä≤-deoxy-Œ≤-L-guanylyl-(3,Ä≤,Üi5,Ä≤)-2,Ä≤-deoxy-5-fluoro-	C19H23FN7O11P	575.4	C	-8.4
6737	C(O[C@@H]1[C@H](OC(c2ccc(OC)cc2)(c2ccc(OC)cc2)c2ccccc2)[C@@H](COC(c2ccc(OC)cc2)(c2ccc(OC)cc2)c2ccccc	1899098-65-7	6H-Purin-6-one, 2-amino-1,9-dihydro-9-[4-(triphenylmethoxy)-3-[(triphenylmethoxy	C48H43N5O3	737.89	C	-8.4
6738	O=c1n(c2c(c(cc(n2)c2ccccc2)c2ccco2)c(=O)n1c1ccccc1)[C@H]1[C@H](OC(=O)c2ccccc2)[C@H](OC(=O)c2ccccc2)[	190319-80-3	Pyrido[2,3-d]pyrimidine-2,4(1H,3H)-dione, 3,7-bis(4-chlorophenyl)-5-(2-furanyl)-	C49H33Cl2N3O10	894.71	C	-8.4
6739	C(#N)[C@]1(O[C@H](COP(=O)(Oc2ccccc2)NC(C(=O)OCC)(C)C)[C@@H](O)[C@H]1O)c1n2c(cc1)c(N)ncn2	1911578-92-1	Not Yet Assigned	C27H33N6O8P	600.56	C	-8.4
6740	[C@@H](C(=O)N[C@@H](Cc1ccccc1)C[C@@H]([C@H](Cc1ccccc1)NC(=O)COc1c(C)cccc1C)O)(C(C)C)N1C(=O)NC(=O)CC1	192725-38-5	1(2H)-Pyrimidineacetamide, N-[(1S,3S,4S)-4-[[2-(2,6-dimethylphenoxy)acetyl]aminc	C37H44N4O6	640.77	C	-8.4
6741	C[C@]12[C@H]([C@H]3[C@@H]([C@]4(C)[C@H](CC3)CCCC4)CC2)CC[C@@H]1[C@@H](CCC(=O)NCCNCCNCCN)C	193898-72-5	Adenosine, 1,Ä≤-[3-(aminocarbonyl)-1H-1,2,4-triazol-1-yl]-3,Ä≤-O-benzoyl-1,Ä≤-d	C54H47N10O17P	1138.98	C	-8.4
6742	C(=O)([C@@H](NC(=O)OC)C(C)C)N1[C@H](c2[nH]c3c4c(cc(cc4)c4cc(C)c(cc4)c4[nH]c(nc4)[C@H]4N(C(=O)[C@@H](	1949770-68-6	Carbamic acid, N-[(1S)-1-[[(2S)-2-[7-[3-hydroxy-4-[2-[(2S)-1-[(2S)-2-[(methoxyca	C42H50N8O7	778.9	C	-8.4
6743	C(=O)([C@@H](NC(=O)OC)C(C)C)N1[C@H](c2[nH]c3c4c(cc(cc4)c4cc(OCCN5CCOCC5)c(cc4)c4[nH]c(nc4)[C@H]4N(C(	1949770-70-0	Carbamic acid, N-[(1S)-1-[[(2S)-2-[7-[3-cyclopropyl-4-[2-[(2S)-1-[(2S)-2-[(metho	C45H54N8O6	802.96	C	-8.4
6744	Clc1c2c(c3c(nc([nH]3)[C@H]3N(C(=O)[C@@H](NC(=O)OC)C(C)C)CCC3)c1)ccc(c2)c1ccc(cc1)c1[nH]c(nc1)[C@H]1N	1949770-71-1	Carbamic acid, N-[(1S)-1-[[(2S)-2-[7-[4-[2-[(2S)-1-[(2S)-2-[(methoxycarbonyl)ami	C48H61N9O8	892.05	C	-8.4
6745	C(=O)([C@@H](NC(=O)OC)C(C)C)N1[C@H](c2[nH]c3c4c(cc(cc4)c4ccc(cc4)c4[nH]c(nc4)[C@H]4N(C(=O)[C@@H](NC(	1949770-77-7	Carbamic acid, N-[(1S)-1-[[(2S)-2-[7-[4-[2-[(2S,4R)-4-(acetylamino)-1-[(2S)-2-[(	C44H53N9O7	819.95	C	-8.4
6746	O(C(=O)/C=C(/C=C/C=C(/C=C/C1=C(C)CCCC1(C)C)\C)\C)[C@@H]1[C@H](n2c3c(nc2)c(N)ncn3)O[C@H](CO)[C@H]1O	197165-28-9	Retinamide, N-(9-Œ≤-D-arabinofuranosyl-9H-purin-6-yl)-	C30H39N5O5	549.66	C	-8.4
6747	N(C(=O)C)[C@H]1[C@H]([C@@H]([C@@H](CO)O)O)OC(=C[C@@H]1NC(=N)NC(=O)Cn1cc(nn1)C1=CCCCC1)C(=O)O	1982351-38-1	D-glycero-D-galacto-Non-2-enonic acid, 5-(acetylamino)-2,6-anhydro-4-[[[[2-(4-cy	C22H33N7O8	523.54	C	-8.4
6748	N(C(=O)C)[C@H]1[C@H]([C@@H]([C@@H](CO)O)O)OC(=C[C@@H]1NC(=N)NC(=O)Cn1cc(nn1)c1ccc(Cl)cc1)C(=O)O	1982351-49-4	D-glycero-D-galacto-Non-2-enonic acid, 5-(acetylamino)-2,6-anhydro-3,4,5-trideox	C23H29N7O8	531.52	C	-8.4
6749	N(C(=O)C)[C@H]1[C@H]([C@@H]([C@@H](CO)O)O)OC(=C[C@@H]1NC(=N)NC(=O)Cn1cc(nn1)c1ccc(F)cc1)C(=O)O	1982351-50-7	D-glycero-D-galacto-Non-2-enonic acid, 5-(acetylamino)-2,6-anhydro-4-[[[[2-[4-(4	C22H26ClN7O8	551.94	C	-8.4
6750	O(COC(=O)c1ccc(N(=O)=O)cc1)c1c2n(N([C@H]3N(C2=O)CCOC3)[C@H]2c3c(CSc4c2cccc4)c(F)c(F)cc3)ccc1=O	1985606-55-0	Carbonic acid, (12aR)-12-[(11S)-7,8-difluoro-6,11-dihydrodibenzo[b,e]thiepin-11-	C28H25F2N3O6S	569.58	C	-8.4
6751	O(C(=O)OCC(C)(C)C)c1c2n(N([C@H]3N(C2=O)CCOC3)[C@H]2c3c(CSc4c2cccc4)c(F)c(F)cc3)ccc1=O	1985606-93-6	Carbonic acid, (12aR)-12-[(11S)-7,8-difluoro-6,11-dihydrodibenzo[b,e]thiepin-11-	C31H31F2N3O6S	611.66	C	-8.4
6752	O(C(=O)Oc1c(C(=O)OC)cccc1)c1c2n(N([C@H]3N(C2=O)CCOC3)[C@H]2c3c(CSc4c2cccc4)c(F)c(F)cc3)ccc1=O	1985607-40-6	Propanoic acid, 3-[[[[(12aR)-12-[(11S)-7,8-difluoro-6,11-dihydrodibenzo[b,e]thie	C31H29F2N3O8S	641.64	C	-8.4
6753	O(C(=O)OC(C(=O)OC(C)(C)C)(C)C)c1c2n(N([C@H]3N(C2=O)CCOC3)[C@H]2c3c(CSc4c2cccc4)c(F)c(F)cc3)ccc1=O	1985607-57-5	Propanoic acid, 2-[[[[(12aR)-12-[(11S)-7,8-difluoro-6,11-dihydrodibenzo[b,e]thie	C29H25F2N3O8S	613.59	C	-8.4
6754	N(C(=O)c1ccc(OC)cc1)c1cc2cc(C34CC5CC(C4)CC(C3)C5)[nH]c2cc1	1987854-61-4	Benzamide, N-(2-tricyclo[3.3.1.13,7]dec-1-yl-1H-indol-5-yl)-	C25H26N2O	370.49	C	-8.4
6755	C(F)(F)c1n(c2c(n1)cccc2)c1nc(nc(n1)N1CCOCC1)N1C[C@H](C(=O)O)CCC1	2001083-69-6	3-Piperidinecarboxylic acid, 1-[4-[2-(difluoromethyl)-1H-benzimidazol-1-yl]-6-(4	C21H23F2N7O3	459.45	C	-8.4
6756	C(=O)([C@@H](NC(=O)OC)C(C)C)N1[C@H](c2[nH]c(c(n2)C2CC2)c2c3c(c(cc2)c2cc4c(cc2)c2c(cc4)nc([nH]2)[C@H]	2007901-48-4	Carbamic acid, N-[(1S)-1-[[(2S,4S)-2-[5-[2,3-dihydro-7-[2-[(2S,5S)-1-[(2S)-2-[(m	C48H60N8O6	845.04	C	-8.4
6757	C(=O)([C@@H](NC(=O)OC)C(C)C)N1C2C(C2)C[C@H]1c1[nH]c2c(n1)ccc1c2ccc(c1)c1c2c(c(cc1)c1[nH]c(nc1)[C@H]1	2007901-81-5	Carbamic acid, N-[(1S)-1-[[(2S,5R)-2-[7-[2,3-dihydro-7-[2-[(2S,4S)-1-[(2S)-2-[(m	C47H52F6N8O6	938.96	C	-8.4
6758	C(=O)([C@@H](NC(=O)OC)C(C)C)N1[C@H](c2[nH]c3c4c(cc(cc4)c4c5c(cc4)c4[nH]c(nc4)[C@H]4N(C(=O)[C@@H](N	2007901-86-0	Carbamic acid, N-[(1S)-1-[[(2S,5S)-2-[5-[2,3-dihydro-7-[2-[(3S)-2-[(2S)-2-[(meth	C51H64N8O6	885.1	C	-8.4
6759	C(=O)(C(=O)Nc1c(C)cc(N(=O)=O)cc1)c1c(C23CC4CC(C3)CC(C2)C4)[nH]c2c1cccc2	2014403-83-7	1H-Indole-3-acetamide, N-(4-chloro-2-methylphenyl)-Œ±-oxo-2-tricyclo[3.3.1.13,7]	C27H27ClN2O2	446.97	C	-8.4
6760	C(=O)(C(=O)Nc1cc(C(C)(C)C)on1)c1c([nH]c2c1cccc2)C12CC3CC(C2)CC(C1)C3	2014403-95-1	1H-Indole-3-acetamide, Œ±-oxo-N-5-pyrimidinyl-2-tricyclo[3.3.1.13,7]dec-1-yl-	C24H24N4O2	400.47	C	-8.4
6761	O=c1n([C@@H]2O[C@H](COC(=O)CCC(=O)Oc3c(OC)ccc(C(=O)c4ccc(N(=O)=O)cc4)c3)C=C2)cc(C)c(=O)[nH]1	2015224-13-0	Butanedioic acid, 1-[[(2S,5R)-5-(3,4-dihydro-5-methyl-2,4-dioxo-1(2H)-pyrimidiny	C28H25FN2O9	552.5	C	-8.4
6762	S(Cc1cc2c(oc1=O)c(OC)ccc2)c1n(c2c(n1)c(=O)nc[nH]2)[C@@H]1O[C@H](CO)[C@@H](O)[C@H]1O	2021230-51-1	Not Yet Assigned	C20H17FN4O7S	476.43	C	-8.4
6763	P(=O)(C(C(=O)O)C)(OCC(F)(F)F)OCC(F)(F)F	1000699-46-6	4-Morpholinecarboxamide, N-[(1S)-2-oxo-1-(phenylmethyl)-2-[[(1S,2E)-1-(phenylmet	C30H33N3O5S	547.67	A/B	-8.3
6764	O[C@H]1[C@@H](O[C@H](COC(=O)CCO[C@H]2[C@H](NC(=O)C)[C@@H](O)[C@@H](O)[C@@H](CO)O2)[C@H]1O)n1nc(C(=O)	1002334-75-9	1H-1,2,4-Triazole-3-carboxamide, 1-[2,3-bis-O-[3-(Œ≤-D-galactopyranosyloxy)-1-ox	C26H40N4O19	712.61	A/B	-8.3
6765	O[C@H]1[C@@H](O[C@H](COCC(=O)NCC(NC(=O)CO[C@@H]2O[C@H](CO)[C@H](O)[C@H](O)[C@H]2O)CNC(=O)COC[C@H]2O[	1002334-79-3	1H-1,2,4-Triazole-3-carboxamide, 1-[5-O-[2-[[2-[[2-(Œ≤-D-galactopyranosyloxy)ace	C29H47N7O20	813.72	A/B	-8.3
6766	O[C@H]1[C@@H](O[C@H](COC(=O)[C@H](CO[C@@H]2O[C@H](CO)[C@H](O)[C@H](O)[C@H]2O)C)[C@H]1O)n1nc(C(=O)N)n	1002334-94-2	1H-1,2,4-Triazole-3-carboxamide, 1-[5-O-[3-[[(2S)-2,6-bis[[3-(Œ≤-D-galactopyranc	C35H57N7O21	911.86	A/B	-8.3
6767	[Si](OC[C@H]1O[C@H]([C@H](O)[C@@H]1O)n1nc(C(=O)N)nc1)(C(C)(C)C)(c1ccccc1)c1ccccc1	1002335-16-1	1H-1,2,4-Triazole-3-carboxamide, 1-[5-O-[[3-[[2,3,4,6-tetrakis-O-(phenylmethyl)-	C46H52N4O13	868.92	A/B	-8.3
6768	O([Si](CC)(CC)CC)[C@H]1[C@@H](O[C@H](CN)[C@H]1O[Si](CC)(CC)CC)n1nc(C(=O)N)nc1	1002335-26-3	1H-1,2,4-Triazole-3-carboxamide, 1-[5-O-[3-[[2-(acetylamino)-2-deoxy-3,4,6-tris-	C42H49N5O13	831.86	A/B	-8.3
6769	[C@@H](Cc1ccccc1)(NC(=O)[C@H](CCN1CCOCC1)NC(=O)N(Cc1nc(C(C)C)sc1)C)CC[C@H](Cc1ccccc1)NC(=O)OCc1cncs1	1004316-87-3	2,7,10,12-Tetraazatridecanoic acid, 9-(2-aminoethyl)-12-methyl-13-[2-(1-methylet	C36H47N7O4S2	705.93	A/B	-8.3
6770	[C@H](Cc1ccccc1)(CC[C@H](Cc1ccccc1)NC(=O)OCc1cncs1)NC(=O)[C@@H](NC(=O)N(Cc1nc(C(C)C)sc1)C)COC(C)(C)C	1004318-18-6	2,7,10,12-Tetraazatridec-4-enoic acid, 12-methyl-9-(1-methylethyl)-13-[2-(1-meth	C37H46N6O4S2	702.93	A/B	-8.3
6771	[C@H](Cc1ncn(C)c1)(C(=O)N[C@@H](Cc1ccccc1)CC[C@H](Cc1ccccc1)NC(=O)OCc1cncs1)NC(=O)N(Cc1nc(C(C)C)sc1)	1004318-20-0	2,7,10,12-Tetraazatridecanoic acid, 9-(hydroxymethyl)-12-methyl-13-[2-(1-methyle	C35H44N6O5S2	692.89	A/B	-8.3
6772	C(Cc1ccccc1)(CCC(Cc1ccccc1)NC(=O)OCc1cncs1)NC(=O)C(NC(=O)N(Cc1cncs1)C)C(C)O	1004318-66-4	2,7,10,12-Tetraazatridecanoic acid, 9-(2-amino-2-oxoethyl)-12-methyl-13-[2-(1-me	C36H45N7O5S2	719.92	A/B	-8.3
6773	O[C@H]1[C@H](n2c3c(nc2)c(=O)nc(NCCSC)[nH]3)O[C@H](COP(=O)(O)O)[C@H]1O	1007552-45-5	5,Ä≤-Guanylic acid, N-(3-phenylpropyl)-	C19H24N5O8P	481.4	A/B	-8.3
6774	C(=O)([C@@H](NC(=O)OC)C(C)C)N1[C@@H](CCC1)c1[nH]c(cn1)c1ccc(cc1)c1ccc(cc1)c1[nH]c(nc1)[C@H]1N(C(=O)[	1007884-52-7	Carbamic acid, N-[(1S)-1-[[(2S)-2-[5-[4,Ä≤-[2-[(2S)-1-[(2S)-2-[(methoxycarbonyl)	C46H54N8O6	814.97	A/B	-8.3
6775	C[C@@]1(N(C(=O)[C@@H](NC(=O)OC)C(C)C)CCC1)c1[nH]c(cn1)c1ccc(cc1)c1ccc(cc1)c1[nH]c(nc1)[C@H]1N(C(=O)[	1009113-48-7	Carbamic acid, N-[(1S)-2-[(2S)-2-[5-[4,Ä≤-[2-[(2S)-1-[(2S)-2-[(methoxycarbonyl)a	C40H48N8O7	752.86	A/B	-8.3
6776	C(=O)([C@@H](Nc1cncnc1)C(C)C)N1[C@@H](CCC1)c1[nH]c(cn1)c1ccc(cc1)c1ccc(cc1)c1[nH]c(nc1)[C@H]1N(C(=O)	1009114-17-3	Carbamic acid, N-[(1S)-1-methyl-2-[(2S)-2-[5-[4,Ä≤-[2-[(2S)-1-[(2S)-3-methyl-1-c	C41H47N9O4	729.87	A/B	-8.3
6777	C(=O)([C@H](Cc1cnn[nH]1)NC(=O)OC)N1[C@@H](CCC1)c1[nH]c(cn1)c1ccc(cc1)c1ccc(cc1)c1[nH]c(nc1)[C@H]1N(C	1009114-20-8	Carbamic acid, N-[(1S)-2-methyl-1-[[(2S)-2-[5-[4,Ä≤-[2-[(2S)-1-[(2S)-3-methyl-1-	C42H50N10O4	758.91	A/B	-8.3
6778	C(=O)([C@H](Cc1cccnc1)NC(=O)OC)N1[C@@H](CCC1)c1[nH]c(cn1)c1ccc(cc1)c1ccc(cc1)c1[nH]c(nc1)[C@H]1N(C(=	1009114-72-0	Carbamic acid, N-[(1S)-2-[(2S)-2-[5-[4,Ä≤-[2-[(2S)-1-[(2S)-2-[(methoxycarbonyl)a	C43H49N9O6	787.91	A/B	-8.3
6779	C(=O)([C@@H](NC(=O)OC)C(C)C)N1[C@@H]([C@@H](O)CC1)c1[nH]c(cn1)c1ccc(cc1)c1ccc(cc1)c1[nH]c(nc1)[C@H]1	1009115-81-4	Carbamic acid, N-[(1S)-1-[[(2S)-2-[5-[4,Ä≤-[2-[(2S)-4,4-difluoro-1-[(2S)-2-[(met	C40H48F2N8O6	774.86	A/B	-8.3
6780	C(=O)([C@@H](N(C(=O)OC)C)C(C)C)N1[C@@H](CCC1)c1[nH]c(cn1)c1ccc(cc1)c1ccc(cc1)c1[nH]c(nc1)[C@H]1N(C(=	1009116-78-2	Carbamic acid, N,N,Ä≤-[[1,1,Ä≤-biphenyl]-4,4,Ä≤-diylbis[1H-imidazole-5,2-diyl-(	C38H46N8O6	710.82	A/B	-8.3
6781	C(=O)([C@@H](NC(=O)OC)[C@@H](C)O)N1[C@@H](CCC1)c1[nH]c(cn1)c1ccc(cc1)c1ccc(cc1)c1[nH]c(nc1)[C@H]1N(C	1009116-92-0	Carbamic acid, N,N,Ä≤-[[1,1,Ä≤-biphenyl]-4,4,Ä≤-diylbis[1H-imidazole-5,2-diyl-(	C40H50N8O8	770.87	A/B	-8.3
6782	C(=O)([C@H](NC(=O)OC)C)N1[C@@H](CCC1)c1[nH]c(cn1)c1ccc(cc1)c1ccc(cc1)c1[nH]c(nc1)[C@H]1N(C(=O)[C@H](	1009117-01-4	Carbamic acid, N,N,Ä≤-[[1,1,Ä≤-biphenyl]-4,4,Ä≤-diylbis[1H-imidazole-5,2-diyl-(	C36H42N8O6	682.77	A/B	-8.3
6783	C(=O)([C@H](N(CC)CC)c1ccccc1)N1[C@@H](CCC1)c1[nH]c(cn1)c1ccc(cc1)c1ccc(cc1)c1[nH]c(nc1)[C@H]1N(C(=O)	1009117-30-9	Carbamic acid, N,N,Ä≤-[[1,1,Ä≤-biphenyl]-4,4,Ä≤-diylbis[1H-imidazole-5,2-diyl-(	C40H46N8O6	734.84	A/B	-8.3
6784	C(=O)([C@H](N(CC)CC)c1ccccc1)N1[C@@H](CCC1)c1[nH]c(cn1)c1ccc(cc1)c1ccc(cc1)c1[nH]c(nc1)[C@H]1N(C(=O)	1009117-61-6	Carbamic acid, N-[(1S)-1-[[(2S)-2-[5-[4,Ä≤-[2-[(2S)-1-[(2R)-2-(diethylamino)-2-p	C45H54N8O4	770.96	A/B	-8.3
6785	C(=O)(C(NC(=O)OC)C(C)C)N1[C@H](CCC1)c1[nH]c(cn1)c1ccc(cc1)c1ccc(cc1)c1[nH]c(nc1)[C@H]1N(C(=O)C(NC(=O	1009119-60-1	Carbamic acid, N,N,Ä≤-[[1,1,Ä≤-biphenyl]-4,4,Ä≤-diylbis[1H-imidazole-5,2-diyl-(	C40H50N8O6	738.88	A/B	-8.3

6786	O=C1[C@@H]2[C@@H](c3c(C(O)[C@H]2CO1)cc1c(c3)OCO1)c1cc(OC)c(O)c(OC)c1	101440-03-3	2-Naphthalenesulfonic acid, 8-[2-(4-cyanophenyl)diazenyl]-5-hydroxy-	C17H11N3O4S	353.35	A/B	-8.3
6787	C(=O)(NC(CC1CCC1)C(=O)C(=O)N)[C@@H]1[C@@H]2[C@@H](C2(C)C)CN1C(=O)[C@@H](NC(=O)NC(C)(C)C)C(C)(C)C.C(C	1015080-45-1	Cyclopenta[c]pyrrole-1-carboxamide, (2S)-2-cyclohexyl-N-(2-pyrazinylcarbonyl)gly	C36H53N7O6.C22H23F4NO2		A/B	-8.3
6788	O(c1c(CCOC)cc(C#N)cc1C)c1nc(Nc2ccc(C#N)cc2)ncc1Br	1022904-35-3	Benzonitrile, 4-[[5-bromo-2-[(4-cyanophenyl)amino]-4-pyrimidinyl]amino]-3-methyl	C22H19BrN6	447.33	A/B	-8.3
6789	C(#CC1CC1)[C@@]1(C(F)(F)F)c2c(N(CCCC(=O)NCc3ccc(C(=O)ON4C(=O)CCC4=O)cc3)C(=O)O1)ccc(Cl)c2	1023756-54-8	Benzoic acid, 4-[[[4-[(4S)-6-chloro-4-(2-cyclopropylethynyl)-2-oxo-4-(trifluorom	C26H22ClF3N2O5	534.91	A/B	-8.3
6790	[C@@H](C(=O)N[C@H](C[C@@H]([C@H](Cc1ccccc1)NC(=O)COc1c(C)cccc1C)OCC(=O)NC(C)(C)C)Cc1ccccc1)(C(C)C)N1	1023756-58-2	Acetic acid, 2-[(1S,3S)-1-[(1S)-1-[[2-(2,6-dimethylphenoxy)acetyl]amino]-2-pheny	C40H52N4O7	700.86	A/B	-8.3
6791	O(C(=O)C)[C@H]1[C@@H](O[C@H](COC(=O)CCCCC)[C@H]1OC(=O)C)n1c2c(nc1)c(=O)nc(N)[nH]2	1024591-78-3	Guanosine, 2,Ä≤,3,Ä≤-diacetate 5,Ä≤-benzoate	C21H21N5O8	471.42	A/B	-8.3
6792	N(C(=O)Nc1cc(N(=O)=O)ccc1)c1nc(nc(NC(=S)NCCc2cccs2)n1)N1CCOCC1	1025364-96-8	Urea, N-[4-(4-morpholinyl)-6-[[[[2-(2-thienyl)ethyl]amino]thioxomethyl]amino]-1,	C21H23N9O4S2	529.6	A/B	-8.3
6793	N(C(=O)Nc1ccc(N(=O)=O)cc1)c1nc(nc(NC(=S)NCCc2cccs2)n1)N1CCOCC1	1025365-00-7	Urea, N-[4-(4-morpholinyl)-6-[[[[2-(2-thienyl)ethyl]amino]thioxomethyl]amino]-1,	C21H23N9O4S2	529.6	A/B	-8.3
6794	N(C(=O)Nc1cc(C)ccc1)c1nc(nc(NC(=S)NCCc2cccs2)n1)N1CCOCC1	1025365-17-6	Urea, N-(2-methylphenyl)-N,Ä≤-[4-(4-morpholinyl)-6-[[[[2-(2-thienyl)ethyl]amino]	C22H26N8O2S2	498.62	A/B	-8.3
6795	[Si](OC[C@H]1O[C@H](CS1)n1c(=O)nc(NCCCCOC(=O)Cl)cc1)(C(C)(C)C)(c1ccccc1)c1ccccc1	1026080-51-2	Methanone, [4-[(3S)-4-[(1R)-2-methoxy-1-[4-(trifluoromethyl)phenyl]ethyl]-3-meth	C27H36F3N5O2	519.6	A/B	-8.3
6796	[C@H](C(=O)N/C(=C/CS(=O)(=O)c1ccccc1)/CCc1ccccc1)(NC(=O)N1CCN(C)CC1)CC(C)C	1026901-43-8	Not Yet Assigned	C20H22N5O9PS	539.46	A/B	-8.3
6797	N(CCCNCCC(C)(C)C)c1c2c(ncc1)cc(Cl)cc2	1027739-58-7	Carbamic acid, N-[[3-[4-[(2-ethyl-5,7-dimethyl-3H-imidazo[4,5-b]pyridin-3-yl)met	C33H42N4O4S2	622.84	A/B	-8.3
6798	C(=CC(=O)CC(=O)C)c1cc(O)c(O)cc1	1032827-67-0	5,Ä≤-Guanylic acid, N-(1-oxo-3-phenyl-2-propen-1-yl)-	C19H20N5O9P	493.36	A/B	-8.3
6799	C(C[C@H](NC(=O)C1CC(C(F)(F)C(Cl)([2H])[2H])([2H])[2H])c1ccccc1)N1[C@@H]2C[C@@H](C[C@H]1CC2)n1c(C(C)C	1033698-66-6	Benzamide, N-[(1S)-3-[(3-exo)-3-[3-(methyl-d3)-5-(1-methylethyl)-4H-1,2,4-triazo	C29H32D5N5O		A/B	-8.3
6800	N(c1c(C)cc(/C=C/C#N)cc1C)c1nc(Nc2ccc(C#N)cc2)nc(COP(=O)(OCC)OCC)c1	1037445-36-5	Benzonitrile, 4-[[4-[[4-[(1E)-2-cyanoethenyl]-2,6-dimethylphenyl]amino]-6-[[(2-t	C28H25N7S	491.61	A/B	-8.3
6801	N(c1c(c(COC)nc(Nc2ccc(C#N)cc2)n1)c1cccnc1)c1c(C)cc(/C=C/C#N)cc1C	1037451-89-0	Benzonitrile, 4-[[4-[[4-[(1E)-2-cyanoethenyl]-2,6-dimethylphenyl]amino]-6-(metho	C30H27N7O2	517.58	A/B	-8.3
6802	C(=C(C#N)C)c1c(Nc2c(C)cc(/C=C/C#N)cc2C)nc(Nc2ccc(C#N)cc2)nc1COC	1037452-79-1	2-Propenamide, 3-[4-[[4-[[4-[(1E)-2-cyanoethenyl]-2,6-dimethylphenyl]amino]-2-[(4-cy	C27H25N7O2	479.53	A/B	-8.3
6803	C(=C\c1ccc(C(F)(F)F)cc1)/c1c(Nc2c(C)cc(/C=C/C#N)cc2C)nc(Nc2ccc(C#N)cc2)nc1COC	1037452-81-5	Benzonitrile, 4-[[4-[[4-[(1E)-2-cyanoethenyl]-2,6-dimethylphenyl]amino]-5-(2-cya	C28H25N7O	475.54	A/B	-8.3
6804	O=c1n2c(-c3c(C2)cc2c(n3)ccc(OP(=O)(C(=O)O)O)c2)cc2c1COC(=O)[C@@]2(CC)O	1038983-64-0	Phosphinecarboxylic acid, 1-[2-[(2S,4S)-4-[(3-amino-2,3,6-trideoxy-Œ±-L-lyxo-hex	C28H30NO15P	651.51	A/B	-8.3
6805	N(S(=O)(=O)CC[Si](C)(C)C)(C(=O)C)[C@@H]1[C@H](CC(=C[C@H]1OC(CC)CC)C(=O)OCC)N1C(=O)c2c(C1=O)cccc2	1041187-72-7	Hexanoic acid, 6-[[5-[(3aS,4S,6aR)-hexahydro-2-oxo-1H-thieno[3,4-d]imidazol-4-yl	C38H47N3O11S	753.86	A/B	-8.3
6806	C(NC(=O)Oc1cc([C@@H](N(C)C)C)ccc1)(C)C12CC3CC(C2)CC(C1)C3	1046462-11-6	Guanosine, 1-[(11-cyclopropyl-6,11-dihydro-6-oxo-5H-dipyrido[3,2-b:2,Ä≤,3,Ä≤-e]	C25H25N9O5	531.52	A/B	-8.3
6807	[C@@H](Cc1ccccc1)(NC(=O)[C@@H](NC(=O)N(Cc1nc(C(C)C)sc1)C)CC(=O)NN1CCCC1)CC[C@H](Cc1ccccc1)NC(=O)OCc1	1051461-68-7	2,7,10,12-Tetraazatridecanoic acid, 12-methyl-13-[2-(1-methylethyl)-4-thiazolyl]	C41H48N8O5S2	797	A/B	-8.3
6808	C(Cc1ccccc1)(NC(=O)C(CCn1ccnc1)NC(=O)N(Cc1nc(C(C)C)sc1)C)CCC(Cc1ccccc1)NC(=O)OCc1cncs1	1051464-42-6	2,7,10,12-Tetraazatridecanoic acid, 12-methyl-13-[2-(1-methylethyl)-4-thiazolyl]	C40H49N7O4S2	755.99	A/B	-8.3
6809	C[C@]12[C@H]([C@H]3[C@@H]([C@]4(C)[C@H](CC3)C[C@H](OC=O)CC4)CC2)CC[C@@H]1[C@@H](CCC(=O)OC)C	105275-93-2	Cytidine, guanylyl-(3,Ä≤,Ü5,Ä≤)-2,Ä≤-deoxy-	C19H25N8O11P	572.42	A/B	-8.3
6810	S(=O)(=O)(O)c1c2c(c(S(=O)(=O)O)cc(/N=N/c3cc(S(=O)(=O)O)c(C=Cc4c(S(=O)(=O)O)cc(/N=N/c5c(N)c6c(c(S(=O)	1053055-78-9	Not Yet Assigned	C34H28N6O18S6	1001.01	A/B	-8.3
6811	O(C(=O)NC(Cc1ccccc1)C(CN(S(=O)(=O)c1ccc(OC)cc1)CC(CCCCBr)(C)C)O)C1C2C(OC1)OCC2	1057218-31-1	Phosphonic acid, P-[[[4-[[6-amino-5-bromo-2-[(4-cyanophenyl)amino]-4-pyrimidinyl	C34H32BrN6O4P	699.53	A/B	-8.3
6812	N(C(=O)C1Oc2c(N(C(=O)C)C1)cccc2)C12CC3CC(C2)CC(C1)C3	1062227-15-9	Benzeneacetic acid, 3,4-dimethoxy-, (5R,5aR,8aR,9R)-5,5a,6,8,8a,9-hexahydro-8-ox	C32H32O11	592.59	A/B	-8.3
6813	C[C@]1(F)[C@@H](O[C@H](COP(=O)(Oc2ccccc2)N[C@H](C(=O)OC(C)C)C)[C@H]1O)n1c(=O)[nH]c(=O)cc1	1064684-43-0	L-Alanine, N-[(2,Ä≤R)-2,Ä≤-deoxy-2,Ä≤-fluoro-2,Ä≤-methyl-P-phenyl-5,Ä≤-uridylyl]	C23H31FN3O9P	543.48	A/B	-8.3
6814	C[C@]1(F)[C@@H](O[C@H](COP(=O)(Oc2ccc(F)cc2)N[C@H](C(=O)OCCCC)C)[C@H]1O)n1c(=O)[nH]c(=O)cc1	1064684-53-2	L-Alanine, N-[(2,Ä≤R)-2,Ä≤-deoxy-2,Ä≤-fluoro-2,Ä≤-methyl-P-(4-methylphenyl)-5,Ä	C23H31FN3O9P	543.48	A/B	-8.3
6815	C[C@]1(F)[C@@H](O[C@H](COP(=O)(N[C@H](C(=O)OC2CCCC2)CC)Oc2ccccc2)[C@H]1O)n1c(=O)[nH]c(=O)cc1	1064684-88-3	L-Alanine, N-[(2,Ä≤R)-2,Ä≤-deoxy-2,Ä≤-fluoro-2,Ä≤-methyl-P-phenyl-5,Ä≤-uridylyl]	C23H29FN3O9P	541.46	A/B	-8.3
6816	C(NC(=O)c1nnc(C)[nH]1)(C)(C)c1nc(C(=O)NCc2ccc(F)cc2)c(O)c(=O)n1C	1064707-05-6	4-Pyrimidinecarboxamide, N-[(4-fluorophenyl)methyl]-1,6-dihydro-5-hydroxy-1-meth	C20H20FN5O5	429.4	A/B	-8.3
6817	N(C(CCCN(CC)CC)C)c1c2c(nc3c1cccc3)cc(Cl)c(N(=O)=O)c2	1071752-50-5	1-Naphthalenesulfonic acid, 4-[(1E)-2-(2-hydroxy-1-naphthalenyl)diazenyl]-	C20H14N2O4S	378.4	A/B	-8.3
6818	Clc1n(c2c(n1)c(=O)nc(N)[nH]2)[C@@H]1O[C@H](COP(=O)(OP(=O)(OP(=O)(O)O)O)O)[C@@H](O)[C@H]1O	1071796-43-4	Guanosine 5,Ä≤-(tetrahydrogen triphosphate), 8-iodo-	C10H15IN5O14P3	649.08	A/B	-8.3
6819	O(C[C@H]1O[C@H](C=C1)n1c(=O)[nH]c(=O)c(C)c1)P1(=O)Oc2c(cc(CCCCC(=O)OCc3ccccc3)cc2)CO1	1072329-81-7	Thymidine, 5,Ä≤-O-[6-(4-carboxybutyl)-2-oxido-4H-1,3,2-benzodioxaphosphorin-2-y	C22H25N2O9P	492.42	A/B	-8.3
6820	[C@@H](NC(=O)N1CCN(C)CC1)(C(=O)N[C@H](C=CS(=O)(=O)c1ccccc1)CCc1ccccc1)CCCNC(=O)N	1076088-79-3	1-Piperazinecarboxamide, N-[(1S)-3-[(aminocarbonyl)amino]-1-[[[(1S)-1-(2-phenyle	C28H38N6O5S	570.7	A/B	-8.3
6821	C(=O)([C@H](Cc1cnc[nH]1)NC(=O)[C@@H](NC(=O)[C@H](Cc1ccc(O)cc1)NC(=O)[C@@H](NC(=O)OCc1ccccc1)C(C)C)[C	108039-30-1	Alanine, [1-[N-[N-[N-(N-carboxy-L-valyl)-L-tyrosyl]-L-leucyl]-L-histidyl]-L-prol	C49H62N8O10	923.06	A/B	-8.3
6822	N(C(=O)OC(OC(=O)c1ccccc1)C(=O)O[Si](C)(C)C)(C(CCCN(CC)CC)C)c1c2c(ncc1)cc(Cl)cc2	1084710-25-7	Acetic acid, 2-(benzoylthio)-2-[[[(7-chloro-4-quinolinyl)[4-(diethylamino)-1-met	C35H38ClN3O5S	648.21	A/B	-8.3
6823	N(=C1c2c(N(CN3CCOCC3)C1=O)ccc(OC(F)(F)F)c2)NC(=S)NC	1084893-28-6	Hydrazinecarbothioamide, 2-[1,2-dihydro-1-methyl-2-oxo-5-(trifluoromethoxy)-3H-i	C17H12F3N5O4S	439.37	A/B	-8.3
6824	C[C@]12[C@H]3[C@@H]([C@]4(C)[C@@H](CC3)C(C)(C)[C@H](O)CC4)CC[C@]1(C)[C@@H]([C@H](C)[C@H]1OC(=O)[C@@H	1084893-42-4	Hydrazinecarbothioamide, 2-[1,2-dihydro-1-(4-morpholinylmethyl)-2-oxo-5-(trifluo	C21H19F3N6O5S	524.47	A/B	-8.3
6825	C(=O)([C@@H](NC(=O)OC)C(C)C)N1[C@@H](CCC1)c1[nH]c(cn1)c1ccc(cc1)c1ccc(cc1)c1[nH]c([C@H](NC(=O)[C@@H]	1087346-62-0	Carbamic acid, N-[(1S)-2-[(2S)-2-[5-[4,Ä≤-[2-[(1R)-1-[[(2S)-2-[(methoxycarbonyl)	C36H44N8O6	684.78	A/B	-8.3
6826	O=c1n([C@@H]2O[C@H](COP(=O)(OP(=O)(OP(=O)(O)O)O)O)[C@H](N=[N+]=[N-])C2)cc(C)c(=O)[nH]1	108988-58-5	1,3-Naphthalenedisulfonic acid, 8,8,Ä≤-[thiobis[(4-methyl-o-phenylene)azo]]bis[	C34H28N6O12S5.4Na		A/B	-8.3
6827	N(Cc1ccc(cc1)c1ccccn1)(C[C@@H]([C@H](Cc1ccccc1)NC(=O)[C@@H](NC(=O)OC([2H])([2H])[2H])C(C)(C)C)O)NC(=	109253-07-8	2-Naphthalenesulfonic acid, 8-[2-(3-acetylphenyl)diazenyl]-5-hydroxy-	C18H14N2O5S	370.38	A/B	-8.3
6828	O=c1n([C@@H]2O[C@H](COC(=O)CC[C@H](NC(=O)CCNC(=O)[C@H](COC(=O)CCCCCCCCCCCCC)NC(=O)C)C(=O)NCC(=O)O)[C	1099829-63-6	L-Glutamic acid, N-[4-(3,Ä≤-deoxy-3,Ä≤-fluorothymidin-5,Ä≤-O-yl)-1,4-dioxobutyl	C37H44FN11O15S		A/B	-8.3
6829	C(c1cc2c(OP(OC[C@H]3O[C@H](C=C3)n3c(=O)[nH]c(=O)c(C)c3)OC2)cc1)c1cc(CO)c(O)cc1	1099829-79-4	Not Yet Assigned	C23H24N3O7PS	517.49	A/B	-8.3
6830	O=c1n([C@@H]2O[C@H](COC(=O)c3ccc(C)cc3)[C@@H](OC(=O)CCCCC)C2)cc(C(F)(F)F)c(=O)[nH]1	110073-57-9	Thymidine, Œ±,Œ±,Œ±-trifluoro-, 5,Ä≤-hexanoate 3,Ä≤-(4-methylbenzoate)	C24H27F3N2O7	512.48	A/B	-8.3
6831	C(NC(=O)c1nnc(C)o1)(C([2H])([2H])[2H])(C([2H])([2H])[2H])c1nc(C(=O)NC(c2ccc(F)cc2)([2H])[2H])c(O)c(=	1100750-92-2	4-Pyrimidinecarboxamide, N-[(4-fluorophenyl)methyl-d2]-1,6-dihydro-5-hydroxy-1-(	C20H13D8FN6O5		A/B	-8.3
6832	O=c1n(cc(C(F)(F)F)c(=O)[nH]1)[C@@H]1O[C@H](COC(OCc2ccccc2)C(C)(C)C)[C@@H](O)C1	110604-83-6	Thymidine, Œ±,Œ±,Œ±-trifluoro-3,Ä≤-O-[2-methyl-1-(phenylmethoxy)propyl]-	C21H25F3N2O6	458.43	A/B	-8.3
6833	O[C@H]1[C@H](n2c3c(nc2)c(=O)nc[nH]3)O[C@H](CO)[C@H]1OP(=O)(OC)O	110604-87-0	Thymidine, Œ±,Œ±,Œ±-trifluoro-5,Ä≤-O-[1-(1-phenylethoxy)ethyl]-	C20H23F3N2O6	444.4	A/B	-8.3
6834	C(#Cc1ccc(C(F)(F)F)cc1)c1n(nc(C(=O)N)n1)[C@@H]1O[C@H](CO)[C@@H](O)[C@H]1O	1106736-01-9	1H-1,2,4-Triazole-3-carboxamide, 5-[2-(4-methoxyphenyl)ethynyl]-1-Œ≤-D-ribofura	C17H18N4O6	374.35	A/B	-8.3
6835	O[C@@H]1[C@H](n2c3c(nc2)c(N)ncn3)O[C@H](COC(=O)[C@@H](C)N2C(=O)c3c(C2=O)cccc3)[C@H]1O	111392-78-0	9H-Purin-6-amine, 9-[5-O-[[2-(6-amino-9H-purin-9-yl)-2-(2-hydroxyethoxy)ethoxy]h	C19H25N10O9P	568.44	A/B	-8.3
6836	O=c1n([C@@H]2O[C@H](COP(=O)(OP(=O)(OC[C@H]3O[C@H](CO)[C@@H](O)[C@H](O)[C@H]3O)O)O)[C@@H](O)C2)cc(C)c	1114549-13-1	Guanosine 5,Ä≤-(trihydrogen diphosphate), 2,Ä≤-deoxy-, P,Ä≤,Ü7-ester with 2,6-	C17H27N5O15P2	603.37	A/B	-8.3
6837	[C@@H](C(CC)CC)(NC(=O)C)[C@H]1[C@H](NC(=N)N)C[C@H](C(=O)O)[C@H]1O.O	111590-59-1	6H-Purin-6-one, 2-amino-1,9-dihydro-9-[3-hydroxy-4-[[(4-methoxyphenyl)diphenylme	C31H31N5O4	537.61	A/B	-8.3
6838	C(N1c2c(/C(=N\N(C(=S)NC)C)/C1=O)cccc2)c1c(Cl)cccc1Cl	112246-55-6	Furo[3,Ä≤,4,Ä≤:6,7]naphtho[2,3-d]-1,3-dioxol-6(5aH)-one, 5,8,8a,9-tetrahydro-9-	C21H20O8	400.38	A/B	-8.3
6839	C(#Cc1c(C(F)(F)F)cccc1)c1n(nc(C(=O)N)n1)[C@@H]1O[C@H](CO)[C@@H](O)[C@H]1O	1123187-66-5	1H-1,2,4-Triazole-3-carboxamide, 5-[2-(4-chlorophenyl)ethynyl]-1-Œ≤-D-ribofuran	C16H15ClN4O5	378.77	A/B	-8.3
6840	C(#Cc1cn(C)cn1)c1n(nc(C(=O)N)n1)[C@@H]1O[C@H](CO)[C@@H](O)[C@H]1O	1123187-68-7	1H-1,2,4-Triazole-3-carboxamide, 5-[2-(1-pyrenyl)ethynyl]-1-Œ≤-D-ribofuranosyl-	C26H20N4O5	468.46	A/B	-8.3
6841	C(C(c1ccccc1)([2H])[2H])([C@]1(CCC)CC(=C([C@H](CC)c2cc(NS(=O)(=O)c3ccc(C(F)(F)F)cn3)ccc2)C(=O)O1)O)(	1126619-17-7	Not Yet Assigned	C31H28D5F3N2O5S		A/B	-8.3
6842	C(Cc1ccccc1)[C@]1(CCC)CC(=C([C@H](CC)c2cc(NS(=O)(=O)c3c(c(c(C(F)(F)F)c(n3)[2H])[2H])[2H])ccc2)C(=O)O	1126619-25-7	Not Yet Assigned	C31H29D4F3N2O5S		A/B	-8.3
6843	C(C([C@](NC(=O)C1CCC(F)(F)CC1)(c1ccccc1)[2H])([2H])[2H])(N1[C@H]2C[C@H](C[C@@H]1CC2)n1c(C(C)C)nnc1C)	1126811-23-1	Cyclohexane-1,2,2,3,3,5,5,6,6-d9-carboxamide-d, 4,4-difluoro-N-[(1S)-3-[(3-exo)-	C29D41F2N5O		A/B	-8.3
6844	N(=Nc1c(O)cc(N=Nc2cc(S(=O)(=O)O)ccc2)c(C)c1)c1c2c(ccc1O)cccc2	112685-63-9	Benzenesulfonic acid, 4-[2-[5-hydroxy-4-[2-(2-hydroxy-1-naphthalenyl)diazenyl]-2	C23H18N4O5S	462.48	A/B	-8.3
6845	C(=O)(CNC(=O)OC)N1CCC2(C[C@H]1c1[nH]c(cn1)c1ccc(cc1)c1ccc(cc1)c1[nH]c(nc1)[C@H]1N(C(=O)CNC(=O)OC)CC3(	1129634-51-0	Carbamic acid, N,N,Ä≤-[[1,1,Ä≤-biphenyl]-4,4,Ä≤-diylbis[1H-imidazole-5,2-diyl-(	C44H54N8O6	790.95	A/B	-8.3
6846	C(=O)([C@@H](NC(=O)OC)C(C)C)N1[C@@H](CCC1)c1[nH]c(C)n1)c1ccc(cc1)c1ccc(cc1)c1[nH]c(nc1)[C@H]1N(C(=	1129636-14-1	Carbamic acid, N-[(1S)-2-[(2S)-2-[5-[4,Ä≤-[2-[(2S)-1-[(2S)-2-[(methoxycarbonyl)a	C37H44N8O6	696.8	A/B	-8.3
6847	C(c1c([nH]c(n1)[C@H]1N(C(=O)[C@@H](NC(=O)OC)C(C)C)CCC1)c1ccc(cc1)c1ccc(cc1)c1[nH]c(nc1)[C@H]1N(C(=O)	1129636-29-8	Carbamic acid, N,N,Ä≤-[[1,1,Ä≤-biphenyl]-4,4,Ä≤-diylbis[(4-methyl-1H-imidazole-	C42H54N8O6	766.93	A/B	-8.3
6848	C(#CC1(C(C1([2H])[2H])([2H])[2H])[2H])[C@@]1(C(F)(F)F)c2c(N(C(=O)O1)[2H])c(c(c(Cl)c2[2H])[2H])[2H]	1132640-84-6	L-Valine, N-[(9H-fluoren-9-ylmethoxy)carbonyl]-, 2-[(2-amino-1,6-dihydro-6-oxo-9	C28H30N6O6	546.57	A/B	-8.3
6849	O=C1c2c(N(c3c(N1)c(C)ccn3)C1CC1)ncc(CCCBr)c2	1139875-25-4	6H-Dipyrido[3,2-b:2,Ä≤,3,Ä≤-e][1,4]diazepin-6-one, 11-cyclopropyl-5,11-dihydro-	C18H20N4O2	324.38	A/B	-8.3
6850	[Si](OC[C@H]1O[C@H](CS1)n1c(=O)nc(NCCCN)cc1)(C(C)(C)C)(c1ccccc1)c1ccccc1	1139875-90-3	6H-Dipyrido[3,2-b:2,Ä≤,3,Ä≤-e][1,4]diazepin-6-one, 11-cyclopropyl-5,11-dihydro-	C18H18N4O	306.36	A/B	-8.3
6851	O=c1n(cc(CCC)c(=O)[nH]1)[C@H]1C[C@H](N=[N+]=[N-])[C@@H](CO)O1	114007-98-6	Furo[3,Ä≤,4,Ä≤:6,7]naphtho[2,3-d]-1,3-dioxol-6(5aH)-one, 5-[4-(acetyloxy)-3,5-d	C23H22O9	442.42	A/B	-8.3
6852	O=c1n([C@@H]2O[C@H](COP(=O)(O)O)[C@@H](O)C2)cc(F)c(=NO)[nH]1	114300-71-9	Guanosine, 2,Ä≤-deoxy-N-phenyl-	C16H17N5O4	343.34	A/B	-8.3
6853	C(N1c2c(C(=NNC(=S)Nc3ccc(OC)cc3)C1=O)cc(Cl)cc2)c1ccccc1	1161792-76-2	Hydrazinecarbothioamide, 2-[1,2-dihydro-5-methyl-2-oxo-1-(phenylmethyl)-3H-indol	C24H22N4OS	414.52	A/B	-8.3
6854	O=c1n([C@@H]2O[C@H](COP(=O)(Oc3ccc(Br)cc3)Oc3cccnc3)[C@@H](N=[N+]=[N-])C2)cc(C)c(=O)[nH]1	1162085-51-9	5,Ä≤-Thymidylic acid, 3,Ä≤-azido-3,Ä≤-deoxy-, 4-chlorophenyl 3-pyridinyl ester	C21H20ClN6O7P		A/B	-8.3
6855	N(CCCCNC(=O)CCC)c1c2c(cc(Cl)cc2)ncc1	117124-51-3	Carbonic acid, 4-(5,5a,6,8,8a,9-hexahydro-9-hydroxy-10-methoxy-6-oxofuro[3,Ä≤,4,	C30H28O11	564.54	A/B	-8.3
6856	N(CCCCNC(=O)C(C)C)c1c2c(ncc1)cc(Cl)cc2	1172131-68-8	4-Pyrimidinecarboxamide, 5-(benzoyloxy)-N-[(4-fluorophenyl)methyl]-1,6-dihydro-1	C27H25FN6O6	548.52	A/B	-8.3
6857	O(CC(F)(F)F)c1nc(nc(NCc2ccc(C)o2)n1)N1CCOCC1	1174117-08-8	Phenol, 4-[[4-[(2-furanylmethyl)amino]-6-(4-morpholinyl)-1,3,5-triazin-2-yl]amir	C18H20N6O3	368.39	A/B	-8.3
6858	O=c1c2c(n(cc1C(=O)O)C1CC1)cc(c(F)c2)N1CCN(Cc2cn(c(=O)[nH]c2=O)[C@H]2C[C@H](N=[N+]=[N-])[C@@H](CO)O2)	1174398-28-7	3-Quinolinecarboxylic acid, 7-[4-[[1-(3-azido-2,3-dideoxy-Œ≤-D-erythro-pentofura	C26H29FN8O7		A/B	-8.3
6859	O(C)c1c2n(cc(C(=O)O)c(=O)c2cc(F)c1N1CC(C)N(Cc2cn(c(=O)[nH]c2=O)[C@H]2C[C@H](N=[N+]=[N-])[C@@H](CO)O2	1174398-30-1	3-Quinolinecarboxylic acid, 7-[4-[[1-(3-azido-2,3-dideoxy-Œ≤-D-erythro-pentofura	C27H30F2N8O7		A/B	-8.3
6860	C([C@H](OC[P@@]1(=O)O[C@@H](CCO1)c1cc(Cl)ccc1)C)n1c2c(nc1)c(N)ncn2	1174716-08-5	9H-Purin-6-amine, 9-[(2R)-2-[[(2R,4S)-4-(3-chlorophenyl)-2-oxido-1,3,2-dioxaphos	C18H21ClN5O4P	437.82	A/B	-8.3
6861	C(C)(N)C12CC3C(O)C(C1)CC(C2)C3	1178091-85-4	1-Naphthalenesulfonic acid, 2,2,Ä≤-[[1,1,Ä≤-biphenyl]-4,4,Ä≤-diylbis[(1E)-2,1-d	C32H24N6O6S2	652.7	A/B	-8.3
6862	O=C1[C@H]2[C@@H](c3c([C@H](O)[C@H]2CO1)cc1c(c3)OCO1)c1ccc(OC)cc1	1179324-24-3	Furo[3,Ä≤,4,Ä≤:6,7]naphtho[2,3-d]-1,3-dioxol-6(5aH)-one, 5,8,8a,9-tetrahydro-9-	C20H18O6	354.35	A/B	-8.3
6863	C(=O)([C@@H](NC(=O)OC)C(C)C)N1[C@H](c2[nH]c3-c4c(cc(cc4)c4cc5c(-c6c(CCC5)nc([nH]6)[C@H]5N(C(=O)[C@@H	1180669-82-2	Carbamic acid, N-[(1S)-2-methyl-1-[[(2S)-2-[3,4,5,6-tetrahydro-8-[4-[2-[(2S)-1-[	C43H55N9O6	793.95	A/B	-8.3
6864	C(=O)([C@@H](NC(=O)OC)C(C)C)N1[C@H]2[C@H](C2)C[C@H]1c1[nH]c2-c3c(CCc2n1)cc(cc3)c1cc2c(-c3c(CC2)nc([n	1180670-03-4	Carbamic acid, N,N,Ä≤-[(4,4,Ä≤,5,5,Ä≤-tetrahydro[7,7,Ä≤-bi-1H-naphth[1,2-d]imic	C44H54N8O6	790.95	A/B	-8.3
6865	C(=O)([C@@H](NC(=O)OC)C(C)C)N1[C@H]2[C@H](C2)C[C@H]1c1[nH]c2-c3c(CCc2n1)cc(cc3)c1cc2c(cc1)c1c(cc2)nc	1180670-19-2	Carbamic acid, N,N,Ä≤-[[7,7,Ä≤-bi-1H-naphth[1,2-d]imidazole]-2,2,Ä≤-diylbis[(2S	C44H50N8O6	786.92	A/B	-8.3
6866	[Si](O[C@@H]1[C@@H](CO[Si](C(C)(C)C)(c2ccccc2)c2ccccc2)O[C@H](C1)n1c(=O)[nH]c(=O)c(C)c1)(C(C)(C)C)(c	1180670-57-8	Carbamic acid, N-[(1S)-2-[(1R,3S,5R)-3-[7-[3-fluoro-4-[2-[(1R,3S,5R)-2-[(2S)-2-[	C46H51FN8O7	846.94	A/B	-8.3
6867	N(CCN1CCOCC1)c1nc(nc(NCCCNc2c3c(ncc2)cc(Cl)cc3)n1)N1CCOCC1	1190366-32-5	1,3-Propanediamine, N1-(7-chloro-4-quinolinyl)-N3-[4-(4-methyl-1-piperazinyl)-6-	C24H32ClN9O	498.02	A/B	-8.3
6868	C(OCCOC(C)C)n1c2c(nc1)c(=O)nc(N)[nH]2	1190884-56-0	4-Pyrimidinecarboxamide, N-[(4-fluorophenyl)methyl]-1,4,5,6-tetrahydro-1-(methyl	C20H18D3FN6O5		A/B	-8.3
6869	[C@@H](C(OC([2H])([2H])[2H])([2H])[2H])(N1[C@@H](C)CN(CC1)C1(C)CCN(C(=O)c2c(C)ncnc2C)CC1)c1ccc(C(F)(	1191293-09-0	Methanone, (4,6-dimethyl-5-pyrimidinyl)[4-[(3S)-4-[(1R)-2-(methoxy-d3)-1-[4-(tri	C28H32D6F3N5O2		A/B	-8.3
6870	C(NC(=O)C(=O)OCC)(C)(C)c1nc(C(=O)NCc2ccc(F)cc2)c(O)c(=O)n1C	1193687-87-4	4-Pyrimidinecarboxamide, 1,6-dihydro-5-hydroxy-1-methyl-2-[1-methyl-1-[[(5-methy	C20H22N6O5	426.43	A/B	-8.3
6871	C[C@]1(O)[C@H](n2c3c(nc2)c(=O)nc(N)[nH]3)O[C@H](COP(=O)(OCCSC(=O)C(CO)(C)C)O)[C@H]1O	1202498-76-7	Guanosine, 2,Ä≤-C-methyl-, 5,Ä≤-[P,Ä≤,P,Ä≤,Ä≤,P,Ä≤,Ä≤-trihydrogen N-(phenylmet	C18H25N6O13P3	626.34	A/B	-8.3
6872	O(COC(=O)OCCOC)c1c2n(cc(C(=O)NCc3c(F)cc(F)cc3)c1=O)C[C@H]1N(C2=O)[C@H](C)CCO1	1206101-98-5	Carbonic acid, [[(4R,12aS)-9-[[[(2,4-difluorophenyl)methyl]amino]carbonyl]-3,4,6	C23H23F2N3O8	507.44	A/B	-8.3
6873	O[C@H]1[C@@H](O[C@H](COS(=O)(=O)N)[C@H]1O)n1nc(C(=O)N)nc1	1206102-03-5	Carbonic acid, [[(4R,12aS)-9-[[[(2,4-difluorophenyl)methyl]amino]carbonyl]-3,4,6	C28H32F2N4O9	606.57	A/B	-8.3
6874	C(=O)(NC(CC1CCC1)C(C(=O)N)O)[C@@H]1[C@@H]2[C@@H](C2(C)C)CN1C(=O)[C@H](CNC(=O)OC(C)(C)C)NC(=O)NC(C)(C	1208118-97-1	Cyclopenta[c]pyrrole-1-carboxamide, 2-[(2S)-2-[[(2S)-2-cyclohexyl-2-[(2-pyraziny	C39H55N7O7	733.9	A/B	-8.3
6875	C([C@H](OCP(=O)(O)O)C)n1c2c(nc1)c(N)ncn2.N(C1CCCCC1)C1CCCCC1	1215020-87-3	Uridine, 5,Ä≤-O-[bis(4-methoxyphenyl)phenylmethyl]-2,Ä≤-deoxy-5-[(1E)-2-(9H-flu	C54H57N4O8P	921.03	A/B	-8.3
6876	C=C1C(n2c3c(nc2)c(=O)nc(N)[nH]3)C[C@H](O)[C@H]1CO	1217513-42-2	Not Yet Assigned	C29H35D6F2N5O2		A/B	-8.3
6877	C(#N)C1=C(N)Oc2c(C1c1cn(c(=O)[nH]c1=O)[C@@H]1O[C@H](CO)[C@@H](O)C1)c(=O)n(C)c(C)c2	1217597-34-6	Uridine, 5-(2-amino-3-cyano-7-methyl-5-oxo-4H,5H-pyrano[4,3-b]pyran-4-yl)-2,Ä≤-(	C23H22N4O10	514.44	A/B	-8.3
6878	C(N1c2c(C(=NNC(=S)N)C1=O)ccc(Br)c2)N1CCN(CC1)c1c2c(cc(C(F)(F)F)cc2)ncc1	1218813-20-7	Hydrazinecarbothioamide, 2-[6-chloro-1,2-dihydro-2-oxo-1-[[4-[7-(trifluoromethyl	C24H21ClF3N7OS	547.98	A/B	-8.3
6879	O=C1[C@@H]2[C@@H](c3c([C@H](OC(=O)CCC(=O)NCCc4ccc(OC)cc4)[C@H]2CO1)cc1c(c3)OCO1)c1cc(OC)c(OC)c(OC)c1	1222083-93-3	Butanoic acid, 4-oxo-4-[[(2R)-2-phenylpropyl]amino]-, (5R,5aR,8aR,9R)-5,5a,6,8,8	C35H37NO10	631.67	A/B	-8.3
6880	C(#CC1CC1)C1(C(C)(C)C)c2c(NC(=O)O1)ccc(Cl)c2	1222400-41-0	Not Yet Assigned	C32H48N6O4	580.76	A/B	-8.3
6881	[C@H]([C@@H](CO)O)(O)[C@H]1[C@H](NC=O)[C@@H](NC(=N)N)C=C(C(=O)O)O1	1222518-93-5	Not Yet Assigned	C35H45FN6O4	632.77	A/B	-8.3
6882	C(#N)C1=C(N)Oc2c(C1c1cc(C(F)(F)F)ccc1)ccn1c2ncc1	1225373-01-2	7H-Imidazo[1,2-a]pyrano[2,3-c]pyridine-8-carbonitrile, 9-amino-2-methyl-7-(3,4,5	C21H20N4O4	392.41	A/B	-8.3
6883	C(#N)C1=C(N)Oc2c(C1c1cc(CC=C)c(O)c(OC)c1)ccn1c2nc(C)c1	1225373-15-8	7H-Imidazo[1,2-a]pyrano[2,3-c]pyridine-8-carbonitrile, 9-amino-7-[4-hydroxy-3-me	C21H18N4O3	374.39	A/B	-8.3
6884	C(=O)([C@H](NC(=O)OC(C)(C)C)C(C)C)N1[C@@H](CCC1)c1[nH]c(cn1)[C@@H]1CC[C@@H](CC1)c1ccc(cc1)c1[nH]c(nc1	1228662-82-5	Cholan-24-oic acid, 3-[(3,Ä≤-azido-3,Ä≤-deoxy-5,Ä≤-thymidylyl)oxy]-12-hydroxy-,	C44H62N9O12P		A/B	-8.3
6885	C(=O)([C@@H](NC(=O)OC)C(C)C)N1[C@H](c2[nH]c(C34CCC(CC3)(CC4)c3ccc(cc3)c3[nH]c(nc3)[C@H]3N(C(=O)[C@@H	1228668-06-1	Carbamic acid, N-[(1S)-2-[(2S)-2-[5-[4-[trans-4-[2-[(2S)-1-[(2S)-2-[(methoxycarb	C36H48N8O6	688.82	A/B	-8.3
6886	O=C1[C@@H]2[C@H](c3c([C@H](O)[C@H]2CO1)cc1c(c3)OCO1)c1cc(OC)c(OC)c(OC)c1	122871-77-6	Furo[3,Ä≤,4,Ä≤:6,7]naphtho[2,3-d]-1,3-dioxol-6(5aH)-one, 5,8,8a,9-tetrahydro-9-	C22H22O8	414.41	A/B	-8.3
6887	C(=O)([C@@H](NC(=O)OC)[C@@H](C)O)N1[C@@H](CCC1)c1[nH]c(cn1)c1ccc(C#Cc2ccc(cc2)c2[nH]c(nc2)[C@H]2N(C(	1228966-10-6	Carbamic acid, N-[(1R)-2-[(2S)-2-[5-[4-[2-[4-[2-[(1S)-1-[[(2R)-2-[(methoxycarbor	C48H48N8O6	832.94	A/B	-8.3
6888	C(=O)([C@H](NC(=O)OC)C(C)C)N1[C@@H](CCC1)c1[nH]c(cn1)c1ccc(C#Cc2ccc(cc2)c2[nH]c([C@@H](NC(=O)[C@H](N	1228966-11-7	Carbamic acid, N,N,Ä≤-[1,2-ethynediylbis[4,1-phenylene-1H-imidazole-5,2-diyl-(2S	C40H46N8O8	766.84	A/B	-8.3
6889	C(=O)([C@H](C(C)C)N(C)C)N1[C@@H](CCC1)c1[nH]c(cn1)c1ccc(C#Cc2ccc(cc2)c2[nH]c([C@@H](NC(=O)[C@@H](NC(	1228966-87-7	Carbamic acid, N,N,Ä≤-[1,2-ethynediylbis[4,1-phenylene-1H-imidazole-5,2-diyl-(2S	C38H42N8O6	706.79	A/B	-8.3
6890	N(C(=O)C)[C@H]1[C@H]([C@@H](C(=O)NC)O)OC(=C[C@@H]1NC(=N)N)C(=O)O	1236003-11-4	2,4-Pyrimidinediamine, N2-(4-methyl-2-pyridinyl)-N4-(2,4,6-trimethylphenyl)-	C19H21N5	319.4	A/B	-8.3
6891	C(N(C(=O)N[C@H](C(=O)N[C@@H](Cc1ccccc1)C[C@@H]([C@H](Cc1ccccc1)NC(=O)OCc1cnco1)O)C(C)C)C)c1nc(sc1)C1	1236406-38-4	2,7,10,12-Tetraazatridecanoic acid, 4-hydroxy-9,12-dimethyl-13-[2-(1-methylethyl	C35H44N6O6S	676.83	A/B	-8.3
6892	[C@H](Cc1ccccc1)(C[C@@H]([C@H](Cc1ccccc1)NC(=O)OCc1ccns1)O)NC(=O)[C@@H](NC(=O)N(Cc1nc(C=C(C)C)C)sc1	1236406-42-0	2,7,10,12-Tetraazatridecanoic acid, 13-(2-cyclohexyl-4-thiazolyl)-4-hydroxy-12-m	C40H52N6O5S2	761.01	A/B	-8.3
6893	[C@H](Cc1ccccc1)(C[C@@H]([C@H](Cc1ccccc1)NC(=O)OCc1cncs1)O)NC(=O)[C@H](NC(=O)N(Cc1nc(C(C)(C)C)sc1)C)	1236406-58-8	2,7,10,12-Tetraazatridecanoic acid, 4-hydroxy-12-methyl-9-(1-methylethyl)-13-[2-	C38H48N6O6S	716.89	A/B	-8.3
6894	[C@H](Cc1ccccc1)([C@@H]([C@@H]([C@H](Cc1ccccc1)NC(=O)OCc1cncs1)O)O)NC(=O)[C@@H](NC(=O)N(Cc1nc(C(C)C)	1236406-85-1	2,7,10,12-Tetraazatridecanoic acid, 13-(2-cyclobutyl-4-thiazolyl)-4-hydroxy-12-m	C38H48N6O5S2	732.95	A/B	-8.3
6895	[C@H](Cc1ccccc1)(C[C@@H]([C@H](Cc1ccccc1)NC(=O)OCc1ccns1)O)NC(=O)[C@@H](NC(=O)N(Cc1nc(CCc2ccccc2)sc1	1236406-90-8	2,7,10,12-Tetraazatridecanoic acid, 4-hydroxy-12-methyl-9-(1-methylethyl)-8,11-c	C39H51N7O5S2	762	A/B	-8.3
6896	[C@H](Cc1ccccc1)(C[C@@H]([C@H](Cc1ccccc1)NC(=O)OCc1cnco1)O)NC(=O)[C@@H](NC(=O)N(Cc1nc(CCc2ccccc2)sc1	1236407-01-4	2,7,10,12-Tetraazatridecanoic acid, 4-hydroxy-12-methyl-9-(1-methylethyl)-8,11-c	C37H48N6O5S2	720.94	A/B	-8.3
6897	[C@H](Cc1ccccc1)(C[C@@H]([C@H](Cc1ccccc1)NC(=O)OCc1cncs1)O)NC(=O)[C@H](NC(=O)N(Cc1nc(CC(C)C)sc1)C)C(	1236407-03-6	Not Yet Assigned	C39H50N6O6S	730.92	A/B	-8.3
6898	[C@H](Cc1ccccc1)([C@@H]([C@@H]([C@H](Cc1ccccc1)NC(=O)OCc1ccno1)O)O)NC(=O)[C@@H](NC(=O)N(Cc1nc(C(C)C)	1236407-09-2	2,7,10,12-Tetraazatridecanoic acid, 13-[2-[(2-ethoxy-4-thiazolyl)methyl]-4-thiaz	C40H49N7O6S3	820.06	A/B	-8.3
6899	[C@H](Cc1ccccc1)(C[C@@H]([C@H](Cc1ccccc1)NC(=O)OCc1cnco1)O)NC(=O)[C@@H](NC(=O)N(Cc1nc(CC)sc1)C)C(C)C	1236407-17-2	2,7,10,12-Tetraazatridecanoic acid, 13-[2-(cyclohexylmethyl)-4-thiazolyl]-4-hydr	C41H54N6O5S2	775.03	A/B	-8.3
6900	C(N(C(=O)N[C@H](C(=O)N[C@@H](Cc1ccccc1)C[C@@H]([C@H](Cc1ccccc1)NC(=O)OCc1cnco1)O)C(C)C)C)c1nc(sc1)C1	1236407-51-4	2,7,10,12-Tetraazatridecanoic acid, 4-hydroxy-12-methyl-9-(1-methylethyl)-8,11-c	C42H48N6O6S	764.93	A/B	-8.3
6901	[C@H](Cc1ccccc1)(C[C@@H]([C@H](Cc1ccccc1)NC(=O)OCc1ccns1)O)NC(=O)[C@@H](NC(=O)N(Cc1nc(CC2CCCC2)sc1)C	1236408-57-3	2,7,10,12-Tetraazatridecanoic acid, 13-(2-cyclopentyl-4-thiazolyl)-4-hydroxy-12-	C39H50N6O5S2	746.98	A/B	-8.3
6902	[C@H](Cc1ccccc1)(C[C@@H]([C@H](Cc1ccccc1)NC(=O)OCc1ccno1)O)NC(=O)[C@@H](NC(=O)N(Cc1nc(CC2CCCCC2)sc1)	1236409-35-0	2,7,10,12-Tetraazatridecanoic acid, 4-hydroxy-9,12-dimethyl-13-[2-(1-methylethyl	C35H44N6O5S2	692.89	A/B	-8.3

6903	C(N(C(=O)N[C@H](C(=O)N[C@@H](Cc1ccccc1)C[C@@H]([C@H](Cc1ccccc1)NC(=O)OCc1ccns1)O)C(C)C)C)c1nc(sc1)C1	1237482-18-6	2,7,10,12-Tetraazatridecanoic acid, 4-hydroxy-12-methyl-9-(1-methylethyl)-13-[2-	C38H50N6O5S2	734.97	A/B	-8.3
6904	C(N(C(=O)N[C@H](C(=O)N[C@@H](Cc1ccccc1)C[C@@H]([C@H](Cc1ccccc1)NC(=O)OCc1cncs1)O)C(C)C)C)c1nc(sc1)C1	1237482-20-0	2,7,10,12-Tetraazatridecanoic acid, 13-[2-(3-cyclohexen-1-yl)-4-thiazolyl]-4-hyd	C40H50N6O5S2	758.99	A/B	-8.3
6905	N(CC(=O)[C@H](Cc1ccccc1)C[C@@H](CN1[C@H](C(=O)NC(C)(C)C)CN(Cc2n(C)ccc2)CC1)O)[C@H]1c2c(C[C@H]1O)cccc	1237988-98-5	Not Yet Assigned	C38H51N5O4	641.84	A/B	-8.3
6906	N(C(=O)[C@H](Cc1ccccc1)C[C@@H](CN1[C@H](C(=O)NC(C)(C)C)CN(Cc2ccco2)CC1)O)[C@H]1c2c(C[C@H]1O)cccc2	1237989-08-0	Not Yet Assigned	C37H51N5O4	629.83	A/B	-8.3
6907	C[C@]12[C@H]([C@H]3[C@H](CC2)[C@]2(C)C(=CC3)C[C@@H](OC(=O)CP(=O)(O)O)CC2)CC=C1n1c2c(nc1)cccc2	1238382-70-1	Androsta-5,16-dien-3-ol, 17-(3-pyridinyl)-, 3-(2-phosphonoacetate), (3Œ≤)-	C26H34NO5P	471.53	A/B	-8.3
6908	C(=O)([C@@H](NC(=O)OC)C(C)C)N1[C@@H](CCC1)c1[nH]c(cn1)c1ccc(cc1)c1ccc(C(=O)NC[C@H]2N(C(=O)[C@@H](NC(	1240427-43-3	Carbamic acid, N-[(1S)-2-[(2S)-2-[[[[4,Ä≤-[2-[(2S)-1-[(2S)-2-[(methoxycarbonyl)a	C35H43N7O7	673.76	A/B	-8.3
6909	C(=O)([C@H](NC(=O)OC)CC)N1[C@H](c2[nH]c(c3cc4c(-c5c(C4)cc(cc5)c4[nH]c(nc4)[C@H]4N(C(=O)[C@H](NC(=O)O	1240880-47-0	Carbamic acid, N,N,Ä≤-[9H-fluorene-2,7-diylbis[1H-imidazole-5,2-diyl-(2S)-2,1-py	C39H46N8O6	722.83	A/B	-8.3
6910	C(=O)([C@H](NC(=O)OC)C(C)C)N1[C@H](c2[nH]c(c3cc4c(-c5c(C4)cc(cc5)c4[nH]c(nc4)[C@H]4N(C(=O)[C@H](NC(=	1240880-69-6	Carbamic acid, N,N,Ä≤-[9H-fluorene-2,7-diylbis[1H-imidazole-5,2-diyl-(2S)-2,1-py	C41H50N8O8	782.88	A/B	-8.3
6911	C(=O)([C@H](NC(=O)OC)C(C)C)N1[C@@H](CCC1)c1[nH]c(cn1)[C@H]1CC[C@@H](CC1)c1ccc(cc1)c1[nH]c(nc1)[C@H]1	1240887-65-3	Carbamic acid, N-[(1S)-2-hydroxy-1-[[(2S)-2-[5-[4-[trans-4-[2-[(2S)-1-[(2S)-3-hy	C40H56N8O8	776.92	A/B	-8.3
6912	C(=O)([C@@H](NC(=O)OC)C(C)C)N1[C@H](c2[nH]c3c(n2)ccc(c3)c2ccc(cc2)c2ccc(cc2)c2[nH]c(nc2)[C@H]2N(C(=O	124118-11-2	2-Naphthalenesulfonic acid, 5,5,Ä≤-(2-nitro-4,4,Ä≤-biphenylylenebisazo)bis[6-am	C32H23N7O8S2	697.7	A/B	-8.3
6913	C(=O)([C@H](NC(=O)OC)c1ccccc1)N1[C@@H](CCC1)c1[nH]c(cn1)C1CCC(CC1)C1CCC(CC1)c1[nH]c(nc1)[C@H]1N(C(=O	1241404-86-3	Carbamic acid, N-[(1R)-2-[(2S)-2-[5-[4-[4-[2-[(2S)-1-[(2R)-2-[(methoxycarbonyl)a	C46H52N8O6	812.96	A/B	-8.3
6914	O[C@H]1[C@@H](n2c3c(nc2)c(N)ncn3)O[C@@H](COS(=O)(=O)NC(=O)[C@H](CCCNC(=N)N)N)[C@@H]1O	1241941-85-4	9H-Purin-6-amine, 9-[5-O-[[[(2S)-2-amino-4-(hydroxyamino)-1,4-dioxobutyl]amino]s	C14H20N8O9S	476.42	A/B	-8.3
6915	C(OC[C@@H](C(=O)N[C@H](C(=O)N[C@H](C(=O)N[C@@H](Cc1cnc[nH]1)C(=O)N[C@H](C(=O)N[C@H](C(=O)N[C@H](C(=O	1246392-13-1	L-Arginine, L-valylglycyl-L-alanyl-1-[(11-cyclopropyl-6,11-dihydro-6-oxo-5H-dipy	C80H112N24O24	1793.89	A/B	-8.3
6916	O(CCOC)c1c(ccc(c1)c1[nH]c(nc1)[C@H]1N(C(=O)[C@@H](NC(=O)OC)C2CCCCC2)CCC1)c1c(OCCOC)cc(cc1)c1[nH]c(nc	1246559-43-2	Carbamic acid, N,N,Ä≤-[[2,2,Ä≤-bis(2-methoxyethoxy)[1,1,Ä≤-biphenyl]-4,4,Ä≤-diy	C50H66N8O10	939.11	A/B	-8.3
6917	C(=O)([C@@H](NC(=O)OC)C)N1[C@@H](CCC1)c1[nH]c(cn1)C1=CCC(CC1)C1CCC(=CC1)c1[nH]c(nc1)[C@H]1N(C(=O)[C@	1246852-24-3	Carbamic acid, N,N,Ä≤-[[1,1,Ä≤-bi-3-cyclohexen]-4,4,Ä≤-diylbis[1H-imidazole-5,2	C40H58N8O6	746.94	A/B	-8.3
6918	O=c1n([C@H]2C[C@H](OCC)[C@@H](CO)O2)cc(C)c(N)n1	1246852-30-1	Carbamic acid, N-[(1S)-2-[(2S)-2-[5-[4-[4-[2-[(2S)-1-[(2S)-2-[(methoxycarbonyl)a	C36H46N8O6	686.8	A/B	-8.3
6919	C[C@]1(F)[C@@H](O[C@H](COP(=O)(N[C@H](C(=O)OC)C)OCC)[C@H]1O)n1c(=O)[nH]c(=O)cc1	1250829-93-6	L-Alanine, N-[(2,Ä≤R)-2,Ä≤-deoxy-2,Ä≤-fluoro-2,Ä≤-methyl-P-phenyl-5,Ä≤-uridylyl	C25H27FN3O9P	563.47	A/B	-8.3
6920	O(c1nc(Nc2ccc(C#N)cc2)nc(N)c1Br)c1c(C)cc(C#N)cc1C.P(=O)(O)(O)O	1253183-21-9	Benzonitrile, 4-[[6-amino-5-bromo-2-[(4-cyanophenyl)amino]-4-pyrimidinyl]oxy]-3,	C20H15BrN6O.HNO3		A/B	-8.3
6921	C(=O)([C@@H](NC(=O)OC)C(C)C)N1[C@@H]([C@@H]2C[C@H]1CC2)c1[nH]c(cn1)c1cc2c(-c3c(COC2)cc(cc3)c2[nH]c(n	1256384-41-4	Carbamic acid, N,N,Ä≤-[(6,7-dihydro-6-methyl-5-oxo-5H-dibenz[c,e]azepine-3,9-diy	C43H53N9O7	807.94	A/B	-8.3
6922	C(=O)([C@@H](NC(=O)OC)C(C)C)N1[C@@H](CCC1)c1[nH]c(cn1)c1ccc(cc1)c1ccc(cc1)c1[nH]c(CN2C(=O)[C@H](N)CC	1256385-55-3	Carbamic acid, N-[(1S)-1-[[(2S)-2-[5-[4,Ä≤-(1H-imidazol-5-yl)[1,1,Ä≤-biphenyl]-	C29H32N6O3	512.6	A/B	-8.3
6923	N(C(=O)[C@@H](NC(=O)OC)C(C)C)C1(CCC1)c1[nH]c(cn1)c1ccc(cc1)c1ccc(cc1)c1[nH]c(nc1)[C@H]1N(C(=O)[C@@H]	1256386-71-6	Carbamic acid, N-[(1S)-1-[[(2S)-2-[5-[4,Ä≤-[2-[1-[[(2S)-2-[(methoxycarbonyl)amir	C39H48N8O6	724.85	A/B	-8.3
6924	C(=O)([C@@H](NC(=O)OC)C(C)C)N1CC2(C[C@H]1c1[nH]c(cn1)c1cc3c(-c4c(C3(F)F)cc(cc4)c3cc4c(cc3)nc([nH]4)[	1256388-44-9	Carbamic acid, N-[(1S)-1-[[(2S)-2-[5-[4,Ä≤-[2-[(1R)-2-hydroxy-1-[[(2S)-2-[(metho	C38H48N8O7	728.84	A/B	-8.3
6925	C(=O)([C@@H](NC(=O)OC)C(C)C)N1[C@@H](CC(=C)C1)c1[nH]c(cn1)c1ccc(cc1)c1cc2c(cc1)cc(cc2)c1[nH]c(nc1)[C	1256392-14-9	Carbamic acid, N-[(1S)-1-[[(6S)-6-[5-[4-[6-[2-[(6S)-5-[(2S)-2-[(methoxycarbonyl)	C48H56N8O6	841.01	A/B	-8.3
6926	C(=O)([C@@H](NC(=O)CC)C(C)C)N1CC2(C[C@H]1c1[nH]c(cn1)c1ccc(cc1)c1ccc(cc1)c1[nH]c(nc1)[C@H]1N(C(=O)[C	1256392-84-3	Carbamic acid, N-[(1S)-2-methyl-1-[[(2S)-2-[5-[4-[2-[4-[2-[(2S)-1-[3,3,3-trifluc	C40H43F3N8O6	788.81	A/B	-8.3
6927	C(=O)([C@@H](NC(=O)OC)C(C)C)N1C(c2c(C1)cccc2)c1[nH]c(cn1)c1ccc(cc1)c1ccc(cc1)c1[nH]c(nc1)[C@H]1N(C(=	1256393-09-5	Carbamic acid, N-[2-[(2S)-2-[5-[6-[4-[2-[(2S)-1-[(2S)-2-[(methoxycarbonyl)amino]	C47H50N8O6	822.95	A/B	-8.3
6928	O(Cc1ccccc1)c1c(OCc2ccccc2)cc(C(=O)OC[C@H]2O[C@H]([C@H](O)[C@@H]2O)n2nc(C(=O)N)nc2)cc1OCc1ccccc1	1257423-91-8	1H-1,2,4-Triazole-3-carboxamide, 1-[5-O-(4-nitrobenzoyl)-Œ≤-D-ribofuranosyl]-	C15H15N5O8	393.31	A/B	-8.3
6929	C(Cc1ccccc1)[C@]1(CCCNC(=O)OCCOCCOCCOCCOCCOC)CC(=C([C@H](CC)c2cc(NS(=O)(=O)c3ccc(C(F)(F)F)cn3)ccc2)C	1258520-33-0	Carbamic acid, N-[3-[(2S)-3,6-dihydro-4-hydroxy-6-oxo-2-(2-phenylethyl)-5-[(1R)-	C35H40F3N3O8S	719.77	A/B	-8.3
6930	C(=O)([C@@H](NC(=O)OC)C(C)C)N1[C@@H](CCC1)c1[nH]c(cn1)c1ccc(cc1)c1cc2c(cc1)cc(cc2)c1[nH]c(nc1)[C@H]1	1261070-50-1	Carbamic acid, N-[2-[(2S)-2-[5-[6-[4-[2-[(2S)-1-[(2S)-2-[(methoxycarbonyl)amino]	C43H50N8O6	774.91	A/B	-8.3
6931	C[C@]1(F)[C@H](n2c3c(nc2)c(=O)nc(N)[nH]3)O[C@H](COP(=O)(OP(=O)(O)O)O)[C@H]1O	1261254-21-0	5,Ä≤-Guanylic acid, 2,Ä≤-deoxy-2,Ä≤-fluoro-2,Ä≤-methyl-, (2,Ä≤R)-	C11H15FN5O7P	379.24	A/B	-8.3
6932	O[C@H]1[C@H](n2c3c(nc2)c(=O)nc(NCC(=O)O)[nH]3)O[C@H](COP(=O)(O)O)[C@H]1O	1261432-22-7	Not Yet Assigned	C22H18N6		A/B	-8.3
6933	O[C@@H]1[C@H](n2c3c(nc2)c(N)ncn3)O[C@H](COP(=O)(Oc2ccccc2)Oc2ccccc2)[C@H]1O	126168-30-7	9H-Purin-6-amine, 9-[5-O-[bis(phenylmethyl)phosphinyl]-Œ≤-D-arabinofuranosyl]-	C24H26N5O5P	495.47	A/B	-8.3
6934	N(=C\1/c2c(N(CN3CCN(CC3)c3cc4c(cc3F)c(=O)c(C(=O)O)cn4CC)C1=O)ccc(F)c2)\NC(=S)NOC	1265623-12-8	3-Quinolinecarboxylic acid, 1-ethyl-6-fluoro-7-[4-[[(3Z)-5-fluoro-2,3-dihydro-3-	C26H25F2N7O5S	585.58	A/B	-8.3
6935	N(=C\1/c2c(N(CN3CCN(CC3)c3cc4c(cc3F)c(=O)c(C(=O)O)cn4CC)C1=O)ccc(C)c2)\NC(=S)NO	1265623-13-9	3-Quinolinecarboxylic acid, 1-ethyl-6-fluoro-7-[4-[[(3Z)-5-fluoro-2,3-dihydro-3-	C27H27F2N7O5S	599.61	A/B	-8.3
6936	O(C)c1c2n(cc(C(=O)O)c(=O)c2cc(F)c1N1CC(C)N(CN2c3c(/C(=N/NC(=S)NOC)/C2=O)cc(Cl)cc3)CC1)C1CC1	1265623-27-5	3-Quinolinecarboxylic acid, 7-[4-[[(3Z)-5-chloro-2,3-dihydro-3-[2-[(hydroxyaminc	C29H29ClFN7O6S	658.1	A/B	-8.3
6937	O=c1n([C@@H]2O[C@H](CO)[C@@H](S)C2)cc(C)c(=O)[nH]1	1268619-57-3	Phosphonic acid, P-[[(1R)-2-[4-(benzoylamino)-2-oxo-1(2H)-pyrimidinyl]-1-[[bis(4	C43H43N4O11P	822.8	A/B	-8.3
6938	C(=O)([C@@H](NC(=O)OC)C(C)C)N1C[C@@]2(C[C@H]1c1[nH]c(cn1)c1ccc(cc1)c1ccc(cc1)c1[nH]c(nc1)[C@H]1N(C(=	1272655-37-4	Carbamic acid, N-[(1S)-1-[[(2S)-2-[5-[4,Ä≤-[2-[(3S)-2-[(2S)-2-[(methoxycarbonyl)	C45H58N8O8	838.99	A/B	-8.3
6939	C(=O)([C@@H](NC(=O)OC)C(C)C)N1[C@@H](CC2(C1)CCOCC2)c1[nH]c(cn1)c1ccc(cc1)c1ccc(cc1)c1[nH]c(nc1)[C@H]	1272655-83-0	Carbamic acid, N-[(1S)-1-[[(2S)-2-[5-[4,Ä≤-[2-[(3S)-8,8-difluoro-2-[(2S)-2-[(met	C45H56F2N8O6	842.97	A/B	-8.3
6940	C(F)(F)C1C2CC3(N)CC1CC(C2)C3	1272656-56-0	Carbamic acid, N-[(1S)-1-[[(2S)-2-[5-[4,Ä≤-[2-[(7S)-2,2-difluoro-6-[(2S)-2-[(met	C43H52F2N8O6	814.92	A/B	-8.3
6941	C(=O)([C@@H](NC(=O)OC)C(C)(C)O)N1[C@@H]([C@@H]2C[C@H]1CC2)c1[nH]c(cn1)c1ccc(cc1)c1ccc(cc1)c1[nH]c(nc	1273589-63-1	Carbamic acid, N-[(1S)-1-cyclopropyl-2-[(2S)-2-[5-[4,Ä≤-[2-[(1R,3S,4S)-2-[(2S)-2	C42H48N8O6	760.88	A/B	-8.3
6942	C(=O)([C@@H](NC(=O)OC)C(C)C)N1[C@@H]([C@@H]2C[C@H]1CC2)c1[nH]c(cn1)c1ccc(C=Cc2ccc(cc2)c2[nH]c(nc2)[C	1273591-77-7	Carbamic acid, N-[(1S)-1-[[(2S)-2-[5-[4-[trans-4-[2-[(1R,3S,4S)-2-[(2S)-2-[(meth	C42H58N8O6	770.96	A/B	-8.3
6943	C([C@H](OP(=O)(CCl)O)CO)n1c2c(nc1)c(N)nc(N)n2	127852-92-0	Butanediamide, N1-[3-[2-[[(1,1-dimethylethyl)amino]carbonyl]-1-piperidinyl]-2-hy	C34H44N6O5	616.75	A/B	-8.3
6944	N(=C1c2c(N(CCNc3c4c(cc(Cl)cc4)ncc3)C1=O)ccc(Br)c2)NC(=S)N	1283084-54-7	Hydrazinecarbothioamide, 2-[1-[2-[(7-chloro-4-quinolinyl)amino]ethyl]-1,2-dihydr	C22H21ClN6OS	452.96	A/B	-8.3
6945	N(c1nc(nc(n1)N1CCOCC1)N1CCOCC1)c1nc(cs1)c1ccc(N(=O)=O)cc1	1285535-40-1	1,3,5-Triazin-2-amine, N-[4-(4-chlorophenyl)-2-thiazolyl]-4,6-di-4-morpholinyl-	C20H22ClN7O2S	459.95	A/B	-8.3
6946	C(=C\C(=O)CC(=O)CCc1ccccc1)/c1cc(OC)c(O)cc1	128589-94-6	1-Naphthalenesulfonic acid, 4-[2-(2-hydroxy-1-naphthalenyl)diazenyl]-, ion(1-)	C20H13N2O4S		A/B	-8.3
6947	C[C@]12[C@@]3(C)[C@@H]([C@@H](C(=C)CC[C@@H](C(=C)C)O)CC3)CC[C@@H]1[C@]1(C)[C@@H](CC2)C(C)(C)[C@@H](O	1287768-02-8	Furo[3,Ä≤,4,Ä≤:6,7]naphtho[2,3-d]-1,3-dioxol-6(5aH)-one, 9-[[2,3-dideoxy-4,6-bi	C42H42O11	722.78	A/B	-8.3
6948	C([C@H](OCP(=O)(OCCCOCCCCCCCCCCCCCCCCCC)O)C)n1c2c(nc1)c(N)ncn2.[KH]	13011-56-8	2,7-Naphthalenedisulfonic acid, 4-[2-[4-[2-(4-amino-1-naphthalenyl)diazenyl]-1-r	C30H21N5O6S2.2Na		A/B	-8.3
6949	O=[11c]1n([C@@H]2O[C@H](CO)[C@@H](O)C2)cc(C)c(=O)[nH]1	130322-38-2	Furo[3,Ä≤,4,Ä≤:6,7]naphtho[2,3-d]-1,3-dioxole-5,8-dione, 5a,6,8a,9-tetrahydro-9	C22H20O8	412.39	A/B	-8.3
6950	C(=O)([C@@H](NC(=O)OC)C(C)C)N1[C@@H](C[C@H](C)C1)c1[nH]c(cn1)c1cc2c(cc1)cc(cc2)c1ccc(cc1)c1[nH]c(nc1	1306734-01-9	Carbamic acid, N-[(1S)-1-[[(2S)-2-[5-[4-[6-[2-[(2S)-1-[(2S)-2-[(methoxycarbonyl)	C46H52N8O6	812.96	A/B	-8.3
6951	S(=O)(=O)(N1[C@H](C(=S)OC)CCC1)c1cc2c(cc1)NC(=O)C2=O	1310453-16-7	1H-Indole-2,3-dione, 5-[[(2S)-2-(1,3-dithian-2-yl)-1-pyrrolidinyl]sulfonyl]-	C16H18N2O4S3	398.52	A/B	-8.3
6952	C(=O)([C@@H](NC(=O)OC)C(C)C)N1[C@@H](CC(=C)C1)c1[nH]c(cn1)c1c(C)c(C)c(cc1)c1c(C)c(C)c(cc1)c1[nH]c(nc	1311398-23-8	Carbamic acid, N-[(1S)-1-[[(2S)-2-[5-[4,Ä≤-[2-[(2S)-1-[(2S)-2-[(methoxycarbonyl)	C43H54N8O6	778.94	A/B	-8.3
6953	C(=O)([C@@H](NC(=O)OC)C(C)C)N1[C@@H](CC(=C)C1)c1[nH]c(cn1)c1c(Cl)cc(cc1Cl)c1cc(Cl)c(c(Cl)c1)c1[nH]c(	1311398-27-2	Carbamic acid, N-[(1S)-1-[[(2S)-2-[5-[3,3,Ä≤-dichloro-4,Ä≤-[2-[(2S)-1-[(2S)-2-[	C41H48Cl2N8O6	819.78	A/B	-8.3
6954	C(=O)([C@@H](NC(=O)OC)C(C)C)N1[C@@H](C2CCC1CC2)c1[nH]c(cn1)c1ccc(cc1)c1ccc(cc1)c1[nH]c(nc1)[C@H]1N(C	1311398-29-4	Carbamic acid, N-[(1S)-1-[[(2S)-2-[5-[4,Ä≤-[2-[(1R,3S,4S)-2-[(2S)-2-[(methoxycar	C43H52N8O6	776.92	A/B	-8.3
6955	C(=O)([C@@H](NC(=O)OC)C(C)C)N1[C@@H](CC(=C)C1)c1[nH]c(cn1)c1cc2c(cc1)cc(C=Cc1[nH]c(nc1)[C@H]1N(C(=O)	1311398-58-9	Carbamic acid, N-[(1S)-1-[[(2S)-2-[5-[2-[6-[2-[(2S)-1-[(2S)-2-[(methoxycarbonyl)	C41H52N8O6	752.9	A/B	-8.3
6956	C(=O)([C@@H](NC(=O)OC)C(C)C)N1[C@@H](CCC1)c1[nH]c(c(Br)n1)c1ccc(cc1)c1ccc(cc1)c1[nH]c(nc1Br)[C@H]1N(	1312335-95-7	Carbamic acid, N,N,Ä≤-[[1,1,Ä≤-biphenyl]-4,4,Ä≤-diylbis[(5-chloro-1H-imidazole-	C42H48Cl2N8O6	831.79	A/B	-8.3
6957	C(=O)([C@@H](NC(=O)OC)C1CCOCC1)N1[C@H]2[C@H](C2)C[C@H]1c1[nH]c(c(Cl)n1)c1ccc(cc1)c1ccc(cc1)c1[nH]c(n	1312336-06-3	Carbamic acid, N,N,Ä≤-[[1,1,Ä≤-biphenyl]-4,4,Ä≤-diylbis[(5-chloro-1H-imidazole-	C42H44Cl2N8O6	827.75	A/B	-8.3
6958	C(=O)([C@@H](NC(=O)OC)C1CCOCC1)N1[C@H]2[C@H](C2)C[C@H]1c1[nH]c(cn1)c1ccc(cc1)c1ccc(cc1)c1[nH]c(nc1Cl	1312336-07-4	Carbamic acid, N-[(1S)-2-[(1R,3S,5R)-3-[5-chloro-4-[4,Ä≤-[2-[(1R,3S,5R)-2-[(2S)-	C44H51ClN8O7	839.38	A/B	-8.3
6959	C(=O)([C@@H](NC(=O)OC)C1CCOCC1)N1[C@@H](CC[C@@H]1C)c1[nH]c(c(Cl)n1)c1ccc(cc1)c1ccc(cc1)c1[nH]c(nc1Cl	1312336-16-5	Carbamic acid, N-[(1S)-2-[(1R,3S,5R)-3-[5-chloro-4-[4,Ä≤-[5-chloro-2-[(1R,3S,5R)	C44H50Cl2N8O8	889.82	A/B	-8.3
6960	C(=O)([C@@H](NC(=O)OC)C(C)C)N1[C@@H](C[C@H](C)C1)c1[nH]c(c(Cl)n1)c1ccc(cc1)c1ccc(cc1)c1[nH]c(nc1)[C@	1312336-24-5	Carbamic acid, N-[(1S)-2-[(2S,5S)-2-[5-[4,Ä≤-[5-chloro-2-[(2S,5S)-1-[(2S)-2-[(me	C46H57ClN8O8	885.45	A/B	-8.3
6961	C(=O)([C@@H](NC(=O)OC)C(C)C)N1[C@H](c2[nH]c(c(n2)c2ccc(cc2)c2ccc(cc2)c2[nH]c(nc2)[C@H]2N(C(=O)[C@@H]	1312336-54-1	Carbamic acid, N-[(1S)-1-[[(2S,5S)-2-[7-[4-[5-chloro-2-[(2S,5S)-1-[(2S)-2-[(meth	C44H53ClN8O6	825.39	A/B	-8.3
6962	C(=O)([C@@H](NC(=O)OC)C1CCOCC1)N1[C@H]2[C@H](C2)C[C@H]1c1[nH]c(c(F)n1)c1ccc(cc1)c1ccc(cc1)c1[nH]c(nc	1312336-69-8	Carbamic acid, N-[(1S)-2-[(1R,3S,5R)-3-[5-[4,Ä≤-[5-cyano-2-[(1R,3S,5R)-2-[(2S)-2	C47H53N9O8	871.98	A/B	-8.3
6963	C(=O)([C@@H](NC(=O)OC)[C@H]1CCCOC1)N1[C@H]2[C@H](C2)C[C@H]1c1[nH]c(c(Cl)n1)c1ccc(cc1)c1ccc(cc1)c1[nH	1312336-71-2	Carbamic acid, N,N,Ä≤-[[1,1,Ä≤-biphenyl]-4,4,Ä≤-diylbis[(5-chloro-1H-imidazole-	C46H52Cl2N8O8	915.86	A/B	-8.3
6964	C(=O)([C@@H](NC(=O)OC)[C@H]1CCCOC1)N1[C@H]2[C@H](C2)C[C@H]1c1[nH]c(c(Cl)n1)c1ccc(cc1)c1ccc(cc1)c1[nH	1312336-75-6	Carbamic acid, N-[(1S)-2-[(1R,3S,5R)-3-[5-[4,Ä≤-[5-chloro-2-[(1R,3S,5R)-2-[(2S)-	C46H53ClN8O8	881.41	A/B	-8.3
6965	C(=O)([C@@H](NC(=O)OC)C1CCOCC1)N1[C@H]2[C@H](C2)C[C@H]1c1[nH]c(c(n1)c1ccc(cc1)c1ccc(cc1)c1c(nc([nH]1	1312337-02-2	Carbamic acid, N,N,Ä≤-[[1,1,Ä≤-biphenyl]-4,4,Ä≤-diylbis[(5-cyclopropyl-1H-imida	C48H58N8O6	843.02	A/B	-8.3
6966	C(=O)([C@@H](NC(=O)OC)C(C)C)N1[C@@H](C[C@H](C)C1)c1[nH]c(cn1)c1ccc(cc1)c1ccc(cc1)c1[nH]c(nc1)[C@H]1N	1312338-34-3	Carbamic acid, N-[(1S)-2-[(2S,4S)-2-[5-[4,Ä≤-[2-[(2S,4S)-1-[(2S)-2-[(methoxycarl	C44H56N8O7	808.96	A/B	-8.3
6967	C(=O)([C@@H](NC(=O)OC)C(C)C)N1[C@H](c2[nH]c(c3c4c(c(cc3)c3ccc(cc3)c3[nH]c(nc3)[C@H]3N(C(=O)[C@@H](NC	1312608-34-6	Carbamic acid, N-[(1S)-1-[[(2S)-2-[5-[4-[7-[2-[(2S)-1-[(2S)-2-[(methoxycarbonyl)	C42H52N10O6	792.93	A/B	-8.3
6968	C(=O)([C@@H](NC(=O)OC)C(C)C)N1[C@H](c2[nH]c(c3c4c(c(cc3)c3ccc(cc3)c3[nH]c(nc3)[C@H]3N(C(=O)[C@@H](NC	1312608-45-9	Carbamic acid, N-[(1S)-1-[[(2S)-2-[5-[4-[2,3-dihydro-7-[2-[(2S)-1-[(2S)-2-[(meth	C43H54N8O6	778.94	A/B	-8.3
6969	C[C@]1(c2[nH]c(c3c4c(c(cc3)c3ccc(cc3)c3[nH]c(nc3)[C@@]3(C)N(C(=O)[C@@H](NC(=O)OC)C(C)C)CCC3)ncs4)cn2	1312608-72-2	Carbamic acid, N-[(1S)-1-[[(2S)-2-[5-[4-[7-[2-[(2S)-1-[(2S)-2-[(methoxycarbonyl)	C43H53N9O6	791.94	A/B	-8.3
6970	O(Cc1ccc(F)cc1)c1c2c(oc(c(O)c2=O)c2ncc(Cl)cn2)ccc1	1312892-05-9	4H-1-Benzopyran-4-one, 5-[(4-fluorophenyl)methoxy]-3-hydroxy-2-(5-methoxy-2-pyri	C21H15FN2O5	394.35	A/B	-8.3
6971	O(Cc1ccc(F)cc1)c1c2c(oc(c(O)c2=O)c2nnc(Cl)[nH]2)ccc1	1312892-13-9	4H-1-Benzopyran-4-one, 5-[(4-fluorophenyl)methoxy]-3-hydroxy-2-(5-methoxy-1H-1,2	C19H14FN3O5	383.33	A/B	-8.3
6972	O(Cc1ccc(F)cc1)c1c2c(oc(c(O)c2=O)c2[nH]c(cn2)c2ccccc2)ccc1	1312892-19-5	4H-1-Benzopyran-4-one, 2-(5-chloro-1H-imidazol-2-yl)-5-[(4-fluorophenyl)methoxy]	C19H12ClFN2O4	386.76	A/B	-8.3
6973	O(Cc1ccc(F)cc1)c1c2c(oc(c(O)c2=O)c2ncc(Cl)cn2)c(C)cc1	1312892-54-8	4H-1-Benzopyran-4-one, 5-[(4-fluorophenyl)methoxy]-3-hydroxy-2-(5-methoxy-2-pyri	C22H17FN2O5	408.38	A/B	-8.3
6974	O(C)c1c2c(c(OCc3ccc(F)cc3)cc1)c(=O)c(O)c(o2)c1ncc(C)cn1	1312893-33-6	4H-1-Benzopyran-4-one, 5-[(4-fluorophenyl)methoxy]-3-hydroxy-8-methoxy-2-(2-pyri	C21H15FN2O5	394.35	A/B	-8.3
6975	O(C)c1c2c(c(OCc3ccc(F)cc3)cc1)c(=O)c(O)c(o2)c1ncc(Cl)cn1	1312893-35-8	4H-1-Benzopyran-4-one, 5-[(4-fluorophenyl)methoxy]-3-hydroxy-8-methoxy-2-(5-meth	C22H17FN2O6	424.38	A/B	-8.3
6976	O(Cc1ccc(F)cc1)c1c2c(oc(c(O)c2=O)c2[nH]c(Cl)nn2)c(OC)cc1	1312893-45-0	4H-1-Benzopyran-4-one, 5-[(4-fluorophenyl)methoxy]-3-hydroxy-8-methoxy-2-(5-meth	C20H16FN3O6	413.36	A/B	-8.3
6977	O(Cc1ccc(F)cc1)c1c2c(oc(c(O)c2=O)c2[nH]c(C)cn2)c(OC)cc1	1312893-48-3	4H-1-Benzopyran-4-one, 5-[(4-fluorophenyl)methoxy]-3-hydroxy-2-(1H-imidazol-2-yl	C20H15FN2O5	382.34	A/B	-8.3
6978	O(Cc1ccc(F)cc1)c1c2c(oc(c(O)c2=O)c2[nH]cc(C)n2)c(Cl)cc1	1312893-98-3	4H-1-Benzopyran-4-one, 8-chloro-5-[(4-fluorophenyl)methoxy]-3-hydroxy-2-(1H-imic	C19H12ClFN2O4	386.76	A/B	-8.3
6979	O(Cc1ccc(F)cc1)c1c2c(oc(c(O)c2=O)c2[nH]cc(Cl)n2)c(Cl)cc1	1312894-00-0	4H-1-Benzopyran-4-one, 8-chloro-5-[(4-fluorophenyl)methoxy]-3-hydroxy-2-(5-methc	C20H14ClFN2O5	416.79	A/B	-8.3
6980	O(Cc1ccc(F)cc1)c1c2c(oc(c(O)c2=O)c2[nH]c(cn2)c2ccccc2)cc(C)c1	1312895-01-4	4H-1-Benzopyran-4-one, 2-(5-chloro-1H-imidazol-2-yl)-5-[(4-fluorophenyl)methoxy]	C20H14ClFN2O4	400.79	A/B	-8.3
6981	O(Cc1ccc(F)cc1)c1c2c(oc(c(O)c2=O)c2ncc(Cl)cn2)c(C)c(C)c1	1312895-35-4	4H-1-Benzopyran-4-one, 5-[(4-fluorophenyl)methoxy]-3-hydroxy-2-(5-methoxy-2-pyri	C23H19FN2O5	422.41	A/B	-8.3
6982	O(Cc1ccc(F)cc1)c1c2c(c(OC)c(C)c1)oc(c(O)c2=O)c1ncc(C)cn1	1312895-83-2	4H-1-Benzopyran-4-one, 5-[(4-fluorophenyl)methoxy]-3-hydroxy-8-methoxy-7-methyl-	C22H17FN2O5	408.38	A/B	-8.3
6983	O(C)c1c2c(c(OCc3ccc(F)cc3)cc1C)c(=O)c(O)c(o2)c1ncc(OC)cn1	1312895-84-3	4H-1-Benzopyran-4-one, 5-[(4-fluorophenyl)methoxy]-3-hydroxy-8-methoxy-7-methyl-	C23H19FN2O5	422.41	A/B	-8.3
6984	O(C)c1c2c(c(OCc3ccc(F)cc3)cc1C)c(=O)c(O)c(o2)c1ncc(cn1)c1ccccc1	1312895-86-5	4H-1-Benzopyran-4-one, 2-(5-chloro-2-pyrimidinyl)-5-[(4-fluorophenyl)methoxy]-3-	C22H16ClFN2O5	442.82	A/B	-8.3
6985	O(Cc1ccc(F)cc1)c1c2c(c(OC)c(C)c1)oc(c(O)c2=O)c1[nH]c(C)cn1	1312895-98-9	4H-1-Benzopyran-4-one, 5-[(4-fluorophenyl)methoxy]-3-hydroxy-2-(1H-imidazol-2-yl	C21H17FN2O5	396.37	A/B	-8.3
6986	O(Cc1ccc(F)cc1)c1c2c(c(OC)c(C)c1)oc(c(O)c2=O)c1[nH]c(OC)cn1	1312895-99-0	4H-1-Benzopyran-4-one, 5-[(4-fluorophenyl)methoxy]-3-hydroxy-8-methoxy-7-methyl-	C22H19FN2O5	410.4	A/B	-8.3
6987	O(Cc1ccc(F)cc1)c1c2c(c(OC)c(C)c1)oc(c(O)c2=O)c1[nH]c(Cl)cn1	1312896-00-6	4H-1-Benzopyran-4-one, 5-[(4-fluorophenyl)methoxy]-3-hydroxy-8-methoxy-2-(5-meth	C22H19FN2O6	426.39	A/B	-8.3
6988	O(Cc1ccc(F)cc1)c1c2c(c(OC)c(C)c1)oc(c(O)c2=O)c1[nH]c(cn1)c1ccccc1	1312896-01-7	4H-1-Benzopyran-4-one, 2-(5-chloro-1H-imidazol-2-yl)-5-[(4-fluorophenyl)methoxy]	C21H16ClFN2O5	430.81	A/B	-8.3
6989	O(Cc1ccc(F)cc1)c1c2c(oc(c(O)c2=O)c2[nH]c(OC)cn2)cc(OC)c1	1312897-60-1	4H-1-Benzopyran-4-one, 5-[(4-fluorophenyl)methoxy]-3-hydroxy-7-methoxy-2-(5-meth	C21H17FN2O5	396.37	A/B	-8.3
6990	O(Cc1ccc(F)cc1)c1c2c(oc(c(O)c2=O)c2ncc(cn2)c2ccccc2)c(C)c(OC)c1	1312897-97-4	4H-1-Benzopyran-4-one, 2-(5-chloro-2-pyrimidinyl)-5-[(4-fluorophenyl)methoxy]-3-	C22H16ClFN2O5	442.82	A/B	-8.3
6991	O(Cc1ccc(F)cc1)c1c2c(oc(c(O)c2=O)c2ncc(C)cn2)c(Cl)c(OC)c1	1312898-95-5	4H-1-Benzopyran-4-one, 8-chloro-5-[(4-fluorophenyl)methoxy]-3-hydroxy-7-methoxy-	C21H14ClFN2O5	428.8	A/B	-8.3
6992	O(Cc1ccc(F)cc1)c1c2c(oc(c(O)c2=O)c2[nH]c(cn2)c2ccccc2)c(Cl)c(OC)c1	1312899-13-0	4H-1-Benzopyran-4-one, 8-chloro-2-(5-chloro-1H-imidazol-2-yl)-5-[(4-fluorophenyl	C20H13Cl2FN2O5	451.23	A/B	-8.3
6993	O(Cc1ccc(F)cc1)c1c2c(oc(c(O)c2=O)c2ncc(Cl)cn2)c(C)c(Cl)c1	1312901-06-6	4H-1-Benzopyran-4-one, 7-chloro-5-[(4-fluorophenyl)methoxy]-3-hydroxy-2-(5-methc	C22H16ClFN2O5	442.82	A/B	-8.3
6994	O(Cc1ccc(F)cc1)c1c2c(c(OC)c(Cl)c1)oc(c(O)c2=O)c1[nH]c(C)nn1	1312901-64-6	4H-1-Benzopyran-4-one, 7-chloro-5-[(4-fluorophenyl)methoxy]-3-hydroxy-8-methoxy-	C19H13ClFN3O5	417.77	A/B	-8.3
6995	O(Cc1ccc(F)cc1)c1c2c(c(OC)c(Cl)c1)oc(c(O)c2=O)c1[nH]c(OC)cn1	1312901-70-4	4H-1-Benzopyran-4-one, 7-chloro-5-[(4-fluorophenyl)methoxy]-3-hydroxy-8-methoxy-	C21H16ClFN2O5	430.81	A/B	-8.3
6996	O(Cc1ccc(F)cc1)c1c2c(oc(c(O)c2=O)c2ncc(Cl)cn2)c(Cl)c(Cl)c1	1312902-06-9	4H-1-Benzopyran-4-one, 7,8-dichloro-5-[(4-fluorophenyl)methoxy]-3-hydroxy-2-(5-m	C21H13Cl2FN2O5	463.24	A/B	-8.3
6997	O(Cc1ccc(F)cc1)c1c2c(c(c(Cl)c1)c1ccccc1)oc(c(O)c2=O)c1ncc(Cl)cn1	1312902-56-9	4H-1-Benzopyran-4-one, 7-chloro-5-[(4-fluorophenyl)methoxy]-3-hydroxy-2-(5-methc	C27H18ClFN2O5	504.89	A/B	-8.3
6998	O(Cc1ccc(F)cc1)c1c2c(c(c(c1)c1ccccc1)c1ccccc1)oc(c(O)c2=O)c1ncc(C)cn1	1312905-66-0	4H-1-Benzopyran-4-one, 5-[(4-fluorophenyl)methoxy]-3-hydroxy-7,8-diphenyl-2-(2-p	C32H21FN2O4	516.52	A/B	-8.3
6999	O(Cc1ccc(F)cc1)c1c2c(c(c(c1)c1ccccc1)c1ccccc1)oc(c(O)c2=O)c1[nH]c(OC)nn1	1312905-77-3	4H-1-Benzopyran-4-one, 5-[(4-fluorophenyl)methoxy]-3-hydroxy-2-(3-methyl-1H-1,2,	C31H22FN3O4	519.52	A/B	-8.3
7000	O(Cc1ccc(F)cc1)c1c2c(c(c(c1)c1ccccc1)c1ccccc1)oc(c(O)c2=O)c1[nH]c(cn1)c1ccccc1	1312905-84-2	4H-1-Benzopyran-4-one, 2-(5-chloro-1H-imidazol-2-yl)-5-[(4-fluorophenyl)methoxy]	C31H20ClFN2O4	538.95	A/B	-8.3
7001	O=c1c2c(oc(c1O)c1ncc(C)cn1)ccc(OCc1ccc(F)cc1)c2	1312906-15-2	4H-1-Benzopyran-4-one, 6-[(4-fluorophenyl)methoxy]-3-hydroxy-2-(2-pyrimidinyl)-	C20H13FN2O4	364.33	A/B	-8.3
7002	Oc1c(oc2c(c1=O)cc(OCc1ccc(F)cc1)cc2)c1[nH]c(OC)cn1	1312906-30-1	4H-1-Benzopyran-4-one, 6-[(4-fluorophenyl)methoxy]-3-hydroxy-2-(5-methyl-1H-imic	C20H15FN2O4	366.34	A/B	-8.3
7003	O=c1c2c(oc(c1O)c1ncc(cn1)c1ccccc1)c(C)cc(OCc1ccc(F)cc1)c2	1312906-67-4	4H-1-Benzopyran-4-one, 2-(5-chloro-2-pyrimidinyl)-6-[(4-fluorophenyl)methoxy]-3-	C21H14ClFN2O4	412.8	A/B	-8.3
7004	O=c1c2c(oc(c1O)c1[nH]cc(C)n1)c(C)cc(OCc1ccc(F)cc1)c2	1312906-79-8	4H-1-Benzopyran-4-one, 6-[(4-fluorophenyl)methoxy]-3-hydroxy-2-(1H-imidazol-2-yl	C20H15FN2O4	366.34	A/B	-8.3
7005	O(C)c1c2c(c(=O)c(O)c(o2)c2[nH]c(OC)nn2)cc(OCc2ccc(F)cc2)c1	1312907-25-7	4H-1-Benzopyran-4-one, 6-[(4-fluorophenyl)methoxy]-3-hydroxy-8-methoxy-2-(3-meth	C20H16FN3O5	397.36	A/B	-8.3
7006	Clc1c2c(cc(OCc3ccc(F)cc3)c1)c(=O)c(O)c(o2)c1ncc(OC)cn1	1312907-65-5	4H-1-Benzopyran-4-one, 8-chloro-6-[(4-fluorophenyl)methoxy]-3-hydroxy-2-(5-methy	C21H14ClFN2O4	412.8	A/B	-8.3
7007	O=c1c2c(oc(c1O)c1[nH]cc(C)n1)c(Cl)cc(OCc1ccc(F)cc1)c2	1312907-79-1	4H-1-Benzopyran-4-one, 8-chloro-6-[(4-fluorophenyl)methoxy]-3-hydroxy-2-(1H-imic	C19H12ClFN2O4	386.76	A/B	-8.3
7008	O=c1c2c(oc(c1O)c1nnc(C)[nH]1)cc(C)c(OCc1ccc(F)cc1)c2	1312908-74-9	4H-1-Benzopyran-4-one, 6-[(4-fluorophenyl)methoxy]-3-hydroxy-7-methyl-2-(1H-1,2,	C19H14FN3O4	367.33	A/B	-8.3
7009	O=c1c2c(oc(c1O)c1nnc(Cl)[nH]1)cc(C)c(OCc1ccc(F)cc1)c2	1312908-76-1	4H-1-Benzopyran-4-one, 6-[(4-fluorophenyl)methoxy]-3-hydroxy-2-(5-methoxy-1H-1,2	C20H16FN3O5	397.36	A/B	-8.3
7010	O=c1c2c(oc(c1O)c1[nH]cc(C)n1)cc(C)c(OCc1ccc(F)cc1)c2	1312908-79-4	4H-1-Benzopyran-4-one, 6-[(4-fluorophenyl)methoxy]-3-hydroxy-2-(1H-imidazol-2-yl	C20H15FN2O4	366.34	A/B	-8.3
7011	Cc1c2c(cc(OCc3ccc(F)cc3)c1C)c(=O)c(O)c(o2)c1ncc(Cl)cn1	1312909-28-6	4H-1-Benzopyran-4-one, 6-[(4-fluorophenyl)methoxy]-3-hydroxy-2-(5-methoxy-2-pyri	C23H19FN2O5	422.41	A/B	-8.3
7012	O(C)c1c2c(cc(OCc3ccc(F)cc3)c1C)c(=O)c(O)c(o2)c1[nH]c(C)cn1	1312909-91-3	4H-1-Benzopyran-4-one, 6-[(4-fluorophenyl)methoxy]-3-hydroxy-2-(1H-imidazol-2-yl	C21H17FN2O5	396.37	A/B	-8.3
7013	O(C)c1c2c(cc(OCc3ccc(F)cc3)c1C)c(=O)c(O)c(o2)c1[nH]c(cn1)c1ccccc1	1312909-94-6	4H-1-Benzopyran-4-one, 2-(5-chloro-1H-imidazol-2-yl)-6-[(4-fluorophenyl)methoxy]	C21H16ClFN2O5	430.81	A/B	-8.3
7014	Clc1c2c(cc(OCc3ccc(F)cc3)c1C)c(=O)c(O)c(o2)c1ncc(Cl)cn1	1312910-28-3	4H-1-Benzopyran-4-one, 8-chloro-6-[(4-fluorophenyl)methoxy]-3-hydroxy-2-(5-methc	C22H16ClFN2O5	442.82	A/B	-8.3
7015	Clc1c2c(cc(OCc3ccc(F)cc3)c1C)c(=O)c(O)c(o2)c1[nH]c(Cl)nn1	1312910-38-5	4H-1-Benzopyran-4-one, 8-chloro-6-[(4-fluorophenyl)methoxy]-3-hydroxy-2-(5-methc	C20H15ClFN3O5	431.8	A/B	-8.3
7016	Cc1c(c2c(cc1OCc1ccc(F)cc1)c(=O)c(O)c(o2)c1[nH]c(OC)nn1)c1ccccc1	1312910-87-4	4H-1-Benzopyran-4-one, 6-[(4-fluorophenyl)methoxy]-3-hydroxy-7-methyl-2-(3-methy	C26H20FN3O4	457.45	A/B	-8.3
7017	O=c1c2c(oc(c1O)c1ncc(cn1)c1ccccc1)cc(OC)c(OCc1ccc(F)cc1)c2	1312911-29-7	4H-1-Benzopyran-4-one, 2-(5-chloro-2-pyrimidinyl)-6-[(4-fluorophenyl)methoxy]-3-	C21H14ClFN2O5	428.8	A/B	-8.3
7018	O=c1c2c(oc(c1O)c1nnc(Cl)[nH]1)cc(OC)c(OCc1ccc(F)cc1)c2	1312911-38-8	4H-1-Benzopyran-4-one, 6-[(4-fluorophenyl)methoxy]-3-hydroxy-7-methoxy-2-(5-meth	C20H16FN3O6	413.36	A/B	-8.3
7019	O=c1c2c(oc(c1O)c1[nH]c(OC)cn1)cc(OC)c(OCc1ccc(F)cc1)c2	1312911-42-4	4H-1-Benzopyran-4-one, 6-[(4-fluorophenyl)methoxy]-3-hydroxy-7-methoxy-2-(5-meth	C21H17FN2O5	396.37	A/B	-8.3

7020	O=c1c2c(oc(c1O)c1[nH]cnn1)cc(Cl)c(OCc1ccc(F)cc1)c2	1312911-62-8	4H-1-Benzopyran-4-one, 7-chloro-6-[(4-fluorophenyl)methoxy]-3-hydroxy-2-(5-pheny	C26H16ClFN2O4	474.87	A/B	-8.3
7021	O=c1c2c(oc(c1O)c1nnc(Cl)[nH]1)cc(Cl)c(OCc1ccc(F)cc1)c2	1312911-78-6	4H-1-Benzopyran-4-one, 7-chloro-6-[(4-fluorophenyl)methoxy]-3-hydroxy-2-(5-metho	C19H13ClFN3O5	417.77	A/B	-8.3
7022	Cc1c2c(cc(OCc3ccc(F)cc3)c1OC)c(=O)c(O)c(o2)c1ncc(OC)cn1	1312912-11-0	4H-1-Benzopyran-4-one, 6-[(4-fluorophenyl)methoxy]-3-hydroxy-7-methoxy-8-methyl-	C23H19FN2O5	422.41	A/B	-8.3
7023	Cc1c2c(cc(OCc3ccc(F)cc3)c1OC)c(=O)c(O)c(o2)c1[nH]c(Cl)nn1	1312912-34-7	4H-1-Benzopyran-4-one, 6-[(4-fluorophenyl)methoxy]-3-hydroxy-7-methoxy-2-(5-meth	C21H18FN3O6	427.38	A/B	-8.3
7024	O(C)c1c2c(cc(OCc3ccc(F)cc3)c1OC)c(=O)c(O)c(o2)c1[nH]cnn1	1312913-18-0	4H-1-Benzopyran-4-one, 6-[(4-fluorophenyl)methoxy]-3-hydroxy-7,8-dimethoxy-2-(5-	C28H21FN2O6	500.47	A/B	-8.3
7025	O(C)c1c2c(cc(OCc3ccc(F)cc3)c1OC)c(=O)c(O)c(o2)c1nnc(C)[nH]1	1312913-30-6	4H-1-Benzopyran-4-one, 6-[(4-fluorophenyl)methoxy]-3-hydroxy-7,8-dimethoxy-2-(1H	C20H16FN3O6	413.36	A/B	-8.3
7026	O(C)c1c2c(cc(OCc3ccc(F)cc3)c1OC)c(=O)c(O)c(o2)c1[nH]c(OC)nn1	1312913-32-8	4H-1-Benzopyran-4-one, 6-[(4-fluorophenyl)methoxy]-3-hydroxy-7,8-dimethoxy-2-(3-	C21H18FN3O6	427.38	A/B	-8.3
7027	O(C)c1c2c(cc(OCc3ccc(F)cc3)c1OC)c(=O)c(O)c(o2)c1[nH]c(nn1)c1ccccc1	1312913-36-2	4H-1-Benzopyran-4-one, 2-(5-chloro-1H-1,2,4-triazol-3-yl)-6-[(4-fluorophenyl)met	C20H15ClFN3O6	447.8	A/B	-8.3
7028	O(C)c1c2c(cc(OCc3ccc(F)cc3)c1Cl)c(=O)c(O)c(o2)c1[nH]c(C)cn1	1312913-85-1	4H-1-Benzopyran-4-one, 7-chloro-6-[(4-fluorophenyl)methoxy]-3-hydroxy-2-(1H-imic	C20H14ClFN2O5	416.79	A/B	-8.3
7029	O(C)c1c2c(cc(OCc3ccc(F)cc3)c1Cl)c(=O)c(O)c(o2)c1[nH]c(OC)cn1	1312913-87-3	4H-1-Benzopyran-4-one, 7-chloro-6-[(4-fluorophenyl)methoxy]-3-hydroxy-8-methoxy-	C21H16ClFN2O5	430.81	A/B	-8.3
7030	O(C)c1c2c(cc(OCc3ccc(F)cc3)c1Cl)c(=O)c(O)c(o2)c1[nH]c(cn1)c1ccccc1	1312913-91-9	4H-1-Benzopyran-4-one, 7-chloro-2-(5-chloro-1H-imidazol-2-yl)-6-[(4-fluorophenyl	C20H13Cl2FN2O5	451.23	A/B	-8.3
7031	Clc1c2c(cc(OCc3ccc(F)cc3)c1OC)c(=O)c(O)c(o2)c1ncc(OC)cn1	1312914-12-7	4H-1-Benzopyran-4-one, 8-chloro-6-[(4-fluorophenyl)methoxy]-3-hydroxy-7-methoxy-	C22H16ClFN2O5	442.82	A/B	-8.3
7032	Clc1c2c(cc(OCc3ccc(F)cc3)c1OC)c(=O)c(O)c(o2)c1ncc(cn1)c1ccccc1	1312914-16-1	4H-1-Benzopyran-4-one, 8-chloro-2-(5-chloro-2-pyrimidinyl)-6-[(4-fluorophenyl)me	C21H13Cl2FN2O5	463.24	A/B	-8.3
7033	Clc1c2c(cc(OCc3ccc(F)cc3)c1OC)c(=O)c(O)c(o2)c1[nH]c(C)cn1	1312914-40-1	4H-1-Benzopyran-4-one, 8-chloro-6-[(4-fluorophenyl)methoxy]-3-hydroxy-2-(1H-imic	C20H14ClFN2O5	416.79	A/B	-8.3
7034	Clc1c2c(cc(OCc3ccc(F)cc3)c1Cl)c(=O)c(O)c(o2)c1ncc(C)cn1	1312914-55-8	4H-1-Benzopyran-4-one, 7,8-dichloro-6-[(4-fluorophenyl)methoxy]-3-hydroxy-2-(2-p	C20H11Cl2FN2O4	433.22	A/B	-8.3
7035	O(C)c1c(c2c(cc1OCc1ccc(F)cc1)c(=O)c(O)c(o2)c1[nH]c(C)nn1)c1ccccc1	1312915-35-7	4H-1-Benzopyran-4-one, 6-[(4-fluorophenyl)methoxy]-3-hydroxy-7-methoxy-8-phenyl-	C25H18FN3O5	459.43	A/B	-8.3
7036	O=c1c2c(cc(c(OCc3ccc(F)cc3)c2)c2ccccc2)oc(c1O)c1[nH]c(cn1)c1ccccc1	1312916-49-6	4H-1-Benzopyran-4-one, 2-(5-chloro-1H-imidazol-2-yl)-6-[(4-fluorophenyl)methoxy]	C25H16ClFN2O4	462.86	A/B	-8.3
7037	Cc1c2c(cc(OCc3ccc(F)cc3)c1c1ccccc1)c(=O)c(O)c(o2)c1ncc(cn1)c1ccccc1	1312916-85-0	4H-1-Benzopyran-4-one, 2-(5-chloro-2-pyrimidinyl)-6-[(4-fluorophenyl)methoxy]-3-	C27H18ClFN2O4	488.89	A/B	-8.3
7038	O(Cc1ccc(F)cc1)c1c(c(C)c2c(c1)c(=O)c(O)c(o2)c1[nH]cnn1)c1ccccc1	1312916-86-1	4H-1-Benzopyran-4-one, 6-[(4-fluorophenyl)methoxy]-3-hydroxy-8-methyl-7-phenyl-2	C33H23FN2O4	530.55	A/B	-8.3
7039	O(C)c1c(c(OCc2ccc(F)cc2)cc2c1oc(c(O)c2=O)c1[nH]c(Cl)nn1)c1ccccc1	1312917-44-4	4H-1-Benzopyran-4-one, 6-[(4-fluorophenyl)methoxy]-3-hydroxy-8-methoxy-2-(5-meth	C26H20FN3O6	489.45	A/B	-8.3
7040	O(C)c1c(c(OCc2ccc(F)cc2)cc2c1oc(c(O)c2=O)c1[nH]c(C)cn1)c1ccccc1	1312917-47-7	4H-1-Benzopyran-4-one, 6-[(4-fluorophenyl)methoxy]-3-hydroxy-2-(1H-imidazol-2-yl	C26H19FN2O5	458.44	A/B	-8.3
7041	O(C)c1c(c(OCc2ccc(F)cc2)cc2c1oc(c(O)c2=O)c1[nH]c(cn1)c1ccccc1)c1ccccc1	1312917-50-2	4H-1-Benzopyran-4-one, 2-(5-chloro-1H-imidazol-2-yl)-6-[(4-fluorophenyl)methoxy]	C26H18ClFN2O5	492.88	A/B	-8.3
7042	Clc1c2c(cc(OCc3ccc(F)cc3)c1c1ccccc1)c(=O)c(O)c(o2)c1ncc(C)cn1	1312917-82-0	4H-1-Benzopyran-4-one, 8-chloro-6-[(4-fluorophenyl)methoxy]-3-hydroxy-7-phenyl-2	C26H16ClFN2O4	474.87	A/B	-8.3
7043	O(Cc1ccc(F)cc1)c1c(c(c2c(c1)c(=O)c(O)c(o2)c1ncc(OC)cn1)c1ccccc1)c1ccccc1	1312918-33-4	4H-1-Benzopyran-4-one, 6-[(4-fluorophenyl)methoxy]-3-hydroxy-2-(5-methyl-2-pyrim	C33H23FN2O4	530.55	A/B	-8.3
7044	O=c1n([C@@H]2O[C@H](COC(=O)CCC(=O)Oc3ccc(/C(=N/O)/c4ccc(Cl)cc4)cc3)C=C2)cc(C)c(=O)[nH]1	1314187-83-1	Thymidine, 2,Ä≤,3,Ä≤-didehydro-3,Ä≤-deoxy-, 5,Ä≤-[4-[4-[(3E)-3-(hydroxyimino)bu	C24H27N3O8	485.49	A/B	-8.3
7045	O[C@H]1[C@H](n2c3c(nc2)c(=O)nc[nH]3)O[C@H](CO)C1	1314187-84-2	Thymidine, 2,Ä≤,3,Ä≤-didehydro-3,Ä≤-deoxy-, 5,Ä≤-[4-[4-[(Z)-(4-chlorophenyl)(hy	C27H24ClN3O8	553.95	A/B	-8.3
7046	O=c1n([C@H]2C[C@@H]([Se]c3ccccc3)[C@@H](CO)O2)cc(C)c(=O)[nH]1	131933-44-3	Thymidine, 3,Ä≤-Se-phenyl-3,Ä≤-seleno-	C16H18N2O4Se	381.29	A/B	-8.3
7047	N(=Nc1c(O)c2c(c(S(=O)(=O)O)c1)cccc2)c1c2c(c(N=Nc3c(C)cc(cc3)c3cc(C)c(N=Nc4c(O)c5c(c(S(=O)(=O)O)c4)cc	131933-47-6	2,4(1H,3H)-Pyrimidinedione, 1-(2-deoxy-3-Se-phenyl-3-seleno-Œ≤-D-threo-pentofura	C16H18N2O4Se	381.29	A/B	-8.3
7048	C(CC(=O)C[C@H](CCc1ccc(O)cc1)O)c1cc(O)c(O)cc1	132-34-3	2,7-Naphthalenedisulfonic acid, 3-amino-4-[2-[4,Ä≤-[2-(2-amino-6-sulfo-1-naphtha	C34H28N6O9S3	760.82	A/B	-8.3
7049	N(c1c(C)c(c(/C(=C(/C#N)\[2H])/[2H])c(c1C)[2H])[2H])c1nc(Nc2c(cc(C#N)cc2[2H])[2H])ncc1	1331866-68-2	Benzonitrile, 4-[[6-[[4-[(1E)-2-cyanoethenyl-1,2-d2]-2,6-di(methyl-d3)phenyl-3,5	C22H6D12N6		A/B	-8.3
7050	C(#CC1CC1)[C@]1(C(F)(F)F)c2c(NC(=O)O1)ccc(Br)c2	1332592-15-0	Not Yet Assigned	C29H40N4O4	508.65	A/B	-8.3
7051	C(=O)(OC)c1cc(c2ccc(C[C@@H]([C@@H](CN(S(=O)(=O)c3ccc(OC)cc3)CC(C)C)O)NC(=O)O[C@H]3CCOC3)cc2)ccc1	1335386-72-5	Carbamic acid, N-[(1S,2R)-1-[(4,Ä≤-chloro[1,1,Ä≤-biphenyl]-4-yl)methyl]-2-hydro	C32H39ClN2O7S	631.18	A/B	-8.3
7052	C(=O)([C@@H](NC(=O)OC)C(C)C)N1[C@@H](CC[C@H]1C)c1[nH]c(cn1)c1ccc(C#Cc2ccc(cc2)c2[nH]c(nc2)[C@H]2N(C(	1337532-47-4	Carbamic acid, N,N,Ä≤-[1,2-ethynediylbis[4,1-phenylene-1H-imidazole-5,2-diyl(2,5	C42H46N8O6	758.86	A/B	-8.3
7053	C(=O)([C@@H](NC(=O)OC)C(C)C)N1[C@H](C=C(C)C1)c1[nH]c(cn1)c1ccc(C#Cc2ccc(cc2)c2[nH]c(nc2)[C@@H]2N(C(=	1337532-63-4	Carbamic acid, N,N,Ä≤-[1,2-ethynediylbis[4,1-phenylene-1H-imidazole-5,2-diyl[(2S	C46H58N8O6	819	A/B	-8.3
7054	O(CC=C)[C@H]1[C@H](n2c3c(nc2)c(=O)nc(N)[nH]3)O[C@H](CO)[C@H]1O	1337533-21-7	Carbamic acid, N,N,Ä≤-[1,2-ethynediylbis[4,1-phenylene-1H-imidazole-5,2-diyl(2,5	C44H50N8O6	786.92	A/B	-8.3
7055	O(c1c(C(F)(F)F)ccn(Cc2n(C)c(=O)[nH]n2)c1=O)c1cc(C(F)F)cc(C#N)c1	1338226-03-1	Benzonitrile, 3-[[5-bromo-1-[(4,5-dihydro-4-methyl-5-oxo-1H-1,2,4-triazol-3-yl)m	C17H10BrClF3N5O3	504.65	A/B	-8.3
7056	O=c1n([C@@H]2O[C@H](COP(=O)(O)O)[C@@H](O)C2)cc(F)c(=O)[nH]1	1339955-42-8	Benzenepropanamide, Œ±-amino-N-[(1S)-2-[(1S,3S,5S)-3-cyano-2-azabicyclo[3.1.0]he	C27H34N4O4	478.58	A/B	-8.3
7057	C(=O)(OC)c1c(CCCCCCCCCCCCCCC)cc(O)cc1O	1342797-87-8	Furo[3,Ä≤,4,Ä≤:6,7]naphtho[2,3-d]-1,3-dioxol-6(5aH)-one, 5,8,8a,9-tetrahydro-5-	C28H26O10S	554.57	A/B	-8.3
7058	O=c1n([C@@H]2O[C@H](CO)SC2)ccc(N)n1	1346799-59-4	2,7,10,12-Tetraazatridecanoic acid, 4-hydroxy-12-methyl-13-[2-(1-methylethoxy)-4	C37H48N6O6S2	736.94	A/B	-8.3
7059	O(C(=O)N[C@@H](Cc1ccc(N(C)C)cc1)[C@@H](CN(S(=O)(=O)c1ccc(OCP(=O)(OCC)N(C)C)cc1)CC(C)C)O)[C@@H]1[C@H]	1347069-23-1	Spiro[3-azabicyclo[3.1.0]hexane-6,1,Ä≤-cyclopropane]-2-carboxamide, N-[3-amino-1	C37H53N5O6	663.85	A/B	-8.3
7060	C(=O)(NC(C)(C)C)[C@H]1N(C[C@H]([C@@H](NC(=O)c2c(C)c(O)ccc2)CSC2CCCCC2)O)C[C@@H]2[C@H](C1)CCCC2	1348247-59-5	Not Yet Assigned	C50H64N6O8	877.08	A/B	-8.3
7061	C([C@@H](OCP(=O)(O)O)CO)N1C(=O)NC(N)C=C1	1348482-26-7	2,7,10,12-Tetraazatridecanoic acid, 13-[2-(dimethylamino)-4-thiazolyl]-4-hydroxy	C36H47N7O5S2	721.93	A/B	-8.3
7062	P(=O)(COc1ccc(C[C@@H]([C@@H](CN(S(=O)(=O)c2ccc(N)cc2)CC(C)C)O)NC(=O)OC2CCOC2)cc1)(c1ccc(O)cc1)c1ccc(	1348884-84-3	Not Yet Assigned	C38H59N7O6	709.92	A/B	-8.3
7063	O(CC(=O)N[C@@H](Cc1ccccc1)[C@H](C[C@H](Cc1ccccc1)NC(=O)[C@H](C(C)C)N1C(=O)NCC=C1)O)c1c(SC)cccc1SC	1349520-05-3	2,5,6,10,13-Pentaazatetradecanedioic acid, 3-(2,2-dimethylpropyl)-8-hydroxy-12-(	C38H52N6O7	704.86	A/B	-8.3
7064	C([C@H]([C@H](Cc1ccccc1)NC(=O)[C@@H](NC(=O)c1nc2c(cc1)cccc2)C(=O)N)O)N1[C@H](C(=O)NC(C)(C)C)C[C@H]2[	1349752-23-3	[1,1,Ä≤-Biphenyl]-2-sulfonamide, 4,Ä≤-[(2-ethyl-5,7-dimethyl-3H-imidazo[4,5-b]p	C28H33N5O5S	551.66	A/B	-8.3
7065	C(C(OCP(=O)(OP(=O)(OP(=O)(O)O)O)O)C)N1c2c(NC1)c(N)ncn2	1349834-40-7	Not Yet Assigned	C33H50N6O7	642.79	A/B	-8.3
7066	C([C@H]([C@H](Cc1ccccc1)NC(=O)[C@@H](NC(=O)c1nc2c(cc1)cccc2)CC(=O)N)O)N1[C@H](C(=O)NC(C)(C)C)CC2C(C1	1349843-40-8	1,6-Heptadiene-3,5-dione, 4-hydroxy-1,7-bis(3,4,5-trihydroxyphenyl)-, (1E,6E)-	C19H16O9	388.32	A/B	-8.3
7067	C(n1c2c(nc1CC)c(C)cc(C)n2)c1ccc(cc1)c1c(S(=O)(=O)NC(=O)OC(C)(C)C)ccc(CC(C)C)c1	1350067-27-4	2,7,10,12-Tetraazatridecanoic acid, 4-hydroxy-12-methyl-9-(1-methylethyl)-8,11-c	C39H52N6O5S2	749	A/B	-8.3
7068	O=c1n([C@@H]2O[C@H](COC(=O)CCC(=O)OC(COC(=O)CCCCCCCCCCCCCCC)COC(=O)CCCCCCCCCCCCCCC)C=C2)cc(C)c(=O)[n	1350627-74-5	Guanosine, 8-[(1E)-2-[4-(trifluoromethyl)phenyl]ethenyl]-	C19H18F3N5O5	453.37	A/B	-8.3
7069	O=C1[C@@H](n2c3c(nc2)c(=O)nc(N)[nH]3)C[C@H](O)[C@H]1CO	1350713-68-6	Not Yet Assigned	C22H18N4O8	466.4	A/B	-8.3
7070	C(=O)([C@@H](NC(=O)OC)C(C)C)N1[C@H](c2[nH]c(c3c4c(c(cc3)c3c5c(c(cc3)c3[nH]c(nc3)[C@H]3N(C(=O)[C@@H](	1350718-55-6	Carbamic acid, N,N,Ä≤-[[1,1,Ä≤-binaphthalene]-4,4,Ä≤-diylbis[1H-imidazole-5,2-d	C48H54N8O6	838.99	A/B	-8.3
7071	C(=O)([C@@H](NC(=O)OC)C(C)C)N1[C@@]2(C[C@H](C1)CC2)c1[nH]c(cn1)c1c2c(c(cc1)c1ccc(cc1)c1[nH]c(nc1)[C@	1350718-84-1	Carbamic acid, N-[(1S)-1-[[(2S)-2-[5-[4,Ä≤-[2-[(1S,4R)-2-[(2S)-2-[(methoxycarbon	C42H52N8O6	764.91	A/B	-8.3
7072	C(=O)([C@@H](NC(=O)OC)C(C)C)N1[C@H](c2[nH]c(c3c4c(c(cc3)c3c5c(c(cc3)c3[nH]c(nc3)[C@H]3N(C(=O)[C@@H](	1350719-36-6	Carbamic acid, N,N,Ä≤-[[4,4,Ä≤-bibenzofuran]-7,7,Ä≤-diylbis[1H-imidazole-5,2-di	C44H50N8O8	818.92	A/B	-8.3
7073	C(=O)([C@@H](NC(=O)OC)C(C)C)N1[C@H](c2[nH]c(c3c4c(c(cc3)c3c5c(c(cc3)c3[nH]c(nc3)[C@H]3N(C(=O)[C@@H](	1350719-40-2	Carbamic acid, N,N,Ä≤-[[7,7,Ä≤-bi-1H-indole]-4,4,Ä≤-diylbis[1H-imidazole-5,2-di	C44H52N10O6	816.95	A/B	-8.3
7074	C(=O)([C@@H](NC(=O)OC)CC#CC)N1[C@@H](CCC1)c1[nH]c(cn1)c1ccc(cc1)c1cc2c(cc1)cc(cc2)c1[nH]c(nc1)[C@H]1	1351332-82-5	Carbamic acid, N-[(1S)-1-[[(2S)-2-[5-[6-[4-[2-[(2S)-1-[(2S)-2-[(methoxycarbonyl)	C44H50N8O6	786.92	A/B	-8.3
7075	C(=O)([C@@H](NC(=O)OC)C(C)C)N1C[C@@]2(C[C@H]1c1[nH]c(cn1)c1ccc(cc1)c1ccc(cc1)c1[nH]c(nc1)[C@H]1N(C(=	1351852-68-0	Carbamic acid, N-[(1S)-1-[[(2S,4S)-4-cyclopropyl-2-[5-[4,Ä≤-[2-[(5S,8S)-7-[(2S)-	C46H58N8O7	835	A/B	-8.3
7076	C(=O)([C@@H](NC(=O)OC)C(C)C)N1C[C@@]2(C[C@H]1c1[nH]c(cn1)c1ccc(cc1)c1ccc(cc1)c1c(CO)nc([nH]1)[C@H]1N	1351852-75-9	Carbamic acid, N-[(1S)-1-[[(2S)-2-[5-[4,Ä≤-[2-[(5S,8S)-7-[(2S)-2-[(methoxycarbon	C45H58N8O8	838.99	A/B	-8.3
7077	C(=O)([C@@H](NC(=O)OC)C(C)C)N1C[C@@]2(C[C@H]1c1[nH]c(cn1)c1c(F)cc(cc1)c1cc(F)c(cc1)c1[nH]c(nc1)[C@H]	1351852-79-3	Carbamic acid, N-[(1S)-1-[[(2S)-2-[5-[4,Ä≤-[5-chloro-2-[(5S,8S)-7-[(2S)-2-[(meth	C43H53ClN8O7	829.38	A/B	-8.3
7078	C(=O)([C@@H](NC(=O)OC)C(C)C)N1C[C@@]2(C[C@H]1c1[nH]c(cn1)c1c(C(F)(F)F)cc(cc1)c1cc(C(F)(F)F)c(cc1)c1[	1351852-85-1	Carbamic acid, N-[(1S)-2-methyl-1-[[(2S)-2-[5-[2,2,Ä≤,3,3,Ä≤-tetraethyl-4,Ä≤-[2	C51H70N8O7	907.15	A/B	-8.3
7079	C(=O)([C@@H](NC(=O)OC)C(C)C)N1CC2(C[C@H]1c1[nH]c(cn1)c1ccc(cc1)c1ccc(cc1)c1[nH]c(nc1)[C@H]1N(C(=O)[C	1351853-19-4	Carbamic acid, N-[(1S)-1-[[(2S)-2-[5-[4,Ä≤-[2-[(3S,6S)-1,1-difluoro-5-[(2S)-2-[	C42H50F2N8O6	800.89	A/B	-8.3
7080	C(=O)([C@@H](NC(=O)OC)C(C)C)N1[C@@H](C[C@@H](C1)C1CC1)c1[nH]c(cn1)c1ccc(cc1)c1ccc(cc1)c1[nH]c(nc1)[C	1351855-78-1	Carbamic acid, N-[(1S)-1-[[(2S,4R)-4-cyclopropyl-2-[5-[4,Ä≤-[2-[(1R,3S,4S)-2-[(2	C45H56N8O6	804.98	A/B	-8.3
7081	C(=O)([C@@H](NC(=O)OC)C(C)C)N1CC2(C[C@H]1c1[nH]c(cn1)c1ccc(cc1)c1ccc(cc1)c1[nH]c(nc1)[C@H]1N(C(=O)[C	1351855-87-2	Carbamic acid, N-[(1S)-1-[[(2S,4R)-4-cyclopropyl-2-[5-[4,Ä≤-[2-[(1R,3S,5R)-2-[(2	C44H54N8O6	790.95	A/B	-8.3
7082	C(=O)([C@@H](NC(=O)OC)C(C)C)N1[C@@H](C=C(C)C1)c1[nH]c(C#Cc2cc3c(cc2)cc(cc3)c2[nH]c(nc2)[C@H]2N(C(=O)	1352392-10-9	Carbamic acid, N-[(1S)-1-[[(2S)-2-[5-[2-[6-[2-[(2S)-1-[(2S)-2-[(methoxycarbonyl)	C43H52N8O6	776.92	A/B	-8.3
7083	C(C(OCP(=O)(O)O)C#C)n1c2c(nc1)c(N)ncn2	1352476-95-9	4-Pyrimidinecarboxamide, N-[(4-fluorophenyl)methyl]-1,4,5,6-tetrahydro-1-methyl-	C20H21FN6O5	444.42	A/B	-8.3
7084	C(=O)(n1c(=O)n(cc(C)c1=O)[C@@H]1O[C@H](CO)C=C1)C1(C)CCCC1	1353143-21-1	2,4(1H,3H)-Pyrimidinedione, 3-(cyclopentylcarbonyl)-1-(2-deoxy-Œ≤-L-erythro-pent	C16H19F3N2O6	392.33	A/B	-8.3
7085	O=c1n(cc(C(F)(F)F)c(=O)n1C(=O)OCc1ccccc1)[C@@H]1O[C@H](CO)[C@@H](O)C1	1353372-57-2	Thymidine, 2,Ä≤,3,Ä≤-didehydro-3,Ä≤-deoxy-3-[(phenylmethoxy)carbonyl]-	C18H18N2O6	358.35	A/B	-8.3
7086	O(C(=O)n1c(=O)n(cc(C)c1=O)[C@@H]1O[C@H](CO)C=C1)C1(C)CCCCC1	1353441-66-3	1(2H)-Pyrimidinecarboxylic acid, 3-(2-deoxy-Œ≤-L-erythro-pentofuranosyl)-3,6-dih	C17H21F3N2O7	422.35	A/B	-8.3
7087	O[C@H]1[C@@H](O[C@H](COP(=O)(N[C@@H](Cc2ccc(O)cc2)C(=O)OCC)Oc2ccc(N(=O)=O)cc2)[C@H]1O)n1nc(C(=O)N)nc	1353639-70-9	L-Tyrosine, N-[hydroxy(4-nitrophenoxy)phosphinyl]-, methyl ester, 5,Ä≤-ester wit	C24H27N6O12P	622.48	A/B	-8.3
7088	O[C@H]1[C@@H](O[C@H](COP(=O)(N[C@@H](Cc2ccccc2)C(=O)OC)Oc2ccc(N(=O)=O)cc2)[C@H]1O)n1nc(C(=O)N)nc1	1353639-71-0	L-Tyrosine, N-[hydroxy(4-nitrophenoxy)phosphinyl]-, ethyl ester, 5,Ä≤-ester with	C25H29N6O12P	636.5	A/B	-8.3
7089	C([C@H](NP(=O)(Oc1ccc(N(=O)=O)cc1)OC[C@H]1O[C@H]([C@H](O)[C@@H]1O)n1nc(C(=O)N)nc1)C(=O)OC)c1c2c([nH]	1353639-74-3	L-Proline, 1-[hydroxy(4-nitrophenoxy)phosphinyl]-, phenylmethyl ester, 5,Ä≤-este	C26H29N6O11P	632.52	A/B	-8.3
7090	C(F)(F)c1n(c2c(n1)cccc2)c1nc(nc(NC(=O)c2ccccc2)n1)N1CCOCC1	1359951-93-1	1,3,5-Triazin-2-amine, N-[2-(cyclopentylsulfinyl)ethyl]-4-[2-(difluoromethyl)-1H	C22H27F2N7O2S	491.56	A/B	-8.3
7091	C(F)(F)c1n(c2c(n1)cccc2)c1nc(nc(NC2CCCCC2)n1)N1CCOCC1	1359952-72-9	1,3,5-Triazin-2-amine, 4-[2-(difluoromethyl)-1H-benzimidazol-1-yl]-6-(4-morpholi	C20H23F2N7O2	431.44	A/B	-8.3
7092	C(F)(F)c1n(c2c(n1)cccc2)c1nc(nc(Nc2c(OC)cccc2)n1)N1CCOCC1	1359953-53-9	1,3,5-Triazin-2-amine, 4-[2-(difluoromethyl)-1H-benzimidazol-1-yl]-6-(4-morpholi	C20H23F2N7O2	431.44	A/B	-8.3
7093	C(F)(F)c1n(c2c(n1)cccc2)c1nc(nc(N(C)C2CCN(C)CC2)n1)N1CCOCC1	1359955-66-0	4-Piperidinol, 4-[[[4-[2-(difluoromethyl)-1H-benzimidazol-1-yl]-6-(4-morpholinyl	C22H28F2N8O2	474.51	A/B	-8.3
7094	C(F)(F)c1n(c2c(n1)cccc2)c1nc(nc(N(C)c2ccc(OC)cc2)n1)N1CCOCC1	1359956-34-5	1,3,5-Triazin-2-amine, 4-[2-(difluoromethyl)-1H-benzimidazol-1-yl]-N-methyl-6-(4	C21H20F2N8O	438.43	A/B	-8.3
7095	O=c1n([C@@H]2O[C@H](COP(=O)(OCCCCCCCCCCCCCC)O)[C@@H](N=[N+]=[N-])C2)cc(C)c(=O)[nH]1	1363821-21-9	Thymidine, thymidylyl-(3,Ä≤,Üí5,Ä≤)-4-deoxy-	C20H27N4O11P	530.42	A/B	-8.3
7096	O(CCOC)c1c(c(OCCOC)cc(c1)c1[nH]c(nc1)[C@H]1N(C(=O)[C@@H](NC(=O)OC)C(C)C)CCC1)c1ccc(cc1)c1[nH]c(nc1)[	1367573-33-8	Carbamic acid, N-[(1S)-1-[[(2S)-2-[5-[4-[2,3,5,6,8,9-hexahydro-14-[2-[(2S)-1-[(2	C46H60N8O10	885.02	A/B	-8.3
7097	C(=O)([C@@H](NC(=O)OC)C(C)C)N1[C@H]2[C@H](C2)C[C@H]1c1[nH]c(c(Cl)n1)c1ccc(cc1)c1ccc(cc1)c1[nH]c(nc1)	1373165-76-4	Carbamic acid, N-[(1S)-1-[[(2S,4S)-2-[5-chloro-4-[4,Ä≤-[2-[(1R,3S,5R)-2-[(2S)-2-	C42H51ClN8O7	815.36	A/B	-8.3
7098	O=c1n([C@@H]2O[C@H](CO)[C@@H](O)C2)cc(CC#C)c(=O)[nH]1	1374152-22-3	1-Piperazinecarboxamide, N-[(1S)-1-[[[(1S)-3-[(4-fluorophenyl)sulfonyl]-1-(2-phe	C29H39FN4O4S	558.71	A/B	-8.3
7099	O[C@H]1[C@@H](O[C@@H](CO)[C@H]1N=[N+]=[N-])n1c(=O)[nH]c(=O)c(C)c1	1375881-15-4	Carbamic acid, N,N,Ä≤-[[1,1,Ä≤:4,Ä≤,1,Ä≤,Ä≤:4,Ä≤,Ä≤,1,Ä≤,Ä≤,Ä≤-quaterphenyl]-4	C52H58N8O6	891.07	A/B	-8.3
7100	C(=O)([C@H](NC(=O)OC)c1ccccc1)N1[C@@H](CC(=C)C1)c1[nH]c(cn1)c1ccc(cc1)c1cc2c(cc1)cc(cc2)c1[nH]c(nc1)	1377037-91-6	Carbamic acid, N-[(1S)-1-[[(2S)-2-[5-[6-[4-[2-[(2S)-1-[2-(dimethylamino)-2-pheny	C47H52N8O4	792.97	A/B	-8.3
7101	C(=C1c2c(-c3c1cc(cc3)c1[nH]c(nc1)[C@H]1N(C(=O)[C@@H](NC(=O)OC)C(C)C)CCC1)ccc(c2)c1[nH]c(nc1)[C@H]1N(	1377040-05-5	Carbamic acid, N-[(1S)-1-[[(2S)-2-[5-[3-[6-[2-[(2S)-1-[(2S)-2-[(methoxycarbonyl)	C44H52N8O6	788.93	A/B	-8.3
7102	C(=O)([C@@H](NC(=O)OC)C(C)C)N1[C@@H]([C@@H]2C[C@H]1CC2)c1[nH]c2c(n1)ccc1c2ccc(c1)c1ccc(cc1)c1[nH]c(n	1377042-08-4	Carbamic acid, N-[(1S)-1-[[(2S)-2-[2,Ä≤-[(1S,3S,4R)-2-[(2S)-2-[(methoxycarbonyl)	C46H52N8O6	812.96	A/B	-8.3
7103	C(=O)([C@@H](NC(=O)OC)C(C)C)N1[C@H]2[C@H](C2)C[C@H]1c1[nH]c2c(n1)ccc1c2ccc(c1)c1ccc(cc1)c1[nH]c(nc1C	1377043-10-1	Carbamic acid, N-[(1S)-2-[(1R,3S,5R)-3-[7-[4-[2-[(1R,3S,4S)-2-[(2S)-2-[(methoxyc	C47H54N8O7	842.98	A/B	-8.3
7104	C(=O)([C@@H](NC(=O)OC)C(C)C)N1[C@H]2[C@H](C2)C[C@H]1c1[nH]c(cn1)c1c(F)cc(cc1)c1cc2c(cc1)c1c(cc2)nc([	1377043-47-4	Carbamic acid, N-[(1S)-1-[[(2S)-2-[7-[4-[5-chloro-2-[(1R,3S,4S)-2-[(2S)-2-[(meth	C44H51ClN8O6	823.38	A/B	-8.3
7105	C(=O)([C@@H](NC(=O)OC)C(C)C)N1[C@H](c2[nH]c3c4c(cc(cc4)c4cc5c(cc4)c4c(cc5)nc([nH]4)[C@H]4N(C(=O)[C@@	1377043-72-5	Carbamic acid, N-[(1S)-1-[[(2S)-2-[2,Ä≤-[(2S,4R)-1-[(2S)-2-[(methoxycarbonyl)am	C46H54N8O7	830.97	A/B	-8.3
7106	C(=O)([C@@H](NC(=O)OC)C(C)C)N1[C@H](c2[nH]c(c3cc4c5-c6c(cc(cc6OC4)c4[nH]c(nc4)[C@H]4N(C(=O)[C@@H](NC	1377045-94-7	Carbamic acid, N-[(1S)-1-[[(2S,4S)-2-[2,Ä≤-[(1R,3S,5R)-2-[(2S)-2-[(methoxycarbon	C47H54N8O7	842.98	A/B	-8.3
7107	C(=O)([C@@H](NC(=O)OC)C(C)C)N1[C@H](c2[nH]c3c4c(cc(cc4)c4cc5c(cc4)c4c(cc5)nc([nH]4)[C@H]4N(C(=O)[C@@	1377047-25-0	Carbamic acid, N-[(1S)-1-[[(2S)-2-[2,Ä≤-[(2S,4S)-4-[(difluoromethoxy)methyl]-1-	C46H52F2N8O7	866.95	A/B	-8.3
7108	N(=Nc1cc(C)c(N=Nc2ccc(C(=O)O)cc2)cc1)c1c2c(c(N=Nc3ccc(O)cc3)cc1)ccc(S(=O)(=O)O)c2	1378293-75-4	2-Naphthalenesulfonic acid, 5-[2-(4-hydroxyphenyl)diazenyl]-8-[2-[3-methyl-4-[2-	C29H22N6O7S2	630.65	A/B	-8.3
7109	N(=Nc1cc(C)c(N=Nc2ccc(C(=O)O)cc2)cc1)c1c2c(ccc(S(=O)(=O)O)c2)c(N)cc1	1378293-82-3	Benzoic acid, 4-[2-[4-[2-[4-[2-(4-hydroxyphenyl)diazenyl]-7-sulfo-1-naphthalenyl	C30H22N6O6S	594.6	A/B	-8.3
7110	N(=Nc1cc(C)c(N=Nc2ccc(S(=O)(=O)O)cc2)cc1)c1c2c(ccc(S(=O)(=O)O)c2)c(N)cc1	1378293-93-6	Benzoic acid, 4-[2-[4-[2-(4-amino-7-sulfo-1-naphthalenyl)diazenyl]-2-methylpheny	C24H19N5O5S	489.5	A/B	-8.3
7111	C(CC(CC(CCc1ccc(O)cc1)O)O)c1cc(O)c(O)cc1	137965-14-1	9H-Purin-6-amine, 9-[3,5-bis-O-(phenylmethyl)-Œ≤-D-arabinofuranosyl]-	C24H25N5O4	447.49	A/B	-8.3
7112	C(=O)([C@H](NC(=O)OC)C(C)C)N1[C@H](CCC1)c1[nH]c(cn1)[C@H]1CC[C@@H](CC1)[C@H]1CC[C@@H](CC1)c1[nH]c(nc	1380289-73-5	Carbamic acid, N-[(1R)-1-[[(2R)-2-[5-[6-[4-[2-[(2R)-1-[(2R)-2-[(methoxycarbonyl)	C42H48N8O6	760.88	A/B	-8.3
7113	C(=O)([C@@H](NC(=O)OC)C(C)C)N1[C@@H](CCC1)c1[nH]c(cn1)c1ccc(C#CC#Cc2[nH]c(nc2)[C@H]2N(C(=O)[C@@H](NC	1380292-75-0	Carbamic acid, N-[(1S)-1-[[(2R)-2-[5-(4,Ä≤-cyclohexyl[1,1,Ä≤-biphenyl]-4-yl)-1H-	C32H40N4O3	528.68	A/B	-8.3
7114	C(=O)(C=Cc1cc(O)ccc1)c1c(OC)cc(OC)c(C(=O)C=Cc2cc(O)ccc2)c1	1383782-58-8	2-Propen-1-one, 1,1,Ä≤-(4,6-dimethoxy-1,3-phenylene)bis[3-(2-hydroxyphenyl)-	C26H22O6	430.45	A/B	-8.3
7115	C(=O)([C@@H](NC(=O)OC)C(C)C)N1[C@H](c2[nH]c(c(n2)c2ccccc2)c2ccc(cc2)c2ccc(cc2)c2c(nc([nH]2)[C@H]2N(C	1388224-01-8	Benzonitrile, 4-[[4-[[4-[(1E)-2-cyanoethenyl]-2,6-dimethylphenyl]amino]-2-pyrimi	C22H18N6.xC7H5NO3S		A/B	-8.3
7116	N(C(=O)C)[C@H]1[C@H]([C@@H]([C@@H](CO)O)O)OC(=C[C@@H]1NC(=N)N)C(=O)O.N	1391054-30-0	[9,9,Ä≤-Bi-6H-dipyrido[3,2-b:2,Ä≤,3,Ä≤-e][1,4]diazepine]-6,6,Ä≤-dione, 11,11,Ä≤	C30H26N8O2	530.58	A/B	-8.3
7117	N(CC(=O)N(C)C)c1nc(nc(n1)N1CCOCC1)c1cc(C(=O)N)ccc1	1391924-88-1	Glycine, N-acetyl-N-[4-[3-(acetylamino)phenyl]-6-(4-morpholinyl)-1,3,5-triazin-2	C19H22N6O5	414.42	A/B	-8.3
7118	Nc1nc(nc(n1)N1CCOCC1)c1cc(OC)ccc1	1391924-94-9	1,3,5-Triazine, 2-(6-fluoro-3-pyridinyl)-4-(4-morpholinyl)-6-(1-piperazinyl)-	C16H20FN7O	345.37	A/B	-8.3
7119	N(CC(=O)N(C)C)c1nc(nc(n1)N1CCOCC1)c1ccc(=O)[nH]c1	1391925-08-8	Acetamide, 2-[[4-(3-cyanophenyl)-6-(4-morpholinyl)-1,3,5-triazin-2-yl]amino]-N,N	C18H21N7O2	367.4	A/B	-8.3
7120	N(CC(=O)N)c1nc(nc(n1)N1CCOCC1)c1cc(OC)ccc1	1391925-46-4	Acetamide, 2-[[4-(3-hydroxyphenyl)-6-(4-morpholinyl)-1,3,5-triazin-2-yl]amino]-	C15H18N6O3	330.34	A/B	-8.3
7121	N(CC(=O)N(C)C)c1nc(nc(n1)N1CCOCC1)c1ccc[illegible]	1391925-49-7	Glycine, N-[4-[3-(hydroxyamino)phenyl]-6-(4-morpholinyl)-1,3,5-triazin-2-yl]-	C15H18N6O4	346.34	A/B	-8.3
7122	N(CC(=O)O)(C)c1nc(nc(n1)N1CCOCC1)c1cc(O)ccc1	1391925-53-3	Alanine, N-[4-(3-hydroxyphenyl)-6-(4-morpholinyl)-1,3,5-triazin-2-yl]-2-methyl-	C17H21N5O4	359.38	A/B	-8.3
7123	N(C(CO)(C)C)c1nc(nc(n1)N1CCOCC1)c1c2c([nH]nc2)ccc1	1391925-98-6	1,3,5-Triazin-2-amine, 4-(1H-indazol-4-yl)-6-(4-morpholinyl)-	C14H15N7O	297.32	A/B	-8.3
7124	N(C(C(=O)N)C)c1nc(nc(n1)N1CCOCC1)c1c2c([nH]nc2)ccc1	1391925-99-7	1-Propanol, 2-[[4-(1H-indazol-4-yl)-6-(4-morpholinyl)-1,3,5-triazin-2-yl]amino]-	C18H23N7O2	369.42	A/B	-8.3
7125	C[C@]1(F)[C@@H](O[C@H](CO[P@@](=O)(Oc2ccc(Br)cc2)N[C@H](C(=O)OC(C)C)C)[C@H]1O)n1c(=O)[nH]c(=O)cc1	1394157-26-6	L-Alanine, N-[[P(R),2,Ä≤R]-P-(4-bromophenyl)-2,Ä≤-deoxy-2,Ä≤-fluoro-2,Ä≤-methyl	C22H28BrFN3O9P	608.35	A/B	-8.3
7126	N(c1c(C)cc(/C=C/C#N)cc1C)c1nc(Nc2ccc(C#N)cc2)ncc1.S(=O)(=O)(C)O	1399384-91-8	Benzonitrile, 4-[[4-[[4-[(1E)-2-cyanoethenyl]-2,6-dimethylphenyl]amino]-2-pyrimi	C22H18N6.C7H8O3S		A/B	-8.3
7127	O=C1c2n(C[C@H]3N1[C@H](C([2H])([2H])[2H])C(C(O3)([2H])[2H])([2H])[2H])cc(C(=O)NC(c1(F)cc(F)cc1)([2H	1407166-91-9	2H-Pyrido[1,Ä≤,2,Ä≤:4,5]pyrazino[2,1-b][1,3]oxazine-2,3,4-d3-9-carboxamide, N-[	C20H9D10F2N3O5		A/B	-8.3
7128	O=C1c2n(C[C@]3(N1[C@](C)C(C(O3)([2H])[2H])([2H])[2H])([2H])[2H])cc(C(=O)NC(c1(F)cc(F)cc1)([2H])[2H	1407167-08-1	2H-Pyrido[1,Ä≤,2,Ä≤:4,5]pyrazino[2,1-b][1,3]oxazine-2,3,4-d3-9-carboxamide, N-[	C20H11D8F2N3O5		A/B	-8.3
7129	O=C1c2n(C([C@]3(N1[C@](C)C(C(O3)([2H])[2H])([2H])[2H])([2H])[2H])([2H])[2H])cc(C(=O)NC(c1(F)cc(F)cc	1407167-14-9	2H-Pyrido[1,Ä≤,2,Ä≤:4,5]pyrazino[2,1-b][1,3]oxazine-2,3,12-d3-9-carboxamide, N-	C20H7D12F2N3O5		A/B	-8.3
7130	O=C1c2n(C([C@]3(N1[C@H](C)C(C(O3)([2H])[2H])([2H])[2H])([2H])[2H])([2H])[2H])cc(C(=O)NC(c1(F)cc(F)cc1)([2	1407167-15-0	2H-Pyrido[1,Ä≤,2,Ä≤:4,5]pyrazino[2,1-b][1,3]oxazine-2,3,4,12-d4-9-carboxamide,	C20H9D10F2N3O5		A/B	-8.3
7131	C(OC[C@@H]1[C@@H](OC(=O)c2ccccc2)[C@@H](OC(=O)c2ccccc2)[C@@H](O1)n1nc(C(=O)N)nc1)(c1ccc(OC)cc1)(c1ccc	140891-08-3	1H-1,2,4-Triazole-3-carboxamide, 1-[3-O-benzoyl-5-O-[bis(4-methoxyphenyl)phenylm	C36H34N4O8	650.68	A/B	-8.3
7132	C(OC[C@@H]1[C@@H](OC(=O)c2ccccc2)[C@@H](OC(=O)c2ccccc2)[C@@H](O1)n1nc(C(=O)N)nc1)(c1ccc(OC)cc1)(c1ccc	140891-38-9	1H-1,2,4-Triazole-3-carboxamide, 1-[5-O-benzoyl-3-O-[(1,4-dihydro-1-methyl-3-pyr	C27H31N5O8	553.56	A/B	-8.3
7133	O(C(=O)c1ccccc1)[C@H]1[C@@H](O[C@@H](CO)[C@H]1OC(=O)C1=CN(C)C=CC1)n1nc(C(=O)N)nc1	140891-42-5	1H-1,2,4-Triazole-3-carboxamide, 1-[2-O-benzoyl-3-O-[(1,4-dihydro-1-methyl-3-pyr	C22H23N5O7	469.45	A/B	-8.3
7134	S(=O)(=O)(O)c1cc2c(ccc(N=Nc3cc(S(=O)(=O)O)c(C=Cc4c(S(=O)(=O)O)cc(N=Nc5c(N)c6c(cc5)cccc6S(=O)(=O)O)cc4)	140942-33-2	2-Naphthalenesulfonic acid, 6,6,Ä≤-[1,2-ethenediylbis[(3-sulfo-4,1-phenylene)az	C34H26N6O12S4.4Na		A/B	-8.3
7135	[C@H](C(=O)N1[C@H]2[C@H](C2)C[C@H]1c1[nH]c(cn1)c1ccc(cc1)c1ccc(cc1)c1[nH]c(nc1)[C@@H]1N(C(=O)[C@@H](N	1412813-73-0	Carbamic acid, N,N,Ä≤-[[1,1,Ä≤-biphenyl]-4,4,Ä≤-diylbis[1H-imidazole-5,2-diyl-(	C48H54N8O8	870.99	A/B	-8.3
7136	C(=O)(C(NC(=O)OC)C1CC(=C)C1)N1[C@H](CC[C@@H]1C)c1[nH]c(cn1)c1ccc(cc1)c1ccc(cc1)c1[nH]c(nc1)[C@@H]1N(	1412816-64-8	Carbamic acid, N,N,Ä≤-[[1,1,Ä≤-biphenyl]-4,4,Ä≤-diylbis[1H-imidazole-5,2-diyl[(	C48H58N8O6	843.02	A/B	-8.3

7137	O[C@H]1[C@@H](O[C@H](C(=O)N[C@H](Cc2ccccc2)C(=O)NCc2cc(on2)c2cc(OC)ccc2)[C@H]1O)n1nc(C(=O)N)nc1	1414793-77-3	Not Yet Assigned	C19H20N6O7	444.4	A/B	-8.3
7138	C(=O)([C@@H](NC(=O)OC)C(C)C)N1[C@H](c2[nH]c(c3c4cc(c(c3)CCc3ccc(CC4)cc3)c3ccc(cc3)c3[nH]c(nc3)[C@H]3	1415119-53-7	Carbamic acid, N,N,Ä≤-[bicyclo[1.1.1]pentane-1,3-diylbis[4,1-phenylene-1H-imida	C45H56N8O6	804.98	A/B	-8.3
7139	C(=O)(Nc1sc(N(=O)=O)cn1)c1c(OC(=O)C)c(N(=O)=O)cc(N(=O)=O)c1	1415566-88-9	Benzamide, 2-(benzoyloxy)-5-methoxy-N-(5-nitro-2-thiazolyl)-	C18H13N3O6S	399.38	A/B	-8.3
7140	C(N(NC(=O)[C@@H](NC(=O)OC([2H])([2H])[2H])C(C([2H])([2H])[2H])(C([2H])([2H])[2H])C([2H])([2H])[2H])C	1415634-41-1	2,5,6,10,13-Pentaazatetradecanedioic acid, 3-(1,1-dimethylethyl)-8-hydroxy-12-(2	C38H47D5N6O8		A/B	-8.3
7141	N(c1nc(nc(NC(=S)Nc2c(OC)cccc2)n1)N1CCOCC1)n1c(SC)nnc(C(C)(C)C)c1=O	1418543-24-4	Urea, N-[4-[[6-(1,1-dimethylethyl)-3-(methylthio)-5-oxo-1,2,4-triazin-4(5H)-yl]a	C22H28N10O3S	512.59	A/B	-8.3
7142	N(n1c(SC)nnc(C(C)(C)C)c1=O)c1nc(nc(NC(=S)Nc2ccc(OC)cc2)n1)N1CCOCC1	1418543-93-7	Urea, N-(4-bromophenyl)-N,Ä≤-[4-[[6-(1,1-dimethylethyl)-3-(methylthio)-5-oxo-1,2	C22H27BrN10O3S	591.48	A/B	-8.3
7143	O=c1n([C@@H]2O[C@H](CO)C(=C2)C)ccc(=O)[nH]1	1418554-35-4	Urea, N-[4-[[6-(1,1-dimethylethyl)-3-(methylthio)-5-oxo-1,2,4-triazin-4(5H)-yl]a	C22H27FN10O3S	530.58	A/B	-8.3
7144	C(=O)(OCC)C1=C(N)OC(=CC1c1cc(N(=O)=O)ccc1)c1[nH]c2c(n1)cccc2	1426813-47-9	4H-Pyran-3-carboxylic acid, 2-amino-6-(1H-benzimidazol-2-yl)-4-(3-hydroxyphenyl)	C21H19N3O4	377.39	A/B	-8.3
7145	C(=O)(OCC)C1=C(N)OC(=CC1c1cc(OC)ccc1)c1[nH]c2c(n1)cccc2	1426813-89-9	4H-Pyran-3-carboxylic acid, 2-amino-6-(1H-benzimidazol-2-yl)-4-(2-methylphenyl)-	C22H21N3O3	375.42	A/B	-8.3
7146	C(=O)(OCC)C1=C(N)OC(=CC1c1ccc(OCC)cc1)c1[nH]c2c(n1)cccc2	1426814-14-3	4H-Pyran-3-carboxylic acid, 2-amino-6-(1H-benzimidazol-2-yl)-4-(3-chlorophenyl)-	C21H18ClN3O3	395.84	A/B	-8.3
7147	C(=O)(OCC)C1=C(N)OC(=CC1c1cc(C)ccc1)c1[nH]c2c(n1)cccc2	1426814-72-3	4H-Pyran-3-carboxylic acid, 2-amino-6-(1H-benzimidazol-2-yl)-4-(3-fluorophenyl)-	C21H18FN3O3	379.38	A/B	-8.3
7148	O[C@H]1[C@H](n2c3c(nc2)c(=O)nc(N)[nH]3)O[C@H](COP(=O)(OCC#C)O)[C@H]1O	1428470-49-8	Diphosphonic acid, P,P,Ä≤-bis[[(1R)-2-(6-amino-9H-purin-9-yl)-1-methylethoxy]met	C30H34N10O7P2	708.6	A/B	-8.3
7149	O(C(=O)[C@H](C(C)C)N)c1c(OC)cc(CCC(=O)/C=C(/CCc2cc(OC)c(OC(=O)[C@H](C(C)C)N)cc2)\O)cc1	1429402-73-2	L-Proline, 2,2,Ä≤-[[(3Z)-3-hydroxy-5-oxo-3-heptene-1,7-diyl]bis(2-methoxy-4,1-ph	C31H38N2O8	566.64	A/B	-8.3
7150	C(#N)[C@]1(O[C@H](CO[P@@](=O)(Oc2ccccc2)N[C@H](C(=O)OCc2ccccc2)C)[C@@H](O)[C@@]1(C)O)c1n2c(cc1)c(N)n	1431932-95-4	L-Alanine, N-[(S)-hydroxyphenoxyphosphinyl]-, phenylmethyl ester, 6-ester with 2	C29H31N6O8P	622.57	A/B	-8.3
7151	O=c1n([C@H]2O[C@@H](CO)[C@H](O)C2)cc(C=CBr)c(=O)[nH]1	143224-34-4	Butanediamide, N1-[(1S,2R)-3-[[[(1,1-dimethylethyl)amino]carbonyl](2-methylpropy	C33H44N6O5	604.74	A/B	-8.3
7152	O=C1[C@@H]2[C@@H](c3c([C@@H](OCCCO)[C@H]2CO1)cc1c(c3)OCO1)c1cc(OC)c(O)c(OC)c1	143247-84-1	Furo[3,Ä≤,4,Ä≤:6,7]naphtho[2,3-d]-1,3-dioxol-6(5aH)-one, 9-(cyclohexyloxy)-5,8,	C27H30O8	482.52	A/B	-8.3
7153	O[C@H]1[C@H](n2c3c(nc2)c(NCC)ncn3)O[C@H](CO)[C@H]1O	1435477-82-9	Benzonitrile, 2-amino-4-[[4-[(2,4,6-trimethylphenyl)amino]-2-pyrimidinyl]amino]-	C20H20N6	344.41	A/B	-8.3
7154	N(C(=O)[C@H](Cc1ccccc1)C[C@@H](CN1[C@H](C(=O)NC(C)(C)C)CN(Cc2c(SC)ccnc2)CC1)O)[C@H]1c2c(C[C@H]1O)ccc	1438395-52-8	D-erythro-Pentonamide, 2,3,5-trideoxy-N-[(1S,2R)-2,3-dihydro-2-hydroxy-1H-inden-	C37H49N5O5	643.82	A/B	-8.3
7155	O=c1n([C@@H]2O[C@H](CO)[C@@H](O)C2)cc(C=CCN)c(=O)[nH]1	1438819-50-1	D-erythro-Pentonamide, 2,3,5-trideoxy-N-[(1S,2R)-2,3-dihydro-2-hydroxy-1H-inden-	C37H48N6O5S2.C36H47N5O4		A/B	-8.3
7156	[C@H](Cc1ccccc1)(C[C@@H]([C@H](Cc1ccccc1)NC(=O)OCc1cccnc1)O)NC(=O)[C@@H](NC(=O)N(Cc1cscn1)C)C(C)C	144142-00-7	2,4,7,12-Tetraazatridecan-13-oic acid, 9-hydroxy-2-methyl-5-(1-methylethyl)-3,6-	C36H44N6O5S	672.84	A/B	-8.3
7157	[C@H](Cc1ccccc1)(C[C@@H]([C@H](Cc1ccccc1)NC(=O)OCc1cccnc1)O)NC(=O)[C@@H](NC(=O)N(Cc1csc(N)n1)C)C(C)C	144142-12-1	2,4,7,12-Tetraazatridecan-13-oic acid, 9-hydroxy-2-methyl-5-(1-methylethyl)-1-(2	C37H46N6O5S	686.86	A/B	-8.3
7158	N(C(=O)C)[C@H]1[C@H]([C@@H]([C@@H](CO)O)O)OC(=C[C@@H]1NC(=N)NC(=O)c1ccc(C(C)(C)C)cc1)C(=O)O	1442563-02-1	D-glycero-D-galacto-Non-2-enonic acid, 5-(acetylamino)-2,6-anhydro-4-[[(benzoyla	C19H24N4O8	436.42	A/B	-8.3
7159	N(C(=O)C)[C@H]1[C@H]([C@@H]([C@@H](CO)O)O)OC(=C[C@@H]1NC(=N)NC(=O)Cc1cc2c(cc1)cccc2)C(=O)O	1442563-06-5	D-glycero-D-galacto-Non-2-enonic acid, 5-(acetylamino)-2,6-anhydro-3,4,5-trideox	C20H26N4O8	450.44	A/B	-8.3
7160	[C@H](OC)([C@H]1[C@H](NC(=O)C)[C@@H](NC(=N)N)C=C(C(=O)O)O1)[C@@H]1OC(CCCCCCC)(OC)OC1	144269-19-2	2-Naphthalenesulfonic acid, 8,8,Ä≤-[[1,1,Ä≤-biphenyl]-4,4,Ä≤-diylbis(azo)]bis[7	C32H24N6O8S2	684.7	A/B	-8.3
7161	N(Cc1ccc(cc1)c1scc(C)c1)C12CC3CC(C2)CC(C1)C3	1443121-68-3	2-Quinolinemethanamine, N-tricyclo[3.3.1.13,7]dec-1-yl-	C20H24N2	292.42	A/B	-8.3
7162	C(CC(COC(=O)C)COC(=O)C)n1c2c(nc1)cnc(NC(=O)CCC/C=C\C/C=C\C/C=C\C/C=C\C/C=C\CC)n2	1443545-43-4	Thymidine, 3,Ä≤-azido-3,Ä≤-deoxy-, 5,Ä≤-[N-[2-[[(5Z,8Z,11Z,14Z,17Z)-1-oxo-5,8,1	C33H47N7O6		A/B	-8.3
7163	C[C@]1(F)[C@@H](O[C@@](CO[P@](=O)(Oc2cc3c(cc2)cccc3)N[C@H](C(=O)OC(C)C)C)(F)[C@H]1O)n1c(=O)[nH]c(=O)	144356-85-4	[1,1,Ä≤-Biphenyl]-3,3,Ä≤-dicarboxylic acid, 4,4,Ä≤-bis[(2-aminobenzoyl)amino]-	C28H22N4O6	510.5	A/B	-8.3
7164	C[C@]1(F)[C@@H](O[C@@](CO[P@](=O)(Oc2c3c(ccc2)cccc3)N[C@H](C(=O)OC(C)C)C)(F)[C@H]1O)n1c(=O)[nH]c(=O)	1443648-64-3	L-Alanine, N-[[P(S),2,Ä≤R]-2,Ä≤-deoxy-2,Ä≤-fluoro-4,Ä≤-C-fluoro-2,Ä≤-methyl-P-2	C26H30F2N3O9P	597.5	A/B	-8.3
7165	S(c1n(c2c(n1)c(=O)nc(N)[nH]2)[C@@H]1O[C@H]2[C@H]([C@H]1O)OP(=O)(O)OC2)c1c(O)cccc1	144509-90-0	Guanosine, 8-[(3-chlorophenyl)thio]-, cyclic 3,Ä≤,5,Ä≤-(hydrogen phosphate)	C16H15ClN5O7PS	487.81	A/B	-8.3
7166	S(c1n(c2c(n1)c(=O)nc(N)[nH]2)[C@@H]1O[C@H]2[C@H]([C@H]1O)OP(=O)(O)OC2)c1cc(OC)ccc1	144509-98-8	Guanosine, 8-[(4-fluorophenyl)thio]-, cyclic 3,Ä≤,5,Ä≤-(hydrogen phosphate)	C16H15FN5O7PS	471.36	A/B	-8.3
7167	O[C@H]1[C@@H](O[C@H](COP(=O)(OP(=O)(OP(=O)(OC)O)O)O)[C@H]1O)n1c(=O)nc(N)cc1	1445947-74-9	1H-1,2,4-Triazole-3-carboxamide, 5,Ä≤,5,Ä≤,Ä≤,Ä≤-O-(oxydiphosphinico)bis[1-Œ≤-D	C16H24N8O15P2	630.35	A/B	-8.3
7168	N(c1c2c(c(O)c(CN3CC(C)CC(C)C3)c1)CCCC2)c1c2c(ncc1)cc(Cl)cc2	1449239-42-2	1-Naphthalenol, 4-[(7-chloro-4-quinolinyl)amino]-5,6,7,8-tetrahydro-2-[(2-methyl	C26H30ClN3O	435.99	A/B	-8.3
7169	O=C1[C@@H]2[C@@H](c3c([C@H](OC(=O)Cc4c5c(ccc4)cccc5)[C@H]2CO1)cc1c(c3)OCO1)c1c(Cl)c(OC)c(OC)c(OC)c1	1449601-77-7	Benzeneacetic acid, (5R,5aR,8aR,9S)-9-(2-chloro-3,4,5-trimethoxyphenyl)-5,5a,6,8	C30H27ClO9	566.98	A/B	-8.3
7170	O=C1[C@@H]2[C@@H](c3c([C@H](OC(=O)c4cc(Cl)ccc4)[C@H]2CO1)cc1c(c3)OCO1)c1c(Br)c(OC)c(OC)c(OC)c1	1449602-05-4	Benzoic acid, 4-fluoro-, (5R,5aR,8aR,9S)-9-(2-bromo-3,4,5-trimethoxyphenyl)-5,5a	C29H24BrFO9	615.4	A/B	-8.3
7171	O=C1[C@@H]2[C@@H](c3c([C@H](OC(=O)Cc4ccc(Br)cc4)[C@H]2CO1)cc1c(c3)OCO1)c1c(Br)c(OC)c(OC)c(OC)c1	1449602-12-3	Benzoic acid, 4-cyano-, (5R,5aR,8aR,9S)-9-(2-bromo-3,4,5-trimethoxyphenyl)-5,5a,	C30H24BrNO9	622.42	A/B	-8.3
7172	CN1C(C23CC4(CC(C3)CC(C2)C4)c2ccccc2)CN=C1C	1449651-16-4	1H-Imidazole, 4,5-dihydro-2-methyl-5-(3-phenyltricyclo[3.3.1.13,7]dec-1-yl)-	C20H26N2	294.43	A/B	-8.3
7173	C(=O)([C@@H](NC(=O)OC)C(C)C)N1[C@H](c2[nH]c(c3cc4OCC5(CC5)COc5c(-c4cc3)ccc(c5)c3[nH]c(nc3)[C@H]3N(C(	1449762-28-0	Carbamic acid, N,N,Ä≤-[(7,8-dihydro-7,7-dimethyl-6H-dibenzo[f,h][1,5]dioxonin-3,	C51H66N8O8	919.12	A/B	-8.3
7174	N(c1c2c(ncc1)cc(Cl)cc2)c1c(O)c(CN2C(C)CCCC2)cc(CN2C(C)CCCC2)c1	145431-57-8	Phenol, 2-[(7-chloro-4-quinolinyl)amino]-4,6-bis(1-piperidinylmethyl)-	C27H33ClN4O	465.03	A/B	-8.3
7175	N(c1c2c(ncc1)cc(Cl)cc2)c1c(O)c(CN2CC(C)CC(C)C2)cc(CN2CC(C)CC(C)C2)c1	145431-60-3	Phenol, 2-[(7-chloro-4-quinolinyl)amino]-4,6-bis[(4-methyl-1-piperidinyl)methyl]	C29H37ClN4O	493.08	A/B	-8.3
7176	N(CCNc1c2c(ncc1)cc(Cl)cc2)c1nc(nc(NCCCCO)n1)N1CCOCC1	1454695-07-8	1,3,5-Triazine-2,4-diamine, N2-[4-[(7-chloro-4-quinolinyl)amino]butyl]-6-(4-morp	C26H29ClN8O	505.01	A/B	-8.3
7177	C(C)C1c2c3c(C(O1)C1=NCCN1)cccc3ccc2	1465783-89-4	1-Piperazinecarboxamide, 4-methyl-N-[(1S)-2-oxo-2-[[(1S,2E)-1-(2-phenylethyl)-3-	C32H38N4O4S.ClH		A/B	-8.3
7178	N(c1c(C)cc(/C=C/C#N)cc1C)c1nc(Nc2ccc(C#N)cc2)ncc1.C(=O)(CC)C.Cl	1469779-07-4	Benzonitrile, 4-[[4-[[4-[(1E)-2-cyanoethenyl]-2,6-dimethylphenyl]amino]-2-pyrimi	C22H18N6.C3H6O.ClH		A/B	-8.3
7179	[C@H](Cc1ccccc1)(C(=O)N[C@@H](CCc1ccccc1)CCS(=O)(=O)c1ccccc1)NC(=O)N1CCN(C)CC1	1469779-28-9	Benzonitrile, 4-[[4-[[4-[(1E)-2-cyanoethenyl]-2,6-dimethylphenyl]amino]-2-pyrimi	C22H18N6.C6H10O.ClH		A/B	-8.3
7180	C(OC1COP(=O)(CNC(C(=O)OCC)c2ccc(O)cc2)OC1)n1c2c(nc1)c(=O)nc(N)[nH]2	1473358-99-4	Benzeneacetic acid, Œ±-[[[5-[(2-amino-1,6-dihydro-6-oxo-9H-purin-9-yl)methoxy]-2	C20H25N6O7P	492.42	A/B	-8.3
7181	C(OC[C@H]1N(C(=O)OC(C)(C)C)[C@H]([C@H](OC(=O)[C@@H](NC(=O)OC(C)(C)C)C(C)C)[C@@H]1OC(=O)[C@@H](NC(=O)	1474023-28-3	L-Leucine, N-[(1,1-dimethylethoxy)carbonyl]-, [(3aR,4R,6S,6aS)-6-(4-azido-5H-pyr	C30H44N8O8		A/B	-8.3
7182	C(=O)(c1cc2c([nH]1)ccc(NS(=O)(=O)CCCN1CCCCC1)c2)N1CCN(CC1)c1c(NC(C)(C)C)cccn1	147540-34-9	1-Piperidinepropanesulfonamide, N-[2-[[4-[3-[(1-methylethyl)amino]-2-pyridinyl]-	C29H41N7O3S	567.75	A/B	-8.3
7183	C(n1c2c(nc1CC)c(C)ccn2)c1ccc(cc1)c1c(S(=O)(=O)NC(=O)OCCC)cccc1	148401-74-5	Carbamic acid, [[4,Ä≤-[(2-ethyl-7-methyl-3H-imidazo[4,5-b]pyridin-3-yl)methyl][1	C25H26N4O4S	478.56	A/B	-8.3
7184	C(n1c2c(nc1CC)c(C)ccn2)c1ccc(cc1)c1c(S(=O)(=O)NC(=O)OCc2ccccc2)cccc1	148401-76-7	Carbamic acid, [[4,Ä≤-[(2-ethyl-7-methyl-3H-imidazo[4,5-b]pyridin-3-yl)methyl][1	C25H24N4O4S	476.55	A/B	-8.3
7185	C(=O)([C@@H](NC(=O)OC)[C@H](CC)C)N1[C@@H](C[C@H](COC)C1)c1[nH]c(cn1)c1ccc(cc1)c1ccc(cc1)c1[nH]c(nc1)	1491957-15-3	Carbamic acid, N-[(1S)-2-[(2S,4S)-2-[5-[4,Ä≤-[2-[(2S,5S)-1-[(2S)-2-[(methoxycarb	C45H58N8O8	838.99	A/B	-8.3
7186	O=c1n(cc([C@H](OC)C)c(=O)[nH]1)[C@@H]1O[C@H](CO)[C@@H](O)C1	1501307-18-1	2H-Indol-2-one, 1,3-dihydro-5-[(4-hydroxy-4-methyl-1-piperidinyl)sulfonyl]-	C14H18N2O4S	310.37	A/B	-8.3
7187	N(c1c(C)cc(/C=C/C#N)cc1C)c1nc(Nc2ccc(C#N)cc2)ncc1.C(=CC(=O)O)c1cc(OC)c(O)cc1	1502822-68-5	Benzonitrile, 4-[[4-[[4-[(1E)-2-cyanoethenyl]-2,6-dimethylphenyl]amino]-2-pyrimi	C22H18N6.xC2H2O4		A/B	-8.3
7188	N(c1c(C)cc(/C=C/C#N)cc1C)c1nc(Nc2ccc(C#N)cc2)ncc1.C(=O)(O)c1c(O)cccc1	1502822-69-6	2-Propenoic acid, 3-(4-hydroxy-3-methoxyphenyl)-, compd. with 4-[[4-[[4-[(1E)-2-	C22H18N6.C10H10O4		A/B	-8.3
7189	N(c1c(C)cc(/C=C/C#N)cc1C)c1nc(Nc2ccc(C#N)cc2)ncc1.[C@H](C(=O)O)(O)c1ccccc1	1502822-72-1	Acetic acid, 2-hydroxy-, compd. with 4-[[4-[[4-[(1E)-2-cyanoethenyl]-2,6-dimethy	C22H18N6.C2H4O3		A/B	-8.3
7190	C(OCCOC(=O)C)n1c2c(nc1)c(=O)nc[nH]2	150557-91-8	Inosine, 2,Ä≤-deoxy-2-[4-[2-(trifluoromethyl)phenyl]-1-piperazinyl]-	C21H23F3N6O4	480.44	A/B	-8.3
7191	C(=CC(=O)c1c(O)cc(OC)cc1)c1c(OC)ccc(C(=O)c2ccccc2)c1	151467-41-3	Carbamic acid, [[3-[4-[(2-ethyl-5,7-dimethyl-3H-imidazo[4,5-b]pyridin-3-yl)methy	C29H36N4O4S2	568.75	A/B	-8.3
7192	C(n1c2c(nc1CC)c(C)cc(C)n2)c1ccc(cc1)c1c(S(=O)(=O)NC(=O)OCCCC)sc(CC(C)C)c1	15147-27-0	Chalcone, 5-benzoyl-2,Ä≤-hydroxy-2,4,Ä≤-dimethoxy-	C24H20O5	388.41	A/B	-8.3
7193	C(OCCOP(=O)(N[C@@H](C(=O)OCC(C)(C)C)C)N[C@H](C(=O)OCC(C)(C)C)C)n1c2c(nc1)c(=O)nc(N)[nH]2	1526922-07-5	5,8-Dioxa-3-aza-4-phosphanonanoic acid, 9-(2-amino-1,6-dihydro-6-oxo-9H-purin-9-	C28H34N7O8P	627.59	A/B	-8.3
7194	C[C@@]12[C@@H]3[C@H]([C@H]4[C@](C)(CC3)[C@@H]([C@@H](CCCC(C)C)C)CC4)CC[C@H]1[C@H](CC=C)[C@H](O)CC2	1527517-44-7	5,Ä≤-Guanylic acid, mono[1,2-dihydro-3,6-dimethyl-2-oxo-4-pyridinyl] ester	C17H21N6O9P	484.36	A/B	-8.3
7195	C(=O)(NC(CC1CCC1)C(=O)C(=O)NCC=C)[C@@H]1[C@@H]2[C@@H](C2(C)C)CN1C(=O)[C@@H](NC(=O)N[C@@H](CN1C(=O)CC	1528752-99-9	3-Azabicyclo[3.1.0]hexane-2-carboxamide, 3-[(2S)-2-[[[[(1R)-1-[(4,4-dimethyl-2,6	C38H64N6O7	716.95	A/B	-8.3
7196	C(N1c2c(cc(S(=O)(=O)N3CCC(C)CC3)cc2)C(=O)C1=O)c1ccccc1	1529814-96-7	1H-Indole-2,3-dione, 5-[(2-methyl-1-piperidinyl)sulfonyl]-	C14H16N2O4S	308.35	A/B	-8.3
7197	C(n1c2c(nc1CC)c(C)cc(C)n2)c1ccc(cc1)c1c(S(=O)(=O)NC(=O)CCCCC)ccc(C(C)C)c1	154551-75-4	Hexanamide, N-[[5-butyl-4,Ä≤-[(2-ethyl-5,7-dimethyl-3H-imidazo[4,5-b]pyridin-3-y	C33H42N4O3S	574.78	A/B	-8.3
7198	C(n1c2c(nc1CC)c(C)cc(C)n2)c1ccc(cc1)c1c(S(=O)(=O)NC(=O)COCCCC)ccc(CC(C)C)c1	154551-97-0	Acetamide, 2-butoxy-N-[[5-butoxy-4,Ä≤-[(2-ethyl-5,7-dimethyl-3H-imidazo[4,5-b]py	C33H42N4O5S	606.78	A/B	-8.3
7199	C(n1c2c(nc1CC)c(C)cc(C)n2)c1ccc(cc1)c1c(S(=O)(=O)NC(=O)COCCCC)ccc(COC)c1	154552-00-8	Acetamide, 2-butoxy-N-[[4,Ä≤-[(2-ethyl-5,7-dimethyl-3H-imidazo[4,5-b]pyridin-3-y	C31H38N4O4S2	594.79	A/B	-8.3
7200	C(n1c2c(nc1CC)c(C)cc(C)n2)c1ccc(cc1)c1c(S(=O)(=O)NC(=O)COCC)ccc(C(C)C)c1	154552-15-5	Acetamide, N-[[5-butyl-4,Ä≤-[(2-ethyl-5,7-dimethyl-3H-imidazo[4,5-b]pyridin-3-y]	C31H38N4O4S	562.72	A/B	-8.3
7201	C(n1c2c(nc1CC)c(C)cc(C)n2)c1ccc(cc1)c1c(S(=O)(=O)NC(=O)COCC)ccc(OCC(F)(F)F)c1	154552-27-9	Acetamide, N-[[5-[(diethylamino)methyl]-4,Ä≤-[(2-ethyl-5,7-dimethyl-3H-imidazo[4	C32H41N5O4S	591.76	A/B	-8.3
7202	C(n1c2c(nc1CC)c(C)cc(C)n2)c1ccc(cc1)c1c(S(=O)(=O)NC(=O)NCCC)ccc(C(C)C)c1	154552-35-9	[1,1,Ä≤-Biphenyl]-2-sulfonamide, 5-butyl-N-[(butylamino)carbonyl]-4,Ä≤-[(2-ethy	C32H41N5O3S	575.76	A/B	-8.3
7203	C(=O)([C@@H](NC(=O)OC)C(C)C)N1[C@H](c2[nH]c(c3c4c(C5CC4CC5)c(cc3)c3ccc(cc3)c3[nH]c(nc3)[C@H]3N(C(=O)	1560638-84-7	Carbamic acid, N-[(1S)-2-methyl-1-[[(2S)-2-[5-[4-[1,2,3,4-tetrahydro-8-[2-[(2S)-	C45H56N8O6	804.98	A/B	-8.3
7204	C(=O)([C@@H](NC(=O)OC)C(C)C)N1[C@H](c2[nH]c(c3c4c(c(cc3)c3ccc(cc3)c3[nH]c(nc3)[C@H]3N(C(=O)[C@@H](NC	1560640-20-1	Carbamic acid, N-[(1S)-1-cyclohexyl-2-[(2S)-2-[5-[8-[4-[2-[(2S)-1-[(2S)-2-cycloh	C52H66N8O6	899.13	A/B	-8.3
7205	CN1C2c3c(C1CC2)c(ccc3c1[nH]c(nc1)[C@H]1N(C(=O)[C@@H](NC(=O)OC)C(C)C)CCC1)c1ccc(cc1)c1[nH]c(nc1)[C@H]	1560649-18-4	Carbamic acid, N-[(1S)-1-[[(2S,4S)-4-fluoro-2-[5-[8-[4-[2-[(2S,4S)-4-fluoro-1-[(	C45H54F2N8O6	840.96	A/B	-8.3
7206	N(c1c2c(cc(Cl)cc2)ncc1)c1c(O)c(CN(CC)CC)cc(Cl)c1	1578257-14-3	1H-1,2,4-Triazole-3-carboxamide, 1-[5-O-[(2R,4S)-4-(3-chlorophenyl)-2-oxido-1,3,	C23H28ClN4O10P	586.92	A/B	-8.3
7207	O=c1n([C@@H]2O[C@H](COP(=O)(OC[C@@H]3[C@@H](N=[N+]=[N-])C[C@@H](O3)n3c(=O)[nH]c(=O)c(C)c3)COc3ccccc3	1579273-87-2	Thymidine, 3,Ä≤-azido-3,Ä≤-deoxy-, 5,Ä≤,5,Ä≤,Ä≤,Ä≤-[P-(methoxymethyl)phosphona	C22H29N10O10P		A/B	-8.3
7208	O=c1n([C@@H]2O[C@H](COP(=O)(OC[C@@H]3[C@@H](N=[N+]=[N-])C[C@@H](O3)n3c(=O)[nH]c(=O)c(C)c3)CF)[C@@H](	1579273-88-3	Thymidine, 3,Ä≤-azido-3,Ä≤-deoxy-, 5,Ä≤,5,Ä≤,Ä≤,Ä≤-[P-(phenoxymethyl)phosphona	C27H31N10O10P		A/B	-8.3
7209	O(C(=O)N[C@@H](Cc1ccccc1)[C@@H](CN(S(=O)(=O)c1ccc(NC(=O)OC(C)(C)C)cc1)CC(C)C)O)[C@@H]1[C@H]2[C@@H](O	157968-65-5	Furo[3,Ä≤,4,Ä≤:6,7]naphtho[2,3-d]-1,3-dioxol-6(5aH)-one, 5,8,8a,9-tetrahydro-9-	C32H40O9	568.65	A/B	-8.3
7210	C(=O)([C@@H](NC(=O)OC)C(C)C)N1[C@H](c2[nH]c(c3c4c(C5(CC4)CCCC5)c(cc3)c3ccc(cc3)c3[nH]c(nc3)[C@H]3N(C	1588403-98-8	Carbamic acid, N-[(1S)-1-[[(2S,4S)-4-fluoro-2-[5-[4-[4,Ä≤-[2-[(2S,4S)-4-fluoro-	C47H58F2N8O6	869.01	A/B	-8.3
7211	N(C(=O)Cn1cc(CCCC)nn1)C12CC3CC(C2)CC(C1)C3	159734-61-9	Guanosine 5,Ä≤-(tetrahydrogen triphosphate), 2,Ä≤-deoxy-N-(2-methyl-1-oxopropyl	C14H22N5O14P3	577.27	A/B	-8.3
7212	O=C1[C@@H]2[C@@H](c3c([C@@H](OCC#C)[C@H]2CO1)cc1c(c3)OCO1)c1cc(OC)c(OCC#C)c(OC)c1	1600500-12-6	Furo[3,Ä≤,4,Ä≤:6,7]naphtho[2,3-d]-1,3-dioxol-6(5aH)-one, 5,8,8a,9-tetrahydro-9-	C31H29N3O8	571.58	A/B	-8.3
7213	O=c1c2c(n(cn2)[C@H]2O[C@@H](CO)CC2)[nH]cn1	160956-62-7	9H-Purin-6-amine, 9-[5-O-[[[6-(Œ≤-D-galactopyranosyloxy)hexyl]oxy]hydroxyphosph	C22H36N5O13P	609.52	A/B	-8.3
7214	N(c1c(C)cc(/C=C/C#N)cc1C)c1nc(Nc2ccc(C#N)cc2)c(F)cn1	1609687-04-8	Benzonitrile, 4-[[4-[(4-cyanophenyl)amino]-5-fluoro-2-pyrimidinyl]amino]-3,5-dim	C20H15FN6	358.37	A/B	-8.3
7215	O=c1c2c(n(cn2)[C@@H]2C(=C)[C@H](COP(=O)(Oc3ccccc3)N[C@H](C(=O)OCC)C)[C@@H](O)C2)[nH]c(N)n1	1610358-00-3	Glycine, N-[[[(1R,3S,5S)-3-(2-amino-1,6-dihydro-6-oxo-9H-purin-9-yl)-5-hydroxy-2	C23H29N6O7P	532.49	A/B	-8.3
7216	O=c1c2c(n(cn2)[C@@H]2C(=C)[C@H](CO[P@](=O)(Oc3ccccc3)N[C@H](C(=O)OCC)C)[C@@H](O)C2)[nH]c(N)n1	1610358-05-8	L-Alanine, N-[(R)-[[(1R,3S,5S)-3-(2-amino-1,6-dihydro-6-oxo-9H-purin-9-yl)-5-hyd	C23H29N6O7P	532.49	A/B	-8.3
7217	C(NP(=O)(OP(=O)(OP(=O)(OC[C@H]1O[C@H]([C@H](O)[C@@H]1O)n1c(=O)[nH]c(=O)c(I)c1)O)O)O)c1n2n(c(=O)c1C)c	161056-98-0	5,Ä≤-Uridylic acid, 5-iodo-, mono[(2,5,6-trimethyl-1,7-dioxo-1H,7H-pyrazolo[1,2-	C19H22IN4O11P	640.28	A/B	-8.3
7218	[C@H](C(=O)N1[C@@H]2[C@@H](C2)C[C@H]1C#N)(N)C12CC3(OC(=O)[C@@H](C)N)CC(C2)CC(C1)C3	1616609-07-4	L-Alanine, 3-[(1S)-1-amino-2-[(1S,3S,5S)-3-cyano-2-azabicyclo[3.1.0]hex-2-yl]-2-	C21H30N4O3	386.49	A/B	-8.3
7219	[C@H](C(=O)N1[C@@H]2[C@@H](C2)C[C@H]1C#N)(N)C12CC3(OC(=O)[C@H](CS)N)CC(C2)CC(C1)C3	1616609-12-1	D-Aspartic acid, 1-[3-[(1S)-1-amino-2-[(1S,3S,5S)-3-cyano-2-azabicyclo[3.1.0]hex	C22H30N4O5	430.5	A/B	-8.3
7220	[C@H](C(=O)N1[C@@H]2[C@@H](C2)C[C@H]1C#N)(N)C12CC3(OC(=O)[C@H](NC)C)CC(C2)CC(C1)C3	1616609-40-5	L-Alanine, N-methyl-, 3-[(1S)-1-amino-2-[(1S,3S,5S)-3-cyano-2-azabicyclo[3.1.0]h	C22H32N4O3	400.51	A/B	-8.3
7221	C(=O)(N[C@@H](CC1CCC1)C(=O)C(=O)N)[C@@H]1[C@@H]2[C@@H](C2(C)C)CN1C(=O)[C@@H](NC(=O)NC1(CCCCC1)c1cccc	1616695-99-8	3-Azabicyclo[3.1.0]hexane-2-carboxamide, N-[(1S)-3-amino-1-(cyclobutylmethyl)-2,	C36H53N5O7S	699.9	A/B	-8.3
7222	[Sn](CCCC)(CCCC)(CCCC)c1cn(c(=O)[nH]c1=O)[C@H]1O[C@@H](CO)[C@H](O)C1	1616949-74-6	1(2H)-Pyrimidineacetamide, N-[(1S,3S,4S)-4-[[2-(2,6-dimethylphenoxy)acetyl]aminc	C37H46F2N4O5	664.78	A/B	-8.3
7223	O=c1n([C@@H]2O[C@H](COP(=O)(Nc3cc(C(=O)Nc4cc(C(=O)Nc5cc(C(=O)NCCC(=N)N)n(C)c5)n(C)c4)n(C)c3)OC3CCCCC	1620662-77-2	5,Ä≤-Thymidylic acid, 3,Ä≤-azido-3,Ä≤-deoxy-, 5,Ä≤-[cyclohexyl N-[5-[[[5-[[[5-[	C37H46N13O9P		A/B	-8.3
7224	C[C@]12[C@@H]3[C@](C)([C@@]4(C)C(=CC3=O)[C@H]3[C@@](C)(CC4)CC[C@@](C(=O)O)(C)C3)CC[C@H]2C(C)(C)C(=O)	1620662-80-7	5,Ä≤-Thymidylic acid, 2,Ä≤,3,Ä≤-didehydro-3,Ä≤-deoxy-, 5,Ä≤-[cyclohexyl N-[5-[	C37H48N11O9P	821.82	A/B	-8.3
7225	N(C(=O)[C@H](Cc1cccnc1)C[C@@H](CN1[C@H](C(=O)NC(C)(C)C)CN(Cc2cc3c(s2)ccs3)CC1)O)[C@H]1c2c(C[C@H]1O)c	162105-29-5	1-Piperazinepentanamide, 4-[(4-bromo-2-thienyl)methyl]-N-(2,3-dihydro-2-hydroxy-	C34H44BrN5O4S	698.71	A/B	-8.3
7226	CC1=C2C3=CC(O)(O2)CCCCCCCCCCCCCC3=C(O)C1=O	1623104-81-3	Carbamic acid, N-[(1R)-2-[[(1S)-1-cyano-2-phenylethyl]amino]-1-(3-hydroxytricycl	C26H35N3O4	453.57	A/B	-8.3
7227	[N+](#N)c1c2c(c(N=Nc3cc4c(cc3)cc(S(=O)(=O)O)cc4O)cc1)ccc(S(=O)(=O)O)c2	1628452-95-8	2-Naphthalenesulfonic acid, 6-[2-(4-amino-6-sulfo-1-naphthalenyl)diazenyl]-4-hyc	C20H15N3O7S2	473.48	A/B	-8.3
7228	O=c1n(ccc(N)n1)[C@H]1O[C@@H](COP(=O)(Oc2ccc(F)cc2)NCCNC(=O)CCC/C=C\C/C=C\C/C=C\C/C=C\C/C=C\CC)SC1	1628917-74-7	Phosphoramidic acid, N-[2-[[(5Z,8Z,11Z,14Z,17Z)-1-oxo-5,8,11,14,17-eicosapentaer	C40H52N5O6PS	761.91	A/B	-8.3
7229	Nc1c2c(cc(S(=O)(=O)O)c1N=Nc1c(C)cc(cc1)c1cc(C)c(N=Nc3c(N)cc(O)cc3)cc1)cc(S(=O)(=O)O)cc2O.[NaH]	1630943-04-2	L-Leucine, N-[[[(2R,5S)-5-(1,6-dihydro-6-oxo-9H-purin-9-yl)tetrahydro-2-furanyl]	C24H31N6O9P	578.51	A/B	-8.3
7230	N(c1nc(nc(Nc2nc(OC)cc(OC)n2)n1)N1CCOCC1)c1c(Cl)cc(Cl)c(Cl)c1	1631751-11-5	1,3,5-Triazine-2,4-diamine, N2-(3-chlorophenyl)-N4-(4,6-dimethoxy-2-pyrimidinyl)	C19H21ClN8O3	444.87	A/B	-8.3
7231	N(c1nc(nc(Nc2nc(OC)cc(OC)n2)n1)N1CCOCC1)c1c(OC)cccc1	1631751-12-6	1,3,5-Triazine-2,4-diamine, N2-(4,6-dimethoxy-2-pyrimidinyl)-6-(4-morpholinyl)-N	C19H19Cl3N8O3	513.77	A/B	-8.3
7232	O[C@@H]1[C@H](n2c3c(nc2)c(N)ncn3)O[C@@H]([C@@H](C)O)[C@@H]1O	1631751-16-0	1,3,5-Triazine-2,4-diamine, N2-(3-chloro-4-fluorophenyl)-N4-(4,6-dimethoxy-2-pyr	C19H20ClFN8O3	462.87	A/B	-8.3
7233	O(C(=O)C)[C@H]1[C@@H](O[C@H](COC(=O)C)[C@H]1OC(=O)C)n1c(/C=C/c2c(C)cccc2)nc(C(=O)N)n1	1632295-91-0	1H-1,2,4-Triazole-3-carboxamide, 5-[(1E)-2-(3-methylphenyl)ethenyl]-1-(2,3,5-tri	C23H26N4O8	486.47	A/B	-8.3
7234	O=C1[C@@H]2[C@@H](c3c([C@H](OC(=O)c4cc(N(=O)=O)ccc4N)[C@H]2CO1)cc1c(c3)OCO1)c1cc(OC)c(OC)c(OC)c1	1633020-36-6	Benzoic acid, 2,3-dimethoxy-, (5R,5aR,8aR,9R)-5,5a,6,8,8a,9-hexahydro-8-oxo-9-(3	C31H30O11	578.56	A/B	-8.3
7235	O(P(=O)(OC[C@H]1O[C@H]([C@H](O)C1)n1c2c(nc1)c(N)ncn2)O)[C@H]1[C@@H](O[C@H](COP(=O)(O[C@H]2[C@@H](O[C	1637769-17-5	Adenosine, N-benzoyl-3,Ä≤-O-benzoyl-5,Ä≤-O-[(4-methoxyphenyl)diphenylmethyl]-P-	C99H86N16O26P2	1977.78	A/B	-8.3
7236	[C@@H](C(CC)CC)(NC(=O)C)[C@H]1[C@H](NC(=N)N)C[C@H](C(=O)OCCCCC(=O)OC[C@H]2N[C@H]([C@H](O)[C@@H]2O)c2	1638370-21-4	Cyclopentanecarboxylic acid, 3-[(1S)-1-(acetylamino)-2-ethylbutyl]-4-[(aminoimir	C30H47N9O8	661.75	A/B	-8.3
7237	C=C1[C@@H](n2c3c(nc2)c(=O)nc(N)[nH]3)C[C@H](O)[C@H]1COP(=O)(Oc1cc2c(cc1)OCO2)N[C@H](C(=O)OC(C)C)C	1638769-96-6	L-Alanine, N-[(S)-[[(2S,5R)-5-(4-amino-2-oxo-1(2H)-pyrimidinyl)-1,3-oxathiolan-2	C19H23N4O9PS	514.45	A/B	-8.3
7238	C=C1[C@@H](n2c3c(nc2)c(=O)nc(N)[nH]3)C[C@H](O)[C@H]1CO[P@](=O)(Oc1cc2c(cc1)OCO2)N[C@H](C(=O)OC(C)C)C	1638769-98-8	L-Alanine, N-[(R)-[[(1R,3S,5S)-3-(2-amino-1,6-dihydro-6-oxo-9H-purin-9-yl)-5-hyd	C25H31N6O9P	590.52	A/B	-8.3
7239	C(=O)([C@@H](NC(=O)N(C)C)C(C)C)N1[C@@H](C=CC1)c1[nH]c(cn1)c1ccc(cc1)c1ccc(cc1)c1[nH]c(nc1)[C@H]1N(C(	1639870-99-7	Carbamic acid, N-[(1S)-1-[[(2S)-2,5-dihydro-2-[5-[5-[4-[2-[(2S)-1-[(2S)-2-[(meth	C44H50N8O6	786.92	A/B	-8.3
7240	C(=O)([C@@H](NC(=O)N(C)C)C(C)C)N1[C@@H](CCC1)c1[nH]c(cn1)c1ccc(cc1)c1ccc(cc1)c1[nH]c(nc1)[C@H]1N(C(=	1639871-06-9	Carbamic acid, N-[(1S)-1-[[(2S)-2-[5-[4,Ä≤-[2-[(2S)-2,5-dihydro-1-[(2S)-3-methyl	C40H49N9O5	735.87	A/B	-8.3
7241	O=c1n(cc(C(F)(F)F)c(=O)n1C(=O)c1ccccc1)[C@@H]1O[C@H](COCOC(C)(C)C)[C@@H](OCC#C)C1	164059-78-3	Thymidine, 3-benzoyl-Œ±,Œ±,Œ±-trifluoro-5,Ä≤-O-[(phenylmethoxy)methyl]-3,Ä≤-O-2	C28H25F3N2O7	558.5	A/B	-8.3
7242	S(c1nc(nc(Nc2ccc(C)cc2)n1)N1CCOCC1)c1oc(nn1)c1ccc(F)cc1	1642573-88-3	Benzonitrile, 4-[[4-[[4-[(1E)-2-cyanoethenyl]-2,6-dimethylphenyl]amino]-6-[[3-(4	C29H32N8O	508.62	A/B	-8.3
7243	N(c1nc(nc(Oc2ccc(C#N)cc2)n1)N1CCOCC1)c1cc(Br)ccc1	1643332-92-6	Benzonitrile, 4-[[4-[(3-methoxyphenyl)amino]-6-(4-morpholinyl)-1,3,5-triazin-2-y	C21H20N6O3	404.42	A/B	-8.3
7244	C(=O)([C@@H](NC(=O)OC)C(C)C)N1[C@@H](C[C@@H](C)C1)c1[nH]c(cn1)c1ccc(cc1)c1ccc(cc1)c1[nH]c(nc1)[C@H]1	1645563-33-2	Carbamic acid, N,N,Ä≤-[[1,1,Ä≤-biphenyl]-4,4,Ä≤-diylbis[1H-imidazole-5,2-diyl[(	C42H54N8O6	766.93	A/B	-8.3
7245	C(=O)([C@@H](NC(=O)OC)C(C)C)N1[C@@H](C[C@@H]2[C@H]1CCC2)c1[nH]c(cn1)c1ccc(cc1)c1ccc(cc1)c1[nH]c(nc1)	1645563-48-9	Carbamic acid, N,N,Ä≤-[[1,1,Ä≤-biphenyl]-4,4,Ä≤-diylbis[1H-imidazole-5,2-diyl[(	C46H58N8O6	819	A/B	-8.3
7246	N(C(=O)[C@H](Cc1ccccc1)C[C@@H](CN1[C@H](C(=O)NC(C)(C)C)CN(Cc2cc(O)c(C)cc2)CC1)O)[C@H]1c2c(C[C@H]1O)c	164725-35-3	1-Piperazinepentanamide, N-(2,3-dihydro-2-hydroxy-1H-inden-1-yl)-2-[[(1,1-dimeth	C38H51N5O6	673.84	A/B	-8.3
7247	C(P(=O)([O-])[O-])C(=O)[O-]	164927-34-8	Thymidine, P-deoxy-P-fluorothymidylyl-(3,Ä≤,Üí5,Ä≤)-	C20H26FN4O11P	548.41	A/B	-8.3
7248	[C@H](Cc1ccccc1)(C[C@@H]([C@H](Cc1ccccc1)NC(=O)OCc1cnc(C)s1)O)NC(=O)[C@@H](NC(=O)N(Cc1nc(C(C)C)sc1)C	165315-11-7	2,4,7,12-Tetraazatridecan-13-oic acid, 10-hydroxy-2-methyl-5-(1-methylethyl)-1-[	C37H48N6O6S	704.88	A/B	-8.3
7249	[C@H](Cc1ccccc1)(C[C@@H]([C@H](Cc1ccccc1)NC(=O)OCc1cncs1)O)NC(=O)[C@@H](NC(=O)N(CCc1nc(C(C)C)sc1)C)C	165315-33-3	2,7,10,12-Tetraazatetradecanoic acid, 12-[(2-cyclopentyl-4-thiazolyl)methyl]-4-h	C40H52N6O5S2	761.01	A/B	-8.3
7250	C(=O)([C@@H](NC(=O)OC)C(C)C)N1C[C@@]2(C[C@H]1c1[nH]c(cn1)c1ccc(cc1)c1cc3c(cc1)c1c(cc3)nc([nH]1)[C@H]	1653967-08-8	Carbamic acid, N-[(1S)-1-[[(2S,3aS,6aS)-hexahydro-2-[5-[4,Ä≤-[2-[(3S,5S)-2-[(2S)	C51H66N8O8	919.12	A/B	-8.3
7251	O=c1n([C@@H]2O[C@H](COP(=O)(C(O)c3ccc(C)cc3)OC[C@@H]3[C@@H](N=[N+]=[N-])C[C@@H](O3)n3c(=O)[nH]c(=O)c	166318-74-7	Thymidine, 3,Ä≤-azido-P,3,Ä≤-dideoxy-P-[hydroxy(4-methoxyphenyl)methyl]thymidyl	C28H33N10O11P		A/B	-8.3
7252	C(OCCOC(=O)c1cc(CN(CCCC)CCCC)ccc1)n1c2c(nc1)c(=O)nc(N)[nH]2	166762-85-2	Benzoic acid, 4-[[bis(1-methylethyl)amino]methyl]-, 2-[(2-amino-1,6-dihydro-6-ox	C22H30N6O4	442.51	A/B	-8.3
7253	O=C1[C@@H]2[C@@H](c3c([C@H](OC(=O)CCC(=O)OC(COC(=O)CCC(=O)O[C@H]4c5c([C@H]([C@@H]6[C@@H]4COC6=O)c4cc	1669412-78-5	1-Propanaminium, N-[2-(1,1-dimethylethoxy)-2-oxoethyl]-2,3-bis[4-[[(5R,5aR,8aR,9	C63H72NO24		A/B	-8.3

7254	O(C(=O)N[C@@H](Cc1ccccc1)[C@@H](CN(S(=O)(=O)c1cc2c(cc1)nc(NC(C)C)o2)CC(C)C)O)[C@@H]1[C@H]2[C@@H](OC1	1672661-44-7	Carbamic acid, N-[(1S,2R)-2-hydroxy-3-[[[2-[(1-methylethyl)amino]-6-benzothiazol	C31H40F2N4O7S2	682.8	A/B	-8.3
7255	O(C(=O)N[C@@H](Cc1cc(OC)ccc1)[C@@H](CN(S(=O)(=O)c1cc2c(cc1)nc(NC(C)C)o2)CC(C)C)O)[C@@H]1[C@H]2[C@@H]	1672661-46-9	Carbamic acid, N-[(1S,2R)-2-hydroxy-1-[(4-methoxyphenyl)methyl]-3-[[[2-[(1-methy	C32H42F2N4O9S	696.76	A/B	-8.3
7256	O(C(=O)N[C@@H](Cc1ccccc1)[C@@H](CN(S(=O)(=O)c1cc2c(cc1)nc(NC1CCN(CC1)C1CCCC1)s2)CC(C)C)O)[C@@H]1[C@H	1672661-49-2	Carbamic acid, N-[(1S,2R)-3-[[[2-(cyclopropylamino)-6-benzothiazolyl]sulfonyl](2	C31H38F2N4O7S2	680.78	A/B	-8.3
7257	O(C(=O)N[C@H]([C@@H](CN(S(=O)(=O)c1cc2c(cc1)nc(N(C)C)s2)CC(C)C)O)Cc1ccccc1)[C@@H]1[C@H]2[C@@H](OC1)O	1672661-51-6	Carbamic acid, N-[(1S,2R)-2-hydroxy-3-[(2-methylpropyl)[[2-(methylsulfonyl)-6-be	C29H35F2N3O9S3	703.79	A/B	-8.3
7258	O[C@H]1[C@@H](O[C@H](CO)[C@H]1O)n1cc(CC=CN)c(=O)[nH]c1=O	1681061-58-4	Guanosine 5,Ä≤-(tetrahydrogen triphosphate), N-(2-methylpropyl)-	C14H24N5O14P3	579.29	A/B	-8.3
7259	C(#Cc1ccccc1)C1(C(Cl)(F)F)c2c(NC(=O)O1)ccc(Cl)c2	168834-34-2	Benzonitrile, 2-[2-[6-chloro-1,4-dihydro-2-oxo-4-(trifluoromethyl)-2H-3,1-benzox	C18H8ClF3N2O2	376.72	A/B	-8.3
7260	C(n1c2c(nc1CC)c(C)cc(C)n2)c1ccc(cc1)c1c(S(=O)(=O)NC(C)(C)C)ccc(CC)c1	169281-48-5	Phosphoramidic acid, [[4,Ä≤-[(2-ethyl-5,7-dimethyl-3H-imidazo[4,5-b]pyridin-3-yl	C27H33N4O5PS	556.61	A/B	-8.3
7261	C(n1c2c(nc1CC)c(C)cc(C)n2)c1c(F)cc(cc1)c1c(S(=O)(=O)NC(=O)OCCCC)ccc(CCC)c1	169281-61-2	Carbamic acid, [[4,Ä≤,Ä≤-[(2-ethyl-5,7-dimethyl-3H-imidazo[4,5-b]pyridin-3-yl)m	C34H36N4O4S	596.74	A/B	-8.3
7262	[C@@H](Cc1ccccc1)(NC(=O)N1CCOCC1)C(=O)N[C@@H](CCc1ccccc1)/C=C/C=C/S(=O)(=O)c1ccccc1	170111-57-6	4-Morpholinecarboxamide, N-[2-[[3-(2-naphthalenylsulfonyl)-1-(2-phenylethyl)-2-p	C35H37N3O5S	611.75	A/B	-8.3
7263	C(n1c2c(nc1CC)c(C)cc(C)n2)c1ccc(cc1)c1c(S(=O)(=O)NC(=O)OCCCC)ccc(Cc2cc(OC)ccc2)c1	17095-37-3	1-Naphthalenesulfonic acid, 3,3,Ä≤-[[1,1,Ä≤:4,Ä≤,1,Ä≤,Ä≤-terphenyl]-4,4,Ä≤,Ä≤-	C38H28N6O6S2	728.8	A/B	-8.3
7264	N(C(CCCl)CC)c1c2c(ncc1)cc(Cl)cc2	170969-73-0	Carbamic acid, [[4,Ä≤-[(2-ethyl-5,7-dimethyl-3H-imidazo[4,5-b]pyridin-3-yl)methy	C36H40N4O5S	640.79	A/B	-8.3
7265	C(OC[C@@H]1[C@@H](OS(=O)(=O)C)[C@H](O)[C@@H](O1)n1c2c(nc1)c(N(C)C)ncn2)(c1ccccc1)(c1ccccc1)c1ccccc1	171045-53-7	L-Valine, N-[(phenylmethoxy)carbonyl]-, 2-[[2-(acetylamino)-1,6-dihydro-6-oxo-9H	C23H28N6O7	500.5	A/B	-8.3
7266	S(=O)(=O)(c1cc2c(cc1)NC(=O)C2)N1CC(C)C(O)CC1	171284-29-0	Inosine, 3,Ä≤-azido-3,Ä≤-deoxy-P-[[4-(2,2-dimethyl-1-oxopropoxy)phenyl]methyl]t	C32H38N9O11P		A/B	-8.3
7267	C[C@@]12[C@@H]3[C@H]([C@H]4[C@](C)(CC3)[C@@H](C(CCCCC#C)C)CC4)[C@H](O)C[C@@H]1CC(OC)(OC)CC2	1712964-47-0	2H-Indol-2-one, 1,3-dihydro-5-[(4-hydroxy-3-methyl-1-piperidinyl)sulfonyl]-	C14H18N2O4S	310.37	A/B	-8.3
7268	C(CC(=O)OCC)c1c2c(N(c3c(C(=O)N2)cccn3)C2CC2)ncc1	172326-24-8	6H-Dipyrido[3,2-b:2,Ä≤,3,Ä≤-e][1,4]diazepin-6-one, 11-cyclopropyl-5,11-dihydro-	C22H20N4O	356.42	A/B	-8.3
7269	C(CC(COC(=O)C)(CO)C)n1c2c(nc1)cnc(N)n2	172645-18-0	Benzoic acid, 3-phenoxy-, 4-(2-amino-1,6-dihydro-6-oxo-9H-purin-9-yl)-2,2-bis(hy	C24H25N5O6	479.49	A/B	-8.3
7270	N(C(=O)[C@H](Cc1ccccc1)C[C@@H](CN1[C@H](C(=O)NC(C)(C)C)CN(Cc2nc(C(C)C)sc2)CC1)O)[C@H]1c2c(C[C@H]1O)c	172649-44-4	1-Piperazinepentanamide, N-(2,3-dihydro-2-hydroxy-1H-inden-1-yl)-2-[[(1,1-dimeth	C34H45N5O4S	619.82	A/B	-8.3
7271	N(C(=O)[C@H](Cc1ccccc1)C[C@@H](CN1[C@H](C(=O)NC(C)(C)C)CN(Cc2nc(N(C)C)sc2)CC1)O)[C@H]1c2c(C[C@H]1O)c	172649-51-3	1-Piperazinepentanamide, N-(2,3-dihydro-2-hydroxy-1H-inden-1-yl)-2-[[(1,1-dimeth	C35H47N5O4S	633.84	A/B	-8.3
7272	O=c1n(c2c([nH]1)c(=O)nc(N)[nH]2)[C@H]1[C@H](O)[C@H]2[C@H](O1)COP(=O)(C)O2	173366-68-2	Guanosine, cyclic 3,Ä≤,5,Ä≤-(P-phenylphosphonate)	C16H16N5O6P	405.3	A/B	-8.3
7273	O=c1n([C@@H]2O[C@H](COP(=O)(OC[C@@H]3[C@@H](N=[N+]=[N-])C[C@@H](O3)n3c(=O)[nH]c(=O)c(C)c3)OCc3ccc(C#	173606-88-7	Thymidine, 3,Ä≤-azido-P-[(4-chlorophenyl)methyl]-3,Ä≤-deoxythymidylyl-(5,Ä≤,Üí5	C27H30ClN10O10P		A/B	-8.3
7274	O=c1n([C@@H]2O[C@H](COP(=O)([C@H](O)c3ccc(N(=O)=O)cc3)OC[C@@H]3[C@@H](N=[N+]=[N-])C[C@@H](O3)n3c(=O)	173658-36-1	Thymidine, 3,Ä≤-azido-P,3,Ä≤-dideoxy-P-[hydroxy(3-nitrophenyl)methyl]thymidylyl	C27H30N11O12P		A/B	-8.3
7275	[C@@H](P(=O)(OC[C@@H]1[C@@H](N=[N+]=[N-])C[C@@H](O1)n1c(=O)[nH]c(=O)c(C)c1)OC[C@@H]1[C@@H](N=[N+]=[N	173658-38-3	Thymidine, 3,Ä≤-azido-P,3,Ä≤-dideoxy-P-[(2,4-dinitrophenyl)hydroxymethyl]thymid	C27H29N12O14P		A/B	-8.3
7276	[C@@H](C(C)(C)C)(C1=C(O)CC(CCC)(CCC)OC1=O)c1cc(NS(=O)(=O)c2ccc(C(F)(F)F)cn2)ccc1	174484-29-8	2-Pyridinesulfonamide, N-[3-[1-(5,6-dihydro-4-hydroxy-2-oxo-6,6-dipropyl-2H-pyra	C28H35F3N2O5S	568.65	A/B	-8.3
7277	C(n1c2c(nc1CC)c(C)cc(C)n2)c1ccc(cc1)c1c(S(=O)(=O)NC(=O)c2cc(OCc3ccccc3)ccc2)cccc1	174844-68-9	Benzamide, N-[[4,Ä≤-[(2-ethyl-5,7-dimethyl-3H-imidazo[4,5-b]pyridin-3-yl)methyl]	C31H30N4O4S	554.66	A/B	-8.3
7278	O=c1c2c(n(cn2)[C@@H]2O[C@H](COP(=O)(OP(=O)(OC[C@@H]3[C@@H](N=[N+]=[N-])C[C@@H](O3)n3c(=O)[nH]c(=O)c(	175923-28-1	Thymidine 5,Ä≤-(trihydrogen diphosphate), 3,Ä≤-azido-3,Ä≤-deoxy-, P,Ä≤,Üí5,Ä≤-e	C19H24N10O13P2		A/B	-8.3
7279	N(C(=O)c1ccc(F)cc1)c1n2c(nccc2nn1)N1CCOCC1	1778635-63-4	Benzamide, N-[5-(4-morpholinyl)-1,2,4-triazolo[4,3-c]pyrimidin-3-yl]-4-(trifluor	C17H15F3N6O2	392.34	A/B	-8.3
7280	C(O[P@@](=O)(Oc1ccccc1)N[C@@H](C(=O)OC(C)C)C)[C@H]1C2([C@H]([C@H](F)[C@@H]1O)n1c3c(nc1)c(=O)nc(N)[nH	1781231-56-8	D-Alanine, N-[(R)-[[(4R,5R,6R,7R)-7-(2-amino-1,6-dihydro-6-oxo-9H-purin-9-yl)-6-	C25H32FN6O7P	578.53	A/B	-8.3
7281	C(O[P@@](=S)(Oc1ccccc1)N[C@@H](C(=O)OC(C)C)C)[C@@]1(C)C2([C@H]([C@H](O)[C@@H]1O)n1c3c(nc1)c(=O)nc(N)	1781232-61-8	D-Alanine, N-[(R)-[[(4R,5R,6S,7R)-7-(2-amino-1,6-dihydro-6-oxo-9H-purin-9-yl)-6-	C25H32FN6O6PS	594.6	A/B	-8.3
7282	C(OP(=O)(OP(=O)(OP(=O)(O)O)O)S)[C@H]1C2([C@H]([C@](C)(O)[C@@H]1O)n1c3c(nc1)c(=O)nc(N)[nH]3)CC2	1782095-87-7	Thiotriphosphoric acid ((HO)2P(O)OP(O)(OH)OP(O)(OH)(SH)), OP,Ä≤,Ä≤-[[(4R,5R,6S,	C14H22N5O12P3S	577.34	A/B	-8.3
7283	C(C=CC)(N)C12CC3CC(C2)CC(C1)C3	178478-40-5	2H-Naphth[1,8-cd]isothiazole, 2-[[1-[2-(4-methyl-1-piperazinyl)-2-tricyclo[3.3.1	C33H46N4O2S	562.81	A/B	-8.3
7284	O=c1n([C@H]2C[C@H](OP3(=S)OCCS3)[C@@H](CO)O2)cc(C)c(=O)[nH]1	1790733-28-6	2H-Indol-2-one, 5-[(4,4-dimethyl-1-piperidinyl)sulfonyl]-1,3-dihydro-	C15H20N2O3S	308.4	A/B	-8.3
7285	C([C@@H](OCP(=O)(O)O)C([2H])([2H])[2H])(n1c2c(nc1)c(N)ncn2)([2H])[2H]	1795707-69-5	4-Piperidinecarboxamide, 1-[(2,3-dihydro-2-oxo-1H-indol-5-yl)sulfonyl]-	C14H17N3O4S	323.37	A/B	-8.3
7286	C(=O)([C@@H](NC(=O)OC)[C@H](CC)C)N1[C@H](c2[nH]c(c3cc4C5(c6c(-c4cc3)ccc(c6)c3[nH]c(nc3)[C@H]3N(C(=O)	1800210-24-5	Carbamic acid, N,N,Ä≤-[spiro[9H-fluorene-9,3,Ä≤-oxetane]-2,7-diylbis[1H-imidazo	C43H52N8O7	792.92	A/B	-8.3
7287	C(=O)([C@@H](NC(=O)OC)C1CC1)N1[C@H](c2[nH]c(c3cc4C5(c6c(-c4cc3)ccc(c6)c3[nH]c(nc3)[C@H]3N(C(=O)[C@@H	1800210-28-9	Carbamic acid, N,N,Ä≤-[spiro[9H-fluorene-9,3,Ä≤-oxetane]-2,7-diylbis[1H-imidazo	C45H56N8O7	820.98	A/B	-8.3
7288	C[C@]1(F)[C@@H](O[C@H](CO[P@](=O)(Oc2ccc(SCCC)cc2)N[C@H](C(=O)OC(C)C)C)[C@H]1OC(=O)C)n1c(=O)[nH]c(=O	1800478-64-1	L-Alanine, N-[[P(S),2,Ä≤R]-3,Ä≤-O-acetyl-2,Ä≤-deoxy-2,Ä≤-fluoro-2,Ä≤-methyl-P-	C28H37FN3O10P	625.58	A/B	-8.3
7289	N(=Nc1c2c(c(S(=O)(=O)O)cc1)cccc2)c1c2c(cc(S(=O)(=O)O)cc2S(=O)(=O)O)ccc1N.[NaH]	1800502-98-0	Carbamic acid, N-[(1S)-1-[[(2S)-2-[5-[4,Ä≤-[2-[(2S)-1-[(2S)-2-[(methoxycarbonyl)	C56H62N8O8	975.14	A/B	-8.3
7290	O(P(=O)(N[C@H](C(=O)OC(C)C)C)CO[C@@H](Cn1c2c(nc1)c(N)ncn2)C)c1c2c(ccc1)CCCC2	1802665-25-3	L-Alanine, N-[[[(1R)-2-(6-amino-9H-purin-9-yl)-1-methylethoxy]methyl][(5,6,7,8-t	C24H33N6O5P	516.53	A/B	-8.3
7291	C([C@H](OCP(=O)(O)O)CO)n1c(=O)[nH]c(=[N])cc1	18031-63-5	2-Naphthalenesulfonic acid, 8,8,Ä≤-[p-phenylenebis(azo)]bis[7-hydroxy-	C26H18N4O8S2	578.57	A/B	-8.3
7292	C(OP(=S)(Oc1ccccc1)N[C@H](C(=O)OC(C)C)C)[C@@]1(C(F)F)O[C@H]([C@@H](O)[C@@H]1O)n1c2c(nc1)c(N)ncn2	1803134-19-1	L-Alanine, N-(hydroxyphenoxyphosphinyl)-, 1-methylethyl ester, 5,Ä≤-ester with 9	C23H29F2N6O8P	586.48	A/B	-8.3
7293	C(=O)([C@@H](NC(=O)OC)C(C)C)N1[C@H](CCC1)c1[nH]c(cn1)c1ccc(cc1)c1[nH]c(nc1)[C@H]1N(C(=O)[C@@H](NC(=O	1808295-76-2	Acetamide, N,N,Ä≤-[[1,1,Ä≤-biphenyl]-4,4,Ä≤-diylbis[1H-imidazole-5,2-diyl[(2S)-	C40H46N8O4	702.84	A/B	-8.3
7294	C(=O)([C@@H](NC(=O)OC)C(C)C)N1[C@@H](C2CC1CC2)c1[nH]c(cn1)c1ccc(cc1)c1ccc(cc1)c1ccc(cc1)c1[nH]c(nc1)	1808296-00-5	Carbamic acid, N-[(1S)-1-[[(2S)-2,5-dihydro-2-[5-[4,Ä≤-[2-[(3S)-2-[(2S)-2-[(meth	C42H50N8O6	762.9	A/B	-8.3
7295	C(=O)([C@@H](NC(=O)OC)C(C)C)N1[C@@H](CCC1)c1[nH]c(cn1)c1ccc(cc1)c1ccc(cc1)c1[nH]c(nc1)[C@H]1N(C(=O)[	1808296-07-2	Carbamic acid, N-[(1S)-1-[[(2S)-2-[5-[4-[(1E)-2-[4-[2-[(3S)-2-[(2S)-2-[(methoxyc	C44H54N8O6	790.95	A/B	-8.3
7296	O=C1[C@@H]2[C@@H](c3c([C@H](OC(=O)C(C)N4C(=O)C5C(C4=O)C4OC5CC4)[C@H]2CO1)cc1c(c3)OCO1)c1cc(OC)c(OC)c	1809325-31-2	2H-Isoindole-2-acetic acid, octahydro-Œ±-methyl-1,3-dioxo-, (5R,5aR,8aR,9R)-5,5a	C33H35NO11	621.63	A/B	-8.3
7297	O=C1[C@@H]2[C@@H](c3c([C@H](OC(=O)[C@H](C)N4C(=O)C5C(C4=O)C4OC5CC4)[C@H]2CO1)cc1c(c3)OCO1)c1cc(OC)c(	1809325-34-5	4,7-Epoxy-2H-isoindole-2-acetic acid, octahydro-Œ±-methyl-1,3-dioxo-, (5R,5aR,8a	C33H33NO12	635.61	A/B	-8.3
7298	C(CC(=O)/C(=C(/CCc1cc(OC)c(O)cc1)\O)/N=Nc1ccc(C(=O)NCCCCCCNC(=O)OC(C)(C)C)cc1)c1cc(OC)c(O)cc1	1820598-82-0	Furo[3,Ä≤,4,Ä≤:6,7]naphtho[2,3-d]-1,3-dioxol-6(5aH)-one, 5,8,8a,9-tetrahydro-5-	C21H20O7	384.38	A/B	-8.3
7299	C(=C(\CCc1cc(OC)c(O)cc1)/O)(\N=Nc1ccc(C(=O)NCC(=O)NCCCCCCNC(=O)OC(C)(C)C)cc1)/C(=O)CCc1cc(OC)c(O)cc1	1820599-59-4	Carbamic acid, N-[6-[[4-[2-[(1E)-2-hydroxy-4-(4-hydroxy-3-methoxyphenyl)-1-[3-(4	C39H50N4O9	718.84	A/B	-8.3
7300	O[C@H]1[C@H](n2c3c(nn2)c(N[C@@H]2CCOC2)nc(Cl)n3)C[C@H](COC(C(=O)OCC)P(=O)(O)O)[C@H]1O	1821044-50-1	Acetic acid, 2-[[(1R,2R,3S,4R)-4-[5-chloro-7-[[[(3R)-tetrahydro-3-furanyl]methyl	C19H28ClN6O9P	550.89	A/B	-8.3
7301	C([C@@H]([C@@H](COC(P(=O)(O)O)C(=O)O)O)O)n1c(=O)[nH]c(=O)c(C)c1	1826078-93-6	Acetic acid, 2-[2-[4-[(3-benzoyl-3,4-dihydro-5-methyl-2,4-dioxo-1(2H)-pyrimidiny	C19H20N5O9P	493.36	A/B	-8.3
7302	[Si](OC[C@@H]1O[C@@H](CS1)n1c(=O)nc(NC(=O)c2cccnc2)cc1)(C(C)(C)C)(c1ccccc1)c1ccccc1	18268-54-7	1-Naphthalenesulfonic acid, 4-[2-(2-hydroxy-1-naphthalenyl)diazenyl]-	C20H14N2O4S	378.4	A/B	-8.3
7303	O[C@@H]1[C@H](n2c3c(nc2)c(N)nc(F)n3)O[C@H](COP(=O)(NCc2ccccc2)OCCSC(=O)C(CO)(C)C)[C@H]1O	1831054-63-7	9H-Purin-6-amine, 2-fluoro-9-[5-O-[(R)-[2-[(3-hydroxy-2,2-dimethyl-1-oxopropyl)t	C32H38FN6O10PS	748.72	A/B	-8.3
7304	Nc1c2c(n(cn2)[C@H]2[C@@H](O)[C@H]3[C@H](O2)CO[P@@](=O)(N[C@H](C(=O)OCC)CC(C)C)O3)nc(C)n1	1831055-18-5	L-Alanine, N-(hydroxyphenoxyphosphinyl)-, 1-methylethyl ester, 5,Ä≤-ester with 9	C23H31N6O8P	550.5	A/B	-8.3
7305	C(COCP(=O)(OCCN=[N+]=[N-])O)n1c2c(nc1)c(N)ncn2	1831055-61-8	L-Alanine, N-(S)-phosphono-, 1-ethyl ester, cyclic (P,Üí3,Ä≤,5,Ä≤)-ester with 2	C23H26FN6O9P	580.46	A/B	-8.3
7306	[P@@](=O)(Oc1ccccc1)(NC(C(=O)OC)(C)C)OC[C@H]1O[C@H](C=C1)n1c(=O)[nH]c(=O)c(C)c1	184031-38-7	L-Phenylalanine, N-(2,Ä≤,3,Ä≤-didehydro-3,Ä≤-deoxy-P-phenyl-5,Ä≤-thymidylyl)-,	C29H34N3O8P	583.57	A/B	-8.3
7307	O=c1n([C@@H]2O[C@H](COP(=O)(Oc3cc(C(F)(F)F)ccc3)N[C@H](C(=O)OC)C)[C@@H](N=[N+]=[N-])C2)cc(C)c(=O)[nH	184031-69-4	L-Tryptophan, N-(2,Ä≤,3,Ä≤-didehydro-3,Ä≤-deoxy-P-phenyl-5,Ä≤-thymidylyl)-, met	C28H29N4O8P	580.53	A/B	-8.3
7308	C(C(OC)(C)C)n1c2c(nc1)c(N)ncn2	1849626-35-2	Phosphonic acid, P-[[(1R)-2-(6-amino-9H-purin-9-yl)-1-methylethoxy]methyl]-, mix	FN6O5.C20H15BrN6O.C9H14N5O4P.C8H10FN3O3S		A/B	-8.3
7309	C(OC[C@@H]1[C@@H](OC(=O)C)C[C@@H](C1=C)n1c2c(nc1)c(=O)nc(NC(=O)OC(C)(C)C)[nH]2)(c1ccccc1)(c1ccccc1)c	1860842-88-1	Carbamic acid, N-[6,9-dihydro-9-[(1S,3R,4S)-4-hydroxy-2-methylene-3-[(triphenylm	C36H37N5O5	619.71	A/B	-8.3
7310	C(OC[C@@H]1[C@@H](OC(=O)CCC)C[C@@H](C1=C)n1c2c(nc1)c(=O)nc(NC(=O)OC(C)(C)C)[nH]2)(c1ccccc1)(c1ccccc1	1860842-90-5	Carbamic acid, N-[6,9-dihydro-9-[(1S,3R,4S)-2-methylene-4-(1-oxopropoxy)-3-[(tri	C39H41N5O6	675.77	A/B	-8.3
7311	C(OC[C@@H]1[C@@H](OC(=O)CC(CC)C)C[C@@H](C1=C)n1c2c(nc1)c(=O)nc(NC(=O)OC(C)(C)C)[nH]2)(c1ccccc1)(c1cc	1860842-91-6	Butanoic acid, (1S,2R,4S)-4-[2-[[(1,1-dimethylethoxy)carbonyl]amino]-1,6-dihydro	C40H43N5O6	689.8	A/B	-8.3
7312	O=C1[C@@H]2[C@@H](c3c([C@H](O[C@H]4[C@H](OC(=O)c5ccccc5)[C@H](OC(=O)c5ccccc5)[C@@H](COC(=O)c5ccccc5)	1883269-82-6	Furo[3,Ä≤,4,Ä≤:6,7]naphtho[2,3-d]-1,3-dioxol-6(5aH)-one, 5,8,8a,9-tetrahydro-9-	C49H44O15	872.86	A/B	-8.3
7313	O=c1n([C@@H]2O[C@H](COP(=O)(OP(=O)(OP(=O)(OCc3ccc(OC(=O)CCCCCCC)cc3)OCc3ccc(OC(=O)C)cc3)O)O)C=C2)cc(	1883329-00-7	Benzoic acid, 4-methyl-, 4-[3-[[4-(acetyloxy)phenyl]methoxy]-9-[(2S,5R)-5-(3,4-d	C34H35N2O17P3	836.57	A/B	-8.3
7314	O=c1c(c2c3c(n(C)cc3)ccc2)cn(c(=O)[nH]1)[C@@H]1O[C@H](CO)[C@@H](O)[C@H]1O	1883514-12-2	Not Yet Assigned	C16H16N4O6	360.32	A/B	-8.3
7315	O=C1C2=C(C(=O)C(=C1O)CCCCCCCCCCC)OC(C)(C)[C@@H]1[C@@H]2C[C@@H](C)CC1	1883514-28-0	Not Yet Assigned	C18H17N3O6	371.34	A/B	-8.3
7316	C[C@]12[C@H]([C@H]3[C@@H]([C@]4(C)[C@H](C[C@@H]3O)C[C@H](N)CC4)CC2)CC[C@@H]1[C@@H](CCC(=O)NCCS(=O)(=	1892521-80-0	Not Yet Assigned	C28H34D4F3N5O2		A/B	-8.3
7317	O[C@@H]1[C@H](n2c3c(nc2)c(N)ncn3)O[C@H](C(O)O)[C@H]1O	1898690-95-3	2,4-Pyrimidinediamine, N4-[(4-methylphenyl)methyl]-N2-(2,4,6-trimethylphenyl)-	C21H24N4	332.44	A/B	-8.3
7318	C(OC[C@@H]1[C@@H](OC(=O)c2ccccc2)[C@@H](O)[C@@H](O1)n1nc(C(=O)N)nc1)(c1ccc(OC)cc1)(c1ccccc1)c1ccccc1	190141-29-8	1H-1,2,4-Triazole-3-carboxamide, 1-[2,3-di-O-benzoyl-5-O-[(4-methoxyphenyl)diphe	C42H36N4O8	724.76	A/B	-8.3
7319	N(c1nc(nc(Nc2ccc(C#N)nc2)n1)N1CCOCC1)c1ccc(OC)cc1	1907643-85-9	2-Pyridinecarbonitrile, 5-[[4-(4-morpholinyl)-6-(phenylamino)-1,3,5-triazin-2-yl	C19H18N8O	374.4	A/B	-8.3
7320	N(c1nc(nc(Nc2ccc(C#N)nc2)n1)N1CCOCC1)c1c(C)cc(C)cc1C	1907643-94-0	2-Pyridinecarbonitrile, 5-[[4-[(3-methoxyphenyl)amino]-6-(4-morpholinyl)-1,3,5-t	C20H20N8O2	404.43	A/B	-8.3
7321	C(CC(=O)C=CC=Cc1cc(O)c(O)cc1)c1cc(O)c(O)cc1	1907909-60-7	2,4-Pyrimidinediamine, N4-(1-phenylethyl)-N2-(2,4,6-trimethylphenyl)-	C21H24N4	332.44	A/B	-8.3
7322	C(#N)[C@]1(O[C@H](CO[P@](=O)(Oc2ccccc2)N[C@H](C(=O)OC)C)[C@@H](O)[C@H]1O)c1n2c(cc1)c(N)ncn2	1911578-85-2	Not Yet Assigned	C30H33N6O8P	636.59	A/B	-8.3
7323	C[C@@]12[C@@H]3[C@H]([C@H]4[C@](C)(CC3)[C@@H]([C@@H](CCCC(=O)O)C)CC4)C[C@@H](O)[C@@]1(O)C[C@H](O)CC	1911579-04-8	Not Yet Assigned	C15H19N6O8P	442.32	A/B	-8.3
7324	Nc1c2c(n(cn2)[C@H]2[C@H](O)[C@H](O)[C@@H](C)O2)[nH]c(=NN)n1	1923662-72-9	Benzonitrile, 4-[[4-methyl-6-[[2-methyl-6-(1-methylethyl)phenyl]amino]-2-pyrimid	C22H23N5	357.45	A/B	-8.3
7325	[C@@H](C(=O)N[C@@H](Cc1ccccc1)C[C@@H]([C@H](Cc1ccccc1)NC(=O)COc1c(C)cc(N)cc1C)O)(C(C)C)N1C(=O)NCC1	192725-24-9	1-Imidazolidineacetamide, N-[(1S,3S,4S)-4-[[2-(2,6-dimethylphenoxy)acetyl]amino]	C36H46N4O4S	630.84	A/B	-8.3
7326	C(=C\c1ccc(F)cc1)/c1n(nc(C(=O)N)n1)[C@@H]1O[C@H](CO)[C@@H](O)[C@H]1O	1927839-22-2	1H-1,2,4-Triazole-3-carboxamide, 5-[(1Z)-2-(4-fluorophenyl)ethenyl]-1-Œ≤-D-ribo	C16H17FN4O5	364.33	A/B	-8.3
7327	C(=C\c1ccc(C(F)(F)F)cc1)\c1n(nc(C(=O)N)n1)[C@@H]1O[C@H](CO)[C@@H](O)[C@H]1O	1927839-28-8	1H-1,2,4-Triazole-3-carboxamide, 5-[(1E)-2-(2-fluorophenyl)ethenyl]-1-Œ≤-D-ribo	C16H17FN4O5	364.33	A/B	-8.3
7328	C(=O)([C@@H](NC(=O)OC)C(C)C)N1[C@@H](CCC1)c1[nH]c(cn1)c1c(c(c(c(c1[2H])[2H])c1c(c(c(c(c1[2H])[2H])c1	1939212-01-7	Carbamic acid, N,N,Ä≤-[[1,1,Ä≤-biphenyl]-4,4,Ä≤-diyl-2,3,5,6-d4-bis[1H-imidazol	C40H40D10N8O6		A/B	-8.3
7329	C(=O)([C@@H](NC(=O)OC)C(C)C)N1[C@@H](CCC1)c1[nH]c(c(n1)[2H])c1ccc(cc1)c1ccc(cc1)c1[nH]c(nc1[2H])[C@H	1939212-13-1	Carbamic acid, N,N,Ä≤-[[1,1,Ä≤-biphenyl]-4,4,Ä≤-diylbis[1H-imidazole-5,2-diyl-4	C40H42D8N8O6		A/B	-8.3
7330	C(Nc1[nH]c2c(c(=O)n1)ncn2COC(COC(c1ccccc1)(c1ccccc1)c1ccccc1)COC(=O)[C@@H](NC(=O)OCc1ccccc1)C(C)C)(c	1942024-79-4	2,7,10,12-Tetraazatridecanoic acid, 4-hydroxy-12-methyl-9-(1-methylethyl)-13-[2-	C37H48N6O5S2.C27H22Cl2N4O		A/B	-8.3
7331	[Se]([C@@H]1[C@H](O[C@H](CO)[C@H]1O)n1c(=O)[nH]c(=O)cc1)c1ccccc1	194672-81-6	2,4(1H,3H)-Pyrimidinedione, 1-(2-Se-phenyl-2-seleno-Œ±-D-ribofuranosyl)-	C15H16N2O5Se	383.26	A/B	-8.3
7332	O=c1c2c(n(cn2)[C@H]2O[C@@H](COP(=O)(Oc3ccccc3)N[C@@H](C(=O)OC(C)C)C)C[C@@H]2O)[nH]c(N)n1	1946763-32-1	Not Yet Assigned	C14H20N6O8S	432.41	A/B	-8.3
7333	O[C@@H]1[C@H](n2c3c(nc2)c(NCc2ccc(C(F)(F)F)cc2)ncn3)O[C@H](CO)[C@H]1O	1947401-72-0	9H-Purin-6-amine, 9-Œ≤-D-arabinofuranosyl-N-[[2-(trifluoromethyl)phenyl]methyl]-	C18H18F3N5O4	425.36	A/B	-8.3
7334	N(Cc1c(O)cc(I)cc1)c1c2c(n(cn2)[C@@H]2O[C@H](CO)[C@@H](O)[C@@H]2O)ncn1	1947402-10-9	Phenol, 2-[[(9-Œ≤-D-arabinofuranosyl-9H-purin-6-yl)amino]methyl]-6-iodo-	C17H18IN5O5	499.26	A/B	-8.3
7335	N(Cc1c(O)cc(Br)cc1)c1c2c(n(cn2)[C@@H]2O[C@H](CO)[C@@H](O)[C@@H]2O)ncn1	1947402-14-3	Phenol, 2-[[(9-Œ≤-D-arabinofuranosyl-9H-purin-6-yl)amino]methyl]-6-bromo-	C17H18BrN5O5	452.26	A/B	-8.3
7336	N(Cc1c(O)cc(F)cc1)c1c2c(n(cn2)[C@@H]2O[C@H](CO)[C@@H](O)[C@@H]2O)ncn1	1947402-18-7	Phenol, 2-[[(9-Œ≤-D-arabinofuranosyl-9H-purin-6-yl)amino]methyl]-6-fluoro-	C17H18FN5O5	391.35	A/B	-8.3
7337	C(=O)([C@@H](NC(=O)OC)C(C)C)N1[C@@H]([C@@H]2C[C@H]1CC2)c1[nH]c2c(n1)ccc1c2ccc(c1)c1ccc(cc1)c1[nH]c(n	1949770-78-8	Carbamic acid, N-[(1S)-1-[[(2S)-2-[7-[4-[2-[(2S,4R)-4-[(methoxycarbonyl)amino]-1	C44H53N9O8	835.95	A/B	-8.3
7338	O=c1n([C@@H]2O[C@H](COP3(=O)Oc4c(CO3)cccc4OC)[C@@H](N=[N+]=[N-])C2)cc(C)c(=O)[nH]1	195885-81-5	Thymidine, 3,Ä≤-azido-3,Ä≤-deoxy-5,Ä≤-O-(6-methoxy-2-oxido-4H-1,3,2-benzodioxap	C18H20N5O8P		A/B	-8.3
7339	C(C(=O)O)([C@@]1(N(C(=Nc2c1c(c(c(c2F)[2H])[2H])[2H])N1CCN(CC1)c1cc(c(OC([2H])([2H])[2H])c(c1[2H])[2	1961283-81-7	4-Quinazoline-5,6,7-d3-acetic-Œ±,Œ±-d2 acid, 8-fluoro-3,4-dihydro-4-d-2-[4-[5-(m	C29H9D19F4N4O4		A/B	-8.3
7340	C(C(=O)O)([C@@H]1N(C(=Nc2c1c(c(c(c2F)[2H])[2H])[2H])N1CCN(CC1)c1c(c(OC)c(c(c1[2H])[2H])[2H])[2H])c1c	1961283-99-7	4-Quinazoline-5,6,7-d3-acetic-Œ±,Œ±-d2 acid, 8-fluoro-3,4-dihydro-2-[4-(5-methox	C29H13D15F4N4O4		A/B	-8.3
7341	C(C(=O)O)([C@@H]1N(C(=Nc2c1cccc2F)N1C(C(N(C(C1([2H])[2H])([2H])[2H])c1c(c(OC)c(c(c1[2H])[2H])[2H])[2	1961284-06-9	4-Quinazolineacetic-Œ±,Œ±-d2 acid, 8-fluoro-3,4-dihydro-2-[4-[5-(methoxy-d3)pher	C29H8D20F4N4O4		A/B	-8.3
7342	C(C(=O)O)([C@@H]1N(C(=Nc2c1cccc2F)N1CCN(CC1)c1c(c(OC)c(c(c1[2H])[2H])[2H])[2H])c1c(OC([2H])([2H])[2H	1961284-11-6	4-Quinazolineacetic-Œ±,Œ±-d2 acid, 8-fluoro-3,4-dihydro-2-[4-[5-(methoxy-d3)pher	C29H16D12F4N4O4		A/B	-8.3
7343	C(C(=O)O)([C@@H]1N(C(=Nc2c1cccc2F)N1CCN(CC1)c1c(c(OC)c(c(c1[2H])[2H])[2H])[2H])c1c(OC)c(c(c(C(F)(F)F	1961284-12-7	4-Quinazolineacetic-Œ±,Œ±-d2 acid, 8-fluoro-3,4-dihydro-2-[4-(5-methoxyphenyl-2,	C29H16D12F4N4O4		A/B	-8.3
7344	C(C(=O)O)([C@@H]1N(C(=Nc2c1c(c(c(c2F)[2H])[2H])[2H])N1C(C(N(C(C1([2H])[2H])([2H])[2H])c1cc(OC([2H])(	1961284-32-1	4-Quinazoline-5,6,7-d3-acetic-Œ±,Œ±-d2 acid, 8-fluoro-3,4-dihydro-2-[4-[3-(metho	C29H6D22F4N4O4		A/B	-8.3
7345	C(C(=O)O)([C@@H]1N(C(=Nc2c1c(c(c(c2F)[2H])[2H])[2H])N1CCN(CC1)c1cc(OC)ccc1)c1c(OC([2H])([2H])[2H])c(	1961284-37-6	4-Quinazoline-5,6,7-d3-acetic-Œ±,Œ±-d2 acid, 8-fluoro-3,4-dihydro-2-[4-[3-(metho	C29H17D11F4N4O4		A/B	-8.3
7346	C(C(=O)O)([C@@H]1N(C(=Nc2c1cccc2F)N1CCN(CC1)c1cc(OC)ccc1)c1c(OC)c(c(c(C(F)(F)F)c1[2H])[2H])[2H])([2H	1961284-46-7	4-Quinazolineacetic-Œ±,Œ±-d2 acid, 8-fluoro-3,4-dihydro-2-[4-(3-methoxyphenyl)-1	C29H20D8F4N4O4		A/B	-8.3
7347	C(C(=O)O)([C@@]1(N(C(=Nc2c1c(c(c(c2F)[2H])[2H])[2H])N1CCN(CC1)c1c(c(OC([2H])([2H])[2H])c(c(c1[2H])[2	1961284-52-5	4-Quinazoline-5,6,7-d3-acetic-Œ±,Œ±-d2 acid, 8-fluoro-3,4-dihydro-4-d-2-[4-[5-(m	C29H12D16F4N4O4		A/B	-8.3
7348	C(C(=O)O)([C@@H]1N(C(=Nc2c1c(c(c(c2F)[2H])[2H])[2H])N1C(C(N(C(C1([2H])[2H])([2H])[2H])c1cc(OC)ccc1)(	1961285-08-4	4-Quinazoline-5,6,7-d3-acetic-Œ±,Œ±-d2 acid, 8-fluoro-3,4-dihydro-2-[4-(3-methox	C29H12D16F4N4O4		A/B	-8.3
7349	C(C(=O)O)([C@@H]1N(C(=Nc2c1cccc2F)N1C(C(N(C(C1([2H])[2H])([2H])[2H])c1cc(OC)ccc1)([2H])[2H])([2H])[2	1961285-18-6	4-Quinazolineacetic-Œ±,Œ±-d2 acid, 8-fluoro-3,4-dihydro-2-[4-(3-methoxyphenyl)-1	C29H15D13F4N4O4		A/B	-8.3
7350	C(C(=O)O)[C@@H]1N(C(=Nc2c1cccc2F)N1C(C(N(C(C1([2H])[2H])([2H])[2H])c1c(c(OC)c(c(c1[2H])[2H])[2H])[2H	1961285-53-9	4-Quinazolineacetic acid, 8-fluoro-3,4-dihydro-2-[4-(5-methoxyphenyl-2,3,4,6-d4)	C29H10D18F4N4O4		A/B	-8.3
7351	C(C(=O)O)[C@@H]1N(C(=Nc2c1c(c(c(c2F)[2H])[2H])[2H])N1CCN(CC1)c1cc(OC([2H])([2H])[2H])ccc1)c1c(OC)c(c	1961285-88-0	4-Quinazoline-5,6,7-d3-acetic acid, 8-fluoro-3,4-dihydro-2-[4-[3-(methoxy-d3)phe	C29H16D12F4N4O4		A/B	-8.3
7352	C(C(=O)O)[C@@]1(N(C(=Nc2c1c(c(c(c2F)[2H])[2H])[2H])N1CCN(CC1)c1cc(OC([2H])([2H])[2H])ccc1)c1c(OC([2H	1961286-65-6	4-Quinazoline-5,6,7-d3-acetic acid, 8-fluoro-3,4-dihydro-4-d-2-[4-(3-methoxypher	C29H16D12F4N4O4		A/B	-8.3
7353	C(C(=O)O)[C@@H]1N(C(=Nc2c1c(c(c(c2F)[2H])[2H])[2H])N1C(C(N(C(C1([2H])[2H])([2H])[2H])c1cc(OC)ccc1)([	1961287-08-0	4-Quinazoline-5,6,7-d3-acetic acid, 8-fluoro-3,4-dihydro-2-[4-[3-(methoxy-d3)phe	C29H14D14F4N4O4		A/B	-8.3
7354	C(OCCOP(=O)OC(CC(=O)OC)c1ccc(OC(=O)C(C)(C)C)cc1)n1c2c(nc1)c(=O)nc(N)[nH]2	197897-81-7	Benzenepropanoic acid, Œ≤,Œ≤,Ä≤-[[[2-[(2-amino-1,6-dihydro-6-oxo-9H-purin-9-yl)	C38H48N5O14P	829.79	A/B	-8.3
7355	N(c1c(CC)cccc1CC)c1nc(Nc2c(C#N)cccc2)ccn1	1980794-21-5	2-Pyridinecarboxylic acid, 6-bromo-, (5R,5aR,8aR,9R)-5,5a,6,8,8a,9-hexahydro-8-c	C28H24BrNO9	598.4	A/B	-8.3
7356	N(C(=O)C)[C@H]1[C@H]([C@@H]([C@@H](CO)O)O)OC(=C[C@@H]1NC(=N)NC(=O)Cn1cc(nn1)C1CCCCC1)C(=O)O	1982351-37-0	D-glycero-D-galacto-Non-2-enonic acid, 5-(acetylamino)-2,6-anhydro-3,4,5-trideox	C25H33N7O8	559.57	A/B	-8.3
7357	C[C@]1(F)[C@@H](O[C@H](CO[P@](=O)(Oc2ccccc2)N[C@H](C(=O)OC(C)C)C)[C@H]1OC(=O)Cn1c2c(cc1)cccn2)n1c(=O	1982359-82-9	L-Alanine, N-[[P(S),2,Ä≤R]-2,Ä≤-deoxy-2,Ä≤-fluoro-2,Ä≤-methyl-3,Ä≤-O-[1-oxo-4-	C33H39FN5O10P	715.66	A/B	-8.3
7358	O=C1[C@@H]2[C@@H](c3c([C@H](OC(=O)c4c(OC)nccc4)[C@H]2CO1)cc1c(c3)OCO1)c1cc(OC)c(OC)c(OC)c1	1983131-61-8	3-Pyridinecarboxylic acid, 6-bromo-, (5R,5aR,8aR,9R)-5,5a,6,8,8a,9-hexahydro-8-c	C28H24BrNO9	598.4	A/B	-8.3
7359	O=C1[C@@H]2[C@@H](c3c([C@H](OC(=O)c4cc(Cl)ncc4)[C@H]2CO1)cc1c(c3)OCO1)c1cc(OC)c(OC)c(OC)c1	1983131-64-1	2-Pyridinecarboxylic acid, 4-chloro-, (5R,5aR,8aR,9R)-5,5a,6,8,8a,9-hexahydro-8-	C28H24ClNO9	553.94	A/B	-8.3
7360	N(c1c(CC)cccc1C)c1nc(Nc2ccc(C)cc2)ncc1	1984648-37-4	Benzonitrile, 2-[[2-[(4-cyanophenyl)amino]-4-pyrimidinyl]amino]-	C18H12N6	312.33	A/B	-8.3
7361	O=C1c2n(N([C@H]3N1CCOC3)[C@H]1c3c(CSc4c1cccc4)c(F)ccc3)ccc(=O)c2O	1985605-76-2	1H-[1,4]Oxazino[3,4-c]pyrido[2,1-f][1,2,4]triazine-6,8-dione, 12-[(11S)-6,11-dih	C25H23N3O4S	461.53	A/B	-8.3
7362	Fc1c2[C@H](N3n4c(C(=O)N5[C@H]3COC[C@@H]5C)c(O)c(=O)cc4)c3c(SCc2ccc1)cccc3	1985605-86-4	1H-[1,4]Oxazino[3,4-c]pyrido[2,1-f][1,2,4]triazine-6,8-dione, 12-[(11S)-8-fluoro	C25H22FN3O4S	479.52	A/B	-8.3
7363	O=C1N2[C@H](N(n3c1c(O)c(=O)cc3)[C@H]1c3c(CSc4c1cccc4)c(F)c(F)cc3)CO[C@H](C(F)(F)F)C2	1985605-87-5	1H-[1,4]Oxazino[3,4-c]pyrido[2,1-f][1,2,4]triazine-6,8-dione, 12-[(11S)-10-fluor	C25H22FN3O4S	479.52	A/B	-8.3
7364	O(COC(=O)OC)c1c2n(N([C@H]3N(C2=O)[C@@H](C)COC3)[C@H]2c3c(CSc4c2cccc4)cc(F)cc3)ccc1=O	1985606-59-4	Carbonic acid, [[(12aR)-12-[(11R)-10-chloro-6,11-dihydrodibenzo[b,e]thiepin-11-y	C27H24ClN3O7S	570.01	A/B	-8.3
7365	O(COC(=O)OC(C(=O)OC(C)(C)C)(C)C)c1c2n(N([C@H]3N(C2=O)CCOC3)[C@H]2c3c(CSc4c2cccc4)c(F)c(F)cc3)ccc1=O	1985607-02-0	Carbonic acid, [[(12aR)-12-[(11S)-7,8-difluoro-6,11-dihydrodibenzo[b,e]thiepin-1	C32H29F2N3O9S	669.65	A/B	-8.3
7366	O(C(=O)OC1CCOCC1)c1c2n(N([C@H]3N(C2=O)CCOC3)[C@H]2c3c(CSc4c2cccc4)c(F)c(F)cc3)ccc1=O	1985607-15-5	Carbonic acid, (12aR)-12-[(11S)-7,8-difluoro-6,11-dihydrodibenzo[b,e]thiepin-11-	C31H29F2N3O7S	625.64	A/B	-8.3
7367	O(C(=O)O[C@@H]1[C@H]2[C@@H](OC1)OCC2)c1c2n(N([C@H]3N(C2=O)CCOC3)[C@H]2c3c(CSc4c2cccc4)c(F)c(F)cc3)cc	1985607-26-8	Carbonic acid, 1-acetyl-3-azetidinyl (12aR)-12-[(11S)-7,8-difluoro-6,11-dihydroc	C30H26F2N4O7S	624.61	A/B	-8.3
7368	O(COC(=O)OC(C)(C)C)c1c2n(N([C@H]3N(C2=O)CCOC3)[C@H]2c3c(CSc4c2cccc4)c(F)c(F)cc3)ccc1=O	1985607-36-0	Cyclopentaneacetic acid, 1-[[[[[(12aR)-12-[(11S)-7,8-difluoro-6,11-dihydrodibenz	C34H33F2N3O9S	697.7	A/B	-8.3
7369	O(C(=O)O[C@@H](C(=O)OC)C)c1c2n(N([C@H]3N(C2=O)CCOC3)[C@H]2c3c(CSc4c2cccc4)c(F)c(F)cc3)ccc1=O	1985607-56-4	Propanoic acid, 2-[[[[(12aR)-12-[(11S)-7,8-difluoro-6,11-dihydrodibenzo[b,e]thie	C29H25F2N3O8S	613.59	A/B	-8.3
7370	N(C(=O)[C@@H](Cc1ccccc1)C[C@H](CN1[C@H](C(=O)NC(C)(C)C)CNCC1)O)[C@H]1c2c(C[C@H]1O)cccc2	1985868-86-7	Benzonitrile, 4-[[4-[(2,5-dimethylphenyl)amino]-6-methyl-2-pyrimidinyl]amino]-	C20H19N5	329.4	A/B	-8.3

7371	N(c1c(C#N)cccc1)c1nc(Nc2c(C)cccc2C)ncc1	1987622-96-7	Benzonitrile, 2-[[4-[(2-ethyl-6-methylphenyl)amino]-2-pyrimidinyl]amino]-	C20H19N5	329.4	A/B	-8.3
7372	N(C(=O)c1c(Cl)cccc1)c1cc2cc(C34CC5CC(C4)CC(C3)C5)[nH]c2cc1	1987854-63-6	Benzamide, 4-chloro-N-(2-tricyclo[3.3.1.13,7]dec-1-yl-1H-indol-5-yl)-	C25H25ClN2O	404.93	A/B	-8.3
7373	N(C(=O)Nc1ccc(C#N)cc1)c1cc2cc(C34CC5CC(C4)CC(C3)C5)[nH]c2cc1	1987854-65-8	3-Pyridinecarboxamide, 2-chloro-N-(2-tricyclo[3.3.1.13,7]dec-1-yl-1H-indol-5-yl)	C24H24ClN3O	405.92	A/B	-8.3
7374	N(C(=O)Nc1nc2c(s1)cccc2)c1cc2cc(C34CC5CC(C4)CC(C3)C5)[nH]c2cc1	1987854-78-3	Urea, N-[2-(2-thienyl)ethyl]-N,Ä≤-(2-tricyclo[3.3.1.13,7]dec-1-yl-1H-indol-5-yl)	C25H29N3OS	419.58	A/B	-8.3
7375	N(Cc1ccc(cc1)c1[nH]nnn1)(C[C@@H]([C@H](Cc1ccccc1)NC(=O)[C@@H](NC(=O)OC)C(C)(C)C)O)NC(=O)[C@@H](NC(=O	198904-13-1	2,5,6,10,13-Pentaazatetradecanedioic acid, 3,12-bis(1,1-dimethylethyl)-8-hydroxy	C35H51N9O7	709.84	A/B	-8.3
7376	N(c1c(C)cc(C)cc1C)c1nc(Nc2cc(C#N)ccc2)nc(C)c1	1990562-63-4	Benzonitrile, 4-[[4-[(2,4-dimethylphenyl)amino]-6-methyl-2-pyrimidinyl]amino]-	C20H19N5	329.4	A/B	-8.3
7377	N(CCCC(CCl)C)c1c2c(cc(Cl)cc2)ncc1	1990588-82-3	Benzonitrile, 3-[[4-methyl-6-[(2,4,6-trimethylphenyl)amino]-2-pyrimidinyl]amino]	C21H21N5	343.42	A/B	-8.3
7378	N(c1nc(Nc2c(C)cccc2C)nc(C)c1)c1c(C#N)cccc1	1991964-50-1	2,4-Pyrimidinediamine, 6-methyl-N2-phenyl-N4-(2,4,6-trimethylphenyl)-	C20H22N4	318.42	A/B	-8.3
7379	N(c1c(CC)cccc1C)c1nc(Nc2ccc(C#N)cc2)nc(C)c1	1992226-48-8	Benzonitrile, 2-[[2-[(2,6-dimethylphenyl)amino]-6-methyl-4-pyrimidinyl]amino]-	C20H19N5	329.4	A/B	-8.3
7380	C(F)(F)c1n(c2c(n1)cccc2)c1nc(nc(n1)N1CCOCC1)N1C[C@@H](C(=O)O)CCC1	2001083-68-5	4-Piperidinecarboxylic acid, 1-[4-[2-(difluoromethyl)-1H-benzimidazol-1-yl]-6-(	C21H23F2N7O3	459.45	A/B	-8.3
7381	[C@@H]([C@H](Cc1ccccc1)NC(=O)COc1c(C)cccc1C)(C[C@H](Cc1ccccc1)NC(=O)[C@H](C(C)C)N1C(=O)NCCC1)OC(=O)[	2001083-78-7	3-Piperidinecarboxylic acid, 1-[4-[2-(difluoromethyl)-1H-benzimidazol-1-yl]-6-(	C21H23F2N7O3	459.45	A/B	-8.3
7382	O[C@H]1[C@H](n2c3c(nc2)c(=O)nc(N)[nH]3)O[C@H](COP(=O)(OP(=O)(O[C@@H]2O[C@H](CO)[C@@H](O)[C@H](O)C2)O	2004706-85-6	Not Yet Assigned	C20H26N8O21P4	838.36	A/B	-8.3
7383	C(=O)([C@@H](NC(=O)OC)C(C)C)N1[C@H](c2[nH]c3c4c(cc(cc4)c4c5c(c(cc4)c4[nH]c(nc4)[C@H]4N(C(=O)[C@@H](N	2007900-85-6	Carbamic acid, N-[(1S)-1-[[(2S,5S)-2-[5-[2,3-dihydro-7-[2-[(2S,5S)-1-[(2S)-2-[(	C48H60N8O6	845.04	A/B	-8.3
7384	O(C[C@H]1O[C@H](C=C1)n1c(=O)[nH]c(=O)c(C)c1)P1(=O)Oc2c(CO1)cc(OC)cc2	2011008-74-3	L-Alanine, N-[[P(S),2,Ä≤R]-2,Ä≤-deoxy-2,Ä≤-fluoro-3,Ä≤-O-[(4-methoxyphenyl)meth	C30H37FN3O10P	649.6	A/B	-8.3
7385	C(=O)(C(=O)Nc1c(C)cc(C)cc1)c1c([nH]c2c1cccc2)C12CC3CC(C2)CC(C1)C3	2014403-81-5	1H-Indole-3-acetamide, N-(4-methoxyphenyl)-Œ±-oxo-2-tricyclo[3.3.1.13,7]dec-1-yl	C27H28N2O3	428.52	A/B	-8.3
7386	C(=O)(C(=O)Nc1cc(C(C)(C)C)on1)c1c([nH]c2c1cccc2)C12CC3CC(C2)CC(C1)C3	2014403-95-1	1H-Indole-3-acetamide, Œ±-oxo-N-5-pyrimidinyl-2-tricyclo[3.3.1.13,7]dec-1-yl-	C24H24N4O2	400.47	A/B	-8.3
7387	O=c1n([C@@H]2O[C@H](COC(=O)COc3c(OC)ccc(C(=O)c4cc(OC)c(OC)c(OC)c4)c3)C=C2)cc(C)c(=O)[nH]1	2015224-16-3	Butanedioic acid, 1-[5-(2-chlorobenzoyl)-2-methoxyphenyl] 4-[[(2S,5R)-5-(3,4-dih	C28H25ClN2O9	568.96	A/B	-8.3
7388	S(Cc1cc2c(oc1=O)cccc2)c1n(c2c(n1)c(=O)nc[nH]2)[C@@H]1O[C@H](CO)[C@@H](O)[C@H]1O	2020089-26-1	Carbamic acid, N-[(1S)-1-[[(2S)-2-[5-[7-[2-[(2S)-4,4-difluoro-1-[(2S)-2-[(methox	C44H50F4N8O6	862.91	A/B	-8.3
7389	[C@H](Cc1ccccc1)([C@H](C[C@H](Cc1ccccc1)NC(=O)OCc1cccnc1)O)NC(=O)[C@@H](NC(=O)N(Cc1nc(C(C)(C)C)sc1)C	202816-26-0	2,7,10,12-Tetraazatridecanoic acid, 5-hydroxy-12-methyl-9-(1-methylethyl)-13-[2-	C39H50N6O5S	714.92	A/B	-8.3
7390	O[C@H]1[C@@H](O[C@H](COCC(=O)NCCNC(=O)CO[C@@H]2O[C@H](CO)[C@H](O)[C@H](O)[C@H]2O)[C@H]1O)n1nc(C(=O)N	1002334-76-0	1H-1,2,4-Triazole-3-carboxamide, 1-[5-O-[3-[[2-(acetylamino)-2-deoxy-Œ≤-D-galact	C19H29N5O12	519.46	C	-8.3
7391	O(Cc1ccccc1)[C@@H]1[C@@H](NC(=O)CCC(=O)OC[C@H]2O[C@H]([C@H](O)[C@@H]2O)n2nc(C(=O)N)nc2)C(OCc2ccccc2)	1002336-72-2	D-Galactopyranoside, phenylmethyl 2-[(3-carboxy-1-oxopropyl)amino]-2-deoxy-3,4,	C48H53N5O13	907.96	C	-8.3
7392	C(OC(=O)COCCO[C@H]1[C@H](OCc2ccccc2)[C@@H](OCc2ccccc2)[C@@H](OCc2ccccc2)[C@@H](COCc2ccccc2)O1)[C@@H]	1002336-73-3	D-Galactopyranoside, phenylmethyl 2-[(3-carboxy-1-oxopropyl)amino]-2-deoxy-3,4,	C46H51N5O12	865.92	C	-8.3
7393	[C@@H](Cc1ccccc1)(NC(=O)[C@H](CCN1CCOCC1)NC(=O)N(Cc1nc(C(C)C)sc1)C)CC[C@H](Cc1ccccc1)NC(=O)OCc1cncs1	1004316-87-3	2,7,10,12-Tetraazatridecanoic acid, 9-(2-aminoethyl)-12-methyl-13-[2-(1-methylet	C36H47N7O4S2	705.93	C	-8.3
7394	C(=O)([C@@H](NC(=O)OC)C(C)C)N1[C@@H](CCC1)c1[nH]c(cn1)c1ccc(cc1)c1ccc(cc1)c1[nH]c(nc1)[C@H]1N(C(=O)[	1007884-52-7	Carbamic acid, N-[(1S)-1-[[(2S)-2-[5-[4,Ä≤-[2-[(2S)-1-[(2S)-2-[(methoxycarbonyl)	C46H54N8O6	814.97	C	-8.3
7395	N(=C1c2c(N(CN(CC)CC)C1=O)cccc2)NC(=S)Nc1ccccc1	100847-02-7	Hydrazinecarbothioamide, 2-[1,2-dihydro-2-oxo-1-[(4-phenyl-1-piperazinyl)methyl]	C26H26N6OS	470.59	C	-8.3
7396	C(=O)([C@@H](NC(=O)OC)C(C)C)N1[C@@H](CCC1)c1[nH]c(cn1)c1ccc(cc1)c1ccc(cc1)c1[nH]c(nc1)[C@H]1N(C(=O)C	1009113-44-3	Carbamic acid, N-[(1S)-2-methyl-1-[[(2S)-2-[5-[4,Ä≤-[2-[(2S)-1-[2-[[(1S)-1-methy	C39H50N8O4	694.87	C	-8.3
7397	C(=O)([C@@H](NC(=O)OC)C(C)C)N1[C@@H](CCC1)c1[nH]c(cn1)c1ccc(cc1)c1ccc(cc1)c1[nH]c(nc1)[C@H]1N(C(=O)C	1009113-45-4	Carbamic acid, N-[(1S)-2-methyl-1-[[(2S)-2-[5-[4,Ä≤-[2-[(2S)-1-[2-[(1-methylethy	C38H48N8O4	680.84	C	-8.3
7398	C(=O)([C@@H](Nc1cccnc1)C(C)C)N1[C@@H](CCC1)c1[nH]c(cn1)c1ccc(cc1)c1ccc(cc1)c1[nH]c(nc1)[C@H]1N(C(=O)	1009114-11-7	Carbamic acid, N-[(1S)-1-[[(2S)-2-[5-[4,Ä≤-[2-[(2S)-1-[(2R)-2-[(4,5-dihydro-1H-	C41H52N10O4	748.92	C	-8.3
7399	C(=O)([C@H](Cc1ccc(O)cc1)NC(=O)OC)N1[C@@H](CCC1)c1[nH]c(cn1)c1ccc(cc1)c1ccc(cc1)c1[nH]c(nc1)[C@H]1N(	1009115-00-7	Carbamic acid, N-[(1S)-1-[[(2S)-2-[5-[4,Ä≤-[2-[(2S)-1-[(2S)-3-(4-hydroxyphenyl)-	C44H50N8O7	802.92	C	-8.3
7400	C(=O)([C@@H](NC(=O)OC)C(C)C)N1[C@@H](C[C@@H](F)C1)c1[nH]c(cn1)c1ccc(cc1)c1ccc(cc1)c1[nH]c(nc1)[C@H]1	1009115-64-3	Carbamic acid, N-[(1R)-1-[[(2S,4R)-4-fluoro-2-[5-[4,Ä≤-[2-[(2S,4R)-4-fluoro-1-[	C42H52F2N8O6	802.91	C	-8.3
7401	C(=O)([C@@H](NC(=O)OC)C1CC1)N1[C@@H](CCC1)c1[nH]c(cn1)c1c(F)cc(cc1)c1ccc(cc1)c1[nH]c(nc1)[C@H]1N(C(=	1009116-44-2	Carbamic acid, N,N,Ä≤-[(3-fluoro[1,1,Ä≤-biphenyl]-4,4,Ä≤-diyl)bis[1H-imidazole-	C40H49FN8O6	756.87	C	-8.3
7402	C(=O)([C@@H](NC(=O)OC)C(C)C)N1[C@@H](CCC1)c1[nH]c(cn1)c1c(F)cc(cc1)c1cc(F)c(cc1)c1[nH]c(nc1)[C@H]1N(	1009116-58-8	Carbamic acid, N-[(1S)-1-cyclopropyl-2-[(2S)-2-[5-[3,3,Ä≤-difluoro-4,Ä≤-[2-[(2S	C40H46F2N8O6	772.84	C	-8.3
7403	C(=O)([C@H](NC(=O)OCC)C)N1[C@@H](CCC1)c1[nH]c(cn1)c1ccc(cc1)c1ccc(cc1)c1[nH]c(nc1)[C@H]1N(C(=O)[C@H]	1009116-61-3	Carbamic acid, N,N,Ä≤-[(2,2,Ä≤-difluoro[1,1,Ä≤-biphenyl]-4,4,Ä≤-diyl)bis[1H-imi	C40H48F2N8O6	774.86	C	-8.3
7404	C(=O)([C@@H](N(C(=O)OC)C)C(C)C)N1[C@@H](CCC1)c1[nH]c(cn1)c1ccc(cc1)c1ccc(cc1)c1[nH]c(nc1)[C@H]1N(C(=	1009116-78-2	Carbamic acid, N,N,Ä≤-[[1,1,Ä≤-biphenyl]-4,4,Ä≤-diylbis[1H-imidazole-5,2-diyl-(	C38H46N8O6	710.82	C	-8.3
7405	C(=O)([C@@H](NC(=O)OC)[C@H](C)O)N1[C@@H](CCC1)c1[nH]c(cn1)c1ccc(cc1)c1ccc(cc1)c1[nH]c(nc1)[C@H]1N(C(	1009116-95-3	Carbamic acid, N,N,Ä≤-[[1,1,Ä≤-biphenyl]-4,4,Ä≤-diylbis[1H-imidazole-5,2-diyl-(	C38H46N8O8	742.82	C	-8.3
7406	C(=O)([C@H](NC(=O)OC)C1CC1)N1[C@@H](CCC1)c1[nH]c(cn1)c1ccc(cc1)c1ccc(cc1)c1[nH]c(nc1)[C@H]1N(C(=O)[C	1009117-12-7	Carbamic acid, N,N,Ä≤-[[1,1,Ä≤-biphenyl]-4,4,Ä≤-diylbis[1H-imidazole-5,2-diyl-(	C38H46N8O6	710.82	C	-8.3
7407	C(=O)([C@H](N(CC)CC)c1ccccc1)N1[C@@H](CCC1)c1[nH]c(cn1)c1ccc(cc1)c1ccc(cc1)c1[nH]c(nc1)[C@H]1N(C(=O)	1009117-57-0	Carbamic acid, N-[(1R)-1-[[(2S)-2-[5-[4,Ä≤-[2-[(2S)-1-[(2R)-2-(diethylamino)-2-	C44H52N8O4	756.93	C	-8.3
7408	C(=O)([C@H](N(CC)CC)c1ccccc1)N1[C@@H](CCC1)c1[nH]c(cn1)c1ccc(cc1)c1ccc(cc1)c1[nH]c(nc1)[C@H]1N(C(=O)	1009117-61-6	Carbamic acid, N-[(1S)-1-[[(2S)-2-[5-[4,Ä≤-[2-[(2S)-1-[(2R)-2-(diethylamino)-2-	C45H54N8O4	770.96	C	-8.3
7409	C(=O)([C@H](NC(=O)OC)c1ccccc1)N1[C@@H](CCC1)c1[nH]c(cn1)c1ccc(cc1)c1ccc(cc1)c1[nH]c(nc1)[C@H]1N(C(=O	1009117-66-1	Carbamic acid, N-[(1R)-2-[(2S)-2-[5-[4,Ä≤-[2-[(2S)-1-[(2R)-2-(diethylamino)-1-o	C43H50N8O4	742.91	C	-8.3
7410	C(=O)([C@@H](NC(=O)OC)C(C)C)N1[C@@H](CCC1)c1[nH]c(cn1)c1ccc(cc1)c1ccc(cc1)c1[nH]c(nc1)[C@H]1N(C(=O)[	1009117-78-5	Carbamic acid, N-[(1S)-1-[[(2S)-2-[5-[4,Ä≤-[2-[(2S)-1-[(2S)-2-[(methoxycarbonyl)	C39H48N8O6	724.85	C	-8.3
7411	C(=O)([C@@H](NC(=O)OC)C(C)C)N1[C@@H](CCC1)c1[nH]c(cn1)c1ccc(cc1)c1ccc(cc1)c1[nH]c(nc1)[C@H]1N(C(=O)C	1009117-84-3	Carbamic acid, N-[(1R)-2-[(2S)-2-[5-[4,Ä≤-[2-[(2S)-1-[(2S)-2-[(methoxycarbonyl)	C43H48N8O6	772.89	C	-8.3
7412	C(=O)([C@@H](NC(=O)OC)C1CC1)N1[C@@H](CCC1)c1[nH]c(cn1)c1ccc(cc1)c1ccc(cc1)c1[nH]c(nc1)[C@H]1N(C(=O)[	1009117-97-8	Carbamic acid, N-[(1S)-1-[[(2S)-2-[5-[4,Ä≤-[2-[(2S)-1-[(2S)-4-amino-2-[(methoxy	C39H47N9O7	753.85	C	-8.3
7413	C(=O)([C@H](N(CC)CC)C)N1[C@@H](CCC1)c1[nH]c(cn1)c1ccc(cc1)c1ccc(cc1)c1[nH]c(nc1)[C@H]1N(C(=O)[C@@H](	1009118-01-7	Carbamic acid, N-[(1S)-2-[(2S)-2-[5-[4,Ä≤-[2-[(2S)-1-[(2S)-2-[(methoxycarbonyl)	C39H48N8O6	724.85	C	-8.3
7414	C(=O)([C@@H](NC(=O)OC)C(C)(C)O)N1[C@@H]2[C@@H](C2)C[C@H]1c1[nH]c(cn1)c1ccc(cc1)c1ccc(cc1)c1[nH]c(nc1	1009118-22-2	Carbamic acid, N,N,Ä≤-[[1,1,Ä≤-biphenyl]-4,4,Ä≤-diylbis[1H-imidazole-5,2-diyl-(	C42H50N8O6	762.9	C	-8.3
7415	C(C(=O)O)(N)C12CC3(C)CC(C)(C1)CC(C2)C3	1009120-58-4	Carbamic acid, N,N,Ä≤-[[1,1,Ä≤-biphenyl]-4,4,Ä≤-diylbis[1H-imidazole-5,2-diyl-(	C43H56N8O6	780.95	C	-8.3
7416	C(C)(C)(F)[C@@H]1n2c3c4c(nc(N)c3nc2C(CCC)OC1)cccc4	1010105-81-3	8H-[1,4]Oxazino[4,Ä≤,3,Ä≤:1,2]imidazo[4,5-c]quinolin-6-amine, 8-ethyl-11-(1-flu	C18H21FN4O	328.38	C	-8.3
7417	O=c1c2c(n(c(n2)[2H])[C@@H]2O[C@H](CO)[C@@H](O)C2)[nH]c(N)n1	1013423-59-0	2,7,10,12-Tetraazatridecanoic acid, 4-hydroxy-12-methyl-9-(1-methylethyl)-13-[2-	C37H48N6O5S2.C31H33F3N2O5S		C	-8.3
7418	C(=O)(NC(CC1CCC1)C(=O)C(=O)N)[C@@H]1[C@@H]2[C@@H](C2(C)C)CN1C(=O)[C@@H](NC(=O)NC(C)(C)C)C(C)(C)C.C(C	1015080-25-7	3-Azabicyclo[3.1.0]hexane-2-carboxamide, N-[3-amino-1-(cyclobutylmethyl)-2,3-di	C27H45N5O5.C19H17NO3		C	-8.3
7419	N(c1nc(Nc2ccc(C#N)cc2)nc(Br)c1)c1c(C)cc(C#N)cc1C	1025826-55-4	2-Propen-1-one, 1-[5-[(2,3-dihydroxyphenyl)methyl]-4-hydroxy-2-methoxyphenyl]-3-	C23H20O5	376.4	C	-8.3
7420	O=c1n(cc(C)c(=O)[nH]1)[C@H]1C(=C[C@@H](CO)O1)O	1025971-16-7	Benzonitrile, 4-[[9-[4-[(1E)-2-cyanoethenyl]-2,6-dimethylphenyl]-6-methyl-9H-pur	C24H19N7	405.45	C	-8.3
7421	O=C1[C@@H]2[C@@H](c3c([C@@]([C@@H](C(C)C)c4ccccc4)(O)[C@H]2CO1)cc1c(c3)OCO1)c1cc(OC)c(OC)c(OC)c1	1027093-36-2	Furo[3,Ä≤,4,Ä≤:6,7]naphtho[2,3-d]-1,3-dioxol-6(5aH)-one, 5,8,8a,9-tetrahydro-9-	C19H16O5	324.33	C	-8.3
7422	C(C[C@H](NC(=O)c1ccccc1)c1ccccc1)(N1[C@@H]2C[C@@H](C[C@H]1CC2)n1c(C(C)C)nnc1C([2H])([2H])[2H])([2H])	1033698-65-5	Benzamide, N-[(1S)-3-[(3-exo)-3-[3-(methyl-d3)-5-(1-methylethyl)-4H-1,2,4-triaz	C29H34D3N5O		C	-8.3
7423	O=c1c2c(n(cn2)[C@H]2[C@H](O)[C@H]3[C@H](O2)COP(=O)(O)OP(=O)(O)O3)[nH]c(NC)n1	1034784-77-4	Guanosine, cyclic 2,Ä≤,3,Ä≤-(P,P,Ä≤,P,Ä≤,Ä≤-trihydrogen triphosphate)	C10H14N5O13P3	505.17	C	-8.3
7424	N(CCCCCCCN(CCO)CCO)c1c2c(ncc1)cc(Cl)cc2	1034784-93-4	Guanosine, N-methyl-, cyclic 2,Ä≤,3,Ä≤-(P,P,Ä≤,P,Ä≤,Ä≤-trihydrogen triphosphate	C11H16N5O13P3	519.19	C	-8.3
7425	N(c1c(C)cc(/C=C/C#N)cc1C)c1nc(Nc2ccc(C#N)cc2)nc(C(=O)NCc2ccco2)c1	1037444-89-5	4-Pyrimidinecarboxamide, 6-[[4-[(1E)-2-cyanoethenyl]-2,6-dimethylphenyl]amino]-2	C29H24N8O	500.55	C	-8.3
7426	N(c1nc(Nc2ccc(C#N)cc2)nc(CNCCc2ccc(S(=O)(=O)N)cc2)c1)c1c(C)cc(/C=C/C#N)cc1C	1037445-06-9	Benzonitrile, 4-[[4-[[4-[(1E)-2-cyanoethenyl]-2,6-dimethylphenyl]amino]-6-[[[(2,	C29H31N7O2	509.6	C	-8.3
7427	N(c1c(/C=C/c2ccc(OC)cc2)c(COC)nc(Nc2ccc(C#N)cc2)n1)c1c(C)cc(/C=C/C#N)cc1C	1037452-85-9	Benzonitrile, 4-[[4-[[4-[(1E)-2-cyanoethenyl]-2,6-dimethylphenyl]amino]-6-(metho	C33H30N6O	526.63	C	-8.3
7428	N(C1scc(Br)c1)C12CC3CC(C2)CC(C1)C3	103908-26-5	C.I. Mordant Violet 16, trisodium salt	C25H20O9.3Na		C	-8.3
7429	C(C12CC3CC(C2)CC(C1)C3)(N1CCN(C)CC1)(c1ccccc1)c1ccccc1	104061-29-2	1,4,6-Heptatrien-3-one, 1,7-bis(3,4-dihydroxyphenyl)-5-hydroxy-	C19H16O6	340.33	C	-8.3
7430	O=c1n([C@@H]2O[C@H](CO)[C@H](O)C2)cc(COC)c(=O)[nH]1	1046144-69-7	Thymidine, 2,Ä≤-deoxy-P-methylcytidylyl-(3,Ä≤,Üí5,Ä≤)-	C20H28N5O11P	545.44	C	-8.3
7431	C(Oc1c2c(n(cn2)[C@@H]2O[C@H](CO)[C@H](O)C2)nc(N)n1)c1c2c(N(c3c(C(=O)N2)cccn3)C2CC2)ncc1	1046462-09-2	Cytidine, 3-[(11-cyclopropyl-6,11-dihydro-6-oxo-5H-dipyrido[3,2-b:2,Ä≤,3,Ä≤-e][	C24H25N7O5	491.5	C	-8.3
7432	O(C(CC)C)[C@@H]1[C@@H](NC(=O)C)[C@H](N)CC(=C1)C(=O)O	1052076-34-2	Not Yet Assigned	C28H36N2O10S	592.66	C	-8.3
7433	C[C@]12[C@H]([C@H]3[C@@H]([C@]4(C)[C@H](CC3)C[C@H](OC=O)CC4)CC2)CC[C@@H]1[C@@H](CCC(=O)OC)C	105275-93-2	Cytidine, guanylyl-(3,Ä≤,Üí5,Ä≤)-2,Ä≤-deoxy-	C19H25N8O11P	572.42	C	-8.3
7434	O=c1n(ccc(N)n1)[C@@H]1O[C@H](COP(=O)(OC(=O)O)O)CC1	1053612-93-3	Not Yet Assigned	C15H17N10O7P	480.33	C	-8.3
7435	C(=O)(NC(CC1(F)CCC1)C(=O)C(=O)N)C1[C@@H]2[C@@H](C2(C)C)CN1C(=O)[C@@H](NC(=O)NC(C)(C)C)C(C)(C)C	1056467-68-5	3-Azabicyclo[3.1.0]hexane-2-carboxamide, N-[3-amino-1-[(cis-3-hydroxycyclobutyl)	C27H45N5O6	535.68	C	-8.3
7436	O=C1[C@@H]2[C@@H](c3c([C@H](O)[C@H]2CO1)cc1c(c3)OCO1)c1cc(OC)c(O)c(O)c1	10566-04-8	Guanosine, guanylyl-(5,Ä≤,Üí5,Ä≤)-	C20H25N10O12P	628.45	C	-8.3
7437	O(C(=O)NC(Cc1ccccc1)C(CN(S(=O)(=O)c1ccc(OC)cc1)CC(OCP(=O)(O)O)(C)C)O)C1C2C(OC1)OCC2	1057217-77-2	Benzonitrile, 4-[[4-amino-6-(4-amino-2,6-dimethylphenoxy)-5-bromo-2-pyrimidinyl]	C19H17BrN6O	425.28	C	-8.3
7438	C(=C\C(=O)CC(=O)/C=C/c1ccccc1)/c1cc(O)c(O)cc1	1061609-19-5	Imidogen, [2-[2-[4,Ä≤-[2-(1-amino-4-sulfo-2-naphthalenyl)diazenyl][1,1,Ä≤-biphe	C32H20N6O6S2		C	-8.3
7439	O=C1[C@@H]2[C@@H](c3c([C@H](OC(=O)/C=C/c4c(OC)cccc4)[C@H]2CO1)cc1c(c3)OCO1)c1cc(OC)c(OC)c(OC)c1	1062226-99-6	2-Propenoic acid, 3-phenyl-, (5R,5aR,8aR,9R)-5,5a,6,8,8a,9-hexahydro-8-oxo-9-(3,	C31H28O9	544.55	C	-8.3
7440	C[C@]1(F)[C@@H](O[C@H](COP(=O)(Oc2ccccc2)N[C@@H](C(=O)OCCCC)C)[C@H]1O)n1c(=O)[nH]c(=O)cc1	1064684-69-0	L-Alanine, N-[[P(R),2,Ä≤R]-2,Ä≤-deoxy-2,Ä≤-fluoro-2,Ä≤-methyl-P-phenyl-5,Ä≤-uri	C25H33FN3O9P	569.52	C	-8.3
7441	C(OC(=O)C)(OC(=O)C)c1c2c(COP(=O)(OC[C@H]3O[C@H](C=C3)n3c(=O)[nH]c(=O)c(C)c3)O2)cc(C)c1	1072917-28-2	Thymidine, 2,Ä≤,3,Ä≤-didehydro-3,Ä≤-deoxy-5,Ä≤-O-[6-(1,1-dimethylethyl)-8-formy	C22H25N2O8P	476.42	C	-8.3
7442	[C@@H](NC(=O)N1CCN(C)CC1)(C(=O)N[C@H](C=CS(=O)(=O)c1ccccc1)CCc1ccccc1)CCCNC(=N)NN(=O)=O	1076088-61-3	1-Piperazinecarboxamide, N-[(1S)-3-[[imino(nitroamino)methyl]amino]-1-[[[(1S)-1-	C28H38N8O6S	614.72	C	-8.3
7443	C(=O)([C@H](Cc1cnc[nH]1)NC(=O)[C@@H](NC(=O)[C@H](Cc1ccc(O)cc1)NC(=O)[C@@H](NC(=O)OCc1ccccc1)C(C)C)[C	108039-30-1	Alanine, [1-[N-[N-[N-(N-carboxy-L-valyl)-L-tyrosyl]-L-leucyl]-L-histidyl]-L-prol	C49H62N8O10	923.06	C	-8.3
7444	C(=O)([C@@H](NC(=O)OC)C)N1[C@@H](CCC1)c1[nH]c(cn1)c1ccc(cc1)c1ccc(cc1)c1[nH]c([C@@H](NC(=O)[C@@H](NC	1087346-55-1	Carbamic acid, N-[(1S)-1-[[(2S)-2-[5-[4,Ä≤-[2-[(1S)-1-[[(2S)-2-[(methoxycarbony	C40H52N8O6	740.89	C	-8.3
7445	N(Cc1ccc(cc1)c1ccccn1)(C[C@@H]([C@H](Cc1ccccc1)NC(=O)[C@@H](NC(=O)OC([2H])([2H])[2H])C(C)(C)C)O)NC(=	109253-07-8	2-Naphthalenesulfonic acid, 8-[2-(3-acetylphenyl)diazenyl]-5-hydroxy-	C18H14N2O5S	370.38	C	-8.3
7446	O[C@H]1[C@H](n2c3c(nc2[3H])c(=O)nc[nH]3)O[C@H](COP(=O)(OP(=O)(O)O)O)[C@H]1O	1093679-47-0	Guanosine 5,Ä≤-(trihydrogen diphosphate), P,Ä≤-(6-deoxy-Œ±-D-altropyranosyl) es	C16H25N5O15P2	589.34	C	-8.3
7447	O=c1n([C@@H]2O[C@H](COP3(=O)Oc4c(CO3)cc(Cc3cc5c(cc3)OP(=O)(OC[C@H]3O[C@H](C[C@@H]3F)n3c(=O)[nH]c(=O)	1099829-88-5	Not Yet Assigned	C29H36N4O7P2S	646.63	C	-8.3
7448	C(NC(=O)c1nnc(C)o1)(C([2H])([2H])[2H])(C([2H])([2H])[2H])c1nc(C(=O)NCc2ccc(F)cc2)c(O)c(=O)n1C	1100750-81-9	4-Pyrimidinecarboxamide, N-[(4-fluorophenyl)methyl]-1,6-dihydro-5-hydroxy-1-meth	C20H18D3FN6O5		C	-8.3
7449	C(NC(=O)c1nnc(C([2H])([2H])[2H])o1)(C)(C)c1nc(C(=O)NC(c2ccc(F)cc2)([2H])[2H])c(O)c(=O)n1C([2H])([2H]	1100750-91-1	4-Pyrimidinecarboxamide, N-[(4-fluorophenyl)methyl]-1,6-dihydro-5-hydroxy-1-(met	C20H9D12FN6O5		C	-8.3
7450	C(NC(=O)c1nnc(C)o1)(C([2H])([2H])[2H])(C([2H])([2H])[2H])c1nc(C(=O)NCc2c(c(F)c(c2[2H])[2H])[2H])[2	1100750-95-5	4-Pyrimidinecarboxamide, N-[(4-fluorophenyl-2,3,5,6-d4)methyl]-1,6-dihydro-5-hy	C20H14D7FN6O5		C	-8.3
7451	C(=O)([C@@H](NC(=O)OC)C(C)C)N1[C@H](c2[nH]c(c(CO)n2)c2ccc(cc2)c2ccc(cc2)c2[nH]c(nc2)[C@H]2N(C(=O)[C@	1129636-50-5	Carbamic acid, N-[(1S)-1-[[(2S)-4,4-difluoro-2-[5-[3,Ä≤-fluoro-4,Ä≤-[2-[(2S)-1-	C40H47F3N8O6	792.85	C	-8.3
7452	[Si](OC[C@H]1O[C@H](CS1)n1c(=O)nc(NCCCN)cc1)(C(C)(C)C)(c1ccccc1)c1ccccc1	1139875-90-3	6H-Dipyrido[3,2-b:2,Ä≤,3,Ä≤-e][1,4]diazepin-6-one, 11-cyclopropyl-5,11-dihydro-	C18H18N4O	306.36	C	-8.3
7453	C(=O)(N[C@@](C(C1(C(C(C1([2H])[2H])([2H])[2H])([2H])[2H])[2H])([2H])[2H])(C(=O)C(=O)N)[2H])[C@@H]1[C	1158082-92-8	3-Azabicyclo[3.1.0]hexane-2-carboxamide, N-[(1S)-3-amino-1-(cyclobutylmethyl)-2,	C27H30D15N5O5		C	-8.3
7454	OC1C(n2c3c(nc2)c(NCC=C)ncn3)OC(CO)C1O	1164118-04-0	Thymidine, [P(S)]-P-thiothymidylyl-(3,Ä≤,Üí5,Ä≤)-5,Ä≤-thio-	C20H27N4O10PS2	578.55	C	-8.3
7455	N(=C\1/c2c(N(C)C1=O)ccc(S(=O)(=O)N1CCOCC1)c2)\NC(=S)Nc1c(CC)cccc1	1164492-51-6	Hydrazinecarbothioamide, 2-[1,2-dihydro-1-methyl-5-(4-morpholinylsulfonyl)-2-ox	C21H23N5O4S2	473.57	C	-8.3
7456	O=C1N2c3c(/C/1=N/NC(=S)N)c(C)ccc3C(=CC2(C)C)C	1164493-89-3	Hydrazinecarbothioamide, 2-[1,2-dihydro-1-methyl-5-(4-morpholinylsulfonyl)-2-ox	C21H23N5O4S2	473.57	C	-8.3
7457	N(=C\1/c2c(N(C)C1=O)ccc(S(=O)(=O)N1CCOCC1)c2)\NC(=S)Nc1cc(C)c(C)cc1	1164513-40-9	Hydrazinecarbothioamide, 2-[1,2-dihydro-1-methyl-5-(4-morpholinylsulfonyl)-2-ox	C21H23N5O4S2	473.57	C	-8.3
7458	C(N1c2c(/C(=N/NC(=S)Nc3c(C)c(Cl)ccc3)/C1=O)cccc2)N1CCOCC1	1164536-73-5	Hydrazinecarbothioamide, 2-[1,2-dihydro-1-methyl-5-(4-morpholinylsulfonyl)-2-ox	C22H25N5O4S2	487.6	C	-8.3
7459	C(=O)(OCC)c1c2c(n(CC)c1CSc1ccccc1)cc(Br)c(O)c2CN(C)C	116688-15-4	2,7-Naphthalenedisulfonic acid, 4-[2-(2,4-dimethylphenyl)diazenyl]-, sodium salt	C18H16N2O6S2.2Na		C	-8.3
7460	O[C@H]1[C@H](n2c3c(nc2)c(=O)nc(N)[nH]3)O[C@H](CO)[C@H]1OP(=O)(O)O	117-32-8	1,3-Naphthalenedisulfonic acid, 7-hydroxy-8-[2-[4,Ä≤-[2-(4-hydroxyphenyl)diazen	C30H24N4O8S2	632.66	C	-8.3
7461	Cc1n(c2nc(nc(n2)N2CCOCC2)c2cc(O)ccc2)c2c(n1)cccc2	1174126-22-7	1,3,5-Triazin-2-amine, 4-ethoxy-6-(4-morpholinyl)-N-[3-(trifluoromethyl)phenyl]-	C16H18F3N5O2	369.34	C	-8.3
7462	C([C@H](OCP(=O)(OCCCOCCCCCCCCCCCCCCCCC)O)C)n1c2c(nc1)c(N)ncn2.Cc1c2c(N(c3c(C(=O)N2)cccn3)C2CC2)ncc1	1174386-11-8	Phosphonic acid, P-[[(1R)-2-(6-amino-9H-purin-9-yl)-1-methylethoxy]methyl]-, mor	C28H52N5O5P.C20H15BrN6O		C	-8.3
7463	C(=O)([C@@H](NC(=O)OC)C1CCOCC1)N1[C@H](c2[nH]c(c3cc4c(-c5c(cc(cc5)c5[nH]c(nc5)[C@H]5N(C(=O)[C@@H](NC	1179150-20-9	Carbamic acid, N,N,Ä≤-[(5,7-dihydrodibenz[c,e]oxepin-3,9-diyl)bis[1H-imidazole-	C42H52N8O7	780.91	C	-8.3
7464	C(=O)([C@@H](NC(=O)OC)C(C)C)N1[C@H](c2[nH]c3-c4c(cc(cc4)c4ccc(cc4)c4c(CCC(F)(F)F)nc([nH]4)[C@H]4N(C(	1180669-94-6	Carbamic acid, N-[(1S)-1-[[(2S)-2-[4,5-dihydro-7-[4-[2-[(2S)-1-[(2S)-2-[(methoxy	C42H52N8O6	764.91	C	-8.3
7465	C(=O)([C@@H](NC(=O)OC)C(C)C)N1[C@H]2[C@H](C2)C[C@H]1c1[nH]c2c(n1)ccc1c2ccc(c1)c1ccc(cc1)c1[nH]c(nc1)	1180670-15-8	Carbamic acid, N-[(1S)-1-[[(2S)-2-[7-[4-[2-[(2S)-1-[(2S)-2-[(methoxycarbonyl)ami	C42H50N8O6	762.9	C	-8.3
7466	O(S(=O)(=O)C(F)(F)F)[C@H]1[C@H]2[C@H](O[C@H]1n1c3c(nc1)c(N)ncn3)CO[Si](C(C)C)(C(C)C)O[Si](C(C)C)(C(C	118535-09-4	Naphtho[2,3-c]furan-1(3H)-one, 9-(1,3-benzodioxol-5-yl)-3a,4,9,9a-tetrahydro-4-	C21H20O7	384.38	C	-8.3
7467	N(=C1c2c(N(CN3CCOCC3)C1=O)ccc(C)c2)NC(=S)NC1CCCCC1	118989-37-0	Hydrazinecarbothioamide, 2-[1-[[4-(4-chlorophenyl)-1-piperazinyl]methyl]-1,2-dih	C26H31ClN6OS	511.08	C	-8.3
7468	C(C(OCP(=O)(OCC)OCC)([2H])[2H])(n1c2c(nc1)c(N)ncn2)([2H])[2H]	118989-49-4	Hydrazinecarbothioamide, 2-[5-chloro-1-[[4-(4-chlorophenyl)-1-piperazinyl]methyl	C26H30Cl2N6OS	545.53	C	-8.3
7469	O=C1[C@@H]2[C@@H](c3c([C@@H](OC(=O)[C@@H](NC(=O)OC[C@H]4O[C@H](C=C4)n4c(=O)[nH]c(=O)c(C)c4)C)[C@H]2C	1191242-92-8	Glycine, N-carboxy-, 1-[(3aR,4S,9R,9aR)-1,3,3a,4,9,9a-hexahydro-6,7-dimethoxy-1-	C36H39N3O14	737.71	C	-8.3
7470	Nc1nc2n(cnc2cn1)[C@@H]1O[C@H](CO)C=C1	1192411-95-2	Pyrimidine, 5-[4-[(3S)-4-[(1R)-2-methoxy-1-[4-(trifluoromethyl)phenyl]ethyl]-3-	C28H40F3N5O	519.65	C	-8.3
7471	C(NC(=O)C(=O)OCC)(C)(C)c1nc(C(=O)NCc2ccc(F)cc2)c(O)c(=O)n1C	1193687-87-4	4-Pyrimidinecarboxamide, 1,6-dihydro-5-hydroxy-1-methyl-2-[1-methyl-1-[[(5-methy	C20H22N6O5	426.43	C	-8.3
7472	O=c1n([C@@H]2O[C@H](COP(=O)(OP(=O)(OP(=O)(O)O)O)O)[C@@H](O)C2)cc(CCCl)c(=O)[nH]1	119659-51-7	Naphthionic acid, 3,3,Ä≤-(3,3,Ä≤-dibromo-4,4,Ä≤-biphenylylenebisazo)di-	C32H22Br2N6O6S2	810.49	C	-8.3
7473	N(S(=O)(=O)c1ccc(N)cc1)(C[C@H]([C@H](Cc1ccccc1)NC(=O)O[C@H]1CCOC1)O)CC(C)C.C([C@H](OCP(=O)(O)O)C)n1c	119697-63-1	Naphthionic acid, 3,3,Ä≤-(2,2,Ä≤-dibromo-4,4,Ä≤-biphenylylenebisazo)di-	C32H22Br2N6O6S2	810.49	C	-8.3
7474	C[C@]1(O)[C@H](n2c3c(nc2)c(=O)nc(N)[nH]3)O[C@H](COP(=O)(OCCSC(=O)C(CO)(C)C)O)[C@H]1O	1202498-76-7	Guanosine, 2,Ä≤-C-methyl-, 5,Ä≤-[P,Ä≤,P,Ä≤,Ä≤,P,Ä≤,Ä≤-trihydrogen N-(phenylmet	C18H25N6O13P3	626.34	C	-8.3
7475	O(COC(=O)OCCN1CCOCC1)c1c2n(C[C@H]3N(C2=O)[C@H](C)CCO3)cc(C(=O)NCc2c(F)cc(F)cc2)c1=O	1206102-01-3	Carbonic acid, [[(4R,12aS)-9-[[[(2,4-difluorophenyl)methyl]amino]carbonyl]-3,4,	C24H26F2N3O12P	617.45	C	-8.3
7476	O[C@H]1[C@@H](O[C@H](COS(=O)(=O)N)[C@H]1O)n1nc(C(=O)N)nc1	1206102-03-5	Carbonic acid, [[(4R,12aS)-9-[[[(2,4-difluorophenyl)methyl]amino]carbonyl]-3,4,	C28H32F2N4O9	606.57	C	-8.3
7477	C(=O)(N[C@H](C(=O)C(=O)NC1CC1)CCC)[C@H]1N(C(=O)[C@@H](NC(=O)[C@@H](NC(=O)c2cnccn2)C2CCCCC2)CCC(=O)C=	1208118-95-9	Cyclopenta[c]pyrrole-1-carboxamide, (2S)-2-cyclohexyl-N-(2-pyrazinylcarbonyl)gly	C36H50N8O7	706.83	C	-8.3
7478	C(=O)(N[C@H](C(=O)C(=O)NC1CC1)CCC)[C@H]1N(C(=O)[C@@H](NC(=O)[C@@H](NC(=O)c2cnccn2)C2CCCCC2)CNS(=O)(=	1208119-19-0	Cyclopenta[c]pyrrole-1-carboxamide, (2S)-2-cyclohexyl-N-(2-pyrazinylcarbonyl)gly	C36H52N8O7	708.85	C	-8.3
7479	O=C1[C@@H]2[C@@H](c3c([C@H](OC(=O)CCCSSCCCC(=O)N[C@@H]4c5c(-c6c(CC4)cc(OC)c(OC)c6OC)ccc(SC)c(=O)c5)[	1213264-37-9	Hexanedioic acid, 1,6-bis[(5R,5aR,8aR,9R)-5,5a,6,8,8a,9-hexahydro-8-oxo-9-(3,4,	C50H50O18	938.92	C	-8.3
7480	O=c1n(cc(C)c(=O)n1CCCn1c(=O)n(cc(C)c1=O)[C@H]1C[C@H](N=[N+]=[N-])[C@@H](CO)O1)[C@H]1C[C@H](N=[N+]=[N	1215171-14-4	Thymidine, 3-[[4-[[3-(3-azido-2,3-dideoxy-Œ≤-D-erythro-pentofuranosyl)-3,6-dihy	C28H31N7O8		C	-8.3
7481	C(NC(=O)C[C@H](NC(=O)[C@H](Cc1ccccc1)NC(=O)C)C(=O)N[C@@H](Cc1ccccc1)C(=O)O)(C)C12CC3CC(C2)CC(C1)C3	1217551-21-7	L-Phenylalanine, N-[(1,1-dimethylethoxy)carbonyl]-L-phenylalanyl-N-(1-tricyclo[	C39H52N4O7	688.85	C	-8.3
7482	[C@H](C(=O)N[C@@H](Cc1ccccc1)C[C@H]([C@H](Cc1ccccc1)NC(=O)COc1c(C)cccc1C)O)(C(C)C)N1C(=O)NCCC1	1217819-15-2	Not Yet Assigned	C31H29D4F3N2O5S		C	-8.3
7483	O=C1[C@@H]2[C@@H](c3c([C@H](OC(=O)CCC(=O)NCCCCCNC(=O)Cc4c5c(n(C(=O)c6ccc(Cl)cc6)c4C)ccc(OC)c5)[C@H]2	1222083-95-5	Butanoic acid, 4-[[[4-[[[5-(dimethylamino)-1-naphthalenyl]sulfonyl]amino]phenyl]	C45H45N3O12S	851.92	C	-8.3
7484	[C@@H](C(=O)N[C@@H](Cc1ccccc1)C[C@@H]([C@H](C(c1ccccc1)([2H])[2H])NC(=O)COc1c(C([2H])([2H])[2H])cccc	1224729-64-9	1(2H)-Pyrimidine-4,5,6-d3-acetamide, N-[(1S,3S,4S)-4-[[2-(2,6-dimethylphenoxy)ac	C37H40D8N4O5		C	-8.3
7485	C(#N)C1=C(N)Oc2c(C1c1c(O)cccc1)ccn1c2ncc1	1225373-09-0	7H-Imidazo[1,2-a]pyrano[2,3-c]pyridine-8-carbonitrile, 9-amino-7-(1,3-benzodiox	C19H14N4O3	346.34	C	-8.3
7486	C(OC(=O)[C@H](Cc1cnc[nH]1)NC(=O)OC(C)(C)C)c1c2c(N(c3c(C(=O)N2)cccn3)C2CC2)ncc1	1225376-15-7	L-Tryptophan, 2-[(11-cyclopropyl-6,11-dihydro-6-oxo-5H-dipyrido[3,2-b:2,Ä≤,3,Ä≤	C26H24N6O3	468.51	C	-8.3
7487	C(=O)([C@@H](NC(=O)OC)C(C)C)N1[C@@H](CCC1)c1[nH]c(cn1)[C@H]1CC[C@H](CC1)c1ccc(cc1)c1[nH]c(nc1)[C@H]1	1228668-00-5	Carbamic acid, N-[(1S)-1-[[(2S)-2-[5-[4-[trans-4-[2-[(2S)-1-[(2S)-2-[(methoxycar	C40H56N8O6	744.92	C	-8.3

7488	C(=O)(/C=C/c1cc(O)c(O)cc1)c1c(OC)cc(O)c(CCC(C)(C)O)c1O	1228673-64-0	2-Propen-1-one, 1,1,Ä≤-(2,4-dihydroxy-6-methoxy-1,3-phenylene)bis[3-(4-hydroxyph	C25H20O7	432.42	C	-8.3
7489	C(=O)([C@@H](NC(=O)OC)C(C)C)N1[C@@H](CCC1)c1[nH]c(cn1)c1ccc(C#Cc2ccc(cc2)c2[nH]c([C@@H](NC(=O)[C@@H]	1228966-02-6	Carbamic acid, N-[(1R)-2-[(2S)-2-[5-[4-[2-[4-[2-[(1S)-1-[[(2R)-2-[(methoxycarbor	C47H46N8O6	818.92	C	-8.3
7490	C(=O)([C@H](NC(=O)OC)c1ccccc1)N1[C@@H](CCC1)c1[nH]c(cn1)c1ccc(cc1)c1ccc(C#Cc2[nH]c(nc2)[C@H]2N(C(=O)	1228966-26-4	Carbamic acid, N,N,Ä≤-[1,4-phenylenebis[2,1-ethynediyl-1H-imidazole-5,2-diyl-(2S	C44H42N8O6	778.85	C	-8.3
7491	C(=O)([C@@H](NC(=O)OC)C(C)C)N1[C@@H](CCC1)c1[nH]c(cn1)c1ccc(cc1)c1ccc(C#Cc2[nH]c(nc2)[C@H]2N(C(=O)[C	1228966-44-6	Carbamic acid, N-[(1R)-2-[(2S)-2-[5-[2-[4,Ä≤-[2-[(2S)-1-[(2R)-2-(dimethylamino)-	C48H48N8O4	800.95	C	-8.3
7492	C(=O)([C@@H](NC(=O)OC)C(C)C)N1[C@@H](CCC1)c1[nH]c(cn1)c1ccc2c(cc1)cc(C#Cc1[nH]c(nc1C)[C@H]1N(C(=O)[C@	1228968-00-0	Carbamic acid, N-[(1S)-2-[(2S)-2-[5-[6-[2-[2-[(2S)-1-[(2S)-2-[(methoxycarbonyl)a	C43H46N8O6	770.88	C	-8.3
7493	C(NC(=O)[C@H](Cc1ccc(O)cc1)N)(C(=O)NCC(=O)N[C@@H](Cc1ccccc1)C(=O)NCC(=O)O)C12CC3CC(C2)CC(C1)C3	1229574-79-1	L-Methionine, L-tryptophyl-2-tricyclo[3.3.1.13,7]dec-1-ylglycylglycyl-L-phenylal	C39H50N6O6S	730.92	C	-8.3
7494	C(=C1C=C(C(=O)ON)C(=O)C=C1)(c1cc(C(=O)O)c(O)cc1)c1cc(C(=O)O)c(O)cc1	1233224-95-7	2-Pyridinesulfonamide, N-[3-[(1R)-1-[(6R)-5,6-dihydro-4-hydroxy-2-oxo-6-(2-pheny	C28H27F3N2O5S	560.58	C	-8.3
7495	S(=O)(=O)(NC(=O)OCCCC)c1c(cc(CC(C)C)s1)c1ccc(C(=O)n2cc(C(F)(F)F)nc2)cc1	1236352-56-9	4-Pyrimidinecarboxamide, N-[[4-(fluoro-18F)phenyl]methyl]-1,6-dihydro-5-hydroxy-	C20H21FN6O5		C	-8.3
7496	[C@H](Cc1ccccc1)(C[C@@H]([C@H](Cc1ccccc1)NC(=O)OCc1cncs1)O)NC(=O)[C@@H](NC(=O)N(Cc1nc(CC)sc1)C)C(C)C	1236407-24-1	2,7,10,12-Tetraazatridecanoic acid, 4-hydroxy-12-methyl-9-(1-methylethyl)-8,11-c	C38H45N7O5S3	776	C	-8.3
7497	N(C(=O)[C@H](Cc1ccccc1)C[C@@H](CN1[C@H](C(=O)NC(C)(C)C)CN(Cc2c(C)ccs2)CC1)O)[C@H]1c2c(C[C@H]1O)cccc2	1237988-63-4	D-erythro-Pentonamide, 2,3,5-trideoxy-N-[(1S,2R)-2,3-dihydro-2-hydroxy-1H-inden-	C37H46N4O4S2	674.92	C	-8.3
7498	N(CC(=O)[C@H](Cc1ccccc1)C[C@@H](CN1[C@H](C(=O)NC(C)(C)C)CN(CCCN2CCCCC2)CC1)O)[C@H]1c2c(C[C@H]1O)cccc	1237988-69-0	D-erythro-Pentonamide, 2,3,5-trideoxy-N-[(1S,2R)-2,3-dihydro-2-hydroxy-1H-inden-	C35H46N4O4S	618.83	C	-8.3
7499	N(CC(=O)[C@H](Cc1ccccc1)C[C@@H](CN1[C@H](C(=O)NC(C)(C)C)CN(Cc2ccccn2)CC1)O)[C@H]1c2c(C[C@H]1O)cccc2	1237988-91-8	Not Yet Assigned	C38H51N5O4	641.84	C	-8.3
7500	C(F)(F)c1n(c2c(n1)cccc2)c1nc(nc(N[C@H]2CC[C@H](NC(=O)C)CC2)n1)N1CCOCC1	1240024-75-2	1,3,5-Triazin-2-amine, N-[(trans-4-aminocyclohexyl)methyl]-4-[2-(difluoromethyl)	C22H28F2N8O	458.51	C	-8.3
7501	C(F)(F)c1n(c2c(n1)cccc2)c1nc(nc(N[C@H]2CC[C@H](NCC(C)C)CC2)n1)N1CCOCC1	1240029-86-0	Ethanesulfonamide, N-[trans-4-[[4-[2-(difluoromethyl)-1H-benzimidazol-1-yl]-6-(4	C23H30F2N8O3S	536.6	C	-8.3
7502	C(=O)([C@@H](NC(=O)OC)C)N1[C@@H](CCC1)c1[nH]c(cn1)c1ccc(cc1)c1ccc([C@H](NC(=O)[C@H]2N(C(=O)[C@@H](NC	1240427-40-0	L-Prolinamide, N-(methoxycarbonyl)-L-valyl-N-[(1S)-1-[4,Ä≤-[2-[(2S)-1-[(2S)-2-[	C40H53N7O7	743.89	C	-8.3
7503	Nc1c(C23CC4CC(C3)CC(C2)C4)n[nH]c1C	1240427-50-2	L-Prolinamide, N-(methoxycarbonyl)-L-valyl-N-[[4,Ä≤-[2-[(2S)-1-[(2S)-2-[(methoxy	C39H51N7O7	729.86	C	-8.3
7504	N(C(=O)c1ccc(/C=C/C=C/c2ccc(C(=O)Nc3c(C(=O)O)cccc3)cc2)cc1)c1c(C(=O)O)cccc1	1240502-71-9	Benzoic acid, 2,2,Ä≤-[(1E)-1,2-ethenediylbis(4,1-phenylenecarbonylimino)]bis-	C30H22N2O6	506.51	C	-8.3
7505	C(=O)([C@@H](NC(=O)OC)C1CC1)N1[C@H](c2[nH]c(c3cc4c(-c5c(C4)cc(cc5)c4[nH]c(nc4)[C@H]4N(C(=O)[C@@H](NC	1240880-59-4	Carbamic acid, N,N,Ä≤-[9H-fluorene-2,7-diylbis[1H-imidazole-5,2-diyl-(2S)-2,1-py	C41H50N8O6	750.89	C	-8.3
7506	C(=O)([C@H](NC(=O)OC)C1CC1)N1[C@@H](CCC1)c1[nH]c(cn1)[C@H]1CC[C@@H](CC1)c1ccc(cc1)c1[nH]c(nc1)[C@H]1	1240887-61-9	Carbamic acid, N-[2-[(2S)-2-[5-[4-[trans-4-[2-[(2S)-1-[2-[(methoxycarbonyl)aminc	C38H52N8O6	716.87	C	-8.3
7507	C(=O)([C@@H](Cc1ccccc1)NC(=O)OC)N1[C@@H](CCC1)c1[nH]c(cn1)[C@H]1CC[C@@H](CC1)c1ccc(cc1)c1[nH]c(nc1)[	1240888-13-4	Carbamic acid, N-[(1S)-2-[(2S)-2-[5-[4-[trans-4-[2-[(2S)-1-[(2S)-2-[(methoxycarb	C46H54N10O6	842.98	C	-8.3
7508	C(=O)([C@H](NC(=O)OC)c1ccccc1)N1[C@@H](CCC1)c1[nH]c(cn1)c1ccc(/C=C/CCc2[nH]c(nc2)[C@H]2N(C(=O)[C@H](	1240891-93-3	Carbamic acid, N-[(1R)-2-[(2S)-2-[5-[4-[[(1S,2S)-2-[[2-[(2S)-1-[(2R)-2-[(methoxy	C48H56N8O6	841.01	C	-8.3
7509	C(Nc1c2c(n3c(n1)c(S(=O)(=O)c1ccc(CC)cc1)nn3)ccs2)(C)C12CC3CC(C2)CC(C1)C3	1246179-14-5	Thieno[2,3-e][1,2,3]triazolo[1,5-a]pyrimidin-5-amine, 3-[(4-methylphenyl)sulfony	C26H29N5O2S2	507.67	C	-8.3
7510	C(=O)(NC)c1c2c(oc1c1ccc(F)cc1)cc(N(CCOC(=O)CCC(=O)OC[C@@H]1[C@@H]3[C@H]([C@@H](O1)n1nc(C(=O)N)nc1)OC	1246280-92-1	Not Yet Assigned	C34H37FN6O12S	772.75	C	-8.3
7511	O=c1n([C@@H]2O[C@H](CO)[C@@H](O)C2)cc([Te])c(=O)[nH]1	1246559-92-1	Carbamic acid, N,N,Ä≤-[7,10,15,18-tetraoxatricyclo[17.3.1.12,6]tetracosa-1(23),2	C48H64N8O10	913.07	C	-8.3
7512	C[C@]1(F)[C@@H](O[C@H](COP(=O)(Oc2ccccc2)N[C@H](C(=O)Oc2ccccc2)C)[C@H]1O)n1c(=O)[nH]c(=O)cc1	1250829-92-5	L-Alanine, N-[(2,Ä≤R)-2,Ä≤-deoxy-2,Ä≤-fluoro-2,Ä≤-methyl-P-phenyl-5,Ä≤-uridylyl	C25H33FN3O9P	569.52	C	-8.3
7513	C(=O)([C@@H](NC(=O)OC)C(C)C)N1[C@@H]([C@@H]2C[C@H]1CC2)c1[nH]c(cn1)c1ccc(cc1)c1ccc(cc1)c1[nH]c(nc1)[	1256382-61-2	3-Pyridinecarboxamide, 1,4-dihydro-4-oxo-1,6-diphenyl-N-(1-tricyclo[3.3.1.13,7]d	C30H32N2O2	452.59	C	-8.3
7514	O=C1c2c(-c3c(cc(cc3)c3[nH]c(nc3)[C@H]3N(C(=O)[C@@H](NC(=O)OC)C(C)C)CCC3)CN1C)ccc(c2)c1[nH]c(nc1)[C@H	1256383-98-8	Carbamic acid, N,N,Ä≤-[(9,10-dihydro-2,7-phenanthrenediyl)bis[1H-imidazole-5,2-d	C42H52N8O6	764.91	C	-8.3
7515	C(=O)([C@@H](NC(=O)OC)C(C)C)N1[C@@H](CCC1)c1[nH]c(cn1)c1ccc(C#CC#Cc2ccc(cc2)c2[nH]c(nc2)[C@H]2N(C(=O	1256385-99-5	Carbamic acid, N-[(1S)-1-[[(2S)-2-[5-[4,Ä≤-[2-[[(3S)-3-amino-2-oxo-1-piperidiny	C35H42N8O4	638.76	C	-8.3
7516	C(=O)([C@@H](NC(=O)OC)C(S(=O)(=O)C)(C)C)N1[C@@H](CCC1)c1[nH]c(cn1)c1ccc(cc1)c1ccc(cc1)c1[nH]c(nc1)[C	1256386-77-2	Carbamic acid, N-[(1S)-1-[[(2S)-2-[5-[4,Ä≤-[2-[1-[[(2S)-2-[(methoxycarbonyl)amir	C40H50N8O6	738.88	C	-8.3
7517	C(=O)([C@@H](NC(=O)OC)C(C)C)N1CC2(C[C@H]1c1[nH]c(cn1)c1cc3c(-c4c(C3(F)F)cc(cc4)c3[nH]c(nc3)[C@H]3N(C	1256391-48-6	Carbamic acid, N-[(1S)-2-methyl-1-[[(2S)-2-[5-[4,Ä≤-[2-[(2S)-1-[(2S)-5,5,5-trif	C40H47F3N8O6	792.85	C	-8.3
7518	C(=O)([C@@H](NC(=O)OC)C(C)C)N1CC2(C[C@H]1c1[nH]c(cn1)c1ccc(cc1)c1ccc(cc1)c1[nH]c(nc1)[C@H]1N(C(=O)[C	1256392-84-3	Carbamic acid, N-[(1S)-2-methyl-1-[[(2S)-2-[5-[4-[2-[4-[2-[(2S)-1-[3,3,3-trifluc	C40H43F3N8O6	788.81	C	-8.3
7519	C(=O)(NC(CC1CCC1)(C(=O)C(=O)N)[2H])[C@@H]1[C@@H]2[C@@H](C2(C)C)CN1C(=O)[C@@H](NC(=O)NC(C([2H])([2H])	1256750-78-3	3-Azabicyclo[3.1.0]hexane-2-carboxamide, N-[3-amino-1-(cyclobutylmethyl-d2)-2,3-	C27H24D21N5O5		C	-8.3
7520	C(=O)(NC(C(C1CCC1)([2H])[2H])C(=O)C(=O)N)[C@@H]1[C@@H]2[C@@H](C2(C)C)CN1C(=O)[C@@H](NC(=O)NC(C([2H])	1256751-07-1	3-Azabicyclo[3.1.0]hexane-2-carboxamide, N-[3-amino-1-(cyclobutylmethyl-d2)-2,3-	C27H33D12N5O5		C	-8.3
7521	C(=O)(NC(C(C1CCC1)([2H])[2H])C(=O)C(=O)N)[C@@H]1[C@@H]2[C@@H](C2(C)C)CN1C(=O)[C@@H](NC(=O)NC(C)(C)C)	1256751-14-0	3-Azabicyclo[3.1.0]hexane-2-carboxamide, N-[3-amino-1-(cyclobutylmethyl-d2)-2,3-	C27H33D12N5O5		C	-8.3
7522	O[C@H]1[C@@H](O[C@H](COC(=O)c2ccc(N)cc2)[C@H]1O)n1nc(C(=O)N)nc1	1257423-92-9	1H-1,2,4-Triazole-3-carboxamide, 1-[5-O-[3,4,5-tris(phenylmethoxy)benzoyl]-Œ≤-D-	C36H34N4O9	666.68	C	-8.3
7523	C(F)(F)c1n(c2c(n1)cccc2)c1nc(nc(Nc2ccccn2)n1)N1CCOCC1	1261281-19-9	1,3,5-Triazin-2-amine, 4-[2-(difluoromethyl)-1H-benzimidazol-1-yl]-6-(4-morpholi	C21H20F2N8O	438.43	C	-8.3
7524	C(F)(F)c1n(c2c(n1)cccc2)c1nc(nc(Nc2cncc2)n1)N1CCOCC1	1261281-20-2	1,3,5-Triazin-2-amine, 4-[2-(difluoromethyl)-1H-benzimidazol-1-yl]-6-(4-morpholi	C20H18F2N8O	424.41	C	-8.3
7525	O=[13C]1[15N]([C@H]2O[C@@H](CO)SC2)CC(F)C([15NH2])[15NH]1	1261364-88-8	Not Yet Assigned	C37H42D6N4O5		C	-8.3
7526	N(C(CCCN(CCC(=O)OC)CC)C)c1c2c(ncc1)cc(Cl)cc2	126456-73-3	Furo[3,Ä≤,4,Ä≤:6,7]naphtho[2,3-d]-1,3-dioxol-6(5aH)-one, 9-[(4,6-O-ethylidene-Œ	C30H34O13	602.58	C	-8.3
7527	N(=N\c1ccc(cc1)c1ccc(/N=N/c2ccc(OS(=O)(=O)c3ccc(C)cc3)cc2)cc1)/c1c2c(cc(S(=O)(=O)O)c1N)cc(S(=O)(=O)O	1266618-98-7	1-Naphthalenesulfonic acid, 2,2,Ä≤-[[1,1,Ä≤-biphenyl]-4,4,Ä≤-diylbis(2,1-diazen	C32H24N6O6S2.2Na		C	-8.3
7528	Cc1c2c(CO[P@](=O)(OC[C@H]3O[C@H](C=C3)n3c(=O)[nH]c(=O)c(C)c3)O2)cc(C)c1	1270965-83-7	Thymidine, 2,Ä≤,3,Ä≤-didehydro-3,Ä≤-deoxy-5,Ä≤-O-[(2S)-6,8-dimethyl-2-oxido-4H-	C19H21N2O7P	420.35	C	-8.3
7529	O=c1n([C@@H]2O[C@H](CO[P@@]3(=O)Oc4c(CO3)cccc4C)[C@@H](N=[N+]=[N-])C2)cc(C)c(=O)[nH]1	1270965-84-8	Thymidine, 2,Ä≤,3,Ä≤-didehydro-3,Ä≤-deoxy-5,Ä≤-O-[(2R)-6,8-dimethyl-2-oxido-4H-	C19H21N2O7P	420.35	C	-8.3
7530	O=C1[C@@H]2[C@@H](c3c(C([C@H]2CO1)[2H])cc1c(c3)OCO1)c1cc(OC)c(OC)c(OC)c1	1273148-08-5	Carbamic acid, N-[(1S)-1-[[(2S)-2-[5-[4,Ä≤-[2-[(2S,3S,8S)-7-[(2S)-2-[(methoxyca	C44H56N8O8	824.96	C	-8.3
7531	C(=O)([C@@H](NC(=O)OC)C(C)C)N1[C@@H]([C@@H]2C[C@H]1CC2)c1[nH]c(cn1)c1ccc(cc1)c1ccc(cc1)c1[nH]c(nc1)[	1273589-77-7	Carbamic acid, N-[(1S)-2-[(2S)-2-[5-[4,Ä≤-[2-[(1R,3S,4S)-2-[(2S)-2-[(methoxycarb	C46H56N8O8	848.99	C	-8.3
7532	C(=O)([C@@H](NC(=O)OC)C(C)C)N1[C@@H]([C@@H]2C[C@H]1[C@@H](O)C2)c1[nH]c(cn1)c1ccc(cc1)c1ccc(cc1)c1[nH	1273591-19-7	Carbamic acid, N-[(1S)-1-[[(2S)-2-[5-[4,Ä≤-[2-[(3S)-2-[(2S)-2-[(methoxycarbonyl)	C43H54N8O6	778.94	C	-8.3
7533	C(=O)([C@@H](NC(=O)OC)C(C)C)N1[C@@H](C2CCC1C=C2)c1[nH]c(cn1)c1ccc(cc1)c1ccc(cc1)c1[nH]c(nc1)[C@H]1N(	1273591-25-5	Carbamic acid, N-[(1S)-1-[[(2S)-2-[5-[4,Ä≤-[2-[(1S,3S,4R,6S)-6-methoxy-2-[(2S)-2	C43H54N8O7	794.94	C	-8.3
7534	C(=O)([C@@H](NC(=O)OC)C(C)C)N1[C@@H]([C@@H]2C[C@H]1CC2)c1[nH]c(cn1)c1c(F)c(F)c(cc1)c1c(F)c(F)c(cc1)c	1273591-45-9	Carbamic acid, N-[(1S)-1-[[(2S)-2-[5-[3,3,Ä≤-difluoro-4,Ä≤-[2-[(1R,3S,4S)-2-[(2	C42H50F2N8O6	800.89	C	-8.3
7535	C(=O)([C@@H](NC(=O)OC)C(C)C)N1[C@@H](CCC1)c1[nH]c(cn1)c1ccc(cc1)c1ccc(C(=O)NC[C@@H]2N(C(=O)[C@@H](NC	1275018-58-0	Carbamic acid, N-[(1S)-1-[[(2S)-2-[5-[4,Ä≤-[[[(2S)-1-[(2S)-2-[(methoxycarbonyl)	C40H53N7O7	743.89	C	-8.3
7536	N(CC(CN)C)(C)c1c2c(ncc1)cc(Cl)cc2	1275032-50-2	Carbamic acid, N-[(1S)-1-[[(2S)-2-[[[4-[trans-4-[2-[(2S)-1-[(2S)-2-[(methoxycarb	C38H57N7O6	707.9	C	-8.3
7537	N(=C\1/c2c(NC1=O)cccc2)\Nc1nc(nc(NCc2cc(Cl)ccc2)n1)N1CCOCC1	1279692-13-5	1H-Indole-2,3-dione, 3-[2-[4-(4-morpholinyl)-6-[(phenylmethyl)amino]-1,3,5-triaz	C22H22N8O2	430.46	C	-8.3
7538	O=c1n(cc(C[Se]C(C)C)c(=O)[nH]1)[C@@H]1O[C@H](CO)[C@@H](O)C1	1279692-18-0	Benzaldehyde, 2-hydroxy-, 2-[4-[[(3-chlorophenyl)methyl]amino]-6-(4-morpholinyl)	C21H22ClN7O2	439.9	C	-8.3
7539	N(c1nc(nc(n1)N1CCOCC1)N1CCOCC1)c1nc(cs1)c1ccc(N(=O)=O)cc1	1285535-40-1	1,3,5-Triazin-2-amine, N-[4-(4-chlorophenyl)-2-thiazolyl]-4,6-di-4-morpholinyl-	C20H22ClN7O2S	459.95	C	-8.3
7540	N(CCCN)(CC(C)C)c1c2c(ncc1)cc(Cl)cc2	129232-80-0	Guanosine, 5,Ä≤-[4-(bromomethyl)benzoate]	C18H18BrN5O6	480.27	C	-8.3
7541	C(=O)([C@@H](NC(=O)OC)CC)N1[C@@H](C[C@H](C)C1)c1[nH]c(cn1)c1cc2c(cc1)cc(cc2)c1ccc(cc1)c1[nH]c(nc1)[C	1306734-13-3	Carbamic acid, N-[(1S)-1-[[(2S,4S)-2-[5-[4-[6-[2-[(2S,4S)-1-[(2S)-2-[(methoxycar	C46H56N8O6	816.99	C	-8.3
7542	S(=O)(=O)(N1[C@H](C(=S)OC)CCC1)c1cc2c(cc1)NC(=O)C2=O	1310453-16-7	1H-Indole-2,3-dione, 5-[[(2S)-2-(1,3-dithian-2-yl)-1-pyrrolidinyl]sulfonyl]-	C16H18N2O4S3	398.52	C	-8.3
7543	C(=O)([C@@H](NC(=O)OC)C(C)C)N1[C@@H](C2CCC1CC2)c1[nH]c(cn1)c1ccc(cc1)c1ccc(cc1)c1[nH]c(nc1)[C@H]1N(C	1311398-29-4	Carbamic acid, N-[(1S)-1-[[(2S)-2-[5-[4,Ä≤-[2-[(1R,3S,4S)-2-[(2S)-2-[(methoxyca	C43H52N8O6	776.92	C	-8.3
7544	C(=O)([C@@H](NC(=O)OC)C(C)C)N1[C@@H](CC(=NOC)C1)c1[nH]c(cn1)c1ccc(cc1)c1ccc(cc1)c1[nH]c(nc1)[C@H]1N(	1311398-32-9	Carbamic acid, N-[(1S)-1-[[(2S)-2-[5-[4,Ä≤-[2-[(2S)-1-[(2S)-2-[(methoxycarbonyl)	C42H50N8O6	762.9	C	-8.3
7545	C(=O)([C@@H](NC(=O)OC)C(C)C)N1[C@@H](CC(=C(F)F)C1)c1[nH]c(cn1)c1ccc(cc1)c1ccc(cc1)c1[nH]c(nc1)[C@H]1	1311398-39-6	Carbamic acid, N-[(1S)-1-[[(2S,4S)-4-fluoro-2-[5-[4,Ä≤-[2-[(2S)-1-[(2S)-2-[(meth	C41H49FN8O6	768.88	C	-8.3
7546	C(=O)([C@@H](NC(=O)OC)C(C)C)N1[C@@H](CC(=C)C1)c1[nH]c(cn1)c1ccc(C#Cc2[nH]c(nc2)[C@H]2N(C(=O)[C@@H](N	1311398-47-6	Carbamic acid, N-[(1S)-1-[[(2S)-2-[5-[2-[4-[2-[(2S)-1-[(2S)-2-[(methoxycarbonyl)	C37H48N8O6	700.83	C	-8.3
7547	C(=O)([C@@H](NC(=O)OC)C(C)C)N1[C@@H](CCC1)c1[nH]c(c(Cl)n1)c1ccc(cc1)c1ccc(cc1)c1[nH]c(nc1Cl)[C@H]1N(	1312335-97-9	Carbamic acid, N-[(1S)-1-[[(2S)-2-[5-[4,Ä≤-[5-chloro-2-[(2S)-1-[(2S)-2-[(methoxy	C40H49ClN8O6	773.32	C	-8.3
7548	C(=O)([C@@H](NC(=O)OC)C1CCOCC1)N1[C@H]2[C@H](C2)C[C@H]1c1[nH]c(c(Cl)n1)c1ccc(cc1)c1ccc(cc1)c1[nH]c(n	1312335-98-0	Carbamic acid, N,N,Ä≤-[[1,1,Ä≤-biphenyl]-4,4,Ä≤-diylbis[(5-chloro-1H-imidazole-	C40H48Cl2N8O6	807.77	C	-8.3
7549	C(=O)([C@@H](NC(=O)OC)C1CCOCC1)N1[C@H]2[C@H](C2)C[C@H]1c1[nH]c(c(Cl)n1)c1ccc(cc1)c1ccc(cc1)c1[nH]c(n	1312336-39-2	Carbamic acid, N-[(1S)-2-[(1R,3S,5R)-3-[5-[4,Ä≤-[5-chloro-2-[(1R,3S,5R)-2-[(2S)-	C47H53ClF2N8O7	915.42	C	-8.3
7550	C(C=C)c1c([nH]c(n1)[C@H]1N(C(=O)[C@@H](NC(=O)OC)C(C)C)CCC1)c1ccc(cc1)c1ccc(cc1)c1[nH]c(nc1)[C@H]1N(C	1312336-57-4	Carbamic acid, N-[(1S)-1-[[(2S)-2-[4-cyclopropyl-5-[4,Ä≤-[2-[(2S)-1-[(2S)-2-[(me	C43H54N8O6	778.94	C	-8.3
7551	C(=O)([C@@H](NC(=O)OC)[C@H]1CCCOC1)N1[C@H]2[C@H](C2)C[C@H]1c1[nH]c(c(Cl)n1)c1ccc(cc1)c1ccc(cc1)c1[nH	1312336-71-2	Carbamic acid, N,N,Ä≤-[[1,1,Ä≤-biphenyl]-4,4,Ä≤-diylbis[(5-chloro-1H-imidazole-	C46H52Cl2N8O8	915.86	C	-8.3
7552	C(=O)([C@@H](NC(=O)OC)C(C)C)N1[C@H]2[C@H](C2)C[C@H]1c1[nH]c(c(n1)c1ccc(cc1)c1ccc(cc1)c1[nH]c(nc1)[C@	1312336-98-3	Carbamic acid, N,N,Ä≤-[[1,1,Ä≤-biphenyl]-4,4,Ä≤-diylbis[(5-cyclopropyl-1H-imida	C46H58N8O6	819	C	-8.3
7553	C(=O)([C@@H](NC(=O)OC)C1CCOCC1)N1[C@H]2[C@H](C2)C[C@H]1c1[nH]c(c(n1)c1ccc(cc1)c1ccc(cc1)c1[nH]c(nc1)	1312337-00-0	Carbamic acid, N-[(1S)-1-[[(1R,3S,5R)-3-[4-cyclopropyl-5-[4,Ä≤-[2-[(1R,3S,5R)-2-	C45H54N8O6	802.96	C	-8.3
7554	C(=O)([C@@H](NC(=O)OC)C(C)C)N1[C@H](c2[nH]c(c3c4c(c(cc3)c3ccc(cc3)c3[nH]c(nc3)[C@H]3N(C(=O)[C@@H](NC	1312608-36-8	Carbamic acid, N-[(1S)-1-[[(2S)-2-[5-[4-[4-[2-[(2S)-1-[(2S)-2-[(methoxycarbonyl)	C43H53N9O6	791.94	C	-8.3
7555	Cc1cc2c(ccc(c2s1)c1ccc(cc1)c1[nH]c(nc1)[C@H]1N(C(=O)[C@@H](NC(=O)OC)C(C)C)CCC1)c1[nH]c(nc1)[C@H]1N(C	1312608-39-1	Carbamic acid, N-[(1S)-1-[[(2S)-2-[5-[4-[4-[2-[(2S)-1-[(2S)-2-[(methoxycarbonyl)	C42H50N8O6S	794.96	C	-8.3
7556	C(=O)([C@@H](NC(=O)OC)C(C)C)N1[C@H](c2[nH]c(c3c4c(c(cc3)c3ccc(cc3)c3[nH]c(nc3)[C@H]3N(C(=O)[C@@H](NC	1312608-40-4	Carbamic acid, N-[(1S)-1-[[(2S)-2-[5-[4-[4-[2-[(2S)-1-[(2S)-2-[(methoxycarbonyl)	C43H52N8O6S	808.99	C	-8.3
7557	C(=O)([C@@H](NC(=O)OC)C(C)C)N1[C@H](c2[nH]c(c3c4c(c(cc3)c3ccc(cc3)c3[nH]c(nc3)[C@H]3N(C(=O)[C@@H](NC	1312608-57-3	Carbamic acid, N-[(1S)-1-[[(2S)-2-[5-[4-[7-[2-[(2S)-1-[(2S)-2-[(methoxycarbonyl)	C42H51N9O6S	809.98	C	-8.3
7558	C(=O)([C@@H](NC(=O)OC)C(C)C)N1[C@H](c2[nH]c(c3c4c(c(cc3)c3ccc(cc3)c3[nH]c(nc3)[C@H]3N(C(=O)[C@@H](NC	1312608-60-8	Carbamic acid, N-[(1S)-1-[[(2S)-2-[5-[4-[7-[2-[(2S)-1-[(2S)-2-[(methoxycarbonyl)	C43H52N8O7	792.92	C	-8.3
7559	C(=O)([C@@H](NC(=O)OC)C(C)C)N1[C@H](c2[nH]c(c3c4c(c(cc3)c3ccc(cc3)c3[nH]c(nc3)[C@H]3N(C(=O)[C@@H](NC	1312608-63-1	Carbamic acid, N-[(1S)-1-[[(2S)-2-[5-[4-[7-[2-[(2S)-1-[(2S)-2-[(methoxycarbonyl)	C41H49N9O6S	795.95	C	-8.3
7560	C(=O)([C@@H](NC(=O)OC)C(C)C)N1[C@@H](CCC1)c1[nH]c(cn1)c1c(N)c(N)c(cc1)c1ccc(cc1)c1[nH]c(nc1)[C@H]1N(	1312610-46-0	Carbamic acid, N-[(1S)-1-[[(2S)-2-[5-[4-[7-[2-[(2S)-1-[(2S)-2-[(methoxycarbonyl)	C40H48N10O6S	796.94	C	-8.3
7561	C(=O)([C@@H](NC(=O)OC)C(C)C)N1[C@@H](CCC1)c1[nH]c(cn1)c1c(O)c(N)c(cc1)c1ccc(cc1)c1[nH]c(nc1)[C@H]1N(	1312610-47-1	Carbamic acid, N,N,Ä≤-[[2,3-diamino[1,1,Ä≤-biphenyl]-4,4,Ä≤-diyl]bis[1H-imidazo	C40H52N10O6	768.9	C	-8.3
7562	O(Cc1ccc(F)cc1)c1c2c(oc(c(O)c2=O)c2[nH]c(nn2)c2ccccc2)c(C)cc1	1312892-66-2	4H-1-Benzopyran-4-one, 2-(5-chloro-1H-1,2,4-triazol-3-yl)-5-[(4-fluorophenyl)met	C19H13ClFN3O4	401.78	C	-8.3
7563	O(Cc1ccc(F)cc1)c1c2c(oc(c(O)c2=O)c2nnc(Cl)[nH]2)c(Cl)cc1	1312893-95-0	4H-1-Benzopyran-4-one, 8-chloro-5-[(4-fluorophenyl)methoxy]-3-hydroxy-2-(5-metho	C19H13ClFN3O5	417.77	C	-8.3
7564	O(Cc1ccc(F)cc1)c1c2c(c(cc1)c1ccccc1)oc(c(O)c2=O)c1ncc(OC)cn1	1312894-34-0	4H-1-Benzopyran-4-one, 5-[(4-fluorophenyl)methoxy]-3-hydroxy-2-(5-methyl-2-pyrim	C27H19FN2O4	454.45	C	-8.3
7565	O(Cc1ccc(F)cc1)c1c2c(c(cc1)c1ccccc1)oc(c(O)c2=O)c1ncc(cn1)c1ccccc1	1312894-36-2	4H-1-Benzopyran-4-one, 2-(5-chloro-2-pyrimidinyl)-5-[(4-fluorophenyl)methoxy]-3-	C26H16ClFN2O4	474.87	C	-8.3
7566	O(Cc1ccc(F)cc1)c1c2c(c(cc1)c1ccccc1)oc(c(O)c2=O)c1[nH]c(C)cn1	1312894-48-6	4H-1-Benzopyran-4-one, 5-[(4-fluorophenyl)methoxy]-3-hydroxy-2-(1H-imidazol-2-yl	C25H17FN2O4	428.41	C	-8.3
7567	O(Cc1ccc(F)cc1)c1c2c(oc(c(O)c2=O)c2[nH]c(OC)nn2)cc(C)c1	1312894-94-2	4H-1-Benzopyran-4-one, 5-[(4-fluorophenyl)methoxy]-3-hydroxy-7-methyl-2-(3-methy	C20H16FN3O4	381.36	C	-8.3
7568	O(Cc1ccc(F)cc1)c1c2c(oc(c(O)c2=O)c2[nH]cc(C)n2)cc(C)c1	1312894-98-6	4H-1-Benzopyran-4-one, 5-[(4-fluorophenyl)methoxy]-3-hydroxy-2-(1H-imidazol-2-yl	C20H15FN2O4	366.34	C	-8.3
7569	O(Cc1ccc(F)cc1)c1c2c(oc(c(O)c2=O)c2[nH]c(nn2)c2ccccc2)c(C)c(C)c1	1312895-46-7	4H-1-Benzopyran-4-one, 2-(5-chloro-1H-1,2,4-triazol-3-yl)-5-[(4-fluorophenyl)met	C20H15ClFN3O4	415.8	C	-8.3
7570	O(Cc1ccc(F)cc1)c1c2c(oc(c(O)c2=O)c2[nH]c(cn2)c2ccccc2)c(C)c(C)c1	1312895-51-4	4H-1-Benzopyran-4-one, 2-(5-chloro-1H-imidazol-2-yl)-5-[(4-fluorophenyl)methoxy]	C21H16ClFN2O4	414.81	C	-8.3
7571	O(Cc1ccc(F)cc1)c1c2c(c(c(C)c1)c1ccccc1)oc(c(O)c2=O)c1ncc(OC)cn1	1312896-84-6	4H-1-Benzopyran-4-one, 5-[(4-fluorophenyl)methoxy]-3-hydroxy-7-methyl-2-(5-methy	C28H21FN2O4	468.48	C	-8.3
7572	O(Cc1ccc(F)cc1)c1c2c(c(c(C)c1)c1ccccc1)oc(c(O)c2=O)c1[nH]c(cn1)c1ccccc1	1312897-01-0	4H-1-Benzopyran-4-one, 2-(5-chloro-1H-imidazol-2-yl)-5-[(4-fluorophenyl)methoxy]	C26H18ClFN2O4	476.88	C	-8.3
7573	O(Cc1ccc(F)cc1)c1c2c(oc(c(O)c2=O)c2[nH]c(cn2)c2ccccc2)cc(OC)c1	1312897-62-3	4H-1-Benzopyran-4-one, 2-(5-chloro-1H-imidazol-2-yl)-5-[(4-fluorophenyl)methoxy]	C20H14ClFN2O5	416.79	C	-8.3
7574	O(Cc1ccc(F)cc1)c1c2c(oc(c(O)c2=O)c2[nH]c(C)nn2)c(C)c(OC)c1	1312898-04-6	4H-1-Benzopyran-4-one, 5-[(4-fluorophenyl)methoxy]-3-hydroxy-7-methoxy-8-methyl-	C20H16FN3O5	397.36	C	-8.3
7575	O(Cc1ccc(F)cc1)c1c2c(c(OC)c(OC)c1)oc(c(O)c2=O)c1ncccn1	1312898-13-7	4H-1-Benzopyran-4-one, 5-[(4-fluorophenyl)methoxy]-3-hydroxy-7-methoxy-8-methyl-	C27H21FN2O5	472.46	C	-8.3
7576	O(C)c1c(c2c(c(OCc3ccc(F)cc3)c1)c(=O)c(O)c(o2)c1ncccn1)c1ccccc1	1312899-14-1	4H-1-Benzopyran-4-one, 8-chloro-5-[(4-fluorophenyl)methoxy]-3-hydroxy-7-methoxy-	C26H18ClFN2O5	492.88	C	-8.3
7577	O(C)c1c(c2c(c(OCc3ccc(F)cc3)c1)c(=O)c(O)c(o2)c1ncc(C)cn1)c1ccccc1	1312899-45-8	4H-1-Benzopyran-4-one, 5-[(4-fluorophenyl)methoxy]-3-hydroxy-7-methoxy-8-phenyl-	C27H19FN2O5	470.45	C	-8.3
7578	O(C)c1c(c2c(c(OCc3ccc(F)cc3)c1)c(=O)c(O)c(o2)c1nnc(Cl)[nH]1)c1ccccc1	1312899-57-2	4H-1-Benzopyran-4-one, 5-[(4-fluorophenyl)methoxy]-3-hydroxy-7-methoxy-2-(5-meth	C26H20FN3O6	489.45	C	-8.3
7579	O(C)c1c(c2c(c(OCc3ccc(F)cc3)c1)c(=O)c(O)c(o2)c1[nH]c(cn1)c1ccccc1)c1ccccc1	1312899-63-0	4H-1-Benzopyran-4-one, 2-(5-chloro-1H-imidazol-2-yl)-5-[(4-fluorophenyl)methoxy]	C26H18ClFN2O5	492.88	C	-8.3
7580	O(Cc1ccc(F)cc1)c1c2c(c(c(Cl)c1)c1ccccc1)oc(c(O)c2=O)c1ncc(OC)cn1	1312902-55-8	4H-1-Benzopyran-4-one, 7-chloro-5-[(4-fluorophenyl)methoxy]-3-hydroxy-2-(5-methy	C27H18ClFN2O4	488.89	C	-8.3
7581	O(Cc1ccc(F)cc1)c1c2c(c(c(Cl)c1)c1ccccc1)oc(c(O)c2=O)c1[nH]c(OC)cn1	1312902-70-7	4H-1-Benzopyran-4-one, 7-chloro-5-[(4-fluorophenyl)methoxy]-3-hydroxy-2-(5-methy	C26H18ClFN2O4	476.88	C	-8.3
7582	O(Cc1ccc(F)cc1)c1c2c(c(c(Cl)c1)c1ccccc1)oc(c(O)c2=O)c1[nH]c(cn1)c1ccccc1	1312902-72-9	4H-1-Benzopyran-4-one, 7-chloro-2-(5-chloro-1H-imidazol-2-yl)-5-[(4-fluorophenyl	C25H15Cl2FN2O4	497.3	C	-8.3
7583	O(Cc1ccc(F)cc1)c1c2c(oc(c(O)c2=O)c2[nH]c(C)cn2)cc(c1)c1ccccc1	1312903-19-7	4H-1-Benzopyran-4-one, 5-[(4-fluorophenyl)methoxy]-3-hydroxy-2-(1H-imidazol-2-yl	C25H17FN2O4	428.41	C	-8.3
7584	Cc1c2c(c(OCc3ccc(F)cc3)cc1c1ccccc1)c(=O)c(O)c(o2)c1ncc(C)cn1	1312903-54-0	4H-1-Benzopyran-4-one, 5-[(4-fluorophenyl)methoxy]-3-hydroxy-8-methyl-7-phenyl-2	C27H19FN2O4	454.45	C	-8.3
7585	Cc1c2c(c(OCc3ccc(F)cc3)cc1c1ccccc1)c(=O)c(O)c(o2)c1ncc(Cl)cn1	1312903-56-2	4H-1-Benzopyran-4-one, 5-[(4-fluorophenyl)methoxy]-3-hydroxy-2-(5-methoxy-2-pyri	C28H21FN2O5	484.48	C	-8.3
7586	O(Cc1ccc(F)cc1)c1c2c(c(C)c(c1)c1ccccc1)oc(c(O)c2=O)c1[nH]c(OC)cn1	1312903-72-2	4H-1-Benzopyran-4-one, 5-[(4-fluorophenyl)methoxy]-3-hydroxy-8-methyl-2-(5-methy	C27H21FN2O4	456.46	C	-8.3
7587	Cc1c2c(c(OCc3ccc(F)cc3)cc1c1ccccc1)c(=O)c(O)c(o2)c1[nH]c(cn1)c1ccccc1	1312903-76-6	4H-1-Benzopyran-4-one, 2-(5-chloro-1H-imidazol-2-yl)-5-[(4-fluorophenyl)methoxy]	C26H18ClFN2O4	476.88	C	-8.3
7588	O(C)c1c2c(c(OCc3ccc(F)cc3)cc1c1ccccc1)c(=O)c(O)c(o2)c1ncc(C)cn1	1312904-24-7	4H-1-Benzopyran-4-one, 5-[(4-fluorophenyl)methoxy]-3-hydroxy-8-methoxy-7-phenyl-	C27H19FN2O5	470.45	C	-8.3
7589	O(C)c1c2c(c(OCc3ccc(F)cc3)cc1c1ccccc1)c(=O)c(O)c(o2)c1[nH]c(OC)nn1	1312904-42-9	4H-1-Benzopyran-4-one, 5-[(4-fluorophenyl)methoxy]-3-hydroxy-8-methoxy-2-(3-meth	C26H20FN3O5	473.45	C	-8.3
7590	O(Cc1ccc(F)cc1)c1c2c(c(Cl)c(c1)c1ccccc1)oc(c(O)c2=O)c1[nH]c(OC)nn1	1312905-20-6	4H-1-Benzopyran-4-one, 8-chloro-5-[(4-fluorophenyl)methoxy]-3-hydroxy-2-(3-methy	C25H17ClFN3O4	477.87	C	-8.3
7591	O(Cc1ccc(F)cc1)c1c2c(c(Cl)c(c1)c1ccccc1)oc(c(O)c2=O)c1[nH]c(Cl)nn1	1312905-22-8	4H-1-Benzopyran-4-one, 8-chloro-5-[(4-fluorophenyl)methoxy]-3-hydroxy-2-(5-metho	C25H17ClFN3O5	493.87	C	-8.3
7592	Clc1c2c(c(OCc3ccc(F)cc3)cc1c1ccccc1)c(=O)c(O)c(o2)c1[nH]c(nn1)c1ccccc1	1312905-24-0	4H-1-Benzopyran-4-one, 8-chloro-2-(5-chloro-1H-1,2,4-triazol-3-yl)-5-[(4-fluorop	C24H14Cl2FN3O4	498.29	C	-8.3
7593	Clc1c2c(c(OCc3ccc(F)cc3)cc1c1ccccc1)c(=O)c(O)c(o2)c1[nH]c(cn1)c1ccccc1	1312905-32-0	4H-1-Benzopyran-4-one, 8-chloro-2-(5-chloro-1H-imidazol-2-yl)-5-[(4-fluorophenyl	C25H15Cl2FN2O4	497.3	C	-8.3
7594	O(Cc1ccc(F)cc1)c1c2c(c(c(c1)c1ccccc1)c1ccccc1)oc(c(O)c2=O)c1ncc(cn1)c1ccccc1	1312905-69-3	4H-1-Benzopyran-4-one, 2-(5-chloro-2-pyrimidinyl)-5-[(4-fluorophenyl)methoxy]-3-	C32H20ClFN2O4	550.96	C	-8.3
7595	O=c1c2c(oc(c1O)c1[nH]c(cn1)c1ccccc1)c(C)cc(OCc1ccc(F)cc1)c2	1312906-82-3	4H-1-Benzopyran-4-one, 2-(5-chloro-1H-imidazol-2-yl)-6-[(4-fluorophenyl)methoxy]	C20H14ClFN2O4	400.79	C	-8.3
7596	O(C)c1c2c(c(=O)c(O)c(o2)c2nnc(C)[nH]2)cc(OCc2ccc(F)cc2)c1	1312907-24-6	4H-1-Benzopyran-4-one, 6-[(4-fluorophenyl)methoxy]-3-hydroxy-8-methoxy-2-(1H-1,2	C19H14FN3O5	383.33	C	-8.3
7597	O=c1c2c(oc(c1O)c1ncc(C)cn1)c(Cl)cc(OCc1ccc(F)cc1)c2	1312907-64-4	4H-1-Benzopyran-4-one, 8-chloro-6-[(4-fluorophenyl)methoxy]-3-hydroxy-2-(2-pyrim	C20H12ClFN2O4	398.77	C	-8.3
7598	O=c1c2c(c(cc(OCc3ccc(F)cc3)c2)c2ccccc2)oc(c1O)c1ncc(OC)cn1	1312908-15-8	4H-1-Benzopyran-4-one, 6-[(4-fluorophenyl)methoxy]-3-hydroxy-2-(5-methyl-2-pyrim	C27H19FN2O4	454.45	C	-8.3
7599	O=c1c2c(c(cc(OCc3ccc(F)cc3)c2)c2ccccc2)oc(c1O)c1ncc(cn1)c1ccccc1	1312908-17-0	4H-1-Benzopyran-4-one, 2-(5-chloro-2-pyrimidinyl)-6-[(4-fluorophenyl)methoxy]-3-	C26H16ClFN2O4	474.87	C	-8.3
7600	O=c1c2c(oc(c1O)c1ncc(C)cn1)cc(C)c(OCc1ccc(F)cc1)c2	1312908-64-7	4H-1-Benzopyran-4-one, 6-[(4-fluorophenyl)methoxy]-3-hydroxy-7-methyl-2-(2-pyrim	C21H15FN2O4	378.35	C	-8.3
7601	O=c1c2c(oc(c1O)c1ncc(cn1)c1ccccc1)cc(C)c(OCc1ccc(F)cc1)c2	1312908-67-0	4H-1-Benzopyran-4-one, 2-(5-chloro-2-pyrimidinyl)-6-[(4-fluorophenyl)methoxy]-3-	C21H14ClFN2O4	412.8	C	-8.3
7602	O=c1c2c(oc(c1O)c1[nH]c(nn1)c1ccccc1)cc(C)c(OCc1ccc(F)cc1)c2	1312908-77-2	4H-1-Benzopyran-4-one, 2-(5-chloro-1H-1,2,4-triazol-3-yl)-6-[(4-fluorophenyl)met	C19H13ClFN3O4	401.78	C	-8.3
7603	O=c1c2c(oc(c1O)c1[nH]c(OC)cn1)cc(C)c(OCc1ccc(F)cc1)c2	1312908-80-7	4H-1-Benzopyran-4-one, 6-[(4-fluorophenyl)methoxy]-3-hydroxy-7-methyl-2-(5-methy	C21H17FN2O4	380.37	C	-8.3
7604	Cc1c2c(cc(OCc3ccc(F)cc3)c1C)c(=O)c(O)c(o2)c1ncc(OC)cn1	1312909-27-5	4H-1-Benzopyran-4-one, 6-[(4-fluorophenyl)methoxy]-3-hydroxy-7,8-dimethyl-2-(5-m	C23H19FN2O4	406.41	C	-8.3

7605	Cc1c2c(cc(OCc3ccc(F)cc3)c1C)c(=O)c(O)c(o2)c1ncc(cn1)c1ccccc1	1312909-29-7	4H-1-Benzopyran-4-one, 2-(5-chloro-2-pyrimidinyl)-6-[(4-fluorophenyl)methoxy]-3-	C22H16ClFN2O4	426.82	C	-8.3
7606	Cc1c2c(cc(OCc3ccc(F)cc3)c1C)c(=O)c(O)c(o2)c1[nH]c(Cl)nn1	1312909-38-8	4H-1-Benzopyran-4-one, 6-[(4-fluorophenyl)methoxy]-3-hydroxy-2-(5-methoxy-1H-1,2	C21H18FN3O5	411.38	C	-8.3
7607	Cc1c2c(cc(OCc3ccc(F)cc3)c1C)c(=O)c(O)c(o2)c1[nH]c(nn1)c1ccccc1	1312909-39-9	4H-1-Benzopyran-4-one, 2-(5-chloro-1H-1,2,4-triazol-3-yl)-6-[(4-fluorophenyl)met	C20H15ClFN3O4	415.8	C	-8.3
7608	Cc1c2c(cc(OCc3ccc(F)cc3)c1C)c(=O)c(O)c(o2)c1[nH]c(Cl)cn1	1312909-43-5	4H-1-Benzopyran-4-one, 6-[(4-fluorophenyl)methoxy]-3-hydroxy-2-(5-methoxy-1H-imi	C22H19FN2O5	410.4	C	-8.3
7609	Cc1c2c(cc(OCc3ccc(F)cc3)c1C)c(=O)c(O)c(o2)c1[nH]c(cn1)c1ccccc1	1312909-44-6	4H-1-Benzopyran-4-one, 2-(5-chloro-1H-imidazol-2-yl)-6-[(4-fluorophenyl)methoxy]	C21H16ClFN2O4	414.81	C	-8.3
7610	Clc1c2c(cc(OCc3ccc(F)cc3)c1C)c(=O)c(O)c(o2)c1ncc(OC)cn1	1312910-27-2	4H-1-Benzopyran-4-one, 8-chloro-6-[(4-fluorophenyl)methoxy]-3-hydroxy-7-methyl-2	C22H16ClFN2O4	426.82	C	-8.3
7611	Clc1c2c(cc(OCc3ccc(F)cc3)c1C)c(=O)c(O)c(o2)c1ncc(cn1)c1ccccc1	1312910-29-4	4H-1-Benzopyran-4-one, 8-chloro-2-(5-chloro-2-pyrimidinyl)-6-[(4-fluorophenyl)me	C21H13Cl2FN2O4	447.24	C	-8.3
7612	Clc1c2c(cc(OCc3ccc(F)cc3)c1C)c(=O)c(O)c(o2)c1[nH]c(OC)nn1	1312910-37-4	4H-1-Benzopyran-4-one, 8-chloro-6-[(4-fluorophenyl)methoxy]-3-hydroxy-7-methyl-2	C20H15ClFN3O4	415.8	C	-8.3
7613	Clc1c2c(cc(OCc3ccc(F)cc3)c1C)c(=O)c(O)c(o2)c1[nH]c(nn1)c1ccccc1	1312910-39-6	4H-1-Benzopyran-4-one, 8-chloro-2-(5-chloro-1H-1,2,4-triazol-3-yl)-6-[(4-fluorop	C19H12Cl2FN3O4	436.22	C	-8.3
7614	Clc1c2c(cc(OCc3ccc(F)cc3)c1C)c(=O)c(O)c(o2)c1[nH]c(OC)cn1	1312910-42-1	4H-1-Benzopyran-4-one, 8-chloro-6-[(4-fluorophenyl)methoxy]-3-hydroxy-7-methyl-2	C21H16ClFN2O4	414.81	C	-8.3
7615	Clc1c2c(cc(OCc3ccc(F)cc3)c1C)c(=O)c(O)c(o2)c1[nH]c(cn1)c1ccccc1	1312910-44-3	4H-1-Benzopyran-4-one, 8-chloro-2-(5-chloro-1H-imidazol-2-yl)-6-[(4-fluorophenyl	C20H13Cl2FN2O4	435.23	C	-8.3
7616	O=c1c2c(oc(c1O)c1[nH]c(OC)nn1)cc(OC)c(OCc1ccc(F)cc1)c2	1312911-37-7	4H-1-Benzopyran-4-one, 6-[(4-fluorophenyl)methoxy]-3-hydroxy-7-methoxy-2-(3-meth	C20H16FN3O5	397.36	C	-8.3
7617	O=c1c2c(oc(c1O)c1ncc(C)cn1)cc(Cl)c(OCc1ccc(F)cc1)c2	1312911-54-8	4H-1-Benzopyran-4-one, 7-chloro-6-[(4-fluorophenyl)methoxy]-3-hydroxy-2-(2-pyrim	C20H12ClFN2O4	398.77	C	-8.3
7618	O=c1c2c(oc(c1O)c1[nH]c(nn1)c1ccccc1)cc(Cl)c(OCc1ccc(F)cc1)c2	1312911-80-0	4H-1-Benzopyran-4-one, 7-chloro-2-(5-chloro-1H-1,2,4-triazol-3-yl)-6-[(4-fluorop	C18H10Cl2FN3O4	422.19	C	-8.3
7619	Cc1c2c(cc(OCc3ccc(F)cc3)c1OC)c(=O)c(O)c(o2)c1[nH]c(C)nn1	1312912-30-3	4H-1-Benzopyran-4-one, 6-[(4-fluorophenyl)methoxy]-3-hydroxy-7-methoxy-8-methyl-	C20H16FN3O5	397.36	C	-8.3
7620	Cc1c2c(cc(OCc3ccc(F)cc3)c1Cl)c(=O)c(O)c(o2)c1ncc(C)cn1	1312912-55-2	4H-1-Benzopyran-4-one, 7-chloro-6-[(4-fluorophenyl)methoxy]-3-hydroxy-8-methyl-2	C21H14ClFN2O4	412.8	C	-8.3
7621	Cc1c2c(cc(OCc3ccc(F)cc3)c1Cl)c(=O)c(O)c(o2)c1ncc(OC)cn1	1312912-57-4	4H-1-Benzopyran-4-one, 7-chloro-6-[(4-fluorophenyl)methoxy]-3-hydroxy-8-methyl-2	C22H16ClFN2O4	426.82	C	-8.3
7622	Cc1c2c(cc(OCc3ccc(F)cc3)c1Cl)c(=O)c(O)c(o2)c1[nH]c(nn1)c1ccccc1	1312912-81-4	4H-1-Benzopyran-4-one, 7-chloro-2-(5-chloro-1H-1,2,4-triazol-3-yl)-6-[(4-fluorop	C19H12Cl2FN3O4	436.22	C	-8.3
7623	O(C)c1c2c(cc(OCc3ccc(F)cc3)c1OC)c(=O)c(O)c(o2)c1[nH]cnn1	1312913-18-0	4H-1-Benzopyran-4-one, 6-[(4-fluorophenyl)methoxy]-3-hydroxy-7,8-dimethoxy-2-(5-	C28H21FN2O6	500.47	C	-8.3
7624	Clc1c2c(cc(OCc3ccc(F)cc3)c1OC)c(=O)c(O)c(o2)c1[nH]c(C)nn1	1312914-30-9	4H-1-Benzopyran-4-one, 8-chloro-6-[(4-fluorophenyl)methoxy]-3-hydroxy-7-methoxy-	C19H13ClFN3O5	417.77	C	-8.3
7625	Clc1c2c(cc(OCc3ccc(F)cc3)c1Cl)c(=O)c(O)c(o2)c1ncc(C)cn1	1312914-55-8	4H-1-Benzopyran-4-one, 7,8-dichloro-6-[(4-fluorophenyl)methoxy]-3-hydroxy-2-(2-p	C20H11Cl2FN2O4	433.22	C	-8.3
7626	Clc1c2c(cc(OCc3ccc(F)cc3)c1Cl)c(=O)c(O)c(o2)c1[nH]c(C)cn1	1312914-85-4	4H-1-Benzopyran-4-one, 7,8-dichloro-6-[(4-fluorophenyl)methoxy]-3-hydroxy-2-(1H-	C19H11Cl2FN2O4	421.21	C	-8.3
7627	Clc1c2c(cc(OCc3ccc(F)cc3)c1Cl)c(=O)c(O)c(o2)c1[nH]c(cn1)c1ccccc1	1312914-91-2	4H-1-Benzopyran-4-one, 7,8-dichloro-2-(5-chloro-1H-imidazol-2-yl)-6-[(4-fluoroph	C19H10Cl3FN2O4	455.65	C	-8.3
7628	O(C)c1c(c2c(cc1OCc1ccc(F)cc1)c(=O)c(O)c(o2)c1[nH]c(Cl)nn1)c1ccccc1	1312915-39-1	4H-1-Benzopyran-4-one, 6-[(4-fluorophenyl)methoxy]-3-hydroxy-7-methoxy-2-(5-meth	C26H20FN3O6	489.45	C	-8.3
7629	O(C)c1c(c2c(cc1OCc1ccc(F)cc1)c(=O)c(O)c(o2)c1[nH]c(OC)cn1)c1ccccc1	1312915-47-1	4H-1-Benzopyran-4-one, 6-[(4-fluorophenyl)methoxy]-3-hydroxy-7-methoxy-2-(5-meth	C27H21FN2O5	472.46	C	-8.3
7630	Cc1c2c(cc(OCc3ccc(F)cc3)c1c1ccccc1)c(=O)c(O)c(o2)c1ncc(cn1)c1ccccc1	1312916-85-0	4H-1-Benzopyran-4-one, 2-(5-chloro-2-pyrimidinyl)-6-[(4-fluorophenyl)methoxy]-3-	C27H18ClFN2O4	488.89	C	-8.3
7631	O(Cc1ccc(F)cc1)c1c(c(C)c2c(c1)c(=O)c(O)c(o2)c1[nH]c(Cl)nn1)c1ccccc1	1312916-94-1	4H-1-Benzopyran-4-one, 6-[(4-fluorophenyl)methoxy]-3-hydroxy-2-(5-methoxy-1H-1,2	C26H20FN3O5	473.45	C	-8.3
7632	Clc1c2c(cc(OCc3ccc(F)cc3)c1c1ccccc1)c(=O)c(O)c(o2)c1ncc(Cl)cn1	1312917-84-2	4H-1-Benzopyran-4-one, 8-chloro-6-[(4-fluorophenyl)methoxy]-3-hydroxy-2-(5-metho	C27H18ClFN2O5	504.89	C	-8.3
7633	Clc1c2c(cc(OCc3ccc(F)cc3)c1c1ccccc1)c(=O)c(O)c(o2)c1ncc(cn1)c1ccccc1	1312917-85-3	4H-1-Benzopyran-4-one, 8-chloro-2-(5-chloro-2-pyrimidinyl)-6-[(4-fluorophenyl)me	C26H15Cl2FN2O4	509.31	C	-8.3
7634	O(Cc1ccc(F)cc1)c1c(c(c2c(c1)c(=O)c(O)c(o2)c1[nH]cnn1)c1ccccc1)c1ccccc1	1312918-36-7	4H-1-Benzopyran-4-one, 6-[(4-fluorophenyl)methoxy]-3-hydroxy-7,8-diphenyl-2-(5-p	C38H25FN2O4	592.61	C	-8.3
7635	O[C@H]1[C@H](n2c3c(nc2)c(=O)nc[nH]3)O[C@H](COP(=O)(OP(=O)(OP(=O)(O)O)O)O)[C@H]1O	1319715-08-6	2H-Pyrido[1,Ä≤,2,Ä≤:4,5]pyrazino[2,1-b][1,3]oxazine-9-carboxamide, N-[(2,4-difl	C37H48N4O5.C20H19F2N3O5		C	-8.3
7636	O=c1n([C@H]2O[C@@H](C(=O)O[C@H]3[C@H](C(C)C)CC[C@@H](C)C3)SC2)cc(F)c(NC(=O)C)n1	1321849-66-4	Carbamic acid, N-[(1S)-2-[(1R,3S,4S)-3-[5-[4,Ä≤-[2-[(1R,3S,4S)-2-[(2S)-2-[(metho	C43H52N8O6	776.92	C	-8.3
7637	N(=C1c2c(N(Cc3ccc(C)cc3)C1=O)ccc(Br)c2)NC(=S)Nc1ccc(Cl)cc1	1323295-89-1	Hydrazinecarbothioamide, 2-[5-bromo-1-[(4-fluorophenyl)methyl]-1,2-dihydro-2-oxo	C22H15BrClFN4OS	517.8	C	-8.3
7638	N(S(=O)(=O)c1ccc(N)cc1)(C[C@H]([C@H](Cc1ccccc1)NC(=O)O[C@H]1CCOC1)O)CC(C)(C)C	1332605-79-4	Not Yet Assigned	C35H45N5O4	599.76	C	-8.3
7639	O=c1n([C@@H]2O[C@H](COP(=O)(Oc3ccc(N(=O)=O)cc3)N[C@H](C(=O)OC)C(C)C=C2)cc(C)c(=O)[nH]1	1333491-90-9	Benzeneacetic acid, Œ±-[[[[(2S,5R)-5-(3,4-dihydro-5-methyl-2,4-dioxo-1(2H)-pyrim	C26H27N4O11P	602.49	C	-8.3
7640	[C@H](C(=O)OC(OC(=O)C1=C[C@@H](OC(CC)CC)[C@H](NC(=O)C)[C@@H](N=C(NC(=O)OC(C)(C)C)NC(=O)OC(C)(C)C1)	1338226-05-3	Benzonitrile, 3-chloro-5-[[1-[(2,5-dihydro-5-oxo-1H-1,2,4-triazol-3-yl)methyl]-1	C16H9ClF3N5O3	411.72	C	-8.3
7641	[C@@H](Cc1ccccc1)(C[C@@H]([C@@H](Cc1ccccc1)NC(=O)OCCc1cncs1)O)NC(=O)[C@@H](NC(=O)N(Cc1nc(C(C)C)sc1)C	1347074-06-9	3-Azabicyclo[3.1.0]hexane-2-carboxamide, N-[3-amino-1-(cyclobutylmethyl)-2,3-dic	C35H51N5O6	637.81	C	-8.3
7642	C(C(C)C)c1n(C2CC3N(CC[C@H](NC(=O)C4CCC4)c4ccccc4)C(C2)CC3)c(C)nn1	1347825-92-6	Cyclobutanecarboxamide, N-[(1S)-3-[3-(3-cyclobutyl-5-methyl-4H-1,2,4-triazol-4-y	C28H39N5O	461.64	C	-8.3
7643	C(OC(CO)CO)n1c2c(nc1)C(=O)NC(N)N2	1347883-56-0	Carbamic acid, N-[(1S,2R)-2-hydroxy-3-[[[4-[[(4-hydroxyphenyl)nitrophosphinyl]me	C34H41N4O14PS	792.75	C	-8.3
7644	C(=O)(N[C@@H](CC1CCC1)C(=O)C(=O)NCc1sc(c(C(=O)N(C)C)n1)c1ccccc1)[C@@H]1[C@@H]2[C@@H](C2(C)C)CN1C(=O)	1348364-52-2	Butanediamide, N1-[(1R,2S)-3-[(3S,4aS,8aS)-3-[[(1,1-dimethylethyl)amino]carbonyl	C38H52N6O4	656.86	C	-8.3
7645	C(C(OCP(=O)(O)O)C)n1c2c(nc1N)c(N)nc(N)n2	1348833-85-1	Carbamic acid, N-[[4,Ä≤-[(2-ethyl-5,7-dimethyl-3H-imidazo[4,5-b]pyridin-3-yl)met	C31H37FN4O4S	580.71	C	-8.3
7646	P(=O)(COc1ccc(C[C@@H]([C@@H](CN(S(=O)(=O)c2ccc(N)cc2)CC(C)C)O)NC(=O)OC2CCOC2)cc1)(c1ccc(O)cc1)c1ccc(	1348884-84-3	Not Yet Assigned	C38H59N7O6	709.92	C	-8.3
7647	C(n1c2c(nc1CC)c(C)cc(C)n2)c1ccc(cc1)c1c(S(=O)(=O)NC(=O)OCCOCC)ccc(CC(C)C)c1	1349743-90-3	Hydrazinecarboxylic acid, 1-[(1S,3S,4S)-3-hydroxy-5-phenyl-1-(phenylmethyl)-4-[[	C41H55N7O7S2	822.05	C	-8.3
7648	C([C@H]([C@H](Cc1ccccc1)NC(=O)[C@@H](NC(=O)c1nc2c(cc1)cccc2)C(=O)N)O)N1[C@H](C(=O)NC(C)(C)C)C[C@H]2[	1349752-23-3	[1,1,Ä≤-Biphenyl]-2-sulfonamide, 4,Ä≤-[(2-ethyl-5,7-dimethyl-3H-imidazo[4,5-b]p	C28H33N5O5S	551.66	C	-8.3
7649	O=c1n(ccc(N)n1)[C@H]1O[C@@H](COC(=O)CCC(=O)OCC(COC(=O)[C@H]([C@H](CC)C)N)O)SC1	1350161-86-2	Carbamic acid, N-[(1S,2R)-2-hydroxy-3-[[[4-[[hydroxy(phenylmethoxy)phosphinyl]me	C35H45N2O12PS	748.78	C	-8.3
7650	O=c1n([C@@H]2O[C@H](COC(=O)CCC(=O)OC(COC(=O)CCCCCCCCCCCCCCC)COC(=O)CCCCCCCCCCCCCCC)C=C2)cc(C)c(=O)[n	1350627-74-5	Guanosine, 8-[(1E)-2-[4-(trifluoromethyl)phenyl]ethenyl]-	C19H18F3N5O5	453.37	C	-8.3
7651	C(=O)([C@@H](NC(=O)OC)C(C)C)N1[C@H](c2[nH]c(c3c4c(c(cc3)c3c5c(c(cc3)c3[nH]c(nc3)[C@H]3N(C(=O)[C@@H](	1350718-44-3	Carbamic acid, N,N,Ä≤-[(2,2,Ä≤-dimethyl[4,7,Ä≤-bibenzothiazole]-4,Ä≤,7-diyl)bis	C44H52N10O6S2	881.08	C	-8.3
7652	C(=O)([C@@H](NC(=O)OC)C(C)C)N1[C@H](c2[nH]c(c3c4c(c(cc3)c3c5c(c(cc3)c3[nH]c(nc3)[C@H]3N(C(=O)[C@@H](	1350718-46-5	Carbamic acid, N,N,Ä≤-[(2,2,Ä≤-dimethyl[4,7,Ä≤-bibenzo[b]thiophene]-4,Ä≤,7-diyl	C46H54N8O6S2	879.1	C	-8.3
7653	C(=O)([C@@H](NC(=O)OC)C(C)C)N1[C@@]2(C[C@H](C1)CC2)c1[nH]c(cn1)c1c2c(c(cc1)c1ccc(cc1)c1[nH]c(nc1)[C@	1350718-84-1	Carbamic acid, N-[(1S)-1-[[(2S)-2-[5-[4,Ä≤-[2-[(1S,4R)-2-[(2S)-2-[(methoxycarbor	C42H52N8O6	764.91	C	-8.3
7654	C[C@@]1(N(C(=O)[C@@H](NC(=O)OC)C(C)C)CCC1)c1[nH]c(cn1)c1c2c(c(cc1)c1c3c(c(cc1)c1[nH]c(nc1)[C@@]1(C)N	1350719-46-8	Carbamic acid, N,N,Ä≤-[[1,1,Ä≤-biphenyl]-4,4,Ä≤-diylbis[1H-imidazole-5,2-diyl-(	C40H38F12N8O6	954.76	C	-8.3
7655	[C@H](Cc1ccccc1)(C[C@@H]([C@H](Cc1ccccc1)NC(=O)OCC(=O)C=O)O)NC(=O)[C@@H](NC(=O)N(Cc1nc(C(C)C)sc1)C)C	1350800-76-8	3H-Cyclopropa[4,5]pyrrolo[1,2-a]pyrazin-3-one, 6-amino-1,1a,4,6a,7,7a-hexahydro-	C18H25N3O2	315.41	C	-8.3
7656	C(=O)([C@@H](NC(=O)OC)C(C)C)N1[C@@H](CCC1)c1[nH]c(cn1)c1cc(C#N)c(cc1)c1cc2c(cc1)cc(cc2)c1[nH]c(nc1)[	1351332-54-1	Carbamic acid, N-[(1S)-1-[[(3R)-3-[5-[6-[4-[2-[(2S)-1-[(2S)-2-[(methoxycarbonyl)	C44H52N8O6S	821	C	-8.3
7657	C(=O)([C@@H](NC(=O)OC1CCOCC1)C(C)C)N1C[C@@]2(C[C@H]1c1[nH]c(cn1)c1ccc(cc1)c1ccc(cc1)c1[nH]c(nc1)[C@H	1351851-13-2	Carbamic acid, N-[(1S)-1-[[(5S,8S)-8-[5-[4,Ä≤-[2-[(2S)-1-[(2S)-2-[(methoxycarbor	C44H56N8O7	808.96	C	-8.3
7658	C(=O)([C@@H](NC(=O)O[C@@H]1CCOC1)C(C)C)N1C[C@@]2(C[C@H]1c1[nH]c(cn1)c1ccc(cc1)c1ccc(cc1)c1[nH]c(nc1)	1351851-14-3	Carbamic acid, N-[(1S)-1-[[(5S,8S)-8-[5-[4,Ä≤-[2-[(2S)-1-[(2S)-2-[(methoxycarbor	C47H60N8O8	865.03	C	-8.3
7659	C(=O)([C@@H](NC(=O)OC)C(C)C)N1C[C@@]2(C[C@H]1c1[nH]c(cn1)c1c(C(F)(F)F)cc(cc1)c1cc(C(F)(F)F)c(cc1)c1[	1351852-85-1	Carbamic acid, N-[(1S)-2-methyl-1-[[(2S)-2-[5-[2,2,Ä≤,3,3,Ä≤-tetraethyl-4,Ä≤-[2	C51H70N8O7	907.15	C	-8.3
7660	C(=O)([C@@H](NC(=O)OC)C(C)C)N1[C@@H](C[C@@]2(C1)CCCO2)c1[nH]c(cn1)c1ccc(cc1)c1ccc(cc1)c1[nH]c(nc1)[C	1351852-93-1	Carbamic acid, N,N,Ä≤-[[1,1,Ä≤-biphenyl]-4,4,Ä≤-diylbis[1H-imidazole-5,2-diyl-(	C46H58N8O8	851	C	-8.3
7661	C(=O)([C@@H](NC(=O)OC)C(C)C)N1CC2(C[C@H]1c1[nH]c(cn1)c1ccc(cc1)c1ccc(cc1)c1[nH]c(nc1)[C@H]1N(C(=O)[C	1351853-19-4	Carbamic acid, N-[(1S)-1-[[(2S)-2-[5-[4,Ä≤-[2-[(3S,6S)-1,1-difluoro-5-[(2S)-2-[	C42H50F2N8O6	800.89	C	-8.3
7662	C(=O)([C@@H](NC(=O)OC)c1ccccc1)N1[C@@H](C[C@@H](C1)C1CC1)c1[nH]c(cn1)c1ccc(cc1)c1ccc(cc1)c1[nH]c(nc1	1351854-17-5	Carbamic acid, N-[(1S)-2-[(2S)-2-[5-[4,Ä≤-[2-[(2S,4R)-4-cyclopropyl-1-[(2S)-2-[	C47H58N8O8	863.01	C	-8.3
7663	C(=O)([C@@H](NC(=O)OC)C(C)C)N1[C@@H](C[C@@H](C1)C1CC1)c1[nH]c(cn1)c1c(F)cc(cc1)c1cc(F)c(cc1)c1[nH]c(	1351855-62-3	Carbamic acid, N-[(1S)-1-[[(2S)-2-[5-[4,Ä≤-[5-chloro-2-[(2S,4R)-4-cyclopropyl-1-	C43H53ClN8O6	813.38	C	-8.3
7664	C(=O)([C@@H](NC(=O)OC)C(C)C)N1[C@@H](C[C@@H](C1)C1CC1)c1[nH]c(cn1)c1c(F)c(F)c(cc1)c1c(F)c(F)c(cc1)c1	1351855-64-5	Carbamic acid, N-[(1S)-1-[[(2S,4R)-4-cyclopropyl-2-[5-[3,3,Ä≤-difluoro-4,Ä≤-[2-	C43H52F2N8O6	814.92	C	-8.3
7665	C(=O)([C@@H](NC(=O)OC)C(C)C)N1[C@@H](C[C@@H](C1)C1CC1)c1[nH]c(cn1)c1c(C)c(C)c(cc1)c1c(C)c(C)c(cc1)c1	1351855-66-7	Carbamic acid, N-[(1S)-1-[[(2S)-2-[5-[4,Ä≤-[2-[(2S,4R)-4-cyclopropyl-1-[(2S)-2-	C45H58N8O6	806.99	C	-8.3
7666	C(=O)([C@@H](NC(=O)OC)C(C)C)N1[C@@H](C[C@@H](C1)C1CC1)c1[nH]c(cn1)c1c(CC)c(CC)c(cc1)c1c(CC)c(CC)c(cc	1351855-67-8	Carbamic acid, N-[(1S)-1-[[(2S)-2-[5-[4,Ä≤-[2-[(2S,4R)-4-cyclopropyl-1-[(2S)-2-	C47H62N8O6	835.05	C	-8.3
7667	C(=O)([C@@H](NC(=O)OC)C(C)C)N1[C@@H](C[C@@H](C1)C1CC1)c1[nH]c(cn1)c1c(Cl)cc(cc1)c1cc(Cl)c(cc1)c1[nH]	1351855-70-3	Carbamic acid, N-[(1S)-1-[[(2S)-2-[5-[4,Ä≤-[2-[(2S,4R)-4-cyclopropyl-1-[(2S)-2-	C45H52F6N8O6	914.93	C	-8.3
7668	C(=O)([C@@H](NC(=O)OC)C(C)C)N1[C@@H](C[C@@H](C1)C1CC1)c1[nH]c(cn1)c1c(Cl)cc(cc1Cl)c1cc(Cl)c(c(Cl)c1)	1351855-74-7	Carbamic acid, N-[(1S)-1-[[(2S,4R)-4-cyclopropyl-2-[5-[3,3,Ä≤-dichloro-4,Ä≤-[2-	C43H52Cl2N8O6	847.83	C	-8.3
7669	C(=O)([C@@H](NC(=O)OC)C(C)C)N1[C@@H](C=C(C)C1)c1[nH]c(cn1)c1cc2c(cc1)cc(cc2)c1ccc(cc1)c1[nH]c(nc1)[C	1352391-95-7	Carbamic acid, N-[(1S)-1-[[(2S)-2-[5-[4-[6-[2-[(2S)-2,5-dihydro-1-[(2S)-2-[(meth	C45H52N8O6	800.94	C	-8.3
7670	C(=O)([C@@H](NC(=O)OC)C(C)C)N1[C@@H](C=C(C)C1)c1[nH]c(cn1)c1cc2c(cc1)cc(C#Cc1[nH]c(nc1)[C@H]1N(C(=O)	1352391-96-8	Carbamic acid, N-[(1S)-1-[[(2S)-2-[5-[4-[6-[2-[(2S)-2,5-dihydro-1-[(2S)-2-[(meth	C46H52N8O6	812.96	C	-8.3
7671	C(CCC[C@@H](CCc1ccc(O)cc1)O)c1cc(O)c(O)cc1	1353639-75-4	L-Tryptophan, N-[hydroxy(4-nitrophenoxy)phosphinyl]-, methyl ester, 5,Ä≤-ester w	C26H28N7O11P	645.51	C	-8.3
7672	C(=O)([C@@H](NC(=O)OC)C(C)C)N1[C@H]2[C@H](C2)C[C@H]1c1[nH]c(cn1)c1c2c(c(cc1)c1ccc(cc1)c1[nH]c(nc1)[C	1359868-94-2	Carbamic acid, N-[(1S)-2-[(2S,5S)-2-[5-[4-[7-[2-[(2S,5S)-1-[(2S)-2-[(methoxycarb	C46H56N10O8S	909.06	C	-8.3
7673	C(=O)([C@@H](NC(=O)OC)[C@H]1C[C@@H](C)O[C@@H](C)C1)N1[C@H](c2[nH]c(c3c4c(c(cc3)c3ccc(cc3)c3[nH]c(nc3	1359868-97-5	Carbamic acid, N-[(1S)-2-[(2S,5S)-2-[5-[4-[7-[2-[(2S,5S)-1-[(2S)-2-[(methoxycarb	C47H58N10O8	891.03	C	-8.3
7674	C(F)(F)c1n(c2c(n1)cccc2)c1nc(nc(NCCS(=O)(=O)N2CCOCC2)n1)N1CCOCC1	1359953-58-4	Cyclohexanol, 4-[[4-[2-(difluoromethyl)-1H-benzimidazol-1-yl]-6-(4-morpholinyl)-	C21H25F2N7O2	445.47	C	-8.3
7675	C(=O)([C@@H](NC(=O)OC)C(C)C)N1[C@@H](C[C@H](C(F)(F)F)C1)c1[nH]c(c(F)n1)c1ccc(cc1)c1ccc(cc1)c1[nH]c(n	1361249-39-9	Carbamic acid, N,N,Ä≤-[[1,1,Ä≤-biphenyl]-4,4,Ä≤-diylbis[(5-fluoro-1H-imidazole-	C42H46F8N8O6	910.85	C	-8.3
7676	O=c1n([C@@H]2O[C@H](COP(=O)(OCCCCCCCCCCCCCCC)O)[C@@H](N=[N+]=[N-])C2)cc(C)c(=O)[nH]1	1363821-21-9	Thymidine, thymidylyl-(3,Ä≤,Ü'5,Ä≤)-4-deoxy-	C20H27N4O11P	530.42	C	-8.3
7677	N(C(C)C)c1c(nccc1)N1CCN(C(=O)c2cc3c([nH]2)ccc(N=[N+]=[N-])c3)CC1	136817-59-9	Methanesulfonamide, N-[2-[[4-[3-[(1-methylethyl)amino]-2-pyridinyl]-1-piperaziny	C22H28N6O3S	456.56	C	-8.3
7678	C(=O)([C@@H](NC(=O)OC)C(C)C)N1[C@@H](CCC1)c1[nH]c(c(F)n1)c1ccc(cc1)c1ccc(cc1)c1[nH]c(nc1)[C@H]1N(C(=	1373165-54-8	Carbamic acid, N-[(1S)-2-[(2S)-2-[5-[4,Ä≤-[2-[(2S,4S)-1-[(2S)-2-[(methoxycarbony	C39H48N8O6S	756.91	C	-8.3
7679	O[C@H]1[C@H](c2c3c([nH]c2)c(N)ncn3)N[C@H](CO)[C@H]1O.Cl	1373206-59-7	Carbamic acid, N-[(1S)-2-methyl-1-[[(2S)-2-[5-[4,Ä≤-[2-[(2S,4S)-4-(methylthio)-2	C34H41N7O3S	627.8	C	-8.3
7680	C(=O)([C@@H](NC(=O)OC)C(C)C)N1[C@@H]([C@@H]2C[C@H]1CC2)c1[nH]c(cn1)c1ccc(cc1)c1cc2c(cc1)c1c(cc2)nc([	1377036-84-4	Carbamic acid, N-[(1S)-2-[(2S)-2-[5-[9,10-dihydro-7-[2-[(1R,3S,4S)-2-[(2S)-2-[(m	C48H58N8O8	875.02	C	-8.3
7681	C(=O)([C@@H](NC(=O)OC)C(C)C)N1[C@@H]([C@@H]2C[C@H]1CC2)c1[nH]c2-c3c(CCc2n1)cc(cc3)c1cc2c(cc1)c1c(cc2	1377040-91-9	Carbamic acid, N-[(1R)-2-[(2S)-2-[5-[4-[6-[2-[(2S)-1-[(2S)-3-ethyl-2-[(methoxyca	C49H54N8O6	851	C	-8.3
7682	C(=O)([C@@H](NC(=O)OC)C(C)C)N1[C@@H]([C@@H]2C[C@H]1CC2)c1[nH]c(cn1)c1cc(C#CC)c(cc1)c1ccc(cc1)c1[nH]c	1377041-62-7	Carbamic acid, N-[(1S)-2-[(1R,3S,4S)-3-[5-[4-[2-[(2S)-1-[(2S)-2-[(methoxycarbony	C49H56N8O7	869.02	C	-8.3
7683	Fc1c2c(c3c(nc([nH]3)[C@H]3N(C(=O)[C@@H](NC(=O)OC)C(C)C)CCC3)cc2)ccc1c1c(F)c2c(cc1)c1c(cc2)nc([nH]1)[	1377045-04-9	Carbamic acid, N-[(1S)-1-[[(2S)-2-[7-[4-[2-[(2S,4S)-1-[(2S)-2-[(methoxycarbonyl)	C44H54N8O7	806.95	C	-8.3
7684	C(=O)([C@@H](NC(=O)OC)C(C)C)N1[C@H](c2[nH]c3c4c(cc5-c6c(COc5c4)cc(cc6)c4[nH]c(nc4)[C@H]4N(C(=O)[C@H]	1377048-90-2	Carbamic acid, N-[(1S)-1-[[(2S,4S)-2-[2,Ä≤-[(2S,4S)-1-[(2S)-2-[(methoxycarbonyl)	C47H56N8O7	845	C	-8.3
7685	Fc1c2c(c3c(nc([nH]3)[C@H]3N(C(=O)[C@@H](NC(=O)OC)C(C)C)CCC3)cc2)ccc1c1cc2c(cc1)c1c(cc2)nc([nH]1)[C@H	1377049-84-7	Carbamic acid, N-[(1R)-2-[(2S,4S)-2-[5-[1,11-dihydro-2-[(2S,5S)-1-[(2S)-2-[(meth	C49H54N8O8	883	C	-8.3
7686	C(=O)([C@@H](NC(=O)OC)C(C)C)N1[C@@H](CCC1)c1[nH]c(cn1)c1ccc(cc1)c1sc(C#CC#Cc2[nH]c(nc2)[C@H]2N(C(=O)	1380291-99-5	Carbamic acid, N-[(1S)-1-[[(2S)-2-[5-[4-[5-[2-[2-[(2S)-1-[(2S)-2-[(methoxycarbor	C40H48N8O6S	768.92	C	-8.3
7687	C(=O)([C@@H](NC(=O)OC)C(C)C)N1[C@@H](CCC1)c1[nH]c(cn1)c1ccc(C#CC#CC#Cc2[nH]c(nc2)[C@H]2N(C(=O)[C@@H]	1380293-14-0	Carbamic acid, N,N,Ä≤-[(trans,trans)-[1,1,Ä≤-bicyclohexyl]-4,4,Ä≤-diylbis[1H-im	C40H62N8O6	750.97	C	-8.3
7688	C(=O)([C@@H](NC(=O)OC)C(C)C)N1[C@@H](C[C@H](N=[N+]=[N-])C1)c1[nH]c(cn1)c1ccc(cc1)c1ccc(cc1)c1[nH]c(n	1388224-11-0	Carbamic acid, N,N,Ä≤-[[1,1,Ä≤-biphenyl]-4,4,Ä≤-diylbis[[5-(2-thienyl)-1H-imida	C48H54N8O6S2	903.12	C	-8.3
7689	O=C1C2(C(C(C(=O)/C=C(\O)/C3C4(N5C(C3c3cc(OC)c(O)cc3)CCCC5)c3c(NC4=O)ccc(N(=O)=O)c3)C3N2CCCC3)c2cc(OC	1389335-20-9	Spiro[3H-indole-3,3,Ä≤(2,Ä≤H)-indolizin]-2(1H)-one, 5-chloro-1,Ä≤-[(2Z)-3-[5-ch	C47H46Cl2N4O8	865.8	C	-8.3
7690	N(C(Cc1ccccc1)C(=O)N)c1nc(nc(n1)N1CCOCC1)c1cc(O)ccc1	1391924-56-3	Ethanone, 2-[[4-(3-hydroxyphenyl)-6-(4-morpholinyl)-1,3,5-triazin-2-yl]amino]-1-	C20H27N7O5S	477.54	C	-8.3
7691	C(OCCCCN)n1c2c(c(=O)nc(N)[nH]2)nc1	1391926-65-0	Benzamide, 4-[[4-(6-amino-3-pyridinyl)-6-(4-morpholinyl)-1,3,5-triazin-2-yl]amir	C21H24N8O2	420.47	C	-8.3
7692	N(c1nc(nc(NCCNC(=O)c2ccc(cc2)C2OOC(OO2)c2ccc(C)cc2)n1)N1CCOCC1)c1c(C)cccc1	1393922-44-5	Benzamide, N-[2-[[4-[(4-methylphenyl)amino]-6-(4-morpholinyl)-1,3,5-triazin-2-yl	C32H35N7O6	613.66	C	-8.3
7693	O=C1c2n(C([C@H]3N1[C@](C([2H])([2H])[2H])(C(C(O3)([2H])[2H])([2H])[2H])[2H])([2H])[2H])cc(C(=O)NCc1c	1407167-20-7	2H-Pyrido[1,Ä≤,2,Ä≤:4,5]pyrazino[2,1-b][1,3]oxazine-2,3,4,12-d4-9-carboxamide,	C20H8D11F2N3O5		C	-8.3
7694	O=C1c2n(C[C@H]3N1[C@](C([2H])([2H])[2H])(C(C(O3)([2H])[2H])([2H])[2H])[2H])cc(C(=O)NCc1c(F)cc(F)cc1)	1407167-22-9	2H-Pyrido[1,Ä≤,2,Ä≤:4,5]pyrazino[2,1-b][1,3]oxazine-12-d-9-carboxamide, N-[(2,4	C20H17D2F2N3O5		C	-8.3
7695	O=C1c2n(C[C@H]3N1[C@](C)(C(C(O3)([2H])[2H])([2H])[2H])[2H])cc(C(=O)NCc1c(F)cc(F)cc1)c(=O)c2O	1407167-24-1	2H-Pyrido[1,Ä≤,2,Ä≤:4,5]pyrazino[2,1-b][1,3]oxazine-2,3-d2-9-carboxamide, N-[(2	C20H12D7F2N3O5		C	-8.3
7696	O=C1c2n(C[C@H]3N1[C@H](C)C(C(O3)([2H])[2H])([2H])[2H])cc(C(=O)NCc1c(F)cc(F)cc1)c(=O)c2O	1407167-25-2	2H-Pyrido[1,Ä≤,2,Ä≤:4,5]pyrazino[2,1-b][1,3]oxazine-2,3,4-d3-9-carboxamide, N-[	C20H14D5F2N3O5		C	-8.3
7697	O=C1c2n(C[C@H]3N1[C@](C([2H])([2H])[2H])(CCO3)[2H])cc(C(=O)NCc1c(F)cc(F)cc1)c(=O)c2O	1407167-26-3	2H-Pyrido[1,Ä≤,2,Ä≤:4,5]pyrazino[2,1-b][1,3]oxazine-2,3-d2-9-carboxamide, N-[(2	C20H15D4F2N3O5		C	-8.3
7698	O=C1c2n(C[C@H]3N1[C@H](C([2H])([2H])[2H])CCO3)cc(C(=O)NCc1c(F)cc(F)cc1)c(=O)c2O	1407167-27-4	2H-Pyrido[1,Ä≤,2,Ä≤:4,5]pyrazino[2,1-b][1,3]oxazine-4-d-9-carboxamide, N-[(2,4-	C20H15D4F2N3O5		C	-8.3
7699	O=C1c2n(C([C@H]3N1[C@H](C([2H])([2H])[2H])C(C(O3)([2H])[2H])([2H])[2H])([2H])[2H])cc(C(=O)NCc1c(F)cc	1407167-30-9	2H-Pyrido[1,Ä≤,2,Ä≤:4,5]pyrazino[2,1-b][1,3]oxazine-4-d-9-carboxamide, N-[(2,4-	C20H18DF2N3O5		C	-8.3
7700	O=C1c2n(C([C@H]3N1[C@](C)(C(C(O3)([2H])[2H])([2H])[2H])[2H])([2H])[2H])cc(C(=O)NCc1c(F)cc(F)cc1)c(=O	1407167-32-1	2H-Pyrido[1,Ä≤,2,Ä≤:4,5]pyrazino[2,1-b][1,3]oxazine-2,3,12-d3-9-carboxamide, N-	C20H10D9F2N3O5		C	-8.3
7701	O=C1c2n(C([C@H]3N1[C@H](C)C(C(O3)([2H])[2H])([2H])[2H])([2H])[2H])cc(C(=O)NCc1c(F)cc(F)cc1)c(=O)c2O	1407167-34-3	2H-Pyrido[1,Ä≤,2,Ä≤:4,5]pyrazino[2,1-b][1,3]oxazine-2,3,4,12-d4-9-carboxamide,	C20H12D7F2N3O5		C	-8.3
7702	O=C1c2n(C([C@H]3N1[C@](C([2H])([2H])[2H])(CCO3)[2H])([2H])[2H])cc(C(=O)NCc1c(F)cc(F)cc1)c(=O)c2O	1407167-36-5	2H-Pyrido[1,Ä≤,2,Ä≤:4,5]pyrazino[2,1-b][1,3]oxazine-2,3,12-d3-9-carboxamide, N-	C20H13D6F2N3O5		C	-8.3
7703	O=C1c2n(C([C@H]3N1[C@H](C([2H])([2H])[2H])CCO3)([2H])[2H])cc(C(=O)NCc1c(F)cc(F)cc1)c(=O)c2O	1407167-38-7	2H-Pyrido[1,Ä≤,2,Ä≤:4,5]pyrazino[2,1-b][1,3]oxazine-4,12-d2-9-carboxamide, N-[(	C20H13D6F2N3O5		C	-8.3
7704	O=C1c2n(C([C@H]3N1[C@](C)(CCO3)[2H])([2H])[2H])cc(C(=O)NCc1c(F)cc(F)cc1)c(=O)c2O	1407167-40-1	2H-Pyrido[1,Ä≤,2,Ä≤:4,5]pyrazino[2,1-b][1,3]oxazine-12-d-9-carboxamide, N-[(2,4	C20H14D5F2N3O5		C	-8.3
7705	O=C1c2n(C([C@]3(N1[C@H](C)CCO3)[2H])([2H])[2H])cc(C(=O)NCc1c(F)cc(F)cc1)c(=O)c2O	1407167-41-2	2H-Pyrido[1,Ä≤,2,Ä≤:4,5]pyrazino[2,1-b][1,3]oxazine-4,12-d2-9-carboxamide, N-[(	C20H16D3F2N3O5		C	-8.3
7706	O=C1c2n(C[C@]3(N1[C@](C([2H])([2H])[2H])(C(C(O3)([2H])[2H])([2H])[2H])[2H])[2H])cc(C(=O)NCc1c(F)cc(F	1407167-42-3	2H-Pyrido[1,Ä≤,2,Ä≤:4,5]pyrazino[2,1-b][1,3]oxazine-12-d-9-carboxamide, N-[(2,4	C20H16D3F2N3O5		C	-8.3
7707	O=C1c2n(C[C@]3(N1[C@H](C)C(C(O3)([2H])[2H])([2H])[2H])[2H])cc(C(=O)NCc1c(F)cc(F)cc1)c(=O)c2O	1407167-45-6	2H-Pyrido[1,Ä≤,2,Ä≤:4,5]pyrazino[2,1-b][1,3]oxazine-2,3,4-d3-9-carboxamide, N-[	C20H13D6F2N3O5		C	-8.3
7708	O=C1c2n(C[C@]3(N1[C@](C([2H])([2H])[2H])(CCO3)[2H])[2H])cc(C(=O)NCc1c(F)cc(F)cc1)c(=O)c2O	1407167-46-7	2H-Pyrido[1,Ä≤,2,Ä≤:4,5]pyrazino[2,1-b][1,3]oxazine-2,3-d2-9-carboxamide, N-[(2	C20H14D5F2N3O5		C	-8.3
7709	O=C1c2n(C[C@]3(N1[C@H](C([2H])([2H])[2H])CCO3)[2H])cc(C(=O)NCc1c(F)cc(F)cc1)c(=O)c2O	1407167-47-8	2H-Pyrido[1,Ä≤,2,Ä≤:4,5]pyrazino[2,1-b][1,3]oxazine-4-d-9-carboxamide, N-[(2,4-	C20H14D5F2N3O5		C	-8.3
7710	O=C1c2n(C[C@]3(N1[C@](C)(CCO3)[2H])[2H])cc(C(=O)NCc1c(F)cc(F)cc1)c(=O)c2O	1407167-48-9	2H-Pyrido[1,Ä≤,2,Ä≤:4,5]pyrazino[2,1-b][1,3]oxazine-9-carboxamide, N-[(2,4-difl	C20H15D4F2N3O5		C	-8.3
7711	O=C1c2n(C[C@]3(N1[C@H](C)CCO3)[2H])cc(C(=O)NCc1c(F)cc(F)cc1)c(=O)c2O	1407167-49-0	2H-Pyrido[1,Ä≤,2,Ä≤:4,5]pyrazino[2,1-b][1,3]oxazine-4-d-9-carboxamide, N-[(2,4-	C20H17D2F2N3O5		C	-8.3
7712	O=C1c2n(C([C@]3(N1[C@H](C([2H])([2H])[2H])C(C(O3)([2H])[2H])([2H])[2H])[2H])([2H])[2H])cc(C(=O)NCc1c	1407167-50-3	2H-Pyrido[1,Ä≤,2,Ä≤:4,5]pyrazino[2,1-b][1,3]oxazine-9-carboxamide, N-[(2,4-difl	C20H18DF2N3O5		C	-8.3
7713	O=C1c2n(C([C@]3(N1[C@](C)(C(C(O3)([2H])[2H])([2H])[2H])[2H])[2H])([2H])[2H])cc(C(=O)NCc1c(F)cc(F)cc1	1407167-51-4	2H-Pyrido[1,Ä≤,2,Ä≤:4,5]pyrazino[2,1-b][1,3]oxazine-2,3,12-d3-9-carboxamide, N-	C20H9D10F2N3O5		C	-8.3
7714	O=C1c2n(C([C@]3(N1[C@H](C)C(C(O3)([2H])[2H])([2H])[2H])[2H])([2H])[2H])cc(C(=O)NCc1c(F)cc(F)cc1)c(=O	1407167-52-5	2H-Pyrido[1,Ä≤,2,Ä≤:4,5]pyrazino[2,1-b][1,3]oxazine-2,3,4,12-d4-9-carboxamide,	C20H11D8F2N3O5		C	-8.3
7715	O=C1c2n(C([C@]3(N1[C@H](C([2H])([2H])[2H])CCO3)[2H])([2H])[2H])cc(C(=O)NCc1c(F)cc(F)cc1)c(=O)c2O	1407167-54-7	2H-Pyrido[1,Ä≤,2,Ä≤:4,5]pyrazino[2,1-b][1,3]oxazine-4,12-d2-9-carboxamide, N-[(	C20H12D7F2N3O5		C	-8.3
7716	O=C1c2n(C([C@]3(N1[C@](C)(CCO3)[2H])[2H])([2H])[2H])cc(C(=O)NCc1c(F)cc(F)cc1)c(=O)c2O	1407167-55-8	2H-Pyrido[1,Ä≤,2,Ä≤:4,5]pyrazino[2,1-b][1,3]oxazine-12-d-9-carboxamide, N-[(2,4	C20H13D6F2N3O5		C	-8.3
7717	[C@@H](Cc1ccccc1)(NC(=O)[C@H](CCN1CCOCC1)NC(=O)N(Cc1nc(C(C)C)sc1)C)CC[C@H](Cc1ccccc1)NC(=O)OCc1cncs1	1407167-56-9	2H-Pyrido[1,Ä≤,2,Ä≤:4,5]pyrazino[2,1-b][1,3]oxazine-4,12-d2-9-carboxamide, N-[(	C20H15D4F2N3O5		C	-8.3
7718	C(=O)([C@@H](NC(=O)OC)C1CCC(=O)CC1)N1[C@H]2[C@H](C2)C[C@H]1c1[nH]c(cn1)c1ccc(cc1)c1ccc(cc1)c1[nH]c(n	1412812-59-9	Carbamic acid, N,N,Ä≤-[[1,1,Ä≤-biphenyl]-4,4,Ä≤-diylbis[1H-imidazole-5,2-diyl[(	C50H62N8O8	903.08	C	-8.3
7719	[C@H](C(=O)N1[C@@H](CC[C@@H]1C)c1[nH]c(cn1)c1c(C)cc(cc1)c1ccc(cc1)c1[nH]c(nc1)[C@H]1N(C(=O)[C@@H](NC	1412813-87-6	Carbamic acid, N,N,Ä≤-[[1,1,Ä≤-biphenyl]-4,4,Ä≤-diylbis[1H-imidazole-5,2-diyl[(	C48H52F6N8O6	950.97	C	-8.3
7720	[C@H](C(=O)N1[C@@H](CC[C@@H]1C)c1[nH]c(cn1)c1c(F)cc(cc1)c1ccc(cc1)c1[nH]c(nc1)[C@H]1N(C(=O)[C@@H](NC	1412814-65-3	Carbamic acid, N,N,Ä≤-[(3-methyl[1,1,Ä≤-biphenyl]-4,4,Ä≤-diyl)bis[1H-imidazole-	C51H64N8O8	917.1	C	-8.3
7721	C(=O)([C@@H](NC(=O)OC)C(C)C)N1[C@H](CCC1)c1[nH]c(cn1)c1ccc(cc1)c1ccc(cc1)c1[nH]c(nc1)[C@H]1N(C(=O)[C	1417333-60-8	Carbamic acid, N,N,Ä≤-[[1,1,Ä≤-biphenyl]-4,4,Ä≤-diylbis[1H-imidazole-5,2-diyl-(	C40H50N8O6	738.88	C	-8.3

7722	C(COCP1(=O)OCC(OCc2ccccc2)CO1)n1c2c(nc1)c(N)ncn2	1417393-17-9	9H-Purin-6-amine, 9-[2-[[(4S,6S)-2-oxido-4,6-diphenyl-1,3,2-dioxaphosphorinan-2-	C23H24N5O4P	465.44	C	-8.3
7723	N(n1c(SC)nnc(C(C)(C)C)c1=O)c1nc(nc(NC(=S)Nc2cc(Cl)ccc2)n1)N1CCOCC1	1418553-55-5	Urea, N-[4-[[6-(1,1-dimethylethyl)-3-(methylthio)-5-oxo-1,2,4-triazin-4(5H)-yl]a	C23H30N10O4S	542.61	C	-8.3
7724	C(N1c2c(C(=NNC(=S)N(CC=C)CC=C)C1=O)cc(C)cc2)N1CCOCC1	1426583-59-6	Hydrazinecarbothioamide, 2-[5-fluoro-1,2-dihydro-1-(4-morpholinylmethyl)-2-oxo-3	C26H24FN5O2S	489.56	C	-8.3
7725	C(=O)([C@@H](NC(=O)OC)C(C)C)N1[C@@H](CCC1)c1[nH]c(cn1)c1ccc(cc1)c1ccc(C(=O)N)cc1	1426938-64-8	Carbamic acid, N-[(1S)-2-methyl-1-[[(2S)-2-[5-(4,Ä≤-methyl[1,1,Ä≤-biphenyl]-4-y	C27H32N4O3	460.57	C	-8.3
7726	S(=O)(=O)(O)c1c2c(c(S(=O)(=O)O)cc(N=Nc3c(N)cc(N)c(C)c3)c2)ccc1	1431635-33-4	1,5-Naphthalenedisulfonic acid, 3-[2-(2,4-diamino-3-methylphenyl)diazenyl]-	C17H16N4O6S2	436.46	C	-8.3
7727	O=c1n([C@H]2O[C@@H](CO)[C@H](O)C2)cc(C=CBr)c(=O)[nH]1	143224-34-4	Butanediamide, N1-[(1S,2R)-3-[[[(1,1-dimethylethyl)amino]carbonyl](2-methylpropy	C33H44N6O5	604.74	C	-8.3
7728	OC1c2c(C(=C3C1COC3)c1cc(OC)c(OC)c(OC)c1)cc1c(c2)OCO1	1448449-11-3	Guanosine 5,Ä≤-(trihydrogen diphosphate), P,Ä≤-(6-deoxy-Œ±-D-galactopyranosyl)	C16H25N5O15P2	589.34	C	-8.3
7729	N(c1c2c(c(O)c(CN3C(C)CCCC3)c1)CCCC2)c1c2c(ncc1)cc(Cl)cc2	1449239-41-1	1-Naphthalenol, 4-[(7-chloro-4-quinolinyl)amino]-2-[(3,5-dimethyl-1-piperazinyl)	C26H31ClN4O	451	C	-8.3
7730	C[C@]1(F)[C@@H](O[C@H](CO[P@](=O)(OCc2c(C)cccc2)N[C@H](C(=O)OC(C)C)C)[C@H]1O)n1c(=O)[nH]c(=O)cc1	1449738-06-0	1,2-Ethanediamine, 1-(3-cyclopentyltricyclo[3.3.1.13,7]dec-1-yl)-N1-phenyl-	C23H34N2	338.53	C	-8.3
7731	C(=O)([C@@H](NC(=O)OC)C(C)C)N1[C@H](c2[nH]c(c3cc4c(-c5c(cc(cc5)c5[nH]c(nc5)[C@H]5N(C(=O)[C@@H](NC(=O	1449762-05-3	Carbamic acid, N,N,Ä≤-[(7,8-dihydro-6H-dibenzo[f,h][1,5]dioxonin-3,11-diyl)bis[1	C43H54N8O8	810.94	C	-8.3
7732	Cc1c(C23CC4CC(C3)CC(C2)C4)[nH]c(n1)c1ccco1	145031-90-9	1H-Imidazole, 4-methyl-2-phenyl-5-tricyclo[3.3.1.13,7]dec-1-yl-	C20H24N2	292.42	C	-8.3
7733	N(CCNc1c2c(ncc1)cc(Cl)cc2)c1nc(nc(NCCCCO)n1)N1CCOCC1	1454695-07-8	1,3,5-Triazine-2,4-diamine, N2-[4-[(7-chloro-4-quinolinyl)amino]butyl]-6-(4-morp	C26H29ClN8O	505.01	C	-8.3
7734	N(c1nc(nc(Nc2ccc(C#N)cc2)n1)N1CCOCC1)c1c(C)cc(/C=C\C#N)cc1C	1458666-14-2	Benzonitrile, 4-[[4-[[4-[(1E)-2-cyanoethenyl]-2,6-dimethylphenyl]amino]-6-(4-mor	C25H24N8O	452.51	C	-8.3
7735	C([C@@H](OCP(=O)(OP(=O)(O)O)O)CO)n1c2c(nc1)c(N)ncn2	1458666-15-3	Benzonitrile, 4-[[4-[[4-[(1Z)-2-cyanoethenyl]-2,6-dimethylphenyl]amino]-6-(4-mor	C25H24N8O	452.51	C	-8.3
7736	N(C(=O)N)C12CC3(CC(C2)CC(C3)C1)c1ccc(C)cc1	1459693-44-7	Glycine, N-[[[3-(4-methylphenyl)tricyclo[3.3.1.13,7]dec-1-yl]amino]carbonyl]-	C20H26N2O3	342.43	C	-8.3
7737	N(c1c2c(ncc1)cc(Cl)cc2)c1cc(cc(CN2CC(C)CCC2)c1O)c1ccc(Cl)cc1	146298-43-3	[1,1,Ä≤-Biphenyl]-4-ol, 4,Ä≤-chloro-3-[(7-chloro-4-quinolinyl)amino]-5-(1-piper	C27H25Cl2N3O	478.41	C	-8.3
7738	O[C@]12[C@](C)([C@@]3(C)[C@H](CC2)[C@]2(C)[C@@H](CC3)C(C)(C)C(=O)CC2)CC[C@]2(C(=O)O)[C@H]1CC(C)(C)CC	1467662-11-8	Uridine, 2,Ä≤-deoxy-5-iodo-3,Ä≤-O-(2,3,5-tri-O-benzoyl-Œ≤-D-ribofuranosyl)-	C35H31IN2O12	798.53	C	-8.3
7739	N(C(C)C)c1c(nccc1)N1CCN(C(=O)c2cc3c([nH]2)ccc(NS(=O)(=O)CCN2CCCCC2)c3)CC1	147539-88-6	1-Pyrrolidineethanesulfonamide, N-[2-[[4-[3-[(1-methylethyl)amino]-2-pyridinyl]-	C27H37N7O3S	539.69	C	-8.3
7740	N(C(C)C)c1c(nccc1)N1CCN(C(=O)c2cc3c([nH]2)ccc(NS(=O)(=O)N(C)C)c3)CC1	147540-36-1	4-Morpholinepropanesulfonamide, N-[2-[[4-[3-[(1,1-dimethylethyl)amino]-2-pyridir	C29H41N7O4S	583.75	C	-8.3
7741	C(n1c2c(nc1CC)c(C)cc(C)n2)c1ccc(cc1)c1c(S(=O)(=O)NC(=O)OCc2ccccc2)cccc1	148401-85-8	Carbamic acid, [[4,Ä≤-[(2-ethyl-5,7-dimethyl-3H-imidazo[4,5-b]pyridin-3-yl)methy	C25H26N4O4S	478.56	C	-8.3
7742	C(n1c2c(nc1CC)c(C)cc(C)n2)c1ccc(cc1)c1c(S(=O)(=O)NC(=O)NCCC)cccc1	148401-90-5	[1,1,Ä≤-Biphenyl]-2-sulfonamide, N-[(ethylamino)carbonyl]-4,Ä≤-[(2-ethyl-5,7-di	C26H29N5O3S	491.61	C	-8.3
7743	C(n1c2c(nc1CC)c(C)cc(C)n2)c1ccc(cc1)c1c(S(=O)(=O)NC(=O)NCC=C)cccc1	148401-92-7	[1,1,Ä≤-Biphenyl]-2-sulfonamide, N-[[(cyclopropylmethyl)amino]carbonyl]-4,Ä≤-[(	C28H31N5O3S	517.64	C	-8.3
7744	O=c1n(cc([C@H](OC)C)c(=O)[nH]1)[C@@H]1O[C@H](CO)[C@@H](O)C1	1501307-18-1	2H-Indol-2-one, 1,3-dihydro-5-[(4-hydroxy-4-methyl-1-piperidinyl)sulfonyl]-	C14H18N2O4S	310.37	C	-8.3
7745	N(c1c(N(=O)=O)cnc(Nc2c(C)cc(/C=C/C#N)cc2C)n1)c1ccc(C#N)cc1	1504553-90-5	Benzonitrile, 4-[[6-[[4-[(1E)-2-cyanoethenyl]-2,6-dimethylphenyl]amino]-9H-purir	C23H18N8	406.44	C	-8.3
7746	N(c1c2c(n(Cc3ccc(OC)cc3)cn2)nc(Nc2ccc(C#N)cc2)n1)c1c(C)cc(/C=C/C#N)cc1C	1504556-70-0	Benzonitrile, 4-[[2-[[4-[(1E)-2-cyanoethenyl]-2,6-dimethylphenyl]amino]-5-nitro-	C22H17N7O2	411.42	C	-8.3
7747	C(N1c2c(C(=NNC(=S)Nc3ccc(C)cc3)C1=O)cc(Cl)c(Cl)c2)N1CCN(C)CC1	1507380-63-3	Hydrazinecarbothioamide, 2-[4,5-dichloro-1,2-dihydro-1-[(4-methyl-1-piperazinyl)	C22H24Cl2N6OS	491.44	C	-8.3
7748	O=C1N(c2c3c(c4c(n2)cccc4)n(CC(C)C)cn3)c2c1cc(I)cc2	1508303-91-0	7-Azabicyclo[4.2.0]octa-1,3,5-trien-8-one, 3-chloro-7-[1-(2-methylpropyl)-1H-imi	C21H17ClN4O	376.84	C	-8.3
7749	C(C(C)C)n1c2c(c(Nc3c(C(=O)O)ccc(Cl)c3)nc3c2cccc3)nc1	1508303-95-4	Benzoic acid, 5-bromo-2-[[1-(2-methylpropyl)-1H-imidazo[4,5-c]quinolin-4-yl]amir	C21H19BrN4O2	439.31	C	-8.3
7750	[C@H](NC(=O)OC12CC3CC(C2)CC(C1)C3)(C(=O)O)C12CC3CC(C2)CC(C1)C3	150891-70-6	Butanoic acid, 2-[[5,5a,6,8,8a,9-hexahydro-8-oxo-9-(3,4,5-trimethoxyphenyl)furo[	C26H28O9Se	563.46	C	-8.3
7751	O=c1n([C@@H]2O[C@H](CO)[C@@H](O)C2)cc(C#C[Si](C)(C)C)c(=O)[nH]1	151345-35-6	Uridine 5,Ä≤-(tetrahydrogen triphosphate), 5-[(1E)-3-[[[3,Ä≤,6,Ä≤-bis(dimethyla	C37H40N5O18P3	935.66	C	-8.3
7752	O[C@H]1[C@H](n2c3c(nc2)c(=O)nc(N)[nH]3)O[C@H](CO)[C@H]1NS(=O)(=O)NC[C@H]1O[C@H]([C@H](O)[C@@H]1O)n1c	1513874-58-2	Guanosine, 2,Ä≤-deoxy-3,Ä≤-de(phosphinicooxy)guanylyliminosulfonylimino-(3,Ä≤,Ü	C20H26N12O8S	594.56	C	-8.3
7753	C(=O)(N[C@@H](C)C12CC3CC(C2)CC(C1)C3)[C@H]1C2c3c(C([C@H]1C(=O)N[C@H](C(=O)OC)C)c1c2cccc1)cccc3	153543-85-2	L-Alanine, N-[[9,10-dihydro-12-[[(1-tricyclo[3.3.1.13,7]dec-1-ylethyl)amino]carb	C34H40N2O4	540.69	C	-8.3
7754	O=c1n([C@H]2O[C@@H](CO[Si](C(C)(C)C)(C)C)CC2)ccc(N)n1	153543-87-4	L-Alanine, N-[[9,10-dihydro-12-[[(1-tricyclo[3.3.1.13,7]dec-1-ylethyl)amino]carb	C34H40N2O4	540.69	C	-8.3
7755	O(C(OC(=O)C1CC1)C)c1c(C(=O)NCc2ccc(F)cc2)nc(C(NC(=O)c2nnc(C)o2)(C)C)n(C)c1=O	1538604-20-4	Propanoic acid, 2-methyl-, 1-[[4-[[[(4-fluorophenyl)methyl]amino]carbonyl]-1,6-c	C26H31FN6O7	558.56	C	-8.3
7756	C(n1c2c(nc1CC)c(C)cc(C)n2)c1ccc(cc1)c1c(S(=O)(=O)NC(=O)CCc2ccccc2)ccc(CN2CCOCC2)c1	154552-66-6	Benzenepropanamide, N-[[5-[(diethylamino)methyl]-4,Ä≤-[(2-ethyl-5,7-dimethyl-3H-	C37H43N5O3S	637.83	C	-8.3
7757	C(n1c2c(nc1CC)c(C)cc(C)n2)c1ccc(cc1)c1c(S(=O)(=O)NC(=O)c2c(OCC)cccc2)ccc(OC)c1	154552-97-3	Benzamide, 2-ethoxy-N-[[4,Ä≤-[(2-ethyl-5,7-dimethyl-3H-imidazo[4,5-b]pyridin-3-y	C36H40N4O4S	624.79	C	-8.3
7758	C(n1c2c(nc1CC)c(C)cc(C)n2)c1ccc(cc1)c1c(S(=O)(=O)NC(=O)c2c(OCC)cccc2)ccc(SCC)c1	154553-09-0	Benzamide, 2-ethoxy-N-[[4,Ä≤-[(2-ethyl-5,7-dimethyl-3H-imidazo[4,5-b]pyridin-3-y	C35H38N4O5S	626.77	C	-8.3
7759	C(n1c2c(nc1CC)c(C)cc(C)n2)c1ccc(cc1)c1c(S(=O)(=O)NC(=O)c2ccccc2)ccc(CN(C)C)c1	154553-16-9	Benzamide, N-[[5-butoxy-4,Ä≤-[(2-ethyl-5,7-dimethyl-3H-imidazo[4,5-b]pyridin-3-y	C34H36N4O4S	596.74	C	-8.3
7760	C(n1c2c(nc1CC)c(C)cc(C)n2)c1ccc(cc1)c1c(S(=O)(=O)NC(=O)c2ccccc2)ccc(CSC)c1	154553-18-1	Benzamide, N-[[4,Ä≤-[(2-ethyl-5,7-dimethyl-3H-imidazo[4,5-b]pyridin-3-yl)methyl]	C31H30N4O4S	554.66	C	-8.3
7761	C(n1c2c(nc1CC)c(C)cc(C)n2)c1ccc(cc1)c1c(S(=O)(=O)NC(=O)NCCc2ccccc2)ccc(CC(C)C)c1	154553-55-6	[1,1,Ä≤-Biphenyl]-2-sulfonamide, 5-butoxy-4,Ä≤-[(2-ethyl-5,7-dimethyl-3H-imidaz	C36H41N5O4S	639.81	C	-8.3
7762	O(C(=O)Nc1ccc(S(=O)(=O)N(C[C@H]([C@H](Cc2ccccc2)N)O)CC(C)C)cc1)[C@H]1[C@@H]2[C@H](OC1)OCC2	1546918-97-1	Carbamic acid, N-[4-[[[(2R,3S)-3-[[[[(3R,3aS,6aR)-hexahydrofuro[2,3-b]furan-3-yl	C34H45N3O11S	703.8	C	-8.3
7763	C(OCCO)n1c2c(c(=O)nc(N[C@@H]3O[C@H](CO)[C@@H](O)[C@H](O)[C@H]3O)[nH]2)nc1	1555813-42-7	Carbamic acid, N-[(1S)-1-[[(2S)-2-[5-[4,Ä≤-[2-[(2S,4S)-4-hydroxy-1-[(2S)-2-[(met	C40H50N8O7	754.87	C	-8.3
7764	CN1C2c3c(C1CC2)c(ccc3c1[nH]c(nc1)[C@H]1N(C(=O)[C@@H](NC(=O)OC)C2CCCCC2)CCC1)c1ccc(cc1)c1[nH]c(nc1)[C	1560639-47-5	Carbamic acid, N-[(1S)-2-methyl-1-[[(2S,4S)-4-methyl-2-[5-[4-[1,2,3,4-tetrahydro	C47H61N9O6	848.04	C	-8.3
7765	C(=O)([C@@H](NC(=O)OC)C(C)C)N1[C@H](c2[nH]c(c3c4c(c(cc3)c3ccc(cc3)c3[nH]c(nc3)[C@H]3N(C(=O)[C@@H](NC	1561047-25-3	Carbamic acid, N-[(1S)-2-methyl-1-[[(2S,4S)-4-methyl-2-[5-[4-[1,2,3,4-tetrahydro	C48H62N8O6	847.06	C	-8.3
7766	N(c1c2c(c(O)c(CN3CCN(C(C)C)CC3)c1)CCCC2)c1c2c(ncc1)cc(Cl)cc2	156893-84-4	1-Naphthalenol, 4-[(7-chloro-4-quinolinyl)amino]-5,6,7,8-tetrahydro-2-[(4-propyl	C27H33ClN4O	465.03	C	-8.3
7767	N(c1c2c(c(O)c(CN3CCN(CCCC)CC3)c1)CCCC2)c1c2c(ncc1)cc(Cl)cc2	156893-85-5	1-Naphthalenol, 4-[(7-chloro-4-quinolinyl)amino]-5,6,7,8-tetrahydro-2-[[4-(1-met	C27H33ClN4O	465.03	C	-8.3
7768	N(c1c2c(c(O)c(CN3CCN(C(CC)C)CC3)c1)CCCC2)c1c2c(ncc1)cc(Cl)cc2	156893-87-7	1-Naphthalenol, 4-[(7-chloro-4-quinolinyl)amino]-5,6,7,8-tetrahydro-2-[[4-(2-met	C28H35ClN4O	479.06	C	-8.3
7769	C(=O)([C@@H](NC(=O)OC)C(C)C)N1CC2(C[C@H]1c1[nH]c(cn1)c1ccc(cc1)c1ccc(cc1)c1[nH]c(nc1)[C@H]1N(C(=O)[C	1571138-40-3	Carbamic acid, N-[(1S)-1-[[(2S)-2-[5-[4,Ä≤-[2-[(7R)-6-[(2S)-2-[(methoxycarbonyl)	C42H53N9O6	779.93	C	-8.3
7770	S(=O)(=O)(O)c1c2c(cc(S(=O)(=O)O)c1)ccc(N=Nc1cc(N)cc1)c1ccccc1)c2	157392-84-2	Thymidine 5,Ä≤-(trihydrogen diphosphate), P-methyl P,Ä≤,P,Ä≤-diphenyl ester	C23H26N2O11P2	568.41	C	-8.3
7771	O=c1n([C@@H]2O[C@H](COP(=O)(OC[C@@H]3[C@@H](N=[N+]=[N-])C[C@@H](O3)n3c(=O)[nH]c(=O)c(C)c3)COC)[C@@H]	1579273-86-1	Thymidine, 3,Ä≤-azido-3,Ä≤-deoxy-, 5,Ä≤,5,Ä≤,Ä≤,Ä≤-(P-phenylphosphonate)	C26H29N10O9P		C	-8.3
7772	O=C1[C@@H]2[C@@H](c3c([C@H](OCc4cn(nn4)c4cc(Cl)c(F)cc4)[C@H]2CO1)cc1c(c3)OCO1)c1cc(OC)c(OC)c(OC)c1	1600500-27-3	Furo[3,Ä≤,4,Ä≤:6,7]naphtho[2,3-d]-1,3-dioxol-6(5aH)-one, 9-[[1-(4-chlorophenyl)	C31H28ClN3O8	606.02	C	-8.3
7773	[C@H](C(=O)N1[C@@H](CC[C@@H]1C)c1[nH]c(cn1)c1ccc(cc1)c1ccc(cc1)c1[nH]c(nc1)[C@H]1N(C(=O)[C@@H](NC(=O	1608494-93-4	Carbamic acid, N,N,Ä≤-[[1,1,Ä≤-biphenyl]-4,4,Ä≤-diylbis[1H-imidazole-5,2-diyl[(	C54H70N8O8	959.18	C	-8.3
7774	C[C@@]12[C@@H]3[C@H]([C@H]4[C@](C)(CC3)[C@@H]([C@@H](CCCC(C)C)C)CC4)C(=O)C=C1[C@H](O)[C@H](CC2)N1CCC	1609273-15-5	5,Ä≤-Adenylic acid, anhydride with P-(5,Ä≤-guanylylmethyl)phosphonic acid (1:1)	C21H29N10O16P3	770.43	C	-8.3
7775	C(OCCOP(=O)(OCOC(=O)OC(CC)C)OCOC(=O)OC(CC)C)n1c2c(nc1)c(=O)nc(N)[nH]2	1610585-44-8	2,4,6,8-Tetraoxa-5-phosphanonanedioic acid, 5-[2-[(2-amino-1,6-dihydro-6-oxo-9H-	C18H28N5O12P	537.41	C	-8.3
7776	O=C1[C@@H]2[C@@H](c3c([C@@H](O)[C@H]2CO1)cc1c(c3)OCO1)c1cc2c(cc1)OCO2	1610886-41-3	Furo[3,Ä≤,4,Ä≤:6,7]naphtho[2,3-d]-1,3-dioxol-6(5aH)-one, 5-(1,3-benzodioxol-5-y	C20H16O7	368.34	C	-8.3
7777	C(=O)([C@@H](NC(=O)OC)C(C)C)N1[C@@H](CCC1)c1[nH]c(cn1)c1ccc(cc1)c1ccc(cc1)c1[nH]c(nc1)[C@H]1N(C(=O)[	1616889-56-5	1-Piperazinecarboxylic acid, 4-[(1S)-1-[5-[4,Ä≤-[2-[(2S)-1-[(2S)-2-[(methoxycarb	C40H50N8O6	738.88	C	-8.3
7778	C(=O)([C@@H](NC(=O)OC)C(C)C)N1C(CN(C(=O)C)CCC1)c1[nH]c(cn1)c1ccc(cc1)c1ccc(cc1)c1[nH]c(nc1)[C@H]1N(C	1616889-58-7	Carbamic acid, N-[(1S,2R)-2-hydroxy-1-[[(2S)-2-[5-[4,Ä≤-[2-[(2S)-1-[(2S)-2-[(met	C39H48N8O7	740.85	C	-8.3
7779	N(C(=O)[C@H](Cc1cccnc1)C[C@@H](CN1[C@H](C(=O)NC(C)(C)C)CN(Cc2cc3c(s2)ccs3)CC1)O)[C@H]1c2c(C[C@H]1O)c	162105-29-5	1-Piperazinepentanamide, 4-[(4-bromo-2-thienyl)methyl]-N-(2,3-dihydro-2-hydroxy-	C34H44BrN5O4S	698.71	C	-8.3
7780	[C@H](C(=O)N[C@H](Cc1ccccc1)C(=O)O)(N)C12CC3(O)CC(C2)CC(C1)C3	1623021-24-8	1(2H)-Pyrimidineacetamide, N-[(1S,3S,4S)-4-[[2-(2,6-dimethylphenoxy)acetyl]aminc	C37H48N4O5	628.8	C	-8.3
7781	[C@@H](C(=O)N[C@H](Cc1ccccc1)C(=O)OC)(NC(=O)OC(C)(C)C)C12CC3(O)CC(C2)CC(C1)C3	1623104-69-7	L-Phenylalanine, (2S)-N-[(1,1-dimethylethoxy)carbonyl]-2-(3-hydroxytricyclo[3.3.	C26H36N2O6	472.57	C	-8.3
7782	[C@H](C(=O)N[C@@H](Cc1ccccc1)C#N)(NC(=O)OC(C)(C)C)C12CC3(O)CC(C2)CC(C1)C3	1623104-76-6	L-Phenylalaninamide, (2S)-N-[(1,1-dimethylethoxy)carbonyl]-2-(3-hydroxytricyclo[	C26H37N3O5	471.59	C	-8.3
7783	N(c1nc(nc(Nc2c(O)cccc2)n1)N1CCOCC1)c1nc(OC)cc(OC)n1	1631751-08-0	Phenol, 2-[[4-[(4,6-dimethoxy-2-pyrimidinyl)amino]-6-(4-morpholinyl)-1,3,5-triaz	C19H21N9O6	471.43	C	-8.3
7784	O=C1[C@@H]2[C@@H](c3c([C@H](OC(=O)c4c(OC)c(OC)ccc4)[C@H]2CO1)cc1c(c3)OCO1)c1cc(OC)c(OC)c(OC)c1	1633020-35-5	Benzeneacetic acid, Œ±-[(3,4-dimethoxyphenyl)methylene]-4-methoxy-, (5R,5aR,8aR,	C40H38O12	710.72	C	-8.3
7785	O=C1[C@@H]2[C@@H](c3c([C@H](OC(=O)/C(=C/c4cc(OC)c(OCc5ccccc5)cc4)/c4cc(OC)c(OC)cc4)[C@H]2CO1)cc1c(c3	1633020-39-9	2-Propynoic acid, 3-phenyl-, (5R,5aR,8aR,9R)-5,5a,6,8,8a,9-hexahydro-8-oxo-9-(3,	C31H26O9	542.53	C	-8.3
7786	O=C1[C@@H]2[C@@H](c3c([C@H](OC(=O)/C(=C/c4cc(OC)c(OC)cc4)/c4cc(OC)c(OC)cc4)[C@H]2CO1)cc1c(c3)OCO1)c1	1633020-45-7	Benzeneacetic acid, 4-methoxy-Œ±-[(3,4,5-trimethoxyphenyl)methylene]-, (5R,5aR,8	C41H40O13	740.75	C	-8.3
7787	O(P(=O)(OC[C@H]1O[C@H]([C@H](OC(=O)c2ccccc2)C1)n1c2c(nc1)c(NC(=O)c1ccccc1)ncn2)OCCc1ccc(N(=O)=O)cc1)	1637769-10-8	Adenosine, 1,Ä≤-[3-(aminocarbonyl)-1H-1,2,4-triazol-1-yl]-3,Ä≤-O-benzoyl-1,Ä≤-d	C67H59N10O16P	1291.22	C	-8.3
7788	C(OC[C@@H]1[C@@H](OC(=O)c2ccccc2)[C@@H](OP(=O)(OC[C@@H]2[C@@H](OC(=O)c3ccccc3)[C@@H](OP(=O)(OC[C@H]3	1637769-13-1	Adenosine, 1,Ä≤-[3-(aminocarbonyl)-1H-1,2,4-triazol-1-yl]-3,Ä≤-O-benzoyl-1,Ä≤-d	C47H43N10O15P	1018.88	C	-8.3
7789	O[C@H]1[C@H](c2c3c([nH]c2)c(N)ncn3)N[C@H](COC(=O)C[C@@H](C(=O)OCCCCOC(=O)[C@@H]2[C@@H](O)[C@@H]([C@H	1638370-27-0	L-Aspartic acid, 1-[3-[[[(1S,2S,3R,4R)-3-[(1S)-1-(acetylamino)-2-ethylbutyl]-4-[	C33H52N10O10	748.83	C	-8.3
7790	C(=O)(O)[C@]12[C@@H]([C@@H]3[C@@](C)(CC1)[C@@]1(C)[C@H](CC3)[C@]3(C)[C@@H](CC1)C(C)(C)NC(=O)CC3)[C@H	1638513-48-0	Not Yet Assigned	C23H19N5OS	413.49	C	-8.3
7791	C(=O)([C@@H](NC(=O)OC)C(C)C)N1[C@@H](CCC1)c1[nH]c(cn1)c1ccc(cc1)c1ccc(cc1)c1[nH]c(nc1)[C@H]1N(C(=O)[	1639870-50-0	Carbamic acid, N-[(1S)-1-[[(2S)-2-[5-[4,Ä≤-[2-[(2S)-1-[[1-[(methoxycarbonyl)amir	C39H46N8O6	722.83	C	-8.3
7792	C(=O)([C@@H](NC(=O)OC)C1CCCC1)N1[C@@H](C=CC1)c1[nH]c(cn1)c1ccc(cc1)c1ccc(cc1)c1[nH]c(nc1)[C@H]1N(C(	1639871-22-9	Carbamic acid, N-[(1S)-1-cyclopentyl-2-[(2S)-2,5-dihydro-2-[5-[4,Ä≤-[2-[(2S)-1-	C42H50N8O6	762.9	C	-8.3
7793	C(=O)([C@@H](NC(=O)OC)C1CCCCC1)N1[C@@H](CCC1)c1[nH]c(cn1)c1ccc(cc1)c1ccc(cc1)c1[nH]c(nc1)[C@H]1N(C(=O	1639871-27-4	Carbamic acid, N-[(1S)-1-cyclohexyl-2-[(2S)-2-[5-[4,Ä≤-[2-[(2S)-1-[(2S)-2-cyclop	C45H54N8O6	802.96	C	-8.3
7794	N(c1nc(nc(Sc2oc(nn2)c2ccc(F)cc2)n1)N1CCOCC1)c1cc(Cl)ccc1	1642584-25-5	Ethanone, 1-[4-[[4-[[5-(4-fluorophenyl)-1,3,4-oxadiazol-2-yl]thio]-6-(4-morpholi	C23H20FN7O3S	493.51	C	-8.3
7795	C(=O)([C@@H](NC(=O)OC)C(C)C)N1[C@H](c2[nH]c(c3cc4c(-c5c(C4)cc(cc5)c4[nH]c(nc4)[C@H]4N(C(=O)[C@@H](NC	1645563-55-8	Carbamic acid, N,N,Ä≤-[(9,10-dihydro-2,7-phenanthrenediyl)bis[1H-imidazole-5,2-(	C44H56N8O6	792.97	C	-8.3
7796	C(c1c2c(c(C)nc1)OC(C)(C)OC2)N1C[C@@H](C(=O)NC(C)(C)C)N(C[C@H](C[C@H](C(=O)N[C@H]2c3c(C[C@H]2O)cccc3)	164725-17-1	1-Piperazinepentanamide, N-(2,3-dihydro-2-hydroxy-1H-inden-1-yl)-2-[[(1,1-dimeth	C40H54N6O5	698.89	C	-8.3
7797	N(C(=O)[C@H](Cc1ccccc1)C[C@@H](CN1[C@H](C(=O)NC(C)(C)C)CN(Cc2ccncc2)CC1)O)[C@H]1c2c(C[C@H]1O)cccc2	164725-24-0	1-Piperazinepentanamide, N-(2,3-dihydro-2-hydroxy-1H-inden-1-yl)-2-[[(1,1-dimeth	C36H53N5O5	635.84	C	-8.3
7798	N(C(=O)[C@H](Cc1ccccc1)C[C@@H](CN1[C@H](C(=O)NC(C)(C)C)CN(Cc2c(C)nc(C)cc2)CC1)O)[C@H]1c2c(C[C@H]1O)c	164725-37-5	1-Piperazinepentanamide, N-(2,3-dihydro-2-hydroxy-1H-inden-1-yl)-2-[[(1,1-dimeth	C38H51N5O4	641.84	C	-8.3
7799	N(C(=O)C(Cc1ccccc1)CC(CN1C(C(=O)NC(C)(C)C)CN(C(CO)c2ccccc2)CC1)O)C1c2c(CC1O)cccc2	164725-49-9	1-Piperazineacetic acid, 4-[5-[(2,3-dihydro-2-hydroxy-1H-inden-1-yl)amino]-2-hyc	C39H50N4O6	670.84	C	-8.3
7800	N(C(=O)[C@H](Cc1ccccc1)C[C@@H](CN1[C@H](C(=O)NC(C)(C)C)CN(CC2CCCCC2)CC1)O)[C@H]1c2c(C[C@H]1O)cccc2	164725-65-9	1-Piperazinepentanamide, N-(2,3-dihydro-2-hydroxy-1H-inden-1-yl)-4-[(2,3-dihydrc	C40H52N4O5	668.86	C	-8.3
7801	S(=O)(=O)(O)c1c2c(c(N)c(N=Nc3ccc(cc3)c3ccc(N)cc3)c1)cccc2	1649477-45-1	1-Naphthalenesulfonic acid, 4-amino-3-[2-[4,Ä≤-[2-(1-nitro-4-sulfo-2-naphthaleny	C32H22N6O8S2	682.68	C	-8.3
7802	C(=O)([C@@H](NC(=O)OC)C(C)C)N1C[C@@]2(C[C@H]1c1[nH]c(cn1)c1ccc(cc1)c1cc3c(-c4c(CCC3)nc([nH]4)[C@H]3N	1653967-01-1	Carbamic acid, N-[(1S)-1-[[(2S,3aS,6aS)-hexahydro-2-[5-[4,Ä≤-[2-[(3S,5S)-2-[(2S)	C47H60N8O7	849.03	C	-8.3
7803	N(C(=O)[C@H](Cc1ccccc1)C[C@@H](CN1[C@H](C(=O)NC(C)(C)C)CN(Cc2scc(Br)c2)CC1)O)[C@H]1c2c(C[C@H]1O)cccc	165946-08-7	1-Piperazinepentanamide, 4-[(2,4-difluorophenyl)methyl]-N-(2,3-dihydro-2-hydroxy	C37H46F2N4O4	648.78	C	-8.3
7804	O=c1n([C@@H]2O[C@H](COP(=O)(C(O)c3ccc(C#N)cc3)OC[C@@H]3[C@@H](N=[N+]=[N-])C[C@@H](O3)n3c(=O)[nH]c(=O	166318-77-0	Thymidine, 3,Ä≤-azido-P-[(4-chlorophenyl)hydroxymethyl]-P,3,Ä≤-dideoxythymidyly	C27H30ClN10O10P		C	-8.3
7805	N(N)C12CC3CC(C2)CC(C1)C3	167773-40-2	Benzamide-carbonyl-11C, N-[[4,Ä≤-[(2-ethyl-5,7-dimethyl-3H-imidazo[4,5-b]pyridir	C30H28N4O3S		C	-8.3
7806	O=C1[C@@H]2[C@@H](c3c([C@H](OC(=O)N4CCC(CCOc5cc(ccc5)c5c6c(sc(C(=O)NCC)c6)nc(N)n5)CC4)[C@H]2CO1)cc1c	167978-37-2	2,7-Naphthalenedisulfonic acid, 5-amino-3-[2-[4,Ä≤-[2-(2,4-diaminophenyl)diazeny	C30H27N7O7S2.2Na		C	-8.3
7807	N(=Nc1ccccc1)c1n(c2c(n1)c(=O)nc(N)[nH]2)[C@@H]1O[C@H](CO)[C@@H](O)[C@H]1O	168701-74-4	Inosine, 2,Ä≤-deoxy-2-(3-phenyl-1-triazenyl)-	C16H17N7O4	371.35	C	-8.3
7808	C(n1c2c(nc1CC)c(C)cc(C)n2)c1ccc(cc1)c1c(S(=O)(=O)NC(=O)OCCCC)ccc(CC)c1	169281-51-0	[1,1,Ä≤-Biphenyl]-2-sulfonamide, 5-ethyl-4,Ä≤-[(2-ethyl-5,7-dimethyl-3H-imidazo	C25H28N4O2S	448.58	C	-8.3
7809	C(n1c2c(nc1CC)c(C)cc(C)n2)c1ccc(cc1)c1c(S(=O)(=O)NC(=O)OCCCC)ccc(C(C)C)c1	169281-57-6	Carbamic acid, [[4,Ä≤-[(2-ethyl-5,7-dimethyl-3H-imidazo[4,5-b]pyridin-3-yl)methy	C33H41N5O4S	603.77	C	-8.3
7810	[C@@H](Cc1ccccc1)(NC(=O)N1CCOCC1)C(=O)N[C@@H](CCc1ccccc1)/C=C/C=C/S(=O)(=O)c1ccccc1	170111-57-6	4-Morpholinecarboxamide, N-[2-[[3-(2-naphthalenylsulfonyl)-1-(2-phenylethyl)-2-p	C35H37N3O5S	611.75	C	-8.3
7811	[C@H](Cc1ccccc1)(C(=O)N[C@H](/C=C/S(=O)(=O)c1ccccc1)CCCCN)NC(=O)N1CCOCC1	170111-94-1	4-Morpholinecarboxamide, N-[2-oxo-2-[[1-(2-phenylethyl)-5-(phenylsulfonyl)-2,4-p	C33H37N3O5S	587.73	C	-8.3
7812	N(C(=O)[C@H](Cc1ccccc1)C[C@@H](CN1[C@H](C(=O)NC(C)(C)C)CN(Cc2cccnc2)CC1)O)[C@H]1c2c(C[C@H]1OC(=O)CCC	170227-69-7	1-Piperazinepentanamide, N-[2-(benzoyloxy)-2,3-dihydro-1H-inden-1-yl]-2-[[(1,1-c	C43H51N5O5	717.9	C	-8.3
7813	N(C(CCC1)CC)c1c2c(ncc1)cc(Cl)cc2	170969-73-0	Carbamic acid, [[4,Ä≤-[(2-ethyl-5,7-dimethyl-3H-imidazo[4,5-b]pyridin-3-yl)methy	C36H40N4O5S	640.79	C	-8.3
7814	C[C@@]12[C@@H]3[C@H]([C@H]4[C@](C)(CC3)[C@@H](C(CCCC#C)C)CC4)[C@H](O)C[C@@H]1CC(OC)(OC)CC2	1712964-47-0	2H-Indol-2-one, 1,3-dihydro-5-[(4-hydroxy-3-methyl-1-piperidinyl)sulfonyl]-	C14H18N2O4S	310.37	C	-8.3
7815	O=C1[C@@H]2[C@@H](c3c([C@H](OC(=O)c4cccs4)[C@H]2CO1)cc1c(c3)OCO1)c1cc(OC)c(OC)c(OC)c1	1715004-92-4	1H-Indole-3-acetic acid, 2-methyl-, (5R,5aR,8aR,9R)-5,5a,6,8,8a,9-hexahydro-8-ox	C33H31NO9	585.6	C	-8.3
7816	N(C(=O)[C@H](Cc1ccccc1)C[C@@H](CN1[C@H](C(=O)NC(C)(C)C)CN(Cc2nc(C(C)C)cs2)CC1)O)[C@H]1c2c(C[C@H]1O)c	172649-46-6	1-Piperazinepentanamide, N-(2,3-dihydro-2-hydroxy-1H-inden-1-yl)-2-[[(1,1-dimeth	C37H51N5O4S	661.9	C	-8.3
7817	N(C(=O)[C@H](Cc1ccccc1)C[C@@H](CN1[C@H](C(=O)NC(C)(C)C)CN(Cc2cnco2)CC1)O)[C@H]1c2c(C[C@H]1O)cccc2	172649-47-7	1-Piperazinepentanamide, N-(2,3-dihydro-2-hydroxy-1H-inden-1-yl)-2-[[(1,1-dimeth	C37H51N5O4S	661.9	C	-8.3
7818	O=c1n([C@@H]2O[C@H](COP(=O)(OC[C@@H]3[C@@H](N=[N+]=[N-])C[C@@H](O3)n3c(=O)[nH]c(=O)c(C)c3)OCc3ccc(C#	173606-88-7	Thymidine, 3,Ä≤-azido-P-[(4-chlorophenyl)methyl]-3,Ä≤-deoxythymidylyl-(5,Ä≤,Ü5	C27H30ClN10O10P		C	-8.3
7819	O=c1n([C@@H]2O[C@H](COP(=O)([C@@H](O)c3ccc(C)cc3)OC[C@@H]3[C@@H](N=[N+]=[N-])C[C@@H](O3)n3c(=O)[nH]c	173658-20-3	Thymidine, 3,Ä≤-azido-P,3,Ä≤-dideoxy-P-[hydroxy(4-methoxyphenyl)methyl]thymidyl	C28H33N10O11P		C	-8.3
7820	O=c1n([C@@H]2O[C@H](COP(=O)([C@@H](O)c3ccc(C#N)cc3)OC[C@@H]3[C@@H](N=[N+]=[N-])C[C@@H](O3)n3c(=O)[nH	173658-23-6	Thymidine, 3,Ä≤-azido-P-[(4-chlorophenyl)hydroxymethyl]-P,3,Ä≤-dideoxythymidyly	C27H30ClN10O10P		C	-8.3
7821	O=c1n([C@@H]2O[C@H](COP(=O)([C@H](O)c3ccc(C#N)cc3)OC[C@@H]3[C@@H](N=[N+]=[N-])C[C@@H](O3)n3c(=O)[nH]	173658-33-8	Thymidine, 3,Ä≤-azido-P-[(4-chlorophenyl)hydroxymethyl]-P,3,Ä≤-dideoxythymidyly	C27H30ClN10O10P		C	-8.3
7822	O=c1n([C@@H]2O[C@H](COP(=O)([C@H](O)c3cc(N(=O)=O)ccc3)OC[C@@H]3[C@@H](N=[N+]=[N-])C[C@@H](O3)n3c(=O)	173658-35-0	Thymidine, 3,Ä≤-azido-P,3,Ä≤-dideoxy-P-[hydroxy(2-nitrophenyl)methyl]thymidylyl	C27H30N11O12P		C	-8.3
7823	[C@@H](C(C)(C)C)(C1=C(O)C[C@@](CCc2ccccc2)(CCC)OC1=O)c1cc(NS(=O)(=O)c2ccccn2)ccc1	174484-22-1	2-Pyridinesulfonamide, N-[3-[1-[5,6-dihydro-4-hydroxy-2-oxo-6-(2-phenylethyl)-6-	C32H38N2O5S	562.72	C	-8.3
7824	[C@@H](C(C)(C)C)(C1=C(O)CC(CCC)(CCC)OC1=O)c1cc(NS(=O)(=O)c2cc(C(F)(F)F)ccn2)ccc1	174484-35-6	2-Pyridinesulfonamide, N-[3-[1-(5,6-dihydro-4-hydroxy-2-oxo-6,6-dipropyl-2H-pyra	C28H35F3N2O5S	568.65	C	-8.3
7825	C(Cc1ccccc1)C1(CCc2ccccc2)CC(=C(C(CC)c2cc(NS(=O)(=O)c3ccc(C#N)cn3)ccc2)C(=O)O1)O	174484-43-6	2-Pyridinesulfonamide, 5-amino-N-[3-[1-[5,6-dihydro-4-hydroxy-2-oxo-6-(2-phenyle	C32H39N3O5S	577.73	C	-8.3
7826	C(Cc1ccc(F)cc1)C1(CCC)CC(=C(C(CC)c2cc(NS(=O)(=O)c3ccc(C(=O)N)cn3)ccc2)C(=O)O1)O	174484-47-0	2-Pyridinesulfonamide, 5-cyano-N-[3-[1-[5,6-dihydro-4-hydroxy-2-oxo-6,6-bis(2-ph	C36H35N3O5S	621.75	C	-8.3
7827	C(Cc1ccccc1)[C@@]1(CCC(F)(F)F)CC(=C([C@@H](C(C)(C)C)c2cc(NS(=O)(=O)c3ccc(C#N)cn3)ccc2)C(=O)O1)O	174590-14-8	2-Pyridinesulfonamide, 5-cyano-N-[3-[1-[5,6-dihydro-4-hydroxy-2-oxo-6-(2-phenyle	C33H34F3N3O5S	641.7	C	-8.3
7828	C(Cc1ccccc1)[C@@]1(CCC(F)(F)F)CC(=C([C@@H](C(C)(C)C)c2cc(NS(=O)(=O)c3ccc(N)cn3)ccc2)C(=O)O1)O	174590-18-2	2-Pyridinesulfonamide, 5-amino-N-[3-[1-[5,6-dihydro-4-hydroxy-2-oxo-6-(2-phenyle	C32H36F3N3O5S	631.71	C	-8.3
7829	O=c1c2c(n(cn2)[C@@H]2O[C@H](COP(=O)(OP(=O)(OC[C@@H]3[C@@H](N=[N+]=[N-])C[C@@H](O3)n3c(=O)[nH]c(=O)c(	175923-16-7	Inosine 5,Ä≤-(trihydrogen diphosphate), 2,Ä≤,3,Ä≤-dideoxy-, P,Ä≤,Ü5,Ä≤-ester w	C19H23FN6O13P2	624.36	C	-8.3
7830	O=C1C2(C(C(C(=O)/C=C(\O)/C3C4(N5C(C3c3cc(OC)c(O)cc3)SCC5)c3c(NC4=O)ccc(N(=O)=O)c3)C3N2CSC3)c2cc(OC)c	1779528-84-5	Not Yet Assigned	C43H38Cl2N4O8S2	873.82	C	-8.3
7831	C(OP(=O)(OP(=O)(OP(=O)(O)O)O)O)[C@H]1C2([C@H]([C@](C)(F)[C@@H]1O)n1c3c(nc1)c(=O)nc(N)[nH]3)CC2	1782095-18-4	Triphosphoric acid, P-[[(4R,5R,6S,7R)-7-(2-amino-1,6-dihydro-6-oxo-9H-purin-9-yl	C14H22N5O13P3	561.27	C	-8.3
7832	C(CN1CCC(CN2c3c4c(S2(=O)=O)cccc4ccc3)CC1)(C12CC3CC(C2)CC(C1)C3)N1CCCC1	178477-46-8	1-Piperidineethanamine, 4-[(1,1-dioxido-2H-naphth[1,8-cd]isothiazol-2-yl)methyl]	C29H39N3O2S	493.7	C	-8.3
7833	C([C@@H](OCP(=O)(O)O)C([2H])([2H])[2H])(n1c2c(nc1)c(N)ncn2)([2H])[2H]	1795707-69-5	4-Piperidinecarboxamide, 1-[(2,3-dihydro-2-oxo-1H-indol-5-yl)sulfonyl]-	C14H17N3O4S	323.37	C	-8.3
7834	O(C(=O)N[C@@H](Cc1cc(ccc1)c1cc(OC)cc(OC)c1)[C@@H](CN(S(=O)(=O)c1ccc(OC)cc1)CC(C)C)O)[C@@H]1[C@H]2[C@	1800006-51-2	Carbamic acid, N-[(1S,2R)-1-[(3,Ä≤,5,Ä≤-dimethyl[1,1,Ä≤-biphenyl]-3-yl)methyl]-	C34H44N2O7S	624.79	C	-8.3
7835	C(=O)(CCC1CCCC1)n1c(ncc1c1ccc(cc1)c1ccc(cc1)c1n(C(=O)CCC2CCCC2)c(nc1)[C@H]1N(C(=O)[C@@H](NC(=O)OC)C(	1800502-85-5	Carbamic acid, N-[(1S)-1-[[(2S)-2-[1-(2-cyclohexylacetyl)-5-[4,Ä≤-[1-(2-cyclohex	C56H74N8O8	987.24	C	-8.3
7836	S(=O)(=O)(O)c1c2c(c(N)c(N=Nc3ccc(N(=Nc4ccc(S(=O)(=O)O)cc4)=O)cc3)c1)cccc2	1800609-69-1	1,3-Naphthalenedisulfonic acid, 7-amino-8-[2-(4-sulfo-1-naphthalenyl)diazenyl]-	C20H15N3O9S3	537.54	C	-8.3
7837	O(C(C)(C)C)c1c(C(=O)NCc2ccc(F)cc2)nc(C(NC(=O)c2nnc(C)o2)(C)C)n(C)c1=O	1801912-40-2	Carbonic acid, 4-[[[(4-fluorophenyl)methyl]amino]carbonyl]-1,6-dihydro-1-methyl-	C28H27FN6O7	578.55	C	-8.3
7838	C[C@]1(Cl)[C@@H](O[C@H](COP(=O)(Oc2ccccc2)N[C@@H](C(=O)OC(C)C)C)[C@H]1O)n1c(=O)[nH]c(=O)cc1	1802921-99-8	Carbamic acid, N-[(1S)-1-[[(2S,4S)-4-(methoxymethyl)-2-[5-[4-[1,2,3,4-tetrahydro	C48H62N8O7	863.06	C	-8.3

7839	C(F)(F)[C@@]12[C@H]([C@@H](F)[C@@H](O1)n1c3c(nc1)c(=O)nc(N)[nH]3)OP(=S)(OC(C)C)OC2	1803128-92-8	Guanosine, 2,Ä≤-deoxy-4,Ä≤-C-(difluoromethyl)-2,Ä≤-fluoro-, cyclic 3,Ä≤,5,Ä≤-(I	C14H17F3N5O6P	439.28	C	-8.3
7840	[C@@H](COC)(N1[C@@H](C)CN(CC1)C1(C)CCN(C(=O)c2c(C)ncnc2C)CC1)c1ccc(C(F)(F)F)cc1	1803132-99-1	4-Pyrimidinecarboxamide, N-[(4-fluorophenyl)methyl]-1,6-dihydro-5-hydroxy-1-meth	C20H21FN6O5		C	-8.3
7841	C(=O)([C@@H](NC(=O)OC)C(C)C)N1C(CCC1)c1[nH]c(cc1)c1ccc(cc1)c1ccc(cc1)c1[nH]c(nc1)[C@H]1N(C(=O)[C@@H]	1807681-28-2	Carbamic acid, N-[(1S,2R)-2-hydroxy-1-[[(2S)-2-[5-[4,Ä≤-[5-[(2S)-1-[(2S)-2-[(met	C40H49N7O7	739.86	C	-8.3
7842	C(=O)([C@@H](NC(=O)OCCF)C(C)C)N1[C@@H](CCC1)c1[nH]c(cn1)c1ccc(cc1)c1ccc(cc1)c1[nH]c(cc1)C1N(C(=O)[C@	1807681-38-4	Carbamic acid, N-[(1S)-2-[(2S)-2-[5-[4,Ä≤-[5-[1-[(2S)-2-[(methoxycarbonyl)amino]	C44H49N7O6	771.9	C	-8.3
7843	C(=O)([C@@](NC(=O)OC)(C(C([2H])([2H])[2H])(C([2H])([2H])[2H])[2H])[2H])N1[C@@H](C2CC1CC2)c1[nH]c(cn1	1808295-87-5	Carbamic acid, N-[(1S)-1-[[(2S)-2-[[5-[4,Ä≤-[2-[(2S)-1-[(2S)-2-[(methoxycarbonyl	C41H52N8O6	752.9	C	-8.3
7844	C(=O)([C@@H](NC(=O)OC)C(C)C)N1[C@@H](CCC1)c1[nH]c(cn1)c1ccc(cc1)c1ccc(cc1)c1[nH]c([C@@H](NC(=O)[C@@H	1808295-95-5	Carbamic acid, N-[(1S)-1-[[(2S)-2-[5-[4,Ä≤-[2-[(7S)-6-[(2S)-2-[(methoxycarbonyl)	C43H55N9O6	793.95	C	-8.3
7845	C(=O)([C@@H](NC(=O)OC)C(C)C)N1[C@@H](CCC1)c1[nH]c(cn1)c1ccc(cc1)c1ccc(cc1)c1[nH]c(nc1)C1N(C(=O)[C@@H	1808295-97-7	Carbamic acid, N-[(1S)-1-[[(2S)-2-[5-[4,Ä≤-[2-[(S)-cyclopentyl[[(2S)-2-[(methoxy	C42H54N8O6	766.93	C	-8.3
7846	C(=O)([C@@H](NC(=O)OC)C(C)C)N1C(CCCCC1)c1[nH]c(cn1)c1ccc(cc1)c1ccc(cc1)c1[nH]c(nc1)[C@H]1N(C(=O)[C@@	1808295-98-8	Carbamic acid, N-[(1S)-1-[[(2S)-2-[5-[4,Ä≤-[2-[hexahydro-1-[(2S)-2-[(methoxycarb	C42H54N8O6	766.93	C	-8.3
7847	O=C1[C@@H]2[C@@H](c3c([C@H](OC(=O)[C@H](Cc4ccccc4)N4C(=O)C5C(C4=O)CCCC5)[C@H]2CO1)cc1c(c3)OCO1)c1cc(	1809321-41-2	2H-Isoindole-2-acetic acid, octahydro-1,3-dioxo-Œ±-(phenylmethyl)-, (5R,5aR,8aR,	C39H39NO11	697.73	C	-8.3
7848	O=C1[C@@H]2[C@@H](c3c([C@H](OC(=O)CCCCC(=O)OC(COP(=O)(OCC(C(=O)O)N)O)COC(=O)CCCCC(=O)O[C@H]4c5c([C@H	1825370-96-4	Octanedioic acid, 1,1,Ä≤-[1-[[[(2,3-dihydroxypropoxy)hydroxyphosphinyl]oxy]methy	C66H79O28P	1351.29	C	-8.3
7849	O=c1n([C@@H]2O[C@H](CO[Si](C(C)(C)C)(C)C)C=C2)cc(C)c(=O)[nH]1	1830312-76-9	Carbonic acid, [[(3R,12aS)-12-[(11R)-7,8-difluoro-6,11-dihydrodibenzo[b,e]thiepi	C28H25F2N3O7S	585.58	C	-8.3
7850	[C@@H](NC(=O)N1CCN(C(C)CC1)(C(=O)N[C@H](/C=C/S(=O)(=O)c1ccccc1)CCc1ccccc1)CCCCNC(=N)N	1838684-32-4	1-Piperazinecarboxamide, N-[(1S)-3-[[(aminoiminomethyl)amino]oxy]-1-[[[(1S,2E)-1	C28H39N7O5S	585.72	C	-8.3
7851	[C@@H](Cc1ccccc1)(NC(=O)N1CCN(CC2CC2)CC1)C(=O)N[C@@H](CCc1ccccc1)/C=C/S(=O)(=O)c1ccccc1	1851276-84-0	1-Piperazinecarboxamide, N-[(1S)-2-oxo-2-[[(1S,2E)-1-(2-phenylethyl)-3-(phenylsu	C37H40N4O4S	636.8	C	-8.3
7852	C[C@]1(F)[C@@H](O[C@H](CO[P@](=O)(Oc2ccccc2)N[C@H](C(=O)O)C)[C@H]1O)n1c(=O)[nH]c(=O)cc1	185905-37-7	Cytidine, 2,Ä≤-deoxy-P-methylguanylyl-(3,Ä≤,Ü15,Ä≤)-P-thiothymidylyl-(3,Ä≤,Ü15,	C30H40N10O16P2S	890.71	C	-8.3
7853	C(OC[C@@H]1[C@@H](OC(=O)CC(CC)C)C[C@@H](C1=C)n1c2c(nc1)c(=O)nc(NC(=O)OC(C)(C)C)[nH]2)(c1ccccc1)(c1cc	1860842-91-6	Butanoic acid, (1S,2R,4S)-4-[2-[[(1,1-dimethylethoxy)carbonyl]amino]-1,6-dihydro	C40H43N5O6	689.8	C	-8.3
7854	O=C1[C@@H]2[C@@H](c3c([C@H](OC(=O)CN4c5c(C(=O)C4=O)ccc(Br)c5)[C@H]2CO1)cc1c(c3)OCO1)c1cc(OC)c(OC)c(O	1864833-33-9	1H-Indole-1-acetic acid, 5-bromo-2,3-dihydro-2,3-dioxo-, (5R,5aR,8aR,9R)-5,5a,6,	C32H26BrNO11	680.45	C	-8.3
7855	O=C1[C@@H]2[C@@H](c3c([C@H](OC(=O)CCCC[C@H]4SC(SCC4)c4ccc(Cl)cc4)[C@H]2CO1)cc1c(c3)OCO1)c1cc(OC)c(OC	1875123-90-2	1,3-Dithiane-4-pentanoic acid, 2-(2-chlorophenyl)-, (5R,5aR,8aR,9R)-5,5a,6,8,8a,	C37H39ClO9S2	727.28	C	-8.3
7856	O=C1[C@@H]2[C@@H](c3c([C@H](OC(=O)CCCC[C@H]4SC(SCC4)c4cc(O)c(OC)cc4)[C@H]2CO1)cc1c(c3)OCO1)c1cc(OC)c	1875123-94-6	1,3-Dithiane-4-pentanoic acid, 2-(3-bromophenyl)-, (5R,5aR,8aR,9R)-5,5a,6,8,8a,9	C37H39BrO9S2	771.73	C	-8.3
7857	O=c1n(cc(F)c(N)n1)[C@H]1[C@H](O)C[C@@H](CO)O1	1882869-27-3	Thymidine, 3,Ä≤-[hydrogen N-(di-1-pyrrolidinylmethylene)phosphoramidate]	C19H30N5O7P	471.44	C	-8.3
7858	O=c1n([C@@H]2O[C@H](COP(=O)(OP(=O)(OP(=O)(OCc3ccc(OC(=O)CCCCCCC)cc3)OCc3ccc(OC(=O)C)cc3)O)O)[C@@H](N	1883329-09-6	Not Yet Assigned	C34H33F3N5O17P3		C	-8.3
7859	C(=O)(O)c1c2c(sc1NC(=O)c1c(CNc3ccc(C[C@@H]([C@@H](CN(S(=O)(=O)c4cc5c(cc4)ncs5)CC4CCC4)O)NC(=O)O[C@@H	1887206-40-7	Benzo[b]thiophene-3-carboxylic acid, 2-[[2-[[[4-[(2S,3R)-4-[(6-benzothiazolylsul	C49H57N5O10S3	972.2	C	-8.3
7860	C(=O)(c1[nH]c2c(c1)cc(NS(=O)(=O)C)cc2)N1CCN(CC1)c1c(N)c(O)ccn1	188780-36-1	Sulfamic acid, N-[2-[4-[[5-[(methylsulfonyl)amino]-1H-indol-2-yl]carbonyl]-1-pip	C19H22N6O6S2	494.54	C	-8.3
7861	C(=O)(c1[nH]c2c(c1)cc(NS(=O)(=O)C)cc2)N1CCN(CC1)c1c(N)c(OS(=O)(=O)O)ccn1	188780-37-2	Methanesulfonamide, N-[2-[[4-(3-amino-4-hydroxy-2-pyridinyl)-1-piperazinyl]carbo	C19H22N6O4S	430.48	C	-8.3
7862	C(#N)[C@]1(O[C@H](COP(=O)(Oc2ccccc2)N[C@H](C(=O)OC2CCCCC2)C)[C@@H](O)[C@H]1O)c1n2c(cc1)c(N)ncn2	1911578-90-9	Not Yet Assigned	C26H31N6O8P	586.53	C	-8.3
7863	N(c1c(CC)cccc1CC)c1nc(Nc2ccc(C#N)cc2)cc(C)n1	1923559-48-1	Benzonitrile, 2-[[2-[(4-cyanophenyl)amino]-6-methyl-4-pyrimidinyl]amino]-	C19H14N6	326.35	C	-8.3
7864	C(Nc1[nH]c2c(c(=O)n1)ncn2COC(COC(c1ccccc1)(c1ccccc1)c1ccccc1)COC(=O)[C@@H](NC(=O)OCc1ccccc1)C(C)C)(c	1942024-79-4	2,7,10,12-Tetraazatridecanoic acid, 4-hydroxy-12-methyl-9-(1-methylethyl)-13-[2-	C37H48N6O5S2.C27H22Cl2N4O		C	-8.3
7865	[C@](N)(c1cc2c(n3c(nc2c2cc(Cl)ccc2)nnn3)cc1)(c1n(C)cnc1)c1ccc(Cl)cc1.[C@H](Cc1ccccc1)(C[C@@H]([C@H](	1942697-59-7	2-Naphthalenesulfonic acid, 5-[(1Z)-2-(3,4-dicyanophenyl)diazenyl]-6-hydroxy-	C18H10N4O4S	378.36	C	-8.3
7866	N(C(=O)CCNC(=N)N)C12CC3CC(C2)CC(C1)C3	194602-72-7	Benzenepropanamide, Œ±-[(aminoiminomethyl)amino]-N-tricyclo[3.3.1.13,7]dec-1-yl-	C20H28N4O	340.46	C	-8.3
7867	O=c1n([C@@H]2O[C@H](COP3(=O)Oc4c(CO3)cc(OC)cc4)[C@@H](N=[N+]=[N-])C2)cc(C)c(=O)[nH]1	195885-80-4	Thymidine, 3,Ä≤-azido-3,Ä≤-deoxy-5,Ä≤-O-(2-oxido-4H-1,3,2-benzodioxaphosphorin-	C17H18N5O7P		C	-8.3
7868	C(C(=O)OC)C1N(C(=Nc2c1cccc2F)N1CCN(CC1)c1cc(OC([2H])([2H])[2H])ccc1)c1c(OC)ccc(C(F)(F)F)c1	1961287-47-7	4-Quinazolineacetic acid, 8-fluoro-3,4-dihydro-2-[4-[3-(methoxy-d3)phenyl]-1-pip	C29H22D6F4N4O4		C	-8.3
7869	C(C(=O)OC)C1N(C(=Nc2c1cccc2F)N1CCN(CC1)c1cc(OC)ccc1)c1c(OC([2H])([2H])[2H])ccc(C(F)(F)F)c1	1961287-67-1	4-Quinazolineacetic acid, 8-fluoro-3,4-dihydro-2-[4-[3-(methoxy-d3)phenyl]-1-pip	C29H25D3F4N4O4		C	-8.3
7870	C(C(=O)OC)C1N(C(=Nc2c1cccc2F)N1CCN(CC1)c1cc(OC([2H])([2H])[2H])ccc1)c1c(OC([2H])([2H])[2H])ccc(C(F)(	1961287-77-3	4-Quinazolineacetic acid, 8-fluoro-3,4-dihydro-2-[4-(3-methoxyphenyl)-1-piperazi	C29H25D3F4N4O4		C	-8.3
7871	O=c1c2c(n(cn2)[C@@H]2O[C@H](COP(=O)(OC[C@@H]3[C@@H](N=[N+]=[N-])C[C@@H](O3)n3c(=O)[nH]c(=O)c(C)c3)O)	197900-97-3	Inosine, 3,Ä≤-azido-3,Ä≤-deoxythymidylyl-(5,Ä≤,Ü15,Ä≤)-2,Ä≤,3,Ä≤-dideoxy-, 5,Ä	C30H34N13O11P		C	-8.3
7872	N(C(=O)C)[C@@H]1[C@@H](NC(=N)NC(=O)CCCn2cc(nn2)c2ccccc2)C=C(C(=O)O)O[C@H]1[C@@H]([C@@H](CO)O)O	1982351-35-8	D-glycero-D-galacto-Non-2-enonic acid, 5-(acetylamino)-2,6-anhydro-3,4,5-trideox	C23H29N7O8	531.52	C	-8.3
7873	N(C(=O)C)[C@H]1[C@H]([C@@H]([C@@H](CO)O)O)OC(=C[C@@H]1NC(=N)NC(=O)CCCCn1cc(nn1)c1ccccc1)C(=O)O	1982351-36-9	D-glycero-D-galacto-Non-2-enonic acid, 5-(acetylamino)-2,6-anhydro-3,4,5-trideox	C24H31N7O8	545.54	C	-8.3
7874	N(C(=O)C)[C@H]1[C@H]([C@@H]([C@@H](CO)O)O)OC(=C[C@@H]1NC(=N)NC(=O)Cn1cc(nn1)C1CCCCC1)C(=O)O	1982351-37-0	D-glycero-D-galacto-Non-2-enonic acid, 5-(acetylamino)-2,6-anhydro-3,4,5-trideox	C25H33N7O8	559.57	C	-8.3
7875	N(C(=O)C)[C@H]1[C@H]([C@@H]([C@@H](CO)O)O)OC(=C[C@@H]1NC(=N)NC(=O)Cn1cc(nn1)c1ccc(OC)cc1)C(=O)O	1982351-45-0	D-glycero-D-galacto-Non-2-enonic acid, 5-(acetylamino)-2,6-anhydro-3,4,5-trideox	C25H31N7O10	589.55	C	-8.3
7876	N(C(=O)C)[C@H]1[C@H]([C@@H]([C@@H](CO)O)O)OC(=C[C@@H]1NC(=N)NC(=O)Cn1cc(nn1)c1ccc(N)cc1)C(=O)O	1982351-46-1	D-glycero-D-galacto-Non-2-enonic acid, 5-(acetylamino)-2,6-anhydro-3,4,5-trideox	C23H29N7O9	547.52	C	-8.3
7877	N(c1nc(Nc2ccc(C#N)cc2)cc(C)n1)c1c(C)cc(C)cc1C	1984820-41-8	Benzonitrile, 2-[[6-methyl-2-[(2,4,6-trimethylphenyl)amino]-4-pyrimidinyl]amino]	C21H21N5	343.42	C	-8.3
7878	Cc1c2[C@H](N3n4c(C(=O)N5[C@H]3COCC5)c(O)c(=O)cc4)c3c(SCc2ccc1)cccc3	1985605-75-1	1H-[1,4]Oxazino[3,4-c]pyrido[2,1-f][1,2,4]triazine-6,8-dione, 12-[(11S)-6,11-dih	C25H20F3N3O4S	515.5	C	-8.3
7879	O(COC(=O)OC)c1c2n(N([C@H]3N(C2=O)CCOC3)[C@H]2c3c(CSc4c2cccc4)c(Cl)ccc3)ccc1=O	1985606-43-6	1H-[1,4]Oxazino[3,4-c]pyrido[2,1-f][1,2,4]triazine-6,8-dione, 7-[(acetyloxy)meth	C27H24ClN3O6S	554.01	C	-8.3
7880	O(C(=O)C(C)C)c1c2n(N([C@H]3N(C2=O)CCOC3)[C@H]2c3c(CSc4c2cccc4)c(F)c(F)cc3)ccc1=O	1985606-46-9	1H-[1,4]Oxazino[3,4-c]pyrido[2,1-f][1,2,4]triazine-6,8-dione, 12-[(11S)-7,8-difl	C27H23F2N3O5S	539.55	C	-8.3
7881	O(COC(=O)C1(CC(=O)OC)CCCC1)c1c2n(N([C@H]3N(C2=O)CCOC3)[C@H]2c3c(CSc4c2cccc4)c(F)c(F)cc3)ccc1=O	1985607-17-7	Carbonic acid, (12aR)-12-[(11S)-7,8-difluoro-6,11-dihydrodibenzo[b,e]thiepin-11-	C32H33F2N3O6S	625.68	C	-8.3
7882	O(P(=O)(NC1(C(=O)OC)CC1)OCC)c1c2n(N([C@H]3N(C2=O)CCOC3)[C@H]2c3c(CSc4c2cccc4)c(F)c(F)cc3)ccc1=O	1985607-21-3	Not Yet Assigned	C31H33F2N4O8PS	690.65	C	-8.3
7883	O(C(=O)OC1CN(C(=O)C)C1)c1c2n(N([C@H]3N(C2=O)CCOC3)[C@H]2c3c(CSc4c2cccc4)c(F)c(F)cc3)ccc1=O	1985607-25-7	Carbonic acid, (12aR)-12-[(11S)-7,8-difluoro-6,11-dihydrodibenzo[b,e]thiepin-11-	C28H23F2N3O7S	583.56	C	-8.3
7884	O(P(=O)(OC(C(=O)OC)(C)C)OCC)c1c2n(N([C@H]3N(C2=O)CCOC3)[C@H]2c3c(CSc4c2cccc4)c(F)c(F)cc3)ccc1=O	1985607-30-4	Carbonic acid, (12aR)-12-[(11S)-7,8-difluoro-6,11-dihydrodibenzo[b,e]thiepin-11-	C31H30F2N4O6S	624.65	C	-8.3
7885	O=C1[C@@H]2[C@@H](c3c([C@H](OC(=O)c4c5c(n(C)n4)cccc5)[C@H]2CO1)cc1c(c3)OCO1)c1cc(OC)c(OC)c(OC)c1	1989611-39-3	Benzo[b]thiophene-2-carboxylic acid, (5R,5aR,8aR,9R)-5,5a,6,8,8a,9-hexahydro-8-c	C31H26O9S	574.6	C	-8.3
7886	C(=O)([C@@H](NC(=O)OC)C(C)C)N1[C@@H](CC2(C1)OCCO2)c1[nH]c(cn1)c1c2c(c(cc1)c1cc3c(cc1)c1c(cc3)nc([nH]	2007901-76-8	Carbamic acid, N-[(1S)-1-[[(2S)-2-[5-[7-[4,5-dihydro-2-[(2S)-1-[(2S)-2-[(methoxy	C46H58N8O7	835	C	-8.3
7887	O(C)c1c2c(COP(=O)(OC[C@H]3O[C@H](C=C3)n3c(=O)[nH]c(=O)c(C)c3)O2)ccc1	201165-98-2	Thymidine, 2,Ä≤,3,Ä≤-didehydro-3,Ä≤-deoxy-5,Ä≤-O-(6-methoxy-2-oxido-4H-1,3,2-be	C18H19N2O8P	422.33	C	-8.3
7888	C(=O)(C(=O)NCCc1cccnc1)c1c(C23CC4CC(C3)CC(C2)C4)[nH]c2c1cccc2	2014403-73-5	1H-Indole-3-acetamide, N-[(4-fluorophenyl)methyl]-Œ±-oxo-2-tricyclo[3.3.1.13,7]c	C27H27FN2O2	430.51	C	-8.3
7889	C(=O)(C(=O)NCCc1cccs1)c1c([nH]c2c1cccc2)C12CC3CC(C2)CC(C1)C3	2014403-74-6	1H-Indole-3-acetamide, Œ±-oxo-N-[2-(3-pyridinyl)ethyl]-2-tricyclo[3.3.1.13,7]dec	C27H29N3O2	427.54	C	-8.3
7890	C(=O)(C(=O)Nc1c(F)cc(Br)cc1)c1c([nH]c2c1cccc2)C12CC3CC(C2)CC(C1)C3	2014403-89-3	1H-Indole-3-acetamide, N-(3-chloro-4-fluorophenyl)-Œ±-oxo-2-tricyclo[3.3.1.13,7]	C26H24ClFN2O2	450.93	C	-8.3
7891	O=C1[C@@H]2[C@@H](c3c([C@@H](SCC)[C@H]2CO1)cc1c(c3)OCO1)c1cc(OC)c(OC)c(OC)c1	2015224-40-3	Acetic acid, 2-[5-(2-chlorobenzoyl)-2-methoxyphenoxy]-, [(2S,5R)-5-(3,4-dihydro-	C26H23ClN2O8	526.92	C	-8.3
7892	O=c1n([C@H]2C/C(=N\OC(=O)CC)/[C@@H](CO)O2)cc(C)c(=O)[nH]1	2016-86-6	2-Naphthalenesulfonic acid, 6-hydroxy-5-[2-(4-sulfophenyl)diazenyl]-, ion(2-)	C16H10N2O7S2		C	-8.3
7893	C([C@H](OCP(=O)(OCOC(=O)OC(C)C)OCOC(=O)OC(C)C)C)n1c2c(nc1)c(N)ncn2.C(=C\C(=O)O)/C(=O)O	2021230-56-6	Not Yet Assigned	C21H21N5O7S	487.49	C	-8.3
7894	O[C@H]1[C@@H](O[C@H](COC(=O)NCCNC(=O)CO[C@@H]2O[C@H](CO)[C@H](O)[C@H](O)[C@H]2O)[C@H]1O)n1nc(C(=O)N)	1002334-83-9	1H-1,2,4-Triazole-3-carboxamide, 1-[5-O-[2-[[2-[bis[3-[[2-(Œ≤-D-galactopyranosyl	C34H58N8O20	898.87	A/B	-8.2
7895	O[C@H]1[C@@H](O[C@H](COC(=O)c2ccc(O[C@@H]3O[C@H](CO)[C@H](O)[C@H](O)[C@H]3O)cc2)[C@H]1O)n1nc(C(=O)N)	1002334-96-4	1H-1,2,4-Triazole-3-carboxamide, 1-[5-O-[3-(Œ≤-D-galactopyranosyloxy)-2,2-dimeth	C19H30N4O12	506.46	A/B	-8.2
7896	O[C@H]1[C@@H](O[C@H](COC(=O)c2ccc(CO[C@@H]3O[C@H](CO)[C@H](O)[C@H](O)[C@H]3O)cc2)[C@H]1O)n1nc(C(=O)N	1002334-97-5	1H-1,2,4-Triazole-3-carboxamide, 1-[5-O-[4-(Œ≤-D-galactopyranosyloxy)benzoyl]-Œ	C21H26N4O12	526.45	A/B	-8.2
7897	O(Cc1ccccc1)[C@@H]1[C@@H](OCc2ccccc2)[C@H](OCC(C(=O)OC[C@H]2O[C@H]([C@H](O)[C@@H]2O)n2nc(C(=O)N)nc2)	1002336-50-6	1H-1,2,4-Triazole-3-carboxamide, 1-[5-O-[2,2-dimethyl-1-oxo-3-[[2,3,4,6-tetrakis	C49H56N4O13	908.99	A/B	-8.2
7898	C(OC(=O)COCCO[C@H]1[C@H](OCc2ccccc2)[C@@H](OCc2ccccc2)[C@@H](OCc2ccccc2)[C@@H](COCc2ccccc2)O1)[C@@H]	1002336-73-3	D-Galactopyranoside, phenylmethyl 2-[(3-carboxy-1-oxopropyl)amino]-2-deoxy-3,4,6	C46H51N5O12	865.92	A/B	-8.2
7899	[C@H](Cc1ccccc1)(CC[C@H](Cc1ccccc1)NC(=O)OCc1cncs1)NC(=O)[C@@H](NC(=O)N(Cc1nc(C(C)C)sc1)C)CCNC(=O)C	1004316-88-4	2,7,10,12-Tetraazatridecanoic acid, 12-methyl-13-[2-(1-methylethyl)-4-thiazolyl]	C40H53N7O5S2	776.02	A/B	-8.2
7900	[C@H](Cc1ccccc1)(CC[C@H](Cc1ccccc1)NC(=O)OCc1cncs1)NC(=O)[C@@H](NC(=O)N(Cc1nc(C(C)C)sc1)C)CCNC	1004317-61-6	2,7,10,12-Tetraazatridecanoic acid, 9-(2-hydroxyethyl)-12-methyl-8,11-dioxo-3,6-	C33H40N6O5S2	664.84	A/B	-8.2
7901	C(Cc1ccccc1)(CCC(Cc1ccccc1)NC(=O)OCc1cncs1)NC(=O)C(NC(=O)N(Cc1nc(C(C)C)sc1)C)C(C)C	1004318-37-9	2,7,10,12-Tetraazatridecanoic acid, 12-methyl-9-(1-methylethyl)-13-[2-(1-methyle	C37H50N6O3S2	690.96	A/B	-8.2
7902	C(Cc1ccccc1)(NC(=O)C(NC(=O)N(Cc1nc(C(C)C)sc1)C)CCNS(=O)(=O)C)CCC(Cc1ccccc1)NC(=O)OCc1cncs1	1004318-57-3	2,7,10,12-Tetraazatridecanoic acid, 9-[2-(acetylamino)ethyl]-12-methyl-13-[2-(1-	C38H49N7O5S2	747.97	A/B	-8.2
7903	C(Cc1ccccc1)(CCC(Cc1ccccc1)NC(=O)OCc1cncs1)NC(=O)C(NC(=O)N(Cc1cncs1)C)CCO	1004318-86-8	2,7,10,12-Tetraazatridecanoic acid, 9-(1H-imidazol-5-ylmethyl)-12-methyl-13-[2-(	C38H46N8O4S2	742.95	A/B	-8.2
7904	[C@H](Cc1ccccc1)(CC[C@H](Cc1ccccc1)NC(=O)OCc1cncs1)NC(=O)C(NC(=O)N(Cc1nc(C(C)C)sc1)C)(C)C	1004319-16-7	2,7,10,12-Tetraazatridecanoic acid, 12-methyl-9-(1-methylethyl)-13-[2-(1-methyle	C37H50N6O3S2	690.96	A/B	-8.2
7905	C(=O)([C@@H](NC(=O)OC)C(C)C)N1[C@@H](CCC1)c1[nH]c(cn1)c1cc(CO)c(cc1)c1ccc(cc1)c1[nH]c(nc1)[C@H]1N(C(	1007885-23-5	Carbamic acid, N-[(1S)-1-[[(2S)-2-[5-[2,Ä≤-(aminocarbonyl)-4,Ä≤-[2-[(2S)-1-[(2S	C41H51N9O7	781.9	A/B	-8.2
7906	C(=O)([C@H](NC(=O)OC)c1ccccc1)N1[C@@H](CCC1)c1[nH]c(cn1)c1ccc(cc1)c1ccc(cc1)c1[nH]c(nc1)[C@H]1N(C(=O	1009107-31-6	Acetamide, N,N,Ä≤-[[1,1,Ä≤-biphenyl]-4,4,Ä≤-diylbis[1H-imidazole-5,2-diyl-(2S)-	C40H50N8O4	706.88	A/B	-8.2
7907	C(=O)([C@@H](NC(=O)OC)C1CCOCC1)N1[C@@H](CCC1)c1[nH]c(cn1)c1ccc(cc1)c1ccc(cc1)c1[nH]c(nc1)[C@H]1N(C(=	1009113-84-1	Carbamic acid, N-[(1S)-1-[[(2S)-2-[5-[4,Ä≤-[2-[(2S)-1-[(2S)-2-[(methoxycarbonyl)	C43H54N8O7	794.94	A/B	-8.2
7908	C(=O)([C@@H](NC(=O)COC)C(C)C)N1[C@@H](CCC1)c1[nH]c(cn1)c1ccc(cc1)c1ccc(cc1)c1[nH]c(nc1)[C@H]1N(C(=O)	1009113-88-5	Propanamide, N-[(1S)-2-methyl-1-[[(2S)-2-[5-[4,Ä≤-[2-[(2S)-1-[(2S)-3-methyl-1-ox	C42H54N8O4	734.93	A/B	-8.2
7909	C(=O)([C@@H](NC(=O)OC)C(C)C)N1[C@@H](CCC1)c1[nH]c(cn1)c1ccc(cc1)c1ccc(cc1)c1[nH]c(nc1)[C@H]1N(C(=O)[	1009114-23-1	Carbamic acid, N-[(1S)-2-[(2S)-2-[5-[4,Ä≤-[2-[(2S)-1-[(2S)-2-[(methoxycarbonyl)a	C40H47N11O6	777.87	A/B	-8.2
7910	C(=O)(N1[C@@H](CCC1)c1[nH]c(cn1)c1ccc(cc1)c1ccc(cc1)c1[nH]c(nc1)[C@H]1N(C(=O)[C@@H](NC(=O)OC)C)CCC1)	1009114-60-6	Carbamic acid, N-[(1S)-1-[[(2S)-2-[5-[4,Ä≤-[2-[(2S)-1-[[cis-4-(diethylamino)cycl	C44H58N8O4	762.98	A/B	-8.2
7911	C(=O)([C@H](Cc1ccc(O)cc1)NC(=O)OC)N1[C@@H](CCC1)c1[nH]c(cn1)c1ccc(cc1)c1ccc(cc1)c1[nH]c(nc1)[C@H]1N(	1009114-92-4	Carbamic acid, N-[(1S)-1-[[(2S)-2-[5-[4,Ä≤-[2-[(2S)-1-[(2S)-3-[4-(aminomethyl)ph	C45H53N9O6	815.96	A/B	-8.2
7912	C(=O)([C@@H](NC(=O)OC)C1CC1)N1[C@@H](CCC1)c1[nH]c(cn1)c1c(F)cc(cc1)c1cc(F)c(cc1)c1[nH]c(nc1)[C@H]1N(	1009116-54-4	Carbamic acid, N-[(1S)-1-cyclopropyl-2-[(2S)-2-[5-[3-fluoro-4,Ä≤-[2-[(2S)-1-[(2S	C40H47FN8O6	754.85	A/B	-8.2
7913	C(=O)([C@@H](NC(=O)OC)[C@H](C)O)N1[C@@H](CCC1)c1[nH]c(cn1)c1ccc(cc1)c1ccc(cc1)c1[nH]c(nc1)[C@H]1N(C(	1009116-95-3	Carbamic acid, N,N,Ä≤-[[1,1,Ä≤-biphenyl]-4,4,Ä≤-diylbis[1H-imidazole-5,2-diyl-(	C38H46N8O8	742.82	A/B	-8.2
7914	C(=O)([C@@H](NC(=O)OC)C(C)C)N1[C@H](CCC1)c1[nH]c(cn1)c1ccc(cc1)c1ccc(cc1)c1[nH]c(nc1)[C@H]1N(C(=O)[C	1009117-26-3	Carbamic acid, N,N,Ä≤-[[1,1,Ä≤-biphenyl]-4,4,Ä≤-diylbis[1H-imidazole-5,2-diyl-(	C40H50N8O6	738.88	A/B	-8.2
7915	C(=O)([C@H](NC(=O)OC)c1ccccc1)N1[C@@H](CCC1)c1[nH]c(cn1)c1ccc(cc1)c1ccc(cc1)c1[nH]c(nc1)[C@H]1N(C(=O	1009117-67-2	Carbamic acid, N-[(1R)-2-[(2S)-2-[5-[4,Ä≤-[2-[(2S)-1-[(2R)-2-(dipropylamino)-1-c	C45H54N8O4	770.96	A/B	-8.2
7916	C(=O)([C@H](N(CC)C)c1ccccc1)N1[C@@H](CCC1)c1[nH]c(cn1)c1ccc(cc1)c1ccc(cc1)c1[nH]c(nc1)[C@H]1N(C(=O)[	1009117-81-0	Carbamic acid, N-[(1S)-1-[[(2S)-2-[5-[4,Ä≤-[2-[(2S)-1-[(2S)-2-cyclopropyl-2-[(me	C40H48N8O6	736.86	A/B	-8.2
7917	C(=O)([C@H](N(CC)CC)COC)N1[C@@H](CCC1)c1[nH]c(cn1)c1ccc(cc1)c1ccc(cc1)c1[nH]c(nc1)[C@H]1N(C(=O)[C@@H	1009117-94-5	Carbamic acid, N,N,Ä≤-[[1,1,Ä≤-biphenyl]-4,4,Ä≤-diylbis[1H-imidazole-5,2-diyl-2	C40H50N8O6	738.88	A/B	-8.2
7918	O=c1c2c(n(c(n2)[2H])[C@@H]2O[C@H](CO)[C@@H](O)C2)n(c(N([2H])[2H])n1)[2H]	1013021-41-4	Guanosine 5,Ä≤-(trihydrogen diphosphate), P,Ä≤-(4-amino-3,4,6-trideoxy-Œ±-D-ara	C16H26N6O13P2	572.36	A/B	-8.2
7919	O=C1[C@@H]2[C@@H](c3c([C@@H](O)[C@H]2CO1)cc1c(c3)OCO1)c1cc(OC)c(OC(=O)C(Cl)Cl)c(OC)c1	1016316-67-8	2-Naphthalenesulfonic acid, 5-[2-(2,3-dimethylphenyl)diazenyl]-6-hydroxy-	C18H16N2O4S	356.4	A/B	-8.2
7920	N(=N\c1ccc(S(=O)(=O)O)cc1)/c1c2c(ccc1O)cccc2	1017018-27-7	2-Naphthalenesulfonic acid, 6-hydroxy-5-[(1E)-2-(4-sulfophenyl)diazenyl]-	C16H12N2O7S2	408.41	A/B	-8.2
7921	S(=O)(=O)(O)c1c2c(c(N=Nc3cc(N=Nc4ccc(cc4)c4ccc(N=Nc5c(N)c(S(=O)(=O)O)cc(C)c5N)cc4)c(N)cc3N)cc1)cccc2	101807-81-2	1-Naphthalenesulfonic acid, 4-amino-3-[2-[4-(2-phenylethenyl)phenyl]diazenyl]-	C24H19N3O3S	429.49	A/B	-8.2
7922	N(c1c(CCC)cc(C#N)cc1C)c1nc(Nc2ccc(C#N)cc2)ncc1Br	1022904-34-2	Benzonitrile, 4-[[5-bromo-2-[(4-cyanophenyl)amino]-4-pyrimidinyl]oxy]-3-methyl-5	C22H18BrN5O	448.32	A/B	-8.2
7923	N(C(CCN(C)C)C)c1c2c(ncc1)cc(Cl)cc2	102345-56-2	4-Quinolinamine, 7-chloro-N-[4-[4-(7-chloro-4-quinolinyl)hexahydro-1H-1,4-diazep	C28H31Cl2N5	508.49	A/B	-8.2
7924	N(C(=O)Nc1ccccc1)c1nc(nc(NC(=S)NCCc2cccs2)n1)N1CCOCC1	1025166-72-6	Benzenesulfonic acid, 2,2,Ä≤-(1,2-ethynediyl)bis[5-[2-(4-amino-1-naphthalenyl)di	C34H24N6O6S2	676.72	A/B	-8.2
7925	O=C1[C@@H]2[C@@H](c3c([C@](C(O)C4C5CCCC4CCC5)(O)[C@H]2CO1)cc1c(c3)OCO1)c1cc(OC)c(OC)c(OC)c1	1026020-95-0	Uridine 5,Ä≤-(tetrahydrogen triphosphate), 2,Ä≤-deoxy-5-(3-nitrophenyl)-	C15H18N3O16P3	589.24	A/B	-8.2
7926	N(C1C2C(=NC(=N1)N)N(C=N2)[C@@H]1C[C@H](CO)C=C1)C1CC1	1026096-20-7	Not Yet Assigned	C35H53N7O6	667.84	A/B	-8.2
7927	O=c1n([C@@H]2O[C@H](CO)[C@@H](O)C2)cc(C=CC#N)c(=O)[nH]1	1026902-82-8	1-Piperazinecarboxamide, 4-methyl-N-[(1S)-3-methyl-1-[[[(1E)-1-(2-phenylethyl)-3	C29H40N4O4S	540.72	A/B	-8.2
7928	N(c1cc(C)c(C#N)c(C)c1)c1nc(Nc2ccc(C#N)cc2)ncc1	1027393-94-7	5H-Dipyrido[3,2-b:2,Ä≤,3,Ä≤-e][1,4]diazepine-3-acetonitrile, 11-cyclopropyl-6,1	C21H22N6O	374.44	A/B	-8.2
7929	O(c1n/c(=N\c2ccc(C#N)cc2)/n(O)c(N)c1Br)c1c(C)cc(C#N)cc1C	1027961-32-5	1-Naphthalenesulfonic acid, 4-amino-3-[(1E)-2-(4,Ä≤-amino[1,1,Ä≤-biphenyl]-4-yl	C22H18N4O3S	418.47	A/B	-8.2
7930	N(c1nc(Nc2ccc(C#N)cc2)nc(CNCCCN2CCOCC2)c1)c1c(C)cc(/C=C/C#N)cc1C	1037445-30-9	Benzonitrile, 4-[[4-[[4-[(1E)-2-cyanoethenyl]-2,6-dimethylphenyl]amino]-6-[[[(te	C28H29N7O	479.58	A/B	-8.2
7931	N(c1c(C)cc(/C=C/C#N)cc1C)c1nc(Nc2ccc(C#N)cc2)nc(C=NO)c1	1037445-40-1	Benzonitrile, 4-[[4-[[(aminocarbonyl)oxy]methyl]-6-[[4-[(1E)-2-cyanoethenyl]-2,6	C24H21N7O2	439.47	A/B	-8.2
7932	N(c1c(c(COC)nc(Nc2ccc(C#N)cc2)n1)c1cccs1)c1c(C)cc(/C=C/C#N)cc1C	1037451-95-8	Benzonitrile, 4-[[4-[[4-[(1E)-2-cyanoethenyl]-2,6-dimethylphenyl]amino]-6-(metho	C29H25N7O	487.56	A/B	-8.2
7933	N(c1c(C(=O)NCCN(C)C)c(COC)nc(Nc2ccc(C#N)cc2)n1)c1c(C)cc(/C=C/C#N)cc1C	1037452-15-5	5-Pyrimidinecarboxamide, N-[2-(acetylamino)ethyl]-4-[[4-[(1E)-2-cyanoethenyl]-2,	C29H30N8O3	538.6	A/B	-8.2
7934	C(=O)(NCC(OC)OC)c1c(Nc2c(C)cc(/C=C/C#N)cc2C)nc(Nc2ccc(C#N)cc2)nc1COC	1037452-29-1	5-Pyrimidinecarboxamide, 4-[[4-[(1E)-2-cyanoethenyl]-2,6-dimethylphenyl]amino]-2	C30H27N7O2S	549.65	A/B	-8.2
7935	O=c1n([C@@H]2O[C@H](CO)[C@H](O)C2)cc(COC)c(=O)[nH]1	1046144-69-7	Thymidine, 2,Ä≤-deoxy-P-methylcytidylyl-(3,Ä≤,Ü15,Ä≤)-	C20H28N5O11P	545.44	A/B	-8.2
7936	C(c1c2c(N(c3c(C(=O)N2)cccn3)C2CC2)ncc1)n1c(=N)c2c(nc1)n(cn2)[C@@H]1O[C@H](CO)[C@@H](O)C1	1046462-05-8	6H-Dipyrido[3,2-b:2,Ä≤,3,Ä≤-e][1,4]diazepin-6-one, 4-[(2-amino-1,6-dihydro-6-ox	C20H17N9O2	415.41	A/B	-8.2
7937	N(=C\1/c2c(N(CCCCCC)C1=O)cccc2)\NC(=S)Nc1ccccc1	1048973-44-9	Hydrazinecarbothioamide, 2-[1-(2-cyclohexylethyl)-1,2-dihydro-2-oxo-3H-indol-3-y	C23H26N4OS	406.54	A/B	-8.2
7938	N(C(=O)[C@H](Cc1ccccc1)C[C@@H](CN1[C@H](C(=O)NC(C)(C)C)CN(Cc2cccnc2)CC1)O)[C@H]1c2c(CC1=O)cccc2	1050328-14-7	D-erythro-Pentonamide, 2,3,5-trideoxy-N-[(1R)-2,3-dihydro-2-oxo-1H-inden-1-yl]-5	C36H45N5O4	611.77	A/B	-8.2
7939	[C@H](Cc1ccccc1)(CC[C@H](Cc1ccccc1)NC(=O)OCc1cncs1)NC(=O)[C@H](CCN1C(=O)CCC1)NC(=O)N(Cc1nc(C(C)C)sc1	1051460-83-3	2,7,10,12-Tetraazatridecanoic acid, 12-methyl-13-[2-(1-methylethyl)-4-thiazolyl]	C38H47N9O4S2	757.97	A/B	-8.2
7940	[C@H](Cc1ccccc1)(CC[C@H](Cc1ccccc1)NC(=O)OCc1cncs1)NC(=O)[C@@H](NC(=O)N(Cc1nc(C(C)C)sc1)C)CCN=C(NC#N	1051460-96-8	2,7,10,12-Tetraazatridecanoic acid, 12-methyl-13-[2-(1-methylethyl)-4-thiazolyl]	C38H48F3N7O4S2	787.96	A/B	-8.2
7941	[C@@H](Cc1ccccc1)(NC(=O)[C@H](Cn1cccn1)NC(=O)N(Cc1nc(C(C)C)sc1)C)CC[C@H](Cc1ccccc1)NC(=O)OCc1cncs1	1051461-46-1	2,7,10,12-Tetraazatridecanoic acid, 12-methyl-13-[2-(1-methylethyl)-4-thiazolyl]	C40H47N7O4S2	753.98	A/B	-8.2
7942	[C@@H](Cc1ccccc1)(NC(=O)[C@H](CCN1CCC(O)CC1)NC(=O)N(Cc1nc(C(C)C)sc1)C)CC[C@H](Cc1ccccc1)NC(=O)OCc1cn	1051462-00-0	2,7,10,12-Tetraazatridecanoic acid, 9-[2-[(3S)-3-hydroxy-1-pyrrolidinyl]ethyl]-1	C40H53N7O5S2	776.02	A/B	-8.2
7943	C(Cc1ccccc1)(NC(=O)C(Cc1ccncc1)NC(=O)N(Cc1nc(C(C)C)sc1)C)CCC(Cc1ccccc1)NC(=O)OCc1cncs1	1051464-51-7	2,7,10,12-Tetraazatridecanoic acid, 12-methyl-13-[2-(1-methylethyl)-4-thiazolyl]	C40H47N7O4S2	753.98	A/B	-8.2
7944	C(Cc1ccccc1)(CCC(Cc1ccccc1)NC(=O)OCc1cncs1)NC(=O)C(CCN1CCC(=O)C1)NC(=O)N(Cc1nc(C(C)C)sc1)C	1051464-75-5	2,7,10,12-Tetraazatridecanoic acid, 12-methyl-13-[2-(1-methylethyl)-4-thiazolyl]	C41H53N7O5S2	788.03	A/B	-8.2
7945	C(Cc1ccccc1)(NC(=O)C(CCN1CCOCC1)NC(=O)N(Cc1cncs1)C)CCC(Cc1ccccc1)NC(=O)OCc1cncs1	1051465-11-2	2,7,10,12-Tetraazatridecanoic acid, 12-methyl-13-[2-(1-methylethyl)-5-thiazolyl]	C40H53N7O5S2	776.02	A/B	-8.2
7946	O=c1n([C@@H]2O[C@H](COP(=O)OCCCCCC)C=C2)cc(C)c(=O)[nH]1	1052538-42-7	Not Yet Assigned	C32H24N6O14S5.Na		A/B	-8.2
7947	[C@H](Cc1ccccc1)(C[C@@H]([C@H](Cc1ccccc1)NC(=O)OCc1cnc(C)s1)O)NC(=O)[C@@H](NC(=O)N(CCc1nc(C(C)C)sc1)	1054621-17-8	2,7,10,12-Tetraazatetradecanoic acid, 4-hydroxy-12-methyl-9-(1-methylethyl)-14-[	C41H56N6O5S2	777.05	A/B	-8.2
7948	O=C1[C@@H]2[C@@H](c3c(C[C@H]2CO1)cc1c(c3)OCO1)c1cc(OC)c(O)c(O)c1	105608-70-6	Furo[3,Ä≤,4,Ä≤:6,7]naphtho[2,3-d]-1,3-dioxol-6(5aH)-one, 5,8,8a,9-tetrahydro-5-	C21H20O7	384.38	A/B	-8.2
7949	C(#CC1CC1)[C@@]1(C(F)(F)F)c2c(cc(CCOCOC)c(Cl)c2)NC(=O)O1	1057218-37-7	Carbamic acid, N-[3-[(2,2-dimethyl-6-phosphonohexyl)[(4-methoxyphenyl)sulfonyl]a	C32H47N2O11PS	698.76	A/B	-8.2
7950	O=c1n([C@H]2O[C@@H](COC(=O)CCCC(=O)OC[C@@H]3O[C@@H](CS3)n3c(=O)nc(NC(=O)C)cc3)SC2)ccc(NC(=O)C)n1	1058191-53-9	Thymidine, 2,Ä≤,3,Ä≤-didehydro-3,Ä≤-deoxy-, 5,Ä≤-[5-[[(2R,5S)-5-[4-(acetylamino	C25H29N5O10S	591.59	A/B	-8.2
7951	N(=Nc1c(C)cc(N)cc1)c1c2c(c(S(=O)(=O)O)ccc2)ccc1	106104-87-4	1-Naphthalenesulfonic acid, 7-[2-(4-amino-2-methylphenyl)diazenyl]-	C17H15N3O3S	341.38	A/B	-8.2
7952	C[C@]1(F)[C@@H](O[C@H](COP(=O)(Oc2ccc(F)cc2)N[C@H](C(=O)OC(C)C)C)[C@H]1O)n1c(=O)[nH]c(=O)cc1	1064684-46-3	L-Alanine, N-[(2,Ä≤R)-2,Ä≤-deoxy-2,Ä≤-fluoro-P-(4-fluorophenyl)-2,Ä≤-methyl-5,Ä	C21H26F2N3O9P	533.42	A/B	-8.2
7953	C[C@]1(F)[C@@H](O[C@H](COP(=O)(Oc2ccc(C)cc2)N[C@H](C(=O)OC(C)C)C)[C@H]1O)n1c(=O)[nH]c(=O)cc1	1064684-49-6	L-Alanine, N-[(2,Ä≤R)-2,Ä≤-deoxy-2,Ä≤-fluoro-P-(4-methoxyphenyl)-2,Ä≤-methyl-5,	C23H31FN3O10P	559.48	A/B	-8.2
7954	C(NC(=O)c1ncccn1)(C)(C)c1nc(C(=O)NCc2ccc(F)cc2)c(O)c(=O)n1C	1064685-47-7	L-Alanine, N-[[P(S),2,Ä≤R]-2,Ä≤-deoxy-2,Ä≤-fluoro-2,Ä≤-methyl-P-phenyl-5,Ä≤-uri	C25H33FN3O9P	569.52	A/B	-8.2
7955	O[C@H]1[C@H](n2c3c(nc2)c(=O)nc(N)[nH]3)O[C@H](COP(=O)O)[C@H]1O	1065478-52-5	Benzenepropanamide, Œ±-[[(2E)-3-(4-chloro-2-fluorophenyl)-1-oxo-2-propen-1-yl]am	C25H25ClFN3O4	485.94	A/B	-8.2

7956	N(c1c(C(C)C)cc(C=CC#N)cc1)c1nc(Nc2ccc(C#N)cc2)ncc1	1066549-60-7	Benzonitrile, 4-[[4-[[4-[(1E)-2-cyano-1-methyl-1-propen-1-yl]-2,3,6-trimethylphe	C25H24N6	408.5	A/B	-8.2
7957	N(=Nc1c2c(c(N=Nc3c(C)cc(N)cc3)cc1)ccc(S(=O)(=O)O)c2)c1c2c(c(S(=O)(=O)O)cc(S(=O)(=O)O)c2)ccc1S(=O)(=O	108075-27-0	1,3,6-Naphthalenetrisulfonic acid, 5-[2-[4-[2-(4-amino-7-sulfo-1-naphthalenyl)di	C28H23N5O12S4	749.77	A/B	-8.2
7958	N(Cc1ccc(cc1)c1ccccn1)(C[C@@H]([C@H](Cc1ccccc1)NC(=O)[C@@H](NC(=O)OC([2H])([2H])[2H])C(C([2H])([2H])	1092540-54-9	2,5,6,10,13-Pentaazatetradecanedioic acid, 3,12-bis[1,1-di(methyl-d3)ethyl-2,2,2	C38H32D20N6O7		A/B	-8.2
7959	N(Cc1ccc(cc1)c1ccccn1)(C[C@@H]([C@H](Cc1ccccc1)NC(=O)[C@@H](NC(=O)OC([2H])([2H])[2H])C(C([2H])([2H])	1092540-55-0	2,5,6,10,13-Pentaazatetradecanedioic acid, 3-(1,1-dimethylethyl)-12-[1,1-di(meth	C38H40D12N6O7		A/B	-8.2
7960	N(C(c1ccc(cc1)c1ccccn1)([2H])[2H])(C[C@@H]([C@H](Cc1ccccc1)NC(=O)[C@@H](NC(=O)OC([2H])([2H])[2H])C(C	1092540-60-7	2,5,6,10,13-Pentaazatetradecanedioic acid, 3-(1,1-dimethylethyl)-12-[1,1-di(meth	C38H35D17N6O7		A/B	-8.2
7961	N(=C1c2c(N(CN3CCCC3)C1=O)ccc(Br)c2)NC(=S)Nc1ccccn1	1094040-88-6	Hydrazinecarbothioamide, 2-[5-bromo-1-[(diphenylamino)methyl]-1,2-dihydro-2-oxo-	C27H21BrN6OS	557.46	A/B	-8.2
7962	C(C)(C)(C)c1c2c(cc(C(=O)C)c1)COP(=O)(OC[C@H]1O[C@H](C=C1)n1c(=O)[nH]c(=O)c(C)c1)O2	1095170-76-5	Thymidine, 5,Ä≤-O-(6-acetyl-8-methyl-2-oxido-4H-1,3,2-benzodioxaphosphorin-2-yl)	C20H21N2O8P	448.36	A/B	-8.2
7963	O(C[C@H]1O[C@H](C=C1)n1c(=O)[nH]c(=O)c(C)c1)P1(=O)Oc2c(cc(C(=C)OC(=O)C)cc2)CO1	1095170-78-7	Thymidine, 5,Ä≤-O-[6-[bis(2-methyl-1-oxopropoxy)methyl]-8-(1,1-dimethylethyl)-2-	C30H39N2O11P	634.61	A/B	-8.2
7964	Cc1c2c(cc(C(=C)OC(=O)C)c1)COP(=O)(OC[C@H]1O[C@H](C=C1)n1c(=O)[nH]c(=O)c(C)c1)O2	1095170-79-8	Thymidine, 5,Ä≤-O-[6-[1-(acetyloxy)ethenyl]-2-oxido-4H-1,3,2-benzodioxaphosphor	C21H21N2O9P	476.37	A/B	-8.2
7965	C(C)(C)(C)c1c2c(cc(C(=C)OC(=O)C)c1)COP(=O)(OC[C@H]1O[C@H](C=C1)n1c(=O)[nH]c(=O)c(C)c1)O2	1095170-80-1	Thymidine, 5,Ä≤-O-[6-[1-(acetyloxy)ethenyl]-8-methyl-2-oxido-4H-1,3,2-benzodioxa	C22H23N2O9P	490.4	A/B	-8.2
7966	C(#Cc1cc(Cl)ccc1)C1(C(F)(F)F)c2c(NC(=O)O1)ccc(C)c2	1096581-08-6	2H-3,1-Benzoxazin-2-one, 4-[2-(2-chlorophenyl)ethynyl]-1,4-dihydro-6-methyl-4-(t	C18H11ClF3NO2	365.73	A/B	-8.2
7967	O[C@@H]1[C@H](O[C@@H](COP2(=O)OC(CCO2)c2ccncc2)[C@@H]1O)n1nc(C(=O)N)nc1	109875-48-1	Inosine, 2-(4-nitrophenyl)-	C16H15N5O7	389.32	A/B	-8.2
7968	O=c1n([C@@H]2O[C@H](COP3(=O)Oc4c(CO3)cc(Cc3cc5c(cc3)OP(=O)(OC[C@H]3O[C@H](C[C@@H]3F)n3c(=O)[nH]c(=O)	1099829-88-5	Not Yet Assigned	C29H36N4O7P2S	646.63	A/B	-8.2
7969	C(NC(=O)c1nnc(C([2H])([2H])[2H])o1)(C)(C)c1nc(C(=O)NCc2ccc(F)cc2)c(O)c(=O)n1C	110073-58-0	Thymidine, Œ±,Œ±,Œ±-trifluoro-, 3,Ä≤-hexanoate 5,Ä≤-(4-methylbenzoate)	C24H27F3N2O7	512.48	A/B	-8.2
7970	C(NC(=O)c1nnc(C)o1)(C([2H])([2H])[2H])(C([2H])([2H])[2H])c1nc(C(=O)NCc2ccc(F)cc2)c(O)c(=O)n1C	1100750-81-9	4-Pyrimidinecarboxamide, N-[(4-fluorophenyl)methyl]-1,6-dihydro-5-hydroxy-1-meth	C20H18D3FN6O5		A/B	-8.2
7971	C[C@]1(O)[C@H](n2c3c(nc2)c(=O)nc(N)[nH]3)O[C@H](COP(=O)(OP(=O)(O)O)O)[C@H]1O	1106011-27-1	5,Ä≤-Guanylic acid, 2,Ä≤-C-methyl-	C11H16N5O8P	377.25	A/B	-8.2
7972	N(C(CCCN(CC)CC)C)c1c2c(nc3c1ccc(Cl)c3)cc(Cl)cc2	1111576-26-1	1,3-Diazaspiro[4.5]decane-3-acetic acid, 7,7,9-trimethyl-2,4-dioxo-, 2-oxo-2-(tr	C25H37N3O5	459.58	A/B	-8.2
7973	C(Cc1ccccc1)[C@]1(CCC)CC(=C([C@](C(C([2H])([2H])[2H])([2H])[2H])(c2cc(NS(=O)(=O)c3ccc(C(F)(F)F)cn3)c	1126619-22-4	Not Yet Assigned	C31H26D7F3N2O5S		A/B	-8.2
7974	O=C1[C@@H]2[C@@H](c3c([C@@H](C(=O)O)[C@H]2CO1)cc1c(c3)OCO1)c1cc(OC)c(OC)c(OC)c1	1126811-29-7	Cyclohexanecarboxamide, 4,4-difluoro-N-[(1S)-3-[(3-exo)-3-[3-methyl-5-(1-methyle	C29H30D11F2N5O		A/B	-8.2
7975	C(=O)([C@@H](NC(=O)OC)CC)N1CC2(C[C@H]1c1[nH]c(cn1)c1ccc(cc1)c1ccc(cc1)c1[nH]c(nc1)[C@H]1N(C(=O)[C@@H	1129634-58-7	Carbamic acid, N,N,Ä≤-[[1,1,Ä≤-biphenyl]-4,4,Ä≤-diylbis[1H-imidazole-5,2-diyl-(	C40H46N8O6	734.84	A/B	-8.2
7976	C(=O)([C@H](NC(=O)OC)C(C)C)N1[C@@H](CC[C@@H]1C)c1[nH]c(cn1)c1ccc(cc1)c1ccc(cc1)c1[nH]c(nc1)[C@H]1N(C	1129634-85-0	Carbamic acid, N,N,Ä≤-[[1,1,Ä≤-biphenyl]-4,4,Ä≤-diylbis[1H-imidazole-5,2-diyl[(	C42H54N8O6	766.93	A/B	-8.2
7977	C(=O)([C@@H](NC(=O)OC)C)N1[C@H](c2[nH]c(c(C)n2)c2ccc(cc2)c2ccc(cc2)c2[nH]c(nc2C)[C@H]2N(C(=O)[C@@H](	1129636-25-4	Carbamic acid, N,N,Ä≤-[[1,1,Ä≤-biphenyl]-4,4,Ä≤-diylbis[(4-methyl-1H-imidazole-	C48H50N8O6	834.96	A/B	-8.2
7978	C(CC)c1c([nH]c(n1)[C@H]1N(C(=O)[C@@H](NC(=O)OC)C)CCC1)c1ccc(cc1)c1ccc(cc1)c1[nH]c(nc1)[C@H]1N(C(=O)[	1129636-41-4	1H-Imidazole-4-acetic acid, 2-[(2S)-1-[(2S)-2-[(methoxycarbonyl)amino]-3-methyl-	C43H54N8O8	810.94	A/B	-8.2
7979	O(C)c1c2n(cc(C(=O)O)c(=O)c2cc(F)c1N1CCN(CN2c3c([C@@](C#CC4CC4)(C(F)(F)F)OC2=O)cc(Cl)cc3)CC1)C1CC1	1134943-89-7	3-Quinolinecarboxylic acid, 7-[4-[[(4S)-6-chloro-4-(2-cyclopropylethynyl)-2-oxo-	C32H27ClF4N4O5	659.03	A/B	-8.2
7980	C(COCP(=O)(CP(=O)(O)O)O)n1c2c(nc1)c(N)ncn2	1135818-17-5	Guanosine 3,Ä≤-(trihydrogen diphosphate), N-(2-methyl-1-oxopropyl)-, 5,Ä≤-(trih	C14H23N5O18P4	673.25	A/B	-8.2
7981	C(Cc1ccccc1)[C@]1(CCC)CC(=C([C@H](CC)c2cc(NS(=O)(=O)c3ccc(C(F)(F)F)cn3)ccc2)C(=O)O1)OC1O[C@H](C(=O)O	1141510-06-6	2-Pyridinesulfonamide, N-[3-[(1R)-1-[(6R)-5,6-dihydro-4-hydroxy-2-oxo-6-(2-pheny	C31H33F3N2O6S	618.66	A/B	-8.2
7982	O=c1n([C@@H]2O[C@H](COP(=O)(NC(=O)[C@@H](NC(=O)C)C)O)C=C2)cc(C)c(=O)[nH]1	1147100-84-2	Thymidine, 2,Ä≤,3,Ä≤-didehydro-3,Ä≤-deoxy-, 5,Ä≤-[hydrogen N-[[(2S)-1-acetyl-2-	C17H23N4O8P	442.36	A/B	-8.2
7983	N(=C1c2c(N(CN3CCCC3)C1=O)ccc(Cl)c2)NC(=S)Nc1ccccn1	1148024-22-9	Hydrazinecarbothioamide, 2-[5-chloro-1-[(diphenylamino)methyl]-1,2-dihydro-2-oxo	C27H21ClN6OS	513.01	A/B	-8.2
7984	O=c1n(ccc(=O)[nH]1)[C@@H]1O[C@H](COP(=O)(C(=O)OCC)OC)[C@@H](O)C1	1153577-03-7	5,Ä≤-Adenylic acid, mono(phosphonomethyl) ester	C11H17N5O10P2	441.23	A/B	-8.2
7985	O=c1c2c(N(C[N]2)[C@@H]2O[C@H](CO)[C@@H](O)C2)[nH]c(N)n1	115561-54-1	Œ≤-D-arabino-Adenosine, P-deoxy-P-methyl-Œ≤-D-arabino-adenylyl-(3,Ä≤,Üí3,Ä≤)-	C21H27N10O9P	594.47	A/B	-8.2
7986	O[C@H]1[C@H](n2c3c(nc2)c(=O)nc(N)[nH]3)O[C@H](COP(=O)(OP(=O)(O[C@H]2O[C@@H](CO)[C@H](O)[C@@H](O)[C@H	1155824-52-4	Guanosine 5,Ä≤-(trihydrogen diphosphate), P,Ä≤-Œ±-D-altropyranosyl ester	C16H25N5O16P2	605.34	A/B	-8.2
7987	C(=O)(N[C@@](CC1(C(C(C1([2H])[2H])([2H])[2H])([2H])[2H])[2H])(C(=O)C(=O)N)[2H])[C@@H]1[C@@H]2[C@@H](	1158083-26-1	3-Azabicyclo[3.1.0]hexane-2-carboxamide, N-[(1S)-3-amino-1-(cyclobutyl-1,2,2,3,3	C27H35D10N5O5		A/B	-8.2
7988	C(OCCOC(=O)[C@@H](NC(=O)[C@@H](NC(=O)OC(C)(C)C)C)C(C)C)n1c2c(nc1)c(=O)nc(N)[nH]2	1160856-93-8	L-Valine, L-phenylalanyl-, 2-[(2-amino-1,6-dihydro-6-oxo-9H-purin-9-yl)methoxy]e	C22H29N7O5	471.51	A/B	-8.2
7989	C(N1c2c(C(=NNC(=S)Nc3ccc(OCC)cc3)C1=O)cccc2)c1ccccc1	1161792-78-4	Hydrazinecarbothioamide, 2-[5-chloro-1,2-dihydro-2-oxo-1-(phenylmethyl)-3H-indol	C23H19ClN4O2S	450.94	A/B	-8.2
7990	O=c1n([C@@H]2O[C@H](COP(=O)(Oc3ccccc3)Oc3ccncc3)[C@@H](N=[N+]=[N-])C2)cc(C)c(=O)[nH]1	1162085-44-0	5,Ä≤-Thymidylic acid, 3,Ä≤-azido-3,Ä≤-deoxy-, phenyl 3-pyridinyl ester	C21H21N6O7P		A/B	-8.2
7991	O=c1n([C@@H]2O[C@H](COP(=O)(Oc3c(Cl)nccc3)Oc3ccccc3)[C@@H](N=[N+]=[N-])C2)cc(C)c(=O)[nH]1	1162085-81-5	5,Ä≤-Thymidylic acid, 3,Ä≤-azido-3,Ä≤-deoxy-, 6-methyl-3-pyridinyl phenyl ester	C22H23N6O7P		A/B	-8.2
7992	O=c1n([C@H]2O[C@H](CO)[C@@H](F)C2)cc(C)c(=O)[nH]1	1162085-87-1	5,Ä≤-Thymidylic acid, 3,Ä≤-azido-3,Ä≤-deoxy-, 5-chloro-3-pyridinyl phenyl ester	C21H20ClN6O7P		A/B	-8.2
7993	N(=C\1/c2c(N(CC=C)C1=O)cccc2)\NC(=S)Nc1ccc(OC)cc1	1164499-18-6	Hydrazinecarbothioamide, 2-[1-(1,3-dihydro-3-oxo-1-isobenzofuranyl)-1,2-dihydro-	C17H12N4O3S	352.37	A/B	-8.2
7994	C(n1c2c(nc1)c(N)ncn2)[C@H]1COP(=O)(O)CO1.[NaH]	1164537-40-9	Hydrazinecarbothioamide, N-(3-chloro-2-methylphenyl)-2-[1,2-dihydro-1-(4-morphol	C21H22ClN5O2S	443.95	A/B	-8.2
7995	N(=C\1/c2c(N(CC=C)C1=O)cccc2)\NC(=S)Nc1cc(C)ccc1	1164554-70-4	2-Propenamide, 3-(2-quinoxalinyl)-N-(1-tricyclo[3.3.1.13,7]dec-1-ylethyl)-, (2E)	C23H27N3O	361.48	A/B	-8.2
7996	O(C(=O)c1ccccc1)[C@@H]1[C@@H](O[C@H](CO)[C@H]1O)n1c2c(nc1)c(N)ncn2	116953-92-5	Thymidine, Œ±,Œ±,Œ±-trifluoro-3,Ä≤,5,Ä≤-bis-O-(phenylmethyl)-	C24H23F3N2O5	476.45	A/B	-8.2
7997	Nc1c2c(n(cn2)[C@@H]2O[C@H](CO)[C@@H](O)[C@@H]2O)nc([18F])n1	1169548-51-9	9H-Purin-6-amine, 9-(2-O-benzoyl-Œ≤-D-arabinofuranosyl)-	C17H17N5O5	371.35	A/B	-8.2
7998	C(=O)(NCc1ccc(F)cc1)c1c(OC(=O)c2ccccc2)c(=O)n(C)c(C(NC(=O)c2oc(C)nn2)(C)C)n1	1172131-66-6	Propanoic acid, 2,2-dimethyl-, 4-[[[(4-fluorophenyl)methyl]amino]carbonyl]-1,6-c	C25H29FN6O6	528.53	A/B	-8.2
7999	O=c1c2c(n(cn2)[C@@H]2O[C@@H](CO)[C@H](O)[C@H]2O)[nH]c(N)n1	117312-00-2	Inosine, 2,Ä≤-deoxy-, 5,Ä≤-benzoate	C17H16N4O5	356.33	A/B	-8.2
8000	Cc1n(c2nc(nc(n2)N2CCOCC2)c2ccc(N)nc2)c2c(n1)cccc2	1174190-90-9	2-Pyrimidinamine, 4-methoxy-5-[4-(2-methyl-1H-imidazol-1-yl)-6-(4-morpholinyl)-1	C16H19N9O2	369.38	A/B	-8.2
8001	C(CCC)c1n(c2c(n1)cccc2)c1nc(nc(n1)N1CCOCC1)c1cnc(N)nc1	1174191-82-2	2-Pyrimidinamine, 5-[4-[2-(1-methylethyl)-1H-benzimidazol-1-yl]-6-(4-morpholinyl	C21H23N9O	417.47	A/B	-8.2
8002	O=c1c2c3c(c(F)c2)N2CCN(C)CC2)OCC(C)n3cc1C(=O)OC[C@@H]1[C@@H](N=[N+]=[N-])C[C@@H](O1)n1c(=O)[nH]c(=	1174398-25-4	Thymidine, 3,Ä≤-azido-3,Ä≤-deoxy-, 5,Ä≤-[1-cyclopropyl-7-(4-ethyl-1-piperazinyl	C29H33FN8O6		A/B	-8.2
8003	S(=O)(=O)(c1cc2c(cc1)NC(=O)C2)N1CCC(CO)CC1	1175938-22-3	4-Piperidinecarbonitrile, 1-[(2,3-dihydro-2-oxo-1H-indol-5-yl)sulfonyl]-	C14H15N3O3S	305.35	A/B	-8.2
8004	OC1c2c(-c3c1cc(cc3)c1[nH]c(nc1)[C@H]1N(C(=O)[C@@H](NC(=O)OC)C(C)C)CCC1)ccc(c2)c1[nH]c(nc1)[C@H]1N(C(	1179149-84-8	Carbamic acid, N,N,Ä≤-[(9-oxo-9H-fluorene-2,7-diyl)bis[1H-imidazole-5,2-diyl-(2S	C41H48N8O7	764.87	A/B	-8.2
8005	C(=O)([C@@H](NC(=O)OC)C(C)C)N1[C@H](c2[nH]c(c3cc4c(-c5c(C4)cc(cc5)c4[nH]c(nc4)[C@H]4N(C(=O)[C@@H](NC	1179149-92-8	Carbamic acid, N,N,Ä≤-[[9-(dimethylamino)-9H-fluorene-2,7-diyl]bis[1H-imidazole-	C43H55N9O6	793.95	A/B	-8.2
8006	C(=O)([C@@H](NC(=O)OC)C(C)C)N1[C@H]2[C@H](C2)C[C@H]1c1[nH]c2-c3c(CCc2n1)cc(cc3)c1ccc(cc1)c1[nH]c(nc1	1180669-98-0	Carbamic acid, N-[(1S)-1-[[(2S)-2-[5-[4-[4,5-dihydro-2-[(2S)-1-[(2S)-2-[(methoxy	C45H55F3N8O6	860.96	A/B	-8.2
8007	C(=O)([C@@H](NC(=O)OC)C(C)C)N1[C@H](c2[nH]c3-c4c(cc(cc4)c4ccc(cc4)c4[nH]c(nc4)[C@H]4N(C(=O)[C@@H](NC	1180670-36-3	Carbamic acid, N-[(1S)-1-[[(2S)-2-[7-[4-[2-[(2S)-4,4-difluoro-1-[(2S)-2-[(methox	C42H46F4N8O6	834.86	A/B	-8.2
8008	C(OC(COP(=O)(O)O)COP(=O)(O)O)n1c2c(nc1)c(=O)nc(N)[nH]2	1185728-95-3	1,4-Benzenedisulfonic acid, 2-[2-[4-[2-(4-amino-7-sulfo-1-naphthalenyl)diazenyl]	C27H21N5O12S4	735.74	A/B	-8.2
8009	C(C#N)C1(CN(S(=O)(=O)c2ccc(F)cc2)C1)n1cc(cn1)c1c2c(ncn1)[nH]cc2	1187594-45-1	3-Azetidineacetonitrile, 1-[(5-methyl-2-thienyl)sulfonyl]-3-[4-(7H-pyrrolo[2,3-d	C19H17N7O2S2	439.51	A/B	-8.2
8010	C(C#N)C1(CN(S(=O)(=O)c2cccnc2)C1)n1cc(cn1)c1c2c(ncn1)[nH]cc2	1187594-51-9	3-Azetidineacetonitrile, 1-[(2-fluorophenyl)sulfonyl]-3-[4-(7H-pyrrolo[2,3-d]pyr	C20H16FN7O2S	437.45	A/B	-8.2
8011	C(C#N)C1(CN(S(=O)(=O)c2ccccn2)C1)n1cc(cn1)c1c2c(ncn1)[nH]cc2	1187594-53-1	3-Azetidineacetonitrile, 1-(3-pyridinylsulfonyl)-3-[4-(7H-pyrrolo[2,3-d]pyrimidi	C19H16N8O2S	420.45	A/B	-8.2
8012	C(C#N)C1(CN(S(=O)(=O)CC)C1)n1cc(cn1)c1c2c(n(CO)cc2)ncn1	1187594-55-3	3-Azetidineacetonitrile, 1-(2-pyridinylsulfonyl)-3-[4-(7H-pyrrolo[2,3-d]pyrimidi	C19H16N8O2S	420.45	A/B	-8.2
8013	O=c1n([C@@H]2O[C@H](COP(=O)(Nc3cc(C(=O)Nc4cc(C(=O)Nc5cc(C(=O)Nc6cc(C(=O)NCCC(=N)N)n(C)c6)n(C)c5)n(C)	1190064-45-9	Thymidine, 2,Ä≤,3,Ä≤-didehydro-3,Ä≤-deoxy-, 5,Ä≤-[1-methylethyl N-[5-[[[5-[[[5-	C34H44N11O9P	781.76	A/B	-8.2
8014	[C@@H](COC([2H])([2H])[2H])(N1[C@@H](C)CN(CC1)C1(C)CCN(C(=O)c2c(C)ncnc2C)CC1)c1ccc(C(F)(F)F)cc1	1191293-13-6	Methanone, (4,6-dimethyl-5-pyrimidinyl)[4-[(3S)-4-[(1R)-2-(methoxy-d3)-1-[4-(tri	C28H34D4F3N5O2		A/B	-8.2
8015	[C@@H](C(OC)([2H])[2H])(N1[C@@H](C)CN(CC1)C1(C)CCN(C(=O)c2c(C)ncnc2C)CC1)c1ccc(C(F)(F)F)cc1	1191293-19-2	Methanone, [4,6-di(methyl-d3)-5-pyrimidinyl][4-[(3S)-4-[(1R)-2-(methoxy-d3)-1-[4	C28H28D10F3N5O2		A/B	-8.2
8016	Nc1nc2n(cnc2cn1)[C@@H]1O[C@H](CO)C=C1	1192411-95-2	Pyrimidine, 5-[[4-[(3S)-4-[(1R)-2-methoxy-1-[4-(trifluoromethyl)phenyl]ethyl]-3-	C28H40F3N5O	519.65	A/B	-8.2
8017	N(C(=O)C)[C@H]1[C@H]([C@@H]([C@@H](CO)O)O)OC(=C[C@@H]1N=C(NC(=O)OC(C)(C)C)NC(=O)OC(C)(C)C(=O)OC(OC	1192538-90-1	Not Yet Assigned	C37H55N5O14	793.86	A/B	-8.2
8018	N(S(=O)(=O)c1ccc(N)cc1)(C[C@H]([C@H](Cc1ccccc1)NC(=O)O[C@H]1CCOC1)O)CC(C)C.C([C@H](OCP(=O)(O)O)C)n1c	119697-63-1	Naphthionic acid, 3,3,Ä≤-(2,2,Ä≤-dibromo-4,4,Ä≤-biphenylylenebisazo)di-	C32H22Br2N6O6S2	810.49	A/B	-8.2
8019	O=C1[C@@H]2[C@@H](c3c([C@H](O)[C@H]2CO1)cc1c(c3)OCO1)C1=CC(=O)C(=O)C(=C1)OCC	119841-82-6	Furo[3,Ä≤,4,Ä≤:6,7]naphtho[2,3-d]-1,3-dioxol-6(5aH)-one, 5-(2-chloro-4,5-dihydr	C20H17ClO8	420.8	A/B	-8.2
8020	O[C@H]1[C@@H](O[C@H](CO)[C@H]1O)n1nc(C(=N)OC)nc1	1203578-22-6	L-Prolinamide, L-valyl-L-prolyl-L-valyl-N-(9-Œ≤-D-arabinofuranosyl-9H-purin-6-yl	C30H45N9O8	659.73	A/B	-8.2
8021	N(CCNc1c2c(ncc1)cc(Cl)cc2)c1nc(nc(n1)N1CCOCC1)N1CCOCC1	1207449-61-3	1,4-Butanediamine, N1-[4-chloro-6-(4-morpholinyl)-1,3,5-triazin-2-yl]-N4-(7-chlo	C20H23Cl2N7O	448.35	A/B	-8.2
8022	C(c1nn(C23CC4CC(C3)CC(C2)C4)nn1)c1nnc(N)[nH]1	1207759-76-9	2H-Tetrazole, 5,5,Ä≤-methylenebis[2-tricyclo[3.3.1.13,7]dec-1-yl-	C23H32N8	420.55	A/B	-8.2
8023	O=c1n(cc(C)c(=O)n1CCCn1c(=O)n(cc(C)c1=O)[C@H]1C[C@H](N=[N+]=[N-])[C@@H](CO)O1)[C@H]1C[C@H](N=[N+]=[N	1215171-14-4	Thymidine, 3-[[4-[[3-(3-azido-2,3-dideoxy-Œ≤-D-erythro-pentofuranosyl)-3,6-dihyd	C28H31N7O8		A/B	-8.2
8024	C(c1cn(c(=O)[nH]c1=O)[C@@H]1O[C@H](CO)[C@@H](O)C1)(c1c(O)cc(C)oc1=O)c1c(O)cc(C)oc1=O	1217597-42-6	Thymidine, Œ±,Œ±-bis(1,2-dihydro-4-hydroxy-6-methyl-2-oxo-3-pyridinyl)-	C22H24N4O9	488.45	A/B	-8.2
8025	N(Cc1ccc(cc1)c1ccccn1)(C[C@@H]([C@H](Cc1ccccc1)NC(=O)[C@@H](NC(=O)OCCOCCOC)C(C)(C)C)O)NC(=O)[C@@H](N	1219092-97-3	2,5,6,10,13-Pentaazatetradecanedioic acid, 3,12-bis(1,1-dimethylethyl)-8-hydroxy	C42H60N6O9	792.96	A/B	-8.2
8026	C(=O)(C=Cc1ccc(OC)cc1)c1c(OC)cc(O)c(C(=O)C=Cc2ccc(OC)cc2)c1	122070-76-2	2-Propen-1-one, 1,1,Ä≤-(2-hydroxy-4-methoxy-m-phenylene)bis[3-(p-methoxyphenyl)-	C27H24O6	444.48	A/B	-8.2
8027	O(C)c1c(OC)cc(CC/C=C/C(=O)CCc2cc(OC)c(O)cc2)cc1	1221411-93-3	Benzamide, N-(2-aminophenyl)-4-[(tricyclo[3.3.1.13,7]dec-1-ylamino)methyl]-	C24H29N3O	375.51	A/B	-8.2
8028	C([C@H]([C@H](Cc1ccccc1)NC(=O)[C@H](NC(=O)c1nc2c(cc1)cccc2)C)O)N1[C@H](C(=O)NC(C)(C)C)C[C@H]2[C@@H](	1222503-31-2	Methanesulfonamide, N-[2,3-dihydro-2-[[4-[4-[(1-methylethyl)amino]-2-pyridinyl]-	C22H30N6O3S	458.58	A/B	-8.2
8029	N(C(=O)C)[C@H]1[C@H](C(=O)C(CO)O)OC(C(=O)O)C[C@@H]1NC(=N)N	1223072-63-6	Not Yet Assigned	C36H48N6O4	628.8	A/B	-8.2
8030	[C@](C(=O)N[C@@H](C(c1c(c(c(c(c1[2H])[2H])[2H])[2H])[2H])([2H])[2H])C[C@@H]([C@](C(c1c(c(c(c(c1[2H])	1224728-97-5	1(2H)-Pyrimidine-4,5,6-d3-acetamide-Œ±-d, N-[(1S,3S,4S)-4-[[2-(2,6-dimethylpheno	C37H17D31N4O5		A/B	-8.2
8031	[C@](C(=O)N[C@@H](C(c1c(c(c(c(c1[2H])[2H])[2H])[2H])[2H])([2H])[2H])C[C@@H]([C@](C(c1c(c(c(c(c1[2H])	1224729-03-6	1(2H)-Pyrimidineacetamide-Œ±-d, N-[(1S,3S,4S)-4-[[2-(2,6-dimethylphenoxy)acetyl-	C37H31D17N4O5		A/B	-8.2
8032	[C@](C(=O)N[C@@H](Cc1ccccc1)C[C@@H]([C@H](Cc1ccccc1)NC(=O)C(Oc1c(C)cccc1C)([2H])[2H])O)(C(C([2H])([2	1224729-18-3	1(2H)-Pyrimidineacetamide-Œ±-d, N-[(1S,3S,4S)-4-[[2-[2,6-di(methyl-d3)phenoxy-3,	C37H29D19N4O5		A/B	-8.2
8033	[C@@H](C(=O)N[C@@H](Cc1ccccc1)C[C@@H]([C@H](Cc1ccccc1)NC(=O)C(Oc1c(C)cccc1C)([2H])[2H])O)(C(C)C)N1C(	1224729-48-9	1(2H)-Pyrimidineacetamide, N-[(1S,3S,4S)-4-[[2-(2,6-dimethylphenoxy)acetyl-2,2-d	C37H38D10N4O5		A/B	-8.2
8034	[C@@H](C(=O)N[C@@H](Cc1ccccc1)C[C@@H]([C@H](C(c1ccccc1)([2H])[2H])NC(=O)COc1c(C([2H])([2H])[2H])cccc	1224729-61-6	1(2H)-Pyrimidineacetamide, N-[(1S,3S,4S)-4-[[2-[2,6-di(methyl-d3)phenoxy]acetyl]	C37H38D10N4O5		A/B	-8.2
8035	Nc1ccc(c2nc(nc(n2)N2CCOCC2)N2CCOCC2)cn1	1225024-08-7	9H-Purin-6-amine, 9-[5-O-[(2S,4R)-2-oxido-4-pyridinyl-1,3,2-dioxaphosphorinan-2-	C18H21N6O7P	464.37	A/B	-8.2
8036	N(C(=O)C)c1ncc(c2nc(nc(n2)N2CCOCC2)N2CCOCC2)cn1	1225037-80-8	Acetamide, N-[5-(4,6-di-4-morpholinyl-1,3,5-triazin-2-yl)-2-pyridinyl]-	C18H23N7O3	385.42	A/B	-8.2
8037	[C@H](OC(=O)CP(=O)(OCC)OCC)(CC=C)c1ccoc1	1225038-00-5	Phenol, 3-[4-(4-morpholinyl)-6-[(2-pyridinylmethyl)amino]-1,3,5-triazin-2-yl]-	C19H20N6O2	364.4	A/B	-8.2
8038	C(#N)C1=C(N)Oc2c(C1C1CCCC1)ccn1c2nc(C)c1	1225373-13-6	7H-Imidazo[1,2-a]pyrano[2,3-c]pyridine-8-carbonitrile, 9-amino-7-(2-furanyl)-	C15H10N4O2	278.27	A/B	-8.2
8039	C(#N)C1=C(N)Oc2c(C1c1ccc(N(C)C)cc1)ccn1c2nc(C)c1	1225373-23-8	7H-Imidazo[1,2-a]pyrano[2,3-c]pyridine-8-carbonitrile, 9-amino-7-phenyl-	C17H12N4O	288.3	A/B	-8.2
8040	N(Cc1ccccn1)c1nc(nc(n1)N1CCOCC1)c1cnc(N)nc1	1225638-96-9	1,3,5-Triazin-2-amine, 4-(6-amino-3-pyridinyl)-6-(4-morpholinyl)-N-(2-pyridinylm	C18H20N8O	364.4	A/B	-8.2
8041	N(Cc1ccccn1)c1nc(nc(n1)N1CCOCC1)c1ccc2c(cc1)[nH]nc2	1225638-97-0	1,3,5-Triazin-2-amine, 4-(2-amino-5-pyrimidinyl)-6-(4-morpholinyl)-N-(2-pyridiny	C17H19N9O	365.39	A/B	-8.2
8042	C(=O)(NC(CC1CC1)C(=O)C(=O)N)[C@@H]1[C@@H]2[C@@H](C2(C)C)CN1C(=O)[C@@H](NC(=O)NC1(C)CCCCC1)C(C)(C)C	1228343-59-6	3-Azabicyclo[3.1.0]hexane-2-carboxamide, N-[3-amino-1-(cyclopropylmethyl)-2,3-di	C28H45N5O5	531.69	A/B	-8.2
8043	O(P(=O)(OC[C@H]1O[C@H]([C@H](O)[C@@H]1O)n1nc(C(=O)N)nc1)NCC(=O)OCc1ccccc1)c1c2c(ccc1)cccc2	1228377-65-8	L-Phenylalanine, N-[hydroxy(1-naphthalenyloxy)phosphinyl]-, phenylmethyl ester,	C34H34N5O9P	687.64	A/B	-8.2
8044	C(=O)([C@@H](NC(=O)OC)C(C)C)N1[C@H](c2[nH]c(c3cc4c(c(ccc4)c4cc(ccc4)c4[nH]c(nc4)[C@H]4N(C(=O)[C@@H](	1228553-46-5	Carbamic acid, N-[(1S)-1-[[(2S)-2-[5-[4-[5-[2-[(2S)-1-[(2S)-2-[(methoxycarbonyl)	C44H52N8O6	788.93	A/B	-8.2
8045	O=c1n([C@H]2C[C@H](OP(=O)(OCCCCCCCCCCCCCCCC)OCCC#N)[C@@H](CO)O2)cc(C)c(=O)[nH]1	1228633-22-4	Carbamic acid, N-[(1S)-2-methyl-1-[[(2S)-2-[5-[4-[6-[2-(2S)-2-pyrrolidinyl-1H-im	C37H41N7O3	631.77	A/B	-8.2
8046	C(=O)([C@H](NC(=O)OC)c1ccccc1)N1[C@@H](CCC1)c1[nH]c(cn1)[C@H]1CC[C@@H](CC1)c1ccc(cc1)c1[nH]c(nc1)[C@	1228667-87-5	Carbamic acid, N-[(1R)-1-[[(2S)-2-[5-[4-[trans-4-[2-[(2S)-1-[(2R)-2-[[(1,1-dimet	C46H68N8O6	829.08	A/B	-8.2
8047	C(=O)([C@@H](NC(=O)OC(C)(C)C)C(C)C)N1[C@@H](CCC1)c1[nH]c(cn1)[C@@H]1CC[C@H](CC1)c1ccc(cc1)c1[nH]c(nc	1228668-01-6	Carbamic acid, N-[(1S)-1-[[(2S)-2-[5-[4-[cis-4-[2-[(2S)-1-[(2S)-2-[(methoxycarbo	C40H56N8O6	744.92	A/B	-8.2
8048	C(=O)([C@@H](NC(=O)OC(C)(C)C)C(C)C)N1[C@@H](CCC1)c1[nH]c(cn1)[C@H]1CC[C@H](CC1)c1ccc(cc1)c1[nH]c(nc1	1228668-02-7	Carbamic acid, N-[(1S)-1-[[(2S)-2-[5-[4-[trans-4-[2-[(2S)-1-[(2S)-2-[[(1,1-dimet	C46H68N8O6	829.08	A/B	-8.2
8049	C(=O)([C@@H](NC(=O)OC)C(C)C)N1[C@H](c2[nH]c(C34CCC(CC3)(CC4)c3ccc(cc3)c3[nH]c(nc3)[C@H]3N(C(=O)[C@H]	1228671-77-9	Carbamic acid, N-[(1S)-2-[(2S)-2-[5-[4-[4-[2-[(2S)-1-[(2S)-2-[(methoxycarbonyl)a	C40H54N8O6	742.91	A/B	-8.2
8050	C(=O)(/C=C/c1ccc(O)cc1)c1c(OC)cc(O)c(C(=O)/C=C/c2ccc(O)cc2)c1O	1228672-14-7	Carbamic acid, N,N,Ä≤-[(1E)-1,2-ethenediylbis[1H-imidazole-5,2-diyl-(2S)-2,1-pyr	C48H48N8O6	832.94	A/B	-8.2
8051	C(=O)([C@H](N(CC)CC)c1ccccc1)N1[C@@H](CCC1)c1[nH]c(cn1)c1ccc(C#Cc2ccc(cc2)c2[nH]c(nc2)[C@H]2N(C(=O)[	1228967-09-6	Carbamic acid, N-[(1S)-2-methyl-1-[[(2S)-2-[5-[4-[2-[4-[2-[(2S)-1-[(2R)-2-phenyl	C47H52N8O4	792.97	A/B	-8.2
8052	C(=O)([C@@H](NC(=O)OC)C(C)(C)O)N1[C@@H](CCC1)c1[nH]c(cn1)c1ccc(C#Cc2ccc(cc2)c2[nH]c(nc2)[C@H]2N(C(=O	1228967-18-7	Carbamic acid, N,N,Ä≤-[1,2-ethynediylbis[4,1-phenylene-1H-imidazole-5,2-diyl-(2S	C42H50N8O8	794.9	A/B	-8.2
8053	O[13C@H]1[13C@H](n2c3c(nc2)c(N)ncn3)O[13C@H]([13CH2]SC)[13C@H]1O	1229433-22-0	2,5,6,10,13-Pentaazatetradecanedioic acid, 3-(1,1-dimethylethyl)-8-hydroxy-12-(1	C34H49N9O7	695.81	A/B	-8.2
8054	O=C1[C@H]2[C@@H](c3c(C[C@@H]2CO1)cc1c(c3)OCO1)c1cc(OC)c(O)c(OC)c1	123287-54-7	Furo[3,Ä≤,4,Ä≤:6,7]naphtho[2,3-d]-1,3-dioxol-6(5aH)-one, 5,8,8a,9-tetrahydro-5-	C21H20O7	384.38	A/B	-8.2
8055	C(N(C(=O)N[C@H](C(=O)N[C@@H](Cc1ccccc1)C[C@@H]([C@H](Cc1ccccc1)NC(=O)OCc1cncs1)O)C(C)C)C)c1nc(sc1)C1	1236363-93-1	Carbamic acid, N-[[5-(2-methylpropyl)-3-[4-[[4-(trifluoromethyl)-1H-imidazol-1-y	C24H26F3N3O5S2	557.61	A/B	-8.2
8056	[C@H](Cc1ccccc1)([C@H]([C@@H]([C@H](Cc1ccccc1)NC(=O)OCc1cncs1)O)O)NC(=O)[C@@H](NC(=O)N(Cc1nc(C(C)C)s	1236406-26-0	Not Yet Assigned	C37H48N6O6S	704.88	A/B	-8.2
8057	[C@H](Cc1ccccc1)(C[C@@H]([C@H](Cc1ccccc1)NC(=O)OCc1cncs1)O)NC(=O)[C@H](NC(=O)N(Cc1nc(/C=C/C)sc1)C)C(	1236406-43-1	2,7,10,12-Tetraazatridecanoic acid, 13-[2-(1,2-dimethyl-1-propen-1-yl)-4-thiazol	C39H50N6O5S2	746.98	A/B	-8.2
8058	[C@H](Cc1ccccc1)(C[C@@H]([C@H](Cc1ccccc1)NC(=O)OCc1ccns1)O)NC(=O)[C@@H](NC(=O)N(Cc1nc(/C(=C/C)/C)sc1	1236406-70-4	Not Yet Assigned	C37H46N6O6S	702.86	A/B	-8.2
8059	[C@H](Cc1ccccc1)(C[C@@H]([C@H](Cc1ccccc1)NC(=O)OCc1cncs1)O)NC(=O)[C@H](NC(=O)N(Cc1nc(Cc2nccs2)sc1)C)	1236407-23-0	2,7,10,12-Tetraazatridecanoic acid, 4-hydroxy-12-methyl-9-(1-methylethyl)-13-[2-	C38H48N6O5S2	732.95	A/B	-8.2
8060	[C@H](Cc1ccccc1)(C[C@@H]([C@H](Cc1ccccc1)NC(=O)OCc1ccno1)O)NC(=O)[C@@H](NC(=O)N(Cc1nc(CC(C)C)sc1)C)C	1236407-91-2	2,7,10,12-Tetraazatridecanoic acid, 13-(2-cyclopropyl-4-thiazolyl)-4-hydroxy-12-	C37H46N6O6S	702.86	A/B	-8.2
8061	C(N(C(=O)N[C@@H](C(=O)N[C@@H](Cc1ccccc1)C[C@@H]([C@H](Cc1ccccc1)NC(=O)OCc1cncs1)O)C(C)C)C)c1nc(sc1)C	1236408-55-1	2,7,10,12-Tetraazatridecanoic acid, 13-(2-cyclopentyl-4-thiazolyl)-4-hydroxy-12-	C39H50N6O5S2	746.98	A/B	-8.2
8062	[C@H](Cc1ccccc1)(C[C@@H]([C@H](Cc1ccccc1)NC(=O)OCc1cncs1)O)NC(=O)[C@@H](NC(=O)CCc1nc(C(C)C)sc1)C(C)C	1236408-62-0	2,7,10,12-Tetraazatridecanoic acid, 13-[2-(cyclopentylmethyl)-4-thiazolyl]-4-hyd	C40H52N6O5S2	761.01	A/B	-8.2
8063	[C@H](Cc1ccccc1)([C@H]([C@H]([C@H](Cc1ccccc1)NC(=O)OCc1cncs1)O)O)NC(=O)[C@@H](NC(=O)N(Cc1nc(C(C)C)oc	1236409-51-0	2,7,10,12-Tetraazatridecanoic acid, 13-(2-ethenyl-4-thiazolyl)-4-hydroxy-12-meth	C36H44N6O5S2	704.9	A/B	-8.2
8064	[C@H](Cc1ccccc1)(C[C@@H]([C@H](Cc1ccccc1)NC(=O)OCc1cnco1)O)NC(=O)[C@H](NC(=O)N(Cc1nc(C(CC)C)sc1)C)C	1237482-24-4	2,7,10,12-Tetraazatridecanoic acid, 4-hydroxy-12-methyl-9-(1-methylethyl)-13-[2-	C38H50N6O5S2	734.97	A/B	-8.2
8065	N(CC(=O)[C@H](Cc1ccccc1)C[C@@H](CN1[C@H](C(=O)NC(C)(C)C)CN(Cc2c(C)nccc2)CC1)O)[C@H]1c2c(C[C@H]1O)ccc	1237988-80-5	Not Yet Assigned	C39H53N5O4	655.87	A/B	-8.2
8066	C(O)C1(CO)O[C@H](C=C1)n1c(=O)[nH]c(=O)cc1	1238950-66-7	2-Naphthalenesulfonic acid, 8-amino-5-[2-[4-(aminocarbonyl)phenyl]diazenyl]-	C17H14N4O4S	370.38	A/B	-8.2
8067	C(=O)([C@@H](NC(=O)OC)CCC)N1[C@H](c2[nH]c(c3cc4c(-c5c(C4)cc(cc5)c4[nH]c(nc4)[C@H]4N(C(=O)[C@@H](NC(=	1240880-57-2	Carbamic acid, N,N,Ä≤-[9H-fluorene-2,7-diylbis[1H-imidazole-5,2-diyl-(2S)-2,1-py	C41H46N8O6	746.85	A/B	-8.2
8068	C(=O)(C(NC(=O)OC)(C)C)N1[C@@H](CCC1)c1[nH]c(cn1)[C@H]1CC[C@@H](CC1)c1ccc(cc1)c1[nH]c(nc1)[C@H]1N(C(=	1240887-58-4	Carbamic acid, N-[(1S)-1-[[(2S)-2-[5-[4-[trans-4-[2-[(2S)-1-[(2S)-2-[(methoxycar	C40H56N8O6	744.92	A/B	-8.2
8069	C(=O)([C@@H](NC(=O)OC)C(C)C)N1[C@@H](CCC1)c1[nH]c(cn1)[C@H]1CC[C@@H](CC1)c1ccc(cc1)c1[nH]c([C@@H](N(	1240890-58-7	Carbamic acid, N-[(1R)-2-[[(1S)-1-[5-[4-[trans-4-[2-[(2S)-1-[2-[(methoxycarbonyl	C39H48N8O6	724.85	A/B	-8.2
8070	C(=O)([C@H](NC(=O)OC)c1ccccc1)N1[C@@H](CCC1)c1[nH]c(cn1)c1ccc(/C=C/CCc2[nH]c(nc2)[C@H]2N(C(=O)[C@H](	1240891-93-3	Carbamic acid, N-[(1R)-2-[(2S)-2-[5-[4-[[(1S,2S)-2-[[2-[(2S)-1-[(2R)-2-[(methoxy	C48H56N8O6	841.01	A/B	-8.2
8071	O[C@@H]1[C@H](n2c3c(nc2)c(N)ncn3)O[C@H](COS(=O)(=O)NC(=O)[C@H](Cc2cnc[nH]2)N)[C@H]1O	1241942-41-5	9H-Purin-6-amine, 9-[5-O-[[[(2S)-2-amino-5-[(aminoiminomethyl)amino]-1-oxopentyl	C16H26N10O7S	502.51	A/B	-8.2
8072	O[C@@H]1[C@H](n2c3c(nc2)c(N)nc(C=CCCCC)n3)O[C@H](COS(=O)(=O)NC(=O)[C@H]([C@H](CC)C)N)[C@H]1O	1241942-43-7	9H-Purin-6-amine, 9-[5-O-[[[(2S)-2-amino-3-(1H-imidazol-5-yl)-1-oxopropyl]amino]	C16H21N9O7S	483.46	A/B	-8.2

8073	O(c1nc(Nc2ccc(C#N)cc2)nc(N)c1Br)c1c(C)cc(C#N)cc1C.C(=O)(N)c1cccnc1	1246956-07-9	Carbamic acid, N-[(1S)-1-[[(2S)-2-[5-[4-[4-[2-[(2S)-1-[(2R)-2-[(methoxycarbonyl)	C40H56N8O6	744.92	A/B	-8.2
8074	[C@@H](NC(=O)[C@@H](NC(=O)N1CCN(C)CC1)CC(C)C)(/C=C/S(=O)(=O)c1ccccc1)c1c2c(ccc1)cccc2	1253912-41-2	1-Piperazinecarboxamide, 4-methyl-N-[(1S)-3-methyl-1-[[[(1R,2E)-1-phenyl-3-(pher	C27H36N4O4S	512.66	A/B	-8.2
8075	O=c1n([C@@H]2O[C@H](CO[P@@](=O)(Oc3c(C)cccc3)N[C@H](C(=O)OC)C)C=C2)cc(C)c(=O)[nH]1	1254939-94-0	L-Alanine, N-[[P(S)]-2,Ä≤,3,Ä≤-didehydro-3,Ä≤-deoxy-P-(2-methylphenyl)-5,Ä≤-thy	C21H26N3O8P	479.42	A/B	-8.2
8076	C(=O)([C@@H](NC(=O)OC)C(C)C)N1[C@@H](CCC1)c1[nH]c(cn1)c1sc(cc1)c1ccc(cc1)c1[nH]c(nc1)[C@H]1N(C(=O)[C	1256385-46-2	Carbamic acid, N,N,Ä≤-[(5,5-dioxido-3,7-dibenzothiophenediyl)bis[1H-imidazole-5,	C40H48N8O8S	800.92	A/B	-8.2
8077	C(=O)([C@@H](NC(=O)OC)C(C)C)N1[C@H](c2[nH]c(c3cc4c(c5c(s4)cc(cc5)c4[nH]c(nc4)[C@H]4N(C(=O)[C@@H](NC(	1256387-72-0	Carbamic acid, N,N,Ä≤-[(2-cyano[1,1,Ä≤-biphenyl]-4,4,Ä≤-diyl)bis[1H-imidazole-5	C41H49N9O6	763.88	A/B	-8.2
8078	O[C@H]1[C@@H](O[C@H](COC(=O)c2cc(O)c(O)c(O)c2)[C@H]1O)n1nc(C(=O)N)nc1	1257423-93-0	1H-1,2,4-Triazole-3-carboxamide, 1-[5-O-(4-aminobenzoyl)-Œ≤-D-ribofuranosyl]-	C15H17N5O6	363.33	A/B	-8.2
8079	C(=O)(Nc1sc(N(=O)=O)cn1)c1c(OC(=O)C=Cc2ccccc2)cccc1	1259483-71-0	Benzamide, 2-(benzoyloxy)-N-(5-nitro-2-thiazolyl)-	C17H11N3O5S	369.35	A/B	-8.2
8080	C(CCCCC)(P(=O)(O)O)C(=O)O	1263191-47-4	3-Quinolinecarboxylic acid, 7-[4-[2-[3-[2-(aminothioxomethyl)hydrazinylidene]-5-	C29H29F2N7O5S	625.65	A/B	-8.2
8081	C(N1c2c(/C(=N/NC(=S)NOC)/C1=O)cc(C)cc2)N1CCN(CC1)c1ccccc1	1265622-96-5	Hydrazinecarbothioamide, 2-[1,2-dihydro-5-methyl-2-oxo-1-[(4-phenyl-1-piperaziny	C21H24N6O2S	424.52	A/B	-8.2
8082	N(=C\1/c2c(N(CN3CCN(CC3)c3cc4c(cc3F)c(=O)c(C(=O)O)cn4CC)C1=O)ccc(Cl)c2)\NC(=S)NOC	1265623-10-6	3-Quinolinecarboxylic acid, 7-[4-[[(3Z)-5-chloro-2,3-dihydro-3-[2-[(hydroxyaminc	C26H25ClFN7O5S	602.04	A/B	-8.2
8083	N(=C\1/c2c(N(CN3CCN(CC3)c3cc4c(cc3F)c(=O)c(C(=O)O)cn4CC)C1=O)ccc(F)c2)\NC(=S)NO	1265623-11-7	3-Quinolinecarboxylic acid, 7-[4-[[(3Z)-5-chloro-2,3-dihydro-3-[2-[(methoxyaminc	C27H27ClFN7O5S	616.06	A/B	-8.2
8084	C(C)n1c2c(c(=O)c(C(=O)O)c1)cc(F)c(c2F)N1CC(C)N(CN2c3c(/C(=N/NC(=S)NO)/C2=O)cc(F)cc3)CC1	1265623-22-0	3-Quinolinecarboxylic acid, 7-[4-[[(3Z)-5-chloro-2,3-dihydro-3-[2-[(methoxyaminc	C28H28ClF2N7O5S	648.08	A/B	-8.2
8085	O=c1n([C@@H]2O[C@H](CO[P@@]3(=O)Oc4c(CO3)cccc4C)[C@@H](N=[N+]=[N-])C2)cc(C)c(=O)[nH]1	1270965-84-8	Thymidine, 2,Ä≤,3,Ä≤-didehydro-3,Ä≤-deoxy-5,Ä≤-O-[(2R)-6,8-dimethyl-2-oxido-4H-	C19H21N2O7P	420.35	A/B	-8.2
8086	C(=O)([C@@H](NC(=O)OC)C(C)C)N1CCC2(CC1c1[nH]c(cn1)c1ccc(cc1)c1ccc(cc1)c1[nH]c(nc1)[C@H]1N(C(=O)[C@@H]	1272656-28-6	Carbamic acid, N-[(1S)-1-[[(2S)-2-[5-[4,Ä≤-[2-[2-[(2S)-2-[(methoxycarbonyl)aminc	C44H57N9O6	807.98	A/B	-8.2
8087	O=C1[C@@H]2[C@@H](c3c(C([C@H]2CO1)[2H])cc1c(c3)OCO1)c1cc(OC)c(OC)c(OC)c1	1273148-08-5	Carbamic acid, N-[(1S)-1-[[(2S)-2-[5-[4,Ä≤-[2-[(2S,3S,8S)-7-[(2S)-2-[(methoxycar	C44H56N8O8	824.96	A/B	-8.2
8088	C(=O)([C@@H](N(C(=O)OC)C)C(C)C)N1[C@@H]([C@@H]2C[C@H]1CC2)c1[nH]c(cn1)c1ccc(cc1)c1ccc(cc1)c1[nH]c(nc	1273589-67-5	Carbamic acid, N-[(1R)-1-[[(2S)-2-[5-[4,Ä≤-[2-[(1R,3S,4S)-2-[(2R)-2-[(methoxycar	C42H52N8O6	764.91	A/B	-8.2
8089	C(=O)([C@@H](NC(=O)OC)C(C)C)N1[C@@H]([C@@H]2C[C@H]1CC2)c1[nH]c(cn1)c1ccc(cc1)c1ccc(cc1)c1[nH]c(nc1)[	1273591-06-2	Carbamic acid, N-[(1S)-1-[[(2S)-4,4-difluoro-2-[5-[4,Ä≤-[2-[(1R,3S,4S)-2-[(2S)-2	C42H50F2N8O6	800.89	A/B	-8.2
8090	C(=O)([C@@H](NC(=O)OC)C(C)C)N1[C@@H]([C@H]2[C@H](OC)C[C@@H]1C2)c1[nH]c(cn1)c1ccc(cc1)c1ccc(cc1)c1[nH	1273591-17-5	Carbamic acid, N-[(1S)-1-[[(2S)-2-[5-[4,Ä≤-[2-[(1S,3S,4S,5R)-5-hydroxy-2-[(2S)-2	C42H52N8O7	780.91	A/B	-8.2
8091	C(=O)([C@@H](NC(=O)OC)C(C)C)N1[C@@H]([C@@H]2C[C@H]1CC2)c1[nH]c(cn1)c1c(Cl)cc(cc1)c1cc(Cl)c(cc1)c1[nH	1273591-50-6	Carbamic acid, N-[(1S)-1-[[(2S)-2-[5-[4,Ä≤-[2-[(1R,3S,4S)-2-[(2S)-2-[(methoxycar	C44H50F6N8O6	900.91	A/B	-8.2
8092	O=c1n(ccc(N)n1)C1OC(COCP(=O)(O)O)CC1	129180-66-1	2-Azocinamine, 3,4,5,6,7,8-hexahydro-N-(phenyltricyclo[3.3.1.13,7]dec-1-ylmethyl	C24H34N2	350.54	A/B	-8.2
8093	C(COCP(=O)(OP(=O)(O)O)O)n1c2c(nc1)c(N)ncn2	129546-34-5	Guanosine, cyclic 3,Ä≤,5,Ä≤-(methylphosphonate) 2,Ä≤-acetate, (S)-	C13H16N5O7P	385.27	A/B	-8.2
8094	O=c1c2c(n(cn2)[C@H]2[C@H](O)[C@H]3[C@H](O2)CO[P@](=O)(C)O3)[nH]c(N)n1	129645-87-0	Guanosine, cyclic 3,Ä≤,5,Ä≤-(methylphosphonate), (S)-	C11H14N5O6P	343.23	A/B	-8.2
8095	S=c1n(nc(s1)c1cc(N(=O)=O)c(OC)cc1)c1nc(nc(n1)N1CCOCC1)N1CCOCC1	1309437-30-6	1,3,4-Thiadiazole-2(3H)-thione, 3-(4,6-di-4-morpholinyl-1,3,5-triazin-2-yl)-5-(3	C19H20N8O4S2	488.54	A/B	-8.2
8096	C(=O)([C@@H](NC(=O)OC)C(C)C)N1[C@@H](CC(=C)C1)c1[nH]c(cn1)c1c(C)cc(cc1)c1cc(C)c(cc1)c1[nH]c(nc1)[C@H	1311398-22-7	Carbamic acid, N-[(1S)-2-methyl-1-[[(2S)-4-methylene-2-[5-[2,2,Ä≤,3,3,Ä≤-tetraf	C41H46F4N8O6	822.85	A/B	-8.2
8097	C(=O)([C@@H](NC(=O)OC)C(C)C)N1[C@@H](CC(=C)C1)c1[nH]c(cn1)c1ccc(cc1)c1ccc(cc1)c1[nH]c(nc1)[C@H]1N(C(	1311398-30-7	Carbamic acid, N-[(1S)-1-[[(2S)-2-[5-[4,Ä≤-[2-[(3S)-2-[(2S)-2-[(methoxycarbonyl)	C44H54N8O6	790.95	A/B	-8.2
8098	C(=O)([C@@H](NC(=O)OC)C(C)C)N1[C@@H](CC(=C(F)F)C1)c1[nH]c(cn1)c1ccc(cc1)c1ccc(cc1)c1[nH]c(nc1)[C@H]1	1311398-39-6	Carbamic acid, N-[(1S)-1-[[(2S,4S)-4-fluoro-2-[5-[4,Ä≤-[2-[(2S)-1-[(2S)-2-[(meth	C41H49FN8O6	768.88	A/B	-8.2
8099	C(=O)([C@@H](NC(=O)OC)C(C)C)N1[C@@H](CC(=C)C1)c1[nH]c(cn1)[C@@H]1CC[C@H](CC1)c1ccc(cc1)c1[nH]c(nc1)[	1311398-48-7	Carbamic acid, N-[(1S)-1-[[(2S)-2-[5-[2-[4-[2-[(2S)-1-[(2S)-2-[(methoxycarbonyl)	C37H46N8O6	698.81	A/B	-8.2
8100	C(=O)([C@@H](NC(=O)OC)C(C)C)N1[C@@H](CC(=C)C1)c1[nH]c(cn1)c1cc2c(cc1)cc(CCc1[nH]c(nc1)[C@H]1N(C(=O)[	1311398-54-5	Carbamic acid, N-[(1S)-1-[[(2S)-2-[5-[4-[2-[4-[2-[(2S)-1-[(2S)-2-[(methoxycarbor	C43H52N8O6	776.92	A/B	-8.2
8101	C(=O)([C@@H](NC(=O)OC)C(C)C)N1[C@H]2[C@H](C2)C[C@H]1c1[nH]c(c(Cl)n1)c1ccc(cc1)c1ccc(cc1)c1[nH]c(nc1C	1312335-94-6	Carbamic acid, N-[(1S)-1-[[(1R,3S,5R)-3-[5-[4,Ä≤-[5-chloro-2-[(1R,3S,5R)-2-[(2S)	C42H49ClN8O6	797.34	A/B	-8.2
8102	C(=O)([C@@H](NC(=O)OC)C1CCOCC1)N1[C@H]2[C@H](C2)C[C@H]1c1[nH]c(c(Cl)n1)c1ccc(cc1)c1ccc(cc1)c1[nH]c(n	1312335-98-0	Carbamic acid, N,N,Ä≤-[[1,1,Ä≤-biphenyl]-4,4,Ä≤-diylbis[(5-chloro-1H-imidazole-	C40H48Cl2N8O6	807.77	A/B	-8.2
8103	C(=O)([C@@H](NC(=O)OC)C1CC1)N1[C@H]2[C@H](C2)C[C@H]1c1[nH]c(c(Cl)n1)c1ccc(cc1)c1ccc(cc1)c1[nH]c(nc1C	1312336-13-2	Carbamic acid, N-[(1S)-2-[(1R,3S,5R)-3-[5-chloro-4-[4,Ä≤-[5-chloro-2-[(1R,3S,5R)	C44H50Cl2N8O7	873.82	A/B	-8.2
8104	C(=O)([C@@H](NC(=O)OC)C(C)C)N1[C@@H](CC[C@@H]1C)c1[nH]c(c(Cl)n1)c1ccc(cc1)c1ccc(cc1)c1[nH]c(nc1Cl)[C	1312336-19-8	Carbamic acid, N-[(1S)-2-[(2S,5S)-2-[5-chloro-4-[4,Ä≤-[5-chloro-2-[(2S,5S)-1-[(2	C44H54Cl2N8O7	877.85	A/B	-8.2
8105	C(=O)([C@@H](NC(=O)OC)C(C)C)N1[C@@H](C[C@H](C)C1)c1[nH]c(c(Cl)n1)c1ccc(cc1)c1ccc(cc1)c1[nH]c(nc1)[C@	1312336-27-8	Carbamic acid, N-[(1S)-2-[(2S,4S)-2-[5-chloro-4-[4,Ä≤-[5-chloro-2-[(2S,4S)-1-[(2	C44H54Cl2N8O7	877.85	A/B	-8.2
8106	C(=O)([C@@H](NC(=O)OC)C(C)C)N1[C@@H](C[C@H](C)C1)c1[nH]c(c(Cl)n1)c1ccc(cc1)c1ccc(cc1)c1[nH]c(nc1Cl)[	1312336-29-0	Carbamic acid, N-[(1S)-2-[(2S,4S)-2-[5-chloro-4-[4,Ä≤-[2-[(2S,4S)-1-[(2S)-2-[(me	C44H55ClN8O7	843.41	A/B	-8.2
8107	C(=O)([C@@H](NC(=O)OC)C(C)C)N1[C@H]2[C@H](C2)C[C@H]1c1[nH]c(c(Br)n1)c1ccc(cc1)c1ccc(cc1)c1[nH]c(nc1)	1312336-43-8	Carbamic acid, N-[(1S)-2-[(1R,3S,5R)-3-[7-[4-[5-chloro-2-[(1R,3S,5R)-2-[(2S)-2-[	C48H53ClN8O8	905.44	A/B	-8.2
8108	C(=O)([C@@H](NC(=O)OC)C(C)C)N1[C@H]2[C@H](C2)C[C@H]1c1[nH]c2c(n1)cc(Cl)c1c2ccc(c1)c1ccc(cc1)c1[nH]c(	1312336-50-7	Carbamic acid, N-[(1S)-1-[[(2S)-2-[5-[4,Ä≤-[5-iodo-2-[(2S)-1-[(2S)-2-[(methoxyca	C40H49IN8O6	864.77	A/B	-8.2
8109	CC1(c2c(nc([nH]2)[C@H]2N(C(=O)[C@@H](NC(=O)OC)C(C)C)CCC2)c2ccc(cc2)c2ccc(cc2)c2[nH]c(nc2)[C@H]2N(C(=	1312336-60-9	Carbamic acid, N-[(1S)-1-[[(2S)-2-[4-(cyclopropylmethyl)-5-[4,Ä≤-[2-[(2S)-1-[(2S	C44H56N8O6	792.97	A/B	-8.2
8110	C(=O)([C@@H](NC(=O)OC)C1CCOCC1)N1[C@H]2[C@H](C2)C[C@H]1c1[nH]c(c(F)n1)c1ccc(cc1)c1ccc(cc1)c1[nH]c(nc	1312336-63-2	Carbamic acid, N-[(1S)-1-[[(1R,3S,5R)-3-[5-[4,Ä≤-[5-fluoro-2-[(1R,3S,5R)-2-[(2S)	C42H49FN8O6	780.89	A/B	-8.2
8111	C(=O)([C@@H](NC(=O)OC)C(C)C)N1[C@H]2[C@H](C2)C[C@H]1c1[nH]c(c(n1)c1ccc(cc1)c1ccc(cc1)c1[nH]c(nc1)[C@	1312336-98-3	Carbamic acid, N,N,Ä≤-[[1,1,Ä≤-biphenyl]-4,4,Ä≤-diylbis[(5-cyclopropyl-1H-imida	C46H58N8O6	819	A/B	-8.2
8112	C(=O)([C@@H](NC(=O)OC)C(C)C)N1[C@H](c2[nH]c(c3c4c(c(cc3)c3ccc(cc3)c3[nH]c(nc3)[C@H]3N(C(=O)[C@@H](NC	1312608-36-8	Carbamic acid, N-[(1S)-1-[[(2S)-2-[5-[4-[4-[2-[(2S)-1-[(2S)-2-[(methoxycarbonyl)	C43H53N9O6	791.94	A/B	-8.2
8113	Cc1c2c(c(ccc2c2ccc(cc2)c2[nH]c(nc2)[C@H]2N(C(=O)[C@@H](NC(=O)OC)C(C)C)CCC2)c2[nH]c(nc2)[C@H]2N(C(=O)	1312608-48-2	Carbamic acid, N-[(1S)-1-[[(2S)-2-[5-[4-[2,3-dihydro-8-[2-[(2S)-1-[(2S)-2-[(meth	C42H52N8O8	796.91	A/B	-8.2
8114	C[C@]1(c2[nH]c(c3c4c(c(cc3)c3ccc(cc3)c3[nH]c(nc3)[C@H]3N(C(=O)[C@@H](NC(=O)OC)C(C)C)CCC3)cc[nH]4)cn2	1312608-74-4	Carbamic acid, N-[(1S)-1-[[(2S)-2-[5-[4-[7-[2-[(2S)-1-[(2S)-2-[(methoxycarbonyl)	C43H53N9O6S	824	A/B	-8.2
8115	Cc1[nH]c2c(ccc(c2c1)c1ccc(cc1)c1[nH]c(nc1)[C@H]1N(C(=O)[C@@H](NC(=O)OC)C(C)C)CCC1)c1[nH]c(nc1)[C@H]1	1312609-57-6	Carbamic acid, N-[(1S)-1-[[(2S)-2-[5-[4-[7-[2-[(2S)-1-[(2S)-2-[(methoxycarbonyl)	C45H56N8O7	820.98	A/B	-8.2
8116	C(=O)([C@@H](NC(=O)OC)C(C)C)N1[C@H](c2[nH]c(c3ccc(c4c5c(c(cc4)c4[nH]c(nc4)[C@@H]4CCCN4)cccc5)cc3)cn2	1312609-62-3	Carbamic acid, N-[(1S)-1-[[(2S)-2-[5-[4-[7-[2-[(2S)-1-[(2S)-2-[(methoxycarbonyl)	C43H53N9O6	791.94	A/B	-8.2
8117	C(=O)([C@@H](NC(=O)OC)C(C)C)N1[C@@H](CCC1)c1[nH]c(cn1)c1c(O)c(N)c(cc1)c1ccc(cc1)c1[nH]c(nc1)[C@H]1N(	1312610-47-1	Carbamic acid, N,N,Ä≤-[[2,3-diamino[1,1,Ä≤-biphenyl]-4,4,Ä≤-diyl]bis[1H-imidazo	C40H52N10O6	768.9	A/B	-8.2
8118	C(OCC[Si](C)(C)C)n1c2c(c(ccc2c2[nH]c(nc2)[C@@]2(C)N(C(=O)[C@@H](NC(=O)OC)C(C)C)CCC2)c2ccc(cc2)c2[nH]	1312610-67-5	Carbamic acid, N,N,Ä≤-[(2-amino-3-hydroxy[1,1,Ä≤-biphenyl]-4,4,Ä≤-diyl)bis[1H-i	C40H51N9O7	769.89	A/B	-8.2
8119	O[C@H]1[C@H](n2c3c(nc2)c(=O)nc(N)[nH]3)O[C@H](C(=O)NC2CCCC2)[C@H]1O	1312883-30-9	Phosphonic acid, P-[[(1S)-1-[[4-(benzoylamino)-2-oxo-1(2H)-pyrimidinyl]methyl]-2	C36H36N3O7P	653.66	A/B	-8.2
8120	O(Cc1ccc(F)cc1)c1c2c(oc(c(O)c2=O)c2ncc(OC)cn2)cc(C)c1	1312894-84-0	4H-1-Benzopyran-4-one, 5-[(4-fluorophenyl)methoxy]-3-hydroxy-7-methyl-2-(5-methy	C22H17FN2O4	392.38	A/B	-8.2
8121	O(Cc1ccc(F)cc1)c1c2c(oc(c(O)c2=O)c2ncc(Cl)cn2)cc(C)c1	1312894-85-1	4H-1-Benzopyran-4-one, 5-[(4-fluorophenyl)methoxy]-3-hydroxy-2-(5-methoxy-2-pyri	C22H17FN2O5	408.38	A/B	-8.2
8122	O(Cc1ccc(F)cc1)c1c2c(oc(c(O)c2=O)c2nnc(C)[nH]2)cc(C)c1	1312894-93-1	4H-1-Benzopyran-4-one, 5-[(4-fluorophenyl)methoxy]-3-hydroxy-7-methyl-2-(1H-1,2,	C19H14FN3O4	367.33	A/B	-8.2
8123	O(Cc1ccc(F)cc1)c1c2c(oc(c(O)c2=O)c2[nH]cc(Cl)n2)cc(C)c1	1312895-00-3	4H-1-Benzopyran-4-one, 5-[(4-fluorophenyl)methoxy]-3-hydroxy-2-(5-methoxy-1H-imi	C21H17FN2O5	396.37	A/B	-8.2
8124	O(Cc1ccc(F)cc1)c1c2c(c(OC)c(C)c1)oc(c(O)c2=O)c1ncc(Cl)cn1	1312895-85-4	4H-1-Benzopyran-4-one, 5-[(4-fluorophenyl)methoxy]-3-hydroxy-8-methoxy-2-(5-meth	C23H19FN2O6	438.41	A/B	-8.2
8125	O(Cc1ccc(F)cc1)c1c2c(oc(c(O)c2=O)c2ncc(cn2)c2ccccc2)cc(OC)c1	1312897-47-4	4H-1-Benzopyran-4-one, 2-(5-chloro-2-pyrimidinyl)-5-[(4-fluorophenyl)methoxy]-3-	C21H14ClFN2O5	428.8	A/B	-8.2
8126	O(Cc1ccc(F)cc1)c1c2c(oc(c(O)c2=O)c2nnc(C)[nH]2)cc(OC)c1	1312897-54-3	4H-1-Benzopyran-4-one, 5-[(4-fluorophenyl)methoxy]-3-hydroxy-7-methoxy-2-(1H-1,2	C19H14FN3O5	383.33	A/B	-8.2
8127	O(Cc1ccc(F)cc1)c1c2c(oc(c(O)c2=O)c2[nH]c(cn2)c2ccccc2)cc(OC)c1	1312897-62-3	4H-1-Benzopyran-4-one, 2-(5-chloro-1H-imidazol-2-yl)-5-[(4-fluorophenyl)methoxy]	C20H14ClFN2O5	416.79	A/B	-8.2
8128	O(C)c1c2c(c(OCc3ccc(F)cc3)cc1OC)c(=O)c(O)c(o2)c1ncc(cn1)c1ccccc1	1312898-48-8	4H-1-Benzopyran-4-one, 2-(5-chloro-2-pyrimidinyl)-5-[(4-fluorophenyl)methoxy]-3-	C22H16ClFN2O6	458.82	A/B	-8.2
8129	O(Cc1ccc(F)cc1)c1c2c(oc(c(O)c2=O)c2ncc(Cl)cn2)cc(Cl)c1	1312900-56-3	4H-1-Benzopyran-4-one, 7-chloro-5-[(4-fluorophenyl)methoxy]-3-hydroxy-2-(5-methc	C21H14ClFN2O5	428.8	A/B	-8.2
8130	O(Cc1ccc(F)cc1)c1c2c(oc(c(O)c2=O)c2[nH]c(OC)cn2)cc(Cl)c1	1312900-70-1	4H-1-Benzopyran-4-one, 7-chloro-5-[(4-fluorophenyl)methoxy]-3-hydroxy-2-(5-methy	C20H14ClFN2O4	400.79	A/B	-8.2
8131	O(Cc1ccc(F)cc1)c1c2c(c(C)c(c1)c1ccccc1)oc(c(O)c2=O)c1[nH]c(C)cn1	1312903-71-1	4H-1-Benzopyran-4-one, 5-[(4-fluorophenyl)methoxy]-3-hydroxy-2-(1H-imidazol-2-yl	C26H19FN2O4	442.44	A/B	-8.2
8132	O(Cc1ccc(F)cc1)c1c2c(c(c(c1)c1ccccc1)c1ccccc1)oc(c(O)c2=O)c1nnc(Cl)[nH]1	1312905-78-4	4H-1-Benzopyran-4-one, 5-[(4-fluorophenyl)methoxy]-3-hydroxy-2-(5-methoxy-1H-1,2	C31H22FN3O5	535.52	A/B	-8.2
8133	O(C)c1c2c(cc(OCc3ccc(F)cc3)c1)c(=O)c(O)c(o2)c1ncc(OC)cn1	1312907-15-5	4H-1-Benzopyran-4-one, 6-[(4-fluorophenyl)methoxy]-3-hydroxy-8-methoxy-2-(5-meth	C22H17FN2O5	408.38	A/B	-8.2
8134	O(C)c1c2c(c(=O)c(O)c(o2)c2nnc(C)[nH]2)cc(OCc2ccc(F)cc2)c1	1312907-24-6	4H-1-Benzopyran-4-one, 6-[(4-fluorophenyl)methoxy]-3-hydroxy-8-methoxy-2-(1H-1,2	C19H14FN3O5	383.33	A/B	-8.2
8135	O=c1c2c(oc(c1O)c1ncc(cn1)c1ccccc1)cc(C)c(OCc1ccc(F)cc1)c2	1312908-67-0	4H-1-Benzopyran-4-one, 2-(5-chloro-2-pyrimidinyl)-6-[(4-fluorophenyl)methoxy]-3-	C21H14ClFN2O4	412.8	A/B	-8.2
8136	O(C)c1c2c(cc(OCc3ccc(F)cc3)c1C)c(=O)c(O)c(o2)c1ncc(C)cn1	1312909-76-4	4H-1-Benzopyran-4-one, 6-[(4-fluorophenyl)methoxy]-3-hydroxy-8-methoxy-7-methyl-	C22H17FN2O5	408.38	A/B	-8.2
8137	O(C)c1c2c(cc(OCc3ccc(F)cc3)c1C)c(=O)c(O)c(o2)c1ncc(cn1)c1ccccc1	1312909-79-7	4H-1-Benzopyran-4-one, 2-(5-chloro-2-pyrimidinyl)-6-[(4-fluorophenyl)methoxy]-3-	C22H16ClFN2O5	442.82	A/B	-8.2
8138	O(C)c1c2c(cc(OCc3ccc(F)cc3)c1C)c(=O)c(O)c(o2)c1[nH]cnn1	1312909-80-0	4H-1-Benzopyran-4-one, 6-[(4-fluorophenyl)methoxy]-3-hydroxy-8-methoxy-7-methyl-	C28H21FN2O5	484.48	A/B	-8.2
8139	O(C)c1c2c(cc(OCc3ccc(F)cc3)c1C)c(=O)c(O)c(o2)c1[nH]c(Cl)nn1	1312909-88-8	4H-1-Benzopyran-4-one, 6-[(4-fluorophenyl)methoxy]-3-hydroxy-8-methoxy-2-(5-meth	C21H18FN3O6	427.38	A/B	-8.2
8140	Cc1c2c(cc(OCc3ccc(F)cc3)c1OC)c(=O)c(O)c(o2)c1[nH]c(C)cn1	1312912-40-5	4H-1-Benzopyran-4-one, 6-[(4-fluorophenyl)methoxy]-3-hydroxy-2-(1H-imidazol-2-yl	C21H17FN2O5	396.37	A/B	-8.2
8141	Cc1c2c(cc(OCc3ccc(F)cc3)c1OC)c(=O)c(O)c(o2)c1[nH]c(Cl)cn1	1312912-44-9	4H-1-Benzopyran-4-one, 6-[(4-fluorophenyl)methoxy]-3-hydroxy-7-methoxy-2-(5-meth	C22H19FN2O6	426.39	A/B	-8.2
8142	O(C)c1c2c(cc(OCc3ccc(F)cc3)c1OC)c(=O)c(O)c(o2)c1ncc(OC)cn1	1312913-12-4	4H-1-Benzopyran-4-one, 6-[(4-fluorophenyl)methoxy]-3-hydroxy-7,8-dimethoxy-2-(5-	C23H19FN2O6	438.41	A/B	-8.2
8143	O(C)c1c2c(cc(OCc3ccc(F)cc3)c1OC)c(=O)c(O)c(o2)c1nnc(Cl)[nH]1	1312913-34-0	4H-1-Benzopyran-4-one, 6-[(4-fluorophenyl)methoxy]-3-hydroxy-7,8-dimethoxy-2-(5-	C21H18FN3O7	443.38	A/B	-8.2
8144	O(C)c1c2c(cc(OCc3ccc(F)cc3)c1OC)c(=O)c(O)c(o2)c1[nH]c(OC)cn1	1312913-42-0	4H-1-Benzopyran-4-one, 6-[(4-fluorophenyl)methoxy]-3-hydroxy-7,8-dimethoxy-2-(5-	C22H19FN2O6	426.39	A/B	-8.2
8145	O(C)c1c2c(cc(OCc3ccc(F)cc3)c1OC)c(=O)c(O)c(o2)c1[nH]c(cn1)c1ccccc1	1312913-46-4	4H-1-Benzopyran-4-one, 2-(5-chloro-1H-imidazol-2-yl)-6-[(4-fluorophenyl)methoxy]	C21H16ClFN2O6	446.81	A/B	-8.2
8146	O(C)c1c2c(cc(OCc3ccc(F)cc3)c1Cl)c(=O)c(O)c(o2)c1ncc(cn1)c1ccccc1	1312913-61-3	4H-1-Benzopyran-4-one, 7-chloro-2-(5-chloro-2-pyrimidinyl)-6-[(4-fluorophenyl)me	C21H13Cl2FN2O5	463.24	A/B	-8.2
8147	O(C)c1c2c(cc(OCc3ccc(F)cc3)c1Cl)c(=O)c(O)c(o2)c1[nH]cnn1	1312913-63-5	4H-1-Benzopyran-4-one, 7-chloro-6-[(4-fluorophenyl)methoxy]-3-hydroxy-8-methoxy-	C27H18ClFN2O5	504.89	A/B	-8.2
8148	O(C)c1c2c(cc(OCc3ccc(F)cc3)c1Cl)c(=O)c(O)c(o2)c1[nH]c(Cl)nn1	1312913-79-3	4H-1-Benzopyran-4-one, 7-chloro-6-[(4-fluorophenyl)methoxy]-3-hydroxy-8-methoxy-	C20H15ClFN3O6	447.8	A/B	-8.2
8149	O(C)c1c2c(cc(OCc3ccc(F)cc3)c1Cl)c(=O)c(O)c(o2)c1[nH]c(nn1)c1ccccc1	1312913-81-7	4H-1-Benzopyran-4-one, 7-chloro-2-(5-chloro-1H-1,2,4-triazol-3-yl)-6-[(4-fluorop	C19H12Cl2FN3O5	452.22	A/B	-8.2
8150	O(C)c1c2c(cc(OCc3ccc(F)cc3)c1Cl)c(=O)c(O)c(o2)c1[nH]c(Cl)cn1	1312913-89-5	4H-1-Benzopyran-4-one, 7-chloro-6-[(4-fluorophenyl)methoxy]-3-hydroxy-8-methoxy-	C21H16ClFN2O6	446.81	A/B	-8.2
8151	Cc1c2c(cc(OCc3ccc(F)cc3)c1c1ccccc1)c(=O)c(O)c(o2)c1ncc(Cl)cn1	1312916-84-9	4H-1-Benzopyran-4-one, 6-[(4-fluorophenyl)methoxy]-3-hydroxy-2-(5-methoxy-2-pyri	C28H21FN2O5	484.48	A/B	-8.2
8152	Clc1c2c(cc(OCc3ccc(F)cc3)c1c1ccccc1)c(=O)c(O)c(o2)c1ncc(cn1)c1ccccc1	1312917-85-3	4H-1-Benzopyran-4-one, 8-chloro-2-(5-chloro-2-pyrimidinyl)-6-[(4-fluorophenyl)me	C26H15Cl2FN2O4	509.31	A/B	-8.2
8153	O(Cc1ccc(F)cc1)c1c(c2c(c(c2c1)c(=O)c(O)c(o2)c1ncc(Cl)cn1)c1ccccc1)c1ccccc1	1312918-34-5	4H-1-Benzopyran-4-one, 6-[(4-fluorophenyl)methoxy]-3-hydroxy-2-(5-methoxy-2-pyri	C33H23FN2O5	546.54	A/B	-8.2
8154	O([C@@H]1[C@@H](COP2(=O)Oc3c(CO2)cccc3)O[C@H](Cl)n1c(=O)[nH]c(=O)c(I)c1)P1(=O)Oc2c(CO1)cccc2	1313031-57-0	Uridine, 5,Ä≤-O-[(2R)-6,8-bis(1,1-dimethylethyl)-5-fluoro-2-oxido-4H-1,3,2-benzc	C24H31FIN2O8P	652.39	A/B	-8.2
8155	O=c1n([C@H]2C[C@H](OP3(=O)Oc4c(CO3)cccc4)[C@@H](CO)O2)cc(I)c(=O)[nH]1	1313031-60-5	Uridine, 3,Ä≤,5,Ä≤-bis-O-[6,8-bis(1,1-dimethylethyl)-5-fluoro-2-oxido-4H-1,3,2-l	C39H51F2IN2O11P2	950.68	A/B	-8.2
8156	C(=O)([C@](NC(=O)OC)(C(C([2H])([2H])[2H])(C([2H])([2H])[2H])[2H])[2H])N1[C@@H](C[C@H](C)C1)c1[nH]c(C	1313207-81-6	Carbamic acid, N,N,Ä≤-[1,4-phenylenebis[2,1-ethynediyl-1H-imidazole-5,2-diyl[(2S	C42H54N8O6	766.93	A/B	-8.2
8157	C(=O)([C@@H](NC(=O)OC)C(C)C)N1[C@@H]([C@@H]2C[C@H]1CC2)c1[nH]c(cn1)c1ccc(cc1)c1ccc(cc1)c1[nH]c(nc1)[	1321849-57-3	Carbamic acid, N-[(1R)-2-[(1R,3S,4S)-3-[5-[4,Ä≤-[2-[(1R,3S,4S)-2-[(2S)-2-[(methc	C42H50N8O6	762.9	A/B	-8.2
8158	O=c1c2c(n(cn2)[C@@H]2O[C@H](CO)CC2)[nH]c(=O)[nH]1	1321928-75-9	1,3-Oxathiolane-2-carboxylic acid, 5-[4-(acetylamino)-5-fluoro-2-oxo-1(2H)-pyrim	C20H28FN3O5S	441.52	A/B	-8.2
8159	N(=C1c2c(N(Cc3ccc(C)cc3)C1=O)ccc(Cl)c2)NC(=S)Nc1ccc(Cl)cc1	1323295-81-3	Hydrazinecarbothioamide, N-(4-chlorophenyl)-2-[5-fluoro-1-[(4-fluorophenyl)methy	C22H15ClF2N4OS	456.9	A/B	-8.2
8160	N(c1c(C)c(c(/C=C/C#N)c(c1C)[2H])[2H])c1nc(Nc2cc(c(C#N)c(c2)[2H])[2H])nc(c1[2H])[2H]	1331866-51-3	Benzonitrile-2,6-d2, 4-[[4-[[4-[(1E)-2-cyanoethenyl]-2,6-di(methyl-d3)phenyl-3,5	C22H8D10N6		A/B	-8.2
8161	N(c1c(C)c(c(/C(=C(/C#N)\[2H])/[2H])c(c1C)[2H])[2H])c1nc(Nc2cc(c(C#N)c(c2)[2H])[2H])ncc1	1331866-75-1	Benzonitrile-3,5-d2, 4-[[6-[[4-[(1E)-2-cyanoethenyl-1,2-d2]-2,6-di(methyl-d3)phe	C22H4D14N6		A/B	-8.2
8162	N(c1c(C([2H])([2H])[2H])c(c(/C(=C(/C#N)\[2H])/[2H])c(c1C([2H])([2H])[2H])[2H])[2H])c1nc(Nc2cc(c(C#N)	1331866-80-8	Benzonitrile-2,6-d2, 4-[[6-[[4-[(1E)-2-cyanoethenyl-1,2-d2]-2-methyl-6-(methyl-c	C22H7D11N6		A/B	-8.2
8163	N(c1c(C([2H])([2H])[2H])c(c(/C(=C(/C#N)\[2H])/[2H])c(c1C)[2H])[2H])c1nc(Nc2c(c(c(C#N)c(c2[2H])[2H])[	1331866-85-3	Benzonitrile-2,3,5,6-d4, 4-[[6-[[4-[(1E)-2-cyanoethenyl-1,2-d2]-2,6-dimethylpher	C22H8D10N6		A/B	-8.2
8164	N(c1c(C([2H])([2H])[2H])cc(/C=C/C#N)cc1C)c1nc(Nc2cc(c(C#N)c(c2)[2H])[2H])nc(c1[2H])[2H]	1331867-00-5	Benzonitrile-2,6-d2, 4-[[6-[[4-[(1E)-2-cyanoethenyl]-2,6-dimethylphenyl]amino]-2	C22H14D4N6		A/B	-8.2
8165	N(c1c(C([2H])([2H])[2H])cc(/C=C/C#N)cc1C)c1nc(Nc2c(c(c(C#N)c(c2[2H])[2H])[2H])[2H])nc(c1[2H])[2H]	1331867-06-1	Benzonitrile-2,3,5,6-d4, 4-[[6-[[4-[(1E)-2-cyanoethenyl]-2,6-dimethylphenyl]amir	C22H12D6N6		A/B	-8.2
8166	N(=Nc1cc(C)c(N=Nc2cc(S(=O)(=O)O)ccc2)cc1)c1c2c(c(S(=O)(=O)O)ccc2)c(N)cc1	1345852-38-1	1-Naphthalenesulfonic acid, 8-amino-5-[2-[7-sulfo-4-[2-(7-sulfo-1-naphthalenyl)c	C30H21N5O9S3	691.71	A/B	-8.2
8167	N(=Nc1c2c(c(N=Nc3ccc(S(=O)(=O)O)cc3)cc1)cccc2)c1c2c(cc(S(=O)(=O)O)cc2)c(N)cc1	1345852-43-8	2-Naphthalenesulfonic acid, 8-[2-(4-amino-7-sulfo-1-naphthalenyl)diazenyl]-5-[2-	C26H19N5O9S3	641.65	A/B	-8.2
8168	C([C@H](OCP(=O)(OC(C)C)O)C)n1c2c(nc1)c(N)ncn2	1346597-32-7	Not Yet Assigned	C29H35ClD6FN5O		A/B	-8.2
8169	C(OCOC(CO)CO)n1c2c(nc1)c(=O)nc(N)[nH]2	1346597-44-1	Cyclohexanecarboxamide, 4,4-difluoro-N-formyl-N-[(1S)-3-[3-[3-methyl-5-(1-methyl	C30H41F2N5O2	541.68	A/B	-8.2
8170	N(C([C@H](Cc1ccccc1)C[C@@H](CN1[C@H](C(=O)NC(C)(C)C)CN(Cc2cccnc2)CC1)O)O)[C@@H]1c2c(C[C@H]1O)cccc2	1346794-02-2	2,7,10,12-Tetraazatridecanoic acid, 4-hydroxy-12-methyl-9-(1-methylethyl)-13-[2-	C38H50N6O5S2	734.97	A/B	-8.2
8171	N(S(=O)(=O)c1ccc(NC(=O)CCCC)cc1)(C[C@H]([C@H](Cc1ccccc1)NC(=O)O[C@H]1CCOC1)O)CC(C)C	1346795-73-0	Not Yet Assigned	C36H49N5O4	615.81	A/B	-8.2
8172	C([C@H]([C@H](Cc1ccccc1)NC(=O)[C@@H](NC(=O)c1nc2c(cc1)cccc2)CC(=O)N)O)N1C2C(CC[C@H]1C(=O)NC(C)(C)C)C	1346796-65-3	2,7,10,12-Tetraazatridecanoic acid, 4-hydroxy-10-methyl-9-(1-methylethyl)-13-[2-	C37H48N6O5S2	720.94	A/B	-8.2
8173	[C@H](Cc1ccccc1)(C[C@@H]([C@H](Cc1ccccc1)NC(=O)OCc1cncs1)O)NC(=O)[C@@H](NC(=O)N(Cc1nc(OC(C)C)sc1)C)C	1346854-16-7	2,7,10,12-Tetraazatridecanoic acid, 4-hydroxy-13-(2-methoxy-4-thiazolyl)-12-meth	C35H44N6O6S2	708.89	A/B	-8.2
8174	[C@H](Cc1ccccc1)(C[C@@H]([C@H](Cc1ccccc1)NC(=O)OCc1ccns1)O)NC(=O)[C@H](NC(=O)N(Cc1nc(C(C)C)sc1)CC)C(	1346953-80-7	12-Oxa-2,5,10-triazatridecanethioic acid, 8-hydroxy-3-(1-methylethyl)-4,11-dioxc	C36H45N5O5S3	723.97	A/B	-8.2
8175	N(C(=O)[C@H](Cc1ccccc1)C[C@@H](CN1[C@H](C(=O)NC(C)(C)C)CN(Cc2cccnc2)CC1)O)[C@H]1c2c(C[C@H]1OC(=O)CCC	1347972-85-3	Not Yet Assigned	C47H67N7O6	826.08	A/B	-8.2
8176	[C@@H](Cc1ccccc1)(C[C@@H]([C@@H](Cc1ccccc1)NC(=O)OCc1cncs1)O)NC(=O)[C@H](NC(=O)N(Cc1nc(CCCCC)sc1)C)C	1348216-62-5	[1,1,Ä≤-Biphenyl]-2-sulfonamide, 4,Ä≤-[(2-ethyl-5,7-dimethyl-3H-imidazo[4,5-b]p	C30H38N4O4S2	582.78	A/B	-8.2
8177	O=c1n([C@H]2O[C@@H](COC(=O)CCC(=O)OCC(OC(=O)[C@H]([C@H](CC)C)N)COC(=O)[C@H]([C@H](CC)C)N)SC2)ccc(N)n	1349409-06-8	3-Azabicyclo[3.1.0]hexane-2-carboxamide, N-[3-amino-1-(cyclopropylmethyl)-2,3-di	C32H48N6O7S	660.82	A/B	-8.2
8178	C([C@H]([C@H](Cc1ccccc1)NC(=O)[C@@H](NC(=O)c1nc2c(cc1)cccc2)CC(=O)N)O)N1[C@H](C(=O)NC(C)(C)C)C[C@@]2	1349431-68-0	Carbamic acid, N-[(1S,2R)-1-[[4-[(butoxyhydroxyphosphinyl)methoxy]phenyl]methyl]	C33H49N2O12PS	728.79	A/B	-8.2
8179	C(CC)c1c(OC)ccc(C(=O)/C=C/c2c(O)cc(O)cc2)c1O	1350107-64-0	1(2H)-Pyrimidineacetamide, N-[(1S,3S,4S)-4-[[2-(2,6-dimethylphenoxy)acetyl]aminc	C33H46N4O6	594.74	A/B	-8.2
8180	Cc1nc2c(ccc(c2s1)c1[nH]c(nc1)[C@H]1N(C(=O)[C@@H](NC(=O)OC)C(C)C)CCC1)c1c2c(c(cc1)c1[nH]c(nc1)[C@H]1N	1350718-43-2	Carbamic acid, N,N,Ä≤-[[4,7,Ä≤-bibenzothiazole]-4,Ä≤,7-diylbis[1H-imidazole-5,2	C42H48N10O6S2	853.02	A/B	-8.2
8181	N(=C1c2c(N(CN3CCOCC3)C1=O)cccc2)n1c(Cc2ccccc2)n[nH]c1=S	1350822-80-8	2H-Indol-2-one, 5-bromo-3-[[1,5-dihydro-3-(phenylmethyl)-5-thioxo-4H-1,2,4-triaz	C23H23BrN6OS	511.44	A/B	-8.2
8182	C(=O)([C@@H](NC(=O)OC)[C@@H](C)O)N1[C@@H](CCC1)c1[nH]c(cn1)c1cc2c(cc1)cc(cc2)c1ccc(cc1)c1[nH]c(nc1)[	1351329-92-4	Carbamic acid, N-[(1R)-1-[[(2S)-2-[5-[4-[6-[2-[(2S)-1-[(2R)-2-[(methoxycarbonyl)	C44H52N8O6	788.93	A/B	-8.2
8183	C(=O)([C@@H](NC(=O)OC)C(C)C)N1[C@@H](CCC1)c1[nH]c(cn1)c1ccc(cc1)c1cc2c(cc1)cc(cc2)c1[nH]c(nc1)[C@H]1	1351332-50-7	Carbamic acid, N-[(1S)-1-[[(2S)-2-[5-[4-[6-[2-[(1S)-1-[[(2S)-2-[(methoxycarbonyl	C42H50N8O6	762.9	A/B	-8.2
8184	C(=O)([C@@H](NC(=O)OC)C(C)C)N1[C@@H](CCC1)c1[nH]c(cn1)c1ccc(cc1)c1cc2c(cc1)cc(cc2)c1[nH]c(nc1)[C@H]1	1351332-53-0	Carbamic acid, N-[(1S)-1-[[(2S)-2-[5-[4-[6-[2-[(2S)-1-[(2S)-2-[(methoxycarbonyl)	C45H54N8O6	802.96	A/B	-8.2
8185	C(=O)([C@H](NC(=O)OC)c1ccccc1)N1[C@@H](CCC1)c1[nH]c(cn1)c1ccc(cc1)c1cc2c(cc1)cc(cc2)c1[nH]c(nc1)[C@H	1351332-55-2	Carbamic acid, N-[(1S)-1-[[(2S)-2-[5-[3-cyano-4-[6-[2-[(2S)-1-[(2S)-2-[(methoxyc	C45H51N9O6	813.94	A/B	-8.2
8186	C(=O)([C@@H](NC(=O)OC)C(C)C)N1[C@@H](C[C@]2(C1)CCCO2)c1[nH]c(c(COC)n1)c1ccc(cc1)c1ccc(cc1)c1[nH]c(nc	1351852-74-8	1H-Imidazole-5-carboxylic acid, 2-[(5S,8S)-7-[(2S)-2-[(methoxycarbonyl)amino]-3-	C45H56N8O9	852.97	A/B	-8.2
8187	C(=O)([C@@H](NC(=O)OC)C(C)C)N1[C@@H](C[C@]2(C1)CCCO2)c1[nH]c(c(CO)n1)c1ccc(cc1)c1ccc(cc1)c1[nH]c(nc1	1351852-77-1	Carbamic acid, N-[(1S)-1-[[(2S)-2-[5-chloro-4-[4,Ä≤-[2-[(5S,8S)-7-[(2S)-2-[(meth	C43H53ClN8O7	829.38	A/B	-8.2
8188	C(=O)([C@@H](NC(=O)OC)C(C)C)N1C[C@]2(C[C@H]1c1[nH]c(cn1)c1ccc(cc1)c1ccc(cc1)c1[nH]c(nc1)[C@H]1N(C(=O	1351853-21-8	Carbamic acid, N-[(1S)-1-[[(2S)-2-[5-[4,Ä≤-[2-[(5S,8S)-7-[(2S)-2-[(methoxycarbor	C43H53N9O7	807.94	A/B	-8.2
8189	C(=O)([C@@H](NC(=O)OC)C(C)C)N1C[C@@]2(C[C@H]1c1[nH]c(cn1)c1ccc(cc1)c1ccc(cc1)c1[nH]c(nc1)[C@H]1N(C(=	1351853-22-9	Carbamic acid, N-[(1S)-1-[[(2S)-2-[5-[4,Ä≤-[2-[(5S,8S)-7-[(2S)-2-[(methoxycarbor	C43H52N8O8	808.92	A/B	-8.2

8190	C(=O)([C@@H](NC(=O)OC)C(C)C)N1C[C@@]2(C[C@H]1c1[nH]c(cn1)c1ccc(cc1)c1ccc(cc1)c1[nH]c(nc1)[C@H]1N(C(=	1351853-24-1	Carbamic acid, N-[(1S)-1-[[(2S)-2-[5-[4,Ä≤-[2-[(3S)-2-[(2S)-2-[(methoxycarbonyl)	C45H59N9O6	822.01	A/B	-8.2
8191	C(=O)(C(NC(=O)OCC)C)N1[C@@H](C[C@@H](C1)C1CC1)c1[nH]c(cn1)c1ccc(cc1)c1ccc(cc1)c1[nH]c(nc1)[C@H]1N(C(	1351853-86-5	Carbamic acid, N-[(1S)-2-[(2S)-2-[5-[4,Ä≤-[2-[(2S,4R)-4-cyclopropyl-1-[(2S)-2-[	C39H46N8O6	722.83	A/B	-8.2
8192	C(=O)([C@@H](NC(=O)OC)C(C)C)N1[C@@H](C[C@@H](C1)C1CC1)c1[nH]c(cn1)c1ccc(cc1)c1ccc(cc1)c1[nH]c(nc1)[C	1351855-47-4	Carbamic acid, N-[(1S)-1-[[(2S,4R)-4-cyclopropyl-2-[5-[4,Ä≤-[2-[(2S,4R)-4-fluor	C43H53FN8O6	796.93	A/B	-8.2
8193	C(=O)([C@@H](NC(=O)OC)C(C)C)N1[C@@H](C[C@@H](C1)C1CC1)c1[nH]c(cn1)c1c(C)c(C)c(cc1)c1c(C)c(C)c(cc1)c1	1351855-66-7	Carbamic acid, N-[(1S)-1-[[(2S)-2-[5-[4,Ä≤-[2-[(2S,4R)-4-cyclopropyl-1-[(2S)-2-	C45H58N8O6	806.99	A/B	-8.2
8194	C(=O)([C@@H](NC(=O)OC)C(C)C)N1[C@@H]([C@@H]2C[C@H]1CC2)c1[nH]c(cn1)c1ccc(cc1)c1ccc(cc1)c1[nH]c(nc1)[	1351855-76-9	Carbamic acid, N-[(1S)-1-[[(2S,4R)-4-cyclopropyl-2-[5-[3,3,Ä≤,5,5,Ä≤-tetrachlor	C43H50Cl4N8O6	916.72	A/B	-8.2
8195	C(=O)([C@@H](NC(=O)OC)C(C)C)N1C[C@]2(C[C@H]1c1[nH]c(cn1)c1ccc(cc1)c1ccc(cc1)c1[nH]c(nc1)[C@H]1N(C(=O	1351856-01-3	Carbamic acid, N-[(1S)-1-[[(2S,4R)-4-cyclopropyl-2-[5-[4,Ä≤-[2-[(5R,8S)-7-[(2S)-	C46H57N9O7	848	A/B	-8.2
8196	C(=O)([C@@H](NC(=O)OC)C(C)C)N1[C@@H](C[C@H](C1)C1CC1)c1[nH]c(cn1)c1ccc(cc1)c1ccc(cc1)c1[nH]c(nc1)[C@	1351856-19-3	Carbamic acid, N-[(1S)-1-[[(2S)-2-[5-[4,Ä≤-[2-[(2S,4R)-4-cyclobutyl-1-[(2S)-2-[	C44H56N8O6	792.97	A/B	-8.2
8197	C(=O)([C@@H](NC(=O)OC)C(C)C)N1[C@@H](C[C@@H](C1)N1CCCC1)c1[nH]c(cn1)c1ccc(cc1)c1ccc(cc1)c1[nH]c(nc1)	1351856-21-7	3-Pyrrolidinecarboxylic acid, 1-[(2S)-2-[(methoxycarbonyl)amino]-3-methyl-1-oxob	C42H52N8O8	796.91	A/B	-8.2
8198	C(OC[C@H](Cn1c(=O)nc(N)cc1)OCP(=O)(OCC)OCC)(c1ccc(OC)cc1)(c1ccc(OC)cc1)c1ccccc1	1351856-27-3	Carbamic acid, N-[(1S)-1-[[(2S)-2-[5-[4,Ä≤-[2-[(2S,4R)-4-cyclopentyl-1-[(2S)-2-	C45H58N8O6	806.99	A/B	-8.2
8199	C(=O)([C@@H](NC(=O)OC)C(C)C)N1[C@@H](C=C(CC)C1)c1[nH]c(cn1)c1ccc(C#Cc2ccc(cc2)c2[nH]c(nc2)[C@H]2N(C(	1352392-54-1	Carbamic acid, N-[(1R)-2-[(2S)-2,5-dihydro-2-[5-[4-[2-[4-[2-[(2S)-1-[(2S)-2-[(me	C46H48N8O6	808.92	A/B	-8.2
8200	C(=O)([C@@H](NC(=O)OC)C(C)C)N1[C@@H](C=C(CC)C1)c1[nH]c(cn1)c1cc2c(cc1)cc(C#Cc1[nH]c(nc1)[C@H]1N(C(=O	1352392-60-9	Carbamic acid, N-[(1S)-1-[[(2S)-2,5-dihydro-2-[5-[6-[2-[2-[(2S)-1-[(2S)-2-[(meth	C40H46N8O6	734.84	A/B	-8.2
8201	Fc1cc(c2nc3c(o2)ccc(N)c3)ccc1c1cn(c(=O)[nH]c1=O)[C@@H]1O[C@H](COP(=O)(OP(=O)(OP(=O)(O)O)O)O)[C@@H](O	1352918-37-6	Uridine 5,Ä≤-(tetrahydrogen triphosphate), 5-[4-(2-benzoxazolyl)-2-fluorophenyl]	C22H21FN3O15P3	679.33	A/B	-8.2
8202	O=c1n(cc(C)c(=O)n1C(=O)OC(CC)C)[C@@H]1O[C@H](CO)C=C1	1353372-62-9	1(2H)-Pyrimidinecarboxylic acid, 3-(2-deoxy-Œ≤-L-erythro-pentofuranosyl)-3,6-dih	C18H17F3N2O7	430.33	A/B	-8.2
8203	O=c1n(cc(C(F)(F)F)c(=O)n1C(=O)OC1CCCC1)[C@@H]1O[C@H](CO)[C@@H](O)C1	1353386-81-8	Thymidine, 3-[(cyclopentyloxy)carbonyl]-2,Ä≤,3,Ä≤-didehydro-3,Ä≤-deoxy-	C16H20N2O6	336.34	A/B	-8.2
8204	C([C@H](OCP(=O)(OCOC(=O)C(C)(C)C)OCOC(=O)C(C)(C)C)C)n1c2c(nc1)c(N)ncn2.C(=O)(C(=O)O)O	1354632-22-6	Furo[3,Ä≤,4,Ä≤:6,7]naphtho[2,3-d]-1,3-dioxol-6(8H)-one, 5-(2,5-dimethoxyphenyl)	C21H16O7	380.35	A/B	-8.2
8205	C(#N)[C@]1(O[C@H](COP(=O)(N[C@H](C(=O)OCC)C)N[C@H](C(=O)OCC)C)[C@@H](O)[C@H]1O)c1n2c(cc1)c(N)ncn2	1355050-12-2	L-Alanine, N-(hydroxyphenoxyphosphinyl)-, ethyl ester, 6-ester with 2-C-(4-aminc	C23H27N6O8P	546.47	A/B	-8.2
8206	O(P(=O)(OC[C@H]1O[C@H]([C@H](OP(=O)(COC[C@H]2O[C@H]([C@@H](O)[C@@H]2O)n2c3c(nc2)c(N)ncn3)O)[C@@H]1O)	1357016-15-9	Adenosine, 5,Ä≤-O-phosphonoadenylyl-(2,Ä≤,Ü∫5,Ä≤)-adenylylmethylene-(2,Ä≤,Ü∫5,Ä	C41H52N20O25P4	1348.87	A/B	-8.2
8207	C(F)(F)c1n(c2c(n1)cccc2)c1nc(nc(NCCO)n1)N1CCOCC1	1359955-67-1	1,3,5-Triazin-2-amine, 4-[2-(difluoromethyl)-1H-benzimidazol-1-yl]-N-methyl-N-(1	C22H28F2N8O	458.51	A/B	-8.2
8208	C(F)(F)c1n(c2c(n1)cccc2)c1nc(nc(NCc2nn(C)cc2)n1)N1CCOCC1	1359955-95-5	1,3,5-Triazin-2-amine, 4-[2-(difluoromethyl)-1H-benzimidazol-1-yl]-6-(4-morpholi	C21H26F2N8O3S	508.54	A/B	-8.2
8209	C(=O)([C@@H](NC(=O)OC)C(C)C)N1[C@@H](C[C@H](C1)n1cc(C(=O)OC)nn1)c1[nH]c(cn1)c1ccc(cc1)c1ccc(cc1)c1[n	1361249-15-1	1H-1,2,3-Triazole-4-carboxylic acid, 1,1,Ä≤-[[1,1,Ä≤-biphenyl]-4,4,Ä≤-diylbis[1	C48H56N14O10	989.05	A/B	-8.2
8210	C(=O)([C@@H](NC(=O)OC)C(C)C)N1[C@@H](C[C@H](C(F)(F)F)C1)c1[nH]c(c(F)n1)c1ccc(cc1)c1ccc(cc1)c1[nH]c(n	1361249-39-9	Carbamic acid, N,N,Ä≤-[[1,1,Ä≤-biphenyl]-4,4,Ä≤-diylbis[(5-fluoro-1H-imidazole-	C42H46F8N8O6	910.85	A/B	-8.2
8211	C(=O)(c1cc2c([nH]1)ccc(N)c2)N1CCN(CC1)c1c(NC(C)C)cccn1	136794-84-8	Furo[3,Ä≤,4,Ä≤:6,7]naphtho[2,3-d]-1,3-dioxol-6(5aH)-one, 5,8,8a,9-tetrahydro-5-	C28H26O8	490.5	A/B	-8.2
8212	O=C1c2n(N([C@@H]3N1CCOC3)[C@H]1c3c(CSc4c1cccc4)cccc3)ccc(=O)c2O	1370244-56-6	1H-[1,4]Oxazino[3,4-c]pyrido[2,1-f][1,2,4]triazine-6,8-dione, 12-[(11R)-10-chlor	C24H20ClN3O4S	481.95	A/B	-8.2
8213	O=c1n(C2O[C@H](CO)[C@@H](O)C2)cc(C)c(=O)[nH]1	137103-31-2	Pyrido[2,3-d]pyrimidine-2,4(1H,3H)-dione, 5-methyl-3-(phenylmethyl)-1-Œ≤-D-ribo	C20H21N3O6	399.4	A/B	-8.2
8214	O(C)c1c2n(cc(C(=O)O)c(=O)c2cc(F)c1N1C[C@@H]2[C@H](C1)CCCN2CCN1c2c(C(=NNC(=S)N)C1=O)cc(F)cc2)C1CC1	1372618-36-4	3-Quinolinecarboxylic acid, 7-[(4aS,7aS)-1-[2-[3-[2-(aminothioxomethyl)hydraziny	C32H34FN7O5S	647.72	A/B	-8.2
8215	C(=O)([C@@H](NC(=O)OC)C(C)C)N1[C@@H](C[C@H](OC)C1)c1[nH]c(c(Cl)n1)c1ccc(cc1)c1ccc(cc1)c1[nH]c(nc1)[C	1373165-67-3	Carbamic acid, N-[(1S)-1-[[(2S)-2-[5-chloro-4-[2-fluoro-4,Ä≤-[2-[(2S,4S)-1-[(2S)	C41H50ClFN8O6S	837.4	A/B	-8.2
8216	C(=O)([C@@H](NC(=O)OC)C(C)C)N1[C@H]2[C@H](C2)C[C@H]1c1[nH]c(cn1)c1ccc(cc1)c1ccc(cc1)c1[nH]c(nc1Cl)[C	1373165-78-6	Carbamic acid, N-[(1S)-1-[[(2S,4S)-2-[5-[4,Ä≤-[5-chloro-2-[(1R,3S,5R)-2-[(2S)-2-	C42H51ClN8O6S	831.42	A/B	-8.2
8217	C(=O)([C@@H](NC(=O)OC)C(C)C)N1[C@H](c2[nH]c(c3cc4c5c(c6c(c4cc3)ccc(c6)c3[nH]c(nc3)[C@H]3N(C(=O)[C@@H	1377035-60-3	Carbamic acid, N,N,Ä≤-[dibenzo[f,h]quinoxaline-6,11-diylbis[1H-imidazole-5,2-diy	C44H50N10O6	814.93	A/B	-8.2
8218	C(=O)([C@@H](NC(=O)OC)C1CCOCC1)N1[C@H](c2[nH]c(c3cc4c5-c6c(cc(cc6CC4)c4[nH]c(nc4)[C@H]4N(C(=O)[C@@H]	1377035-68-1	Carbamic acid, N,N,Ä≤-[2,7-triphenylenediylbis[1H-imidazole-5,2-diyl-(2S)-2,1-py	C46H52N8O6	812.96	A/B	-8.2
8219	C(=O)([C@@H](NC(=O)OC)C(C)C)N1C(c2c(C1)cccc2)c1[nH]c(cn1)c1ccc(cc1)c1cc2c(cc1)cc(cc2)c1[nH]c(nc1)[C@	1377037-22-3	Carbamic acid, N-[(1S)-1-[[(1R,3S,5R)-3-[7-[4-[2-[(1R,3S,4S)-2-[(2S)-2-[(methoxy	C45H52N8O6	800.94	A/B	-8.2
8220	C(=O)([C@@H](NC(=O)OC)C(C)C)N1[C@@H]([C@@H]2C[C@H]1CC2)c1[nH]c2-c3c(CCc2n1)cc(cc3)c1cc2c(cc1)c1c(cc2	1377040-91-9	Carbamic acid, N-[(1R)-2-[(2S)-2-[5-[4-[6-[2-[(2S)-1-[(2S)-3-ethyl-2-[(methoxyca	C49H54N8O6	851	A/B	-8.2
8221	C(=O)([C@@H](NC(=O)OC)C(C)C)N1[C@H](c2[nH]c3c(c4c(c5c3ccc(c5)c3ccc(cc3)c3[nH]c(nc3)[C@H]3N(C(=O)[C@@	1377041-17-2	Carbamic acid, N-[(1S)-1-[[(2S)-2-[9-[4-[2-[(2S)-1-[(2S)-2-[(methoxycarbonyl)ami	C46H52N8O6	812.96	A/B	-8.2
8222	C(=O)([C@H](NC(=O)OC)c1ccccc1)N1[C@@H](CCC1)c1[nH]c(cn1)c1ccc(cc1)c1c(C#CC)cc(cc1)c1[nH]c(nc1)[C@H]1	1377042-35-7	Carbamic acid, N-[(1S)-2-[(2S)-2-[5-[4,Ä≤-[2-[(2S)-1-[(2S)-2-[(methoxycarbonyl)a	C45H54N8O7	818.96	A/B	-8.2
8223	C(=O)([C@@H](NC(=O)OC)C(C)C)N1[C@H]2[C@H](C2)C[C@H]1c1[nH]c2c(n1)ccc1c2ccc(c1)c1cc2c(cc1)c1c(cc2)nc(	1377043-75-8	Carbamic acid, N-[(1S)-1-[[(2S)-2-[2,Ä≤-[(2S,4S)-1-[(2S)-2-[(methoxycarbonyl)am	C46H54N8O7	830.97	A/B	-8.2
8224	C(=O)([C@@H](NC(=O)OC)C1CCOCC1)N1[C@H](c2[nH]c3c4c(cc(cc4)c4ccc5c(cc4)c4c(cc5)nc([nH]4)[C@H]4N(C(=O)[	1377047-55-6	Carbamic acid, N-[(1S)-1-[[(2S)-2-[5-[4-[2-[(2S,4S)-1-[(2S)-2-[(methoxycarbonyl)	C45H56N8O7	820.98	A/B	-8.2
8225	C(=O)([C@@H](NC(=O)OC)C1CCOCC1)N1[C@@H]([C@@H]2C[C@H]1CC2)c1[nH]c2c(n1)ccc1c2ccc(c1)c1ccc(cc1)c1[nH]	1377604-11-9	Carbamic acid, N-[(1S)-1-[[(2S)-2-[5-[4-[2-[(1R,3S,4S)-2-[(2S)-2-[(methoxycarbor	C44H52N8O6	788.93	A/B	-8.2
8226	O=c1n(C2(C[C@H](O)[C@@H](C[3H])O2)[3H])c(c(N)n1)[3H])[3H]	1378293-94-7	2-Naphthalenesulfonic acid, 5-amino-8-[2-[3-methyl-4-[2-(4-sulfophenyl)diazenyl]	C23H19N5O6S2	525.56	A/B	-8.2
8227	C(=O)([C@@H](NC(=O)OC)C(C)C)N1[C@@H](CCC1)c1[nH]c(cn1)c1ccc(C#Cc2ccc(cc2)c2ccccc2)cc1	1380292-00-1	Carbamic acid, N-[(1S)-1-[[(2S)-2-[5-[4-[5-[4-[2-[(2S)-1-[(2S)-2-[(methoxycarbor	C42H48N8O6S	792.95	A/B	-8.2
8228	N(=Nc1ccc(S(=O)(=O)[O-])cc1)c1c2c(c([O-])cc1)cccc2	138152-14-4	Benzenesulfonic acid, 4-[2-(2-hydroxy-1-naphthalenyl)diazenyl]-, ion(2-)	C16H10N2O4S		A/B	-8.2
8229	O(C(=O)C(C)C)[C@H]1[C@@H](O[C@H]2[C@H]1OP(=S)(N(C)C)OC2)n1c2c(nc1)c(=O)nc(N)[nH]2	1382467-82-4	Guanosine, cyclic 3,Ä≤,5,Ä≤-[S-(4-bromo-2,3-dioxobutyl) phosphorothioate]	C14H15BrN5O8PS	524.24	A/B	-8.2
8230	F[C@@H]1[C@@H](O[C@H](COC(=O)C)[C@H]1OC(=O)C)n1cc(CC)c(=O)[nH]c1=O	1383566-40-2	Carbamic acid, N-[(1S)-1-[[(2S)-2-[5-[4,Ä≤-(2-amino-1H-imidazol-5-yl)[1,1,Ä≤-bi	C29H33N7O3	527.62	A/B	-8.2
8231	[C@H](Cc1ccccc1)(CC[C@H](Cc1ccccc1)NC(=O)OCc1cncs1)NC(=O)[C@@H](NC(=O)N1CCCC1)CCN1CCOCC1	1383569-75-2	Carbamic acid, N-[(1R,4R)-4-[[(2S)-4-(4-morpholinyl)-1-oxo-2-[(1-piperidinylcarb	C37H50N6O5S	690.9	A/B	-8.2
8232	O=c1c2c(n(cn2)[C@@H]2O[C@H](CO)[C@@H](O)[C@H]2O)[nH]c(n1)n1cc(CO)nn1	1384519-80-5	Inosine, 2-[4-(4-methoxyphenyl)-1H-1,2,3-triazol-1-yl]-	C19H19N7O6	441.4	A/B	-8.2
8233	O=c1c2c(n(cn2)[C@@H]2O[C@H](CO)[C@@H](O)C2)[nH]c(n1)n1cc(CO)nn1	1384519-86-1	Inosine, 2,Ä≤-deoxy-2-[4-(4-methoxyphenyl)-1H-1,2,3-triazol-1-yl]-	C19H19N7O5	425.4	A/B	-8.2
8234	N(c1c(C)cc(/C=C/C#N)cc1C)c1nc(Nc2ccc(C#N)cc2)ncc1.[C@@H]([C@H](C(=O)O)O)(C(=O)O)O	1388223-99-1	Benzonitrile, 4-[[4-[[4-[(1E)-2-cyanoethenyl]-2,6-dimethylphenyl]amino]-2-pyrimi	C22H18N6.xC4H4O4		A/B	-8.2
8235	C(=O)([C@@H](NC(=O)OC)C(C)C)N1[C@@H](CCC1)c1[nH]c(c(F)n1)c1ccc(cc1)c1ccc(cc1)c1[nH]c(nc1F)[C@H]1N(C(	1388224-21-2	Carbamic acid, N,N,Ä≤-[[1,1,Ä≤-biphenyl]-4,4,Ä≤-diylbis[(5-iodo-1H-imidazole-4,	C40H48I2N8O6	990.67	A/B	-8.2
8236	O=C1C2(C(C(C(=O)/C=C(\O)/C3C4(N5C(C3c3cc(OC)c(O)cc3)CCC5)c3c(NC4=O)ccc(Cl)c3)C3N2CCC3)c2cc(OC)c(O)cc	1389334-79-5	Spiro[3H-indole-3,3,Ä≤-[3H]pyrrolizin]-2(1H)-one, 1,Ä≤,2,Ä≤,5,Ä≤,6,Ä≤,7,Ä≤,7,Ä	C45H44N4O8	768.85	A/B	-8.2
8237	S(=O)(=O)(N)N1CCN(c2nc(nc(n2)N2CCOCC2)c2cnc(N)nc2)CC1	1391924-19-8	5H-1,4-Diazepin-5-one, 1-[4-(2-amino-5-pyrimidinyl)-6-(4-morpholinyl)-1,3,5-tria	C16H21N9O2	371.4	A/B	-8.2
8238	N(CC(=O)N)c1nc(nc(n1)N1CCOCC1)c1cc(C(=O)N)ccc1	1391925-01-1	2-Pyridinamine, 5-[4-(4-morpholinyl)-6-(1-piperazinyl)-1,3,5-triazin-2-yl]-	C16H22N8O	342.4	A/B	-8.2
8239	N(C(CO)(C)C)c1nc(nc(n1)N1CCOCC1)c1cc(O)ccc1	1391925-21-5	Cyclopentanecarboxylic acid, 1-[[4-(3-hydroxyphenyl)-6-(4-morpholinyl)-1,3,5-tri	C19H23N5O4	385.42	A/B	-8.2
8240	N(c1nc(nc(NCCNC(=O)c2ccc(cc2)C2OOC(OO2)c2ccc(C)cc2)n1)N1CCOCC1)c1ccc(C(C)C)cc1	1393922-45-6	Benzamide, N-[2-[[4-[(2-methylphenyl)amino]-6-(4-morpholinyl)-1,3,5-triazin-2-yl	C32H35N7O6	613.66	A/B	-8.2
8241	C(OC(=O)c1cccnc1)[C@H]1O[C@H]([C@H]2[C@@H]1OC(C)(C)O2)n1nc(C(=O)N)nc1	1395899-00-9	Dodecanoic acid, 12-amino-, [(2R,5S)-5-[4-[[bis(4-methoxyphenyl)phenylmethyl]ami	C41H51FN4O6S	746.93	A/B	-8.2
8242	C(OC[C@@H]1[C@@H](OC(=O)C)[C@@H](OC(=O)C)[C@@H](O1)n1nc(C(=O)N)nc1)(c1ccc(OC)cc1)(c1ccc(OC)cc1)c1ccc	139620-93-2	1H-1,2,4-Triazole-3-carboxamide, 1-[2,3-O-cyclopentylidene-5-O-[(1,4-dihydro-1-m	C20H25N5O6	431.44	A/B	-8.2
8243	C(OCCOP(=O)(NCCCCCC)NCCCCCC)n1c2c(nc1)c(=O)nc(N)[nH]2	1400636-38-5	4-Piperidinecarboxylic acid, 1-[(2,3-dihydro-2,3-dioxo-1H-indol-5-yl)sulfonyl]-	C14H14N2O6S	338.34	A/B	-8.2
8244	O(C(=O)C1=CN(C)C=CC1)[C@H]1[C@@H](O[C@H](CO)[C@H]1OC(=O)c1ccccc1)n1nc(C(=O)N)nc1	140891-10-7	1H-1,2,4-Triazole-3-carboxamide, 1-[3-O-benzoyl-2-O-(3-pyridinylcarbonyl)-Œ≤-D-r	C21H19N5O7	453.4	A/B	-8.2
8245	O(C(=O)CCCC)[C@H]1[C@@H](O[C@H](CO)[C@H]1OC(=O)c1cccnc1)n1nc(C(=O)N)nc1	140891-16-3	1H-1,2,4-Triazole-3-carboxamide, 1-[5-O-[bis(4-methoxyphenyl)phenylmethyl]-3-O-(	C40H41N5O9	735.78	A/B	-8.2
8246	N(=Nc1cc(S(=O)(=O)O)c(CCc2c(S(=O)(=O)O)cc(N=Nc3c4c(cc(S(=O)(=O)O)cc4)c(N)cc3)cc2)cc1)c1c2c(cc(S(=O)(	140942-27-4	2,7-Naphthalenedisulfonic acid, 4,4,Ä≤-[1,2-ethenediylbis[(3-sulfo-4,1-phenylene	C34H26N6O18S6.6Na		A/B	-8.2
8247	S(=O)(=O)(O)c1c2c(c(S(=O)(=O)O)cc(N=Nc3cc(S(=O)(=O)O)c(C=Cc4c(S(=O)(=O)O)cc(N=Nc5c(N)c6c(c(S(=O)(=O)	14102-77-3	Isopodophyllotoxin, 3,Ä≤,4,Ä≤,5,Ä≤-tridemethoxy-, (¬±)-	C19H16O5	324.33	A/B	-8.2
8248	C(=O)([C@H](NC(=O)OC)C1CC(=C)C1)N1[C@@H](CC[C@@H]1C)c1[nH]c(cn1)c1ccc(cc1)c1ccc(cc1)c1[nH]c(nc1)[C@H	1412816-49-9	Carbamic acid, N,N,Ä≤-[[1,1,Ä≤-biphenyl]-4,4,Ä≤-diylbis[1H-imidazole-5,2-diyl[(	C46H54N8O6	814.97	A/B	-8.2
8249	[C@H](C(=O)N1[C@@H](CC[C@@H]1C)c1[nH]c(cn1)c1ccc(cc1)c1ccc(cc1)c1[nH]c(nc1)[C@H]1N(C(=O)[C@@H](NC(=O	1412819-62-5	Carbamic acid, N,N,Ä≤-[[1,1,Ä≤-biphenyl]-4,4,Ä≤-diylbis[1H-imidazole-5,2-diyl[(	C46H54N8O6	814.97	A/B	-8.2
8250	C(=O)(NS(=O)(=O)C1CC1)[C@]12[C@H](C1)/C=C\CCCCN(C)C(=O)[C@H]1[C@H](C(=O)N2)C[C@@H](Oc2c3c(nc(c2)c2nc	1414684-55-1	4H-1-Benzopyran-4-one, 2-[3,4-dihydroxy-2-[(2E)-7-hydroxy-3,7-dimethyl-2-octen-1	C25H28O8	456.48	A/B	-8.2
8251	C(OC(CO)CO)n1c2c(nc1)c(=O)nc(NCNc1ccc(S(=O)(=O)Nc3noc(C)c3)cc1)[nH]2	1415032-07-3	Benzenesulfonamide, 4-[[[[6,9-dihydro-9-[[2-hydroxy-1-(hydroxymethyl)ethoxy]meth	C22H31N9O8S	581.6	A/B	-8.2
8252	C(=O)(Nc1sc(N(=O)=O)cn1)c1c(OC(=O)c2ccccc2)c(Cl)cc(Cl)c1	1415566-84-5	Benzamide, 2-(benzoyloxy)-5-chloro-N-(5-nitro-2-thiazolyl)-	C17H10ClN3O5S	403.8	A/B	-8.2
8253	C(=O)(Nc1sc(N(=O)=O)cn1)c1c(OC(=O)C)c(C(C)C)cc(C(C)C)c1	1415566-91-4	Benzamide, 2-(benzoyloxy)-3,5-dinitro-N-(5-nitro-2-thiazolyl)-	C17H9N5O9S	459.35	A/B	-8.2
8254	O[C@H]1[C@@H](O[C@H](COP(=O)(OP(=O)(O[C@H]2O[C@H](CO)[C@H](O)[C@H](O)[C@H]2O)O)O)[C@H]1O)n1cc(C(F)(F	1415916-60-7	Not Yet Assigned	C26H36N2O5	456.57	A/B	-8.2
8255	O[C@H]1[C@@H](O[C@H](COP(=O)(OP(=O)(O)O)O)[C@H]1O)n1cc(C(F)(F)F)c(=O)[nH]c1=O	1415972-95-0	Uridine 5,Ä≤-(trihydrogen diphosphate), 5-(trifluoromethyl)-, P,Ä≤-Œ±-D-glucopy	C16H23F3N2O17P2	634.3	A/B	-8.2
8256	C(OC(CO)C1CC1)n1c2c(nc1)c(=O)nc(N)[nH]2	1416170-12-1	Hydrazinecarbothioamide, 2-[1,2-dihydro-2-oxo-1-(2-phenylethyl)-3H-indol-3-ylide	C31H34N4O10S	654.69	A/B	-8.2
8257	O=c1c2c(n(cn2)[C@@H]2O[C@H](CO)[C@@H](O)C2)[nH]c(NC2O[C@H](CO)[C@@H](O)C2)n1	1416956-41-6	Ethanone, 1-[4-[4-[2-(difluoromethyl)-1H-benzimidazol-1-yl]-6-(4-morpholinyl)-1,	C23H29F2N9O2	501.53	A/B	-8.2
8258	N(c1nc(nc(NC(=S)Nc2ccccc2)n1)N1CCOCC1)n1c(SC)nnc(C(C)(C)C)c1=O	1418311-55-3	Cyclopenta[c]pyrrole-1-carboxamide, (2S)-2-cyclohexyl-N-(2-pyrazinylcarbonyl)gly	C33H48N6O7	640.77	A/B	-8.2
8259	N(n1c(SC)nnc(C(C)(C)C)c1=O)c1nc(nc(NC(=S)Nc2cc(Cl)ccc2)n1)N1CCOCC1	1418553-55-5	Urea, N-[4-[[6-(1,1-dimethylethyl)-3-(methylthio)-5-oxo-1,2,4-triazin-4(5H)-yl]a	C23H30N10O4S	542.61	A/B	-8.2
8260	O=C1C2C(c3c(C(O)C2CO1)cc1c(c3)OCO1)c1ccccc1	14254-91-2	Picropodophyllin, 3,Ä≤,4,Ä≤,5,Ä≤-tridemethoxy-, DL-	C19H16O5	324.33	A/B	-8.2
8261	O=c1n([C@@H]2O[C@H](COP(=O)(Oc3ccccc3)Oc3ccccc3)[C@@H](N=[N+]=[N-])C2)cc(C)c(=O)[nH]1	142629-84-3	5,Ä≤-Thymidylic acid, 3,Ä≤-azido-3,Ä≤-deoxy-, bis(4-nitrophenyl) ester	C22H20N7O11P		A/B	-8.2
8262	N(=C1c2c(N(CN3CCN(CC3)c3cc4c(cc3F)c(=O)c(C(=O)O)cn4CC)C1=O)ccc(C)c2)NC(=S)N(c1ccccc1)c1ccccc1	1426583-84-7	3-Quinolinecarboxylic acid, 7-[4-[[3-[2-[(di-2-propen-1-ylamino)thioxomethyl]hyc	C33H36FN7O4S	645.75	A/B	-8.2
8263	C(=O)(OCC)C1=C(N)OC(=CC1c1c(OC)cccc1)c1[nH]c2c(n1)cccc2	1426813-71-9	4H-Pyran-3-carboxylic acid, 2-amino-6-(1H-benzimidazol-2-yl)-4-(4-fluorophenyl)-	C21H18FN3O3	379.38	A/B	-8.2
8264	C(=O)(OCC)C1=C(N)OC(=CC1c1c(F)cccc1)c1[nH]c2c(n1)cccc2	1426813-84-4	4H-Pyran-3-carboxylic acid, 2-amino-6-(1H-benzimidazol-2-yl)-4-(2-methoxyphenyl)	C22H21N3O4	391.42	A/B	-8.2
8265	C(=O)(OCC)C1=C(N)OC(=CC1c1c(Cl)cccc1)c1[nH]c2c(n1)cccc2	1426813-85-5	4H-Pyran-3-carboxylic acid, 2-amino-6-(1H-benzimidazol-2-yl)-4-(2-fluorophenyl)-	C21H18FN3O3	379.38	A/B	-8.2
8266	C(=O)(OCC)C1=C(N)OC(=CC1c1cc(F)ccc1)c1[nH]c2c(n1)cccc2	1426814-45-0	4H-Pyran-3-carboxylic acid, 2-amino-6-(1H-benzimidazol-2-yl)-4-(4-hydroxyphenyl)	C21H19N3O4	377.39	A/B	-8.2
8267	C(=O)(OCC)C1=C(N)OC(=CC1c1c(N(=O)=O)cccc1)c1[nH]c2c(n1)cccc2	1426814-87-0	4H-Pyran-3-carboxylic acid, 2-amino-6-(1H-benzimidazol-2-yl)-4-(2-bromophenyl)-,	C21H18BrN3O3	440.29	A/B	-8.2
8268	O[C@H]1[C@@H](O[C@H](COP(=O)(Oc2c3c(ccc2)cccc3)N[C@H](C(=O)OC)C)[C@H]1O)n1c(=O)[nH]c(=O)c(I)c1	1427057-12-2	L-Alanine, N-(5-iodo-P-phenyl-5,Ä≤-uridylyl)-, phenylmethyl ester	C25H27IN3O10P	687.37	A/B	-8.2
8269	O=c1n(cc(I)c(=O)n1CC(F)(F)c1ccccc1)[C@@H]1O[C@H](COP(=O)(OP(=O)(O)O)O)[C@@H](O)[C@H]1O	1427676-58-1	Uridine 5,Ä≤-(trihydrogen diphosphate), 3-[2-(2,4-difluorophenyl)ethyl]-5-iodo-	C17H19F2IN2O12P2	670.19	A/B	-8.2
8270	S(=O)(=O)(O)c1c2c(c(S(=O)(=O)O)cc(N=Nc3c(N)cc(N)c(C)c3)c2)ccc1	1431635-33-4	1,5-Naphthalenedisulfonic acid, 3-[2-(2,4-diamino-3-methylphenyl)diazenyl]-	C17H16N4O6S2	436.46	A/B	-8.2
8271	N(C(=O)C)[C@H]1[C@H](OC(CC)CC)C=C(C(=O)OCC)C[C@@H]1N=C(NO)C	1431932-96-5	L-Alanine, N-[(R)-hydroxyphenoxyphosphinyl]-, phenylmethyl ester, 6-ester with 2	C29H31N6O8P	622.57	A/B	-8.2
8272	C(C(C(Cc1ccccc1)NC(=O)C(NC(=O)c1nc2c(cc1)cccc2)CC(=O)N)O)N1C(C(=O)NC(C(=O)NC(Cc2ccccc2)C(=O)NC)C(CC)	143348-07-6	L-Phenylalaninamide, 1-[3-[[4-amino-1,4-dioxo-2-[(2-quinolinylcarbonyl)amino]but	C45H56N8O7	820.98	A/B	-8.2
8273	N(Cc1nc2c(cc1)cccc2)C12CC3CC(C2)CC(C1)C3	1443121-67-2	1H-Indole-4-methanamine, N-tricyclo[3.3.1.13,7]dec-1-yl-	C19H24N2	280.41	A/B	-8.2
8274	C[C@]1(F)[C@@H](O[C@H](COP(=S)(Oc2ccccc2)NC(C(=O)OCC(C)(C)C)C)[C@H]1O)n1c(=O)[nH]c(=O)cc1	1443998-32-0	Alanine, N-[(2,Ä≤R)-2,Ä≤-deoxy-2,Ä≤-fluoro-2,Ä≤-methyl-P(O)-phenyl-P-thio-5,Ä≤-	C25H33FN3O8PS	585.58	A/B	-8.2
8275	O=C1[C@@H]2[C@@H]([C@]3(C)[C@@H](C1)C[C@H](NCCc1cnc[nH]1)CC3)CC[C@@]1(C)[C@H]2CC[C@@H]1[C@@H](CCCC(C	1447333-62-1	1H-1,2,4-Triazole-3-carboxamide, 1-[2,3-bis-O-[(phenylmethoxy)carbonyl]-Œ≤-D-rib	C24H24N4O9	512.47	A/B	-8.2
8276	C(=O)([C@@H](NC(=O)OC)C(C)C)N1[C@@H](CCC1)c1[nH]c(cn1)c1ccc(cc1)c1ccc(cc1)c1[nH]c([C@H](C(C)C)N)nc1	1447832-67-8	1H-Indole-2,3-dione, 5-(1-piperazinylsulfonyl)-	C12H13N3O4S	295.31	A/B	-8.2
8277	C(C(C)C)n1c2c3c(nc(N)c2nc1CCOC)cccc3	144875-34-3	1H-Imidazo[4,5-c]quinolin-4-amine, 2-(methoxyphenylmethyl)-1-(2-methylpropyl)-	C22H24N4O	360.45	A/B	-8.2
8278	O(P(=O)(OP(=O)(O)O)O)[C@H]1[C@@H](O[C@H](CO)[C@H]1O)n1c2c(nc1)c(N)ncn2	1448858-37-4	Benzonitrile, 4-[[5-bromo-4-chloro-6-(2,4,6-trimethylphenoxy)-2-pyrimidinyl]amir	C20H16BrClN4O	443.72	A/B	-8.2
8279	N(c1c2c(c(O)c(CN3CCN(CC3)c3ccccc3)c1)CCCC2)c1c2c(ncc1)cc(Cl)cc2	1449239-44-4	4-Piperidinol, 1-[[4-[(7-chloro-4-quinolinyl)amino]-5,6,7,8-tetrahydro-1-hydroxy	C25H28ClN3O2	437.96	A/B	-8.2
8280	O=C1[C@@H]2[C@@H](c3c([C@H](OC(=O)/C=C/c4ccccc4)[C@H]2CO1)cc1c(c3)OCO1)c1c(Cl)c(OC)c(OC)c(OC)c1	1449601-82-4	Benzoic acid, 4-chloro-3-nitro-, (5R,5aR,8aR,9S)-9-(2-chloro-3,4,5-trimethoxyphe	C29H23Cl2NO11	632.4	A/B	-8.2
8281	O=c1nc(C(F)(F)F)c(N)n1)1O[C@@H](CO)SC1	1449651-22-2	1H-Imidazole, 1-cyclohexyl-5-(3-cyclopentyltricyclo[3.3.1.13,7]dec-1-yl)-4,5-dih	C24H38N2	354.57	A/B	-8.2
8282	C([C@H]OCP(=O)(N[C@H](C(=O)OC(C)C)C)OCOC(=O)OC(C)C)n1c2c(nc1)c(N)ncn2	1449678-54-9	Thymidine, 4-O-[(11-cyclopropyl-6,11-dihydro-6-oxo-5H-dipyrido[3,2-b:2,Ä≤,3,Ä≤-	C25H26N6O6	506.51	A/B	-8.2
8283	C[C@]1(F)[C@@H](O[C@@H](CO[P@@](=O)(OCc2c(C)cccc2)N[C@H](C(=O)OC(C)C)C)[C@H]1O)n1c(=O)[nH]c(=O)cc1	1449756-46-0	L-Alanine, N-[[P(R),2,Ä≤R]-2,Ä≤-deoxy-2,Ä≤-fluoro-2,Ä≤-methyl-P-[(2-methylpheny	C24H33FN3O9P	557.51	A/B	-8.2
8284	C[C@]1(F)[C@@H](CO)(CO1)n1c(=O)nc(N)cc1	1449756-47-1	L-Alanine, N-[[P(S),2,Ä≤R]-2,Ä≤-deoxy-2,Ä≤-fluoro-2,Ä≤-methyl-P-[(2-methylpheny	C24H33FN3O9P	557.51	A/B	-8.2
8285	C(=O)([C@@H](NC(=O)OC)C(C)C)N1[C@@H](c2[nH]c3cc4c(-c5cc(cc5)c5[nH]c(nc5)[C@H]5N(C(=O)[C@@H](NC(=O	1449762-05-3	Carbamic acid, N,N,Ä≤-[(7,8-dihydro-6H-dibenzo[f,h][1,5]dioxonin-3,11-diyl)bis[	C43H54N8O8	810.94	A/B	-8.2
8286	C(C(=O)O)[C@@H]1N(C(=Nc2c1cccc2F)N1CCN(CC1)c1cc(OC)ccc1)c1cc(OC)ccc(C(F)(F)F)c1.[NaH].O	1452177-24-0	4-Quinazolineacetic acid, 8-fluoro-3,4-dihydro-2-[4-(3-methoxyphenyl)-1-piperazi	C29H28F4N4O4.Na		A/B	-8.2
8287	O=c1n([C@@H]2O[C@H](CO)SC2)cc(F)c(=N)[nH]1	1452579-97-3	Carbonothioic dihydrazide, 2-[1,2-dihydro-2-oxo-1-(phenylmethyl)-3H-indol-3-ylic	C23H19N5OS	413.49	A/B	-8.2
8288	N(c1c2c(ncc1)cc(Cl)cc2)c1cc(O)c(CN(CC)C2CCCCC2)cc(CN(CC)C2CCCCC2)c1	145431-64-7	Phenol, 6-[(7-chloro-4-quinolinyl)amino]-3-methyl-2,4-bis(1-piperidinylmethyl)-	C28H35ClN4O	479.06	A/B	-8.2
8289	N(c1c2c(ncc1)cc(Cl)cc2)c1cc(O)c(CN2CCCCC2)ccc1	145431-65-8	Phenol, 2-[(7-chloro-4-quinolinyl)amino]-4,6-bis[(cyclohexylethylamino)methyl]-	C33H45ClN4O	549.19	A/B	-8.2
8290	N(c1nc(Nc2c(C)cc(C#N)cc2)ncc1)c1c(C)cc(C)cc1C	1460306-52-8	Benzonitrile, 4-[[4-[[2-(hydroxymethyl)-4,6-dimethylphenyl]amino]-2-pyrimidinyl]	C20H19N5O	345.4	A/B	-8.2
8291	O=C1[C@@H]2[C@H](c3c(C(=O)c2c2CO1)cc1c(c3)OCO1)c1cc(OC)c(OC)cc1	146237-06-1	Furo[3,Ä≤,4,Ä≤:6,7]naphtho[2,3-d]-1,3-dioxole-5,8-dione, 9-(3,4-dimethoxyphenyl	C21H18O7	382.36	A/B	-8.2
8292	N(c1c2c(ncc1)cc(Cl)cc2)c1ccc(cc(CN2CCCC2)c1O)c1ccc(Cl)cc1	146298-40-0	[1,1,Ä≤-Biphenyl]-4-ol, 4,Ä≤-chloro-3-[(7-chloro-4-quinolinyl)amino]-5-[(diprop	C28H29Cl2N3O	494.46	A/B	-8.2
8293	C(N(NC(=O)[C@@H](NC(=O)OC(C)(C)C)C(C)(C)C)[C@H](Cc1ccccc1)NC(=O)[C@@H](NC(=O)OC)C(=O)C(C)(O)C)c1ccc(	1463472-88-9	2,5,6,10,13-Pentaazatetradecanedioic acid, 3,12-bis(1,1-dimethylethyl)-8-hydroxy	C38H52N6O8	720.85	A/B	-8.2
8294	N(C(=O)C)[C@@H]1[C@@H](NC(=N)NC(=O)[C@@H](C)c2ccc3c(cc2)cc(OC)cc3)C=C(C(=O)O)O[C@H]1[C@@H]([C@@H](CO)	1469338-33-7	Not Yet Assigned	C26H32N4O9	544.55	A/B	-8.2
8295	N(c1c(C)cc(/C=C/C#N)cc1C)c1nc(Nc2ccc(C#N)cc2)ncc1.C(C)Cl	1469759-66-7	1H-Indole-2,3-dione, 5-[[(2S)-2-(fluoromethyl)-1-pyrrolidinyl]sulfonyl]-	C13H13FN2O4S	312.32	A/B	-8.2
8296	N(c1c(C)cc(/C=C/C#N)cc1C)c1nc(Nc2ccc(C#N)cc2)ncc1.O=C1CCCCC1.Cl	1469779-25-6	Benzonitrile, 4-[[4-[[4-[(1E)-2-cyanoethenyl]-2,6-dimethylphenyl]amino]-2-pyrimi	C22H18N6.CHCl3.ClH		A/B	-8.2
8297	C(OP(=O)(O[C@@H]1[C@H](O[C@H](CO)[C@@H]1OC(=O)c1ccccc1)n1nc(C(=O)N)nc1)OCC1clccc(N(=O)=O)cc1)[C@@H]1[C	1469860-67-0	Benzonitrile, 4-[[4-[[4-[(1E)-2-cyanoethenyl]-2,6-dimethylphenyl]amino]-2-pyrimi	C22H18N6.CH4O.ClH		A/B	-8.2
8298	N(C(C)C)c1c(nccc1)N1CCN(C(=O)c2cc3c([nH]2)ccc(NS(=O)(=O)CCc2ccccc2)c3)CC1	147539-94-4	4-Morpholineethanesulfonamide, N-[2-[[4-[3-[(1-methylethyl)amino]-2-pyridinyl]-1	C27H37N7O4S	555.69	A/B	-8.2
8299	N(C(CC)(C)C)c1c(nccc1)N1CCN(C(=O)c2cc3c([nH]2)ccc(NS(=O)(=O)C)c3)CC1	147540-14-5	1-Piperazinesulfonamide, N-[2-[[4-[3-[(1,1-dimethylpropyl)amino]-2-pyridinyl]-1-	C28H40N8O3S	568.73	A/B	-8.2
8300	N(C(C)C)c1c(nccc1)N1CCN(C(=O)c2cc3c([nH]2)ccc(NS(=O)(=O)CCN2CCCCC2)c3)CC1	147540-16-7	1-Piperazinesulfonamide, 4-(1,1-dimethylpropyl)-N-[2-[[4-[3-[(1-methylethyl)amir	C30H44N8O3S	596.79	A/B	-8.2
8301	C(n1cc(nc1CCCC)cccc1)c1ccc(cc1)c1ccc(cc1)S(=O)(=O)NC(=O)OCC)cccc1	148401-55-2	[1,1,Ä≤-Biphenyl]-2-sulfonamide, N-[di-2-propen-1-ylamino)carbonyl]-4,Ä≤-[(2-e	C30H33N5O3S	543.68	A/B	-8.2
8302	C(n1c2nc(C)cc(C)c2n1)c1ccc(cc1)c1c(S(=O)(=O)NC(=O)OCc2ccccc2)cccc1	148401-85-8	Carbamic acid, [[4,Ä≤-[(2-ethyl-5,7-dimethyl-3H-imidazo[4,5-b]pyridin-3-yl)methy	C25H26N4O4S	478.56	A/B	-8.2
8303	N(c1ccc(C#N)cc1)C([C@@H](C(=O)NC(=O)[C@@H](NC(=O)OC(C)C)C(C)C)O)NC(=O)[C@@H](NC(=O)OC(C)C)C(C	14926-00-2	[1,1,Ä≤-Biphenyl]-3,3,Ä≤-dicarboxylic acid, 4,4,Ä≤-bis(benzoylamino)-	C28H20N2O6	480.47	A/B	-8.2
8304	O=c1nc([C@H]2O[C@H](CO[Si](C)(C)C)SC2)c(F)c(N)n1	14997-54-7	Guanosine, 5,Ä≤-[hydrogen P-[[hydroxy(phosphonooxy)phosphinyl]methyl]phosphonate	C11H18N5O13P3	521.21	A/B	-8.2
8305	N(C(C(=O)N)c1c(Cl)cccc1Cl)c1c2c(CCC2=O)cc(C)c1	150796-38-6	Acetamide, N-[1-[(2R,5S)-2-[(benzoyloxy)methyl]-1,3-oxathiolan-5-yl]-5-fluoro-1,	C17H16FN3O5S	393.39	A/B	-8.2
8306	O=C1N(c2c3c(c4c(n2)cccc4)n(CC(C)C)cn3)c2c1cc(I)cc2	1508303-91-0	7-Azabicyclo[4.2.0]octa-1,3,5-trien-8-one, 3-chloro-7-[1-(2-methylpropyl)-1H-imi	C21H17ClN4O	376.84	A/B	-8.2

8307	C(C(C)C)n1c2c(c(Nc3c(C(=O)O)cc(Cl)cc3)nc3c2cccc3)nc1	1508303-96-5	Benzoic acid, 4-chloro-2-[[1-(2-methylpropyl)-1H-imidazo[4,5-c]quinolin-4-yl]ami	C21H19ClN4O2	394.85	A/B	-8.2
8308	O=c1n(cc(COC)c(=O)[nH]1)[C@@H]1C[C@H](N=[N+]=[N-])[C@@H](CO)O1	1514912-73-2	Phosphonic acid, P-[[(1R)-2-(6-amino-9H-purin-9-yl)-1-methylethoxy]methyl]-, bis	C23H24Cl2N5O4P	536.35	A/B	-8.2
8309	O=c1n([C@@H]2O[C@H](COP(=O)(N[C@H](C(=O)OCC(C)(C)C)C)N[C@H](C(=O)OCC(C)(C)C)C)C=C2)cc(C)c(=O)[nH]1	1526921-92-5	Not Yet Assigned	C28H43N4O9P	610.64	A/B	-8.2
8310	O=c1n([C@@H]2O[C@H](COP(=O)(N[C@H](C(=O)OCC(C)(C)C)C)N[C@H](C(=O)OCC(C)(C)C)C)[C@@H](N=[N+]=[N-])C2)	1526921-95-8	L-Alanine, N,N,Ä≤-(3,Ä≤-azido-3,Ä≤-deoxy-5,Ä≤-thymidylylidene)bis-, 1,1,Ä≤-bis	C30H36N7O9P		A/B	-8.2
8311	O[C@H]1[C@@H](O[C@H](COP(=O)(N[C@H](C(=O)OCc2ccccc2)C)N[C@H](C(=O)OCc2ccccc2)C)[C@H]1O)n1nc(C(=O)N)n	1526922-00-8	8-Oxa-3,5-diaza-4-phosphanonanoic acid, 4-[[(2R,5S)-5-(4-amino-2-oxo-1(2H)-pyrim	C28H34N5O8PS	631.64	A/B	-8.2
8312	O(C(OC(=O)OC(C)C)C)c1c(C(=O)NCc2ccc(F)cc2)nc(C(NC(=O)c2nnc(C)o2)(C)C)n(C)c1=O	1538603-90-5	Carbonic acid, ethyl 1-[[4-[[[(4-fluorophenyl)methyl]amino]carbonyl]-1,6-dihydro	C25H29FN6O8	560.53	A/B	-8.2
8313	C(=O)(NCc1ccc(F)cc1)c1c(OC(OC(=O)OCC2CC2)C)c(=O)n(C)c(C(NC(=O)c2oc(C)nn2)(C)C)n1	1538603-93-8	Carbonic acid, 1-[[4-[[[(4-fluorophenyl)methyl]amino]carbonyl]-1,6-dihydro-1-met	C30H31FN6O8	622.6	A/B	-8.2
8314	O(C(OC(=O)OC)C)c1c(C(=O)NCc2ccc(F)cc2)nc(C(NC(=O)c2nnc(C)o2)(C)C)n(C)c1=O	1538603-94-9	Carbonic acid, cyclopropylmethyl 1-[[4-[[[(4-fluorophenyl)methyl]amino]carbonyl]	C27H31FN6O8	586.57	A/B	-8.2
8315	O(C(OC(=O)C1CC1)C)c1c(C(=O)NCc2ccc(F)cc2)nc(C(NC(=O)c2nnc(C)o2)(C)C)n(C)c1=O	1538604-20-4	Propanoic acid, 2-methyl-, 1-[[4-[[[(4-fluorophenyl)methyl]amino]carbonyl]-1,6-d	C26H31FN6O7	558.56	A/B	-8.2
8316	C(n1c2c(nc1CC)c(C)cc(C)n2)c1ccc(cc1)c1c(S(=O)(=O)NC(=O)COCCCC)ccc(CN(C)C)c1	154552-05-3	Acetamide, 2-butoxy-N-[[5-(1,1-dimethylethyl)-4,Ä≤-[(2-ethyl-5,7-dimethyl-3H-im	C33H42N4O4S	590.78	A/B	-8.2
8317	C(n1c2c(nc1CC)c(C)cc(C)n2)c1ccc(cc1)c1c(S(=O)(=O)NC(=O)COCC)ccc(COC)c1	154552-20-2	Acetamide, 2-ethoxy-N-[[4,Ä≤-[(2-ethyl-5,7-dimethyl-3H-imidazo[4,5-b]pyridin-3-	C29H34N4O4S2	566.73	A/B	-8.2
8318	C(n1c2c(nc1CC)c(C)cc(C)n2)c1ccc(cc1)c1c(S(=O)(=O)NC(=O)COCC)ccc(OCC)c1	154552-21-3	Acetamide, 2-ethoxy-N-[[4,Ä≤-[(2-ethyl-5,7-dimethyl-3H-imidazo[4,5-b]pyridin-3-	C29H34N4O5S	550.67	A/B	-8.2
8319	C(n1c2c(nc1CC)c(C)cc(C)n2)c1ccc(cc1)c1c(S(=O)(=O)NC(=O)NCCCC)ccc(CC(C)C)c1	154552-37-1	[1,1,Ä≤-Biphenyl]-2-sulfonamide, 5-butoxy-N-[(butylamino)carbonyl]-4,Ä≤-[(2-eth	C32H41N5O4S	591.76	A/B	-8.2
8320	C(#Cc1ccccc1)[C@@]1(C(F)(F)F)c2c(NC(=O)O1)ccc(Cl)c2	154598-09-1	2H-3,1-Benzoxazin-2-one, 6-chloro-1,4-dihydro-4-(2-phenylethynyl)-4-(trifluorome	C17H9ClF3NO2	351.71	A/B	-8.2
8321	O(C(=O)N[C@@H](Cc1ccccc1)[C@@H](CN(S(=O)(=O)c1ccc(NC(=O)C)cc1)CC(C)C)O)[C@@H]1[C@H]2[C@@H](OC1)OCC2	1546919-06-5	Carbamic acid, N-[(1S,2R)-3-[[[4-[[[[(3S,3aR,6aS)-hexahydrofuro[2,3-b]furan-3-yl	C41H53N3O15S	859.94	A/B	-8.2
8322	N(c1c2c(n(cn2)[C@H]2C[C@@H](COC(=O)CCCCCCC(=O)OC[C@@H]3C[C@@H](C=C3)n3c4c(nc3)c(NC3CC3)nc(N)n4)C=C2	1551302-04-5	Heptanedioic acid, 1,7-bis[[(1R,4S)-4-[2-amino-6-(cyclopropylamino)-9H-purin-9-y	C35H44N12O4	696.8	A/B	-8.2
8323	N(c1c2c(n(cn2)[C@H]2C[C@@H](COC(=O)CCCCCCCCC(=O)OC[C@@H]3C[C@@H](C=C3)n3c4c(nc3)c(NC3CC3)nc(N)n4)C=C	1551302-09-0	Nonanedioic acid, 1,9-bis[[(1R,4S)-4-[2-amino-6-(cyclopropylamino)-9H-purin-9-yl	C37H48N12O4	724.85	A/B	-8.2
8324	Clc1c2c(c3c(cc2)cccc3)nc(CC(P(=O)(OCC)OCC)C(=O)OCC)c1	155213-67-5	2,7,10,12-Tetraazatridecanoic acid, 4-hydroxy-12-methyl-9-(1-methylethyl)-13-[2-	C37H48N6O5S2	720.94	A/B	-8.2
8325	O=C1C2c3c(C1CC2)c(ccc3c1[nH]c(nc1)[C@H]1N(C(=O)[C@@H](NC(=O)OC)C(C)C)CCC1)c1ccc(cc1)c1[nH]c(nc1)[C@H	1560649-85-5	Carbamic acid, N-[(1S)-1-[[(2S)-2-[5-[4-[1,4-dihydro-8-[2-[(2S)-1-[(2S)-2-[(meth	C45H54N8O6	802.96	A/B	-8.2
8326	C([C@H](OC[P@](=O)(Oc1cc2c(cc1)cccc2)NC(C(=O)OC(C)C)(C)C)C)n1c2c(nc1)c(N)ncn2	1571076-29-3	Alanine, N-[(S)-[[(1R)-2-(6-amino-9H-purin-9-yl)-1-methylethoxy]methyl](4-chloro	C22H30ClN6O5P	524.94	A/B	-8.2
8327	C(=O)([C@@H](NC(=O)OC)C(C)C)N1CCC2(C[C@H]1c1[nH]c(cn1)c1ccc(cc1)c1ccc(cc1)c1[nH]c(nc1)[C@H]1N(C(=O)[C	1571138-06-1	Carbamic acid, N-[(1S)-1-[[(2S)-2-[5-[4,Ä≤-[2-[(3S)-2-[(2S)-2-[(methoxycarbonyl)	C44H56N8O8S	857.03	A/B	-8.2
8328	C(=O)([C@@H](NC(=O)OC)C(C)C)N1[C@H](CC2(C1)CCNCC2)c1[nH]c(cn1)c1ccc(cc1)c1ccc(cc1)c1[nH]c(nc1)[C@H]1	1571138-15-2	Carbamic acid, N-[(1S)-1-[[(2S)-2-[5-[4,Ä≤-[2-[(3S)-2-[(2S)-2-[(methoxycarbonyl)	C44H57N9O6	807.98	A/B	-8.2
8329	C(=O)([C@@H](NC(=O)OC)C(C)C)N1CCC2(C[C@H]1c1[nH]c(cn1)c1ccc(cc1)c1ccc(cc1)c1[nH]c(nc1)[C@H]1N(C(=O)[C	1571138-20-9	Carbamic acid, N-[(1S)-1-[[(2S)-2-[5-[4,Ä≤-[2-[(3S)-8-acetyl-2-[(2S)-2-[(methoxy	C46H59N9O7	850.02	A/B	-8.2
8330	Brc1n(c2c(n1)c(=O)nc(N)[nH]2)[C@H]1[C@H](OC(=O)C)[C@H](OC(=O)C)[C@@H](COC(=O)C)O1	1571138-56-1	Carbamic acid, N-[(1S)-1-[[(2S)-2-[5-[4,Ä≤-[2-[(7S)-6-[(2S)-2-[(methoxycarbonyl)	C43H55N9O8S	858.02	A/B	-8.2
8331	O[C@H]1[C@@H](O[C@H](COP2(=O)O[C@H](CCO2)c2ccncc2)[C@H]1O)n1nc(C(=O)N)nc1	1578257-05-2	1H-1,2,4-Triazole-3-carboxamide, 1-[5-O-[(4S)-2-oxido-4-(4-pyridinyl)-1,3,2-diox	C16H20N5O8P	441.33	A/B	-8.2
8332	N(c1c2c(ncc1)cc(Cl)cc2)c1c(O)c(CN2CC(C)CCC2)cc(Cl)c1	157831-63-5	Phenol, 6-[(7-chloro-4-quinolinyl)amino]-3-fluoro-2,4-bis(1-piperidinylmethyl)-	C27H32ClFN4O	483.02	A/B	-8.2
8333	O=c1n([C@@H]2O[C@H](COP(=O)(OCC(COCCCCCCCCCCCCCCCCCC)O)O)[C@@H](N=[N+]=[N-])C2)cc(C)c(=O)[nH]1	157831-92-0	Phenol, 4-chloro-2-[(7-chloro-4-quinolinyl)amino]-6-[(3-methyl-1-piperidinyl)met	C22H23Cl2N3O	416.34	A/B	-8.2
8334	N(CCCN(C)C)c1c2c(cc(Cl)cc2)ncc1	1584693-93-5	Carbamic acid, N-[6,9-dihydro-9-[(1S,3R,4S)-4-hydroxy-2-methylene-3-[(phenylmeth	C24H29N5O5	467.52	A/B	-8.2
8335	C(C/C(=C/CC(P(=O)(OCOC(=O)N(C)C)O)C(=O)OCOC(=O)N(C)C)/C)c1cc2c(cc1)cccc2	159274-29-0	4,6-Dioxa-2-aza-7-phosphanonan-9-oic acid, 7-hydroxy-2-methyl-8-[(2E)-3-methyl-5	C22H28NO7P	449.43	A/B	-8.2
8336	O(CCCCn1ccnc1)[C@H]1[C@H](n2c3c(nc2)c(=O)nc(N)[nH]3)O[C@H](CO)[C@H]1O	160526-97-6	Guanosine, 2,Ä≤-O-[3-(1,3-dihydro-1,3-dioxo-2H-isoindol-2-yl)propyl]-	C21H22N6O7	470.44	A/B	-8.2
8337	O[C@@H]1[C@H](n2c3c(nc2)c(N)ncn3)O[C@H](COP(=O)(OCCCCCCO[C@@H]2O[C@H](CO)[C@H](O)[C@H](O)[C@H]2O)O)[	160956-61-6	9H-Purin-6-amine, 9-[5-O-[[3-(Œ≤-D-galactopyranosyloxy)propoxy]hydroxyphosphinyl	C19H30N5O13P	567.44	A/B	-8.2
8338	O(C(=O)N[C@H]([C@@H](CN(S(=O)(=O)c1ccc(N)cc1)CC(C)C)O)Cc1ccccc1)[C@@H]1[C@@H]2[C@@H]([C@H](OC)CO2)OC	1609687-32-2	Benzonitrile, 4-[[2-[[4-[(1Z)-2-cyanoethenyl]-2,6-dimethylphenyl]amino]-5-fluoro	C22H17FN6	384.41	A/B	-8.2
8339	O=c1c2c(n(cn2)[C@@H]2C(=C)[C@H](COP(=O)(Oc3ccccc3)N[C@H](C(=O)OC3CCCCC3)C)[C@@H](O)C2)[nH]c(N)n1	1610358-03-6	L-Alanine, N-[[[(1R,3S,5S)-3-(2-amino-1,6-dihydro-6-oxo-9H-purin-9-yl)-5-hydroxy	C24H31N6O7P	546.51	A/B	-8.2
8340	C(=O)([C@H](NC(=O)OC)C(C)C)N1[C@@H](CCC1)c1[nH]c(cn1)c1ccc(C#CC#Cc2[nH]c(nc2)[C@H]2N(C(=O)[C@@H](NC(	1610702-57-2	Carbamic acid, N-[(1S)-1-[[(2R)-2-[5-[4-[4-[2-[(2R)-1-[(2S)-2-[(methoxycarbonyl)	C38H46N8O6	710.82	A/B	-8.2
8341	C(OP(=O)(OP(=O)(OP(=O)(O)O)O)O)[C@H]1C[C@H]([C@H]2[C@@H]1OC(C)(C)O2)n1c2c(nc1)c(=O)nc(N)[nH]2	1610702-67-4	Carbamic acid, N-[2-[(2S)-2-[5-[4-[4-[2-[(2S)-1-[2-[(methoxycarbonyl)amino]-2-me	C36H42N8O6	682.77	A/B	-8.2
8342	O=C1[C@@H]2[C@@H](c3c([C@@H](O)[C@H]2CO1)cc1c(c3)OCO1)c1ccc(Br)cc1	1610886-62-8	Furo[3,Ä≤,4,Ä≤:6,7]naphtho[2,3-d]-1,3-dioxol-6(5aH)-one, 5-(4-bromophenyl)-5,8,	C19H15BrO5	403.22	A/B	-8.2
8343	C(OCCOC(=O)[C@@H](NC(=O)Nc1ccc(F)cc1)C(C)C)n1c2c(nc1)c(=O)nc(N)[nH]2	1612850-48-2	L-Valine, N-[[(4-bromophenyl)amino]carbonyl]-, 2-[(2-amino-1,6-dihydro-6-oxo-9H-	C20H24BrN7O5	522.35	A/B	-8.2
8344	O[C@H]1[C@@H](O[C@H](COP(=O)(Oc2ccccc2)N[C@H](C(=O)OCC)C)[C@H]1O)n1c(=O)c(C(=O)N)nc(F)c1	1613031-72-3	Glycine, N-(hydroxyphenoxyphosphinyl)-, 1-methylethyl ester, 5,Ä≤-ester with 6-	C21H26FN4O10P	544.42	A/B	-8.2
8345	[C@H](C(=O)N1[C@@H]2[C@@H](C2)C[C@H]1C#N)(N)C12CC3(OC(=O)[C@@H](C(C)C)N(C)C)CC(C2)CC(C1)C3	1616608-87-7	D-Valine, N-methyl-, 3-[(1S)-1-amino-2-[(1S,3S,5S)-3-cyano-2-azabicyclo[3.1.0]he	C24H36N4O3	428.57	A/B	-8.2
8346	[C@H](C(=O)N1[C@@H]2[C@@H](C2)C[C@H]1C#N)(N)C12CC3(OC(=O)[C@@H](CCC(=O)O)N)CC(C2)CC(C1)C3	1616609-20-1	L-Glutamic acid, 1-[3-[(1S)-1-amino-2-[(1S,3S,5S)-3-cyano-2-azabicyclo[3.1.0]hex	C23H32N4O5	444.52	A/B	-8.2
8347	[C@H](C(=O)N1[C@@H]2[C@@H](C2)C[C@H]1C#N)(N)C12CC3(OC(=O)[C@H]([C@@H](CC)C)N)CC(C2)CC(C1)C3	1616609-21-2	D-Glutamic acid, 1-[3-[(1S)-1-amino-2-[(1S,3S,5S)-3-cyano-2-azabicyclo[3.1.0]hex	C23H32N4O5	444.52	A/B	-8.2
8348	[C@H](C(=O)N1[C@@H]2[C@@H](C2)C[C@H]1C#N)(N)C12CC3(OC(=O)[C@H]([C@H](CC)C)N)CC(C2)CC(C1)C3	1616609-47-2	D-Leucine, 3-[(1S)-1-amino-2-[(1S,3S,5S)-3-cyano-2-azabicyclo[3.1.0]hex-2-yl]-2-	C24H36N4O3	428.57	A/B	-8.2
8349	C(=O)(N[C@@H](CC1CCC1)C(=O)C(=O)N)[C@@H]1[C@@H]2[C@@H](C2(C)C)CN1C(=O)[C@@H](NC(=O)NC1(CS(=O)(=O)c2c	1616695-98-7	3-Azabicyclo[3.1.0]hexane-2-carboxamide, N-[(1S)-3-amino-1-(cyclobutylmethyl)-2,	C30H49N5O5	559.74	A/B	-8.2
8350	C(=O)([C@@H](NC(=O)OC)C(C)C)N1[C@@H](CC2C1CN(C)CC2)c1[nH]c(cn1)c1ccc(cc1)c1ccc(cc1)c1[nH]c(nc1)[C@H]	1616889-49-6	Carbamic acid, N-[(1S)-2-methyl-1-[[(2S)-2-[5-[4,Ä≤-[2-[(2S)-octahydro-1-[(2S)-	C44H57N9O8S	872.04	A/B	-8.2
8351	[C@@H](C(=O)N[C@@H](Cc1ccc(F)cc1)C[C@@H]([C@H](Cc1ccc(F)cc1)NC(=O)COc1c(C)cccc1C)O)(C(C)C)N1C(=O)NCC	1616889-59-8	Carbamic acid, N-[(1S)-1-[[(2S)-2-[5-[4,Ä≤-[2-[4-acetylhexahydro-1-[(2S)-2-[(met	C43H55N9O7	809.95	A/B	-8.2
8352	C([C@H](OC[P@](=O)(NCc1ccccc1)OP(=O)(CO[C@@H](Cn1c2c(nc1)c(N)ncn2)C)O)C)n1c2c(nc1)c(N)ncn2	1619914-56-5	Amidodiphosphonic acid, P,P,Ä≤-bis[[(1R)-2-(6-amino-9H-purin-9-yl)-1-methyletho	C22H35N11O6P2	611.53	A/B	-8.2
8353	O=c1n([C@@H]2O[C@H](COP(=O)(Nc3cc(C(=O)Nc4cc(C(=O)Nc5cc(C(=O)NCCC#N)n(C)c5)n(C)c4)n(C)c3)OC3CCCCC3)C	1620662-78-3	5,Ä≤-Thymidylic acid, 3,Ä≤-azido-3,Ä≤-deoxy-, 5,Ä≤-[cyclohexyl N-[5-[[[5-[[[5-[	C37H49N14O9P		A/B	-8.2
8354	C[C@]1(F)[C@@H](O[C@H](COP(=O)(Oc2ccccc2)N[C@H](C(=O)OC)C)[C@H]1O)n1c(=O)[nH]c(=O)c(c1[2H])[2H]	1621154-29-7	L-Alanine, N-[(2,Ä≤R)-2,Ä≤-deoxy-2,Ä≤-fluoro-2,Ä≤-methyl-P-phenyl-5,Ä≤-uridylyl	C22H27D2FN3O9P		A/B	-8.2
8355	C[C@]1(F)[C@@H](O[C@H](COP(=O)(Oc2ccc(F)cc2)N[C@H](C(=O)OCC(C)C)C)[C@H]1O)n1c(=O)[nH]c(=O)c(c1[2H])[	1621156-06-6	L-Alanine, N-[(2,Ä≤R)-2,Ä≤-deoxy-2,Ä≤-fluoro-P-(4-fluorophenyl)-2,Ä≤-methyl-5,Ä	C21H24D2F2N3O9P		A/B	-8.2
8356	C[C@]1(F)[C@@H](O[C@H](C(OP(=O)(Oc2ccccc2)N[C@H](C(=O)OCC(C)C)C(C)C)C)[C@H]1O)n1c(=O)[nH]c(=O)c(c1[2	1621166-23-1	L-Valine, N-(hydroxyphenoxyphosphinyl)-, ethyl ester, 5,Ä≤-ester with 1-[(2R,5Ga	C24H31D2FN3O9P		A/B	-8.2
8357	[C@@H](C(=O)N[C@H](Cc1ccccc1)C(=O)OC)(NC(=O)OC(C)(C)C)C12CC3(O)CC(C2)CC(C1)C3	1623104-69-7	L-Phenylalanine, (2S)-N-[(1,1-dimethylethoxy)carbonyl]-2-(3-hydroxytricyclo[3.3.	C26H36N2O6	472.57	A/B	-8.2
8358	[C@@H](C(=O)N[C@H](Cc1ccccc1)C(=O)N)(NC(=O)OC(C)(C)C)C12CC3(O)CC(C2)CC(C1)C3	1623104-77-7	Carbamic acid, N-[(1S)-2-[[(1S)-1-cyano-2-phenylethyl]amino]-1-(3-hydroxytricycl	C26H35N3O4	453.57	A/B	-8.2
8359	N(CCCNc1nc(ccn1)N1CCN(CC)CC1)c1nc(nc(n1)N1CCOCC1)N1CCOCC1	1623752-50-0	1,2-Ethanediamine, N1-(4,6-di-4-morpholinyl-1,3,5-triazin-2-yl)-N2-[4-(4-ethyl-1	C23H37N11O2	499.61	A/B	-8.2
8360	[C@H](C(=O)N1[C@H](C#N)C[C@H](NS(=O)(=O)C)C1)(N)C12CC3(O)CC(C2)CC(C1)C3	1624277-73-1	Benzeneacetamide, N-[(3S,5S)-1-[(2S)-2-amino-2-(3-hydroxytricyclo[3.3.1.13,7]dec	C25H32N4O3	436.55	A/B	-8.2
8361	N(C(=O)[C@H](Cc1ccc(O)cc1)C[C@@H](CN1[C@H](C(=O)NC(C)(C)C)CN(Cc2cccnc2)CC1)O)[C@H]1c2c(C[C@H]1O)cccc	162518-58-3	D-erythro-Pentonamide, 2,3,5-trideoxy-N-[(1S,2S,3S)-2,3-dihydro-2,3-dihydroxy-1H	C30H42N4O5	538.68	A/B	-8.2
8362	O(C)c1ccc(c2nc(nc(n2)N2CCOCC2)N2CCNCC2)cc1	1627734-71-7	1,3,5-Triazin-2-amine, 4-(4-methoxyphenyl)-N-(3-methylphenyl)-6-(4-morpholinyl)-	C21H23N5O2	377.44	A/B	-8.2
8363	N(c1nc(nc(n1)N1CCOCC1)N1CCOCC1)c1ccc(C(=O)C=Cc2ccc(Br)cc2)cc1	1628172-06-4	2-Propen-1-one, 1-[4-[(4,6-di-4-morpholinyl-1,3,5-triazin-2-yl)amino]phenyl]-3-(	C29H34N6O6	562.62	A/B	-8.2
8364	C[C@]1(F)[C@@H](O[C@H](COP(=O)(Oc2c(Cc3ccccc3)cccc2)N[C@H](C(=O)OC(C)C)C)[C@H]1O)n1c(=O)[nH]c(=O)cc1	1628724-50-4	L-Alanine, N-[(2,Ä≤R)-P-[1,1,Ä≤-biphenyl]-2-yl-2,Ä≤-deoxy-2,Ä≤-fluoro-2,Ä≤-meth	C28H33FN3O9P	605.55	A/B	-8.2
8365	C=C1[C@@H](n2c3c(nc2)c(=O)nc(N)[nH]3)C[C@H](O)[C@H]1COP(=O)(Oc1ccccc1)NCCNC(=O)CCCCCCC/C=C\CCCCCCCC	1628917-33-8	Thymidine, 3,Ä≤-azido-3,Ä≤-deoxy-, 5,Ä≤-[phenyl N-[2-[[(4Z,7Z,10Z,13Z,16Z,19Z)-	C40H54N7O7P		A/B	-8.2
8366	O=c1c2c(n(cn2)[C@@H]2O[C@H](COP(=O)(OCCNC(=O)C=C)O)[C@@H](O)[C@H]2O)[nH]c(N)n1	1629023-92-2	Benzonitrile, 4,4,Ä≤-(2,4-pyrimidinediyldiimino)bis[3-methyl-	C20H16N6	340.38	A/B	-8.2
8367	C([C@H](OCP(=O)(Cl)Cl)C)n1c2c(nc1)c(N)ncn2	1630726-94-1	1-Naphthalenesulfonic acid, 3,3,Ä≤-[(2,2,Ä≤-dimethyl[1,1,Ä≤-biphenyl]-4,4,Ä≤-di	C34H28N6O6S2.Na		A/B	-8.2
8368	O=C1[C@@H]2[C@@H](c3c([C@H](OC(=O)C#CC)[C@H]2CO1)cc1c(c3)OCO1)c1cc(OC)c(OC)c(OC)c1	1633020-37-7	Benzoic acid, 2-amino-5-nitro-, (5R,5aR,8aR,9R)-5,5a,6,8,8a,9-hexahydro-8-oxo-9-	C29H26N2O11	578.52	A/B	-8.2
8369	C=C1[C@@H](n2c3c(nc2)c(=O)nc(N)[nH]3)C[C@H](O)[C@H]1CO[P@@](=O)(Oc1cc2c(cc1)OCO2)N[C@H](C(=O)OC(C)C)	1638769-97-7	L-Alanine, N-[[[(1R,3S,5S)-3-(2-amino-1,6-dihydro-6-oxo-9H-purin-9-yl)-5-hydroxy	C25H31N6O9P	590.52	A/B	-8.2
8370	O=c1c2c(n(cn2)[C@@H]2O[C@H](COP(=O)(O)O)[C@@H](O)[C@H]2O)[nH]c(NCSc2c(O)cccc2)n1	1638949-50-4	5,Ä≤-Guanylic acid, N-[[(2-phenylethyl)thio]methyl]-	C19H24N5O8PS	513.46	A/B	-8.2
8371	O=c1c2c(n(cn2)[C@@H]2O[C@H](COP(=O)(O)O)[C@@H](O)[C@H]2O)[nH]c(NCSc2ccc(O)cc2)n1	1638949-51-5	5,Ä≤-Guanylic acid, N-[[(2-hydroxyphenyl)thio]methyl]-	C17H20N5O9PS	501.41	A/B	-8.2
8372	C(=O)(C1(NC(=O)OC)CC1)N1[C@@H](CCC1)c1[nH]c(cn1)c1ccc(cc1)c1ccc(cc1)c1[nH]c(nc1)[C@H]1N(C(=O)[C@@H](	1639870-46-4	2H-Isoindole-2-carboxylic acid, 4-fluoro-1,3-dihydro-, (3R,5S)-1-[(2S)-2-[[(1,1-	C52H62FN9O8	960.1	A/B	-8.2
8373	C(=O)([C@@H](NC(=O)OC)C(C)C)N1[C@@H](CC(O)C1)c1[nH]c(cn1)c1ccc(cc1)c1ccc(cc1)c1[nH]c(nc1)[C@H]1N(C(=	1639871-28-5	Carbamic acid, N-[(1S)-1-cyclopentyl-2-[(2S)-2-[5-[4,Ä≤-[2-[(2S)-1-[(2S)-2-cyclo	C44H52N8O6	788.93	A/B	-8.2
8374	O[C@@H]1[C@H](n2c3c(nc2)c(N)nc(F)n3)O[C@H](COP(=O)(Oc2c3c(ccc2)cccc3)NCCNC(=O)CCC/C=C\C/C=C\C/C=C\C/	1642320-06-6	9H-Purin-6-amine, 2-fluoro-9-[5-O-[[[2-[[(5Z,8Z,11Z,14Z,17Z)-1-oxo-5,8,11,14,17-	C38H51FN7O7P	767.83	A/B	-8.2
8375	N(c1c(C)cc(/C=C/C#N)cc1C)c1nc(Nc2ccc(C#N)cc2)nc(NCCCN2CCOCC2)c1	1642573-86-1	Benzonitrile, 4-[[4-[[4-[(1E)-2-cyanoethenyl]-2,6-dimethylphenyl]amino]-6-[3-(4-	C29H31N7O2	509.6	A/B	-8.2
8376	O(c1nc(nc(Nc2ccc(Cl)cc2)n1)N1CCOCC1)c1ccc(C#N)cc1	1643332-89-1	Benzonitrile, 4-[[4-[(4-methoxyphenyl)amino]-6-(4-morpholinyl)-1,3,5-triazin-2-y	C21H20N6O3	404.42	A/B	-8.2
8377	C(=O)([C@@H](NC(=O)OC)C(C)C)N1[C@H](c2[nH]c(c3cc4c(-c5c(C4)cc(cc5)c4[nH]c(nc4)[C@H]4N(C(=O)[C@@H](NC	1645563-55-8	Carbamic acid, N,N,Ä≤-[(9,10-dihydro-2,7-phenanthrenediyl)bis[1H-imidazole-5,2-d	C44H56N8O6	792.97	A/B	-8.2
8378	N(C(=O)[C@H](Cc1ccccc1)C[C@@H](CN1[C@H](C(=O)NC(C)(C)C)CN(CC(=O)OC(C)(C)C)CC1)O)[C@H]1c2c(C[C@H]1O)c	164725-14-8	1-Piperazinepentanamide, N-(2,3-dihydro-2-hydroxy-1H-inden-1-yl)-2-[[(1,1-dimeth	C32H46N4O4	550.73	A/B	-8.2
8379	C[C@]1(F)[C@@H](O[C@H](CO[P@](=O)(Oc2ccccc2)N[C@@H](C(=O)OC(C)C)C)[C@H]1O)n1c(=O)[nH]c(=O)c(F)c1	1650593-60-4	D-Alanine, N-[[P(R),2,Ä≤R]-2,Ä≤-deoxy-2,Ä≤,5-difluoro-2,Ä≤-methyl-P-phenyl-5,Ä≤	C22H28F2N3O9P	547.44	A/B	-8.2
8380	C[C@]1(F)[C@@H](O[C@H](C(O[P@](=O)(Oc2ccccc2)N[C@@H](C(=O)OC(C)C)C)([2H])[2H])[C@H]1O)n1c(=O)[nH]c(=	1651206-59-5	D-Alanine, N-[[P(S),2,Ä≤R]-2,Ä≤-deoxy-2,Ä≤-fluoro-2,Ä≤-methyl-P-phenyl-5,Ä≤-uri	C22H26D3FN3O9P		A/B	-8.2
8381	[C@H](Cc1ccccc1)(C[C@@H]([C@H](Cc1ccccc1)NC(=O)OCc1cncs1)O)NC(=O)[C@@H](NC(=O)N(Cc1nc(C(C)C)oc1)C)C	165315-28-6	2,7,10,12-Tetraazapentadecanoic acid, 4-hydroxy-14-methyl-9-(1-methylethyl)-12-[	C40H54N6O5S2	763.02	A/B	-8.2
8382	C(=O)([C@@H](NC(=O)OC)C(C)C)N1CCC2(C[C@H]1c1[nH]c3c(n1)ccc1c3ccc(c1)c1cc3c(cc1)c1c(cc3)nc([nH]1)[C@H]	1653966-58-5	Carbamic acid, N-[(1S)-1-[[(3S,5S)-3-[7-[4-[2-[(1R,3S,5R)-2-[(2S)-2-[(methoxycar	C47H56N8O7	845	A/B	-8.2
8383	C(=O)([C@@H](NC(=O)OC)C(C)C)N1C[C@]2(C[C@H]1c1[nH]c(cn1)c1ccc(cc1)c1cc3c(cc1)c1c(cc3)nc([nH]1)[C@H]1	1653967-15-7	Carbamic acid, N-[(1S)-1-[[(2S)-2-[7-[4-[2-[(5S,8S)-7-[(2S)-2-[(methoxycarbonyl)	C45H54N8O7	818.96	A/B	-8.2
8384	O=C1N([C@H]2O[C@H](CO)[C@@H](O)C2)CCC(=O)N1	166318-80-5	Thymidine, 3,Ä≤-azido-3,Ä≤-deoxy-P-(phenylmethyl)thymidylyl-(5,Ä≤,Üí5,Ä≤)-3,Ä≤-	C27H31N10O10P		A/B	-8.2
8385	O=c1n([C@H]2O[C@@H](COP(=O)(O)O)[C@H](O)C2)cc(I)c(=O)[nH]1	166586-33-0	Acetic acid, (phenylthio)-, 4-(5,5a,6,8,8a,9-hexahydro-9-hydroxy-6-oxofuro[3,Ä≤,	C29H26O9S	550.58	A/B	-8.2
8386	O=c1c(c2c3c4c5c(cc3)cccc5ccc4cc2)cn(c(=O)[nH]1)[C@@H]1O[C@H](CO)[C@@H](O)C1	1670258-62-4	Carbonic acid, 1-[[4-[[[(4-fluorophenyl)methyl]amino]carbonyl]-1,6-dihydro-1-met	C26H31FN6O9	590.56	A/B	-8.2
8387	O(C(=O)N[C@@H](Cc1cc(OC)ccc1)[C@@H](CN(S(=O)(=O)c1cc2c(cc1)nc(NC(C)C)s2)CC(C)C)O)[C@@H]1[C@H]2[C@@H]	1672661-47-0	Carbamic acid, N-[(1S,2R)-2-hydroxy-1-[(3-methoxyphenyl)methyl]-3-[[[2-[(1-methy	C32H42F2N4O9S	696.76	A/B	-8.2
8388	C[C@]1(F)[C@@H](O[C@H](COP(=O)(Oc2ccccc2)N[C@H](C(=O)OC(C)C)C)[C@H]1O)n1c(=S)[nH]c(=O)cc1	1678507-58-8	L-Alanine, N-[(2,Ä≤R)-2,Ä≤-deoxy-2,Ä≤-fluoro-2,Ä≤-methyl-P-phenyl-4-thio-5,Ä≤-u	C22H29FN3O8PS	545.52	A/B	-8.2
8389	C(O)[C@]12[C@H](C[C@@H](O1)n1c(=O)nc(N)cc1)CCC2	169322-05-8	9H-Purin-6-amine, 9-[5-O-[[(diphenoxyphosphinyl)oxy]hydroxyphosphinyl]-Œ≤-D-arab	C22H23N5O10P2	579.39	A/B	-8.2
8390	[C@H](Cc1ccc(O)cc1)(C(=O)N[C@H](/C=C/S(=O)(=O)c1ccccc1)CCc1ccccc1)NC(=O)N1CCOCC1	170111-44-1	4-Morpholinecarboxamide, N-[1-methyl-2-oxo-2-[[1-(2-phenylethyl)-3-(phenylsulfon	C32H37N3O5S	575.72	A/B	-8.2
8391	N(C(=O)[C@H](Cc1ccccc1)C[C@@H](CN1[C@H](C(=O)NC(C)(C)C)CN(Cc2cccnc2)CC1)O)[C@H]1c2c(C[C@H]1OC(=O)CCC	170227-65-3	Butanedioic acid, mono[1-[[4-(3-carboxy-1-oxopropoxy)-5-[2-[[(1,1-dimethylethyl)	C44H55N5O10	813.93	A/B	-8.2
8392	N(C(=O)[C@H](Cc1ccccc1)C[C@@H](CN1[C@H](C(=O)NC(C)(C)C)CN(Cc2cccnc2)CC1)O)[C@H]1c2c(C[C@H]1OC(=O)c1c	170227-76-6	Benzoic acid, 4-[(diethylamino)methyl]-, 1-[[5-[2-[[(1,1-dimethylethyl)amino]car	C48H62N6O5	803.04	A/B	-8.2
8393	O(c1c(Br)c(N)nc(Nc2ccc(C#N)cc2)n1)c1c(C)cc(C(=O)N)cc1C	170654-76-9	2H-Tetrazole, 5-phenyl-2-tricyclo[3.3.1.13,7]dec-1-yl-	C17H20N4	280.37	A/B	-8.2
8394	S(=O)(=O)(O)c1c2c(c(N)c(N=Nc3ccc(cc3)c3ccc(cc3)c3ccc(N=Nc4c(N)c5c(c(S(=O)(=O)O)c4)cccc5)cc3)c1)cccc2	17095-35-1	1-Naphthalenesulfonic acid, 3,3,Ä≤-[1,4-phenylenebis(azo)]bis[4-amino-	C26H20N6O6S2	576.6	A/B	-8.2
8395	N(=O)(=O)c1c(c2cc(nc(N)n2)c2ccc(Nc3nc(nc(n3)N3CCOCC3)N3CCOCC3)cc2)cccc1	1710363-37-3	2-Propen-1-one, 3-[4-(diethylamino)phenyl]-1-[4-[(4,6-di-4-morpholinyl-1,3,5-tri	C30H37N7O3	543.66	A/B	-8.2
8396	S(=O)(=O)(O)c1c2c(c(S(=O)(=O)O)cc(N=Nc3c(C)cc(N)c(C)c3)c2)cc(N=Nc2c(C)cc(N)c(C)c2)c1	1715638-27-9	2H-Indol-2-one, 1,3-dihydro-5-[(3-hydroxy-3-methyl-1-piperidinyl)sulfonyl]-	C14H18N2O4S	310.37	A/B	-8.2
8397	C(Oc1ccc(N(=O)=O)cc1)c1c2c(N(c3c(C(=O)N2)cccn3)C2CC2)ncc1	172326-14-6	Benzonitrile, 4-[(11-cyclopropyl-6,11-dihydro-6-oxo-5H-dipyrido[3,2-b:2,Ä≤,3,Ä≤	C22H17N5O2	383.4	A/B	-8.2
8398	C(c1c2c(N(c3c(C(=O)N2)cccn3)C2CC2)ncc1)c1c(O)cc(C)cc1	172326-18-0	6H-Dipyrido[3,2-b:2,Ä≤,3,Ä≤-e][1,4]diazepin-6-one, 11-cyclopropyl-5,11-dihydro-	C22H20N4O3	388.42	A/B	-8.2
8399	O=c1n([C@@H]2O[C@H](COP(=O)(Oc3c(F)c(F)c(F)c(F)c3F)N[C@H](C(=O)OC)C)C=C2)cc(C)c(=O)[nH]1	173070-90-1	L-Alanine, N-[2,Ä≤,3,Ä≤-didehydro-3,Ä≤-deoxy-P-[3-(trifluoromethyl)phenyl]-5,Ä≤	C21H23F3N3O8P	533.39	A/B	-8.2
8400	O=c1n([C@@H]2O[C@H](COP(=O)([C@@H](O)c3ccccc3)OC[C@@H]3[C@@H](N=[N+]=[N-])C[C@@H](O3)n3c(=O)[nH]c(=O	173658-21-4	Thymidine, 3,Ä≤-azido-P,3,Ä≤-dideoxy-P-[hydroxy(4-methylphenyl)methyl]thymidyly	C28H33N10O10P		A/B	-8.2
8401	[C@@H](C(C)(C)C)(C1=C(O)C[C@@](CCc2ccccc2)(CCC)OC1=O)c1ccc(NS(=O)(=O)c2ccccn2)ccc1	174484-22-1	2-Pyridinesulfonamide, N-[3-[1-[5,6-dihydro-4-hydroxy-2-oxo-6-(2-phenylethyl)-6-	C32H38N2O5S	562.72	A/B	-8.2
8402	[C@H](CC)(C1=C(O)CC(CCC)(CCC)OC1=O)c1ccc(NS(=O)(=O)c2ccc(C(F)(F)F)ccn2)ccc1	174484-26-5	2-Pyridinesulfonamide, N-[3-[(1S)-1-(5,6-dihydro-4-hydroxy-2-oxo-6,6-dipropyl-2H	C26H31F3N2O5S	540.59	A/B	-8.2
8403	[C@H](C(C)(C)C)(C1=C(O)CC(CCC)(CCC)OC1=O)c1ccc(NS(=O)(=O)c2ccc(C(F)(F)F)cn2)ccc1	174484-28-7	2-Pyridinesulfonamide, N-[3-[1-(5,6-dihydro-4-hydroxy-2-oxo-6,6-dipropyl-2H-pyra	C26H31F3N2O5S	540.59	A/B	-8.2
8404	[C@@H](C(C)(C)C)(C1=C(O)CC(CCC)(CCC)OC1=O)c1ccc(NS(=O)(=O)c2cc(C(F)(F)F)ccn2)ccc1	174484-35-6	2-Pyridinesulfonamide, N-[3-[1-(5,6-dihydro-4-hydroxy-2-oxo-6,6-dipropyl-2H-pyra	C28H35F3N2O5S	568.65	A/B	-8.2
8405	C(Cc1ccccc1)C1(CCc2ccccc2)CC(=C(=C([C@H](C(C)(C)C)c2cc(NS(=O)(=O)c3ccc(C#N)cn3)ccc2)C(=O)O1)O	174484-64-1	2-Pyridinesulfonamide, 5-amino-N-[3-[1-[5,6-dihydro-4-hydroxy-2-oxo-6,6-bis(2-ph	C37H41N3O5S	639.8	A/B	-8.2
8406	O=c1n([C@@H]2O[C@H](COP(=O)OC)[C@@H](O)C2)cc(C)c(=O)[nH]1	174746-72-6	Thymidine, 2,Ä≤,3,Ä≤-didehydro-3,Ä≤-deoxy-5,Ä≤-O-[(2R)-2-oxido-4H-1,3,2-benzodi	C17H17N2O7P	392.3	A/B	-8.2
8407	Nc1c2c(n(cn2)[C@@H]2O[C@H](COP(=O)(OP(=O)(OC[C@H]3O[C@H](CC3)n3c4c(nc3)c(=O)nc[nH]4)O)O)CC2)ncn1	175923-19-0	Adenosine 5,Ä≤-(trihydrogen diphosphate), 2,Ä≤,3,Ä≤-dideoxy-, P,Ä≤,Üí5,Ä≤-ester	C20H26N10O11P2		A/B	-8.2
8408	O=c1n([C@H]2O[C@@H](C(=O)O[C@H]3[C@H](C(C)C)CC[C@@H](C)C3)SC2)cc(F)c(NC(=O)c2ccccc2)n1	1773498-59-1	1,3-Oxathiolane-2-carboxylic acid, 5-[2-oxo-4-[(phenylmethyl)amino]-1(2H)-pyrimi	C25H33N3O4S	471.61	A/B	-8.2
8409	C=C1[C@@H](n2c3c(nc2)c(=O)nc(N)[nH]3)[C@H](F)[C@H](O)[C@H]1CO[P@@](=O)(Oc1ccccc1)N[C@@H](C(=O)OC(C)C	1781230-04-3	D-Alanine, N-[(R)-[[(1R,3R,4R,5R)-3-(2-amino-1,6-dihydro-6-oxo-9H-purin-9-yl)-4-	C24H30FN6O7P	564.5	A/B	-8.2
8410	C=C1[C@@H](n2c3c(nc2)c(=O)nc(N)[nH]3)[C@H](O)[C@H](O)[C@H]1CO[P@@](=S)(Oc1ccccc1)N[C@@H](C(=O)OC(C)C	1781230-09-8	D-Alanine, N-[(R)-[[(1R,2R,3S,4R)-4-(2-amino-1,6-dihydro-6-oxo-9H-purin-9-yl)-3-	C25H32FN6O7P	578.53	A/B	-8.2
8411	C(O[P@@](=O)(Oc1ccccc1)N[C@@H](C(=O)OC(C)C)C)[C@H]1C2([C@H]([C@](C)(F)[C@@H]1O)n1c3c(nc1)c(=O)nc(N)[	1781231-60-4	D-Alanine, N-[(R)-[[(4R,5R,6S,7R)-7-(2-amino-1,6-dihydro-6-oxo-9H-purin-9-yl)-5,	C26H35N6O8P	590.57	A/B	-8.2
8412	C(O[P@@](=S)(Oc1ccccc1)N[C@@H](C(=O)OC(C)C)C)[C@H]1C2([C@H]([C@H](O)[C@@H]1O)n1c3c(nc1)c(=O)nc(N)[nH	1781231-61-5	D-Alanine, N-[(R)-[[(4R,5R,6S,7R)-7-(2-amino-1,6-dihydro-6-oxo-9H-purin-9-yl)-6-	C26H34FN6O7P	592.56	A/B	-8.2
8413	C(O[P@@](=S)(Oc1ccccc1)N[C@@H](C(=O)OC(C)C)C)[C@H]1C2([C@H]([C@H](F)[C@@H]1O)n1c3c(nc1)c(=O)nc(N)[nH	1781232-57-2	D-Alanine, N-[(R)-[[(4R,5R,6R,7R)-7-(2-amino-1,6-dihydro-6-oxo-9H-purin-9-yl)-6-	C25H32FN6O6PS	594.6	A/B	-8.2
8414	C([C@H](OCP(=O)(OCOC(=O)OC(C)C)OCOC(=O)OC(C)C)C)n1c2c(nc1)c(N)ncn2.S(=O)(=O)(O)c1ccccc1	1781232-67-4	D-Alanine, N-[(R)-[[(4R,5R,6S,7R)-7-(2-amino-1,6-dihydro-6-oxo-9H-purin-9-yl)-6-	C26H34FN6O6PS	608.62	A/B	-8.2
8415	C=C1[C@@H](n2c3c(nc2)c(=O)nc(N)[nH]3)[C@H](O)[C@H](O)[C@]1(CO[P@](=O)(Oc1ccccc1)N[C@@H](C(=O)OC(C)C)	1782088-35-0	D-Alanine, N-[(S)-[[(1R,3R,4S,5R)-3-(2-amino-1,6-dihydro-6-oxo-9H-purin-9-yl)-4-	C24H30FN6O7P	564.5	A/B	-8.2
8416	C(O[P@](=O)(Oc1ccccc1)N[C@@H](C(=O)OC(C)C)C)[C@H]1C2([C@H]([C@](C)(F)[C@@H]1O)n1c3c(nc1)c(=O)nc(N)[n	1782089-96-6	D-Alanine, N-[(S)-[[(4R,5R,6S,7R)-7-(2-amino-1,6-dihydro-6-oxo-9H-purin-9-yl)-5,	C26H35N6O8P	590.57	A/B	-8.2
8417	C=C1[C@@H](n2c3c(nc2)c(=O)nc(N)[nH]3)[C@H](O)[C@H](O)[C@H]1COP(=O)(OP(=O)(OP(=O)(O)O)O)O	1782090-68-9	D-Alanine, N-[(S)-[[(4R,5R,6S,7R)-7-(2-amino-1,6-dihydro-6-oxo-9H-purin-9-yl)-6-	C26H34FN6O6PS	608.62	A/B	-8.2
8418	C(CN1CCC(CN2c3c4c(S2(=O)=O)cccc4ccc3)CC1)(N(CC)CC)C12CC3CC(C2)CC(C1)C3	178477-48-0	2H-Naphth[1,8-cd]isothiazole, 2-[[1-[2-(1-pyrrolidinyl)-2-tricyclo[3.3.1.13,7]de	C32H43N3O2S	533.77	A/B	-8.2
8419	C(CN1CCC(CN2c3c4c(S2(=O)=O)cccc4ccc3)CC1)(C12CC3CC(C2)CC(C1)C3)N1CCN(C)CC1	178478-39-2	2H-Naphth[1,8-cd]isothiazole, 2-[[1-[2-(4-morpholinyl)-2-tricyclo[3.3.1.13,7]dec	C32H43N3O3S	549.77	A/B	-8.2
8420	ClC1(Cl)[C@H]2[C@H](O[C@H]1n1c(=O)nc(N)cc1)CO[P@](=O)(OC(C)C)O2	178609-80-8	2,7-Naphthalenedisulfonic acid, 3-amino-6-[2-[4-[2-(2-naphthalenyl)diazenyl]-1-n	C30H21N5O6S2.2Li		A/B	-8.2
8421	S(=O)(=O)(O)c1c2c(c(N)c(N=Nc3ccc(N=Nc4c(N)c5c(c(S(=O)(=O)O)c4)cccc5)cc3)c1)cccc2.[NaH]	179472-46-9	1-Naphthalenesulfonic acid, 3,3,Ä≤-[9H-fluorene-2,7-diylbis(azo)]bis[4-amino-, d	C33H24N6O6S2.2Na		A/B	-8.2
8422	C(OC(C(O)([2H])[2H])([2H])[2H])n1c2c([nH]c(N)nc2=O)nc1	179472-47-0	1-Naphthalenesulfonic acid, 3,3,Ä≤-[1,4-phenylenebis(azo)]bis[4-amino-, disodium	C26H20N6O6S2.2Na		A/B	-8.2
8423	C[C@]1(F)[C@@H](O[C@H](CO[P@](=O)(Oc2ccc(SCCC)cc2)N[C@H](C(=O)OC(C)C)C)[C@H]1O)n1c(=O)[nH]c(=O)cc1	1800478-22-1	L-Alanine, N-[[P(S),2,Ä≤R]-P-[4-(2-cyclopropylethynyl)phenyl]-2,Ä≤-deoxy-2,Ä≤-f	C27H33FN3O9P	593.54	A/B	-8.2

8424	C(=O)(C)n1c(ncc1c1ccc(cc1)c1ccc(cc1)c1n(C(=O)C)c(nc1)[C@H]1N(C(=O)[C@@H](NC(=O)OC)C(C)C)CCC1)[C@H]1N	1800478-80-1	L-Alanine, N-[[P(S),2,Ä≤R]-2,Ä≤-deoxy-2,Ä≤-fluoro-P-[4-(3-fluoro-3-oxetanyl)phe	C25H32F2N3O10P	603.51	A/B	-8.2
8425	S(=O)(=O)(O)c1c2c(c(N)c(N=Nc3ccc(N(=Nc4cc(S(=O)(=O)O)c(C=Cc5c(S(=O)(=O)O)cccc5)cc4)=O)cc3)c1)cccc2	180074-89-9	1-Naphthalenesulfonic acid, 4-amino-3-[2-[4-[1-oxido-2-(4-sulfophenyl)diazenyl]p	C22H17N5O7S2	527.53	A/B	-8.2
8426	C(=O)([C@@H](NC(=O)OC)C(C)C)N1CC2(C[C@H]1c1[nH]c(cn1)c1cc3c(-c4c(C(F)(F)C3(F)F)cc(cc4)c3[nH]c(nc3)[C	1801161-71-6	Carbamic acid, N,N,Ä≤-[(9,9,10,10-tetrafluoro-9,10-dihydro-2,7-phenanthrenediyl)	C44H52F4N8O6	864.93	A/B	-8.2
8427	C(=O)([C@@H](NC(=O)OC([2H])([2H])[2H])C(C)C)N1[C@@H](CCC1)c1[nH]c(cn1)c1ccc(cc1)c1ccc(cc1)c1[nH]c(nc	1801686-51-0	Decanedioic acid, 1,10-bis[(5R,5aR,8aR,9R)-5,5a,6,8,8a,9-hexahydro-8-oxo-9-(3,4,	C54H58O18	995.03	A/B	-8.2
8428	C(NC(=O)c1oc(C)nn1)(C)(C)c1nc(C(=O)NCc2ccc(F)cc2)c(OCOC)c(=O)n1C	1801912-42-4	4-Pyrimidinecarboxamide, N-[(4-fluorophenyl)methyl]-1,6-dihydro-1-methyl-2-[1-me	C23H25FN6O5	484.48	A/B	-8.2
8429	O=c1n([C@H]2C[C@H](OC(=O)OCCSc3ccc(OCC(=O)Nc4cc5c(cc4)nc(C(=O)NCCN(CC)CC)cn5)cc3)[C@@H](CO)O2)cc(I)c	1802555-78-7	Not Yet Assigned	C35H40IN7O10S2	909.77	A/B	-8.2
8430	C([C@H](OC[P@@](=O)(Oc1cc2c(cc1)CCC2)N[C@H](C(=O)OC(C)C)C)n1c2c(nc1)c(N)ncn2	1802665-17-3	L-Alanine, N-[(S)-[[(1R)-2-(6-amino-9H-purin-9-yl)-1-methylethoxy]methyl][(2,3-d	C24H33N6O5P	516.53	A/B	-8.2
8431	C(OP(=O)(Oc1ccc(OC)cc1)Oc1ccc(OC)cc1)[C@@]1(C(F)F)O[C@H]([C@@H](O)[C@@H]1O)n1c2c(nc1)c(N)ncn2	1803134-28-2	9H-Purin-6-amine, 9-[4-C-(difluoromethyl)-5-O-(2-sulfido-4H-1,3,2-benzodioxaphos	C18H18F2N5O6PS	501.4	A/B	-8.2
8432	O=c1n(ccc(N)n1)[C@H]1O[C@@H](CO[P@@H](=O)Nc2nc(C)cs2)SC1	1803255-29-9	Phosphonamidic acid, N-(4-methyl-2-thiazolyl)-, [(1S,4R)-4-[2-amino-6-(cycloprop	C18H23N8O2PS	446.47	A/B	-8.2
8433	O=c1n([C@@H]2O[C@H](COC(=O)CCC(=O)OCc3c4c(OC(CCCCCCCC)OC4)c(C)nc3)[C@@H](N=[N+]=[N-])C2)cc(C)c(=O)[n	1805775-79-4	Thymidine, 3,Ä≤-azido-3,Ä≤-deoxy-, 5,Ä≤-[4-[[2-(1,1-dimethylethyl)-8-methyl-4H-	C27H34N6O9		A/B	-8.2
8434	C(=O)([C@@H](NC(=O)OC)[C@H](CC)C)N1[C@@H](CCC1)c1[nH]c(cn1)c1ccc(cc1)c1ccc(cc1)c1[nH]c(cc1)[C@H]1N(C	1807681-25-9	Carbamic acid, N-[(1S)-1-[[(2S)-2-[5-[4,Ä≤-[2-[(2S)-1-[(2S)-2-cyclohexyl-2-[(met	C44H55N7O6	777.95	A/B	-8.2
8435	C(=O)([C@@H](NC(=O)OC)C(C)C)N1[C@@H](CCC1)c1[nH]c(cn1)c1ccc(cc1)c1ccc(cc1)c1[nH]c(nc1)C1N(C(=O)[C@@H	1808295-97-7	Carbamic acid, N-[(1S)-1-[[(2S)-2-[5-[4,Ä≤-[2-[(S)-cyclopentyl[[(2S)-2-[(methoxy	C42H54N8O6	766.93	A/B	-8.2
8436	O=C1[C@@H]2[C@@H](c3c([C@H](OC(=O)C(Cc4ccccc4)N4C(=O)c5c(C4=O)cccc5)[C@H]2CO1)cc1c(c3)OCO1)c1cc(OC)c	1809249-37-3	L-Alanine, N-[(S)-hydroxyphenoxyphosphinyl]-, 2-ethylbutyl ester, 6-ester with 2	C27H35N6O8P	602.58	A/B	-8.2
8437	O=C1[C@@H]2[C@@H](c3c([C@H](OC(=O)[C@H](C)N4C(=O)C5C(C4=O)CCCC5)[C@H]2CO1)cc1c(c3)OCO1)c1cc(OC)c(OC)	1809321-46-7	2H-Isoindole-2-acetic acid, octahydro-Œ±-methyl-1,3-dioxo-, (5R,5aR,8aR,9R)-5,5a	C33H35NO11	621.63	A/B	-8.2
8438	O[C@H]1[C@H](n2c3c(nc2)c(=O)nc[nH]3)O[C@H](COP(=O)(OP(=O)(O[C@@H]2[C@@H](O)[C@H](O)[C@H](O)[C@H](C)O	181427-77-0	Xanthosine 5,Ä≤-(trihydrogen diphosphate), P,Ä≤-(6-deoxy-Œ≤-L-galactopyranosyl)	C16H24N4O16P2	590.33	A/B	-8.2
8439	O=c1n(ccc(NC(=O)OCC)n1)[C@@H]1O[C@H](CO)CC1	181428-04-6	Guanosine 5,Ä≤-(tetrahydrogen triphosphate), P,Ä≤,Ä≤-(6-deoxy-Œ≤-L-galactopyran	C16H26N5O18P3	669.32	A/B	-8.2
8440	C(=O)([C@@H](NC(=O)OC)C(C)C)N1[C@@H](C[C@H](S(=O)C)C1)c1[nH]c(c(Cl)n1)c1ccc(cc1)c1ccc(cc1)c1[nH]c(nc	1819985-26-6	Carbamic acid, N-[(1S)-1-[[(2S)-2-[5-chloro-4-[4,Ä≤-[5-chloro-2-[(2S,4S)-1-[(2S)	C41H50Cl2N8O6S	853.86	A/B	-8.2
8441	O=C1[C@@H]2[C@@H](c3c(CC2CO1)cc1c(c3)OCO1)c1cc(OC)c(O)c(OC)c1	1819985-27-7	Carbamic acid, N-[(1S)-1-[[(2S)-2-[5-chloro-4-[4,Ä≤-[5-chloro-2-[(2S,4S)-1-[(2S)	C41H50Cl2N8O7S	869.86	A/B	-8.2
8442	C(CC(=O)/C(=C(/CCc1cc(OC)c(O)cc1)\O)/N=Nc1ccc(C(=O)NCC(=O)NCCCCCCN)cc1)c1cc(OC)c(O)cc1	1820599-61-8	Benzamide, N-(6-aminohexyl)-4-[2-[(1E)-2-hydroxy-4-(4-hydroxy-3-methoxyphenyl)-1	C34H42N4O7	618.72	A/B	-8.2
8443	N(C(CCCNCC)CCCNCC)c1c2c(nccl)cc(Cl)cc2	1820599-62-9	Urea, N-(6-aminohexyl)-N,Ä≤-[2-[4-[2-[(1E)-2-hydroxy-4-(4-hydroxy-3-methoxypheny	C36H45N5O8	675.77	A/B	-8.2
8444	N(C[C@H]1COCC1)c1c2c(n(nn2)[C@@H]2C[C@H](COC(C(=O)OCC)P(=O)(O)O)[C@@H](O)[C@H]2O)nc(Cl)n1	1821044-49-8	Acetic acid, 2-[[(1R,2R,3S,4R)-4-[5-chloro-7-[[[(3S)-tetrahydro-3-furanyl]methyl	C19H28ClN6O9P	550.89	A/B	-8.2
8445	O(COC(=O)OC)c1c2n(N([C@H]3N(C2=O)CCOC3)[C@H]2c3c(CSc4c2cccc4)c(F)c(F)cc3)ccc1=O	182959-81-5	1,3,5-Naphthalenetrisulfonic acid, 7-[2-[4-[2-(4-amino-7-sulfo-1-naphthalenyl)di	C30H21N5O15S5	851.84	A/B	-8.2
8446	O=c1n([C@@H]2O[C@H](CO[P@@](=O)(Oc3ccc(CC)cc3)N[C@H](C(=O)OC)C)C=C2)cc(C)c(=O)[nH]1	1839983-41-3	1-Piperazinecarboxamide, N-[(1S)-4-hydroxy-1-[[[(1S,2E)-1-(2-phenylethyl)-3-(2-p	C27H37N5O5S	543.68	A/B	-8.2
8447	O=c1n([C@@H]2O[C@H](CO[P@@](=O)(N[C@H](C(=O)OC)CC)Oc3ccccc3)C=C2)cc(C)c(=O)[nH]1	184031-40-1	L-Alanine, N-(2,Ä≤,3,Ä≤-didehydro-3,Ä≤-deoxy-P-phenyl-5,Ä≤-thymidylyl)-, cycloh	C25H32N3O8P	533.51	A/B	-8.2
8448	N(CCN(C)C)c1c2c(n(C[C@H](OCP(=O)(O)O)C)cn2)ncn1	1843237-87-5	Not Yet Assigned	C15H18N4O7S	398.39	A/B	-8.2
8449	N(S(=O)(=O)c1ccoc1)(C[C@@H]([C@H](Cc1ccccc1)NC(=O)O[C@H]1CCOC1)O)CC(C)C	184490-07-1	Carbamic acid, [3-[[[3-(aminosulfonyl)phenyl]sulfonyl](2-methylpropyl)amino]-2-h	C25H35N3O8S2	569.69	A/B	-8.2
8450	O=c1n([C@@H]2O[C@H](COC(=O)C[C@]3(C)OC[C@@](C)(O)C3)C=C2)cc(C)c(=O)[nH]1	184769-10-6	L-threo-Hexonic acid, 3,6-anhydro-2,4-dideoxy-3,5-di-C-methyl-, 5,Ä≤-ester with	C18H24N2O7	380.39	A/B	-8.2
8451	[C@H](Cc1ccccc1)(C(=O)N[C@H](/C=C/S(=O)(=O)c1ccccc1)CCc1ccccc1)NC(=O)N1CCN(CC)CC1	1851276-99-7	1-Piperazinecarboxamide, 4-(cyclopropylmethyl)-N-[(1S)-2-oxo-2-[[(1S,2E)-1-(2-ph	C35H42N4O4S	614.8	A/B	-8.2
8452	[C@H](Cc1ccccc1)(C(=O)N[C@H](/C=C/S(=O)(=O)c1ccccc1)CCc1ccccc1)NC(=O)N1CCN(CCOC)CC1	1851279-71-4	1-Piperazinecarboxamide, 4-cyclopentyl-N-[(1S)-2-oxo-2-[[(1S,2E)-1-(2-phenylethy	C36H44N4O4S	628.82	A/B	-8.2
8453	N(=C(NC(=O)OC(C)(C)C)NC(=O)OC(C)(C)C)[C@@H]1[C@@H](NC(=O)C)[C@H]([C@@H]([C@@H](CO)O)O)OC(=C1)C(=O)OC	185378-11-4	5,Ä≤-Inosinic acid, 2-(2-phenylethyl)-	C18H21N4O8P	452.36	A/B	-8.2
8454	O=c1c2c(n(cn2)[C@@H]2C(=C)[C@H](CO)[C@@H](OC(=O)CCCC3CCCCC3)C2)[nH]c(N)n1	1860843-18-0	Cyclohexanecarboxylic acid, (1S,2R,4S)-4-(2-amino-1,6-dihydro-6-oxo-9H-purin-9-y	C19H25N5O4	387.43	A/B	-8.2
8455	O=c1c2c(n(cn2)[C@@H]2O[C@H](COP(=O)(O[C@@H]3[C@@H](CO)O[C@@H](NC=O)C3)O)[C@@H](O)C2)[nH]c(N)n1	187340-26-7	Adenosine, thymidylyl-(3,Ä≤,Ü5,Ä≤)-1,Ä≤-de(6-amino-9H-purin-9-yl)-2,Ä≤-deoxy-1	C16H24N3O11P	465.35	A/B	-8.2
8456	O=C1[C@@H]2[C@@H](c3c([C@H](OC(=O)CCCC[C@H]4SC(SCC4)c4c(C)cccc4)[C@H]2CO1)cc1c(c3)OCO1)c1cc(OC)c(OC)	1875123-82-2	1,3-Dithiane-4-pentanoic acid, 2-phenyl-, (5R,5aR,8aR,9R)-5,5a,6,8,8a,9-hexahydr	C37H40O9S2	692.84	A/B	-8.2
8457	O=C1[C@@H]2[C@@H](c3c([C@H](OC(=O)CCCC[C@H]4SC(SCC4)c4ccc(Cl)cc4)[C@H]2CO1)cc1c(c3)OCO1)c1cc(OC)c(OC	1875123-90-2	1,3-Dithiane-4-pentanoic acid, 2-(2-chlorophenyl)-, (5R,5aR,8aR,9R)-5,5a,6,8,8a,	C37H39ClO9S2	727.28	A/B	-8.2
8458	O=c1n([13C@@H]2O[13C@H]([13CH](O)[2H])[13C@@H](O)[13CH2]2)cc(C)c(=O)[nH]1	187533-43-3	5,Ä≤-Xanthylic acid, monoanhydride with imidodiphosphoric acid	C10H16N5O14P3	523.18	A/B	-8.2
8459	C(=O)(c1[nH]c2c(c1)cc(NS(=O)(=O)C)cc2)N1CCN(CC1)c1nc(OS(=O)(=O)O)ccc1N	188780-41-8	Methanesulfonamide, N-[2-[[4-(3-amino-1,6-dihydro-6-oxo-2-pyridinyl)-1-piperazir	C19H22N6O4S	430.48	A/B	-8.2
8460	N(C(C)C)c1c([nH]c(=O)cc1)N1CCN(C(=O)c2cc3c([nH]2)ccc(NS(=O)(=O)C)c3)CC1	188780-42-9	Methanesulfonamide, N-[2-[[4-[3-amino-6-(sulfooxy)-2-pyridinyl]-1-piperazinyl]ca	C19H22N6O7S2	510.54	A/B	-8.2
8461	Nc1c2c(n(cn2)[C@@H]2O[C@H](COP(=O)(O)S)[C@@H](O)[C@H]2O)nc(N)n1	1892543-48-4	Not Yet Assigned	C28H36F3N5O2T2		A/B	-8.2
8462	C(OC[C@@H]1O[C@@H](CS1)n1c(=O)nc(NC(=O)C)cc1)(c1ccc(OC)cc1)(c1ccc(OC)cc1)c1ccccc1	1899098-57-7	1H-1,2,4-Triazole-3-carboxamide, 1-[2,5-bis-O-(triphenylmethyl)-Œ≤-D-ribofuranos	C46H40N4O5	728.83	A/B	-8.2
8463	N(c1c(C)cc(C)cc1C)c1nc(NCc2ccccc2)ccn1	1899537-42-8	2,4-Pyrimidinediamine, N4-[(3-methylphenyl)methyl]-N2-(2,4,6-trimethylphenyl)-	C21H24N4	332.44	A/B	-8.2
8464	C(OC[C@@H]1[C@@H](OC(=O)c2ccccc2)[C@@H](OP(=O)(OC[C@@H]2[C@@H](OC(=O)c3ccccc3)[C@@H](OC(=O)c3ccccc3)	190141-36-7	Adenosine, N-benzoyl-3,Ä≤-O-benzoyl-P-[2-(4-nitrophenyl)ethyl]adenylyl-(2,Ä≤,Üi	C54H47N10O17P	1138.98	A/B	-8.2
8465	O=c1n(c2c(c(cc(n2)c2ccc(Cl)cc2)c2ccco2)c(=O)n1c1ccc(Cl)cc1)[C@H]1[C@H](OC(=O)c2ccccc2)[C@H](OC(=O)c2	190319-79-0	Pyrido[2,3-d]pyrimidine-2,4(1H,3H)-dione, 7-(4-chlorophenyl)-5-(2-furanyl)-3-phe	C49H34ClN3O10	860.26	A/B	-8.2
8466	O=c1n(c2c(c(cc(n2)c2ccccc2)c2ccco2)c(=O)n1c1ccc(Cl)cc1)[C@H]1[C@H](OC(=O)c2ccccc2)[C@H](OC(=O)c2cccc	190319-81-4	Pyrido[2,3-d]pyrimidine-2,4(1H,3H)-dione, 5-(2-furanyl)-3,7-diphenyl-1-(2,3,5-tr	C49H35N3O10	825.82	A/B	-8.2
8467	C(CCCCCCCCCCCCCC)C[C@@H](CCCO)C	190649-40-2	2,7,10,12-Tetraazatridecanoic acid, 13-[2-(1,2-dihydroxy-1-methylethyl)-4-thiazo	C37H48N6O7S2	752.94	A/B	-8.2
8468	C(#N)[C@]1(O[C@H](COP(=O)(Oc2ccccc2)NC(C(=O)OCC)(C)C)[C@@H](O)[C@H]1O)c1n2c(cc1)c(N)ncn2	1911578-92-1	Not Yet Assigned	C27H33N6O8P	600.56	A/B	-8.2
8469	O=C1[C@@H]2[C@H](c3c(C[C@H]2CO1)cc1c(c3)OCO1)c1c(OC)cccc1OC	1913279-95-4	Furo[3,Ä≤,4,Ä≤:6,7]naphtho[2,3-d]-1,3-dioxol-6(5aH)-one, 5-(2,3-dimethoxyphenyl	C21H20O6	368.38	A/B	-8.2
8470	C[C@]1(F)[C@@H](O[C@](COP(=O)(Oc2ccccc2)N[C@H](C(=O)OC(C)C)C)([C@]1(O)[2H])[2H])n1c(=O)[nH]c(=O)c(cl	1922915-55-6	L-Alanine, N-[(2,Ä≤R)-2,Ä≤-deoxy-2,Ä≤-fluoro-2,Ä≤-methyl-P-phenyl-5,Ä≤-uridylyl	C22H27D2FN3O9P		A/B	-8.2
8471	C[C@]1(F)[C@@H](O[C@](C(OP(=O)(Oc2ccccc2)N[C@H](C(=O)OC(C)C)C)[2H])([C@]1(O)[2H])[2H])n1c(=O)[nH]c(=	1922916-01-5	L-Alanine, N-[(2,Ä≤R)-2,Ä≤-deoxy-2,Ä≤-fluoro-2,Ä≤-methyl-P-phenyl-5,Ä≤-uridylyl	C22H24D5FN3O9P		A/B	-8.2
8472	C[C@]1(F)[C@@H](O[C@](C(OP(=O)(Oc2ccccc2)N[C@H](C(=O)OC(C)C)([2H])[2H])([C@]1(O)[2H])[2H])n1c(=O)[	1922916-29-7	L-Alanine, N-[(2,Ä≤R)-2,Ä≤-deoxy-2,Ä≤-fluoro-2,Ä≤-methyl-P-phenyl-5,Ä≤-uridylyl	C22H26D3FN3O9P		A/B	-8.2
8473	C[C@]1(F)[C@@H](O[C@](CO[P@](=O)(Oc2ccccc2)N[C@H](C(=O)OC(C)C)C)([C@H]1O)[2H])n1c(=O)[nH]c(=O)cc1	1922916-53-7	L-Alanine, N-[(2,Ä≤R)-2,Ä≤-deoxy-2,Ä≤-fluoro-2,Ä≤-methyl-P-phenyl-5,Ä≤-uridylyl	C22H25D4FN3O9P		A/B	-8.2
8474	[C@@H](C(=O)N[C@@H](Cc1ccccc1)C[C@@H]([C@H](Cc1ccccc1)NC(=O)COc1c(C)cc(O)cc1C)O)(C(C)C)N1C(=O)NCCC1	192725-26-1	1-Imidazolidineacetamide, N-[(1S,3S,4S)-3-hydroxy-5-phenyl-1-(phenylmethyl)-4-[[	C37H48N4O5	628.8	A/B	-8.2
8475	[C@@H](C(=O)N[C@H](C[C@@H]([C@H](Cc1ccccc1)NC(=O)COc1c(C)cccc1C)O)C(C)C)(C(C)C)N1C(=O)NCCC1	192725-40-9	1,2,4-Triazine-4(1H)-acetamide, N-[(1S,3S,4S)-4-[[2-(2,6-dimethylphenoxy)acetyl]	C36H47N5O5	629.79	A/B	-8.2
8476	C(=C\c1cc(F)ccc1)\c1n(nc(C(=O)N)n1)[C@@H]1O[C@H](CO)[C@@H](O)[C@H]1O	192725-42-1	1(2H)-Pyrimidineacetamide, N-[(1R,3S,4S)-4-[[2-(2,6-dimethylphenoxy)acetyl]aminc	C33H46N4O6	594.74	A/B	-8.2
8477	FC1(F)[C@@H](O[C@H](COC(=O)Cn2cnc(N)c2)[C@H]1O)n1c(=O)nc(NC(=O)OCCCC)c(I)c1	1932677-19-4	Cytidine, N-(butoxycarbonyl)-2,Ä≤-deoxy-2,Ä≤,2,Ä≤-difluoro-5-iodo-, 5,Ä≤-(4-nit	C19H21F2IN6O9	642.31	A/B	-8.2
8478	N(=Nc1ccc(cc1)c1ccc(N=Nc2c(N)c3c(c(S(=O)(=O)O)c2)cccc3)cc1)c1c2c(cc(S(=O)(=O)O)cc2O)ccc1N.[NaH]	193612-20-3	2,7-Naphthalenedisulfonic acid, 4,4,Ä≤-[(3,3,Ä≤-dimethyl[1,1,Ä≤-biphenyl]-4,4,Ä	C34H26N4O14S4	842.85	A/B	-8.2
8479	C(=O)([C@@H](NC(=O)OC([2H])([2H])[2H])C(C)C)N1[C@@H](CCC1)c1[nH]c(cn1)c1c(c(c(c(c1[2H])[2H])c1ccc(cc	1939211-99-0	Carbamic acid, N-[(1S)-1-[[(2S)-2-[5-[4,Ä≤-[2-[(2S)-1-[(2S)-2-[(methoxycarbonyl)	C40H43D7N8O6		A/B	-8.2
8480	C(=O)([C@@H](NC(=O)OC)C(C)C)N1[C@@H](CCC1)c1[nH]c(c(n1)[2H])c1c(c(c(c(c1[2H])[2H])c1c(c(c(c(c1[2H])[	1939212-08-4	Carbamic acid, N,N,Ä≤-[[1,1,Ä≤-biphenyl]-4,4,Ä≤-diyl-2,3,5,6-d4-bis[1H-imidazol	C40H38D12N8O6		A/B	-8.2
8481	C(OC(COC(=O)[C@H](C(C)C)N)CO)n1c2c(nc1)c(=O)nc(N)[nH]2.Cl	194154-40-0	L-Valine, N-[(phenylmethoxy)carbonyl]-, 2-[(2-amino-1,6-dihydro-6-oxo-9H-purin-9	C22H28N6O7	488.49	A/B	-8.2
8482	C(Nc1[nH]c2c(c(=O)n1)ncn2COC(COC(c1ccccc1)(c1ccccc1)c1ccccc1)COC(=O)[C@@H](NC(=O)OC(C)(C)C)C(C)C)(c1	194204-67-6	L-Valine, N-[(phenylmethoxy)carbonyl]-, 2-[[1,6-dihydro-6-oxo-2-[(triphenylmethy	C60H56N6O7	973.12	A/B	-8.2
8483	[Se]([C@H]1[C@H](O[C@H](CO)[C@H]1O)n1c(=O)[nH]c(=O)cc1)c1ccccc1	194672-70-3	Uridine, 2,Ä≤-Se-phenyl-2,Ä≤-seleno-	C15H16N2O5Se	383.26	A/B	-8.2
8484	O[C@@H]1[C@H](O[C@@H](COP(=O)(Oc2ccccc2)N[C@@H](C(=O)OC(C)C)C)C1)n1c(=O)nc(N)cc1	1946820-29-6	D-Alanine, N-(hydroxyphenoxyphosphinyl)-, 1-methylethyl ester, 5,Ä≤-ester with 2	C22H29N6O8P	536.47	A/B	-8.2
8485	O[C@@H]1[C@H](n2c3c(nc2)c(NCc2cc(O)c(OC)cc2)ncn3)O[C@H](CO)[C@H]1O	1947400-92-1	Phenol, 2-[[(9-Œ≤-D-arabinofuranosyl-9H-purin-6-yl)amino]methyl]-6-methoxy-	C18H21N5O6	403.39	A/B	-8.2
8486	O[C@@H]1[C@H](n2c3c(nc2)c(NCc2c(F)c(Cl)ccc2F)ncn3)O[C@H](CO)[C@H]1O	1947401-63-9	9H-Purin-6-amine, 9-Œ≤-D-arabinofuranosyl-N-[[2-fluoro-6-(trifluoromethyl)phenyl	C18H17F4N5O4	443.35	A/B	-8.2
8487	C(=O)([C@@H](NC(=O)OC)C(C)C)N1[C@H](c2[nH]c3c4c(cc(cc4)c4ccc(cc4)c4[nH]c(nc4)[C@H]4N(C(=O)[C@@H](NC(	1949770-75-5	Carbamic acid, N-[(1S)-1-[[(2S)-2-[5-chloro-7-[4-[2-[(2S)-1-[(2S)-3-methyl-2-[[	C48H57ClN8O6	877.47	A/B	-8.2
8488	C(=O)([C@@H](NC(=O)OC)C(C)C)N1[C@@H]([C@@H]2C[C@H]1CC2)c1[nH]c2c(n1)ccc1c2ccc(c1)c1ccc(cc1)c1[nH]c(n	1949770-80-2	Carbamic acid, N-[(1S)-1-[[(2S,4R)-4-methoxy-2-[5-[4-[2-[(1R,3S,4S)-2-[(2S)-2-[(	C45H54N8O7	818.96	A/B	-8.2
8489	O=c1n([C@@H]2O[C@H](COP3(=O)Oc4c(CO3)cccc4)[C@@H](N=[N+]=[N-])C2)cc(C)c(=O)[nH]1	195885-79-1	Thymidine, 3,Ä≤-azido-5,Ä≤-O-(6-chloro-2-oxido-4H-1,3,2-benzodioxaphosphorin-2-	C17H17ClN5O7P		A/B	-8.2
8490	O=c1n([C@@H]2O[C@H](COP3(=O)Oc4c(CO3)cc(C)cc4)[C@@H](N=[N+]=[N-])C2)cc(C)c(=O)[nH]1	195885-82-6	Thymidine, 3,Ä≤-azido-3,Ä≤-deoxy-5,Ä≤-O-(8-methoxy-2-oxido-4H-1,3,2-benzodioxap	C18H20N5O8P		A/B	-8.2
8491	S(=O)(=O)(O)c1c2c(c(N)c(N=Nc3ccc(C(C(Cl)(Cl)Cl)c4ccc(N=Nc5c(N)c6c(c(S(=O)(=O)O)c5)cccc6)cc4)cc3)c1)c	195885-85-9	Thymidine, 3,Ä≤-azido-3,Ä≤-deoxy-5,Ä≤-O-(6,8-dimethyl-2-oxido-4H-1,3,2-benzodio	C19H22N5O7P		A/B	-8.2
8492	C(C(=O)O)([C@@]1(N(C(=Nc2c1c(c(c(c2F)[2H])[2H])[2H])N1C(C(N(C(C1([2H])[2H])([2H])[2H])c1c(c(OC([2H])	1961283-76-0	4-Quinazoline-5,6,7-d3-acetic-Œ±,Œ±-d2 acid-d, 8-fluoro-3,4-dihydro-4-d-2-[4-[5-	C29D28F4N4O4		A/B	-8.2
8493	C(C(=O)O)([C@@]1(N(C(=Nc2c1c(c(c(c2F)[2H])[2H])[2H])N1C(C(N(C(C1([2H])[2H])([2H])[2H])c1c(c(OC([2H])	1961283-77-1	4-Quinazoline-5,6,7-d3-acetic-Œ±,Œ±-d2 acid, 8-fluoro-3,4-dihydro-4-d-2-[4-[5-(m	C29HD27F4N4O4		A/B	-8.2
8494	C(C(=O)O)([C@@]1(N(C(=Nc2c1c(c(c(c2F)[2H])[2H])[2H])N1C(C(N(C(C1([2H])[2H])([2H])[2H])c1c(c(OC)c(c(c	1961283-78-2	4-Quinazoline-5,6,7-d3-acetic-Œ±,Œ±-d2 acid, 8-fluoro-3,4-dihydro-4-d-2-[4-[5-(m	C29H4D24F4N4O4		A/B	-8.2
8495	C(C(=O)O)([C@@]1(N(C(=Nc2c1c(c(c(c2F)[2H])[2H])[2H])N1C(C(N(C(C1([2H])[2H])([2H])[2H])c1c(c(OC)c(c(c	1961283-79-3	4-Quinazoline-5,6,7-d3-acetic-Œ±,Œ±-d2 acid, 8-fluoro-3,4-dihydro-4-d-2-[4-(5-me	C29H4D24F4N4O4		A/B	-8.2
8496	C(C(=O)O)([C@@]1(N(C(=Nc2c1c(c(c(c2F)[2H])[2H])[2H])N1CCN(CC1)c1c(c(OC([2H])([2H])[2H])c(c(c1[2H])[2	1961283-80-6	4-Quinazoline-5,6,7-d3-acetic-Œ±,Œ±-d2 acid, 8-fluoro-3,4-dihydro-4-d-2-[4-(5-me	C29H7D21F4N4O4		A/B	-8.2
8497	C(C(=O)O)([C@@]1(N(C(=Nc2c1c(c(c(c2F)[2H])[2H])[2H])N1CCN(CC1)c1c(c(OC)c(c(c1[2H])[2H])[2H])[2H])c1c	1961283-83-9	4-Quinazoline-5,6,7-d3-acetic-Œ±,Œ±-d2 acid, 8-fluoro-3,4-dihydro-4-d-2-[4-(5-me	C29H12D16F4N4O4		A/B	-8.2
8498	C(C(=O)O)([C@@]1(N(C(=Nc2c1cccc2F)N1C(C(N(C(C1([2H])[2H])([2H])[2H])c1c(c(OC)c(c(c1[2H])[2H])[2H])[2	1961283-86-2	4-Quinazolineacetic-Œ±,Œ±-d2 acid, 8-fluoro-3,4-dihydro-4-d-2-[4-[5-(methoxy-d3)	C29H7D21F4N4O4		A/B	-8.2
8499	C(C(=O)O)([C@@]1(N(C(=Nc2c1cccc2F)N1CCN(CC1)c1c(c(OC)c(c(c1[2H])[2H])[2H])[2H])c1c(OC)c(c(c(C(F)(F)F	1961283-91-9	4-Quinazolineacetic-Œ±,Œ±-d2 acid, 8-fluoro-3,4-dihydro-4-d-2-[4-(5-methoxypheny	C29H15D13F4N4O4		A/B	-8.2
8500	C(C(=O)O)([C@@H]1N(C(=Nc2c1c(c(c(c2F)[2H])[2H])[2H])N1C(C(N(C(C1([2H])[2H])([2H])[2H])c1c(c(OC([2H])	1961283-92-0	4-Quinazolineacetic-Œ±,Œ±-d2 acid, 8-fluoro-3,4-dihydro-4-d-2-[4-(5-methoxypheny	C29H18D10F4N4O4		A/B	-8.2
8501	C(C(=O)O)([C@@H]1N(C(=Nc2c1c(c(c(c2F)[2H])[2H])[2H])N1C(C(N(C(C1([2H])[2H])([2H])[2H])c1c(c(OC)c(c(c	1961283-94-2	4-Quinazoline-5,6,7-d3-acetic-Œ±,Œ±-d2 acid, 8-fluoro-3,4-dihydro-2-[4-[5-(metho	C29H5D23F4N4O4		A/B	-8.2
8502	C(C(=O)O)([C@@H]1N(C(=Nc2c1c(c(c(c2F)[2H])[2H])[2H])N1CCN(CC1)c1c(c(OC([2H])([2H])[2H])c(c(c1[2H])[2	1961283-96-4	4-Quinazoline-5,6,7-d3-acetic-Œ±,Œ±-d2 acid, 8-fluoro-3,4-dihydro-2-[4-(5-methox	C29H8D20F4N4O4		A/B	-8.2
8503	C(C(=O)O)([C@@H]1N(C(=Nc2c1c(c(c(c2F)[2H])[2H])[2H])N1CCN(CC1)c1c(c(OC)c(c(c1[2H])[2H])[2H])[2H])c1c	1961283-98-6	4-Quinazoline-5,6,7-d3-acetic-Œ±,Œ±-d2 acid, 8-fluoro-3,4-dihydro-2-[4-[5-(metho	C29H13D15F4N4O4		A/B	-8.2
8504	C(C(=O)O)([C@@H]1N(C(=Nc2c1cccc2F)N1C(C(N(C(C1([2H])[2H])([2H])[2H])c1c(c(OC)c(c(c1[2H])[2H])[2H])[2	1961284-08-1	4-Quinazolineacetic-Œ±,Œ±-d2 acid, 8-fluoro-3,4-dihydro-2-[4-(5-methoxyphenyl-2,	C29H8D20F4N4O4		A/B	-8.2
8505	C(C(=O)O)([C@@H]1N(C(=Nc2c1cccc2F)N1CCN(CC1)c1c(c(OC([2H])([2H])[2H])c(c(c1[2H])[2H])[2H])[2H])c1c(O	1961284-09-2	4-Quinazolineacetic-Œ±,Œ±-d2 acid, 8-fluoro-3,4-dihydro-2-[4-(5-methoxyphenyl-2,	C29H11D17F4N4O4		A/B	-8.2
8506	C(C(=O)O)([C@@]1(N(C(=Nc2c1c(c(c(c2F)[2H])[2H])[2H])N1CCN(CC1)c1cc(OC([2H])([2H])[2H])ccc1)c1c(OC([2	1961284-17-2	4-Quinazoline-5,6,7-d3-acetic-Œ±,Œ±-d2 acid, 8-fluoro-3,4-dihydro-4-d-2-[4-(3-me	C29H11D17F4N4O4		A/B	-8.2
8507	C(C(=O)O)([C@@]1(N(C(=Nc2c1cccc2F)N1C(C(N(C(C1([2H])[2H])([2H])[2H])c1cc(OC([2H])([2H])[2H])ccc1)([2	1961284-21-8	4-Quinazoline-5,6,7-d3-acetic-Œ±,Œ±-d2 acid, 8-fluoro-3,4-dihydro-4-d-2-[4-(3-me	C29H19D9F4N4O4		A/B	-8.2
8508	C(C(=O)O)([C@@]1(N(C(=Nc2c1cccc2F)N1C(C(N(C(C1([2H])[2H])([2H])[2H])c1cc(OC)ccc1)([2H])[2H])([2H])[2	1961284-23-0	4-Quinazolineacetic-Œ±,Œ±-d2 acid, 8-fluoro-3,4-dihydro-4-d-2-[4-[3-(methoxy-d3)	C29H11D17F4N4O4		A/B	-8.2
8509	C(C(=O)O)([C@@]1(N(C(=Nc2c1cccc2F)N1C(C(N(C(C1([2H])[2H])([2H])[2H])c1cc(OC)ccc1)([2H])[2H])([2H])[2	1961284-24-1	4-Quinazolineacetic-Œ±,Œ±-d2 acid, 8-fluoro-3,4-dihydro-4-d-2-[4-(3-methoxypheny	C29H11D17F4N4O4		A/B	-8.2
8510	C(C(=O)O)([C@@]1(N(C(=Nc2c1cccc2F)N1CCN(CC1)c1cc(OC([2H])([2H])[2H])ccc1)c1c(OC([2H])([2H])[2H])c(c(	1961284-26-3	4-Quinazolineacetic-Œ±,Œ±-d2 acid, 8-fluoro-3,4-dihydro-4-d-2-[4-(3-methoxypheny	C29H14D14F4N4O4		A/B	-8.2
8511	C(C(=O)O)([C@@]1(N(C(=Nc2c1cccc2F)N1CCN(CC1)c1cc(OC)ccc1)c1c(OC([2H])([2H])[2H])c(c(c(C(F)(F)F)c1[2H	1961284-28-5	4-Quinazolineacetic-Œ±,Œ±-d2 acid, 8-fluoro-3,4-dihydro-4-d-2-[4-[3-(methoxy-d3)	C29H19D9F4N4O4		A/B	-8.2
8512	C(C(=O)O)([C@@H]1N(C(=Nc2c1c(c(c(c2F)[2H])[2H])[2H])N1C(C(N(C(C1([2H])[2H])([2H])[2H])c1cc(OC([2H])(	1961284-31-0	4-Quinazolineacetic-Œ±,Œ±-d2 acid, 8-fluoro-3,4-dihydro-4-d-2-[4-(3-methoxypheny	C29H22D6F4N4O4		A/B	-8.2
8513	C(C(=O)O)([C@@H]1N(C(=Nc2c1c(c(c(c2F)[2H])[2H])[2H])N1CCN(CC1)c1cc(OC([2H])([2H])[2H])ccc1)c1c(OC([2	1961284-35-4	4-Quinazoline-5,6,7-d3-acetic-Œ±,Œ±-d2 acid, 8-fluoro-3,4-dihydro-2-[4-(3-methox	C29H12D16F4N4O4		A/B	-8.2
8514	C(C(=O)O)([C@@H]1N(C(=Nc2c1c(c(c(c2F)[2H])[2H])[2H])N1CCN(CC1)c1cc(OC([2H])([2H])[2H])ccc1)c1c(OC)c(	1961284-36-5	4-Quinazoline-5,6,7-d3-acetic-Œ±,Œ±-d2 acid, 8-fluoro-3,4-dihydro-2-[4-[3-(metho	C29H14D14F4N4O4		A/B	-8.2
8515	C(C(=O)O)([C@@H]1N(C(=Nc2c1cccc2F)N1C(C(N(C(C1([2H])[2H])([2H])[2H])c1cc(OC)ccc1)([2H])[2H])([2H])[2	1961284-41-2	4-Quinazolineacetic-Œ±,Œ±-d2 acid, 8-fluoro-3,4-dihydro-2-[4-[3-(methoxy-d3)pher	C29H12D16F4N4O4		A/B	-8.2
8516	C(C(=O)O)([C@@H]1N(C(=Nc2c1cccc2F)N1CCN(CC1)c1cc(OC([2H])([2H])[2H])ccc1)c1c(OC([2H])([2H])[2H])c(c(	1961284-43-4	4-Quinazolineacetic-Œ±,Œ±-d2 acid, 8-fluoro-3,4-dihydro-2-[4-(3-methoxyphenyl)-1	C29H15D13F4N4O4		A/B	-8.2
8517	C(C(=O)O)([C@@]1(N(C(=Nc2c1c(c(c(c2F)[2H])[2H])[2H])N1C(C(N(C(C1([2H])[2H])([2H])[2H])c1c(c(OC([2H])	1961284-47-8	4-Quinazolineacetic-Œ±,Œ±-d2 acid, 8-fluoro-3,4-dihydro-2-[4-(3-methoxyphenyl)-1	C29H23D5F4N4O4		A/B	-8.2
8518	C(C(=O)O)([C@@]1(N(C(=Nc2c1c(c(c(c2F)[2H])[2H])[2H])N1C(C(N(C(C1([2H])[2H])([2H])[2H])c1c(c(OC)c(c(c	1961284-49-0	4-Quinazoline-5,6,7-d3-acetic-Œ±,Œ±-d2 acid, 8-fluoro-3,4-dihydro-4-d-2-[4-[5-(m	C29H7D21F4N4O4		A/B	-8.2
8519	C(C(=O)O)([C@@]1(N(C(=Nc2c1c(c(c(c2F)[2H])[2H])[2H])N1C(C(N(C(C1([2H])[2H])([2H])[2H])c1c(c(OC)c(c(c	1961284-50-3	4-Quinazoline-5,6,7-d3-acetic-Œ±,Œ±-d2 acid, 8-fluoro-3,4-dihydro-4-d-2-[4-(5-me	C29H7D21F4N4O4		A/B	-8.2
8520	C(C(=O)O)([C@@]1(N(C(=Nc2c1cccc2F)N1CCN(CC1)c1c(c(OC([2H])([2H])[2H])c(c(c1[2H])[2H])[2H])[2H])c1c(O	1961284-60-5	4-Quinazolineacetic-Œ±,Œ±-d2 acid, 8-fluoro-3,4-dihydro-4-d-2-[4-(5-methoxypheny	C29H13D15F4N4O4		A/B	-8.2
8521	C(C(=O)O)([C@@]1(N(C(=Nc2c1cccc2F)N1CCN(CC1)c1c(c(OC)c(c(c1[2H])[2H])[2H])[2H])c1c(OC([2H])([2H])[2H	1961284-62-7	4-Quinazolineacetic-Œ±,Œ±-d2 acid, 8-fluoro-3,4-dihydro-4-d-2-[4-[5-(methoxy-d3)	C29H18D10F4N4O4		A/B	-8.2
8522	C(C(=O)O)([C@@]1(N(C(=Nc2c1cccc2F)N1CCN(CC1)c1c(c(OC)c(c(c1[2H])[2H])[2H])[2H])c1c(OC)ccc(C(F)(F)F)c	1961284-63-8	4-Quinazolineacetic-Œ±,Œ±-d2 acid, 8-fluoro-3,4-dihydro-4-d-2-[4-(5-methoxypheny	C29H18D10F4N4O4		A/B	-8.2
8523	C(C(=O)O)([C@@H]1N(C(=Nc2c1c(c(c(c2F)[2H])[2H])[2H])N1C(C(N(C(C1([2H])[2H])([2H])[2H])c1c(c(OC)c(c(c	1961284-66-1	4-Quinazoline-5,6,7-d3-acetic-Œ±,Œ±-d2 acid, 8-fluoro-3,4-dihydro-2-[4-[5-(metho	C29H8D20F4N4O4		A/B	-8.2
8524	C(C(=O)O)([C@@H]1N(C(=Nc2c1c(c(c(c2F)[2H])[2H])[2H])N1C(C(N(C(C1([2H])[2H])([2H])[2H])c1c(c(OC)c(c(c	1961284-67-2	4-Quinazoline-5,6,7-d3-acetic-Œ±,Œ±-d2 acid, 8-fluoro-3,4-dihydro-2-[4-(5-methox	C29H8D20F4N4O4		A/B	-8.2
8525	C(C(=O)O)([C@@H]1N(C(=Nc2c1c(c(c(c2F)[2H])[2H])[2H])N1CCN(CC1)c1c(c(OC([2H])([2H])[2H])c(c(c1[2H])[2	1961284-72-9	4-Quinazoline-5,6,7-d3-acetic-Œ±,Œ±-d2 acid, 8-fluoro-3,4-dihydro-2-[4-[5-(metho	C29H13D15F4N4O4		A/B	-8.2
8526	C(C(=O)O)([C@@H]1N(C(=Nc2c1c(c(c(c2F)[2H])[2H])[2H])N1CCN(CC1)c1c(c(OC)c(c(c1[2H])[2H])[2H])[2H])c1c	1961284-73-0	4-Quinazoline-5,6,7-d3-acetic-Œ±,Œ±-d2 acid, 8-fluoro-3,4-dihydro-2-[4-[5-(metho	C29H16D12F4N4O4		A/B	-8.2
8527	C(C(=O)O)([C@@H]1N(C(=Nc2c1cccc2F)N1C(C(N(C(C1([2H])[2H])([2H])[2H])c1c(c(OC)c(c(c1[2H])[2H])[2H])[2	1961284-78-5	4-Quinazolineacetic-Œ±,Œ±-d2 acid, 8-fluoro-3,4-dihydro-2-[4-[5-(methoxy-d3)pher	C29H11D17F4N4O4		A/B	-8.2
8528	C(C(=O)O)([C@@H]1N(C(=Nc2c1cccc2F)N1CCN(CC1)c1c(c(OC)c(c(c1[2H])[2H])[2H])[2H])c1c(OC)ccc(C(F)(F)F)c	1961284-84-3	4-Quinazolineacetic-Œ±,Œ±-d2 acid, 8-fluoro-3,4-dihydro-2-[4-(5-methoxyphenyl-2,	C29H19D9F4N4O4		A/B	-8.2
8529	C(C(=O)O)([C@@]1(N(C(=Nc2c1c(c(c(c2F)[2H])[2H])[2H])N1C(C(N(C(C1([2H])[2H])([2H])[2H])c1cc(OC([2H])(	1961284-85-4	4-Quinazolineacetic-Œ±,Œ±-d2 acid, 8-fluoro-3,4-dihydro-2-[4-(5-methoxyphenyl-2,	C29H22D6F4N4O4		A/B	-8.2
8530	C(C(=O)O)([C@@]1(N(C(=Nc2c1c(c(c(c2F)[2H])[2H])[2H])N1C(C(N(C(C1([2H])[2H])([2H])[2H])c1cc(OC)ccc1)(	1961284-87-6	4-Quinazoline-5,6,7-d3-acetic-Œ±,Œ±-d2 acid, 8-fluoro-3,4-dihydro-4-d-2-[4-[3-(m	C29H11D17F4N4O4		A/B	-8.2
8531	C(C(=O)O)([C@@]1(N(C(=Nc2c1cccc2F)N1C(C(N(C(C1([2H])[2H])([2H])[2H])c1cc(OC([2H])([2H])[2H])ccc1)([2	1961284-94-5	4-Quinazoline-5,6,7-d3-acetic-Œ±,Œ±-d2 acid, 8-fluoro-3,4-dihydro-4-d-2-[4-(3-me	C29H22D6F4N4O4		A/B	-8.2
8532	C(C(=O)O)([C@@]1(N(C(=Nc2c1cccc2F)N1C(C(N(C(C1([2H])[2H])([2H])[2H])c1cc(OC)ccc1)([2H])[2H])([2H])[2	1961284-98-9	4-Quinazolineacetic-Œ±,Œ±-d2 acid, 8-fluoro-3,4-dihydro-4-d-2-[4-(3-methoxypheny	C29H14D14F4N4O4		A/B	-8.2
8533	C(C(=O)O)([C@@]1(N(C(=Nc2c1cccc2F)N1CCN(CC1)c1cc(OC)ccc1)c1c(OC([2H])([2H])[2H])ccc(C(F)(F)F)c1)[2H]	1961285-01-7	4-Quinazolineacetic-Œ±,Œ±-d2 acid, 8-fluoro-3,4-dihydro-4-d-2-[4-[3-(methoxy-d3)	C29H22D6F4N4O4		A/B	-8.2
8534	C(C(=O)O)([C@@H]1N(C(=Nc2c1c(c(c(c2F)[2H])[2H])[2H])N1C(C(N(C(C1([2H])[2H])([2H])[2H])c1cc(OC([2H])(	1961285-03-9	4-Quinazolineacetic-Œ±,Œ±-d2 acid, 8-fluoro-3,4-dihydro-4-d-2-[4-(3-methoxypheny	C29H25D3F4N4O4		A/B	-8.2
8535	C(C(=O)O)([C@@H]1N(C(=Nc2c1c(c(c(c2F)[2H])[2H])[2H])N1CCN(CC1)c1cc(OC)ccc1)c1c(OC([2H])([2H])[2H])cc	1961285-13-1	4-Quinazoline-5,6,7-d3-acetic-Œ±,Œ±-d2 acid, 8-fluoro-3,4-dihydro-2-[4-[3-(metho	C29H20D8F4N4O4		A/B	-8.2
8536	C(C(=O)O)([C@@H]1N(C(=Nc2c1cccc2F)N1CCN(CC1)c1cc(OC([2H])([2H])[2H])ccc1)c1c(OC)ccc(C(F)(F)F)c1)([2H	1961285-20-0	4-Quinazolineacetic-Œ±,Œ±-d2 acid, 8-fluoro-3,4-dihydro-2-[4-[3-(methoxy-d3)pher	C29H20D8F4N4O4		A/B	-8.2
8537	C(C(=O)O)[C@@]1(N(C(=Nc2c1c(c(c(c2F)[2H])[2H])[2H])N1C(C(N(C(C1([2H])[2H])([2H])[2H])c1c(c(OC([2H])(	1961285-25-5	4-Quinazoline-5,6,7-d3-acetic acid, 8-fluoro-3,4-dihydro-4-d-2-[4-[5-(methoxy-d3	C29H3D25F4N4O4		A/B	-8.2
8538	C(C(=O)O)[C@@]1(N(C(=Nc2c1c(c(c(c2F)[2H])[2H])[2H])N1C(C(N(C(C1([2H])[2H])([2H])[2H])c1c(c(OC)c(c(c1	1961285-27-7	4-Quinazoline-5,6,7-d3-acetic acid, 8-fluoro-3,4-dihydro-4-d-2-[4-[5-(methoxy-d3	C29H6D22F4N4O4		A/B	-8.2
8539	C(C(=O)O)[C@@]1(N(C(=Nc2c1c(c(c(c2F)[2H])[2H])[2H])N1C(C(N(C(C1([2H])[2H])([2H])[2H])c1c(c(OC)c(c(c1	1961285-28-8	4-Quinazoline-5,6,7-d3-acetic acid, 8-fluoro-3,4-dihydro-4-d-2-[4-(5-methoxyphen	C29H6D22F4N4O4		A/B	-8.2
8540	C(C(=O)O)[C@@]1(N(C(=Nc2c1cccc2F)N1C(C(N(C(C1([2H])[2H])([2H])[2H])c1c(c(OC([2H])([2H])[2H])c(c(c1[2	1961285-35-7	4-Quinazolineacetic acid, 8-fluoro-3,4-dihydro-4-d-2-[4-[5-(methoxy-d3)phenyl-2,	C29H6D22F4N4O4		A/B	-8.2

8541	C(C(=O)O)[C@@]1(N(C(=Nc2c1cccc2F)N1C(C(N(C(C1([2H])[2H])([2H])[2H])c1c(c(OC)c(c(c1[2H])[2H])[2H])[2H]	1961285-36-8	4-Quinazolineacetic acid, 8-fluoro-3,4-dihydro-4-d-2-[4-[5-(methoxy-d3)phenyl-2,	C29H9D19F4N4O4		A/B	-8.2
8542	C(C(=O)O)[C@@]1(N(C(=Nc2c1cccc2F)N1C(C(N(C(C1([2H])[2H])([2H])[2H])c1c(c(OC)c(c(c1[2H])[2H])[2H])[2H]	1961285-37-9	4-Quinazolineacetic acid, 8-fluoro-3,4-dihydro-4-d-2-[4-(5-methoxyphenyl-2,3,4,6	C29H9D19F4N4O4		A/B	-8.2
8543	C(C(=O)O)[C@@H]1N(C(=Nc2c1c(c(c(c2F)[2H])[2H])[2H])N1C(C(N(C(C1([2H])[2H])([2H])[2H])c1c(c(OC([2H])(	1961285-43-7	4-Quinazoline-5,6,7-d3-acetic acid, 8-fluoro-3,4-dihydro-2-[4-[5-(methoxy-d3)phe	C29H4D24F4N4O4		A/B	-8.2
8544	C(C(=O)O)[C@@H]1N(C(=Nc2c1c(c(c(c2F)[2H])[2H])[2H])N1C(C(N(C(C1([2H])[2H])([2H])[2H])c1c(c(OC)c(c(c1	1961285-44-8	4-Quinazoline-5,6,7-d3-acetic acid, 8-fluoro-3,4-dihydro-2-[4-[5-(methoxy-d3)phe	C29H7D21F4N4O4		A/B	-8.2
8545	C(C(=O)O)[C@@H]1N(C(=Nc2c1c(c(c(c2F)[2H])[2H])[2H])N1C(C(N(C(C1([2H])[2H])([2H])[2H])c1c(c(OC)c(c(c1	1961285-45-9	4-Quinazoline-5,6,7-d3-acetic acid, 8-fluoro-3,4-dihydro-2-[4-(5-methoxyphenyl-2	C29H7D21F4N4O4		A/B	-8.2
8546	C(C(=O)O)[C@@H]1N(C(=Nc2c1c(c(c(c2F)[2H])[2H])[2H])N1CCN(CC1)c1c(c(OC)c(c(c1[2H])[2H])[2H])[2H])c1c(	1961285-49-3	4-Quinazoline-5,6,7-d3-acetic acid, 8-fluoro-3,4-dihydro-2-[4-(5-methoxyphenyl-2	C29H15D13F4N4O4		A/B	-8.2
8547	C(C(=O)O)[C@@H]1N(C(=Nc2c1cccc2F)N1C(C(N(C(C1([2H])[2H])([2H])[2H])c1c(c(OC([2H])([2H])[2H])c(c(c1[2	1961285-51-7	4-Quinazolineacetic acid, 8-fluoro-3,4-dihydro-2-[4-[5-(methoxy-d3)phenyl-2,3,4,	C29H7D21F4N4O4		A/B	-8.2
8548	C(C(=O)O)[C@@H]1N(C(=Nc2c1cccc2F)N1C(C(N(C(C1([2H])[2H])([2H])[2H])c1c(c(OC)c(c(c1[2H])[2H])[2H])[2H]	1961285-52-8	4-Quinazolineacetic acid, 8-fluoro-3,4-dihydro-2-[4-[5-(methoxy-d3)phenyl-2,3,4,	C29H10D18F4N4O4		A/B	-8.2
8549	C(C(=O)O)[C@@]1(N(C(=Nc2c1c(c(c(c2F)[2H])[2H])[2H])N1C(C(N(C(C1([2H])[2H])([2H])[2H])c1cc(OC([2H])([	1961285-60-8	4-Quinazoline-5,6,7-d3-acetic acid, 8-fluoro-3,4-dihydro-4-d-2-[4-[3-(methoxy-d3	C29H7D21F4N4O4		A/B	-8.2
8550	C(C(=O)O)[C@@]1(N(C(=Nc2c1c(c(c(c2F)[2H])[2H])[2H])N1CCN(CC1)c1cc(OC([2H])([2H])[2H])ccc1)c1c(OC([2H]	1961285-63-1	4-Quinazoline-5,6,7-d3-acetic acid, 8-fluoro-3,4-dihydro-4-d-2-[4-(3-methoxypher	C29H13D15F4N4O4		A/B	-8.2
8551	C(C(=O)O)[C@@]1(N(C(=Nc2c1cccc2F)N1C(C(N(C(C1([2H])[2H])([2H])[2H])c1cc(OC)ccc1)([2H])[2H])([2H])[2H]	1961285-71-1	4-Quinazolineacetic acid, 8-fluoro-3,4-dihydro-4-d-2-[4-[3-(methoxy-d3)phenyl]-1	C29H13D15F4N4O4		A/B	-8.2
8552	C(C(=O)O)[C@@H]1N(C(=Nc2c1c(c(c(c2F)[2H])[2H])[2H])N1C(C(N(C(C1([2H])[2H])([2H])[2H])c1cc(OC([2H])([	1961285-80-2	4-Quinazoline-5,6,7-d3-acetic acid, 8-fluoro-3,4-dihydro-2-[4-[3-(methoxy-d3)phe	C29H8D20F4N4O4		A/B	-8.2
8553	C(C(=O)O)[C@@H]1N(C(=Nc2c1c(c(c(c2F)[2H])[2H])[2H])N1C(C(N(C(C1([2H])[2H])([2H])[2H])c1cc(OC)ccc1)([	1961285-84-6	4-Quinazoline-5,6,7-d3-acetic acid, 8-fluoro-3,4-dihydro-2-[4-(3-methoxyphenyl)-	C29H11D17F4N4O4		A/B	-8.2
8554	C(C(=O)O)[C@@H]1N(C(=Nc2c1c(c(c(c2F)[2H])[2H])[2H])N1CCN(CC1)c1cc(OC([2H])([2H])[2H])ccc1)c1c(OC([2H]	1961285-86-8	4-Quinazoline-5,6,7-d3-acetic acid, 8-fluoro-3,4-dihydro-2-[4-(3-methoxyphenyl)-	C29H14D14F4N4O4		A/B	-8.2
8555	C(C(=O)O)[C@@H]1N(C(=Nc2c1cccc2F)N1C(C(N(C(C1([2H])[2H])([2H])[2H])c1cc(OC([2H])([2H])[2H])ccc1)([2H]	1961285-93-7	4-Quinazolineacetic acid, 8-fluoro-3,4-dihydro-2-[4-[3-(methoxy-d3)phenyl]-1-pip	C29H11D17F4N4O4		A/B	-8.2
8556	C(C(=O)O)[C@@H]1N(C(=Nc2c1cccc2F)N1C(C(N(C(C1([2H])[2H])([2H])[2H])c1cc(OC)ccc1)([2H])[2H])([2H])[2H]	1961285-94-8	4-Quinazolineacetic acid, 8-fluoro-3,4-dihydro-2-[4-[3-(methoxy-d3)phenyl]-1-pip	C29H14D14F4N4O4		A/B	-8.2
8557	C(C(=O)O)[C@@]1(N(C(=Nc2c1c(c(c(c2F)[2H])[2H])[2H])N1C(C(N(C(C1([2H])[2H])([2H])[2H])c1c(c(OC([2H])(	1961286-08-7	4-Quinazoline-5,6,7-d3-acetic acid, 8-fluoro-3,4-dihydro-4-d-2-[4-[5-(methoxy-d3	C29H6D22F4N4O4		A/B	-8.2
8558	C(C(=O)O)[C@@]1(N(C(=Nc2c1c(c(c(c2F)[2H])[2H])[2H])N1C(C(N(C(C1([2H])[2H])([2H])[2H])c1c(c(OC)c(c(c1	1961286-12-3	4-Quinazoline-5,6,7-d3-acetic acid, 8-fluoro-3,4-dihydro-4-d-2-[4-(5-methoxypher	C29H9D19F4N4O4		A/B	-8.2
8559	C(C(=O)O)[C@@]1(N(C(=Nc2c1cccc2F)N1C(C(N(C(C1([2H])[2H])([2H])[2H])c1c(c(OC([2H])([2H])[2H])c(c(c1[2	1961286-23-6	4-Quinazolineacetic acid, 8-fluoro-3,4-dihydro-4-d-2-[4-[5-(methoxy-d3)phenyl-2,	C29H9D19F4N4O4		A/B	-8.2
8560	C(C(=O)O)[C@@]1(N(C(=Nc2c1cccc2F)N1C(C(N(C(C1([2H])[2H])([2H])[2H])c1c(c(OC)c(c(c1[2H])[2H])[2H])[2H]	1961286-24-7	4-Quinazolineacetic acid, 8-fluoro-3,4-dihydro-4-d-2-[4-[5-(methoxy-d3)phenyl-2,	C29H12D16F4N4O4		A/B	-8.2
8561	C(C(=O)O)[C@@]1(N(C(=Nc2c1cccc2F)N1C(C(N(C(C1([2H])[2H])([2H])[2H])c1c(c(OC)c(c(c1[2H])[2H])[2H])[2H]	1961286-26-9	4-Quinazolineacetic acid, 8-fluoro-3,4-dihydro-4-d-2-[4-(5-methoxyphenyl-2,3,4,6	C29H12D16F4N4O4		A/B	-8.2
8562	C(C(=O)O)[C@@]1(N(C(=Nc2c1cccc2F)N1CCN(CC1)c1c(c(OC([2H])([2H])[2H])c(c(c1[2H])[2H])[2H])[2H])c1c(OC	1961286-27-0	4-Quinazolineacetic acid, 8-fluoro-3,4-dihydro-4-d-2-[4-(5-methoxyphenyl-2,3,4,6	C29H15D13F4N4O4		A/B	-8.2
8563	C(C(=O)O)[C@@H]1N(C(=Nc2c1c(c(c(c2F)[2H])[2H])[2H])N1C(C(N(C(C1([2H])[2H])([2H])[2H])c1c(c(OC)c(c(c1	1961286-35-0	4-Quinazoline-5,6,7-d3-acetic acid, 8-fluoro-3,4-dihydro-2-[4-(5-methoxyphenyl-2	C29H10D18F4N4O4		A/B	-8.2
8564	C(C(=O)O)[C@@H]1N(C(=Nc2c1cccc2F)N1CCN(CC1)c1c(c(OC)c(c(c1[2H])[2H])[2H])[2H])c1c(OC([2H])([2H])[2H]	1961286-57-6	4-Quinazolineacetic acid, 8-fluoro-3,4-dihydro-2-[4-[5-(methoxy-d3)phenyl-2,3,4,	C29H21D7F4N4O4		A/B	-8.2
8565	C(C(=O)O)[C@@]1(N(C(=Nc2c1c(c(c(c2F)[2H])[2H])[2H])N1C(C(N(C(C1([2H])[2H])([2H])[2H])c1cc(OC)ccc1)([	1961286-62-3	4-Quinazoline-5,6,7-d3-acetic acid, 8-fluoro-3,4-dihydro-4-d-2-[4-(3-methoxypher	C29H13D15F4N4O4		A/B	-8.2
8566	C(C(=O)O)[C@@]1(N(C(=Nc2c1cccc2F)N1C(C(N(C(C1([2H])[2H])([2H])[2H])c1cc(OC([2H])([2H])[2H])ccc1)([2H]	1961286-80-5	4-Quinazolineacetic acid, 8-fluoro-3,4-dihydro-4-d-2-[4-[3-(methoxy-d3)phenyl]-1	C29H13D15F4N4O4		A/B	-8.2
8567	C(C(=O)O)[C@@]1(N(C(=Nc2c1cccc2F)N1C(C(N(C(C1([2H])[2H])([2H])[2H])c1cc(OC)ccc1)([2H])[2H])([2H])[2H]	1961286-86-1	4-Quinazolineacetic acid, 8-fluoro-3,4-dihydro-4-d-2-[4-(3-methoxyphenyl)-1-pipe	C29H16D12F4N4O4		A/B	-8.2
8568	C(C(=O)O)[C@@]1(N(C(=Nc2c1cccc2F)N1CCN(CC1)c1cc(OC([2H])([2H])[2H])ccc1)c1c(OC([2H])([2H])[2H])ccc(C	1961286-90-7	4-Quinazolineacetic acid, 8-fluoro-3,4-dihydro-4-d-2-[4-(3-methoxyphenyl)-1-pipe	C29H19D9F4N4O4		A/B	-8.2
8569	C(C(=O)O)[C@@H]1N(C(=Nc2c1c(c(c(c2F)[2H])[2H])[2H])N1C(C(N(C(C1([2H])[2H])([2H])[2H])c1cc(OC([2H])([	1961287-07-9	4-Quinazoline-5,6,7-d3-acetic acid, 8-fluoro-3,4-dihydro-2-[4-[3-(methoxy-d3)phe	C29H11D17F4N4O4		A/B	-8.2
8570	C(C(=O)O)[C@@H]1N(C(=Nc2c1c(c(c(c2F)[2H])[2H])[2H])N1CCN(CC1)c1cc(OC([2H])([2H])[2H])ccc1)c1c(OC)ccc	1961287-15-9	4-Quinazoline-5,6,7-d3-acetic acid, 8-fluoro-3,4-dihydro-2-[4-[3-(methoxy-d3)phe	C29H19D9F4N4O4		A/B	-8.2
8571	C(C(=O)O)[C@@H]1N(C(=Nc2c1c(c(c(c2F)[2H])[2H])[2H])N1CCN(CC1)c1cc(OC)ccc1)c1c(OC)ccc(C(F)(F)F)c1	1961287-20-6	4-Quinazoline-5,6,7-d3-acetic acid, 8-fluoro-3,4-dihydro-2-[4-(3-methoxyphenyl)-	C29H22D6F4N4O4		A/B	-8.2
8572	C(C(=O)O)[C@@H]1N(C(=Nc2c1cccc2F)N1C(C(N(C(C1([2H])[2H])([2H])[2H])c1cc(OC)ccc1)([2H])[2H])([2H])[2H]	1961287-29-5	4-Quinazolineacetic acid, 8-fluoro-3,4-dihydro-2-[4-[3-(methoxy-d3)phenyl]-1-pip	C29H17D11F4N4O4		A/B	-8.2
8573	C(C(=O)O)C1N(C(=Nc2c1cccc2F)N1CCN(CC1)c1cc(OC([2H])([2H])[2H])ccc1)c1c(OC([2H])([2H])[2H])ccc(C(F)(F	1961287-35-3	4-Quinazolineacetic acid, 8-fluoro-3,4-dihydro-2-[4-(3-methoxyphenyl)-1-piperazi	C29H20D8F4N4O4		A/B	-8.2
8574	[C@@H](Cc1ccccc1)(NC(=O)[C@H](CCN1CCOCC1)NC(=O)N(Cc1nc(C(C)C)sc1)C)CC[C@H](Cc1ccccc1)NC(=O)OCc1cncs1	1964472-16-9	2,7,10,12-Tetraazatridecanoic acid, 12-methyl-13-[2-(1-methylethyl)-4-thiazolyl]	C40H53N7O5S2.C23H16O6		A/B	-8.2
8575	C[C@]1(F)[C@@H](O[C@H](COP(=O)(Oc2ccccc2)N[C@H](C(=O)OCCCCCC#C)C)[C@H]1O)n1cc(=O)[nH]c(=O)cc1	1977515-11-9	L-Alanine, N-[(2,Ä≤R)-2,Ä≤-deoxy-2,Ä≤-fluoro-2,Ä≤-methyl-P-phenyl-5,Ä≤-uridylyl]	C25H31FN3O9P	567.5	A/B	-8.2
8576	N(C(CCCN(CCOC(=O)c1ccccc1)CC)C)c1c2c(ncc1)cc(Cl)cc2	1977541-79-9	Not Yet Assigned	C17H22N5O14P3	613.3	A/B	-8.2
8577	P(=O)(OC(CC(=O)OC)c1ccc(OC(=O)C)cc1)(OC(CC(=O)OC)c1ccc(OC(=O)C)cc1)OCCOCn1c2c(nc1)c(=O)nc(N)[nH]2	1978408-27-3	2-Cyclopentene-1-methanol, 4-[2-amino-6-(cyclopropylamino)-9H-purin-9-yl]-, 1-be	C21H22N6O2	390.44	A/B	-8.2
8578	N(C(=O)C)[C@H]1[C@H]([C@@H]([C@@H](CO)O)O)OC(=C[C@@H]1NC(=N)NC(=O)Cn1cc(nn1)c1ccc(N)cc1)C(=O)O	1982351-46-1	D-glycero-D-galacto-Non-2-enonic acid, 5-(acetylamino)-2,6-anhydro-3,4,5-trideox	C23H29N7O9	547.52	A/B	-8.2
8579	C[C@]1(F)[C@@H](O[C@H](CO[P@](=O)(Oc2ccccc2)N[C@H](C(=O)OC(C)C)C)[C@H]1OC(=O)CN1CCOCC1)n1cc(=O)[nH]c(	1982351-52-9	D-glycero-D-galacto-Non-2-enonic acid, 5-(acetylamino)-2,6-anhydro-3,4,5-trideox	C27H31N7O9	597.58	A/B	-8.2
8580	O=C1[C@@H]2[C@@H](c3c([C@H](OC(=O)c4cc(Cl)ccn4)[C@H]2CO1)cc1c(c3)OCO1)c1cc(OC)c(OC)c(OC)c1	1983131-63-0	3-Pyridinecarboxylic acid, 6-methoxy-, (5R,5aR,8aR,9R)-5,5a,6,8,8a,9-hexahydro-8	C29H27NO10	549.53	A/B	-8.2
8581	O=C1[C@@H]2[C@@H](c3c([C@H](OC(=O)c4ccc(Br)cn4)[C@H]2CO1)cc1c(c3)OCO1)c1cc(OC)c(OC)c(OC)c1	1983131-67-4	4-Pyridinecarboxylic acid, 2-bromo-, (5R,5aR,8aR,9R)-5,5a,6,8,8a,9-hexahydro-8-c	C28H24BrNO9	598.4	A/B	-8.2
8582	N(c1c(CC)cccc1CC)c1nc(Nc2c(C#N)cccc2)cc(C)n1	1983131-68-5	2-Pyridinecarboxylic acid, 5-bromo-, (5R,5aR,8aR,9R)-5,5a,6,8,8a,9-hexahydro-8-c	C28H24BrNO9	598.4	A/B	-8.2
8583	N(c1c(CC)cccc1CC)c1nc(Nc2ccc(C#N)cc2)ncc1	1984578-07-5	Benzonitrile, 2-[[4-[(2,6-diethylphenyl)amino]-2-pyrimidinyl]amino]-	C21H21N5	343.42	A/B	-8.2
8584	N(c1c(C#N)cccc1)c1nc(Nc2ccc(C#N)cc2)ncc1	1984578-31-5	Benzonitrile, 4-[[4-[[2-methyl-6-(1-methylethyl)phenyl]amino]-2-pyrimidinyl]amir	C21H21N5	343.42	A/B	-8.2
8585	O=C1c2n(N([C@H]3N1CCOC3)[C@H]1c3c(CSc4c1cccc4)c(F)c(F)cc3)ccc(=O)c2O	1985175-56-1	Benzonitrile, 2-[[2-[(3,4-dimethylphenyl)amino]-4-pyrimidinyl]amino]-	C19H17N5	315.37	A/B	-8.2
8586	O(COC(=O)[C@@H](CC(=O)OC)C)c1c2n(N([C@H]3N(C2=O)CCOC3)[C@H]2c3c(CSc4c2cccc4)c(F)c(F)cc3)ccc1=O	1985606-80-1	1H-[1,4]Oxazino[3,4-c]pyrido[2,1-f][1,2,4]triazine-6,8-dione, 12-[(11S)-7,8-difl	C29H23F2N3O6S	579.57	A/B	-8.2
8587	O(P(=O)(N[C@H](C(=O)OC)CC(C)C)OCC)c1c2n(N([C@H]3N(C2=O)CCOC3)[C@H]2c3c(CSc4c2cccc4)c(F)c(F)cc3)ccc1=	1985606-88-9	Butanedioic acid, 2,2-dimethyl-, 1-[[[(12aR)-12-[(11S)-7,8-difluoro-6,11-dihydrc	C32H31F2N3O8S	655.67	A/B	-8.2
8588	O(COC(=O)OC(C(=O)OCC)(C)C)c1c2n(N([C@H]3N(C2=O)CCOC3)[C@H]2c3c(CSc4c2cccc4)c(F)c(F)cc3)ccc1=O	1985607-03-1	Propanoic acid, 2-[[[[[(12aR)-12-[(11S)-7,8-difluoro-6,11-dihydrodibenzo[b,e]thi	C34H35F2N3O9S	699.72	A/B	-8.2
8589	O(P(=O)(N[C@H](C(=O)OC)C(C)(C)C)OCC)c1c2n(N([C@H]3N(C2=O)CCOC3)[C@H]2c3c(CSc4c2cccc4)c(F)c(F)cc3)ccc	1985607-18-8	Cyclopentaneacetic acid, 1-[[[[(12aR)-12-[(11S)-7,8-difluoro-6,11-dihydrodibenzc	C34H33F2N3O8S	681.7	A/B	-8.2
8590	O(C(=O)OCCS(=O)(=O)C)c1c2n(N([C@H]3N(C2=O)CCOC3)[C@H]2c3c(CSc4c2cccc4)c(F)c(F)cc3)ccc1=O	1985607-27-9	Carbonic acid, (12aR)-12-[(11S)-7,8-difluoro-6,11-dihydrodibenzo[b,e]thiepin-11-	C31H27F2N3O8S	639.62	A/B	-8.2
8591	O(P(=O)(OCC)OCC)c1c2n(N([C@H]3N(C2=O)CCOC3)[C@H]2c3c(CSc4c2cccc4)c(F)c(F)cc3)ccc1=O	1985607-33-7	Benzoic acid, 4-(dimethylamino)-, (12aR)-12-[(11S)-7,8-difluoro-6,11-dihydrodibe	C33H28F2N4O5S	630.66	A/B	-8.2
8592	O(C(=O)OCC(C(=O)OC)(C)C)c1c2n(N([C@H]3N(C2=O)CCOC3)[C@H]2c3c(CSc4c2cccc4)c(F)c(F)cc3)ccc1=O	1985607-39-3	Propanoic acid, 2-[[[[(12aR)-12-[(11S)-7,8-difluoro-6,11-dihydrodibenzo[b,e]thie	C31H29F2N3O8S	641.64	A/B	-8.2
8593	O(Cc1ccc(N=[N+]=[N-])cc1)c1c2n(N([C@H]3N(C2=O)CCOC3)[C@H]2c3c(CSc4c2cccc4)c(F)c(F)cc3)ccc1=O	1985607-46-2	Benzoic acid, 2-(acetyloxy)-, (12aR)-12-[(11S)-7,8-difluoro-6,11-dihydrodibenzo[	C33H25F2N3O7S	645.63	A/B	-8.2
8594	O(C(=O)OCC(=O)OCC)c1c2n(N([C@H]3N(C2=O)CCOC3)[C@H]2c3c(CSc4c2cccc4)c(F)c(F)cc3)ccc1=O	1985607-54-2	Benzeneacetic acid, Œ±-[[[[(12aR)-12-[(11S)-7,8-difluoro-6,11-dihydrodibenzo[b,e	C34H27F2N3O8S	675.66	A/B	-8.2
8595	N(c1c(CC)cccc1C)c1nc(Nc2ccc(C#N)cc2)cc(C)n1	1987752-30-6	Benzonitrile, 4-[[2-[(2,6-dimethylphenyl)amino]-6-methyl-4-pyrimidinyl]amino]-	C20H19N5	329.4	A/B	-8.2
8596	N(C(=O)c1ccc(Cl)cc1)c1cc2cc(C34CC5CC(C4)CC(C3)C5)[nH]c2cc1	1987854-62-5	Benzamide, 4-methoxy-N-(2-tricyclo[3.3.1.13,7]dec-1-yl-1H-indol-5-yl)-	C26H28N2O2	400.51	A/B	-8.2
8597	N(C(=O)Nc1c(C)cc(Cl)cc1)c1cc2cc(C34CC5CC(C4)CC(C3)C5)[nH]c2cc1	1987854-68-1	Urea, N-(4-chloro-2-methoxyphenyl)-N,Ä≤-(2-tricyclo[3.3.1.13,7]dec-1-yl-1H-indol	C26H28ClN3O2	449.97	A/B	-8.2
8598	C[C@@]1(F)[C@@H](O[C@](COP(=O)(Oc2ccccc2)N[C@H](C(=O)OC(C)C)C)([C@H]1O)[2H])n1cc(=O)[nH]c(=O)cc1	1987958-40-6	L-Alanine, N-(2,Ä≤-deoxy-2,Ä≤,2,Ä≤-difluoro-P-phenyl-5,Ä≤-uridylyl-4,Ä≤-C-d)-,	C21H25DF2N3O9P		A/B	-8.2
8599	N(Cc1ccc(cc1)c1cnccn1)(C[C@@H]([C@H](Cc1ccccc1)NC(=O)[C@@H](NC(=O)OC)[C@H](CC)C)O)NC(=O)[C@@H](NC(=O	198904-27-7	2,5,6,10,13-Pentaazatetradecanedioic acid, 3-(1,1-dimethylethyl)-8-hydroxy-12-(1	C36H49N7O7	691.82	A/B	-8.2
8600	N(Cc1ccc(cc1)c1ccccn1)(C[C@@H]([C@H](Cc1ccccc1)NC(=O)[C@@H](NC(=O)OC)[C@H](CC)C)O)NC(=O)[C@@H](NC(=O	198904-31-3	2,5,6,10,13-Pentaazatetradecanedioic acid, 3,12-bis(1,1-dimethylethyl)-8-hydroxy	C38H52N6O7	704.86	A/B	-8.2
8601	C(N(C[C@@H]([C@H](Cc1ccccc1)NC(=O)[C@@H](NC(=O)OC)[C@H](CC)C)O)NC(=O)OC(C)(C)C)c1ccc(cc1)c1ccccn1	198905-04-3	12-Oxa-2,5,9,10-tetraazatetradecanoic acid, 3-(1,1-dimethylethyl)-7-hydroxy-13,1	C35H47N5O6	633.78	A/B	-8.2
8602	Cn1c(c2ccc(CN(C[C@@H]([C@H](Cc3ccccc3)NC(=O)[C@@H](NC(=O)OC)C(C)(C)C)O)NC(=O)[C@@H](NC(=O)OC)C(C)(C)	198905-15-6	12-Oxa-2,5,9,10-tetraazatetradecanoic acid, 7-hydroxy-13,13-dimethyl-3-(1-methyl	C34H45N5O6	619.75	A/B	-8.2
8603	N(c1c(C#N)cccc1)c1nc(Nc2ccc(CC)cc2)ncc1	1992586-67-0	Benzonitrile, 2-[[2-[(2-ethyl-6-methylphenyl)amino]-4-pyrimidinyl]amino]-	C20H19N5	329.4	A/B	-8.2
8604	C[C@]1(F)[C@@H](O[C@H](CO[P@](=O)(Oc2ccccc2)N[C@H](C(=O)OC(C)C)C)[C@H]1O)n1cc(=O)[nH]c(=S)cc1	2003228-30-4	L-Alanine, N-[[P(R),2,Ä≤R]-2,Ä≤-deoxy-2,Ä≤-fluoro-2,Ä≤-methyl-P-phenyl-2-thio-5	C22H29FN3O8PS	545.52	A/B	-8.2
8605	C(C(CC)OC)n1c2c(nc1)c(N)ncn2	200705-76-6	2-Naphthalenesulfonic acid, 8-hydroxy-5-[2-(4-sulfophenyl)diazenyl]-	C16H12N2O7S2	408.41	A/B	-8.2
8606	C(=O)([C@@H](NC(=O)OC)C(C)C)N1[C@H](c2[nH]c3c4c(cc(cc4)c4c5c(c(cc4)c4[nH]c(nc4)[C@@H]4N(C(=O)[C@@H](N	2007900-91-4	Carbamic acid, N-[(1S)-1-[[(2S,4S)-4-[(difluoromethoxy)methyl]-2-[5-[2,3-dihydrc	C48H58F2N8O7	897.02	A/B	-8.2
8607	C(=O)([C@@H](NC(=O)OC)C(C)C)N1[C@H](c2[nH]c(c(n2)C2CC2)c2c3c(cc(cc2)c2cc4c(cc2)c2c(cc4)nc([nH]2)[C@H]	2007901-48-4	Carbamic acid, N-[(1S)-1-[[(2S,4S)-2-[5-[2,3-dihydro-7-[2-[(2S,5S)-1-[(2S)-2-[(m	C48H60N8O6	845.04	A/B	-8.2
8608	C[C@]1(F)[C@@H](O[C@H](CO[P@](=O)(Oc2ccccc2)N[C@H](C(=O)OC(C)C)C)[C@H]1OCc1ccccc1)n1c(=O)n(Cc2ccccc2	2011008-48-1	L-Adenosine, N-[[P(S),2,Ä≤R]-2,Ä≤-deoxy-2,Ä≤-fluoro-2,Ä≤-methyl-P-phenyl-3,Ä≤-(	C30H35FN3O11P	663.58	A/B	-8.2
8609	C(=O)(C(=O)NCCc1ccncc1)c1c(C23CC4CC(C3)CC(C2)C4)[nH]c2c1cccc2	2014403-73-5	1H-Indole-3-acetamide, N-[(4-fluorophenyl)methyl]-Œ±-oxo-2-tricyclo[3.3.1.13,7]c	C27H27FN2O2	430.51	A/B	-8.2
8610	C(=O)(C(=O)Nc1ccccc1)c1c(C23CC4CC(C3)CC(C2)C4)[nH]c2c1cccc2	2014403-76-8	1H-Indole-3-acetamide, N-[2-(1H-indol-2-yl)ethyl]-Œ±-oxo-2-tricyclo[3.3.1.13,7]c	C30H31N3O2	465.59	A/B	-8.2
8611	O=c1n([C@@H]2O[C@H](COC(=O)CCC(=O)Oc3c(OC)ccc(C(=O)c4c(Cl)cccc4)c3)C=C2)cc(C)c(=O)[nH]1	2015224-15-2	Butanedioic acid, 1-[[(2S,5R)-5-(3,4-dihydro-5-methyl-2,4-dioxo-1(2H)-pyrimidiny	C29H28N2O10	564.54	A/B	-8.2
8612	O=c1n([C@@H]2O[C@H](COC(=O)COc3c(OC)ccc(C(=O)c4ccc(Br)cc4)c3)C=C2)cc(C)c(=O)[nH]1	2015224-35-6	Acetic acid, 2-[5-(4-fluorobenzoyl)-2-methoxyphenoxy]-, [(2S,5R)-5-(3,4-dihydro-	C26H23FN2O8	510.47	A/B	-8.2
8613	O=c1n([C@@H]2O[C@H](COC(=O)COc3c(OC)ccc(C(=O)c4ccc(N(=O)=O)cc4)c3)C=C2)cc(C)c(=O)[nH]1	2015224-36-7	Acetic acid, 2-[5-(4-bromobenzoyl)-2-methoxyphenoxy]-, [(2S,5R)-5-(3,4-dihydro-5	C26H23BrN2O8	571.37	A/B	-8.2
8614	O=c1n([C@H]2C/C(=N\OC(=O)CC)/[C@@H](CO)O2)cc(C)c(=O)[nH]1	2016-86-6	2-Naphthalenesulfonic acid, 6-hydroxy-5-[2-(4-sulfophenyl)diazenyl]-, ion(2-)	C16H10N2O7S2		A/B	-8.2
8615	O=c1n([C@@H]2O[C@H](COP(=O)(OP(=O)(O[C@@H]3C[C@@H](O)[C@@H](O)[C@H](C)O3)O)O)[C@@H](O)C2)cc(C)c(=O)[	202338-64-5	2-Naphthalenesulfonic acid, 6-hydroxy-5-[2-(4-methylphenyl)diazenyl]-, ion(2-)	C17H12N2O4S		A/B	-8.2
8616	[C@H](Cc1ccccc1)(C[C@@H]([C@H](Cc1ccccc1)NC(=O)OCc1cccnc1)O)NC(=O)[C@@H](NC(=O)N(Cc1nc(CC)sc1)C)C(C)	202816-22-6	2,7,10,12-Tetraazatridecanoic acid, 13-(2-ethyl-4-thiazolyl)-5-hydroxy-12-methyl	C38H48N6O5S	700.89	A/B	-8.2
8617	C(N(C(=O)N[C@H](C(=O)N[C@@H](Cc1ccccc1)C[C@@H]([C@H](Cc1ccccc1)NC(=O)OCc1cncs1)O)C)CC(C)C)c1nc(C(C)C	202816-75-9	2,7,10,12-Tetraazapentadecanoic acid, 4-hydroxy-9-methyl-12-[[2-(1-methylethyl)-	C37H48N6O5S2	720.94	A/B	-8.2
8618	[C@H](C(C)C)(CO)n1c2c(cc(Cc3cc(cc(Cl)c3F)N3S(=O)(=O)CCC3)c(OC)c2)c(=O)c(C(=O)O)c1	1000161-69-2	3-Quinolinecarboxylic acid, 6-[[3-chloro-2-fluoro-5-(4-morpholinyl)phenyl]methyl	C28H32ClFN2O6	547.01	C	-8.2
8619	[C@H](C(C)C)(CO)n1c2c(cc(Cc3cc(c(F)c(Cl)c3F)N3C(=O)CCC3)c(OC)c2)c(=O)c(C(=O)O)c1	1000161-73-8	3-Quinolinecarboxylic acid, 6-[[3-chloro-5-(1,1-dioxido-2-isothiazolidinyl)-2-fl	C26H28ClFN2O7S	567.03	C	-8.2
8620	[C@H](C(C)C)(CO)n1c2c(cc(Cc3cc(cc(Cl)c3F)c3noc(C)n3)c(OC)c2)c(=O)c(C(=O)O)c1	1000161-98-7	3-Quinolinecarboxylic acid, 6-[[3-chloro-2,4-difluoro-5-(4-morpholinyl)phenyl]me	C28H31ClF2N2O6	565.01	C	-8.2
8621	N(c1nc(nc(n1)N1CCOCC1)N1CCOCC1)c1ccc(C(=O)OC)cc1	10021-57-5	Uridine, inosinylyl-(3,Ä≤,Üí5,Ä≤)-	C19H23N6O13P	574.39	C	-8.2
8622	O[C@H]1[C@@H](O[C@H](COC(=O)CCNC(=O)CO[C@@H]2O[C@H](CO)[C@H](O)[C@H](O)[C@H]2O)[C@H]1O)n1nc(C(=O)N)n	1002334-87-3	1H-1,2,4-Triazole-3-carboxamide, 1-[5-O-[(2S)-2-[[2-(Œ≤-D-galactopyranosyloxy)ac	C19H29N5O13	535.46	C	-8.2
8623	O(C(=O)N[C@@H](Cc1ccc(OC)cc1)[C@@H](CN(S(=O)(=O)c1ccc(OC)cc1)CC(C)C)N)[C@@H]1[C@H]2[C@@H](OC1)OCC2	1005324-21-9	Carbamic acid, N-[(1S)-1-[(4-methoxyphenyl)methyl]-3-[[(4-methoxyphenyl)sulfonyl	C29H38N2O9S	590.68	C	-8.2
8624	C(COCP1(=O)OC(CCO1)c1c(F)ccc(F)c1)n1c2c(nc1)c(N)ncn2	1007390-02-4	9H-Purin-6-amine, 9-[2-[[4-(2,4-difluorophenyl)-2-oxido-1,3,2-dioxaphosphorinan-	C17H18F2N5O4P	425.33	C	-8.2
8625	C(=O)([C@H](NC(=O)OC)c1ccccc1)N1[C@@H](CCC1)c1[nH]c(cn1)c1ccc(cc1)c1ccc(cc1)c1[nH]c(nc1)[C@H]1N(C(=O	1007882-36-1	Carbamic acid, N-[(1R)-2-[(2S)-2-[5-[4,Ä≤-[2-[(1S)-1-[[(2R)-2-[(methoxycarbonyl)	C45H46N8O6	794.9	C	-8.2
8626	C(=O)([C@@H](NC(=O)OCc1ccccc1)C(C)C)N1[C@@H](CCC1)c1[nH]c(cn1)c1ccc(cc1)c1ccc(cc1)c1[nH]c(nc1)[C@H]1	1007884-33-4	Carbamic acid, N,N,Ä≤-[(2,2,Ä≤,6,6,Ä≤-tetramethyl[1,1,Ä≤-biphenyl]-4,4,Ä≤-diyl)	C44H58N8O6	794.98	C	-8.2
8627	C(=O)([C@@H](NC(=O)OC)C(C)C)N1[C@@H](CCC1)c1[nH]c(cn1)c1cc(C(=O)N)c(cc1)c1ccc(cc1)c1[nH]c(nc1)[C@H]1	1007885-17-7	[1,1,Ä≤-Biphenyl]-2-carboxylic acid, 4,4,Ä≤-bis[2-[(2S)-1-[(2S)-2-[(methoxycarb	C41H50N8O8	782.88	C	-8.2
8628	O=c1n(c2c(c(=O)[nH]1)c(C)cc(=O)o2)[C@H]1[C@H](OC(=O)C)[C@H](OC(=O)C)[C@@H](COC(=O)C)O1	100891-85-8	Hydrazinecarbothioamide, 2-(1,2-dihydro-1-methyl-2-oxo-3H-indol-3-ylidene)-N-[4-	C21H23N5OS	393.51	C	-8.2
8629	C(=O)([C@H](NC(=O)OC)c1ccccc1)N1[C@@H](CCC1)c1[nH]c(cn1)c1ccc(cc1)c1ccc(cc1)c1[nH]c(nc1)[C@H]1N(C(=O	1009057-21-9	3-Isoquinolinecarboxamide, 2-[3-[(3,4-dihydroxy-2-methylbenzoyl)amino]-2-hydroxy	C33H47N3O5S	597.81	C	-8.2
8630	C(=O)([C@H](NC(=O)C)C(C)C)N1[C@@H](CCC1)c1[nH]c(cn1)c1ccc(cc1)c1ccc(cc1)c1[nH]c(nc1)[C@H]1N(C(=O)[C@	1009107-27-0	Carbamic acid, N,N,Ä≤-[[1,1,Ä≤-biphenyl]-4,4,Ä≤-diylbis[1H-imidazole-5,2-diyl-(	C40H50N8O6	738.88	C	-8.2
8631	C(=O)([C@H](NC(=O)OC)c1ccccc1)N1[C@@H](CC[C@@H]1c1ccccc1)c1[nH]c(cn1)c1ccc(cc1)c1ccc(cc1)c1[nH]c(nc1	1009113-49-8	Carbamic acid, N-[(1S)-1-[[(2S)-2-[5-[4,Ä≤-[2-[(2S)-1-[(2S)-2-[(methoxycarbonyl)	C41H52N8O6	752.9	C	-8.2
8632	C(=O)([C@@H](NC(=O)OC)C1CCOCC1)N1[C@@H](CCC1)c1[nH]c(cn1)c1ccc(cc1)c1ccc(cc1)c1[nH]c(nc1)[C@H]1N(C(=	1009113-72-7	Carbamic acid, N,N,Ä≤-[[1,1,Ä≤-biphenyl]-4,4,Ä≤-diylbis[1H-imidazole-5,2-diyl-(	C42H54N8O6	766.93	C	-8.2
8633	C(=O)([C@@H](NC(=O)OC)C1CCOCC1)N1[C@@H](CCC1)c1[nH]c(cn1)c1ccc(cc1)c1ccc(cc1)c1[nH]c(nc1)[C@H]1N(C(=	1009113-84-1	Carbamic acid, N-[(1S)-1-[[(2S)-2-[5-[4,Ä≤-[2-[(2S)-1-[(2S)-2-[(methoxycarbonyl)	C43H54N8O7	794.94	C	-8.2
8634	C(=O)([C@@H](NC(=O)COC)C(C)C)N1[C@@H](CCC1)c1[nH]c(cn1)c1ccc(cc1)c1ccc(cc1)c1[nH]c(nc1)[C@H]1N(C(=O)	1009113-88-5	Propanamide, N-[(1S)-2-methyl-1-[[(2S)-2-[5-[4,Ä≤-[2-[(2S)-1-[(2S)-3-methyl-1-o>	C42H54N8O4	734.93	C	-8.2
8635	C(=O)([C@H](Cc1ccc(CN)cc1)NC(=O)OC)N1[C@@H](CCC1)c1[nH]c(cn1)c1ccc(cc1)c1ccc(cc1)c1[nH]c(nc1)[C@H]1N	1009114-79-7	Carbamic acid, N-[(1S)-2-[(2S)-2-[5-[4,Ä≤-[2-[(2S)-1-[(2S)-2-[(methoxycarbonyl)a	C41H45N9O6	759.85	C	-8.2
8636	C(=O)(C(NC(=O)OC)(C)C)N1[C@@H](CCC1)c1[nH]c(cn1)c1ccc(cc1)c1ccc(cc1)c1[nH]c(nc1)[C@H]1N(C(=O)C(NC(=O	1009116-74-8	Carbamic acid, N,N,Ä≤-[[1,1,Ä≤-biphenyl]-4,4,Ä≤-diylbis[1H-imidazole-5,2-diyl-(	C38H46N8O6	710.82	C	-8.2
8637	C(=O)([C@@H](NC(=O)OC)C(C)(C)O)N1[C@@H](CCC1)c1[nH]c(cn1)c1ccc(cc1)c1ccc(cc1)c1[nH]c(nc1)[C@H]1N(C(=	1009116-88-4	Carbamic acid, N,N,Ä≤-[[1,1,Ä≤-biphenyl]-4,4,Ä≤-diylbis[1H-imidazole-5,2-diyl-(	C46H46N8O6	806.91	C	-8.2
8638	C(=O)([C@@H](NC(=O)OC)C1CC1)N1[C@@H](CCC1)c1[nH]c(cn1)c1ccc(cc1)c1ccc(cc1)c1[nH]c(nc1)[C@H]1N(C(=O)[	1009117-14-9	Carbamic acid, N,N,Ä≤-[[1,1,Ä≤-biphenyl]-4,4,Ä≤-diylbis[1H-imidazole-5,2-diyl-(	C40H46N8O6	734.84	C	-8.2
8639	C(=O)([C@H](NC(=O)OC)C1CC1)N1[C@H](CCC1)c1[nH]c(cn1)c1ccc(cc1)c1ccc(cc1)c1[nH]c(nc1)[C@@H]1N(C(=O)[C	1009117-28-5	Carbamic acid, N,N,Ä≤-[[1,1,Ä≤-biphenyl]-4,4,Ä≤-diylbis[1H-imidazole-5,2-diyl-2	C40H50N8O6	738.88	C	-8.2
8640	C(=O)([C@H](N(CC)CC)c1ccccc1)N1[C@@H](CCC1)c1[nH]c(cn1)c1ccc(cc1)c1ccc(cc1)c1[nH]c(nc1)[C@H]1N(C(=O)	1009117-58-1	Carbamic acid, N-[(1S)-1-[[(2S)-2-[5-[4,Ä≤-[2-[(2S)-1-[(2R)-2-(diethylamino)-2-p	C44H52N8O4	756.93	C	-8.2
8641	C(=O)([C@H](N(CC)CC)c1ccccc1)N1[C@@H](CCC1)c1[nH]c(cn1)c1ccc(cc1)c1ccc(cc1)c1[nH]c(nc1)[C@H]1N(C(=O)	1009117-62-7	Carbamic acid, N-[(1S)-1-[[(2S)-2-[5-[4,Ä≤-[2-[(2S)-1-[(2R)-2-(diethylamino)-2-p	C46H56N8O4	784.99	C	-8.2
8642	C(=O)([C@H](N(CC)CC)C(C)C)N1[C@@H](CCC1)c1[nH]c(cn1)c1ccc(cc1)c1ccc(cc1)c1[nH]c(nc1)[C@H]1N(C(=O)[C@	1009117-82-1	Carbamic acid, N-[(1S)-1-[[(2S)-2-[5-[4,Ä≤-[2-[(2S)-1-[(2R)-2-(ethylmethylamino)	C44H52N8O4	756.93	C	-8.2
8643	C(=O)([C@@H](NC(=O)OC)[C@H](CO)C)N1[C@@H](CCC1)c1[nH]c(cn1)c1ccc(cc1)c1ccc(cc1)c1[nH]c(nc1)[C@H]1N(C	1009118-11-9	Carbamic acid, N-[(1S)-1-[[(2S)-2-[5-[4,Ä≤-[2-[(2S)-1-[(2S)-3-hydroxy-2-[(methox	C40H50N8O7	754.87	C	-8.2
8644	C(=O)([C@@H](NC(=O)OC(C)(C)C)C(C)C)N1[C@@H](CCC1)c1[nH]c(cn1)c1ccc(cc1)c1ccc(cc1)c1[nH]c(nc1)[C@H]1N	1009120-26-6	Carbamic acid, N-[(1R)-1-[[(2S)-2-[5-[4,Ä≤-[2-[(2S)-1-[(2S)-2-amino-3-methyl-1-c	C38H48N8O4	680.84	C	-8.2
8645	N(CCCC)[C@H]1[C@H]2[C@H]3[C@@](C)([C@@H]([C@@H](CCCC(C)C)C)CC3)CC[C@@H]2[C@]2(C)[C@@H](Cl)C[C@@H](O)	101609-53-4	Benzenesulfonic acid, 4-[2-(2-hydroxy-4-phenanthrenyl)diazenyl]-	C20H14N2O4S	378.4	C	-8.2
8646	N(=N\c1ccc(S(=O)(=O)O)cc1)/c1c2c(ccc1O)cccc2	1017018-27-7	2-Naphthalenesulfonic acid, 6-hydroxy-5-[(1E)-2-(4-sulfophenyl)diazenyl]-	C16H12N2O7S2	408.41	C	-8.2
8647	N(C(CCCN(CC)CC)C)c1c2c(ncc1C)cc(Cl)c(C)c2	1024656-58-3	2H-1,2,3-Triazole, 4-(4-chlorophenyl)-5-phenyl-2-tricyclo[3.3.1.13,7]dec-1-yl-	C24H24ClN3	389.92	C	-8.2
8648	Cc1c2c(n(cn2)c2c(C)cc(/C=C/C#N)cc2C)nc(Nc2ccc(C#N)cc2)n1	1025963-35-2	1,3,4-Oxadiazole-2-carboxamide, N-[1-[(4Z)-4-[[[(4-fluorophenyl)methyl]amino]hyc	C20H21FN6O5	444.42	C	-8.2
8649	O=C1c2c(N(c3c(N1C)c(C)cc(F)n3)C1CC1)ncc(CCOc1c3c(NS(=O)(=O)C3)ccc1)c2	1026294-86-9	Benzonitrile, 4-[[4-[[4-[(1E)-2-cyanoethenyl]-2,6-dimethylphenyl]amino]-5-[(3-th	C27H23N7S	477.58	C	-8.2
8650	N(CCCNCCC(C)(C)C)c1c2c(ncc1)cc(Cl)cc2	1027739-58-7	Carbamic acid, N-[[3-[4-[(2-ethyl-5,7-dimethyl-3H-imidazo[4,5-b]pyridin-3-yl)met	C33H42N4O4S2	622.84	C	-8.2
8651	O(c1nc(Nc2ccc(C#N)cc2)nc(N)c1Br)c1c(C)cc(C#N)cc1C.C(=C\C(=O)O)/C(=O)O	1030633-41-0	Benzonitrile, 4-[[6-amino-5-bromo-2-[(4-cyanophenyl)amino]-4-pyrimidinyl]oxy]-3,	C20H15BrN6O.C7H8O3S		C	-8.2
8652	N(c1c(C)cc(/C=C/C#N)cc1C)c1nc(Nc2ccc(C#N)cc2)nc(C(=O)NCc2ccncc2)c1	1037444-87-3	4-Pyrimidinecarboxamide, 6-[[4-[(1E)-2-cyanoethenyl]-2,6-dimethylphenyl]amino]-N	C26H22N8O	462.51	C	-8.2
8653	N(c1cc(C(=O)c2nccs2)nc(Nc2ccc(C#N)cc2)n1)c1c(C)cc(/C=C/C#N)cc1C	1037444-91-9	4-Pyrimidinecarboxamide, 6-[[4-[(1E)-2-cyanoethenyl]-2,6-dimethylphenyl]amino]-2	C28H23N7O2	489.53	C	-8.2
8654	N(c1c(C)cc(/C=C/C#N)cc1C)c1nc(Nc2ccc(C#N)cc2)nc(CNCCc2ccccn2)c1	1037445-12-7	Benzonitrile, 4-[[4-[[4-[(1E)-2-cyanoethenyl]-2,6-dimethylphenyl]amino]-6-(1H-im	C26H22N8	446.51	C	-8.2
8655	N(c1c(C)cc(/C=C/C#N)cc1C)c1nc(Nc2ccc(C#N)cc2)nc(CN(CC#N)C)c1	1037445-14-9	Benzonitrile, 4-[[4-[[4-[(1E)-2-cyanoethenyl]-2,6-dimethylphenyl]amino]-6-[[[2-(	C30H28N8	500.6	C	-8.2
8656	N(c1nc(Nc2ccc(C#N)cc2)nc(Cl)c1)c1c(C)cc(/C=C/C#N)cc1C	1037445-42-3	Benzonitrile, 4-[[4-[[4-[(1E)-2-cyanoethenyl]-2,6-dimethylphenyl]amino]-6-[(hydr	C23H19N7O	409.44	C	-8.2
8657	N(c1nc(Nc2ccc(C#N)cc2)nc(C(=O)N(OC)C)c1)c1c(C)cc(/C=C/C#N)cc1C	1037445-57-0	4-Pyrimidinecarboxylic acid, 6-[[4-[(1E)-2-cyanoethenyl]-2,6-dimethylphenyl]amir	C23H18N6O2	410.43	C	-8.2

8658	C(=C\c1ccc(C(F)(F)F)cc1)/c1c(Nc2c(C)cc(/C=C/C#N)cc2C)nc(Nc2ccc(C#N)cc2)nc1COC	1037452-81-5	Benzonitrile, 4-[[4-[[4-[(1E)-2-cyanoethenyl]-2,6-dimethylphenyl]amino]-5-(2-cya	C28H25N7O	475.54	C	-8.2
8659	C(OCCOC(=O)[C@H](CO)N)n1c2c(nc1)c(=O)nc(N)[nH]2	1043455-80-6	Benzenesulfonic acid, 4-[2-(2-hydroxy-1-naphthalenyl)diazenyl]-3-methyl-, ion(1-	C17H13N2O4S		C	-8.2
8660	O=C1[C@@H]2[C@@H](c3c(C[C@@H]2C(=O)O1)cc1c(c3)OCO1)c1cc(OC)c(OC)c(OC)c1	1049986-97-1	Benzoic acid, 3,3,Ä≤-[[5-(dihydroxymethylene)-4,6-dioxo-2-cyclohexen-1-ylidene]m	C24H18O12	498.39	C	-8.2
8661	[C@@H](Cc1ccccc1)(NC(=O)[C@@H](NC(=O)N(Cc1nc(C(C)C)sc1)C)CC(=O)NS(=O)(=O)C)CC[C@H](Cc1ccccc1)NC(=O)O	1051461-71-2	2,7,10,12-Tetraazatridecanoic acid, 12-methyl-13-[2-(1-methylethyl)-4-thiazolyl]	C40H52N8O5S2	789.02	C	-8.2
8662	[C@@H](Cc1ccccc1)(NC(=O)[C@H](CCN1CCOCC1)NC(=O)N(Cc1sc(C(C)C)nc1)C)CC[C@H](Cc1ccccc1)NC(=O)OCc1cncs1	1051465-36-1	14-Thia-2,7,13-triazapentadecanoic acid, 12-imino-9-[[[methyl[[2-(1-methylethyl)	C38H50N8O6S3	811.05	C	-8.2
8663	O[C@H]1[C@H](n2c3c(nc2)c(=O)nc(N)[nH]3)O[C@H](COP(=O)(OP(=O)(OP(=O)(OC[C@H]2O[C@H]([C@H](O)[C@@H]2O)	10527-46-5	Guanosine 5,Ä≤-(pentahydrogen tetraphosphate), P,Ä≤,Ä≤,Ä≤,Üí5,Ä≤-ester with ade	C20H28N10O20P4	852.39	C	-8.2
8664	O=c1c2c(n(cn2)[C@H]2C[C@H](OP(=O)(OCCCCCCS)O)[C@@H](CO)O2)[nH]c(N)n1	1052712-16-9	2-Pyridinecarboxamide, 3-amino-N-[4-[(3S)-3-amino-1-piperidinyl]-3-pyridinyl]-6-	C26H34N6O	446.59	C	-8.2
8665	O[C@H]1[C@H](n2c3c(nc2)c(N)ncn3)O[C@H](COP(=O)(OCC)O)[C@H]1O	10547-39-4	4-Quinolinamine, 7-chloro-N-[4-[4-(7-chloro-4-quinolinyl)-1-piperazinyl]-1-methy	C27H29Cl2N5	494.46	C	-8.2
8666	O[C@H]1[C@H](n2c3c(nc2)c(=O)nc[nH]3)O[C@H](COP(=O)(OP(=O)(OP(=O)(O)O)S)O)[C@H]1O	1056646-53-7	Benzonitrile, 4-[[5-bromo-4-[4-[(1E)-2-cyanoethenyl]-2,6-dimethylphenoxy]-2-pyri	C22H16BrN5O	446.3	C	-8.2
8667	O(C)c1c(c(C)c(OC)c2c1cccc2)c1cn(c(=O)[nH]c1=O)[C@@H]1O[C@H](COP(=O)(O)O)[C@@H](O)C1	105785-87-3	Uridine, 2,Ä≤-deoxy-5-(1,4-dihydro-3-methyl-1,4-dioxo-2-naphthalenyl)-	C20H18N2O7	398.37	C	-8.2
8668	C(Cc1ccccc1)[C@]1(CCC)CC(=C([C@H](CC)c2cc(N(S(=O)(=O)c3ccc(C(F)(F)F)cn3)CCOCCOCCOCCOCCOCCOCCOC)ccc2)	1058690-26-8	2-Pyridinesulfonamide, N-[3-[(1R)-1-[(6R)-5,6-dihydro-4-hydroxy-2-oxo-6-(2-pheny	C42H55F3N2O10S	836.95	C	-8.2
8669	C(NC(=O)c1cnco1)(C)(C)c1nc(C(=O)NCc2ccc(F)cc2)c(O)c(=O)n1C	1064707-04-5	2-Pyrimidinecarboxamide, N-[1-[4-[[[(4-fluorophenyl)methyl]amino]carbonyl]-1,6-d	C21H21FN6O4	440.43	C	-8.2
8670	C(OCCOC(=O)[C@H](NC(=O)[C@@H](C(C)C)N)C(C)C)n1c2c(nc1)c(=O)nc(N)[nH]2	1064707-07-8	4-Pyrimidinecarboxamide, N-[(4-fluorophenyl)methyl]-1,6-dihydro-5-hydroxy-1-meth	C20H22FN7O4	443.43	C	-8.2
8671	Cc1c2c(cc(C(OC(=O)C(C)C)OC(=O)C(C)C)c1)COP(=O)(OC[C@H]1O[C@H](C=C1)n1c(=O)[nH]c(=O)c(C)c1)O2	1072917-34-0	Thymidine, 5,Ä≤-O-[(2S)-6-[bis(acetyloxy)methyl]-8-(1,1-dimethylethyl)-2-oxido-4	C26H31N2O11P	578.5	C	-8.2
8672	C(C)(C)(C)c1c2c(cc(C(OC(=O)C(C)C)OC(=O)C(C)C)c1)CO[P@@](=O)(OC[C@H]1O[C@H](C=C1)n1c(=O)[nH]c(=O)c(C)	1072917-36-2	Thymidine, 5,Ä≤-O-[(2R)-6-[bis(2-methyl-1-oxopropoxy)methyl]-8-(1,1-dimethylethy	C30H39N2O11P	634.61	C	-8.2
8673	N(=C1c2c(N(CN3CCOCC3)C1=O)ccc(OC(F)(F)F)c2)NC(=S)Nc1ccc(OC)cc1	1084893-37-7	Hydrazinecarbothioamide, 2-[1,2-dihydro-1-(4-morpholinylmethyl)-2-oxo-5-(trifluo	C22H22F3N5O3S	493.5	C	-8.2
8674	C(NC(=O)c1nnc(C)o1)(C([2H])([2H])[2H])(C([2H])([2H])[2H])c1nc(C(=O)NC(c2ccc(F)cc2)([2H])[2H])c(O)c(=	1100750-88-6	4-Pyrimidinecarboxamide, N-[(4-fluorophenyl)methyl]-1,6-dihydro-5-hydroxy-1-(met	C20H12D9FN6O5		C	-8.2
8675	C(NC(=O)c1nnc(C)o1)(C)(C)c1nc(C(=O)NCc2c(c(c(F)c(c2[2H])[2H])[2H])[2H])c(O)c(=O)n1C([2H])([2H])[2H]	1100750-96-6	4-Pyrimidinecarboxamide, N-[(4-fluorophenyl-2,3,5,6-d4)methyl]-1,6-dihydro-5-hyd	C20H11D10FN6O5		C	-8.2
8676	C(NC(=O)c1nnc(C)o1)(C)(C)c1nc(C(=O)NC(c2c(c(c(F)c(c2[2H])[2H])[2H])[2H])([2H])[2H])c(O)c(=O)n1C	1100750-97-7	4-Pyrimidinecarboxamide, N-[(4-fluorophenyl-2,3,5,6-d4)methyl]-1,6-dihydro-5-hyd	C20H14D7FN6O5		C	-8.2
8677	C(NC(=O)c1nnc(C)o1)(C([2H])([2H])[2H])(C([2H])([2H])[2H])c1nc(C(=O)NC(c2c(c(c(F)c(c2[2H])[2H])[2H])[	1100751-02-7	4-Pyrimidinecarboxamide, N-[(4-fluorophenyl-2,3,5,6-d4)methyl]-1,6-dihydro-5-hyd	C20H8D13FN6O5		C	-8.2
8678	O=c1n([C@@H]2O[C@H](COP(=O)(OP(=O)(OCc3ccc(OC(=O)C)cc3)OCc3ccc(OC(=O)C)cc3)O)[C@@H](N=[N+]=[N-])C2)c	1101448-28-5	Thymidine 5,Ä≤-(trihydrogen diphosphate), 2,Ä≤,3,Ä≤-didehydro-3,Ä≤-deoxy-, P,Ä≤	C28H30N2O14P2	680.49	C	-8.2
8679	C[C@]1(O)[C@H](n2c3c(nc2)c(=O)nc(N)[nH]3)O[C@H](COP(=O)(OP(=O)(O)O)O)[C@H]1O	1106011-27-1	5,Ä≤-Guanylic acid, 2,Ä≤-C-methyl-	C11H16N5O8P	377.25	C	-8.2
8680	O=c1n(cc(C(F)(F)F)c(=O)[nH]1)[C@@H]1O[C@H](COCOCCc2ccccc2)[C@@H](O)C1	110604-79-0	Thymidine, 3,Ä≤-O-[1-[(4-butylphenyl)methoxy]ethyl]-Œ±,Œ±,Œ±-trifluoro-	C23H29F3N2O6	486.48	C	-8.2
8681	O[C@H]1[C@H](n2c3c(nc2)c(=O)nc[nH]3)O[C@H](CO)[C@H]1OP(=O)(OC)O	110604-87-0	Thymidine, Œ±,Œ±,Œ±-trifluoro-5,Ä≤-O-[1-(1-phenylethoxy)ethyl]-	C20H23F3N2O6	444.4	C	-8.2
8682	C(#Cc1ccc(F)cc1)c1n(nc(C(=O)N)n1)[C@@H]1O[C@H](CO)[C@@H](O)[C@H]1O	1106736-04-2	1H-1,2,4-Triazole-3-carboxamide, 1-Œ≤-D-ribofuranosyl-5-[2-[3-(trifluoromethyl)p	C17H15F3N4O5	412.32	C	-8.2
8683	O[C@@H]1[C@H](n2c3c(nc2)c(N)ncn3)O[C@H](COC(=O)[C@H](C)N2C(=O)c3c(C2=O)cccc3)[C@H]1O	111392-86-0	9H-Purin-6-amine, 9-[5-O-[2-(1,3-dihydro-1,3-dioxo-2H-isoindol-2-yl)-1-oxopropyl]	C21H20N6O7	468.42	C	-8.2
8684	O[C@@H]1[C@@H](O[C@H](COP(=O)(OP(=O)(OP(=O)(O)O)O)O)[C@H]1O)n1cc(CCc2ccccc2)c(=O)[nH]c1=O	112066-16-7	Furo[3,Ä≤,4,Ä≤:6,7]naphtho[2,3-d]-1,3-dioxol-6(5aH)-one, 5,8,8a,9-tetrahydro-9-[	C21H20O8	400.38	C	-8.2
8685	C(#Cc1n(nc(C(=O)N)n1)[C@@H]1O[C@H](CO)[C@@H](O)[C@H]1O)c1c2c3c4c(cc2)cccc4ccc3cc1	1123187-67-6	1H-1,2,4-Triazole-3-carboxamide, 1-Œ≤-D-ribofuranosyl-5-[2-[2-(trifluoromethyl)p	C17H15F3N4O5	412.32	C	-8.2
8686	C(=O)([C@@H](NC(=O)OC)C)N1[C@H]2[C@H](C2)C[C@H]1c1[nH]c(cn1)c1ccc(cc1)c1ccc(cc1)c1[nH]c(nc1)[C@H]1N(	1129401-27-9	Benzoic acid, 3,3,Ä≤-carbonylbis[6-[(4-butylbenzoyl)amino]-	C37H36N2O7	620.69	C	-8.2
8687	C(=O)([C@@H](NC(=O)OC)C(C)C)N1[C@H]2[C@H](C2)C[C@H]1c1[nH]c(cn1)c1ccc(cc1)c1ccc(cc1)c1[nH]c(nc1)[C@H	1129633-75-5	Carbamic acid, N,N,Ä≤-[[1,1,Ä≤-biphenyl]-4,4,Ä≤-diylbis[1H-imidazole-5,2-diyl-(	C42H50N8O6	762.9	C	-8.2
8688	C(=O)([C@H](NC(=O)OC)c1ccccc1)N1[C@@H](CCC1)c1[nH]c(c(C)n1)c1ccc(cc1)c1ccc(cc1)c1[nH]c(nc1C)[C@H]1N(	1129636-16-3	Carbamic acid, N-[(1S)-1-[[(2S)-2-[5-[4,Ä≤-[2-[(2S)-1-[(2S)-2-[(methoxycarbonyl)	C41H52N8O6	752.9	C	-8.2
8689	C(CC)c1c([nH]c(n1)[C@H]1N(C(=O)[C@@H](NC(=O)OC)C)CCC1)c1ccc(cc1)c1ccc(cc1)c1[nH]c(nc1)[C@H]1N(C(=O)[	1129636-41-4	1H-Imidazole-4-acetic acid, 2-[(2S)-1-[(2S)-2-[(methoxycarbonyl)amino]-3-methyl-	C43H54N8O8	810.94	C	-8.2
8690	C(=O)([C@@H](NC(=O)OC)C(C)C)N1[C@@H](CC(F)(F)C1)c1[nH]c(cn1)c1ccc(cc1)c1cc(F)c(cc1)c1[nH]c(nc1)[C@H]	1129636-49-2	Carbamic acid, N-[(1S)-1-[[(2S)-2-[5-[4,Ä≤-[2-[(2S)-1-[(2S)-2-[(methoxycarbonyl)	C43H56N8O6	780.95	C	-8.2
8691	C(#CC1CC1)[C@@]1(C(F)(F)F)c2c(N(CN3CCN(CC3)c3ccc(F)cc3)C(=O)O1)ccc(Cl)c2	1134943-82-0	2H-3,1-Benzoxazin-2-one, 6-chloro-4-(2-cyclopropylethynyl)-1,4-dihydro-1-[[4-(4-	C26H25ClF3N3O3	519.94	C	-8.2
8692	C(#CC1CC1)[C@@]1(C(F)(F)F)c2c(N(CN3CCN(CC3)c3cc4n(cc(C(=O)O)c(=O)c4cc3F)C3CC3)C(=O)O1)ccc(Cl)c2	1134943-88-6	3-Quinolinecarboxylic acid, 7-[4-[[(4S)-6-chloro-4-(2-cyclopropylethynyl)-2-oxo-	C31H27ClF4N4O5	647.02	C	-8.2
8693	C(Cc1ccccc1)[C@]1(CCC)CC(=C([C@H](CC)c2cc(N(S(=O)(=O)c3ccc(C(F)(F)F)cn3)O)ccc2)C(=O)O1)O	1141510-04-4	2-Pyridinesulfonamide, N-[3-[(1R)-1-[(6R)-5,6-dihydro-4-hydroxy-2-oxo-6-(2-pheny	C31H33F3N2O5S		C	-8.2
8694	C(Cc1ccccc1)[C@]1(CCC)CC(=C([C@H](CC)c2cc(NS(=O)(=O)c3ccc(C(F)(F)F)cn3)ccc2)C(=O)O1)OC1O[C@H](C(=O)O	1141510-06-6	2-Pyridinesulfonamide, N-[3-[(1R)-1-[(6R)-5,6-dihydro-4-hydroxy-2-oxo-6-(2-pheny	C31H33F3N2O6S	618.66	C	-8.2
8695	C(=O)(N[C@@H](C(C1CCC1)([2H])[2H])C(=O)C(=O)N)[C@@H]1[C@@H]2[C@@H](C2(C)C)CN1C(=O)[C@@H](NC(=O)NC(C(	1158083-12-5	3-Azabicyclo[3.1.0]hexane-2-carboxamide, N-[(1S)-3-amino-1-(cyclobutylmethyl-d2)	C27H33D12N5O5		C	-8.2
8696	C(N1c2c(C(=NNC(=S)Nc3ccc(OC)cc3)C1=O)cc(Cl)cc2)c1ccccc1	1161792-76-2	Hydrazinecarbothioamide, 2-[1,2-dihydro-5-methyl-2-oxo-1-(phenylmethyl)-3H-indol	C24H22N4OS	414.52	C	-8.2
8697	N(=C\1/c2c(N(C)C1=O)ccc(S(=O)(=O)N1CCOCC1)c2)\NC(=S)Nc1c(C)cccc1C	1164536-63-3	Hydrazinecarbothioamide, 2-[1,2-dihydro-1-methyl-5-(4-morpholinylsulfonyl)-2-oxo	C22H25N5O4S2	487.6	C	-8.2
8698	Nc1c2c(n(cn2)[C@@H]2O[C@H](CO)[C@@H](O)[C@@H]2O)nc([18F])n1	1169548-51-9	9H-Purin-6-amine, 9-(2-O-benzoyl-Œ≤-D-arabinofuranosyl)-	C17H17N5O5	371.35	C	-8.2
8699	Cc1n(c2nc(nc(n2)N2CCOCC2)c2cc(O)ccc2)ccn1	1174190-66-9	Benzonitrile, 3-[4-(2-methyl-1H-benzimidazol-1-yl)-6-(4-morpholinyl)-1,3,5-triaz	C22H19N7O	397.43	C	-8.2
8700	C(C)(N)C12CC3C(O)C(C1)CC(C2)C3	1178091-85-4	1-Naphthalenesulfonic acid, 2,2,Ä≤-[[1,1,Ä≤-biphenyl]-4,4,Ä≤-diylbis[(1E)-2,1-d	C32H24N6O6S2	652.7	C	-8.2
8701	C(=O)([C@@H](NC(=O)OC)C(C)C)N1[C@H](c2[nH]c3-c4c(cc(cc4)c4ccc(cc4)c4c(CCC(F)(F)F)nc([nH]4)[C@H]4N(C(	1180669-64-0	Carbamic acid, N-[(1S)-2-methyl-1-[[(2S)-2-[5-[4-[1,4,5,6-tetrahydro-2-[(2S)-1-[	C45H55F3N8O6	860.96	C	-8.2
8702	C(=O)([C@@H](NC(=O)OC)C(C)C)N1[C@H]2[C@H](C2)C[C@H]1c1[nH]c2-c3c(CCc2n1)cc(cc3)c1ccc(cc1)c1[nH]c(nc1	1180669-98-0	Carbamic acid, N-[(1S)-1-[[(2S)-2-[5-[4-[4,5-dihydro-2-[(2S)-1-[(2S)-2-[(methoxy	C45H55F3N8O6	860.96	C	-8.2
8703	C(=O)([C@@H](NC(=O)OC)C(C)C)N1[C@H](c2[nH]c3c4c(cc(cc4)c4ccc(cc4)c4[nH]c(nc4)[C@H]4N(C(=O)[C@@H](NC(	1180670-13-6	Carbamic acid, N-[(1S)-1-[[(2S)-2-[5-[4-[2-[(2S)-4,4-difluoro-1-[(2S)-2-[(methox	C42H50F2N8O6	800.89	C	-8.2
8704	N(C(=O)[C@@H](C[C@@H](CN1[C@H](C(=O)NC(C)(C)C)CN(Cc2cccnc2)CC1)O[Si](C(C)(C)C)(C)C)Cc1ccccc1)[C@@H]1	1185758-21-7	D-erythro-Pentonamide, 2,3,5-trideoxy-N-[(8S,8aS)-3a,8a-dihydro-2,2-dimethyl-8H-	C39H51N5O5	669.85	C	-8.2
8705	C(N1c2c(C(=NNC(=S)NC3CCCCC3)C1=O)cccc2)N1CCN(CC1)c1ccc(Cl)cc1	118989-36-9	Hydrazinecarbothioamide, N-cyclohexyl-2-[1,2-dihydro-2-oxo-1-[(4-phenyl-1-pipera	C26H32N6OS	476.64	C	-8.2
8706	O[C@H]1[C@@H](O[C@H](CO)[C@H]1O)n1nc(C(=N)OC)nc1	1203578-22-6	L-Prolinamide, L-valyl-L-prolyl-L-valyl-N-(9-Œ≤-D-arabinofuranosyl-9H-purin-6-yl	C30H45N9O8	659.73	C	-8.2
8707	O(COC(=O)OCCOP(=O)(O)O)c1c2n(C[C@H]3N(C2=O)[C@H](C)CCO3)cc(C(=O)NCc2c(F)cc(F)cc2)c1=O	1206102-00-2	Carbonic acid, [[(4R,12aS)-9-[[[(2,4-difluorophenyl)methyl]amino]carbonyl]-3,4,6	C24H25F2N3O9	537.47	C	-8.2
8708	C(c1nn(C23CC4CC(C3)CC(C2)C4)nn1)c1nnc(N)[nH]1	1207759-76-9	2H-Tetrazole, 5,5,Ä≤-methylenebis[2-tricyclo[3.3.1.13,7]dec-1-yl-	C23H32N8	420.55	C	-8.2
8709	O[C@@H]1[C@H](n2c3c(nc2)c(NC)nc(Cl)n3)O[C@H](CO)[C@H]1O	121021-43-0	Uridine 5,Ä≤-(trihydrogen diphosphate), 3,Ä≤-azido-2,Ä≤,3,Ä≤-dideoxy-	C9H13N5O10P2		C	-8.2
8710	C(OCCOP(=O)(OCCOCn1c2c(nc1)c(=O)nc(N)[nH]2)O)n1c2c(nc1)c(=O)nc(N)[nH]2	121117-73-5	Phosphoric acid, mono[2-[(2-amino-1,6-dihydro-6-oxo-9H-purin-9-yl)methoxy]ethyl]	C18H23N10O7P	522.41	C	-8.2
8711	C([C@H](OCP(=O)(OCOC(=O)OC(C)C)OCOC(=O)OCCC)C)n1c2c(nc1)c(N)ncn2	1217536-16-7	Not Yet Assigned	C29H35D6F2N5O2		C	-8.2
8712	N(C(=O)[C@H](Cc1ccccc1)C[C@@H](CN1[C@H](C(=O)NC(C)(C)C)CN(Cc2cccnc2)CC1)O)[C@@H]1c2c(C[C@@H]1O)cccc2	1217754-41-0	6H-Furo[3,Ä≤,4,Ä≤:6,7]naphtho[2,3-c]-1,2-dioxol-6-one, 3,5,5a,8,8a,9-hexahydro-	C21H20O8	400.38	C	-8.2
8713	C(N1c2c(C(=NNC(=S)N)C1=O)ccc(Cl)c2)N1CCN(CC1)c1c2c(ncc1)cc(Cl)cc2	1218813-10-5	Hydrazinecarbothioamide, 2-[4-bromo-1-[[4-(7-chloro-4-quinolinyl)-1-piperazinyl]	C23H21BrClN7OS	558.88	C	-8.2
8714	N(Cc1ccc(/C=C/C(=O)NO)cc1)C12CC3(O)CC(C2)CC(C1)C3	1221411-78-4	2-Propenamide, N-hydroxy-3-[4-[(tricyclo[3.3.1.13,7]dec-1-ylamino)methyl]phenyl]	C20H26N2O2	326.43	C	-8.2
8715	O=C1[C@@H]2[C@@H](c3c([C@H](OC(=O)CCC(=O)NCCc4ccc(OC)cc4)[C@H]2CO1)cc1c(c3)OCO1)c1cc(OC)c(OC)c(OC)c1	1222083-93-3	Butanoic acid, 4-oxo-4-[[(2R)-2-phenylpropyl]amino]-, (5R,5aR,8aR,9R)-5,5a,6,8,8	C35H37NO10	631.67	C	-8.2
8716	O=C1[C@@H]2[C@@H](c3c([C@H](OC(=O)CCC(=O)N4CCN(C(=O)Cc5c6c(n(C(=O)c7ccc(Cl)cc7)c5C)ccc(OC)c6)CC4)[C@	1222083-96-6	Butanoic acid, 4-[[5-[[2-[1-(4-chlorobenzoyl)-5-methoxy-2-methyl-1H-indol-3-yl]a	C50H52ClN3O13	938.41	C	-8.2
8717	N(C(=O)C)[C@H]1[C@H](C(=O)C(CO)O)OC(C(=O)O)C[C@@H]1NC(=N)N	1223072-63-6	Not Yet Assigned	C36H48N6O4	628.8	C	-8.2
8718	N(C(=O)[C@H](Cc1ccccc1)C[C@@H](CN1[C@H](C(=O)NC(C)(C)C)CN(Cc2c(S)cccc2)CC1)O)C1c2c(C[C@H]1O)cccc2	1223096-37-4	Not Yet Assigned	C45H63N5O6	770.01	C	-8.2
8719	C(C1(CC1)c1ccccc1)N1C[C@@H](C(=O)NC(C)(C)C)N(C[C@H](C[C@H](C(=O)NC2c3c(C[C@H]2O)cccc3)Cc2ccccc2)O)CC	1223101-05-0	Not Yet Assigned	C33H48FN5O4	597.76	C	-8.2
8720	[C@](C(=O)N[C@@H](C(c1c(c(c(c(c1[2H])[2H])[2H])[2H])[2H])([2H])[2H])C[C@@H]([C@](C(c1c(c(c(c(c1[2H])	1224728-96-4	1(2H)-Pyrimidine-4,5,6-d3-acetamide-Œ±-d, N-[(1S,3S,4S)-4-[[2-[2,6-di(methyl-d3)	C37H8D40N4O5		C	-8.2
8721	[C@](C(=O)N[C@@H](Cc1ccccc1)C[C@@H]([C@H](Cc1ccccc1)NC(=O)C(Oc1c(C)cccc1C)([2H])[2H])O)(C(C([2H])([2	1224729-18-3	1(2H)-Pyrimidineacetamide-Œ±-d, N-[(1S,3S,4S)-4-[[2-[2,6-di(methyl-d3)phenoxy-3,	C37H29D19N4O5		C	-8.2
8722	[C@@H](C(=O)N[C@@H](C(c1c(c(c(c(c1[2H])[2H])[2H])[2H])[2H])([2H])[2H])C[C@@H]([C@](C(c1c(c(c(c(c1[2H	1224729-23-0	1(2H)-Pyrimidine-4,5,6-d3-acetamide, N-[(1S,3S,4S)-4-[[2-[2,6-di(methyl-d3)pheno	C37H24D24N4O5		C	-8.2
8723	[C@](C(=O)N[C@@H](Cc1ccccc1)C[C@@H]([C@](C(c1c(c(c(c(c1[2H])[2H])[2H])[2H])[2H])([2H])[2H])(NC(=O)CO	1224729-28-5	1(2H)-Pyrimidine-4,5,6-d3-acetamide-Œ±-d, N-[(1S,3S,4S)-4-[[2-[2,6-di(methyl-d3)	C37H17D31N4O5		C	-8.2
8724	[C@@H](C(=O)N[C@@H](Cc1ccccc1)C[C@@H]([C@](C(c1c(c(c(c(c1[2H])[2H])[2H])[2H])[2H])([2H])[2H])(NC(=O)	1224729-53-6	1(2H)-Pyrimidine-4,5,6-d3-acetamide, N-[(1S,3S,4S)-4-[[2-(2,6-dimethylphenoxy)ac	C37H34D14N4O5		C	-8.2
8725	[C@@H](C(=O)N[C@@H](Cc1ccccc1)C[C@@H]([C@H](Cc1ccccc1)NC(=O)COc1c(C([2H])([2H])[2H])c(c(c(c1C([2H])(	1224729-57-0	1(2H)-Pyrimidine-4,5,6-d3-acetamide, N-[(1S,3S,4S)-4-[[2-(2,6-dimethylphenoxy)ac	C37H42D6N4O5		C	-8.2
8726	C(#N)C1=C(N)Oc2c(C1c1cccs1)ccn1c2nc(C)c1	1225373-05-6	7H-Imidazo[1,2-a]pyrano[2,3-c]pyridine-8-carbonitrile, 9-amino-7-(2-thienyl)-	C15H10N4OS	294.33	C	-8.2
8727	C(#N)C1=C(N)Oc2c(C1c1cc(OC)c(CC=C)ccc1)ccn1c2nc(C)c1	1225373-16-9	7H-Imidazo[1,2-a]pyrano[2,3-c]pyridine-8-carbonitrile, 9-amino-7-[4-hydroxy-3-me	C22H20N4O3	388.42	C	-8.2
8728	C(=O)([C@@H](NC(=O)OC)C)N1[C@@H](CCC1)c1[nH]c(cn1)[C@@H]1CC[C@H](CC1)c1ccc(cc1)c1[nH]c(nc1)[C@H]1N(C	1228668-03-8	Carbamic acid, N-[(1S)-1-[[(2S)-2-[5-[4-[cis-4-[2-[(2S)-1-[(2S)-2-[[(1,1-dimethy	C46H68N8O6	829.08	C	-8.2
8729	C(=O)([C@@H](NC(=O)OC)C(C)C)N1[C@@H](CCC1)c1[nH]c(cn1)c1ccc(cc1)[C@@H]1CC[C@H](CC1)c1[nH]c(nc1)[C@H]	1228670-66-3	Carbamic acid, N-[(1S)-1-[[(2S)-2-[5-[4-[trans-4-[2-[(2S)-1-[(2R)-2-(dimethylami	C43H56N8O4	748.96	C	-8.2
8730	O=c1c2c(n(cn2)[C@@H]2O[C@H](COP(=O)(OP(=O)([C@@](P(=O)(O)O)(C)F)O)O)[C@@H](O)C2)[nH]c(N)n1	1228897-56-0	5,Ä≤-Guanylic acid, 2,Ä≤-deoxy-, anhydride with P,P,Ä≤-[(1R)-1-fluoroethylidene	C12H19FN5O12P3	537.23	C	-8.2
8731	C(=O)([C@H](NC(=O)OC)c1ccccc1)N1[C@@H](CCC1)c1[nH]c(cn1)c1ccc(C#Cc2ccc(cc2)c2[nH]c(nc2)[C@H]2N(C(=O)	1228964-82-6	Carbamic acid, N,N,Ä≤-[1,2-ethynediylbis[4,1-phenylene-1H-imidazole-5,2-diyl-(2S	C48H62N8O6	847.06	C	-8.2
8732	C(=O)([C@@H](NC(=O)OC)C(C)C)N1[C@@H](CCC1)c1[nH]c(cn1)c1ccc(C#Cc2ccc(cc2)c2[nH]c(nc2)[C@H]2N(C(=O)[C	1228965-86-3	Carbamic acid, N-[(1R)-2-oxo-2-[(2S)-2-[5-[4-[2-[4-[2-[(2S)-1-[(2R)-1-oxo-2-pher	C48H47N7O4	785.93	C	-8.2
8733	C(=O)([C@H](C(C)C)N(C)C)N1[C@@H](CCC1)c1[nH]c(cn1)c1ccc(C#Cc2ccc(cc2)c2[nH]c([C@@H](NC(=O)[C@@H](NC(	1228966-87-7	Carbamic acid, N,N,Ä≤-[1,2-ethynediylbis[4,1-phenylene-1H-imidazole-5,2-diyl-(2S	C38H42N8O6	706.79	C	-8.2
8734	C(=O)([C@@H](NC(=O)OC)C(C)C)N1[C@@H](CCC1)c1[nH]c(C#Cc2c3c(c(C#Cc4[nH]c(nc4)[C@H]4N(C(=O)[C@@H](NC(=	1228968-03-3	Carbamic acid, N-[(1S)-1-[[(2S)-2-[5-[2-[6-[2-[(2S)-1-[(2S)-2-[(methoxycarbonyl)	C41H50N8O6	750.89	C	-8.2
8735	[C@H](Cc1ccccc1)(C[C@@H]([C@H](Cc1ccccc1)NC(=O)OCc1cncs1)O)NC(=O)[C@@H](NC(=O)N(Cc1nc(OC(C)C)sc1)C)C	1236406-35-1	Not Yet Assigned	C41H48N6O7S	768.92	C	-8.2
8736	S(=O)(=O)(N(C[C@H]([C@H](Cc1ccccc1)NC(=O)O[C@H]1COCO1)O)CC(C)C)c1cc2c(cc1)OCC2	1237809-07-2	Not Yet Assigned	C37H46N6O5	654.8	C	-8.2
8737	N(CC(=O)[C@H](Cc1ccccc1)C[C@@H](CN1[C@H](C(=O)NC(C)(C)C)CN(CCc2ccccn2)CC1)O)[C@H]1c2c(C[C@H]1O)cccc2	1237988-97-4	Not Yet Assigned	C37H49N5O4	627.82	C	-8.2
8738	[C@H](Cc1ccccc1)(C[C@@H]([C@H](Cc1ccccc1)NC(=O)OC(C)c1cncs1)O)NC(=O)[C@@H](NC(=O)N(Cc1nc(C(C)C)sc1)C	1237989-15-9	Not Yet Assigned	C37H48ClN5O4	662.26	C	-8.2
8739	C(O)C1(CO)O[C@H](C=C1)n1c(=O)[nH]c(=O)cc1	1238950-66-7	2-Naphthalenesulfonic acid, 8-amino-5-[2-[4-(aminocarbonyl)phenyl]diazenyl]-	C17H14N4O4S	370.38	C	-8.2
8740	C(F)(F)c1n(c2c(n1)cccc2)c1nc(nc(N[C@H]2CC[C@H](NC(=O)CBr)CC2)n1)N1CCOCC1	1240023-37-3	Acetamide, 2-amino-N-[trans-4-[[4-[2-(difluoromethyl)-1H-benzimidazol-1-yl]-6-(4	C23H29F2N9O2	501.53	C	-8.2
8741	C(F)(F)c1n(c2c(n1)cccc2)c1nc(nc(NC2Cc3c(CC2)nc[nH]3)n1)N1CCOCC1	1240023-56-6	1,4-Cyclohexanediamine, N1-[4-[2-(difluoromethyl)-1H-benzimidazol-1-yl]-6-(4-mor	C21H26F2N8O	444.48	C	-8.2
8742	C(F)(F)c1n(c2c(n1)cccc2)c1nc(nc(N[C@H]2CC[C@H](N)CC2)n1)N1[C@H](C)COCC1	1240025-19-7	Acetamide, N-[trans-4-[[4-[2-(difluoromethyl)-1H-benzimidazol-1-yl]-6-(4-morphol	C23H28F2N8O2	486.52	C	-8.2
8743	C(=O)([C@@H](NC(=O)OC)C)N1[C@@H](CCC1)c1[nH]c(cn1)c1ccc(cc1)c1ccc(C(=O)NC[C@H]2N(C(=O)[C@@H](NC(=O)O	1240427-42-2	L-Prolinamide, N-(methoxycarbonyl)-L-valyl-N-[(1R)-1-[4,Ä≤-[2-[(2S)-1-[(2S)-2-[	C40H53N7O7	743.89	C	-8.2
8744	C(=O)([C@H](NC(=O)OC)c1ccccc1)N1[C@@H](CCC1)c1[nH]c(cn1)[C@H]1CC[C@H](CC1)c1ccc(cc1)c1[nH]c(nc1)[C@H	1240890-67-8	Carbamic acid, N-[(1S)-1-[[(2S)-2-[5-[trans-4-[4-[2-[(1S)-1-[[(2S)-2-[(methoxyca	C39H56N8O6	732.91	C	-8.2
8745	C(Nc1c2c(n3c(n1)c(S(=O)(=O)c1ccc(C(C)C)cc1)nn3)ccs2)(C)C12CC3CC(C2)CC(C1)C3	1246179-39-4	Thieno[2,3-e][1,2,3]triazolo[1,5-a]pyrimidin-5-amine, 3-[(4-ethylphenyl)sulfonyl	C27H31N5O2S2	521.7	C	-8.2
8746	NC12CC3C4(C(C1)CC(C3)C2)OCCO4	1247871-28-8	Methanone, (1,1-dioxido-4-thiomorpholinyl)[2-[[(1S)-1-tricyclo[3.3.1.13,7]dec-1-	C22H29F3N4O3S	486.55	C	-8.2
8747	C(=O)([C@H](NC(=O)OC)c1ccccc1)N1[C@@H](CCC1)c1[nH]c(cn1)c1ccc(cc1)c1ccc(cc1)c1ccc(cc1)c1[nH]c(nc1)[C	1250936-29-8	Carbamic acid, N,N,Ä≤-[1,2-ethanediylbis[4,1-phenylene-1H-imidazole-5,2-diyl-(2S	C48H50N8O6	834.96	C	-8.2
8748	C(=O)([C@@H](NC(=O)OC)C(C)C)N1[C@@H](CC[C@@H]1C)c1[nH]c(cn1)c1ccc(C#Cc2ccc(cc2)c2[nH]c(nc2)[C@H]2N(C	1250938-96-5	Carbamic acid, N,N,Ä≤-[(1S,2S)-1,2-cyclopropanediylbis[4,1-phenylene-1H-imidazol	C45H58N8O6	806.99	C	-8.2
8749	C(=O)([C@@H](NC(=O)OC)C(C)C)N1[C@@H](C[C@@H](O)C1)c1[nH]c(cn1)c1ccc(C#Cc2ccc(cc2)c2[nH]c(nc2)[C@H]2N	1250940-92-1	Carbamic acid, N,N,Ä≤-[1,2-ethynediylbis[4,1-phenylene-1H-imidazole-5,2-diyl[(2S	C44H50N8O6	786.92	C	-8.2
8750	C(=O)([C@@H](NC(=O)OC)C(C)C)N1[C@@H](CCC1)c1[nH]c(cn1)c1cc(C#Cc2cc(ccc2)c2[nH]c(nc2)[C@@H]2CCCN2)ccc	1250941-28-6	Carbamic acid, N,N,Ä≤-[1,2-ethynediylbis[3,1-phenylene-1H-imidazole-5,2-diyl-(2S	C42H50N8O6	762.9	C	-8.2
8751	Nc1c2c(n(cn2)[C@@H]2O[C@H](CO)[C@@H](O)[C@@H]2O)nc(n1)c1ccco1	125339-08-4	9H-Purin-6-amine, 9-Œ≤-D-arabinofuranosyl-2-(phenylmethyl)-	C17H19N5O4	357.36	C	-8.2
8752	C(=O)(O[C@H](CCc1cc(OC)c(OC)cc1)c1cc(OCCNC(=O)CCCN2C(=O)N([C@H](C(=O)N[C@@H](Cc3ccccc3)C[C@@H]([C@H]	1255196-41-8	1(2H)-Pyrimidinecarboxylic acid, 3-[(1S)-1-[[[(1S,3S,4S)-4-[[2-(2,6-dimethylpher	C74H96N6O15	1309.59	C	-8.2
8753	C(=O)([C@@H](NC(=O)OC)C(C)C)N1[C@H](c2[nH]c(c3cc4c(c5c(s4)cc(cc5)c4[nH]c(nc4)[C@H]4N(C(=O)[C@@H](NC(	1256387-72-0	Carbamic acid, N,N,Ä≤-[(2-cyano[1,1,Ä≤-biphenyl]-4,4,Ä≤-diyl)bis[1H-imidazole-5	C41H49N9O6	763.88	C	-8.2
8754	C(=O)([C@H](Cc1ccccc1)NC(=O)OC)N1[C@@H](CCC1)c1[nH]c(cn1)c1ccc(cc1)c1cc2c(cc1)cc(cc2)c1[nH]c(nc1)[C@	1256389-48-6	Carbamic acid, N-[(1S)-1-[[(2S,4S)-4-cyano-2-[5-[4-[6-[2-[(2S)-1-[(2S)-2-[(metho	C45H51N9O6	813.94	C	-8.2
8755	C(=O)(NC(CC1CCC1)C(=O)C(=O)N)[C@@H]1[C@@H]2[C@@H](C2(C)C)CN1C(=O)[C@@H](NC(=O)NC(C([2H])([2H])[2H])(	1256751-10-6	3-Azabicyclo[3.1.0]hexane-2-carboxamide, N-[3-amino-1-(cyclobutylmethyl)-2,3-dio	C27H35D10N5O5		C	-8.2
8756	C(=O)([C@@H](NC(=O)OC)C(C)C)N1[C@H](c2[nH]c(c3ccc([C@H]4N([C@H](CC4)c4ccc(cc4)c4[nH]c(nc4)[C@H]4N(C(	1258226-87-7	L-Prolinamide, 2,2,Ä≤-[[(2S,5S)-1-[4-(1,1-dimethylethyl)phenyl]-2,5-pyrrolidined	C50H67N7O8	894.11	C	-8.2
8757	C(=O)([C@@H](NC(=O)OC)C(C)C)N1[C@H](c2[nH]c(c3ccc(C4N(C(CC4)c4ccc(cc4)c4[nH]c(nc4)[C@H]4N(C(=O)[C@@H	1258227-55-2	Carbamic acid, N,N,Ä≤-[[(2R,5S)-1-phenyl-2,5-pyrrolidinediyl]bis[4,1-phenylene-1	C50H61N9O6	884.08	C	-8.2
8758	O=C1[C@@H]2[C@@H](c3c([C@H](O[C@H]4O[C@H]5[C@@H]([C@H](O)[C@H]4O)O[C@H](C)OC5)[C@H]2CO1)cc1c(c3)OCO1	126453-80-3	Furo[3,Ä≤,4,Ä≤:6,7]naphtho[2,3-d]-1,3-dioxol-6(5aH)-one, 9-[[(1R)-4,6-O-ethylide	C30H34O13	602.58	C	-8.2
8759	C(=O)([C@@H](NC(=O)OC)CC)N1[C@@H](CCC1)c1[nH]c(cn1)c1ccc(C#Cc2[nH]c(nc2)[C@H]2N(C(=O)[C@@H](NC(=O)OC	1265531-60-9	Carbamic acid, N-[(1S)-2-[(2S)-2-[5-[4-[2-[2-[(2S)-1-[(2S)-2-[(methoxycarbonyl)a	C32H38N8O6	630.69	C	-8.2
8760	C(N1c2c(/C(=N/NC(=S)NOC)/C1=O)cc(F)cc2)N1CCN(CC1)c1ccc(Cl)cc1	1265623-00-4	Hydrazinecarbothioamide, 2-[1-[[4-(4-chlorophenyl)-1-piperazinyl]methyl]-5-fluor	C20H20ClFN6O2S	462.93	C	-8.2
8761	N(=N\c1ccc(cc1)c1ccc(/N=N/c2c(N)c3c(c(S(=O)(=O)O)c2)cccc3)cc1)/c1c2c(cc(S(=O)(=O)O)cc2O)ccc1N	1266249-29-9	1-Naphthalenesulfonic acid, 4-amino-3-[(1E)-2-[4,Ä≤-[(1E)-2-(2-amino-8-hydroxy-6	C32H24N6O7S2.2Na		C	-8.2
8762	C[C@]12[C@H]([C@H]3[C@@H]([C@]4(C)[C@H](CC3)CCCC4)CC2)CC[C@@H]1[C@@H](CCCOS(=O)(=O)O)C	1270965-90-6	Thymidine, 2,Ä≤,3,Ä≤-didehydro-3,Ä≤-deoxy-5,Ä≤-O-[(2S)-8-methyl-2-oxido-4H-1,3,	C18H19N2O7P	406.33	C	-8.2
8763	C(=O)([C@@H](NC(=O)OC)C(C)(C)O)N1[C@@H]([C@@H]2C[C@H]1CC2)c1[nH]c(cn1)c1ccc(cc1)c1ccc(cc1)c1[nH]c(nc	1273589-63-1	Carbamic acid, N-[(1S)-1-cyclopropyl-2-[(2S)-2-[5-[4,Ä≤-[2-[(1R,3S,4S)-2-[(2S)-2	C42H48N8O6	760.88	C	-8.2
8764	C(=O)([C@@H](NC(=O)OC)C(C)C)N1[C@@H]([C@H]2[C@@H](OC)C[C@@H]1C2)c1[nH]c(cn1)c1ccc(cc1)c1ccc(cc1)c1[n	1273591-27-7	Carbamic acid, N-[(1S)-1-[[(2S)-2-[5-[4,Ä≤-[2-[(1S,3S,4S,5S)-5-hydroxy-2-[(2S)-2	C42H52N8O7	780.91	C	-8.2
8765	C(=O)([C@@H](NC(=O)OC)C(C)C)N1[C@@H]([C@@H]2C[C@H]1CC2=O)c1[nH]c(cn1)c1ccc(cc1)c1ccc(cc1)c1[nH]c(nc1	1273591-29-9	Carbamic acid, N-[(1S)-1-[[(2S)-2-[5-[4,Ä≤-[2-[(1R,3R,4S)-2-[(2S)-2-[(methoxyca	C42H50N8O6	762.9	C	-8.2
8766	C(=O)([C@@H](NC(=O)OC)C(C)C)N1[C@@H]([C@@H]2C[C@H]1CC2)c1[nH]c(cn1)c1ccc(cc1)c1ccc(cc1)c1[nH]c([C@@H	1273591-39-1	Carbamic acid, N-[(1S)-1-[[[(1S)-1-[5-[4,Ä≤-[2-[(1R,3S,4S)-2-[(2S)-2-[(methoxyca	C41H52N8O6	752.9	C	-8.2
8767	C(=O)([C@@H](NC(=O)OC)C(C)C)N1[C@@H]([C@@H]2C[C@H]1CC2)c1[nH]c(cn1)c1ccc(cc1)c1ccc(cc1)c1[nH]c([C@@H	1273591-40-4	Carbamic acid, N-[(1S)-1-[[(1R,3S,4S)-3-[5-[4,Ä≤-[2-[(1S)-1-[[(2S)-2-[(methoxyca	C40H50N8O6	738.88	C	-8.2
8768	O=c1c2c(n(cn2)[C@@H]2O[C@H](COP(=O)(OP(=O)(OP(=O)(O)O)O)O)[C@@H](O)C2)[nH]c(NCCCCCC)n1	127860-13-3	Guanosine, 5,Ä≤-[3-(fluorosulfonyl)benzoate]	C17H16FN5O8S	469.4	C	-8.2
8769	O=c1c2c(c(ncc2)n2c(=O)cccc2)oc(c1c1ccc(cc1)C1(N)CCC1)c1ccccc1	1279087-27-2	4H-Pyrano[2,3-c]pyridin-4-one, 3-[4-(1-aminocyclobutyl)phenyl]-2-phenyl-8-(4H-1,	C26H21N5O2	435.48	C	-8.2
8770	O=c1n(cc([C@H](C(=O)O)O)c(=O)[nH]1)[C@@H]1O[C@H](CO)[C@@H](O)[C@H]1O	1279087-34-1	4H-Pyrano[2,3-c]pyridin-4-one, 3-[4-(1-aminocyclobutyl)phenyl]-8-(1H-imidazol-1-	C27H22N4O2	434.49	C	-8.2
8771	N(Cc1ccccc1)c1nc(nc(N/N=C/c2c(O)cccc2)n1)N1CCOCC1	1279692-16-8	Benzaldehyde, 2-hydroxy-, 2-[4-[[(4-fluorophenyl)methyl]amino]-6-(4-morpholinyl)	C21H22FN7O2	423.44	C	-8.2
8772	N(=Nc1ccc(S(=O)(=O)O)cc1)c1c2c(c(S(=O)(=O)O)ccc2)ccc1O	128098-55-5	Benzenesulfonic acid, 4-[2-(2-hydroxy-1-naphthalenyl)hydrazinyl]-	C16H14N2O4S	330.36	C	-8.2
8773	O=n1c2c(n(c1)[C@@H]1O[C@H](COP(=O)(O)O)[C@@H](O)[C@H]1O)[nH]c(N)nc2=O.[NaH]	1291087-81-4	Carbonic acid, (1R,2S)-1-[[[(4-aminophenyl)sulfonyl](2-methylpropyl)amino]methyl	C34H45N3O11S	703.8	C	-8.2
8774	N(C(=O)CC1(CC(=O)OC)CCCC1)C12CC3CC(C2)CC(C1)C3	1310678-44-4	2-Azaspiro[4.5]decane-2-carboxamide, 3-oxo-N-tricyclo[3.3.1.13,7]dec-1-yl-	C20H30N2O2	330.46	C	-8.2

8775	N(C(=O)NCCC(O)c1ccncc1)C12CC3CC(C2)CC(C1)C3	131107-83-0	1-Naphthalenesulfonic acid, 4-amino-3-[2-(2-methylphenyl)diazenyl]-	C17H15N3O3S	341.38	C	-8.2
8776	C(=O)([C@@H](NC(=O)OC)C(C)C)N1[C@@H](CC(=NN(C)C)C1)c1[nH]c(cn1)c1ccc(cc1)c1ccc(cc1)c1[nH]c(nc1)[C@H]	1311398-33-0	Carbamic acid, N-[(1S)-1-[[(2S)-2-[5-[4,Ä≤-[2-[(2S)-1-[(2S)-2-[(methoxycarbonyl)	C42H51N9O7	793.91	C	-8.2
8777	C(=O)([C@@H](NC(=O)OC)C(C)C)N1[C@@H](C[C@H](C)C1)c1[nH]c(cn1)c1ccc(cc1)c1ccc(cc1)c1[nH]c(nc1)[C@H]1N	1311398-37-4	Carbamic acid, N-[(1S)-1-[[(2S)-2-[5-[4,Ä≤-[2-[(6S)-5-[(2S)-2-[(methoxycarbonyl)	C43H52N8O6	776.92	C	-8.2
8778	C(=O)([C@@H](NC(=O)OC)C(C)C)N1[C@@H](CC(=C)C1)c1[nH]c(cn1)c1cc2c(cc1)cc(C#Cc1[nH]c(nc1)[C@H]1N(C(=O)	1311398-59-0	Carbamic acid, N-[(1S)-1-[[(2S)-2-[5-[2-[6-[2-[(2S)-1-[(2S)-2-[(methoxycarbonyl)	C41H50N8O6	750.89	C	-8.2
8779	O=c1n([C@@H]2O[C@H](CO)[C@@H](Cl)C2)cc(C)c(N)n1	1311398-60-3	Carbamic acid, N-[(1S)-1-[[(2S)-2-[5-[2-[6-[2-[(2S)-1-[(2S)-2-[(methoxycarbonyl)	C41H48N8O6	748.87	C	-8.2
8780	C(=O)([C@@H](NC(=O)OC)C1CCOCC1)N1[C@H]2[C@H](C2)C[C@H]1c1[nH]c(c(Cl)n1)c1ccc(cc1)c1ccc(cc1)c1[nH]c(n	1312336-06-3	Carbamic acid, N,N,Ä≤-[[1,1,Ä≤-biphenyl]-4,4,Ä≤-diylbis[(5-chloro-1H-imidazole-	C42H44Cl2N8O6	827.75	C	-8.2
8781	C(=O)([C@@H](NC(=O)OC)C(C)C)N1[C@@H](C[C@@H](C)[C@@H]1C)c1[nH]c(c(Cl)n1)c1ccc(cc1)c1ccc(cc1)c1[nH]c(	1312336-30-3	Carbamic acid, N,N,Ä≤-[[1,1,Ä≤-biphenyl]-4,4,Ä≤-diylbis[(5-chloro-1H-imidazole-	C42H52Cl2N8O6	835.82	C	-8.2
8782	C(=O)([C@@H](NC(=O)OC)C1CCOCC1)N1[C@H](c2[nH]c(c(CC=C)n2)c2ccc(cc2)c2ccc(cc2)c2[nH]c(nc2)[C@H]2N(C(=	1312336-58-5	Carbamic acid, N-[(1S)-1-[[(2S)-2-[5-[4,Ä≤-[2-[(2S)-1-[(2S)-2-[(methoxycarbonyl)	C43H54N8O6	778.94	C	-8.2
8783	CC1(c2c(nc([nH]2)[C@H]2N(C(=O)[C@@H](NC(=O)OC)C(C)C)CCC2)c2ccc(cc2)c2ccc(cc2)c2[nH]c(nc2)[C@H]2N(C(=	1312336-60-9	Carbamic acid, N-[(1S)-1-[[(2S)-2-[4-(cyclopropylmethyl)-5-[4,Ä≤-[2-[(2S)-1-[(2S	C44H56N8O6	792.97	C	-8.2
8784	C(=O)([C@@H](NC(=O)OC)C1CCOCC1)N1[C@H]2[C@H](C2)C[C@H]1c1[nH]c(c(F)n1)c1ccc(cc1)c1ccc(cc1)c1[nH]c(nc	1312336-69-8	Carbamic acid, N-[(1S)-2-[(1R,3S,5R)-3-[5-[4,Ä≤-[5-cyano-2-[(1R,3S,5R)-2-[(2S)-2	C47H53N9O8	871.98	C	-8.2
8785	O[C@H]1[C@H](n2c3c(nc2)c(=O)nc[nH]3)O[C@H](COP(=O)(F)O)[C@H]1O	131239-75-3	1,4-Benzenedisulfonic acid, 2-[2-(1-amino-4-sulfo-2-naphthalenyl)diazenyl]-, soc	C16H13N3O9S3.3Na		C	-8.2
8786	N(c1c(C([2H])([2H])[2H])cc(/C=C/C#N)cc1C([2H])([2H])[2H])c1nc(Nc2ccc(C#N)cc2)ncc1	1312424-25-1	Benzonitrile, 4-[[4-[[4-[(1E)-2-cyanoethenyl]-2-methyl-6-(methyl-d3)phenyl]aminc	C22H15D3N6		C	-8.2
8787	C(=O)([C@@H](NC(=O)OC)C(C)C)N1[C@H](c2[nH]c(c3c4c(c(cc3)c3ccc(cc3)c3[nH]c(nc3)[C@H]3N(C(=O)[C@@H](NC	1312608-34-6	Carbamic acid, N-[(1S)-1-[[(2S)-2-[5-[4-[7-[2-[(2S)-1-[(2S)-2-[(methoxycarbonyl)	C42H52N10O6	792.93	C	-8.2
8788	C(=O)(CNC(=O)OC)N1[C@H](c2[nH]c(c3c4c(c(cc3)c3ccc(cc3)c3[nH]c(nc3)[C@H]3N(C(=O)CNC(=O)OC)CCC3)cccc4)	1312608-55-1	Carbamic acid, N-[(1R)-1-[[(2S)-2-[5-[4-[4-[2-[(2S)-1-[(2R)-2-[(methoxycarbonyl)	C44H52N8O6	788.93	C	-8.2
8789	Cc1oc2c(ccc(c2c1)c1ccc(cc1)c1[nH]c(nc1)[C@H]1N(C(=O)[C@@H](NC(=O)OC)C(C)C)CCC1)c1[nH]c(nc1)[C@H]1N(C	1312608-59-5	Carbamic acid, N-[(1S)-1-[[(2S)-2-[5-[4-[7-[2-[(2S)-1-[(2S)-2-[(methoxycarbonyl)	C43H52N8O6S	808.99	C	-8.2
8790	Cn1c2c(c(ccc2c2[nH]c(nc2)[C@@]2(C)N(C(=O)[C@@H](NC(=O)OC)C(C)C)CCC2)c2ccc(cc2)c2[nH]c(nc2)[C@H]2N(C(	1312608-75-5	Carbamic acid, N-[(1S)-1-[[(2S)-2-[5-[4-[7-[2-[(2S)-1-[(2S)-2-[(methoxycarbonyl)	C43H53N9O6	791.94	C	-8.2
8791	O(P(=O)(OC[C@H]1O[C@H]([C@H](O)[C@@H]1O)n1nc(C(=O)N)nc1)O)[C@H]1[C@@H](O[C@H](CO)[C@H]1O)n1nc(C(=O)N	1312805-79-0	Adenosine, 1,Ä≤-[3-(aminocarbonyl)-1H-1,2,4-triazol-1-yl]-1,Ä≤-de(6-amino-9H-pu	C16H23N8O12P	550.37	C	-8.2
8792	O(Cc1ccc(F)cc1)c1c2c(oc(c(O)c2=O)c2ncc(C)cn2)c(C)cc1	1312892-52-6	4H-1-Benzopyran-4-one, 5-[(4-fluorophenyl)methoxy]-3-hydroxy-8-methyl-2-(2-pyrim	C21H15FN2O4	378.35	C	-8.2
8793	O(Cc1ccc(F)cc1)c1c2c(oc(c(O)c2=O)c2ncc(OC)cn2)c(C)cc1	1312892-53-7	4H-1-Benzopyran-4-one, 5-[(4-fluorophenyl)methoxy]-3-hydroxy-8-methyl-2-(5-methy	C22H17FN2O4	392.38	C	-8.2
8794	O(Cc1ccc(F)cc1)c1c2c(oc(c(O)c2=O)c2ncc(cn2)c2ccccc2)c(C)cc1	1312892-55-9	4H-1-Benzopyran-4-one, 2-(5-chloro-2-pyrimidinyl)-5-[(4-fluorophenyl)methoxy]-3-	C21H14ClFN2O4	412.8	C	-8.2
8795	O(Cc1ccc(F)cc1)c1c2c(oc(c(O)c2=O)c2[nH]c(OC)nn2)c(C)cc1	1312892-63-9	4H-1-Benzopyran-4-one, 5-[(4-fluorophenyl)methoxy]-3-hydroxy-8-methyl-2-(3-methy	C20H16FN3O4	381.36	C	-8.2
8796	O(Cc1ccc(F)cc1)c1c2c(oc(c(O)c2=O)c2[nH]c(OC)cn2)c(C)cc1	1312892-75-3	4H-1-Benzopyran-4-one, 5-[(4-fluorophenyl)methoxy]-3-hydroxy-8-methyl-2-(5-methy	C21H17FN2O4	380.37	C	-8.2
8797	O(Cc1ccc(F)cc1)c1c2c(oc(c(O)c2=O)c2[nH]c(cn2)c2ccccc2)c(C)cc1	1312892-81-1	4H-1-Benzopyran-4-one, 2-(5-chloro-1H-imidazol-2-yl)-5-[(4-fluorophenyl)methoxy]	C20H14ClFN2O4	400.79	C	-8.2
8798	O(Cc1ccc(F)cc1)c1c2c(oc(c(O)c2=O)c2[nH]c(C)nn2)c(OC)cc1	1312893-43-8	4H-1-Benzopyran-4-one, 5-[(4-fluorophenyl)methoxy]-3-hydroxy-8-methoxy-2-(1H-1,2	C19H14FN3O5	383.33	C	-8.2
8799	O(Cc1ccc(F)cc1)c1c2c(c(cc1)c1ccccc1)oc(c(O)c2=O)c1ncc(C)cn1	1312894-33-9	4H-1-Benzopyran-4-one, 5-[(4-fluorophenyl)methoxy]-3-hydroxy-8-phenyl-2-(2-pyrim	C26H17FN2O4	440.42	C	-8.2
8800	O(Cc1ccc(F)cc1)c1c2c(oc(c(O)c2=O)c2[nH]c(nn2)c2ccccc2)cc(C)c1	1312894-96-4	4H-1-Benzopyran-4-one, 2-(5-chloro-1H-1,2,4-triazol-3-yl)-5-[(4-fluorophenyl)met	C19H13ClFN3O4	401.78	C	-8.2
8801	O(Cc1ccc(F)cc1)c1c2c(oc(c(O)c2=O)c2ncc(C)cn2)c(C)c(C)c1	1312895-33-2	4H-1-Benzopyran-4-one, 5-[(4-fluorophenyl)methoxy]-3-hydroxy-7,8-dimethyl-2-(2-p	C22H17FN2O4	392.38	C	-8.2
8802	O(Cc1ccc(F)cc1)c1c2c(c(OC)c(C)c1)oc(c(O)c2=O)c1ncc(C)cn1	1312895-83-2	4H-1-Benzopyran-4-one, 5-[(4-fluorophenyl)methoxy]-3-hydroxy-8-methoxy-7-methyl-	C22H17FN2O5	408.38	C	-8.2
8803	O(Cc1ccc(F)cc1)c1c2c(c(OC)c(C)c1)oc(c(O)c2=O)c1[nH]c(C)cn1	1312895-98-9	4H-1-Benzopyran-4-one, 5-[(4-fluorophenyl)methoxy]-3-hydroxy-2-(1H-imidazol-2-yl	C21H17FN2O5	396.37	C	-8.2
8804	O(Cc1ccc(F)cc1)c1c2c(oc(c(O)c2=O)c2ncc(C)cn2)c(Cl)c(C)c1	1312896-33-5	4H-1-Benzopyran-4-one, 8-chloro-5-[(4-fluorophenyl)methoxy]-3-hydroxy-7-methyl-2	C21H14ClFN2O4	412.8	C	-8.2
8805	O(Cc1ccc(F)cc1)c1c2c(oc(c(O)c2=O)c2[nH]c(C)cn2)c(Cl)c(C)c1	1312896-48-2	4H-1-Benzopyran-4-one, 8-chloro-5-[(4-fluorophenyl)methoxy]-3-hydroxy-2-(1H-imic	C20H14ClFN2O4	400.79	C	-8.2
8806	O(Cc1ccc(F)cc1)c1c2c(oc(c(O)c2=O)c2[nH]c(OC)cn2)c(Cl)c(C)c1	1312896-49-3	4H-1-Benzopyran-4-one, 8-chloro-5-[(4-fluorophenyl)methoxy]-3-hydroxy-7-methyl-2	C21H16ClFN2O4	414.81	C	-8.2
8807	O(Cc1ccc(F)cc1)c1c2c(c(C)c1)c1ccccc1)oc(c(O)c2=O)c1ncc(cn1)c1ccccc1	1312896-86-8	4H-1-Benzopyran-4-one, 2-(5-chloro-2-pyrimidinyl)-5-[(4-fluorophenyl)methoxy]-3-	C27H18ClFN2O4	488.89	C	-8.2
8808	O(C)c1c(c2c(c(OCc3ccc(F)cc3)c1)c(=O)c(O)c(o2)c1ncc(Cl)cn1)c1ccccc1	1312899-47-0	4H-1-Benzopyran-4-one, 5-[(4-fluorophenyl)methoxy]-3-hydroxy-7-methoxy-2-(5-meth	C28H21FN2O6	500.47	C	-8.2
8809	O(Cc1ccc(F)cc1)c1c2c(oc(c(O)c2=O)c2nnc(Cl)[nH]2)cc(Cl)c1	1312900-66-5	4H-1-Benzopyran-4-one, 7-chloro-5-[(4-fluorophenyl)methoxy]-3-hydroxy-2-(5-methc	C19H13ClFN3O5	417.77	C	-8.2
8810	O(Cc1ccc(F)cc1)c1c2c(oc(c(O)c2=O)c2ncc(C)cn2)c(C)c(Cl)c1	1312901-04-4	4H-1-Benzopyran-4-one, 7-chloro-5-[(4-fluorophenyl)methoxy]-3-hydroxy-8-methyl-2	C21H14ClFN2O4	412.8	C	-8.2
8811	O(Cc1ccc(F)cc1)c1c2c(oc(c(O)c2=O)c2[nH]c(nn2)c2ccccc2)c(C)c(Cl)c1	1312901-17-9	4H-1-Benzopyran-4-one, 7-chloro-2-(5-chloro-1H-1,2,4-triazol-3-yl)-5-[(4-fluorop	C19H12Cl2FN3O4	436.22	C	-8.2
8812	O(Cc1ccc(F)cc1)c1c2c(oc(c(O)c2=O)c2[nH]c(C)cn2)c(C)c(Cl)c1	1312901-19-1	4H-1-Benzopyran-4-one, 7-chloro-5-[(4-fluorophenyl)methoxy]-3-hydroxy-2-(1H-imic	C20H14ClFN2O4	400.79	C	-8.2
8813	O(Cc1ccc(F)cc1)c1c2c(oc(c(O)c2=O)c2ncc(C)cn2)c(Cl)c(Cl)c1	1312902-04-7	4H-1-Benzopyran-4-one, 7,8-dichloro-5-[(4-fluorophenyl)methoxy]-3-hydroxy-2-(2-p	C20H11Cl2FN2O4	433.22	C	-8.2
8814	O(Cc1ccc(F)cc1)c1c2c(oc(c(O)c2=O)c2[nH]c(C)cn2)c(Cl)c(Cl)c1	1312902-19-4	4H-1-Benzopyran-4-one, 7,8-dichloro-5-[(4-fluorophenyl)methoxy]-3-hydroxy-2-(1H-	C19H11Cl2FN2O4	421.21	C	-8.2
8815	O(Cc1ccc(F)cc1)c1c2c(c(C)c(c1)c1ccccc1)oc(c(O)c2=O)c1[nH]c(Cl)cn1	1312903-74-4	4H-1-Benzopyran-4-one, 5-[(4-fluorophenyl)methoxy]-3-hydroxy-2-(5-methoxy-1H-imi	C27H21FN2O5	472.46	C	-8.2
8816	O(C)c1c2c(c(OCc3ccc(F)cc3)cc1c1ccccc1)c(=O)c(O)c(o2)c1ncc(OC)cn1	1312904-26-9	4H-1-Benzopyran-4-one, 5-[(4-fluorophenyl)methoxy]-3-hydroxy-8-methoxy-2-(5-meth	C28H21FN2O5	484.48	C	-8.2
8817	O(C)c1c2c(c(OCc3ccc(F)cc3)cc1c1ccccc1)c(=O)c(O)c(o2)c1[nH]c(nn1)c1ccccc1	1312904-46-3	4H-1-Benzopyran-4-one, 2-(5-chloro-1H-1,2,4-triazol-3-yl)-5-[(4-fluorophenyl)met	C25H17ClFN3O5	493.87	C	-8.2
8818	O(C)c1c2c(c(OCc3ccc(F)cc3)cc1c1ccccc1)c(=O)c(O)c(o2)c1[nH]c(OC)cn1	1312904-51-0	4H-1-Benzopyran-4-one, 5-[(4-fluorophenyl)methoxy]-3-hydroxy-8-methoxy-2-(5-meth	C27H21FN2O5	472.46	C	-8.2
8819	O(C)c1c2c(c(OCc3ccc(F)cc3)cc1c1ccccc1)c(=O)c(O)c(o2)c1[nH]c(cn1)c1ccccc1	1312904-53-2	4H-1-Benzopyran-4-one, 2-(5-chloro-1H-imidazol-2-yl)-5-[(4-fluorophenyl)methoxy]	C26H18ClFN2O5	492.88	C	-8.2
8820	Clc1c2c(c(OCc3ccc(F)cc3)cc1c1ccccc1)c(=O)c(O)c(o2)c1ncc(Cl)cn1	1312905-06-8	4H-1-Benzopyran-4-one, 8-chloro-5-[(4-fluorophenyl)methoxy]-3-hydroxy-2-(5-methc	C27H18ClFN2O5	504.89	C	-8.2
8821	O(Cc1ccc(F)cc1)c1c2c(c(Cl)c(c1)c1ccccc1)oc(c(O)c2=O)c1[nH]c(OC)cn1	1312905-28-4	4H-1-Benzopyran-4-one, 8-chloro-5-[(4-fluorophenyl)methoxy]-3-hydroxy-2-(5-methy	C26H18ClFN2O4	476.88	C	-8.2
8822	O=c1c2c(oc(c1O)c1ncc(C)cn1)ccc(OCc1ccc(F)cc1)c2	1312906-15-2	4H-1-Benzopyran-4-one, 6-[(4-fluorophenyl)methoxy]-3-hydroxy-2-(2-pyrimidinyl)-	C20H13FN2O4	364.33	C	-8.2
8823	Oc1c(oc2c(c1=O)cc(OCc1ccc(F)cc1)cc2)c1[nH]c(OC)nn1	1312906-25-4	4H-1-Benzopyran-4-one, 6-[(4-fluorophenyl)methoxy]-3-hydroxy-2-(3-methyl-1H-1,2,	C19H14FN3O4	367.33	C	-8.2
8824	Cc1c2c(cc(OCc3ccc(F)cc3)c1)c(=O)c(O)c(o2)c1ncc(OC)cn1	1312906-65-2	4H-1-Benzopyran-4-one, 6-[(4-fluorophenyl)methoxy]-3-hydroxy-8-methyl-2-(5-methy	C22H17FN2O4	392.38	C	-8.2
8825	O=c1c2c(oc(c1O)c1ncc(Cl)cn1)c(C)cc(OCc1ccc(F)cc1)c2	1312906-66-3	4H-1-Benzopyran-4-one, 6-[(4-fluorophenyl)methoxy]-3-hydroxy-2-(5-methoxy-2-pyri	C22H17FN2O5	408.38	C	-8.2
8826	O=c1c2c(oc(c1O)c1[nH]cc(C)n1)c(C)cc(OCc1ccc(F)cc1)c2	1312906-79-8	4H-1-Benzopyran-4-one, 6-[(4-fluorophenyl)methoxy]-3-hydroxy-2-(1H-imidazol-2-yl	C20H15FN2O4	366.34	C	-8.2
8827	Cc1c2c(cc(OCc3ccc(F)cc3)c1)c(=O)c(O)c(o2)c1[nH]c(OC)cn1	1312906-80-1	4H-1-Benzopyran-4-one, 6-[(4-fluorophenyl)methoxy]-3-hydroxy-8-methyl-2-(5-methy	C21H17FN2O4	380.37	C	-8.2
8828	O=c1c2c(oc(c1O)c1[nH]cc(C)n1)c(Cl)cc(OCc1ccc(F)cc1)c2	1312907-79-1	4H-1-Benzopyran-4-one, 8-chloro-6-[(4-fluorophenyl)methoxy]-3-hydroxy-2-(1H-imic	C19H12ClFN2O4	386.76	C	-8.2
8829	O=c1c2c(oc(c1O)c1ncc(Cl)cn1)cc(C)c(OCc1ccc(F)cc1)c2	1312908-66-9	4H-1-Benzopyran-4-one, 6-[(4-fluorophenyl)methoxy]-3-hydroxy-2-(5-methoxy-2-pyri	C22H17FN2O5	408.38	C	-8.2
8830	O(C)c1c2c(cc(OCc3ccc(F)cc3)c1C)c(=O)c(O)c(o2)c1ncc(C)cn1	1312909-76-4	4H-1-Benzopyran-4-one, 6-[(4-fluorophenyl)methoxy]-3-hydroxy-8-methoxy-7-methyl-	C22H17FN2O5	408.38	C	-8.2
8831	Clc1c2c(cc(OCc3ccc(F)cc3)c1C)c(=O)c(O)c(o2)c1[nH]c(Cl)nn1	1312910-38-5	4H-1-Benzopyran-4-one, 8-chloro-6-[(4-fluorophenyl)methoxy]-3-hydroxy-2-(5-methc	C20H15ClFN3O5	431.8	C	-8.2
8832	Clc1c2c(cc(OCc3ccc(F)cc3)c1C)c(=O)c(O)c(o2)c1[nH]c(C)cn1	1312910-41-0	4H-1-Benzopyran-4-one, 8-chloro-6-[(4-fluorophenyl)methoxy]-3-hydroxy-2-(1H-imic	C20H14ClFN2O4	400.79	C	-8.2
8833	Clc1c2c(cc(OCc3ccc(F)cc3)c1C)c(=O)c(O)c(o2)c1[nH]c(Cl)cn1	1312910-43-2	4H-1-Benzopyran-4-one, 8-chloro-6-[(4-fluorophenyl)methoxy]-3-hydroxy-2-(5-methc	C21H16ClFN2O5	430.81	C	-8.2
8834	Cc1c(c2c(cc1OCc1ccc(F)cc1)c(=O)c(O)c(o2)c1ncc(Cl)cn1)c1ccccc1	1312910-78-3	4H-1-Benzopyran-4-one, 6-[(4-fluorophenyl)methoxy]-3-hydroxy-2-(5-methoxy-2-pyri	C28H21FN2O5	484.48	C	-8.2
8835	O=c1c2c(oc(c1O)c1ncc(C)cn1)cc(OC)c(OCc1ccc(F)cc1)c2	1312911-26-4	4H-1-Benzopyran-4-one, 6-[(4-fluorophenyl)methoxy]-3-hydroxy-7-methoxy-2-(2-pyri	C21H15FN2O5	394.35	C	-8.2
8836	O=c1c2c(oc(c1O)c1nnc(C)[nH]1)cc(OC)c(OCc1ccc(F)cc1)c2	1312911-36-6	4H-1-Benzopyran-4-one, 6-[(4-fluorophenyl)methoxy]-3-hydroxy-7-methoxy-2-(1H-1,2	C19H14FN3O5	383.33	C	-8.2
8837	O=c1c2c(oc(c1O)c1[nH]c(OC)cn1)cc(OC)c(OCc1ccc(F)cc1)c2	1312911-42-4	4H-1-Benzopyran-4-one, 6-[(4-fluorophenyl)methoxy]-3-hydroxy-7-methoxy-2-(5-meth	C21H17FN2O5	396.37	C	-8.2
8838	O=c1c2c(oc(c1O)c1[nH]cc(Cl)n1)cc(OC)c(OCc1ccc(F)cc1)c2	1312911-43-5	4H-1-Benzopyran-4-one, 6-[(4-fluorophenyl)methoxy]-3-hydroxy-7-methoxy-2-(5-meth	C21H17FN2O6	412.37	C	-8.2
8839	O=c1c2c(oc(c1O)c1nnc(Cl)[nH]1)cc(Cl)c(OCc1ccc(F)cc1)c2	1312911-78-6	4H-1-Benzopyran-4-one, 7-chloro-6-[(4-fluorophenyl)methoxy]-3-hydroxy-2-(5-methc	C19H13ClFN3O5	417.77	C	-8.2
8840	O=c1c2c(oc(c1O)c1[nH]cc(Cl)n1)cc(Cl)c(OCc1ccc(F)cc1)c2	1312911-88-8	4H-1-Benzopyran-4-one, 7-chloro-6-[(4-fluorophenyl)methoxy]-3-hydroxy-2-(5-methc	C20H14ClFN2O5	416.79	C	-8.2
8841	Cc1c2c(cc(OCc3ccc(F)cc3)c1Cl)c(=O)c(O)c(o2)c1ncc(cn1)c1ccccc1	1312912-61-0	4H-1-Benzopyran-4-one, 7-chloro-2-(5-chloro-2-pyrimidinyl)-6-[(4-fluorophenyl)me	C21H13Cl2FN2O4	447.24	C	-8.2
8842	Cc1c2c(cc(OCc3ccc(F)cc3)c1Cl)c(=O)c(O)c(o2)c1[nH]c(Cl)nn1	1312912-79-0	4H-1-Benzopyran-4-one, 7-chloro-6-[(4-fluorophenyl)methoxy]-3-hydroxy-2-(5-methc	C20H15ClFN3O5	431.8	C	-8.2
8843	O(C)c1c2c(cc(OCc3ccc(F)cc3)c1OC)c(=O)c(O)c(o2)c1nnc(C)[nH]1	1312913-30-6	4H-1-Benzopyran-4-one, 6-[(4-fluorophenyl)methoxy]-3-hydroxy-7,8-dimethoxy-2-(1H	C20H16FN3O6	413.36	C	-8.2
8844	O(C)c1c2c(cc(OCc3ccc(F)cc3)c1Cl)c(=O)c(O)c(o2)c1ncc(C)cn1	1312913-55-5	4H-1-Benzopyran-4-one, 7-chloro-6-[(4-fluorophenyl)methoxy]-3-hydroxy-8-methoxy-	C21H14ClFN2O5	428.8	C	-8.2
8845	Clc1c2c(cc(OCc3ccc(F)cc3)c1OC)c(=O)c(O)c(o2)c1ncc(C)cn1	1312914-10-5	4H-1-Benzopyran-4-one, 8-chloro-6-[(4-fluorophenyl)methoxy]-3-hydroxy-7-methoxy-	C21H14ClFN2O5	428.8	C	-8.2
8846	Clc1c2c(cc(OCc3ccc(F)cc3)c1Cl)c(=O)c(O)c(o2)c1ncc(OC)cn1	1312914-57-0	4H-1-Benzopyran-4-one, 7,8-dichloro-6-[(4-fluorophenyl)methoxy]-3-hydroxy-2-(5-m	C21H13Cl2FN2O4	447.24	C	-8.2
8847	Clc1c2c(cc(OCc3ccc(F)cc3)c1Cl)c(=O)c(O)c(o2)c1[nH]c(OC)nn1	1312914-77-4	4H-1-Benzopyran-4-one, 7,8-dichloro-6-[(4-fluorophenyl)methoxy]-3-hydroxy-2-(3-m	C19H12Cl2FN3O4	436.22	C	-8.2
8848	Clc1c2c(cc(OCc3ccc(F)cc3)c1Cl)c(=O)c(O)c(o2)c1[nH]c(nn1)c1ccccc1	1312914-81-0	4H-1-Benzopyran-4-one, 7,8-dichloro-2-(5-chloro-1H-1,2,4-triazol-3-yl)-6-[(4-flu	C18H9Cl3FN3O4	456.64	C	-8.2
8849	Clc1c(c2c(cc1OCc1ccc(F)cc1)c(=O)c(O)c(o2)c1ncc(cn1)c1ccccc1)c1ccccc1	1312915-66-4	4H-1-Benzopyran-4-one, 7-chloro-2-(5-chloro-2-pyrimidinyl)-6-[(4-fluorophenyl)me	C26H15Cl2FN2O4	509.31	C	-8.2
8850	Cc1c2c(cc(OCc3ccc(F)cc3)c1c1ccccc1)c(=O)c(O)c(o2)c1ncc(C)cn1	1312916-82-7	4H-1-Benzopyran-4-one, 6-[(4-fluorophenyl)methoxy]-3-hydroxy-8-methyl-7-phenyl-2	C27H19FN2O4	454.45	C	-8.2
8851	O(Cc1ccc(F)cc1)c1cc(c(C)c2c(c1)c(=O)c(O)c(o2)c1[nH]c(OC)nn1)c1ccccc1	1312916-93-0	4H-1-Benzopyran-4-one, 6-[(4-fluorophenyl)methoxy]-3-hydroxy-8-methyl-2-(3-methy	C26H20FN3O4	457.45	C	-8.2
8852	Cc1c2c(cc(OCc3ccc(F)cc3)c1c1ccccc1)c(=O)c(O)c(o2)c1[nH]c(nn1)c1ccccc1	1312916-95-2	4H-1-Benzopyran-4-one, 2-(5-chloro-1H-1,2,4-triazol-3-yl)-6-[(4-fluorophenyl)met	C25H17ClFN3O4	477.87	C	-8.2
8853	O(Cc1ccc(F)cc1)c1cc(c(C)c2c(c1)c(=O)c(O)c(o2)c1[nH]c(OC)cn1)c1ccccc1	1312916-98-5	4H-1-Benzopyran-4-one, 6-[(4-fluorophenyl)methoxy]-3-hydroxy-8-methyl-2-(5-methy	C27H21FN2O4	456.46	C	-8.2
8854	O(Cc1ccc(F)cc1)c1cc(c(C)c2c(c1)c(=O)c(O)c(o2)c1[nH]c(Cl)cn1)c1ccccc1	1312916-99-6	4H-1-Benzopyran-4-one, 6-[(4-fluorophenyl)methoxy]-3-hydroxy-2-(5-methoxy-1H-imi	C27H21FN2O5	472.46	C	-8.2
8855	Cc1c2c(cc(OCc3ccc(F)cc3)c1c1ccccc1)c(=O)c(O)c(o2)c1[nH]c(cn1)c1ccccc1	1312917-00-2	4H-1-Benzopyran-4-one, 2-(5-chloro-1H-imidazol-2-yl)-6-[(4-fluorophenyl)methoxy]	C26H18ClFN2O4	476.88	C	-8.2
8856	O(Cc1ccc(F)cc1)c1cc(c(Cl)c2c(c1)c(=O)c(O)c(o2)c1ncc(OC)cn1)c1ccccc1	1312917-83-1	4H-1-Benzopyran-4-one, 8-chloro-6-[(4-fluorophenyl)methoxy]-3-hydroxy-2-(5-methy	C27H18ClFN2O4	488.89	C	-8.2
8857	O(Cc1ccc(F)cc1)c1cc(c(Cl)c2c(c1)c(=O)c(O)c(o2)c1[nH]c(Cl)nn1)c1ccccc1	1312917-94-4	4H-1-Benzopyran-4-one, 8-chloro-6-[(4-fluorophenyl)methoxy]-3-hydroxy-2-(5-methc	C25H17ClFN3O5	493.87	C	-8.2
8858	Clc1c2c(cc(OCc3ccc(F)cc3)c1c1ccccc1)c(=O)c(O)c(o2)c1[nH]c(nn1)c1ccccc1	1312917-95-5	4H-1-Benzopyran-4-one, 8-chloro-2-(5-chloro-1H-1,2,4-triazol-3-yl)-6-[(4-fluorop	C24H14Cl2FN3O4	498.29	C	-8.2
8859	O(Cc1ccc(F)cc1)c1cc(c(Cl)c2c(c1)c(=O)c(O)c(o2)c1[nH]c(OC)cn1)c1ccccc1	1312917-98-8	4H-1-Benzopyran-4-one, 8-chloro-6-[(4-fluorophenyl)methoxy]-3-hydroxy-2-(5-methy	C26H18ClFN2O4	476.88	C	-8.2
8860	O(Cc1ccc(F)cc1)c1cc(c(c2c(c1)c(=O)c(O)c(o2)c1ncc(C)cn1)c1ccccc1)c1ccccc1	1312918-32-3	4H-1-Benzopyran-4-one, 6-[(4-fluorophenyl)methoxy]-3-hydroxy-7,8-diphenyl-2-(2-p	C32H21FN2O4	516.52	C	-8.2
8861	C(O)[C@@H]1[C@H](C[C@@H](O1)n1c(=O)[nH]c(=O)c(C)c1)n1cc(CN=[N+]=[N-])nn1	1313031-73-0	Uridine, 5,Ä≤-O-[(2S)-6,8-bis(1,1-dimethylethyl)-5-fluoro-2-oxido-4H-1,3,2-benzc	C24H31FIN2O8P	652.39	C	-8.2
8862	C(OC(=O)CCC(=O)OC[C@H]1O[C@H](C=C1)n1c(=O)[nH]c(=O)c(C)c1)(C)c1c(non1=O)c1ccccc1	1314187-78-4	Uridine, 5-iodo-, 5,Ä≤-[4-[1-(2-oxido-4-phenyl-1,2,5-oxadiazol-3-yl)ethyl] butar	C23H23IN4O11	658.35	C	-8.2
8863	[C@@H](NC(=O)c1ccccc1)(C(=O)N[C@H](C(=O)N[C@H](C(=O)C(=O)NCc1ccc(S(=O)(=O)N)cc1)C(C)C)C)C12CC3CC(C2)	1318775-18-6	L-Alaninamide, (2S)-N-benzoyl-2-tricyclo[3.3.1.13,7]dec-1-ylglycyl-N-[(1S)-1-(1-	C35H44N4O5	600.75	C	-8.2
8864	S(Cc1cc2c(oc1=O)cccc2)c1n(c2c(n1)c(=O)nc(N)[nH]2)[C@@H]1O[C@H](CO)[C@@H](O)[C@H]1O	1328839-28-6	1,3,4-Oxadiazole-2-carboxylic acid, 5-methyl-, 4-[[[(4-fluorophenyl)methyl]aminc	C24H23FN8O7	554.49	C	-8.2
8865	N(Cc1cc(Cl)ccc1)c1nc(nc(NN=Cc2c(O)cccc2)n1)N1CCOCC1	1331749-50-8	Benzaldehyde, 2-hydroxy-, 2-[4-[[(4-fluorophenyl)methyl]amino]-6-(4-morpholinyl)	C21H22FN7O2	423.44	C	-8.2
8866	N(c1nc(nc(NN=Cc2c(C)[nH]cc2)n1)N1CCOCC1)c1ccc(F)cc1	1331749-52-0	Benzaldehyde, 2-hydroxy-, 2-[4-[[(3-chlorophenyl)methyl]amino]-6-(4-morpholinyl)	C21H22ClN7O2	439.9	C	-8.2
8867	N(c1c(C([2H])([2H])[2H])c(c(/C=C/C#N)c(c1C([2H])([2H])[2H])[2H])[2H])c1nc(Nc2c(cc(C#N)cc2[2H])[2H])n	1331866-44-4	Benzonitrile-3,5-d2, 4-[[4-[[4-[(1E)-2-cyanoethenyl]-2-methyl-6-(methyl-d3)pheny	C22H11D7N6		C	-8.2
8868	N(c1c(C)c(c(/C=C/C#N)c(c1C)[2H])[2H])c1nc(Nc2c(cc(C#N)cc2[2H])[2H])nc(c1[2H])[2H]	1331866-45-5	Benzonitrile-3,5-d2, 4-[[4-[[4-[(1E)-2-cyanoethenyl]-2,6-di(methyl-d3)phenyl-3,5	C22H8D10N6		C	-8.2
8869	N(c1c(C([2H])([2H])[2H])c(c(/C=C/C#N)c(c1C([2H])([2H])[2H])[2H])[2H])c1nc(Nc2c(c(c(C#N)c(c2[2H])[2H]	1331866-56-8	Benzonitrile-2,3,5,6-d4, 4-[[4-[[4-[(1E)-2-cyanoethenyl]-2-methyl-6-(methyl-d3)p	C22H9D9N6		C	-8.2
8870	N(c1c(C([2H])([2H])[2H])c(c(/C=C/C#N)c(c1C)[2H])[2H])c1nc(Nc2c(c(c(C#N)c(c2[2H])[2H])[2H])[2H])nc(c1	1331866-58-0	Benzonitrile-2,3,5,6-d4, 4-[[6-[[4-[(1E)-2-cyanoethenyl]-2,6-dimethylphenyl-3,5-	C22H10D8N6		C	-8.2
8871	N(c1c(C([2H])([2H])[2H])c(c(/C(=C(/C#N)\[2H])/[2H])c(c1C)[2H])[2H])c1nc(Nc2ccc(C#N)cc2)nc(c1[2H])[2H	1331866-63-7	Benzonitrile, 4-[[6-[[4-[(1E)-2-cyanoethenyl-1,2-d2]-2,6-dimethylphenyl-3,5-d2]a	C22H12D6N6		C	-8.2
8872	N(c1c(C([2H])([2H])[2H])c(c(/C(=C(/C#N)\[2H])/[2H])c(c1C([2H])([2H])[2H])[2H])[2H])c1nc(Nc2c(c(c(C#N	1331866-83-1	Benzonitrile-2,3,5,6-d4, 4-[[4-[[4-[(1E)-2-cyanoethenyl-1,2-d2]-2-methyl-6-(meth	C22H7D11N6		C	-8.2
8873	N(c1c(C)c(c(/C(=C(/C#N)\[2H])/[2H])c(c1C)[2H])[2H])c1nc(Nc2c(c(c(C#N)c(c2[2H])[2H])[2H])[2H])nc(c1[2	1331866-84-2	Benzonitrile-2,3,5,6-d4, 4-[[4-[[4-[(1E)-2-cyanoethenyl-1,2-d2]-2,6-di(methyl-d3	C22H4D14N6		C	-8.2
8874	N(c1c(C([2H])([2H])[2H])cc(/C=C/C#N)cc1C)c1nc(Nc2ccc(C#N)cc2)nc(c1[2H])[2H]	1331866-88-6	Benzonitrile, 4-[[6-[[4-[(1E)-2-cyanoethenyl]-2,6-dimethylphenyl]amino]-2-pyrimi	C22H16D2N6		C	-8.2
8875	N(c1c(C([2H])([2H])[2H])cc(/C=C/C#N)cc1C([2H])([2H])[2H])c1nc(Nc2cc(c(C#N)c(c2)[2H])[2H])ncc1	1331866-98-8	Benzonitrile-2,6-d2, 4-[[4-[[4-[(1E)-2-cyanoethenyl]-2-methyl-6-(methyl-d3)pheny	C22H13D5N6		C	-8.2
8876	N(c1c(C([2H])([2H])[2H])cc(/C=C/C#N)cc1C([2H])([2H])[2H])c1nc(Nc2cc(c(C#N)c(c2)[2H])[2H])nc(c1[2H])[	1331867-01-6	Benzonitrile-2,6-d2, 4-[[6-[[4-[(1E)-2-cyanoethenyl]-2-methyl-6-(methyl-d3)pheny	C22H11D7N6		C	-8.2
8877	N(c1c(C([2H])([2H])[2H])cc(/C=C/C#N)cc1C)c1nc(Nc2c(c(c(C#N)c(c2[2H])[2H])[2H])[2H])nc(c1[2H])[2H]	1331867-06-1	Benzonitrile-2,3,5,6-d4, 4-[[6-[[4-[(1E)-2-cyanoethenyl]-2,6-dimethylphenyl]amir	C22H12D6N6		C	-8.2
8878	N(c1c(C([2H])([2H])[2H])cc(/C(=C(/C#N)\[2H])/[2H])cc1C([2H])([2H])[2H])c1nc(Nc2c(cc(C#N)cc2[2H])[2H]	1331867-18-5	Benzonitrile-3,5-d2, 4-[[6-[[4-[(1E)-2-cyanoethenyl-1,2-d2]-2-methyl-6-(methyl-c	C22H9D9N6		C	-8.2
8879	C([C@@H]([C@@H](CN(S(=O)(=O)c1ccc(OC)cc1)CC(C)C)O)NC(=O)O[C@H]1CCOC1)c1ccc(cc1)c1ccsc1	1335386-69-0	Carbamic acid, N-[(1S,2R)-1-[(2,Ä≤-fluoro[1,1,Ä≤-biphenyl]-4-yl)methyl]-2-hydro:	C32H39FN2O7S	614.72	C	-8.2
8880	C(=O)([C@@H](NC(=O)OC)C(C)C)N1CC2(C[C@H]1c1[nH]c(cn1)c1ccc(C#Cc3ccc(cc3)c3[nH]c(nc3)[C@@H]3N(C(=O)[C	1337532-52-1	Carbamic acid, N,N,Ä≤-[1,2-ethynediylbis[4,1-phenylene-1H-imidazole-5,2-diyl-(6S	C46H54N8O6	814.97	C	-8.2
8881	C(=O)([C@@H](NC(=O)OC)C(C)C)N1[C@@H](C=C(C)C1)c1[nH]c(cn1)c1ccc(C#Cc2ccc(cc2)c2[nH]c(nc2)[C@@H]2N(C(	1337532-70-3	Carbamic acid, N,N,Ä≤-[1,2-ethynediylbis[4,1-phenylene-1H-imidazole-5,2-diyl[(2F	C44H50N8O6	786.92	C	-8.2
8882	C(=O)([C@@H](NC(=O)OC)C(C)C)N1C(C=C(C)C1)c1[nH]c(cn1)c1ccc(C#Cc2ccc(cc2)c2[nH]c(nc2)C2N(C(=O)[C@@H](	1337532-76-9	Carbamic acid, N,N,Ä≤-[1,2-ethynediylbis[4,1-phenylene-1H-imidazole-5,2-diyl[(2S	C44H50N8O6	786.92	C	-8.2
8883	N(C(=O)C)[C@@H]1[C@H](OC(CC)CC)C=C(C(=O)OC(OC(=O)[C@@H](C(C)C)N)c2ccccc2)C[C@@H]1NC(=N)N	1338231-81-4	2H-Isoindole-2-acetic acid, 1,3-dihydro-Œ±-(1-methylethyl)-1,3-dioxo-, [[[(3R,4F	C45H59N5O12	861.98	C	-8.2
8884	O=c1n([C@@H]2O[C@H](COP(=O)(O)O)[C@@H](O)C2)cc(F)c(=O)[nH]1	1339955-42-8	Benzenepropanamide, Œ±-amino-N-[(1S)-2-[(1S,3S,5S)-3-cyano-2-azabicyclo[3.1.0]he	C27H34N4O4	478.58	C	-8.2
8885	C(=O)(OC)c1c(CCCCCCCCCCCCCCC)cc(O)cc1O	1342797-87-8	Furo[3,Ä≤,4,Ä≤:6,7]naphtho[2,3-d]-1,3-dioxol-6(5aH)-one, 5,8,8a,9-tetrahydro-5-	C28H26O10S	554.57	C	-8.2
8886	[C@H](Cc1ccccc1)(C[C@@H]([C@H](Cc1ccccc1)NC(=O)OCc1cncs1)O)NC(=O)[C@H](NC(=O)N(Cc1nc(OC)sc1)C)C(C)C	1346847-45-7	Not Yet Assigned	C39H53N5O4S	687.93	C	-8.2
8887	O(C(=O)N[C@@H](Cc1ccccc1)[C@@H](CN(S(=O)(=O)c1cc(OCCC=C)ccc1)CCCCC=C)O)[C@@H]1[C@H]2[C@@H](OC1)OCC2	1346988-80-4	2,7,10,12-Tetraazatridecanoic acid, 13-[2-(cyclopentylmethyl)-4-thiazolyl]-4-hyc	C40H52N6O5S2	761.01	C	-8.2
8888	O(C(=O)N[C@@H](Cc1ccccc1)[C@@H](CN(S(=O)(=O)c1cc(OCCCC=C)ccc1)CCCCC=C)O)[C@@H]1[C@H]2[C@@H](OC1)OCC2	1347036-16-1	2,7,10,12-Tetraazatridecanoic acid, 13-(2-cyclohexyl-4-thiazolyl)-4-hydroxy-10-m	C40H52N6O5S2	761.01	C	-8.2
8889	C(C)(C)c1n([C@@H]2C[C@@H]3N(CCC(NC(=O)C4CCC(F)(F)CC4)c4ccccc4)C[C@@H](C2)CC3)c(C([2H])([2H])[2H])nn1	1347562-45-1	Acetamide, 2-butoxy-N-[[4,Ä≤-[(2-ethyl-5,7-dimethyl-3H-imidazo[4,5-b]pyridin-3-y	C34H43N5O4S	617.8	C	-8.2
8890	C(n1c2c(nc1CC)c(C)cc(C(=O)O)n2)c1ccc(cc1)c1c(S(=O)(=O)NC(=O)OCCCC)cccc1	1347625-83-5	Not Yet Assigned	C30H33D10F2N5O		C	-8.2
8891	C(n1c2c(nc1CC)c(C)cc(C)n2)c1ccc(cc1)c1c(S(=O)(=O)NC(=O)OC(C)(C)C)cccc1	1348027-53-1	Not Yet Assigned	C40H55N7O6	729.91	C	-8.2

8892	N(Cc1c(C(C)C)scn1)(C(=O)N[C@H](C(=O)N[C@@H](Cc1ccccc1)C[C@@H]([C@H](Cc1ccccc1)NC(=O)OCc1cncs1)O)C(C)	1348981-13-4	Benzamide, 2-ethoxy-N-[[4,Ä≤-[(2-ethyl-5,7-dimethyl-3H-imidazo[4,5-b]pyridin-3-y	C37H41N5O4S	651.82	C	-8.2
8893	C([C@H]([C@H](Cc1ccccc1)NC(=O)[C@@H](NC(=O)c1nc2c(cc1)cccc2)CC(=O)N)O)N1[C@H]([C@H](NC(C)(C)C)O)C[C@	1349059-15-9	Not Yet Assigned	C36H48N6O4	628.8	C	-8.2
8894	C([C@H](OCP(=O)(O)O)C)n1c2c([nH]c1=S)c(N)nc(N)n2	1349233-15-3	Phosphoramidic acid, N-[[4,Ä≤-[(2-ethyl-5,7-dimethyl-3H-imidazo[4,5-b]pyridin-3-	C41H45N4O5PS	736.86	C	-8.2
8895	O=c1n([C@H]2C[C@H](C#C)[C@@H](CO)O2)cc(C)c(=O)[nH]1	1349525-48-9	[1,1,Ä≤-Biphenyl]-2-sulfonamide, N-[(butylamino)carbonyl]-4,Ä≤-[(2-ethyl-5,7-di	C33H42N6O3S	602.79	C	-8.2
8896	[C@H](Cc1ccccc1)([C@@H]([C@@H]([C@H](Cc1cncs1)NC(=O)OCc1cncs1)O)O)NC(=O)[C@@H](NC(=O)N(Cc1nc(C(C)C)s	1349653-35-5	Carbamic acid, N-[[4,Ä≤-[(2-ethyl-5,7-dimethyl-3H-imidazo[4,5-b]pyridin-3-yl)met	C35H38N4O4S	610.77	C	-8.2
8897	C(=O)(N[C@H](C(=O)C(=O)NC1CC1)CCC)[C@@H]1[C@@H]2[C@H](CN1C(=O)[C@@H](NC(=O)[C@@H](NC(=O)c1ncccn1)C1C	1350075-96-5	Carbamic acid, N-[[4,Ä≤-[(2-ethyl-5,7-dimethyl-3H-imidazo[4,5-b]pyridin-3-yl)met	C32H40N4O4S	576.75	C	-8.2
8898	C(=O)([C@@H](NC(=O)OC)C(C)C)N1[C@@]2(C[C@H](C1)CC2)c1[nH]c(cn1)c1c2c(c(cc1)c1ccc(cc1)c1[nH]c(nc1)[C@	1350718-91-0	Carbamic acid, N,N,Ä≤-[[4,4,Ä≤-bibenzothiazole]-7,7,Ä≤-diylbis[1H-imidazole-5,2	C46H52N10O6S2	905.1	C	-8.2
8899	C(=O)([C@H](C(CC)CC)NC(=O)OC)N1[C@@H](CCC1)c1[nH]c(cn1)c1cc2c(cc1)cc(cc2)c1ccc(cc1)c1[nH]c(nc1)[C@H]	1351331-15-1	Carbamic acid, N-[(1S)-1-cyclopropyl-2-[(2S)-2-[5-[4-[6-[2-[(2S)-1-[(2S)-2-cyclo	C44H48N8O6	784.9	C	-8.2
8900	C(=O)([C@@H](NC(=O)OC)C1CCOCC1)N1C[C@@]2(C[C@H]1c1[nH]c(cn1)c1ccc(cc1)c1ccc(cc1)c1[nH]c(nc1)[C@H]1N(	1351851-16-5	Carbamic acid, N-[(1S)-1-[[(5S,8S)-8-[5-[4,Ä≤-[2-[(2S)-1-[(2S)-2-[(methoxycarbor	C46H58N8O8	851	C	-8.2
8901	C(=O)([C@@H](NC(=O)OC)CC)N1[C@@H](C[C@@H](C1)C1CC1)c1[nH]c(cn1)c1ccc(cc1)c1ccc(cc1)c1[nH]c(nc1)[C@H]	1351853-87-6	Carbamic acid, N-[2-[(2S)-2-[5-[4,Ä≤-[2-[(2S,4R)-4-cyclopropyl-1-[2-[(ethoxycarb	C41H50N8O6	750.89	C	-8.2
8902	C(=O)([C@H](NC(=O)OC)CC)N1[C@@H](C[C@@H](C1)C1CC1)c1[nH]c(cn1)c1ccc(cc1)c1ccc(cc1)c1[nH]c(nc1)[C@H]1	1351853-91-2	Carbamic acid, N-[(1S)-1-[[(2S)-2-[5-[4,Ä≤-[2-[(2S,4R)-4-cyclopropyl-1-[(2S)-2-	C41H50N8O6	750.89	C	-8.2
8903	C(=O)([C@@H](NC(=O)OC)C(C)(C)O)N1[C@@H](C[C@@H](C1)C1CC1)c1[nH]c(cn1)c1ccc(cc1)c1ccc(cc1)c1[nH]c(nc1	1351854-02-8	Carbamic acid, N-[(1S)-1-cyclopropyl-2-[(2S)-2-[5-[4,Ä≤-[2-[(2S,4R)-4-cyclopropy	C43H50N8O6	774.91	C	-8.2
8904	C(=O)([C@H](NC(=O)OC)C(C)C)N1[C@@H](C[C@@H](C1)C1CC1)c1[nH]c(cn1)c1ccc(cc1)c1ccc(cc1)c1[nH]c(nc1)[C@	1351854-03-9	Carbamic acid, N-[(1S)-1-[[(2S)-2-[5-[4,Ä≤-[2-[(2S,4R)-4-cyclopropyl-1-[(2S)-3-h	C43H54N8O8	810.94	C	-8.2
8905	C(=O)([C@@H](NC(=O)OC)C1CCOCC1)N1[C@@H](C[C@@H](C1)C1CC1)c1[nH]c(cn1)c1ccc(cc1)c1ccc(cc1)c1[nH]c(nc1	1351854-13-1	Carbamic acid, N-[(1S)-2-[(2S)-2-[5-[4,Ä≤-[2-[(2S,4R)-1-[(2S)-3-cyano-2-[(metho:	C41H44N10O6	772.85	C	-8.2
8906	C(=O)([C@@H](NC(=O)OC)C(C)C)N1[C@@H](C[C@@H](C1)C1CC1)c1[nH]c(cn1)c1ccc(cc1)c1ccc(cc1)c1[nH]c(nc1)[C	1351855-45-2	Carbamic acid, N-[(1S)-1-[[(2S,4R)-4-cyclopropyl-2-[5-[4,Ä≤-[2-[(2S)-4,4-difluo	C43H52F2N8O6	814.92	C	-8.2
8907	C(=O)([C@@H](NC(=O)OC)C(C)C)N1[C@@H](C[C@@H](C1)C1CC1)c1[nH]c(cn1)c1ccc(cc1)c1ccc(cc1)c1[nH]c(nc1)[C	1351855-47-4	Carbamic acid, N-[(1S)-1-[[(2S,4R)-4-cyclopropyl-2-[5-[4,Ä≤-[2-[(2S,4R)-4-fluor	C43H53FN8O6	796.93	C	-8.2
8908	C(=O)([C@@H](NC(=O)OC)C(C)C)N1[C@@H](C[C@@H](C1)C1CC1)c1[nH]c(cn1)c1c(C)cc(cc1)c1cc(C)c(cc1)c1[nH]c(	1351855-65-6	Carbamic acid, N-[(1S)-1-[[(2S)-2-[5-[4,Ä≤-[2-[(2S,4R)-4-cyclopropyl-1-[(2S)-2-	C43H50F4N8O6	850.9	C	-8.2
8909	C(=O)([C@@H](NC(=O)OC)C(C)C)N1[C@@H](C[C@@H](C1)C1CC1)c1[nH]c(cn1)c1ccc(cc1)c1ccc(cc1)c1[nH]c([C@@H]	1351855-91-8	Carbamic acid, N-[(1S)-1-[[(2S,4R)-4-cyclopropyl-2-[5-[4,Ä≤-[2-[(3S,6S)-1,1-dif]	C45H54F2N8O6	840.96	C	-8.2
8910	C(=O)([C@@H](NC(=O)OC)C(C)C)N1[C@@H](C[C@@H](C1)C1CC1)c1[nH]c(cn1)c1ccc(cc1)c1ccc(cc1)c1[nH]c([C@@H]	1351855-93-0	Carbamic acid, N-[(1S)-1-[[(2S,4R)-4-cyclopropyl-2-[5-[4,Ä≤-[2-[(S)-cyclopropyl	C44H56N8O6	792.97	C	-8.2
8911	C(=O)([C@@H](NC(=O)OC)C(C)C)N1[C@@H](CC2(C1)CCOCC2)c1[nH]c(cn1)c1ccc(cc1)c1ccc(cc1)c1[nH]c(nc1)[C@H]	1351856-04-6	Carbamic acid, N-[(1S)-1-[[(2S,4R)-4-cyclopropyl-2-[5-[4,Ä≤-[2-[(3S,5S)-2-[(2S)-	C47H60N8O7	849.03	C	-8.2
8912	C(=O)([C@@H](NC(=O)OC)C(C)C)N1[C@@H](C=CC1)c1[nH]c(cn1)c1cc2c(cc1)cc(C#Cc1[nH]c(nc1)[C@H]1N(C(=O)[C@	1352392-57-4	Carbamic acid, N-[(1S)-1-[[(2S)-4-cyclopropyl-2-[5-[4-[2-[4-[2-[(2S)-4-cycloprop	C48H54N8O6	838.99	C	-8.2
8913	C([C@H](NP(=O)(Oc1ccc(N(=O)=O)cc1)OC[C@H]1O[C@H]([C@H](O)[C@@H]1O)n1nc(C(=O)N)nc1)C(=O)OC)c1c2c([nH]	1353639-74-3	L-Proline, 1-[hydroxy(4-nitrophenoxy)phosphinyl]-, phenylmethyl ester, 5,Ä≤-este	C26H29N6O11P	632.52	C	-8.2
8914	Oc1c2c(cc(O)cc2cc2c1C(=O)c1c(C2(C)C)c(Cl)c(O)cc1O)c1c(C)cc(O)cc1O	1355359-12-4	Thymidine, 3,Ä≤-azido-3,Ä≤-deoxy-3-[(1E,3Z)-5-[1-[(4-fluorophenyl)methyl]-1H-1,	C24H23FN8O6		C	-8.2
8915	C(C1C(=O)N(N=C1C)c1ccccc1)(c1c(O)n(nc1C)c1ccccc1)c1cn(c(=O)[nH]c1=O)[C@H]1O[C@@H](CO)[C@H](O)C1	1356470-37-5	2,4(1H,3H)-Pyrimidinedione, 1-(3,5-di-O-acetyl-2-deoxy-Œ≤-L-erythro-pentofuranos	C34H34N6O9	670.67	C	-8.2
8916	C(=C\C(=O)c1cc(C(=O)C)c(O)cc1O)/c1c(OC)cc(OC)c(OC)c1	1357165-28-6	1-Naphthalenesulfonic acid, 4-amino-3-[2-[4-(methylthio)phenyl]diazenyl]-, sodiu	C17H15N3O3S2.Na		C	-8.2
8917	N(CCO)c1n(c2c(n1)c(=O)nc[nH]2)[C@@H]1O[C@H](CO)[C@@H](O)[C@H]1O	1358070-37-7	Cyclopenta[c]pyrrole-1-carboxamide, (2R)-2-cyclohexyl-N-(2-pyrazinylcarbonyl)gly	C36H53N7O6	679.85	C	-8.2
8918	C(F)(F)c1n(c2c(n1)cccc2)c1nc(nc(N[C@H]2CC[C@@](C)(O)CC2)n1)N1CCOCC1	1359952-51-4	Benzamide, N-[4-[2-(difluoromethyl)-1H-benzimidazol-1-yl]-6-(4-morpholinyl)-1,3,	C22H19F2N7O2	451.43	C	-8.2
8919	C(F)(F)c1n(c2c(n1)cccc2)c1nc(nc(NCC2CCCO2)n1)N1CCOCC1	1359952-70-7	Cyclohexanol, 4-[[4-[2-(difluoromethyl)-1H-benzimidazol-1-yl]-6-(4-morpholinyl)-	C22H27F2N7O2	459.49	C	-8.2
8920	C(F)(F)c1n(c2c(n1)cccc2)c1nc(nc(Nc2cc3c(cc2)nc[nH]3)n1)N1CCOCC1	1359953-76-6	1H-Benzimidazole, 2-(difluoromethyl)-1-[4-(4-morpholinyl)-6-(4-pyridinylthio)-1,	C20H17F2N7OS	441.46	C	-8.2
8921	C(F)(F)c1n(c2c(n1)cccc2)c1nc(nc(Nc2cc(OC)ccc2)n1)N1CCOCC1	1359956-23-2	1,3,5-Triazin-2-amine, 4-[2-(difluoromethyl)-1H-benzimidazol-1-yl]-N-(4-methoxyp	C22H21F2N7O2	453.44	C	-8.2
8922	C(=O)([C@@H](NC(=O)OC)C(C)C)N1[C@@H](C[C@@H](C1)n1cc(C(=O)OC)nn1)c1[nH]c(cn1)c1ccc(cc1)c1ccc(cc1)c1[	1361249-09-3	Carbamic acid, N-[(1S)-1-[[(2S)-2-(5-[1,1,Ä≤-biphenyl]-4-yl-1H-imidazol-2-yl)-1-	C26H30N4O3	446.54	C	-8.2
8923	C(=O)([C@@H](NC(=O)OC)C(C)C)N1[C@@H](C[C@H](C(F)(F)F)C1)c1[nH]c(c(Cl)n1)c1ccc(cc1)c1ccc(cc1)c1[nH]c(	1361249-40-2	Carbamic acid, N-[(1S)-1-[[(2S,4S)-2-[5-fluoro-4-[4,Ä≤-[2-[(2S)-1-[(2S)-2-[(met]	C41H48F4N8O6	824.86	C	-8.2
8924	C(=O)([C@@H](NC(=O)OC)C(C)C)N1[C@@H](C[C@H](SC)C1)c1[nH]c(c(F)n1)c1ccc(cc1)c1ccc(cc1)c1[nH]c(nc1F)[C	1361249-42-4	Carbamic acid, N-[(1S)-1-[[(2S,4S)-2-[5-[4,Ä≤-[5-chloro-2-[(2S)-1-[(2S)-2-[(met]	C41H48ClF3N8O6	841.32	C	-8.2
8925	O=c1n([C@H]2O[C@H](CO)[C@@H](Cl)C2)cc(C)c(=O)[nH]1	136135-07-4	Triphosphoric acid, P-[[3-(2-amino-1,6-dihydro-6-oxo-9H-purin-9-yl)-2-fluoro-4,5	C11H17FN5O13P3	539.2	C	-8.2
8926	O=c1n([C@@H]2O[C@H](COP(=O)(NCC(=O)OC)OCC)[C@@H](N=[N+]=[N-])C2)cc(C)c(=O)[nH]1	1361474-13-6	Guanosine, 5,Ä≤-[N-[2-(1H-indol-2-yl)ethyl]carbamate]	C21H23N7O6	469.45	C	-8.2
8927	N(C(C)C)c1c(nccc1)N1CCN(C(=O)c2cc3c([nH]2)ccc(NS(=O)(=O)C)c3)CC1	136817-57-7	Methanone, [4-[3-[(1-methylethyl)amino]-2-pyridinyl]-1-piperazinyl](5-nitro-1H-i	C21H24N6O3	408.45	C	-8.2
8928	C(=O)([C@@H](NC(=O)OC)C(C)C)N1[C@@H](C[C@H](OC)C1)c1[nH]c(c(Cl)n1)c1ccc(cc1)c1ccc(cc1)c1[nH]c(nc1)[C	1373165-67-3	Carbamic acid, N-[(1S)-1-[[(2S)-2-[5-chloro-4-[2-fluoro-4,Ä≤-[2-[(2S,4S)-1-[(2S)	C41H50ClFN8O6S	837.4	C	-8.2
8929	O=C1OC2(c3c1cc(cc3)N1C(=O)C(SCCN[C@@H]3[C@@H](NC(=O)C)[C@H](OC(CC)CC)C=C(C(=O)OCC)C3)CC1=O)c1c(Oc3c2	1373244-20-2	1-Cyclohexene-1-carboxylic acid, 4-(acetylamino)-5-[[[(3,Ä≤,6,Ä≤-dihydroxy-3-ox	C37H39N3O10	685.72	C	-8.2
8930	C(=O)([C@@H](NC(=O)OC)C(C)C)N1[C@@H](CCC1)c1[nH]c(cn1)c1ccc(cc1)c1ccc(ccc1)c1ccc(cc1)c1[nH]c(nc1)[C@H	1375881-13-2	Carbamic acid, N,N,Ä≤-[2,5-pyridinediylbis[4,1-phenylene-1H-imidazole-5,2-diyl-	C45H53N9O6	815.96	C	-8.2
8931	C(=O)([C@@H](NC(=O)OC)C(C)C)N1[C@H]2[C@H](C2)C[C@H]1c1[nH]c2c(n1)ccc1c2ccc(c1)c1cc(F)c(cc1)c1[nH]c(n	1377041-55-8	Carbamic acid, N-[(1S)-1-[[(1R,3S,5R)-3-[7-[3-fluoro-4-[2-[(1R,3S,4S)-2-[(2S)-2-	C45H51FN8O6	818.93	C	-8.2
8932	C(=O)([C@H](NC(=O)OC)c1ccccc1)N1[C@@H](CCC1)c1[nH]c(cn1)c1ccc(cc1)c1c(C#CC)cc(cc1)c1[nH]c(nc1)[C@H]1	1377042-35-7	Carbamic acid, N-[(1S)-2-[(2S)-2-[5-[4,Ä≤-[2-[(2S)-1-[(2S)-2-[(methoxycarbonyl)a	C45H54N8O7	818.96	C	-8.2
8933	C(=O)([C@@H](NC(=O)OC)C(C)C)N1[C@H](c2[nH]c(c3cc4c5-c6c(cc(cc6OC4)c4[nH]c(nc4)[C@H]4N(C(=O)[C@@H](NC	1377042-82-4	Carbamic acid, N-[(1S)-2-[(1R,3S,5R)-3-[5-[4-[2-[(1R,3S,4S)-2-[(2S)-2-[(methoxyc	C47H54N8O7	842.98	C	-8.2
8934	C(=O)([C@@H](NC(=O)OC)C(C)C)N1[C@H](c2[nH]c3c4c(cc(cc4)c4c(C#Cc5ccccc5)cc(cc4)c4[nH]c(nc4)[C@H]4N(C(	1377044-89-7	Carbamic acid, N,N,Ä≤-[[7,7,Ä≤-bi-1H-naphth[1,2-d]imidazole]-2,2,Ä≤-diyl[[(2S,4	C48H58N8O8	875.02	C	-8.2
8935	C(=O)([C@@H](NC(=O)OC)C(C)C)N1[C@H](c2[nH]c3c4c(cc(cc4)c4ccc(cc4)c4[nH]c(nc4C)[C@H]4N(C(=O)[C@@H](NC	1377047-28-3	Carbamic acid, N-[(1S)-1-[[(2S)-2-[2,Ä≤-[(2S,4S)-1-[(2S)-2-[(methoxycarbonyl)am:	C45H52N8O6S	833.01	C	-8.2
8936	C(=O)([C@@H](NC(=O)OC)C1CCOCC1)N1[C@@H]([C@@H]2C[C@H]1CC2)c1[nH]c2c(n1)ccc1c2ccc(c1)c1ccc2c(cc1)c1c(c	1377604-14-2	Carbamic acid, N-[(1S)-2-[(2S)-2-[5-[4-[2-[(1R,3S,4S)-2-[(2S)-2-[(methoxycarbony	C46H54N8O7	830.97	C	-8.2
8937	C(=O)([C@H](NC(=O)OC)C(C)C)N1[C@H](CCC1)c1[nH]c(cn1)[C@H]1CC[C@@H](CC1)[C@H]1CC[C@@H](CC1)c1[nH]c(nc	1380289-73-5	Carbamic acid, N-[(1R)-1-[[(2R)-2-[5-[6-[4-[2-[(2R)-1-[(2R)-2-[(methoxycarbonyl)	C42H48N8O6	760.88	C	-8.2
8938	O=c1c2c(n(cn2)[C@@H]2O[C@H](CO)[C@@H](O)[C@H]2O)[nH]c(n1)n1cc(nn1)c1ccc(C)cc1	1384519-78-1	Inosine, 2-(4-phenyl-1H-1,2,3-triazol-1-yl)-	C18H17N7O5	411.37	C	-8.2
8939	O=c1c2c(n(cn2)[C@@H]2O[C@H](CO)[C@@H](O)[C@H]2O)[nH]c(n1)n1cc(CO)nn1	1384519-80-5	Inosine, 2-[4-(4-methoxyphenyl)-1H-1,2,3-triazol-1-yl]-	C19H19N7O6	441.4	C	-8.2
8940	N(c1c(C)cc(/C=C/C#N)cc1C)c1nc(Nc2ccc(C#N)cc2)ncc1.[C@@H]([C@H](C(=O)O)O)(C(=O)O)O	1388223-99-1	Benzonitrile, 4-[[4-[[4-[(1E)-2-cyanoethenyl]-2,6-dimethylphenyl]amino]-2-pyrimi	C22H18N6.xC4H4O4		C	-8.2
8941	C(=O)([C@@H](NC(=O)OC)C(C)C)N1[C@H](c2[nH]c(c(n2)c2ccccc2)c2ccc(cc2)c2ccc(cc2)c2c(nc([nH]2)[C@H]2N(C	1388224-01-8	Benzonitrile, 4-[[4-[[4-[(1E)-2-cyanoethenyl]-2,6-dimethylphenyl]amino]-2-pyrimi	C22H18N6.xC7H5NO3S		C	-8.2
8942	C(O)[C@H]1[C@H](n2c3c(nc2[3H])c(=O)nc(N)[nH]3)C[C@@H]1CO	1388224-22-3	Carbamic acid, N,N,Ä≤-[[1,1,Ä≤-biphenyl]-4,4,Ä≤-diylbis[(5-fluoro-1H-imidazole-	C40H48F2N8O6	774.86	C	-8.2
8943	O=C1C2(C(C(C(=O)/C=C(\O)/C3C4(N5C(C3c3cc(OC)c(O)cc3)CSC5)C(=O)c3c(C4=O)cccc3)C3N2CSC3)c2cc(OC)c(O)cc	1389320-77-7	Spiro[3H-indole-3,5,Ä≤-[3H,5H]pyrrolo[1,2-c]thiazol]-2(1H)-one, 5-chloro-6,Ä≤-[	C43H38Cl2N4O8S2	873.82	C	-8.2
8944	O=C1C2(C(C(C(=O)/C=C(\O)/C3C4(c5c(NC4=O)ccc(N(=O)=O)c5)NC(C3c3cc(OC)c(O)cc3)c3ccccc3)C(N2)c2ccccc2)c	1389335-40-3	Spiro[3H-indole-3,2,Ä≤-pyrrolidin]-2(1H)-one, 3,Ä≤-[(1Z)-3-[1,2-dihydro-3,Ä≤-(4	C41H38N6O12	806.77	C	-8.2
8945	N(C(C(=O)N1CCN(S(=O)(=O)C)CC1)C)c1nc(nc(n1)N1CCOCC1)c1cc(O)ccc1	1391924-42-7	2(1H)-Pyridinone, 5-[4-[[2-[4-(methylsulfonyl)-1-piperazinyl]-2-oxoethyl]amino]-	C19H26N8O5S	478.53	C	-8.2
8946	CN1CCN(c2nc(nc(n2)N2CCOCC2)c2c3c(ccc2)[nH]nc3)CC1	1391925-94-2	Œ≤-Alanine, N-[4-(1H-indazol-4-yl)-6-(4-morpholinyl)-1,3,5-triazin-2-yl]-	C17H19N7O3	369.38	C	-8.2
8947	Nc1sc(c2nc(nc(n2)N2CCOCC2)N2CCNCC2)cn1	1391926-10-5	Benzamide, 3-[[4-(2-amino-5-thiazolyl)-6-(4-morpholinyl)-1,3,5-triazin-2-yl]amir	C19H22N8O2S	426.5	C	-8.2
8948	S(=O)(=O)(O)c1c2c(c(N)c(N=Nc3c(C(=O)O)cc(cc3)c3cc(C(=O)O)c(N=Nc4c(N)c5c(c(S(=O)(=O)O)c4)cccc5)cc3)c1	1393659-92-1	[1,1,Ä≤-Biphenyl]-3,3,Ä≤-dicarboxylic acid, 4,4,Ä≤-bis[2-(1-amino-4-sulfo-2-nap	C34H24N6O10S2.2Na		C	-8.2
8949	N(c1nc(nc(NCCNC(=O)c2ccc(cc2)C2OOC(OO2)c2ccc(C)cc2)n1)N1CCOCC1)c1ccc(OC)cc1	1393922-46-7	Benzamide, N-[2-[[4-[[4-(1-methylethyl)phenyl]amino]-6-(4-morpholinyl)-1,3,5-tri	C34H39N7O6	641.72	C	-8.2
8950	N(c1c(C)cc(/C=C/C#N)cc1C)c1nc(Nc2ccc(C#N)cc2)ncc1.P(=O)(O)(O)O	1399384-88-3	Benzonitrile, 4-[[4-[[4-[(1E)-2-cyanoethenyl]-2,6-dimethylphenyl]amino]-2-pyrimi	C22H18N6.H2O4S		C	-8.2
8951	N(c1c(C)cc(/C=C/C#N)cc1C)c1nc(Nc2ccc(C#N)cc2)ncc1.Br	1399384-89-4	Benzonitrile, 4-[[4-[[4-[(1E)-2-cyanoethenyl]-2,6-dimethylphenyl]amino]-2-pyrimi	C22H18N6.H3O4P		C	-8.2
8952	N(c1c(C)cc(/C=C/C#N)cc1C)c1nc(Nc2ccc(C#N)cc2)ncc1.C(=C\C(=O)O)\C(=O)O	1399384-92-9	Benzonitrile, 4-[[4-[[4-[(1E)-2-cyanoethenyl]-2,6-dimethylphenyl]amino]-2-pyrimi	C22H18N6.CH4O3S		C	-8.2
8953	N(c1c(C)cc(/C=C/C#N)cc1C)c1nc(Nc2ccc(C#N)cc2)ncc1.C(C(=O)O)C(=O)O	1399384-96-3	Ethanesulfonic acid, compd. with 4-[[4-[[4-[(1E)-2-cyanoethenyl]-2,6-dimethylphe	C22H18N6.C2H6O3S		C	-8.2
8954	C1CCOC1.N(c1c(C)cc(/C=C/C#N)cc1C)c1nc(Nc2ccc(C#N)cc2)ncc1.C(=C\C(=O)O)\C(=O)O	1399385-00-2	Benzonitrile, 4-[[4-[[4-[(1E)-2-cyanoethenyl]-2,6-dimethylphenyl]amino]-2-pyrimi	C22H18N6.C6H8O7		C	-8.2
8955	O=C1c2n(C[C@H]3N1[C@](C([2H])([2H])[2H])(C(C(O3)([2H])[2H])([2H])[2H])[2H])cc(C(=O)NC(c1c(F)cc(F)cc1	1407166-90-8	2H-Pyrido[1,Ä≤,2,Ä≤:4,5]pyrazino[2,1-b][1,3]oxazine-12-d-9-carboxamide, N-[(2,4	C20H15D4F2N3O5		C	-8.2
8956	O=C1c2n(C[C@H]3N1[C@](C)(C(C(O3)([2H])[2H])([2H])[2H])[2H])cc(C(=O)NC(c1c(F)cc(F)cc1)([2H])[2H])c(=O	1407166-92-0	2H-Pyrido[1,Ä≤,2,Ä≤:4,5]pyrazino[2,1-b][1,3]oxazine-2,3-d2-9-carboxamide, N-[(2	C20H10D9F2N3O5		C	-8.2
8957	O=C1c2n(C[C@H]3N1[C@H](C)C(C(O3)([2H])[2H])([2H])[2H])cc(C(=O)NC(c1c(F)cc(F)cc1)([2H])[2H])c(=O)c2O	1407166-93-1	2H-Pyrido[1,Ä≤,2,Ä≤:4,5]pyrazino[2,1-b][1,3]oxazine-2,3,4-d3-9-carboxamide, N-[	C20H12D7F2N3O5		C	-8.2
8958	O=C1c2n(C([C@H]3N1[C@H](C([2H])([2H])[2H])C(C(O3)([2H])[2H])([2H])[2H])([2H])[2H])cc(C(=O)NC(c1c(F)c	1407166-98-6	2H-Pyrido[1,Ä≤,2,Ä≤:4,5]pyrazino[2,1-b][1,3]oxazine-9-carboxamide, N-[(2,4-difl	C20H17D2F2N3O5		C	-8.2
8959	O=C1c2n(C([C@H]3N1[C@](C([2H])([2H])[2H])(CCO3)[2H])([2H])[2H])cc(C(=O)NC(c1c(F)cc(F)cc1)([2H])[2H])	1407167-01-4	2H-Pyrido[1,Ä≤,2,Ä≤:4,5]pyrazino[2,1-b][1,3]oxazine-2,3,12-d3-9-carboxamide, N-	C20H11D8F2N3O5		C	-8.2
8960	O=C1c2n(C([C@H]3N1[C@](C)(CCO3)[2H])([2H])[2H])cc(C(=O)NC(c1c(F)cc(F)cc1)([2H])[2H])c(=O)c2O	1407167-03-6	2H-Pyrido[1,Ä≤,2,Ä≤:4,5]pyrazino[2,1-b][1,3]oxazine-12-d-9-carboxamide, N-[(2,4	C20H12D7F2N3O5		C	-8.2
8961	O=C1c2n(C([C@]3(N1[C@H](C)CCO3)[2H])([2H])[2H])cc(C(=O)NC(c1c(F)cc(F)cc1)([2H])[2H])c(=O)c2O	1407167-04-7	2H-Pyrido[1,Ä≤,2,Ä≤:4,5]pyrazino[2,1-b][1,3]oxazine-4,12-d2-9-carboxamide, N-[(	C20H14D5F2N3O5		C	-8.2
8962	O=C1c2n(C[C@]3(N1[C@H](C([2H])([2H])[2H])C(C(O3)([2H])[2H])([2H])[2H])[2H])cc(C(=O)NC(c1c(F)cc(F)cc1	1407167-06-9	2H-Pyrido[1,Ä≤,2,Ä≤:4,5]pyrazino[2,1-b][1,3]oxazine-2,3,4-d3-9-carboxamide, N-[	C20H8D11F2N3O5		C	-8.2
8963	O=C1c2n(C[C@]3(N1[C@](C([2H])([2H])[2H])(CCO3)[2H])[2H])cc(C(=O)NC(c1c(F)cc(F)cc1)([2H])[2H])c(=O)c2	1407167-09-2	2H-Pyrido[1,Ä≤,2,Ä≤:4,5]pyrazino[2,1-b][1,3]oxazine-2,3-d2-9-carboxamide, N-[(2	C20H12D7F2N3O5		C	-8.2
8964	O=C1c2n(C[C@]3(N1[C@H](C)CCO3)[2H])cc(C(=O)NC(c1c(F)cc(F)cc1)([2H])[2H])c(=O)c2O	1407167-12-7	2H-Pyrido[1,Ä≤,2,Ä≤:4,5]pyrazino[2,1-b][1,3]oxazine-4-d-9-carboxamide, N-[(2,4-	C20H15D4F2N3O5		C	-8.2
8965	O=C1c2n(C([C@]3(N1[C@H](C([2H])([2H])[2H])C(C(O3)([2H])[2H])([2H])[2H])[2H])([2H])[2H])cc(C(=O)NC(c1	1407167-13-8	2H-Pyrido[1,Ä≤,2,Ä≤:4,5]pyrazino[2,1-b][1,3]oxazine-9-carboxamide, N-[(2,4-difl	C20H16D3F2N3O5		C	-8.2
8966	O=C1c2n(C([C@]3(N1[C@](C)(CCO3)[2H])[2H])([2H])[2H])cc(C(=O)NC(c1c(F)cc(F)cc1)([2H])[2H])c(=O)c2O	1407167-18-3	2H-Pyrido[1,Ä≤,2,Ä≤:4,5]pyrazino[2,1-b][1,3]oxazine-12-d-9-carboxamide, N-[(2,4	C20H11D8F2N3O5		C	-8.2
8967	O=C1c2n(C([C@]3(N1[C@](C([2H])([2H])[2H])(C(C(O3)([2H])[2H])([2H])[2H])[2H])([2H])[2H])cc(C(=O)	1407167-19-4	2H-Pyrido[1,Ä≤,2,Ä≤:4,5]pyrazino[2,1-b][1,3]oxazine-4,12-d29-carboxamide, N-[(2	C20H13D6F2N3O5		C	-8.2
8968	O=C1c2n(C[C@H]3N1[C@H](C([2H])([2H])[2H])C(C(O3)([2H])[2H])([2H])[2H])cc(C(=O)NCc1c(F)cc(F)cc1)c(=O)	1407167-23-0	2H-Pyrido[1,Ä≤,2,Ä≤:4,5]pyrazino[2,1-b][1,3]oxazine-2,3,4-d3-9-carboxamide, N-[	C20H11D8F2N3O5		C	-8.2
8969	O=C1c2n(C[C@]3(N1[C@H](C([2H])([2H])[2H])C(C(O3)([2H])[2H])([2H])[2H])[2H])cc(C(=O)NCc1c(F)cc(F)cc1)	1407167-43-4	2H-Pyrido[1,Ä≤,2,Ä≤:4,5]pyrazino[2,1-b][1,3]oxazine-2,3,4-d3-9-carboxamide, N-[	C20H10D9F2N3O5		C	-8.2
8970	O=C1c2n(C[C@]3(N1[C@](C)(C(C(O3)([2H])[2H])([2H])[2H])[2H])[2H])cc(C(=O)NCc1c(F)cc(F)cc1)c(=O)c2O	1407167-44-5	2H-Pyrido[1,Ä≤,2,Ä≤:4,5]pyrazino[2,1-b][1,3]oxazine-2,3-d2-9-carboxamide, N-[(2	C20H11D8F2N3O5		C	-8.2
8971	O=C1c2n(C([C@]3(N1[C@](C([2H])([2H])[2H])(CCO3)[2H])[2H])([2H])[2H])cc(C(=O)NCc1c(F)cc(F)cc1)c(=O)c2	1407167-53-6	2H-Pyrido[1,Ä≤,2,Ä≤:4,5]pyrazino[2,1-b][1,3]oxazine-2,3,12-d3-9-carboxamide, N-	C20H12D7F2N3O5		C	-8.2
8972	O(C(=O)c1cccnc1)[C@H]1[C@@H](O[C@H](CO)[C@H]1OC(=O)c1ccccc1)n1nc(C(=O)N)nc1	140891-09-4	1H-1,2,4-Triazole-3-carboxamide, 1-[3-O-benzoyl-5-O-[bis(4-methoxyphenyl)phenylm	C42H37N5O9	755.77	C	-8.2
8973	O(C(=O)c1=CN(C)C=CC1)[C@H]1[C@@H](O[C@H](CO)[C@H]1OC(=O)c1ccccc1)n1nc(C(=O)N)nc1	140891-10-7	1H-1,2,4-Triazole-3-carboxamide, 1-[3-O-benzoyl-2-O-(3-pyridinylcarbonyl)-Œ≤-D-r	C21H19N5O7	453.4	C	-8.2
8974	O[C@H]1[C@@H](O[C@H](C(=O)N[C@H](Cc2ccccc2)C(=O)NCc2cc(on2)c2ccc(OC)ccc2)[C@H]1O)n1nc(C(=O)N)nc1	1414793-77-3	Not Yet Assigned	C19H20N6O7	444.4	C	-8.2
8975	C(OC(CO)CO)n1c2c(nc1)c(=O)nc(NCNc1ccc(cc1)S(=O)(=O)Nc3noc(C)c3)cc1)[nH]2	1415032-07-3	Benzenesulfonamide, 4-[[[[6,9-dihydro-9-[[2-hydroxy-1-(hydroxymethyl)ethoxy]meth	C22H31N9O8S	581.6	C	-8.2
8976	C(OC(CO)C1CC1)n1c2c(nc1)c(=O)nc(N)[nH]2	1416170-12-1	Hydrazinecarbothioamide, 2-[1,2-dihydro-2-oxo-1-(2-phenylethyl)-3H-indol-3-ylide	C31H34N4O10S	654.69	C	-8.2
8977	C(F)(F)c1n(c2c(n1)cccc2)c1nc(nc(n1)N1CCOCC1)N1CCN(C(=O)[C@H](C)N)CC1	1416956-30-3	Acetamide, N-[2-[4-[4-[2-(difluoromethyl)-1H-benzimidazol-1-yl]-6-(4-morpholinyl	C23H27F2N9O3	515.52	C	-8.2
8978	C(=O)([C@@H](NC(=O)OC)C(C)C)N1[C@H](CCC1)c1[nH]c(cn1)c1ccc(cc1)c1ccc(cc1)c1[nH]c(nc1)[C@@H]1N(C(=O)[C@	1417333-58-4	Carbamic acid, N,N,Ä≤-[[1,1,Ä≤-biphenyl]-4,4,Ä≤-diylbis[1H-imidazole-5,2-diyl-(	C40H50N8O6	738.88	C	-8.2
8979	C(=O)([C@@H](NC(=O)OC)C(C)C)N1[C@H](CCC1)c1[nH]c(cn1)c1ccc(cc1)c1ccc(cc1)c1[nH]c(nc1)[C@H]1N(C(=O)[C@	1417333-63-1	Carbamic acid, N,N,Ä≤-[[1,1,Ä≤-biphenyl]-4,4,Ä≤-diylbis[1H-imidazole-5,2-diyl-2	C40H50N8O6	738.88	C	-8.2
8980	N(c1nc(nc(NC(=S)Nc2ccc(Cl)cc2)n1)N1CCOCC1)n1c(SC)nnc(C(C)(C)C)c1=O	1418741-53-3	Thiourea, N-[4-[[6-(1,1-dimethylethyl)-3-(methylthio)-5-oxo-1,2,4-triazin-4(5H)-	C23H30N10O2S2	542.68	C	-8.2
8981	C(=O)(NC)c1c(=O)[nH]cc(F)n1	1418741-56-6	Thiourea, N-[4-[[6-(1,1-dimethylethyl)-3-(methylthio)-5-oxo-1,2,4-triazin-4(5H)-	C26H30N10O2S2	578.71	C	-8.2
8982	C(=O)([C@@H](NC(=O)OC)C(C)C)N1[C@H](c2[nH]c(c3ccc(c4C5CC6(Cc5cc4)c4ccc(cc4)c4[nH]c(nc4)[C@H]4N(C(=	1419385-37-7	Carbamic acid, N-[(1S)-1-[[(2S)-2-[5-[4-[1,Ä≤,3,Ä≤-dihydro-7,Ä≤-[2-[(2S)-1-[(2S	C47H58N8O7	847.01	C	-8.2
8983	C(Nc1nc(NCCNC(=O)C=C)nc(NCC#C)n1)(C)C12CC3CC(C2)CC(C1)C3	1422258-95-4	Benzamide, 2-chloro-5-nitro-N-[2-[[4-(2-propyn-1-ylamino)-6-[(1-tricyclo[3.3.1.1	C27H33ClN8O3	553.06	C	-8.2
8984	C(=C)c1n(c2c(n1)c(N)ncn2)[C@@H]1O[C@H](CO)[C@@H](O)[C@H]1O	1423582-77-7	1-Propanone, 1-[2,6-dihydroxy-3-[(2E)-3-(4-hydroxyphenyl)-2-propen-1-yl]-4-metho	C25H24O6	420.45	C	-8.2
8985	C(N1c2c(C(=NNC(=S)N(CC=C)CC=C)C1=O)cc(F)cc2)N1CCOCC1	1426583-57-4	Hydrazinecarbothioamide, 2-[5-chloro-1,2-dihydro-1-(4-morpholinylmethyl)-2-oxo-3	C26H24ClN5O2S	506.02	C	-8.2
8986	C(N1c2c(C(=NNC(=S)N(CC=C)CC=C)C1=O)cc(C)cc2)N1CCN(CC1)c1ccccc1	1426583-61-0	Hydrazinecarbothioamide, 2-[1,2-dihydro-5-methyl-1-(4-morpholinylmethyl)-2-oxo-3	C27H27N5O2S	485.6	C	-8.2
8987	O(C)c1c2n(cc(C(=O)O)c(=O)c2cc(F)c1N1CC(C)N(CN2c3c(C(=NNC(=S)N(Cc4ccccc4)c4ccccc4)C2=O)cc(F)cc3)CC1)C1CC1	1426583-88-1	3-Quinolinecarboxylic acid, 1-cyclopropyl-7-[4-[[3-[2-(di-2-propen-1-ylamino)th	C35H37F2N7O5S	705.77	C	-8.2
8988	C(=O)(OCC)C1=C(N)OC(=CC1c1ccc(F)cc1)c1[nH]c2c(n1)cccc2	1426813-71-9	4H-Pyran-3-carboxylic acid, 2-amino-6-(1H-benzimidazol-2-yl)-4-(4-fluorophenyl)-	C21H18FN3O3	379.38	C	-8.2
8989	C(=O)(OCC)C1=C(N)OC(=CC1c1ccccc1)c1[nH]c2c(n1)cccc2	1427057-13-3	L-Alanine, N-(5-iodo-P-1-naphthalenyl-5,Ä≤-uridylyl)-, methyl ester	C23H25IN3O10P	661.34	C	-8.2
8990	C[C@]12[C@@]3(C)[C@@H]([C@H](CC3)[C@@]3(C)O[C@@H]1[C@]1(C)[C@@H](CC2)C(C)(	1431852-68-4	Dammar-25-ene-3,12-diol, 20,24-epoxy-, 3-acetate, (3Œ≤,12Œ≤,24R)-	C32H52O4	500.75	C	-8.2
8991	O=c1n([C@@H]2O[C@H](COC(=O)NCC)[C@H](O)C2)cc(C)c(=O)[nH]1	1436864-99-1	2,4,6,8-Tetraoxa-5-phosphanonanedioic acid, 5-[[(1R)-2-(6-amino-9H-purin-9-yl)-1	H18N6.C19H30N5O10P.C8H10FN3O3S.C4H4O4		C	-8.2
8992	C([C@H]([C@H](Cc1ccccc1)NC(=O)[C@@H](NC(=O)c1nc2c(cc1)cccc2)CC(=O)N)OC(=O)CCCCC[C@@H]1O[C@H](CO)[C@@	1438393-27-1	Guanosine 5,Ä≤-(trihydrogen diphosphate), P,Ä≤-(2,6-dideoxy-Œ±-D-arabino-hexopy	C16H25N5O14P2	573.34	C	-8.2
8993	N(C(=O)C)[C@H]1[C@H]([C@@H]([C@@H](CO)O)O)OC(=C[C@@H]1NC(=N)NC(=O)c1ccc(N(=O)=O)cc1)C(=O)O	1442563-03-2	D-glycero-D-galacto-Non-2-enonic acid, 5-(acetylamino)-2,6-anhydro-3,4,5-trideox	C23H32N4O8	492.52	C	-8.2
8994	N(C(=O)C)[C@H]1[C@H]([C@@H]([C@@H](CO)O)O)OC(=C[C@@H]1NC(=N)NC(=O)c1ccc(N(C)C)cc1)C(=O)O	1442563-04-3	D-glycero-D-galacto-Non-2-enonic acid, 5-(acetylamino)-2,6-anhydro-3,4,5-trideox	C19H23N5O10	481.41	C	-8.2
8995	N(C(=O)C)[C@H]1[C@H]([C@@H]([C@@H](CO)O)O)OC(=C[C@@H]1NC(=N)NC(=O)OCSc1cc2c(cc1)cccc2)C(=O)O	1442563-10-1	D-glycero-D-galacto-Non-2-enonic acid, 5-(acetylamino)-2,6-anhydro-3,4,5-trideox	C25H30N4O8	514.53	C	-8.2
8996	C(C(=O)NC(=N)N[C@@H]1[C@H](NC(=O)C)[C@H]([C@@H]([C@@H](CO)O)O)OC(=C1)C(=O)O)C12CC3CC(C2)CC(C1)C3	1442563-11-2	D-glycero-D-galacto-Non-2-enonic acid, 5-(acetylamino)-2,6-anhydro-3,4,5-trideox	C24H28N4O8S	532.57	C	-8.2
8997	N(C(=O)C)[C@H]1[C@H]([C@@H]([C@@H](CO)O)O)OC(=C[C@@H]1NC(=N)NC(=O)CCC1CCOC1)C(=O)O	1442563-19-0	D-glycero-D-galacto-Non-2-enonic acid, 5-(acetylamino)-4-[[[3-(4-aminophenyl)-1	C21H29N5O8	479.48	C	-8.2
8998	C([C@H](OCP(=O)(O)O)C)n1c2c(nc1)c(Cl)nc(N)n2	1443648-71-2	L-Alanine, N-[[P(S),2,Ä≤R]-5-bromo-2,Ä≤-deoxy-2,Ä≤-fluoro-4,Ä≤-C-fluoro-2,Ä≤-me	C26H29BrF2N3O9P	676.4	C	-8.2
8999	N(C1cccs1)(c1nc(nc(C#N)n1)N1CCOCC1)C1CCCCC1	1443830-75-8	1,3,5-Triazine-2-carbonitrile, 4-[cyclohexyl[[4-(3-methyl-1H-pyrazol-1-yl)phenyl	C25H30N8O	458.56	C	-8.2
9000	C[C@]1(F)[C@H](O[C@H](COP(=S)(Oc2ccccc2)NC(C)C(=O)OC(C)C)[C@H]1O)n1ccc(=O)[nH]c1=O	1443998-32-0	Alanine, N-[(2,Ä≤R)-2,Ä≤-deoxy-2,Ä≤-fluoro-2,Ä≤-methyl-P(O)-phenyl-P-thio-5,Ä≤-	C25H33FN3O8PS	585.58	C	-8.2
9001	S(c1n(c2c(n1)c(=O)nc([nH]2)N)[C@@H]1O[C@@H]2[C@H](O1)COP(=O)(O)O2)c1ccc(O)cc1	144509-93-3	Guanosine, 8-[(2-hydroxyphenyl)thio]-, cyclic 3,Ä≤,5,Ä≤-(hydrogen phosphate)	C16H16N5O8PS	469.37	C	-8.2
9002	O=c1n(C2OC(CO)C(O)C2[3H])cc(C)c(=O)[nH]1	144660-67-3	1H-Imidazo[4,5-c]quinoline-2-methanol, 4-amino-Œ±-(4-chlorophenyl)-1-(2-methylpr	C21H21ClN4O	380.87	C	-8.2
9003	C=C1c2ccc(S(=O)(=O)N3CCNCC3)c2)NC1=O	1447746-67-9	Carbamic acid, N-[(1S)-2-[(2S)-2-[5-[4,Ä≤-[2-[(1S)-1-[(2,2-dimethyl-1-oxopropyl	C38H49N7O4	667.84	C	-8.2
9004	O(c1nc(Nc2ccc(C#N)cc2)nc(Cl)c1Br)c1c(C)cc(C)cc1C	1448853-80-2	Thymidine, [P(S)]-thymidylyl-(3,Ä≤-5,Ä≤,Ü≤,Ä≤)-3,Ä≤-azido-3,Ä≤-deoxy-	C20H24N7O10P		C	-8.2
9005	N(c1c2c(c(O)c(CN3CC(C)C3)ccc2)ncc1)c1ccc(Cl)cc1	1449239-42-2	1-Naphthalenol, 4-[(7-chloro-4-quinolinyl)amino]-2-[(2-methyl	C26H30ClN3O	435.99	C	-8.2
9006	O=C1[C@@H]2[C@@H](c3c([C@H](OC(=O)c4cc(N(=O)=O)c(Cl)cc4)[C@H]2CO1)cc1c(c3)OCO1)c1cc(OC)c(OC)c(OC)c1	1449601-81-3	Furo[3,Ä≤,4,Ä≤:6,7]naphtho[2,3-d]-1,3-dioxol-6(5aH)-one, 5-(2-chloro-3,4,5-trime	C29H24ClNO11	597.95	C	-8.2
9007	O=C1[C@@H]2[C@@H](c3c([C@H](OC(=O)c4cc(C)ccc4)[C@H]2CO1)cc1c(c3)OCO1)c1c(Br)c(OC)c(OC)c(OC)c1	1449602-01-0	1-Naphthaleneacetic acid, (5R,5aR,8aR,9S)-9-(2-bromo-3,4,5-trimethoxyphenyl)-5,5	C34H29BrO9	661.49	C	-8.2
9008	C(P(=O)(OCC)OCC)(Cc1cn(Cc2c(N)nc(C)nc2)nn1)C(=O)OCC	1449602-14-5	Benzoic acid, 4-chloro-3-nitro-, (5R,5aR,8aR,9S)-9-(2-bromo-3,4,5-trimethoxypher	C29H23BrClNO11	676.85	C	-8.2

9009	C(n1c2c(nc1CC)c(C)cc(C)n2)c1ccc(cc1)c1c(S(=O)(=O)NC(=O)OCC)cccc1	145004-27-9	Guanosine, 2,Ä≤,3,Ä≤-dideoxy-, 5,Ä≤,ÜíP,Ä≤,Ä≤-ester with thiotriphosphoric aci	C10H16N5O11P3S		C	-8.2
9010	N(CCCNc1nc(nc(Nc2ccc(CC)cc2)n1)N1CCOCC1)c1c2c(ncc1)cc(Cl)cc2	1454694-92-8	1,3,5-Triazine-2,4-diamine, N2-[2-[(7-chloro-4-quinolinyl)amino]ethyl]-6-(4-morp	C24H25ClN8O	476.96	C	-8.2
9011	N(C(=O)NCC(=O)O)C12CC3(CC(C2)CC(C3)C1)c1ccc(C)cc1	145933-68-2	5,Ä≤-Guanylic acid, monoanhydride with 2,6-anhydro-1,7-dideoxy-7-phosphono-D-gly	C17H27N5O14P2	587.37	C	-8.2
9012	N(c1nc(Nc2c(O)cc(C#N)cc2)ncc1)c1c(C)cc(C)cc1C	1460306-52-8	Benzonitrile, 4-[[4-[[2-(hydroxymethyl)-4,6-dimethylphenyl]amino]-2-pyrimidinyl]	C20H19N5O	345.4	C	-8.2
9013	N(c1c2c(ncc1)cc(Cl)cc2)c1cc(cc(CN2CCC(C)CC2)c1O)c1ccc(Cl)cc1	146298-44-4	[1,1,Ä≤-Biphenyl]-4-ol, 4,Ä≤-chloro-3-[(7-chloro-4-quinolinyl)amino]-5-[(3-meth	C28H27Cl2N3O	492.44	C	-8.2
9014	N(C(=O)C)[C@H]1[C@H](OC(CC)CC)C=C(C(=O)OCCCO)C[C@@H]1NC(=O)OC(C)(C)C	1469338-39-3	D-glycero-D-galacto-Non-2-enonic acid, 5-(acetylamino)-2,6-anhydro-3,4,5-trideox	C25H36N4O8	520.58	C	-8.2
9015	N(c1c(C)cc(/C=C/C#N)cc1C)c1nc(Nc2ccc(C#N)cc2)ncc1.O=C1CCCCC1.Cl	1469779-25-6	Benzonitrile, 4-[[4-[[4-[(1E)-2-cyanoethenyl]-2,6-dimethylphenyl]amino]-2-pyrimi	C22H18N6.CHCl3.ClH		C	-8.2
9016	C(OP(=O)(O[C@H]1[C@@H](O[C@H](CO)[C@H]1OC(=O)c1ccccc1)n1nc(C(=O)N)nc1)OCCc1ccc(N(=O)=O)cc1)[C@@H]1[C	1469860-67-0	Benzonitrile, 4-[[4-[[4-[(1E)-2-cyanoethenyl]-2,6-dimethylphenyl]amino]-2-pyrimi	C22H18N6.CH4O.ClH		C	-8.2
9017	O[C@H]1[C@H](n2c(nc3c2[nH]c(N)nc3=O)N2CCCC2)O[C@H](COP(=O)(O)O)[C@H]1O	1474031-11-2	Not Yet Assigned	C14H21N6O9P	448.33	C	-8.2
9018	O=C1c2c(C(=O)N1CCS(=O)(=O)Nc1cc3c(cc1)[nH]c(C(=O)N1CCN(CC1)c1c(NC(C)C)cccn1)c3)cccc2	147540-02-1	Ethanesulfonamide, 2-(dimethylamino)-N-[2-[[4-[3-[(1-methylethyl)amino]-2-pyridi	C25H35N7O3S	513.66	C	-8.2
9019	C(=O)(c1cc2c([nH]1)ccc(NS(=O)(=O)N1CCN(C(CC)(C)C)CC1)c2)N1CCN(CC1)c1c(NC(C)C)cccn1	147540-15-6	Methanesulfonamide, N-[2-[[4-[3-[(1,1-dimethylpropyl)amino]-2-pyridinyl]-1-piper	C24H32N6O3S	484.61	C	-8.2
9020	C(n1c2c(nc1CC)c(C)cc(C)n2)c1ccc(cc1)c1c(S(=O)(=O)NC(=O)OC)cccc1	148401-77-8	Carbamic acid, [[4,Ä≤-[(2-ethyl-7-methyl-3H-imidazo[4,5-b]pyridin-3-yl)methyl][	C30H28N4O4S	540.63	C	-8.2
9021	O=c1n([C@@H]2O[C@H](CO)[C@@H](O)C2)cc(CNOC)c(=O)[nH]1	1485692-24-7	2H-Pyrido[1,Ä≤,2,Ä≤:4,5]pyrazino[2,1-b][1,3]oxazine-9-carboxamide, N-[(2,4-difl	C20H19F2N3O6	435.38	C	-8.2
9022	O=c1n([C@@]2(O[C@](C(O)([2H])[2H])([C@@](O)(C2([2H])[2H])[2H])[2H])[2H])cc(C)c(=O)[nH]1	148715-73-5	Cytidine, 3,Ä≤,3,Ä≤,Ä≤,Ä≤-dithiobis[2,Ä≤,3,Ä≤-dideoxy-	C18H24N6O6S2	484.55	C	-8.2
9023	C(=O)([C@@H](NC(=O)OC)C(C)C)N1[C@@H](C[C@H](COC)C1)c1[nH]c(cn1)c1ccc(cc1)c1ccc(cc1)c1[nH]c(nc1Cl)[C@	1491956-98-9	Carbamic acid, N-[(1S)-1-[[(2S,5S)-2-[5-chloro-4-[4,Ä≤-[5-chloro-2-[(2S,4S)-1-[	C43H54Cl2N8O7	865.84	C	-8.2
9024	C(=O)([C@@H](NC(=O)OC)C1CCOCC1)N1[C@@H](C[C@H](COC)C1)c1[nH]c(cn1)c1ccc(cc1)c1ccc(cc1)c1[nH]c(nc1)[C	1491957-05-1	Carbamic acid, N-[(1S)-1-[[(2S,5S)-2-[5-[4,Ä≤-[5-chloro-2-[(2S,4S)-1-[(2S)-2-[(	C43H55ClN8O7	831.4	C	-8.2
9025	O[C@H]1[C@H](n2c3c(nc2)c(=O)nc(N)[nH]3)O[C@H](COP(=O)(CP(=O)(OP(=O)(O)O)O)O)[C@H]1O	1499175-77-7	4-Morpholinecarboxamide, N-[(1S)-2-[[(1S)-1-[(4-hydroxyphenyl)methyl]-3-(phenyls	C30H33N3O6S	563.66	C	-8.2
9026	N(c1c(C)cc(/C=C/C#N)cc1C)c1nc(Nc2ccc(C#N)cc2)ncc1.[C@H](C(=O)O)(O)c1ccccc1	1502822-72-1	Acetic acid, 2-hydroxy-, compd. with 4-[[4-[[4-[(1E)-2-cyanoethenyl]-2,6-dimethy	C22H18N6.C2H4O3		C	-8.2
9027	C(N1c2c(C(=NNC(=S)Nc3ccc(C)cc3)C1=O)cc(Cl)c(Cl)c2)N1CCCCC1	1507380-61-1	Hydrazinecarbothioamide, 2-[4,5-dichloro-1,2-dihydro-2-oxo-1-(1-piperidinylmethy	C22H23Cl2N5OS	476.42	C	-8.2
9028	C(C(C)C)n1c2c(c(Nc3c(C(=O)O)cc(Br)cc3)nc3c2cccc3)nc1	1508303-94-3	Benzoic acid, 3-bromo-2-[[1-(2-methylpropyl)-1H-imidazo[4,5-c]quinolin-4-yl]amir	C21H19BrN4O2	439.31	C	-8.2
9029	O[C@H]1[C@H](n2c3c(nc2)c(=O)nc(N)[nH]3)O[C@H](CO)[C@H]1N[Si](NC[C@H]1O[C@H](C[C@@H]1O)n1c2c(nc1)c(=O	1513874-15-1	Guanosine, 3,Ä≤-O-diphosphinicoguanylylimino-(3,Ä≤,Üí5,Ä≤)-2,Ä≤,5,Ä≤-dideoxy-	C20H25N11O8	547.48	C	-8.2
9030	C(n1c2c(nc1CC)c(C)cc(C)n2)c1ccc(cc1)c1c(S(=O)(=O)NC(=O)OCCCC)sc(CC(C)C)c1	151488-11-8	Carbamic acid, N-[[3-[4-[(2-ethyl-5,7-dimethyl-3H-imidazo[4,5-b]pyridin-3-yl)met	C30H38N4O4S2	582.78	C	-8.2
9031	C(=O)(NC(C(=O)C(=O)NCC=C)CCCC)[C@@H]1[C@@H]2[C@@H](C2(C)C)CN1C(=O)[C@@H](NC(=O)N[C@@H](CN1C(=O)CCCC1	1528752-79-5	3-Azabicyclo[3.1.0]hexane-2-carboxamide, 3-[(2S)-2-[[[[(1R)-1-[(2,5-dioxo-1-pyrr	C35H56N6O7	672.86	C	-8.2
9032	C(=O)(NC(CC1CCC1)C(=O)C(=O)NCC=C)[C@@H]1[C@@H]2[C@@H](C2(C)C)CN1C(=O)[C@@H](NC(=O)NC(C)(C)C)C(C)(C)C	1528753-12-9	3-Azabicyclo[3.1.0]hexane-2-carboxamide, N-[1-(2-cyclopropylethyl)-2,3-dioxo-3-(	C39H62N6O7	726.95	C	-8.2
9033	O(C(OC(=O)OC)C(C)C)c1c(C(=O)NCc2ccc(F)cc2)nc(C(NC(=O)c2nnc(C)o2)(C)C)n(C)c1=O	1538604-05-5	Carbonic acid, 1-[[4-[[[(4-fluorophenyl)methyl]amino]carbonyl]-1,6-dihydro-1-met	C32H35FN6O8	650.65	C	-8.2
9034	O(C(OC(=O)OCC)C(C)C)c1c(C(=O)NCc2ccc(F)cc2)nc(C(NC(=O)c2nnc(C)o2)(C)C)n(C)c1=O	1538604-06-6	Carbonic acid, 1-[[4-[[[(4-fluorophenyl)methyl]amino]carbonyl]-1,6-dihydro-1-met	C26H31FN6O8	574.56	C	-8.2
9035	C(=O)(NCc1ccc(F)cc1)c1c(OCOC(=O)OC(C)C)c(=O)n(C)c(C(NC(=O)c2oc(C)nn2)(C)C)n1	1538604-08-8	Carbonic acid, cyclohexyl 1-[[4-[[[(4-fluorophenyl)methyl]amino]carbonyl]-1,6-di	C30H37FN6O8	628.65	C	-8.2
9036	O(C(OC(=O)CC)C(C)C)c1c(C(=O)NCc2ccc(F)cc2)nc(C(NC(=O)c2nnc(C)o2)(C)C)n(C)c1=O	1538604-26-0	4-Pyrimidinecarboxamide, 5-[1-(benzoyloxy)-2-methylpropoxy]-N-[(4-fluorophenyl)m	C31H33FN6O7	620.63	C	-8.2
9037	C(n1c2c(nc1CC)c(C)cc(C)n2)c1ccc(cc1)c1c(S(=O)(=O)NC(=O)CCCCC)ccc(C(CC)(C)C)c1	154551-83-4	Hexanamide, N-[[4,Ä≤,Ä≤-[(2-ethyl-5,7-dimethyl-3H-imidazo[4,5-b]pyridin-3-yl)me	C35H38N4O3S	594.77	C	-8.2
9038	C(n1c2c(nc1CC)c(C)cc(C)n2)c1ccc(cc1)c1c(S(=O)(=O)NC(=O)COCC)ccc(C(C)(C)C)c1	154552-24-6	Acetamide, N-[[5-(1,1-dimethylpropyl)-4,Ä≤-[(2-ethyl-5,7-dimethyl-3H-imidazo[4,5	C32H40N4O4S	576.75	C	-8.2
9039	C(n1c2c(nc1CC)c(C)cc(C)n2)c1ccc(cc1)c1c(S(=O)(=O)NC(=O)NCCCC)ccc(c1)c1ccccc1	154552-42-8	[1,1,Ä≤-Biphenyl]-2-sulfonamide, N-[(butylamino)carbonyl]-5-ethoxy-4,Ä≤-[(2-eth	C30H37N5O4S	563.71	C	-8.2
9040	C(n1c2c(nc1CC)c(C)cc(C)n2)c1ccc(cc1)c1c(S(=O)(=O)NC(=O)NCCCC)ccc(C(C)(C)C)c1	154552-44-0	[1,1,Ä≤-Biphenyl]-2-sulfonamide, N-[(butylamino)carbonyl]-5-(1,1-dimethylpropyl)	C33H43N5O3S	589.79	C	-8.2
9041	C(n1c2c(nc1CC)c(C)cc(C)n2)c1ccc(cc1)c1c(S(=O)(=O)NC(=O)c2c(OCC)cccc2)ccc(C(C)C)c1	154552-94-0	Benzamide, N-[[5-butyl-4,Ä≤-[(2-ethyl-5,7-dimethyl-3H-imidazo[4,5-b]pyridin-3-y]	C36H40N4O4S	624.79	C	-8.2
9042	C(n1c2c(nc1CC)c(C)cc(C)n2)c1ccc(cc1)c1c(S(=O)(=O)NC(=O)c2c(OCC)cccc2)ccc(CC(C)C)c1	154552-96-2	Benzamide, N-[[5-butoxy-4,Ä≤-[(2-ethyl-5,7-dimethyl-3H-imidazo[4,5-b]pyridin-3-y	C36H40N4O5S	640.79	C	-8.2
9043	C(n1c2c(nc1CC)c(C)cc(C)n2)c1ccc(cc1)c1c(S(=O)(=O)NC(=O)c2c(OCC)cccc2)ccc(c1)c1ccccc1	154553-01-2	Benzamide, 2-ethoxy-N-[[5-ethoxy-4,Ä≤-[(2-ethyl-5,7-dimethyl-3H-imidazo[4,5-b]py	C34H36N4O5S	612.74	C	-8.2
9044	C(n1c2c(nc1CC)c(C)cc(C)n2)c1ccc(cc1)c1c(S(=O)(=O)NC(=O)c2c(OCC)cccc2)ccc(CN(CC)CC)c1	154553-05-6	Benzamide, N-[[5-[(dimethylamino)methyl]-4,Ä≤-[(2-ethyl-5,7-dimethyl-3H-imidazo	C35H39N5O4S	625.78	C	-8.2
[truncated: 7,536,384 more chars]
